# Transcriptional Variation of Diverse Enteropathogenic *Escherichia coli* Isolates under Virulence-Inducing Conditions

**DOI:** 10.1128/mSystems.00024-17

**Published:** 2017-07-25

**Authors:** Tracy H. Hazen, Sean C. Daugherty, Amol C. Shetty, James P. Nataro, David A. Rasko

**Affiliations:** a Institute for Genome Sciences, University of Maryland School of Medicine, Baltimore, Maryland, USA; b Department of Microbiology and Immunology, University of Maryland School of Medicine, Baltimore, Maryland, USA; c Department of Pediatrics, University of Virginia School of Medicine, Charlottesville, Virginia, USA; University of California, Irvine

**Keywords:** EPEC, *Escherichia coli*, diversity, transcriptome

## Abstract

Enteropathogenic *Escherichia coli* (EPEC) bacteria are a diverse group of pathogens that cause moderate to severe diarrhea in young children in developing countries. EPEC isolates can be further subclassified as typical EPEC (tEPEC) isolates that contain the bundle-forming pilus (BFP) or as atypical EPEC (aEPEC) isolates that do not contain BFP. Comparative genomics studies have recently highlighted the considerable genomic diversity among EPEC isolates. In the current study, we used RNA sequencing (RNA-Seq) to characterize the global transcriptomes of eight tEPEC isolates representing the identified genomic diversity, as well as one aEPEC isolate. The global transcriptomes were determined for the EPEC isolates under conditions of laboratory growth that are known to induce expression of virulence-associated genes. The findings demonstrate that unique genes of EPEC isolates from diverse phylogenomic lineages contribute to variation in their global transcriptomes. There were also phylogroup-specific differences in the global transcriptomes, including genes involved in iron acquisition, which had significant differential expression in the EPEC isolates belonging to phylogroup B2. Also, three EPEC isolates from the same phylogenomic lineage (EPEC8) had greater levels of similarity in their genomic content and exhibited greater similarities in their global transcriptomes than EPEC from other lineages; however, even among closely related isolates there were isolate-specific differences among their transcriptomes. These findings highlight the transcriptional variability that correlates with the previously unappreciated genomic diversity of EPEC.

**IMPORTANCE** Recent studies have demonstrated that there is considerable genomic diversity among EPEC isolates; however, it is unknown if this genomic diversity leads to differences in their global transcription. This study used RNA-Seq to compare the global transcriptomes of EPEC isolates from diverse phylogenomic lineages. We demonstrate that there are lineage- and isolate-specific differences in the transcriptomes of genomically diverse EPEC isolates during growth under *in vitro* virulence-inducing conditions. This study addressed biological variation among isolates of a single pathovar in an effort to demonstrate that while each of these isolates is considered an EPEC isolate, there is significant transcriptional diversity among members of this pathovar. Future studies should consider whether this previously undescribed transcriptional variation may play a significant role in isolate-specific variability of EPEC clinical presentations.

## Abstract (precis)

Recent studies have demonstrated that there is considerable genomic diversity among EPEC isolates; however, it is unknown if this genomic diversity leads to differences in their global transcription. This study used RNA-Seq to compare the global transcriptomes of EPEC isolates from diverse phylogenomic lineages. We demonstrate that there are lineage- and isolate-specific differences in the transcriptomes of genomically diverse EPEC isolates during growth under *in vitro* virulence-inducing conditions. This study addressed biological variation among isolates of a single pathovar in an effort to demonstrate that while each of these isolates is considered an EPEC isolate, there is significant transcriptional diversity among members of this pathovar. Future studies should consider whether this previously undescribed transcriptional variation may play a significant role in isolate-specific variability of EPEC clinical presentations.

## INTRODUCTION

Enteropathogenic *Escherichia coli* (EPEC) is a leading cause of moderate to severe diarrhea among young children, particularly in developing countries (1). EPEC strains are characterized by the presence of the locus of enterocyte effacement (LEE) region and are subclassified as typical EPEC (tEPEC) or atypical EPEC (aEPEC) by the presence or absence of the bundle-forming pilus (BFP), respectively (2–4). The LEE region is a characteristic feature of both EPEC and enterohemorrhagic *E. coli* (EHEC), which includes the O157:H7 and non-O157 EHEC that are a significant cause of foodborne outbreaks in the United States (2, 5).

The LEE region encodes the intimin adherence protein, the translocated intimin receptor protein (Tir), and a type III secretion system (T3SS), which have been identified as major components of the EPEC and EHEC virulence mechanisms (2, 3, 6–8). The intimin and Tir proteins, as well as the T3SS, are involved in attachment to host cells and the translocation of effector proteins that confer changes in the host cell (6, 7). BFP is a type IV pilus that is involved in the localized adherence to the host cell, which is a unique feature that is found in tEPEC and not in aEPEC or EHEC (2, 3).

Global views of bacterial transcriptomes have provided insight into genome-wide virulence gene regulation, as well as the identification of novel virulence factors (9–12). RNA sequencing (RNA-Seq) provides an unbiased high-throughput sequencing approach that can capture the global transcriptional response of an organism under particular growth or environmental conditions (13–16). RNA-Seq has been used to investigate the global transcriptomes of numerous pathogenic bacteria (9, 17–21) and also to examine the transcriptome of different *E. coli* pathovars, specifically EHEC (21–23) and ETEC (9). RNA-Seq has also been used to investigate differences in the transcriptional responses of genomically diverse commensal and environmental *E. coli* isolates during growth under multiple laboratory conditions (24).

The EPEC virulence mechanisms have been extensively characterized for a limited number of EPEC isolates (25–30). However, considering the genomic diversity that has been described in recent years for EPEC isolates (31–33), this raised the issue of whether EPEC isolates have greater variability in the transcription of their virulence mechanisms. To investigate whether there are differences in the global transcriptomes of three frequently studied EPEC reference isolates, we previously used RNA-Seq to identify genes that were coordinately expressed under multiple laboratory growth conditions (34). Included among the isolates examined were the frequently studied E2348/69 and B171 archetype EPEC strains, which have been used in many previous studies to characterize EPEC virulence mechanisms (25, 26, 35–40). The transcriptional study by Hazen et al. included the global transcriptomes of the archetype aEPEC isolate E110019 (27) and of a recently described tEPEC isolate, C581-05, which belongs to a *E. coli* phylogenomic lineage that is different from that of the archetype strains (31, 34). The global transcriptomes of these four EPEC isolates were compared during growth in multiple medium types and three different growth phases (early exponential, late exponential, and early stationary phase) (34). The findings demonstrated that these EPEC isolates exhibited isolate-, medium-, and growth-phase-specific differences in their global transcriptomes (34). Surprisingly, there were also differences in the timing of expression of the LEE genes, the key defining virulence factor, among these four EPEC isolates (34).

The current report describes the application of RNA-Seq to investigate differences in the global transcriptomes of nine phylogenomically diverse EPEC isolates representing eight EPEC lineages from three *E. coli* phylogroups (B2, B1, and A) (34, 41, 42). Among the EPEC isolates analyzed in the current study were E2348/69, B171, C581-05, and E110019 (34). The additional five EPEC isolates in the current study originated from the Global Enteric Multicenter Study (GEMS) (43) and belong to unexplored EPEC phylogenomic lineages. By including GEMS isolates, we were able to consider the genomic variation of contemporary circulating EPEC isolates that have caused illness within the last decade in countries in Africa, where tEPEC is associated with increased lethality among infants with diarrheal illness (44). Inclusion of the more recent and genomically diverse human EPEC isolates is critical for our deeper understanding of the virulence mechanisms of modern isolates that have not been passaged many times in the laboratory, which could potentially introduce mutations that would alter virulence phenotypes. For instance, the EPEC archetype strain, E2348/69, was initially isolated in 1969 and, as we recently described, there are multiple versions of this strain in existence that exhibit genomic differences along with altered growth and virulence phenotypes (45).

This report identifies phylogroup-, lineage-, and isolate-specific differences in the global transcriptomes of genomically diverse EPEC isolates. Overall, this report addresses the biological variation among the isolates of a single *E. coli* pathovar and demonstrates that, while each of these isolates is considered an EPEC isolate, and a great deal is known about some of the isolates, very little is known about the transcriptional diversity of the members of this pathovar or of the *E. coli* species in general.

## RESULTS

### Phylogenomic analysis of the representative EPEC isolates.

**FIG 1:**
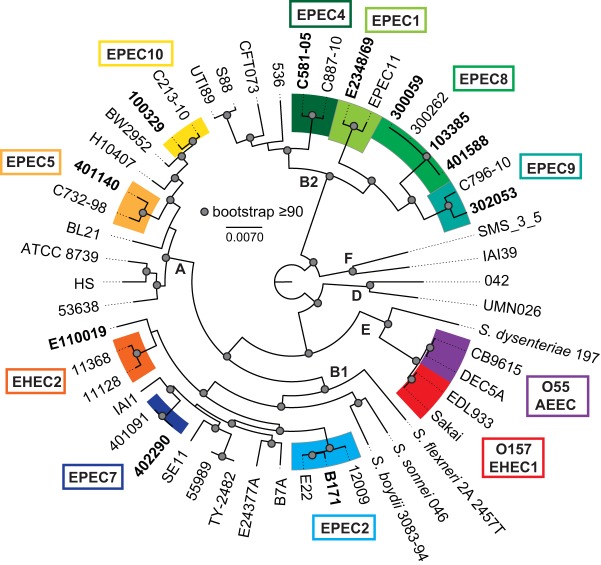
Phylogenomic analysis of representative EPEC isolates. The genome sequences of representative EPEC isolates were compared with those of a reference collection of diverse *E. coli* and *Shigella* isolates that had been sequenced previously and are available in the public domain. The genomes were aligned using Mugsy (95) as previously described (31, 94). A 1.9-Mb aligned region was used to generate a maximum-likelihood phylogeny with 100 bootstrap replicates using RAxML v.7.2.8 (97), and the results were visualized using FigTree v.1.4.2 (http://tree.bio.ed.ac.uk/software/figtree/). The representative EPEC isolates that were analyzed using RNA-Seq are indicated in bold.

Phylogenomic analysis of a collection of *E. coli* isolates classified as EPEC based on the presence of the LEE and/or BFP regions has previously demonstrated that isolates from the EPEC pathovar occur in numerous phylogenomic lineages (31–33). The purpose of this study was to investigate the impact of genomic diversity on the global transcriptional regulons of isolates in diverse phylogenomic lineages during growth under laboratory conditions that promote virulence factor expression (46–48). RNA-Seq was used to analyze the global transcriptomes of nine EPEC isolates (E2348/69, B171, C581-05, 401140, 402290, 401588, 302053, 100329, and E110019) representing eight EPEC phylogenomic lineages and three *E. coli* phylogroups (A, B1, and B2) (Fig. 1; Table 1). The EPEC isolates analyzed in this study included the frequently studied E2348/69 and B171 EPEC reference isolates from the EPEC1 and EPEC2 phylogenomic lineages, respectively, as well as archetype aEPEC isolate E110019 (34) (Fig. 1). Also, we included a previously characterized isolate, C581-05 (31, 34), from the EPEC4 lineage (49). The nonarchetype EPEC isolates from the GEMS (32, 43) were selected as genomic representatives of other diverse EPEC phylogenomic lineages (32). Where available, we selected tEPEC isolates that contained a complete or nearly complete BFP region. These isolates belong to the previously identified EPEC5 phylogenomic lineage or to the newly defined EPEC7, EPEC8, EPEC9, and EPEC10 phylogenomic lineages, which were designated based on phylogenomic analysis of 70 EPEC isolates from the GEMS (32).

**TABLE 1:** Number of shared or unique genes identified using LS-BSR analysis^a^

Isolate ID	Location of isolation	Date of isolation	Phylogenomic lineage^b^	Phylogroup^c^	No. of isolate-specific gene clusters^d^
E2348/69	England	1969	EPEC1	B2	206
B171	United States	1983	EPEC2	B1	131
C581-05	Africa	NK	EPEC4	B2	141
401140	Kenya	2008	EPEC5	A	90
402290	Kenya	2009	EPEC7	B1	169
401588	Kenya	2008	EPEC8	B2	212
302053	Mozambique	2009	EPEC9	B2	53
100329	The Gambia	2008	EPEC10	A	62
E110019	Finland	1987	None	B1	164

a ID, identifier; NK, date of isolation not known.

b The total number of core gene clusters (LS-BSR ≥ 0.8) in all EPEC isolates.

c The numbers of gene clusters with significant similarity (LS-BSR ≥ 0.8) in all genomes of one phylogroup that were divergent (LS-BSR < 0.8, ≥ 0.4) or absent (LS-BSR < 0.4) from genomes of other phylogroups were 44 (phylogroup A), 62 (phylogroup B1), and 128 (phylogroup B2).

d The isolate-specific genes are those identified in one genome with an LS-BSR ≥ 0.8 and in the other genomes with an LS-BSR < 0.4.

### Comparative genomic analysis of the EPEC isolates.

To understand the potential transcriptional variation among the genomically diverse EPEC isolates, we first performed a comparative genomic large-scale BLAST score ratio (LS-BSR) analysis (50). Genomic comparison of the nine EPEC genome sequences identified 2,989 gene clusters that had significant similarity (LS-BSR ≥ 0.8) in all genomes, representing a conserved core EPEC genome (Table 1). The number of phylogroup-specific gene clusters identified among the EPEC isolates ranged from 44 to 128 (Table 1). The phylogroup-specific gene clusters were those identified with significant similarity (LS-BSR ≥ 0.8) in all genomes of each of the *E. coli* phylogroups (A, B1, or B2) that were divergent (LS-BSR < 0.8 and ≥ 0.4) or absent (LS-BSR < 0.4) from the genomes of the other phylogroups. The number of isolate-specific gene clusters that were specific to one of the genomes and were absent (LS-BSR < 0.4) from all other genomes ranged from 53 to 212 (Table 1). These numbers of genes were similar to those identified for other comparisons of *E. coli* (31, 32, 51).

### Characteristics of the RNA-Seq samples.

The global transcriptomes of each of the nine EPEC isolates were characterized using RNA-Seq during growth in lysogeny broth (LB) or Dulbecco’s modified Eagle’s medium (DMEM) to determine the interisolate and interphylogenomic lineage variation. Growth in nutrient-limited DMEM has previously been demonstrated to induce virulence factor expression of EPEC compared to growth in nutrient-rich LB, which does not promote expression of the majority of the primary EPEC virulence factors (46–48). The global transcriptomes were also characterized for three EPEC8 phylogenomic lineage isolates under the same growth conditions (Fig. 1; see also Table S2 in the supplemental material). These comparisons provide insight into the variation of the global transcriptomes of isolates of the same phylogenomic lineage under the same growth conditions. RNA-Seq was performed on a total of 44 RNA samples, generating approximately 2.6 billion Illumina sequence reads across all samples. The number of total reads and number of mapped reads are provided in Table S2. The number of reads that mapped to protein-coding (genic) regions ranged from 5 to 15 million reads per sample (14% to 56% of the total mapped reads), with an average of 10 million reads per sample mapping to protein-coding genes (Table S2). The approximate sequence coverage of mapped reads to each genome across all the samples ranged from 104× to 303×, with an average level of coverage of 202× (Table S2).

10.1128/mSystems.00024-17.4 TABLE S1  Primers used in this study. Download TABLE S1, PDF file, 0.1 MB. Copyright © 2017 Hazen et al. 2017 Hazen et al. This content is distributed under the terms of the Creative Commons Attribution 4.0 International license.

10.1128/mSystems.00024-17.5 TABLE S2  Characteristics of the RNA-Seq generated in this study. Download TABLE S2, PDF file, 0.05 MB. Copyright © 2017 Hazen et al. 2017 Hazen et al. This content is distributed under the terms of the Creative Commons Attribution 4.0 International license.

**FIG 2:**
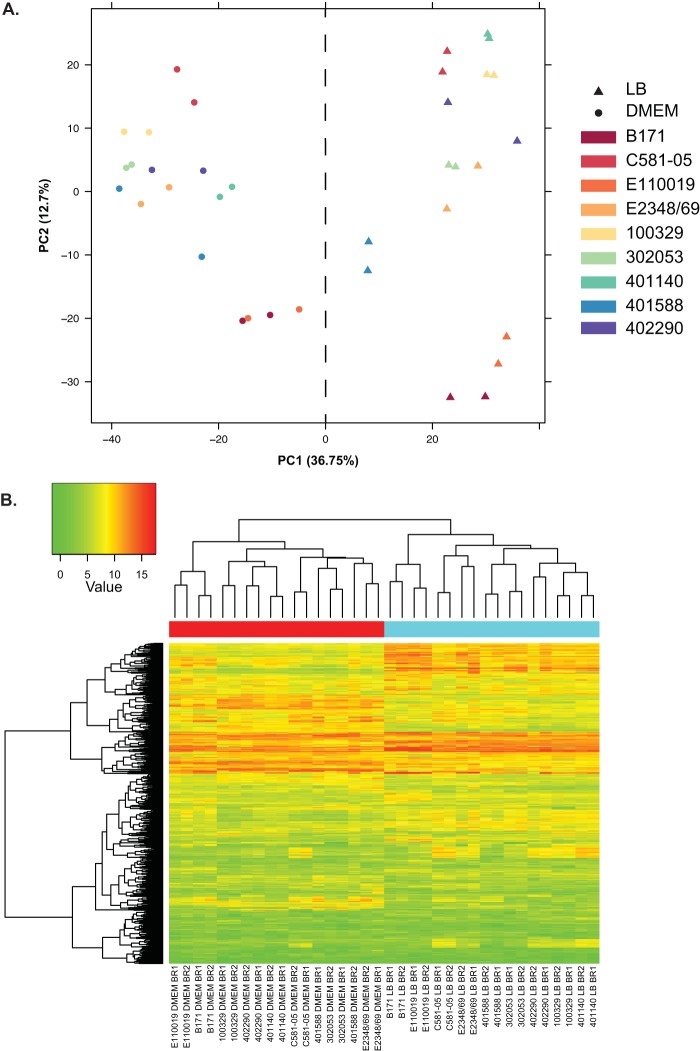
Principal-component analysis and hierarchical cluster analysis of the RNA-Seq samples examined in this study. (A) Principal-component analysis of the expression of gene clusters identified in the EPEC isolates, comparing all RNA-Seq samples analyzed. The first (PC1) and second (PC2) principal components were visualized in a scatterplot to demonstrate the clustering of the strains by gene content and gene expression. The comparative matrix is composed of the maximum normalized expression values for each gene cluster in each of the isolates and under each of the conditions examined for the gene clusters that were present among all isolates. The first component contains 36.75% of the variation, and the second component is responsible for 12.7% of the variation. The samples are colored by isolate, and symbols represent LB or DMEM as indicated in the legend. (B) A heatmap with clustering analysis of the expression values was constructed for the 674 LS-BSR gene clusters that were present in all of the EPEC isolates and had the greatest standard deviations of expression values. The normalized gene expression values were used to compute the standard deviation for each LS-BSR gene cluster across all samples. The heatmap was constructed using the R package gplots v2.11.0. A red label at the top of the heatmap designates the DMEM samples, while a blue label designates the LB samples.

Principal-component analysis of the RNA-Seq reads for each sample of the nine EPEC isolates demonstrated there was a correlation among the biological replicates for each medium type (Fig. 2A). This demonstrated that the medium type had a significant impact on the global transcriptomes of the EPEC, as all of the LB samples (triangles in Fig. 2A) and all of the DMEM samples (circles in Fig. 2A) grouped together (Fig. 2A). Hierarchical cluster analysis of the read counts for 674 gene clusters that were identified in all nine EPEC isolates and exhibited the greatest deviation of expression also demonstrated a similar correlation among the biological replicates and samples of the same medium type (Fig. 2B, red and blue labels).

### Identification of the core EPEC transcriptional regulon.

The total number of genes of each EPEC isolate that exhibited significant differential expression during growth in DMEM compared to LB ranged from 334 to 572 (Table 2), representing between ~6% and 10% of the genome. Among these, there were 145 to 278 genes that exhibited increased expression and 134 to 315 genes that exhibited decreased expression (Table 2). A total of 242 to 394 of the differentially expressed genes of each EPEC isolate were identified as being part of the conserved EPEC core gene set (LS-BSR ≥ 0.8 in all nine of the EPEC isolates) (Table 2). Interestingly, the core genes represented more than half of the total differentially expressed genes of each of the EPEC isolates examined (Table 2); however, there were only 21 conserved core genes that exhibited significant differential expression in all nine of the EPEC isolates (Table S3). The majority of the remaining core genes that were differentially expressed during growth in DMEM compared to LB encoded proteins involved in central metabolism such as glycerol-3-phosphate and biotin biosynthesis (Table S3). This demonstrates that a small number of highly conserved genes comprise the core regulon of genomically diverse EPEC isolates under these virulence-inducing laboratory conditions. Furthermore, this core regulon does not include most of the virulence factors hypothesized to be regulated under these growth conditions.

10.1128/mSystems.00024-17.6 TABLE S3  Differential expression of core, phylogroup-specific, and isolate-specific genes. Download TABLE S3, PDF file, 0.1 MB. Copyright © 2017 Hazen et al. 2017 Hazen et al. This content is distributed under the terms of the Creative Commons Attribution 4.0 International license.

**TABLE 2:** Number of genes that were differentially expressed in the EPEC isolates examined in this study

Isolate ID	Phylogenomic lineage^a^	Phylogroup^a^	LFC ≥ 2^b^	LFC ≤ −2^b^	Total DE genes^c^	No. of DE genes of core clusters^d^	No. of DE genes of phylogroup- specific clusters^e^	No. of DE genes of isolate- specific clusters^f^	Total DE sRNAs^g^
E2348/69	EPEC1	B2	180	251	431	253	18	10	6
B171	EPEC2	B1	220	255	475	249	2	4	10
C581-05	EPEC4	B2	162	235	397	291	12	1	17
401140	EPEC5	A	145	189	334	242	1	3	22
402290	EPEC7	B1	243	268	511	354	1	2	7
401588	EPEC8	B2	267	134	401	253	11	4	12
302053	EPEC9	B2	228	315	543	392	15	0	21
100329	EPEC10	A	278	294	572	394	1	2	30
E110019	None	B1	172	246	418	280	0	7	9

a The phylogenomic lineage and phylogroup are those that have been previously described (Hazen et al. [32], Jaureguy et al. [41], Tenaillon et al. [42]).

b LFC, log_2-fold_ change of the genes that exhibited significant (LFC ≥ 2 or ≤ −2 and FDR ≤ 0.05) differential expression (DE).

c The total number of genes that exhibited significant (LFC ≥ 2 or ≤ −2 and FDR ≤ 0.05) DE.

d The total number of core gene clusters (LS-BSR ≥ 0.8 in all genomes) was 2,989.

e The number of clusters in all EPEC genomes of one phylogroup that were divergent or absent (LS-BSR < 0.8) from EPEC genomes of the other phylogroups were 44 (phylogroup A), 62 (phylogroup B1), and 128 (phylogroup B2).

f The isolate-specific genes are those that were in one genome with an LS-BSR ≥ 0.8 and in the other genomes with an LS-BSR < 0.4.

g The total number of sRNA that were previously investigated in *E. coli* by Raghavan et al. (60) and exhibited significant (LFC ≥ 2 or ≤ −2 and FDR ≤ 0.05) DE in each of the nine EPEC isolates.

**FIG 3:**
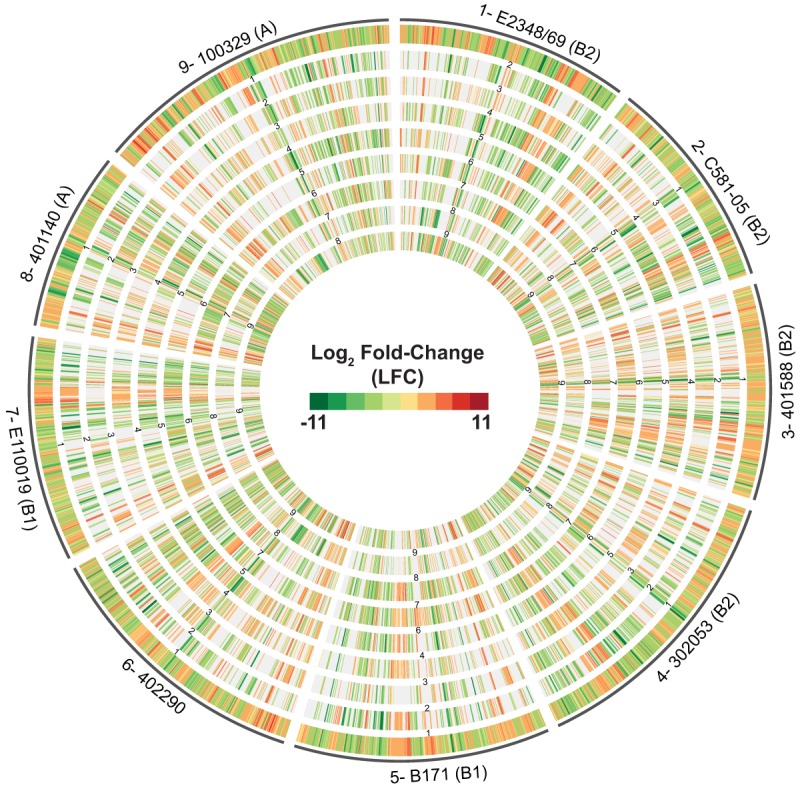
Comparison of the global transcriptomes of nine EPEC isolates. A circular plot of the log_2_-fold-change (LFC) values for genes that exhibited significant differential expression during exponential growth in DMEM compared to LB is shown. The outermost track contains all of the significant LFC values for each of the indicated nine EPEC isolates. The inner tracks are numbered to correspond to the same number of each EPEC isolate in the outermost track. The phylogroup that each EPEC isolate belongs to is indicated in parentheses following the isolate designation. For example, 5-B171 (B1) indicates that data tracks containing B171 data are labeled track 5 in all of the comparisons, while B171 is the isolate designation and B1 the phylogroup designation. The inner tracks contain the LFC values of genes of another of the EPEC isolates belonging to the same LS-BSR gene cluster as the genes in the outermost reference track. The genes that were not identified in the other EPEC isolates or did not exhibit significant differential expression are absent from the inner tracks.

Genes involved in biotin synthesis, including *bioD*, had increased expression in all nine EPEC isolates in both the RNA-Seq and quantitative reverse transcriptase PCR (qRT-PCR) analyses (Table S3; see also Fig. S1 in the supplemental material). Also, other genes involved in central metabolism (*nark*, *glpD*, and *treB*) exhibited decreased expression in all nine EPEC isolates during growth in DMEM compared to LB (Table S3; Fig. S1). Comparison of the LFC values of all differentially expressed genes in each of the EPEC isolates demonstrated there were regions within each genome that exhibited similar expression trends among multiple isolates but not all isolates (Fig. 3).

10.1128/mSystems.00024-17.3 FIG S1  qRT-PCR verification of RNA-Seq expression trends. The expression trends of selected genes that were determined by the use of RNA-Seq to have significant differential expression levels were analyzed using quantitative reverse transcription-PCR (qRT-PCR). The values represent the log_2_ of the relative fold differences (2^−ΔΔ*CT*^) between the results determined with DMEM samples and those determined with LB samples for the nine EPEC reference isolates (Table 1). The fold change value was calculated using two biological replicates for each gene and three technical replicates for each medium type. The error bars denote the standard deviations of the ΔΔ*C*_*T*_ values. The expression trends observed using qRT-PCR were consistent with those determined using RNA-Seq. Data are not shown for the isolates that did not contain a particular gene(s) (*cdtA*, *hmuV chuU*, and *shuA chuA*). Download FIG S1, PDF file, 0.1 MB. Copyright © 2017 Hazen et al. 2017 Hazen et al. This content is distributed under the terms of the Creative Commons Attribution 4.0 International license.

### Phylogroup- and isolate-specific gene expression.

Beyond the core conserved gene clusters, we wanted to identify the gene clusters that were present and transcriptionally active in the specific phylogroups, phylogenomic lineages, or only in individual isolates. In this study, the number of phylogroup-specific genes ranged from 44 to 128, depending on the EPEC isolate (Table 1); however, the number of genes that were phylogroup-specific and also exhibited altered expression ranged from 0 to 18 (Table 2), depending on the isolate. The phylogroup-specific gene clusters that were differentially expressed included genes encoding conserved hypothetical proteins and genes involved in iron acquisition (Table S3). We anticipated that we would find a greater number of genes that were identified as phylogroup specific, but this was not the case, suggesting a more significant contribution of isolate-specific genes to variations in the global transcriptomes.

The number of isolate-specific genes ranged from 53 to 212 in the nine representative EPEC isolates (Table 1) and included several previously characterized virulence genes (Table S3). The T3SS secreted effector gene, *espV*, was identified and found to be differentially expressed only in aEPEC isolate E110019, while the cytolethal distending toxin genes, *cdtAB*, were identified and found to be differentially expressed only in tEPEC isolate 401140 (Table S3). These results highlight the potential contribution of isolate-specific genes to the global transcriptomes of these genomically diverse EPEC isolates. Further investigation is necessary to determine whether particular isolate-specific genes are global transcriptional regulators that can explain differences in the transcription of highly conserved genes among the diverse EPEC isolates.

### Within-lineage conservation and variability of the global transcriptomes of EPEC8 isolates.

**FIG 4:**
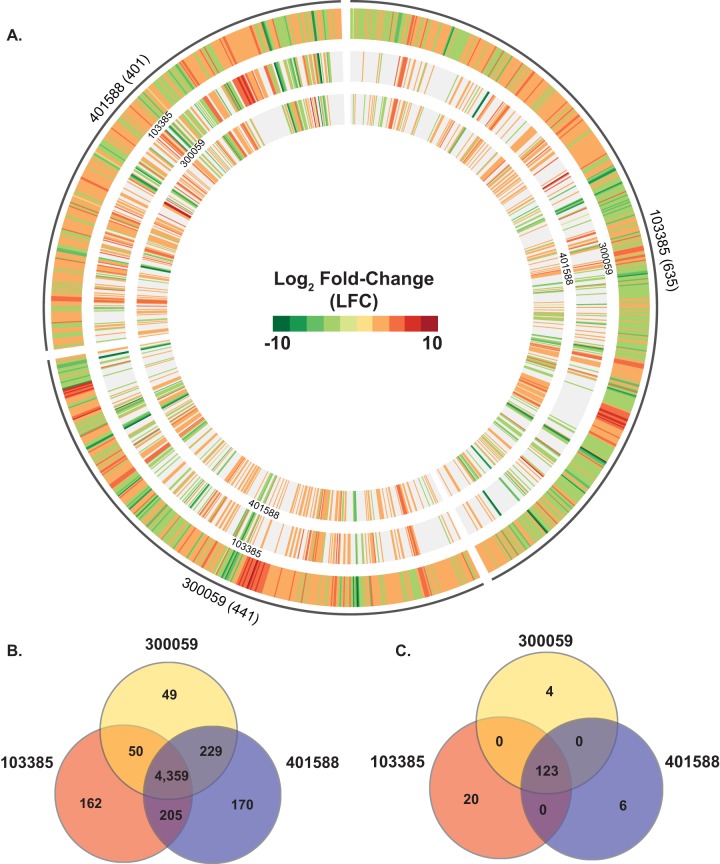
Comparison of the global transcriptomes of multiple EPEC isolates of the EPEC8 phylogenomic lineage. (A) Circular plot of the log_2_-fold-change (LFC) values for genes that exhibited significant differential expression during exponential growth in DMEM compared to LB. The outermost track contains all of the significant LFC values for one of the three EPEC8 isolates. The inner tracks contain the LFC values of genes of another of the EPEC8 isolates belonging to the same LS-BSR gene cluster as the genes in the outermost reference track. The genes that were not identified in the other EPEC8 isolates and/or did not exhibit significant differential expression are absent from the inner tracks. The number of genes that had significant differential expression is indicated in parentheses next to each isolate name. (B) The number of genes that were highly conserved (LS-BSR ≥ 0.8) in all four of the EPEC isolates is indicated in the center. The number of genes that were identified with significant similarity (LS-BSR ≥ 0.8) that also exhibited significant differential expression in two or three EPEC isolates is also designated. The number of isolate-specific genes indicates the genes that were identified with significant similarity in one EPEC isolate and that were divergent or absent from the other two EPEC8 isolates. (C) Venn diagram showing the number of genes differentially expressed for each of the EPEC8 isolates analyzed in this study grown to an OD_600_ of 0.5 in DMEM compared to LB. The number of core genes that were highly conserved (LS-BSR ≥ 0.8) in all three of the EPEC8 isolates that also exhibited significant differential expression in all of the EPEC8 isolates is indicated in the center. There were no genes that were present with significant similarity and also differentially expressed in only two of the three EPEC8 isolates. The number of isolate-specific genes indicates those genes that exhibited significant similarity (LS-BSR ≥ 0.8) that were divergent or absent (LS-BSR < 0.8) from the other two EPEC8 isolates and also exhibited significant differential expression during growth to an OD_600_ of 0.5 in DMEM compared to LB.

We also used RNA-Seq to examine two additional EPEC8 isolates (103385 and 300059) in order to investigate whether there was variation in the global transcriptomes of EPEC isolates within the same phylogenomic lineage (Table S2; Fig. 4). Comparison of the three EPEC8 isolates (103385, 300059, and 401588) using LS-BSR identified 4,359 core gene clusters (Fig. 4B). In contrast, there were only 49, 162, and 170 isolate-specific gene clusters that were present with significant similarity (LS-BSR ≥ 0.8) in only one of the three EPEC8 isolates and were divergent (LS-BSR < 0.8 and ≥ 0.4) or absent (LS-BSR < 0.4) from the other EPEC8 isolates (Fig. 4B). The number of gene clusters that were present with significant similarity (LS-BSR ≥ 0.8) in two of the EPEC8 isolates but divergent in (LS-BSR < 0.8 but ≥ 0.4) or absent from (LS-BSR < 0.4) the third isolate ranged from 50 to 229 (Fig. 4B). These data demonstrate that while the isolates were EPEC and were within the same phylogenomic lineage, they still contained considerable genomic variation. The total number of genes that exhibited significant differential expression in each of the three EPEC8 isolates ranged from 401 to 635 (Fig. 4A). Of these, there were 123 core gene clusters that exhibited significant differential expression in all three of the EPEC8 isolates (Fig. 4C). As anticipated, this number of common and expressed genes is greater than that determined for all of the EPEC isolates but were still relatively few. Among these core and expressed gene clusters were genes encoding predicted proteins involved in metabolism and iron uptake and genes encoding numerous hypothetical proteins (Table S4). There were 4, 6, and 20 gene clusters that were identified in only one of the EPEC8 isolates that also had significant differential expression (Fig. 4C). Among these isolate-specific genes were genes encoding putative proteins involved in iron uptake and also phage-associated genes (Table S4). These findings demonstrate that while there is greater similarity in the global transcriptomes of EPEC isolates belonging to the same phylogenomic lineage, the isolate-specific genes that are unique to each of the isolates contribute to differences in their global transcriptomes.

10.1128/mSystems.00024-17.7 TABLE S4  Differential expression of core, phylogroup-specific, and unique genes from EPEC8 isolates. Download TABLE S4, PDF file, 0.1 MB. Copyright © 2017 Hazen et al. 2017 Hazen et al. This content is distributed under the terms of the Creative Commons Attribution 4.0 International license.

### Differential expression of known EPEC virulence genes.

**FIG 5:**
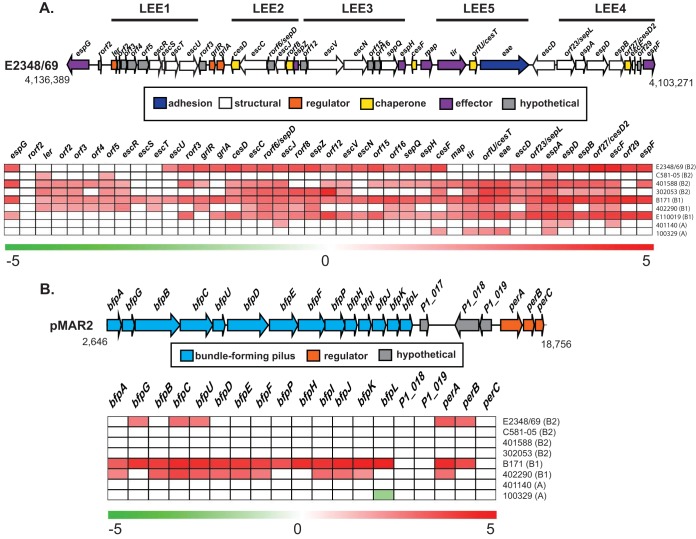
Differential expression analysis of genes carried by the LEE and BFP regions in each of the representative EPEC isolates analyzed. (A) Diagram of the genetic structure and results of differential expression analysis of LEE genes for the nine representative EPEC isolates examined (E2348/69, B171, C581-05, 401588, 401140, 402290, 302053, 100329, and E110019) during exponential growth (OD_600_ = 0.5) in DMEM compared to LB. The values in the heatmap are the significant log_2_-fold-change (LFC) values for LEE genes. (B) Diagram of the gene organization and the RNA-Seq LFC values of genes carried by the BFP operon of the eight EPEC isolates that contained BFP (E2348/69, B171, C581-05, 401588, 401140, 402290, 302053, and 100329). The expression values in the heatmap are significant LFC values for BFP genes.

To determine the transcriptional patterns of known EPEC virulence genes in these genomically diverse EPEC isolates, we compared the trends of expression of the LEE and BFP genes in each of the isolates (Fig. 5). Similar to what was observed with the four archetype EPEC isolates (34), there were differences in the expression levels of LEE genes among the nine EPEC isolates (Fig. 5A). As expected, the majority of the LEE genes exhibited significantly increased expression in DMEM compared to LB for all of the tEPEC isolates belonging to phylogroups B1 and B2 (Fig. 5A). An exception to this trend was EPEC4 isolate C581-05 of phylogroup B2, which showed significant differential expression of only 3 of the 41 LEE genes under these conditions (Fig. 5A). Also, there were only 4 to 5 of the 41 LEE genes that exhibited significant differential expression in the two EPEC isolates from phylogroup A, 401140 and 100329 (Fig. 5A). Interestingly, the *bfp* genes of four of the eight tEPEC isolates, three from phylogroup B2 and one from phylogroup A, did not exhibit significant differential expression under these conditions (Fig. 5B). In contrast, nearly all of the *bfp* and *per* genes exhibited increased expression in EPEC isolates B171 and 402290, both of *E. coli* phylogroup B1 (Fig. 5B). These findings highlight the phylogroup-specific regulation of these important virulence factors.

**FIG 6:**
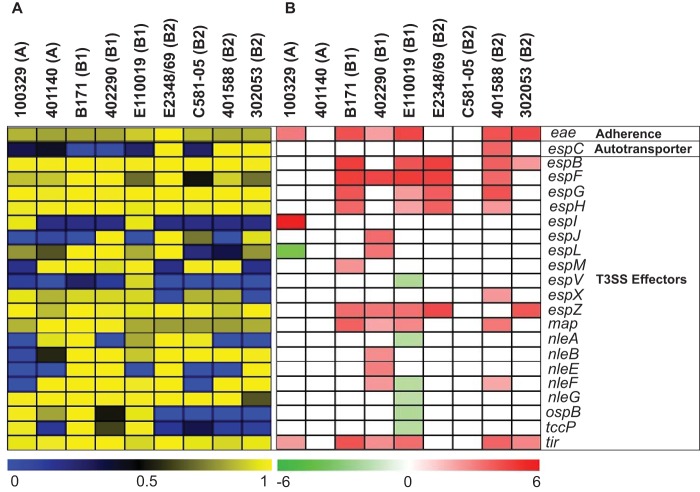
Differential expression analysis of known virulence genes of EPEC. The phylogroup that each EPEC isolate belongs to is indicated in parentheses. (A) Heatmap of LS-BSR values indicating the presence or absence of known EPEC virulence genes in the genomes of each of the EPEC isolates analyzed. Genes present with significant similarity are indicated by yellow, genes with divergent similarity are indicated by black, and genes that are absent are indicated by blue. (B) Heatmap of the log_2_-fold-change (LFC) values for known virulence genes of EPEC that exhibited significant differential expression during exponential growth in DMEM compared to LB. The color gradient indicates decreased expression (green) or increased expression (red) of the virulence genes, while white indicates a gene that either was not present in the isolate or did not exhibit significant differential expression.

In addition to investigating differences in the expression of the LEE and BFP regions, we interrogated differences in expression of other characterized EPEC virulence factors such as autotransporters and other non-LEE secreted effectors (Fig. 6). Of the autotransporter-encoding genes that were detected with significant differential expression in the EPEC genomes, *espC* was identified as having significantly increased expression only in EPEC isolate 401588 (Fig. 6). Also, of the LEE and non-LEE T3SS effectors that were detected in the genomes, more than half had increased expression during growth in DMEM compared to LB (Fig. 6). Only two of the T3SS effectors exhibited discordant trends of differential expression among the EPEC isolates (Fig. 6). These included *espL*, which had increased expression in EPEC isolate 402290 but decreased expression in EPEC isolate 100329, and *nleF*, which had increased expression in EPEC isolates 402290 and 401588 but decreased expression in aEPEC isolate E110019 (Fig. 6). Interestingly, most of the T3SS effectors that had decreased expression (*espV*, *nleA*, *nleF*, *nleG*, *ospB*, and *tccP*) were identified in aEPEC isolate E110019 (Fig. 6). Further analysis is necessary to determine whether these effectors would also exhibit decreased expression in additional aEPEC isolates during growth under these same conditions.

### Semiconserved genes of the global EPEC regulon.

There were additional genes belonging to LS-BSR gene clusters that were identified among many but not all of the EPEC isolates and that were thus not identified as part of the conserved core regulon even though they exhibited similar expression trends in two or more of the EPEC isolates (Table S3; see also Data Set S2 in the supplemental material). For example, there were proteins involved in biosynthesis of flagella that exhibited decreased expression in six of the nine EPEC isolates (Table S5). This is consistent with previous reports that genes encoding flagellar proteins have an expression pattern opposite that of virulence factors under these virulence gene-inducing growth conditions (52–55).

10.1128/mSystems.00024-17.1 DATA SET S1  Fasta file of nucleotide sequences of each LS-BSR gene cluster. Download DATA SET S1, PDF file, 5.6 MB. Copyright © 2017 Hazen et al. 2017 Hazen et al. This content is distributed under the terms of the Creative Commons Attribution 4.0 International license.

10.1128/mSystems.00024-17.2 DATA SET S2  Differentially expressed genes of all the isolates (each represented on a different sheet). Download DATA SET S2, XLSX file, 0.7 MB. Copyright © 2017 Hazen et al. 2017 Hazen et al. This content is distributed under the terms of the Creative Commons Attribution 4.0 International license.

10.1128/mSystems.00024-17.8 TABLE S5  Differential expression of select nonvirulence genes. Download TABLE S5, PDF file, 0.1 MB. Copyright © 2017 Hazen et al. 2017 Hazen et al. This content is distributed under the terms of the Creative Commons Attribution 4.0 International license.

Similar to the differentially expressed conserved core genes, many of the differentially expressed semiconserved core genes encode proteins involved in metabolism, such as the *his* genes encoding predicted proteins for histidine synthesis, which had increased expression in nearly all of the EPEC isolates (Table S5). Among the genes that were conserved in all of the EPEC isolates but had significant differential expression in only a limited number of isolates were genes encoding predicted proteins involved in processes involving resistance to the host such as colanic acid biosynthesis (Table S5). The colanic acid biosynthesis genes had increased expression in EPEC isolates E2348/69 and 100329, which belong to two different phylogroups (B2 and A, respectively) (Table S5). Whether these genes have a unique role in pathogenesis of these EPEC isolates is not yet clear.

There were a total of 2,382 LS-BSR gene clusters that were identified with significant similarity (LS-BSR ≥ 0.8) in one or more of the EPEC isolates that contained a predicted protein domain of secreted or membrane-associated proteins. Among these gene clusters, there were 595 that had significant differential expression in one or more of the EPEC isolates (Data Set S2). Of these, 32 gene clusters were also differentially expressed in one or more of the EPEC isolates analyzed (Table S6). There were 26 gene clusters that had increased expression in DMEM compared to LB, while only six had decreased expression (Table S6). Included among these were genes encoding predicted proteins previously known to be secreted or membrane-associated proteins such as type IV pilus, a sucrose porin, and T3SS proteins (Table S6). There were also nine gene clusters encoding hypothetical proteins that could be further investigated for their role in the virulence mechanism of EPEC (Table S6).

10.1128/mSystems.00024-17.9 TABLE S6  Differential expression of the LS-BSR gene clusters that are only in EPEC and encode proteins with predicted secreted or surface-associated domains. Download TABLE S6, PDF file, 0.05 MB. Copyright © 2017 Hazen et al. 2017 Hazen et al. This content is distributed under the terms of the Creative Commons Attribution 4.0 International license.

### Differential expression of known sRNAs of *E. coli*.

To determine whether there was significant differential expression among small RNAs (sRNAs) in the genomically diverse EPEC isolates, the RNA-Seq reads were mapped to the previously identified *E. coli* sRNAs (56–60) that could be identified in each EPEC genome. The total number of sRNAs that exhibited significant differential expression in the EPEC isolates ranged from 6 to 30 sRNAs per isolate (Table 2). Unlike what we observed for some of the protein-coding genes that exhibited significant differential expression, the sRNAs examined did not have any phylogroup-specific expression trends.

Interestingly, the sRNA DsrA exhibited significant differential expression in only three of the EPEC isolates, all of which were part of phylogroup B2 (Table S7). Similar to some of the other sRNAs, DsrA had an expression trend in EPEC isolate C581-05 that was different from that of other isolates from phylogroup B2 (Table S7). The expression of *dsrA* was decreased in C581-05 in DMEM compared to LB, whereas it was increased in isolates 103385 and 300059 under the same conditions (Table S7). DsrA has been described for its role in regulating a number of global transcriptional pathways, including the RpoS-mediated stress response mechanism (61–63), which then potentially alters the expression of other pathways, such as the LEE region of EHEC and EPEC (64).

10.1128/mSystems.00024-17.10 TABLE S7  Differential expression of known *E. coli* sRNAs. Download TABLE S7, PDF file, 0.05 MB. Copyright © 2017 Hazen et al. 2017 Hazen et al. This content is distributed under the terms of the Creative Commons Attribution 4.0 International license.

The sRNAs GlmY and GlmZ (65) both had increased expression during growth in DMEM compared to LB (Table S7). GlmY had significant differential expression in all of the EPEC isolates, except aEPEC isolate E110019 (Table S7). In comparison, GlmZ had significant differential expression only in EPEC isolate 302053 (Table S7). In *E. coli* K-12, GlmY and GlmZ regulate expression of *glmS*, which encodes the enzyme glucosamine-6-phosphate required for hexosamine metabolism, which generates precursor molecules for the synthesis of amino sugars that are used to make peptidoglycan and lipopolysaccharides (65). Meanwhile, GlmY and GlmZ in EHEC were found to regulate acid resistance, tryptophan metabolism, adhesion, and the expression of non-LEE effectors (22, 66). Previous studies demonstrated that *glmZ* is constitutively expressed (67), whereas the expression of *glmY* is regulated by QseEF in EHEC (66, 68). Our findings demonstrate that *glmY* had increased expression during growth in DMEM compared to LB in EPEC and that *glmZ* expression was also increased, but it was not great enough to be considered significant by our criteria for all but one of the EPEC isolates, 302053 (Table S7).

Another sRNA that had similar expression trends in nearly all of the EPEC isolates was RyhB, which exhibited increased expression in DMEM versus LB in all but three of the EPEC isolates (Table S7). RyhB is an sRNA that regulates iron metabolism, is required for siderophore production by uropathogenic *E. coli* (69, 70), and was previously described as having increased expression during growth in minimal media (60, 71). We observed similar results of increased expression of *ryhB* in minimal media (DMEM) compared to nutrient-rich media (LB) for 8 of the total of 11 EPEC isolates examined (Table S7). Interestingly, genes involved in iron acquisition (*hmuV* and *shuA*) had increased expression in the EPEC isolates belonging to phylogroup B2 (Table S3). To our knowledge, the role of RyhB in regulation of iron metabolism in EPEC has yet to be determined. Future studies are needed to determine the role of these sRNAs in regulating gene expression of EPEC.

## DISCUSSION

Previous studies investigating the transcriptional networks and regulators of EPEC pathogenesis have focused on a limited number of genes in a limited number of isolates (46, 72–76). These genes are typically within pathogenicity islands or within other regions containing known virulence factors or have been described as global regulators of virulence in other bacteria (46, 72–76). In particular, the transcriptional regulation of the LEE and BFP regions has been extensively studied (46, 47, 72–83). Regulation of EPEC virulence mechanisms is known to involve numerous transcription factors, which are influenced by environmental conditions, including cell density (2, 46, 78, 79, 82–86). No previous studies of EPEC virulence had used an unbiased global sequencing approach such as RNA-Seq to identify all genes that are simultaneously expressed under virulence gene-inducing growth conditions.

In addition to investigating a small number of genes at a time, previous studies also investigated EPEC virulence mechanisms using primarily a select few isolates (E2348/69, B171, E22, E110019) (25, 26, 35–40), which represent only 2 EPEC phylogenomic lineages (EPEC1 and EPEC2) of the more than 10 that have been described (31, 32). The results of our previous study demonstrated that there are differences in the global transcriptomes of these frequently studied EPEC isolates during growth under virulence gene-inducing laboratory conditions (34), indicating there were significant differences associated with even the archetype isolates. However, the archetype isolates were isolated in the past and may not represent the modern EPEC isolates.

In the current study, we demonstrated that EPEC isolates representing genomically diverse lineages can have limited conservation with respect to their transcriptional responses under virulence-inducing laboratory growth conditions (see Table S3 in the supplemental material). Our study investigated the global transcriptional response of EPEC under a single growth condition (high-glucose DMEM) that induces the expression of virulence among EPEC isolates (46–48). However, other conditions have also been described that induce EPEC virulence, including growth in static cultures (87) or with different glucose concentrations (88). The focus of our study was on investigating variation in the transcriptional response of genomically diverse EPEC isolates under a single set of conditions known to induce virulence gene expression, and we would anticipate that there would also be significant variation observed if other growth conditions were investigated. Given the considerable genomic diversity identified among the EPEC isolates analyzed, it was not surprising that there were only 21 conserved gene clusters that were present that also exhibited significant differential expression in all nine of the EPEC isolates (Table S3). Nearly all of these genes had known functions associated with central metabolism. In contrast, many of the known EPEC virulence factors were not identified as part of the core EPEC regulon under the examined growth conditions due to genetic diversity or differences in the timing of transcription of these genes (Fig. 5 and 6). This included genes within the LEE and BFP regions that are critical to EPEC virulence (Fig. 5). Many of these genes exhibited phylogroup-specific differences in expression, a result that was also observed in our previous studies (34). Although all the isolates in the current study were identified as EPEC, this study highlighted the variability of the transcriptional responses of these isolates. The underlying regulatory mechanisms of EPEC virulence have been assumed to be similar in all isolates based on the study of a limited number of genes in a limited number of archetype isolates; however, the current study highlighted that this assumption represents an underestimation of isolate-specific genomic content that may contribute to transcriptional differences and variable clinical presentations.

The phylogenomically diverse EPEC isolates analyzed in this study contained unique isolate-specific genomic content that was differentially expressed under the virulence-inducing conditions, representing an isolate-specific transcriptional response (Table S3). The cytolethal distending toxin, identified only in EPEC isolate 401140 of phylogroup A (Table S3; see also Fig. S1 in the supplemental material), represents an accessory virulence factor that has been identified in multiple pathovars of *E. coli*, including some EPEC isolates (89–92), but is absent from many EPEC isolates and is not considered a major component of the EPEC virulence mechanism (4). Comparison of the regulons of multiple EPEC isolates belonging to the same phylogenomic lineage (EPEC8) demonstrated a greater number of conserved genes that were present and also differentially expressed in all three of these isolates (Fig. 4; Table S4). This finding is not surprising, considering that there is greater genomic similarity among these EPEC isolates than among EPEC isolates from different lineages or phylogroups. However, it was interesting that there was unique genomic content in each of these EPEC8 isolates that exhibited differential expression under the virulence-inducing conditions. This finding demonstrates that there was additional diversification of EPEC isolates within the same phylogenomic lineage that is either directly or indirectly linked to EPEC transcriptional regulation.

Further investigation is needed to determine what role, if any, these genes have in coordinating gene expression of EPEC. This study characterized the biological variation among the isolates of a single pathovar and demonstrated that, while each of these isolates is considered an EPEC isolate based on genomic features, they exhibited differences in their global transcriptomes, and very little is known about how the diversity of their transcriptional responses can result in differing clinical presentations. The observed transcriptional variation in responses to host and other signals may be more biologically significant for the observed clinical presentation than the genome content alone. Continued investigation of differences in the global transcriptomes of EPEC isolates in the presence of other pathogenic *E. coli* strains, commensal *E. coli* strains, and other members of the gut microbiome is ongoing and will be required to fully comprehend the importance of this transcriptional variability.

## MATERIALS AND METHODS

### Bacterial isolates and media.

The EPEC isolates examined in this study (Table 1) were previously characterized, and their genome sequences are publicly available (25, 26, 31, 32, 93). The EPEC isolates were grown in lysogeny broth (LB) media (Difco) or in Dulbecco’s modified Eagle’s medium (DMEM) supplemented with 4.5 g/liter of glucose (Gibco).

### Phylogenomic analysis.

The genomes of the EPEC isolates analyzed in this study were compared with 40 previously sequenced *E. coli* and *Shigella* genomes by whole-genome phylogenomic analysis as previously described (31, 94). The genomes were aligned using Mugsy (95), and homologous blocks were concatenated using the bx-python toolkit (https://bitbucket.org/james_taylor/bx-python). The columns that contained one or more gaps were removed using Mothur (96). The resulting 1.98-Mb aligned region from each of the genomes was used to construct a maximum-likelihood phylogeny with 100 bootstrap replicates using RAxML v7.2.8 (97). The phylogeny was constructed using the GTR model of nucleotide substitution with the gamma model of rate heterogeneity and 100 bootstrap replicates. The phylogeny was then visualized using FigTree v1.4.2 (http://tree.bio.ed.ac.uk/software/figtree/).

### Large-scale BLAST score ratio (LS-BSR) analysis.

The nine EPEC isolates (E2348/69, B171, C581-05, 401140, 402290, 401588, 302053, 100329, and E110019) represent eight different EPEC phylogenomic lineages (Table 1) and were examined along with two additional EPEC8 isolates (300059 and 103385). All of these isolates were subjected to comparative genomics using LS-BSR as previously described (31, 50, 98). Briefly, the predicted protein-coding genes of each genome that had ≥80% nucleotide identity to each other were assigned to gene clusters using uclust (99). The representative sequences of each gene cluster were translated and the amino acid sequences were compared to those of each genome using TBLASTN (100) with composition-based adjustment turned off. The bit scores were used to generate a BSR value indicating the detection of each gene cluster in each of the genomes by dividing the score of a gene compared to a genome by the score of the gene compared to its own sequence. The genomes of ED1a, SE11, HS, and K-12 strain MG1655 were also included in the LS-BSR analysis to provide nonpathogenic reference isolates for the identification of conserved *E. coli* genes, as well as pathogen-specific genes. The nucleotide sequences of the gene clusters are included in Data Set S1 in the supplemental material.

The functions of the proteins encoded by each gene cluster were predicted using the Institute for Genome Sciences (IGS) prokaryotic annotation pipeline (101). The presence of protein and functional domains characteristic of membrane-associated or secreted proteins (analyzed using TMHMM, SignalP, and an in-house script that searches for motifs common in outer membrane proteins) or of lipoproteins (analyzed using TMH, SignalP I, and SignalP II]) among the proteins encoded by each gene cluster was predicted using the indicated algorithms and the IGS prokaryotic annotation pipeline (101).

### RNA isolation and sequencing.

The EPEC isolates were grown overnight in LB and were inoculated at 1:100 into 50 ml of LB, or of DMEM supplemented with 4.5 g/liter glucose, in a 250-ml flask. The cultures were grown at 37°C with shaking (225 rpm) to an optical density at 600 nm (OD_600_) of approximately 0.5, corresponding to the exponential-growth phase. Total RNA was isolated and prepared for sequencing from the cell pellet using a Ribopure bacteria kit (Ambion) and treated with Ribopure DNase I to remove contaminating DNA. The samples were then treated with a Turbo DNA-free kit (Ambion) to ensure that all contaminating DNA was removed. RNA samples were verified to be DNA-free by quantitative PCR (qPCR) analysis for the conserved *rpoA* gene as previously described (34). The DNA-free RNA samples were submitted for library construction using an Ovation prokaryotic RNA-Seq system (NuGen) and were sequenced using 100-bp paired-end sequencing at the Institute for Genome Sciences Genome Resource Center on an Illumina HiSeq platform.

### RNA-Seq analyses.

The Illumina reads generated for each RNA sample were analyzed and compared using an in-house Ergatis-based (102) RNA-Seq analysis pipeline as previously described (34). Briefly, the RNA-Seq reads were aligned to the corresponding genome sequences using Bowtie (103), and the number of reads that aligned to the protein-coding regions and intergenic regions was determined using HTSeq (104). The differential expression of each gene across the biological replicates for DMEM compared to LB was determined using DESeq (105). The log_2_-fold-change (LFC) values were calculated for the DMEM samples compared to the LB samples of each EPEC isolate. The gene expression data were then filtered for further analysis using the following criteria: LFC ≥ 2 or ≤ −2 and false-discovery rate (FDR) ≤ 0.05. Genes that met these criteria were identified as having significant differential expression during growth in DMEM compared to LB. The differential expression of the sRNAs was examined by detecting all of the previously identified sRNAs (56–60) in each of the EPEC genomes using BLAST analysis. The differential expression of the identified sRNA regions was then determined as described above for the protein-coding genes.

Circular displays of the differential expression data were generated using Circos v. 0.67-6 (106). The genes that exhibited significant LFC values and belonged to the same LS-BSR gene cluster were aligned in the plots for each of the EPEC isolates. Heat maps of the significant LFC values for the LEE and BFP genes and other virulence factors of each EPEC isolate were generated using MeV (107).

Correlation of the read counts for all of the RNA-Seq samples, including each of the two biological replicates, was examined as previously described (34). Expression values were normalized using DESeq v1.10.1 (105). The conserved LS-BSR gene clusters were used to compute the eigenvectors by principal-component analysis. The first and second principal components were displayed in a scatterplot to visualize the clustering of the RNA-Seq samples by isolate and medium type. The analysis was performed using R statistical package v2.15.2, and the data were visualized using gplots v2.11.0.

The normalized gene expression values were also used to compute the standard deviation for each LS-BSR cluster across all samples, and the 674 LS-BSR gene clusters showing the greatest standard deviations of expression values were used to generate a heatmap of the samples. The heatmap was generated using R statistical package v2.15.2, which in turn used DESeq v1.10.1 for normalization and gplots v2.11.0 for visualization.

### Quantitative reverse transcriptase PCR (qRT-PCR).

The trends of differential expression of selected genes during growth in DMEM compared to LB were verified for the genes listed in Table S1 in the supplemental material using previously described qRT-PCR methods (34, 108). Briefly, RNA was reverse transcribed and primed with random hexamers to generate cDNA using a SuperScript III first-strand synthesis system for RT-PCR (Invitrogen). The cDNA was diluted 1:20 into nuclease-free water (Ambion) before analysis with qPCR was performed. The qPCR was performed on the reverse-transcribed RNA samples by the use of SYBR green master mix (Life Technologies, Inc.) with 10-µl reaction mixtures comprised of the following: 5 µl of 2× SYBR master mix, 1 µl of each of the 5 µM forward and reverse primers (Table S1), 1 µl of nuclease-free water (Ambion), and 2 µl of cDNA diluted 1:20 with nuclease-free water. Triplicate reactions were performed for each cDNA template and primer combination. The reactions were cycled using a 384-well plate on a 7900HT Fast real-time PCR system (Applied Biosystems) and a two-step reaction with an initial incubation performed at 50°C for 2 min and 95°C for 10 min and then 40 cycles of 95°C for 15 s and 60°C for 1 min, followed by a dissociation stage. The cycle threshold (*C*_*T*_) values were calculated using Applied Biosystems software. The *C*_*T*_ values of the biological replicates were averaged, and the standard deviation was calculated. The *C*_*T*_ value for each of the target genes of each sample was normalized by subtracting from it the *C*_*T*_ value of the constitutively expressed RNA polymerase alpha subunit gene, *rpoA*, resulting in the Δ*C*_*T*_ value of a particular gene for each sample. The difference between the expression level of a target gene (ΔΔ*C*_*T*_) in the DMEM samples and its expression level in the LB samples was then calculated by subtracting the Δ*C*_*T*_ of the LB sample from the Δ*C*_*T*_ of the DMEM sample. The fold difference in the levels of expression of a particular gene in DMEM and in LB was determined by calculating the 2^−ΔΔ*CT*^ value. The difference in expression is represented in the figures as the log_2_ (2^−ΔΔ*CT*^) value for each gene in DMEM compared to LB. The error bars indicate the standard deviations of the ΔΔ*C*_*T*_ values. Differences in the exact fold change values between the RNA-Seq and the qRT-PCR data were observed; however, this result was most likely due to the differences in the methodology required for the amplification, library construction, and sequencing involved in RNA-Seq versus a focused and optimized qRT-PCR assay.

### Data availability.

All raw data generated by RNA-Seq analysis have been deposited in the Short Read Archive (SRA) under the accession numbers listed in Table S2, and the expression data were deposited in GEO under accession number GSE73885.
