# Supplementary material for: Transcriptional Variation of Diverse Enteropathogenic Escherichia coli Isolates under Virulence-Inducing Conditions
Source: mSystems. 2017 Jul 25;2(4):e00024-17. doi: 10.1128/mSystems.00024-17 (PMC5527300; doi:10.1128/mSystems.00024-17)
Supplement: TABLE S1 [file sys004172117st4.pdf]

Table S1. Primers used in this study

Gene	Predicted Protein Function	Protein Accession No.[a]	Primer ID	Sequence 5'-3'	Source
rpoA	RNA polymerase, alpha subunit	CAS11106.1	Ec-rpoA-F	TGTAGGCAATACGCTCCACA	(Sahl *et al.* 2012)
			Ec-rpoA-R	GGTTATGTGCCGGCTTCTAC	"
eae	Intimin, attachment protein	YP_002331401.1	eae_361F	TATAGTGCCTTACCTGTCTTAGGTTCGG	(Hazen *et al*. 2015)
			eae_495R	ATAATTTAGAGCCTTGTCATCGGTCGTG	"
narK	nitrite extrusion protein 1	CAS08895.1	narK_47F	CAGATTGGCGACCGGAAGATCCTG	This study
			narK_178R	CCGCAACAGCGCTGAACAACAT	
cdtA	cytolethal distending toxin	AAA18785.1	cdtA_97F	GAATTGGTTGGAATCCCTGGACAAGG	"
			cdtA_241R	GAACAGGTCTGGCCCAAATAGTAAGC	
hmuV/chuU	hemin import ATP-binding protein HmuV/ChuU	CAS11296.1	hmuV_122F	CTACGCTGCTTCGCCAGCTAAC	"
			hmuV_277R	GTACGCTGAATGGAAACGCCATGTG	
shuA/chuA	outer membrane heme receptor ShuA/ChuA	CAS11291.1	shuA_82F	GCTACTGAAACCATGACCGTTACGGC	"
			shuA_223R	CAGGAACATGACGCAGCAAATCAGTGG	
bioD	dethiobiotin synthase	CAS08278.1	bioD_19F	GTCACCGGAACGGATACCGAAG	"
			bioD_211R	TTGCGTAATCCAGCTGCAGGC	
glpD	aerobic glycerol-3-phosphate dehydrogenase	CAS11218.1	glpD_155F	GCCTGCGCTACCTTGAGCACTAT	"
			glpD_311R	CCAATGCGAATCATCCACGCCG	
treB	PTS system trehalose-specific EIIBC component	CAS12114.1	treB_49F	CGAGGTGAGTACAATCGGGATGATGATG	"
			treB_217R	GATGATTGGTTCTGGTCTGGCAGG	

[a]Primers were selected by comparing with the sequences from all EPEC genomes analyzed; however, the protein accession number is provided for the respective gene from the E2348/69 genome or other EPEC genome available in GenBank.

Supplemental References.

1. Sahl JW, Rasko DA. 2012. Analysis of global transcriptional profiles of enterotoxigenic *Escherichia coli* isolate E24377A. Infect Immun **80:**1232-1242.

2. Hazen TH, Daugherty SC, Shetty A, Mahurkar AA, White O, Kaper JB, Rasko DA. 2015. RNA-Seq analysis of isolate- and growth phase-specific differences in the global transcriptomes of enteropathogenic Escherichia coli prototype isolates. Front Microbiol 6.
