# Supplementary material for: Transcriptional Variation of Diverse Enteropathogenic Escherichia coli Isolates under Virulence-Inducing Conditions
Source: mSystems. 2017 Jul 25;2(4):e00024-17. doi: 10.1128/mSystems.00024-17 (PMC5527300; doi:10.1128/mSystems.00024-17)
Supplement: TABLE S2 [file sys004172117st5.pdf]

Table S2. Characteristics of the RNA-Seq generated in this study

Isolate ID	EPEC Phylogenomic Lineage	Phylogroup	Condition[a]	Replicate	Total Reads	Total Reads Mapped	No. of Reads Mapped to Genic Regions (%)[b]	No. of Reads Mapped to Intergenic Regions (%)[c]	Coverage of Reads Mapped to Genic Regions[d]	SRA Accession No.
EPEC of										
E2348/69	EPEC1	B2	LB	1	39,347,430	25,579,676	11,428,999 (44.68)	14,150,677 (55.32)	229	SRX1322473
				2	43,218,880	28,221,680	8,139,133 (28.84)	20,082,547 (71.16)	163	SRX1322474
			DMEM	1	90,223,668	64,341,630	10,191,714 (15.84)	54,149,916 (84.16)	204	SRX1322475
				2	78,280,450	52,692,826	14,627,528 (27.76)	38,065,298 (72.24)	293	SRX1322476
B171	EPEC2	B1	LB	1	50,584,352	28,468,286	14,219,909 (49.95)	14,248,377 (50.05)	284	SRX1322477
				2	44,770,910	23,920,488	13,113,212 (54.82)	10,807,276 (45.18)	262	SRX1322478
			DMEM	1	44,629,450	24,494,208	13,758,397 (56.17)	10,735,811 (43.83)	275	SRX1322479
				2	46,997,680	25,875,712	12,777,427 (49.38)	13,098,285 (50.62)	256	SRX1322480
C581-05	EPEC4	B2	LB	1	68,153,706	45,326,156	9,364,384 (20.66)	35,961,772 (79.34)	187	SRX1322481
				2	86,191,504	57,582,684	12,564,542 (21.82)	45,018,142 (78.18)	251	SRX1322482
			DMEM	1	60,758,124	37,669,542	11,847,071 (31.45)	25,822,471 (68.55)	237	SRX1322483
				2	84,509,240	56,850,714	12,689,079 (22.32)	44,161,635 (77.68)	254	SRX1322484
401140	EPEC5	A	LB	1	98,122,142	62,935,928	8,578,167 (13.63)	54,357,761 (86.37)	172	SRX481602
				2	109,658,950	68,745,070	9,294,333 (13.52)	59,450,737 (86.48)	186	SRX481643
			DMEM	1	143,164,326	93,428,500	13,369,618 (14.31)	80,058,882 (85.69)	267	SRX481650
				2	68,560,186	43,607,642	7,605,173 (17.44)	36,002,469 (82.56)	152	SRX481651, SRX481652
402290	EPEC7	B1	LB	1	41,672,396	25,888,056	5,213,854 (20.14)	20,674,202 (79.86)	104	SRX481833
				2	38,086,026	23,856,680	5,224,613 (21.9)	18,632,067 (78.1)	104	SRX481834
			DMEM	1	43,796,848	27,200,176	6,266,921 (23.04)	20,933,255 (76.96)	125	SRX481835
				2	57,575,912	38,939,098	9,174,051 (23.56)	29,765,047 (76.44)	183	SRX481836
401588	EPEC8	B2	LB	1	60,512,394	41,119,036	9,432,707 (22.94)	31,686,329 (77.06)	189	SRX481847
				2	53,305,308	37,264,176	9,353,308 (25.1)	27,910,868 (74.9)	187	SRX481859
			DMEM	1	48,767,194	31,691,680	9,536,027 (30.09)	22,155,653 (69.91)	191	SRX481860
				2	39,567,402	27,781,490	6,503,647 (23.41)	21,277,843 (76.59)	130	SRX481861
302053	EPEC9	B2	LB	1	46,792,656	29,683,240	11,410,237 (38.44)	18,273,003 (61.56)	228	SRX481725
				2	58,907,128	37,986,902	15,145,378 (39.87)	22,841,524 (60.13)	303	SRX481737
			DMEM	1	53,941,936	34,498,746	13,706,352 (39.73)	20,792,394 (60.27)	274	SRX481793
				2	40,593,700	25,749,842	10,799,484 (41.94)	14,950,358 (58.06)	216	SRX481825
100329	EPEC10	A	LB	1	52,180,888	34,414,562	5,674,961 (16.49)	28,739,601 (83.51)	113	SRX481826
				2	50,848,702	33,921,444	6,075,331 (17.91)	27,846,113 (82.09)	122	SRX481827
			DMEM	1	54,496,792	35,314,084	7,532,494 (21.33)	27,781,590 (78.67)	151	SRX481828
				2	48,423,314	32,160,070	6,046,093 (18.8)	26,113,977 (81.2)	121	SRX481832
E110019	None	B1	LB	1	55,671,402	30,777,290	11,532,251 (37.47)	19,245,039 (62.53)	231	SRX1322485
				2	65,738,936	31,867,460	11,615,689 (36.45)	20,251,771 (63.55)	232	SRX1322486
			DMEM	1	47,416,470	35,651,848	13,045,011 (36.59)	22,606,837 (63.41)	261	SRX1322487
				2	48,395,020	43,749,684	14,568,645 (33.3)	29,181,039 (66.7)	291	SRX1322488
Additional EPEC8 isolates analyzed										
103385	EPEC8	B2	LB	1	65,638,992	48,106,818	11,463,855 (23.83)	36,642,963 (76.17)	229	SRX481653, SRX481654
				2	57,801,512	42,287,912	10,166,014 (24.04)	32,121,898 (75.96)	203	SRX481657, SRX481658
			DMEM	1	63,427,586	46,387,156	10,400,000 (22.42)	35,987,156 (77.58)	208	SRX481659, SRX481660
				2	69,093,890	51,367,842	14,028,558 (27.31)	37,339,284 (72.69)	281	SRX481661, SRX481662
300059	EPEC8	B2	LB	1	43,069,956	28,668,440	6,602,342 (23.03)	22,066,098 (76.97)	132	SRX481708
				2	47,076,358	32,034,222	6,820,086 (21.29)	25,214,136 (78.71)	136	SRX481709
			DMEM	1	40,504,972	27,196,184	6,279,599 (23.09)	20,916,585 (76.91)	126	SRX481716
				2	52,405,042	36,483,322	8,259,824 (22.64)	28,223,498 (77.36)	165	SRX481722

[a]The EPEC isolates were grown to an OD_{600} of 0.5 in either Luria-Bertani (LB) broth or Dulbecco's Modified Eagle's Medium (DMEM) supplemented with high glucose (4.5 g/L).

[b]The genic regions are all protein-coding genes (CDS).

[c]The intergenic regions are all non-protein-coding regions of the genome, including rRNA genes.

[d]The approximate sequence coverage of the reads that mapped to genic regions was calculated as the total number of bases generated (101 bp Illumina reads) divided by an estimated *E. coli* genome size of 5 Gb.
