# Supplementary material for: Transcriptional Variation of Diverse Enteropathogenic Escherichia coli Isolates under Virulence-Inducing Conditions
Source: mSystems. 2017 Jul 25;2(4):e00024-17. doi: 10.1128/mSystems.00024-17 (PMC5527300; doi:10.1128/mSystems.00024-17)
Supplement: TABLE S3 [file sys004172117st6.pdf]

Table S3. Differential-expression of core, phylogroup-specific, and isolate-specific genes

Core Genes[b]

Cluster ID[a]	Gene	Predicted Protein Function	LFC[d]								
			100329	401140	B171	402290	E110019	E2348/69	C581-05	401588	302053
Cluster_22333	*cirA*	colicin I receptor	7.40	4.91	3.23	6.96	4.53	4.16	5.44	2.62	4.71
Cluster_22417	*glpA*	glycerol-3-phosphate dehydrogenase, anaerobic, A subunit	-7.80	-6.51	-7.06	-6.25	-5.52	-6.13	-7.77	-3.31	-9.21
Cluster_22418	*glpB*	glycerol-3-phosphate dehydrogenase, anaerobic, B subunit	-6.73	-6.51	-6.83	-6.88	-5.91	-6.62	-8.18	-3.86	-7.83
Cluster_24491	*treC*	alpha,alpha-phosphotrehalase	-3.30	-5.45	-8.30	-6.95	-7.06	-7.42	-6.46	-7.03	-8.27
Cluster_24492	*treB*	PTS system trehalose-specific EIIBC component	-4.37	-5.65	-7.04	-8.28	-8.16	-8.79	-7.36	-7.09	-9.40
Cluster_25541	*bioD*	dethiobiotin synthase	6.09	3.83	3.82	5.20	4.81	5.10	3.51	4.75	6.57
Cluster_2623	*glpT*	glycerol-3-phosphate transporter	-6.41	-7.54	-9.27	-7.91	-7.91	-8.07	-6.53	-4.32	-9.88
Cluster_2626	*glpC*	glycerol-3-phosphate dehydrogenase, anaerobic, C subunit	-5.44	-5.51	-5.54	-4.76	-5.64	-5.78	-6.03	-3.51	-6.06
Cluster_27245		MauM/NapG ferredoxin-type family protein	-6.11	-2.40	2.38	-2.76	3.19	-2.92	-3.12	-2.60	-6.22
Cluster_28414	*glpD*	aerobic glycerol-3-phosphate dehydrogenase	-2.85	-4.87	-5.86	-4.60	-5.58	-5.68	-5.87	-4.41	-6.39
Cluster_30847	*narK*	nitrite extrusion protein 1	-6.92	-5.92	-2.25	-7.04	-4.52	-6.39	-6.48	-4.32	-6.16
Cluster_33251		RbsD / FucU transport family protein	-6.80	-6.87	-5.32	-7.04	-4.73	-5.62	2.31	-6.44	-4.74
Cluster_33836	*deoC*	deoxyribose-phosphate aldolase	-3.70	-3.57	-3.62	-4.05	-2.65	-3.30	-3.20	-4.17	-3.98
Cluster_34698	*bioC*	malonyl-CoA O-methyltransferase BioC	7.50	2.44	2.47	5.57	4.41	4.55	4.36	4.36	5.83
Cluster_37491	*dtpB*	dipeptide and tripeptide permease B	-3.95	-2.69	-2.37	-3.29	-3.48	-3.72	-3.29	-3.30	-5.06
Cluster_37569	*dppF*	oligopeptide/dipeptide ABC transporter, ATP-binding, C-terminal domain protein	3.52	3.63	2.08	3.65	2.60	3.76	2.16	2.18	2.58
Cluster_23696	*dppD*	oligopeptide/dipeptide ABC transporter, ATP-binding, C-terminal domain protein	3.89	4.14	2.38	4.42	3.12	4.95	4.18	3.32	4.24
Cluster_39493	*bioB*	biotin synthase	6.45	3.94	2.73	6.10	3.85	5.02	5.86	4.76	6.29
Cluster_39494	*bioF*	8-amino-7-oxononanoate synthase	5.65	2.05	2.81	5.84	3.93	4.83	6.02	5.36	6.10
Cluster_40107		nitrate reductase, alpha subunit	-8.34	-6.28	-3.12	-6.45	-5.18	-8.36	-9.75	-5.98	-8.71
Cluster_40648	*purT*	phosphoribosylglycinamide formyltransferase 2	3.81	2.96	4.44	3.94	4.98	2.82	3.49	3.23	2.17

Phylogroup-Specific Genes[c]

Cluster ID	Gene	Predicted Protein Function	LFC[d]
100329 (A)			
Cluster_6463		outer membrane autotransporter barrel domain protein	2.37
401140 (A)			
Cluster_6463		outer membrane autotransporter barrel domain protein	2.66
B171 (B1)			
Cluster_4478		conserved hypothetical protein	2.64
Cluster_4503		conserved hypothetical protein	2.11
402290 (B1)			
Cluster_47272		conserved hypothetical protein	2.20
E110019 (B1)			
None		None	None
E2348/69 (B2)			
Cluster_20380		BAAT / Acyl-CoA thioester hydrolase C terminal family protein	-2.50
Cluster_22955		PfkB carbohydrate kinase family protein	-2.65
Cluster_23655	*hmuV*	hemin import ATP-binding protein HmuV	2.42
Cluster_24280		His Kinase A domain protein	-2.74
Cluster_27665		alkylhydroperoxidase AhpD family core domain protein	3.24
Cluster_27796		PTS system, sucrose-specific IIBC component	-7.00
Cluster_27797	*scrB*	sucrose-6-phosphate hydrolase	-5.67
Cluster_28491		periplasmic binding family protein	5.76
Cluster_28495		FecCD transport family protein	3.75
Cluster_28578	*yiaM*	2,3-diketo-L-gulonate TRAP transporter small permease protein YiaM	-3.96
Cluster_28657		PTS system, Fru family, IIB component domain protein	-2.39
Cluster_29027		bacterial capsule synthesis PGA_cap family protein	-3.28
Cluster_32975	*shuA*	outer membrane heme receptor ShuA	5.00
Cluster_39344		BNR/Asp-box repeat family protein	-2.00
Cluster_41853	*scrY*	sucrose porin	-6.62
Cluster_42553		putative heme utilization radical SAM enzyme HutW	5.12
Cluster_42555		NADH(P)-binding family protein	3.42
Cluster_42637		conserved hypothetical protein	-2.28
C581-05 (B2)			
Cluster_23201		ABC transporter family protein	2.49
Cluster_23655	*hmuV*	hemin import ATP-binding protein HmuV	2.96
Cluster_27665		alkylhydroperoxidase AhpD family core domain protein	5.13
Cluster_27796		PTS system, sucrose-specific IIBC component	-2.11
Cluster_28491		periplasmic binding family protein	4.63
Cluster_28495		FecCD transport family protein	3.27
Cluster_32975	*shuA*	outer membrane heme receptor ShuA	5.20
Cluster_42553		putative heme utilization radical SAM enzyme HutW	4.16
Cluster_42554		conserved hypothetical protein	2.92
Cluster_42555		NADH(P)-binding family protein	3.19
Cluster_43425	*iraD*	anti-adapter protein IraD	-2.62
401588 (B2)			
Cluster_23201		ABC transporter family protein	2.08
Cluster_23655	*hmuV*	hemin import ATP-binding protein HmuV	2.31
Cluster_25294		phage integrase family protein	-2.30
Cluster_27665		alkylhydroperoxidase AhpD family core domain protein	3.60
Cluster_28491		periplasmic binding family protein	2.33
Cluster_28495		FecCD transport family protein	2.17
Cluster_32975	*shuA*	outer membrane heme receptor ShuA	4.57
Cluster_42098		TonB-dependent siderophore receptor family protein	3.19
Cluster_42553		putative heme utilization radical SAM enzyme HutW	2.40
Cluster_42554		conserved hypothetical protein	2.97
Cluster_42555		NADH(P)-binding family protein	2.54
302053 (B2)			
Cluster_19907		periplasmic binding family protein	-2.19
Cluster_23201		ABC transporter family protein	2.43
Cluster_23655	*hmuV*	hemin import ATP-binding protein HmuV	3.01
Cluster_27665		alkylhydroperoxidase AhpD family core domain protein	4.06
Cluster_28495		FecCD transport family protein	2.48
Cluster_29027		bacterial capsule synthesis PGA_cap family protein	-2.15
Cluster_29029		bacterial capsule synthesis PGA_cap family protein	-2.09
Cluster_32975	*shuA*	outer membrane heme receptor ShuA	6.19
Cluster_42098		TonB-dependent siderophore receptor family protein	3.34
Cluster_42553		putative heme utilization radical SAM enzyme HutW	2.02
Cluster_42554		conserved hypothetical protein	2.25
Cluster_42555		NADH(P)-binding family protein	2.53
Cluster_42637		conserved hypothetical protein	-2.09
Cluster_43425	*iraD*	anti-adapter protein IraD	-3.10

Isolate-Specific Genes[c]

Cluster ID	Gene	Predicted Protein Function	LFC[d]
100329 (A)			
Cluster_6731		putative dNA-Binding protein G5P	2.62

Cluster_7611		Eag family protein	2.34
401140 (A)			
Cluster_34158		DDE superendonuclease family protein	-2.51
Cluster_38392		cytolethal distending toxin A/C family protein	3.82
Cluster_38393	*cdtB*	cytolethal distending toxin subunit B	3.80
B171 (B1)			
Cluster_4030		UTRA domain protein	-2.65
Cluster_4031		phosphoenolpyruvate-dependent sugar phosphotransferase system, EIIA 2 family protein	-2.72
Cluster_4032		PTS system, Lactose/Cellobiose specific IIB subunit	-2.88
Cluster_4611		conserved hypothetical protein	2.42
402290 (B1)			
Cluster_46067		glycosyl transferase 2 domain protein	2.37
Cluster_48546		glycosyltransferase like 2 family protein	2.08
E110019 (B1)			
Cluster_10956		conserved hypothetical protein	-2.69
Cluster_11127		conserved hypothetical protein	-2.06
Cluster_12490	*espV*	putative T3SS effector protein EspV	-2.43
Cluster_14838		conserved hypothetical protein	2.33
Cluster_14839		transcriptional regulatory, C terminal family protein	2.10
Cluster_14939	*col*	colicin-E2	2.05
Cluster_14941		lysis family protein	2.16
E2348/69 (B2)			
Cluster_17152		glycosyl transferase 11 family protein	2.43
Cluster_17153		glycosyl transferase 2 family protein	2.86
Cluster_17154		putative membrane protein	3.61
Cluster_17155		bacterial transferase hexapeptide family protein	2.77
Cluster_17156		polysaccharide biosynthesis family protein	3.49
Cluster_17212		mannitol dehydrogenase Rossmann domain protein	-3.93
Cluster_17481		putative predicted protein	3.47
Cluster_17640		putative predicted protein	2.65
Cluster_17644		putative predicted protein	2.68
Cluster_18911		putative predicted protein	-2.82
C581-05 (B2)			
Cluster_48767		conserved hypothetical protein	2.18
401588 (B2)			
Cluster_25495	*rtcB*	tRNA-splicing ligase RtcB	-2.04
Cluster_40811		Mu-like prophage FluMu gp41 family protein	3.21
Cluster_40813		phage major tail tube protein	4.20
Cluster_40830		conserved hypothetical protein	4.15
302053 (B2)			
None		None	None

[a]Cluster IDs are listed once for each comparison group, although there may be more than one gene in a single genome that belonged to the same cluster.
[b]Core genes were those identified in all of the EPEC genomes with an LS-BSR ≥0.8.
[c]Genes identified with significant similarity (LS-BSR ≥0.8) in all of the genomes of one phylogroup (phylogroup-specific) or only In one genome (isolate-specific) that were absent (LS-BSR <0.4) from the other EPEC isolates.
[d]LFC is Log_2 Fold-Change of the DMEM samples compared to the LB samples.
