# Supplementary figures and images for: Transcriptional Variation of Diverse Enteropathogenic Escherichia coli Isolates under Virulence-Inducing Conditions
Source: mSystems. 2017 Jul 25;2(4):e00024-17. doi: 10.1128/mSystems.00024-17 (PMC5527300; doi:10.1128/mSystems.00024-17)

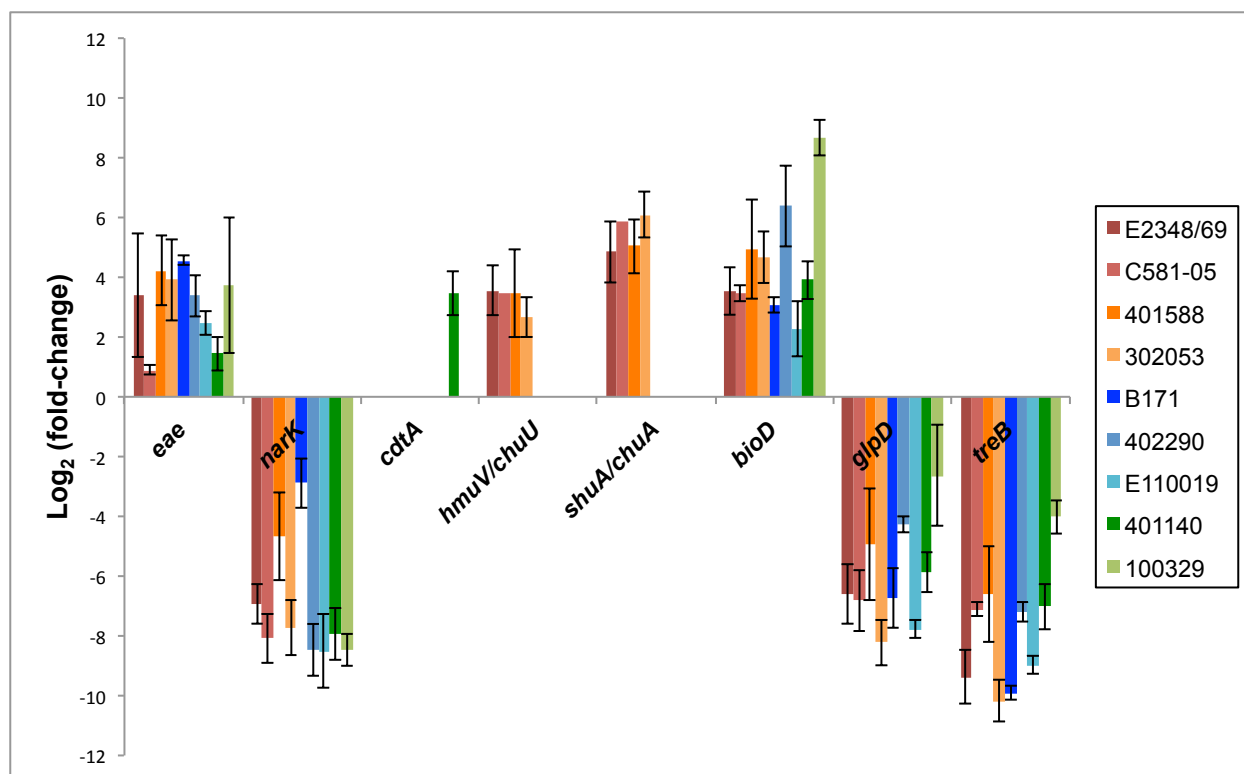

Supplement: FIG S1 [file sys004172117sf3.pdf]
