# Supplementary material for: Transcriptional Variation of Diverse Enteropathogenic Escherichia coli Isolates under Virulence-Inducing Conditions
Source: mSystems. 2017 Jul 25;2(4):e00024-17. doi: 10.1128/mSystems.00024-17 (PMC5527300; doi:10.1128/mSystems.00024-17)
Supplement: TABLE S4 [file sys004172117st7.pdf]

Table S4. Differential-expression of core, phylogroup-specific, and unique genes

		EPEC8 Core Genes[b]			
Cluster ID	Gene	Predicted Protein Function	LFC[a]		
			103385	401588	300059
Cluster_11698		conserved hypothetical protein	3.04	2.53	4.95
Cluster_11754	*osmC*	peroxiredoxin OsmC	4.32	3.53	4.61
Cluster_12188	*yebE*	inner membrane protein YebE	3.39	2.36	3.87
Cluster_12751	*lpxL*	lipid A biosynthesis lauroyl (or palmitoleoyl) acyltransferase family protein	3.38	2.17	2.19
Cluster_13818	*nikD*	nickel import ATP-binding protein NikD	-2.07	-4.26	-3.81
Cluster_13819	*nikE*	nickel import ATP-binding protein NikE	-2.49	-4.16	-3.86
Cluster_16936		conserved hypothetical protein	5.49	3.81	4.76
Cluster_17045	*rcsA*	colanic acid capsular biosynthesis activation protein A	2.18	2.45	5.03
Cluster_18751	*slp*	outer membrane protein Slp	4.18	3.39	3.88
Cluster_19444	*rpiB*	ribose 5-phosphate isomerase B	-3.16	-2.20	-2.82
Cluster_19503		entericidin EcnA/B family protein	5.08	2.83	2.68
Cluster_20470	*tsx*	nucleoside-specific channel-forming protein Tsx	-3.88	-3.19	-2.17
Cluster_2094		conserved hypothetical protein	4.46	4.07	2.94
Cluster_21151		conserved hypothetical protein	2.67	2.33	3.17
Cluster_21209		putative L,D-transpeptidase YcfS	2.85	2.31	3.54
Cluster_21556		conserved hypothetical protein	3.75	2.95	3.74
Cluster_21804	*sufD*	FeS assembly protein SufD	2.58	3.27	3.11
Cluster_21854	*katE*	catalase HPII	3.84	3.15	3.54
Cluster_21905		conserved hypothetical protein	4.38	3.50	3.03
Cluster_22321	*cdd*	cytidine deaminase	-5.04	-4.28	-5.48
Cluster_22333	*cirA*	colicin I receptor	3.79	2.62	3.86
Cluster_22417	*glpA*	glycerol-3-phosphate dehydrogenase, anaerobic, A subunit	-5.10	-3.31	-5.94
Cluster_22418	*glpB*	glycerol-3-phosphate dehydrogenase, anaerobic, B subunit	-3.92	-3.86	-6.36
Cluster_2291	*otsA*	alpha,alpha-trehalose-phosphate synthase	3.19	2.50	2.20
Cluster_22982		L-serine ammonia-lyase	-6.08	-3.82	-3.26
Cluster_23260	*ebgA*	evolved beta-galactosidase subunit alpha	-4.00	-3.05	-2.71
Cluster_23382	*gltD*	glutamate synthase [NADPH] small chain	3.27	3.33	3.54
Cluster_23648	*hmuS*	hemin transport protein HmuS	4.58	3.83	4.20
Cluster_23655	*hmuV*	hemin import ATP-binding protein HmuV	2.58	2.31	3.42
Cluster_23891	*emrD*	multidrug resistance protein D	-2.26	-2.24	-2.02
Cluster_24046	*metE*	5-methyltetrahydropteroyltriglutamate--homocysteine S-methyltransferase	3.71	4.16	4.11
Cluster_24222	*purD*	phosphoribosylamine--glycine ligase	2.28	3.13	3.62
Cluster_24247		exopolysaccharide production YjbE family protein	2.24	2.29	6.28
Cluster_24491	*treC*	alpha,alpha-phosphotrehalase	-6.57	-7.03	-4.43
Cluster_24492	*treB*	PTS system trehalose-specific EIIBC component	-8.44	-7.09	-5.54
Cluster_25515		ybgS-like family protein	3.57	3.44	4.74
Cluster_25537	*bioA*	adenosylmethionine-8-amino-7-oxononanoate transaminase	2.76	2.20	3.69
Cluster_25541	*bioD*	dethiobiotin synthase	4.85	4.75	5.04
Cluster_25677	*poxB*	pyruvate dehydrogenase [ubiquinone]	3.55	3.22	2.53
Cluster_2623	*glpT*	glycerol-3-phosphate transporter	-6.13	-4.32	-3.47
Cluster_26471		peptidase M16 inactive domain protein	3.34	3.41	4.06
Cluster_26472		tonB-dependent Receptor Plug domain protein	2.57	3.21	3.64
Cluster_26602	*sodB*	superoxide dismutase [Fe]	-2.67	-2.60	-3.74
Cluster_27306	*elaB*	protein ElaB	4.55	3.70	4.72
Cluster_27481	*tal*	transaldolase	3.08	2.93	2.69
Cluster_27668		ribonucleoside-diphosphate reductase, alpha subunit	3.37	2.69	3.26
Cluster_28138	*ilvA*	threonine ammonia-lyase	-3.61	-2.37	-3.92
Cluster_28465	*nikC*	nickel ABC transporter, permease subunit NikC	-2.69	-3.48	-4.32
Cluster_28495		FecCD transport family protein	2.65	2.17	3.07
Cluster_28978	*fdxH*	formate dehydrogenase, beta subunit	-3.81	-3.01	-3.59
Cluster_30616		conserved hypothetical protein	3.72	2.77	2.20
Cluster_30847	*narK*	nitrite extrusion protein 1	-3.56	-4.32	-6.73
Cluster_31984		conserved hypothetical protein	3.08	2.51	4.46
Cluster_32906		conserved hypothetical protein	-2.37	-2.86	-2.04
Cluster_32975	*shuA*	outer membrane heme receptor ShuA	5.75	4.57	5.32
Cluster_33251		RbsD / FucU transport family protein	-8.23	-6.44	-6.23
Cluster_34698	*bioC*	malonyl-CoA O-methyltransferase BioC	3.76	4.36	5.35
Cluster_3575	*hybO*	hydrogenase-2 small chain	-3.01	-2.00	-4.86
Cluster_36754		conserved hypothetical protein	5.56	4.23	4.56
Cluster_36949	*serA*	D-3-phosphoglycerate dehydrogenase	2.78	3.48	3.08
Cluster_37491	*dtpB*	dipeptide and tripeptide permease B	-3.04	-3.30	-3.40
Cluster_37583		helix-turn-helix family protein	4.61	4.05	2.64
Cluster_37835		branched-chain amino acid transport system / permease component family protein	-5.36	-4.17	-2.88
Cluster_37837	*rbsK*	ribokinase	-4.33	-3.82	-2.36
Cluster_37959		molybdopterin oxidoreductase Fe_4S_4 domain protein	-4.54	-2.61	-3.20
Cluster_39493	*bioB*	biotin synthase	4.26	4.76	5.55
Cluster_39494	*bioF*	8-amino-7-oxononanoate synthase	4.18	5.36	5.79
Cluster_39851	*ycdZ*	inner membrane protein YcdZ	-2.04	-2.65	-3.00
Cluster_39870		YceI-like domain protein	4.14	3.31	3.74
Cluster_39918	*fhuE*	FhuE receptor domain protein	2.68	2.44	3.51
Cluster_40107		nitrate reductase, alpha subunit	-5.90	-5.98	-9.09
Cluster_40489	*sufC*	FeS assembly ATPase SufC	3.00	3.28	3.25
Cluster_40490	*sufB*	FeS assembly protein SufB	3.19	3.35	2.94
Cluster_40648	*purT*	phosphoribosylglycinamide formyltransferase 2	2.46	3.23	3.59
Cluster_41050	*wcaE*	colanic acid biosynthesis glycosyltransferase WcaE	2.81	2.26	8.61
Cluster_41088	*fbaB*	fructose-bisphosphate aldolase class 1	3.43	3.42	4.74
Cluster_41400	*mntH*	manganese transport protein MntH	3.44	3.80	4.56
Cluster_41462	*tkt*	transketolase	2.69	2.56	3.11
Cluster_41477	*purC*	phosphoribosylaminoimidazolesuccinocarboxamide synthase	2.55	2.78	3.54
Cluster_41489	*purM*	phosphoribosylformylglycinamidine cyclo-ligase	2.21	2.38	4.30
Cluster_41580	*raiA*	ribosome-associated inhibitor A	2.61	2.43	2.41
Cluster_41878	*sdaC*	serine transporter	-5.32	-4.11	-3.35

Cluster_42020	*nupG*	nucleoside permease NupG	-4.88	-4.34	-3.80
Cluster_42098		TonB-dependent siderophore receptor family protein	3.28	3.19	2.27
Cluster_42168		integral membrane, TerC family protein	2.33	2.11	3.79
Cluster_42524	*nikA*	nickel ABC transporter, nickel/metallophore periplasmic binding protein	-2.20	-2.37	-6.81
Cluster_42553		putative heme utilization radical SAM enzyme HutW	2.61	2.40	2.21
Cluster_42554		conserved hypothetical protein	3.16	2.97	2.95
Cluster_42555		NADH(P)-binding family protein	2.37	2.54	3.20
Cluster_42600		conserved hypothetical protein	3.73	3.61	3.15
Cluster_42613	*yiaD*	inner membrane lipoprotein YiaD	-4.96	-3.61	-2.51
Cluster_42868	*ccmA*	heme ABC exporter, ATP-binding protein CcmA	-7.70	-6.70	-6.12
Cluster_42872	*rbsR*	ribose operon repressor	-3.52	-3.67	-2.33
Cluster_43311		conserved hypothetical protein	-3.73	-3.02	-3.02
Cluster_4373	*glpF*	glycerol uptake facilitator protein	-6.76	-5.02	-3.32
Cluster_4600		conserved hypothetical protein	3.99	2.89	3.56
Cluster_50634		30S ribosomal subunit S22 family protein	5.44	4.01	2.84
Cluster_50835		SmpA / OmlA family protein	3.90	2.51	3.06
Cluster_52412		conserved hypothetical protein	3.51	3.03	2.12
Cluster_54045		conserved hypothetical protein	3.53	2.41	2.18
Cluster_54408		conserved hypothetical protein	2.93	2.35	2.29
Cluster_55486	*otsB*	trehalose-phosphatase	3.70	2.86	3.20
Cluster_56230		proteolipid membrane potential modulator family protein	3.90	2.54	3.08
Cluster_56240	*nrdF*	ribonucleoside-diphosphate reductase 2 subunit beta	4.88	3.58	3.82
Cluster_57084		bacterial regulatory, LuxR family protein	5.83	4.47	5.33
Cluster_58724	*dps*	DNA protection during starvation protein	2.90	2.06	2.13
Cluster_59591	*sufE*	cysteine desulfuration protein SufE	3.66	3.39	3.07
Cluster_59641	*ydjM*	inner membrane protein YdjM	-3.12	-2.30	-3.80
Cluster_59696		PrkA AAA domain protein	4.47	3.33	2.90
Cluster_60563	*nrdH*	glutaredoxin-like protein NrdH	2.89	3.47	3.14
Cluster_60948	*ygjG*	putrescine aminotransferase	2.00	2.09	2.63
Cluster_61411		bacterial extracellular solute-binding, 5 Middle family protein	3.91	4.44	4.18
Cluster_61689	*udp*	uridine phosphorylase	-3.65	-3.05	-4.11
Cluster_61749		formate dehydrogenase, alpha subunit	-4.35	-2.95	-3.81
Cluster_63081		endonuclease/Exonuclease/phosphatase family protein	2.63	2.19	2.14
Cluster_64024	*sufA*	FeS assembly scaffold SufA	2.75	3.11	2.93
Cluster_66112		conserved hypothetical protein	-2.97	-2.50	-2.59
Cluster_6908	*sufS*	cysteine desulfurase	3.13	3.19	3.06
Cluster_6913		conserved hypothetical protein	5.73	4.30	3.66
Cluster_8935	*uhpT*	hexose phosphate transport protein	-3.79	-3.15	-2.67
Cluster_8994	*phoU*	phosphate transport system regulatory protein PhoU	2.13	2.09	3.43
Cluster_9089	*rmuC*	DNA recombination protein RmuC	-2.65	-2.30	-2.03
Cluster_9149		formate dehydrogenase, gamma subunit	-3.04	-2.74	-2.85

Isolate-Specific Genes[c]

Cluster ID	Gene	Predicted Protein Function	LFC[a]
103385			
Cluster_14663		ABC transporter family protein	-2.17
Cluster_20007		hypothetical protein	2.37
Cluster_20009		conserved hypothetical protein	2.40
Cluster_20009		conserved hypothetical protein	2.40
Cluster_21067	*rbsK*	ribokinase	-3.26
Cluster_21081		bacterial lipid A biosynthesis acyltransferase family protein	-3.25
Cluster_21082	*msbB*	lipid A biosynthesis (KDO)2-(lauroyl)-lipid IVA acyltransferase	-2.84
Cluster_21085		polysaccharide deacetylase family protein	-3.17
Cluster_21091	*fecD*	Fe(3+) dicitrate transport system permease protein FecD	-3.44
Cluster_21092		ABC 3 transport family protein	-2.78
Cluster_22297		Ead/Ea22-like family protein	2.38
Cluster_24590		cytidine and deoxycytidylate deaminase zinc-binding region family protein	-2.76
Cluster_24591		conserved hypothetical protein	-2.32
Cluster_24667		conserved hypothetical protein	-3.47
Cluster_44764		putative YfdA	2.10
Cluster_45471		periplasmic binding family protein	-2.46
Cluster_45480		conserved hypothetical protein	-3.49
Cluster_4936		glycosyl transferases group 1 family protein	-3.45
Cluster_62139	*fecA*	Fe(3+) dicitrate transport protein FecA	-3.72
Cluster_6675		putative RNA polymerase sigma factor FecI	2.37
401588			LFC[a]
Cluster_39452	*cro*	regulatory protein Cro	2.68
Cluster_40245		conserved hypothetical protein	3.08
Cluster_40811		Mu-like prophage FluMu gp41 family protein	3.21
Cluster_40830		conserved hypothetical protein	4.15
Cluster_50962		pentapeptide repeats family protein	2.41
Cluster_64304		conserved hypothetical protein	3.00
300059			LFC[a]
Cluster_25606		bacteriophage replication gene A family protein	-3.05
Cluster_25965		tetratricopeptide repeat family protein	2.13
Cluster_29501		conserved hypothetical protein	-2.79
Cluster_34728		transposase family protein	-3.52

[a]LFC is Log_2 Fold-Change of the DMEM samples compared to the LB samples.

[b]Genes identified in all three of the EPEC8 genomes with an LS-BSR ≥0.8.

[c]Genes identified with significant similarity (LS-BSR ≥0.8) in only one of the EPEC8 isolates that were absent (LS-BSR <0.4) from the other two EPEC8 isolates.
