# Supplementary material for: Transcriptional Variation of Diverse Enteropathogenic Escherichia coli Isolates under Virulence-Inducing Conditions
Source: mSystems. 2017 Jul 25;2(4):e00024-17. doi: 10.1128/mSystems.00024-17 (PMC5527300; doi:10.1128/mSystems.00024-17)
Supplement: TABLE S5 [file sys004172117st8.pdf]

Table S5. Differential-expression of select non-virulence genes

Cluster ID	Gene	Predicted Protein Function	100329 (A)	401140 (A)	B171 (B1)	402290 (B1)	E110019 (B1)	E2348/69 (B2)
Cluster_42524	*nikA*	nickel ABC transporter, nickel/metallophore periplasmic binding protein	-5.35			-4.20		
Cluster_23623	*nikB*	nickel ABC transporter, permease subunit NikB	-5.45	-2.17		-4.38	2.09	-3.00
Cluster_28465	*nikC*	nickel ABC transporter, permease subunit NikC	-7.22	-3.25		-3.06	2.34	-3.57
Cluster_13818	*nikD*	nickel import ATP-binding protein NikD	-5.49	-3.67		-2.76	2.01	-3.77
Cluster_13819	*nikE*	nickel import ATP-binding protein NikE	-5.50	-3.86		-2.53		-4.43
Cluster_37491	*dtpB*	dipeptide and tripeptide permease B	-3.95	-2.69	-2.37	-3.29	-3.48	-3.72
Cluster_39198	*cusS*	sensor kinase CusS				2.33		
Cluster_39199	*cusR*	transcriptional regulatory protein CusR		2.47		2.22		
Cluster_39200	*cusC*	cation efflux system protein CusC		3.08		3.76		-2.19
Cluster_44152	*cusF*	cation efflux system protein CusF		3.08		4.69		
Cluster_30102	*cusB*	cation efflux system protein CusB		2.56		4.54	3.14	
Cluster_30103	*cusA*	cation efflux system protein CusA				3.31		
Cluster_24888		MerC mercury resistance family protein				-5.21		
Cluster_5690	*entF*	enterobactin synthase component F	5.15		3.25	5.00	2.72	
Cluster_54210	*fepE*	ferric enterobactin (Enterochelin) transport	3.08		2.95	3.84	2.34	
Cluster_5696	*fepB*	ferrienterobactin-binding periplasmic protein	4.21		2.21	2.08		
Cluster_25325	*entC*	isochorismate synthase EntC	6.70	2.44	3.26	4.81	3.26	
Cluster_5698		(2,3-dihydroxybenzoyl)adenylate synthase	6.52	2.27	3.35	4.86	2.81	
Cluster_58522	*entB*	isochorismatase	6.33	2.09	3.33	5.46	2.85	
Cluster_25537	*bioA*	adenosylmethionine-8-amino-7-oxononanoate transaminase	5.17	2.53		4.28		3.51
Cluster_39493	*bioB*	biotin synthase	6.45	3.94	2.73	6.10	3.85	5.02
Cluster_39494	*bioF*	8-amino-7-oxononanoate synthase	5.65	2.05	2.81	5.84	3.93	4.83
Cluster_34698	*bioC*	malonyl-CoA O-methyltransferase BioC	7.50	2.44	2.47	5.57	4.41	4.55
Cluster_25541	*bioD*	dethiobiotin synthase	6.09	3.83	3.82	5.20	4.81	5.10
Cluster_12385	*hisG*	ATP phosphoribosyltransferase				2.19		
Cluster_27059	*hisD*	histidinol dehydrogenase	2.02	2.42		2.50	2.24	
Cluster_41011	*hisC*	histidinol-phosphate transaminase	2.18			2.81		
Cluster_12388	*hisB*	histidine biosynthesis bifunctional protein HisB		2.27				
Cluster_64513	*hisH*	imidazole glycerol phosphate synthase, glutamine amidotransferase subunit	2.22			2.32		2.69
Cluster_46060	*hisA*	1-(5-phosphoribosyl)-5-[(5-phosphoribosylamino)methylideneamino]imidazole-4-carboxamide isomerase	2.40			2.79		
Cluster_2401	*hisF*	imidazole glycerol phosphate synthase subunit HisF	2.35	2.09		2.25		
Cluster_27065	*hisE*	phosphoribosyl-ATP diphosphatase	2.04			2.62		
Cluster_46067		glycosyl transferase 2 domain protein				2.37		
Cluster_35561	*fimH*	protein FimH			-3.59	-3.26	-2.14	
Cluster_40322	*fimG*	protein FimG			-3.55	-3.16		
Cluster_21599	*fimF*	protein FimF			-3.27	-2.83		
Cluster_59417		type VII secretion system (T7SS), usher family protein		-2.72	-2.64	-2.89	-2.01	
Cluster_40325	*focC*	chaperone protein FocC			-2.22	-2.92		
Cluster_1885	*fimA*	type-1 fimbrial protein, A chain			-3.47	-3.93	-2.34	
Cluster_59591	*sufE*	cysteine desulfuration protein SufE	4.83			4.47		
Cluster_6908	*sufS*	cysteine desulfurase	4.80			4.91		
Cluster_21804	*sufD*	feS assembly protein SufD	5.16			4.64		
Cluster_40489	*sufC*	feS assembly ATPase SufC	4.99			4.32		
Cluster_40490	*sufB*	feS assembly protein SufB	5.18			4.22		
Cluster_64024	*sufA*	feS assembly scaffold SufA	5.12	2.00		4.30		
Cluster_6913		conserved hypothetical protein	4.56	2.36	3.65	3.73	2.76	3.44
Cluster_45685		4Fe-4S dicluster domain protein	2.04			2.09		
Cluster_11100	*flgA*	flagellar basal body P-ring biosynthesis protein FlgA	-3.77	-5.79		-3.19		-2.95
Cluster_58981	*flgB*	flagellar basal-body rod protein FlgB	-7.40	-8.66		-4.72	-4.30	-5.80
Cluster_30655	*flgC*	flagellar basal body rod protein FlgC	-6.13	-8.50		-4.01	-4.03	-4.59
Cluster_21169	*flgD*	flagellar basal body rod modification protein	-5.52	-7.79		-4.01	-4.38	-5.52
Cluster_21170	*flgE*	flagellar hook protein FlgE	-4.91	-6.75		-3.47	-3.67	-5.16
Cluster_6341	*flgG*	flagellar basal body rod protein FlgG	-4.52	-6.74		-3.45	-3.69	-3.45
Cluster_35075	*flgH*	flagellar basal body L-ring protein	-4.63	-6.20		-3.17		-3.59
Cluster_26079	*flgI*	flagellar basal body P-ring biosynthesis protein FlgA	-4.40	-6.28		-3.30		-2.86
Cluster_58989	*flgJ*	flagellar rod assembly protein/muramidase FlgJ	-4.70	-6.03		-3.52	-2.23	-2.92
Cluster_21176	*flgK*	flagellar hook-associated protein FlgK		-5.90		-3.32		-2.37
Cluster_17152	*wbiQ*	fucosyltransferase						2.43
Cluster_17153	*wbiP*	glycosyl transferase family protein						2.86
Cluster_17154	*wzy*	O-antigen polymerase						3.61
Cluster_17155	*wbiO*	O-acetyltransferase						2.77
Cluster_17156	*wzx*	O-antigen flippase						3.49
Cluster_22198	*manB*	phosphomannomutase	3.91					3.08
Cluster_17158	*manC*	GDP-mannose pyrophosphorylase						2.36
Cluster_27093	*gmm*	GDP-mannose mannosyl hydrolase	4.62					3.17
Cluster_22202	*fcl*	bifunctional GDP-fucose synthetase: GDP-4-dehydro-6-deoxy-D-mannose epimerase/GDP-4-dehydro-6-L-deoxygalactose reductase	3.37					5.70
Cluster_22203	*gmd*	GDP-D-mannose dehydratase	4.15					5.66
Cluster_27096	*wcaF*	colanic acid biosynthesis acetyltransferase WcaF	3.21					4.31
Cluster_41050	*wcaE*	glycosyl transferase family protein	4.96					2.51
Cluster_41051	*wcaD*	colanic acid biosynthesis protein	3.54					3.25
Cluster_22207	*wcaC*	glycosyl transferase family protein	3.40					4.61
Cluster_27100	*wcaB*	colanic acid biosynthesis acetyltransferase WcaB	2.82					3.58
Cluster_27101	*wcaA*	glycosyl transferase family protein	2.28					4.51
Cluster_27085	*wcaM*	colanic acid biosynthesis protein WcaM	2.34					
Cluster_22194	*wcaL*	colanic acid biosynthesis glycosyltransferase WcaL	2.26					
Cluster_27087	*wcaK*	colanic acid biosynthesis pyruvyl transferase WcaK	2.64					
Cluster_22196	*wzxC*	lipopolysaccharide biosynthesis protein WzxC	2.88					
Cluster_22197		undecaprenyl-phosphate glucose phosphotransferase	3.47					
Cluster_41044		mannose-1-phosphate guanylyltransferase/mannose-6-phosphate isomerase	3.34					
Cluster_41045	*wcaI*	colanic acid biosynthesis glycosyltransferase WcaI	3.77					
Cluster_41055	*wzc*	tyrosine kinase	3.82					3.57
Cluster_27103	*wzb*	tyrosine phosphatase	2.99					4.07
Cluster_41057	*wza*	lipoprotein required for capsular polysaccharide translocation through the outer membrane	3.47					4.47
Cluster_65186	*hypA*	hydrogenase nickel incorporation protein	-3.11					-2.83
Cluster_22903	*hypB*	hydrogenase nickel incorporation protein HypB	-4.00					-3.22
Cluster_8035	*hypC*	hydrogenase assembly chaperone	-3.90					-3.83
Cluster_22905	*hypD*	HypD protein required for maturation of hydrogenases	-2.46					-2.76
Cluster_36807	*hypE*	carbamoyl phosphate phosphatase, hydrogenase 3 maturation protein HypE	-3.22					-2.44
Cluster_18751	*slp*	outer membrane lipoprotein						4.44
Cluster_57084	*yhiF*	DNA-binding transcriptional regulator						5.23
Cluster_23648	*chuS*	heme/hemoglobin transport protein						3.91
Cluster_32975	*chuA*	outer membrane heme/hemoglobin receptor						5.00
Cluster_28491	*chuT*	hemin binding protein						5.76
Cluster_42553	*chuW*	coproporphyrinogen III oxidase						5.12
Cluster_42555	*chuY*	ShuY-like protein						3.42
Cluster_28495	*chuU*	hemin permease						3.75
Cluster_23655	*hmuV*	hemin importer ATP-binding subunit						2.42
Cluster_37569	*dppF*	dipeptide transporter ATP-binding subunit	3.52	3.63	2.08	3.65	2.60	3.76
Cluster_23696	*dppD*	dipeptide transporter ATP-binding subunit	3.89	4.14	2.38	4.42	3.12	4.95
Cluster_61409	*dppC*	dipeptide transporter	4.31	3.70	2.24	3.72	3.06	5.10
Cluster_57117	*dppB*	dipeptide transporter permease DppB	4.85	4.26	2.80	4.95	3.56	4.80
Cluster_61411	*dppA*	dipeptide transporter	4.56	4.52		5.02	2.18	3.44
Cluster_33251	*rbsD*	D-ribose pyranase	-6.80	-6.87	-5.32	-7.04	-4.73	-5.62
Cluster_42868	*rbsA*	D-ribose transporter ATP-binding protein	-6.18	-6.23	-5.95	-7.01	-4.93	-5.83
Cluster_37835	*rbsC*	ribose ABC transporter permease	-3.85	-4.38	-6.09	-4.67	-5.64	-4.82
Cluster_4267	*rbsB*	D-ribose transporter subunit RbsB			-2.93		-4.82	-4.57
Cluster_37837	*rbsK*	ribokinase	-3.74	-2.84	-3.85	-3.05	-3.68	-4.08
Cluster_42872	*rbsR*	transcriptional repressor RbsR	-3.31	-2.76	-3.75	-2.94	-4.05	-2.57
Cluster_25315	*fepA*	ferrienterobactin receptor	7.32	2.47	4.62	7.15	5.16	
Cluster_60311		conserved hypothetical protein						

Cluster_25316	*fes*	enterochelin esterase	6.89		2.88	3.87	2.56	
Cluster_5689		MbtH-like family protein	6.03		3.29	4.11	2.93	
Cluster_30847	*narK*	nitrite extrusion protein 1	-6.92	-5.92	-2.25	-7.04	-4.52	-6.39
Cluster_40107		nitrate reductase, alpha subunit	-8.34	-6.28	-3.12	-6.45	-5.18	-8.36
Cluster_40108	*narH*	nitrate reductase, beta subunit	-6.44	-5.41		-3.56	-3.46	-6.83
Cluster_26242	*narJ*	nitrate reductase molybdenum cofactor assembly chaperone	-6.34	-5.72			-2.01	-5.08
Cluster_40110	*narI*	respiratory nitrate reductase, gamma subunit	-5.48	-3.73				-4.03
Cluster_59591	*sufE*	cysteine desulfuration protein SufE	4.83			4.47		
Cluster_6908	*sufS*	cysteine desulfurase	4.80			4.91		
Cluster_21804	*sufD*	FeS assembly protein SufD	5.16			4.64		
Cluster_40489	*sufC*	FeS assembly ATPase SufC	4.99			4.32		
Cluster_40490	*sufB*	FeS assembly protein SufB	5.18			4.22		
Cluster_64024	*sufA*	FeS assembly scaffold SufA	5.12	2.00		4.30		
Cluster_28854	*ilvB*	acetolactate synthase, large subunit, biosynthetic type				2.72		
Cluster_14164	*ilvM*	acetolactate synthase isozyme 2 small subunit				2.67		
Cluster_28856	*ilvE*	branched-chain amino acid aminotransferase						
Cluster_23994	*ilvD*	dihydroxy-acid dehydratase						
Cluster_55878		NADH-Ubiquinone/plastoquinone (complex I), various chains family protein				-2.37		
Cluster_55878		NADH-Ubiquinone/plastoquinone (complex I), various chains family protein				-2.37		
Cluster_7513	*nuoM*	NADH-quinone oxidoreductase subunit M				-2.80		
Cluster_22451	*nuoL*	NADH-quinone oxidoreductase subunit L				-2.68		
Cluster_12623	*nuoK*	NADH-quinone oxidoreductase subunit K				-2.32		
Cluster_46275	*nuoJ*	NADH-quinone oxidoreductase subunit J				-2.27		
Cluster_41293		NADH-quinone oxidoreductase, chain I family protein	-2.07			-2.21		
Cluster_27318	*nuoH*	NADH-quinone oxidoreductase subunit H				-2.10		
Cluster_27319	*nuoG*	NADH dehydrogenase (quinone), G subunit						
Cluster_17411	*nuoF*	NADH oxidoreductase (quinone), F subunit	-2.21		-2.15			
Cluster_17412	*nuoE*	NADH-quinone oxidoreductase subunit E	-2.09		-2.06			
Cluster_41298	*nuoC*	NADH-quinone oxidoreductase subunit C/D			-2.09			
Cluster_55889	*nuoB*	NADH-quinone oxidoreductase subunit B	-2.01					
Cluster_33836	*deoC*	deoxyribose-phosphate aldolase	-3.70	-3.57	-3.62	-4.05	-2.65	-3.30
Cluster_33837	*deoA*	thymidine phosphorylase	-2.57	-3.17	-4.28	-2.89		-3.14
Cluster_24617	*deoB*	phosphopentomutase	-2.47	-2.35	-3.55	-2.97		
Cluster_5057	*deoD*	purine nucleoside phosphorylase	-2.19		-2.61	-2.23		
Cluster_26043	*csgC*	curli assembly protein CsgC	-3.76	-4.45				
Cluster_24302	*nrfA*	cytochrome c-552	-4.61					-4.99
Cluster_43190	*nrfB*	cytochrome c nitrite reductase, pentaheme subunit	-3.41					-3.81
Cluster_43191	*nrfC*	cytochrome c nitrite reductase, Fe-S protein	-5.06	-2.60		-3.80	2.50	-4.58
Cluster_43192	*nrfD*	cytochrome c nitrite reductase, NrfD subunit	-4.59	-2.00				-2.20
Cluster_29171	*ccmF*	cytochrome c-type biogenesis protein CcmF	-2.94					
Cluster_43194		cytochrome C biogenesis family protein	-2.81				2.60	

[a]LFC is Log_2 Fold-Change of the DMEM samples compared to the LB samples.
