# Supplementary material for: Transcriptional Variation of Diverse Enteropathogenic Escherichia coli Isolates under Virulence-Inducing Conditions
Source: mSystems. 2017 Jul 25;2(4):e00024-17. doi: 10.1128/mSystems.00024-17 (PMC5527300; doi:10.1128/mSystems.00024-17)
Supplement: TABLE S6 [file sys004172117st9.pdf]

Table S6. Differential expression of the LS-BSR gene clusters that are only in EPEC and encode proteins with predicted secreted or surface-associated domains

Cluster ID	Gene	Predicted Protein Function	LFC[a]								
			100329 (A)	401140 (A)	B171 (B1)	402290 (B1)	E110019 (B1)	E2348/69 (B2)	C581-05 (B2)	401588 (B2)	302053 (B2)
Cluster_42784		SdiA-regulated family protein			4.09	2.21	3.92	5.16	2.34	4.42	
Cluster_46985	*espA*	EspA	2.58	2.09	4.95	3.15		4.71			3.29
Cluster_52862		transglycosylase SLT domain protein			3.56		3.60	3.82		3.44	
Cluster_49719		conserved hypothetical protein			3.47	2.11		3.28			
Cluster_42098		TonB-dependent siderophore receptor family protein								3.19	3.34
Cluster_7210		TonB-dependent siderophore receptor family protein	5.86						2.22		
Cluster_7864		putative BfpJ			4.50	2.82					
Cluster_7865		putative BfpK			4.71	2.18					
Cluster_7878		bundlin family protein			3.33	2.73					
Cluster_41670		putative prepilin			4.21	2.92					
Cluster_42765		SepZ family protein					3.33	4.33			
Cluster_45818		putative membrane protein				-5.30					-4.81
Cluster_41853	*scrY*	sucrose porin						-6.62			
Cluster_48965	*traK*	type-F conjugative transfer system secretin TraK	2.40								
Cluster_8246		outer membrane insertion C-terminal signal domain protein	5.82								
Cluster_38581		putative Csi protein			2.39						
Cluster_4032		PTS system, Lactose/Cellobiose specific IIB subunit			-2.88						
Cluster_4033		PTS system sugar-specific permease component family protein			-2.09						
Cluster_40830		conserved hypothetical protein								4.15	
Cluster_41715	*trbB*	type-F conjugative transfer system pilin assembly thiol-disulfide isomerase TrbB	3.64								
Cluster_42607		fimbrial family protein						3.35			
Cluster_45238		conserved hypothetical protein			3.10						
Cluster_48547		polysaccharide biosynthesis/export family protein				2.24					
Cluster_49109		tripartite tricarboxylate transporter TctB family protein						-2.39			
Cluster_51092		major facilitator superfamily protein	5.09								
Cluster_5138	*traK*	type-F conjugative transfer system secretin TraK			2.11						
Cluster_52204		tripartite ATP-independent periplasmic transporters, DctQ component family protein						-2.76			
Cluster_8890		secretion system effector C (SseC) like family protein									2.01
Cluster_38584		conserved hypothetical protein			2.51						
Cluster_38608	*traA*	type IV conjugative transfer system pilin TraA			2.00						
Cluster_5140	*traL*	type IV conjugative transfer system protein TraL			2.12						
Cluster_8906		SepZ family protein									4.00

[a]LFC is Log_2 Fold-Change of the DMEM samples compared to the LB samples.
