# Supplementary material for: Transcriptional Variation of Diverse Enteropathogenic Escherichia coli Isolates under Virulence-Inducing Conditions
Source: mSystems. 2017 Jul 25;2(4):e00024-17. doi: 10.1128/mSystems.00024-17 (PMC5527300; doi:10.1128/mSystems.00024-17)
Supplement: TABLE S7 [file sys004172117st10.pdf]

Table S7. Differential-expression of known *E. coli* sRNAs

sRNA ID	100329 (A)	401140 (A)	B171 (B1)	402290 (B1)	E110019 (B1)	E2348/69 (B2)	C581-05 (B2)	401588 (B2)	103385 (B2)	300059 (B2)	302053 (B2)
arcZ	-1.29										-1.29
c0299						-3.70					
c0343	1.82						-3.48	4.66	4.72	2.31	2.27
csrB	2.44	1.42	2.08	2.77				2.69	4.25	4.53	2.27
csrC			1.94		-1.75						
cyaR		-1.32								-1.22	
dsrA							-1.53		3.19	1.33	
ffs	-1.53				1.87			-1.90		-1.25	-1.98
fnrS									2.05		
gadY							-2.20		1.94		
gcvB		-1.16		-2.36			-2.42				-2.07
glmY	3.71	1.21	2.64	2.98		3.89	1.75	2.87	2.39	3.41	2.73
glmZ											1.46
IS009							-1.98				
IS014	1.81									1.26	1.38
IS018		2.21									
IS019		1.64									
IS022							1.57			1.26	2.88
IS029	2.27				-2.60						
IS042	2.64	1.70		2.68			1.67	3.34	4.72	3.13	1.67
IS116							1.24		-1.34		
IS128	-1.82										
IS141		-1.21									
IS143	-1.54	-1.57		-2.36		-4.27		-3.22	-2.78	-2.68	-1.58
IS166	1.75										
IS173	-1.12								-1.41		
IS174						2.65			-1.25		
isrB	2.62								2.55	1.74	2.29
k2			-2.15							1.61	
k27										1.06	
mgrR	-1.90	-2.63					-3.31	-2.78			
micA							-1.48		1.26		
NC021											1.54
NC048	3.04										3.11
NC087									-2.83		
p1	-1.06								-1.18		
p2									1.43		
psrNd	2.68									3.73	
rnpB		-1.02						-1.19			-1.90
rprA			3.86								
rseX			4.28		2.49						
rttR	-1.56										
rybA	3.26	2.27		2.46			1.57	1.80	1.79	1.84	2.34
rybB									2.21		
rydB	2.97	1.69				3.41		3.01	4.18	3.23	2.86
rydC							-1.25			-1.44	
ryeA									2.21	4.26	
ryfD			-1.97		-3.20						
ryhB	5.19	4.58		3.59		2.60	2.27		2.94	3.33	4.72
ryjA	1.86								1.40		
sgrS	1.95						1.35				
sibA	2.13	1.42									
sibB	1.58							1.81		1.53	
sibC	1.32	1.11	2.02		1.65				1.69		1.65
sibE		1.13	1.98						1.41		
sokC									-1.23		
sokX	-1.64										
spf	-1.79										
sroA									-1.37		
sroC		2.89					1.59			1.77	
sroH					2.01						
ssrA									1.82		
ssrS	1.38	1.23							1.48		
te10		-1.19								1.21	
te21										2.08	1.99
tk5									1.30		
tp2								-4.08			
tp28										1.46	
tp46									-2.71		-1.54
tpke11		-1.56	-2.12		-2.20						
tpke70	1.37	1.62			2.69				1.11	1.82	
tpke85							-2.31				
