# Supplementary material for: Transcriptional Variation of Diverse Enteropathogenic Escherichia coli Isolates under Virulence-Inducing Conditions
Source: mSystems. 2017 Jul 25;2(4):e00024-17. doi: 10.1128/mSystems.00024-17 (PMC5527300; doi:10.1128/mSystems.00024-17)
Supplement: DATA SET S1 [file sys004172117sd1.pdf]

```
>Cluster_57190
ATGGCCAATGTTGAAATCTATACCAAAGAAACCTGCCCGTATTGCCATCGTGCAAAAGCACTGCTGAGCAGCAAGGGCGT
GAGTTTTCAGGAGTTGCCGATCGATGGCAATGCCGCCAAGCGTGAAGAGATGATCAAACGCAGCGGTCGCACCACGGTTC
CCCAGATTTTTATTGACGCACAGCACATTGGCGGCTGTGATGACTTGTATGCATTGGATGCACGTGGTGGACTGGATCCC
CTGCTGAAATAA
>Cluster_13112
ATGAACGGGCGCGCGTGTGGCCCGAATTCGGTTTATCCCCGCTGGCGCGGGGAACACGCGGGCGGATTCAATCTGGCCAA
TATCGACGACGGTTTATCCCCGCTGGCGCGGGGAACACCGCCTGCACGCGACATCAAAATAGTGATTGCTCGGTTTATCC
CCGCTGGCGCGGGGAACACGGGTAAAGGCATTGTTTTAGCCCTGAACCCGCCGGTTTATCCCCGCTGGCGCGGGGAACAC
ACTAAGCATACATAA
>Cluster_46088
ATGGCCAGAACGATGACGGTTGATCTCGGCGATGAGTTGCGTGAATTTATCGAGTCCTTAATTGAATCCGGCGACTATCG
CACCCAAAGCGAAGTGATCCGTGAGTCACTGCGATTACTTCGAGAAAAGCAAGCAGAGTCTCGCCTGCAAGCGTTACGTG
ACATGCTCGCAGAAGGGTTGAGTAGTGGTGAAGCTCAGCCGTGGGAAAAGGACGCGTTCTTACGGAAGGTGAAAGCAGGG
ATAAGGAAATAA
>Cluster_17765
ATGAGCAGATTGCTGTTCCAAAGTGAGAGCAAAACCATGTTGTGGGAGCCAGTGTTATATCGCGTTGACTGGCTTAATTC
GTGCGAACACTGCCAGTGCAGCAAGGGGATTGAAGTTATCAGTCGCTCATTGGCAGGGACATACCTTTACGGCGGTGACC
TGGTTAAGTGTCCGCAGTGTGGCAATCAGGGTGTTATCGAGGCTGATGGTGAAGGCGCTTGGGTTGAATGGGATACAGAG
TGGGAGGACTAA
>Cluster_43434
ATGAAAGAGTATCTGTTTTTATTTCACTCCACGGTCGGCGTCATACAAACCCGCAAGGCGTTGCAGGCAGCGGGCATGAC
TTTTCGCGTCAGCGATATTCCCCGTGATTTACGCGGCGGCTGCGGGTTATGCATTTGGCTGACCTGTCCGCCTGGCGAGG
AAATTCAATGGGTGATCCCTGGGCATACGGAATCAGTTTATTGTCAGCAGGATGGTGGCTGGCGCTGCATTGCACATTAC
GGGATTTCTCCGCGTTAA
>Cluster_48410
ATGCATGATATTTCTGTTTCTGCGCCAGCTCCGGCTGCGCCATTATTTCCTGTGCTGAACCAGCATGAGCGATTTTTACG
TCTTCCCGAGGTGATGCATTTATGCGGCTTGTCCCGCTCAACTATATACGACCTGATAAGCCGCGATGCTTTCCCGAAGC
AGATCCCGCTTGGTGGTAAAAATGTTGCCTGGGCACAGTCAGAGGTAAGCGCATGGATGGCGGAGCGTATAAGTGCCCGC
GGACGGGGATGTGATGCATAA
>Cluster_20779
ATGGAACAAATTAAACTGAGTGTCTGTGGGGTTGATATTGTTTTTGAACCTAATCAGACTGCTTATAATAAACTGATTAA
TGAAATGGCAATGGATAATAAAGTTGCCCCTGCAAATAATTATCTGATGCGTATTGTTACCCCAGAAACGAAAGAAGCAC
TGATTGACGTATTAAAACGTCCGGGTGCAGCACTGCAACTGGTCAGTAAGGTTAATGATATTTACGCGCCGGAACTGGAG
ATTGAAGTAAAAAACTGA
>Cluster_27439
ATGTTCCAGCAAGAAGTTACCATTACCGCTCCGAACGGTCTGCACACCCGCCCTGCTGCCCAGTTTGTAAAAGAAGCTAA
GGGCTTCACTTCTGAAATTACTGTGACTTCCAACGGCAAAAGCGCCAGCGCGAAAAGCCTGTTTAAACTGCAGACTCTGG
GCCTGACTCAAGGTACCGTTGTGACTATCTCCGCAGAAGGCGAAGACGAGCAGAAAGCGGTTGAACATCTGGTTAAACTG
ATGGCGGAACTCGAGTAA
>Cluster_59048
ATGCTTCAAATGTTAACTCTTGAAGAATGGGCTGCTGAAAAATACAGAAGTAATCCTCCAAGTGTGTCCACATTGCGTCG
TTATGCTAAGCAGAATTTATTTTGTCCACCGGCCATGAAACAAGGCCGGTTATGGCGAGTGCGTGAGGACGCCGAGTTAG
TTGGGGAGTTGGTAACACCTGTAATCAAGAAAAATGACTCATTACTTTTGCAACGGATTTTGAGTAATGGCAGCCAGACC
GCGTAA
>Cluster_11841
ATGAAATCAAAAGACACCCTAAAGTGGTTCCCTGCGCAGCTTCCTGAAGTAAGAATTATCCTAGGGGATGCTGTAGTGGA
AGTAGCAAAACAGGGAAGACCTATCAATACCAGAACATTGCTTGATTACATTGAAGGAAACATAAAGAAAAAATCATGGC
TGGATAACAAAGAATTATTACAAACAGCGATATCAGTTCTTAAAGACAACCAAAATTTAAATGGTAAAATGTAA
>Cluster_40484
ATGAAAGCTACTAAACTGGTACTGGGCGCGGTAATCCTGGGTTCTACTCTGCTGGCAGGTTGCTCCAGCAACGCTAAAAT
CGATCAGCTGTCTTCTGACGTTCAGACTCTGAACGCTAAAGTTGACCAGCTGAGCAACGACGTGAACGCAATGCGTTCCG
ACGTTCAGGCTGCTAAAGATGACGCAGCTCGTGCTAACCAGCGTCTGGACAACATGGCTACTAAATACCGCAAGTAA
>Cluster_27754
ATGGTCACAGAATACCGAGGCAATATCGGAGATACTCAGAGCAGCATACGTGGCGAAGAAAATATTGTAGGAAGACAGTA
TTCTGAACTACAAAATCACCATAAAACAGAGGCATTATCTCAGAATAATAAATATAATGAAGAAAAATCGGGTCAGGAAA
GAATGCCCGGGGCTGACAGTCCACAGGAACTGATGAAAAGAGCAAAGGAGTATCAGGATAAGCACAAGCAATAA
>Cluster_64301
ATGTGTAATAACCATTATTCAGATACCACTGTTACGGTAATTAAAACTCTGATGGATGCGCTTATTGACATCTCTGTTAT
TGCAGACAGGGCGCATAAGCACGCCACCAGTGAAACAGAATATGCCGGGACTTTCGTTCCTCATTCACTGGCTGTCATGC
AAATTAGCGCCGACCAGGCGCTGGAAACAGCCAGTAAAATGCTCATGGCTGATGTGCAGGAGGTACATCCATGA
>Cluster_24669
ATGACTATCGAAAATACACCGGAAAACATAAAGAAATTACGTAAAAAAATTGGCCTTACCCAGACAGAGTGCGGTGAGAT
TTTTGGCGTAGGCTTAAGTACATGGCAGAAAAAAGAAGCTAAAACCCACAACCAGCTTAATTTATCGAAAGGTGAATTTG
AGTACCTGTTATTACTTGCAGGGGAGCACCCTGAATACGTCCTGCATAAAAAAGCTGAATCCCGCTCACCGGAGGAACCG
TGA
>Cluster_22100
ATGATTGATGAATTTCATGTGATGTATATGTATAAAAAAATCCAAGCAGAAGCCGCAACCACTGACCTCAAAACACTTGA
ACAGCTTTTAAAAAAGTTCAGGAGCGTGGTTAACGAACGTCGGGAAGAATACTATCAGGAGATTGGTGATAAAAAAGCGC
GTAAGTTGAAAATGAAAAGAGCACAAAGATTACTTGAGAAAATGGCATGTGACCGGGTTTCGCCAGAAGATCTCATTGAC
TGA
>Cluster_60047
ATGGATGTACAGCAGTTTTTTGTCGTTGCCGTTTTTTTCCTTATCCCGATATTTTGTTTCCGCGAAGCATGGAAAGGCTG
GCGCGCAGGCGCGATTGATAAACGGGTTAAAAATGCACCGGAACCGGTGTATGTCTGGCGAGCAAAAAATCCCGGACTCT
TTTTCGCTTATATGGTGGCATATATCGGCTTCGGAATTTTATCTATCGGCATGATTGTTTATCTTATTTTCTATCGTTAA
>Cluster_19629
ATGAGCAAAACTATCGTATTGCTGCCGCCCCGCAAGAATACAGACTGGGCTGCACAATTAAAGCTGATTTCTGAATCGCT
TGAGGTGAGTCAGGCAGACCTCGCCCACGCCTATCAGGTTGATCGTCGGGATATGGGTAAAGCGTATCACGGTGTGCGCA
AACTGCCGGAGCGTTGTGTGCCTGTTCATATGCTGTTGTTAGCGCAGGTACACGACTTTAGAGCATTATCAGGAGAATAA
```

>Cluster_66848
ATGATCACAGGAAATGGAATAAACACTGTAACTGTCAATGGAAAAGTGAAACACATCACAGAACTGGATTATATAACACT
TTGTTTAGAGTGGACAAAACTACGAGAGGAAAACAATCGTCTTTATGAAATTAATAACCAGGCAAACAGAGGGTGGCGCG
GTTTCATCCTAAGATTGATAGGGGTTAACCTGCCAGATAAAAGGACTGAGTTTACACAACGTCTCTTATTGACGCGCAAT
ATTTCGGGTTCGGTCATGAAAAAGTAA
>Cluster_36452
ATGGAATGGCTGGTCAAAAAATCGTGCTGCAATAAACAAGATAACAGACATGTACTTATGCTCTGCGATGCTGGCGGCGC
GATAAAAATGATCGCCGAAGTGAAGAGTGACTTCGCCGTGAAAGTGGGGGATTTACTCTCGCCTTTGCAGAATGCGCTTT
ATTGTATTAATCGTGAAAAGCTGCATACGGTGAAAGTCCTCTCGGCAAGCAGTTATAGCCCCGATGAGTGGGAACGGCAG
TGTAAAGTGGCAGGGAAAACTCAGTAA
>Cluster_62938
ATGAAAACCAAACTTAACGAACTGCTTGAATTCCCTACTCCTTTTACTTACAAAGTTATGGGGCAGGCGTTACCTGAGCT
GGTTGATCAGGTGGTTGAAGTGGTACAGCGCCATGCGCCAGGTGACTACACCCCAACGGTAAAACCAAGCAGCAAAGGCA
ACTACCACTCGGTATCTATCACTATCAACGCCACTCATATCGAGCAGGTTGAAACACTGTATGAAGAACTGGGCAAAATC
GATATTGTCCGCATGGTTCTGTAA
>Cluster_24862
ATGTTTGATGGGCTGTTTATGGATCAGCGAGAAAAGGCCAAATTGTTGGCTTATGTATTAAAAACCGAGTGGAACGCAAA
GCAAAAACACATCGCTTTAGTACTACAGGTATCAGAAGGAACTATCTCTTTGTGGGTGAAAGAGATGTCTTTAAAACATC
AGATTTATAAACTTAGTCAAGAATTACAAGAGGTTAGGGCTTTAGCTCAGGGGCTGGTAAGTTCTGGGCAGATTGAGCAA
AACCCTGGTTTTTTACCTAGTTTCTAA
>Cluster_11165
ATGAGCGAACAGTACCTGATAACGCTCGATGAGTGGAAGCCAAAACGGTTCAGTCTCCCAATAACAAACACTACCCTGGT
GAAATACGGAAAACTAGGATACATCGTTCCAAGACCACAAAAAATTCGTGGGCGTTGGCTGATAGATCGCCGAGCAGTAT
TTGTTGGACCTGGTGAAACAGGAATTGCGCCGGAAATTCATACTGGCGATGATGATGCACTGAAGGAGATTTTAACTCAT
GTCACCGAGGCCACGAAAAAACAGCACTGA
>Cluster_14900
ATGGTACATGACCGCATAGCGGAAGAACTCGAAGCGAAAGGATTTTACCGGAGGGCGGCGGCGCGATGGGGTGAAGTCAT
GATGCTGGTGGAGACAGACAAGGAACGGCATCACATCACGATGCGACGGCTGGAATGTTCCAGGAAGGCACAGAGGCCAC
CGGAGCCGCCGGATAATTACGGAGACCTGAAAAAGGCGGTCGATCGCACTTATGCCGAAATGGGTATGGATGGTGCTGGT
GATGAAATATGGCGAAATTACCAGGACAGATAA
>Cluster_62533
GTGGTAACGAGCAATCAACCACCAGCTCAGGATTATCCCACACTGGAAGACAGCATCATGACAAAAAATATTTCTTTACT
TGATAACCAACTGGTGGATATGCGCTTTATCACAAAGCTAACGGGGCTGACGGATAAATGGTTCTATAAGTTGATAAAAG
ACGAACTGTTCCCGAAACCAATTAAGCTGGGCCGCAGCTCCCGCTGGAAGAAAAGTGAAGTCGAATTATGGTTACAGCAA
CGGATTGCCGATTCACGCGGTCATTGA
>Cluster_12373
ATGCAAATCTATATGCTTTTCCGGCAACAAGCAGGAATCTCAGCGCTGAGGCAGGAATTAGAGGTAAAAACGCCCTACCG
GGCCATGAATCACCCGGTCATTGGAGTAGTAACCAAAGCAGATTTAGCCAGCATGGAACACATATCTTTGGTGAAATGTT
GGTTACGGGAAGCTGGAGCGCACAACGTGTTAGTAACCAGTGCGGTTAATAACAATGGAGTTACAGAACTTTTTGCCTTG
CTGCATACAGAAGAAGGCTGTTGCTAA
>Cluster_66865
ATGGATCAGGAAATGACGTTTTCACTCAGCTATGAACAACTGACCCGGTTTGCGGAAAAACGCATCCGGGAATGCAATCT
GGACAGCCAGGGCGCGATTTATCTCTGCGAGTCAGCCAAAGCCAAAGCCGGAGCCGTGCTGATCTTCTGGCATGAACTGG
CCATAAATGGCTATGCCTCAATGAATGCCATAAAGAGACAGGAGCTCATTGACGCCGACTTTCAGCGCCTCAGAAACCTC
ATCTGGCCGGAGGATGACAGGTAA
>Cluster_10127
ATGTCAATGAATGATACGTATCAACCAATCAATTGTGATGATTACGATAATCTTGAGCTCGCCTGCCAGCATCATTTAAT
GCTGACACTTGAGCTGAAAGATGGCGAAAAATTGCAGGCAAAAGCCAGCGATTTAGTCTCCCGCAAAAATGTGGAGTATC
TGGTCGTCGAGGCCGCTGGCGAAACCCGTGAGCTACGTCTGGATAAAATTACCAGCTTTAGCCACCCGGAAATCGGTACG
GTGGTGGTGAGCGAGTCCTGA
>Cluster_21055
ATGTTTATAGTTCTGAATACTATGGTGTTTTACACGTTCATTCTGGTTATTATACGTAGAAAAGAATTTATCGGGCAACG
CCGAGATGACGCGGCCCCGTTAAACCGTGTAGTGGTAGATGATGAGATCAGATACAGGGATCAACTGACGGTGAGCGCAG
TCCCCACATTTTATCCGGAGTTTACCGCTATTCCCGGCTGCCATTCGTTAGCACAGGCCCGCAAGTACCCTGATTTGCCG
CTGGTTTTGCTTTCCCATTGA
>Cluster_44
ATGACAACGTTCACCGACAAAGAACTGATTAAAGAAATCAAAGAGCGCATCGGCAGCCTGGACGTTCGAGACAATATTGA
ACGCCGTGCTTATGAAATTGCACTGGCATCGCTGGAAGCAGAGCCAGTAGCATGGATGCATGTAAATAACGGCATCGGAA
TACCAGCAATAACAAGGAGTAAAGATGTTGCAGAGAGTTGGTTATCAAAAGGCTGGTATGTCCAACCCTTACATCTAGCC
CAGCCTGCATCAAAGCTATAG
>Cluster_14930
ATGGACGATATTGTTTTTGCGGGGAATAGGGCGCTGTATCTGATTTTAGTTATGTCTGCAGGACCTATTGCAGTTGCGAC
ATTTGTTGGTTTATTGGTAGGGCTTTTTCAAACGGTAACGCAGTTGCAAGAACAGACGTTGCCATTTGGCGTGAAATTAT
TGTGCGTAAGTATTTGCTTTTTTTTAATGAGCGGGTGGTATGGTGAAAAATTATATTCATTTGGTATCGAAATGCTGAAT
TTAGCATTCGCGAGAGGATAG
>Cluster_1534
ATGGCAACTTTGACAAAAAAAGAACGGGCATGGTTGAACGAATTACAGGAAGTTCTTGATCGCTGTCCATCACCGAAAAA
AATTGGCTTTTACACCATTGGCGATAAAAGCATTTACCTGTATGACCTACGCCGCATGGATGAAATCATGGAGGCTCTTG
ATAATCGTTCGTCGATGGATTGGTGTGTTGCTGTTCATGATATGAATGCAGGGTTTGATGAAAAGATTTTGTTCCCCTCA
TCAGTTGAAAGCACTGCGGGTTAA
>Cluster_34175
ATGGGCCATGGCCTTCGGCGGCGGTGTCGGGAAGGTGTGTTGGCCGGACGGATACTGTTGAACTACGTTGTCTGGGGCAA
CGGAAGTGTTTCGGCCCGACTCTGGAACGCGATCCGCAGCGACGACTGGGCAATTCCGCACGTTGGCCTGAGCAGCCTCG
GGGAAATCGTCGTCTGGGCGCGCCCAGACGAGTTTCCGCCAAGGAACATGCAGACAAGCAAGGGGCTGCGGGCACTCGGC
TACAACGTGAGGATCGGTGTTTGA
>Cluster_63891
GTGTCGGAAGTCGAGGCTGAGGTCTTTCTGAAACTCGTAACCACGTCTGCGGTAGTTCTGAATCAGCCATTCGGCCTGTT
CTTCAGTGCATGGATCATGCTGGAACCAGTCAGATTTGAATGCATGAGAACGCCGCCCGTGCCTGCTGGCAAGGTCGGTA
TCAGAATTGTGATGTTTGGTATTGTGCGCCATCGGTTTTCTCTGCTGGCGCAGCAGGTGCCAGTTGTTCAGGCTGGCCTG

TGGATTGTAAACCAGAATACGTAA
>Cluster_57169
ATGAGCGGAAAACCGGCAGCGCGTCAGGGCGACATGACGCAGTATGGCGGTAGCATTGTTCAGGGTTCAGCCGGGGTGCG
CATTGGTGCCCCCACCGGCGTGGCCTGTTCGGTGTGCCCCGGCGGAGTGACGTCCGGCCATCCGGTCAATCCCCTGCTCG
GTGCAAAGGTCCTTCCCGGTGAAACCGACCTTGCCCTGCCCGGCCCGCTGCCGTTCATCCTCTCCCGCACCTACAGCAGT
TATGGCGACGTGAACGGGACCTGA
>Cluster_46970
ATGGCTACCCCAGTTTCACTGATGGATGACCAGATGGTCGACATGGCATTTATCACTCAACTGACCGGCCTGACAGATAA
GTGGTTTTACAGGCTCATCAGGGATGGGGCCTTTCCGGCTCCCATCAAGCTGGGCCGCAGCTCCCGCTGGCTGAAAAGTG
AAGTGGAAGCCTGGCTGCAGGCGCGTATTGCACAGCCCCGCCCGTAA
>Cluster_43643
ATGGCTAAATTACCTCAAGAAAAGCCGAAAGAAGTCATCCAAGCCGGATTTGATGATGTTAAAAAGGCCTATAAAAAAGG
CGCTAAGACTGTAAACGATACCTATGCTGAGCAAGCAGGTCGTGACTTACAATCAGGCGATGGTGAACTAATGGCTACCT
ATCGGCGCAATAAAAAAGCGATAGACAAGTTAGATGCTGAAGGTTGA
>Cluster_41473
ATGGACTGGCTGGCTAAATATTGGTGGATTCTGGTGATTGTCTTTTTGGTAGGCGTCCTGCTGAACGTGATTAAAGATCT
CAAGCGCGTCGACCATAAGAAATTTCTCGCCAACAAGCCGGAGCTTCCCCCGCATCGTGATTTCAACGATAAGTGGGACG
ATGACGACGACTGGCCGAAAAAGGATCAACCGAAGAAGTAA
>Cluster_44655
ATGACTACGACTATTGATAAAAATCAATGGTGTGGACAATTCAAGCGATGCAATGGATGCAAGCTGCAATCGGAATGCAT
GGTTAAGCCTGAAGAAATGTTTCCTGTAATGGAAGATGGGAAATATGTCGATAAATGGGCAATACGAACGACGGCAATGA
TTGCCAGAGAACTTGGTAAACAGAACAACAAAGCTGCCTGA
>Cluster_52021
ATGAAACAAGAAATTATCCTGAAATATGGCAATGAAAAGTTAACACTAACTGAAGATGTGTTGAATGCTACAGCCTACGC
GCTTGGCATCACTCCCACAGAATTAAAGAAACAGTGTGAACGTCTTTATAAGGCTGAAAGCAATAAACCATCTCACCAAG
ATTTGATTAATGCTAAAGCTCAACAAATCAAACAATCTAAGGAAAAATAA
>Cluster_48599
ATGAACACCACTATTTCCAGCCTAATAGCTCTTGAGATCGGACACGTACAAAAATTAGCTGATGAGTGTGTAGCTGACAT
CCTCACCGAGCTACCGAATGAGCAGATTGAGGTTGGTGTGAATGACACAACTGGCTTTATATTCGAACTTAACAACAAAC
GCTTCACGCTTATCAATACCGGCTCCGGGTCTTTAGCCGTCAGAACCTGTTAA
>Cluster_43592
ATGAACGGCATAAAAATGGGGCTGGGGATCACGCCGGGTGAGCACATTATCAGTGCCAACTCTGCGCTCAGCAGAAATAT
CCGGCATTGTTTCTGCCTTTCCTGTCGTGGCCGGTTGATTTTGCAGACAGATGCTCAAGGGGCGTGGTTTGAGCATGATT
TACATGCCCTGAGTGCGCAACAGAAGGCGGCCTTGCAGCCCCTGGATTAA
>Cluster_45264
ATGTGGATGGAGTTCGACAGGGTATCCCCGCTGGGTGATGAGCGCGGGGATATCCGTAATGCACAGATCGTGAAAGCGGT
TTTCGGGGCACAGGGGATGAATGTTGCACTGAAGGACGCCATGCTCTGCTGGGGCGAGGATGAGGATAAGCCGGAGGTGG
ATCCGTTTGCGGCGCTGGAAGACGCGCTGAGCCTTGCAGCAATGTCATAA
>Cluster_64074
ATGAAAGTGAAGGCACTGGAAGGCGACACCGTGGATTCGCTCTGTTTCCGGTACTACGGCACGACGCAGGGCGTCACCGA
AAAGGTGCTGGATGCCAACCCCGGACTCTGTCAGCAGGTATTTCTGGACGCCGGGCAGGACGTGGAGATGCCGGAGCCGG
AGAAGAAGAAACGAGAAATGATTCAGTTGTGGGGGGAGTAG
>Cluster_62117
ATGCCTGAAATCCAGTGTTTATCTATCTTTCCCGCTATATTAACTCTCTCAAGGTCAACCGATATCAACGTACATCTACC
AACATATGTTGGTACAGATGATGGTATTTCCGGTTCGATAATGCTTGTACCAACAGGGAGGGAATACGATGGCATTAACA
GATATCAAAGTCAGAGCAGCCAAGCCAACGGATAA
>Cluster_48782
ATGAACAAGCAGCAGCAAACTGTACTCAACATGGCGGGATTCATAAAAAGCCAGAGCCTGACGCTGCTCGAAAAACTGGA
CGCACTCGATGCTGACGAGCAGGCCACCATGTGTGAGAAGCTGCACGAACTCGCAGAAGAACTCCAGAACAGCATACAGA
CACGCTTTGAAGTAGAAAATAGCACAGAGATATAA
>Cluster_22597
GTGAACACTCGCCAGCTACTGAGCGTCGGTATCGATATCGGCACCACCACCACCCAGGTAATTTTCTCCCACCTGGAGCT
GGTTAACCGTGCGGCGGTGTCGCAGGTGCCGCGCTACGAATTCATCAAACGCGAAATTAGCTGGCAAAGCCCGGTGTTCT
TTACCCCTGTCGATAAACCTGTCTCTTGA
>Cluster_6275
ATGGAAAAAACGCCACCTGCGCGGCCTTTGTCCCTGCCTTCGGCTTTTTGCTCACATGTTCTTTCCGGCGTTATCCCCTGA
TTCTGTGGATAACCGTATTACCGCCTTTGAGTGCGCTGACACCGCTCGCCGCAGTCGAACGACCGAGCGTAGCGAGTCAG
TGAGCGAGGAAGCGGAAGAGCGCCTTATGTGA
>Cluster_1683
ATGCTATGCGTTACCTTTGAATACCACACGGATAAAATGATTCGCTACATCTCCGACCTCCTGATTGAAGGAAACGGATT
CGGTGACATCCATAACTCAAAAGATATCTTCATTCACGCCACCAGCCCAAGCGAAACATTAAAAACAGCAGTTACAACGG
AATGGTTTGAACGAAATAAAGTAGAATTAGGCTATTGGTAG
>Cluster_3005
ATGAGCGAGAAACCCGACGATTTACTCACCCCGGATGAAGTATGCCAAAAGTTAGGTATTACACAGAAAACGCTATGTGA
GTGGAATATTAAGCATCGTCATCGGGCTATCCTGGCACCAATTCGTTTCAGTGCAAAAGTAGTTCGTTATGAGCGCCGAA
ATATCGACGCTTTTATTCAAAAATGTCGCAGCCAGTATTAA
>Cluster_20334
ATGAATAACTCAATTAATGCCCCTCGACTTACGTCCGCACTTCAATTAATCGAGCAAGCAGCGGCTGTCCTGGTTGCTGT
CAGTCTTTCGGCTGAAGAAATGGACGCTGCTGATGTCGTGGATGCGATTAAAGCGTGCTCATCTTTGGTTAACGATGCCC
GTGCAGAGCTGGTAATTCTTGGGGGTGAAAAATGA
>Cluster_4762
ATGGTGATACGCAAAAAGAAGTGCAGGGACTGTGGTAACGCGATTACTCATAATACGGTGTGTTGTCCGTACTGTGGTTC
TGTCGACCCCTTCGGCTATTACCGGAATACGGACAGGATCTTCACAGCCCTGCTGGCGCTGCTGGTTGTGGTTTTGCTGG
TGACATCTGCTGTCAGCATGTATGTGCTGTGGTAG
>Cluster_2898
ATGCGGCGTGAACGCCTTATCCGAACTACGTCCGACAAACAAATTTCGTGCGAATTACCGCTAAAATCGCGTTTTCCTGC
CGACGCACACGTTTGCGTATCATATCAGAAAAAAGGGCCGGATGATTCCAGCCCTGTATTTTTACTTGCTAAACGCAGCC
TGGAAGACAGCTACCAGCGCGTCGTTCTGACTCTGAGTCAGAGTATGACCTTTCGGATCGATGAACTGTAG
>Cluster_51317

ATGCGTGATGATCGTTTTAATGCCCTGAAACAGGAATTTGATGGCGCACCGGAACACGAAGCAGACGCATTGTTGGGCGT
TGCTGACATGATAAAAGCCGCATTTTTTCTTATCAATACCAGTGGCTACAGGTCAGAGGGTGCGGAGATTCTCAATATTG
CGTCGGACTATGCGGAATATGTTGCAGAGGCGCGTTACAGAAGAAAATTCCCGGAGGTGGTGAGCCATGAATGA
>Cluster_53540
ATGAGGGATGATGACCTGGAGCACTTAAGTGCCAATTACTGGATAACCACAGTTCTCTGCGGCCTCTTTTTGGGGATATT
CCTGTTCATGCTGCAACAGCATCAGCGTGGTTTAGCTCTGTATTGTATTGCGGCTCTTTCACTTATTTGCGCCTTTTTTT
CTTTACGCCTTTCCCGACAATTGCAACACATGAAAAAGGAACGGCGAAATATTCGACCTCATTGA
>Cluster_59480
ATGCTTAAAACTGACGCTCTTTTGTATTTCGGTTCAAAAACAAAACTTGCACAAGCAGCAGGTATTCGTTTGGCTTCGCT
TTATAGCTGGAAAGGGGATTTAGTTCCCGAAGGTCGCGCGATGCGTCTACAGGAGGCATCTGGCGGGGAGCTTCAGTATG
ATCCCAAAGTTTATGATGAATATCGTAAGACGAAGCGGGCGGGGCGGTTGAACAATGAAAATCACTCCTGA
>Cluster_17630
ATGCATCAGGATATCAAAGAGTACAGGGCTGGGAACCGTTGCGCAGCGTATTCGCTGGGCGCGTCCAGAGCAGAACAACG
CGGCGATTATGCCGAAGCTGAAAAACTGTGGCGTAAGGCAGCACAAAGCCCGTGTAGTACCCTGCGCCGCATCTGGGCGG
AACACCGGGCAGAGTTCTGTGCCAATGCTCACCTGAAAGGATGGAAGCCGCCTCATGAACGCGAAACGCTTTAA
>Cluster_29447
ATGAGCGAATATTTCAGAATACTTCAGGGACTGCCGGACGGTCCTTTTACCCGCAAACAGGCCGAAGCCGTTGCCGCACA
GTACCGGAATGTCTTTATCGAGGATGATCAGGGAACGCATTTTCGCCTGGTTGTCCGTCAGGATGGCACGTTGATCTGGC
GCTCCTGGAATTTTGAGGACTGTGCCGGGTACTGGATGAACCGGTACATCAGGGATTTCGGGATTCTTAAGTAA
>Cluster_64472
ATGTCAGAGCTTGAACAACTTAAAGATGAACTGGCTTTACTTAAAAAGGAGCTAAAGTCTCACCAGGTAATACTGTCTGC
GTTGGTTGGTCGTATGAGCTACGAGGAGAAATCCGACCTATCAAAGCATGTTGCAAAAGCTAAATCAGAGCTTTATCCGG
GTGATGATTTCAGCCTGGGAATATTCAATAAGTTGCTTCTAGTAACTGATTACAAACCGATTCCCAGAAAGTAG
>Cluster_2971
ATGCCGCATCCGGCATCTTTCCTAACGCCGATCGAGAGAAATCGCCCCCGGTCCGGTAATTGCCAACAGCAGGAATGCGC
CAGCGATACTGACATTCTTCCAGAAATTAATCATATTTGGCCCAACCGCATCGCCGGTCATATCCTAGTAATGGTGACCA
ATCACCGCCGTACCCAGCGTGTAGAAAATAAACAGCACCGCCAGCGGACGGGTGAAAAAGCCAAGCACGATTAA
>Cluster_58211
ATGAGCGCATTCAAACTCCCGGATACATCTCAATCACAGCTCATTTCAACAGCTGAGTTAGCTAAAATCATTAGCTACAA
ATCTCAAACCATTCGTAAATGGCTTTGTCAGGACAAATTGCCTGAGGGGCTACCTCGCCCAAAACAAATCAATGGCCGCC
ATTACTGGTTACGTAAAGATGTCCTCGATTTTATAGATACATTTTCTGTACGAGAAAGTCTGTAA
>Cluster_26136
ATGTTCAAAGATGATGTTCTGCGCTATTTCAAAAAAAAGCGACTAGTAGCTGAGGCTCTTGGAATTTCACATGTGGCTGT
TGTGCGGTGGAAAGCAGTTATTCCCAAACTTCGCGCAATGGAACTGGATGAAATTACTAACGGTGAATTGAAATACAACC
CAGAACTTTACAAGAAGCAGGATAGCACCTCGAACGAAGGAAAGAATGATTCATGA
>Cluster_58475
ATGTTGGGGAGGCAACGCCTCCCGCAACATATCTTTTTCGTAATCAGATTAGAACTGGTAAACCAGACCTACAGCAACGA
TGTCATCAGTGCTTACACCGAGTGCTTTAGGGAAGGTGCGAATAAGCGGGGAAATTCTTCTCGGCTGACTCAGTCATTTC
ATTTCTTCATGTTTGAGCCGATTTTTTCTCCCGTAAATGCCTTGAATCAGCCTATTTAG
>Cluster_11887
TTGTCCCGTAAAATGACAGGAATTGTCAAAACCTTTGACGGCAAAAGCGGCAAGGGTCTTATCACCCCATCCGATGGTCG
TATCGATGTCCAGCTTCATGTTTCAGCGCTCAATCTCCGCGATGCAGAAGAAATTACCACCGGATTACGCGTGGAATTTT
GCCGGATAAATGGTCTGCGTGGCCCTTCAGCTGCCAATGTTTACCTTTCATGA
>Cluster_9194
ATGAAAAAAGATATTCACCCGAAATACGAAGAAATTACTGCTAGCTGCTCTTGCGGTAACGTAATGAAAATCCGCTCCAC
CGTTGGTCATGACCTGAACCTCGACGTGTGCAGCAAGTGCCACCCGTTCTTCACTGGCAAACAGCGTGATGTTGCTACCG
GTGGCCGTGTTGACCGCTTCAACAAGCGTTTCAACATCCCGGGCAGCAAATAA
>Cluster_24756
ATGAACTATGCCGGACATGAAAAACTTCGCGCCGAAGTGGCGGAGGTGGCCAATGCCATGTGCGACCTGCGTACAACCAT
GAATGAGATGGAGCGGCGGTACAGCTTTAATGCTGACACCCTGCCGGAACGTCTGGTGCGTCAGACGTTGTTTCGCGCAA
ACCGCCTCCTGATGGAGGCATATACCGAAATTCTGGAACTGGATTCCTGCTTCAAAGATTGA
>Cluster_10566
GTGAAAAAACTGATGGTGACGATTGGTCCGTTCGAAACAGAAGTTAGTTTTCGTGTTGTTCAGGGGGAGAGTGTACTTGT
TGAAGATGTATTTCATGGAAAATCAACAGGTCCTTATGTAAAAGAATATCTTATCGACGCCACGGATGAAAATATTGAGG
TGGTGTACGATTCCGTCAATCACCCTGATTTGATCATTAAGGCAAAATTGAAGCCACTTTATTGA
>Cluster_26970
ATGGTTAAAAGAGAAATAAAAGCAAAGACAACGTTTATCCGTTTACCAGAAGTAATAAAACGCACCGGATTTGGAAAAAC
GTGGATTTACGAACTGATTAAAGCCGGACGGTTTCCATCGCAGGTGAAAACAGGGATAAGAGCGGTGGCATTCATTGAAA
GTGAGATTGATGAATGGATTGACGCGACTATTCGAAACTCACGCCAGAACGCTGCATAA
>Cluster_59903
ATGAAAATTATCACCCGTGGTGAAGCCATGCGTATTCACCAACAACATCCGACATCCCGTCTTTTTCCGTTCTGTACCGG
TAAATACCGCTGGCACGGCAGTGCTGAAGCGTATACCGGTCGTGAAGTGCAGGATATTCCCGGTGTGCTGGCCGTGTTTG
CTGAACGCCGTAAGGACAGTTTTGGTCCGTATGTCCGGCTGATGAGCGTCACCCTGAACTGA
>Cluster_17521
ATGCGAAACAAAGGCTTTAATCCACCTGATACACACAAAGAAGCTAAGCGTTTGCGCTTCCTTCGTTCCATTGATGAAAG
AACTCAAATCTCTTTTGTGAAAGTTGCCAGAACTGAGCTTCTGAAGGCTGAGGCGAGGGCGTTGCTTCCGTCTCTACCAA
AAGAGGAGGGATATACGTTCATTCCAAACGCATTTCTGGAAAAGCTGCTCAAAGAAGACATATCCGTAAGTCAGTTTAAC
AATGTTCTTAAGGTCTTTCGTCAAGGCAGGTAG
>Cluster_34960
ATGGATTATTCACAGTTAAGTGATTTTGAAATTAACAGAATGGTAGGAGACATAATTTTTAAAGGCCTTTGGGCAAGTAA
ACCGGAAACATCAGGGAATAACACCAACAAATGGTATTACGGAAATGCTGATACAACTTTTGAGCCATTAAATCATTTGC
CTGACTACTGCAATGATCCGAGCGCTTCATGGCCGATTATTGAGAAATACAGGATTTCTATCTTAGACCAGTTAACTGAA
TGGTGTGTGGATGCAAAAGGCGTAAGCCCAATATTTGATACCAGACCTCTCCGCGCCGCCATGATTGTCTTTCTCCTGAT
GCAGGAGGCCAATAATGCTTAG
>Cluster_24992
ATGAGCATTACCACAATCAACAGCACCATGCAGGCGCAAATCATGCAGGACGTGCAGCGGATGCAGGCCAACGCCCAGGC
GCCGGTGCTTCCCGCCATGACCTTCAGTTCCACCGACCCGGACGTTTCGTTTAACCGCATTATGAGCGGTGCGCTCGGTC
ACGTTGACCAGTTCCAGCAGGTGGCGGAGCAGCAGCAAACGGCTGTCGACACGGGCAAAAGTGATGACCTCGCCGGGGCG
ATGATCGCCAGCCAGCAGGCGTCGCTCTCGTTCTCCGCGCTGGTGCAGGTGCGCAACAAAATTGCCACCGGATTTAACGA

TCTGATGAGCATGTCGATCTAA
>Cluster_67098
ATGAAAGGTGGAGCAATGGAGAACAGATTATCCGGACAGAAGGGGGTGTTTTTTTTCTCAAAGAATAAAGGACATAAGCT
TTTGCCGGCAGAGTTGCCGGAAGAAAAATTCTGGCTGCTTGCAGAGATATCGCCGATTCGCAGTGAAAAAGTGCTTTATG
CGCTGAGAGATTATCTGGTTCTGGGATACGAGCGCAAAGAGGTTTGTGCGCGATACGATGTGTCGTCAAGTTATTTTTCT
ATAGCGCTGGGGCGGATATCGCACGTAAACAGAGTTGTGTATTCGCTGGCACCTTATTATGCAGATCCGGGCAATGATTT
TTTTGACAGACCGTATTAA
>Cluster_14937
ATGAACATAGGGCGACTGCGTGACAGGGTAACGATTCAGACCCTGAAACAGACCAGGGATATAACCGGCGAAATACTCGA
AACGTGGGTGGACGGTCACACACTCTGGGCAAGCGTGAACATGATCAGCAGCAAGGAGGCCATTTCATCGGGTGCAGAAC
TGGCGACTGGAACGGTAAGGATATGGATACGCTACAGGAAGGACATCAACGCCACCAGCCGGATAAAGGTCAATACGGGG
CCGCTGGCGGGGCGTGTACTGAATATCATCGGGCAGCCGCTGCCGGATGCCGCCAGGACACGCCTTGAAATTCTTTGTCG
TGAGGGCGCGGAAAAATGA
>Cluster_42802
ATGAAAATTTCCCGCCTCGGAGAAGCGCCCGATTACCGCTTCTCGCTGGCTAATGAGCGTACTTTTCTGGCGTGGATCCG
TACTGCACTAGGATTTCTGGCGGCGGGGGTTGGGCTTGATCAACTTGCGCCAGATTTCGCCACGCCCGTCATCCGCGAAC
TGCTGGCGTTGTTGTTGTGTCTGTTTTCCGGCGGGTTAGCGATGTATGGCTACTTGCGCTGGTTACGCAATGAAAAAGCG
ATGCGCCTGAAAGAGGACCTGCCCTATACCAACAGCTTATTAATCATTAGCTTAATATTGATGGTCGTGGCAGTGATCGT
CATGGGACTGGTGTTGTATGCCGGATAG
>Cluster_55598
ATGATCTCACTCCCATCAGGTACCCGTATCTGGCTCGTTGCCGGCGTTACCGATATGCGTAAATCCTTCAACGGACTGGG
AGAACAGGTACAACATGTGCTGAATGATAATCCCTTCTCCGGTCACCTGTTTATCTTCCGTGGCCGACGGGGTGACACCG
TCAAAATTCTTTGGGCTGATGCTGATGGTCTGTGCCTGTTCACCAAACGCCTGGAGGAAGGCCAGTTTATCTGGCCTGCG
GTACGTGACGGCAAGGTATCCATTACCCGCTCGCAACTGGCAATGCTCCTCGATAAGCTGGACTGGCGTCAGCCAAAAAC
ATCCAGCCGTAACTCACTGACAATGTTGTAA
>Cluster_51325
ATGATTGAAAGAGATAGTCAGCTACGAGAGCTATTAGATATCGATGACAAAATGAGTTTAAGCGAGCGCCTTGTTGATAT
GGATCAGGTAGTGGAGCTTACAACCCTTAGCCGTCGCACATTGCTAAACCTTGAGGCTCGCGGAGAGTTCCCCGAACGCG
TACAGGTTACGAAAGGGCGCAAGGCATGGTATTTAAGCGAGGTTGTTGCGTGGATTAACAACATTCCGCGCGCATCAAAT
TATTGCCTTGTTCCCACCCCCGAAAAACCTGATGCGGCGTTATGCCTCAAGATTGAACGCGCCAGGCGTAATGCCCTGAA
AGGACGCAATAAGTTGATTGGTTGA
>Cluster_2735
ATGAAACGATGGATATTTTCATTGCTGGCATTAGTGTCTGTTGGTGTAAGTGCAAACACCATAACGATGCAATGCGGCAA
CTTTCGTATGGATGCGATCCCTGATTCATTATTTAAAATCAATGGCGAAACAGTAACGTCCCAAAAAGTAAAAATGTTGG
GTAAAGACGGTACAGGCATGCAGATCAAAATGGACCTGATGCCTGCCAAAGATGGCAACAATTATGGGTTCGAGTATATC
CATCGACCTGGCACTAAAACGCGTTTCCTGAACGTCCAGTTGTTGCAGAACAGCATGGATGCTCCGAAAATTATTGGCTC
TTTCCCGTGTAAGAAAGTTGTTGGTTGA
>Cluster_3554
GTGTTTTTAGGAACAAAATCCGTAGGTGAATATGCTCTTAATATTCTTGGTCAAAATGTTTCAAGAGTCACAACAGGGAA
AAAACCTTATGATATATTATTCCTGCACGAAGCTACAAAACAAGATTTTGATAAAAAGAAAACAGAGTTTACCTTTCCTG
GTGCAAACAGAAGTTATCTACAATCAAGTAACACCGATGTTGCTGCCGCAGCAGCTATAAGTATTGCGGCAACAGAGATG
AAAACTATCCTACCAAAGGATTTAACACCAGAAAAATACAATAAAATTTATCTGCCAGGGGATGGTTCTGCTGGCCTACT
GATCCTACCCACGTAA
>Cluster_614
ATGAGTCATTCTACCGATCACAGCGGCGCGTCCCATGGCAGCGTAAAAACCTACATGACAGGCTTTATCCTGTCGATCAT
TCTGACGGTGATTCCGTTCTGGATGGTGATGACAGGGGCTGCCTCTCCGGCCGTAATTCTGGGAACAATCCTGGCAATGG
CAGTGGTACAGGTTCTGGTGCATCTGGTGTGCTTCCTGCACATGAATACCAAATCAGATGAAGGCTGGAACATGACGGCG
TTTGTCTTCACCGTGCTAATCATCGCTATCCTGGTTGTAGGCTCCATCTGGATTATGTGGAACCTCAACTACAACATGAT
GATGCACTAA
>Cluster_39185
ATGCAACGCTGCAACGGATTTTTGATTTCTGGCATCGCCCTTACGGCAGGAGCGCCACTGTGTATTAGCTGGTTCGGTCT
TGTCGGCTTCTATTTTGGCGCATTGTCACTGGTAGCTGCGGCGGTCATTTCCTGGATAGTCTATTTCTGGCTCATTTATT
TAGTACTCACCCGCCCCCATAACCAACGTTTTGTAGAAAGATTTATATTGATTGTCGGTATTTGCTTTTTTCCATGGAGC
ATTGTGTTGATATGGGCATTCTTTGCCAGTTCAGCCAGAGCGCATAATCAACACGCAGAACAAATTAAAAACTCAACAAC
TGACGGTTAA
>Cluster_48707
ATGGGTTCATGTGCAGCTCCATCAGCAAAAGGGGATGATAAGTTTATCACCACCGACTATTTGCAACAGTGCCATATGGG
GCAAATGGTGGTCACCATCCTGTCGGCTGTGGCACAGGCTGAACGCCGGAGGATCCTAGAACGCACGAATGAGGGCCGAC
AGGAAGCAAAGCTGAAAGGAATCAAATTTGGCCGCAGGCGTACCGTGGACAGGAACGTCGTGCTGACGCTTCATCAGAAG
GGCACTGGTGCAACGGAAATTGCTCATCAGCTCAGTATTGCCCGCTCCACGGTTTATAAAATTCTTGAAGACGAAAGGGC
CTCGTGA
>Cluster_14889
ATGCCATATGCCAGCAATATAAGTATTGCGGTATTAGATGATAATGTATTGGAAAGTCAGGCTATTGAAACGTTGTCAGC
GATAGGTCTTTCTTATGAAAAATATAAAACAGCTAACAATGTGTATTTCAATATCATTGGTACGTTAAGTGACAGTGAAT
TAAATAAAATTAATAACTATGTTGACGAATATTATAAACAATGGGGTAAACAATATGTAAGATTTAATGTGAATTTGAAA
AATCAGGACACAAATAATAGTTCATTTAGCTACGGAGATAACCGATTCGAGAAGTCTCAAGGTAGCAACTGGACATTTCA
GGAATAA
>Cluster_32369
ATGGAGTTACTGGAAGCTCTGGCTGAAGCTGGTGTAGCTATTGGCAACTTCTTCAGTAATGACCTGATGACCGCCTATGG
TCGCCGCCAGCTTAACAAACGAGCTGAGGCTGAGCGTATCGCTAAAGAGCAGCGGCTTCAGGCTGAGCGTATGCGTGAAG
AGAACATGACCGATGCAGAGTGGCAGAAAGAGCTACAGCGTCGCGAACAGGTCAAAGCTGACCGCCGGACCTACGGTGAG
AACCTCCGCAGTGCTACCCATTCCGCTGGTCGCTCCCGTGCTTCTATGGTCGCTGACATTGAGTCCACGGACAACTGGAT
GGACAGCCTGTAA
>Cluster_51050
ATGAACCCTTATATTTATCTTGGTGGCGCAATACTTGCAGAGGTCATTGGTACAACCTTAATGAAGTTTTCAGAGGGGTT
TACACGGTTATGGCCCTCTGTTGGCACAATTATTTGCTATTGTGCATCATTCTGGTTATTAGCTCAGACGCTGGCTTATA
TACCTACAGGGATTGCTTATGCTATCTGGTCAGGAGTCGGTATCGTTCTGATTAGTTTGCTTTCCTGGGGATTTTTTGGT
CAACGACTGGACCTGCCAGCTATCATAGGCATGATGTTGATTTGTGCCGGTGTGTTAGTCATTAACTTGTTGTCACGAAG
TGCACCGCATTAA

```
>Cluster_60542
ATGAACACTCTACCTGCTACAATTTCGCAGGCGGCGAAGCCCTGCCTGTCGCCAGTGGCTGTCTGGCAAATGCTACTGAC
ACGCCTGCTGGAACAACACTATGGCCTGACACTGAACGACACGCCGTTCAGTGATGAAACTGTTATTAAGGAACATATCG
ATGCTGGTATCACTCTGGCCGATGCAGTCAATTTTCTGGTGGAAAAGTACGAACTGGTACGTATCGATCACAGAGGATTT
TCGTGGCAACAACAGTCTCCATATATTTCCGTAGTAGATATTCTGCGAGCAAGGCGCTCTACCGGCTTGCTAAAAACTAA
TGTGAAATAA
>Cluster_28184
ATGTACGAAGCTCTTTTAGTAGTTTTCCTTATTGTGGCAATTGGCCTTGTTGGTCTGATCATGCTGCAGCAAGGTAAAGG
CGCTGATATGGGAGCCTCCTTCGGAGCAGGCGCTTCCGCTACGCTGTTTGGTTCAAGTGGTTCTGGTAACTTCATGACCC
GCATGACGGCGCTGCTGGCAACGTTATTCTTCATCATCAGTCTGGTGCTGGGTAACATCAATAGCAACAAAACCAATAAA
GGTAGCGAATGGGAAAACCTGAGTGCGCCGGCCAAAACCGAACAAACTCAGCCAGCTGCTCCGGCTAAGCCGACCAGCGA
TATCCCGAACTAA
>Cluster_16388
ATGGCTGAGCTTCGTACACTCCAGAGCAGAATCAAAACACTGAATACCCGACGGGTGAATATTCTGAAGGGGGAGCAGCG
TCGTGTCAGTGGCAGTGCACGTGTTTCCCTCAAGCGTCATATCTGGCTGAGGGACGCCGGGCAGTGCTGTATCTGTGGTC
GTGTGGTTGACCTCTGTGACAGTGAACTCGATCACCGAATTGCACTTCAGTTCGGTGGTGGTAATGAGGAGACGAATCTT
TGGACGCTCTGTGCCGAATGCCATCGACAAAAGTCTGCTCGTGAAGCGGCGAGTGGTATGCCGGACCCGACGCTGCCGGA
GGTGTCCGGAGGTAGTGGCAGAGAGGACGACATCATCGGACTGTAA
>Cluster_60934
ATGGATATTGTATTTATAGAGCAACTTTCGGTAATCACCACTATTGGTGTTTACGACTGGGAACAGACCATCGAACAGAA
GTTAGTGTTCGATATCGAAATGGCGTGGGATAACCGTAAAGCGGCGAAAAGTGATGATGTGGCGGATTGCCTCAGTTACG
CTGACATTGCAGAAACGGTGGTCAGCCACGTCGAGGGGGCGCGTTTTGCGCTGGTGGAACGCGTGGCTGAAGAGGTGGCG
GAGCTGCTGTTAGCACGCTTCAACTCGCCGTGGGTGCGTATCAAACTCAGCAAGCCAGGCGCAGTGGCGCGGGCGGCGAA
TGTTGGCGTAATCATTGAGCGTGGCAATAATCTGAAAGAAAATAATTAA
>Cluster_48901
ATGGCTACAGTCCCAACTTCCCCATTCCTTAAAATCGAAGTCGTCAACGGCAAAGCTGTTATTTTCTCCCTGCATGTTGC
CTGCCACTTTAAGCGCATGCACCAGAACATCGTCGACAAAATCGAGTATCTGAACTGCTCGCGCGAATTTTTTACCCGCA
ACTTCATACCGGGTACTTACCACATCTATGGTGACTCCCTGCGTGGTTATTACATCACCCTTGATGGCCTGATGATGCTT
CAGCTTGGGTTAAGTCTACGCACAATGCGGTACTACGAGAGCTGCATTGAAGCATTCCATGAGGCTGAAACCAGCCTGAA
TCACACTGCTTTTCGCCGTAATCAATGGGAGGTACGCCCATGA
>Cluster_179
ATGATTCTCGCCAACGACTTTCTTGAATACCTGCTCAACACAGAACGTGATCTTGCCGCTCGCGTGCGTGATCGTTATGA
CATGTACCTGAAATCCCTGCCTGTACCGCAGCTCGCTGACGGAAAGATTGTTATTGATGGTCGCTACATGATTGACAGCC
ACGAGGGAAATTACAGGCTTTACCGCATTGAAGGTGGCACCCCGTCCGTTATTGGCATTTACCAGCGCCCATCCTCTGCA
ATCGTCGATGTGATTGCCGACAGCATCCGCATCACACATCGCCATGCCGACACAGAAGACACCGTGCTGGAAATTCAGCG
GCTGGCTACAGTCTGCCGCGACACCCTGAATGGCATGACGAAGTAA
>Cluster_28670
ATGAAGGATTATCTCTGTAAAAAGTTATTCAACCGGCTTTCCGGTACCCTGGTGATCAGAGCGCGCTGCGGCAATAACAT
TACTGGCCTGGCATGTTGCAATATTCTTTATCCCTCGCCCCGATACAGCGGTCAGCTGCATATTAAAGAGCTGTATGTTT
CTCAGTGCGATCGAAATAAGGGCACGGGTAAAGCGATAATGCGCTTTATAGCCCGGCTTGCGCTTGAACAGGAATGCCTT
AGCCTTAGCTGGAACGCTGAAAAATCCAACCCCGGCGCTAACCGTTTTTATCAGGCTCTGGGAGGCAGAATAAATGATCA
TATCGTCAATTATTACCTCCACGGGGAGAGCCTGAGCAAACTGGCCTCAGGCATTTGA
>Cluster_51798
ATGAAAAAGAAAGTAATCAGCGGGCTGTTTCTGATGTTATGGATAGCGCTGTTAATCGCCGCAATGGTGTATCCGCAGGG
GATTTTTCCGGTACTGGCAGCATCTGGTGTCTGGGTAGCCTGTCTGCTGACATGGGCAGTAATTCCGGTATCACTGGCTG
CGTTAATTAAGAACGGCCCGCTCTGGCAGGAGTTGAGGGCATCTTTGCTGAAGACAATTACCCGAAAAGAAAACGTATTT
ATCAGCTGGATGATGCGATTGCTGATTGTCGTCAGTCTCGCCTGGACGGGGTGGGCTCTTACCCTGGTCTTTTATCTACT
GACCGTTATTGCCTTCTGGAACACCCGTAATCAGATGGCGCAACAGGTAGCAGCATGA
>Cluster_13002
ATGAAAAAGGATCTGAAAACGCTGGCGCTGGCCAGACTGTCAGGGTTTCGTCATAAAACGGTGAAGGTGCCGGAATGGGG
TAATGTCAGCGTGGTGCTGCGGGAGCCTTCGGCAGAGGCCTGGTATCTGTGGCAGGAAGTGCTCAATGGTGATGGAGAGG
ATGACGATACCCTGTCGGTGGTGGCGAAAACCCGCCGTAACCTGGAAGCGGATGTGACGCTGTTCTGCGATGTCCTGTGT
GATACGGATCTGCAGCGGGTGTTCGCTCCGGACGACCGTGAGCAGGTGCTGGCCGTCTATGGTCCGGTACATGCCCGGTT
GCTGCGTCAGGCACTGGAACTGATCGCTGATGCAGAGTCGGCCAGAAAAAAGTAG
>Cluster_20596
GTGATTGATTTGGGTAAATTTAACTTACTGGATATTCGCCGCCACACAATGTCGAAAACATTCAGTATCAAGACAACAAA
ATCGCAAAATGACCAGTGTACCGACGGATTTAAGGTCAGCTCCTCGTTTTATACCGAAAAAACGTTGGTTGAAGAAGATA
AAGCATTGTTGATTGGTAATGGTCTGAAGCTACGTATACTGGATGAACATGCCTCGCCCTATATCTTCAACAAATACAGC
GAATATGCCGATTTCACCAGTGGCATGTTGATCTACGAAAAAACCTATACTGCTGAACTTTCGTCCATTGCAGGCGCCCC
CATCGAGGCTGGGCCCTTCGATACAGTGGTGCTTTTTAAGATTAACTATAACTGA
>Cluster_25074
ATGATTATTGATTTAGACGCTCTTTTTCCAAGTCGAAAGACGTTTACAGATCTGGTGGCTTATTGTACTGATCCGTTTGC
CTCTGTAGAAAGCAAGGTTTTTGCTTCTCTTCCTGCTGATGTTGAATGCGGAGACTTGATCACCAGCACTGGCGCTAAAT
ATGAATCAGGAGATGATATCTATGTTGTTATGAGTGAATTTGTAACGGCTGGAGACAACAAGCCTGTAGATGTGCTACGT
AGCAATGCTGGGCTTGTATGCATCAAAGCTGATGCCCTGAATGCTGTAAGTGAAGCAGCAAAAACAGCATTAATTAAAAA
AGGCTTCCAGCTTGAAGGCTTTAATACGGTTTTCACTTCTTAA
>Cluster_42530
ATGATCAAACTGAAGACGCCAAACTCTATGGAGATTGCCGGGCAACCTGCTGTTATCACTTACGTGCCAGAACTGAATGC
ATTTCGTGGCAAATTCCTGGGCTTGTCAGGTTATTGTGATTTTGTCTCTGACAGCATTCAGGGGCTGCAAAAAGAAGGGG
AGCTTTCCCTGCGAGAGTATCTTGAGGATTGTAAAGCTGCGGGTATTGAGCCGTATGCGCGTACAGAAAAGATTAAGACC
TTTACATTACGCTATCCAGAATCGCTGAGTGAACGCCTTAATAATGCGGCTGCGCAGCAACAAGTCTCTGTAAATACTTA
TATTATTGAAACGCTAAATGAGCGTTTGAATCATTTATAA
>Cluster_38608
ATGAATTTATCCTTATCTGAAGGCGGTCGCCCCGCGTCTGTAAAAAACCGTGCATGGCATTATTGCCAGATGGCATGGCG
TGGAGTGTGCACCCCAAAAGCACTGTCCCGTCTGGCTGCGTTCTCTCCGCTGCTGTTACTGGGGATGGGGCAGATGGCTC
ATGCCACTGACCTGCTGGCCGGGGGCAAGAACGATGTAAAAGCGACCTTTGGTGCCGACTCATTCGTCATCATGTGTATT
TATATCGCTGAAATCGTTATCGGTGTTGCGACGTATATCCGAACCAAGAACCTGCTGATTCTGCTGGGACTCGTCGTGGT
TATTGTCTTCACCACCGTTGGCCTGACATTCGTCAACTGA
>Cluster_20043
```

ATGAAAAAAAAACGCTAATAATCCATACTCCAAATTTCGTAATGGCGTAGAACGCCATGTACACCACGTCGCTACCAGTGC
ATCACGTAGTAACAGTCGCTATAACCTGAACGAGACGCACGCAACACCGGATGGCCACGCTGTAAAACAAATCGGCGAGC
ATGCCTGGCTGATTGAGAAAGCTGGAATCGTGGTCCACAAATGCCCACGCAATCCATTTACCGGAAACCGCATTTTTGCA
TTGAACTGCGGCGACAATCACTTCGGGCAGGATTTCACATTATACGAAGCACTTCGCACGGTTGATCGTCTGCTTCGCGG
GCAAAGTTTTATTAAACAGGCTGATTTATAA
>Cluster_28721
ATGAATCTTTTAGTTAAAAGAAACGTTGAAGAGTTTTTAAGATTGTTGGGAAATGATTTTCATTTATTTGATAATCGTGT
TGAAATTGATTTTAATGACTTTTCTTTTTTTATTGAAATAATTGATAATAATGTTTTTGTTACGTTTGCTTTAGAGTATA
ATGAAAACGCATTTTTCTCTTTCTTTAGTGCCCTTGCTCCTGAGCGTACGCAGGGAGTGATTGAACATATTTTTGTCTAC
GATAATAAATTGTGCTTAAGTTGTTTGTTAACCAATATCGATGTTTTTTTTCTAATGAATACTTTTCAACAGCATGTGCA
GATTATTGAGCGCGTTCGCAGGATGACATCATGA
>Cluster_53897
ATGAACGAATATCAGTTTGTGCTTCCATACCCGCCGTCGGTGAACACCTACTGGCGAAGACGGGGAAGCCAATACTACAT
CAGCGCTAAAGGCCAGAAATACCGAAAAGATGTACAGCAAATAATCCGCCAACTCAAGTTAGACATTTTCACCAAATCAA
GACTCCGCATCAAAGTCATCGCAGACGTTCCAGACTCCCGCCGCCGCGACCTCGACAACATCCTGAAAGGTTTACTCGAC
TCCCTTATCCACGCCGGATTTGCGGAAGACGACGAGCAATTCGATGACATTCGCGTAATTCGTGGCGTGAAAGTACCAGG
CGGAAGGCTTGGAATAAAAATCACCGAACTGGAGAACGCATGA
>Cluster_30056
ATGAATGAATTTGAGAAGATTTTTAATGAAATGAATCTGGACCGGGCATTGCTACCGATCCTTTTTCGTTCGAACCGGTC
AACAGTGTGGAAGTATTTAAGCGGCGATAGTACGGCTCCTGCCTCAGCTATGTCACTGATTATGCTGTTGCAGTTAATCC
AGAAACGAAACCCCGATCTATTAGCCGAATGGTTAACGCTTTCTGATTTCACCATTCCGCCAGAAGTTTACCTGGACCAG
CCTGATTACTGGAAAGGGTGGGTGTATACCCAACACAAGGTCAATAAAAACGTCCTGGAATATCTAAAAAAACATTATCC
GGATGAAGACCAAAAAAGTATGGGTAAAGGCCGGGAAGAGTAA
>Cluster_48616
ATGTCCATGAAAATGGGTGACCTAGCAAAGCGCAAAGAGCCTGATGCACCGGCTAAGAACACAACTCCTTTGCGCCAACC
AGTCAGACCACAGGGACGCCCGACTCGTGGCAAAGAGAAAATTAAAAGCCGCACAATGTCACTGGAAGACGAATACTTCG
AACTGCTGGAGATGATGAAGTTCATCCCTCGCTTCGAGAAGTTCACTCGTTCTGACGTGATTCGAGCAGCCATTTTCCAT
CTGGCAGAGAAGTCACCGCAGGAAATCGAGGACATCGTGAAGCTTAACGAGGCGATCACAGCTGCCGACGTTACGATGCG
TACCGATGAAATTAAACGAGAGTTGATGAAGAAAGGTTGA
>Cluster_58603
ATGTATTATGGCGCGCTATCCATCCGGGCAGAGGCCTGGCTGATTGTTTCCCCCGAAGTCACCAAGATCATGGCCAAAGA
ACAAACGGACCGTACGACATTAGATCTGTTCGCGCACGAGCGTCGACCGGGACGACCGAAAACTAATCCGCTTTCGCGCG
ATGAACAGCTGCGTATTAATAAACGCAACCAGCTAAAACGCGACAAAGTACGTGGCCTTAAGCGTGTCGAACTGAAGCTG
AACGCGGAAGCTGTCGAGGCGCTGAACGAGCTGGCGGAGTCGCGCAATATGAGCCGTAGCGAACTGATCGAAGAGATGTT
AATGCAGCAACTGGCGGCGCTGCGTAGTCAGGGCATCGTTTAA
>Cluster_11134
GTGCGTAATAAAAAAGCCCCTCAGACCGTCTCAGCGCGTCATGACGCCCGTGAACACCTCAGCATTGAGGCTTACCATAA
GCTCAACCGCGCCAGCGCCGTATCCCAGTTTGTTGGGGGTGATTTGATTCACCGTGAACTCTCCGGCCTGCATCAGCTCT
ACATTCCGCATATTTTCAGCTACCTGAATGAAGATATTGATTTTGTGCTGAATGAGCTGAAAGCCAAAGGCCTGTGCCGC
GATTTTCTCGCCCAGCAGAAAGACCGGGGAGACAGGACGCATGTTTGA
>Cluster_35772
ATGATTAACGATATTCTGACCGAAGACCGTCGTCTGGTCATCCTGCGTTCCCTGATGGACTGCAATAACGAAGCAAACGA
ATCCATTCTTCAGGACTGTCTGGATGCATACGGACACAACGTTTCCCGCGATGTGGTTCGCGGCCAGATTGACTGGCTGG
CAGAGCAGCAACTGGTCACCGTGGAAAACCTGCGCGGCTTTTATGTCGTAACGCTGACCTCCCGAGGTCAGGATGTGGCA
GAAGGTCGTGCCCGCGTTGCCGGTGTCAAGCGTCCTCGTCCCCGTGCATGA
>Cluster_16050
ATGTCAAATCAAACCGGACAGCTTGACACACCTTCACGCATCCTGCGTATGGCTGAACTTACCCGGCTGCTGGGTATATC
CAGGTCCACGATTTACGTGAAAATAAATCCGGCCTCGAAATACCACGACCCTACCTTTCCCAAACCGATAAGGCTTGGAA
GTGCATCCGTTGGCTGGCGGGCATCTGCCATCGATGAGTGGCTGACGTTACATATGGCACCAGCCTGGTCTGAACCTGAA
AACAAACAAAAGGAACTTAATGATGACGACAAAACTATCACTCCCTGA
>Cluster_5026
ATGCCAATGGAGTTTGAATGGGATGAAAACAAGGCCAAAAGTAATCGGGTGAAACATGGCATCCGCTTTGAAGATGCGGT
GTTATTGTTTGATGATCCACAACATTTGTCACAGCAAGAACGCATAGAAAACGGTGAGTATCGCTGGCAGACAATCGGCC
TGGTTTACGGCATCGTGGTTATTCTGGTTGCGCATACCATCCGTTTCGAAAGCGGAAATGAAATTATTCGAATCATCAGT
GCACGAAAAGCAGATCGTAAAGAGAGGAATCGCTATGAGCATGGTTAA
>Cluster_44992
ATGTTTTGTGTGATTTATCGAAGCAGCAAGCGTGACCAGACCTATTTATATGTCGAAAAAAAAGACGATTTTTCGCGTGT
TCCTGAGGAACTGATGAAAGGTTTTGGTCAGCCTCAGTTAGCGATGATTCTGCCGTTGGATGGGCGGAAGAAACTGGTCA
ATGCTGATATTGAAAAGGTTAAACTGGCATTAACCGAGCAAGGTTACTATTTGCAGTTACCGCCACCACCCGAAGATTTG
CTGAAGCAACATCTTTCCGTCATGGGGCAGAAAACAGACGACACTAACAAATAA
>Cluster_28744
ATGAAGCCAACGATGCTACTCATGATTACCGTATTCCTGATTTTTCCAGCCATTTCTCAGGCCGAGTCGCCATTCAGTTC
TCTACAGTCGGCAAAAGAGAAAACCACGGTATTGCAAGATTTACGTAAAATTTGCACGCCACAGGCGTCATTATCCGATG
AAGCGTGGGAAAAGTTAATGTTGTCAGATGAAAACAATAAACAACATATACGCGAGGCCATCGTGGCGATGGAACGTAAT
AATCAGAGCAATTATTGGGAAGCGTTGGGTAAGGTAGAATGCCCGGATATGTAA
>Cluster_63638
ATGGTGAATTTACGTAAAGCGGCTAAAGGCCAGATGTGCCAGATCAGAATCCCTGGCTACTGCAATCACAATCCCGAAAC
CTCTGTGCTGGCGCATTACAGGCTGGCGGGGACGTGCGGAACAGCGACAAAACCACACGATATGCAGGCGGCGATAGCCT
GTAGCTCATGCCACGATCTAATCGACGGGCGGGTAAAAACCAGCGATTACACCAAAGAAGAATTGCGCCTGATGCATGCT
GAAGGGGTTTTTCGCACACAAGAAATCTGGAGAAAGAAAGGTCATTTATGA
>Cluster_2094
ATGGCCAGTGGCGATCTTGTCCGTTATGTCATAACCGTCATGTTGCATGAGGATACATTGACTGAAATTAACGAGTTGAA
TAATTACCTGACTCGCGACGGTTTTTTGCTCACCATGACGGATGATGAGGGAAATATCCATGAGCTGGGGACTAACACTT
TTGGACTTATCAGTACCCAAAGTGAAGAAGAAATTAGAGAACTGGTTTCGGGGCTTACCCAAAGTGCAACCGGCAAAGAT
CCTGAAATCACCATCACGACCTGGGAGGAATGGAATAGCAACAGAAAATAA
>Cluster_35316
ATGGAAGAATTAAGAATATTTCTCAATTCTCTTTCGTCAGATGAACAGCGTATGTTTGCATGCGAGTGTGGTACCAGCAT
CGGTTATCTAAGAAAGGCATTGAGTAAAGGTCAAGTGTTAGGGGCATCGTTATGTGTCCTTATTGAGCGAGCCAGTAATG
GTGAAGTTACACGTCAGCAACTAAGGCCTTTTGATTGGATGAATATTTGGCCCGAGCTGGAAGATACCAAAACGTTAACA

CAACCACTTTCTAGGAGCTTGATTCATGAAAATCAAGCATGA
>Cluster_4503
ATGTCATTTGAGAAAAAAGAGCAGAATGAGGTAAAAATCTTTGGCTGTGTATTCAGTCTGGTAATCATCCAAGGAAAGCT
TATCAGAAGGGTGATGACTCCAATTAGTAGAACATATATTTTCGATAAAAGCACCGATGACTTTTTCATGAAGTTCTACA
GAACGCGAGAAACAGATGGTTCTGCGGGCCAGACGTTTGATTCGGGTTCTCAGGGTCAGATTGTTACGCTCAATGTGCTG
AGTAAAAATCTTACCAGTCAGATGCTTCTCCTTCGGTAA
>Cluster_66857
ATGAGCGGCCTCATTTCTTATTCTGAGTTACAACAGTCCGCACCGCTGCCGGTAGCTATTGACTATCCGGCTGCACTAGC
CCTGCGTCAGATGGCTCTGATCCAAGGCAAACTGCCAAAATATCTGCTGGCACCGGAAGTCAGCGCCCTGCACCATTATG
TTCCGGATCTGCATCGCAGGATGCTGCTGGCTACCCTGTGGAACACCTACATCTGTATTAACGAAGCGCTGGCATTGACC
CTGAGTGATTTTTCTCTGGTGCCGCCTTATCACTGTTGA
>Cluster_4606
ATGAACACCTTGTTTTTACTGATGGCTGAATTCAATACCCCAAACATTGAGCTGTCAGCTGTATGCCAAAAGTATTTCGG
TATGAGCCCTAACACAGCAGAAGCGAAAGCAAATGCATGCCAGTTGCCGATCCCGACTTATCGTGTTGGTACATCACAGA
AAGCAAAGCGCTGCATCAACATTCAGGATCTTGCTGAATATATAGATAAACGGCGTGAAGAAGGCAGAATTGAATGGGAG
AGGGTAAGAACAATAGGAAAATAAATAACTAA
>Cluster_17895
ATGGATAAAACTAACGAAAATATCGTCACTCTGATTAAACCCATTAAGCGCGGTGAGCAGGTTATTACTGACGTCACCCT
GTTAAAACCGTGTGCCGGAACCCTTCGCGGTGTCAGCCTGGCATCAGTCGCAAACTCTGACGTCGATGCGCTGATTAAAG
TGCTGCCGCGCATGACCATGCCGTCGCTGACCGAGCAGGAAGCCGCCGCACTGGAGCTGCCCGACCTGCTGTCGTTTGCC
GGTAAGGTGGTCGGTTTTTTGTCACCGAGTTAG
>Cluster_29453
ATGAATTACGATGAAATAACGAAAATCACTGCAGAACGTATTAGCGACTACATGACTGAAGCGGTTAATACAGACTCTAA
ATCTGTTGCGGAAATGTTTCATAACGCTGCATGGGGAGTGCTATCATTGTGGTTTGAACTCGTCACAAAAATAGATCTGG
ATATACATAAAAAGAACAGGTATGCAAGTTATGATTTTAGAAGAAAAATCGAAATGCAGCATGAAGAGTTTCAGAAAATG
ACAGAGCGTGAGCAAGTGCCTTTATTGAAATTGCCTGAATAA
>Cluster_7335
ATGAATAAGTATTGGCTGTCAGGAATTATTTTTCTTGCCTATGGATTGGCTTCTCCCGCGTTCTCTTCAGAGACTGCAAC
ATTAACCATCAATGGCAGAATTAGTTCCCCTACATGCAGTATGGATGTTGTTAATAATCATCTTCAGCAACGTTGTGGGC
AGTTGTTACAACGAGTTGAAACCAACTATCGTGCTTCTTCTACTGCAAAAGGCGTTACGACAGAAGTGGTAACTGTGGGT
AATAACGACAAACGCAAAATCGTGTTAAATCGCTACGATTAA
>Cluster_15960
ATGAGTATCAATGAGTTAGAGTCTGAGCAAAAAGATTGGGCGTTATCAATGTTGTGCAGATCCGGTGTCTTGTCTCCATG
CAGACATCACGAAGGTGTTTATGTAGATGAAGGTATAGATATAGAGTCGGCATACAAATATTCCATGAAGGTTTATAAGT
CTAATGAAGACAAATCCCCATTCTGCAATGTGCGAGAAATGACTGATACCGTGCAAAATTATTATCACGAGTACGGTGGA
AACGATACTTGCCCTCTCTGTACAAAACATATAGATGATTAA
>Cluster_34291
TTGCTGCAAAGTCGCAATGATCATTTACGCCAAACGGCATTGCGCAACGCACATACCCCGGCGTTGTTGTTAACGACATT
GACTGAGCCTCAGGATCGGTCGCTGGCTATTAATAATCCACAGCTGGCTGCCGATGTGAAAACGGCGTGGTTAAAAGAGG
ATCCATCATTACTCTTATTTGTCGAACAACCCGATCTTTCGCTGTTACGTGATTTAGTGAAAACCGGGGCAACGCGGAAA
ATTCGCAGTGAAGCGCGTCACCGGCTTGAGGAAAAACAATGA
>Cluster_23066
ATGCAGCCAAACGACATCACTTTTTTTCAACGTTTCCAGGATGACATTCTGGCTGGGCGTAAAACCATCACCATCCGCGA
CGAGTCTGAATCGCACTTCAAAACGGGTGATGTGCTTCGTGTCGGACGGTTTGAAGATGACGGTTATTTTTGCACGATTG
AAGTCACCGCAACCTCAACCGTAACGCTGGATACGCTGACGGAAAAACATGCAAAGCAGGAAAATATGACCCTGACTGAA
CTGAAAAAGGTCATTGCCGACATCTATCCCGACCAGACACAGTTTTATGTGATTGAATTTAAATGCCTTTAA
>Cluster_24436
ATGACCAACCGTCTGGTGTTGTCCGGCACCGTGTGCAGGACTCCCCTTCGAAAGGTCAGTCCATCAGGAATTCCTCACTG
CCAGTTCGTGCTTGAGCATCGTTCTGTGCAGGAGGAAGCCGGCTTTCACCGGCAGGCGTGGTGCCAAATGCCCGTTATTG
TTAGCGGACACGAAAACCAGGCCATTACTCACAGTATAACGGTCGGCAGTCGCATAACCGTTCAGGGGTTCATTTCATGC
CATAAGGCAAAGAACGGACTGAGCAAAATGGTTTTGCATGCCGAGCAGATTGAATTGATAGATTCTGGAGACTAG
>Cluster_64470
GTGAGCGTTTTTAAATGGGAAAGCGGACAAAATGAACCAAAAGGTCAGAACTTGCTGGCTTTGGCCAATGCACTTAAGTG
TTCTGCCACATGGCTGCTTTTTGGTGACGAAGACCAGGCGCCAACTCCGGTAGACGAATTACCAACAGAGCTGGATTCAC
GTCAAAAAAGATTGTTAGAGTTATTTGATTCATTACCCGAGTCAGAAAAAGAAAAGCAACTAATTGAATTAGAAGATAAA
GTTGAAAACTTTAATCGTTTATTTGAAGAGTTACTGGTTACCAGAAAGAAACTAACCCCCATCAAAAAATAA
>Cluster_3230
ATGGGTAAACTAACGCTGCTGTTGCTGGCTATTCTGGTCTGGCTACAGTATTCGCTGTGGTTCGGTAAGAACGGTATACA
TGACTATACCCGCGTCAATGATGATGTGGCGGCACAGCAAGCTACAAACGCGAAACTTAAAGCGCGAAACGATCAACTTT
TTGCCGAAATTGACGATCTCAATGGCGGCCAGGAGGCGCTCGAAGAGCGTGCGCGTAATGAACTCAGCATGACCAGGCCG
GGCGAAACTTTTTATCGTCTGGTGCCTGACGCGTCGAAGCGCGCACAGTCTGCGGGGCAAAACAATCGATAA
>Cluster_41625
ATGCTTCTGACAATGGAAGAGATTAAAGCCCAACTCCGGCTGGATGAGGATTTCGATGCTGATGACCGCCATCTGCAACT
GCTGGCCTGTGCGGCACAAAAGCGGACGGAAACGTATCTGAACCGGAAGCTCTATGCACCGGATGAAACCATTCCGGACA
GCGATCCGGACGGGCTGCACCTGCCGGATGATATTCGTCTGGGGATGCTGATGCTTATCAGCCATTTTTACGAAAACCGC
TCGTCGGTTACGGAAGTGGAGAAACTCGACATGCCGCAGAGTTTTGGCTGGCTTGTCGGCCCGTACAGGTACTTTCCGCA
ATGA
>Cluster_38480
ATGGAAAAGCGAACAAAAGAGATCAAAATCCGGCTCACCGAAACTGAGCATCAGCGACTGCTTGATCGCTGTGACCGGAC
TCATCTTGCAGAGTGGCTGCGTCAGCTTGGTCTGGGAGAACACACCTCACGCAAGCGCCGTGTGCCTGATGTGGCGCCCG
AACTTCTGCGACAGGTCAGCGGGATTGGTAACAACCTGAACCAGATCGCCCGTCGCCTGAACCAGACTGATTCCCTGACG
CCTTCTGAACGTGCCTCGCTGCTCGTCGTACTAACCAGCCTTGACCGTCAGCTTGGCGACCTGCTGGAGCAGAACCGTGA
TCGTTAA
>Cluster_6634
ATGTTTAAAAAAGGCTTACTTGCTCTGGCACTGGTGTTTTCACTGCCCGTTTTCGCCGCTGAACACTGGATCGATGTTCG
TGTTCCAGAGCAGTATCAGCAAGAGCACGTTCAGGGGGCCATCAATATTCCCCTGAAGGAAGTGAAAGAGCGCATTGCCA
CCGCCGTTCCGGATAAAAACGACACCGTGAAAGTGTATTGCAATGCCGGACGCCAGTCAGGGCAAGCAAAAGAGATCCTT
AGCGAGATGGGATATACCCACGTTGAGAACGCCGGTGGCCTGAAAGACATCGCAATGCCGAAGGTCAAAGGTTAA
>Cluster_7925

ATGAAAACACCCTTACCGCCCGTCTTACGCGCTGCCCTTTACCGTCGCGCTGTCGCCTGTGCCTGGCTGACCGTGTGCGA
ACGTCAGCACCGCTACCCGCATCTCACCCTTGAGTCACTGGAGGCGGCCATCGCCGCTGAGCTGGAAGGCTTTTATCTGC
GCCAGCACGGTGAGGAAAAAGGGCGTCAGATAGCCTGTGCCCTGCTGGAAGATTTAATGGAATCCGGCCCCCTGAAGGCC
GCGCCGTCGCTGTCCTTTCTCGGGCTGGTTGTGATGGATGAACTCTGTGCCCGTCACATAAAAGCGCCGGTACTGCACTG
A
>Cluster_63881
GTGCGGCGACGGGCTTCGATATCAGGAGACGATGATGGAAAAACAGAAAACAAACCGATTGTAATTGGTGCTGATGCTGC
TCCGTTTAAGTTTGAGTTGTCTCAACTGGTGGAGATGCGCATCAGTGATGAATGGGGTGAGGTTAAAGCCCGTGCGCAGT
ATGCGGATGGCGAAAACCAGTACTTGATCCACTACAAAGCAGCTGACGGTCGCGCCACGACGGAGTGGTTTGGTGAGTCA
ATGCTGGAAGCAACAGAAGATGCTCGTCATCCGGGCTGTCCGGTATTTGCCTTCATGAAATTACCGTAA
>Cluster_17529
ATGGGATTTAAAGGTACTGAAGGTAAGTGGGAAATAATGATGGATGGGGATGAGATTAAAATCATCCAGGCAGACTCTCT
TGAAAATGGCACAGGCTGGCGTTCGTATATTGCAATCTGTGAGGAAGTTCAATGTATTGAAGATGCCAATCTAATAGCGG
CAGCACCTGACCTTCTCGAAGCACTTCAGTTATTACTTAATCAATCCAAAAATAGAACAACGACAACATATCCAGAATGG
TATGGAGCTGTTAATAAAGGTCTTGCAGCAATCAGAAAAGCTCTTGGGGATAAGTAA
>Cluster_17648
ATGGAAAGTAGCCTTGCCGGTATCGTGAACGTCTGGCTGTGTCTCGCCATTGTGCTGGGGTTGTTTGTGTATCGCCGCCA
CGGTGCGGCGCATAAACCGATGATTACCTGGCTGGCGTACTGGCTGATGCTCGGCTACATCATCATCCCGTTCCGCTGGC
TGTCCGGTACATACACGCACTCCAGCTGGCTGGTTGTGGCGCTGAATCTGGTTTTCTGTGCGCTTATCGTGTGGGCGCAC
GGGAATTTGTCAAAAATCCTTTCGTTACTGAGGCATCCACATGAAATCAAAAGATGA
>Cluster_24798
GTGATCGGCTTCCGCAATGTTGATATGGCGGAACTGCTTATCCACACCCTGACTGAGCGCGTAGCTATAACTGAAGGCCA
TCTTTTCCGGGTGGCTTTTGCGATATTTTTTCAGCTCAGTCAGGCTGCTGAATTCCAGGACGTCCCACTCTTCCCCGGTG
TTTTTTTCTTCCCGGCAACAAGAAGCGTGTACGACGCGGTGTTAACGTGGATATTTCGGGTACTCATCCTGCTTTCTCCT
CTCTCTGGTTTTCAGCCCCCATCATCTGTTGAGTTTCGGGAGCCGCGGTTTTGA
>Cluster_58158
ATGCATCGAATTCTCGCTGAAAAATCGGTCAATATCACTGAGTTACGTAAAAACCCAGCTAAATACTTTATTGATCAACC
GGTTGCGGTTCTTTCTAATAATCGCCCCGCAGGATATCTCTTAAGTGCCAGCGCATTCGAAGCGTTAATGGACATGCTTG
CTGAACAAGAGGAGAAAAAGCCCATAAAGGCGCGCTTCCGTCCAAGTGCTGCAAGATTAGAGGAAATTACACGCCGCGCT
GAACAATATCTTAATGATATGACGGATGATGATTTCAATGACTTTAAGGAATAA
>Cluster_10482
ATGAGTGCTGGCAAGGGAGTGTTGCTCGTCATCTGTCTGTTATTTCTGCCGTTGAAGTCTGCGCTTGCCCTGAACTGCTA
CTTTGGTTCATCCGGTGGCTCGGTAGAAAAATCAGAAGCGATTCAACCTTTTGCTGTACCAGGCAATGCCAAACTCGGCG
ATAAGATTTGGGAATCTGACGATATTAAAATTCCCGTCTATTGTGACAACAATACCAACGGCAATTTTGAAAGTGAGCAT
GTTTATGCCTGGGTAAACCCCTACCCGGGGTGCAGGATCGCTATTATCAACTCGGCGTAA
>Cluster_17754
ATGATGGATATCGACGACGAAGGACGCAAAGCAGAGCTTGACGCCAAACAGCAACTTCTGGCTCAGCGTGATATCGACGA
CATCAAGTTCGTTATGGGTAGCGAGCAAGGCCGCCGCGTTATCTGGTCGATGCTGGAGAGGGGAAAGGTGTTCTCACCAT
GCTTCGCTGGTGATTCGCACTTAACTGCATTCAACGAAGGGCAGCGCAACCTGGCACTGGCATTGTTTCAGCGCGTCATG
GCGCACTGTCCTGAACAGTATCTGAAAATGGCCGCTGAGTCTCAGGAGAAAGACAAATGA
>Cluster_40610
ATGCCTGCTGTAATAGATAAAGCCCTGGATTTCATTGGTGCCATGGATGTATCAGCGCCAACACCAAGTTCGATGAATGA
AAGTACGGCGAAGGGAATCTTTAAATATTTAAAAGAACTGGGAGTACCCGCGAGTGCCGCTGATATTACGGCGCGAGCTG
ACCAGGAAGGCTGGAACCCTGGTTTCACGGAAAAAAATGGTTGGATGGGCAAAAAAAATGGAGTCTGGTGAACGCTCTGTG
ATTAAAAATCCTGAATACTTTTCAACATATATGCAGGAAGAACTAAAAGCACTGGTTTGA
>Cluster_14896
ATGAGAACATATTTATCTGGCTTGACTGCCAGCGGTTATGCACACCCCCAAATTATCCCCGGCGCTATTTATCTGGATAA
GAACGGTAACAGAGTAACGGTAAAAGAACTGATGTTTGACCGTGTGTATTTTATCCGTGATGGCTATTCATTTCTTAGTT
CGCTGAACGTGGAGATCTTTATTAGCAGATTCAGGCGGGAAATCCCGCTTTCCAGAAATAACCATGTGTCATGTATGGAT
GTTGATAAAAAACTACAGGAACTAAAAAACATGATTGCCGCGTGGAGAGAGCAGAAATGA
>Cluster_24627
GTGGCTCAATTCGTTTATACCATGCATCGTGTCGGCAAAGTTGTTCCGCCGAAACGTCATATTTTGAAAAACATCTCTCT
GAGTTTCTTCCCTGGGGCAAAAATTGGTGTCCTGGGTCTGAACGGCGCGGGTAAGTCCACCCTGCTGCGCATTATGGCGG
GCATTGATAAAGACATCGAAGGTGAAGCGCGTCCGCAGCCAGACATCAAGATTGGTTATCTGCCGCAGGAACCGCAGCTG
AACCCGGAACACACCGTGCGTGAGTCCATTGAAGAAGCGGTTTCTGAAGTGGTTAACGCCCTGAAACGCCTGGATGAAGT
GTATGCGCTGTACGCCGATCCGGATGCCGATTTTGACAAGCTGGCCGCTGAACAAGGCCGTCTGGAAGAGATCATTCAGG
CTCACGACGGTCACAACCTGAACGTTCAGCTGGAGCGTGCGGCGGATGCGCTGCGTCTGCCGGACTGGGACGCGAAAATC
GCTAACCTCTCCGGTGGTGAGCGTCGTCGCGTGGCGTTGTGCCGCCTGCTGCTGGAAAAACCAGACATGCTGCTGCTCGA
CGAACCGACCAACCACCTGGATGCCGAATCCGTGGCGTGGCTGGAACGCTTCCTGCACGACTTCGAAGGCACCGTGGTGG
CGATTACCCACGACCGTTACTTCCTCGATAACGTTGCGGGCTGGATCCTCGAACTGGACCGCGGTGAAGGTATTCCGTGG
GAAGGTAACTACTCCTCCTGGCTGGAGCAGAAAGATCAGCGTCTGGCGCAGGAAGCTTCACAAGAAGCTGCGCGTCGTAA
GTCGATTGAGAAAGAGCTGGAGTGGGTACGTCAAGGTACTAAAGGCCGTCAGTCGAAAGGTAAAGCACGTCTGGCGCGCT
TTGAAGAACTGAACAGCACCGAATATCAGAAACGTAACGAAACCAACGAACTGTTTATTCCACCTGGACCGCGTCTGGGC
GACAAAGTGCTGGAAGTCAGCAATCTGCGTAAATCCTATGGCGATCGTCTGCTGATTGACTCCCTGAGTTTCTCGATCCC
GAAAGGGGCGATCGTCGGGATCATCGGTCCGAACGGTGCGGGTAAATCGACGCTGTTCCGTATGATCTCTGGTCAGGAAC
AGCCGGACAGCGGCACCATCACTTTGGGTGAAACGGTGAAACTGGCATCGGTTGATCAGTTCCGTGACTCAATGGATAAC
AGCAAAACCGTTTGGGAAGAAGTTTCCGGCGGGCTGGATATCATGAAAATCGGCAACACCGAGATGCCAAGCCGCGCCTA
CGTTGGCCGCTTTAACTTTAAAGGGGTTGATCAGGGTAAACGCGTTGGCGAACTCTCCGGCGGTGAGCGCGGTCGTCTGC
ATCTGGCGAAGCTGCTGCAGGTTGGCGGCAACATGCTGCTGCTCGACGAACCAACCAACGACCTGGATATCGAAACCCTG
CGCGCGCTGGAAAACGCCCTGCTGGAGTTCCCGGGCTGCGCGATGGTTATCTCGCACGACCGTTGGTTCCTCGACCGTAT
CGCCACGCACATTCTGGATTACCAGGATGAGGGTAAAGTTGAGTTCTTCGAAGGTAACTTTACCGAGTACGAAGAGTACA
AGAAACGCACGCTGGGCGCAGACGCGCTGGAGCCGAAGCGTATCAAGTACAAGCGTATTGCGAAGTAA
>Cluster_42037
ATGGTTACCTGGACCCAAATGTATATGCCGATGGGAGGACTGGGGCTATCCGCTCTGGTCGCCCTGATCCCGATAATATT
CTTCTTCGTTGCACTCGCGGTATTACGTCTGAAAGGACATGTCGCCGGAGCAATAACCCTTATATTATCTATTCTGATTG
CGATATTCGCCTTTAAAATGCCGATTGATATGGCATTTGCTGCTGCGGGCTATGGCTTTATTTATGGTTTATGGCCTATT
GCGTGGATTATTGTCGCGGCGGTATTCCTGTATAAATTAACCGTTGCCAGCGGCCAGTTCGATATTATCCGCAGCTCGGT
TATCTCCATCACCGACGATCAGCGTTTGCAGGTGTTACTGATTGGTTTCTCCTTTGGTGCGCTGCTGGAAGGGGCGGCTG
GCTTTGGCGCACCGGTGGCGATTACCGGTGCTCTACTGGTGGGCCTGGGCTTCAAACCGCTATACGCGGCGGGGCTGTGT

CTGATTGCCAACACTGCGCCGGTGGCGTTTGGTGCGTTGGGTGTGCCAATTCTGGTCGCCGGTCAGGTAACGGGAATCGA
TCCGTTCCACATTGGCGCAATGGCGGGACGTCAGTTACCGTTCCTGTCGGTTCTTGTACCGTTCTGGCTGGTAGCAATGA
TGGACGGCTGGAAAGGGGTGAAAGAGACGTGGCCAGCGGCGCTGGTTGCTGGGGGAAGCTTCGCTGTCACGCAGTTCTTT
ACCTCTAACTATATTGGTCCGGAACTGCCGGATATTACTTCGGCGCTGGTGAGTATCGTCTCACTCGCTTTATTCCTTAA
AGTCTGGCGGCCGAAAAATACCGAAACGGCAATCAGCATGGGACAATCCGCAGGAGCGATGGTGGTGAATAAACCATCTT
CTGGCGGTCCCGTGCCTTCAGAATATAGTCTGGGGCAAATCATTCGTGCGTGGTCACCGTTTTTAATCTTAACGGTGCTG
GTCACCATCTGGACCATGAAGCCGTTTAAAGCGTTATTTGCTCCGGGCGGCGCGTTTTATTCACTGGTGATTAATTTCCA
GATCCCTCATTTGCATCAACAAGTGTTGAAAGCGGCACCCATTGTCGCCCAACCAACGCCAATGGATGCGGTGTTTAAAT
TCGACCCCCTCTCTGCTGGTGGCACCGCTATTTTTATTGCGGCGATTATCTCGATCTTCATCCTCGGCGTGGGGATCAAG
AAAGGTATTGGTGTCTTTGCCGAAACGCTAATTAGTCTGAAGTGGCCGATACTGTCGATTGGCATGGTGCTGGCATTTGC
CTTCGTGACCAACTATTCCGGCATGTCCACCACGTTGGCGCTGGTACTGGCTGGTACAGGCGTGATGTTCCCGTTCTTCT
CACCGTTCCTCGGCTGGCTGGGCGTATTCCTTACCGGCTCGGACACTTCTTCAAACGCCCTGTTTGGTTCACTGCAATCG
ACCACGGCGCAGCAAATCAACGTCTCTGACACCCTGCTGGTGGCAGCAAACACCAGCGGCGGCGTAACTGGCAAGATGAT
CTCCCCGCAATCTATCGCCGTGGCCTGCGCCGCGACGGGCATGGTGGGTCGAGAATCTGAACTGTTCCGCTACACCGTGA
AGCACAGTCTGATTTTTGCCAGCGTTATCGGCATTATCACGCTGTTGCAGGCGTATGTGTTTACCGGGATGTTAGTTTCG
TAA
>Cluster_42570
ATGCTCAATCAGAAAATTCAAAACCCTAATCCAGACGAACTGATGATCGAGGTCGATCTCTGCTATGAGCTGGACCCGTA
TGAATTAAAACTGGATGAGATGATCGAGGCAGAACCGGAACCCGAGATGATTGAAGGGCTGCCCGCCTCTGATGCGCTGA
CGCCTGCCGATCGCTATCTCGAACTGTTCGAGCATGTTCAGTCGGCGAAAATTTTCCCCGACAGTAAAACCTTTCCCGAC
TGCGCACCCAAAATGGACCCGCTGGATATTTTAATCCGCTACCGTAAAGTGCGCCGTCATCGTGATTTTGACTTGCGCAA
GTTTGTTGAAAACCACTTCTGGCTGCCGGAGGTCTACTCCAGCGAGTATGTATCGGACCCGCAAAATTCCCTGAAAGAGC
ATATCGACCAGCTGTGGCCGGTGCTAACCCGTGAACCACAGGATCACATTCCGTGGTCTTCTCTGCTGGCGCTGCCGCAG
TCATATATTGTCCCGGGCGGCCGTTTTAGCGAAACCTACTATTGGGATTCCTATTTCACCATGCTGGGGCTGGCGGAAAG
TGGTCGGGAAGATTTGCTGAAATGCATGGCCGATAACTTCGCCTGGATGATCGAAAACTACGGCCACATCCCCAACGGCA
ACCGCACCTATTATTTGAGCCGCTCGCAACCACCGGTTTTTGCGCTGATGGTGGAGTTGTTTGAAGAAGATGGTGTACGC
GGCGCGCGCCGCTATCTCGACCACCTGAAAATGGAATATGCCTTCTGGATGGACGGTGCAGAATCGTTGATCCCTAATCA
GGCCTATCGCCATGTTGTGCGGATGCAGGACGGCTCGCTGCTCAACCGTTACTGGGACGATCGCGACACGCCGCGTGACG
AATCCTGGCTTGAGGACGTTGAAACCGCGAAACATTCTGGTCGCCCGCCCAACGAGGTGTACCGCGATTTACGTGCGGGG
GCGGCCTCCGGTTGGGATTACTCTTCCCGTTGGCTGCGTGATACTGGTCGTCTGGCGAGCATTCGTACCACCCAGTTCAT
CCCCATCGATCTGAATGCCTTCCTGTTTAAACTGGAGAGCGCCATCGCCAACATCTCGGCGCTGAAAGGCGAGAAAGAGA
CAGAAGCACTGTTCCGCCAGAAGGCCAGTGCCCGTCGCGATGCGGTAAACCGTTACCTCTGGGATGATGAAAACGGCATC
TACCGCGATTACGACTGGCGACGCGAGCAACTGGCGCTGTTTTCCGCTGCCGCCATTGTGCCGCTCTATGTCGGTATGGC
GAACCATGAACAGGCCGATCGTCTGGCAAACGCCGTGCGCAGCCGGTTACTGACACCTGGCGGGATTCTGGCAAGCGAGT
ACGAAACCGGTGAACAGTGGGATAAACCCAACGGCTGGGCACCGTTACAATGGATGGCAATTCAGGGATTTAAGATGTAC
GGCGATGACCTTCTGGGTGATGAAATCGCGCGCAACTGGCTGAAGACGGTGAATCAGTTCTATCTGGAACAGCACAAACT
GATCGAGAAATACCATATTGCCGATGGTGTTCCTCGCGAAGGCGGCGGTGGCGAGTATCCGTTGCAGGATGGGTTTGGCT
GGACTAACGGTGTGGTACGCCGTTTAATTGGTTTGTACGGCGAACCATAA
>Cluster_21620
ATGACGAAGACTTCACATTCAAAGATCTATTCAACTGAGAGCGGTATCACCGGGGATGTTTTTTTTAAAACCCATCCCCT
TCACTTTACAAAAGAACAGCTTGAATGGATTGAAAATATTCAGCTCCCTAAACGATTGTTTTTAATAAGTGCAACAGAAT
TTGATGACCGATATTTATATCTACATGATGATTGGCGATACAGATTCTCCGGTAATAATTTGGCTATATTATTCTCTACT
GGTAAATACTTTTGTGATTTATCACCAGTTTCACTCAAGCTCTTGAAATGCTTAACGATCGCGTACATTAATGAGAACTC
GGCAAGTGTTGCTGATAAATTTGCTCAGTTTGTCGCAACAACGTTCAAACAAATTGAGGTTATCACTCGCGAAAGCCTTA
TTGAAAAAATGCAAATGCTAGTTGCGAAGACTGAGCGAAATCACTCAGACAGCAGTCAGTTCTATTACACCCTCTATGCG
TTACGAAAACTTGATCGTAATGGATTCTTCGAAAGTAAGGATGACAGTGATGAGCTGGAAGATCTATTGCTGGAAGTGCC
ACGCCCTAGGAACAATAACTGGGCTCGCTACCAAAACCTAGACCTCGTTATACCAGAAGAAGTATGCCTAATGATAGAAA
ATGGCATTCAACGTTGGGCATCTAAGCTATGTCCAAAAATTGATGTTGAAGAAAATAAAGACATTCATCTGGAAACTGTA
AGGAAAACCATTAAAATTGATACATTACGGGACTGCATTATTATTGGCATAGTTTATTACATTGGGGCCAGACCTGTACA
GATCGGTAAACTTTCCGGAGGCGATTTTATCGTTGATACCGAGAATAAGTACGGGATGCGTTATTCAGTACGTGTGCCAT
ATGCCAAAAAGTCAAAACTAACTATCGATCGAGTTCGAGTTGCAATTCCGGAAGAACTTGGAAAGTTAATCTTACTGTAT
AAGTACCTAGTTGGAATTGGTGATACCGACCCAATGTTTTCCATCATAGTATCTTCAATGAAGATGGTGGAAGCGTCATT
AAAGAAAATGCTTTTCAGATTTTCACCACAAGAGATTCAGGAGGCAGTAAAAAACGATGATTATCAACTACCTGTATATA
CCGCAAGCCTCTTTCGTCACAACGTTGGTCACTCAATGGCAATGAGTGGTGCCAGTGCTCCTGAGATAGCTTATATATTG
GGACAATCCTCTTATGTTGTTGCAGATCGATACATTGCAGCCACCCCTGAGCTGGCTGATATTCGTGAGGCAGCTTTGGG
TCGCAACCCTGTGTTTAAGAACATGATGGCACTCATGTTGACTGGGAATTTAGTTCACAGCAGCGAATGGGAAGGTCGCA
ATGTTGCAGGGTCAATTGGTGGGCAACTTCATTACCATCTCGGTGGATGTAATTATGAAGAAGACATTTGCCCCTTTGCA
CAGGGTCGTGGATGTTACGGTTGCCTGTATTTCAAACCATTTATAGATGGAAACCACAAAAAAGTATTTCTTTCGCTGAA
TGATGAAATCCAAAATGTCCGTGATGTTGCCGATGATGCTGGCATTTTAAATCATCCACTTCTCAAGGAATTAGTGCGGC
GGAAACAGCATGTAAATCAGGTTATGGCTCGCATTGAAATGGCAAACGTCCGAAGAGCTATTTAA
>Cluster_49155
GTGGGCGATATTGTTTACCTGAGAATAATCGGTGAGAAGCAGGGTGATATTTCTTCAGGCTGTGGGACGTATGCGTCGGT
TGGTAATCGCTGGCAGGTTGGCCATGAAGATGAAATTTTTGCCTTTGCACTCACCAACGCCATTACCAGTACTGGTAAAG
GCGTTAATCTACAGGGGCTACAATTTTGCAAACTCATTGATAAAAGCTCACCGCTACTGTCTAATGCCATCAATCAGAAT
GAGCGGTTATTTATTGAAATCGATTTGTATCGTATAAATAAAAGCGGGCGCTGGGAACGGTATTATTATATTCAGCTAAG
AAATGCTTCATTAACTGCTATTCATGTAAACATTTCTGACAATAATCTTCCTACCGAATGTGTAAATGTTAATTATGACT
ACATATTATGCAAACATCTAATAGCCAATACGGAATTTGACTGGTTGGCCTTTCCTGCTGGCTATAATAGCTTATTTATT
CCACCTAAAAGCCCACCTGCCAGTAATCTTAACCCTGAGCCGCTACCAGTTGTTAACCTTCCACTCTCTCCACCAGCGGT
TAAACCGGTCTATGCCAAATCCTGTCTGAAGGAGAAGGGATGTACAGATGCCGGAACGGCAGAAGAACCCGCTGAAAACT
TCGGGCAAGTAGCGATTTTTGCTCTGCCAGTGGTTGATGACTGCTGTGGATACCACCATCCGGAGGCTAACGATGTTGGG
CAACCCGCAGAAGCTCAAACCATGCTACTGTTTCCGGGTAGTGTGTTGGCGGCTCAAATATGGGGAAAATGGTCGCTCAG
TGGCATACTCAGTGCAACCCGCGGCTCTTACATCGGTGCGTTGGCATCTGCTTTGTATATTCCCTCTGCGGGCGAGGGCA
GTGCTCGCGTGCCCGGACGTGATGAGTTCTGGTATGAGGAAGAACTGCGGCAGAAAGCACTAGCAGGCAGTACCGCCACC
ACCCGGGTACGTTTTTTCTGGGGAACTGACATTCACGGCAAGCCTCAGGTGTATGGTGTTCATACGGGTGAAGGTACGCC
GTATGAAAACGTCCGCGTGGCGAACATGCAGTGGAACGAGCAGACGCAGCGTTATGAATTTACCCCCGCTCACGATGTCG
ATGGCCCCCTGATTACCTGGACGCCGGAAAATCCGGAACATGGGAATGTTCCGGGCCATACCGGTAACGACAGGCCGCCG
CTGGATCAGCCCACCATTCTGGTGACGCCGATTCCGGACGGCACCGATACCTATACCACGCCGCCATTCCCGGTTCCTGA
TCCGAAAGAATTCAACGATTATATTCTGGTTTTTCCGGCGGGATCCGGTATTAAGCCCATCTATGTTTACCTGAAGGAGG
ATCCGCGAAAGCTGCCTGGTGTTGTAACAGGGCGCGGCGTCCCGCTTTCACCAGGAACTCGCTGGCTGGATATGTCGGTA

TCCAATAACGGCAACGGCGCACCAATCCCGGCGCATATTGCTGATAAATTGCGCGGACGGGAGTTTAAAACCTTTGATGA
GTTTCGCGAGGCGCTGTGGCTGGAGGTGAGTAAAGACTCAGTGTTATTGGCGCAGTTTATAAAAAGTAATCAGAATAACG
TTAGTCAAGGATATTCCCCCTATGTCCCTGAGGAGGGGTATTATTACGGACCAAATGAGATAGTTAAGAAATTCCAGATA
CATCATGTAGTTGCTATAGAACATGGTGGTGGAGTGTATGATATTGATAATTTTAGGATTGTTACGCCCCGACTACATGA
TGAAATTCACTACAGGAGATAA
>Cluster_57530
ATGATTAAAAAAGCTTCGCTGCTGACGGCGTGTTCTGTCACAGCCTTTTCCGCTTGGGCACAGGATACCAGCCCGGATAC
TCTCGTCGTTACTGCTAACCGTTTTGAACAGCCGCGCAGCACTGTGCTTGCACCAACCACCGTTGTGACCCGTCAGGATA
TCGACCGCTGGCAGTCGACCTCGGTCAATGATGTGCTGCGCCGTCTTCCGGGCGTCGATATCACCCAAAACGGCGGTTCA
GGTCAGCTCTCATCTATTTTTATTCGCGGTACAAATGCCAGTCATGTGTTGGTGTTAATTGATGGCGTACGCCTGAATCT
GGCGGGGGTGAGTGGTTCTGCCGACCTTAGCCAGTTCCCTATTGCGCTTGTCCAGCGTGTTGAATATATCCGTGGCCGC
GCTCCGCTGTTTATGGTTCCGATGCAATAGGCGGGGTGGTGAATATCATCACGACGCGCGATGAACCCGGAACGGAAATT
TCAGCAGGGTGGGGAAGCAATAGTTATCAGAACTATGATGTCTCTACGCAGCAACAACTGGGGGATAAGACACGGGTAAC
GCTGTTGGGCGATTATGCCCATACTCATGGTTATGATGTTGTTGCCTATGGTAATACCGGAACGCAAGCGCAGACAGATA
ACGATGGTTTTTTAAGTAAAACGCTTTATGGCGCGCTGGAGCATAACTTTACTGATGCCTGGAGCGGCTTTGTGCGCGGC
TATGGCTATGATAACCGTACCAATTATGACGCGTATTATTCTCCCGGTTCACCGTTGCTCGATACCCGTAAACTCTATAG
CCAAAGTTGGGACGCCGGGCTGCGCTATAACGGCGAACTGATTAAATCACAACTCATTACCAGCTATAGCCATAGCAAAG
ATTACAACTACGATCCCCATTTTGGTCGTTATGATTCGTCGGCGACGCTCGATGAGATGAAGCAATACACCGTCCAGTGG
GCAAACAATGTCATCGTTGGTCACGGTAGTATTGGTGCGGGTGTCGACTGGCAGAAACAGACTACGACGCCGGGTACAGG
TTATGTTGAGAATGGATATGATCAACGTAATACCGGCATCTATCTGACCGGGCTGCAACAAGTCGGCGATTTTACCTTTG
AAGGCGCAGCACGCAGTGACGATAACTCACAGTTTGGTCGTCATGGAACCTGGCAAACCAGCGCCGGTTGGGAATTCATC
GAAGGTTATCGCTTCATTGCTTCCTACGGGACATCTTATAAGGCACCAAATCTGGGGCAACTGTATGGCTTCTACGGAAA
TCCGAATCTGGACCCGGAGAAAAGCAAACAGTGGGAAGGCGCGTTTGAAGGCTTAACCGCTGGGGTGAACTGGCGTATTT
CCGGATATCGTAACGATGTCAGTGACTTGATCGATTATGATGATCACACCCTGAAATATTACAACGAAGGGAAAGCGCGG
ATTAAGGGCGTCGAGGCGACCGCCAATTTTGATACCGGACCACTGACGCATACTGTGAGTTATGATTATGTCGATGCGCG
CAATGCGATTACCGACACGCCGTTGTTACGCCGTGCTAAACAGCAGGTGAAATACCAGCTCGACTGGCAGTTGTATGACT
TCGACTGGGGTATTACTTATCAGTATTTAGGCACTCGCTATGATAAGGATTACTCATCTTATCCTTATCAAACCGTTAAA
ATGGGCGGTGTGAGCTTGTGGGATCTTGCGGTTGCGTATCCGGTCACCTCTCACCTGACAGTTCGTGGTAAAATAGCCAA
CCTGTTCGACAAAGATTATGAGACAGTCTATGGCTACCAAACTGCAGGACGGGAATACACCTTGTCTGGCAGCTACACCT
TCTGA
>Cluster_22072
GTGCAGCACGAGACAAAAATGGAAAACCAGAGCTGGTTGAAAAAACTCGCACGCCGCCTGGGGCCTGGTCATGTCGTTAA
TCTCTGCTTTATCGTGGTATTGCTTTTTTCCACCTTGCTCACCTGGCGTGAAGTGGTGGTGCTGGAAGATGCCTATATCT
CCAGCCAGCGTAATCATCTGGAAAACGTAGCCAACGCGCTCGATAAGCATTTGCAGTATAACGTCGACAAACTGATCTTT
TTGCGTAATGGCATGCGCGAAGCTCTCGTGGCGCCACTGGATTTCATCTCTCTGCGTAATGCCGTAACCGAGTTCGAACA
GCATCGCGACGAGCACGCCTGGCAAATCGAACTCAACCGACGGCGCACCCTGCCAGTCAATGGCGTATCGGATGCATTAG
TCAGCGAGGGGAATCTCCTGTCTCGCGAAAATGAAATCCTCGACAATGAAATTACCGCTGCACTGGAAGTTGGTTACTTG
CTGCGACTGGCGCACAACTCCTCGTCGATGGTTGAACAGGCGATGTATGTCTCGCGTGCTGGATTTTACGTTTCGACGCA
GCCGACCTTGTTTACGCGCAATGTACCAACGCGTTATTACGGCTATGTCACCCAACCCTGGTTTATCGGTCATTCGCAAC
GAGAAAATCGTCACCGCGCGGTACGCTGGTTCACTTCGCAACCGGAACACGCCAGCAATACTGAACCGCAGGTTACCGTC
AGCGTTCCGGTAGACAGTAATAACTACTGGTATGGCGTGCTGGGGATGAGTATTCCCGTGCGTACTATGCAGCAATTTTT
AAGAAACGCCATCGATAAAAACCTCGATGGTGAGTATCAGCTCTATGACGGTAAGCTGAGATTTTTGACCTCTTCCAATC
CTGACCATCCAACAGGGAATATTTTTGATCCTCGTGAACTGGCCTTGCTGGCGCAGGCAATGGAACATGACACGCGGGGC
GGCATTCGTATGGACAGTCGCTATGTTAGCTGGGAACGTCTGGACCATTTCGACGGTGTGCTGGTGCGCGTCCATACGCT
AAGCGAAGGCGTGCGCGGCGATTTCGGCAGTATCAGCATTGCATTAACCCTGCTGTGGGCGCTCTTTACCACCATGTTAC
TCCTCTCCTGGTATGTGATTCGCCGGATGGTCAGCAACATGTATGTTCTGCAAAGCTCGTTGCAGTGGCAGGCGTGGCAC
GACACCTTAACGCGTTTATATAACCGTGGCGCACTGTTCGAAAAAGCCCGTCCGCTCGCTAAATTGTGTCAGACGCACCA
ACATCCATTTTCTGTCATTCAGGTCGATCTTGACCATTTTAAAGCGATTAATGACCGCTTTGGTCATCAGGTGGGCGACC
GTGTTCTTTCTCATGCTGCCGGATTAATTAGCAGTTCCTTACGTGCGCAGGACGTTGCCGGGCGGGTCGGTGGTGAGGAG
TTTTGTGTGATTCTGCCAGGCGCGAATCTGACGCAGGCTGCGGAGGTCGCAGAACGTATTCGCCTGAAGTTAAATGAAAA
AGAGATGCTGATCGCCAAGAGTACAACGATACGCATCAGTGCCTCGCTGGGGGTAAGCAGCAGCGAGGAAACCGGTGATT
ATGATTTTGAACAACTCCAGTCATTGGCCGACCGTCGGCTTTACCTCGCAAAACAGGCCGGGCGCAATCGGGTATTCGCG
AGCGATAACGCTTAA
>Cluster_20507
GTGCGATTATTTGCTCAATTAAGCTGGTATTTCCGTCGGGAATGGCGTCGCTATCTCGGGGCTGTCGCCTTGCTTGTCAT
TATCGCGATGCTGCAACTGGTTCCGCCAAAAGTGGTTGGTATTGTTGTTGATGGCGTGACAGAACAACACTTTACTACCG
GGCAGATCCTGATGTGGATCGCCACCATGGTGCTGATTGCCGTTGTGGTTTATCTCCTGCGTTACGTCTGGCGCGTATTG
CTGTTTGGTGCGTCTTATCAACTGGCTGTTGAACTGCGTGAAGATTATTACCGCCAGCTAAGCCGGCAGCATCCTGAGTT
TTACCTGCGTCATCGCACGGGTGACCTCATGGCTCGTGCGACCAATGACGTCGATCGCGTCGTGTTTGCCGCCGGAGAAG
GGGTGCTGACGCTGGTGGATTCACTGGTAATGGGCTGCGCTGTGTTGATTATGATGTCTACGCAAATTAGCTGGCAGTTG
ACCTTATTTGCCCTGTTGCCGATGCCAGTGATGGCGATCATGATCAAGCGCAACGGCGATGCCTTGCATGAACGCTTTAA
GCTGGCACAGGCGGCATTTTCCAGTCTTAATGACCGCACCCAGGAAAGCCTCACCAGTATCCGCATGATCAAAGCCTTTG
GTCTGGAAGATCGTCAGTCGGCGTTATTTGCCGCGGATGCCGAAGATACCGGCAAAAAAAACATGCGGGTGGCGCGTATT
GATGCTCGTTTCGACCCGACCATCTATATCGCGATTGGTATGGCGAACTTGCTGGCGATTGGCGGCGGTAGTTGGATGGT
GGTACAGGGCAGTTTAACGCTGGGCCAGCTCACCAGTTTTATGATGTATTTAGGTCTGATGATTTGGCCAATGCTGGCGC
TGGCATGGATGTTTAACATTGTGGAACGTGGCAGCGCGGCGTATAGCCGAATTCGTACCATGCTGGCCGAAGCGCCAGTC
GTTATTGATGGTAGTGATAAGGTGCCAGAAGGTCGTGGTGAACTGGATGTGAATATTCGCCAGTTTACGTATCCGCAGAC
TGACCATCCTGCGCTGGAAAACGTCAATTTCGCCCTGAAACCCGGTCAGATGGTAGGTATTTGCGGGCCGACTGGTTCCG
GCAAAAGTACCCTGTTGTCGCTCATTCAGCGTCATTTCGACGTCAGCGAGGGGGATATCCGCTTTCATGATATTCCTCTG
ACGAAGTTACAACTCGATAGCTGGCGTAGCCGCCTGGCGGTGGTCAGCCAGACACCGTTCCTTTTTTCCGACACCGTAGC
GAACAACATCGCGTTGGGTTGCCCGAATGCCACCCAGCAAGAGATTGAGCATGTCGCGCGGTTAGCCAGCGTGCATGACG
ATATTTTGCGTTTACCGCAAGGTTACGATACAGAAGTGGGCGAGCGCGGCGTGATGCTTTCCGGTGGGCAAAAACAGCGT
ATCTCCATTGCTCGTGCGTTATTAGTCAACGCGGAAATCCTCATCCTTGATGATGCGCTTTCGGCAGTAGACGGACGCAC
AGAGCACCAGATCCTGCATAACCTGCGTCAGTGGGGGCAGGGAAGAACGGTAATCATCAGTGCCCATCGCCTTTCTGCAC
TGACGGAAGCCAGTGAAATTATTGTGATGCAGCACGGACATATCGCCCAGCGTGGCAATCATGATGAGCTGGCGCAACAA
AGTGGCTGGTATCGCGATATGTATCGCTATCAACAACTGGAGGCGGCGCTCGACGACGCTCCGGAAATTCGCGAGGAGGC
CATCGATGCGTAG
>Cluster_60083
ATGACGCAAACTCTGTTAGCGATTGAAAATTTGTCGGTGGGTTTTCGCCATCAGCAAACCGTACGTACAGTAGTCAATGA

TGTTTCACTACAGATTGAGGCTGGCGAAACGCTGGCGCTGGTGGGTGAGTCAGGTTCAGGCAAAAGCGTTACCGCGCTGT
CAATTTTACGCCTGCTCCCTTCCCCGCCGGTTGAATATCTCTCCGGCGATATTCGTTTTCATGGCGAATCGCTGCTTCAC
GCCAGCGATCAAACGTTGCGCGGTGTACGCGGTAATAAGATCGCCATGATTTTTCAGGAACCGATGGTGTCGTTAAATCC
ATTGCATACCCTGGAAAAACAGCTTTATGAAGTGCTTTCACTCCACCGCGGGATGCGTTCGGGAAGCGGCTCGTGGCGAAA
TTCTTAACTGCCTTGATCGCGTTGGTATCCGCCAGGCGGCAAAACGGCTGACAGATTATCCGCATCAGCTCTCCGGCGGC
GAACGGCAGCGGGTGATGATTGCGATGGCGCTGTTAACGCGACCGGAATTATTAATTGCCGATGAACCGACCACCGCACT
GGACGTCTCTGTCCAGGCGCAGATTTTACAGCTGTTGCGCGAACTGCAAGGCGAGCTGAATATGGGCATGCTGTTTATTA
CTCATAACCTCAGCATTGTCAGAAAACTGGCCCACCGCGTGGCGGTAATGCAAAACGGTCGCTGTGTCGAGCAAAATTAC
GCCGCTACGCTATTTGCATCACCCACTCATCCTTACACACAAAAGCTACTCAACAGTGAACCGTCAGGCGATCCAGTGCC
GTTGCCAGAACCTGCCTCAACGTTGCTGGATGTTGAACAGCTTCAGGTTGCCTTCCCCATTCGCAAAGGGATTTTGAAGC
GCATTGTGGATCATAATGTGGTGGTGAAAAACATCAGTTTTACGCTACGAGCGGGTGAAACACTGGGTTTAGTGGGCGAG
TCCGGTTCCGGGAAAAGTACGACGGGACTGGCGCTGCTGCGACTGATTAATTCTCAGGGCAGCATCATCTTTGACGGTCA
GCCACTGCAAAATTTAAATCGCCGCCAGCTGTTACCTATTCGTCATCGCATTCAGGTGGTATTTCAGGATCCAAACTCCT
CGCTCAACCCACGACTCAACGTTTTGCAGATTATTGAGGAAGGCTTACGGGTTCACCAGCCGACGCTTTCTGCCGCACAA
CGCGAACAACAAGTGATAGCCGTGATGCATGAAGTGGGATTAGATCCTGAAACACGCCACCGTTATCCGGCGGAGTTCTC
TGGTGGTCAGCGACAACGTATTGCGATTGCCAGGGCATTAATTCTTAAGCCCTCGCTGATCATACTTGATGAACCGACAT
CATCACTCGACAAAACGGTACAGGCGCAAATATTGACGCTATTGAAATCATTGCAACAAAAGCATCAACTGGCCTATTTG
TTTATCAGCCACGATTTGCACGTTGTCCGCGCGTTATGTCATCAGGTTATCATACTGCGACAAGGGGAAGTAGTGGAACA
AGGACCGTGCGCGCGCGTGTTTGCCACACCGCAGCAGGAGTATACGCGTCAGCTACTGGCGTTGAGCTGA
>Cluster_6480
ATGGAGATCTCCTGGGGCCGCGCGCTATGGCGCAATTTTTTGGGCCAGTCCCCCGACTGGTACAAACTCGCCCTCATCAT
TTTCTTAATCGTAAACCCGTTAATTTTCCTCATCAGCCCTTTCGTCGCGGGCTGGTTGCTGGTAGCGGAATTTATTTTCA
CTCTGGCGATGGCCCTGAAATGCTACCCGCTGCTCCCCGGTGGTCTGTTGGCTATCGAAGCGGTATTCATCGGCATGACC
AGCGCGGAACACGTCCGTGAAGAGGTGGCGGCAAATCTTGAAGTCTTGCTGTTACTGATGTTTATGGTGGCGGGTATCTA
TTTTATGAAACAGCTGTTGCTGTTCATATTTACCCGTTTGCTGTTAAGCATTCGCTCCAAAATGCTGCTGTCGCTCTCTT
TTTGCGTGGCGGCTGCGTTCCTCTCCGCGTTCCTCGATGCCTTAACCGTCGTGGCGGTGGTGATCAGCGTTGCAGTCGGT
TTTTATGGTATTTATCACCGCGTAGCCTCTTCCCGTACCGAAGACACCGACCTGCAAGACGATAGTCATATCGACAAGCA
TTACAAAGTGGTTCTGGAACAGTTCCGTGGCTTTCTGCGTAGCCTGATGATGCATGCCGGTGTCGGCACCGCATTAGGCG
GCGTAATGACCATGGTGGGCGAACCACAGAACCTGATCATCGCTAAAGCGGCTGGCTGGCATTTTGGCGATTTCTTCCTG
CGCATGTCGCCGGTGACCGTTCCGGTTCTGATTTGTGGCCTGTTAACCTGCCTGCTGGTAGAGAAGCTGCGTTGGTTTGG
CTACGGTGAAACGCTGCCGGAGAAAGTCCGCGAAGTGTTGCAACAGTTTGACGATCAAAGCCGCCACCAGCGTACCCGTC
AGGATAAAATCCGTCTGATTGTCCAGGCGATTATTGGCGTCTGGCTGGTGACTGCGCTGGCGTTGCATCTGGCGGAAGTT
GGCTTGATTGGTTTGTCAGTCATTATTCTGGCAACATCATTGACCGGTGTCACCGATGAGCATGCTATCGGTAAAGCCTT
CACCGAATCTCTGCCATTCACCGCACTGTTGACGGTGTTTTTCTCGGTAGTCGCGGTGATTATCGACCAACAACTGTTTT
CGCCAATTATTCAGTTTGTGTTGCAGGCATCGGAACATGCTCAGCTGTCGCTGTTCTATATTTTCAACGGTCTGCTGTCA
TCCATTTCGGATAACGTCTTCGTGGGGACGATTTATATCAACGAAGCGAAAGCGGCAATGGAAAGTGGCGCTATCACGTT
GAAGCAATACGAGCTGCTGGCGGTCGCCATTAATACCGGTACCAATCTGCCCTCCGTCGCTACGCCGAACGGTCAGGCTG
CGTTCCTGTTCCTGCTGACCTCTGCACTCGCGCCATTGATTCGCCTCTCTTATGGCCGCATGGTGTGGATGGCCCTGCCT
TACACCCTCGTCCTGACACTCGTCGGCTTGCTCTGCGTCGAGTTTACGCTTGCCCCTGTAACCGAATGGTTTATGCAAAT
GGGCTGGATAGCAACGCTTTGA
>Cluster_38717
ATGGATATCATTGGCGGACAACATCTACGTCAAATGTGGGACGATCTGGCGGACGTTTACGGTCATAAAACGGCGCTGAT
TTGTGAATCCAGCGGCGGAGTCGTTAACCGGTATAGTTATCTTGAGTTAAATCAGGAGATTAACCGCACGGCAAACCTGT
TTTATACGCTGGGGATTCGCAAAGGCGACAAGGTTGCTCTACATCTCGACAACTGCCCGGAATTTATCTTTTGCTGGTTC
GGGCTGGCAAAAATTGGTGCGATTATGGTGCCGATTAACGCCCGCCTGTTACGCGAGGAAAGCGCGTGGATCCTGCAAAA
TAGCCAGGCGTGCCTGCTGGTGACCAGTGCGCAATTCTATCCTATGTATCAACAGATTCAGCAGGAAGATGCCACTCAGT
TGCGGCACATTTGCCTGACAGATGTGGCACTTCCCGCTGATGATGGCGTGAGTTCGTTTACTCAACTGAAAAATCAACAA
CCTGCCACCTTGTGCTATGCACCGCCGCTATCGACTGACGATACGGCGGAAATTCTCTTCACCTCCGGCACCACCTCCCG
ACCGAAAGGTGTGGTGATTACCCATTACAACCTGCGCCTCGCTGGATATTACTCCGCCTGGCAGTGTGCACTGCGTGACG
ATGACGTCTACCTGACGGTAATGCCTGCGTTTCATATCGATTGCCAGTGTACTGCGGCGATGGCGGCGTTTTCTGCCGGG
GCCACCTTTGTGCTGGTCGAGAAATACAGCGCCCGCGCCTTCTGGGGACAGGTGCAGAAGTACCGCGCCACCATTACCGA
ATGTATTCCGATGATGATCCGTACGTTGATGGTACAGCCGCCTTCAGCGAACGATCGGCAACACCGCCTGCGGGAAGTGA
TGTTTTATCTCAACTTGTCGGAGCAGGAAAAAGACGCATTTTGTGAACGCTTCGGCGTTCGCTTGCTGACGTCTTATGGG
ATGACGGAAACCATTGTGGGCATTATCGGCGATCGCCCTGGCGATAAACGACGCTGGCCGTCGATTGGTCGGGCGGGGTT
TTGCTACGAAGCGGAGATCCGCGACGATCACAATCGCCCGCTCCCGGCAGGTGAGATCGGTGAAATCTGTATTAAAGGCG
TACCAGGGAAAACCATCTTCAAAGAGTATTTTCTCAACCCGAAAGCCACTGCGAAAGTACTGGAAGCCGATGGCTGGCTA
CATACCGGCGATACCGGATACCGCGACGAAGAGGGCTTTTTTTATTTCGTCGATCGCCGCTGCAATATGATCAAACGCGG
GGGCGAGAATGTCTCCTGCGTGGAGCTGGAAAATATCATCGCCGCGCACCCGAAAATTCAGGACATCGTGGTTGTGGGTA
TTAAAGATTCGATACGCGATGAAGCCATCAAAGCATTTGTGGTGCTGAATGAAGGTGAAACATTGAGCGAAGAGGAATTT
TTCCGCTTCTGCGAACAAAATATGGCGAAATTTAAAGTGCCCTCTTATCTGGAGATCAGAAAAGATCTGCCACGTAATTG
CTCGGGGAAAATAATTAGAAAGAATCTGAAATAA
>Cluster_104
ATGGGCCGTATTCTGGATATTTCCGGGCAGCCGTTTGATTTTGACGATGAAATGCAGACCCGCAGCGATGAGCTGGCAAT
GGTCATGAAGCGTACGCAGGAGCATCCTTCCAGTGGTGTGACGCCGAACCGGGCCGCGCAGATGCTGCGTGATGCCGAAC
GCGGTGATCTGACAGCACAGGCTGACCTGGCCTTTGATATGGAAGAAAAGGACACCCACCTTTTTTCCGAACTGAGCAAA
CGCCGCCTTGCCATTCAGGCGCTGGAATGGCGCATCGCTCCGGCACGTGATGCCAGCGCACAGGAGAAAAAAGACGCTGA
CATGCTGAATGAATATCTGCATGATGCGGCGTGGTTTGAAGATGCCCTGTTTGATGCCGGTGATGCCATTCTCAAGGGCT
ATTCCATGCAGGAGATTGAATGGGGCTGGCTGGGGAAAATGCGCGTGCCGGTGGCGCTGCATCATCGCGATCCGGCGCTG
TTCTGCGCGAACCCCGACAATCTGAACGAATTACACCTGCGTGATGCGTCTTATCACGGGCTGGAGCTGCAACCGTTCGG
CTGGTTTATGCACCGGGCCAAATCCCGCACCGGGTATGTCGGCACCAATGGCCTTGTGCGCACGCTTATCTGGCCTTTTA
TTTTTAAAAACTATTCCGTGCGTGATTTTGCAGAGTTTCTGGAAATTTACGGACTCCCCATGCGCGTCGGTAAATATCCG
ACCGGCTCCACGAACCGCGAGAAAGCCACGCTGATGCAGGCCGTGATGGATATCGGGCGACGTGCCGGTGGCATTATTCC
GATGGGGATGACGCTGGATTTTCAGAGCGCCGCAGATGGTCAGTCAGACCCCTTTATGGCGATGATTGGCTGGGCAGAAA
AAGCGATTTCAAAAGCCATTCTGGGCGGCACCCTGACCACAGAGGCTGGCGATAAAGGCGCACGGTCGCTGGGTGAAGTG
CATGATGAGGTTCGCCGGGAAATCCGTAACGCGGATGTGGGCCAGCTTGCCCGCAGTATTAACCGTGATCTGATTTATCC
CCTGCTGGCACTGAACAGTGACAGCACCATTGATATTAACCGCCTGCCGGGCATTGTGTTTGATACCAGTGAAGCGGGTG
ATATTACGGCACTGTCTGACGCCATTCCGAAACTGGCGGCGGGAATGCGTATTCCGGTCTCCTGGATACAGGAAAAACTC
CATATTCCACAGCCGGTCGGTGACGAAGCCGTGTTTACTGCTCAACCGGTTGTCCCGGATAACGGTCCGCAGAAAGAAGC
CGCATTATCCGCTGATGATATCCCGCAGGAGGATGATGTCGACCGGATGGGCGTTTCACCGGAAGACTGGCAGCGTTCGG

TTGATCCCCTGCTGAAACCCGTCATTTTTTCTGTGCTGAAAGATGGCCCGGAAGCCGCCATGAACAAAGCCGCTTCATTA
TATCCGCAGATGGATGATGCAGAACTTATCGACATGCTGACCCGTGCCATTTTTGTTGCCGATATATGGGGGCGTCTGGA
TGCCGCAGCAGACCATTGA
>Cluster_10080
ATGTGCAGGAAATATGCGCATGTTGTGGACGCCCTGCAAGGGAATTTCCCGTATGCAACAGACCCGGCGGGAAACCGGCT
GCCGGACCCGGAACTGCATCCGGACAGCACGCTCACGGCGTGGCCGGATAACCGCATCGCGGAAGATGCGCACTATGTCT
ATCGCTACGATGAATACGGCAGGCTGGCGGAGAAGACGGACCGTATCCCGGAAGGGGTTATCCGGATGCACGACGAGCGC
ACCCACCACTATCACTACGACAGCCAGCACCGCCTGGTGTTCTACACGCGGATACAGCACGGCGAACCACAGGTGGAGAG
CCGGTACCTCTATGACCCGCTGGGCCGCCGGACGGGAAAACGGGTATGGCGGCGGGAGCGTGACCTGACGGGGTGGATGT
CGCTGTCGCGTAAACCGGAGGTGACATGGTACGGGTGGGACGGTGACAGGCTGACGACGATACAGACCGGCACCACGCGT
ATCCAGACGGTATACCAGCCGGGGAGCTTCGCGCCGCTCATCCGCATCGAAACAGACAACGGTGAACGGGAGAAAGCGCA
GTGCCGCAGCCTGGCGGAGAAGTTACAGCAGGAAGGGAGCGAAGACGGTCACGGTGTGGTGTTCCCGGCTGAACTGGTGG
GGCTGCTGGACAGGCTGGAGGGGGAAATCCGGGCAAACTGCGTGAGCAGTGAAAGCCGCCAGTGGCTTGCGCAGTGCGGG
CTGACGGTGGAGCGGCTGGCGGCACAGATAGAGCCGGTTTATTTGCCTGAGCGAAAAATACATCTTTATCACTGTGACCA
CCGGGGCCTGCCGCTGGCGCTTATCAGTGAAGACGGCAATACGGCGTGGAGCGCGGAATATGATGAATGGGGCAACCAGC
TTAATGAGGAGAACCCGCATCACCTGCACCAGCCGTACCGGCTGCCGGGGCAGCAGTATGATAAGGAGTCGGGGCTGTAC
TATAACCGTCACCGGTACTACGATCCGTTGCAGGGGCGGTATATCACCCCGGACCCGATTGGGTTGAGAGGTGGATGGAA
TATGTATCAGTATCCGTTGAATCCCATACAAGTGATAGACCCAATGGGGTTAGATGCGATTGAGAATATGACATCAGGTG
GACTAATTTATGCCGTATCTGGTGTACCTGGATTGATTGCTGCAAACAGCATTACTAACAGTGCTTACCAGTTCGGTTAT
GATATGGATGCTATTGTTGGCGGAGCTCATAATGGGGCCGCCGATGCAATGAGACATTGTTACTTGATGTGTCGAATGAC
TAAGACATTTGGATCAACAATAGCTGACGTGATAGGTAAAAATCATGAGGCGGCAGGGGATAGACAAGGTCAGCCAGCTA
AAGAAAGAATCATGGATCTTAAAAATAACACTGTCGGTATTGCTTGTGGCGATTTTTCTGCCAAATGTAGCGATGCATGT
ATTGAAAAATATAACACTGGGCAACTCTTCGGGTTAGATGGTATAAAAGCAGATAATCCAATAAAAGCAAAGCAAGGGAG
TTCAGATGCTTCAAATTATTAG
>Cluster_6257
ATGAGTCAGAAATACCTCATTCGCATCGCAGAGCTGGAAAGGTTGCTCTCTGAGCAGGCTGAAGCCCTCCGTCAGAAAGA
CCAACAACTGAGTCTGGTTGAAGAGACGGAGGCCTTCCTGCGTTCTGTGCTGGCACGTGCCGAAGAAAAGATCGAAGAAG
ATGAACGGGAAATAGAACATCTGCGGGCTCAGATAGAAAAACTGCGCCGGATGCTGTTCGGTACCCGTTCTGAAAAACTG
CGTCGTGAAGTTGAACAGGCTGAGGCCCTGCTGAAGCAACGCGAACAGGACAGCGATCGTTACAGTGGGCGAGAGGATGA
CCCGCAGGTTCCCCGCCAGTTGCGACAGTCACGCCATCGTCGTCCGTTACCGGAGCATCTGCCCCGCGAAATAAACCGCC
TGGAGCCTGAAGAAAGCTGTTGCCCGGAGTGTGGCGGTGAGCTGGATTATCTGGGGGAAGTCAGTGCAGAACAACTGGAA
CTGGTGAGCAGCGCCCTGAAAGTGATCCGCACAGAACGGGTAAAAAAAGCCTGTACAAAATGTGACTGCATCGTTGAAGC
ACCGGCACCATCCCGTCCGATAGAGCGTGGTATCGCGGGCCCGGAGTTACTTGCCCGCGTGTTAACGGGAAAATACTGCG
AACATCTGCCACTGTATCGTCAGAGTGAAATCTTTGCCCGCCAGGGTGTCGAACTGAGCCGTGCCTTACTCTCCAACTGG
GTTGATGCGTGCTGCCAGTTAATGACTCCGCTGAATGATGCCCTGTACAGTTATGTGATGAACACCCGCAAGGTTCACAC
TGATGACACACCAGTAAAAGTACTGGCACCGGGCAGGAAGAAGGCGAAAACAGGATATATTTGGACGTATGTCCGGGATG
ACCGAAATGCCGGTTCGCCAGAACCTCCGGCGGTCTGGTTCGCCTACTCACCGGACCATCAGGGTAAACATCCGGAGCAA
CACCTTCGTCCCTTCCGGGGTATCCTGCAGGCAGATGCGTTCGCAGGTTACGATCGGCTGTTCAGTGCCGAACGTGAAGG
CGGCGCGTTGACGGAAGCAGGATGCTGGGTTCATGCACGGCGCAAAATCCACGATGTATATATCAGTACCAAAAGCGCGA
CGGCGGAAGAAGCACTGAAACTAATCGGTGAACTGTACGCCATTGAGCACGAAATACGCGGGTTGCCGGTGTCTGAACGC
CTGGCGGTCAGGCAAATGCAGAGTAAACCGCTACTGACTTCCCTGTATAAGCTGATGCAGGAGAAAGAACACACGTTATC
GAAAAAATGCCGTCTGAGAGATGCGTTCCGGTATATCAGGAAGCACTGGGTTGCGTTGTGCAACTTCTGTGATGACGGTC
TGGCGGAGGCGGACAATAACACAGCGGAAAGAGCGCTTCGTGCAGTCTGTCTCGGAAAGAAAAATTCTTATGACCTTTGT
CAAATATTAAGCCCAAAAAGACCTTACGCAGCTCCGGGAGCTTCGTTATATCGCGGATTAAAGAACCTGATACAGTCAGA
CTGTATCTTGGATTTTACAAACTCTTATTCGGGGATTTTAGCCCCATGA
>Cluster_51983
ATGAAACAAAAGAAAGCCTGGAGTTTTTTTCAGAGCCTGGGGAAGGCATTTATGTATCCCATTGCTCTGCTAAGTGTATG
TGGCATGATGCTAGGGCTGGGAAGTGGTTTAGCCAGTGATGATATGGCAAAGTTAATTCCATTTCTGGCTATTCCAATAA
TTAAAACCATACTTGATTTCATTGTTAGTCTTGGTTTGTTTGCCTTTGTTAATTTACCTGTATTGTTTGCGATAGCGATT
CCCTTAGGATTATTAAAAGATAAAGAGGATAAAGCCTATGGTGCTTTTTCTGGCTTAATTGGTTTTATGGCGATGCATCT
GGGAACGAACTTTTATCTTAAACAGCACGACTTATTGGTCGTTGCTGACCAAATGTCGACACATGGGCAAACCATCATTC
TGGGGATCCAGTCCTACAATACCAGCGTGTTGGGGGGAATTGTTGCTGGGTTATTAGTCGCCAGCATGTATAAAAAGATC
GTTAATTTACGCATTCCTGAATCGTTAGGTTTTTATAGCGGCCCACGTCTGGTGCCTATCATTACACTGATTGTGATGAG
TGGATTTGGTTTGATCATTCCTTTTATCTGGCCGCCGTTTTTCAATCTTTTCATGCTCATTGGCCACTGGATTTCAACTT
CCGGTCCTGTTGGTTATTTCTTCTATGCAGTTGCCGAACGCGTGACGATTCCTTTTGGCTTAAACCATCTGGTGACGTCA
GTTTTCCGCTTTACGCCAATCGGCGGTTCGGCTGTGATTGGCGGCGAAGAATATTACGGCACCCTGAACATGTTTATGGC
ATACGTCAAAGAGAATGCGGTCATTCCGCTGGATTTGGCGGGGAAAATGGAGCAGGGCAAACTGATGATTCAGTATGGTC
TGGCTGGTGCTGCGCTGGCGATGTATCGCACTGCTCATGCTCAAAACAGAAAGGCTATCAAAGCATTGCTTATTTCCGGG
GTGCTTACGGTGATTATTGGCGGCGTCAGCGAACCGATTGAGTTTCTGTTCTTATTTGTCAGTCCACTGCTGTTTGTCTT
CCATGCCTTTATGAATGGATTCGCTAACATGGTTCTGCCATATATGGGGGTGAAGATGGGATTTACTGGTGATCTGATTC
AATTTATTAGCTTTGGCGTATTGCGTGGCACAAGAACAGGTTGGCCGATCGCGGTGTGTGTCGAAGTGGCCTATTTCTTC
ATTTATTACTTTGTGTTCCGTTGGACCATCCTTAAATTTAACCTGATGACCGTAGGCCGTGAAGAGTCCAGTCCTGTTAC
GCTGAACGCTCACGAAGATACGGCTATAGCGGATATCCCAACTCCTGATAAATCAGAGCTGCAAGCGGCGGAGCAGATGG
TTAAGGCACTTGGTGGTAAAGAGAATATTAAGTCACTGGATAATTGCGTAACTCGTTTACGTTTAACAATCGCAGATATG
AGATTGATTGACGAAGTTGCAATAAAAAGAGCTGGCGGGATTGCGGTTGTAAAACTTGATCAAAATACCCTACAAGTCAT
TATCGGCACTAAAGTCATCGCCCTGCGTCGGGATATGGATAACTATATGGGGATATACTGA
>Cluster_3243
ATGAACTCGTTTTCACTTCTGACAACCCCGTGGTTGCCCGTTCGTTTTAAAGACGGAACGACAGGCAAACTGGCACCAGT
CGATCTGGCTGATGAAAATGTTGTCGATATCGCTGCGCCGCGGGCAGATCTCCAGGGGGCGGCCTGGCAGTTTTTGTTGG
GGCTACTACAAACCAGCTTCGCGCCAAAAGATCAACGTCGTTGGGATGATATCTGGGAAGACGGACTGGAAGCTGAAAAG
CTAAGGGAAGCATTGCTGTCTTTAGAACATGCTTTTCAGTTTGGGCCAGATTCCCCTTCATTTATGCAGGATTTTGATGA
ATTAAAAGTTAAGGCAACTTCTATTGCTTCGCTTCTTCCTGACGCACCAGGAAAACAAACAAAAGAACGAAATACAGACC
ATTTTATTAAGCGAGACACAACTCAGCATCTGTGCCTGCATTGTGTGCCTTTAGCGCTTTTTTCAATCCAGTTGAATGCA
CCTATAGGTGGACGCGGTTACTATCCTGGGCTACGTGGCGGAGGGCCTCTGACGACACTTATAGAATTGCTGGAGTATCA
GGGCAATCAACAAACCCCTTTGTGGCGAAAGCTGTGGCTAAACGTAATGCCGCAAGATGAAGCCGATTTACCTTTACCAA
AAACCTTTGATGACCTGGTCTTTCCATGGCTTGCACCGACGCGTACCAGCGAACTGGACGGTGCTGTGGTAACGGATGAA
CAAGTCAATAAACTTCAGGCCTACTGGGGAATGCCACGACGTATACGTATTGATTTTAAGACAACCTCAATAGGTAACTG
CGACATTTGCGGTAGGCAGAGCGATGCTCTTTTGGGCTTGATGTCACTCAAAAACTATGGTGTTCAATACGTCATGTGGC
GCCATCCTCTAACACCTTACCGTCTGCCGCTTAAAGAGGGTGGAGATTTTTACTCAGTTAAACCACAACCTGGTGGTTTA

ATCTGGCGCGACTGGTTAGGCCTCATAGAGGTAGGTAACTCAAAAAACAATACTGAACTCCCCGCACAGGTCGTGAAACT
TTTGAATGCCAGCAATCTAAAGCAGACAAGGGTGGGCTTATGGGGATTTGGTTTTGATTTTGAAGATATGAAGGTTCGTT
GTTGGTATGAACACCATTTCCCGCTGTTGTTAAACAAAAAAGAAGACCTAATACCTAAGTTGCGTCTGGCAGCGCAAGCG
GCATCACACATTCTAAGCTTGTTACACCGAGCGTTGAAAGAAGCATGGTTCTCTGAGAAGAAAACCACAAAGCTTGATTT
CGGTTTTGTGGATATTGACTTCTGGAACAAAACTCAGCATCGATTCCTGAGATTAGTGCGCAAAATTGAAGAAGGTCAGG
ATCCGTACGAATTACTCAGTAAATGGCAAAAGGAAATGTGGTTATTCGCGCGTCAGGATTTTGACGATCGTGTCTTTACC
AATCCATATGAGCCCGTTGATTTGGAACGCGTCATGACCGCACGCAAGAAATATTTTACAACATCAGCGGAGAAGCAAAA
TGCTAACGCCGCCAGGGAGAAAAAGCAGGAGGCTGCTGAATGA
>Cluster_34583
ATGTTAGATATAGTCGAACTGTCGCGCTTACAGTTTGCCTTGACCGCGATGTACCACTTCCTTTTTGTGCCACTGACGCT
CGGTATGGCGTTCCTGCTGGCCATTATGGAAACGGTCTACGTCCTCTCCGGCAAACAGATTTATAAAGATATGACCAAGT
TCTGGGGCAAGTTGTTTGGTATCAACTTCGCTCTGGGTGTGGCTACCGGTCTGACCATGGAGTTCCAGTTCGGGACTAAC
TGGTCTTACTATTCCCACTATGTAGGGGATATCTTCGGTGCGCCGCTGGCAATCGAAGGTCTGATGGCCTTCTTCCTCGA
ATCCACCTTTGTAGGTCTGTTCTTCTTCGGTTGGGATCGTCTGGGTAAAGTTCAGCATATGTGTGTCACCTGGCTGGTGG
CGCTCGGTTCAAACCTGTCCGCACTGTGGATTCTGGTTGCGAACGGCTGGATGCAAAACCCCAATCGCGTCCGATTTCAAC
TTTGAAACTATGCGTATGGAGATGGTGAGCTTCTCCGAGCTGGTGCTTAACCCGGTTGCTCAGGTGAAATTCGTTCACAC
TGTAGCGTCTGGTTATGTGACTGGCGCGATGTTCATCCTCGGTATCAGCGCATGGTATATGCTGAAAGGTCGTGACTTCG
CCTTCGCTAAACGCTCCTTTGCTATCGCTGCCAGCTTCGGTATGGCTGCTGTTCTGTCTGTTATTGTTCTGGGTGATGAA
TCCGGCTACGAAATGGGCGACGTGCAGAAAACCAAACTGGCTGCTATTGAAGCCGAGTGGGAAACGCAACCTGCGCCTGC
TGCCTTTACTCTGTTCGGCATTCCTGATCAGGAAGAGGAGACGAACAAATTTGCGATTCAGATCCCTTACGCACTGGGCA
TCATTGCAACGCGTTCCGTGGATACCCCGGTTATCGGCCTGAAAGAGCTGATGGTGCAGCATGAAGAACGCATTCGTAAC
GGGATGAAGGCGTACTCTCTGCTCGAACAACTGCGTTCTGGTTCTACCGACCAGGCGGTTCGTGACCAGTTCAATAGCAT
GAAGAAAGACCTCGGTTACGGTCTGCTGCTGAAACGCTATACGCCAAACGTGGCTGATGCGACTGAAGCGCAGATTCAAC
AGGCAACCAAAGACTCCATCCCGCGTGTAGCGCCGCTGTACTTTGCGTTCCGTATCATGGTGGCGTGTGGCTTCCTGCTT
CTGGCAATCATCGCGCTCTCTTTCTGGAGTGTCATCCGCAACCGCATTGGCGAGAAAAAATGGCTTCTGCGCGCCGCGCT
GTACGGTATTCCGCTGCCGTGGATTGCTGTAGAAGCGGGCTGGTTCGTGGCTGAATATGGCCGCCAACCGTGGGCTATCG
GTGAAGTGCTGCCGACAGCTGTGGCGAACTCGTCACTGACCGCAGGCGATCTCATCTTCTCAATGGTGCTGATTTGCGGC
CTGTATACCCTGTTCCTGGTGGCAGAATTGTTCTTAATGTTCAAGTTTGCACGCCTCGGCCCAAGCAGCCTGAAAACCGG
TCGCTATCACTTTGAGCAGTCTTCCACGACTACTCAGCCGGCACGCTAA
>Cluster_29184
ATGAGCGCGCTCAACTCCCTGCCATTACCGGTGGTCAGACTGCTGGCGTTCTTTCATGAAGAGTTAAGCGAGCGGCGACC
AGGGCGCGTGCCACAGACGATGCAACTCTGGGTAGGTTGCCTGCTGGTGATTCTGATCTCGATGACTTTTGAGATCCCTT
TTGTGGCGTTATCACTGGCAGTGCTGTTTTACGGTATTCAGTCGAACGCGTTTTACACCAAATTTGTGGCAATCTTGTTT
GTGGTCGCCACGGTGCTGGAGATCGCCAGCCTGTTTTTGATCTACAAATGGTCATATGGCGAACCGTTGATCCGATTGAT
CATTGCCGGGCCGATCCTGATAGGCTGTATGTTTTTGATGCGCACCCATCGGTTGGGGCTGGTCTTTTTCGCCGTCGCCA
TTGTCGCTATTTACGGGCAAACCTTCCCCGCCATGCTCGACTATCCGGAAGCGGTCGTGCGCTTAACGCTGTGGTGTATC
GTTGTTGGCCTCTATCCAACCTTGCTGATGACGTTAATCGGCGTGCTGTGGTTTCCCAGTCGTGCCATTACGCAGATGCA
TCAGGCGCTTAATGATCGGCTTGATGATGCCATTAGCCACCTGACGGACAGCCTCGCACCGCTACCCGAAACGCGGATTG
AAAGAGAGGCGCTGGCGCTGCAAAAACTCAATGTCTTTTGCTTCGCGGACGATGCCAACTGGCGAACCCAGAGCGCATGG
TGGCAAAGCTGCGTGGCAACGGTAACCTACATTTACTCGACACTGAATCGCTACGATCCCACCTCTTTTGCTGATTCTCA
GGCAATTATTGAATTCCGACAAAAATTAGCTTCAGAAATCAACAAGATACAGCATTCCATTACCGAAGGTCAGTGCTGGC
AAAGCGACTGGCGGATCAGTGAAAGTGAAGCAATGGCGGCACGGGAATGTAACCTGGAGAATATTTGCCAGACGCTATTA
CAGCTTGGTCAGATGGACCCGAATACGCCGCCAACGCCCGCAGCCAAACCGCCATCAATGGTCGCCGATGCTTTTACCAA
TCCAGACTATATGCGCTACGCGGTAAAAACGCTGCTCGCCTGTTTGATCTGTTACACCTTCTACAGCGGCGTGGACTGGG
AAGGCATTCACACCTGTATGCTGACCTGCGTGATCGTCGCTAATCCAAATGTCGGTTCGTCGTACCAGAAGATGGTGCTG
CGTTTTGGTGGGGCCTTTTGCGGCGCGATTCTGGCGCTGTTATTCACGCTACTGGTCATGCCCTGGCTGGACAATATTGT
CGAATTGCTGTTTGTGCTGGCACCGATTTTCCTGTTGGGCGCATGGATTGCCACCAGCTCTGAACGCTCTTCTTATATCG
GCACACAGATGGTGGTCACCTTCGCGCTCGCCACGCTCGAAAACGTTTTTGGCCCGGTGTACGACCTGGTGGAAATTCGC
GATCGCGCTCTGGGTATCATCATTGGTACCGTGGTGTCCGCGGTGATTTACACCTTTGTCTGGCCTGAAAGCGAAGCGCG
CACGCTGCCGCAAAAACTGGCTGGCGCGCTGGGTATGTTGAGTAAAGTAATGCGGATCCCACGCCAGCAGGAAGTCACGG
CTCTGCGCACTTATCTGCAAATTCGCATAGGTCTGCATGCGGCGTTTAATGCCTGTGAAGAGATGTGCCAACGCGTGGTG
CTGGAGCGTCAACTGGACAGCGAAGAACGCGCATTACTGATTGAACGTTCGCAAACGGTTATTCGTCAGGGCCGCGATAT
TCTTCACGCCTGGGATGCCACCTGGAACTCGGCGCAGGCGCTGGATAACGCACTACAGCCGGACAGAGCAGGTCAGTTTG
CCGACGCCCTGGAGAAATACGCCGCCGGTCTGGCAACCGCACTCAGCCGTTCTCCTCAAATAACGCTTGAAGAAACACCC
GCCTCGCAGGCCATTCTGCCCCACCTTATTAAAACAGGAGCAACACGTCTGCCAGCTTTTCGCCCGCTTGCCAGACTGGAC
AGCCCCGGCATTAACGCCCGCCACGGAACAGGCACAAGGAGCCACGCAATGA
>Cluster_53941
ATGCAAACCCATCATGATTTACCTGTTTCAGGCGTATCCGCAGGGGAAATTGCCTCCGAGGGTTACGATCTGGACGCCCT
GCTGAACCAGCATTTTGCTGGTCGTGTGGTGCGTAAAGATCTCACCAAGCAACTCAAGGAAGGGGCAAACGTCCCGGTGT
ATGTGCTGGAGTATCTGCTCGGCATGTACTGCGCCTCTGACGATGACGACGTGGTCGAGCAAGGGTTGCAAAACGTTAAG
CGTATTCTGGCTGATAACTATGTGCGCCCGGATGAAGCAGAGAAAGTGAAGTCGCTGATCCGCGAGCGTGGTTCGTACAA
AATCATCGATAAAGTGTCGGTGAAGCTAAACCAGAAAAAAGACGTTTACGAAGCCCAGCTTTCTAACCTCGGCATCAAAG
ACGCGCTGGTGCCATCGCAGATGGTTAAAGACAACGAGAAGCTACTAACGGGCGGTATCTGGTGCATGATTACCGTCAAC
TATTTCTTTGAAGAAGGGCAGAAGACTTCGCCCTTCTCATTGATGACGCTTAAGCCTATCCAGATGCCGAATATGGATAT
GGAAGAAGTGTTCGATGCGCGTAAACACTTTAACCGTGATCAGTGGATCGATGTGCTGCTGCGCTCAGTGGGTATGGAGC
CCGCCAATATTGAGCAACGCACCAAATGGCACCTTATCACCCGTATGATCCCGTTCGTGGAGAACAACTATAACGTTTGC
GAGCTGGGGCCGCGTGGCACCGGTAAAAGCCATGTGTATAAAGAGTGTTCTCCTAACTCTCTGTTAGTTTCTGGCGGGCA
AACGACCGTTGCCAACTTGTTCTACAACATGGCCAGTCGCCAGATCGGCCTGGTTGGCATGTGGGATGTGGTAGCGTTCG
ACGAAGTCGCGGGGATCACTTTCAAAGATAAAGACGGCGTGCAAATCATGAAAGATTACATGGCGTCAGGATCTTTCTCT
CGCGGCAGAGATTCGATTGAAGGTAAAGCGTCGATGGTTTTCGTCGGCAACATCAATCAAAGCGTAGAGACTCTCGTTAA
AACCAGCCATTTGCTGGCGCCATTTCCGGCTGCGATGATTGATACTGCATTTTTCGACCGCTTTCATGCCTATATTCCCG
GTTGGGAAATCCCCAAAATGCGCCCGGAATTTTTTACCAACCGTTACGGGCTGATTACGGATTATCTCGCTGAATATATG
CGCGAAATGCGCAAACGCAGTTTCTCTGATGCGATTGATAAATTCTTTAAGCTGGGTAACAACCTCAACCAGCGTGACGT
TATTGCCGTTCGACGTACCGTGTCGGGGTTGTTAAAACTCATGCATCCCGATGGCGCGTACAGCAAAGAAGATGTGCGAG
TCTGCCTGACCTATGCGATGGAAGTTCGTCGCCGCGTGAAAGAGCAACTTAAAAAACTGGGCGGTCTGGAGTTCTTCGAT
GTGAACTTTAGCTACATCGACAACGAAACGCTGGAAGAGTTTTTTGTGAGCGTACCGGAACAGGGCGGCAGCGAACTTAT
TCCTGCCGGAATGCCAAAGCCGGGTGTTGTGCATCTGGTCACTCAGGCAGAAAGCGGCATGACCGGGCTGTATCGTTTTG
AAACACAGATGACTGCCGGTAATGGTAAGCATAGTGTATCGGGTCTGGGTTCAAATACCTCCGCGAAAGAAGCTATCCGC
GTCGGTTTCGATTACTTCAAAGGCAATTTGAATCGGGTAAGCGCGGCCGCGAAATTCTCCGATCATGAATATCACCTTCA

TGTCGTTGAACTGCATAATACTGGCCCAAGCACCGCAACCAGTCTTGCTGCGCTTATCGCTTTATGTTCGATATTGCTGG
CAAAACCGGTGCAGGAACAGATGGTGGTGTTGGGCAGTATGACGCTTGGTGGGGTAATTAACCCGGTGCAGGATCTTGCC
GCCAGTTTACAGCTCGCCTTCGACAGCGGTGCAAAACGGGTTCTGTTGCCGATGTCCTCGGCTATGGATATTCCAACGGT
TCCGGCAGAGTTATTTACCAAGTTTCAGGTGAGTTTTTACTCAGACCCGGTTGATGCTGTTTATAAGGCGCTGGGTGTGA
ATTAA
>Cluster_58200
ATGACCATTGAGAAAATTTTCACCCCGCAGGACGACGCGTTTTATGCGGTGATCACCCACGCGGCGGGGCCGCAGGGCGC
TCTGCCGCTGACCCCGCAGATGCTGATGGAATCTCCCAGCGGCAACCTGTTCGGCATGACGCAGAACGCCGGGATGGGCT
GGGACGCCAACAAGCTCACCGGCAAAGAGGTGCTGATTATCGGCACTCAGGGCGGCATCCGCGCCGGAGACGGACGCCCA
ATCGCGCTGGGCTACCACACCGGGCATTGGGAGATCGGCATGCAGATGCAGGCGGCGGCGAAGGAGATCACCCGCAATGG
CGGGATCCCGTTCGCGGCCTTCGTCAGCGATCCGTGCGACGGGCGCTCGCAGGGCACGCACGGTATGTTCGATTCCCTGC
CGTACCGCAACGACGCGGCGATCGTGTTTCGCCGCCTGATCCGCTCCCTGCCGACGCGGCGGGCGGTGATCGGCGTAGCG
ACCTGCGATAAAGGGCTGCCCGCCACCATGATTGCGCTGGCCGCGATGCACGACCTGCCGACTATTCTGGTGCCGGGCGG
GGCGACGCTGCCGCCGACCGTCGGGGAAGACGCGGGCAAGGTGCAGACCATCGGCGCGCGTTTCGCCAACCACGAACTCT
CCCTGCAGGAGGCCGCCGAACTGGGCTGTCGCGCCTGCGCCTCGCCGGGCGGCGGGTGTCAGTTCCTCGGCACGGCGGGC
ACCTCGCAGGTGGTCGCGGAGGCGCTGGGTCTGGCGCTGCCGCACTCCGCGCTGGCGCCGTCCGGGCAGGCGGTGTGGCT
GGAGATCGCCCGCCAGTCGGCGCGCGCGGTCAGCGAGCTGGATAGCCGCGGCATCACCACGCGGGATATCCTCTCCGATA
AAGCCATCGAAAACGCGATGGTGATCCACGCGGCGTTCGGCGGCTCCACCAATTTACTGCTGCACATTCCGGCCATCGCC
CACGCGGCGGGCTGCACGATCCCGGACGTTGAGCACTGGACGCGCATCAACCGTAAAGTGCCGCGTCTGGTGAGCGTGCT
GCCCAACGGCCCGGACTATCACCCGACCGTGCGCGCCTTCCTCGCGGGCGGCGTGCCGGAGGTGATGCTCCACCTGCGCG
ACCTCGGCCTGCTGCATCTGGACGCCATGACCGTGACCGGCCAGACGGTGGGCGAGAACCTTGAATGGTGGCAGGCGTCC
GAGCGCCGGGCGCGCTTCCGCCAGTGCCTGCGCGAGCAGGACGGCGTAGAGCCGGATGACGTGATCCTGCCGCCGGAGAA
GGCAAAAGCGAAAGGGCTGACCTCGACGGTCTGCTTCCCGACGGGCAACATCGCTCCGGAAGGTTCGGTGATCAAGGCCA
CGGCGATCGACCCGTCGGTGGTGGGCGAAGATGGCGTATACCACCACACCGGCCGGGTGCGGGTGTTTGTCTCGGAAGCG
CAGGCGATCAAGGCGATCAAGCGGGAAGAGATTGTGCAGGGCGATATCATGGTGGTGATCGGCGGCGGGCCGTCCGGCAC
CGGCATGGAAGAGACCTACCAGCTCACCTCCGCGCTAAAGCATATCTCGTGGGGCAAGACGGTGTCGCTCATCACCGATG
CGCGCTTCTCGGGCGTGTCGACGGGCGCCTGCTTCGGCCACGTGTCGCCGGAGGCGCTGGCGGGCGGCCGATTGGCAAG
CTGCGCGATAACGACATCATCGAGATTGCCGTGGATCGTCTGACGTTAACTGGCAGCGTGAACTTCATCGGCACCGCGGA
CAACCCGCTGACGCCGGAAGAGGGCGCGCGCGAGCTGGCGCGGCGGCAGACGCACCCGGACCTGCACGCCCACGACTTTT
TGCCGGACGACACCCGGCTGTGGGCGGCACTGCAGTCGGTGAGCGGCGGCACCTGGAAAGGCTGTATTTATGACACCGAT
AAAATTATCGAGGTAATTAACGCCGGTAAAAAAGCGCTCGGAATTTAA
>Cluster_41115
ATGAATTCACGACGTCCGGAATTATTAGAACTGACACCACAGGCACTGACGGCGTTAAGCAATGCCGGTTTTGTTAAGCG
CAGCCTTAAGGAACTGGAAAATGGCAACGTCCCGGAAATCAGTCATGAGAACGGCGCTTTAATCGCCACCTTCAGTGACG
GTGTCCGTACCCAGCTGGCGAACGGCCAGGCACTGAAAGAGGCTCAGTGCACCTGCGGGGCCAGCGGCATGTGCCGTCAT
CGCGTGATGCTGGTGTTAAGTTATCAACGACTTTGTGCCACCGCCCAGCCTACGGAAAAAAAAGAAGAAGAGTGGGATCC
GGCAATCTGGCTGAAAGAATTGGCTACCCTGCCCGATGCCACCCGCAAACGCGCGCAGGCGCTGGTCGCTAAAGGCATCA
CCATTGAGTTGTTCTGTGCGCCTGGCGAAATTCCCTCTGCCCGATTACCGATGAGCGATGTGCGTTTTTATTCCCGCAGC
AGCATTCGCTTCGCACGCTGTGATTGCATTGAAGGCACACTTTGCGAACATGTCGCACTGGCTGTGCAAGCCTTCGTCGA
GGCCAAAACTCAGCAAGCGGAATTTACTCATTTAATCTGGCAGATGCGCAGTGAACACGCCACATCATCTGATGATCCGT
TTGCCAGCGAAGAAGGCAAAACGTGTCGTCAATATGTTCAGCAATTAAGCCAGGCATTATGGCTGGGCGGGATAAGTCAA
CCGCCAATCCATTACGAGGCCGCTTTCAGTCGCGCGCAGCAAGCGGCGGAACGCTGCAACTGGCGATGGGTGAGTGAATC
GCTACGGCAATTGCGCGCCAGTGTTGATGCCTTCCACGCCCGTGCCAGCCACTACCATGCCGGAGAATGCTTACGTCAGC
TTGCGGCATTAAACAGTCGATTAAATTGCGCACAAAAGATGGCGCGGCGCGACAGTGTTGGTGAAGTTCCTCCTGTGCCC
TGGCGCACGGTCGTTGGCGCTGACATTGCCGGAGAAGCAAAACTTGATCATCTGCGGCTGGTGTCTTTAGGTATGCGTTG
CTGGCAGGATATTGAGCAGTATGGTTTACGCATCTGGTTTACCGATCCCGACACAGGAAGTATTTTGCATCTTTCGCGTA
GTTGGCAGAGAAGCGAACAGGAAAACTCACCGGCAGCGACGCGTCGGCTGTTTAGTTTTCAGGCTGGCGCACTGGCAGGT
GGGCAAATTGTTTCACAAGCTGCAAAACGCAGTGCCGATGGCGAGCTGCTGTTAGCGACCCGTAACCGCTTGAGTAGTGT
TGTTCCACTGTCGCCAGACGCCTGGCAAATGTTGAGCGCGCCGTTGCGCCAGCCGGGCATTGTGGCTTTGCGGGAATATT
TACGCCAGCGTCCCCCCGCCTGCATACGACCACTCAACCAGGTCGATAACTTATTTATTCTGCCGGTAGCGGAGTGTATT
TCGCTCGGTTGGGATAGCAGCCGCCAGACACTGGATGCGCAGGTAATCAGCGGCGAAGGGGAAGATAATGTGCTGACGTT
ATCCCTTCCGGCATCTGCCTGTTCTCCTTTTGCCGTTGAACGCATGGCGGCGCTTTTGCAACAAACAGACGACCCCGTGA
GCCTGGTTTCTGGCTTTGTCAGTTTTGTAGAGGGGCAATTGACACTGGAACCACGGGTGATGATGACAAAAACCCGCGCC
TGGGCGCTGGACGCAGAAACTGCGCCTGTGGCACCGCTACCTTCTGCCAGCGTTCTGCCTGTGCCGTCTACCACTCATCA
GTTGCTGATGCGCTGCCAGGCGTTACTTATTCAACTGCTCCATAACGGCTGGCGCTATCAGGAACAGAGTGTTATTGGTC
AGGCAGAGTTGCTGGCGAATGACCTCACCGCGGTGGGTTTTTATCGGCTGGCACATGTGTTGGGACAATTTCGTAATACA
GAAAGCGAGGCACGGGTAGAAGCAATGAATAACGGTGTTTTGCTTTGCGAACAATTATTCCCCATGCTTCAGCAATAA
>Cluster_7534
GTGTCCCGTATTATTATGCTGATCCCTACCGGAACCAGCGTCGGTCTGACCAGCGTCAGCCTTGGCGTGATCCGTGCAAT
GGAACGCAAAGGCGTTCGTCTGAGCGTTTTCAAACCTATCGCTCAGCCGCGTACCGGTGGCGATGCGCCCGATCAGACTA
CGACTATCGTGCGTGCGAACTCTTCCACCACGACGGCCGCTGAACCGCTGAAAATGAGCTACGTTGAAGGTCTGCTTTCC
AGCAATCAGAAAGATGTGCTGATGGAAGAGATCGTCGCAAACTACCACGCTAACACCAAAGACGCTGAAGTCGTTCTGGT
TGAAGGTCTGGTCCCGACACGTAAGCACCAGTTTGCCCAGTCTCTGAACTACGAAATCGCTAAAACGCTGAATGCGGAAA
TCGTCTTCGTTATGTCTCAGGGCACTGACACCCCGGAACAGCTGAAAGAGCGTATCGAACTGACCCGCAACAGCTTCGGC
GGTGCCAAAAACACCAACATCACCGGCGTTATCGTTAACAAACTGAACGCACCGGTTGATGAACAGGGTCGTACTCGCCC
GGATCTGTCCGAGATTTTCGACGACTCTTCCAAAGCTAAAGTAAACAATGTTGATCCGGCGAAGCTGCAAGAATCCAGCC
CGCTGCCGGTTCTCGGCGCTGTGCCGTGGAGCTTTGACCTGATCGCGACTCGTGCGATCGATATGGCTCGCCACCTGAAT
GCGACCATCATCAACGAAGGCGACATCAATACTCGCCGCGTTAAATCCGTCACTTTCTGCGCACGCAGCATTCCGCACAT
GCTGGAGCACTTCCGTGCCGGTTCTCTGCTGGTGACTTCCGCAGACCGTCCTGACGTGCTGGTGGCCGCTTGCCTGGCAG
CCATGAACGGCGTAGAAATCGGTGCCCTGCTGCTGACTGGCGGTTACGAAATGGACGCGCGCATTTCTAAACTGTGCGAA
CGTGCTTTCGCTACCGGCCTGCCGGTATTTATGGTGAACACCAACACCTGGCAGACCTCTCTGAGCCTGCAGAGCTTCAA
CCTGGAAGTTCCGGTTGACGATCACGAACGTATCGAGAAAGTTCAGGAATACGTTGCTAACTACATCAACGCTGACTGGA
TCGAATCTCTGACTGCCACTTCTGAGCGCAGCCGTCGTCTGTCTCCGCCTGCGTTCCGTTATCAGCTGACTGAACTTGCG
CGCAAAGCGGGCAAACGTATCGTACTGCCGGAAGGTGACGAACCGCGTACCGTTAAAGCAGCCGCTATCTGTGCTGAACG
TGGTATCGCAACTTGCGTACTGCTGGGTAATCCGGCAGAGATCAACCGTGTTGCAGCGTCTCAGGGTGTAGAACTGGGTG
CAGGGATTGAAATCGTTGATCCAGAAGTGGTTCGCGAAAGCTATGTTGGTCGTCTGGTCGAACTGCGTAAGAACAAAGGC
ATGACCGAAACCGTTGCCCGCGAACAGCTGGAAGACAACGTGGTGCTCGGTACGCTGATGCTGGAACAGGATGAAGTTGA
TGGTCTGGTTTCCGGTGCTGTTCACACTACCGCAAACACCATCCGTCCGCCGCTGCAGCTGATCAAAACTGCACCGGGCA
GCTCCCTGGTATCTTCCGTGTTCTTCATGCTGCTGCCGGAACAGGTTTACGTTTACGGTGACTGTGCGATCAACCCGGAT

CCGACCGCTGAACAGCTGGCAGAAATCGCGATTCAGTCCGCTGATTCCGCTGCGGCCTTCGGTATCGAACCGCGCGTTGC
TATGCTCTCCTACTCCACCGGTACTTCTGGTGCAGGTAGCGACGTAGAAAAAGTTCGCGAAGCAACTCGTCTGGCGCAGG
AAAAACGTCCTGACCTGATGATCGACGGTCCGCTGCAGTACGACGCTGCGGTAATGGCTGACGTTGCGAAATCCAAAGCG
CCGAACTCTCCGGTTGCAGGTCGCGCTACCGTGTTCATCTTCCCGGATCTGAACACCGGTAACACCACCTACAAAGCGGT
ACAGCGTTCTGCCGACCTGATCTCCATCGGGCCGATGCTGCAGGGTATGCGCAAGCCGGTTAACGACCTGTCCCGTGGCG
CACTGGTTGACGATATCGTCTACACCATCGCGCTGACTGCGATTCAGTCTGCACAGCAGCAGTAA
>Cluster_38818
GTGACTATTAAATCCACTAATCATCTTACCCACATCGCCACGTTTTGCGCTCTGCTGTATAGCAATAGTGCTTTATGCGC
TGAACTCGTCGAATATGACCATACATTTCTGATGGGAAACGATGCTTCAAATATCGACCTTAGTCGGTATACAGAAGGCA
ACCCGACACGACCAGGCATTTACGATGTCAGTGTGTATATTAACGATCAACCCATCATGAGCCAAAGCATTGCTTTTACT
GTTATTGAAGGTAAAAAGAACGCTCAGGCCTGCATTACGCAGAAAAATTTATTGCAATTTCATATCAGTTCACCAGACAA
GAATAGCGGAAAAGCCATTTTGCTTAAGCGAGACGACGATCTTGGTGATTGCCTCAATCTGGCTGAAATGATCTCTCAAT
CATCCATTCGTTACGATGTTAACGATCAACGCCTGGATATCGACGTCCCCCAGGCATGGATTATGAAGAATTATCAAAAC
TATGTTGATCCCTCTTTGTGGGAAAATGGCATTAATGCAGCCATGCTTTCCTACAACCTCAACGGATATCACAGTGAAAG
TCCAGGCAGAACAAATGACAGTATCTACGCGGCATTCAACGGTGGGATAAACTTAGGTGCCTGGCGGTTACGTGCCTCGG
GTAACTACAACTGGATGACCAATGTCCATAGCGATTACGATTTTCAGAATCGCTATTTACAACGCGATCTTGCGTCACTA
CGTTCACAACTGGTTATTGGTGAATCTTACACGACCGGCGAGACCTTCGATTCAGTCAGCATTCGCGGTATTCGCTTATA
CAGTGACAGTCGAATGCTGCCTCCGGTATTAGCCAGTTTTGCGCCAATCATTCATGGCGTAGCGAATACCAATGCCAAAG
TAACCGTTATGCAAAATGGTTATAAAATCTATGAAACGACGGTGCCGCCAGGGGCCTTTGCCATTGACGATCTTAGCCCG
TCAGGTTATGGCAGCGATCTCATTGTGACTATCGAAGAAGCTGATGGCACAAAACGGACGTTCTCCCAGCCATTTTCATC
TGTTGTGCAAATGCTGCGCCCTGGCGTTGGTCGCTGGGATATTAGCGCCGGTCAGGTTTTAAAAGACAGTATCCAGGATG
AGCCTAATTTATTTCAAGCCAGCTACTACTATGGTTTGAATAACTACCTGACAGGATATACCGGTATTCAGCTAACTGAT
AATAACTATACCGCCGGATTGCTGGGGCTTGGTATGAATACCCCCGTGGGGGCTTTTTCTGTCGATGTTACTCACTCAAA
TGTCAGCATTCCGGACGATAAAACCTATCAAGGACAAAGCTACCGTATTTCCTGGAACAAGTTATTTGAAAATACCAGTA
CATCATTAAATATCGCGGCCTATCGCTACTCCACCCAGCATTATCTGGGCCTCAATGATGCGCTGACGCTGATTGATGAA
GTAGAACATCCTGAGCAAGATCTTGAGCCTAAATCCATGCGCAATTACTCGCGCATGAAAAATCAGGTCACGGTCAGTAT
CAACCAACCGCTGAAATTTGAGAAAAAGGAATCGCCGTGGGGAACGCTGTCAGGTTCAGCCTCAGCAAGCAGTGATAATA
GTCGCCAATTCTCACTCAATACTGATGGTGGATTTGTGTTACATAGCGGCGGGCTGACGTTCAGTAATGACAGTTTTAGT
GATTCCGACACTCTGGCTGTCATTCAGGCGCCAGGAGCAAAAGGCGCTCGTATTAACTACGGAAACAGCACCGTAGACCG
CTGGAGTTATGGTGTGACCAGCGCCCTTTCGCCATACCATGAAAATCGTATCGCACTGGACATTAACGATCTGGAAAACG
ATGTTGAATTGAAAAGCACCAGTACTGTCGCCATCCCGCGCCAGGGAGCCGTCGTCTTTGCTGATTTTGAAACAGTTCAG
GGACAATCGGCAATTATGAATATCGTGCGAAGCGATGGTAAAAATATTCCATTTGCGGAGGATATTTATGATGAACAAAA
CAATATCATCGGCAACGTAGGTCAGGGTGGTCAAGCCTTTGTTCACGGCATCGAACAGGAAGAAAATATACGAATTACCT
GGATTGAAGAGGGCAAACCAGTCAGTTGTTTTGCTCATTACCAACAAAACACGGCATCAGAAAAAATAGCACAATCTATT
ATTCTTAACGGGCTTCGTTGTCAGATACAGTAA
>Cluster_58859
ATGAATTCTCTGTTTGCCAGTACGGCCCGTGGGCTGGAAGAGCTGTTAAAAACTGAACTGGAAAACCTGGGGGCCGTTGA
ATGCCAGGTGGTTCAGGGTGGGGTCCATTTCAAGGGCGACACACGGCTTGTTTACCAGAGCCTGATGTGGAGCCGCCTGG
CCTCGCGTATTATGTTGCCGCTGGGCGAGTGTAAGGTTTACAGCGATTTAGACCTCTATCTCGGTGTTCAGGCGATCAAC
TGGACAGAGATGTTTAATCCTGGCGCGACCTTCGCTGTCCACTTCAGTGGTTTGAATGACACCATCCGCAACAGTCAGTA
CGGCGCGATGAAAGTGAAAGACGCGATCGTCGATGCTTTCACGCGGAAAAATCTGCCGCGTCCAAATGTTGATCGCGATG
CGCCGGATATCCGCGTTAACGTCTGGCTGCATAAAGAAACCGCCAGTATCGCTCTTGATCTGAGTGGTGATGGTTTACAT
CTGCGTGGCTATCGCGATCGTGCTGGTATTGCGCCGATCAAAGAAACCCTGGCAGCCGCTATTGTGATGCGATCCGGCTG
GCAGCCAGGAACACCGCTGCTCGATCCGATGTGTGGTTCCGGTACGTTGCTGATTGAAGCAGCGATGCTGGCGACCGATC
GCGCACCAGGCTTGCACCGTGGGCGTTGGGGCTTTAGCGGCTGGGCGCAGCATGATGAAGCTATCTGGCAGGAAGTGAAA
GCGGAAGCGCAAACTCGCGCCCGTAAAGGCCTGGCTGAGTATAGCTCTCATTTTTACGGTTCGGACAGCGACGCACGGGT
GATTCAACGTGCACGCACTAACGCCCGTCTTGCGGGGATTGGTGAACTGATCACCTTTGAGGTGAAAGATGTCGCGCAAC
TGACCAATCCGCTGCCGAAAGGGCCGTACGGCACAGTGTTGAGCAACCCGCCATACGGGGAACGTCTGGACAGCGAACCG
GCGCTGATTGCGCTGCATAGCCTGCTGGGTCGGATCATGAAAAACCAGTTTGGTGGCTGGAATCTCTCTTTGTTTAGTGC
CTCGCCGGATCTGCTAAGCTGCTTGCAGCTGCGTGCAGACAAACAGTACAAGGCGAAAAACGGCCCGCTGGACTGCGTAC
AGAAAAATTACCACGTTGCCGAAAGCACTCCAGACAGCAAACCGGCGATGGTAGCGGAAGACTACACCAACCGCCTGCGT
AAGAACCTCAAAAAATTCGAGAAGTGGGCTCGCCAGGAAGGGATTGAATGTTACCGCCTGTATGACGCCGATCTGCCAGA
ATATAACGTTGCCGTTGACCGTTATGCCGACTGGGTGGTGGTGCAGGAGTATGCGCCGCCAAAAACTATTGATGCTCACA
AAGCGCGTCAGCGTCTGTTCGATATTATCGCTGCAACCATTTCGGTACTGGGGATTGCGCCAAACAAACTGGTGCTGAAA
ACCCGTGAACGCCAGAAGGGCAAAAACCAATACCAGAAACTGGGCGAGAAGGGCGAGTTTCTTGAAGTTACCGAATATAA
CGCTCACTTGTGGGTGAACCTGACGGATTATCTCGATACTGGTCTGTTCCTCGATCACCGCATCGCCCGTCGTATGCTCG
GTCAGATGAGCAAAGGCAAAGATTTCCTCAACCTGTTCTCTTATACCGGCAGCGCCACCGTGCATGCGGGATTAGGCGGT
GCACGCAGCACCACCACCGTGGATATGTCGCGTACTTATCTGGAATGGGCAGAACGCAACCTGCGTCTGAATGGCCTGAC
CGGGCGTGCGCATCGCCTGATTCAGGCCGATTGCCTGGCGTGGCTGCGTGAGGCAAATGAACAGTTCGATCTGATCTTTA
TCGATCCGCCAACCTTCTCTAACTCAAAACGAATGGAAGATGCGTTTGATGTTCAGCGCGATCATCTGGCGCTGATGAAA
GATTTGAAACGTCTGCTGCGTGCAGGTGGACGATCATGTTCTCGAACAACAAACGTGGCTTCCGTATGGATCTCGACGG
CCTGGCAAAACTGGGACTGAAAGCACAAGAAATTACGCAAAAAACGCTCTCCCAGGATTTCGCCCGTAACCGCCAGATCC
ACAACTGCTGGCTGATTACCGCAGCCTGA
>Cluster_25057
ATGGCAGCAAATGTAATCTTACCGGAAGGTTTTGTGCTCGATGAACAACCTGATAACTCACAGTTTCCTGATAGTTCACA
GCTTCCTGATGGTTTTGTGCTTGATGCCCAACCAGAACAGCAGCAATCACCTTTGGTTTCACCAGAGGAAAATTCCAGAC
AGGAAAATGTTGTTAATAATGCTAACGGTTTCGACCGTTTTATGTATGGCGTTCTCAGTGGATTGATGGATGTTGGTAAA
GGTGTTGGCCTGTTTCAGGATATGACACCAGAAGAGCAAGCCGCAATTCAGTCTCTACAGCAGAAGTTAGCGGCAAAACC
ATCAACCGCACAAGATGTTGGTGAGTTCGTTGGACAAGCAGCGCCATTTGTTAGTGGTGGTGGGATTATTTCTCAGGTTC
CCAAAGGGGCGGCAAGACTGGCTGCCGCCGCAGGGCTTGGTGCTGGAGAAGGGGCTATTGTAGCCAATGGAACAAATAGC
GATGTTGCTTCCGGCGCTGCTATTGGCGCTGTGGCTGGCCCTGTAGCCGAACTTGCTGGCCCTGCAATTAATAAAATAGC
TGGGAAATTAACCAAGAAGAATGTTTCTTCGATTCCAGATATTGAGGGGATTACTCCTGCCGAAGAGAAAATTCGTAAGT
TATCTGCACAAGGCAACCCAAATCTTGCCTCTTCGCTTGAAGGGCTAGATCTAAAACCTCAGAAAGAAATTGTTGAGGCT
GCAAATAGGCTTCATGTTGATTCAGTTCTTCCTTCTCATTTATCAGGAAATGAGCAATACCAAGCTGTAGAGCAAGCCCT
AAAGTCACGGACAGGATCAGCGTTAAAAGCTCAAGAAGATAAAGCAATAAGCGAATTAGCCTCTAGTGCTGGCAAGATAA
TCGATGATGTTGCTGGTGCGCCAGATGCGTTAGCTATGAGCGAAAAATATATTTATCAGATAAATAGTAGAATGGAAGCA
CTAAAAAACCGTAGTGATCAGTTATATAAACGTGTTGATTCCGCTCTATCACCAAGAACAAAAGTCGAAGCAAATAACAC
AAGCACTATGCTGGAAAAAATCGCTGATGATTTAGGAGGGTGGGATAATCTTGACCCAATAGAAAAGCGAGTTTTTACAG
CAATAAACCCCGGCCCAGATAGAATCCTAACGTATGCAAATTTAAACAGGCAAAGGCGTTTAGTTGGTGATGCCCTAAAT

AAAAAGCAGGGGCCATACAAAGATGCTGATAAAGAAGCGCTATCAAGACTATATGGAGCTTTAGCTGAAGACCAAAAATC
TGTCTTATCCAATACTGGATACTTGCGAGATTTCGAAGTCGCACAACGCCTTGTAGCTATGAGAAAAAACTTAGAAAGGC
AAATGGTAAATCTTCGCGGGAAAACACTTAATGGGGATATAGCGTATAAAGTAACGAATTCTCTTCAATCTATGGCAAAA
GGTGATGCGAGAGGGTTTCGTGAGATTATGCAAAATACTCCATCAAGAACATTGCGAAGGGAGCTAGTAGGAACAGCATT
AAGAGATATGCTTTCCTCTGGGAAGCGTGGGGCCGACTTTAATCCATCTGGCTTTGCAGATTGGTGGCAAAACTTGCAAC
ACAGTGGACAGCTAAAGACTTTGGCTGAACATTTGCCAAAAGAAACAATGAGCGGACTATATGATGTATACAAAGTTGCC
AGAGCAATAAAAAATGCTAAAGCGCATGAAATATCTACTGGTAAGCTGAATGAGTTTGTAAACAGATTTAATCGGGTAAC
AGCTCCATACGAACTAGCAGCAAAACACATTCAAAAAATAGGCACGATGGTAGGCGCTCATTTTGGATCGCTTGGAGCCA
TTGCTGGGGCTGATATTGGAGCAAGATTGGCGAGTAAAGCTCGATTAGCTGGTGGAGCAGGGGCGGCTGATGCTGCGGAT
AAATTAATTGCCTCGCCAGCCTTCCAGAGCGCAGTAAAAAATCTACACGGAAAAGCGCCTGGGCACATTGTTGATTCACG
TATCAGGCGCTCCCCTCAATGGAAGAGTTTTTTAACTCTCTTCCTGAACAGGAAAAACGGACCATCGCCAGACTGGGCA
TCATTACTTGGCTATCTAATGATCCAGATAATGACTAG
>Cluster_24628
GTGGAAAAAGCCAAACACGTTACCTGGCGGTTGTTGGCTGCCGGTGTCTGTCTGCTGACGGTCAGCAGCGTGGCGCGAGC
CGACTCACTGGATGAGCAGCGTAGCCGTTACGCGCAAATTAAGCAGGCCTGGGATAATCGACAAATGGATGTGGTCGAAC
AAATGATGCCTGGACTGAAAGATTACCCACTTTATCCCTACCTGGAATACCGCCAGATCACTGATGACCTGATGAATCAA
CCGGCGGTGACGGTCACTAACTTTGTTCGCGCTAACCCCACGCTTCCTCCCGCTCGCACGCTGCAATCTCGTTTCGTCAA
TGAACTGGCGCGGCGTGAAGACTGGCGTGGCTTGTTAGCCTTTAGCCCGGAAAAACCCGGAACTACCGAAGCGCAATGTA
ATTACTACTATGCGAAATGGAACACCGGGCAGAGTGAAGAAGCCTGGCAAGGGGCGAAAGAGCTGTGGCTAACCGGCAAG
AGCCAGCCTAACGCCTGTGACAAGTTATTTAGCGTCTGGCGTGCGTCAGGTAAACAAGATCCGCTGGCGTATTTAGAGCG
TATCCGTCTGGCGATGAAAGCGGGTAACACCGGCCTGGTAACAGTGCTGGCAGGGCAGATGCCTGCCGATTACCAGACTA
TCGCTTCGGCAATCATTTCACTGGCGAACAATCCTAATACGGTACTGACCTTCGCGCGTACAACCGGCGCGACCGATTTT
ACCCGTCAAATGGCGGCGGTGGCGTTTGCCATTGTGGCGCGGCAGGATGCTGAAAATGCACGGCTGATGATCCCGTCACT
TGCTCAGGCACAGCAGCTTAATGAAGATCAGATTCAGGAGCTGCGCGATATCGTCGCCTGGCGTTTGATGGGCAACGATG
TCACCGACGAGCAGGCGAAATGGCGCGATGACGCGATTATGCGCTCGCAATCTACTTCGCTTATTGAACGCCGTGTGCGA
ATGGCGCTTGGCACCGGCGATCGTCGCGGCCTGAATACCTGGCTGGCGCGTCTGCCTATGGAAGCGAAAGAGAAAGATGA
ATGGCGTTACTGGCAGGCGGATTTACTGCTGGAACGCGGACGTGAAGCTGAAGCAAAAGAGATTTTGCATCAACTCATGC
AACAGCGTGGTTTCTACCCGATGGTTGCTGCACAACGCATCGGCGAAGAGTATGAGCTGAAGATTGATAAAGCGCCGCAG
AATGTTGACAGCGCCCTGACTCAGGGGCCGGAGATGGCGCGCGTGCGCGAGTTGATGTACTGGAATCTCGATAACACCGC
GCGTAGCGAGTGGGCCAATCTGGTGAAGAGCAAGTCAAAAACAGAGCAGGCTCAACTGGCTCGGTATGCTTTCAATAACC
AATGGTGGGATCTTAGCGTTCAGGCAACGATCGCCGGGAAGCTGTGGGATCATCTGGAAGAGCGATTCCCGCTGGCCTAC
AAAGATCTTTTCAAACGCTACACCAGTGGTAAGGAGATCCCGCAAAGCTATGCGATGGCGATTGCCCGTCAGGAGAGTGC
CTGGAATCCGAAAGTGAAATCACCGGTAGGGGCCAGCGGCCTGATGCAGATTATGCCTGGTACCGCGACCCATACGGTGA
AGATGTTCTCTATTCCCGGTTATAGCAGCCCTGGGCAATTGCTGGATCCGGAAACGAACATCAACATTGGCACCAGTTAC
CTGCAATATGTTTATCAGCAGTTTGGCAATAATCGTATTTTCTCCTCAGCAGCTTATAACGCCGGACCAGGGCGGGTGCG
AACTTGGCTTGGCAACAGCGCAGGGCGTATCGACGCAGTGGCATTTGTCGAGAGTATTCCATTCTCCGAGACGCGCGGTT
ATGTGAAAAACGTGCTGGCTTATGACGCTTACTACCGCTATTTCATGGGGGATAAACCGACGTTGATGAGCGCCACGGAA
TGGGGACGTCGTTACTGA
>Cluster_38386
ATGTCGATTAAACGTTATGATGTGGTCAGGGCAGCCTCGCCGTCAGATCTGGCGGAAAAACTGACACACAAACTGAAAGA
GGGCTGGCAGCCATACGGCGGACCGGTTGCCATTACGCCGTACACACTTATGCAGGCGGTGGCCATTGAAGGCGAGCCAC
AGGTAGGTCCTTTATCTGAGCCGGACTGGTATTACGTGGTTGTGCTTGCCGGACAGTCCAACGGCATGGCTTACGGTGAA
GGGCTTCCGTTACCGGATTCTTACGATGCTCCGGATCCGCGCATTAAACAACTGGCACGCCGCAGCACGGTAACCCCGGG
TGGCGAGAGTTGTGCGTATAACGACATCATCCCGGCTGACCATTGCCTGCATGATGTGCAGGATATGAGTACGCTGAATC
ATCCGAAGGCAGACCTGAGCAAAGGGCAGTACGGCTGTGTCGGACAGGGCTTACATATTGCCAAAAAACTGCTTCCGTAT
ATCCCGAATAACGCGGGGATCCTGCTGGTACCATGCTGTCGTGGTGGTTCGGCATTCACCCAGGGCGCGGAGGGGACATT
CAGTGCGGACGCGGGGGCCAGCCAGGATTCGGCGCGCTGGGGTGTGGGTAAACCGTTATATCAGGACCTGATTGCGCGCA
CTAAAGCTGCATTACAGAAGAACCCGAAAAATGTGTTGCTGGCGGTGTGCTGGATGCAGGGAGAGTTTGACATGAGCGCC
GCCACCCACGCACAGCAACCTGCGCTGTTTACAGCCATGCTGAAGCAGTTTCGTGCTGACCTCACTGTGTTTAACGCGCA
GTGTCATGGTGGCAGTGCTGTAAATGTGCCGTGGATTTGTGGTGACACGACGTATTACTGGAAAAATACATACGCTACCC
AGTACAACACCATTTACGGGGCGTACAAAAACAGGGAGAGTGAGGGCGTTTATTTTGTGCCCTTCATGACAGACGGTAAC
GGCGTCAATACCGCCACTAACGCGCCGGCAGAAGATCCGGATATTCCGGCATCAGGATATTACGGTGCGGCATCGAGAAC
GAATGGAAACCAGGTATCATCAAACCGCCCGACACATTTCAGTTCATGGGCGCGCAGGAGCATTATTCCGGATCGTCTGG
CAACCGCTATTCTGAACGCAGCCGGGCGCACCTCCGCCTTCATCAGTGGTAAGGCACCGGAAATCAAACCCTCGCCCGGC
GGCAACACGCCATCGGGTCCGTCTGCAGATACGTCCGTTCGCACAATCTCCCTGCTGCCGGCAGCCGGAGAGGCTGCTGC
GCAGGGCTGGAGCATTAAGGATGGCGGAATTCAGTTGTCAGATGGTGTATTTAAGATCACCAAGCAGAGCAATAAAACCT
GGTCCCTGACGCATCCGGTGGATGACGCAGTCTCCCTGCTGACACGGGGTGGCAGACTGAGCTGTAAGTTTCGACTGTCA
GGCGCACTGACCAACAATCAGTTCGGTCTGGGAATTTATCTGTATACCGATGTAGCGTTACCTGACGTCGTGGCGATGAC
GGGTACCGGTAACCCGTTCCTGATGTCGTTCTTCACCCAGACCACAGACGGCAAACTGAATCTGATGCATCACAGGAAAG
CCGGAAACACGAAGCTGGGGGAGTTCGGCGATTACGGTAACGACTGGCAGACGCTGGAGCTGGTGTTCACCGCCGGCAGT
GCCACGGTTACTCCGAAACTGAATGGAGTGGCTGGCCCGGCATTCCAGGTTATAAAAGACAGTCTGACACTGGGACTGAA
TGCGCTGACGCTGACGGATGTTACAAAAAATGCAGCGTATGGCGTTGAGATAGAAAGTCTGGTGCTGGAGATAAATGCAC
CGGCAGCATAA
>Cluster_39086
ATGAGTTTTGATATTGCCAAATACCCGACCCTGGCACTGGTCGACTCCACCCAGGAGTTACGACTGTTGCCGAAAGAGAG
TTTACCGAAACTCTGCGACGAACTGCGCCGCTATTTACTCGACAGCGTGAGCCGTTCCAGCGGGCACTTCGCCTCCGGGC
TGGGCACGGTCGAACTGACCGTGGCGCTGCACTATGTCTACAATACCCCGTTTGACCAGTTAATCTGGGATGTGGGGCAT
CAGGCTTATCCGCATAAAATTTTGACCGGGCGCCGCGACAAAATCGGCACCATCCGTCAGAAAGGTGGCCTGCACCCGTT
CCCGTGGCGCGGCGAAAGCGAATATGACGTATTAAGCGTCGGGCATTCATCAACCTCCATCAGTGCCGGAATTGGGATCG
CAGTTGCTGCCGAGAAAGAAGGCAAAAATCGCCGCACCGTCTGTGTCATTGGCGATGGCGCGATTACTGCTGGCATGGCG
TTTGAAGCGATGAATCACGCGGGCGATATCCGTCCTGATATGCTGGTGGTTCTCAACGACAATGAAATGTCGATTTCCGA
AAATGTTGGCGCGCTCAACAACCATCTGGCGCAGCTGCTTTCCGGTAAGCTTTACTCTTCACTGCGCGAAGGCGGAAAAA
AAGTTTTCTCTGGCGTGCCACCCATTAAAGAGCTGCTCAAACGTACCGAAGAACATATTAAAGGCATGGTAGTGCCTGGC
ACGTTGTTTGAAGAGCTGGGCTTTAACTACATCGGCCCGGTGGACGGTCACGATGTGCTGGGGCTTATCACCACGCTTAA
GAACATGCGCGACCTGAAAGGCCCGCAGTTCCTGCATATCATGACCAAAAAAGGTCGTGGTTATGAACCGGCAGAAAAAG
ACCCAATCACCTTCCACGCCGTGCCTAAATTTGATCCCTCCAGCGGTTGTCTGCCGAAAAGTAGCGGCGGTTTACCAAGC
TATTCAAAAATCTTTGGCGACTGGTTGTGCGAAACCGCAGCGAAAGATAACAAGCTGATGGCGATTACTCCGGCGATGCG
TGAAGGTTCCGGCATGGTCGAGTTTTCACGTAAATTCCCGGATCGCTACTTCGACGTGGCAATTGCCGAGCAACACGCAG
TGACCTTTGCTGCGGGTCTGGCGATTGGAGGCTACAAACCCATTGTTGCGATCTACTCCACCTTCCTGCAACGCGCCTAT

GATCAGGTGCTGCATGACGTGGCGATTCAAAAGCTTCCGGTCCTGTTCGCCATCGACCGTGCGGGCATTGTTGGTGCTGA
CGGTCAAACCCACCAGGGCGCTTTTGATCTCTCTTACCTGCGCTGCATACCGGAAATGGTCATTATGACCCCGAGCGATG
AAAACGAATGTCGCCAGATGCTCTATACCGGCTATCACTATAACGATGGCCCGTCCGCGGTGCGCTACCCGCGCGGCAAC
GCAGTTGGCGTGGAACTGACGCCACTGGAAAAACTGCCAATTGGCAAAGGCATTGTGAAGCGTCGTGGTGAGAAACTGGC
GATCCTAAACTTTGGTACGCTGATGCCAGAAGCGGCGAAAGTCGCTGAATCGCTGAACGCTACGCTGGTCGATATGCGTT
TCGTGAAACCGCTTGATGAAGCGTTAATTCTGGAAATGGCCGCCAGCCATGAAGCGCTGGTTACCGTAGAAGAAAACGCC
ATTATGGGTGGTGCAGGCAGCGGCGTGAACGAAGTGCTGATGGCCCATCGTAAACCAGTACCCGTGCTGAACATTGGTCT
GCCGGACTTCTTTATTCCACAAGGAACTCAGGAAGAAATGCGCGCCGAACTCGGCCTCGATGCCACCGGTATGGAAGCCA
AAATCAAAGCCTGGCTGGCATAA
>Cluster_19623
ATGCCAACAAACCTACGCCGCCTGCTTGCGCAGGCTGCCGACGCTGACCCGCTCAATACTGCGGACGCAGATGTACAGCA
AATGATCGCTGACGGGATTGTTATTCGACGTTCCAAGATATGCCCTGTAAAGCGTAAACCCGTTAAGGCGGCATACAACA
CAAAGACCGAAGAGTTTTATTACAATGCAGATGGCTCCGCGATGACCTTGCGGGAGTGGCGAACGCTGCACGGTGTTCAA
CCGAAGAAACAAAAACAGCGCTCTGACACTGGGTTGCGGCAAGTCATCCAGCCCGGCGACGCGATTCACTGCCGACGCGC
TGAGATTGAGCAATGGCTTAATAAGAAGCCCGCTCGTTATGTTGACCCGCTGCCGCCAGATTTTCCCGGTGTTCGCGTAG
ACGATGCCGCATTGAAACCGCGCGAGCACGCCGTGCCGGAAGCGACAACCGGACAGAATGCTTTTCTCAAAGCCTGCCGC
GAGTTCCTGATTTGCGATGAGAAGACCATGCGCCGTATCAGTCGGGGCATGAACCAAATGGAAGCTATGCGCAACGCAGA
TTTCAAACGCTACGGAGACTCAGGGGAGTTTCGTCAATTGTGGTGTGGAGAGTCGAAGCCTACCGAAGATAGATTTAATA
GTTCTAATCATCATTGTGACCCGAACGGCTTGGCTGCTTCGCCGTCTGTGCCGCCACTGAATACTGCACCGTATGAGTTC
CGCCCGCTGGCTCGCTGTGACCGAAACGCACGCGTTAGCCTGCTGCCACCTGATGGGCTGGAGTGGAGGCCGGGGCAGTT
GTCTGTTGCTGATGCGATCTTTTCCCGTGGCAGTGATGTGCTTGCCGTACTGCCGACCGGGTATGGCAAAAGCGCCGCCT
TCACTTATCCATTGATTTATGAGTCCCGCGCTGGCATTGGCCTGTCTGTAATCGTGGTTCCTACTAATACCCTTAAGCGT
CAGATTCTCCGCCAGTTGCGGGAGGCTGGCATTCACGCTGTGGCCTTTGGCGATAAGCTACCGAGGCACCGCAACGCACA
ACAACTGGCGGGACTAGCAGCCGGGGCGTTCCGGGTTGCTGTTGTTTCGCCTGAACATCTGGTTCCGTCGAGTCAGGTTC
TCGCGGCGCTGATGGCTTATGGCGTTGATCGTTTTATTATCGATGAGGCACACACGGTCAAAGACTGGGATTTCCGCCCA
GCTTACGACGCAATGCAGCACACACTACAGAATGCTTTTCCCTCGGCTCAGCGCTGCGTGTTTACGGCTACTCTACTACG
AGAAAATGAGCGCGGTTTAATGGAGGCGCTGGGATGCCGTGGCGATTACGTTACATTCCGTGCTGATGTTGCTCGCGAGA
ATATCCAGTTGATTCCGGTCAGGCAGTGCCGCCAGCGATACCGCGCGGCTGTTGGTCTGGTCGAGGCTCTGCCAGAGGAA
GAGAAGACGCTCATTCTAGTCAATGACCTTAAGACGTTGCGGGCGTACCTGAAACTCTTCGGCGACCGGGCGCGCCCATA
TCACTCGAAGACGATGCACCTGAGCAACGAGGAACGCCACGCAAACGCTGAGTGGTTTGCCTCTGCGCCGGGCGCGATTC
TGATTGCTACTAAAGCCTTCGGGCTGGGTGCTGATGTTCCTGATATCCGTAACGTTGTCGTGACATACGTTCCCGATGAC
TTGCCTGATCTCGTTCAGATGATTGGACGAGCCGGGCGCGATGGGGAACCTGCCCGCGCTTTTCTGCTGTGGTCATCGCT
GGCGCTGGATGTTGCTCCTGATGAGCTTGCTTACTTCTACCGCCCTGAAAACTGTAACTGGCAGTACATCGCTAAATACC
TCTGA
>Cluster_23994
ATGCCTAAGTACCGTTCCGCCACCACCACTCATGGTCGTAATATGGCGGGTGCTCGTGCGCTGTGGCGCGCCACCGGAAT
GACCGACGCCGATTTCGGTAAGCCGATTATCGCGGTTGTGAACTCGTTCACCCAATTTGTACCGGGTCACGTCCATCTGC
GCGATCTCGGTAAACTGGTCGCCGAACAAATTGAAGCGGCTGGCGGCGTTGCCAAAGAGTTCAACACCATTGCGGTGGAT
GATGGGATTGCCATGGGCCACGGGGGGATGCTTTATTCACTGCCATCTCGCGAACTGATCGCTGATTCCGTTGAGTATAT
GGTCAACGCTCACTGCGCCGACGCCATGGTCTGCATCTCTAACTGCGACAAAATCACCCCGGGGATGCTGATGGCTTCCC
TGCGCCTGAATATTCCGGTGATCTTTGTTTCCGGCGGCCCGATGGAGGCCGGGAAAACCAAACTGTCCGATCAGATCATC
AAGCTCGATCTGGTTGATGCGATGATCCAGGGCGCAGACCCGAAAGTTTCTGACTCCCAGAGCGATCAGGTTGAACGTTC
CGCCTGTCCAACCTGCGGATCTTGCTCCGGGATGTTTACCGCTAACTCTATGAATTGCCTGACCGAAGCACTGGGCCTGT
CGCAGCCGGGCAACGGCTCGCTGCTGGCGACCCACGCCGATCGCAAGCAACTGTTCCTCAATGCCGGTAAACGCATTGTT
GAATTGACCAAACGTTACTACGAGCAAAACGACGAAAGTGCACTGCCGCGCAATATTGCTAGCAAAGCGGCGTTTGAAAA
CGCCATGACGCTGGATATCGCGATGGGCGGATCGACTAACACCGTACTTCACCTGCTGGCGGCGGCACAGGAAGCGGAAA
TCGACTTCACCATGAGTGATATCGATAAGCTCTCCCGCAAAGTACCGCAGCTGTGTAAAGTCGCGCCGAGCACCCAGAAA
TACCATATGGAAGATGTTCACCGTGCTGGTGGTGTTATCGGTATTCTCGGCGAACTGGATCGCGCGGGGTTACTGAACCG
TGATGTGAAAAACGTACTTGGCCTGACGTTGCCGCAAACACTGGAACAATACGACATCGTCGTGACCCAGGATGATGCGG
TAAAAAATATGTTCCGCGCTGGCCCTGCGGGCATTCGTACTACACAGGCATTCTCGCAGGATTGCCGTTGGGATTCTCTC
GATGACGATCGCGCCAATGGCTGTATCCGCTCGCTGGAACACGCCTACAGCAAAGACGGTGGTCTGGCGGTGCTGTACGG
TAATTTCGCAGAAAACGGCTGCATCGTTAAAACCGCGGGCGTCGATGACAGCATCCTCAAATTCACCGGCCCGGCGAAAG
TGTACGAAAGCCAGGATGACGCGGTAGAAGCGATTCTCGGCGGCAAAGTTGTCGCTGGCGATGTAGTGGTGATCCGGTAC
GAAGGCCCGAAAGGCGGTCCGGGGATGCAGGAAATGCTCTACCCAACCAGCTTCCTGAAATCAATGGGTCTCGGCAAAGC
CTGTGCGCTGATCACCGACGGTCGTTTCTCTGGCGGCACCTCTGGCCTTTCTATCGGCCACGTCTCACCGGAAGCGGCAA
GCGGCGGCAGCATTGGCCTGATTGAAGACGGCGACCTGATCGCCATCGACATTCCGAACCGTGGTATTCAGTTACAGGTA
AGCGATGCCGAACTGGCGGCGCGTCGTGAAGCGCAGGAAGCTCGTGGCGACAAAGCCTGGACGCCGAAAAACCGTGAACG
CCAGGTTTCCTTTGCGCTGCGTGCCTACGCCAGCCTGGCAACCAGCGCCGACAAAGGTGCGGTGCGCGATAAATCGAAAC
TGGGGGGTTAA
>Cluster_29445
ATGCTGCCTGATGAACTTGAGAAAAGATTAGATGGAATTGAGGATGCTTCAAAAAAAGGGTATCCAGTACGTAATCTATA
CAAAATTGCGTGTGAGCCCGGACTGTGGCTGCAAGCCTACGTGAATATACGGGCCAATGCAGGTGCACTGACTCAAGGTG
TATCAACAGATACCATTGATGGCTTCTCTATCGAAAGAGCAGAGAAGCTGGCAAAAACATTGCGGAGTAGAGAATACGTA
GCGAAACCTGTACGCAGAGTTCAGATCCCAAAAAAGGACGGAAAAACCAGACCGCTGGGGATTCCAGGTGGAGACGATAA
ACTGGCGCAAGAAGTAGCAAGAATTATTCTGGAAAGGGTTTATGAACCAGTATTCTCTGAACACTCTCATGGATTCAGAC
CAAAACGTTCGTGTGAAACTGCGCTCAGATCTATTCGTCCAGTCTGGAACGGAGTTAAATGGATCGTGGATGTTGATATC
AAAGGTTTCTTTGACAATATCCCCCACAAACTATTGCTGAATACACTGGCAGAGAAAATTAAGGATAAGAATTTCCTGAA
ATTAATCGAACAGTGGCTGAAGGCAGGATATGTGGACAATTGGAAATACCATCGCTCATATTCTGGCACGCCACAAGGTG
GGATAATTTCTCCCTTACTGGCAAATATATACCTCGACAAACTGGACCGTTTTGTAGAACAGAACTTAATTCCTGCATAT
ACAAGCGGTACAAAACGAAGAGCGAATCCAGATATGAACAGGCTGGCCCATAAGATTCATAAACTACGAAAGAAAGTAGA
CGGTATGGCAACTGATTCTGAATCAGAAAAAGAAGCCATAAAGAAAGAAATTGAACGACTGCTTGAAGAAAAACGTTCTA
TACCATCTCAGGTCATGAACGATCCAAACTTCAGGCGTATGTACTATGTTCGATATGCTGATGACTTCGTAATTGGTGTC
ATCGGCTCGAAGAAAGATGCTGAACATATATCAAGACAGGTCAGAAACTTTATTACTACTTCACTGGGATTGGAAGTTAA
TGAAGCAAAGACCAGGATCCGCCATATCTCAGAAGGAGTTAATTTTTTAGGCTATGAAATCAGACAGGCAGATGCAAAGA
AATTGCTGAAGCAGAAAATGCAAGGGCGGCACGCTCTCCGGAGATCCACCACAGGAATTGTGCAGCTCTTCGTTCCTGAC
AATATAGCCGCTAAATTTTGTCACCAAAAGAAATATGGCTGTTATGAGAATGTGAAGGCTGTACATCGTTCAAGTCTACA
AAATCTCAGTGAAGCTGAAATTGTTCTGACTTTCAACGCGGAAATGCGCGGATTGGCGAACTACTACAGTCTGGCGCTGG
ACATGAAATATAAATTATCAAAATTATATTTCATATGGCAAATAAGCCTTTTTAAGACCCTAGCGAATAAACGACGTAGT
TCAGTGAATAAAGTCGCCAAATCGCTACGGCAAAATAATGGAGATCTGGCTATTACCGTTCAGGCCAAAAATGGATCCCG

AAAGATTGAGGTTTTCAAACTCAAACATGTCAATCGCAACCGAACAACGATTGTCGATACGGAACCGAGAACCGCACATA
TCACAACCCGCACAACAGAGATCATGCGTCGATTAGGGGCAAGGATCTGCGAGTATTGTGCGAAGACTGGGCGCTGCGAA
GTACATCATGTCAGAAAGCTAAAAGACCTGAAGAAAGGTCGTAAGGCCGGATATAAACCCAGCCTGTGGCAACTAATGAT
GATCGCACGCCGCCGGAAAACGATGATTTTGTGCGCAAGTTGCCACGACAAGTTACATAGCGGAAAACTACCCGATCTGA
GACAGGAAAAGGGAAGAATACTCCTGCAACCACCTGTCTCAAGTGGGGATTCTGCTAAATGA
>Cluster_16391
GTGACTCTTAAACGGGCCTGTTCCCTGCTGACGGTGAAATCCTTCAGTGAGGATGAACGAGTGATCACCGGGATTGCGTC
AACGCCTTCTCCGGATCGGGATGGTGACATCCTGGAGCCGGAGGGCGCGGAGTTTGGCAGTGCGATCCCGTTTCTCTGGC
AGCATGACCATTCCCGCCCGGTGGGGCAGTGTACGGTGCGCCGGGTCAGCGAAGGGCTGGAAATCACGGCAACACTGGTG
AAGCCCGTACCGGATATGCCGTCGCAACTGGCTGCCCGGCTGGATGAGGTCTGGGCGGCCATTAAGACCGGGCTGGTCAG
GGGGCTGTCCGTGGGCTTCCGTCCCCATGAATACACCTTTCTGGACGGAGGCGGACTGCATTTTCTGCGCTGGGAACTGA
TGGAGGTGTCTGCCGTCACCGTGCCCGCGAATGCGGAATGCACCATCCGGACCATTAAATCTTACGACCGCCCGTTTTCT
GCCGCGTCCGGCAACCGGAAACCGGTGGTGAAAATCGCATCTTCTGCCGGCGCTGCGGCACAGTCAACAACCGTTTTTCA
TAAGGAAAAGACCATAATGAATATTGGCGAACAGATTAAAAGTTTTGAAAACAAGCGTGCAGCGCTGGCAGCCTCCCTTG
AGGAGGTCATGACCAAAGCCGCAGAGGAAGGGCGCACGCTGGATGTGGAGGAGGAAGAGCATTACGACAACACCGCAGCG
GAAATCCGTCAGGTGGATGCGCACCTGAAGCGCCTGCGTGAACTGGAAGCCGGTAAGGCCGCCACGGCGCAGCCGGTGAA
ACAGGCCGGTAACGGGAATGTGGCCGCGGTGGCTTCTGCGCCGGTGATCCGTGTGGAGCAGAAACTGGATAAGGGGATTG
GCTTCGCCCGCTTTGCCAAATCGCTGGCTGCGGCTAAAGGCGTCCGATCTGAAGCCCTGGAAGTGGCCCGTCGTCAGTAT
CCGGATGACAGTCGTCTGCATCATGTCCTGAAATCGGCAGTGGGCGCGGGGACCACCACGGATCCGCAGTGGGCAGGCAG
CCTGTCTGAATATCAGGAATACGCACAGGACTTTATTGATTACCTGCGTCCGCAGACCATTATCGGGCGATTTGGTCAGG
GCGGGATCCCTGCACTTCGTCAGGTGCCGTTCAATATCCGTGTGCACGCCCAGGTGTCCGGCGGTGCTGCCGGCTGGGTG
GGTGAGGGTAAGGCAAAACCCCTGACGAAGTTTGATTTTGAATCCATCACCTTCAGTCATGCGAAGGTGTCGGCCATTGC
GGTACTGACGGAAGAATTGATCCGTTTTTCCAGTCCGGCTGCTGATGCACTGGTCCGTAATGCGCTGGCGGAAGCGGTGG
TGGCGCGTCTGGATACAGACTTTGTGGACCCGAAAAAAGCCGCAGTGGCAGATGTCTCCCCGGCGTCCATCACCCATGAT
GTGAAGGGCACGGCATCAACCGGTAACCCGGATGCGGATGCAGAGGCTGCGTTTGGACAGTTTGTGGCAGCAAACCTGCA
GCCCACCGGTGCGGTCTGGCTGATGTCCAGCACCAATGCCCTGGCACTGTCCATGCGTAAAAATGCGCTGGGTCAGAAGG
AATACCCGGACATGACCCTGCTGGGTGGCTCCTTCCAGGGGCTGCCGGTGATTGTCTCCCAGTACGTGGGTGACCAGCTG
GTGCTGGTGAATGCCCCGGATATTTATCTGGCGGATGACGGAGGCGTGGCAGTGGATATGTCCCGCGAGGCATCACTGGA
AATGCAGTCTGAGCCGACCGGCGACAGTACCACGCCGTCCCCGGTGGAGCTGGTTTCCATGTTCCAGACAGGCAGCGTGG
CCATCCGTGCGGAGCGCTGGATCAACTGGCGTCGTCGCCGTACCGCGGCGGTGGCGGTGATCACCGGAGTGAACTACGGC
AGTGCGTCCGGCGGCTGA
>Cluster_38031
ATGATTAAAAAAGCTTCGCTGCTGACGGCGTGTTCTGTCACAGCCTTTTCCGCTTGGGCACAGGATACCAGCCCGGATAC
TCTCGTCGTTACTGCTAACCGTTTTCAGCAGCCAGTAAATACTGTCCTTGCACCTACTGATATTGTTACCCGTGAGGATA
TTCAACGCTGGCAGTCGAAAGACCTCAACGATGTTATGAGTCATCTCCCCGGTGTAAATATCTCTCAAACTGGTGGAATG
GGGACGAGTTCTTCTCTTTATGTTCGTGGCACTGAATCGCGCCATGTTTTAGTGTTGATTGACGGAGTGCCGATGGCTCG
TGCCGGTATCGACAATGCAATTGATATTAGCCAGTTCCCGGTATCACTTGTACAGCGTGTTGAATATATTCGTGGGCCGC
GTTCTGCCGTTTATGGTTCTGGTGCCATCGGAGGTGTGGTGAACATTATTACAATGAGTAATGATGAGAAAGCTCAGATC
AACGCAGGAATGGGTACAAATGGTTATCAAGCCTATGATGGGGTAATAAATAAACGTTTTGGCGATACGATGGTAACAGC
AGCAGGTGCTTACCAGACGACCAAAGGTTTTAACATTCAGCCTGGCTCACCATACAGTGGTGATAGCGATCGCGATGGAT
ATCGCAACAAACTTTTCTGGGGGGGGCTGCAGCATAAGTTTAACGATAACTTCTCGGGGTTCTTCCGTGGTTACAGCTAC
ACAGCTAATAATGATTACGACCAGGGCTCGATGTATGGTTCTGCTACTGGCAACGAAGAAGCTCAGAATTACAACCAGTC
CTGGGATACTGGCCTACGCTATAACTCCGGCATCTATTCGTCTCAGTTAATTGCTAACTATCAACGACTCAAGAATTATA
ACTACACAAATAATCTTGGTCGTTATGCGGGAGATGCCACCCTGGATGACATGGAACAGCGTTATATTCAGTGGGGTAAT
AGCATTGAGGTAGGGCATGGTGCAATTAGCGGCGGGATCGATTGGAAACAGGAAAAACTGACGTCCAGCAGCATTACAAA
ATCAGATGATTACGAACGCGATACCACCGGGTTATACCTGACTGGACAGCAACAGATTTCCAATGTGACTTTGGAAGCTT
CTGGCCGAGAAGATCATGATGAACAGTTTGGCTGGCATGGTACCTGGCAGACAGCTGCTGGCTGGGAGTTTATTGATGGC
TACCAGGCAACACTTTCTTACGGTACCAGTTTTCTTTCGCCTTCTCTTGGACAGCAATACGGGGCTGCTCGCTTTGCTTC
AATTTATGGTCCTGGTATTGCAGCCAACCCAAATTTGAAGCCAGAAGAATCTAAACAGTGGGAGATTGGAATTGAGGGGG
TAACTGGGACTCTGGATTGGCGGCTCTCAGGATATCGTTATGAAATTCAGAACCTGATTGATTATAAAAACATAAATAAT
GTCAACCAGTACATTAATGTGAAATCAGCCACGATTAAAGGGCTGGAATGGACGGGAAATATTACAACCGGTCCTGTTGA
ACATCGCTTGACGTTACAATATGTTGACCCGCGCGATGATGAAACAGATAAAGTGCTGTACCGTCGGGCAAAACAGCAGG
TTAAGTACGAGTTGACTGGACAAGCTTATGGTTTCGAATGGGACGTTTCTTACCAATATATAGGTCAACGATACGACAAC
GCTTATGACGAGACCTCTGGCTCATCACATACAGTTAAATTAGGCGGTGTGAGCTTGTGGGATCTTGCGGTTGCATATCC
GGTCACCTCTCATCTGACAGTTCGTGGTAAAATAGCCAACCTGTTCGACAAAGATTATGAGACAGTCTATGGCTACCAAA
CTGCAGGACGGGAATACACCTTGTCTGGCAGCTACACCTTCTGA
>Cluster_43754
ATGGCTATCGAAATCAAAGTACCGGACATCGGGGCTGATGAAGTTGAAATCACCGAGATCCTGGTCAAAGTGGGCGACAA
AGTTGAAGCCGAACAGTCGCTGATCACCGTAGAAGGCGACAAAGCCTCTATGGAAGTTCCGTCTCCGCAGGCGGGTATCG
TTAAAGAGATCAAAGTCTCTGTTGGCGATAAAACCCAGACCGGCGCACTGATTATGATTTTCGATTCCGCCGACGGTGCA
GCAGACGCTGCACCTGCTCAGGCAGAAGAGAAGAAAGAAGCAGCTCCGGCAGCAGCACCAGCGGCTGCAGCGGCAAAAGA
CGTTAACGTTCCGGATATCGGCAGCGACGAAGTTGAAGTGACCGAAATCCTGGTGAAAGTTGGCGATAAAGTTGAAGCTG
AACAGTCGCTGATCACCGTAGAAGGCGATAAAGCGTCTATGGAAGTTCCGGCTCCGTTTGCTGGCACCGTGAAAGAGATC
AAAGTGAACGTGGGTGACAAAGTGTCTACCGGCTCGCTGATTATGGTCTTCGAAGTCGCGGGTGAAGCAGGCGCGGCAGC
TCCGGCGGCTAAACAGGAAGCGGCTCCGGCAGCGGCCCCTGCACCAGCGGCTGGCGTGAAAGAAGTTAACGTTCCGGATA
TCGGCGGTGACGAAGTTGAAGTGACCGAAGTGATGGTGAAAGTGGGCGACAAAGTTGCCGCTGAACAGTCACTGATTACC
GTAGAAGGCGACAAAGCTTCTATGGAAGTTCCGGCTCCGTTTGCAGGCGTCGTGAAGGAACTGAAAGTCAACGTTGGCGA
TAAAGTGAAAACTGGCTCGCTGATTATGATCTTCGAAGTTGAAGGCGCAGCGCCTGCGGCAGCTCCTGCGAAACAGGAAG
CGGCAGCGCCGGCTCCGGCAGCAAAAGCTGAAGCTCCGGCAGCAGCACCGGCTGCGAAAGCGGAAGGCAAATCTGAATTT
GCAGAAAACGACGCTTACGTTCACGCGACTCCGCTGATCCGCCGTCTGGCACGCGAGTTTGGCGTTAACCTGGCGAAAGT
GAAGGGCACTGGCCGTAAAGGTCGTATCCTGCGCGAAGACGTTCAGGCTTACGTGAAAGAAGCTATCAAACGTGCAGAAG
CAGCTCCGGCGGCGACTGGCGGCGGTATCCCAGGCATGCTGCCGTGGCCGAAGGTGGACTTCAGCAAGTTTGGTGAAATC
GAAGAAGTGGAACTGGGCCGTATCCAGAAAATTTCTGGTGCGAACCTGAGCCGTAACTGGGTGATGATCCCGCATGTTAC
TCACTTCGACAAAACCGATATCACCGAGCTGGAAGCGTTCCGTAAACAGCAGAACGAAGAAGCGGCGAAACGTAAGCTGG
ATGTGAAGATCACCCCGGTTGTCTTCATCATGAAAGCCGTTGCTGCAGCTCTTGAGCAGATGCCTCGCTTCAACAGTTCG
TTGTCGGAAGACGGTCAGCGTCTGACCCTGAAGAAATACATCAACATCGGTGTGGCGGTGGATACCCCGAACGGTCTGGT
TGTTCCGGTATTCAAAGACGTCAACAAGAAAGGCATCATCGAGCTGTCTCGCGAGCTGATGACTATTTCTAAGAAAGCGC
GTGACGGTAAGCTGACTGCGGGCGAAATGCAGGGCGGTTGCTTCACCATCTCCAGCATCGGCGGCCTGGGTACTACCCAC
TTCGCGCCGATTGTGAACGCGCCGGAAGTGGCTATCCTCGGCGTTTCCAAGTCCGCGATGGAGCCGGTGTGGAATGGTAA

AGAGTTCGTGCCGCGTCTGATGCTGCCGATTTCTCTCTCCTTCGACCACCGCGTGATCGACGGTGCTGATGGTGCCCGTT
TCATTACCATCATTAACAACACGCTGTCTGACATTCGCCGTCTGGTGATGTAA
>Cluster_39204
GTGAAAAACGCGTCAACCGTATCGGAAGATACTGCGTCGAATCAAGAGCCGACGCTTCATCGCGGATTACATAACCGTCA
TATTCAACTGATTGCGCTGGGTGGCGCAATTGGTACTGGTCTGTTTCTTGGCATTGGCCCGGCGATTCAGATGGCGGGTC
CGGCTGTATTGCTGGGCTACGGCGTCGCCGGGATCATCGCTTTCCTGATTATGCGCCAGCTCGGCGAGATGGTGGTCGAA
GAGCCGGTATCCGGTTCATTTGCCCACTTTGCCTATAAATACTGGGGACCGTTTGCGGGCTTCCTCTCTGGCTGGAACTA
CTGGGTAATGTTCGTGCTGGTGGGAATGGCAGAGCTGACCGCTGCGGGCATCTATATGCAGTACTGGTTCCCGGATGTTC
CAACGTGGATTTGGGCTGCCGCCTTCTTTATTATCATCAACGCCGTTAACCTGGTGAACGTGCGCTTATATGGCGAAACC
GAGTTCTGGTTTGCGCTGATTAAAGTGCTGGCGATCATCGGTATGATCGGCTTTGGCCTGTGGCTGCTGTTTTCTGGGCA
CGGCGGTGAGAAAGCCAGTATCGATAACCTCTGGCGCTACGGCGGTTTCTTTGCCACCGGCTGGAATGGACTGATTTTGT
CGCTGGCGGTAATTATGTTCTCCTTCGGCGGTCTGGAGCTGATTGGGATTACTGCGGCTGAAGCGCGCGATCCGGAAAAA
AGCATCCCTAAAGCAGTGAATCAGGTGGTGTATCGCATCCTGCTGTTTTATATCGGTTCACTGGTGGTTTTACTGGCGCT
CTACCCGTGGGTGGAAGTGAAATCCAACAGTAGCCCGTTTGTGATGATTTTCCATAATCTCGACAGCAATGTGGTGGCTT
CTGCGCTGAACTTCGTCATTCTGGTGGCATCGCTGTCGGTGTATAACAGCGGGGTTTACTCTAACAGCCGCATGCTGTTT
GGCCTTTCTGTGCAGGGTAATGCGCCAAAGTTTTTGACTCGCGTCAGCCGTCGCGGCGTGCCGATTAACTCGCTGATGCT
TTCCGGAGCGATCACTTCGCTGGTGGTGTTAATCAACTATCTGCTGCCGCAAAAAGCGTTTGGTCTGCTGATGGCGCTGG
TGGTAGCAACGCTGCTGTTGAACTGGATTATGATCTGCCTGGCGCATCTGCGTTTTCGCGCGGCGATGCGACGTCAGGGG
CGTGAAACACAATTTAAGGCGCTGCTCTATCCGTTCGGCAACTATCTTTGCATCGCCTTCCTCGGCATGATTTTGCTGCT
GATGTGCACGATGGATGATATGCGCCTGTCAGCGATTCTGCTGCCGGTGTGGATTGTGTTCCTGTTTGTGGCGTTTAAAA
CATTGCGTAGGAAAGTGAATTAA
>Cluster_37817
ATGGCTACTGGAAAGATTGTCCAGGTAATCGGCGCCGTAGTTGACGTCGAATTCCCTCAGGATGCCGTACCGCGCGTGTA
CGATGCTCTTGAGGTGCAAAATGGTAATGAGCGTCTGGTGCTGGAAGTTCAGCAGCAGCTCGGCGGCGGTATCGTACGTA
CCATCGCAATGGGTTCCTCCGACGGTCTGCGTCGCGGTCTGGATGTAAAAGACCTCGAACACCCGATTGAAGTCCCGGTA
GGTAAAGCGACTCTGGGCCGTATCATGAACGTACTGGGTGAACCGGTCGACATGAAAGGCGAGATCGGTGAAGAAGAGCG
TTGGGCGATTCACCGCGCAGCACCTTCCTACGAAGAGCTGTCAAACTCTCAGGAACTGCTGGAAACCGGTATCAAAGTTA
TCGACCTGATGTGTCCGTTCGCTAAGGGCGGTAAAGTTGGTCTGTTCGGTGGTGCGGGTGTAGGTAAAACCGTAAACATG
ATGGAGCTCATTCGTAACATCGCGATCGAGCACTCCGGTTACTCTGTGTTTGCGGGCGTAGGTGAACGTACTCGTGAGGG
TAACGACTTCTACCACGAAATGACCGACTCCAACGTTATCGACAAAGTATCCCTGGTGTATGGCCAGATGAACGAGCCGC
CGGGAAACCGTCTGCGCGTTGCTCTGACCGGTCTGACCATGGCTGAGAAATTCCGTGACGAAGGTCGTGACGTTCTGCTG
TTCGTTGACAACATCTATCGTTACACCCTGGCCGGTACGGAAGTATCCGCACTGCTGGGCCGTATGCCTTCAGCGGTAGG
TTATCAGCCGACCCTGGCGGAAGAGATGGGCGTTCTGCAGGAACGTATCACCTCCACCAAAACTGGTTCTATCACCTCCG
TACAGGCAGTATACGTACCTGCGGATGACTTGACTGACCCGTCTCCGGCAACCACCTTTGCGCACCTTGACGCAACCGTG
GTACTGAGCCGTCAGATCGCGTCTCTGGGTATCTACCCGGCCGTTGACCCGCTGGACTCCACCAGCCGTCAGCTGGACCC
GCTGGTGGTTGGTCAGGAACACTACGACACCGCGCGTGGCGTTCAGTCCATCCTGCAACGTTATCAGGAACTGAAAGACA
TCATCGCCATCCTGGGTATGGATGAACTGTCTGAAGAAGACAAACTGGTGGTAGCGCGTGCTCGTAAGATCCAGCGCTTC
CTGTCCCAGCCGTTCTTCGTGGCAGAAGTATTCACCGGTTCTCCGGGTAAATACGTCTCCCTGAAAGACACCATCCGTGG
CTTTAAAGGCATCATGGAAGGCGAATACGATCACCTGCCGGAGCAGGCGTTCTACATGGTCGGTTCCATCGAAGAAGCTG
TGGAAAAAGCCAAAAAACTTTAA
>Cluster_56416
ATGGACTTAGAAAATAAATTCTCATATCATTTTCTTGAGGGATTAACGCTCACGGAAGATGGAATTCTTACTCAAGGAAA
TGAGCAAGTTTATATTCCACAGAAAGAGTTAGGTGTATTAATAGTATTACTTGAATCCGCTGGTCATGTTGTACTGAAAG
ATATGATCATCGAATCAGTATGGAAAAATATTATTGTTAGTGACGAGTCCCTGACAAGATGTATCTATTCTTTGCGCTGC
ATTTTTGAAAAAATTGGCTATGATCGTTGCATAGAAACAATCTACCGGAAAGGTTATCGTTTCAGCGGGCAGGTTTTCAA
AACTAAAATTAATGAAGATAATACTTCAGACTATTCCATAGCTATATTCCCTTTCACTACTTCATTGAATACACTGGATC
CATTAATACTTAATCAGGAATTAGTGCAAATCATTTCAAATAAAAAAATCGATGGTCTCTATACCTATCCGATGGCTGCG
ACAAATTTTTGTAATGATCACATATCTCAAAATTCATTCTTGAGCAGATTCAAGCCAGATTATTTCGTTACAGGAAGAAT
AAACCAGAATAATGCAGTGAACACTTTATACATTGAGTTGATCGACGCTAAAAACCTTTTCCTCATCGCCAGTAATCATC
TCCCTGTTGATGAACTACATAATACATCACAATTTATTATAGATAATATCCTTCAAACGGTACATAAACCAGAACGATCT
GTAAGATTAGCTAAGCAGGACCAAGGATATAAGAATCATTATTTATCAGATGAAATGTTAGCCGGAAAGAAAGAACTTTA
CGACTTCACCCCTGAAAGCATTTACAGGGCCATGACTATATTTGATAGATTACAAAATAAAAGTGATATACAGACGCTAA
AAACAGAATGTTATTGCCTTCTAGCGGAATGCCATATGTCTTTGGCACTTCATGGAAAAAGTGAACTTGAACTTGCTGCT
CAAAAAGCATTAGAGCTTTTAGATTATGTATCAGACATAACCACTGTCGATGGAAAAATTTTAGCTATTATGGGACTGAT
AACTGGTCTGTCTGGACAAGCAAAAGTATCTCATATCTTATTTGAACAGGCTAAGATACACTCAACTGATATAGCCTCTC
TCTACTACTATAGGGCACTAGTCCACTTTCATAATGAAAAAATTGAAGAGGCAAGGATTTGTATAGACAAATCACTACAA
CTCGAACCCAGAAGACGAAAAGCAGTTGTGATAAAAGAATGTGTAGATATGTATGTGCCTAACCCGCTCAAAAACAACAT
TAAACTCTACTATAAAGAAACTGAGAGTGAAAGCCATCGAGTTATAATTGACAACATTTTGAAATTAAAGCAGCTGACGA
GAATTTGTATGCGATAA
>Cluster_57881
ATGAGCGACACCTCACCTGCCATACCGGAGAGTATCGATCCGGCGAATCAGCATAAAGCGCTGACTGCCGGACAACAGGC
GGTTATTAAGAAGCTATTTCGCCGCCTGATCGTCTTTCTGTTCGTGCTGTTTATCTTCTCGTTCCTTGATCGCATCAACA
TCGGCTTTGCCGGACTCACGATGGGACGCGACCTCGGTCTGAGCGCCACCATGTTTGGCCTCGCTACCACCCTGTTCTAC
GCCGCTTATGTCATCTTCGGCATTCCCAGCAACATTATGCTGAGTATTGTCGGTGCGCGGCGCTGGATCGCCACCATCAT
GGTGCTTTGGGGCATCGCCTCTACTGCCACCATGTTTGCCACTGGCCCCACCAGCTTGTACGTACTGCGTATACTGGTTG
GCATTACCGAAGCCGGCTTTCTGCCTGGCATTCTGCTGTATTTAACCTTCTGGTTTCCAGCCTATTTCCGCGCCCGTGCC
AACGCCTTGTTTATGGTTGCAATGCCGGTAACGACAGCGTTGGGATCGCTCGTTTCCGGCTACATTTTGTCGCTGGATGG
CGTAATGGCATTAAAAGGCTGGCAGTGGCTGTTTTTGCTGGAAGGCTTCCCGTCGGTATTACTCGGCGTCATGGTGTGGT
TCTGGCTTGATGACTCACCGGACAAAGCTAAGTGGCTGACGAAAGAAGACAAAAAATGCCTGCAAGAGATGATGGATAAC
GATCGTCTGACGCTGGTTCAGCCAGAGGGAGCCATCAGCCACCACGCCATGCAACAACGCAGCATGTGGCGGGAGATCTT
CACTCCGGTGGTGATGATGTATACCCTGGCGTATTTCTGCCTGACCAACACACTTAGTGCGATCAGCATCTGGACACCGC
AGATCCTGCAAAGCTTTAATCAGGGCAGCAGTAATATCACCATCGGCCTGCTGGCCGCCGTACCGCAGATTTGTACCATT
CTCGGGATGATCTACTGGAGCCGTCACTCAGATCGCCGCCAGGAACGAAGGCATCACACCGCCCTTCCTTATTTGTTCGC
TGCCGCGGGTTGGTTACTGGCTTCGGCAACTGATCACAACATGATCCAGATGCTGGGGATCATTATGGCTTCGACCGGAT
CATTCAGCGCAATGGCGATTTTCTGGACAACACCTGATCAGTCCATCAGCCTGCGGGCACGAGCGATCGGTATTGCGGTG
ATCAACGCCACTGGCAACATTGGCTCAGCGTTAAGTCCGTTTATGATCGGCTGGTTGAAAGATCTGACCGGCAGCTTTAA
CAGTGGATTGTGGTTTGTTGCCGCGCTGCTGGTGATTGGTGCGGGGATTATCTGGGCAATTCCAATGCAGTCCTCCCGTC
CGCGAGCGACCCCGTAA
>Cluster_38839

ATGAAAACTGATACTCCCTCTTTAGAAACACCGCAGGCCGCGCGCCTGCGACGCAGACAACTGATTCGCCAACTTCTTGA
GCGCGATAAAACCCCGTTAGCCATTTTGTTTATGGCGGCAGTCGTCGGCACGCTTGTCGGGCTGGCTGCGGTTGCTTTTG
ACAAAGGTGTCGCCTGGTTGCAGAACCAACGTATGGGGGCACTGGTACATACTGCTGATAATTATCCGCTTCTGTTAACC
GTCGCTTTTCTCTGTTCGGCGGTGCTGGCGATGTTCGGCTACTTTTTGGTGCGCAAATACGCGCCGGAAGCAGGTGGTTC
GGGGATCCCGGAAATTGAAGGGGCGCTGGAAGATCAACGTCCCGTTCGCTGGTGGCGTGTGTTGCCGGTGAAATTCTTTG
GCGGGCTGGGAACACTCGGCGGAGGCATGGTGTTGGGGCGCGAAGGGCCAACCGTGCAGATAGGCGGCAATATCGGGCGG
ATGGTACTCGATGTTTTCCGCCTGAAAGGTGACGAAGCTCGTCATACGCTGTTGGCAACCGGTGCTGCTGCGGGGCTGGC
TGCGGCCTTTAACGCGCCGCTGGCGGGTATTTTGTTTATTATCGAAGAGATGCGTCCGCAGTTTCGCTATACGTTAATTT
CGATTAAAGCGGTATTTATTGGCGTCATTATGTCGACCATTATGTACCGGATTTTTAATCATGAAGTTGCGTTGATTGAC
GTCGGTAAACTTTCTGATGCGCCGCTTAATACGCTGTGGCTTTATCTGATCCTCGGTATTATTTTTGGCATTTTCGGCCC
TATTTTTAATAAATGGGTGCTGGGGATGCAGGATTTGCTGCACCGTGTGCACGGTGGCAATATTACCAAATGGGTACTGA
TGGGTGGTGCGATTGGCGGTCTGTGTGGATTGCTGGGGTTTGTGGCACCAGCAACGTCGGGCGGCGGTTTTAACCTGATT
CCTATCGCTACCGCGGGGAATTTCAGCATGGGAATGCTGGTGTTTATCTTCGTCGCGCGGGTCATTACCACCTTACTTTG
CTTCTCTTCCGGCGCGCCGGGCGGTATTTTTGCACCGATGCTGGCGCTGGGTACTGTGCTGGGAACCGCTTTCGGAATGG
TTGCCGTTGAGTTGTTTCCGCAATATCACCTTGAGGCGGGGACGTTTGCTATTGCCGGAATGGGGGCGTTACTGGCGGCA
TCTATTCGCGCGCCGTTAACGGGGATCATTCTGGTTCTGGAAATGACCGATAACTACCAGCTCATTTTGCCAATGATTAT
TACCGGTCTTGGCGCAACACTATTAGCACAATTTACCGGTGGGAAACCGCTATACTCGGCGATTCTTGCGCGCACGCTGG
CAAAACAGGAAGCTGAGCAACTGGCGCGAAGCAAGGCCGCATCAGCCCGCGAGAATACTTGA
>Cluster_27052
ATGATGAATGACGGTAAGCAACAATCTACCTTTTTGTTTCACGATTACGAAACCTTTGGAACGCACCCCGCGTTAGATCG
CCCTGCACAGTTCGCAGCCATTCGCACTGATAACGAATTCAATGTCATCGGCGAACCCGAAGTCTTTTACTGCAAGCCCG
CGGATGACTATTTACCCCAGCCTGGAGCAGTATTAATTACCGGTATTACCCCGCAGGAGGCACGGGCGAAAGGAGAAAAC
GAAGCCGCGTTTGCCGCCCGTATTCACTCGCTTTTTACCGTACCGAAGACCTGTATTCTGGGCTACAACAATGTGCGTTT
CGACGACGAAGTCACACGCAACGTTTTTTATCGTAATTTCTACGATCCTTACGCCTGGAGCTGGCAGCATGATAACTCGC
GCTGGGATTTACTGGATGTTATGCGTGCCTGTTATGCCCTACGCCCGGAAGGAATAAACTGGCCTGAAAATGATGACGGT
CTACCGAGCTTTCGCCTTGAGCATTTAACCAAAGCGAATGGTATTGAACATAGCAACGCCCACGATGCGATGGCTGATGT
GTACGCCACTATTGCGATGGCGAAACTGGTAAAAACGCGTCAGCCACGCCTGTTTGATTATCTCTTTACCCATCGTAATA
AACACAAACTGATGGCGTTGATTGATGTTCCGCAGATGAACCCCCTGGTTCACGTTTCCGGAATGTTTGGGGCATGGCGC
GGCAATACCAGCTGGGTGGCACCGCTGGCGTGGCATCCTGAAAATCGCAATGCCGTAATTATGGTGGATTTGGCAGGAAA
CATTTCGCCATTACTGGAGCTGGATAGCGACACATTGCGCGAGCGTTTATATACCGCAAAAGCCGATCTTGGCGATAACG
CCGCCGTTCCGGTTAAGCTGGTGCATATCAATAAATGTCCGGTACTGGCCCAGGCGAATACGCTACGCCCGGAAGATGCC
GACCGACTGGGAATTAATCGTCAGCATTGCCTCGATAACCTAAAAATTCTGCGTGAAAATCCGCAAGTGCGTGAAAAAGT
GGTAGCGATATTCGCGGAAGCCGAACCGTTTACACCTTCAGATAACGTGGATGCACAGCTTTATAATGGCTTTTTCAGTG
ACGCCGATCGTGCAGCAATGAAAATTGTGCTGGAAACCGAGCCGCGTAATTTACCGGCACTGGATATCACTTTTGTTGAT
AAACGAATTGAAAAACTGTTGTTCAATTATCGAGCGCGCAACTTCCCGGGGACGCTGGATTATGCCGAGCAGCAACGCTG
GCTGGAGCACCGCCGCCAGGTCTTCACGCCAGAGTTTTTGCAGGGCTACGCTGATGAATTGCAGATGCTGGCACAACAGT
ATGCTGATGACAAAGAGAAAGTGGCGCTGTTAAAAGCGCTTTGGCAGTACGCGGAAGAGATCGTCTGA
>Cluster_39352
ATGCGTGCGTTTAGTGGCAAACGTAGTACGCTGGCGCTGGCTATCGCCGGTGTTACAGCTATGTCGGGCTTTATGGCAAT
TCCGGAGGCTCGCGCCGAAGGATTCATCGACGATTCAACCTTAACCGGCGGTATCTATTACTGGCAGCGTGAACGCGACC
GTAAAGATGTTACCGACGGCGATAAATACAAAACCAACCTTTCTCACTCCACCTGGAACGCCAACCTCGATTTTCAGTCT
GGTTATGCTGCTGATATGTTCGGCCTTGATATTGCCGTGTTTACGGCGATTGAAATGGCGGAAAACGGCGACAGCTCTCA
CCCGAACGAAATCGCGTTTTCAAAAAGTAATAAAGCCTATGACGAAGACTGGTCCGGCGATAAAAGCGGTATAAGCCTGT
ATAAAGCAGCGGCCAAATTTAAATACGGTCCGGTTTGGGCGAGGGCAGGTTATATTCAGCCAACCGGTCAGACGCTGTTA
GCGCCACACTGGAGCTTTATGCCGGGTACTTATCAGGGGGCGGAGGCCGGGGCGAATTTTGATTACGGCGATGCTGGTGC
GTTGAGTTTCTCCTACATGTGGACCAACGAATACAAAGCACCGTGGCATCTGGAAATGGATGAGTTTTATCAGAACGATA
AAACCACCAAAGTTGATTATCTACACTCCCTTGGGGCGAAGTACGACTTCAAAAATAACTTCATACTGGAAGCGGCGTTT
GGTCAGGCCGAAGGGTATATCGATCAATATTTTGCCAAAGCCAGCTACAAATTTGATATCGCCGGTAGCCCGTTAACCAC
CAGCTACCAGTTCTATGGTACGCGCGATAAAGTTGACGATCGCAGCGTCAACGATCTTTATGACGGCACCGCCTGGCTGC
AGGCGTTGACCTTTGGTTACCGGGCGGCTGACGTAGTGGATTTGCGCCTCGAAGGTACCTGGGTTAAGGCTGACGGTCAG
CAAGGATACTTCCTGCAACGTATGACTCCAACCTACGCTTCCTCGAACGGTCGCCTGGATATCTGGTGGGATAACCGTTC
CGACTTCAACGCCAACGGCGAAAAAGCGGTCTTCTTCGGTGCGATGTATGACCTGAAAAACTGGAATCTTCCAGGCTTCG
CCATCGGCGCTTCCTACGTTTACGCATGGGATGCTAAACCCGCTACCTGGCAGAGCAATCCGGATGCGTACTACGACAAA
AACCGGACTATTGAAGAGTCTGCCTACAGCCTGGATGCGGTCTACACCATTCAGGACGGTCGCGCCAAAGGCACGATGTT
CAAACTGCACTTCACCGAATACGACAACCACTCCGACATCCCAAGCTGGGGCGGTGGTTACGGCAACATCTTCCAGGATG
AGCGTGACGTGAAATTTATGGTAATCGCACCATTCACCATCTTCTGA
>Cluster_55643
ATGTTACTTCCTGTAATTATGGCTGGCGGTACCGGCAGTCGTCTCTGGCCGATGTCTCGCGAGCTTTACCCGAAACAGTT
CCTCCGTCTGTTCGGGCAGAACTCCATGCTGCAGGAAACCATTATCCGACTCTCGGGCCTTGAAATCCATGAACCGATGG
TCATCTGTAACGAAGAGCATCGTTTCCTGGTGGCTGAACAGCTGCGCCAGCTCAATAAGCTGTCGAATAACATTATACTT
GAGCCGGTCGGGCGCAACACCGCGCCGGCCATCGCCCTGGCGGCCCTTCAGGCCACCCGCTACGGTGACGACCCGCTGAT
GCTGGTCCTCGCCGCCGACCATATCATCAACAATCAGTCGGCCTTCCACGACGCCATCCGCGTCGCCGAGCAGTATGCCG
ATGAAGGCCATCTGGTCACCTTCGGTATCGTGCCGAACGCCCCGGAAACCGGCTACGGCTACATCCAGCGCGGCGTGGCG
CTCACCGACAGCGCCCACGCCCCGTACCAGGTGGCCCGCTTCGTGGAGAAGCCGGACCGCGAGCGCGCCGAGGTCTACCT
CGCCTCCGGGGAGTACTACTGGAACAGCGGCATGTTTATGTTCCGTGCCAAAAAATACCTCTCTGAGCTGGCCAAATACC
GTCCGGATATCCTGGAAACCTGCCAGGCTGCGGTGAATGCCGCCGATAATGGCAGCGATTTCATCAATATCCCACATGAT
ATTTTCTGCGAGTGCCCGGACGAGTCCGTGGACTACGCCGTTATGGAGAAAACCGCTGATGCGGTGGTGGTCGGTCTCGA
TGCTGACTGGAGCGACGTTGGCTCCTGGTCCGCACTATGGGAGGTCAGCCCGAAAGACGGGCAGGGTAACGTTCTCAGCG
GTGACGCGTGGGTGCACAACAGCGAAAACTGCTACATCAACAGCGACGAGAAGCTGGTGGCGGCCATCGGTGTGGAGAAC
CTGGTGATTGTCAGCACCAAGGACGCCGTGCTGGTGATGAACCGCGAGCGCTCCCAGGACGTGAAGAAGGCGGTCGAGTT
CCTCAAGCAGAACCAGCGCAGCGAGTACAAGCGCCACCGTGAGATTTACCGTCCCTGGGGCCGCTGCGACGTGGTGGTCC
AGACCCCGCGCTTCAACGTCAACCGCATCACGGTGAAACCTGGCGGTGCCTTCTCGATGCAGATGCACCACCATCGCGCT
GAGCATTGGGTTATTCTCGCCGGCACCGGTCAGGTGACGGTCAACGGTAAGCAGTTCCTGTTGTCCGAGAACCAGTCCAC
CTTTATTCCGATTGGCGCCGAGCACTGCCTGGAAAACCCTGGCTGTATTCCGCTGGAAGTGCTGGAGATCCAGTCGGGGT
CGTACCTCGGCGAGGACGACATTATTCGTATTAAAGACCAGTATGGTCGTTGCTAA
>Cluster_42977
ATGTCTGTACAGCAAATCGACTGGGATCTGGCCCTGATCCAGAAATATAACTATTCCGGGCCACGATACACCTCGTACCC
GACCGCGCTGGAGTTTTCAGAAGACTTCGGCGAACAGGCGTTTTTACAAGCCGTGGCGCGCTATCCTGAGCGCCCATTAT
CTCTCTACGTACATATTCCGTTCTGCCATAAGCTTTGTTACTTCTGCGGTTGCAATAAGATTGTTACTCGCCAGCAGCAC

AAGGCCGATCAGTATCTGGATGCGCTGGAGCAAGAAATCGTCCATCGTGCACCGCTGTTCGCCGGGCGTCACGTCAGCCA
ATTGCACTGGGGCGGCGGAACGCCGACGTATCTGAATAAAGCGCAAATCAGCCGTCTGATGAAGCTGCTGCGCGAAAACT
TCCAGTTCAATGCCGATGCGGAGATCTCGATCGAAGTCGATCCGCGGGAAATCGAACTGGATGTACTCGATCATTTACGC
GCCGAAGGCTTTAATCGCCTGAGCATGGGCGTGCAGGACTTCAACAAAGAAGTGCAACGTCTGGTTAACCGCGAGCAGGA
TGAAGAGTTCATCTTTGCACTGCTTAACCATGCGCGTGAGATTGGCTTTACCTCCACCAACATCGACCTGATTTACGGTC
TGCCGAAACAGACGCCGGAAAGTTTTGCCTTTACCCTGAAACGTGTGGCAGAGCTGAACCCCGATCGTCTGAGCGTCTTT
AACTACGCGCATCTGCCAACTATTTTTGCCGCCCAACGCAAAATCAAAGATGCTGACTTGCCGAGTCCGCAACAAAAACT
CGATATCTTGCAGGAAACCATCGCCTTCCTGACACAATCGGGCTATCAGTTTATCGGTATGGATCACTTTGCTCGCCCTG
ATGACGAGCTGGCAGTGGCCCAGCGCGAAGGCGTGTTGCATCGTAACTTCCAGGGCTATACCACTCAGGGCGATACCGAT
TTGCTGGGGATGGGCGTTTCCGCCATCAGCATGATTGGCGACTGCTACGCGCAGAACCAGAAAGAGTTGAAGCAGTACTA
TCAGCAAGTGGATGAACAAGGCAATGCGCTGTGGCGTGGTATTGCGCTAACGCGTGATGACTGTATTCGCCGCGATGTGA
TTAAGTCGCTCATCTGCAACTTCCGTCTGGATTACGCTCCCATTGAGCAACAGTGGGATTTGCACTTCGCTGATTACTTT
GCGGAAGATCTCAAGCTGCTCGCCCCGTTAGCAAAAGATGGGCTGGTGGATGTAGATGAGAAGGGGATTCAAGTGACGGC
GAAAGGTCGCTTGCTGATCCGCAACATTTGCATGTGCTTTGATACCTATCTGCGCCAGAAAGCGCGGATGCAGCAGTTCT
CACGGGTGATTTAA
>Cluster_47347
ATGGCTAAACAACCGGGATTAGATTTTCAAAGTGCCAAAGGTGGCTTAGGCGAGCTGAAACGCAGACTGCTGTTTGTTAT
CGGTGCGCTGATTGTGTTCCGTATTGGCTCTTTTATTCCGATCCCTGGTATTGATGCCGCTGTACTTGCCAAACTGCTTG
AGCAACAGCGAGGCACCATCATTGAGATGTTTAACATGTTCTCTGGTGGTGCTCTCAGCCGTGCTTCTATCTTTGCTCTG
GGGATCATGCCGTATATTTCGGCGTCGATCATTATCCAGCTGCTGACGGTGGTTCACCCAACGTTGGCAGAAATTAAGAA
AGAAGGGGAGTCTGGTCGTCGTAAGATCAGCCAGTACACCCGTTACGGTACTCTGGTGCTGGCAATATTCCAGTCGATCG
GTATTGCTACCGGTCTGCCGAATATGCCTGGTATGCAAGGCCTGGTGATTAACCCAGGCTTTGCATTCTACTTCACCGCT
GTTGTAAGTCTGGTCACAGGAACGATGTTCCTGATGTGGTTGGGCGAACAGATTACTGAACGAGGTATCGGCAACGGTAT
TTCAATCATTATCTTCGCCGGTATTGTCGCGGGACTCCCGCCAGCCATTGCCCATACTATCGAGCAAGCGCGTCAAGGCG
ACCTGCACTTCCTCGTGTTGCTGTTGGTTGCAGTATTAGTATTTGCAGTGACGTTCTTTGTTGTATTTGTTGAGCGTGGT
CAACGCCGCATTGTGGTAAACTACGCGAAACGTCAGCAAGGTCGTCGTGTCTATGCTGCACAGAGCACACATTTACCGCT
GAAAGTGAATATGGCGGGGGTAATCCCGGCAATCTTCGCTTCCAGTATTATTCTGTTCCCGGCGACCATCGCGTCATGGT
TCGGGGGCGGTACTGGTTGGAACTGGCTGACAACAATTTCGCTGTATTTGCAGCCTGGGCAACCGCTTTATGTGTTACTC
TATGCGTCTGCAATCATCTTCTTCTGTTTCTTCTACACGGCGTTGGTTTTCAACCCGCGTGAAACAGCAGATAACCTGAA
GAAGTCCGGTGCATTTGTACCAGGAATTCGTCCGGGAGAGCAAACGGCGAAGTATATCGATAAAGTAATGACCCGCCTGA
CCCTGGTTGGTGCGCTGTATATTACCTTTATCTGCCTGATCCCGGAGTTCATGCGTGATGCAATGAAAGTACCGTTCTAC
TTCGGTGGGACCTCACTGCTTATCGTTGTTGTCGTGATTATGGACTTTATGGCTCAAGTGCAAACTCTGATGATGTCCAG
TCAGTATGAGTCTGCATTGAAGAAGGCGAACCTGAAAGGCTACGGCCGATAA
>Cluster_55951
ATGCACTCTCAAATCTGGGTTGTGAGCACGCTGCTTATCAGCATCGTGTTAATTGTACTGACCATCGTGAAGTTCAAATT
CCACCCGTTTCTGGCGCTGTTGCTGGCCAGCTTCTTCGTGGGAACGATGATGGGCATGGGGCCACTGGATATGGTAAATG
CTATTGAAAGTGGAATTGGCGGAACGCTGGGGTTCCTCGCAGCGGTTATCGGCCTTGGCACGATACTGGGAAAAATGATG
GAAGTATCCGGGGCCGCAGAAAGAATTGGTCTGACACTTCAACGCTGCCGCTGGCTTTCAGTTGATGTCATTATGGTGCT
GGTTGGCCTGATTTGTGGCATCACGCTGTTTGTTGAAGTGGGCGTCGTGCTATTGATTCCTCTGGCTTTTTCAATTGCCA
AAAAAACCAATACCTCATTATTAAAGCTTGCCATTCCGCTATGTACCGCATTGATGGCAGTGCACTGCGTGGTTCCTCCA
CATCCGGCTGCTTTATATGTTGCCAATAAGCTGGGCGCAGATATCGGTTCGGTGATCGTCTACGGTTTGCTGGTTGGGCT
GATGGCATCACTGATCGGTGGCCCACTTTTCCTTAAATTTCTGGGTCAACGACTGCCCTTTAAACCTGTACCCACAGAGT
TTGCAGATCTCAAAGTTCGCGATGAAAAAACACTACCGTCATTAGGCGCAACGTTATTCACCATACTGCTACCCATTGCG
CTGATGTTGGTTAAAACGATTGCCGAATTGAATATGGCGCGTGAGAGTGGTTTGTATATCTTGGTTGAGTTTATTGGCAA
CCCTATCACTGCCATGTTTATCGCCGTGTTTGTCGCCTATTATGTGTTGGGTATACGCCAGCATATGAGCATGGGGACGA
TGCTCACACATACGGAAAATGGCTTCGGTTCTATTGCTAATATTTTGCTGATTATCGGGGCCGGAGGCGCATTCAACGCC
ATTTTAAAAAGCAGCAGTCTCGCTGATACGCTGGCAGTTATTCTCTCCAATATGCATATGCACCCGATTCTTCTGGCCTG
GTTAGTGGCTCTTATTCTGCATGCGGCAGTGGGCTCCGCTACCGTGGCAATGATGGGGGCAACGGCAATTGTTGCACCCA
TGCTGCCGCTTTATCCCGACATCAGCCCGGAAATTATTGCGATTGCTATCGGTTCAGGTGCAATTGGCTGCACGATCGTT
ACGGACTCGCTTTTCTGGCTAGTGAAGCAATATTGCGGCGCTACGCTCAATGAAACATTTAAATACTATACGACAGCGAC
ATTTATCGCTTCAGTCGTCGCTCTGGCGGGCACATTCCTGCTGTCATTTATCATCTAA
>Cluster_29086
ATGACGCACGATAATATCGATATTCTGGTGGTGGATGATGACATTAGCCACTGCACTATTTTGCAGGCTTTGCTGCGCGG
CTGGGGCTATAACGTCGCGCTGGCGAACAGCGGGCGACAGGCGCTGGAGCAGGTGCGGGAACAGGTTTTTGATCTTGTGC
TTTGCGATGTGCGAATGGCAGAGATGGACGGCATCGCCACGCTCAAAGAGATCAATGCGTTAAATCCGGCAATTCCGGTA
CTGATTATGACCGCGTACTCCAGCGTCGAGACAGCGGTAGAGGCGTTGAAAACCGGGGCGCTGGATTATCTCATCAAGCC
GCTGGATTTCGATAACCTGCAGGCGACGCTGGAAAAGGCGCTCGCACATACGCACAGTGTTGACGCTGAAACGCCTGCGG
TGTCTGCCAGCCAGTTCAGTATGGTCGGTAAAAGCCCGGCGATGCAACACCTGCTCAGTGAAATCGCCCTCGTCGCGCCA
TCGGAAGCCACGGTGCTGATCCACGGCGATTCCGGCACCGGTAAAGAGCTGGTCGCCAGGGCGATTCACGCCAGTAGCGC
ACGGAGTGAAAAACCACTGGTAACGCTCAACTGTGCGGCACTCAACGAATCCTTGCTGGAATCTGAATTGTTCGGTCACG
AAAAAGGGGCGTTTACCGGAGCCGACAAACGGCGGGAAGGGCGCTTTGTTGAGGCGGACGGCGGCACGCTGTTTCTCGAT
GAAATTGGCGATATCTCGCCGATGATGCAGGTTCGTCTGCTGCGTGCGATTCAGGAGCGCGAAGTTCAGCGTGTCGGCAG
CAACCAGACCATCTCGGTTGATGTCCGGCTGATTGCGGCGACTCATCGCGATCTTGCCGCAGAGGTGAATGCCGGGCGTT
TTCGCCAGGATCTCTACTATCGCCTGAACGTGGTGGCGATTGAAGTTCCGTCGCTGCGTCAGCGGCGGGAAGATATTCCT
CTGCTGGCTGGCCATTTTCTTCAGCGCTTTGCCGAACGTAATCGCAAGGCGGTAAAAGGCTTTACGCCCCAGGCGATGGA
TCTGCTGATTCATTACGACTGGCCGGGAAATATTCGTGAACTGGAAAACGCGGTGGAACGGGCGGTGGTGCTGCTGACCG
GGGAATATATTTCCGAACGCGAGCTGCCGCTGGCGATTGCCAGTACGCCGATCCCGCCGGTACAAAGTCAGGATATTCAG
CCGCTGGTGGAAGTCGAAAAAGAAGTGATTCTGGCGGCACTGGAGAAAACGGGCGGCAACAAAACCGAAGCCGCCCGTCA
GTTAGGGATCACGCGTAAAACGCTATTGGCAAAACTGTCGCGTTAG
>Cluster_60763
ATGTTGATTCTGAAGAATGTCACTGCGGTACAGCTACACCCGGCAAAAGTGCAGGAAGGCGTTGATATCGCCATCGAAAA
CGATGTGATTGTCGCTATCGGCGATGCCCTGACGCAACGCTACCCCGACGCCAGCTTCAAAGAGATGCATGGCCGGATTG
TGATGCCAGGGATTGTCTGCTCGCACAACCATTTTTACTCGGGGCTTTCCCGCGGAATTATGGCAAACATCGCCCCTTGC
CCGGATTTCATCTCAACGCTGAAAAATCTCTGGTGGCGGCTCGATCGCGCCCTTGATGAAGAGTCGCTCTATTACAGCGG
ACTGATTTGTTCCCTGGAAGCGATTAAGAGCGGATGTACATCGGTTATCGATCACCATGCCTCTCCGGCGTATATCGGCG
GGTCGCTCTCCACATTGCGCGACGCATTTTTAAAAGTTGGCCTGCGCGCGATGACCTGTTTTGAAACTACTGACCGTAAC
AACGGCATCAAAGAGTTGCAGGAAGGTGTAGAAGAAAACATCCGTTTCGCCCGTTTGATTGATGAGGCGAAGAAAGCGAC
AAGCGAGCCGTATCTGGTGGAAGCACATATCGGTGCTCACGCGCCGTTTACCGTGCCGGATGCCGGTCTGGAGATGCTGC
GTGAAGCCGTGAAAGCCACAGGCCGTGGTTTGCATATTCACGCTGCGGAAGACCTTTACGACGTTTCCTACAGTCACCAC

TGGTACGGCAAAGACCTGCTGGCACGACTGGCGCAATTCGATCTCATCGACAGCAAAACGCTGGTCGCTCATGGGCTGTA
CTTGTCGAAAGATGACATCACCCTACTCAATCAGCGCGATGCGTTCCTGGTGCATAACGCCCGTTCAAACATGAACAACC
ATGTCGGCTACAACCATCACCTTAGCGACATCCGCAATCTGGCGTTGGGAACGGACGGCATTGGTTCGGACATGTTTGAA
GAGATGAAATTTGCCTTCTTTAAACATCGCGATGCGGGTGGTCCGCTGTGGCCTGACAGTTTTGCCAAAGCCCTGACTAA
CGGTAACGAACTGATGAGCCGCAACTTTGGCGCGAAATTTGGGCTTCTGGAAGCCGGTTACAAAGCTGATTTAACCATTT
GCGATTACAACTCGCCGACGCCGCTGCTGGCAGACAATATCGCCGGGCATATCGCTTTCGGTATGGGCTCAGGCAGCGTT
CACAGCGTGATGGTCAATGGTGTGATGGTCTATGAAGACCGTCAGTTTAACTTCGATTGCGATTCCATTTATGCACAAGC
CAGAAAAGCCGCTGCCAGTATGTGGCGTCGGATGGATGCGCTGGCATAA
>Cluster_22161
ATGTCGCATAACGTTACTCCAAACACCTCTCGCGTGGAATTGCGTAAAACGCTTACGTTAGTTCCGGTTGTAATGATGGG
TCTAGCCTATATGCAGCCGATGACGCTGTTTGATACATTTGGTATCGTTTCAGGCCTCACGGATGGTCATGTGCCGACAG
CCTATGCGTTCGCATTGATTGCGATCCTGTTTACGGCTCTGAGCTACGGGAAGCTGGTTCGCCGCTATCCTTCTGCTGGC
TCTGCATACACTTACGCCCAGAAATCCATTAGCCCGACTGTCGGCTTTATGGTGGGTTGGTCTTCTCTGCTCGACTATCT
GTTCGCGCCGATGATCAACATTCTGCTGGCGAAAATCTATTTTGAAGCTCTGGTGCCTTCCATTCCATCGTGGATGTTTG
TGGTGGCGCTGGTGGCCTTTATGACCGCCTTTAACCTGCGTAGTCTGAAATCCGTAGCGAACTTCAACACTGTAATCGTC
GTGTTGCAGATAGTGCTGATCGCGGTGATTCTGGGCATGGTTGTTTATGGCGTATTTGAAGGTGAAGGCGCTGGTACGCT
GGCGAGCACTCGTCCGTTCTGGTCTGGCGATGCGCATGTTATCCCGATGATTACCGGGGCGACAATCCTGTGCTTCTCCT
TTACCGGCTTTGACGGCATCAGCAACCTGTCGGAAGAAACCAAAGATGCAGAGCGCGTGATCCCGCGTGCGATTTTCCTG
ACCGCGCTGATCGGCGGCATGATCTTCATCTTTGCAACTTACTTCCTGCAGCTGTACTTCCCGGATATCTCTCGCTTTAA
AGATCCGGATGCGTCACAGCCTGAAATCATGCTGTACGTTGCGGGTAAAGCGTTCCAGGTAGGCGCGCTGATCTTCTCCA
CCATTACCGTACTGGCGTCCGGTATGGCGGCGCACGCAGGCGTAGCGCGTCTGATGTACGTAATGGGTCGTGACGGCGTG
TTCCCGAAAAGCTTCTTCGGTTATGTACACCCGAAATGGCGTACTCCGGCGATGAACATCATCCTGGTTGGCGCGATTGC
CCTGCTGGCAATCAACTTCGACCTGGTAATGGCTACGGCGCTGATTAACTTTGGTGCGTTGGTGGCGTTCACCTTCGTTA
ACCTGTCGGTCATCTCGCAGTTCTGGATCCGTGAGAAGCGTAACAAGACGCTGAAAGATCACTTCCAGTATCTGTTCCTG
CCGATGTGTGGTGCGCTGACCGTTGGTGCGCTGTGGGTTAACCTGGAAGAAAGCTCAATGGTTCTGGGTCTGATCTGGGC
TGCTATCGGTCTGATTTACCTGGCTTGCGTAACCAAGAGTTTCCGTAACCCGGTTCCGCAGTACGAAGACGTAGCGTAA
>Cluster_25196
ATGAACGATTATAAAATGACGCCAGGTGAGCGGCGCGCGACCTGGGGTTTAGGGACCGTATTTTCATTACGCATGCTGGG
CATGTTCATGGTTCTGCCGGTTCTGACCACGTACGGCATGGCTCTGCAAGGTGCCAGCGAAGCATTAATCGGTATTGCCA
TTGGTATTTATGGTCTGACTCAGGCCGTTTTTCAGATTCCGTTTGGCCTGCTTTCAGACCGCATTGGTCGCAAACCATTA
ATTGTCGGTGGGCTGGCGGTGTTTGCCGCCGGTAGCGTTATCGCTGCGCTCTCTGACTCCATCTGGGGAATTATTCTGGG
CCGGGCGCTACAAGGCTCCGGTGCGATTGCTGCTGCCCGTTATGGCGCTGCTTTCCGATCTCACGCGCGAACAAAACCGCA
CCAAAGCGATGGCGTTTATCGGCGTGAGCTTTGGCATTACCTTTGCCATTGCGATGGTGCTTGGCCCGATCATCACTCAC
AAACTTGGGCTGCACGCGCTGTTCTGGATGATCGCTATTCTGGCAACAACCGGCATTGCGTTGACCATTTGGGTTGTGCC
CAACAGTAGCACTCACGTACTTAATCGTGAGTCCGGAATGGTGAAAGGCAGTTTCAGTAAAGTGCTGGCGGAACCGCGGC
TGCTGAAACTCAACTTTGGCATTATGTGTCTGCATATTTTGCTGATGTCGACCTTTGTTGCCCTGCCCGGACAGATGGCT
GATGCGGGGTTACCGGCGGCTGAACACTGGAAGGTCTACCTGGCAACGATGTTAATCGCCTTTGGCTCGGTCGTGCCTTT
CATTATCTACGCTGAAGTTAAGCGCAAAATGAAGCAAGTCTTTGTCTTTTGCGTCGGGTTGATCGTGGTTGCGGAAATTG
TGTTGTGGAACGCACAAACGCAGTTCTGGCAACTGGTGGTCGGCGTGCAGCTTTTCTTTGTGGCGTTTAATTTGATGGAA
GCCCTTCTGCCTTCACTTATCAGTAAAGAGTCGCCAGCAGGTTACAAAGGTACGGCGATGGGTGTTTACTCCACCAGCCA
GTTTCTTGGCGTGGCGATTGGCGGTTCGCTGGGTGGCTGGATTGACGGCATGTTTGACGGTCAGGGCGTATTTCTCGCTG
GCGCAATGCTGGCCGCAGTGTGGCTGGCAGTCGCCAGTACCATGAAAGAACCGCCGTATGTCAGCAGCTTGCGCATTGAA
ATCCCGGCGAACATTGCCGCAAACGAGGCGTTAAAAGTGCGTTTGCTGGAAACTGAAGGCATCAAAGAAGTGTTGATTGC
AGAAGAAGAACATTCAGCTTATGTGAAAATCGACAGCAAAGTGACGAATCGCTTTGAGGTTGAACAGGCAATTCGCCAGG
CATAA
>Cluster_43346
ATGTTAAACAGTATTTTAGTCATACTCTGTTTGATCGCTGTAAGTGCGTTCTTCTCGATGTCCGAGATCTCGCTTGCCGC
CTCACGCAAAATCAAACTTAAACTGCTGGCTGATGAAGGCAATATAAATGCCCAACGCGTTCTGAATATGCAGGAAAATC
CCGGCATGTTCTTTACCGTGGTCCAAATCGGTCTGAACGCAGTGGCGATTCTCGGCGGTATCGTCGGTGATGCGGCATTT
TCTCCAGCTTTTCACAGCCTGTTCTCCCGCTATATGTCGGCAGAACTCTCTGAGCAACTGAGCTTTATTCTCTCTTTCTC
GTTAGTGACTGGCATGTTTATCCTGTTTGCGGATTTAACCCCGAAACGCATCGGTATGATTGCGCCAGAAGCTGTGGCTT
TGCGTATCATCAACCCGATGCGCTTCTGCCTGTACGTTTGCACCCCGCTGGTGTGGTTCTTCAACGGACTGGCGAACATA
ATCTTCCGTATTTTCAAACTGCCAATGGTACGTAAAGATGACATCACTTCTGATGACATCTACGCGGTAGTGGAAGCCGG
TGCGCTGGCGGGCGTGTTACGTAAACAGGAACACGAGCTGATTGAAAACGTCTTTGAGCTGGAATCCCGTACCGTTCCGT
CTTCAATGACACCGCGTGAAAACGTGATTTGGTTTGATCTCCACGAAGATGAGCAAAGTCTGAAGAATAAGGTGGCGGAA
CATCCGCACTCTAAGTTCCTCGTCTGTAATGAAGATATTGACCACATCATCGGCTATGTCGATTCTAAAGACCTGCTGAA
CCGCGTGCTGGCTAACCAAAGCCTGGCACTGAACAGCGGCGTACAAATTCGCAATACGCTGATTGTGCCGGATACGTTAA
CCCTTTCAGAGGCGTTGGAAAGTTTTAAAACCGCAGGTGAAGACTTCGCGGTGATCATGAATGAGTACGCACTGGTAGTG
GGGATCATCACCCTCAACGACGTGATGACCACGCTGATGGGCGATCTGGTCGGTCAGGGGCTGGAAGAGCAGATTGTCGC
CCGTGATGAGAACTCATGGCTGATAGATGGCGGCACTCCGATTGACGACGTCATGCGCGTGCTGGATATTGACGAGTTCC
CGCAGTCGGGCAACTACGAAACCATCGGCGGCTTTATGATGTTTATGCTGCGTAAGATCCCGAAACGCACCGATTCGGTG
AAATTCGCCGGCTACAAATTTGAAGTGGTGGATATCGATAACTACCGCATCGACCAGCTGCTGGTGACCCGAATCGACAG
CAAAGCCACCGCCCTTTCGCCAAAACTGCCTGACGCTAAAGATAAAGAAGAAAGCGTCGCATAA
>Cluster_27998
TTGGCGGGTTTAAACATCAAACATTGGTTTGCAGATGGCGCGTTTCGCACCATTATTCGCAATAGCGCCTGGTTAGGCTC
CAGTAATGTCGTGAGCGCTTTGCTGGGTCTGTTGGCGCTCTCGTATGCCGGTAAAGGGATGACGCCCGCCATGTTTGGCG
TACTGGTAATTGTGCAATCGTACACCAAGTCGATCAGCGATTTTATTAAGTTTCAGACATGGCAGCTGGTGGTTCAGTAC
GGAACGCCGGCCTTGACCAACAATAATCCGCAGCAATTTCGCAATGTTGTCTCATTTTCCTTCTCGCTGGATATCGTCAG
CGGCGCGGTGGCGATTGTCGGCGGCATTGCCTTACTGCCATTCCTTTCCCATTCATTAGGTCTGGATGACCAAAGCTTTT
GGCTGGCGGCGCTCTATTGCACGCTCATTCCTTCAATGGCTTCCTCCACGCCGACCGGCATTCTGCGTGCGGTAGATCGC
TTCGATCTAATTGCTGTACAGCAGGCGACGAAACCTTTTCTGCGCGCAGCGGGGAGCGTCGTAGCCTGGTATTTTGACTT
TGGTTTTGCGGGTTTTGTTATTGCCTGGTACGTGTCGAATCTGATTGGCGGCACCATGTACTGGTGGTTTGCCGCGCGCG
AATTACGCCGCAGAAATATCCATAACGCCTTCAAATTGAATCTGTTTGAGTCTGCCCGACACATTAAAGGCGCGTGGAGT
TTTGTCTGGTCAACCAACATTGCCCACTCCATCTGGTCGGCGCGTAACTCGTGCAGCACAGTGTTGGTGGGGATCGTGTT
AGGACCCGCTGCCGCCGGGTTATTTAAAATCGCCATGACATTCTTCGATGCCGCCGGAACGCCAGCGGGTTTACTGGGCA
AAAGTTTTTACCCAGAGGTGATGCGTTTAGATCCACGCACCACCAGACCGTGGTTGTTAGGCGTGAAGTCCGGTTTACTG
GCGGGCGGAATCGGTATTCTGGTGGCGCTCGCGGTGTTGATCGTCGGCAAGCCGCTCATTTCGCTGGTGTTTGGCGTTAA
GTATCTCGAAGCGTATGACCTGATTCAGGTGATGCTGGGCGCAATTGTGATTTCGATGCTGGGCTTCCCACAGGAGTCAT
TGCTATTGATGGCGGGAAAACAGCGCGCTTTTCTCGTGGCGCAAACCATCGCCTCAATCGGCTACATCGTATTGTTGTTC

ATGTTCTGTCATCTGTTTGGCGTGCTGGGGGCTGCATTTGCTTACTTCGGTGGGCAATGTCTGGATGTGGTGTTCTCGCT
AATTCCGACCTTAAAGGCATTTTTTCAGCGCCATTCCTTGCTTTATAACTCAACCGGAGAGAAATCCTGA
>Cluster_62596
ATGGAAACTTTCCTTTCCCTTCGTCATATCAACAAAACGTTTCATGCCACGCGAGCGCTGCGCGATGTTTCTCTCGACTT
TATGTCGGGTGAAGTGCACTGCCTTGCCGGGCAAAACGGCTGTGGTAAATCGACGTTGATTAAAATTATGTCCGGTGTTT
ATCGCCCGGATGAGGGAGCGGAAATTACGCTTGGTGGGAAAAACTGGTCAAAGCTGACACCCGCCGCTTCGGTGGCGCAG
GGGATTCAGGTGATTTATCAGGACCTCTCTTTATTTCCTAACCTGAGTGTCTGGGAGAATATCGCCGTGAATCACTATCA
CCACGGTCTGTTTGTTAACCGCCGTCGTCTGCGTGAGGTGGCGCAGGCGGCAATGACCAGTATTAACGTCACATTGCCAC
TGGATACACTCGTTTCTGAGCTTTCAATTGCCCGCTGTCAGCTGGTGGCTATTTGCCGTGCACTGGCGCAGGACGCACGG
TTGATCGTGATGGATGAACCAACCGCTTCGCTGACCCATCAGGAAGTGCAAGGGTTATTACAGGTGGTGCATCAATTGCG
TGAACGCGGGATCTGCGTGGTCTTCGTCAGTCATCGTCTGGAAGAAGTGATGGAAGTTTCCGACCGTATTTCAGTGCTGA
AAGATGGTGAGCTGGTCGGGACTTTTCCGGCTGCAGAGATGACCACAAAACAACTCGGTTTCCTGATGACCGGCCAGGAG
TTTGAATATCAGGTGCGAGAGTTGTGGCAGGGAAAATCCAGCACCCCGGTGCTGGAGGTGCGTAATTTAAGTCGTCATGG
GGAATATCTAAATATTAACTTGCGGGTGGAAGCGGGCGAAGTGGTATCGATTGTTGGCCTGCTTGGTGCGGGGCGCACGG
AATTATGTCTGAGTCTGTTTGGTATGACCCGACCGGATGCTGGCGAGATCCTCATCAACGGCCAGCCGGTTACACTGCAT
AGTAACCAGGACGCTATCCGCCACGGTATTGGTTATGTTTCGGAAGATCGCATGTCGCGCGGTTTGGTGATGGCGCAATC
TATTGAAGACAACATCATTAGCACTGTTTTTCACAAGGTAAAAGATCGCTTCGGCTTTCTGAGTGAAACTAAAGTTCGTG
ACCTGGTGGACAGGCTAATCAAGGCATTGACCATCAAAGTCTCTGATCCTCATCTTCCGGTGAATACGCTTTCTGGTGGT
AACGCGCAGCGAGTATCGATCGCCAAATGGCTGGCGATTGGGCCACGACTGTTGATTCTCGATAGCCCGACAGTCGGTGT
TGATATCGCCAACAAAGCAGGAATATACGGCATTATCAGCGATCTGGCGGCTCATGGCATTGCGGTATTGATGATCTGCG
ATGAAATTGAAGAAGCGTGGTATCAAAGCCACCGGATTTTGGTAATGCAAAAAGGTCAGATCACCCATAGCTTCCTGCCT
GACAGCAGTTCTCAGGCCAGGATTGCGGAGGTGGTAAATGGCTGA
>Cluster_42175
ATGCAATACATCAAGATCCATGCGCTGGATAACGTCGCGGTTGCTTTAGCGGATTTGGCTGAAGGCACAGAAGTCAGTGT
CGATAACCAGACTGTTACGCTGCGCCAGGATGTTGCTCGTGGACATAAATTTGCGTTAACGGATATCGCAAAAGGGGCCA
ACGTCATTAAATATGGCCTGCCGATTGGTTATGCATTGGCGGATATTGCGGCGGGGGAACACGTTCACGCCCACAATACG
CGCACGAATCTGAGCGATCTGGATCAGTATCGCTATCAACCTGATTTTCAGGATCTGCCTGCGCAAGCGGCAGATCGTGA
AGTGCAGATCTATCGTCGCGCGAACGGCGATGTCGGGGTGCGTAATGAGCTGTGGATCCTGCCAACCGTGGGCTGTGTCA
ACGGCATCGCGCGGCAGATCCAAAATCGTTTCCTGAAAGAGACCAACAACGCTGAAGGTACCGACGGCGTGTTCCTCTTC
AGCCACACCTACGGCTGCTCACAACTGGGCGACGATCACATCAATACTCGCACCATGCTGCAAAACATGGTGCGCCACCC
GAACGCGGGCGCAGTGCTGGTGATTGGTCTGGGCTGTGAAAACAACCAGGTTGCCGCATTCCGTGAAACGTTGGGCGATA
TCGATCCTGAACGCGTTCATTTCATGATCTGCCAACAGCAGGATGATGAAATCGAGGCCGGGATCGCGCATTTGCATCAG
TTGTATAACGTGATGCGCAACGACAAGCGCGAGCCGGGCAAACTTAGCGAACTGAAGTTTGGTCTGGAGTGCGGCGGTTC
TGACGGTCTTTCCGGTATTACCGCCAACCCAATGCTCGGTCGTTTCTCTGACTACGTGATTGCTAACGGTGGTACTACCG
TACTGACCGAAGTGCCGGAGATGTTTGGTGCAGAGCAGTTGCTGATGGACCATTGCCGTGACGAAGCAACGTTTGAAAAA
CTGGTCACCATGGTCAACGACTTCAAACAGTACTTTATTGCCCATGACCAGCCGATCTACGAAAACCCATCACCGGGGAA
CAAAGCGGGCGGTATCACTACGTTGGAAGACAAATCCCTCGGCTGTACCCAGAAAGCGGGTTCCAGTGTCGTGGTTGACG
TGCTGCGTTACGGCGAGCGTCTGAAAACGCCGGGGCTGAACTTGTTAAGTGCGCCGGGTAACGATGCCGTAGCGACCAGC
GCCCTGGCGGGTGCGGGCTGCCATATGGTGCTGTTCAGTACTGGTCGTGGCACGCCGTATGGTGGATTTGTGCCGACGGT
GAAAATCGCCACCAACAGTGAACTGGCGGCGAAGAAAAAACACTGGATCGACTTTGACGCGGGTCAGCTGATCCACGGTA
AAGCGATGCCGCAGTTGCTGGAAGAATTTATCGACACCATCGTTGAGTTTGCCAATGGTAAACAAACCTGCAACGAGCGT
AACGACTTCCGCGAACTGGCGATCTTCAAAAGCGGCGTAACGCTATAA
>Cluster_22196
ATGAGCCTACGTGAAAAAACCATCAGCGGCGCGAAGTGGTCGGCGATTGCTACGGTGATCATCATCGGCCTCGGGCTGGT
GCAGATTACTGTGCTGGCGCGGATTATCGACAACCATCAGTTCGGTTTGCTTACCGTGTCGCTGGTGATTATCGCGCTGG
CAGATACGCTTTCTGACTTCGGTATCGCTAACTCCATTATTCAGCGAAAAGAAATCAGTCACCTTGAACTCACCACGTTG
TACTGGCTGAACGTCGGGCTGGGGATCGTGGTGTGCGTGGCGGTGTTTTTGTTGAGTGATGCCATTGGTGACGTGCTGAA
TAACCCGGACCTGGCACCGTTGATTAAAACATTGTCGCTGGCGTTTGTGGTGATCCCCCACGGGCAGCAGTTCCGCGCGT
TGATGCAAAAGGAGCTGGAGTTCAACAAAATTGGCATGATCGAAACCAGCGCGGTGCTGGCGGGCTTTACTTTTACGGTG
GTTAGCGCCCATTTCTGGCCGCTGGCGATGACCGCGATCCTCGGTTATCTGGTCAATAGTGCGGTAAGAACGCTGCTGTT
TGGCTACTTTGGCCGCAAAATTTATCGCCCCGGTCTGCATTTCTCGCTGGCGTCGGTGGCACCGAACTTACGCTTTGGTG
CCTGGCTGACGGCGGACAGCATCATCAACTATCTCAACACCAACCTTTCAACGCTGGTGCTGGCACGTATTCTCGGCGCG
GGCGTGGCTGGGGGATACAACCTGGCGTACAACGTGGCCGTTGTGCCACCGATGAAGCTGAACCCAATCATCACCCGCGT
GTTGTTTCCGGCATTCGCCAAAATTCAGGACGACACCGAAAAGCTGCGCGTTAACTTCTACAAGCTGTTGTCGGTAGTGG
GGATTATCAACTTTCCGGCGCTGCTCGGGCTAATGGTGGTGTCGAATAACTTTGTACCGCTGGTATTTGGCGAAAAGTGG
AACAGCATTATTCCGGTGCTGCAATTGCTGTGTGTGGTGGGTCTGCTGCGTTCGGTGGGTAACCCGATTGGTTCGCTGCT
GATGGCGAAAGCGCGGGTCGATATCAGCTTTAAATTCAACGTATTCAAAACCTTTCTGTTTATTCCGGCGATTGTTATTG
GTGGGCAGATGGCGGGCGCAATCGGCGTCACGCTCGGCTTCCTGCTGGTGCAGATTATCAACACCATTCTGAGTTACTTC
GTGATGATTAAACCGGTTCTTGGTTCCAGTTATCGCCAGTACATCCTGAGTTTATGGCTGCCGTTTTATCTCTCGCTGCC
GACGCTGGTGGTCAGTTATGCGCTGGGCATTGTGCTGAAAGGGCAACTGGCGCTGGGGATGCTGCTGGCGGTGCAAATAG
CCACGGGTGTGCTGGCGTTTGTGGTGATGATTGTGCTGTCGCGCCATCCGCTGGTGGTGGAAGTGAAGCGTCAGTTTTGT
CGCAGCGAAAAAATGAAAATGCTTTTACGGGCGGGGTGA
>Cluster_6015
ATGAGCACAAAATTTTATACCCTGCTGACGGATATTGGCGCGGCGAAACTTGCCAGCGCCGCCGCGCTCGGTGTGCCTTT
AAAAATTACCCATATGGCGGTCGGCGATGGCGGAGGGACATTGCCAACGCCGGACGCAAAGCAGACTGCACTGGTAAATG
AGAAACGCCGGGCTGCGCTGAATATGCTCTATATCGACCCGCAGAACAGCAGCCAGATTATTGCTGAACAGGTGATCCCT
GAAAACGAGGGCGGTTGGTGGATACGTGAAGTGGGCCTGTTTGATGAGTCCGGAGCATTGATTGCCGTGGGCAACTGCCC
GGAAAGCTATAAGCCGCAACTGGCTGAAGGCAGCGGGCGCACCCAGACCGTGCGCATGGTGCTGATTACCAGCAGCACGG
ACAATATCACCCTGAAAATCGACCCTGCCGTAGTGCTGGCAACCCGCAAGTATGTGGATGACAAGGTACTGGAGCTGAAA
GTGTACGTGGATGACCTGATGGCAAAACATCTTGCCGCACAGGACCCACATTCACAGTATGCGCCAAAAGCCAGCCCGAC
ATTTACCGGAACCCCCAAAGCGCCAACGCCAGCGGCAGGGAATAACACCACGCAGGTTGCGACCACCGCGTTTGTTCAGG
CGGCACTGACGGCCCTTATTAATGGTGCGCCAGCCACGCTGGACACGCTAAAAGAAATAGCCGCAGCCATTAACAATGAT
CCGAATTTCAGTACCACCATTAACAATGCGCTGGCACTGAAAGCGCCGTTGTCGAGTCCGGCACTCACCGGAACGCCAAC
AGCACCTACTGCGGCACAGTCGGTCAACAATACACAGATTGCCACCACGGCTTTTGTGAAATCGGCGATTGCGGCAATGG
TGGGTTCTGCACCTGCTGCACTGGATACACTGAACGAACTGGCGGCGGCACTGGGGAATGATCCGAACTTTGCCACGACA
ATGCTTAATGCGCTGGCAGGTAAACAACCGCTGGACAATACGCTGACTAATTTGAGTGGAAAGGATGTAGCTGGTCTTCT
CGCATACCTTCAATTGGGAGAAGCGGCAAAACGGGATGTCGGAACCGGTAATAATCAACTCCCGGACATGTCCGCATTTG
GTATGTCGCGAAACGGACAAACTGCCTGGGATATTCTCCCAAATGGTATGATTCGACAGGCTGGCACTGTGACACTGACA
CCAGTTGGTAACTTCAACGCGCAGGTGCTTGGTGGGGTGACGTACTACACCCATTACTACAGGGTTAATTTCCCCAGACA

ATTCCCAAATGCACAAATAGCAACGCTGGCAACACTAGCGAGCTCAGAGTTTTCGAAACAGACAACAATGGCCGGAAAAT
CACTGTCAACGCATCGCGATACTGATTCTGGCGCTGACGTATCAAGAACGCGATTCACTGTGGCGTATACGACACCCAAT
CTCGGCGAAACACCGACACTGCATTTTGAAGCAATAGGATACTAA
>Cluster_27897
ATGTCTGAACAACACGCACAGGGCGCTGACGCGGTAGTCGATCTTAACAATGAACTGAAAACGCGTCGTGAGAAGCTGGC
GAACCTGCGCGAGCAGGGGATTGCCTTCCCGAACGATTTCCGTCGCGATCATACCTCTGACCAATTGCACGCAGAATTCG
ACGGTAAAGAGAACGAAGAACTGGAAGCGCTGAACATCGAAGTCGCCGTTGCTGGCCGCATGATGACCCGCCGTATTATG
GGTAAAGCGTCTTTCGTTACCCTGCAGGATGTGGGTGGCCGCATTCAGCTGTACGTTGCCCGTGACGATCTGCTGGAAGG
CGTTTACAACGAGCAGTTCAAAAAATGGGACCTCGGCGATATCCTCGGCGCGAAAGGTAAACTGTTCAAAACCAAAACTG
GCGAACTGTCTATCCACTGTACCGAGCTGCGTCTGCTGACCAAAGCACTGCGTCCGCTGCCGGATAAATTCCACGGCTTG
CAGGATCAGGAAGCGCGCTATCGTCAGCGTTATCTGGATCTCATCTCTAACGATGAATCCCGCAACACCTTTAAAGTGCG
CTCGCAGATCCTCTCTGGTATTCGCCAGTTCATGGTGAACCGCGGCTTTATGGAAGTTGAAACGCCGATGATGCAGGTGA
TCCCAGGCGGTGCCGCTGCGCGTCCGTTTATCACTCACCATAACGCGCTGGATCTCGACATGTACCTGCGTATCGCGCCG
GAACTGTACCTCAAGCGTCTGGTGGTCGGTGGCTTCGAGCGTGTATTCGAAATCAACCGTAACTTCCGTAACGAAGGTAT
TTCCGTACGTCATAACCCAGAGTTCACCATGATGGAACTCTATATGGCTTACGCAGATTACAAAGATCTGATCGAGCTGA
CCGAATCGCTGTTCCGTACTCTGGCACAGGATATTCTCGGTAAGACGGAAGTGACCTACGGCGACGTAACGCTGGACTTC
GGTAAACCGTTCGAAAAACTGACCATGCGTGAGGCTATCAAGAAATATCGCCCGGAAACCGACATGGCGGATCTGGATAA
CTTCGACTCTGCGAAAGCGATTGCTGAATCTATCGGCATCCACGTTGAGAAGAGCTGGGGTCTGGGCCGTATCGTTACCG
AGATCTTCGAAGAAGTGGCAGAAGCACATCTGATTCAGCCGACCTTCATTACTGAATATCCGGCAGAAGTTTCTCCGTTG
GCGCGTCGTAACGACGTTAACCCGGAAATCACAGACCGCTTTGAGTTCTTCATTGGTGGGCGTGAAATCGGTAACGGCTT
TAGCGAGCTGAATGACGCGGAAGATCAGGCGCAGCGCTTCCTGGATCAGGTTGCCGCGAAAGATGCAGGTGACGACGAAG
CGATGTTCTACGACGAAGATTATGTCACCGCACTGGAACATGGCTTACCGCCGACAGCAGGTCTGGGAATTGGTATCGAC
CGTATGGTAATGCTGTTCACCAACAGCCATACCATCCGCGACGTTATTCTGTTCCCGGCGATGCGTCCGGTAAAATAA
>Cluster_7513
ATGTTACTACCCTGGCTAATATTAATTCCCTTTATTGGCGGCTTCCTGTGCTGGCAGACCGAACGCTTTGGCGTCAAGGT
GCCACGCTGGATCGCGCTGATCACCATGGGATTGACGCTGGCGCTGTCGCTGCAACTGTGGTTGCAGGGCGGTTATTCAC
TGACGCAATCCGCCGGAATTCCGCAGTGGCAGTCTGAATTCGACATGCCGTGGATCCCGCGTTTTGGTATCTCTATTCAT
CTCGCCATTGACGGGCTGTCGCTGCTGATGGTCGTGCTGACCGGTCTGCTCGGTGTGCTGGCGGTACTATGTTCGTGGAA
AGAGATCGAAAAATATCAGGGCTTCTTCCACCTCAACCTGATGTGGATCCTGGGCGGCGTTATCGGCGTGTTCCTTGCCA
TCGACATGTTCCTGTTCTTCTTCTTCTGGGAAATGATGCTGGTGCCGATGTACTTCCTGATCGCACTGTGGGGGCATAAA
GCCTCTGACGGTAAAACGCGTATCACGGCGGCAACCAAGTTCTTCATTTACACCCAGGCGAGTGGCCTGGTGATGTTGAT
TGCCATCCTGGCGCTGGTTTTTGTTCACTACAATGCGACCGGCGTCTGGACCTTCAACTATGAAGAGCTGCTGAATACGC
CAATGTCCAGTGGTGTGGAATACCTGTTAATGCTGGGCTTCTTCATCGCCTTCGCAGTCAAAATGCCGGTGGTTCCGCTG
CATGGCTGGCTGCCGGATGCGCACTCCCAGGCTCCGACCGCCGGTTCCGTTGACCTCGCGGGGATCTTGCTGAAAACTGC
CGCTTACGGTTTGCTGCGTTTCTCCCTGCCGCTGTTCCCGAACGCGTCGGCAGAGTTCGCGCCAATTGCTATGTGGCTGG
GTGTTATCGGCATCTTCTACGGTGCGTGGATGGCCTTCGCCCAGACCGATATCAAACGTCTGATCGCCTACACCTCGGTT
TCCCACATGGGCTTCGTGCTGATTGCTATCTACACCGGCAGCCAGTTGGCCTACCAGGGCGCGGTAATCCAGATGATTGC
GCACGGTTTGTCGGCGGCGGGTCTGTTTATTCTTTGTGGTCAGCTTTATGAACGTATCCATACCCGCGACATGCGCATGA
TGGGCGGTCTGTGGAGCAAGATGAAATGGCTGCCAGCACTGTCGCTGTTCTTTGCGGTGGCAACGCTTGGGATGCCTGGC
ACCGGTAACTTCGTCGGCGAATTTATGATTCTGTTCGGCAGCTTCCAGGTTGTACCGGTGATTACCGTTATCTCTACCTT
TGGGCTGGTCTTTGCATCTGTTTATTCGCTGGCGATGTTACATCGCGCTTACTTCGGTAAAGCGAAAAGCCAGATTGCCA
GCCAGGAACTGCCAGGGATGTCGCTGCGTGAGCTGTTTATGATCCTGTTGCTGGTGGTGCTGCTGGTACTGCTGGGCTTC
TATCCGCAGCCGATTCTGGATACCTCGCACTCCGCGATTGGCAATATCCAGCAGTGGTTTGTTAATTCCGTTACTACTAC
AAGGCCGTAA
>Cluster_28476
ATGCTACATTTGTTTGCTGGCCTGGATTTGCATACCGGGCTGTTATTATTGCTTGCACTGGCTTTTGTGCTGTTCTACGA
AGCCATCAATGGTTTCCATGACACAGCCAACGCCGTGGCAACCGTTATCTATACCCGCGCGATGCGTTCTCAGCTCGCCG
TGGTTATGGCGGCGGTGTTCAACTTTTTGGGTGTTTTGCTGGGTGGTCTGAGTGTTGCCTATGCCATTGTGCATATGCTG
CCGACGGATCTGCTGCTTAATATGGGATCGTCTCATGGCCTTGCCATGGTGTTCTCTATGTTGCTGGCGGCGATTATCTG
GAACCTGGGTACCTGGTACTTTGGTTTACCTGCATCCAGCTCTCATACGCTGATTGGCGCGATCATCGGGATTGGTTTAA
CCAATGCGTTGATGACCGGGACGTCAGTGGTGGATGCACTCAATATCCCGAAAGTATTAAGTATTTTCGGTTCTCTGATC
GTTTCCCCTATTGTCGGCCTGGTGTTTGCTGGCGGTCTGATTTTCTTGCTGCGCCGCTACTGGAGCGGCACCAAGAAACG
CGCCCGTATCCACCTGACCCCAGCGGAGCGTGAAAAGAAAGACGGCAAGAAAAAGCCGCCGTTCTGGACGCGTATTGCGC
TGATCCTTTCCGCTATCGGCGTGGCGTTTTCGCACGGCGCGAACGATGGTCAGAAAGGCATTGGTCTGGTTATGTTGGTA
TTGATTGGCGTCGCACCAGCAGGCTTCGTGGTGAACATGAATGCCACTGGCTACGAAATCACCCGTACCCGTGATGCCAT
CAACAACGTCGAAGCTTACTTTGAGCAGCACCCTGCGCTGCTGAAACAGGCTACCGGTGCTGATCAGTTAGTACCGGCTC
CGGAAGCTGGCGCAACGCAACCTGCGGAGTTCCATTGCCATCCGTCGAATACCATTAACGCGCTCAACCGCCTGAAAGGC
ATGTTGACTACTGATGTGGAAAGCTACGACAAGCTGTCGCTTGATCAACGTAGCCAGATGCGCCGCATTATGCTGTGCGT
TTCTGACACTATCGATAAAGTGGTGAAGATGCCTGGTGTGAGTGCTGACGATCAGCGTCTGTTGAAGAAACTGAAGTCCG
ACATGCTTAGCACCATCGAGTATGCGCCGGTGTGGATCATCATGGCGGTCGCGCTGGCGTTAGGTATCGGTACGATGATT
GGCTGGCGTCGTGTGGCAACGACTATCGGTGAGAAAATCGGTAAGAAAGGCATGACCTACGCTCAGGGGATGTCTGCCCA
GATGACTGCGGCAGTGTCTATCGGCCTGGCGAGTTATACCGGGATGCCGGTTTCCACTACCCACGTACTCTCCTCTTCTG
TCGCGGGGACGATGGTGGTAGATGGCGGCGGCTTACAGCGTAAAACCGTAACCAGTATTCTGATGGCCTGGGTATTTACC
CTTCCGGCTGCGGTACTGCTTTCCGGCGGGCTGTACTGGCTCTCCTTGCAGTTCCTGTAA
>Cluster_20656
GTGAGACGTAATCTTTCACACATTATTGCCGCAGCATTCAATGAACCGCTGCTTCTGGAGCCCGCCTATGCGCGGGTTTT
CTTTTGCGCGCTCGGGCGCGAGATGGGGGCATCAAGTCTTTCGGTACCACAGCAGCAGGTACAGTTTGATGCTCCCGGAA
TGCTGGCTGAAACGGACGAGTACATGGCCGTAGGTAAACGACCGGCCCGTGTTTACCGGGTGGTGAACGGTATTGCTGTA
CTGCCGGTGACCGGCACGCTGGTGCACCGGCTGGGGGGTATGCGGCCATTTTCCGGAATGACAGGCTATGACGGCATTGT
CGCCTGTCTTCAGCAGGCAATGGCGGATAGCCAGGTGCGGGGCGTACTGCTGGACATTGACAGTCCGGGCGGGCAGGCCG
CCGGCGCGTTTGACTGCGCTGACATGATTTACCGCCTCCGTCAGCAGAAGCCGGTCTGGGCACTGTGCAATGACACGGCC
TGCTCTGCAGCCATGCTGCTGGCGTCGGCCTGCTCCCGACGGCTGGTTACCCAGACATCCCGTATCGGCTCCATTGGCGT
GATGATGAGCCATGTCAGCTATGCCGGTCATCTGGCGCAGGCCGGTGTTGATATCACGCTGATTTACTCAGGGGCGCACA
AGGTGGATGGCAATCAGTTTGAAGCCTTACCGGCAGAGGTTCGCCAGGACATGCAGCAGCGCATTGATGCGGCGCGCCGG
ATGTTTGCCGAAAAAGTGGCCATGTTTACCGGTCTGTCTGTTGATGCCGTCACGGGAACAGAGGCCGCCGTTTTTGAAGG
TCAGTCCGGCATTGATGCCGGGCTGGCGGATGAATTAGTCAATGCGTCGGATGCCATCAGTGTGATGGCCACGGCGCTGA
ACAGTAATGTCAGAGGAGGCACTATGCCGCAATTAACTGCAACGGAAGCCGCCGCGCAGGAGAACCAGCGAGTGATGGGG
ATCCTGACATGCCAGGAAGCGAAAGGACGTGAACAGCTTGCCACGATGCTGGCAGGACAACAGGGCATGAGCGTTGCACA
GGCCCGGGCGATTCTGGCCGCGGCGGCACCGCAGCAGCCGGTGGCATCCACGCAGAGTGAAGCCGATCGCATTATGGCGT

GTGAAGAAGCGAACGGTCGTGAACAACTGGCGGCAACGCTGGCGGCGATGCCGGAGATGACGGTGGAAAAAGCCCGCCCG
ATCCTGGCTGCTTCACCGCAGGCGGATGCCGGACCCTCACTCCGTGATCAGATCATGGCACTGGATGAGGCAAAAGGGGC
TGAGGCGCAGGCTGAACAGCTGGCTGCCTGCCCGGGAATGACTGTGGAGAGCGCCCGGGCTGTGCTGGCTGCGGGATCAG
GTAAGGCAGAACCGGTCTCTGCATCCACAACCGCCCTGTTTGAACGCATCATGGCGAACCATTCACCGGCAGCGGTACAG
GGTGGCGTGCCACAGACGTCAGCAGACGGTGATGCGGACGTGAAAATGCTCATGGCCATGCCATGA
>Cluster_36956
ATGGAAACTCAGTGGACAAGGATGACCGCCAATGAAGCGGCAGAAATTATCCAGCATAACGACATGGTGGCATTTAGCGG
CTTTACCCCGGCGGGTTCGCCGAAAGCCCTACCCACCGCGATTGCCCGCAGAGCTAACGAACAGCATGAGGCCAAAAAGC
CGTATCAAATTCGCCTTCTGACGGGTGCGTCAATCAGCGCCGCCGCTGACGATGTACTTTCTGACGCCGATGCTGTTTCC
TGGCGTGCGCCATATCAAACATCGTCCGGTTTACGTAAAAAGATCAATCAGGGCGCGGTGAGTTTCGTTGACCTGCATTT
GAGCGAAGTGGCGCAAATGGTCAATTACGGTTTCTTCGGCGACATTGATGTTGCCGTCATTGAAGCATCGGCACTGGCAC
CGGATGGTCGAGTCTGGTTAACCAGCGGGATCGGTAATGCGCCGACCTGGCTGCTGCGGGCGAAGAAAGTGATCATTGAA
CTCAATCACTATCACGATCCGCGCGTTGCAGAACTGGCGGATATTGTGATTCCTGGCGCGCCACCGCGGCGCAATAGCGT
GTCGATCTTCCATGCAATGGATCGCGTCGGTACCCGCTATGTGCAAATCGATCCGAAAAAGATTGTCGCCGTCGTGGAAA
CCAACTTGCCCGACGCCGGTAATATGCTGGATAAGCAAAATCCCATGTGCCAGCAGATTGCCGATAACGTGGTCACGTTC
TTATTGCAGGAAATGGCGCATGGGCGTATTCCGCCGGAATTTCTGCCGCTGCAAAGTGGCGTGGGCAATATCAATAATGC
GGTAATGGCGCGTCTGGGGGAAAACCCGGTAATTCCTCCGTTTATGATGTATTCGGAAGTGCTACAGGAATCGGTGGTGC
ATTTACTGGAAACCGGCAAAATCAGCGGGGCCAGCGCCTCCAGCCTGACAATCTCGGCCGATTCCCTGCGCAAGATTTAC
GACAATATGGATTACTTTGCCAGCCGCATTGTGTTGCGTCCGCAGGAGATTTCCAATAACCCGGAAATCATCCGTCGTCT
GGGCGTCATCGCTCTGAACGTCGGCCTGGAGTTTGATATTTACGGGCATGCCAACTCAACACACGTAGCCGGGGTCGATC
TGATGAACGGCATCGGCGGCAGCGGTGATTTTGAACGCAACGCGTATCTGTCGATCTTTATGGCCCCGTCGATTGCTAAA
GAAGGCAAGATCTCAACCGTCGTGCCAATGTGCAGCCATGTTGATCACAGCGAACACAGCGTCAAAGTGATCATCACCGA
ACAAGGGATCGCCGATCTGCGCGGTCTTTCCCCGCTTCAACGCGCCCGCACTATCATTGATAATTGTGCACATCCTATGT
ATCGGGATTATCTGCATCGCTATCTGGAAAATGCGCCTGGCGGACATATTCACCACGATCTTAGCCACGTCTTCGACTTA
CACCGTAATTTAATTGCAACCGGCTCGATGCTGGGTTAA
>Cluster_14531
ATGTCAAACAACATTCGTATCGAAGAAGATCTGTTGGGTACCAGGGAAGTTCCAGCTGATGCCTACTATGGTGTTCACAC
TCTGAGAGCGATTGAAAACTTCTATATCAGCAACAACAAAATCAGTGATATTCCTGAATTTGTTCGCGGTATGGTAATGG
TTAAAAAAGCCGCAGCTATGGCAAACAAAGAGCTGCAAACCATTCCTAAAAGTGTAGCGAATGCCATCATTGCCGCATGT
GATGAAGTCCTGAACAACGGAAAATGCATGGATCAGTTCCCGGTAGACGTCTACCAGGGCGGCGCAGGTACTTCCGTAAA
CATGAACACCAACGAAGTGCTGGCCAATATCGGTCTGGAACTGATGGGTCACCAGAAAGGTGAATATCAGTACCTGAACC
CGAACGACCATGTTAACAAATGTCAGTCCACTAACGACGCCTACCCGACCGGTTTCCGTATCGCAGTTTACTCTTCCCTG
ATTAAGCTGGTAGATGCGATTAACCAACTGCGTGAAGGCTTTGAACGTAAAGCTGTCGAATTCCAGGACATCCTGAAAAT
GGGTCGTACCCAGCTGCAGGACGCAGTACCGATGACCCTCGGTCAGGAATTCCGCGCTTTCAGCATCCTGCTGAAAGAAG
AAGTGAAAAATATCCAACGTACCGCTGAACTGCTGCTGGAAGTTAACCTTGGCGCAACAGCAATCGGTACTGGTCTGAAC
ACGCCGAAAGAGTACTCTCCGCTGGCAGTGAAAAAACTGGCTGAAGTCACTGGCTTCCCATGCGTACCGGCTGAAGACCT
GATCGAAGCGACCTCTGACTGCGGCGCTTATGTTATGGTTCACGGCGCGCTGAAACGCCTGGCTGTGAAGATGTCCAAAA
TCTGTAACGACCTGCGCTTGCTCTCTTCTGGTCCACGTGCCGGCCTGAACGAGATCAACCTGCCGGAACTGCAGGCGGGC
TCTTCCATCATGCCAGCTAAAGTAAACCCGGTTGTTCCGGAAGTGGTTAACCAGGTATGCTTCAAAGTCATCGGTAACGA
CACCACTGTTACCATGGCAGCAGAAGCAGGTCAGCTGCAGTTGAACGTTATGGAGCCGGTCATTGGCCAGGCCATGTTCG
AATCCGTTCACATTCTGACCAACGCTTGCTACAACCTGCTGGAAAAATGCATTAACGGCATCACTGCTAACAAAGAAGTG
TGCGAAGGTTACGTTTACAACTCTATCGGTATCGTTACTTACCTGAACCCGTTCATCGGTCACCACAACGGTGACATCGT
GGGTAAAATCTGTGCCGAAACCGGTAAGAGTGTACGTGAAGTCGTTCTGGAACGCGGTCTGTTGACTGAAGCGGAACTTG
ACGATATTTTCTCCGTACAGAATCTGATGCACCCGGCTTACAAAGCAAAACGCTATACTGATGAAAGCGAACAGTAA
>Cluster_25192
ATGAAGTTTATCATTAAATTGTTCCCGGAAATCACCATCAAAAGCCAATCTGTGCGCTTGCGCTTTATAAAAATCCTTAC
CGGGAACATTCGTAACGTTTTAAAGCACTATGATGAGACGCTCGCTGTCGTCCGCCACTGGGATAACATCGAAGTTCGCG
CAAAAGATGAAAACCAGCGTCTGGCTATTCGCGACGCCCTGACCCGTATTCCGGGTATCCACCATATTCTCGAAGTCGAA
GACGTGCCGTTTACCGACATGCACGATATTTTCGAGAAAGCGTTGGTTCAGTATCGCGATCAGCTGGAAGGGAAAACCTT
CTGCGTACGCGTGAAGCGCCGTGGCAAACATGATTTTAGCTCGATTGATGTGGAACGTTACGTCGGCGGCGGTTTAAATC
AGCATATTGAGTCCGCGCGCGTGAAGCTGACCAATCCGGATGTGACTGTCCATCTGGAAGTGGAAGACGATCGCCTCCTG
CTAATTAAAGGCCGCTACGAAGGTATTGGCGGTTTCCCGATCGGCACCCAGGAAGATGTGCTGTCGCTCATTTCTGGTGG
TTTCGACTCCGGCGTTTCCAGCTATATGTTGATGCGTCGCGGCTGCCGCGTGCATTACTGCTTCTTTAACCTTGGCGGCG
CGGCGCATGAAATTGGCGTGCGTCAGGTGGCGCATTATCTGTGGAACCGTTTTGGCAGCTCCCACCGCGTGCGTTTTGTC
GCCATTAATTTCGAACCGGTCGTCGGGGAAATTCTCGAGAAAATCGACGACGGTCAGATGGGCGTTATCCTCAAACGTAT
GATGGTGCGTGCCGCATCTAAAGTGGCTGAACGTTACGGCGTACAGGCGCTGGTCACCGGCGAAGCGCTCGGCCAGGTGT
CCAGCCAGACGCTGACCAACCTACGCCTGATTGATAACGTCTCCGATACGCTGATCCTGCGTCCGCTGATCTCTTACGAC
AAAGAGCACATCATCAACCTGGCTCGCCAGATTGGCACCGAAGACTTTGCCCGCACGATGCCGGAATATTGCGGCGTGAT
TTCCAAAAGCCCGACGGTGAAAGCGGTTAAATCGAAGATTGAAGCGGAAGAAGAGAAGTTCGACTTCAGCATTCTCGATA
AAGTGGTGGAGGAAGCGAATAACGTTGATATCCGCGAAATCGCCCAGCAGACCGAGCAGGAAGTGGTGGAAGTGGAAACC
GTCAATGGCTTCGGCCCGAACGACGTGATCCTCGATATCCGTTCTATCGATGAACAGGAAGATAAGCCACTGAAAGTCGA
AGGGATTGATGTGGTTTCTCTGCCGTTCTATAAACTGAGCACCAAATTTGGCGATCTCGACCAGAGCAAAACCTGGCTGT
TGTGGTGTGAGCGCGGGGTGATGAGCCGCCTGCAGGCGCTCTATCTGCGCGAGCAGGGCTTTAACAATGTGAAGGTGTAT
CGCCCGTAA
>Cluster_65203
ATGGCATTTGATGATTTACGCAGCTTTTTACAGGAGCTTGATGACCACGGCCAGTTACTGAAAATCAGCGAAGAAGTGAA
CGCCGAGCCGGATCTGGCAGCAGCAGCTAACGCCACCGGGCGTATCGGCGACGGCGCGCCCGCGCTGTGGTTTGATAATA
TTCGCGGCTTTACCGATGCCCGCGTGGCGATGAACACCATCGGTTCCTGGCAGAACCACGCGATTTCCCTCGGCCTGCCG
CCAAATGCCCCGGTTAAAAAGCAGATTGATGAGTTTATCCGCCGCTGGGATAACTTCCCGATTGCCCCGGAGCGCCGCGC
CAATCCAGCCTGGGCGCAGAACACCGTTGATGGCGACGAGATCAACCTGTTCGATATCCTGCCGCTGTTTCGTTTAAACG
ATGGCGATGGCGGTTTCTATCTCGACAAAGCGTGCGTGGTTTCCCGCGATCCGCTCGACCCGGATAACTTCGGCAAGCAG
AACGTCGGCATCTACCGCATGGAAGTGAAGGGCAAGCGTAAGCTCGGCCTGCAACCGGTGCCGATGCACGATATCGCCCT
GCATCTGCATAAAGCAGAAGAGCGCGGTGAAGATCTGCCGATTGCGATCACGCTCGGTAACGATCCGATCATCACGCTGA
TGGGGGCCACGCCGCTGAAATATGATCAGTCCGAGTACGAAATGGCAGGCGCGCTGCGTGAAAGCCCGTACCCGATCGCC
ACCGCCCCGTTGACCGGTTTTGATGTGCCGTGGGGTTCAGAAGTGATCCTCGAAGGGGTCATCGAAAGCCGTAAACGCGA
AATCGAAGGGCCGTTCGGTGAGTTTACCGGGCACTACTCCGGCGGGCGTAACATGACCGTGGTGCGCATCGATAAAGTCT
CTTACCGCACCAGGCCGATTTTCGAATCGCTGTACCTCGGTATGCCGTGGACCGAAATCGACTACCTGATGGGGCCAGCC
ACCTGCGTGCCGCTGTATCAGCAGCTGAAAGCCGAGTTCCCTGAAGTGCAGGCGGTAAACGCCATGTACACCCATGGCCT
GCTGGCGATTATCTCCACCAAAAAACGCTACGGCGGCTTTGCCCGCGCGGTGGGCCTGCGCGCAATGACCACGCCGCATG

GTCTGGGCTACGTGAAGATGGTGATTATGGTCGATGAAGACGTTGACCCGTTCAACCTGCCGCAGGTGATGTGGGCGCTC
TCCTCGAAAGTGAACCCGGCAGGGGATTTGGTGCAGTTGCCGAATATGTCCGTGCTGGAACTCGATCCAGGCTCAAGCCC
TGCGGGGATCACCGACAAGCTGATTATCGACGCCACTACGCCTGTCGCCCCGGACAACCGTGGTCACTACAGCCAACCGG
TGGTGGATTTACCGGAAACCAAAGCCTGGGCTGAAAAACTGACCGCTATGCTGGCTGCACGTAAATAA
>Cluster_32589
ATGAAAGTAACGCTGCCAGAGTTTGAACGTGCAGGAGTGATGGTGGTTGGTGATGTGATGCTGGATCGTTACTGGTACGG
TCCCACCAGCCGTATCTCGCCGGAAGCGCCGGTGCCCGTGGTTAAAGTGAATACCATCGAAGAACGTCCGGGCGGCGCGG
CTAACGTGGCGATGAATATCGCTTCTCTCGGTGCTAATGCACGCCTGGTCGGGTTGACGGGCATTGACGATGCGGCGCGC
GCGCTCAGTAAATCTCTGGCCGACGTCAACGTCAAATGCGACTTTGTTTCTGTACCGACGCATCCGACCATCACCAAATT
ACGGGTACTTTCCCGCAACCAACAACTGATCCGTCTGGATTTCGAAGAAGGTTTCGAAGGGGTTGATCCGCAGCCGCTGC
ACGAGCGGATTAATCAGGCACTGAGTTCGATTGGCGCGCTGGTGCTTTCTGACTACGCCAAAGGTGCGCTGGCAAGCGTA
CAGCAGATGATCCAACTGGCGCGTAAAGCGGGTGTTCCGGTGCTGATTGATCCAAAAGGTACCGATTTTGAGCGCTACCG
CGGCGCTACGCTGTTAACGCCGAATCTCTCGGAATTTGAAGCTGTTGTCGGTAAATGTAAGACCGAAGAAGAGATTGTTG
AGCGCGGCATGAAACTGATTGCCGATTACGAACTCTCGGCTCTGTTAGTGACCCGTTCCGAACAGGGTATGTCGCTGCTG
CAACCGGGTAAAGCGCCGCTGCATATGCCAACCCAGGCGCAGGAAGTGTATGACGTTACCGGTGCGGGCGACACGGTGAT
TGGCGTCCTGGCGGCAACGCTGGCAGCGGGTAATTCGCTGGAAGAAGCCTGCTTCTTTGCCAATGCGGCGGCTGGCGTAG
TGGTCGGCAAACTGGGGACATCCACGGTTTCGCCGATCGAGCTGGAAAACGCTGTACGTGGACGTGCAGATACCGGCTTT
GGTGTGATGACCGAAGAGGAACTGAAGCTTGCTGTAGCGGCAGCGCGTAAACGCGGTGAAAAAGTGGTGATGACCAACGG
TGTCTTTGACATCCTGCACGCCGGGCACGTCTCTTATCTGGCAAATGCCCGTAAACTGGGTGACCGTTTGATTGTCGCTG
TGAACAGCGATGCCTCCACCAAACGGCTGAAAGGGGATTCCCGCCCCGTTAACCCGCTCGAACAGCGTATGATTGTGCTG
GGCGCACTGGAAGCGGTCGACTGGGTGGTGTCGTTTGAAGAGGACACGCCGCAGCGCTTGATCGCCGGGATCTTGCCAGA
TCTGCTGGTGAAAGGCGGCGACTATAAACCAGAAGAGATTGCCGGGAGTAAAGAAGTCTGGGCCAACGGCGGCGAAGTGC
TGGTGCTCAACTTTGAAGACGGTTGCTCGACGACCAACATCATCAAGAAGATCCAACTGGATAAAAAAGGCTAA
>Cluster_43010
ATGACCTTTCGCAATTGTGTCGCCGTCGATCTCGGCGCATCCAGTGGGCGCGTGATGCTGGCGCGTTACGAGCGTGAATG
CCGCAGCCTGACGCTGCGCGAAATCCATCGTTTTAACAATGGCCTGCATAGCCAGAACGGTTATGTCACCTGGAATGTGG
ATAGCCTGGAAAGTGCCATTCGCCTTGGATTAAACAAGGTGTGCGAGGAAGGGATTCGTATCGATAGCATTGGGATTGAT
ACTTGGGGCGTGGACTTTGTGCTGCTCGACCAACAGGGTCAGCGTGTGGGCCTGCCCGTTGCTTATCGCGATAGCCGCAC
CAATGGCCTAATGGCGCAGGCACAACAGCAACTCGGCAAACGCGATATTTATCAACGTAGCGGCATCCAGTTTCTGCCCT
TCAATACGATCTATCAGCTACGTGCACTGACAGAGCAGCAACCAGAACTTATTCCACACATTGCTCACGCTTTGCTGATA
CCGGATTACTTTAGTTATCGCCTGACCGGCAAGATGAACTGGGAATATACCAACGCCACGACCACGCAACTGGTCAATAT
CAATAGCGACGACTGGGACGAGTCGCTACTGGCGTGGAGCGGTGCCAACAAAGCCTGGTTTGGTCGCCCGACGCATCCGG
GTAATGTCATAGGTCACTGGATTTGCCCGCAGGGTAATGAGATTCCGGTGGTCGCCGTTGCCAGCCATGATACCGCCAGC
GCGGTTATCGCCTCGCCGTTAAACGGTTCACGCGCCGCTTATCTCTCTTCTGGCACCTGGTCATTGATGGGCTTCGAAAG
CCAGACGCCATTTACCAATGACACGGCGCTGGCAGCCAATATCACCAACGAAGGCGGGGCGGAAGGTCGCTATCGGGTGC
TGAAAAATATTATGGGCTTATGGCTGCTTCAGCGAGTGCTTCAGGAGCGGCAAATCAACGATCTTCCGGCGCTTATCGCC
GCAACACAGGCACTTCCGGCCTGCCGCTTCATCATCAATCCCAATGACGATCGCTTTATTAATCCTGACGAGATGTGCAG
CGAAATTCAGGCTGCGTGTCGGGAAACGGCGCAACCGATCCCGGAAAGTGATGCTGAACTGGCGCGCTGTATTTTCGACA
GTCTGGCGCTGCTGTATGCCGATGTGTTGCATGAGCTGGCGCAGCTGCGTGGTGAAGATTTCTCGCAACTGCATATTGTC
GGCGGCGGCTGCCAGAACACGCTGCTCAACCAGCTGTGTGCCGATGCCTGCGGTATTCGGGTGATCGCCGGGCCTGTTGA
AGCCTCGACGCTCGGCAATATCGGCATCCAGTTAATGACGCTGGATGAACTCAACAATGTGGATGATTTCCGTCAGGTCG
TCAGCACTACCGCGAATCTGACCACCTTTACCCCTAATCCTGACAGTGAAATTGCCCACTATGTGGCGCAGATTCACTCT
ACACGACAGACAAAGGAGCTTTGCGCATGA
>Cluster_61187
ATGTCTACGAGAAGAGAAGTTATTCTTTCCTGGTTGTGTGAGAAACGTCAAACCTGGCGTCTATGCTATTTGTTGGGTGA
GGCTGGAAGTGGAAAAACCTGGCTGGCGCAGCAACTGCAAAAAGATAAACATCGCCGTGTGATTACTTTAAGCCTCGTTG
TTTCCTGGCAAGGTAAGGCCGCATGGATCGTTACCGACGATAACGCGGCTGAACAGGGCTGCCGTGACAGCGCCTGGACG
CGAGATGAGATGGCGGGGCAATTACTGCATGCGCTTCATCGAACTGATAGTCGCTGCCCTCTTATTATTATTGAAAATGC
TCACCTGAATCACCGCCGCATACTTGATGATCTCCAACGCGCAATATCTCTTATTCCTGACGGTCAATTTTTGCTGATCG
GCAGACCCGATCGAAAAGTCGAACGTGATTTTAAAAAACAGGGCATTGAACTTGTCTCGATAGGACGTCTGACGGAGCAC
GAACTTAAGGCAAGCATCCTTGAAGGTCAGAATATTGACCAACCCGATCTCCTTTTAACCGCCAGAGTTCTGAAACGGAT
AGCTTTATTATGTCGGGGCGATCGCAGAAAGCTGGCGCTTGCCGGCGAAACAATTCGCTTATTACAGCAAGCCGAGCAGA
CCAGCGTTTTCACAGCAAAACAATGGCGAATGATTTACCGCATATTGGGTGATAACCGCCCCCGGAAAATGCAGCTTGCC
GTGGTGATGAGTGGAACCATTATCGCTCTTACCTGTGGTTGGCTACTGCTGTCCTCTTTTACAGCTACGTTACCCGTTCC
TGCGTGGCTGATACCCGTAACGCCAGTAGTTAAACAAGATATGACTAAGGATATTGCTCATGTGGTGATGCGCGATAGTG
AGGCGCTAAGCGTGTTATACGGCGTATGGGGGTACGAGGTCCCAGCTGATTCGGCCTGGTGCGATCAGGCAGTAAGAGCG
GGGCTGGCATGTAAAAGTGGGAATGCTTCTTTACAAACATTGGTAGATCAGAATTTGCCCTGGATCGCATCGCTTAAAGT
CGGGGACAAAAAATTGCCTGTTGTGGTCGTTCGCGTCGGTGAGGCTAGCGTTGATGTCCTCGTAGGTCAGCAAACGTGGA
CGCTGACACATAAATGGTTTGAGTCGGTATGGACGGGTGATTATCTTCTGTTGTGGAAAATGTCGCCGGAAGGGGAGAGT
ACCATTACGCGTGATAGCAGCGAGGAAGAGATTCTCTGGCTGGAAACGATGTTAAACCGCGCATTGCATATTTCGACAGA
ACCTTCGGCGGAGTGGCGTCCCTTGTTGGTAGAAAAAATAAAGCAATTTCAAAAAAGCCACCACTTGAAAACTGACGGTG
TTGTTGGCTTTTCAACGTTAGTACATCTCTGGCAGGTGGCAGGGGAGAGTGCCTACTTATATCGGGATGAAGCAAATATT
TCCCCGGAAACAACAGTGAAGGGGAAATGA
>Cluster_55878
ATGACAATAACTCCACAAAACCTGATCGCACTGCTACCGTTGCTGATCGTCGGCTTGACGGTGGTGGTTGTGATGCTCTC
CATTGCGTGGCGACGCAATCATTTCCTCAACGCTACGCTCTCGGTTATTGGGCTTAACGCGGCGCTGGTTTCGCTCTGGT
TTGTTGGCCAGGCGGGCGCTATGGACGTTACGCCGCTGATGCGCGTTGATGGTTTCGCCATGCTTTACACCGGGCTGGTA
TTGTTGGCGAGCCTCGCCACCTGTACTTTCGCCTACCCGTGGCTTGAAGGCTATAACGACAACAAGGATGAGTTCTACCT
GTTGGTGTTAATTGCCGCGCTGGGCGGGATCCTGCTGGCGAATGCCAACCATCTGGCGTCTCTGTTCCTCGGTATCGAAC
TGATCTCTTTGCCGCTGTTTGGCCTGGTCGGTTACGCTTTCCGCCAGAAACGTTCACTGGAAGCCAGTATCAAATACACC
ATCCTTTCTGCCGCAGCGTCTTCTTTCCTGCTGTTTGGTATGGCGCTGGTGTATGCGCAGTCTGGCGACCTGTCGTTTGT
CGCGTTGGGTAAAAACCTTGGCGACGGTATGCTCAACGAGCCGCTGTTGCTGGCAGGTTTCGGCCTGATGATTGTTGGCC
TCGGCTTCAAACTCTCTCTGGTGCCGTTCCACCTGTGGACGCCAGACGTATACCAGGGCGCGCCTGCGCCGGTTTCCACT
TTCCTGGCGACGGCGAGCAAAATCGCTATCTTCGGTGTGGTGATGCGTCTGTTCCTCTACGCACCGGTGGGTGACAGCGA
AGCGATTCGCGTGGTGCTGGCGATTATCGCCTTTGCCTCCATCATCTTCGGTAACCTGATGGCGCTGAGCCAGACCAATA
TCAAACGTCTGCTCGGTTACTCATCTATCTCTCACCTCGGCTATCTGCTGGTAGCGCTGATTGCGCTGCAAACCGGCGAG
ATGTCGATGGAAGCGGTAGGGGTTTACCTGGCCGGTTATCTGTTCAGCAGCCTCGGCGCGTTCGGCGTGGTCAGCCTGAT
GTCCAGCCCGTATCGTGGCCCGGATGCTGATTCCCTGTTCTCTTACCGCGGTCTGTTCTGGCATCGTCCGATCCTCGCGG
CAGTGATGACGGTGATGATGCTGTCTCTGGCCGGTATCCCGATGACGCTGGGCTTTATCGGTAAGTTCTACGTGCTGGCG

GTCGGTGTCCAGGCACACTTGTGGTGGCTGGTGGGTGCCGTGGTTGTCGGTTCGGCAATCGGCCTCTACTACTACCTGCG
CGTGGCGGTGAGCCTGTATCTTCACGCCCCGGAACAACCGGGTCGCGATGCACCATCAAACTGGCAGTACAGCGCGGGCG
GTATCGTTGTGCTGATCTCTGCACTGTTGGTACTGGTGCTGGGTGTATGGCCACAACCGCTGATTAGCATTGTGCGTTTG
GCAATGCCGCTGATGTAA
>Cluster_24547
ATGACTACTATTGTTGACAGCAATCTGCCGGTTGCCCGCCCGTCATGGGATCATTCTCGTCTGGAATCACGCATTGTGCA
TCTCGGTTGCGGGGCGTTTCACCGCGCGCACCAGGCGCTGTATACCCATCATCTGCTGGAAAGTACCGACAGCGACTGGG
GTATCTGCGAAGTTAACCTGATGCCAGGCAACGACCGCGTGCTGATCGAAAACCTGAAAAAACAGCAACTGCTGTACACC
GTGGCGGAAAAAGGCGCGGAAAGCACCGAGCTGAAAATTATCGGTTCGATGAAAGAAGCGCTGCATCCGGAAATCGACGG
TTGCGAAGGTATTCTCAACGCGATGGCGCGTCCGCAGACGGCGATTGTCTCTCTGACGGTCACGGAAAAAGGCTACTGCG
CTGATGCGGCAAGCGGTCAGCTGGATCTCAATAACCCGCTGATCAAGCACGATCTCGAAAACCCGACTGCGCCGAAGTCC
GCGATTGGTTACATCGTAGAAGCCCTGCGTCTGCGTCGTGAAAAAGGGCTGAAAGCATTTACGGTGATGTCCTGCGACAA
CGTGCGTGAAAACGGTCATGTGGCGAAGGTTGCCGTTCTTGGGCTGGCTCAGGCGCGTGACCCGCAACTGGCGGCATGGA
TTGAAGAAAACGTCACCTTTCCGTGCACCATGGTTGACCGCATCGTTCCGGCGGCGACGCCAGAAACCTTACAGGAAATT
GCTGACCAGCTGGGTGTTTACGACCCGTGCGCCATTGCCTGCGAACCGTTCCGTCAGTGGGTAATTGAAGATAACTTCGT
TAATGGTCGTCCGGACTGGGATAAAGTCGGTGCGCAGTTTGTTGCGGATGTTGTACCGTTCGAAATGATGAAGCTGCGTA
TGCTGAACGGTAGCCACTCTTTCCTGGCGTACCTCGGTTACCTCGGCGGCTATGAAACTATTGCCGACACCATGACTAAC
CCGGCTTATCGCAAAGCGGCCTTTGCCCTGATGATGCAGGAACAAGCGCCAACGCTGTCGATGCCGGAAGGTACGGACCT
GAACGCCTATGCGACGCTGCTGATCGAGCGTTTCAGCAACCCGTCTCTGCGTCACCGTACCTGGCAGATTGCAATGGACG
GCAGCCAGAAGTTACCGCAGCGTCTGCTGGACCCGGTGCGTCTGCACCTGCAAAACGGCGGTAGCTGGCGTCACCTGGCG
CTGGGCGTGGCGGGCTGGATGCGCTACACCCAGGGCGTGGATGAGCAGGGTAATGCTATTGACGTGGTTGACCCGATGCT
GGCGGAGTTCCAGAAGATCAACGCGCAGTATCAGGGTGCAGACCGCGTGAAAGCACTGCTGGGCCTGAGCGGTATCTTTG
CCGATGATCTGCCGCAGAACGCCGACTTTGTTGGCGCGGTGACGGCGGCATATCAGCAGCTGTGTGAGCGCGGTGCGCGC
GAGTGTGTGGCTGCGCTGTAA
>Cluster_11031
ATGATGCGCAAAAAGTATATGCCCCGGGCTCTTGGTCCGCTGCTTCTTGTCGTGCTGTCACCAGCTGTCGCCCAGCAAAA
CGATGATAATGAGATCATAGTGTCTGCCAGCCGCAGCAATCGAACTGTAGCGGAGATGGCGCAAACCACCTGGGTTATCG
AAAATGCCGAACTGGAGCAGCAGATTCAGGGCGGTAAAGAGCTGAAAGACGCACTGGCTCAGTTAATCCCCGGCCTTGAT
GTCAGCAGCCAGAGCCGAACCAACTACGGTATGAACATGCGTGGCCGCCCGCTGGTTGTCCTGATTGACGGTGTGCGCCT
CAACTCTTCACGTTCCGACAGCCGACAACTGGACTCTGTCGATCCTTTTAATATCGACCATATTGAAGTGATCTCCGGCG
CGACGGCCCTGTACGGTGGCGGGAGTACCGGAGGGTTGATCAACATCGTGACCAAAAAAGGCCAGCCGGAAACCATGATG
GAGTTTGAGGCTGGCACAAAAAGTGGCTTTAACAGCAGTAAAGATCACGATGAGCGCATTGCCGGTGCTGTCTCCGGCGG
AAATGACCATATCTCCGGACGTCTTTCCGTGGCATATCAGAAATTTGGCGGCTGGTTTGACGGTAACGGCGATGCCACCC
TGCTTGATAACACCCAGACCGGCCTGCAGCACTCCAATCGGCTGGACATCATGGGAACCGGTACGCTGAACATCGATGAA
TCCCGGCAGCTTCAACTGATAACGCAGTACTATAAAAGTCAGGGGGACGACAATTACGGGCTTAATCTCGGGAAAGGCTT
TTCCGCCATCAGCGGGAGCAGCACACCATACGTCAGTAAGGGGCTGAATTCTGACCGCATTCCCGGCACTGAGCGGCATT
TGATCAGCCTGCAGTACTCTGACAGTGATTTCCTGAGACAGGAACTGGTCGGTCAGGTTTACTACCGCGATGAGTCGTTG
CGGTTCTACCCGTTCCCGACGGTAAATGCGAATAAACAGGCGACGGCTTTCTCCTCGTCACAGCAGGATACCGACCAGTA
CGGCATGAAACTGACTCTGAACAGCCAACTTATGGACGGCTGGCAAATCACCTGGGGGCTGGATGCTGAGCATGAGCGCT
TTACCTCCAACCAGATGTTCTTCGATCTGGCTCAGGCAAGTGCTTCCGGAGGGCTGAACAACCATAAGATTTACACCACC
GGGCGCTATCCGTCATATGACATCACCAATCTGGCGGCCTTCCTGCAATCCAGCTATGACATTAATGATATTTTTACCGT
CAGCGGTGGCGTACGCTATCAGTATACTGAGAACAGGGTAGATGATTTTATCGACTACACGCAGCAACAGAAGATTGCTG
CCGGGAAGGCGATATCTGCCGACGCCATTCCTGGTGGTTCGGTAGATTACGATAACTTTCTGTTCAATGCTGGTCTGCTG
ATGCACATCACCGAACGTCAGCAGGCATGGTTCAATTTTTCCCAGGGGGTGGCATTGCCGGATCCGGGGAAATATTATGG
TCGCGGCATCTATGGTGCAGCAGTGAACGGCCATCTTCCCCTGACAAAGAGCGTGAACGTCAGCGACAGTAAGCTGGAAG
GCGTGAAAGTCGATTCTTATGAACTGGGCTGGCGCTTTACCGGTGACAACCTGCGGACTCAAATCGCAGCATATTACTCG
CTTTCCAATAAGAGCGTGGAAAGGAATAAAGATCTGACCATCAGTGTGAAGGACGACAGGCGCCGTATTTACGGCGTGGA
AGGTGCGGTGGACTACCTGATCCCGGATACTGACTGGAGTACCGGTGTGAACTTCAATGTGCTGAAAACCGAGTCGAAAG
TGAACGGTCAATGGCAAAAATATGACGTGAAGGAATCAAGTCCATCGAAAGCGACAGCTTACATTAGCTGGGCGCCGGAA
CCGTGGAGTCTGCGTGTGCAGAGCACCACTTCTTTCGACGTAAGCGATGCAGAGGGTAACGATATTAATGGTTACACTAC
CGTCGATTTTATCAGTAGTTGGCTGCTTCCGGTGGGAACACTCAGCTTCAGCGTTGAGAACCTCTTCGACCGTGACTATA
CCACTGTCTGGGGACAGCGTGCACCTCTGTACTACAGCCCGGGTTACGGCCCTGCTTCACTGTACGACTACAAAGGCCGG
GGCCGAACCTTTGGTCTGAACTACTCAGTGCTGTTCTGA
>Cluster_44668
ATGCAGTTATTTTCGCCATCTTGGTATTGTGGAAATACCGACATCCATAGCTTTGGTGAGCTCTGTTCAGGTGGCCAAAT
TCAGAAAACCATTACGGAGGAAGAAGGCGATGGCTAA
>Cluster_60490
ATGGAGCTGAAATCAGTCGAAGATACCAGCTGGCTGCAACTGTTTATTAAAAACACAGCACTGTGCAAACACGAAAGTGG
ACGTATACGGTGTGACGCCTGCCCGGTGCCGGAAGGTTAA
>Cluster_35159
ATGTCGGATGATATATCACTGGCAATGGAAGGTGCGCTGGCTGTTGTTGCTGTTGTGGGCGTTTACTGCCTGGTTGTGTT
TTTGATGGATCGACTAGGGAACTGA
>Cluster_57449
ATGGTTGCCAATATTAATCTCATTAAACAAGAAAGTTATTCTGTTGTTAATTTAGAAAAACAACTTTCCCGAACTGTTAC
CAACAAAATTATAACGATGAGCAAGTAA
>Cluster_30807
ATGCCTTATCCGGCCTACAAAGTATCGCAAATTCAATACATTGCAAAAAAAACACGTAGGCCTGATAAGCGTAGCGCATC
AGGCAATTATACGTTTGTCATCCGTCTGGAAGGATTATCGCCCCTGAATGGCTAA
>Cluster_9815
ATGAGGGTATCAATCTCCAACCTTGCCTGGGAAGTTACCGAAGATGTAGAAGTTGCTAATCTATTGTCTGCATTTAACAT
TGACGCGATTGATATCGCTCCCGGAAAGTATTTTCCGGAGCCTACTAACGCGACTAGGTAG
>Cluster_39335
ATGGTATTTCACAAATTAACCGGAAAGGACGTGATGCAATACCGCAAGATATCGCTGGCTGACCGTTTTGTATTATGGGC
CAGCGCTATAACTACACTGATTTTTATATTCATAGCCTTATTTAATTAA
>Cluster_8040
ATGATACCGGAGTTAATCATTTCAGGGGAAGAAGCGAACGGCGAGTTCTTGATGCTGGTTCCGGGAAAGATGCTGTTTTG
CAGCTTTTGCGTGATGCTTATGAAGCAAGTACGCAAAATTGTATCGGTTGGCTAA
>Cluster_17214
ATGCTGGAGAAGGTGTCTGAGATTATAGATTCCGGTGACACCACGGTGCGGCGCATTGAGATCTCACCGGAATTGATCCC

CAGTCTGAATGTGGTTGCTTGA
>Cluster_57182
ATGAACAAAATATTTAAAGTTATCTGGAACCCTGCCACAGGGAATTATACTGTTACCAGCGAAACGGCAAAAAGCCGTGG
CAAGAAATCTGGGCGCAGTAAGCTGTTAATTTCTGCGCTGGTTGCGGGTGGGTTGTTGTCATCGTTTGGGGCGCTGGCTG
ATAATTACGACGGTCAGGGCGTTGATTACGGCGATGGCTCAGCTAGTGACGGCTGGGTTGCTATCGGTAAAGGTGCAAAA
GCAAATATTTTTTTAAACAACGCTGGTGCCAGTACCGCTTTAGGTTATGACGCTATAGCTGAAGGCCAATATAGCTCTGC
CATCGGCTCAAAAACCCATGCTATTGGTGGTGCATCAATGGCCTTTGGGGTTAGTGCAATCTCTGAAGGTGACAGGAGTA
TCGCGCTGGGTGCATCGTCGTATTCATTCGGCCAATACTCAATGGCCCTTGGTCGTTATTCCAAAGCGCTGGGTAGATTG
TCTATAGCTATGGGGGATAGCTCCAAAGCGGATGGAGCAAACGCCATTGCGCTGGGAAATGCCGCTAAGGCGGCTGGTAT
TATGAGCATCGGTCTCGGTGATAATGCCAATGCGTCACAAGATTATGCTATGGCGCTGGGAGCAGAAAGCGAAGCCGCTG
AAAATGCGACCGCTATCGGCAATAAAGCGCATGCAAAAGGAGTGAATAGCATCGCGTTGGGTAATGGCAGCCAGGCTCTG
GCAGATAGTGCAATTGCTATTGGCCAGGGCAACAAGGCTAACGGCGCTGATGCTATCGCTCTGGGTAATGGTAGCCAGTC
GAGTGGCTTAAACGCTATTGCCTTAGGTAAGGCCAGTGTTGTAACTGGCGATAATAGTCTTGCGCTTGGGAGCAATACCA
ATGCCAACGGTATTAACTCTGTCGCGCTGGGCGCAGGTTCTATTGCGGATCAAGACGATAGCGTTTCTGTCGGCAGTGAC
TCATTACAACGCAAGATCGTTAATGTAAAAAATGGCACGATCAAGGCTGATAGCCACGACGCCATTAATGGCTCACAGCT
TTATGCCATCAGCGACTCGGTGGCAAAACGGCTTGGCGGGGGTTCATCGGTAAACGTTGACGACGGTACTGTTAAAGCAC
CAACCTACAATTTAAAAAATGGTAACAAAAATAACGTTGGCGATGCGCTCACTGTACTCGATCAATTCACCCTGCAATGG
GATCAAAACAGAGACAAATACAGCGCCGCTCATGGTAGCTCAACTGCCAGCGTAATCACCGATGTGGCGGATGGCGCAGT
TTCAGATTCCAGTAAAGACGCGGTTAACGGTTCACAACTGAAAGCCACCAATGATGATGTTGAGACCAATACCACCAATA
TCGCTACCAATACCGGAAATATTGCAACTAACACTGCAAATATTGCTACCAACACCACCAATATCACTAATTTGACAGAT
ACTGTTGGCGACCTTAAAGATGATGCCCTGCTCTGGAATGGCACTGCATTCAACGCCGCTCATGGTACGGAAACCACCAG
CACCATCACCAACGTGAAAGCTGGTACCCTTTCGGATGACAGTACTGACGCGGTTAATGGCTCGCAACTGAAAGACACTA
ACGATAACGTGGCGACCAACACCACCAATATCGCTAGCAACACGGCTAACATTGCCACTAACACCAGTAATATTGCCGAT
AACACTGCCAACATTGCTACCAACACCAGTAATATTGCCGATAACACTGCCAACATTGCGACCAACACCAGCAATATTGC
AGGTAACACTGCCAACATTGCTACCAATACCACTAATATCGCGGCTAACACCACAAGCATAAATAGCCTGAACACGTCTG
TGGATGCTCTTGAACAGGATGCTATGCTCTGGAACGGCACTGCATTCAACGCCGCTCATGGTACGGAAACCACCAGCACC
ATCACTAATGTGAAAGCTGGCACCCTTTCGGATGACAGTACTGACGCGGTTAATGGCTCGCAACTGAAAGCTACTAACGA
TAACGTGGCAACCAACACCACCAATATCGCCAGCAATACGGCTAACATTGCGACCAACACCGCCAATATTAATACTCTCA
ATACGTCGATCGATACCCTTGAGCAGGATGCAATACTCTGGAACGGAACAGCGTATAGCGCCGCGCACGGTACAGAAACA
GCCAGCACTATTACCAACGTTAAAGCAGGTACTTTATCTGAAAATAGTACGGACGCAGTTAACGGTGCGCAGCTGAACGC
GACTAACGCGAATGTGGCGACCAACACCACCAATATTGCTACTAACACCGCCAGCATTAATACTCTGAATACATCCATTG
ACGCTCTGGAACAGGATGCGCTGCTTTGGGATGGCACTGCATTCAGTGCAGCGCATGGTGCAAACAAAGATGCCAGCAAA
ATCACCAATGTCCTGGCAGGGACTGTATCATCCGCCAGTACTGATGCCATTAATGGCAGCCAGCTTCATGGGTTGAGCAG
TTCGATCGCCACCTATCTTGGTGGTGGTGCCACTGTGAGCGATTCTGGCGTATTTAGCGGCCCTACCTATAACATTGATG
GTAATGATTACACCAATGTTGGTGCTGCACTTGACGCCATTAACACCTCACTTAGCGACTCACTCGGCGATGCCCTCCTT
TGGGATAGCACGACTGGCGCATTTAGTGCCAAACACGGTTCTACCGCCAGCGTAATCACTAACGTCGCAGACGGTGCAGT
CTCTGACTCTAGCTCTGATGCTGTGAATGGTTCACAACTGTACGATGTAAGCAACTCTGTTGTCGATGTTCTGGGTGGTG
GTGCTGGCGTGAATACGGATGGCAGCATCAGTGCACCAACGTACACCATTGCTAACACTGATTACGATAATGTCGGCGAT
GCCCTAAACGCGCTTGATACCACTCTTGACGATGCGATGCTATGGGATGCTACCGCAGGTGAAAATGGTGCCTTCAGTGC
CAGCCACGATGGCAGTGCCAGCAAAATAACTAACGTCGCGGCAGGAACAATTTCTGACACCAGCACCGATGCGGTTAATG
GGGCTCAACTCCACGGCGTTAGCAGTTCCGTCGCTGAGGCTCTTGGTGGTGGTGCGGCGGTGAATTCTGACGGCAGCATC
AGCGCACCGACTTACACCATTGCTGATACCGACTATACCAACGTCGGCGATGCGATGAATGCAATTGACTCAACCCTGGA
TAACGCCTTGCTTTGGGACGCTACCGCAGGTGAAAACGGTGCGTTTAACGCCAGCCATGATGGCAAAGCCAGTGTCATCA
CCAACGTAGCCAATGGTCAGATTAACGAAACCAGCACCGACGCGGTTAACGGTTCCCAGTTAAATGCCACGAATATGTTG
ATCCAGAATATTGCCGGTGATACCAGCGAAAGCTATATCACAGAGAACGGTGAAGGTATCAACTATGTGCGTACTAACGA
CAGCGGCTTAGTCTTTGAGGATGCCAGTGCTACGGGTGTGGGCGCTACAGCTGTAGGTTATAACTCCGTCGCATCAGGCG
ATAGCAGTGTGGCAATTGGACAGAACAGCAGCAGTACTATTGAATCTGGAATTGCTCTGGGCAGCAGTTCTGTGTCTAAC
CGTGTCATCCTTCAGGGCTCCCGTGATACCAGCGTAACCGAAGATGGCGTAGTCATTGGTTATAACACCAGTGATGGCGA
ACTGCTTGGCGCGTTGTCAATTGGTGATGACGGTAAATATCGTCAAATCATCAACGTCGCCGATGGTTCCGAAGCCCATG
ACGCCGTTACGGTTCGCCAGTTGCAGAATGCGATTGGTGCTGTAGCAACGACACCGTCCAAATACTTCCATGCTAATTCA
ACGGAAGAAGATTCACTGGCTGTCGGTGAAGACTCACTGGCAATGGGTGCGAAAACCATTGTTAATGGTGATGCCGGGAT
CGGTATTGGCCTGAACACTCTGGTGTTAACTGATGCAATCAACGGTATTGCTATCGGTAGCAACGCGAGTGCAAATCATG
CAAACAGTATTGCGATGGGTAGTGGTTCCCAGACCACCCGTGGTGCGCAGACTGACTACACCGCCTACAACATGGACGCG
CCGCAGAATTCTGTCGGTGAATTCTCTGTCGGCAGCGAAGACGGTCAACGTCAGATCACCAACGTCGCGGCTGGTTCAGC
GGATACCGATGCGGTTAACGTAGGTCAGTTGAAAGTCACTGATGAGCGCGTAGCGCAAAATACCCAGAGCATTACTAACC
TGAACAATCAGGTCACTAATCTGGATACTCGCGTTACTAATATCGAAAACGGTATTGGCGACATTGTCACCACCGGTAGC
ACCAAGTACTTCAAGATCAACACCGATGGCGTAGATGCCAACGCCCAGGGTAAAGATAGCGTTGCTATTGGTTCTGGTTC
CATTGCTGCCGCTGACAACAGCGTCGCACTGGGTACCGGTTCCGTTGCAGATGAAGAAAATACAATCTCTGTAGGTTCTT
CCACTAACCAACGCCGTATTACTAACGTTGCCGCAGGTAAAAATGCTACCGATGCTGTTAACGTTGCGCAGTTGAAGTCT
TCTGAGGCGGGCGGCGTGCGTTACGACACCAAAGCTGATGGTTCTATCGACTATAGCAATATCACCCTCGGTGGCGGCAA
CGGTGGTACGACTCGTATCAGCAACGTCTCCGCTGGCGTCAACAACAACGACGCGGTGAACTACGCGCAGTTGAAGCAAA
GCGTGCAGGAAACGAAGCAATACACCGATCAGCGGATGGTTGAGATGGATAACAAACTGTCTAAAACCGAAAGCAAGTTG
AGTGGTGGTATCGCTTCTGCAATGGCAATGACCGGTCTGCCGCAGGCTTATACACCGGGTGCCAGCATGGCTTCTATTGG
TGGCGGTACTTACAACGGTGAATCGGCAGTTGCTTTAGGTGTATCGATGGTGAGCGCCAATGGTCGTTGGGTCTACAAAT
TACAAGGTAGTACCAATAGCCAGGGTGAATACTCCGCCGCACTCGGTGCCGGTATTCAGTGGTAA
>Cluster_38585
ATGCTCTCCATTTCTTCCATTAAAGGTGATGCGGGCTACTACAGTCATGAAGACAACTACTACGCCAGCGGTTCACTGGA
CTCCCGCTGGATGGGGGAGGGTGCGGAGAAACTGGGACTGAAGGGGGAAGTCGCCAGCGCCGATATGGATGCTGTGCGTC
AGGGTAAACTTCCGGACGGTTCAGATCTCTCCCGTATGGTAAATGGTGTGAATAAGCACCGCAGCGGCTACGACCTGACG
TTTTCTGCCCCCAAAAGTGTCTCCGTCATGGCGCTGGTCGGGGAAGACCGACGCTTTATTGAGGCGCATAACCGGGCTGT
AGCGGTGGTGATGCAGGAGGTTGAGAAGCTGGTCAGCGCGCGCATTACAGAAGAGGGGAAAACGGAAACAGTACTGACCG
GCAACATGGTGGCCGCACTCTATAACCATGATACGTCCCGTGACCTCGATCCGCAGTTGCATACCCACGCTCTGGTCTTT
AACGCCACGTTTGCCGGTGAAAAAATGGCGGTCGCTGGCCAGTGATACGCGCATGAAAACGGGTTTCTCCGAGAATCTGTA
TGCCACCAAGATAGCGCTGGGAAACCTGTATCGTTCTGCTTTACGTGAAGACGTTGAGTCGATGGGATTTGAAACTGTTG
CGGCCGGAAAGAATGGTCTGTGGGAGCTGAAGGATGTCCCTGTCGATGTTTTTTCCTCACGTAGTCAGACTATCCGGGAG
GCCGCCGGCCCGGACGCGTCTGCCAAATCCCGCGACGTTGCCGCGCTCGATACCCGTCAGGCCAAAGCCTGGGCCGACCC
GGAGCTGCTGAAAGCTGACTGGCGCCGGCGGCTGGCCGATGAAAAATTTGATATCGATGGCTACATCAGACAGGCGCAGG
CGCGCGTGGAGGTTCCTGGTGCTGTTGCAGGTGGACATAGCGGTATACATGCACCGGACCTGTCGGGAGGCGCGTCTTCC
GGCGAGATTGCTGACGAGCAGGTGCGGAAAGCTGTCAGCGACACGATTTCGGCGTTGTCGGAGAAAAAGGTCCAGTTCAC

CTGGTCGGAGATGCTGGCCGGTACGGTCAACCGTCTGCCTTCCGCACCCGGTCTGTTTGAGCAGGCCCGGGCCGGCATCG
ATGCCGCGATTGAAGGGCAGCGTCTTATCCCTCTGGACCGTGAGAAGGGGATATTCACCTCTGATATCCACCTGCTGAAT
GAACTCAGCGTCCATCAGCTGGCGCGCACGGCCATTCAGGAACAGACCGTGCTGCTCTTTCCTGAACGGGCACAGGAACG
CGATATACCGGCAGGTGATGCTGTCTCTGTTCTGACGCAGGACAAAAGCCCGGTGGCCATTCTCAGTGGTCGCGGAGGCG
CGCAGGCCCTGCGTGAGCGCACGGAAGACGTGGCCATGATGGCCCGTTCACAGGGCCGTGAGGTGATGGTGATTGCAGCC
GACGGTCGCAGTGGCCAGTTTATGTCAGAAAGTCCGCATCTGGCAGGCCATGTGATGCTGCGCTCGCAGATGAACGCTGA
CACCGTATTGCCGGTGCAGGGAACGGTCATCGTTGACCGCGCGGAGCGGCTGTCGCTGAAAGAGACGGTGCTGCTGCAGG
AAAAGGCGCTCAGCGCCGGGGCGCAGCTGATTTTTATGGATACGGAGAACCGGCAGGGAACGGGGAATGCCCTGTCTGTG
CTGAAAGAAGCCGGTATTCCGCAGTACCGGTTTTATAGCACCCAACTACCGGAGGTGCGACTGGTCAGCGAGGCGGACAA
GCGAAGCCGTTACAGCCAGCTGGCACAGGACTATGTGCGCCTGTCGGCGGAAGGGCGGGATGTGGTGGCCCAGGTGACCG
GTGCCCGGGAGCAGCAGCAACTGACGGATATCATTCGCGAGTCACGGCGTGAGGCCGGAGAACTGGGCCGGGAGCAGATG
ACACTGCAGGTACTGGAGCCTGTGTGGCTGGACAGCAAAACCCGCCATCAGCGTGATAACTACCGGGCCGGGATGGTGAT
GGAGCATTGGGACGCTGAGAAGAAAACGATGACACGGCACACCATTGACCGGGTGGCCGAAGCCACCAATAGCCTGGTAT
TGCAGGGAGAAGACGGCAGTCGCCTGACGCTGAAGGTCTCGCAGCTTGACGGCAGCTGGAGCCTGTACCGCAGCCGGACG
CTGGCAGTTTCAGAGGGGGACCGCGTCAGGGCGCTGGGGCGTGAGCTGAAGGGGGCCATCAAAGCGAAGGAGCAGTTCAC
GGTGACTGCTCTGGAAAACGGTGCGATACGATTGCGCAGTGGCGACCGGGAGCTGCGTCTGCCCACTGAGCGGGCCGTGA
AGCTGGCCCACGATTATGTGGAGGGGACCGGTGCCGGAACGAGTGCTTCCCGGACAGTGCTGGCCGCGGTCGGGCCACGA
AGTCTGAATAAACAGGTCCTGAACGCGCTGGCGCAGAGTGGCGGTGACATCCGGATTTATACGCCACTGGAGGCCGGGCA
GGCCGCGCGTAAAGTAGAGAATGTGTCTTCTGTCCGGCTTGCCAGTGACCAGGTCCGCCAGTCGACTGGCGAGACAAATC
TGGATACCGCCATACAGGCCAGCCGGGAGCGGCTGATGTCGGATGCAGAGCAGGCGGTGAATCTGGCCATCCCGCGCGCA
CAGCAGGGACAGGTGTATCTCAGCGAAATTGCCCTGCTGTCAGAAGCGGTAAAATCTGGCCAGCCGCTGGCTGATGTCCG
GACAGCAATCGCCCGGCAGGTGAACAGCGGGGAGCTGATTCAGCTGGATTCGGTTTCCGGGGTGGGAAACAGGGTGCTTG
TTCCGCGTGTGGCGTATGAGATGGAAAAGAGCATCATCCGCCATATTGCCGAGGGTAAGGATGCCGTTCAGCCCCTGATG
GCGCTGACACCTGCGTCTGTGCTGGCCGGTTTGACGGCAGGCCAGCGGGAGGCCACGCGGACAGTGCTGGAAAACACCGA
CCGCTTTATGGCCATTCAGGGCTACGCCGGGGTGGGGAAAACCACCCAGTTCCGGGCGGTGATGGGTGCCCTGAACACCC
TGTCTGAATCCGTGCGTCCGCAGGTCATCGGCCTGGGACCCACGCACCGGGCCGTGCATGAGATGCGTGAGGCCGGGGTG
GAGGCACGAACGCTCGCCAGTTTCCTGAGTGAAACCCGCCAGGCCATTCAGGCCGGGGAAACTCCTGACTTTCGCAACGT
CCTGTTCCTGACTGATGAGAGCTCGATGGTGGGCAACCGTGATATGGCGGAGCTCTACCAGCTGGTTGCCGCCGGTGGCG
GGCGAATGGTCTCCAGCGGGGACACCGCGCAGTTGCAGGCTGTCTCCACCGGGCAGCCGTTCCGTCTGGTACAACAGCGC
AGCGCCATCGATACGGTGGTGATGCAGGAGATTGTCCGTCAGACCCCGGCGCTGCGCCCGGCAATTGAAAGCATTATCGC
CGGTCAGGTGGAGACATCCCTTCGCCAGGTGGGTGACGTCAGCCCGCTACAGGTACCACGTCAGGAAGGAGCCTGGGTGC
CGGAAAAGTCCGTTATGGAAATCCGTGCGCCGAAAAAGGGCCAGGAGCAGGACACGCCTGCCGCCGGTGAACAGACGCTG
ACACCAGAGCAGTTGTCACTGGTCCGGACCGACATCATTGAAGCCATCCGGGATGACTGGATGGGGCGAACCCCCGAGGC
GCAGCAGCAGACGCTGGTTGTGGCAGAACTCAACGCCGATCGTCATGCCATCAATGCTGCCATTCATGCCGCCCGGCATG
AGAAAGGCGATACAGGTGCTGAAGAACGCACGTTCACCGTTCTGGAGCCGCTCCGGGTGCCTGACAACGCCCTGCGTGCG
GCTGAAACTTTTGCGGAATACACCGGCGCCGTGGCCATGATGAACGAGCGCTACTGGACGGTGGCGGAGGTGAATACTCA
GGACGCTGTGGTCACGCTGCGGAATGCTGACGGGGAGTCGGTGCTGCTCTCTCCTCAGCAGAACACCGCTCAGGATATTT
CCCTATTCACACCCCGCGAACTGACGGTCAGTCAGGGGGACCGGGTGCGCTTTACCCGCTCGGACACCGACCGCGGCTAT
GTGGCCAACAGTCTGTGGGAGGTGGCCGGGTTCACTGACGACGGGGCCATCCGCTTCCGCCAGGGAGAGCAGGAGAAAAT
CGTCGACCCGCAGGCCATGACGGAGGACCGTCATATTGACCTCGCCTACGCCCTGACGGTGTATGGCGTGCAGGGGGCCA
GTGAACGTTTTGCCATTGCCCTGACCGGTACCGAAGGTGGCAGGAAAAGAATGGCGTCCCTGGAGTCCACTTATGTGACG
CTGTCACGTGCGAAAGAGCATGTCCAGGTCTACACGGACAACCTGGAGGACTGGCAGCAACAGGTCCGGCAGTCTGACGG
CGGCAAAACTGCCCACGACCTGCTGCACGAGAAAAGTGACCGCGAGTCCGACACCGGTAACCGTCTGCTGGCTACCGCAT
CGCGGCTGGATAAAACGGCGCTGGGCCGCCGGGTGCTGGCGGAAAACGGTCTGGAGGGTGAAACGATGGCCCGCTTTATC
GCGGCAGGCAAAAAGTATCCGGCACCGTATGTGGCTCTTCCTGTGTGGAACCGACACGGAAAAGAAGCCGGGGCGCTGCT
GACGGAAATTCGTATTGAGGATGATGGAATGCGCGTGGTGCTCAGCGAAGAGTCCCGGCTACGCGCTGGTGAAGATGCGC
AGTTCGCCGGCCTGCAGGCCAGCCGTAACGGACAGACACTCATTGCTGATGATGCACAGACGGCGCTGCGCCTGGCACAG
GAGAACCCGGAGAGTGGGGTGGTGATACGCCTGCACGGTGAAGAACGGTTGCTCAATGCAGCCCGCCTGACCGGGGGACG
AATCACGGAGCCTGATGAGGTGGCACGGACGGTGCGCAGTGTGGCGGAGGCTGAATCGGTGGCGAAAGCGGAAGACCCCA
TTACGCTGCCTCCGGATGAGCAGCAGAAGCTGGCGGAGGCGCAGGAGAAAGCCGCCCGTGAGCTGGCGGAGCAGGCCCGG
CGTGAACTGTTGTCGGTGGCACCGCAGAAAGATGAAGGCAAACGTGACCCGCTGTTATCTGCCGATGATGAACGTCGCCT
GCGTGACAATTCAGAACGGGAAATCCGGGAGCTGGACGAGGCTGTCCGGGAGGCGGTTGCCGATGGTCGTGGTCCCGCC
AACAGGCACGTGAACAGATACTGCGTACTGAACGTGAGTGGGTGGTGAATATGCCGGAAAAAGAAATAGAGATGGAAAAG
ACACTCGGTGGCGACTGA
>Cluster_22691
ATGATGGAAATTCTGCGTGGTTCGCCTGCATTGTCGGCATTCCGAATCAACAAACTGCTGGCACGTTTTCAGGCTGCCAG
GCTCCCGGTTCACACTATTTACGCCGAGTATGTCCATTTTGCTGACCTCAATGCGCCGTTAAACGATGATGAGCACGCAC
AACTTGAACGCCTGCTGAAATATGGCCCGGCACTCGCCAGCCACGCCCCGCAAGGCAAACTCCTGCTGGTGACCCCGCGT
CCTGGCACCATCTCTCCCTGGTCTTCGAAAGCGACCGATATTGCCCATAACTGCGGGCTGCAACAGGTTAACCGCCTTGA
GCGCGGCGTTGCTTACTATATAGAAGCCGGTACGCTGACCAATGAACAATGGCAGCAGGTTACCGCTGAACTGCACGACC
GCATGATGGAAACGGTCTTTTTTGCTTTAGATGATGCAGAGCAGCTGTTTGCACACCATCAACCGACTCCGGTCACCAGC
GTTGATTTGCTGGGGCAGGGCCGTCAGGCGCTAATCGACGCTAACCTGCGTCTTGGCCTGGCACTGGCGGAAGATGAAAT
CGACTATCTGCAGGATGCTTTCACAAAGCTTGGGCGTAACCCGAACGACATCGAGCTGTATATGTTCGCTCAGGCGAACT
CTGAGCACTGCCGCCACAAAATTTTTAACGCCGACTGGATTATCGACGGTGAACAGCAGCCGAAATCGCTGTTTAAGATG
ATCAAAAACACCTTCGAAACCACGCCGGATCACGTTCTCTCTGCTTATAAAGATAACGCCGCCGTCATGGAAGGGTCTGA
AGTGGGCCGCTACTTTGCTGACCACGAAACGGGCCGCTACGATTTCCATCAGGAACCGGCGCATATTCTGATGAAAGTTG
AAACCCACAACCACCCGACGGCGATTTCTCCGTGGCCGGGCGCGGCGACCGGTTCCGGCGGTGAAATCCGCGATGAAGGT
GCCACCGGACGTGGCGCAAAGCCGAAAGCTGGTCTGGTTGGTTTCTCCGTATCCAACCTGCGAATTCCTGGCTTCGAACA
GCCGTGGGAAGAAGATTTCGGTAAACCTGAGCGCATTGTTACCGCGCTGGACATCATGACCGAAGGCCCGCTGGGCGGTG
CGGCGTTTAACAACGAATTTGGTCGCCCGGCACTGAACGGCTACTTCCGTACCTATGAAGAAAAAGTGAACAGCCACAAT
GGCGAAGAGCTGCGCGGTTATCACAAACCGATCATGCTGGCGGGTGGGATCGGCAACATTCGCGCCGATCACGTACAAAA
AGGCGAGATCAACGTTGGTGCGAAGCTGGTCGTTCTCGGCGGCCCGGCAATGAACATCGGTCTTGGCGGCGGCGCGGCGT
CTTCCATGGCATCTGGTCAGTCTGATGCCGACCTCGACTTCGCTTCCGTACAGCGCGACAACCCGGAGATGGAGCGTCGC
TGCCAGGAAGTGATCGACCGTTGCTGGCAGCTTGGTGATGCCAACCCAATCCTGTTTATCCACGACGTTGGCGCTGGCGG
TCTTTCTAACGCCATGCCGGAACTGGTGAGCGACGGCGGGCGCGGCGGTAAATTCGAACTGCGCGATATCCTGAGCGACG
AACCGGGCATGAGCCCGCTGGAAATCTGGTGTAACGAATCCCAGGAACGCTACGTGCTGGCGGTTGCTGCCGATCAGTTA
CCGCTGTTTGACGAACTGTGTAAGCGTGAGCGCGCGCCATACGCGGTGATTGGCGAGGCCACCGAAGAACTGCATCTTTC
TTTGCACGATCGTCATTTTGATAATCAGCCGATCGATCTGCCGCTGGACGTACTGCTTGGTAAAACGCCGAAGATGACCC
GCGAAGTACAAACGCTGAAAGCGAAGGGTGATGCGCTGGTTCGTGAAGGGATTACCATTGCTGACGCGGTGAAACGCGTG

CTGCACCTGCCGACCGTGGCGGAGAAAACTTTCCTGGTGACCATTGGTGACCGCAGCGTAACCGGCATGGTAGCGCGCGA
TCAGATGGTGGGTCCGTGGCAGGTGCCGGTCGCTAACTGCGCGGTCACTACCGCCAGCCTCGACAGTTACTACGGTGAAG
CGATGGCAATTGGCGAACGTGCGCCAGTTGCGCTGCTGGATTTCGCCGCTTCTGCCCGTCTGGCGGTCGGTGAAGCGTTA
ACCAACATCGCCGCAACACAAATTGGCGATATCAAACGCATTAAACTTTCCGCCAACTGGATGGCGGCGGCAGGCCACCC
TGGTGAAGATGCTGGCCTGTATGAAGCCGTTAAAGCTGTGGGTGAAGAACTTTGTCCGGCGCTGGGCCTGACGATTCCGG
TGGGTAAAGACTCTATGTCGATGAAAACCCGCTGGCAGGAAGGTAACGAAGAGCGCGAAATGACGTCGCCACTGTCGCTG
GTGATTTCTGCATTTGCCCGCGTGGAAGATGTACGTCACACCATTACGCCGCAGCTTTCTACCGAAGATAACGCACTGTT
GCTGATTGATTTGGGCAAAGGCAATAACGCGTTGGGCGCAACGGCGCTGGCGCAGGTTTATCGTCAGCTTGGCGACAAAC
CGGCAGATGTACGCGATGTCGCGCAACTGAAAGGCTTTTATGACGCGATTCAGGCGCTGGTTGCACAGCGTAAGCTGCTC
GCGTACCACGACCGCTCGGATGGCGGCCTGCTGGTAACGCTGGCGGAAATGGCGTTTGCTGGTCATTGTGGCATTGACGC
AGATATCGCCTCTCTGGGTGACGATCGCCTGGCGGCGTTGTTTAACGAAGAACTGGGTGCGGTGATTCAGGTTCGTGCCG
CTGACCGTGAAGCGGTCGAGTCCGTACTGGCGCAGCATGGACTTGCGGATTGCGTCCATTATGTAGGGCAGGCGGTTTCC
GGTGACCGTTTTGTGATTACCGCCAACGGGCAGACTGTATTCAGCGAAAGCCGCACTACGTTGCGTGTCTGGTGGGCAGA
AACTACCTGGCAGATGCAGCGCCTGCGTGACAACCCGGAGTGTGCCGACCAGGAGCACCAGGCGAAATCAAACGACGCCG
ATCCGGGCCTGAATGTGAAACTGTCGTTCGATATCAACGAAGATGTGGCAGCACCGTATATTGCCACTGGTGCGCGTCCG
AAAGTTGCCGTGCTGCGTGAGCAGGGGGTGAACTCGCATGTTGAAATGGCGGCAGCTTTCCACCGTGCAGGCTTCGATGC
AATCGATGTGCATATGAGCGACCTGCTGGCTGGACGCACGGGCCTGGAGGATTTCCACGCTCTGGTAGCGTGCGGCGGAT
TCTCCTACGGTGACGTGCTGGGTGCTGGTGAAGGCTGGGCGAAGTCTATCCTGTTCAATGACCGTGTACGCGATGAGTTT
GCAACCTTCTTCCACCGTCCGCAAACGCTGGCGCTGGGGGTATGTAACGGTTGCCAGATGATGTCTAATCTGCGCGAACT
GATCCCAGGTAGTGAGTTGTGGCCACGTTTTGTGCGCAATACCTCCGATCGCTTTGAAGCGCGTTTCAGCCTGGTTGAAG
TAACCCAAAGCCCGTCGCTGCTATTGCAGGGGATGGTGGGCTCGCAAATGCCGATTGCTGTCTCTCATGGTGAAGGGCGC
GTGGAAGTGCGTGATGCGGCGCATCTGGCGGCACTGGAAAGCAAAGGGCTGGTGGCACTGCGCTATGTCGATAACTTCGG
CAAAGTCACTGAAACCTACCCGGCTAACCCGAACGGTTCTCCGAATGGTATTACGGCGGTCACGACTGAAAGTGGTCGAG
TCACTATTATGATGCCGCACCCGGAACGTGTTTTCCGTACTGTCAGCAACTCCTGGCATCCGGAAAACTGGGGCGAGGAT
GGGCCGTGGATGCGTATTTTCCGCAATGCACGTAAGCAATTGGGATAA
>Cluster_5375
ATGAGCGGAAAACCGGCGGCGCGGCAGGGTGACATGACCCGCAAGGGACTGGATATTGTGCAGGGTTCAGCAGGTGTGCT
GATAGGTGCGCCGACGGGAGTGGCCTGCTCGGTATGTCCCAACAGCCCGATAGAGGAGCAGAAGGGCCACCCGGTGAACC
CGGTGCTGGGTGCGAAGGTGCTGCCGGGCGAGACGGACCTTGCGCTGCCCGGCCCGTTGCCGTTTATTCTTTCCCGCGCT
TACAGCAGCTACCGGACCAGGACGCCCGCGCCGGTGGGGGTGTTCGGTCCCGGCTGGAAAGCGCCCTTCGATATCCGCTT
ACAGATACGCGATGAAGGACTGATACTCAACGACAGCGGCGGCAGGAGCATTCACTTTGAGCCGCTGTTTCCCGGCGAGA
TAAGCTACAGCCGCAGCGAGTCGTTCTGGCTGGCGCGGGGCGGGGTGGCGACGCAGCACAGCTCGCAGCCGTTAAGCGCG
CTCTGGCAGGTGCTGCCGGAAGATGTTCGCCTGAGTCCGCATGCGTACCTGGCAGCAAACAGCCTTCAGGGGCCGTGGTG
GATACTCAGCTGGCCGGAGCGGGTGCCGGAGGTGGACGAGGTGCTGCCGCCGGAGCCGCCCGCATACCGGGTGCTGACGG
GCGTGGTGGACGGCTTCGGGCGGACGCTGGCTTTCCACCGCGAGGCCGCAGGCGAACTGGCTGGCGAAATCACCAGGGTG
ACGGACGGCGCGGGTCGCCGTTTTCACCTGGCGCTGAGCACACAGGCGCAGCGGGCGGAAGCCTTCCGTAAACAGCGCGA
GTCTTCCTTATCTTCTCCGGCCGGTCCCCGTTCTGTTTCCTCTTCTTCTTCAGCTTTCCCCGACACGCTGCCCGCCGGTA
CAGAATACGGTGCCGATAACGGTATCCGGCTGGAGGCGGTATGGCTGACACACGACCCGGCATACCCGGATGAACAGCCG
ACTGCGCCGCTGGCGCGCTACACGTACACGGCCAGCGGAGAACTGCGGGCGGTGTATGACCGCAGCGGGACGCAGGTTCG
CGGGTTTACTTATGATGCGGAGCACGCCGGGCGGATGGTGGCGCACCATTATGCGGGGAGGCCGGAGAGCCGCTACCGGT
ATGATGATACCGGTCGGGTGACGGAGCAGGTCAACCCGGAGGGGCTGGACTACCGCTTTGAGTACGGAGAGCGCCGGGTA
ATCATCACGGACAGTCTGAACCGGCGGGAGGTGCTGTACACGGAGGGCGAAGGTGGCCTGAAGCGGGTGGTGAAAAAGGA
GCATGCGGACGGGAGCATCACCCGCAGCGAGTATGATGAGGCGGGGAGGCTGAAGGCGCAGACGGATGCGGCGGGGCGGC
GGACGGAGTACAGCCTGCATATGGCGTCGGGTGCGGTGACGGCGGTGACGGGGCCGGACGGCAGGACGGTACGGTATGGC
TATAACAGCCAGCGGCAGGTGACGTCGGTGACGTACCCGGACGGGCTTCGCAGCAGCCGGGAGTATGATGAGAAGGGAAG
GCTGGCGGCGGAGACCTCACGCAGCGGTGAGACGACGAGTTACAGCTATGATGACCCGGCGAGTGAGCTGCCGACAGGGA
TAGAGGATGCGACGGGCAGTACAAAACAGATGGCATGGAGCCGTTACGGTCAACTGCTGGCCTTTACGGACTGCTCGGGG
TACACGACGCGGTATGAGTACGACTGGTACGGTCAACAAATCGCCGTTCACCGGGAGGAAGGCATCAGCACTTACAGCAG
TTATAACCCGCGTGGGCAACTGGTCAGTCAGAAGGATGCGCAGGGGCGGGAGACCCGTTATGAGTACAGTACCGCAGGCG
ACCTCACCGCTATCATTGCCCCGGACGGTAGCCGCAGTGAGACACAGTATGATGCGTGGGGTAAAGCCATCAGCACCACG
CAGGGCGGTCTGACGCGCAGCATGGGGTATGACGCTGCCGGGCGCATCACCGTGCTGACCAACGAGAACGGCAGCCAGTC
CACGTTCCGGTATGACCCGGTGGACAGGCTGACTGAACAGCGCGGCTTTGACGGGCGGACGCAACGTTACCATTATGACC
TGACCGGAAAACTCACGCAGAGTGAAGACGAGGGGCTTATCACCCTCTGGCATTACGATGCGTCAGACCGTATCACCCGG
CGCACGGTGAACGGTGAACCGGCGGAGCAGTGGCAGTATGACGACCACGGCTGGCTGACAGAAATCAGCCACCTGAGTGA
AGGCCACCGTGTGGCGGTCCACTACGGCTATGACGATAAAGGCCGCCTGACGGGCGAACGGCAGACGGTGGAGAACCCGG
AGACGGGGGAGATGCTGTGGGAGCATGAGACGGGGCATGCGTACAGCGAACAGGGGCTGGCGACCCGTCAGGAGCCGGAC
GGTCTGCCGCCGGTGGAGTGGCTGACGTATGGCAGCGGTTATCTTGCGGGGATGAAGCTGGGCGGAACACCGCTGGTCGA
GTACACGCGGGACAGGCTGCACCGTGAGACGGCCCGCAGCTTCGGCGGGGAGGCATATGAACTTGCCACCGCCTGGAATA
CCAGCGGCCAGCTCCGGAGCAGGCACCTGAACCTTCCGCAGCTTGACCGTGACTACGACTGGAACGACAACGGACAGCTA
ATCCGCATCAGCGGCCCGCAGGAGAGCCGGGAGTACCGTTACAGCGACACGGGAAGGCTGACGGGCGTCCACACCACGGC
GGCGAACCTGGATATCGATATCCCGTATGCGACGGACCCGGCAGGAAACCGGCTGCCGGACCCGGAACTGCACCCGGACA
GCACGCTCACGGCATGGCCGGATAACCGCATCGCGGAAGATGCGCACTATGTCTACCGCCACGATGAATACGGCAGGCTG
GCGGAGAAGACGGACCGCATCCCGGAAGGGGTGATACGGATGCACGACGAGCGGACCCACCACTACCACTACGACAGCCA
GCACCGCCTGGTGTTCTACACGCGGATACAGCACGGCGAACCACAGGTGGAGAGCCGGTACCTCTATGACCCGCTGGGCC
GCCGGACGGGAAAACGGGTATGGCGGCGGGAGCGTGACCTGACGGGGTGGATGTCGCTGTCGCGTAAACCGGAGGTGACA
TGGTACGGGTGGGACGGTGACAGGCTGACGACGATACAGACCGGCACCACGCGTATCCAGACGGTATACCAGCCGGGGAG
CTTCACGCCGCTCCTGAGAATCGAAACAGAGAATGGCGAACAGGCGAAGGCGCGGCACCGGAGCCTGGCGGAGGTGTTGC
AGGAGGACACGGGTGTGACGCTACCGGCGGAGCTGGCGGTGATGCTGGGAAGGCTGGAGCGGGAGCTGCGTGCGGGAGCG
GTGAGTGCGGAGAGTGAGGCATGGCTTGCGCAGTGCGGGCTGACGGCGGAACAGATGGCCGCGCAGATGGAGGATGCATA
CATCCCGGAGCGGAGGCTGCATCTTTACCACTGTGACCACCGTGGACTGCCGCAGGCGCTCATCACCCCGGAAGGCGAAA
CGGCGTGGTGCGGGGAGTATGACGAGTGGGGGAACCAGCTTAATGAGGAGAACCCGCATCACCTGTACCAGCCGTACCGT
TTGCCGGGGCAGCAGTATGATGAGGAGTCGGGACTGTATTACAACCGTCACCGGTACTACGATCCGTTGCAGGGGCGGTA
TATCACCCAGGATCCGATAGGGTTGAAGGGGGGAATAAATCTATACACGTATCCACTGGTACCAATACGTTACACTGATC
CACTGGGGTTAGAACGAGTTATAAGTGTATATGGGCCTCCCGCTCCAGACCGGGCAGGTGCTGAAACTCCTCTTGTTCTT
ACTGATATGACTGGAGGAGTGACTATTTATTATGATCCTGAAACAGGTGATTCAATGACTTTTGATAGTAGCAATAGAAT
TGATCGTCGTTCTCAACGTGGTGCTGGTGATCCATATACAGGTGAAGTCGTTGGTTGTGAGACAAATGAATCAGGAATAT
CGGCAGCATATGGCACAACGAAGATCTATACTACAGATACTAGAGCAAGATGGCTACATGGTGGGGATCATCGTTGCGC
GATCCTTATGCTCCTCGACAAGGCTGGAAACCAACTATGGGATGTACCCGTGCACAGAATGAAGATGTAGATGAGTTGTG
TAAGAAAGTGACAAGTTGGATGTATTCGCATCCTGGAGAGAGAATTAGATATGAACGTTTTAAGACACGTTAA

>Cluster_11468
ATGAAGATGATAAAAACGGACTGGAAATTTTTGCTGGTCTGGCTGATTCCGTTTTTATGGGTGGTTGCCCGGTTAATTAC
TGCTATTAAGGGGTAA
>Cluster_25105
ATGAGTGAACAAATCAAACAGGATATCGATCTGATTGAGATTTTATTTTATCTGAAGAAAAAGGTAGATGAATACCAACA
AAACTCTGGGCAGTAA
>Cluster_51096
ATGATTTCTGGCGCACCATCTCAGGATTCGCTGTTACCGGACAACCGCCACGCGGCTGATTACCAACAATTACGCGAGCG
GCTCATACAGGAACTGAATTTAACGCCGCAGCAGTTACATGAAGAGAGCAACCTGATCCAGGCCGGCCTGGATTCCATAA
GATTGATGAGATGGTTACACTGGTTTCGTAAAAATGGCTACCGCCTTACCCTTCGCGAGCTGTATGCCGCCCCCACGCTG
GCGGCATGGAACCAGTTAATGCTCAGCCGGTCGCCGGAGAACGCGGAAGAAGAAACGCCGCCCGACGAATCATCCTGGCC
GAACATGACCGAAAGTACCCCCTTCCCATTGACGCCAGTACAGCACGCCTACCTGACGGGCCGCATGCCGGGGCAGACGC
TTGGCGGCGTGGGTTGCCACCTGTATCAGGAGTTTGAAGGCCATTGTCTGACGGCGTCGCAGCTGGAGCAGGCCATCACG
ACCTTGCTGCAACGCCACCCAATGCTGCATATCGCCTTTCGCCCCGACGGGCAGCAGGTCTGGCTACCGCAACCTTACTG
GAACGGCGTCACCGTTCATGATTTACGCCATAACGACGCTGAAAGCCGCCAGGCCTATCTGGACGCACTGCGCCAGCGCC
TGAGCCACCGTCTTTTACGCGTGGAAATCGGCGAAACGTTTGATTTTCAGCTGACGCTCTTGCCGGACAATCGCCACCGC
CTACATGTCAATATTGACCTGCTGATTATGGATGCCTCCAGCTTTACGCTTTTCTTCGATGAGCTTAACGCCCTGCTGGC
CGGAGAATCGCTGCCGGCTATCGACACCCGCTATGATTTCCGCTCGTATTTGCTGCACCAGCAGAAGATCAATCAACCAC
TGAGAGACGACGCGCGCGCTTACTGGCTGGCGAAAGCATCGACGCTTCCCCCCGCGCCCGTCTTGCCGCTGGCCTGCGAA
CCCGCCACGCTACGTGAAGTCCGTAATACCCGACGCCGCATGATTGTCCCGGCAACACGCTGGCACGCCTTTAGCAACCG
GGCCGGCGAGTATGGCGTGACGCCGACAATGGCACTGGCGACCTGTTTTTCTGCCGTGCTGGCTCGCTGGGGCGGCCTGA
CGCGTCTGCTGCTTAACATCACCTTATTCGACCGCCAGCCGCTGCACCCAGCGGTTGGCGCGATGCTTGCCGACTTCACC
AATATTCTTCTGCTGGATACCGCCTGCGATGGCGATACCGTCAGCAACCTGGCGCGTAAAAACCAGCTCACGTTTACGGA
GGACTGGGAGCATCGCCACTGGTCCGGCGTCGAATTACTCCGTGAACTCAAACGCCAGCAGCGCTACCCCCACGGCGCCC
CGGTGGTATTTACCAGCAATCTGGGGCGTTCCCTCTACAGCAGCCGCGCAGAATCGCCGTTGGGCGAGCCGGAATGGGGC
ATCTCGCAAACGCCGCAGGTCTGGATAGATCATCTGGCGTTCGAGCATCACGGCGAGGTCTGGCTACAATGGGACAGCAA
CGACGCGCTGTTCCCTCCGGCGTTAGTCGAAACATTGTTCGACGCCTACTGCCAGTTGATTAACCAACTCTGCGATGACG
AAAGCGCCTGGCAAAAGCCGTTCGCAGATATGATGCCCGCCAGCCAGCGCGCGATACGCGAACGGGTCAACGCCACCGGC
GCCCCCATTCCCGAAGGCTTGCTGCATGAAGGCATTTTCCGTATCGCTCTGCAACAGCCGCAGGCGCTGGCGGTAACGGA
CATGCGTTATCAGTGGAATTATCATGAGCTGACAGACTATGCCCGCCGTTGCGCGGGCAGGTTAATCGAGTGCGGGGTTC
AGCCCGGCGATAATGTGGCTATCACGATGTCGAAAGGCGCAGGACAACTTGTTGCGGTTCTGGCCGTCCTGCTGGCCGGG
GCGGTTTACGTTCCGGTTTCGCTGGATCAGCCTGCCGCACGGCGCGAGAAAATCTACGCTGACGCCAGCGTCCGGCTGGT
GCTCATTTGTCAGCACGACGCCAGCGCCGGGTCAGACGATATTCCCGCCCTTGCCTGGCAGCAGGCCATTGAGGCGGAGC
CGATCGCCAACCCGGTAGTACGCGCCCCCACGCAACCGGCCTACATTATCTACACCTCCGGCTCTACCGGTACGCCGAAA
GGGGTAGTCATTTCTCACCGGGGAGCGCTTAACACCTGTTGCGATATCAATACCCGCTATCAGGTTGGCCCGCATGACAG
GGTGCTGGCCCTCTCCGCCCTACATTTTGATTTATCGGTTTACGACATTTTTGGCGTACTGCGCGCGGGCGGCGCGCTGG
TGATGGTGATGGAAAATCAACGGCGCGATCCTCACGCATGGTGTGAGCTGATCCAGCGCCATCAGGTCACGCTCTGGAAC
AGCGTCCCGGCGCTGTTCGATATGCTGCTGACCTGGTGTGAAGGTTTCGCCGACGCCACGCCGGAAAACCTGCGCGCAGT
GATGCTTTCCGGCGACTGGATCGGGCTTGACCTCCCCGCCCGTTATCGGGCCTTCCGGCCACAAGGACAATTTATCGCGA
TGGGCGGTGCCACCGAGGCGTCTATCTGGTCTAACGCCTGCGAAATTCACGACGTCCCCGCCCACTGGCGCTCCATCCCT
TACGGTTTTCCGCTAACCAACCAACGCTACCGGGTGGTGGATGAACAGGGCCGGGACTGCCCTGACTGGGTGCCGGGTGA
ATTATGGATTGGCGGCATTGGGGTCGCGGAAGGCTATTTCAACGATCCCCTGCGTAGCGAGCAGCAATTTTTGACGCTCC
CGGACGAGCGCTGGTATCGCACCGGCGATCTCGGCTGCTACTGGCCAGATGGCACAATCGAGTTCCTCGGTCGTCGCGAC
AAGCAGGTCAAAGTCGGAGGATATCGCATCGAGCTGGGCGAAATCGAAAGCGCGCTCAGCCAGCTGGCGGGGGTGAAACA
AGCAACCGTTCTGGCGATGGCGAAAAAGAAAAAACGCTGGCGGCATACGTTGTTCCTCAGGGCGAGGCTTTTTGCGTTA
CCGATCATCGGAACCCGGCACTGCCGCAGGCGTGGCACACGCTTGCGGGAACGTTGCCCTGTTGCGCCATCTCGCCAGAG
ATCTCCGCAGAACAGGTAGCCGATTTCCTTCAGCATCGCCTGCTAAAACTGAAGCCGGGTCACACCGCTGGCACCGATCC
TCTCCCCCTGATGAACTCACTCGCTATCCAGCCGCGCTGGCAGGCCGTGGTGGAACGCTGGTTAGCATTTCTGGTGACAC
AACGGCGACTGAAGCCCGCTGCTGAAGGTTATCAGGTCTGCGCTGGTGAAGAACGCGAGGATGAGCACCCGCACTTCAGC
GGACATGATTTAACGTTATCGCAAATTCTTCGCGGTGCCCGTAACGAACTGTCGTTACTGAACGACGCGCAGTGGTCGCC
GGAAAGCCTGGCCTTTAACCATCCGGCCAGCGCCCCGTATATTCAGGAACTGGCGACAATTTGCCAACAGCTTGCACAGC
GCTTACAGCGCCCGGTACGCCTGCTTGAGGTGGGAACCCGCACTGGCCGCGCCGCAGAATCGCTGTTAGCACAGCTCAAC
GCCGGACAGATTGAGTATGTCGGGCTTGAGCAGAGCCAGGAGATGCTGCTGAGCGCCCGGCAGAGGCTCGCCCCCTGGCC
TGGCGCCCGTCTGTCCCTCTGGAATGCAGACACGCTGGCGGCGCACGCTCACTCGGCGGACATTATCTGGCTTAATAACG
CCCTGCATCGTCTGCTGCCGGAAGATCCCGGGCTCCTTGCGACATTACAACAGCTTGCCGTTCCCGGCGCGCTGCTCTAC
GTGATGGAGTTTCGCCAGTTAACGCCGTCCGCCCTACTCAGCACGCTCCTGTTAACCAATGGGCAGCCGGAGGCCTTGCT
GCATAACAGCGCCGACTGGGCGGCATTATTTAGCGCGGCCGGCTTCAACTGTCAGCATGGCGATGAGGTCGCGGGGTTAC
AACGCTTCCTCGTACAATGTCCTGACAGGCAGGTGCGCCGCGATCCCCGTCAACTTCAGGCCGCCCTAGCCGGGCGTCTG
CCGGGGTGGATGGTGCCGCAACGGATCGTCTTCCTCGACGCCTTACCGCTGACGGCTAACGGGAAAATTGACTACCAGGC
GCTGAAGCGTCGTCATACCCCTGAAGCGGAAAACCCGGCCGAAGCGGATTTACCCCAAGGCGACATTGAAAAACAGGTTG
CCGCCCTCTGGCAGCAACTCTTATCAACTGGCAATGTCACCAGAGAAACCGACTTCTTCCAGCAAGGCGGCGATAGCCTG
CTGGCGACCCGTCTGACCGGGCAACTTCATCAGGCAGGTTATGAAGCGCAATTAAGCGACCTGTTTAATCATCCCCGGCT
GGCGGATTTTGCCGCCACGCTGCGGAAAACCGACGTCCCGGTAGAACAACCATTCGTCCACTCCCCTGAAGATCGCTACC
AGCCCTTTGCGCTTACCGACGTGCAGCAGGCTTACCTGGTGGGGCGTCAGCCGGGCTTTGCCCTGGGCGGCGTCGGCTCA
CATTTCTTTGTTGAATTTGAAATTGCCGATCTGGATCTCACCCGGTTGGAGACGGTCTGGAACCGATTAATCGCCCGCCA
CGATATGCTACGCGCCGTCGTGCGTGATGGACAGCAACAGGTGCTGCTCGAACAGACGCCTCGCTGGGTAATACCCGCACACA
TCCTCCATACGCCTGAAGAGGCGTTGCAGGTGCGCGAAAAACTGGCCCATCAGGTACTCAACCCCGAAGTCTGGCCGGTA
TTCGATCTCCAGGTCGGTTACGTTGACGGGATGCCCGCCCGCCTGTGGCTGTGTCTGGATAACCTGTTACTTGACGGGCT
GAGCATGCAGATTCTGTTGGCGGAGCTGGAACATGGCTACCAATATCCGCAACAGTTGCCTCCGCCGCTACCCGTCACCT
ACAGGGATTACCTGCAACAACCCGCGATCCAGTCGCTTAACGCAGATTCTCTGGCATGGTGGCAGGCGCAACTTGATGAT
ATTCCTCCGGCTCCTGCATTGCCGCTGCGCTGCTTGCCTCAGGAGGTTGAAACACCGCGCTTCGCTCGCCTGAACGACGT
ACTCGACAGCACACGCTGGCATCGGCTGAAAAAACGGGCGGCTGACGCCCATCTCACCCCGTCAGCCGTGCTGTTGTCCG
TGTGGTCAACCGTGCTCTCTGCATGGAGCGCGCAGCCTGAGTTCACGCTTAATCTTACGCTTTTCGACAGGCGACCACTG
CACCCGCAAATCAACCAGATTCTGGGCGATTTCACCTCGCTGATGCTGCTGAGCTGGCATCCCGGCGAAAGCTGGCTGCA
CAGCGCGCAGTCACTACAGCAGCGGCTGAGCCAGAACCTCAACCACCGCGATGTGTCAGCCATCCGCGTGATGCGTCAAC
TGGCGCAACGGCAAAACGTGCCAGCCGTTCCGATGCCCGTCGTCTTTACCAGCGCGCTGGGCTTTGAGCAGGATAACTTC
CTCGCCCGGCGTAATCTGCTCAAACCGGTCTGGGGCATCTCCCAGACGCCGCAGGTCTGGCTCGATCACCAGATTTATGA
ATCCGAAGGCGAACTGCGCTTTAACTGGGATTTTGTCGCCGCGCTGTTTCCTGCCGGGCAGGTGGAGCGCCAGTTTGAAC
AGTATTGCGCATTGCTAAACCGAATGGCCGAGGATGAAAGCGGCTGGCAACTGCCGCTCGCCGCGCTGGTGCCTCCCGTT

AAACACGCAGGGCAATGCGCAGAGCGCTCACCGCGCGTATGCCCTGAGCACTCTCAGCCACACATTGCGGCGGACGAGAG
CACCGTCAGCCTGATTTGCGACGCCTTCCGCGAGGTGGTTGGCGAGTCTGTCACGCCCGCAGAAAACTTCTTTGAGGCGG
GCGCAACGTCGCTGAATCTGGTGCAACTGCACGTTTTGTTACAACGTCACGAATTTTCCACCCTGACGTTGCTTGACCTC
TTCACCCACCCTTCTCCTGCTGCCCTGGCCGATTATCTGGCCGGCGTCGCCACGGTGGAGAAAACAAAACGACCTCGCCC
TGTTCGCCGTCGTCAGCGGCGGATATCGCGCGAAGCAAACTGA
>Cluster_7244
ATGACTGGTGGAATGCAGACTAGACCAATAACCAAATTCTCTAAATTAGAACAAATGGTTATTAATGAGGAGTTCGATAC
AAAAGAATAA
>Cluster_64450
ATGAATATTCAAGAATCTATAGGCGAACTACCGGAAACATGCCGGGCTGTTATCAAGCGTAAGGACGGACACATCGTTGG
TGTGCGTGTTCTGACTGATGATGAACGGATTGCCAGCCTGATGGCGTTTCTCGAGTTGGCAGAAATAGCTGGATATACTA
TTACACCCCCTGACGCGTAA
>Cluster_41194
ATGGCAACGAAAGCAGTTAATGGAAAATCCAAAAAACTTGAGGCACGAGTCCCCCATGCAATAGCTGACGCTGTAGAAAA
TTTAAAGGAAGAAGGCGAAAGCACTGGGCAATTTATCGTTTCGGCACTTGAAGGCGAGATCAAACGCCGCCAGCGCCGCA
AGGCCAAAGAACCAGAGTAA
>Cluster_40330
ATGAAATATACACCGGTTGGCGTTGATATCGCAAAACATGTCATTCAGGTTCACTTCATCAATGAGCACACAGGTAAAGT
GGTAGTTAAGCAGATTCGCCGTCAGGATTTTCTGACATTTTTCAGTAACCGGGAGCCTTGCCTGATTGGCATGGAGGCCT
GTGGGGGCTCTCAGCACTGA
>Cluster_14905
ATGAATATAATGAAACGTTTTTTTATGTGATGTCCCTGAACTGGATACTGACGTTTGTTGATAAACATATCACCTCCGG
ATGGAGCAGGAAGAGAAAGCTAATAACCCAGCTTATTATTTTAATCACGGGAATTACCTTATTTAAGGTCTTAGGTGGAA
TCAGTCAGAGCTGGTTGTGA
>Cluster_6260
ATGTTAACTGGGGCTTTTCTCTATCTGCCGTTGGTGTCCATGCCCGAGGCAGATAGCCTCAAGCACCCGCAGTCATTCTA
CTTAACTAAGATTTCCCCGCAAACCGTTTTTGTCCAGCACAGTAAATATCCAACTAAACCAATGGAGTTCGCTGTATTTA
CCGCCAGTATTCAATGCACATGA
>Cluster_44649
ATGTACAAGAAAGATGTTATCGACCACTTCGGAACCCAGCGTGCGGTAGCTAAAGCGTTAGGCATTAGCGATGCAGCAGT
CTCTCAGTGGAAGGAAGTCATCCCAGAGAAAGACGCCTATCGACTGGAAGTCGTTACAGCTGGCGCCCTGAAGTATCAAG
AAAGTGCTTATCGCAAAGCGGCATAA
>Cluster_49823
ATGGATCATCGTCTGCTTGAAATCATTGCCTGCCCGGTTTGCAACGGAAAACTTTGGTATAACCAGGAAAAACAAGAGCT
TATTTGCAAACTGGACAACCTGGCTTTCCCGCTGCGCGATGGCATTCCAGTGTTGCTGGAAACGGAAGCCCGCGTGCTGA
CTGCTGATGAGAGTAAATCATGA
>Cluster_21917
ATGGGCAAAGCAACGTATACCGTGACCGTCACCAATAACAGCAATGGCGTTTCTGTCGATTATGAAACAGAGACGCCGAT
GACTTTGCTGGTGCCAGAAGTGGCGGCTGAAGTGATAAAAGATCTGGTGAATACCGTGCGTTCTTATGACACGGAAAACG
AACATGATGTTTGTGGTTGGTAA
>Cluster_7611
ATGTCTTGTCCAAAATGCGGTTCTGGAAATATTGCAAAAGAAAAAACAATGCGTGGATGGTCTGATGATTATGTGTGCTG
CGATTGCGGATACAACGACTCTAAAGACGCATTTGGAGAGCGTGGTAAAAACGATTTTGTCAAAATTAATAAAGAACGCG
AAGGCAACGAAAAAAGCTAA
>Cluster_17773
ATGCAGTGGAAAATCATCAACGGTTGGTACTGCGTTACAGCTTGCGGGCTGATGAGCTGGAAGTTCCGCACGCTGGGTGA
AGCAATGAACTGGGTATTCGTCAGCAAGCTGGCGGTAAAAACAGAAATGGATATGGGGGTGAGCAAGTGA
>Cluster_38820
ATGTTTTTTAATACTAAACATACCGCAGCATTGTGCTTAGCGAGCTGTATGGCTTTTAGCTCATCAGCTATTGCAGACAT
CGTAATTTCAGGTACACACGTAGTTTATAAAAGCGAACAAAAAAAGCTTCAATGTACGTTTGGAAAATAA
>Cluster_33812
ATGAGTTTAGTTATCGCAGATAAGATCTCTTATTCGGTTGATGATTTATTAAAAATAAAATCATTAATTTGGCTGCGGTC
ATTTTTAATTGTCAATACAGATAAAAACCAAGTGTTGACGCAGAATGGTGGTATCCATTAA
>Cluster_29478
ATGGCTGATTTTGGTTCAACTAAATACAACGTCGGTTTTGAAGAATGGCATGAACTGTTAATGGGTAATGACTCCAACTT
ATTGATAGTGTTTTATGTTCAGATAATGCCCGATGACTTTGTCATGCAGCTCCACCGATTTTGA
>Cluster_50035
ATGTCACTTGCAACCACAGTGAAGGAAAGCAAGTTACAGAGACGCATGTACACGCAGCAGGCGTTAATGTATCGCCAGAA
GGGAGATCGTGAAGGTGTTCGCGTATTTTTAAATGCGGCAAAGACTGAAGTATTAAATCAGCGTTATTTCCTTGGGCCGT
GTCCATTCTGA
>Cluster_15656
ATGAATCACAAACAATTCGTACTTATAGTTCTTGATAACATCGAACTGTTAGTTCATTATTATCACCATCAGCAGGACGC
TGGCAGCCAAACGGAACAGATTGGCAGGCTCTTTAACTTCGATGGTGCGCTGACAAAGCGCGAACAGATACCAAACGAGA
TGGGTTTGGGGTGA
>Cluster_34968
ATGCCTTCGATGATAGCGATAATTTTACTCATCATCCTTCACATCTGGCTCTGTAGACAGGGTGGTGATCACTTCTGGAG
TAAATCCAGATTAAACATCTCATTGCTGATGCTTGATATTGAGCATCTGGCGCGCAGTAAGGGGCTGCGTTGA
>Cluster_37337
ATGACAATGCTACTGCCGCTCTTCATTCTGGTTGGTTTTATTGCAGGTTATTTTGTTAATGCCATCGCCTATCACCTCTC
GCCGTTGGAGGATAAAACGGCGTTAACGTTTCGCCAGGTATTGGTTCATTTCAGGCAAAAAAATATGCCTGGCATGATAC
AGTGCCCCTGA
>Cluster_47107
GTGGAACAGATGTATAAAAAATTAAAATTAACCACAATAAGCGAATTGATTAAAAATATTTATTGTTCATTATCCGTTCT
CATCATTGGTTGTGCGTCAGCTTATGCCGTTGAATTCAACAAAGATTTAATCGAAGCCGAAGATCGTGAAAACGTTAACC
TTTCCCAATTTGAAACTGATGGCCAATTACCCGTAGGTAAATACTCATTAAACGCTCTTATTAATAATAAAAGGACGCCA
ATCCATCTGGACCTGCAATGGGTATTAATTGATAACCAAACTGCAGTTTGTCTGACTCCAGAGCAATTAACATTATTAGG
CTTTACTGATGAGATTATTGAAGTGGCTCAGCAAAACCTGATCGATGGTTGTTACCCTATCGAAAAAGAAAAACAAATTA
CAACTTATCTCGATAAAGGGAAAATGCAATTATCCATATCTGCACCTCAGGCATGGTTAAAATACAAAGATGCAAACTGG
ACGCCTCCTGAACTTTGGGATCATGGTATTGCTGGGGCATTTCTTGACTACAATTTATATGCCTCTCATTATGCACCACA

TCAGGGCGATAATTCACAAAATATAAGTTCCTATGGGCAGGCTGGGGTTAATCTTGGGGCCTGGCGCCTGCGTACTGATT
ACCAGTACGATCAGTCATTTAACAATGGCAAAAGCCAGGCGAACAACCTGGATTTTCCGCGTATTTATTTGTTTCGCCCA
ATCCCAGCAATTAATGCAAAACTAACTATAGGTCAATACGATACTGAATCCTCTATTTTCGACTCTTTCCATTTTTCTGG
CGTTTCGTTGAAAAGCGATGAGAATATGTTGCCGCCTGATCTACGTGGTTACGCACCGCAAATCACGGGTGTTGCACAAA
CGAATGCAAAGGTCACTGTCTCACAGAACAACCGTATTATTTATCAAGAAAATGTTCCTCCTGGCCCATTTGCTATTACT
AATTTATTCAATACATTACAGGGGCAACTGGACGTCAAGGTTGAAGAAGAGGACGGGCAAGTCACGCAATGGCAAGTTGC
ATCTAATAGTATTCCTTATCTGACGCGTAAAGGGCAGATTCGCTACACCACTGCTATGGGTAAACCGACCAGCGTTGGTG
GTGATTCCTTACAACAACCCTTCTTCTGGACTGGTGAATTCTCATGGGGTTGGCTGAACAATGTATCCCTGTATGGTGGT
TCAGTTTTAACAAACCGTGATTATCAATCTCTGGCTGCCGGCGTTGGCTTTAACCTTAACTCATTGGGTTCATTATCTTT
TGATGTCACACGATCTGATGCTCAGTTGCATAATCAGGATAAAGAAACGGGTTATAGCTACCGCGCTAACTATTCAAAAC
GTTTTGAATCTACCGGTAGCCAGCTCACTTTCGCTGGTTACCGTTTCTCTGATAAAAACTTTGTGACAATGAATGAATAT
ATCAATGACACTAACCATTACACGAATTATCAGAATGAAAAAGAGAGTTATATTGTCACGTTTAACCAGTATCTTGAATC
ATTAAGGTTAAATACATACGTAAGTTTGGCTCGTAATACTTACTGGGACGCCAGCAGTAATGTGAATTATTCATTATCAC
TTAGCCGCGATTTTGATATCGGCCATTAAAAAACGTCTCCACTTCTCTAACATTTAGCCGAATAAACTGGGAAGAAGAC
AACCAGGATCAACTGTACCTAAATATTTCGATTCCCTGGGGAACCAGTAGAACATTGAGCTATGGTATGCAACGAAATCA
GGATAACAAGATTTCGCATACTGCTTCGTGGTATGACTCTTCCGATCGAAATAATTCCTGGAGCGTTTCTGCTTCAGGCG
ACAATGATGAATTCAAAGATATGAAAGCGTCACTACGCGCCAGTTATCAGCATAATACCGAGAACGGTCGTCTCTATCTC
TCCGGGACATCACAGCGAGACAGTTATTATTCTCTGAATGCCAGTTGGAATGGTTCATTCACGGCGACTCGTCACGGAGC
CGCTTTCCATGACTATAGTGGTAGTGCTGACTCGCGTTTTATGATCGACGCAGATGGTGCAGAAGATATTCCGTTGAACA
ATAAACGCGCGGTAACTAATCGGTATGGCATCGGAGTTATTCCATCAGTCAGCAGTTACATAACAACATCATTAAGCGTT
GATACCCGAAATCTTCCAGAAAATGTGGATATCGAAAACTCGGTTATCACCACCACCTTAACCGAAGGTGCTATTGGCTA
CGCCAAACTTGATACCCGCAAGGGCTATCAAATCATGGGGGTTATTCGCCTGGCAGATGGTAGTCACCCACCACTGGGGA
TTAGCGTAAAAGATAAAACCAGCCATAAAGAATTAGGACTGGTTGCTGATGGCGGCTTTGTATACCTCAACGGCATTCAG
GATGACAGCAAACTTACTTTACGCTGGGGTGATAAATCCTGTTTTATTCAACCACCCAATAGCAGCAACTTAACCACCGG
AACGGTTATTTTACCGTGTATTAGCCAAAATTAA
>Cluster_24023
TTGTACCTCTATATTGAGACTCTGAAACAGAGACTGGATGCCATAAATCAATTGCGTGTGGATCGCGCGCTTGCTGCTAT
GGGGCCTGCATTCCAACAGGTCTACAGTCTACTGCCGACATTGTTGCACTATCACCATCCGCTAATGCCGGGTTACCTTG
ATGGTAACGTTCCCAAAGGCATTTGCCTTTACACGCCTGATGAAACTCAACGCCACTACCTGAACGAGCTTGAACTGTAT
CGTGGAATGTCAGTACAGGACCCACCGAAAGGTGAGCTTCCAATTACTGGTGTATACACCATGGGCAGCACCTCGTCCGT
AGGGCAAAGTTGTTCCTCTGACCTGGATATCTGGGTCTGTCATCAATCCTGGCTCGATAGCGAAGAGCGCCAGTTGCTAC
AACGTAAATGTAGTCTGCTGGAAAGCTGGGCCGCCTCGCTGGGTGTGGAAGTCAGCTTCTTCCTGATTGATGAAAACCGC
TTCCGTCATAATGAAAGCGGCAGCCTGGGGGGCGAAGATTGTGGCTCCACCCAGCATATACTGCTGCTTGACGAATTTTA
TCGTACCGCCGTGCGTCTCGCCGGTAAGCGTATTCTGTGGAATATGGTGCCGTGCGACGAAGAAGAGCATTACGACGACT
ATGTGATGACGCTTTACGCGCAGGGCGTGCTGACGCCAAATGAATGGCTGGATCTTGGTGGCTTAAGCTCGCTTTCTGCT
GAAGAGTACTTTGGTGCCAGCCTTTGGCAGCTCTACAAGAGTATCGATTCCCCATACAAAGCGGTGCTGAAAACACTGCT
GCTGGAAGCCTATTCCTGGGAATACCCGAACCCACGTCTGCTGGCGAAAGATATCAAACAGCGTTTGCACGACGGCGAGA
TTGTATCGTTTGGCCTCGATCCATACTGCATGATGCTGGAGCGTGTTACTGAATATCTGACGGCGATTGAAGATTTCACC
CGTCTGGATTTAGTGCGTCGCTGCTTCTATTTAAAAGTGTGCGAAAAGCTCAGCCGTGAACGCGCCTGTGTAGGCTGGCG
TCGCGCAGTGTTGAGCCAGTTAGTGAGCGAGTGGGGTTGGGACGAAGCTCGTTTGGCAATGCTCGATAACCGCGCCAACT
GGAAGATTGATCAGGTGCGTGAGGCGCACAACGAGCTGCTCGACGCGATGATGCAGAGCTACCGTAATCTGATCCGCTTT
GCGCGTCGCAATAACCTTAGCGTCTCCGCCAGTCCGCAGGATATCGGCGTGCTGACGCGTAAACTGTACGCTGCGTTTGA
AGCATTACCGGGTAAAGTGACGCTGGTAAACCCGCAGATTTCGCCCGATCTCTCGGAACCGAATCTGACCTTTATTTATG
TACCGCCGGGCCGCGCCAACCGTTCAGGTTGGTATCTGTATAACCGCGCGCCAAATATTGAGTCAATCATCAGCCATCAG
CCGCTGGAATATATAATCGCTACCTGAATAAACTAGTGGCGTGGGCATGGTTTAACGGCCTGCTGACCTCTCGCACCCGTTT
GTATATTAAAGGTAACGGCATTGTCGATTTGCCTAAGTTGCAGGAGATGGTCGCAGACGTGTCGCACCATTTCCCGTTGC
GCCTGCCTGCACCGACGCCAAAAGCGCTCTACAGCCCGTGTGAGATCCGCCATCTGGCGATTATCGTTAACCTGGAATAT
GACCCGACGGCAGCCTTCCGCAATCAGGTGGTGCATTTTGATTTCCGTAAGCTGGATGTCTTCAGCTTTGGCGAGAATCA
AAATTGCCTGGTGGGTAGCGTTGACCTGCTGTATCGCAACTCGTGGAACGAAGTGCGTACGCTGCACTTCAACGGTGAGC
AGTCGATGATTGAAGCCCTGAAAACCATTCTCGGCAAAATGCATCAGGACGCCGCACCGCCAGATAGTGTGGAAGTCTTC
TGTTATAGCCAGCATCTGCGCGGGTTAATCCGTACTCGCGTGCAGCAACTGGTTTCTGAGTGTATTGAACTGCGTCTTTC
CAGCACCCGCCAGGAAACCGGGCGCTTCAAGGCGCTGCGCGTCTCCGGTCAAACCTGGGGCTTGTTCTTCGAACGGCTGA
ATGTCTCGGTACAGAAACTGGAAAACGCCATCGAGTTTTATGGCGCTATTTCGCATAACAAACTGCACGGCCTGTCAGTG
CAGGTTGAAACCAATCACGTCAAATTACCGGCGGTGGTGGACGGCTTTGCCAGCGAAGGGATCATCCAGTTTTTCTTCGA
AGAAACGCAAGACGAGAATGGCTTTAATATCTACATTCTCGACGAAAGCAACCGGGTTGAGGTGTATCACCACTGCGAAG
GCAGCAAAGAGGAATTGGTGCGTGACGTCAGTCGCTTCTACTCGTCTTCGCACGACCGTTTCACCTACGGCTCAAGCTTT
ATCAACTTTAACTTGCCGCAGTTCTATCAGATTGTGAAGGTTGATGGTCGTGAACAGGTGATTCCGTTTCGCACCAAATC
TATCGGTAACATGCCACCTGCCAATCAGGAGCATGACGCGCCGCTGTTGCAGCAGTATTTTTCTTGA
>Cluster_29036
ATGGCCCTGATTGTGGAATTTATTTGTGAGCTACCTAACGGCGTCCATGCGCGTCCGGCAAGCCACGTTGAAACGCTGTG
TAATACTTTTTCATCACAAATTGAGTGGCATAACCTGCGCACTGACCGCAAGGGCAACGCCAAAAGCGCCCTTGCGCTGA
TTGGCACCGATACGCTGGTGGGCGATAACTGCCAGTTACTGATTTCCGGGGCCGACGAACAGGAAGCGCACCAGCGTTTA
AGCCAATGGCTGCGCGATGAATTCCCCCACTGCGACGCACCGCTGGCGGAAGTAAAATCTGACGAACTGGAACCACTGCC
GGTTTCACTGACCAATCTGAATCCGCAAATTATCCGTGCCCGCACCGTGTGCAGCGGTAGCGCAGGCGGCATTCTGACGC
CGATCTCTTCTTTAGATCTCAATGCGCTGGGTAATCTTCCCGCTGCCAAAGGCGTTGACGCCGAGCAATCCGCGCTGGAA
AACGGCCTGACGCTGGTACTGAAAAACATTGAGTTTCGTCTGCTGGATAGCGACGGTGCTACCAGCGCGATTCTGGAAGC
GCACCGATCCCTGGCTGGCGATACTTCCCTGCGCGAACATTTACTGGCAGGCGTCAGCGCCGGATTAAGCTGCGCCGAAG
CAATTGTCGCCAGTGCAAATCACTTTTGCGAAGAGTTCGCCCGTTCCAGCAGCAGCTACCTGCAAGAACGTGCGCTGGAC
GTACGCGACGTCTGCTTCCAGTTACTCCAGCAAATCTACGGTGAGCAACGCTTCCCGGCACCGGGCAAACTGACGCAGCC
CGCCATTTGTATGGCTGATGAACTGACCCCCAGCCAGTTCCTCGAACTGGATAAAAATCACCTCAAAGGATTGTTGCTCA
AAAGCGGCGGCACCACCTCACATACGGTGATCCTTGCCCGTTCGTTCAACATTCCGACGCTGGTTGGTGTGGATATTGAT
GCCCTCACTCCGTGGCAGCATCAAACGATTTATATCGACGGCAACGCTGGGGCGATTGTGGTTGAGCCAGGGGAAGCCGT
AGCTCGTTATTATCAGCAGGAAGCCCGCGTACAGGACGCCCTGCGTGAGCAACAGCGTGTCTGGCTGACCCAACAAGCCC
GTACTGCTGACGGTATCCGCATTGAAATTGCTGCTAACATCGCTCACTCCGTGGAAGCGCAGGCCGCATTCGGCAATGGT
GCGGAAGGCGTTGGTTTATTCCGCACTGAAATGCTCTATATGGATCGCACCAGTGCACCGGGCGAAAGCGAGCTGTACAA
CATTTTTTGTCAGGCGCTGGAGTCCGCCAACGGACGCAGCATTATTGTGCGCACTATGGATATTGGCGGTGACAAACCCG
TTGATTATCTGAACATTCCCGCAGAAGCTAACCCGTTCCTCGGTTATCGCGCCGTGCGTATTTATGAAGAGTACGCGTCG
TTATTTACCACACAACTACGGTCGATTCTCCGTGCCTCCGCTCACGGCAGCCTGAAAATCATGATCCCGATGATCTCCTC
AATGGAAGAGATCTTATGGGTAAAAGAAAAACTGGCAGAAGCCAAACAGCAACTACGTAACGAACACATTCCGTTTGATG

AGAAGATCCAGCTCGGCATTATGCTGGAAGTGCCGTCGGTGATGTTCATCATCGATCAATGCTGCGAAGAGATTGATTTC
TTTAGTATCGGTAGTAATGACCTGACGCAATATCTGCTGGCAGTGGATCGCGATAACGCTAAGGTTACTCGTCACTATAA
CAGCCTGAATCCAGCATTCTTGCGGGCGCTCGATTACGCCGTGCAGGCGGTGCATCGCCAGGGCAAATGGATTGGTCTGT
GCGGTGAACTGGGAGCGAAAGGTTCCGTGCTGCCGTTGCTGGTCGGTTTAGGGCTGGATGAACTCAGCATGAGCGCACCA
TCAATTCCGGCGGCGAAAGCGCGGATGGCGCAACTTGATAGCCGTGAGTGCCGCCAGTTGCTCAACCAGGCAATGGCCTG
CCGTACTTCGCTGGAAGTGGAACACCTGCTGGCGCAATTCCGCATGACCCAACAGGACGCACCGCTGGTCACCGCCGAGT
GCATCACACTGGAAAGCGACTGGCGCAGCAAAGAAGAAGTGCTCAAAGGCATGACCGATAACCTGCTGCTGGCGGGCCGC
TGCCGCTATCCGCGTAAACTGGAAGCCGACTTGTGGGCGCGCGAGGCCGTTTTCTCTACCGGACTGGGCTTTAGTTTCGC
CATTCCCCACAGTAAATCGGAACACATTGAGCAATCCACCATCAGCGTAGCGCGTCTGCAAGCGCCAGTGCGCTGGGGCG
ATGATGAAGCGCAATTCATCATTATGTTAACCCTGAACAAACATGCTGCGGGCGATCAGCACATGCGCATTTTCTCGCGC
CTCGCTCGTCGCATCATGCACGAAGAATTCCGTAACGCGCTGGTTAACGCCGCCTCTGCCGACGCTATCGCCAGCCTGCT
GCAACATGAACTGGAACTGTAA
>Cluster_66962
ATGAAGTATTCTAAGCTGTTCCTGTCCGTTGGGTTGGCTTTGGTTACATTATCTGGTTGGGGGCGAACCTATACGTTTGA
TCCTTCGTTAGTTGAAAGCAGTGGAGGTGATAGTGTAGATGTTTCATTATTTAATCAGGGTCTTCAATTGCCGGGAGAAT
ACTTTGTTAGTATTTTCGTTAATGGTGAAAAGGTAGGCTCTGACAATATTAATTTTCGTATAGAAAATCATAATGGCGAA
GATACATTATCACCGTGTTTGAACGCAGACCAGCTAACTAAGTATGGGATTGATATCCATAAATACTCAGATTTGTTTAA
TGCTGGTCCAGAGCAATGTGCTAATTTATGGGCGATCCCGCAGGCTGATATTCAGTTTGATTTTAATCAGCAAAAACTTT
CATTGCTTCTGCCAACGCAGGCTCTTTTACCGAAATTAAATGGAATTGCTCCTGAGCAATTGTGGGATGATGGTATTCCT
GCATTGTTTATGAATTATCAAACGAATATGCAGCAAAGAGAATACCAGGGAGCCTATAAGTCGCATGACGAGTCATATTA
TGCGCAGTTACAACCAGGACTGAATATTGGCCCCTGGCGTTTTCGTAGCGCCGCGTCATGGCAAAAGGAACAGGGCTGGC
AACGTTCATATATCTATGCTGAGCGCGGGCTAAATACTATAAAAGGCCGTTTGACATTAGGGGAGTCATATTCTGATGGC
AGTATCTTTGACAGTATTCCGTTTACTGGCGGAAAGCTGGCTTCAGATGAAACGATGCTCCCTTATGACCAATGGAGTTT
TTCGCCAGTCATTCGTGGTGTGGCGCGTACACAGGCCAGAGTTGAGGTTCAGCAGAATGGTTACACCGTGAGTAATGACC
TTATTCCATCAGGTCCTTTTGAATTAACAAACCTTCCTCTGGGAGGTGGAAGCGGTGACCTGAAAGTCATTGTTCATGAG
AGTGACGGAACTCAACAAGTATTTACTGTTCCATATGACACGCCTGCCGTTGCATTACGACAGGGATATTTCGAATATTC
GGTCATGGGAGGAGAGTATCGCCCTGCTAATGATGCCGTGCAAACGACACCTGTAGGTGCTCTTGAAATGAAGTACGGGT
TACCGTGGAACCTCACACTATATGGAGGGCTTCAAGGGGCCGGTAATTACCAAGCTGCTGCTTTAGGAATCGGTTCTTTA
TTAGGTGATTTTGGTGCTTTATCTGCTGATGTTGTTCAATCAAATAGCAAAAAGGATAATCAGCAAAAGGAAAGCGGTCA
GCGTTGGCGTGTCAGATACAATAAATCGCTGGATAGTGGTACGTCGGTCAACATCGCAAGTGAGGAATATGCGACCGAAG
GATTCAATACCTTGTCTGATACGTTAAATACGTATTGTAAGCCTGATGCCGGAAACATTTGTTACTCTGATTATAAGAAA
CCCAAGAACAAGGTCAATCTTAGTATCAGTCAGACAACAGACGGTTGGGGAACTTTCAATTTCAATGGTTACCGACAAAA
CTACTGGAATGATAAATCAACAACAACGTCTTTTACTGCTGGATATAGTCGGATGTTTGATAGCGGGATTTCTTTAAATG
TTAATTTATCGAAAACCCAAAATATCGATAAAAACGGGAAGAAAACAAATGATCGGCTAACTAGCCTTTGGCTGAGTTTC
CCTCTGAGTCGCTGGTTAAGTAATAGTTCGGTAAATGCAAACTATCAAATGACATCAGATACCCGAGGCGATTCCATGCA
TGAGTTTGGGGTATACGGTGACGCATTTAATCGTCAACTACATTGGGATCTCCGGGAGCGTTATAGAGATAATGCTTCAG
ATAATAAAGCATCCAGCGCGTTAAGTCTGAACTATCGTGGTACTTACGGAGAATTACGAGGCAATTATAGTTATGATAAA
AAACAACGGCAGCTTGGTATAGGAATTAATGGAAATATTGTCGCTACTCAGTATGGAATAACAGCAGGACAAAGCTCGGG
AGACACCATGGCATTAGTTCAGGCTCCGGGAGTAGATGGTGCTTCTGTAGGTTACTGGCCCGGTATGAAAACTGATTTCA
GGGGATATACCAGTTATGGATATTTAACACCTTACCGGGAAAATAATATTGACATTAACCCGGTGACATTGCCAAAGAAT
GCAGAGATATCGCAGACATCTACGCGTGTTGTTCCTACAAAAGGAGCCGTTGTTTTAGCTAAATTTGATACACGTATCGG
TGGGCGACTGTTATTACAATTGAAACGTTCTGATAATAAGCCAGTACCTTTTGGCTCTGTTGCTACTGTTGAAGGCCAGG
CATCCAGTTCTGGGATTGTTGGGGATAACAGTCAGGTATATTTAACAGGCGTTCCCAAAGAAGCTACTGTAAAGATTCAA
TGGGGTAAAGATAAAACACAGTCATGTCATGCCAGAGTATTATTACCTGAAGATGTTAATACGACGGGAATATATAATTT
AACAGCAGTATGTACTCTTAATGGTTAA
>Cluster_43066
ATGAACGAACAATATTCCGCATTGCGTAGTAATGTCAGTATGCTCGGCAAAGTGCTGGGAGAAACCATCAAGGATGCGTT
GGGAGAACACATTCTTGAACGCGTAGAAACTATCCGTAAGTTGTCCAAATCTTCACGCGCTGGCAATGATGCTAACCGCC
AGGAGTTGCTCACCACCTTACAAAATTTGTCGAACGACGAGCTGCTGCCCGTTGCGCGTGCGTTTAGTCAGTTCCTGAAC
CTGGCCAACACCGCTGAGCAATACCACAGCATTTCGCCGAAAGGCGAAGCTGCCAGCAACCCGGAAGTGATCGCCCGCAC
CCTGCGTAAACTGAAAAACCAGCCGGAACTGAGCGAAGACACCATCAAAAAAGCAGTGGAATCGCTGTCGCTGGAACTGG
TCCTCACGGCTCACCCAACCGAAATTACCCGTCGTACACTGATCCACAAAATGGTGGAAGTGAACGCCTGTTTAAAACAG
CTCGATAACAAAGATATCGCCGACTACGAACACAACCAGCTGATGCGCCGCCTGCGCCAGTTGATCGCCCAGTCATGGCA
TACCGATGAAATCCGTAAACTGCGCCCAAGCCCGGTAGATGAAGCCAAATGGGGCTTTGCCGTAGTAGAAAACAGCCTGT
GGCAAGGCGTACCGAATTACCTGCGCGAACTGAACGAACAACTGGAAGAGAACCTCGGCTACAAACTGCCCGTCGAATTT
GTTCCGGTCCGTTTACCTCGTGGATGGGCGGTGACCGCGACGGCAACCCGAACGTCACTGCCGATATCACCCGCCACGT
CCTGCTACTCAGCCGCTGGAAAGCCACCGATTTGTTCCTGAAAGATATTCAGGTTCTGGTTTCTGAACTGTCGATGGTTG
AAGCCACCCCTGAACTGCTGGCGCTGGTTGGCGAAGAGGGTGCCGCAGAACCGTATCGCTATCTGATGAAAAACCTGCGT
TCTCGCCTGATGGCGACACAGGCATGGCTGGAAGCGCGCCTGAAAGGCGAAGAATTGCCAAAACCAGAAGGTCTGCTGAC
ACAAAACGAAGAACTGTGGGAACCGCTCTACGCTTGCTACCAGTCACTTCAGGCGTGTGGCATGGGTATTATCGCCAATG
GCGATCTGCTCGACACCCTGCGCCGCGTGAAATGTTTCGGCGTACCGCTGGTGCGTATTGATATCCGTCAGGAGAGCACG
CGTCATACCGAAGCATTAGGCGAACTGACCCGCTACCTCGGTATCGGCGATTATGAAAGCTGGTCAGAAGCCGACAAACA
GGCGTTCCTGATCCGCGAACTGAACTCCAAACGTCCGCTTCTACCACGCAACTGGCAACCAAGCGCCGAAACGCGCGAAG
TGCTCGATACCTGCCAGGTGATTGCCGAAGCGCCGCAAGGCTCCATTGCCGCCTACGTGATCTCGATGGCGAAAACGCCG
TCCGACGTGCTGGCTGTCCACCTGCTGCTGAAAGAAGCGGGTATCGGGTTTGCGATGCCGGTTGCTCCGCTGTTTGAAAC
CCTCGATGACCTGAACAACGCCAACGATGTCATGACCCAGCTGCTGAATATCGACTGGTATCGCGGCCTGATTCAGGGCA
AACAGATGGTGATGATTGGCTATTCCGACTCAGCAAAAGATGCGGGCGTGATGGCAGCTTCCTGGGCGCAATATCAGGCA
CAGGATGCATTAATCAAAACCTGCGAAAAAGCGGGTATTGAGCTGACGTTGTTCCACGGTCGCGGCGGTTCCATTGGTCG
CGGCGGCGCACCTGCCCATGCGGCGCTGCTGTCACAACCGCCAGGAAGCCTGAAAGGTGGCCTGCGCGTGACCGAACAGG
GCGAGATGATCCGCTTTAAATATGGTCTGCCAGAAATCACCGTCAGCAGCCTGTCGCTGTACACCGGGGCGATTCTGGAA
GCCAACCTGCTGCCACCGCCTGAGCCGAAAGAGAGCTGGCGTCGTATTATGGATGAACTGTCTGTTATCTCTTGCGATGT
CTACCGCGGCTACGTACGTGAAAACAAAGATTTTGTGCCTTACTTCCGCTCCGCTACGCCGGAACAAGAATTGGGTAAAC
TGCCGTTGGGTTCACGTCCGGCGAAACGTCGCCCGACCGGCGGCGTCGAGTCACTGCGCGCTATTCCGTGGATTTTCGCC
TGGACGCAAAACCGCCTGATGCTCCCCGCCTGGCTGGGTGCAGGTACGGCGCTGCAAAAAGTGGTCGAAGATGGCAAACA
GAGCGAGCTGGAAGCCATGTGCCGCGATTGGCCATTCTTCTCGACGCGTCTCGGCATGCTGGAGATGGTCTTCGCCAAAG
CAGACCTGTGGCTGGCGGAATACTATGACCAACGCCTGGTAGACAAAGCACTGTGGCCGTTAGGTAAAGAGTTACGCAAT
CTGCAAGAAGAAGACATCAAAGTGGTGCTGGCGATTGCCAACGATTCTCACCTGATGGCCGATCTGCCGTGGATTGCAGA
GTCTATTCAGCTACGGAATATTTACACCGACCCGCTGAACGTATTGCAGGCCGAGTTGCTGCACCGCTCCCGCCAGGCAG
AAAAAGAAGGCCAGGAACCGGATCCTCGCGTCGAGCAGGCGTTAATGGTCACTATTGCCGGGATTGCGGCAGGTATGCGT

AATACCGGCTAA
>Cluster_58096
ATGAATACCCTTCCAGAACAGTACGCAAACACCGCTCTCCCCACCCTGCCCGGTCAACCGCAAAATCCATGCGTCTGGCC
CCGTGATGAATTAACCGTCGGTGGGATAAAAGCCCATATCGATACTTTCCAGCGTTGGCTGGGTGATGCCTTTGACAATG
GGATCTCTGCAGAACAGTTGATTGAGGCGCGCACCGAGTTTATCGACCAGCTCCTGCAACGATTATGGATTGAAGCGGGA
TTCAGCCAGATTGCCGACCTGGCATTGGTCGCCGTCGGTGGCTACGGTCGTGGCGAGCTGCATCCACTTTCAGACGTCGA
TTTACTGATTTTAAGCCGTAAAAAGCTCCCGGACGATCAGGCGCAAAAAGTGGGCGAGCTGTTAACGCTGCTCTGGGATG
TAAAGCTGGAAGTCGGTCATAGCGTGCGCACGCTTGAAGAGTGCATGCTGGAAGGGTTATCGGATTTAACCGTCGCCACC
AATTTAATCGAATCCCGCTTATTAATTGGCGATGTTGCGCTGTTCCTCGAACTGCAAAAACATATTTTCAGCGAAGGATT
CTGGCCTTCCGACAAGTTCTACGCGGCGAAAGTTGAAGAACAGAACCAGCGCCATCAGCGTTACCATGGCACCAGCTACA
ACCTTGAACCAGACATCAAAAGCAGCCCTGGCGGCTTGCGCGATATCCACACTCTGCAATGGGTGGCCCGCCGTCATTTT
GGCGCAACATCGCTGGATGAAATGGTCGGGTTTGGCTTCTTAACCTCAGCGGAGCGGGCGGAATTAAACGAATGTCTGCA
TATATTGTGGCGTATTCGCTTTGCCCTGCATCTGGTCGTCAGCCGTTACGATAATCGCCTGTTATTCGATCGCCAGCTTA
GCGTCGCCCAGCGTCTGAATTACAGTGGTGAAGGTAACGAACCGGTCGAGCGGATGATGAAGGATTACTTCCGCGTTACA
CGCCGCGTCAGTGAACTCAACCAGATGCTGCTGCAACTGTTCGATGAAGCCATCCTCGCCCTTCCCGCCGACGAAAAACC
ACGTCCAATCGACGATGAGTTTCAGCTACGCGGTACGCTAATCGACCTGCGTGATGAAACACTATTTATGCGCCAGCCGG
AAGCCATCTTGCGTATGTTCTACACCATGGTGCACAACAGTGCGATCACCGGCATTTACTCCACCACGCTGCGCCAGTTA
CGCCATGCCCGTCGCCATCTGCAACAACCGCTGTGTAATATTCCGGAAGCACGAAAACTGTTTTTGAGCATTCTGCGTCA
CCCCGGAGCGGTGCGGCGCGGGCTATTGCCAATGCATCGCCATAGCGTGCTCGGCGCGTATATGCCGCAATGGTCGCATA
TCGTCGGGCAGATGCAGTTTGATCTGTTCCACGCCTACACGGTGGATGAACATACTATCCGCGTGATGCTGAAACTGGAG
AGTTTTGCCAGTGAAGAAACGCGCCAGCGCCATCCGTTGTGTGTGGACGTCTGGCCGCGCCTGCCGTCAACTGAGCTGAT
TTTCATCGCCGCGCTGTTTCACGATATCGCCAAAGGACGCGGCGGCGACCACTCCATTCTCGGTGCTCAGGATGTAGTGC
ATTTTGCCGAACTCCACGGGCTGAACTCACGCGAAACACAGCTGGTCGCCTGGCTGGTTCGCCAGCACCTGTTGATGTCG
GTGACCGCCCAACGCCGCGATATTCAGGACCCGGAAGTCATCAAGCAGTTTGCCGAAGAAGTGCAAACGGAAAATCGTCT
GCGCTATCTGGTATGCCTGACTGTGGCTGACATTTGCGCCACCAACGAAACGCTGTGGAATAGCTGGAAGCAAAGTCTGT
TGCGTGAGCTCTACTTTGCCACCGAAAAGCAGCTACGACGCGGAATGCAAAACACGCCGGATATGCGCGAACGGGTTCGC
CATCACCAACTCCAGGCACTGGCACTACTGCGCATGGATAACATCGACGAAGAGGCGCTGCACCAAATTTGGTCACGCTG
TCGTGCTAACTATTTTGTCCGCCATAGCCCAAATCAACTGGCCTGGCATGCCCGCCATTTATTACAGCATGATTTAAGCA
AACCGCTGGTATTGCTTAGCCCGCAGGCTACGCGTGGAGGCACCGAGATTTTTATCTGGAGCCCGGACCGCCCTTATCTG
TTTGCCGCCGTCTGTGCCGAATTAGACCGCCGCAATTTAAGTGTTCACGACGCACAAATTTTCACCACTCGCGACGGTAT
GGCGATGGATACCTTTATCGTGCTGGAACCCGATGGCAACCCGCTGTCCGCAGATCGTCATGAGGTTATTCGGTTTGGTC
TGGAGCAAGTACTGACGCAAAGTAGCTGGCAGCCACCGCAGCCCCGTCGCCAACCCGCCAAATTACGCCATTTTACTGTT
GAAACCGAAGTAACGTTTTTGCCGACCCATACCGACCGCAAATCGTTCCTCGAACTGATCGCCCTCGACCAACCTGGACT
GCTGGCGCGAGTCGGGAAAATTTTTGCCGATCTGGGAATTTCGCTTCATGGTGCCCGAATTACAACCATTGGCGAGCGAG
TAGAAGATTTATTCATAATTGCCACCGCTGACCGGCGTGCGCTTAATAACGAGTTGCAGCAGGAAGTGCATCAGCGGTTG
ACAGAGGCCCTCAATCCAAACGATAAAGGGTGA
>Cluster_150
ATGTCCCAGCCGGTTGGTGATCTTGTTATTGACCTTAGTCTGGATGCGGTCCGTTTCGATGAGCAGATGAGCCGGGTAAG
GCGTCATTTTTCAGGACTGGATACTGACGCCAGAAAAACCGCCAGTGCTGTTGAACAGGGCCTGAGCCGCCAGGCGCTGG
CTGCACAAAAAGCCGGGATTTCCGTCGGGCAGTATAAAGCGGCCATGCGAACCCTGCCCGCACAGTTTACGGATATCGCC
ACGCAGCTTGCCGGTGGTCAGAATCCCTGGCGGATCCTGCTGCAACAGGGCGGTCAGGTGAAGGACTCCTTCGGCGGGAT
GATCCCCATGCTCAGGGGGCTTGCCGGTGCGATCACCCTGCCGATGGTCGGGGTCACCTCGCTGGCGGTGGCGACAGGTG
CGCTGGCGTACGCCTGGTACCAGGGGGATTCCACGCTTTCAGCGTTTAATAAAACCCTGGTTCTTTCCGGTAATCAGTCC
GGACTGACTGCCGATCGTATGCTGACTCTCTCAAGAGCCGGGCAGGCAGCAGGGCTGACGTTTAACCAGGCGAGAGAGTC
ACTGGCAGCCCTGGTGAATGCCGGTGTGCGTGGTGGTGAACAGTTTGATGCCATCAACCAGAGTGTCGCGCGTTTTGCGT
CTGCATCCGGTGTGGAGGTGGATAAAGTCGCTGAAGCCTTCGGGAAGCTGACCACTGACCCGACGTCGGGACTGATGGCG
ATGGCGCGCCAGTTCCGTAACGTGACGGCAGAGCAGATTGCGTATGTTGCGCAGCTGCAGCGTTCCGGTGATGAGGCCGG
GGCCTTACAGGCGGCGAACGAGGCCGCAACGAAAGGGTTTGATGACCAGACCCGCCGCCTGAAAGAGAACATGGGCACGC
TGGAGACCTGGGCAGACAGGACTGCACGGGCATTCAAATCCATGTGGGATGCGGTGCTGGATATTGGTCGTCCTGATACC
GCGCAGGAGATGCTGATTAAGGCAGAGGCTGCGTTTAAGAAAGCAGACGACATCTGGAATCTGCGCAAGGATGATTATTT
TGTTAACGATGAAGCGCGGGCGCGTTACTGGGATGATCGTGAAAAGGCCCGTCTTGCGCTTGAAGCCGCCCGAAAGAAGG
CTGAGCAGCAGACTCAACAGGACAAAAATGCGCAGCAGCAGAGCGATACCGAAGCGTCACGGCTGAAATATACCGAAGAG
GCGCAGAAGGCTTACGAACGGCTGCAGACGCCGCTGGAGAAATATACCGCCCGTCAGGAAGAACTGAACAAGGCACTGAA
AGACGGGAAAATCCTGCAGGCGGATTACAACACGCTGATGGCGGCGGCGAAAAAGGATTATGAAGCGACGCTGAAAAAGC
CGAAACAGTCCGGCGTGAAGGTGTCTGCGGGCGATCGTCAGGAAGACAGTGCTCATGCTGCCCTGCTGACGCTTCAGGCT
GAACTCCGGACGCTGGAGAAGCATGCCGGAGCAAATGAGAAAATCAGCCAGCAGCGCCGGGATTTGTGGAAGGCGGAGAG
TCAGTTCGCGGTACTGGAGGAGGCGGCACAACGTCGCCAGCTGTCCGCACAGGAGAAATCCCTGCTGGCGCATAAAGATG
AGACGCTGGAGTACAAACGCCAGCTGGCTGCACTTGGCGATAAGGTCACGTATCAGGAGCGTCTGAATGCGCTGGCGCAG
CAGGCGGATAAATTCGCACAGCAGCAACGGGCAAAACGGGCCGCCATTGATGCGAAAAGCCGGGGGCTGACTGACCGGCA
GGCAGAACGGGAAGCCACAGAACAGCGCCTGAAGGAACAGTATGGCGATAATCCGCTGGCGCTGAATAACGTCATGTCAG
AGCAGAAAAAGACCTGGGCGGCTGAAGACCAGCTTCGCGGGAACTGGATGGCAGGCCTGAAGTCCGGCTGGAGTGAGTGG
GCGGAAAGTGCGACGGACAGTTTTTCGCAGGTAAAAAGCGTGGCCACGCAGACCTTTGACGGTATTGCACAGAATATGGC
AGCGATGCTGACCGGCAGTGAGCAGAACTGGCGCAGCTTCACCCGTTCCGTGCTGTCCATGATGACAGAAATTCTGCTTA
AGCAGGCAATGGTGGGGATTGTCGGGAGTATCGGTAGCGCCATTGGCGGGGCTGTTGGTGGCGGCGCATCCGCATCAGGC
GGTACAGCCATTCAGGCAGCTGCGGCGAAATTCCATTTTGCAACCGGAGGATTTACGGGAACCGGCGGCAAATATGAGCC
AGCGGGAATTGTTCACCGCGGTGAATTCGTCTTCACGAAGGAGGCAACCAGACGGATTGGCGTGGGGAATCTCTACCGGC
TGATGCGCGGCTATGCCACCGGCGGTTATGTCGGTACACCGGGCAGCATGGCGGACAGCCGGTCGCAGGCGTCCGGGACG
TTTGAGCAGAATAACCATGTGGTGATTAACAACGACGGCACGAACGGTCAGATAGGGCCACAGGCACTGAAGGCTGTTTA
TGACGTAGCCCGTAAGGCGGCAATGGATGTTGTGACCGGGCAGATGCGCGATGGTGGTCTGTTCTCCGGAGGTGGACGAT
GA
>Cluster_63232
ATGTATAGAACTCATCGACAACACAGCCTGTTAAGCTCTGGTGGAGTGCCATCGTTTATTGGTGGGCTGGTGGTGTTTGT
GTCGGCAGCGTTCAATGCACAAGCTGAAACCTGGTTCGATCCTGCTTTTTTCAAAGATGATCCCTCAATGGTGGCCGATT
TGTCTCGTTTCGAAAAAGGACAAAAAATAACGCCAGGGGTTTATCGTGTCGATATTGTTCTGAATCAGACAATTGTAGAT
ACGCGCAACGTCAATTTTGTTGAGATAACGCCAGAGAAGGGGATTGCCGCCTGTTTGACGACTGAAAGCCTGGATGCAAT
GGGTGTTAATACTGATGCGTTTCCGGCTTTTAAACAACTGGACAAACAAGCGTGTGTGCCATTGGCGGAGATTATTCCGG
ATGCCAGCGTAACGTTTAATGTGAATAAACTCCGTCTGGAAATTTCAGTACCGCAAATCGCCATCAAAAGTAATGCCCGT
GGTTATGTCCCCCCTGAACGTTGGGATGAAGGATCAACGCGCTATTACTGGGATATTCGTTTAGCGGGGCTAACAGTAT
TCATAGCAGCGCAGACAGTGATTCTGGCGACAGCTATTTTCTGAATTTAAACAGTGGCGTTAATTTAGGCCCATGGAGAT

TGCGCAACAATTCAACATGGAGTCGCAGTAGTGGCCAAACCGCAGAATGGAAGAATCTCAGCAGCTATTTGCAGCGGGCG
GTTATTCCACTAAAAGGCGAACTGACCGTTGGTGATGATTATACTGCAGGCGATTTTTTCGATAGTGTCAGCTTTCGTGG
TGTGCAGCTGGCGTCAGATGACAACATGCTGCCAGACAGCCTGAAAGGGTTTGCGCCTGTGGTGCGTGGTATCGCCAAAA
GCAATGCCCAGATAACGATTAAGCAAAATGGTTACACCATTTACCAAACTTATGTATCGCCTGGTGCTTTTGAAATTAGT
GATCTCTATTCCACGTCGTCGAGCGGTGATTTGTTAGTTGAAATCAAAGAAGCGGACGGCAGCGTCAATAGCTACAGCGT
ACCGTTTTCCAGCGTGCCATTACTCCAGCGTCAGGGGCGAATCAAATACGCGGTGACACTGGCGAAATACAGAACCAATA
GTAATGAACAGCAGGAGAGCAAATTTGCCCAGGCCACGTTGCAGTGGGGCGGACCGTGGGGAACGACATGGTATGGTGGT
GGACAATATGCTGAATATTACCGTGCCGCCATGTTTGGTCTGGGATTTAACCTTGGCGATTTCGGAGCAATTTCGTTCGA
TGCGACCCAGGCGAAGAGTACGCTGGCAGACCAAAGCGAACATAAAGGTCAGTCATATCGTTTTCTGTATGCCAAAACGC
TCAACCAATTGGGCACTAATTTTCAATTGATGGGCTATCGCTATTCGACGTCGGGTTTCTACACCCTTTCCGACACCATG
TATAAACATATGGATGGCTACGAATTTAATGACGGTGATGATGAAGATACGCCGATGTGGTCGCGTTATTACAATTTGTT
TTACACCAAACGTGGCAAACTGCAGGTCAATATCTCCCAGCAATTAGGCGAGTACGGTTCGTTTTATTTAAGTGGTAGCC
AGCAAACTTACTGGCATACCGATCAACAGGATCGGCTATTACAGTTTGGCTACAACACGCAAATTAAAGATCTCTCGCTG
GGGGTTTCCTGGAACTACAGTAAGTCCCGTGGTCAACCTGATGCTGATCAGGTGTTTGCACTAAATTTTTCCCTGCCGCT
CAATCTGTTGCTCCCCAGAAGTAATGATAGCTATACCAGGAAAAAAAATTACGCCTGGATGACCTCTAACACCAGTATCG
ATAACGAAGGGCACATTACACAAAACCTGGGTTTAACGGAGACACTACTCGATGACGGTAACCTGAGCTACAGCGTGCAA
CAGGGATATAACAGCGAGGGGAAAACGGCTAATGGTAGCGCCAGCATGGACTACAAAGGGGCGTTTGCAGATGCCCGAGT
GGGCTACAACTACAGCGATAACGGCAGTCAACAACAACTGAACTACGCTCTTTCAGGCAGTTTAGTTGCCCATTCACAGG
GCATTACCCTGGGGCAATCGCTGGGGGAAACTAACGTGCTGATTGCAGCACCAGGCGCAGAGAATACTCGTGTGGCGAAC
AGCACCGGGCTGAAAACTGACTGGCGCGGATATACCGTTGTCCCCTATGCCACTTCTTATCGGGAAAATCGAATCGCACT
TGATGCGGCATCGTTAAAACGTAACGTGGATCTTGAAAATGCAGTAGTCAACGTGGTTCCCACCAAAGGGGCGTTGGTTC
TGGCGGAGTTCAATGCCCATGCGGGAGCAAGGGTATTAATGAAAACATCAAAGCAGGGTATACCGCTGCGTTTTGGCGCG
ATAGCGACGCTGGACGGCATACAGACTAATAGCGGCATTATTGATGATGATGGCTCGCTCTATATGTCCGGTTTGCCCGC
TCAGGGAGCGATAACTGTACGCTGGGGCGAAGCTCCCGATCAAATTTGTCATATCAGTTACCAGCTTACCGAACAACAAA
TTAACTCTGCGATTACGCGAATGGATGCCATATGCAGATAA
>Cluster_54540
ATGAAAACGAAAATCCCTGATGCGGTATTGGCTGCTGAGGTGAGTCGCCGTGGTTTGGTAAAAACGACAGCGATCGGCGG
CCTGGCAATGGCCAGCAGCGCATTAACATTACCTTTTAGTCGGATTGCGCACGCTGTCGATAGCGCCATTCCAACAAAAT
CAGACGAAAAGGTTATCTGGAGCGCCTGTACAGTTAACTGTGGTAGTCGCTGCCCGCTACGTATGCACGTCGTGGACGGT
GAAATCAAATATGTCGAAACGGACAATACCGGCGATGACAATTACGACGGCCTGCACCAGGTTCGCGCCTGCCTGCGTGG
GCGTTCCATGCGTCGCCGTGTCTACAATCCGGACCGCCTGAAATATCCGATGAAACGAGTCGGGGCGCGCGGTGAAGGCA
AATTCGAGCGCATTAGCTGGGAAGAAGCCTACGACATCATCGCGACCAATATGCAGCGCCTGATCAAAGAGTACGGCAAC
GAGTCTATCTATCTGAACTATGGCACCGGTACGCTGGGCGGCACCATGACCCGCTCCTGGCCGCCGGGAAATACCCTGGT
CGCGCGGCTGATGAACTGCTGCGGCGGCTATCTGAACCATTACGGCGACTACTCCTCCGCGCAAATTGCGGAAGGTTTGA
ACTATACCTACGGCGGCTGGGCAGATGGCAACAGCCCGTCGGATATCGAAAACAGTAAGCTGGTAGTGCTGTTTGGTAAT
AACCCTGGCGAAACGCGAATGAGTGGCGGTGGGGTGACTTACTATCTTGAACAGGCACGCCAGAAATCTAATGCCCGCAT
GATCATCATCGATCCGCGCTATACCGACACCGGTGCCGGGCGCGAAGATGAGTGGATCCCTATTCGTCCGGGAACAGATG
CCGCACTGGTTAACGGTCTGGCGTACGTCATGATCACTGAAAACCTGGTGGATCAGGCATTCCTCGATAAATATTGCGTT
GGCTACGATGAGAAAACCCTGCCAGCCAGTGCGCCGAAAAATGGCCACTATAAAGCTTATATTCTGGGTGAAGGGCCAGA
TGGCGTGGCTAAAACGCCGGAATGGGCCTCGCAAATCACTGGTGTTCCGGCAGACAAAATCATCAAATTGGCTCGTGAAA
TCGGTAGTACCAAACCGGCGTTTATCAGCCAGGGATGGGGCCCGCAGCGTCACGCTAACGGTGAAATCGCAACCCGTGCT
ATCTCGATGCTGGCGATTCTGACCGGTAACGTTGGTATTAACGGAGGCAACAGCGGCGCGCGTGAAGGTTCATACAGCTT
ACCGTTTGTCCGTATGCCGACCTTGGAAAACCCGATCCAGACCAGCATTTCGATGTTTATGTGGACCGATGCCATTGAAC
GTGGCCCGGAAATGACGGCGCTGCGTGATGGTGTACGCGGGAAAGATAAGCTGGATGTGCCGATCAAAATGATCTGGAAC
TATGCCGGTAACTGCCTGATTAACCAGCATTCTGAAATCAACCGTACCCATGAAATCCTTCAGGATGATAAGAAGTGCGA
GCTGATTGTGGTTATCGACTGCCACATGACCTCATCGGCGAAATATGCTGACATCCTGCTGCCTGACTGCACCGCTTCCG
AACAGATGGACTTTGCGCTGGATGCATCCTGCGGGAATATGTCTTACGTGATTTTCAACGATCAGGTGATTAAACCGCGC
TTTGAATGTAAGACCATCTATGAAATGACCAGCGAACTGGCAAAACGTCTTGGCGTTGAGCAACAGTTTACTGAAGGCCG
TACCCAGGAAGAGTGGATGCGGCATCTGTATGCCCAGTCGCGGGAAGCGATTCCTGAACTGCCAACGTTTGAAGAGTTCC
GCAAGCAGGGGATCTTTAAAAAGCGCGACCCACAAGGGCATCACGTTGCTTATAAAGCCTTCCGTGAAGATCCGCAGGCA
AACCCACTGACTACGCCATCGGGCAAAATTGAGATTTATTCGCAGGCGCTGGCTGACATTGCCGCTACCTGGGAATTGCC
TGAAGGCGATGTGATCGATCCACTGCCGATCTACACGCCGGGCTTTGAAAGTTATCAGGATCCGCTGAACAAACAGTATC
CGCTGCAGCTTACAGGTTTCCACTATAAATCTCGCGTTCACTCAACTTACGGCAACGTTGATGTGCTGAAAGCGGCTTGC
CGTCAGGAAATGTGGATCAACCCGCTTGATGCCCAAAAACGCGGTATCCACAACGGCGATAAAGTCAGGATCTTTAACGA
TCGTGGTGAGGTTCATATTGAGGCGAAAGTGACGCCACGAATGATGCCGGGTGTGGTCGCACTGGGTGAAGGTGCCTGGT
ATGACCCGGATGCAAAACGTGTCGATAAGGGTGGTTGTATTAACGTACTGACCACTCAACGTCCGTCTCCTCTCGCTAAG
GGGAATCCGTCACATACAAACCTTGTTCAGGTTGAAAAGGTGTAA
>Cluster_31841
ATGAATCAGAATCTGCTGGTGACAAAGCGCGACGGTAGCACAGAGCGCATCAATCTCGACAAAATCCATCGCGTTCTGGA
TTGGGCGGCAGAAGGACTGCATAACGTTTCGATTTCCCAGGTCGAGCTGCGCTCCCACATTCAGTTTTATGACGGTATCA
AGACCTCTGACATCCACGAAACTATTATCAAGGCTGCCGCAGACCTGATCTCCCGTGATGCGCCGGATTATCAGTATCTC
GCCGCACGTCTGGCGATCTTCCACCTGCGTAAAAAAGCCTACGGTCAGTTTGAGCCGCCTGCGCTGTACGACCATGTGGT
GAAAATGGTCGAGATGGGCAAATACGATAATCATCTGCTGGAAGACTACACGGAAGAAGAGTTCAAGCAGATGGACACGT
TTATCGATCACGACCGTGATATGACCTTCTCTTATGCTGCCGTTAAGCAGCTGGAAGGCAAATATCTGGTACAGAACCGC
GTGACCGGCGAAATCTACGAAAGCGCCCAGTTCCTTTATATTCTGGTTGCCGCGTGCCTGTTCTCGAACTACCCGCGTGA
AACGCGCCTGCAATACGTGAAGCGTTTTTACGACGCGGTTTCCACATTTAAAATTTCGCTGCCGACGCCAATCATGTCCG
GCGTGCGTACCCCGACTCGTCAGTTCAGCTCATGCGTGCTGATCGAGTGCGGCGACAGCCTGGATTCCATTAACGCCACC
TCCAGCGCGATTGTTAAATACGTTTCCCAGCGTGCCGGGATCGGCATCAACGCCGGGCGTATTCGTGCGCTGGGTAGCCC
GATTCGCGGTGGCGAAGCGTTCCATACCGGTTGTATTCCGTTCTACAAACATTTCCAGACAGCGGTGAAATCCTGCTCTC
AGGGCGGTGTGCGCGGCGGTGCGGCAACGCTGTTCTACCCGATGTGGCATCTGGAAGTGGAAAGCCTGCTGGTGTTGAAA
AACAACCGTGGTGTGGAAGGCAACCGCGTGCGTCATATGGACTACGGGGTTCAAATCAACAAACTGATGTATACCCGTCT
GCTGAAAGGTGAAGATATCACTCTGTTCAGCCCGTCCGACGTACCGGGGCTGTACGACGCGTTCTTCGCCGATCAGGAAG
AGTTTGAACGTCTGTATACCAAATATGAGAAAGACGACAGCATCCGCAAGCAGCGTGTGAAAGCCGTTGAGCTGTTCTCG
CTGATGATGCAGGAACGTGCATCTACCGGTCGTATCTATATTCAGAACGTTGATCACTGCAATACCCATAGCCCGTTTGA
TCCGGCCATCGCGCCAGTGCGCCAGTCTAACCTGTGCCTGGAAATCGCCCTGCCGACCAAACCGCTGAACGACGTCAACG
ACGAGAACGGTGAAATCGCGCTGTGTACGCTGTCTGCTTTCAACCTGGGCGCAATTAACAGCCTGGATGAACTGGAAGAG
CTGGCAATTCTGGCAGTACGCGCACTCGACGCGCTGCTGGATTATCAGGATTACCCGATCCCGGCCGCCAAACGTGGAGC
GATGGGTCGTCGTACGCTGGGTATTGGTGTGATCAACTTCGCTTACTACCTGGCGAAGCACGGTAAACGCTACTCCGACG
GCAGCGCCAACAACCTGACGCATAAAACCTTCGAAGCCATTCAGTATAACCTGCTGAAAGCGTCTAATGAGCTGGCGAAA

GAGCAAGGCGCGTGCCCGTGGTTTAACGAAACCACTTACGCGAAAGGGATCCTGCCGATCGATACCTATAAGAAAGATCT
GGATGCCATCGCTAATGAGCCGCTGCATTACGACTGGGAAGCACTGCGTGAGTCAATCAAAACGCACGGCCTGCGTAACT
CCACGCTTTCTGCCCTGATGCCGTCCGAGACTTCTTCGCAGATCTCTAACGCCACTAACGGTATTGAACCGCCGCGCGGT
TACGTCAGCATCAAAGCGTCGAAAGACGGTATTCTGCGCCAGGTGGTGCCGGACTACGAGCATCTGCACGACGCCTATGA
GCTGCTGTGGGAAATGCCGGGTAACGATGGCTACCTGCAACTGGTGGGTATCATGCAGAAATTTATCGATCAGTCGATCT
CTGCTAACACCAACTACGATCCGTCACGTTTCCCGTCAGGAAAAGTGCCGATGCAGCAGTTGCTGAAAGACCTGCTCACC
GCCTACAAATTCGGGGTCAAAACGCTGTATTATCAGAACACCCGTGACGGTGCTGAAGACGCACAAGACGATCTGGTGCC
GTCAATCCAGGACGATGGCTGCGAAAGCGGCGCATGTAAGATCTGA
>Cluster_32885
ATGATGAATGATTCGTTCTGCCGCATTATTGCGGGTGAAATTCAGGCGCGCCCGGAACAGGTTGACGCTGCCGTTCGCCT
GCTTGACGAAGGGAATACCGTGCCGTTTATCGCACGTTATCGTAAGGAAATCACCGGCGGTCTGGATGACACGCAGCTGC
GTAATCTGGAAACGCGTCTGAGCTATCTGCGCGAGCTGGAAGAGAGACGTCAGGCGATCCTCAAGTCCATTTCCGAGCAA
GGCAAACTCACCGATGATCTGGCGAATGCCATCAACGCCACCCTAAGCAAAACCGAACTCGAAGACCTCTACCTGCCCTA
CAAACCTAAACGCCGCACCCGTGGGCAAATCGCCATTGAAGCAGGGCTTGAGCCGTTGGCCGATCTGCTGTGGAGCGATC
CGTCACACACGCCAGAAGTCGCCGCTGCACAATATATTGATGCCGATAAAGGCGTGGCAGATACCAAAGCCGCGCTGGAC
GGCGCGCGCTATATCCTGATGGAACGGTTTGCCGAAGATGCCGCGCTGCTGGCGAAAGTGCGTGATTATCTGTGGAAGAA
CGCGCATTTGGTTTCTACGGTGGCGAGCGGTAAAGAAGAGGAAGGGGCGAAATTCCGCGACTATTTCGATCATCACGAAC
CGCTGTCCACGGTGCCTTCTCACCGCGCGCTGGCGATGTTCCGTGGTCGTAACGAAGGCATACTTCAGCTTTCGCTGAAT
GCCGATCCGCAGTTCGAAGAACCGCCGAAAGAGAGCTATTGCGAGCAAATCATCATGGATCACCTTGGCCTGCGCCTGAA
CAATGCCCCAGCGGATAGCTGGCGCAAAGGTGTGGTGAGCTGGACGTGGCGCATCAAGGTGTTGATGCATCTGGAAACTG
AACTGATGGGCACCGTGCGCGAACGCGCGGAAGATGAAGCAATCAACGTCTTTGCCCGTAACCTGCATGATCTGCTGATG
GCGGCCCCTGCCGGGCTGCGTGCAACGATGGGCCTCGATCCGGGTCTGCGTACTGGGGTAAAAGTGGCGGTAGTCGATGC
AACTGGCAAACTGGTGGCGACCGATACCATTTATCCGCATACCGGGCAGGCCGCAAAAGCAGCGATGACCGTTGCTGCGC
TGTGTGAAAAGCATAATGTTGAACTGGTGGCGATCGGCAACGGTACAGCTTCTCGCGAAACCGAACGTTTCTATCTCGAC
GTGCAGAAGCAGTTCCCGAAAGTGACCGCGCAGAAAGTGATCGTCAGCGAAGCTGGCGCGTCGGTTTACTCGGCTTCCGA
GCTGGCAGCACAGGAATTCCCGGATCTCGACGTTTCGCTGCGTGGCGCGGTGTCTATCGCCCGTCGTTTGCAGGATCCGC
TGGCGGAGCTGGTGAAAATCGATCCGAAATCTATCGGCGTAGGTCAGTATCAGCATGACGTCAGCCAGACGCAGCTGGCA
CGCAAACTGGATGCAGTAGTAGAAGACTGCGTAAACGCCGTTGGCGTCGATCTCAACACCGCTTCTGTTCCTCTGTTAAC
CCGCGTGGCGGGCCTGACGCGCATGATGGCGCAAAACATCGTAGCCTGGCGCGATGAGAACGGCCAGTTCCAGAACCGTC
AGCAACTGTTAAAAGTGAGCCGTCTGGGGCCGAAAGCCTTCGAGCAGTGCGCGGGTTTCTTGCGCATTAACCACGGTGAT
AACCCGCTGGACGCTTCTACCGTTCACCCGGAAGCCTATCCGGTGGTGGAACGCATTCTGGCAGCAACACAGCAGGCACT
GAAAGATCTGATGGGTAACAGCAGCAAACTGCGTAACCTGAAAGCATCTGACTTTACTGATGAGAAATTCGGTGTGCCGA
CGGTAACCGACATCATCAAAGAGCTGGAAAAACCGGGTCGCGATCCGCGTCCGGAATTTAAAACCGCTCAGTTTGCCGAT
GGCGTCGAGACAATGAACGACCTGCAACCGGGTATGATCCTCGAAGGTGCAGTGACCAACGTCACCAACTTTGGCGCGTT
TGTCGATATTGGCGTGCATCAGGACGGCCTGGTTCACATCTCTTCATTGTCGAACAAGTTTGTGGAAGATCCGCATACCG
TGGTGAAAGCGGGCGACATTGTGAAGGTGAAAGTGCTGGAAGTGGATCTTCAGCGTAAACGTATCGCCCTGACCATGCGT
CTGGACGAGCAGCCTGGCGAAACCAACGCCCGTCGCGGCGGCGGTAATGAACGCCCGCAAAACAACCGCCCGGCAGCCAA
AACGCGTGGGCGTGAAGCGCAGCCTGCCGGTAACAGCGCGATGATGGATGCGCTGGCGGCGGCAATGGGCAAAAAACGTT
AA
>Cluster_42082
ATGAGCTCTATCTCCCTGATCCAACCGGATCGCGACCTGTTCTCCTGGCCGCAGTACTGGGCCGCCTGTTTTGGACCGGC
ACCGTTTTTGCCGATGTCACGTGAAGAGATGGATCAACTTGGCTGGGATAGCTGCGACATCATTTTGGTTACTGGCGACG
CGTATGTCGATCACCCAAGCTTCGGGATGGCGATTTGCGGTCGTATGCTGGAAGCGCAGGGCTTTCGCGTCGGGATCATC
GCTCAGCCAGACTGGAGCAGTAAGGACGATTTCATGCGTCTGGGTAAACCGAATCTGTTTTTCGGCGTTACTGCTGGCAA
CATGGACTCGATGATCAACCGCTATACCGCCGATCGCCGTTTACGTCATGACGATGCCTACACGCCAGATAATGTCGCGG
GTAAGCGTCCGGATCGCGCCACACTGGTTTATACCCAACGTTGTAAAGAGGCGTGGAAAGATGTGCCGGTGATCCTCGGC
GGTATTGAGGCCAGCCTGCGCCGTACCGCGCACTATGATTACTGGTCCGATACCGTGCGCCGTTCCGTGTTGGTGGATTC
GAAAGCCGACATGCTGATGTTTGGCAATGGCGAGCGTCCGCTGGTGGAAGTGGCGCACCGTCTGGCGATGGGCGAGCCGA
TTAGTGAAATCCGCGATGTGCGTAATACCGCGATTATCGTAAAAGAGGCGTTGCCTGGCTGGAGCGGTGTGGATTCCACC
CGTCTTGATACCCCAGGGAAAATCGACCCAATCCCGCATCCGTATGGCGAAGATTTGCCGTGCGCGGATAACAAACCGGT
AGCTCCGAAAAAGCAGGAAGCCAAAGCTGTAACCGTGCAGCCACCACGCCCGAAACCGTGGGAAAAAACCTACGTGTTGC
TGCCTTCTTTCGAGAAAGTGAAGGGCGATAAAGTGCTGTACGCCCATGCTTCGCGCATTCTGCACCACGAAACTAACCCA
GGCTGCGCCCGCGCATTGATGCAAAAACATGGCGACCGCTATGTATGGATCAACCCGCCTGCTATTCCGCTTTCTACCGA
AGAGATGGATAGCGTCTTTGCGCTGCCGTACAAGCGCGTGCCGCATCCGGCTTACGGTAATGCCCGAATTCCGGCTTACG
AAATGATTCGTTTCTCGGTCAACATTATGCGTGGCTGCTTTGGCGGCTGCTCTTTCTGTTCTATTACCGAGCACGAAGGG
CGCATTATTCAGAGCCGTTCCGAAGATTCGATTATTAATGAGATCGAAGCGATCCGCGACACTGTTCCAGGTTTTACGGG
CGTGATTTCCGATCTCGGTGGGCCTACTGCCAACATGTATATGTTGCGTTGCAAATCGCCACGCGCTGAGCAAACCTGCC
GTCGTTTGTCGTGCGTTTATCCGGATATTTGTCCGCATATGGACACTAACCACGAACCGACGATCAACCTCTATCGCCGT
GCGCGCGATCTGAAAGGCATTAAAAAGATCCTGATTGCCTCTGGTGTGCGTTATGACATCGCCGTAGAAGATCCGCGCTA
TATCAAAGAGCTGGCGACCCATCACGTCGGCGGTTATCTGAAGATTGCCCCGGAACATACCGAAGAAGGGCCGTTATCGA
AGATGATGAAGCCGGGCATGGGCAGCTATGACCGCTTTAAAGAGCTGTTCGATACTTACTCGAAACAGGCAGGTAAAGAA
CAGTATCTGATCCCGTATTTCATCTCCGCGCACCCGGGTACGCGTGATGAAGATATGGTGAATCTGGCGCTATGGCTGAA
AAAGCATCGCTTCCGCCTCGACCAGGTGCAGAACTTCTATCCGTCGCCGCTGGCTAACTCGACCACCATGTATTACACCG
GCAAAAACCCGCTGGCGAAGATTGGTTATAAGAGTGAAGACGTCTTCGTACCGAAGGGCGACAAGCAGCGTCGCCTGCAT
AAAGCGTTGTTGCGTTACCACGATCCGGCAAACTGGCCGTTAATCCGCCAGGCGCTGGAAGCAATGGGTAAAAAGCATCT
GATTGGCAGCCGTCGCGATTGCTTAGTGCCTGCGCCAACCATTGAAGAGATGCGTGAGGCTCGTCGCCAGAACCGCAATA
CCCTTCCGGCGTTGACTAAACATACGCCGATGGCGACCCAGCGTCAGACGCCTGCTACGGCAAAAAAAGCGTCGTCTACG
CAATCTCGTCCGGTGAATGCTGGTGCGAAGAAACGCCCTAAAGCGGCGGTTGGACGTTAA
>Cluster_22609
ATGGATGACCAGTTAAAACAAAGTGCACTTGATTTCCATGAATTTCCAGTTCCAGGGAAAATCCAGGTTTCTCCAACCAA
GCCTCTGGCAACGCAGCGCGATCTGGCGCTGGCCTACTCACCAGGCGTTGCCGCACCTTGTCTTGAAATCGAAAAAGACC
CGTTAAAAGCCTACAAATATACCGCGCGCGGTAACCTGGTAGCGGTGATCTCTAACGGTACGGCGGTGCTGGGGTTAGGC
AACATTGGCGCGCTGGCAGGCAAACCGGTGATGGAAGGCAAGGGCGTTCTGTTTAAGAAATTCGCCGGGATTGATGTATT
TGACATAGAAGTTGATGAACTCGACCCGGACAAATTTATTGAAGTCGTCGCTGCGCTCGAACCGACCTTTGGCGGCATCA
ACCTCGAAGACATTAAAGCGCCAGAATGTTTCTATATTGAACAGAAACTGCGCGAGCGGATGAATATTCCGGTATTCCAC
GACGATCAGCACGGCACGGCAATTATCAGCACTGCCGCCATCCTCAACGGCTTGCGCGTGGTGGAGAAAAACATCTCCGA
CGTGCGGATGGTGGTTTCTGGCGCGGGTGCCGCAGCAATCGCCTGTATGAACCTGCTGGTAGCGCTGGGTCTGCAAAAAC
ATAACATCGTGGTTTGCGATTCAAAAGGCGTTATCTATCAGGGCCGTGAGCCAAACATGGCGGAAACCAAAGCCGCATAC
GCGGTGGTGGATGACGGTAAACGTACCCTCGATGATGTGATTGAAGGCGCGGATATTTTCCTCGGCTGTTCCGGCCCGAA

AGTGCTGACCCAGGAAATGGTGAAGAAAATGGCCCGTGCGCCAATGATCCTGGCGCTGGCGAACCCGGAACCGGAAATTC
TGCCGCCGCTGGCGAAAGAAGTGCGTCCGGATGCCATCATCTGTACCGGCCGTTCCGACTACCCGAACCAGGTGAACAAC
GTCCTTTGCTTCCCGTTTATCTTCCGTGGCGCGCTGGACGTTGGCGCAACCGCCATCAACGAAGAGATGAAACTGGCGGC
GGTACGCGCAATTGCAGAACTGGCCCATGCGGAACAGAGTGAAGTGGTGGCTTCAGCTTATGGCGATCAGGATCTGAGCT
TTGGTCCGGAATACATCATTCCAAAACCGTTTGATCCGCGCTTGATCGTTAAGATCGCTCCTGCGGTCGCTAAAGCCGCG
ATGGAGTCGGGCGTGGCGACGCGTCCGATTGCTGATTTCGACGTCTACATCGACAAGCTGACGGAGTTCGTTTACAAAAC
CAACCTGTTTATGAAGCCGATTTTCTCCCAGGCTCGCAAAGCGCCGAAGCGCGTCGTTCTGCCGGAAGGGGAAGAGGCGC
GCGTTCTGCATGCCACTCAGGAACTGGTAACGCTGGGGCTGGCGAAACCGATCCTTATCGGTCGTCCGAATGTGATCGAA
ATGCGCATTCAGAAACTGGGCTTGCAGATCAAAGCGGGTGTTGATTTTGAGATCGTCAATAACGAATCCGATCCGCGCTT
TAAAGAGTACTGGACCGAATACTTCCAGATCATGAAGCGTCGCGGTGTCACTCAGGAGCAGGCGCAGCGTGCGCTGATCA
GTAACCCGACAGTGATCGGCGCGATCATGGTTCAGCGTGGCGAAGCCGATGCAATGATTTGCGGTACGGTGGGCGATTAT
CATGAACACTTTAGCGTGGTGAAAAATGTCTTTGGTTATCGCGATGGCGTTCACACCGCAGGTGCAATGAACGCGCTGCT
GTTGCCGAGTGGTAACACCTTTATTGCCGATACCTACGTCAATGATGAGCCTGATGCAGAAGAGCTGGCGGAGATCACCT
TTATGGCGGCAGAAACTGTTCGTCGTTTTGGTATTGAGCCGCGTGTGGCTCTGTTGTCGCACTCCAACTTTGGTTCTTCT
GACTGCCCGTCGTCGAGCAAAATGCGTCAGGCGCTGGAACTGGTCAGGGAACGTGCACCAGAACTGATGATTGATGGTGA
AATGCACGGCGATGCAGCGCTGGTGGAAGCGATTCGCAACGACCGTATGCCGGACAGCCCTTTGAAAGGTTCCGCTAATA
TTCTGGTGATGCCGAACATGGAAGCTGCCCGCATTAGTTACAACTTACTGCGCGTTTCCAGCTCGGAAGGTGTGACTGTC
GGTCCGGTGCTGATGGGCGTGGCGAAACCGGTTCACGTGTTAACGCCGATCGCATCTGTACGTCGTATCGTCAACATGGT
GGCGCTGGCCGTGGTTGAAGCGCAAACCCAACCGCTGTAA
>Cluster_21574
ATGACCAACCACTGGGTGGATATCAAAAACGCTAACGTCGTAATGGTGATGGGCGGTAACGCTGCTGAAGCGCATCCCGT
CGGTTTCCGCTGGGCGATGGAAGCGAAAAACAACAACGATGCAACCTTGATCGTTGTCGATCCCCGTTTTACGCGTACCG
CTTCTGTGGCGGATATTTACGCACCTATTCGTTCCGGTACGGACATTACGTTCCTGTCTGGCGTTTTGCGCTACCTGATC
GAAAACAACAAAATCAACGCCGAATACGTTAAGCATTACACCAACGCCAGCCTGCTGGTGCGTGATGATTTTGCTTTCGA
AGACGGTCTGTTCAGCGGCTACGACGCTGAAAAACGTCAATACGATAAGTCGTCCTGGAACTATCAGTTCGATGAAAACG
GCTATGCGAAACGCGATGACACGCTGACTCATCCGCGCTGTGTATGGAACCTGCTGAAAGCGCACGTTTCCCGCTACACG
CCGGACGTAGTAGAAAACATTTGCGGTACGCCAAAAGCCGACTTCCTGAAAGTGTGTGAAGTGCTGGCCTCCACCAGCGC
ACCGGATCGCACAACCACCTTCCTGTACGCGCTGGGCTGGACGCAGCACACCGTGGGTGCGCAGAACATCCGTACGATGG
CGATGATCCAGTTGCTGCTCGGTAACATGGGTATGGCCGGTGGCGGCGTGAACGCATTGCGTGGTCACTCCAACATTCAG
GGCCTGACTGACTTAGGCCTGCTCTCTACCAGCCTGCCAGGTTATCTGACGCTGCCGTCAGAAAAACAGGTTGATTTGCA
GTCGTATCTGGAAGCGAACACGCCGAAAGCGACGCTGGCTGATCAGGTGAACTACTGGAGCAACTATCCGAAGTTTTTCG
TTAGCCTGATGAAATCTTTCTACGGCGATGCCGCGCAGAAAGAGAACAACTGGGGCTACGACTGGCTGCCGAAGTGGGAC
CAGACCTACGACGTCATCAAGTATTTCAACATGATGGATGAAGGTAAAGTCACCGGTTATTTCTGCCAGGGCTTTAACCC
GGTTGCGTCCTTCCCGGACAAAAACAAAGTGGTGAGCTGCCTGAGCAAACTGAAGTACATGGTGGTTATCGATCCGCTGG
TGACTGAAACCTCTACCTTCTGGCAGAACCACGGCGAGTCGAACGATGTCGATCCGGCATCTATTCAGACTGAAGTATTC
CGTCTGCCTTCGACCTGCTTTGCTGAAGAAGATGGTTCTATCGCTAACTCCGGTCGCTGGTTGCAGTGGCACTGGAAAGG
TCAGGACGCGCCGGGCGAAGCACGTAACGACGGCGAAATTCTGGCGGGTATCTACCATCATCTGCGCGAGCTGTACCAGG
CCGAGGGTGGTAAAGGCGTAGAACCGCTGATGAAGATGAGCTGGAACTACAAGCAGCCGCACGAACCGCAGTCTGACGAA
GTGGCGAAAGAGAACAACGGCTATGCGCTGGAAGATCTCTATGACGCTAATGGCGTGCTGATTGCGAAGAAAGGTCAGTT
GCTGAGTAGCTTTGCGCATCTGCGTGATGACGGTACAACCGCATCTTCTTGCTGGATCTACACCGGTAGCTGGACCGAGC
AGGGCAACCAGATGGCTAACCGCGATAACTCCGACCCGTCCGGTCTGGGGAATACGCTGGGATGGGCCTGGGCGTGGCCG
CTCAACCGTCGCGTACTCTACAACCGTGCTTCGGCGGATATCAACGGTAAACCGTGGGATCCGAAACGGATGCTGATCCA
GTGGAACGGTAGCAAGTGGACGGGTAACGATATTCCGGACTTCGGCAATGCCGCACCGGGTACGCCAACCGGGCCGTTTA
TCATGCAGCCGGAAGGGATGGGACGCCTGTTTGCCATCAACAAAATGGCGGAAGGTCCGTTCCCGGAACACTACGAGCCA
ATTGAAACGCCGCTGGGCACTAACCCGTTGCATCCGAACGTGGTGTCTAACCCGGTTGTTCGTCTGTATGAACAAGACGC
GCTGCGGATGGGTAAAAAAGAGCAGTTCCCGTATGTGGGTACGACCTATCGTCTGACGGAGCACTTCCACACCTGGACCA
AGCACGCATTGCTCAACGCAATTGCTCAGCCGGAACAGTTTGTGGAAATCAGCGAAACGCTGGCGGCGGCGAAAGGCATT
AATAATGGCGATCGTGTCACTGTCTCCAGCAAGCGTGGCTTTATCCGCGCGGTGGCTGTGGTAACGCGCCGTCTGAAACC
GCTGAATGTAAACGGTCAGCAGGTTGAAACGGTGGGTATTCCAATCCACTGGGGCTTTGAGGGTGTCGCGCGTAAAGGTT
ATATCGCTAACACTCTGACGCCGAATGTCGGTGATGCAAACTCGCAAACGCCGGAATATAAAGCGTTCTTAGTCAACATC
GAGAAGGCGTAA
>Cluster_25629
ATGACCACACTAAAACTGGATACGCTCAGCGACCGCATTAAAGCGCACAAAAATGCGCTGGTGCATATTGTGAAACCGCC
GGTCTGTACCGAACGAGCACAACACTATACCGAGATGTATCAACAACATCTCGATAAGCCGATCCCGGTACGTCGCGCGC
TGGCACTGGCGCATCACCTGGCGAATCGCACCATCTGGATCAAGCACGATGAGTTGATCATTGGCAACCAGGCAAGCGAA
GTTCGCGCCGCGCCGATCTTCCCGGAATATACCGTCTCCTGGATCGAAAAAGAGATTGATGATCTGGCAGATCGTCCCGG
TGCTGGTTTTGCGGTGAGCGAAGAGAACAAACGTGTTCTGCATGAAGTGTGCCCGTGGTGGCGCGGTCAGACCGTACAGG
ATCGCTGCTACGGCATGTTTACCGATGAGCAAAAAGGTCTGCTGGCGACCGGCATCATTAAAGCGGAAGGCAATATGACC
TCCGGCGATGCGCACCTGGCGGTCAATTTCCCGCTACTGCTGGAAAAAGGTCTTGATGGTCTGCGTGAGAAAGTGGCGGA
ACGTCGCTCGCGCATCAATCTGACGGTGCTGGAAGATCTGCACGGCGAGCAGTTCCTGAAAGCGATTGATATCGTGCTGG
TGGCAGTCAGTGAACACATTGAACGTTTCGCTGCCCTGGCGCGTGAAATGGCCGCGACCGAACCCCGCGAAAGCCGTCGC
GATGAACTGCTGGCGATAGCAGAAAACTGCGATCTTATCGCCCACCAGCCGCCGCAGACTTTCTGGCAGGCGCTGCAACT
GTGTTACTTCATCCAGTTGATATTGCAGATCGAATCTAACGGTCACTCGGTATCGTTTGGTCGTATGGACCAGTATCTCT
ATCCATACTATCGCCGTGACGTTGAACTGAACCAGACACTGGATCGCGAACACGCCATCGAGATGCTGCATAGCTGCTGG
TTGAAATTGCTGGAAGTGAACAAAATCCGCTCCGGCTCACACTCAAAAGCCTCTGCGGGAAGTCCGCTGTATCAGAACGT
CACCATTGGCGGGCAAAATCTGGTTGATGGTCAACCGATGGACGCGGTAAATCCACTCTCTTATGCGATCCTCGAATCTT
GCGGTCGCCTGCGTTCCACTCAGCCTAACCTCAGCGTGCGCTACCACGCGGGAATGAGCAACGATTTCCTCGATGCCTGT
GTTCAGGTGATCCGCTGTGGTTTCGGGATGCCGGCGTTCAACAACGATGAAATCGTCATCCCGGAATTTATTAAACTCGG
TATTGAACCGCAGGACGCTTACGACTACGCAGCGATTGGTTGTATCGAAACCGCCGTCGGTGGCAAATGGGGCTATCGCT
GTACCGGCATGAGCTTTATCAACTTCGCCCGCGTGATGCTGGCGGCGCTGGAAGGCGGTCGTGATGCCACCAGCGGCAAA
GTGTTCCTGCCACAAGAAAAAGCGTTGTCAGCGGGTAACTTCAACAACTTCGATGAAGTAATGGACGCATGGGATACGCA
AATCCGTTACTACACGCGTAAATCAATCGAAATCGAATATGTCGTCGACACCATGCTGGAAGAGAACGTACACGATATTC
TCTGCTCGGCGCTGGTGGATGACTGTATTGAGCGAGCGAAAAGTATCAAGCAAGGCGGCGCGAAATATGACTGGGTTTCT
GGCCTGCAGGTCGGCATTGCTAACCTCGGCAACAGCCTGGCGGCAGTGAAGAAACTGGTATTTGAACAGGGCGCGATTGG
TCAGCAACAGCTGGCTGCCGCACTGGCGGATGACTTCGACGGCCTGACTCACGAGCAGCTGCGTCAGCGGCTGATTAACG
GTGCGCCTAAGTACGGCAACGACGATGATACTGTCGATACGCTGCTGGCTCGCGCTTATCAGACCTATATCGACGAACTA
AAACAGTACCATAACCCGCGCTATGGTCGTGGTCCGGTTGGCGGCAATTATTACGCGGGTACGTCATCTATCTCCGCTAA
CGTACCCTTTGGCGCGCAGACGATGGCAACGCCGGACGGACGTAAAGCGCATACCCCGCTGGCAGAAGGCGCTAGTCCGG
CCTCCGGTACTGACCATCTCGGCCCAACGGCGGTCATTGGCTCAGTGGGTAAACTGCCTACGGCCGCGATTCTCGGCGGC

GTGCTGCTCAACCAGAAACTGAATCCAGCAACGCTGGAGAACGAATCTGACAAGCAGAAACTGATGATACTGCTACGCAC
CTTCTTTGAAGTGCATAAAGGCTGGCATATTCAGTACAACATCGTTTCCCGCGAAACGCTGCTGGAAGCGAAAAAACATC
CGGATCAGTACCGCGATCTGGTAGTGCGTGTCGCGGGCTATTCCGCCTTCTTCACCGCGCTCTCTCCAGACGCTCAGGAC
GATATCATCGCCCGTACTGAACATATGCTGTAA
>Cluster_50962
ATGCTCCCCACGACAAATATCCCTGTAAATTCTGGAATAATATCTTTTGAAAGTCCTGTAGATTCACCATCTAACGAGGA
TATCAAAGTTGCACTCGAAAAGTGGTGTACTGAGGGTGATTTCAGCGAATCTCGTCATGAGGTTGCATCAAAAATACTTG
ATGTTATAAGTACTGATGGAGAGACTTTATCAATCAGTGAGTCAATAACAACATTACCAGACTTGCTTCCTGGTTCTCTG
AAAGAGCTGGTTTTGAATGGATGTACAGAGCTTAAATCGATAAACTATCTACCACCCGACTTATCTTCATTAAGTATGGT
TGGATGCTCATCATTAGAGGCTATAAATTGCAACATACCTGGCTCTGTTGATAATTTGTCCTTATGCCATTGTCGTTCTT
TGAAACATATAGAAGGAACCTTTCCTGAGGCGCTCAGGAATTCCGTATATTTAAATGGCTGTAATTCATTAAATGAATCG
CAACGTCAATTCCTTGAATATGATGTCAGTCAAGGCCGTGCCTGCCTGAGCAAAGCTGAGCTTACTGCTGACTTAATGTG
GTTGTCTGCTAACAGAACGGGTGAAGAGCCTGCTGAACAATTAAATTATTCTGGATGTGACTTGTCAGATCTAAGTCTTG
CAGGGCTGAATTTATCATCAGTAATTTTTTCTGATTCCAGATTTGATAATACTGATCTTAGTGGCTGTAACCTGTCAGGT
GCGTCTCTGAAAAACACATCTTTCAAGAGCTCTGTTCTGGAGGAGAGTGATTTATCTTTCGCTGATTTGGCAAACAGCAC
AATCAGCGCAGCGTTTGATAGCGCTAATTTTAGTGGTGCCAATCTTAACAATGCGTCGTTTAAAGGATCTTCATATATAC
AAAACTCTCCAATTTTGAAAGATGCTGACTTAACAGGAACCATTATAATTCCTGGAATGGTTTTAAGTGGTGCTATCTTA
GGTGATGTAAAGGAGTTCTTTGGTGAAAAAAATAACAGTATTCATTTAGGAGGGTGTTATATAGATCTGTCTGGCATACA
GGAAGATATGCTAACTGCATTGGATAATTATACACAATTAAATAAATCAATTTTATTGACCATAAATACATCTGATGAGA
AGTATAACCATCAAAAAGTAATGGCAGCTGAGGAACTTATCAAAAAAATACCTCTTGATGAATTAGCGGCGTTCCAACCC
TATGTTAAGATGTCTTTGGCTGATTCATTTAGTATTCATCCTTATTTGAATAACACATATATACAACAATGGCTAGAGCC
TATATGTGATAACTTCTTTGATATTGTAATCTCTTGGTTTAATAATTCAATAATGATGTATATGGAGAATGGTAGTTTAT
TGCAGGCAGGAATGTATTTTGAGCGGCATCCTAGTGCGATGGTATCTTATAGTAGTTCATTTATACAAATCGTAATGAAT
GGTTCACAGCGTGATGGAATGCAGGAACGATTTAGGAGGCTCTATGAAATCTATTTAAAAAATGAAAAAGTTCACCCTAT
CACACAGCAGAGTGATTTTGGGTTGTGCGATGGATCTGGGAAACCTGACTGGGATGATGACTCAGATTTAGCTTATAACT
GGGTTTTGTTATCATCAAAGGATGATGGTATGGCAATGATGTGTTCTTTAAGTCATATGGTTGATATGTTATCTCCTAAT
ACATCAACTAACTGGATGTCATTTTTTTTATATAAGGATGGAGAAATTCAGAATACATTTGGGTATTCATTGAGTAATCT
ATTTTCTGAATCATTTCCAATTTTCAGTATTCCTTATCATAAATCTTGTTCACAGAATTTCGTTTCTGGTATTCTGGATA
TACTCATTTCTGATAATGATCTCAAAGAGAAGTTTATTGTAGTACTTAATTCAAATAAATCAGATTGTAAACTTATTGCT
GATGACCAGCAAAGGAAACTTGCTCATGTTTGGGCGCCATTTCTTGATGGCTGGGAATTAAGTGCACAGCATATAAATAT
GATTATGGGGAGTCATGTGTTGAAGGATATGTCTCCAAGAAAACAGGCTGAGATATTATTTTGTTTAGGCGGTGTTTTCT
GTAAATACTCATCGAGCGATATGTTTGGTACAGAGTATGACTCTCCTGAGATTCTACGTCGATATGCAAATGGATTGATT
GAGCAAGCTTATAAAATAGATTCGAAGGTATTTGGCTCACTTCATTATTATAATGATATTTTAGACAGGTTACAGGGAAG
GGACAATGCTTTTTCTTGTACCTCTGTGTTAACTGATATGCTAACAGAACATGCAAGAAAATATTTTCCTGAAATATTTT
CATTGTATTACCCTGTTGCATGGCGTTAA
>Cluster_42463
ATGTCACAACCTATTTTTAACGATAAGCAATTTCAGGAAGCGCTTTCACGTCAGTGGCAGCGTTATGGCTTAAATTCTGC
GGCTGAAATGACTCCTCGCCAGTGGTGGCTGGCAGTGAGTGAAGCACTGGCCGAAATGCTGCGTGCTCAGCCATTCGCCA
AACCGGTGGCGAATCAGCGACATGTTAACTACATCTCAATGGAGTTTTTGATTGGTCGTCTGACGGGCAACAACCTGTTG
AATCTCGGCTGGTATCAGGATGTACAGGATTCGTTGAAGGCTTATGACATCAATCTGACTGACCTGCTGGAAGAAGAGAT
CGACCCGGCGCTGGGTAACGGTGGTCTGGGCCGTCTGGCGGCGTGCTTCCTCGATTCAATGGCAACTGTTGGTCAGTCTG
CGACGGGTTACGGTCTGAACTATCAATATGGTTTGTTCCGCCAGTCTTTTGTGGATGGCAAACAGGTTGAAGCGCCGGAT
GACTGGCATCGCAGTAACTACCCGTGGTTCCGCCACAATGAAGCACTGGATGTGCAGGTAGGGATTGGCGGTAAAGTGAC
GAAAGACGGACGCTGGGAGCCGGAGTTTACCATTACCGGTCAAGCGTGGGATCTCCCCGTTGTCGGCTATCGTAATGGCG
TGGCGCAGCCGCTGCGTCTGTGGCAGGCGACGCATGCGCATCCGTTTGATCTGACTAAATTTAACGATGGTGATTTCCTG
CGTGCCGAACAGCAGGGCATCAACGCGGAAAAACTGACCAAAGTTCTCTATCCAAACGACAACCATACTGCCGGTAAAAA
GCTGCGCCTGATGCAGCAATACTTCCAGTGTGCCTGCTCGGTAGCGGATATTTTGCGTCGCCATCATCTGGCGGGGCGTA
AACTGCACGAACTGGCGGATTATGAAGTTATTCAGCTGAACGATACCCACCCAACTATCGCGATTCCAGAACTGCTGCGC
GTGCTGATCGATGAGCACCAGATGAGCTGGGATGACGCCTGGGCCATCACCAGTAAAACTTTTGCTTACACCAACCATAC
CCTGATGCCAGAAGCGCTGGAACGCTGGGATGTGAAACTGGTGAAAGGCTTACTGCCGCGCCACATGCAGATTATTAACG
AAATTAATACACGCTTTAAAACGCTGGTGGAAAAAACCTGGCCGGGCGATGAAAAAGTGTGGGCCAAACTGGCGGTGGTA
CACGACAAACAAGTGCATATGGCGAACCTGTGTGTGGTTGGCGGTTTCGCGGTAAACGGTGTTGCGGCGCTACACTCGGA
TCTGGTAGTGAAAGATCTGTTCCCGGAATATCACCAGCTATGGCCGAACAAATTCCATAACGTCACCAACGGTATTACCC
CACGTCGCTGGATCAAACAGTGCAACCCGGCACTGGCGGCTCTGTTGGATAAATCACTGAAAAAAGAGTGGGCTAACGAT
CTCGATCAACTGATCAATCTGGAAAAATTCGCTGATGATGCGAAATTCCGTCAGCAATATCGCGAGATCAAGCAGGCGAA
TAAAGTCCGTCTGGCAGAGTTTGTGAAAGTTCGTACCGGTATTGAGATCAATCCACAGGCGATTTTCGATATTCAGATCA
AACGTCTGCATGAGTACAAACGCCAGCACCTGAATCTGCTGCATATTCTGGCGTTGTACAAAGAAATTCGTGAAAACCCG
CAGGCTGATCGCGTACCGCGCGTCTTCCTCTTTGGCGCGAAAGCGGCACCGGGCTACTACCTGGCGAAAAACATTATCTT
TGCGATCAACAAAGTGGCTGACGTGATCAACAACGATCCGCAGGTTGGCGACAAATTGAAGGTGGTGTTCCTGCCGGATT
ATTGCGTTTCGGCGGCGGAAAAACTGATCCCGGCGGCGGATATCTCCGAACAAATTTCGACGGCAGGTAAAGAAGCTTCC
GGTACCGGCAATATGAAACTGGCGCTCAGTGGTGCGCTTACTGTCGGTACGCTGGATGGTGCGAACGTTGAAATCGCCGA
GAAAGTTGGTGAAGAAAATATCTTTATTTTTGGTCATACCGTGGAACAAGTGAAGGCAATTCTGGCCAAAGGCTACGACC
CGGTGAAATGGCGGAAGAAAGATAAGGTGCTGGACGCAGTATTGAAAGAGCTGGAAAGCGGCAAATACAGCGACGGCGAT
AAGCATGCCTTCGACCAGATGCTGCACAGTATCGGCAAACAGGGCGGCGATCCGTATCTGGTGATGGCGGATTTCGCAGC
CTATGTAGAGGCACAAAAGCAGGTGGATGTGCTGTACCGCGACCAGGAGGCCTGGACTCGCGCGGCGATCCTCAATACCG
CCCGCTGCGGTATGTTTAGCTCGGATCGCTCTATTCGCGATTATCAGGCTCGTATCTGGCAGGCAAAACGCTAA
>Cluster_44686
ATGGCAGTAAAGATTTCAGGTGTACTGAAAGACGGCACAGGAAAACCGGTACAGAACTGCACAATCCAGCTGAAAGCAAA
ACGTAACAGCACCACGGTGGTGGTGAACACGCTGGCCTCAGAAAATCCGGATGAAGCCGGGCGTTACAGCATGGACGTTG
AGTACGGGCAGTACAGCGTTATTCTGTTGGTGGAGGGATTCCCGCCGTCACATGCCGGGACCATCACCGTGTATGAAGAT
TCCCGACCCGGTACGCTGAATGATTTTCTCGGTGCCATGACGGAGGATGATGCCCGTCCTGAGGCACTGCGCCGTTTTGA
ACTGATGGTGGAAGAGGTGGCGCGTAACGCGTCCGCGGTGGCACAGAACACGGCAGCCCGCGAAGAAGTCAGCCAGCGATG
CCAGCACATCAGCCCGTGAGGCGGCAACCCATGCGACTGATGCTGCAGGCTCAGCACGCGCAGCCAGCACGTCAGCCGGA
CAGGCCGCGTCGTCGGCTCAGTCAGCATCTTCCAGCGCAGGAACGGCATCAACAAAGGCCAGTGAAGCATCGAAAAGTGC
TGCTGCTGCAGAGTCCTCAAAAAGCGCGGCAGCTACCAGTGCCGGTGCGGCGAAAACGTCAGAAACGAATGCGGCAGCGT
CACAACAATCAGCAGCCACTTCTGCATCCGCCGCGACCACGAAGGCGTCAGAAGCAGCCACCTCAGCCCGGGATGCGGCG
GCCTCAAAAGAGGCAGCGAAATCATCAGAAACGAACGCATCATCAAGCGCCAGTAGTGCCGCTTCCTCGGCAACGGCGGC
AGGCAATTCCGCGAAGGCGGCAAAGACGTCTGAGACAAACGCCAGGTCTTCTGAAACGGCAGCGGGACAGAGCGCCTCGG
CTGCGGCAGGCTCAAAAACAGCGGCTGCGTCGTCTGCCAGTGCCGCGTCAACAAGTGCCGGGCAGGCCTCAGCCAGTGCC

ACCGCCGCCGGAAAATCGGCAGAAAGCGCCGCATCGTCTGCTTCAACAGCCACAACGAAGGCTGGCGAAGCCACTGAACA
GGCCAGCGCAGCAGCAAGGTCTGCTTCCGCAGCGAAGACATCCGAGACGAACGCGAAAGCGTCGGAAACCAGCGCAGAAT
CCTCAAAAACGGCTGCCGCATCGTCCGCCAGTTCGGCGGCGTCATCGGCATCATCGGCGTCTGCTTCAAAAGATGAGGCG
ACCAGACAAGCGTCCGCAGCGAAGGGCAGCGCCACGACGGCATCCACGAAGGCGACAGAGGCAGCTGGCAGTGCGACGGC
GGCAGCACAGAGCAAAAGTACGGCGGAATCCGCGGCAACGCGCGCCGAGACAGCGGCAAAACGGGCAGAGGATATTGCAT
CCGCCGTGGCGCTTGAGGATGCAAGTACGACGAAAAAGGGGATAGTACAGCTCAGCAGTGCGACCAACAGTACGTCTGAA
ACGCTGGCGGCAACGCCAAAGGCAGTAAAATCAGCCTATGACAATGCAGAGAAACGTCTGCAGAAAGACCAGAACGGCGC
TGATATACCCGATAAGGGACGCTTCCTGAACAACATTAACGCGGTCAGTAAAACAGACTTTGCTGATAAGCGTGGTATGC
GTTATGTGCGGGTTAACGCTCCTGCAGGTGCAACATCTGGAAAATATTACCCTGTTGTTGTTATGCGTTCTGCTGGCTCA
GTAAGCGAACTGGCATCAAGGGTCATTATCACCACGGCAACGCGAACCGCAGGCGATCCGATGAATAACTGCGAGTTTAA
CGGATTTGTTATGCCTGGTGGCTGGACTGACAGGGGGCGTTATGCTTATGGAATGTTCTGGCAATATCAAAACAATGAAC
GAGCCATCCACTCAATAATGATGAGTAATAAGGGCGATGATTTGCGCTCTGTGTTCTATGTTGATGGCGCTGCTTTCCCT
GTTTTTGCGTTTATCGAAGATGGCCTGTCAATATCCGCACCTGGTGCTGATCTCGTTGTTAATGATACGACCTATAAGTT
TGGGGCAACAAATCCGGCGACTGAATGTATCGCGGCGGACGTTATCCTTGATTTTAAGAGTGGGCGTGGTTTTTATGAGT
CTCATTCGTTAATCGTTAACGATAACTTGTCGTGCAAAAAACTTTTTGCCACAGACGAAATTGTAGCGCGTGGTGGTAAT
CAGATTCGAATGATAGGTGGGGAGTATGGGGCATTATGGCGTAATGATGGCGCTAAAACTTACCTGCTGCTTACCAATCA
AGGTGATGTTTATGGTGGCTGGAATACATTAAGACCGTTTGCTATTGATAACGCAACCGGCGAACTGGTTATTGGAACCA
AACTGTCCGCAAGTCTGAACGGTAATGCATTAACAGCAACAAAGCTGCAAACGCCAAGACGGGTTTCTGGTGTTGAGTTT
GATGGTTCCAAAGATATTACTTTAACCGCCGCGCATGTGGCTGCTTTTGCCAGAAGGGCAACGGATACATATGCCGATGC
GGATGGTGGCGTTCCCTGGAATGCCGAATCAGGCGCTTATAATGTCACCCGCTCTGGCGACAGCTATATTCTGGTTAACT
TCTATACCGGAGTCGGAAGTTGCCGGACCTTGCAGATGAAGGCGCATTACAGAAATGGTGGTCTGTTCTACCGTTCTTCA
AGAGACGGTTATGGTTTTGAGGATGACTGGGCAGAAGTTTATACCTCGAAAAATCTTCCACCAGAAAGCTACCCAGTCGG
CGCACCAATCCCGTGGCCATCAGATACCGTTCCGTCTGGTTATGCCCTGATGCAGGGGCAGACTTTTGACAAATCTGCTT
ACCCGAAACTTGCAGCCGCTTATCCGTCAGGCGTGATCCCTGATATGCGTGGCTGGACGATTAAGGGCAAACCTGCCAGT
GGTCGGGCCGTATTGTCTCAGGAACAGGACGGCATTAAATCGCATACCCACAGCGCCAGCGCATCCAGTACAGATTTGGG
GACGAAAACCACATCGTCGTTTGATTACGGCACTAAATCCACGAATAACACTGGTGCGCATACCCATAGTTTAAGTGGCA
GCACGAATGCAGCTGGTAATCACAGCCATAGAGATGGCCGTCGATTTAACCCCAGTGTTTTAAAGATACTTATCAATAT
GGTTATACAAGCTCAGGTCAAAATACCTGGGGTGTACAAGGCTCAGTAGGTATGTCTACGGGGTGGTTAGCGAATACCAG
TACAGATGGTAATCATAGCCATTCACTGTCCGGCACAGCAGCATCTGCAGGTGCACACGCGCATACTGTCGGTATTGGTG
CTCATACGCACTCCGTTGCGATTGGTTCACATGGACACACCATCACCGTTAACGCTGCTGGTAACGCGGAAAACACCGTC
AAAAACATCGCATTTAACTATATTGTGAGGCTTGCATAA
>Cluster_27459
ATGGCGATCCAGACTTATGGACTGAGTTGGTGGGGTCAGCAGTGGTTAAACGCGCTGATGCATATTGATGCCGACAACCG
CTTACCGCGCGGTCGTACCTACGCGAATAAAGGGGCGGTGCAAAAGCTTGATATCAAGGACGGTCACATCCGTGCGCAAG
TGCGGGGATCGTACATTTATACGGTTGAAATTACCGTTCCACCGTTTTCCGGGCAGGAAAAAGAACGCCTGCTGGATGCG
CTGGTCAACGATCCGCTGATTATCTCGCAGTTATTAAATCGCGAACTTTCGCCAACGGTGCTGGATCTGGCGAATGAGAC
GAATATCAAGATTTTCCCCGCGAGCTGGAAAGATCTTCATATGGATTGCAACTGCCCCGACTGGGCCGTGCCCTGTAAAC
ATCTGGCCGCAGTGATTTATCTGATCAGTCGCCAGATCGACAGCGATCCGTTTCTGGTTTTTGCGCTACATGGTCTGGAT
TTGCCGACCGCATTGCGCCAGCGTGGAATGGACATCGACAAAAAGATCTTTATCACTCCCCCCTCGCTGTCAGCGCTATT
CGCAAATCAACCTTTTGTTCATGAGGAAAAAGCGAATCAGGGGCTGGATTTTACCCTGATCCCTGCTTTACACCACGATC
TGTTGCGACTGTTGCCCGATAATCCGGTTTTTTATCCCTTTGGTGATTTTCATCAGGTTTACAGCCAACAACTGGCACGC
CTTGTTCGTTATGGGGAGCAATTGCAAAAAATTGTTCCTGAATCAGAGAGCCAGATGCAGTGGGCGCTGGGAGCAACCGA
ATCCCCGATGCTCAGAATTGACGGGGATTACCGCTGTTGCGTGCAGGGTATTACGCATCTTAAAGATCTCTCCACGCTGC
TGGGGGAGCTGAAAAATTTGACCCCGCGACGCTTGCAGATGTTGCAACCTTCCGTCGCAGGTTTACACCTGGCCTGGCAA
TTCGCTGTTCATCTGCTGGCGAAAGGCGCAGTTATGCCACAGGTTTTTTCCTGTGAAAGTGAGACGCTGGTTCGCTGGCT
TCCAGCTACGCTGGATGTAACGGTCGATAATTTGCTCGAACAGGTCGCCCGACAATTGCCGGACGGGTTGATCTCTTTTA
ACGATTGGGCGCTTACCGGCAAGCGGCAGGCTATAACCTTGTGCTCGCTGATGCTTGATCGGTTGGTACAGTCGGTTCCC
CTGAAGAAAGGCGATACGCCATATGACAAGCTCTTTTTCCTCACCGGGCGGGTGACATTTGACTCGCGACGGGAAAGCGC
CAGCGCCGCCAGTATCGCACAATGGCTGTCGCGCCTGACGCTGGTGCATCGCGAGCAGCCGCCAGTGATTATGCTGGATG
AAGCCGGGGAAGACTTTGCGCTGCATCTGGCGGTGAAAATAACGGACCAGCCCGCGCCAGTGATGCTGGCGGATATTCTG
TGGCAAAAAATGCTACGAAAATACACGGATGGCCGTCCTGCAAACGCTGGCGATGTTGGCGGAATTCTACCCTCCGTTGTG
TGAGTATCTGCGTGCCGGAGGGCGGCAGGCGCTGCGCCTCACCGCACAAACGTTGCCGGATTTTCTCTTTACCGCGTTGC
CCGTTATTCGCATGCTGGGCATTCAGGCGTTGCTGCCGAAAGCGCTGGATAAAATATTACGCCCCAGACTCAGCATGCAG
GTGAAATCATCCTCCGGCGCGGGAGGCAGCAGCGGGTTTCAACTGGGAAATCTGTTCGATTTTGACTGGCGCATTGCGTT
GGGAGACAGGCAACTGACGGTTACTGAATTTGAAACGCTCATTGACAGTGCCGAAGGGATTGTCCGCTTCAAAGAACAGT
TTATCTATCTTGACCCTGACGAAATCGCCCGACTGCGCAATAAGCTGTTAAATCCACCGGACATTAAAGGCATGGCGCTA
ACGCAGACGGCGTTGTCCGGTGAGTACGCAGGCACGCCGATTATGCTGGATAGCGCGGCGCGTGAGCAACTTCGGCAATT
GACCTCGGTTGGTATGCCCTCTCAGCCCGTTTCTCTTAACGCCACGCTGCGGCCTATCAGCAAACGGGTTTTGCCTGGC
TGACGCATAACCTGGCGCTGGGGATCGGCGGCATCCTGGCCGATGATATGGGCCTCGGGAAAACGCTTCAGCTTATCGCT
GTCATTCAGCATCAAAAAGAGACAGGCGCGCTGGCGGAGCGTAAGGTGCTGATTATCATGCCGACCACCTTGTTGACTAA
CTGGCAACGAGAAATCGCTCGTTTCGCCCCAACGTTAACGACGGCGATATACCACGGCGGCAAGCGCGCGTTGTCCGATG
AGATGCCGGATATCCTGCTCACCACTTACGGCGTGGTGCGTAGCGATGCCGCAACGTTAAAGAAACAGCACTGGCGTCTG
GTGGCGGCGGATGAGGCACAAAATCTAAAAAATCCGGCCTCGGCACAGACGAAGGCTGCAAAATCAATTCCTGCCGATGC
GTTTATTGCTCTAAGCGGTACGCCGGTGGAAAACCGTCTTGCAGAATACTGGAGCATTATGGACTTTGCTCTGCGTGGCT
ATCTGGGGCCGCTAAAAACGTTTATTTCGGAGATTGAGAAACCGATCCAGATGGATCGTAATCAACAGGTTGCTGAACGT
TTTCGCCGCATAACCGCGCCATTTTTGCTGCGTCGGCTGAAATCGGACAAAACCATCATCAGCGATCTGCCCGAAAAAAT
TGAAACCGACCGTTTCTGTGAACTGGCAACTAAACAGGCGGCAATTTATGCGTCCGTCGTGCGCGAAGGGATGCAGGTGA
TCCGTGGGGAGTCTGATGCCTTCCAGCGTCAGGGGCTGGTGCTGCAAATGATTATGGCGTTGAAGCAGGTCTGTAACCAT
CCGGCGCACTATCTGAAAAATAAGCAGATGGATGCGGCGCTTTCTGGCAAGGCGCAACTGTTATTGGATCTTCTGGACAG
CATTGAAGAGACAACAGAAAAGGTGCTGATTTTTACCCAGTTCAAAGAGATGGGCGAACTACTGCAAAGCTGGCTTACCG
CGCGTTATGGCGAACAACCCCTGTTTTTACACGGCGGCTTAAGCCGCAAAAAGCGCGATGAGCAGGTCGAACGTTTTCAA
AATGACCGTTCTGTACGCCACTTTATTCTGTCGCTGAAAGCTGGTGGTACGGGGCTGAATCTGACCGCCGCCAGCCAGGT
GATTCATTACGATTTATGGTGGAACCCGGCGGTGGAAGCGCAGGCCACCGACCGCGCCTATCGTATCGGCCAGCAGCGTA
ATGTGCAGGTGCATCGCCTGTTGACCCGTGAGACGTTTGAAGAGCGGATCAACGACATGATCAGAAATAAAAAAGCGCTG
GCGGAGATGACTGTCGGCAGCGGCGAGCAGTGGTTGGGTAAGATGACGGATGATGAACTGGAAGAGCTGTTCAGGGCAGG
CCAGGATTAG
>Cluster_34426
ATGGCAGTAAAGATTTCAGGTGTACTGAAAGACGGCACAGGAAAACCGGTACAGAACTGCACAATCCAGCTGAAAGCAAA
ACGTAACAGCACCGCGGTGGTGGTGAACACGCTGGCCTCAGAAAATCCGGATGAAGCCGGGCGTTACAGCATGGACGTTG

AGTACGGTCAGTACAGCGTTATTCTGTTGGTGGAAGGATTCCCGCCGTCACATGCCGGGACCATCACCGTGTATGAAGAT
TCCCGACCCGGTACGCTGAATGATTTTCTCGGTGCCATGACGGAGGATGATGCCCGTCCGGAGGCACTGCGCCGTTTTGA
ACTGATGGTGGAAGAGGTGGCGCGTAACGCGTCCGCGGTGGCACAGAACACGGCAGCCCGCGAAGAAGTCAGCCAGTGATG
CCAGCACATCTGCCAGTGAGGCGGCAACCCATGCGACTGATGCTGCAGGCTCAGCACGCGCAGCCAGCACGTCAGCCGGA
CAGGCCGCGTCGTCGGCTCAGTCAGCGTCTTCCAGCGCAGGAACGGCATCAGCAAAGGCCACTGAAGCATCAAAAAGTGC
TGCCGCTGCAGAGTCCTCAAAAAGCGCGGCAGCTACCAGTGCCAGTGCCGCGAAAACGTCAGAAACGAATGCTGCAGCGT
CACAACAATCAGCAGCCACTTCTGCATCCACCGCGACCACGAAAGCGTCAGAAGCTGCCACCTCAGCCCGGGATGCGTCG
GCTTCAAAAGAGGCGGCAAAATCATCAGAAACGAACGCATCCTCGAGCGCCAGTAGCGCAGCTTCCTCGGCAACAGCGGC
AGGAAATTCCGCGAAGGCGGCAAAGACGTCCGAGACGAACGCCAGGTCTTCTGAAACGGCAGCGGGACAGAGTGCCTCAG
CTGCGGCAGGCTCAAAAACAGCGGCTGCATCATCTGCCAGTGCCGCGTCAACAAGTGCCGGGCAGGCCTCAGCCAGTGCC
ACCGCTGCCGGAAAGTCGGCAGAAAGTGCTGCATCGTCTGCTTCAACAGCCACAACGAATGCTGGCGAAGCCGCTGTACA
GGCCAGCGCAGCAGCGAGGTCTGCTTCCGCAGCGAAGACATCCGAAACGAACGCGAAAGCGTCGGAAACCAGCGCAGAAT
CCTCAAAAACGGCTGCCGCATCGTCCGCCAGTTCGGCGGCGTCATCGGCATCATCTGCGTCTGCTTCAAAAGATGAGGCG
ACCAGACAGGCGTCAGCAGCGAAGGGCAGCGCCGCGACGGCATCCACGAAAGCAACAGAGGCGGCAGGCAGTGCGACGGC
AGCATCTCAGAGCAAAACTGCTGCTGAATCCGCTGCGACCCGTGCGGAAGCTGCTGCTGATCGTGCTGAAGAGATTGCCG
GTGCAGTTGCGATGGAAGACGCAAGCCTTACAACTAAAGGTGTTGTGAAACTTAGCAGTGCTGTTGATAGCACCAGTGAA
TCGCTGGCCGCAACGCCAAAAGCAGTTAAAGCAGCCAATGACAATGCGAATAGCAGGGTGCCATCTAACCGAAAAGTTAA
CGGAAAAGCACTGACTGCGGATATAACATTAACGCCGAAAGATATTGGTACTTTAAATTCAGTAACGATGTCTTTCTCTG
GCGGGGCTGGGTGGATCAAACTGGCTACGGTTACCATGCCACAAGCGAGTTCCATCGTTTACATCGCATTGATTGGTGGC
GCTGGTTACAACGTCGGTTCCCCACATCAGGCAGGCATTTCAGAACTGGTTCTACGAGCAGGCAATGGAAACCCCAAAGG
AATTACCGGGGCTTTGTGGAAGCGTACAGCCGTCGGATTAACGAATTTCGCCTGGATCAACACATCCGGCGATACATATG
ATATTTACGTTGAGATTGGCAATTATGCGACTCGTGTAAATATCCATTGGGATTGTACTGCAAATGCGTCAGTTTCTGTT
TATACCTCGCCAACATATTCAGCGAGTAAGCCTTCCAGCGTTACCTATGGTGTTGTTTATACGATGTATAGCTCACATCA
GAAACCTACGCCGTTAGATATTGGAGCACTGCCAACAACCGGAGGAACAGTTTCAGGTCCGTTGTCTGTTACAGGTGGAT
TAACTGGTTCATTGAATGGTAATGCAAGTACAGCCACGAAATTGCAGACGGCAAGATCTATCGGTGGAGTTGTTTTCGAC
GGTTCTGCAAATATTAACCTTCCAGGTGTAAACACTACGGGTAATCAGAACACCACTGGTAATGCTGCAACTGCTACAAA
ACTTCAGACTGCAAGAAAAATATCCGGTGTTCCATTTGATGGTTCTACTGATATCACTTTAACCGCCGCGCATGTGGCTG
CTTTTGCCAGAAGGGCAACGGATACATATGCCGATGCGGATGGGGGCGTTCCATGGAATGCCGAATCAGGCGCTTACAAT
GTCACCCGCTCTGGCGACAGCTATATTCTGGTTAACTTCTATACCGGAGTCGGAAGTTGCCGGACCTTGCAGATGAAGGC
GCATTACAGAAATGGTGGTCTGTTCTACCGTTCTTCAAGAGACGGTTATGGTTTTGAAAGTGGTTGGGCGCAGCTTTACA
CCTCGGCTCATCCTCCTGCAGAGTTTTATCCAGTCGGTGCACCAATCCCGTGGCCATCAGATACCGTTCCGTCTGGTTAT
GCCCTGATGCAGGGGCAGACTTTTGACAAATCTGCCTACCCGAAACTTGCAGCCGCTTATCCGTCAGGCGTGATCCCTGA
TATGCGTGGCTGGACGATTAAGGGCAAACCTGCCAGTGGTCGCGCCGTATTGTCTCAGGAACAGGACGGCATTAAATCGC
ACACCCACAGCGCCAGCGCATCCAGTACGGATTTGGGGACGAAAACCACATCGTCGTTTGATTACGGAACCAAATCCACG
AATAACACCGGGGCGCATACCCATAGTATTAGCGGGACTGCAAATAGTGCCGGTGCGCACCAACACGAGAGTTCCGGTGC
ATTTGGTGGCACGAACACGAGCATTTTCCCTAATGGTTATACCGCGATTTCAAATCTAAGCGCGGGGATTATGAGCACAA
CAAGCGGTAGTGGCCAGACTCGTAATGCAGGGAAGACATCATCAGATGGTGCTCATACCCACTCGCTGTCCGGCACTGCT
GCAAGCGCAGGCGCACATGCACATACTGTCGGTATTGGTGCTCATACGCACTCCGTTGCGATTGGCTCACATGGACACAC
CATCACCGTTAACGCTGCGGGTAACGCGGAAAACACCGTCAAAAACATCGCATTTAACTATATTGTGAGGCTTGCATAA
>Cluster_3308
GTGGTTCCGGGATTAAACCGTCTCCGGCCCCGTTCTGTTTCAGGGCCTGCCATCGTCATGACCGTGGCTGTTGTGGTTGC
GCTTTGCACGTTTATTCACCGTAAACCGGCATTGCTGGGCGTTGAACCGGGTAGCTCACAGCAAATGTACTGGATGGCTG
GCGTGATTGCCGCAGGCGTGGTCTTTTTGATTATCATGGGGGTTTATCATCTGCTGAACCGCCACGCTGCCGTACAAAAC
TTTGAACTCCGCAGACAACTTGCCGCCGGAGATGACTATCGTGCGGTGACGACAAAGAAAGCCGACAGCCGTCATCCGGC
AATGGTGGAACTCGCCGCCTTCCTGCGCGACCGCTACGGTCCGTTCTGGCGGCGTAACGTCCGCCTGCTGCTGGTGACCG
GCGAGCCTGAACAGGCAGAAGCCATCGCGCCGGGGCTGACCGGGCAACACTGGCTGGAGGGCGACCACACGGTGCTGATA
TATGGCGGCAGGCCATCAGCGGAGCCTGATGTCACACTGCTGACCGCCTTAAAAAAACTGCGCCGCAGCCGTCCGCTGGA
CGGCATCATCTGGGCGCTGACAGAAGAACAGAGCCGCCAGACAGCGCAACTCGACAAAGGCTGGCGCGAACTGATAAACG
GCGGTAAGCGACTCGGTTTTCAGGCCCCACTCTATTTGTGGCAGGTCTGTGACAACGGTGATTATCAGGCCGGACGCCCC
CTGCAAAGCGTCGGCTGCCTGCTGCCGGAACGCTGCACCCCGGAACAACTGACCGCCATGCTGGAAGCTCACACCCTGCC
GCTGACGGAACAGGGCATGTCGCAACTGCTGGCTGATAACCGCCATGATTTCTGCTGCGTCTGGCCCATACCCTTGCAG
AGCGGGGTATCGCTCACTGGCAAAGCGTCCTGAAACCGCTGCTGGCAGGCGGCGCATTCTCTTCCCTGCGCCTGCGTGGT
CTGATGTTCAGCCCGCCGCTTACCGCCGTACCGGAGGCCGCACCTCATGCGTGGCTGCCGTCACAGGTCTGGGCGGGCGT
GACCGGCGATAACGCACGGGGGCGTGCAGTGGGCTTTCCGTGGCTACGCGCCGCGCTGATGTCCGCTGTCTGCGTGCTGG
CAATATGGGGGGCCGAAATGACGACCTCTTTCTTCGCCAACTGCGCTCTTGTTCAGGAAACCGGTATCCAGACGGCGCGG
GCGCTCGATACCCGCCTGCCGCTGGCAGAACAACTGGTGGCGCTGCATACCCTGCAGGGCGAACTGGAACGCCTGCAATA
TCGTATCCGCGAAGGTGCGCCGTGGTATCAGCGTTTTGGCCTTGAACGTAACCAACAACTGCTCGCCGCCGCCTTTCCCG
GCTATGCGCAGGCGGCAAACCGGCTGGTGCGCGACGTGGCCGTTGACCATCTGCAACAGCAACTGAACGCCTTTGTTGCC
CTGCCGCCCAACAGTCCTCAGCGTACCGCCACCGGTGAACAACGCTATAAGCAGCTTAAGGCATTGCTGATGACTTCCCG
CCCGGAAAAGGCCGACGCTGCCTTTTTCAGTACCACGCTGATGGCAGACAGCCTGCGCTACGAGAATATCCCGGAAGGTG
TGCAGCAGAGCGTGTTTCCGTCACTGCTGACCTTCTGGATGGCGAACCTGCCGGAACACCCGCAGTGGAAAACCTCGCCG
CCACCGGAACTGACCGGCGCGGTGCGTAAAATCCTGCTGCGCCAGATTGGTGTGCGTAATGCTGAAAACACTCTCTACCA
GAACGTACTGCAACAGGTGTCCCGCAACTACGCCGATATGACGCTGGCGGACATGACCGGGGATACCCTCACCGAATCTC
TTTTCAGTACGGAACAGACGGTGCCGGGGATGTTCACCCGTCAGGCGTGGGAAGGACAGGTCAGGGAAGCCATCGAGCAG
GTGGTGACGGCGCGGCGCGAGGAAATCGACTGGGTGCTCAGCGACCGCCAGCAGGATACCTCTGCGGATATATCGCCGGA
TACGCTGCGTAACCGTCTCACCTCACGCTACTTTACCGACTTCACCGGAAGCTGGCTGGCGTTTCTCAACAGCATTCGCT
GGAAAAAGGAAGACTCGCTCTCCGGCATTCTCGACCAGCTGACACTGATGGCCGATGCCCGTCAGTCGCCGCTGATTGCG
CTGACCGATACCCTCGCCTGGCAGGCGGCGGCAGGCAGGGAAAACCGTGGCCTGTCAGACTCGCTGGCGAAATCGGCACA
GGAACTGTTTAACGGCAAGGAGAAAACGCCGCAGCAATCCCGTGAAGGCGACGTGCCTGTCGGGCCGCTGGATAAAACCT
TCGCTCCGCTGCTGCGGTTGCTGGGCGATAAAGCCGGAGGCGGCGACAGCCAGCTGAGTCTGCAGACCTACCTCACCCGC
GTTACCCGTGTGCGTCTCAAACTGCAACAGGTGACCAACGCCCCCGACCCGCAGGAAATGACGCAACAGCTGGCACAGAC
GGTACTGCAGGGCAAAACCGTTGACCTCACCGACACCCGCGACTACGGACGGTTAATCGCCGCCAGTCTGGGCGAGGAAT
GGAGTGGCTTCGGTCAGGCGCTGTTCGTCCGCCCGGTGGAGCAGTCGTGGCGGCAGGTGCTGACGCCTGCGGCGGACAGC
CTGAACCGCCAGTGGCAGCGGGCGATTGTCAGCCACTGGAATCAGGACTTCGCTGGTCGCTACCCGTTCAAAGCCTCACA
GAACGATGCCTCCCTCCCCCTGCTGGCGCAGTATCTGCGCGATGACGGGCGCATCAACCAGTTTATTGCCGCCAACCTTT
CCGGCGTTCTGAAACGTGAAGGCCGCTACTGGGTGGCGGACGCCATGAACACGCAGGGGCTGACGGTCAACCCGGACTTT
ATCCGCGCCCTGAACCGCCTGCGCGACGTGGCTGACACCGCCTTTGCCAGCGGCGATGCCGGAATACATTTTGAACTGCA
GGCAAAACCGGCCCGAGACGTGATGAAGACACATCTGGTGATTGACGGGCAGGAACTGGAGTATTTCAACCAGAAAGAAC
GCTGGCAGCGTTTCAACTGGCCGGATGAACAGTGGCAACCCGGCGCATCGCTGAGCTGGACCAGTACGCAGGCGATGGAA

CGCATACTGGCGGATTACCGGGGGAGCTGGAGCCTTATTCGCCTGCTGGAACAGGCGCAGGTGACACCGGTGGACAGCAG
CACCTTTAAAGTGGTGTGGAAAGCGCAGGACGGCCTGCCGCTGAATTACCTGCTGCGGGTTGAACAGGGTAAAGGGCCGC
TGGCGCTGCTGGAACTGAAAAACTTCCGCCTGCCGGGACAGGTATTTCTGACCGGCAGGAGTATGAAAGATGCGGAAGAA
TACGGGGAAGACGCCGATGAGTGA
>Cluster_44095
GTGCCGGGTGCTGAGGACGTACTGCCAGCGCCGCTGCCGCCGTACCGGGTGCTTACCGGGCTGGCAGACCGTTTCGGGCG
GACGCTGACGTACCGGCGTGAGGCCGCAGGCGACCTGGCCGGGGAAATCACCGGCGTGACGGACGGTGCCGGGCGTGAGT
TCCGTCTGGTACTGACCACACAGGCACAGCGGGCGGAAGAGGCCCGCACCTCTTCGCTATCTTCTTCTGACAGTTCCCGC
CCTCTCTCAGCCTCACCGTTCCCCGACACACTGCCCGGTACCGAATACGGCCCCGACAGGGGCATCCGTCTTTCGGCGGT
GTGGCTGATGCACGACCCGGCATACCCGGAAAGCCTGCCCGGTGCGCCACTGGCGCGGTACACGTATACGGAAGCCGGTG
AACTGCTGGCGGTATATGACCGCAGCAATACGCAGGTGCGCGCTTTCACGTATGACGCGCAGCACCCGGGCCGGATGGTG
GCGCACCGTTACGCGGGAAGGCCGGAGATGCGCTACCGCTACGACGATACCGGGCGGGTGGTGGAGCAGCTGAACCCGGC
AGGCCTGAGTTACCGCTACCAGTATGAGCAGGACCGCATCACCGTCACGGACAGCCTGAACCGGCGTGAGGTGCTGCATA
CAGAAGGCGGGGCCGGGCTGAAGCGGGTGGTGAAAAAAGAACTGGCGGACGGCAGCGTCACGCACAGCGGGTATGACGCG
GCAGGAAGGCTCACGGCGCAGACGGACGCGGCGGGACGGCGGACAGAGTACGGTCTGAATGTGGTGTCCGGCGATATCAC
GGACATCACCACACCGGACGGGCGGGAGACGAAATTTTACTATAACGACGGGAACCAGCTGACGGCGGTGGTGTCCCCGG
ACGGGCTGGAGAGCCGCCGGGAATATGATGAACCGGGCAGGCTGGTATCGGAGACATCGCGCAGCGGGGAGACAGTACGC
TACCGCTACGATGACGCGCACAGTGAGTTACCGGCGACGACAACGGATGCGACGGGCAGCACCCGGCAGATGACGTGGAG
CCGCTACGGGCAGTTGCTGGCGTTCACTGACTGCTCGGGCTACCAGACCCGCTATGAATACGACCGCTTCGGCCAGATGA
CGGCGGTTCACCGCGAGGAAGGTATCAGCCTTTACCGCCACTATGACAACCGTGGCCGGTTAACCTCGGTGAAAGACGCA
CAGGGCCGTGAAACGCAGTATGAGTACAACGCCGCAGGCGACCTGACTGCCGTTATCACCCCGGACGGCAACCGGAGCGA
GACACAGTACGATGCGTGGGGAAAGGCGGTCAGCACCACGCAGGGCGGGCTGACGCGCAGTATGGAGTACGATGCTGCCG
GACGTGTCATCAGTCTGACTAACGAGAACGGCAGCCACAGCGACTTCAGCTACGATGCGCTGGACCGGTTGGTACAGCAG
GGCGGCTTTGACGGGCGGACGCAACGTTATCATTATGACCTGACCGGAAAACTCACGCAGAGTGAAGATGAGGGGCTTGT
CACCCTCTGGTACTACGATGAATCGGACCGTATCACTCACCGCACGGTGAACGGCGAACCGGCAGAGCAGTGGCAGTATG
ATGACCACGGCTGGCTGACAGACATCAGCCACCTGAGCGAAGGCCACCGTGTCGCTGTCCACTACGGCTACGACGATAAG
GGCCGCCTGACCGGCGAACGGCAGACGGTGGAGAACCCGGAGACGGGGGAACTGCTGTGGCAGCATGAGACAACACACGC
CTACAACGAACAGGGGCTGGCAAACCGCGTCACGCCGGACAGCCTGCCGCCGGTGGAGTGGCTGACCTACGGCAGCGGTT
ATCTGGCGGGCATGAAGCTTGGCGACACGCCGCTGCTGGAGTACACGCGCGACCGCATGCACCGGGAGACGGTGCGCAGC
TTTGGCAGCATGGCAGGCAGTAATGCCGCATATAAACTGACCAGCACATACACCCCCGCAGGCCAGTTACAGAGCCAGCA
CCTGAACAGCCTGGTGTATGACCGTGACTACGGGTGGAATGACAACGGCGACCTGGTGCGCATCAGCGGCCCGCGACAGA
CGCGGGAATACGGCTACAGCGCCACGGGCAGGCTGGAGAGCGTGCGTACCCTCGCACCGGACCTGGACATCCGCATCCCG
TATGCCACCGACCCGGCGGGAAACCGCCTGCCGGACCCGGAGCTGCACCCGGACAGTACCCTCACGGCGTGGCCGGATAA
CCGCATCGCAGAGGATGCGCACTATGTTTACCACTATGATGAATACGGCAGACTGACGGAGAAGACGGACCTTATCCCGG
CGGGTGTTATCCGGACGGACGACGAGCGGACCCACCACTACCACTACGACAGCCAGCACCGTCTGGTGTTCTACACGCGG
ATACAGCATGGCGAGCCACTGGTCGAGAGCCGCTACCTCTACGACCCGCTGGGACGGCGAATGGCGAAACGGGTATGGCG
GCGGGAGCGTGACCTGACGGGGTGGATGTCGCTGTCGCGTAAACCGGAGATGACGTGGTACGGGTGGGACGGCGACCGGC
TGACGACGGTACAGACCGACACCACGCGTATCCAGACGGTATATCAGCCGGGGAGCTTCGCGCCGCTCATCCGCATCGAA
ACAGACAACGGCGAGCGGGAGAAAGCGCAGCGCCGCAGCCTGGCGGAGAAGCTGCAGCAGGAAGGGAGCGAGGACGGGCA
CGGCGTGGTATTTCCGGCTGAACTGGTGCGGCTGCTGGACAGGCTGGAGGAAGAAATCCGGGCAGACCGCGTGAGCAGTG
AAAGCCGGGCGTGGCTTGCGCAGTGCGGGCTGACGGTGGAGCAACTGGCCAGACAGGTGGAGCCGGAATACACACCGGCG
CGAAAAGTTCATTTTTACCACTGCGACCACCGGGGCCTGCCGCTGGCGCTCATCAGCGAAGACGGCAATACGGCGTGGCG
CGGGGAGTATGATGAATGGGGCAACCAGCTTAATGAAGAGAACCCGTATTACCTGCACCAGCCATACCGTCTGCCGGGGC
AGCAGCATGATGAGGAATCAGGGCTGTACTATAACCGGAACCGGTACTATGACCCGCTACAGGGGAGGTATATTACACAA
GACCCCATTGGGCTGGAGGGGGGATGGAGTCTGTATGCGTATCCGCTGAATCCGGTGAATGGTATTGATCCATTAGGGTT
AAGTCCCGCAGATGTAGCGCTAATAAGAAGAAAAGATCAACTAAACCATCAAAGAGCATGGGATATATTATCTGATACTT
ATGAAGATATGAAGAGATTAAATTTAGGTGGGACTGATCAATTTTTCCATTGTATGGCATTTTGTCGAGTGTCTAAATTA
AATGACGCTGGTGTTAGCCGATCGGCGAAAGGGCTGGGTTATGAAAAAGAGATTAGAGATTACGGGTTAAATCTGTTCGG
TATGTACGGCAGAAAAGTAAAGCTATCCCATTCTGAAATGATTGAAGATAATAAAAAAGACTTGGCTGTAAATGACCATG
GGTTGACATGTCCATCAACAACAGATTGCTCAGATAGATGTAGTGATTATATTAATCCAGAGCATAAAAAAACGATAAAG
GCTTTACAAGATGCTGGCTATCTCAAGTAA
>Cluster_29327
ATGAGTTTATGGAAAAAAATCAGCCTCAGCGTGGTTATCGTTATCTTACTGTTGCTGGGATCGGTGGCGTTTCTGGTGGG
CACCACCAGCGGCCTGCATCTGGTATTTAAAGCGGCGGATCGCTGGGTACCAGGACTGGATATTGGCAAAGTCACCGGCG
GCTGGCGCGATCTCACCTTGTCTGACGTTCGTTATGAGCAGCCAGGCGTGGCGGTAAAAGCGGGCAATCTGCATCTGGCG
GTCGGGCTTGAGTGCCTGTGGAACAGTAGCGTTTGTATTAATGACCTGGCGCTGAAAGACATTCAGGTCAACATCGACAG
TAAAAAAATGCCTCCTTCTGAACAGGTTGAAGAAGAGGAAGATAGCGGCCCCGCTGGATCTCTCTACGCCGTATCCCATCA
CCCTGACACGGGTGGCGCTGGACAACGTCAACATCAAGATTGATGACACCACGGTATCGGTGATGGACTTCACCTCCGGC
CTGAACTGGCAGGAGAAAACCCTGACCCTGAAACCGACGTCGCTGAAAGGCCTGTTGATTGCTTTGCCGAAAGTGGCGGA
AGTGGCGCAGGAAGAAGTGGTCGAACCGAAAATTGAAAATCCGCAGCCGGATGAAAAGCCGCTCAGCGAAACGCTGAAAG
ATCTCTTTTCTCGCCCGGTATTGCCGGAAATGACCGACGTGCATCTGCCGCTCAACCTGAACATTGAAGAGTTTAAGGGC
GAGCAACTGCGCGTGACGGGCGACACGGACATCACCGTGCGCACCATGCTGCTGAAAGTGAGCAGTATCGACGGCAATAC
CAAACTGGACGCGCTGGATATCGACTCCAACCAGGGGATTGTCAACGCCAGCGGCACGGCGCAGCTATCAGACAACTGGC
CGGTGGATATCACCCTCAACAGCACACTGAACGTGGAGCCGTTGAAAGGTGAAAAGGTGAAGCTGAAAGTGGGCGGCGCG
CTGCGCGAACAGCTGGAGATTGGCGTTAACCTTTCCGGTCCGGTGGATATGGATTTACGCGCCCAGACGCGACTGGCGGA
AGCTGGATTGCCACTCAACGTGGAAGTGAACAGCAAACAGCTTTACTGGCCGTTCACTGGTGAGAAGCAGTATCAGGCGG
ATGATCTGAAACTGAAACTTACCGGCAAAATGACCGATTACACGCTCTCTATGCGTACGGCAGTGAAGGGACAGGAGATC
CCGCCCGCGACTATTACCCTCGACGCGAAAGGTAATGAACAGCAGGTCAATCTCGACAAACTCACCGTCGCGGCGCTGGA
AGGGAAAACTGAACTCAAGGCGTTGCTCGACTGGCAGCAGGCCATTAGTTGGCGCGGTGAGCTAACGCTTAACGGTATTA
ACACCGCCAAAGAGATCCCGGAGTGGCCGTCGAAACTCAATGGCTTGATTAAAACCCGCGGTAGCCTGTACGGCGGCACC
TGGCAGATGGAGGTGCCAGAACTGAAGCTGACCGGTAACGTCAAACAGAACAAAGTGAACGTTGACGGCACGCTGAAAGG
CAACAGTTATATGCAGTGGATGATCCCTGGGCTGCATCTGGAACTGGGGCCAAACAGTGCCGAAGTGAAAGGCGAGCTGG
GGGTAAAAGATCTCAATCTTGATGCCACCATCAACGCGCCGGGGCTGGATAACGCGCTGCCGGGGCTTGGCGGTACAGCG
AAAGGGCTGGTGAAAGTACGCGGCACGGTGGAAGCGCCACAACTACTGGCAGATATCACCGCGCGCGGCCTGCGCTGGCA
GGAACTTTCCGTGGCGCAGGTTCGCGTGGAAGGCGACATTAAATCCACCGATCAGATCGCCGGGAAACTCGACGTACGCG
TTGAGCAAATTTCGCAACCGGATGTAAATATCAACCTCGTCACCCTGAATGCCAAAGGCAGTGAAAAGCAGCACGAGCTA
CAGTTGCGGATTCAGGGCGAGCCTGTCTCCGGGCAGCTTAATCTGGCAGGAAGTTTTGATCGCAAAGAAGAACGCTGGAA
GGGAACTCTTAGCAATACCCGCTTCCAGACGCCGGTCGGCCCGTGGTCGCTGACCCGCGATATTGCGCTGGATTACCGCA
ATAAGGAGCAAAAAATCAGCATCGGGCCACACTGCTGGCTTAACCCGAATGCGGAACTGTGCGTGCCGCAAACTATCGAT

GCGGGTGCCGAAGGGCGTGCGGTGGTGAATCTCAACCGCTTCGACCTGGCCATGCTGAAACCATTTATGCCAGAAACCAC
TCAGGCCAGCGGTATCTTCACGGGTAAAGCGGACGTTGCCTGGGACACCACGAAAGAGGGGCTGCCGCAGGGCAGTATCA
CCCTTTCGGGGCGTAATGTGCAGGTGACGCAAACCGTCAATGATGCGGCGCTGCCCGTGGCGTTTCAGACGCTGAATCTG
ACGGCGGAGTTGCGTAACAACCGTGCCGAATTGGGCTGGACCATCCGCCTGACCAATAACGGCCAGTTTGATGGTCAGGT
GCAGGTGACCGATCCGCAAGGCCGCCGTAATCTTGGTGGCAACGTCAATATCCGTAACTTCAACCTTGCGATGATAAACC
CCATCTTTACCCGTGGGGAAAAAGCAGCGGGGATGGTGAGTGCCAACTTGCGTCTGGGTGGTGATGTGCAAAGCCCGCAG
TTGTTTGGTCAGCTTCAGGTTACGGGGGTGGATATCGACGGCAACTTTATGCCGTTTGATATGCAACCAAGCCAGCTTGC
GGTCAACTTTAACGGTATGCGCTCGACGCTTGCAGGTACCGTGCGGACCCAGCAGGGTGAAATCTACCTGAACGGTGATG
CCGACTGGAGCCAGATTGAAAACTGGCGGGCGCGGGTAACAGCGAAGGGCAGTAAAGTGCGTATCACCGTGCCGCCGATG
GTACGAATGGATGTATCACCAGATGTTGTATTCGAGGCTACACCAAACCTGTTTACCCTCGATGGTCGCGTGGATGTTCC
GTGGGCGCGCATCGTGGTGCACGATCTGCCGGAAAGCGCAGTAGGCGTTTCCAGCGATGTGGTGATGCTTAACGATAACC
TGCAACCGGAAGAGCCGAAAACGGCGTCGATTCCGATTAACAGTAACCTGACTGTCCACGTTGGCAACAATGTGCGCATT
GACGCCTTTGGCCTGAAAGCGCGGCTGACGGGCGATCTCAACGTCGTTCAGGACAAACAAGGGCTGGGCCTGAACGGGCA
GATCAACATCCCTGAAGGGCGCTTCCATGCCTATGGTCAGGATCTGATTGTGCGTAAGGGTGAGCTATTGTTCTCTGGTC
CGCCAGATCAACCGTATCTTAATATCGAAGCTATTCGTAACCCGGATGCTACAGAAGACGACGTCATCGCCGGAGTTCGC
GTCACTGGTCTGGCGGACGAACCGAAAGCGGAGATCTTCTCTGACCCGGCGATGTCGCAACAAGCTGCATTGTCTTATTT
GCTACGTGGACAAGGGCTGGAGAGCGATCAGAGCGACAGTGCGGCAATGACCTCGATGCTGATTGGCCTGGGGGTTGCGC
AAAGTGGCCAGATTGTGGGTAAAATCGGCGAGACGTTTGGCGTAAGCAATTTAGCGCTCGACACCGAGGGAGTTGGCGAC
TCCTCCCAGGTGGTGGTCAGCGGCTATGTATTGCCAGGCCTGCAAGTGAAATACGGCGTGGGTATATTTGACTCTATAGC
AACACTCACGTTACGTTATCGCCTGATGCCTAAGCTATATCTGGAAGCCGTGTCTGGTGTAGACCAGGCACTGGATTTGC
TCTATCAGTTCGAGTTTTAG
>Cluster_34992
ATGGGTAAAGGCAGCAGTAAGGGGCATACCCCGCGCGAAGCGAAGGACAACCTGAAGTCCACGCAGCTGCTGAGTGTGAT
CGATGCCATCAGCGAAGGGCCGGTTGAAGGTCCGGTGGATGGATTAAAAAGCGTGCTGCTGAACAGTACGCCGGTGCTGG
ACAGTGAGGGGAATACCAATATATCCGGCGTCACGGTGGTGTTCCGGGCCGGTGAGCAGGAGCAGACACCGCCGGAGGGA
TTTGAATCCTCCGGCTCCGAGACGGTGCTCGGTACAGAAGTGAAATATGACACGCCGATCACCCGCGCCATCACGTCGGC
AAACATCGACCGACTGCGCCTGACCTTCGGTGTGCAGGCACTGGTGGAAACCACCTCAAAGGGGGACCGGAATCCGTCGG
AAGTTCGCCTGCTGGTTCAGATACAGCGTAATGGTGGCTGGGTGACGGAAAAAGACATCACCATTAAGGGCAAAACCACC
TCGCAGTATCTGGCCTCGGTGGTGGTGGATAACCTGCCGCCGCGCCCGTTTAATATCCGGATGCGCAGAATGACGCCGGA
CAGCACCACAGACCAGCTGCAGAACAAAACGCTCTGGTCGTCATACACCGAAATCATCGATGTGAAACAGGGCTACCCGA
ACACGGCACTGGTCGGCGTAAAGGTGGATTCGGAGCAGTTCGGCAGCCAGCAGGTGAGCCGTAATTATCATCTTCGCGGG
CGTATTCTGCAGGTGCCGTCGAACTATAACCCGCAGACGCGGCAATACAGCGGTATCTGGGACGGAACGTTAAAACCGGC
ATACAGCAACAACCCGGCCTGGTGTCTGTGGGATATGCTGACCCACCCGCGCTACGGCATGGGGAAACGTCTTGGTGCGG
CGGATGTGGATAAATGGGCGCTGTATGTCATCGGCCAGTACTGCGACCAGTCGGTGCCGGACGGCTTTGGCGGCACGGAG
CCGCGCATTACCTGTAATGCGTACCTGACCACACAGCGTAAGGCGTGGGATGTGCTCAGTGATTTCTGCTCGGCGATGCG
CTGTATGCCGGTATGGAACGGGCAGACGCTGACGTTCGTGCAGGACCGACCATCGGATAAGGTGTGGACCTATAACCGCA
GTAATGTGGTGATGCCGGATGATGGTGCGCCGTTCCGCTACAGCTTTAGCGCCCTGAAGGACCGCCATAATGCCGTTGAG
GTGAACTGGATTGACCCGAATAACGGCTGGGAGACGGCGACAGAGCTTGTTGAGGATACGCAGGCCATTGCCCGTTACGG
TCGTAATGTCACGAAGATGGATGCTTTTGGTTGTACCAGTCGGGGGCAGGCACACCGCGCCGGGCTGTGGCTGATTAAAA
CGGAACTGCTGGAAACGCAGACCGTGGACTTCTGCGTGGGCGCAGAAGGGCTTCGCCATGTGCCGGGCGATGTTATTGAA
ATCTGTGATGATGACTATGCCGGTATCAGCATCGGTGGTCGTGTGCTGGCGGTGAACAGCCAGACCCGGACGCTGACGCT
CGACCGTGAAATCACGCTGCCATCCTCCGGTACCACGCTGATAAGCCTGGTTGACGGGCAGGGTAATCCGGTGAGCGTGG
AGGTCCAGTCCGTCACCGACGGCGTGAAGGTGAAAGTGAGCCGTGTTCCTGACGGCGTTGCTGAATACAGCGTGTGGGGG
CTGAAGTTGCCGACGCTGCGCCAGCGCCTGTTCCGCTGCGTGAGTATCCGTGAGAACGACGACGGCACGTATGCCATCAC
TGCCGTGCAGCATGTACCGGAAAAAGAAGCCATCGTGGATAACGGGGCGCACTTTGACGGCGACCAGAGCGGCACGGTGA
ATGGTGTCACGCCGCCAGCGGTGCAGCACCTGACCGCCGAAGTCACCGCAGACAGCGGGGAATATCAGGTGCTGGCGCGC
TGGGACACGCCGAAGGTGGTGAAGGGCGTGAGCTTCCTGCTTCGCCTGACCGTGGCAGCGGATGACGGCAGTGAGCGGCT
GGTCAGCACGGCCCGGACGACGGAAACCACATACCGCTTCAGGCAACTGGCGCTGGGGCGTTACACGCTGACAGTCCGGG
CGGTAAATGCGTGGGGGCAGCAGGGCGATCCGGCGTCGGTATCGTTCCGGATTGCCGCACCGGCAGCGCCTGTCACTATT
GAACTGATACCGGGGTATTTTCAGATAACAGCGGTCCCGAAACTGGCTGTATATGACCCGACGGTGCAGTTTGAGTTCTG
GTTCTCGGAAAAGCGGATTGCGGATATCAGGCAGGTTGAAACCACAGCCCGCTATCTTGGCACGGCGCTGTACTGGATAG
CTGCCAGTATCAATATCAAGCCGGGCCATGATTATTATTTTTACATCCGCAGTGTGAACACCGTTGGCAAATCGGCATTT
GTGGAGGCTGTTGGCCAGCCGAGTGATGATGCATCCGGCTATCTGGATTTTTTCAAAGGAGAGATAGGGAAAACCCATCT
GGCTCAGGAGTTGTGGACGCAGATTGATAACGGTCAGCTTGCGCCTGACCTGGCTGAAATCAGGACGTCCATTACGGATG
TCAGCAATGAAATCACACAGACCGTCAATAAGAAACTGGAAGACCAGAGTGCAGCGATCCAGCAGATACAGAAAGTTCAG
GTTGATACAAATAATAACCTGAACAGCATGTGGGCTGTGAAGCTGCAGCAGATGCAGGACGGACGCCTTTATATCGCGGG
TATTGGTGCCGGTATTGAAAACACCCCTGACGGCATGCAGAGTCAGGTGCTGCTGGCGGCAGACAGGATTGCGATGATTA
ATCCTGCGAATGGCAACACAAAGCCGATGTTTGTTGGGCAGGGCGATCAGATATACATGAATGAAGTGTTCCTGAAATAT
CTGACGGCTCCCACCATTACCAGCGGCGGTAATCCTCCGGCATTTTCCCTGACACCGGACGGGCGACTGACGGCGAAAAA
TGCGGATATCAGTGGCAGTGTGAATGCGAACTCCGGGACGCTCAATAACGTCACGATAAATGAGAACTGTCAGATTAAGG
GGAAACTGTCCGCCAACCAGATTGAAGGCGATATTGTCAAAACGGTCAGCAAGTCTTTCCCCCGCACGAGCACTTATGCC
AGCGGCACCATTACGGTCACGATTAGTGATGATCAGAAGTTTGACCGACAGGTCATGATCCCGGCTCTGCTGTTTAAAGG
AAGCAGGAAGGAAAATTATGGCAGTAATAATCAACAGTCTTATGTTTATTCTGTATGCCGTTTGCAGGTAACGAAAAACG
GGACAGAAATTTTTAATCAGTCAACAACGGATGCTCCGGCGGTTTTTTCTTCCGTTATTGATATGCCTGCGGGGCAGGGA
ACGTTGACGTTAAAGTTTACGGTCTCTTCTTCAATGGTCAATAACTGGACACCGACAACCAGTATCAGCGATTTGCTTGT
TGTGGTGATGAAGAAATCCACCGCAGGTATCACGATTAGCTGA
>Cluster_41379
ATGAAGTTTTTACCCTATATTTTTCTTCTCTGTTGTGGTCTTTGGTCGACCATAAGTTTCGCAGACGGAAATTACATCGA
ATATCGTGGCATCAGTAGTAACAACCGTGTCACACTTGATCCACTACGTCTGAGCAACAAGGAATTACGTTGGCTAGCGA
GCAAAAAAAATCTTGTGATTGCAGTACACAAGTCCCAAACAGCTACGTTGTTGCATACCGATTCGCAGCAACGGATTCGT
GGTATTAATGCTGATTATTTAAATCTTTTAAAAAGAGCGTTAAATATCAAATTAACACTCCGGGAATACGCAGATCATCA
AAAAGCAATGGACGCGCTGGAAGACGGTGAAGTTGATATAGTGTTATCACATTTAGTTGCTTCGCCGCCTCTTAATGATG
ACATTGCTGCAACTAATCCTCTGATAATTACCTTTCCGGCGCTGGTCACTACCCTTCACGATTCAATGCGACCGCTTACC
TCATCAAAACCAGTAAATATTGCTCGAGTAGCAAATTATCCCCCAGACGAGGTAATTCATCAATCATTTCCAAAAGCAAC
AATTATCTCTTTTACAAATTTATATCAGGCATTAGCATCCGTCTCAGCCGGACAGAATGATTACTTTATTGGTAGTAACA
TCATTACCAGCAGTATGATTTCCCGTTATTTACTCACTCCTTAAATGTAGTGAAGTATTATAACTCACCGCGTCAATAT
AACTTTTTATTAACCAGAAAAGATTCAATCGTTCTTAATGAAGTACTCAATCGATTTGTTGATGCTTTAACAAATGAAGT
TCGCTATGAAGTATCACAAAATTGGCTTGATACAGGAAACCTGGCCTTTCTGAACAAACCATTAGAACTCACTGAACATG
AAAAACAGTGGATTAAGCAGCATCCCGATTTAAAGGTGCTGGAAAATCCTTACTCGCCACCCTATTCTATGACAGATGAA

ACTGGCTCAGTTCGGGGCGTGATGGGTGATATTCTTAATATTATTACCTTGCAAACAGGTTTAAATTTTTCTCCGATCAC
CGTTTCACACAATATTCATGCGGGGACACAGCTTAACCCCGGTGGATGGGATATATTGCCCGCCGCTATTTATAGTGAAG
ATCGAGAAAATAATGTTTCATTTGCTGAAGTCTTCATAACAACGCCTTACGTTTTTGTCATGCAAAAAGCGCCTGACAGT
GAACAAACATTAAAAAAAGGAATGAAAGTTGCCATTCCATATTATTATGAGCTTCATTCGCAATTAAAAGAGATGTATCC
GGAGGTAGAGTGGATAAAAGTCGATAACGCCAGCGCTGCATTTCACAAGGTCAAGGAAGGTGAACTTGATGCTCTGGTCG
CGACACAGTTAAATTCGCGTTACATGATCGACCATTACTATCCTAATGAACTTTATCATTTTCTTATTCCCGGCGTTCAG
AATGCATCACTTTCGTTCGCTTTTCCTCGCGGAGAACCGGAACTTAAGGATATTATTAATAAAGCACTGAATGCAATTCC
CCCAAGCGAAGTTCTGCGCCTGACCGAAAAATGGATTAAAATGCCCAATGTGACCATTGACACATGGGACCTCTATAGCG
AGCAGTTTTATATTGTTACGACATTATCCGTTTTATTAGTTGGCAGTAGCCTTTTATGGGGATTCTACCTGTTACGCTCA
GTTCGTCGTCGTAAAGTCATTCAGGGTGATTTAGAAAACCAAATATCATTCCGGAAAGCACTCTCGGACTCCTTACCGAA
TCCAACTTATGTTGTAAACTGGCAAGGTAATGTCATTAGTCATAATAGTGCATTTGAACATTATTTCACTGATGATTACT
ACAAAAATGCAATGTTGCCATTAGAAAACAGTGAATCCCCCTTTAAAGATGTTTTTTCTAATACGCATGAAGTCACAGCA
GAAACGAAAGAAAACCGAACAATATACACACAGGTATTTGAAATTGATAATGGCATCGAGAAAAGATGCATTAATCACTG
GCATACCTTATGTAATCTGCCAGCAAGCGAACATGCTGTTTATATTTGTGGTTGGCAAGATATTACCGAGACGCGTGATT
TAATTCATGCACTCGAAGTAGAGAGAAATAAAGCGATCAATGCAACTGTCGCAAAAAGCCAGTTTCTGGCAACAATGAGT
CACGAAATAAGAACACCAATAAGCTCCATTATGGGCTTCCTGGAACTACTGTCGGGTTCTGGTCTTAGCAAGGAACAACG
GGTGGAGGCGATTTCACTTGCCTACGCCACCGGACAATCACTCCTCGGCTTAATTGGTGAAATCCTTGATGTCGACAAAA
TTGAATCGGGTAACTATCAACTTCAACCACAATGGGTCGATATCCCTACTTTAGTCCAGAACACTTGTCACTCTTTCGCT
GCGATTGCTGCAAGCAAATCGATCGCATTAAGTTGCAGCAGTACGCTTCCTGAACGCTACCTGGTTAAGATCGACCCTCA
GGCGTTTAAGCAGGTCTTATCTAATTTACTGAGTAATGCTCTCAAATTTACTACCGAGGGGGCAGTAAAAATTACGACCT
CCCTGGGTCACATTAATGACAACCACGCTGTTATCAAAATGACGATTATGGATTCTGGAAGTGGATTATCGCAGGAAGAA
CAACAACAACTGTTTAAACGCTATAGCCAAACAAGTGCAGGTCGTCAGCAAACAGGTTCTGGTTTAGGCTTAATGATCTG
CAAAGAATTAATTAAAAACATGCAGGGCGATTTGTCATTAGAAAGTCATCCAGGCATAGGAACGATATTTACGATCACAA
TCCCGGTAGAAATTACCCAACAAGTGGCGGCTGTCGAGGCAAAAGCAGAACAACCTATCACACTACCTGAAAAGTTGAGC
ATATTAATCGCGGATGATCATCCGACCAACAGGCTATTACTCAAACGCCAGCTAAATCTATTAGGATATGATGTTGATGA
AGCCACTGATGGTGTGCAAGCGCTACACAAAGTCAGTATGCAACATTATGATCTGCTTATTACTGACGTTAATATGCCGA
ATATGGATGGTTTTGAGTTGACTCGCAAACTCCGTGAGCAAAATTCTTCCTTACCCATCTGGGGGCTTACAGCCAACGCA
CAGGCTAACGAACGTGAAAAAGGGTTAAATTGCGGCATGAACTTATGTTTGTTCAAACCGTTGACTCTGGATGTACTGAA
AACACATTTAAGTCAGTTACACCAGGTTGCGCATATTGCACCTCAGTATCGCCACCTTGATATCGAGGCCCTGAAGAATA
ATACGGCGAATGATCTACAACTGATGCAGGAGATTCTCATGACTTTCCAGCATGAAACGCATAAAGATCTACCCGCTGCG
TTTCATGCACTAGAAGCTGGCGATAACAGAACTTTCCATCAGTGTATTCATCGCATCCACGGTGCGGCTAACATCCTGAA
TTTGCAAAAGTTGATTAATATTAGCCACCAGTTAGAAATAACACCTATTTCAGATGACAGTAAGCCTGAAATTCTTCAGT
TGCTGAACTCTGTAAAAGAACACATTGCAGAGCTGGACCAGGAGATTACTGTTTTCTGTCAGCAAAATGACTAA
>Cluster_51780
ATGAGTGATAACAACCTGCGTCTGCAGGTCATTCTTAATGCGGTTGACAAACTCACCCGCCCATTCCGTGCTGCACAGGC
CAGTTCGAAAGAGCTGGCTGGCGCAATTCAGAATACCAGAAACAGCCTCAAAGAACTGAATAAGCAGGCTGGCAGAATTG
ATGAGTTTCGCAAGACGCGCTCGCAACTAGCCATAACAGCCAACAACCTGAACGCAGCCCGCGAAGAGGCGGCAAAACTC
GCCACACAATTTGCTGCCACTAACAGGCCAACCGCCGCGCAGGCAAAGTTATTCAGTCAGGCCAAAACACGAGTACAGGA
ACTTCAGCAGACCTATAACGGCTTGGTGGGGGCGGTCCAGAGACAACGTCAGGCACTTAAAGAATCAGGGATTGATACCA
GGCAACTCAGTAGTGCCCAGCGAGAACTTAAGAAAAATGCTGAAGAAACAAGGCAGGCACTGGAGGGCCAGCAAAAAGCA
CTTAAACGTCTGGGTGAACAACAGGCACGGATGAACGCTGCCAGAGAACAATACTCAAGACGGCTTGAAGTGCGCGATCG
TATCGCAGGAGCCGGAGCCACCACCACGGCTGCAGGGCTGGCAATGGGCGCGCCAGTGATGGCGGCGGTAAAAAGCTATA
CCAGCATGGAAGATGCCATGAAAGGTGTGGCAAAGCAGGTCAATGGTCTGCGTGACGATAATGGCAACCGCACTGCGCGT
TTTTACGAAATGCAGGATGCCATCAAGGCTGCCAGCGAACAGTTGCCGATGGAAAACGGTGCGGTGGACTTCGCTGCACT
GGTTGAAGGTGGTGCGCGCATGAACGTGGCAAACCCTGACGACAGCTGGGAAGACCAGAAACGTGACCTGCTGGCCTTCG
CCAGTACGGCAGCAAAGGCGGCAACAGCCTTTGAGCTGCCAGCGGATGAACTGTCAGAAAGTCTGGGGAAAATCGCCCAG
CTCTACAAAATACCTACCCGCAATATTGAACAGCTCGGCGATGCGCTGAACTATCTGGATGATAACGCCATGTCGAAAGG
GGCGGACATCATTGATGTGATGCAACGTCTGGGCGGTGTGGCTGACCGTCTGGATTATCGTAAAGCGGCGGCACTGGGTT
CCACCTTTCTGACACTGGGCGCTGCGCCGGAGGTTGCAGCCAGTGCAGCAAACGCGATGGTGCGTGAATTGTCCATTGCC
ACCATGCAAAGCAAGAGTTTCTTTGAAGGGATGAATCTGCTGAAACTCAATCCTGAAGTGATTGAAAAGCAGATGACGAA
GGATGCGATGGGAACCATCCAGCGTGTACTGGAGAAGGTGAACGCACTGCCGCAGGACAAGCGTCTGTCTGCCATGACCA
TGTTGTTTGGTAAAGAGTTTGGCGATGACGCGGCGAAACTGGCAAACAACCTGCCGGAACTACAGCGCCAGCTAAAACTG
ACAGCGGGCAATGATGCGCTCGGTTCGATGCAGAAAGAATCCGACATCAACAAAGACTCACTTTCTGCTCAGTGGTTGCT
GGTCAAAACCGGAGCGCAGAACACCTTCAGCAGCCTGGGCGAAACGCTGCGCCAGCCGCTGATGGATATTCTGTACACGG
TGAAAAGCGTCACGGGGGCGTTGCGCCGCTGGGTGGAAGCTAACCCGGAACTGACAGGCACACTGATGAAAGTAGTGGCT
GTTGTGGCTGCGGTTACCGTAGGCCTCGGCACCTTAGCGGTGGCGCTGGCTGCAGTGCTGGGGCCGCTGGCAGTGATCCG
TCTGGGATTCTCTGTGCTGGGTATCAAAACGTTACCTTCCGTTACGGCAGCAGTAACTCGAACCAGCAGCGCGTTGTCCT
GGCTGGCTGGCGCACCACTGGCACTGCTGCGACGCGGGCTTGCTTCATCGGGCAACGCAGCGGGTTTACTTACTGCTCCG
TTGTCGTCTTTGCATCGCACGGTATCACTGATGGGGAATGTCCTGAAAACTGTAGCAGGTGCGCCGGTTGCACTTTTGCG
GTCTGGATTATCCGGTTTACGTGCTGTTGCTGTGATGTTTATGAATCCACTGGCAGCACTACGCGGTGGGCTGGCTGCCG
CAGGCGCGGTGCTGCGTGTGCTCGCATCCGGCCCGCTGGCGATGCTGCGCGTTGCCCTGTATGCCATATCTGGTCTGTTA
GGTGCTCTGCTCAGTCCGATAGGTCTTGTGGTTACTGCACTGGCGGGCGTGGCGCTGGTTGTCTGGAAATACTGGCAACC
CATCACCGCATTTCTCGGTGGCGTGGTGGAAGGATTCAAAGCGGCGGCAGGTCCCATCAGTGCAGCGTTCGAACCGCTTA
AGCCCGTGTTCCAGTGGATTGGCGACAAAGTGCAGGCGCTGTGGGGCTGGTTTACTGATCTGCTGACGCCCGTTAAGTCG
ACCTCTGCCGAACTGCAGAGCGCAGCGGCAATGGGGCGGCGATTCGGGGAGGCACTGGCGGAAGGGCTGAATATGGTCAT
GCATCCGCTGGACTCCCTGAAATCCGGCGTTTCCTGGTTGCTGGAGAAGCTCGGCATTGTCAGTAAAGAGGCCGCAAAGG
CAAAACTGCCGGAAAGCGTGACGCGTCAGCAACCTGCGACGGTGAATGCAGACGGTAAAGTGATGATGCCATCGGGTGGT
TTTCCATCATGGGGATATGGCTTTGCGGGGATGTATGACAGCGGCGGGTATATCCCGCGCGGGCAGTTTGGCATCGTCGG
TGAAAACGGGCCGGAAATTGTTAACGGCCCGGCAAATGTGACCAGCCGGAGAAATACAGCTGCACTGGCTGCCGTTGTTG
CCGGAATGATGGGCGTTGCTGCCGCGCCAGCAGAGCTTCCACCGTTGCACCCTTTGGCACTTCCCGCGAAAGGTGGAGAA
GCAATTGTGAGTCGCGCAGCCACTGTGCCGCTCGTTCAACGGATTGAGGCACCGACGCAGATCATCATTCAGACGCAGCC
AGGACAAAGTGCGCAGGATATTGCGCGGGAGGTGGCACGCCAGCTTGATGAACGTGAACGCAGGCTGAAGGCAAAAGCCA
GGAGTAACTACAGCGATCAGGGGGGATACGACGCATGA
>Cluster_37925
ATGGTTCAGATCCCCCAAAATCCACTTATCCTTGTAGATGGTTCATCTTATCTTTATCGCGCATATCACGCGTTTCCCCC
GCTGACTAACAGCGCAGGCGAGCCGACCGGTGCGATGTATGGTGTCCTCAACATGCTGCGCAGTCTGATCATGCAATATA
AACCGACGCATGCAGCGGTGGTCTTTGACGCCAAGGGAAAAACCTTTCGTGATGAACTGTTTGAACATTACAAATCACAT
CGCCCGCCAATGCCGGACGATCTGCGTGCACAAATCGAACCCTTGCACGCGATGGTTAAAGCGATGGGACTGCCGCTGCT
GGCGGTTTCTGGCGTAGAAGCGGACGACGTTATCGGTACTCTGGCGCGCGAAGCCGAAAAAGCCGGGCGTCCGGTGCTGA

TCAGCACTGGCGATAAAGATATGGCGCAGCTGGTGACGCCAAATATTACGCTTATCAATACCATGACGAATACCATCCTC
GGACCGGAAGAGGTGGTGAATAAGTACGGCGTGCCGCCAGAACTGATCATCGATTTCCTGGCGCTGATGGGTGACTCCTC
TGATAACATTCCTGGCGTACCGGGCGTCGGTGAAAAAACCGCGCAGGCATTGCTGCAAGGTCTTGGCGGACTGGATACGC
TGTATGCCGAGCCAGAAAAAATTGCTGGGTTGAGCTTCCGTGGCGCGAAAACAATGGCAGCGAAGCTCGAGCAAAACAAA
GAAGTTGCTTATCTCTCATACCAGCTGGCGACGATTAAAACCGACGTTGAACTGGAGCTGACCTGTGAACAACTGGAAGT
GCAGCAACCGGCAGCGGAAGAGTTGTTGGGGCTGTTCAAAAAGTATGAGTTCAAACGCTGGACTGCTGATGTCGAAGCGG
GCAAATGGTTACAGGCCAAAGGGGCAAAACCAGCCGCGAAGCCACAGGAAACCAGTGTTGCAGACGAAGCACCAGAAGTG
ACGGCAACGGTGATTTCTTATGACAACTACGTCACCATCCTTGATGAAGAAACACTGAAAGCGTGGATTGCGAAGCTGGA
AAAAGCGCCGGTATTTGCATTTGATACCGAAACCGACAGCCTTGATAACATCTCTGCTAACCTGGTCGGGCTTTCTTTTG
CTATCGAGCCAGGCGTAGCGGCATATATTCCGGTTGCTCATGATTATCTTGATGCGCCCGATCAAATCTCTCGCGAGCGT
GCACTCGAGTTGCTAAAACCGCTGCTGGAAGATGAAAAGGCGCTGAAGGTCGGGCAAAACCTGAAATACGATCGCGGTAT
TCTGGCGAACTACGGCATTGAACTGCGTGGGATTGCGTTTGATACCATGCTGGAGTCCTACATTCTCAATAGCGTTGCCG
GGCGTCACGATATGGACAGCCTCGCGGAACGTTGGTTGAAGCACAAAACCATCACTTTTGAAGAGATTGCTGGTAAAGGC
AAAAATCAACTGACCTTTAACCAGATTGCCCTCGAAGAAGCCGGACGTTACGCCGCCGAAGATGCAGATGTCACCTTGCA
GTTGCATCTGAAAATGTGGCCGGATCTGCAAAAACACAAAGGGCCGTTGAACGTCTTCGAGAATATCGAAATGCCGCTGG
TGCCGGTGCTTTCACGCATTGAACGTAACGGTGTGAAGATCGATCCGAAAGTGCTGCACAATCATTCTGAAGAGCTCACC
CTTCGTCTGGCTGAGCTGGAAAAGAAAGCGCATGAAATTGCAGGTGAGGAATTTAACCTTTCTTCCACCAAGCAGTTACA
AACCATTCTCTTTGAAAAACAGGGCATTAAACCGCTGAAGAAAACGCCGGGTGGCGCGCCGTCAACGTCGGAAGAGGTAC
TGGAAGAACTGGCGCTGGACTATCCGTTGCCAAAAGTGATTCTGGAGTATCGTGGTCTGGCGAAGCTGAAATCGACCTAC
ACCGACAAGCTGCCGCTGATGATCAACCCGAAAACCGGGCGTGTGCATACCTCTTATCACCAGGCAGTAACTGCAACGGG
ACGTTTATCGTCAACCGATCCTAACCTGCAAAACATTCCGGTGCGTAACGAAGAAGGTCGTCGTATCCGCCAGGCGTTTA
TTGCGCCAGAGGATTATGTGATTGTCTCAGCGGACTACTCGCAGATTGAACTGCGCATTATGGCGCATCTTTCGCGTGAC
AAAGGCTTGCTGACCGCATTCGCGGAAGGAAAAGATATCCACCGGGCAACGGCGGCAGAAGTGTTTGGTTTGCCACTGGA
AACCGTCACCAGCGAGCAACGCCGTAGCGCGAAAGCGATCAACTTTGGTCTGATTTATGGCATGAGTGCTTTCGGTCTGG
CGCGGCAATTGAACATTCCACGTAAAGAAGCGCAGAAGTACATGGACCTTTACTTCGAACGCTACCCTGGCGTGCTGGAG
TATATGGAACGCACCCGTGCTCAGGCGAAAGAGCAGGGCTACGTTGAAACGCTGGACGGACGCCGTCTGTATCTGCCGGA
TATCAAATCCAGCAATGGTGCTCGTCGTGCAGCGGCTGAACGTGCAGCCATTAACGCGCCAATGCAGGGAACCGCCGCCG
ACATTATCAAACGGGCGATGATTGCCGTTGATGCGTGGTTACAGGCTGAGCAACCGCGTGTACGTATGATCATGCAGGTA
CACGATGAACTGGTATTTGAAGTTCATAAAGATGATGTTGATGCCGTCGCGAAGCAGATTCATCAACTGATGGAAAACTG
TACCCGTCTGGATGTGCCGTTGCTGGTGGAAGTGGGGAGTGGCGAAAACTGGGATCAGGCGCACTAA
>Cluster_12050
ATGGATAACATTCATAGCTGCAGTTCAATTAGTCAAAATCATACCGGAACAGGCCATAATGTTGCTGGTAACTTAATTAT
ATACAATAGTGATAATGTTGATTATGCGACTGAGTTAGCGATTGCTGTTTTAGTCGGTTGCTGGGATGAAAAAAATAATG
GTGATATTGATATAATTACAAGCCTTATTGGCGAGTCATATGACAGTTGGATTCGAAAATTAAGAATAATTGAAGGGATG
CAAGATAGTCCTTTGATTCATGAGAGAGGTTCTTGGAGTTTTAAAGATAGAATACAATCATTTCAAACGGTATCGTCAAG
ATTATTCGATGATCATTTGGATTTGTTTCGGACGACTGTTGTATCCGTTTTTAAAACTATCGACCCACAATTTGAACTGG
CACCTGAAGAGCGTTATGCAGCAGTAATTTATGGGAAGGTTTTACCACACAGCCGATTAATAAGGAAAGGACTATCCGAA
GGGTTGGCATTAGTTGCAACAAAGCAAGAGTTACTTACTAATTGCTCAAAATATAAAGGGCAATATTGCGCCTCAAGTGT
GGTTAAAGAGGTTTTTTCGGCTTCTAGCTGGCAATTATGGGCTAGTACGCAAGATATTCAAGTTATGCTTGCGGAAAGTG
CTCCTGACTGTTTTATCGACGAAGTGGAAAATGCTGCATCTCACCAAGACAAGCCATTTGATTCACTTTTTGCACAGGAA
GGTATCGGTGGTATTTCAGGTCGGAATTATATGACAGGACTGCTATGGGCTATTGAAGGATTAGCTTGGGCGCCTAACTA
TTTATCTCGCTCTTTAGTAATATTAGGGGAGCTGGATAGCCATGATCCTGGTGGCAATTGGGCTAACAGGCCACTTAACT
CGATAATCAACATATTGTTACCATGGCTTCCTCATACTACAGCTGATATAGATAGACGTATTGCTGCTTTCAACGCATTG
GCAAGGGAGTGGCCCGATACAGCATGGCGTGTTTTAGTTCAGTTGCTACCCAACAACACGCAAGTAACATCTGGCACACA
TATTCCAACTTTTAGAAACTTTATTCCTAACGGTTTTAATAAAAGGCCATCGGGTGATGAGTACCGTACTCAGATTGAAA
TATACACTCAATTAACAATTGAATTGGCAAGTAAATCATCATTAAGACTTGTTGATTTAGTTGAAAATATTGGTAGATTA
GCGCCATTTAAATTCGATGATGCAATTAAACTATTGTATGATTTTTCTAAAAAAACAGATAGTGCTGAATTGAAGTTAGC
GGTGTGGGAAAAATGTTTAGAAGTTTATAACAAAAACAAAAGATTCTCAGGTGCTTATTGGGCAATGCAACCAGAACAAA
ATGAAAAAATCAAAGATGTTTCTACAGTCTTGAAGCCCGAAAATCCTATGCTTTATAGCAAGCGACTTTTTGCACAAAAC
AATTACTTCCTATTTGATGAAAATGGAAACTGGCGGGACCAAGAAGAAAGGTTGACAAAAGAACGAGGGCAATCAATATA
CGAAATATATGCTATTAATGGTATTGATGGAGTCTTAGAATTCTCTCTATCTATTGAAAGGCCTGAGTTTGTTGGTAGTA
GCCTGGTCGCAGAAGATCTAGAAATTCCTTTAGATATACTGAAAAAACTTATTGTTAGTGATATTAAAAAGGAAAATTCT
CTTGCTTCTGGTTATGTACAGGCTAAAAACTACAGAGAAGGAAAATCATGGGTGTTGACAGTTATTAATGACTGGAGTGC
TCAAGAAAAGATTGCTTTTTTTCTTTTGTTACCGTTTAAAAAAGAAACATGGGATGCATTAAACGCTTCATTTGATGATT
TTCCTTTCGAGTATTGGAAAAATACTCTTGTTAATGCATACAGCTGTGATTCAACGGAAGAAATTTATTTTGCTATAGAC
TGCTTGATTAAAGTAGACCGTCCCTTGATGGCAATAGATTGTTTATCTAAAATTTTGCATATCGATAATTCAGTTGATAG
CAGTAAGGTTGTGCTTGCTCTATTGCAATCAATTAGAACCACAGAAAATATTGAGCGATTTGATATTCACTCATTTAATG
AACTTGTGAATTATATTCAAAATGATAACACTGTTTCAGATGACGACTTAGTAAAGATTGAATGGGCATACCTACCTGTA
ATTAACAGGGGGGCTAATGATTCGTTGCATCCTCAAGTATTAGAGAAAAAACTTGCAAGTGAACCCAGTTTTTTTTGTGA
AGTCATTCAATATGCGTATCGATCAGAATTTCAAAAAGCAAGTAAAGAACTATCTGAAAGTGAAATAAATATTGCCAAAA
ACTCACTCTATTTACTTGATGATTGGAAAAAGATTCCGGGTGTTGATGCTGAAGGCAAGTTTGATGTCAATGCGTTTAAT
AACTGGTTTGATTTTGTCCAAACAGAATGCGAAAAGTCAGGCCATTTAGATTTTGCTTATCATAGAATTGGAAGCATATT
AATTTTTAGCCCTGCAAATGACGAGCATTGGATACTTCCAGAACTGGCCGAGTTTTTGAATAGACGTGAACTTGATAAAG
TGCGTACTGGTTATAAGAATGCGATAATAAATTCGCGTGGTGTCTACATTGTTGACCCAGAAGGTAAACCGGAATTGGAG
TTGGCCCAACAATATCACACTAAGTCTGATGCAATGGAACTTCTAGGCTTTCATCGATTTGCCCGAATTCTTCGAGAGTT
AGCTCATGAATACCAAGCGGAAGCTGAGTTGATAATAGAACAGCACAGTAAAAAAAAGATTGCTGAGATTTAG
>Cluster_56755
ATGACAGATGTAACGATTAAAACGCTGGCCGCAGAGCGACAGACCTCCGTGGAACGCCTGGTACAGCAATTTGCTGATGC
AGGTATCCGGAAGTCTGCTGACGACTCTGTGTCTGCACAAGAGAAACAGACTTTGATTGACCACCTGAATCAGAAAAATT
CAGGCCCGGACAAATTGACGCTGCAACGTAAAACACGCAGCACCCTTAACATTCCTGGTACCGGTGGAAAAAGCAAATCG
GTACAAATCGAAGTCCGCAAGAAACGCACCTTTGTGAAACGCGATCCGCAAGAGGCTGAACGCCTTGCAGCGGAAGAGCA
AGCGCAGCGTGAAGCGGAAGAGCAAGCCCGTCGTGAGGCAGAAGAATCGGCTAAACGCGAGGCGCAACAAAAAGCTGAAC
GTGAGGCCGCAGAACAAGCTAAGCGTGAAGCTGCTGAACAAGCGAAACGTGAAGCTGCGGAAAAAGACAAAGTGAGCAAT
CAACAAGACGATATGACTAAAAACGCCCAGGCTGAAAAAGCCCGCCGTGAGCAGGAAGCTGCAGAGCTCAAGCGTAAAGC
TGAAGAAGAAGCGCGTCGTAAACTCGAAGAAGAAGCACGTCGCGTTGCTGAAGAAGCACGTCGTATGGCGGAAGAAAACA
AATGGACTGATAACGCGGAACCGACTGAAGATTCCAGCGATTATCACGTCACTACTTCTCAACATGCTCGCCAGGCAGAA
GACGAAAGCGATCGTGAAGTCGAAGGCGGCCGTGGCCGTGGTCGTAACGCGAAAGCAGCGCGTCCGAAGAAAGGCAACAA
ACACGCTGAATCAAAAGCTGATCGTGAAGAAGCACGCGCAGCAGTACGTGGCGGTAAAGGCGGAAAACGTAAAGGTTCTT
CGCTGCAGCAAGGCTTCCAGAAGCCTGCTCAGGCCGTTAACCGTGACGTTGTGATCGGCGAAACTATCACCGTTGGCGAA

CTGGCGAACAAGATGGCGGTTAAAGGCTCTCAGGTCATCAAAGCGATGATGAAACTGGGCGCAATGGCAACCATCAACCA
GGTTATCGATCAGGAAACCGCACAGCTGGTTGCTGAAGAGATGGGCCATAAAGTTATCCTGCGTCGTGAAAACGAGCTGG
AAGAGGCGGTAATGAGCGACCGTGACACGGGTGCTGCGGCTGAACCGCGCGCGCCGGTTGTGACCATCATGGGTCACGTT
GACCACGGTAAAACCTCTCTGCTGGACTACATTCGTTCAACGAAAGTGGCCTCTGGCGAAGCGGGCGGCATTACCCAGCA
CATTGGTGCATACCACGTTGAAACTGAAAACGGCATGATCACCTTCCTGGACACCCCGGGGCACGCCGCGTTTACTTCAA
TGCGTGCTCGTGGTGCGCAGGCAACGGACATCGTAGTCCTGGTTGTTGCTGCCGACGACGGTGTGATGCCGCAGACCATC
GAAGCAATCCAGCACGCGAAAGCGGCGCAGGTACCGGTGGTGGTTGCAGTGAACAAGATCGATAAACCAGAAGCTGATCC
GGATCGCGTTAAGAACGAACTCTCCCAGTACGGCATCCTGCCGGAAGAGTGGGGCGGTGAAAGCCAGTTCGTACACGTAT
CTGCGAAAGCGGGTACCGGTATCGATGAACTGCTGGACGCTATCCTGCTGCAGGCGGAAGTTCTGGAGCTGAAAGCGGTA
CGTAAAGGTATGGCGAGCGGTGCGGTTATCGAATCCTTCCTCGATAAAGGTCGTGGTCCGGTTGCTACCGTTCTGGTACG
TGAAGGTACTCTGCACAAGGGCGATATCGTTCTGTGTGGCTTCGAATACGGTCGTGTTCGTGCGATGCGTAACGAACTGG
GTCAGGAAGTGCTGGAAGCGGGTCCGTCCATTCCGGTGGAAATCCTCGGCCTGTCCGGCGTACCGGCTGCGGGTGATGAA
GTTACCGTTGTACGTGACGAGAAGAAAGCGCGTGAAGTTGCACTCTATCGTCAGGGTAAATTCCGCGAAGTTAAACTGGC
GCGTCAGCAGAAATCTAAACTCGAGAACATGTTCGCCAACATGACCGAAGGCGAAGTTCACGAAGTGAATATCGTCCTGA
AGGCAGACGTACAGGGTTCTGTCGAAGCGATCTCCGACTCCTTGCTGAAACTGTCTACTGACGAAGTTAAAGTGAAGATC
ATCGGTTCTGGCGTAGGTGGTATCACCGAAACCGACGCCACCCTGGCTGCGGCGTCCAACGCCATCCTGGTTGGCTTTAA
CGTACGTGCTGATGCCTCTGCACGTAAAGTGATTGAAGCGGAAAGCCTGGATCTGCGTTACTACTCCGTCATCTATAACC
TGATTGACGAAGTGAAAGCGGCGATGAGCGGTATGCTGTCTCCGGAACTGAAACAGCAGATTATCGGTCTGGCGGAAGTT
CGTGACGTGTTCAAATCGCCGAAATTTGGTGCCATCGCAGGCTGTATGGTTACCGAAGGTGTGGTTAAACGTCACAACCC
GATCCGCGTTCTGCGTGACAACGTGGTTATCTACGAAGGCGAGCTGGAGTCCCTGCGCCGCTTCAAAGATGACGTTAACG
AAGTCCGTAACGGTATGGAATGTGGTATCGGCGTTAAGAACTACAACGACGTCCGCACTGGCGATGTGATCGAAGTATTC
GAAATCATCGAGATCCAACGTACCATTGCTTAA
>Cluster_51540
ATGACAACACAGACGGTTACGCAGATTTCAGCCGCAGCGCGGGGAAAATGGCCCGTCATTCTGCAGATGCTGCGTATTGA
TGTACCTGAAAACGGCAGGCACGGTCCCTGCCCGAAATGTGGGGGCAAGGATCGCTTCCGCCTCGATGACCTCGACGGGC
GCGGGACGTGGATTTGCAGCCAGTGCGGCAATGGTGATGGTCTGGATCTGGTTAAGCTCATAACCGGTTACGGCATCAGA
AAGGCAGCGCAGGAGGTGGCGCAGGTGCTTAATGTGCCGGATGTGCAGGAACTGCCCGTTAAGCCCGCCAGGCAAAAAGC
CCCTAAACGCGATATGAGCCTTACCGTGGCGGCCCTGATGAAAGAGAGTCACACGGGAGAAAGCCCCTATCTGACCGGAA
AAGGGTTCGCCGGATACCCGGCTCCCTGACCGGGAGCGTGCAGCATATCAGTGGTAAGGATTTCCCTGCCGGTTCCCTG
CTGTTACCGCTCACGACGAACGCCGGAGCCGTGACCGGTGCACAGCTTATCGCCCCGACGGGTGAAAAAAGCATACTGCC
CGGCAGCACGATGAAAGGCGCGTTTGTGGCGCTCAGCCCGTTACCGTCTGAACCGCCGGTACAGGTGGTGATTACCGAAG
GTTACGCCACGGCGCTGACGGTAAGCCAGCTCACCGCCGGATGCGTAGTGGCGGCCATATCGGCGGGCAACCTGCCCAAC
GTCGCACAGGCGCTGCGGGCACGCTGGCCTGAGGTAAAAATTATCATCGCCGGTGATAACGATTTTCAGGACGGGGGCGA
GAATCCCGGCAGATCCTTTGCTGAACGGGCGGCAAAATCAGTCGGTGGCTGGGTGACGCTGCCACCGGGAGAAATTAAAG
CTGACTGGAATGACTTCCACCGTGAGCACGGTATTACACGTGCCCGTGAAGCCTTTCGTAACGGTCTTGTACTGTGCGGA
GAAGGCCGCACGCAGTTGCCGCACGGGTTCCGTCTTACCCAGGAATATCTCTGGTATGAAAAACAGGTACAGCGCAACGG
TGAGACGGAGATCCAGAACGTCAAAATATGCAACCCGTTGCGCGTGACGGCAATCACCTGCGATGCTGATGGCGGTAATT
TCGGGCGACTGCTGGAGTGGGAAGATACATGGGGAGAGCGCCGCCGCTGGGCGATGCCGATGGAAATGCTCAGTGGTAGC
GGTGAGGAACTACGCCGGGTACTGCTGGTTAACGGGCTGTCCTATATCAGCACCACCGGTGAGGCCCGCGCCCGCCTGAT
GGAATATATCTCGCTGTGTAAACCGGAACGCCGCGTGACCTGCGTCAGCCGCACTGGCTGGCATGGTCAGGTTTACGTCC
TGCAGGATGAGGTCAGCGGTGAAGGTGCAGAGGGTGTCATTCTCCAGACCACTTCCGTGCAGGGGCGCGATTTCCGTGTG
TCAGGTACAACGGAGGAATGGCGGGAGCACGTTTCCCGTTACTGCACCGGTAACTCCCGCGTGGCGTTTGCCGTCAGTCT
GGCCTTTGCTGCACCCCTGTTACGGCTGGTTGGGATGGACGGCGGCGGCTACCACCTCAAGGGGGAATCGACTGACGGTA
AGACCACCACCATGAAAGCGGCAACCTCCGTTTGTGGCGGGCCTGATTACTGGCAGACGTGGCGGGCAACCGGCAACGCG
CTGGAGGGATGCGCCAGCCGCCGCAACGATGCCGCCATGATGCTCGATGAGATCCGGGAGGTGGACGGACGCGAGGCGGG
TAATATCGCCTACATGCTGGCAAACGGTCAGGGCAAGGGCCGTGCCGGTACGGACGGTGAGCTGCGCACCCGTAAGCAGT
GGCGGCTGCTGTTCTTTTCAACCGGCGAGCTGTCACTGACCGAACATGCGGCAAAAGCCGGTGAGCGTACCTTTGCCGGG
ATGGAAGTCAGGATGATCCAGATCCCCAGCGATTCCGGGAAATTTGGCGTATTTGAGGAGCTGCACGGCTTCGACAGCGG
CAAGGCTCTGGCGGAACATCTGGAGTGGGCCACGTCCAGCTACTACGGTTCGCCGTTCAGGGAGTGGCTGAAAGCCCTGA
CCGCTGACCTTAACGGGCTGACGGCACAGGCAAAGTCACTGATGAAGGAATATACAGCCGCCCTGACCCCGAAAGATGCG
GGTAATCAGGTAGGCCGGGCTGTGAACCGCTTTGCACTGGTGGCGATGGCGGGCGAGCTGGCAACCCGGCTGGGTATTAC
CGGCTGGCCTGAGGGTGAAGCGCTGCGGGCAACCCGCGTCTGCCTGAACGCATGGCTGAAAGATCGCGGGCACACCGCCA
ATCAGGAAGATATTGCCGCACTGGAGCAGGTACGCAGCTTCTTTACCGCGAATCAGTACAGCCGCTTTGCTGACTGGCAC
GACGAGCGTAACCGCCCCGGCAATATGGTGGGCTGGCGCAGGGTGGAGAAAGGCAGTACCGCGCAGGGCACGGAGGCCGT
CACCACGTTCTACGTCATGCCGTCCGGCTGGAAAGAAATCTGCCGGGGATTTGACCCGCGCAAGGTGGCGCGTCTGTGCG
CGGATCGTGGATACCTGCTGCCCTCCACCGATGGCAAGCTGCAGACAACCATTCGCCCGCCAGAGATGAATCCCCGCAGA
CTCTATGTCTTCAACAGCGAGGTGCCGGGTTAA
>Cluster_748
ATGGCAGTAAAGATTTCAGGTGTACTGAAAGACGGCACAGGAAAACCGGTACAGAACTGCACAATCCAGCTGAAAGCAAA
ACGTAACAGCACCACGGTGGTGGTGAACACGCTGGCCTCAGAAAATCCGGATGAAGCCGGGCGTTACAGCATGGACGTTG
AGTACGGGCAGTACAGCGTTATTCTGTTGGTGGAAGGATTCCCGCCGTCACATGCCGGGACCATCACCGTGTATGAAGAT
TCTCAACCCGGTACGCTGAATGATTTTCTCGGTGCCATGACGGAGGATGATGCCCGTCCGGAGGCACTGCGCCGTTTTGA
ACAGATGGTGGAAGAGGTGGCGCGTAACGCGTCCGTGGTGGCACAGAACACGGCAGCCGCGAAGAAGTCAGCCAGTGATG
CCGGCACATCTGCCCGTGAGGCGGCAACCCATGCGACTGATGCTGCAGATTCAGCACGTGCAGCCAGCACGTCAGCCGGA
CAGGCCGCTTCGTCGGCTCAGTCAGCGTCTTCCAGCGCAGGAACGGCATCAACAAAGGCCACTGAAGCGGAAAAAAGTGC
TGCCGCTGCAGAGTCCTCAAAAAGCGCGGCAGCTACCAGTGCCGCTGCCGCGAAAACGTCAGAAACGAATGCTGCAGCGT
CACAACAATCAGCCGCCACTTCTGCATCCACCGCGACCACGAAAGCGTCAGAAGCTGCCACCTCAGCCCGGGATGCGTCG
GCTTCAAAAGAGGCGGCAAAATCATCAGAAACGAACGCATCCTCGAGCGCCAGTAGCGCAGCTTCCTCGGCAACGGCGGC
AGCAAATTCCGCGAAGGCGGCAAAAAACGTCCGAGACGAACGCCAGGTCTTCTGAGACGGCAGCGGGACAGAGCGCCTCAG
CTGCGGCAGGCTCAAAAACAGCGGCTGCGTCGTCTGCCAGTGCCGCGTCAACAAGTGCCGGGCAGGCCTCAGCCAGTGCC
ACCGCTGCCGGAAAATCGGCAGAAAGCGCCGCATCGTCCGCTTCAACAGCCACAACGAAGGCTGGCGAAGCCACTGAACA
GGCCAGCGCAGCAGCGAGGTCTGCTTCCGCAGCGAAGACATCCGAGACGAACGCGAAAGCGTCGGAAACCAGCGCAGAAT
CCTCAAAAACGGCTGCCGCATCGTCCGCCAGTTCGGCGGCGTCATCGGCATCATCTGCGTCTGCTTCAAAAGATGAGGCG
ACCAGACAGGCGTCAGCAGCAAAGGGCAGCGCCACGACGGCATCCACGAAGGCGACAGAGGCAGCTGGCAGTGCGACGGC
GGCAGCTCAGAGCAAAAGTACGGCGGAATCCGCGGCAACGCGCGCCGAGACAGCGGCAAAACGGGCAGAGGATATTGCAT
CCGCCGTGGCGCTTGAGGATGCGAGCACGACGAAAAAGGGGATAGTACAGCTCAGCAGTGCGACTAACAGCACTTCCGAG
TCACTGGCGGCAACGCCAAAAGCCGTTAAGGCCGCGTATGAGCTGGCTAACGGGAAATACACCGCACAGGATGCAACGAC
AGCACAGAAAGGGATAGTTCAGCTCAGCAGCGCGACCAACAGCACATCTGAAATGCTGGCGGCAACGCCAAAGTCGGTAA
AGGCAGCCTATGACCTTGCTAACGGGAAATATACTGCTCAGGACGCTACGACAGCACAAAAAGGAATTGTCCAGCTCAGT

AGTGCAACCAACAGCGCATCTGAAACGCTTGCCGCGACACCGAAAGCAGTGAAAGCAGCTAATGATAATGCGAATGGTCG
GGTACCTTCTGCCCGTAAGGTGAATGGTAAGGCGCTTTCAGCGGATATAACACTGACGCCGAAAGATATTGGTACGCTTA
ACTCAACAACAATGTCATTCAGCGGTGGTGCTGGTTGGTTCAAATTAGCAACGGTAACCATGCCACAGGCGAGTTCTGTT
GTTTCAATTACGTTGATTGGTGGCGCGGGATTTAACGTGGGGTCACCTCAACAGGCAGGTATATCTGAACTTGTTTTGCG
TGCAGGTAATGGTAATCCGAAGGGGATTACTGGTGCTTTATGGCAGCGCACATCGACAGGGTTTACAAATTTTGCCTGGG
TCAATACATCTGGTGATACTTACGATATTTACGTTGCAATCGGAAATTATGCGACTGGTGTAAATATTCAATGGGATTAT
ACCAGTAATGCCAGCGTGACTATTCATACGTCACCAGCATATTCTGCTAATAAGCCGGAAGGGTTAACGGACGGTACAGT
TTATTCACTCTATACACCATCAGAGCAGTTTTATCCGCCTGGCGCACCAATCCCGTGGCCATCAGATACCGTTCCGTCTG
GTTATGCCCTGATGCAGGGGCAGACTTTTGACAAATCCGCTTACCCGAAACTTGCAACCGCTTATCCGTCAGGCGTGATC
CCTGATATGCGTGGCTGGACGATTAAGGGCAAACCTGCCAGTGGTCGGGCCGTATTGTCTCAGGAACAGGACGGCATTAA
ATCGCACACCCACAGCGCCAGTGCATCCAGTACGGATTTGGGGACGAAAACCACATCGTCGTTTGATTACGGCACTAAAT
CCACGAATAACACCGGGGCGCATACGCACAGTGTGAGCGGTACAGCCGCAAGTGCCGGAAACCATACTCATAGTGTCACA
GGCGCATCAGCAGTCAGCCAGTGGTCACAAAATGGGTCAGTACATAAGGTAGTGTCTGCGGCCAGTGTGAATACAAGTGC
TGCAGGAGCGCACACTCATAGTGTCAGCGGCACAGCTGCATCTGCAGGTGCTCACGCACATACTGTCGGTATTGGTGCTC
ATACGCACTCTGTTGCGATTGGCTCACATGGACACACCATCACCGTTAACGCTGCTGGTAACGCGGAAAACACTGTCAAA
AACATCGCATTTAACTACATTGTGAGGCTTGCATAA
>Cluster_64261
ATGGCAGTAAAGATTTCAGGTGTACTGAAAGACGGCACAGGAAAACCGGTACAGAACTGCACAATCCAGCTGAAAGCAAA
ACGTAACAGCACCACGGTGGTGGTGAACACGCTGGCCTCAGAAAATCCGGATGAAGCCGGGCGTTACAGCATGGACGTTG
AGTACGGGCAGTACAGCGTTATTCTGTTGGTGGAGGGATTCCCGCCGTCACATGCCGGGACCATCACCGTGTATGAAGAT
TCCCGACCCGGTACGCTGAATGATTTCTCGGTGCCATGACGGAGGATGATGCCCGTCCTGAGGCACTGCGCCGTTTTGA
ACTGATGGTGGAAGAGGTGGCGCGTAACGCGTCCGCGGTGGCACAGAACACGGCAGCCGCGAAGAAGTCAGCCAGCGATG
CCAGCACATCAGCCCGTGAGGCGGCAACCCATGCGACTGATGCTGCAGATTCAGCACGTGCAGCCAGCACGTCAGCCGGA
CAGGCCGCTTCGTCGGCTCAGTCAGCGTCTTCCAGCGCAGGAACGGCATCAACAAAGGCCACTGAAGCCTCAAAAAGTGC
TGCCGCTGCAGAGTCCTCAAAAAGCGTGGCAGCTACCAGTGCCGGTGCGGCGAAAACGTCAGAAACGAATGCCGCAGCGT
CAGAACAATCAGCCGCCACTTCTGCATCCACCGCGACCACGAAAGCGTCAGAAGCTGCCACCTCAGCCCGGGATGCGGCG
GCCTCAAAAGAGGCAGCGAAATCATCAGAAACGAACGCATCCTTGAGCGCCAGTAGCGCAGCTTCCTCGGCAACGGCGGC
AGGAAATTCCGCGAAGGCGGCAAAAACGTCCGAGACGAACGCCAGGTCTTCTGAGACGGCAGCGGGACAGAGCGCCTCAG
CTGCGGCAGGCTCAAAAACAGCGGCTGCGTCGTCTGCCAGTGCCGCGTCAACAAGTGCCGGGCAGGCCTCAGCCAGTGCC
ACCGCCGCCGGAAAATCGGCAGAAAGCGCCGCATCATCCGCTTCAACAGCCACAACGAAGGCTGGCGAAGCCACTGAGCA
GGCCACGGCAGCAGCGAGGTCAGCTTCCGCAGCGAAGACATCCGAAACGAACGCGAAAGCGTCGGAAACCCGTGCAGAAT
CCTCAAAAACGGCAGCGGCATCGTCCGCCAGTTCGGCGGCGTCATCGGCATCATCGGCGTCTGCTTCAAAAGATGAGGCG
ACCAGACAGGCGTCAGCAGCGAAGGGCAGCGCCACGACGGCATCCACGAAGGCGACAGAGGCTGCTGGTAGTGCGAAGGC
AGCAGCTCAGAGCAAAAGTACGGCGGAATCTGCAGCAACGCGCGCTGAGACAGCGGCAAAACGGGCAGAGGATATTGCAT
CCGCCGTGGCGCTTGAGGATGCGAGCACGACGAAAAAGGGGATAGTACAGCTCAGTAGTGCGACTAACAGCACTTCCGAG
TCACTGGCGGCAACACCAAAAGCCGTTAAGGCCGCGTATGACCTGGCTAGCGGGAAATACACCGCACAGGATGCAACGAC
AGCACAGAAAGGGATAATCCAGCTAAGCAGCGCGACCAACAGCACGTCTGAAACGCTGGCGGCAACGCCAAAGGCAGTAA
AAGCAGCCAATGACAATGCTGAGAAACGTCTGCAGAAAGATCAGAACGGTGCGGATATCCCTGGCAAAGACACCTTTACG
AAAAATATTGGTGCCTGCCGTGCCTTCGGTGGGTCAGTAAGCACAACAACAGGAAACTGGACGACTGCACAGTTTATCGA
GTGGCTGGATTCTCAGGGAGCATTTAACCATCCATACTGGATGTGCAAGGGTTCCTGGTCTTATGGCAATAATAAAATCA
TTACTGATACTGGCTGCGGTAATATTCATCTCGCCGGAGCTGTCATTGAAGTAATGGGGATAAAGTCAGCGATGACGATC
CGCATTACCACTCCGACCACATCCAGCGGTGGCGGAACAACCAACGCCCAGTTTACCTATATTAATCACGGAACAGATTA
TTCACCTGGCTGGCGAAGGGACTATAACTCCAGAAATAAGCCAACGGCATCAGAGATCGGGGCGCTACCGTCAGGTGGAA
CAGCAGTATCATCAGTTAATCTGGCTTCAAAAGGTCGGGTAGCCGCGCTGACAGACAATACGCAGGGGGCAGCAGGTCTT
GAGTTATACGAGGTGTATAACAACGGATATCCAACAACGTATGGAAATATCATTCACCTGAAAGGGATGACAGCCGTTGG
CGAAGGTGAGTTACTCATCGGCTGGAGTGGTACAAGCGGTGCTCATGCTCCGGCATTTATTCGTTCACGACGGGATACGA
CCGACGCAAACTGGTCGCCGTGGGCGCAGCTTTACACCTCGGCTCATCCTCCTGCAGAGTTTTATCCAGTCGGTGCACCA
ATCCCGTGGCCATCAGATACCGTTCCGTCTGGTTATGTCCTGATGCAGGGGCAGACTTTTGACAAATCTGCATACCCGAA
ACTTGCAGCCGCTTATCCGTCAGGCGTGATCCCTGATATGCGTGGCTGGACGATTAAGGGCAAACCTGCCAGTGGTCGGG
CCGTATTGTCTCAGGAACAGGACGGCATTAAATCGCACACCCACAGCGCCAGCGCATCCAGTACGGATTTGGGTACGAAA
ACCACATCGTCGTTTGATTACGGCACTAAATCCACGAATAACACTGGGGCGCATACCCATAGTGTTAGCGGGACTGCAAA
TAGTGCCGGTGCGCACCAACACAAGAGTTCCGGTGCATTTGGTGGCACGAACACGAGCATTTTCCCTAATGGTTATACCG
CGATTTCAAATCTAAGCGCGGGGATTATGAGCACAACAAGCGGTAGTGGCCAGACTCGTAATGCAGGGAAGACATCATCA
GATGGTGCTCATACCCACTCGCTGTCCGGCACTGCTGCAAGCGCAGGCGCACATGCACATACTGTCGGTATTGGTGCTCA
TACGCACTCCGTTGCGATTGGCTCACATGGACACACCATCACCGTTAACGCTGCTGGTAACGCGGAAAACACCGTCAAAA
ACATCGCATTTAACTATATTGTGAGGCTTGCATAA
>Cluster_26900
ATGAAGACTCTTGATATCCGGGTCGCTTTCAGCGCCGTTGACAGGCTGACCCGGCCTGCCGAAAACGCCCGCCGCCTGAT
GGGGCAGTTTGGTGACTCCATCCAGCGAACGCAGGGGGCGATCAAAAATCTCGAGCGTCAGGCGCGATCATTTGAGCGCG
CCCGCGACGCTGTCAGTAAAGCGGATGCGGGTATCGTGAAAGCACGACGCCAGCTTAACGCCCCTTAATCAGTTACAACGC
ACGGGTACAGTGCTCAGCGAAAAACAACAAAAGCTGATGCAGCAGTTAAGCACCCGGCTTGAACGCCTGAATGAATCGCG
CACACGGGAAATTCAGAAAATGCGGGAGCTTGGCGGAGAGCTGAAACGCCACGGCATTTCCCTGACAGGCAGCGATAACA
CCATCCAGCAGGCCATCAGACGCACCGAACAGTACAACAACCAGCTTGAACGCGAACGGCAGGCGCTTGCGCGTGTAACG
CGGGCGCGTGAGCGGTATTCGCGCGCGCAGGAAACCGCGGGAAAACTGAAAACAGGTGGTGCGCTGGCAATTGGTGCGGC
AGCGGCGGGCGGCTATGCTGCCGGGCGTTTTTTGCAGCCTGCGATCGGGTTCGGCAAAGAGATGTCCCGCGTTCAGGCAC
TGACGCGAATCGACAAAAACAGCCCGCAGTTTAAGGCGCTGCGTGAGCAGGCGTTAAAACTTGGCTCTGAAACACAGTTT
ACTGCGAGTGATGCCGCCAGTGGGCAGAGCTTTCTGGCAATGGCTGGTTTTACTCCGCAGGCCATTCAGGCCGCATTGCC
CGGTGTTCTTAATATGGCGCTGGCAGGTGGCGTCGAACTCGGCGAGACGGCGGATATAGGCTCCAATATCCTCACACAGT
TCAACCTGACAGCCGATCAAATGGACCGGGTGGGCGATACGCTGACAGCAGCATTTACCCGGACCAATACTGATTTACGC
GCGCTGGGCGAAACCATGAAGTATACCGGTCCGGTTGCCGCAAAACTTGGTATCAGTCTTGAAGAAGCGGCGGCCATGGC
CGGGATGCTTGCCAATAATGGTCTTCGCGGAAGCGATGCTGGCACGGCCATGCGCGCAAGTCTGTCCCGCCTTGCATCAC
CGCCAAAAGCTGCGGCTGATGCACTGAAAGAGCTGGGGGTGTCAGTTGCTGACGCCAGAGGCAAAATGCGCCCGATGGAG
GATGTGCTGCTTGATCTCTATAAGGCGACACAAAAATACGGACAGGTGGACCAGGTCTCCTTTTTCAAGGACATCGCCGG
AGAAGAGGCGTTCGTTGGTTTGCAGACGCTTGTTGCGGCGGCTGGTTCAGGAGAGCTGCAAAAACTGACCAGAGAATTGC
AGGGGGCAAGGGGAGAGGCCGATCGCGTTGCAAAAGTAATGGCCGATAATCTTGATGGGGACCTGAAAAATCTCGACAGC
GCATGGGAAGGTCTTCGTATTCGCATCAGTGATCTGGTTGACGGTCCGCTGCGTTCTGTCACGCAGTGGCTCACGCGGGT
GCTTGAAAAAATCACCTCGCTGGCGCAGGCCCATCCGGTACTGACACGCCAGCTACTGATAGCAGGCGGTGCGTTGCTGG
CAATGACTGCAACGATTGGCTCGTTGTCGCTGGTTATTGGGGTGCTTTACGGAAAGCTGGCCACCCTGCGTCTTGGTTTT
GACATTCTTACCCGGTCAATGAATGTCGTCAGGGTGTTGCCTGCGCTGTGGGGAATGCTGACGGGTTCCGTTTCTTTGCT

GGGAGGCGCTATCGGGGCGCTGTTCAGTCCGGTTGGTCTTATCGTGGCTGCGCTTGCCGGAGCTGCCGTTCTTATCTGGA
AATACTGGGATCCCATCAGGGCATTTTTTGCCGGGGTGTTCAGCGGGATTATGGAAAGGCTGAACCCGTTGCGTGAAACC
TTTGAACGGTTTGGCCCTGTTTTTGACGCAATCGGAAGCGGGATCAGCCAGGTGTTTAACTGGTTTAAATCGCTGCTGTC
ACCGATGGAGTCCAGCAAGGAAACGCTGGATAAATGTACCAGTGCTGGCGAGATATTCGGTAACGTTCTTGGTGGCGCGT
TACAGCTTGTTCTGACGCCTGCAAAAATGTTGCTGGATACGCTGGCGTGGATACTTGAAAAACTCGGTGTTCTTCCGGAT
GAAGCGGAAAGGGCGAGAAAGAAAATCGAAGACGCACAGCGTGCGGCCATTCTTCAGGACAAGGTTGCCTTGCTTCAGGG
GGACCTGGCGAAAATCAATCCGCCGAAGCCTGTGGAAAATGGCAATGGCACCGGAGGTGATAACCCCAAAGACAACAAAC
CGCTCACAGACAGCAATACCGGTACGCTACGCAGACTCAGCAAAATTGCTGATAACACAGGTAAGCTGGTTGATGTGACG
AAAAAACGCATTGGCCCCGGCGATATTGTCTTTAAGAACCTGCCCCGCGCACTTGCTGTTCGTGGGGAGTGGCAGGAGCG
GAAGATTGCGCAGGTCAGTAAGCCTGCCCCCGCAATTAATATCACACCCGTGGTTCCGGCTCCGCTGCCTCCGGCGCTGG
TCCCTGTTGTTGCGGCCAGCTCCCGCCCGGTGGCGGAGGCCATACGATCGCCAGTGGCATCAGTTCCTGCAACTTCCCGT
AACCGTGAGCCTGTTGCCTCCGGATTTGGCGGTGAAATTCATGTTCATCTGCATAACGTTGTTACACAGAATCCCCGCGA
ACTGGCGAAACTGGTCGGTGAAATGGTCAGGGCAGAAATGGAACGGCGCGCCCGTGCCGGGCGTGGCAGTTTTTACGATA
AAGATTGA
>Cluster_41964
ATGACACAGACGTTAAGCCAGCTTGAAAACAGCGGCGCTTTTATTGAACGCCATATCGGACCGGACGCCGCGCAACAGCA
AGAAATGCTGAATGCCGTTGGTGCACAATCGTTAAACGCGCTGACCGGCCAGATTGTGCCGAAAGATATTCAGCTTGCGA
CACCACCGCAGGTTGGCGCACCGGCGACCGAATACGCCGCACTGGCAGAACTCAAGGCTATTGCCAGTCGCAATAAACGC
TTCACGTCTTACATCGGCATGGGTTACACCGCCGTGCAGCTACCGCCGGTTATCCTGCGTAACATGCTGGAAAATCCGGG
CTGGTATACCGCGTATACTCCGTATCAACCTGAAGTCTCCCAGGGCCGCCTTGAAGCACTGCTCAACTTCCAGCAGGTAA
CGCTGGATTTAACCGGGCTGGATATGGCCTCTGCTTCTCTTCTTGACGAAGCCACCGCTGCCGCCGAAGCAATGGCGATG
GCGAAACGCGTCAGCAAACTGAAAAATGCCAACCGCTTCTTTGTTGCTTCTGACGTTCATCCGCAAACGCTGGATGTGGT
CCGTACTCGTGCCGAAACCTTTGGTTTTGAAGTGATTGTCGATGACGCGCAAAAAGTGCTCGACCATCAGGACGTCTTCG
GCGTGCTGTTACAGCAGGTCGGCACTACTGGTGAAATTCACGACTACACTGCGCTTATTAGCGAACTGAAATCACGCAAA
ATTGTGGTCAGCGTTGCCGCCGATATTATGGCGCTGGTGCTGTTAACTGCGCCAGGTAAACAGGGCGCGGATATTGTTTT
TGGTTCGGCGCAACGCTTCGGCGTGCCGATGGGCTATGGTGGCCCACACGCGGCATTCTTTGCGGCGAAAGATGAATACA
AACGCTCAATGCCGGGCCGTATTATCGGTGTATCGAAAGATGCAGCTGGCAACACCGCTCTGCGCATGGCGATGCAGACT
CGCGAGCAACATATTCGTCGTGAGAAAGCGAACTCCAACATTTGTACTTCCCAGGTACTGCTGGCAAACATTGCCAGCCT
GTATGCCGTTTATCACGGTCCGGTTGGCCTGAAACGTATCGCTAACCGCATCCACCGTCTGACCGATATCCTGGCGGCGG
GCCTGCAACAAAAAGGTCTGAAGCTGCGCCATGCGCACTATTTCGATACTTTGTGTGTGGAAGTGGCCGACAAAGCGGGC
GTGCTGGCGCGTGCCGAAGCGGCTGAAATCAACCTGCGTAGCGATATTCTGAACGCGGTTGGGATCACCCTTGATGAAAC
AACCACGCGCGAAAACGTGATGCAGCTTTTCAGCGTGCTGTTGGGCGATAACCACGGGCTGGACATCGACACACTGGACA
AAGACGTGGCTCACGACAGTCGCTCTATTCAGGCTGCGATGCTGCGCGACGACGAAATCCTCACCCATCCGGTGTTTAAT
CGCTACCACAGCGAAACCGAAATGATGCGCTATATGCACTCGCTGGAGCGTAAAGATCTGGCGCTGAATCAGGCGATGAT
CCCGCTGGGTTCCTGCACCATGAAACTGAACGCCGCCGCCGAGATGATCCCGATCACCTGGCCGGAATTTGCCGAACTGC
ACCCGTTCTGCCCGCCAGAACAGGCTGAAGGTTATCAGCAGATGATCGCGCAGCTGGCTGACTGGCTGGTGAAACTGACC
GGTTACGACGCCGTTTGTATGCAGCCGAACTCTGGCGCACAGGGCGAATACGCGGGCCTGCTGGCGATTCGTCATTATCA
TGAAAGCCGCAACGAAGGGCATCGCGATATCTGCCTGATCCCGGCTTCTGCGCACGGCACTAACCCGGCTTCCGCGCATA
TGGCAGGAATGCAGGTGGTGGTTGTGGCGTGTGATAAAAATGGCAACATCGATCTGACTGATCTGCGCGCGAAAGCGGAA
CAGGCGGGCGATAACCTCTCCTGTATCATGGTGACCTATCCTTCTACCCACGGCGTGTATGAAGAAACGATCCGTGAAGT
GTGTGAAGTCGTGCATCAGTTCGGCGGTCAGGTTTACCTTGATGGCGCGAACATGAACGCTCAGGTTGGCATCACCTCGC
CGGGCTTTATTGGTGCGGACGTTTCCCACCTTAACCTGCATAAAACTTTCTGCATTCCGCATGGCGGTGGTGGTCCGGGT
ATGGGACCGATCGGTGTGAAAGCGCATCTGGCTCCGTTTGTACCGGGTCATAGCGTGGTGCAAATCGAAGGCATGTTAAC
CCGTCAGGGCGCGGTTTCTGCGGCACCGTTCGGTAGTGCCTCTATCCTGCCAATCAGCTGGATGTACATCCGCATGATGG
GCGCAGAAGGGCTGAAAAAAGCAAGCCAGGTGGCAATTCTTAACGCCAACTATATTGCCAGCCGTCTGCAGGATGCCTTC
CCGGTGCTGTATACCGGTCGCGACGGTCGCGTGGCGCACGAATGTATTCTCGATATTCGCCCGCTGAAAGAAGAAACCGG
CATCAGCGAGCTGGATATTGCCAAGCGCCTGATCGACTACGGTTTCCACGCGCCGACCATGTCGTTCCCGGTGGCGGGTA
CGCTGATGGTTGAACCGACTGAATCTGAAAGCAAAGTGGAACTGGATCGCTTTATCGATGCGATGCTGGCTATCCGCGCA
GAAATTGACCAGGTGAAAGCCGGTGTCTGGCCGCTGGAAGATAACCCGCTGGTGAATGCGCCGCACATTCAAAGCGAACT
GGTCGCCGAGTGGGCGCATCCGTACAGCCGTGAAGTTGCAGTATTCCCGGCAGGTGTGGCAGACAAATACTGGCCGACCG
TGAAACGTCTGGATGATGTTTACGGCGACCGTAACCTGTTCTGCTCCTGCGTACCGATTAGCGAATACCAGTAA
>Cluster_9096
ATGTTTGATATCGGCGTTAATTTGACCAGTTCGCAATTTGCGAAAGACCGTGATGATGTTGTAGCGTGCGCTTTTGACGC
GGGAGTTAATGGGCTACTCATCACCGGCACTAACCTGCGTGAAAGCCAGCAGGCGCAAAAGCTGGCGCGTCAGTATTCGT
CCTGTTGGTCAACGGCGGGCGTACATCCTCACGACAGCAGCCAGTGGCAAGCTGCGACTGAAGAAGCGATTATTGAGCTG
GCCGCGCAGCCAGAAGTGGTGGCGATTGGTGAATGTGGTCTCGACTTTAACCGCAACTTTTCGACGCCGGAAGAGCAGGA
ACGCGCTTTTGTTGCCCAGCTACGCATTGCCGCAGATTTAAACATGCCGGTATTTATGCACTGTCGCGATGCCCACGAGC
GGTTTATGACATTGCTGGAGCCGTGGCTGGATAAACTGCCTGGTGCGGTTCTTCATTGCTTTACCGGCACACGCGAAGAG
ATGCAGGCGTGCGTGGCGCATGGAATTTATATCGGCATTACCGGTTGGGTTTGCGATGAACGACGCGGACTGGAGCTGCG
GGAACTTTTGCCGTTGATTCCGGCGGAAAAATTACTGATCGAAACTGATGCGCCGTATCTGCTCCCTCGCGATCTCACGC
CAAAGCCATCATCCCGGCGCAACGAGCCAGCCCATCTGCCCCATATTTGCAACGTATTGCGCACTGGCGTGGAGAAGAT
GCCGCATGGCTGGCTGCCACCACGGATGCTAATGTCAAAACACTGTTTGGGATTGCGTTTTAG
>Cluster_1728
ATGGGTTTTCTTTCCGGTAAGCGCATTCTGGTAACCGGTGTTGCCAGCAAACTATCCATCGCCTACGGTATCGCTCAGGC
GATGCACCGCGAAGGAGCTGAACTGGCATTCACCTACCAGAACGACAAACTGAAAGGCCGCGTAGAAGAATTTGCCGCTC
AATTGGGTTCTGACATCGTTCTGCAGTGCGATGTTGCAGAAGATGCCAGCATCGACACCATGTTCGCTGAACTGGGGAAA
GTTTGGCCGAAATTTGACGGTTTCGTACACTCTATTGGTTTTGCACCTGGCGATCAGCTGGATGGTGACTATGTTAACGC
CGTTACCCGTGAAGGCTTCAAAATTGCCCACGACATCAGCTCCTACAGCTTCGTTGCAATGGCAAAAGCTTGCCGCTCCA
TGCTGAATCCGGGTTCTGCCCTGCTGACCCTTTCCTACCTTGGCGCTGAGCGCGCTATCCCGAACTACAACGTTATGGGT
CTGGCAAAAGCGTCTCTGGAAGCGAACGTGCGCTATATGGCGAACGCGATGGGTCCGGAAGGTGTGCGTGTTAACGCCAT
CTCTGCTGGTCCGATCCGTACTCTGGCGGCCTCCGGTATCAAAGACTTCCGCAAAATGCTGGCTCATTGCGAAGCCGTTA
CCCCGATTCGCCGTACCGTTACTATTGAAGATGTGGGTAACTCCGCGGCATTCCTGTGCTCCGATCTCTCTGCCGGTATC
TCCGGTGAAGTGGTCCACGTTGACGGCGGTTTCAGCATCGCTGCAATGAACGAACTCGAATTGAAATAA
>Cluster_8832
ATGATTGTTTTACCCAGACGCCTGTCAGACGAGGTTGCCGATCGTGTGCGGGCGCTGATTGATGAAAAAAACCTGGAAGC
GGGCATGAAGTTGCCCGCTGAGCGCCAACTGGCGATGCAACTCGGCGTATCACGTAATTCACTGCGCGAGGCGCTGGCAA
AACTGGTGAGTGAAGGCGTGCTGCTCAGTCGACGCGGCGGCGGGACGTTTATTCGCTGGCGTCATGACACATGGTCGGAG
CAAAACATCGTCCAGCCGCTAAAAACACTGATGGCCGATGATCCGGATTACAGTTTCGATATTCTGGAAGCCCGCTACGC
CATTGAAGCCAGCACCGCATGGCATGCGGCAATGCGCGCCACACCTGGCGACAAAGAAAAGATTCAGCTTTGCTTTGAAG

CAACGCTAAGTGAAGACCCGGATATCGCCTCACAAGCGGACGTTCGTTTTCATCTGGCGATTGCCGAAGCCTCACATAAC
ATCGTGCTGCTGCAAACCATGCGCGGTTTCTTCGATGTCCTGCAATCCTCAGTGAAGCATAGCCGTCAGCGGATGTATCT
GGTGCCACCGGTTTTTTCACAACTGACCGAACAACATCAGGCTGTCATTGACGCCATTTTTGCCGGTGATGCTGACGGGG
CGCGTAAAGCAATGATGGCGCACCTTAGTTTTGTTCACACCACCATGAAACGATTCGATGAAGATCAGGCTCGCCACGCA
CGGATTACCCGCCTGCCCGGTGAGCATAATGAGCATTCGAGGGAGAAAAACGCATGA
>Cluster_60591
ATGAATCAGGTTGCCGTTGTCATCGGTGGTGGGCAAACCTTAGGCGCGTTCCTGTGCCACGGTCTGGCTGCCGAGGGGTA
TCGCGTCGCGGTTGTCGATATTCAGAGCGACAAAGCCGCAAATGTGGCACAAGAAATTAACGCCGAATATGGTGAAAGTA
TGGCGTACGGTTTTGGTGCTGACGCCACTAGCGAGCAAAGCGTTCTGGCGCTCTCTCGTGGGGTAGATGAAATCTTTGGT
CGCGTGGATTTGCTGGTCTACAGCGCCGGAATAGCAAAAGCAGCCTTTATCAGCGACTTCCAGCTCGGCGATTTTGACCG
TTCGCTACAGGTGAATCTGGTGGGTTATTTCCTGTGTGCGCGTGAATTTTCGCGTTTGATGATCCGCGACGGGATTCAGG
GGCGCATTATTCAGATCAACTCGAAATCCGGCAAAGTGGGCAGCAAACACAACTCTGGCTACAGCGCAGCGAAATTTGGT
GGCGTCGGGCTGACTCAATCACTGGCGCTGGATCTGGCGGAGTACGGCATTACGGTGCATTCACTGATGCTCGGTAACCT
GCTGAAATCGCCGATGTTCCAGTCACTGTTGCCACAATACGCGACCAAGCTGGGTATCAAACCGGATCAAGTCGAGCAGT
ATTACATCGACAAAGTACCGCTCAAACGCGGCTGCGATTATCAAGATGTGCTGAATATGCTGCTGTTCTACGCCAGTCCT
AAGGCGTCGTACTGCACCGGACAGTCGATCAATGTCACCGGCGGTCAGGTGATGTTCTGA
>Cluster_41028
ATGATAATCGATGAAGCTGAATCTGCCGAATCAAGTCATCCTGTTGTTTCTGTTATTCTGCCAGTTAATAAAAAAAACCC
TTTTCTTGATGAGGCAATAAATAGTATTTTATCGCAAACATTTTCGTCATTCGAGATAATAATAGTTGCAAATTGTTGTA
CGGATGATTTTTATAATGAGTTGAAACACAAAGTTAATGACAAAATTAAGTTGATTCGTACAGATATTGCTTATTTACCG
TACTCATTAAATAAAGCCATCGATTTGTCCAATGGTGAGTTTATTGCAAGGATGGATTCCGATGATATTTCTCATCCTGA
TAGATTCACTAAACAAGTTGATTTTTTAAAAAATAATCCTTATGTGGATGTCGTCGGTACTAATGCAATATTTATTGATG
ATAAAGGTCGAGAAATAAACAAAACAAAGCTACCTGAAGAAAATTTGGATATTGTAAAAAACTTACCGTATAAATGTTGC
ATTGTTCATCCATCTGTAATGTTTAGGAAGAAAGTAATCGCTTCAATGGGCGGTTATATGTTTTCAAACTATTCTGAGGA
TTATGAGTTATGGAATAGATTAAGTTTAGCAAAAATAAAATTTCAAAATTTACCGGGATATTTATTCTATTACAGGTTGC
ATGAAGGTCAGTCAACTGCTAAAAAAAAATTTGTATATGGTTATGGTAAATGATTTGGTAATAAAGATGAAATGCTTTTTT
TTGACAGGTAATATCAACTATCTCTTCGGAGGGATTAGAACTATTGCTTCTTTTATATACTGTAAATACATAAAGTGA
>Cluster_39775
ATGAACAAAACGCTAATCGCCGCAACTGTGGCAGGGATAGTTTTACTCGCTTCAAATGCTCAGGCACAAACCGTACCGGA
AGGCTATCAGCTACAGCAAGTGCTCATGATGAGCCGCCATAACTTACGTGCGCCGCTGGCGAACAATGGCAGTGTGCTGG
AACAGTCGACGCCGAATAAATGGCCTGAATGGGACGTCCCCGGTGGGCAGCTCACCACTAAAGGGGGCGTGCTCGAAGTG
TATATGGGCCATTATATGCGTGAATGGCTGGCAGAACAGGGGATGGTGAAATCAGGGGAATGCCCGCCACCGGACACCGT
TTATGCCTATGCCAATAGTCTGCAACGCACCGTCGCGACCGCGCAGTTCTTTATTACTGGCGCATTCCCTGGGTGTGATA
TTCCTGTTCATCACCAGGAAAAAATGGGCACTATGGACCCCACCTTTAACCCAGTGATCACCGATGATTCCGCCGCATTC
AGTGAACAGGCGGTGGCGGCAATGGAGAAAGAGCTCAGCAAACTCCAGCTTACCGACAGCTACAAGCTACTGGAACAAAT
CGTTAACTATAAAGATTCTCCTGCGTGTAAAGAGAAGCAACAATGTTCGCTTGTAGATGGCAAAAATACCTTTAGCGCGA
AGTATCAACAAGAACCGGGCGTTTCCGGGCCGCTGAAAGTCGGCAACTCGCTGGTAGATGCGTTTACTTTGCAATACTAC
GAAGGTTTTCCGATGGATCAGGTGGCCTGGGGAGAAATCAAATCTGACCAGCAGTGGAAGGTGTTGTCGAAGCTGAAAAA
CGGTTACTAG
>Cluster_45182
ATGATGGAATTCATCCTCAGTCATGTAGAAAAGGGCGTGATGACACTAACGCTCAACCGCCCGGAACGCCTGAACAGTTT
TAATGATGAGATGCACGCACAACTGGCAGAGTGCCTGAAACAGGTCGAGCGCGACGACACTATCCGTTGCCTGTTACTTA
CAGGTGCCGGGCGCGGATTTTGTGCTGGTCAGGATCTTAACGATCGTAACGTTGATCCCACAGGCCCCGCACCGGATTTA
GGGATGTCAGTTGAACGTTTCTATAACCCACTGGTACGTCGCCTGGCAAAACTGCCAAAACCGGTGATCTGTGCAGTCAA
TGGCGTGGCGGCAGGGGCTGGCGCAACACTGGCACTGGGATGCGACATCGTTATTGCTGCCCGTTCAGCAAAATTCGTCA
TGGCGTTTAGTAAGTTAGGTTTAGTACCCGATTGCGGTGGAACCTGGTTACTGCCACGCGTTGCTGGGCGAGCGCGCGCC
ATGGGGCTGGCACTGCTGGGGAATCAACTGAGTGCTGAACAGGCGCACGAATGGGGGATGATCTGGCAGGTTGTTGATGA
TGAAACGCTGGCAGATACCGCGCAACAGCTGGCACGGCATCTGGCGACACAACCGACATTTGGTCTTGGACTTATCAAGC
AAGCGATAAATAGCGCTGAAACCAATACGCTCGATACGCAACTGGATCTGGAACGTGACTATCAGCGACTTGCCGGACGG
AGCGCAGATTATCGTGAAGGTGTCAGTGCGTTCCTGGCTAAACGCTCACCGCAGTTCACGGGGAAATAG
>Cluster_61500
ATGGTTGAATTAAAAGAGCCGCTTGCCACACTTTGGCGTGGTAAAGATGCTTTTGCAGAGGTCAAAAAACTGAACGGCGA
GGTCTTTCGTGAACTGGAGACTCGTCGTACATTACGCTTTGAACTGTCCGGGAAAAGCTATTTTCTTAAATGGCACAAGG
GGACGACATTAAAAGAGATTATAAAAAATCTACTCTCATTGCGGATGCCCGTTTTGGGCGCAGACAGAGAGTGGCACGCT
ATTCATCGCCTGAGTGATGTTGGCGTTGATACAATGAAGGGCATTGGGTTTGGCGAAAAAGGGTTAAATCCATTAACTCG
CGCATCATTTATTATTACCGAAGATCTCACTCCCACAATTAGCCTTGAAGATTATTGTGCCGATTGGGCAGTCAACCCGC
CTGATATACGTGTTAAGCGTATGCTGATCGCACGTGTAGCAACTATGGTGCGTAAAATGCATACTGCAGGGATAAATCAC
CGCGACTGTTACATTTGCCACTTTTTGCTTCATTTGACACTCTTGAAAGCTGGCGTGACCAGAAGCAAATTATTTCTGT
TCTGCATCGGGCACAGATACGTGCAAAAGTACCGCGCCGCTGGCGCGATAAAGACCTGATTGGTTTATATTTCTCATCAA
TGAATATTGGCCTGACGCAAAGAGATATCTGGCGATTTATGAAGGTTTATTTTGGGATGCCTTTACGCAAAATATTGTCT
CTTGAACAGAATTTATTGAACATGGCAAGCGTTAAGGCCGAGCGTATTAAAGAGCGAACACAACGCAAAGGATTATAA
>Cluster_1688
ATGATTTACTCTAATCAGATTCGTTACAGTGGCAAAGATGGCGTTTACTTCCACTTAAGAGATAATGTAGATGCCTTCTT
AACTTTATCAGCAACTGAAAATATGGAATTTGATAGCCCTATGCAGGTGACTACACAGAACATGCAATCAGGGCAAACCG
TCACAGATAATGTACAAAGAGCACCCAGAACAATCACTATCAGTGGTGTCGTTGTAGTTGGCTATGAAGGAAGCCTATTA
TTAACTCGTCAGGGTCAATTAGTAGAAAACTTCATTGACACTCTTGAAAGCTGGCGTGACCAGAAGCAAATTATTTCTGT
CATCTGTAAAGACGGTATTAAAATTGATGATTCCATTATTACGAGTTTTAAAGCCTCTAAAGATGTTGGTATTTCAAATG
GTCTAAGAATCCAGCTAACTTTTCAGGAAATTAACTTCAAAGCTATTGTAGGGCAAACTGATGTTTCTGCTGCTACTGGA
AAGACCGCTACCACAAATGATGGTGGCGCTACTTCTAAGAAGAATGCGGGTAACGTGACTACTGAACGCAATAACGCACC
TATGCTGATGTGTCAGGAACTGTACAGTTACTCAGCATCTGAGCTATCTGATGAAGCGCTAAGGGTCCGTAGCAATTGCT
CTAAAAATGTCACGGTGAAAGATGGTGTAAGCACCTTCACAGCCTCAGCTAATGAGCAGGCTTCAAAAGTGCTTAAGTCG
GGTAATGCTTTACGGAAGTTTTCTGTGAATCCGAATAAGAAGGGCACTTATTGA
>Cluster_10143
ATGTTGTCTGCTACTCAACCACTTAGCGAAAAATTGCCAGCGCATGGCTGCCGTCATGTTGCGATCATTATGGACGGCAA
TGGTCGCTGGGCAAAAAAGCAAGGGAAGATTCGTGCCTTTGGGCATAAAGCCGGGGCAAAATCCGTCCGCCGGGCTGTCT
CTTTTGCGGCCAACAACGGTATTGAGGCGTTAACGCTGTATGCCTTTAGTAGTGAAAACTGGAACCGACCAGCGCAGGAA
GTCAGTGCGTTAATGGAACTGTTTGTGTGGGCGCTCGATAGCGAAGTAAAAAGTCTGCACCGACATAACGTGCGTCTGCG
TATTATTGGCGATACCAGTCGCTTTAACTCGCGTTTGCAAGAACGTATTCGTAAATCTGAAGCGCTAACAGCCGGGAATA
CCGGTCTGACGCTGAATATTGCGGCGAACTACGGTGGACGTTGGGATATAGTCCAGGGAGTCAGGCAACTGGCTGAAAAG

GTGCAGCAAGGAAACCTGCAACCAGATCAGATAGATGAAGAGATGCTAAACCAGCATGTCTGTATGCATGAACTGGCCCC
TGTAGATTTAGTAATTAGGACTGGGGGGGAGCATCGCATTAGTAACTTTTTGCTTTGGCAAATTGCCTATGCCGAACTTT
ACTTTACAGATGTTCTCTGGCCCGATTTCGATGAACAAGACTTTGAAGGGGCGTTAAATGCCTTTGCTAATCGAGAGCGT
CGTTTCGGCGGCACCGAGCCCGGTGATGAAACAGCCTGA
>Cluster_7947
ATGGCAACCCCCGCACAGCGTCACGCGATGCGGGTCTCGGCTGTCAGGGCATCGCAGCGGGATAACGCCCCGCTGCGTCA
TGCCTCACCTTACGAGCAAATGCTCGTCAAGCTGGCCGCAGACCGCAGGACGCTATCAGCCATTCGTTCTAAAGAGCGCA
AAGCGGATAAAAAACGCGAATTACTCCCGCTGTACCTGCCGTGGGTCGCTGGCGTACTGGAAAGCGGCACCGGCGCACAG
GATGACATTCTGATGACGGTGATGCTCTGGCGTCTGGATGCGGGGATATCACCGGCGCTGTTGAGATTGCGCGCTATGC
CCTGCGTTTTGGCCTGTCGATGCCGGAAAACCATTCCCGCCCCGCGCCTTACATGCTGGCCGAAGAGGTCGCGCTCGCGG
CTCTCCGCGCCCGTATCGGCGGTGAGCCGGTCGACGCTGCACAACTGCTCGAGGTCATCGGGCTGACCGATGCCGCCGAT
ATGCCTGACGAAGTGCGCGCCCGACTGCATAAGGTCACCGGTCTGACCCTGCGTGATGCCGGTCAGCTTCCTGATGCACT
GGCGCACCTGCAACGCGCCTTACAGCTCGACACCCACGCCGGGGTAAGAAAAGACATTGAGACCCTCACTCGTGAGCTGA
ACCCGAAACCGGTCGCCGTCAAAAAAACGGCGCCGAAAGCCGCGAAAAAGGCACCCGCGAAAAAACAAGATTCACCGGTG
AAACGAGGGCGGGGACGCCCGAAGAAAGTCGCCGGTTAA
>Cluster_48618
ATGGCACCTACACTAGAACGCACATACTCTAAAAATCTTTATGAGTTTCCTCACCGTGGGGAAACTCGCGTTTCACGTTT
TGGCTACCTCATCAACGAAGCATCGCTTTTCAAAATATCCGAAATAACCATTATCGAGCCTGATGATGATATCTGTCTGT
ATATCCTGATGGAGAAAGTTGGAGCACGAGATCAGGGTGAACTGATGGACTTCATTCTGGACAGAGGCGAAGATGGAATG
TCTGATTCGGACATTATTCAAGCAATTCTGCGTTCCGACATGCTCGATCAGAGCAGAAACACGATAGCAGGCAGAATTGC
ATTGCGTGAATACACATTCATCGAGGATGGGGTAGAGATTGACTGCTATCAGATTGCAGGTGTTGAGACAGAAAGAGCAA
TAAGGCAAAGGGGACTGTGCAACCTCACGTATCGCTTCCTCCTTCATTGGTACGAACATCTTGTCTGTGATTACAATCAG
ACCATTCCTGGCGCAAAAATCTGGGCCGGTCCATTAATGCGAACAGGTGATGTAAGAATTTACAACGCAAAAACTGAGAC
GTTCGAGGATGTGTTAGGCGAATATGGGATGGGAAAAGAAACCGGCTTTTTGCCGTGGAACAGAGGATTGTTACTTGATC
CAGAATTAAGCTCTTGGTTTCCAAATAAGGTGCAAGTTAACGTTGAGAAATTTATCGTACTTATCATCTCCCGCAAAACA
AGAACCCCAGTTGGTTTATATCTAAAAGATTAA
>Cluster_24063
ATGGTGGATAAGTCACAAGAAACGACGCACTTTGGTTTTCAGACCGTCGCGAAGGAACAAAAAGCGGATATGGTCGCCCA
CGTTTTCCATTCCGTGGCATCAAAATACGATGTCATGAATGATTTGATGTCATTCGGTATTCATCGTTTGTGGAAGCGAT
TCACGATTGATTGCAGCGGCGTACGCCGTGGGCAGACCGTGCTGGATCTGGCTGGTGGCACCGGCGACCTGACAGCGAAA
TTCTCTCGCCTGGTCGGAGAAACTGGCAAAGTGGTCCTTGCTGATATCAATGAATCCATGCTCAAAATGGGCCGCGAGAA
GCTGCGTAATATCGGTGTGATTGGCAACGTTGAGTATGTTCAGGCGAACGCTGAGGCGCTGCCGTTCCCGGATAACACCT
TTGATTGCATCACCATTTCGTTTGGTCTGCGTAACGTCACCGACAAAGATAAAGCACTGCGTTCAATGTATCGCGTGCTG
AAACCCGGCGGCCGCCTGCTGGTGCTTGAGTTCTCGAAGCCAATTATCGAGCCGCTGAGCAAAGCCTATGATGCATACTC
CTTCCATGTGCTGCCGCGTATTGGCTCACTGGTCGCGAACGACGCCGACAGCTACCGTTATCTGGCAGAATCCATCCGTA
TGCATCCCGATCAGGATACCCTGAAAGCCATGATGCAGGATGCCGGATTCGAAAGTGTCGACTACTACAATCTGACGGCA
GGGGTTGTGGCGTTGCATCGTGGTTATAAGTTCTGA
>Cluster_22560
ATGAAAAAACATCTTTTTACTCTGACACTCTCTTCTGTATTGGCGATACCAGCAGTATCCCATGCAGAATTTAAAGGCGG
TTTTGCTGATATAGGTATTCATTATCTTGACTGGACTAGCAGAACCACTGAGAAATCGTCGACCAAATCACACAAAGATG
ATTTTGGATATCTGGAACTGGAAGGCGGCGCTAACTTTAGTTGGGGAGAAATGTACGGGTTCTTCGACTGGGAAAACTTT
TATAATGATCGTCATGATAAACCGGGTAGCGAGCAGCGTTATACATTTAAAAATACCAACCGTATTTACCTGGGTGACAC
CGGATTTAATCTCTACTTGCATGCGTATGGCACCTACGGTTCTGCGAATCGTGTGAATTTCCATGACGATATGTTCCTTT
ACGGGATCGGCTACAATTTCACCGGCAGCGGTTGGTGGTTCAAACCGTTCTTTGCAAAACGCTATACAGACCAAACCTAT
TATACCGGCGATAACGGTTATGTCGCCGGTTGGGTTGCAGGTTACAGCTTTATGCTGGGCAGTGAGAAATTCACCCTCAC
CAACTGGAACGAGTACGAGTTTGATCGTGATGCCACCTACGCGGCAGGTAATGGCGGCAAAGAAGGTCTGAACGGTGCCG
TCGCACTTTGGTGGAATGCAACTTCACACATCACCACAGGGATTCAGTATCGTTATGCGGATGACAAACTGGGCGAAGAT
TTTTACCAGGATGCGATAATCTACTCCATCAAATTTAATTTCTGA
>Cluster_62113
ATGAACGATCTATTTTCACTGGCAGGAAAAAATATCTTGATTACCGGTTCAGCACAGGGCATTGGCTTTTTACTGGCAAC
CGGCCTGGGTAAATATGGCGCACAAATAATTATTAATGATATTACTGCCGAACGCGCAGAACTTGCTGTAGAAAAACTCC
ACCAGGAGGGTATTCAGGCCGTTGCCGCACCTTTTAATGTTACTCATAAACATGAAATTGATGCCGCCGTTGAACATATC
GAAAAGGACATCGGCCCCATTGATGTGCTGGTGAATAACGCCGGTATCCAGCGCCGTCATCCTTTTACTGAGTTCCCTGA
ACAAGAGTGGAATGATGTGATCGCAGTAAACCAGACCGCCGTGTTCCTGGTATCGCAAGCGGTAACTCGTCACATGGTTG
AACGCAAGGCAGGTAAAGTTATTAATATTTGCTCGATGCAAAGCGAACTGGGACGTGACACCATCACCCCTTATGCCGCA
TCGAAAGGGGCGGTAAAAATGCTCACCCGCGGCATGTGCGTCGAGCTGGCGCGCCACAATATTCAGGTCAACGGTATTGC
GCCGGGCTATTTCAAAACAGAAATGACTAAAGCACTGGTTGAGGACGAAGCCTTCACCGCCTGGTTGTGCAAACGGACCC
CCGCCGCACGCTGGGGAGATCCGCAGGAACTGATTGGTGCTGCGGTGTTCCTTTCTTCAAAAGCCTCTGATTTCGTAAAC
GGCCACCTGTTGTTTGTTGATGGCGGCATGTTAGTGGCTGTTTAA
>Cluster_40077
GTGACTTTGCTTGCCGTCCGTCATGCGTCTCTGGGTTACTCCCGCCATCCGGTGCTACGCGACGTGTCCTTCACCCTGTC
GCAAGGAACAATTTGTTGTCTGTTAGGAGCCAATGGTTGCGGCAAAACCACACTCATGCGCTCCATTCTTGGCGTTATAC
CGTTATTAAAAGGCGAGATCTTACTGGATAGCCTCCCTGTGCAGACACTCAGCCATCGCCAGCGCGCGCAGGCTATTGCC
TGGGTGCCGCAGGCGCACGACGGCATTTTTGCTTTTAGTGTGCTGGATATGGTGTTGATGGGCCTGGCCCCCACCATAGG
CGCTTTTTCCGTTCCCGGTAAGCAAGAACGCCTGAAAGCAACGGAGCAACTGGAAAAGCTGGGCATTTTGCATCTGGCGA
CACGACGCTGGAATACGCTTAGCGGCGGTGAACGTCAGCTGGCTTTAATCGCCCGTGCACTCGTCCAACAACCGCGCTTG
TTATTACTGGATGAGCCTGCCTCCAGCCTCGATTTCGGTCATCAGATCCAACTTCTGGACACGCTGGCGCAGCTCAAAAA
TAACGGCATGACAATGTTAATGTCGACTCATCATCCGCTCCATGCTAACGCCATTGCCGACAGCATTATCCAGGTAGAGC
CAGATGGACGAGTAACACAAGGCTTACCGACCGAACAGTTAACAACGAAAAAACTGGCTGCCCTGTACCGGGTTTCTGCC
GACCAGATTCACCATCACCTCTCTGCAATTAAGCACTAA
>Cluster_60167
ATGATCCTGCACGCGCAGGCAAAACACGGAAAACCAGGTTTACCCTGGCTGGTGTTTTTGCACGGTTTTTCCGGCGATTG
CCACGAATGGCAAGAAGTGGGCGAGGCGTTTGCCGACTACTCACGGTTGTATGTTGATCTCCCAGGTCACGGTGGTTCGG
CGGCGATTAGCGTCGATGGATTTGATGATGTCACCGACTTACTGCGTAAAACCTTGGTTAGTTACAACATCCTTGACTTC
TGGCTGGTGGGGTACTCGCTTGGTGGACGGGTGGCGATGATGGCGGCTTGCCAGGGGCTGGCGGGGCTTTGTGGGGTTAT
TGTCGAAGGCGGGCATCCGGGGCTGCAAAATGCTGAACAACGTGCGGAACGTCAGCGTTCCGATCGCCAATGGGTGCAGC
GTTTTCTCACAGAACCGTTAACGGCGGTATTTGCCGACTGGTATCAACAGCCTGTTTTTGCCTCACTCAATGACGATCAA
CGCCGGGAGCTGGTGGCGCTGCGCAGCAACAATAATGGCGCAACGCTTGCCGCCATGCTGGAGGCGACTTCTCTCGCCGT
CCAGCCTGATTTACGTGCTAACCTTAGCGCCCGCACATTTGCGTTTTATTATTTATGTGGTGAACGTGACAGCAAATTCC

GCGCCCTGGCGGCGGAACTGGCTGCCGACTGCCATGTCATTCCTCGCGCCGGACATAACGCGCATCGGGAAAATCCCGCT
GGCGTAATCGCAAGTCTGGCGCAGATCTTGCGTTTCTGA
>Cluster_8602
ATGAAAACAGGTATGTTTACCTGCGGCCACCAGCGGCTGCCTATTGAACACGCATTTCGTGATGCAAGCGAGCTGGGTTA
CGACGGCATCGAAATTTGGGGCGGTCGCCCGCACGCGTTCGCGCCGGACTTAAAAGCGGGCGGCATCAAACAAATCAAGG
CGCTGGCGCAGACGTATCAGATGCCGATTATCGGCTATACGCCAGAAACCAACGGCTATCCGTATAACATGATGCTGGGC
GATGAACATATGCGTCGCGAAAGCCTCGACATGATCAAGCTGGCGATGGATATGGCAAAAGAGATGAACGCGGGTTATAC
GCTGATTTCCGCGGCCCACGCGGGCTATCTCACGCCACCTAATGTTATCTGGGGACGGCTGGCAGAGAACCTAAGCGAAC
TGTGTGAGTACGCGGAAAACATCGGCATGGATCTAATCCTCGAACCGTTAACGCCGTATGAATCGAACGTCGTGTGTAAT
GCTAATGATGTGCTTCATGCGCTGGCGCTGGTGCCTTCGCCGCGCCTGTTCAGCATGGTCGACATTTGCGCGCCGTATGT
CCAGGCGGAACCGGTGATGAGTTATTTCGACAAACTGGGCGATAAATTACGTCATCTGCATATTGTCGACAGCGACGGGG
CCAGCGACACGCATTACATTCCTGGAGAAGGCAAAATGCCGCTGCGGGAACTGATGCGCGATATTATTGAGCGGGGCTAT
GAGGGTTACTGTACGGTGGAGCTGGTGACGATGTATATGAACGAGCCCAGACTCTATGCCCGCCAGGCGCTGGAACGCTT
TCGCGCGCTGCTGCCGGAGGATGAGAGATGA
>Cluster_10740
ATGAGAAGAGTCTTCTGGCTGATCGCTGCCGCTCTGTTACTGGCAGGGTGTGCAGGCGAAAAAGGCATTGTCGAGAAAGA
GGGATATCAGCTTGATACCCGACGCCAGGCGCAGGCGGCGTATCCGCGCATTAAAGTGCTGGTGATCCACTACACCGCAG
ATGATTTTGATAGCTCGCTGGCGACACTGACCGATAAGCAGGTCAGCTCGCATTATCTGGTCCCTGCGGTACCACCGCGA
TACAACGGTAAACCGCGCATCTGGCAACTGGTGCCGGAACAAGAACTGGCCTGGCATGCGGGGATTAGCGCCTGGCGCGG
GGCAACGCGCCTTAACGACACCTCTATTGGCATTGAGCTGGAAAACCGTGGCTGGCAAAAATCGGCCGGAGTGAAATATT
TTGCCCCGTTTGAACCGGCACAGATTCAGGCGCTTATTCCACTGGCGAAAGATATTATTGCCCGTTATCACATCAAGCCG
GAAAACGTAGTGGCACATGCGGATATCGCACCGCAGCGCAAAGACGATCCGGGGCCATTATTTCCCTGGCAGCAACTGGC
GCAGCAGGGGATTGGTGCCTGGCCGGATGCGCAGCGGGTTAACTTTTACCTTGCCGGGCGCGCGCCGCACACACCGGTAG
ATACCGCGTCATTGCTGGAGCTTTTGGCGCGCTACGGTTATGACGTTAAACCTGATATGACACCGCGCGAGCAGCGGCGC
GTGATTATGGCATTCCAGATGCATTTCCGCCCGACGTTATATAACGGCGAAGCGGATGCAGAAACTCAGGCGATTGCCGA
AGCATTGCTGGAGAAATACGGGCAGGATTAG
>Cluster_13603
TTGACATCATTATTCAATCGGTTAACGGGAAAAGCGGTTAGCCGGACAGCTTTCGTCGAACACCTTGGTCAGGAAGTTGT
ACAACATCATCCAAACTGGAAAGTCATGATTTCGACTGACCACAAATTGATGCGCATTGATACGGGTCACCAAACGGTGG
AAATCACCCTTGATGATGCCTACAAACGTTATCAAGCCGCACCAGACCCGCTGCAAGAAGTGGCTGCAGATATGCTGAAA
CAGCTTGCAGAAATGCTGCCGACCAGGCGCTGTATTGTTCCGGTGATTAAAGAAAAAGCCTGGCTGGATACCGTTCGCCA
ACAGCTTAGCCAGTATGAGTCTGATCCAGAGGTCCTCAACGCCTCGCTGGATTTTGCCTGGGAAGACTTTAACGATGAGT
TAATTATCTTGTTTGCTGAAGATGGTAGCGAAACCATGCGCTATCTGATGCGCTACGAAGTGACCATTGACCGCGAACAA
GCCATCAGTAATTTGAAGCAAATATTGCCGGAAGTTACGGCTGAAATCGTTGATACAGGCGCAAACGATGGAAAAGTTGC
CGTTATCTCCGCAGGGGGTACTTATGAGCCCAGTCTGATGCTCATTGACGATCTTTGGGTGCAATCACCCGATCCTGAAT
GCGGCGACCTGTTAGTGACCGTTCCCTCCAGAGGAAAACTGTTTTTCACCCACAATGATCAGCCAGCAAATCTCAGCGCA
TTACGACAGCTAACAGAACAATGCTTCCGTGAGGATGACCATCCTCTGACAACACAGATATTCATTCGCCGCAACGGTCA
CTTTTATCCCTGGGTAGAAACCTTGCATTAA
>Cluster_61287
ATGTTGATGATTACCTCTTTTGCTAACCCCCGCGTGGCGCAGGCGTTTGTTGATTACATGGCGACGCAGGGTGTTATCCT
CACGATTCAACAACATAACCAAAGCGATGTCTGGCTGGCGGATGAGTCCCAGGCCGAGCGCGTACGGGCGGAGCTGGCGC
GTTTTCTCGAAAACCCGGCAGATCCGCGTTATCTGGCGGCGAGCTGGCAGGCAGGCCATACCGGCAGTGGCCTGCATTAT
CGCCGTTATCCTTTCTTTGCCGCCTTGCGTGAACGCGCAGGTCCGGTAACCTGGGTGATGATGATCGCCTGCGTGGTGGT
GTTTATTGCCATGCAAATTCTCGGCGATCAGGAAGTGATGTTATGGCTGGCCTGGCCATTCGATCCAACACTGAAATTTG
AGTTCTGGCGTTACTTCACCCACGCGTTAATGCACTTCTCGCTGATGCATATCCTCTTTAACCTGCTCTGGTGGTGGTAT
CTCGGCGGTGCGGTGGAAAAACGCCTCGGTAGCGGTAAGCTAATTGTCATTACGCTTATCAGCGCCCTGTTAAGCGGCTA
TGTGCAGCAAAAATTCAGCGGGCCGTGGTTTGGCGGGCTTTCTGGCGTGGTGTATGCGCTGATGGGCTACGTCTGGCTAC
GTGGCGAACGCGATCCGCAAAGTGGCATTTACCTGCAACGTGGGTTAATTATCTTTGCGCTGATCTGGATTGTCGCCGGA
TGGTTTGATTTGTTTGGGATGTCGATGGCGAACGGAGCACACATCGCCGGGTTAGCCGTGGGTTTAGCGATGGCTTTTGT
TGATTCGCTCAATGCGCGAAAACGAAAATAA
>Cluster_9153
GTGAAAAAAACACAGCGAAAAGAAATCGAAAATGTGACAAATATCACAGGTGTTCGTCAAATTGAGTTATGGCGGCGTGA
TGATTTACAACACCCACGGTTAGATGAGGTCGCGGAAGAAGTTCCCGTTGCGCTGGTCTACAACGGCATTTCGCATGTGG
TGATGATGGCGTCGCCCAAAGACCTTGAGTACTTTGCGCTCGGTTTTTCGCTTTCCGAGGGGATTATCGAAAGTCCGCGC
GATATCTTCGGCATGGATGTCGTTCCTTCCTGTAATGGTCTTGAAGTACAAATTGAGCTTTCCAGCCGCCGCTTTATGGG
GTTGAAGGAGCGCCGCCGGGCGCTGGCGGGACGTACGGGCTGTGGCGTATGCGGCGTGGAGCAACTTAATGACATCGGAA
AACCGGTGCAGCCGCTACCGTTCACCCAGACGTTTGATCTCAACAAACTGGATGATGCATTACGTCATCTCAACGATTTT
CAGCCAGTGGGGCAACTGACTGGTTGTACTCACGCCGCTGCCTGGATGTTGCCATCTGGCGAACTGGTCGGCGGGCATGA
AGACGTGGGTCGCCATGTGGCGCTGGACAAACTGTTAGGCCGCCGGTCACAAGAAGGGGAAAGCTGGCAGCAAGGTGCGG
TACTGGTTTCCAGTCGTGCCAGTTATGAAATGGTGCAAAAGTCGGCGATGTGCGGCGTAGAGATTTTGTTTGCGGTGTCT
GCCGCGACCACGCTTGCTGTAGAAGTGGCTGAGCGCTGTAATCTGACACTGGTAGGTTTTTGTAAACCGGGTCGGGCAAC
GGTTTATACCCATCCGCAGCGTTTGAGCAATTAA
>Cluster_25451
GTGCGGATAACGGAAACCGAGTTTGCCAGCGATCCGAAAACCCCGGTATTGAGCGCCCTAATTGCGGACATCATCCGGTT
GCAGACGGCCATTCCATGCCAGCCCGTTACGGTTTCTCAGCTCAGCCATTTGTCTTATGAGCAACCGTGGATTAGCGTGA
TAGATGCGCAGACGGACAGCGACCTGGATCGCATCGCTGCCGCTGTGATGCAGGCTAAACAACCGCTGCTGCTGGTCGGC
TCCGCTGGTATTTGTGATGCCGTTGCCCGGCGCTCCGCGATTATGCCGCCACCCACCGTGCTGGCCATTATTGGTTCAAT
GAGCGAAATCGCTCAACGGCAGATCGCCACACTTCATTCACACCCACGTATCACACAGATATACGTCGATGTTGAACACA
TTCTGGCAGGCAATGCCAGTGACTATGACGCGCGTATTGTGCAGGCCTTGCAGAAAGGCGACCACTGCATTGTCCACACC
TGTAACGATTCTGTAGCAAGGCATCAGATCGACACCCTTTGCCAGCGTTGGCAGATGAGTCGCGCTGCACTTGGGGAAAA
AATTTGTCGGTTTCTTGGCGAACTTACCCGGCAAGTTCTGCTTCGCACAATGCCCGATGCGCTTTATCTCTCTGGTGGTG
ATGTCGCAATGGCAACAGCCAGTGCGTTAGGCGCGACCGGATTTCGTATTACGGGGAAGGTGGCGCAATGCGTTCCATAC
GGTCACTTTCTGGGCGGTGTCTGGTCCCGTTCGGTGATGACGAAAGCCGGTGGGTTTGGCGATGAGACCACGTTGCATCA
GGTTCTGAATTTTATCGAGGAGAAGTGTAGTGAATAA
>Cluster_39852
ATGCAGCGCTTATTTCTTTTGGTTGCCGTCATGTTACTGAGCGGATGCTTAACCGCCCCGCCTAAAGAAGCCGCCAGACC
GACATTAATGCCTCGTGCTCAGAGCTACAAAGATTTGACCCATCTGCCAGCGCCGACGGGTAAGATCTTTGTTTCGGTAT
ACAACATTCAGGACGAAACCGGGCAATTTAAACCCTACCCGGCAAGTAACTTCTCCACTGCTGTTCCGCAAAGCGCCACG
GCAATGCTGGTCACGGCATTGAAAGATTCTCGCTGGTTTATACCGCTGGAGCGCCAGGGCTTACAAAATCTGCTTAACGA

GCGCAAGATTATTCGTGCGGCACAAGAAAACGGCACAGTTGCCATTAATAACCGAATCCCGCTGCAATCTTTAACGGCGG
CAAATATCATGGTTGAAGGTTCGATTATCGGTTATGAAAGCAACGTCAAATCGGGCGGGGTTGGGGCAAGATATTTTGGG
ATCGGTGCCGACACGCAATACCAGCTCGATCAGATTGCCGTGAACCTGCGCGTCGTCAATGTGAGTACCGGTGAGATCCT
CTCCTCGGTGAACACCAGTAAGACGATACTTTCTTATGAAGTTCAGGCCGGGGTTTTCCGCTTTATTGACTACCAGCGCT
TGCTGGAAGGGGAAGTGGGTTACACCTCGAACGAACCTGTTATGCTGTGCCTGATGTCGGCTATCGAAACAGGGGTCATT
TTCCTGATTAATGATGGTATCGACCGTGGTCTGTGGGATTTGCAAAATAAAGCAGAACGGCAGAATGACATTCTGGTGAA
ATACCGCCATATGTCGGTTCCACCGGAATCCTGA
>Cluster_14917
ATGGCATGGGGAGGCCGCAGGGTAAGTAAGGGCGCGGTTATCTATATCGCTGGCGAAGGCAGTATGGGCGTAAAACGCCG
CGTTAAGGCGTGGGAGATAACCCACGACAAGGTGGTTACGGATTTGTGCATCGTTAACGCGCCTGTCTTCCCTGCATCGC
CTGACTATGTGGAGCAGGTTATCAGGACTGCCGGACTTGTTAAGAGCAGAACGGGCGAAAATGTGCGGCTGATTGTGATC
GACACGCTGGCCCGTTGCTTTGGTGGGAATGATGAAAATGATTCCCGTGATATGGGCGCATTTATCCAGGGGTGTGACGC
GATAAAGCAGGCCACAGGGGCCACGGTACTGGTGGTTCACCATTCCGGCAAGGATGAAACAAAGGGGGCGCGAGGTTCCA
GCGCATTCCGCGCTGCACTGGATGCAGAGTACCGAATCAGCCGGGAAAATTCAGACGTTACAGCACTGGTGGCGGCGTGT
ACGAAGATGAAAGACGCAGAGGAGCCAAAAGAGAGCGCATACGACCTTAAAAGCGTGGAGGTGTTTACCGATACCGATGG
CGAAGAGATTGTGTCCATGGTGGTGATTGACGTTCCCCGCGCCCCTGCTGAACTGGAGCGCATAGAGGAGGCCGGGAACA
AGACGGAAAATCACACGGCTTTATGGGGTTGTATCCGGTCACGTATACAGCATGGTGATAAATGCACGATCCCGCTGCTA
CGTGATGACATGAAAAAGCTGGGATATGACACAAAACACCTTAAACGATGGTTAGCCAAACTGGAAAAAGACGGCGTGAT
TTGCGTCGATGGGGATGATGTTCACCCACTGTAA
>Cluster_27636
ATGTCTCAGAACTTAGACGCAACCGCAATTAATCAAATCCATGCCCTTATTTCTGCTCAGGGTGTTAATGAAATTATCAG
TAAGATTGGTGCCGATGCTGTGGCATTGCCTGAGAATTTCCGCATTCATGATCTGGAAAAATTTAATTTAAATCGTTTCC
GTTTCCGTGGTGCGCTTTCCACTGCCAGCATCGATGACTTTACCCGTTATTCTAAAGATCTTGCAGATGAAGGCACCCGC
TGCTTTATCGATGCCGATAATATGCGTGCCGTCAGTGTGCTTAACCTGGGTACTATTGATGAACCAGGTCACGCAGATAA
CACCGCCACTCTCAAACTGAAAAAGACAGCACCGTTCTCTGCTCTGTTGTCTGTTAATGGCGAGCGTAACTCCCAGAAAT
CACTGGCAGAATGGATTGAAGACTGGGCCGACTACCTTGTGGGCTTTGATGCTAATGGTGACACCATTCAGGCAACAAAA
GCGGCTGCGGCGGTCCGTAAAATCACGATTGAAGCAAACCAGACTGCTGATTTTGAAGATAATGACTTCAGCGGCAAACG
CTCCCTGATGGAGTCTGTCGAAGCGAAGACCAAAGACATTATGCCAGTGGCATTTGAGTTTAAATGCGTTCCGTTTGAAG
GTCTGAAAGAACGTCCGTTTAAATTACGCCTCAGCATTATCACTGGCGATCGTCCGGTACTGGTTCTGCGCATTATTCAG
CTGGAAGCGGTGCAGGAAGATATGGCTAACGAATTTCGTGATCTGCTTGTTGAGAAATTCAAGGACAGCAAAGTAGAAAC
CTTTATTGGGACTTTCACCGCCTGA
>Cluster_2968
ATGAGCGATCTGCATAACGAGTCCATTTTTATTACCGGCGGCGGATCGGGATTAGGGCTGGCGCTGGTCGAGCGATTTAT
CGAAGAAGGCGCGCAGGTTGCCACGCTGGAACTGTCGGCGGCAAAAGTCGCCAGTCTGCGTCAGCGATTTGGCGAACATA
TTCTGGCGGTGGAAGGTAACGTGACCTGTTATGCCGATTATCAACGCGCGGTCGATCAGATCCTGACTCGTTCCGGCAAG
CTGGATTGTTTTATCGGCAATGCAGGCATCTGGGATCACAATGCCTCACTGGTTAATACTCCCGCAGAGACGCTCGAAAC
CGGCTTCCACGAGCTGTTTAACGTCAACGTACTCGGTTACCTGCTGGGCGCAAAAGCCTGCGCTCCGGCGTTAATCGCCA
GTGAAGGCAGCATGATTTTCACACTGTCAAATGCCGCCTGGTATCCTGGCGGCGGTGGCCCGCTGTACACCGCCAGTAAA
CATGCCGCAACCGGACTTATTCGCCAACTGGCTTATGAACTGGCACCGAAAGTGCGGGTGAATGGCGTCGGCCCGTGTGG
TATGGCCAGCGACCTGCGCGGCCCACAGGCGCTCGGGCAAAGTGAAACCTCGATAATGCAGTCTCTGACGCCGGAGAAAA
TTGCCGCCATTTTACCGCTGCAATTTTTCCCGCAACCGGCGGATTTTACGGGTCCGTATGTGATGTTGGCATCGCGGCGC
AATAATCGCGCATTAAGCGGTGTGATGATCAACGCTGATGCGGGTTTAGCGATTCGCGGCATTCGCCACGTAGCGGCTGG
GCTGGATCTTTAA
>Cluster_27354
ATGTCCGACCAGCAACAACCGCCAGTTTATAAAATTGCGCTGGGCATTGAGTACGACGGCAGTAGGTATTACGGCTGGCA
ACGGCAGAATGAAGTCCGCAGTGTGCAGGAGAAGCTGGAAAAGGCGCTCTCCCAGGTGGCGAACGAACCCATCACCGTCT
TCTGCGCCGGGCGTACTGACGCTGGTGTACACGGTACCGGGCAGGTTGTGCATTTCGAAACGACCGCGCAGCGCAAAGAC
GCTGCGTGGACGTTGGGCGTAAATGCGAATTTACCTGGTGACATCGCCGTGCGTTGGGTTAAAGCTGTACCTGATGATTT
TCATGCCCGATTTAGCGCCACGGCTCGCCGTTATCGCTACATCATCTACAATCATCGGCTGCGCCCGGCGGTACTGAGTA
AAGGGGTAACCCATTTTTACGAACCGCTGGACGCTGAACGGATGCATCGGGCTGCGCAATGCTTGCTGGGCGAGAATGAT
TTCACCTCGTTCCGTGCGGTGCAGTGTCAGTCCCGGACCCCGTGGCGCAACGTTATGCATATTAATGTCACGCGTCACGG
TCCTTATGTGGTGGTAGATATCAAAGCGAATGCCTTTGTACATCATATGGTCAGGAATATTGTCGGCAGCCTGATGGAAG
TCGGTGCCCACAACCAGCCGGAGAGCTGGATAGCAGAGTTGCTGGCGGCAAAGGACAGAACGCTGGCGGCAGCAACGGCA
AAAGCGGAAGGGCTATATCTGGTCGCGGTGGATTACCCTGACCGATATGATCTTCCAAAACCGCCAATGGGCCCGCTATT
TCTGGCGGACTAA
>Cluster_28924
ATGGCAACAACACAACAATCTGGATTTGCACCTGCTGCATCGCCTCTCGCTTCGACCATCGTTCAGACTCCGGACGACGC
GATTGTGGCGGGCTTCACCTCTATCCCTTCACAAGGGGATAACATGCCTGCTTACCATGCCAGACCAAAGCAAAGCGATG
GCCCACTGCCAGTGGTCATTGTAGTGCAGGAAATTTTTGGCGTGCATGAACATATCCGCGACATTTGTCGCCGTCTGGCG
CTGGAGGGGTATCTGGCTATCGCACCTGAACTTTACTTCCGCGAAGGCGATCCGAATGATTTTGCCGATATCCCCACGTT
GCTTAGCGGTCTGGTAGCAAAAGTGCCTGACTCGCAGGTGCTGGCCGATCTCGATCATGTCGCCAGTTGGGCTTCCCGCA
ACGGCGGCGATGTTCATCGTTTAATGATCACCGGATTCTGCTGGGGTGGACGTATCACCTGGCTGTATGCCGCGCATAAT
CCACAGCTAAAAGCCGCAGTGGCGTGGTACGGCAAACTGACGGGCGATAAGTCGCTGAATTCACCGAAACAACCTGTTGA
TATCGCAACCGATCTTAACGCTCCGGTTCTCGGCTTATATGGCGGCCAGGATAACAGCATTCCGCAAGAGAGCGTGGAAA
CGATGCGCCAGGCGCTGCGGGCAGCAAACGCGAAAGCAGAGATTATCGTGTACCCGGATGCAGGGCATGCGTTCAACGCC
GATTATCGTCCGAGCTATCATGCCGAGTCTGCGAAAGACGGCTGGCAGCGAATGTTGGAATGGTTTACACAGTATGGTGT
TAAAAAGTAA
>Cluster_60349
ATGAGTTACGAAATCAAAATTTGCGACATCCTCAAAGGCGCTGCGATGGAAGGACAATATAAGGGCGCTCAACGTGGGGC
CAAATGCGAGGAGATCGCAAACGAGTTAACTCGCCGGGGAGTTAAAAACAACAAGGGAGAGGTTATTACTAAGGGCGGCG
TTAGCCATTGGCTGGAAGGTAGACGGGAGCCAAATTTCGACACTCTGGCTGAACTGTGCGACATGTTCGGCGTTTATGCC
CTGATGCCGATGCGTGGCGGTAAATGGATTCGAGTTCACCCGGAAGACCGTGGAGAAATGGAATTACGCGAGGCTGTAGC
TGAACGAGATGCTATTATCGACGACCTGAAGGCCCGTATCGCTGAGCTTGAGGCGGCACTAGCAAATAAACAAGTCCCAG
CAGAAGCGGAAGAGATGGGAGGTGAAAAAGTGGAAGAAGTGGCAGCAGAGCAAGCGCCCAACGATGAAAAAGAGATGGGA
GCTAAGGAGTGGGTTAACCCGAACCCGAAAAAATACAGCGTCGGCATGTTGTGTCAGGTACTGGCTGCAATGGGCGGCGA
ATACCTGGGCAACAACGCAGGGTTACAGCAGAAGATCACTGTGTTGGATAACGACGGCAACCGGAAGCCAATCAGTAACG
GCGCGTTTTATCGACTGATTGAGCAGGCCAAAGGGAGAGGATTGATTAGCGTTGAACAGGAAATCAAACACAAGAAAGAC
GAAAACGGCAACCAGATCGGCAAAGGCAAGAAAGGTGACAAGCTGATAACTTTGCTTCCCAACTGGATTGATAAGCTGGG
AGACGAATAA

>Cluster_21216
ATGCTGTCGCGTCGGGGTCATCGGTTAAGTCGTTTTCGTAAAAATAAACGCCGCCTGCGCGAGCGTTTGCGTCAGCGTAT
TTTTTTCAGAGATAAAGTGGTGCCGGAAGCAATGGAAAAACCAAGAGTACTCGTACTGACAGGGGCTGGGATTTCTGCGG
AATCAGGTATTCGTACCTTTCGCGCCGCAGATGGCCTGTGGGAAGAACATCGGGTTGAAGATGTGGCAACGCCGGAAGGT
TTTGATCGCGATCCTGAACTGGTGCAAACGTTTTATAACGCCCGTCGTCGACAGTTGCAGCAGCCAGAAATTCAGCCTAA
CGCTGCACATCTTGCGCTGGCGAAACTGCAAGATGCTCTCGGCGATCGCTTTTTGCTGGTGACGCAGAATATCGACAACC
TTCATGAGCGTGCCGGTAACACCAATGTTATTCATATGCATGGGGAACTGCTGAAAGTACGTTGTTCGCAAAGTGGCCAG
GTCCTCGACTGGACCGGAGACGTTACGCCAGAAGATAAATGCCATTGTTGCCAGTTCCCGGCACCCTTGCGCCCGCACGT
AGTGTGGTTTGGCGAAATGCCACTCGGCATGGATGAAATTTATATGGCGTTGTCGATGGCCGATATTTTCATTGCCATTG
GTACTTCGGGGCATGTTTATCCGGCGGCTGGGTTTGTTCACGAAGCGAAACTGCATGGCGCGCACACCGTGGAACTGAAT
CTTGAACCGAGTCAGGTTGGTAATGAATTTGCCGAGAAATATTACGGTCCGGCAAGCCAGGTGGTGCCAGAGTTTGTTGA
AAAGTTGCTGAAGGGATTATAA
>Cluster_9067
ATGCAGTTCTCGAAAATGCATGGCCTTGGCAACGATTTTATGGTCGTCGACGCGGTAACGCAGAATGTCTTTTTTTCACC
GGAGCTGATTCGTCGCCTGGCTGATCGGCACCTGGGGGTAGGGTTTGACCAACTGCTGGTGGTTGAGCCGCCGTATGATC
CTGAACTGGATTTTCACTATCGCATTTTCAATGCTGATGGCAGTGAAGTGGCGCAGTGCGGCAACGGTGCGCGCTGCTTT
GCCCGTTTTGTGCGTCTGAAAGGACTGACCAATAAGCGTGATATCCGCGTCAGCACCGCCAACGGGCGGATGGTTCTGAC
CGTCACCGATGATGATCTGGTCCGCGTAAATATGGGCGAACCCAACTTCGAACCTTCCGCCGTGCCGTTTCGCGCTAACA
AAGCGGAAAAGACCTATATTATGCGCGCCGCCGAGCAGACAATCTTATGCGGCGTGGTGTCGATGGGAAATCCGCATTGC
GTGATTCAGGTCGATGATGTCGATACCGCGGCGGTAGAAACGCTTGGTCCTGTTCTGGAAAGCCACGAGCGTTTTCCGGA
GCGCGCCAATATCGGTTTTATGCAAGTGGTTAAGCGCGAGCATATTCGTTTACGCGTTTATGAGCGTGGGGCAGGAGAAA
CCCAGGCCTGCGGCAGCGGCGCGTGTGCGGCGGTTGCAGTAGGGATTCAGCAAGGTTTGCTGGCCGAAGAAGTACGCGTG
GAACTCCCCGGCGGTCGTCTTGATATCGCCTGGAAAGGTCCGGGTCACCCGTTATATATGACTGGCCCGGCGGTACATGT
CTACGACGGATTTATTCATCTATGA
>Cluster_9842
ATGCATGATGCAAACATCCGCGTTGCCATCGCGGGAGCCGGGGGGCGTATGGGCCGCCAGTTGATTCAGGCGGCGCTGGC
ATTAGAAGGCGTGCAGCTGGGCGCGGCGCTGGAGCGCGAAGGATCTTCTTTACTGGGCAGCGACGCCGGTGAGCTGGCTG
GTGCAGGGAAAACAGGCGTTACCGTGCAAAGCAGCCTCGATGCGGTAAAAGATGATTTTGATGTGTTTATCGATTTTACC
CGTCCGGAAGGTACGCTGAACCATCTCGCTTTTTGTCGCCAGCATGGCAAAGGGATGGTGATCGGCACTACGGGGTTTGA
CGAAGCCGGTAAACAGGCGATTCGTGACGCCGCCGCCGATATTGCGATTGTCTTTGCCGCCAATTTTAGCGTTGGCGTTA
ACGTCATGCTTAAGCTGCTGGAGAAAGCAGCCAAAGTGATGGGTGACTACACCGATATCGAAATTATTGAAGCACATCAT
AGACATAAAGTTGATGCGCCGTCAGGCACCGCACTGGCAATGGGAGAGGCGATCGCCCACGCCCTTGATAAAGATCTGAA
AGATTGCGCGGTCTACAGTCGTGAAGGCCACACCGGTGAACGTGTGCCTGGCACCATTGGTTTTGCCACCGTGCGTGCAG
GTGACATCGTTGGCGAACATACCGCGATGTTTGCCGATATTGGCGAGCGTCTGGAGATCACCCATAAGGCGTCCAGCCGC
ATGACATTTGCTAACGGCGCGGTAAGATCGGCTTTGTGGTTGAGTGGTAAGGAAAGTGGTCTTTTTGATATGCGAGATGT
GCTTGATCTCAATAGTTTGTAA
>Cluster_60237
ATGAAAATACTACGATGGTTGTTCGCTCTGGTCATGCTTATTGCTACTACAGAAGCTATGGCTGCCGGGCATAGCGTTGA
CGTTTACTACGGTTACAACGGTGATTCGCGTAATATTGCGACTTTTAATTTAAAAATAATGATGCCATCCGCAGTGTACG
TTGGTGAATATAAAAGTAGTCAATGGCTCATGACTGGGGAAATATTGCAAAATGTGAGCTGGAGTGGTCCTCCTCCTGCC
CCTTCGGTTAAGCTGATTGGCTACCATCAAAATATCAACAAAGCAAGTTGTCCTGGATTACCGTCTGGGTGGAACTGCGG
GTATTATACATTTGAGGTTATTGTCTCTGCAGAGATTGAATCTTACTTTTCTTGTCCATGGTTGGTTATTATGAATGACT
CTGAAGCCTCTCCAGGGGGCGTGACGTATCAGGGGCCTGATAGTCACGACACGATTTGTCCATCCGTTTCTGTTCAACCT
TATGACGTTTCATGGAATGAGAATTATGTCAGTAAGTCAAAACTGCTCACCCTGCAGTCGACGGGCGGTGTGGTTGAGAA
AACGCTTTCAACGTATTTGATGAAAGATGGGAAGTTGTGCGACAGTACGCAGATGAATGAAACAGGTGGTTACTGCCGCT
GGGTTGCACAGATGATAACTTTTACCGCGTCGGGATGTGACAAGGCGGAAGTCTCGGTCACGCCAAATCGACACCCTATA
ACGGACAAACAGTTACACGATATGGTCGTGCGTGTTGATACCAGCAGTATGCAGCCAATCGATTCTACTTGCCGGTTCCA
GTACATACTGAATGAGTTGTAG
>Cluster_58573
ATGGACATGGAGTCGCAAAAAATATTGTTTGCACTTTCCACTCCGATGGAAATACGCAATGAATGCTGTTTACCTTCGCA
TTCATCACCCAAAATGTATTTAGGTACATGCTTTTTTGACCTTTCTTCCTCATGGGGAATTGATGACCGTGATGACCTGC
TGCGCACAATTCACCGAATGATCGATAACGGTCATGCCGCTCGGTTGGCAGGGTTTTATCACCGCTGGTTTCGTTATTCG
CCATGTGAATGGCGTGACTATCTTGCTGAACTAAATGAGCAAGGTCAGGCTTATGCGCAATTTGTCGCCAGCACCGCCGA
ATGCTGTGGCGAAGGGGGAATCAAAGCCTGGGATTATGTCCGGATGGGTTTTTAAGCCGAATGGGCGTACTTAATAACT
GGTTGAGCGAAGAAGAAAGTCTATGGATCCAGTCGCGCATTCATCTACGGGCTCTACGTTATTATAGTAACTGGCGGCAA
TACTTTGCCGGTTATACCTTTGGTCGGCAATATTGGCAGTCTCCCGAAGATGATCATCTGCCTTTATTACGCGAATTCTT
AGCGCGTAAAGAATACGACGATTCCGGCAATGATATGTTTTATCAATTATTTGCCAGTGATGATGCGTATTACCCTACCT
TGTCCTGGCAACCATTGGCTTACTATTCTGCATGCCCGGAAACGCTTAAGGATATGAGCGACCTATGA
>Cluster_12327
ATGTCAACATGGGTCACTATAACGGAAGCAGTTGAGATCACTACCAAAGCCATAAAACAAAAAATTACTCCTAGCGATAT
TTATCGTCATGCCTTGAGTGGCAACATCCTACTATCGGTCTATTTCCAGTCTCCTGTGATACTTAAGAAGATACAAACTT
TTAATGGAAAAATAAAATTCAGGCAGTTTGTGGGAGACCTACTTGATAAACTATGCATGCTTGACAGGGACGGTTTTATT
TATGGTCAAAATTTAAGACTCTGTACAGAAGCTAGATATATTTGTCCTGTGCAACAAATTATAGATACCCCCCTGTTACG
CAAACTCAACCAGTTTCGTACGTTTGTTCGTAATGTCCGCCCGGGTGATGAACTGGATGTCCCGGCACAAGTTAGTGAAA
AAAATTTAACCCCGCCGCCGGGTAACAGCAGTGGCAACCTTGAGCAACAGATAGCCAGTACTTCACAGCTAATTGGATCT
CTGCTCGCCGAAGATATGAACAGCGAGCAAGCGGCAAACATAGCACGTGGATGGGCCTCTTCTCAGGCTTCAGGGGTAAT
GACAGACTGGTTAAGCCGCTTCGGTACCGCAAGAATCACGCTGGGCGTGGATGAAGATTTCAGCCTGAAGAACTCCCGTG
ACGGCAACCCTCGAAAATGGCGATTCGATGCAACAAACAGTGAACTATGTGCCGAACGTCACGAATGCTGA
>Cluster_52594
ATGAAATTCAATTTATCTAATTTATCCGCAGTATTACTGGCATCAGGTATGCTGATGTCTACTGCGGTAACCGCAGCACC
CGGCGATGCAACACAATTTGGTGGGGCGGATACTGACTGGAGCACCGTTGATTATCCCAGGCTCACTGATATGGATGACA
ACGTTGATTCAATGGGGGGGAAAATCCGCTTTACTGGCCGTGTAGTGAAAGCTACCTGTAAGGTCGCAACCGATTCAAAA
CAGATTGAAGTTGTCCTGCCGGTTGTGCCTTCCAACCTTTTCACTGGTATCGACGTAGAAGCACAGGGGGCGAGCAACCA
GACCGATTTCAATATTAATCTGACCGAATGTAGCAATACAGATGATCAGAAAATTGAGTTCCGTTTTACCGGTACTGCAG
ATAGCGCTAATAAAACGCTCGCTAACGAAGTAGAAGGATCAACGGATGCTGACAACAGCGGCAATGCGGGGGCGACTGGT
GTAGGGATTCGAATTTACTCCAAAGGTACGACGAATAATGGTCTGATTAACCTGAATACTACTGCGGCAGAGGGTAGCGC
CTCCACCGCCGCTTATACAATTCCAGGAAATGCTACGACCCATGATTTCAGCGCGGCCTTTACTGCAGGTTATGCTCAAA
ACGGTAGCACTGTTGCACCTGGTGTAGTTAAGTCAACAGCAAGTTTTGTTGTGCTGTACGAGTAA
>Cluster_43605

ATGACGAGTTCGCAGCCCGCCGGATGGACGGCGGCCGAGTTGGCGCAGGCGGCGGCGCGCGGACAGCTTGACCTGCATTA
CCAGCCGCTGGTCGATCTGCGCGATCACCGGATCGCTGGCGCGGAAGCGTTGATGCGCTGGCGGCATCCGAGGCTTGGCC
TGTTGCCGCCCGGCCAGTTCCTGCCGCTGGCCGAGTCGTTCGGCCTGATGCCGGAAATAGGCGCGTGGGTGCTGGGCGAG
GCCTGTCGCCAGATGCACAAGTGGCAAGGACCGGCATGGCAACCGTTCCCGTCTTGCCATCAATGTGTCCGCCAGCCAGGT
TGGGCCAACGTTCGACGACGAGGTAAAGCGGGTGCTGGCCGATATGGCCCTGCCCGCCGAGCTTCTGGAGATCGAACTGA
CCGAATCGGTCGCATTCGGCAATCCAGCCCTGTTCGCCAGTTTCGACGCCTTGCGCGCCATCGGCGTGCGCTTCGCCGCC
GACGACTTCGGCACCGGCTATTCCTGCCTGCAACATCTGAAATGCTGCCCCATCACCACATTGAAAATCGACCAATCCTT
TGTCGCCAGGCTCCCGGATGATGCCCGTGACCAAACTATCGTGCGGGCGGTGATCCAGCTCGCGCACGGGCTGGGCATGG
ATGTCATTTTCAGAAGACGACTGCACCAGTTGATTGGGCGTAATGGCTGTTGTGCAGCCAGCTCCTGA
>Cluster_60077
ATGATTAAAAATTTGCCGCAAATAGTGTTGTTGAATATTGTCGGCCTCGCGCTGTTTCTTTCCTGGTATATCCCCGTTAA
TCATGGATTCTGGTTGCCGATTGATGCGGATATTTTTATTTCTTTAATCAGAAACTGGTCGAAAGTAAGGCCTTTTTGT
GGCTGGTTGCATTGACCAACAATCGCGCCTTCGACGGTTGTTCACTGCTGGCGATGGGTATGTTGATGCTGAGTTTCTGG
CTGAAAGAAAACGCCCCTGGCAGACGACGTATCGTGATTATTGGTCTGGTCATGCTATTAACTGCAGTGGTATTAAACCA
GCTGGGTCAGGCATTAATTCCTGTAAAACGGGCCAGCCCAACATTGACTTTTACCGATATTAACCGCGTCAGCGAACTGC
TCTCTGTTCCCACGAAAGATGCCTCACGAGATAGCTTTCCCGGCGATCACGGCATGATGCTGCTTATTTTTTCGGCATTC
ATGTGGCGTTATTTCGGCAAAGTTGCAGGCCTTATCGCCCTTATTATTTTTGTGGTTTTTGCATTTCCCAGAGTAATGAT
TGGCGCACACTGGTTTACTGACATCATTGTCGGTTCGATGACCGTGATATTGATCGGTTTGCCCTGGGTGTTGCTGACGC
CATTAAGTGATCGATTAATCACCTTTTTTGACAAATCACTACCAGGAAAAAACAAACATTTCCAAAACAAATAA
>Cluster_40969
GTGGGACGTAAATGGGCCAATATTGTTGCTAAAAAAACGGCTAAAGACGGTGCAACGTCTAAAATTTATGCAAAATTCGG
TGTAGAAATCTATGCTGCTGCCAAACAAGGTGAACCCGATCCAGAATTAAACACATCTTTAAAATTCGTTATTGAACGTG
CAAAGCAGGCACAAGTTCCAAAGCACGTTATTGATAAAGCAATTGATAAAGCCAAAGGCGGCGGAGATGAAACGTTCGTA
CAGGGACGTTATGAAGGCTTTGGTCCTAATGGCTCAATGATTATTGCTGAGACATTGACTTCAAATGTTAACCGTACGAT
TGCTAACGTTCGCACAATTTTCAATAAAAAAGGCGGCAATATCGGAGCGGCAGGTTCTGTCAGCTATATGTTTGACAATA
CGGGTGTGATTGTATTTAAAGGAACAGACCCTGACCATATTTTTGAAATTTTGCTTGAAGCTGAAGTTGATGTTCGTGAT
GTAACTGAAGAAGAAGGTAATATTGTTATTTATACTGAACCTACTGACCTTCATAAAGGAATCGCGGCGCTAAAAGCGGC
TGGAATCACTGAGTTCTCAACAACAGAATTAGAAATGATTGCTCAAACTGAAGTTGAGCTTTCCCCAGAAGATTTAGAAA
TCTTTGAAGGGCTTGTTGATGCCCTTGAAGATGACGACGATGTACAAAAAGTTTATCATAACGTCGCAAATCTCTAA
>Cluster_24995
ATGACCATTGAAACACTGCGCGGGCGCTATCGCCTGCATCGATTTCCGCCGCGCCAGCGTGCGGTGACCGGAGAACCGCT
CACCGCCAGCATTAGCGCCGCCGATTACCAGCGTCAGTTGATGGACGGTTTTCAGGAAGGGCTGCAAAAAGGGTTTGCTC
AGGGAATGACCGAAGGTCAGGAGCAGGGCTTTAGCGAAGGTCATCAACAAGGTTTTGCCGAGGGGCGACGTCAGGGCTAC
ACCGAAGGCAGCCTCGCCGGGCAGCAGGAAGGGCGCAAACAGTTTGTTGAGGCGGCGCAGCCGCTGGAAGCCATCAGCGG
AAAAGTGAACGACTTTCTCGCCCATATCGAACGCAAACAGCGGGAAGATTTGCTGCAACTGGTGGAAAAAGTCACCCGCC
AGGTGATCCGCTGCGAACTGGCGCTGCAACCGACGCAGTTACTGGCGCTGGTGGAAGAGGCGCTGGCGGCCTTCCCGACG
ATGCCGGAGACCCTGCAAGTGATGCTGAGTACGGAAGAGTTTAACCGCCTGCGCGACGCGGTGCCGGAAAAAGTCAGCGA
ATGGGGGCTGACGCCTTCGCCGGATCTGCCGCCGGGGGAATGCCGAGTGATTACCGACAAATCTGAACTGGATATCGGCT
GTGAGCATCGCCTTGAACAATGCATGACCGCGCTGCAAGAGACGCTGACGCCGGAGTCACAGGGTGAGTGA
>Cluster_21117
ATGCGTCTGAAGGCACTGGCCTCCCTGGTGCCCCTGGAAAAAATATCCTGGATCGCGGGCAGTGAACGCGCATGTTTATT
TTCCTGGTATCTGTTGATGCAGTTTATCCAGAATAACAGGACAAAAATAAGCGCAGACTTGCTTCAAAAGAATAAACTGT
ATCTTAAAGAAGAGTATTTAGAAGGGAATGCCTTCCCGTCGGACTCATCGACACAGTTCAGACAAATACTCAGAGTTCTC
GATATTCTTTCTGATAAGAATTTGCGGGATGAGTGGATAATCCAGACCAAAGACAGATGGATACGGGCATTTAAATCAAA
GAGTCCTTTCAGTTACCTCCTCCCGGAGAACGAACATGAATGCATATGGACATGGAATTATTTGAAGGGAAAAAATATTG
CACTGGAAAAACTGGCCAGTTTTCCTGATTCTGCTGATATTTACCACGCAATTCACCTTTCATTTGACATATGGGTTACC
TGTCCACTCACCTCACCTGACGATATAAAAAATTTCAGAAACAGTTTCAATAAGGCCAAGGCGCAGCGCAAGTACAAAAA
AATGCAGGAAGATAAAGTCAATGTACAGTTTTTTCTTGACGCAGAAACCAGGGCGCAGCTCAAAGAATTATCGAGGGTCA
GAAGACTCAGTACCGGCGAAATGCTGCATGACCTGATTGTTGAAGAGTATAAAAGGTACCGACATTCCAGATAA
>Cluster_28678
ATGAACCCATTCAAAGGCCGGCATTTTCAGCGTGACATCATTCTGTGGGCCGTACGCTGGTACTGCAAATACGGCATCAG
TTACCGTGAGCTGCAGGAGATGCTGGCTGAACGCGGAGTGAATGTCGATCACTCCACGATTTACCGCTGGGTTCAGCGTT
ATGCGCCTGAAATGGAAAAACGGCTGCGCTGGTACTGGCGTAACCCTTCCGATCTTTGCCCGTGGCACATGGATGAAACC
TACGTGAAGGTCAATGGCCGCTGGGCGTATCTGTACCGGGCCGTCGACAGCCGGGGCCGCACTGTCGATTTTTATCTCTC
CTCCCGTCGTAACAGCAAAGCTGCATACCGGTTTCTGGGTAAAATCCTCAACAACGTGAAGAAGTGGCAGATCCCGCGAT
TCATCAACACGGATAAAGCGCCCGCCTATGGTCGCGCGCTTGCTCTGCTCAAACGCGAAGGCCGGTGCCCGTCTGACGTT
GAACACCGACAGATTAAGTACCGGAACAACGTGATTGAATGCGATCATGGCAAACTGAAACGGATAATCGGCGCCACGCT
GGGATTTAAATCCATGAAGACGGCTTACGCCACCATCAAAGGTATTGAGGTGATGCGTGCACTACGCAAAGGCCAGGCCT
CAGCATTTTATTATGGTGATCCCCTGGGCGAAATGCGCCTGGTAAGCAGAGTTTTTGAAATGTAA
>Cluster_20967
ATGCCACATCACATTGTTATTGTTGAAGATGAGCCGGTTACCCAGGCGCGATTACAATCTTACTTCACTCAGGAGGGGTA
TACCGTTTCCGTTACGGCGAGCGGTGCCGGGCTGCGGGAAATTATGCAGAATCAGCCGGTGGATTTGATTCTGCTGGATA
TCAATTTGCCCGATGAAAATGGCCTGATGTTAACCCGCGCCCTGCGAGAACGCTCAACGGTGGGGATTATTCTGGTTACC
GGACGCAGCGATCGGATTGACCGTATTGTTGGGCTGGAAATGGGCGCAGACGATTACGTCACCAAACCGCTGGAACTGCG
CGAACTGGTGGTACGAGTGAAAAACTTGCTCTGGCGAATCGACCTCGCGCGACAGGCGCAACCGCACACTCAGGACAACT
GCTATCGCTTTGCCGGTTATTGCCTGAATGTGTCGCGCCATACGCTGGAGCGGGATGGCGAGCCGATTAAACTGACCCGC
GCAGAGTATGAAATGCTGGTGGCATTTGTGACTAATCCGGGCGAAATTCTCAGCCGTGAACGTCTGCTGCGTATGCTCTC
TGCGCGTCGGGTGGAAAACCCTGACCTGCGCACCGTCGATGTGTTAATTCGTCGTTTACGCCATAAACTCAGTGCGGATT
TACTGGTGACACAACATGGCGAAGGTTATTTCTTAGCCGCTGATGTGTGCTGA
>Cluster_40055
ATGTCAAACACGCCAATCGAGCTTAAAGGCAGTAGCTTCACTTTATCTGTGGTTCATCTGCATGAGGCAGAACCTAAGGT
TATCCATCAGGCGCTGGAAGACAAAATCGCTCAGGCCCCCGCATTTTTAAAACATGCCCCCGTTGTACTCAACGTCAGTG
CACTGGAAGACCCGGTAAACTGGTCAGCGATGCATAAGGCGGTTTCGGCAACTGGTTTGCGGGTTATTGGCGTAAGTGGC
TGCAAAGATGCGCAACTTAAAGCCGAAATTGAAAAGATGGGGCTGCCTATCCTGACGGAAGGAAAGGAAAAAGCGCCGCG
TCCAGCCCCCGCACCGCAGGCTCCAGCGCAAAATACAACGCCGGTCACAAAAACGCGTTTAATTGATACCCCAGTGCGTT
CCGGTCAGCGTATTTATGCTCCACAATGTGATCTGATTGTTACAAGCCACGTTAGCGCTGGGGCCGAATTGATTGCCGAT
GGTAACATTCATGTCTATGGCATGATGCGCGGTCGTGCGCTGGCAGGGGCAAGTGGTGACCGGGAAACGCAAATATTTTG
TACGAACCTGATGGCGGAACTGGTGTCCATCGCAGGTGAATACTGGCTGAGTGATCAAATCCCAGCAGAATTTTATGGCA
AAGCGGCACGACTGCAGTTAGTCGAAAACGCTTTGACCGTTCAACCGTTAAATTGA

>Cluster_39484
ATGTTATATGCATTTAAGCTGGGCAGAAAACTGCGCGGCGAGGAACCTTATTGCCCTGAAAAAGGCGGGAAAGGTGGTAG
CTCTGATAAAAGCGCAAAGTATGCCGCAGAAGCTCAGAAGTATGCAGCAGACCTGCAAAATCAGCAGTGGCAGACGATCA
TGAAAAACCTTGCTCCGTTCACGCCTCTTGCGGAGCAGTATGTTAACCAGCTTCAGAACCTTTCCAGTTTAGAAGGTCAG
GGGCAGGCACTTAATCAGTATTACAACTCTCAGCAGTATAAAGACCTTGCAGGTCAGGCTCGTTACCAGAGTCTTGCTGC
TGCGGAGGCTACGGGAGGACTTGGTTCGACAGCCACAAGCAATCAACTGGCTACGATCGCGCCGACTCTCGGTCAGTCTT
GGTTATCAAACCAGATGAGCAATTACAACAATCTGGCAAACGTTGGGCTTGGTGCTCTGCAAGGTCAGGCAAACGCCGGG
CAGACGTACGCCAACAACATGAGCAGCATTGCACAGCAAAGCGCAGCTCTTGCCGCTGCTAATGCCAACAAACCATCAAG
TCTTCAGACAGCAATTAGTGGCGGAACGTCTGGTGCGATTGCCGGTGCAGGTCTTGCCAGCCTTTTGGGAACATCAACAC
CTTGGGGCGCTGGCATTGGTGCTGGTATCGGATTGCTTGGCTCGTTGTTTTAA
>Cluster_23269
ATGCTGCGCTTATTTGCCCACCTTCTTCTCCGTATTTGTTTTTCTCGCCGGACTCTTAAAATTGGCTGTTTATTGTTGCT
TATAGCAGGAGCGACCATTTTTATTGCCGATCGCGTTATGGTCAACGCCAGCAAGCAGCTGACCTGGAGTGATGTCAACG
CCGTTCCGGCGCGGAACGTGGGTTTATTGCTGGGGGCGAGGCCGGGTAATCGCTACTTTACCCGACGTATTGATAGCGCC
GCAGAGTTGTACCACGCCGGAAAAGTGAAATGGCTATTGGTGAGCGGCGATAATGGACGTAAAAATTACGATGAAGCGTC
AGGAATGCAGCAAGCCTTGATCGCTAAAGGCGTACCGGCAAAAGTGATCTTCTGCGACTATGCCGGATTCTCAACGCTTG
ATTCGGTGGTGCGTGCCAACAAGGTGTTTAGTGAAAACCATATCACCATTATTTCGCAAGAATTTCATAACCAACGCGCC
ATCTGGCTGGCAAAACAGTACGGCATCGACGCTATCGGCTTTAACGCGCCAGACCTCAATATGAAGCACGGTTTTTATAC
TCAGTTACGGGAGAAACTCGCCAGAGTCAGCGCGGTGATTGACGCAAAAATCCTTCATCGCCAGCCGAAATATCTCGGGC
CATCGGTCATGATTGGGCCGTTTAGTGAGCATGGCTGTCCGGCGAAAGAATAA
>Cluster_22623
ATGAAACATGACCATTTTGTTGTTCAAAGCCCGGATAAACCAGCACAACAGTTGTTACTGCTTTTCCACGGTGTCGGGGA
TAACCCGGTGGCAATGGGGGAAATAGGCAGCTGGTTTGCGCCGCTGTTTCCTGATGCGCTGGTGGTGAGTGTCGGTGGCG
CGGAGCCGAGCGGTAATCCGGCGGGGCGTCAGTGGTTTTCGGTGCAGGGTATTACGGAAGATAATCGCCAGGCGCGTGTG
GATGCCATCATGCCGACGTTTATTGAAACGGTACGCTACTGGCAGAAACAAAGCGGAGTAGGTGCTAACGCCACGGCGCT
AATTGGTTTCTCACAAGGCGCGATTATGGCGCTGGAGAGCATTAAAGCCGAACCGGGCCTTGCTTCGCGCGTCATTGCCT
TTAACGGACGCTACGCGAGCCTGCCGGAAACCGCGTCTACAGCCACGACAATTCACCTGATTCATGGCGGTGAAGATCCT
GTTATCGATCTGGCGCATGCGGTTGCGGCGCAGGAAGCGTTAATCAGTGCCGGTGGTGACGTGACGCTGGATATCGTGGA
GGATTTAGGCCATGCGATCGACAATCGCAGTATGCAATTTGCCCTGGATCACTTGCGTTATACCATTCCGAAACATTATT
TCGACGAAGCGCTAAGTGGCGGTAAGCCTGGCGATGATGATGTGATTGAAATGATGTAA
>Cluster_24128
ATGCAGATAAAACGCTCGATTGAGAAAATCCCGGGGGGGATGATGCTCGTCCCGCTATTCCTTGGCGCACTGTGCCACAC
CTTCTCGCCGGGGGCGGGGAAATATTTTGGATCATTCACCAACGGGATGATTACCGGTACGGTGCCCATTCTGGCGGTGT
GGTTTTTTTGCATGGGGGCGTCAATAAAATTAAGCGCGACGGGAACGGTACTGCGTAAATCCGGTACGCTGGTGGTAACT
AAAATTGCCGTCGCGTGGGTGGTTGCGGCAATTGCCTCGCGCATTATTCCGGAACATGGTGTTGAAGTTGGATTCTTTGC
CGGACTTTCAACGCTGGCGCTGGTGGCGGCGATGGATATGACCAACGGCGGACTTTACGCTTCCATCATGCAGCAGTACG
GCACAAAAGAAGAAGCTGGGGCATTTGTGTTGATGTCGTTGGAGTCCGGGCCGCTCATGACGATGATTATTCTGGGCACT
GCCGGAATTGCCGCTTCCAGTTCCGCAGGGGCCGCGGTAGCGACACCTGTGCTGATTGCAGAAATGGTGCCTGCGTTTAA
ACCTATGGCTCCGGCAGCAACTTCGCTGGTAGCGACGGCGGTCATTGTGACTTCGATTCTGGTGCCAATTCTTACCTCTA
TCTGGTCACGTAAAGTCAAAGCCAGAGCGGCGAAAATCGAAATTTTAGGTACGGTGAAATAA
>Cluster_10574
ATGAAAGAGATGATTTCTGTCGATCATGAAATATCCATGAGTAGTCTGGATTTTCTGAATAACATTATTAATCCAGCCCG
GGCAGAAGCCGGAGAAGTCCCTCATGAACCGCGTAAGTTTCTTGCAAAAATTGAGGATGAGCTAGAGCTTGATGGAACCG
GAAAAAAAATTCCGGTTAAACAATAACCAGACAAGAACGGCATACTATGATCTGGATTTTGACCAGATGATGCTCGTTGGC
ATGAGGGAGTCAAAGGCCGTTCGTCGTTCTGTGCTGGCAAGACTCAAAGCGATGCATGGTATTCAGATCCCCCGGACTTT
ACCTGAGGCGTTGCGATTTGCGGAAAAACTGGCTGAACAGAAAGCAGTGCTGGAAAATCAACTGGCAATAGCAGCGCCGA
AGGCTGAATTTGTTGATAACTATGTTGAAGCATCTGGTCTGATGGGATTCCGGGAAGTTGCTAAGTTACTCGGTATCAAA
GAAACCGATTTCCGGCTGTTTTTGTTGGAGAACGGAATAATGTATCGCCTCGCTGGAAAAATGACGCCTTACTCGCATCA
CCTGGATGCGGGGCGGTTTAGCGTGAAAACGGGCGAGGCGGCAATGGTCATGCTTTTACGCAGGTTAAATTCACCCCAA
AAGGTGTTCAGTGGATTGCTGGTCTGCTTGCTGCATGGAGAGCTACCGCAGCATGA
>Cluster_21169
ATGTCCATTGCGGTAACCACCACCGATCCGACGAATACCGGCGTCAGTACCACCAGCAGTAGTTCGCTCACGGGCAGCAA
CGCCGCAGATTTACAAAGCAGTTTTCTGACTTTGTTGGTGGCGCAGCTGAAAAACCAGGACCCGACCAATCCAATGGAAA
ACAACGAGCTGACGTCGCAATTGGCACAAATCAGCACGGTCAGTGGGATTGAAAAACTCAATACCACGCTCGGATCTATT
TCCGGACAGATTGATAACAGCCAGTCGTTACAGGCCAGTAACCTGATTGGTCACGGCGTGATGATCCCCGGTACCACTGT
TCTTGCGGGAACCGGCAGTGAAGAAGGGGCTGTGACCACGACCACGCCGTTTGGTGTTGAGTTGCAACAGGCAGCAGACA
AAGTGACGGCCACCATCACCGATAAAAACGGCGCGGTCGTCCGTACCATTGATATCGGTGAACTGACTGCCGGAGTCCAC
AGTTTCACCTGGGACGGTACGTTGACTGACGGCAGCACTGCGCCGAACGGTTCTTACAATGTAGCGATTAGCGCCAGTAA
CGGTGGTACACAACTGGTTGCCCAACCGCTGCAGTTTGCTCTGGTGCAGGGGGTGATCCGCGGCAACAACGGTAATACGC
TGGATCTTGGCACTTACGGCACCACTACCCTCGACGAAGTACGGCAGATAATTTAA
>Cluster_8813
ATGTTAATTAATCGCAATATTGTGGCGTTATTTGCGTTGCCTTTTATGGCAAGCGCAACTGCTTCTGAATTATCCATTGG
TGCTGGTGCGGCTTATAATGAATCGCCTTATCGCGGTTATAATGAAAATACGAAGGCAATTCCGCTGATTAGTTATGAAG
GTGATACTTTTTATGTTCGTCAGACCACGTTAGGTTTTATTCTGTCGCAAAGTGAAAAAAATGAACTTAGCCTGACCGCA
TCCTGGATGCCGCTGGAATTTGACCCTACCGATAATGACGATTATGCCATGCAACAGCTTGATAAGCGTGATAGTACGGC
TATGGCGGGGGTTGCCTGGTATCACCACGAGCGTTGGGGAACCGTGAAAGCCTCTGCAGCTGCGGACGTTCTGGATAACA
GCAACGGCTGGGTGGGGGAGCTATCGGTATTCCACAAAATGCAGATAGGTCGTCTGTCGCTGACACCTGCGCTGGGCGTT
CTCTATTATGACGAGAATTTCAGTGACTATTACTATGGCATTTCAGAGAGTGAGTCCCGTCGTAGCGGTCTGGCAAGTTA
TTCCGCGCAGGATGCCTGGGTGCCCTATGTCAGCCTGACGGCAAAATACCCGATAGGAGAGCACGTCGTATTGATGGCGA
GCGCAGGATACAGCGAGCTGCCGGAAGAGATTACCGACAGCCCGATGATTGATCGTAATGAGAGTTTTACCTTTGTCACC
GGGGTGAGCTGGCGTTTTTAA
>Cluster_1286
ATGACCACACCAAATCCACTGGCAAAGACGAAAGGTGCGGGGACGACGTTCTGGATGTATACCGGCAACGGCGACGCATT
TGCGAACCCTTTGTCGGACACTGACTGGCTGCGTCTTGCGATGGTGAAGGATCTGCAACCTGGCGAAATGACCGCTGATG
CAGAAGATGATACTTATCTCGATGATGAAGATGCAGACTGGAAAACGACAACCCAGGGGCAGAAATCAGTCGGTGATACT
TCGGCGACGCTGGCCTGGCGTCCGGGTGACAGCGGGCAGAAAAAACTGGTTCAGTTGTTCGACTCCGGTGAAGTCTGCGC
GTTTCGTATCAAATATCCCAACGGTACTGTTGATGTTTTCCGTGGCTGGCTGAGTTCACTGGGTAAAACCATTGCCTCAA
AAGACGTGATGACCCGCACTGTGAAAATCAGCGGTGTGGGGCGTCCGTATCTGGCAGAGGAAGGCACTGAAACAGTGAGC
GTTACCGGGCTGACGGTGGCACCGGCATCTGCCAGTGTAAAAGTGGGAGCAACCACCACGCTGACCTTTACAGTAAAACC

TGACGGAGCCAGTGACAAAGCGATCAGTGTGCATTCGTCAGATCCACAGACTGCCACGGTGACCCTGAACGGGCTTGTAG
CCACGGTGAAAGGCGTGAAGCAGGGCAGTGTCAGCATTGTGGGCATGACCGCTGACGGGAATTTTGTGGCTGTGGCTGCG
GTGACTGTCAGCGCCGCAGGTTAA
>Cluster_7813
ATGTCACAGTCTACATCCGTGCTTCGTCGTAATGGATTTACTTTTAAACAGTTTTTTGTTGCTCACGATCGCTGTGCGAT
GAAAGTGGGAACGGATGGTATTTTATTGGGCGCATGGGCACCGGTGGCTGGGGTAAAACGTTGCCTTGATATCGGCGCGG
GTAGCGGGTTGCTGGCATTAATGCTGGCGCAGCGAACCGATGACAGCGTGATGATTGATGCAGTTGAACTGGAAAGTGAA
GCTGCCGCGCAGGCGCAGGAAAATATCAACCAGTCCCCGTGGGCAGAGCGGATTAACGTCCATACGGCGGATATTCAGCA
GTGGATCACACAGCAGACAGTACGCTTCGATTTAATCATCAGTAACCCACCTTACTATCAGCAGGGAGTAGAGTGCTCGA
CACCTCAACGGGAACAGGCTCGCTATACCACTACGCTTGATCACCCATCGTTGCTGACCTGTGCGGCGGAGTGCATTACC
GAAGAGGGATTTTTCTGCGTAGTGCTGCCAGAGCAGATTGGTAATGGTTTTACGGAGCTGGCATTAAGCATGGGCTGGCA
TTTACGTTTGCGGACGGATGTGGCGGAAAACGAAGCGCGACTGCCGCATCGGGTGCTGCTGGCATTCTCCCCGCAGGCGG
GAGAATGCTTTAGCGATCGCTTAGTCATTCGTGGGCCAGACCAGAACTATTCCGAAGCGTATACGGCGCTGACCCAGGCT
TTTTATCTGTTTATGTAA
>Cluster_24980
ATGCGTAAAATCGCATTAATTCTTGCGATGTTGTTGATCCCATGCGTTTCGTTTGCCGGTTTGCTGGGCAGCAGTAGCTC
GACCACGCCAGTGAGCAAAGAGTATAAGCAGCAGTTGATGGGATCCCCTGTCTACATCCAGATCTTCAAGGAAGAACGTA
CGCTCGATCTCTACGTCAAAATGGGCGAGCAATATCAACTGCTCGACAGCTATAAAATCTGTAAATATTCCGGCGGCTTA
GGGCCAAAACAGCGTCAGGGCGATTTCAAAAGCCCGGAAGGGTTTTATAGCGTCCAGCGTAATCAGTTAAAACCAGACAG
CCGTTACTACAAAGCGATTAATATCGGTTTCCCCAATGCCTATGACCGTGCGCATGGTTATGAAGGGAAATACCTGATGA
TTCACGGCGATTGTGTTTCCATCGGCTGCTACGCGATGACCAATCAGGGTATTGATGAGATCTTCCAGTTCGTTACTGGT
GCGCTGGTGTTTGGTCAGCCGAGCGTGCAAGTGAGTATTTATCCGTTCCGCATGACCGACGCCAATATGAAGCGCCATAA
ATATTCCAACTTTAAGGACTTCTGGGAGCAACTGAAGCCGGGCTACGACTACTTTGAGCAAACCCGTAAGCCACCGACCG
TTTCTGTGGTCAATGGCCGTTACGTGGTCAGCAAGCCGTTAAGCCACGAAGTTGTGCAGCCACAACTGGCATCAAACTAC
ACGCTCCCCGAGGCAAAATAA
>Cluster_63630
ATGAAAAATATTGCGACAGGCGATGTTCTTGAACGTATCCGCAGACTGGCCCCGTCACATGTAACCGCGCCATTCAAGAC
GGTAGCGGAGTGGCGCGAGTGGCAACTTTCCGAAGGCCAGAAACGTTGTGAGGAGATCAACCGTCAGAATCGTCAGTTGC
GGGTGGAAAAAATTCTGAATCGCTCTGGCATCCAGCCATTGCACCGCAAATGCTCGTTTTCGAATTACCAGGTGCAGAAC
GAAGGGCAGCGATACGCGTTGAGTCAGGCGAAATCCATCGCTGATGAACTGATGACCGGGTGTACAAATTTTGCGTTCAG
CGGAAAACCTGGTACCGGGAAGAACCACTTAGCGGCAGCTATCGGGAATCGCCTGCTGAAAGACGGTCAGACAGTGATTG
TGGTTACCGTGGCTGATGTTATGAGTGCCCTGCACGCCAGCTATGACGATGGGCAGTCAGGCGAAAAATTTTTGCGGGAA
CTGTGCGAAGTGGATCTGCTGGTTCTTGATGAAATTGGCATTCAGCGCGAGACGAAAAACGAGCAGGTGGTACTGCACCA
GATTGTTGATCGCCGGACAGCGTCGATGCGCAGCGTGGGGATGCTGACAAACCTGAACTATGAGGTCATGAAAACATTGC
TCGGCGAGCGGATATTGGATCGCATGACCATGAACGGCGGGCGATGGGTGAATTTTAACTGGGAGAGCTGGCGTCCGAAT
GTCGTCCAGCCAGGAATTGCGAAGTAA
>Cluster_41486
GTGGTAAACTTCTCGCGATTTTGTGAAATCCTGGTTGAGGTATCTCTGAACACACCGGCACAACTCTCTTTGCCACTTTA
TCTTCCTGACGACGAAACCTTTGCAAGTTTCTGGCCGGGGGATAACTCCTCTTTACTGGCCGCGCTGCAAAACGTGCTGC
GTCAGGAACATAGCGGTTACATCTATCTCTGGGCACGCGAAGGCGCGGGGCGCAGCCATCTGCTGCACGCGGCTTGCGCG
GAATTGTCACAGCGTGGCGATGCGGTGGGCTATGTCCCGCTGGATAAACGCACCTGGTTTGTTCCGGAAGTGCTCGACGG
TATGGAGCATTTGTCGCTGGTCTGTATTGACAACATTGAGTGTATTGCCGGCGATGAGTTGTGGGAGATGGCGATTTTCG
ATCTCTACAATCGAATTCTGGAATCGGGCAAAACACGGTTGTTGATCACCGGCGATCGTCCACCGCGGCAGTTGAATCTG
GGATTACCGGATCTCGCGTCGCGTCTCGACTGGGGGCAGATCTACAAATTGCAGCCACTTTCTGATGAAGATAAGTTGCA
GGCGCTACAGTTACGCGCGCGTTTGCGTGGTTTTGAACTGCCGGAAGATGTGGGGCGTTTCTTGCTGAAGCGGCTCGACA
GAGAAATGCGCACGTTGTTTATGACGCTGGATCAGCTGGATCGTGCGTCGATTACCGCGCAACGCAAGCTGACCATTCCG
TTTGTGAAAGAAATTCTGAAGTTGTAG
>Cluster_5259
ATGCAGACCCAGGTCCTTTTTGAACATCCACTAAATGAAAAAATGCGTACATGGCTGCGCATTGAGTTTTTGATTCAGCA
ACTCACCGTTAATTTACCCATCGTTGACCACGCTGGCGCGCTGCATTTCTTCCGTAATGTCAGTGAATTACTGGATGTTT
TCGAGCGCGGCGAAGTCCGCACTGAGCTGTTGAAAGAACTTGACCGGCAGCAACGTAAACTCCAGACCTGGATTGGCGTG
CCTGGCGTGGACCAGAGCCGTATTGAAGCATTAATTCAGCAGTTAAAAGCGGCGGGGAGCGTATTAATTTCCGCGCCGCG
TATCGGGCAATTTCTGCGTGAAGATCGTTTGATTGCTCTGGTGCGTCAGCGACTGAGCATCCCAGGCGGCTGTTGCAGCT
TTGATTTACCTACATTGCACATTTGGCTGCATCTACCCCAGGCGCAGCGCGACAGCCAGGTAGAAACCTGGATTGCCAGC
CTGAACCCGCTCACCCAGGCACTTACCATGGTGCTGGATTTAATTCGCCAGTCGGCCCCCTTCCGTAAACAAACCAGCCT
GAATGGTTTTTATCAGGATAACGGTGGCGATGCCGACTTGCTGCGCCTGAATCTGTCGCTCGATTCACAGCTTTATCCGC
AAATTTCCGGACATAAGAGCCGTTTTGCCATTCGTTTTATGCCGCTGGACACTGAAAACGGACAGGTACCGGAACGTCTG
GATTTCGAACTGGCCTGTTGCTAA
>Cluster_52586
ATGGAATTAGTTGATACGTTATTTGCAAGCCTCTCCGGTACTGAACCATTCACTGGCGTGGATATTACGATTGCCAACTG
CAAATCGACATACTGGGATGAAGGCATTGTTCAGCAACTCATTAACCAGGTCCTGGATGAAGGGGAAAAGTTCGCCGGTG
CTGCCGGTCTTGAGGGATTATCTCGATATGATGTCACATTAAATATTGGTTTAACGTCCAGCAATGTATGGCCGGGATTT
TCCCTCGATACCGCCACCATTAGCCGTCTTTGTGCCTGTGGTGCTGACTTTGGATTTGACCTCTATATCAGTGATGTACC
AGATGTTCAATGTGATCTGAACACGACAAATGACTTCACCGTACAATTTACGGCGATGCTCAACCCGGATGAGCGGGTGA
TCATAGCAAAGCGTCCGCTTAAAAAATGTGACGCCTGGATTGAGGATGTTTATATCTTTCAGGTGTTTAAAGAAGCCTGG
CAATTTCAAAACGGTAACAGTTTACGCGGATTTCGCGATAAGCAGGCTGAACTGAAACTCTACGCGCGTCATTACTCAGT
GGAAAATTGCACAGAGGAAAGCTGTTGGGACTGTAATTATTGCATTCGGCCCAGCTTTTCCCTCTCTCGTAGTGCCATTA
TTCGCCTGAATGCGGCGAATGCTCGTTTTGTTTATCAACCGTTTACACGCGATCAGCGGGCGAGGGGTAATTATGCAACC
CAGCCCCGCTATGCAGGCATTAATTGA
>Cluster_24249
ATGATTAAACAAACTATTGTCGCGTTGCTTTTGAGTGCGGGGGCGTCATCAGTCTTCGCGGCGGGAACCGTCAAGGTGTT
CAGCAATGGCAGCAGTGAGGCTAAAACGCTGACGGGCGCAGAGCATTTAATCGATCTGGTAGGCCAACCGCAGCTGGCAA
ACAGCTGGTGGCCTGGTGCGGTGATTAGCGAAGAGCTGGCCACGGCGGCAGCATTGCATCAGCAGCAGGCGTTGCTGACA
CGGCTGGCAGAACTGGCTGCAGACTCCAGCGCCGACGATGCCGCCGCGATTAACGCCTTACGCCAGCAAATTCAGGCGTT
GAAAGTGACGGGCAGGCAAAAAATCAATCTTGATCCTGATATCGTCCGCGTTGCCGAACGCGGTAACCCGCCGTTGCAGG
GCAACTACACTCTGTGGGTCGGAACGCCGCCGTCCACGGTCACGTTGTTCGGGCTTATCAGTCGTCCTGGCAAGCAGTCA
TTCACTCCAGGTCGCGACGTGGCGAGCTATCTCTCTGACCAGAGCCTGCTCAGCGGTGCGGATCGCAGCTACGCGTGGGT
GGTTTACCCGGACGGACGCACGCAAAAAGCGCCGGTGGCTTACTGGAACAAGCGTCACGTAGAGCCGATGCCCGGCAGCA
TTATTTATGTTGGCCTCGCGGACTCCGTCTGGAGTGAGACACCTGATGCCCTTAACGCCGACATTCTTCAGACTCTGACG

CAGCGGATACCCCAATAA
>Cluster_54812
ATGGTCATTAAGGCGCAAAGCCCGGCGGGTTTCGCGGAAGAGTACATTATTGAAAGTATCTGGAATAACCGCTTCCCTCC
CGGGACTATTTTGCCCGCAGAACGTGAACTTTCAGAATTAATTGGCGTAACGCGTACTACGTTACGTGAAGTGTTACAGC
GTCTGGCACGAGATGGCTGGTTGACCATTCAACATGGCAAGCCGACGAAGGTGAATAATTTCTGGGAAACTTCCGGTTTA
AATATCCTTGAAACACTGGCGCGACTGGATCACGAAAGTGTGCCGCAGCTTATTGATAATTTGCTGTCGGTGCGTACCAA
TATTTCCACTATTTTTATTCGCACCGCGTTTCGTCAGCATCCCGATAAAGCGCAGGAAGTGCTGGCTACCGCTAATGAAG
TGGCCGATCACGCCGATGCCTTTGCCGAGCTGGATTACAACATATTCCGCGGCCTGGCGTTTGCTTCCGGCAACCCGATT
TACGGTCTGATTCTTAACGGGATGAAAGGGCTGTATACGCGTATTGGTCGTCACTATTTCGCCAATCCGGAAGCGCGCAG
TCTGGCGCTGGGCTTCTACCACAAACTGTCGGCGTTGTGCAGTGAAGGCGCGCACGATCAGGTGTACGAAACAGTGCGTC
GCTATGGGCATGAGAGTGGCGAGATTTGGCACCGGATGCAGAAAAATCTGCCGGGTGATTTAGCCATTCAGGGGCGATAA
>Cluster_48548
GTGAAAGTTGTCGGCAAAACACTGTCTGAAATCCGTAATTTAGTCAGCAGTAAACTGGCTACCTATATTGAATCCCCACA
AGTGGATGTCAGTATTGCGGCATTCCGCTCACAAAAAATGTATGTTTCTGGTGAAGTCGCTAAACCAGGTACATTGCCAA
TTACCAATATCCCTCTGACCGTTCTGGAAGCCTTTAATAACGCGGGTGGCCTCACCGAAAAAGCCGATTGGGATAACGTC
GTGTTGACCCGTAATGGCAAAGAAATTAAACTCTCATTGCAATCATTGGTACAGTATGGCGATATGACCCAAAACTATCT
AATGATCCCAGGTGATACACTTTATGTACCACGCAACGACGGGCAAAAAGTATTCGTGATGGGTGAAGTTGGTCAGCCAA
ATACCCTAACTATTGACCGTGCAGGTATGAGTATCACTGAAGCACTGAGTAAAGCAGGGGGGACCGATCAAACAACCGCT
AATGCAACCGGTGTGTTTGTTATCCGTCCAATTAAAAACCAAACCTCTGGTGATAAAGAACTTGATGCGTTGCTACCGAA
AAAAATGGCAGCAGTGTATCAGTTAGACTTATCTGATGCGACCGCAATGGTGATGGGAACTGAGTTTAAGCTACAACCAT
ACGATCTGGTTTATGTTACCGCCGCTCCGGTTGTGCGTTGGAACCGTCTGATCACTCAGTTATTACCAACTATCCGATAA
>Cluster_10843
ATGTCAACCATAGCAACATCACTTGGTAGTAACATTGGTTACATGAAAAACACTATCAGCGAACGTATTCGGAATCGTCG
AAAAGACGTTGGATTAACCCAACAGCAGGTTGCGAAAGCAATCGGCATATCTCGTGTATCCGTAACAAAATGGGAAAATG
GCTCTTCAAAACCTGACGGTGAGAATTTGTATCTACTGTCAAAATTGCTTTCCAAATCTCCTGAATGGATTCTTTATGGA
AAGGACGGTCACGATAAAGCCGATGATCTGCGTCTGAATCAGTACCCTTACATTAGTGACAACATCGCCCGGTTGCCCGT
TTTAACGTGGGAACAGGCTGGTTATTGGGATATGAGTTGTCCAGTAACCGAGATTCCTGGTATTAAGAATTGGGTTGATG
TCATGACAAAAACCGCTGAAAACTCTTTTTTATTGCATGTTGAGGGAGATGCGATGACAAACTCTAACGGCCTCCCAACC
ATCCCCGACGGATCTACCGTGCTGATCACACCATGCTCAAGTAACATTAGAGAACTGGTGGGAAAAATAATCTTAATCCA
ATTGGAAGGAACGCCAAACGTAACACTAAAAAAAGTTGCGATTGACGGACCAAACATCTATCTGTTGTCACTGAATCCGC
TTTACAAACCCATCGAACTGAATGGTGGTTACACCATTAAAGGTAAAGTTTCACAAATACATCAATACTTAGACTGA
>Cluster_33300
ATGCGTTTTTACCGGCCTTTGGGGCGCATCTCGGCGCTCACCTTTGACCTGGATGATACCCTTTACGATAACCGTCCGGT
GATTTTGCGCACCGAGCGAGAGGCGCTTACCTTTGTGCAAAATTATCATCCGGCGCTGCGCAGCTTCCAGAATGAAGATC
TGCAACGCCTGCGCCAGGCGGTACGGGAAGCGGAACCCGAGATCTATCACGACGTGACGCGCTGGCGTTTTCGTTCGATT
GAACAAGCGATGCTCGACGCCGGGCTGAGTGCCGAAGAAGCCAGTGCAGGCGCACATGCAGCAATGATCAACTTTGCCAA
ATGGCGCAGCCGGATCGACGTCCCGCAGCAAACCCACGACACCTTAAAACTGCTGGCGAAGAAATGGCCGCTGGTGGCGA
TCACCAACGGTAACGCCCAGCCGGAACTGTTTGGTTTGGGGCATTATTTTGAGTTTGTGCTGCGCGCTGGCCCGCACGGG
CGCTCAAAACCGTTCAGCGATATGTACTTCCTGGCGGCGGAAAAACTCAACGTGCCGATCGGCGAGATCTTACATGTCGG
GGACGATCTCACCACCGATGTGGGGGGCGCGATCCGTAGTGGAATGCAGGCCTGTTGGATCAGACCGGAAAATGGCGATC
TGATGCAAACCTGGGACAGCCGTTTACTGCCGCATCTGGAAATTTCCCGGTTGGCATCTCTGACCTCGCTGATATAA
>Cluster_20786
ATGTGGCGTGAAGCGCGTCTGGCTTTCACAGACTCACTGGCCGCGCTGAATTGTTCCGTTGTTCCGGCGCATCCGTGGAT
AAACGGTCTGGGGCAACAGACGGATAACGGGGCATACCTCAGCCCGGTGAATGCCGTCCGCTATTTTGCTGAAAGGCTGG
CCGGAACGGGCGGGAATGCCGATGTGATGATCATGATGGTGACAGGCCAGACGCATGAGAATTTTATGACCCGTCTCAAC
GGTCTGGTCGATGTTTTTCCGGCACCGGCATTCACGCAGGTCAAGCGCCTGGCACAATCCGCAGCGACGCTGGCCATCGA
AAAAATGCAGATCCCCGCTAAAACCGGGGCGGGGTTGCCGGTGGCCATTCCGCTGTCTGTGCCAACCAGCAGGGTGGCAT
TATCTGCGGCGGCTGTCAGCCAGGCACAAAAAGCGGCCAGTGCGGGATTCAGCCTGGACGGACTAAAGCAGCAACTGGGC
GAGTTCGCGCAGCTGCGTGACAGCCTGATCAGTGATGTGGCCAGCGGGCTGAATGATTTGCAGGGGAAAAGTGCCAGAGC
ATGGGTGTTTACCAGCACCGGCGATACAGCCACCACGCTGCTGGAACTGGTAAAGGATATCCCGCAACCGTCAGCCGTCT
ATACAGCGGCGGTGATGCTGGTCGGTGACAATCTGGATGGAATAAAGGGAATGATTCATGACTTCGATCCCAACGCTGGC
GCTTAA
>Cluster_6274
ATGAAAATACTCAATTCGAATCTCATAACAAACATATCATCAACAATAAGAAATCACCTTCTAACATCATCCAAAAATAT
AAAAACACACCCATCACCAAACATAGACGTAACAAGTCATCGTCGTTTATTCCAGAAAGACGGAATAGGATTGAGCCAAA
CAGGAAAAGCAACACAACTTGTCATTATGTCGCATGGTGGATGGAAAGAGATCTCCCATCCACAAACACTATTCACGCGT
CAAAAAGGCGATGGATGGACTGTAGTCCCCAAAAACCTAAGACTTGATTTTTATACAAAAGACAACGACTTCACAAAGGG
ACTAAGCGTTCTTTCAGAGGTAAACAAACGCCATACTGAAGCTCAAAAAGGGTTAACACCATCACTAAACATAAGCAAAG
ATGACCTAAAAGTTCTAGCGAACGCGCGCAATATACCTCAATCAAAATTAGAAGAAGAAATGATGTCACAAGCCATATAT
AGAAAAGAATACGCAACAAGTAATGAGAAGATAAAAAACTATGCTCTATATAGCCATGAACAAGCCCAAAATATTATACA
AAGACATCAAGATGGCTTAGGAAACAAAAATATTGATATAGCATTTATAACTGATAAAAATCATAAGAAGCACCTTTCTG
ACATATTTAAAGTAATAAATGAATCAGGAGTAAAATATGATGTAATTCACTTTGGCGCTTGCCGAGTAAATAGAGAGGAG
AGATAA
>Cluster_49304
GTGAATGTCAATTTCTTTGTCACCTGTATTGGTGACGCCCTGAAATCAAGAATGGCACGAGACTCCGTGCTGCTACTGGA
AAAACTCGGCTGTCGCGTAAATTTCCCGGAGAAACAGGGATGCTGCGGTCAGCCTGCGATCAATAGTGGTTATATCAAAG
AAGCGATTCCAGGGATGAAAAATCTGATCGCCGCACTGGAGGATAACGACGATCCCATTATTTCACCGGCTGGCTCTTGC
ACCTATGCCGTAAAAAGTTACCCGACGTATCTGGCGGATGAACCTGAATGGGCATCACGTGCCGAAAAGGTTGCCGCGCG
TATGCAGGATCTCACCTCTTTTATTGTTAATACATTAGGGGTAGTCGATGTAGGTGCCAGTTTGCAAGGGAGAGCGGTGT
ATCACCCATCTTGTAGCCTGGCCCGTAAGCTGGGGGTGAAGGACGAACCACTTACGCTGCTGAAAAATGTGCGTGGACTG
GAGCTGTTGACCTTTGCTGAACAGGATACCTGCTGCGGATTTGGCGGCACGTTCTCGGTCAAAATGGCCGAAATATCCGG
CGAGATGGTGAAAGAAAAGGTTGCGCACCTGATGGAAGTCCGCCCTGAGTATTTAATTGGTGCTGACGTGAGTTGCCTGC
TGAACATCAGTGGACGATTACAACGGGAAGGGCAGAAAGTCAAAGTGATGCATATTGCTGAAGTGCTGATGAGCCGCTGA
>Cluster_39389
ATGGGACGGAAACCCTTATACCGACAGATTGCTGATCGCATTCGTGAGCAAATTGCGCGTGGCGAGCTTAAACCTGGCGA
TGCGTTACCCACTGAATCAGCGCTACAAACAGAGTTTGGCGTCAGCCGGGTTACGGTGCGTCAGGCTTTGCGCCAGTTAG
TTGAACAGCAGATCCTCGAAAGTATTCAGGGCAGCGGGACTTACGTAAAAGAAGAGCGGGTCAATTACGATATTTTTCAG
TTAACCAGTTTTGATGAAAAACTGACGGACCGTCACGTCGATACACACAGTGAAGTTCTGATATTCGAAGTGATTCCGGC
TGACGATTTTCTTCAGCAACAGCTACAAATCACCCCACAGGATCGTGTGTGGCATGTGAAGCGCGTGCGCTATCGCAAGC

AAAAGCCAATGGCGCTGGAAGAAACCTGGATGCCGCTTGCGTTGTTCCCGGATCTCACCTGGCAGGTCATGGAAAATTCG
AAATATCACTTTATTGAAGAAGTGAAGAAAATGGTTATCGATCGTAGCGAACAGGAAATCATTCCTCTGATGCCAACCGA
AGAAATGAGCCGTCTGCTTAATATCAGCCAGACTAAGCCGATTCTTGAAAAGTATCGCGTGGATATTTAGTTGATGGCC
GGGTGTTTGAATATAGCCGCAACGCTTTTAATACCGATGACTATAAATTTACCCTGATAGCGCAGCGAAAATCATCGCGA
TAA
>Cluster_23968
GTGGATACGGAATTGTTAAAAACTTTCCTGGAAGTTAGCCGAACGCGTCACTTTGGTCGAGCGGCTGAATCGCTCTATCT
GACCCAATCAGCCGTGAGCTTTCGAATCAGACAACTGGAAAATCAACTGGGTGTGAATCTTTTCACCCGCCACAGAAACA
ATATCCGTTTAACCGCTGCCGGAGAAAAGCTTCTTCCTTATGCAGAAACGCTCATGAGCACCTGGCAGGCCGCCCGCAAG
GAGGTGGCGCATACCTCACGACATAATGAGTTTCTATCGGTGCCAGCGCCTCGCTGTGGGAGTGTATGCTTAATCAGTG
GCTGGGACGTTTGTATCAAAATCAGGATGTCCATACAGGCTTACAGTTCGAAGCGCGAATTGCCCAACGGCAGTCTCTGG
TAAAACAGTTACATGAACGCCAGCTTGATCTTCTTATTACAACTGAAGCCCCAAAATGGACGAATTTTGTAGTCAGTTG
CTGGGGTATTTCACTTTAGCGCTTTATACCAGTGCCCCTTCTAAACTAAAGGGAGATCTTAACTATCTGCGACTAGAGTG
GGGGCCAGATTTTCAACAGCATGAGGCAGGTTTGATCGGTGCTGACGAAGTTCCCATTCTGACAACCAGTTCTGCTGAAC
TGGCACAGCAACAGATTGCGATGCTTAATGGTTGCACCTGGCTACCCGTCAGTTGGGCACGTAAAAAAGGCGGCCTGCAT
ACCGTTGTCGATAGCACAACACTTTCACGGCCGCTTTATGCCATATGGCTACAAAATAGCGATAAAAATGCGTTGATTCG
CGATCTATTGAAAATTAACGTGCTGGATGAAGTGTATTAA
>Cluster_56223
ATGAATGCACTGACCGCCGTACACAATAACGCTGTCGATTCAGGCCAGGACTATAGCGGATTCACTCTCATCCCGTCGGC
GCAATCCCCGCGTCTGCTGGAACTCACCTTCACCGAACAGACGACCAAACAGTTTCTCGAACAGGTTGCCGAATGGCCCG
TGCAGGCGCTGGAGTACAAATCGTTTCTGCGTTTTCGGGTAGGCAAAATTCTTGACGATCTGTGTGCGAATCAGCTGCAA
CCATTGCTGTTGAAGACCCTGCTAAACCGTGCTGAAGGTGCGCTGTTGATCAATGCGGTGGGTATCGATGATGTGGCGCA
GGCAGATGAGATGGTGAAGCTGGCAACGGCGGTAGCGCATCTGATTGGTCGCTCAAATTTCGATGCGATGAGCGGTCAGT
ATTACGCGCGTTTCGTGGTGAAAAATGTCGATAACTCAGACAGCTATCTGCGTCAGCCGCACCGCGTGATGGAACTGCAC
AACGACGGCACGTATGTCGAAGAGATCACCGATTACGTGCTGATGATGAAAATCGATGAGCAAAACATGCAGGGTGGAAA
TTCGTTGCTGCTGCATCTCGATGACTGGGAACATCTGGACCACTATTTCCGCCACCCGCTGGCGCGTCGCCCAATGCGCT
TTGCCGCGCCGCCGAGTAAAAACGTCAGCAAAGATGTTTTCCATCCGGTGTTCGACGTTGATCAACAGGGTCGCCCGGTG
ATGCGTTATATCGACCAGTTCGTCCAGCCAAAAGACTTCGAAGAAGGCGTGTGGTTGAGCGAGCTTTCGGACGCCATTGA
AACCAGCAAAGGCATTCTTTCTGTGCCCGTTCCCGTTGGCAAATTCCTGTTGATTAACAACCTGTTCTGGCTGCACGGTC
GCGACCGCTTTACTCCGCACCCGGATCTGCGCCGTGAACTGATGCGTCAGCGTGGCTATTTCGCTTACGCCACTCACCAC
TACCAGACGCATCAGTAG
>Cluster_16878
ATGAAAAAGATACCTTTAGGCACAACGGATATAACGCTTTCGCGAATGGGGTTGGGGACATGGGCCATTGGCGGCGGTCC
TGCATGGAATGGCGATCTCGATCGACAAATATGCATTGATACGATTCTTGAAGCCCATCGTTGCGGCATTAACCTGATTG
ATACTGCGCCAGGATATAACTTTGGCAATAGTGAAGTTATCGTTGGTCAGGCGTTAAAAAAACTGCCCCGTGAACAGGTT
GTAGTAGAAACCAAATGCGGCATTGTCTGGGAACGAAAAGGGAGTTTATTCAACAAAGTTGGCGATCGGCAGTTGTATAA
AAACCTTTCTCCGGAATCTATCCGCGAAGAGGTAGAAGCCAGCTTGCAACGTCTGGGTATTGATTACATCGATATCTACA
TGACGCACTGGCAGTCGGTGCCGCCATTTTTTACGCCGATAGCTGAAACTGTCGCAGTGCTTAATGCGTTAAAGGCCGAA
GGGAAAATTCGCGCTATAGGCGCTGCTAACGTCGATGCTGACCATATCCGCGAGTATCTGCAACATGGTGAACTGGATAT
TATTCAGGCGAAATACAGTATCCTCGACCGGGCAATGGAAAACGAACTGCTGCCGCTATGTCGTGATAATGGCATTGTGG
TACAGGTCTACTCTCCGCTAGAGCAGGGATTGTTGACCGGGACCATCACGCGTGATTACGTTCCGGGCGGCGCTCGGGCA
AATAAAGTCTGGTTCCAGCGTGAAAACATGCTGAAAGTGATTGATATGCTTGAACAGTGGCAGCCACTCTGTGCTCGTTA
TCAGTGCACAATTCCCACTCTGGCACTGGCGTGGATATTAAAACAGAGTGATTTAATCTCCATTCTTAGTGGGGCTACTG
CGCCGGAACAGGTACGTGAAAATGTCGCGGCACTGAATATCAACTTATCGGATGCAGACGCAACATTGATGAGGGAAATG
GCAGAGGCCCTGGAGCGTTAA
>Cluster_62639
ATGACAGACTTAATCCAACGCCCTCGTCGCCTGCGCAAATCTCCTGCGCTGCGCGCTATGTTTGAAGAGACAACACTTAG
CCTTAACGACCTGGTGTTGCCGATCTTTGTTGAAGAAGAAATTGACGACTACAAAGCCGTTGAAGCCATGCCAGGCGTGA
TGCGCATTCCAGAGAAACATCTGGCACGCGAAATTGAACGCATCGCCAACGCCGGTATCCGTTCCGTAATGACTTTTGGC
ATCTCTCACCATACCGATGAAACCGGCAGCGACGCCTGGCGGGAAGATGGACTGGTGGCGCGTATGTCGCGCATCTGCAA
GCAGACTGTGCCAGAAATGATCGTCATGTCAGACACTTGTTTCTGTGAATACACTTCTCACGGTCACTGCGGTGTGCTGT
GCGAGCATGGCGTCGACAACGACGCGACTCTGGAAAATTTAGGCAAGCAAGCCGTGGTTGCAGCTGCTGCAGGCGCAGAC
TTCATCGCCCCTTCCGCCGCGATGGACGGCCAGGTACAGGCGATTCGTCAGGCGCTGGACGCTGCAGGCTTTAAAGATAC
GGCGATTATGTCGTATTCGACCAAGTTCGCCTCTTCCTTTTATGGCCCGTTCCGTGAAGCTGCCGGAAGCGCATTAAAAG
GCGACCGCAAAAGCTATCAGATGAACCCAATGAACCGTCGCGAGGCGATTCGTGAGTCACTGCTGGATGAAGCCCAGGGC
GCAGACTGCCTGATGGTTAAACCTGCTGGAGCGTACCTCGACATCGTGCGTGAGCTGCGTGAACGTACTGAATTGCCGAT
TGGCGCGTATCAGGTGAGCGGTGAGTACGCGATGATTAAGTTCGCCGCGCTGGCGGGTGCTATAGATGAAGAGAAAGTCG
TGCTCGAAAGCTTAGGTTCAATTAAGCGTGCGGGTGCGGATCTGATTTTCAGCTACTTTGCGCTGGATTTGGCTGAGAAG
AAGATTCTGCGTTAA
>Cluster_10317
ATGGCATGTGGCGAGTTCTCCCTGATTGCCCGTTATTTTGACCGTGTAAGAAGTTCTCGTCTTGATGTCGAACTGGGCAT
CGGCGACGATTGCGCTCTTCTCAATATCCCCGAGAAACAGACCCTGGCGATCAGCACTGATACGCTGGTGGCGGGCAACC
ACTTCCTCCATGATATCGATCCTGCTGATCTGGCGTATAAAGCACTGGCGGTGAACCTAAGCGATCTGGCAGCGATGGGG
GCCGATCCGGCCTGGCTGACGCTGGCATTAACCTTACCGGACGTAGACGAAGCGTGGCTTGAGTCCTTCAGCGACAGTTT
GTTTGATCTTCTCAATTATTACGATATGCAACTCATTGGCGGCGATACCACGCGTGGGCCATTATCAATGACGTTGGGTA
TCCACGGCTTTGTTCCGATGGGACGAGCCTTAACGCGCTCTGGAGCGAAACCGGGTGACTGGATCTATGTGACCGGTACA
CCGGGCGATAGCGCCGCCGGGCTGGCGATTTTGCAAAACCGTTTGCAGGTTGCCGATGCTAAAGATGCGGACTACTTGAT
CAAACGTCATCTCCGTCCATCGCCGCGTATTTTACAGGGACAGGCACTGCGCGATCTGGCAAATTCAGCTATCGATCTCT
CTGACGGTCTGATTTCCGATCTCGGGCATATCGTGAAAGCCAGCGACTGCGGCGCACGTATTGACCTGGCATTGCTGCCG
TTTTCTGATGCGCTTTCTCGCCATGTTGAACCGGAACAGGCGCTGCGCTGGGCGCTCTCTGGCGGTGAAGATTACGAGTT
GTGTTTCACTGTGCCGGAACTGAACCGTGGCGCGCTGGATGTGGCTCTCGGACACCTGGGCGTACCGTTTACCTGTATCG
GGCAAATGACCGCCGATATCGAAGGGCTTTGTTTTATTCGTGACGGCGAACCTGTCACGTTTGACTGGAAAGGATATGAC
CATTTTGCCACGCCATAA
>Cluster_32459
ATGAAAATGAGGTCATTTACCCGCTCATTAGTCTGCGCATCCTTGCTGGCTCTGGTTAGCACTGGCGTAAACGCCACAGA
AAAAGTGACGTTAAAGTTAGCACACAACCTGGAAAGAAGTCATGTTGTACATCAGAGTTTTGAAGAGCTGGCGAAAGAGG
TAAAACAGCTGTCAAAAGGTAATATGGTGATCCGTATTTATCCCAGCAGCCAGATGGGTAATGCGCGAGAAACTATGGAA
TTGTTGCAAAATGGCGCACTGGATATGACCAAAGGTTCCGCCAGTGATCTGGAGTCTTTTGATAATATTTATGCTATCTA
TAATTTGCCGTTCTTATTTAAAGATCAGGCTCACTTCAACAAAGTTGTTTTTGGTGAAGTTGGTAAAGAAATTATGGACT

CAACAAAAGACAAAGGCTTTTTTGCTTTATCAGCTTATGTAGCGGGTACACGCAGTTTCTATGCGAAAAAACCGATTACT
AAGCCAGAAGATCTTAAAGGGCTGAAAATTCGTGTTCAGCCAAGCCCGACCACCATCAAAATGATTGAACTGATGGGTGG
CTCACCCACTCCAATTTCATTTGGCGAAGTTTATACTGCGATGCAGCAAGGTGTAGTAGATGGCGCAGAAAATAACGTGC
CTTCCTGGGTGCAGACTCGACATATCGAAATTGCCAAAGTCTTTTCTGAAGATGAACATGCCTCAATTCCTGACTTTTTG
GTTATCTCCACCAAAACATGGAATAAATTAACCCCAGAACAGCAACAAATCCTCGAAACAGCGGCGAAGAAATCCGAAGC
TTATCAACAAAAATTGTGGGAAAAAATTGACGCTGACACACGTGCTCAGGCGAAAGCTATGGGTGGTGAAATCGTTAAGG
TTGATAAAGCCCCATTCCGTGCCGCAGTTCAACCTCTGTTTGACGACTTCAAAAAAGATCCAAAACAAGCTGCTTTGTTA
GAGAAATTCGATAACGCTGCTCAATAA
>Cluster_28259
ATGACAGAGAAAACTCTTGCCAGTCCGAAACCTTCGGAAATTCCCATTGCAAATAATTTTAAAAAAGTCTTCAGGCAATA
TGGTGGTATCCTCAGTGGAATGGTGGTGTTAATCATTCTGTTCAGTTTTATTAATGACAGTTTTTTTACCGCCAATAACA
TTACTAATATTATTCTTCAGGTTTCGATAATTGCAATTACCGCCTATGGAATGACTTATGTGCTTTTGCTTGGCGATATT
GATTTATCGGTCGGTTCAACCATTGCATTGATTGGCACATTTGCTGCGCTTGGGGCTTCATGGGGAATTCCATTTGTTTT
ATTGGTCCCATTATCCATTATTGCAGCACTGGCTCTGGGGATGATAAATGGTGGCTTGACGGCTATAGCGGGGATCCCTT
CGTTTATTGTCACCGTTGCTACTATGGGGATCTTCCGTGGTATCGCTTATATAGTGACTGATGGTATGCCCATCATGATT
AAAGATGATGCCTTTCTGGCGCTGGGGAATGGAGAATTTCTCTATATACCTATTCCTATATGGATATTAATCATCTTATT
ACTGATAAACCACTTCATTCTTACTAAAACAACCTTTGGACGAAAAATTTATATCACTGGTGGTAATAAAGAAGCGGCAA
TTTATTCAGGGATAAATGTCACCCGCTTGAAAATTAAAGTCTTTATGATTACAGCCGTGCTGGCAGGTATCAGCGGGATG
ATCCTTGCCTCACGCCTCTATTCCGGGCAACCGAATTCCGCGTTGAGCTACGAACTGGATGCTATTGCCGCAGCTGTACT
GGGAGGCACCAGCCTGAACGGCGGCTATGGAACCGTGGTGGGGACGGTGATCGGCGCATTAACCATCGGCGTTATTAATA
ATGGCATGAACTTAATGAATGTTCCTTATTTTTACCAGATGGTGGTTAAAGGATTAGTCATTCTTGTAGCCGTTTATTTT
GACGTAAGAAATAAAAGAAAACGCAGCTAA
>Cluster_4390
ATGCAGATAAAACGCTCGATTGAGAAAATCCCGGGGGGGATGATGCTCGTCCCGCTATTCCTTGGCGCACTGTGCCACAC
CTTCTCGCCGGGGGCGGGGAAATATTTTGGATCATTCACCAACGGGATGATTACCGGTACGGTGCCCATTCTGGCGGTGT
GGTTTTTTTGCATGGGGGCGTCAATAAAATTAAGCGCGACGGGAACGGTATTGCGTAAATCCGGTACGCTGGTGGTAACA
AAAATTGCCGTCGCGTGGGTGGTTGCGGCAATTGCCTCGCGCATTATTCCGGAACATGGTGTTGAAGTTGGATTCTTTGC
CGGACTTTCAACGCTGGCGCTGGTGGCGGCGATGGATATGACCAATGGCGGACTTTACGCTTCCATCATGCAGCAGTACG
GCACAAAAGAAGAAGCTGGGGCATTTGTGTTGATGTCGTTGGAGTCCGGGCCGCTCATGACGATGATTATTCTGGGCACT
GCAGGGATTGCCTCGTTTGAACCGCATGTTTTCGTCGGCGCAGTATTACCGTTCCTGGTGGGCTTTGCCCTGGGGAACCT
TGACCCTGAATTACGAGAATTTTTCAGCAAAGCGGTGCAAACGCTGATTCCATTCTTTGCCTTCGCGCTGGGCAATACCA
TTGATTTGACTGTGATTGCCCAGACTGGTTTGCTGGGGATCCTGTTGGGTGTGGCAGTAATTATCGTGACCGGTATTCCG
TTGATTATCGCCGATAAATTGATTGGCGGTGGCGATGGCACTGCCGGAATTGCCGCTTCCAGTTCCGCAGGGGCCGCGGT
AGCGACACCTGTGCTGATTGCAGAAATGGTGCCTGCGTTTAAACCGATGGCTCCGGCAGCAACTTCGCTGGTAGCGACGG
CGGTCATTGTGACTTCGATTCTGGTGCCAATTCTTACCTCTATCTGGTCACGTAAAGTCAAAGCCAGAGCAGCGAAAATC
GAAATTTTAGGTACGGTGAAATAA
>Cluster_37921
ATGAATAACAGCGCTTTTACTTTCCAGACACTACACCCGGATACCATCATGGACGCTCTGTTTGAGCATGGGATCCGGGT
GGATTCCGGTCTTACCCCGCTTAACAGCTATGAAAACCGTGTCTATCAATTTCAGGACGAAGATCGTCGACGTTTTGTCG
TCAAATTTTATCGCCCTGAACGTTGGACAGCCGATCAAATCCTCGAAGAACATCAATTTGCGTTGCAGCTGGTAAATGAT
GAAGTTCCGGTCGCAGCACCTGTGGCCTTTAACGGTCAGACTTTATTGAATCATCAGGGATTTTATTTCGCTGTTTTTCC
AAGCGTCGGTGGTCGCCAGTTCGAAGCTGATAATATCGATCAGATGGAAGCGGTTGGGCGTTATTTAGGGCGTATGCACC
AGACGGGGCGCAAACAGCTTTTTATCCATCGCCCGACCATCGGTTTGAACGAATATCTCATTGAGCCACGCAAGCTGTTT
GAGGACGCTACACTGATACCTTCCGGGTTGAAAGCGGCATTCCTGAAAGCGACAGATGAGCTGATTGCCGCCGTTACAGC
ACACTGGCGGGAAGATTTCACCGTTCTGCGGCTACATGGAGACTGCCACGCCGGGAATATTCTCTGGCGCGATGGTCCAA
TGTTTGTTGATCTGGATGATGCACGTAATGGTCCAGCCGTTCAGGATTTGTGGATGTTGCTCAATGGCGATAAAGCCGAG
CAGCGGATGCAACTGGAAACTATTATTGAAGCTTATGAAGAATTTAGCGAGTTCGACACCGCTGAAATCGGACTGATTGA
ACCTTTACGCGCCATGCGTTTGGTTTATTATCTTGCCTGGCTAATGCGGCGTTGGGCTGATCCCGCGTTCCCGAAAAATT
TCCCGTGGTTAACCGGGGAAGATTACTGGCTGCGACAGACGGCGACTTTTATAGAACAGGCAAAAGTTCTACAAGAACCC
CCTTTGCAATTAACACCTATGTATTAA
>Cluster_40159
ATGAAATTACAACAACTTCGCTATATTGTTGAGGTGGTCAATCATAACCTGAATGTCTCATCAACAGCGGAAGGACTTTA
CACATCACAACCCGGGATCAGTAAACAAGTCAGAATGCTGGAAGACGAGCTAGGCATTCAAATTTTTTCCCGAAGCGGCA
AGCACCTGACGCAGGTAACGCCAGCAGGACAAGAAATAATTCGTATCGCTCGCGAAGTCCTGTCGAAAGTCGATGCCATA
AAATCGGTCGCCGGAGAGCACACCTGGCCGGATAAAGGCTCGCTGTATATCGCCACCACGCATACCCAGGCACGCTATGC
GTTACCAAACGTCATCAAAGGCTTTATTGAGCGTTATCCTCGCGTTTCTTTGCATATGCACCAGGGCTCGCCGACACAAA
TTGCTGATGCCGTCTCTAAAGGCAATGCCGATTTCGCGATTGCCACAGAAGCGCTGCATCTGTATGAAGATTTAGTGATG
TTACCGTGCTACCACTGGAATCGGGCTATTGTAGTCACTCCGGATCACCCGCTGGCAGGCAAAAAAGCCATTACCATTGA
AGAACTGGCGCAATATCCGTTGGTGACATATACCTTCGGCTTTACCGGACGCTCAGAACTGGATACTGCCTTTAACCGCG
CAGGGTTAACGCCGCGTATCGTCTTCACGGCAACGGATGCTGACGTCATTAAAACTTACGTCCGGTTAGGGTTGGGGGTA
GGGGTTATTGCCAGCATGGCGGTGGATCCGGTCGCCGATCCCGACCTGGTGCGCGTTGATGCTCACGATATCTTCAGCCA
CAGTACAACCAAAATTGGTTTTCGCCGTAGTACTTTTTTGCGCAGTTATATGTATGATTTCATTCAGCGTTTTGCACCGC
ATTTAACGCGTGATGTCGTTGATGCGGCTGTCGCATTGCGCTCTAATGAAGAAATTGAGGCCATGTTTAAAGATATAAAA
CTGCCGGAAAAATAA
>Cluster_44468
ATGAATACACTTGAACCTGCTGCCCAGTCGAAACCGCCGGGCGGATTTAAGCTGTGGTTGTCGCAGCTGCAAATGAAGCA
TGGGCGCAAACTGGTCATTGCGTTGCCATATATCTGGTTGATCTTGCTGTTTCTGCTGCCATTTCTGATTGTCTTTAAAA
TAAGCCTGGCGGAGATGGCGCGCGCTATTCCACCTTATACCGAACTGATGGAGTGGGCTGACGGGCAACTGTCCATCACT
CTTAATCTCGGTAATTTTCTGCAACTGACCGACGATCCGCTCTATTTCGATGCTTATCTCCAGTCGCTACAGGTGGCTGC
GATTTCGACATTTTGCTGTTTACTGATCGGCTATCCGCTGGCGTGGGCGGTGGCGCACAGTAAGCCTTCGACCCGTAATA
TTTTATTACTACTGGTGATCCTGCCGTCGTGGACCTCGTTTCTGATCCGCGTTTATGCCTGGATGGGAATATTAAAAAAC
AACGGTGTGCTGAATAATTTTCTGCTGTGGCTGGGGGTTATCGATCAACCGCTGACCATTCTTCATACCAATCTGGCAGT
TTATATTGGCATTGTTTACGCCTACGTGCCGTTTATGGTACTGCCGATTTATACCGCGTTGATTCGTATTGATTATTCGC
TGGTGGAAGCAGCGCTGGATCTCGGTGCACGACCGCTGAAAACGTTCTTTACTGTGATCGTGCCGCTGACTAAAGGTGGG
ATTATTGCCGGATCGATGCTGGTGTTTATCCCGGCTGTGGGCGAGTTTGTGATCCCGGAACTGCTCGGTGGCCCGGACAG
CATCATGATCGGGCGCGTGCTATGGCAAGAGTTTTTCAATAACCGCGACTGGCCGGTGGCCTCGGCGGTAGCGATCATCA
TGTTGCTGCTGCTAATTGTGCCGATAATGTGGTTCCACAAACACCAGCAAAAAAGCGTGGGAGAACACGGATGA
>Cluster_35086
ATGTATACGAAGATTATTGGTACTGGCAGCTATCTGCCCGAACAAGTGCGGACAAACGCCGATTTGGAAAAAATGGTGGA

CACCTCTGACGAGTGGATTGTCACTCGTACCGGTATCCGCGAACGCCACATTGCCGCGCCAAACGAAACCGTTTCAACCA
TGGGCTTTGAAGCGGCGACACGCGCAATTGAGATGGCGGGCATTGAGAAAGACCAGATTGGCCTGATCGTTGTGGCAACG
ACTTCTGCTACGCACGCTTTCCCGAGCGCAGCTTGTCAGATTCAAAGCATGTTGGGCATTAAAGGTTGCCCGGCATTTGA
CGTTGCAGCAGCCTGCGCAGGTTTCACCTATGCATTAAGCGTAGCCGATCAATACGTGAAATCTGGGGCGGTGAAGTATG
CTCTGGTCGTCGGTTCCGATGTACTGGCGCGCACCTGCGATCCAACCGATCGTGGGACTATTATTATTTTTGGCGATGGC
GCGGGCGCTGCGGTGCTGGCTGCCTCTGAAGAGCCGGGAATCATTTCCACCCATCTGCATGCCGACGGTAGTTATGGTGA
ATTGCTGACGCTGCCAAACGCCGACCGCGTGAATCCAGAGAATTCAATTCATCTGACGATGGCGGGCAACGAAGTCTTCA
AGGTTGCGGTAACGGAACTGGCGCACATCGTTGATGAGACGCTGGCGGCGAATAATCTTGACCGTTCTCAACTGGACTGG
CTGGTTCCGCATCAGGCTAACCTGCGTATTATCAGTGCAACGGCGAAAAAACTCGGTATGTCTATGGATAATGTCGTGGT
GACGCTGGATCGCCACGGTAATACCTCTGCGGCCTCTGTCCCGTGCGCGCTGGATGAAGCTGTACGCGACGGGCGCATTA
AGCCGGGGCAGTTGGTTCTGCTTGAAGCCTTTGGCGGTGGATTCACCTGGGGCTCCGCGCTGGTTCGTTTCTAG
>Cluster_39194
ATGCGGGCGAAACTTCTGGGAATAGTCCTGACAACCCCTATTGCGATCAGCTCTTTTGCTTCTACCGAGACTTTATCGTT
TACTCCTGACAACATAAATGCGGACATTAGTCTTGGAACTCTGAGCGGAAAAACAAAAGAGCGTGTTTATCTAGCCGAAG
AAGGAGGCCGAAAAGTCAGTCAACTTGACTGGAAATTCAATAACGCTGCAATTATTAAAGGGGCAATTAATTGGGATTTG
ATGCCCCAGATATCTATCGGGGCTGCTGGCTGGACAACTCTCGGTAGCCGAGGTGGCAATATGGTCGATCGGGACTGGAT
GGATTCCAGTAACCCCGGAACCTGGACGGATGAAAGTAGACACCCTGATACACAACTCAATTATGCCAACGAATTTGATC
TGAATATCAAAGGCTGGCTCCTCAACGAACCCAATTACCGCCTGGGACTCATGGCCGGATATCAGGAAAGCCGTTATAGC
TTTACAGCCAGAGGTGGTTCCTATATCTACAGTTCTGAGGAGGGATTCAGAGATGATATCGGCTCCTTCCCGAATGGAGA
AAGAGCAATCGGCTACAAACAACGTTTTAAAATGCCCTACATTGGCTTGACTGGAAGTTATCGTTATGAAGATTTTGAGC
TAGGTGGCACATTTAAATACAGCGGCTGGGTGGAAGCATCTGATAACGATGAGCACTATGACCCAGGAAAAAGAATCACT
TATCGCAGTAAAGTCAAAGACCAAAATTACTATTCTGTTGCAGTCAATGCAGGTTATTACGTAACACCTAACGCAAAAGT
TTATGTTGAAGGCGCATGGAATCGGGTTACGAATAAAAAAGGTAATACTTCACTTTATGATCACAATGATAACACTTCAG
ACTACAGCAAAAATGGTGCAGGCATAGAAAACTATAACTTCATCACTACTGCTGGTCTTAAGTACACCTTTTAA
>Cluster_34497
GTGGATAGTAATAATCAAATTGAACCCTGTTTAAGCAGAAAGTCGTCAGAAGGTAAACCACAAATATTTACAACCTTACG
AAATATTGATCTTAACCTTCTGACTATTTTTGAAGCTGTATATGTACATAAAGGGATCGTTAATGCAGCGAAAGTGCTTA
ATCTGACCCCCTCGGCAATCAGTCAGTCTATTCAGAAACTGCGCGTTATATTCCCTGACCCATTGTTTATTCGCAAAGGC
CAGGGTGTCACTCCTACCGCATTTGCGATGCATCTACATGAGTATATCAGTCAGGGCCTTGAGTCCATTCTTGGCGCGCT
GGATATCGAAGGAAGCTATGATAAGCAACGAACGATAACTATTGCGACCACTCCCTCGGTCGGAGCACTGGTCCTTCCTG
TCATCTATCGGGCGATTAAAACTCACTATCCGCAGCTTTTACTGCGCAACCCGCCCATCAGCGACGCGGAAAACCAACTC
AGTCAGTTTCAAACCGATCTCATCATCGATAACATGTTTTGCACCAATCGTACGGTGCAACATCATGTTCTGTTCACCGA
CAATATGGTGTTAATTTGCCGCGAGGGAAATCCACTACTCTCTTTAGAAGATGACAGAGAGACTATCGACAACGCTGCGC
ATGTACTTCTGTTACCGGAAGAGCAAAATTTCAGCGGTCTGCGGCAGAGAGTTCAAGAAATGTTTCCGGACCGGCAAATC
AATTTCACCAGCTACAACATTTTGACAATCGCTGCACTGGTTGCCAACAGTGACATGTTAGCGATTATTCCCAGCCGTTT
TTATAACCTGTTTAGCCGCTGCTGGCCGCTGGAAAAACTGCCTTTTCCGTCCTTAAATGAGGAGCAAATAGATTTCTCTA
TCCACTACAACAAATTCAGCCTGCGTGATCCCATCCTGCACGGAGTAATAGATGTTATCCGTAATGCATTTTGA
>Cluster_54956
ATGAGAACCGTTAAGGTATTCGAGGAAGCCTGGCCCTTACATACCCCGTTTGTGATTGCCCGGGGAAGTCGCAGTGAAGC
GCGCGTGGTGGTGGTTGAACTGGAAGAAGAGGGTATTAAAGGCACCGGCGAATGCACGCCGTATCCGCGTTATGGGGAAA
GTGATGCCTCGGTAATGGCGCAAATTATGAGCGTCGTGCCGCAACTAGAGAAAGGGCTGACACGGGAGGAGTTGCAAAAA
ATTCTCCCTGCCGGCGCAGCACGTAATGCGCTGGATTGTGCATTGTGGGATCTGGCCGCGCGAAGACAGCAGCAATCGCT
GGCTGATTTGATCGGCATAACGCTTCCCGAGACAGTTATCACTGCACAGACGGTTGTCATCGGTACGCCTGATCAGATGG
CCAATAGTGCATCAACACTCTGGCAGGCAGGCGCGAAATTACTGAAAGTGAAGCTGGATAACCATCTTATCAGTGAGCGG
ATGGTGGCAATTCGCACAGCTGTGCCCGATGCGACGCTGATCGTTGATGCAAATGAATCCTGGCGTGCAGAAGGGTTGGC
GGCGCGTTGCCAGCTATTGGCGGATTTAGGCGTTGCGATGCTTGAACAACCGCTTCCTGCGCAGGACGATGCGGCGCTGG
AGAATTTTATTCATCCGTTGCCGATTTGTGCTGATGAAAGTTGTCATACTCGTAGCAATTTGAAGGCGCTGAAAGGGCGC
TATGAGATGGTTAATATTAAGCTCGATAAAACCGGGGGTCTGACGGAAGCGCTGGCGCTGGCGACTGAAGCGCGTGCACA
AGGTTTCAGTCTGATGCTGGGCTGCATGTTGTGTACCTCTCGTGCCATTAGCGCCGCTTTACCGCTGGTGCCGCAGGTCA
GTTTCGCCGATCTTGACGGACCGACCTGGCTGGCGGTAGATGTGGAACCGGCGCTTCAGTTCACGACGGGCGAATTGCAT
CTTTAG
>Cluster_39447
ATGAGTGAAGTAACAGATTTAGTTGTTATTGAAAAAGCAAATGCAATGACTGTATTTCAGTCTGCCGACCAGATTGAAGA
AATCCTTCAAAAGGTTGAACGTGAAGTTATGTCCTTTGTGCCTGATATCACAACGGCAAAGGGCAGAAAGGAGATCGCTT
CTCTGGCGTATAAAGTTGCGCAGACGAAAACATATCTCGATGGTCTTGGCAAAGACCTTGTTGCTGAACTGAAGGAAATT
CCAAAGCTAATTGATGCCAACCGCAAGACAGTGCGCGATCGCCTTGATGAGCTGAAAGCCAAGGCGCGCCAGCCTCTTAC
TGATTATGAGGAAGAACAGGCGCGGATTAAAGCCGAAGAAGAAGCTAAGGCAGAAGCTGAAGCTCTCGCAAAGCAAATTG
AGTCTGACCATGAAATAGCTATTTTGATGGATCGCGAATTTGACCGCCAAAGAGAAGAGGCAAGACTCAAAGCGGAGCAG
GAAAAGCGAGAGCATGAAGAACGCTTAAAAAGAGAAGCTGAAGAGAAAGCCAGAGCAGAAGCCGAAGCAAAGGCAAAAGC
CGAAATTGAAGCAGCAGCAAGGCGAGAAGCAGAAGCTAAGGCCGCAGCAGAACGTGCAGAGCGTGAACGCATTGAAGCCG
AGCAACGAGCACAGCGCGAAGCAAAAGAGGCAGCAGAACGAGCTGAAAGAGAAAAGCAGGCAGCAATTGAAGCAGAACGC
AGAAAAGCACAGGAGGAGGCTGAACGAATCCGTCGCGAGGCTGAAGCAAAAGAGCAAGCCAGAATAGCAGAAGAAAAAAG
AATCAAGGACGAAGAAGAGCGTAGAGCAAAGGATAAAGCTCACCGGAAAGAAGTGAATAACAAAATACTTGCTGACCTTA
TCAAGGTTGGTGCATCAGAAGATGTTGCTAAAAATATCATAACAGCCATCGTAAAAGGCGAAGTATTCGCAACAAAAATA
ACCTACTAA
>Cluster_42319
ATGAGTAAGGTCTTCACAATTGGAGAAATTCTGGTTGAAATTATGGCGAGTAAAATCGGCCAGCCATTTGATCAACCAGG
TATCTGGAACGGCCCTTATCCCAGTGGTGCGCCCGCTATTTTTATCGACCAGGTGACACGTCTTGGCGTCCCGTGCGGCA
TCATTAGCTGCGTGGGGAATGATGGCTTTGGTGATATTAATATACATCGTCTGGCTGCTGACGGTGTGGACATCAGGGGG
ATTTCAGTCTTACCTCTGGAAGCCACCGGTAGTGCCTTTGTTACTTATCATAACTCTGGCGATCGCGATTTTATTTTTAA
TATCAAGAATGCCGCTTGCGGTAAATTATCGGCACAGCATGTTGATGAAAATATTCTGAAGGATTGTACCCATTTTCATA
TTATGGGTTCATCCCTGTTTTCATTCCATATGGTAGACGCAGTAAAAAAAGCAGTAACCATCGTTAAAGCAAATGGTGGC
GTTATTTCTTTCGACCCGAATATTCGTAAAGAGATGCTCGATATTCCAGAAATGCGCGATGCCCTCCACTTTGTTCTTGA
GTTAACAGATATCTACATGCCCAGCGAGGGCGAAGTGTTGCTCTTGTCGCCACACTCGACACCGGAGCGTGCAATCGCTG
GTTTTCTTGAAGAAGGTGTTAAAGAGGTGATTGTGAAGCGCGGCAATCAAGGAGCCAGTTACTACTCCGCGAATGAGCAG
TTTCATGTTGAGAGCTATCCGGTGGAAGAAGTTGACCCGACCGGTGCGGGAGACTGTTTTGGTGGCGCATGGATTGCCTG
CCGCCAGCTAGGGTTTGACGCCCATCGGGCATTACAATATGCCAATGCCTGCGGTGCACTGGCGGTAACCCGACGTGGCC
CAATGGAGGGAACGTCTCGCCTTATGGAAATCGAAACGTTCATCCAGCGCCATGACATGTCCATTCGGGAAGCAGCACAA
TGA

>Cluster_25583
ATGGACTATCGCAAAATCATTAAAGAGATCGGGCGCGGGAAAAACCACGCGCGCGATTTAGACCGGGATACTGCGCGCGG
TCTGTATGCCCATATGCTCAATGGTGAAGTCCCTGACCTTGAGTTGGGCGGCGTATTGATTGCGCTACGTATCAAAGGGG
AAGGGGAAGCAGAGATGCTCGGCTTTTACGAAGCCATGCAAAATCACACCATCAAGCTGACGCCGCCTGCAGGCAAGCCG
ATGCCGATTGTCATCCCCAGTTACAACGGCGCGCGTAAACAGGCCAACCTGACGCCGTTGCTGGCGATTCTGCTGCACAA
ACTCGGTTTTCCTGTGGTGGTTCACGGGGTTAGCGAAGATCCAACCCGCGTGCTGACCGAAACCATTTTCGAACTGATGG
GTATTACGCCAACGTTGCACGGCGGGCAGGCGCAGGCGAAACTCGACGAACATCAACCTGTCTTTATGCCTGTCGGCGCG
TTTTGCCCGCCGCTGGAAAAACAACTGGCGATGCGCTGGCGGATGGGCGTGCGTAACAGTGCACATACCCTGGCGAAACT
GGCGACGCCATTTGCCGAAGGTGAGGCACTGCGTCTTTCCAGCGTTTCGCATCCGGAATACATTGGACGCGTCGCGAAGT
TCTTTAGTGATATCGGCGGGCGGGCGCTACTAATGCATGGTACTGAAGGTGAAGTGTATGCTAATCCGCAGCGCTGCCCG
CAGATCAATCTCATTGACCGTGAAGGGATGCGGGTACTGTATGAAAAACAGGGCACTGCTGGTAGCGAGTTACTGCCACA
AGCAAAAGATCCGGAAACCACGGCGCAGTGGATTGAGCGTTGCCTTGCTGGCAGCGAACCGATTCCCGAATCGCTGAAAA
TCCAGATGGCTTGCTGCCTGATGGCTACGGGTGAAGCGGCAACTATCAGCGACGGCCTGGCGCGCGTTAATCAGGCATTT
TAA
>Cluster_43230
ATGAACGCTAAGATAATTGCCTCGCTGGCCTTCACCAGCATGTTCAGCCTCAGCACCCTGTTAAATCCGGCATACGCCGA
AGAGCAGGAAAAGGCGCTGAATTTCGGCATTATTTCAACGGAATCACAGCAAAACCTGAAACCGCAATGGACGCCGTTCT
TACAGGATATGGAGAAGAAGCTGGGCGTAAAGGTCAACGCCTTCTTTGCCCCGGACTACGCGGGCATTATCCAGGGGATG
CGCTTCAATAAAGTGGATATCGCCTGGTACGGCAACCTGTCGGCGATGGAAGCGGTGGATCGCGCCAACGGTCAGGTCTT
CGCCCAGACGGTCGCGGCGGATGGATCGCCGGGTTACTGGAGCGTGTTGATCGTCAACAAAGATAGCCCGATCAACAACC
TGAACGATCTGCTGGCGAAGCGGAAAGATCTCACCTTCGGCAATGGCGATCCTAACTCTACCTCCGGCTTCCTCGTCCCC
GGTTACTACGTCTTCGCTAAAAACAATATCTCCGCCAGCGACTTTAAGCGCACCGTCAACGCCGGGCATGAAACCAACGC
CCTCGCCGTCGCTAACAAGCAAGTGGATGTCGCCACCAACAACACCGAAAACCTCGACAAGCTGAAAACCTCCGCGCCGG
AGAAGCTGAAAGAACTGAAGGTGATCTGGAAATCACCGCTGATCCCTGGCGATCCGATCGTCTGGCGCAAAAATCTTTCC
GAAACCACTAAAGACAAGATCTACGACTTCTTTATGAATTACGGCAAAACGCCGGAAGAGAAAGCGGTGCTGGAGCGCCT
GGGCTGGGCGCCGTTCCGCGCCTCCAGCGACTTGCAACTGGTGCCGATTCGTCAGCTCGCACTGTTTAAAGAGATGCAAA
GCGTGAAGGACAACAAAGGGCTGAACGAACAGGACAAACTGGCAAAAACCACCGCCATTCAGGCGCAACTGGATGATTTG
GACCGCCTGAACAACGCGTTAAGCGCAATGAGTTCGGTGAGTAAAGCGGTGCAGTAA
>Cluster_49922
ATGGCGCGTGCCGGATTTGAAAAAACCGAACGTGATAATGTCTACAACGGGATGACCCTGCGTGAATATGCCCGTATGTC
ACTGACTGAACGGGGTATTGGGGTTTCCAGTTATAACCCGATGCAGATGGTCGGTGCGGCGTTCACACACAGTACGTCTG
ACTTCGGTAATATTCTGCTGGATGTTGCGAACAAAGCCATTCTGCAGGGCTGGGAAGATGCTCCTGAAACCTATGAACAG
TGGACGCGGAAAGGTCAGTTGTCTGATTTTAAAATTGCCCATCGTGTGGGTATGGGGGGCTTCAGTGCTCTGCGTCAGGT
GCGTGAAGGGGCGGAATATAAATACGTCACCACCGGAGATAAACAGGCCACTATTGCACTGGCGACCTATGGCGAGCTGT
TCAGTATCACCCGTCAGGCCATTATCAATGATGATCTGAATATGCTGACCGATGTCCCGATGAAGCTGGGCCGTGCGGCG
AAATCCACTATTGCCGATCTGGTTTATGCCATTCTGACGTCTAACCCGAAAATCTCCACAGATAATGTAAGTCTGTTCGA
TAAAGCGAAACATGCAAACGTACTGGAGAGCGCTGCAATGGACGTGGCATCGCTGGATAAAGCCCGCCAGTTGATGCGCG
TTCAGAAAGAGGGGGAGCGTCATCTGAATATTCGTCCTGCGTTCGTACTGGTACCGACGGCGATGGAGTCTGTTGCTAAC
CAGGTCATTCGCTCCTCAAGTGTCAAGGGGGCTGACATTAACGCCGGTATTATTAACCCGGTGAAAGATTTTGCGACCGT
TATTGCAGAGCCTCGTCTTGATGATAACAGCCAGACCACCTTCTACCTGGCTGCGTCAAAAGGCTCCGATACGATTGAAG
TGGCTTATCTCAACGGTGTGGATACGCCATATATTGATCAGATGGAGGGCTTCAGTGTGGATGGCGTGACAACGAAAGTG
CGTATTGACGCCGGTGTCGCGCCAGTTGATCACCGCGGTCTGGTGAAATGTACGGCGTAA
>Cluster_41427
ATGGCCGTTAACTTACTGAAAAAGAACTCACTCGTGCTGGTTGCTTCTCTGCTACTGGCGGGCCATGTACAGGCAACGGA
ACTGCTGAACAGTTCTTATGACGTCTCCCGCGAGCTGTTTGCCGCCCTGAACCCGCCGTTTGAGCAACAATGGGCAAAAG
ATAACGGTGGCGACAAACTGACGATTAAACAATCTCATGCCGGATCATCAAAACAGGCGCTGGCAATTTTGCAGGGTTTA
AAAGCCGACGTTGTCACTTATAACCAGGTGACCGACGTACAAATCCTGCACGATAAAGGCAAGCTGATCCCAGCGGACTG
GCAGTCGCGCCTGCCGAATAATAGCTCGCCGTTCTACTCCACCATGGGCTTCCTGGTGCGTAAGGGCAACCCGAAGAATA
TCCACGACTGGAACGACTTGGTGCGCTCCGATGTGAAGCTGATTTTCCCGAACCCGAAAACGTCGGGTAATGCGCGTTAC
ACCTACCTTGCGGCATGGGGCGCAGCGGACAAAGCCGACGGTGGTGATAAAGCCAAAACCGAACAGTTTATGACCCAGTT
CCTGAAAAACGTTGAAGTGTTCGATACCGGCGGTCGTGGCGCGACCACCACTTTTGCCGAGCGCGGGCTGGGCGATGTAC
TGATTAGCTTTGAATCGGAAGTGAACAACATTCGCAAACAGTATGAAGCGCAAGGCTTTGAAGTGGTGATTCCGAAAACC
AATATTCTGGCGGAATTCCCGGTGGCGTGGGTCGATAAAAACGTGCAGGCCAACGGTACGGAAAAAGCAGCAAAAGCTTA
CCTGAACTGGCTCTACAGCCCGCAGGCGCAAACCATCATCACCGACTATTACTACCGCGTAAATAACCCGGAAGTCATGG
ACAAACTGAAAGACAAATTCCCGCAGACCGAACTGTTCCGCGTGGAAGACAAATTTGGCTCCTGGCCGGAAGTGATGAAA
ACCCACTTCACCAGCGGCGGCGAGTTAGACAAGCTGTTAGCGGCGGGGCGTAATTAA
>Cluster_54794
GTGAATGAGGAGGATAACAGCTCAGAGGGAAATTCAGAATCAACAGAAACGCCTACCCCCGTCCTGCGCCCCGAAGCTGG
AAGTTACGTCGCTAACCTGGCAGCTGCTAACACTCTTTTTGTTATGCGTCTGAACGACCGTGCGGGTGAAACGCGCTACA
TCGATCCTGTAACTGAACAGGAGCGTTCAAGCCGACTTTGGCTACGTCAAATTGGCGGGCATAATGCCTGGCGTGACAGC
AACGGACAGTTGAGAACGACCTCGCATCGCTACGTCTCGCAGTTAGGGGGCGATCTGTTAACCGGTGGTTTTACCGATAG
TGACAGTTGGCGTTTGGGAGTGATGGCTGGTTATGCCCGCGACTACAACTTAACTCATTCCAGCGTGTCGGATTATCGTT
CGAAAGGGAGTGTCAGAGGCTATAGCGCAGGGCTGTATGCCACCTGGTTTGCCGATGACATCAGTAAAAAAGGCGCATAC
ATTGACTCCTGGGCGCAATATAGCTGGTTTAAAAACTCGGTGAAAGGGGATGAATTAGCCTATGAATCCTATAGCGCGAA
AGGTGCAACCGTCTCGCTGGAAGCGGGTTACGGTTTTGCCCTGAATAAATCCTTTGGTCTGGAAGCGGCGAAATATACGT
GGATCTTCCAGCCACAGGCACAGGCTATCTGGATGGGCGTCGATCATAATGCGCACACGGAAGCCAATGGCTCACGTATT
GAGAATGACGCAAATAACAACATCCAGACCCGACTCGGCTTCCGCACCTTTATTCGTACTCAGGAGAAAAACAGCGGTCC
GCACGGTGACGACTTTGAACCTTTTGTTGAAATGAACTGGATCCATAACAGTAAAGATTTTGCTGTCTCAATGAACGGTG
TGAAAGTCGAACAAGATGGGGCGAGTAATTTGGGGGAAATTAAACTTGGCGTAAATGGCAACCTGAATCCAGCGGCCAGC
GTCTGGGGAATGTGGGCGTGCAGCTGGGTGATAATGGCTACAATGACACCGCAGTGA
>Cluster_48467
ATGTCTGCATTTACCCCGGCAAGTGAAGTCTTGCTGCGTCACAGTGATGATTTCGAACAAAGCCGTATTCTGTTTGCCGG
AGACTTACAGGATGACCTGCCCGCGCGTTTAGATACCGCGGCCAGCCGTGCTCATACCCAGCAATTCCACCACTGGCAGG
TATTAAGCCGCCAGATGGGGGATAACGCCCGTTTCAGTCTGGTCGCCACGGCGGATGACGTCGCAGATTGCGATACGCTG
ATTTACTACTGGCCGAAGAACAAACCGGAAGCCCAGTTCCAGTTGATGAATTTACTTTCTCTGCTGCCGGTGGGGACGGA
TATTTTTGTCGTTGGCGAGAACCGCAGCGGCGTGCGCAGCGCCGAGCAGATGCTGGCAGATTATGCGCCGTTGAATAAAG
TCGACAGCGCTCGTCGCTGTGGCCTCTATTTTGGTCGTCTGGAAAAACAGCCGGTATTTGATGCCGATAAATTCTGGGGC
GAATACAGCGTCGATGGCCTGACGGTCAAAACGCTGCCTGGCGTGTTTAGCCGCGACGGTCTGGATGTCGGTAGCCAGTT
GCTGCTCTCGACGTTAACTCCGCACACGAAAGGTAAAGTGCTGGATGTCGGCTGTGGCGCGGGGGTGCTTTCAGTTGCCT

TTGCGCGCCATTCGCCGAAAATTCGTCTCACCTTGTGCGATGTCTCTGCGCCAGCGGTAGAAGCCAGCCGCGCAACACTT
GCGGCCAACTGTGTTGAAGGTGAAGTCTTTGCCAGCAACGTCTTTTCCGAGGTGAAAGGTCGTTTTGATATGATCATCTC
CAACCCGCCGTTCCACGATGGGATGCAAACCAGCCTGGATGCGGCGCAAACGCTGATTCGCGGCGCGGTGCGTCATCTTA
ATAGCGGCGGCGAGCTGCGAATTGTAGCGAACGCCTTCCTGCCTTACCCGGACGTGCTGGATGAGACATTTGGCTTCCAT
GAAGTTATCGCGCAAACCGGGCGCTTCAAGGTGTATCGCGCCATTATGACCCGCCAGGCGAAGAAAGGTTAA
>Cluster_40409
ATGGCAAAGCCGATCATCACGCTCAATGGCCTAAAAATCGTCATTATGTTGGGAATGCTGGTCATTATTCTCTGCGGTAT
CCGTTTTGCCGCCGAGATCATCGTGCCGTTTATTCTCGCATTATTTATTGCTGTTATTCTTAACCCGCTGGTGCAACACA
TGGTCCGCTGGCGTGTGCCGCGTGTACTGGCAGTGTCAATTTTGATGACCATCATCGTGATGGCGATGGTGTTGCTGTTA
GCTTATCTGGGTTCCGCGCTCAATGAGTTGACGCGGACGTTACCGCAATATCGCAACTCTATTATGACGCCACTGCAAGC
GATTGAACCGTTGTTGCAACGCGTAGGGATTGATGTCTCAGTTGACCAACTGGCGCATTACATTGATCCGAACGCGGCGA
TGACGTTGCTCACCAACTTATTGACGCAGTTATCTAATGCCATGTCATCAATATTTTTATTGCTGCTGACGGTGCTGTTT
ATGTTGCTCGAAGTGCCACAATTGCCCGGAAAATTTCAGCAAATGATGGCGCGTCCGGTTGAAGGGATGGCGGCGATTCA
GCGTGCGATTGACTGTGTGTCTCATTATCTGGTGCTGAAAACAGCCATCAGCATCATCACCGGCCTGGTCGCCTGGGCGA
TGCTCGCCGCACTCGATGTTCGCTTCGCTTTTGTCTGGGGATTGCTGGCCTTTGCGCTTAATTACATCCCGAATATTGGT
TCAGTTCTCGCGGCAATCCCCCCTATCGCTCAGGTACTGGTGTTTAATGGCTTCTACGAAGCGTTGCTGGTGCTGGCGGG
ATATCTGCTGATTAATCTGGTCTTCGGCAATATTCTGGAGCCACGCATCATGGGGCGTGGGCTGGGGCTTTCCACATTGG
TGGTATTTTTGTCGTTGATTTTTTGGGGATGGTTGTTAGGACCGGTGGGTATGCTGCTTTCCGTGCCGTTGACAATTATT
GTCAAAATTGCGCTTGAACAAACAGCGGGAGGTCAAAGCATCGCCGTTCTGTTAAGCGATCTCAATAAAGAGTGA
>Cluster_4767
ATGGTCACTTTTGAGACAGTTATGGAAATTAAAATCCTGCACAAGCAGGGAATGAGTAGCCGGGCGATTGCCAGAGAACT
GGGGATCTCCCGCAATACCGTTAAACGTTATTTGCAGGCAAAATCTGAGCCGCCAAAATATACGCCGCGACCTGCTGTTG
CTTCACTCCTGGATGAATACCGGGATTATATTCGTCAACGCATCGCCGATGCTCATCCTTACAAAATCCCGGCAACGGTA
ATCGCTCGCGAGATCAGAGACCAGGGATATCGTGGCGGAATGACCATTCTCAGGGCATTCATTCGTTCTCTCTCGGTTCC
TCAGGAGCAGGAGCCTGCCGTTCGGTTCGAAACTGAACCCGGACGACAGATGCAGGTTGACTGGGGCACTATGCGTAATG
GCCGCTCACCGCTTCACGTGTTCGTTGCTGTTCTCGGATACAGCCGAATGCTGTACATCGAATTCACTGACAATATGCGT
TATGACACGCTGGAGACCTGCCATCGTAATGCGTTCCGCTTCTTTGGTGGTGTGCCGCGCGAAGTGTTGTATGACAATAT
GAAAACTGTGGTTCTGCAACGTGACGCATATCAGACCGGTCAGCACCGGTTCCATCCTTCGCTGTGGCAGTTCGGCAAGG
AGATGGGCTTCTCTCCCCGACTGTGTCGCCCCTTCAGGGCACAGACTAAAGGTAAGGTGGAACGGATGGTGCAGTACACC
CGTAACAGTTTTTACATCCCACTAATGACTCGCCTGCGCCCGATGGGGATCACTGTCGATGTTGAAACAGCCAACCGCCA
CGGTCTGCGCTGGCTGCACGATGTCGCTAACCAACGAAAGCATGAAACAATCCAGGCACGTCCCTGCGATCGATGGCTCG
AAGAGCAGCAGTCCATGCTGGCACTGCCTCCGGAGAAAAAGAGTATGACGTGCATCTTGATGAAAATCTGGTGAACTTC
GACAAACACCCCCTGCATCATCCACTCTCCATCTACGACTCATTCTGCAGAGGAGTGGCGTGA
>Cluster_15798
ATGGGAAAGAGTAAAAAAAACCGCGTAGCGGCGATGAATCAGATCCAGCATAAAAGCCAGACTTCAGCCGAAGCATTCAG
CTTCGGCGATCCCGTTCCTGTTCTGGACCGCCGTGAACTGCTGGACTATGTGGAATGCGTACAGACAGATCGATGGTATG
AGCCGCCAGTGAGTTTTGACGGGCTGGCGCGCACCTTTCGCGCCGCCGTGCATCACAGCTCACCGATTGCGGTGAAATGC
AATATTCTGACCAGTACCTACATCCCTCACCCGCTGCTCAGCCAGCAGGCTTTTTCACGTTTTGTGCAGGACTATCTGGT
ATTTGGTAACGCCTACCTGGAGAAACGCACGAACCGCTTCGGTGAAGTTATCGCCCTTGAGCCTGCTCTGGCAAAATACA
CCCGACGCGGGTTAGACCTGGATACCTACTGGTTTGTGCAATACGGTATGACAACCCAGCCGTATCAGTTCACGAAAGGC
AGCATTTTTCATCTGATGGAACCGGACATCAACCAGGAGATCTACGGCCTGCCAGGTTATCTTTCTGCCATTCCGTCAGC
CCTGCTCAACGAGTCCGCCACGCTGTTCCGCCGGAAGTATTACATTAACGGCAGTCATGCAGGCTTCATCATGTACATGA
CCGATGCCGCGCAGAACCAGGAGGATGTGAACAACCTCCGCAATGCGATGAAAAGCGCCAAAGGCCCTGGCAACTTCCGC
AACCTGTTTATGTACTCGCCTAACGGCAAAAAGGACGGGCTTCAGATCATCCCGTTGTCAGAAGTCGCGGCGAAGGATGA
ATTTCTTAACATCAAGAACGTGAGTCGGGATGACATGATGGCAGCGCATCGTGTGCCGCCACAAATGATGGGGATAATGC
CTAATAATGTTGGGGGATTTGGGGATGTAGAAAAAGCTAGTAAGGTATTTGTGAAAAATGAATTATTACCATTACAAAAA
AGAATGAAAGAATTTAATCATTGGAGCGGTGAAGAAATTATAAAATTCGAGAGATATCAAATTTAG
>Cluster_29355
ATGTCCGGGTTTTATCATAAGCATTTCCTGAAATTACTCGATTTCACGCCAGCTGAACTTAACAGCCTGCTGCAGTTAGC
CGCGAAGCTGAAAGCTGATAAGAAAAGCGGTAAAGAAGAAGCCAAACTCACTGGTAAAAACATCGCGCTCATCTTCGAAA
AAGACTCGACCCGTACCCGATGCTCTTTCGAAGTTGCCGCATATGACCAGGGTGCTCGCGTTACTTATCTCGGCCCAAGC
GGTAGTCAGATTGGTCATAAAGAGTCGATTAAAGACACTGCTCGCGTGCTTGGTCGCATGTATGACGGTATTCAGTATCG
CGGCTATGGTCAGGAGATTGTCGAAACACTGGCGGAATACGCTGGCGTGCCGGTATGGAACGGTCTGACCAATGAGTTTC
ACCCCACGCAGTTGCTGGCGGATCTCCTCACCATGCAGGAGCATTTACCCGGTAAAGCATTCAACGAAATGACGCTGGTC
TATGCAGGTGACGCACGTAACAACATGGGCAATTCGATGCTCGAAGCTGCGGCGCTTACCGGTCTGGATTTGCGTCTGGT
CGCGCCACAGGCATGCTGGCCGGAAGCTGCGCTGGTTACGGAATGCCGCGCTCTGGCACAGCAAAATGGCGGGAATATTA
CGCTAACGGAAGATGTAGCCAAGGGAGTAGAAGGTGCTGACTTTATCTATACCGATGTGTGGGTGTCGATGGGGGAAGCA
AAAGAGAAATGGGCAGAACGGATTGCATTACTGCGTGATTATCAGGTGAACAGCAAGATGATGCAGTTGACCGGTAACCC
GGAGGTCAAATTCCTCCACTGCCTGCCCGCTTTTCATGACGACCAGACGACGCTTGGTAAGAAAATGGCGGAAGAGTTTG
GCCTACATGGAGGAATGGAAGTGACTGATGAGGTCTTCGAATCTGCCGCCAGCATTGTTTTTGATCAGGCGGAAAACCGC
ATGCATACCATCAAAGCGGTGATGGTCGCGACGCTCAGTAAATTAAATAATTAA
>Cluster_22192
ATGAACGATAACGTTTTGCTCATAGGAGCTTCCGGATTCGTAGGAACCCGACTACTTGAAACGGCAATTGCTGACTTTAA
TATCAAGAACCTGGACAAACAGCAGAGCCACTTTTATCCAGAAATTACACAGATTGGCGATGTTCGCGATCAACAGGCAC
TCGACAAGGCGTTAGCCGGTTTTGACACTGTTGTGCTACTGGCAGCGGAACACCGCGATGACGTCAGCCCTACTTCTCTC
TATTATGATGTCAACGTTCAGGGTACCCGCAATGTGCTGGCGGCCATGGAAAAAAATGGCGTTAAAAATATCATCTTTAC
CAGTTCCGTTGCTGTTTATGGTTTGAACAAACACAACCCTGACGAAAACCATCCACACGATCCTTTCAACCACTACGGCA
AAAGTAAGTGGCAGGCAGAGGAAGTGCTGCGTGAATGGTATAACAAAGCACCAACAGAACGTTCATTAACCATCATCCGT
CCTACCGTTATCTTCGGTGAACGCAACCGCGGTAACGTCTATAACTTGCTGAAACAGATTGCTGGCGGCAAGTTTATGAT
GGTAGGCGCAGGGACTAACTATAAGTCCATGGCTTATGTTGGAAACATTGTTGAGTTTATCAAGTACAAACTGAAGAATG
TTGCCGCAGGTTATGAGGTTTATAATTACGTTGATAAGCCAGACCTGAACATGAACCAGTTGGTTGCTGAAGTTGAACAA
AGCCTGAACAAAAAGATCCCTTCTATGCACTTGCCTTACCCACTAGGAATGCTGGGTGGATATTGCTTTGATATCCTGAG
CAAAATTACGGGAAAAAAATACGCTGTCAGCTCTGTGCGCGTGAAAAAATTCTGCGCAACAACACAGTTTGACGCAACGA
AAGTGCATTCTTCAGGTTTTGTGGCACCGTATACGCTGTCGCAAGGTCTGGATCGAACTCTGCAGTATGAATTCGTTCAT
GCCAAAAAAGACGACATAACGTTTGTTTCTGAGTAA
>Cluster_57826
ATGCGCAATCACAGAATTTCTTTACAGGATATCGCTACGCTGGCTGGCGTAACAAAAATGACCGTGAGTCGTTATATCCG
CTCGCCGAAAAAGGTGGCAAAGGAAACAGGCGAGCGCATCGCCAAAATTATGGAAGAGATCAATTATATCCCTAATCGCG
CTCCCGGCATGTTGTTGAATGCACAAAGTTATACCCTTGGTATCTTGATTCCGTCATTTCAAAATCAACTCTTCGCTGAC

ATTCTCGCGGGAATAGAATCCGTCACCTCTGAGCATAATTATCAGACCCTTATTGCTAATTACAATTACGATCGCGATTC
CGAAGAAGAGTCGGTGATTAACCTGCTCTCTTATAACATTGACGGGATTATACTTTCGGAAAAATATCACACCATCAGGA
CGGTGAAATTTCTGCGTTCAGCCACCATTCCGGTCGTGGAATTGATGGATGTACAGGGAGAACGGCTGGATATGGAGGTC
GGTTTTGATAATCGGCAGGCCGCTTTTGACATGGTGTGTACCATGCTGGAGAAGCGAGTCAGGCACAAAATTCTGTATCT
CGGTTCCAAAGATGACACCCGCGATGAACAGCGTTATCAGGGGTATTGCGATGCGATGATGCTGCATAATCTTTCCCCAT
TACGCATGAATCCACGCGCCATCTCATCCATTCATTTAGGAATGCAGTTAATGCGTGATGCCCTTAGCGCAAACCCGGAT
TTAGACGGTGTATTTTGCACCAATGACGATATTGCGATGGGTGCGCTTCTGCTGTGCCGCGAGCGAAACCTCGCCGTGCC
AGAACAAATTTCCATTGCGGGTTTTCATGGGCTGGAGATTGGCAGGCAAATGATCCCAAGCCTCGCCAGCGTAATTACTC
CCCGTTTTGATATTGGTCGGATGGCCGCCCAAATGTTGCTGAGCAAAATTAAAAATAACGATCACAACCACAACACTGTT
GACCTCGGGTATCAGATTTATCAAGGCAACACGCTTTAG
>Cluster_42872
TTGGCTACAATGAAAGATGTTGCCCGCCTGGCGGGCGTTTCTACCTCAACAGTTTCTCACGTTATCAATAAAGATCGCTT
CGTCAGTGAAGCGATTACAGCCAAAGTTGAAGCGGCGATTAAAGAACTCAATTACGCGCCATCAGCTCTGGCGCGTAGCC
TCAAACTCAATCAAACACATACCATTGGCATGTTGATCACTGCCAGTACCAATCCTTTCTATTCAGAACTGGTGCGTGGC
GTTGAACGCAGCTGCTTCGAACGCGGTTATAGTCTTGTCCTTTGCAATACCGAAGGCGATGAACAGCGGATGAATCGTAA
TCTGGAAACGCTGATGCAAAAACGCGTTGATGGCTTGCTGTTACTGTGCACCGAAACGCATCAGCCTTCGCGTGAAATCA
TGCAACGTTATCCGACAGTGCCTACCGTGATGATGGACTGGGCTCCGTTCGATGGCGACAGCGATCTTATTCAGGATAAC
TCGTTGCTGGGCGGAGACTTAGCAACGCAATATCTGATCGATAAAGGTCATACCCGTATTGCCTGTATTACCGGTCCGCT
GGATAAAACTCCGGCGCGCCTGCGGTTGGAAGGTTATCGGGCGGCGATGAAACGTGCGGGTCTCAACATTCCTGATGGCT
ATGAAGTCACTGGTGATTTTGAATTTAACGGCGGGTTTGACGCCATGCGCCAACTGTTATCACATCCGCTGCGTCCTCAG
GCTGTCTTTACCGGAAATGACGCTATGGCTGTTGGCGTTTACCAGGCGTTATATCAGGCAGAGTTACAGGTTCCGCAGGA
TATCGCGGTGATTGGCTATGACGATATCGAACTGGCAAGCTTTATGACGCCACCATTAACAACTATCCACCAACCGAAAG
ATGAACTGGGGGAGCTGGCGATTGATGTACTCATCCATCGGATAACCCAGCCGACCCTTCAGCAACAACGATTACAACTT
ACTCCGATTCTGATGGAACGTGGTTCGGCTTAG
>Cluster_62521
ATGAAATGTCTGGCAATTGCCGATCTTTTTATCAACAAAGCGATGATGGATGCAGGTCTTAATGCGCTCAGGGATAAAGG
TATCGACGTTGAAGTTCGTGAATGGTCGCATGATTCGATCGAAAAATTGCAGGAGGATAATCTGCGGGTCGAGCAGGAAG
GCGCAGAAGCGGTTACGTTACCCGCAGCCTTACTTGAGGGTGCTGACGACGTTGAAATCCTGATAACCCAGTTTGCTCCG
GTCAATACGGCAGTGCTGGATAAACTGCCAAAGCTCAAATACATCGGGGTATTGCGAGGGGGTGTTGAAAACGTCAACCA
GCAGGTCGCACTGGCCCGGGGAATTGAGGTAATGAACACGCCGGGGCGCAATGCCCGCAGCGTGGCGGAATTTACGGTAG
GGATGATGCTGGCCGAAATGCGCAATATTGCCCGTTCTCATGATGCGTTACGCGATAAGTTCTGGCGTAAAGACAGCCCG
AATCATCAGGCTATTCCAGAGCTTGGCGGTAAGGTTGTCGGTCTGGTTGGCCTGGGTCATATTGCTCAACTCGTCGCGGG
TTTTCTTAGCGCCTTCGGCACGGAAATTATCTTTTATGATAAGTACGTCTCCGGCCACGAACGCTATGAGAAAGTCGATA
CCCTTGACGAGCTGGTAACACGGGCTGACGTTGTCTCACTCCATGCCCGTATGACGCCGGAAACCGAAAACCTGATCAAC
GCACACCATTTCGGGTTAATGAAAAGCAGCGCCATTATTGTTAACACCGCGCGCTCCGGGCTAATCAATGAACGCGATCT
GATTGACGCTCTACAGACCGGAAAAATCATGGGGGCGGCGCTGGATACTTTCGACGATGAGCCGTTACCTGACGACAGCG
CGTTTTATTTACTGAATAATGTCACCATCACCCCGCATATTGCGGGCAGTACGCTCGACGCCTTCAGTAATTCACCCAAA
CTATTTGCTGAGATCTTGCTGAAGAAACTCAGTTAA
>Cluster_54182
ATGAAGATAATCTGCAGATTATTATTGGCGATGGCATGTTTGTGGTTAACAAACATATCCTGGGCTACTGTTTGTGCAAA
TAGTACTGGCGTAGCAGAAGATGAACACTATGATCTCTCAAATGTCTTTAATAGCACCAATAACCAGCCAGGGCAGATTG
TTGTTTTACCGGAAAAATCCGGCTGGGTAGGTGTGTCAGCAATTTGTCCACCCGGCACGCTGGTGAATTATACATACCGT
AGTTATGTCACCAACTTTATTGTTCAGGAAACTATCGATAATTATAAATATATGCAATTATATGATTATCTATTAGGTGC
GATGAGTCTGGTTGATAGTGTTATGGATATTCAGTTCCCCCCGCAAAATTATATTCGGATGGGAACAGATCCTAACGTTT
CGCAAAACCTTCCATTCGGGGTGATGGATTCTCGTTTAATATTTCGTTTAAAGGTTATTCGTCCCTTTATTAACATAGTG
GAGATCCCCAGACAGGTGATGTTTACCGTGTATGTGACATCAACGCCTTACGATCCGTTGGTTACACCTGTTTATACCAT
TAGTTTTGGTGGCCGGGTTGAAGTACCGCAAAACTGCGAATTAAATGCCGGGCAGATTGTTGAATTTGATTTTGGTGATA
TCGGCGCATCGTTATTTAGTGCGGCAGGGCCGGGTAATCGACCTGCTGGTGTCATGCCGCAAACCAAGAGCATTGCGGTC
AAATGTACGAATGTTGCTGCGCAGGCTTATTTAACAATGCGTCTGGAAGCCAGTGCCGTTTCTGGTCAGGCGATGGTGTC
GGACAATCAGGATTTAGGTTTTATTGTCGCCGATCAGAACGATACGCCGATTACGCCTAACGATCTCAATAGCGTTATTC
CTTTCCGTCTGGATGCAGCTGCGGCAGCCAATGTCACACTTCGCGCCTGGCCTATCAGTATTACCGGTCAAAAACCGACC
GAGGGGCCGTTTAGCGCGCTGGGGTATTTACGCGTCGATTATCAATGA
>Cluster_33358
ATGGATAAAATTACCCAGGTTTTATTTTCAGATATTGGGAAAGTCACCACTCAATATGTTGAAGTACCACACCAGCAACT
TAAACCGCACGAAGTACGGATTGCGCCCGTGTTCTACGGGATATGCGGTTCGGATCTGCATGTTCTGAAAGGCGGTCATC
CGTTTGCCAAACCGCCTGTCGTCCCCGGTCATGAAATTGCAGCGCGCGTTACGGAAGTGGGCAGCGACGTTAAAAATGTA
CAGCCGGGCGATCATGTTGTGGTCGATCCCATCATGGCTTGCATGGAATGCCGAGCCTGCAAAGCAGGACGTTTTAATCT
TTGTGAACCACCGCAGGTTGCTGGTTTTCGCGCACCGGGCTTTGCTCGCTCACAACACATTGTTCCTGCGCGTAATTGCC
ATGTCGCACCAGCCTCTTTACCGCTAAAAGTGTTGGCCTTTGCCGAACCGGCGGCTTGTGCTCGTCACTGCGTTAACCGA
ATGCCGAAAGCTTCTCTGGAAAGCGTACTGGTGATTGGTGCCGGAACGATAGGCTTATCCATCGTGCAGGCACTGCGCAT
TATGGGGGCAGGTAAGATTACCGTGATTGAACCTGACGCTGCCAAACGCGCGCTGGCGTTAAAGCTGGGCGCAGCAGAAG
TTTGGGCACCAGGTGAGCTGGCCGCAGATGTGAGATTTACGGGGGCGATTGATGTCGTTGCAGCGCAGGCCACGCTTAAC
GATGCATGTACCCGTGTATATGCCGGAGGCACCGTCGTGTGCATGGGCGTACCAAGTGGGCCGCGTGAAATACCATTACC
GATGATGCAACGTTTCGAGCGTGACTTGCTCAACTCTGGCATGTACATCCCTGAAGATTTCGATGCTGTTATCGAATGGC
TGGCGGATGGGCGGTTTGATACCAGTGAACTGGTTACCGATTTATTTGCCATTGAGGATGCAGCGGCGGCATTCGAACGC
GCGCAGCAAAATGACTCCATAAAGGTCATGCTGCAATTTGCGCCGGAATGA
>Cluster_21092
ATGACCGCGATAAAACACCCGATGCTGCTGTGGGGGCTTCCCGTTGCAGCACTTATTATTATTTTCTGGCTGAGTCTGTT
TTGCTACTCGGCCATTCCTGTTTCCGGAGCAGATGCAACCCGCGCCCTGCTGCCTGGACACACGCCAACGCTACCAGAAG
CGCTGGTGCAAAACCTTCGTTTGCCACGAAGCCTGGTCGCCGTTCTGATCGGCGCAAGCCTGGCGCTCGCGGGCACGCTG
CTGCAAACCCTGACCCACAACCCAATGGCCTCTCCTTCACTGCTCGGCATTAACAGCGGCGCGGCGCTGGCTATGGCGCT
TACCAGCGCGCTGAGTCCGACGCCGATTGCAGGCTATTCTCTGTCGTTCATCGCGGCATGCGGGGGCGGCGTGAGCTGGC
TGCTGGTCATGACCGCAGGAGGCGGATTTCGTCATACCCATGACAGAAACAAACTGATCCTCGCGGGTATCGCGCTGTCG
GCCTTTTGTATGGGCCTTACCCGCATCACCCTGCTGCTGGCCGAAGATCATGCTTACGGCATCTTTTACTGGCTGGCAGG
CGGAGTGTCCCACGCCCGCTGGCAGGATGTCTGGCAGCTCTTGCCGGTGGTGGTCACTGCAGTCCCTGTCGTGTTACTGC
TGGCGAATCAACTGAACCTGCTCAACCTCAGCGACAGCACCGCCCATACGCTGGGAGTGAACCTGACGAGGCTACGTTTG
GTCATCAATATGTTAGTGCTGCTTCTGGTTGGTGCGTGCGTCAGCGTGGCAGGTCCGGTGGCGTTTATCGGTCTGCTGGT
GCCACATCTGGCGCGCTTCTGGGCAGGCTTCGATCAGCGCAACGTACTGCCAGTGAGCATGCTGCTGGGGGCCACGCTGA
TGCTGCTGGCAGATGTACTCGCACGCGCGCTGGCCTTCCCCGGAGATCTGCCCGCAGGCGCAGTGCTGGCGCTGATTGGC

AGCCCTTGCTTTGTCTGGCTTGTGAGGAGGCGAGGATGA
>Cluster_37569
ATGAGTACGCAAGAGGCCACCCTGCAACAACCGCTGTTGCAGGCTATCGACCTGAAAAAACATTATCCGGTGAAGAAAGG
CATGTTCGCGCCGGAACGTCTGGTTAAAGCGCTGGATGGCGTTTCGTTTAACCTTGAACGTGGCAAAACGCTGGCAGTAG
TGGGCGAATCTGGCTGCGGTAAATCGACCCTCGGTCGGTTGCTGACGATGATTGAAATGCCCACCGGTGGCGAGCTGTAT
TACCAGGGGCAGGATCTGCTTAAGCACGATCCGCAGGCGCAGAAGCTGCGTCGGCAGAAAATCCAGATCGTCTTCCAGAA
CCCTTACGGTTCGCTGAATCCGCGTAAAAAAGTCGGGCAAATTCTTGAAGAGCCGCTGCTGATCAACACCAGCTTAAGCA
AAGAACAGCGTCGGGAAAAAGCCCTGTCGATGATGGCGAAAGTCGGCCTGAAAACCGAGCACTATGACCGCTATCCGCAT
ATGTTCTCCGGCGGTCAGCGTCAGCGTATCGCCATCGCCCGTGGTCTGATGCTCGACCCGGATGTGGTGATTGCCGATGA
ACCGGTTTCCGCGCTGGATGTTTCAGTGCGCGCGCAGGTGCTGAATCTGATGATGGATTTGCAGCAGGAGTTGGGGCTGT
CTTATGTCTTTATCTCCCACGACCTGTCGGTGGTGGAGCACATTGCTGATGAAGTGATGGTGATGTACCTGGGCCGCTGC
GTGGAGAAGGGAACGAAAGACCAAATCTTCAATAACCCGCGCCATCCGTACACTCAGGCGCTACTTTCCGCGACGCCGCG
CCTGAACCCGGACGATCGCCGCGAGCGCATCAAGCTCAGCGGTGAACTACCAAGCCCACTGAATCCACCGCCGGGTTGCG
CCTTCAACGCCCGCTGTCGTCGGCGCTTCGGCCCCTGCACCCAGTTGCAGCCGCAGCTAAAAGACTACGGCGGTCAACTG
GTAGCTTGTTTTGCTGTTGATCAGGATGAAAATCCGCAGCGTTAA
>Cluster_3555
ATGAAGTATGTCTTTATTGAAAAACATCAGGCTGAGTTCAGCATCAAAGCAATGTGCCGCGTGCTCCGGGTGGCCCGCAG
CGGCTGGTATACGTGGTGTCAGCGGCGGACAAGGATAAGCACGCGTCAGCAGTTCCGCCAACACTGCGACAGCGTTGTCC
TCGCGGCTTTTACCCGGTCAAAACAGCGTTACGGTGCCCCACGCCTGACGGATGAACTGCGTGCTCAGGGTTACCCCTTT
AACGTAAAAACCGTGGCGGCAAGCCTGCGCCGTCAGGGACTGAGGGCAAAGGCCTCCCGGAAGTTCAGCCCGGTCAGCTA
CCGCGCACACGGCCTGCCTGTGTCAGAAAATCTGTTGGAGCAGGATTTTTACGCCAGTGGCCCGAACCAGAAGTGGGCAG
GAGACATCACGTACTTACGTACAGATGAAGGCTGGCTGTATCTGGCAGTGGTCATTGACCTGTGGTCACGTGCCGTTATT
GGCTGGTCAATGTCGCCACGCATGACGGCGCAACTGGCCTGCGATGCCCTGCAGATGGCGCTGTGGCGGCGTAAGAGGCC
CCGGAACGTTATCGTTCACACGGACCGTGGAGGCCAGTACTGTTCAGCAGATTATCAGGCGCAACTGAAGCGGCATAATC
TGCGTGGAAGTATGAGCGCAAAAGGTTGCTGCTACGATAATGCCTGCGTGGAAAGCTTCTTTCATTCGCTGAAAGTGGAA
TGTATCCATGGAGAACACTTTATCAGCCGGGAAATAATGCGGGCAACGGTGTTTAATTATATCGAATGTGATTACAATCG
GTGGCGGCGGCACAGTTGGTGTGGCGGCCTCAGTCCGGAACAATTTGAAAACCAGAACCTCGCTTAG
>Cluster_41593
ATGCAACGGTTGGAGCAGCGTTCACCTGACGCTATACTGCTTCTCTTTCTTATTGCTCAAACTGTCGACATTACTATGCC
CGTTTTTGCTCTGCTCGCGCTTGTCGCCTACTCCGTCAGTCTTGCGCTGATTGTTCCCGGTCTGCTGCAAAAAAACGGCG
GCTGGCGGCGCATGGCTATTATTTCTGCGGTCATCGCGCTGGTCTGCCACGCAATCGCTCTGGAAGCCCGCATCCTGCCC
GACGGTGATAGCGGACAAAACCTCAGCCTGCTGAACGTTGGTTCATTGGTCAGTTTGATGATCTGTACGGTAATGACCAT
TGTGGCTTCTCGCAATCGTGGCTGGTTGTTGCTACCCATTGTCTATGCCTTTGCGCTTATCAACCTGGCGCTGGCGACCT
TTATGCCCAATGAATACATCACCCATCTGGAAGCAACGCCGGGGATGCTGGTGCACATTGGCTTATCGCTTTTTTCCTAT
GCCACGCTAATTATCGCTGCCCTGTACGCGCTGCAACTGGCGTGGATTGATTACCAACTGAAGAACAAGAAGCTGGCGTT
TAACCAGGAAATGCCTCCATTGATGAGTATCGAGCGTAAAATGTTCCATATCACGCAGATTGGCGTGGTGCTGCTAACGC
TCACTCTTTGCACTGGCCTGTTCTACATGCACAACTTATTTAGCATGGAAAATATCGACAAGGCCGTGCTCTCTATCGTG
GCGTGGTTTGTCTATATTGTGCTGCTGTGGGGACATTATCATGAAGGATGGCGTGGACGCCGCGTCGTCTGGTTTAACGT
TGCGGGTGCGGTCATTCTGACACTGGCCTACTTCGGTAGCCGAATTGTCCAGCAGTTAATCAGCTAA
>Cluster_58182
ATGAATAATTCTGAAGGTTTGAAGTCGTTTCAGCAATCGCTTGCTGATCTGCCGCAATGGGTATCAGAGCGGCTTCTGCA
GCAAATTAACCAGTTAACTAACTACGAGCCAGTGATCGGCATTATGGGTAAAACCGGTGTTGGGAAGAGCAGTCTGTGCA
ATGCCCTGTTTGCCGGAGATATATCACCGGTCAGCGATGTGGCGGCCTGTACACGTGAGCCACTGCGCTTTCGCCTGCAA
GTCGGCGACCGCTATATAACGCTGATGGATCTGCCCGGCGTGGGCGAAAGTGGCGCTCGCGATACCGAGTATGCTGCGCT
GTACCGCGAACAGCTTCCTCGCCTCGACCTGGTACTGTGGCTGATTAAGGCCGATGACCGGGCGCTGACGGTGGATGAAC
ATTTTTATCATCAGGTGATTGGAGAGGTATACCGGCATAAGGTGCTGTTTGTTATCAGCCAGTCGGATAAGGCCGAACCT
ACCAGCGGTGGGGACAGTTGTCCACGGCGCAGAAGCAGAATATCAGCCGCAAAATCTGCCTGCTGCATGAGCTGTTCCA
GCCCGTCCATCCGGTGTGTGCTGTGTCGGTCCGTCTACAATGGGGGCTTAAGGTGATGGCCGAGCGGATGATTAAGTGCC
TGCCACGTGAGGCCACCAGCCCGGTGGTGTCGCAACTCCATCCTTCCTTTCGCACGACGGTAGTCCGGGAACAGGCTCGT
AGCGATTTTGGTGAAACCGTCGGGGCCGTACTCGATAGCATCAGTGCCTTTCCCCTGATACCCGCCCCGGTGCGGGCCGT
CATTCAGGCCGTGCGCACCACGGTGGTGTCAGTGGCCCGCGCCGTCTGGGATTTCTTCTTCTGA
>Cluster_7945
ATGGCTAAAAAAATTTCGAAATGGTTTCGCATCGGCGTCGAGGGTGACACCTGCGACGGTCGCGTCATCAGTGCGACCGA
TATTCAGGAAATGGCCGACGGCTTTGACCCGCGCGTCTACGGCTGTCGCATTAACCTCGAGCATATCCGCAGCGTTATTC
CCGACAGCCCGTTTGCCGCTATGGCGACGTCACGGAAGTGAAAGCAGAAGTGATTGACGACGATTCGGCGCTCAACGGC
AAGCTGGCGCTGTTCGGCAAAATTGCCCCGCTCGACAACCTGCTCGCGATGCTGGCGAAAGGCCAGAAAGTTTACACCTC
GATGGAAATTCGCCCGAACTTTGCCAACACCGGCAAATGTCACCTCATCGGGCTGGCGGTAACCGACGACCCGGCGAGCC
TCGGTACCGAGTACCTGCAATTCTGTTCCCGCGCACAACAAAACCCACTGGCCGGGAAGAAAGACCAGCCGGGCGACCTG
TTCTCAGTGGCAACCCTTGCCGAGCTGGAATTCGAAGACCTGCCCGACACCCTGCTGACCAAACTCAGCGACACCGTGAA
AGGCATCTTCAGCCGTAAACAGACTGACGACGATGCGCGTTTCGGTGATGTACATGAAGCCGTGACCGCCATTGCCGAAC
GGGTGCAGACCGGCGGCGAAAGCGCTGAGGTGCGTTTCAGTGCCATTGAAACCGAACTCGCCGACGTCAAAAAAGCGCTG
GCTGAACAGGCCGACGCCACCTCGCAGCAATTCAGCACTATCAAAACCACGCTGGAGCACACCGAAGACAAAACGCAGCC
GCGCCGCAAGTTAAGCGCCGGTGGCGACGGTGATTCGTCTGGCTCCACGCTGACCGACTGCTAA
>Cluster_21944
ATGATGCGAATCGCGCTCTTCCTGCTAACGAACCTGGCCGTAATGGTCGTTTTCGGGCTGGTACTGAGCCTGACAGGGAT
ACAGTCGAGCAGCGTTCAGGGGCTGATGATCATGGCCTTGCTGTTCGGTTTTGGTGGTTCCTTCGTTTCGCTTCTGATGT
CCAAATGGATGGCATTACGATCTGTTGGCGGGGAAGTGATCGAGCAACCGCGTAACGAAAGGGAACGTTGGCTGGTCAAT
ACGGTAGCAACCCAGGCTCGTCAGGCGGGGATCGCTATGCCGCAAGTGGCTATCTACCATGCGCCGGACATCAACGCTTT
TGCAACCGGTGCGCGCCCGTGATGCCTCTCTGGTTGCTGTCAGCACCGGTTTGCTGCAGAACATGAGCCCGGATGAAGCCG
AGGCGGTAATTGCTCATGAAATCAGCCACATCGCCAATGGTGATATGGTCACCATGACGCTGATTCAGGGCGTGGTGAAC
ACCTTCGTTATCTTTATTTCCCGTATTCTGGCGCAGCTTGCTGCGGGTTTTATGGGCGGAAATCGTGATGAAGGTGAAGA
GAGCAACGGCAACCCGCTGATCTACTTTGCGGTTGCAACGGTTCTGGAACTGGTGTTTGGTATTCTGGCGAGCATTATCA
CCATGTGGTTCTCGCGTCATCGTGAATTCCACGCCGATGCGGGTTCAGCAAAACTGGTTGGTCGCGAGAAAATGATTGCC
GCGCTGCAGCGCCTGAAAACCAGCTATGAACCGCAAGAAGCAACCAGCATGATGGCTTTCTGCATTAACGGTAAGTCGAA
ATCGCTCAGTGAGTTGTTCATGACTCACCCGCCGCTGGATAAACGTATTGAAGCTCTGCGTACGGGTGAATACCTGAAGT
AA
>Cluster_67121
ATGCTTCAGACTATGATTATCAATGATATCCTCATATGGATTGAGAATCACATAGAAGATGGGATTATAATTGAAGATGT
CGTGAATCTTTCTGGTTACAGCAGAAGGTATATCCAGTATTTATTTAAGAAAGAGACCGGCCTTTCTCTGGGAGGGTATT

TAAAACGGAGGCGTCTGTGCAGAGGTGCAATGTATGTTAGATTAACAACAATGCGAATGTTAGATATTGCTGTCCGACTT
CGCTTTGATTCACAGCAATCTTTTAGTCGTGAATTTAAAAAAGTGTTCAACTGCCCTCCTTATATTTACAGAACGAGAGA
TTACTGGGACCTGTCCTGTCTGTGTCCTCCATGGGAATATAATATGACAGGTATCCCGAAAGTTGATTTTGTTCGACTCC
CTCAATTATCATATTCCGGTCATATGTTCTCATATAATGAGAATATCCTGGGGACAAGGGGGGAGGCTGTAAGAGAAAAG
AATATTACAAATGACATGAGATTATATTCTGAAAATTTTCTCTACATTACTTACTACTCTCCCTCAATAAAAAATAGCGA
AAATGTTTTTATCAAAACATTTTCTTGTCCGCAAGAAAATGAAGAAAGAAAATGCAACAACCAAAATATATCAATTCCCG
CAGGAGAGTACCTTAAAACTTACTTTAAAGGGAACATTGCATCGCTTCATAAATTCATCAGGCAAGTGTATTTTGTTATA
TTACCAGAATATAAAATGGCCCGTAGAAGGGGGGGCGATATAGAATATTTTGATATATGTGAATACAGAAACAATGATAA
AAAACCTTACGTTAATTTGCAATTTTTTGTCCCTGTTATACCGCTCAGCGATATAATGGCAAAAAATGATGGTGTGGCCA
CTTGA
>Cluster_20761
ATGGCAGTAAAAACTCCGCTTAAGTGGGTGGGCAGCAAAGCCCGCCTTATGCCGCAGTTGCTTCCCCATCTGCCGGAGGG
TAAGCGCCTGATAGAACCTTTTGCCGGTTCCTGTGCTGTCATGATGAATACGGATTATGACGAGTATCTGATAGCTGATG
TGAATCCTGACCTTGTTAATCTGTATAAGACGATGGCTTATCACACTGATGCATTGCTCATTGAGCTGGAGGCTTTATTT
TCTGCCGGTTCATTAGGTGATGAAGAAAACCGGGCAATTTTCTATTACGCGGTACGTGATGCCTTCAACCACTCCGGCAA
ATCCTTTGGTTCTGAATCTGTGGAAGCTGCGGCGCGTTTTCTTTATCTGAACCGTCACTGCTTCAATGGCCTGTGCCGAT
ACAATCGCCGTGGCCAGTTCAATGTTCCGTTTGGTAAGTACAAAAAAACCTACTTTCCTGCTGATGAAATCCGCGCTTTT
GCAGAAAAAGCGAAACGTGCAACGTTCATTACTGCCCACTATTCAGAAACGCTTGATTTGGTAAGGGACGGGCATGACGT
TGTTTATTGTGATCCGCCTTACCTGACTGATACCGACAATTTCACCGCGTACCATGAGCGCGGTTTTTCTCACATGGATC
AGGGGCGGCTGGCGCGTAAGCTGCGCCGCCTGGCTGAGCGTGGGATTAAGGTTGTCGCGTCAAACAGCGATCTGGAAATG
GTGCATTACCTGTATGCAGGATTTGACGCTGTGCGGGTAAATGCCCCACGCAGTGTGGGTGCAGCAGCTGCAAGCCAGAA
AGTTGCCGCAGAGCTGATTCTTAAGTCACCTGCAAACCCGGCGATCGGCGTTAGGGCGGTGGTGGCATGA
>Cluster_42209
ATGGCATCTAATCAAACCACCCTGCCGAACGTCAGCGAAAATGAAGAAACACTGCTGACTGGCGTCAATGAAAACGTGTA
TGAAGATCAGAGTATTGGCGCGGAGCTGACGAAAAAAGATATCAACCGCGTCGCCTGGCGTTCCATGCTGTTACAAGCGT
CTTTTAACTACGAACGTATGCAGGCTTCCGGCTGGCTGTACGGCCTGCTGCCAGCTCTGAAAAAGATCCACACTAATAAA
CGCGACCTGGCGCGCGCCATGAAGGGGCATATGGGTTTCTTCAATACCCATCCGTTTCTGGTGACGTTTGTTATCGGCAT
TATCCTTGCGATGGAGCGTTCTAAGCAGGACGTTAACAGTATTCAGAGCACCAAAATTGCCGTCGGTGCGCCGCTCGGCG
GGATTGGCGATGCGATGTTCTGGCTAACGCTACTGCCGATTTGTGGCGGGATAGGTGCAAGCCTCGCGCTACAAGGCTCT
ATTCTTGGCGCTGTCGTCTTTATTGTGCTGTTCAACGTGGTACACCTGGGGCTGCGTTTTGGTCTGGCGCATTATGCTTA
TCGCATGGGCGTGGCGGCGATTCCACTCATTAAAGCGAATACCAAAAAAGTCGGCCATGCGGCATCTATCGTTGGGATGA
CGGTAATCGGCGCGCTGGTGGCAACCTATGTTCGTTTAAGCACCACGCTGGAAATCACCGCGGGCGACGCAGTGGTTAAG
TTACAGGCTGATGTTATCGACAAACTGATGCCAGCCTTCTTACCGCTGGTCTACACCCTGACCATGTTCTGGCTGGTACG
CCGCGGCTGGAGTCCGCTGCGCCTGATTGCAGTGACAGTGGTTCTCGGCATCGTCGGTAAATTCTGCCATTTCCTTTAA
>Cluster_41845
ATGAATATCGCAATTGTGACAATAAATCAGGAACATGCCGCAATGGCGGGCTGGCTGGCGGCGCAGGATTTCAGCGGCAG
CACACTAACGCATTGGCAAATAGAACCACAGCCGATGGTTGCGGAACAGGTGCTGGATGCGCTGGTGGAACAGTGGCAAC
GAACGCCTGCGGAGGTTGTCCTTTTTCCTCCGGGCGCGTTTGGCGATGAACTGGCTACCCGGCTGGCCTGGCGTTTACAT
GGTGCAAGCATCTGCCAGGTGACTTCGTTAGATATTCCAACCGTAAGCGTGCGCAAATCCCACTGGGGAAATGCGCTAAC
GGCGACGTTGCAAACAGAAAAACGCCCGCTATGCCTTTCGCTGGCACGTCAGGCGGGAGCAGATAAAAACGCCACGTTGC
CCTCTGGGATGCAGCAACTGAACATTGTCCCCGGCGCGCTTCCAGACTGGCTTATCAGCGTCGAAAACCTGAAAAATGTT
ACCCGTGACCCATTAACCGAGGCCAGACGTGTGCTGGTGGTGGGACAGGGCGGAGAGGCAGATAACCAGGAAATCGCTAT
GCTGGCAGAAAAGATGGGAGCCGAAGTGGGCTACAGTCGGGCACGAGTGATGAACGGTGGCGTTGATGCCGAAAAAGTGA
TTGGTATTTCCGGACATCTGCTGGCACCTGAGGTGTGTATTGTTGTTGGCGCTTCCGGCGCAGCAGCGTTAATGGCGGGC
GTACGCAATAGCAAGTTTGTGGTGGCGATTAATCATGACGCCAGTGCAGCGGTATTTTCACAGGCAGATGTTGGCGTGGT
TGATGACTGGAAAGTGGTGCTGGAGGCACTGGTGACAAACATTCACGCAGATTGCCAATAA
>Cluster_50000
ATGGCTGATTCGAATCTCAACACCCCTGTTATTGTGCAGGCGACGCGGCTCGATACATCAATCCTTCCACGCAATATCTT
CTCGCAGTCGTATCTGCTTTACGTTATCGCACAGAGCACTGATGTTGGTAACGTGGCTAACAAGGCCAACGAGGCCGGAC
AGGGCGCTTATGATGCACAGGTCAGGAACGATGAGCAGGATGTGATTCTCGCTGACCATGAGCAGCGAATTTCTGCTGCG
GAAGCAACGCTTGTTAATCATGAGGAGCGAATCAGCCAGGCAGAATCAACTCTTCAGGAACATGAAACGCGAATCGCTCA
GAATGAAAGCGATATTGCGTCGCTTGATACCAGAGTTCAGTCGCTGGAGTCGCAGGTTTCAGACCATGAATCGCGCATCG
ATTCTCTGGAGTATGCCACTACTCGCAAGAAGTCAGAGGTTGTTTACTCTGGCGTATCAGTAACCATCCCGACAGCGCCG
ACCAACCTTGTTAGCCTGCTGAAAACGCTCACGCCGTCATCCGGCTCGTTGGCACCATTCTTCGACACCGTTAACAACAA
GATGGTTGTGTTCAACGAGAACAAAACCTTGTTCTTCAAGCTGTCGATCGTCGGGACGTGGCCCAGCGGAACCGCCAACA
GGTCAATGCAGCTAACCTTTTCCGGCTCTGTTCCTGACACACTGGTAAGCAGTCGCAACTCGGCGACAACAACCGACAAC
ATCCTGTTAGCTACGTTCTTCAGCGTGGATAAAGACGGATTTCTTGCCACAAATGGCAGTACGTTAACCATCCAGTCAAA
TGGTGCGGCGTTTACTGCCACAAACATCAAGATAATCGCGGAGCAGTAA
>Cluster_25095
ATGCAAAAAGTAAAACTGAACAACGGTATTGAAATGCCCCTGTTGGGCTTTGGTGTATTTCAGATGACCGATGCCGCTGA
ATGCGAAAGAGCGGTCATTGACGCTATTAACACCGGTTATCGACTGATTGACACCGCCGCCTCCTATCAAAATGAAACCC
AGGTCGGGAACGCACTGAAACGAAGCGGTATCGCCCGTAACGAACTCTTTGTCACCACAAAACTCTGGTTGCAGGATACC
AGTTACGAAGGGGCCAAAGCCCAGTTCGAACGCTCTCTGAATCGACTGCAACTTGATTACGTTGACCTGTATTTGATTCA
CCAGCCATACGGCGATGTCCACGGAGCCTGGCGTGCCATGGAAGAGCTGCAACAGGCCGGTAAAATTCGCGCTATTGGCG
TCAGTAACTTCCATCCTGACCGACTGGCTGACCTTATCGCTTTTAATAACGTCGTTCCGGCGGTAAACCAGGTTGAAGTT
AACCCTTTCAATCAGCAGCTACAGGCCGTTCCCTGGATGCAAAGTCGTGGCATTCAGCCAGAAGCCTGGGCACCGTTTGC
GGAAGGGAAAAATGGTCTGTTCCAGCATCCTGTTCTCACGGCAATTGGCGAGAAGTATGGCAAAAGCGTGGGCCAGGTTG
TTCTGCGCTGGATCTACCAACGAGGCGTTGTTTCACTGGCAAAATTAGTGCGAAAAGAACGCATGGAAGAGAACATCAAC
ATTCTCGATTTTGAACTCAGCCCTGAAGATATGCTGCAAATTACCGCCCTGGATACCGCAACCAGCGCGTTCTTCTCTCA
CCGCGATCCCGCAATGGTGGAATGGCTGACTGGCCGCAAACTTGATGTTTAA
>Cluster_61363
ATGCTCAGTTATCGCCACAGCTTTCACGCTGGCAACCACGCCGACGTCCTTAAACATACCGTTCAGAGCCTGATCATCGA
GTCGCTGAAAGAGAAAGATAAACCGTTTCTCTATCTCGACACCCACGCAGGGGCCGGGCGTTATCAGTTAGGCAGCGAAC
ATGCCGAGCGTACCGGCGAATATCTCGAAGGCATCGCCCGTATCTGGCAGCAGGACGATTTGCCCGCAGAACTGGAGGCG
TACATCAATGTGGTAAAACACTTCAACCGTAGCGGTCAGTTGCGTTACTACCCCGGTTCGCCGTTGATTGCTCGCCTGCT
ACTGCGTGAACAGGACAGCCTGCAACTGACCGAACTGCACCCGAGCGATTACCCGTTGTTGCGTTCTGAATTTCAGAAAG
ATAGCCGTGCGCGTGTCGAAAAAGCCGACGGTTTCCAGCAGCTTAAGGCCAAACTGCCGCCGGTTTCCCGCCGTGGTTTA
ATCCTTATCGACCCGCCGTATGAAATGAAAACTGACTATCAAGCGGTGGTCAGCGGGATAGCAGAAGGTTACAAACGTTT
CGCCACTGGTATTTACGCACTGTGGTATCCGGTGGTGCTGCGTCAGCAAATTAAGCGCATGATCCACGATCTGGAAGCGA

CCGGTATTCGCAAAATTCTGCAAATTGAACTGGCGGTACTGCCAGACAGCGATCGCCGTGGCATGACCGCTTCCGGCATG
ATTGTGATTAACCCGCCGTGGAAACTGGAACAACAGATGAATAACGTGCTGCCGTGGCTGCACAGCAAACTGGTTCCGGC
AGGCACCGGGCACGCCACCGTAAGCTGGATCGTGCCGGAGTAA
>Cluster_45853
ATGCATCATATTAAAACAGCCGCAGATCTTAATTGTTTTTAAATTCAATGGTTGCCGTTTCGCAGCCAACTGTTGATAA
AATCATTTTTGGTGCTGAAGCGACACGTATTAATAAAATTGGTACCTGCTGGATTGCCTCAATGGATGTTTTAAGAAAAG
CTGTTTTTGAAGGTGTCAATATCATTATTACTCATGAACCTACATTTTATTCTTATGCTGATCTGGAGGGAGAAGATCTT
GAGTTTTCATGGGCCAGAAAAGTAATGGGTTATACTCACGGTGAACTCAGTTATTTAAAAATAATTGAGCAAAAAAAGGA
GTTTATGCATAAAAATAATTTAGTAATCATTCGTTGCCATGATGTCATGGATCGAGAACCTACGTTCGGAATGTCCAAGG
CTTTGGCTCATCAGTTAGAATTGGATGTTACAAATATTGTTGCTTCTGATGATATGTACCATGTTTATGCTATAGAACCT
GACAGTGCCATTAATATTACCAAACGTTTTGCCAAAAATTTGAAAATTTATAATCTTCCCGGTATTCAATTTTATGGAGA
TAAAGCCAGAGTAGTCAGAACTGTTGGGATAGGGGCTGGCTGTTTTTGTGATCCAATACAGTATATGGAATATCAGGCTG
ATTATTATATTACGATTAACGATTCAATCAAAACATGGGTGCAGACACAATACTCAAAAGATAGTGGATTACCGATGGCA
GTTATTGGACATAGTGTTGCAGAGGAAGCGGGTATGCGACGTCTTGCAAGCTATTTAGATCTCCATTCAGGCTATCCCTG
CATTCATTTTACAGGGGGCTGTGATTATGATTGGATTGAATGA
>Cluster_10984
ATGGATATCTCGCTTCTCAGTACCACCAGTGACCCTGAACAGCTTCGCGCTCTGGCAATTGCCATGGTGCAAAAAGTCAT
GGCTGAGAATGCAGAACTTCAGAACCGTATCCGCATCCTGGAAGAGCAGATGAAGCTCGCACGCCAGCAGCGTTTCGGTA
AAAAATGCGAATCGCTGGCCGGGATGCAGCGTTCCCTGTTCGAAGAGGATGTTGACGCGGATATCGCAGAAATCTCGGCG
CATCTGGACAAACTGCTTCCTCAGACCGGGGACGAAGAAAAAACAACAACACGTCCTGTTCGTAAGCCCTTGCCTTCACC
TCTTCCCCGTGCTGAAAAGGTTATCCCGCCAGCTGAGGAACGTTGTCCTGACTGTGATGCCCCCCTGCACTTCATTCGCG
ATGAAGTCAGCGAGAAGCTGGAATATATTCCGGCACAGGTCGTGGTCAACCGCTATATCCGCCCGCAGTACAGCTGTCCC
TGCTGTGAGAAAGTGTTCAGCGGAAAAATGCCGGCACATATCCTTCCGAAAAGTGCTGTTGAGCCATCGGTCATCGCACA
GGTTGTCATCAGCAAATATACCGACCATCTGCCCCTGTATCGCCAGCAGCACATTTTCTCCCGAATGGGGGTGGAGCTCC
CCGTCAGTACAATGGCGGATATGGTCGGTGTTGCTGGCGCTGCACTGGCCCCGCTGGCAAAATTGTTACGCCACGAGCTT
CTGACACGGGATGTTATTCATGCAGATGAAACGTCCCTGAGGCTCCTTGATACCCGGAAAGGCGGTAAATCCTGCTCCGG
CTGGCTGTGGGCTTACGTCAGCGGAGAAAGAGTAAGCGTCAACGGAGCACCGTATTGA
>Cluster_28654
ATGTTAGTTTCGATGAAAGAATTACTGCAACCTACCCGCCAGCACGGCTTTGCCATTGGCGCATTTAATGTGGCGGATAA
CTGTTTCCTTCGCGCAGTCGTGGATGAAGCAGAAGCGACAAATACTCCGGCGATTATTGCCATCCATCCCAGCGAACATG
ATTTGTGGGCGATGCCTTTTTCGCTCACGTTCGCGAAATTACCCAGCGTAGTCCGGTCCCGTTTGTACTGCACCTCGAT
CATGGTGCCTCTGTAGAGCATGTTTTGCGTGCGATTCGCTGCGGATTTACCTCAGTAATGATCGACGGTTCGCTGCTGCC
GTATGAAGAAAACGTGGTGCTGACCGCAGAAGTGGTTCACCTCGCTCACGCGGTTGGCGTGTCGGTTGAGGGTGAACTGG
GAACCATCGGACAAACAGGAACGTCGGTAGAAGGTGGTGTTTCACAAGTGACCTACACAGATCCGGCGCAGGCCGCCGAT
TTTATTGCTCGTACAGGTGCCGACACACTGGCTGTGGCGATTGGTACAGCGCATGGCATCTATCCAAAAGGGATGCAGCC
GAAGCTGCAAATGGACATTTTGCGCGACATTGCCGGACGTATTGATATTCCGCTGGTGCTACACGGTGGTTCGGCAAACC
CGGACGCGGAGATTGCTGAATCAGTTACGCTCGGCGTCGGCAAAATCAATATCTCCAGCGATATGAAATACGCCTATTTC
CAGAAAGTGCGTGAAATTCTCGCTAAAGAGAGCTGGTGGGACCCGAACGTCATCTATCCGGATGCGATTAACGCGGCGCG
GGAAGTCATTCGCCACAAAATGAAACTGTTCGGTTCGCTCGGTAAAGCATCACTGTACTAA
>Cluster_53956
ATGGATGCCCTTAGCCGTTTGTTGATGCTTAACGCTCCACAAGGAACGATCGATAAGAATTGCGTGTTAGGAAGTGACTG
GCAGCTTCCACATGGTGCCGGGGAATTATCGGTTATTCGTTGGCATGCGTTAACGCAAGGAGCGGCGAAGCTGGAAATGC
CGACGGGAGAGATTTTACATTACGCCCGGGAAATGTGGTCCTGCTACCACAAAATTCAGCTCATCGCCTGAGTCATGTC
GATAATGAATCGACCTGTATTGTCTGCGGCACTCTTCGGTTGCAACATTCGGCGCGTTATTTTTTAACGTCTTTGCCGGA
AACGCTGTTTTTAGCACCGGTTAACCACAGCGTGGAATATAACTGGCTGAGGGAGGCGATCCCGTTTTTACAACAGGAAT
CCAGATCGGCGATGCCGGGAGTGGATGCACTGTGTAGCCAGATCTGCGCTACATTCTTTACCCTCGCGGTGCGTGAGTGG
ATTGCACAGGTTAATACAGAGAAAAACATTCTCAGTTTGCTTCTGCATCCACGTCTTGGTGCGGTAATACAGCAAATGCT
GGAAATGCCAGGACACGCCTGGACCGTCGAATCGCTGGCCAGCATCGCCCACATGTCTCGGGCAAGTTTTGCCCAGCTTT
TCCGTGATGTTTCCGGAACCACGCCGCTGGCTGTATTAACAAAGTTGCGTCTACAAATAGCGGCCCAGATGTTTTCCCGG
GAAATGCTCCCTGTTGTGGTGATCGCTGAGTCAGTAGGCTATGCCAGTGAATCATCTTTTCACAAGGCGTTTGTCCGCGA
GTTTGGTTGTACTCCGGGAGAATATCGGGAAAGGGTCAGACAGCTTGCACCCTGA
>Cluster_54462
ATGAGCACCATCCTGAAATGGGCGGGAAATAAAACCGCCATTATGTCCGAACTGAAAAAACATCTTCCTGCTGGCCCGCG
ACTGGTTGAACCTTTCGCGGGGTCCTGTGCTGTGATGATGGAGACGGATTATCCCAGCTATCTGGTTGCGGATATTAATC
CTGATTTAATCAACCTCTATAAAAAGGTTGCCGCTGATTGTGAATCGTTTATATCTCGCGCCAGAGTTTTATTTGAGATC
GCAAACAGGGAGGTGGCTTATTACAACATAAGGCAGGAGTTTAATTACTCAACTGAAATTACTGATTTCATGAAAGCGGT
ATATTTCCTGTATCTCAATCGTCACGGTTACCGTGGTTTATGTCGCTATAACAAGAGCGGGCATTTCAACATTCCCTACG
GTAATTATAAAAATCCGTATTTCCCTGAAAAAGAAATTCGCGCATTTGCAGAAAAGGCCCAGCGAGCAACGTTTATCTGC
GCCAGCTTTGATGAAACGCTGGCGATGTTGAAGGCGGGAGATGTGGTGTATTGCGATCCGCCGTATGACGGTACGTTTTC
CGGCTATCACACTGATGGTTTCACTGAAGATGACCAGTATCACCTGGCATCCGTTCTTGAACATCGGTCATCAGAAGGAC
ATCCGGTCATTGTTTCTAACAGTGACACATCCCTGATCCGTTCGCTGTATCGCAATTTTACTCACCACTATATCAAGGTA
AAACGCAGCATCGGTGTGGCAGCTGGCGAGGGTAAATCAGCAACAGAAATCATTGCTGTTTCCGGGCCGCGCTGCTGGAT
GGGATTTGATTATTCGCGTGGCGTGGATAGTTCTGCCGTGTACGGAGTACGTGCATGA
>Cluster_27832
ATGTCTAAACGATTACCACCGCTAAATGCCTTACGAGTTTTTGATGCCGCAGCACGCCATTTAAGTTTCACTCGCGCAGC
AGAAGAGCTTTTTGTGACCCAGGCCGCAGTAAGTCATCAAATCAAGTCTCTTGAGGATTTTCTGGGGCTAAAACTGTTCC
GCCGCCGTAATCGTTCACTCCTGCTGACCGAGGAAGGGCAAAGCTATTTCCTCGATATCAAAGAGATATTTTCGCAATTA
ACCGAAGCGACGCGTAAACTCCAGGCCCGTAGCGCTAAGGGGGCGTTGACGGTCAGTTTACTCCCCAGTTTCGCCATTCA
TTGGTTGGTTCCGCGACTTTCCAGCTTTAATTCAGCTTATCCGGGAATTGACGTTCGAATCCAGGCGGTTGATCGTCAGG
AAGATAAGCTGGCGGATGATGTTGATGTGGCGATATTTTATGGTCGGGGCAACTGGCCGGGGCTACGGGTGGAAAAACTG
TACGCCGAATATTTATTGCCGGTGTGCTCGCCGCTACTGCTGACTGGCGAAAAACCCTTGAAGACCCCGGAAGATCTGGC
TAAACATACGTTATTACATGATGCGTCACGCCGTGACTGGCAGACATATACCCGACAGTTGGGGTTAAATCATATCAACG
TTCAGCAAGGGCCAATTTTTAGCCATAGCGCCATGGTGCTGCAAGCGGCTATCCACGGGCAGGGAGTGGCGCTGGCAAAT
AACGTGATGGCGCAATCTGAAATCGAGGCCGGACGTCTTGTTTGCCCGTTTAATGATGTTCTGGTCAGTAAAAACGCTTT
TTATCTGGTTTGTCATGACAGCCAGGCAGAACTGGGTAAAATAGCCGCCTTTCGCCAATGGATCCTGGCGAAAGCCGCTG
CTGAACAAGAAAAAATTCCGCTTTCGTTATGAACAATAA
>Cluster_7031
ATGAATAATTTACCGCTGCTGAATGATTTGCGCGTCTTTATGCTGGTGGCTCGCCGGGCTGGTTTTGCCGCCGTGGCGGA
AGAACTGGGCGTTTCACCGGCGTTCGTCAGCAAGCGCATCGCCTTGCTGGAGCAAACGCTAAACGTGGTGTTGTTACACC

GCACCACCCGCCGCGTCACCATTACCGAAGAAGGAGAGCGGATTTATGAATGGGCGCAGCGGATTTTGCAGGATGTCGGT
CAGATGATGGACGAACTTTCTGACGTGCGTCAGGTGCCGCAGGGGATGCTGCGTATTATCAGCAGCTTTGGTTTTGGACG
ACAGGTGGTGGCTCCGGCGTTATCAGCGCTGGCGAAGGCGTATCCGCAACTGGAATTACGTTTTGATGTGGAAGACCGAC
TGGTCGATTTGGTCAATGAAGGTGTCGATCTCGATATTCGCATTGGCGATGACATCGCCCCTAATTTAATCGCCCGCAAA
CTGGCGACCAATTACCGTATTCTCTGCGCGTCGCCGGAATTTATTGCGCAACATGGCGCGCCGAAACATTTAACCGATCT
TTCTGCATTGCCGTGCCTGGTCATTAAAGAGCGCGATCATCCTTTTGGTGTCTGGCAATTACGCAATAAAGAAGGCCCCC
ATGCGATTAAAGTCACCGGCCCACTGTCGTCTAATCATGGAGAAATTGTCCATCAGTGGTGTCTCGATGGGCAGGGGATT
GCTCTGCGTTCCTGGTGGGATGTTAGCGAAAACATTGCCAGTGGTCATTTAGTGCAAGTGCTACCGGAATATTACCAGCC
AGCGAACGTCTGGGCCGTTTATGTTTCAAGGCTGGCGACGTCAGCGAAAGTGCGGATAACGGTAGAATTTTTACGCCAGT
ATTTTGCCGAGCACTACCCGAATTTTTCACTGGAGCATGCCTGA
>Cluster_43420
ATGAAACGAGTTATTACCCTGTTTGCTGTACTGCTGATGGGCTGGTCGGTAAATGCCTGGTCATTCGCCTGTAAAACCGC
CAATGGTACCGCAATCCCTATTGGCGGTGGCAGCGCCAATGTTTATGTAAACCTTGCGCCTGCCGTGAATGTGGGGCAAA
ACCTGGTCGTGGATCTTTCGACGCAAATCTTTTGCCATAACGATTACCCGGAAACCATTACAGATTATGTCACACTGCAA
CGAGGCTCGGCTTATGGCGGCGTGTTATCTAATTTTTCCGGGACCGTAAAATATAGTGGCAGTAGCTATCCATTTCCTAC
CACCAGTGAAACGCCGCGGGTTGTTTATAATTCGAGAACGGATAAGCCGTGGCCGGTGGCGCTTTATTTGACGCCTGTGA
GCAGTGCGGGCGGGGTGGCGATTAAAGCTGGCTCATTAATTGCCATGCTTATTTTGCGACAGACCAACAACTATAACAGC
GATGATTTCCAGTTTGTGTGGAATATTTACGCCAATAATGATGTGGTGGTGCCTACTGGCGGCTGCGATGTTTCTGCTCG
TGATGTCACCGTTACTCTGCCGGACTACCCTGGTTCAGTGCCGATTCCTCTTACCGTTTATTGTGCGAAAAGCCAAAACC
TGGGGTATTACCTCTCCGGCACAACCGCAGATGCGGGCAACTCGATTTTCACCAATACCGCGTCGTTTTCACCCGCGCAG
GGCGTCGGCGTACAGTTGACGCGCAACGGTACGATTATTCCAGCGAATAACACGGTATCGTTAGGAGCAGTAGGGACTTC
GGCGGTAAGTCTGGGATTAACGGCAAATTACGCACGTACCGGAGGGCAGGTGACTGCAGGGAATGTGCAATCGATTATTG
GCGTGACATTTGTTTATCAATAA
>Cluster_803
ATGGCCAATCTCTACGACTTGAAAAAGTTCGACTTAAACCTTCTTGTCATATTTGAGTGTATTTACCAGCATTTAAGCAT
TAGCAAAGCTGCTGAATCACTATATATCACCCCATCAGCCGTGAGCCAGTCGTTACAACGCTTACGTGCGCAATTTAATG
ACCCTTTATTCATTCGCTCAGGAAAAGGTATCGCGCCGACGACAACAGGGTTGAATCTGCATCACCATCTTGAGAAAAAC
CTCAAAGGCCTTGAACAAACAATTAATATAGTCAATAAGTCCGAACTAAAGAAAAACTTTATTATTTACGGCCCTCAGCT
CATTTCTTGCTCTAATAATAGTATGCTGATCCGCTGCCTACGGCAAGATGCCTCTGTCGAGATTGAGTGCCATGATATCC
TCATGTCAGCAGAGAATGCTGAAGAACTGTTGGTTCACCGCAAAGCCGATCTGGTGATCACCCAAATGCCAGTGATCAGC
CGTTCCGTCATTTGCATGCCATTGCATACAATTCGCAATACTCTAATTTGTAGTAATAAGCATCCGCGAATTACTGACAA
TAGCACTTATGAACAAATTATGGCAGAAGAGTTCACCCAATTAATATCCAAATCAGCAGGAGTTGATGATATTCAAATGG
AAATAGACGAGAAGTTTATGAATCGCAAAATTTCATTTCGTGGTTCCTCTTTGCTGACAATCATTAATAGCATTGCAGTT
ACAGATTTACTGGGAATAGTCCCCTATGAACTTTATAATTCCTACCGTGACTTTCTTAATTTAAAAGAGATAAAACTAGA
ACATCCGTTACCTTCAATAAAGCTTTACATTTCCTATAATAAATCTTCTTTAAATAATTTGGTGTTCTCCCGATTTATTG
ACCGTCTAAATGAGAGTTTTTGA
>Cluster_20680
ATGAAGAATATCGAGACAAAATGGTTATACGATTTCCTGACACTGGAAGCCTGTCGCCATTTTTCTCAGGCGGCAAAAGA
GCGTAATCTTTCGCAACCCGCATTCAGCCGACGAATTAAGGCGCTGGAGGCAGCGATCGGCGTTGTTTTATTCGATCGCA
CGACCACCCCTTTACAATTAACCGAAGAGGGCAAGCTCTTTCATTCCCAGACTCGCAGCTTATTACAGCAGCTTGAATGC
AATTTGGGGGAGTTAAACGGCCAGAGTTTACTCGGCGTTCCCAACATTAAAATTGCAGCCGCGCACTCACTATCCCTGAG
CGTATTACCCAAACTGGTGCATTCACTTACCGCCTATGGCGGAGAATTTGTCTATCATGTTGAAGCAATTGATGTTGTCC
AGGCCGTAAATACGTTACGCGAAGGCAAGAGTGACTTTATTATTTCGTTTCGCGATGAAGATCTTATGCAATCGCCTTTT
TGCTGCCTGAAACTGTTTGAATCTGAGTTGTACCCGGTATGTGCCGCCGACGCACAGGGGCACCCTGTATTCGATATCAC
TCAGCCCCAGGTTCCTTTGCTCAACTATACCGCCACATCTTATATGGGCAGGTTGGTCAATCGTCATCTGGCAGAGGTTG
GAGGTATTACCGGGCGCACGATATTTATCTCTTCGATGAGCGAGTTATTAAAGAATATGGCGCTCAACGGATACGGCATT
GCCTGGTTACCGATCTGGTCGATCGTCGATGAACTACAGACCAAACGTCTGATATGCCTTGATGCTGCTAAATTGACAAT
CCCCATTCAGGCTTATATTTACCGAATGAACACCCGACTGAATAGAACCGCCGAGAACCTCTGGCGTATTTTACAGAAAC
ATATGCCAGACGATTTAATCCAGCAGATATCAATGGAAGAGCCAGCACGCCGCAATTGA
>Cluster_21492
ATGGCTATGTATCAGAACATGCTCGTTGTTATCGATCCTAACCAGGACGACCAACCAGCATTGCGGCGAGCTGTTTATTT
ACATCAACGGATTGGTGGCAAAATTAAAGCCTTTTTGCCGATCTATGACTTCTCATACGAAATGACCACCCTGCTCTCTC
CGGACGAACGTACCGCTATGCGTCAGGGCGTCATCAGCCAGCGTACAGCCTGGATCCACGAGCAGGCAAAATATTATCTC
AATGCTGGCGTTCCCATTGAAATTAAAGTGGTCTGGCATAACCGTCCTTTCGAAGCCATCATTCAGGAAGTGATCAGCGG
CGGGCACGATTTGGTGCTAAAAATGGCGCACCAACATGACCGTCTGGAAGCGGTGATTTTTACGCCAACGGACTGGCATC
TGTTACGCAAATGCCCAAGCCCCGTGTGGATGGTGAAAGACCAGCCGTGGCCGGAAGGGGGTAAGGCGCTGGTGGCGGTG
AATCTCGCCAGTGAAGAGCCGTACCATAATGCGCTCAATGAAAAACTGGTCAAAGAGACGATCGAACTGGCAGAACAAGT
CAACCATACCGAAGTTCATCTGGTTGGCGCTTATCCGGTAACGCCAATTAATATCGCGATTGAACTGCCGGAATTTGACC
CGAGCGTTTATAACGATGCCATTCGTGGCCAACATTTGCTGGCAATGAAAGCTCTGCGGCAGAAATTCGGCATTAATGAA
AACATGACGCACGTAGAAAAAGGTCTGCCCGAAGAGGTGATTCCTGATTTGGCGGAGCACTTACAGGCGGGGATTGTGGT
TCTGGGCACGGTCGGACGCACCGGTATTTCAGCAGCATTCCTTGGCAACACGGCGGAACAGGTGATTGATCATCTTCGCT
GCGACCTGCTGGTCATTAAACCTGACCAGTATCAGACACCCGTTGAACTGGATGACGAAGAAGACGATTAA
>Cluster_47269
ATGCAGTTACAGAAATTAGTCAATATGTTTGGTGGTGATCTCACCCGTCGTTATGGGCAAAAGGTGCATAAGCTGACGCT
ACACGGCGGTTTTAGCTGCCCTAACCGTGACGGTACCATCGGGCGTGGTGGCTGCACATTCTGTAATGTTGCCTCGTTTG
CCGATGAAGCGCAGCAGCATCGTTCCATTGCCGAGCAACTGGCGCACCAGGCGAATTTAGTTAACCGCGCTAAACGCTAT
CTGGCCTACTTTCAGGCGTATACCAGCACCTTTGCGGAAGTTCAGGTGCTGCGTTCTATGTATCAGCAGGCGGTGAGCCA
GGCCAATATTGTCGGTTTGTGTGTTGGTACCCGCCCGGACTGCGTGCCGGATGCGGTGCTGGATCTGCTTTGCGAATATA
AGGACCAGGGTTACGAAGTGTGGCTGGAGCTGGGGCTACAAACCGCCCACGACAAAACACTGCATCGCATCAACCGCGGC
CATGATTTTGCCTGTTATCAGCGTACAACCCAGCTGGCACGTCAGCGCGGGCTGAAGGTGTGTTCCCACTTAATTGTCGG
CCTGCCTGGCGAAGGGCAGGCTGAATGTTTGCAAACGCTGGAGCGAGTGGTTGAAACCGGCGTGGACGGCATAAAGCTGC
ATCCGCTGCATATTGTGAAAGGCAGCATTATGGCGAAAGCCTGGGAAGCGGGGCGTTTGAACGGTATTGAACTGGAGGAT
TACACGCTCACTGCCGGAGAAATGATTCGCCATACGCCACCGGAAGTGATCTACCACCGCATTTCCGCCAGCGCCCGTCG
CCCGACGCTGCTTGCTCCTCTGTGGTGCGAAAATCGATGGACGGGAATGGTCGAGCTGGATCGCTATCTGAATGAACATG
GCGTACAGGGATCGGCGCTGGAACGTCCGTGGATACCTCCAACGGAGTAG
>Cluster_41137
ATGCCGAAATTTCGAGTTTCTTTATTTAGCCTGGCCCTGATGCTGGCTGTGCCTTTTGCACCGCAGGCCGTTGCGAAAAC
GGCAGCCGCTACCACCGCGTCACAACCGGAAATTGCCTCCGGTAGCGCGATGATTGTTGATCTGAATACCAACAAAGTGA
TCTATTCGAACCACCCGGATCTGGTGCGTCCGATTGCGTCTATCAGCAAATTAATGACCGCGATGGTTGTGCTGGATGCA

CGACTGCCGCTGGATGAAAAACTAAAAGTGGATATCAGCCAGACGCCGGAGATGAAAGGGGTCTATTCGCGCGTACGACT
GAACAGCGAAATCAGCCGTAAAGATATGCTGTTGCTGGCGCTGATGTCTTCAGAAAACCGCGCGGCGGCAAGCCTTGCGC
ACCATTATCCCGGTGGTTACAAAGCCTTTATTAAGGCGATGAATGCGAAAGCGAAATCGCTCGGAATGAACAACACGCGC
TTTGTTGAACCAACCGGATTGTCGGTGCATAACGTTTCAACCGCCCGTGACTTAACCAAACTACTTATTGCCAGCAAACA
ATATCCGTTGATCGGGCAGTTAAGTACTACCCGGGAAGATATGGCAACCTTCTCTAACCCGACGTATACGCTGCCGTTCC
GCAATACTAACCATCTGGTGTATCGTGATAACTGGAATATTCAGTTAACCAAAACCGGTTTTACCAATGCGGCGGGCCAT
TGTCTGGTGATGCGCACGGTTATCAATAATAAACCGGTGGCGCTGGTAGTGATGGACGCGTTTGGCAAATATACCCATTT
TGCCGATGCCAGCCGCCTGCGTACCTGGATTGAAACCGGTAAAGTGATGCCTGTTCCGGCAGCAGCGTTGAGCTATAAAA
AACAAAAAGCCGCCCAAATGGCGGCGGCGGGGCAGACGGCACAGAACGATTAA
>Cluster_30581
ATGGCAATTGATATCTTTCAGTCACTTAAAAATTGCGTATTCGACCTTCAGCGCGCTGACGTTCAGAACTATCAGCAACC
TCTTAAGCAATTGGCCCGGCTCTTGAACTCAGAGAATCTGCAGTCAGTTAATGCACATCTGACCCGCAATGTTGAACTTG
ATACCTTTCTGGCCAGGAGTGAAGATACTGAATCCAGCATGGCTGGTTCCGCTGTTCTACAGTGGCCTGATGAGCCTGCT
GATATTTTGGGCCTGAAGTTGCTGCTGATCGAGAAAATGGCAGACGATAACAATTTTTCCTTTAATTTCTGTCATACATT
TTTCTATGATCGAAACATTATTGAATCCATTCGAAAATTCACGTCGTCATTAGTTGCCCCGTTCGTGCGCGATTACCAGT
TGTATGTGGAAAACCAACACGATCCTGAGCCTGCAGAGTTTCGGCCGGTTTCCCGAAAAATTTTTATTGTTCATGGCCAT
GATAATGATGCCCTTCAGTCTGTTGCCCGTTTTATATCCCGGATAGGTCTTGAGGAAATCATTCTTTCAGAACGTCCTGA
CGGCTCCAGGACGATTATCGAGAAATTTGAAGCGGAGTCAGGGGATGTCAGTTTTGCCATCGTGCTGATGACGCCCGACG
ATAGCGGCAGTGCTCTGGCATCTGAAAGCACACGTCTCCGCGCACGCCAGAATGTCCTGTATGAGCTCGGATATTTCGCC
GGGAAGCTTGGACGCGGGAAAGTACTTGTACTCAGGAAGGGAGATATCGAAATCCCTTCAGATCTTGCCGGGGTTCTCTA
TACCGAGCTTGATGAGCATGGTGGATGGAAACGCAAACTGCTGAGTGAGCTTGCGTATGCCGGTGTTCCTTTTGACAAGG
ATAAAGCATTAAGCGCCTGA
>Cluster_2683
ATGACAATATCGACAACTTCCACGCCGCATGATGCGGTATTTAAATCTTTTTTACGCCATCCAGACACCGCGCGGGATTT
TATTGATATTCATCTTCCCGCGCCGCTGCGCAAACTGTGTGATTTAACGACGCTTAAACTGGAACCAAACAGTTTTATTG
ATGACGACCTGCGGCAATATTATTCCGACCTCTTGTGGTCTGTGAAAACGCAGGAGGGAGTGGGTTATATTTATGTAGTG
ATAGAGCACCAAAGTAAGCCGGAAGAATTAATGGCTTTTCGCATGATGCGTTATTCCATTGCGGCAATGCAAAACCATCT
TGATGCGGGCTATAAAGAGCTTCCATTGGTGATCCCGATGCTGTTTTATCATGGTTGCAGAAGTCCTTATCCTTATTCAC
TCTGCTGGCTTGATGAATTTGCCGAGCCTGCTATAGCCCGCAAAATATATTCATCAGCGTTTCCGTTGGTGGATATTACC
GTGGTGCCGGATGACGAGATTATGCAACACCGCAAAATGGCGCTGTTGGAGTTAATTCAGAAACATATTCGTCAGCGCGA
TCTGTTGGGATTAGTCGACCAAATTGTTTCGCTGCTAGTTACAGGGAACACTAATGACAGACAGCTAAAAGCCCTGTTTA
ATTACGTATTACAAACAGGGGATGCCCAGCGTTTTCGTGCATTTATTGGTGAGATAGCGGAACGCGCACCACAAGAAAAG
GAGAAACTGATGACCATTGCTGACAGATTACGTGAAGAAGGCGCAATGCAGGGCAAACACGAAGAAGCCCTGCGTATTGC
TCAGGAGATGCTGGATAGAGGTTTAGACAGAGAGTTAGTTATGATGGTGACCCGACTTTCACCAGACGATCTTATCGCGC
AAAGCCACTAA
>Cluster_50005
ATGGACCAGATGGCAGAAAACACACCAGAAGTTGAAATCGAAACCGACGCGTCAGAGCAGATTCCTGATGATGTCGAACT
GGCTGAAGAAGTCGAAACAGCAGATGGCAGTGAGTCCTCAGGAAATGATGCAGAGGAAGCTACTGACACTGATGACGACG
AATCAGAACAGGAATTCTACTTTGGTGACGAAAAGCTGGATTCGCCAACCAGCGAAGATGGCGCAGAGCATGGACTGGTA
AAACACCTGCGCAAGACGATTAAAGAGAAAGACCGTGAGCTGAAAGAGCTGATGCGTCAGTCTCAGAAACCCGTCGAGCA
GCAGCCGGTAATCACTCAACCACCGCGAATGCCAAAACTGGATGATGAGGACATCGGTTTCGATGAAGAAATCTATCAGC
AACGCATGGCTAAGTGGGCAGAGGATAACGGCAAATACCAGGAGCAAGTACGAGAGCGGAAACGAGAGGAAGAGGCGCGT
ACCGCAACGCTTCAGCAGAAAGCAGCCAATTACATGCATAGAGTAAAAGCACTGAAAGTGGCTGGTTACCAGGATGCAGA
GCAGGCTGTACGCGAAGATGTTCCTGTTCACATTCAGGACATGATCCTTCTTGAGTCAGAGAAGCCGGAAATCGTTGTTC
TGGCACTCGGTCGCAACGCTGAACTGCGCAAGCAACTGGCAGAAGCTACCAACTCCGTAGCAATTGGTCGTCTGCTGGAA
CGTATCGAATCGAAGGCCAGAATCATGCCAAAAGCAAAAACCACGGCAGCCACAACCCCGACAGTTAAGGGGAGCAACGG
CGCAGTAATCAATAACCTCGACAAATTGAAAGCCAAGGCGCTGGAAACTGGTGACTGGACGCCGTATTTCGCCGCTAAAA
AGGCAAAAAAAATAA
>Cluster_34166
ATGAATGAGCAAACACCGAATCCCAATGCGACGAACGAAGGAATAAACGAACAGGCCGCGGTAAGCAGCCTTCCTGTCAG
TCCAGAGTCCAAGCCTGAAGTCGTCACGGAGGTACAGCCTGAGGTTCAGAAAGAAACGGACTCGCAGGCGGCAGACAAAC
GTAAACAAGTCCTCGATGAGGCAGTCTCGGCCTTGTCGCTGACCAAATCAGCGCTGGCCGCACTTGACGGCAAGGACACT
GCACGCGCATTGGCAACGCTGGCCGAAGTGACGGGAAAGCTGGAGCTGATCGTCGCGCGCGAACCCACGCTCGCCCTGGC
CGGCGTTGATGTGCGCACCATCGTGCACGACTTGTTCGCCAACACGGAAACCATTGAGGCGATGACCGACGAGGCGCTGG
ATGCCCTCAAACATGGCGAGGTGCAACAGGCTCGCCACGTGCTGGCTTTGCTGGCCAGTGAAATCGTGATCACGGTCACC
AACATCCCTTTGGCGTCCTATCCCGCAGCCGTGAAGGCAGCCGTGCCGCTGATCGATCAGGGCAAGATCGAAGAAGCCAA
AGCCGCGCTGCAGTCAGCGCTCAGCACGCTGGTCGAGGAGCGCAGCGTGCTTCCGCTGCCCGTCCTGCGTGCGAAGCTGC
TCCTGAAGCGCGCCGAGCCTCTGGTAGAGATGGTCAGCGCAGCGAAGCATCCAATGAGCGCCTGGAGACATTGTTGAAC
GAAGCCCGGCAGCAGTTGGAAATGGCAGAACTGCTGGGCTATGGAAAAAGGAAGGACTTTGAGCCCCTGTACGCTGAACT
CAAGAAAATCAAGGAAAAGACGGGAGGGGGGAGGCTTCGGAAAAGGCTGGCTCGATGAAGTCAAGGCAAAACTGTCCAAGT
TGTTCTGA
>Cluster_42069
ATGCCGCGTTTAACCGCCAAAGATTTCCCACAAGAGTTGCTGGATTACTACGACTATTACGCTCACGGGAAAATCTCGAA
ACGTGAGTTCCTCAACCTTGCGGCGAAGTATGCGGTGGGCGGGATGACGGCATTAGCGCTGTTTGATTTGCTCAAGCCAA
ATTATGCGCTGGCGACTCAGGTAGAGTTTACCGACCCGGAAATTGTTGCTGAGTACATCACGTATCCCTCGCCAAATGGT
CACGGCGAGGTACGGGGTTATCTGGTGAAGCCCGCAAAAATGAGCGGCAAAACGCCAGCCGTGGTGGTGGTGCATGAGAA
TCGTGGACTGAATCCGTATATCGAAGATGTGGCACGGCGAGTGGCGAAGGCGGGGTATATCGCCCTGGCACCTGACGGCT
TAAGTTCCGTTGGAGGTTATCCGGGAAATGATGATAAAGGTCGTGAGCTGCAACAGCAGGTTGATCCAACCAAACTGATG
AATGATTTCTTTGCCGCAATTGAGTTTATGCAACGCTATCCGCAAGCGACAGGCAAAGTGGGTATTAGCGGATTTTGCTA
TGGCGGGGGCGTATCGAACGCGGCAGCAGTCGCGTATCCGGAACTGGCCTGCGCGGTGCCGTTTTATGGGCGTCAGGCAC
CCACTGCCGATGTGGCGAAGATTGAAGCGCCTTTACTGCTCCACTACGCTGAACTGGATAGCCGAATCAACGAGGGCTGG
CCCGCTTACGAGGCGGCGTTGAAAGCCAATAATAAGGTCTATGAGGCGTATATATATCCGGGGGTTAATCACGGATTCCA
TAATGATTCCACGCCCCGTTATGACAAATCTGCCGCCGATCTTGCCTGGCAAAGGACGCTGAAATGGTTCGATAAATATC
TCTCCTGA
>Cluster_23080
ATGAAACGCCCGGACTACAGAACATTACAGGCACTGGATGCGGTGATACGTGAACGAGGATTTGAGCGCGCGGCACAAAA
GCTGTGCATTACACAATCAGCCGTCTCACAGCGCATTAAGCAACTGGAAAATATGTTCGGGCAGCCGCTGTTAGTGCGTA
CCGTACCGCCGCGCCCGACGGAACAAGGGCAAAAACTGTTGGCGCTACTACGCCAGGTGGAGCTGCTGGAAGAAGAGTGG
CTGGGCGATGAACAAACCGGTTCGACTCCGCTGCTGCTTTCACTGGCGGTCAACGCCGACAGTCTGGCGACGTGGTTGCT

TCCGGCGCTGGCTCCTGTGTTGGCTGATTCGCCTATCCGCCTCAACTTGCAGGTAGAAGATGAAACCCGCACTCAGGAAC
GTCTGCGCCCGCGGCGAAGTGGTCGGCGCGGTGAGTATTCAACATCAGGCGCTGCCGAGTTGTCTTGTCGATAAGCTCGGT
GCGCTCGACTATCTGTTCGTCAGCTCAAAACCGTTTGCCGAAAAATATTTCCCTAACGGCGTAACGCGTTCGGCATTATT
GAAAGCACCTGTGGTCGCCTTTGACCATCTTGACGATATGCACCAAGCCTTTTTGCAGCAAAACTTCGATCTGCCCCCAG
GCAGCGTACCGTGCCATATCGTTAATTCTTCAGAAGCGTTCGTACAACTTGCTCGCCAGGGCACCACCTGCTGTATGATC
CCGCACCTGCAAATCGAGAAAGAACTGGCCAGCGGTGAACTGATTGACTTAACGCCGGGGCTATTTCAACGCCGGATGCT
CTACTGGCATCGCTTTGCTCCTGAAAGCCGCATGATGCGTAAAGTCACTGATGCGTTGCTCGATTATGGCCACAAAGTCC
TTCGTCAGGATTAA
>Cluster_27452
ATGTCTCAGGTTCAGAGTGGCATTTTGCCAGAACATTGCCGCGCGGCGATTTGGATCGAAGCCAACGTGAAAGGGGAAGT
TGACGCCCTGCGTGCGGCCAGTAAAACATTTGCCGACAAACTGGCAACTTTTGAAGCGAAATTCCCGGACGCGCATCTTG
GTGCGGTGGTTGCCTTTGGTAACAATACCTGGCGCGCTCTGAGCGGCGGCGTTGGGGCCGAAGAGCTGAAAGATTTTCCG
GGCTACGGTAAAGGTCTTGCACCGACGACCCAGTTCGATGTGTTGATCCACATTCTTTCTCTGCGTCACGACGTGAACTT
CTCTGTCGCTCAGGCGGCGATGGAAGCCTTTGGTGACTGCATTGAAGTGAAAGAAGAGATCCACGGCTTCCGTTGGGTTG
AAGAGCGTGACCTGAGCGGCTTTGTTGACGGTACAGAAAACCCGGCGGGTGAAGAGACGCGCCGCGAAGTGGCTGTTATC
AAAGACGGCGTGGATGCGGGCGGCAGCTATGTGTTTGTGCAGCGTTGGGAGCACAACCTGAAGCAGCTCAATCGGATGAG
CGTTCACGATCAAGAGATGATGATCGGGCGCACCAAAGAGGCCAACGAAGAGATTGACGGCGACGAACGTCCGGAAACCT
CTCACCTGACCCGCGTTGATCTGAAAGAAGATGGCAAAGGGCTGAAGATTGTTCGTCAGAGCCTGCCGTACGGCACCGCC
AGTGGCACTCACGGTCTGTACTTCTGCGCCTACTGCGCGCGTCTGCATAACATTGAGCAGCAACTGCTGAGCATGTTTGG
CGATACCGATGGTAAGCGTGATGCGATGCTGCGTTTCACCAAACCGGTAACCGGCGGCTATTACTTCGCACCGTCGCTGG
ACAAGCTGATGGCGCTGTAA
>Cluster_21566
ATGAAACCGCAGGTTTACCACGTCGATGCTTTTACCTCTCAACCGTTTCGCGGCAATTCTGCCGGTGTTGTTTTCCCCGC
CGATAATCTCAGCGAAGCGCAAATGCAGCTTATCGCCCGCGAGTTAGGTCATTCGGAAACTGCTTTTCTGCTGCACAGCG
ACGACAGCGATGTGCGCATCCGCTACTTTACGCCAACGGTTGAAGTTCCGATTTGTGGTCACGCGACAGTAGCTGCGCAC
TATGTACGTGCAAAGGTGTTAGGTTTAGGAAATTGCACGGTCTGGCAAACATCGCTGGCAGGAAAACATCGTGTGACTAT
AGAAAAGCAAAATGATGATTATCGTATTTCGCTGGACAAGGTACTCCAGGCTTTGAGCCACCGCTGGAAGGTGAAACAC
GTGCAGCGATTATCAACGCGCTTCATCTCACTGAGGACGATATTCTGCCAGGCTTGCCAATTCAGGTAGCAACAACAGGA
CACTCAAAAGTGATGATCCCGTTGAAACCAGAAGTGGATATCGACGCCCTTTCGCCTGATCTTGCTGCGCTGACCGCTAT
CAGCAAACAGATTGGCTGCAATGGTTTCTTCCCGTTCCAGATCCGCCCAGGCAAAAGCGAAACCGACGGTCGCATGTTCT
CGCCTGCGATTGGTATTGTGGAAGAGCCGGTGACCGGGAATGCCAATGGTCCGATGGGAGCATGGTTAGTACATCACAAC
GTATTATCCCACGATGGCAACGTGTTGCGTGTTAAAGGCCATCAGGGGCGCGCATTGGGGCGTGATGGTGTAATTGATGT
GACGGTGACAATTCGAGATAACCAACCGGAGAAAGTTACCATTTCCGGCACGGCGGTGATTCTATTCCATGCTGAATGGG
CCATTGATTTTTGA
>Cluster_1246
ATGATAAATAGCATTAATTCTTTTTTTTCCAGCATTCCTCGCAGTATATCAAGTGTTACGCGTAACAGTAGCTTCACTGC
CTCACAGCACAAAAGCACACCTAACACGGTAAAAACCAGCTCACCTCTTTCTCCCAGCAACAGTCCTGCCAGTGCAACAA
CTATTTTTAAGGTAAAAAATTCTTATACGGAATCAGGACTTCAACGTTCCACATCTTATACTCAGTCAAGTATAGAAAAA
AACGCTTTACATCGGCCTCTACCTGATGTGGCCAAACGCCTAGTGCAGCATCTTGCAGAGCATGGCATTCAACCAGCCCG
GAATATGGCTGAACATATTCCTCCAGCACCTAACTGGCCTGCGCCAACGCCGCCGGTACAAAATGAACAATCAAGACCTC
TGCCTGATGTGGCTCAGCGTCTGATGCAGCATCTTGCAGAGCATGGCATTCAACCAGCCCGGAATATGGCTGAACATATT
CCTCCGGCACCTAACTGGCCTGCGCCAACGCCGCCAGTACAAAATGAACAATCAAGACCTCTGCCTGATGTGGCTCAGCG
TCTGGTGCAGCATCTTGCAGAGCATGGCATTCAACCAGCCCGGAATATGGCTGAACATATTCCTCCGGCACCTAACTGGC
CTGCGCCAACGCCGCCAGTACAAAATGAACAATCAAGACCTCTGCCTGATGTGGCTCAGCGTCTGGTGCAGCATCTTGCA
GAGCATGGCATTCAACCAGCCCGGAATATGGCTGAACATATTCCTCCGGCACCTAACTGGCCTGCGCCAACGCCGCCAGT
ACAAAATGAACAATCAAGACCTCTGCCTGATGTGGCTCAGCGTCTGGTGCAGCATCTTGCAGAGCATGGCATTAATACAT
CTAAGCGCTCGTGA
>Cluster_66717
ATGGCTAAACCCCGTTTCACTAACGAACAAATTGCTGAAATTTTACAGCAGTCAAAAGAGGGGGCGTCTAATAAGGAGCT
ATGTGAACACTATCAATTTAGCGTTAGCACGCTGCGGCGCTGGCAAGAACAGCACGCTGACGGCATCAGAAGCGAATTAA
AAAAAACAGAATCCAAAGCACAAATCGTCTTTCTGGTTTTCTTTGCTATCGCTATTCTACTGACTCTTATATTTGACAAA
CCTACAGGTGGATGGGTAATTCCGCCTTTATTAATCTATTGTGTTTATTATATCCGCCAGTATCGCAACATCTCTGGCAG
ACATATTAAAAAAGAAGATATCTATCTCTCTCGTTCAGTAAATAACAGCTATAGCGCCCTTTATAATTTGAGCTGGACGT
TTATTTGTTTTTTTATTTTTGCAGTAATTTATTTTTTTATTCAGGTGTTTTCGTAA
>Cluster_44936
GTGAACCTCCCACAAGACGGTATCAAATTGCATCGCGGTAACTTCACCGCTATCGGTCAGCAGATCCAGCCTTATCTGGA
GGACGGAAAATGCTTTCGCATGGTGCTTAAACCGTGGCGCGAGAGACGCAGTCTTTCCCAGAATGCACTCAGCCACATGT
GGTACAGCGAAATCAGTGAATACCTCATCAGCAGGGGTAAAACGTTCGCCACTCCAGCTTGGGTAAAAGATGCTCTCAAA
CACACATATCTCGGTTATGAAACCAAAGACCTGGTTGATGTCGTAACCGGTGATATCACCACTATCCAGTCGTTACGCCA
TACCTCCGATCTTGATACCGGAGAGATGTATGTCTTCCTGTGTAAGGTTGAAGCCTGGGCGGTGAATATTGGCTGCCACC
TGACTATTCCGCAGAGCTGCGAGTTCCAGCTGCTCCGCGACAAGCAGGAGGCGTAA
>Cluster_17488
ATGGAATTCGAAATCAAAGGCGTGAAATATCGCGCGGCAAAACTCAGCGTTTTTGATCAGCTGAAAGTGACCCGCAAACT
TCTGCCGGTGCTGGCAGGAATGATGTCAGATTTCGGGAGCATTCGCTCCCGTTTGCCTGCCGACGGCAAAATCGACACCG
TGAAATTCGAGCAGTTAAAACCGGTGTTTGAAGCCATGCTCCCGCGTATCGCTGAGGAACTGTCTTCCCTGACTGAAGAA
GATACCAACGCGATTATTCATCCTTGCCTGGCCGTAGTGTCACGTAAGCACATGGACGGATGGACGCCGGTATTCAACAG
CGGTCAGCTGATGTTCGATGATATCGACCTGCTAACCATGCTGCAGCTGGTGGCGCGGGTGGTCGCCGATTCGCTGGGAA
ATTTTTTGCCTACACCCCTTACCAGCACGACGCAGAGCCTGCAACAGGGCTGA
>Cluster_20775
ATGAGTAAACGTATTTCGGGTATGTCGTTTGATACCTATCTTGATGGCGATCTGATCCATATCGAGAAAATCACACTCGA
TATCACGGATAACAGCGCCGCCGCCCAGACCCGTGGTGTGCCGGATGGCCATGTTGATGGTGATGTGGCCGCAGAGGGAG
AAATTGAAGTCAGTTCCAAAGTGCTGGGCGTACTGACGGCTAAAGCCCGCGCCGCAGGTTCGTGGCGTGGTATTCCGCCG
CTGGATTTTCTTTTCTATGCCAAAGCGGGCAGCGAAGAAATGAAAGTGGAATCGTTCGGTAACAAACTCCAGCTGAGTAA
TCTGCTGGATATCGATCCAAAGGGCGGCGGTGTGGCGACGCACAAAATCAAATATTTTGTGACCAGTCCCAAGTTCGTGA
ACATCAACGGTGTGCCGTATCTGGAAGCGGAAGCCACGGAAAACCTGATCGGATAA
>Cluster_64861
ATGAAACGGATCTCGCTTATTTTGCTGTGGGGCTTTTGCTCGATGGCACTCTCAAACGTCTCTTTTCACGGTTATTTAGT
GCAGCCGCCTAACTGCACGATTAGCAACGCTCAAACCATTGAAATAACTTTTCAGGATGTGCTTATTGATGACATTAATG
GCAGCAATTATGAGCAAACCGTGCCGTATTCCATTACCTGCGATACGGCGGTTCGCGACCCATTAATGGAAATGACCTTG

AGCTGGAGTGGCACACCAAGCGATTTTGATAATGCTGCAGTAAGCAGCAATATCACTGGTTTAGGCATTCAGCTCAAACA
GGCAGGGCAGAGTTTTACGATCAATACGCCACTTGTGGTGAATGAAACCGATTTACCGGTGTTAACGGCTGTGCCAGTAA
AAAAGAGTGGTGTCGTTCTGCCAGAGGCGGACTTTGAGGCCTGGGCCACGTTACAGGTAGATTACCAATGA
>Cluster_106
GTGAGTCTTGATATGAACGTTGCCGTGGATGTGCGACGTATTCAGCTTGCACTGGATGAACTGGGAACCGTTACACGTGA
CCGCGCCATTCCCCGTGTGATGGCTGCGGCGCTGCTGTCATCCACGGAACAGGCATTTGAACGGCAGGCAGACCCGGATA
CAGGGAAAGGCTGGGAAGCGTGGAGCGATTCGTGGCAGGCATGGCGTCAGGATCATGGTTTTGTTCCGGGCAGTATCCTT
ACCCTGCATGGCGATCTGGCACGCAGCATTACCACCGATTATGGACAGGATTATGCCCTGATTGGTTCGCCAAAAATTTA
TGCTGCCATCCATCAGTGGGGCGGTACACCCGATATGGCACCCCGCCCGGCAGGCGTTCCTGCGCGTCCTTATATGGGGC
TGGATAAGACCGGGGAACAGGAAATTTTTGATGCCATCAGAAAACGCGTCAGCGCCGCTCTGAGGCAATAA
>Cluster_21832
ATGCGTTTCTGCCTTATTTTGATCACAGCACTGTTTCTGGCCGGGTGTAGCCACCATAAAGCACCGCCGCCAAATGCCAG
ACTTTCTGATTCGATTACCGTTATTGCCGGTTTGAACGACCAGCTACAAAGCTGGCATGGCACGCCGTATCGTTATGGTG
GCATGACGCGGCGCGGTGTGGACTGTTCGGGATTTGTGGTTGTGACGATGCGCGATCGTTTCGATTTGCAGCTGCCCCGT
GAAACCAAGCAACAAGCCTCTATTGGCACGCAAATTGATAAAGACGAGTTGCTGCCAGGTGACCTGGTCTTTTTCAAAAC
GGGTTCCGGACAAAATGGTTTGCATGTAGGTATTTACGATACCAACAACCAATTTATCCACGCCTCTACCAGCAAGGGAG
TGATGCGTTCCTCACTTGATAATGTCTATTGGCAGAAAAATTTCTGGCAGGCGAGACGAATCTAG
>Cluster_34242
ATGATTCAGTCACAAATTAACCGCAATATTCGTCTTGATCTTGCCGATGCCATTTTGCTCAGCAAAGCTAAAAAAGATCT
CTCATTTGCCGAGATTGCCGACGGCACCGGTCTGGCAGAAGCCTTTGTAACCGCGGCTTTGCTGGGTCAGCAGGCGCTTC
CTGCCGACGCCGCCCGCCTGGTCGGGGCGAAGCTGGATCTCGACGAAGACTCCATTCTACTGTTGCAGATGATTCCACTG
CGTGGCTGCATTGATGACCGTATTCCAACTGACCCAACGATGTATCGTTTCTATGAAATGTTGCAGGTGTACGGTACAAC
CCTGAAAGCGTTGGTTCATGAGAAATTTGGCGATGGCATTATTAGCGCGATTAACTTCAAACTCGACGTTAAGAAAGTGG
CGGACCCGGAAGGTGGCGAACGTGCGGTCATCACCTTAGATGGTAAATATCTGCCGACCAAACCGTTCTGA
>Cluster_2752
GTGGGCCAGGTGATGCAATCCATCATCGCCGAACAGGTCAAGTACATCAAATCGCTCCCCCTCGAGGCGGCTGACAGGGT
GTACGACATCCAGAATCGGGCGACAGAAGCTGTTGTGACCGGTGGGAGAGCAGAATATTTTGCTAAAGAAATAGCCGCAT
CGGGTGATATAGCAAAGTCCAGAGCTGACCTGATTGCCCGTACTGAACTTGGACGTGCAACCGGCGCGCTGGATCAGGCG
CGTGCGCTGTCAATTGGTTCGAATAGTTATATCTGGCGTACAGCCGAAGATGGTGACGTCAGGCATTCTCATCGGGAAAT
GGAAGGTAAATTTGTCGAATGGGGCAAACCTCCAACGCTTGACGGCATGACAGGTCACGCTGGCGAGCTCCCGAATTGTC
GCTGTTATAAAGAAATCGTTTTTCCCACCTCCCAATCTTATCCCGCCTGA
>Cluster_22813
ATGAGTACCGTTTATCACCGTCCGGCTGACCCTTCAGGCGATGACAGTTACGTGCGACCGTTGTTTGCCGATCGTTGCCA
GGCCGGTTTTCCTTCGCCTGCCACTGATTATGCTGAGCAGGAACTGGATCTGAACAGCTATTGCATCAGCAGACCTGCAG
CCACCTTCTTTCTGCGCGCCAGCGGTGAATCGATGAACCAGGCTGGCGTGCAGAATGGTGATCTGCTGGTAGTGGACAGG
GCCGAAAAACCACAACACGGGGACATCGTTATCGCTGAGATCGACGGTGAGTTCACCGTCAAACGACTGCTGTTGCGCCC
ACGCCCGGCACTGGAGCCGGTTTCAGACAGCCCGGAGTTCCGCACACTGTATCCGGAAAACATCTGTATTTTTGGTGTTG
TCACTCACGTGATACACAGGACGCGGGAGTTACGCTGA
>Cluster_17750
ATGATCCTCGACAAACTGTTGATGTTCTCCGAAGCGCAGGCGGTTACGGCTTCCGCAGCTTCTACTGATGTAATCGATCT
CGGTCCAATTGACGGTACCCGTCGTGATATCGGAGTTGGTTACCCGCTGGAGTTCTGGGCAAACGTGAATACCACTGCAA
CCGCAGGAGGTGCTGCAACCCTGAACGTTCAGTTGCAGACCAGCCCGGATAACTCCACCTGGACCACGCTGTACGATAGC
GGCACGTTGGCGCTAGCGGCGTTGACTGCTGGAAAGCGCCTGTTTTCCGCGAAAGTTCCGGCAGGTGTCCAGCGTTATCT
GCGTGTTAACTATGTGGTCGGTACTGGTCCGCTGACGGCCGGTGCTTTCACCTCGGGAATTAACCTGGATGTTGATAACA
ACAGCCCGTACTACCCGATTCGTTCAAAAGTGACTGGCTAA
>Cluster_19
GTGGTCTACAAAAAGGGTTTATATCTTAAGTTTAACCCAGAGATAGTAGACCAGCCTATAGTATATAGGCTGGTAAAGGA
TTATGATTTAGTTTTTAACATTCTTAGGGCGGTAATCACTCCTGGTAAAGAAGGTATCATGATCTTAGAGTTAGAAGGCA
CCCCTGAAAAACTAAGAGAAGGATTAAACTATCTTAGAGAGGTAGGGGTTGAAGTCAAGTCTTTAGAACAACAGATCATC
AAAAATGAGGAAAAATGTATTCATTGTGGAAACTGTACTGCTGCATGTCCAACAGGGGCTCTTTATGTAGAAAAAGATAC
CTTTTATATAAGGTTTAACCCTGAAAAATGCACTGCTTGTGAGTTTTGTGTGGCAGTTTGTATCACCAAAGCTATGGAGG
TTCACGTAGAAAGTGAGGTAGAAAGTTTTACCTAA
>Cluster_56905
ATGCGCGCAACGGATAAGCAACGCGGTTTTACATTACTGGAAATTATGGTGGTGATCGTCATTATCGGCGTACTTGCCAG
CCTGGTGGTTCCTAACCTAATGGGCAATAAAGAAAAGGCGGATAAGCAAAAAGCCGTCAGCGATATTGTGGCGCTGGAAA
ACGCCCTTGATATGTACAAACTCGACAATCATCACTATCCAACCACAAATCAGGGGCTTGAATCCTTAGTAGAAGCGCCG
ACACTGCCACCGCTGGCCGCAAACTATAACAAGGAAGGTTATATCAAGCGTCTGCCTGCCGATCCCTGGGGCAATGATTA
TGTCCTCGTTAATCCTGGTGAACATGGTGCATACGATCTGCTTTCAGCAGGGCCCGATGGTGAAATGGGAACCGAGGACG
ACATCACCAACTGGGGTTTGAGCAAGAAGAAAAAGTAA
>Cluster_46837
ATGGGGTCCGAACTCGTAAAATGGGTTAAATCGCATAAGATAGATGCTCATATTATTACATTTGTAGCAAAGATGCCGTA
TATTGATTCAATAAAATTGCTTGAAGCAGGTGCGAAAGGATGCGTATGGAAAACCAGTCACCCGGCGAAACTAAATCGTG
CTATTGATTCGATTAGTAATGGTTACACTTATTTTGATAGTGTACATATGGATTGTGAAAAAATATCTTCCAGGTATTCC
TCTGATAATCAACTTACAAATCGTGAGTCAGAAATATTACAACTCATAGCTGATGGTAAAACAAACAAAGAAATCGCTAA
TTTCTTGCAGTTAAGCAGAAAAACAGTCGAAACTCATAGACTCAATATCATGAAGAAATTAGATGTTCACAGTGGTATCG
AGTTGATCAAAACAGCTTTACGTATGGGTGTTTGCACTATATAA
>Cluster_22090
ATGAGTAAACTGGACTGGTTCAATAACGTAGAAAATCGCCCACATCCTTGGGAAGGTATGGGGCTGACATGGTTGACAAC
AGCACTAAATGTCCCTGATGTAAAAGCAGCTATTAATTTTTACACTGAAGTAATGAAGATGGTGGTAATTTCTGAACTTG
AGGGTGATGGTGGTGATGTATTATTTGCTCGTATCCGTTACCGTGGCACAAACTTCACCATAAATAAGGAAGGATGGGAT
TCCGACCTGAAAAGCCCAAATACTTCGGGAGCTATGCCTCCCTTTATTTTTTATATGTATGTAGATGATATGATCACTCT
GGTAAATGAAATGATTGAAGCTGGCTCGACTGTTCTAAATGAACCTGCTGAAATGTTCTGGGGTGATTTAAAAGCCCGTG
TGAAAGACCCATTTGGTTTTATCTGGGATATCGCACAGAAACTGTAA
>Cluster_17891
ATGAATTACATATATTCCGCGACTACAAACTCTTTCTATCCGCTGGAGATGAAAGAGGATTACACTCAAGCTGACTCATG
GCCAGATGATGCTGTTGAAGTTGATGAGCAAGTGTATATTGAGTTTTCCGGATTACCGCCGAAAGGAAAAATCCGTATCG
CTGGAGAAAATAGTTTTCCTGCATGGACTGAAATTCCACCACCAACACATGAGGAACAGATTGCTGCAGCCGAACTTGAA
AAGCAGCAACTGATTAATCAGGCCAACGATTATATGAACAGTAAACAATGGCCTGGTAAAGCGGCTATTGGTCGTCTGAA
AGGTGAGGAACTGGCGCAATATAATTTGTGGCTGGATTATCTGGACGCACTGGAACTGGTCGATACTTCCGGTGCGCCAG

ATATTGAATGGCCTACGCCTCCGGCAGTTCAGGCCAGATGA
>Cluster_49007
ATGAACGAATATGTTTATAGCGCAAGGCATAATGCTTTTTTCCCTGTGGATATGATTGATAAATATAAATCAGAGGGATG
GGATTTATCAGACGCTAAGGAAGTAAATCAAAATATTATCAGTGAGTTTATGGCTGAACCGCCACAAGGAAAAATCCGTA
TTGCCGGAGATGATGGGCTGCCTGCGTGGGCAGATATTCCTCTACCCACGTATGAAGAACTTATTGAAATTACTGAATCA
GAAAGACAGCTGCTAATTAACCAGGCCAACGAATACATGAACAGTAAGCAATGGCCCGGTAAAGCCGCTATTGGTCGTCT
GAAAGATGATGAACTGGCGCAATATAATTTGTGGCTGGATTATCTGGACGCACTGGAAATGGTCGATACTTCCGGTGCGC
CAGATATTGAATGGCCTACGCCTCCGGCAGTTCAGGCCAGATGA
>Cluster_50965
ATGTCATCGTATATGGGGTTACCAACAAATCAGCACGATGCTCTTGAGAATATTCAGCTCTTAGTCGGGGCTGGTGCACT
ACTGTATAGCAGCAATAACCCTGAGTTAATTGAACTTGCCAGCTCCATCCTGGCTGTAACTCAAAAATATAGCCTCACAG
CGTCGCGTTTATGCAAACAAAATTTAGCTATTAATTCCCAGAATCGGATTAGCACCCAACGCGAAGCCTGCGGCTTAACA
ACAGCCGAACTCGCCAGGCTGCTCGATCTCGATGAAGAAATTATCATCCAGTGGGAGAGCGGAGAGTATGAACCAACTAT
CAGTATGCTTATCCCACTGGCAAATATTCTTGGCTGCGATCCGATGTGCCTGTTAACTGGTGAGGTTACTCCTCCGGAGC
AACCAAAAAGTGAGGAGCAGCAACACCATGACGCATCTCAACAAGTTTGCCCCTTATCTCGCGAAGCTCTTCTGCGGAAG
AACCAATACCAATGGTGA
>Cluster_24872
GTGACGCGAGGTAAGGATGGCAGCGCACATCCGCATTTTCACTGTCTGCTGATGGTGCAACCTTCTTGGTTTAAAGGGAA
GAACTACGTTAAGCACGAACGTTGGGTAGAACTCTGGCGCGATTGCTTGCGGGTGAACTATGAGCCGAATATCGATATTC
GGGCAGTAAAAACTAAGACAGGTGAGGTTGTGGCCAACGTTGCCGAGCAACTGCAAAGCGCGGTTGCTGAAACGCTGAAA
TACTCCGTTAAACCGGAAGATATGGCAAACGATCCTGAGTGGTTTCTTGAGCTGACGCGGCAGCTTCACAAGCGCCGTTT
TATCTCGACCGGTGGGGCGCTAAAAAACGTCCTCCAGTTGGATCGAGAAACCAATGAGGATCTTGTCATTGCCGACGATG
TAGGGGATGGCACTGATGACGGGAAGCGGACGGCGTTTGTCTGGGATTCAGGTAAACGGCGTTACAAACGCGCCCCTGAG
AAGGATAAATCGGATTAA
>Cluster_18185
ATGCTGCAACAACAAGCGATGAAAAACCTGAAAAACCGCCAGCGCGCGGCAAAGATTGGCGTACTGTTTCAGGAAGCGGA
AAATCAGCTTTTTCACAGCACCGTTGCTGACGAAATCGCATTTGGTTTGATACTGCAAAAATGCCCGGCGGATGAAATCA
CTCAACGTACCAATGCTGCCCTGCAATGCTGTCAGTTAGCAGACACTGCCAGCGCGCATCCGTTAGATCTGCATAGTGCG
CAGCGACGGATGGTCGCCGTCGCCTGCCTGGAAGCACTCTCTCCGCCACTATTGCTACTGGACGAGCCGAGTCGGGATTT
TGACGAAAATTGGTTGAGCGTGTTTGAAAGCTGGCTGGAAAAGTGCCGCCAGCGAGGCACGAGCGTCGTAGCAATCAGTC
ATGACGCCGCATTTACCCGGCGTCATTTCTCTCGTGTGGTGCGACTGGAAGATGGTGTAATCAGGAACGTTAATCCATCA
GACGATATTCACCCGTAG
>Cluster_11730
ATGTGTTACTGGACCAGTAGTTGTCCTTTAGATAGCCCACATGAGAATGATAATTCGTCAGGGAGTCAGCCGAATGTTGG
GAAGGATTTGACGGATGAAGAGAAGAACTGCCTGGCCGGCGGCAGTACGGGAAGCCCGGGAGGATGGGGACCAGAAGATG
AGAATAGCGCAAGGGATCGTATGCAGCATGAACGGTATAAAGATGAATTGCGACAGAAAATGGGAAAACCCGACGTTACA
GATTCAAATTTGAAAAATATTATAGACGATCTTTATCGGCCAAATGCAAAAGTAGGAAGTGGAAGCACAGCAGATGCTGT
TCGTTATGAATTGGCCACAGGTGAAAAAGTTGGTGGTAGAGGACATGTATTGAAAGCACAGGATTATACAAGGGCTTTAC
AGGATTGGTTGGATAAGAATCCAACAGCATCTCCAGGGGATCGAGCAGCAGCAGAAAACGTTCTTCAGGATTTACAGAAT
GCTTTGGGAGGCAAGTAA
>Cluster_16058
ATGATTAATCCTGTTACTAATACTCAGGGTGTGTCCCCTATAAATACTAAACATGCTGAACATGTGGTGAAAAATATTTA
CCCGGAAATTAAACATGATTACTTTAATGAATCACCCAATATAGATGATAAGAAGTATATATCCGGAAAACGCCCAATGG
GGCAGTTTTCTGTAGATAGTTTATACAATCCTGATTTACATGCATTATGTGAGCTTCCGGACATTTGTTGTAAGATCTTC
CCTAAAGAAAATAATGATTTTTTATACATGGTTGTTGTGTACAGAAATGACAGCCCTCTAGGAGAACAACGGACAAATAG
ATTTATAGAATTATATAATATAAAAAGAGATATCATGCAGGAATTAAATTATGAGTTACCAGATTTAAAGGCAGTAAAAT
CTGAAATGATTATTGCACGTGAAATGGGAGAAATCTTTAGCTACATGCCTGTGGAAATAAACAGTTATATGAAATACATA
AATAATAAATTTGCTAAAATAGAGTAG
>Cluster_39442
ATGGCAAGCAGAGGCGTAAATAAGGTGATCATTATTGGTCGCCTTGGGCATGATCCAGAAATCAGATATTCACCATCAGG
AACGGCATTTGCAAACCTTACAGTTGCTACGTCAGAACAATGGCGTGATAAGCAAACTGGAGAGCAAAAGGAGCAGACGG
AGTGGCACCGTGTGGTAATGAGCGGGAAACTGGCAGAAATTGCCAGCGAATATCTGCGAAAAGGCTCAGAGGTTTATCTT
GAAGGCAAATTGCGGACAAGAAAATGGCAGGATCAAAGCGGACAGGATCGGTTCACTACAGAAGTTATCGTGGGCGTTGG
TGGAGCCATGCAAATGCTTGGTGGCAAGCAAGGAAGCAATGAACAGTCTTCACCTCAGCGAAATAACGGCCAGCAACAAA
GACAGCAATCTCAGCAGCATGGGAATTACGGCGAACCACCTATGAACTTCGACGATGAAATCCCCTTTGCACCAGTAACT
CTCCCCTTCCCTCGTCACGCTATTCACGCAATTTAA
>Cluster_35768
ATGGCGGGGATACCCAAAAAACTGAAAGCCGCACTGCTGGCGGCAACGATTGCCGGTGGCGGTGTGGGTGGTTATCAGGA
AATGACCCGTCAGTCTCTGATCCATCTGGAAAATATCGCTTATATGCCTTACCGCGATATTGCCGGTGTGCTTACGGTCT
GTGTGGGACATACCGGGCCGGATATCGAAATGCGCCGTTACAGCCATGCGGAGTGTATGGCTCTTCTCGACGGTGATTTA
AAACCGGTTTATGCGGCAATCGATCGTCTTGTCAGGGTGCCGCTGACGCCATATCAGAAAACGGCGCTGGCAACGTTTAT
TTTCAACACCGGTGTTACCGCATTCAGTAAATCAACGCTGCTGAAAAAACTCAATGCCGGTGATTACGCCCGGAGCGCGTG
ACCAGATGGCCCGCTGGGTGTTTGCCGCAGGCCATAAATGGAAAGGACTGATGAACCGTCGCGAAGTGGAAATGGCTATC
TGGAATATCAGGGGGGGCGGATGA
>Cluster_52126
ATGATATTTAACCGCCTGAAACTTGCGGTAGACCGCGTCATTGCCGCGTTTTCTGTCGCCCGTAATGCTTGCTCTGGTTGT
ATGCGTAGTCTGGCAAGTATTTAGCCGCTACATACTTAACCAGCCAAGTACATTAACCGATGAGCTGGCACGCTTTTTGA
TGATTTGGGTAGGATTATTAGGTGCTGCATATACCGTCGGCGCGCAGCGACATCTCTCTATTGATTTATTTGCTCTCGCC
CTGAATAAGCGCAAACAATTACTTCTTAGTATAGTCATTAACTTTCTCATTTAGGCTTTGCCGGTTCGGTCATTGTGAC
TGGTGGCCTGAAATTAATTGATAAAACGCTGGCAACATCACAAGTATCTGCCGCGATGCAAATACCGATGGGATACGTAT
ATATCATTCTGCCATTAAGTGGAGTGGTAATGATGTTTTATGCTTTGTGTTTTATTAATCAAAGCATTCAACAACTGAAA
CAACCTGCGCAGGAGGCCAGTTAA
>Cluster_57845
ATGTTTGCTCTGGCCGGAAATAAATGGAATACCACGCTGCCCGGCGGAAATATGCAATTTCAGGGCGTCATTATTGCGGA
AACTTGCCGGATTGAAGCCGGTGATAAACAAATGACGGTCAATATGGGGCAAATCAGCAGTAACCGGTTTCATGCGGTAG
GGGAAGATAGCGCACCGGTGCCTTTTGTTATTCATTTACGGGAATGTAGCACGGTGGTGAGTGAACGTGTGGGTGTGGCG
TTTCACGGTGTCGCGGATGGTAAAAATCCGGATGTGCTTTCCGTGGGAGAGGGGCCAGGGATAGCCACCAATATTGGCGT
AGCGTTGTTTGATGATGAAGGAAACCTCGTACCGATTAATCGTCCTCCAGCAAACTGGAAACGGCTTTACTCAGGCTCTA
CTTCGCTACATTTCATCGCCAAATATCGTGCTACCGGGCGTCGGGTTACTGGCGGCATCGCCAATGCCCAGGCCTGGTTC

TCTTTAACCTATCAGTAA
>Cluster_22158
ATGAACTACGAGATTAAGCAGGAAGATAAACGTACCGTTGCAGGTTTCCATCTTGTTGGCCCGTGGGAGCAGACGGTAAA
GAAAGGTTTTGAGCAGTTGATGATGTGGATAGATAGCAAAAATATTGTGCCGAAGGAGTGGGTTGCTGTCTATTACGACA
ATCCAGATGAAACACCCGCCGAAAAATTACGCTGCGACACCGTCGTGACGGTACCGAATAACTTTACGCTCCCCGAAAAC
AGTGAGGGCGTCATTCTGACAGAAATTTCAGGTGGTCAGTATGCGGTGGCGGTGGCTCGTGTAGTCGGTGATGATTTTGC
TAAACCCTGGTATCAGTTCTTTAATAGCCTCTTGCAGGACAGTGCTTATGAAATGTTACCAAAGCCCTGCTTCGAGGTTT
ACTTGAACAATGGCGCGGAAGATGGGTACTGGGATATCGAAATGTATGTTGCGGTGCAGCCAAAACATCACTAA
>Cluster_52746
ATGAACCGGTTTATTATTGCGGATGCGACGAAATGTATCGGTTGCCGTACCTGTGAAGTGGCTTGCGCAGTGTCGCATCA
GGAGAATCAGGATTGCGCTGCGTTGTCACCAGACGAGTTTATTTCCCGTATTCGTGTCATTAAAGACCACAGCTGGACCA
CGGCAGTAGCCTGTCATCAGTGTGAAGATGCACCGTGCGCGAATGTCTGCCCTGTTGACGCGATAAGCCGCGAACATGGG
CATATTTTCGTTGAACAATCACGTTGCATTGGCTGTAAAAGCTGTATGCTGGCTTGCCCGTTTGGTGCGATGGAGGTCGT
TTCTTCGCGCAAAAAGGCGAGGGCGATTAAGTGCGATCTGTGCTGGCATCGGGCGACGGGGCCGGCCTGTGTTGAAGCCT
GCCCGACAAAGGCGTTGCAGTGCATGGATGTCGAGAAAGTGCAGCGGCATCGGCTACGGCAGCAGCCTGTTTGA
>Cluster_8002
ATGGTGCATCCGGGAGGATTACTCGCCTTGCGGCTCGCCCCTTCGGGCCGTTGCTAAAGCAACGTTATCATCCCTAGTGCC
TGCGGTTAATTCCGCTACCTCTTCGATACCTTCATTGCTGAAGATTTCGGAGAATATGGTGCATCCGGGAGGATTCGAAC
CTCCGACCGCTCGGTTCGTAGCCGAGTACTCTATCCAGCTGAGCTACGGATGCATCGGGAAATTTATTTACTGCTGATA
TTGATACCGCTACTAAAGCCATATCAAGTAAGAGATGGTGCATCCGGGAGGATTCGAACCTCCGACCGCTCGGTTCGTAG
CCGAGTACTCTATCCAGCTGAGCTACGGATGCATCGGGAAACTTACTTTACTGCAGATTTTTTGATACCGCTACTAAAGC
CGTATCAAGTAAGAGATGGTGCATCCGGGAGGATTACTCGCCTTGCGGCTCGCCCCTTCGGGCCGTTGCTAA
>Cluster_13222
ATGTTTGGGCTTTTTAAGAAAAAGGCAAAAGCGGAAAGGTGTTATGATGTAACACGCATTCAATACGAGCTGGTTGTAAT
GTCATCTAAGCTGATAAAAAAACAGACCGATATGTTCAAAGTAATAAAAGGGGAGCGAGAGGAGGCTTTAGGAGACCCTT
TATTATCAGCAGGGTATATAACGCCATGGGTCATTGGATATTGCACTGGTCTCCTGGATGCAGTGACTCAGACGGAGGCT
AACAGGATGGCATTTACATCAGAAATGCTTGGGCTTCTTTTTGCTGCAGTTTTCGGTGATGATAAGTCTCTTGAGGCCAT
GGGAACTTATATAGCCTGTAACGAAGCCTCCTCACCAGACCATGGATTTCATGTTACGTATGAGCTTTATATGTCTGGTC
TAGCGAAAGCGTTTAATGAGGTATGTGAGGATAAACCTGCGATAGGACTTCTGAACTATTTAATGCAGGATTGA
>Cluster_53824
ATGGCAAATATAAGTTATTTATCTTTGAGTGGAGAGACACAAGGGCTGATTTCAGCAGGTTGCTCAACGCTTGATTCTGT
CGGAAACAAAGCGCAGCCAGAACATAAAGATCAGATCATGGTTTATGCGTTAATGCATTCCATCTCAAGAAGTCAAAATG
TCAATCATCATGAATTAATTATCACCAAACCGGTTGATAAATCCTCACCATTGCTGGCAAAAGCACTTAGTGACAATGAA
AAAATGGCAATATGTGAATTTATACTCTACCGAACCAGTAAAGCGGGTATTTACCAGCCTTATTATAAAATAAATCTGTC
AAAAGCCCGAATATCAAGTATTGATTTTGTCACACCGCATGCTGTCCTGGAAAAAGAACTGGAACCACAGGAGAGAATTG
CCTTTATTTATGAAGATATTTCCTGGGAGCATACGTTAGCCGGTACGAATGCAATGAGTAAATGGCAGGATCGTGTACAG
TAA
>Cluster_10955
ATGGTGATACTTACAGCAACTCGCTCGGGCGAAGCCAGGGGAATGCGCTGGGCTGAAATTGATTTTCATAAGCGGGTATG
GACTATACCTGCAGAAAGAATGAAAGCCAGGATACAGCATCGTGTTCCTTTATCCCGGCAGGCTATTTACATTCTGGAAA
ATATACGTGGCCTGCATGATGAACTGGTGTTCCCTTCACCCAGAAAGCAGCAGATCCTTTCCGATATGGTGTTGACAAGT
TTTCTGCGTAAAAAGAAAGCCGTCAGTGACATTCCGGGGCGAGTTGCCACGGCACATGGTTTTCGCTCAACATTCAGGGA
CTGGTGTAGCGAACAGGGGTATTCGCGGGATCTGGCGGAAAGGGCGCTCGCTCATACGCTGAAAAATAAGGTTGAGGCGG
CATATCATCGTACTGATCTACTGGAGCAGCGTGTACCGATGATGCAGGCATGGGCGGATTATGTGATGTCTCAAATTGTG
AATAAATAA
>Cluster_1569
ATGGACGGCGAGCTGAAAAATATGAAGTTAAATATTAATCAACTGGCAGCCCTTTCAGGTCTGCACCGGCAGACTGTTGC
CGCCAGAATGGCGGATGTTCCTCTTGCACCAGGCAGTAATGAAAAGAAAAAAATGTATCTCCTGACGGATTTGATTATTT
CGTTGCTGGAAAAACCACCGGCTTCCGAAGATGAAGAGATGAACCCACATGATCGGAAGGCATGGTATCAGTCCGAGCGC
GAGCGTCTTAAATTTCAGCATGAAACTGTTCAGCTTGTGCCAGTCAGTGATGTCAGGCGGTCCTTTTCTGTCGTGGTGAA
AGCGATAGTTCAGGTACTGGAAACCTGGCCTGACCGGCTGGAGAGGGACAGGGGGTGGACCGCATCACAACTGAATGAAG
TACAGATTGTGGTTGATGAGATCCGCGATACACTGGAAAAGGCAGTCATTGACTGTTGTGATGAGGCCGATATGTGA
>Cluster_17462
ATGAAACGACTCTCGTTAATTTTATTATCCGCTTTTTGCACCATAACGCATGCCGCACCGGAAGATATCACCTTCACGGG
TACGCTGATCGAACCTCCGGTCTGCACCGTCAGCAATGGTGATGATATTGAGATCCAGTTTATCGACGTCATTATCGACA
ATATTGACGGGGTTAATTACCGCAAAGATGTTCCGTACCAGATAACGTGTGACCCTGATATTACCGATGACGCCTGGGTA
ATGACGCTAACCTGGACCGGTACACAGACCAACTATAACGATGCGGCGATTCAGACGGATGTCACGGGATTAGGGATTGA
ACTCCAGCAAAACGGTCAGCCCTTTAAGCTGAATACCCCGCTTAACGTGACACCCGCCAACCTGCCGAAGTTGAAGGCCG
CACCTGTAAAAGCTTCCGATGCCACGCTGACTGACGGTAACTTTTCCGCTTACGCCACATTGCAGGTGGATTATCAATGA
>Cluster_19535
ATGCATATACTGGATTCACTTCTTGCCTTTAGCGCTTATTTTTTTATTGGCGTGGCGATGGTGATTATTTTCCTGTTTAT
CTACTCCAAAATTACACCGCACAACGAATGGCAGTTAATCAAAAACAATAACACCGCGGCGGCACTGGCGTTCAGCGGAA
CATTGTTGGGGTACGTGATCCCCTTATCCAGTGCGGCAATCAATGCGGTGAGTATTCCAGACTATTTCGCATGGGGCGGG
ATTGCTCTGGTGATTCAATTACTCGTTTTTGCTGGCGTCAGGCTTTATATGCCCGCATTAAGCGAAAAAATTATTAATCA
CAATACCGCAGCAGGAATGTTTATGGGAACCGCCGCGCTGGCTGGCGGTATTTTTAACGCAGCTTGTATGACATGGTAA
>Cluster_63267
ATGAGCAACGACACGCCATTTGATGCGTTGTGGCAACGAATGCTGGCGCGCGGCTGGACGCCAGTCAGTGAATCCCGTCT
TGACGACTGGCTTACGCAAGCGCCAGACGGCGTGGTGTTATTAAGCAGTGACCCGAAACGCACGCCAGAGGTCAGCGATA
ATCCGGTAATGATTGGCGAATTACTGCGCGAGTTTCCCGACTATACATGGCAGGTGGCGATTGCTGACCTTGAGCAGAGC
GAAGCCATCGGCGATCGCTTTGGCGTCTTTCGCTTTCCTGCCACTTTAGTGTTTACCGGCGGAAACTATCGCGGCGTGCT
GAATGGTATTCACCCGTGGGCGGAACTGATAAACCTGATGCGCGGGCTTGTCGAACCGCAGCAGGAGCGTGCCTCATGA
>Cluster_23320
ATGGCTACAGTACCAACAAGGTCAGGTAGTCCCCGCCAGTTAACCACCAAACAGACCGGCGATGCGTGGGAAGCACAAGC
GCGTCGCTGGCTGGAAGGCAAAGGACTACGGTTTATCGCCGCTAACGTGAACGAGCGTGGCGGCGAGATCGATCTGATAA
TGCGTGAAGGCCGGACCACCATTTTTATCGAGGTGCGTTACCGCCGCTCTGCGCTTTATGGCGGCGCGGCAGCCAGTGTG
ACCCGCAGCAAACAACACAAATTATTACAGACTGCCCGCTTGTGGCTCGCGCGTCATAATGGGAGTTTTGATACTGTGGA
TTGCCGGTTCGATGTGGTAGCCTTCACCGGGAATGAGGTTGAGTGGATTAAGGATGCCTTTAATGACCACTCATAA
>Cluster_54345
GTGAATACAACGCTGTTTCGATGGCCGGTTCGCGTCTACTATGAAGATACCGATGCCGGTGGTGTGGTGTACCACGCCAG

TTACGTCGCTTTTTATGAAAGAGCACGCACAGAGATGCTGCGTCATCATCACTTCAGTCAGCAGGCGCTGATGGCTGAAC
GCGTTGCCTTTGTGGTACGTAAAATGACGGTGGAATATTACGCACCTGCGCGGCTCGACGATATGCTCGAAATACAGACT
GAAATAACATCAATGCGTGGCACCTCTTTGGTTTTCACGCAACGTATTGTCAACGCCGAGAATACTTTGCTGAATGAAGC
AGAGGTTCTGGTTGTTTGCGTTGACCCACTCAAAATGAAGCCTCGTGCGCTTCCCAAGTCTATTGTCGCGGAGTTTAAGC
AGTGA
>Cluster_24960
ATGCGTGCTTTACCGATCTGTTTAGTAGCACTCATGCTAAGCGGCTGTTCCATGTTAAGCAGATCCCCTGTCGAACCCGT
TCAAAGCACTGCACCCCAGCCGAAAGCGGAGCCTGCAAAACCGAAAGCGCCGCGCGCCACGCCGGTCCGAATTTATACCA
ATGCAGAAGAATTAGTCGGCAAACCGTTCCGCGATCTCGGTGAAGTCAGTGGCGACTCTTGCCAGGCCTCTAATCAGGAC
TCTCCGCCGAGCATTCCAACCGCACGTAAGCGGATGCAAATCAACGCCTCGAAAATGAAAGCCAATGCGGTATTACTGCA
TAGCTGCGAAGTCACCAGCGGTACGCCAGGTTGCTATCGTCAGGCTGTATGTATCGGTTCTGCGCTTAACATCACGGCGA
AATGA
>Cluster_20996
ATGACGAAAGATGAACTGATTGCCCGTCTCCGCTCCCTGGGTGAACAACTGAACCGTGATGTCAGCCTGACGGGGACGAA
AGAAGAACTGGCGCTCCGTGTGGCAGAGCTGGAAGAGGAGCTTGATGACACGGATGACGCTGCGGGTCAGGATACCCCTC
CCGGCCCGGAAAATGTGCTGACCGGGCATGAAAATGAGGTGGTATCAGCGCAGCCGGACACCGTGATTCAGGATACGGCT
GAACTGGCCACGGTCGTGGCACTGGTGACGCTGCATACTGATGCACTTCACGCCACGCGGGATGAACCTGTGGCATTTGT
GCTGCCGGGAACGGCATTTCGTGTCTCTGCCGGTGTGGCAGCCGAAATGACAGAACGTGGCCTGGCCAGAATGCAATAA
>Cluster_41606
ATGCCATTTGCTCGTTATTTCTGCATTTTCATAAACGTTGGTTTGGGAGAAGCGGCAAAACGGAATGTAGGTAACGAGCA
AAACCAAATTCCGGATATGGCGGCGTTTGCCAGTTCACTTTCATCAACGGGGTTTCAAAAACTCCCTTCAGGTCTGATTA
TTCAGTGGGGTATTGTCAGTGGAGCATCAAACTATACGGTGACTTACCCGGTAACATTCCCAAATCGTTCACTTGCGCTG
TTGGCTGTGCCACATACAACGTCGGTGGCTGGTATATCTGCAATGGGCATAGCGAACTGTTCTGATATCAGCAAATCACA
GTTCTATATAATTGTTGGCGGTATATCTCAGGGAGAAATTGTCAAATATGAAAGGTCCTGTTTTTGGGTAGCAATCGGTG
TATAG
>Cluster_10618
ATGATCGACAACCGCACCGCCAGCACCATTGACCAGGCATTACAGAAACATGATACACCCGTCGGCCCGTTATTTTTTGT
AACACGCCACGGAAGAACAAAAAAATGCCTCACCCGAAAAACGGCAATTCGTTACCTGGCATTCTTTATGACCACCCGCG
CTTTTGAACGTTCAGGATTCCGACAACGCTATCCTGACAAGCGTTTTATCTTCAACAGGAATGAGATATGGAAACGTGGA
GAATCAACCACAGAGTATACCCGCGCACACCAGCGAACAATCAGACGACTGCGCAGACTCATCGCCAGGAAACAGTATAC
AGAAAAAATGGTTCAGAAAATATGACACATGGAGCGCCGGATATTACGAACTGATGGCAACAAAACCATTCTGA
>Cluster_63658
ATGGGGCTTCGTGAAGAGATTCAGTCAGAGGTTGCCGCTGCTTTTGATGAGGATTTAGCGGACGCCGTGAGTGATTTTTC
TGGTTCTTACGTTACGCACCGGCACTGGGATCCTGTGACGGAAACTGGCGGCGAATCCACCGCAATCTATACCGGGCGAG
GCGTGTTGACACGTTATAAGCTGGGCAGAATTGATGGGATTAATATTTTGCATGGTGACCTTAAATTAACCGCTCTGGTA
TGCGAGGTTACGGATAAACCAGCTGTCGACCATATTATTGAGATTTATGATCCTGTATCAAGGCAGTTACAACGATACGA
GGTAATTACAGCAAGTGTAGATCCTTCCGCATCAGTTTACTCTATCCAGTTAAGGAGAGCGTAA
>Cluster_30371
ATGTTCGTTGACAGACAGCGAATCGATCTGCTGAACCGGTTGATCGACGCACGCGTTGACCTCGCCGCATACGTGCAACT
GAGGAAGGCAAAAGGATACATGTCCGTCAGCGAAAGCAATCATCTACGAGATAACTTTTTTAAACTGAATCGCGAACTGC
ACGATAAATCGCTGCGGTTGAATCTTCATCTGGATCAGGAAGAGTGGAGTGCTCTTCATCATGCTGAAGAAGCATTAGCG
ACAGCCGCAGTATGTTTGATGAGTGGGCACCATGATTGCCCGACTGTTATTACCGTCAACGCCGATAAGCTTGAAAATTG
TCTGATGAGCTTAACGCTGAGTATCCAGAGCCTGCAGAAGCACGCCATGCTTGAGAAGGCCTGA
>Cluster_63659
ATGGCAAAGGCATGGGATATAGAACCGTCAATATTTGCCGGGATGATTGAGGAAGATGTAGGGCTGAAGATTCGCTACAT
CGCTATTCAGATTCTTACTGCTATTGATATTGCTGCTCCGGTTGATACCGGACGTTTCAGAAATAACAACCTGGTGTCGT
TACAGCATCCCGATTTTGGTATATCTGATAACGTGGATCCGAACGGTACGATTGCTGTTCAGCGTGGGATCGGGGTTATT
TCGAAAGCTGCAAATTACGGAGTTATTTATATCCAGAATAACCTTCCTTATGCAGAGGCTCTTGAAAACGGTCATTCACA
ACAAGCGCCAACTGGCGTGTATGCCAACGCTTTTCATGGTGTTTTACAGGCTTACAAATGA
>Cluster_4466
ATGTCTGCAGAGTTCATGGTAATATGTAAAAAAATATTGTTTAGGAACTGTGTCATTGTATCTTTGTTTGTTTTTACATA
CAACACATGGGCGCAGTGTAATAATAATATTAAAATAATGCGCAAGTATGAAAGTGAAGGTAAATATACCGTTAGAAATT
TGGTTAAAAATAAAGCTATAGCATTGGAATTAGCTGAGATATATGTTAAGAATCGTTATGGACAGGATGCCGCAGAAGAA
GAAAAACCATACGAAATCACTGAGTTAACAACAAGTTGGGTTGTTGAAGGTACCATTCACTCAGACCAAATTGCTGGTGG
GGTTTTTATTATAGAAATAGGCAAAAATGATGGGAGAATTCTGAATTTTGGCCACGGAAAATAA
>Cluster_61209
ATGCGTTTTGCCATCGTGGTGACCGGGCCAGCATACGGTACGCAACAGGCGAGTAGTGCTTTTCAGTTTGCGCAGGCGCT
GATAGCAGATGGCCATGAGTTAAGCAGCGTCTTTTTCTATCGGGAAGGGGTCTATAACGCTAACCAATTGACCTCTCCGG
CAAGTGACGAATTTGACCTCGTACGGGCCTGGCAACAACTGAATGCGCAACATGGTGTGGCGCTGAATATCTGCGTAGCG
GCAGCATTACGCCGTGGCGTTGTTGATGAAACGGAGGCCGGAAGACTGGGGCTGGCTTCGTCAAACCTTCAGCAGGGATT
TACCTTAAGCGGACTTGGGGCGCTGGCGGAAGCCTCGCTGACCTGTGACAGGGTGGTACAGTTCTGA
>Cluster_24878
ATGAAGCTGATCCTGCCTTTTCCGCCCAGCGTGAACACGTACTGGCGACACCCCAACAAAGGGGCGTTTGCTGGTAAGAG
CCTGATAAGCGCGGCGGGGCGAAAATTCCAGAGCGCGGCGTGCGCAGCAATAGTTGAGCAGTTACGTCGTCTGCCGAAAC
CAACGTCGGCACCTGCTTCAGTGGAGATCGTGTTGTTTCCTCCGGATAACCGGATCCGCGATCTGGACAACTATAACAAG
GCGCTGTTTGACGCCCTGACCCACGCGGGTGTGTGGGAAGACGACAGTCAGGTGAAAAGAATGCTGGTGGAGTGGGGACC
GGTTATCCCGGAAGGGAAGGTCGAGATCGCTATCAGTAAGTACGAGAAACCGGCGGGTGCAGCCGCCTGA
>Cluster_48977
ATGTCCGATCCGATTTCCGGTACTGGGCTGGCTGGTGGTGCCCTGACGGGTGCCAGTGTTTATGGACTGCTGACCGGAAC
TGATTACGGCGTTGTATTTGGCGCATTTGCAGGGGCTGTATTCTACATAGCAACAGCTGCAGATCTGAGTGCATCGCGCC
GACTGGCATATTTTATCGTGTCATATATTGCCGGGATTCTTTGCTCTGGGTTGGTTGGCTCCAAGCTGGCGAACTTGACC
GGATACAGTGATAAACCTCTGGATGCTATTGGTGCCGTAATCGTCTCTGCTTTAGCCGTTAAAATCCTGACGTTCCTGAA
TAATCAGGATATCGGCTCGCTGGTGGCGCTCATAACGCGCCGGGGAGGTTCAGGTGGAGCTAAATGA
>Cluster_53512
ATGACCCATGAATACCAGCGACAAAGCCTGGAACAACTAATTACTCAGCAAACAGCAACTCCCAGAAGCGCCGCCGTTGT
ATTTAATGAGAACCAAACCCTCTGGCAAACACTTGGCTTTAGCCAGTCTCAAGTTTCGCTATGGCTTGCCTCATTGCCTC
AAAGTAATCATCTAAAAGGGCCCACAACGGCAACGTACCAGGTTATACCTGATATCGCGGCACATTTGGTATCATTGTTG
CATCAAGCTGGTGGGCGCATGCCCCTTGCACAAGTACTGAAAAAGCTCCCTGCTGGTATTACGACCAGTGAACAGCAGAT
ACGCAAACTGGCACAGAAGCACGCGCAGATAGAGATTAAAGGCCCCTTGCTGGTACTGATAAATTAG

>Cluster_57081
ATGAGCAACATTACCATTTATCACAACCCGGCCTGCGGCACGTCGCGTAATACGCTGGAGATGATCCGCAACAGCGGCAC
AGAACCGACCATTATCCATTATCTGGAAACTCCGCCAACGCGCGATGAACTGGTCAAACTCATTGCCGATATGGGATTT
CCGTACGCGCGCTGCTGCGTAAAAACGTCGAACCGTATGAGGAGCTGGGCCTTGCGGAAGATAAATTTACTGACGATCGG
TTAATCGACTTTATGCTTCAGCACCCGATTCTGATTAATCGCCCGATTGTGGTGACGCCGCTGGGAACTCGCCTGTGCCG
CCCTTCAGAAGTGGTGCTGGAAATTCTGCCAGATGCGCAAAAAGGCGCATTCTCCAAGGAAGATGGCGAGAAAGTGGTTG
ATGAAGCGGGTAAGCGCCTGAAATAA
>Cluster_38553
ATGCCGGCCTTTGAATGGGTTCATGTGCAGCTCCATCAGCAAAAGGGGATGATAAGTTTATCACCACCGACTATTTGCAA
CAGTGCCGCCGATATGATCCAACTGATAAAGGAATTTGACGCTCAGGGCGTGGCAGTCCGGTTCATTGATGACGGGATCA
GTACCGACGGTGATATGGGGCAAATGGTGGTCACCATCCTGTCGGCTGTGGCACAGGCTGAACGCCGGAGGATCCTAGAA
CGCACGAATGAGGGCCGACAGGAAGCAAAGCTGAAAGGAATCAAATTTGGCCGCAGGCGTACCGTGGACAGGAACGTCGT
GCTGACGCTTCATCAGAAGGGCACTGGTGCAACGGAAATTGCTCATCAGCTCAGTATTGCCCGCTCCACGGTTTATAAAA
TTCTTGAAGACGAAAGGGCCTCGTGA
>Cluster_40988
GTGAAGCAATCACTGGGATTACTTGAAGTCAGTGGTCTGGCACTCGCCATCAGTTGCGCAGATGCTATGGCAAAAGCCGC
GTCCATCACGCTGGTTGGCCTTGAGAAGACCAATGGCTCAGGCTGGACGCTCATCAAAATCACCGGTGACGTCGCCTCGG
TTAATGCGGCGATCGCGACGGGAGCCAGTTTCGCCGGGCAGCGAAATGGCCTTGTCGCACACAAAGTGATTGCCCGTCCG
GGAGACGGCATTCTGCCCCACACAACTGAAGCATTGACTGTGACGGTTGAAACAAGCGAAACACCAGCAACAACCGTCAT
CTCAGAGATTATCAGTTGTAATTTGTGTCTTGACCCGGCATGTCAGCGGCAAAAAGGTGAGCCCCGCTCACTTTGCCTGT
ACGCCAATAAGCGAGGGGAGTAG
>Cluster_37293
ATGTATCGCATTGGTGAGCTGGCAAAAATGGCGGAAGTAACACCCGACACGATTCGTTATTACGAAAAACAGCAGATGAT
GGAGCATGAAGTGCGTACTGAAGGTGGGTTTCGCCTATATACCGAAAGCGATCTCCAGCGATTGAAATTTATCCGCCATG
CCAGACAACTAGGTTTCAGTCTGGAGTCGATCCGCGAGTTGCTGTCGATCCGCATCGATCCTGAACACCATACCTGTCAG
GAGTCAAAAGGCATTGTGCAGGAAAGATTGCAGGAAGTCGAAGCACGGATAGCCGAGTTGCAGAGTATGCAGCGTTCCTT
GCAACGCCTTAACGATGCCTGTTGTGGGACTGCTCATAGCAGTGTTTATTGTTCGATTCTTGAAGCTCTTGAACAAGGGG
CGAGTGGCGTTAAGAGTGGTTGTTGA
>Cluster_12059
GTGAGTCTGAGAGAAGCTCTGGAGAAAGCAGAAGAAGCCGGAGTAGACTTAGTCGAGATCAGCCCTAACGCCGAGCCGCC
GGTTTGTCGTATAATGGATTACGGCAAATTCCTCTATGAAAAGAGCAAGTCTTCTAAGGAACAGAAGAAAAAGCAAAAAG
TTATCCAGGTTAAGGAAATTAAATTCCGTCCTGGTACAGATGAAGGCGACTATCAGGTAAAACTCCGCAGCCTGATTCGC
TTTCTCGAAGAGGGTGATAAAGCCAAAATCACGCTGCGTTTCCGCGGTCGTGAGATGGCGCACCAGCAAATCGGTATGGA
AGTGCTTAATCGCGTGAAAGACGATTTGCAAGAACTGGCAGTGGTCGAATCCTTCCCAACGAAGATCGAAGGCCGCCAGA
TGATCATGGTGCTCGCTCCTAAGAAGAAACAGTAA
>Cluster_24997
ATGCGACAAATCATCGACACCCTGGCGCAGTTGCAGCGCCTGCGGGATAAATCGGTCAAAGACAAAACCGTCGAGCTGGC
AAAGCAAAAACAGATTTGTGCGGGTTATGACAACAATATCAAAGCATTAGGCTACCTGGTGGATAAAACATCTGCGGGCG
CGGCAGCGTCGGTAGAATCGCTGAAAAACGTCAGCGACTACAAAGGCACCTTGCGCAAGGTGATCGCCTGGCAGGAGCAG
GAAAAAACGCTGGCTAACATCAAAGCCACGCGAATGCAGAAAAACCTGACGGCGGCGGCGTGCGAAGAGAAAGTAGTGGC
CCTGACGCTGGACGACAAACGCCACGAGCAGCAGGAAAGCGCCACGGCGAAAGCGCAAAAAGCGGTGGATGACATCGCCG
TCCAGTGCTGGCTGCGGCATAAGTTAGCGGAGTAG
>Cluster_9525
ATGGCTCAAGTTATTAATGAAATGGATGTTCCGTCCCATTCGTTTGTTTTTCATGGTACAGGTGAGAGATATTTTCTTAT
TTGTGTGGTGAATGTGTTGTTAACGATTATAACGCTAGGTATCTATTTACCATGGGCATTAATGAAATGTAAGCGTTATC
TCTATGCTAATATGGAGGTTAACGGACAACGATTTTCTTATGGGATTACCGGTGGGAATGTTTTTGTTAGTTGTCTTGTT
TTTGTTTTTTTCTATTTCGCAATCTTAATGACAGTGTCAGCAGATATGCCGCTTATTGGCTGTGTTTTGACTTTGTCACT
GTTGGTTTTGCTTATATTTATGGCAGCAAAAGGACTGCGTTATCAGGCCTTGATGACTAGTCTCAACGGCGTAAGATTTA
GTTTTAATTGCTCTATGAAAGGGTTCTGGTGA
>Cluster_62122
ATGTCAGTGTATGACTATCCCGTACCAACCACACCGTGGCTGAATACGGCACCCGGTTTATTTATTGACGATTATACCTC
GACAGCCTCCAGTACAGTGTCTTCCCTGAGCCGGACACTGATTTATGACTATGAGCAGAACCCTGATTCCGGCAACAATG
TGGTGGCGCTGGCAGCAAAAGCAGGATACAGCACATGGTGGATATCCAATCAAGGAAAACTGGGGGAGCATGACACACGC
ATCTCTGTTATTGCTTCTGATGCGGAGCATGCCACTTTCCTCAAGAAAGGCAGCTTCGCTTCCCGTAAAACAGATGACAA
ACTGTTGTTACAGGAAACAGAACGTGCGCTGGCGGATACATCCTCTCCGAAGATAATTTTCCTGCACATGATGGGTTCTC
ATCCAAATCCGTGTGACAGCCTTAACTCCTAG
>Cluster_22989
ATGCTGAAAACAATTTCGCCGTTAATTTCTCCCGAACTATTGAAAGTGCTGGCAGAGATGGGGCATGGAGATGAAATTAT
TTTTTCCGATGCTCACTTTCCCGCCCATTCGATGGGACCGCAGGTGATCCGCGCTGATGGCCTGTTGGTGAGCGACTTGC
TCCAGGCGATTATCCCGTTATTTGAGCTGGACAGTTATGCACCACCGCTGGTGATGATGGCGGCGGTAGAAGGTGACACT
CTCGATCCTGAAGTAGAACGACGTTATCGTAATGCGCTTTCACTACAAGCCCCCTGTCCTGACATCATCCGCATCAATCG
TTTTGCGTTTTATGAACGGGCGCAAAAAGCCTTTGCGATCGTTATCACAGGCGAACGAGCGAAGTACGGGAATATTCTTT
TAAAAAAAGGGGTAACACCGTAA
>Cluster_1556
ATGGCTCAGGTTGCCATTTTTAAACAAATATTCGATAAAGTGCGAAATAATTTAAACTATCACTGGTTTTATTCTGAACT
AAAACGTCACAATGTCTCACATTACATTTACTATTTAGCCACAGAGAATATTCATCTTGTTCTTGAAAACGATAATACGG
TTTTAATAAAAGGACAGGGTAAGGTTGTAAATGTAAGATTTTCAAAAAATAAATGCCTTATAGAAGCCACCTTAAAAGGA
TTCAAATCAGGAGAGTTATCATTTTACGAATACAGGAAAAATCTTGCTACAGCAGGGGTTTTCAGATGGATTACAAATAT
CCACGAAAACAAAAGGTATTACTATACCTTTGATAATTCATTACTCTTTACTGAGAACATTCAGAACACTACACAAATAT
TTCCGCACTAA
>Cluster_38519
ATGTTTTTTATTGAGAATGAAGGTCAGGCTGTCGCCAGAACGGATTACTGGCAGTCTGTACAGGCGCAGGCCGGATATGT
CTACCTCAGCTGGAATGCCGGCGCAGCCAGGCTGCTTGTCCCGGATGCGGCAAAACATTTACTCAGGGAGATGCGGGGAG
CTGAGTACGTCATCATCAGTAAGGGAACGGTGCATGGCCGCGATGCGCTGGAACTGGTATTTGAAGACGGCAGCGATGCG
CCGTTCGTGATCCACATGCTGAGTGAGCAGTGCGATCGCCTGCTCCCCGAAAACAACCTGGGAGGGGGTTTTGTTGTCAC
CGTCTGGACGCGTGGCGGTAACCAGCTCCGTTATCCGGGAAAGTACCGGGTTGTGGAAAACCTGCCCGACGTTTCCCCGT
GGAGTGAACACTGA
>Cluster_64810
ATGACAAATCAGCAGGATTTATTTGAACATGATCCGGCTGTAAGCCAGTTAATGGATCATATCGACAATATACCGGCTCC

TGAGCAGGAGGCCCGCTGGCCCCGCGCGCTGGTTGAGCTGGTTGACGTTCTGGAAACAGAACTGAAACGTCAGGGCGTGG
ATGATGCACGGAGCATAGCCCGTAAACAGGTGATGTCCTTATCATGGTTCCTGGGGGGGCGGCAGTATTACATCCCCCGG
GGTGATGCACTTCTGGCAGCGTTGCGGGATGATCTGATTTACTGCCAGTTTAACGGTCGGAACATAGAGGAGCTGCGGCG
TGAACATCGTCTGTCACAACCGCAGATTTACAAGATTATTGCCCGGCAGAGAAAACTTCATTCACGGCGACATCAGCCCG
ATTTATTCTGA
>Cluster_20360
ATGGCAAAAAGAAAAAACAATGTTGTTAAGAAGATCGGTGATTCAGCTTCTCTTCTAAGTAAACCTAAATCTATTCTAAG
ACGTAAAGACTTTAAAGAGCTAGTTACGCTCACCAAGCATAATAGCGCCCCTGGCGAATGGAAAACAGAGATCATAGAAC
ATTCTCCCTCTGTGCCTTGTGGAGATGAATATATCGCATTACAAGAAATCTTATCTTCAACACCTGGCGTATTCTGGAAA
CCTAGAAAAAGAAAAGAGTATATTGTTGATAGCTCTGATTTACGCAAATACCAGATTTTAGGATTTGAAGATTATAATCA
CTACGTTGGTTATCTCGCCACCAATGGCCTAAATAATTTAGTTCCTGAATTCCAGATTCTGGATAATGTTGATCACTATG
GAGACTTTTAA
>Cluster_29871
ATGAAGAGGATTATTGGCGTCGTTGCTGGCGCTATATTGTTATCTGGGTGCGCAACTATTGTTGGTGACGAAACGCAGCT
TGTGCAAGTGAACAGCAATCCTTCCGGTGCGAGCTTTAAGGTAAAAGACGAATCAGGCGTGATTGTTGCGCAAGGTAAGA
CCCCGCAAGGAGTAACTCTTGCCAAGTCAGATGGTAGTTATTTTGGCAAAAGAGCTACCAGATCACTATGGAGAAGGAT
GGGTACGAACCAGTTACCCTACCAATCAAAGCCAATGCTAATGGTTGGTATATTGGTGGAAATCTTGTGTTTGGTGGGTT
AATTGGTTGGCTTGCTGTAGATCCATTTAATGGTGGGATGTATACCTTGAAGCCTAAAGAGGCAAATGCATCCCTTATAC
CGTCAACAAAGCAAGACTAA
>Cluster_992
ATGAACGTCAAAATCCGAAATGAAATTCAGGCATTAATCCGAATTCAGGAACGCAATAACAACGGTGGTGAATTACGCGA
GTTTATTTGCGCCCGGAAAGTTGATGATTGTGGTGAAAAGACTTACCTGATTGCTTTCGACCATTACAGCATCTGTGCAC
GTTATTGCGGCGAAAGCATATCCCGCGCCATTGCATTTGGCGGTGCGTTCAACGTGGATTTATGGGAGTACGTCATGGAC
CGGGAATACATCTGCGCATCAGACCCTGAAGCCCGTGAAATGTGGCAGCGTATCTGGCGCGATTACCGGTTAATGGCAAA
AGGCTGGGCGCGCTGCTGTTATTCCTCGCTTGCCCTGAAAGCGGTTCAGTTATCGCTGCGGCATATTCCGGCATCACTGC
GCGAGCCTCTGCTGTACTGA
>Cluster_25760
ATGAAAGAAACCGATATTGCAGGCATTTTAACGTCCACTCACACCATTGCGCTGGTGGGGGCAAGCGACAAACCCGATCG
TCCCAGCTATCGCGTGATGAAATATCTACTCGACCAGGGGTATCACGTCATTCCTGTTTCGCCAAAAGTTGCCGGCAAAA
CACTGCTGGGCCAGCAGGGGTATGGCACGCTGGCAGACGTTCCAGAAAAAGTCGATATGGTCGATGTTTTCCGGAATTCA
GAAGCGGCGTGGGGCGTAGCGCAGGAAGCGATCGCCATCGGCGCGAAAACCCTCTGGATGCAACTGGGGGTGATTAATGA
GCAAGCGGCAGTACTGGCTCGGGATGCCGGATTAAATGTAGTGATGGATCGCTGCCCGGCTATTGAGATCCCTCGCCTGG
GCCTGGCCAAATAA
>Cluster_9738
ATGACGGATTATCAGCCATACAGGAAAGGAACTGTGCTTGCCCCAACTGGACCATGCAATCATCTTCATGTGATTTGTAA
TGATCCTGTTTATTACCCCGTTAACGATTGTTATTGTGTTTTAGTTGTTAATATTTCTAGTATCAAGGATGGTGTCCCCC
ACGATCCGTCTTGCGTCTTGAATTCTGGTGATCATCGCTTTATCAAGCATCCAAGTTATGTTGTTTACGCTGAAGCTATA
ATTTGGCGAGTGGATAACATGGTTAGAAAGCAGCGATCGGGTGAGATTTCTGTTCATGATGATATGCCAGAAGCTACATT
CAATAGAATTCTGGACGGTTTTGATATCTCTGATGAAGTTACGCCAAAGAACCTTAAATTTAAAAATAAATATTGCGTAT
CATCTATTGATGATGAGTAA
>Cluster_58726
ATGAAAAAAATTGCATGTCTTTCAGCACTGGCCGCAGTTCTGGCTTTCACCGCAGGTACTTCCGTAGCTGCGACTTCTAC
TGTAACTGGCGGTTACGCACAGAGCGACGCTCAGGGCCAAATGAACAAAATGGGCGGTTTCAACCTGAAATACCGCTATG
AAGAAGACAACAGCCCGCTGGGTGTGATCGGTTCTTTCACTTACACCGAGAAAAGCCGTACTGCAAGCTCTGGTGACTAC
AACAAAAACCAGTACTACGGCATCACTGCTGGTCCGGCTTACCGCATTAACGACTGGGCAAGCATCTACGGTGTAGTGGG
TGTGGGTTATGGTAAATTCCAGACCACTGAATACCCGACCTACAAACACGACACCAGCGACTACGGTTTCTCCTACGGTG
CGGGTCTGCAGTTCAACCCGATGGAAAACGTTGCTCTGGACTTCTCTTACGAGCAGAGCCGTATTCGTAGCGTTGACGTA
GGCACCTGGATTGCCGGTGTTGGTTACCGCTTCTAA
>Cluster_42555
ATGACACCGTGGCTACTCTTTGGCGCAGGCGGAAAAGGTGTGGGTGCCAGAACGCTTGAACTGGCGCTGGCGGAACAACG
TCCGGTCGTTGCTGTCATTCGTCATGCCGATGCCGCCACGAAACTGGCGCAACAAGGCGTACAGGTTTTTACAGGCGACG
CCTGTGATGCCAGCGTAGTCGCGGCTGCTTGCCGCGCAGCTGGCCCGGATGCGCTTATCATCTCAACAATGGGCGGAGCT
CAGGATTATCTGGCGCACAGAACAGTGATTGACGAGGCCGAGAAAGCAGGCATCACACGCATGATTCTGGTGACGTCACT
GGGATGCGGCGATAGCTGGCCGTTTTTATCAGAGCGGGCAAAGGCTGCGTTTGGTCAGGCGGTACGGGAAAAAACGCTGG
CGGAAAGCTGGTTGCAAACCAGCCAGCTTGATTATGCCATTCTCCGCCCTGGCGGTCTGCTTGATGGTGCGGCAACGGGC
AAAGCACAACGGATACAAAATCAGGAGTGTCATGGTTTTGTTAACCGTGCTGATGTCGCCGCACATATCCATGAACTGGC
AAATGCGCCAGCACTTAACCAGCAGGTCTACAGTCTGATTGAACCGGACCTGAAACCGGCGTGA
>Cluster_59060
ATGCATCGTATAGACACGAAAACCGCGCAGAAGGATAAGTTCGGCGCGGGTAAGAACGGTTTTACCCGTGGTAACCCCCA
GACCGGCACGCCTGCCACCGATCTGGATGATGACTACTTTGACATGTTGCAGGAGGAACTTTGCAGCGTGGTGGAGGCAT
CCGGTGCCAGCCTGGAGAAGGGGCGGCACGACCAGTTACTTACCGCACTTCGCGCGCTGCTGTTAAGCCGCAAGAATCCG
TTTGGCGATATCAAATCGGATGGCACTGTGCAAACGGCTCTCGAAAACCTTGGTTTGGGAGAAGGAGCAAAACTCAATGC
AGCAACGGCTACATTAGGACGCACCGGTTTCATAGCTATACCGGTTATGATTGGTGGTATTGAGCAATCAGTAATCATTC
AGTGGGGGTGGAATGCCGCAAAAGCATCTGCCTCTGGGGGGGATGGAAATACAGTTGTATTCCCGGTTGCGTTTAATAAT
GCCTGTGTTGCCGTTGTTGCAAATTATGACAATGTCAGCGCACCTATCAATGCAGTGGCAACGGGGGGATATACAACCAC
TTCGTTTTTATTACGGTGCGCAGCTCAAACGGGTAGTTATTACTATAACTGGATTGCTATTGGGTATTAA
>Cluster_66923
ATGTGTACAATTCATATCACCCCACGCTGCCGGAGACCTGCCAGAGGGCAGGTATTTCTTAAAAAACTTGAGGTAAAAGG
TGACAGTTTCTCATTCACTCTGCCGTCACGTGTTTCAGGTTACATCCGGCCCCCAATCCCAGAGTGATATACGCCTCGG
ATGCCAGAACACAGGGAAAGGTTACAGGCATGCGGCTGTTGTTGCCGTCCAGCACAGATGAGCTGATAACAACGGCAATA
TGGCAGGTTTCTGTCCCGGATGAAGCACCATTCGACGTTCAGCATGTCATCCAGTGGATACTCCCTTCACCGCTGGCTTC
GCTGATGTCCGATGACACCTGGTTATGGCCATCCGTTCCCGGAACGCCGGCACTGAGTGCCGGTGACTGGCCTGTTATGG
ATACTGACCCTTCCACCTTACGGCGCGTCAGACGTCGTTCTTTCAGCATCATCGATAACCGGAACAGGAAAGGTTATATC
ACCAGGCGATATCAGTGTTTCCCACTGGCCCCACTACCTGAAGACAGAAAATACGACAGCCTTCCTGACCTTGTGCTCCA
CCCCGAGCAGGAGACAATAAAATGCAAAGAAAAACATTACTCGCAGCCCTTATAG
>Cluster_43880
ATGATCTTGCTACAACTCTCCTCAGCGCAGGGGCCGGAAGAATGTTGTCTCGCAGTGAAAAAGGCGCTGGAGCGATTAAT
TAAAGAAGCTGCCCGACAAGACGTCGCGGTAACGGTGTTGGAAACAGAAACGGGCCGCTACTCTGACACGCTGCGTTCAG
CTCTGGTTTCTCTGGATGGCGATAACGCATGGGCATTAAGCGAAAGCTGGTGCGGCACTATTCAGTGGATTTGTCCGAGT

CCGTATCGGCCTCATCATGGGCGCAAAAACTGGTTTCTGGGCATTGGGCGTTTTACCGCTGATGAGCAGGAACAATCGGA
TGCAATCCGTTATGAGACGTTGCGTTCGTCGGGGCCGGGCGGTCAACATGTCAATAAAACCGACTCGGCGGTACGCGCCA
CGCATTTGGCATCCGGTATTAGCGTGAAGGTTCAGTCAGAGCGTAGTCAGCATGCTAACAAGCGGCTGGCACGATTGCTG
ATTGCCTGGAAGCTGGAGCAGCAGCAACAGGAAAATAGCGCGGTGCTGAAATCGCAGCGGCGAATGTTCCATCACCAGAT
TGAACGTGGCAACCCGAGACGGACATTTACAGGGATGGCTTTTATCGAAGGATAA
>Cluster_15640
ATGACCACTATTACCAGAGAACAGGCACAGAAAATTATTGAAGCAGCCGATGAGGTTATTAGTGCGCTTGCCGGAACTAA
CGAGGATGTTCACCCTGGTAGCGATAACATGCTACGCCTGTGGGATGACCTGAATGACCGTTACGCGCCCCCTGAAGTTG
TGCGTGAGCTGGCACGAATTGCACTGGCATCACTGGAAGCAGAGCCTGTAAGCCAAACTTACAACTTGCCAGAATTAATC
GAAGGGATGGAAGTTTCCATTGATGTAAGCACTTGTGATGCTGATTTAGGTAATCGCTATTTCGGTACCGTCACCGAGGC
GTTAGAACTTGATACAGCCAAGAATGGTTACATCCTCCTAGTTCAGGACGCAGAGCCAAACTTCGATGTAAATGGCAACT
CTCCGGTAATTCCGGGCGGTTGGATAAGCTGTAGTGAGCGAATGCCGGATAGCAAAACAGCCGTTCTTGTTGCCAGGGAG
TTTGACAGGAAAGGTGACTGGCGAATGAAATGGGCGACTTACATCCCGGGGCATCCTGACGCTAATGATGGGTGGGTAAT
ACCTGGTGCGTCGTGGATACCATCACACTGGATGCCGCTACCAGAACCGCCGCAGGAGGTTAACCATGGCTAA
>Cluster_4144
TTGACTAATTTGAATTATCAACAGACGCATTTTGTGATGAGTGCGCCTGATATTCGCCACCTACCTTCCGATACCGGAAT
TGAAGTGGCTTTTGCAGGCCGTTCCAACGCAGGTAAATCCAGCGCGCTGAACACGCTGACTAACCAGAAAAGCCTGGCTC
GTACCTCAAAAACCCCAGGGCGCACCCAGCTTATCAACCTGTTTGAAGTGGCTGACGGCAAGCGTCTGGTTGACTTGCCT
GGGTACGGTTATGCGGAAGTCCCGGAAGAGATGAAGCGCAAATGGCAGCGTGCGCTCGGCGAATACCTCGAAAAACGTCA
GAGCCTGCAAGGTCTGGTGGTGCTAATGGATATTCGCCATCCGCTGAAAGATTTGGATCAGCAGATGATTGAGTGGGCGG
TAGACAGCAATATCGCCGTTCTGGTGCTGCTGACCAAAGCGGACAAACTGGCAAGCGGCGCACGTAAAGCGCAATTGAAT
ATGGTGCGTGAAGCTGTACTGGCGTTTAACGGTGATGTGCAGGTTGAAACGTTTTCTTCGTTGAAGAAACAAGGCGTGGA
CAAGCTGCGGCAGAAACTGGATACCTGGTTTAGCGAGATGCAGCCTGTAGAAGAAACGCAGGACGGCGAATAA
>Cluster_47111
ATGATTTTATTCGCCGACTATAATACCCCTTATCTTTTCGCTATTTCCTTTGTACTGTTGATCGGCCTTTTAGAAATTTT
AGCGCTTATCTGCGGTCACATGCTCTCCGGTGCTCTTGATGCACATCTTGACCATTATGATTCCATAACCACAGGTCATA
TTAGCCAGGCACTTCATTATCTCAATATCGGAAGATTGCCAGCTCTCGTCGTTCTCTGCTTGCTGGCTGGTTTTTTTGGT
CTTATTGGCATCCTGTTGCAGCATGCCTGCATCATGGTCTGGCAATCACCACTGTCGAACCTGTTCGTGGTTCCTGTCAG
TTTACTGTTTACGATTATTGCGGTGCATTATACCGGTAAAATAGTTGCGCCCTGGATTCCACGCGACCATAGTTCAGCTA
TTACAGAAGAAGAATATATTGGTAGCATGGCATTAATTACCGGTCATCAAGCCACTTCAGGTAATCCCTGCGAAGGAAAA
CTCACCGATCAATTTGGCCAAATACATTATTTACTACTGGAGCCTGAAGAAGGAAAATTTTTCACTAAAGGAGACAAAGT
GTTAATTATTTGCCGACTTTCTGCAACGCGATATTTGGCGGAAAACAATCCCTGGCCACAAATATTGTAA
>Cluster_5667
ATGAAACCAACGTCGGTGATCATTATGGATACTCATCCTATCATCAGAATGTCTATTGAAGTTCTGTTGCAAAAAAACAG
TGAATTGCAGATTGTCCTGAAAACGGATGATTATCGCATAACCATCGATTATCTCCGAACCCGTCCTGTTGATTTAATCA
TTATGGATATAGACTTGCCCGGAACAGACGGTTTTACCTTCCTGAAAAGGATCAAACAAATCCAGAGCACAGTGAAAGTG
TTATTTTTATCATCGAAATCAGAATGCTTTTATGCTGGCAGAGCGATACAAGCTGGTGCTAACGGTTTTGTCAGTAAATG
CAATGATCAGAATGATATTTTTCATGCCGTTCAGATGATCCTCTCCGGATACACGTTTTTTCCCAGCGAAACGCTTAACT
ATATAAAAAGCAATAAATGTAGTACGAATAGTTCAACGGTCACTGTGCTATCTAATCGTGAAGTGACCATATTACGTTAT
CTGGTTAGCGGATTATCTAATAAAGAAATTGCCGATAAGTTATTACTTAGCAATAAAACAGTTAGTGCGCATAAATCTAA
TATTTATGGCAAGCTAGGTTTGCATTCAATTGTAGAGCTTATCGACTACGCCAAATTATACGAATTAATATAA
>Cluster_6070
ATGAAAAAAATTGCCATCACCTGTGCATTACTCTCAAGCTTAGTAGCAAGCAGCGTTTGGGCTGATGCCGCAAGCGATCT
GAAAAGCCGCCTGGATAAAGTCAGCAGCTTCCACGCCAGCTTCACACAAAAAGTGACTGACGGTAGCGGCGCGGCGGTGC
AGGAAGGTCAGGGCGATCTGTGGGTGAAACGTCCAAACTTATTCAACTGGCATATGACACAACCTGATGAAAGCATTCTG
GTTTCTGACGGTAAAACACTGTGGTTCTATAACCCGTTCGTTGAGCAAGCTACGGCAACCTGGCTGAAAGATGCCACCGG
TAATACGCCGTTTATGCTGATTGCCCGCAACCAGTCCAGCGACTGGCAGCAGTACAATATCAAACAGAATGGCGATGACT
TTGTCCTGACGCCGAAAGCCAGCAATGGCAATCTGAAGCAGTTCACCATTAACGTGGGACGTGATGGCACAATCCATCAG
TTTAGCGCGGTGGAGCAGGACGATCAGCGCAGCAGTTATCAACTGAAATCCCAGCAAAATGGGGCTGTGGATGCAGCGAA
ATTTACCTTCACCCCGCCGCAAGGCGTCACGGTAGATGATCAACGTAAGTAG
>Cluster_10993
ATGGTTTCATTAGTTAAGAACCAGACGGTATCACTCAGCAAAGAATCATCTGCATTAAGCCAGCTTCATTTCGGTCTCGG
TTGGGATCCGGTTAAGAAAAAAGGTCTTCTTGGAGGATTGTTTGGAGGCAACGACTCAATCGATCTCGATGCTGGCTGTG
TATTAATGGACAGCACAGGTAAAACAATTGACACCATCTGGTTCCGCAAACTGAAATCCACCTGCGGCGCTGTTGTGCAC
AGTGGCGATAACCTGACCGGTGAGGGTGACGGTGACGATGAAGTGATTAAGGTCAACCTCTCCCGCCTGCCTGCAAACGT
TGAATACCTGGCGTTTACGGTAAACAGCTTCCGTGGTCAGTCGTTCAATGACGTCGAAAACGCTTTCTGCCGCGTTGTCG
ATCAAACCGGCAAAGAGCTGGCTCGCTACAAGCTGACTGAGCAGGGCTCACATACCGGGATCGTTATTTCTTCGCTGCGC
CGTAATAACGGCAACTGGGACTTTACCGCCCTTGGCCATGCCTGTCGCGGCCGCACAATTGACGATATGCACTCCGATAT
CGTTTCAGCGGTTATTCGCTAA
>Cluster_66940
ATGATGAAACCACGTTCTTCTTATTCAAAAACAGCATTCATTCTTTTATTTTCTGTTTTCCTGGTAGCGGCAGTGACAAA
AGCAAAAAGCTCGCTTCCCGATATCACACTGGAACAGGCAAAAGAAATTAATGCTGATAACACTGTTATTTTCCTTTTCC
GCCATGGAGAACGTTGCGACCGTTCAGATATGCCCTGCTATTCGGATAAGAGCGGAATCACTATTACCGGTACAGAGAAA
GCACAGCAGGAAGGTATTAAGTTCGCAACAATCTTCAGTGAATATGATATTTACTCCAGCAATGCGGTAAGAACCATACA
AACTGCAAAATTCTTCTCCGGTAAAGAACCCGTCGTTATGGACAGTTTATCAAACTGCAATAATGATCTTTATAAGACGC
TTGAATCAATCGCAAGAAAATCTCATAAAAGGAACATAGTAATAATGACACATAATCATTGTTTATCATTTCTGGCCAGA
GATAGGCTCGGCAAGAAATTCAAACCGGCGTACCTTGATGCCCTGATCATGCATTACGATGGTACCCGGCTCATACTTGA
CGGAAAATACAACAAAGAGGCATAG
>Cluster_10595
GTGAACACGGCAGCGGAAGCCATCCGTGCCCTGTCGATGCAGATGCCTGGATTCCGCCGTCAGATGAACGAAGGCTGGTA
CCAGATACGTATTCGCGGTGAGGACACGGCACCGGAGGCGGTGTACGCCCGTCTTCACGAACAGCTGGGTGAGGGAACGA
TCATCCACATTGTGCCGCGACTGGCCGGGGCCGGAAAAGGTGGACTGCAGATTGTGCTGGGGGCGGCAGCCATCGTGGGC
TCTTTCTTCACTGCCGGAGGCTCGATGGCGTTATGGGGTACAGCCCTGAGTGCCGGTGGTTTTTCTGCCACCACGATGCT
GTTTTCACTGGGTGCCAGTATGATTCTGGGCGGTGTGGCCCAGATGCTGGCCCCGAAGGCAAAAACACCGGATTACCGCG
CAACGGATAACGGCAGACAGAACACGTATTTTTCGTCACTGGACAACATGATTGCCCAGGGTAACCCGATGCCGGTGCCT
TACGGTGAAATGCTGGTTGGCTCCCGGCGAATCTCCCAGGACATCAGCACCCGTGATGAAGGCGGGGGCGGAAAGGTCGT
GGTTATCGGGCGGCAGGGGTAA
>Cluster_17663
ATGGTGCGGGAAGTCCGCACCTATGCCGGTGAACTGGATGAAGACCCGGGCCGGATTGTTCGCAGCCTTCCGGCAGCCTG

GGTGACGTTCGGCGGCATCGCGAAAACCGAACGCTATTCCACGTCACGCCAGAAATACATTGCCACCGGACGCTTTGTGG
TTGTGGTGGGTGATTACAACACCCGCAGCGAACAGAGCGCCAGACAGGGAGGCACCGTCCGGGATGAAGTCGGGACAAAC
CAGCTGGTTGAATCCGTCCGTCGCCTGCTGACCGGTCAGGATTTGGGGCTGGAGATTGATTATTTCGAACCCGGGCGCGT
CAGGACGCTGTTTAACACCGGCGTGGCAGAGCGGGCAATGTCCGTGTTTGCCTGTGAGTTTGATACCCGCTGGGTGGAGC
ACGCGCTGGAGAACGGCAAATGGCCGGAGCGCGGTGCAGAAGCGGATATGGTTTTCAACCGTTACCACGGCCGTCTGTCA
GACCCCGACCCGGATTTACTGCGAATTGGTACGCAATACGGCGCAGATATCTGTGGTCTGGATGAATTAAGAGAGCAACA
GCATGAACAAAATGAAGGTTAA
>Cluster_2405
ATGCCTTTCTCTACGGATGATTCCGTTGATCACCCCGTATCTCTGTATGCGGCAACCAAAAAAGCCAACGAACTGATGGC
CCATACTTATTCCCATTTGTACGGATTGCCCGCAACCGGGTTGCGTTTCTTTACCGTGTACGGCCCCTGGGGTCGTCCGG
ATATGGCACTGTTTAAGTTCACCAAAGCAATGCTGGAAGGGAAAAGTATTGACGTCTATAACTACGGCAAAATGAAGCGT
GACTTTACCTATATCGATGATATCGCAGAAGCAATCATTCGCCTGCAGGATGTCATTCCACACGCAGACACACACAATGGAC
CGTTGAAACAGGCACGCCAGCAGCCAGCATTGCCCCCTATCGCGTCTACAATATCGGCAATAGTTCACCGGTTGAGCTCA
TGGACTATATCCAGGCTTTGGAAGATGCGCTGGGTATTGAAGCAAAGAAAAACATGCTACCGCTACAGCCTGGTGATGTG
CTGGAAACCAGCGCGGACACTAAAGCACTGTATGAGGTAATTGGTTTTACGCCTGAAACCACGGTCAAAGATGGGGTGAA
GAACTTTGTTAACTGGTACCGTGATTTCTACAAAGTTTGA
>Cluster_46582
ATGATAAGTAAAGCTGTATTAAATAATGAACTCATCGCCACAAAGGCCGGAGACATTACCGTTTATAATTATGATGGTGA
AACACGGGAATATATTTCCACATCAACTGAATATCTTGCTGTGGGTGTTGGTATCCCGGCATGTTCCTGTTTAGATGCTC
CTGGCTCATATAAAGCTGGTTATGCAATTTGCCGTTCTGCAGATTTTAACTCATGGGAATATGTGCCAGACCATCGCGGT
GAAATCGTCTATAGCACCGAAACAGGAGAATCAAAAGAAATCACAGTTCCGGGTGATTACCCTGAAAATACAACCACTAT
CGCCCCGTTAACGTCATACGATAAATGGGATGGTGAGAAATGGGTGACCGATACTGAGGCACAGCATAACGCCGCAGTAG
ACGCGGCAGAAGTACGGCGCCAGTCACTGATTGATGCTGCTATGGCTTCCATTAGTCTGATTCAGCTGAAATTGCAGGCC
GGGCGGAAGCTGACGCAGGCAGAAACAACCCGGCTTAACGCTGTGCTGGATTACATTGACGCGGTGACTGCAACAGATAC
CAGCACCGCGCCGGATGTCATCTGGCCTGAACTACCGGAGGCGTAG
>Cluster_10602
GTGCGTCATGTAGCAAGACACAATAACCCTGAAACAATCATTGCCGGGGAGCGTCACCTAAGGGTCCATCATATCCCTGG
GTTAGATGGTTTTCAAGTGGAGGCGATTCCAGGTAGTCTTCTTGACCGTTTTGGTGATAGAAGAAGAACGCATCGTGAAC
TCGCTTTAGCGCTGGAAACACAGCTTAACGGAGGAAGAACTTTTCTGCAGGCAGTCTCTGCTTATATAGCAGAGGCCCGT
GCCAATCCGCAAGTTCCAGAAAGACCAGAGCATCCGATGTATAGCCGAATTAGCTCTTGTTCATTCACCGTTAACCCTGA
CGATTTGAAATGCGGTGAGTTACATCTGACTTGTCCGATAACATTATGTGTCCCTGGAAAAGGTATTTTTGTCAGAAATG
CGAGTGATTCTGATATCTGCTCTCTATATGACCCAGATGCACTGAAGGAAATGGTCCATCACAACGCTAGCCATCCTTTC
AGCCGTGAGCCCTTGACTCCAAGAATGATTGTCCACAAAAATGATTGCTATTTTAATGAGGAAAAACAACAGTTCTGCGT
AAAATCTTCCTCCGGTGAGGATACGTATCTTTAA
>Cluster_33168
ATGACAATTTTTAATAAAATAGACTATAACTATTATAAATTAATTAATTATTATCCAATCATGATGGTTCATGATGAATGGCT
AGGTGATTTAACCGGTGTCAATCAGGTCAGTTTTCGGCGTTTAAGAGAAACTAGTTCTACTCGTAATCAACTCAATAAGA
TATTGCGTCAGGAAATACAAGATAAAATTTCTGGTGTGGAATTATCTGACATAAATAAGGAAGGTTTTTTATATCAGTCA
ATTGGGAAAATAAGATTGCTTGCTCTAAGTAGTGCATTATTTGACATTCAATGCCCTGATTATATTTTCTCACGATTATA
TCGAGAAACTCTGATTAGAGAGATAGGATATCAAAATGTGAAGCAGTTATCATTCTATTGGCAGGGAGGGCAATGTAAAC
CAGAATATGGTGAAGAACGGTTTTGTGCTGAGTTAATAAAATACGGTGCAGGAAATTTGGAGTGGCTATTTGCTGATAAT
CCTTTATGGACCATAGTCAAATATCTTTTACCGAAAAGTGGAGAAATAAAACCAACTCATATTAATGATTTATTTCTTAA
CAGACTTAATAAAATTTTATTGCCTTATGAAACTTTATGA
>Cluster_43016
ATGCGATATCCGGTTGATGTATACACAGGCAAGATTCAGGCTTATCCCGAAGGCAAACCCAGCGCAATTGCCAAAATCCA
GGTTGATGGTGAGTTGATGCTGACGGACCTGGGGCTGGAAGGTGACGAACAGGCGGAGAAAAAGATCCACGGCGGGCCAG
ACAGAGCGCTGTGTCATTATCCTCGTGAGCATTATCTCTACTGGGCGCGGGAATTTCCGGAACAGGCGGAGCTGTTTGTT
GCGCCTGCGTTTGGCGAAAATCTCTCAACCGACGGTCTGACGGAAAGTAATGTTTATATTGGTGATATTTTCCGCTGGGG
AGAGGCATTAATTCAGGTCAGCCAGCCACGCTCACCTTGCTATAAACTCAATTACCATTTTGATATCAGCGATATAGCGC
AGTTGATGCAAAACACGGGTAAAGTGGGGTGGTTGTATAGCGTAATTGCACCAGGGTTGGTATCTGCTGATGCGCCGCTG
GAGTTGGTTTCCCGTGTCAGCGATGTGACCGTGCAGGAGGCTGCCGCCATCGCATGGCATATGCCGTTTGATGACGATCA
GTATCACCGTTTACTCTCCGCCGCAGGGTTATCGAAAAGCTGGACGCGAACGATGCAAAAGCGCCGACTTAGCGGCAAGA
TCGAAGATTTTTCCCGCCGTTTGTGGGGAAAATAA
>Cluster_27151
ATGAAAGGATTATTAGCTTTACTCATTTCTTCTATGGTCCTTCCTGCACATGCCGGTATTGTTATCTACGGGACGCGTAT
TATTTACCCGGCAGAAAATAAAGAAGTGATGGTGCAGTTGATGAATCAGGGAAACCGTTCTTCGCTGCTGCAGGCGTGGA
TTGATGATGGCGATACGTCGTTACCACCAGAAAAAAATTCAGGTTCCTTTCATGTTAACGCCACCAGTGGCAAAAATAGGG
GCAAATTCCGGGCAGCAAGTAAAAATCAAAATTATGCCGAATAAACTACCCACTAATAAAGAGAGTATTTTTTTGTCTGAA
TGTTCTGGATATTCCACCAAATAGTCCAGAACAAGAAGGTAAGAATGCACTGAAGTTTGCGATGCAAAACAGAATTAAGT
TGTTTTACCGGCCAGCGGGTATTGCTCCGGTAAATAAAGAGACCTTTAAAAAGTTACTGGTAAATCGCAGTGGCAATGGA
TTGGTGATAAAAAATGACTCAGCTAATTGGGTGACGATTTCGGATGTCAAAGCTAATAATGTTAAAGTCAATTATGAAAC
TATTATGATTGCCCCCTTAGAAAGTCAGAGTGTTAATGTCAAAAGTAATAATGCAAATAACTGGCATCTGACCATTATCG
ATGACCATGGCAACTATATTAGTGACAAAATTTAA
>Cluster_48673
ATGATGCATAAATCAGTAGCCGGTGAGTTTCAGAAGGAAGTCGATAATACTACCGTTCTATTGGACGATATTTTAAGCAT
TCTCGCGCTTCTTGAGGCTGGCGATTGGTCAGAACATTGCACTAAAACGGAGTTAGGCGGTCGGCTTGAAAGAGAGATTA
CACGACTTATTGGTGATGCGCAGGAAGCTACAGTCACTAGTTATGAGTTAATCGCTGAAGCCTGGCGTTTGATGGATGGA
CAAGATCCTAAAACCAGCGATTGGCATAGCAAGGCTTCGAAGTATTTAAATTCCGATAGGGTAGAAAAAGTTGATGATGT
GAAACCGAAGCCAGTAGATCACGGTTTCCGCGATAACTGCGAATGCTCTAGTTGCCAGACCACGGCCCGTATTTGTTCCG
AATTGACAGATAAGTCCAGCCTAATCTACGAAGTTAATGTAGGCGGTAATACATGGGTCGAATGCACAAGAGCTGCATAC
GTAAGAGCAAAAGACAAGGGTGAATTAACCAGAGTTGTTGCCCATCACCCAAATAATGAGCTTAAAGATCACCAGATTAG
AGAACTGGTGAATGAGTTGCGGGATATTGCGGTTCAGTACCACGGAACGCAGCAATTGCGGGAGAAAATTGCCAGAGCAG
TGAACAACTCAATTAGATCAGAAAGTATGTAG
>Cluster_10267
GTGGATAAGGTTTTAGATTCAGCCCTCCTTTCTTCGGCAAATAAAAGAAAGGGTATCCTTGCTATTGGCGCACATCCTGA
CGATATAGAATTAGGCTGTGGCGCATCGCTTGCTCGTCTTGCGCAAAAAGGAATTTATATCGCAGCAGTGGTGATGACTA
CCGGCAACTCTGGTACAGATGGAATAATCGATCGCCATGAAGAATCGCGCAACGCCCTAAAGATATTAGGGTGCCACCAA
ACTATTCATCTTAATTTTGCTGACACCCGCGCTCATTTACAGCTCAATGATATGATTTCCGCCCTGGAAGACATCATTAA
AAATCAAATTCCTTCTGATGTTGAAATCATGCGGGTATATACCATGCATGATGCCGACCGCCATCAGGATCATCTCGCTG

TTTATCAAGCTTCAATGGTTGCCTGCCGCACTATTCCACAAATTCTCGGCTACGAAACCCCAAGTACCTGGCTTTCATTT
ATGCCTCAGGTTTTGAATCCGTTAAAGAAGAATATTTCACAGTCAAACTTGCTGCATTAAAAAAACATAAAAGCCAGGA
ACGACGTGATTATATGCGTCATGATCGCCTGCGTGCAGTTGCACAATTTCGCGGCAACAGGTCAATAGCGATCTGGGTG
AAGGCTTTGTAATTCATAAAATGATTCTTTGA
>Cluster_956
ATGACACCGGACATTATCCTGCAGCGTACCGGGATCGACGTGAGAGCTGTCGAACAGGGGGATGATGCGTGGCACAAATT
ACGGCTCGGCGTCATCACAGCTTCAGAAGTTCATAACGTAATAGCAAAACCCCGCTCCGGAAAAAAGTGGCCTGACATGA
AAATGTCCTACTTCCACACCCTGCTTGCTGAGGTTTGCACCGGTGTGGCTCCGGAAGTTAACGCTAAAGCGCTGGCCTGG
GGAAAACAGTACGAGAACGACGCCAGAGCCCTGTTTGAGTTCACTTCCGGCGTGAATGTTACTGAATCACCGATCATCTA
TCGCGACGAAAGTATGCGTACCGCCTGCTCTCCAGATGGTTTATGCAGTGACGGCAACGGCCTTGAACTGAAATGCCCGT
TTACCTCCCGGGATTTCATGAAGTTCCGGCTCGGTGGTTTCGAGGCCATAAAGTCAGCTTACATGGCCCAGGTGCAGTAC
AGCATGTGGGTGACGCGAAAAGATGCCTGGTACTTTGCCAACTATGACCCGCGTATGAAGCGTGAAGGACTGCATTATGT
CGTGGTTGAGCGGGATGAAAAGTACATGGCGGGTTTTGACGAGATGGTGCCGGAGTTCATCGAAAAAATGGACGAGGCAC
TGGCTGAAATTGGTTTTGTATTTGGGGAGCAATGGCGATGA
>Cluster_58855
TTGGCGCTCTTGATTTTCGCCGTTTTATCGCTGCTGGTGGCGGGTGAATTGCAGGCAGGAGTGGTGGTCGGCGGAACGCG
ATTTATCTTTCCGGCAGACAGAGAATCGATATCTATTTTACTGACTAATACCAGTCAGGAATCCTGGCTTATTAACAGTA
AAATCAACCGCCCAACGCGTTGGGCCGGGGGGGAAGCGTCGACAGTGCCAGCACCATTACTGGCCGCTCCGCCACTTATT
CTCCTGAAGCCCGGTACGACAGGCACGTTGCGCTTGCTGAGAACGGAAAGCGACATCTTGCCTGTGGATCGCGAAACGCT
ATTTGAGTTAAGCATTGCCAGCGTGCCATCCGGCAAAGTTGAAAATCAGAGCGTAAAAGTGGCGATGCGCTCGGTATTTA
AACTGTTCTGGCGACCCGAAGGTTTGCCGGGCGACCCGCTGGAAGCTTATCAACAATTACGCTGGACACGGAATTCGCAG
GGTGTACAACTCACTAACCCAACGCCTTATTACATTAACCTGATTCAGGTGAGTGTAAATGGTAAAGCGTTAAGTAATGT
GGGAGTTGTGCCGCCTAAAAGCCAGCGTCAGACAAGCTGGTGTCAGGCAATCGCACCGTGTCATGTCGCCTGGCGGGCGA
TAAATGATTACGGCGGGTTGAGTGCAAAGAAGGAGCAAAATCTGCCCTGA
>Cluster_40110
ATGCAATTCCTGAATATGTTCTTCTTTGATATCTACCCGTACATAGCCGGGGCGGTCTTCCTGATTGGTTGCTGGCTGCG
TTATGACTACGGGCAGTACACATGGCGTGCGGCGTCCAGCCAGATGCTGGATCGCAAAGGGATGAACCTCGCGTCGAACC
TGTTCCATATCGGTATTCTGGGGATTTTTGTCGGTCACTTCTTCGGTATGCTGACGCCGCACTGGATGTATGAAGCCTGG
CTGCCGATTGAAGTGAAACAGAAAATGGCGATGTTTGCTGGTGGTGCCAGCGGCGTACTGTGTCTGATTGGCGGCGTGCT
GTTGTTGAAACGTCGTCTGTTCAGCCCACGCGTGCGTGCAACGACTACCGGAGCGGATATCCTGATCCTGTCGCTACTCG
TTATCCAGTGCGCGCTGGGCCTGCTGACCATTCCGTTCTCTGCTCAGCATATGGACGGTAGCGAGATGATGAAACTGGTT
GGCTGGGCGCAGTCGGTAGTGACCTTCCACGGTGGCGCTTCTCAACATCTTGATGGCGTGGCGTTTATCTTCCGTCTGCA
CCTGGTGTTGGGGATGACGTTATTCCTGCTGTTCCCGTTCTCGCGTCTGGTACACATCTGGAGCGTCCCGGTGGAGTATC
TGACGCGTAAGTATCAGCTGGTGCGCGCTCGTCACTAA
>Cluster_10403
ATGACAGCTCCATTAACCCTATTGATCGTTGAGGACGAAACGCCGCTGGCAGAGATGCATGCGGAATATATTCGTCACAT
TCCCGGATTCAGTCAGATATTACTGGCGGGAAACCTGGCGCAGGCCCGAATGATGATCGAGCGTTTTAAGCCGGGGCTAA
TCTTGCTCGATAACTATCTTCCTGACGGCAGAGGGATTAATTTATTGCATGAACTGGTGCAGGCGCATTATCCCGGCGAC
GTGGTGTTTACCACTGCAGCCAGCGATATGGAAACGGTGTCTGAAGCCGTACGTTGTGGTGTATTTGATTATCTCATTAA
GCCCATTGCCTATGAACGGCTGGGGCAAACGCTAACCCGTTTCCGCCAGCGTAAACATATGCTGGAAAGTATTGATAGCG
CCAGCCAGAAGCAAATTGATGAGATGTTTAATGCCTATGCTCGCGGTGAACCTAAAGACGAACTGCCGACCGGCATTGAT
CCCTTGACGCTAAACGCGGTGCGAAAACTGTTTAAAGAGCCTGGTGTGCAACATACGGCAGAAACGGTGGCGCAGGCACT
GACCATCAGTCGCACCACTGCCAGGCGTTATCTTGAATATTGCGCCAGCCGCCATCTGATTATTGCTGAAATTGTTCACG
GCAAAGTTGGCAGACCGCAACGCATATACCACAGTGGGTGA
>Cluster_58239
ATGCGTGAACAAATCAAACAGGATATCGATCTGATTGAGATTTTATTTTATCTGAAGAAAAAGATTCGTGTTATCCTTTT
TATTATGGCTATATGTATGGCTATGGTGCTGTTGTTTCTGTATATCAATAAAGACAATATAAAAGTGATTTACAGCCTAA
AAATAAACCAGACAACGCCAGGTATACTTGTTAGCTGTGATAGCAATAATAATTTTGCCTGTCAGACTACAATGACTGAA
GATGTTATTCAGCGAATTACTACATTTTTTCACACCAGCCCAGATGTCAAGAACAGAGAAATAAGGCTGGAATGGTCAGG
AGATAAGAGAGCTTTACCAACTGCTGAAGAGGAAATATCTCGCGTGCAGGCCTCTATTATCAAATGGTATGCGTCAGAAT
ATCATAATGGCAGGCAAGTTCTCGATGAGATACAAACGCCTTCAGCAATTAACAGTGAGCTTTATACAAAAATGATATAC
CTGACCAGGAACTGGTCATTGTATCCGAACGGTGATGGCTGTGTAACTATAAGCTCACCAGAAATAAAAAATAAATACCC
TGCTGCCATTTGCCTGGCTCTGGGATTTTTTCTAAGCATTGTAATTTCTGTAATGTTTTGCCTTGTCAAAAAAATGGTAG
ATGAATACCAACAAAACTCTGGGCAGTAA
>Cluster_29224
ATGACCCGCACACTCAAGCCGTTAATTCTTAACACCGGCGCACTGGCGCTAACGTTAATCCTGATTTATACCGGCATTTC
GGCCCATGACAAACTCACCTGGCTGATGGAAGTGACACCGGTGATTATTGTCGTGCCGCTACTGCTTGCCACCGCTAAAC
GTTATCCGTTAACGCCGCTGCTCTATACGCTCATTTTCTTTCACGCCATCATCCTGATGGTCGGCGGCAATACACCTAC
GCGAAAGTCCCCGTTGGTTTTGAGGTACAGGAATGGCTGGGGTTGAGCCGTAATCCGTATGACAAACTGGGGCATTTTTT
CCAGGGGCTGGTGCCTGCACTGGTGGCACGAGAGATCCTTGTACGCGGGATGTACGTTCGCGGGCGTAAAATGGTGGCGT
TTTTGGTCTGTTGTGTCGCGCTGGCGATAAGCGCGATGTATGAGTTAATCGAGTGGTGGGCAGCGCTGGCAATGGGCCAG
GGCGCAGATGATTTTCTCGGTACTCAAGGCGACCCGTGGGATACGCAATCCGATATGTTCTGCGCACTACTGGGCGCATT
AACGACGGTGATATTCCTCGCCCCGCTTTCATTGCCGCCAGCTACGGCGCTTTGGCTTAATCACCAGAGCGCCGGAAGCGA
TTACCCCTTAA
>Cluster_50168
ATGTTTAATGAAGTCCATAGTATTCATGGTCATACATTATTGTTGATCACTAAACCTTCTTTGCAGGCGACAGCTCTCTT
GCAGCACCTTAAACAATCGCTGGCAATTACAGGAAAATTACATAATATTCAACGTTCTCTGGACGATATATCTTCAGGCT
CTATTATTCTTCTGGATATGATGGAAGCGGATAAAAAGCTTATCCATTATTGGCAGGATACTTTGAGCAGGAAAAACAAC
AATATCAAAATATTGTTGCTAAATACGCCTGAAGATTACCCGTACCGCGACATTGAAAACTGGCCTCATATCAACGGCGT
TTTTTATGCCATGGAGGATCAAGAACGTGTTGTCAATGGGTTGCAAGGCGTCCTGCGCGGCGAATGCTACTTTACGCAAA
AGCTTGCCAGTTACCTGATTACGCATTCAGGTAACTATCGTTATAACAGCACGGAATCAGCCCTCCTTACTCATCGGGAA
AAAGAGATCCTGAATAAGCTGCGTATCGGCGCGTCTAATAACGAGATCGCTCGTTCGTTGTTCATCAGCGAAAATACGGT
TAAAACGCATCTTTATAATCTTTTCAAGAAGATAGCCGTAAAAAACCGGACACAAGCGGTTTCCTGGGCAAACGATAACC
TCAGGCGATAA
>Cluster_11021
ATGGGGGATTCTGTGACACCGGAGATAATTGGCAACATGATTCGTCAGTACTTCAGCCAGGAACGAGCAGAAGAAGAGAC
CATACAGGCCCTTAATCACCTGCGCAGAGTGCTTCATGAAGTCAGTCCTTTTGCACATGAGCCAGTGGACTGTGTGCTGT
GGGTAAAAGCAGATGAGGTGGTGGCCAATGATTACAATCCTAATGTCATGGCGCCGGGTGAGAAGAAACTGCTGAAGCAG
TCACTGGAGAAAGATGGTTTCACACAACCGGTGGTGGTGTCTGAGGAAAAGAACCATTACCTGGTGGTGGATGGTTTTCA

TCGCCAGGTGCTGGGCAGGGAAGCAGGTACGGGAAAACGTCTGAAGGGCTGGCTGCCGGTGGTCTGTATTAATCCGGAGA
GAAAGGGACAGGCCTCACGTATTGCTGCGACCATCCGACATAACCGGGCCAGGGGCAAACATCAGATAACCTCAATGTCT
GATATTGTCAGGGACCTGTCGCGCCTGGGCTGGACGAATGAACGCATTGGTACGGAGCTGGGTATGGACCAGGATGAAGT
TCTCAGATTGAAACAGATTAGCGGGCTGACGGAGCTGTTTCAGGAAGAGGATTTCAGCCCGGCATGGACGGTTCGCTGA
>Cluster_53873
GTGAATAACCGCTTTTACATGATGTGCTTGCGTGAAACTGTGGGTAATAACGCCTCATTCCATTGCCATAACGGCAATGG
TTACAGTTCTGATATCGATCGCGCTCATGTTTACACGCTGGAAGAAGCCCAAAAAGCCTGGAATTGTGGACGAGATATCG
ATCAGCCTGTTTGCGCTGATAGCGTGGATGCAATGGCTGTGTGGCACGTTGATTGCCAGTACATCCCCACAGAAAGCCTG
ATTGAGTCAGATTGCACTGCGTATGTGGCCTACAAAAAAGGTAGCTGGAACGGCAACGATGTTTACTGGCTTCAACACGG
TGGATTGCCAACAGATGACTTCAGTAAAGCGACCATCTTTAGCGTCGCCAACAAAAACGAACCAGGAATAGTTTGGTTGC
CATTTTCCATTGCTGATGCAGCAAAGCGCCGGACGTTCAATATCAATAACTTTAACCGCAGAACAATGGTTCAGGGCGCA
GGTTTGGTCATGCCTGACTGGTTGAAAGAGCAGAACAGAAGAAAGAAGTCGCGAAGCGGGAAGGTGCGTTGGAATTGTCC
GCATTGCGGAAAAATCACCTGGCAGTACAGCCCATATGATTTTGAAGGCTGTAGTGATTACAACTGTGAGGGATGGCGAG
TATGA
>Cluster_55917
GTGGCAAGTAAGTTTCAGAATCGGTTAGTGGGCACGATCGTGCTGGTGGCGCTGGGGGTGATTGTACTTCCAGGGCTGCT
GGACGGGCAGAAAAAACATTATCAGGATGAGTTCGCGGCTATCCCGCTGGTGCCGAAAGCGGGCGATCGTGATGAGCCTG
ATATGATGCCAGCCGCCACCCAGGCGTTACCGACGCAGCCGCCGGAAGGCGCAGCGGAAGAGGTGCGGGCAGGTGATGCC
GCAGCACCGTCGCTCGATCCGGCCACTATTGCAGCCAATAACACCGAGTTTGAACCGGAACCTGCACCGGTCGCCCCACC
GAAGCCGAAACCGGTGGAGCCGCCTAAACCAAAGGTTGAAGCACCACCTGCGCCGAAGCCAGAACCGAAGCCGGTCGTGG
AAGAAAAAGCTGCACCGACGGGTAAAGCCTATGTTGTGCAACTGGGTGCGCTGAAAAATGCCGATAAAGTGAATGAGATT
GTCGGTAAGCTGCGCGGTGCCGGTTATCGGGTTTATACGTCGCCATCCACGCCAGTGCAGGGTAAAATTACCCGTATTCT
GGTTGGGCCGGATGCCTCGAAAGATAAGCTGAAAGGTTCGCTGGGTGAGTTGAAGCAACTTTCTGGCTTAAGTGGCGTGG
TAATGGGCTATACGCCGAATTAA
>Cluster_26926
ATGTGTGGACGCTTTGCCCAATCCCAAACGCGTGAAGATTACCTTGCGCTTCTCGCGGAAGATATTGAACGCGATATTCC
CTATGATCCCGAACCCATTGGCAGATACAACGTCGCGCCGGGAACCAAAGTCCTTCTGCTCAGTGAACGTGATGAACACC
TTCATCTGGATCCGGTTTTCTGGGGATATGCTCCCGGATGGTGGGATAAACCGCCGCTGATTAACGCCCGCGTAGAAACT
GCCGCAACCAGTCGTATGTTTAAACCGCTCTGGCAACATGGTCGGGCAATCTGTTTTGCCGATGGCTGGTTTGAGTGGAA
AAAAGAAGGCGACAAAAAACAGCCTTATTTTATCTATCGCGCTGACGGACAACCTGTTTTTATGGCCGCGATAGGTAGCA
CACCGTTTGAGCGTGGTGACGAAGCCGAAGGATTTTTGATCGTCACTGCGGCGGCAGATCAAGGTCTGGTAGATATTCAT
GACCGCCGCCCGCTGGTTCTGTCGCCAGAAGCTGTACGGGAATGGATGCGGCAAGAGGTTGGCGGTAAAGAAGCCTCAGA
AATCGCGGCCAGTGGCTGTGTTACAGCAAACCAGTTCACCTGGCACCCTGTGTCGCGCGCGGTGGGTAATGTCAAAAACC
AGGGCGCGGAGTTAATTCAACATGTTTGA
>Cluster_20252
ATGTCTGAGCCGATGATGTGGCTGCTGGTTCGTGGCGTATGGGAAACGCTGGCAATGACCTTCGTATCCGGTTTTTTTGG
CTTTGTGATTGGTCTGCCGGTTGGCGTTCTGCTTTATGTCACGCGTCCGGGGCAAATTATTGCTAACGCGAAGCTGTATC
GTACCGTTTCTGCGATTGTGAACATTTTCCGTTCCATCCCGTTCATTATCTTGCTGGTATGGATGATTCCGTTTACCCGC
GTTATTGTCGGTACATCGATTGGTTTGCAGGCAGCGATCGTTCCATTAACCGTTGGTGCAGCACCGTTTATTGCCCGTAT
GCTCGAGAACGCTCTGCTGGAGATCCCAACCGGGTTAATTGAAGCTTCCCGCGCAATGGGTGCCACGCCGATGCAGATCG
TCCGTAAGGTGCTGTTACCGGAAGCGCTGCCGGGTCTGGTAAATGCGGCAACTATCACCCTGATTACCCTGGTTGGTTAT
TCCGCGATGGGTGGTGCAGTCGGTGCCGGTGGTTTAGGTCAGATTGGCTATCAGTATGGCTACATCGGCTACAACGCGAC
GGTGATGAATACGGTACTGGTATTGCTGGTCATTCTGGTTTATTTAATTCAGTTCGCAGGCGACCGCATCGTCCGGGCTG
TCACTCGCAAGTAA
>Cluster_23909
ATGAAGTTGAATTTTAAGGGATTTTTTAAGGCTGCCGGTTTGTGTTCTTTGGCATTTGCGTTGACCGGATGTATCACCTG
GGGATTGGTTTCCAATACTGCAAGCCGTCCACATGCCGAGCAATGGCGGTCAGACACGATTAAGGGTTTATCGCTGGCAG
AAGATAGTAACGGTACGAAAGGCTATGTTTTTGTTGGTGAATCGTTGGACTATCTGTTGACCACCGGCGGCGGTGAGGTG
GTTAAAATGCTGAACGATCCGGCAATTCATGGTGAAAGAATCACCGTTTCTGATAACGCGAAATTTATTTTAAGCAGCAG
TAATAAGAAATTCTCTGGTGCTATTACGCTCTATTACGACTGGAACAACGAAGAAGATAAGGCACTGGCAACACAATATG
GTTTTATTTGCGATACCCGGCGTTGTACATGGATACTGGACGGCCTGAAAGGCTCTATCCACCAGAAAAATAAAAAAGCG
GATTACTCAAACGTGATGGTTTTCCATCAACCCTTTACGGTGGGATTTTATGAGTACAAAACAACAGATGGCATCCCGAG
CGGCCTCGCTAACGCGCTGCTGCCAATCACTCTTACGCTGGATATAGTAACGTCACCACTGCAGTTCCTGATTTTATGCA
CTACCAAAAATTGTTAA
>Cluster_29150
ATGGCCAGCAGAGGCGTAAACAAGGTTATTCTCGTTGGTAATCTGGGTCAGGACCCGGAAGTACGCTACATGCCAAATGG
TGGCGCAGTTGCCAACATTACGCTGGCTACTTCCGAATCCTGGCGTGATAAAGCGACCGGCGAGATGAAAGAGCAGACTG
AATGGCACCGCGTTGTGCTGTTCGGCAAACTGGCAGAAGTGGCCAGCGAATATCTGCGTAAAGGTTCTCAGGTTTATATC
GAAGGCCAGCTGCGTACCCGCAAATGGACCGATCAATCCGGCCAGGATCGCTACACCACAGAAGTCGTGGTGAACGTTGG
CGGCACCATGCAGATGCTGGGTGGTCGTCAGGGTGGTGGCGCTCCGGCAGGTGGTAATATCGGTGGTGGTCAGCCGCAGG
GCGGTTGGGGTCAGCCTCAGCAGCCGCAGGGTGGCAATCAGTTCAGCGGCGGCGCGCAGTCTCGCCCGCAGCAGTCCGCC
CCGGCAGCGCCGTCTAACGAGCCGCCGATGGATTTTGATGACGACATTCCGTTCTAA
>Cluster_3659
GTGCAACGGACAAAGCCTGAAATAACAAAAGGAGAGTTTTTTCATAGCATTTATAAATCGCATATCAAATATAAATATGA
TGTTCTCGATAGAAAGATATTTCCTCATGAAAGCACCAGAAATGCTATGGGAGTTGCAGAGAAAAAGGGAATTAAAGAGA
ATGCGACCTTAATGCTGGAATATTATAAAGTTGAGAAGGCAATTTGTATTTACACGAACAGAAAAGTTTCCCATACCCTA
AATCGTGCAGGAGGGTTTTATAAAACCATTCTGATTAAAACCAGTGTTTTTGGGGATTATTTTTTTGACTTTTGCAATTC
AGTTTGCCTGCAGATAGATGAGTTGATTGAGTATGGTACGAAAGAAACAGTGAGGCGACATCAGATTCGTAGTACAGGAT
TTTGTACTTTTCATATTCCGATTTTTTATATCAATAATAAGGCTGTAATAGTGCCTGTTCTGAGAACAGAGGAAGTTTCA
CAAAGTTCCAGAACAGGTGGAGATGTTATTATTATTAACCCATTTGAAGATGAATGA
>Cluster_17086
ATGAAGAAGATTTTACTCGTTGCTGGCGCAGCTCTGGCACTGGCGGGATGTGGTGAGAAAGGTGAGTTTGAGAAAGCGAT
TAATGCCAAGATTGGGCAGGGCAAATACTGCTTCTCGCTGGATAACAATAACACCAGCTTCCCCATCCGTCTTGCCAAGC
CACTACGTGATTCGACTGGCACAGGGACCAATTCAGTGATCCTCGACGGTTTTATTGAGCAGGGCCTGATGGTTTTTGAA
CAGGGCTATGACTCGAACGTATTAGGGATAACAGAGGAAGGCAAAAAAGCCAAAGTCTGGAGCACAGCAGACGGTGCCTG
TGTTGGTCGTCGTGCTGTTGATGAGATTAAGGAATGGACGGAGCCGGGAAACGGTAATCAGAAAGTAGTTCGCGTGACTT
ATACATGGAAGTTGGTTGACGTTCCGGGCTGGATTGACAAAAAAGCCTTCGCCAGCGTTAAAGGAATGAATGATTCTGCT
GATGGTGCCATGAATCTTGTTAAAACCAGCAATGGCTGGAGAGCCAATTAA
>Cluster_64085

ATGATGCACTTAAAAAACATTACTGCTGGCAACCCTAAAACAAAAGAGCAATACCAGCTAACAAAGCAATTTAACATCAA
ATGGCTTTATTCAGATGATGGAAAAAACTGGTATGAGGAACAAAAGAATTTCCAGCCAGACACTTTGAAAATGGTCTATG
ACCATAACGGCGTTATTATTTGTATTGAAAAGGATGGTTCAGCAATTAATCCGGAAGGCGCAAGCGTCGTTGAATTACCT
GATATTACAGCAAATCGCCGGGCTGATATTTCGGGGAAATGGTTGTTCAAAGATGGCGTAGTGATAAAGCGAACTTATAC
CGAGGAAGAGCAGAGGCAACAAGCGGAAAATGAAAAGCAAAGCCTGTTGCAACTTGTCAGGGATAAAACCCAGCTATGGG
ACTCACAGCTACGGCTGGGCATCATTTCCGACGAGAATAAACAAAAATTAACCGAGTGGATGCTCTATGCGCAGAAAGTC
GAATCTACAGACACCTCCAGCCTGCCAGTAACGTTTCCCGAACAACCAGAATGA
>Cluster_11080
ATGAGACGTGGCATTTCCTACATCCGGTTTTCAAGTGAACGACAACTAAAAGGGGACTCTGTACGCAGACAATCTAAGCT
GGTTACTGACTGGCTTGATAAGAACCCTGAGTTTTACTTGGATTCATCCTTGAGCTTTAAAGATTTGGGGAAAAGTGCTT
TTAGTGGTAAGCACCTGAAAGGTGGTCTAGGTGACTTTCTAACCGCTATCGAGAAAGGGCTTGTTAAAGCTGGTGATACG
CTACTAATCGAGAGTTTAGACCGTTTGTCACGTCAGGATATTGATATTGCTTCTGAGCTATTACGCCGTATCTTAAGGGC
TGGTGTAGATGTAGTCACCCTGTCCGATGGTGAACATTACACAAAGGAATCTCTTAAAGACCCGCAATCCCTGATTAAAA
GCATTCTGATAATGCAACGAGCGCATGAGGAATCATTACGTAAATCAGAGCGTGTACAGGCTGCATGGAACCGTAAAAAA
GAACTCATCTCTGAGGGTATCAAGATTTCTAGGCGTTGTCCTGCATGGCTTAAGCTTAATGATGACTGA
>Cluster_2997
ATGAAACTGAAGCTGAACAATGTTCGTCTGGCCTTCCCGTCTCTGTTTGAAGCTAAAACTGTAAACGGCGAAGGCGATCC
GCGTTTCTCCGCAGTATTTTTAATGTCTCCCAAACACCCACAACTGGAAGAAATCCGTAAAGCTATGAAGCAGGTAGCGA
AGGAAAAAATGGGGAGAGAAGTGGGAGCCCATTTATAACCAGCTGGAGAAAAAACTCAATCTGTGCCTGCATGATGGTGAT
GAAAAAGCAGAGTATGAAGGCTTCCCCGGTAATTTCTTCCTGAATGCTGCTAACAAAGCGCGCCCAGCTGTTCTTGATCG
CGATCGTTCGCCACTTATTCAGGCTGACGGACGTCCCTATGCAGGGTGCTATGTAAACGCCGTTATCGATATCTGGGCAC
AGGACAATAATTTCGGTAAACGCATTAATGCCTCACTCGGCGGAGTCCAGTTCCTGCGAGACGGCGATGCATTCGCTGGC
GGCGGAGTGGCAAGCGCTGACGATTTCGACGATATCAGCGAAGGTGCTGATGCTGAAGCACTAATTTAA
>Cluster_4604
ATGAATCCGTTTAACGCCATTACTTTTGCTGCATTGTGCGGGCCTCTGGCTTGTCCTGCTGCTATGGCGCAGGAGTTCAT
CATCCAGCCAGCCCCTGTGATTGCTAAACCTTTTGAGTACTCTCCTTCTGTTGAGGAATTCTCACGCAAAATGGAAGAGG
GTAAGGAGATCTTGCAAAAGTTAACAATTGCAGCAGATGACTACTATATCTGTTTGATTGATCTTAATAGCCAAGATGCT
CGCGAGTTTGTTTCCAAAAACGGGACAGATACGACAGAGGCATGCGAAATGTTCCTGCGTGCTTTTGAAGAAGAAGTGAA
GCGTACAATTGAATCGCCTCTGCCTGAATTTATCAGGTCAGAACTGAAGGTTTACTGGCGTCATATTGCTAAAGCTCGCT
CATCTGTCACTCGCCTTAACAATTACATAAAGAGCATATTTAAAGAAACTGTTACTTTTAGCGGGCGCGCGGATCTTGCT
GGAATTGCAGCACTAGCTAGTCACACATCAAATAAGCTAAAATCGATGCAATTTCACTAA
>Cluster_62156
ATGACAGTACACCACTTCACTTTTCATATCACAGATAAAAGTGATGCCAGTGATATCCGGGAAGTCGAAACCCGCGCATT
TGGCTTCAGCAAAGAAGCCGATTTAGTGGCCTCTTTACTTGAGGATGAAAGCGCGCGCCCCGCGTTATCGCTGCTAGCCC
GGTATGAAGGCAAAGCCGTCGGGCATATTTTATTTACCCGGGCAACCTTCAAAGGAGAAATGGATTCACCGTTGATGCAC
ATCCTCGCTCCCCTGGCGGTCATACCGGAATACCAAGGGATGGGCGTGGGAGGACGGTTGATACGCACTGGCATCGAGCA
CCTGAGGTTGATGGGCTGCCAGACGGTTTTTGTTCTCGGCCACGCCACCTATTACCCCCGACATGGGTTCGAACCCTGTG
CCGGAGACAAAGGCTATCCGGCACCTTATCCCATCCCGGAGGAGCACAAAGCCTGCTGGATGATGCAATCACTCACCGCT
CAGCCCATGACCCTGACCGGCCACATCCGGTGCGCAGACCCTGATGAAACCGGAGCACTGACGTGA
>Cluster_86
ATGGTTGATGCAAAAATTCTGAATGGTGTCAGCACATTATTACGGGCTTACGGACGCCTGACCTGCGGAGTTCTGGCTGA
AAAAATGAATATGCTGCCCTCGTCAATGGTGTATTTCCTGCGTGATGCGGTTGATGCCGGAGTGCTCACCGAATGCAACG
GATTTTATGACGTTCCGCGCCCGCGTCCGACGCCGCCTGTAAGACGAAACGCAACTGAGCAGCCTGCTGTTGATGATGCG
GTGTGGTGCAACTGGCGTCGCTCATTACCCTGGGTGGAAGGTAATACCATCCCCGCGCTGGCAAAAGAGTTTGCGACGGG
CGTGCTGACCTGTGAGTCAGTTCACATTGTTGCTGAGGTGGATAACAGAATGTGCGAACAAGGAATGCCCCGTTTTGTGA
TGGCTTATATCGATATCCGGCTGGGGCGTTTTATTTGCAGTTCCAGCGCCTGGAATATCACCGACCATGTACTGCGTTAT
CTCATTCTTGATTGTTCTCCGGCTCCCGCAGCGGTGCAGGAGGTGGCGTGA
>Cluster_62871
ATGAACGAAACATATGTAATTGAACCACTAGAATTTATATTTACAGATTCCTTCTTTAGAGGGTTGCATACTAATACTGG
GTTGAAATGCATTGTTATTAAAGATATAAATGATGCATGGAAATACGCTTTTGAACAAAACCTCTCATCAGGTTTTAAAG
TTTGGAACGATATTATTGAATTGGAGCGTTCCCGACTAAGGAGCAATGATGAATTTCAGGAGGCATATAAATTTGTTAGT
GATTATTTAAAGACATTGCAAGTTAATCACTCTTCAAATTTTCTGGAGTATAGAAAGAAAAAAATAAAAAAAAACAAATAA
TAAACTTGATGACTTTATTTTTTCCGATGCTAGAGATGATTCTTTTTTTGTATTAAGTACTATTGCAATAAATAGATATT
TAAATAATTATATTAAAGATTCATTCCTTGAGGATGTCTTTAAGCTTTATAAACTTGGGGGGTGGCCGTGTGGGCTAAAG
GGACATGATATTCTCGTCTTTGATCCGATGGTGCTTAATTAA
>Cluster_58995
ATGCAAAAGGTAAAATTACCCCTGACTCTCGATCCGGTTCGTACGGCTCAAAAACGCCTTGATTACCAGGGTATCTATAC
CCCTGATCAGGTTGAGCGCGTCGCCGAATCCGTAGTCAGTGTGGACAGTGATGTGGAATGCTCCATGTCGTTCGCTATCG
ATAACCAACGTCTCGCAGTGTTAAACGGCGATGCGAAGGTGACGGTAACGCTCGAGTGTCAGCGTTGCGGGAAGCCGTTT
ACTCATCAGGTCTACACAACGTATTGTTTTAGTCCTGTGCGTTCAGACGAACAGGCTGAAGCACTGCCGGAAGCGTATGA
ACCGATTGAGGTTAACGAATTCGGTGAAATCGATCTGCTTGCAATGGTTGAAGATGAAATCATCCTCGCCTTGCCGGTAG
TTCCGGTGCATGATTCTGAACACTGTGAAGTGTCCGAAGCGGACATGGTCTTTGGTGAACTGCCTGAAGAAGCGCAAAAG
CCAAACCCATTTGCCGTATTAGCCAGCTTAAAGCGTAAGTAA
>Cluster_56082
ATGAAATTTAAAAAAATGTCTTCTGCCTGTGGCAATGTTAGCGTCATTCACTCTGGCAGGATGCCAGTCAAATGCTGACGA
TCATGCTGCCGATGTTTATCAAACCGATCAACTGAATACCAAACAAGAAACTAAAACCGTTAATATTATTTCCATTCTTC
CCGCAAAAGTTGCCGTAGACAACTCCCAAAATAAACGGAACGCACAAGCCTTCGGCGCGCTTATTGGCGCAGTCGCTGGC
GGTGTTATCGGCCACAACGTCGGGTCTGGCAGCAATTCCGGAACGACGGCAGGTGCAGTTGGCGGCGGAGCTGTAGGCGC
GGCAGCGGGTTCTATGGTGAATGATAAAACCTTAGTGGAAGGTGTTTCTTTAACCTATAAGGAAGGCACCAAAGTGTATA
CCTCTACCCAGGTGGGTAAAGAGTGCCAGTTTACGACAGGTTTAGCCGTTGTTATTACCACGACGTATAACGAAACGCGT
ATTCAGCCAAATACCAAATGTCCTGAAAAGAGCTAA
>Cluster_60501
ATGCGTTTTTCTCACCGACTGTTCTTACTTCTGATTCTCCTGCTGACGGGAGCACCAATACTCGCTCAGGAGCCTTCTGA
CGTTGCGAAAAACGTGCGTATGATGGTTTCCGGTATTGTCAGTTACACCCGCTGGCCTGCGCTATCAGGGCCACCAAAGT
TATGTATCTTTTCATCTTCACGTTTTAGTACCGCCCTACAAGAGAATGCCGCGACATCTTTGCCTTATCTGCCTGTCATC
ATTCACACTCAACAAGAGGCGATGATTTCAGGCTGCAATGGTTTTTATTTTGGCAACGAGTCACCGACATTCCAGATGGA
ATTAACCGAACAATATCCGTCAAAGGCGTTATTATTAATTGCCGAACAAAATACCGAGTGCATTATTGGTAGCGCCTTTT
GTCTGATCATCCACAATAATGACGTCAGATTTGCCGTAAACCTGGATGCCTTATCGCGTAGCGGTGTAAAGGTCAACCCG
GATGTCTTAATGCTCGCACGGAAGAAAAATGATGGATAA

>Cluster_60574
ATGGATAGTTCGTTTACGCCCATTGAACAAATGCTAAAATTTCGCGCCAGCCGCCACGAAGATTTTCCTTATCAGGAGAT
CCTTCTGACTCGTCTTTGCATGCACATGCAAAGCAAGCTGCTGGAGAACCGCAATAAAATGCTGAAGGCTCAGGGGATTA
ACGAGACGTTGTTTATGGCGTTGATTACGCTGGAGTCTCAGGAAAACCACAGTATTCAGCCTTCTGAATTAAGTTGTGCT
CTTGGATCATCCCGTACCAACGCGACGCGTATTGCCGATGAACTGGAAAAACGCGGTTGGATCGAACGTCGTGAAAGCGA
TAACGATCGCCGCTGCCTGCATCTGCAATTAACGGAAAAAGGTCACGAGTTTTTGCGCGAGGTTTTACCACCGCAGCATA
ACTGCCTGCATCAACTCTGGTCCGCGCTCAGCACAACAGAAAAAGATCAGCTCGAGCAAATCACCCGCAAATTGCTCTCC
CGTCTCGACCAGATGGAACAAGACGGTGTGGTTCTCGAAGCGATGAGCTAA
>Cluster_177
ATGATGGATAAAGTTGTGGTATTAGGGATAGGGAATGTTTTACTTGCAGACGAAGGGATAGGAGTTTTAGCAGTTAAGGT
TTTGGAAGAGTTTTTTGAAGTTCCTTCTGAAAAGGTGGAGCTTGTTGACGGAGGAGTAGGGAGTTTTCAACTACTTCCTT
ACATAGAAAAAGCCAAAAGACTTTTGGTAATAGATGCCATCGCAAAAGGAAGTCCTCCTGGCACCCTTCACAAATTTACT
AAAGACACCCTACCTAAACAGATTCTTGAAAAACTCTCCATCCACGAGGTAAGTTTTTTAGACATACTCACCTTAGCTGA
GATGAGAGGTTTTTCTCCTGAGGAGCTTGTGGTGTTAGGGTTAGAACCTGAAAGCCTTGAGATGAGGTATGAACTTTCTC
CTTCAGTAAAGGCTAATTTTATCCCTTTGATTGAAGCGGTGGTTTCTCAACTTAAAGATTGGGATATAGAGCTTGTCCCA
AAGAAAGACCTCCGTCGTTGGGAGGAACTTTTCTTTGTAGATAGACCTTAA
>Cluster_34010
ATGATTATGGCTTTTTTACTCTTAAAAATCAATCACTCAGCGCAATTGCACAAAAATAATTTGCAATTTTGCTTGACTCG
CTTTTTTGTCATGGCGTATCGTTTGCCCGCACCTCATAAAGCGGGTGCCGGGTTTAGCAGCCTGATTGTAAGAGCGGGTA
GCCGCTATATTTCCGTAATGCGGTTTTTTTGTGCCCGTAATTCCACCCTACCCCGTATTATGGCGGGGCGTAATGGGGGA
GCCTTTGTGCTCGCTGGTTTCTCTTACGCCAGTCTGCTAACCCTGTTACGTCCCGCCACCATGTTTAGCAGCGTGCTGGC
GAGACTCCGTAAATCAGTAAGAGGAGCCGCCAAAATGGCTATATCAACACGCCCTGATTTTATCTGGCGCTTTATGCAGT
GCCACGGTAAAAATATCCGCCTTCATACCGTTACCGCTGCCAGTGAGCGCGAAGCGCGCGCCATGCTTCCGGCATCCCGT
CTGGTCTTTGTTGCCCGTATCCGTGTCCGGGAGGCGTGCCATGTTTAA
>Cluster_83
ATGATGCACTTAAAAAATATTACTGCTGGCAACCCTAAAACAAAAGAACAATACCAGCTAACTGAAAAAGCGGGCGTGGT
ATGGCTCTTTTGCGAAGATGGCAAAAACTGGTATGAGGAACAAAAGAATTTTCAGCCAGACACCATAAAAATTGCTTACG
ATGAGAACAACATCATCGTTGCTGTCAGTAAAGACGTTTCAACTATTAATCCTGAAGGGCTAAGCATAGTTGAAGTTCCT
GATATTACGGCAAATCGTCGCGCAGACAATAACGGAAAATGGATGTTTCTGTATGGTAAAGTAGTAAAGAGGGAATATAC
AGAACAGGAACTGCAACAACAGGCCGAATCACAAAAGGCCGCGTTGCTTTCTGAAGCAGAAAGCGTTATTCAGCCACTGG
AACGCGCTGTCAGGCTGAACATGGCAACAGATGAGGAGCGTAGCCGACTGGAAGCATGGGAGCGCTACAGTGTTCTGGTC
AGCCGTGTGGATACGGCAAATCCTGAATGGCCACAAAAGCCTGAATAA
>Cluster_45956
GTGCTGTCTTACTTAATGGCAATTCACTTTGTTTTATTTGGGGACTCTACTAATTTAAAAAACTTCTGGAAATATGAAGT
AATTCGGCGGAAACGTATGGATATCTGGAGGCTTTTAAGAGAGAAAAAACAGCGTAACCGGAATTTCCTTTTCTGGTGGC
GGTTGGCTAACGAAATGTATATTAATGGTAATAAATTACATAAGAAAGCAGCCAAAAAGTTAAACAGTAAAATAATTAAC
AAATTTGGTTGTGAAATTGGATTGGGCGCAAATATTGGAAAAGGGTTAACAATTCCCCATCATGCTGGAATTGTTGTTCA
TTTTGCTGTTGATGCTGGTGAAAATCTGGTGTTACGACAGAATACTACCATTGGACAGATAGATGGTGACATGCCTGGTT
CAAGAGTAAAAATTGGTAGTAACGTTGATATCGGAGCTAATTGTTGCATCATTGGATTATCACGTAAAATTGGGGATAAT
GTAAAAATAGGTGCAATGTCTTTTATAAATAAAGATATACCATCGAACTGCACATATATAACTAAAAAGAGCGGTGTTGT
ATTGTATAAATAG
>Cluster_67032
ATGTGGAAGCAGGCCCTGACGGGCGTGTTGCTGATTGCCACCAGTGTACAGGCGTCCACGCTGGAAGAGCTGCGCCAGCT
TGAAGCGCACAAAACAACGCCGGGTAAGGCCAGCCAGATACAGGCAGTTGCCACTTCTTCATCACCTGTGGCTCCTGCAG
CCCCTGAACGCCGGGATTACCGTCTGCCGGACGGACGGGTAGTCAATCTGAAGAACTACAAACTGGTGTTGTTCATGCAG
TCACGCTGTGGTTACTGCCAGCAGTTTGACCCGTTACTGAAGCGTGTAACCGCACAGTTGGGGCTGGATGTTTTCCCGTA
TACCCTGGACGGTATGGGCGATGTGTCTTTTCCGGATGCCATTCCGGCAACAGAAGCTGTGATTCGTGATATGTGGAGCA
ATATGCGACCGGTGACCCCGGCAGCGTTCCTGGTGGAAGTGAATACCCTGAAAACACTGCCATTGCTGTATGGCATTGTG
GATGAGCAGACGCTCAGGCTGCGTATTGATGAGGGGCTGGGATTTGCGAATGCTCCCGGAACGGGAGTAAAGAGTAAAAC
AGGAAGTGAAAAATGA
>Cluster_25496
ATGGAACTTCGAAATAAGAAATTAACCCATGACGAATTCATGACCGAGCGGCAACAGGTACTGAAAACCTGGGAAACCGG
CAAGGATGTTGAAAACTTTGAAGATGGCGTTAAATACCAACAAACCATTCCTGAGCATAAACGCTTCTCTCTTGCCCTGC
TGAAAGCAGACAAGGAAGGTAAAACTCTTAGCCAACCGCGCGCAGGCGTGGCGCTGATGGACGAACACATCGAACTACTG
AAAACACTACAAGAAGAGTGCGATCTGCTGCCGAGCACCATCGATGCATATACCCGTCTGAATCGGTACGAAGAAGCGGC
TGTAGGGATTAAAAAATCCATCGAAGCCGGAACCTCTAAACTGAACGGTTTACCGGTCGTTAACCATGGCGTCGCAGCTT
GCCGTCGCCTCACCGAAACTCTGCAAAAACCGCTACAGATCCGCCACGGCACGCCAGATGCGCGTCTGCTGGCTGAAATC
TCGATGTGTATACATGAATATACTCTAAATAAGTCAAGTTGCAGGAAGGGAACAAATAATGAATTTCCAGAAGCTTACAT
AAGTAAGTGA
>Cluster_52590
ATGCCGGGCAAGCAAATGCTCTGTTGTATTTGATATCGATTATCAGTGAGGGAGATATGAAGATATTCATTAGTTTATT
TTTGTTTATAATATCAACAAGTTCTTTTGCTGATGATATCACCCATGCCGGAGTAGTTCGTATTGAAGGGTTAATTACCG
AAAAAACCTGCATTATTTCTGATGAGTCAAAAAATTTTACAGTTAATATGCCAGACGTACCCAGTAGTTCGGTAAGGAGT
GCAGGAGACGTTACTGAAAAGGTTTATTTTTCCATAACGTTAACCCGCTGTGGTAGTGATGTTGGCAACGCTTATATAAA
GTTTACCGGTAATACAGTTTCAGAAGATGCCAGTTTATATAAGCTGGAAGAAGGCTCGGTAGAGGGGCTTGCACTCACGA
TTTTTGATAAGAATAAAGGTAGTATTAGTAATGATGTTAAAAGCATCGGTTTTTCACTTACACCATCAGTTGATAATATA
TTACATTTTTTTGCGGCTTACAAAGCATTAAAAAATAATGTCCAACCAGGGGATGCCAATGCGTCAGTATCGTTTATTGT
CACCTATGATTAA
>Cluster_45808
GTGGAATACCGTAGCCTGACGCTTGATGATTTTTTATCTCGCTTTCAACTTTTGCGCCCGCAAATTAACCGGGAAACCCT
AAATCATCGTCAGGCTGCCGTGTTAATCCCCATCGTCCGTCGACCGCAACCGGGGTTGTTGCTGACTCAGCGTTCAATTC
ATCTGCGTAAACACGCTGGACAAGTGGCATTCCCTGGAGGTGCAGTCGATGACACGGACGCATCAGTTATCGCCGCCGCG
CTGCGCGAAGCTGAAGAAGAGGTCGCTATACCGCCTTCCGCCGTTGAAGTTATCGGCGTGCTGCCGCCCGTCGATAGCGT
AACTGGCTATCAGGTAACCCCAGTGGTCGGCATTATCCCGCCCGATCTGCCGTATCGCGCCAGTGAAGATGAAGTCTCGG
CGGTGTTTGAAATGCCGCTCGCCCAGGCATTACATCTGGGTCGTTATCACCCTTTAGATATCTACCGCCGTGGCGATTCA
CATCGGGTATGGCTGTCCTGGTACGAACAGTATTTTGTATGGGGAATGACCGCAGGCATAATTCGTGAGCTGGCGCTGCA
AATTGGTGTGAAACCCTGA
>Cluster_48757
ATGCAATACGATGAATTCCAGGCTGAAGCAACAGCCCATGGTATACGAACTGGCAGTATGACGATTGATTATCACGACGC

CATACGTCGTCTGGATGCCGGAGAATTCGATACTCCTAATGTGCGAGGTTTACGTATCCTTCAGTGTCTGGCGCAAGCCG
ACGAAGCAGGATTACTGGGTAAACTTCCGGTTGAGATGAAGGTTGCTCAGTGGCGATGGTTGTATGTGACGACATTCATC
AACGAAGAAGAAGACAAGAACGGCACAATTGATATCCTGAATGAACACGGAACAACTGAACACGCCGTGGTATATAACGG
GATGTATGGGTTTATGACGATATATCCCGGCCCCATTCGATTTGCCTTACAACAGTATATTGAATGGAATTTAATTCAAA
AATACGGCGAAGCTGAAGGAATGGGAAGAGCGCTGTTTCTTTATCAGAAAATGCTCACTACTTCCCCTGATAAAGGTTTC
ATTCTTTCAGATATGGGTCGAGAAGGGCTTGAAATCCTTCTGGATGAAATTATTAACGAAATGAATACTCATGGCATGCA
ATCCGAAACAGATATTAAGTAA
>Cluster_10997
ATGAGTGTTTCTCTTTCCAAAGGCGGGAACGTCTCCCTGAGTAAAGCAGCTCCGTCAATGAAAAATGTCCTGGTGGGCCT
TGGCTGGGATGCGCGTTCAACAGACGGTCAGGACTTTGACCTGGATGCTTCAGCATTCCTGCTGGCCTCAAACGGCAAAG
TGCGCGGCGATTCAGATTTCATCTTCTATAACAACCTGACGTCATCCGACGGTTCCGTAACGCACACCGGCGATAACCGC
ACCGGTGAGGGCGATGGTGATGATGAATCGCTGAAAATTAAACTGGACGCCGTCCCGTCTGAAGTTGACAAGATCATCTT
CGTTGTGACCATCCACGATGCTCAGGCTCGTCGCCAGAGCTTTGGTCAGGTATCCGGTGCGTTTATTCGTCTGGTTAATG
ACGATAACCAGACTGAAGTCGCTCGCTACGATCTGACCGAAGATGCGTCCACTGAGACTGCCATGCTGTTCGGCGAGCTG
TATCGCCACAATGGTGAGTGGAAATTCCGCGCAGTAGGCCAGGGTTATGCTGGTGGTCTGGCATCTGTATGTGCTCAGTA
CGGCATTAACGCGTCCTGA
>Cluster_48975
ATGATTAATCCTTCTGAAGTTGGCAAATCAGGTGAAATGGTTCGCCTTCGTACTCTGGAAAGTATCTGGATACAAGGCAA
GTTGCGCATGTGGGGCCGCTGGTCTTATATCGGCGGTGGTAGTGGTGGAAACATGTTTAACCAGCTTCTGGCATCCGGGA
AAATCACCAAGACAGCTATCAATGAAGCGCTACGCCGGATGAAGATAGCGGGTATCACCAAGCCTGAACTTGAAGCGTAC
CTGCGTGAAATTCTCAACAGCAAAAATAAAAGCGGCCTGGCGTTCTGTTCAGATGAAGAAGGGTTGTTAGTGGACGGTGT
CATTGCTTCAGTACTGATGAATGATGACTACCGATCGCTCTATAGCGTGATTGTTGACCGGTATCGTCTTCGTAAGAGCA
AGTTGCAGATGGCCAATGAACTGCAGGCTAAACATCCTGACTGGCCGCTGATCACCTGTCGTCGACGCATTGACACTTGG
CTAAGTCTTGCAGAATCGATCCTGTACGCTCCAGTTTGTGACGCATTCGGCACAAATAGCGACAGATTTAAGTTGCAGAG
TGAGCAAGAAAGTGCTTAA
>Cluster_28681
ATGACACACGAGTACAACCCCTTCTGGCAACAACGTATCCGTGAGACGGTGCTGCGCGCACTGGATGTTCATCCCCGCCT
GACGGCATTGCGGGTTGACCTGCGTCTCCCGGATGTACCGGCAGCAACGGACGCAGCTGTGATATCCCGCTTCATCAATG
CCCTGAAAGCCCGAATCAACGCTTACCAGAAGCGTAAGCGCCGTGAAGGTAAACGCGTACATCCCACGACCCTGCATTAC
GCCTGGGCCCGGGAATTCGGGGAGCTTAAAGGCAAAAAACACTATCACCTCCTGCTGCTGGTCAACCGGGATACCTGGTG
TCGTGCCGGCGATTACCGCGCTCCGAGATCACTGGCCGGGATGATTAAACAGGCCTGGTGCAGTGCCCTGGGGGTGGATG
CCGGGCCTTATGCCACGTTGGCGCATTTTCCTGACACGTCGGCGGTGTGGCTGGAACGCGATGATGACACTGGCTTTCAG
CAGGTGCTGGAACGTGCTGACTATCTGGCGAAGGAACATACCAAAGCTCACGCCACTGGTGAGCGCAACTTTGGCTGCAG
TCGTAGCTGA
>Cluster_64816
GTGAATCGCACATCGCTTATTAAATTAATCCATGTCGCCCGTCGGGAGCTACAACTCGACGACGACACCTACCGTGCCTT
TCTGATGCAGAACACGGGCAAAATCAGTTGCCGTGAGCTGACCGTCGCGCAACTGGAGCTGGTGCTCGATGCCATGAAAG
AACGCGGTTTTAAGAAGCAGAATAAACACCCACGCCGTCGCTTTAATGGGCATGTAACGCCCCACGAAAAGGTATTAAAA
ATCTGGCAGCAAATGGCAGAAGACGGTTTTATTGCCGATGGCAGTGATACAGCACTGGATAAATACGTCGAACGACTGAC
AGCCAGACGTAATGGCGGACAGGGTGTTTCCACGCTGGCGTGGTGTCACGGAGAATCGTTACAAATTGTGCTTGAAACCC
TGAAACAGTGGCACATCCGCTGTATCCGTGAAGCCTTTTCCCATTATGGGCTCCCGTTGCCGGTCAGCCCGTCCGGGCGT
GAACTGCGTGGCTATGATGCCATGACAGCAGCTTATGCCCGTGCAAGAAAAACACGGAGACTGGCACAATGA
>Cluster_61216
ATGTCTCAGCCAGCGAAAGTTTTGCTGCTGTATGCCCATCCGGAATCTCAGGACTCGGTGGCAAACCGGGTACTGCTTAA
ACCGGCCACGCAGCTCAGCAATGTTACCGTGCACGACCTTTACGCGCACTATCCCGATTTTTTATTGATATCCCCCGTG
AGCAGGCATTACTGCGCGAGCACGAGGTGATTGTCTTTCAGCATCCTCTTTATACCTATAGCTGCCCGGCGCTACTGAAA
GAGTGGCTGGACCGGGTATTAAGTCGTGGTTTTGCCAGCGGGCCGGGAGGAAACCAACTGGCGGGAAAGTACTGGCGTAG
CGTGATTACCACCGGCGAGCCGGAAAGTGCTTACCGTTATGACGCGCTGAATCGCTACCCGATGAGCGATGTGCTGCGCC
CCTTTGAACTGGCGGCGGGCATGTGCCGGATGCATTGGTTAAGTCCCATCATTATTTACTGGGCGAGACGGCAAAGCGCA
CAGGAGCTGGCGAGCCACGCCAGAGCCTACGGTGACTGGCTGGCAAATCCGCTGTCTCCAGGAGGCCGCTGA
>Cluster_40007
ATGAACGTCAATAAGAAAAAACTGGCCGATATTTTTGGCGTTGATGTCAGGACCATCACCGCCTGGCAGAGTCAGGGGTT
ACCACTAGTTTCTGGTGGAGGGAAAGGGACTGAATCAGTTTTTGATACAACTGCTGCCATTCAGTGGTATGCGCAGAGGG
AATCTGATATTGAAAACGAAAAACTCCGTAAAGAGATCGAGGATTTGAGGGCTGCCAGCGAATCAGACCTTCAGCCCGGC
ACCATTGATTACGAACGTTACCGACTGACGAAGGCACAGGCCGATGCACAGGAGCTGAAAAATGCTCGTGAGGAAGGCCT
TGTCCTCGAGACGGAGTTATTTACCTACATCTTTCAGCGAGTGGCACAGAATATATCAGGGATCCTTGTCCGTGTCCCTC
AGACACTGCAGCGTAAATACCCTGATATATCACCCGCACATCTTGATGCTGTGAAAACTGAAATCGCGAAAGCATCCGAT
GTGGCTTCTGAAGCCGGTGAGAATGTGCGCAGGTGGATTGATGATTTCAGACGAACTGAGGGCGGCTAA
>Cluster_64857
ATGAAAAAGAAAACAACACTTAGCGAGGAGGACCAGGCTCTGTTTCGCCAGTTGATGGCGGGGACTCGCAAGATTAAGCA
GGACACGATTGTCCACCGACCGCAGCGTAAAAAAATCAGCGAAGTGCCGGTGAAACGCTTGATCCAGGAGCAGGCTGATG
CCAGCCATTATTTCTCCGATGAGTTTCAGCCGTTATTAAATACCGAAGGTCCGGTGAAATATGTTCGCCCGGATGTCAGC
CATTTTGAGGCGAAGAAACTGCGCCGCGGCGATTATTCACCGGAATTGTTTTTGGATTTACACGGTCTGACGCAGCTGCA
GGCCAAGCAGGAACTGGGCGCGTTGATTGCCGCCTGTCGCCGCGAACATGTGTTTTGTGCCTGCGTGATGCATGGGCACG
GGAAGCATATTTTGAAGCAACAAACACCGCTGTGGCTGGCACAACATCCGCATGTGATGGCATTTCACCAGGCACCGAAA
GAGTACGGCGGTGATGCGGCTTTGTTGGTGTTGATTGAAGTGGAAGAGTGGTTGCCGCCGGAGTTGCCCTGA
>Cluster_94
ATGCCTGAGTTTTCCCCTGCATTCCTTCATTCGCTGAATTTCGTTATACGCCCTGATGTTGAAGGTGGCTATGTTAATGA
TCCTGCCGACCGTGGCGGCGAAACGAAATACGGTATTTCTGACCGTCGCGACGGCGTTATTGATGGCAAAACCGATGTTA
ACGGTGACGGAAAACCTGATACCCGCATTAAGGATTTAACCCGCGAGCAGGCGGCGCAGATCTACTGGCGTGATTACTGG
CTGGCGGCAAAGTGTAACGAATGGCCTGATGGCGTTTCCTTATTCGTTTTTGATGCTGCCGTTCAGCACGGTGTTAAACG
CGCGATTCAGATCCTCCAGGCGGCAGCAGGTGTTGATGATGATGGCATCGTCGGTAAAAACACACGCGCAGCCGTTCTGC
GTGCCGATACTGACTGGCTGCTGTTGCAGTGCTTCCTGCGTCGTTCCCGCTATTACGCAGGGATTATCAAATCCAGTGCC
TCGCAGGGGAAATATCTGAATGGCTGGTTTAACCGTCTGGATCTGCTCGCCAGCGCCTGTCGGGAGGTTCTGCATGGGTA
A
>Cluster_53636
ATGCATGAACTTAACGAGCAAAACATCCAGTATTTGACTGCCCTTAATATTAATATACATAAAATGTTGCTATCAAATAT
AACAATAGAAAAGAGCGACCTTTCATATGGGTATTATTTTGGTTGTGTATTATCCAATATATTATGTTTTGAAAGCGACT
TATCAAACACCATCTTTTCAAATGGAGAAATTAATAATCTCTTCATTAAAAAAAGCAACATATTTGGAGCATCATTCACT

AATACCAGAATAAAAAATTTGCTCTGTGAAGATATTATGCCTGGTCGATGGACCACTCAACTTGTCAATAAGCACTTAGG
TTACAGATACACAGGAGTATTCAAAACCCTGGCAAGTATTGATGACAAACCGTCCCGTTTCGAAATTTTGATACCATTGG
TACAAACTTTAGTTCGCGACAATGTAAAGCTAAATAATGATGTGTATAAAGAACTTAATAAGTTTATGCACGATTACGAC
AAAACATCATCAGAAATGAGAAAATACCTTAAAAGCATCAACGAGTGTATGTTCCTTATGAAAAACATAGCTCATCAAAA
TTAA
>Cluster_11240
ATGGTGAATAACATCACGGAGATGCTGAACGGAAATAAAGTGGAATGGCGCAGCTGGGCCTCATCAGTGCTGCAGGAAAT
ATCAAAAGTTCTTATGAATGCCGCGATTGTCAACGGAATTAAGACGGCGGCAAACGGTATGTCCGGTGCGGGAGGATTTC
TCGGCAGCATTGGTGACTGGCTGGGCGGGGCGGTGGCCAATGCAAAAGGCGGCGTGTATACCTCGGCAAACCTGAGTGCG
TACAGCAACAGTATTGTGGATACGCCCACGTACTTTGCCTTTGCAAAAGGGGCCGGGCTGATGGGGGAGGCCGGACCTGA
AGCCATTATGCCTCTGACCCGGGCGGCGGATGGCTCGCTGGGTGTGCGAGCGGTGGGTAGTATGTACGGCAGTGCGGGCC
TGGTGTATTCCCCGGTCTACCATATCGCCATTCAGAATGACGGGACTAATGGCCAGATAGGGCCGGAGGCGGCAGGCAGT
CTTGTGCAGCTGATTGACCAGCGGGTGCAGGCGGTGATGCTGTCCATGCGACGTGACGGAGGAATGCTGAGTGGCTGA
>Cluster_64457
ATGAACGGAATATCAAAAATCATCTTAGCACTGACCGCTGGATTTACGCTGTCCGGATGTGTAAGCCATACCCAAAAAAA
TTCACAACCAGATCCGGCTGCAATCGAGGCGGCAAAACTTGCGCAGTACACCGATTCACAGCTTTGCCAGGCAGAAGATA
ATATAGAGCCATTGCTCAACCTTAATCCATCATCTCATCCTGAATTTAATGAAAATTATCGCCGCGCCCAGGCTATTAAA
AAGGTGATCGCCTCTCGTAATTTAAGCTGCTACCGCCTGAAAATGGAGAGGGCAGAACAGGCCACACCAGCACGCCCGTT
AAGCCCACGACAAAAACAAGCGAGGGCATCCGCTGTTTGCCAGGCTTACGCCCAGCGTACCCGATTTGCGGATCCGACAG
TCACATTTCAGATGTGCGGGAAAGGATTCCAGTCTACTTCGGCAAAATGTAAGGAAGATTTGGCGAGTTTTGAACGTGAG
TCTCAGAAACTTAGAGGTACGGCCAGAGCGGAATATGTTGAAATTAGCAGTGCATTTCGGGCAGGCTGTACTTTAAAATA
A
>Cluster_32006
GTGAAGAAAATCATTTGTCTTGTCGTTACGCTGCTAATGACACTCCCCGCTTATGCGAAGTTAACGGCCCATGAAGAAGC
CCGTATCAACGCCATGCTGGAGGGATTAGCACAGAAAAAGGATTTGATATTTGTGCGTAACGGTGATGAACATACCTGCG
ATGAAGCGGTTTCTCATCTGCGACTGAAGCTGGGCAATACCCGTAATCGCATTGATACCGCCGAGCAGTTTATTGACAAG
GTGGCCTCGTCGTCATCGATTACCGGGAAGCCGTATATCGTGAAGATGCCCGGTAAGAGCGATGAGAACGCACAGCCTTT
TTTACATGCGTTAATTGCGCAGACGGATAAAACGGTGCCTGCGCAATAA
>Cluster_3358
ATGTTCAGAATCGAACCAAATTTAATTAAAGCTATCGCTCTTGTTGAAAGCAATCTAAAAAAGGATAGCATAGGCAAGAA
CAGAGATAAGAATAACAATATAAAGAGTTTAGATTATTGGTTAATGCAGATAAACCAGATGCATATTCCCCTGTTAAAAA
AACGTGGAATAATAAAAGATGAACGAGATTTGTTAGATAACCCTTGTCTGAACATTAAAATAGGAACCGAGATTTTATAT
AACCATTTTTCACGTTGTGGAGTAACGTGGCAATGCCTGGGGACTTATAATGCGGGTTTTGCAATGGATAACCAAAAAAA
GAGACAGCAATATGCCCCAAAATATATATTGTATATACCAGGCTTAATGAACTAG
>Cluster_8825
ATGTTTCTAAACTATTTCGCGTTGGGAGTGCTGATCTTCGTCTTTCTGGTGATTTTTTATGGAATCATCGCGATACATGA
CATCCCTTATCTGATTGCCAAAAAGCGCAACCATCCCCATGCCGACGCTATTCATACGGCGGGCTGGGTGAGCCTGTTTA
CTCTGCATGTTATCTGGCCGTTTCTGTGGATCTGGGCGACGCTCTATCAACCGGAGCGTGGCTGGGGTATGCAGTCACAT
GTTGCGTCGCAGGAGAAAGCGACTGACCCGGAAATCGCCGCACTTTCTGACCGAATTTCCCGGCTGGAGCATCAACTCGC
CGCCGAGAAAAAGACTGACTATTCCACGTTCCCGGAGATCTAA
>Cluster_62369
ATGGATTACGAATTTCTGCGCGATATTACCGGAGTGGTAAAGGTGCGTATGTCCATGGGGCATGAAGTGGTCGGGCACTG
GTTTAATGAAGAGGTGAAAGAAAACCTGGCCTTGCTTGATGAAGTGGAACAAGCAGCGCACGCACTGAAAGGTAGCGAAC
GGTCCTGGCAACGGGCAGGGCATGAATACACCCTGTGGATGGACGGTGAAGAGGTGATGGTTCGCGCCAATCAACTGGAA
TTCGCTGGCGATGAAATGGAAGAGGGGATGAACTACTACGACGAAGAAAGCCTGTCGCTATGCGGCGTTGAGGATTTTCT
GCAGGTCGTGGCGGCTTACCGCAATTTCGTGCAGCAGAAGTAA
>Cluster_64432
ATGAGCTACTACGACGACCTCCGGGCCGGCGACGAGGAGACGATTCGTGAGTGGGGACGGCCAGTCAAATTACGCGGTAA
AGCTGACCCCATTATCGCTATTTTCAACGAGCCTTACGCGCGCGTTGACGTTCCTCACGCTGGTTTTATTACCGGCACAG
TAACGAGCCTGACGGCGCTCTCTGACGACGTTGCTGGCGTTGTTGCGCGTGACGTCGTCCAGGTCCCAAAGCAGCGCAGT
ATCGCTGCTGACGGCTCTGTTACCTGGTCTGGCTGGACCGATTACGTGGTTAAAGAGCCTCAACCGGATGGCATCGGTCT
CACGAATATTTTTTTAGAGCCTCACACGTCCAGCGAAAACAGCGAGTATTCAAAATACTAA
>Cluster_48681
ATGTTTGGCATTGACGCACAGCGTATAGCCGCTTTTGCAAAAAGCCCTCTTGATAATCCCTTGTCTCGTAGTGAGCAAAT
GGAGCTGGCAAGGCTTTTTCTTCACATTCAAAAACAGGCAGACATTTTCAATAACATGCCTAATCAACCTATTCTGGATG
GTCACATCCAGATGGTCATTAACAGTCATGAGAAAGGCTGGGCTGCAATCGTCCCCTGCACAATTACATACAAGTTGGCG
AAAGAGGTTCAAGAGTTTCGAAAAGCCAGTGTCGAATCGGAGTCTACCAAAGCCGCAATAAACACTCTTATTCGCATGGG
GTTCACATGGACCGGTGGAGCCTACTGGCAAGCACCTCACCCCATTTTATCCGGCAATTAG
>Cluster_45215
ATGGAATTTAAAGATTTACCAGTACCATTCCAGGAAATGGCATCGAATGTGGTTCGCTCTCAACTGGCGACTCTTGACCT
GAGTACCGTAGAAAAAGAAACCATCGATACTATATCCGGTAACGTGCGTCGTGCCTTTATCGGTCTGTACGAAGAGAAGC
AGCTCTCTGATAACCAGGATTTACATGAAAAATACTTCCTGGAATTAATGGACATCATTAATAAAGGATTTGGCTTGTTA
ATGAAAAAGAAAGGGATTCGAATAGCTCCCCTTGAAAATCATTTTACAGCGAGCAGTATTAATTCCTGTGATTTAAAGCA
TCACACATCCGATGGGAAAGTTGAATCAAACAACAAAATATCAATTAATCATTAA
>Cluster_35511
ATGAAAACTATCGTTTATAACGTCAATGACGACACTCTGGATGGCAACGACACCATTGTTTCCGTGGCGTCATGCACCAC
TAACTGTCTTGCGCCGATGGCCAAAGCCTTGCATGACAGTTTCGGGATAGAAGTCGGCACGATGACGACCATTCATGCCT
ATACTGGCACCCAGTCACTGGTGGATGGCCCGCGTGGTAAAGATTTACGTGCTTCACGCGCAGCGGCAGAAAATATCATT
CCCCACACTACGGGGGCGGCAAAAGCCATTGGTCTGGTGATCCCGGAACTGAGCGGCAAACTGAAAGGTCATGCGCAACG
CGTGCCGGTGAAAACAGGTTCGGTCACTGAACTGGTATCGATTCTCGGAAAAAAGTGA
>Cluster_1467
ATGCGTATAGAGTTTGTTTTGCCTTACCCGCCAACGGTGAACACCTACTGGCGACGTCGTGACAACACATATTTTGTATC
AAAAGCCGGTGAGCGTTATCGCCGGGATGTGGCGCTAATTGTTCGCGAGCAGCGGCTGAAATTAAACCTGTCCGGACGGC
TGGCGATAAAGATTATTGCAGAGCCACCGGATAAGCGTCGTCGTGACCTGGACAATATCCTGAAAGCACCACTGGATGCG
CTGACGCATGCCGGACTTCTCATAGACGACGAGCAGTTTGATGAAATTAATATTGTGCGCGGTCAGCTCGTTCCTGGGGG
GCGGCTGGGCGTGAAGATTTACAAAATTGAGAGTGAGTGA
>Cluster_40638
ATGATGAAAAAAAGTATTCTGGCGTTTCTGTTACTCACCAGTTCTGCAGCGGCGCTGGCTTCACCGCAGGTGATTACCGT
CAGCCGTTTTGAAGTGGGTAAAGACAAATGGGCGTTTAATCGCGAAGAGGTGATGCTGACTTGCCGACCAGGTAATGCTT

TGTATGTCATTAACCCAAGTACCCTCGTGCAGTATCCTTTAAACGATATCGCACAAAAGGAAGTTGCCAGTGGGAAGACT
AAAGCCCAACCCATTTCGGTGATTCAGATTGATGATCCTAACAATCCCGGCGAAAAAATGAGTCTGGCACCGTTTATAGA
GCGAGCTGAAAAACTCTGTTAA
>Cluster_18414
ATGACGGGGATAACCATATTTTATGGCGATAACATCATTCGTTATGTGGTTAATACAAAAAAGGGGCTGAGACTATATTT
CAAGCAATTACCGGATAATTACCAGGCAAAATTTGAGTTAAATTTAATGAGTAAGTTCTCAAATTTTATTATAAACAAAC
CATTTTCAGCGATAAATAATGCGGCACGTCACATTTTTTCACGCTATTTGTTGGAGAACAAACATTTATTTTATCAATAT
CTTAAAATTTCGAATACAGGTATTGATCATCTCGAACAATTGATTAACGTCAACCTTTTCTCTTCTGACAGAATGTCATT
TTGTGAATGCAATCGTTTTCCATAA
>Cluster_51324
ATGGCTACTCATTACACTGAACTCATGGCTGGCACTGAAGCACTGGTTACTACGTTGGGGATATTTTCAGCTAATAAAGG
GGTAATTCCTGCATTTACGCCACTGATGCAAGAAGATGCAACTGGTGCACTGGTGGTATGGGATGGAACGAGCGCAGGCA
AAGCTGTTTATGTTTCCGCTGTACAAATCGATACAGCGACAAAAACGCAGGCTCAGGTTTATAAAACCGGCGTCTTAAAT
GTTGATGCTCTGAACTGGCCTGAGTCTGTAACACAACTATCAGCAAAAGTTGCTGCGTTTGTTGGCTCAGGTATTTCTGT
TCAGCCGCTGGCACGTGTGTGA
>Cluster_55985
ATGGCCACTATCTCTTCAACTTCAATTCCTTCCATACAAACACAGAGTAGTAACAGGGCATCACAAGGTGGCGATGTTGC
TTCACAAATCGCGCGGATAAGTCAGCAAATCATCAAACTCACTCAACAAATTAAAGAGATTGTAGACACTAGCGGCAGTG
CTGAAGACAAGCAAAAACAGGCCGAATTGATTCAACAGCAAATCACCCTACTGGAAACACAACTGGCGCAGTTGCAGAGA
CAGCAGGCAGAAAAGGCGCAGGAGAAAGAGCAGCGTTTGTCTCTGAATGTATCCCTTCTAAATCCTGTTGAAAAAACGAC
TCATATCGATATCTATATCTGA
>Cluster_22620
ATGGTTACACTTTACGGCATCAAAAATTGTGACACAATTAAAAAGGCTCGCCGTTGGCTGGAAGCCAATAACATCGACTA
TCGTTTTCATGATTACCGCGTCGATGGATTGGACAGCGAATTATTGAACGGTTTTATCAACGAATTAGGCTGGGAAGCGT
TACTCAACACCCGTGGTACAACCTGGCGTAAACTGGACGAAACCACCCGCAATAAAATCACCTATGCGGCCTCTGCGGCG
ACATTAATGACTGAAATGCCTGCAATTATCAAACGTCCATTGCTCTGCGCGCCTGGTAAGCCTATGCTGCTGGGTTTCAG
TGAATCCAGTTATCAGCAATTTTTCCATGAGGTGTAG
>Cluster_10620
ATGACACATGAACCCATTAATACATATCGTCGCCGTATAGCTGTTGCGGCACTCCATCGAATAAAACGTAAAACAGGTGG
TAATCTGCTTATTGTTGACCTTCCGGATGGGAACATTACGACCATAGAAATAACTGAACAGTTTATAAACCAGTTGCTGT
TACGCTTCGAAGGTATTACCCGTGGTGAATTGGGCCGGGTGGAGGGTGAAACCGAAATCCACACTGCATACCAGAATGCT
ATCGGGATTAATCAACATACTGAATACCTGACTGAAACCGGAAAGTTAATTATAGACAACCTTTTTCAAGAGGTTATTGA
TTACGCGAAAGAAAAATATATCAGCGGAGGAATTAACTGA
>Cluster_49655
ATGCAAAACAGTGGAGATACTCAACTGGCAAGAAAATATAATCGCGAAGGTGAAGCTGTTTATAAAACCGGTCAACTTGA
GCAAGCCATTCAACTCTTCCAACAGGCAACAGAATTAGACGGCAATTACGGACAGGCATTTAGTAACCTGGGGCTGGCGT
ATCAGAAAAATGGCAACATCGCGGAAGCAATTTGGGCAAATCGTAAAGCGATCTCGTTGGCCAGTGGAGCAAATGCGGCG
ACAACGCGCGCTAACTCCTATTATAACATTGCCAAAATTTATGAAACTGCTGGACAGGACGCTGATGCATTGCAGCATTA
TCAACTCGCTCTTCACGATGCCATTTTATAA
>Cluster_11178
ATGAGCGAGATTAACTATCAGGAGCTGCGTGTTGAGCTGGGAGCAGCAAAAAAACGCATAGAAGAGCTGGAGGCTAATCG
TGTGGTGCTTGAGGTGGAAAATGAGAGGTTGAAACACGCAATGGCCGTAGCCCTTGAGCATATATCCGTCACAGATGCAG
GACAGGCAGGTGTCGCTGCAATGATTATCTATGATGCCATGTACCACAGTGAAAAAACTGACCCCTACGCTTTTCTGGCA
GAATTGCGGGCGCAGGGTGTGGAGATGGTGCGCGAACATCCAGCAATCAAACTTTGCTCTTTGACGCACATATGTGACGA
GTTAGTCGCAGAGCTTCGCAAAGGAGGCAACAAGTGA
>Cluster_19930
ATGAAAATTAAGGGCATCAGTAAGGCTGTATTGTTGCTTGCACTGCTAACTTCCACGAGCTTTGCTGCGGGTAAGAATGT
CAATGTTGAGTTCAGAAAAGGCCATAGCAGCGCTCAGTACTCCGGCGAAATAAAGGGATACGATTACGATACATATACCT
TCTATGCCAAAAAAGGTCAGAAAGTGCATGTGAGTATCTCTAATGAAGGCGCTGATACTTACCTCTTCGGGCCAGGAATC
GATGATTCTGTTGATTTATCCAGATATTCCCCAGAACTCGATAGTCACGGACAATACTCACTCCCTGCTTCTGGCAAATA
TGAGCTGCGAGTGCTGCAAACCCGAAACGACGCCCGTAAAAACAAAACTAAAAAATATAACGTCGATATTCAAATTAAGT
AA
>Cluster_32200
ATGTCCGTAATGTTACAAAGTTTAAATAACATTCGCACCCTCCGTGCGATGGCTCGCGAATTCTCCATTGACGTTCTTGA
AGAAATGCTCGAAAAATTCAGGGTTGTCACTAAAGAAAGACGTGAAGAAGAAGAACAACAGCAGCGTGAACTGGCAGAGC
GCCAGGAAAAAATTAGCACCTGGCTGGAGCTGATGAAAGCTGACGGAATTAACCCGGAAGAGTTATTGGGTAATAGCTCT
GCTGCGGCACCACGCGCTGGTAAAAAACGCCAGCCGCGTCCGGCGAAATATAAATTCATCGATGTTAACGGTGAAACTAA
AACCTGGACCGGTCAGGGCCGTACACCGAAGCCAATCGCTCAGGCGCTGGCAGAAGGTAAATCTCTCGACGATTTCCTGA
TCTAA
>Cluster_11723
ATGGAAATGCTTGGGAAGATATTAGTGATAATTATTTCCATGATAATTATATCTTGCGATTATTATAATGCTGGCATGGA
CTATCAAGATAATATAGCCCTCGAGCAAGTCGTAACATGGGATTCTGATAATATATTCACACCTAATATCTTTGAACATA
ATGGAAGATGTTTTATGAAGAAAAATGAAACATGGATGTTATTAAATGGAGATGCAACACCAAACATTAGAACAATGAGC
AATAAGAAAATTGAATGCCTGGAGAAGAGTGAAGTAGAATGGGCTAGTAGGGTTTGCGACAGTATCACAGACAAGGAAAG
GGAAAATCTGAAGGCATTAATATGTAGCAAACCAGCTAAATCAGTTAATACAGGCAAAAGACCAGCGTTAGGATATTGA
>Cluster_35313
ATGGAATTTAAAGATTTACCAGTACCATTCCAGGAAATGGCATCGAATGTGGTTCGCTCTCAACTGGCGACTCTTGACCT
GAGTACCGTAGAAAAAGAAACCATCGATACTATATCCGGTAACGTGCGTCGTGCCTTTATAGGTCTGTATGAAGAGAAGC
GCCTATTCGGCGGACAGAATTCGCCTGAAAACAAGAATCAAGCAAATGATGAGAAGCTGAAACACATTATCGCCTTACTT
TTGGAAGACGCAAAACGTCTACAGCAACTGGAACCAAATGCAGGCACAGAGGCCCGCATTTGGATTGCCATGAAATCACT
CAAATGTGAAAGCAGTGATTATTTCAAAACAACAATTAAAACTACTCAACTTTCGGGAGAGCTACTGAAGAAATTGCCAT
AA
>Cluster_46067
ATGATAGCTATGCATGACTGGTGGTTAGCCGTGACTGCAAAACTTTTTGGTCGAATCCATTTTGATAATACTCAAACGAT
TCTTTATCGACAACATCAGGGCAATGTATTAGGTGCAAAATCATCAGGTATGATGCGTTTTATTCGATTAGGATTAAATG
GGCAAGGGATTTCGCGTGTAGTATCTTTTAGAAAAAAAGTTTGTGCGCAAAATAAGCTTCTTTTACATGTCTATGATAAA
GATTTAAATCTTGAGCAAAAAAAATCTATCAGGCTTGTAATTGAGGGCCTTAAAGAGAACTCTTCAATTTCTGACCTTTT
AAAATGTTTCTATCATGGTAGCTATATGCAAGGTTTTAAACGTAATCTTGCCTTAATATATTCAGTTCTTTACACAAAAA
AAAGAAGATAG

>Cluster_19631
ATGAGTACCTGTAATTTCCATGCCGTGGTGCGTGTCGCTGACGTGCGCCCGGATTTCGCCTTGCCGCTGGGTAGATGCGA
CCACATTTACCCTGAGCGTGTGCAGCACGTTTTCAATCCAGACGAAGCGGTTTATATCGTTGCCCGGTCTGCCACATCCG
GCACCCGCACATCTGGCGTTAGCCGTGGGCTTTCCCCTGTGCTGCCGCCGAAGATGGCCGAGCGGATGCGCGAACGTGAA
CGGCACGAGAAAAACCGAGTCAGACCCCGCAAGGTGATCACTGAGGCGATACGCGCTGACATTATCGATCGCTCTGCAAA
AGGTCAGAAGAATCGTTACATTGCAAGCAAGGTTGGTGTGTCTGAAACCTCTGTGCGGAAGGTGTTGAACATGGCGCGTA
TGAGTGCGCACTAG
>Cluster_11788
ATGATATTTAGCAGTACGAAGAATCTGGAAACCAACATAAAGGCGAACCTACATACCTTCGATAATCGTGACGATGTTGC
AGGATTCATTGAGGCGTCGTTAGACGATGGATTATTCGAAGACATAGCCGCTGCGTTCGAAGATATCAGTAAAAATGAAG
GGCCATCGCAAGCGACTGAAATGGTGCATAGGCACGCGGTGCTGCATCCATTGAAATTGGGCTCCTGCATGAGAGACGTA
AACTTGTGGGGATGCCCTTATCGAATGAAGTGTCAGGCATTGAAGCCCTGTGAGCATTTCACGTTAACAGGTCGTATTGA
TGAATACTCCACAATTGAAGCCAAAGGTCGTGCATTAAACGAGGCTACGTTTGCTTTTGAACAGTATACTGTAGTGGTCA
AGTAA
>Cluster_14965
ATGAAAGATAACATCGTGGATTTTCAACGTATCAGGAGAGACCTGGGCGCTCATTCAACAGATGTTGCAAATGATGCCGT
TTATTCTGACAAAAAGAAAACATCCAGGTTCTTTTATCTGCTGACACCTGTCAGATACCTTCTGGCTGCTTCCCTTTATC
TGGTTGTTGTTATTCCATTGGGGATTCTTGCGGGATTTCGTTTCTTGTTGCAGTTTTTAGGCGGTATCGCAGTGATGGGG
TTAACTATCGCCTGGTACATGGGTGCAGATGTCAGTGGTTACATTGTTCTTGCTGCATGGATCGCCTTAACTGTTTGTGG
TTGCGCAGAGCAGATTTTTGAATGGTGGATCGAAAGCCGTTTTGCGTTCCGTCTTTTTGGTATCGGGAGAACGCCGGAAG
AATGA
>Cluster_46439
ATGCTGAAATTCATGCTGGATACCAATATCTGTATTTTCACTATTAAGAATAAACCAGTACATGTCAGAGAGCGGTTTAA
CCTGAATTCTGGCCGGATGTGTATAAGTTCTGTAACATTGATGGAGCTTATCTATGGGGCAGAAAAAAGTCAGATGCCAG
AACGCAATTTGGCGGTTATTGAAGGTTTCGTATCTCGTTTGGTAGTGCTTGATTATGATAGTGCTGCTGCGACGCATACC
GGGCAAATCAGGGCCGAGCTTGCCCGGCAGGGAAGGCCTGTTGGACCTTTTGACCAGATGATAGCGGGACATGCTCGCAG
TCGCGGTTTGATTGTGGTTACCAACAATACCCGAGAGTTTGAGCGAGTGGCGGGAATCAGACTAGAAGACTGGAGCTAA
>Cluster_16081
ATGGGATTCAATGACCAGCCCAAAAACCTGAAAAAATCCATTAACGATAAATATAACAGAAGGGTAAAGTCACTTTCTCG
GCATCTCGAGGATTTTATTTATCATGCAGAAATGAAACTGGACGAATCTATTTCTGGAAGGATTTATCGCAGAAGCGAGA
TTGAGTCAATTGTTGAGAAAGTAAAAGAATTGATGGCTATCCTTCCGGCTTCAGATGAACCTCCTTCAATTAATTTTGAT
ATTCTTGAAATCATTGAGAATGCAACAGAAGAAACTCAGCGCATTTTATCAGAAGGACAAAAATTCTTCGACAACCTCTC
TCCTGAAGAATTGGCAGAAATGATATCAACATTCCAACAAAATAAAAAAGATAGTGAATCTGAGTGA
>Cluster_26875
ATGATTGACGCTCATGACTTCACAAGATGGGTGCGCACACAGGACACCCGTCTGGCTCCCGTTCTTCAGGGGTTATTTGA
TCTCTACATCCGTGGTCGTGACAACAGAGCACGCACCACAAAACCGGAGAATGCAGACACCCCTTTATTTCACAGTAGACG
ACTGCTACCGCGTGGATTTCACACCACACGGACTGGCGTTACACTGCCTGACACCGCACGGCGAATCACTGCTGGCGTAT
TACGACTCCCCGGCCTCCGTATTTGCAGCAATGCTGGCGCATCGCACTGCTGGCGGGTGTGCCTCGCTGAGTGAATACAC
CGCTGAATTTAACCGCCTTTCCGCCCTCTTCTCGCAGGAGTGGCAGCGCGTGACGGGATACCAGCCATGA
>Cluster_44941
ATGCGTGATATTCAGATGGTTCTTGAGCGTTGGGGAGCGTGGGCGGCTAATAATCATGAAGATGTGACCTGGTCGTCCAT
TGCCGCCGGTTTTAAGGGATTAATTACTTCAAAAGTAAAATCTCGCCCGCAATGTTGTGACGATGACGCGATGATTATTT
GCGGGTGCATGGCCCGTCTGAAAAAGAACAACAGCGATTTGCACGATTTATTAGTAGATTATTATGTAGTCGGTATGACA
TTCATGTCACTGGCAGGTAAGCATTGCTGCTCTGATGGTTATATCGGGAAAAGGTTACAGAAGGCTGAGGGCATAATTGA
AGGGATGTTAATGGCATTAGATATCCGGTTAGAGATGGATATCGTTGTTAATAACTCTAATTAA
>Cluster_126
TTGGTTAAAAAAGGATTAACCTCAGATAGAAAAGCATTAGTTTTATTGATAGGCACCAACCCTCTGGCAAACTATGTAAT
TCCTCTTTATTTTAAGACATTTCATAACTATAGAAATTTCGTTTTTATTTTTTCTGAAGATAACCAAGTACAAAAAGGTA
CCCAAATTTATGCTGAAAGGGTAAAAAATCTTCTTCATCTACACCCCGACCAGGTATTTTTAGTTCCGCTAAAGGACATA
GGTAATGTTCAGCAGATTCTTGAAGACCTTGATACGGTAGGTATATGGGAACAATTTGATGAAGTCCATCTAAACTATAA
ACCTGGGACCAAACCCATGGTAGTTGGGGTCTATAACTTTTTAAAGAATTTTTATCAGACCTAA
>Cluster_35641
ATGTCTGAACCCTTATCCGGTTCCGGTACGGCTGCGGCGCTCGGCGGGGCGACGGTATTCGGGCTGTTTACCGGAACGGA
TTTCGGGATTGTGTTTGGTGCATTCGCCGGGGCGTTGTTCGTGGCCACGATGCCGCAGGCGTTTTCAGGCTGGCGAGTGG
CAGCACATTTTCTGGTGTCATTTATTGTTGGCGTGCTGGGTGCACGTGCGTTGTCAGCCTGGGTTGCATCAAAAACAGGT
TATGACGGTACATCGGCGGATGCACTGTGTGCGGTGCTGGTGTCAGTGATGTCGGTAAAGATTCTGGCATTCATCCACCA
GCAGGATATTGCAGCGCTGATATCCGGTTTGTTCTCCCGCCTGCGGAGTGGAGGTGGTGGCAATGCCAGATAA
>Cluster_56607
ATGAAATGTCCGGTTTGCCACCAGGGAGAAATGGTTTCTGGCATTAAAGATATTCCATACACCTTCCGTGGACGAAAAAC
AGTATTGAAAGGTATCCACGGTTTATATTGTGTCCATTGCGAAGAGAGCATCATGAATAAAGAAGAGTCAGATGCTTTCA
TGGCGCAAGTAAAGGCATTTCGGGCTTCGGTGAATGCCGAAACAGTGGCACCTGAATTTATAGTGAAGGTTCGAAAAAAG
CTCTCTCTTACCCAAAAAGAGGCAAGCGAAATTTTTGGGGGAGGTGTAAATGCGTTTTCGCGTTACGAAAAAGGCAATGC
CCAACCTCATCCTTCCACAATCAAACTTTTACGTGTTCTGGATAAGCATCCAGAACTATTGAATGAAATCCGTTAA
>Cluster_39854
ATGAAACGTTATTTACGCTGGATTGTGGCGGCAGAATTTCTGTTCGCCGCAGGGAATCTTCACGCCGTTGAGGTAGAAGT
CCCGGGATTGTTAACTGACCATACTGTTTCATCTATTGGCCATGATTTTTACCGAGCCTTTAGTGATAAATGGGAAAGTG
ACTATACGGGTAACTTAACGATTAATGAAAGGCCCAGTGCACGATGGGGAAGCTGGATCACAATAACGGTGAATCAGGAC
GTTATTTTCCAGACTTTTTTATTTCCGTTGAAAAGAGACTTCGAGAAAACTGTCGTCTTTGCACTGATTCAAACTGAAGA
AGCACTAAATCGTCGCCAGATAAATCAGGCGTTATTAAGTACGGGCGATTTGGCGCATGATGAATTTTAA
>Cluster_63660
ATGACGTTTACTGAAATCAGAAATACGGTTATTTCCAGAATGACGGCACAGACGATTATTGATGGAAAAGACGTATTGTA
TCCGAACGCGCCAACGTTCGATCCTTCCGGTAAGTTAATCTGGGCGCGGTTAAGTAATATTCCCGGGCAGGCTGGAGTTA
ATGAAATTGGCGCGGGGCCGATTGTTTATCGCACGGGGATAATCATTATTCAGTTATTTGTCCCCGCAGGTTCTGGTTCA
AAACTCATTACTGAGACAGCCGATAAATTGCGGGAACTGTTTGAGTTTCAGGATGACGATCGTCTCAGTTACCAGGCTGT
TTCCTCAATAGCCGTTGGCGAAAAGAATGGCTGGTTCCAGCTTAATCTTCAAATTCCATATCGCGCGCTCTAG
>Cluster_28122
ATGTCGAAAGAACACACTACGGAACATCTGCGTGCTGAGTTGAAATCCCTTTCCGATACGCTGGAAGAGGTGCTTAGCTC
ATCTGGCGAGAAGTCAAAAGAAGAGTTGAGTAAGATTCGTAGCAAAGCGGAGCAGGCACTGAAACAGAGCCGTTATCGCC
TGGGTGAAACCGGTGATGCCATTGCCAAACAAACCCGTGTCACGGCGGCGCGTGCCGATGAGTATGTGCGTGAAAATCCG

TGGACGGGCGTGGGCATTGGCGCAGCAATCGGTGTAGTGCTCGGCGTTCTGCTGTCGCGTCGTTAA
>Cluster_20010
ATGAAGAATTCTTTTGAGCTGATAAGTACCGATCCAGTCAAGTTCAACACATGGTCGATAAAGTCAAAACTCATAATCAT
AATCAGAAAGCTGATGATTGATAACAATTGGTCTCAATCTGAAGCTGCAGAAAAACTGGGTATCAGCCAACCCAGAGTGA
GTAACATGCTCAACGGGAAGTTGGACAAATTCTCGGTCGACACACTATTAGAAATAGTGTTCAAGATGGGATACAAACTG
GACATGGATTTCACCCCACTAAACACCGAGTCACCACTGACAATGGTTGTAAAAAAGGCGATGGTGTAA
>Cluster_54489
ATGAGCGATAAGCAGACTGAAAAGACTATTAAACTGGATACTCCCATCATGCGCGGTAAAACAGAAATTACCGAAATTGT
GCTGCGTAAACCGCAGTCCGGTGCGCTGCGCGGCACACGCCTGCAGGCCATTATGGATATGGATGTGAACGCGATGATGA
CCGTGATCCCCCGCATCTCCAGTCCGGCACTGACTGCACAGGAAATCGCAGAGATGGACCCGGCAGATCTCACTGCCATG
TCGGTTGAGGTTGTCACTTTTTTGTTGAAGAAGTCGGTGCTTGCCGGTTTACCGACAGCCTGA
>Cluster_35760
ATGAACAATGAAGATAAATTTACTCCCCTTAATATTGATAACGTCATGGCTGAAAAAGGGATGCTGGAACGTGTTCGCGC
TATCGTTGAATATGGTATTAAGCATAATCTCACCGCCAGAGAAGTCCGTGACATTATCAACCGCGAAATGAATCGTCTGG
AAACAGTAGTGGCACTGCAAAATGAAACAGCCCGCGAGGAGTACATCCGCCGCAGGCTGGGGTTATCAGACCAGGATATT
GTTACTGACGCTCATGTGTTTGAGGCTTTTGAGATCCGTCAGCATCTGGGTCTAACGAACTGA
>Cluster_9948
ATGGAAAAAGAAGATTCATTTCCCCAACGCGTCTGGCAAATCGTCGCCGCTATTCCCGAAGGCTATGTCACCACTTACGG
TGATGTGGCGAAACTGGCGGGATCGCCCCGCGCCGCGCGCCAGGTGGGCGGTGTGTTAAAGCGTCTCCCTGAAGGCAGCA
CCTTACCCTGGCACCGGGTGGTTAATCGCCACGGCACAATTTCGCTAACCGGACCGGATTTACAGCGTCAGCGACAGGCA
TTACTGGCAGAAGGTGTGATGGTATCGGGAAGCGGGCAAATCGACTTGCAGCGTTATCGCTGGAACTACTAA
>Cluster_60541
ATGAGCAACACCACATGGGGCCTGCAGCGAGATATCACGCCGCGCCTGGGAGCACGTCTGGTGCAGGAGGGCAACCAGCT
GCACTATCTGGCTGACCGGGCCAGTATCACCGGTAAGTTTAGTGACGCCGAATGTCCTAAGCTGGATGTGGTATTTCCAC
ATTTTATCAGCCAGATAGAGTCGATGCTGACCACTGGTGAACTGAATCCCCGCCATGCCCAATGCGTCACCCTGTACCAC
AACGGTTTTACCTGCGAAGCCGATACTCTTGGTAGTTGCGGCTACGTATACATCGCTGTTTACCCCACTCAACGCTAA
>Cluster_52367
ATGTACGCGGTTTTCCAAAGTGGTGGTAAACAACACCGAGTAAGCGAAGGTCAGACCGTTCGCCTGGAAAAGCTGGACAT
CGCAACTGGCGAAACTGTTGAGTTCGCTGAAGTGCTGATGATCGCAAACGGTGAAGAAGTCAAAATCGGCGTTCCTTTCG
TTGATGGCGGCGTAATCAAAGCTGAAGTTGTTGCTCACGGTCGTGGCGAGAAAGTTAAAATCGTTAAGTTTCGTCGTCGT
AAACACTATCGTAAGCAGCAGGGCCATCGTCAGTGGTTCACTGATGTGAAAATTACTGGCATCAGCGCCTAA
>Cluster_44026
ATGCCTGGAAAAACAGCCACTCTACCCGATGTCGATAAAACGCTGAAAAATGCGCGGGTCGAACTCAAAACTAGTCCCGA
CGCCAAAAACAAACTTCGTGAAGCTGCGCAAGCCGTTGGGGTAGATTTAAGCGCTTTTATTTTGAGCGCTGCTATGGAAC
GCGCCGAAAGTGTGCTTGATAACCAACGTCGCCGTGAGCTTTCGAATCAAAGCTGGGAACTGATGAACCAACTCATCGCT
GAACCTGCTCAACCGACGCTCGCCCTCAAGGCGTTAATGAAAAGGAAAAACAGCGATGGTCGACAAGCATGA
>Cluster_35771
ATGGATTTGATTTCAGTTTTAGCGTTATGGCCTTACCTGTTGCCTGTTGTGGCCGGTGGGGCCGTCTGGGCAATGCGTCG
TAGTTTTGCCAGCACTGAGCGTGTGGAGCGTCTTGAAAACCGGATGACCGAAATGGAAACCCGCTACGCCAGCATCCCTG
GCACAGAAGATGTACACGAAATGCGCCTGCGAATTGCAGAGCTGTCCGGCGATATCCGGGTGCTGTCCCAGCGGGTGCAG
TCATTTTCCCATCAACTGGAGCTGCTGCTTGAAAACGCAGTAAACCGGAGTAATTCATGA
>Cluster_4801
ATGAATATTCGTCCATTGCATGATCGCGTGATCGTCAAGCGTAAAGAAGTTGAAACTAAATCTGCTGGCGGCATCGTTCT
GACCGGCTCTGCAGCGGCTAAATCCACCCGCGGCGAAGTGCTGGCTGTCGGCAATGGCCGTATCCTTGAAAATGGCGAAG
TGAAGCCGCTGGATGTGAAAGTTGGCGACATCGTTATTTTCAACGATGGCTACGGTGTGAAATCTGAGAAGATCGACAAT
GAAGAAGTGTTGATCATGTCCGAAAGCGACATTCTGGCAATTGTTGAAGCGTAA
>Cluster_44946
ATGAAAAAAAATGTTGTTTTCTGCCGCTCTGGCAATGCTTATTACAGGATGTGCTCAACAGACGTTTACTGTTGGAAACAA
ACCGACAGCAGTAACACCAAAGGAAACCATCACCCATCATTTCTTCGTTTCGGGAATTGGACAGGAGAAAACTGTTGATG
CAGCCAAAATTTGTGGCGGCGCAGAAAATGTTGTTAAAACAGAAACCCAGCAAACATTCGTAAATGGATTTCTCGGTTTT
ATTACTTTAGGCATTTATACTCCGCTGGAAGCGCGTGTGTATTGCTCACAATAA
>Cluster_36001
ATGAACGAAGTTGTAAATTCAGGCGTGATGAACATTGCGTCTTTGGTTGTATCGGTGGTGGTTCTTCTTATCGGGCTCAT
CTTGTGGTTTTTTATCAATCGTGCCAGTTCTCGGACTAACGAACAGATTGAACTGCTTGAGGCGTTGCTGGATCAGCAAA
AACGTCAAAATGCACTGTTACGTCGTTTGTGCGAAGCAAACGAACCTGAGAAAGCAGATAAAAAGACCGTTGAGAGTCAA
AAATCGGTTGAAGACGAAGATATTATTCGTCTGGTCGCCGAACGATAA
>Cluster_49156
ATGCGTGATTTAAAAGATAAAATATATTTTTACACAGAAGGTGAATTTCTTGAAATGCTTGAGGAGATAGTAAATGCAAC
TAGCAAAGATAAATCCCTAAAAGGGAAAAAATTAGAAAAATATCTCGATACCTTAGTGGATCATTTCATTAAAATTACTG
AACATCCTAAAAAGGGAGATCTCATTTTCTATCCTAATTCCCAAGAAGATGGTGAACCTGAAAACATCCTGAAAATCGTC
AAAGAATGGCGGCGTTCACAGGGGCTACCCTTGTTTAAAGACTCTAAATAA
>Cluster_41652
ATGTTAAAGCAGGTGTATGAAATGAAAGTCCAGATTTTAAACAATAACTGTGAAGTCGTTTGGTCATACGACATAGCCGC
CCCTGTAGATCAGAGCGGCGATAGCTGGACCAATGGGAAACATCAGATTATGGCTGGAGTTGTGTTCTCTTTACGCCGTG
CTTTGGAACAGGCTGAAGTATTTCCATCAGACCCTGAATGGAAATGGCCTTTTTCTATTTGTCCAAATTCGGAGAGCACA
TTTCAGAAAATTGGTCAGAAAGTCGCACTCGAAGAGCATCAGCCAACTGTTTCCTGA
>Cluster_43253
ATGGAAGGTAAAAACAAGTTCAATACTTATGTTGTTTCTTTTGATTATCCATCATCTTATTCCTCAGTGTTCTTAAGGTT
AAGATCATTGATGTATGATATGAATTTCTCCTCTATCGTGGCTGATGAATATGGGATACCACGACAATTGAATGAAAACT
CCTTCGCAATAACGACATCGTTAGCCGCAAGTGAAATCGAAGATTTAATCAGGCTCAAATGCTTAGACTTACCGGATATT
GATTTTGACCTCAGCATTATGACAGTTGATGACTATTTCCGTCAGTTTTACAAGTAG
>Cluster_49157
ATGTTTAAAGAAAAGCTCGAAGATTATACCGAGGAGGAATTTCTTAATTTTTTAGGCGGATTGAGATCAACTATGAAAGA
TGGAAAGCCCTTAAAAGGTAAGGAGCTAGAAATGTACTGGGATTCATTAGTAGATCATTTTATTGAAATTACACAGCATC
CGTCAGGGAGCGATCTAATTTTCTACCCTAAAAGTCAAGGAGATGATAAGCCTGAAAACATCCTGAAAATTGTCAAAGAA
TGGCGGCGTTCACAGGGGCTACCCTTGTTTAAAGACTCTAAACAAAGTAGATAA
>Cluster_40255
ATGTGCACTATTGTTGATGCCGTCGCGAGTGTCACACATATCATGCAGGAAATCGCCACCGCCTCGGATGAACAAAGTCG
AGGCATAACGCAGGTTAGCCAGGCGATTTCTGAAATGGATAAGGTGACGCAACAGAATGCTTCTCTGGTAGAAGAGGCCT

CAACGGCAGCGGTGTCCCTTGAAGAACAGGCGGCACGATTAACTGAGGCGGTGGACGTATTCCGTCTGAACAAACATTCT
GTGTCGGCAGAACCTCGCGGAGCTTGTGAACCAGTTAGTTTTGCTACGGTGTGA
>Cluster_14499
ATGTCATTACCACACTGCCCAAAATGCAACTCCGAATACACTTACGAAGATAACGGCATGTACATCTGCCCGGAATGTGC
CTACGAATGGAACGACGCAGAACCTGCACAGGAAAGCGACGAGCTGATCGTTAAAGATGCTAACGGCAATCTGCTGGCTG
ACGGCGACAGCGTTACCATCATTAAAGATCTGAAGGTGAAAGGTAGCTCTTCGATGCTGAAAATTGGCACCAAAGTGAAA
AACATCCGCCTGGTTGAAGGCGACCATAACATCGATTGCAAAATCGACGGTTTTGGTCCGATGAAACTGAAATCTGAGTT
TGTGAAAAAGAACTGA
>Cluster_13148
ATGGTAAGCCGATACGTACCCGATATGGGCGATCTGATTTGGGTTGATTTTGACCCGACAAAAGGTAGCGAGCAAGCTGG
ACATCGTCCAGCTGTTGTCCTGAGTCCTTTCATGTACAACAACAAAACAGGTATGTGTCTGTGTGTTCCTTGTACAACGC
AATCAAAAGGATATCCGTTCGAAGTTGTTTTATCCGGTCAGGAACGTGATGGCGTAGCGTTAGCTGATCAGGTAAAAAGT
ATCGCCTGGCGGGCAAGAGGAGCAACGAAGAAAGGAACAGTTGCCCCAGAGGAATTACAACTCATTAAAGCCAAAATTAA
CGTACTGATTGGGTAG
>Cluster_9829
ATGTCTCGAGCGTCATATAACAACCAGGCGCGTGTGCACAATGGCTACCATTATCCAAGAAGTATTTTGACGGCTTTACG
TTCACGTATCTCTTTCCCGCGTTTTGTTGATGAATTTCGTGATTGCGTTGATTCAGACTTTGATAAGTATTATTTAATTA
GCGAAATACTGGGGAAAGTTTCGGCCAGCCAATTCCGCATGTTCTGTGACCGAATTGGTGCAGACTGTGATTATGCTCCA
GCGAAAATACAGAACATGGTTAATCCAAAAATGATTAATGCATGTTTTACGGTTAAAGAGTTTGCCTTTGATGCCTTTAA
GTTGCGCGGGTGA
>Cluster_24716
ATGAAAAAGTTATCTTTTAAAGAAAAACTGGAAATAGTGAGAAACATCATAAGAGAGTCATTATCAGGTAATGCGGCTTT
TGCTTTACTGGTGTATTCATTATTGCACTCCCTCCCCATTAAAACCTTTGCTGATTTATTTGTGACGATACTGTTATTTA
TAGCGATTATCGCTGTTGTGATATGGCTATTTCTAATCATCTATGTTTATTTTTGTGAGTTATTCAGAAGTCACTGGATA
GCAGTCTGGTTTATCCTCTGGTCTGTGGTAATCAACCTCACTATTGGATTTGGCATTTATGACAAATTAGCACCAGTCAT
TTCACTAATGAAATAA
>Cluster_24565
ATGACTGACACGCATTCTATTGCACAACCGTTCGAAGCAGAAGTCTCCCCGGCAAATAACCGTCAATTAACCGTGAGTTA
TGCGAGCCGCTACCCGGATTACAGCCGTATTCCCGCCATCACCCTGAAAGGTCAGTGGCTGGAAACCGCCGGTTTTGCCA
CCGGCACGGCGGTAGATGTCAAAGTGATGGAAGGCTGTATTGTCCTCACCGCCCAACCACCCGCCGCCGAAGAGAGCGAA
CTGATGCAGTCACTGCGCCAGGTGTGCAAGCTGTCGGCGCGTAAACAAAAGCAGGTGCAGGAGTTTATTGGGGTGATTAG
TAGCAAAACGCCACGCTAA
>Cluster_32530
ATGCATGAGTTGTCGCTTTGCCAGAGCGCCGTTGAAATTATCCAACGGCAGGCGGAGCAGCACGATGTTAAGCGCGTCAC
CGCCGTGTGGCTGGAAATTGGCGCACTCTCCTGCGTTGAGGAGAGCGCCGTCCGTTTTAGTTTTGAAATTGTCTGCCACG
GAACGGTAGCGCAAGGGTGCGATTTACATATCGTCTATAAACCCGCCCAGGCCTGGTGTTGGGATTGCAGCCAGGTGGTG
GAGATTCATCAGCACGATGCGCAGTGTCCGATCTGTCACGGCGAGCGGTTGCGTGTCGATACCGGCGATTCGCTAATCGT
CAAAAGTATTGAAGTTGAATAA
>Cluster_11929
ATGGGGCTGGTAATCAAAGCCGCGCTCGGTGCGCTGGTAGTGCTGTTGATTGGTGTTTTAGCAAAAACGAAAAATTATTA
TATCGCCGGGCTGATTCCACTTTTCCCGACCTTTGCGCTTATCGCGCATTATATTGTTGCCAGCGAACGCGGCATTGAAG
CCTTACGCGCAACCATCATTTTTAGTATGTGGTCGATTATTCCCTATTTTGTTTACCTGGTGTCGCTGTGGTATTTCACC
GGAATGATGCGCTTGCCCGCCGCTTTTGTTGGTTCTGTTGCGTGTTGGGGGATAAGCGCATGGGTGTTGATTATATGCTG
GATAAAGCTGCATTAA
>Cluster_48941
ATGAGTTTATGTTTTGACAGAAAAACACTGGAAGCTATCAGAGACATTATCCGGGAAACAATGTTAGGCAATGTTGCCAT
TGTCTTACTGATAAACATTTCACCGGACTACATGGTACAAAAGAACTGTCCATCAATTATCATACTCTCTGTTTTTATAT
TAACAATAATATGCATTGAACTCTGGATGTTCCTCTCTGTGATAAATTACTTGTCCGCGTTACGAATAAGAAAAGCAAAA
CTTGCACTTATAATATTTATTCTCTGGATGTGTCTTATTCTCGCATTAACTATTCGTTATTTTATTATATTTTCTGATGC
ATGGAGCAACCAGTAG
>Cluster_20994
ATGACGAGCAAAGAAACCTTTACCCATTACCAGCCGCTGGGCAACAGTGACCCGGCTCATACCGCAACCGCGCCCGGCGG
ATTGAGTGCGAAAACGCCTGCAATGACCCCGCTGATGCCGGATACCTCCACCCGTAAGCTGGTTGCGTGGGATGGCACCA
CTGACGGTGCTGCTGTTGGCATTCTGGCGGTTGCTGCTGACCAGACCAGCACCACGCTGACGTTCTACAAGTCCGGCACG
TTCCGTTATGAGGATGTGCTCTGGCCGGATGCTGCCAGCGACGAGACGAAAAAACGGACCGCGTTTGCCGGAACGGCAAT
CAGCATCGTTTAA
>Cluster_2700
ATGGCATCTTTGTCAAGCATGGGTGAAAAATTTCGCCTGATTCGTGAAGCAGAAGGTATGACCCGCCAGGAGTTTGCGGA
TTGTGTTGGGCTGCCTTATGGGACGGTAACTAACTATGAGGTGAGGGGGAAGCAAGTAACAGAGGGTGCCTTACTTAAGG
TTACGAAACACCCTAAATTTAAGAAGTATGCTTATTGGTTATCAACAGATGAAACGATGCCGGAGGTCGGGCAAATTTCT
CCGGCTCTCTCTCTTGATGGTTCCTGCAATTCGGAGGGAGATCGCGTTTCAACCGAAACAATCCAAAAATTATCCCGCTG
A
>Cluster_42595
ATGATCTGCTCAGGACCACAAAATCTGGCTCAAGAGTTGAAAGCGATCGGCGACCAGCTCCAGGAGTTGCAGAAGAAACT
TATTCAGGGGCCGAATATTAGTCAACCACGCCCCCTCCTGACCATTGAGACTCCCCGTCATTTGGGGGAACAACTGAACG
CACGTCGCAAAGAATTGGGCATTGATTTATACACACTTGAACTACAAACGGGGATCTCAACGTCCACGTTAAAGCGACTT
TTTAAGGATCCTGAACAGGTTAAATTCGGCAGTGTGTTCGCGGTGGCGAATGTTCTTGGAGTGAAACTATGCATCGACGA
GTGA
>Cluster_12666
ATGATCCATAAATCAAGCCTTGGAGAAAAACTTCGCCTGATTCGAGAGGCAGAGGGATTGTCACGTAGAGAGATGGAAGA
GGTGACAGGGGTATCTCAATACAATCTCAAAAATTATGAAATATTGGGAAGAATGATACCTGGAGAAACGTTACTCCTGA
TTTTGAATCATCCTCGTTTTCGGAAGTATTCGGATTGGGTGATGTTTAATCAAACTAATGCTGCGACGGGGCAGATTGCT
CCGCCTCTCTCTCTTGATGGCTTCTTCGATTCGGAGGGCGATCAGGTTTCAACCGAAACAAACCAAAAATCACCCCGCTA
A
>Cluster_16234
GTGAGACGTAATCTTTCACACATTATTGCCGCAGCATTCAATGAACCGCTGCTTCTGGAGCCCGCCTATGCGCGGGTTTT
CTTTTGCGCGCTCGGGCGCGAGATGGGGGCAGCAAGTCTTTCGGTACCACAGCAGCAGGTACAGCTTGATGCTCCCGGAA
TGCTGGCTGAAACGGACGAGTACATGGCCGGAGGTAAACGACCGGCCCGTGAGCACAACCGGGCGATGTGCTGCTGTGCT
GTTTTGGTTCATCGGTGCCGAATCATGCCGCCATTTACTGTGGTGACGGCGAGCTGCTGCACCATATTCCTGAACAACTG

A
>Cluster_43311
ATGGTTAGCAGGAAACGTAATAGCGTTATTTACCGGTTTGCCAGTTTATTATTGGTGTTGATGTTAAGTGCCTGTAGCGC
ACTGCAAGGTACGCCACAGCCAGCACCGCCAGTAACGGACCATCCGCAAGAGATTCGCCGCGACCAGACGCAAGGGTTAC
AGCGAATAGGTAGCGTAAGCACGATGGTTCGGGGTTCTCCGGATGACGCATTAGCAGAAATCAGAGCGAAAGCTGTCGCT
GCAAAAGCTGATTATTACGTTGTCGTCATGGTGGACGAAACCATCGTGACAGGACAGTGGTATTCACAAGCCATTTTGTA
CCGTAAATAA
>Cluster_26546
ATGGCGCAGTTTGAATGGGTTCACGCCGCCTGGCTGGCATTGGCAATCGTGCTGGAAATCGTTGCTAACGTCTTTTTGAA
ATTTTCTGACGGCTTTCGTCGCAAAATATTTGGCTTGCTCTCTCTGGCGGCGGTGCTGGCTGCCTTTAGTGCGCTTTCTC
AGGCCGTTAAAGGGATCGACTTGTCTGTCGCTTATGCATTGTGGGGCGGGTTTGGTATTGCCGCCACGTTAGCCGCAGGT
TGGATCTTGTTTGGTCAAAGGTTAAATCGTAAAGGCTGGATTGGCCTGGTGCTGCTGTTGGCTGGAATGATCATGGTGAA
ACTTGCCTGA
>Cluster_49936
ATGCCTACCGCAATCTGCGACGAAAACAAAAAGCGTTATCCCGCAAGCGCGAACCGTAAAAAGGCCAGAATCCGTCTGGC
AGACTTACACGAACGGGTAGCGAATGCTCGTGCTGATTTTCAGCATAAACTTTCTCGTACAATTGTTGACGAAAACCAAG
CGGTAATTGTTGAGACGCTGAAAACAGCCAACATGATGAAAAACCACAATCTAGCAAGAGCGATAGGTGATGCAGGCTGG
CATAGCGTCATCACAACGCTGGAGTACAAAGCTGCGGGAAGAGGCATTCACCTAGTAAAACTCGACCAATGGTTTGATAA
ATAA
>Cluster_24676
ATGGAAAACTACGCTAAATTTGTTGCTACTGAAATACTGAAACAACTCGGTGGAAATCGTTTCATAGCGATGACAGGGGC
AACAAAATTTGCTTATTTCGATGAAAATGGTGAGTGTGGCCTTTTCTTTCGTTTGCCTTCAAACTTTGCAATGAAGGGCA
TTAATTTAGTTAAAATCAAATTAACATTTTCTGATACATACCTGGTGACATTTTTCCGTATCCGTGGAACCACAAAAAAA
GAAATATCAAAATTTGATAACATTTATTGTGATCAGCTTGAAAGTTTGTTTAGCGAAGAAACCGGACTTGCAACCAGATT
TTAA
>Cluster_51670
ATGAAGCAGGATAAAAGACGCGGTCTGACCCGGATCGCATTAGCGCTGGCACTGGCAGGTTATTGTGTGGCACCTGTGGC
GCTGGCTGAAGACAGTGCCTGGGTCGACAGCGGCGAAACCAATATTTTCCAGGGGACCATTCCGTGGCTCTATTCGGAAG
GGGGAAGTGCTACGACAGATGCCGACCGTGTAACGTTGACTTCTGATCTAAAAGGCGCTCGCCCGCAAGGCAGTGAAACG
GACAAGCGTCTTTACTCGGGTGATAAATTAACCGTAAGTTGGGAAATTGGTGATACCGAAGGCGACGTGGATCTTGGAGG
ATTGGGCGATAACGCGAAAACTATCGATACTATCCGCTGGATGAGCTACAAGGATGCGCAGGGGGGGATCCAAAAGAGCT
GGCAACGAAGGTGA
>Cluster_66998
TTGACGTTTTGCGGGGACCTGCCGTTTGTTCCCTCCGGTATTAACCATCACCCGTTTGAGAACCTTCGCCGCATGGGGCA
TCACTGCCACGGCCGGAAACGACTGTCCGGGGGCAGACCGCAAAAGTTTTTGCCTGCTTTTGGGCAAAAAGTTTTGCGGG
ATGTGCATTTCACCCCTGGACTGGCGGAGACGGTTGTGGCTGTCTTTGCCCCCGGCGACAGGGGCTTAAACAGGTGTATG
GCACCGGACACAACAACGGGCAGGGACCCGCCGCAACGACGAAGAGCGCGTGCGCGAAACGGCGTTGCCCCTTACATCCC
GGCGGCCTTTTGCCGGGATGCCAGGAGCGCAGGCCACGGCGCGACAGAGGCGGCGCGACCGGAACGGTCGCTGCAAGCCG
GGGGTTTTGACGCATCGGACCGTAGCGACAGCCCGAAACCGCGCCAGCGGGTTCGGCGGAGACTGGCAGGTGCGCCAGCA
GCTGCCTGTCGGAGTCGGGCCTGA
>Cluster_55476
ATGACCGGTATGACGAATGTAACAAAGCTGGCCAGCGAGCCGTCAGGCCAGGAATTTCTGGTATTTACCCTTGGTGATGA
AGAGTACGGTATTGATATCCTGAAAGTGCAGGAGATCCGTGGCTACGATCAGGTAACACGGATTGCGAACACGCCAGCGT
TTATCAAAGGCGTCACGAATCTGCGCGGCGTTATTGTGCCGATTGTTGACTTACGAATTAAGTTCAGCCAGGTGGATGTG
GACTATAACGACAACACGGTAGTTATCGTCCTGAATCTCGGACAGCGGGTGGTCGGCATCGTGGTTGACGGCGTCTCAGA
CGTGCTTTCATTGACGGCGGAGCAAATTCGTCCGGCACCGGAATTTGCCGTGACGCTTTCAACAGAATATCTCACTGGAC
TGGGCGCACTGGGCGACCGGATGTTGATTCTGGTGAACATCGAAAAACTGCTGAACAGCGAAGAGATGGCGCTGTTAGAT
AGCGCGGCGTCAGAAGTGGCGTAA
>Cluster_39648
ATGACAGTGATGACGCTCAATCTCGTTGAAAAACAGCCAGCAACTATGCGCCGGATAATTGGTAAGCATCTGGCCGTCCC
TCGCTGGCAGGAGACATGCGATTATTATAATCAGATGATGGAACGCGAACGGCTAACGGTTTGCTTTCATGCGCAGTTAA
AACAGCGTCACGCAACGATGCGTTTTGAAGAAATGAACGACGTCGAACGTGAACGACTGGTATGTGCAATTGATGAATTG
CGCGGGGCATTCTCAAAACGCCGTCAGGTTGGCGCAAGTGAGTATGCATATATTAGTTTTTTAACAGTCAGTCAGCGTCG
CACTTTATTTATGCACGCACGACTGACAGAAAAAGAATTTAACCAGCCATACTGGCGAATTAATGAAGAGTCATGTTACT
GGCGTGATGCTTTATTCCGTGCATTACGTGAATTATTCAGCCTGTTTGAGTATGCACCGACAATTCTGACGTCGGTAAAA
CCAGAGCAATATCTGCATTAA
>Cluster_48678
ATGAGAGAGAAAATCAAGAACCCGGTCGTCGTATTGTATAAACGGGAAACCAGCGATTCTTATGCGGTATCCATCACTGA
TGGAAGCCAGAACATGCACGATGGTCTGCTAATGGCCTCCGTATCTCCTGATGATTCTGACTACCCTTTCGCCACTTTCG
CTATGGTTGGTTACTACATGGCTGCCGAAATTGAGAAGTTGCGAGCGCAGAGAGACGCATTAGCGGCGGAGAATGCAGCC
CTGAAAGAATCTGAGCGAGCATTCGATGCGATGTGTGCCGAGGAACACGGAGATAATTGGGTTAGCGAATTAACGGAGAC
TCCAGCCACCGATGCTTTTCTGGCTGAAGTACGTGCGCAGGGCGTGGATATGGCTCGTAACGCGATGATTGATTTTGTTG
ATGGTGAAGTTGGGCCAAACAAGAACGTTCCGGGGCTGATTAGAGGCGCAGAGATATGCGTAAGTATTGCTGAACAGCTT
CGTAAAGGAGTCATCCAGTGA
>Cluster_3526
ATGTCGTCATCGTTATCAGGAATAATACTCAATACTTCGCTGACAAGTCACCATATGTCATGGGGCAAGTTTAGCAGAAC
TCTTCATGTATTGAATGAAGATATCGTAGATGAAATTCAGCTTAAAGCCCGAACACAACAACGCCACCATCGAGTATATT
CAGAAATGGCGGATTACTCCGTTGTCTCCTTTCGGGGCAAAGAATATTCTGTTCGTTTTATTAAAGATTACAAAACAGAT
AATTATGTGTACAAAGTACAAAAGATGACTCAGGAAAGTGGGTGTCTCTCAAGATTATTTAGCTTTCTTTCTGGCGGTGT
GACTAAGGCTTTGGAAAGTAAACTAAATGAGAGGCATATCACTCCACTTTCTCCAACCTGGTTTCCTCGAACTCCACTGG
AGGGGGTCTTAACGGAAAGAGGATTATCTTCTTTGTTACGTCGCGTACAGTCTACTGAAAACCTTGATTATAGTAATGCT
ATGATATCAAACTCTTTCTCTAACTCCGTAGTGTGA
>Cluster_51245
ATGGCGTTAACACACCACGAACTCTGTCAGATTGCGTACAAGTTCCTTAAGCGCAACGGGTTCAAGGTTTGCTTTCATGA
CCGCTTTGTTGCTGTAACCAGTACCGGAGAACAGCCAGATGCTATGGGATTCAGAAATTCAGCATCATGCCTGATAGAGG
CGAAGTGTTCTCGTGCTGACTTGTTGGCAGATAGAAAAAAGCGTTTCCGTAAAAATCCCTCACTTGGCATGGGCGACTGG
CGATTCTTTATTAGTGAGCCGGAAATTATTTCAGTTGAGGATTTACCTCCCGGCTGGGGATTACTTCACGTTGTTAACGG
AAGAGTACGGAAAGTACATGGATGGCCCAGGGGTAATTGCTGTTGGGGTAATCCTGACGATAAGCCATTTACTGGGAATA
AGCAGGTTGAATGCGATTACATGTTATCTGCATTAAGGCGCATGGAGTTGAGAGGGCACCTTAATGAAATATATGACGGT

GTGATTGTTAATAAGAAAGAAGGAAACGCGGCATGA
>Cluster_12132
ATGACTGAAATGGCTAAAGGAAGCGTGACGCATCAGCGGTTAATCGCTTTATTATCACAAGAAGGTGCTAACTTCCGCGT
CGTTACCCATGAAGCGGTAGGTAAATGTGAAGCGGTGTCAGAAATTCGTGGCACCGCTTTAGGCCAGGGAGCTAAGGCAC
TGGTATGTAAAGTCAAAGGCAATGGCGTAAATCAACATGTTCTGGCGATCCTCGCTGCCGATCAACAGGCCGATCTGAGC
CAACTCGCCAGCCATATAGGCGGATTACGCGCTTCGTTGGCCAGCCCGGCGGAAGTCGATGAACTCACAGGCTGTGTCTT
CGGCGCGATCCCCCCTTTCAGCTTCCATCCAAAACTCAAACTGGTTGCCGACCCTCTACTCTTCGAACGATTTGATGAAA
TCGCCTTCAATGCAGGGATGCTGGATAAATCCGTTATTTTGAAAACCACGGATTATTTGCGCATTGCGCAACCAGAACTG
CTCAATTTCCGCCGCACTGCGTAA
>Cluster_64092
ATGGCTGTACCAAAACATCTTCGCTTTTTTACGTTGTTTGTGGATGGTGAAAACGAAGTGGGTAAAGTGACATCCGTCAC
GCCGCCCAAACTGACGCGCAAAACCGACAGCTATCGTGGTGGTGGCATGATGGGGGCGGTAAGTATTGATCTCGGTCTGG
ACGACTCCGCGCTTGATGCGAGTTTTGTTATGGGGGGAGCTGTTCGTGCGCTGTTCCTTAAATATGGCGGCACGATTGAC
GGCACGCTGCTGCGTTTTGCGGGTGAATACTACACCGATGCAGAAAGCGATCTGTATGAAATCGAGATGCGCGGACGTGT
GACGGAAATTGATATGGGGGAAGCCAAACAGGGCGAAGCCACATCACACACTTACGCTGTCAAAAACACCTACTACAAGC
TGAGCGTTAACGATCGCCCGCTGTGGGAAATCGACCTGTTGAACCACATCTACCGGAAGGACGGCAAGGACATTGTGCCT
GACCGTATCCGTTCCGCGCTTGGGCTTGGCTGA
>Cluster_66901
ATGTCTGTTGTTGCACCTGCTGTATACGTTGGAACCTGGCACAAATACAACTGTGGAAGCATCGCCGGACGCTGGTTTGA
CCTGACCACGTTTGATGATGAGCGCGACTTTTTCGCCGCCTGCCGTGCTCTTCACCAGGATGAAGCCGATCCTGAACTGA
TGTTTCAGGATTATGAGGGATTCCCGGGGAATATGGCCTCTGAATGCCATATCAACTGGGCCTGGGTTGAAGGCTTCCGC
CAGGCACGGGATGAAGGCTGCGAAGAGGCTTATCGTCTCTGGGTGGAGGATACCGGTGAGACGGATTTTGACACCTTCCG
CGATGCCTGGTGGGGCGAGGCTGACAGTGAGGAGGCTTTTGCGGTTGAGTTCGCCAGTGATACCGGCCTGCTGGCTGACG
TGCCGGAGACGGTGGCGCTCTATTTTGACTATGAGGCGTATGCGCGGGATTTATTCCTGGACTCCTTCACCTTTATTGAC
GGTCATGTGTTCCGTCGGTGA
>Cluster_14211
ATGAAAAGCAAACTCCAGAAAATTATATTATGTTTATTTCTTCTATGCTGCATCTATAATTTATGGACATTGCGTCCTGT
ACAGATTTTGTATACATACTCTGATGCTGGCAATTCAGTCTTTCTGGTCGTTGATCATCTGCCATGGACTGACAGCGATA
AAATAAATTGGTATCTGAAGCATCAGAATGAAATTAAAAATCAACATCCCCTACCAGAAGGTAGTTGGCATACCTGGTAT
GTCATTGATATCGGTAATGGCTTCACTGACTATAAAAAATATATCGAAGGGCCGTATGAAGATCTCTACTGTTTCCCGAC
AATTAAGAGCAATGATAATTGCATCGTGAAAAATTACTTAATGGTTATTAATGAATACCCTTATCGTAACACTCATATTG
GTATTAACGATTTTACTGAGTATCAATTAACACAAGAAAATAAAATAGAGCGAGTTTTCAATCCGCATGACTTTAAATAA
>Cluster_4148
ATGCAAAAACGGGCGATTTATCCGGGTACTTTCGATCCCATTACCAATGGTCATATCGATATCGTGACGCGCGCCACGCA
AATGTTCGATCACGTTATTCTGGCGATTGCCGCCAGCCCCAGTAAAAAACCGATGTTTACCCTGGAAGAGCGTGTGGCGC
TGGCACAGCAGGCAACCGCGCATCTGGGGAACGTGGAAGTGGTCGGGTTTAGTGATTTGATGGCGAACTTCGCCCGTAAT
CAACACGCTACGGTGCTGATTCGTGGCCTGCGTGCGGTGGCGGATTTTGAATATGAAATGCAGCTGGCGCATATGAATCG
CCACTTAATGCCGGAACTGGAAAGTGTGTTTCTGATGCCGTCGAAAGAGTGGTCGTTTATCTCTTCATCGTTGGTGAAAG
AGGTGGCGCGCCATCAGGGCGATGTCACCCATTTCCTGCCGGAGAATGTCCATCAGGCGCTGATGGCGAAGTTAGCGTAG
>Cluster_39826
ATGCAACAGCTTTCCTTTCTGCCCGGAGAGATGACGCCCGGCGAGCGCAGCCTCATTCAACGGGCCCTGAAAACCCTGGA
CCGCCATCTTCATGAACCCGGCGTGGCCTTCACCTCCACCCGTGCGGCACGGGAATGGCTGATTCTGAACATGGCGGGAC
TGGAGCGTGAAGAGTTCCGGGTGCTGTATCTGAACAACCAGAATCAGCTGATTGCCGGTGAAACCCTCTTCACCGGCACC
ATCAACCGCACGGAAGTCCATCCCCGGGAAGTGATTAAACGCGCCCTGTATCACAATGCCGCTGCTGTGGTGCTGGCACA
CAATCACCCGTCCGGTGAAGTCACACCCAGTAAGGCAGACCGGCTTATCACCGAACGTCTGGTACAGGCTCTGGGCCTGG
TGGATATCCGGGTGCCGGACCATCTGATAGTCGGTGGCAACCAGGTTTTCTCCTTTGCCGAACATGGTCTGCTTTAA
>Cluster_30051
GTGAACAGGGAGCCGCCCGGTTTTTGTCTGCTCTCGAAAACTGGAATCGCCCGCGCTCTGGCCTGGTATCTCCCTGATTC
CTATCTCTGCGTTCTGCTCGACGGACACCATAAGGCTACTGCCGCCGCGCTGGAAGGGCGACCGCTCAAAACACTGGTGC
TCAGCACGGCAACCCATTTTAATGATGAGCAACAAACCCTGGTATTTCCTGGCGGGGAATGTCTGCACAAAACAGAGCTG
CAATACCACATACCAAAACTCACCGCGTGCAAAACATTACCGCCCAACGCATGGGAGAGTTTCGGCCCTGATAAGCACAT
CAGCCCATCCGAAACCTGGTCAGGCCCCCTCACCTTCCGGTTGATGGCGCAAAACCGAACGCCGCAAGCAGATGCTTTTT
TTCTGGATTTTGCCATCAATGATGACGGGGAACGTCCGGAGCTGACGAAAATAATGGACGACTATTTTCGCAAGCCTTAA
>Cluster_35178
ATGGCTGAGATTGTTGAAGGAGTGCATTACCTTACGGTTGATGATCTTGTTGAAATCAATCATTCCCTAATTGAATTACA
GACGCCAGATGAGCCCGTTGGTGTTCTGAGTCCAGATAACTTAAGTTCTTCTCAGGCCCGTCCCAGCATGGTTCGATGGT
ATGAACAGACTAATGACATGTTTGTACTGGCATCGGTATTGATTGAAAGTCTGATTCAGAATCATCCGTTTGCTAATGCG
AACAAACGAACAGCTATGATGGCTGGTTACGTCTTCTTGTTGAATGGCTATGAGTTAACAGCACCAGGCGATGAAAT
CGTGGAAATGGCAGAGGGACTGGCCTGCAAAACCTATACTCGAGAAGATCTCGAGAACTGGTTGTGTTATTGGTCTCGCG
CGTATGACAGCCGGGAATTATGTAAAACAAGCGCAACTATTGTTTTGTATGAAACTATCAAGCTTAAAATAGAACAGCAA
AACTAA
>Cluster_27331
ATGGAAATGACCAACGCGCAACGTCTTATTTTGTCTAACCAGTACAAGATGATGACTATGCTCGATCCGGCAAATGCTGA
ACGTTACCGTCGCCTGCAAACAATCATTGAGCGTGGTTATGGATTACAGATGCGTGAACTGGATCGCGAGTTTGGCGAGC
TGAAAGAAGAAACCTGCCGCACTATCATCGACATTATGGAGATGTATCATGCGTTGCATGTTTCCTGGTCTAATTTGCAT
GACCAGCAATCCATCGATGAACGTCGTGTCACCTTCCTCGGCTTTGACGCCGCCACTGAAGCACGTTACCTCGGTTATGT
CCGCTTTATGGTTAATGTGGAAGGGCGCTATACCCATTTTGACGCTGGAACTCACGGTTTTAACGCCCAGACGCCAATGT
GGGAAAAAATATCAGCGCATGCTTAATGTGTGGCATGCCTGCCCGCGTCAGTACCATTTGAGCGCCAACGAAATTAATCAA
ATTATTAACGCCTGA
>Cluster_32373
ATGTCTCAACAAGAATACACTATCCATATCTTCGGTCAGGACCTCACCAGTCAAGACGGATTGTTCCGATTGGATGAAAT
TCGCCGGGTCGGTATTCAGATTGGTAAGGTAGAAGACACCGAGAAGACAATGGTTAAGCGTTTCGCTCAGACCAAAGTCG
GGCGCTATCTTATGGGGCAGAAAAACCGCCGCATAAAGAAAGTTGATATGGGTCGCCTCGGTACGTCATGGCTTGCTGAC
CCCACGTCCGCCGTCAAGTTTGGCGAGTGGCTGGACTTTGAGTACGGAATAGCTGTAGCGGATGCCTTTGTGTCTATGAC
CTCCAGTGCCAGCGTACAGGCCGCTGTAGCATCTTCGGACGACATCACGCCGGACTCTAAGGCATTCATTAAGAAGCACA
CGGTATCGCACTACAGGGCTGGACAACTCGTCTCGAAAGAGGAGTTTGAGGAGCTACGTGTCGGTCGCTGTGAGTGGAGA
CTTTAA
>Cluster_22297
GTGAGCGAAATTAATTACCAGGCACTGCGTGAGGTGGCGGAACGTGCAATTCCAGCAATGGAACGCCTGTTAATGTTGCC

AGCTGATGATGACTTGTTAAGTGAACAGGAACTTAAAGATTACGGTGTGGATATTGATGCGCTCAACGCCTTCAAATTTC
TGACCGGACCAGAAACCGTGCTGGCACTGCTGGATGAACGGAAAGAAACCTGCAATACATCAAAAGCCGCGATCAGGAG
AACGAGGATATTGCGCTAACGGTAGGGAAGCTGCGCGTTGAGCTTGAAGCAGAAAAACAGCGGGCAAAAGTTCTATTTAT
GGAAAATGCTCGGCTTAAGTCAGGCATAGCCGGTCTGATACACCTCGGTATTCGATATGCAGATGTTGAGGTCATGAAAA
TTGCTGGAGATGCCCAGCTTTCTACCCCATGCACTGACAGCATCATAAACAGCATTGCAACAGGCATTCGCATCAAAGGA
GAGTGA
>Cluster_39032
ATGGCAAAACTTACCTTACAAGAGCAGTTGCTCAAAGCGGGACTAGTCACCAGCAAAAAAGCGGCGAAGGTCGAGAGAAC
GGCAAAAAAATCGCGTGTTCAGGCGCGTGAAGCTCGGGCGGCGGTAGAAGAAAATAAAAAGGCACAGCTTGAGCGTGATA
AACAGCTTAGCGAGCAACAAAAACAAGCGGCGTTGGCGAAAGAATATAAAGCCCAGGTGAAGCAGCTCATTGAAATGAAC
CGAATTACCATTGCCAATGGCGATATTGGTTTTAACTTCACTGACGGCAATCTGATTAAGAAGATTTTTGTTGATAAGCT
CACGCAAACGCAGTTGATTAATGGTCGTCTGGCGATTGCTCGCTTGTTGGTCGATAACAATAGCGAAGGTGAATACGCCA
TTATTCCCGCCAGCGTTGCCGATAAAATTGCTCAGCGTGATGCCAGCAGTATTGTGTTGCACAGCGCGCTCAGCGCCGAA
GAGCAGAATGAAGATGATCCGTATGCCGACTTCAAAGTGCCTGATGATTTGATGTGGTAA
>Cluster_46747
ATGAGTATTGTAAAAGAGGAGCACAAAGCCACGCTGCGTAAATGGCGCGAAGAGTTGCAGGCTAAACGCGGCAATCGTGC
AAGTCTGCGGCGTAGCACAACGGTTAATGATGTTTGCTTGTCAGAAGGGTTTCGTTCTCTGCTGATGCAGACCCACACTT
TGTGGAAAATTGAAGCACAAGAGTGGCGTTTTACCGCACTGGCGCTGGTCGCCGCAGTTAGCGCCAATGTAAAAGCTATT
GATGAACGGCAGCCTTTTGCCGCGCAGCTGGCGGCGGTAATGTCAAAGGGGCGTTTTACTCGTCTGTCGGCAGTAAAAAC
ACCGGATGAATTACTGCGCCAGTTACGCCGCGCAGTCAGATTATTAAACGGTTCCGTCAATCTTGACTCTCTGGCAGAGG
GGGTCTTCCGTTGGTGCCAGGAGAGCGACGACCTGCTCAATCATCACCGCCGCCAGCAGCGGCCAACCGAATTTATCCGT
ATCCGCTGGGCGCTGGAATATTACCAGGCTGGCGACGCCGATAACGAACAAAATCAATAA
>Cluster_2258
GTGAGCGAAATTAATTACCAGGCACTGCGTGAGGTGGCGGAACGTGCAATTCCAGCAATGGAACGCCTGTTAATGTTGCC
AGCTGATGATGATTTGTTAAGTGAACAGGAACTTAAAGATTACGGTGTGGATATTGATGCGCTCAATGCCTTCAAATTTC
TGACCGGACCAGAAACCGTGCTGGCACTACTGGATGAACGGAAAGAAACCAGCAATACATCAAACGCCGCGACCAGGAG
AACGAGGATATTGCGCTTACGGTTGGGAAGCTGCGCGTTGAGCTTGAAGCAGCAAAATCAAAACTCAACGAGCAGCGCGA
GTATTACGAGGGAGTTATCTCTGATGGGTGCAAGCGTATTGCTGAACTGGAAGCACGGGAAGTTCAATTACCGACTCGCT
ACGACCTTCGATATGGACACCCGATAAATGCAGATGAGCGACAAGTCATGATACCTAAAGAAAATGGCAGTTGGCTTTAC
CTGATTGACCTAGAACACGCATTACGCGTCGCTGACATTCGTATCAAAGGAGAGTGA
>Cluster_55936
ATGAAAACAGCACACATTTATTCACCCGGTTATGCGCTGTTAGCAGGCCTGCTGCTGACCGCCAGCACGGGGGCACGGGC
AGTCGATAACAACCTGCATTTTTACGGCAACCTGCTGAGTAAATCCTGCACGCTGGTGGTGGATGGTGCCAATCTGGCAG
AAATCCATTTTCCCACCGTCAGCAACCGGGATTTGATAGTCGCCGGACAGTCTGTGCATCTGCCGGTTGTGTTTAAGCTG
AAAGACTGCAAAGGGCCAGCGGGCTACAACGTCAAAGTCACGCTGAGCGGCACCGAAGACAGCGGTCAGCCGGGTTTTCT
GGCAGTCGATGACACCTCCACCGCGCAGGGCGTGGGGATAGGGATGGAAACGCTCGACGGCAAGCGGGTGGATATTAACA
ACACTACCGGGGCGACTTTTGCGCTTACCAACGGCAACAATGATATTAATTTCCGGGCCTGGATCCAGGCGAAAAGCGGT
CGCGATGTGACGATCGGTGAATTCACTGCCAGCCTGACCGCTACCTTCGAGTATATTTAA
>Cluster_17753
ATGATCCGTAAACGCATGATCAGGCTGAGAAACGATGGTAACTCGACCATTGCAGACTTTTATGCAGTGGATGAAGAGCC
GCAGACATTTCAGATGGACATATTTAACTGGACTGGATCTCAGGTTGTGGCTGATGCCGCTTTTCAGAACTTCTTTTCAC
TTTCCGGGATCGTTAAAGCTGCTGACGGTAACGCGAATCTCAGCATCAATTCGGGAAATCTTATATTCCCTGCTCAGGAA
GCGCCGAGCCAGGTTCTTTTCAGTACCCGTATTAACGGTACCGTTGGTGGTGCGTCAGGAACGGCAAGGGAATGGATGAT
TCAGACCAGGCGCCCAAATGGCGACATCGTCGGATCCGAGTCAAGCGTGAAGGTGAACGGAACCAGCATCAGTAACCGTG
ACGATGTACTTGCCAGTTTCACCATGAATGAACTTGATCCGTTCACCGTCAGCGGAATTCAGGTCGGCCTTTCAAATGAA
TCAGGCCAGACCATCACGTTGACCTCTGTATCTGTCAGGGTTCAACGCGTCATCAGCATTGATTAA
>Cluster_20787
ATGACTTCGATCCCAACGCTGGCGCTTAATGGTGAGGCTATCCAGCTGAAAAATATGCGCGTGACCGTCTCGCAGCAGTT
TCAGGATAAAGACCAGTCCGGCCAGACAAGTGCCACAACCAAATCAGAACAGGGGGCAAAAGGGAAAGAGCTGCGTATCA
GTGGCGAAATTCCGTTTAAAAGCCCGGAGATCCTGAAGCGTATTTTCGAACTGGCCAGCGCCACGGATGCCGGAGGCAAC
CGCCAGAAATACCGCGTGGCGCATGAAGTGGCCAGAGCCGTGAATTTTCGTGAGGCGACATTCAGCGGAATGCTTGATGC
CCCGCAGCAGGACGGGAAAATGGCCTGGCTGGTCACGTTTACGCTGGCAGAACATATCAGCGTGCAGGAAAAGCGGGAAG
CCAGGGCAACAGGCAAAACGTCTGCCAAAAAAACAGACGGCCGGTAATGCGGGACAATCCGGTGGCCAGAGTGCCGGGGAA
GATGAAGAAAAACTGACGTGGTTTGAAAAACGGGTACTGAAACCCGTCAATGATGCTTTGGGTTAA
>Cluster_24671
ATGAACTCTTTTTTTGAACAATATCACCCTGTCTTTGAAGTTGTCTGCCGTATCCTGGGGAACGGCTGGCGCGTGAACAC
ACTTGATGATTGTCCGTCTCGTATAAAACTGACGTCACCGCAGTTTAAAAATTACTCTGTGCATATTCGGATGGAAAAAG
ACCGATTTTCTGTTGTGGGGAGTGTGGATAGTCGTTCCTGGAGTAGTCCGTATCATGTTTGCACGTTATCCAGGAAGCGG
AATCCCGTTGATATAGCAGCCAATATCGAGCGAAAAATTTTGCTAAATGCTTCGCAGGAGGTGTTACAGGCGATTGAGTA
TGAGAAACGCCAGGCGGCAAAAAAGGACGAAATTCTGATCCTGAAAGGTATGTTATCGCAACTTGTCCAGCTTGAAAGCT
GGTATGGGGCATTGACAGGCTTTAAAGCTGAAAATGGATTGAACGGTAAAGTCACCGAGCAGGGCGAGCGTTATGATTTG
CAGATTAGGGGTTTGAGTATAGATCAACTTGTTAAAATTACAGGATATTTGAAACAGTTATGA
>Cluster_6128
ATGCGAATTAAACCAGACGATAACTGGCGTTGGTATTACGATGAAGAGCACGATCGAATGATGCTCGATTTAGCCAATGG
TATGCTATTTCGCTCACGTTTTGCGCGCAAGATGTTGACTCCAGATGCCTTTTCTCCCGCAGGCTTTTGCGTTGACGACG
CCGCGCTCTATTTCTCTTTTGAAGAAAAGTGCCGCGATTTTAATTTATCTAAAGAACAAAAAGCCGAACTGGTGCTGAAT
GCACTGGTAGCAATTCGCTATCTCAAACCGCAAATGCCGAAAAGCTGGCATTTTGTTTCCCATGGTGAAATGTGGGTGCC
CATGCCGGGCGATGCGGCCTGCGTTTGGTTGAGCGATACTCACGAGCAGGTCAATTTGCTGGTCGTTGAATCTGGCGAAA
ATGCCGCACTATGCCTGCTGGCACAACCCTGCGTTGTTATTGCGGGTCGCGCGATGCAGTTGGGCGACGCCATTAAAATC
ATGAACGACAGGCTGAAACCGCAGGTTAATGTTGACAGCTTCAGCCTCGAACAGGCAGTCTAA
>Cluster_41656
ATGTCATTTATTAAAACTTTTTCCGGGAAGCATTTTTATTATGACAGGATAAATAAAGACAACATCGATATTAACGATAT
CGCGGTTTCCCTTTCAAATATCTGTCGCTTTGCCGGCCATCTTTCACACTTCTACAGCGTCGCCCAACATGCGGTGCTTT
GCAGCCAGCTGGTACCGCAGGAATTTGCTTTTGAAGCGTTAATGCATGATGCAACAGAAGCGTATTGCCAGGACATCCCG
GCTCCACTGAAACGCCTTCTTCCTGACTATAAACGGATGGAAGAAAAAATAGACGCCGTAATCCGTGAGAAATACGGGTT
ACCCCCGGTTATGAGTACACCCGTGAAATATGCCGATCTCATCATGCTGGCAACCGAACGCCGCGATCTCGGGCTTGATG
ATGGCTCTTTCTGGCCTGTGCTGGAAGGTATCCCGGCAACAGAGATGTTCAACGTGATTCCACTGGCTCCAGGCCATGCC
TACGGGATGTTTATGGAACGCTTTAACGAATTATCGGAGTTACGCAAATGCGCATAA
>Cluster_430

ATGAACAGGAAGGCGTTTCTGGCCTGCGTGCTGATGTGCGTCATATTAACCGGCTGCGAAACGGCGAAAAAAATCAGTGA
GGTGATCAAAAACCCGGATATCCAGGTCGGGAGCCTGAAGGAGCAACCTTCGGAAATAACCGTCACCCTGCTGACCGAAC
CGGACACCAATACCAATGCGGAAGGTGAATCAGCCGCCGTGGATGTGCAACTGGTGTACCTGACTGACGACTCAAAATTG
CTGGCAGCCGACTATGACCAGATAGCCAGTACGCCGCTACCGGATGTGCTGGGTAAAAACTATATCGACCACCAGGATTT
CAACCTGTTGCCGGACACCATCAAAACCCTGCCGCCGGTAAAGCTGGATGAGAAAACGCAGTTTATTGGTGTCGTGGCCT
ATTTCTCCGATGACCAGGCCACTGAATGGAAACAAATCGAGACGGTGGAAGGAACCGGCCACCATTACCGCCTGCTGGTG
CATGTACGTCAAAGCAGCATCGAAATGAAAAAAGAGGATGAATAA
>Cluster_14964
ATGATATTTAACCGTAATAAAAAACGAGATGAACAACAGAATTCTTCTGTTGTTTTGCCATCAACTGAAAATGTATTCTC
TGATTTTATTAAGAACAATCCAGCTTTGACAACCAATGCTTTTACAGCCATTGGGGAGGTTGATTGTTTTACTAAAGTAC
CCTTACTCTCAAATAAGGAATCAGAGTTTTTAGGCATTCTTTCACGTAATATTATACCTGATTATTCCGTTTTTCCCAAA
ATACGACTCATTGAATTTGTTCGTCCTCATGGTAGCGAGGAAGCTGTAAAAGAATTAATCCGTGAAGTGCAGAATATAAC
CTGTGATTTTGTCCTGAAATCATATGATGAAACAGTCCTTGCTGTTATCCAGTTTGGTGAAACTGCAACCGTCAGACAGC
AAAAGAAAAAACTGATTATCCAGCGTGTGTGTGCTATGACAGAAATTTCTTTTTTCGAATTTAAAAATACTGTCGATATG
ATGGGGAGCGAAGACTTTTGTCAGTTCCTGCGAGATGATGAAGAGTAA
>Cluster_14047
ATGATAATGAATACTTTCAGAAAAGGACTTGATTTACTTCTCGGCAGCGTTTGTTGTTTAATGCTGGCTATTATGGTCGG
TATTGCTTGCTGGCAAGTTATTAGCCGTTACATCCTGGATGTACCTAGTACCCTTACAGAAGAAATGCTGCGATTCTCAC
TGGTATGGGTATCTATGCTAGGAATGGCTTTCGTTGTTGGGAAAAAACAGCATATTAGTTTAACAATATTACTGGATAAA
GCTCCCACAACATTACGCAGGTGGTGGGAGATTATTCTACAGATTATTTTATTGCCTTTTCTGTTTGGTATTAATTAT
CGGAGGCTTAAGGATATCGGTGGTTTCCGCATTACAGGTATCTCCTGCCCTAGGCATTTCAATGTCCAAGATTTATTACG
CACTCCCTGTTTCTGGAGTGCTAATTATTATCTATAGTTTGTTAAATATTATTGAGTCACTAAAAGATAAAAGTTCAACA
ACGGATGCTACTTTACATTCTTTAGAGAAATCACATGACTGA
>Cluster_55579
GTGGCACTGGGGCGAAAACTGGAATCGCCGACGCCGTTCACCGTCAACTCAGTGGGCTCTGCTGGCGCCAGAAAGAACAA
CCTCCGCGCAAAAGTCTTTGTGCGCGATGTCGCCGCTGAATATCTCGAACCCTTTGAATTTGGTGGTGAACATAAACTGA
ACAGCCAGGCGCTGCTCAATCCAAAGAACATCAAACTGAACAAATACGGCAACATGCCGCGAAATAAGCTGTCGCAGTTG
AAAGCGAAGCCGAATGTGTTCGTAGGTGAGGTCAATGGTGTTGATGCTGTCTGGCAGAGGCGTAAACCGAAGAAGGCGAA
AAAGAAACGAGCCCGGCGCTCAGCGAATGGTACGCGCAGACCAAAGCGGAAACAGCGTGCTCCTAAGCTGCTGGTGAGGT
TTGGTGATGCTCTACCTGTTACGCCTACGCTTGACTACATGAACCGCTCACGGTCAATGGCAGCCGGCTTAATGCCCGGC
GCGCTGAGTAGGGCGATTGAGGAAGCGATTCGAACGGCAAAATGA
>Cluster_22912
ATGCCATCTACACGCTATCAAAAAATCAATGCCCATCACTATCGCCATATATGGGCCGTTGGTGATATTCATGGTGACTA
TCAATTATTACAATCCCGCTTACATCAACTCTCTTTTTGCCCTGAAACCGACTTACTTATTTCTGTCGGCGATAATATTG
ATCGTGGGCCGGAGAGTCTTAACGTGCTGCGCCTGCTAAACCAGCCCTGGTTTACATCGGCTAAAGGCAACCACGAAGCG
ATGGCGCTTGAGGCATTCGAAACTGGCGATGGAAATATGTGGCTTGCCAGCGGTGGTGACTGGTTTTTCAATTTAAATGA
TTCAGAGCAACAAGCGGCAATAGATCTGTTGCTGAAATTTCATCACCTTCCACATATTATTGAAATCACTAACGACAACA
TAAAATATGTCATCGCACATGCAGATTATCCGGGGAAGGAGTATCAGTTTGGCAAAGAAATAGCGGAGAGCGAATTACTC
TGGCCTGTTGATCGTGTGCAGAAATCACTTAATAGCGAATTACAAAAATAA
>Cluster_24907
ATGTCTGAACCGCAACGGCTGTTCTTTGCTATCGACTTACCTGCAGAAATCCGCGAACAGATTATCCACTGGCGCGCCAC
ACAGTTCCCACCTGAGGCGGGACGTCCGGTCGCCGCCGATAATTTGCATCTGACTCTGGCATTTTTAGGCGAAGTGAGCG
CCGAGAAAGAGAAGGCGCTTTCTCTTTTAGCCGGGCGGATTCGTCAACCGGGTTTCACACTCACACTTGATGACGCCGGT
CAATGGCTGCGTTCGCGTGTGGTGTGGTTAGGGATACGTCAGCCACCACGCGGCTTAATCCAGCTGGCGAATATGCTCCG
TTCACAGGCTGCCCGCAGCGGTTGTTTTCAAAGCAATCGTCCGTTTCATCCACATATTACCTTATTGCGCGACGCCAGCG
AGGCGGTGACAATCCCGCCGCCAGGTTTTAACTGGTCGTATACGGTGACGGAGTTCACCCTTTACGCCTCCTCGTTTGCC
CGTGGACGCACACGCTACACGCCGCTAAAACGCTGGGCGCTAACGCAATAA
>Cluster_9190
GTGACAACTATAGTAAGCGTACGCCGTAACGGCCATGTGGTCATCGCTGGTGATGGTCAGGCCACGTTGGGCAATACCGT
AATGAAAGGCAACGTGAAAAAGGTCCGCCGTCTGTACAACGACAAAGTCATCGCGGGCTTTGCGGGCGGTACTGCGGATG
CTTTTACGCTGTTCGAACTGTTTGAACGTAAACTGGAAATGCATCAGGGCCATCTGGTCAAAGCCGCCGTTGAGCTGGCA
AAAGACTGGCGTACCGATCGCATGCTGCGCAAACTTGAAGCACTGCTGGCAGTCGCGGATGAAACTGCATCGCTTATCAT
CACCGGTAACGGTGACGTGGTGCAGCCAGAAAACGATCTTATTGCTATCGGCTCCGGCGGCCCTTACGCCCAGGCTGCGG
CGCGCGCGCTGTTAGAAAACACTGAACTTAGCGCCCGTGAAATTGCTGAAAAGGCGTTGGATATTGCAGGCGACATTTGC
ATCTATACCAACCATTTCCACACCATCGAAGAATTAAGCTACAAAGCGTAA
>Cluster_21599
ATGAAATACAATAACGTTATTTTCCTCGGTTTATGTCTGGGGTTAACCACCTATTCTGCTTTATCCGCAGATAGCGTTAT
TAAAATTAGCGGGCGCGTTCTCGATTATGGCTGCACAGTCTCATCGGATTCGCTTAATTTACCGTAGATCTCCAAAAAA
ACAGTGCCAGACAATTTCCAACGACCGGTAGCACAAGTCCAGCCGTCCCTTTTCAGATTACGTTAAGTGAATGCAGCAAA
GGGACAACGGGGGTTCGGGTTGCATTTAACGGTATTGAGGATGCAGAAAATAATACTCTGTTGAAACTGGATGAAGGAAG
CAATACGGCCTCCGGTTTGGGTATAGAAATACTGGACGGAAATATGCGTCCGGTGAAATTGAATGACCTTCATGCCGGGA
TGCAGTGGATCCCATTGGTACCAGAACAGAACAATATTTTGCCTTACTCCGCTCGTTTGAAGTCAACTCAGAAGTCCGTC
AATCCAGGACTGGTGAGCGCTTCGGCAACCTTTACCCTTGAATTTCAATAA
>Cluster_17757
ATGAGTAAAGACCTGTTCGACATGAAGCGCCTTCCGGTTGACCGGGTTGCTGCGAGAGTTGTTGGTAAGGGCGTTGATTG
GACGCCAAATAAAGTTATTCAAACTGGTGACAGCGATCTTCCGTTGCCAGTATTTCCTGCATATAACATTAAAAACCCGC
AACAACGACGCGAAGTGCAAGCAATGATCGGAAGAAAGAGCGGGAGATTGACAATAATTGGTCTCGCGGTCACACAGGGA
GCAAAAAAATAAAGGGGCTCGCTATGTTGCTCGTTGTGTTTGCGGGATTTATACGTATCGCAGAGCAAAAAATTTCAAGAA
TAATTCTGATGATTTTGACGGATGCGAGCGCTGCAGAGAACTGTTGTTTCTCAAAAGGGAAGAAGTTAAACGCAGAACGG
GAAAATGGGTTGACTGGAAAGAGCTGATGTAA
>Cluster_24805
ATGAAAACTGAACTGACCCTGAATGCCCTGCAGTCCATGAACGCACAGGAATATGAAGATATCCGTGCTGCGGGAAGCGA
TATGCGCCGTAATCTCACTCACGAGGTGATGCGTGAAGTGGACGCACCGGCTAACTGGATGATGAATGGCGAGTATGGCA
GTGAGTTCGGGGGCTTTTTCCCCGTCCAGGTTCGTTTCACGCCAGCCCACGAACGTTTCCACCTGGCATTATGTTCGCCG
GGAGACGTCTCTCAGGTCTGGGTGCTGGTTCTGGTGAATGCCGGTGGTGAGCCGTTTGCGGTGGTTCAGGTGCAGCGGCG
GTTTGCACCGGAAGCTGTCAGCCATTCACTGGCACTGGCGGCGTCACTGGATGCGCAGGGGTACAGCGTTAACGACATCA
TTCATATCCTGATGGCGGAAGGAGGTCAGGCATGA
>Cluster_8247
ATGCCGGCAGAGCAGCGGCTTGCTGAACGTCAGCGAAAAACGAAACCGCTGTTGAAATCCCTGGAAAGCTGGTTGCGTGA

AAAGATGAAGACCCTGTCGCGACACTCAGAGTTGGCGAAGGCGTTCGCGTACGCACTTAACCAGTGGCCGGCACTGACGT
ACTATGCGAACGATGGCTGGGTGGAAATCGACAACAACATCGCTGAAAATGCCCTGCGGGCGGTCAGTCTGGGTCGTAAA
AACTTCCTGTTCTTCGGCTCTGACCATGGTGGTGAGCGGGGAGCGCTACTGTACAGCCTGATCGGGACGTGCAAACTGAA
TGACGTGGATCCAGAAAGCTACCTTCGCCATGTGCCTGGCGTCATAGCAGACTGGCCGGTCAACCGGGTCAGCGAACTGC
TTCCGTGGCGCATAGCACTGCCAGCTGAATAA
>Cluster_48594
ATGAACACCGATATTTACGAGAAAATAATGTCTGATCTCGAGTTTGACAGAGACAATCTGGAAGACGTCTGGCGTAAACA
ACCTCGCCTGTTGATGGAGTATGGTTCAAAGCTCGCCCATGCAGAAAGAAGTGTCGCAGAGGCAAAACTTAACCTTGAAG
CTGTTGAAGCAAAGCTATACGACACAGAGCGTAAAAACTTGAGTATGAACGGCATTAAGTTTAACGAATCTGTACTAGAC
GCTAAGGTTAAAACAAACCCACAGTATCTGTCTAAACGGCAGAAGTTGGATGAAGCACGGCACATCGCAGACATATACAA
ACATGCTGTCGCCGCCTTTTCGCATCGCCGAGACATGATCGTTCAGGCGTCGAAGATGGCTATCGTTGAATTAGAGCGAT
TAGGCTCTGAACGCTTTATTACTCCCCGTTGA
>Cluster_48829
ATGGCATGGCACACAGTTCAAGAGACACTGAAATTAACAGGAAAGTCGCGCTCGCAATTATATCGTGACATGGCTAAAGG
CCTTGTCAGCTATCGCACAGGAAAAAATGATCGCCGGGAGTTCGAGACCAGTGAACTGATTCGGGCCTATGGCGAGCTGC
GCACTTCTGAGACACCAAAAAGGCACAAGGAGGGACACTCCAAATCTGTCGTTAGCCAGGCGGAAAATGCTCAGCTAGAC
GTCATTCAGCAGAAACTGGACGCTCTGCAGCAGACTGTTACCCTAATGCTTGAAGACAAGATGGCGCGGGAAGCAGAAAA
CCGCCTGCGAAATGAGGAGAGGGAACAACTACAGGCCGAAATTAGCCGTCTGGCTGACGCGCTGGCACAGGAAAAAAAGC
GGGGATTCTGGTCGCGGCTGTTTCGCTCCGGTTCAGAGTAA
>Cluster_45623
ATGACCACCACAACGCCACAACGTATTGGAGGCTGGTTGCTTGGCCCGTTGGCCTGGCTTTTAGTCGCTCTGTTGAGTAC
GACGCTGGCGTTGTTGCTGTATACCGCTGCGTTATCTTCTCCGCAAACATTTCAAACGCTCGGCGGACAAGCACTGACGA
CACAAATCTTATGGGGCGTTTCTTTTATTACCGCTATCGCCATGTGGTATTACACACTGTGGTTGACCATCGCTTTCTTT
AAACGTCGGCGTTGTGTTCCTAAACACTATATTATCTGGCTGCTTATTTCCGTATTGCTGGCAGTAAAAGCATTCGCTTT
TTCACCCGTGGAAGATGGCATAGCCGTTCGCCAGTTGCTGTTTACTTTGTTGGCAACCGCATTGATCGTCCCCTATTTCA
AACGCTCGTCGCGGGTAAAAGCGACGTTTGTGAATCCGTAA
>Cluster_55036
ATGCGAGGGACAGTCGAAATTATGCGTTATCCCGTCACTCTTACACCCGCGCCGGAAGGCGGTTATATGGTTTCTTTTGT
GGATATCCCTGAAGCGTTGACGCAGGGCGAAACTGTCGCTGAAGCGATGGAAGCGGCAAAAGATGCTTTACTGACCGCAT
TTGATTTTTATTTTGAAGATAACGAGCTTATCCCTTTACCTTCGCCATTAAATAGTCATGATCACTTTATTGAAGTGCCT
TTGAGCGTCGCCTCTAAGGTATTGCTGTTAAATGCTTTTTTACAGTCAGAAATCACTCAGCAAGAGTTAGCCAGGCGAAT
TGGCAAACCTAAACAGGAGATTACTCGCCTATTTAACTTGCATCATGCGACAAAAATCGACGCCGTCCAGCTCGCGGCAA
AGGCGCTTGGCAAAGAGTTATCGCTGGTGATGGTTTAA
>Cluster_19624
ATGCCTGTTACCGAACATGCGCAGCGTCGTGCTGCGCGTGCGGATGCGCTCATCACCGCACAACTAGCCTTTGCCCTGAA
TGGGTACAACTGGGATGAACTGGCCGAACCTCATCGCAAGACCCTTAATCGTGAGGCCATCATTCGCGAGGTATCTACAC
CGCTGGAGCGATATTGCCTGCTGAATGATTGCGAGCCGTGGGAGTGTCTGGCCGCTGTACTGAACCGCATGAAAAGGCCG
GATGGCTCGCGTCGCTGGAAAGCCGTCCGGTTGTCCGACCGGGTTTTCAATGGCCGGGTGCATCCAGTGCGCAAGACTCC
TGATGAAGTTTACGCCGCCATTGCCCGCCGCTTGTTCCTGCCGAAGCCTGTGCATCAAGAAGTGATACAGCACATCCGTG
CGCTTGGCTTGTTACCAGTTGGAGATAACCACCAATGA
>Cluster_20758
ATGGCGATTAGCCAGGAACAACAAAAGCGCGGACTGGAACATTTAAAACAGATCCGCCGCAAATACTTCAGTGAAAGCAG
CGAGGCCGCTGAATGGTGGGACAACCTGACACCGGAGTGGCGCGGCGTGGTGCTTCATGCCGCTGCGGTTACTTCTGGCG
CCAGGGCATTTAAAGCCCACTTAAGCAAGTGCTGCTGGCGTGAGCTTTATGAACGCCTGGGCTATCGGGACATGATTGTG
CTGCGCCAGGGCATTTCACGGGCGCGGTTAACGTTTGATGGTTTCGGCAGTTTACGTGACAGCGATTTTTCGAAGCGCAC
CGCGAACCGTCCGATCAAGAAGGTACATCCAATTTATAGCAGTAGCGGGGTGCAGATGGTAATCGCGCCTCATATCGTCC
AAAAGTTGCAGCAACAGGGGAATCACTAA
>Cluster_37733
ATGAAACTGGCCCTGACGCTGGAAGCCGACAGCATTAATGCGCAGGCGCTGAACATGGGGCGCATTGTCGTTGACGTCGA
TGGTATTAAGCTCGCTGAACTGATTAACAAAGTCGCTGAAAACGGTTATTCACTCCGCGTGGTGGATGAACATGACGCTA
CCGAAACACCGGCGACTTCTGCCAGCCCTCATCAGTTGCCGGAAGACAATACAGTGATACAGAGCGTGATGCCATTAACC
ACTGAAACAGCGCTGGATATCCTCATTGCGTGGCTGCAGGACAATATTGACTGCGAATCGGAAATTATCTTTGACAACGA
TGAGGACAAAACGGATTCGGCAGCACTGTTGCCCTGTATTGAACAGGCCAGGGAGGATATCCGTACCCTGCGCCAACTGC
AGCTTCTGTACCAGAACCGGTGA
>Cluster_63887
ATGTTTGGTATTTTCAAAAAGAAAACCCGCAAGGCCATTACCGAAGTGAAGAAGATGGAGAACCGCGATGCAGTGGAGGC
GACCGTCTGGGGTGCGTATTCCATAGCATTCGCCGACGGCACCTGTGATGCGAAAGAAATCGCTGTGCTGGAGAAAACCA
TTGCAGCACTTCCTGCCTTTGCGCCGTTCTCGGGTGAGATTGCACAAATGAGTGCAAATATCCGCGCCCGTTATGAAGCG
TCACCGCGTTCTGCCAATGCCGAAGCCCTTCGTCAGCTGGCTGATGTTGCCGGTACTGATGATGCAGTTAACGTGCTGTG
TCTGTGTCTGGATATTGCTGACCAGGACGGCATCGGTCCTGATGAAGAAGTGCAGCTCAAGAAAATTGCACAGGCGTTGC
AGTTGCCGCTGGAGCAGTACCTGTGA
>Cluster_35315
ATGGACAAATATGAATTTAGACGACAGCAACTCATCAAAATTCGTGATGAGAAATGCGATGGTAAAGCGGTTAACGTGGC
CAGAAAGATCGGGCGCGAGCCTTCTTATGTATCAAGAATGTTGTACCCAGAGGGGAAAAAGGGAAAAAAACGGATCGCTG
ATGATATGGTGGAGATTATCGAAGAGTCCTTTGGGTTACCCCGGGGATGGATGGATGGTATCGTTTCATCATCAACGAAC
ACAGCCTCCAGTTATGAAACAAGGGTTCTAACGCCACGACAACGTATTTTTTAGATCTCTTAGACGAACTGCCAGAAAG
TGAAGCGGATAAATTATTAAAAACTCTTGAAGAGAAAAAACAGTATTACAATATGATCTACGAAGAAATCCGTAAAAAGA
AAGCACAAAACGCATCATAG
>Cluster_63626
ATGGTTCATGAAGATAAAGCGCGTAAAGAGTTCGCCAGTAGGCTTGCGCTAGCCTGTGAAAACGCTGGTTATGAACAACA
TGGAAGGCAGGCAGAAATTGCCCGTCGAATGAAATTAACACCAAAAGCGGTTAGCAAATGGTTTAATGGTGAAACAATTC
CTCGCCGAGAGAAATTAAGGGAATTAGCAACACTCATTGGAACAACACCAACCTATCTTTTGGGAGAGGATACAGAAGAA
AGTGGACAGATACGTTTCTATCAGGAGTTAAATCCAAGACAAAAAATCATCATTGACCTTCTGGACGAGCTCCCTGACAG
TGAGACAGATGAACTTTTAAAAACTCTTGAGGAGAAAAAACAGAAGTACAATGCAATTTACGAAGAGTTAGCACGAAAGA
AAAAACAAAAAGCCTCTTAA
>Cluster_3459
TTGACCACAACTGTACCTATTCATCCGTTACCTGAAATTCTGCTGCTAACTCACGGCGGCTGGGGGCTGCAGCTCTGCAA
CAGTCTGCGCATGGTGATGGGCGAAATCGAAGGCGTGACGGAAATCGCGCTGATGCCCGTCGATACGCTGGGCGAGTTTT
ACCAGCGCGTCGAAGCCGTGGTGAAAACCATGCCGGAAGGTTCGCTTATCGTGACCGATTTTATCGGCGGCACCACCTCT

AACGTGGCGGCGCGGCTGAGCGCCGATTATCCGGTGGCGGTGATCTCCGGGCTTAATGCCTCGCTGCTGCTGGAGGCGCT
GGACAAGCGCGAACATGGCCCACTGACCGCCTGCGTGGCCGATCTGGTGGAAGCTGGGCGCAACAGTTGCCTGGACGTCG
TCTCCCATATTCGTCAATTACAACAGTAA
>Cluster_3315
ATGTCGACACCTTCCTTATATGAAATGCTGACGTTCAGTTTCAGCGGCGAGTTACCACTGGAACAGGTCAGCGAGCGGGA
TCAGCTTATCCTTTCCGTGATGGACAATATGCAACGCATTATCAACTGTCGGGCAGGCACACTGGCGCATTTGCCGGATT
ACGGTCTGCCGGATCTCAGTCTGATCCATCAGGGTATGGTTGCGGGTATTCATGGCCTGATGCGTCAGATTGAAGAAACG
CTGCTGCGTTATGAACCACGCCTGAGTCAGATACAGGTGGAGTTACTCCCCCAGCCCCGTCCGGGGTATCTTAATTACCT
GATCCACGCGCAGCTTCCCGATACCGGCTGGATACGCTTTGATGGCGTGTTTTCTCCGGAAGGACGAATTGTTCTGCGTC
ATCTCAAACAGCAGGAGCGGGCGTACTGA
>Cluster_54478
ATGAACCGGTTGCTGCTGGTTGTGCTGGCGTTATTACTGGCGGCGCTGGGCTGGCAGACGTGGCGGCTGGCTGATGCCAG
CCAGACCATCAGCACGCAGGCAGACGAGCTGCAGAGCAAAAGCCAGGCACTGGCAAAGAGCAACAGCCAGCTTATCAGCC
TGTCCATTCTGACTGAAACCAATAACCGGGAGCAGGCGCGGCTCTATGCCGAAGCAGAACAGACCAGCGCACAGCTGAGA
CAACGACAACGCCGGATCGAGGAACTGAAACGTGAGAACGAGGATTTACGCCGCTGGGCTGATACTCCTTTGCCTGCTGA
CATTATCCGGCTGCGGGAACGTCCGGCACTCACCGGAGGTGCAGCTTACCGTCAGTGGTTGTCCGCGAGTGACGCCGTGT
CGGCTGGAGCAGGCAGCACCGCGCACTAA
>Cluster_34620
ATGAAATTAGACCAGCAATATCTAAAAGATCTACTTATCGCATTCGAAAAAACTTATGGCCCTGACACGATGCTTAGTGA
ACTAGAGGATAATGGCTTTAATAGATATGACCAAAATTTTATTTTCCATATGCGATTATTATGTGACTACGAATTAATAG
TTAGGGTTGATGGAAAACCTGGGTTCGGTCATATAATGTCCAACGAGTTAGGGGAAGGTGTTGAATATAGTTGGATCGAA
GTACCACTGAGGTTGACAGCAAGAGGGCATGATTTTATTGCTGACTTACGTCAAAAGGAGGTCTGGCAAGCTATAAAAAC
AAACTTTAAGGATGAGGGGATTAGTACTCTTATGAGTGTTTCAAAATCACTAGCAAAAGGCTTTGCAAGAAAAAAAAATAA
AAGATATTACAGGAATAGATATTGAATAA
>Cluster_54473
ATGAAGTTTGTTGCGCTAGAACAGGCACCGGAACAGGCGGAAATCATCAGAAATACGCCGTTCTGGCCTGATGTGGACCT
GTCGGAGTTTCGCAGCGTGATGCGCACTGACGGCACGGTGACGCAGCCGCGTTTAAAGCAGGTTGCGCTGTCGGCAATTT
CGGAGGTCAACGCAGAGCTGTATGAGTTTCGCAGACGTCAGCAGATGCTGGGGTATGTGTCGCTGGCTGAGGTTCCGGCT
GAACAGCTGGACGGCAAAAGTGAGCGCATTCAGCACTATTTCAACGCGGTTTACTGCTGGGCACGCGCCATGCTCAACGA
ACGTTACCAGGACTATGACGCCACGGCATCCGGTGTGAAGCGGGGCGAAGAACTGGCAGAAGCCAGCGGTGATTTGTGGC
GTGACGCCCGCTGGGCCATCAGCCGGGTGCAGGATGCGCCGCACTGCACAGTGGAGCTTATCTGA
>Cluster_14970
ATGAGCGAACTGACTAAAGAAGATGAATACGGCATTATCAGCCGGACTATGATGAATATTCGTTCATTGCGTGTTTTTGC
CCGTGAGATTGATTTTGAGCAGTTGCTCGAAATGCAGGAAAAGCTCAACGTTGTTATTGAAGAACGTCGTGAAGATGCTG
AACGTGAAGCGGCTGAACGCCAGGAACTTGAAGCCAAACGTCAGCAGGCCATTGAATACATCATATCTTTAGGTCTTGAT
CCCGAATCACTCCTTGCTCCTGTAACAGCTGATACAGTGAAAACTAGGCGGAAAGCCAAAGGCGGAGTAAGAAAGGCTAA
ATACCGCTTTAAGGATGAAAATGGCGAGATCAGAGAATGGTCTGGTAATGGCAAGCGTCCGCTTGCTTTACAGAAGCTGC
TTGATGAGGGCCATTTCATGGAAGATTTCCTCATTGACAAACTGCAACCTGGGCAGGTTGAATAA
>Cluster_34150
ATGATAACACAGACGCAGCACGACCTCGCACCCGCTAACCAACCCGAATTTGAACTGACCGTCACGCCGGTCCCCGATGA
ACAGCGTATCGATTTCTGGCCACAGTACTTTGGCGCTATCCCGCAGTGGCTACTCCTGGAGCCGCATATCTTCGCCTGGA
TGGACCGCTTCTGTGAGGGCTACAGCGGTGGTATCTGGTCGTTCTACACCCTCAGCAATGGCGGCGCATTTATGTCCCCC
GAGCCTGACAACGATGAGACATGGCGTCTGTTTAACTGCCTGAACGGTAACGATGCCCAAATGAGTGCAGAAGCAGCAGG
TATTGCTGTCTGCCTGATTGCGTATAGCCACCATGCCTGCCGTACAGAATGTGACGCGATGACCGCACACTATTACCGCC
TGCGGGAGTATGCCATGCAACATCCAGAGGCTCACGCCATTCTGCGTATTATCGACTGA
>Cluster_17745
ATGATTAAAGCTCAGATCGTACCCGCTACAGCAGAGCATATCGAAGCCATTATTCCGCTTGTTCGCCAGGCTGATATCGA
TGAGTTTCTGGCAACCAACGGTTGGAGTCCGCGCCGCGTGCTGGAGACCGGTCTACGCACGTCAACATTTTGCTGTGCCG
GATTGATTAACGGTGAAGTAGTGACTGTCTTTGGCGTAGCACCGGCATCGATGATCGGCGGCAGTGGTATCCCATGGCTG
GTGGGCACTGACGCGCTGGAGAAATACCAGCGTACTTTCCTGCGTCGGTGCGGAAAAGTGGTCAATGCAATGCTGACTGT
TTACCCGTATCTTGAAAATTATGTTGATGCACGCAACCACACAGCGCGCATCTGGCTTCACTGGCTGGGATTCACCATCG
ATGAACCTCAGCCATACGGCATTAAAAACCTACCGTTTCACCGTTTCCACATGGAGAGAAAATAA
>Cluster_29599
ATGCATAAATCAGAAGCTGAAAAAATAGATTCTATTCTGAATGAACATCAACAGATATATCGCAGACATAACCGAATAGA
TAACATCCTGGCGTTTTGTGCGTGTATTGCATGGGTTCCCTGTATTATTGCTGCGTTCGGCGTTGATGGTATATATCTGA
AAATCCTGTTTGCAGTCCTTGCTTGCTGTGGTGCGGTATGCTTTTTTATTTTCTTCTTCACCCTCCTGCCGGAATCACTC
ATGGTCCTCAGCCGTCAGAGCCTGCTTCAGCTTATGCGTCTGACTGAAGACGTACCGGATGCGAGACAGGAACTGCTTAA
CCGTCTGCTGTCCGGTAAAAAACTGACTGGTCGGGATGAAAAGGATATCCGGCGTCTCTGGCAGGAGAAAGTGGATGCAA
TGCAGGAGTCTGCCACCCGTCAGCGTGAACAGGATACTATTCGGAAGTTTACTGAAGGGATTAAATCCGAATGA
>Cluster_33854
ATGAAATACAAGCATTTAATCCTGTCTTTAAGCCTGATAATGCTGGGGCCATTGGCTCATGCAGAAGAGATTGGTTCGGT
CGACACCGTATTTAAAATGATCGGCCCGGATCACAAAATTGTTGTTGAAGCCTTTGACGATCCCGATGTGAAAAATGTCA
CCTGTTATGTGAGCCGGGCGAAAACCGGTGGTATTAAAGGGGGATTGGGCCTGGCAGAAGATACCTCCGATGCGGCCATT
TCTTGTCAACAAGTCGGGCCGATTGAACTGTCGGATCGTATTAAAAACGGCAAAGCTCAGGGCGAGGTAGTATTCAAAAA
ACGCACGTCCCTGGTCTTTAAGTCGTTACAGGTCGTGCGCTTTTATGACGCTAAACGCAACGCGCTCGCTTATCTGGCTT
ACTCCGACAAAGTTGTAGAAGGTTCGCCGAAAAACGCGATTAGCGCGGTTCCTGTCATGCCGTGGCGGCAATAA
>Cluster_17054
ATGGCCGACGTTCACGATAAGGCCACCCGCAGCAAAAATATGCGCGCGATTGCCACGCGTGATACGGTGATTGAGAAGCG
CCTCGCCAGTCTGTTAACCGGGCAGGGCCTGGCATTTCGCGTTCAGGACGCCAGTCTGCCCGGACGCCCGGATTTTGTCG
TTGATGAATATCGCTGCGTGATATTTACCCACGGCTGCTTCTGGCATCATCATCACTGCTATCTGTTTAAAGTGCCTGTG
ACTCGAACCGAGTTCTGGCTGGAGAAGATAGGTAAAAATGTTGAGCGGGATCGCCGCGATATCAATCGCTTGCAGGCACT
CGGCTGGCGTGTACTGATTGTCTGGGAGTGTGCGTTACGTGGGCGCGAGAAGCTGACGGATGAAGCGCTTACCGAGCGTC
TGGAAGAGTGGATCTGCGGCGAAGGTGCCAGCGCGCAGATCGACACGCAGGGGATTCATTTACTCGCTTGA
>Cluster_39825
ATGAAAACATTGTCTCAGAACACCACCGCTTCGGCCTGTGCACCGGAGACTGGCCTGCAACAACTGGTTGCCACCATTGT
CCCTGATGAACAGCGCATCAGCTTCTGGCCGCAGCATTTTGGCCTCATTCCACAGTGGGTCACCCTGGAACCCCGTGTCT
TCGGCTGGATGGACCGTCTTTGCGAAAACTACTGCGGTGGTATCTGGAATCTGTACACCCTGAACAACGGCGGGGCATTT
ATGGCACCCGAACCGGATGACGATGATGACGAAACATGGGTACTGTTCAATGCCATGAACGGTAACCGCGCTGAAATGAG
CCCGGAAGCCGCCGGCATTGCTGCCTGTCTGATGACGTACAGTCATCATGCCTGTCGTACGGAGTGTTATGCCATGACGG

TCCATTATTACCGGCTGCGGGATTACGCCCTGCAGCATCCGGAATGCAGCGCCATTATGCGCATCATCGACTGA
>Cluster_22974
ATGGCGGAAATTGGTATTTTTGTCGGCACCATGTACGGGAATTCACTGTTAGTGGCCGAAGAAGCGGAAGCGATTCTGAC
CGCGCAGGGCCACAAAGCAACGGTATTTGAAGATCCTGAATTAAGCGACTGGCTGCCCTATCAGGATAAGTATGTTCTGG
TGGTTACGTCCACGACCGGGCAGGGCGACCTTCCTGATAGCATTGTGCCACTCTTTCAGGGAATCAAAGATAGTCTGGGC
TTCCAGCCGAATCTGCGTTATGGCGTGATTGCGCTCGGCGACAGTAGTTATGTGAATTTCTGCAATGGCGGCAAACAGTT
CGATGCCTTGTTGCAGGAACAGAGCGCTCAGCGGGTGGGAGAAATGCTGTTGATTGACGCCAGTGAGAACCCGGAACCGG
AAACGGAATCAAATCCGTGGGTTGAACATTGGGGCACGATGTTGTCCTGA
>Cluster_6733
ATGGGCAACAATGAGCATGAAGTTATGGCGGACAAACAGAGAGCAGAACGTATAAAAAAGGCAATTCTGGAACACTCGAC
TTATGAAGAACTGGCCGAGAAGACTGGCATTAGCGTAAGTACGCTGGTCAGGATAGCGTCCGGCAAAACTGAACCTAAAT
TCAGCGATATAATTCAGATAGCTAAAATTACTGGCGCAGATTTGAACACCTTAGCTTATGGCTATGCTCTTGATGTTAAA
GAAGAAGCTACTGAGCGAAAGCTGATAACTTCTGCGGACGGATATACTGATGAAGAAACCACGAACGCGCATAATTTCAT
CGTGTGGAACATCAGAACACTGGAGAAGCAAGATATTCTGGCACTTGCAAGGCAAGTTTCTGCATTAAGTTCATATACTT
ACAGTACAAAAATGTTTATGAAAAAAGCATTGTCAGGCGAAGAGTAA
>Cluster_10958
ATGTATAATGCTAATCCCAATTACGAAATGGACTTCATGATTCTGAAAGACGTGAATGAACACATGGAAGGTCTGTTTCA
ACGTTTTTCTAAACTGTTACCTTTCAGAATTGATTTTGCCTACAGGAAAGACACGCCCAGTTTTGGTCATTCCTGTAGGC
ACAGTATGTGTATTGAAATGCACCGCTTACTTAGTGAAACCCAAACGATGCTGGCGGGATATTACTGGGTAATGGAATAT
ACGTCAAACAAGGGACTTCATATTCATTTTATCGGCTACCTGGATGGGCAACGTCACAACAAATCTTATCGGATATCCAG
GCAACTGGGAGATATCTGGAGGCGGATCACGGAAGGGGAGGGTTATTTTCACCTGTGCAGGGCTAAAGACAAATACCCGG
TGCGTATCGATCACGTAATTCACTATAGTGATAAATCTGCGGTATGA
>Cluster_6491
GTGATTCAGAAAATAAATATTTTAGACAGTAGCCAGTCAGGCGTTAAAAATGGGGCGGATAGTGAGGCGGTTTTGGCTGC
GCTTATTGAACATTTAGAATTGATCAATCCTTCTGGCAGATTAAGCCAAAATACCCGTTCGGCAATGCTTCAGTTACGCG
AAGAGGAATGGTCTGAATTCTTTTTTTGGTTGCTAAATTCTCTGGAGTGTCTGGATTATGTGATTATTAATTTAACTCCA
GAAAGCAAAAAAACGTTGATGAGTGAACACCGTAACAATATTCAGGTAGCAATTGATGCTTTATATAGCCAACGTCGGCG
TAAATCGCCGGGTGATGAAAGTGAAACACTTACCCGTAGAAATGACGCTATCTTCGGCAACCACGTATGGCAAACATTTG
CGCAATACTTCCCGCCAGGACTTGAAAAACCGTCTGTGTAG
>Cluster_11531
ATGTTACCCATTAGCTCATCTAATAGTGTTTCACGGCCTCAGAATGAGGCCCACTCCACCTCAGCACAACAAGTGTGTGT
GCAAAAAACAAACAGAACTATAAAAATAACACAACCATTACAGAGACCAAAACTGACTTTAAATTTTGATATAAACCCCT
CCCCCTGCATCGGTAGGGGGCTTGCAGGAGAAGTATTTAAGGATCCAGATAATCCTGGATATGTTATCAAACGTTATAAT
AAAGCGAATTCTAGTGATGCGCAAAAAGAATGTCATTTATTTAACACATATTATGGTGAAGGCAGTGCTGAATTATTTAC
TGAAAAAGGACGTACCTGTTTACGTATGCTGGAGGTTCCTGGTGTACCATTAACGAGAATGAGTCAGTTTCCTGAAAATG
CCAACAAAATGTTTCTTGATATGATCATTGACATGGGGAGAGAGTAA
>Cluster_63662
ATGTTTATTCTTAATCAGAAAATTATCATTGGTGGAGAACGCTGGTTTACGCCAATGAAGGACTTAAAACCTGTAGACGG
GTTAAAACTGTTGGTGGCAAGCAGCGATAACGATCAGTATCGCTCCCGTAATGCATTAATCCGTCGCCACATTGAGAAAA
TGGATGCCAGTTTGCACGTCGGAACGAAGGAGTTTGATATTTCAAAGGTTTCCGAGGTGGATTCTGTTGATGATTTACTC
ATTGATAATGCCGCTCGTTATCTGCTGAAAGACTGGAAAGGGGTTGGTGAACTGGTTAATGGTGTTGAGGTTGCACTGGA
ATATACGGCAGAACGAGGGATCGCGCTGCTTAAGCAGAATCCAGAGTTGTACTGGCAGATCCTTGCAGAAGCAGCCAGCA
TCGCCCAGGGTAAAGAGCAGCAGAAGCAGGATACGATAAAAAAGCCATAG
>Cluster_54400
ATGCAAGACACCATCATAAATTTCTACAGCACCAGCGATGACTACGGTGATTTTTCTAACTTTGCCGCCTGGCCCATCAA
GGTTGACGGGAAAACCTGGCCCACCTCAGAACACTATTTTCAGGCACAAAAATTCCTTGATGAAAAATACCGCGAAGAGA
TCCGTCGGGTTTCTTCCCCTATGGTCGCCGCACGCATGGGGCGCGATCGTTCTAAGCCTCTGCGTAAAAACTGGGAGTCG
GTCAAAGAACAGGTGATGCGAAAAGCACTTCGCGCTAAATTCGAACAGCATGCAGAACTGCGTGCGCTCTTGCTGGCAAC
CGCGCCCGCAAAACTGGTTGAGCATACGGAAAACGATGCTTACTGGGGAGACGGTGGTCATGGTAAGGGCAAGAATCGAC
TGGGCTACCTTTTAATGGAGTTGCGCGAACAATTGGCTATAGAGAAGTAA
>Cluster_43462
ATGAACACGATTTCTTCCCTCGAAACGACTGATTTACCAGCGGCTTTCCACATTGAACAACGCGCCCACACCTTTCCGTG
GAGTGAAAAAAACCTTTGCCAGCAACCAGGGCGAGCGCTATCTCAACTTTCAGTTAACGCAAAACGGCAAAATGGCGGCGT
TTGCGATTACGCAAGTGGTGCTGGATGAAGCTACATTGTTCAATATTGCGGTCGATCCTGACTATCAGCGTCAGGGACTG
GGAAGGGCGCTGCTGGAACATCTGATCGACGAACTGGAAAAACGCGGCGTGGCGACGCTATGGCTGGAAGTCCGTGCCTC
AAATGCTGCCGCCATTGCCCTGTACGAAAGTTTAGGCTTTAACGAGGCGACGATTCGCCGCAATTACTACCCCACCACGG
ACGGTCGCGAAGACGCCATCATCATGGCGTTACCAATCAGTATGTAA
>Cluster_40849
ATGCAGAAAAAAACGCCTGATACAGCTTATCCATATTGCCCGTAATGAACTGGGTATGGATGAAGACACCTACCGCCAGAT
GTTACAGGGGCTGACCGGTAAAGCCTCAACCAAAGGAATGGATACCACACAACTAAACTGCGTGCTGGAATCCATGAAAA
AGAAAGGCTTTCGCGTTAAACCAGCCAGAAAAGCCAGCTCCGGTTTACCGCTGGATAACCATCCGCAGTCCAGGAAAATT
CGTGCGCTATGGCTTGAAATGGCTGCTGCTGGCATTGTTCGTGACCGTTCAGAAAATGCATTAGCGAGGTGGATCAAGCG
GGAAACGGGCATCAGCGCCCTGCGCTGGCTCAGTACTGAACAGGCAAGCAGTGTTATTGAGAAACTGAAGAAGTGGCAGC
GCAGAGCTGCGGGAGTCAAACATGAGCGACCTGAATCAGTTTCGAAGTAA
>Cluster_42307
GTGAGGCGATTGCCGGGGATTTTACTGCTTACTGGAGCCGCGCTCGTTGTGATCGCTGCCCTGCTGGTTAGCGGCCTGCG
TATTGCTTTACCGCATCTTGACGCCTGGCGTCCGGAAATCCTCAACAAAATAGAATCCGCGACTGGCATGCCGGTAGAAG
CCAGTCAGCTCTCAGCCAGCTGGCAGAATTTTGGTCCGACGCTTGAAGCGCACGACATCCGTGCAGAATTAAAAGATGGC
GGCGAATTTTCGGTTAAACGCGTTACTCTGGCGCTGGATGTCTGGCAGAGCCTGTTACATATGCGCTGGCAGTTTCGCGA
CCTCACTTTCTGGCAGCTGCGTTTTCGCACTAACACTCCTATCACCAGCGGCGGTGGTAATGATAGCCTGGAAGCCAGTC
ACATCAGCGATCTGTTTCTTCGTCAATTTGACCATTTCGATCTCCGCGACAGTGAAGTCAGTTTCCTGACGCCATCCGGT
CAGCGCGCCGAGCTGGCGATCCCACAGCTCACCTGGCTGAACGATCCACGTCGACACCGTGCGGAAGGCCTGGTAAGCCT
CTCCAGCCTTACCGGACAGCACGGCGTGATGCAGGTGCGTATGGATTTGCGCGATGATGAGGGGTTGTTAAGCAATGGTC
GCGTCTGGCTCCAGGCGGATGACATCGACCTGAAGCCGTGGCTCGGTAAATGGATGCAGGACAATATTGCGCTGGAAACG
GCACAGTTCTCCCTTGAAGGCTGGATGACGATCGACAAAGGCGATGTAACCGGCGGTGACGTCTGGCTGAAACAGGGCGG
TGCCAGCTGGTTGGGCGAGAAGGAAACACATACGCTGTCGGTGGATAATCTGACTGCGCATATTACGCGTGAAAATCCGG
GATGGCAGTTCTCTATTCCCGATACACGGATCACGATGGACGGCAAACCCTGGCCGAGCGGAGCATTGACGCTGGCCTGG
ATACCGGAACAGGACGTTGGCGGCAAAGACAATAAACGCAGTGACGAACTCCGGATTCGCGCCAGTAATCTGGAGCTGGC
AGGCCTGGAGGGCGTACGCCCGCTGGTCGCGAAACTTTCACCTGCACTGGGTGATGTTTGGCGCTCCACACAACCGAGCG

GCAAGATTAACACTCTGGCGCTGGATATCCCGCTTCAGGCGGCAGACAAGACCCGTTTTCAGGCATCGTGGAGCGATCTG
GCCTGGAAGCAATGGAAATTATTACCGGGTGCGGAACACTTCTCCGGGACGCTTTCCGGCAGCGTAGAAAATGGTTTGCT
TACTGCGTCGATGAAGCAGGCAAAGATGCCTTACGAAACGGTATTCCGTGCGCCACTGGAAATCGCCGACGGCCAGGCAA
CGATAAGCTGGCTGAACAATGACAAAGGTTTCCAGCTGGATGGGCGTAATATTGACGTTAAAGCCAAAGCCGTCCATGCG
CGCGGCGGTTTTCGTTACCTGCAACCTGCTAACGATGAACCCTGGCTGGGTATTCTGGCTGGCATCAGTACCGATGATGG
TTCACAAGCCTGGCGCTATTTCCCGGAAAACTTGATGGGTAAAGACCTGGTTGATTACTTAAGTGGCGCGATTCAGGGCG
GTGAAGCGGATAACGCGACGCTGGTTTATGGTGGCAATCCGCAACTCTTCCCCTATAAACACAACGAAGGTCAGTTTGAA
GTGCTGGTGCCGCTGCGCAACGCGAAGTTTGCCTTCCAGCCGGACTGGCCTGCATTAACTAACCTTGATATTGAACTGGA
CTTTATTAACGACGGTTTATGGATGAAAACCGATGGCGTTAATCTGGGCGGCGTGCGCGCGAGTAATCTCACCGCAGTGA
TCCCTGACTACTCAAAAGAAAAACTGCTGATTGACGCTGACATTAAAGGTCCGGGTAAAGCCGTTGGCCCTTACTTTGAT
GAGACACCGCTGAAAGATTCCCTGGGTGCGACCCTGCAAGAACTCCAGCTCGACGGCGATGTGAATGCTCGCTTACATCT
TGATATCCCGCTGAACGGCGAGTTGGTAACCGCGAAAGGTGAAGTGACGCTGCGTAATAACAGTCTGTTTATCAAACCAC
TCGACAGCACCCTGAAAAATTTGAGCGGTAAATTCAGCTTTATCAATGGCGATCTGCAAAGTGAACCACTGACAGCAAGC
TGGTTTAATCAGCCGTTGAACGTGGATTTTTCCACCAAGGAAGGGGCAAAAGCCTACCAGGTAGCGGTAAACCTCAACGG
TAACTGGCAACCGGCGAAAACCGGCGTTCTGCCTGTAGCGGTGAACGAAGCATTGAGTGGCAGCGTGGCGTGGGATGGTA
AAGTGGGCATTGATCTGCCTTATCATGCTGGCGCGACCTATAACGTAGAGCTGAACGGCGATTTGAAGAATGTGAGCAGT
CACTTACCTTCACCGTTAGCCAAACCTGCGGGTGAACCACTACCGGTAAACGTTAAGGTTGATGGCAATCTCAACAGCTT
TGAATTAACCGGACAGGCTGGTGCGGATAACCATTTCAATAGCCGCTGGTTGCTCGGTCAAAAGCTGACGCTCGATCGTG
CTATTTGGGCGGCAGACAGTAAAACGCTCCCGCCGTTGCCGGAACAAAGTGGCGTTGAACTCAATATGCCGCCGATGAAT
GGTGCCGAGTGGCTGGCCCTGTTCCAGAAAGGCGCGGCGGAGAGTGTCGGTGGTGCGAGCGAGTTCCCACAACACATAAC
GTTACGTACGCCTATGTTGTCGCTGGGAAATCAGCAATGGAATAACCTGAGTATTGTTTCGCAACCGACGGCAAATGGCA
CCCTGGTTGAGGCGCAAGGGCGTGAAATCAACGCCACGCTGGCGATGCGTAATAACGCACCGTGGCTGGCGAATATCAAA
TATCTTTATTACAACCCAAGCGTGGCGAAAACTCGTGGTGATTCAACACAGTCATCACCTTTCCCGACAACGGAACGCAT
TAACTTCCGTGGCTGGCCAGACGCACAAATACGATGCGCAGAGTGCTGGTTCTGGGGGCAAAAATTCGGTCGTATTGACA
GTGATATCACCATTTCTGGCGATACGTTAACGCTGACTAATGGACTGATTGATACTGGTTTTTCGCGGCTCACTGCCGAT
GGTGAATGGGTTAACAATCCGGGGAATGAACGTACCTCGCTGAAAGGAAAACTGCGCGGGCAGAAAATTGATGCCGCCGC
AGAATTTTTTGGTGTCACGACGCCCATACGCCAGTCGTCATTTAATGTGGATTACGATTTACACTGGCGCAAAGCACCCT
GGCAGCCAGATGAGGCGACGTTGAATGGCATCATTCATACTCAACTGGGTAAAGGCGAAATTACCGAAATCAATACCGGA
CATGCCGGGCAATTGCTGCGCTTATTGAGCGTAGATGCCCTGATGCGTAAGCTGCGTTTTGATTTCAGAGACACTTTTGG
CGAAGGGTTCTATTTTGACTCCATTCGCAGCACCGCGTGGATTAAAGACGGCGTTATGCACACCGACGACACGCTGGTGG
ATGGCCTGGAAGCGGACATCGCCATGAAAGGGTCGGTAAATCTGGTACGTCGCGACCTGAATATGGAAGCAGTTGTCGCA
CCAGAGATTTCTGCGACGGTGGGCGTGGCTGCGGCCTTTGCGGTTAACCCCATTGTTGGCGCGGCAGTGTTTGCCGCCAG
TAAAGTGCTGGGTCCGCTGTGGAGCAAAGTCTCCATTTTGCGCTATCACATTTCGGGTCCGCTGGACGATCCGCAAATCA
ACGAAGTGTTGCGACAACCGCGTAAAGAAAAAGCGCAATGA
>Cluster_21521
ATGACAGAACAACAAAAATTGACCTTTACTGCCTTGCAGCAGCGGCTGGATTCGCTGATGCTGCGTGACAGACTGCGTTT
TTCTCGCCGTCTGCACGGCGTGAAGAAGGTTAAAAATCCTGATGCACAACAGGCCATTTTCCAGGAGATGGCGAAAGAGA
TTGACCATGCGGCAGGGAAAGTCCTGCTGCGTGAAGCGGCACGACCGGAAATTACTTACCCGGACAATTTGCCGGTTAGT
CAGAAAAAGCAGGACATTCTCGAAGCGATTCGTGACCACCAGGTGGTGATCGTCGCCGGGGAAACGGGTTCCGGTAAAAC
GACTCAGCTACCGAAAATCTGTATGGAGCTGGGGCGCGGGATTAAAGGGCTGATCGGCCATACCCAGCCGCGTCGGCTGG
CGGCGCGAACGGTGGCGAACCGTATTGCGGAAGAGCTAAAAACGGAGCCGGGCGGTTGCATCGGTTACAAAGTGCGTTTC
AGCGATCACGTCAGTGATAACACGATGGTCAAGCTGATGACCGACGGTATCCTGCTGGCAGAGATCCAGCAAGACCGCCT
GCTGATGCAGTACGACACCATCATTATTGACGAAGCGCACGAACGCAGCCTGAATATCGATTTTCTCCTCGGCTATTTGA
AAGAGTTGCTGCCGCGGCGTCCTGACCTAAAAATCATTATCACTTCCGCGACTATCGACCCGGAACGCTTTTCGCGCCAC
TTTAATAATGCGCCGATTATCGAAGTCTCCGGTCGGACCTATCCGGTGGAAGTGCGCTATCGCCCGATTGTTGAAGAAGC
CGATGATACCGAGCGCGATCAGTTGCAGGCGATTTTTGACGCCGTAGATGAACTGAGCCAGGAAAGCCCTGGCGACATTC
TGATCTTTATGAGCGGCGAGCGAGAAATTCGCGATACCGCCGATGCGCTGAACAAGCTGAACTTACGCCATACCGAAATC
TTGCCGCTTTATGCGCGGCTTTCCAACAGCGAACAGAACCGCGTGTTCCAGTCGCACAGCGGGCGACGCATTGTACTGGC
GACCAACGTCGCGGAAACCTCGCTGACCGTACCGGGTATTAAATACGTTATCGACCCAGGTACAGCGCGTATCAGCCGCT
ACAGCTATCGCACCAAAGTGCAGCGTTTGCCGATTGAGCCAATTTCGCAGGCTTCAGCTAATCAGCGTAAAGGCCGCTGC
GGCCGTGTGTCAGAAGGGATCTGTATTCGTCTCTATTCCGAAGACGATTTCCTCTCGCGCCCGGAGTTTACCGACCCAGA
AATTCTGCGTACTAACCTGGCCTCGGTTATTTTGCAGATGACCGCACTGGGCTTGGGCGATATCGCCGCATTCCCGTTTG
TCGAAGCACCGGATAAACGCAATATCCAGGATGGCGTGCGTCTGCTCGAAGAGCTGGGCGCGATCACCACTGATGAACAG
GCCAGCGCCTATAAACTGACGCCGCTCGGTCGCCAACTCTCGCAGTTGCCTGTCGACCCACGTCTGGCGCGTATGGTGCT
GGAAGCGCAGAAACATGGCTGCGTGCGTGAGGCGATGATTATCACTTCCGCGCTCTCTATTCAGGACCCGCGTGAGCGTC
CGATGGATAAACAGCAGGCATCTGACGAAAAACATCGTCGCTTCCACGACAAAGAGTCTGACTTCCTCGCGTTTGTGAAT
CTGTGGAATTATCTTGGCGAGCAGCAAAAGGCGCTTTCTTCAAACGCCTTCCGTCGCCTGTGTCGTACTGATTATCTCAA
CTATCTGCGCGTGCGCGAATGGCAGGATATCTACACCCAGTTGCGCCAGGTGGTGAAAGAACTTGGCATTCCGGTTAACA
GCGAACCGGCGGATTATCGCGAAATTCACATTGCGTTGCTGACCGGTTTGCTTTCGCATATCGGTATGAAAGATGCCGAT
AAACAAGAATATACCGGCGCACGTAACGCGCGTTTCTCCATCTTCCCCGGTTCTGGTTTATTCAAAAAGCCGCCGAAATG
GGTAATGGTGGCGGAACTGGTAGAAACCAGCCGCCTGTGGGGACGCATTGCTGCGCGGATCGACCCGGAATGGGTGGAGC
CAGTTGCTCAGCATTTGATTAAACGCACCTACAGCGAACCGCACTGGGAACGGGCGCAGGGCGCGGTGATGGCAACGGAA
AAAGTCACTGTTTATGGTTTGCCGATTGTTGCCGCGCGCAAGGTCAACTACAGCCAGATCGATCCGGCGTTATGTCGTGA
ACTCTTTATTCGCCACGCGCTGGTGGAAGGTGACTGGCAGACGCGTCACGCATTCTTCCGTGAAAACCTGAAACTACGGG
CGGAAGTAGAAGAGCTGGAACACAAATCACGTCGCCCGCGATATTCTGGTTGATGACGAAATGCTGTTTGAGTTCTACGAC
CAGCGCATCAGCCACGATGTGATTTCCGCACGTCACTTCGACAGCTGGTGGAAAAAAGTCAGCCGCGAAACGCCTGATTT
GCTCAACTTTGAAAAGAGCATGTTGATCAAAGAAGGGGCGGAAAAAATCAGCAAGCTGGATTATCCGAACTTCTGGCATC
AGGGCAATCTCAAGCTGCGTTTGAGTTATCAGTTTGAGCCGGGGGCAGATGCTGACGGAGTGACCGTACATATTCCGCTG
CCGCTGCTTAACCAGGTTGAGGAGAGTGGGTTTGAGTGGCAGATCCCCGGTCTGCGTCGTGAGCTGATCATCGCCCTGAT
TAAATCGCTGCCGAAGCCGGTACGCCGTAATTTTGTGCCCGCGCCAAACTACGCCGAAGCGTTTTTAGGCCGCGTCACAC
CGCTGGAATTGCCGTTGCTCGACAGCCTTGAACGAGAGTTACGGCGGATGACTGGCGTCACCGTTGACCGCGAAGACTGG
CACTGGGATCAGGTGCCCGATCACCTGAAAATCACTTTCCGCGTGGTGGATGACAAAAACAAGAAGCTAAAAGAAGGGCG
CTCATTACAGGATCTGAAAGACGCCCTGAAAGGCAAAGTGCAGGAAACGCTGTCTGCCGTGGCGGATGACGGTATCGAGC
AGAGCGGTTTACATATCTGGAGTTTTGGTCAACTTCCGGAAAGTTACGAGCAGAAGCGTGGCAACTACAAAGTGAAGGCG
TGGCCAGCGCTGGTGGATGAGCGTGACAGCGTAGCGATCAAACTGTTTGATAACCCGCTGGAACAGAAGCAGGCAATGTG
GAACGGTCTTCGCCGTTTACTGCTGCTAAATATTCCATCGCCGATCAAGTACTTGCACGAAAAGCTGCCAAACAAAGCCA
AGCTGGGGCTGTACTTTAACCCGTATGGCAAAGTGCTGGAGCTGATCGATGACTGTATCTCCTGCGGTGTGGATCAACTG
ATCGACGCCAATGGTGGCCCGGTCTGGACGGAAGAAGGCTTTGCTGCGCTGCATGAAAAAGTCCGCGCTGAACTGAACGA
CACGGTGGTGGATATTGCGAAGCAGGTCGAGCAAATCCTCACTGCGGTGTTCAATATCAACAAACGTCTGAAAGGGCGGG

TGGATATGACCATGGCGCTGGGGCTTTCTGACATTAAAGCGCAGATGGGCGGGCTGGTGTATCGCGGTTTTGTCACTGGT
AATGGCTTCAAACGGCTGGGCGACACGCTGCGTTATTTGCAGGCGATTGAAAAGCGACTGGAAAAACTGGCGATTGATCC
ACATCGCGACCGTGCTCAGATGCTGAAAGTCGAAAACGTCCAGCAGGCGTGGCAGCAATGGATCAACAAACTACCGCCTG
CACGTCGTGAGGATGAAGACGTGAAAGAGATCCGCTGGATGATAGAAGAACTGCGCGTTAGTTACTTCGCTCAACAACTT
GGCACGCCTTATCCGATTTCGGATAAGCGTATTTTGCAGGCGATGGAGCAGATTAGCGGTTAA
>Cluster_33660
ATGAAACGACATCTGAACACCAGCTACAGGCTGGTATGGAATCACATTACGGGCGCTTTCGTGGTTGCCTCCGAACTGGC
CCGCGCACGGGGTAAACGTGGCGGTGTGGCGGTTGCACTGTCTCTTGCCGTGGTCACGTCACTCCCGGTGCTGGCTGCTG
ACATCGTTGTGCACCCGGGTGAAACAGTGAATGGCGGAACACTGGTAAACCATGACAACCAGTTTGTATCCGGAACAGCT
GATGGCGTGACTGTCAGTACCGGGCTTGAGCTGGGGCCGGACAGTGACGAAAACACCGGCGGGCAATGGATAAAAGCGGG
TGGCACAGGCAGAAACACCACTGTCACCGCAAATGGTCGTCAGATTGTGCAGGCAGGAGGAACTGCCAGTGATACGGTTA
TTCGTGATGGCGGAGGGCAGAGCCTTAACGGACTGGCGGTGAACACCACGCTGGATAACAGAGGTGAGCAGTGGGTACAC
GGGGGAGGGAAAGCTGACGGTACAATTATTAACCAGGAGGGTTACCAGACCATAAAACATGGCGGACTGGCAACCGGAAC
CATCGTCAACACCGGTGCAGAAGGTGGTCCGGAGTCTGAAAATGTGTCCAGCGGTCAGATGGTCGGAGGGACGGCTGAAT
CCACCACCATCAACAAAAATGGCCGGCAGGTTATCTGGTCTTCGGGGATGGCACGGGACACCCTCATTTACGCCGGTGGT
GACCAGACGGTACACGGAGAGGCACATAACACCCGGCTGGAGGGGGGTAACCAGTATGTACACAACGGTGGCACGGCAAC
AGAAACGCGGATAAACCGTGATGGCTGGCAGGTGATTAAGGAAGGAGGAACTGCCGCGCATACCACCATCAACCAGAAAG
GAAAGCTGCAGGTGAATGCCGGCGGTAAAGCGTCTGATGTCACCCAGAACACGGGCGGTGCACTGGTTACCAGTACAGCT
GCAACCGTCACCGGCACAAACCGCCTGGGAGCATTCTCCGTTGTGGCGGGTAAGGCTGATAATGTCGTACTGGAAAATGG
CGGACGTCTGGATGTGCTGAGCGGACACACAGCCACTAATACCCGTGTGGATGATGGCGGAACGCTGGATGTCCGCAACG
GTGGTGCCGCCACCACCGTATCCATGGGGAATGGCGGTGTACTGCTGGCCGATTCCGGTGCCGCTGTCAGTGGTACCCGG
AGCGACGGAAAGGCATTCCGTATCGGAGGCGGTCAGGCGGATGCCCTGATGCTGGAAAAAGGCAGTTCATTCACGCTGAA
CGCCGGTGATACGGCCACGGATACCACGGTAAATGGCGGACTGTTCACCGCCAGGGGCGGCACACTGGCGGGCACCACCA
CGCTGAATAACGGCGCCATACTTACCCTTTCCGGGAAAACGGTGAATAACGATACCCTGACCATCCGTGAAGGTGATGCA
CTCCTGCAGGGAGGCGCTCTTACCGGTAACGGCAGGGTGGAAAAATCAGGAAGTGGCACACTCACTGTCAGCAACACCAC
ACTCACCCAGAAAACCGTCAACCTGAATGAAGGCACGCTGACGCTGAACGACAGTACCGTCACCACGGATGTCATCGCTC
AGCGCGGCACGGCCCTGAAGCTGACCGGCAGCACAGTGCTGAACGGTGCCATTGACCCCACGAATGTCACCCTGACATCC
GGTGCCACCTGGAATATCCCCGATAACGCCACGGTTCAGTCGGTGGTGGATGACCTCAGCCATGCCGGACAGATTCATTT
CACCTCCGCCCGCACAGGGAAGTTCGTTCCGACAACCCTGCAGGTGAAAAACCTGAACGGGCAGAATGGCACCATCAGCC
TGCGTGTACGCCCGGATATGGCACAGAACAATGCTGACAGACTGGTCATTGACGGTGGCAGGGCAACCGGAAAAACCATC
CTTAACCTGGTGAACGCCGGCAACAGTGGTACGGGGCTGGCGACCACCGGTAAGGGTATTCAGGTGGTTGAGGCCATTAA
CGGTGCCACCACGGAGGAAGGGGCCTTTGTCCAGGGGAATATGCTGCAGGCCGGGGCCTTTAACTACTCCCTCAACCGGG
ACAGTGATGAGAGCTGGTATCTGCGCAGTGAAGAACGTTATCGTGCTGAAGTCCCCCTGTACGCCTCCATGCTGACACAG
GCAATGGACTATGACCGGATTCTGGCAGGCTCCCGCAGCCATCAGACCGGTGTAAACGGTGAAAATAACAGCGTCCGTCT
CAGCATTCAGGGTGGCCATCTCGGTCACGATAACAACGGCGGTATTGCCCGTGGGGCCACGCCGGAAAGCAGCGGCAGCT
ATGGCTTCGTCCGCCTGGAGGGTGACCTGCTGAGAACAGAGGTTGCCGGTATGTCTGTGACCGCAGGGGTATATGGTGCT
GCAGGCCATTCTTCCGTTGATGTTAAGGATGATGACGGCTCCCGCGCCGGCACGGTCCGGGATGATGCCGGCAGCCTGGG
CGGATATATGAACCTGACACACACCTCCTCCGGCCTGTGGGCTGACATTGTGGCACAGGGAACCCGCCACAGTATGAAAG
CGTCATCGGGCAATAACGACTTCCACGCACGGGGCCGGGGCTGGCTGGGCTCACTGGAAACCGGTCTGCCCTTCAGTATC
ACTGATAACCTGATGCTGGAGCCACGGCTGCAGTACACCTGGCAGGGGCTTTCGCTGGATGACGGTAAGGACAACGCCGG
TTATGTGAAGTTCGGGCATGGCAGTGCACAGCATGTGCGTGCCGGTTTCCGTCTGGGCAGCCACAACGATATGACCTTTG
GTGAAGGCACCTCATCCCGTGCCCCCCTGCGTGACAGTGCAAAACACAGTGTGCGTGAACTGCCGGTGAACTGGTGGGTA
CAGCCTTCCGTTATCCGCACCTTCAGCTCCCGGGGTGATATGCGTGTGGGGACCTCCACCGCAGGCAGCGGGATGACGTT
CTCTCCCTCACAGAATGGCACTTCACTGGATCTGCAGGCCGGACTGGAAGCCCGTGTCCGGGAAAATATCACCCTGGGCG
TTCAGGCCGGTTATGCCCACAGCATCAACGGCAGCAGCGCTGAAGGTTATAGCAGTCAGGCCACGCTGAATGTGACCTTC
TGA
>Cluster_22683
ATGACTCCAGTAAAAGTGTGGCAAGAGCGCGTTGAGATCCCGACCTATGAAACCGGGCCGCAGGATATACATCCCATGTT
CCTGGAAAATCGCGTTTATCAGGGATCGTCCGGCGCGGTTTATCCCTACGGCGTGACCGATACGCTGAGCGAGCAGAAAA
CCCTGAAATCCTGGCAGACGGTGTGGCTGGAAAACGACTACATCAAAGTGATGATCCTGCCGGAACTGGGCGGTCGGGTG
CATCGCGCATGGGATAAAGTGAAACAGCGCGATTTTGTCTATCACAATGAAGTCATTAAACCTGCGCTGGTGGGGCTGCT
GGGACCGTGGATCTCCGGCGGGATTGAGTTTAACTGGCCGCAACACCATCGCCCGACCACCTTTATGCCCGTTGATTTCA
CCCTCGAAGCCCATGAAGACGGCGCACAGACGGTGTGGGTAGGCGAAACGGAGCCGATGCATGGTTTACAGGTGATGACA
GGTTTCACCCTGCGCCCTGACCGGGCGGCGCTGGAAATCGCCAGCCGCGTCTATAACGGCAACGCCACGCCGCGTCATTT
CTTGTGGTGGGCCAACCCGGCAGTGAAAGGGGGTGAAGGGCATCAGAGCGTCTTCCCGCCGGATGTAACGGCGGTGTTTG
ATCACGGCAAACGGGCCGTCTCCGCTTTCCCCATCGCCACCGGCACTTACTACAAAGTGGACTACTCCGCCGGAGTGGAC
ATCTCTCGCTATAAAAATGTGCCAGTGCCGACTTCGTATATGGCCGAAAAATCGCAGTACGATTTTGTTGGCGCATGGTG
TCACGATGAAGATGGCGGTTTGCTACACGTTGCCAACCACCACATCGCGCCAGGTAAAAAACAGTGGAGCTGGGGACACA
GTGAATTCGGCCAGGCGTGGGATAAGAGCCTGACCGACAATAACGGCCCATATATCGAACTGATGACCGGTATTTTTGCC
GATAACCAGCCTGATTTTACCTGGCTTGATGCGTACGAAGAGAAGCGTTTCGAGCAGTATTTCCTGCCTTACCATTCTCT
GGGCATGGTGCAAAATGCCTCCCGCGATGCGGTGATAAAACTCCAGCGTAGTGAGCGGGGGATTGAGTGGGGGCTGTATG
CCATCTCTCCGTTGAACGGATACCGCCTGGCGATCCGCGAAATCGGCAAATGCAACGCGTTACTTGATGATGCCGTGGCA
CTGATGCCAGCGACCGCCATCCAGGGCGTGTTGCACGGTATCAATCCTGACAGACTAACCATTGAGCTCTCCGATGCCGA
CGGCAATATCATACTGAGTTATCAGGAACATCAGCCGCAAGAGTTGCCGTTGCCGGACGTCGCCAAAGCGCCACTGTCAG
CACAAGACATTACCAGTACAGATGAAGCCTGGTTTATCGGTCAGCATCTGGAGCAATATCATCACGCCAGCCGTTCACCG
TTCGATTACTACCTGCGCGGCGTGGCGCTGGATCCGCTGGATTATCGCTGTAACCTGGCGCTGGCGATGCTGGAATATAA
CCGCGCCGATTTCCCGCAAGCGGTGGCGTATGCCACTCAGGCGCTGAAACGCGCACATGCGCTGAACAAAAATCCGCAGT
GCGGACAGGCCAGTTTGATTCGCGCCAGTGCTTACGAACGTCAGGGACAATATCAACAAGCCGAAGAGGATTTCTGGCGG
GCGGTCTGGAGCGGCAATAGTAAAGCAGGCGGCTATTATGGTCTGGCACGACTGGCGGCGCGTAATGGTCACTTCGATGC
TGGTCTGGATTTTTGCCAACAAAGTCTTCGCGCCTGTCCAACCAACCAGGAAGTGCTTTGCCTGCATAATCTGCTGCTGG
TGTTAAGTGGTCGTCAGGACAACGCGCGTTTGCAGCGCGAGAAACTGCTGCGCGATTATCCGCTGAACGCCACTTTGTGG
TGGCTGAAATGGTTCGATGGTCGTAGCGAATCAGCCCTCGCGCAGTGGCGAGGTCTTTGTCAGGGACGCGACGTTAACGC
CCTGATGACCGCCGGGCAACTGATTAACTGGGGAATGCCCACCCTGGCGGCAGAGATGCTGAACGCACTGGACTGCCAGC
GCACACTGCCGCTTTACCTGCAAGCCAGCTTGCTGCCGAAAGCTGAACGCGGCGAACTGGTCGCAAAAGCCATTGATGTC
TTCCCACAGTTTGTCCGTTTCCCGAATACGCTGGAAGAAGTAGCGGCGCTGGAGAGTATCGAAGAGTGCTGGTTTGCCCG
GCATTTACTGGCCTGCTTCTACTACAACAAGCGTAGCTACGGTAAAGCCATTGCCTTATGGCAGCGTTGCGTAGAGATGT
CGCCGGAGTTTGCCGACGGCTGGCGCGGGTTGGCGATCCATGCGTGGAATAAGCAGCACGATTATGAGCTGGCCGCGCGT
TATCTTGATAATGCTTATCAGCTTGCGCCGCAGGATGCACGTCTGCTTTTCGAACGGGATTTGCTGGATAAGTTAAGCGG
TGTCACACCGGAGAAACGACTGGCGCGTCTGGAGAATAATCTGGAAATTGCGCTGAAACGCGACGACATGACCGCAGAAC

TGCTCAATTTGTGGCATCTCACTGGTCAGGCAGACAAAGCGGCGGACATTCTCGCCACGCGCAAATTCCACCCGTGGGAA
GGTGGGGAAGGGAAGGTCACCAGCCAGTTTATCCTCAACCAGTTATTACGCGCCTGGCAGCATCTTGATGCCAGACAGCC
GCAGCAGGCCAGCGAACTGCTTCATGCCGCGCTGCATTATCCGGAGAATTTAAGCGAAGGCCGTTTACCGGGGCAAACTG
ACAACGACATCTGGTTCTGGCAGGCAGTATGCGCCAACGCGCTGGGCGATGAAACTGAAGCGACGCGTTGTTTACGTCTG
GCGGCGACCGGCGATCGCACCATTAACATCCACAGTTATTACAACGATCAGCCGGTTGATTATCTCTTCTGGCAAGGAAT
GGCGCTGCGACTGCTGGGCGAACAACAAACTGCACAGCAACTGTTTAGTGAAATGAAACAGTGGGCACAAGAGATGGCGA
AAACCAGTATCGAAGCGGATTTCTTTGCCGTCTCGCAGCCTGACTTGTTGTCGCTGTATGGCGATTTACAACAGCAGCAT
AAAGAAAAATGCCTGATGGTGGCGATGCTGGCGGCCGCGGGATTAGGGGAGGTTGCGCACTATGAATCTGCTCGCGCTGA
ATTGATGGCGATTAATCCAGCCTGGCCGAAAGCGGCATTATTCACCACCGTGATGCCTTTTATTTTTAGCTACGTTCACT
AA
>Cluster_55583
GTGAAACAAACGTTAATTGTCAGTTTTGTCGGCGACTCGAGTACCGCGCAGGTTGATCTGCAGAAGTCGAAAAATGAAGT
GGTCGCTGACGGCAATGATAGCGCCACAATGACCGCGACAGTCCGGGATGCAAAAGGCAACCTGCTCAATGACGTCAAGG
TCACTTTCAATGTTAATTCAGCAGAGGCGAAACTGAGCCAAACCGAAGTGAATAGCCACGACGGGATCGCCACAGCTACG
CTGACCAGTTTGAAAAATGGTGATTATAGGGTTACGGCCTCTGTGAGCTCTGGTTCCCAGGCTAATCAACAGGTGAATTT
TATCGGTGATCAAAGTACTGCAGCCCTGACCCTCAGTGTGCCTTCAGGTGATATCACCGTCACCAACACAGCTCCGCTAC
ATATGACTGCAACCTTGCAGGATAAAAATGGCAACCCACTAATAGATAAAGAAATCACCTTCTCTGTGCCAAACGACGTC
GCAAGTCAGTTCTCAATTAGCAACGGAGGAAAAGGCATGACGGATAGTAACGGGGTTGCAATCGCCTCCCTGACCGGCAC
GTTAGCGGGCACGCATATGATCACGGCTCGTCTGGCTAACAGCAATGTCAGCGATACACAGCCAATGACGTTTGTGGCGG
ATAAAGACAGAGCGGTTGTCGTTCTGCAAACATCGAAAGCGGAAATCATTGGGAATGGCGTGGATGAGACAACTCTGACA
GCAACAGTGAAAGATCCGTCGAATCATCCGGTGGCGGGAATAACGGTGACCTTCACCATGCCACAGGACGTTGCGGCAAA
CTTTACCCTCGAAAATAACGGCATTGCCATCACCCAAGCCAATGGGGAAGCGCATGTCACGCTGAAAGGTAAAAAAGCGG
GTACGCATACGGTTACCGCAACGCTGGGTAATAACAATACCAGTGATTCGCAGCCGGTAACGTTTGTGGCGGACAAAACC
TCGGCTCAGGTTGTCCTGCAGATGTCAAAAGATGAGATCACAGGTAATGGCGTCGATAACGCAACGCTAACTGCAACGGT
TAAAGATCAGTTCGACAATGAGGTGAATAATCTTCCGGTAACATTCAGCTCAGCCTCTTCAGGACTCACCCTGACCCCGG
GAGTAAGTAATACCAATGAGTCTGGCATCGCGCAGGCCACTCTCGCAGGCGTTGCCTTTGGTGAGCAGACGGTCACTGCA
TCACTGGCTAATAATGGTGCCAGCGACAACAAAACTGTGCATTTTATTGGCGACACAGCAGCGGCAAAAATTATCGAGTT
GACGCCTGTCCCAGACAGCATAATCGCCGGTACCCCGCAGAACAGCTCCGGCAGCGTCATCACCGCCACAGTCGTTGATA
ATAATGGCTTTCCGGTGAAAGGTGTGACTGTGAACTTCACCAGCAGAACAAACTCTGCCGAAATGACGAATGGCGGCCAA
GCCGTAACGAACGAACAGGGTAAGGCTACCGTCACTTATACCAATACCCGCTCCTCGATAGAATCAGGAGCGAGACCGGA
TACCGTTGAGGCCAGTCTGGAAAATGGTAGCTCCACGCTTAGCACATCAATTAATGTCAACGCTGATGCGTCTACGGCAC
ATCTCACCTTGCTACAGGCACTTTTTGATACAGTCTCCGCAGGCGACACTACCAATCTGTATATTGAGGTGAAGGATAAT
TACGGCAACGGTGTACCCCAGCAGGAGGTAACCCTCAGAGTATCACCAAGTGAAGGCGTGACCCCCAGTAATAACGCTAT
ATATACTACCAACCACGACGGCAATTTTTACGCAAGCTTTACCGCTACAAAAGCCGGGGTTTATCAAGTGACGGCAACCC
TCGAAAATGGCGATTCGATGCAACAAACAGTGACCTATGTGCCGAACGTCGCGAATGCCGAAATCACGCTGGCAGCCTCG
AAGGATCCGGTGATTGCCGACAATAACGATCTCACGACACTAACAGCAACAGTCGCTGATACAGAGGGCAATGCGATAGC
CAACACTGAAGTAACATTTACTCTGCCGGAAGATGTGAAGGCGAACTTCACGCTGAGCGATGGCGGTAAAGCGATTACTG
ATGCTGAAGGCAAAGCGAAAGTCACGCTGAAAGGTACAAAAGCAGGCGCTCATACTGTTACAGCATCGATGACTGGCGGT
AAGAGTGAGCAGTTGGTGGTGAACTTTATTGCGGATACGCTCAGTGCGCAGGTTAATCTTAACGTTACCGAGGACAACTT
TATCGCCAATAACGTTGGGATGACCACACTTCAGGCAACAGTGACTGATGGAAACGGCAACCCGTTAGCCAATGAGGCGG
TGACATTCACGCTACCGGCAGACGTGAGCGCAAGCTTCACTCTCGGACAAGGCGGTTCCGCCATTACTGATATCAACGGC
AAGGCTGAAGTTACACTGAGCGGTACAAAATCCGGCACCTACCCCGTGACAGTTAGCGTGAACAATTATGGTGTCAGTGA
TACGAAACAGGTGACTTTGATTGCCGATGCTGGTACCGCAACACTAGCCTCCTTAACCTCTGTATACTCATTCGTCGTCA
GCACGACCGAGGGCGCAACCATGACGGCAAGCGTCACTGACGCTAACGGCAACCCGGTAGAAGGCATAAAAGTTAATTTC
CGCGGAACCTCCGTCACGCTAAGCAGCACCAGCATTGAAACGGATGATCGGGGTTTCGCTGAAATTCTTGTGACAAGCAC
CGAGGTCGGACTGAAAACAGTTTCAGCCTCTCTGGCAGATAAACCTACTGAAGTCATCTCGCGATTACTGAATGCCAGTG
CAGATGTTAATTCTGCGACGATTACCAGTCTGGAGATACCGGAAGGTCAGGTAATGGTCGCACAAGACGTAGCAGTTAAA
GCTCACGTTAACGACCAGTTTGGCAACCCGGTTGCGCATCAACCCGTGACATTCAGTGCAGAGCCATCCTCGCAAATGAT
CATCAGCCAGAATACGGTCTCTACTAATACGCAGGGTGTAGCCGAGGTCACCATGACGCCCGAAAGAAACGGTTCGTATA
TGGTGAAAGCATCCCTGCCGAATGGAGCCTCACTTGAGAAACAACTGGAGGCTATTGATGAAAAACTGACACTCACGGCG
TCCAGTCCGCTTATCGGTGTCTATGCCCCTACAGGCGCTACTCTGACGGCAACGCTAACCTCTGCAAATGGCACTCCAGT
GGAGGGTCAGGTCATCAACTTTAGCGTAACGCCAGAAGGGGCGACGTTAAGTGGCGGAAAAGTGAGAACTAACTCTTCAG
GTCAGGCTCCAGTCGTTTTGACCAGCAATAAAGTCGGTACATATACGGTGACTGCATCTTTCCATAACGGCGTAACAATA
CAGACACAGACAACCGTGAAAGTCACTGGCAACTCAAGCACCGCCCATGTTGCTAGCTTTATCGCTGATCCATCGACTAT
CGCCGCCACCAACACTGATTTAAGTACCTTAAAGGCAACGGTTGAGGATGGCAGTGGTAACCTGATCGAAGGTCTCACTG
TGTACTTCGCCTTAAAAAGCGGCTCTGCCACATTAACGTCATTAACAGCGGTGACCGATCAAAACGGAATCGCGACAACA
AGCGTGAAAGGAGCGATGACAGGTAGCGTCACGGTAAGCGCAGTCACGACCGCTGGTGGAATGCAAACAGTAGATATAAC
GCTGGTGGCTGGCCCGGCAGACACCTCGCAGTCCGTCCTTAAGAGCAATCGGTCATCACTGAAAGGGGACTATACCGATA
GTGCTGAATTACGTCTTGTTCTGCACGATATATCAGGCAATCCGATCAAAGTTTCTGAAGGGATGGAATTTGTGCAATCA
GGTACTAACGTGCCCTATATAAAAATTAGCGCAATTGATTACAGTCTAAATATCAACGGTGATTACAAAGCCACTGTTAC
AGGCGGCGGAGAGGGTATCGCAACGCTGATCCCTGTATTGAATGGTGTTCATCAAGCTGGTCTGAGTACCACAATACAAT
TCACTCGAGCAGAAGACAAAATAATGAGCGGTACAGTATCAGTCAATGGTACTGACCTACCGACAACTACATTCCCTTCG
CAGGGGTTCACCGGGGCGTATTATCAGTTGAATAATGACAACTTTGCCCCAGGAAAAACGGCGGCTGATTATGAGTTTTC
AAGCTCTGCCTCCTGGGTCGATGTTGATGCTACCGGTAAAGTGACATTTAAAAATGTCGGCAGCAATTGGGAAAGGATTA
CGGCGACGCCAAAATCAGGAGGCCCTAGCTATGTATACGAAATCCGTGTGAAGAGTTGGTGGGTGAACGCCGGCGATGCT
TTCATGATATACAGCCTTGCTGAAAATTTTTGCAGTAGCAATGGCTACACACTTCCCCGTGCAGACCATTTAAACCATAG
TCGTTCCCGAGGCATCGGGTCACTGTACAGTGAATGGGGAGATATGGGGCATTACACGACTGACGCTGGTTTTCAATCAA
ATATGTATTGGTCATCTAGTCCCGCAAACTCAAGCGAACAATACGTAGTTTCCCTGGCAACAGGTGATCAAAGCGTATTT
GAAAAGCTTGGGTTTGCTTATGCGACATGTTATAAAAACATCTGA
>Cluster_6882
ATGGCAGATAATCCAGACCCTTCATCGCTCCTGCCGGACGTGTTTTCACCGGCGACCCGCGACTGGTTTCTTCGCGCCTT
TAAACAGCCGACCGCTGTCCAGCCGCAAACCTGGCATGTGGCGGCGCGAAGCGAACATGCGCTGGTGATTGCACCGACCG
GCTCCGGGAAAACGCTGGCAGCATTTCTCTACGCCCTCGATCGGCTCTTCCGCGAAGGCGGCGAAGATACCCGCGAGGCG
CATAAGCGTAAAACCTCACGCATCCTCTATATTTCACCGATAAAAGCCCTGGGCACCGACGTTCAGCGCAACTTGCAGAT
CCCGTTGAAGGGTATTGCCGATGAACGGCGGCGGCGCGGCGAAACGGAAGTCAATCTTCGCGTAGGGATCCGTACTGGCG
ATACGCCTGCACAGGAACGCAGCAAACTCACCCGTAATCCGCCGGATATTCTGATCACCACACCCGAATCACTCTATCTG
ATGCTGACCTCCCGCGCGCGCGAAACGCTACGCGGCGTCGAAACGGTAATTATTGATGAAGTCCACGCGGTAGCGGGCAG
TAAACGTGGTGCGCATCTGGCGTTAAGTCTGGAGCGGCTCGATGCGCTGCTCCACACCTCAGCACAGCGAATTGGCCTTT
CTGCCACTGTGCGCTCAGCCAGCGATGTGGCAGCATTTCTTGGTGGCGATCGCCCGGTTACGGTAGTCAACCCGCCCGCA

ATGCGCCATCCGCAGATACGAATTGTCGTACCGGTCGCCAATATGGATGATGTCTCATCGGTCGCCAGCGGCACCGGCGA
AGACAGCCATGCCGGCCGGGAAGGCTCCATCTGGCCATATATTGAAACGGGTATCCTTGATGAAGTGTTGCGCCATCGCT
CGACCATTGTCTTTACTAATTCGCGGGGGCTGGCGGAAAAACTGACGGCACGATTAAATGAGCTTTACGCCGCACGCTTA
CAGCGTTCCCCGTCTATCGCCGTTGATGCGGCCCATTTCGAGTCGACCTCCGGCGCAACCTCTAACCGTGTACAAAGTAG
CGACGTTTTTATTGCCCGCTCACACCACGGCTCCGTCTCTAAAGAACAACGAGCAATCACCGAACAGGCGCTGAAATCGG
GTGAATTACGCTGCGTGGTCGCAACCTCCAGTCTTGAACTGGGGATTGATATGGGCGCGGTGGATCTGGTGATTCAGGTG
GCAACGCCGCTTTCTGTTGCCAGTGGGTTACAACGCATTGGTCGCGCCGGACATCAGGTTGGCGGTGTATCTAAAGGGCT
GTTTTTCCCCCGTACCCGGCGTGATTTAGTCGATTCCGCAGTCATTGTAGAGTGTATGTTCGCAGGCAGGCTGGAAAACC
TGACACCACCGCATAATCCTCTCGACGTCCTTGCGCAGCAAACCGTTGCCGCCGCGGCGATGGATGCATTACAGGTAGAC
GAATGGTACTCCCGCGTACGCCGTGCCGCACCGTGGAAAGATCTGCCAAGACGTGTTTTTGACGCCACGCTGGATATGCT
TTCCGGGCGCTATCCCTCTGGCGATTTTTCTGCTTTTCGCCCCAAACTGGTCTGGAACAGGGAGACCGGGATATTGACCG
CCCGACCTGGCGCTCAATTGTTGGCGGTTACCAGCGGCGGCACCATTCCGGATCGTGGCATGTATAGCGTGTTATTACCC
GAAGGTGAAGAAAAGGCCGGTTCGCGGCGGGTGGGTGAACTGGATGAGGAGATGGTATATGAGTCGCGGGTGAACGACAT
TATCACTCTCGGCGCTACCTCATGGCGGATCCAGCAAATCACCCGCGATCAGGTGATTGTGACTCCTGCTCCGGGTCGTT
CTGCCCGGCTCCCCTTCTGGCGTGGTGAAGGTAACGGACGTCCGGCTGAATTAGGCGAGATGATCGGCGATTTTCTTCAT
TTGCTGGCGGATGGCGCGTTCTTTTCCGGGACTATTCCCCCGTGGCTGGCAGAAGAAAATACGATCGCCAATATTCAGGG
GTTGATTGAGGAGCAGCGCAACGCGACGGGCATCGTTCCGGGGAGTCGCCATCTGGTCCTCGAACGGTGCCGTGATGAAA
TTGGTGACTGGCGTATTATTTTGCACTCTCCCTATGGAAGACGGGTGCATGAACCCTGGGCGGTGGCGATTGCCGGGCGA
ATACATGCGCTATGGGGCGCTGACGCGTCGGTGGTCGCCAGTGATGACGGCATTGTTGCACGTATTCCTGACACCGATGG
TAAATTGCCCGATGCCGCGATTTTTTGTTTGAACCAGAAAAGTTGCTGCAAATTGTCCGCGAGGCGGTAGGCAGCTCGG
CACTTTTCGCCGCCCGTTTTCGCGAATGCGCCGCGCGGGCATTATTAATGCCGGGGCGCACTCCGGGCCATCGCACCCCG
CTTTGGCAACAACGGCTGCGCGCCAGTCAGTTGCTGGAAATCGCTCAGGGATATCCGGATTTTCCGGTCATTCTCGAAAC
CCTACGCGAATGTCTGCAAGATGTTTATGATCTTCCCGCACTGGAACGTTTGATGCGTCGCCTGAACGGTGGCGAAATTC
AAATATCCGATGTAACGACCACTACGCCCTCGCCTTTCGCCACAAGTTTATTGTTCGGCTATGTCGCGGAATTTATGTAC
CAGAGCGACGCCCCGCTGGCAGAGCGCCGGGCATCCGTACTGTCGCTGGACAGCGAGTTACTGCGCAATCTACTCGGACA
GGTCGATCCGGGGGAATTACTCGACCCGCAGGTCATTCGCCAGGTGGAAGAAGAGTTGCAACGACTGGCTCCTGGCAGAA
GAGCGAAAGGTGAAGAAGGATTGTTCGACCTGCTGCGCGAACTGGGGCCAATGACCGTTGAAGACCTGGCGCAACGGCAT
ACAGGCAGCAGTGAAGAGGTTGCGTCGTATCTGGAAAATCTTCTTGCAGTAAAACGAATCTTCCCAGCGATGATTAGCGG
ACAGGAGCGTCTTGCCTGTATGGATGATGCCGCCAGGCTGCGTGATGCCCTCGGCGTACGACTACCAGAGTCATTGCCAG
AGATTTATTTACATAGAGTCAGTTACCCGCTTCGCGACCTCTTTCTGCGCTATCTCCGGGCTCATGCTCTGGTCACGGCT
GAACAACTGGCTCATGAGTTTAGTCTCGGTATTGCCATTGTCGAAGAGCAGCTTCAGCAACTGCGTGAACAGGGTCTGGT
GATGAATCTGCAACAAGACATCTGGGTGAGCGATGAAGTATTTCGTCGTCTGCGTTTGCGCTCGCTGCAAGCCGCCAGAG
AAGCGACGCGTCCCGTTGCAGCCACGACCTATGCGCGATTGCTGCTGGAACGTCAGGGCGTATTACCCGCCACCGATGGT
AGCCCGGCGCTCTTTGCCTCAACATCGCCAGGCGTTTATGAGGGCGTAGATGGCGTGATGCGGGTGATCGAACAGCTTGC
CGGAGTCGGTTTACCCGCCTCACTCTGGGAAAGCCAGATCCTGCCTGCCCGCGTACGCGACTATTCGTCAGAAATGCTCG
ATGAATTACTGGCAACCGGTGCGGTTATCTGGTCGGGGCAAAAAAAACTGGGTGAAGATGACGGCCTGGTGGCACTGCAT
CTACAGGAATATGCTGCAGAATCGTTCACTCCCGCCGAAGCGGATCAGGCGAATCGTTCGGCGCTGCAACAAGCGATAGT
CGCTGTTCTGGCTGACGGAGGAGCCTGGTTTGCACAACAAATCAGCCAACGGATACGCGACAAAATCGGCGAATCGGTTG
ATCTCTCTGCCCTGCAAGAGGCGTTATGGGCGCTGGTCTGGCAAGGCGTCATCACCAGCGACATTTGGGCACCGTTACGC
GCCCTCACCCGCAGCAGTTCCAACGCACGCACCTCAACTCGCCGCAGTCACCGGGCTCGTCGTGGACGTCCTGTCTATGC
GCAACCCGTCTCGCCGCGGGTATCTTACAACACACCAAATCTGGCTGGACGCTGGTCGTTATTGCAGGTGGAGCCACTAA
ACGATACCGAAAGGATGCTGGCGCTGGCGGAAAATATGCTCGACCGCTACGGCATCATCAGTCGTCAGGCGGTGATAGCC
GAAAATATCCCTGGCGGGTTTCCATCGATGCAAACGCTTTGTCGAAGTATGGAAGACTCCGGGCGAATTATGCGAGGTCG
TTTTGTAGAAGGTCTGGGTGGCGCGCAATTCGCTGAACGTCTGACTATTGACCGATTGCGCGATCTGGCGACACAAGCCA
CGCAAACGCGCCACTATACACCAGTGGCGCTCTCTGCCAACGATCCGGCTAATGTGTGGGGAAATCTTCTGCCCTGGCCT
GCACATCCGGCAACGCTGGTTCCAACGCGTCGGGCGGGTGCGCTGGTGGTCGTTTCTGGCGGCAAATTGTTACTCTATCT
GGCGCAAGGTGGCAAAAAAATGCTGGTCTGGCAGGAAAAAGAGGAATTACTCGCCCCAGAGGTTTTCCACGCGCTGACTA
CCGCACTGCGTCGCGAACCACGGCTGCGCTTTACGCTAACAGAAGTGAATGATCTACCGGTCCGGCAAACGCCGATGTTT
ACGCTGCTGCGTGAGGCGGGATTTTCAAGTTCGCCACAAGGGCTGGATTGGGGATAG
>Cluster_50614
ATGGAAGAGGCATTCTGGGCGGCAAGACAGGGTGATGCCCTGCTGCATACGTCGTTTATGGCAGATGTACTGGGGGCGGT
GCTGGAGGTGGCCGCAAATGTCGTTATTGATGCCCTCATTGTCGGCACAACCTCGTTACTGGCGGCGTCAGTGGGGATCA
CGATGGGGTGCACAGCCGTCGTGCTGACCGGTTTTGTGGCGGGTGTGGCGATGGTGTACACCGGCGTGACAGAAAAGGTC
AGCGAAGCCTGTTCAGCCCTCGCAGATATGCTTTTCCCGCCTCAGACTGAAGGTTACATCCTCACCGGCTCTGGTGACAC
ATGGATTAACAGCAAACCTGCCGCGCGGGCAGCGGCGACTGCGGCATCGCGGCAGGACATTGAGGCGCAGGAAGCGCAGG
CGAAGGCGGAACAGGAAGAAGCACAGCGTCAGGAGGAGGCCAGAACGTTCCGGGATGTGGCCGGGGAATATCTGGAAATG
GCGGGGGCCATAGCCCCGGCAGTTAACCCGGTGACAGGGCCTGTGCTGATGTCCCGGGCGCTGCACGGCCAGCTCAGTAC
GGAAGAAGGGCGTTCTGAAATGGTGGATGGCGTGACACATTTTGTGTCGGAACTATGGCAGCCCACGGTGGCCGGTGCGG
CTCCTGCCTCCACCCTGACTCCTGACGATAAAATCGACTGCCATAAACATCCCTCCTCTCTGACGCAGTTACTCGCACAA
AAAAAAGCCGCCTTTCTGGATGACCCTCCGGGAACGCTCCTTAATCTGTTGAGTCCGGACGGTATGCTGGAAACCGCCTT
TCAGGGAGTGAATGCGGTTATTGGCAGCATCAGTAATCTGTTCAAAGGCGATGACGAACCGCCCGCGGCGGAATATATCG
CGGAAGGCGCGCGGGATGTGCGTATCAACAGCCAGCCGGCAGCACGCAGCGGGGCACGCTGTACCTGCGAGGCCAGGGTG
GTGAATGCGCCGGGGAACGGGGCGTTTGTGTCACCGGATGTGCGCATTGGCGGTCCGTTACTGGTGGTCAGGGATATCAG
AAGCGGCAGGTCGCAGATAACCCTGGTGTCAACCGTGGCGCTGATGTTTCTTCGCCCCGGGTAAAATGCTTTCTGAAATCG
CCTGTTTTGCTGCCGGTATCGGGATGAGCATTCTGACGCAGAAGGCCGCCACAGCCCTTCCGCATCCGGTCAGTGCCGCC
ACCGGGGCCAAATACCTGGCCGGTGATGATGACTTCGATTTCAGTCTGCCGGGCCACTTTCCGCTGGACTGGCAGCGCGT
GTACAGCAGTCGTGACGAACGCACGGAAGGCATGTTTGGTCAGGGCTGGAGTGTAATGTATGAAGTCAGCCTCGTGCGCA
CACCGGGTAGCGCAGACGAAAACTGCATGACCTTTGTGTCGGGTATGGGACGACGCCTGGAGATGGAAGCCGTACTGCCG
GGGGGCGGGTTTTACAGCCCCGGCGAAGGACTGGCGGTACGGCGGGGTGAGCAGGGGCACTGGCTTATCAGCAGCGATGA
TGGACAGTTTTTTCTGTTTGAGGCCGACCCGCACCACCCGCAGCGCCAGCGGCTGAAAATGCTGGGCGACCGCAACAGCA
ACAGTCTGAATCTGTACTACGACGACCGGGGACGCATCACTGAAATCAGCGGCGAGCAGCAGCGTCCGTGCATCCGTCTG
TATTACGAACTGGCGGCGCATCCCCGGCGGGTGACACAGATTTATCAGCATTTCCCTGATACGACGCCTTTACTGCTGCG
TCGTTACCGCTATGACGGGGCCGGCGACCTGAACGGCGTGTACGACAGCACGGGGCACCTGCTGCGTGAGTTTGCTTACG
ACGAAAACCACTGCATGACGCTGCACCGCCAGCCGGGCGGCGAAGGGTATTATTACCAGTGGGGCTGGTATGAAGGGCCG
GATGACGCGGCATGGCGGGTGACGGGGCATCACACGGACAGCGGTGAACAGTACCGGCTGGCGTGGAGCCTGGCGGCACG
CAGGCTGTGCGTGACGGACGGGCTGGGGCGCACGCGTTATCATCAGTGGGACGCGCAGAATCAGGTGACGGCGTACCAGG
ACGAAGCCGGACAGGTGACGACGTTCCGCTGGAGCGACGAAGAACGGCTGCTGCTGGGAATGACGGATGCGCAGGGCGGC
AAATGGCGCTATGTGTATGACCGGCAGGGGCATATCACGGAGACGCACGACCCGCTGGGACGGGTGGAGCAGGCGCAGTG
GCACCCGGTGTGGCATCAGCCGGAAACGGAGGTGGACGCGGCGGGAAACAGCTGGCGCTGTGTGTACGATGAACGCGGCA

ACCTTCTTGCCGTGACCGACCCGCTGCAGCAGAACACCCGGTATCAGTATGACCGCCACGGTCAGGTGGTGCAGATAACG
GATGCGCGCGGGGGAATAAATACCTCCAGTGGAATGAAGACGGGCAGCTCATGCGGCACACGGACTGCTCCGGCTCGCA
GACGGCGTGGTTCTACGACGAACGCACGCGGCTCATCAGAATGACGGATGCGGAGAGCCACAGCACGCGCTACGGGTATG
ACGACAGCGGCCACCTGACGGAGGTGATACTGGCGGACGGCCGCACGGTGAATTACCAGTCGGACGCGGCGGGCAGGCTG
GTGAAGTACACCAGCCCGATGGGGAGGATAACGCGCTGGCAGCGTGACGGGCAGGGCCGGGTGCGCAGCCGGACTGACGC
GACGGGACGCAGGACGGCGTTCGGGTACGACGCTTACGGGCGTCTGGTCACGCTGACAAACGAGAACGGAGAAAGCTACC
GGTTCCGCCATGACGTGCTGGACCGTCTGGCGGAGCAGATAAACCCGGACGGCTGCCGCCAGGCGTACCGCTACAACGCG
CTGAACGCGGTCACGGAAGTGGTGTTCACCGGGGACCGGGGCGGTGAAATCCGTCACCGGCTGGCGCGTGACGCGGCGGG
CCGGCTCACGGCAAAGGAGACAGCCGGTAGCCGTACGGAATATGTCCATGATGCGGCAGACCAGCTACTGGAAATCCGCC
GCCGGAGGCCGGATGCCGGGGAAACGGACGCCCCGGAGATTATCCGTTTCAGTTACGACAGACTGGGGCGGATGCTGAGC
GAGGAGACGGCGCAGGGCATCCTGACCCATCAGTACGATGAACTCAGTAACCGGACTGCCACCACCTTTCCGGACGGCAG
AACGCAGCGCCATCTGTACTACGGCAGCGGCCATCTCCAGCAGATAAATCTGGACCGGGAGGTCATCAGCGAGTTCACGC
GCGACCACCTGCACCGTGAAGTGCTCAGAAGTCAGGGACGACTGAGCACGCGGCAGCTTTACGACCCCGGCGGGCGACTG
AAGCGCAGGGAGACATACAGCGGGATGCGCGGGGTGGTCCCGGAGACGTTCACGGACCGGCAGTACAGCTACAGCGGCGA
GGATGAACTGCTGAAGACGCGGCACAGCCGGCGGGGGGAAACGGACTACTTCTATGACACCACGGGGCGCATCACGGCGT
GTCGCTCGGAGGATGAGGGGTATCTGGCAAGCTGGGAGTACGACGCGGCGGGCAACCTGCTGGACAGGCGCGCGGGGGA
AGGGCCACGGCAGAAAACAGCGTGGTGCCGTTCAACCGGCTGATGTCTTACCGTGGTGTGCACTACCGTTATGACGAATT
TGGCCGTGCGGTGGAAAAGGAGGGGCCTCAAGGAACCGTTGTCACAGTTAAATCAAAACAGGAAGCCCATGATTTATTGA
TGGAGGCCTTTCCTGATGCTCAAAAAGTAAGAGGGATCGGTTCGCAGGATGCCGCCGGTATTAGAAAAAAACATAAGATG
GAACAATTTAAGAAAAGAGATGGTACTGTCCGTTATAGGAAAGATTACCCTATTGATTCTAATACTGGTCGTGTATATGG
TCATGATGACCCTAAAGGCACAGGGCATGGTTCTTTGCCTCATATAAATATTAAACGTTCTGATGGGACAATGGTGAGGA
TAGATATAGATGGTTGA
>Cluster_48634
ATGTCACAAAACGTAGAGTTTATCCTGTCGCTGGAAGACAAACAGTTTACAGCGTCAATCGATCGTGCGGGGAAACTGCT
TACTCGATTTGGTGAGCAGGTAACAAAGCCTGCTCAAAAAATCCAAACCTTTGAACGCTCTTTGGGTTCGGTCGCCCGTA
TCATTCGCGTTCTGGAAAGCAAGCTCGATTCTACGGCAGATAAACTACAGGATGTAGCTGCCGGTTTTGAGCTTGCTTCA
GATGCTACGCGTAAAATGCGTGGTAACATCACCAGCCTCAATTCTGGTCTTAAAGCCCTGATTGAGCGCGTCGATACGAC
AACTTCTTCAGTAGATAAACTCACCGCGTCATTACGTAAAGTGCAATCAGAGCTAAATGATTTCTCTGATTGGTCGACCT
ATGCAAGCAAAAGCGCAAGCCGCTTTGGTACGGAGGTCAAAGAGGCCTCTGCGTCCGTGAGTGGCATGAATACGCGCCTT
AATACCACGACGAAGCGACTCAGTAATTGGGGTGTCACAACGAGCCAGGCTGCCGAGGGACTGAAAAAGGTTCGTGAGCA
GATGGACGAAGTTATCGGACGTCAGCAACTGATTAGCAAGCCGGTACGCGTTCGTACATCTGGCAGTGGCGATGGTGGCA
GCGGGCGGCGTAGCGGCTCTGGCCATAGCGGTAAAAGTAACGAAGGAGGTATGTTCTCTGGCCTTCGTGGCAACATTTTC
CTGCTTGGCGAGATCGGAGATGCAGCCAGAACGGTTACTGACATCATGTTTGGATGGCAGAAGCCTATTGTTGAAGCTGC
GGCCGAAATGGAACGTATGCGGGTGATGCTTCGTGGGTTGAATAAGGAGAAGTCTAACCCTGGCCAGGCTGCCGCCGATG
ACATGAAGTACATCGTAGACATGGCTCAAAATGCGCCGTTCGCGATGCAGGCGTTAACAGATTCCTTCGTGAAGTTTCGT
TCTGCTGGCTTAGATCCAACCGATGGCTCTCTGAAGGCGCTGGTGGACTCCGTTGCTCGTTTTGGTGGTGATAGTGAGCT
GTTGAAACGTGCGGCTGTGGCCGTTCAGCAGATGTCCGGTAAGGGCGTCGTGTCAATGGAAGAGCTACGTCAGCAATTAG
GTGAAGCCGTTCCTAACGCGATGCAGGCAATGGCAGACGCCGCAGGCATCACTATGGGGGAACTGACTAAAGCCGTTGCC
AGCGGTACGGTTGAGGCAAAACAGGCGTTGTCTCTGATGTTTGTTGGTCTGCGTGCGGAGAATGAGAACGCAGCGAAAGA
CATGATGCAAACCTACACAGGTGCGCTGGCGCAACTTCAGACGTCATTCACGTTATTTGCTGATCGGGTTGGTCAGGCCG
GATATCTGGATTCTCTATCGAAGGGGATGAAAGAACTGGCTTCAATCATGAATAGCGCCGAAGGGATTTCGTTTGCTAAT
TCTTTAGGTTCGGGGTTAACGACGGCAATCGATGGGTTGCGTCAGCTTGCTCAATGGTTAGCAAAGAACCAAGAGCTGGT
AATTAATCTCGGTAAGGTCGTGGCCGCGATGGTTGCGTTCAAACTGATGCGTGCAGGGATTATGGGCGTAGTTGGTGCTG
GCGGCCAGATGGTTAGTACCTTCGCCACGATGGCAACCGCCATACAGACTCCATTTAACCTCGGCGCTACAGCAGTAACT
CGATTCAATCGTGCGGCACGTATGGGGTTGGCTCCGATCCCCTCTCTCATTTTCGCCATCCGTGGGGCGATTACGGGGCT
GAAAGGCGCTTTTGCAGGATTAACGGCGTTCATTGCAGCAAACCCTATAGGGGCAGCATTTACAGTTGCCACTGTAGCTG
TTGCTGGCCTAATCACGTACATGACCATGCTCCGCAGCGAAACTTCAAAGGTCGTTGACGAGATTAGGAAAATACCAGAA
GCGATGACAGCGGCCAAACGTGCACAAATGGCCGACCGCGCAGAAAAGCTCGAAAAGCAGATTCTTGCTGATCAACGAGC
ATTAAAAACTGGTGAGGGTGTTAATTACGTATCTAACTCTGCCGGTGTTATTACTCACAAAGAGTCGAAGGCTGACATCG
AAGCACGCCTGAAAAAAAATCAGGAAGAGTATCAAAAGATAACCGGCACGATAGCTCTTGGTGACGGCGCTGTAGCAAAG
CGTTTGGCTAAAGAGGCTGCCGAATCTCAGATTGAGAAAATTCGAGCGGAAAACCAGATTTTCGCGGCAACATTCGTGAA
AGCCCGGCAGGAGGCTCTGGATAAGATCCAGAAAATCAATGATGACGGTTCACTTTCAGATGACGAAAAGAACAAGCTAT
TGGCACCGTTACGTGAAACGGTAAACAAAAGCTATCTGGAGCCTGCGCAAAAACTGGTTGATTCACTTTCTTCTCGTAAG
AATGCTACCGAGAAGCAAATAGCGACTCTTAATGATCAGCTTGAAAAAGCCAAAAAAGATGGCAATACCGAGCAAATCCA
GAAACTGCAAGGCAGTATTCGTGGTTATCAGGAGCATTTGGAAGCCGTTGCTCAGGAACTGACACAGGCAGAGTTCGAGA
GAGATAACGCGGCCAAAACTGGTAAGGGCGTAATGTCAAACCAGGGGACGGTTCTTGGGTTAGGTACAACTGATAAAGCT
GCTCAGAAGGCGCTGGCGCAATATATGCGAAACCAGATGGATTCTGCGACTTATCAGCGTACTTTGCCTGATGGCACTCC
GATGAAGGACTTCGAAGGTAAGCCGATTATTGGGCCTAAACAACTCAAGACGCAGCTTAATTTGCAGAAAGCATCCAGTG
CCAGCTCTCTGGAGAAAATGAGCGATGAAGAGCGTGCCGCAGCCATTGCCGCACTGACTAAAGCTCGTGAACAGGATGCC
GCAGCCGCCGAGAAAGCAGCCCAACGATCAGCTAATGCCTCGCAGCGTGCGGCCAAGAAAGAACAGGCAGCGCAACAGAA
ACTGGCGGCCGGATATCAGAAGGCTCTGGATAAAGCCGATCAGCTTATGGGGCAAATGGGTGAAAGCTCTAAGGCTACGG
TATCGTTTGATCAGTCTCTTCGCGATACAACGAAATCGCTAACTGATTTGGCTAATGCGGTTCCTAACGAGTTCATCACT
CAAGAGATGATCGACAAGGCGAAAAAACGTCTTGAAGACCTCAAAAATGCGACACCTGAATATCGCGAGATGTTTAATCG
CCGCAATGTTGAGCAGATGATCTCCACTTGGGCACCGGAGGCGGATTCCATTATTAGTGCTGGCTATACGCCGTCTCGTG
AAGAGAAAGTTGCTGATTTCGAAGACACCTACAACCGCAATCTCAAAGCGTTGATAGAACTTCGTGATAAGGCGTCTGAT
CCTAAAGTCGTGGCGCTTTATACAAAGAAAATCAATCAACTTATTGCTGCTGGCAATACCGCGCTTATTAAAGAGACGGG
GACTGCGACGCAGAAGTTGGCACTGGAATACGAAAACCTGGCAGAGCAGATTGAAAGCACCTGGACTGATTTGTTTAGTG
GCTTAACTGATGTCCTGACTGATTTCGTTATTAACGGGAAGATGAGCTTCTCCAGCCTATCTCAGTCCATTTTGAAAGAT
ATCACCAATATGGTCGTGAAGTCGCAAATCACGCTGCCTCTAATGAACATGTTGGGGATGGGAACCACCGCAGCTGGTAG
TTCACAGAGTGGCAATTTGCTGACCGGGGTTGCTTCTGCCGTTGCCAATCAAGGTGTACGAATGGGCAACACTGTTAATG
GCGACAAGTCGGTAGGAGAAGCCACGAAGGAGACGTCCAGTTCGGTAACTGGATTGGGGCAAACAACACAGCAGACCACC
AGCGCAATTGGCACAGCAACAAATGCGATTGGTAGCTGGGTATCGGGGCTATTTGATAGCACCGAAGCCAAAGATGCTGA
GACAAAAGCAGTGAAGGACTCCATCTTCTCGATGCAGAATCTCAGCTCTGTTACCGGCGCTCTGTCAGCCGCGTTTGCAA
TGCTTGGAGCTAATGCTTCCGGCTCTGGTAATAAGTGGTTGAATTTCGGCGCGACAGTTGCATCTGGTTTGGTTTCAGTA
TGGGCTGGTGGTGGATTCGATGGATTGATGTCCGGTTCTTCTGGCTCAACTTCCGGCTTCAACAATCTGACCAGTTCTGC
TGCCGATGGCACGAATGGTATTCCTGCAATTCCGAAGTTTGCAAATGGTGGCATATTTGGAAAAGACGGCGTGATCCCGC
TCCGGGCATACCAGAAAGGCGGTATCGCCAACTCACCTCAATTGGCGTTGTTTGGGGAAGGCGATATGAATGAGGCGTAT
GTTCCATTGCCTGATGGTCGAACAATCCCTGTAACGCTCAGTACCGATGGTATGAGTGGAGGAGGAAATGTTCTTTCTCC
GGTATCAATTGAGATCAACGTCCATAGTGACGGTAGCACAACTGAATCCGGCGATACAGAAAGCATATGGAACAATGCCG

CTCAACGGATGAAAGCAATCGCGCTTGAGACTATCGCTCAAGAGAAACGCCCTGGCGGATCACTCAACCCAAACACTCAA
CGTAACTAA
>Cluster_4985
ATGACTCATCACACACACACCATTGCTGAATCCAATAACTTTATCGTCCTTGATAAGTATATCAAAGCTGAGCAAACAGG
CGACAGCTATCAGAGCGAATCGGACCTGGAACGTGAACTGATTCAGGACCTACAAAATCAGGGCTATGAATTTATAGCCG
TAAAATCTCAGTCGGCGATGCTGGCCAATGTTCGACAACAGCTTCAGAACCTCAATGGTGTGGTATTTAATGACAGTGAG
TGGCGGCGTTTCACGGAGCAGTATCTGGATAACCCGAGCGATGGCATTCTGGATAAGACCCGTAAAATCCATATCGACTA
TATTTGCGACTTTATTTTTGATGACGAACGCCTTGAGAACATCTATTTGATAGATAAAAAGAATCTCATGCGCAATAAGG
TGCAGATTATCCAGCAGTTTGAACAGGCGGGTTCTCATGCTAACCGTTATGACGTCACGATCCTGGTTAATGGCTTGCCA
CTGGTGCAAATCGAACTGAAAAAGCGCGGCGTGGCGATTCGTGAGGCCTTTAACCAGATACATCGTTACAGCAAAGAGAG
TTTTAACAGCGAAAATTCTCTGTTTAAGTATCTGCAACTGTTTGTCATTTCCAACGGCACCGATACCCGTTATTTTGCCA
ACACGACAAAGCGCGATAAAAACAGTTTTGACTTCACCATGAATTGGGCGAAATCAGACAACACGCTGATTAAAGACCTC
AAAGACTTTACCGCTACCTTTTTCCAGAAACATACTCTGCTTAATGTCTTAGTGAACTACAGCGTGTTCGATATTAGCCA
AACGCTACTGGTGATGCGACCGTACCAGATTGCCGCCACTGAGCGTATCTTATGGAAAATCAACAGCTCCTATAAAGCGA
AAAACTGGTCTACCCCCGAAAGCGGTGGCTTTATCTGGCACACAACCGGTTCTGGTAAAACCCTGACCAGCTTTAAAGCC
GCACGTCTGGCAACAGAACTGGACTTTATTGATAAAGTTTTCTTTGTGGTCGACAGGAAAGACCTCGATTACCAGACCAT
GAAGGAATACCAGCGTTTTTCGCCAGACAGCGTCAACGGCTCGGAAAATACCGCAGGTCTTAAACGAAATCTGGATAAGG
ATGATAACAAAATCATCGTCACCACTATTCAGAAACTTAATAACCTGATGAAAGCAGAAAGCGACCTGCCTGTATACAAT
CAGCAAGTGGTGTTTATATTTGATGAATGTCACCGCAGCCAGTTTGGAGAAGCGCAGAAAAACCTGAAGAAGAAATTCAA
ACGCTATTATCAGTTTGGTTTTACCGGTACCCCTATTTTCCCGGAAAACGCTTTAGGTTCAGAAACGACCGCCAGCGTAT
TTGGTCGTGAATTGCATTCATATGTTATTACCGATGCGATTCGTGATGAAAAAGTGCTCAAATTCAAGGTGGACTATAAC
GATGTGCGGCCACAGTTTAAGTCTTTAGAGACAGAAACCGACGAGAAAAAACTGAGTGCGGCTGAAAATCAGCAGGCGTT
TCTTCATCCCATGCGTATACAGGAAATTACGCAATATATTCTGAATAACTTCCGCCAGAAGACCCACCGTACTTTCCCAG
GTTCCAAAGGCTTTAATGCCATGCTGGCAGTGAGTAGCGTGGATGCCGCGAAAGCCTATTACGCGACGTTTAAACGGTTA
CAAGAGGAAGCAGCTAATAAATCGGCCACCTATAAACCGCTGCGTGTTGCGACAATCTTCTCCTTTGCCGCCAATGAAGA
ACAAAATGCCATTGGTGAAATTTCCGATGAAACTTTTGATACCAGCGCAATGGACAGCAGTGCTAAAGAGTTTCTTGACG
CTGCAATTCGTGAGTATAACAGCTATTTTAAAACTAACTTCAGCACCGACAGTAACGGTTTTCAGAACTACTATCGTGAT
TTAGCCCAACGGGTTAAAAATCAGGATATCGATCTGTTAATTGTCGTTGGGATGTTTTTAACCGGCTTCGACGCTCCAAC
ATTGAACACGCTATTCGTCGATAAAAAACTTGCGTTTTCACGGCCTGATGCAGGCATTCTCCCGCACCAACCGCATTTATG
ACGCTACTAAAACCTTCGGTAACATCGTCACTTTCCGGGATCTGGAACGCTCAACCATTGATGCCATAACGCTGTTTGGT
GATAAAAAATACCAAAAATGTGGTGTTAGAAAAGAGTTATGCAGAGTATATGGAAGGCTTTACAGATGCTGCCACTGGTGA
AGCTAAACGCGGCTTTATGGCAGTAGTTTCAGAACTGGAACAACGGTTCCCTGACCCTGCCAGTATTGAAAGTGAAAAAG
AGAAGAAAGACTTCGTAAAACTGTTTGGTGAATACCTGCGTGCCGAGAACATCCTGCAAAACTATGATGAATTTGCCACG
CTGAAAGCCCTGCAACAAATCGATCTTAGCGATCCTGTTGCGGTAGAAAAAATTCAAAGCAGAACATTATGTGGATGATGA
AAAGTTCGCTGAATTGCAAATGATTCGTCTCCCTGCTGAACGCAAGATTCAGGATTATCGTTCTGCCTATAACGATATTC
GTGACTGGCAGCGCCGTGAGAAAGAAGCTGACAAAAAAGAAAAATCAACCACTGACTGGGATGACGTGGTTTTTGAAATC
GATTTGCTGAAGTCTCAGGAAATAAACCTGGATTATATCCTTGGACTGATTTTCGAACACAACAGACAAAACAAAGGTAA
GGGCGAAATGACCGAAGAGGTCAAACGCTTAATTCGTTCAAGCCTTGGGAACCGTGCTAAAGAAGGATTGGTGGTCGATT
TTATTCAGCAAACGAACCTGGATGATTTGCCGGATAAAGCCAGCATCATTGACGCGTTTTTTACCTTTGCTCAACGCGAA
CAGCAACGTGAAGCAGCAGCATTGATCAAAGAAGAAAATCTCAATGAAGAGGCGGCGAAACGCTATATTCGCACGTCTTT
AAAACGCGAATACGCAACCGAAAATGGCACGGAATTAAACGAGACATTGCCAAAACTCAGTCCGTTAAATCCGCAATATA
AGACGAAAAAACAGACGGTTTTCCAGAAGATCGTCGCGTTTATTGAGAAGTTTAAAGGTGTAGGCGGGCAGATATAA
>Cluster_61202
ATGAAATTAAATATATTTACTAAATCTATGATTGGTATGGGGCTGGTGTGTTCCGCTCTGCCAGCATTGGCAATGGAAGC
ATGGAATAACCAACAAGGTGGTAATAAATATCAGGTTATTTTCGATGGCAAAATTTATGAAAATGCCTGGTGGGTTTCTT
CTACAAATTGCCCGGGAAAAGCGAAAGCAAATGATGCAACTAACCCGTGGCGTTTAAAGCGTACCGCAACAGCTGCTGAA
ATTAGTCAGTTTGGCAATACACTTTCCTGCGAAAAGAGCGGCAGCTCATCTTCTTCAAATTCAAATACGCCTGCATCCAA
TACGCCGGCTAATGGCGGTTCGGCTACACCAGCACAGGGCACTGTTCCGTCTAATTCTTCTGTAGTTGCCTGGAATAAAC
AGCAGGGCGGTCAGACCTGGTATGTCGTCTTTAATGGTGCGGTATATAAAAATGCCTGGTGGGTAGCCTCTTCTAACTGT
CCGGGTGATGCGAAAAGCAATGATGCCAGCAACCCATGGCGTTATGTTCGTGCCGCTACGGCAACGGAAATCTCAGAAAC
CAGTAATCCACAGTCCTGTACTTCAGCACCACAGCCTTCACCGGATGTGAAACCGGCACCGGACGTTAAACCGGCTCCTG
ATGTTCAGCCAGCCCCAGCTGATAAGTCAAACGACAACTATGCTGTAGTAGCCTGGAAAGGTCAGGAAGGTTCTTCTACA
TGGTACGTTATCTATAACGGCGGCATTTATAAGAACGCCTGGTGGGTAGGCGCGGCAAATTGCCCAGGCGATGCGAAAGA
AAACGATGCCAGTAACCCATGGCGTTATGTTCGCGCGGCAACGGCAACAGAAATCAGCCAGTATGGTAACCCTGGCTCCT
GTTCCGTTAAGCCGGATAATAATGGCGGTGCTGTGACTCCGGTTGATCCAACTCCGGAAACACCGGTGACCCCAACTCCG
GATAACAGCGAGCCATCAACACCAGCGGATAGCGTTAACGATTACTCATTGCAAGCGTGGAGCGGCCAGGAAGGTAGCGA
AATTTACCATGTTATTTTCAATGGTAATGTTTACAAGAACGCCTGGTGGGTTGGGTCTAAAGATTGCCCACGGGGTACCA
GCGCTGAAAACTCCAATAACCCATGGCGTCTCGAGCGTACAGCTACCGCTGCGGAATTGAGTCAGTACGGTAACCCGACT
ACCTGTGAAATTGATAACGGCGGCGTCATTGTTGCGGATGGTTTCCAGGCCAGCAAAGCGTACAGCGCGGACAGCATCGT
AGATTATAACGATGCACATTATAAAACTTCTGTCGATCAAGACGCATGGGGCTTTGTCCCGGGCGGCGATAACCCGTGGA
AGAAATACGAACCGGCGAAAGCATGGTCCGCATCCACTGTGTACGTGAAAGGTGATCGCGTTGTTGTTGATGGGCAGGCT
TATGAAGCGCTGTTCTGGACGCAAAGTGACAACCCTGCTCTGGTGGCGAACCAAAACGCCACCGGTAGCAATAGCCGCCC
GTGGAAGCCGTTAGGTAAGGCTCAGAGCTATAGCAACGAAGAGCTGAATAATGCGCCGCAGTTTAATCCAGAAACGCTTT
ATGCCAGCGATACGCTGATTCGCTTTAACGGTGTGAACTACATTTCTCAGAGTAAAGTGCAGAAAGTTTCTCCTTCTGAC
AGCAACCCGTGGCGTGTTTTTGTTGACTGGACCGGAACCAAAGAGCGCGTAGGTACGCCGAAGAAAGCGTGGCCGAAACA
CGTTTATGCACCGTATGTCGACTTTACGCTGAATACGATCCCGGATCTGGCTGCGCTGGCTAAGAATCATAACGTCAACC
ACTTCACGCTGGCGTTTGTGGTGAGTAAAGATGCGAACACCTGTCTGCCGACATGGGGTACCGCTTACGGTATGCAGAAT
TACGCTCAGTACAGCAAAATCAAAGCTCTGCGTGAGGCTGGCGGCGATGTGATGCTGTCTATCGGTGGTGCTAACAACGC
TCCGCTGGCTGCTTCCTGTAAGAACGTAGACGATCTGATGCAGCATTATTATGACATCGTTGATAACCTGAACCTCAAAG
TCCTGGACTTCGATATCGAAGGCACCTGGGTTGCGGATCAGGCATCTATTGAACGTCGTAACCTTGCTGTGAAGAAAGTG
CAGGATAAATGGAAGTCAGAAGGCAAAGATATTGCTATCTGGTACACCTTGCCAATTCTGCCGACTGGCCTGACGCCGGA
AGGGATGAATGTCCTGAGCGATGCCAAAGCGAAAGGTGTTGAGCTGGCGGGTGTGAACGTGATGACAATGGACTACGGTA
ACGCGATTTGTCAGTCTGCAAATACCGAAGGCCAGAACATTCACGGTAAGTGTGCAACGTCTGCGATTGCCAACCTGCAT
TCACAATTGAAAGGCCTCCATCCCAATAAGAGCGATGCAGAAATTGACGCTATGATGGGTACCACGCCGATGGTTGGCGT
GAACGACGTTCAGGGCGAGGTGTTCTATCTCTCTGATGCTCGTCTGGTCATGCAGGATGCGCAGAAGCGTAATCTCGGTA
TGGTTGGTATCTGGTCAATCGCGCGCGACCTGCCGGGCGGCACTAACCTGTCTCCGGAATTCCACGGCCTGACTAAAGAA
CAGGCACCGAAGTACGCATTTAGCGAAATCTTCGCGCCGTTTACTAAGCAATAA
>Cluster_30548
ATGGCCCGCGAGGTGGAGAAAACCCGCCAGCGCATGGAGGCGCTGAGCCAGAAAATGCGCGAGGAACAGGCGCAGGCCAT

GGCTCTGGCGGAGGCTCAGGATAAAGCGGCTGCTGCGTTTTATCGTCAGATTGACAGTGTGAAACAGGCCAGTGCGGGGC
TGCAGGAATTACAGCGTATTCAGCAGCAGATCCGACAGGCCAGAAACAGTGGCGGGATTGGTCAGCAGGATTATCTGGCG
CTGATTTCTGAGGTTACGGCGAAAACCCGTGTTCTTACACAGGCTGAGGAAGAGGCTACCCGACAGAAAGTGGCGTTTAT
CCGTCAGCTTAAAGAGCAGGCAACCCGCCAGAATCTTTCTTCTTCTGAGTTGCTTCGTGCTAAGGCTGCCCAGCTGGGGG
TAAGTAGTGCTGCAGAAGTGTATATCCGCAAAATGGAGCAGGCAGGAAAAGCCACGCATTCGCTGGGGCTGAAAAGTGCA
GCGGCCCGCCAGGAGATAGGCGTTCTGATAGGTGAACTGGCTCGCGGCAATTTAGGTGCGCTGAGGGGATCCGGGATAAC
GCTGGCTAACCGTGCCGGATGGATAGACACACTGATGTCACCGAAAGGCATGATGCTGGGCGGGGTTATTGGCGGTATTG
CCGCGGCCGTCTATGGTCTGGGTAAAGCCTGGTATGACGGTCAGAAGGAGGGGGAAGAATTTAACCGCCAGTTGTCGCTG
ACGGGGCATTATGCCGGAGTCACTGCCGGGCAGCTGTGGACGCTTAGTCGTGCTATTTCCGGGAATGGTATCACGCAACA
TGCTGCAGCCGGTGCGCTGGCTCAGGTGGTGGGGAGTGGTGCATTTCGTGGAAACGATATCGGTATGGTGGCGAGAGCTG
CCGCACAGATGGAGCGATCGGTTGGCCAGTCGGTCAGCGATACCATAAATCAGTTTAAGCGGCTGAAGGATGATCCTGTA
AATGCCGCGAAGGCTCTGGACAATGAGCTGCATTTCTTACTGCCACTCAGCTTGAGCAGATACGTGTCCTTGGGGATCA
GGGACGGTCCAGTGATGCTGCACGGATAGCCATGTCTGCACTGGCAGAGGAAACCGGTCGGCGTACTGCGGATATTGATA
ATAACCTCAATGCGCTTGGCAGTACGCTGAAGTATCTGTCTGATTTGTGGAGTCGTTTCTGGGATGCGGCCATGAATATT
GGTCGTGAAGACTCGCTGGATGAACAGATTGCCGCTTTACAGGAGAAAGTGTCGCGGGCGAAAAGACTCCCCTGGACGGC
ATCATCTTCTCAGGTTGAATACGATCAGCAGCGTCTTAACGATCTTCAGGAGAAAAAACGCCAGAAGGATTTGCAGGATG
CAAAAGAGCAGGCAGAGCGGAATTATCAGGAGCAACAGAAACGACGTAATGCTGAAAATGCTGCACTGAACCGGATGAAT
GAAACGGAAGCAGCACGACATCAGCGTGAAATTGCGCGTATTAATGCCATGCAGTACGCCGACCAGGCTGTCAGGGATGC
GGCGATACAACGTGAAAATGAACGTTACGAGAAAGCCCTGGCATCCGGTAAGAAAAAAACACGCGAAACCCGTAATGATG
AGGCCACCCGGTTATTGCTGCAGTACAGTCAGCAACAGGCACAGGTGGAAGGACAGATTGCTGCTGCCAGACAGTCAGCA
GGCATTGCCACGGAAAGGATGACAGAAGCGCATAAACAGCTTCTGGCTCTGCAGCAGCGCATCAGCGACCTGGACGGGAA
AAAACTGACGGAAGATGAAAAGAGTGTGCTGGCCCGTAAAGATGAACTGATTCAGGCACTGACGCTGCTGGATGTAAAAC
AGCAGGAGCTTCAGAAACAGACGGCACTCAACGAGCTGAAGAAAAAAACAATTCAGCTGACCAGTCAACTGGCTGAAGAA
GAGCTCGCTCAGCGTCAGCAACATGACCTGGATATCGCCACGGTGGGTATGGGTGATCAGCAGCGGCAGCGATATCAGGT
ACAACTGAGTCTTCGCCAGAAATACCAGCAACAGCTGGAGCAGTTGAGGCGGGATAGTGAGCAGAAAGGGACATATAACA
CGGATGACTACAGAAAGGCCGAGCAGGCGCTGACGGAGAGCCTGAACCGACAACTGAATGAGAATCGCCGTTACTGGCAA
CAGCTTGAAGTTGTGCAGGGTAACTGGAAAAACGGAGTCCTGCGTGCATTTCAGGATTTTACCGTGGATGCAGACAATAC
GGCAGGAACAGCAGAACAGGTGTTCTCGTCAGCCTTCAGCAACATGGGAAATGGCCTGGCAACTTTTGTCACTACCGGCA
AACTCAACTTCAAATCCTTCACCTCTTCGGTGCTGTCAGATATGGCGAAAATCCTGGCGCAGGCAACCATGATGAAATCG
ATAAAAGGGATTGGCAGTGTACTGGGATTTGATCTCAGCAGCCTTTCCCTGAATGCCAATGGGGGATTTATCAGTCTGC
TGATTTGAGTCGTTACAGTGGCACGGTGGTTAACCGTCCGACGTTTTTTGCTTTTGCAAAAGGCGCGGGTGTGATGGGGG
AAGCTGGACCTGAAGCCATTCTGCCACTGCGTCGTGGTGCTGACGGTAAGCTGGGGGTTGTGGCGGATATTGGTGGTTCA
GGTATGGCGATGTTTGCCCCGCAGTACAACATCGAGATCAATAACGATGGCACGAACGGGCAGATAGGTCCGGCTGCCCT
GAAGGTGGTTTATGACCTTGGGAAAAAAGCGGCAGCGGACTTTATGCAACAGCAGGCCCGTGATGGTGGTCGGTTAAGTG
GAGCATATCGGTAA
>Cluster_3303
ATGACCGGAAATCACCCCGCCGCGCTGCTGCGTCGCCTTAACCCGTACTGTGCACGGGCGCTGGACGCTGCCGCCTCACT
GTGTCAGACCCGCGCCCATGCGGAGATAACCATTGAACACTGGCTGCTGAAACTGCTGGAGCAGGGAGAAGGCGATATCA
CGGTGATTGCCCGCCGCTATGAATGGGATATCGACACGCTCTGGCAGTCTCTGCTGGCACATCTGGACACCTTACCCCGC
TCGGTCCGCGAACGTCCGCAGCTTTCTGAACCCCTGACGGCGCTTATCAGGCAGGCGTGGCTGATTGCCTCGCTGGAAGG
CGACGACCCGCAAATCCGCAGTCAGCACCTGCTGATGGCGCTGACAGAAAAACCGATGCTGCCCGCCTGTAATGACCTGT
GGGTATTGCTGAGCCTGAGCCGCGTGCAGCTTGAGCGGCTGCGCCCCCTGCTGGATGCGCAGTCGGATGAATGTCCGGCA
CGTCAGCCACAGGCCACCGAACCGCTGACATCCGCGCAACCAGAGTCGGCAACGACGGACGCACCGGCAAAAACGCTGAC
GGGGAAACAGGATGACGCCCTGCTGGCGGTACTTAACCGCTTTACCGAAGACGTGACGGAAAAAGCCCGCAGCGGGCGAA
TCGACCCGGTATTCGGGCGCGACACCGAAATTCGCCAGATGGTCGATATCCTCTCCCGTCGCCGCAAAAACAACCCGATT
CTGGTGGGAGAACCGGGCGTGGGCAAAACCGCGCTGGTGGAAGGGCTGGCGCTGCGTATCACCGAAGGCAACGTGCCGGA
CAGCCTGAAAACGGTGCATATCCGCACACTGGACCTCGGTCTGTTACAGGCTGGCGCGGGCGTTAAAGGTGAATTTGAGC
AGCGACTGAAAAACGTGATTGATGCAGTACAGAAATCACCGGAGCCGGTACTGCTGTTTATTGATGAAGCCCATACCATT
ATCGGCGCGGGTAATCAGGCGGGCGGCGCGGATGCGGCGAACCTGCTGAAACCGGCGCTGGCAAGGGGCGAGCTGCGCAC
CATCGCCGCCACAACGTGGAGCGAATACAAACAGTATTTTGAGCGCGACGCCGCGCTGGAACGCCGCTTCCAGATGGTGA
AAGTCGACGAGCCGGATGATGACACCGCCAGCCTGATGCTGCGGGGACTCAAGGCCCGCTATGCACAGCACCACGGCGTA
CATATGCTGGACAGCGCCATTCAGACCGCCGTGCGCCTGTCGCGCCGCTATCTGACCGGACGCCAGTTGCCGGACAAGGC
GGTTGATTTGCTGGATACCGCCGGGGCAAGAGTCCGCATGAGCCTCGACACCCTGCCGGAACCGTTGACGCAGCTTCAGG
CGCGTCTGACCGCGCTGGATATTGAGCGGGAAGCGATTGAGCAGGACAGCGTGTTTTATCCCGAAACCAGCCCGGAGCGG
CTGGCGGAACTGACCGATTTGCGTGATGAGCTACAGGCAGAAGCCGGGCATCTGGAAGCGCAGTATCAGCAGGAAAAGCG
GCTTGCGCAGCAGATTATGGCGTTGCGTCAGGAAGGGGCAGACAGCACTGATTTGCAACAGCAACTGCGAACGCATCAGG
GCTTTGCACCGCTGCTGGCGCTGGATGTGGACGCCCCGCGCCGTCGCCACTGTGGTGGCGGACTGGACCGGCATCCCGCTC
TCTTCCCTGCTCAGGGACGAGCAGAGCGACCTGCTCAGTATGGAACAAAGCCTTGAAAACCGCGTTGTCGGGCAAAGCCC
GGCGCTCTGCGCCATCGCACAGCGGCTGCGGGCGGCTAAAACCGGCCTCACGCCGGAGAACGGCCCGCAGGGGGTATTCC
TGCTGACCGGCCCCAGCGGCACCGGTAAAACCGAAACTGCGCTCACACTGGCCGACACCCTGTTTGGCGGTGAAAAATCC
CTTATCACCATCAACCTCTCCGAATATCAGGAGCCGCATACCGTCTCCCAGCTTAAGGGGTCTCCTCCGGGCTACGTTGG
CTACGGTCAGGGCGGCGTACTCACCGAAGCCGTTCGCAAACGCCCGTACAGCGTGGTGCTGCTCGACGAAGTGGAAAAGG
CGCATCGCGACGAGATGAACCTGTTCTATCAGGTATTTGATCGGGGCTTTATGCGCGACGGCGAAGGCCGGGAAATTGAC
TTCCGCAACACCGTGATTCTGATGACGGCTAATCTGGGCAGTGACCACATCATGCAGCTGCTGGAGGAAAAACCGGACGC
CACGGACGCAGACCTGCATGAACTGCTGTACCCCCTGCTGCGCGAGCACTTCCAGCCTGCACTGATGGCGCGTTTTCAGA
CGGTGATTTACCGCCCTCTGGGACAGGAAGCGATGCGCGCCATTGTGGAAATGAAACTGGCGCAGGTGGTTCGTCGTCTT
CGTCAGCACTATGGGCTGGAAACGGAAATCAACGACAGCCTGTATGACGCGCTGACCGCCGCCTGCCTGCTGCCGGACAC
CGGGGCGCGTAATATCGACAGCCTGCTGAACCAGCAAATCCTGCCGGTCTTAAGCCAGCAGTTGCTGGCGCAACAGGCCG
CGCACCGTAAACCAGCCCAGCTACGGCTTGGCTGGGATGAGGAAGACGGGATTGTACTGGAGTTCGCTACGGAAGAGATG
CAATAA
>Cluster_60486
ATGAGTCAGCGAGGACTGGAAGCACTACTGCGACCAAAATCGATAGCGGTAATTGGCGCGTCGATGAAACCCAATCGCGC
AGGTTACCTGATGATGCGTAACCTGCTGGCGGGAGGCTTTAACGGACCGGTACTCCCGGTGACGCCAGCCTGGAAAGCGG
TGTTGGGTGTGTTGGCCTGGCCGGATATTGCCAGCTTGCCCTTTACACCCGACCTTGCGGTTTTATGTACCAATGCCAGC
CGTAATCTTGCTCTTCTGGAAGAGCTCGGCGAGAAAGGCTGTAAAACCTGCATTATTCTTTCCGCCCCGGCATCGCAACA
CGAAGATCTCCGCGCCTGCGCCCTGCGCCATAACATGCGCCTGCTTGGACCAAACAGTCTGGGTTTACTGGCTCCCTGGC
AAGGTCTGAATGCCAGCTTTTCGCCTGTGCCGATTAAACGCGGCAAGCTGGCGTTTATTTCGCAATCGGCTGCCGTCTCC
AACACCATCCTCGACTGGGCGCAACAGCGTAAGATGGGCTTTTCCTACTTTATTGCGCTCGGCGACAGCCTGGATATCGA
CGTTGATGAATTGCTTGACTATCTGGCACGCGACAGTAAAACCAGCGCCATCCTGCTCTATCTCGAACAGTTAAGCGACG

CGCGACGCTTTGTTTCGGCGGCCCGTAGTGCCTCGCGTAATAAACCGATTCTGGTGATTAAAAGCGGACGTAGCCCGGCG
GCACAGCGACTGCTCAACACGACGGCAGGAATGGACCCGGCATGGGATGCGGCTATTCAGCGTGCCGGTTTGTTGCGGGT
ACAGGACACCCACGAGCTGTTTTCGGCGGTGGAAACCCTTAGCCATATGCGCCCGCTACGTGGCGACCGGCTGATGATTA
TCAGCAACGGTGCTGCGCCTGCCGCGCTGGCGCTGGATGCCTTATGGTCACGCAATGGCAAGCTGGCAACGCTAAGCGAA
GAAACCTGCCAGAAACTGCGCGATGCACTGCCAGAACATGTGGCAATATCTAACCCGCTCGATCTACGCGATGACGCCAG
CAGTGAGCACTATATTAAAACGCTGGATATTCTGCTCCACAGCCAGGATTTTGACGCGCTGATGGTTATTCATTCGCCCA
GCGCCGCTGCTCCCGCAACAGAAAGCGCGCAAGTATTAATTGAAGCGGTAAAGCATCATCCCCGCAGCAAATATGTCTCT
TTGCTGACGAACTGGTGCGGCGAGCACTCCTCGCAAGAGGCACGACGTTTATTCAGCGAAGCCGGGCTGCCGACCTACCG
TACCCCGGAAGGAACCATCACTGCTTTTATGCATATGGTGGAGTACCGGCGTAATCAGAAGCAACTACGCGAAACGCCGG
CGTTGCCCAGCAATCTGACTTCCAATACCGCAGAAGCGCATCTTCTGTTGCAACAGGCGATTGCCGAAGGGGCTACGTCG
CTCGATACCCATGAAGTTCAGCCCATCCTGCAAGCGTATGGCATGAACACGCTCCCTACCTGGATTGCCAGCGATAGCAC
CGAAGCGGTGCATATTGCCGAACAGATTGGTTATCCGGTGGCGCTGAAATTGCGTTCGCCGGATATTCCACATAAATCGG
AAGTTCAGGGCGTCATGCTTTACCTGCGTACAGCCAATGAAGTCCAGCAAGCGGCGAACGCTATTTTCGATCGCGTAAAA
ATGGCCTGGCCACAGGCGCGGGTCCACGGCCTGTTGGTGCAAAGTATGGCTAACCGTGCTGGCGCTCAGGAGTTGCGGGT
TGTGGTTGAGCACGATCCGGTTTTCGGGCCGTTGATCATGCTGGGTGAAGGCGGTGTGGAGTGGCGTCCTGAAGATCAAG
CCGTCGTCGCACTGCCGCCGCTGAACATGAACCTGGCCCGCTATCTGGTTATTCAGGGGATCAAAAGTAAAAAGATTCGT
GCGCGCAGTGCGCTACGCCCATTGGATGTTGCAGGCTTGAGCCAGCTTCTGGTGCAGGTTTCCAACTTGATTGTCGATTG
CCCGGAAATTCAGCGTCTGGATATTCATCCTTTGCTGGCTTCTGGCAGTGAATTTACCGCGCTGGATGTCACGCTGGATA
TCTCGCCGTTTGAAGGCGATAACGAGAGTCGGCTGGCAGTGCGCCCTTATCCGCATCAGCTGGAAGAATGGGTAGAATTG
AAAAACGGTGAACGCTGCTTGTTCCGCCCGATTTTGCCAGAAGATGAGCCACAACTTCAGCAATTCATTTCGCGAGTCAC
CAAAGAAGATCTTTATTACCGCTACTTTAGCGAGATCAACGAATTTACCCATGAAGATTTAGCCAACATGACACAGATCG
ACTACGATCGGGAAATGGCGTTTGTAGCGGTACGACGTATTGATCAAACGGAAGAGATCCTCGGCGTCACGCGTGCGATT
TCCGATCCTGATAACATCGATGCCGAATTTGCTGTACTGGTTCGCTCGGATCTCAAAGGGTTAGGCTTAGGTCGACGCTT
AATGGAAAAGTTGATTACCTATACGCGAGATCACGGACTACAACGTCTGAATGGTATTACGATGCCAAACAATCGTGGCA
TGGTGGCGCTAGCCCGCAAGCTCGGGTTTAACGTTGATATCCAGCTCGAAGAGGGGATCGTTGGGCTTACGCTAAATCTT
GCCCAGCGCGAGGAATCATGA
>Cluster_67035
ATGAGTATGGTGGATACCGTTTATGTCATGAGTGGCGGTGAGTTCTTCCAGGCGGCATTTAATGGCGTGGCCACGCTTAT
CGGCACATCCACATGGGACTCCTTGTTTCATATAGTGGATTCTTCTCCGCGATGGCACTTTTCTTCGTTTATATCCGGG
GGCATGACCCGAAGGAAGTGCTGAAGTTTATTGCGGTGTTCATTCTGATAACCAGTGTGCTGGTTATTCCGAAACGTACC
GTGCATATCATCGATATGACGAATCCCACACTGGTGCTGCGGGTGGATAATGTGCCGACCGGGCTGTCGGTACCGTTCCG
GTTTATCACCAGTATTGGTGGCTCCCTGACGAAAATTTATGACTCCGTGTTCCATACGCCGGATTCCGTGGCATACAGCA
AAACCGGTATGTTGTTTGGTGCCACCCTGATGGCCAACACCACGGATGTGGTGGCACAGAACGGTGACCTGGCCATGCTG
CTGAGCGAATATGTACAGCAGTGTGTTATCGGCGACATTATGCTGACACACAAATACAGCATGGCTGAACTGATGCAGTC
ATCTGACCCTTATGAAATCATCTTCCGCAAACCCAGCCCACTGCGTGGCGTGATTGTTCCTCGTGGCAACAGCCTGGCGC
AGGCAGGATTCCAGACCTGCGAGGCGCTGGCCAACAATGTGCTGAAGCGTGAGCTGAAAGTGGATACGACAAAGGGCGGA
AAGACCTGGGATTATTATGTTAACCGCTTCATTGGCCCCCGTGCCAGCGCCGATACGCTGTTCGGTCTGATGATGGCGGA
CAGCTATGGCTTCTACTATCAGGGCGGACGGGATGCCAGTGAAATCCTGCGCCAGAATGTGGTGATGAATGCCATAAAAC
AGGGTATCACCACCCATACGGCAGCCAGCGGCAACGTCGCCAGTCTGGTGAATATGGCGGACCAGTCATCGAACAGCAAA
ATGCGCCTCAGCTGGGCGGCGTCAGGTGGTCTGGCAGCCACATTTGTGCCGGTGATGCATACTGTCCTGATGGCCATGCT
GGTCGGGATGTTTCCGATAATCATTCTGCTGGCCACCATTCACGGTTTGACACTGCCGGTACTGAAAGGCTATGTGTTCT
CCCTTGTTTATCTGCAGAGCTGGCCACCACTGTATGCCATTCTGAACTACGCGATGGCGTTTTATCTGAAAGGCGAAACG
AAGGGGATGAATTTCTCCCTGTCGAATCTGAACACCATCCAGCAGACTCACAGTGATATCGGGTTGATGGCAGCCTGGCT
GTCAAACTCAATTCCGTTTATTGCTGCCGGTCTGGTGTTTGGTTTGTGGCGCGTGGTTTCGCAGGCAGGGAACTATCTGG
GCTCATCCATTAACAGCACAGCCTCGTCTGCTGCCTCTCAGGCTGCAGACGGTACGTGGGCGTTTAACAACATGCAGATG
GAGAATGTCTCCGGCTTTAAATGGGACACCAACCAGTCCGTGCGTGACGGTCAGATGCTGACCCAGCATGCCAGCGGTGC
GACAACAACAAAAACAGCGGGTGGTGGCATTCTGCATGATGGTAAATCGGCGATTTCCAGTCTGGCGACGGATATTCAGT
TTGGTCGCATGCTCAGCAGCAGCTACCAGGCGCAACAGCGTGAAGCGGAAAGCCAGGTGCAGAGTCTCAGTAACAGCATC
AGTCATGGCAGCCAGCTGGTCGGCAGTCAGTTGACGCAGTGGGCGCAGCAGCGGGGCAACAGCGATACAGTGGTAAGCGG
TGCGGACAGCAGCCGGGCCACCACACTGACGCAGGCCATCAACAAACTGAACAGCGTGGCTGATTCACTGGCAAAACGTG
CCGGTATCAGTACTTCCGAAGCGGTTACGGAGGTACTGAATAAATCACAGAGCGGTGATTTTACTGCCGGTGGACATGGC
GAAGCCGGTATTGATACCAGGAAAAATATTCTCGGAAAAGCTGTTGAGTTAACAACCGGGGCCAGTGCCAGTGGAAAGCT
GTATACCGACATTAGCTATAAAGGTTCGGATCGGTCATCGCATGGTATTAATTCAGATATCAGCTCAAAAACAGGGCATG
ACCGTAGCCTGACAGCGCAGGAAACAAAAGATGTGCGTGATGCCATGGATGTCATTAATAACCATCGCACGACGGATAAC
AGCAGTCATACTGACAATGCCTCTTCGTCCCTGTCTGAGCAGCTGGCCAGCAACCTGAGCGATTTGCAGAGCCAGATTAA
GCAGTATAATGATGCACTTAACCGCAGCCATGAATATGCTCAGATGGCCAGTTATGCGGAAAACAACAGTGCAAGTATTA
ACAGCAACTACAGTCAGGAGTTTGTTAACTATGTGCATCAGTCTGCTCCGGGACGTGCCACGACCGTGCTGACCGATGCC
GGAAATCCGTCACTGCGGGCGGAGCGGGAACAACTGGCGCAGGCATTTGTGGATGAGAAACTGAAACCCCAGCTGGAGCA
GGAATTCAGGGAAAACCGGTTGCGGGCTTCAGAAAGTATGGGCAGCGTCGGGCCAGTCGTGGATATGCAGAGTGACCTGA
AGCGAGCGTATGCAGAGAATTTTGCTGACATGCAGCAACGGGCTTCCGGCGCATCGGTTCAGGGTGCAGAGCAGATCCAG
AAACAGGTGCGTGATACCCGTCAGACTGTGGAATCACAGGTGCTGAAAAATGAGCAGGAAATGGCATCATCCCAACAGGA
ACTGAAGACACAACGGAACACGCTTGCAGAGCAGCATAAGGAGAGTGAAGGAGCATATAACGAAGCATTGAAGCAGGAGC
GCTCAGAACAGAAATTTGGATGGAACTCAGATAAAGATGAAGAGAATATGCAACGTCTGAGGGATGCTGCAAAGTGGAAG
GATAAATAA
>Cluster_9811
ATGCCGCGTCGCTTGATCCTCTCGGCCACGGAGCGGGACACCCTGCTTGCGCTGCCGGAAAGCCAGGATGACCTGATCCG
CTACTACACCTTCAACGACTCCGACCTGTCGCTGATCCGCCAGCGACGCGGCGACGCCAACCGCCTCGGCTTCGCCGTGC
AGCTCTGCCTGCTGCGCTACCCCGGTTACGCGCTGGGAACCGACAGCGAGCTGCCCGAGCCGGTCATCCTGTGGGTGGCG
AAGCAAGTCCAGGCCGAGCCGGCGAGCTGGGCAAAGTACGGCGAGCGCGACGTGACCCGTCGCGAGCATGCCCAGGAACT
GCGCACCTACCTGCAACTGGCCCCGTTCGGCCTGTCCGACTTCCGCGCCCTGGTGCGCGAGCTAACCGAGCTGGCCCAGC
AGACCGACAAAGGCTTGCTGCTGGCCGGTCAGGCCCTGGAGAGCCTACGGCAGAAACGACGCATCCTGCCGGCGCTGAGC
GTGATTGACCGGGCCTGCTCGGAAGCCATTGCGCGAGCCAATCGGCGGGTCTACCGCGCCCTGGTCGAACCACTCACGGA
CTCGCATCGGGCCAAGCTGGACGAGCTGTTGAAGCTCAAGGCCGGCAGCAGCATCACCTGGTTGACCTGGCTGCGCCAGG
CACCGCTGAAACCCAACTCTCGGCACATGCTGGAACACATCGAGCGGCTGAAGACATTTCAGTTGGTGGACTTGCCCGAA
GGCCTGGGCCGGCACATCCACCAGAACCGCCTGCTCAAGCTGGCCCGCGAGGGTGGGCAGATGACGCCCAAAGACCTCGG
TAAGTTCGAGCCGCAGCGCCGCTACGCGACCCTGGCCGCCGTGGTGCTGGAGAGCACCGCGACCGTGATCGATGAGCTGG
TCGATCTGCATGACCGCATCCTGGTCAAGCTGTTCAGCGGCGCGAAGCACAAGCATCAGCAGCAGTTCCAGAAGCAGGGC
AAGGCGATCAACGACAAGGTGCGCCTGTACTCCAGGATCGGCCAGGCGCTGCTGGAAGCGAAGGAAAGCGGCAGCGACCC
CTATGCCGCCATCGAGGCGGTGATTCCCTGGGACGAGTTCACCGAGAGCGTCAGCGAGGCCGAGCTGCTGGCCCGGCCGG

AAGGCTTCGACCACCTGCACCTGGTCGGCGAGAACTTCGCCACCCTGCGCCGTTACACGCCGGCCTTGCTGGAGGTGCTG
GAACTGCGCGCCGCGCCGGCCGCGCAAGGCGTGCTGGCAGCCGTGCAGACCCTGCGTGAGATGAACGCCGACAACCTGCG
CAAGGTGCCGGCCGATGCACCCACGGCCTTCATCAAGCCGCGCTGGAAGCCGCTGGTGATCACCCCGGAAGGCCTCGACC
GGAAATTCTACGAAATCTGCGCCCTGTCCGAGCTGAAGAACGCCCTGCGCTCCGGCGACATCTGGGTCAAGGGCTCGCGG
CAGTTCCGCGACTTCGACGACTACCTGCTGCCGGCCGAGAAGTTCGCCGCACTCAAGCGCGAGCAGGCCCTGCCCCTGGC
GATCAACCCGAACAGCGACCAGTACCTGGAAGAGCGTTTGCAGCTGCTGGACGAGCAGTTGGCCACCGTCACCCGCCTGG
CCAAGGACAACGAGCTGCCCGATGCCATCCTCACCGAGTCAGGGCTGAAAATCACCCCGCTGGATGCGGCGGTGCCGGAT
CGGGCGCAGGCGCTGATCGACCAAACCAGCCAGTTACTGCCGCGCATCAAGATCACCGAACTGCTGATGGACGTGGACGA
CTGGACGGGCTTCAGCCGCCACTTCACCCACTTGAAGGACGGGGCCGAGGCCAAAGACAGGACGTTGCTGCTGTCCGCAA
TCCTCGGTGATGCGATCAACCTCGGGCTGACCAAGATGGCCGAGTCGAGCCCCGGCCTGACCTACGCCAAGCTGTCCTGG
CTGCAAGCCTGGCACATCCGCGACGAAACCTATTCGGCGGCCTTGGCCGAGCTGGTCAACCACCAGTATCGCCACGCCTT
TGCCGCCCACTGGGGCGACGGCACGACCTCATCCTCCGATGGCCAGCGCTTCCGCGCGGGTGGCCGGGGCGAGAGCACCG
GGCACGTCAACCCGAAGTACGGTAGCGAGCCGGGACGGCTGTTCTATACCCATATCTCCGACCAGTACGCGCCGTTCAGC
ACCCGCGTGGTGAATGTCGGCGTCCGCGATTCCACCTATGTGCTCGACGGCCTGCTGTACCACGAGTCCGACCTGCGGAT
CGAGGAGCACTACACCGACACGGCCGGCTTCACCGATCACGTCTTTGCCCTGATGCACCTGCTAGGCTTCCGCTTCGCGC
CGCGCATCCGCGACCTCGGCGAAACCAAGCTGTACGTGCCGCAGGGCGTGCAAGCCTACCCGACGTTGCGCCCGCTGATC
GGCGGCACCCTGAACATCAAGCACGTGCGTGCCCACTGGGACGACATCCTGCGCCTGGCCAGCTCGATCAAGCAGGGCAC
CGTCACCGCCTCGCTGATGCTGCGCAAGCTCGGCAGCTACCCGCGCCAGAACGGACTGGCCGTGGCCCTGCGCGAGCTGG
GCCGGATCGAGCGCACGCTGTTCATCCTGGACTGGCTGCAAAGTGTTGAACTGCGCCGCCGCGTGCATGCCGGCCTGAAC
AAAGGTGAGGCGCGCAACTCGCTGGCCAGGCGGTGTTCTTCAACCGCCTTGGGGAAATCAGGGATCGGAGCTTCGAGCA
GCAGCGCTACCGGGCCAGCGGCCTCAACCTGGTGACGGCGGCTATCGTGCTGTGGAACACGGTGTACCTGGAACGCGCCA
CCCAGGGGTTGGTCGAGGCCGGCAAGCCGGTGGACGGCGAGCTGCTGCAATTCCTGTCGCCGCTGGGCTGGGAGCACATC
AACCTAACCGGCGATTACGTCTGGCGGCAGAGCCGCAGACTGGAAGACGGGAAGTTTCGGCCCTTACGGATGCCCGGAAA
ACCTTAG
>Cluster_31473
ATGACGGCAGAGTACATCAGGGACTGGCAACAACCGCGCCACGCAGTGGGGCGTGAAGGAACGGGGATCCCCGCTCCTGA
ATCCGCGCTTTCCTCCTGGCTGGATGCCTACCGGGCAGAGAACGAGCGCCGCCAGGAAATGGCTGATGCGGCGTTCTCCG
CCACGCCGCTGGGCAACCTGATTAATAAAAGCCTAGACGCACAGGAAAAACAGGACAAAACAATCACACTGGCAGGAGAC
GCCAGAAAACAGGCACGCGGCGCGGTAGATGAGGCCATGGCCTCACTGCGCCTGCTGCCGTCCTATCTGCGCGATCCGCT
TATTCGCCACCTCTCCTTCCTGCGCAAAAAACAGGAAGCCGATCGTCAGCAAGGAAAAAACGCCAGGCAGGCAGAACGCT
ATGCGCGTGGGACCCTGCGCAAAATATTCGAACGTCTGGAGCGCACCGATCACCGCTGGCTGACATCGGGTTATCGCTCC
CTTGCCGGACGTGAACGCCTGGACGATTTGATTTACCTGCCGCAACTCAACAAACACCAGATACAGACGCTGGCCACCAT
GACGGCGGCGATGTTCAGCAGCACCTTCGAAAAACTCTGCGATGGTTTTGGCGCGACTGATGGCGAGCTGACCATGGATG
TAACGCTGAAGGCGTATCAGATGCTGGCCCGCATGGCGTTACACTTACACGCCATGCCTCCGCATTATGACGCACTGACA
ACAGACAAAGACCGGAGGCACGAACCGGACACAGAACTGCTGCCGGGCGCAATCCTTCGCCTGACCTGTGCGGAATGGTG
GAAACGCAAACTGTGGCTGTTACGTTGCGAGTGGAGAGAAGAACAACTCCGCGCCGCCTGTCTAGTTTCCAGAAAAACAT
CGCCCTATCTGAGCCAGGACGCGTTAAGCGAGTTTCGCGCACAGCGCGAGAAAACACGCGATTTCCTGAAAAGTTTCATG
CTGGAAAATGAAGACGGGTTCACGATTGATCTCGAGACGGTGTATTACGCGGGAGTAAGTAACCCGGTTCACCGTAAGGC
AGAAATGATGGCCACCATGAAGGGGCTGGAACTTCTGGCCGAAGCCCGTGGCGACAAAGCGGTGTTTCTGACTGTCACCT
GCCCGTCAAAATACCACGCCACAACGGAGAACGGTCATCCGAATCCCAAATGGAACGGGGCCACAATGCGCGACTCCAGC
GATTACCTGGTTAACACGTTTTTTGCGGCGGTCCGCAAAAAACTGAACCGCGACGACCTGCGCTGGTATGGCATCCGCAC
GGTGGAGCCTCATCATGACGGCACCGTGCACTGGCATATGATGGTCTTTGCACATCCGGAAGAAATCGACACCATTGTGT
CCCATACCCGCGATATTGCCATTCAGGAAGACCGCCACGAGCTGGGCAATGATATTACTCCGCGCTTTAAGGTGGAGTAT
GTCGACGGCTCAAAAGGCACGCCAACCAGCTACATCGCCACCTACATCGGAAAGAACCTGGACAGCCGCGCCGTGGATGG
CATCGACCCGAAAACGGACAAGCCACGCGTGGACCACGAAACCGGAAAATCAATGGCCGAGAGCGTGGAACGCGCCATCG
GCTGGGCGCGTCTTCACCGCGTCCGCCAGTTCCAGTTCTTTGGTATCCCCTCCCGTCAGGTATGGCGTGAACTCCGCCGC
CTTGCCAGTCAGATGGCCCGCAACCCGGAAGGTCCACAACGTCTGGAAAATGACGCAATGGATGCGGTACTCGCTGCCGC
TGATGCCGGGTGTTTTGCCACCTACATTGAGAAACAGGGTGGCGTACTTGTTCCACGCAAAGACTACCTGATTCGCACCG
CCTACGACCTCGCAGAAGAGCTGAACGATTACGGCGAGCAAAGCGTACAGATTTACGGGATCTGGTCGCCACAAATCGGG
GAATCTTCCCGCGTGTGCACGCACCCGGATAACTGGAAGCTGGTAAGACGTAAACCGGAAGCGGAAGACAGCGCCCGCGA
AAATGGTTTTGACCTTCAGGGCGGCCCTGCCGCCCCTTGGACTCGTGGCAATAACTGTCCCCGTGTACAGGAAACGGACA
ACAACGGGACAGAACAGCCGGAAGAACGGCCAGCACCGTGGCCGCAGCTCCCTGACGGCGTTGAAGTGAACGAATGGATG
CGCTCACTGAAACGGCACGAACGCCGGGCGCTGATGCGTTCGCTTCGTGACAAACAGGCAAAAAACAGCAGTGATGAAAT
GCAGAGCTGGACACAGAGCCGCAAACAGCAGCGGCCTTTGCCTGATAACCACGAATTACTCGCTAAAGAATGGCGGGAGT
CTGCTGAATCTCTCGGCCTGCATATCGGTGAACAGCAGATGCAGCACCTGTTACGGGGCGGCAGTCTGTACGTTGACGGC
AGCATCATTGCACCGCAGGGATTTGAAATTGTACGCAAACCGGATACCCGACCGGACAGCCGGATCACGCAGCTCTGGCA
GCGCCTGAGCCGTAACCACGGCGTAAGCAGCACGGAGATCCGCCATAACCCGGTCGCCAGCTATCTGGAACAGCTGGGGG
CATCAGACCCCGAAGCCGCCGCACGCCTGGCATCCACACTTCAGCAAGACCAGAACACCATGAAAACACCCGTTACCGTG
CTTTCTGACATGCTGCGCGCCATCCGCGACGCAGAGCACGCACAGAGAATCAGTGAAACCACTGAACGCGCCCGCCGCAA
AGCAGACCTGCTGCGAGGTGGCCTGACCAGTGGAAACAAAAAACAGACAGAAACGGGATTCACAAATCCCGTAAATGAGC
AAAAAACGCGCCGCGATATATGA
>Cluster_23235
ATGAAGCCGCTCTCTTCACCGTTACAGCAGTACTGGCAGACCGTTGTTGAGCGGCTGCCAGAGCCTTTAGCCGAGGAATC
ACTTAGCGCACAGGCGAAGTCAGTACTTACTTTCAGTGATTTTGTGCAGGACAGCGTGATTGCGCATCCAGAGTGGCTGA
CGGAACTGGAAAGCCAACCGCCGCAGGCCGACGAATGGCAGTATTACGCTGCATGGTTGCAGGAGGCGCTCAGTAATGTG
AGTGACGAAGCCGGGTTAATGCGCGAGCTGCGGCTATTCCGGCGGCGCATTATGGTGCGCATCGCCTGGGTGCAAACGCT
GGCACTGGTTACTGAAGAGAGCATATTGCAGCAGCTCAGCCATCTGGCGGAGACGCTGATTGTTGCGGCGCGTGACTGGC
TGTATGACGCCTGCTGCCGCGAATGGGGAACGCCGTGCAATGCGCAGGGCGAAGCGCAACCGCTGCTGATTTTAGGCATG
GGTAAGCTGGGCGGTGGGGAACTGAATTTCTCCTCAGATATCGATCTGATTTTTGCCTGGCCGGAACATGGTTGTACCCA
GGGCGGACGCCGGGAACTGGATAACGCACAGTTTTTTACCCGCATGGGGCAGCGGCTGATTAAAGTGCTGGATCAACCAA
CGCAGGATGGTTTCGTCTATCGCGTTGATATGCGGCTGCGTCCGTTTGGTGAAAGTGGCCCGCTGGTGCTGAGTTTTGCG
GCGCTGGAAGATTATTACCAGGAGCAGGGGCGCGACTGGGAGCGTTACGCGATGGTCAAGGCGCGGATTATGGGGGATAG
CGACGGCGTCTATGCTAACGAGTTGCGTGCGATGCTGCGTCCGTTTGTTTTCCGTCGTTACATCGATTTCAGCGTGATTC
AGTCGCTGCGCAACATGAAAGGAATGATTGCCCGTGAAGTGCGTCGACGCGGTTTGACCGACAACATCAAACTTGGCGCG
GGTGGCATTCGTGAAATTGAATTTATCGTCCAGGTATTCCAGCTTATTCGCGGCGGGCGCGAGCCGTCGCTGCAATCGCG
CTCTTTACTACCAACGCTCAGCGCTATTGCCGAGCTGCATCTGCTTTCTGAAAACGATGCTGAACAATTGCGAGTGGCGT
ATCTGTTCCTGCGGCGTCTGGAAAACCTGCTGCAAAGCATTAACGACGAACAAACCCAGACGCTTCCTTCTGATGAGCTT
AACCGTGCGCGGCTGGCGTGGGCGATGGACTTTGCTGACTGGCAGCAACTGACCGGGGTGCTGACCGCACATATGACCAA
TGTGCGCCGGGTGTTTAATGAATTGATTGGCGACGACGAAAGTGAAACTCAGGAAGAGTCGCTGTCGGAACAGTGGCGTG

AGCTGTGGCAGGACGCGTTGCAGGAAGATGACACTACACCAGTGCTGGCGCATCTTAGCGAGGACGATCGCAAACAGGTG
CTGACGCTGATTGCCGATTTCCGCAAAGAGCTGGATAAGCGCACAATCGGGCCACGCGGACGTCAGGTGCTCGACCATCT
GATGCCACATCTGCTAAGTGATGTCTGTGCGCGTGAAGACGCTGCTGTCACGCTGTCGCGCATTACCGCCTTGCTGGTGG
GGATTGTTACTCGCACCACTTATCTTGAGCTGCTTAGTGAATTCCCTGCGGCGCTTAAACATTTGATTTCTCTGTGTGCT
GCATCGCCGATGATTGCCAGCCAGCTAGCGCGTTATCCATTATTGCTGGATGAATTGCTCGATCCAAATACCCTCTACCA
ACCAACGGCGACGGATGCCTACCGTGATGAACTGCGCCAGTATTTGCTGCGCGTGCCGGAAGATGACGAAGAACAACAGC
TTGAGGCGCTGCGTCAGTTCAAACAGGCGCAGCTGTTACGCATTGCGGCAGCGGATATCGCCGGTACGCTACCGGTAATG
AAAGTGAGCGATCACTTAACCTGGCTGGCAGAAGCCATGATTGATGCCGTCATTCAGCAGGCGTGGGTTCAAATGGTTGC
CCGCTACGGTAAGCCAAATCACCTGAACGAACGCGAAGGGCGTGGTTTTGCGGTGGTCGGCTACGGCAAGCTGGGTGGCT
GGGAGTTAGGCTACAGTTCCGATCTTGACCTGATCTTCCTCCATGATTGCCCAATGGATGCGATGACTGACGGCGAGCGG
GAAATCGACGGGCGACAGTTTTATCTGCGTCTGGCGCAACGCATTATGCATCTGTTCAGTACGCGAACCTCTTCCGGCAT
TTTGTATGAAGTGGATGCACGACTGCGTCCGTCCGGGGCAGCGGGAATGCTGGTCACATCCGCAGAAGCATTTGCTGATT
ATCAGAAAAACGAGGCCTGGACGTGGGAGCATCAGGCGCTGGTGCGTGCGCGTGTAGTGTACGGCGATCCGCAGCTCACC
GCGCACTTTGACGCAGTACGCCGGGAGATTATGACGCTGCCGCGCGAAGGCAAAACCCTGCAAACAGAAGTGCGGGAAAT
GCGCGAGAAAATGCGTGCCCATCTCGGCAATAAACATCGCGATCGCTTTGATATCAAAGCCGATGAAGGCGGAATTACCG
ATATCGAATTTATTACTCAATATCTGGTGTTGCGCTACGCTCATGAAAAACCGAAGTTAACGCGCTGGTCAGACAACGTG
CGCATTCTGGAACTACTGGCGCAAAACGACATTATGGAAGAGCAGGAAGCGATGGCGCTGACCCGTGCTTACACAACGCT
GCGCGATGAACTTCATCATCTGGCATTACAGGAATTGCCGGGCCATGTGTCGGAGGATTGCTTCACCGCAGAGCGTGAAC
TGGTGCGGGCAAGCTGGCAGAAGTGGCTGGTGGAAGAATGA
>Cluster_12157
ATGTTTGCAGGTTTACCTTCACTCACCCATGAACAGCAGCAAAAAGCTGTCGAGCGGATCCAGGAACTGATGGCACAAGG
GATGAGCAGCGGCCAGGCAATCGCGCTGGTGGCAGAAGAGCTGCGTGCCAACCACTCCGGCGAGCGAATCGTGGCGCGTT
TTGAGGATGAAGACGAGTAA
>Cluster_35973
ATGCTTCAAATCCCACAGAATTATATTCATACGCGCTCAACGCCTTTCTGGAATAAACAAACTGCACCTGCCGGAATATT
CGAACGTCATCTTGATAAAGGAACGCGCCCGGGGGTTTACCCCATAAGCGCTAACTTAAGGGTTGAACCATCTGAAGAAT
GCGACGCCTCGGTGCCTCGTTAA
>Cluster_16052
ATGACTCCGTTACAGTTTATAGGTATAGGTAAATATGATCTTTTCTGTATGGAACAATCTTCTTATGGTGTGTGGGTATG
TTCTGTATACCATTATTCTTTTATGCTACTGATTAAATACCCGTGGTCTCTCTCGGCTTTACTTAATGGTTCGACAATAT
TAATAGCGATGTAG
>Cluster_14925
GTGTCAATACACAAAAACGAAAGGCGTGGTAATCCTCCGTTCCAGTTCCGTTTAGATCCTGACTTACGGGAAAAGATGGA
AAAGGCACAACAACAGGACGGCGATGATTCTCTTGCTGCCTGGATAAAGCGAATAATCCGCAAAGAATTACAGCAACGCG
GCATAGAACCCAAAGGATAA
>Cluster_38479
ATGTCAAACCAATTAACCGTTATAGACGGGTATGCGGAAAGAGAGGCGACTAACGATAACAATGCCGAAAATCTCAACAT
TTCTTATATATGTGGCAATGTAGTGGATTTTTTTGTAAACTTTCAAGAAAAAGAAACAATAAATTCATTTGATAAAATAC
TAAAGAAAGAAATAATACTTGATTAG
>Cluster_1835
ATGAAAAAATATATATTTTTAGTTTTATTTTTATTGTGTGGTTGTTCTCCAGTGGACATAGAGCCAGCTAGACAAGAAGT
TAATATCTTTCGTGGTTTATATCAGACAGGGAAATACAGTGATATATATAAAATCACTTCAAGTGATTTTCAGACTGCCA
CATCTGAAAAAAATTTATCGACATAA
>Cluster_43560
GTGAGCAAGATTGACTATCAGGCACTGCGCGAGGCGGCGGAACAGGCAACGCAAGATGAATGGGTAGCATATATTTTGCC
GGGTCATAACGGCATTTATCCTGCGCGCACGTCTGAGGGTAGGCATTGTGCGGATACTTTATTGACTGGCCTGGCGTCTG
TCAGGGGCGGGAGAGCATCAACATGA
>Cluster_60147
ATGACTATTGCTGAACGGCTGCGGCAGGAAGGACATCAAATTGGCTGGCAGGAAGGTAAATTAGAAGGTTTGCATGAACA
AGCCATTAAAATTGCTTTGCGCATGCTGGAACAGGGCTTTGATCGTGACCAGGTGCTCGCGGCCACCCAGCTAAGCGAAG
CCGATCTGGCAGCGAATAACCACTAA
>Cluster_5625
ATGAAACGTGTTGCAATGTCGGCGCTGGTATTGCGGAAATCAGCCCTGTCCCTGGTGCTGGCGACATTACCGTTCATCTT
CCTGCTGACCCTCTCCGGTCAGCCAGCAGATTGTAGCTCTTTCCTGACTGATTCGTCATTCCATTCACATTGTTTATAA
>Cluster_28397
ATGACAGATGCAGAAAGAGAATATCTTATTAGTTACCTGATTGGTTATTTATTGGGTCGACGCCTGGAAGCCCTGGATAT
CGCCAAAAGAATGCTGTAA
>Cluster_30110
ATGCGGAAGTTCTGGAATGTTTCTTTTTTTGGTGATGGAGAACTGATGGTGACTGAAGAAGTTTGGCTACTTTTGCAATG
TGACAAGTTATGGCAGAGCTGGCTGGTGGCGAAGAATTTTGACGATTGA
>Cluster_4779
ATGAGACTGCCAGAGAAAGTTCTTTTTCCTCCTGTCACTAGTGGCCTGTCAGGGCAGGAAAAACAAAAAAAACCGAAGAG
CATTACCGGATTTCAGGAAAATTATCAACGCAATATCAGGCCAATCAAAACAGCATCAGAAGCCCGACTACGCTTCTTTG
ATAAAATGGTTTCGAAAGAAAACTCTCTGGAAGATGTTGTTTCTTTAGGTGAAATGATTCAGAAGGAAATTTATGGGCAT
GAACAAAGAACATTTTCACCAGTTCATCATACAGGTAACTGGAAATCATCATTGTTACACAACGCGCTCCTTGGTCTGGC
AAATGTTTATAATGGCTTACGGGAAACAGAATACCCTAACACTTTCAACAGAGATGGTATAAAAAGTACTAACTCTTTTA
GAGATAACTTATTGACAAAAACAAGAACTCCCAGAGATAATTTTGAGGAAGGAATAAAACATCCTGAACATGCAACAATA
CCATATGACAACGACAATGAAAGTAATAAATTGCTAAAAGCAGGAAAGATAGCTGGTAACAATAACGAGCTGTTGATGGA
AATAAAAAAGGAATCCCAAAGCGACCATCAAATCCCCCTGTCAGATAAGTTCCTGAAAAGGAAAAAACGATCTCCTGTAG
CTGAAGATAAAGTTCAAAACTCGTTAACACCAGAAAATTTTGTTCAGAAAATTTCACTTAGTGATGAGCTTAAAACAAAA
TATGCAAATGAAATTATAGAGATAAAAAGAATAATGGGAGAATACAATCTTTTACCGGATAAAAACAGTCGTAATGGTCT
AAAACTTCTACAGAAGCAAGCTGATTTACTAAAAATAATCATGGAGGATACCTCTGTTACAGAAAATACCTTTAAAAACA
TAGAGATGGCTATAACAGATATAAAAAGGGAGTACTATTCGCATACAGTTGATATTGAGAAAAATATTCATGCCATATGG
GTTGCGGGTTCCCCACCTGAAAGCATTTCGGACTATATTAAGACTTTTCTCAAAACCTACAAAGAGTTTACTTACTATCT
TTGGGTTGATGAAAAAGCATTTGGAGCCGCGAAATTTACCAGTGTCTTAAAACAAATTGCATTTGATTTAGCATGCAGAA
CTATACAGCAGAATACTCCACAAAAAAAATATTGATTTTATTAATCTATATAATGAAATAAGGAAGAAATACAACAACAAC
CCATCGGGACAACAAGAGTACTTAAACAAACTCAGAGAGCTTTATGCTACTTATCAAAAAATATCCACCCCTCTGAAACA
CATGTTTAATTCATTTTTTTTAGAAAACATGATTAAACTTCAGGATAATTTCTTCAACTATTGCATTGTCAAAGGTGTTA
CAGAGATTAATGATGAGTTACGAATAAACTACCTTAAAAATGTAATAAAACTGTCAGACGATGACATTGGTAATTATCAG

AAAACAATTAACGACAATAAAGATAGAGTAAAAAAACTAATTCTTGATTTACAGAAACAATTTGGTGAAAACCGCATTTC
AATTAAAGATGTTAACTCTTTAACCTCTCTTTCAAAATCAGAAAATAATCACAATTATCAAACTGAAATGTTGCTTCGAT
GGAACTATCCTGCCGCCTCAGACCTGCTCAGGATGTATATCCTTAAAGAGCATGGTGGTATTTATACAGACACAGATATG
ATGCCTGCATACTCTAAACAAGTAATTTTTAAAATTATGATGCAGACAAACGGAGATAATCGTTTCTTGGAAGATTTGAA
ACTACGTCGTGCAATATCTGATGGTGTATTAAGATATGTTAATAACCAAAATATTGATGAAGTTAACTATAATGAAATCA
GTGATGCAGATAAAAACATTATCAAGAAGATATTAACAGAAATATCTAAAATGCCAGAAGATAGTATTTTTACTAAGATC
AATACAAGGATTCCTCGAGACACAATGCCCATCCTTCGTCGTTATCACCTATGGCCTGATGGATGGAATATTCGTGGGCT
CAATGGATTCATGCTATCACATAAAGGTAGTGAAGTGATTGATGCTGTTATCGCAGGCCAGAATCAGGCTTACAGGGAAC
TAAGAAGAATAAGAGATAATATTCATAGTGAAATATACTTCAAACAAACTGATGAATTGTCCTCACTTCCAGATACAGAC
AAAATTGGAGGGATTCTGGTAAAAAAATACCTTTCAGGAAGTCTTTTTTCAAAATTCAGACAAGATACTATTATCCCGGA
GGCATTGAGCACCCTCCAGATATCAGGTCCTGACTTGATTCAAAGAAAGATGTTGCAATTTTTCAGGAGTAGAGGGGTGT
TAGGTGAAGAGTTCATTAATGAAAGAAAACTGAGTGATAAAGCTTATATTGGTGTCTACAAAACAACTGGCACAGGGAAA
TATGACTGGTTAACCCCTGAATCGATCGGCGTTAATGATGTCACGCCCGCAGATGAAAGTACCTGGTGTATAGGAAAAGG
CCGGTGTGTTGATGACTTCCTGTTCAAAGATGTTTCAACACTAAAAACAGAAAATCTTCCAGAATTATTCTTAACAAAAA
TAGATACTGATACGTTTTCTCTCAGTGGTCAACCAAAACCAAGAAAGATCTGCAAAAAAAAAATACAAGACCTTACTGTA
CGTTATAATGAGCTAATTGACTCGTCGACTATCGACTTTAAAAATCTATATGAAATAGATCAAATGCTCCATATGATTAT
GCTAGAGATGAATGATGATATAGCCAAAAGGTCATTGTTTTCATTGCAAGTTCAAATAGCCGAAAAAATTCGGAGGATGA
CCATTCCTGTAGACAATATAATTAACATCTATCCTGATCTACATAAAAAAAATGACAATGATCTGAGTATGTCCATAAAA
GGCTTTCTTGCGAGTAATCCACATACAAAAATAAATATTCTTTATAGCAATAAGACTGAGCATAATATTTTTATAAAGGA
TTTATTCTCCTTCGCAGTTATGGAAAATGAGTTAAGAGACATTATCAATAACATGAGCAAAGATAAGACTCCTGAAAACT
GGGAAGGGAGGGTAATGTTACAAAGATATCTCGAATTAAAAATGAAAGATCATCTTAGTTTGCAATCTTCTCAGGAAGCA
AATGAGTTTCTTGAAATATCTACTTTTATTTATGAGAATGATTTCTTGAGAGAAAAGATTGAAGCAGTAAAAAACAAAAT
GAATTCTCACGAACTTTATTTTGAAAAAATAAAAAAAGAACAAAACACATGGCAGGATCTGTCCACAAAAGAACAAAAAT
TACAGCTTATTAAAGCATTGAAAGAAATTTCAGGAAATACAGAGAAGGACTCTCATTACGATAGACTTCTTGATGCTTTT
TTTAAAAAACATAATGAAAATATTCATAATAAAATACAAAGAATAAAAGACGAGTTCAAGGAATACTCCCGTGTAGCCAT
TCATAATATAGATAAAGTTATATTTAAAGGGCAAACACTGGATCGTCTTTATCATGAAGGATATGTATTTTCTGATATCA
ATACCTTGTCTCGTTATACACTACACGGACTAGGAATAACTGGTGTACATACTGAAGAAAACCTGCTACCAGCTCCTTCA
TCGTCCTTAATTAATATATTGAAAGAACATTATAATGAAGATGAAATTAGTGCGAAATTACCACTAGCATATGATTACAT
TTTAAATAAAAAAGAATCAAGCTCTATTCCTGTTGAAATTTTGAACAAACTTTCAGAGTTACCACCACATGAACTACTCA
CACCTGTTCTTGGCCAGAGTGTTAATCCTCTGGGCATGGGCTACTCATCCGATAATGGAAAAATCACAGAGCAAGTAATA
GTCAGTGGAGCTGATGGATTTGATAATCCCATATCTGGACTTATATATACCTATCTTGAAGATCTATATAACATCCATGT
AAGGATGCGAGAAGGTACACTAAATTCACAGAATCTTCGTCAGCTTCTGGAAAACTCTGTTTCTTCATGCTTTTTGACTG
AGCAAAGTATTAATAAATTACTTAGTGAGGCAGAAAAAAGGCCTTATCAGTCTTTAACAGAAATACATCAGCATCTCACA
GGATTACCAACTATTGCCGATGCAACCCTTTCATTACTTTCTGTTGGATTACCTGGTACGGGAAAACTATTGCGCAGGGA
GCAGGACTATGGGCGTCCACCAGTTACAGCAATTCAGGATTCCACATTTGTACTCCCTTATAATTTCAAAGGTATTGGTT
TTAACGATAACATTATATCCTCTGCACCTGTAGCCTCCTCATTACATTTTATCGCTGAACATGCGAAATATACTTTATTG
TCATGGCCTGAGTTTTATCGTCATCATGCACAGCGATGGTTCGAAATGGCTAAAGGATATGGAAGCCAGAATATTGATTT
TCACCCTCAGTCTCTATTGGTAACCCAAGAAGGACGCTGTATGGGATTAGCCTTACTTTATTTACAGACTGAAGATACTG
CTCATTATAGCATTCTCCAGGAAAACCTAATGACTGTGAGTGCACTTCATCAGACCAGTAATCGCGATAAGTTGCCACTG
TCCAAAGATGATAATTCCTTGATGACAAGAACTTATAGTCTGATTGAAATGCTACAGTATCAGGGAAACAAATATATTAC
CAACGAATCGCTACTACATAAGACCGCATGGAACCAAGAAAGAATAACTTTATTATTCAATGAAAAAGGAGTTAAGCGAG
CCCTGATAAGCACGCCTAATCATACTCTGGTTCTGCAACAACTGGAGGATATTTACCGGCTCACTGATCCAAATTTTGGG
CATGCAGATTTCCTTTCACCTATAGATGCTCTGAAGTTTATTGAGGCTATGATACAATTAACTCCAACACTTCAGGAATA
TTATGGCCTATTAAACAAAGACATTAATAAACATATACAAGTACATTATGCCGAATCAGATATGGTCTGGAATAAGCTTC
TGCCAGAAAATGATGCTGGACTGAGCACCAGAATTCAGCACACCACCACCGACCGTCTGGCGAATCTGGCTGAACCAGTC
GCTGTTGCAGGTATCTCCCTGCCAGTAAAAACACTTTATGATATCGGAGCCACCCTTGACGGTCGGCGCATCACCTCTCC
TCCAACATCGGAGCAAATCCCTTCTCTGCGTCTCAACGGTGATGTTCTGAATGATTATCTGTCCCGCACAGTTCTGACTC
CAGAACAGGCTGATAACATAAGAAAAATACTGCACACTCAGGGAATACGCAGCGGTACCCGTCCCATAGATCCGGAGATG
ATTCGTGGGACGCAGGATGACCTAGTTTCGTCACAGACTCGTCTGCAAAGGCAAGCAACACGGGTTAAACAGCAACTCGC
CGGTGTACTTGATACTCTGCAACAGCACTTCCAGAACATTCCACGTTCATCCGGTCGTCATCTTTCTGTAGAGAATATTG
AGCTGGCTGATATCGGAAGTGGGCGTTTCAACCTTCAAATTCGAGATGGAGAAACATTGCATACCACTTCTGTGGAAGTA
CCGGAAGTTGTGTCCCGTTTTCAGAAACTTTCTACCATGCTTTCAGCCCTGCCTGCCAGTGGAATCATGGATTTCGACCT
CGGCATGAGTGTGGTCGGAGTCGTCCAGTATGCCCGCCTGCTACAGCAGGGGCACGAAGACAGTACCCTAGCCAAGATAA
ATCTAGCTATGGATATCAAGCAACTTTCCGAAGCAACTCTCGGCAGCATGATTCAGATTGCCGGGAATAAGTTTCTCAAT
ACAGAAGGAATCCAGGGGTTCAGACTGGAAAGCGCCGTCGCTGAAGGTATGCGCTCAGTAGCAACCCGTACCGGAGGCAC
AATGGGGAAAGCCCTTTCTGCCAGTGCCCGTGTTCTTGAACTGCCTGTACTGGAAACAGTTCTGGGGACATGGAACCTGT
ACAACAGCGTCATTCAGCTCCAGCAAGCTACATCTTATTCTGAGACAATGGCTGCCCGGGTACAGATTGCGTTTGATTCC
ATTTCTCTGGGATTAACTGCCGCTTCGGTAGCTTTCCCGCCACTAATTATTGCCGCTGGCCCCATTGCGGCTATTGGTAT
GGGAGCTTCCAGTATTGCACGTAATGTGGCACGGAAAGAAGAACGGCATACACAATGGCTGGAATATAAAAAATTCCTGA
CTGATGGCAGTAAACACATTGTTGTGGCCTCTCCGGAAAGAGGTCTGCTGGATTTCTCCGGAAACAAAGTTTTTGGAAAA
ATGGTGCTGGATCTGCGTCAGTCTCCTCCTCTCTTGCATGGAGAAAGCTCTTTTAACGCTGACCGCAAAATCGGTCATCG
TCCGGATCTGGGGGACTGGCAAATTCGTGAGAAGGTGGGGTATGCCAACAGTATCAGTCCCTACTCTTCTCTGGCGCACG
GTTACGCCAACAGTAAATGGCCACGAACAATACCGAAAATTCCCTCGGGAGATAGACTGGCCCCCTGA
>Cluster_3495
ATGATCCCGTTACCTTCCGGGACCAAAATTTGGCTGGTTGCCGGTATCACCGATATGAGAAATGGCTTCAACGTAAGCGG
CTCGCCAGAACCGTATTGA
>Cluster_66851
ATGAGCTGGAAGCGGAGCCTGAAGGTCGGCGATCTCGACGACGGCCAGAAACTGGAAATGACCTGCGTGCGCTGCTGCCA
TGTCCACTACCTGACGCCGGTGATGGTGATGGCCTCGCCGGAGCGGGAATTTCGGCACTGTTGCAAATAG
>Cluster_11023
ATGAAAGTGAATGGTGTTACTACATCAAAATATGAAGGATGGCGAGGGACGTTTCAGTTATATAGATATAAAACGGTAGT
TCTGGTGCACCCCAAAGGATATCTGGCAGGGGAAATGATTCATCGCCAGGCCCTAACGTCGTCTTGTCAAGATTAA
>Cluster_48785
ATGGAAGCCGATAAATATATAATGATAATCAAATACTTATCAGACAACTCTCACAAACTAACCCCGCTTGTCTTGGCGGT
TTCGGTTCTTGTCGCCTTTCTATCAATCCTGTTTATTGCAATGCGCATGGGGTAA
>Cluster_44940
ATGTTTGAGTTTTATATGGCAGAACTTCTTCGCCACCGCTGGGGGCATCTGCGCTTATATCGTTTCCCCGGTTCTGTTTT
GACCGATTACCGAATACTGAAGAATTACGCCAAAACCCTGACAGGAGCAGGAGTATGA
>Cluster_10564

TTGCAGAATGCATCACTTAACGGAGAGATCGATAACGCACTGGACGCTTATTTTTCGAAAAATGGCGGCGCAGGCGGTAA
TCGTCCCGACGTAAAACTCCTTGTTAAGGACAAATACGGCAAGCAGTATCCCGTTCTCATCGAGTATAAAGGCTACAAGG
ACAGGCTTATCAGACTTGGCAGCAACGGTATTATTGAGAACAAAGATGCCAGAAAAGAATGGAATTTTAAAAATATTAAT
GGATACGCGGTGAATGGCGCAGTCCATTATGCCAACGCACTTCTTCAGTTCACCAATTACCCTGATATTATTGCTATTGG
TATGACCGGCTGGCGCGATGATGGTACAGGCGAACTGCATCATGAAATCGGCGTGTGGTACGTATCAAAGAATAATTTAG
GAGCCGGACAGAAGGTTGGTGAATTCACCGACTTATCGTTCCTTGCTGACAAAAATGTTGATGGATTTCTCAACAAGATA
AAGCTGCTTAATCTTCCTCCCGAAGAACTGGAAAAAATCAAGGCCAGCAAAGAGGAAGAGATAGACACGCGACTTTCCCG
TCTGAATAACGATATTTACCAGAATGAAAAGGGGCTTGGTGAAAGCGATCGTGTTTACCTTGTTGTGGCTACCGTTATTG
CAACGTTGGGGATTCCGGGAAAGTTAGCACCGCTGGATAAAAAAGAACTGACCTCTTCAACGGAGGAGGACTTGCGTGAT
GGTGATATTATTTTCAGGAAAATAAGGAATTTTTTAAGGCTGAAAGCTGTTCCTGAAACAAAAAGAGAAATGATTTTGCG
CTCGTTGCAAAATACGCTGTGGACTGAAAACATTAACAAACCAGTCAACGGTGAAAGCCAGTTAAAGCGCGTGTTTGTTA
AAGTTGTTGATGATCTTGGGGAGTATTACAAAATTGGACTGACCACCGATTTTACGGGCAAACTGTTTAATGAGATGTAT
CGCTGGCTTGGATTTACGCAGGACAAGCTCAATGACGTCGTTCTGACACCACCTTATGTTGCCACATTGCTGGCACGACT
CGCCAGAGTAAACAAAGATTCCTATGTGTGGGATTTTGCTACAGGTTCTGCGGGATTGCTTGTGGCTGCAATGAATGAAA
TGCTCATTGACGCCAGAGAAAATATTCACTCACCGAACGAACTACAACTTAAGGAAGCGCAAATCAAGGCTGAACAGCTT
CTTGGCCTTGAGGTGTTATCCAGTATTTATATGCTGGCCATCCTGAATATGATTTTGATGGGGGATGGTAGCTCGAATAT
CCTCAATAAAGACTCGCTGGCCGATTTTGACGGCAAGTATGGATTTGGGAAAACAGGAGAAAAATTCCCTGCGGACGCAT
TTATTCTCAATCCTCCGTATTCAGCTAAAGGTAACGGCATGATTTTCGTGCAGAAGGCGCTGTCGATGATGGATAAAGGC
TATGCTGCAGTGATTATCCAGAGTTCAGCTGGCACAGGAAAGGCTACGGAGTACAACAAAAAAATACTCAAGGAAAATAC
CTTGCTGGCAAGCATCAAAATGCCTGCGGATCTGTTTATTGGTAAATCAAGCGTTCAGACGTATATTTACGTTTTTCAGG
TAAAAATTCCGCATAACGCGAAACAGGCTGTTAAGTTTATCGACTTTTCCAATGACGGCTACGCTCGCTCTAATCGCAAA
AAGGCAAGAAACAATCTTGTTGATGCCGATCGTGCAAAAGAGCGTTATCAGGAGGTTGTGGATCTGGTTCACTTCGGGAA
AGGTTGCCTTAATATCTTTACGGAGGATGAATATTTTGAGGGAACAATCGATCCTGATAGTGGTGAGGACTGGAATCAGA
CCAGGCCAGTAGATGCTAGGCCAACGCTGGAGGATTTCAAAAAGACGGTAGGTGATTACCTTGCATGGAGGTCTCGCAA
TTACTGAAAAAACAAGGTGAAAACAATTTTGCGGGAAAGTAA
>Cluster_21771
ATGAGTACCACAACACGAAAATTTAAAACCATTATCACCGATACGGGTGCCAAAAAATTAGCGCAGGCAGCCGCACCAGA
TGGTAAACCCGTCCGCCTGACACATATGGCCGTGGGTGACGGCGGCGGTACTTTACCCACGCCAGACAGTAAGCAGACCC
GTCTTGTGCGTGAGGTGTGGCGACATACCGTTAATCGCGTCTTCCTGGACGCAACACATCAGAACCGCATTATTGCGGAG
CTGGCTATTCCTCCAGAAACGGGCGGATTCTGGATCCGGGAAATTGGTGTGTTTGATGAGCACGGCGATTTAATCGCGGT
AGGCAATACTGCCGAAAGTTACAAGCCAGCCGTTGCCGAAGGGTCCGGTCGTGCACAGACATTTCGCACCATTCTGACCG
TATCCAGCACTGCCACCGTGGCACTTACCGTGGATAACACCATGGTGATGGCCACAGTGGATTACGTGGATGACAAACTG
AAAGAGCATGAACAGTCACGACGTCACCCGGACGCCTCGCTGACCGCAAAAGGCTTTGTTCAACTCAGTAGCGCCACTAA
CAGCGTGTCTGAAACACAAGCTGCAACGCCGAAAGCAGTAAAGGACGCGTATGACCTTGCTAACGGGAAATATACTGCGC
AGGATGCCAGCACGACGCGAAAAGGCCTTGTTCAGCTCAGTAGCGCCACCAATAGCGATTCTGAAACGCTGGCGGCAACG
CCAAAGGCGGTAAAGGCAGCATATGACCTTGCTAACGGGAAATATACCGCACAGGACGCTACCACAGAGCGAAAAGGTCT
TGTCCAGCTAAGTAGTGCCACCAACAGCGATTCTGAAACGCTGGCGGCAACGCCAAAGGCGGTAAAGGCAGCATATGACC
TTGCTAATGGCAAATACACCGCACAGGATGCAACAACAGCACAAAAAGGGATAGTCCAGCTTACTAGTGCAACCAACAGC
ACGTCTGAAACGCTTGCCGCGACTCCGAAAGCGGTGAAAGCTGCTAATGACAATGCAAACGGGCGTGTGCCCTCTGGACG
TAAGGTTAATGGCAAACCACTGACCAATGATGTCAATGTTACATCACAGGATATTTTTAACGATCAGAGTATTGAGATTG
GTGCAAACCAGAATCTGGATAATTACAAAACGCCGGGGCTGTACCATCAGCCTGCGAATGCCAATACAAGTGCAGCGCTG
AAATACCCAGAGAATCTTGCCGGTACACTGGTTGTGCTTAAAAGTGCCGGGATAACACAGATTTACTATGTGTATAACAC
ATCCAGAAGCTATACCCGCAGCCAGTACTCAACGGGTGACTGGACAGCATGGACGCCGCAGGATTCATTTCCTGTAGGTG
CGGCGATTCCGTGGCCTTCTGATTCAGTGCCTACAGGCTATGCCGTTATGCAGGGGCAGACTTTTGATAAAACAACCTAT
CCCCTGCTTGCAGCAGCTTATCCCTCTGGTGTACTCCCCGATATGCGTGGCTGGACAATCAAGGGCAAGCCCGCAAGTGG
TCGAGCCGTATTGTCTCAGGAACAGGATGGCATTAAATCGCACACCCACAGTGCCAGCGCATCCAGTACGGATTTGGGTA
CGAAAACCACATCGTCGTTTGATTACGGTACTAAATCAACGAATAACACAGGTGCACATACCCACAATGTATCTGGTACT
GCAAATAGTGCTGGCGCACATACTCATACCGTTCCATTAAGGAGACCAAACAGTGGAGGTATGAATTTCGACTGGCTTGA
TGGTGCATCAAGTGGCACGGTCGTGGGGAATGGAACAGTGCCTTCTTCTGGTGCACACACCCACTCAGTATCAGGCACTG
CTGCAAGTGCAGGGGCGCATGCACATACAGTTGGTATTGGCGCTCATACGCACTCTGTTGCGATTGGTTCACATGGACAT
ACCATCACCGTTAACGCTGCTGGTAACGCGGAAAACACCGTTAAAAACATCGCATTTAATTATATTGTGAGGCTTGCATA
A
>Cluster_20823
ATGACGCCCTTGCTCGAATTAAAGGATATTCGTCGCAGCTATCCTGCCGGTGATGAGCAGGTTGAGGTGCTGAAGGGCAT
CAGCCTCGATATTTATGCCGGTGAGATGGTGGCGATTGTCGGCGCTTCGGGTTCCGGTAAATCGACCCTGATGAATATTC
TCGGCTGCCTGGATAAGGCCACCAGCGGCACCTATCGCGTCGCCGGTCAGGATGTTGCCACGCTGGACGCCGATGCGCTG
GCGCAACTGCGCCGCGAGCATTTCGGCTTTATTTTCCAGCGTTACCATTTGCTTTCGCATTTAACCGCCGAGCAGAACGT
TGAAGTACCCGCCGTCTATGCCGGTCTTGAGCGGAAACAGCGGCTGCTTCGCGCCCAGGAGTTACTGCAACGACTGGGGC
TGGAAGACCGTACAGAGTATTACCCGGCACAGCTTTCGGGTGGCCAGCAACAGCGCGTCAGCATCGCGCGGGCATTGATG
AACGGCGGTCAGGTAATTCTTGCCGATGAACCGACCGGCGCACTGGACAGCCATTCTGGCGAAGAGGTGATGGCGATCCT
GCATCAGCTGCGCGATCGCGGGCATACGGTGATTATCGTCACCCACGATCCGCAGGTTGCTGCTCAGGCCGAGCGGGTGA
TCGAAATTCGCGACGGCGAAATTGTGCGCAATCCTCCCGCCGTTGAAAAAGTGAATGCTACTGGCGGAACGGAACCTGTT
GTCAACACGGCGTCTGGCTGGCGGCAGTTTGTCAGCGGTTTTAACGAGGCGCTGACGATGGCATGGCGGGCGCTGGCAGC
GAATAAAATGCGTACTTTTCTGACCATGCTGGGGATTATTATCGGTATTGCGTCGGTGGTTTCCATTGTCGTGGTGGGCG
ACGCCGCTAAACAAATGGTGCTGGCGGATATTCGTTCTATTGGTACGAATACTATTGATGTCTATCCCGGGAAAGATTTT
GGCGATGACGATCCGCAATATCAGCAGGCGCTGAAGTACGACGACTTAATCGCCATCCAAAAACAACCGTGGGTTGCCTC
TGCCACGCCCGCCGTCTCGCAAAACCTGCGCCTGCGTTATAACAATGTTGATGTGGCTGCCAGTGCCAATGGCGTGAGCG
GCGATTATTTTAATGTCTATGGCATGACCTTCAGTGAAGGGAACACCTTTAATCAGGAGCAGCTGAACGGTCGTGCGCAG
GTCGTGGTTCTCGACAGTAATACTCGCCGCCAGCTTTTCCCCCATAAAGCAGATGTGGTTGGCGAGGTGATTCTGGTCGG
CAATATGCCCGCCAGAGTCATTGGTGTGGCGGAAGAAAAACAGTCGATGTTTGGTAGCAGTAAAGTGCTGCGTGTCTGGC
TGCCATACAGCACGATGTCCGGGCGAGTTATGGGGCAGTCGTGGCTGAACTCCATTACTGTCAGGGTGAAAGAAGGCTTT
GACAGCGCTGAGGCGGAACAGCAACTCACGCGTTTACTTTCACTGCGCCACGGTAAGAAGGATTTCTTTACCTGGAACAT
GGATGGCGTCTTGAAAACCGTTGAAAAGACCACACGTACTTTACAACTGTTTCTGACGCTGGTTGCGGTGATTTCGCTGG
TGGTGGGCGGTATTGGTGTAATGAATATTATGCTGGTGTCAGTGACCGAGCGGACGCGGGAAATTGGCATTCGCATGGCT
GTAGGTGCGCGAGCAAGCGATGTTTTGCAACAGTTTCTGATCGAAGCCGTACTGGTTTGCCTGGTTGGTGGCGCTTTGGG
AATAACACTGTCACTGTTAATTGCTTTCACCTTGCAGCTTTTCTTACCCGGCTGGGAGATTGGTTTTTCACCGTTGGCAC
TGCTGCTGGCGTTTCTTTGCTCGACGGTCACCGGGATTTTATTTGGCTGGTTACCCGCACGAAATGCGGCACGACTGGAT
CCAGTAGATGCTCTGGCACGAGAGTAA
>Cluster_57832

ATGTCCCAGCGTTATAACCTCTATCGTCGAACCAGCGGCATTTATGTTGTCCGCATTAGCGTTCCCCAACGTTTTCGTCG
ATACGCAGGACAGTGTGAGATTCACACTTCAACAGGCACTCATGATCTTCATGAAGCAAAGCAGAAGTCCGCGCTCCTGT
TGGCTGTCTGGTATCAGACCTTACAAGAGTATGAACAATTGGATTACCGAACTTTAAGTGACTGCGCCCCGCTGCTTGCT
GGTGAGGGGATGATCTCGCTTTCTAACTTTGCTCAGTCAATCGAGTTGCCTATATCGCAATTGATTCGAGAGGTGATTAA
TCGTAACCTCCCGGTATTCTGGCTGGCGACTGGTCAGTTCGGTTTCTATGTTGATGAATTTAATGCAGTAGAGCGGGAAC
CCGGTGCAAAACGAGAAAAACAGTCTGATGATGAAAAGGATCAACCTAAAGAAGTCATCATTCTCAATAGCGCGTTTGAG
CTGGGTATCGAGAGCTTCGCAAATGGTTATCTCCGCCCCTTCAATCCCCGGCATACTTTAGATTGTCTGTTGAGCGCTGG
AGTATCCGAAGGAGAGGCTGCATTTCGAACTAGTGGTGATAACCAAAGTGGAGGTTGGTTCTTCGATTTACCCGGCGTAG
ATATAACTGCTGATAGCCTCTTGATTAGCAAAGTTCATGCTGAAGGCCTTCGACTTACATGGCTGGTTAAGACCACGCCA
CCAGCAGTTAGCATTCACCCTGCCGTGCCTCTTGTCGCCCCTGTTATCGCTAACGAATATGTTCACCGCAAACATTACAA
TGAAAACTTGTCATGGCTTCGTGAAGAGTATTTGAAACATCGGCGTAAGGGCAAGGTATCAGAAGCGGCGCTCCGCGATA
TTCGCTATTACTTCGATTTGATGATCGAAGTGATGGGGATATTCAGTTGGAAGATTTCGACCGTGATTTCCTCCGGGCT
TATGAGAGCAAGTTGCGCACAATTCCTGCTAACCGTAATTTGATGAAAGGTAAGCACGGGGTTAAGACGCTGGATGAGTT
AATCGCCAAAGCGGCAGAATGTGGCGATAAACTGATGACAGAAGAGTCTGTCAAAAAGTATATCAACGGCCTTTATGGTG
CAATGGAGTGGGCTGTTGATGACGGTAAGTTTCTGAAATCGCCATGCGACAACTTTTTCCCTCCCGATGACAAAGGTGAG
CGAGAGCAGGATCACACTGACATATTTGAACCGCATGAAATTAAGGCAATTTTTTCGCAACCGTGGTTTGTGGCTGGAAC
TGTTGAACGTAATGCGCAAGGGCGATTCCATCAATATTGCCCGTTTCACTATTGGGCGCCGTTGTTGGGCTTGATGACGG
GGGCAAGGGTTAACGAGATTGCACAGTTAATGCTGGACGATGTTCTGGCAGATGACGGCGTTTATTACCTGAACCTTGAA
AGCGATAGCGAAAACGGAAAGAAACTAAAAAACGCCCAATCCCGCCGCAAGATTCCGGTTCATTCTACGCTGATTGAACT
CGGTTTTATCGAGTATGTGGATGCGTTGAAAGCTGCCGGGTATGACCGTCTTTTTCCCGAGCTTAAACCACATAAAACTA
AAGGCTATGGTAGGCCGGTTTCCGCATGGTTCAATGAATCATTGCTTGCGGGTCGATTAAAACTTGAAAGAGACAGAAGC
AAATCTTTCCACTCTTTCCGGCATTCTGTTTCAACTTTGCTTAAAGAGAAGGGTGTTAGTTCGGAACTGCGTGGGCAGCT
ACTTGGGCATGTGCGCGGCAAAACAGAAACTGAAGTGCGATACAGCAAAGATTTAAAACCGGTTCACATGGTTGAGGTTG
TCGAAAAGATTGATTTTTCTTTGCCCGAGATAGCGAGATTCAACATTCCTGATGGGCTGGATGCTGTAAGTGATGCGCTG
CGAAGAAAGCGTGGCAAACAAACAGGTTGA
>Cluster_61362
ATGACGAATCCGTTACTGACTCCCTTTGAATTGCCTCCGTTTTCTAAAATTCTCCCGGAACATGTCGTTCCAGCCGTGAC
TAAGGCATTGAACGACTGCCGCGAAAACGTGGAGCGCGTAGTAGCGCAAGGGGCACCGTACACCTGGGAAAATCTCTGCC
AGCCGCTGGCGGAAGTGGACGATGTGTTGGGGCGTATCTTCTCCCCGGTCAGCCACCTGAACTCGGTGAAAAATAGCCCG
GAACTGCGTGAAGCCTACGAACAAACCCTGCCGCTGCTGTCGGAATACAGCACCTGGGTAGGGCAACATGAAGGGCTGTA
TAAAGCGTATCGCGACCTGCGCGATGGCGATCATTACGCCACGCTGAACACGGCGCAGAAGAAAGCGGTTGATAACGCAC
TGCGCGACTTTGAACTCTCTGGCATAGGTCTGCCGAAAGAGAAACAGCAGCGTTACGGCGAAATTGCGACCCGTCTTTCT
GAACTGGGCAACCAGTACAGCAACAACGTCCTCGATGCGACAATGGGCTGGACCAAACTCGTTACCGACGAAGCGGAGCT
GGCGGGGATGCCAGAAAGCGCGCTGGCTGCGGCAAAAGCCCAGGCCGAAGCGAAAGAGCTGGAAGGTTATTTGCTGACGC
TGGATATCCCAAGCTACTTGCCGGTAATGACCTACTGCGACAACCAGGCTCTGCGTGAAGAGATGTATCGCGCTTACAGC
ACCCGCGCCTCCGATCAAGGCCCAAACGCCGGTAAATGGGATAACAGCAAGGTGATGGAAGAGATCCTCGCGCTGCGTCA
CGAACTGGCGCAACTGCTGGGCTTTGAAAACTACGCCTTTAAATCCCTTGCCACTAAAATGGCAGAAAACCCGCAGCAGG
TGCTGGATTTCTTAACCGATCTGGCAAAACGCGCGCGTCCACAAGGCGAAAAAGAGCTGGCGCAATTGCGTGCCTTTGCC
AAAGCCGAATTTGGCGTCGATGAGTTGCAGCCGTGGGATATCGCTTACTACAGCGAAAAACAAAAACAGCACCTCTACAG
CATCAGTGACGAACAGCTGCGTCCGTACTTCCCGGAAAACAAAGCGGTTAACGGCCTGTTTGAAGTGGTTAAGCGTATTT
ACGGCATCACCGCTAAAGAGCGTAAAGATGTTGATGTCTGGCATCCGGATGTACGTTTCTTCGAACTGTATGACGAAAAT
AACGAACTGCGCGGTAGCTTCTACCTCGATCTGTATGCCCGTGAAAACAAGCGCGGCGGGGCGTGGATGGATGACTGCGT
AGGCCAGATGCGTAAAGCTGATGGTTCTTTGCAAAAACCGGTCGCGTATTTGACTTGTAACTTCAACCGCCCGGTAAATG
GTAAACCGGCGCTGTTCACTCACGACGAAGTGATCACCCTCTTCCACGAGTTCGGTCACGGCCTGCACCATATGCTGACC
CGCATCGAAACCGCTGGTGTTTCCGGTATCAGCGGTGTGCCGTGGGATGCGGTCGAACTGCCGAGTCAGTTTATGGAAAA
CTGGTGCTGGGAGCCGGAGGCGCTGGCGTTTATCTCTGGTCACTATGAAACCGGCGAACCGCTGCCGAAAGAGTTGCTGG
ATAAAATGCTGGCGGCGAAGAACTACCAGGCGGCGCTGTTTATTCTGCGTCAGCTGGAGTTCGGCCTGTTTGATTTCCGC
CTTCATGCCGAGTTCCGCCCGGATCAGGGGGCAAAAATCCTCGAAACTCTGGCAGAAATCAAGAAACTGGTTGCCGTGGT
GCCATCTCCGTCCTGGGGCCGTTTCCCGCACGCTTTCAGCCATATTTTCGCCGGTGGTTATGCCGCAGGTTACTACAGCT
ACCTGTGGGCTGACGTACTGGCGGCAGATGCTTTCTCGCGCTTTGAGGAAGAGGGCATTTTCAACCGTGAAACCGGGCAG
TCGTTCCTCGACAACATTCTGAGCCGTGGCGGTTCAGAAGAGCCGATGGATCTGTTCAAACGCTTCCGTGGTCGTGAACC
GCAGCTGGATGCGATGCTGGAGCATTACGGCATTAAGGGCTGA
>Cluster_33769
ATGGTTCAGGATTCATCAGCATTAGCCCCCTTCTTTCGTGCTCAAATTGAAAAAAATAATCAATACAATTCAGTTTGGGG
CAAACACTACCGCCAGCTTGTAGAACAGATTCAGGCCGAAAAACCAAACTTTTCTGAAGCCACGATTCGGCAGGTATGGT
ATGAACGTGACAATGGTGTCTCCAGCCTGATGCAAGGAGGGATGTCATTGGTTGAATTTGAAAATGCACAAAAAGACCTT
CTAACGCTCACCCGTCAGATAGCAGAAGAGTGTACACAAGAAAATTATAATCGGGTCATTGAACGCCTTATCAAACTTAA
AGCGGACGGAGTATTAAATAAGGTATATAGGGCTCTTTGTAATCGTGCTTTTGCAGCAATATACCCATCAAAAATTAGCA
GTGTCGTGAATGTTAATAATTTTTTAGTTGCTACAATTATTGCAACAACCATTTCCAGCTAGGTCTTTCCGGAGAACGG
GAGTGGCTGGCTCGCAATGTTGATCTTAAAGAAACGCTAAATCGTGCGCTGGGTGATGATATCGACCCTATTGAGCTGAA
TATGTCACTTTGGCATCTCTATGCGTATGAAATTCAGGATAAAAATGCTATCGGTTTAGTTGGAAGCGAAAGTGCTTTAC
AAGAAAATGAGCAAGAAGATGATGTTATAAGTTCGCCATTGCCTAAAAACACAATTCTCTATGGCCCGCCAGGTACAGGT
AAAACATATCGTACTATTGAGATTGCTGTACAAATTTGTGATCCGATCGCTTACTCCCTGCAGGAGGGTAAGGATGAGAG
TGAAAAACGTCGCGAGTTGAAAAAGATTTATAATCAGCTTATCAAAGATAATCGAGTGCGTTTTATCACCTTTCATCAGA
GCTTTGGTTATGAAGAGTTTATTGAAGGTTTAAGAGCTGAGACTACTGATGACGGTAATGTAAAATACGAAATAAAGGCA
GGTATTTTTAAACAGATATGCGAGGATGCCGCCTTTGGTCATGCTGGTGTGCAGGAAATGTTGGATAGCGCACTTACTCG
CCTACAAGAACGTTTATCTGAAGGTGAAAATATCACGCTGGAAACACAACAGGGTAAGGCCTTCCAGCTGACGTATAAAA
GTCAAACCAACTTTGGAATTTTCCCGTCACAATCGAAAAAAGAGGATTTAGGACAGGGTTACAATGCCTATTTGAAGGAT
ATTCGGCAAGTTTATCAGGATCCACGAGCAAAAGTACATAATCCATCTTATGTACGCAGTATCCTTAACTATTTAATTAA
TCAACGTTATCTGCCTCTGAATACTCAGAGCGTAGCTTCAGAAAAAAAGGAAAACTATGTTCTCATTATTGATGAAATAA
ATCGGGGTAATATTTCAAAAATTTTTGGTGAACTAATTACCCTTATTGAGCCATCTAAACGGGCTGGTGAGCCAGAATCG
CTTAGCGTATTGCTGCCATACTCTTCCACTCCATTTAGTGTACCGAATAACCTTTATTTAATTGGTACCATGAACACTGC
TGATCATTCGCTTACTGCACTTGATACTGCACTACGTCGCAGATTTGATTTTGAAGCGATGCTGCCAGATATTACTGTTC
TGAAAGATACTGTCATTAAAGGTATTGATCTGCCTCGTCTATTGCAGACGCTTAACGATCGCATCCAGGTTTTATACGAT
AGAGAACATATGCTGGGGCATGCTTTTTTTATCCCTGTTGTGCAGGCAAAAGAAGACGAAGCTCTGGCATTTGAAAGATT
CAAACGGATTATGCGAAATAAGATACTACCATTGCTGGAAGAATATTTTTACAATGACTGGCAAAAAATTCGCATGGTAC
TGGCTGATAACCAAAAAGAGGAAGTACCAGATCTTCAATTTGTACGTGAGGTGAAATACCAGAAAAAGTATGCCGATCTC
TTTGGTGATAACGGAACAGATGACTTGGGAACCAGTTTTCACCTGGCAGCAGAGGATGACAACGTTTGGGACAATCCAAT
CGCCTGGCAGCAAATTTATGCACCTCAAAAAGAAAAACGCAGGAGTGCGGAATGA

>Cluster_25606
ATGAAAACGGAATCAGCCATCCTTACAGCGTATAACGCCATCGACGCGGCATCCGTTGCCCTTGGTCGTCCCTGCCTGAC
GCTGCCCGAACGTGAGCATTTTCGGACTGAGCGCGATGTTGCCACCCGTAACCTTGCCACGCTTTCCCGCCAGTATCGTG
ATGAGGTCGGGACCTTATCTGCACAGCTCGCATTGCCACGTAACACACAGGTTGACCGTGTGATTTACGGAATTAACTGC
ACGGATTTATCTCTCGCTGACAGGGGCAGGGCTGCCGGACTGGTATCACAGTTTGGCTCGTTTTCGTTCACCGTGGCGCA
TGCTTTCTGCCGTCTGGTTTACCAGCTCGGTGTGAAAAATGCGCTGATGGCGCTTGAGTCCGCGCACCGTTTTGCTTTCT
TCAATCAGGACGGAGACCCTTATGAATTCTCGCTTTTCTGCACTGATGAGCAGATTGCTGAGGTGGCTGATTCGGTTGTT
CGTGACTGCCTTGTTTCTCGGGCCATTTATAGTGATGTACCAGAGCATCACCTTCTCAATGTGATGGCCTATTTTCAGGC
CATCTCCGGCTTTGATTTCGCCCCCGTTTATGCCACTTATCATGAGCGTTACGGCGATGAGGGATTGCTTAAGCGTCTGA
CTGATTTGGCTTTTGTCACCCGTTTCCTGCGGGTTATCCGTGACCAGCGGGTCAATGAGGTCTGTCGCATGCTCGGCATA
CTGAACCGCAACGCGCCTTATATTTCTGACTGGCATCGTGACCTGTTTGCTGTCCGCAGTAAACGCGTGAAAAAGTATCT
CCGGTCCTCCGGTGTCTTTGATGCGTTTAATGAGCTGGTCTGTACGCTGGAAGATGCGCATAACGCCTCTGTTTCGAATC
CGAAAAACCGGATTGCTGAACTCTGCGTTCGTGGAAAGGCCGTCTGTGAACTGTCCGAAGATATGGGGCTGTCCGGCTAC
TTCATCGTGCTCACCACGCCGTCACGTTTTCACCCGACGACCAGTTTTAAGGTTGCCGGAAAATGGCATTCTCGTCCGAA
TAAAAAATGGTGGGAAGCAGGTTGCCCGACCGTTAAGGATTCGCACGCCTGGCTGAATACCGTCTGGCGTCGGGTCTGCC
GCAGACTGGATAAAGCCGGTATTCAGATACCCGGCCTGCGCACGGTCGAGCCTCATGCTGACGGTACAACGCACTGGAAT
TTCCTGATTTACTGCAATCCCCATGAGAGCGCAACGGTGCTGGCCATTTTCCGTGAAGAGGCCATGCGCGATGAGCCGGA
TGAAAAGGGGGCGAAAGAGCACCGTATTCGTATTGAGGCTATTGACCCTGAAAAGGGTGATGGTTTCCGTTACATCGTGA
AGTACATCACCAAAATGGCGGGTGATGTCAGTGCGGATGGTATTACCGCCCTGAATGACCGTTATTCTGCCCGTTCATTC
TGTGATGCCGTCAGCCGTGCCGCCTGCTGGCAGAAAGCAACGCGCCTTCGCCTGTTCCAGTTCTTTGGCGTTCCCTCCGT
TACGGCTTACCGCCAGATGCGCAGCTTCCGCGCACCGCTTGAGGCGCATCATATCAATATGCAGCAGTTCACGCCGCAGC
AGGTCGCCGAACTGGAAGCCATCCGCATGGCCTGTGATGCAGGCGATTTCCGGACCTATATCCTGCTGAACGGCGGTTTC
TTCTGTTCTGAACGTCTGCTTCGTCCGTTTTACGTTCAGCCGCAGGAAGGTGGTAAACCCCGTTTTAACCGTTACGGTGA
ACCCTGCGCACCGGTGATTTCCGGTTTCATGTTTGGCCCTGTTCCGGTCATAACCCGTTTTATGGGCTGTGTTGTCCGCC
GTATGACCCCCGCAGAAAAAAATCCGCGCAGAGGAAGTCAGAAGCGGCAGCAGCTATGAATCTGTGTTTATTTCGTCGGCT
TCACGACACCGCACGACGCGCTCCCGCGCGGCGGGTGGGGGCGGCGAAGCCGACCCTTGGACTTGTGACAATAACTGTCC
CTGA
>Cluster_41462
ATGTCCCGAAAAGACCTTGCCAATGCGATTCGCGCACTCAGTATGGATGCGGTACAAAAAGCCAATTCTGGTCATCCCGG
CGCGCCGATGGGCATGGCTGATATTGCCGAAGTGCTGTGGAATGATTTTCTTAAACATAACCCCACCGACCCAACCTGGT
ATGACCGTGACCGCTTTATTCTTTCCAACGGTCACGCGTCGATGCTGCTCTACAGTTTGCTGCATCTGACCGGTTACGAC
CTGCCGCTGGAAGAGCTGAAGAACTTCCGTCAGTTGCATTCGAAAACCCCTGGTCACCCGGAAATCGGCTATACGCCAGG
TGTTGAAACCACCACCGGTCCTCTTGGACAAGGTTTGGCAAACGCCGTCGGGCTGGCGATAGCAGAGCGTACGCTGGCGG
CGCAATTTAATCAGCCGGATCATGAAATTGTCGATCACTTCACCTATGTGTTTATGGGCGACGGCTGCCTGATGGAAGGT
ATCTCTCACGAAGTCTGTTCGCTGGCAGGCACGCTGGGGCTGGGCAAGCTGATTGGTTTTTACGATCACAACGGTATTTC
TATTGATGGGGAAACCGAAGGCTGGTTTACCGACGATACCGCAAAACGATTTGAAGCCTATCACTGGCATGTGATCCACG
AGATCGACGGTCACGATCCACAGGCGGTGAAGGAAGCGATCCTTGAAGCGCAAAGCGTGAAAGATAAGCCGTCGCTGATT
ATCTGCCGTACGGTGATTGGCTTTGGTTCGCCGAATAAAGCAGGTAAGGAAGAGGCGCACGGCGCACCACTGGGGGAAGA
AGAAGTGGCGCTGGCACGGCAAAAACTGGGCTGGCACCATCCGCCATTTGAGATCCCTAAAGAGATTTATCACGCCTGGG
ATGCCCGCGAAAAAGGCGAAAAAGCGCAGCAGAGCTGGAATGAGAAGTTTGCCGCCTATAAAAAGGCTCATCCGCAACTG
GCAGAAGAGTTTACCCGTCGGATGAGCGGCGGTTTGCCGAAGGACTGGGAGAAAACGACTCAGAAATATATCAATGAGTT
GCAGGCGAATCCGGCGAAAATCGCTACCCGTAAGGCTTCGCAAAATACGCTTAACGCTTACGGGCCGATGCTGCCTGAGT
TGCTCGGCGGTTCGGCGGATCTGGCTCCCAGCAACCTCACCATCTGGAAAGGTTCTGTTTCGCTGAAGGAAGATCCGGCG
GGCAACTACATTCACTACGGGGTGCGTGAATTTGGTATGACCGCCATCGCCAACGGCATCGCGCACCACGGTGGCTTTGT
GCCGTATACCGCGACGTTCCTGATGTTTGTTGAATACGCCCGTAACGCCGCGCGGATGGCGGCACTGATGAAAGCGCGGC
AGATTATGGTTTATACCCACGACTCAATTGGTCTGGGCGAAGATGGTCCGACGCACCAGGCTGTTGAGCAACTGGCTAGT
CTGCGCTTAACGCCAAATTTCAGCACCTGGCGACCGTGCGATCAGGTGGAAGCGGCGGTTGGCTGGAAGCTGGCGGTTGA
GCGCCACAACGGACCGACAGCACTGATCCTCTCCAGGCAGAATCTGGCACAGGTAGAACGAACACCGGATCAGGTTAAAG
AGATTGCTCGTGGCGGTTATGTGCTGAAAGACAGCGGCGGTAAGCCAGATATTATTCTGATTGCCACCGGTTCAGAGATG
GAAATCACCCTGCAAGCGGCAGAGAAATTAGCGGGAGAAGGTCGCAATGTTCGCGTGGTTTCCCTGCCCTCGACCGATAT
TTTCGATGCCCAGGATGAGAAATATCGGGAGTCGGTACTGCCTTCAAATGTTTCTGCCCGCGTGGCAGTAGAAGCGGGTA
TTGCCGATTACTGGTACAAATATGTTGGCCTGAAAGGGGCAATTGTCGGGATGACAGGTTATGGGGAATCAGCTCCGGCG
GATAAGCTGTTCCCGTTCTTTGGCTTTACCGCCGAGAATATTGTGGCAAAAGCGCATAAAGTACTGGGAGTGAAAGGTGC
CTGA
>Cluster_27236
ATGATGCCAGAAATTGGTAACGGGCTGCTGTGTCTGGCGCTAGGAATTGCGCTGCTGCTGTCTGTGTATCCGCTATGGGG
CGTGGCGCGCGGGGACGCGCGCATGATGGCGTCTTCCCGCTTGTTTGCCTGGCTGCTGTTTATGTCTGTGGCTGGCGCAT
TTCTGGTGCTGGTCAATGCTTTCGTGGTCAACGACTTTACCGTCACCTATGTTGCCAGCAACTCCAATACCCAGCTTCCG
GTGTGGTATCGCGTGGCGGCTACCTGGGGCGCGCATGAAGGCTCGCTCCTGCTGTGGGTGCTACTGATGAGCGGCTGGAC
CTTTGCGGTAGCGATTTTTAGTCAGCGTATTCCGCTGGATATTGTTGCCCGCGTACTGGCGATAATGGGGATGGTCAGTG
TCGGCTTTTTGCTGTTCATTCTCTTTACCTCTAACCCGTTCTCACGCACGCTGCCGAATTTCCCGATTGAAGGGCGTGAT
CTTAACCCACTGTTGCAGGATCCGGGGCTGATCTTCCATCCGCCTCTGCTTTATATGGGGTACGTGGGTTTCTCGGTGGC
GTTTGCTTTTGCCATTGCTTCTTTGCTGAGTGGGCGTCTGGACAGCACTTATGCGCGTTTTACTCGTCCGTGGACGCTGG
CTGCGTGGATCTTCCTGACGCTCGGCATTGTGCTCGGTTCCGCATGGGCCTATTACGAACTCGGCTGGGGCGGCTGGTGG
TTCTGGGATCCGGTAGAAAACGCCTCGTTTATGCCGTGGCTGGTGGGGACTGCGCTGATGCACTCACTGGCGGTCACTGA
ACAACGCGCCAGCTTCAAAGCGTGGACATTACTGCTGGCAATCAGTGCCTTCTCGTTGTGTCTACTGGGGACTTTCCTGG
TGCGTTCCGGCGTGCTGGTATCGGTACACGCGTTTGCGTCTGATCCGGCGCGCGGTATGTTTATCCTCGCCTTTATGGTG
CTGGTGATTGGCGGTTCGCTGCTGCTGTTTGCCGCGCGTGGACACAAAGTTCGCTCACGCGTAAACAATGCGCTGTGGTC
GCGGGAATCTTTGCTGTTAGCGAACAACGTTTTGCTGGTCGCCGCGATGCTGGTGGTGTTGCTGGGGACGTTGCTGCCGC
TGGTGCACAAGCAACTGGGACTGGGCAGTATTTCGATTGGCGAACCGTTCTTCAACACCATGTTTACCTGGTTGATGGTG
CCGTTTGCGCTACTGCTTGGTGTCGGCCCCTCTGGTGCGCTGGGGACGCGATCGCCCACGTAAAATCCGCAATTTGTTGAT
TATCGCCTTCATCTCTACGCTGGTGCTGTCGCTGCTGTTGCCGTGGCTGTTCGAAAGCAAAGTTGTGGCGATGACAGTGC
TCGGCCTGGCAATGGCCTGCTGGATTGCGGTGCTGGCAATTGCGGAAGCTGCGCTGCGTATTTCACGCGGCACGAAAACC
ACCTTCAGTTATTGGGGGATGGTGGCGGCTCACCTGGGGCTGGCAGTGACAATTGTTGGCATTGCTTTTAGTCAGAACTA
TAGCGTTGAGCGTGATGTGCGCATGAAGTCCGGCGATAGCGTCGATATTCATGAATATCGCTTCACCTTCCGTGATGTCA
AAGAGGTGACCGGTCCGAACTGGCGTGGCGGTGTGGCGACTATCGGCGTAACGCGAGATGGCAAGCCGGAAACAGTGCTG
TATGCAGAAAAACGTTATTACAACACTGCCGGGTCGATGATGACCGAAGCGGCGATTGACGGTGGCATCACGCGTGACCT
GTACGCGGCACTCGGTGAAGAGCTGGAAAACGGCGCGTGGGCCGTGCGTCTTTACTACAAACCATTTGTTCGCTGGATTT
GGGCGGGCGGGCTGATGATGGCGTTGGGCGGACTGCTGTGTCTGTTTGATCCTCGCTATCGTAAGCGCGTGAACCCGCAG

AAAACTGCGCCGGAGGCTGCATGA
>Cluster_29379
ATGTTAGAGCTAAACGTGGCACGCCTCTCGAACGTTGTCACCTTCGAAGTTAACCGACCATCATTAGAGAAAGCAAAAGC
AGAAGTAGAAAAACTTCGTAAACAAATGGCGGCGGTGAAAGATATAGAATTGCGTGTTAAGACACATAAACAATCAACCG
CTAAAGCAAAAAAAGACGTAGACGATATTGCTAAAAAGCAAGCACAGGCAGATAAAGCCAATGCTAAAGCACAATTAGCC
GCTCAACGTGTAGTTGCAAGAGAACAAAAAGCCCTGGCAGCACGGAAAGAGAAGGCTGAATTAAAGCTTTTAGACGTTGG
AGCAAGCATTAGCGCATTGCACCGTCTTTCTGTAGCGGAACAATATAAAGCTATTGCACAGGCTAGAGAAATTGCCCTCC
AATATGAAAGAGGCGCTATTAGTCTGGCACGTATGAATAGCCAAATGAAACGCCTACAACAGCAACAACGTAAGATTAAT
GGCAATCGTAACGCTCACGCTAAAGCCTATGCACCTGTTAAGGGTAGCGGAGTATTGGCGGCGGTGCTGCGGCTGTTAT
GCTGGGCGGCTTAGGCGCTGGCGCTGCATATATGGCTATGAGCAAAGCCTCTGAATTTGTTACTAACAGCTTTGCGAATG
CTGAAACGCTGGGTGAATTGTATAGCCGTGCCAAGCTGGGCGGTGTAGACGTTAACCAGATGAACAATATTGAGCAATGG
GCGTATAAAAACGGCGTTGATTCAATGATGGGCGAACAAGGTAAACGTAAGTATCTCGATCAGATGAAAGATGTGCGTGA
ACGTGCAACCAAATCTTATGATGAAGCTGAATATGTAGTTGATAAGAAAACTGGTAAAGGAGAATGGAAGGGCGGCGATA
GCGGCATCAATACTTTAATGAATGAAGGATTCCTAACTAAAAAGGATCTAAAAGACTTTGCTGATAATCCGGCTGGTCTG
GTTAGTAAAGCAGTTCAGGGAATGGTAGCTAAAGGCTATTCAGATGCACAAATCGGTAGTCGTCTGGAAGATCTGGCTGA
TGATTTGATGCTCACCTCTAAATACTGGACACGCTCCGCTAAAGAAGTGGAACAATCAGCACGAGAACAACGAGAATCAG
GGCGCTGGGTAACACAGGCACAACAGGAAAGCGTTATTAAATTCCGTGAACTGAATAATGCACTTAGTGGGCTTTCTGAC
TCACAAGGGATCGCCTTTGTTGATGGCTTTATGAAATCTCTTGATCCGAAAGTGATTGAAGAGTTTAAAAAGAGCATGGT
TGCAATGCTGCCAATGTTTACCAAATTAGGCGAGGCTGTAGGTGGTCTGCTTAATGCGATCATGAAAACTATCAACTGGC
TGATGAAAAACGACGAGAAGACGGCAGCTATTCAGAAGAATTTAGGTGATGCACCGCCACTATCTAACGAAGGAATGAAG
CAGAATCTTTCTAATCTCACTCCTGATCAATATAAAGGCGCTGGCACCGGAACAATTAAGCCAGATAACAGCAATTCCCT
GGTTAACACTATTAAAAGCTGGTTTATGCCAGAAGAAACGGTAGGTGGGGCGCAGGCTGTAAATCAATACAGTCTCGAAG
GACAAAACATTGCAAATCTGAAACAGAGTGCAGCACAACAAGCTTTCAAGGCTCCTTCTTACACCTTTGCACCTGTTATT
AACTTCAATCCAGAATTACAAGTAAATGCAGAAGTGCCGCTAACTATCGAATCAGACACCGGACGATTGAGCGAATTTAT
CGACTTCAAATCAAAGGCATCTTCCGCTGAGTTTAGCAAGCTATTAACGCTTGGTGTTATGTCAGGCGGGTCAACCTATT
AA
>Cluster_20906
ATGTTGCTTAATATGATGTGTGGTCGTCGGCTGTCGGCAATCAGTTTGTGCCTGGCCGTAACATTCGCTCCACTGTTCAA
TGCGCAGGCCGATGAGCCTGAAGTAATCCCTGGCGACAGCCCGGTGGCTGTCAGTGAACAGGGCGAGGCACTGCCGCAGG
CGCAAGCCACGGCAATAATGGCGGGGATCCAGCCATTGCCTGAAGGTGCGGCTGAAAAAGCCCGCACGCAAATCGAATCT
CAATTACCCGCAGGTTATAAGCCGGTTTATCTTAACCAGCTTCAATTGTTGTATGCCGCACGCGATATGCAACCCATGTG
GGAAAACCGTGATGCTGTTAAAGCCTTCCAGCAACAGCTGGCAGAGGTGGCGATTGCCGGTTTCCAGCCGCAGTTTAATA
AATGGGTAGAGTTACTGACCGATCCTGGTGTTAACGGGATGGCACGCGACGTGGTGCTCTCTGATGCGATGATGGGCTAT
CTCCATTTCATTGCAAATATTCCGGTCAAAGGCACTCGCTGGCTATATAGCAGTAAACCTTATGCGCTTTCAACGCCGCC
GCTTTCGGTGATTAACCAATGGCAGCTGGCGCTGGATAAAGGTCAATTGCCTACGTTTGTTGCAGGACTGGCACCGCAGC
ATCCGCAATATGCGGCGATGCATGAATCGTTACTGGCCTTACTCAGTGACACCAAACCGTGGCCCCAATTGACCGGCAAA
GCAACGTTGCGCCCAGGACAGTGGAATAACGACGTACCGGCGTTGCGCGAAATATTGCAACGCACAGGCATGTTGGATGG
GGGGCCGAAAATTACTCTACCTGGCGATGACACGCCAACTGACGCGGTAGTCAGCCCATCCGCTGTTACTGTTGAAACAG
CAGAAACTAAGCCGATGGACAAGCAAACTACGTCTCGTAGTAAACCTGCGCCTGTCGTTCGTGCCGCCTACGATAATGAA
CTGGTGGAAGCCGTTAAACGTTTTCAGGCATGGCAAGGATTGGGGGCAGATGGTGCTATTGGCCCGGCAACGCGTGACTG
GTTAAACGTAACGCCCGCCCAACGTGCTGGCGTGTTGGCACTCAACATCCAGCGATTGCGCTTGCTGCCAACAGAGCTTT
CTACCGGGATCATGGTTAACATTCCGGCCTATTCGCTGGTCTACTATCAGAACGGCAATCAGGTGCTGGATTCGCGAGTC
ATTGTCGGTCGCCCCGATCGCAAAACGCCGATGATGAGTAGTGCCCTTAACAACGTGGTGGTAAACCCGCCGTGGAACGT
CCCACCAACTCTGGCACGCAAAGATATTCTGCCGAAAGTGCGCAATGATCCTGGGTATCTCGAAAGCCATGGCTATACAT
TGATGCGCGGCTGGAACAGCAGAGAAGCGATTGACCCATGGCAGGTTGACTGGTCTACAATCACGGCCTCGAATTTACCG
TTCCGCTTCCAGCAGGCTCCAGGCCCACGGAACTCACTGGGGCGCTATAAATTCAATATGCCGAGTTCAGAGGCCATTTA
TTTGCATGACACGCCGAACCACAATCTGTTCAAGCGTGATACACGTGCATTGAGCTCAGGCTGTGTACGAGTGAATAAAG
CTTCCGATCTGGCGAATATGCTGTTGCAGGATGCAGGATGGAATGACAAACGTATTTCTGATGCGCTGAAGCAGGGCGAT
ACGCGTTACGTCAATATTCGGCAGTCGATTCCGGTGAATCTCTACTATCTGACGGCCTTTGTTGGTGCAGATGGTCGTAC
CCAGTATCGTACAGATATTTACAATTATGATCTGCCTGCGCGATCCAGCTCGCAAATCGTATCGAAAGCGGAACAATTAA
TCAGGTAA
>Cluster_20767
ATGGCTAAATACTCAGAAGAACTAAAAGGCGTTGTCCGCGCACTTTATTTGCGCCGCTATACGCCAAAAGAAATTGCATC
AGAATTAAATCTGCCGAATGCGCGGATCGTTTACTACTGGGCTGAGAAATACAGCTGGGCGGATTTACTCAGCTTTGAAA
GTACAGAGGAGGCAATTGAACGCCGCTACCAGCTGCTGGCCAGCCGCGATAATAAAACCGATCTCGACCTGAAAGAAATG
GACATGCTGATTGCCCATGCGACAAAGCTGCGTGCTCAAAGCAATAAGCATAAAGAAAAAATGGCCAGCGGCCAGAGTAA
CAGTCAGGCAGCTGCGCGGGACAGCAACAGCGATGAACCGCGCCCCAAACGCAAAAACAGGAAAAACGATATTTCTTCTC
TGACTCAGGCGGATTTTGACACCTGGGCGGAAGAACATCTTTTTGAATACCAGAAACACCTGCGCCGGAATATTGGCCAG
CAGGTCAGGAACATCCTTAAAAGCCGCCAGATCGGTGCCACCTGGTACTTTGCATTTGAAGCCTTTGAAAACGCGGTCAT
GACAGGCGATCCGCAAATCTTCCTGTCTGCCTCCAAAGTCCAGGCGGAATACTTCCGGTCTTACATCGTCAACATTGCAG
AACAGTATTTTGGCATCACGCTGACCGGCAACCCGATCCGCCTCAGCAACGGCGCTGAACTGCGTTTTCTCTCAACCAAC
AAAAACACGGCCCAGTCCTACAGTGGCCACCTGTACTGTGACGAATATTTCTGGGTGCCTAACTTCGCCAGGCTTAACGA
AGTGGCCAGCGCAATGGCCACCCATGACAAATGGCGCACCACCTACTTTTCAACGCCATCGGCCAAAACGCACCAGGCCT
ACCCGTTCTGGACGGGTGAGGAATGGAAACAGGGCAGCAAAAAACGCGCGGCCGCTCAGTTCCCGTCTTTTGATGAAATG
CGCAACGGCGGACGGCTTTGCCCGGATGGTCAGTGGCGCTATGTCATCACGATGGAGGATGCCATTGCGGGCGGCTTCAA
CCTGGCCAACATCGAGAAGCTGCGCAACCGCTACAACACCGCCACCTTCAACATGCTTTATATGTGCGTGTTCGTGGACA
GCAAGGATTCTGTATTCAGTTTTTCAGACCTGGAAGCCTGCGGCGTGGAGGTGGACACCTGGCAGGATCACAACCCGGAC
GCTGCGCGGCCATTTGGTGACAGGCCGGTATGGGGAGGATTTGACCCTGCACGCAGCGGCGATTTGTCGTGTTTTGTGAT
TATTGCCCCGCCGATGTACGCCGCAGAGAAATTCCGCGTTCTGAAGGTCATTAACTGGAAGGGCATGAACTTCCGCTATC
AGGCCAGGCAGATCGAACTCCTGTTTAAAAAATACAACTTCACCTATCTGGGAGTGGACGTTACCGGTATTGGCCAGGGT
GTTTTTGACAACATCCAGCATTTTGCCATGCGCGTGGCCGTCGCCATTCGTTACGACATGAACACGAAAAATCAGCTGGT
ACTGAAAGCGGCGGACGTGGTGGAAAGCCAGCGTATCGAATGGGACAAAAACCTGAAAGAGATCCCGGCCAGCTTTATGG
CTGTACGCCGCACCACCACGCAAAGCGGTAACGCCATGACATTTGTCGCTGACCGCAGCCAGGACACAGGCCACGCGGAG
GCTTTCTGGGCGATTACCCACGCCCTGCATAACGAACCACTCAACTACGAAAATAAACCGAAATCCCGCTGGGGTGTAAG
GAAAGAGGCTGCATGA
>Cluster_63957
ATGTTACGTCCTGTAGAAACCCCAACCCGTGAAATCAAAAAACTCGACGGCCTGTGGGCATTCAGTCTGGATCGCGAAAA
CTGTGGAATTGATCAGCGTTGGTGGGAAAGCGCGTTACAAGAAAGCCGGGCAATTGCTGTGCCAGGCAGTTTTAACGATC
AGTTCGCCGATGCAGATATTCGTAATTATGCGGGCAACGTCTGGTATCAGCGCGAAGTCTTTATACCGAAAGGTTGGGCA

GGCCAGCGTATCGTGCTGCGTTTCGATGCGGTCACTCATTACGGCAAAGTGTGGGTCAATAATCAGGAAGTGATGGAGCA
TCAGGGCGGCTATACGCCATTTGAAGCCGATGTCACGCCGTATGTTATTGCCGGGAAAAGTGTACGTATCACCGTTTGTG
TGAACAACGAACTGAACTGGCAGACTATCCCGCCGGGAATGGTGATTACCGACGAAAACGGCAAGAAAAAGCAGTCTTAC
TTCCATGATTTCTTTAACTATGCCGGGATCCATCGCAGCGTAATGCTCTACACCACGCCGAACACCTGGGTGGACGATAT
CACCGTGGTGACGCATGTCGCGCAAGACTGTAACCACGCGTCTGTTGACTGGCAGGTGGTGGCCAATGGTGATGTCAGCG
TTGAACTGCGTGATGCGGATCAACAGGTGGTTGCAACTGGACAAGGCACTAGCGGGACTTTGCAAGTGGTGAATCCGCAC
CTCTGGCAACCGGGTGAAGGTTATCTCTATGAACTGTGCGTCACAGCCAAAAGCCAGACAGAGTGTGATATCTACCCGCT
TCGCGTCGGCATCCGGTCAGTGGCAGTGAAGGGCGAACAGTTCCTGATTAACCACAAACCGTTCTACTTTACTGGCTTTG
GTCGTCATGAAGATGCGGACTTGCGTGGCAAAGGATTCGATAACGTGCTGATGGTGCACGACCACGCATTAATGGACTGG
ATTGGGGCCAACTCCTACCGTACCTCGCATTACCCTTACGCTGAAGAGATGCTCGACTGGGCAGATGAACATGGCATCGT
GGTGATTGATGAAACTGCTGCTGTCGGCTTTAACCTCTCTTTAGGCATTGGTTTCGAAGCGGGCAACAAGCCGAAAGAAC
TGTACAGCGAAGAGGCAGTCAACGGGGAAACTCAGCAAGCGCACTTACAGGCGATTAAAGAGCTGATAGCGCGTGACAAA
AACCACCCAAGCGTGGTGATGTGGAGTATTGCCAACGAACCGGATACCCGTCCGCAAGGTGCACGGGAATATTTCGCGCC
ACTGGCGGAAGCAACGCGTAAACTCGACCCGACGCGTCCGATCACCTGCGTCAATGTAATGTTCTGCGACGCTCACACCG
ATACCATCAGCGATCTCTTTGATGTGCTGTGCCTGAACCGTTATTACGGATGGTATGTCCAAAGCGGCGATTTGGAAACG
GCAGAGAAGGTACTGGAAAAAGAACTTCTGGCCTGGCAGGAGAAACTGCATCAGCCGATTATCATCACCGAATACGGCGT
GGATACGTTAGCCGGGCTGCACTCAATGTACACCGACATGTGGAGTGAAGAGTATCAGTGTGCATGGCTGGATATGTATC
ACCGCGTCTTTGATCGCGTCAGCGCCGTCGTCGGTGAACAGGTATGGAATTTCGCCGATTTTGCGACCTCGCAAGGCATA
TTGCGCGTTGGCGGTAACAAGAAAGGGATCTTCACTCGCGACCGCAAACCGAAGTCGGCGGCTTTTCTGCTGCAAAAACG
CTGGACTGGCATGAACTTCGGTGAAAAACCGCAGCAGGGAGGCAAACAATGA
>Cluster_28593
ATGTCATCCGATATTAAGATCAAAGTGCAAAGCTTTGGTCGTTTCCTCAGCAACATGGTGATGCCAAATATCGGCGCGTT
TATCGCGTGGGGTATCATCACCGCGTTATTTATTCCAACAGGGTGGTTACCGAACGAAACGCTAGCGAAGCTGGTCGGGC
CGATGATCACTTATCTCCTGCCGCTGCTGATCGGTTATACCGGTGGTAAGCTGGTAGGCGGCGAACGTGGCGGCGTAGTC
GGTGCCATCACCACCATGGGCGTTATCGTCGGCGCAGACATGCCGATGTTCCTCGGTTCTATGATTGCAGGTCCGCTGGG
CGGCTGGTGCATTAAGCACTTCGACCGCTGGGTAGACGGTAAGATCAAATCCGGTTTTGAAATGCTGGTGAATAACTTCT
CCGCAGGCATCATCGGGATGATCCTCGCTATTCTGGCATTTCTCGGCATTGGCCCGGTTGTTGAAGCCCTGTCTAAAATG
CTGGCGGCGGGCGTTAACTTCATGGTTGTCCATGACATGCTGCCGCTGGCGTCTATCTTTGTTGAACCGGCGAAGATCCT
GTTCCTCAACAACGCTATTAACCACGGTATCTTCTCGCCGCTGGGTATTCAGCAGTCCCATGAACTGGGTAAATCCATCT
TCTTCCTGATTGAAGCTAACCCAGGTCCAGGTATGGGCGTGCTGCTGGCGTACATGTTCTTTGGTCGTGGTAGCGCTAAA
CAGTCTGCGGGCGGTGCGGCAATCATCCACTTCCTGGGGGGTATCCACGAAATCTACTTCCCGTATGTGCTGATGAATCC
GCGTCTGATTCTGGCCGTTATCCTCGGCGGTATGACTGGCGTGTTCACGCTGACTATCCTGGGCGGTGGGCTGGTTTCTC
CGGCATCTCCGGGTTCTATCCTTGCTGTACTGGCGATGACGCCAAAAGGTGCTTACTTCGCTAACATCGCGGGTGTGTGT
GCGGCGATGGCAGTCTCCTTCGTTGTCTCTGCCATTCTGCTGAAAACCAGCAAAGTGAAAGAAGAAGATGATATTGAAGC
TGCTGCACGTCGCGTTCAGGAAATGAAAGCTGAGTCTAAAGGCGCATCTCCGCTGTCTGCGGGCGATGTGACTAACGACC
TGAGCCACGTACGTAAAATCATCGTTGCCTGTGATGCCGGTATGGGTTCCAGTGCGATGGGTGCAGGCGTGCTGCGTAAG
AAAATTCAGGATGCAGGTCTGTCGCAGATTTCGGTCACTAACAGCGCGATCAACAACCTGCCGCCAGATGTGGACCTGGT
CATCACTCACCGTGACCTGACCGAGCGCGCTATGCGCCAGGTTCCGCAGGCACAGCATATTTCGCTGACCAACTTCCTCG
ACAGCGGCCTGTACACCAGCCTGACCGAACGTCTGGTTGCTGCCCAGCGCCATACTGAAAACGAAGTAAAAGTGAAAGAC
AGTCTGAAAGACAGCTTTGACGATTCCAGTGCTAACCTGTTCAAGCTAGGCGCGGAGAACATCTTCCTCGGTCGCAAAGC
GGCCACCAAAGAAGAAGCGATTCGTTTTGCTGGCGAGCAACTGGTGAAAGGCGGTTACGTTGAGCCGGAATACGTTCAGG
CGATGCTGGATCGTGAAAAACTGACCCCGACTTATCTGGGTGAGTCTATCGCGGTGCCACACGGTACGGTTGAAGCGAAA
GATCGCGTACTGAAAACGGGCGTGGTGTTCTGCCAGTATCCGGAAGGCGTGCGCTTCGGTGAAGAAGAAGATGACATTGC
CCGTCTGGTGATTGGTATTGCTGCCCGTAACAACGAGCACATTCAGGTTATCACCAGCCTGACCAATGCACTGGATGATG
AGTCCGTCATCGAGCGTCTGGCACACACCACCAGCGTGGATGAAGTGCTGGAATTGCTGGCAGGTCGTAAGTAA
>Cluster_28187
TTGAGTGACATGGCGAAAAACCTAATACTCTGGCTGGTCATTGCCGTTGTGCTGATGTCAGTATTCCAGAGCTTTGGGCC
CAGCGAGTCTAATGGCCGTAAGGTGGATTACTCTACCTTCCTACAAGAGGTCAATAACGACCAGGTTCGTGAAGCGCGTA
TCAACGGACGTGAAATCAACGTTACCAAGAAAGATAGTAACCGTTATACCACTTACATTCCGGTTCAGGATCCGAAATTA
CTGGATAACCTGTTGACCAAGAACGTCAAGGTTGTCGGTGAACCGCCTGAAGAACCAAGCCTGCTGGCTTCTATCTTCAT
CTCCTGGTTCCCGATGCTGTTGCTGATTGGTGTCTGGATCTTCTTCATGCGTCAAATGCAGGGCGGCGGTGGCAAAGGTG
CCATGTCGTTTGGTAAGAGCAAAGCGCGCATGCTGACGGAAGATCAGATCAAAACGACCTTTGCTGACGTTGCGGGCTGC
GACGAAGCAAAAGAAGAAGTTGCTGAACTGGTTGAGTATCTGCGCGAGCCGAGCCGCTTCCAGAAACTCGGCGGTAAGAT
CCCGAAAGGCGTCCTGATGGTCGGTCCTCCGGGTACCGGTAAAACGCTGCTGGCGAAAGCGATTGCAGGCGAAGCGAAAG
TTCCGTTCTTTACTATCTCCGGTTCTGACTTCGTCGAAATGTTCGTCGGTGTGGGTGCATCCCGTGTTCGTGACATGTTC
GAACAGGCGAAGAAAGCGGCACCGTGTATCATCTTTATCGATGAAATCGACGCCGTAGGCCGCCAGCGTGGCGCAGGCTT
GGGCGGTGGTCACGATGAACGTGAACAGACTCTGAACCAGATGCTGGTTGAGATGGATGGTTTTGAAGGTAACGAAGGTA
TCATCGTTATCGCTGCGACTAACCGTCCGGACGTTCTCGACCCAGCCCTGCTGCGTCCTGGCCGTTTCGACCGTCAGGTT
GTGGTTGGCTTGCCAGATGTTCGCGGTCGTGAGCAGATCCTGAAAGTTCACATGCGTCGCGTACCATTGGCACCCGATAT
CGACGCGGCAATCATTGCCCGTGGTACTCCTGGTTTCTCCGGTGCTGACCTGGCGAACCTGGTGAACGAAGCGGCACTGT
TCGCTGCTCGTGGCAACAAACGCGTAGTTTCGATGGTTGAGTTCGAGAAAGCGAAAGACAAAATCATGATGGGTGCGGAA
CGTCGCTCCATGGTGATGACGGAAGCGCAGAAAGAATCGACGGCTTACCACGAGGCGGGTCATGCGATTATCGGTCGCCT
GGTGCCGGAACACGATCCGGTGCACAAAGTAACGATTATCCCGCGCGGTCGTGCGCTGGGTGTGACCTTCTTCTTGCCTG
AAGGTGATGCAATCAGCGCCAGCCGTCAGAAACTGGAAAGTCAGATCTCCACGCTGTACGGTGGTCGTCTGGCGGAAGAG
ATCATCTACGGGCCGGAACATGTTTCTACCGGGGCGTCCAACGATATTAAAGTTGCGACCAACCTGGCGCGTAACATGGT
TACCCAGTGGGGCTTCTCTGAGAAACTCGGTCCGCTGCTGTATGCGGAAGAAGAAGGTGAAGTGTTCCTCGGCCGTAGCG
TAGCGAAAGCGAAACATATGTCTGATGAAACTGCACGTATCATCGACCAGGAAGTGAAAGCACTGATTGAGCGTAACTAT
AATCGTGCGCGTCAGCTTCTGACCGACAATATGGATATTCTGCATGCGATGAAAGATGCTCTCATGAAATATGAGACTAT
CGACGCACCGCAGATTGATGACCTGATGGCACGTCGCGATGTACGTCCGCCAGCGGGCTGGGAAGAACCAGGCGCTTCTA
ACAATTCTGGCGACAATGGTAGTCCAAAGGCTCCACGTCCGGTTGATGAACCGCGTACGCCGAACCCGGGTAACACCATG
TCAGAGCAGTTAGGCGACAAGTAA
>Cluster_26709
ATGCGAACCCTTTGGCGATTTATTGCCGGATTTTTTAAATGGACGTGGCGTCTGCTGAATTTCGTCCGTGAAATGGTACT
TAACCTGTTCTTTATTTTTCTCGTGCTGGTTGGTGTGGGGATCTGGATGCAGGTCAGTGGTGGTGATTCGAAAGAAACGG
CCAGTCGTGGCGCACTGCTGCTGGACATTTCTGGTGTGATCGTCGATAAACCCGACAGTTCTCAGCGGTTTAGTAAATTA
AGCCGCCAGCTGCTTGGTGCCAGTTCCGATCGTCTGCAGGAAAACTCACTGTTTGATATCGTCAACACCATTCGCCAGGC
GAAGGAGGACCGCAATATCACCGGTATTGTGATGGATCTGAAAAACTTCGCAGGCGGCGACCAGCCGTCTATGCAGTACA
TCGGCAAAGCTCTGAAAGAGTTTCGTGACAGCGGGAAACCGGTTTATGCCGTTGGCGAGAACTACAGCCAGGGGCAATAT
TATCTCGCCAGTTTCGCCAATAAAATTTGGCTGTCTCCGCAAGGCGTGGTTGATCTGCACGGCTTTGCCACCAACGGTCT

GTACTACAAATCGTTACTGGATAAGCTGAAAGTTTCCACCCATGTGTTCCGCGTGGGTACGTATAAATCTGCCGTTGAAC
CGTTTATTCGTGATGATATGTCGCCGGCAGCCCGCGAAGCTGACAGCCGCTGGATTGGTGAGCTGTGGCAAAACTATCTG
AATACTGTTGCCGCTAACCGCCAGATCCCTGCTCAGCAGGTATTCCCTGGCGCGCAAGGGTTGCTTGAGGGTTTAACCAA
AACCGGTGGCGATACCGCGAAATATGCGCTGGAAAACAAGCTGGTCGATGCACTGGCATCGAGTGCCGAAATCGAAAAAA
CTCTGACCAAAGAGTTCGGCTGGAGTAAAACTGATAAAAATTATCGCGCCATCAGTTATTACGATTACGCATTGAAAACG
CCGGCAGATACCGGTGACAGTATCGGTGTCGTCTTCGCTAATGGCGCAATTATGGATGGCGAGGAAACTCAGGGGAATGT
TGGCGGTGATACCACTGCGGCACAAATCCGCGACGCTCGCCTTGACCCGAAAGTGAAAGCGATTGTCCTGCGTGTTAATA
GCCCAGGCGGCAGCGTTACCGCGTCTGAAGTGATTCGCGCTGAACTGGCAGCAGCCCGGGCAGCGGGTAAGCCTGTGGTT
GTATCGATGGGTGGCATGGCGGCATCTGGTGGTTACTGGATTTCCACACCAGCTAATTACATTGTGGCTAACCCCAGCAC
CCTGACCGGTTCTATCGGTATCTTCGGCGTGATCACCACCGTAGAAAATAGTCTGGATTCGATTGGTGTTCATACTGATG
GTGTCTCAACTTCACCGCTGGCAGATGTTTCTATCACCAGGGCACTGCCGCCGGAAGCGCAGCAGATGATGCAGTTAAGC
ATTGAGAATGGCTATAAACGCTTTATCACGCTGGTTGCTGATGCGCGTCATTCAACGCCGGAGCAGATTGATAAAATCGC
CCAGGGTCACGTCTGGACCGGTCAGGATGCAAAAGCTAACGGGCTGGTCGATAGTCTCGGGGATTTCGATGATGCGGTCG
CCAAAGCAGCAGAGCTGGCAAAAGTGAAACAGTGGCATCTGGAATACTACGTTGATGAACCGACCTTCTTCGACAAAGTG
ATGGACAACATGTCTGGTTCTGTCCGGGCAATGTTGCCAGATGCGTTCCAGGCCATGTTACCTGCACCGCTGGCCTCGGT
AGCCTCTACCGTTAAAAGTGAAAGCGACAAGCTGGCCGCGTTTAACGACCCACAAAACCGTTATGCGTTTTGCCTGATCT
GCGCCAACGTGCGTTAA
>Cluster_57551
ATGTCTGCAACAAAACTGACCCGCCGCGAACAACGCGCCCGGGCCCAACATTTTATCGACACCCTGGAAGGCACCGCCTT
TCCCAACTCAAAACGCATTTATATCACTGGCACACACCCCGGCGTGCGCGTGCCGATGCGTGAGATCCAGCTTAGCCCGA
CGCTAATTGGCGGTAGCAAAGAACAGCCGCAGTACGAAGAAAACGAAGCGATTCCGGTCTACGACACCTCCGGCCCGTAT
GGTGATCCGCAGATTGCCATTAACGTGCAGCAAGGGCTGGCAAAACTACGCCAGCCGTGGATCGATGCGCGCGGCGATAC
CGAAGAACTTACCGTGCGCAGTTCCGATTACACTAAAGCGCGGCTGGCAGATGATGGCCTCGACGAACTGCGTTTTAGCG
GTGTACTAACACCAAAACGCGCCAAAGCAGGACGCCGTGTCACCCAACTGCACTACGCCCGCCAGGGCATCATCACGCCG
GAAATGGAATTCATCGCCATCCGCGAGAATATGGGCCGCGAGCGCATCCGTAGCGAGGTTTTACGCCACCAGCATCCGGG
AATGAGCTTTGGCGCACATCTGCCGGAAAATATCACTGCGGAATTTGTCCGTGATGAAGTTGCTGCCGGACGTGCGATTA
TCCCGGCCAACATTAATCATCCGGAATCGGAGCCGATGATTATTGGTCGCAATTTCCTGGTAAAAGTTAACGCCAATATC
GGCAACTCGGCGGTCACCTCTTCCATCGAAGAAGAAGTGGAAAAGCTGGTATGGTCCACGCGCTGGGGAGCGGATACGGT
GATGGATCTCTCCACCGGTCGCTATATTCACGAAACCCGCGAGTGGATTTTGCGTAACAGCCCGGTGCCGATCGGTACAG
TGCCGATCTACCAGGCGCTGGAGAAGGTTAACGGGATCGCCGAAGATCTTACCTGGGAAGCGTTCCGCGACACGCTGCTG
GAACAGGCCGAGCAAGGTGTGGATTACTTCACTATCCATGCGGGCGTACTGCTGCGCTATGTGCCGATGACCGCGAAACG
CCTGACCGGTATCGTCTCTCGCGGCGGTTCGATTATGGCGAAATGGTGCCTCTCCCATCATCAGGAAAATTTCCTCTATC
AACACTTCCGCGAAATTTGTGAAATCTGTGCCGCTTATGACGTTTCGCTGTCGCTGGGCGACGGTCTGCGCCCCGGTTCT
ATTCAGGACGCCAACGATGAAGCGCAGTTTGCCGAGCTGCATACGCTGGGCGAACTGACCAAAATTGCCTGGGAATATGA
CGTGCAGGTGATGATTGAAGGCCCAGGTCACGTGCCGATGCAGATGATCCGCCGCAATATGACCGAGGAGTTAGAGCACT
GCCACGAAGCGCCGTTTTACACTCTGGGGCCGCTAACTACCGATATTGCGCCGGGCTATGACCACTTCACGTCGGGGATT
GGTGCGGCGATGATTGGCTGGTTTGGCTGCGCGATGCTCTGTTACGTAACGCCAAAAGAGCATCTGGGTCTGCCTAATAA
AGAAGATGTTAAGCAGGGGCTTATCACCTATAAGATTGCCGCCCACGCCGCTGACCTGGCGAAAGGGCATCCGGGCGCGC
AAATTCGCGATAACGCCATGTCGAAAGCCCGCTTCGAATTTCGCTGGGAAGACCAGTTTAATCTGGCCCTCGACCCGTTT
ACCGCCCGCGCTTATCACGATGAAACCCTGCCGCAAGAGTCAGGTAAAGTCGCCCATTTTTGCTCCATGTGTGGGCCGAA
ATTCTGTTCGATGAAAATCAGCCAGGAAGTGCGTGATTACGCCGCCACGCAAACTATTGAAATGGGAATGGCGGATATGT
CGGAGAACTTCCGTGCCAGAGGCGGAGAAATCTACCTGCGTAAGGAGGAAGCGTGA
>Cluster_24414
ATGTCACAAGATCCTTTCCAGGAACGCGAAGCTGAAAAATACGCGAATCCCATCCCTAGTCGGGAATTTATCCTCGAACA
TTTAACCAAACGTGAAAAACCGGCCAGCCGTGATGAGCTGGCGGTAGAACTGCACATTGAAGGCGAAGAGCAGCTTGAAG
GCCTGCGTCGCCGCCTGCGCGCGATGGAGCGCGATGGTCAACTGGTCTTCACTCGCCGTCAGTGCTATGCGCTGCCGGAA
CGCCTCGACCTGGTGAAAGGTACCGTTATTGGCCACCGTGATGGCTACGGCTTTCTGCGAGTTGAAGGGCGTAAAGATGA
TTTGTATCTCTCCAGCGAGCAGATGAAAACCTGCATTCATGGCGATCAGGTGCTGGCTCAGCCGCTGGGCGCTGACCGTA
AAGGTCGTCGTGAAGCGCGTATTGTCCGCGTACTGGTGCCAAAAACCAGCCAGATTGTTGGTCGCTACTTCACCGAAGCG
GGCGTCGGCTTTGTGGTTCCTGACGATAGCCGTCTGAGCTTCGATATCTTAATCCCGCCCGATCAGATCATGGGCGCGCG
GATGGGCTTTGTGGTGGTGGTTGAACTGACTCAACGCCCGACTCGCCGTACCAAAGCGGTGGGTAAAATCGTCGAAGTGC
TGGGCGATAATATGGGCACCGGCATGGCGGTTGATATTGCTCTGCGTACCCATGAAATTCCGTACATCTGGCCGCAGGCT
GTTGAGCAACAGGTTGCCGGGCTGAAAGAAGAAGTGCCGGAAGAAGCAAAAGCGGGCCGTGTCGATCTGCGCGATTTACC
GCTGGTCACCATTGATGGCGAAGATGCCCGTGACTTTGACGATGCAGTTTACTGTGAGAAAAAACGCGGCGGTGGCTGGC
GTTTGTGGGTCGCAATTGCCGACGTCAGCTACTATGTGCGTCCGCCAACGCCGCTGGACAGAGAAGCGCGTAATCGTGGT
ACGTCGGTGTACTTCCCTTCGCAGGTTATCCCGATGCTGCCGGAAGTGCTCTCTAACGGCCTGTGCTCGCTCAACCCGCA
GGTAGACCGCCTGTGTATGGTGTGCGAGATGACGGTTTCGTCGAAAGGCCGCCTGACTGGCTACAAATTCTACGAAGCGG
TGATGAGCTCTCACGCGCGTCTGACCTACACCAAAGTCTGGCATATACTGCAAGGCGATCAGGATCTGCGCGAGCAGTAT
GCGCCGCTGGTTAAGCATCTCGAAGAGTTACATAACCTCTATAAAGTGCTGGATAAAGCCCGTGAAGAACGCGGTGGGAT
CTCATTTGAGAGCGAAGAAGCGAAGTTCATTTTCAACGCTGAACGCCGTATTGAACGTATCGAACAGACTCAGCGTAACG
ACGCCCACAAGTTAATTGAAGAATGCATGATTCTGGCGAATATCTCGGCGGCGCGTTTCGTTGAGAAAGCCAAAGAACCG
GCACTGTTCCGTATTCACGACAAGCCGAGCACCGAAGCGATTACCTCTTTCCGTTCAGTGCTGGCGGAGCTGGGGCTGGA
ATTGCCGGGCGGTAACAAGCCGGAACCGCGCGACTATGCAGAGTTACTGGAATCAGTTGCCGATCGTCCTGATGCAGAAA
TGCTGCAAACCATGCTTTTGCGCTCGATGAAACAGGCGATTTACGATCCGGAAAACCGTGGTCACTTCGGTCTGGCATTG
CAGTCCTATGCGCACTTTACTTCGCCGATTCGTCGTTATCCTGACCTGACGTTGCACCGCGCCATTAAATATCTGCTGGC
GAAAGAGCAGGGGCATCAGGGCAACACCACTGAAACCGGCGGCTACCATTATTCGATGGAAGAGATGCTGCAACTGGGTC
AGCACTGTTCGATGGCGGAACGTCGTGCCGATGAAGCAACGCGCGATGTGTCTGACTGGCTGAAGTGTGACTTCATGCTC
GACCAGGTAGGTAACGTCTTTAAAGGCGTAATTTCCAGCGTCACTGGCTTTGGCTTCTTCGTCCGTCTGGACGACTTGTT
CATTGATGGTCTGGTCCATGTCTCTTCGCTGGACAATGACTACTATCGCTTTGACCAGGTAGGGCAACGCCTGATGGGTG
AATCCAGCGGCCAGACTTATCGCCTGGGCGATCGCGTGGAAGTTCGCGTTGAAGCGGTTAATATGGACGAGCGCAAAATT
GACTTTAGCCTGATCTCCAGCGAACGCGCACCGCGCAACGTCGGTAAAACGGCGCGCGAGAAAGCGAAAAAAGGCGATGC
AGGTAAAAAAGGCGGCAAGCGTCGTCAGGTCGGCAAAAAGGTCAACTTTGAGCCAGACAGCGCCTTCCGCGGCGAGAAAA
AACCGAAGCCGAAAGCAGCGAAGAAAGACGCGAGAAAAGCGAAAAAGCCATCGGCGAAAACGCAGAAAATAGCCGCAGCG
ACCAAAGCGAAGCGTGCGGCGAAGAAAAAAGTGGCAGAGTGA
>Cluster_58636
GTGAATATTTATCGACTCTCTTTTGTATCCTGCCTGGTCATGGCGATGCCTTGCGCAATGGCGGTCGAATTCAATCTGAA
TGTTCTCGATAAATCAATGCGCGACCGCATTGATATTTCATTATTAAAGGAAAAAGGAGTCATTGCTCCCGGTGAATATT
TTGTTAGCGTTGCGGTGAATAACAACAAAATCAGTAATGGGCAAAAAATTAACTGGCAAAAAAAGGGTGACAAAACCATT
CCATGCATCAATGATTCACTGGTCGATAAATTTGGTTTAAAACCAGATATCCGTCAGTCCTTGCCACAGATAGATCGGTG

TATTGATTTCAGTTCCCGACCTGAAATGCTCTTCAATTTCGATCAAGCCAATCAGCAACTGAATATTAGTATTCCGCAAG
CCTGGCTGGCGTGGCACTCAGAAAACTGGGCTCCCCCCTCTACATGGAAAGAAGGTGTTGCCGGTGTCCTGATGGATTAC
AACTTGTTTGCCAGCAGCTACCGCCCACAGGACGGCAGCAGCAGCACTAACCTGAATGCCTACGGTACCGCCGGAATTAA
CGCCGGGGCATGGCGCTTACGCAGTGATTACCAGCTTAATAAGACCGATAGCGAAGATAACCATGACCAGTCAGGCGGAA
TATCGCGCACCTATCTTTTTCGTCCATTACCGCAATTAGGCTCTAAGTTAACCCTCGGCGAAACCGATTTCAGTTCCAAT
ATTTTCGATGGTTTTTCTTATACCGGCGCGGCACTGGCGAGTGACGATCGAATGTTACCGTGGGAGCTGCGTGGCTACGC
CCCACAAATTAGCGGTATTGCACAGACCAATGCCACGGTGACGATCAGTCAATCAGGCCGCGTCATTTACCAGAAAAAAG
TCCCGCCAGGCCCGTTTATTATTGATGACCTCAATCAGTCTGTTCAGGGCACGCTGGATGTCAAAGTGACGGAAGAAGAT
GGTCGGGTGAACAATTTCCAGGTTTCGGCAGCATCGACGCCCTTCCTGACTCGCCAGGGACAGGTTCGCTATAAATTGGC
CGCGGGTCAGCCACGGCCTTCCATGTCACATCAAACTGAAAATGAAACCTTTTTAGCAATGAAGTTTCCTGGGGGATGC
TCTCAAACACCTCGCTGTACGGCGGCCTGCTGATTTCTGATGATGACTACCATTCTGCCGCAATGGGTATCGGGCAAAAT
ATGCTGTGGCTTGGCGCACTGTCCTTTGATGTCACCTGGGCCAGTAGCCATTTTGATACTCAGCAGGACGAGCGGGGCTT
AAGCTACCGTTTTAATTACAGCAAACAAGTGGATGCCACCAACAGCACGATTTCGCTCGCCGCTTATCGCTTCTCAGATC
GTCATTTTCACAGCTACGCCAACTATCTGGATCACAAATACAACGACAGCGATGCGCAGGACGAAAAACAGACGATCAGC
TTATCCGTGGGCCAACCGATTACCCCACTAAACCTCAATCTTTACGCCAACCTGCTACATCAAACCTGGTGGAATGCAGA
CGCCTCCACGACCGCCAACATCACAGCAGGTTTTAATGTTGATATTGGTGACTGGAGAGATATCTCGATTTCGACGTCAT
TCAATACGACCCACTACGAAGATAAAGATCGCGACAACCAGATTTATCTGTCGATTTCGCTCCCCTTCGGTAACGGTGGT
CGGGTTGGCTATGACATGCAAAACAGTAGCCACAGCACCATACACCGCATGTCGTGGAACGATACGCTGGATGAACGTAA
TAGCTGGGGCATGTCTGCCGGACTGCAATCCGATCGTCCGGACAATGGAGCCCAGGTGAGCGGTAACTATCAGCACCTGA
GTTCAGCGGGTGAGTGGGATATTTCTGGTACCTATGCCGCCAGTGATTACAGTTCCGTCAGCAGCAGCTGGAGCGGTTCT
TTCACCGCAACCCAATATGGTGCAGCATTTCATCGCCGCAGCTCCACCAATGAACCACGCCTGATGGTCAGCACCGATGG
CGTGGCAGATATTCCGGTTCAGGGCAATCTCGACTACACCAACCATTTTGGCATTGCGGTGGTGCCGTTGATTTCCAGTT
ACCAGCCTTCCACCGTGGCGGTGAACATGAATGACTTACCCGACGGCGTAACAGTTGCAGAAAACGTCATCAAGGAAACA
TGGATTGAAGGCGCGATAGGTTACAAATCACTGGCTTCCCGTTCCGGTAAAGACGTTAACGTCATCATACGCAACGCCAG
CGGTCAGTTCCCTCCCCTCGGTGCGGATATCCGCCAGGATGACAGCGGCATTAGCGTGGGTATGGTTGGCGAGGAAGGAC
ATGCCTGGTTAAGCGGTGTCGCTGAAAATCAACTGTTTACCGTGGTCTGGGGTGAGCAAAGCTGCATTATTCATCTGCCA
GAACGTCTGGAAGACACGACCAAACGCCTGATTTTACCTTGTCATTAA
>Cluster_28776
ATGTCGAATTCTTATGACTCCTCCAGTATCAAAGTCCTGAAAGGGCTGGATGCGGTGCGTAAGCGCCCGGGTATGTATAT
CGGCGACACGGATGACGGCACCGGTCTGCACCACATGGTATTCGAGGTGGTAGATAACGCTATCGACGAAGCGCTCGCGG
GTCACTGTAAAGAAATTATCGTCACCATTCACGCCGATAACTCTGTCTCCGTACAGGATGACGGGCGCGGCATTCCGACC
GGTATTCACCCGGAAGAGGGCGTATCGGCGGCGGAAGTGATCATGACCGTTCTGCACGCAGGCGGTAAATTTGACGATAA
CTCCTATAAAGTGTCCGGCGGTCTGCACGGCGTTGGTGTTTCGGTAGTAAACGCCCTGTCGCAAAAACTGGAGCTGGTTA
TCCAGCGCGAGGGTAAAATTCACCGTCAGATCTACGAACACGGTGTACCGCAGGCCCCGCTGGCGGTTACCGGCGAGACT
GAAAAAACCGGCACCATGGTGCGTTTCTGGCCAAGCCTTGAAACCTTCACCAATGTGACCGAGTTCGAATATGAAATTCT
GGCGAAACGTCTGCGTGAGTTGTCGTTCCTCAACTCCGGCGTTTCCATTCGTCTGCGCGACAAGCGCGATGGCAAAGAAG
ACCACTTCCACTATGAAGGCGGCATCAAGGCGTTCGTTGAATATCTGAACAAGAACAAAACGCCGATCCACCCGAATATC
TTCTACTTCTCCACCGAAAAAGACGGTATTGGCGTCGAAGTGGCGTTGCAGTGGAACGATGGCTTCCAGGAAAACATCTA
CTGCTTTACCAACAACATTCCGCAGCGTGACGGCGGTACTCACCTGGCAGGCTTCCGTGCGGCGATGACCCGTACTCTGA
ACGCCTACATGGACAAAGAAGGCTACAGCAAAAAAGCCAAAGTCAGCGCCACCGGTGACGATGCGCGTGAAGGCCTGATT
GCGGTCGTTTCCGTGAAAGTGCCGGACCCGAAATTCTCCTCACAGACCAAAGACAAACTGGTTTCTTCTGAGGTGAAATC
GGCGGTTGAACAGCAGATGAACGAACTGCTGGCGGAATACCTGCTGGAAAACCCAACCGACGCGAAAATCGTGGTCGGCA
AAATTATCGATGCTGCCCGTGCCCGTGAAGCTGCGCGTCGCGCGCGTGAAATGACCCGCCGTAAAGGTGCGCTGGATTTA
GCTGGCCTGCCGGGCAAACTGGCAGACTGCCAGGAACGCGATCCGGCGCTTTCCGAACTGTACCTTGTGGAAGGGGACTC
CGCGGGCGGCTCTGCGAAGCAGGGGCGTAACCGCAAGAACCAGGCGATTCTACCGCTGAAGGGTAAAATCCTCAACGTCG
AGAAAGCGCGCTTCGATAAGATGCTCTCTTCTCAGGAAGTGGCGACGCTTATCACCGCGCTTGGTTGTGGTATCGGTCGT
GACGAGTACAACCCGGACAAACTGCGTTATCACAGCATCATCATCATGACCGATGCGGACGTCGACGGCTCGCACATTCG
TACGCTGCTGTTGACCTTCTTCTATCGTCAGATGCCGGAAATCGTTGAACGTGGTCACGTCTACATCGCTCAGCCGCCGC
TGTACAAAGTGAAGAAAGGTAAGCAGGAACAGTACATTAAAGACGACGAAGCGATGGATCAGTACCAGATCTCTATCGCG
CTGGATGGCGCAACGCTGCACACCAACGCCAGTGCACCGGCGCTGGCTGGCGAAGCGTTAGAGAAACTGGTGTCTGAGTA
CAACGCGACGCAGAAAATGATCAACCGCATGGAGCGTCGTTATCCGAAAGCAATGCTGAAAGAGCTTATCTATCAGCCGA
CGCTGACGGAAGCCGATCTCTCTGATGAGCAGACCGTTACCCGCTGGGTGAATGCGCTGGTCAGCGAACTGAACAACAAA
GAACAGCACGGCAGCCAGTGGAAGTTTGATGTCCACACCAATGCCGAACAAAACCTGTTCGAGCCGATTGTTCGCGTGCG
TACCCACGGTGTGGATACTGACTATCCGCTGGATCACGAGTTTATCACCGGTGGTGAATATCGTCGTATCTGCACGCTGG
GTGAGAAACTGCGTGGCTTGCTGGAAGAAGATGCATTTATCGAACGTGGCGAACGTCGTCAGCCGGTAGCCAGCTTCGAG
CAGGCGCTGGACTGGCTGGTGAAAGAGTCCCGTCGCGGCCTCTCCATCCAGCGTTATAAAGGTCTGGGCGAGATGAACCC
GGAACAGCTGTGGGAAACTACCATGGACCCGGAAAGCCGTCGTATGTTGCGCGTTACCGTTAAAGATGCGATTGCTGCCG
ACCAGTTGTTCACCACGCTGATGGGCGACGCCGTTGAACCGCGCCGTGCGTTTATTGAAGAGAACGCTCTGAAAGCGGCG
AATATCGATATTTAA
>Cluster_5328
ATGGCGATGAAAAAGTTGCTCATAGCGTCGCTGCTGTTTAGCAGCGCCACCGTATACGGTGCTGAAGGGTTCGTAGTGAA
AGATATTCATTTCGAAGGCCTTCAGCGTGTCGCCGTTGGTGCGGCCCTCCTCAGTATGCCGGTGCGCACAGGCGACACGG
TTAATGATGAAGATATCAGTAATACCATTCGCGCTCTGTTTGCTACCGGCAACTTTGAGGATGTTCGCGTCCTTCGTGAT
GGTGATACCCTTCTGGTTCAGGTAAAAGAACGTCCGACCATTGCCAGCATTACTTTCTCCGGTAACAAATCGGTGAAAGA
TGACATGCTGAAGCAAAACCTCGAGGCTTCTGGTGTGCGTGTGGGCGAATCCCTCGATCGCACCACCATTGCCGATATCG
AGAAAGGTCTGGAAGACTTCTACTACAGCGTCGGTAAATATAGCGCCAGCGTAAAAGCTGTCGTGACCCCGCTGCCGCGC
AACCGTGTTGACCTAAAACTGGTGTTCCAGGAAGGTGTGTCAGCTGAAATCCAGCAAATTAACATTGTTGGTAACCATGC
TTTCACCACCGACGAACTGATCTCTCATTTCCAACTGCGTGACGAAGTGCCGTGGTGGAACGTGGTAGGCGATCGTAAAT
ACCAGAAACAGAAACTGGCGGGCGACCTTGAAACCCTGCGCAGCTACTATCTGGATCGCGGTTATGCCCGTTTCAACATC
GACTCTACCCAGGTCAGTCTGACGCCAGATAAAAAAGGTATTTACGTCACGGTGAACATCACCGAAGGCGATCAGTACAA
GCTTTCTGGCGTTGAAGTGAGCGGCAACCTTGCCGGGCACTCCGCTGAAATTGAGCAGCTGACTAAGATCGAGCCGGGTG
AGCTGTATAACGGCACCAAAGTGACCAAGATGGAAGATGACATCAAAAAGCTTCTCGGTCGCTATGGTTATGCCTATCCG
CGCGTACAGTCGATGCCCGAAATTAACGATGCCGACAAAACCGTTAAATTACGTGTGAACGTTGATGCGGGTAACCGTTT
CTACGTGCGTAAGATCCGTTTTGAAGGTAACGATACCTCGAAAGATGCCGTCCTGCGTCGCGAAATGCGTCAGATGGAAG
GTGCATGGCTGGGGAGCGATCTGGTCGATCAGGGTAAGGAGCGTCTGAATCGTCTGGGCTTCTTTGAAACTGTCGATACC
GATACCCAACGTGTTCCGGGTAGCCCGGACCAGGTTGATGTCGTCTACAAGGTAAAAGAGCGCAACACCGGTAGCTTCAA
CTTTGGTATTGGTTACGGTACTGAAAGTGGCGTGAGCTTCCAGGCTGGTGTGCAGCAGGATAACTGGTTAGGTACAGGTT
ATGCTGTTGGTATCAACGGGACCAAAAACGATTACCAGACCTATGCTGAACTGTCGGTAACCAACCCGTACTTCACCGTA
GATGGCGTAAGCCTCGGTGGTCGTCTCTTCTATAATGACTTCCAGGCAGATGACGCCGACCTGTCCGACTATACCAACAA

GAGTTATGGTACAGACGTGACGTTGGGCTTCCCGATTAACGAATATAACTCGCTGCGTGCAGGTCTGGGTTATGTACATA
ACTCCCTGTCCAACATGCAGCCTCAGGTTGCGATGTGGCGTTATCTGTACTCTATGGGTGAACATCCGAGCACCTCTGAT
CAGGATAACAGCTTCAAAACGGACGACTTCACGTTCAACTATGGTTGGACCTATAACAAGCTTGACCGTGGTTACTTCCC
GACAGATGGTTCACGTGTCAACCTGACCGGTAAAGTGACCATTCCTGGATCGGATAACGAATACTACAAAGTGACGTTAG
ACACGGCGACTTATGTGCCGATCGATGACGATCACAAATGGGTTGTTCTGGGGCGTACCCGCTGGGGTTATGGTGATGGT
TTAGGCGGCAAAGAGATGCCGTTCTACGAGAACTTCTATGCCGGTGGTTCCAGCACCGTGCGTGGCTTCCAGTCCAATAC
CATTGGTCCGAAAGCAGTTTACTTCCCGCATCAGGCCAGTAATTATGATCCGGACTATGATTACGAATGTGCGACTCAGG
ACGGCGCGAAAGACCTGTGTAAATCGGATGATGCTGTAGGCGGTAACGCCATGGCGGTTGCCAGCCTCGAGTTCATCACC
CCGACGCCGTTTATTAGCGATAAGTATGCTAACTCGGTTCGTACTTCCTTCTTCTGGGATATGGGTACCGTTTGGGATAC
AAACTGGGATTCCAGCCAATATTCTGGATATCCGGACTATAGTGATCCAAGCAATATCCGTATGTCTGCGGGTATCGCAT
TACAATGGATGTCCCCATTGGGGCCGTTGGTGTTCTCCTACGCCCAGCCGTTCAAAAAGTACGATGGAGACAAGGCAGAA
CAGTTCCAGTTTAACATCGGTAAAACCTGGTAA
>Cluster_53940
TTGCAAAATCAGGACTTTATTGCTGGCCTTAAAGCTAAATTTGCCGAACATCGCATCGTTTTCTGGCACGATCCCGATAA
ACGTTTTATTGAGGAACTGGAACAGCTCAAGCTTGAAAACGTCACGCTAATCAACATGACCCACGCGTCACAGCTGGCGG
TGAAAAAACGCATCGAGATGGATGAGCCAGAACAGCAGTTCCTGCTGTGGTTCCCCCATGATGCGCCGCCTCATGAACAA
GACTGGCTGCTGGATATCCGCCTTTACAGCAGCGAATTCCATGCCGATTTTGCCGCCATCACCCTGAACACGCTGGGCAT
TCCCCAGCTTGGCCTGCGCGAGCATATTCAGCGACGCAAGGCCTTCTTCAGCACTAAACGTACGCAGGCGCTGAAAAACC
TGGTGACGGAACAGGAAGATGAAACCTCGCTGGATAAGAAAATGATTGCGGTGATCGCTGGCGCAAAGACCGCGAAAACC
GAAGACATTTTGTTTAACCTGATTACCCAGTACGTTAACCAACAAACAGAAGACGACAGCGAACTGGAAAACACGCAGGC
GATGTTGAAACGCCACGGTCTGGACCCGGTATTGTGGGAAATGCTCAACCACGAAATGGGCTACCAGGCAGAGGAGCCAT
CGCTGGAAAACCTGCTCCTGAAACTGTTTTGTACCGATCTCTCTGCCCAGGCCGACAGACAGCAGCGCGCCTGGCTGGAA
AAAAATGTCCTGCTGACGCCATCCGGCAGAGCATCCGCCCTGGCATTTATGGTGACCTGGCGTGCCGATCGTCGCTATAA
AGAGGCTTATGACTACTGCGCTCAGCAAATGCAGGCGGCCCTGCGCCCGGAAGATCATTACCGACTCAGTTCGCCGTATG
ATTTGCACGAATGCGAAACCACCCTCAGCATCGAACAAACCATTATTCATGCGCTGGTAACGCAACTGCTGGAAGAGAGC
ACCACGCTCGATCGGGAAGCCTTTAAAAAACTGCTCTCTGAACGCCAGAGCAAATACTGGTGCCAGACACAACCAGAGTA
TTACGCCATCTATGACGCGCTGCGCCAGGCTGAGCGTTTGCTGAATCTGCGCAATCGCCACATCGATGGTTTCCACTACC
AGGACAGCGCCACCTTCTGGAAAGCCTACTGCGAAGAACTGTTCCGCTTCGACCAGGCTTATCGCCTGTTTAATGAATAT
GCCCTGCTGGTTCACAGCAAAGGGGCGATGATCCTCAAGAGCCTGGATGATTATATCGAGGCGCTCTACAGCAACTGGTA
TCTGGCAGAATTAAGCCGTAACTGGAACGAAGTGCTGGAAGCGGAAAATCGTATGCAGGCGTGGCAAATCCCCGGCGTGC
CGCGTCAGAAGAACTTTTTCAATGAGGTGGTGAAGCCGCAGTTCCAAAATCCGCAAATCAAACGCGTGTTCGTGATAATT
TCCGATGCCCTGCGTTATGAAGTGGCGGAGGAGCTGGGGAATCAAATCAATACCGAGAAACGCTTTACCGCAGAACTGCG
CTCGCAGCTCGGCGTGCTCCCCAGCTACACCCAACTGGGAATGGCGGCATTGCTGCCCCATGAACAACTTTGCTATCAAC
CAAGTAACGGCGACATCGTTTATGCTGATGGGCTGTCGACCTCGGGTATTCCTAACCGCGATACCATTCTGAAGAACTAT
AAGGGAATGGCGATAAAATCGAAGGACCTTCTGGAGTTAAAAAATCAGGAAGGGCGGGACCTTATTCGCGATTACGAAGT
GGTGTATATCTGGCATAACACGATTGATGCCACCGGTGACACGGCATCCACGGAAGATAAAACCTTCGAAGCGTGCCGCA
CGGCGGTGACTGAACTGAAAGATTTAGTCACCAAGGTAATCAACCGTCTCCACGGCACACGCATTTTTGTTACGGCGGAT
CACGGTTTCCTGTTCCAGCAACAGGCGCTTTCGGTTCAGGATAAAACCACTCTGCAAATTAAGCCGGAAAACACCATCAA
GAACCACAAACGCTTTATTATCGGCCATCAGCTTCCCGCCGATGATTTTTGCTGGAAAGGAAAGGTGGCGGATACCGCAG
GCGTGAGCGACAACAGCGAGTTCCTTATTCCGAAAGGGATCCAGCGCTTCCATTTCTCTGGCGGCGCGCGCTTCGTTCAT
GGCGGCACCATGTTGCAGGAGGTTTGCGTTCCGGTATTGCAGATAAAAGCCCTGCAAAAAACCGCCGCAGAAAAACAGCC
GCAGCGCCGCCCGGTGGATATTGTCGCTTACCATCCCATGATTAAGCTGGTGAACAATATCGATAAAGTGAGCCTGTTGC
AGACGCATCCGGTGGGCGAACTTTATGAACCGCGTATCCTGAACATTTACATTGTCGATAACGCCAACAATGTGGTCTCG
GGCAAAGAGCGCATCAGCTTTGACAGCGATAACAGCACCATGGAAAAACGCGTACGCGAAGTTACGCTGAAGCTGATTGG
CGCTAACTTCAACCGTCGCAATGAGTACTGGTTGATACTGGAAGACGCACAAACGGAAACGGGGTATCAGAAGTACCCGG
TCATTATCGATCTGGCGTTCCAGGATGATTTCTTCTAA
>Cluster_67020
GTGACAGAGAACGTACCCCATGAACAATCTGAAGTGAAAGGCTGGCAGGTGTTGTTATCGCAACTCATGGGCGGCTGGAA
AAAACCGGACGGCAGCCAGCAGGCACAGCAAATCCTGGATGAGATGGACTATCCCTCTTTCGCCAGCCTGCTTCCGTACC
GTCAGTACGACCCGGACAGCGAGCTGTTTATCAATGCAAAAACCGCCGGTTTTGTCCTGGAATGCCGGCCCCTGTCCGGG
GCCAACGAAGCTATCAGTAAGTCACTGGATTATTTTTTGCGGGACAAAATCCCCCGCAAAATTCCGCTGACGTTCACCCT
CGTGGGGTCACCGTGTGTCCGCCCGATGCTGGAGCACGGGCTGGCAGACTTCCGCTGGCAGGGGAAGCGGGCGGATGAAT
TCAATGCCATTACCCGTGCCTTCTACGAGCGGGCGGCGCTGACACAGTTCGCCAACAAGAAAGGATACCCACTGACCTTG
CGTCATTACCGTCTCTTTGTCTCCTATGCCGAAGAAGTAGGCAGCCTGACGGAGCAGAAAAAGACGGAACTGAACCAGGT
GGCCAACCGGGTGGTGGCCGGCCTGGCGTCGGCGGGGCTGTGGTCGCAGCGGGTGGATAAAAAGGGGCTGGTTTCCCTCG
TGCGCGAACTGGCGAATTTTCGCCACGGCAATATTGAGCCACCGAATGGCAGTGTTGCGGAGTTTGAGCCGCTGAACACC
GAATGTGTCGACCGCTCCCTGCGGCTGATGCTGCATCCGGACCGGATTGAGCAGAGTCTGCAGTCGCGTCGCGGCGGCGA
ACGTGAGCGCACACGCATTCTTAATTTCATGCTGGAAAAAATCCCGAGAAACCGTATGCCCTGTGGCAGATGGGGGACA
ATATCAGCAACGTCACGGACCCGCTGTGCACCATTTCCTGCCCGTTTGTGATGACGCTGGCGCTGGAAGTGGAAGAGCAG
GCCGGTACCCAGCGGGAGGCCAACCGCAAATTTATCCAGCTGGACAAAAAGGCGAACTCGCCCTTTGCGAAGTTTGTACC
GTCGGTGAAAAAGCAGGCTGCGGAGTGGGGCGGGCTGCGGGAATCCCTCAGCACCAACCAGGATTCACTGGCGCGTTTTT
ACTTTAACGTCACGCTGTTCTGTGAAGACAGGGATGAAGTGGCGCTGGAAACGGAGATGACGGTGCTGAACACCTTCCGG
CGTAACGGGCTGTCAATGTACATGCCGGAGTTTATGCAGCTGCGTAATTACCTCTCCGTCTTTCCGTTCATGATGCCGGA
AGGGCTGTGGAGTGATATCTGCCGTACAAATGCCATCCAGCGGGCGCGGGCCAGCAATGTGGCCAATATTCTGCCGGTGG
TGGCGGATAACCAGGTCTGCCTGCAGGGGCTGCCCATACCGTCTTACCGCAACCAACTTTCTTTTTTGAACCTGTTCGAC
CGCAATGCGGGGCTGGATACGGATAACAACAATCTCTCCGTCACCGGTACCTCCGGGGGCGGTAAATCATTCCTGATGCA
GGGCATCATTCGGCAGGTGCTGGACAGCGGTGGCAGGGCCTGGGTGTTTGATATGGGGGACTCCTACAAGGGGCTCTGCC
GTAATGTGGGCGGGGTGTATCTGAACGCGCTCGACCTGCGCTTCAACCCCTTTGCCAACATCAAGGATATTACGGCGTCT
GCCGAAAGTATCCGTAACCTGCTGGTCGTGCTGGCTAACCCGTCCGGTAACTGCGACGACACCTTTATGTCGCTGTTGCT
GAAAGCGGTGCATGAAGTCTGGTTGCGCAAAAAGAACAGCGCACGGATTGACGATATCGTGCACTACATGCGGAAAGCGC
TGGAGCAGCCGGAATACCGCGATACCACCACCGTGCGTTCACGCATGGATGAGATTATCGTCGGTCTGGAAAAGTACTGC
TCCTGGGGACTTTATGGCGAGTACTTCAACAGTGAAAAGCCGTCGCTGGATGACAACGTGCGTTTCTCCGTGCTGGAGAT
GGGCGAGCTGAAGGACAAGCCTGACCTGCTGGCTGCCGTGATGTTCTCTATGATGATTTACGTTGAGCAGCGGATGTATC
TCTCCGACCGTCAGCAACACAAGGTCTGTCTGATTGATGAGGCCTGGAAGCTGCTGTCGAAGGACAACAAGCGGGCCGAG
GACTTTATCGAGAACGGGTACCGCACGGCGCGTAAATACAACGGGGCTTATATCACCATCACCCAGGGGATTGAAGACTT
CGACGGGCAGAAAGCCAGTGGCGCGGCGAAAGCGGCCTGGGCGAACTCCTCCTTCAAAATCATCCTGCGTCAGAACCGCG
ATGCGTTCCGTAAATACTGCCTGGATAACCCGGACCAGTTTAACCGGTTTGAACGGAGGTCATTGAGGGCTTTCCGGCG
GCACGTGAGGCGCATTTCAGTTCCTTCATGCTGCGTTACGGCGGGCAGGTTTCCTTCCATCGTCTGCTGCTCGACCCCAT
CAGCCGGGTGATGTTCTCGTCTGACGGTGAAGATTTTGACTACCGTGAAGCCGGACTCCGTGCCGGTAAAGATATCCACG

ACATCATCGGAGAACTGGCCTGGCGTAAGTTCCCGCAGGAAATGGAGACACTGACGGAATGGATAAAACACAATCACTGA
>Cluster_24909
ATGGCCGGGAATGACCGCGAGCCAATTGGACGCAAAGGGAAACCGACGCGTCCGGTCAAACAAAAGGTAAGCCGTCGTCG
TTACGAAGATGACGATGATTACGACGATTATGATGACTATGAGGATGAAGAACCGATGCCGCGCAAAGGTAAGGGCAAAG
GCAAAGGGCGTAAGCCTCGTGGCAAACGCGGCTGGCTATGGCTGCTGCTAAAACTGGCTATCGTTTTTGCCGTGCTGATC
GCCATTTACGGCGTTTATCTCGATCAAAAAATTCGTAGCCGTATTGATGGCAAGGTCTGGCAATTGCCCGCGGCAGTTTA
TGGCCGAATGGTCAACCTTGAGCCAGACATGACCATCAGCAAGAACGAGATGGTGAAGCTGCTGGAGGCGACCCAGTATC
GCCAGGTGTCGAAAATGACCCGTCCTGGCGAGTTTACCGTACAGGCCAACAGCATTGAGATGATCCGCCGTCCGTTTGAT
TTCCCGGACAGCAAAGAAGGGCAGGTGCGCGCGCGTCTGACCTTTGATGGCGATCATCTGGCGACGATCGTCAATATGGA
GAACAACCGTCAGTTCGGTTTCTTCCGTCTTGATCCGCGCCTGATCACCATGATTTCTTCGCCAAACGGTGAGCAGCGTC
TGTTTGTGCCGCGCAGTGGTTTCCCGGATTTGCTGGTGGATACTTTGCTGGCGACAGAAGACCGTCATTTCTACGAGCAT
GATGGGATCAGTCTCTACTCAATCGGACGTGCGGTGCTGGCAAACCTGACTGCCGGACGCACTGTGCAGGGGGCGAGTAC
GCTGACGCAACAGTTGGTTAAAAACCTGTTCCTCTCCAGCGAGCGTTCTTACTGGCGTAAAGCGAACGAAGCCTACATGG
CGCTGATCATGGACGCGCGTTACAGCAAAGACCGTATTCTTGAGCTGTATATGAACGAGGTGTATCTCGGTCAGAGCGGC
GACAACGAAATCCGCGGCTTCCCGCTGGCGAGCTTGTATTACTTTGGTCGCCCGGTAGAAGAATTGAGCCTCGATCAGCA
GGCGCTGTTGGTGGGTATGGTGAAAGGGGCGTCCATCTACAACCCGTGGCGTAACCCGAAACTGGCGCTGGAGCGACGTA
ACCTGGTACTACGTTTGCTGCAACAGCAACAAATTATTGATCAAGAACTCTACGACATGCTGAGTGCGCGTCCGCTGGGT
GTTCAGCCGCGCGGTGGGGTGATCTCTCCTCAGCCAGCCTTTATGCAGCTGGTGCGTCAGGAGCTGCAGGCGAAATTAGG
CGATAAGGTAAAAGATCTCTCCGGCGTGAAGATCTTCACTACCTTTGACTCGGTGGCCCAGGACGCGGCAGAAAAAGCCG
CCGTGGAAGGCATTCCGGCACTGAAGAAACAGCGTAAGTTGAGCGATCTTGAAACCGCGATTGTGGTTGTTGACCGCTTT
AGCGGTGAAGTTCGTGCGATGGTCGGTGGCTCTGAGCCGCAGTTTGCGGGCTACAACCGTGCGATGCAGGCGCGTCGTTC
GATTGGTTCTCTTGCAAAACCAGCGACTTATCTAACGGCCTTAAGCCAGCCGAAAATCTATCGTTTGAATACGTGGATTG
CCGATGCGCCAATCGCGCTGCGTCAGCCGAATGGCCAGGTCTGGTCACCGCAGAACGATGATCGTCGTTATAGCGAAAGT
GGCAGGGTGATGCTGGTAGATGCGTTGACCCGTTCGATGAACGTGCCGACGGTGAATCTGGGCATGGCGTTGGGGCTGCC
TGCGGTTACGGAGACCTGGATTAAACTGGGTGTGCCGAAAGATCAACTGAATCCTGTTCCAGCAATGTTGTTGGGGGCGT
TGAACTTAACGCCAATCGAAGTGGCGCAGGCCTTCCAGACTATCGCCAGCGGTGGTAATCGTGCTCCGCTTTCCGCGCTA
CGTTCAGTCATCGCAGAAGACGGCAAGGTGTTGTATCAGAGCTTCCCGCAGGCGGAACGCGCTGTTCCAGCGCAGGCGGC
GTATCTGACACTATGGACCATGCAGCAGGTTGTACAACGCGGTACGGGTCGTCAGCTTGGGGCGAAATACCCGAACCTGC
ATCTGGCAGGGAAAACAGGGACTACCAACAATAACGTAGATACCTGGTTTGCGGGCATTGATGGGAGCACGGTGACCATC
ACCTGGGTCGGCCGTGATAACAACCAGCCGACCAAACTGTATGGAGCCAGCGGGGCAATGTCGATTTATCAGCGTTATCT
GGCTAACCAGACGCCAACGCCGCTAAATCTTGTTCCGCCAGAAGATATTGCAGATATGGGCGTGGACTACGACGGCAACT
TTGTTTGCAGCGGTGGTATGCGTGTCTTGCCGGTCTGGACCAGCGATCCGCAATCGCTGTGCCAGCAGAGTGAGATGCAG
CAGCAACCGTCAGGCAATCCGTTTGATCAGTCTTCTCAGCCGCAGCAACAGCCGCAACAGCAACCTGCTCAACAGGAGCA
GAAAGACAGCGACGGCGTGGCTGGCTGGATCAAGGAGATGTTTGGTAGTAATTAA
>Cluster_28545
ATGTCATGGATGGTCGTTTCGCGCACTTACACTTCTTTTTTTCCCTTTTCTTTATCTGTATTGGCGCTTACGGTTGCAGG
CTCTTTTTCTGCGACAGCTGGAAAATTTAATCCGCGTTTTCTGGAAGATACCGCAGGGATCAACCAGCATGTTGACCTTT
CCATGTACGAAACTGACCATGGCGCGCAGTTGCCTGGAACATATCGCGTTTCTTTAATCGTCAATGAACAAAAAATGGAA
ACGCGCACTCTGGAGTTTAAGGCGGCTACAGAATCCCAACGAAAGGAGATGGGGGAGTTTCTCATACCTTGTTTAAGCCG
GACACAACTGGCCGATATGGGCGTTCGTGTAGACAGTTTTTCAGCACTTAACCTTATACCAGCAGAAGCCTGTGTGGCGT
TTAATGAAATTATCCCTCAGGCGACCAGTCATTTTGATTTTAGCGAACAAAAACTGGTGATGAGTTTTCCTCAGGCGGCA
ATGCAGCAGGTTGCTCGCGGTACAGTACCTGAATCTCGCTGGGACGATGGTGTGCCCCGCGTTGCTTCTTGATTACAGTTT
CAGCGGTAGTAACAGTTCTCATGATACAAAAAGTTATAATCGCTACATTGATGAAAATGGTAATCATCACCAGGATAAAA
ACGAAACTAGCCAGACTAACGATAGCTACTACTTAAGTATGCGAAGCGGCCTTAACCTTGGGGCATGGCGTCTGCGAAAT
TACAGTAACTGGAGCTATAGTAACGGCGAGAAGCAGTGGGACAATATTGGCACCTATGTAACACGGGCCATTGTTCCGCT
GAAAGCGCAACTGACATTGGGTGATACAGCAACACCTTCAGATATTTTTGACAGTGTGCAGATGCGCGGGGTATTACTGG
CCTCTGATGAAGAAATGTTGCCAGACAGCCAACGTGGTTTTGCTCCGGTAGTTCGAGGGATAGCGAAAAGTAACGCGGAA
GTCAGTATTGAACAAAATGGTTACGTGATTTATCGAGCATTTGTCCAGCCAGGTGCGTTTGAAATTAACGATTTATACCC
CACAGCGAACAGCGGTGATTTAACCGTAATTGTTAAAGAGGCTGATGGTAGTGAACAAAGATTTATTCAGCCATTCTCTT
CAGTCGCTGTTTTCCAACGTGAAGGGCATCTCAAATATAGCCTTTCTGCTGGTGAATACCGTGCTGGTAATTACGATAGC
GCCAACCCACGCTTTGGTCAGTTTAATGCGATGTATGGTTTACCGTTTGGTATGACCACCTATGGTGGCGCATTAATTTC
TAAGGATTACAATGCATTTGCTCTGGGATTGGGAAAAAACTTTGGCTCTATTGGTGCAATATCGGTTGATATTACTCAGG
CAAAAAGTACATTAAATAATAATGCAACCGATCAGGGACAATCGTACCGCTTTCTTTATTCAAAAAGCTTTGCCAGCGGC
ACAGATTTCCGTTTATTGGGTTACAAGTATTCTACCAGTGGTTTTTATACCTTCCAGGAAGCAACAGATGTGCGTAGCGG
TGCTGACAGCGATTACGGCCGTTACCATAAGCGTAGTGAAATTCAGGGTAATTTGACACAGCAATTGGGCACCTATGGGT
CGGTTTATTTCAATATGACCCAGCAGGATTACTGGAATGATGACGGTAAGCGCCTTTCTTTGGCTACCGGGTACAACGGC
CGTATTGGACGTGTTAATTACAGTATTGCTTACAGTTGGAATAAAAGCCCTGAATGGGATGAAAACGACCAGTTATGGTC
ATTTAATATTTCTATTCCGTTTGGTCGGGCATGGAGCAACTACCGCGTGACAACCGATCAGGATGGCCGCACGATACAAC
AGCTCGGCGTCAATGGCACTCTGTTAGAAGATCGCAATCTTAGCTATAACGTTCAGGAAGGGTATTCCAGTAATGGTGTT
GGTAATAGCGGTAATGCCAGTTTGGCGTACCAGGGCGGCGCCGGTAATATCAGCGTGGGTTACAGCTATGGTAAAGACTA
CCAGCAGACTAACTACAGCCTGCGTGGTGGCATTGTAGCGCACAGTGAAGGGATCTCGCTTTCCCAACCTTTGGGTGAAA
CAATTGCTATAGTTTCAGCACCAGGTGCCCGGGGGGCGAAAGTATTAAATAATAGTGGTGTGAGTGTTGACTGGCAAGGT
AATGCGGTCGTTCCTTATCTGAGTATTTACCGTGAAAATGATGTGTCCATTCGCAGTGAAACGTTGAATGACAGTGTTGA
TATGAACAGCGCATTCCAGACAATTGTGCCAACTCGAGGAGCTGTCGTACGGGCGCATTTCGATACCCGTGTGGGTTATC
GAGTTCTTATGACATTAATTCGGCAAAATGGAGTGAGCGTCCCCTTTGGTGCAACTGCAACGCTGGTTTCAGATACAACT
GAACAGATTAGCGGTATTGTTGGTGAAGATGGTCAGTTATATATCAGTGGGATGCCTAAAACAGGGAATGTGAAGATCGT
ATGGGGTAAAGATACGTCACAACAATGCGTTGCGAAATATGAACTACCGGTTGAGGAAAAAAATTCCGGTATTATTTCCG
TGACAGCAAATTGTCAATAA
>Cluster_15792
ATGAGTCATGATGATATGAGCAACTCTAGCGGCTTTAACGAGGCCGCTGCAGCATTTTCATGGAACGGCCCGAAAAAGGC
CATTAACCCTTATCTGGATCCGGCGGAAGTTGCGCCGGAGTCTGCACTTTCAAACATGATCACTCTGTACGCTGCCGATA
ACGAGCAGGAACAGCTGCGCCCGCGATGCACTGAGTGAGCAGGTCTGGGAGCGTTATTTCTTTAATGAATCCCGTGATCCT
GTCCAGCGCGAAATGGAGCAGGATAATCTCATTAGTCGGGCAAAGCTGGCGCATGATCAGCAGCGTTTTAACCCGGACAT
GGTCATTCTGGCGGACGTCAGCGCCCAGCCCACCCACATCAGCAAGCCGCTGATGCAACGTATCGAATACTTCAGCAGCC
TGGGCAGGCCAAAGGCTTATTCCCGCTATTTGCGTGAGACGATTAAGTCATGTCTGGAGCGACTGGAGTATGTACGCGAC
AGTCAGCTATCCGCTTCTTTCCGTTTTATGGCAAGCCATCAAGGGCTTGAGGGCCTGCTGATCCTGCCTGAAATGAGTCA
GGATCAGGTAAAACGCCTGTCCACCTTGGTAGCAGCGCATATGAGCATGTGTCTTGAGGCCGCTTGTGGTGATTTGTATG
CCTCCGATGACGTTAAGCCAGAAGAAATCCGCAAGACATGGGAAAAGGTGGCAGCGGAAACCCTGCGTCTGGATGTTATC
CCGCCTGCGTTTGAGAAACTCCGTCGGAAAAGAAACCGCCGCAAACCCGTGCCCTATGAACTCATACCGGGTTCGCTGGC

ACGTATGCTTTGCGCCGACTGGTGGTATCGAAAATTGTGGAAGATACGTTGCGAATGGCGGGAAGAGCAGTTGCGTGCTG
TCTGCCTGGTCAGCAAAAAAGCATCTCCCTATGTCAGCTATGAAGCAGTGACGCATAAACGTGAGCAGCGCCGCAAGTCG
CTGGAGTTTTTCCGTTCTCATGAACTGGTGAACGAAGACGGCGACACGCTGGATATGGAAGACGTGGTAAACGCCAGCAG
CAGCAACCCGGCGCACCGCCGCAATGAGATGATGGCCTGTGTTAAAGGTCTGGAGCTTATCGCGGAAATGCGTGGTGACT
GCGCCGTTTTCTACACCATTACCTGTCCGTCACGTTTCCATTCTACGCTCAATAACGGCAGACCAAACCCAACCTGGACA
AATGCGACGGTAAGACAAAGCAGCGATTATCTGGTCGGCATGTTTGCTGCATTTCGTAAGGCTATGCACAAAGCCGGGTT
GCGCTGGTATGGCGTGCGGGTGGCTGAGCCGCACCATGACGGTACTGTGCACTGGCATCTCATGTGTTTCATGCGCAAAA
AAGACCGCCGCGCCATTACTGCATTGTTGCGTAAGTTTGCCATCCGTGAAGACCGCGAGGAACTGGGCAATAACACGGGT
CCACGCTTTAAGTCTGAGCTGATAAACCCGCGCAAAGGAACGCCGACAAGCTACATCGCGAAATACATCAGTAAGAACAT
TGACGGACGTGGTCTGGCTGGCGAGATCAGCAAGGAAACGGGTAAATCCCTGCGTGATAACGCCGAATACGTTAATGCCT
GGGCGTCTCTGCATCGTGTTCAGCAGTTCCGCTTCTTTGGTATTCCGGGGCGTCAGGCGTACCGTGAACTTCGCTTGCTG
GCTGGTCAGGCGGCAAGGCAACAGGGTGACAAAAAAGCAGGTGCGCCGGTACTGGATGACCCGCGCCTTGATGCCATCCT
GGCTGCCGCTGATGCTGGCTGTTTTGCCACCTACATCATGAAGCAGGGCGGCGTACTGGTTCCCCGCAAATATCACCTTA
TCAGAACAGCTTATGAAATTAACGAAGAGCCGACCGCTTATGGCGATCACGGTATTCGTATTTATGGCATCTGGTCACCC
ATTGTACAGGGCAAGATCTGCACTCATGCAATGAAATGGAAAATGGTTCGTAAGGTCGTTGACGTTCAGGAGGCGGCAGC
CGACCAGGGCGCTTGCGCCCCTTGGACTCGTGGCAATAACTGTCCCCTTGCTGAAAATTTGAACCGACAGGAGAAAGATA
AATCAGCTGATGGGGACCCCAGAACGGACATTACCCGCATGGATGACAAGGAGTTACACGATTACCTGCACAGTATGAAC
AAAAAGGAGCGCCGGGAACTGGCTGCAAGGTTACGTCTGGTGAAACCGAAACGGCGTAGAGACTACAAACAGCGAATTAC
AGACCATCAACGACAGCAGCTCGTCTATGAACTGAAATCCAGAGGATTTGATGGCAGCGAGAAAGAGGTCGATTTACTCC
TTCGCGGCGGCAGTATTCCGTCAGGAGCTGGCCTGCGTATCTTCTATCGGAACCAGCGTTTGCAGGAAGATGATAAGTGG
CGGAACCTGTATTAA
>Cluster_53511
ATGGCAAAAGAAGCAAAAAAACAAGACCCGCAACAGCAGCTAGAAGCACTGTTCAATAAACTGGTTAAACAGCACTCCGA
GTTTACTCAGCCATTGGTCGAATTACCCGAGCATGAATTCGCCAAGGCGGGTCTCATTCAAGGCCTGGTTGAAGGCAACT
TAACGCCACCAGTCATGTTGATTGCGCTGACAGAAGGCCATTGGGAATACGCCGCTGACACTGCAAAGTTTGATGATTTA
GCCTATCAGCTGTTAAGTGATATCGAAGGTGACTGGCCAGTATATGCCGTTGTGGACGATGGCTTGAACCAACGTATTTT
GGCCTTGTTCGGTGCCGATGGTTCTGACGGTACCCACGGTGCAGATACCCTGCCAGGTCTGGAAGAATTACGTAGCTTTG
ATCGCATGGAACGTGATCCAACTTTTCGCTGGTCGATGCGTGTTTATACACGCTTAATGCAGCGATTTGATGCCTTTCAT
GAAAATGTTTATCGCGTTACCAAAGACAAGGTAAACGACAAGAACGACATTATTGAGGAAGTGGCTAAACTTCTGTTTTT
AGAAACTTTCCGCTTGCATCACGATGAAGACCTTACCTTTAAAGATGATGAGGGAAATACCCTGCGTTTTCGTGATGTCT
TTGATTGGCAATATGTGGAGAGCCACGGCGATAAAGCCGTGGCCCAAATCAAAGCCGCCTTTGAGGAATTTAAAAACCAC
GAAAACTACGTTGTGATCTCAGATGATGGCTCACGTAACCCCATCTTTAGTAAAGAAACACACCTGCGGCTGTCTGTTGC
GAAAAACTATCAGGATTTGCTGGAAGCAATACAAAACCTGGGGCCGGTAAAAACCAACGATGGCAAAATCGCTAAAGAAC
ACGGCACGTTGGCTGATGTGTCAGGAGATTTACTCGGCAGAGTGTTTGATGTATTCCTACGCGCTAACTTTGAATCCAAA
GGTGGCCTTGGCGTTTATTTAACACCAAACCCAGTAAAACAGGCCATGCTTGAAATTGCTATGCACGACATCGACGATGA
CGATGAAATGCGATCGCGCTTAGCCAATGGCGATTTTCGTTTCTGCGACCCCACCTGTGGCTCCTTTGGTTTTGGCTCGG
TGGCGTTAAGCCAGATTGATAAATGGATCGATTTTAAGCTGGTGCTGGCCGACGATAAAAAAGAATCATTGAAACAAAAA
TTACGCGACTGTGCATTTACCGGTGCCGATGCTGCACCACGCATGGTAATGCTGGCCCGCGTGAATATGGCATTGCAAGG
TGCCCCCAAAGCGCAGATTTTCTATACCGACAACTCACTCACTACTAACGCCCTGAAGCCGAATAGTTTTGACCTTATTT
GCACCAACCCGCCGTTCGGTACGCCTAAATTTGATAAAGGCAAAAATGGTCAAAACTCAAAAGCCAATTACGAAGCCAAT
ATGGAGCAAGTGCTCGGTGGTTTTCGCCCGACCAAAAAAGTCGTTGATAGCTACAACGCCTGGTATGACCACGTGAAAAT
GAAGTGGCAGGATATCGGTGATTTAGAGCTGGATGAAAACGGCGAGCCTAAATGGGCGGGCTATCGTACCGATTTGCGCA
GAACGGGCGGTACTGATAAAAAGCCGTTTTACAGCCTGCAGCCTACAACCGCAGGGCTCGCGCTGGGTAGCAAGCCCGAT
AGCAAGGGCAACTGGCAACCGGTAGGTGCCACTATTGACCCTGCAGTACTTTTTATCGACCGCTGCCTGCAACTGCTTAA
ACCCGGAGGTCGCCTGTTAATAGTGCTGCCTGATGGTATTTTATGTAACTCCGGCGACCGTTATGTCCGTGAGTACATTA
TGGGGAAAAAAGACGAAAAAACCGGCGAGTTTGTTGGCGGTAAAGCTATTGTAAAAGCAGTAATTTCATTGCCGAGCGAC
TGCTTTAAACTCTCCGGCACGGGGGCCAAAACCTCGATTTTGTATTTACAAAAACGCCATGCTAACCCCAACCAGCCTGA
GCAATTTTTACCCGAGCCACAAACCGACGTATTTATGGCAGTGGCTGAAACCTTGGGCTACGTGGTGAAAAATAATATTG
AAGACTATAACGCTGGTGTCGCAAACGATCTGGATAAGATTGTATCGGCTTATAAGCGGGGTGTGTAA
>Cluster_49989
ATGACAGATTCGATAAATGCCAATGTTGTAGTGAGTATGCCTTCGCAACTCTTCACTATGGCGCGTTCTTTTAAAGCCGT
AGCCAATGGTAAAATTTATATCGGTAAAATTGACACTGACCCGGTAAACCCTGAAAACCAGATTCAGGTTTATATAGAGA
ACGAAGACGGCTCTCACGTTCCTGTTTCGCAACCAATCATCATTAACGCTGCTGGTTATCCGGTATATAACGGACAAATT
GCCAAATTCGTTACTGTACAGGGCCATTCTATGGCTGTTTATGATGCATACGGTGCGCAGCAGTTTTATTTCCCTAATGT
GTTGAAGTATGAGCCGGATCAATTAAGGTTGGAGCTTGAATCATCTGGTGGAGCAGGATTGATAGGTGGTGTTTCGAAGC
CTATTACTTTTTTTGGTGCTATTGGTGATGGATTAACAAACAATACGCAGGCATTTAACGATGCAGAGATATTAGAATTC
AATGACATCTACATTCCTGATGGTGTTTATAATGTAAATAATATTAACTTAAATAAGAGATATTGGGGGCCTGGCATAGT
AGTGATTAATTACCAGACTGAATATTCTGGCTCTGGTGTTAACGACATGACCGTGTCAGGAATATACAATGGACAGCAAG
ATCTAAATGCAATTGTAAGAATAAAAGAAGCTTCAAATCCTGATAAGCTTGAATTCAGTACAGATAATGGAGTTACATGG
CATGACACGATAGACGTGTACAATCCAATCGATGACAGTGTATCTCCGCAACCAATCGTAATACTTGCAGGCGGGGTGGA
TTTTTTCGTGAGTGGATTGAAGATAAACTTTTCTTCAACCACTGGACATACAGTAAATGACTCATGGTCATTTTTCATTC
CTTCCAATCCATCAGTAATAAACACGGGAAGTGGCAGCATCGTTAAATCAGGAAGCGTTATTTTTAATGTTAGCGGAAAC
AATGATACTAATACTTCCGCTGGAAAAGACTCATTAGGCGGTGGAAACGTAGGAGCTAACAATACCGCATATGGGTGGAA
GAGTTTGCACTCAAACACTACAGGATACGCAAATACCGCGTCTGGGATTCAGTCTATGGAAAACAATAAGACTGGTAAAA
ACAATTGTGCATACGGTGCTGATTCTCTAAGAGCTAACGTATCTGGTTCTGATAACGTAGCGGTTGGGGTCTTTGCGTCC
GGAGCAAACACAACCGGGTACGGTAACACTGGACTTGGTAACGATGCCAATAGGTACAATGAAACAGGATTCGGAAATAC
CGCAGCGGGTGTGCAGGCACTGTACCACAACAAGGCCGGTAATGAGAACACAGCTTTCGGTGAATACGCATTACGCGGAG
GAAATTCCAGCCTACCTACGGGAACATCCATTCAGTATTCCGTCGCTGTAGGAGCTAAAGCCGGTTTCAACGCGTTAGGT
AATAATAATACAGCTATAGGTTACGAGGCGCTATATTCTGCATCAGGATCTGATAATGTTGGTATTGGATTCGATGCAGG
GTTTAAAGTAACGGCTGGTGCTTTCAATGTATTTTTAGGTTCTAATTCTGGTAACGGGTCAGAGCAGGCTACAGATGTGA
CAAACTGCGTCCTTATCGGAGATAATACAAAATCAACCGGAAGTAACGCAGTAGCTATCGGTAGTGGGGTAATTGCCGCT
CAGAATACTGTAGCTATAGGTAATAGTGAGCATGAATTATTCTTGCATTATGGGAAATTTATACCACAAATTGATAATGC
TTTTGATATTGGTGGGCCGGATGCGAGATATAAAACAATATATGCAACTACAGGAATGATAAATACATCAAATGAAATGT
TGAAAACAAAACTAACAGACATTGATGTTGCTGAAAAAAAAGCTGCACTAGAAATAAAAGATAATATTAAAAAGTTTAAG
ATGCTGGATTCTGTTGAGGTAAAGGGATTTGATGGTGCTAGGTGGCATTTTGGTGTTGGAGCTCAAACTGTTGGTAATAT
AATGAGGAAGCACGGACTTGATCCTAGTAAGTATGGGTTTTGGTGTCACGATAAAACGCATCGCGAACACATGACTGGAA
ATATAGATATCTTTTGTGAAAGTTATGGTATAAGATATGACGAACTTGCAATGTTTATATTGGCATCAATATCTTTATGT
GAATAA
>Cluster_61390

ATGAGCAAGGCAGGCAAAATAACCGCTGCGATTTCAGGGGCTTTCTTGTTGTTGATTGTCGTGGCGATCATTTTGATTGC
AACTTTTGACTGGAATCGACTCAAACCGACTATCAACCAGAAAGTCTCTGCGGAGTTGAATCGTCCGTTCGCTATCCGTG
GCGATCTGGGCGTGGTGTGGGAGCGGCAAAAACAAGAAACTGGCTGGCGCAGCTGGGTGCCGTGGCCCCATGTACACGCG
GAAGACATCATTCTTGGCAATCCACCGGATATTCCCGAAGTCACGATGGTGCATTTGCCACGCGTAGAGGCAACGCTGGC
CCCGCTGGCGCTGCTGACCAAAACGGTCTGGCTGCCGTGGATCAAGCTCGAAAAGCCCGACGCGCGCCTGATTCGCCTCT
CTGAAAAGAACAATAACTGGACGTTTAATCTTGCCAACGATGATAACAAAGACGCGAATGCAAAGCCGTCGGCATGGTCG
TTTCGGCTGGATAATATTCTTTTCGATCAAGGGCGGATCGCCATTGATGACAAAGTAAGCAAAGCGGATCTGGAGATTTT
TGTTGATCCCTTAGGCAAGCCGCTGCCGTTCAGCGAAGTTACTGGATCGAAAGGTAAAGCGGATAAAGAAAAGGTGGGCG
ATTACGTTTTTGGCCTGAAGGCGCAGGGACGATATAACGGTGAACCGCTCACGGGTACGGAAAAATAGGCGGTATGCTG
GCGCTGCGTGGCGAAGGGACGCCGTTTCCGGTACAGGCTGATTTCCGCTCTGGTAACACCCGTGTTGCTTTTGATGGCGT
CGTGAATGACCCAATGAAGATGGGCGGTGTCGATTTACGGCTTAAATTTTCTGGCGATTCACTGGGTGATCTCTATGAAC
TGACGGGCGTTCTGCTGCCCGATACCCCGCCGTTTGAAACGGATGGTCGGCTGGTAGCGAAAATCGACACTGAAAAATCG
TCGGTCTTTGATTATCGCGGTTTTAATGGGCGAATTGGTGATAGCGATATCCACGGTTCTCTGGTCTACACCACCGGAAA
GCCACGACCAAAACTGGAAGGTGATGTCGAGTCGCGGCAATTGCGGCTGGCGGACCTGGGACCGTTGATTGGCGTTGATT
CCGGGAAAGGGGCAGAAAAGTCGAAACGGTCTGAACAGAAGAAGGGCGAAAAAAGCGTTCAGCCTGCGGGCAAAGTGCTG
CCTTATGACCGCTTCGAAACCGATAAATGGGACGTTATGGATGCCGATGTTCGCTTCAAAGGGCGGCGCATTGAGCATGG
CAGTAGCCTGCCGATTAGCGATCTTTCTACTCATATCATCCTCAAAAATGCTGACCTGCGCCTGCAACCGCTGAAATTTG
GCATGGCGGGCGGCAGCATTGCGGCGAATATTCATCTGGAAGGCGATAAAAAGCCGATGCAGGGGCGGGCAGATATTCAG
GCTCGTCGACTGAAACTGAAAGAACTGATGCCCGATGTGGAACTGATGCAGAAGACGCTGGGGGAAATGAACGGTGACGC
GGAACTACGCGGTAGCGGTAACTCGGTGGCGGCACTTTTAGGCAACAGTAACGGCAACCTGAAACTGTTGATGAATGACG
GGCTGGTGAGCCGCAACCTGATGGAGATTGTTGGGCTGAATGTCGGCAACTACATTGTCGGTGCGATATTTGGTGATGAT
GAGGTGCGGGTGAACTGCGCGGCGGCGAATCTGAATATTGCCAACGGCGTGGCGCGCCCGCAGATTTTTGCTTTCGATAC
TGAGAACGCGTTGATTAATGTTACCGGCACGGCAAGTTTTGCTTCGGAACAGCTGGATTTGACTATTGATCCGGAGAGTA
AAGGAATTCGGATTATCACACTGCGTTCGCCGCTGTATGTGCGGGGACGTTTAAAATCCGCAGGCTGGGGTGAAAGCC
GGACCGCTGATTGCCCGTGGTGCTGTTGCTGCGGCACTGGCAACGCTGGTAACACCGGCGGCGGCGTTACTGGCACTGAT
CTCACCTTCCGAAGGGGAGGCTAATCAGTGTCGGACGATTTTGTCGCAGATGAAGAAGTGA
>Cluster_9400
ATGGATGAAAATGCTTTAGGGTTTGCCTCATACTGGCGCAACTCGCTTGCGGATGCTGAGTCAGGAAAGGGCAGTTTTAA
ACGGAAAGACGCCCAAAATTTCACTCACTGGCATGGGATAGCGGCGGGACGTCTTGACGAAGCGATTGTCAGTAAATTTT
TTGAGGGAGAAAAAGACGATGTCGAAACGGTCGATGTCATCTTGCGCCCAAAAGTTTATTTCCGGTTACTGCAGCATGGT
AAGGACCGTTCCGCAGGCGCGCCTGATATTGTTACCCCGATAGTGACGCCAGCCTTGCTAAGCCGTGAGGGTTTTTTATA
TCCGACGCCAGCGACCTCCATTCCCAGAGACCTGCTTGAACCTTTGCCAAAAGGAGCATTTTCGATTGGTGAGATTGGGC
AGTATGACAAATACAAGACGACCCATACCACGTTCTCTATCAACTTTGATGACAGCGTTGATAAGACTGCCGAAACGGAT
GAAGAACGGGAAGCACGATATGCCGCCTTGCAGCAGGAGTGGCGTCAATATCTGTATGACTCAGAGAGGCTACTGAAGAG
CGTTGCCGGCGACTGGATTGAAAAACCTGAGCAATATGAACTCGCTGAGCACGGTTATATTGTTAAAACGGCTCAATCTG
GCGGTGCCAGTTCCCATATCCTTTCTCTTTATGATCACCTGCTTGTTTGCAATAAGGATGTGCCGCTCTTCAATCGCTTC
GCCTCGCGAGAGGTTCATGCTGCAGAGTCTTTGCTGGCCCCAGGAGCAAAATTCAGCGACAGGCTTGGACACTCCGGAGA
TAAGTTTCCGCTGGCAAAGGCTCAGCGCGATGCCTTAAGCCATTTTCTGGATGCAAGACATGGCGATATCCTTGCTGTTA
ATGGCCCTCCGGGAACCGGAAAAACCACGCTGGTGCTTTCTATCATCGCCACGCAGTGGGCCAGAGCGGCTCTCGAAAAA
TCTGAGCCTCCGGTTATTATCGCGACTTCAACGAATAACCAGGCTGTAACGAACATTATTGAGGCATTCGGGAAAGACTT
TTCGCAAGGTTCAGGTGCGATGGCCGGGCGATGGTTGCCAGAGCTGAAAAGCTTCGGTGCTTATTTTCCCTCAAGCAGTC
GTAAAGCTGAGGCAGCCAAAAAAATATCAAACTGAAGATTTCTTCAACCAGGTTGAGTCAAAAGAGTATGTAGAGGATGCA
CTGCTGTTTTATCTGGAAAAGGCTAAGGCAGCCTTTCCTGGAAAAGAGTGTTCATCCCCTGAAAAGGTCATTGAACTCTT
GCATGGTCAGTTGGCAGCAAAATCTGAGCAACTGATAAGACTGAACGCAACATGGCAAACGTTAAGCCAGATTCGGGCTG
CGCGTGAGCTTATTGCTAATGATATTGAGCAATATCTCGATAATTTAAATAAATTACTTTCCGGACAAGAACAAAAAGTC
ACTCTACTGAAGAGTGCTAAAACGGAATGGAAAAAATATCGCGCCGGTGAATCACTGATCTATTCATTATTTTCCTGGCT
CCCGGCGGTTCGCAATAAGCGACAGTACCAAATACAGCTGTTTCTCGAAGATAAATTAGGCGCGCTGATTGCAGGAAATC
AGTGGTCTGATCCTGAAACTATCGAACGTAATATTGATGGGCTGCTCAATTCCGCTGAGCGCGAGCAAACAACATACCGG
CAGCAGATTGACTCCGCCCATGAAATCGTTCTTAAAGAACAGCAGGCGGTTCAGGAGTGGCAGAGGCTGGCATTTGATTT
AGGGTATGAGGGCGACGAGGAACTGAGCTTCTCACAGGCCGATGAACTGGCTGATACGCAGATTCGCTTCCCTGCATTTT
TACTGACGACTCACTACTGGGAAGGTCGTTGGCTGATGGATATGGCCAGCATTGATGATCTGCAGGACGAGAAGAAGAAA
AAAGGTGCTAAAGGGGTAACCGCCCGTTGGCAACGTCGAATGAAACTCACGCCATGTGTGGTGATGACATGCTATATGCT
GCCCGGTAATATGCAGATAAGTGAGCACAAAGGACAACGTAAATTCGAGAAAAGTTATTTGTATGATTTTGGGTAG
>Cluster_27696
ATGGCAAAAAACACATCTTGCGGTGTCCAACTGCGTATTCGTGGCAAAGTGCAGGGCGTCGGTTTTCGTCCGTTTGTCTG
GCAACTGGCACAGCAATTAAATCTTCACGGCGATGTCTGTAATGACAGCGATGGCGTAGAAGTCCGGCTGCTGGAAGAGC
CGGAAACGTTTCTTGTTCAATTGCATCAGCATTGTCCGCCGCTGGCGCGTATTGATAGCGTCGAGCGTGAGCCGTTTATC
TGGTCACAACTGCCCACTGAGTTCACCATCCGCCAGAGCGCGGGTGGCGTCATGAATACGCAAATTGTTCCCGATGCCGC
CACTTGCCCTGCTTGCCTTGCCGAAATGAATACCCCAGGCGAACGACGCTGGCGTTATCCGTTTATCAACTGTACCCACT
GTGGCCCGCGTTTCACCATTATTCGCGCCATGCCTTACGACCGCCCGTTTACCGTGATGGCGGCGTTTCCGCTGTGTCCG
GCTTGTGACAAAGAGTACTGTGACCCGCTCGATCGTCGCTTCCACGCCCAGCCGGTGGCCTGCCCGGAGTGTGGCCCCCA
TCTTGAATGGGTAAGTCATGGTGAACATGCAGAACAAGAGGCGGCATTACAGGCAGCTATCGCACAGTTAAAAATGGGCA
ACATTGTCGCCATCAAAGGGATTGGCGGATTTCATCTTGCCTGCGATGCACGTAACAGTAACGCGGTGGCGACACTTCGG
GCGCGCAAACATCGCCCGGCGAAACCGCTGGCGGTCATGTTGCCAGTGGCTGACGGTTTACCAGACGCTGCGCGCCAGTT
GCTTACCACGCCCGCCGCGCCGATTGTGCTGGTGGATAAAAAATATGTACCTGAGCTTTGTGATGATATCGCCCCTGACC
TTAACGAAGTCGGGGTAATGTTGCCTGCGAACCCGCTCCAGCATTTGCTGTTACAGGAACTGCAATGCCCGCTGGTGATG
ACCTCCGGCAACCTGAGCGGTAAACCACCGGCTATCAGCAACGAACAGGCGCTGGCGGATTTGCAGGGCATTGCCGACGG
ATTCTTGATACATAACCGCGACATCGTGCAGCGGATGGATGATTCGGTGGTGCGCGAAAGCGGCGAAATGCTGCGCCGTT
CGCGGGGGTATGTGCCGGATGCGCTGGCTTTGCCTCCGGGCTTTAAAAATGTTCCGCCTGTGCTGTGTCTCGGTGCGGAT
CTGAAAAATACCTTCTGCCTGGTGCGCGGCGAACAAGCGGTGTTGAGTCAGCATCTGGGCGATTTAAGTGACGATGGCAT
CCAGATGCAGTGGCGCGAAGCGTTACGCCTGATGCAAAACATCTACGATTTCACTCCGCAATACGTTGTGCATGACGCGC
ATCCGGGCTATGTCTCCAGCCAGTGGGCACGCGAAATGAATCTGCCGACGCAAACGGTGCTGCATCATCATGCCCACGCA
GCGGCGTGTCTGGCAGAACACCTCTGGCCTCTGGATGGCGGAGATGTCATTGCGTTGACGCTCGACGGTATTGGTATGGG
GAAGAACGGCGCTTTGTGGGGCGGCGAGTGCCTGAGGGTGAACTATCGCGAATGCCAGCACCTGGGCGGCTTGCCCGCAG
TGGCGCTTCCGGGGGGCGATTTGGCTGCAAAGCAGCCGTGGCGAAACCTGCTGACGCAGTGCTTGCGTTTTGTGCCGGAG
TGGCAGAGTTATCCCGAAACGGTAAGTGTGCAACAGCAAAACTGGAGCGTGCTGGCGCGGGCCATTGAGCGTGGAATCAA
TGCACCACTGGCGTCATCGTGTGGGCGGTTGTTCGATGCCGTAGCGGCGGCGCTGGGCTGTGCGCCAGCCACGTTAAGTT
ATGAAGGTGAAGCGGCTTGTGCTCTGGAGGCGCTGGCGACTTCGTGCCACGGCGTGACGCATCCGGTGACGATGCCGCTG
GTGGACAATCAACTGGATCTCGCCACTTTCTGGCAGCAGTGGCTGAACTGGCAGGCTCCGGTTAATCAACGCGCGTGGGC

GTTTCATGATGCGCTGGCGCAGGGTTTTGCCGCGTTGATGCGTGAGCAGGCCACGATGCGTGGTATCACTACGCTGGTAT
TTAGCGGCGGAGTTATTCATAACCGTTTGCTGCGTGCACGTCTGGCGCATTATCTTGCTGATTTTACGTTGCTCTTCCCG
CAGAGTTTACCGGCGGGCGATGGTGGATTGTCTCTGGGGCAGGGGGTTATTGCTGCGGCGCGCTGGGGGGCGGGGAAAGT
CCAGAACGGATAA
>Cluster_26472
ATGAAGCGAGTTCTTATTCCTGGCGTCATTTTATGTGGCGCTGATGTGGCGCAGGCCGTCGATGACAAAAATATGTACAT
GCATGTTTTTGAAGAGATGACGGTCTATGCTCCTGTCCCTGTACCCGTAAACGGCAACACGCATTACACCAGTGAAAGCA
TCGAGCGTTTACCGACCGGGAATGGCAATATCAGCGATCTGCTGAGAACCAACCCTGCGGTACGCATGGATTCAACGCAA
AGTACCTCATTGAACCAGGGAGATATTCGCCCGGAGAAAATCTCTATTCACGGTGCGTCGCCCTACCAGAATGCCTATTT
GATTGACGGTATTAGTGCAACGAATAACCTGAACCCAGCGAATGAGTCCGATGCCAGTAGTGCAACCAATATTAGCGGGA
TGTCACAGGGGTATTATCTTGATGTCAGCTTACTGGACAATGTGACGCTTTATGACAGTTTTGTGCCGGTTGAATTCGGT
CGCTTCAATGGCGGGGTAATTGATGCAAAGATCAAACGCTTCAACGCTGATGATAGCAGCGTGAAACTGGGTTATCGCAC
TACGCGTTCGGACTGGTTAACATCGCATATCGATGAGAATAACAAGAGCGCATTTAATCAAGGTTCTTCAGGAAGTACTT
ATTACTCTCCAGATTTTAAAAAGAACTTTTATACCTTGTCGTTTAATCAGGAACTCGCTGATAACTTTGGCGTTACCGCT
GGTTTATCGCGCCGCCAGTCTGATATCACCCGCGCGGATTATGTTTCGAATGACGGCATTGTCGCCGGCAGGGCACAGTA
TAAAAACGTTATCGATACTGCATTGAGCAAATTTACCTGGTTTGCCAGCGACCGCTTTACCCACGATTTAACCTTAAAAT
ATACTGGCTCCAGCCGTGATTATAATACCAGCACCTTCCCGGAGTCTGATCGCGAAATGGGTAATAAATCCTATGGTCTG
GCATGGGATATGGATACCCAGCTCGCATGGGCCAAACTGCGTACCACCGTTGGTTGGGATCATATTAGTGATTATACCCG
TCACGATCATGACATCTGGTACACCGAACTTTCATGTACATATGGTGATATTTCAGGGCGTTGCACCCGTGGCGGATTAG
GACACATTTCCCAGGCTGTAGATAATTACACCTTCAAAACACGCCTGGACTGGCAAAAATTCACCGTGGGTAATGTTTCG
CATCAACCCTACTTCGGCGCGGAATACATCTATTCCGATGCATGGACTGAACGCCATAACCAGTCTGAATCCTATGTGAT
TAATGCTGCCGGAAAGAAAACTAACCATACCATTTACCATAAAGCTAAAGGCAGCCTGGGAATTGACAACTACACGCTGT
ATATGGCGGATCGCATTAGCTGGCGTAATGTGTCGTTAATGCCCGGCGTGCGGTATGACTATGATAACTATCTGTCAAAC
CACAATATCTCCCCGCGTTTTATGACGGAATGGGATATTTTTGCTGATCAAACCTCAATGATTACCGCAGGTTATAACCG
TTACTATGGCGGGAATATTCTTGATATGGGATTACGTGATATCCGCAATAGCTGGACGGAATCGGTATCAGGCAATAAAA
CCCTGACGCGTTATCAGGATTTGAAAACGCCTTATAACGATGAACTGGCAATGGGATTGCAGCAAAAAATCGGTAAGAAC
GTTATTGCGCGCGCAAACTATGTTTACCGTGAAGCGCATGATCAAATCAGCAAAAGCAGTCGTACCGACAGCGCGACTAA
AACCACCATTACTGAATATAACAACGACGGCAAAACCAAAACGCATTCGTTCAACCTCAGTTTTGAACTGGCCGAACCCC
TGCATATCAGCCAGGTAGATATTAACCCGCAAATTGTCTTTAGCTATATCAAGAGCAAGGGCAACTTGTCATTAAACAAT
GGTTATGAGGAGAGCAATACCGGTGATAACCAGGTGGTTTATAACGGTAATCTGGTCTCTTACGATAGCGTTCCAGTGGC
GGATTTTAATAATCCACTAAAGATCTCCTTAAACATGGATTTCACGCATCAACCGAGCGGGTTGGTGTGGGCGAATACGC
TGACCTGGCAAGAAGCGCGTAAAGCTCGCATTATCCTGGGTAAGACGAATGCGCAATACATCAGCGAATATTCAGATTAC
AAGCAGTATGTTGACGAGAAACTGGATAACAGCCTGACCTGGGACACCCGCTTGTCCTGGACGCCACAATTTCTGAAACA
ACAAAACCTGACGATCAGTGCCGATATTCTCAATGTACTGGATAGCAAAACCGCTGTTGATACAACGAATACCGGCGTGG
CGACCTACGCCAGTGGCCGTACTTTCTGGCTTGATGTCAGCATGAAATTTTAA
>Cluster_25464
ATGTCAAAATTAAAAATTGCGGTTAGTGATTCTTGCCCGGACTGTTTTACCACGCAGCGAGAATGTATCTATATTAATGA
AAACAGTAATATCGATGTGGCGGCAATAGTTTTATCGCTCAACGATGTTACACGCGGAAAACTCGATGAAATCGATGCCA
CGGGTTATGGCATCCCGGTATTTATTGCTACTGAAAATCAAGAACGTGTACCCGCAGAGTATTTGCCCCGTATTTCGGGT
GTCTTTGAGAATTGCGAATCGCGACGAGAATTTTATGGTCGTCAGTTAGAAACCGCAGCCAGCCATTATGAAACTCAACT
GCGCCCGCCCTTCTTCCGCGCACTGGTCGATTATGTCAATCAGGGTAACAGCGCGTTTGATTGCCCCGGTCATCAGGGCG
GCGAATTTTTCCGTCGCCATCCGGCGGGGAATCAGTTTGTGGAATACTTTGGTGAGGCGCTGTTCCGTGCTGACTTGTGC
AACGCGGATGTGGCAATGGGCGATCTGCTGATTCACGAAGGCGCGCCATGCATTGCACAGCAACATGCGGCAAAAGTGTT
TAATGCCGATAAAACATACTTCGTTCTAAATGGCACTTCATCTTCTAACAAAGTGGTTTTAAACGCCCTGTTAACACCGG
GGGATCTGGTGCTGTTTGACCGTAATAACCATAAATCTAATCACCACGGAGCGTTGCTACAGGCTGGCGCAACACCAGTT
TATCTGGAAACCGCACGCAACCCGTATGGCTTTATCGGTGGTATTGATGCCCACTGCTTCGAAGAAAATTACCTGCGCGA
ACTGATTACAGAAGTCGCACCGCAGCGGGCAAAAGAGGCGCGTCCTTTCCGCCTCGCCGTTATTCAGCTAGGCACTTACG
ACGGTACGATTTATAACGCCCGTCAGGTGGTGGATAAAATTGGTCATCTGTGTGACTACATCCTCTTTGACTCCGCATGG
GTCGGCTATGAGCAGTTTATCCCGATGATGGCGGACTGTTCACCACTGTTGCTGGATCTTAATGAGAACGATCCGGGTAT
TCTGGTTACGCAATCTGTGCATAAACAGCAGGCTGGTTTTTCCCAGACTTCACAAATCCATAAAAAAGACAGCCATATCA
AAGGGCAACAGCGTTATGTACCGCACAAACGCATGAACAACGCCTTTATGATGCATGCCTCCACCAGCCCGTTCTATCCG
CTGTTTGCCGCACTGGATATCAACGCCAAAATGCATGAAGGTGTCAGCGGTCGTAATATGTGGATGGATTGTGTAGTAAA
TGGCATTAATGCCCGCAAACTGATCCTCGATAACTGTCAGCATATTCGTCCATTCGTACCTGAACTGGTGGATGGTAAAC
CCTGGCAGTCGTATGAAACAGCGCAAATTGCGGTTGATCTGCGCTTCTTCCAGTTTGTACCAGGTGAGCACTGGCATTCT
TTTGAAGGCTATGCAGAGAATCAATACTTTGTCGATCCGTGCAAACTGTTGCTGACAACCCCAGGTATTGATGCACGTAA
CGGCGAATATGAAGCGTTCGGTGTACCCGCGACGATTCTTGCTAACTTCCTGCGCGAAAATGGCGTAGTGCCGGAAAAAT
GCGATCTTAACTCCATCCTCTTTCTGCTGACTCCGGCAGAAGATATGGCCAAACTTCAGCAACTTGCTGCTCTGCTGGTA
CGCTTCGAAAAACTGCTTGAGTCCGACGCGCCATTATCAGAAGTACTGCCTTCCATCTACAAACAGCATGATGAGCGCTA
CACCGGTTATACCCTGCGTCAGTTGTGTCAGGAAATGCATGATTTGTATGCCCGTCACAACGTGAAACAACTGCAAAAAG
AGATGTTTCGTAAGGAACACTTCCCACGTGTCAGCATGAATCCACAAGAAGCCAACTACGCCTACTTACGCGGTGAAGTG
GAGCTGGTTCGTCTGCCGGATGCAGAAAGCCGTATCGCTGCCGAAGGTGCGCTTCCTTATCCTCCGGGTGTGCTGTGTGT
TGTTCCTGGTGAAATCTGGGGTGGTGCTGTTCTGCGTTACTTCAGCGCTCTGGAAGAAGGCATCAACCTGCTGCCAGGTT
TTGCACCGGAGCTGCAGGGTGTCTATATCGAAGAACATGATGGTCGTAAGCAAGTTTGGTGCTATGTCATCAAGCCTCGT
GATGCGCAAAGCGCCCTGTTGAAAGGGGAAAAATTATGA
>Cluster_8128
ATGCTCACTCGCCTGCGCGAAATAGTCGAAAAGGTAGCCAGCGCACCACGCCTGAATGAGGCGTTAAATATTCTGGTTAC
CGACATCTGTCTTGCGATGGATACCGAGGTCTGTTCGGTCTACCTGGCCGATCATGATCGACGTTGTTACTACCTGATGG
CGACCCGGGGGCTGAAAAAACCACGCGGTCGCACTGTAACGCTCGCGTTTGATGAAGGGATCGTCGGCCTGGTTGGCAGG
CTGGCGGAACCGATAAACCTTGCAGATGCGCAAAAGCACCCCAGCTTCAAATACATCCCCTCCGTAAAAGAAGAACGTTT
CCGCGCGTTTTTAGGCGTACCAATTATTCAACGTCGCCAGTTGCTTGGTGTACTGGTGGTACAGCAACGAGAGTTGCGCC
AGTATGACGAAAGTGAAGAATCCTTCCTGGTGACGCTTGCCACCCAGATGGCAGCTATTCTTTCTCAGTCGCAGTTGACT
GCCTTGTTTGGGCAATATCGCCAGACGCGAATCCGCGCATTACCGGCAGCACCTGGTGTGGCGATTGCCGAAGGCTGGCA
GGATGCCACGTTACCTTTAATGGAACAGGTGTATCAGGCATCAACGCTGGATCCGGCTCTGGAACGCGAACGACTGACCG
GGGCGCTGGAAGAAGCGGCAAACGAGTTTCGCCGCTACAGCAAACGCTTTGCCGCCGGTGCACAAAAAGAAACGGCGGCT
ATTTTCGATCTTTACTCGCACCTGCTTTCGGACACCCGGCTGCGTCGCGAATTGTTTGCCGAGGTTGATAAAGGCTCGGT
GGCAGAGTGGGCGGTAAAAACGGTCATTGAAAAATTTGCCGAACAGTTTGCCGCGCTAAGTGATAACTATCTCAAAGAGC
GGGCTGGCGATTTACGTGCGCTGGGTCAGCGATTGCTGTTTCATCTTGATGACGCTAATCAAGGGCCGAACGCCTGGCCG
GAACGTTTCATTCTGGTGGCAGATGAACTGTCAGCGACAACGCTTGCTGAGCTGCCCCAGGATCGCTTAGTCGGTGTTGT
CGTGCGAGATGGCGCAGCCAACTCCCATGCTGCGATCATGGTACGTGCGCTGGGGATCCCTACCGTGATGGGCGCGGATA

TTCAGCCTTCGGTGCTGCATCGTAGGACGCTGATCGTTGATGGCTATCGCGGTGAATTGCTGGTCGATCCGGAGCCGGTA
CTGCTGCAAGAATATCAGCGGCTAATTAGTGAAGAGATTGAGCTTAGCCGTCTGGCGGAAGATGACGTCAATTTACCCGC
GCAGTTAAAAAGCGGTGAGCGTATAAAAGTCATGCTCAATGCTGGTTTAAGCCCGGAACATGAAGAAAAACTGGGCAGCC
GTATTGATGGCATCGGTCTTTATCGCACTGAAATCCCATTCATGCTGCAAAGTGGTTTCCCGTCGGAAGAAGAACAGGTG
GCGCAGTATCAGGGGATGCTGCAAATGTTCAATGATAAACCCGTCACCTTGCGTACGCTGGATGTCGGAGCAGATAAGCA
GCTGCCTTACATGCCGATCAGCGAAGAGAATCCATGCCTGGGTTGGCGTGGGATTCGCATTACGCTCGATCAGCCGGAGA
TCTTCTTGATCCAGGTGCGGGCGATGCTGCGTGCTAATGCCGCTACGGGCAACCTGAATATTCTGTTGCCGATGGTCACA
AGCCTCGATGAAGTTGATGAAGCACGCCGCCTGATTGAACGTGCCGGACGTGAAGTCGAGGAGATGATCGGTTACGAAAT
TCCCAAACCACGTATCGGCATCATGCTGGAAGTGCCGTCAATGGTATTTATGCTGCCGCATCTGGCAAAGCGGGTCGATT
TCATCTCTGTTGGCACCAACGATCTGACTCAATACATTCTGGCCGTTGATCGCAACAATACCCGGGTGGCGAACATTTAT
GACAGTCTTCATCCTGCAATGTTACGAGCTCTGGCGATGATCGCCCGGGAAGCGGAAATACATGGAATCGATCTCCGTTT
GTGCGGTGAAATGGCGGGCGATCCCATGTGCGTGGCAATCCTCATTGGGCTTGGGTATCGCCATCTGTCTATGAACGGAC
GTTCTGTAGCGCGGGCAAAATACCTGCTGCGGCGCATTGATTATGCCGAAGCAGAAAATCTTGCGCAGCGTAGTCTGGAA
GCGCAACTGGCGACCGAAGTTCGCCATCAGGTTGCAGCCTTTATGGAGCGTCGCGGCATGGGCGGGCTGATTCGCGGAGG
GTTATAG
>Cluster_43405
ATGGCCCTAACGGATACTAAAGTTCGCTCTGCGAAACCTGAGGAGAAAGAGTATTCACTTGTTGACGGCGACGGCATGTG
TAGTGGTCAACAAAAACTGGCCACCGCTTTAGAGTTTTTCCAGTATCGGTTTTCCGATTCATTTGGTGGCAACCCACCGT
TATATTCGTGCGGCCTGAGCGCGTTGTAA
>Cluster_25465
ATGCAGAAACCCTTATCAGTCCGGTGGCTGATAATACAAGGGTTAACAGAGAATGAGCACTGCGGAATACAGGCATATGC
CTGTAATACAGAGAGTCAGGAAGACCTGTACGCTGAAGTGCCCTTAGTCGGTAGACTAACGGGCATTGAAGAAGTGAAAG
TCGAAGCTATTGCGATGAGCAAACTTCATAATATGAGTTGTCCGCCTTATATGGGGCATAGTGCGGCTGTTATTTTCCAT
TTAATTTACCTTTCCTGA
>Cluster_49935
ATGGTCGCTATCCATGAAGCACTGGCGAAATGCAGTGCGGAAAACCCCGTGTTTTACGATGATGAAGTGGATATTCACCT
CAATCCCAAAATTGGCGCAGACTGGCAGTTACGTGGGCAGCAAAAGCGCGTTGTGACGCCGGGCCAGAATGAAAAGTACT
ATCTTGCCGGTGCGCTGCACAGCGGTACGGGAAAAGTGAGCTACGTTGGTGGAAACAGTAAAGGTTCGTCCTTATTTATC
ATTCTGTTAAAAATCTGA
>Cluster_23967
ATGACGTCAAGTCATCATGGCCCTTACGACCAGGGCTACACACGTGCTACAATGGCGCATACAAAGAGAAGCGACCTCGC
GAGAGCAAGCGGACCTCATAAAGTGCGTCGTAGTCCGGATTGGAGTCTGCAACTCGACTCCATGAAGTCGGAATCGCTAG
TAATCGTGGATCAGAATGCCACGGTGAATACGTTCCCGGGCCTTGTACACACCGCCCGTCACACCATGGGAGTGGGTTGC
AAAAGAAGTAGGTAG
>Cluster_41173
ATGGAATGCCGTCCGGGTTGCGGGGCGTGTTGCACTGCCCCTTCAATTTCCAGCCCTATTCCCGGTATGCCAGATGGCAA
GCCCGCCAATACGCCCTGCATTCAGCTTGATGAACAGCAACGCTGTAAAATTTTCACCTCGCCACTGCGACCGAAAGTCT
GTGCAGGCTTACAAGCCAGCGCTGAAATGTGCGGTAACTCGCGTCAGCAGGCAATGACCTGGCTTATCGATCTGGAAATG
CTGACCGCGCCTTAA
>Cluster_11780
ATGCGTAAAGCCCGATTCACCGACCACCAGATCATTGCCGTTCTGAAGTCCGTCGAAGCCGGACGTACCGTCAAGGATGT
CTGCCGCGAAGCCGGGATCTCTGAGGCCTCGTATTACAACTGGAAAGCGAAGTTCGGCGGTATGGAAGCCTCTGATATCA
AAAAGATGAAAGACCTGGAAGATGAGAACCGGCGTTTAAAGCAGATGTTTGCCGACCTGAGCCTTGAATGCCGGGCACTG
AAAGACGTCATAGAAAAAAAGCTTTAA
>Cluster_44978
ATGCTTGAAGATACTACAATTCATAATGCAATAACTGATAAAGCGTTAGCAAGTTACTTTCGCAGTTCGGGTAATTTGTT
AGAAGAAGAGTCAGCAGTGTTAGGGCAGGCTGTCACCAATTTAATGCTTTCAGGCGATAATGTTAATAATAAAAATATTA
TCTTAAGTCTGATACACTCCCTGGAAACAACAAGTGATATTCTCAAAGCTGATGTGATTAGAAAAACACTGGAAATCGTG
TTGCGATACACAGCTGATGATATGTAA
>Cluster_67076
ATGATTACTCATAACCTGAAAAATGTCGGATATCTGATTAATATATTTATCAGCCTACTCTTCACCGAAATGGTGGTACC
CGTAATGCTAGATATCCTCCAGATCCTGACTGGCTTTAGGTGTGAGTTTAACGGTTTTCACCGGAAGCCCTCGCGCCAGC
TTTCACTTTTTTCAGAAACGCATTTTTTTCCCACGACACAGGTTCACCGCTGCTCAAGCCTTCTGCCAGTAAATCCCGCA
GGACACCTGGATACGTGA
>Cluster_39435
ATGATGGATGTAAAAGAGAAGGTTTTGCAGGTGATGCGTTCCCGTGCTGCCCTGCAAGAGAAAGCTCTCGGCGGGGAATA
TCCATTCACGATAGCAACCTGGAATCTGCGGTTGGCAATGGAGAAGGAATTTCCTGATGAAGAATGGCGTTCGGCAGATT
TGCGCAAAATTCTTATGGAGCTGGCTAAAGACGGAGCAGTATCCAAAGATACCTATGCCAGCCGGATTGGTCAGGCGGTA
TGGAGACTGGAGGTGCGGTAA
>Cluster_40073
ATGGGAATTATTGCCTGGATTATTTTTGGCCTGATAGCCGGTATTATCGCCAAGCTAATCATGCCGGGGCGTGATGGTGG
TGGATTTTTCCTGACCTGTATTCTTGGGATAGTCGGTGCGGTGGTCGGCGGCTGGCTGGCGACCATGTTTGGCATTGGCG
GCTCCATCAGCGGTTTTAATTTGCACAGCTTCCTGGTGGCGGTGGTGGGCGCTATCCTGGTGCTGGGCGTATTCCGCCTC
CTGCGAAGAGAATAA
>Cluster_14003
ATGGTTTTTAACAGCATCTTTGTTATACAAGGGGGCATTGAGGATATCAGGAAAAATCTCAAAATCGGTGCGGACAGGAA
ACTACGTGGGCAGCAAAAACGTATGGTAACGCCGGGAAATGCAACGCCCACCAGTCGGGCTAATCCACCACTCCAATCGT
GGTTCGCAGTACTGCACACACGATTACCGGGCTATACAGGAGCGGTTTGGTCTGAAAACATCCATGCCGCGTAA
>Cluster_9146
ATGAACTCTCTGGCAGGTATAGATATGGGCAGAATTTTACTCGATTTATCGAATGAGGTGATTAAGCAACTTGATGATCT
CGAGGTGCAGCGTAATCTTCCTCGTGCGGACCTATTAAGGGAAGCGGTAGATCAATACCTGATAAATCAATCGCAAACAG
CAAGAACCAGTGTTCCTGGCATCTGGCAAGGGTGTGAGGAAGATGGTGTCGAATATCAGCGTAAGCTGCGCGAGGAATGG
TAA
>Cluster_40738
ATGGCAGACCGCAAACAGCACCGCGCTATCGCGGAGCGTCGTCACATCCAGACTGAAATCAACCGCAGACTTTCCCGCGC
ATCACGCGTCGCGCAAATCATGCACATCAATATGCTGCATGAGCGCAGCCACGCACTATCAAACATTTATTCCGCCTCTG
TTTTCAGCTATCTGGCGGATGATCTGCACGAGCTTCAACAGCTCATCCAGCAGCAAAACAAACTCCATTAA
>Cluster_24665
ATGAGTTCAGAAGTAATTAAGCAAATTCAGAAAATTCAAGATCGCGGCATTATCATCTATTCAAAGTTTAGAGCGGCAGA

ATTTGATCAGGATGACGTTTACAGAGAGTCATATTTTCTGGTCGTAGAGTTTAACGAGTTAATAGCAGAAAACATCATTC
ATGACGAAAAAATGGTAGATCAAACGGCTTGTATTCTTCATGAGCTTAGAAGAATCGCTATAGAAGGGAAATAA
>Cluster_29600
ATGAATAAGCTGGTTTCTGATGGCAGCGTCAAAAAAATAAATTATCCGGTGCTGTATGAGTCCGGAATAACACCTCCGTT
ATGTGAAGTCAGTGCACCAGAACCTGATGCGGGGGGAAAACGCATTGTGGCATATGTTTATAAATCTTCACGGAATACGG
TTTTTGAAAATCCTGACATTGTAAAAACCTGTACGGTCAGGGATTTGAAGAATGATTTTGTGAACTGCGATGAGAAAGGT
GAGGAACAATGA
>Cluster_40815
ATGAAAGCATACCGCTATTCCGGCCCGGCCAGCGGCGTCACGCTGTCGGACGGAACCGAAATCCTGCTCTGGCCGGGGAA
GGCGGTTTCCCTGCCGGAGGAGCATGACTACGTGAAGGTACTGGTGGCGCTGAAGCATCTGACGCCGGTACCAGAAGATA
CTAAACCCGCCGTCACACCGGCTGTGCAGTCACCAAAGCGCAGAAGCAGCAGTGACAGCGAAGTGAAAACGGAGGACGCC
CATGGCAGCTAA
>Cluster_63854
ATGTCCCATATATGCCCCAAAGGTGCTCCATGCAAAATATCTGGCGTAGTCTTCTTTCTTGCCAGTAGTTTTAGAGCTTT
CACAATCCAGTCTCCTCCGCTAAAATTGATCACGGAAGACCTTCATATTAGAACTTGCTATACTCAAAAGCTAAAAGCCC
GACTGTTGACGCAGCCGGGCTTTCATTTTTCAGATACAAAAAAACCGCTCATGGCGGCTTATTCATTACTCAACGTCAAG
CGCTCGAATTGA
>Cluster_10962
ATGGAATCGTTTGTACAGGATTCACCATTTTATAGTGGAAGAGATCTTTATTGGTTACGTCCTAAAGTTGAACTGACTCT
GGAAGAGAAATTGTACTACTGCTCTTGTATAAGAAGAAACAGACACAAATACAGCTACGGGCGTCAGGCAAACCGAACGC
TGAAGAATTTACTGGTACCGTCATTAGATTCTGTTCCGGCATGGGTATATGGTGTGACAGGGAAAATAATATCTGAATTG
TCTGAACGTTGA
>Cluster_11071
ATGAGCAAACACACTTTAACCAGAGTTACAAAGACAGCCTTTGATGAACTGAACCGAATCAAAAACATTACAGGTCTGCC
ACACTCAGCAGTGATGAAGCTTGCAATTAACCGTGATATTAACGAACAAGATTTGCTTAATAGTCGCTTAGAGGCTGCAA
GACACCATATCAGGATTAAGCAAGATGACCTTGATAGTCTGAAATCCTTAAATGCTTTTGGACTGGATTTAGCAAGGTTA
CTAAGTATCAAAATTCACAAGCTGTCTCTGGAGGTCTGA
>Cluster_20521
ATGAACAACGCATTAACCCTGTTTAAAGGACTATTGATCGCCGCGATTGTGGCGGTGGATGGTTGGGCGCTGTTTCATGT
GGTCAGCTATACCCTGCATAACTGCCATGATGGGCTGGCGGCAACTTTGATGATTGGTGTGGGCAGCGTTGGTGTACTGT
TGCTGACCGCACTGGCAGGCTGGTTATTTAGCCCGGCCATTTCCCTGCTGGCCTTCCTGTTCGCCCTTGTCGGCAGCATC
ACCGAATGGGTACTCAACCGCCGCCGTTCTCACGCTTAA
>Cluster_38490
ATGAACAGATTACGCTTTGAAGTGAAGAATAATCAGGGCTGCTTCATTTTCCCGGAAACCTGGTATGAATCACTACTGGA
GAAGTTTGACGAACTGCTTGACGCCTTTGACGAGGATGAGCTCAGCGACAGGTCTTATATCAATGCCCTGAAGCATATCA
CACAACAGGAACCGGATTTTATTGATGCGTATGCATACATGGCATATGCCTTTCTGGAGCAGGAGACACCGCGAAAAGCG
CTGAATATTGCACTCAAAGGACTGGCGGCAGGTATATGA
>Cluster_23839
ATGGTTAATGATATTTCTGCTAATAAGATACTGGTGTGGGCGGCTGTAGCTGCGGCAAACCATAAGCTTCCCAAATATGC
GGAAGCTATCCTGAATGTATTCCCACAAATTATACCTGATAAAAAAGATATCGCACATTTAGAATTTATTATCTTATTTG
GATTAAATAGAAAAAATGATGCGATAAAGGCTTTGGAGGGCTGTATGGATGATGAGTCGAGTCAGTTATTGTACAGTTTG
GTCCATGAGAATGTTAGTGGTTGGGTACGAGGATTTTAA
>Cluster_41354
ATGAGTAAATGTAGTGCTGATGAAACCCCCGGTTTGCTGCTGTATGGATGTTGGCACCATTATGGACAACTCCGACTGCAC
CGCGTCTTACAGCCGTGTGTTTGCAAACCGCGCAGAAGCAGAACAAACGCTGGCGGCGCTGACTGAAAAAGCCCGTAGCG
TGGAATCCGAACCGTGCAAAATCACCCCGACTTTCACTGAAGAGTCTGACGGTGTGCGCCTGGATATCGATTTCACTTTT
GCCTGCGAAGCTGAAATGCTGATCTTCCAGCTGGGTCTGCGTTAA
>Cluster_10531
ATGACCAGTCAACTCATCCCCGTATTCAACGGCACTATCGCCAACGAAACCACTCTTCTCGTTAATGCTCGCGATTTACA
TACTTTTCTTGGGGTAGGTAAACGCTTTGCATCGTGGATTACAGAACGCATTGCTGAATATGGTTTCGTTGAAAATCAGG
ACTATATTTTGGTTTCCCCAAATCGGGAAATCAAAGGTCGAGGAGGCGATCGCCGTAGCAAATACTATCACCTCACCCTC
GACACAGCCAAAGAGCTTGCGATGGTTGACGACTGCGTCCAATACTGA
>Cluster_28861
ATGGCAAAAACAGCAGCAGCACTGCATATCCTTGTAAAAGAAGAGAAACTGGCTCTGGATCTTCTCGAGCAGATCAAAAA
CGGTGCCGATTTCGGCAAGCTGGCGAAGAAACACTCCATTTGCCCGTCAGGCAAACGCGGCGGTGATTTGGGTGAATTCC
GCCAGGGTCAAATGGTTCCGGCGTTCGATAAAGTGGTTTTCTCTTGTCCGGTACTGGAGCCAACCGGCCCGCTGCATACC
CAGTTCGGTTACCACATCATTAAGGTGCTGTACCGTAACTAA
>Cluster_1579
ATGTCTGATCTGTTTACGCGAATGTGTTGCCGGATGGACGGGGCGACCGTTCGGGTGATGGGCAAACAGGCGGAGATTAA
CGGCGTCGTGTATGACGTGATGCCGGAGGAAGAGTCCGCGGAGATGGGGGCGCTTTCGGGCAGCCAGTTGTCACTGGTGG
TGTTTTCAGCCCGGTACCGTCCGGCCCGTCATGATGTTGTTGTGTTTGCGGGGCGCACACTGACGGTGACCCGTTATGAC
ACGTACAACGGTAAACCCCGGATTTTTGTCGAACAGGAATGA
>Cluster_51560
ATGAAAGTAAACTTAATGCTTTTTAGCTTATTTTTATTTGTCTCTATTATGGCATGCAATGTTTTTGCATTTTCTATTTC
TGGTGGTGGTAGTGAGGCGAGCTATAAAGAGACAGAAAAAAACGTCAGTGATGACGACAACGCATTCTACAAAACTTCAGC
CATCACAGGCGATTTTGTTTAAGATGAGAGAAGATGCACCACCATTAAACCTCACAGAAGACATCACGCCTACTTATCCG
ACAAAGGCGAATTATCTCATTCATCCTTTGCGATAG
>Cluster_10087
GTGTTGCAGCGTGAACAACTGGGGTCATGGAAAGAAAGGAGCGACTTAGAAAGAGAGCTTAATACCTGGATCCGTCAGTA
TGTTGCCGACCAGGAAAACCCGCCTGCAGACGTGCGCAGCCGCAAACCTCTGCGCGCTGCCAAAGTTGAGGTTATGGATG
TAGAAGGCGAACCCGGCTGGTACCAGGTTGCGCTAAGCGTGAGACCTCATTTTAAATTTATGGGGGCAAATTTTGAGCTT
TCCCTGGTTGGCCGGTTAGACAGGGAGTAA
>Cluster_14848
ATGGCACAGGTTAATATGAGTTTAAGAATCGACGCTGAACTGAAGGATGCTTTTATGGCCGCTGCAAAAAGCATGGACCG
TAATGGCTCTCAGTTAATTCGGGATTTTATGCGCCAGACCGTTGAACGGCAGCATAATACTTGGTTCCGTGACCAGGTTG
CGGCAGGACGTCAGCAACTCGAACGCGGCGATGTGCTTCCCCATGACATGGTCGAATCCTCTGCTGCTGCATGGCGGGAT
GAAATGAGCAGGAAGGTCGCCGGTAAATGA
>Cluster_7872
ATGGAAATAAGAGATAAAAAGGCGAAGTACTTGGAAGAAAAAGGGTTTTATAGACGAGCTGCAGATCGCTGGGCAGAGAT

AATGGTGCTTTTATCTAGTGACGCTGAGAGAAAATTGGCAGCTCAAAAGCGTGCATTCTGCATTAATAAATCTCTACGTA
ATAATCTGCAAGCAGATAACTATAGCGACATAAAACAAGGAGTTTATAAAGCATATAAGAAAATGGGGCTTGTAAATGAA
AAAATTTTTAGAAACTACAAGGAAAATTAA
>Cluster_64825
ATGGCAAAAGTAATCTTTGAGTTTAACAATACAGAAAATATTGAGTTTCAGGAAAATGGAGGTTTTGCCGTAAATATGAA
TGTGCGACTGGAAGAATCCTCACCTGAGGACCAGACCGGGCCACATGACGTAATGGCAGGCATCATCAAGAGTATGGCTC
CTGAAATCATTAAAAAAGCGACCGAGGAGTTGTTGAAATCAGCCAGGGAGCTTGGGCTGGAAGCTGAGGGGGAACTATTC
AGCTATAACCCAAATGCAGCCAGACATTAA
>Cluster_20412
ATGAAAATAATCTCTAAAATGTTAGTCGGTGCGTTAGCGTTTGCCGTTACCAATGTCTATGCCGCAGAATTAATGACCAA
AGCGGAATTTGAAAAGGTTGCATCGCAATATGAAAAAATAGGTGATATTTCAACCAGTAATGAAATGTCGACCGCAGATG
CGAAAGAAGATTTGATCAAAAAAGCCGATGAAAAAGGGGCTGATGTGTTGGTACTGACCTCTGGTCAAACTGACAATAAG
ATCCACGGCACGGCAAATATTTATAAGAAGAAGTAA
>Cluster_39681
ATGAACAAAGAAAATGTGATTACCCTGGACAATCCGGTCAAACGTGGTGAGCAGGTTATCGAACAAGTCACGCTGATGAA
ACCCAGTGCCGGGACGCTGCGCGGTGTCAGTCTGGCTGCGGTCGCGAACTCCGAAGTCGATGCACTGATTAAGGTGCTGC
CGCGCATGACGGCACCGATGCTGACCGAGCAGGAAGTCGCCGCACTGGAACTGCCTGACCTTGTGGCGCTGGCCGGTAAG
GTGGTCGGTTTTTTGTCGCCGAACTCGGTGCAGTGA
>Cluster_67031
ATGACACGCGAACGTTTTACTGAAAATCTGCTGATGTATCCGGGAATGGCGCTGATGGTTGCCTCCGTTATCTGGTTTTA
TCTGGCCGGTCTTCTGTCCCTGCCTGCAGAGGCTGTCAGTGATGAACTGGCATACGCCTTGTACCAGATGACGCTGGCTC
GTGATGCACTGGCGATATTTGTTATCGGGGCGACGATGGGATTATCCGGTCTGGGGCTGGCTGCTTTTCATGCCTGGAAG
AAATGGCACGCAGCACCGGCAGGAGAGCAGTGA
>Cluster_7836
ATGGCCAGAGTTAATATTCATTGTCCCCGTTGTCAGTCAGCTCAGGTTTACCGCCATGGTCAGAACCCTAAAGGCCGTGA
CAGACTTCGCTGCCGTGACTGCCACCGTGTGTTTCAGCTCACTTATACTTATCAAGCACGTAAGCCGGGTATGAAAGAGC
TGATCACTGAAATGGCCTTTAATGGTGCCGGGGTTCGCGATACCGCCAGGACACTGAAAATTGGTAGTAACACCGTCATC
CGGACTTTAAAAAACTCGCGCCAAAGCGAATAA
>Cluster_23987
ATGAGAAATTATACCAGACACCCGCAAGGGCGGGACTCGTACAACCTGAATAAATACATCTGGCGTTTTATCGCCCTGAG
CACGGCACAACCGCGCGTGATTACCATTGAGGCCACCAGCGAACAGGAAGCGCGTCAGCAATCTCCTGATGGCTGCGTGA
TGGTATTCGCTGCCCGTATTCGCCAGGGGGAAACCTATGCACAATAA
>Cluster_34623
ATGAAAAAAATCAACACTAATTTTAGGGCTTACGTTAATTGTCTCATCTCAAATACCATCGGCAATGGCAAAAAATGAATC
AAAACTTTGGGTTGTTGTTGATCGAACGGAAAGACATACCTGCCCTTCAAGTAAATGTGGAGTGGCTGGGAAACTATTTT
TCAGGGAAGGCGTAGATTTTCTAGAAAAAAAGGTGAATGGGTTCGTATAA
>Cluster_36429
ATGAATTTTATTCGACAGGGATTAGGCATCGCCTTACAACCAGAGTTAACGCTGAAAAGCATTGCAGGTGAATTGTGTTC
CGTTCCTCTCGAACCAACTTTCTATCGACAGATTTCGTTGCTGGCTAAAGAAAAGCCGGTAGAAGGCAGTCCACTGTTTT
TACTACAAATGTGCATGGAACAATTAGTGGCGATTGGAAAAATTTGA
>Cluster_44968
ATGAACGTGAATACTGAGATTTTGAAACTCTGGCAATCTAGGGGCGAAAATGAGCGCACCGGATTCGATGCGGAGAGGGT
TGCAGATATTGCCTGGGAGCAGGGATTACAACTTCACCGCAACCGGCAGGAACATTACCAGCGAGTGATGTACCTCATAG
GCCATCGCAATATTGATCCTACCCACGTAATATGGACACAGGCCTAA
>Cluster_20762
ATGATGGCGGAAATAATCACCGGCATTTATGCCTTGTTTGCCTGTATCACTTTCGTTTTTCTCTTATGGGACGAATGCAA
GGATGAATTTGATCACGATGCTGAGGACTTAGGGTTAATCACTCTGAGTGCGGTTTTCTGGCTACCGCTGGTAGCAGCCG
TACTTGTGGCCTTCCTGATTGATTTCTGGCGGCAGTGGGTTAACCGTGGATGA
>Cluster_17788
ATGGAAGTACAGTGTCCGACCTGTCATCAGTTCATTGAATTATACGGTGATCGTACATATTGTGAGCATTGCCAACAGAA
TGTGGTATTTCAGGCGTTGTGCCCGGACTGCCACCAACCGTTACAGATATTAAAAGCCTGCGGTGCGGTGGATTACTTCT
GCCAGCATGGCCACGGTTTGATCTCTAAAAAGCGTGTGGAATTTGTGCCGGTTTAG
>Cluster_30150
ATGTCTAAGATTAAAGGTAACGTTAAGTGGTTTAATGAGTCCAAAGGATTCGGTTTCATTACTCCGGAAGACGGCAGCAA
AGACGTGTTCGTACACTTCTCTGCAATCCAGACTAATGGTTTTAAAACTCTTGCTGAAGGTCAGCGCGTAGAGTTCGAAA
TCACTAACGGTGCCAAAGGCCCTTCTGCTGCAAACGTAATCGCTCTGTAA
>Cluster_26498
ATGATAACGTTTTCTATGGATGAAAGTGGCTATACGGGTTATGACTTACTCCACAAAGAGCAAAAATTCCAAGGCGCGTC
ATCAATATTAATAAACCATGATGACGCCAGTCGTCTGATAAAAGAATATTTCCCCAAACTTCAAGCCGATGAATTGAAGT
ACAGCTCGCTGAAAAGAAGAGATTCCAACCGCAAGCGTTTTGCAACGGTCTAA
>Cluster_21529
ATGGAGCGCTTCTTCAGGAGTCTGAAAAACGAGTGGATACCGGTGACGGGTTACATGAACTTCAGCGATGCTGCCCATGA
AATAACGGACTATATCGTTGGGTATTACAACGCGCTCAGGCCGCACGAATATAACGGTGGGTTGCCACCAAATGAATCGG
AAAACCGATACTGGAAAAACTCTAAAGCGGTGGCCAGTTTTTGTTGA
>Cluster_47
ATGGCAACCAACAACCGTAAAGCAAAGATTCTGTTAGTTCGCAGAAACGATCCAGGCGTATGGCAGTGGGTGAGACTCAG
CAACCGACGAATGGGGTTGATGAAATATTACGGGATGATGGATTGCGGTTTTTGCAAAAAGCCCAGCGCGGCGCAAAACC
GCTGGAAAAACCACTTTCGCATTAAAGGAGAGTGA
>Cluster_25044
ATGCTGGCTACTACCCCTACCCAAAAACCGCAATTTATCTGGATTATCGCCGCAGTTCGCCGCGATTGCCCGACAATTAC
CGCCAAAATTCATCATATTGCTGCCGAGTCTGAACGCGATGCTCGCCGTTCTCTGGTGCGCGATCACATCTGCTTTTTTG
CTGGTCGTATCCGCATGGAGGTGGCACATGATTAA
>Cluster_66926
ATGTCTGCCTGGAGTCCGGAGCACATAACGTTACGACGATCAGAAAACGGCAATGTGATTGCCCACGACAGTCGGGACGA
TCAGGAATTCATTTTCGCAGAATGCTGTTCCGATGAAATCGCAAACATCATTCTGGAAGCCGTGAAACGTTACACCAGTA
CGGAGCCTGCTCATGCAACACAGCATTAA
>Cluster_10621
ATGAAAACTCCATTAAATATTCTTGAGGAAGTGGCAGCACAAATAAAAAAGAATACATCAATGCTTGAATTTATATTTAA
GAATTCGCCCGACTCAGGAGAGACAGACGATTATTTATGTTGCCTCATTCGCTCCATGAATAAAACCTGTGAAATGGCTT

ACGCGTATATCGACACACTGCGCAACGAATAA
>Cluster_4768
GTGTGGATCACTATTGCACCGTTCGTGACACCAGGGGGCTGCATTGTTCATTTCCCTGCTAATGGACGTGACATCCTGGA
TATTAACGATCCTGATTTCCCTGAGCAGTATCAGCGCTTATTGGCACGTCTGGATTATCTGACGAAGCTCGACACTAAAG
CATCAGGCCAGCGTAACTCTGGCTACAGCGGTTTTTGA
>Cluster_32098
ATGGGACTTAAGTGGACCGATAGCCGCGAAATTGGCGAAGCACTGTACGATGCGTATCCTGATATTGATCCGAAAACGGT
TCGATTCACCGATATGCATCAGTGGATTTGCGCTCTGGAAGATTTCGACGACGACCCGCAGGCATCGAACGAGAAAATCC
TCGAAGCGATTTTGTTGGTCTGGCTGGACGAAGCCGAGTGA
>Cluster_59412
ATGCATGCGACCACAGTGAAAAACAAAATCACGCAAAGAGACAACTATAAAGAAATCATGTCTGCAATTGTGGTTGTCTT
ATTACTGACACTTACGTTGATAGCCATTTTTTCGGCAATTGATCAGCTGAGTATTTCAGAAATGGGTCGCATTGCAAGAG
ATCTTACACATTTCATTATCAATAGTTTGCAAGGCTGA
>Cluster_1548
ATGCTAAAACAGCAGGATATGACCGAAACCGCCAGAGTGGTGTTTAATGAATTAAGCGTCACCGAACCGGCGACCGCCGG
GGAAATTGCGCAGAACACTTATCTTTCACGCGAACGCTGCCAGTTAATACTGACCCAGCTTGTTATGGCGGGTCTGGCAG
ATTATCAGTTCGGTTGTTACAGACGCCTTCCTCAGTGA
>Cluster_20757
ATGAAAGCGTTTGTTACTTACCTGAAAAAAGAATCACCAGCCATGCAACGACCAAGCGGTTCAACGGGCTGGATAGAACT
GCCGAACGGTCAGCGCTGGAATCCTGGCCACACGTATAAATTCAATGCGCATGAGTCTGTTCGAATGAAAGGCGAGGTGG
TTTTACGTTTCTTGCCTACAAAAGTCCGTCGCCTGGTTGGGTGTATAGGGGGGCGCTATGGCGATTAG
>Cluster_51318
ATGAATGAAATCCCTTTCGATGTTCTTATTCATTCTGAAAACGCATTAATCCGCGCAAAGGAAATGGAGGCATTATTACT
TAAGTTAATGGAAGTGCCGGAAAATGGCGATGAATCATATCCCATGATGTTTTCTGCCGTGCACACATTATTAACGCCAG
TAATTAACGAATTAAATACGGTGATGGCAATTCACGAGAATAATAAAACGCATCACACCGGAGAATAA
>Cluster_39651
ATGCCGGACAACGTAGATTTTATTCAGGAACAACAGGCTGAATTACTGGAGCGCCAGATTAACGCGGCAAGGGTAAAACA
TTGCGGTGCTTCTGCGCTGGTTTGCGAAGAGTGTGACGCGCCAATACCTGCTGCCCGTCGTGCGGCTTATCCGTCAGCCA
CGCGTTGTGTTTCCTGTCAGTCAGTCTTTGAAGCAAAAAACAAACATTACCGGAGAACGGCATGA
>Cluster_2249
ATGACCCTTTATGAAATATTAAAAATTCAATTTAAAACCAATGCCGCTATTGGTCGCAGGTTCCCAAAGAAAGGAAGGCC
TCGTGGCAGTCAAGGTGTTGGAAAGTGGAAAACGCGAGGTGTTCCGGAGGATGTTGCCATTCTTTGTCATCTGGATCCGA
GCATTCCATATACACACCCAAGTCTAGCGAATACAGAAGATGACAAGCCCACAGGAGACCAACAATGA
>Cluster_44930
ATGAACCCAGCTATCAAAACAGCGATCAATATCGTTGGTTCACAAAAGAAACTGGGCGCTGCCTGCGAAGTTTCACAGCA
GGCCGTCTATAAGTGGCTTCACAACAAAGCAAAGGTATCCCCTGAACATGTCGGCAGCATTGTTACGGCTACTGGTGGAA
TAGTGAAGGCATACCAGATTCGCCCGGATCTTCCGAAGTTGTTTCCACATACCGAAAAGAACGCAGCTTAA
>Cluster_41975
ATGCATTTCGCTCAGCGAGTTCGCGCCCTGGTTGTGTTGAATGGTGTGGCGTTATTGCCTCAGTTTGCCTGTAAACAGGG
GCTTGCTAACGGGGAACTGGTGCGCCTGTTCGCACCGTGGAGCAGCATCCCCAGACCGTTGTATGCTTTATTTGCGGGGC
GAAAGGGGATGCCCGCCATTGCGCGATATTTTATGGATGAGTTAACCACCCGGCTTGCCAACGGGGTCTGA
>Cluster_12379
ATGGCGATTAAAAAGCTGGATGTGGTGACGCAGGTCTGTCCGTTTCCACTCATAGAAGCAAAAGCAGCACTGGCAGAAAT
GGCCAGCGGCGATGAGTTGGTAATTGAGTTTGATTGCACCCAGGCAACTGAAGCAATCCCACAGTGGGCGGCAGAAGAAG
GACATGCCATCACCGATTATCAGCAGATTGGCGATGCAGCGTGGAGCATCACCGTTCAAAAAGCCTGA
>Cluster_35751
ATGTGTATTAAAGCTGAAAAATATATTGAATGGGTTAAACACTGTCAGTGCCACGGGGTGCCGCTGACGACATATAAATG
CCCCGGATGTGGTGAACAGATTATGACGCAATGCTCACCCGAAAAGGAAATTCGGGATTCCATGACATGTTGTCCGTGGT
GCAGCGCAGTTTTCTTTAAACAGGTAAAAGGCGCGAAGGTAAAAGCCAGCGCGGTTATTCAGAATCAATAA
>Cluster_59824
ATGAGTACGCCTGATTTTTCTACTGCCGAGAATAATCAAGAACTGGCAAATGAAGTCTCCTGCCTGAAAGCGATGCTGAC
GCTGATGCTGCAGGCGATGGGACAAGCTGACGCGGGCCGCGTGATGTTAAAGATGGAAAAACAGCTTGCGCTGATCGAAG
ACGAAACCCAGGCTGCAGTATTTTCCAAAACGGTTAAGCAAATTAAACAAGCCTACCGTCAGTAA
>Cluster_26238
ATGAGCAATAACCTTAATGAATGTGACGATACATTCTGGAATGGCAGTATTCTCGGCTATTGGCTGAAGTATACCCATAC
CCGGAAAGAGTTACTCCTTATTTGCCGTGTGGTTAGTCGCTTTACATCGGTAAGGGTAGGGATTTTACAGCACCGTGAAA
AATCTCATAATTTTTATGAAATCACTGTACTCACTATGGATAATGATAAATATCAATGA
>Cluster_35850
ATGAGCAGGAGAAGAATCACTCGCAGACATCACCGCACACACCTGAATTCCTCAGCAACGCTAAAGGCACTTATTCAAAG
CGAGATCGGTGATTTCTTCGCGGGAGTTGGCTCACTAGGTGAACCAGAAACACCAGAAGCGATGCAGCGTGAGCTCATGA
TACGCATAGATAACACTTTTGATTTCTTCTACAGCATGCACGGAATTAAACAGAAATGA
>Cluster_7607
ATGACACCTCAGCAAGAAAACGCCCTTCGCAGTATTGCCCGTCAGGCTAATTCTGAAATCAAAAAAGCCAGACAGCAGTT
TCCGGATAAAAACGTCGATGACATTTGCCGTAGCGTACTGAAGAAGCACCGCGAAACGGTAACGCTGATGGGATTCACAC
CGACTCATTTAAGCCTGGCGATCGGCATGTTAAACGGCGTCTTTAAGGAACGGTGA
>Cluster_61212
ATGCAGGATTTATCATTGGAAGCACGCCTGGCAGAGCTGGAGAGCCGACTGGCTTTTCAGGAAATCACCATTGAAGAACT
GAACGTCACGGTGACCGCTCATGAAATGGAGATGGCGAAACTGCGCGATCATCTGCGTCTGCTGACCGAGAAGTTAAAAG
CCAGCCAGCCGTCGAACATCGCGTCGCAGGCTGAAGAAACGCCACCGCCACATTATTGA
>Cluster_11783
ATGTATAAGAATAGATTTCCGAAAAGAGATAATAACCACATCATAGAAACTTTTTCGTACAAGGTTTTTAGTAACTCGAT
CCCTAATCAATGGATAATAAGAGATTTAACTAAACGAGACTATGGGACAGATGTTTTAATTGAGTACGTCACAGATAGTG
GTGATGTAACCGGCTATAGTGGATGTAACAGGCAGCACAAATCTGCTGCCCTCGGGAAATAA
>Cluster_20755
ATGAATCAACACATTTCAATCGCAATTACAACTCCACATGTAACTATTGAACGCTATAGTGAACTTACTGGTTTGTCGGT
GGAGACTATCAACGACATGTTGGCCGATGGACGGCTCATAAGGCATCGACTACGCAAAGATAAAAAGCGTGAAAAAGTGA
TGATTAACATCGCAGCCATGACAATAGATGCACTTTCTAACAGCAACGTAATCATTAGTTAG
>Cluster_32083
ATGTTTCTCTGCTGCTTGATTACATATTCTTACGAGCAGGTAGCAAGATGTGACGGTGAGGCTCAGCGTTTCAGGCTGTG

TTTTACCTACTTAGCAGAAAAATGTTCGGCAAAAAGTTGCGATGAAGAAGATGGCATTAATAAGATCACAGAAATCGAGC
AAATGATTAAGCAATGGCATCAGTTACTGCCGCGTTTATTATTACTCACTATATATCAATAA
>Cluster_59913
ATGCAGCGTGGGCGTTTTTGCCTGACAGGTGGTTTTCGCGATATGTATATCGTGAAAAATAATCGCATGTTTTACGCCTT
GCTGATTGCAATTTCGGTACAAAGCGTGGGGGTTTTTGCGTTAATTCAGGCGGGCCTACTGACTTACGAAGCCGGGGCGT
TCCCGTGGCTTGGTACTGTTATAGGTGGGTATATCTTCGGGCTGGGAATTGTTCTGGCGGGTGGATGTGCCACCGGGACC
TGGTATCGCGCGGGTGAGGGATTGATCGGCAGTTGGATCGCGCTTTTCACTTATATGGTGATGAGTGCGGTGATGCGTTC
TCCACATGCCAGTGGTTTAAATCAAACCTTGCAGCACTACAGTACTGAACATAACTCTATTGCTGAGACTTTTAATTTGT
CTGTGTGGCCGTTGGTTGCCGTTTTGCTGGTGATAACGCTCTGGGTGGTGATGAAAGAGTTGAAGAAGCCAAAACTCAAA
GTCGCGACCTTACCGCCGCGCCGAACCGGGATCGCTCATATTCTGTTTGAAAAACGCTGGCATCCCTTTGTCACAGCTGT
ACTCATCGGTTTGATTGCGCTTTTAGCCTGGCCCCTGAGTGAAGCAACCGGACGCATGTTTGGGTTAGGAATCACTTCCC
CAACGGCCAATATTCTGCAATTTCTGGTCGCGGGTGACATGAAATACATTAACTGGGGCGTTTTCCTGGTGTTAGGGATC
TTCGTGGGGTCATTTATTGCAGCCAAAGCGAGCCGTGAGTTCCGCGTTCGCGCAGCTGATGCACAAACAACATTACGTAG
CGGGTTAGGTGGTGTACTGATGGGCTTCGGTGCCAGTATTGCAGGTGGTTGCTCTATCGGTAATGGACTGGTTATGACTG
CAATGATGACCTGGCAGGGCTGGATTGGCCTTGTATTTATGATTCTCGGAGTCTGGACTGCGTCCTGGCTTGTGTATGTT
CGACCGCAGCGTAAGGCGCGACTGGCAACTGCTGCGGCAAATTAA
>Cluster_19392
ATGAAGCCTTTTGCAACGGGCCTGGTGGTTGGTAAATTCGCGCCTTTGCATTGTGGTCATGAAAAGTTGATCAATACCGC
ACTGGCGCAATGTGAAGAACTGTTCATCATCAGTTATTCCGTGCCGGAAATGCCTGACTGTGAACCTGAAAAGCGCCTGA
CGTGGCTGCAAGTGCGCTTCCCGCAAGCGACGATTTTGGTACTGACGCCAGAGCTTGTTGCGCGTTATAACTTACCTGCC
ATACCGCATAACGATGCCGATGCCGACATCCACCGTCATTATGTGGCGACGCTGTGCCTGCAGATATTACGCTGTCGTCC
ACATGCTGTATTTACTGCAGAAGACTATGGCGATGGCTTTGCTAACGTGCTGGCGCGGCGTTTTGCCCAACCCGTTGAAC
ACGTTCGAATGGCGCGCCCAGTTGGCGATGAAGCGCCTTCGGGAACGCTTATTCGTTCCGATGTTCACCGCTATCGCTAC
ATGTTGGCGAATGATGTTTATTACAGTTTCGTTCGCCGTATCTGTCTGCTTGGTGGCGAATCTACCGGTAAAGCACGCT
TTCAAAAGCGCTGGCGGATGGGCTTGATACGGTGTATGTCGCTGAATTTGGCCGCGACTATTGGGAAGAAAAAAACGGAA
TCTTAACCGCGGATGATTTGTTGCATATCGCCTGCGAACAGGTACGCCGGGAACAACAAGCAGAAGCTAATCACTATCTT
ATATGTGACACGTCGCCACTCACGACCTTGTTCTATGCCCTTGATCAATATGGTCATGCTCCGCAAGAACTTCATCAACT
GGCGGAGCGGGAATACTCTCTGGTGGTGCTTTGCGGCGCAGAATTTCCCTTCGTGCAGGATGGCACCCGGCAAGGAGAGG
TATTTCGCGCACGGCAACAGGTGTGGTATGAACAGGAATTGTCGCGCAGGAACATTCCTTATCTTTCGGTTTCGGGGCCA
CTGCAAGAGCGAGTGGAGCAAATTCTTCGCCAACTTCCCGACTGA
>Cluster_59535
ATGATTGATACCACCCTGCCATTAACTGATATCCATCGCCACCTTGATGGCAACATTCGTCCCCAGACCATTCTTGAACT
TGGCCGCCAGTATAATATCTCGCTTCCTGCACAATCCCTGGAAACACTGATTCCCCACGTTCAGGTCATTGCCAACGAAC
CCGATCTGGTGAGCTTTCTGACCAAACTTGACTGGGGCGTTAAAGTTCTCGCCTCTCTTGATGCCTGTCGCCCGCGTGGCA
TTTGAAAACATTGAAGATGCAGCCCGTCACGGCCTGCACTATGTCGAGCTGCGTTTTTCACCAGGCTACATGGCAATGGC
ACATCAGCTGCCTGTAGCGGGTGTTGTCGAAGCGGTGATCGATGGCGTACGTGAAGGTTGCCGCACCTTTGGTGTGCAGG
CGAAGCTTATCGGCATTATGAGCCGGACCTTCGGCGAAGCCGCCTGTCAGCAAGAGCTGGAGGCCTTTTTAGCCCACCGT
GACCAGATTACCGCACTTGATTTAGCCGGTGATGAACTTGGTTTCCCGGGAAGTCTGTTCCTTTCTCACTTCAACCGCGC
GCGTGATGCGGGCTGGCATATTACCGTCCATGCAGGCGAAGCTGCCGGGCCGGAAAGCATCTGGCAGGCGATTCGTGAAC
TGGGTGCGGAGCGTATTGGACATGGCGTAAAAGCCATTGAAGATCGGGCGCTGATGGATTTTCTCGCCGAGCAACAAATT
GGTATTGAATCCTGTCTGACCTCCAATATTCAGACCAGCACCGTAGCAGAGCTGGCTGCACATCCGCTGAAAACGTTCCT
TGAGCATGGCATTCGTGCCAGCATTAACACTGACGATCCCGGCGTACAGGGAGTGGATATCATTCACGAATATACCGTTG
CCGCGCCAGCTGCTGGGTTATCCCGCGAGCAAATCCGCCAGGCACAGATTAATGGTCTGGAAATGGCTTTCCTCAGCGCT
GAGGAAAAACGCGCACTGCGAGAAAAAGTCGCCGCGAAGTAA
>Cluster_48107
ATGTTGAATACGCTGATTGTGGGTGCCAGCGGCTACGCTGGCGCAGAGCTAGTGACCTATGTAAATCGCCATCCGCATAT
GAACATAACCGCTTTGACTGTTTCAGCGCAAAGCAATGATGCGGGAAAGTTAATCTCCGATTTGCATCCGCAGCTAAAAG
GCATCGTTGATCTGCCGTTGCAGCCGATGTCGGATATCAGCGAGTTTAGCCCAGGGGTGGACGTAGTGTTTCTCGCCACC
GCCCATGAAGTTAGCCACGATTTAGCGCCGCAGTTTCTTGAAGCGGGCTGCGTGGTGTTCGACCTTTCCGGCGCGTTTCG
TGTTAACGACGCCACCTTCTATGAAAAATATTACGGCTTTACCCATCAATACCCGGAACTGTTGGAACAGGCAGCCTACG
GTCTGGCGGAGTGGTGCGGTAATAAATTAAAAGAAGCGAATTTGATTGCGGTGCCGGGCTGTTATCCGACGGCGGCACAG
CTGGCGCTGAAACCGTTGATTGATGCCGATCTTCTTGACCTCAATCAGTGGCCGGTGATCAACGCCACCAGCGGCGTGAG
CGGTGCAGGGCGTAAAGCGGCCATTTCAAACAGCTTTTGTGAAGTTAGCCTGCAACCGTATGGCGTCTTTACTCATCGCC
ATCAACCAGAGATCGCCACACACCTCGGTGCTGACGTTATCTTCACCCCACATCTGGGCAATTTCCCGCGCGGCATTCTC
GAAACCATTACCTGCCGCCTGAAATCGGGTGTGACCCAGGCGCAAGTCGCGCAAGTGTTACAGCAGGCGTATGCCCATAA
ACCGCTGGTGCGGCTGTATGACAAAGGCGTTCCGGCGCTGAAAAATGTCGTTGGGCTGCCATTTTGCGATATCGGGTTTG
CCGTTCAGGGCGAGCATCTGATTATTGTGGCGACCGAAGACAACTTACTGAAAGGCGCGGCGGCACAAGCGGTACAGTGC
GCCAATATTCGTTTCGGCTATGCGGAAACGCAGTCTCTTATTTAA
>Cluster_55922
ATGTCTGAAGGCTGGAACATTGCCGTCCTGGGCGCAACTGGCGCTGTGGGCGAAGCCCTGCTTGAAACGCTGGCTGAACG
TCAGTTCCCGGTTGGGGAAATTTATGCACTGGCACGTAACGAAAGCGCAGGCGAACAACTGCGCTTTGGTGGTAAGACAA
TCACCGTGCAGGATGCCGCTGAATTCGACTGGACGCAGGCGCAGCTGGCATTTTTTGTCGCAGGCAAAGAAGCTACCGCT
GCCTGGGTTGAAGAAGCGACCAACTCAGGTTGCCTGGTGATCGACAGCAGTGGATTGTTTGCTCTCGAACCCGACGTACC
GCTGGTGGTGCCGGAAGTAAACCCGTTTGTACTGACAGATTACCGGAACCGGAATGTCATCGCCGTACCAGACAGTCTGA
CCAGCCAGCTGCTGGCGGCACTGAAACCGTTAATCGATCAGGGCGGTTTATCACGTATCAGCGTTACCAGCCTGATTTCA
GCCTCCGCCCAGGGCAAAAAAGCGGTCGATGCGTTAGCGGGGCAGAGTGCGAAATTGCTCAACGGCATTCCGATTGACGA
AGAAGATTTCTTCGGGCGTCAGCTGGCGTTCAACATGCTGCCGTTACTGCCGGATAGCGAAGGTAGCGTGCGTGAAGAAC
GTCGTATCGTTGACGAAGTACGCAAAATCCTGCAGGACGAAGGGCTGATGATTTCGGCTAGCGTCGTCCAGGCACCGGTA
TTCTACGGTCATGCCCAGATGGTCAACTTTGAAGCTCTGCGTCCACTGGCAGCAGAAGAAGCGCGTGATGCGTTTGTTCA
AGGCGAAGATATTGTGCTCTCTGAAGAGAACGAATTCCCAACTCAGGTAGGTGATGCTTCGGGTACGCCGCATCTTTCTG
TTGGCTGCGTGCGTAATGACTACGGTATGCCGGAGCAAGTCCAGTTCTGGTCGGTGGCCGATAACGTTCGCTTTGGCGGC
GCGCTGATGGCAGTAAAAATCGCCGAGAAACTGGTGCAGGAGTATCTGTACTAA
>Cluster_17162
ATGAAAAATGTTGGTTTTATTGTTACAAAATCAGAAATTGGTGGTGCACAAACATGGGTAAATGAAATATCTAACCTTAT
TAAAGAGGAATGTAATATATTTCTTATTACATCTGAAGAAGGATGGCTCACACATAAAGATGTCTTTGCCGGAGTTTTTG
TCATACCAGGTATTAAAAAATATTTTGACTTCCTTACATTGTTTAAATTGAGAAAAATTTTAAAAGAAAATAACATTTCA
ACGTTAATAGCAAGTTCTGCTAATGCCGGAGTTTATGCCAGGTTAGTTCGATTACTAGTCGACTTTAAATGTATTTATGT
TTCGCATGGATGGTCTTGTTTATATAATGGTGGTCGCCTAAAATCAATTTTTTGCATTGTGGAAAAATACCTTTCTTTAT
TAACTGATGTTATATGGTGTGTTTCCAAAAGTGATGAAAAAAAGGCAATTGAGAATATTGGTATAAAAGAACCAAAGATA

ATCACAGTATCGAATTCAGTGCCTCAGATGCCGAGATGTAATAATAAACAACTCCAGTATAAGGTTCTGTTTGTTGGTAG
GTTAACACACCCTAAGCGCCCCGAGTTGTTAGCGAATGTAATATCGAAAAAGCCCCAGTATAGCCTCCATATCGTAGGAG
GGGGGGAAAGGTTAGAATCATTGAAGAAACAATTCAGTGAATGTGAAAATATTCATTTTTTGGGTGAGGTCAATAATTTT
TATAACTATCATGAGTATGATTTATTTTCACTGATATCCGATAGTGAAGGTTTGCCTATGTCAGGCCTTGAGGCTCACAC
AGCTGCAATACCACTCCTGTTAAGTGATGTGGGCGGATGTTTTGAATTAATTGAGGGTAATGGGTTACTTGTGGAAAATA
CTGAAGACGACATTGGATATAAATTGGATAAAATATTCGATGACTATGAAAATTATCGGGAACAGGCAATTCGTGCCTCC
GGGAAATTTGTTATCGAGAACTATGCTTCAGCATATAAAAGCATTATTTTAGGTTGA
>Cluster_40111
GTGGCTCGACCTAATCAACCGACGATCTCCGCGGCGTTAAAAATAATCGCCACGGGGATCGCCTTACCCCCTGATCAAAT
CACTTCTCAACAACTGGATGTTCAGTTAAGTAAGCCAGTTGGCTATGTTGAGCGACGTTCCGGTATTTGCTGGCGCTATT
TTGCCCGCAATGACGCCAGCCAGGCCGAACTGGCGGCAGAGGCTTTACATAATGCGTTAGTAGCGGCAGCCTTAAATCCT
TCTGATATTAATCTGCTAATTTGTGCATCGGCCATTGCCGTACAGGCATTACCCTGTAGCGCAATCATATTCTTAAAGC
CGCAGCCATGCCCGCAGGCGTTGCCGGGTTTGATATCAACAGTAGCTGTGTGAGCTTTATTTCCGCTCTTGAAGTCGCTG
CCGGATTATTAAACTGTGGAGCATATCGGCGCATTGCCATCGTTTCGGCAGATATTGCTTCACGCGGTATCGACTGGGAT
CATGAAGAGTCTTCATTAATTTTTGGTGATGGCGCGGCGTGCGCGATTGTTGAGCGGGGCGATGGGCGCAGCGGGATTAT
CGCTAGCCTGATCGAAATGTATCCCGAAGGCAGCGAACTGTGTGAAATACGCGCCGGCGGTACGCGGCGTAATCCTCGTT
CTGGTGTGAGTGACAGCGACTTTCTTTTTCATATGCAGGGGAAACCGCTGTTTCGTTTTGCTTCTTCGCTTATTGAAGAT
TATTTTTCTCGGTTGCTGCAAAAAAGCAGTCTGCAACTGGATGATATCGGCACGGTCGTTCCACATCAGGCCAGTCATCT
TTCCCTTGAACATATGCGTAAACGGCTGCGAGTGCCGGAATCTGCGCTCATCGATGTGTATCGTTTTACGGCAATCAGG
TCGCGGCCTCTATCCCCACGGCGCTGCATGAAGCGGTGATTAGCGGTCGTTTCACAGCGGATAAACCTGTAATGTTGATT
GGCACGGCTGCGGGGTTAACGCTGGCTGGCATGGTGCTTTTACCGTGA
>Cluster_59773
ATGATTGAAGCAGACCGTCTGATTTCTGCCGGTACCACTTTGCCGGAAGATGTAGCAGATCGCGCCATTCGCCCCAAATT
ACTGGAAGAGTATGTTGGTCAGCCGCAGGTTCGTTCACAGATGGAGATTTTCATCAAAGCAGCGAAACTGCGCGGCGATG
CCCTCGATCATTTGTTGATTTTTGGTCCTCCGGGGTTGGGTAAAACTACGCTTGCCAACATTGTCGCCAATGAAATGGGC
GTTAATTTACGCACGACTTCTGGTCCGGTGCTGGAAAAGGCGGGCGATTTGGCTGCGATGCTCACTAACCTTGAACCGCA
TGACGTGCTGTTTATTGATGAGATCCACCGTCTATCGCCAGTTGTTGAAGAAGTGCTGTACCCGGCAATGGAAGACTACC
AACTGGATATCATGATTGGTGAAGGTCCGGCGGCACGCTCCATTAAAATTGATTTGCCGCCGTTTACCCTGATTGGTGCA
ACCACGCGCGCAGGTTCGCTGACATCACCGTTGCGCGACCGTTTTGGTATTGTGCAACGTCTGGAGTTTTATCAGGTGCC
GGATCTGCAATATATCGTCAGTCGCAGCGCACGCTTTATGGGGCTTGAGATGAGTGATGACGGCGCGCTGGAAGTTGCTC
GTCGCGCTCGCGGTACGCCGCGCATTGCCAACCGTCTGCTGCGTCGAGTGCGTGATTTCGCCGAAGTGAAGCACGATGGC
ACCATCTCGGCAGATATCGCTGCTCAGGCGCTGGATATGTTGAATGTCGATGCTGAAGGTTTCGATTATATGGACCGCAA
ATTGTTGCTGGCGGTAATCGATAAGTTCTTTGGTGGACCTGTAGGTCTGGATAACCTGGCGGCAGCCATTGGCGAAGAAC
GTGAAACCATTGAGGATGTGCTGGAACCTTATTTGATTCAGCAAGGCTTTTTGCAGCGTACACCGCGTGGGCGTATGGCG
ACGACGCGGGCGTGGAATCACTTTGGCATAACGCCGCCAGAAATGCCGTAA
>Cluster_53606
ATGAGCCTGTTAATGACATCCCAGCCCATTGTGATAAATCGTGATCTTGCATGCCGTATTGGTCTGAATGAGGCAATTGT
GTTGCAGCAGCTTCATTACTGGCTGAATGAAACGAATTCAGGCACTGAGCATGGCGGAATTCGCTGGGTTTATAACACGA
CAGAACAGTGGCTGGAGCAGTTTCCGTTCTGGTCAGAGTCCACTCTGAAACGCACATTTGCAAGCCTGAAATCACTTGGG
GTTTTGCGTCGCGAGCAACTCAATAAATCGAAGCGTGACATGACCAATTTCTACACGATCAACTATGAAAGTGAGCTTTT
AGAAGAGGTCAAAGTGAACGAATCCATCAGGTCAAAATGCACTTCTCCATCGGGTCAAAGTGACCTGATGGATGGGCGCA
AAATGACACGATCCATTGGTTCAAAACGACACGCTGTCATCGGGTCAAAATGGCCCAATGATCTTACAGAGAATACAACA
GAGATTACTACAGAGAATAAAACCTCTTCTCGTCCGGACGCTTCGCAACCGGACACGCAGGTGGCTGAACAGGATTTTTT
AACTCGCCATCCTGATGCGGTTGTATTCAGCCCTAAAAAGCGCCAGTGGGGAACGCAGGATGATTTGACCTGCGCACAGT
GGCTCTGGAAAAAAATCATCGCCCTGTACGAGCAGGCCGCCGAATGTGACGGCGAGGTGGTTCGTCCCAAAGAACCGAAC
TGGACAGCCTGGGCAAACGAAATTCGCCTGATGTGTGTGCAGGATGGTCGTAATCATAAACAAATCTGCGAGATGTACAG
CCGCGTCAGTCGCGATCCGTTCTGGTGCCGTAACGTGCTCAGCCCGTCGAAGCTGCGGGAAAAATGGGATGAGCTTTCCC
TGCGCTTATCGCCGTCCGTCAGCACGTACACCGAAAAACGCGAAGACCCGTACTTCAAATCCAGTTACGACAACGTGGAC
TACAGCCAGATCCCGGCAGGATTCAGGGGGTGA
>Cluster_47884
ATGGCTGCAACCAAGCCTGCTTTTAACCCACCGGGTAAAAAGGGCGACATAATTTTCAGCGTGCTGGTAAAACTGGCGGC
GCTGATTGTGCTATTGATGTTGGGTGGCATTATTGTCTCTCTGATCATCTCCTCCTGGCCGAGCATTCAGAAATTTGGTC
TGGCTTTCCTATGGACCAAAGAGTGGGATGCACCGAACGATATCTACGGGGCGCTGGTGCCGATCTACGGTACGTTGGTG
ACTTCGTTTATCGCGCTGCTGATCGCCGTCCCGGTGAGTTTCGGTATCGCCCTGTTCCTGACTGAGCTTGCGCCTGGCTG
GCTGAAACGCCCGCTGGGTATCGCCATTGAGCTGCTGGCAGCCATTCCAAGTATCGTTTACGGCATGTGGGGCCTGTTTA
TCTTTGCGCCGCTGTTCGCCGTTTACTTTCAGGAGCCGGTCGGCAATATCATGTCGAATATCCCGATTGTTGGCGCGCTG
TTCTCTGGCCCCGCATTTGGTATCGGTATCCTCGCGGCAGGCGTGATCCTCGCCATCATGATTATTCCGTACATTGCGGC
GGTAATGCGTGATGTGTTCGAACAAACCCCGGTGATGATGAAAGAGTCGGCCTACGGTATTGGCTGCACCACCTGGGAAG
TTATCTGGCGTATCGTTCTTCCGTTCACCAAAAATGGTGTTATCGGCGGCATCATGCTGGGGCTGGGCCGCGCGCTCGGT
GAAACCATGGCGGTGACCTTTATCATCGGTAACACCTACCAGCTCGACAGCGCCTCGCTGTATATGCCGGGCAACAGTAT
CACCTCTGCGCTGGCGAACGAATTTGCGGAAGCGGAATCCGGTCTGCACGTTGCCGCACTGATGGAACTGGGCCTGATCC
TGTTTGTGATTACCTTCATCGTCCTCGCCGCATCGAAGTTTATGATTATGCGCCTGGCTAAGAATGAGGGGGCACGCTAA
>Cluster_24022
ATGATAATGACGGTAACAAGCATGTTAGACAATGTTTTAAGAATTGCCACACGCCAAAGCCCACTTGCACTCTGGCAGGC
ACACTATGTCAAAGACAAGTTGATGGCGAGCCATCCGGGCCTGGTCGTTGAACTGGTACCGACGGTGACGCGCGGCGATG
TGATTCTTGATACGCCGCTGGCGAAAGTAGGCGGGAAAGGCTTATTTGTGAAAGAGCTGGAAGTCGCGCTCCTGGAAAAT
CGCGCCGATATTGCCGTTCATTCAATGAAAGATGTGCCCGTTGAATTCCCGCAAGGTCTGGGGCTGGTCACCATTTGCGA
GCGCGAAGATCCCCGCGATGCCTTTGTATCCAACACCTATGACAGTCTGGATGCGCTACCGGCAGGCAGTATCGTCGGGA
CGTCCAGTTTACGTCGCCAGTGCCAACTGGCTGAACGCCGCCCGGATCTGATTATCCGCTCCCTGCGCGGCAACGTCGGC
ACTCGTCTGAGCAAACTGGATAACGGTGAATATGATGCCATCATTCTTGCCGTAGCCGGACTAAAACGTTTAGGTCTGGA
GTCCCGCATTCGCGCCGCGTTGCCACCCGAGATTTCTCTTCCGGCGGTAGGACAAGGTGCGGTGGGCATTGAATGCCGCC
TTGATGATACACGCACTCGCGAGCTGCTTGCCGCGCTGAATCACCACGAAACTGCACTGCGCGTTACCGCAGAACGCGCC
ATGAATACCCGTCTCGAAGGCGGATGTCAGGTGCCAATTGGTAGCTACGCCGAGCTTATTGATGGCGAAATCTGGCTGCG
TGCGTTGGTCGGTGCGCCGGACGGTTCGCAGATTATTCGCGGTGAACGCCGCGGTGCGCCCGCAAGATGCCGAACAAATGG
GGATTTCGCTGGCAGAAGAGCTACTGAATAACGGCGCGCGCGAGATCCTCGCTGAAGTCTATAACGGAGACGCCCCGGCA
TGA
>Cluster_43352
ATGTGGAAACGCTTACTTGTAGTCTCTGCAGTCTCGGCAGCCATGTCGTCTATGGCGTTGGCCGCTCCATTAACCGTAGG
ATTTTCGCAGGTCGGATCGGAATCCGGCTGGCGCGCCGCAGAAACCAATGTGGCGAAAAGTGAAGCCGAAAAACGCGGTA

TCACACTGAAAATTGCCGATGGTCAGCAAAAGCAGGAAAACCAGATTAAAGCGGTACGTTCCTTCGTCGCACAAGGGGTG
GATGCGATCTTTATCGCTCCGGTGGTGGCGACGGGTTGGGAACCGGTATTAAAAGAGGCGAAAGATGCCGAAATCCCGGT
CTTCTTGCTTGACCGTTCCATCGATGTGAAAGACAAATCTCTCTATATGACCACTGTCACCGCCGACAACATCCTCGAAG
GCAAGTTGATTGGTGACTGGCTGGTAAAAGAAGTGAATGGCAAACCATGCAACGTGGTGGAGCTGCAGGGCACCGTTGGG
GCCAGCGTCGCCATTGACCGTAAGAAAGGCTTTGCCGAAGCCATTAAGAATGCGCCAAATATCAAAATTATCCGCTCGCA
GTCAGGTGACTTCACCCGCAGCAAAGGCAAAGAAGTGATGGAGAGCTTTATCAAAGCGGAAAACAACGGCAAAAACATCT
GCATGGTTTACGCCCATAACGATGACATGGTGATTGGTGCAATTCAGGCAATTAAAGAAGCGGGCCTGAAACCGGGTAAA
GATATTCTGACAGGCTCTATCGACGGCGTGCCGGACATCTACAAAGCGATGATTGATGGCGAAGCGAACGCCAGCGTTGA
ACTGACGCCGAACATGGCAGGTCCTGCTTTCGATGCGCTGGAGAAATACAAAAAAGACGGCACCATGCCTGAAAAGCTGA
CGTTAACCAAATCCACCCTTTACCTGCCTGATACCGCAAAAGAAGAGTTAGAGAAGAAGAAAAATATGGGGTATTGA
>Cluster_21209
ATGATCAAAACGCGTTTTTCTCGCTGGCTAACGTTTTTTACGTTCGCCGCTGCCGTGGCGCTGGCGCTACAGGCAAAAGC
CAACACCTGGCCGCTGCCGCCAGCGGGCAGTCGTCTGGTTGGCGAAAACAAATTTCATGTGGTGGAAAATGACGGTGGTT
CTCTGGAAGCCATCGCCAAAAAATATAACGTCGGCTTTCTCGCTCTGTTACAGGCTAACCCCGGCGTTGATCCTTACGTA
CCGCGCGCGGGCAGCGTGTTAACGATCCCGTTGCAAACCCTACTTCCAGATGCGCCGCGCGAAGGCATTGTGATCAATAT
TGCGGAACTGCGTCTCTATTACTACCCGCCGGGTAAAAATTCGGTAACCGTGTATCCCATCGGCATTGGTCAGTTAGGTG
GTGACACGCTGACACCGACAATGGTGACCACCGTTTCAGACAAACGTGCAAACCCAACCTGGACGCCAACGGCAAACATC
CGCGCCCGTTATAAAGCACAGGGAATTGAGTTGCCTGCGGTAGTGCCGGCTGGACCGGATAATCCAATGGGCCATCATGC
GATTCGTCTGGCGGCCTATGGCGGCGTTTATTTGCTTCATGGTACGAACGCCGATTTCGGCATTGGCATGCGGGTAAGTT
CTGGCTGTATTCGTCTGCGGGATGACGATATCAAAACACTCTTTAGCCAGGTCACCCCAGGTACCAAAGTAAATATCATC
AACACCCCGATTAAAGTCTCTGCCGAACCAAACGGTGCGCGTCTGGTTGAAGTACATCAGCCGCTGTCAGAGAAGATTGA
TGACGATCCGCAGCTGCTGCCAATTACGCTGAATAGCGCAATGCAATCATTTAAAGATGCAGCACAAACTGACGCTGAAG
TGATGCAACATGTGATGGATGTCCGTTCCGGGATGCCGGTGGATGTCCGCCGTCATCAAGTGAGCCCACAAACTCTGTAA
>Cluster_66931
ATGCAATCACGTTATTTACTGAGCACACTGCTCCTGGTGTGCAGCGCCGCCACGTCTGCAGATAACGCAGGATGGCAGAA
CGCACGCACACCACAAACGACAAATTCTGCCAGCGATCACGTTCAGAATGCCTCCCAAAATACCGGAGGTGTTCCAGCAA
CACTGGTAAAAGGTGAGCTGCCTGCCCCGGGCAGGCCAGCCCGCTGGTACAGGATGCGGCAAGACTTGATTCTGAGCTC
TCCGCCGATGAAATTCGCTCACTACGCAGTCTCATGGCGGATAACGAGCGGGCCATCAATGCGCCCATCACCAGTGTTGT
ACCCCGTATCAGCTCTCTGACAGTTAATCTGTCCCCCGGTGCCAGCCTTCCTCTTGTTCGCACAGCCATGAACAACCTGA
GCGTGGTGACATTTACCGATATCAACGGTTCACCATGGCCACAATCCGATCCTCCTTATAACGCGGCACCAAAACTCTTT
GATGTGCAGTACAACGGGAACATGGTGACAGTCACACCATTGCGGCCATGGGCATCCGGAAACATTTCTGTCTACCTGAA
AGGTCTGTCGGTACCGGTCATCCTGAACGTAACCAGTGGCGAAACAGATACGCCGTCATCCAGTCAAGAAATGGACAGTC
GTCTGGATCTGCGAATACCACGGCAGGGACCAACCAGCCCGGTTGTCTCCATCCCGACGGATAAAATCGCCCTGCATGAT
GCAACATTACAGGCATTTCTTGATGGTATTCCTCCCCGGGACCCTTCAGTTAAGAGGCTGAAATTCACAGGTAATGTCCC
GGATACAACGATCTGGCAACACGGCGATGACCTTCTGGTTCGTTCCCGGGCGATGTTGCGCGATGAATTTGAACAGACCT
TGTCCTCCGCCGACGGAACTCATCTCTGGAAGCTTCCCGTCACCCCACTACTGACTTTCTCTGTGAATGGCCAGTCCGTT
CACGTGACACCAGAACTGGAGTAA
>Cluster_48854
ATGAACCGTTATGTAACAGGACTGGCGCTGATGTTGTTATGCCTGACCGGGACGGCCAGGGCCGCTTCGGCTGAATGTGA
AGGCAATTTTGTCAATCCAATAACGGATATCTGCTGGGAGTGTATTTTTCCGGTAACCATTGGCAATGTTCCGGTCGCCA
AAGGACGTCAGCCGGATACGCCGAATCCTTCCATGCCGATCCAGTTTTGCCCGATGGGCATTTTATACCGGGTGGGGCTG
GCCATTGGTTACTGGGAGCCGATGGCGCTGACGGATGTGACACGCTCCCCTTACTGCATGGTGAATCTGGGGGGCTTTAA
TATCAATATTGGTAATGTGGGTAGCGGCAATGGTGGTCAGGAAAGCCGCGCCAAACCGGGGGCCTTCTACCATGTTCACT
GGTACAAATACCCGTTGACCTACTGGCTGAACATTATCACGTCGATGGGCTGCCTGCAGGGTGGCGACCTGGATATTGGT
TATCTGTCGGAACTTGATCCCATGTGGGACAGCAGCTCTCTCAGCATGATTATCCAGCCGGAAGCGATTCTCTTTGGCAA
CATCATCGCCCAGGGAGCCTGTGCCGCTGATGCCGTGGCCAGTCTGGTGAGTAAACCCATCGACGCCCTTTTCTGGTGTG
CCGGCAGTCAGGGCAGTATGTACCCGTTTAACGGCTATGTTTCTAACGAATTCAGTCCGTTACAGACGTCACTGTTACTC
AGTGAGCGCATGGCGTTCAAGCTTCACCGCCAGGGCATGATCATGAACAGCATCGGTGAAAACAGGGCTGTCTGCTTTGA
ATATCCATCCCCGATCATCCCCAAAAGCCGCTGGCGATATCACATGGTCAACAAGTATCCCGATGCCGGACAGTGTCATC
CGCTGGGGCGTAGCGTAACCCGCTGGGAAGCGGGCAAGAACATGCCCAATGACCGGCGTAACTTTGGGTATCTCTTCTGG
CGTAAGCGCAACTGCGTCTTTCTGTAA
>Cluster_10410
ATGAGTAAACCCATTGTGATGGAACGCGGTGTTAAATACCGCGATGCCGATAAGATGGCCCTTATCCCGGTTAAAAACGT
GGCAACAGAGCGCGAAGCCCTGCTGCGCAAGCCGGAATGGATGAAAATCAAGCTTCCAGCGGACTCTACACGTATCCAGG
GCATCAAAGCCGCAATGCGCAAAAATGGCCTGCATTCTGTCTGCGAGGAAGCCTCCTGCCCTAACCTGGCGGAATGCTTC
AACCACGGCACAGCAACGTTTATGATCCTCGGCGCTATTTGTACCCGCCGTTGTCCGTTCTGTGATGTTGCCCACGGTCG
CCCGGTAGCTCCTGATGCCAATGAACCAGTGAAACTGGCGCAGACCATTGCCGATATGGCGCTGCGTTATGTGGTTATCA
CCTCCGTTGACCGTGATGACCTGCGCGATGGCGGTGCCCAGCACTTTGCGGATTGCATTACTGCCATTCGGGAAAAAAGC
CCGCAAATCAAAATTGAAACTCTGGTGCCGGATTTCCGTGGTCGTATGGATCGTGCTCTGGATATTCTGACTGCAACGCC
ACCAGATGTGTTCAACCATAACCTGGAAAACGTACCGCGTATTTACCGTCAGGTACGGCCTGGTGCAGATTACAACTGGT
CGCTGAAGCTGCTGGAACGCTTTAAAGAAGCGCATCCGGAAATCCCGACCAAGTCTGGTCTGATGGTGGGACTGGGTGAA
ACCAATGAAGAAATTATTGAGGTAATGCGCGACCTGCGCCGTCATGGTGTGACGATGTTAACGCTGGGGCAATATTTGCA
GCCAAGCCGCCATCACCTGCCGGTTCAACGTTACGTTAGCCCGGATGAGTTCGACGAAATGAAAGCCGAAGCGCTGGCGA
TGGGCTTTACCCATGCTGCATGCGGTCCGTTTGTCCGCTCTTCTTACCACGCCGATTTGCAGGCGAAAGGGATGGAAGTT
AAGTAA
>Cluster_61453
ATGACAAAATTACAACTTAAGTACCGGGAGTTAAAAATTATCTCGGTAATCGCTGCCAGTGAAAATATCAGCCATGCCGC
GACTGTACTTGGCATCGCACAGGCCAACGTCAGCAAATATCTTGCTGATTTTGAATCAAAAGTGGGTTTAAAAGTCTTTG
ACCGAACCACCCGGCAGCTGATGCTCACACCTTTTGGCACCGCGCTGTTACCGTACATCAACGACATGCTGGACAGAAAC
GAGCAACTTAATAATTTTATTGCAGATTATAAGCATGAGAAACGTGGTCGGGTCACCATCTACGCCCCAACCGGCATAAT
TACCTATTTATCCAAACATGTAATCGATAAAATTAAAGATATCGGTGATATCACCTTATCGCTAAAAACCTGCAATCTTG
AGCGTAATGCCTTTTATGAAGGCGTCGAATTTCCTGATGATTGTGATGTTTTAATCAGCTACGCTCCACCAAAAGATGAA
TCGCTGGTTGCCAGTTTTATTACCCAATATGCCGTAACCGCTTATGCCAGCCAGCGCTATCTTGAGAAACATCCCATTAG
CCGTCCTGACGAACTCGAACATCACTCCTGTATTTTGATCGACTCGATGATGATCGACGACGCGAATATCTGGCGCTTCA
ACGTCGCCGGGAGTAAAGAGGTGCGGGATTATCGCGTGAAGGGAAATTACGTTTGCGACAACACGCAATCTGCGCTGGAG
CTGGCACGAAATCACCTGGGGATTGTGTTTGCGCCAGACAAAAGTGTGCAGAGCGACCTGCAAGACGGCACGCTGGTGCC
CTGCTTTCAGCAACCCTATGAATGGTGGCTGGATCTGGTGGCTATCTTCCGTAAACGGAATACCAGCCCTGGCGGGTCC
AGTATGTGCTGGATGAAATGCTGCGTGAAATTCGCCACCAGCTTGCTCAGTCGCAGCAACTGCGGCCTGAACAAGCGGCG
GAAAGCGAAGATTAA

>Cluster_35940
ATGAAAAAAGTTTATTACAAAACCATCCTGGGAGCGAGAAGTATTCTTTTAATGGCTGGGAAATATTTAATAGTAATTT
TGAACGGATGATTAAAGAAAATAAGGCCATGCTGCTTTGTAAGTGGGGGTTTTATTTAACATGTGTTGTCGCTGTAATGT
TTGTGTTCGCAGCGATAACATCCAACGGTTTGAATGAAAGAGGCCTGATTACCGCGGGATGCTCTTTTCTTTATCTATTA
ATTATGATGGGGCTTATTGTTCGGGCCGGTTTTAAAGCAAAAAAAGAACAACTGCATTATTATCAGGCTAAAGGTATTGA
GCCGCTCAGTATCGAGAAGTTACAGGCGCTACAATTGATCGCACCTTATCGATTCTATCATAAGCAATGGTCTGAAACGC
TGGAGTTCTGGCCGCGAAAGCCTGAACCTGGCAAAGATACCTTCCAATATCATGTGCTTCCTTTTGACTCGATCGATATC
ATAAGTAAAAGACGCGAGTCTTTAGAGGATCAATGGGGTATCGAAGATAGCGAAAGTTATTGTGCCTTAATGGAGCATTT
TCTTTCTGGCGACCATGGAGCCAATACCTTTAAAGCAAACATGGAGGAAGCCCCAGAGCAGGTTATCGCCTTGTTGAATA
AATTTGCTGTTTTTCCCTCAGACTATATCTCTGATTGCGCTAATCATAGCTCCGGTAAATCCTCGGCGAAGCTAATATGG
GCGGCGGAATTATCATGGATGATCTCGATATCAAGCACAGCTTTTCAAAACGGGACAATTGAAGAAGAACTGGCCTGGCA
TTATATAATGCTTGCTTCTCGAAAGGCGCACGAGTTGTTCGAAAGCGAAGAAGATTATCAAAAAAATAGTCAAATGGGAT
TTCTTTACTGGCATATCTGCTGCTATCGCAGAAAGTTAACGGATGCTGAACTTGAAGCGTGTTATCGTTACGACAAGCAG
TTTTGGGAGCACTACAGTAAGAAATGCCGTTGGCCCATAAGAAATGTTCCGTGGGGAGCATCATCCGTTAAATACTCATA
A
>Cluster_42678
ATGAAAATACTGGTGATCGGCCCGTCTTGGGTTGGCGACATGATGATGTCGCAAAGTCTCTATCGCACGCTCCAGGCGCG
CTATCCCCAGGCGATAATCGACGTGATGGCACCGGCCTGGTGCCGTCCGTTATTATCGCGGATGCCGGAAGTTAACGAAG
CCATCCCTATGCCCCTCGGTCACGGGGCGCTGGAAATCGGCGAACGCCGCAAGCTGGGTCATAGTCTGCGCGAAAAACGC
TACGACCGTGCCTACGTCTTACCCAACTCCTTCAAATCCGCGTTAGTGCCTTTCTTCGCGGGGATCCCTCATCGCACCGG
CTGGCGCGGTGAAATGCGCTACGGTTTACTCAACGATGTGCGCGTGCTCGATAAAGAAGCCTGGCCGCTAATGGTGGAAC
GCTATGTTGCGCTGGCCTATGACAAAGGCGTAATGCGTACCGCACAAGATCTACCAAAACCGCTGTTATGGCCGCAGTTG
CAGGTGAGCGAAGGTGAAAAATCATATACCTGTAATCAATTTTCGCTTTCATCAGAACGTCCGATGATTGGTTTTTGCCC
GGGGGCGGAGTTTGGTCCGGCAAAACGCTGGCCACACTACCACTATGCTGAGCTGGCAAAGCAGCTGATTGATGAAGGTT
ATCAGGTGGTTCTGTTTGGCTCGGCGAAAGATCATGAAGCGGGCAATGAGATTCTTGCCGCTTTGAATACCGAGCAGCAG
GCATGGTGTCGGAATCTGGCGGGAGAAACACAGCTTGATCAAGCGGTTATCCTGATTGCAGCCTGTAAAGCCATTGTTAC
TAACGATTCTGGTCTGATGCACGTTGCGGCGGCGCTCAATCGTCCGCTGGTTGCCCTGTATGGACCAAGCAGCCCGGACT
TCACACCACCGCTATCCCATAAAGCACGCGTGATCCGCCTGATTACCGGCTATCACAAAGTGCGTAAAGGTGACGCTGCG
GAGGGTTATCACCAGAGCTTGATCGACATTACTCCCCAGCGCGTACTGGAAGAACTCAACGCGCTATTGTTACAAGAGGA
AGCCTGA
>Cluster_40553
ATGATGGTCATCCGTCCCGTTGAATGCACTGATGTCTCAGCGTTAATGCAGCTTGCCAGCAAAACGGGCGGCGGCCTGAC
GTCGCTTCCCGCCAATGAAGCCACGCTTTCGGCGCGTATCGAAAGGGCAATCAAAACCTGGCAAGGCGAACTGCCCAAAA
GTGAGCAGGGCTATGTGTTCGTGCTGGAAGATAGCGAGATAGGCACAGTGGCGGGGATTTGTGCCATTGAGGTGGCAGTT
GGGCTGAACGATCCCTGGTACAACTATCGCGTCGGCACGTTGGTTCACGCCTCAAAAGAGCTGAATGTCTATAACGCATT
GCCGACGCTGTTTCTCAGTAACGATCACACCGGCAGCAGCGAGCTGTGCACGCTGTTTCTCGACCCGGAGTGGCGCAAAG
AGGGCAACGGCTATTTGCTGTCGAAATCGCGCTTTATGTTTATGGCGGCTTTTCGCGACAAGTTTAATGACAAAGTGGTT
GCTGAAATGCGCGGGGTGATTGACGAACACGGCTATTCACCGTTCTGGCAAAGCCTCGGTAAACGCTTCTTTTCGATGGA
TTTTAGCCGCGCCGATTTTCTCTGCGGCACCGGGCAAAAGGCGTTTATTGCAGAACTGATGCCGAAACATCCGATCTATA
CCCACTTTTTATCCCAGGAAGCCCAGGACGTCATCGGTCAGGTACATCCGCAAACCGCGCCTGCCCGTGCGGTGCTGGAG
AAAGAAGGTTTTCGCTACCGTAACTATATCGACATCTTTGACGGCGGGCCGACACTTGAATGTGATATCGACCGCGTGCG
CGCCATCCGTAAAAGTCGGCTGGTGGAAGTGGCAGAAGGGCAGCCTGCGCAGGGCGATTTTCCGGCCTGCCTGGTCGCCA
ATGAAAATTATCACCATTTCCGCGTGGTGCTGGTGCGTACCGATCCAGCGACCGAGCGTTTGATTTTAACCGCCGCACAA
CTGGATGCCCTCAAATGCCACGCCGGGGAGCGCGTTCGTCTGGTGCGCCTGTGCGCAGAGGAGAAAACAGCATGA
>Cluster_64434
ATGAGCGATTCGTTTACTACGTCAGAACTGATTACCGCAACGCAGCAGGTATTTAAGTTCAATCCGTTGTTTTTAAGATT
GTTTTTCCGTGAGACCTACACGTTTACGAGCGAAGAGGTTTTTCTGGATAAAATCCCGGGCAAAGTCAATATGGCGGTAT
ATTGCGCCCCGATGATCACCGGCAAAGTTGACCGCACTCGCGGCTATTCAACGAACCACTTTAAACCGGGTTACACGAAA
CCGAAACACACGATCAATCCGAATATGAGCATCAAGCGCGCCGCCGGTGAGCAAATTGGCCAGCCAGAAACGCCGGTCGA
ACGTCGTGCAAAAATCATCATGCAGAATTTGCTCGACGAGGAGCTCAGCATCAGCCAGCTCGAAGAGTTCCAGGCAGTGC
AGGCGGTTCTGTACGGTAAATACACCGTTTCCGGCAGCAATATCGAGACCTATGAGATCGACATGAGCCGCAGCGCGACG
AATAACGTCACTCAGTCCGGTTCGACCGCCTGGTCGACTCAGGACGCGGAAACGTATGACCCGAGCGACGATATTGAATC
CTATGCAGACCTCGCCTCCGGTGCGGTTAACGTGATCATCATGGACGGTAAAGCCTGGAAGCAGCTTAAACGCTTTAAAA
AATTCTGGACGGCACTGGATACGCGCCGCGGCTCAAACAGTCAGCTCGAAGTCGCGCTGAAAAACCTGGGCGATGTCGTT
AGCTTTAAGGGCTACTACGGCGACACGGCGCTGTTTGTCTACAAAGGGCAATACATCGACCCGGTAACAGGCACTGAAAC
GCGTTATATGCCGGATAACACGATGATCCTTGGCAACACAAAAAAACCGCGGGCTCCGCACTTATGGCGCAATTCAGGACG
AAGACGCGCTGAAAGAGGCTATTTGCGAAGCGACGCGCTATCCAAAAGTCTGGACCACTACCGGTGATCCGGCAGTGACG
CAAACAATGACGCAATCCGCGCCAGCAATGGTCCTCACGGACGCCGACGCGTTCGTTGTCGTAAAAATCGCGTAA
>Cluster_26577
ATGGTATTCAGAATAGCTAGCTCCCCTTATACCCATAACCAGCGCCAGACGTCGCGCATTATGCTGTTGGTATTGCTCGC
AGCCGTGCCAGGAATCGCAGCGCAACTGTGGTTTTTTGGTTGGGGTACTCTCGTTCAGATCCTGTTGGCGTCGGTCAGTG
CTCTTCTGGCCGAAGCTCTCGTCCTCAAACTACGCAAGCAGTCGGTAGCTGCAACGTTGAAAGATAACTCAGCATTGCTG
ACAGGCTTATTGCTGGCGGTAAGTATTCCCCCCCTCGCGCCATGGTGGATGGTCGTGCTGGGTACGGTGTTTGCGGTGAT
CATCGCTAAACAGTTGTATGGCGGTCTGGGGCAAAACCCGTTTAATCCGGCAATGATTGGTTATGTGGTCTTACTGATCT
CCTTCCCGGTGCAGATGACCAGTTGGTTACCGCCACATGAAATTGCGGTCAACATCCCTGGTTTTATCGACACCATACAG
GTTATTTTCAGTGGTCATACCGCCAGTGGTGATATGAACACGCTACGCTTAGGTATTGATGGCATTAGTCAGGCGAC
ACCGCTGGATACATTTAAAACCTCTGTCCGTGCCGGTCATTCGGTTGAAGAGATTATGCAATATCCGATCTACAGCGGTA
TTCTGGCGGGCGCTGGTTGGCAATGGGTAAATCTCGCCTGGCTGGCTGGCGGCGTGTGGTTGCTATGGCAGAAAGCGATT
CGCTGGCATATTCCCCTCAGCTTCTTAGCAACGCTGGCGTTATGCGCAACGTTGGGCTGGTTGTTCTCACCAGATACTCT
GGCAGCACCGCAAATTCATCTGTTGTCCGGTGCGACCATGCTCGGCGCATTCTTTATTTTGACTGACCCGGTTACCGCTT
CTACGACCAATCGTGGTCGTCTTATTTTCGGCGCGCTGGCGGGCTTATTAGTCTGGTTGATCCGCAGTTTCGGCGGCTAT
CCTGACGGCGTGGCTTTTGCCGTCCTGCTGGCGAACATCACGGTTCCTCTGATCGATTACTACACGCGTCCGCGCGTCTA
CGGCCATCGCAAAGGGTAA
>Cluster_11228
ATGACAGAGCGTGAACTGTCCATTATCCGTGCGCTGGGTGAAGAATTCTCCACGGTGCTGGCGGATTTACAGCGCACATT
TGAGGGGAAGATGGCCTCGCAGGCACAAGCGTTTGAAGAGAAACTGACTTCCCTGTCGGCGGTATTACAGAAGCATGTGA
CGGTGGATGAGGTGCGTCCGGTTCTGCAGGCGATGGTGGATGACGCTGTGGGGGCCATTTCGGTACCGCGTGATGGTCGT
GATTATGATCCGGATGTACTGCAGCAGGCGGTGAATGATGCGGTCGGGAAAATACCGGTACCGACGGACGGCAAAAGTAT
CACCCCCGATGATGTGCGTCCGATGCTTGAACAGATGGTGAAGGAGGCGGTAAGCCATATCCCTGTTCCGCGTGACGGTC

GTGACTACGATCCGGAAGTACTGCAGAAGGCGGTGAATGATGCGGTCGCAAATATTCCACAGCCGGCGGACGGTAAAAGT
CTCACCCCGGATGATGTGCGTCCGATGCTTGAACAGATGGTGAAGGAGGCGGTAAGCCATATTCCTGTTCCGCGTGATGG
TCGTGACTACGATCCGGATGTTCTGCAGAAGGCGGTTCTGGATGCGGTGAGTGCCCTGCCGGCTCCGCAGGACGGCGTG
ATGCCACGGCACTGGAAATACTCCCCGCCATTGACGATCAAAAATCCTTTCCCCGGGGCACGTATGCCACACACCAGGGC
GGACTCTGGCGGGCGTATGAAAAAACGCACAGGATGCGGGGATGGGAATGCCTGGTTGACGGGGTGGCGGATATTGACGT
CAGTATGACGGGGGAGCGGTTGTTCTCTGTGGTGATCCGGCAGAGCAGTGGCCAGCGTACGGAAAAAACATTTTCCCTGC
CGGTGATGCTCTACCGCGGTGTGTTCAGAGCCGGTGAAACCTACCACCCAGGCGATACGGTGACATGGGGGGGCTCGCTG
TGGCACTGCAACAGTATGACCGGTGATAAACCCGGAGAAGCTCATTCATCAGGCTGGACCCTGGCTGCCAAACGGGGACG
GGATGCTGGAGGCGGAAAATGA
>Cluster_34945
ATGTCAGCGATTGATCAACCTGCGCAATATGTTATCAAGTCAGAAGACGACTTTGTATCTTTTGTAACAGAAATGCTCGG
AAGGAAGGATATCGACTCAAGCGACTTCACATTCCCTAACGTAGTTTCAGTGGATGGCCAAAGATCAATATTAATGTAA
AAGGCGATCCAAATAGATATAATTCTTCATTGACTGCGTCAATGCTTTTTGGTATGGCAGAGCTTACGCATGAAATACAA
AAAGCATTTACGGTAGTCAGTCACAGCACTCATAATCGGCAGAAGCTAAAAGATGCAGAGAAAGGTTTGCTGGATATTGT
CTACCGAATAAGCGAAGGCTCAAGCCAAGCGGATGGTGACTCAGATCCGATTGTAAACGGAGTGGTGACAGTGTTTACTC
AAGCGATAGGCAAAATGACAGGAAGGCAAGCACTATGCGCCGTCGCCGCTATCGTCTTAGCTGCCAGCACGGTAGGCTAT
AAGTGGATCAATGAGTACTACGAAACACAGCGACATGACCAAGATTCTCAGGTGCAATTGGTGGAAAAAAGCACCAAAGC
GGTAAACGAAGCACAAGACAATGTGCTCAAGTTGTTGATTAGCGGTCAAACAAACATTAGCAGAGAGGTTCTGGCTCACG
GCGAGGATGGTAAAAGCAAGTTGCTTAAAAAGCTTGCTCAAGACTCGTCAGTTGAGAGGGTAACAATTGGTCAGAGAGTG
GTAAATAGAGAACAGTTGAATACCTTAAACCAAAGACAATCAATTGATCGAAAAAAGGAAACTAGAAGAGATAACTTTTA
TGTCACGGGCGTTCGTCGCTCGGGAGAAACAAATCAGGACATAAATATTGACGTCATAAGGGTTTCAAATGGTGAATCTT
TCACCATAAAAACCTCAGCCGACATAACATCTACTGATGAGCTTCTTGAGTTTTTGAATGCTGTAGCCAAAGAATCAACT
GTTGAAATTTCATACCTAGAGGTTGTAGAAAATGGGCACATTTCAACAGGGCAATTGATAAATATATTCCCCAGCGAACC
AGAGTGA
>Cluster_7582
ATGCTACCATCTATTTCAATCAACAATACCAGCGCAGCTTACCCAGAATCCATCAATGAAAATAACAATGATGAAGTTAA
TGGATTAGTACAAGAGTTCAAAAACCTTTTTAATGGTAAGGAAGGAATAAGCACCTGTATTAAACATCTACTTGAGCTTA
TAAAAAACGCCATACGAGTAAACGACGATCCTTATAGATTTAATATTAATAATTCCTCAGTTACTTATATTGATATTGAC
TCCAATGATACAGACCATATTACTATTGGTATCGACAACCAAGAACCAATAGAATTACCTGCGAACTATAAAGACAAAGA
ACTCGTCCGTACTATCATTAATGACAACATAGTTGAGAAGACTCATGATATCAATAACAAGGAAATGATCTTCAGCGCAT
TAAAAGAAATATATGATGGAGATCCTGGTTTTATCTTCGATAAGATATCACACAAACTCAGACATACGGTAACGGAATTT
GATGAGAGCGGGAAAAGCGAACCAACGGACTTATTTACCTGGTACGGTAAAGATAAAAAAGGCGACTCTCTCGCTATTGT
AATTAAAAATAAAAACGGAAATGATTACTTATCTCTCGGTTACTACGATCAGGACGACTACCACATTCAAAGAGGAATTC
GTATTAATGGTGATAGTCTCACCCAATATTGTAGTGAAAACGCCAGGAGTGCCTCAGCGTGGTTTGAAAGCAGTAAAGCT
ATCATGGCAGAATCATTTGCAACTGGTTCCGATCATCAGGTTGTAAACGAGCTCAACGGGGAAAGACTGAGAGAACCAAA
CGACGTTTTTAAACGTTATGGTCGAGCAATAAGATATGATTTTCAAGTGGACGATGCAAAATATAAATGCGACCATCTAA
AAGAAATAGTTTCTACTTTAGTCGGTAACAAAATTAACGTTGGCCATTCTCAAAAAATATATAAGCATTTTAAGGATCTC
GAAGGTAAAATTGAAGAAAGGCTTCAAAATCGCCAGGCTGAATATCAAAATGAAATTAATCAACCATCTGCGCCAGGTGT
TAATTTTGATGATATTTAA
>Cluster_39325
ATGCCAGCAAATGAACCTGCAAATGCAATCGTTGTAAATCATGTGGTTATTCACCTTTTAGATAAAGAGCCGCATGGGGT
GACATCCTTACAGCTTAGCCCAGCAGAGAGTGGCGTCACCGCAGCATCACAACGCTTAATAGATGATATCTGTGGGAAAT
ATGCAGGGCGAATGGGGAAAGGTTATGGCCATTTTGAAGATGACCCTGACAATTACCCGATGCAAACAATCGTATCAGAC
TATTTAGCTCAAGAGCATCCTGATGATTTTTACCGTAGCTCCTGCCGCATGATTCGTCATCTGGAATCTCGGATTCAAAC
AACACCACTCGCTACTGGTGGCTATGTTCTATTCGCCCACATTACGATGAATCAACACGAGCATATTTTAGTTGCCATGT
TAAGCCCCACGACGGGAACTGCGGTTAACGATGCCTTTGCTATCCAGGAAAGTGTCTACCTTGATATCGACAAGCTACGT
GTGGCGGGGCGTATTGACATTACGTCCTGGCAATCAGGTGCCGAACGTTATATAAGTTTCCTGAAAGGGAGCAATGCTGT
ATCTGGTTACTTTAAGCAGTTCCTGGGCTGTAACGATGTTTTGATCGCCAAGCGTGAAACAGAGAAATTGCGGGATGCGT
TGAAAGAGTTTGCGACTGAGCAAGGGCTTGACCCCGCACGTCGAGAGGCTTTTCTGCAAACGGCCCATGACCATTTGAAG
CAACTGAGCAAAGATGGTGAGCCTGTAGATCTGACAACCTTTGTGAACGCAGTCTGGCCACAAGAACCAGAACTGCTATC
GACTAAGCTGGCCAACCAAGAACTGGAACTGTCGGATGGTTTTGTCCCAGATGGACGCGTACTAAAAGGAATCATTTCCT
TTAAAGGAAAGTCCAATCATTGGGAGCTAAAGTTTGATCGTGCTGGTCTTCAAGATGGTTCAATTGAGTATAATCGTGAT
CAGGATGTAATCATTCTAAGGAATATTCCTGATAGCTTCAGAGAAGAGATACTGGATGAGGTTGAGGAGTGA
>Cluster_40387
ATGAAAAGCATTTTAATTGAAAAACCGAATCAACTGGCAATTATCGAACGCGAAATACCCACCCCGTCAGCGGGTGAAGT
ACGAGTAAAAGTGAAACTTGCCGGAATTTGTGGTTCAGATAGCCATATTTATCGTGGGCATAATCCTTTTGCGAAATATC
CGCGCGTCATTGGTCATGAATTCTTTGGCGTCATTGATGCAGTGGGTGAAGGCGTGGAAAGCGCCAGAGTCGGTGAACGT
GTTGCTGTCGATCCGGTGGTCAGCTGTGGGCATTGCTATCCGTGCTCTATAGGTAAACCGAACGTTTGTACGACACTGGC
TGTATTAGGTGTGCACGCTGACGGTGGTTTCAGTGAATATGCCGTGGTTCCTGCAAAAAATGCGTGGAAAATTCCTGAAG
CAGTGGCCGATCAATATGCGGTAATGATCGAACCTTTTACCATTGCGGCTAACGTAACCGGACATGGTCAACCGACTGAA
AATGATACCGTTCTGGTTTATGGTGCCGGTCCAATCGGCCTGACGATCGTTCAGGTATTAAAAGGCGTCTATAACGTTAA
AAATGTGATTGTTGCCGATCGCATTGATGAACGACTGGAAAAAGCGAAAGAGAGCGGGGCAGACTGGGCGATTAATAACA
GCCAGACACCGCTTGGCGAGAGTTTCGCTGAAAAAGGCATCAAGCCGACTTTAATTATCGATGCCGCTTGTCATCCTTCT
ATCTTGAAAGAGGCCGTAACGCTGGCTTCTCCAGCGGCACGTATTGTATTGATGGGCTTCTCCAGTGAACCGTCTGAAGT
GATTCAGCAAGGAATTACCGGAAAAGAACTCTCTATTTTCTCTTCACGCTTAAATGCAAATAAATTCCCGGTCGTTATCG
ACTGGTTAAGTAAAGGGTTAATTAAACCAGAAAAACTAATTACCCATACGTTTGATTTCCAGCATGTTGCGGATGCCATT
AGTTTATTTGAACAGGATCAAAAACATTGCTGCAAAGTCTTACTCACTTTTTCTGAATAA
>Cluster_1864
GTGACCTTCTGGAGTATTTTACGCCAACGCTGCTGGGGGCTGGTGCTCGTGGTGGCGGGCGTCTGCGTGATTACGTTTAT
TATCTCGCATCTGATCCCTGGCGATCCGGCGCGGTTACTGGCGGGTGACCGCGCCAGCGATGCTATCGTGGAAAATATTC
GCCAGCAACTGGGACTGGACCAGCCACTGTATGTACAGTTTTACCGCTACGTCAGCGACCTGTTTCATGGTGACCTGGGA
ACATCCATTCGTACCGGGCGTCCGGTGCTGGAAGAGTTGCGTATATTTTTCCCGGCGACGCTGGAGCTGGCTTTTGGTGC
CCTGCTGCTGGCACTCCTGATTGGCATCCCGCTGGGCATACTCTCTGCAGTCTGGCGAAATCGCTGGCTGGATCATCTGG
TGCGAATAATGGCCATTACCGGAATCTCCACACCTGCGTTCTGGCTTGGGCTGGGCGTCATTGTGCTGTTTTATGGTCAT
CTGCAAATTCTTCCCGGCGGCGGAAGGCTTGATGACTGGCTGGATCCACCAACGCACGTTACCGGCTTTTATCTGCTCGA
TGCGCTGCTTGAAGGCAACGGTGAAGTCTTCTTCAATGCGTTGCAACATCTCATCTTACCGGCATTAACGCTGGCGTTCG
TTCACCTGGGAATTGTCGCTCGCCAGATCCGCTCAGCGATGCTGGAACAATTGAGTGAAGACTACATTCGTACCGCCCGG
GCCAGCGGCTTGCCCGGCTGGTATATCGTTTTATGTTATGCGCTACCCAATGCGTTGATCCCATCGATTACCGTATTGGG
TTTGGCGCTGGGCGATTTGTTGTATGGCGCAGTGCTCACCGAAACCGTTTTTGCCTGGCCCGGAATGGGTGCATGGGTAG

TAACATCAATACAGGCGCTCGACTTCCCGGCAGTGATGGGCTTTGCCGTCGTGGTTTCATTTGCTTATGTGCTGGTCAAC
CTGGTGGTGGATTTGCTCTATTTGTGGATTGATCCGCGTATCGGACGTGGAGGTGGTGAATGA
>Cluster_13282
ATGACCGTACGCGTAGCGATAAATGGCTTCGGTCGCATCGGGCGTAATGTGGTTCGTGCTTTGTATGAATCCGGACGCCG
GGCGGAAATTACCGTGGTGGCAATCAACGAACTGGCGGATGCTGCGGGCATGGCGCATTTGTTGAAATATGACACCAGCC
ATGGCCGTTTTGCATGGGAAGTACGACAGGAACGCGATCAACTTTTTGTTGGTGATGACGCCATCCGCGTATTGCATGAA
CGTTCACTGCAATCGCTCCCCTGGCGTGAACTTGGCGTTGATGTAGTCCTCGACTGCACCGGCGTATATGGCTCCCGCGA
GCATGGCGAAGCGCATATTGCCGCCGGGGCCAAAAAAGTGCTCTTTTCACATCCTGGCAGTAACGATCTCGACGCGACCG
TTGTTTACGGCGTCAATCAGGATCAACTTCGTGCGGAACACCGCATCGTTTCTAACGCTTCCTGTACCACGAATTGCATA
ATTCCCGTCATCAAATTGTTAGATGATGCGTACGGTATTGAGTCCGGCACTGTGACCACAATTCACTCCGCCATGCACGA
TCAACAGGTTATTGATGCATACCATCCTGACCTGCGTCGCACCCGGGCAGCCAGCCAGTCGATCATTCCGGTCGATACTA
AACTGGCCGCCGGTATCACACGATTTTTCCGCAATTTAACGATCGCTTTGAAGCGATTGCGGTACGTGTGCCAACCATA
AATGTGACGGCAATCGATTTAAGCGTGACGGTGAAGAAACCTGTAAAAGCCAATGAAGTCAACCTGTTGCTGCAAAAAGC
AGCACAAGGTGCATTTCATGGTATAGTTGACTATACGGAATTGCCGTTGGTCTCTGTAGATTTTAACCACGATCCGCACA
GTGCCATTGTCGATGGCACCCAAACCCGGGTCAGTGGCGCACACCTGATCAAAACGTTGGTCTGGTGCGATAACGAATGG
GGCTTTGCTAACCGAATGCTCGACACGACGTTAGCTATGGCTACTGTTGCTTTCAGGTAA
>Cluster_51155
ATGTCTATCGAATTTAAAAATAAGGTTTTAATGATTACGGGCGGAACAGGATCTTTCGGAAATACGGTTCTCAAACGATT
TCTAGAGACGGATATTAAAGAAATTGTCATTTTCTCAAGAGATGAAAAAAAACAGGAAGATATGCGAATCGCTTTGAATA
ATAAAAAACTAAAATTTGTTATTGGTGATACTCGCTGTTATGAAAGTATAAATGAAGCTATGAGAGGCGTAGATTATGTT
TTTCATGCTGCAGCCCTGAAACAAGTGCCATCGTGCGAATTCTATCCAATGGAGGCCTTAAAGACAAATGTCATTGGGAC
AGAAAACGTATTGAATGCTGCAATTAATAATGAAGTAAAAAAAGTTGTTTTGTTAAGTACAGACAAGGCTGTATATCCTA
TTAATGCAATGGGAATATCGAAAGCAATGGCGGAAAAGATTTTAGTTGCTAAATCACGGATCGTTGGAAGTAGTGGACCA
ATTATGTGTGCTACCAGATATGGTAATGTAATGGCGTCAAGAGGCTCCGTTATTCCTCTTTTTGTCGAAAAAATTAAGCA
AGGCAAACCATTAACCATTACAGATCCTAATATGACTCGGTTTTTAATGTCTTTAGAGGATTCTGTTGACTTGGTTCTCC
ATGCTTTTGCCTATGGACAGCAAGGTGACATTTTTGTTCAAAAAGCTCCTGCAAGTACCGTTAATGAATTATCTGTTGCG
CTATGTGATATTTTTAATTCGCAGTTGAATTATAAAATAATCGGGACCAGACACGGAGAAAAACTTTATGAAACACTAGT
ATCAAGAGAGGAAATGGCAAAAGCACAAGATATGGGGCGCTACTTTAGAATACCGGCAGATAATAGGGATTTAAATTATG
ATAAGTATATAATATCAGGAAAGCCGGAAACTAATAATATAGATGATTATACATCGCACAATACAACAAGATTAAATGTG
GAAGAAATTAAAGATCTATTGTTGACTTTAGATTACATAAAATATGAGATGAGTTATTGA
>Cluster_54453
ATGGCTGTACGAAAACTCACCACAGGAAAATGGCTTTGCGAATGTTACCCCGCCGGACGTAGTGGGCGTCGTGTGCGTAA
ACAATTCGCCACCAAAGGCGAAGCACTGGCTTTTGAGCGTCACACGATGGAAGAAACCGAAGCAAAGCCCTGGCTGGGTG
AATCAGTGGATCGTCGAACACTGAAAGACGTGGTTGAGCTATGGTTCAAACTACATGGTAAATCACTGACTGCTGGGCAG
CATGTCTATGACAAATTGCTGCTGATGGTTGACGCTCTGGGCAATCCCCTTGCAACTGATCTCACATCTAAAATGTTTGC
CCACTATCGAGATAAACGCCTGACAGGTGAGATCTACTTCAGCGAGAAATGGAAGAAAGGAGCAAGCCCGGTCACCATTA
ACCTGGAGCAAAGCTATCTAAGTAGTGTTTTTAGCGAACTATCCCGCCTGGGCGAATGGTCGTATCCGAACCCACTGGAG
AACATGCGAAAATTCACCATCGCAGAAAAAGAGATGGCATGGCTTACCCATGAGCAGATTGTTGAATTGCTGGCTGATTG
CAAACGTCAGGACCCAATTCTGGCACTGGTAGTTAAGATATGCTTAAGCACAGGCGCACGTTGGCGTGAAGCCGTAAATC
TTACCCGCTCACAGGTGACCAAATACCGAATTACCTTTGTAAGAACGAAGGGGAAGAAAAACAGAAGCATCCCTATCAGT
AAAGAGCTTTACGAAGAGATCATGGCGCTTGATGGGTTCAATTTCTTTACAGACTGCTATTTTCAATTTTTATCCGTGAT
GGAAAAAACGTCTATCGTGCTCCCTCGCGGTCAACTGACACACGTTCTGCGCCATACGTTTGCGGCGCACTTCATGATGT
CGGGTGGAAATATCCTGGCCTTACAAAAAATTCTCGGGCATCACGATATAAAAATGACTATGCGTTACGCACATCTGGCA
CCGGATCACCTGGAAACTGCATTACGGTTTAATCCGCTGGCAACACTACCAACATCAATAGCAAGTTTTTGA
>Cluster_45374
ATGATCGCTGAACTGTTTACAAATAATGCGCTTAATCTGGTCATTATTTTCGGTAGCTGCGCAGCATTGATTCTGATGAG
CTTTTGGTTTCGCCGCGGAAATCGTAAAAGAAAAGGTTTTTATTCCATGCGGTGCAATTTTTAATCTACACCATAATTA
TCAGTGCTGTTGGTAGCATTATTAATTATGTTATAGAAAACTATAAACTCAAATTTATCACCCCAGGCGTTATCGATTTT
ATCTGTACGTCCCTGATTGCGGTTATTCTGACGATTAAGCTATTTTTGCTGATTAATCAGTTTGAAAAACAGCAGATTAA
AAAAGGTCGCGATATCACCAGTGCGCGGATTATGTCGCGCATTATCAAAATCACCATTATTGTGGTGCTTGTTCTGCTTT
ATGGCGAACATTTCGGCATGAGCCTTTCTGGCTTGCTGACCTTTGGTGGTATTGGTGGTCTGGCTGTCGGTATGGCCGGT
AAAGATATTCTGAGTAATTTCTTTTCCGGGATCATGCTCTATTTCGACCGTCCTTTCAGTATTGGCGACTGGATCCGTTC
ACCGGACAGAAATATCGAAGGTACAGTAGCGGAAATTGGCTGGCGAATTACCAAAATTACGACCTTTGATAATCGTCCAT
TGTACGTACCGAACTCGCTGTTTTCGTCGATCAGCGTAGAAAACCCAGGACGAATGACCAACCGCCGCATTACCACGACC
ATTGGTTTACGTTATGAGGATGCGGCAAAAGTGGGCGTTATTGTCGAAGCCGTACGTGAGATGCTGAAAAATCACCCGGC
CATCGACCAGCGACAAACCTTACTGGTTTATTTCAACCAGTTTGCCGACTCGTCATTGAATATTATGGTTTATTGCTTTA
CCAAAACCACGGTATGGGCTGAATGGCTTGCTGCACAGCAAGACGTTTATTTGAAGATTATCGATATTGTGCAGTCACAC
GGCGCGGACTTTGCCTTCCCCAGCCAGACGCTGTATATGGATAACATTACACCGCAGGAACAGGGGCGCTAA
>Cluster_19907
ATGTCACTAACCAGACGGAGGTTTACACAGATTCTTGCGTCGACATTGTTCCTGCATCATCTGCCGTCCTTTGCACAATC
AGTCAAATTCTGGGCCTCACGGACGCTTCCCGAAGCCCAAAACATTACACGCATCGTCAGCGCAGGCGCGCCCGCCGATT
TACTATTGCTGGCTGTCGCGCCAGAAAAAATGGTCGGTTTTTCCTCTTTTGATTTTGCCCGTCAGGCATTAATTCCATTG
CCAGAGCACATTCGCCAGTTCCCCAGGCTGGGACGACTCGCCGGGCGCGCCAGCACACTCTCGCTGGAAGGGCTTATGGC
GTTACATCCCGATTTGGTCGTTGATTGCGGCAATACGGATGAAACCTGGATCTCCCAGGCACGGCAGGTTAGCGAACAGA
CACAAATACCCTGGTTATTGCTTAACGGGAAACTGGAACAATCAGCAGAACAGTTAACAACGCTTGGCAAAACGTTAGGC
GAAGAGCACCGCGCCGCAGAACAAGCCAATCTCGCCAGCCGCTTCGTTGGTGAAGCTCAGGCATTCGCCACCTCACCCGC
CGCTAATCTCAGCTTTTATGCTGCGCGCGGTCCTCGAGGGCTGGAAACGGGCTTACAGGGTTCGTTGCATACCGAGGCGG
CGGAATTATTAGGTTTGCACAACGTCGCGCAAATAGCCGATCGCCACGGTCTGACACAGGTTTCCATGGAAAATCTCCTG
CGCTGGCAGCCGGATATTATTCTGGTTCAGGAGGCCGTTACTGCAGATTTTATTCGTCGTGATCCTCTCTGGCAGGGCGT
GAAAGCGGTTGCGGAACAACGCATCTTATTTTTAAGTGGCCTGCCCTTTGGCTGGCTGGATGCCCCGCCGGGAATCAACC
GTCTTCTGGGATTACGCAGACTTCACGCCTGGCTGGATCCCGCCATCAATCGCCAGTTTAAAAGTGACATACAGCATTAC
GCCCAACTGTTCTGGCATTGTTCACTCAGTGACGCCGACTATCAAAAATTGGTGGCGAGCTAA
>Cluster_20966
ATGCGCGTACTGCTATTTTTACTTCTTTCCCTTTTCATGTTATCGGCATTTTCGGCTGATAACCTGTTGCGCTGGCATGA
TGCGCAGCATTACACGGTGCAAGCCTCTACGCCGCTTAAAGCCAAACGCGCATGGAAACTGTGCGCGCTTTATCCCAGCC
TGAAAGATTCATACTGGTTATCGTTGAACTATGGTATGCAGGAGGCTGCTCGCCGCTACGGTGTGGATTTAAAAGTGCTG
GAGGCAGGCGGCTACAGCCAGTTGGCTACGCAGAAAGCACAAATCGACCAGTGTAAACAGTGGGGCGCAGAGGCCATTTT
GCTCGGTAGTAGCACGACGTCATTTCCCGATCTGCAAAAGCAGGTAGCAAGTCTGCCGGTGATTGAACTGGTGAATGCTA
TTGATGCTCCCCAGGTGAAAAGCCGCGTTGGTGTGCCCTGGTTTCAGATGGGCTATCAACCAGGGCGATATCTGGTGCAA

TGGGCGCACGGTAACCCACTGAACGTGCTGTTGATGCCCGGCCCCGATAACGCCGGGGGCAGTAAGGAGATGGTAGAGGG
GTTTCGCGCAGCCATTGCCGGAAGCCCGGTACGTATTGTTGATATTGCGCTCGGTGATAACGATATTGAAATCCAGCGTA
ACCTGTTGCAGGAGATGCTGGAACGCCATCCAGAAATCGACGTCGTTGCCGGAACGGCCATTGCGGCAGAGGCGGCAATG
GGGGAAGGGCGCAACCTGAAAACGCCGCTTACCGTGGTGTCGTTTTATCTTTCACATCAGGTGTATCGTGGGTTGAAGCG
GGGAAGAGTGATTATGGCAGCCAGCGATCAAATGGTCTGGCAGGGGGAACTGGCGGTTGAGCAGGCTATCAGGCAATTAC
AGGGGCAATCGGTGTCTGATAATGTCAGCCCACCGATTTTAGTCATGACGCCGAAAAATGCCGACCGCGAACATATCCGC
CGCTCGCTGTCACCAGGGGGATTTCGTCCGGTTTATTTTTATCAGCACACATCAGCGGCTAAGAAATAA
>Cluster_59940
ATGAAAATGCGTAAAGGTATTATTTTAGCGGGTGGTTCTGGTACACGTCTTTATCCTGTGACTATGGCTGTCAGTAAACA
GCTATTACCTATTTATGATAAACCGATGATCTATTACCCGCTCTCTACACTGATGTTGGCGGGTATTCGCGATATTTTGA
TTATCAGTACACCTCAGGATACTCCTCGTTTTCAACAATTGCTGGGTGACGGTAGCCAGTGGGGCCTGAATCTTCAGTAC
AAAGTGCAACCTAGCCCAGATGGCCTCGCGCAGGCATTTATCATCGGTGAAGAGTTTATTGGTGGTGATGATTGTGCTTT
GGTTCTTGGTGATAATATCTTTTACGGTCACGATCTGCCGAAGCTAATGGAGGCCGCTGTTAACAAAGAAAGTGGTGCAA
CGGTATTTGCCTATCACGTTAATGATCCAGAACGCTATGGTGTCGTTGAGTTTGATAAAAACGGTACGGCAATCAGTCTG
GAAGAAAAACCGTTAGAACCAAAGAGTAATTACGCCGTTACAGGTCTGTACTTTTATGATAACGACGTGGTTCAGATGGC
GAAAAACTTGAAGCCGTCTGCACGTGGTGAGTTAGAAATTACAGATATTAACCGTATTTATCTTGAGCAGGGACGTCTGT
CTGTCGCGATGATGGGGCGTGGCTACGCGTGGCTGGACACGGGGACTCATCAGAGTCTGATAGAAGCAAGTAATTTTATT
GCGACAATTGAAGAGCGCCAGGGATTGAAGGTTTCCTGTCCTGAAGAGATTGCATTTCGTAAAGGTTTTATTGATGTTGA
GCAAGTAAGAAAATTAGCTGTACCACTAATAAAGAATAATTATGGGCAGTATCTTTATAAAATGACGAAGGATTCAAATT
AA
>Cluster_22321
ATGCATCCACGTTTTCAAACCGCTTTTGCCCAACTTGCGGATAACTTGCAATCTGCACTGGAACCTATTCTGGCAGACAA
GTACTTCCCCGCTTTGTTGACCGGGGAGCAAGTCTCATCGCTGAAGAGCGCAACGGGGCTGGACGAAGACGCGCTGGCAT
TCGCACTACTTCCGCTGGCGGCAGCCTGTGCGCGTACGCCATTGTCGAATTTTAATGTTGGCGCAATTGCGCGCGGTGTG
AGCGGAACCTGGTATTTTGGTGCCAATATGGAATTTATTGGTGCGACAATGCAGCAAACCGTTCATGCCGAACAAAGCGC
GATCAGCCACGCCTGGCTGAGTGGTGAAAAAGCGCTTGCAGCGATCACCGTTAACTACACGCCTTGTGGTCACTGCCGTC
AGTTTATGAATGAACTGAACAGCGGTCTGGATCTGCGTATTCATCTGCCGGGACGCGAGGCACACGCGCTGCGTGACTAT
CTACCGGATGCCTTTGGGCCGAAAGATCTGGAGATTAAAACGCTGCTGATGGACGAGCAGGATCACGGCTATGCGCTGAC
TGGTGATGCGCTTTCTCAGGCGGCGATTGCGGCGGCAAACCGTTCGCACATGCCTTACAGTAAGTCGCCAAGCGGTGTCG
CGCTGGAATGTAAAGACGGTCGTATTTTCAGCGGCAGTTACGCTGAAAACGCCGCATTCAACCCGACTCTGCCACCGTTG
CAGGGAGCGTTAATTCTATTGAATCTCAAGGGTTATGATTACCCGGATATCCAGCGCGCGGTTCTGGCAGAAAAAGCCGA
TGCGCCGTTGATTCAGTGGGATGCCACCTCCGCAACGCTGAAAGCTCTCGGCTGTCACAATATCGACCGCGTGCTTCTCG
CTTAA
>Cluster_5762
ATGAGCGACGACAATTCACACAGTAGTGACACGATAAGCAACAAGAAGGGATTTTTCTCCCTGTTACTCAGCCAACTTTT
CCACGGTGAACCGAAAAACCGTGACGAACTGCTGGCGCTGATCCGTGATTCCGGGCAGAACGACCTTATCGACGAAGATA
CGCGCGATATGCTCGAAGGGGTGATGGACATCGCAGACCAACGCGTTCGCGACATCATGATCCCCCGCTCCCAGATGATT
ACCCTGAAACGCAACCAGACGCTGGACGAATGTCTTGATGTCATCATCGAGTCCGCCCACTCACGTTTCCCGGTGATTAG
CGAAGACAAAGATCACATTGAAGGGATTCTGATGGCGAAAGACTTGCTGCCGTTTATGCGCAGCGATGCTGAAGCCTTCA
GCATGGACAAAGTGTTACGTCAGGCGGTTGTCGTTCCTGAAAGTAAGCGCGTAGACCGGATGCTGAAAGAGTTTCGCTCT
CAGCGTTACCACATGGCGATCGTTATTGACGAATTCGGTGGGGTTTCCGGTCTGGTGACCATTGAAGACATCCTGGAACT
GATTGTTGGTGAGATTGAAGACGAGTATGACGAAGAAGATGATATCGACTTCCGTCAGCTGAGTCGTCATACCTGGACCG
TACGCGCACTGGCTTCCATTGAAGACTTCAACGAAGCGTTCGGCACCCACTTTAGCGATGAAGAAGTCGACACTATCGGT
GGTCTGGTGATGCAGGCATTTGGGCATCTTCCGGCGCGTGGCGAAACTATCGACATCGACGGTTACCAGTTCAAAGTGGC
GATGGCCGACAGTCGGCGTATTATTCAGGTTCATGTCAAAATCCCGGATGACTCACCCCAGCCGAAGCTGGATGAATAA
>Cluster_29040
ATGACATCATCCGCCGGACAGCGCATCAGCTGTAACGTTGTGGAAACGCGCCGCAATGATGTGGCGCGCATTTTCAACAT
TCAGCGTTATTCACTGAATGACGGCGAGGGCATTCGTACGGTGGTCTTTTTTAAAGGCTGTCCACATCTTTGCCCGTGGT
GTGCTAATCCGGAGTCGATCTCCGGCAAAATCCAGACTGTACGCAGAGAGGCGAAATGTCTGCACTGTGCGAAATGTTTG
CGTGATGCGGATGAATGCCCCTCCGGTGCGTTTGAACGGATTGGTCGCGATATCAGCCTTGACGCTCTGGAACGGGAAGT
GATGAAAGATGACATTTTCTTTCGCACGTCCGGCGGCGGCGTCACGCTTTCTGGCGGCGAAGTGTTAATGCAGGCGGAGT
TTGCTACTCTTTTTTTACAGCGGCTGCGGTTGTGGGGTGTGTCATGCGCCATCGAAACTGCCGGAGACGCGCCAGCCAGC
AAGCTGTTACCGCTGGCGAAATTGTGCGATGAAGTGTTGTTCGATTTAAAAATTATGGACGCGACTCAGGCGCGGGATGT
GGTGAAGATGAACCTGCCACGCGTGCTGGAGAATCTGCGTTTGCTGGTGAGTGAGGGTGTCAACGTGATCCCGCGTTTAC
CGCTGATCCCCGGTTTCACGCTCAGTCGGGAGAATATGCAGCAGGCTCTGGACGTGCTGATCCCGCTGAATATCAGGCAG
ATCCATCTGTTACCGTTTCATCAGTACGGCGAACCGAAATACCGCCTGCTGGGGAAAACATGGTCGATGAAAGAGGTGCC
TGCGCCGTCGTCAGCTGATGTGGCAACGATGCGCGAAATGGCAGAACGGGCCGGATTTCAGGTTACCGTGGGAGGTTAA
>Cluster_31444
GTGCTTATCTTATTAGGTTACCTGGTTGTTCTCGGTACAGTTTTCGGCGGTTATTTGATGACCGGTGGAAGCCTTGGAGC
ACTCTATCAACCCGCTGAACTGGTGATTATTGCCGGTGCAGGGATTGGGTCGTTTATCGTCGGCAATAATGGAAAAGCGA
TTAAAGGCACGCTGAAGGCGCTGCCGTTGCTGTTTCGTCGCTCTAAATACACCAAAGCAATGTATATGGATCTGCTGGCT
CTGCTTTATCGGTTGATGGCGAAATCGCGGCAGATGGGGATGTTTTCGCTGGAACGCGATATTGAAAATCCCCGTGAGAG
CGAGATCTTCGCCAGCTACCCACGCATCCTTGCGGATAGCGTCATGCTTGAATTTATCGTCGATTATCTGCGCCTGATTA
TCAGCGGTCACATGAACACCTTCGAAATCGAAGCCCTGATGGATGAAGAGATTGAGACGCACGAAAGCGAGGCAGAAGTC
CCGGCGAACAGTCTGGCGCTGGTCGGGGATTCCCTTCCGGCGTTTGGTATTGTTGCGGCTGTAATGGGGGTCGTTCACGC
GTTAGGTTCAGCCGATCGTCCTGCCGCCGAGCTGGGGGCGCTTATCGCACATGCGATGGTGGGGACTTTCCTCGGCATTT
TATTGGCTTACGGATTTATTTCCCCATTAGCGAGTGTTTTACGTCAGAAAAGCGCCGAAACCAGCAAAATGATGCAGTGC
GTCAAAGTCACACTGCTATCTAATCTGAACGGTTACGCACCGCCTATCGCCGTTGAGTTTGGTCGCAAAACGCTCTATTC
CAGCGAACGTCCGTCGTTTATTGAGCTGGAAGAGCATGTGCGTGCGGTGAAAAATCCGCAACAACAGACGACAACCGAGG
AAGCATGA
>Cluster_23060
GTGAAACAGGATCTGGCACGCATCGAGCAGTTTCTTGATGCTCTGTGGCTGGAAAAAAATCTGGCTGAAAATACGTTGAA
CGCTTACCGTCGCGATCTGTCAATGATGGTGGAGTGGTTGCATCACCGCGGGTTGACGCTGGCGACGGCGCAAAGTGACG
ATTTGCAGGCATTACTGGCAGAACGGCTGGAGGGCGGGTATAAAGCCACCAGCTCAGCGCGTTTGCTGAGTGCAGTGCGC
CGATTGTTCCAGTATCTTTATCGCGAAAAGTTTCGTGAAGATGATCCCAGTGCGCATCTCGCTTCACCGAAATTGCCCCA
GCGTTTGCCAAAAGATTTAAGCGAAGCGCAGGTCGAACGTTTATTACAGGCACCATTAATTGATCAGCCACTGGAGCTAC
GCGATAAAGCCATGCTTGAAGTGTTGTATGCTACCGGACTGCGTGTCTCTGAACTGGTCGGGCTGACAATGAGTGATATC
AGCCTGCGTCAGGGCGTAGTGCGGGTCATTGGTAAAGGCAACAAAGAGCGCCTGGTGCCGTTAGGTGAAGAGGCGGTTTA
CTGGCTGGAAACCTATCTGGAACATGGGCGTCCGTGGCTGTTGAATGGTGTGTCAATTGACGTGTTGTTTCCCAGCCAGC

GTGCGCAGCAGATGACGCGACAGACCTTCTGGCACCGCATTAAACATTATGCCGTGCTGGCGGGTATCGACAGCGAAAAG
CTGTCACCGCATGTGTTGCGTCACGCTTTTGCCACTCACTTATTAAATCATGGTGCGGATTTGCGCGTGGTGCAGATGCT
ACTGGGCCACAGCGATCTCTCCACCACGCAAATTTATACGCATGTCGCTACCGAGCGTCTGCGGCAACTTCATCAACAGC
ATCACCCGCGGGCGTGA
>Cluster_8928
ATGTGCGATATCATTGATAACTGGTTTTCAGATCGCTTTGCAGAAGGCGCCACGACCAAAAATGTTAATACTATGGCTCC
ATTTCTAACCCTGGCTTATCGCTACGAAGACACTGGCAGACCGGAGTATTTGCCCTGGCTTGATAGTTGGGCTGAGTGGG
CTATGCATACTATGCCGCGTACCAACCATAACGGTATGCAGCATATTACCCTGGCGGAAGAGAACGATCAGCAAATGTGG
GATGACACGTTGATGATGACCGTCCTGCCGCTGGCGAAAATAGGGAAATTGCTGAACCGTAATGAATATGTTGAAGAAGC
TGTTTATCAGTTTCTGCTACACGTGCAAAATTTACAGGATAGAACAACGGGCCTGTGGTTTCATGGCTGGAGTTACGATG
GATGCCATAACTTTGCCAATGCCAGATGGGCTCGTGGTAACAGCTGGCTGACGATTGTGATCCCTGACTTTTTAGAATTG
CTTAATTTGCCGGAAAACAACTCGGTACGTCGGTATCTTGTTCAGGTATTACATACGCAAATAGAGGCGCTGGCAAAATG
CCAGGATGAGAGCGGGTTATGGCATACGTTGCTGGACGATCCAAATTCATATCTGGAATCATCGGCTACCGCAGGTTTTG
ACTATGGCATCCTGAAGGCGGTACGCAAGCATTATGTTGCCCCCCTGTATGCCGAGGTAGCCGAGAAGGCAATTCGCGGG
ATCGTGAAAAATATTTCTCCGGAGGGGGAGCTTTTGCAGACATCTTTCGGTACCGGGATGGGAGCAAATCTTGATTTTTA
TCGCGAGATCCCGCTGACATCCATGCCCTACGGTCAGGCAATGGCAGTGTTGTGTCTGACAGAATATCTGCGTAAATATT
TTTAA
>Cluster_8271
ATGTCTCATTTAAAAGACCCGACCACGCAGTATTACACTGGTGAATATCCCAAACAGAAACAACCGACGCCAGGCATCCA
GGCGAAGATGACACCGGTACCGGATTGCGGCGAGAAAACCTATGTTGGTAGCGGTCGCCTGAAAGATCGTAAAGCACTGG
TGACAGGGGGCGATTCCGGAATAGGTCGCGCTGCCGCCATCGCTTACGCGCGTGAAGGGGCTGACGTGGCGATCAGTTAT
CTTCCCGTGGAAGAAGAAGACGCTCAGGATGTGAAAAAGATCATTGAAGAATGCGGACGCAAAGCCGTTCTGCTGCCAGG
CGATTTAAGCGATGAGAAATTTGCCCGTTCGCTGGTTCACGAAGCGCACAAGGCGTTAGGCGGGCTGGATATTATGGCGC
TGGTCGCCGGGAAACAGGTTGCCATTCCGGATATTGCAGACCTCACCAGCGAACAGTTTCAAAAGACCTTTGCCATTAAC
GTTTTCGCGCTGTTCTGGCTAACCCAGGAAGCGATCCCCCTGCTACCGAAAGGTGCAAGTATCATCACCACTTCGTCAAT
CCAGGCATACCAGCCAAGTCCGCATTTACTGGACTATGCGGCTACGAAGGCGGCGATTCTGAACTACAGCCGTGGCTTGG
CAAAACAGGTCGCGGAGAAAGGTATTCGGGTGAATATTGTCGCGCCAGGCCCGATCTGGACAGCACTGCAAATTTCCGGC
GGACAAACGCAGGATAAGATCCCGCAGTTTGGTCAGCAAACGCCGATGAAACGTGCGGGGCAACCGGCGGAACTGGCCCC
TGTATATGTTTATCTGGCAAGTCAGGAGTCGAGCTACGTCACCGCAGAAGTGCACGGCGTGTGCGGCGGCGAGCATTTAG
GTTAA
>Cluster_4787
ATGGCTAAGTTCGCCGAGCATCTGCCGCTGTACCGTCAGTCGGAAATCTATGCCCGCCAGGGCGTGGAGCTGCACCGCAA
TACGATGGGGCGCTGGGTTGACATCATGGGAGAGCAGCTTCGCCCGCTGTATGATGAACTGAAGCACTATGTGCTGATGC
CGGGTAAAGTGCATGCCGATGACACGCCGGTAAATGTACTGGAGCCGGGTCAGGGTAAAACCCGTACCGGACGGCTGTGG
GTCTATGTTCGTGACGATCGCAACGCCGGTTCGACCATGCCGGCAGCGGTGTGGTTCTCATACTCTCCCGACCGCAAAGG
CATCCACCCACAGCAACATCTGGCGGACTACAGAGGTATCCTGCAGGCCGATGCATATGCGGGTTACAATGCTCTTTACG
AAAGCGGTCAGGCAACCGAAGCGGCTTGTATGGCACATGCCCGACGCAAGATCCACGATGTACATGTCCGCCATCCAACG
ACAGTAACGGGAGAAGCGCTCCGTCGTATCGGGGAACTGTACGCTATCGAGGCTGAGATCCGCGGCAGTCCGGCAGAAGA
GCGACTGGCGGTCAGAAAAGCCAGAACGGTACCGCTAATGCAGTCGTTGTATGAGTGGCTCCAGGGGCAGATGAACACGC
TGTCGCGCCACTCGGATACAGCGAAAGCGTTCACCTATCTGCTGAAGCAATGGGACGCTCTGAACGAATACTGCCGCAAT
GGCTGGGTGGAGATCGACAATAACCTGTGTGAAAACGCCCTCCGGGTAGTTGCACTGGGGCGGCGTAACTACCGGACGTG
GTTTCTGCCAGGTAAAGGCAACGGAACAAAAGAATCATGGTTTTTCCGGAACCGGCCACACCATGAATGA
>Cluster_30752
ATGAAAAAAGAATTGTTTGGACGTTGCCCGTTTGATCTTGACGAAGATGTAAAAAAAATAGCCGCGATAAATAAATATAT
CCATGCAAAATTTGAGGAGCATAAAAATAAAATAGACAACCTAAAGGGTGTGGAACAAAAATTGATGGTCTTACAGTACT
TTAATGTACTGAATGAACTTAGTAATCAGGCTATCTCTTCTTTGAATATAGGAGCCTTTTCAGCATCGGAAGTATTAGCT
AGGGTTATAATAGAACAGGCTGCCAATCAATTTTATATAGCAATAGATGATGGCGAAAATGCACAAGCATTACTCAAGGC
TAGTAAGAAATTAGTTCATAGTAATGGAAAACGGTGGCTGGAGTGTTTAAAATCCAAAGGAATGACTAATCCAGCAGCAA
ATGAAAGGATTCGCATGGGAAAAGAACTAACTGATCTATTTAATAGATTGTGGCCAAATACACCTGAATATCCAGGAACC
AGAAAACTTTTTGAATTAATTGGCTGGGAAACACACTATCATGCCCATTACGTTCCCCTTTGTGATTCAATACATACATT
CTCTGATGATATGGTAAATATAGTATCTCTATATAATGCAATCCAAAGTGATGAAAATACTGCTATCGAATTCATTCTTG
CAGTTAAACAAGAAAATAAACGGCTGGCAATTTATAATTTTGTAATTGCTACGGCCCTTCGGTGTGAGGCCTTAGTAAAT
GTATTTAACTCTTTAGGTTATCAGGATATTATAACAGAAATGACACCAACAATAGATACAGTCAACCAGACTATAATTCG
ATATGATGATTTTGATCATTCCAGAATTGATGATTGTCAATAA
>Cluster_39603
ATGAATAATTTACCGGTGGTTCGTTCGCCCTGGCGGATTGTGATTTTGCTGTTGGGCTTCACCTTTCTCTACGCGCCAAT
GCTGATGCTTGTTATCTATTCGTTTAACAGCTCGAAGTTAGTGACGGTGTGGGCCGGCTGGTCAACACGCTGGTATGGCG
AATTATTACGCGATGACGCAATGATGAGTGCGGTTGGTTTAAGTCTGACAATTGCGGCCTGTGCGGCGACGGCGGCGGCG
ATCCTCGGGACTATTGCGGCGGTCGTGCTGGTGCGCTTTGGCAGGTTTCGCGGATCAAATGGTTTTGCCTTCATGATCAC
CGCACCGCTGGTAATGCCAGATGTCATCACGGGTTTGTCGCTGTTGTTGTTATTCGTCGCGCTTGCTCATGCTATTGGCT
GGCCTGCGGACCGCGGTATGCTCACCATCTGGCTGGCGCATGTCACTTTCTGTACGGCTTATGTGGCAGTCGTTATTTCG
TCGCGTCTGCGGGAACTGGATCGCTCGATAGAAGAAGCCGCGATGGATCTCGGTGCGACGCCGCTGAAAGTGTTTTTTGT
CATTACGCTACCGATGATCATGCCCGCGATCATTTCTGGCTGGTTACTGGCTTTTACTTTGTCGCTTGATGATCTGGTGA
TCGCCAGCTTTGTTTCCGGGCCGGGAGCCACCACGTTACCGATGCTGGTCTTTTCCAGCGTGCGGATGGGGGTGAATCCG
GAAATCAACGCCCTGGCAACATTGATCCTCGGCGCGGTCGGAATTGTCGGATTTATCGCCTGGTATCTGATGGCGCGCGC
AGAAAAACAGCGGATACGCGACATCCAGCGTGCAAGACGTGGCTGA
>Cluster_25042
ATGAACATCGAAAAAAGCAGATTAATTTCTGAGGCCGCCCCTCATCTGAACGCCTCTCTGGGCACAATTAACGGTAATGA
ATTTGCCGCAATTGTCCCGGTTATTCCTGGTCACATCGGTGGGCGTGAAACCAATATTGTTAGCGCAAAAGCGTTACACA
AAGCGTTGGGCGTGGGAAAAGACTTCTCTACATGGATCACTGATCGCATCTCTGAATATGACTTCACCATTGGGCACGAT
TACTCAGTCCATAAAACAATTTCCCCAAATTTGGGGAAAAGCCCGAATGGCACGGCTTACAGCAAGATTAAGCAGTCTGG
CAGACCCGGCAAAGACTATCTGTTAAGTGTCGGAATGGCGAAAGAGCTGGCAATGATCGAACGCAATGATCAGGGTCGCG
CTATCCGTCGTTATTTCATCCAGTGCGAGGAAGAATTACAGCGTAGCGTGCCTGAAATCGCCGCCCGCTATCGTCGCCAG
CTAAAAGCCCGTATCAGTGCCGCAAACAACTTTAAGCCAATGTGCGATGCGCTGAATATGGCCCGTGCCGAGCAGGGGAA
AACGACGCAGCAACACCACTACACAAACGAGAGCAATATGATTTCTCGTATCGTTCTTGGAGGGCTAACTGCTAAGCAGT
GGGCGCGGATAAATGGCTATTCTGGCGAACCTCGCGACCATATGAACGCAGAACAGCTTGAGCACCTCTCATATCTCGAA
AGCACCAATATCACGTTAATTGATATGGGCATGGAATATGAGCAGCGCAAAGGAGAACTCACCCGCCTGTCGCAACGCTG
GCTCGCCAAGCGTCTGGAGGCGGTCCATGTTTAA
>Cluster_12684

ATGGCTAATGAAAAAAAGACATCCCGCAAAAAGTTTCGCGTGGCTGTCTCCGGATCAACTGTTGATGGCCGTGAAATCAG
TCCGGTGCATCTGCGTGAAGCCGCCGAGAACTTCAACCCGGATGTTTACGCTGCCCGCGTGAACGTTGAGCACTATCTCT
CGCCATGCCCGTCAAGCGAATTTTCCGCAATGGGCGATGTCACCGCACTGAGTACGGAAGACATTACGGAAGGTCCGCTG
GCCGGACGTACTGCGCTGTATGCAGAAATCGAACCGACCGAGCGCATGAAGCAGCTTGTCGCGGACGGCAAGAAAATCTA
TTCCAGTATCGAACTGCACCCGCAGTTCTCCGTTAACGGGCGCGCCTATCTGGTCGGGCTGGCGATGACCGACACCCCGG
CAAGCCTGGGCACTGAGCGCCTGAAATTCACGGCACAGCAACGTCAGGCGGTGATGACGTTCAACAGTGTCCAGGGTGAA
GCACCGCTCATTTCCGAAGCCATCGAGTCTGAAATCATCGAAATGGCAGAACAACGCCAGGAAGAAGGCACCCAGTGGTT
TAACCGCGTAATGGGGATTATTGGTCGTGGCCGCAAAGCGGATGACGCCAGTTTCTCCCGTATTCAGGAAGCGGTGGAAG
GCGTCGCAACGTCACAGGCCGACATTATCGACCGTTTTAATGTGCTGGAAACCCGCCATCAGCAGGACCGCCAGAAAATC
ACGTCACTGACCACAGAGCTGACAGCACTGAAGGAAAAACTGCGCACGCAGGACGGCGATCCGCAGAACCGCTTCACCGC
AACGGGCGCAGCCTCCGACCAGCTGGCTGACTTCTGA
>Cluster_38390
ATGAAAGACATTAGCCTTCCTCCCCCCCCGACGTCCGCGTCCTGTCTGACGGGGGCCATATCTGTAAATACTGAAGCTGT
ATTATCTCCCATGCAACACACTTCAGCCTTACATGTAAGATATTTTGCTTCCCTGTGCTCACAGAATCTCAAAGCTAATG
TACTGCTAAATAGTGATGACCACGAAGTACCTATACATCAGAAAAATCCTGCTGCAATAATGCAAAATATCGACTCTAAC
ATCAAACAGATGGCAACAGACTGGGGGATGTCGATTGAGGAGGTTGAGGTTATTATAGGGCGAGAGAAAGGCATTGTGGA
ACCCTCCTGCGGAGTTACCGCTAATGCCATTATGAAACTATTTCTGGACAAGGATGGCTTCAGTTACTGCTTTGAAAATG
AACAGACACTATCGCTCGAGCAGCTTCAGGAGCGCCTGTCCTGTATGCCTGAATGTAAGAGCTTTGTATTACGTGTTAAT
GATGGTGCGCTTGGTCATGCTTACATTGTCGATATTCCCAAAGGAGAAAACTCTTGTCGTCTTGCATTCTTGTATCAGTC
AGATTTAGGAGAGGGCGTCACCAGAAAGTTAAGATTTGAGGACTGGATGACGCATAAAGCATTGACTCCGATTTTGCTGG
ATGATATTTGCAATTACTTCTCCTGTATGTCTCAAAATAAGACAGATTTGGAGCAGATTGCAACGTTATTTGATATTGAT
GGAAATGTTAAAATGTTACGAAAGGAAAATATTCAATATCAAAAATATGATAATTTTAGTTTCCAGTTGTTTGAGTATGA
CACCGATAACATTGAAAAAAATATTGAGATAATAAAATCACTATGTGGTTAG
>Cluster_22242
ATGTACGTGGTATCGACAAAGCAGATGCTGAACAACGCGCAGCGCGGCGGTTATGCGGTTCCGGCATTCAATATTCACAA
TCTCGAAACGATGCAAGTGGTGGTGGAAACCGCTGCCAGCATGCATGCGCCGGTCATCATCGCCGGAACGCCCGGCACAT
TTACTCATGCCGGTACAGAAAATCTGATGGCGCTGGTCAGCGCGATGGCGAAGCAATATCACCATCCACTGGCGATTCAT
CTCGACCATCACACGAAATTTGACGATATCGCTCAGAAGGTTCGTTCTGGCGTGCGCTCAGTCATGATTGACGCCTCGCA
TTTGCCTTTTGCGCAAAATATTTCACGGGTCAAAGAGGTGGTGGATTTTTGCCATCGCTTTGATGTCAGCGTCGAAGCGG
AGCTGGGGCAACTTGGCGGCCAGGAAGATGATGTGCAAGTCAATGAAGCCGATGCTTTTTATACCAACCCCGTTCAGGCG
CGTGAATTTGCCGAGGCAACCGGGATTGATTCCCTGGCGGTCGCCATCGGCACGGCTCACGGTATGTATGCCAGAGCACC
GGCGCTTGATTTTTCTCGACTGGAGAACATTCGCCAGTGGGTGAATTTACCGCTGGTGCTGCATGGCGCGTCAGGATTAT
CGACTAAGGATATTCAGCAAACCATCAAACTGGGGATATGCAAAATCAACGTTGCAACGGAGCTGAAAAATGCCTTCTCG
CAGGCGTTAAAAAATTACCTGACCGAGCACCCCGAAGCGACCGATCCCCGTGATTATTTGCAGTCGGCTAAATCTGCAAT
GCGCGATGTGGTGAGCAAAGTGATTGCCGATTGTGGCTGCGAGGGCAGGGCATAA
>Cluster_56155
ATGAACGGCTTACTTCGTATCCGTCAGCGTTACCAGGGGCTTGCCCAGAGTGATAAAAAACTGGCGGATTATCTGCTGCT
ACAACCTGATACGGCGCGCCATTTAAGCTCTCAGCAACTGGCCAACGAAGCCGGAGTCAGTCAGTCCAGCGTCGTGAAGT
TCGCGCAAAAACTCGGCTATAAAGGTTTTCCGGCGCTTAAGTTGGCGTTGAGTGAAGCGCTGGCAAGCCAGCCGGAATCA
CCCTCCGTGCCCATTCATAACCAAATCCGCGGTGATGATCCGTTACGGCTGGTCGGCGAAAAACTGATTAAAGAAAATAC
TGCCGCTATGTACGCAACGCTAAACGTTAATAGTGAAGAGAAACTGCATGAATGCGTAACAATGTTGCGCTCTGCGCGGC
GGATAATTCTGACCGGTATTGGCGCTTCGGGTCTGGTGGCGCAAAACTTTGCCTGGAAGCTGATGAAGATTGGCTTCAAT
GCTGCCGCAGTGCGCGATATGCATGCGCTACTCGCAACAGTACAGGCGTCGTCCCCTGACGATCTGTTATTAGCCATTTC
CTACACCGGTGTACGACGCGAGTTAAACCTGGCGGCAGATGAGATGCTGCGAGTGGGCGGAAAAGTGCTGGCGATTACCG
GCTTTACTCCGAATGCCCTGCAACAGCGTGCTTCTCATTGCCTGTATACCATTGCCGAAGAACAAGCGACAAACAGTGCT
TCAATCTCTGCTTGTCACGCTCAGGGAATGTTAACGGATTTGCTGTTCATTGCGCTGATTCAGCAGGATCTGGAACTGGC
ACCAGAACGTATTCGTCATAGTGAAGCGCTGGTGAAAAAACTGGTCTGA
>Cluster_26956
ATGACTGTTCAAACAAGTAAAAACCCGCAGGTCGATATTGCTGAAGATAATGCATTCTTCCCTTCAGAATATTCGCTTAG
CCAATATACGAGTCCCGTCTCTGATCTTGATGGCGTGGACTATCCAAAACCGTATCGCGGTAAACATAAAATTCTGGTGA
TCGCCGCGGACGAACGTTATTTGCCGACCGATAACGGAAAACTGTTCTCGACCGGTAACCATCCGATTGAAACGTTGCTG
CCGTTGTATCATCTCCATGCTGCAGGTTTCGAATTCGAAGTGGCGACCATTTCCGGTCTGATGACCAAGTTTGAATACTG
GGCTATGCCGCACAAAGATGAAAAAGTAATGCCATTCTTTGAACAGCATAAATCGTTATTCCGCAATCCGAAGAAACTCG
CGGAGGTTGTCGCCAACCTCAACGCTGATAGCGAATATGCAGCAATCTTTGTTCCTGGTGGGCATGGCGCACTTATTGGT
TTACCAGAAAGCCAGGACGTGGCTGCCGCTTTACAGTGGGCAATCAAAAATGACCGTTTTGTTATCTCCCTTTGCCACGG
TCCGGCGGCTTTTCTGGCACTTCGCCGCAGCGATAACCCACTGAATGGTTATTCTATTTGCGCATTCCCAGACGCCGCAG
ACAAACAAACGCCAGATATTGGCTATATGCCGGGTCATCTCACCTGGTACTTCGGCGAAGAGCTGAAGAAAATGGGCATG
AACATCATTAATGACGACATCACCGGGCGAGTACATAAGGACCGTAAACTTCTCACCGGCGACAGTCCTTTCGCAGCGAA
TGCGTTGGGTAAACTGGCGGCGCAGGAAATGCTGGCAGCTTACGCGAGTTAA
>Cluster_8426
ATGAGTCGTCCTCGTCGTCGCGGTCGCGACATTAACGGCGTTTTGTTGCTGGATAAACCTCAGGGTATGTCCAGCAACGA
TGCGCTGCAAAAAGTGAAACGTATATATAACGCCAACCGTGCCGGGCATACCGGTGCGCTGGACCCGCTGGCGACCGGCA
TGTTGCCGATTTGCCTCGGGGAAGCGACGAAGTTTTCCCAGTATCTGCTGGACTCCGACAAACGCTATCGGGTCATTGCG
CGTCTTGGACAGCGTACCGATACTTCTGATGCCGACGGACAGATCGTTGAAGAACGTCCGGTAACCTTTAGCGCAGAGCA
GCTGGCAGCGGCACTGGATACTTTCCGTGGCGATATCGAACAGATCCCTTCGATGTATTCAGCACTGAAATATCAGGGCA
AAAAACTGTACGAATATGCGCGTCAGGGCATTGAAGTTCCGCGTGAAGCGCGTCCGATTACCGTTTATGAATTGCTGTTT
ATTCGCCATGAAGGCAATGAGCTGGAGCTGGAAATTCACTGCTCAAAAGGCACTTATATCCGCACCATCATTGATGACCT
GGGTGAAAAACTCGGCTGTGGCGCGCATGTTATTTACCTGCGCCGTCTGGCGGTAAGTAAATATCCGGTTGAACGGATGG
TGACCCTGGAGCACCTGCGTGAACTTGTTGAGCAAGCTGAACAGCAGGATATTCCAGCCGCGGAGTTACTTGATCCATTA
CTGATGCCAATGGACAGTCCAGCTTCGGACTATCCGGTGGTGAATCTTCCGTTAACGTCTTCTGTTTACTTCAAAAATGG
TAACCCGGTTCGTACATCTGGTGCGCCACTGGAAGGACTGGTTCGCGTCACGGAAGGTGAGAACGGCAAATTTATCGGTA
TGGGCGAAATTGACGATGAAGGCCGCGTTGCGCCTCGTCGCCTGGTGGTTGAATACCCGGCGTAA
>Cluster_25500
ATGAGTCCTGATAATATCGGTTCGGCTGTCAATCGCTTCTCAGGTTGGCCATCTAATGATCCTCTAAAAACAGATAACGA
ATCAATGACATCACCCGTGACTGTGCAGGTAATAAAGATGATTGCCGCCATCCTTGACCCATATCGGATAGTCGTTGGAA
TCGATACACTTTATACAACGCAGCGCGAAACATTTACCTCAGAGAGTCAACAGCTCATTGAATCAGCATTGCAAACACTT
CATGTGATAAATTTAAATAACCTGGAAACAAATCTCGATGAGGACGGTCACTATCTGGCTTTTGGATATAACGTTGACAC
TCCTGACAGAATAACCGTTTCTGCTTATAATGAAAAAAAGCCTGACTGCTATGGCCAATGTCTTTTAATACAGGATGACG
AAACAGACTTATTTAACCTGATAAAAGTTAAAATTTTTGAAGCCAGGCATAATTATGTCGCTCAATTGATGAGCGATCCG

GAATTTATGTTCAAATTTAGTTATAGCGCTCAACAAAATAGATTAGAACCTCATCTTCTGGTTCCCACTTATTTCCCTCT
AAAAACAAACGAACCGGTTACGCAAGAGGATGTACTGTTACTTTATCGTTTCTTTAAAATGGATGGTGATTTTAATATAT
TAACATCAGATGAATACATGAGTATCATGACACCTCTGATGAAGTGTGAGCGCAGCATCCACGACCACAACAGGTATGTG
ACGGGCAAAGATACCCTTCTTCTGGACTATCCTCCTTCTGGTAACCAGATATATTTCCATATTTTTCCTGACGAATCAGC
AACACTAGTCCTTTATTTTTCAAACAATGACATTGAATGTTTCGTCTTTGAAAGAGATATTCCGAGCCAGTATCGCTTTT
TCAAAATGTTTACCAATCTGGCACTGGTTATTGATCAGTTAACCGATGCCAATCAAATACTTTAA
>Cluster_33138
ATGAATACTATTGATAATACTCAAGTAACGATGGTTAATTCCGCTTCGGAGAGTACGACCGGCGCTTCCAGTGCAGTTGC
CGCATCTGCTTTATCAATTGATTCATCTCTGCTTACTGATGGTAAGGTTGATATTTGTAAGCTGATGCTGGAAATTCAAA
AACTCCTCGGCAAGATGGTGACTCTATTGCAGGATTACCAACAAAAACAATTGGCGCAAAGCTATCAGATTCAGCAGGCC
GTTTTTGAGAGCCAGAATAAAGCTATTGAGGAAAAAAAAAGCCGCGGCAACCGCTGCTTTGGTTGGCGGGATTATTTCATC
AGCATTGGGGATCTTAGGTTCTTTTGCAGCAATGAACAACGCGGCTAAAGGGGCTGGTGAGATTGCTGAAAAAGCAAGCT
CTGCATCTTCAAAGGCTGCTGGTGCGGCTTCTGAGGTTGCAAATAAAGCTCTGGTCAAGGCTACGGAAAGTGTTGCTGAT
GTCGCAGAGGAGGCATCCAGTGCGATGCAGAAAGCGATGGCCACAACAACGAAAGCAGCCAGCCGTGCATCTGGCGTTGC
AGATGATGTTGCGAAAGCCTCTGACGTTGCTGAAGATCTTGCAGACGCCGCCGAGAAGACAAGCAGAATCAATAAGTTGT
TGAATGCCGTAGATAAACTGACCAATACCACAGCATTTGTTGCCGTGACCAGTCTTGCTGAAGGTACGAAAACGTTGCCA
ACAACAATATCTGAGTCCGTCAAATCGACTCATGAGGTTAATGAACAACGTGCGAAGTCGCTGGAAAACTTCCAGCAGGG
GAATCTGGAGCTGTATAAACAAGACGTTCGCAGAACGCAGGATGATATCACGACCCGTCTGCGTGATATAACGTCCGCTG
TCCGCGATCTCCTTGAGGTCCAGAATCGTATGGGGCAATCGGGTCGCTTAGCTGGGTAA
>Cluster_27207
ATGCGCGAAAAGGATTATGTCGTAATTATAGGCTCAGCGAATATTGATGTCGCCGGATATTCACATGAATCATTAAATTA
TGCGGATTCAAATCCAGGTAAAATAAAATTTACGCCTGGTGGGGTAGGGCGCAATATTGCACAAAACCTGGCGTTGCTGG
GTAATAGAGCTTGGCTACTGAGCGCCGTAGGCAGTGATTTTTATGGCCAATCGCTGCTAACGCAAACCAATCAATCTGGC
GTTTATGTCGATAAATGCCTGATTGTGCCGGGAGAAAATACGTCGAGCTATTTATCATTACTCGATAATACCGGTGAAAT
GCTGGTTGCTATAAATGACATGAATATTAGCAACGCTATTACAGCTGAATATCTCGCACAGCACCGTGAATTTATTCAGA
GGGCAAAGGTCATTGTCGCGGACTGTAATATCAGTGAAGAGGCACTGGCATGGATTCTGGATAATGCTGCCAACGTACCC
GTATTTGTCGATCCGGTTTCAGCGTGGAAATGTGTCAAAGTGCGCGACCGTCTAAATCAAATCCACACTCTCAAGCCAAA
CCGCCTTGAAGCGGAAACCCTGAGTGGGATTGCGCTGTCAGGGCGTGAAGATGTGGCAAAAGTTGCTGCCTGGTTCCATC
AACATGGCCTGAACCGACTGGTATTGAGCATGGGCGGCGATGGCGTTTATTACAGCGATATCAGCGGTGAAAGTGGCTGG
TCTGCGCCGATCAAAACCAATGTTATTAATGTTACCGGAGCGGGCGATGCCATGATGGCGGGGCTTGCTTCGTGTTGGGT
AGACGGAATGCCGTTTGCCGAATCTGTTCGTTTCGCACAGGGATGTTCGTCAATGGCGCTCTCCTGTGAATACACCAATA
ACCCCGATTTATCGATTGCCAACGTTATATCGTTAGTGGAGAACGCAGAATGTCTGAATTAA
>Cluster_6676
ATGAATCCTTTGTTAACCGATTCCCGCCGTCAGGCGCTGCGTTCAGCTTCCCACTGGTATGCCGTGCTAAGCGGCGAGCG
CGTCAGCCCACAACAGGAAGCGCGCTGGCAACAGTGGTATGAACAGGATCAGGATAACCAGTGGGCCTGGCAGCAGGTTG
AAAACCTGCGCAACCAGCTTGGCGGTGTGCCTGGCGACGTTGCCAGCCGGGCGTTGCACGATACCCGCCTCACCCGCCGT
CACGTGATGAAAGGATTACTGCTGTTGCTCGGCGCTGGCGGAGGCTGGCAGCTCTGGCAGTCGGAAACCGGCGAAGGTCT
GCGGGCAGATTACCGCACCGCCAAAGGCACAGTCAGCCGTCAGCAACTGGAAGATGGCTCCCTGCTCACGCTGAATACCC
AAAGCGCGGCGGATGTGCGTTTTGATGCGCATCAGCGCACCGTCCGGCTCTGGTACGGTGAAATCGCCATTACTACCGCG
AAAGATGCCCTGCAACGCCCCTTCCGTGTCCTGACCCGTCAGGGCCAGCTCACTGCTTTAGGGACAGAATTTACCGTCCG
CCAGCAGGATAATTTCACGCAGCTTGACGTTCAGCAGCACGCTGTGGAAGTGCTTCTCGCCAGTGCCCCCGCGCAAAAAC
GCATCGTGAACGCTGGTGAAAGCCTGCAGTTCAGCGCCTCTGAGTTTGGCGCAGTGAAACCGCTGGATGACGAGAGTACA
AGCTGGACGAAGGACATCCTGAGCTTCAGCGATAAACCGCTGGGTGAGGTGATAGCCACGCTAACCCGTTACCGCAACGG
CGTGCTGCGCTGCGATCCCGCCGTTGCCGGGCTGCGCCTGAGCGGGACGTTCCCGCTGAAAAATACCGATGCGATCCTGA
ACGTTATCGCGCAAACGCTTCCCGTTAAAATTCAGTCTATTACGCGGTACTGGATAAACATTTCACCACTGTAA
>Cluster_10988
ATGAAAAATCGTCTTTCTCCCTGGAATCTGGGAGCCACGCTATACATGCCTGCAACACGGGAAGATATTGCTGATGCCGT
CCTGCACGGGAAAATCCCGGGCTTGCGCTCTCTGGTGATTTGCCTGGAGGATGCTGTCAGTGAAGCAGACATCCCCGTCG
CCCTCAAAAATCTGGAGCACCTGCTGCACGAGCTCAGTAACAGCATGAGTAGCCTGGGTAAAAATGACTGGCCTCTGGTG
TTCATTCGCCCCCGGCATGCCGAAATGGGCAGATGGTTAAAAGCGCATTATGATCTTTCCGCTGTCGACGGGTTTGTGCT
GCCTAAATTTACCCTCAGTTCGTTAGCTGAATGGTGGGATATCATGGTCGGGACTCACCTGTGCATGATGCCAACGCTGG
AAACAGAAGATGTCTTTGACGTGGTTCAGATGCGCGAGCTGGCTACCCGTCTGGAGGAACATCCCTGCCACGACCGCATT
ATTGCGCTTCGAATCGGCGGCAACGATCTTATGAACGTTGTGTCGCTTCGCCGTCCCCGGGACCTGACGCTGTATGACAG
CCCGATGGGCTACGTCATCAAAATGCTCGTTTCGGTCTTCGGCCCGCGTGATTTTGCCCTGACCGCGCCTGTATGTGAGC
ATATTGACGATCATGCCGTTATGGCCAGAGAGCTGGCTCTTGATATGGCACACGGGCTTGTTGGGAAAACGGCCATTCAC
CCGGGTCAGATAGAGGTCATTCAAAACGCGCTGATGGTCACTCAGGGTGAGCATTCTGACGCTCTGCGGATCCTGAACTC
TACCCAGGCCGTGTTTAAGTCGCAGGGAGCAATGTGTGAACCCGCCACACATCGCCGCTGGGCGGCTGGCATTCTGGACA
GGGCTCGTTTTATGGGTTACAGAACGAGCAAAGCGCTGATGGAATCAGATTACTTACCGTGACCCAGCATCATTAA
>Cluster_29690
ATGCCAGAGGTACAAACAGATCATCCAGAGACGGCGGAGTTAAGCAAGCCACAGCTACGCATGGTCGATCTCAACTTATT
AACCGTTTTCGATGCCGTGATGCAGGAGCAAAACATCACCCGTGCCGCTCATGCTCTGGGAATGTCGCAACCTGCGGTCA
GTAACGCTGTTGCACGCCTGAAGGTGATGTTTAATGACGAGCTTTTTGTTCGTTATGGCCGTGGTATTCAACCGACTGCT
CGCGCATTTCAACTTTTTGGCTCAGTTCGTCAGGCATTGCAACTAGTACAAAATGAATTGCCTGGTTCAGGTTTTGAACC
CGCGAGCAGTGAACGTGTATTTCATCTTTGTGTTTGCAGCCCGTTAGACAGCATTCTGACCTCGCAGATTTATAATCACA
TTGAGCAGATTGCGCCAAATATACATGTTATGTTCAAGTCTTCATTAAATCAGAACACTGAACATCAGCTGCGTTATCAG
GAAACGGAGTTTGTGATTAGTTATGAAGACTTCCATCGCCCTGAATTTACCAGTGTGCCATTATTTAAAGATGAAATGGT
GTTGGTAGCCAGCAAAAATCATCCAACAATTAAAGGCCCGTTACTGAAACATGATGTTTATAACGAACAACATGCGGCGG
TTTCGCTCGATCGTTTCGCATCATTTAGTCAAACCTGGTATGACACGGTAGATAAGCAAGCCAGTATCGCGTATCAGGGC
ATGGCAATGATGAGCGTACTTAGCGTGGTGTCGCAAACGCATCTGGTCGCTATTGCGCCGCGTTGGTTGGCTGAAGAGTT
CGCTGAATCCTTAGAGTTACAGGTATTACCGCTGCCGTTAAAACAAAACAGCAGAACCTGTTATCTCTCCTGGCATGAAG
CTGCCGGGCGCGATAAAGGCCATCAGTGGATGGAAGAGCAATTAGTCTCAATTTGCAAACGCTAA
>Cluster_56215
ATGATAACTGACATTCCAACAAAGACTGATTTTTATACAATGGCTGACCACATGGTAAATGAGGCTTGGGAAAAAAATTGC
CGATTTAGCTTATGATTACAGAGATATAAACCTTTGGAATAGTTATTGTAAAACGTCTGAATTCTATACAGAATCAGATA
TTAAAAATTTAGAGCATTATTGGTTATTTGCTAGACCAAAATTGATTACATCTTTTAATTTGATACTTCAATCTATTGAA
TTCAGACTAAAAGGACTTATCGTCGAAATATCACCTTATCTACTTATCACGAATGCAACTCGCAATACACCTAAACCTGA
TAGTGAAGGTAACATTTCATTTTCAGACTTCCACACATTAGACGCCCAAGATTTAATTAAAGTATATAATACTTTCGCCT
CTAAATCACTGCCTGATGACTTTATTACTTGGTTTAATAGTATGCGCATTCTAAGAAATCGTTTTATGCATACGGTTGAT
AAAAAAACAGATATAATGCCAGAGCTAATTTTCACATCAGTTGTACGTGCTCACAACTATCTAAATACTGAATCAGCACA

CTGGATATGGCATAGGTATAAATATAAAGCAATACATTCCTCTGGCGGCATTAATATAGGATTAGATGAAGACGAGGATG
AAGAATTTTCAGGTATTGTATGGGCTATGCTTCAAACTCATTATGAGTTCACTGCAGCCATTTCCGCATGTTCAAAGGAA
TCAGCAAAAAAATTCTTTGGCTACACTAAAAATGATTCAGGAGATACTAACTCAAAAGAAAGTTTCTATTGCAGAAAATG
TGTTTCTGTTATGGAAAAAAGCTGGAACTTTGATAGTAAACATCTTGACTCTGCTTTAGAAACGGTTCAGTACAACCGAA
GTAAGGGGGCTTACATCTGTGCATTTTGTTTAGATGAGCAGAAAACAAAACCCATAGATATATGGTAA
>Cluster_64054
ATGCGCCCTGAGGGGCGTAAAGGCAAACGCATTCGCAAAAAATTTAAAACGAAATCGGATGCAGTTTTATATGAGCGGTG
GGTGCTGGCGCAACAGCATAACAATGAGTGGAAAGGAAACTCTATTGATCGCCGTCCTCTGTCAGTGCTTATTGACTTGT
GGTGGAAATACCACGGCCAGCTAATGAAGTCAGGGCATAACACGCGCCTTAAATTGCTGCGCTTGAGTGAAGCAATGGAT
GACCCGTGCGTGCATAAACTTAATACAACGATGCTCACCGAGCTACGTGTGTCCAGGATAGAGCAGGGGATACAGCCCAG
CACCATAAATCGAGAGATTGGGGCGTTAAGCGCGATGTTTACCGCACTCATCTCATCCGGCCATTTTCTTAACGATAACC
CCGTTCAAGGCCTTAAAGGAATGAAGGTTAACGAGCGCGAAATGGGATATCTGAGTAAGTCTGAATGTGTTCAGTTGCTG
GATGCACTGGCTGAAAATCCCGATGAACGACTGGCTGTCGAAATCCTTCTGTCGACCGGGGCGCGATGGGGCGAGGTAGC
GGCACTGGAGCAGCGCCGTGTTCTTCATTGTCGAATCACTTTTTCAAAAACGAAGAACAGCAAAAACCGTACCGTTCCTA
TTTCTGAAAGCCTGTTTGAAAAGATCAAAAAACGGGGCGGGAAACTGGTGTTTCCGACGCTGGATTATTCATTGGTTCGC
GATGTCATCAAAACGGTCGCACCTGATGTTCCTGACGGCCAGGCTGTTCATGCGCTGCGCCACACCTTCGCCAGTCATTT
CATGATGAACGGCGGCAATATTCTGACGCTCCAGAAAATTCTGGGGCACGCAAAGATTCAGACAACGATGATTTATGCCC
ATCTTGCGCCGGATTACCTGCAGGATGCGGTGAGGTTTAATCCACTAGGAGGTGCTTAA
>Cluster_28997
ATGAATCAATCTTATGGACGGCTGGTCAGTCGGGCGGCGATTGCTGCGACGGCGATGGCTTCGCTGCTATTGCTGATTAA
AATTTTTGCATGGTGGTATACCGGGTCGGTGAGTATTCTCGCCCGCGCTGGTGGATTCGCTGGTGGATATCGGCGCGTCGT
TGACGAATTTACTGGTGGTGCGATATTCCCTGCAACCTGCCGACGATAATCACTCGTTTGGTCACGGTAAAGCTGAGTCT
CTCGCGGCGCTGGCGCAAAGTATGTTTATCTCCGGTTCGGCACTATTCCTGTTTTTGACGGGTATTCAACATCTGGTATC
TCCAACACCGATGACAGATCCAGGCGTCGGGGTTATCGTGACAATTGTGGCGCTAATTTGTACGATTATCCTTGTCTCGT
TTCAGCGTTGGGTGGTGCGCCGGACGCAAAGCCAGGCGGTGCGGGCTGATATGCTACATTACCAGTCTGATGTTATGATG
AACGGCGCAATTCTGCTGGCGCTGGGGTTGTCCTGGTACGGCTGGCATCGCGCCGATGCTCTGTTTGCATTGGGGATCGG
CATCTATATTTTATATAGCGCGTTACGCATGGGATATGAGGCGGTACAGTCATTACTGGATCGCGCATTGCCTGATGAGG
AACGGCAAGAAATTATTGATATCGTGGCTTCCTGGCCGGGTGTTAGCGGCGCTCACGATCTTCGCACGCGGCAGTCAGGG
CCGACCCGCTTTATTCAGATTCATTTGGAAATGGAAGACTCTCTGCCTTTGGTTCAGGCACATATGGTGGCGGATCAGGT
AGAGCAGGCTATTTTACGGCGTTTTCCGGGATCGGATGTGATTATCCATCAGGACCCCTGTTCCGTCGTACCCAGGGAGG
GTAAACGGTCTATGCTTTCATAA
>Cluster_45471
ATGTTGGCATTTATCCGTTTTCTTTTTGCAGGCCTGCTGCTGGTGATCAGCCACGCCTTTGCCGCCATGGTTCAGGACGA
ACACGGCACGTTTACACTCGAAAAAACGCCACAACGGATTGTGGTGCTGGAACTCTCGTTCGCCGATGCGCTGGCCGCCG
TGGACGTCAGCCCGATCGGTATTGCCGACGATAACGATGCAAAACGCATCCTGCCCGAAGTGCGTGCGCACCTGAAACCG
TGGCAGTCCGTCGGAACGCGCGCGCAGCCGAGCCTGGAAGCCATTGCCGCTCTGAAACCAGACCTGATCATTGCCGACAG
CAGTCGCCATGCGGGGGTTTACATCGCCTTGCAGCAAATCGCGCCGGTACTGCTGCTTAAGTCCCGCAACGAAACCTACG
CTGAAAATTTGCAATCTGCGGCTATCATCGGCGAAATGGTGGGTAAAAAGCGAGAGATGCAGGCACGTCTGGAACAACAT
AAAGAGAGGATGGCGCAGTGGGCCAGCCAGCTTCCCAAAGGGACACGCGTGGCCTTTGGCACATCACGCGAACAGCAATT
CAACCTGCATACTCAGGAGACCTGGACCGGCAGCGTGCTGGCCTCTCTGGGGCTGAACGTTCCCGCTGCGATGGCGGGTT
CGTCCATGCCGTCCATCGGCCTGGAGCAACTGCTGGCGGTCAATCCTGCCTGGCTGCTGGTTGCCCACTATCGCGAAGAG
AGCATTGTTAAACGCTGGCAACAAGATCCGCTCTGGCAGATGTTAACCGCCGCGCAGAAGCAGCAGGTTGCTTCGGTCGA
CAGTAACACCTGGGCGCGGATGCGCGGTATTTTTGCTGCAGAGCGTATTGCCGCTGACACGGTAAAAATCTTCCACCATC
AGCCGCTTACCGTTGTGAAATGA
>Cluster_60242
ATGCCGTGGGGCGACAACAGTACCGTTAGCTATAACGGCAACTATGGCAGTGGGACGGACAGCAGCCAGGTCGGTTATTT
TAGCCGTGTCGATGACGCGACTCACTATCAGTTGAACATCGGCACCAGTGACAAACACACCAGCGTTGATGGCTACTACA
GCCATGATGGTTCGCTGGCGCAGGTTGACCTCAGTGCGAACTACCATGAAGGGCAATACACCTCTGCGGGCTTGTCGTTA
CAGGGCGGCGCAACGCTTACTACCCACGGCGGCGCACTTCACCGTACCCAGAATATGGGCGGGACACGCCTGCTGATTGA
TGCCGATGGCGTTGCCGATGTTCCGGTGGAAGGTAACGGGGCTGCTGTTTATACCAATATGTTTGGTAAAGCCGTCGTTT
CTGACGTTAACAATTATTATCGCAATCAGGCGTATATCGACCTCAACAAATTGCCGGAAAACGCCGAAGCAACCCAGTCG
GTGGTGCAAGCCACGCTAACTGAAGGAGCCATTGGCTACCGCAAATTTGCCGTCATCAGTGGTCAAAAAGCGATGGCGGT
GCTGCGTTTACAAGATGGCAGCCATCCACCGTTTGGCGCAGAAGTAAAAAATGATAACGAGCAGACAGTGGGCCTTGTCG
ATGATGACGGTAGTGTCTATCTGGCTGGGGTGAAACCTGGCGAACATATGAGTGTGTTCTGGAGTGGTGTTGCGCATTGC
GATATCAACCTGCCGGACCCGCTGCCTGCCGATCTGTTTAACGGCTTGTTACTGCCATGCCAGCATAAAGGCAACGTCGC
GCCGGTTGTTCCTGATGACATAAAGCCTGTCATTCAGGAGCAGACGCAACAGGTGACACCCACCGATCCCCCTGTTTCTG
TTTCAGCTAACCAATAA
>Cluster_46092
ATGGCAGAATTTCCCGCCAGCTTACTGATTCTTAATGGCAAAAGTACTGACAATCTACCCTTGCGCGAAGCAATTATGCT
GTTGCGTGAGGAAGGAATGACGATCCATGTGCGGGTCACCTGGGAGAAAGGCGATGCCGCACGATATGTAGAGGAGGCCC
GGAAGTTGGGCGTCGCAACGGTGATTGCCGGTGGTGGCGATGGCACCATTAATGAAGTTTCTACGGCGTTGATTCAGTGT
GAGGGGGATGACATACCCGCGCTGGGAATTTTGCCATTAGGAACCGCCAATGATTTTGCCACCAGTGTAGGGATTCCTGA
GGCACTGGATAAGGCGCTGAAACTGGCAATTGCCGGTAACGCCATTGCGATAGATATGGCGCAGGTCAACAAACAAACCT
GTTTTATTAATATGGCGACAGGCGGATTTGGGACGCGTATTACCACAGAAACGCCGGAAAAATTAAAAGCCGCGCTGGGT
GGCGTCTCTTACATCATTCATGGCTTAATGCGCATGGATACTCTGCAACCGGACCGTTGTGAAATCCGCGGTGAAAACTT
TCACTGGCAAGGTGACGCCCTGGTCATTGGTATTGGTAACGGGCGTCAGGCCGGTGGCGGTCAGCAATTGTGTCCGAACG
CGTTAATTAACGATGGCTTGCTGCAACTGCGCATTTTTACCGGCGATGAAATTCTTCCGGCTCTCGTATCAACCTTAAAA
TCTGACGAAGATAACCCGAATATTATCGAAGGCGCTTCGTCGTGGTTTGATATACAAGCCCCACACGAAATCACTTTTAA
TCTTGATGGCGAACCGTTGAGCGGACAAAACTTTCATATTGAAATACTTCCGGCGGCGTTGCGTTGTCGATTACCACCAG
ATTGTCCATTATTGCGTTAA
>Cluster_42303
GTGAAAACACTAATAAGAAAATTCTCCCGTACGGCCATCACGGTCGTATTAGTCATTCTGGCCTTCATCGCAATTTTTAA
TGCCTGGGTCTATTACACCGAATCCCCGTGGACGCGTGACGCGCGCTTTAGCGCTGACGTCGTTGCGATCGCGCCGGACG
TTTCTGGACTCATTACCCAGGTGAATGTTCATGATAACCAGCTGGTGAAAAAAGGACAGGTACTGTTCACCATCGACCAG
CCGCGTTATCAAAAGGCGCTTGAGGAAGCGCAAGCCGATGTTGCTTATTATCAGGTACTGGCACAGGAGAAACGCCAGGA
GGCCGGACGTCGTAACCGTCTCGGTGTGCAGGCGATGTCTCGCGAAGAGATCGACCAGGCCAACAACGTACTACAAACAG
TTCTGCATCAGTTAGCGAAAGCGCAGGCGACCCGCGATCTGGCAAAACTGGATCTTGAACGTACAGTCATCCGCGCGCCA
GCAGATGGCTGGGTGACCAACCTCAACGTCTATACCGGTGAGTTTATTACTCGAGGATCAACGGCGGTTGCGCTGGTGAA
ACAGAACTCCTTCTATGTACTGGCCTATATGGAAGAAACTAAGCTGGAAGGGGTGCGCCCTGGGTATCGTGCGGAGATCA

CGCCGCTTGGCAGTAACAAAGTGCTGAAAGGGACTGTTGATAGTGTTGCCGCAGGGGTCACCAATGCCAGCAGCACGCGT
GACGACAAAGGGATGGCGACCATCGACTCTAACCTTGAATGGGTACGTTTGGCGCAACGTGTTCCGGTACGTATTCGTCT
CGACAACCAGCAAGAGAACATCTGGCCTGCGGGCACCACTGCTACGGTGGTAGTCACTGGCAAACAAGATCGCGACGAAA
GTCAGGATTCGTTCTTCCGTAAAATGGCTCATCGCCTGCGTGAGTTTGGTTAA
>Cluster_40219
ATGCAAGAAAATCAACAAATTACAAAGAAAGAACAATACAACCTGAACAAATTACAAAAACGTCTGCGTCGTAACGTGGG
CGAAGCCATTGCTGACTTCAATATGATTGAAGAAGGCGATCGCATCATGGTTTGCCTCTCCGGGGGTAAAGACAGCTATA
CCATGCTGGAGATTCTGCGCAATTTGCAGCAAAGCGCGCCAATCAATTTTTCGCTGGTGGCTGTTAACCTCGATCAAAAG
CAACCGGGCTTCCCGGAACACGTTCTGCCCGAGTATCTTGAAACGCTGGGCGTTGAGTACAAGATTGTTGAAGAGAACAC
TTACGGTATCGTGAAAGAGAAGATTCCGGAGGGCAAAACCACTTGCTCACTGTGTTCTCGTCTTCGTCGCGGTATCCTTT
ATCGCACCGCAACGGAACTGGGGGCGACGAAGATCGCGCTGGGTCACCATCGTGACGATATCCTGCAAACGCTGTTCTTA
AATATGTTCTACGGTGGCAAGATGAAGGTATGCCTCCGAAACTGATGAGCGATGATGGCAAACATATCGTGATTCGTCC
GCTGGCCTACTGCCGCGAGAAAGATATTCAGCGCTTTGCCGATGCAAAAGCGTTCCCGATTATTCCGTGCAACCTGTGCG
GTTCTCAGCCTAACCTGCAACGTCAGGTAATTGCTGACATGTTGCGTGACTGGGATAAACGTTATCCGGGGCGTATCGAG
ACGATGTTCAGCGCAATGCAGAATGTGGTGCCGTCGCATCTGTGCGATACCAACCTGTTCGATTTCAAAGGCATTACCCA
CGGTTCTGAAGTGGTAACGGGGGTGATCTGGCGTTTGATCGCGCAGAGATCCCACTACAACCAGCGGGCTGGCAGCCTG
AAGAAGATGAAAATCAGTTGGATGAGTTACGGCTGAATGTGGTTGAAGTGAAATAA
>Cluster_28914
ATGTTCATCTCAGACGAAACGCTTCGTGTTATTCATCTGGTCGCCAGACACCAGAGCATTACCACCGCAGCCGAGCAGTT
AAATAAAGTCCCTTCCGCCATCAGTTACACTATCAAGAAAGCGGAAGAGAGCCTGGGCGTTGAACTGTTCATTCGTAAGG
GACGCTATATTGAATTATCGCCAGCGGGAAGTTACTTCATCGAACATAGCAAAACTATTCTTGGCGATCTGGAAGCATTA
AAGCGCAACACAGTGCTGATTCACGATGGTATTGAGCGTGAACTGACAATTGCGGTAAACAATATTATTCCTGGTAATTT
GCTGGTGGCATTTATTCGAGATTTTGAACGGCAATTCACATCAACAACACTGACGGTAGATCTTGAGGTATACAACGGCT
GCTGGGATGCGCTGTACAGTAAGCGTGCTGATCTGGTCTACGGTGCTCCTCATGCAGTACCAAGCAGTGAAGGCATCATT
AGCGAACCGGTCGGACAAATGGAATGGGATTTTGTCGTCAGCCCACTCCACCCACTGGCAGCGAAGCGGCATCCATTAGA
AAACAGTGAATTGCGTCATTATCCAGCGGTTTGTATCCGCGATACCTCCGTTAACTTCCCCCCGATGCAAGCCTGGTTGT
TGGAAGGACAAAAACCCATTTTTGTTCCTGACTTCGCTACGGCAATCGCCTTAATAGAACAAAACGTCGGTATTGGTTAT
ATTCCACATCATCTGGCATTGCCGTTATTGAACAGCGGTAAATTGCTTAAAAAGCCAATGCGCGAACATAAACACGCCAC
CAAGTTATTTCTTGCCGCTCGTTCCGATGGTATGGGCAAAGCCTGTCAGTGGTGCATAGAATATTTAAGAAACCCGCAAT
TAATGACACGATTTGTATTTAATTGA
>Cluster_39674
ATGCCGATTATCGACCTGAACCAGCTACCCGCACCGGATGTGGTCGAGGAGCTGGACTTTGAAACCATTCTTGCCGAACG
CAAGGCGACACTGATTTCCCTTTACCCGGAAGACCAGCAGGAGGCGGTTGCCCGTACCCTGACGCTGGAATCCGAGCCTC
TCCTCAAACTGCTGGAGGAAAATGCTTATCGTGAGCTTATCTGGCGTCAGCGTGTGAATGAGGCCGCACGGGCGGTAATG
CTGGCCTGTGCCGCCGGTAATGACCTTGATGTGATTGGTGCCAATTACAACACCACGCGCCTGATTATCACCCCGGCAGA
TGATTCGACCATCCCGCCGACACCGGCAGTGATGGAGTCTGACACCGATTATCGTCTGCGTATTCAGCAGGCGTTTGAGG
GTTTAAGCGTCGCCGGGTCGGTGGGTGCCTATCAGTATCATGGTCGCAGTGCCGACGGGCGCGTCGCGGATATCTCTGTC
ACCAGTCCGTCTCCGGCCTGCGTCACCATCTCTGTGCTGTCACGTGAAAATAACGGCGTCGCATCCGAAGACCTGCTGGC
CGTGGTGCGTAACGCCCTTAATGGCGAGGACGTCAGACCGGTGGCCGACCGCGTGACCGTGCAGTCTGCCGCCATTGTTG
AATACCAGATAAACGCCACGCTTTACCTTTACCCTGGTCCCGAAAGCGAACCCATCCGCGCGGCCGCCGTGAAAAAACTG
GAAGCATATATCACGGCACAGCACCGGCTGGGGCGCGACATCCGTCTGTCTGCCATTTATGCCGCTTTGCATGTGGAAGG
TGTGCAGCGTGTCGAACTGGCTGCACCACTGGCCGACATCGTGCTCAACAGTACGCAGGCGTCTTTCTGTACCGAATACC
GCGTCGTGACCGGAGGCTCGGATGAGTGA
>Cluster_46066
ATGAAAAAAATTGCTATTATCGGTACTGTTGGCATACCAGCATCATATGGCGGATTTGAAACATTAGTTGAAAATTTAAC
AAGATACAATTCCTCGGGAGTTGAATATAATGTTTTTTGTTCATCGTTTCACTACAAATCCCCCCAAAAAAAACATAATG
GGGCTCGTTTAATTTATATTCCGCTTAAAGCCAATGGATGGCAGAGCATTGCGTATGACATAATTTCGTTAGCATATTCT
ATTTTTTTGAAGCCTGATGTGATTCTGATATTAGGGGTTTCTGGTTGTTCATTTTTGCCTTTCTTCAAACTCTTAACACG
CGCTAAGTTTATTACTAATATTGATGGCTTGGAATGGCGAAGAGATAAATGGAATTCAAAAGTGAAACGATTCTTAAAAT
TTTCAGAAAAAATCGCAGTTCAATATTCGGATGTCGTTATTACGGATAATGAGGCAATTTCTGAGTACGTTTTTAACGAG
TATAATAAAGATAGCCGAGTTATTGCCTATGGAGGGGATCATGCATGGTTAAATACTGAGGATGTATTTACAACAAGAAA
TTATAAAAGCGATTACTACCTTTCTGTATGTCGTATCGAACCCGAAAACAATGTAGAATTAATTTTAAAAACATTTTCAA
AGCTAAAATATAAAATAAAATTTATTGGAAATTGGAATGGCAGCGAGTTCGGAAAGAAACTTAGGCTGCATTATTCTAAC
TATCCAAATATTGAAATGATTGATCCGATTTATGATCTTCAACAATTATTTCACTTACGAAATAATTGCATGGGATATAT
ACATGGTCATTCGGCTGGAGGAACAAACCCTTCTTTAGTCGAGGCAATGCATTTTAGTAAACCTATATTTGCATATGATT
GTAAGTTTAATAGGTACACTACTGAAAATGAAGCATGTTATTTTTCTAATGAATCTGACCTCGCAGAGAAAATCATAATG
CATTGTGAGCTATCATTAGGTGTCTCTGGCACGAAAATGAAAGAAATTGCTAACCAGAAATACACTTGGAGACGAATAGC
AGAAATGTATGAGGATTGCTATTAA
>Cluster_49106
ATGTATCGGGATACCCGTCGTGAACATATTGTCCGGTTACTTCGCAACAAGGATGAGTGTAGTGTTAGCGATTTAGCTAA
CTATTTTTCTGTTACCAAAGAGACGATTCGGACAGATTTAGCGTGGTTGCAAAAGCGTGGTGTAGTGAGTCGTCATCATG
GAGGCGTCTCATTGAAAAAACATTTAATGCAAAATGCGCTTTCGCAACAAGAGTATGTCGATATGAGTTTGCTTATCAAG
CAACAACAGCAGGGTATTGGCTATTTAACCTCGCTTGATGATAAAGGACGCCGAATGGTAGGTAAAGTTTGTGTGTTAGG
ATCATTTAACGTCGATATTGTGGCGAAAGTGTATCGCTTCCCGAATGAAGGCGAAACGTTAGTGGCCAGGGAAACTACCA
TGGGGCCGGGGGGTAAAGGCGCGAATCAGGCACTAGCCGCTCATCGGGCAGGTGCGCAAATACACTTCGCCTGTAAGATT
GGTCGCGATCAGTTTAATCATTTTGCCCGCAATCATATTGAGTCCATTGGTATTGATTCATTTACTTTTTATGAGTCTGA
ACTATCTACCACCGGTTGTGCGGTCATTTATGTAAATGATGAAGGTGAAAACATGATTGCTATGTCTCCAGGGGCAAATC
ATGAATTAAGTGATAACGATATTATTCAACTTTCTCACTTTATAGCCGAGTCCGATGTATTCATTGTACAGATGGAAAAT
AATCTTGCCGCTACTCAACTGGCATTAAAATGTGCTCAAAAAATGCAGGTGACAACGATTTTGAATCCTGCACCGTGGTC
CAGTGATGTCGCCACATTATTACCTTTTGTTGATATTGTGACGCCAAATGAAACCGAAGCATCGGCGATGTCAGGTATGG
TAATTGACAATATTTCTGATGCTGTGAAGGCTGCGAAGCATATTTATAATGCCGGGCAATGTTCAGTAATTATTACGATG
GGAAAACGAGGTGCATTAATTTATGACGGGCAACAATGTGCGCATATTCCGGCATTTTCGGCAGTCGCTGTAGATACAAC
AGGTGCGGGTGATGCGTTTAATGGCGCGCTCGCCGCCGCACTGGCTAAGGGAGAATCACTTGTTAAAGCAGCCTGGTATG
CCAGCGCTTTCGCTTCGCTGGCAGTAGAAAAAGAAGGGGCAGCCAATATGCCTGATATCTCTCTGGTGGATGCCAGAATT
CAGCAGCAGAATGTTGCCATTCAGACTTTCTGA
>Cluster_60519
ATGGCAAGAAAAACCAAGCCGTTAACTGATACGGAAATCAAAGCCGCCAAACCTAAAGATGCCGATTACCAGCTTTATGA
CGGTGACGGGCTTACTCTGTTAATCAAGTCCAGTGGCAGTAAGCTTTGGCAATTCCGTTACTATCGGCCTTTGACCAAGC
AGCGAACCAAACAGAGCTTCGGTGCCTATCCTGCCGTCTCGCTTTCTGATGCACGTAAACTCAGAGCCGAATCTAAAGTT

TTATTGGCGAAAGACATTGATCCTCAGGAACATCAGAAAGAACAGGTGAGGAATTCTCAAGAGGCCAAAACCAATACCTT
CTTGTTAGTTGCCGAGCGTTGGTGGAATGTGAAGAAAACCAGCGTAACAGAGGACTATGCCGACGATATCTGGCGCTCGC
TTGAGAGAGATATTTTCCCGGCAATCGGTGATATCAGTATCACTGAGATTAAGGCTCATACTCTGGTTAAAGCAGTTCAG
CCGGTTCAGGCCAGAGGTGCATTAGAGACTGTTCGCCGCCTTTGTCAGCGTATTAACGAAGTCATGATTTATGCGCAGAA
CACAGGCCTGATTGATGCTGTTCCTAGTGTAAATATCGGAAAAGCTTTCGAGAAACCGCAAAAGAAAAACATGCCAAGCA
TCCGGCCGGATCAACTTCCGCAGCTAATGCACACCATGCGTACGGCAAGTATCAGCATGTCCACAAGATGCCTGTTCATG
TGGCAACTTCTAACCATCACCCGCCCTGCCGAAGCTGCTGAGGCTCGATGGGATGAGATCGATTTCAATGCTAGCGAATG
GAAAATTCCTGCAGCTCGAATGAAGATGAACCGGGACCATACGGTTCCACTATCTGATGGGGCTCTTGCTATTCTGGAAA
TGATGAAGCCTCTCAGTGGTGGCCGAGAATTTATCTTTCCTAGCCGTATCAAGCCCAACCAACCAATGAATAGCCAAACA
GTGAATGCAGCACTCAAGCGTGCTGGCTTAGGAGGTGTACTTGTTTCACACGGCTTGCGTTCTATCGCCAGTACGGCACT
CAATGAGGAAGGATTTCCACCTGATGTCATTGAAGCAGCGCTTGCTCATGTAGACAAAAATGAGGTGCGTCGCGCTTATA
ACCGCAGTGATTATCTTGAGCAACGTCGTCCGATGATGCAATGGTGGGCTGATCTCGTAAAAGCAGCAGATAGTGGTAGC
ATCGTTTTAACTCATTTGAGCAAAATTCGTCTTGTCGGATAA
>Cluster_64135
ATGTCTCAGCCAATTACGCGTGAAAACTTTGATGAATGGATGATACCTGTTTACGCTCCGGCACCCTTTATACCGGTACG
TGGCGAAGGTTCGCGCTTGTGGGATCAGCAGGGGAAAGAGTATATCGACTTCGCGGGTGGCATTGCGGTGAACGCGCTGG
GCCATGCGCATCCGGAACTGCGTGAAGCGCTGAACGAACAGGCGAGTAAGTTCTGGCATACCGGCAACGGTTACACCAAC
GAGCCGGTACTGCGACTGGCGAAAAAATTGATCGACGCCACGTTTGCCGATCGCGTCTTCTTTTGTAACTCCGGTGCGGA
AGCCAACGAAGCGGCGCTAAAACTGGCGCGTAAATTCGCTCACGACCGCTACGGCAGCCATAAGAGCGGCATCGTGGCGT
TCAAAAATGCGTTTCATGGTCGCACGCTGTTTACTGTCAGTGCGGGTGGGCAGCCAGCCTATTCACAGGATTTTGCGCCA
CTGCCGGCGGATATTCGTCATGCTGCATATAACGATATTAACTCTGCCAGCGCGCTGATTGACGACTCTACCTGTGCGGT
GATTGTCGAACCCATCCAGGGGGAAGGCGGTGTGGTGCCAGCCAGCAACGCGTTTTTACAAGGTCTGCGTGAATTGTGTA
ACCGCCACAATGCGCTGTTGATTTTTGATGAAGTACAAACCGGCGTCGGGCGCACCGGGGAACTGTATGCCTATATGCAC
TACGGCGTGACGCCTGATCTGTTAACTACCGCCAAAGCGCTGGGCGGCGGTTTCCCGGTCGGTGCGTTGTTGGCAACCGA
AGAGTGCGCCCGCGTGATGACCGTTGGCACTCATGGCACCACCTATGGCGGTAATCCGCTGGCCTCGGCGGTGGCAGGCA
AAGTGCTGGAGCTCATCAACACACCAGAGATGCTTAATGGCGTTAAACAGCGTCACGACTGGTTTGTTGAGCGTCTTAAT
ACTATTAATCACCGCTATGGTTTGTTCAGTGAAGTTCGCGGCTTAGGTTTGCTGATTGGCTGTGTACTGAATGCCGATTA
CGCCGGGCAAGCGAAACAGATCTCTCAGGAAGCGGCGAAAGCAGGCGTGATGGTACTGATTGCGGGTGGCAACGTGGTGC
GTTTTGCGCCTGCGCTCAATGTCAGCGAAGAAGAGGTGACGACCGGACTGGATCGCTTTGCAGCTGCTTGCGAACACTTT
GTTAGCCGAGGTTCATCATGA
>Cluster_11207
ATGAATGAACGTGAACTGTCCATTATCCGTGCGCTGGGTGAAGAATTTTCCGCAGTGCTGGCGGATTTACAGCGCACATT
TGAGGGGAAAATAGCTGCGCAGGCACAAACGTTTGAAGAAAAACTGGCGTCCCTGTCTGTGGTATTACAGAAGTGCGTGA
CGGGCGATGATATGCGTCCGATGCTTGAGCAGATGGTGAAGGAGGCGGTAAGCCATATCCCTGTTCCGCGTGACGGTCGT
GACTACGATCCGGAAGTACTGCAGAAGGCGGTGAATGATGCGGTCGCAAATATTCCACAGCCGGCGGACGGTAAAAGTCT
CACCCCGGATGATGTGCGTCCGATGCTTGAACAGATGGTGAAGGAGGCGGTAAACCATATTCCTGTTCCGCGTGACGGTC
GTGACTACGATCCGGATGTTCTGCAGAAGGCGGTGAATGATGCGGTCGCAAATATTCCACAGCCGGCGGATGGTAAAAGT
CTCACCCCGGATGATGTGCGTCCGATGCTTGAGCAGATGGTGAAAGAGGCCGTAAGCCATATCCCTGCTCCGCGTGACGG
TCGTGATTACGATCCGGAAGTACTGAAGCTGGCGGTGAATGATGCGGTCGGGAGAATTCCGGTACCGGCGGACGGCAAAA
GTATCACCCCCGATGATGTGCGTCCGATGCTTGAGCAGATGGTGAAAGAGGCTGTAAGCCATATCCCTGCTCCGCGTGAT
GGTCGTGACTACGATCCGGATGTTCTGCGGAAGGCGGTTCTGGAGGCGGTGAATGCCCTGCCGGCTCCGCAGGACGGGCG
TGATGCCACGGCTCTGGAAGTGCTCCCCGCCATTGACGATCAAAAATCCTTTCCCCGGGGCACGTATGCCACACACCTGG
GAGGATTCTGGCGGGCGTATGAAAAAACGCACGGGATGCGGGGATGGGAATGCCTGGTTGACGGGGTGGCGGATATTGAC
GTCAGTATGACGGGGGAGCGGTTGTTCTCTGTGGTGATCCGGCACAGCAGTGGCCAGCGTACGGAAAAAACATTTTCCCT
GCCGGTGATGCTCTACCGCGGTGTGTTCAGAGCCGGTGAAACCTACCACCCCGGCGATACGGTGACGTGGGGGGCTCGC
TGTGGCACTGCAACAGTATGACCGGTGATAAACCCGGAGAAGCTCATTCATCAGGCTGGACCCTGGCTGCAAAACGTGGA
CGGGATGCAGGAGGCGGAAAATGA
>Cluster_48404
ATGAAGCTCAACGCCAGACAGGTCGAGACTGCAAAGCCAAAAGACAAAACCTACAAAATGGCCGATGGCGGCGGTTTGTA
TCTCGAGGTTTCGACCAAGGGGTCTAAATACTGGCGCATGAAATACAGACGGCCCTCTGACAAAAAAGAGGATCGCCTCG
CTTTTGGTGTTTGGCCTACGGTGACGCTTGCTCAGGCAAGAGCTAAGCGCGATGAAGCTAAAAAGCTATTAGTACAGGGC
ATTGACCCAAAAGCCGAACAGAAAGAAGCTCAAGCCGAGAATTCAGGGGCGTATACTTTCGAAACTATTGCTCGCGAATG
GCACGCCAGCAACAAGCGATGGAGTGAAGACCATCGATCGCGCGTTCTTCGCTATCTTGAGCTTTATATCTTTCCTCATA
TCGGTTCGTCCGACATTCGCCAGCTCAAAACCAGTCACCTGTTAGCCCCGATTAAAAAAGTTGATGCAAGTGGTAAACAC
GATGTTGCTCAGCGCCTGCAACAGCGCGTCACGGCCATAATGCGTTACGCCGTACAAAACGATTACATCGACTCAAATCC
AGCCAGTGATATGGCCGGTGCGCTATCTACAACCAAAGCGCGACATTACCCCGCTTTACCCTCTAGCCGCTTCCCTGAGT
TTCTTGCTCGTCTTGCTGCATATCGTGGCCGCATAATGACACGGATCGCGGTCGAGCTTTCCTTACTGACCTTTGTGCGC
TCTAGTGAACTACGTTTTGCGCGCTGGGATGAATTCGACTTCGATAAATCTCTTTGGCGTATACCTGCAAAGCGTGAAGA
AATTAAAGGCGTTCGTTACTCGTATCGTGGCATGAAGATGAAAGAGGAGCATATAGTTCCTCTTAGTCGACAGGCGATGA
TTTTGTTAGAGCAGCTTAAGCAGATTAGTGGTGATAAAGAGCTACTTTTTCCAGGGGATCATGACGCAACAAAGGTTATG
AGTGAAAACACAGTGAACAGCGCTTTACGTGCAATGGGCTATGACACGAAAACCGAAGTCTGTGGGCATGGGTTTAGGAC
GATGGCGCGTGGTGCATTGGGTGAGTCAGGGTTATGGAGCGATGACGCGATAGAAAGGCAGCTGAGTCATTCAGAACGAA
ATAGTGTGCGTGCGGCATATATTCATACGTCTGAACACTTGGATGAACGTCGATTGATGGTTCAATGGTGGTCTGATTAT
ATAGATGCAACTAAGAAACAAATGATTACTCCTTATGAGTTTGCTAAAAAAACTTAA
>Cluster_24122
ATGACCACTCAACTGGAACAGGCCTGGGAGCTTGCGAAACAGCGTTTCGCGGCAGTAGGGATTGATGTCGAGGAGGCACT
GCGCCAACTTGATCGTTTACCCGTTTCAATGCACTGCTGGCAGGGCGATGATGTTTCCGGTTTTGAAAACCCGGAAGGTT
CGCTGACCGGTGGTATTCAGGCTACTGGTAATTATCCGGGCAAAGCGCGTAATGCCAGCGAGCTGCGTGCCGATCTGGAA
CAGGCTATGCGGCTGATTCCGGGGCCGAAACGGCTTAATTTACATGCCATCTATCTGGAATCAGACACGCCAGTCTCACG
TGACCAAATCACACCAGAGCACTTCAAAAACTGGGTTGAATGGGCGAAAGCCAATCAGCTCGGTCTGGATTTTAACCCCT
CCTGTTTTTCGCATCCGCTAAGCGCCGATGGCTTTACGCTTTCCCATGCCGACGACCGCATTCGCCAGTTCTGGATTGAC
CACTGTAAAGCCAGCCGCCGTGTTTCAGCCTATTTTGGTGAGCAACTCGGCACACCGTCGATGATGAACATCTGGATCCC
GGATGGCATGAAAGATATTACCGTTGACCGTCTCGCCCCACGCCAGCGTTTGCTGGCAGCACTGGATGAAGTGATCAGCG
AGAAGCTAAACCCTGCGCACCATATCGACGCCGTTGAGAGCAAATTGTTTGGCATTGGCGCGGAGAGCTACACGGTTGGC
TCCAATGAGTTTTACCTGGGGTATGCCACCAGCCGCCAGACGGCGCTGTGCCTGGACGCCGGGCATTTCCACCCGACTGA
AGTGATTTCCGACAAGATTTCCGCCGCCATGCTGTATGTGCCGCAGTTGCTGCTGCACGTCAGCCGTCCGGTTCGCTGGG
ACAGCGATCACGTAGTACTGCTGGATGATGAAACCCAGGCGATTGCCAGTGAGATTGTTCGTCACGATCTGTTCGACCGG
GTGCATATCGGCCTCGACTTCTTCGATGCCTCTATCAACCGCATTGCCGCGTGGGTCATTGGTACACGCAATATGAAAAA
AGCCCTGCTACGTGCGTTACTGGAACCTACCGCTGAACTACGCAAGCTGGAATCGGCGGGCGATTACACTGCGCGTCTGG

CGCTACTGGAAGAGCAGAAATCGTTGCCGTGGCAGGCGGTATGGGAAATGTATTGTCAGCGTCACGATACGCCAGCGGGT
AGCGAATGGCTGGAGAACGTGCGGGCTTATGAGAAAGAAATTTTGAGTCGCCGCGGGTAA
>Cluster_36272
ATGAACATTCAACCGATCGTACAATCCGGAATTACCACACAAAACAATCAACATCATCACACAGAACAAACATCCCCAAC
ACAAATACCGCAATCCGAATTACCTCATGGATGCGAAACGGGATTTGTTGTTAATATTCCAGAGGATATACAGCAACACA
TTCCGGAATGCGGTGAAACAACAGCTCTGTTGGGTTTAATAAGAGATAAAGGTCTACTCTCAGGGTTAGACGCATATCTT
GCTCCTCACCTTGAAGAAGGCTCGGCAGGAAAAAAAGCATTGGATATGTTTGGTTTATTCAATGTTACTCAGATGGCATT
AGAAATACCCAGCACCGTTCCTGGTATCTCTGGTAAATATGGTGTTCAGATGAACATCGTAAAACCAGATATTCACCCCA
CAACCGGGAATTATTTTTTACAGTTATTCCCCCTACATGATGAGGTGGGAATAAACTTCGGTGCGCTCCCAGGACCACTC
CAGAAGTTTCTCTCTCAAAATCCCAATCCATGGTTTGGAGTAACAGCTCAAGTTGTTCGTAACCACGGCATAGAACTACC
TATAGTCAAAACAGATAATGGATGGAAACTAGTTGGAGAAACACCACTTACACCTGAAGGGCCTAAAGCAAATTATACGG
AAGAATGGGTCATCAGACCTGGTGAGTTTGACTTAAAGTATGGCAGCACTCCGTTGCAGAGCACTTTAGGTTTAGAATTT
GGAGCTCATTTTAAATGGGATTTAGATAATCCTAATACCAAATATGCCATTCTTACCAATGCACTTGCGAATGCAGCTGG
TGCTGCAGGGGGATATACTCTATCTCAATATACAGGAACAGACGCAATGCTGAGCCCCCATGTTGGCTCGATGGCCGGAC
AGGCTTTGGGGCATGCTGTACAATGCTACACTCCAAGATTAAAGCCAGACACTATTTTATGGTGGGCAGGTACGACTCTG
GGGCTGGCAGATTTAAACAAAGCCGAGACAGGTGAAGGCAGATTCACTGACTATCCTCGTATATGGTGGCACGCAAGAGA
AGGTGCCATCTTCCCTAATAAAGAAGATATTGCCCGTGCAACAGGGGCTGATATACGAGCTATGGAAGAAGGTATCCCTG
TTGGACAGCGGCATCCAAATCCAGAGGATGTGGTAATCGATATCGAAAGCAATGGCTTACCACATCATAATCCATCAAAT
CATGTTGATATCTTTGATATAATCCAAGAAACAAGAGTCTAA
>Cluster_48647
ATGGCAATCCCGTCCTCGCTCAGTCTTGTGCAACTGCATTCTGGACAGATGAAAGTCTTCCAGTCTCCGCATCGATTTAA
AGTTGTTTGTGCTGGTCGACGCTGGGGAAAATCCCGGTTGTCGATCTCCACTATTATTCGTGCGGCGGCAAAGGAAAAAA
AGCAAAGGGTCTGGTATGTCGCTCCTACTTACCAGATGGCTCGCCAGATTTTGTGGGACGATCTACAGGAAGTTCTGCCT
CGTAAGTGGGTTAGGAAAAAGAACGACACCACGATGACAATCGTGTTGAAGAACGGTTCGGAGATCGCCCTCAAAGGTGC
TGATAAGCCTGACACTCTGCGCGGCGTAGCGTTGCATTTTGTAGTGCTTGATGAATTTCAGGATATGAAGGCTGACACCT
GGTACAAGGTGTTACGACCTACTCTTTCATCGACACGCGGCGGTGCACTTATCATTGGTACGCCAAAAGGTTTCTCGGAA
TTTCACAAACTGTGGACTATAGGCCAGAACGTAGAGCTGCAAAGAAAGGGACAGTGGAAGAGCTGGCAGTTTGTAACTGC
CGATTCTCCGTTTGTGCCTACGGCGGAAATTGAAGCTGCTAAGAACGATATGGACCCGAAATCGTTCGCTCAGGAGTACC
TGGCGAGCTTTGAGAACATGTCCGGGCGCGTTTACTACCCGTTCGATCGTAACGTGCATGTAAAACCGCTTCAGTTCAAC
CCTCGGTTGCCTATATGGGTAGGGCAGGACTTCAACATTGACCCGATGTCTTCAGTAATTTTGCAACCTCAGCCAAATGG
TGAGCTATGGGCAATTGATGAATTGGTGCTCTTTTCCTCTAACACGGCAGAGGTTTGTGATGAGCTTGAGAGACGCTTCT
GGCGCTGGAAATCACAGATAACGGTATTTCCAGATCCGGCAGGTGCTTATCGCCAACATGCTCGCGGGGAGTCTGACGTC
GACATATTCAAAGAGAAGGGATTCTTACGTGTCGATTATTCGAAAAAGCACCCGCCAATTGCGGATCGTGTTAATGCTGT
TAACCGAATGCTGATGACCGCATCTGGAGATATCCGGTTGTATATCGATCCGAAGTGCAAGCATTTGATTGATTCACTGG
AAAAAGTCATCTACAAGCCTGGAACACGAGATATGGATAAGACAGGTGGCATTGAGCATAGTGCAGACGCATTGGGCTAT
CCAGTACATCGTAGGTATCCAGTCAAAAACCGTGTTATTCTTGGTGGTTCTAGATAG
>Cluster_17498
ATGAAATATTTTTTCAATACCCGGCTGGGGGAAACCCGCTATCAGCTGGCTGACGGCTCGCTGCTGTGCAAAGACGTGCC
GATAGGTCGAACGGGTAAGCAGCTCTACGGCGCTGCCGATCTGCCAAACCTCAAACCCGACAAGCTCGGTGAGATAGTCG
TAACGCGTTCTCCTGAGCAGGTATTCCATCCGGCCACGCTCGCCTCATTCGAAGGGATGAGCATCACGATCCTGCATCCT
GAAGATGAAAACGGGAATGTGCGGCTGGTAAATCCCGAGAACTGGAAAGAGCTTGCTGTCGGGCATCTTCAGAATGTGCG
GCGCGGGACTGGTAACCAGTCTGATTTGATGCTGGCTGACCTTATCGTCAAAGACGAAAGCGCTATTCAGCTTATCGAAG
ATGGCCTGCGTGAAGTGTCGTGTGGCTATGACGCGGAGTACGAGCAGACCGAGCCAGGTAAAGCCGAGCAGGTCGATATT
ACCGGAAACCATGTGGCTCTTGTCCCTAAAGGCAGAGCCGGAAATCGTTGTGCAATTGGAGACAGAGACACAATGGCAAA
TCAAAAGAAAAGCTGGTGGACCCGCATGCGCACGGCCATCAAAACGGGTGACGCTGACACCATGAACGAACTGGTGGAGT
CGGCTCCCGCATCGGTTACAGGAGATGAGGGGGATTTGCCGCAAGGCGTTAATCTCAACATCAACCTGTCCCCGCAGCAA
CCGCTACCGGACAAAGCACCAGAGATGGGCGGAGGTCCAACCGGCGACAGTGATGATGACCTCAAAACATTACTGAAAGC
CCTGCTGGCTAAGCTGGAAGGAAATGCGACGGGCGATAACGACAATAAGCCTGACGATAATCCGACCGGTGACGGCGAGG
ACGATGAAGAGGAAACCACGATTACTGGTGACTCAGCCTGGCGTGCCGAAGTTATCGTTCCGGGTATCGATCTGAGCCGT
AAGATGAAACCGACCGCGTTCAAACGTGAGGTTCTGGCTTCTGCTGACAAAACGCTGGTTCGCCAGATAGTCGGTGATGC
GGATATCCGCAAATTACCGAAACAATCGGTCGACATGGCGTTTAATGCCGTGTCTGAAATTGCCAAAGGGCGAAACACCC
GCGCCACCACCGGCGATGCACAGCGCCTAAACATGGGCATGACCAGTATCGCTTCCCTGAACAAACAAAACGCTGAATTC
TGGGCAAACCGTAAAGGGTAA
>Cluster_28374
ATGCTGATACCCGGCGTACAGGCGGCAAAGCCGCAAAAAGTGACGCTGATGGTGGATGACGTTCCGGTAGCTCAGGTGTT
GCAGGCGCTGGCTGAACAGGAGAAGTTGAACCTGGTGGTTTCGCCAGACGTCAGCGGTACGGTGTCGTTACATTTAACTG
ACGTTCCCTGGAAGCAGGCACTACAAACTGTAGTGAAAAGCGCCGGATTGATAACGCGCCAGGAGGGCAACATTCTCTCG
GTGCATTCCGTTGCCTGGCAGAATGACAATATCGCCCGCCAGGAGGCGGAGCAGACGCGGGCGCAGGCAAATCTGCCGCT
GGAAAATCGCAATATTACTCTGCAATACGCCGACGCCGGAGAGCTGGCGAAAGCGGGGGAGAAGCTACTGAGTGCCAAAG
GGAGTATGACCGTCGATAAACGCACCAATCGCCTTTTGCTGCGAGATAACAAAACGGCGTTAAGCACGCTTGAACAGTGG
GTATCGCAAATGGATCTGCCGGTCGGGCAGGTTGAGCTGTCGGCGCATATTGTCACCATTAATGAAAAAAGTTTGCGTGA
GTTAGGTGTGAAATGGACGCTGGCCGATGCGCAACAAGCTGGTGGCGTTGGGCAAGTCACCACGCTTGGCAGCGACCTCT
CCGTAGCGACGGCGACAACGCATATCGGTTTTAACATTGGACGCATCAACGGACGTTTACTGGATCTTGAGCTTTCCGCG
CTCGAACAAAAACAGCAGCTGGACATTATCGCCAGTCCGCGTCTGTTGGCCTCACATCTTCAGCCTGCCAGCATTAAACA
GGGGAGCGAAATTCCATATCAGGTTTCCAGCGGGGAAAGTGGCGCGACGTCGGTGGAATTTAAAGAGGCCGTCCTGGGGA
TGGAAGTTACGCCCACGGTGTTACAAAAGGTCGTATCCGGCTGAAATTACACATCAGCCAGAACGTTCCGGGACAGGTG
CTACAGCAGGCAGATGGCGAAGTGCTGGCGATTGATAAGCAGGAGATCGAAACGCAGGTCGAGGTCAAAAGCGGAGAAAC
GTTGGCGCTGGGCGGCATTTTTACCCGTAAAAATAAATCGGGTCAGGATAGCGTACCGTTGCTTGGCGACATTCCCTGGT
TCGGGCAATTATTTCGTCATGACGGAAAAGAAGATGAACGACGCGAGTTAGTGGTGTTTATCACGCCACGACTGGTTTCC
AGTGAGTAA
>Cluster_53930
ATGAAACTTAATGCGCGACAGGTAGACGCAGCTAAACCCAGAGAGAAAGCCTATAAGCTGGCAGATGGTGCTGGTTTGTA
TCTTGAGGTTGTTCCCTCTGGTTCCAGATACTGGCGGATGAAATATCGCTTCAATGGAAAAGAGAAGCGTATGGCCTTTG
GTGTCTATCCGGCAGTGTCCCTTGCACAAGCGAGGGCACTGCGTGACGACGCCAAGAAAAAGCTTGCCGAAGGTATCGAT
CCATCGCTTGCCAAGAAAGAAGAAAAGCTGGTTCGAGATGTGCAGCTAAATAATACGTTTCAGGCTGTGGCACTTGAATG
GCATGGAACGAAGGTGAGCCGATGGTCAGAAGGTTATGCCTCGGACATTATCGAAGCCTTTAATAAAGATATTTTTCCCT
ATATTGGCCAACAACCGGTGAATGAAATCAAACCGCTGGTTCTGCTTAATGTGCTGCGTCGAATTGAAGCCGTGGCGCG
ACAGAGAAGGCCAAGAAGGTTCGCCAGCGTTGCAGCGAAGTCTTTCGTTACGCCATCGTAACCGGTCGTGCGGAATACAA
TCCTGCAGCGGATCTTACCAGCGCGATGTCAGGGCATGAATCGAAGCATTATCCCTTCCTTACTGTTGAGGAGTTACCAG

ACTTTTTTAAAGCTCTCGCAGGCTACACAGGAAGCCCGTTAGTTGTTCTTGCGGCACGTCTGCTGATCCTCACGGAGTT
CGCACTGGCGAACTCCGAGGTGCTTTCTGGAGTGAGTTTGATCTTGAAAAAGCGGTGTGGGAGATACCTGCCGAGCGTAT
GAAGATGAAACGGCCTCACCTTGTCCCCCTCTCTACCCAAGCGCTGGAAATCGTACAGCAACTCAAAGTGATGTCAGGGC
AATATCCACTGGTGTTCCCGGGACGTAATGATCCCCGCAAGACGATGAGTGAAGCGAGTATTAATCAGGTTTTTAAGCGG
ATTGGATATACGGGGAGGGTAACGGGGCATGGTTTCCGTCACACGATGAGTACGATTTTGCATGAGGAAGGTTTCAATAC
AGCGTGGATTGAAACCCAGCTGGCTCACGTTGATAAAAATGCGATTCGTGGGACGTATAACCATGCGTTGTATTTGGAAG
GACGTAGGGAGATGATGCAGTGGTATGCGGACTATATAAACGCTAATCATAATTCATGTATTTCTTTGCTGGTGAACGCT
ACAAAGTAG
>Cluster_21170
ATGGCCTTTTCTCAAGCGGTTAGCGGATTAAACGCTGCCGCCACCAACCTCGATGTTATTGGCAACAATATCGCCAACTC
CGCCACCTACGGCTTTAAATCAGGCACGGCCTCTTTTGCCGATATGTTTGCCGGTTCGAAAGTGGGACTGGGGGTAAAAG
TTGCTGGTATCACTCAGGACTTTACCGATGGCACGACCACCAACACCGGGCGTGGTCTGGACGTTGCTATCAGCCAGAAC
GGTTTTTTCCGTCTGGTAGACAGCAACGGTTCGGTGTTCTACAGCCGTAACGGACAATTTAAGCTGGATGAAAACCGTAA
CCTGGTGAATATGCAAGGTTTACAGCTGACGGGTTACCCGGCAACCGGTACGCCGCCGACTATTCAGCAAGGGGCGAATC
CGACCAATATTTCGATTCCGAATACCCTGATGGCAGCGAAAACTACCACCACGGCGTCGATGCAGATCAACCTGAATTCC
AGTGATCCGCTTCCCTCTGTTAACGCATTTGATGCCAGCAATGCGGATAGCTATAACAAAAAAGGTTCGGTGACTGTTTT
CGATAGTCAGGGTAATGCTCATGACATGAGCGTGTACTTTGTGAAGACCGGGGATAATAACTGGCAGGTCTACACCCAGG
ATAGCAGTGATCCAACAGGTACAGCTGAGCCTGCAATGACGCTGGTGTTTAATGCCAATGGCGTTCTGACCTCAGATCCA
ACAGCGAATATTTCCACCGGCGCAATTAACGGCGCAGATCCCGCCACGTTTAGCCTGAGTTTCCTCAACTCCATGCAGCA
AAATACCGGTGCTAACAACATTGTGGCAACCACCCAGAACGGCTACAAACCGGGCGATCTGGTGAGTTATCAAATCAATG
ATGACGGTACGGTTGTCGGCAACTATTCCAACGAACAAACCCAACTGCTGGGGCAGATTGTACTGGCGAACTTTGCCAAC
AACGAAGGTCTGGCATCCGAAGGCGACAACGTCTGGTCCCGCTACGCAATCTTCTGGCGTGGCGCTGTTAGGGACAGCCGG
GACGGGCAACTTTGGCACCCTGACCAACGGTGCGCTGGAAGCGTCTAACGTCGATCTCAGTAAAGAACTGGTCAATATGA
TCGTTGCTCAGCGTAACTATCAGTCTAACGCCCAGACCATCAAAACCCAGGACCAGATCCTCAATACGCTGGTTAACTTA
CGCTAA
>Cluster_28649
ATGTTTCATCTCGATACTTTAGCAACGCTTGTTGCCGCAACGCTGACGTTGCTGCTCGGGCGCAAGTTGGTCCATTCCGT
CTCCTTTTTGAAAAAATACACCATACCGGAACCTGTTGCGGGTGGTTTATTAGTGGCGCTGGCGCTGCTGATACTGAAAA
AAAGCATGGGCTGGGAAGTCAACTTTGATATGTCCCTGCGCGATCCGTTGATGCTGGCTTTCTTCGCCACCATTGGCCTG
AACGCCAACCTTGCCAGTCTGCGAGCTGGTGGGCGTGTGGTGGGGGTTTTCCTGATTGTGGTTGTTGGCCTGTTGGTGAT
GCAAAATGCCATTGGCATTGGTATGGCTAGCCTGTTAGGGCTTGATCCGCTGATGGGGCTGCTGGCCGGTTCTATTACGC
TTTCCGGCGGTCACGGTACGGGTGCTGCATGGAGTAAATTGTTCATTGAACGTTATGGCTTCACCAACGCGACAGAAGTG
GCGATGGCCTGTGCAACGTTCGGTTTGGTGCTGGGCGGCTTGATTGGCGGTCCGGTGGCGCGCTATCTGGTGAAACACTC
CACCACACCGAACGGTATTCCGGATGACCAGGAAGTTCCGACTGCGTTCGAAAAGCCGGATGTGGGCCGCATGATCACCT
CGCTGGTGCTGATTGAAACTATCGCGCTGATTGCTATCTGCCTGACAGTGGGGAAAATTGTTGCGCAACTTTTGGCTGGT
ACTGCTTTTGAACTACCGACCTTCGTCTGTGTACTATTTGTTGGCGTGATTCTGAGCAACGGTCTGTCAATGATGGGCTT
TTACCGCGTCTTTGAGCGAGCGGTATCCGTGCTGGGTAACGTAAGCCTGTCGTTGTTCCTGGCGATGGCGCTGATGGGGC
TGAAACTGTGGGAGCTGGCTTCGCTGGCGTTGCCGATGCTGGCGATTCTGGTGGTACAGACCATCTTCATGGCGTTGTAT
GCCATCTTCGTTACCTGGCGCATGATGGGCAAAAACTACGATGCGGCAGTGCTGGCTGCGGGTCACTGTGGTTTTGGCCT
CGGTGCAACGCCAACAGCAATCGCCAACATGCAGGCGATCACTGAACGCTTTGGCCCGTCGCACATGGCGTTCCTGGTCG
TGCCGATGGTCGGTGCGTTCTTTATCGATATCGTCAATGCGCTGGTGATTAAGCTGTATTTGATGTTGCCGATTTTTACG
GGTTAA
>Cluster_23977
ATGTGGTGGCCTTTTAGTCGTAAAAAAAGCGATCTGCGTAACCTGTCCATTGATGATTTTCTGGCGTTGTCTGGTGTACC
AAATACCGGATCCGGAGAATATGTTTCTGCCGGGACGGCTGAATCATTGCCTGCTGTGATGAACGCGGTTTCTGTCATCG
CTGAGGCGGTGGCCACGATGCCGTGTTACCTGTATCTGGTACGCAATGATAAGGGCAGAGAGGCGCGGGAATGGCTGGAC
AGTCACCCGGTAGATATTCTGCTGAATGAGCAGCCTAATTCGTGCCTGACACCTTACCAGTTTAAACGCACAATGATGCG
TCACTGCCTGCTGAACGGTAACGCCTATGCGGTTATTGAGTGGGGGCGGGACGGGCAGCCAAAATCACTTCATCCTTATG
CGCCGGGGTGTGTTGTACCGGAACGCACAGGCACACACAAATACCGCTATACCATCACCGAACCCTGTACAGGAACGGTG
CGCACGTATTTACAGGAAGAAGTTCTGCATCTCCGCTATGCCTCGGATGATGGCTTTCTGGGGCGTTCCCCCGTCACGAT
TTGCCGTGAGGCGCTGGGGCTTGGCCTTGCTCAACAGCGTCACGGAGCCAGCATTATGAAAGATGGCATGATGGCGGCAG
GGATTATCACGTCAGGTGAATGGCTGGACGGCGTGAAAGGTAAACAGGCATTAGACGCACTGGAACGCTACAAGGGGGCG
AAAAATGCCGGAAAAACGCCAATCCTTGAAGGGGGCATGGATTACAGGCAGCTGGGGATGAGTAACCAGGATGCGGAATG
GCTGGCCTCCCGTCGCTTCTCCATTGAAGACATCGCCAGGATGTTCAACGTGTCGCCGATTTTTCTTCAGGAATACAGCA
ACAGCACCTACAGCAATTTCAGTGAGGCGAGCCGCGCATTTCTGACCATGACAATGCGCCCGTGGCTGGCGAACTTCGAA
CAGCAAATCAAGGCCGCTTTGCTGGTGACTTCTCCCGTACCTGGTACCCGTTATCTGGTTGAGTTTGATTCAGCCGATTT
ACTGCGCGCCACACCCACCGAACGTTATGCCACGTATGAGAAAGGGATTAAGAGCGGGATCATGAATCCGAACGAAGCTC
GCGAACGCGAGGGTATGCCGCCGCGTGAAGGTGGTGATGAGTTCAGCCAGGCATGGAAACAGACTGTGGAAATTAAAGGG
AGAAAAGATGAGTGA
>Cluster_41303
ATGTCCCCCATTGAAAAATCCAGCAAATTAGAGAATGTCTGTTATGACATCCGTGGTCCGGTGCTGAAAGAAGCAAAACG
CCTGGAAGAAGAAGGTAACAAGGTACTGAAACTGAACATCGGCAACCCTGCCCCGTTCGGTTTTGACGCGCCAGATGAAA
TTCTCGTTGACGTGATACGCAACCTGCCTACCGCTCAAGGGTATTGCGATTCCAAAGGTCTTTACTCCGCGCGTAAAGCC
ATCATGCAGCACTATCAGGCTCGTGGCATGCGTGATGTTACCGTGGAAGATATTTACATCGGCAATGGTGTATCGGAGCT
TATCGTTCAGGCAATGCAGGCATTGCTGAACAGCGGGGACGAAATGTTGGTTCCTGCACCTGATTACCCACTATGGACCG
CGGCGGTTTCGCTCTCCAGCGGTAAAGCGGTGCATTATCTTTGCGATGAATCCTCTGACTGGTTCCCGGACCTCGATGAT
ATTCGCGCTAAAATTACGCCTCGTACGCGTGGGATCGTTATTATCAACCCAAATAACCCAACCGGCGCGGTATATTCCAA
AGAGCTTTTAATGGAGATTGTGGAGATTGCACGTCAGCATAATCTCATTATCTTCGCCGATGAAATTTATGACAAAATTC
TCTACGACGACGCTGAGCATCACTCCATTGCACCGCTGGCACCTGACCTGCTGACCATTACCTTTAACGGACTGTCGAAA
ACGTACCGCGTTGCAGGCTTCCGTCAGGGGTGGATGGTGTTGAACGGGCCGAAAAAACACGCCAAAGGTTACATCGAAGG
TCTGGAAATGCTGGCTTCAATGCGCCTGTGTGCTAACGTTCCTGCACAACACGCCATTCAGACCGCACTGGGTGGTTATC
AGAGCATCAGTGAATTTATTACCCCTGGCGGTCGTCTTTATGAGCAGCGTAACCGCGCGTGGGAATTGATCAACGATATT
CCGGGCGTTTCCTGCGTGAAACCTCGTGGTGCGCTGTATATGTTCCCGAAAATCGACGCCAAACGCTTTAACATTCACGA
CGATCAGAAAATGGTGCTGGATTTCCTGTTGCAGGAAAAAGTCCTGTTGGTGCAAGGGACGGCATTCAACTGGCCGTGGC
CGGATCACTTCCGCATTGTCACGCTACCGCGTGTCGATGATATCGAGCTGTCTTTGAGCAAGTTCGCGCGTTTCCTTTCT
GGTTATCATCAGCTGTAA
>Cluster_34185
ATGACGACCCGTACCTCCGCATCTTACCGCCTGACGTTGACCGTTGGGCTTTGCTTTTTAGTTGCCCTGATGGAAGGGCT
GGATTTACAGGCCGCCGGTATCGCTGCCGTCGGTATCGCCCACGCGTTTGCGCTGGATAAAATGCAGATGGGCTGGATCT

TCAGTGCGGGCATTCTCGGGCTGCTGCCCGGCGCGCTGGTGGGCGGCATGCTGGCCGACCGCTATGGCCGCAAGCGTATT
CTCATTGCCTCTGTGGCGCTGTTCGGCCTGTTCTCGCTGGCGACGGCGATTTCCTGGGATTTCTCATCGCTGGTGTTTGC
CCGTCTGATGACCGGCGTCGGGCTGGGCGCGGCGTTGCCGAATCTGATCGCGCTGACTTCCGAGGCGGCGGGCCCGCGTT
TTCGCGGTACGGCGGTGAGCCTGATGTACTGCGGTGTGCCGATTGGCGCGGCGCTGGCGGCAGCGCTCGGGTTTAGCGGC
CTGGCCTCCGCCTGGCAAACGGTGTTCTGGGTAGGCGGGGTGATCCCGCTGCTGCTGGTGCCGCTGTTGATGCGCTGGCT
GCCGGAATCTCAGGTTTATACCCAACAAACGCAGGTGGCCGCCGCACCGTTGCGCGCCCTGTTTGCGCCAGCGACGGCGA
GTGCCACGCTGCTGCTGTGGTTGTGCTATTTCTTCATCCTGCTGGTGGTGTATATGCTGATCAACTGGCTGCCGATGCTG
CTGGTGGAGCAGGGCTTTCGGCCATCGCAGGCGGCAGGCGTAATGTTTGCTCTGCAAATTGGCGCGGCCAGCGGCACGTT
AATTCTCGGGGCGTTAATGGATAAACTGCGCCCGGTGGTGATGTCGCTGCTGATCTACACCGGGATGTTGGCTTCACTGC
TGGCGCTGGGTACGGTGTCGTCATTGAGCGGCATGCTAATGGCGGGATTTGTGGCGGGGATGTTCGCCACCGGCGGGCAA
AGTGTGTTGTATGCGCTGGCGCCGCTGTTTTACAGCACGCAGATCCGCGCCACAGGCGTGGGAACCGCCGTCGCAGTCGG
GCGTCTGGGGGCGATGAGCGGTCCGCTGCTGGCAGGGAAAATGTTAGCGCTCGGTACCGGAACGGTAGGGGTGATGGCGG
CTTCTGCGCCGGGCATTTTGCTGGCAGGCCTGGCGGTATTTATTCTGATGAGCCGCAAATCACAGATGCCGATCCCGGCC
AGCGGTGCTTAA
>Cluster_36172
ATGAAAAGAAAAGTTCTGGCAATGCTGGTCCCGGCGTTATTAGTTGCTGGCGCAGCAAATGCGGCTGAAATCTATAATAA
AGATGGTAATAAACTGGATTTGTACGGAAAAGTAGCGGGCCTGCACTACTTCTCTGATGATGCCGGCAGCGATGGCGACA
AGTCATATGCCCGTATCGGTTTCAAAGGTGAAACTCAGATCGCTGACCAGTTCACTGGTTATGGTCAGTGGGAATACAAC
CTGCAAGCAAATGGCACAGAAGGCGATGGAGATAACTCTGCTACCCGTCTGGCTTTCGCAGGTTTAGGTTTTGGTCAGAA
TGGTACTTTCGATTATGGTCGTAACTACGGTGTCGTATATGACATCGAAGCATGGACCGATATGCTGCCAGAATTTGGTG
GCGATACCTATGCTGGTGCCGACAATTTCATGAACGGTCGTACCAACGGTGTTGCAACCTACCGTAACAATGGCTTCTTT
GGTCAGGTTGATGGCTTGAACTTTGCGCTGCAATATCAGAGTAATAATGAAAGTGGCAGTGACAGTCTCTTTGGCCAAGA
AGGATCTGGCAGCGGAGATGGCCGTAGCCTTGCTAAAGAAAATGGCGATGGTTTCGGTATGTCTACTTCCTACGACTTCG
ACTTCGGTTTAAGTCTGGGTGCTGCTTACTCTAATTCTGACCGTTCCAATAACCAAGCTGCTCGTGGGTATGGAGATGGC
AATCATTTCTATGGTAATAGCTATGCAGGTGGTGGTACTGCAGAAGCGTGGACTGTTGGTGCTAAATACGATGCCAACAA
TGTATATTTAGCAGCAATGTATGCAGAGACTCGCAATATGACCGCTTATGGTAGCAGCGACTCTCATTCAGCCGATGGGA
AACTTGGCGGCGGTGGTATCGCTAACAAAACTCAGAACTTTGAAGTTGTTGCTCAGTACCAATTTGATTTCGGCCTGCGT
CCTTCAATTGCTTACCTCCAATCTAAAGGTAAGGATTTAGGTGGTCAAGAAATGTACAACAATGGTAATTATCATTATAC
CAATAAAGATCTGGTTAAATACGTTGAGGTTGGTGCAACCTATTACTTTAACAAAAACATGTCCACCTATGTTGATTACA
AAATCAACCTGCTGGACAACGACGATGACTTCTACGCAAACAACGGCATCGCTACCGATGACATCGTTGCTGTGGGCTTA
GTCTACCAGTTCTAA
>Cluster_34406
ATGTTCAATTTTGCAGTCAGCCGCGAAAGCCTGTTATCAGGATTTCAGTGGTTTTTCTTTATTTTTTGCAACACGGTTGT
GGTTCCTCCTACGCTACTTTCTGCTTTTCAGTTGCCGCAAAGTAGCCTGCTTACGCTCACGCAATATGCTTTTCTTGCTA
CCGCACTGGCCTGCTTCGCTCAGGCGTTTTGCGGTCATCGTCGCGCTATTATGGAAGGGCCAGGTGGCCTGTGGTGGGGA
ACCATCCTTACTATCACCCTTGGTGAAGCATCGCGCGGGACACCGATCAACGATATCGCCACCAGCCTGGCAGTGGGGAT
TGCACTCTCCGGCGTGCTGACGATGTTGATTGGTTTTAGCGGATTAGGCCATCGCCTGGCACGGTTATTTACGCCGTCGG
TGATGGTCTTGTTTATGTTGATGCTGGGCGCGCAGCTGACCACTATCTTTTTCAAAGGTATGCTCGGGCTGCCGTTTGGC
ATAGCCGACCCGAATTTTAAAATTCAGTTACCGCCGTTCGCGCTCTCGGTGGCGGTGATGTGCCTGGTACTGGCGATGAT
TATCTTCCTGCCGCAACGTTTTGCCCGTTATGGCCTGCTGGTCGGCACCATAACCGGCTGGTTGTTGTGGTACTTTTGCT
TTCCTTCTTCGCACTCGCTCTCCGGTGAGTTGCACTGGCAGTGGTTCCCGCTCGGCAGTGGCGGTGCTTTGTCGCCGGGA
ATTATTCTGACGGCGGTGATTACAGGTCTGGTAAATATCAGCAATACCTACGGTGCGATTCGGGGCACGGATGTTTTTTA
TCCGCAGCAGGGCGCAGGGAATACGCGTTATCGTCGTAGCTTTGTGGCGACCGGATTTATGACGCTGATAACCGTACCGC
TGGCGGTAATTCCATTTTCACCGTTTGTTTCATCCATTGGTTTATTAACCCAGACTGGCGATTACACGCGGCGTTCGTTT
ATTTATGGCAGCGTTATTTGCCTGCTGGTGGCGCTGGTTCCTGCACTCACGCGACTGTTTTGCAGTATCCCTTTACCCGT
GAGTAGTGCGGTCATGCTGGTTTCTTATCTGCCTTTACTCTTTTCCGCGCTGGTGTTTAGCCAGCAAATAACGTTTACCG
CTCGCAATATTTATCGACTCGCATTGCCGTTATTTGTCGGCATATTTTTAATGGCATTACCGCCTGTGTATCTGCAAGAC
CTTCCATTAACGCTTCGTCCTCTGCTCAGTAACGGCTTATTGGTCGGGATTTTACTGGCTGTTCTTATGGATAACCTTAT
TCCGTGGGAACGCATCGAATAA
>Cluster_39925
TTGACTACGCCATTGAAAAAAATTGTGATTGTCGGCGGCGGTGCTGGTGGGCTGGAAATGGCAACACAGCTGGGGCATAA
GCTGGGACGCAAGAAAAAAGCCAAAATCACGCTGGTCGATCGTAACCACAGCCACCTGTGGAAACCACTGCTGCACGAAG
TGGCGACTGGCTCGCTTGATGAAGGCGTCGATGCGTTGAGCTATCTGGCCCATGCGCGCAATCATGGTTTCCAGTTCCAA
CTGGGTTCCGTCATTGATATTGATCGTGAAGCGAAAACAATCACTATTGCAGAACTGCGCGACGAAAAAGGTGAATTGCT
GGTTCCGGAACGTAAAATCGCCTATGACACCCTGGTAATGGCGCTGGGTAGCACCTCCAACGATTTCAATACGCCAGGTG
TCAAAGAGAACTGCATTTTCCTCGATAACCCGCACCAGGCGCGTCGCTTCCACCAGGAGATGCTGAATCTGTTCCTGAAA
TACTCCGCCAACCTGGGCGCGAATGGCAAAGTGAACATTGCGATAGTCGGCGGCGGCGCGACGGGTGTAGAACTCTCCGC
TGAATTGCACAACGCGGTCAAGCAACTGCACAGCTACGGCTATAAAGGTCTGACCAACGAAGCCCTGAACGTAACGCTGG
TAGAAGCGGGCGAACGTATTTTGCCTGCATTACCGCCACGTATCTCTGCTGCGGCCCATAATGAGTTAACGAAACTTGGC
GTTCGCGTGCTGACGCAAACCATGGTCACCAGTGCTGATGAAGGCGGCCTACATACTAAAGATGGCGAATATATTGAGGC
TGATCTGATGGTGTGGGCAGCCGGGATCAAAGCGCCAGACTTCCTGAAAGATATCGGTGGTCTTGAAACCAACCGTATCA
ACCAGCTGGTGGTGGAACCGACGCTGCAAACCACTCGCGATCCAGACATATACGCTATTGGCGACTGCGCGTCATGCCCG
CGTCCGGAAGGGGGCTTTGTTCCGCCGCGCGCTCAGGCCGCACATCAGATGGCAACCTGCGCAATGAACAACATTCTGGC
GCAGATGAACGGTAAGCCGCTGAAAAATTATCAGTATAAAGATCACGGTTCGCTGGTATCGCTGTCGAACTTTTCCACAG
TCGGTAGCCTGATGGGTAACCTGACGCGCGGCTCAATGATGATTGAAGGACGAATTGCGCGCTTTGTATACATCTCGCTG
TACCGAATGCACCAGATTGCGCTGCATGGTTACTTTAAAACCGGATTAATGATGCTGGTGGGGAGTATTAACCGCGTTAT
CCGCCCACGTTTGAAGTTGCATTAA
>Cluster_55640
ATGAGTATCAAAGTTCAGCACGTCGGCAAGGCGTATAAATATTATCCATCTAAATGGAACCGGGTCATTGAGAAACTACT
GCCGGGCGATAAGCCGCGGCACAGCAAGAAATGGGTATTGAAAGATATCAATTTCAGCATTGAACCTGGGGAAGCGGTCG
GCATTGTTGGGGTGAACGGCGCAGGTAAAAGTACGTTACTGAAGCTGCTGACTGGCACCACTCAGCCCACCAAAGGTAGC
ATTGAAATCCAGGGGCGCGTCGCCCGCGCTGCTGGAGCTGGGCATGGGCTTCCATCCTGACTTTACCGGCCGGCAGAACGT
GTATATGTCCGGGCTGATGATGGGCCTGGGCCGGGAAGAGATCGAGCGCTTAATGCCGGAGATCGAAGCCTTCGCCGATA
TCGGCGACTACATTGAAGAGCCCGTACGCATCTATTCCAGCGGGATGCAAATGCGCCTGGCGTTCGCTGTCGCCACGGCC
TCCCGGCCGGATATTCTGATCGTCGATGAAGCGCTTTCCGTTGGTGATTCCCGTTTTCAAGCGAAGTGCTATGCCCGTAT
TGCGGACTTTAAAAAGCAGGGCACCACGCTGCTGCTGGTCTCACACAGCGCCGGGGATATCGTCAAACACTGCGACCGCG
CCATTTTCCTCAAAAATGGTGATATCTGTATGGACGGCACCGCCCGTGACGTGACCAACCGTTATCTGGATGAGCTGTTT
GGAAAAGCCGACAAAAACAGCGCGCCAAAAAGCGAAACGGCAACCTCGTCAGCCAGCGGCGAAAGTCAGATGTCTCTCGA
TGAGATTGAAGATGTGTACCACACGCGCCCAGGCTATCGTCCTGAAGAATACCGTTGGGGGCAGGGGGGCGCAAAAATCA

TTGATTATCACATCCAGAGCGCCGGGGTTGATTTTCCTCCCTCACTGACGGGCAATCAGCAGACCGATTTTCTGATGAAG
GTCGTGTTTGAATACGATTTTGATTGCGTGGTGCCAGGCATCCTGATTAAGACCCTCGATGGCTTATTCCTCTACGGAAC
CAACTCTTTCCTCGCCTCTGAAGGGCGGGAAAATATTTCGGTTTCCCGCGGCGATGTTCGGGTCTTTAAATTTAGCCTTC
CGGTTGATTTAAATAGTGGCGATTACCTGCTGTCATTTGGTATCTCTGCCGGCAACCCGCAGACGGATATGACGCCGCTG
GACAGACGTTACGACTCCATTATTTTACATGTGACCAAGAGCATGGATTTCTGGGGTGTCATCGATCTTAAGTCGTCCTT
TACTAGCTACCAATGA
>Cluster_40067
ATGCGAGTTGTCATACTGGGAAGTGGTGTGGTAGGCGTTGCCAGCGCCTGGTACTTAAATCAGGCAGGACATGAGGTCAC
CGTCATTGATCGGGAGCCGGGGGCCGCCCTGGAAACCAGTGCCGCGAATGCCGGGCAAATCTCCCCCGGATATGCAGCAC
CGTGGGCGGCACCAGGTGTGCCTTTAAAAGCGATTAAATGGATGTTCCAGCGCCATGCGCCGCTGGCGGTTCGTCTCGAC
GGTACGCAGTTCCAGTTGAAATGGATGTGGCAAATGTTACGTAACTGCGACACCAGCCACTACATGGAAAACAAAGGGCG
GATGGTGCGTCTGGCGGAATACAGCCGTGATTGCCTGAAAGCATTGCGCGCCGAAACCAATATTCAGTATGAAGGGCGTC
AGGGTGGGACGCTGCAACTGTTCCGTACCGAACAACAGTATGAAAATGCGACCCGCGATATCGCCGTGCTGGAAGATGCC
GGCGTACCGTACCAGCTGCTGGAATCCAGCCGTCTGGCGGAAGTGGAGCCCGCGCTGGCAGAAGTGGCGCACAAACTGAC
GGGGGGGCTCCAGCTGCCCAATGATGAAACCGGAGACTGCCAGCTGTTTACCCAGAATCTGGCGCGGATGGCGGAGCAGG
CGGGGGTTAAATTCCGCTTTAATACGCCCGTTGACCAACTGCTTTGCGACGGCGAGCAAATCTACGGCGTGAAGTGTGGT
GATGAAGTGATTAAGGCCGATGCGTATGTGATGGCGTTTGGTTCTTACTCGACGGCGATGCTCAAAGGCATTGTTGATAT
TCCGGTGTATCCGCTGAAAGGCTACTCGCTGACCATTCCAATTGCGCAGGAAGATGGTGCGCCGGTATCCACTATTCTTG
ATGAAACCTACAAAATCGCCATTACCCGTTTCGATAACCGCATTCGTGTCGGCGGAATGGCGGAAATTGTTGGCTTTAAC
ACCGAGCTGTTGCAACCGCGTCGTGAAACGCTGGAGATGGTGGTTCGCGATCTCTATCCACGCGGCGGTCATGTTGAGCA
GGCGACTTTCTGGACTGGCCTGCGCCCGATGACGCCAGACGGCACGCCGGTTGTCGGGCGTACACGCTTTAAAAATCTGT
GGCTGAATACCGGTCACGGCACGCTCGGCTGGACGATGGCTTGCGGTTCCGGTCAGTTGTTAAGCGATCTGCTCTCGGGG
CGCACGCCAGCGATCCCGTATGAAGATCTAAGCGTAGCGCGCTACAGCCGTGGATTTACGCCATCACGCCCGGGCCATTT
ACATGGCGCACACAGCTAA
>Cluster_39795
GTGACAGCAGAGCTGCGTAATCTCCCGCATATTGCCAGCATGGCCTTTAATGAGCCGCTGATGCTTGAACCCGCCTATGC
GCGGGTTTTCTTTTGTGCGCTTGCAGGCCAGCTTGGGATCAGCCGCCTGACAGATGCGGTGTCCGGTGACAGCCTGACTG
CCGGAGAGGCACCCGCGACGCTGGCGTTATCCGGTGATGATGACGGACCACGACAGGCCCGCAGTTATCAGGTCATGAAC
GGCATCGCCGTGCTGCCGGTTTCCGGCACGCTGGTCAGCCGGACGCGGGCGCTGCAGCCGTATTCGGGGATGACCGGTTA
CAACGGCATTATCGCCCGTCTGCAACAGGCTGCCAGCGATCCGATGGTGGACGGCATTCTGCTCGATATGGACACGCCCG
GCGGGATGGTGGCGGGGGCATTTGACTGCGCTGACATCATCGCCCGTGTGCGTGACATAAAACCGGTATGGGCGCTGGCC
AACGACATGAACTGCAGTGCAGGTCAGTTGCTTGCCAGTGCCGCCTCCCGGCGTCTGGTCACGCAGACCGCCCGGACAGG
CTCCATCGGCGTCATGATGGCTCACAGTAATTACGGCGCTGCGCTGGAGAAACAGGGTGTGGAAATCACGCTGATTTACA
GCGGCAGCCATAAGGTGGATGGCAACCCCTACAGCCATCTTCCGGATGATGTCCGGGAAACACTGCAGTCCCGGATGGAT
GCAACCCGCCGGATGTTTGCGCAGAAGGTGTCGGCATATACCGGCCTGTCCGTGCAGGCCGTGCTGGATACCGAGGCTGC
AGTATACAGCGGTCAGGAGGCCATTGATGCCGGACTGGCTGATGAACTTGTTAACAGCACCGATGCGATCACCGTCATGC
GTGATGCACTGGATGCACGTAAATCCCGTCTCTCAGGAGGGCGAATGACCAAAGAGACTCAATCAACAACTGTTTCTGCC
ACTGCTTCGCAGGCTGACGTTACTGACGTGGTTCCAGCGACGGAGGGCGAAAACGCCAGCGCGGCGCAGCCGGACGTGAA
CGCGCAGATCACCGCAGCGGTTGCGGCAGAAAACAGCCGCATTATGGGGATCCTCAACTGTGAGGAGGCCCACGGACGTG
AAGAACAGGCACGCGTGCTGGCAGAAACCCCCGGAATGACCGTGGAAACGGCCCGCCGCATTCTGGCCGCAGCACCACAG
AGTGCACAGGCGCGCAGTGACACTGCGCTGGATCGTCTGATGCAGGGGGCACCGGCACCGCTGGCTGCAGGTAACCCGGC
ATCCGATGCCGTTAACGATTTGCTGAACACACCAGTGTAA
>Cluster_58213
ATGGCGCTTTCAAGACAAAAATTTACCTTCGAAAGACTTCGCAGATTCACCTTACCGGAAGGGAAAAAACAAACTTTTCT
TTGGGATGCAGATGTAACAACCCTGGCATGCCGAGCAACTAGCGGAGCAAAAGCCTTTGTATTCCAAAGCGTATATGCGG
GGAAAACCCTTCGCATGACTATTGGCAACATTAACGACTGGAAGATTGATGATGCGAGAGCCGAGGCAAGACGGTTACAA
ACATTGATCGATACAGGGATAGATCCACGAATTGCTAAGGCTGTAAAAATCGCAGAAGCAGAATCCCTGCAGGCAGAATC
ACGTAAAACAAAAGTGACTTTCTCCGTCGCCTGGGAAGACTATCTTCAAGAATTGAGAACCGGTATCAGTGCAAAAACTA
AACGCCCATATTCTACTCGATACATTGCCGATCACATTAACTTGTCCAGTCGTGGAGGCGAAAGTAAAAAAAGAGGCCAA
GGCCCGACTTCGGCTGGACCATTGGCTAGTTTGCTCAACCTGCCGTTATCGGAGCTAACCCCAGATTACATAGCAGCGTG
GCTGAGTACAGAAAGGCAAAATAGACCTACCGTCACTGCTCACGCTTATCGCCTACTACGTGCTTTCATCAAATGGAGTA
ATTATCAGAAAAAAATATCAAGGGATCATTCCTGGCGATCTGGCACAAGATTACAACGTAAGAAAAATGGTTCCCGTGTCA
GCGAGTAAAGCTGATGATTGCCTGCAAAAGGAACAACTAAAAAGCTGGTTTAGTGCCGTGCGTAGCCTCAATAATCCTAT
TGCATCGGCCTATCTCCAAGTACTTTTGCTCACTGGTGCTCGGCGTGAAGAAATTGCGTCGCTTCGCTGGTCAGACGTAG
ATTTCAAATGGTCAAGCATGCGAATTAAAGACAAGATCGAAGGTGAACGTATCATCCCTCTCACTCCTTATGTTTCTGAA
TTGTTAAATGTACTAGCGCAATCCCCAAATTCTGACGTAAATAAGGAGGGTTGGGTTTTCAGAAGTAACAGTAAAAGTGG
CAAAATTATTGAGCCGCGTTCAGCGCACAACAGAGCATTAGTGCTGGCTGAGTTACCACATATCAGCCTTCACGGTTTAC
GTCGTAGTTTTGGTACTTTGGCCGAGTGGGTTGAAGTTCCCACTGGTATTGTTGCTCAAATTATGGGACACAAACCCAGC
GCTCTTGCCGAAAAACACTATCGCCGTCGTCCGTTAGATCTGTTACGAAAATGGCACGAGAAAATTGAGACATGGATCTT
AAATGAAGCAGGTATTACCATAAAAAAACAACGTTGATATGCGTTGA
>Cluster_36596
ATGAAACGGACAATGCTCTATCTTTCTCTGCTGGCTGTTAGTTGTAGCGTCAGCGCCGCAAAATACCCTGTTCTGACAGA
AAGTTCGCCAGAGAAAGCAGGGTTCAACGTCGAACGGCTTAACCAGATGGATCGCTGGATTAGCCAGCAAGTGGACGCCG
GTTATCCCGGCGTAAACCTGCTGATCATTAAAGATAATCAGATTGTGTATCGCAAAGCCTGGGGAGCGGCGAAAAAGTAC
GATGGCAGCGTGTTGATGGAACAGCCTGTCAAAGCCACCACCGGGACGCTGTATGACCTGGCCTCAAACACCAAAATGTA
CGCCACGAACTTCGCCCTGCAAAAGCTGATGAGCGAAGGCAAACTGCATCCTGACGATCGGATTGCGAAATATATTCCGG
GGTTTGCCGATAGTCCAAATGACACCATTAAAGGCAAAAACACCCTGCGGATTTCTGACCTGCTGCATCACAGCGGCGGT
TTCCCGGCAGATCCGCAATACCCGAATAAAGCGGTCGCGGGCGCGTTATATTCCCAGGATAAAGGCCAGACGCTGGAGAT
GATCAAGCGCACGCCGCTAGAGTATCAGCCCGGCAGCAAGCATATTTACAGTGATGTCGATTATATGCTGCTTGGATTTA
TCGTCGAGTCGGTCACCGGTCAGCCGCTTGACCGCTATGTTGAAGAGTCGATTTATCGCCCGCTCGGCCTGACACATACG
GTATTTAACCCGCTGCTGAAAGGCTTTAAACCGCAACAAATTGCCGCCACGGAATTGAACGGCAATACCCGCGATGGCGT
GATCCATTTCCCGAATATCCGCACCTCCACTCTCTGGGGTCAGGTGCACGATGAAAAAGCCTTTTATTCGATGGGCGGCG
TTTCCGGGCACGCAGGTTTGTTTTCCAATACCGGCGATATTGCGGTGTTAATGCAAACGATGCTGAACGGCGGGGGCTAT
GGCGATGTGCAGCTGTTCAGTGCGGAAACGGTGAAGATGTTCACCACCAGCTCTAAGGAAGATGCCACTTTTGGCCTCGG
CTGGCGCGTGAATGGTAATGCCACCATGACGCCGACGTTTGGCACGCTGGCAAGCCCGCAAACCTACGGTCACACTGGCT
GGACCGGAACGGTGACCGTTATCGATCCGGTGAATCATATGGCGATTGTGATGTTAAGCAACAAGCCACATTCGCCGGTT
GCCGATCCGCAAAAGAATCCCAATATGTTCGAAAGCGGTCAGCTGCCGATTGCAACGTATGGTTGGGTAGTTGATCAGGT
GTATGCGGCGTTAAAGCAAAAGTAA
>Cluster_22264

ATGGACAAGGAATTACCGTGGCTGGCGGATAACGCGCAACTGGAACTGAAATATAAAAAGGGTAAAACACCACTCAGCCA
TCGCAATTGGCCGGGTGAACCGGTGCCTGTTATCACTGAAAGCATCATCCAGACACTGGGTGATGAATTGCTACAAAAAG
CTGAGAAGAAAAAAAACATTGTCTGGCGTTATGATAATTTTTCACTGGAGTGGCAGTCCGCCATCACGCAGGCCATCAAC
TTGATCGGCGAACACAAACCCTCAATCCCGGCCCGGACAATGGCGGCGCTAGCCTGTATCGCGCAAAATGATAGCCAACA
GTTGCTCGACGAAATCGTCCAACAAGAGGGACAGGAATATGCGACTGAGGTGGTGATTGCACGCCAGTTTATTGCGCGGT
GTTATGAGAGTGATCCTCTGGTAGTGACATTGCAGTATCAGAACGAGGACTATGGCTATGGTTATCGCTCAGAAACCTAT
AACGAATTCGATCTCCGATTGCGTAAGCATCTCTCTCTGGCAGAGGAAAGCTGCTGGCAGCGTTGCGCCGACAAACTCAT
TGCCGCAGTGCCAAAGATACCGAAAGTTCGCCGTCCTTTTATTGCACTGCTCCTCCCGGAAAAACCCGAAATCGCCAACG
AGCTGGTAAGCCTTGAATGTTCGCAAACTCATTTTCACTCAAAAGAGTGGTTAAAAGTTGTTGCTGATGATCCTAAAGCG
GTGAAAGAACTCGCACGTTACTGGAGTCAGGATATATTTAGCGATCGAGAAGCCAGCTACATGTCGCATGAAAACCGCTT
CGGCTACGCGGCCTGCGCCGCCCTTTTGCGCGAACAAGGACTGGCATCCATTCCACGCCTGGCAATGTATGCCCATAAAG
AAGATTGTGGCAGTCTGCTGGTACAAATTAACCATCCGCAGGTCATCCGCACCTTGCTGTTAGTTGCTGATAAAAACAAA
CCCAGCCTGCAACGTGTGGCTAAATACAGTAAAAACTTCCCCCATGCGACGCTCGCCGCACTGGCAGAACTGCTGGCGTT
AAAAGCCCCCCCTGCGCGCCCTGGTTATCCAATCATCGAAGACAAAAAGCTGCCTGCACAGCAAAAAACACGTGATGAAT
ACTGGCATACGCTGTTACAAACGCTGATGGCATCGCAGCCACAACTGGCAGAAGAGGTGATGCCGTGCTTAAGTACACAA
GCCCAGGCAGTGGTGAATGGTTATTTATCGGCATCGCCAAAACCGGTTATTGATAGCACCGATAACAGCAGTCTGCCTGA
AATCCTCGTTTCACCACCGTGGCGTAGTAAGAAAAAATGA
>Cluster_66950
ATGGCTGATGAAGATATCAACCCGGTAGTGCTTCTCGCAGATCCTAAAGTAAATCATCGCGTGTGGGCTGCCTGTCTTAA
ATGGACTCCTGTCGTAAAAAAACAGCGCGTCCCCTCACATCATAAACACAAGTCTCATGTAAAACCCAGACGGCTTACCT
CCCTGAAAGTGACTGTAGGCAGCAGAAATTCCCGTGGAAAGATCAGCCGCCTTACCGGCACAGGAATACTGACCAGGCCA
GAACGCAATCATTATTTTTCCCTGGCACTGGCTTTCTGCTCATGGGTCAGAAATGGATACGGTGTCTTCCGGTACAGCGA
TAAGGAGCTCTTATTCCTTGCTTCCATTAATGGCCAGCCTGCCGTTATGGCTGACTTATCAGGAAATGATGCTGATGTTG
CTCAAAAGGTCAGTCTTTTTCTGACGATGAACGAAGAGCCCCCCGAAAAATGGCAGGTCGTATCTCCTCTGGAACATCCT
GATAACTGGGAAAGTATTATTACCCGGTTATCATCAGCAGATCTCCGCCGTTGCAAACTTACTGTCGGCAATCGCAGCAA
ATTCACACTGCCTGCCGTACTGTTTTTAGTAGCAGCCGGCGCAGGCACTGTATTCTGGATGACTCAGCCAGAGCCGGATG
TCGTACCGACGCCCGAGGAGATAGCTGCACGGGCAAGATTGCAGTTTAAAAAGCCCGATCCACCGCCAGAACTCCCGCAT
CCATGGGCATCACAACCTGTAATCAGTGATTTTCTGAAAGCCTGCGCGGACCTTCGAAAACCATCACCGGTCGCACTGGA
AGGCTGGAAACTTACCGGTGGAACATGTACTCCCGAGACATTCACACTGATTTACGAACGACAGCCAGGAGGAACTATTG
AAGGCTTTCTGGCAAGAAGTAAAGAAATCTTTAATGTTATTCCTGACTTTAACCTTAAAGATGGTGCACGCCTGGCTTCT
GTAACCCGGCCACTGCCTTCCCTGCCCCGCAGGGATGAGGCAGTCCCTACCCCCTCAGAACAACTGATGCGGGTGTTCAC
GTGGTTTCAGAAAAAACAGTTAACGCCGGCCATTAATGAAATAGCCATACCGGAACCATTACCAGGTAACGATGGTGAAG
CAGCCCCTGTTCAGAAATGGAAAGAATATCAGTTCAGTTTATCAACGCCTGTAAATCCTGATGAGTTGTTCCCTTTATTT
CAGGATACCGGCGTTCGTCTGAGCAATATCCATTTTGAATTAAACGGCGGTACTTTCAGTTATTCCTCAGAAGGTCATAT
TTATGCGTCCAGGTAA
>Cluster_22653
GTGGCAAAAAACATTCAAGCCATTCGCGGCATGAACGATTACCTGCCTGGCGAAACGGCCATCTGGCAGCGCATTGAAGG
CACACTGAAAAACGTGCTCGGCAGCTACGGTTACAGTGAAATCCGCTTGCCGATTGTAGAGCAGACCCCGCTATTCAAAC
GTGCGATTGGTGAAGTCACCGACGTGGTTGAAAAAGAGATGTACACCTTTGAGGATCGCAATGGCGACAGCCTGACTCTG
CGCCCTGAAGGGACGGCGGGCTGTGTACGCGCCGGCATCGAGCATGGTCTTCTGTACAATCAGGAACAGCGTCTGTGGTA
TATCGGGCCTATGTTCCGTCACGAGCGTCCGCAGAAAGGGCGTTATCGTCAGTTCCATCAGTTGGGCTGCGAAGTTTTCG
GTCTGCAAGGTCCGGATATCGACGCCGAACTGATTATGCTCACTGCCCGCTGGTGGCGCGCGCTGGGTATTTCCGAGCAC
GTAACTCTTGAGCTGAACTCTATCGGTTCGCTGGAAGCTCGTGCGAATTACCGCGATGCGCTGGTGGCATTCCTTGAGCA
GCATAAAGAAAAGCTGGACGAAGACTGCAAACGCCGCATGTACACTAACCCGTTGCGCGTACTCGATTCAAAAAATCCGG
AAGTGCAGGTGCTTCTCAACGACGCTCCGGCATTAGGCGATTATCTGGACGAGGAATCTCGTGAGCACTTTGCCGGTCTG
TGCAAACTGCTTGAGAGCGCGGGGATCGCTTACACCGTAAACCAGCGTCTGGTGCGCGGTCTGGATTACTATAACCGTAC
CGTTTTCGAGTGGGTGACTAACAGCCTCGGTTCCCAGGGCACCGTGTGTGCAGGTGGTCGTTATGACGGTCTGGTGGAAC
AACTGGGTGGTCGTGCAACACCGGCAGTCGGTTTTGCGATGGGCCTCGAACGTCTTGTATTGTTAGTTCAGGCCGTTAAT
CCGGAATTTAAAGCCGATCCTGTTGTCGATATATACCTGGTGGCTTCAGGTGCTGACACACAATCTGCGGCTATGGCATT
AGCAGAGCGTCTGCGTGATGAATTACCGGGCGTAAAACTGATGACCAACCACGGCGGCGGCAACTTTAAGAAACAGTTTG
CCCGTGCTGATAAATGGGGTGCCCGCGTTGCTGTGGTGCTGGGTGAGTCTGAAGTGGCTAACGGCACAGCAGTAGTGAAG
GATTTGCGCTCTGGTGAGCAAACTGCAGTTGCGCAGGATAGCGTAGCCGCGCATTTGCGCACGTTACTGGGTTAA
>Cluster_21071
ATGATTTCACGCTGGAAATGGATGCTGAAGCAGACAATTAAAAAACTATGGTTCAGGGCAACGTTATTCGCAATTGTCGC
GATAATAACGGCCCTTTTATCAATTCTTTTTAAATCAATGATACCTGAGTCGGTTTCCGTGAAGGTTGGTGCGGAAGCAG
TCGATAACATTCTGAACATACTGGCATCGAGTATGCTGGCAGTGACCACATTTTCGCTGAGTATCATGGTCACAGCCTAC
GGTTCAGCCACTACTAATGTGACTCCCAGAGCTACGCGTTTAGTTGTTGAAGACGTCACCACACAAAATGTACTGGCCAC
CTTCATCGGTTCTTTTCTCTTCAGTCTGGTAGGGATTATTGCCCTCAATATGGGAGCTTATGGAGAAAGGGGGAGAGTCA
TTTTATTCATTGTCACACTGGTTGTCATTGCCTTAATCCTCATCACATTGCTTCGCTGGATACAGCATTTGACCTCTCTG
GGGAGGGTTGGTGAGACAACGGCACAAGTAGAACAGGCGGCCATCGAAACATTTATTGCGAGAGCAAGAAATCCCTGTCT
CGGTGGATATCCATGGCTTGAGAACAATGAACAGCCGAAAGGAACGGTTGCAGTTTATCCGAAGAAGATTGGCTATGTTG
AATATATTGATATGGTGAAACTTAGCAAGCTGCTGACCAATGATCCCCGTCATGTATACCTCGTGGCGCAGCCAGGCAGT
TTCATACATCCGTCCATGCCAGTTTTGTACCTGAGTCAGGGCCAGGAGTCGTCAATCAACACCGATTTACTTGAGACGAT
TATTGTCTCGGATGCACGTTCATTTGCTCAGGATCCTCGATTTTGTCTTAGCGTCATGGCCGAAATAGCCTGCCGGGCCC
TTTCCCCTGCAGTGAACGATCCTGGAACCGCCATTGATGTCATTGGCAGAGGTGTTCGTATACTTTCCACTTACGCGCAG
AATAAATCTGATGAAATAGAAGTGAAATATCCTTCAGTACATGTTGCACCACTTCAGAATAACGATCTACTGGAAGACTT
TTTCTCACCTGTCGCGCGCGATGGTGCCAGTATGAGGGAGATTCAGATAAGAGTCCTTAAAGGACTGTCGATGCTGAGTA
ACGGTTGGCCTGAGATATTTGCTGAAGCCGCACATACCCTGGCATTTGAAACATTAGAGCATGCAACTCGTGCTGACCAT
ATAGATTCTGATAGATATCTAATAAAATCAACCTATTATAATTTATTTAGTGGCGAATATTCTAATAAAAAAACATAG
>Cluster_22418
ATGCGCTTTGATACTGTCATTATGGGCGGCGGCCTCGCCGGATTACTCTGTGGCCTGCAACTGCAAAAACACGGCCTGCG
CTGTGCCATTGTCACTCGTGGTCAAAGCGCGCTGCATTTCTCATCTGGATCGCTGGATTTGCTGAGCCATCTGCCGGATG
GTCAACCGGTGACAGACATTCACAGTGGACTGGAATCTTTGCGTCAGCAGGCACCTGCCCATCCTTACACTCTTCTCGGG
CCACAACGCGTGCTCGATCTCGCTTGCCAGGCGCAGGCATTAATCGCTGAAAGCGGTGCGCAATTGCAGGGCAGCGTAGA
TCTGGCTCACCAGCGGATTACACCGCTCGGCACTCTGCGCTCAACCTGGCTAAGTTCGCCAGAAGTCCCTGTCTGGCCGC
TGCCCGCGAAGAAAATATGTGTAGTGGGAATTAGCGGCCTGATGGATTTTCAGGCGCATCTTGCGGCGGCTTCATTGCGC
GAATTGGACCTTAAAGTTGAAACCGCTGAAATTGAACTGCCGGAACTGGATGTGCTGCGCAATAACGCCACCGAGTTCCG
GGCGGTGAATATTGCCCGCTTCCTTGATAATGAAGAAAACTGGCCGTTGTTACTTGATGCGCTTATTCCGGTCGCCAATA
CCTGCGAAATGATCCTGATGCCCGCCTGCTTCGGCCTGGCCGATGACAAACTGTGGCATTGGTTGAATGAAAAACTACCT

TGTTCATTGATGCTTTTGCCAACGCTGCCGCCTTCCGTGCTGGGCATTCGTCTGCAAAACCAGTTACAGCGCCAGTTTGT
GCGCCAGGGTGGCGTGTGGATGCCGGGCGATGAAGTGAAAAAAGTGACCTGTAAAAATGGTGTAGTGAATGAAATCTGGA
CGCGCAATCACGCCGATATCCCGCTGCGCCCACGATTTGCGGTGCTTGCGAGCGGCAGTTTCTTTAGCGGCGGACTGGTG
GCAGAACGTGACGGCATTCGCGAGCCGATTCTCGGCCTTGATGTGCTACAAACCGCTACGCGGGGTGAATGGTATAAGGG
CGATTTTTTTGCGCCGCAGCCGTGGCAGCAGTTCGGTGTAACCACTGATGAGGCGCTACGCCCGTCGCAGGCGGGGCAAA
CCATTGAGAACCTGTTTGCCATCGGTTCGGTGCTGGGCGGATTTGATCCCATCGCCCAGGGATGCGGCGGCGGTGTTTGT
GCCGTCAGTGCTTTACATGTCGCTCAACAGATTGCCCAACGCGCAGGAGGCCAACAATGA
>Cluster_4287
ATGAGCAAAACACATTTAACAGAACAGAAGTTTTCCGACTTCGCCCTGCATCCGAAGGTTGTAGAAGCCCTTGAAAAAAA
AGGGTTTCATAACTGTACGCCCATTCAGGCACTGGCCCTTCCGCTGACGCTGGCGGGTCGTGACGTAGCCGGGCAGGCGC
AAACCGGTACCGGGAAAACGATGGCGTTTCTTACGTCAACGTTTCATTATCTTCTCTCTCATCCTGCGATTGCCGATCGC
AAGGTGAATCAACCACGTGCCTTAATTATGGCACCGACGCGTGAACTTGCCGTGCAGATCCATGCCGACGCAGAACCGCT
GGCGGAAGCTACCGGCCTGAAGCTGGGTCTGGCTTACGGTGGTGATGGCTACGACAAACAGCTGAAAGTGCTGGAAAGCG
GCGTTGACATTCTGATTGGCACTACGGGGCGTTTAATTGACTACGCCAAGCAGAACCACATTAACCTCGGTGCCATTCAG
GTGGTGGTACTGGACGAAGCCGATCGCATGTACGATCTGGGCTTTATTAAAGATATCCGCTGGCTGTTCCGCCGTATGCC
GCCTGCAAACCAGCGCCTCAACATGCTGTTCTCCGCCACGCTTTCGTACCGGGTACGTGAACTGGCGTTCGAGCAGATGA
ACAATGCCGAATATATTGAAGTGGAACCGGAACAGAAAACGGGCCACCGTATTAAAGAAGAGCTTTTCTACCCTTCTAAC
GAAGAAAAAATGCGTTTGCTGCAAACGCTGATCGAAGAAGAGTGGCCAGACAGAGCGATTATTTTCGCCAATACCAAACA
CCGTTGTGAAGAGATCTGGGGCCACCTGGCGGCAGATGGTCATCGTGTCGGTTTGTTGACAGGCGATGTCGCGCAGAAAA
AACGTCTGCGTATTCTTGATGAATTTACCCGTGGCGATCTGGATATTCTGGTTGCCACCGACGTTGCCGCGCGTGGTTTG
CATATTCCGGCAGTGACGCACGTCTTTAACTACGATTTACCCGATGACTGTGAAGATTACGTTCACCGTATTGGTCGTAC
AGGTCGCGCAGGCGCAAGCGGTCACTCTATCAGCCTGGCGTGTGAAGAGTATGCATTGAATTTGCCTGCTATTGAGACCT
ATATTGGTCACTCAATTCCGGTAAGCAAATACAATCCGGACGCATTGATGACCGATCTGCCAAAACCGCTGCGCCTCACG
CGCCCACGTACAGGCAATGGTCCGCGTCGTACTGGCGCTCCGCGTAATCGTCGTCGTTCAGGTTAA
>Cluster_48211
ATGCGCTATAATGGTTTAAATAATATGTTTTTCTCTCTTTGCCAGATTAACGATAACCACTCTTTCACAAGTTCATCACA
TACAAAGAAAACAAAATCATATAATTACAGCAAACATCATAAAAACACGTTAATTGACAATAAAGCCCTCTCTCTTTTCA
AGATGGATGATCATGAAAAAGTGATAGGTTTGATTCAGAAAATGAAAAGAATTTATGATAGTTTACCATCAGGAAAAATC
ACGAAGGAAACGGACAGGAAAATACATAAACATTTTATAGATATAGCTTTATATGCAAATAATAAATGTGACGATAGAAT
TACGAGAAGAGTTTACCTTAGTAAAGAAAAGGAAGTATCCATTAAGGTGGTATATTTTATAAATAATGTCGCCGTCCATA
ATAATACTATCGAAATTCCACAGACAGTAAATGGTGGTTACGATTTTTCACACCTTAGCCTGAAAGGTATCGTGATTAAA
GATGAAGATTTATCCAATTCGAATTTTGCAGGTTGCAGACTACAAAACGCTATTTTCCAGGACTGTAATATGTATAAAAC
GAATTTTTATTACGCCATAATGGAAAAAATACTTTTTGATGATTGTATTCTCGATGACTCAAATTTCGCTCAGATAAAAA
TGGCCGACGGAACTCTAAATGCATGCTCCGCTATGCATGTTCAATTCTACAATGCAGCAATGAATAGAGCCAATATTAAA
AATACCTTTCTTGACTATTCAAATTTTTATATGGCGTACATGGCTGAGGTAAATCTTTATAAAGTAATAGCGCCATATGT
TAATTTATTTAAAGCCGACCTTAGTTTCTCTAAACTCGATTTAATTAACTTTGAACATGCTGATCTGTCTCGCGTCAATC
TGAACAAAGCAATCCTCCAGAATATAAACTTAATTGATAGCAAACTCTTTTGTACGTGGCTAACAAATACATTCCTCGAA
ATGGTTATATGTACCGACTCTAATATGGCTAATGTTAATTTTAATAATGCCAATTTAAGCAACTGCCATTTCAACTGTTC
TATTTTAACAAAAGCCTGTATGTTTAATACCCGTCTCTATCGGGTTAATTTTGATGAGGCTAGCGTCCAGGGAATGGGCA
TTTCCATACTCCGTGGGGAGGAAAATATCCCCATTGATAGTGATACCCTGGTAACACGACAGAAATTCTTTGAAGAAGAT
TGTACCTCTCATACTGGCATGTCACAAACTGAGGATAATATTAATGCAGTCGCTATGAAGATTACTGCAGATATTATGCA
ACACGCAGATTGA
>Cluster_45352
ATGATTAAATCAAAAATCGTGCTGTTATCAGCACTGGTTTCCTGCGCCCTGATTTCAGGCTGTAAAGAAGAAAATAAAAC
GAATGTATCCATCGAATTTATGCATTCTTCGGTGGAGCAGGAGCGCCAGGCCGTTATCAGTAAATTGATTGCCCGTTTTG
AAAAAGAAAACCCTGGCATCACCGTTAAGCAAGTGCCCGTGGAAGAAGATGCCTATAACACTAAAGTCATTACTCTTTCA
CGTAGCGGTTCGCTGCCGGAAGTGATCGAAACCAGCCATGACTACGCCAAAGTGATGGACAAAGAGCAGCTTATCGATCG
CAAAGCGGTTGCCACAGTCATCAGTAACGTTGGTGAAGGCGCGTTTTACGATGGCGTACTGCGTATTGTGCGTACCGAAG
ATGGTAGCGCATGGACCGGTGTTCCTGTCAGCGCCTGGATTGGCGGTATCTGGTATCGCAAAGATGTGCTGGCAAAAGCG
GGGCTTGAGGAGCCGAAAAACTGGCAACAGCTGCTGGACGTTGCACAGAAACTGAATGACCCGGCGAATAAAAAATACGG
CATTGCGCTGCCTACAGCAGAAAGCGTGTTGACGGACAATCCTTCTCCCAGTTTGCGTTATCCAACCAGGCTAACGTCT
TTAACGCCGAAGGCAAAATCACCCTTGATACACCAGAGATGATGCAGGCACTGACCTATTACCGCGACCTTGCTGCCAAC
ACTATGCCGGGTTCTAACGACATCATGGAAGTGAAAGACGCCTTTATGAACGGCACCGCGCCGATGGCGATTTACTCCAC
CTATATCCTTCCGGCTGTGATTAAAGAAGGCGACCCGAAAAACGTCGGTTTCGTGGTGCCAACCGAGAAAAACTCTGCGG
TCTACGGCATGTTGACCTCGCTGACCATTACCGCCGGGCAAAAGACCGAAGAGACGGAAGCAGCTGAAAAATTTGTCACC
TTTATGGAGCAGGCAGACAACATTGCCGACTGGGTGATGATGTCGCCAGGTGCTGCGCTGCCGGTGAATAAAGCGGTTGT
CACTACCGCCACCTGGAAAGACAACGACGTTATTAAGGCGCTGGGTGAACTACCGAATCAGCTAATCGGTGAACTGCCAA
ATATTCAGGTCTTTGGTGCAGTAGGGGATAAAAACTTTACCCGCATGGGTGATGTGACGGGTTCTGGCGTGGTGAGCTCC
ATGGTGCATAACGTCACCGTGGGTAAAGCCGATCTCCCTGGCACGCTGCAAGCGAGCCAGAAAAAGCTGGATGAACTGAT
CGAACAGCACTAA
>Cluster_29674
ATGAAGAACTGGAAAACGCTGCTTCTCGGTATCGCCATGATCGCGAATACCAGTTTCGCTGCCCCCCAGGTAGTCGATAA
AGTCGCAGCCGTCGTCAATAACGGCGTCGTGCTGGAAAGCGACGTTGATGGATTAATGCAGTCGGTAAAACTGAACGCTG
CTCAGGCAAGGCAACAACTTCCTGATGACGCGACGCTGCGCCACCAAATCATGGAGCGTTTGATCATGGATCAAATCATC
CTGCAGATGGGGCAGAAAATGGGAGTGAAAATCTCCGATGAGCAGCTGGATCAGGCGATTGCTAACATCGCGAAACAGAA
CAACATGACGCTGGATCAGATGCGCAGCCGTCTGGCTTACGATGGTCTGAACTACAACACCTATCGTAACCAGATCCGCA
AAGAGATGATTATCTCTGAAGTGCGTAACAACGAGGTGCGTCGTCGCATCACCATCCTGCCGCAGGAAGTCGAATCCCTG
GCGCAGCAAGTGGGTAACCAAAACGACGCCAGCACTGAGCTGAACCTGAGCCACATCCTGATCCCGCTGCCGGAAAACCC
GACCTCTGATCAGGTGAACGAAGCGGAAAGCCAGGCGCGCGCGATTGTCGATCAGGCGCGTAACGGCGCTGATTTCGGTA
AGCTGGCGATTGCTCATTCTGCCGACCAGCAGGCGCTGAACGGCGGACAGATGGGCTGGGGCCGTATTCAGGAGTTGCCG
GGGATCTTCGCCCAGGCATTAAGCACCGCGAAGAAAGGCGACATTGTTGGCCCGATTCGTTCCGGCGTTGGCTTCCATAT
TCTGAAAGTTAACGACCTGCGCGGCGAAAGCAAAAATATCTCGGTGACCGAAGTTCATGCTCGCCATATTCTGCTGAAAC
CGTCGCCGATCATGACTGACGAACAGGCCCGCGTGAAACTGGAACAGATTGCTGCCGATATCAAGAGTGGTAAAACGACT
TTTGCTGCCGCAGCGAAAGAGTTCTCTCAGGATCCAGGCTCTGCTAACCAGGGCGGTGATCTCGGCTGGGCTACACCAGA
TATTTTCGATCCGGCCTTCCGTGACGCCCTGACCCGCCTGAACAAAGGTCAAATGAGTGCACCGGTTCACTCTTCATTCG
GCTGGCATTTAATCGAACTGCTGGATACCCGTAATGTCGATAAAACCGACGCGGCACAGAAAGATCGTGCATACCGCATG
CTGATGAACCGTAAGTTCTCGGAAGAAGCAGCAAGCTGGATGCAGGAACAACGTGCCAGCGCCTACGTTAAAATCCTGAG
CAACTAA
>Cluster_5609

ATGATGAACACGGAAGGTAATAACGGTAACAAACCTCTCGGTCTATGGAACGTCGTTTCCATCGGCATTGGGGCAATGGT
GGGGGCGGGGATCTTCGCGCTGCTGGGGCAGGCTGCATTGCTAATGGAAGCCTCGACCTGGGTCGCCTTTGCTTTTGGCG
GTATTGTGGCGATGTTTTCCGGTTATGCCTATGCGCGTCTGGGGGCGAGCTATCCCAGCAATGGCGGCATTATCGACTTC
TTTCGTCGCGGATTAGGCAACGGCGTCTTTTCGCTGGCGCTCTCGTTACTGTACCTGTTGACGCTGGCGGTGAGCATCGC
CATGGTCGCCCGTGCTTTTGGCGCTTATGCCGTGCAGTTTTTGCATGAAGGCAGCCAGGAGGAGCACCTTATTTTGCTCT
ACGCGTTGGGGATCATTGCGGTGATGACGCTTTTCAACTCCTTAAGCAACCATGCGGTAGGGCGGCTGGAAGTGATCCTC
GTCGGCATTAAAATGATGATCCTGTTATTGCTGATTATTGCCGGTGTCTGGTCGCTGCAACCGGCGCATATTTCCGTCTC
TGCGCCCCCCAGCTCCGGTGCGTTCTTCTCCTGTATTGGGATAACTTTCCTTGCCTATGCGGGCTTTGGCATGATGGCGA
ACGCGGCGGATAAAGTGAAAGATCCGCAGGTCATTATGCCACGGGCGTTTCTGGTGGCGATTGGCGTTACCACGTTGCTT
TATATCTCGCTGGCACTGGTTTTGCTTAGCGATGTATCGGCATTAGAGTTAGAAAAATATGCCGATACCGCCGTAGCGCA
GGCTGCTTCTCCGCTGCTCGGGCATGTGGGTTATGTGATCGTCGTCATCGGCGCTTTACTGGCGACGGCTTCAGCCATTA
ACGCGAACCTGTTCGCCGTGTTTAACATCATGGACAACATGGGCAGCGAACGCGAACTGCCGAAGCTAATGAATAAATCC
CTGTGGCGGCAGAGTACCTGGGGCAACATTATTGTCGTGGTGTTGATTATGCTGATGACGGCGGCACTGAATTTAGGCTC
ACTCGCCAGCGTTGCCAGCGCCACCTTTTTGATTTGCTACCTGGCGGTGTTTGTGGTGGCGATCCGCCTGCGTCATGATA
TTCACGCCTCGTTGCCGATTCTTATCGTTGGTACGTTGGTGATGTTGTTGGTGATCGTTGGCTTTATCTACAGTCTGTGG
TCCCAGGGTAGCCGTGCGTTGATATGGATTATTGGCTCACTCTTACTCAGCCTTATTGTGGCAATGGTCATGAAGCGCAA
TAAAACCGTATAA
>Cluster_39605
ATGCAGTGCGCACTTTACGACGCGGGTCGCTGTCGTTCCTGTCAGTGGATAACGCAGCCGATTCCAGAGCAACTCTCCGC
TAAAACCGCCGATCTTAAAAATCTGCTCGCTGATTTTCCGGTTGAGGAATGGTGTGCGCCGGTGTCAGGCCCGGAACAAG
GGTTTCGTAATAAAGCCAAAATGGTGGTGAGTGGTAGCGTTGAAAAACCACTGCTCGGTATGCTGCATCGGGATGGTACA
CCGGAAGACCTTTGTGACTGCCCGCTTTATCCAGCCTCATTTGCGCCCGTTTTTGCGGCGCTAAAACCGTTTATCGCCCG
TGCGGGTTTAACGCCTTACAACGTGGCGCGTAAGCGTGGTGAACTGAAATACATTCTGCTGACTGAAAGCCAGAGCGATG
GCGGCATGATGCTGCGTTTTGTACTGCGTTCTGATACCAAACTGGCGCAACTGCGTAAGGCGCTGCCGTGGTTACAGGAA
CAATTACCGCAGCTGAAAGTTATTACCGTCAATATTCAGCCGGTACATATGGCGATTATGGAAGGGGAGACGGAGATCTA
CCTGACCGAACAACAGGCACTGGCAGAGCGTTTTAATGACGTACCGCTGTGGATCCGTCCGCAAAGTTTCTTCCAGACTA
ATCCGGCGGTCGCCAGCCAGCTGTACGCCACTGCGCGCGACTGGGTACGACAGCTGCCGGTTAAACATATGTGGGATCTG
TTCTGCGGTGTGGGTGGCTTTGGTTTACACTGCGCGACGCCTGACATGCAGTTAACCGGGATCGAAATTGCGCCAGAGGC
CATTGCCTGTGCAAAGCAGTCAGCCGCTGAACTGGGCTTAACGCGTTTGCAATTTCAGGCGCTGGACTCTACGCAGTTTG
CCTCCGCCCAGGGGGAAGTGCCGGAGCTGGTACTGGTTAACCCGCCGCGCCGCGGCATCGGTAAACCGCTGTGTGATTAT
CTCTCAACGATGGCACCGCGTTTTATTATCTACTCCAGCTGTAACGCCCAAACTATGGCGAAAGATATCCGCGAACTGCC
TGGTTATCGCATCGAACGGGTACAGCTTTTTGATATGTTCCCGCACACCGCGCACTATGAAGTGCTGACGCTGCTGGTGA
AGCAATAA
>Cluster_52588
ATGCGTATACATACTTATTGGTATAGAAGATATTTCATTTTATTGATTATTATATTTTCAAATGTTCTTTCTTCTATTGC
TAATGCTGAAGATATGGGGCGAGAACGTGCATATTGTTATCCGGGTTCACCGAGTAATAATACTACGCCTGCATCCTTTT
CTTATAATTTTGGTACTATAGTGGTTTCTGATGTCAACAAAAATGCGCCTGGCACTGTATTGCCATCACAAATCTGGAAG
GTTGGAACCTATAAGGCTTATTGTAATTCTCTTGATGATTATGAGATTTACTTCAGTGCTGTCTCTGGAATAGATCCGTC
TGGTGCCAGTGGTGATCATCAAGGGAGTGATGTATTTATTCCACTCACCCATGAAATATCTGTCTCTACTCATATAAAAC
TTTATAATCAGAATGGCACAATGACAGATAAAATTGTGCCATTCGAAAATTATAACACCAATTATCCGGGGGACAGAAGC
AAACCATCTAATTGGGCATCAGATACTGAAGGATATATTAAAATCAGGATTGATAAAAAAATTATATCTGATGTTTCATT
AAGTAACGTATTATTGGTGTCATTATATGTCAGCCAGATCCCTACCGAACATGGTCCTATCCCTGTCTTTAATGCCTACA
TAGGAAACTTAAATATTCAGGTTCCGCAAGGTTGCACTATTAATGAGGGTACGAGTTTTACTGTTAATATGCCGGATGTG
TGGGCCAGTGAATTGAGCCGGGCTGGTGCCGGAGCGAAGCCCGCTGGTGTTACTCCTGTAGCAACAACTATTCCGATTAA
TTGTACGAATAAAGATACAGATGCGGTAATGACGTTGGTATTCGACGGTAACATTTCCGCCACACGTGATACCAATGGGA
AACAAAGTATTATTCAGGCACAGGATAATCCTGATGTTGGTATTATGATTATGGATAGTCAGCAAAACTCCGTAGATTTA
AATGCTCTGGCAACATCAGTAGGCGTTCCGTTCAGATTGGTGGAAAACCAGGCTGCTTCTGCACAAACTGCTGATGTTAC
TTTCCTGACTTTACCTGTTAGTACAACTGGAAAAGTGCCAGCGGCAGGTCGTTATAATGCACTGGCTATATTGCGAGTGG
AATATCAGTAA
>Cluster_20921
GTGGCGACATTAACCCGGCTTTTTATTCATCCTGTTAAATCGATGCGCGGCATTGGTCTTACACATGCTCTGGCAGATGT
CAGTGGTCTGGCCTTCGATCGCATCTTTATGATCACGGAACCTGACGGTACGTTTATTACCGCTCGCCAGTTTCCCCAGA
TGGTACGGTTTACTCCTTCACCCGTGCATGATGGCTTGCATCTCACCGCCCCAGATGGCAGTAGTGCATATGTTCGTTTT
GTTGATTTCGCCACACAAGACGCACCAACCGAAGTTTGGGGCACACATTTTACCGCGCGAATTGCACCAGACGCGATCAA
CAAATGGCTAAGTGGATTTTTCTCCCGCGAAGTGCAGTTGCGCTGGGTGGGGCCACAAATGACGCGGCGCGTGAAACGCC
ACAACACTGTACCCCTGTCATTTGCTGATGGCTATCCTTACCTTCTTGCTAACGAAGCCTCGTTACGTGATCTCCAACAA
CGTTGTCCGGCCAGTGTAAAAATGGAGCAATTCCGCCCCAATCTGGTGGTTTCCGGCGCGTCAGCCTGGGAAGAAGATAG
CTGGAAAGTGATTCGCATTGGTGATGTGGTGTTTGATGTGGTTAAACCTTGTAGCCGCTGTATTTTCACCACCGTCAGTC
CAGAAAAAGGGCAAAAACATCCGGCAGGCGAACCATTAAAAACATTGCAATCTTTCCGCACTGCCCAGGATAACGGCGAT
GTCGATTTTGGTCAGAATTTAATTGCCCGTAATAGCGGCGTAATTCGCGTTGGCGATGAGGTGGAAGTTCTGGCAACGGC
TCCGGCTAAAATTTACGGCGCAGCCGCCGCTGATGATACCGTCAACATCACGCAACAACCGGACGCAAATGTAGATATTG
ACTGGCAGGGACAGGCATTTCGTGGAAATAACCAGCAGGTGTTGCTGGAGCAATTAGAAAATCAGGGAATTCGTATCCCT
TATTCTTGCCGCGCGGGCATTTGTGGAAGTTGCCGTGTTCAGCTTTTAGAAGGCGAAGTCACGCCGCTGAAAAAATCAGC
AATGGGCGATGATGGCACCATTCTTTGCTGTAGCTGTGTACCGAAGACTGCACTTAAGTTGGCGCGTTAG
>Cluster_2417
ATGAATAAAGTGGCATTAATTACTGGTATCACTGGGCAAGATGGCTCCTATTTGGCAGAATTATTGTTAGAAAAAGGTTA
TGAAGTTCATGGTATTAAACGCCGTGCATCTTCATTTAATACTGAGCGAGTGGATCACATCTATCAGGATTCACATTTAG
CTAATCCTAAACTTTTTCTACACTATGGCGATTTGACAGATACTTCCAATCTGACCCGTATTTTAAAAGAAGTTCAACCA
GATGAAGTTTACAATTTGGGGGCGATGAGCCATGTAGCGGTATCATTTGAGTCACCAGAATACACTGCTGATGTTGATGC
GATAGGAACATTGCGTCTTCTTGAAGCTATCAGGATATTGGGGCTGGAAAAAAAGACAAAATTTTATCAGGCTTCAACTT
CAGAGCTTTATGGTTTGGTTCAAGAAATTCCACAAAAAGAGACTACGCCATTTTATCCACGTTCGCCTTATGCTGTTGCA
AAATTATATGCCTATTGGATCACTGTTAATTATCGTGAGTCTTATGGTATGTTTGCCTGCAATGGTATTCTCTTTAACCA
CGAATCACCTCGCCGTGGCGAGACCTTTGTTACTCGTAAAATAACACGCGGGATAGCAAATATTGCTCAAGGTCTTGATA
AATGCTTATACTTGGGAAATATGGATTCTCTGCGTGATTGGGGACATGCTAAGGATTATGTCAAAATGCAATGGATGATG
CTGCAGCAAGAAACTCCAGAAGATTTTGTAATTGCTACAGGAATTCAATATTCTGTCCGTGAGTTTGTCACAATGGCGGC
AGAGCAAGTAGGCATAGAGTTAGCATTTGAAGGTGAGGGAGTAAATGAAAAAGGTGTTGTTGTTTCGGTCAATGGCACTG
ATGCTAAAGCTGTAAACCCGGGCGATGTAATTATATCTGTAGATCCAAGGTATTTTAGGCCTGCAGAAGTTGAAACCTTG
CTTGGCGATCCTACTAATGCGCATAAAAAATTAGGATGGAGCCCTGAAATTACATTGCGTGAAATGGTAAAAGAAATGGT
TTCCAGCGATTTAGCAATAGCGAAAAAGAACGTCTTGCTGAAAGCTAATAACATTGCCACTAATATTCCGCAAGAATAA

>Cluster_24985
ATGGCGGATAGCAGCAGCGAAGAAAAAACAGAGAAACCCTCGGCGCAAAAGCTGCGTAAAGCGCGCGAAGAGGGGCAACT
GCCGCGCTCGAAGGATATGGGGCTGGCGGCGAGCCTGTTTGCGGCGTTTGTGGTGATCTCCAGCAGCTTCCCGTGGTATG
CCGATTTTGTGCGCGAGAGTTTTATCAGCGTGCATCAGTACGCGCAGGAGATTAATAACCCGGATGTGATCGGCCAGTTT
CTGCGCCATCACCTGTTAATTCTCGGCAAGTTTATTCTTACCTTGCTGCCGATGCCCGCCGCCGCGCTGCTCTCCTCGCT
GGTGCCCGGCGGCTGGCTGTTCTTGCCGAAAAAAATCCTCCCGGATTTCAGCAAAATTAGCCCGTTAAAAGGGATTGGGC
GACTGTTTTTCAGTGAGCATCTGGCGGAAACGGGAAAGATGACAGTGAAGTCGGTGGTGGTGCTGGTGATGCTGTGGATT
AGCCTGCGCAATAACTTTGCCGCCTTTCTCGGCCTGCAGGCACTGCCGTTCAAACTGGCGATGAATGATGGGTTATCGCT
CTACGCCAGCGTGATGCGCAACTTTGTCATCCTGTTTATCTTCTTTGCGTTAATCGACGTGCCGCTGGCGAAAGCCCTGT
TCACCAAAGGGCTGAAGATGACCAAACAGGAGCTGAAAGAAGAGTACAAAAATCAGGAAGGCAAGCCGGAAGTGAAGGCG
CGCGTGCGGCGGCTGCAACGGCAACTGGCGATGGGGCAAATTCGCAAAGTGGTGCCGAAAGCCAACGTGGTGATCACCAA
CCCGACCCACTACGCCGTGGCGTTGCAGTATGACCAGTCGCGCGCCGCCGCGCCGTTTGTGGTGGCGAAAGGCACCGATG
AAATCGCCCTCTATATTCGCCAGGTCGCCACTGAGAATCAGGTGGAAGTGGTCGAGTTCCCGCGACTGGCGCGCTCGGTT
TACTACACCACCCAGGTGAACCAGCAAATTCCGTTTCAGCTTTACCGGGCCATCGCCCACGTTCTGACCTACGTCCTGCA
AATGAAACACTGGCGCGAGGGCGCACAGCCGCGCCCGGCCCTGAACAGACATATTTCCATTCCCAAAGAGGTGCTTAAAC
TGGATGGCGAAAACAACTAA
>Cluster_38719
ATGGATTTTAATTTAAATGATGAGCAGGAACTGTTTGTCGCCGGTATCCGCGAACTGATGGCCAGCGAAAACTGGGAGGC
CTATTTTGCCGAGTGCGACCGTGACAGCGTCTACCCGGAACGTTTTGTCAAAGCCCTGGCGGATATGGGTATCGACAGTC
TGCTGATCCCGGAAGAACACGGTGGTCTGGACGCGGGGTTTGTTACTCTCGCCGCCGTGTGGATGGAACTGGGTCGTCTG
GGAGCGCCAACTTACGTGCTGTATCAGTTACCGGGCGGGTTTAACACCTTCCTGCGTGAAGGTACGCAAGAGCAGATCGA
CAAGATCATGGCTTTCCGCGGCACCGGTAAGCAGATGTGGAACTCAGCAATTACCGAACCGGGCGCAGGCTCCGACGTGG
GTAGCCTGAAAACGACTTATACCCGTAGAAATGGTAAGATTTATCTTAATGGTAGTAAGTGTTTTATTACCAGTAGCGCC
TACACCCCGTACATCGTGGTGATGGCGCGCGACGGGGCTTCTCCGGACAAACCTGTCTACACCGAATGGTTTGTTGATAT
GAGCAAACCGGGCATCAAAGTGACCAAACTTGAAAAGCTCGGTCTGCGTATGGATAGCTGCTGTGAAATCACTTTTGACG
ACGTGGAACTGGACGAGAAAGACATGTTCGGTCGGGAAGGTAACGGCTTTAACCGCGTCAAAGAAGAGTTCGACCATGAA
CGTTTCCTGGTAGCCCTGACCAACTACGGTACGGCGATGTGCGCCTTTGAAGATGCGGCGCGCTACGCCAACCAGCGTGT
GCAGTTTGGCGAGGCTATTGGTCGTTTCCAGTTGATTCAGGAAAAATTCGCCCACATGGCGATCAAATTAAACTCCATGA
AAAACATGCTGTATGAAGCAGCGTGGAAAGCAGACAACGGCACCATCACCTCTGGCGATGCAGCGATGTGCAAATACTTC
TGCGCCAATGCGGCATTTGAAGTGGTGGATAGCGCAATGCAGGTGCTGGGCGGTGTCGGGATTGCGGGCAACCACCGCAT
CAGCCGCTTCTGGCGTGACCTGCGTGTAGACCGCGTTTCCGGAGGATCTGACGAAATGCAGATCCTGACGCTGGGTCGCG
CGGTGCTGAAGCAATACCGCTAA
>Cluster_22270
ATGTCTGAACTGAACGATCTTCTGACCACCCGTGAGCTACAACGCTGGCGATTAATTCTTGGCGAAGCGGCAGAAACGAC
GCTTTGTGGGCTGGATGACAACGCCCGGCAGATAGACCACGCGCTGGAGTGGCTGTATGGGCGCGATCCTGAACGGCTCC
AGCGTGGTGAACGCTCTGGTGGATTAGGTGGCTCAAATCTCACCACCCCTGAGTGGATCAACATTATTCACACGCTGTTT
CCGCAACGGGTGATCGAGCGGCTGGAAAGCGATGCCGTGCTGCGCTACGGCATTGAAGATGTGGTGACGAATCTCGACGT
GCTGGAACGTATGCAGCCTTCTGAAAGCCTGCTACGCGCCGTTTTGCACACTAAACATCTGATGAATCCCGAAGTACTGG
CTGCCGCCCGCCGGATAGTGCACCAGGTTGTTGAAGAGATTATGGCGCGACTGGCAAAGGAAGTTCGTCAGGCTTTTTCT
GGAGTACGCGATCGCCGTCGCCGCTCATTTATTCCACTGGCGCGAAACTTTGATTTCAAAAGTACTCTGCGCGCCAACCT
GCAACACTGGCACCCGCAACACGGCAAGTTGTATATCGAATCCCCCCGCTTTAACAGCCGCATTAAACGCCATAGCGAAC
AATGGCAACTGGTCCTACTGGTTGATCAAAGCGGATCAATGGTGGACTCGGTGATCCACTCCGCAGTGATGGCGGCCTGT
TTATGGCAGTTACCCGGTATTCGTACCCATCTGGTGGCGTTTGACACCAGCGTCGTCGATCTCACGGCAGACGTTGTCGA
TCCGGTAGAATTATTAATGAAAGTGCAATTAGGCGGCGGGACCAATATCGCCAGTGCCGTGGAGTATGGTCGGCAACTTA
TTGAACAACCAGCGAAAAGCGTCATTATTCTCGTGAGCGATTTTTATGAAGGTGGGTCATCATCATTGCTGACGCATCAG
GTGAAAAAGTGTGTCCAGAGCGGCATCAAAGTGCTGGGGTTGGCAGCGCTCGACAGCACCGCAACGCCTTGCTATGACCG
CGATATGGCCCAGGCGCTGGTTAATGTCGGCGCACAAATAGCCGCAATGACACCGGGCGAGCTGGCTTCCTGGCTTGCGG
AGAATCTTCAGTCATGA
>Cluster_42011
ATGGTTAAATTACCGCCGCTGAGTCTCTACATTCACATCCCGTGGTGCGTGCAGAAATGCCCGTACTGCGATTTCAACTC
TCACGCGTTGAAAGGAGAAGTGCCGCACGACGATTATGTTCAGCATCTGCTTAACGATCTGGACAACGATGTGGCTTACG
CTCAGGGCCGTGAAGTAAAGACAATCTTTATTGGCGGTGGTACGCCGAGCCTGCTTTCCGGCCCGGCGATGCAAACGCTG
CTGAACGGCGTACGTGCGCGTTTGCCGCTGGCAGCGGATGCAGAAATTACGATGGAAGCGAACCCTGGTACGGTTGAAGC
CGATCGCTTTGTTGATTATCAGCGTGCTGGTGTGAACCGCATCTCCATTGGCGTGCAGAGTTTTAGCGAAGAAAAGCTGA
AACGACTTGGGCGCATTCATGGCCCGCAAGAAGCGAAACGAGCGGCAAATCTGGCGAGCGGTTTAGGGTTACGTAGCTTT
AACCTCGATTTGATGCATGGGCTGCCGGATCAATCACTGGAAGAGGCGCTTGGCGATCTACGCCAGGCTATTGAACTGAA
TCCGCCGCATCTTTCCTGGTATCAACTGACCATCGAACCTAATACGCTGTTTGGCTCTCGCCCTCCTGTACTGCCGGACG
ATGACGCGCTGTGGGATATTTTCGAACAGGGGCATCAGTTATTAACCGCAGCGGGTTATCAGCAATATGAAACTTCCGCT
TACGCCAAACCAGGTTATCAGTGCCAGCACAATCTCAACTACTGGCGATTTGGCGACTACATTGGTATTGGCTGCGGCGC
GCACGGCAAAGTGACCTTCCCGGATGGGCGCATTCTGCGTACCACTAAAACGCGTCATCCGCGTGGTTTTATGCAAGGAA
GGTATCTGGAAAGCCAGCGTGATGTCGAAGCCGCAGATAAGCCGTTTGAGTTCTTTATGAATCGCTTCCGTCTGCTGGAG
GCCGCGCCGCGCGTGGAGTTTAGCCAGTATACTGGCCTTTCTGAAGAAGTTATTCGCTCTCAGTTAGACGAGGCTATCGC
TCAGGGTTATCTCACAGAATGTGCGGATTACTGGCAGATAACGGAACATGGGAAGTTGTTTTTAAATTCGCTGCTGGAGC
TTTTTCTGGCTGAGTAA
>Cluster_48583
ATGTCCACAAAAAATAAAAAAGGTAAAAATAACAAAGAAGTAGAAGATAATCTCGACAACTTTGAAGAAGGTTCCCTTGA
ATTGTACACAGGGGAACTTGTCCCTAATAGCAACAATACAGTCCAACCAATTGCGTTGATGCGGCTGGGGTTGTTCGTGC
CCACGCTCAAGGGGACAAAATACAGCAAGCGTAACAAGCCAAATGAGATCGATGCTTCAAAGGAGCTTGTTCAGCTCGAA
GTAGCCCGTTCTGAAGGCTATTCCGATATCAAGATAACCGGTCCTCGTCTCGACATGGATCATGATTTCAAAACGTGGGT
GGGCGTTGTGCGTTCGCTGGCAGAATACGGCGAACCTAACGGTCGTGTCGAGTTAAGCATCACGAAATTTGCGAAGTTTT
GTGGCTATCCATCGTCACAGATCCGCAAAACACTCCGCGACCGGCTTACAAACAGTCTTTTGAAGATCATGCGTACAACT
CTGTCTTTCCAGAGAACGTATGAAGAAAAGAACGTCGACGGCTCTAACAAGATCTCGCTTCTGATGGTGCACCTCATCAA
TAGCGTGGACTACAACGAGCAGAAAGATACGGTGGTGTTCTATGCAGAACCGAAGCTGGCCGAACTATATCGCTTTGACC
ATAAGGTTCTTTTGCAGTTAAAGGTTATCAATAAGCTACCACGCAAAGAAACAGCACAGGCTCTGTACACCTTCATCGAA
AGTCTTCCAACCAAGCCTGCGCCGGTATCGCTTGCTCGATTGCGTGCGCGACTCAATTTGAGTAGCAGGAATGTCAGCTC
GCAGAATCAGACTATACGTAACGGCTTAAAAGCTCTCCAAGATTTGGGCTATCTCGAATACAGCGAGATTAAGCGTGGGC
GGTCGATCTATATCCAGATTCACAGCCGCAATCCAAAACTCAAAGTCGCACCGCCAAAACCTGAAGACATCGAACCCAAA
AAACCAGATGAGAAAGCTGGGGAAATTGATGCCAAACAGAACATTATCAACAAGATAACCGAACTTTCGCAGAATTTGAC
GCCTGAAAATATCAAGATGATTGAGATCCTTTCCAATAGTCTCAAACTGCTTTGA

>Cluster_3313
ATGGACAGAGCATCACAGCCAGCGCGTACCGGGCTGACCGACCGGCTGGCCCCGGATATTGCGCGGATCAATTTTTACCG
TTTTTGCCAGTTTCTGGAACAGAGCCAGCAGAACGCGCCGCTGGGCAGTACCGACAACCCCGCCACCGATGCGGTGCGCT
TTCGTCCCCATCCCGGAATGGGCTTTCCGGTCAGCGAGTTAAAGAACGTTGAACGTGACGCGGGCAATCCCAACGTGACA
CCCACGGTGCGCACCACCTTTCTCGGCCTGTACGGCGTGGATTCGCCGCTGCCAACCGCCTATCTGGACTACATTACCCA
GCGTCATGACGGACATGACGCAGTGATGGCGTTTCTCGATATTTTTAACCATCGCTTTATCACCCAGTATTACCGCATCT
GGCGCAAATATAACTACCCGGCGTCGTTTGAAGCAGGTGCTATGGATGACATTTCCCGTTGTCTGCTGGGACTCATCGGG
CTGGGTATTCCCGGCAGCGAGAACCATATCGCCACGCCGGTATCCCGTTTTCTGGCCCTGCTCAGCGTGATGCGCCTGCC
GACGCGCACGGCGGAAGGCGTCACGGCACTGGTGGGACTGCTTGCCCCGCTGACGAAAGCGACGGTGGTGCCTCACGATC
CGCAGCCTGTTATTCTCCCGGCCCCTGCGGGTCTGTCAAAAAATTCACGCATCAGCCTGAAAACGCGAACGCTGCTGGGG
CGTACCGGCACCGACGTCAACAGCCAGTTGCTGCTGAAACTGTATACCGAAGATGCCGCAGAAGCACGTGGCTGGCTACC
TGGCGGGCAGCTGCACAGCGATTTGCTGGTGCTGCTGCGCGTTTATCTGGGCTGGCGCTGTCAGGCCCGTCTGCAACTGA
CGCTGCCCGTCAGCCTGTTGCCTGCTGCCCGGCTGGGAAAACAGCGCGTACAGATTAGCCGTACCGGCATCCTTCGCGCC
TCCTTTGCCGCGCCCGCGACCGGAACAGTGACGGTCAGTCTGGGGCGTTATCAGGGATTAATACCCGCTTTATCCATTCG
TAACAGGGAGAGTATGACTCATGTTTCCTATTCGTTTTAA
>Cluster_64294
ATGAAAAAATTAACAGTGGCAATCTCTGCTGTAGCAGCATCAGTATTGATAGCTATGTCCGCTCAGGCAGCTGAGATTTA
TAACAAAGACAGCAATAAATTAGATCTGTACGGAAAAGTTAATGCCGGGCACTACTTCTCCTCTAACGATAAACACGATG
GTGACAAAACTTATGCTCGTCTGGGATTCAAAGGTGAAACCCAAATCAACGATAAACTGACTGGTTTCGGTCAGTGGGAA
TATGAATTCAAAGGCAACCATGCTGAATCTCAAGGTTCCTCCGCAGACAAAACCCGTCTTGCGTTTGCTGGTCTGAAATT
CGGCGACTACGGCTCCATCGATTATGGTCGTAACTACGGTGTAGCATACGATATCGGTGCATGGACTAACGTTCTGCCAG
AATTCGGTGGCGATACCTGGACTCAAACAGATGTATTCATGACTGATCGCACCACAGGTGTTGCAACTTATCGTAACAAC
GACTTATTTGGTCTGGTTGATGGTCTGAACTTTGCTGCTCAGTATCAGGGTAAAAATGACCGCACTGACGTAACTCAAGC
TAATGGTGATGGTTTCGGTTTCTCCACTACTTATGAGTATGAAGGATTCGGCGTAGGTGCAACCTATGCTAAATCTGACC
GCACTAATAATCAGGTTATCTACGGTAACAACAGCCTGAATGCATCTGGTCAAAATGCTGAAGTATGGGCAGCTGGTCTG
AAATATGATGCGAGCAACATCTATCTGGCTACTACCTATTCTGAAACCCAAAACATGACTGTTTTTGGTAAAAACCATAT
TGCCAACAAAGCACAAAACTTCGAAGTAGTTGCACAATATCAGTTCGACTTCGGTCTGCGTCCGTCCGTTGCTTACCTGC
AATCTAAAGGTAAAGACCTTGGCGTTTTTGGTGACCAAGATTTAGTCAAATATGTTGATGTAGGTGCAACCTATTATTTC
AACAAAAATATGTCAACTTTTGTTGATTACAAAATCAACCTACTTGACAAAAATGATTTCACCAGAAATCTTGATATAAA
CGCCGACGATATTGTTGCTGTCGGTATGGTTTACCAGTTCTAA
>Cluster_43225
ATGTACGTTGCCGTGAAAGGGGGCGAAAAGGCGATCGACGCCGCCCACGCCCTGCAAGAGAGCCGACGCCGGGGCGATAC
TGATTTGCCTGAACTGAGCGTCGCCCAGATTGAACAGCAGCTTAACCTCGCGGTAGATCGCGTGATGACCGAAGGCGGTA
TTGCCGACCGCGAGCTGGCCGCGCTGGCGCTGAAACAGGCCAGCGGCGATAACGTCGAAGCGATTTTCCTGTTGCGCGCC
TACCGCACCACGCTGGCGAAGCTGGCGGTCAGTGAGCCGCTCGACACCACCGGGATGCGTCTAGAACGGCGTATTTCAGC
AATTTATAAAGACATTCCCGGCGGCCAACTGCTTGGCCCCACCTACGACTACACCCATCGCCTGCTCGATTTCACCCTGC
TGGCAAACGGCGAAACGCCAACGCTGACCACCGCTGACAGCGAACAGCAACCGTCGCCACACGTCTTCAGCCTGCTGGCG
CGTCAGGGGCTGGCGAAGTTTGAAGAAGATAGCGGCGCACAACCGGACGACATCACCCGCACGCCGCCGGTTTATCCCTG
TTCGCGTTCTTCCCGTCTACAACAATTAATGCGCGGTGACGAAGGCTATTTGTTGGCGCTGGCCTACTCCACCCAACGCG
GTTACGGGCGCAATCACCCGTTCGCAGGCGAAATTCGCAGTGGCTATATCGATATATTTATTGTGCCGGAAGAGCTGGGA
TTTGCGGTGAACGTCGGCGAATTGTTGATGACCGAGTGTGAAATGGTCAACGGTTTTATCGACCCACCGGATGAGCCGCC
GCACTTCACGCGCGGCTACGGGCTGGTGTTCGGCATGAGCGAGCGCAAAGCGATGGCAATGGCGCTGGTTGACCGCGCTC
TGCAAGCCCCGGAGTACGGCGAGCACGCGACAGGCCCGGCGCAGGATGAAGAGTTCGTGCTGGCGCATGCCGACAACGTC
GAAGCCGCAGGCTTTGTCTCGCACCTTAAACTCCCGCACTACGTCGATTTCCAGGCCGAACTGGAGCTACTCAAACGTCT
GCAACAGGAGCAGAACCATGGCTAA
>Cluster_64520
ATGCAAAAAGTTTCAATTTTGATTGCCGATATTACTTGTGTAGGTGGCATAGAACGCGTGATTTGCTTATTAGCCAACGC
GTTAGCTAATTATGGGTTTAAGGTGGAAATTATATCCTTGTACCGTACTAATGCTAACATCAATTACGCTTTAACTAAAG
ATATAAAAGTTACTTTTATAAATAAACTAAAATATTCAGGGAAGCCTGGGGGATATTTTCGATTATTTAAACATATTCTG
AGTGCTGTGAAACTCAATAAACATCTATTGGCTAATAAAAATTCATTTTTCATAATAAATACATTTCCAATGGCTTTTAT
TTCAATGCCTTTTATTCTTTTTTCGAAAAATACGTGTGTGTCGTTGAACATGTCTATCATTCATACTATTCAAAGCCTATAC
AATATATACGACAGTTTATTTATAAGTTTATCACTACAATTGTGGCGCTTACGGATACGGATGCGGAAAAATATAAGCGT
GGGCATCGTCATGTAGTTAAAATCCCAAATCCATTAAGTTTTGTAGAATGTGGGAAAGTACTCTGAGAAATCATAAGAT
AATTGCAGTTGGTAGGCTGGAATATCAAAAAGGTTTTGATTTGCTAATTCAGGCTTTTGCACGAGCATCAAAAGATACAG
ATTGGTCATTAGATATTTATGGCGATGGTACATTAAGAAAGGAATTAGAAGAGATTATTCAGTTTAATGAAATTTCTAAT
ATAAATTTATTAGGGAATGTAAGTAATATTGATGAAATTTATAAGGATTATTCATTGTTTGTATTTTCTTCAAGGTTTGA
AGGGTTTGGAATGGTGCTACTGGAGGCCATGAGGGCTGGTTTGCCATGCATTTCTTTCAACTGCCCCACAGGACCCGCAG
AGATATTTGATAATGGAGAATATGGTATTCTGGTAGATAATGGCAATATTGACGAGTTATCTAATGTAATGAAAATGTTT
ATGGATAGTTTTGAGTTGCGTTCAAAGTTTTCTAAACTATCTATAATCCGAACAGAGAACTATACACTAGATAAAATATT
GCCATTGTGGGTGGATTTAATAAATAGAAATTAA
>Cluster_55564
ATGCTTGGCCTAATAAGGTTTTTTTTGGCATCGTGCGTCATTGCTTTTCACTTAACGGCTCGCATACCTGCACTTGGCAA
CTTTGCAGTGAATTGCTTCTATGTTATTAGTGGTTTTTTAATAACATACATTTTGCATGAAACATACAAGTTCAACTTCT
CTATGTTTTGGAAAAATAGAATACTGAGGTTATTCCCGGCATATATATTTTTTCTCGTCATGGGCTTTCTTATTATAAAA
TTAATTCCATCCGCAAAGGAATTCCACTCAAACTGGACAGGCAATTTCTTACCTGGAGATTTATTAGGAAACCTCTTAAT
CTTCCCCTGGGCTTTCCTTTCTGATAATGCTGTAGCAAATCCATTCGGTGCCTTTTCATCCATTTATCACTTTGCTATTG
ATGGTAATAGATTCAGAATAGTCACATCATCATGGTCAGTGGGTGTTGAGATAACATGTTATTTTTTATTGTGGCTTTTC
ATAGCCCGAAATAAATTTACAGCCATTACGTCGATTCTGCTTTCACTGCTTTATCATGCTTACGTATATGTAGTACACCA
TTCTTTTGATATGGCGTATTTCCCATTCCTTGCTGCAACACTTCCTTTTAGCATGGGTTCTCTGGGTTATTTTGCCCATA
GAAAGTTCAAAGCTATGTATCTTTCTCCACACAAAGCTTTTTTAATAACATTTATTTGCATCGGGATTTTTATTACCAAT
TGGCATTTATATACAATTAATCCATTAGGTCAGTACAACATAATACTTTACTACACCAATAATGTAATAGCATTGTTTAC
AACCCTGGTATTATTAAAAATAAAGACAAACATACATCTTGAGAAAATACTCAAGTGGTTTGGTGACCTGGCCTATCCAA
TTTTTCTTTGTCAATATTTTGGTGGATTTCTAGCATGGCTTGCTATTGGTGGTGAAAATCGTGGCTTATCCATCTTTTTA
TTAGGCTACCCAATATCAATTGCACTTGGGATCGTTTGTGTAATATTAATAGACAAGCCACTAATTAAAATTAGAGCAAA
AATAAGGGCGGACGCTCAAAGCAAAAATAATCAGGAAAACTCTTCAAGGTAA
>Cluster_23070
ATGGCACAACAGACTCCTTTGTACGAACAACACACGCTTTGCGGCGCTCGCATGGTGGATTTCCACGGCTGGATGATGCC
GCTGCATTACGGTTCGCAAATCGACGAACATCATGCGGTACGTACCGATGCCGGAATGTTTGATGTGTCACATATGACCA
TCGTCGATCTTCGCGGCAGCCGCACCCGGGAGTTTCTACGTTATCTGCTGGCGAACGATGTGGCGAAGCTCACCAAAAGC

GGCAAAGCCCTTTACTCGGGGATGTTGAATGCCTCTGGCGGTGTGATAGATGATCTCATCGTCTACTACTTTACTGAAGA
TTTCTTCCGCCTCGTTGTTAACTCCGCCACCCGCGAAAAAGACCTCTCCTGGATTACCCAACACGCTGAACCTTTCGGCA
TCGAAATTACTGTTCGTGATGACCTTTCCATGATCGCCGTACAAGGGCCGAATGCGCAGGCAAAAGCTGCCACACTGTTT
AATGACGCCCAGCGTCAGGCGGTGGAAGGGATGAAACCGTTCTTTGGCGTGCAGGCGGGCGATCTGTTTATTGCAACCAC
CGGTTACACCGGTGAAGCGGGCTACGAAATTGCGCTGCCCAATGAAAAAGCGGCCGATTTCTGGCGCGCGCTGGTGGAAG
CTGGCGTTAAGCCATGTGGCCTGGGCGCGCGTGACACGCTGCGTCTGGAAGCGGGCATGAATTTGTATAGCCAGGAGATG
GACGAAACTATTTCTCCTTTAGCCGCCAATATGGGCTGGACTATCGCCTGGGAACCGGCAGATCGTGACTTTATCGGTCG
TGAAGCTCTGGAAGCGCAGCGTGAACATGGCACAGAAAAACTGGTTGGTCTGGTGATGACCGAAAAAGGCGTGCTGCGTA
ATGAACTGCCGGTACGTTTTACTGATGCGCAGGGCAACCAGCATGAAGGCATTATCACCAGCGGTACTTTCTCCCCAACG
CTGGGTTATAGCATTGCGCTGGCGCGCGTGCCGGAAGGTATTGGTGAAACGGCGATTGTGCAAATTCGCAACCGTGAAAT
GCCGGTTAAAGTGACGAAACCTGTTTTTGTGCGTAACGGCAAAGCCGTCGCGTGA
>Cluster_11125
TTGCTTATCAATCCCGATGGCTCGAAAGGCTGGCGTTTCCGTTTCCGGTTTGCAGGGAAAGCGCGGTTAATGTCATTTGG
CAGCTACGATTTAGTAAGCCTCGCAGAAGCACGTGAAAAGCGTGATATCGCCCGTAAGCAGGTTGCTAATGGCATTGACC
CGGTAGAGGAACGCAAAGCTTTAAGACTCGCCCAAAAGTTATCAACAGAAAATTCTTTCGAAGCAATATGTCGAGAATGG
CATACCAACAAAGCTGACCGCTGGACTGTGGCCTATCGAGAAGAAATCATTAAGACTTTTGAGCAAGATGTGTTCCCGTT
CATTGGTAAACGCCATATCAGTGAAATCAAACCATTAGAACTACTTGAAGTATTGCGACGAATAGAGAAACGTGGAGCAC
TAGAGAAAACACGTAAGGTACGTCAGAGATGCGGTGAGGTTTATCGTTACGCAATCATAACTGGTCGCGCGGAATACAAT
CCTGCTCCTGATTTAGCCATCGCTCTGGCCGTTCCTAAGCCAAAACATCATCCATTTCTATCTGCCGAAGAATTACCTCA
TTTTATTCGGGATCTCGAAGCGTATACCGGTAGCATTATCACCAAAAATGCTACGAAGATAGTTATGCTAACTGGTGTAA
GAACGCAGGAGATGCGCTTTGCTACGTGGAAAGAAGTAGACCTCGAAAAAGGTATATGGGAGATACCAGCAGAACGTATG
AAAATGCGTAGGCCGCACATTGTTCCTTTATCGACTCAGGTAGTTGACCTTTTTAAACAGCTCAAACCTATTACCGGAAA
TTATCCTTACATTTTTGCCGGTAGGAACAACCGCAGCAAACCGATCTCGAAAGAAAGCGTATCGCAAGTGATTGAGTTAC
TTGGTTATAAAGGACGTGCTACAGGTCACGGTTTTAGGCATACAATGTCGACAATTCTGCACGAGCAAGGGTTTGATAGT
GCATGGATTGAGATACAGCTAGCCCATGTAGATAAAAATACTATTCGAGGCACATATAATCATGCTATATACTTGAAAAA
TAGGCGAATAATGCTACAGTGGTATTCTGATAAATTAATGAACTGA
>Cluster_55570
ATGAGTACGGATAATGATCAGGATATTGTCTCCCTGACAGTGGGCGGAAAAATCATTGAAGGATGGGACTCTGTGCGGGT
GACCCGTGGCATTGAGCGTTTTCCCTCCGATTTTGACCTGGGACTGATGGATTATTTTCCGGGAAGTGACCAGAAGCAGC
TGGTAAAGGAAGGGATGCCTTGTCAGGTAAAGCTTGGTAACGACCTGGTAGTGACGGGGTATGTTGATGACTGGTCGCCT
GCTATTTCCCGTTCGCGCCATGAAGTCAGAGCCTCAGGCCGCAGTAAGTGCGCAGATCTCGTTGATTGCTCTGCTGAATG
GCCAAATAACGTCATCAATAACAGTAATGCGCTTGATATAGCCTCCCGCCTCGCATCCCATTACAACATTGGCGTAAGTA
CAGACGTTGATGATCTGGTGAAAGTTCCGCAATTTTCCCTGAACTGGGGGGAATCGCCACAGGAAATACTTGATCGTGTC
TCCCGCTGGTCGGCATTGCTTTATTACGATCAGCCTGACGGTAACTTATTTCTGACCCGGGTGGGGACAAAGCGCGCGGC
CAGCGGTATCGCAGAAGGGGTAAATATCGAACAGGCTTATTACCGCCGTTCGATGGCTGACCGCTTTTCTGATTACGTCG
GCGTATCGATGAGCATTTCGCCGATTGCAGGATTCTCGCCAGATACGGCTTATGATTCGGTGACGCTGGCGACGGCCCGC
GATCCTGAGGCGGCCAGTATGCGTTACCGAAAACGGATCATTATTGTTGAAAGTACACTGATGGCTTCACAACAGGCTCA
GCGAGCGATTGACTGGGAGATGAACCGCCGATACGGCCGTTCCCGTCAGTTAACGGTGACGATTGATTCCTGGCGCGATA
AATCCGGGAAATTATGGGAGCCGAATACGCTTATCCCGGTCAACATCCCGAGTCTGCAGCTGCCTGACACCGAATTGCTT
ATCGCAGAGGTGACTTATATCAGAGACAGTGACGGCACCCATGCGCGCCTTTATCTGATGCCCCCGGAGGCTTTCGCTGT
CCAGCCTTATGCTTTCTACCAGCAAATACCCGGACTAAACCAATGA
>Cluster_21060
ATGTTTGATTTCGACAAAATCATTGAGCGTCAAAGTGATAAGTGCCGTAAATGGGACCATACCTTTGTTTGCTCGCGTTT
CGGTGACGTCCCGGAGTCCTTTATCCCCCTATGGATAGCCGATATGGATTTCACCTCACCACCTGCGGTGATTGACGGTT
TCCGGCGCATCGTGGAGCACGGCACCTTTGGTTATACCTGGTGCTTTGACGAATTCTACGACGCGGTCATTGCCTTCCAG
CGCAAACGTCATCAGGTTGAGGTGGAAAAGTCGTGGATCACGTTGACCTACGGCACCGTATCCACGCTGCACTACACGGT
TCAGGCATTCTGCAAACCGGGTGACAGCGTGATGATGAACACGCCGGTCTACGATCCTTTTGCGATGGCGGCACAGCGCC
AGGGCGTGCAGGTACTGGCTAACCCGCTGCGCGTGGAGGAAAACCGCTATCAGCTTGATTTTAATCTGATAGAAGAACAG
CTCAAAACCCACCGTCCAACGCTGTGGTTCTTCTGCTCGCCACATAACCCGTCCGGCAGGATCTGGCGCGAGGAAGAAAT
ACGCCAGGTGTCCGATCTCTGTCAACGCTACGGCACGATTCTGGTGGTCGATGAGGTTCACGCTGAACACATTCTGGATG
GCAAATTCGCCAGTTGTCTCACCTCTGGCTGTGCCGCCCAGGACAACCTGATCGTGCTCACATCGCCCAACAAAGCGTTC
AATTTGGGCGGGCTGAAAACCTCCTACTCCATGATTCCAGACGACTCGCTGCGCCAGCGCTTCCGCCAGCAGCTCGAGAA
GAACTCCATTACCTCGCCCAATTTGTTCGGGGTATGGGGAATCATTCTGGCCTATCAACACGGTCTGCCCTGGCTCGACG
CGCTGAACGGTTATCTGCAAGGCAACGCCCGGTATCTGGCGGATGCCCTCCAGACCCACTTCCCGGCGTGGAAGATGATG
AACCCGGAATCGTCGTATCTGGCGTGGATAGACGTAAGCGCGGATGAGCGTAGCGCAACGCAGCTAACCCAACATTTCGC
ACGGCAGGCAGGCGTGGTCATAGAAGACGGCAGCCACTATGTACAAAACGGCGAAAACTACCTGCGGATTAATTTTGGCA
CCCAGCGCTACTGGCTGGAGCAGTCCATTAACCGAATGCTGAAAAATGACAAATAA
>Cluster_6813
ATGACGCTTGATGAGTTTATGGCGCTGGCCGGCACATCGAACACGGGGGCGGGTGAGTACGTATCATCGGGGACAGCGGA
ATCACTGCCCGCCGTCATGAACGTCGTGACGGTCATCTCTGAGGCGGTGGCTACCATGCCGTGTTACCTGTACCTGGTAC
GCAATGAGAAGGGGAAGGAGGCCCGCGAGTGGCTTGATTCTCATCCGGTCGATCACATCCTCAACGAGCGCCCGAACGCG
TGGCAAACCCCCTACCAGTTTAAGCGAATGATGATCCGCCACTGCCTGTTAAACGGTAATGCTTATGCGGTGATTCGGTG
GGGGCGTGATGGTTTTCCGGTGGCTTTACATCCTTACCCGCCGCAGTCGGTGAACGTGGAGCAGACAGGTGAGCATAACT
GGCGCTACTGCATCACTGACGCCTACACCGGAAACACCCGCAACTATTTACCGTGGGAGGTTCTCCACCTTCGTTACTCC
ACAGATGACGGTTTTATGGGGCGCTCACCTGTAACCATCTGCCGCGAATCGCTGGGGCTTGGGCTGGCCCAACAACGCCA
CGGCGCGAGCGTGATGCGTGATGGCATGATGGCGGCAGGGGTTATCACGTCAGGCGAATGGCTGGACGGCGTGAAGGGCA
AACAGGCATTAGCCGCACTGGAACGCTACAAAGGGGCCAGAAACGCCGGAAAAAACGCCCATCCTTGAAGGGGGTATGAGC
TATCAGCAGCTGGGCATGAGTAATCAGGATGCTGAATGGCTGGCCTCCCGTCGCTTCACCATTGAAGACATCGCCCGAAT
GTTCAACGTCTCGCCGATTTTTTTGCAGGAATACAGCAACAGCACCTACAGCAATTTCAGCGAGGCAAGCCGCGCATTTC
TCACCATGACGATGCGCCCGTGGCTGGCGAACTTTGAGCAGCAGATAAAAAACGCCCTGCTGGTGGCCTCACCTGTACCT
GGTATCCGGTATCAGGTGGAGTTTGACAGCGCGGACCTGTTACGGGCCACACCTGGCGAACGCTTTGCCACCTATGAACG
CGGCATCAAATCCGGCGTTATGTGCCCGAACGAAGCCCGCGAACGTGAAGGGCTGTCCCCGCGTGATGGTGGTGATGAGT
TCAGCCAGGCATGGAAACAGGAAGTAAAAATCAGCGAGGGAGAAAAACCGGAATGA
>Cluster_55645
ATGAAAATTACTATTTCCGGTACAGGTTATGTTGGTTTATCGAACGGTGTCCTGATTGCGCAAAACCACGAAGTGGTTGC
TCTGGATATCGTACAGGCCAAAGTGGATATGCTTAACCAGAAGATCTCTCCGATTGTGGATAAAGAGATTCAGGAATATC
TGGCAGAAAAACCGTTAAATTTCCGTGCCACCACGGACAAGCACGACGCCTATCGTAATGCCGACTACGTGATCATTGCG
ACGCCGACCGATTACGATCCCAAAACCAACTACTTCAACACCTCTACAGTGGAAGCGGTTATTCGCGATGTCACAGAGAT
CAACCCGAACGCGGTGATGATCATTAAATCGACCATCCCGGTGGGGTTCACCCGCGACATCAAAGAACGTTTAGGGATTG

ATAATGTTATTTTCTCTCCTGAGTTCCTGCGTGAAGGCCGTGCGCTGTACGACAACCTGCACCCATCGCGCATTGTTATT
GGTGAGCGCTCTGCGCGTGCCGAGCGTTTCGCAGACCTGCTGAAAGAAGGCGCGATTAAGCAGGATATCCCGACCCTGTT
TACCGACTCCACTGAAGCGGAAGCGATCAAACTGTTCGCGAACACCTATCTGGCGCTGCGTGTTGCCTATTTCAATGAGC
TCGACAGCTATGCTGAAAGCCAGGGGCTGAACAGCAAGCAGATTATCGAAGGGGTATGCCTGGATCCGCGTATCGGCAAC
CACTACAACAACCCGTCCTTTGGCTATGGCGGCTACTGCCTGCCGAAAGATACCAAGCAGCTGCTGGCGAACTACGAATC
GGTCCCGAACAATATCATCGCGGCTATCGTGGATGCTAACCGTACCCGTAAAGACTTTATCGCGGATTCTATTCTCGCCC
GTAAGCCGAAAGTGGTGGGTGTGTATCGCCTGATCATGAAGAGTGGTTCGGACAACTTCCGTGCTTCTTCTATTCAGGGC
ATTATGAAGCGCATCAAGGCGAAAGGTATTCCGGTTATTATCTATGAACCGGTGATGCAGGAAGATGAGTTCTTTAACTC
CCGCGTCGTGCGCGACCTGGATACCTTCAAACAAGAGGCGGATGTGATCATCTCTAACCGTATGGCGGAAGAGCTGGCGG
ATGTGGCGGACAAGGTATACACCCGCGATCTGTTTGGTAACGATTAA
>Cluster_21452
ATGCTGGAGTTGTTGTTTCTGCTTTTGCCTGTAGCCGCTGCCTATGGCTGGTATATGGGCCGCAGAAGTGCGCAACAAAA
CAAGCAAGATGAAGCCAACCGCTTGTCGCGTGATTACGTAGCGGGGGTTAACTTCCTGCTTAGTAATCAACAGGATAAAG
CGGTAGACCTGTTTCTCGATATGCTTAAAGAGGATACAGGTACCGTTGAAGCCCACCTTACGCTCGGAAACCTGTTCCGT
TCGCGTGGCGAAGTTGATCGCGCTATTCGCATCCATCAGACCCTAATGGAAAGCGCCTCGCTGACCTATGAACAGCGGCT
TTTGGCGATTCAACAACTGGGGCGTGATTACATGGCTGCCGGGTTATACGACCGCGCGGAAGACATGTTCAATCAGCTGA
CAGATGAAACTGACTTCCGCATTGGCGCGCTGCAACAGTTGCTACAAATCTACCAGGCTACCAGCGAGTGGCAGAAAGCA
ATTGATGTTGCCGAACGCCTGGTGAAGCTGGGTAAAGATAAACAGCGCGTCGAAATTGCCCATTTCTACTGTGAGTTAGC
TCTGCAGCATATGGCCAGCGACGATCTCGATCGTGCCATGACTTTGCTAAAAAAAGGTGCGGCGGCAGATAAAAACAGCG
CCCGCGTATCCATCATGATGGGACGCGTGTTTATGGCGAAAGGAGAATACGCCAAAGCCGTCGAAAGTTTGCAACGTGTC
ATATCCCAGGACAGAGAACTGGTCAGCGAAACGCTGGAAATGCTGCAAACATGCTATCAGCAGTTGGGTAAAACTGCCGA
ATGGGCAGAATTCCTGCAGCGCGCGGTGGAAGAGAACACCGGTGCCGATGCTGAATTGATGCTTGCGGATATTATCGAAG
CGCGCGACGGTAGTGAGGCCGCACAGGTCTATATTACGCGTCAGCTTCAGCGTCATCCGACCATGCGTGTGTTCCATAAG
CTAATGGATTACCACTTAAATGAAGCGGAAGAAGGGCGTGCCAAAGAGAGTCTGATGGTGCTGCGTGACATGGTTGGCGA
GAAGGTGCGTAGTAAGCCTCGTTATCGCTGCCAGAAATGTGGTTTTACCGCATACACCCTCTACTGGCATTGTCCGTCTT
GTCGGGCCTGGTCAACCATTAAACCGATTCGCGGTCTTGATGGCCTGTAA
>Cluster_22402
ATGAAAAATTGTGTCATCGTCAGTGCGGTACGTACTGCTATCGGTAGTTTTAACGGTTCACTCGCTTCCACCAGCGCCAT
CGACCTGGGGGCGACAGTAATTAAAGCCGCCATTGAACGTGCAAAAATCGATTCACTACACGTTGATGAAGTGATTATGG
GTAACGTGTTGCAAGCCGGACTGGGGCAAAATCCGGCACGTCAGGCGCTGCTAAAAAGCGGACTGGCTGAAACGGTGTGC
GGATTCACGGTCAATAAAGTGTGTGGTTCGGGTCTTAAAAGTGTGGCGCTTGCCGCCCAGGCAATTCAGGCAGGTCAGGC
GCAGAGCATTGTGGCGGGGGGTATGGAAAATATGAGTTTAGCGCCCTACTTACTCGATGCAAAAGCACGCTCTGGTTATC
GTCTTGGAGACGGACAGGTTTATGACGTAATCCTGCGCGATGGTCTGATGTGCGCCACCCATGGTTATCATATGGGGATT
ACCGCCGAAAACGTGGCTAAAGAGTACGGAATTACCCGTGAAATGCAGGATGAACTGGCGCTACATTCACAGCGTAAAGC
GGCAGCCGCAATTGAGTCCGGTGCTTTTACAGCCGAAATCGTCCCGGTAAATGTTGTCACTCGGAAGAAAACCTTCGTCT
TCAGTCAAGACGAATTCCCGAAAGCGGATTCTACGGCTGAAGCGTTAGGCGCATTGCGCCCGGCCTTCGATAAAGCAGGA
ACAGTCACCGCCGGGAACGCGTCTGGCATTAACGACGGTGCTGCCGCTCTGGTGATTATGGATGAATCTGCGGCGCTGGC
AGCAGGCCTTCCCCCCCTGGCTCGCATTAAAAGTTATGCCAGCGGTGGCATGCCCCCCGCATTGATGGGTATGGGGCCAG
TACCTGCCACGCAAAAAGCGTTACAACTGGCGGGGCTGCAACTGGCGGATATTGATCTCATTGAGGCTAATGAAGCATTT
GCTGCACAGTTCCTTGCCGTTGGGAAAACCCTGGGCTTTGATCCTGAGAAAGTGAATGTCAACGGCGGGGCCATCGCGCT
CGGACATCCTATCGGTGCCAGTGGTGCGCGTATTCTGGTCACACTATTACATGCAATGCAGGCACGCGATAAAACGCTGG
GGCTGGCAACACTATGCATTGGTGGCGGCCAGGGAATTGCGATGGTGATTGAGCGGTTGAATTAA
>Cluster_11026
ATGTCACCTGGGATCGAAGATACGCCCCAAAAACCACTGTCCTGCTGGCCACTTGCGTTCAGTGCCGGGCTTCTCGGTAT
CGGGCAGAACGGTCTGCTGGTTGTGCTCCCTGTTCTGGTCATACAGACAAATCTGAGTCTGTCTGTATGGGCTGCCCTGC
TGATGCTTGGCTCAATGCTGTTTCTGCCATCGTCCCCGTGGTGGGGAAAGCAAATTTCCCGTACTGGCAGTAAACCTGTG
GTGTTGTGGGCTCTGGGAGGATATGGCATAAGCTTTACCCTGCTAGGGCTGGGAAGCGTGCTGATGGCTACCAGCGCCAT
AACAACAGCGGTGGGACTGGGAATATTAATCATCGCCCGGATCGCCTACGGGCTGACCGTGTCGGCAATGGTGCCAGCCT
GTCAGGTCTGGGCATTGCAGAGAGCAGGAGAAGGGAATCGCATGGCCGCTCTGGCAACCATCAGCTCCGGCCTGAGTTGC
GGCAGGCTATTCGGGCCGCTGTGCGCGGCAGCAATGCTGGCCATTCATCCTCTGGCGCCACTGGGGCTGCTGATGGCAGC
CCCAGTGCTGGCGCTGCTGATGCTGCTGCGGTTGCCCGGCACACCACCACAGCCCACACCGGAGCGCAAGAGCGTCAGTC
TGAAGCGGGATTGTCTGCCTTATCTGCTTTGCGCAATATTACTGGCTGCGGCGGTGAGCATGATGCAGCTTGGACTTTCG
CCTGCCCTTACTCGCCAGTTCGCCACGGATACCACCGCCATTAGCCAACAGGTGGCCTGGCTGTTGGGTCTGTCCGCAGT
AGCTGCGCTTATCGCGCAGTTCGGGGTACTCCGTCCGCAGCGCCTGACTCCGGTGGCCCTGCTCCTGAGTGCCGGAGTGT
TGATGAGTGGTGGTCTGGCTATCATGCTCTCCGAACAGCTATGGTTGTTTTACCCGGGCTGTGCAGTGCTGTCATTTGGA
GCAGCTCTGGCAACACCCGCTTATCAACTTCTACTGAATGATAAGCTGGCTGATGGCGCAGGCGCGGGCTGGCTCGCTAC
CAGTCACACACTTGGCTATGGACTGTGCGCTTTGCTGGTGCCGCTGGTATCGAAAACAGGTGTCGCAATCGCGCTGATTA
TGGCGGCATTATTTGCAACTATATTATTTACCATTGTGTCTGTATTTATCTGGCATTACCGTGCTATCAAATAA
>Cluster_23169
ATGAGTCATGATCCACAACCGCTGGGCGGCAAAATCATCAGTAAACCGGTCATGATTTTTGGACCGTTAATCGTCATCTG
TATGCTCCTGATTGTGAAGCGTCTGGTGTTCGGTCTGGGCTCTGTCTCCGACCTGAACGGCGGCTTCCCGTGGGGCGTGT
GGATCGCGTTTGACCTGCTGATCGGTACCGGCTTTGCCTGTGGCGGCTGGGCGCTGGCGTGGGCAGTATACGTCTTTAAC
CGTGGGCAATACCATCCGCTGGTGCGTCCGGCGCTGTTGGCGAGTCTGTTTGGTTATTCACTGGGTGGCTTGTCGATCAC
TATCGACGTGGGTCGCTACTGGAACCTGCCGTACTTCTACATTCCGGGTCACTTCAACGTGAACTCGGTACTGTTCGAGA
CGGCGGTCTGTATGACCATCTACATCGGTGTGATGGCACTGGAGTTTGCTCCGGCATTGTTTGAACGTCTGGGCTGGAAG
GTGTCACTCAAACGTCTGAACAAGGTGATGTTCTTCATCATCGCGCTTGGTGCGCTGCTGCCAACTATGCACCAGTCTTC
AATGGGGTCGCTGATGATCTCGGCGGGCTACAAGGTGCATCCGCTGTGGCAAAGCTATGAAATGTTGCCGCTGTTCTCGC
TGCTGACGGCGTTCATCATGGGCTTCTCGATTGTCATCTTTGAAGGTTCGTTGGTGCAGGCGGGTCTGCGTGGCAACGGT
CCGGATGAAAAGAGTCTGTTCGTTAAGCTGACCAACACCATCAGTGTGTTGCTGGCGATTTTCATCGTGCTGCGCTTTGG
CGAGCTTATCTATCGCGACAAGCTGTCGTTAGCGTTTGCCGGTGACTTCTACTCCGTGATGTTCTGGATTGAAGTCCTGC
TGATGCTCTTCCCGCTGGTCGTTCTGCGTGTGGCGAAGTTGCGTAATGATTCCCGCATGCTGTTCCTGTCAGCACTGAGT
GCACTGTTAGGTTGTGCAACCTGGCGTCTGACCTATTCGCTGGTGGCATTCAACCCGGGTGGCGGTTACGCCTACTTCCC
GACCTGGGAAGAACTGTTGATTTCTATTGGTTTTGTGGCTATTGAGATTTGCGCTTATATCGTACTCATTCGTCTACTGC
CGATACTTCCTCCTTTAAAACAAAACGATCATAATCGTCATGAGGCGAGCAAAGCATGA
>Cluster_24556
ATGCCTTCGTCCACGCATCCCGTAGAACGCTTTTCTTTCAGCACCGCGCTTTTCGGGATGCTGGTTCTGACCTTAGGTAT
GGGTTTAGGCCGCTTCCTTTATACGCCTATGTTGCCCGTCATGATGGCGGAAGGATCGTTTTCATTTAGCCAGCTCTCGT
GGATTGCCAGCGGCAACTATGCGGGGTATCTGGCTGGCAGTCTGCTATTTTCTTTTGGCGCATTTCACCAGCCATCGCGC
CTGCGCCCATTTCTGTTGGCTTCTGCCCTGGCGAGCGGGTTGTTGATCCTCGCAATGGCATGGTTGCCGCCATTTATTCT

GGTGTTATTGATTCGCGTCCTGGCGGGTGTCGCCAGCGCCGGAATGCTGATTTTTGGTTCGACGCTGATTATGCAGCACA
CGCGCCATCCTTTTGTGCTGGCAGCTTTGTTTTCTGGCGTTGGCATTGGCATCGCACTGGGCAATGAATATGTTCTGGCA
GGCCTGCATTTTGACCTCTCCTCGCAAACGTTATGGCAAGGTGCCGGGGCGCTTTCTGGCATGATGCTGATTGCACTTAC
GCTTTTAATGCCCTCGAAAAAACACGCCATCACACCAATGCCATTGGCAAAAACGGAGCAACAGATAATGAGCTGGTGGT
TACTGGCTATTCTGTACGGCCTGGTGGGATTTGGTTATATCATCGTCGCGACTTACCTGCCGCTCATGGCGAAAGATGCA
GGCTCACCATTGTTAACCGCCCATCTCTGGACGTTAGTCGGCTTATCAATTGTGCCTGGTTGCTTTGGCTGGCTATGGGC
AGCAAAACGTTGGGGGGCTTTGCCCTGCCTGACGGCGAATTTGCTGGTGCAGGCTATTAGTGTGTTGCTTACTCTCGCCA
GCGACTCGCCTCTCTTGCTTATCATCAGCAGTATTGGTTTTGGCAGTACCTTTATGGGCACGACATCTCTGGTGATGACT
ATCGCCCGCCAACTGAGCGTACCGGGAAATCTCAATCTTTTAGGCTTTGTGACGCTCATTTACGGCATTGGGCAAATTCT
TGGCCCGGCGCTGACCAGTATGCTTGGCAATGGAACATCGGCGCTTGCCAGCGCCACTCTCTGCGGCGCGGCGGCGTTGT
TTATTGCTGCACTTATCAGCACAGCGCAATTATTTAAGCTGCAAGTGGTCACTTCATGA
>Cluster_41024
ATGATTAACTACCCATTAGCAAGTAGCACTTGGGATGATTTAGAATATAAAGCAATCCAATCAGTTTTAGACTCTAAAAT
GTTCACGATGGGGGAGTATGTAAAACGATACGAAACCCAATTTGCAAAGACTTTTGGAAGTAAATATGCCGTGATGGTGA
GTTCAGGGTCTACAGCAAATTTATTAATGATTGCTGCATTATTTTTCACAAAAAAACCGAGATTAAAAAAGGGGGATGAA
ATAATAGTTCCTGCTGTTTCTTGGTCAACTACTTATTATCCGTTGCAACAATATGGATTGAGGGTGAAGTTTGTTGATAT
CGATATTAATACCTTAAATATTGATATTGAAAGTTTGAAAGAAGCTGTAACAGATAGTACAAAAGCAATACTCACGGTAA
ATTTATTAGGTAATCCTAATAACTTCGATGAAATTAATAAAATTATTGGAGGTAGAGATATTATCTTATTGGAAGATAAC
TGTGAGTCTATGGGAGCAACCTTTAATAATAAATGCGCCGGCACTTTTGGTCTGATGGGAACATTTAGCTCTTTCTATTC
TCATCATATAGCTACCATGGAAGGGGGATGCATTGTCACTGATGATGAAGAAATTTATCATATCTTGCTGTGCATTCGAG
CTCATGGTTGGACGCGTAATTTACCGAAAAAAAATAAAGTTACAGGTGTGAAGAGTGATGATCAATTTGAAGAGTCATTC
AAATTCGTTTTACCCGGCTATAATGTTAGGCCATTGGAAATGAGTGGTGCAATAGGCATAGAACAATTAAAAAAATTGCC
AGGATTTATTTCTGTTAGAAGAAAAAATGCAGAGTATTTTCTTGAGAAATTCAAGGACCATCCATACCTGGACGTGCAAC
AGGAAACTGGCGAGAGTAGCTGGTTTGGTTTCTCATTTATCATAAAAAAAGATTCTGGTGTCATTAGGAAACAACTTGTT
GAAAATTTAAATTCTGCTGGTATTGAGTGTAGACCAATTGTCACTGGTAACTTTCTGAAAAATACAGATGTTCTTAAATA
TTTCGACTATACCGTTCATAACAATGTAGATAACGCTGAGTATTTAGATAAAAATGGTCTTTTTGTTGGAAATCATCAGA
TAGAACTATTTGATGAGATTGATTATCTCAGAGAGGTTTTGAAATGA
>Cluster_6815
ATGCCGAGAATAATCGAATTACGCCAGCAGAAAACCGCCATTAAAAATCAGATGCGCGACATGCTGGAGAACGCGGAAAA
AGAAAACCGCAGTCTTAACGATGCTGAGGGCGCAAAATTTGACGAATTACGCGCTAAAGCTGAATCCCTCGATAAAGACA
TTTCCCGCCTTGAAGCCATTGCAGACGAAGAGCGCAGCAAGCCAGGTAAAAGCAGTCAGACCACTGACCCCGCAGAACTA
CGCAACTACATCCTGACAGGTGAAACCCGCGCATTAAGTACAGGCGTTCCCGCTGATGGTGGTTATACCGTTATCCCCGA
GCTGAACACCGAAATCATGCGAATGCTGGCGGATGAATCCACCATGCGCCGCATCTGTACCGTGAAGAAAATCAGCAGCA
ACGAGTTTAAGCAGCTTGTTTCCGCTGGCGGTGCGACCGTTAACCACGGTGAAGAGGGTAAGGCACGCGAACAGACCAGC
ACCCCGCAGATTAACGAGGTGAGCATTAAGCTGTATCCGGTCTATGCGTACCCGCGCACCACACAGGAAATCGTGGATTT
TTCCGATGTGGACATCCTTTCATGGCTGACGGGTGAGATTGGCGACACCTTCACGGAAACCGAAGAAAGCGATCTGGTTG
TGGGCGACGGTGACAAAAAAGCAAAAGGTTTTTTATCCGTACCCCGTGCAGAGAAGAACGACAAAGAGCGTGATTTTGGT
ACGTTGCAGGTAATTAAACCTTCCGAATCTCTGGCGTGGACATCTGCGGACCCGCTGATCGACCTGAAATTTGCATTACG
TAAAAAATACCGCAAAAACGCGGTCTGGGTGGTTAACTCCACGACGGCGGCAAAACTTCAGAAGGTGAAGAACGCGAACG
GCGATTACATCTGGCGCGACCGTTTACAGGCGGGTGATCCTGATACGTTGCTGGGGCTTCCGGTGGAGTATCTGGAATTT
ATGCCGGATAACGTTATCGCCCTGGGTGACTTCAAACGCGGTTATTACATCGTTGATCATGAAACGGGCGTGCGCACCAG
ACCGGACAACCTGACAGAGCCGGGCTTTATCAAAATTTTCACGCAGAAATATTTAGGCGGTGGCGTGGTGGATTCGAACG
CTATAAAGATTCTGGAACTGCCGCAGGACGACGATTAA
>Cluster_22309
GTGACTTATTTCCGTATTAATCCTGTTCTGGCGCTGCTGCTGTTGCTGACGGCAATCGCAGCGGCGCTGCCGTTTATCAG
TTACGCACCTAATCGTTTAGTTTCGGGTGAGGGGCGTCATCTCTGGCAGCTGTGGCCGCAAACTCTCTGGATGCTGGTGG
GCGTTGGTTGCGCCTGGCTGACAGCCTGTTTTATTCCCGCTAAAAAAGGCAGCATTTTTGCACTCATTCTGGCGCAATTC
GTCTTCGTATTGCTGGTGTGGGGAGCTGGAAAGGCGGCGACACAACTGGCGCAAAATGGTAGTGCGCTGGCGCGTACCAG
CCTCGGAAGTGGTTTCTGGCTGGCTGCGGCGCTGGCACTGCTGGCCTGTAGCGATGCCATCCGCCGAATCTCCACGCATC
CGCTGTGGCGCTGGTTGTTGCATATGCAGATTGCCATTGTTCCGCTGTGGTTGCTGTACTCCGGCACGCTTAACGATCTC
TCGTTAATGAAAGAATACGCCAACCGTCAGGATGTGTTTGACGACGCGCTGGCACAACATCTGACGTTGCTGTTTGGTGC
GGTGCTGCCTGCGTTAGTGATTGGTGTGCCGTTGGGCATCTGGTGCTACTTTTCCACCGCGCGGCAGGGGACGATTTTTT
CTCTGCTGAATGTCATTCAGACCGTGCCTTCGGTAGCGCTCTTTGGCCTGTTGATTGCGCCGCTTGCCGCGCTGGTTACG
GCCTTTCCGTGGCTGGGAACGCTCGGCATAGCAGGAACCGGAATGACACCCGCACTGATTGCACTGGTGCTCTATGCCCT
GTTACCGCTGGTGCGCGGCGTGGTAGTCGGCTTGAACCAGATCCCGCGCGATGTGCTGGAGAGCGCCAGAGCGATGGGGA
TGAGCGGGGCGCAGCGTTTCCTGCATGTTCAGTTACCGCTGGCGTTACCGGTATTTCTGCGCAGCCTGCGGGTGGTGATG
GTGCAAACCGTTGGCATGGCGGTGATTGCGGCGTTAATCGGCGCAGGCGGTTTTGGTGCGCTGGTTTTCCAGGGGCTGCT
AAGCAGTGCCATTGATTTAGTGTTGCTGGGGGTGATTCCGGTAATTGTTCTGGCGGTGCTTACCGACGCGCTGTTCGATT
TGCTTATCGCACTGCTGAAGGTGAAACGTAATGATTGA
>Cluster_55637
ATGAAAATTATTTTTGCTACTGAGCCAATTAAATACCCATTAACGGGCATCGGTCGGTATTCCCTGGAGCTGGTTAAGCG
GCTGGCGGTTGCCCGTGAAATCGAAGAGCTGAAGCTGTTTCACGGCGCGTCGTTTATCGACCAGATCCCCCAGGTGGAGA
ATAAAAGCGATAGCAAAGCCAGCAATCATGGTCGTCTGTCGGCGTTTCTGCGCCGCCAGCCGCTGCTGATTGAGGCGTAT
CGCCTGCTGCATCCGCGGCGCCAGGCGTGGGCATTGCGCGATTACAAAGACTACATTTACCATGGTCCCAATTTCTATCT
GCCGCATCGCCTGGAACGCGCCGTGACCACGTTTCATGACATCTCCATTTTTACCTGCCCGGAATATCATCCAAAAGATC
GGGTTCGCTATATGGAGAAGTCCCTGCACGAGAGCCTGGATTCGGCGAAGCTGATCCTGACCGTCTCCGACTTCTCGCGC
AGTGAAATCATCCGCCTGTTCAACTATCCGGCGGATCGGATCGTCACCACCAAGCTGGCCTGCAGCAGCGACTATATTCC
GCGCAGCCCGGCGGAGTGCCTGCCGGTCCTGCAGAAATATCAGCTGGCGTGGCAGGGGTATGCGTTATATATCGGCACCA
TGGAGCCGCGTAAAAATATCCGCGGTCTGCTGCAGGCCTATCAGCTGCTGCCGATGGAGACCCGCATGCGCTACCCGCTG
ATCCTCAGCGGTTATCGCGGATGGGAAGACGATGTGCTGTGGCAGTTAGTCGAGCGCGGTACGCGTGAAGGGTGGATCCG
TTACCTGGGCTATGTCCCGGATGAAGACCTGCCTTATCTGTACGCGGCGGCCAGAACCTTTGTTTATCCCTCCTTCTATG
AGGGATTCGGTTTACCTATTATTGAAGCGATGTCTTGCGGTGTGCCGGTAGTATGTTCCAATGTCACTTCTTTGCCTGAG
GTGGTTGGCGATGCCGGCCTCGTTGCCGATCCTAATGATGTAGACGCGATTAGCGCACATATTTTGCAGAGCCTGCAGGA
TGATAGCTGGCGGGAAATCGCCACCGCGCGCGGTCTTGCCCAGGCGAAACAGTTTTCGTGGGAGAACTGTACGACCCAGA
CCATTAACGCCTATAAATTACTCTAA
>Cluster_51148
ATGAACATTGATTCAAAAAAAACGCTATTGTCAATAGCGATACCTACAAAGAACAGACATAAAACGCTACTAATTACTCT
TGATAATATGCTTCGCCGAATGGATGACTCTGTGGAATTTGTCATTTGTGATAACTCTGATGAGGAGCTTCCTTCGTCTC
TACCTTTATTTTTGGATAAACGCATTAAATACTTTTATTCCTCAAAAACTATGTCAATTGTCGATAATACAGAAAATGCA

TTAAGTCATTGTACAGGGGAATTTGTATGTTTTATCGGAGATGATGATTTTGTATCACCATATATAATAAATTATCTTAA
AGAGATTCTCCATACTAATATAGATGCTGTATCATACCAAGGCGGTTATTATTGGTGGAAGGATATTGACTTTAATCGGA
AAAGCTATTATGAACAGGCTGGCGCGTTTTGGTATCCTTCTGCTACGTTTGAGCCTCGGTTAATTCGTGCTGATGAATCA
CTTCGTGCTCTGCTTAGCGGAGGTGGGCTTTCAGTTGGTCTACTACCAAGGTTATACCACGGAATCGTGAGAAGGTTTGT
TTTAGATAATTTGAAAAAGAAAACTGGTAGATATGTTCACGGAGCTTCGCCTGATATATCGTTGGCGGTAAGCCTTGCTT
TGTTAGGTGTTAACACATTTTATATAAGTACACCATTAACAATTTTTGGCGCCTCAAAGAAAAGTGGTGGTGGATGGACT
GCCGAAAACAAGCACTATGGTAGTATAAGAGAACAGCAGCATATACCTCAATATACTAAGGATAATTGGTCTAGTTTCTT
GCCTGATGTATGGTCTGAGCACACCATATATCCTCAGTCTATTATTGAGGTCTATGATAGTTTTAATAAGGAATGCATAC
TAAATCTTGATATGTTTTATGCTTCTCTATATATAAATGAACCTCATCTTCGTAAAATTGTAACTCCTTTTATTATTAAG
TATTATAAACGGCATCCCAATAAATTATTCTTTTTTATACCAGCTCTAATCAGAAAAAAATGTGGAGATATTAAGCGTAA
ATTTGAATCAAAATTCAAGTGCAGAAAGTTTTATGTTTATCATTCTCTTGAAGTAAGTGATGTTATGGATAAATTAGAAC
AATTGCAAACAAAGAGAGATACTTTATGA
>Cluster_17656
ATGCCGACAACTGACGATGTTGACCTGGGTTATGCGTACACCCTGAAGCCGGAAGAAGCGATTAAGTATTTCGAAAGCAA
GGGATACGTCATCGGCTTCCGTTGGCACGATGTGAAGGACATCGCACACGCCCGGGCGTTCACGGTGGCGGGCGTGCTGA
AACTGGATGTTCTGAAGGATATCCGTGATGGCCTGACGGCGGCACTGGCTGACGGCGGGACGTTCCGGGAGTTTGCTGCA
CAACTGGAACCACTGCTGGAATCAAAAGGCTGGCTGGGTAAAAAACTGATTGTGGACGAGGATACCGGCGAACTGCACGG
CAGACAGCTGACGCCGCGCCGGTTGCGCACGATATTTGATACCAATATTCAGTCGTCCTACAACGCCGGGCGTTATCAGC
AACAGATGGCGAACGTGGCTGACCGGCCTTATTTTGAGCGGGTGGCGGTGATGGACCTTCACACCCGCCCGAAACACGCC
GCCCTGAACGGCTTTACCGCCCGGGCGGATGACCCGGTCTGGGAGTATTTCTACGCGCCTGACGGATACGGATGCCGCTG
CCGTATCCGGGCGCGTTCGGCGTCAGATGTGGAGAAATATGGCCTGACGGTGCAGAGCAGCGAGGGGCGACTGGTTGAGG
TTGAACAGGAATATGGCCAGCCGGGCCAGACCATCAGAACAATGGGGCTGAAGATGCCGGACGGTTCCGTGTATACCGCC
GACCCGGGCTTCGGATTTAATCCCGGTCGGGTGGCATGGCAGCCGGAACTGGAAAAATACGATTACCGCAGCGCCCGCCA
GTATGTCACCGGCACCCTGACCGGGCCGGATTTTGCACGGGGGCTGGCGAACGTCAGCGAACTGGATGCGCGCCAGCGAT
ATCCGCTGGCCATCCGTTCACCGGAACAGGTCGCCGCCACGGGCGCTGCACGGCAGACGGTAAACCTTACGGCTGACGTC
ATGAAGCGCCTCAGTGCAGCAAACACGCCCCCGACTGCCGCCGACTATGCGCTGATGCAGCAGACCATCGAACGGGCGGA
GCATGTCACGCAGGACGGTAACGCGTGGCGGTATGCGTTACAGTCAGGCGACCGCTGGTCGGTGGTGACGGTTGAGGATG
ACGTGCTGACAGACTGGGTTATGCAGGACACCCCGGAGGCATCATGA
>Cluster_2412
ATGATTACATACCCACTTGCTAGTAATACTTGGGATGAATATGAGTATGCAGCAATACAGTCAGTAATTGACTCAAAAAT
GTTTACCATGGGTAAAAAGGTTGAGTTATATGAGAAAAATTTTGCTGATTTGTTTGGTAGCAAATATGCCGTAATGGTTA
GCTCTGGTTCTACAGCTAATCTGTTAATGATTGCTGCCCTTTTCTTCACTAATAAACCAAAACTTAAAAGAGGTGATGAA
ATAATAGTACCTGCAGTGTCATGGTCTACGACATATTACCCTCTGCAACAGTATGGCTTAAAGGTGAAGTTTGTCGATAT
CAATAAAGAAACTTTAAATATTGATATCGATAGTTTGAAAAATGCTATTTCAGATAAAACAAAAGCAATATTGACAGTAA
ATTTATTAGGTAATCCTAATGATTTTGCAAAAATAAATGAGATAATAAATAATAGGGATATTATCTTACTAGAAGATAAC
TGTGAGTCGATGGGCGCGGTCTTTCAAAATAAGCAGGCAGGCACATTCGGAGTTATGGGTACCTTTAGTTCTTTTTACTC
TCATCATATAGCTACAATGGAAGGGGGCTGCGTAGTTACTGATGATGAAGAGCTGTATCATGTATTGTTGTGCCTTCGAG
CTCATGGTTGGACAAGAAATTTACCAAAAGAGAATATGGTTACAGGCACTAAGAGTGATGATATTTTCGAAGAGTCGTTT
AAGTTTGTTTTACCAGGATACAATGTTCGCCCACTTGAAATGAGTGGTGCTATTGGGATAGAGCAACTTAAAAAGTTACC
AGGTTTTATATCCACCAGACGTTCCAATGCACAATATTTTGTAGATAAATTTAAAGATCATCCATTCCTTGATATACAAA
AAGAAGTTGGTGAAAGTAGCTGGTTTGGTTTTTCCTTCGTTATAAAGGAGGGAGCTGCTATTGAGAGGAAGAGTTTAGTA
AATAATCTGATCTCAGCAGGCATTGAATGCCGACCAATTGTTACTGGGAATTTTCTCAAAAATGAACGTGTTTTGAGTTA
TTTTGATTACTCTGTACATGATACGGTAGCAAATGCCGAATATATAGATAAGAATGGTTTTTTTGTCGGAAACCACCAGA
TACCTTTGTTTAATGAAATAGATTATCTACGAAAAGTATTAAAATAA
>Cluster_61197
ATGCCTGAATCGTTGATGGTCATACGTTCTTCCTCGACGCTGCGTAAGCATTGGGAATGGATGACGTTCTCCGCAGATAG
CGTATCTTCGGTACATACGCTCACGGACGATCTTCCGCTGGAGTCGCTGGCCGATCAGCCCGGTGCCGGAAATGTTCATC
TTTTGATCCCACCAGAGGGCCTTTTATATCGTTCCCTCACGCTGCCCAACGCCAAATACAAACTCACCGCACAAACATTA
CAATGGCTGGCAGAGGAGACGTTACCCGACAACACCCAGGACTGGCACTGGACGGTTGTCGATAAACAAAACGAAAGTGT
CGAGGTGATAGGCATTCAGTCGGAGAAACTCAGTCGCTATCTTGAGCGTTTACATACTGCCGGACTCAACGTGACGCGGG
TTTTGCCAGATGGCTGCTATCTTCCCTGGGAAGTAGATAGCTGGACTCTGGTTAACCAGCAAACAAGCTGGCTCATTCGC
AGTGCCGCACATGCCTTCAACGAACTGGATGAACACTGGTTACAGCATCTTGCTGCCCAGTTTCCGCCTGAGAATATGCT
TTGTTATGGTGTAGTACCTCACGGCGTTGCAGCGGCCAATCCACTTATCCAGCATCCTGAAATACCATCACTGAGTTTGT
ATTCTGCCGATATTGCATTCCAGCGCTATGACATGCTGCATGGTATTTTTCGTAAACAGAAAACTGTAAGCAAATCGGGA
AAATGGCTCGCCCGCCTGGCAGTAAGCTGTCTCGTTCTCGCCATTCTTTCTTTTGTTGGCAGCCGGAGCATCGCGCTCTG
GCATACGCTTAAGATTGAAGATCAACTTCAGCAACAACAGCAAGAAACCTGGCAACGTTATTTCCCGCAGATCAAACGCA
CCCACAATTTTCGTTTTTACTTTAAGCAGCAACTCGCTCAGCAATATCCTGAAGCTGTGCCGTTGCTCTATCATTTACAA
ACACTTCTGCTGGAACACCCTGAACTGCAGTAATGGAGGCGAACTATAGCCAGAAGCAAAAGTCGCTGACCTTAAAAAT
GAGTGCTAAGAGTGAAGCTAATATCGATCGTTTCTGTGAGTTAACACAGTCCTGGCTTCCGATGGAAAAAACAGAAAAAG
ATCCGGTCAGTGGTGTATGGACAGTAAGGAACTCAGGGAAATGA
>Cluster_26348
ATGGCTAGAGTCTCTCTTTCATGGGCATTGATTCTTGGTCTTTTAGCCGGTATCGGCCCGATGTGTACCGATCTTTATTT
GCCGGCTTTGCCGGAGATGTCTGAGCAACTGGCGGCAACCACGACCATAACGCAATTAACCCTGACCGCATCACTGATTG
GTCTTGGCGTCGGACAACTGTTATTTGGCCCTCTGAGCGACAAAATAGGGCGTAAACGTCCGTTAATCTTGTCGTTGTTA
TTGTTTATTGTTTCTTCCATTTTGTGCGCGACAACGAACAATATTTACTGGCTGGTGGTCTGGCGTTTTATTCAAGGGAT
CGCGGGGGCGGGTGGTTCGGTGCTCTCTCGTTCTATTGCTCGTGACAAATATCAGGGAGTAACGTTGACCCAGTTTTTTG
CGCTGTTAATGACGGTGAATGGCCTGGCACCGGTGTTGTCACCAGTGCTGGGCGGGTACATTGTTAGCACTTTTGACTGG
CGCACTTTATTCTGGGTAATGGCTGAAATTAGCACCGTACTGTTGCTGGGCTGCCTGTTATTTATTAATGAGACCTTGCC
AGAAAATAAAAGGGGCTCATCATTGCTATTAACCGGACGAAGCGTGGTGCAGAACCGCCGTTTTATGCGCTTTTGCCTGA
TTCAAAGTTTTATGCTGGCCGGTTTGTTTGCATATATCGGCTCTTCGTCGTTCGTGTTGCAGAAGGAATTTGGCTTTAGT
CCAATGCAATTTAGCCTGGTGTTTGGCCTTAACGGCATCGGACTTATCATTGCTTCATGGATCTTCTCGCGCCTGGCGCG
ACGGATTAACGCGATGACATTGTTGCGAGGTGGCCTGATAGCGGCAATTTTGTGTGCATTGCTCACGGTCTTATGCGCAT
GGGTACAATTGCCCATTCCGGCACTGGTGGCATTATTTTTCACCATCGCATTTTGTAGCGGCATCGGCACTGTTGGCGGG
GCAGAGGCTATGAGTGCAGTAGGGACGCAGGAATCTGGAACGGCGTCTGCGTTGATGGGGATGAGCATGTTTGTCTTCGG
CGGTATAGCCGCGCCATTGTCGGGAATTGGCGGAGAAACACTGTTAAAAATGAGTCTGGCAATTACGGTGTGTTATACGC
TGGCATTGCTGGTTGCTCTCACCAGAATCGACAATCAAAAGTAA
>Cluster_15644
GTGAGCAAAATTGACCATCAGGCACTGCGTGAGGCGGCAGAGCAGGCAATGCATGACGACTGGGGATTTGACGCGGACCT
TTTCCATGAGCTGGTAACACCATCGATTGTGCTGGAACTGCTGGATGAACAGGAAAGAAACCAGCAATACATCAAACGCC

GCGACCAGGAGAACGAGGAGATTGCGCTTACGGTTGGGAAGCTGCGTGTTGAGCTTGAAGCAGCAGAGAACAACCTTATT
GATAGTGAATGCCATGTTGCTGAACTGGAAGAAGCTCTACGCGATAAGCAGGCGTTACTTGAAGCCTCAGAAAAGCGCAA
CGCAAAATTACAAAGCGAGAATGCATACATCCGCAACCGGTACAAAGAACTGGACCTATTAATCGGGAAAAACATTCTGG
TCATGCAGGCTGCCATTATCGAATGGCAGGCAACTGGCGACGCTAAGAGCGGACTAGCATGGATTTATAACACACTGTTT
GGCCCTGGCGAATTACCGGACGAATCTGAGAAAGATGCTCAGGCCTACTTTAATCGCAAATATGCACCGATTGACGAAAA
GCTTATGGCGCTTCACAAGTGGTTTTGGGAACAAAGTGAAGCCGAGCGCGCCGCTGGCATTCGCATCAAAGGAGAGTGA
>Cluster_7956
ATGACAACATTATCACGTATACAAGAAATTTCACGCCTCCTTCGCAACCTGATACGCACCGGCGTCGTCGTCGAAGTTGA
CCTCGATGACGGGCGCTGTCGCGTCCAGACTGGCGGCATTGTTACCGACTGGCTTCAGTGGCTGACTACGCGCGCCGGTC
GCTCACGTGTCTGGTGGGCTCCGTCTGCGGGTGAGCAGGTTTTACTGCTGGCCGTCGGTGGTGAGCTCGACACCGCTTTT
GTGTTGCCCGGTATTTTCTCCGATGACCACCCCGGGCCGTCGGCATCGGCTGATGCATTTCACATCACCTTTCCTGATGG
GGCTGTTATCGAGTACGAGCCGGAGACCGGCGCACTGACTGTGAGCGGCATTAAAACCGCAGATGTTACGGCGTCGGATT
CCATCACCGCGACGGTACCGCTGGTGACGGTGAAAGCCGAGACCCGCATCACCCTCGAAACCCCGGAAGTGGTCTGCACC
AACAAACTGACGACGGCGACGCTTGAGGTGCAACAAGGCGGCACCATGCGCGGAAACATCGAACACATCGACGGTACGTT
TAAATCAAACGGCGTTCAGGTCGACAACCACGGTCACGGCGGTGTGCAAAGGGGTGGAGCCTGGACGGAGGGCACGAAAT
GA
>Cluster_28201
ATGTTTAAACGTTTAATGATGGTCGCTTTGCTGGTGATTGCACCGCTGAGTGCGGCAACCGCGGCAGACCAGACCAATCC
GTATAAGCTGATGGACGAGGCGGCGCAGAAAACGTTCGATCGCCTGAAGAATGAGCAACCGCAAATTCGGGCTAACCCGG
ATTATCTGCGTACCATTGTTGATCAGGAACTGTTGCCATACGTACAGGTGAAATACGCCGGTGCGCTGGTGCTGGGCCAG
TATTACAAGAGTGCAACCCCAGCTCAACGTGATGCTTACTTTGCCGCTTTCCGTGAGTACCTGAAGCAGGCTTACGGTCA
GGCGCTGGCGATGTATCACGGTCAAACCTATCAGATTGCGCCAGAACAGCCGCTGGGTGATAAAACCATTGTGCCGATTC
GCGTTACCATTATTGACCCGAATGGCCGCCCGCCGGTGCGTCTGGACTTCCAGTGGCGTAAAAACTCCCAGACGGGCAAC
TGGCAGGCTTACGACATGATTGCTGAAGGCGTCAGTATGATCACCACCAAACAAAACGAGTGGGGAACGCTGCTGCGTAC
CAAAGGTATCGACGGCCTGACTGCACAACTGAAATCGATTTCTCAACAGAAAATCACTCTGGAAGAGAAAAAATAA
>Cluster_5130
ATGTACCGACGTCTGCTCATACTGCTTGCCCTGTCCGGCATGGTACAGGCAAAAAACCTGGGCACATGGGGGGAGATGTA
CCCCATTGCCGAGCAGGACATGCTGACCACCATTCAGACCCGGCTGAAAGCAATGGAGGCCAGTGGTGAGATGGCCCGGG
AGCAGGAAGCATTCAAACAGCGTGTCATAGAAAACACCCTGCGTCCTAAGCCGGTGGAAGGGCTGACGTTGGCGCAGGAA
AACACCACGCACTATATCGATCCATCGCTGACGGTCAGTGAAGATCTGAAAGACCATCAGGGGCGGGTGTTTGCCCGTAA
GGGACAGGTGATTAATCCGCTTGATACGGTGCCCTTTACCGACACGCTGTATTTTATTGATGCGGATAATCCGCAGCAGC
TGGCGTGGATGAAGAAGCAGAATCCCGGCACGCTGACGTACCGCGTCATACTGGTCAATGGGGATGTTCGAGAAGCGACC
AATGCCCTGAATACACGCATTTACTTCGATCAGGATGGCTCGCTCAGCCGGAAGTTTGAACTCAAAACCATCCCAGCCCG
TGTGACGCTGGCTGAAGATCGTCGCCGTCTGCGGGTGGATTCGTTTGCACTGGATGTGCCGGAGGTGAAGCCATGA
>Cluster_48571
ATGGATTACCGGATTGGGATCACTGGTGCTCAGGGCAGTGGGAAAACAACCCTGGCAAAATATATCGACAAACATTACGG
AATCCCTTACGTGGATGCTGGTGTCGGAAGTTTGATGAGCCGACTCGGTGTTCGAGTAGGTGAGTCTATGCCTCTATATG
AGCGGCTTCAGATTCAAATGGAAATAGCAAAGCATATAGAGCTACTTACGCGTGGTGTTGAAGGCTTTGTTATCGATCGC
ACACCTGCTGATGTTATGGCCTACACGTTGGATTTGGTCGGCCATACCAATGAAGATCGATGTATTGAGTTAGCCCTCGA
TATCGAAAAGTTTTGCCACAAAACTGCTATTTCAAACTTTAACGCCATTGCTGGCCTACGCCCGGGGGTCGCTCTCTCAG
AGCGAGATTACTTGCGGTCACAACGAGCATCATTAGACCGTCTGTATGTCGCTCGTATTGATGCGTTGATGTGCGGGGAA
CTGACAAAAATTCACCTGCATCCGCAAAGGGGGGATCTGCAAACCTTCGTCGTTTCCAACCGATATCGCACGGTTGAAGC
AAGAGCCAGATCAGTGATGAGAATGCTAGATAACGCTGTAGAAAAGATAGAAAACCGGTTCTGTGGCCGAGTGACCGTTC
ATTAG
>Cluster_54120
ATGGCACGAAAAACCAAACAAGAAGCGCAAGAAACGCGCCAACACATCCTCGATGTGGCTCTACGTCTTTTCTCACAGCA
GGGGGTATCATCCACCTCGCTGGGCGAGATTGCAAAAGCAGCTGGCGTTACGCGCGGTGCAATCTACTGGCATTTTAAAG
ACAAGTCGGATTTGTTCAGTGAGATCTGGGAACTGTCAGAATCCAATATTGGTGAACTAGAGCTTGAGTATCAGGCAAAA
TTCCCTGGCGATCCACTCTCAGTATTAAGAGAGATATTAATTCATGTTCTTGAATCCACGGTGACAGAAGAACGGCGTCG
ATTATTGATGGAGATTATATTCCACAAATGCGAATTTGTCGGAGAAATGGCTGTTGTGCAACAGGCACAACGTAATCTCT
GTCTGGAAAGTTATGACCGTATAGAACAAACGTTAAAACATTGTATTGAAGCGAAAATGTTGCCTGCGGATTTAATGACG
CGTCGCGCAGCAATTATTATGCGCGGCTATATTTCCGGCCTGATGGAAAACTGGCTCTTTGCCCCGCAATCTTTTGATCT
TAAAAAAGAAGCCCGCGATTACGTTGCCATCTTACTGGAGATGTATCTCCTGTGCCCCACGCTTCGTAATCCTGCCACTA
ACGAATAA
>Cluster_28638
ATGAAACCATATCAGCGCCAGTTTATTGAATTTGCGCTTAGCAAGCAGGTGTTAAAGTTTGGCGAGTTCACGCTGAAATC
CGGGCGCAAAAGCCCCTATTTCTTCAACGCCGGGCTGTTTAATACCGGGCGCGATCTGGCACTGTTAGGCCGTTTTTACG
CTGAAGCATTGGTGGATTCCGGCATTGAGTTCGATCTGCTGTTTGGCCCTGCTTACAAAGGGATCCCGATTGCCACCACT
ACCGCCGTGGCGCTGGCGGAGCATCATGACCTTGACCTGCCGTACTGCTTTAACCGCAAAGAGGCAAAAGACCACGGTGA
AGGCGGTAATCTGGTTGGTAGCGCGTTACAAGGACGCGTAATGCTGGTAGATGATGTGATCACCGCAGGAACGGCGATTC
GCGAGTCGATGGAGATTATTCAGGCTAATGGCGCGACGCTGGCTGGCGTATTGATTTCGCTTGATCGTCAGGAACGCGGG
CGCGGCGAGATTTCGGCGATTCAGGAAGTTGAGCGTGATTACAACTGCAAAGTGATCTCTATCATCACCCTGAAAGACCT
GATAGCTTACCTGGAAGAGAAGCCAGAAATGGCGGAACATCTGGCGGCGGTTAAGGCCTATCGCGAAGAGTTTGGCGTTT
AA
>Cluster_48680
ATGTCTAGTATTTCCGAAGCTCATATGTTCGCCGCTGGCGCTCATGGTGGCGTTGGTCAGAAAAGAAAATATACCGGCGA
AGACTACATCAACCACCCGGTTGCTGTCCGAGAGATTGTTGCATTGCATGACGGAACCGTGGAGATGCAAATCGCGGCGC
TTCTGCATGATGTGGTCGAAGACACCCATGTAACCATAGAGATGGTTCGTGATCACTTCGGTGAGCGCGTAGCTGAAATG
GTTCAGGCTCTGACCAACATTGCAAGGCCAGAAGACGGCAATCGAATACAGCGTTTCATCATCAATGTCCTGGAGCTGGA
GCAAAATCTCGACATGCAGACGCGCATGATCAAGCTGGCTGACCTGCTCGACAATACCTCTTCTATCGTGAGTCGCGATC
CCGAGTTTTCAGCTGTCTACCTCGCAGAGAAAGAACTGATGCTGGACGTACTCTTTCACGGCAAAGAGATTGGTGCCAAC
GCTGATGTTGTCGAGTACATGGAGAGGATAGGAATAGAACACCCGTTGCTATTAAGCGCAATGGCGAAAGTCACTGAAGG
TATTGCGCTTCTGAAACCGGTACACATCAAACGTTATGAAAAACACAAATCGCTGATCTGGAGCGCGTGGGAGGCTGCAT
GA
>Cluster_1570
GTGAATCAGGTGAACGAGAGCCATAGCCGCGCATCCGATATCTGGCGCGAAGTGGCCTCGCTGTTTCGCCCACCCAGCCG
GTTACCAGTAGCGGAAGCCATCAGGCGTTATATGCGGGTACCACGGGGAGCCAATACTTCCGGTCCGTGGGAGTCATCGT
TGACGCCCTATATGATTGACCCCATTAATACATTATCAGCCCGTGAATATGACGCGGTGGTGTTTGTGGGACCTGCGCGA
ACCGGGAAAACCGAAGGGCTGATTGATGGCTGGATTGTGTACGGCATCATCTGTGATCCGGCGGATATGCTGGTGGTGCA

GATGACTGAGACGAAGGCGCGTGAGCATTCCAGAACGCGTCTTTCCAGAACGTTTCGCCACAGTCCGGAGGTCAGCAAGC
GCCTCAGTCCTTCCCGTAATGACAACAACGTCCACGATAAAATGTTTCTTGACGGCTCCTTCCTGAAGATTGGCTGGCCG
TCGATCACTGTATTTTCTTCTTCGGATTACCGTAAGCGTGCAGCAAGAACCGTATTGACGGGGATGTGTTATTCAGTCGG
CAGTGCTACGCGCCAGGGGAGCAGTTCGCCGACCCGGTTTATCGGCCAGTCGGCTATGACGTCAAGGACATAG
>Cluster_58994
ATGGCTATCATCAATACGCTATATCTGATGGAAAAAAATATGCCTAAACTTATTTAGCCTCCACCTCGCCATGGCGTCG
CGCTCTTCTGGAAAAACTGCAAATCTCTTTTGAATGTGCAGCACCAGAGGTCGACGAAACCCCACGCAGCGACGAATCAC
CGCGACAGTTGGTGCTTCGACTGGCACAAGAAAAAGCGCAATCTCTGGCGTCACGTTATCCGGATCATTTAATTATTGGT
TCAGACCAGGTATGTGTTCTTGATGGCGAAATCACCGGTAAACCGTTAACGGAAGAAAATGCCCGTCTGCAATTACGCAA
AGCCAGCGGCAATATCGTCACCTTCTATACTGGACTGGCGTTGTTTAATTCGGCGAATGGGCATCTGCAAACAGAAGTGG
AGCCTTTTGACGTCCATTTCCGTCACCTGAGCGAGGCGGAGATTGATAATTACGTGCGTAAAGAGCATCCCCTGCACTGC
GCGGGTAGCTTTAAGAGTGAAGGATTTGGCATTACGCTGTTTGAGCGTTTAGAGGGGCGTGATCCTAACACGCTGGTTGG
TTTACCGCTAATCGCGCTGTGTCAGATGTTACGTCGGGAAGGGAAAAACCCGCTGATGGGATAG
>Cluster_20435
ATGTTATCTGTAGTTAAACCTCTTCAGGAATTTGGTAAGCTCGATAAATGTTTGTCCAGATACGGTACGCGCTTCGAGTT
TAATAATGAAAAGCAAGTTATATTTTCCAGTGATGTCAATAGCGAAGATACTTTCGTTATTTTAGAGGGCGTTATCTCTT
TGCGTAGAGAAGAAAACGTACTTATCGGTATTACACAGGCTCCTTATATTATGGGGCTGGCTGATGGTTTAATGAAAAAC
GATATACCATACAAATTAATATCAGAAGGAAACTGTACGGGATATCATTTGCCAGCCAAACAAACCATTACTCTTATTGA
ACAAAATCAACTCTGGCGAGACGCTTTTTACTGGTTAGCCTGGCAAAATAGAATTCTGGAATTACGCGACGTTCAGCTCA
TTGGGCATAATTCCTACGAACAAATCCGCGCGACGTTATTATCAATGATTGACTGGAATGAAGAATTGCGATCACGTATT
GGCGTGATGAATTATATCCATCAACGTACACGTATATCGCGTTCTGTCGTTGCGGAAGTTCTCGCTGCTTTGCGTAAAGG
CGGCTATATCGAAATGAATAAAGGCAAACTGGTCGCTATCAACCGTTTGCCTTCAGAGTATTAA
>Cluster_9166
ATGAGCTATACCCTGCCATCCCTGCCGTATGCTTACGATGCCCTGGAACCGCACTTCGATAAGCAGACCATGGAAATCCA
CCACACCAAACACCATCAGACCTACGTAAACAACGCCAACGCGGCGCTGGAAAGCCTGCCAGAATTTGCCAACCTGCCGG
TTGAAGAGCTGATCACCAAACTGGACCAGCTGCCAGCAGACAAGAAAACCGTACTGCGCAACAACGCTGGCGGTCACGCT
AACCACAGCCTGTTCTGGAAAGGTCTGAAAAAAGGCACCACCCTGCAGGGTGACCTGAAAGCGGCTATCGAACGTGACTT
CGGCTCCGTTGATAACTTCAAAGCAGAATTTGAAAAAGCGGCAGCTTCCCGCTTTGGTTCCGGCTGGGCATGGCTGGTGC
TGAAAGGCGATAAACTGGCGGTGGTTTCTACTGCTAACCAGGATTCTCCGCTGATGGGTGAAGCTATTTCTGGCGCTTCC
GGCTTCCCGATTATGGGCCTGGATGTGTGGGAACATGCTTACTACCTGAAATTCCAGAACCGCCGTCCGGACTACATTAA
AGAGTTCTGGAACGTGGTGAACTGGGACGAAGCAGCGGCACGTTTTGCGGCGAAAAAATAA
>Cluster_47973
ATGTTGATGTTATTTCTCACCGTCGCCATGGTGCACATTGTGGCGCTTATGAGCCCCGGTCCCGATTTCTTTTTTGTCTC
TCAGACCGCTGTCAGTCGTTCCCGTAAAGAAGCGATGATGGGCGTGCTGGGCATTACCTGCGGCGTAATGGTTTGGGCTG
GGATTGCGCTGCTTGGCCTGCATTTGATTATCGAAAAAATGGCCTGGCTGCATACGCTGATTATGGTGGGCGGTGGCCTG
TATCTCTGCTGGATGGGTTACCAGATGCTACGCGGTGCACTGAAAAAAGAGGCGGTTTCTGCACCTGCGCCACAGGTCGA
GCTGGCGAAAAGTGGGCGCAGTTTCCTGAAAGGTTTACTGACCAATCTCGCTAATCCGAAAGCGATTATCTACTTTGGCT
CGGTGTTCTCATTGTTTGTCGGTGATAACGTTGGCACTACCGCGCGCTGGGGCATTTTTGCGCTGATCATTGTCGAAACG
CTGGCGTGGTTTACCGTCGTTGCCAGCCTGTTTGCCCTGCCGCAAATGCGCCGTGGTTATCAACGTCTGGCGAAGTGGAT
TGATGGTTTTGCCGGGGCGTTATTTGCCGGATTTGGCATTCATTTGATTATTTCGCGGTGA
>Cluster_10825
ATGAAAAGCCTCTCCTATAAGCGGATCTATAAATCACAAGAATACCTGGCAACGTTGGGCACAATTGAATACCGATCATT
GTTTGGCAGTTACAGCCTGACCGTTGACGACACGGTGTTTGCGATGGTTTCTGATGGTGAGTTGTATCTTCGGGCTTGTG
AGCAAAGTGCACAGTACTGTGTAAAACATCCGCCTGTCTGGCTGACATATAAAAAGTGTGGCCGATCCGTTACCCTCAAT
TACTATCGGGTTGATGAAAGTCTATGGCGAAATCAACTGAAGCTGGTGCGTCTGTCGAAATATTCTCTCGATGCAGCGCT
GAAAGAGAAAAGCACGCGCAATACCCGGGAAAGACTGAAAGATTTGCCCAATATGTCTTTTCATCTGGAAGCGATTCTCG
GGGAGGTGGGGATTAAGGATGTACGGGCGTTACGTATACTTGGGGCAAAAATGTGTTGGTTGCGACTGCGGCAGCAAAAC
AGTCTGGTGACAGAAAAGATTCTGTTTATGCTTGAAGGTGCCATTATCGGCATTCATGAAGCTGCGCTCCCGGTGGCACG
CCGCCAGGAGCTTGCAGAATGGGCTGACTCTCTTACGCCGAAACAGGAGTTTCCTGCGGAACTTGAGTAA
>Cluster_65789
ATGATTGGTTTAGTCGGTAAAAAAGTGGGTATGACCCGTATCTTCACAGAAGACGGCGTTTCTATCCCAGTAACCGTAAT
CGAAGTTGAAGCAAACCGCGTTACTCAGGTTAAAGACCTGGCTAACGATGGCTACCGTGCTATTCAGGTGACCACCGGTG
CTAAAAAAGCTAACCGTGTGACCAAGCCTGAAGCTGGCCACTTCGCTAAAGCTGGCGTAGAAGCTGGCCGTGGTCTGTGG
GAATTCCGCCTGGCTGAAGGCGAAGAGTTCACTGTAGGTCAGAGCATTAGCGTTGAACTGTTTGCTGACGTTAAAAAAGT
TGACGTAACTGGCACCTCTAAAGGTAAAGGTTTCGCAGGTACCGTTAAGCGCTGGAACTTCCGTACCCAGGACGCTACTC
ACGGTAACTCCTTGTCTCACCGCGTTCCGGGTTCTATCGGTCAGAACCAGACTCCGGGCAAAGTGTTCAAAGGCAAGAAA
ATGGCAGGTCAGATGGGTAACGAACGTGTAACCGTTCAGAGCCTTGACGTAGTACGCGTTGACGCTGAGCGCAACCTGCT
GCTGGTTAAAGGTGCTGTCCCGGGTGCAACCGGTAGCGACCTGATCGTTAAACCAGCTGTGAAGGCGTAA
>Cluster_17045
ATGTCAACGATTATTATGGATTTATGTAGTTACACCCGACTAGGTTTAACCGGGTATCTGTTGAGTAGAGGGGTTAAAAA
AAGAGAAATCAACGACATTGAAACCGTTGATGACCTTGCCATAGCTTGTGATTCACAGCGCCCCTTCAGTGGTGTTTATTA
ATGAGGACTGTTTCATCCACGATGCTTCTAACAGTCAGCGCATCAAGCACATCATTAATCAACATCCCAATACGTTATTT
ATCGTTTTTATGGCAATTGCCAATGTTCATTTTGATGAATATCTATTGGTCAGAAAAAAATTTATTGATCAGTTCTAAATC
GATTAAACCGGAATCTCTCGACGATATCCTTGGCGATATTCTGAAAAAAGAGACAACGATAACCTCGTTTTTAAATATGC
CGACGTTATCATTGAGCCGAACCGAATCGAGTATGTTGCGAATGTGGATGGCAGGTCAGGGAACCATTCAAATCTCCGAC
CAAATGAATATCAAAGCCAAGACCGTTTCATCGCATAAAGGTAATATTAAACGTAAGATCAAAACGCATAATAAACAGGT
TATCTACCATGTCGTTCGACTGACGGATAATGTGACTAATGGTATTTTTGTCAACATGCGCTAA
>Cluster_11997
ATGAACCCGGTTGATCGTCCACTATTAGATATTGGCTTAACGCGATTAGAATTCTTGCGTATATCAGGAAAAGGCCTTGC
TGGTCTAACAATTGCCCCTGCGTTACTGTCACTTTTGGGTTGCAAACAGGAAGATATTGATAGCGGCACAGTAGGGTTGA
TAAATACACCCAAAGGGGTGCTGGTGACCCAGCGAGCGCGATGCACGGGTTGCCACCGCTGTGAAATCTCTTGTACCAAC
TTCAACGATGGCTCAGTAGGGACATTCTTCTCCCGTATCAAAATCCACCGCAATTATTTCTTTGGCGACAACGGGGTTGG
CTCTGGCGGCGGCCTGTATGGCGATCTCAACTATACCGCGGACACCTGCCGTCAATGCAAAGAGCCGCAATGCATGAACG
TCTGCCCGATTGGTGCGATTACCTGGCAGCAGAAAGAAGGCTGTATTACCGTCGATCATAAACGCTGTATTGGCTGTAGC
GCCTGTACTACGGCATGCCCGTGGATGATGGCCACCGTAAATACCGAAAGTAAAAAATCCTCGAAATGTGTGTTATGCGG
TGAATGCGCAAACGCCTGCCCAACAGGGGCGTTAAAAATTATCGAGTGGAAAGATATTACTGTGTGA
>Cluster_59051
ATGAAAACAATCCATGACATCCGGCGGTCTAACGCCAGAAAACTGAGAGATGGTGTTGGCGGGAATTCTTCCTTTGCCAC
TATGATTGATCGCGAGCCAACCCAGACCAGCAGGTTTATGGGAGATGGTGCTACTAAAAATATCGGTGACAGCATGGCAC

GACACATCGAAAAATGTTTCGACCTGCCTGTAGGATGGCTCGATCAAGAACACCAGACAACGAACATCACAAAAAAACCT
GATGTTTCAATCACTAATAAACAAATCACATTAGTCCCTGTCATATCATGGGTACAGGCCGGAGCATGGAAAGAAGTTGG
ATATTCTGAGGTTGATTTGAGCACAGCAGAAACGTATCCCTGCCCTGTACCCTGTGGGGAAATGACTTATATCTTGCGGG
TAATAGGTGATTCAATGATTGATGAGTACCGCCCGGGAGACATGATTTTTGTCGATCCTGAAGTACCTGCCTGCCACGGT
GACGACGTTATTGCATTGATGCACGATACAGGTGAAACCACCTTCAAAAGGTTGATAGAAGATGGGACACAGCGTTATCT
CAAAGCGTTAAACCCAAACTGGCCTGAGCCTTACATTAAGATCAACGGTAATTGCTCTATAATTGGTACTGTGATTTTCT
CAGGAAAACCAAGAAGATACAAAATCAAAGCCTAA
>Cluster_25377
ATGTACGAGTTTGACTGGAGTTCCATTGTCCCTTCCCTGCCATATCTGCTGGATGGGCTGGTGATCACCCTGAAAATCAC
CGTCACGGCAGTGGTGATTGGTATTTTGTGGGGCACCATGCTGGCGGTGATGCGCTTGTCCAGCTTTGCGCCCGTAGCGT
GGTTTGCCAAAGCCTACGTTAACGTATTCCGCTCGATTCCTTTAGTTATGGTTTTGCTGTGGTTTTACCTGATCGTACCG
GGTTTTCTGCAAAACGTGCTGGGATTATCACCAAAAAATGATATCCGTTTAATTTCGGCGATGGTGGCGTTTTCGATGTT
TGAAGCGGCGTACTATTCAGAGATTATCCGTGCTGGTATTCAAAGTATTTCTCGTGGTCAGTCGAGCGCCGCGCTGGCGT
TGGGAATGACTCACTGGCAGTCGATGAAACTGATTATTCTGCCGCAGGCGTTCCGCGCGATGGTGCCGCTGTTGCTCACT
CAGGGCATCGTACTGTTTCAGGATACCTCACTGGTGTATGTGTTATCTCTGGCCGATTTCTTCCGTACCGCCTCAACCAT
TGGTGAGCGTGATGGTACGCAGGTTGAGATGATCCTGTTTGCCGGATTTGTTTATTTCGTTATTAGCCTTAGCGCGTCGT
TGTTGGTCAGCTACTTGAAAAGAAGGACAGCATAA
>Cluster_43191
ATGACCTGGTCTCGTCGCCAGTTTCTCACCGGCGTCGGCGTGCTGGCAGCCGTCAGCGGCACCGCAGGACGCGTGGTGGC
GAAAACGTTGAATATCAATGGGGTGCGTTACGGCATGGTGCATGACGAGTCGTTATGCATCGGCTGTACCGCCTGTATGG
ATGCTTGTCGGGAAGTGAACAAGGTGCCGGAAGGCGTCTCGCGCCTGACGATTATTCGTAGCGAGCCGCAGGGCGAATTT
CCTGATGTGAAATATCGCTTCTTCCGTAAGTCTTGCCAGCACTGCGATCATGCGCCGTGCGTTGACGTTTGCCCGACCGG
TGCGTCTTTTCGCGACGCTGCCAGTGGGATTGTCGATGTTAATCCGGATCTCTGCGTCGGTTGTCAGTACTGCATCGCCG
CCTGTCCGTACCGCGTGCGCTTTATCCATCCGGTCACGAAGACGGCGGACAAATGCGATTTCTGCCGTAAGACCCATTTG
CAGGCCGGTAAGCTGCCCGCGTGCGTTGAGGCTTGCCCGACGAAGGCGCTGACGTTTGGCAATCTGGACGATCCCAGCAG
TGAGATTTCGCAACTGCTGCGCCAGAAGCCCACTTACCGCTACAAGCTGGCGCTGGGAACCAAACCGAAGCTGTACCGCG
TACCGTTTAAATACGGGGAGGTGAGCCAATGA
>Cluster_41857
ATGCAGTACCCGATTAACGAGATGTTCCAGACCCTGCAAGGTGAGGGTTACTTTACCGGCGTCCCCGCCATTTTTATTCG
TTTACAGGGATGTCCGGTTGGCTGTGCCTGGTGCGACACCAAACACACCTGGGAAAAGCTTGAGGATCGGGAAGTCTCCC
TTTTCAGCATTCTGGCGAAGACCAAAGAGAGTGATAAGTGGGGGGCGGCGAGCAGTGAAGATTTGCTGGCTGTCATTGGC
CGTCAGGGATACACCGCGCGTCACGTGGTGATTACGGGTGGTGAGCCTTGCATTCATGATTTGCTGCCACTGACTGACCT
GCTCGAAAAGAACGGTTTTAGCTGCCAGATCGAAACCAGTGGTACTCATGAGGTACGCTGCACACCGAATACCTGGGTTA
CCGTATCGCCAAAGCTGAACATGCGCGGCGGCTATGAAGTATTGTCGCAAGCCCTGGAGCGAGCTAACGAAATCAAACAC
CCAGTTGGACGCGTACGTGATATCGAAGCACTGGATGAATTGCTTGCGACGCTGACCGATGATAAACCGCGAGTCATTGC
ACTTCAGCCAATTAGCCAGAAGGAAGATGCTACGCGTTTATGTATCGATACCTGCATTGCGCGCAACTGGCGTTTGTCGA
TGCAGACACATAAATATTTGAATATTGCCTGA
>Cluster_3443
ATGGTTACGTTAGAACAGTTCCGTTATTGCCCGACACACTCAACGCATCCTCCGTTCTGTTATGACTTTCACTATGTCAA
ACCGGGGATGGTCGCCATCTTTGGTGACAACGGCAGTGGCAAAAGTACGCTGGCACAGCTGATGGCGGGCTGGTATCCGG
ACTTTCTGCCAGGAGAGATAACAGGCACGGGGACGCTCCTCGGTACGCCCATTGGGCATCTCCCGCTTAATGAACAATCC
GCCACCATCCAGCTCGTCCAGCAATCGCCCTATTTGCAGCTCTCCGGCTGTACCTTTAGCGTGGAAGAAGAAGTTGCCTT
TGGGCCAGAAAATCTGTGTCTTGCTGAAGCAGAGATTATGGCGCGGATTGATGCCGCCCTGACTCTGACCGAGTGCCAGC
CGTTGCGCCATCGTCACCCGGCAACACTCTCCGGTGGAGAGACACAGCGCGTGGTCATCGCCTGCGCCATCGCGATGCAG
CCAAAGCTCTTAATTCTTGATGAAGCCTTTAGCCGCCTGACTCCGCAGGCCAGCGAAATGCTGTTGCAGCGCTTACAACA
CTGGGCGTTTGAACGTGGTTCGCTGATTATTCTTTTTGAACGTCATCGCACACCTTTCCTGAACTACTGCCAGCAAGCGT
GGCAATTGCAAAACGGAGCGCTCCAGCCACTATGTTAA
>Cluster_53872
ATGACAATTGACTATCAGGCACTACGTGAGGCGGCGGAACGTGCAATTCCAGCAATGGAACGCCTGTTAATGTTGCCAGC
TGATGATGATTTGTTAAGTGAACAGGAACTTAAAGATTACGGTGTGGATATTGATGCGCTCAACGCCTTCAAATTTCTGG
CCGGACCAGAAACCGTGCTGGCACTACTGGATGAACGGGAAAGAAACCAGCAATACATCAAACGCCGTGACCAGGAGAAC
GAGGATATTGCTCTTACGGTTGGGAAGCTGAGAGTTGAGCTGGAAGGCAAAGACAGCAAAATAGCCAATCTTACCGCCGA
ACGCGATGCTCTTCGTGAAGGTGAGATGGGCGACGCTAGGCATAGCAACACACGGGCCGCAGCTGATATCTACTTCCAAC
TGGTCGAGGAGTGCGAAATTCCTGCTGGCGGATCTCTGGTCGAGTACGTTGACGATATGCGCGAGAAGTTGGAAGCCGCA
GAGAAGCGCATAGCAGAGTTAGAAAGTGATTCTCAGGCACAAAAGTTAGTTGAAGCAATCATTGTTGCGATAGAAAACGA
ACAGGAACGTCTTTTTGATGAAGATTACCTAATGGATTCGAAAGAATGCATTGACGTAATTCGTGAAGAAGTAAAGCGAT
GGAATGATTCCCGCACCGCTGGCATTCGCATCAAAGGAGAGTGA
>Cluster_18226
ATGAATTCCCAGACAAAAAAAGATATTCCCTGCTTCCGTTCTTATTTGCCTGATGCTCTGCGTTTAAGATTTGAAGATAA
ACTGACCATCCGGGCCATCGCTCAGCGTCTAGGTCTCAGTCATTCCACAATACATACGCTTTTTCAGCGATTTATTGCAT
CCGGTATCGCATGGCCATTGCCCGATTCAGTTTCATTAGCTCAGCTTGACGCCATCCTTTATGCCAACAGAAAGAAGGAA
TTAACAGAGCCTCAAATCAGCGAAGGCACATGGCGAAAAGAACGGCGAGCCAGCTACAGCCGTGAATTTAAGGTCCGTCT
GGCTAAGCAGGCATTACAGCCCGGTGCTGTTGTTGCCCGGATCGCCAGAGAGCACGGTATCAATGATAACCTGCTGTTTA
AATGGAAAAGCCAGTACGAGGACGGCTTACTGAGCGATGATGACATACAGGAATGCATGCCTGTCCCGGTGGCTCTGACT
GATACGCCAGAGCCGACCAGACCAGTTACAAATCCCTTCTGGCGTAACAAGCCTGATGAGTGCCCTGAGAGTGATCCCGG
AAACGTCCCACGGTGCGAGCTGCATCTTAAATCAGGTGTGGTAAAACTGTTTGACCCTCTCACTCCGGAAATGTTACGGG
CGCTAATCCGCGAAATGAAAGGAGGTACCCGATGA
>Cluster_24529
ATGGCAATGGGTCCGTTAACCATTGGTCGCCCGACTGAGGCGAAAATCAACAGCACAAGCAACGATAGCAACGGTAGAAC
AACTAAAGGGGGTAACGGCTTTGCGATCATTACCTCTAATCTCGGCAGTGGTGCAAATGCTGCTTACAATGACTACCAGG
CATTATCATTTGATAGCGTGGAATCGACGAGAGTTAGCAGAAATGCAGACGTTACAAGCTATGCTGTCGAATCAGGATCA
GAAGTTAGTGATCATGTTCAAATACGGAATAACAAGTTCACATTACAAGGGCGTATCTCTGAAACCGTATTGAAGCTCAA
TCCTGATATGATTAAGAATGCTGGAATTAACGGCAATCGACGTATGCTTATGCTTGAATACCTAAATCAGTTGATGGACA
GTCGCCAACCTTTCCTACTTGTCACTGAGTTAAAAAATTATGACAACGTTGTGTTAGTTGGCATGTCTTACGCAGAAGAA
GCAAGCGAAAGCCTATTGTTTACTCTTGATTTTGAGCAAATCCGTCTTGTCTCTAAAGCAACTACAAGCGCAATTGCGGT
AAAAACAGCACCAAATAAATCTGTAGGGGGACAGGTTAAAATGCAGGTAAATACAACAGAACAGAAAAATCAAAAAAGCC
CAGGGCAAGACGTTGTAACGCCAGTATTTAAACAATAG
>Cluster_26913
ATGCAAGAAAGTATACAACTGGTTATTGATTCCTTGCCGTTCCTGTTGAAAGGGGCTGGGTATACCCTGCAACTCAGTAT

CGGCGGCATGTTTTTTGGCTTACTGCTAGGGTTTATTCTCGCGCTGATGCGCCTGTCGCCAATCTGGCCCGTGCGCTGGC
TGGCGCGTTTTTATATCTCTATTTTTCGTGGTACGCCGCTCATCGCACAACTGTTTATGATCTATTACGGCTTGCCGCAG
TTTGGCATCGAACTAGATCCCATTCCGTCAGCAATGATTGGTCTGTCGCTAAACACTGCCGCCTATGCTGCCGAAACGCT
GCGTGCCGCCATTTCTTCTATTGATAAAGGTCAGTGGGAAGCTGCTGCCAGTATTGGTATGACGCCGTGGCAGACCATGC
GTCGCGCGATTTTGCCACAGGCGGCGCGGGTGGCGTTGCCGCCGCTGTCGAACAGCTTTATCAGCCTGGTAAAAGATACG
TCGCTGGCCGCGACAATCCAGGTGCCGGAGCTGTTCCGTCAGGCGCAGTTGATTACCTCGCGCACGCTGGAGGTGTTCAC
CATGTATCTGGCGGCTTCGCTCATCTACTGGATTATGGCGACTGTGTTATCGACGTTGCAGAACCATTTTGAGAATCAAC
TTAATCGCCAGGAGAGGGAACCCAAATGA
>Cluster_25308
ATGGATATCATTTCTGTCGCCTTAAAGCGTCATTCCACTAAGGCATTTGATGCCAGCAAAAAACTTACCCCGGAACAGGC
CGAGCAGATCAAAACTCTACTGCAATACAGCCCATCCAGCACCAACTCCCAGCCGTGGCATTTTATTGTTGCCAGCACGG
AAGAAGGTAAAGCGCGTGTTGCCAAATCCGCTGCCGATAATTACGTGTTCAACGAGCGTAAAATGCTTGATGCCTCGCAC
GTCGTGGTGTTCTGTGCAAAAACCGCGATGGACGATGCCTGGCTGAAGCTGGTTGTTGACCAGGAAGATGCCGATGGCCG
CTTTGCCACGCCGGAAGCGAAAGCCGCGAACGATAAAGGTCGCAAGTTCTTCGCCGATATGCACCGTAAAGATCTGCATG
ATGATGCAGAGTGGATGGCAAAACAGGTTTATCTCAACGTCGGTAACTTCCTGCTGGGCGTGGCGGCGCTGGGTCTGGAC
GCGGTGCCCATCGAAGGTTTTGACGCCGCCATCCTCGATGAAGAATTTGGTCTGAAAGAGAAAGGCTACACCAGTCTGGT
AGTTGTTCCGGTGGGTCATCACAGCGTTGAAGATTTTAACGCTACGCTGCCGAAATCTCGTCTGCCGCAAAACATCACCT
TAACCGAAGTGTAA
>Cluster_50029
ATGAAATGGTATGAACTGGCTAGATCCAGAATGAAAGAACTCGGCATAACTCAAGAGAAGTTAGCTGAAGAGCTTGGTAT
GACGCAGGGTGGAATTGGTCACTGGTTGCGCGGATCTCGTCATCCATCTCTTGACGAGATTGGTGTGGTGTTTAAATACC
TTGGTATTGATAACGTCTCATTCAACCACGACGGTACATTTTCACCTGTTGGCGAATACTCATCTGCCCCCGTTAAAAAA
CAATATGAGTACCCTGTTTTTTCTCATGTTCAGGCCGGGATGTTCTCGCCTGAGCTTAGAACCTTTACCAAAGGCGATGC
GGAGAGATGGGTCAGCACAACCAAAAAAGCCAGTGATTGTGCGTTTTGGCTTGAAGTTGAAGGTAATTCCATGACCGCAC
CAACAGGATCCAAGCCAAGCTTTCCTGACGGGATGTTAATTCTCGTTGACCCTGAGCAGGCTGTTGAGCCAGGTGATTTC
TGCATAGCCAGACTTGGTGGTGACGAGTTTACCTTCAAGAAACTGATCAGGGATAGCGGTCAGGTGTTCCTACAACCACT
AAACCCGCAATATCCAATGATCCCATGCAATGATAGCTGTTCCGTAGTAGGGAAAGTTATCGCCAGCCAGTGGCCTGAAG
AGACATTTAGTTAA
>Cluster_64986
ATGACCGGTTCTATGATCGTAAATAATCTGGCGGGACTGATGATGCTGACATCGCTGTTTGTGATTAGCGTCAAAAGCTA
TCGCCTGTCATGCGGATTTTACGCCTGCCAGTCGCTGGTGCTGGTGTCTATTTTCGCCACTCTCTCGTGCCTGTTCGCCG
CAGAGCAACTGCTGATCTGGTCCGCCAGCGCCTTTATCACCAAAGTGCTGCTGGTACCGTTAATCATGACTTACGCTGCA
CGAAATATTCCCCAGAACATCCCGGAAAAAGCGTTATTCGGTCCGGCAATGATGGCACTGCTCGCGGCGTTAATTGTCCT
GCTTTGCGCATTTGTCGTTCAGCCCGTGAAGCTACCGATGGCTACCGGGCTGAAACCGGCGCTGGCGGTAGCGTTAGGTC
ATTTTCTGCTTGGCCTGCTGTGCATTGTCAGCCAGCGCAATATCCTGCGGCAAATTTTTGGTTACTGCCTGATGGAAAAC
GGCTCCCATCTGGTGCTGGCGCTTCTTGCCTGGCGAGCACCGGAACTGGTGGAAATAGGTATCGCTACCGACGCCATCTT
CGCCGTCATTGTGATGGTGTTACTGGCAAGAAAAATATGGCGTACCCACGGCACGCTGGACGTGAACAACTTGACCGCGC
TGAAGGGATAA
>Cluster_204
ATGGCTGCTGCACTCCTCTTAAACGCTCTCATGAAAAAAGAAAGAGAAATATTCCTTAGAGATAGTATTGATAATAAAGC
TAATGGTTACTATGAAAGACAACTTGCCTGTTTCTTAGGTAATCTCGGTATCTCTGTCCCAAGAGATAGAAAATCTGAAT
TCAGACCTGCTATTCTTCCTCCTGAATGGCAAAAAGCTGATGAATCCTTCCAGGACTTTATCCTTAACCTCGTTCTCCAA
AGCTACTCCCCTAATAAAATCAAAGCCCTCTTGCAATCTATGAAACTCCCCTACTCCCCAGAACAAATAGAAGAAATTAA
AGAAGAATTGTATAACCAAGCCAAAGAATTAAAAACCAAAGAATTGCCAGAAAATTTGTTTGCTATGTTTATAGACGCTT
ATCATACTCAGATAAAAGATACCGAAGCCAACAGAATCAGAAAAGCAGTTATTTATAATATCATCGGAATAGATATGGAG
GGAAGAAAAAATTTACTTTCTTATTACATTTATTTGGTTCAGAGACGAAGGAGGACTGGCTTCAGATACTTAATGATTT
GATAAAGAGGGGAGTTAAGAGGGTTATGGTAATAGGGAGTGATGATTTTCCTGGCCTTACTCAAGCCATAAAAGCCCTTT
TTTTTTCCTGA
>Cluster_66919
ATGAAAGCTAAAAAAGAAACAACAGACAGATTTCCAACCTGGTGGCTATTTTATTATGTATTAAGAAAAGCTTATTTCTT
TCTTGGAATACCTTTTTTTCTGTTCTGCGCATTAGGCTTTACCGAAATGCTATGTTCTGATCGTTATTTCGGAAATAAGG
TCGAAGATTATGTTGTCACTTTTGGTAGTTGGTTCTTATTACTTGCGCCAGGCATATGGATGTACTCACGTGCAAAAACA
AGGAGAGAAAAAATAAGAAAAGTTGTACAAACAATAAAAGAGTCAGGCTTCTACTCTCCAGAGAAGGGGTATGAAGGATT
ATCTTTAACTCAGGGAGCTTATTTCGGTATTGACCTTAAAAATGGCACAATGTTGTATGTCCGTATTTATCCAGGCAACA
TTATGGATGTTATCGGTTTTGACATTCATAATTTCACTCGTACTGTAACAGACGACAAAACACTGGAAATTCATACGAAA
TACATCAACCTGCCAATGGTTCCTATTCCATCATGGTGTACTCATCCAGAAACCGCATCAAACACCATGCACGCGATGGC
AAGTCGAGGATATGATTACCCTGTCGACTTTCCTCGCCTGATTCAGGAAAAGCGTAAGGAGTGGGAGCAGATTGCAGGCA
TACCTGTAGCTGAAGTATTTTAA
>Cluster_34753
ATGGAACTGTATCTGGATACTTCAGACGTTGTTGCGGTGAAGGCGCTGTCACGTATTTTTCCGCTGGCGGGTGTGACCAC
TAACCCAAGCATTATCGCCGCGGGTAAAAAACCGCTGGATGTTGTGCTTCCGCAACTTCATGAAGCGATGGGCGGTCAGG
GGCGTCTGTTTGCCCAGGTAATGGCTACCACTGCTGAAGGGATGGTGAATGACGCACGTAAATTGCGTTCTATTATTGCG
GATATCGTGGTGAAAGTTCCGGTGACCGCCGAGGGGCTGGCAGCTATTAAGATGTTAAAAGCGGAAGGGATTCCAACGCT
GGGAACCGCGGTATATGGCGCAGCACAAGGGCTGCTGTCGGCGCTGGCAGGTGCGGAATATGTTGCGCCTTACGTTAATC
GTATTGATGCTCAGGGCGGTAGCGGCATTCAGACTGTGACCGACTTACACCAGTTATTGAAAATGCATGCGCCGCAGGCG
AAAGTGCTGGCAGCGAGTTTCAAAACCCCGCGTCAGGCGCTGGACTGCTTACTGGCAGGATGTGAATCAATTACGCTGCC
ACTGGATGTGGCACAACAGATGATTAGCTATCCGGCGGTGGAAGCCGCTGTGGCGAAGTTTGAACAGGACTGGCTGGGAG
CGTTTGGCAGAACGTCGATTTAA
>Cluster_32375
ATGGCTGAGTATTATCCGGCGGTGTTTGATGGTAAAGCTTTTAATTGCCCGCACTGTGGTGTTTATTCCAAGCAAAGCTG
GCGGTCAATGGTGGGAGTTGCGCCTCATTCTACTTATATGGAAACTGATTTCCACTTATCATATTGTCAACATTGCTATG
AAAATGCTTATTGGTATAAAGGCAATATGATGATTCCTGCGGCTGGTAGTGTTGAGCTGCCTAATCCTGACATGCCAGAA
GACTGTAAGTCCGACTACATGGAAGCCAGAAGCATCATTAATCTGTCACCTAAGGGCGCTGCCGCACTCCTGCGCTTAAG
TCTTCAAAAGCTGATGGTCCATCTCGGTGAGCCGGGGAAGCATATCGACACGGACATTAAGTCCCTTGTAGCTAAAGGGC
TGTCTCCACTGGTCCAGCGAGCTGCTGACATCTGTCGTATTGTTGGCAATCAGGCGGTACATCCGGGTGAAATTAACATT
GATGACGACCCTCAGTTGGCGCATGGTCTCTTTAAGCTGCTGAACATTATTGTCACTGAGCAGATTACTCGACCGAAAGA
AGTCGAAGCCATGTTTAACAGTATGCCGGAACGGGCGCTTAAGGGCATTGAAGACCGGGACCGGAAAGCGAGGGAACAGC
AACAGGCCGCTAACGAGTAG
>Cluster_55889

ATGGATTATACGCTCACCCGCATAGATCCCAACGGTGAGAACGACCGTTACCCCCTGCAAAAGCAGGAGATCGTAACCGA
CCCTCTGGAGCAAGAAGTTAACAAAAACGTGTTTATGGGCAAGCTCAATGACATGGTTAACTGGGGTCGTAAAAACTCAA
TTTGGCCGTATAACTTCGGTCTTTCCTGCTGTTACGTTGAGATGGTGACTTCGTTTACCGCGGTGCATGACGTGGCGCGT
TTTGGCGCAGAAGTATTGCGTGCTTCGCCGCGTCAGGCTGACCTGATGGTGGTTGCAGGAACCTGCTTTACCAAAATGGC
ACCGGTTATTCAGCGTCTGTATGACCAGATGCTGGAACCAAAATGGGTTATCTCAATGGGTGCCTGTGCCAACTCTGGTG
GTATGTACGATATTTATTCCGTTGTGCAGGGCGTCGATAAATTCATCCCGGTTGATGTGTATATCCCGGGCTGCCCGCCG
CGTCCTGAAGCGTACATGCAGGCACTGATGCTGTTGCAGGAATCTATCGGCAAAGAACGTCGTCCGCTCTCCTGGGTGGT
TGGCGATCAGGGCGTTTATCGCGCCAATATGCAATCAGAGCGCGAACGCAAGCGCGGTGAACGCATTGCCGTAACCAACC
TGCGTACACCTGACGAGATTTAA
>Cluster_6224
ATGGTCATTGTCCCGATTGTTAATTGTTTCCGGGCTTTCCGGAGTACATTTTACTATCATGTCAGACGGCTTGCTGCTCC
TGACCGGTATCAGTCTGCCAGAGCGCTTGTGCTGAAAATTTATCACCAGCACAAAGGCCGCTATGGTTACAGACGCATCA
GGCTGGCATGTCGTAACGCGGGGGTTTTACTTAATGGTAAAACCGTCAGAAAACTGATGAAGGAACTGGGTATTAGCAGC
CTGATCCGCGTTAAAAAATACCGTTCATATAAAGGAGAACAAGGACGAATTTGCGATAATCTCCTGAAACGCAATTTTGA
TGCAAAACGCCCTAACGAGAAGTGGGTTACAGATGTCACTGAATTTAAAGTCAATGGCAAGAAGCTGTATTTATCGCCCA
TCATGGATCTTTATAATGGGGAAATAATTTCCTATAACCTCGCTACTCGTCCTCAACCTTCAATGGTGCAGACTATGCTT
ACGGATGCGCTGAAACAGCTGTCGAAAGATGAACACCCCATACTGCACAGCGATCAAGGTTGGCAATATCAGATGTCCCG
GTGGCAACGATGA
>Cluster_17674
ATGAATACGGTTGATTTATTTCGTGCCATGTTACCACCGGTCAGCTATGACCCGAACGGGAAATATATTTCAGCAGAACT
GAACGCAGAAGCAGCCATTATGGATGAAGTAAAAGCGTCAGCTGCACGAGTGCTTGCATCTGTCACACCATTTTACGCAT
CCATGACGCTGTCTGACTGGGAACGAGTGTATGGAGTAATACCCCGCGACGGTGCCACACAGCAGGAACGCCGAGAAAAT
ATTCTTATAAAGATGGCTGCAACTGGTGGGTTATCTATCCCTTATTTAAACAACTTGCAGCCAGCCTCGGGTACACCAT
AACAATTACTGAACTTCATCCATTCAGAGCGGGGATAAACCGATGTGGTGATCGTCTCTATATAACAGATGCCCGATGGA
TTTGGCAGGTTAATGTCACAGGAAGCAGAACGCCAGCCTACAGATTCAGAACCGGAGTATCTGCTGCCGGGGAGCCGTTA
CTTTCCTTTGGTGCCCCTGTACTGGAAGAAACATTTAAAGACCTTAAACCCGCTTTTACTTATTGCCATTTCACATATAA
GGAGGAGCAGTAA
>Cluster_37577
ATGGAACGTTGCGGCTGGGTGAGTCAGGACCCGCTTTATATTGCCTACCATGATAATGAGTGGGGCGTGCCTGAAACTGA
CAGTAAAAAACTGTTCGAAATGATCTGCCTTGAAGGGCAGCAGGCTGGATTATCGTGGATCACCGTCCTCAAAAAACGCG
AAAACTATCGCGCCTGCTTTCATCAGTTCGATCCGGTGAAGGTCGCAGCAATGCAGGAAGAGGATGTCGAAAGACTGGTA
CAGGACGCCGGGATTATCCGCCATCGAGGGAAAATTCAGGCAATTATTGGTAATGCGCGGGCGTACCTGCAAATGGAACA
GAACGGCGAACCGTTTGTCGACTTTGTCTGGTCGTTTGTAAATCATCAGCCACAGGTGACACAAGCCACAACGTTGAGCG
AAATTCCCACATCTACGTCCGCCTCCGACGCCCTATCTAAGGCACTGAAAAAACGTGGTTTTAAGTTTGTCGGCACCACA
ATCTGTTACTCCTTTATGCAGGCATGTGGGCTGGTGAATGATCATGTGGTTGGCTGCTGTTGCTATCCGGGAAATAAACC
ATGA
>Cluster_51243
ATGCCATTAACCTCAGATATTGGATCACGTTCATTTAATCTTGGGCTGGAGGTTGTTCGTGCCCGAATTGTAGCCAATGA
GCGCGGAGATATTACAGTCGGTGGTGAAACTGTCAGTATTGTGTATGATTCTATTAATGGGCGCTTTTCATCCAGTGGCG
GTAATGGCGGATTGCTTTCTGAGTTATTGCTTTTGGGATTTAATACTGGTCCTCGAGCCCTTGGTGAGAGAATGCTAAGT
ATGCTTTCAGACTCAGGTGAAGCACAATCGCAAGAGAGTATTCAGGACAAAATATCTCAATGTAAGTTTCCTGTTAGTTC
AGGAAATTTCCAGTGCCCGCTTGAGGCTATTCAATGTCCAATTACACTGGAGCAGCCTGAAAAAGGTATTTTTGTGAAGA
ATTCAGATGGTTCAGATGTATGTACTTTATTTGATGCCGCTGCATTTTCTCGTTTGACTGGTGAAGGCTTACCCCACCCA
CTGACCCGGGAGCCAATAACGTCATCAATGATTGTAAGTCAAGAACAATGTATTTATGATCAAGCCAAAGGAAACTTTGT
TATAAAATAA
>Cluster_40803
ATGACTGAGCCATTACAACTCCCGCCACCGCTTGAGGGTGATATCAGTCTCAGGACGCTGGGAAGACTGGCAGGACGGCT
GGATAACATCGACCTGAGCGTACTGATGGTCTTTCTCGTCGATATCGTCGACAGTTCCGCGCTGCCATGGCTGGGCGAGC
AGTTCTCACTGTCTGGCGATGGCTGGGAGCTGGCGGAATCGGACGATGTTCGCCGCATGCTTATAAAAGCAGCCATCGAA
CTGCACCGGTATAAAGGGACGCCGTGGTCAATCCGGGAAGTTATCCGCCGTTTTGGCTTTGGTGAAGTGGATCTGATTGA
AGGCACAGGTCGTCTCAGTTACGACGGCAATCGCAGCTATAACGGACTGTTTGTTCATGGAGATGCAGCCGCCTGGGCAG
TTTATCGCGTTATTCTGAAACAGCCCATTACTAACGATCAGGCCGCGATGCTGCGTCAGACGCTGGCTGCATTTGCACCG
GCCCGCTGCCATCTGGCCAGCCTGGAGTATCAGTCTGTGGCCATTCGCTATAACAATACCGCCATCCATGATGGCAGTTA
TAACCACGGGAGCAGTTGA
>Cluster_11556
ATGCCAGGAACAGTAAACTCCTGTGGTTTTGGATTTAGCATTACAAAAGCCCCCTATTCTTCTGGACAAAAACCAGTTAT
AGATGGTTTCTTTTTAGGCGCGAGGAGAATCTCATTCTCTTATCCTCGACTGGAAAGCGAGTTAATACAGTGCATTAACC
TAAAAAATGAAGGAAAAAAGAATGAGTGGATGAAGGAGGAGAGTATTTGTTTTGTGTCGCGGGATGTCAATAAACTCCTG
GATATGTTTGCTAAAAACAACCAGACAAACATACCTAAGGGGGTCCGGGAGCGGGTTTTTCAGCTCGCAAGTTTTTATTG
CGGTTTCTCGTTGGATGCAAGATGCGCCCAGGCATCCACTCATCACATGATTTTAAATAGTCAGTATTTTCAGAAAAAAA
TGGATACTCTTTTGACTTCAGTAGATATAAATGTCAGAAATCAGTGTGTTCGTACAGCGCTAAGCAGTCTTGCGGATACT
TTTTTTGAGAATAATGTTAACAATATAGATATGAATAAATTACGTGACAGGGTTCATAATACTATTGTGCAGGAGGTTCA
GAGAAATTTAAAATATGTTTAA
>Cluster_38443
ATGAATATAATTCTGCCGATAAAAGATATATTTATTATTGCGGGAAACATAGTTTTTAGCGTTTTATACAATGAACATCT
TGAGTTCCCATGCCGCTGCCAATTACTATCTTCAAATTCTGAAGTTATGCAAGAGTTATATATTGAAAAAGAGTTATTTA
TTAAACGCACTACGGAAAATGATTGTCGTGCTTTAGTGTTAAGAGGAACATTGGAATACTTATTTGATGAAATTATTGCT
GGCGAGTGTGCATTGGCCTTATTACAAAAAGAAATAAAAAAGATTAATCCAATATGTAGAACGGGACAATACCCATCTTT
TAACAATGAACTTCGTAATTTTTATCTTAGAAATAATGCTACATTATATGAGGTATATCATGAGGTAAAAAATGTTAACA
AGGGAGTTGGCCTGGTGGGAGATAATGGTATCGTCCTGGTAATTAGAAGTTCCAATTCCAAAAATACACTTGAGGAATTT
CATGAATTCTTTGGAAACTTCAAACCTAATAATTCAACAGCTCGAGTTGAAGTGATTGATCAATCCGTTTTAGAACCTTT
TTGGAGATTTTAA
>Cluster_59804
ATGAGTGAAAAAAGCATTGTTCAGGAAGCGCGGGATATTCAGCTGGCAATGGAATTGATCACCCTGGGCGCTCGTTTGCA
GATGCTGGAAAGCGAAACACAGTTAAGTCGCGGACGCCTGATAAAACTTTATAAAGAACTGCGCGGAAGCCCACCGCCGA
AAGGCATGCTGCCATTCTCAACCGACTGGTTTATGACCTGGGAACAAAACGTTCATGCTTCGATGTTCTGTAATGCATGG
CAGTTTTTACTGAAAACCGGTTTGTGTAATGGCGTCGATGCGGTGATCAAAGCCTACCGTTTATACCTTGAACAGTGCCC
ACAAGCAGAAGAAGGACCACTGCTGGCATTAACCCGTGCCTGGACATTGGTGCGGTTTGTTGAAAGTGGATTACTGCAAC
TTTCCAGCTGCAACTGCTGCGGCGGCAATTTTATTACCCACGCTCACCAGCCTGTTGGCAGCTTTGCCTGCAGCTTATGT

CAACCGCCATCCCGGGCAGTAAAAAGACGTAAACTTTCCCAGAATCCTGCCGATATTATCCCACAACTGCTGGATGAACA
GAGAGTACAGGCTGTTTAA
>Cluster_26987
ATGCTGTATAGACTGGGATGGCCTAAGGGAGAGATCACCAGAAAAATTGAGGCTATGCTGAATTCATCGACAGTGAGCAC
GACTATAGAAAGAGGATCTGTTCGTTCGAACAGACCTGATTTACCTCCAGTGGATTATGCGCAGCCGGAGTTACCGCCAG
CGGATTATACTCAGTCAGCGTTGCTGAGGCTTAGCAACAACAAATCACCCGTGCTAGGTAACGTTATTGGTAAAGGTGGT
AATGCTGTCGTGTATGAAGATATGGAAAATACAACAAAAGTGTTGAAGATGTTTACTATATCTCAAAGCCATGAAGAGGT
GACAAGCGAAGTTCGTTGTTTCAACCAGTATTATGGTGCCGGGAGTGCAGAGAAAATATATGGCGATAATAGAGATATTA
TTGGTATTAGAATGGATAAAATAAATGGAGAATCGCTTTTAAATATTTCGTCCTTGCCAGCACAGGCTGAGCATGCTATT
TATGATATGTTTGAGAGACTGGAAAAAAAGGAATTCTTTTTGTTGATACAACAGAAACAAATGTTTTATATGATCGTGTG
A
>Cluster_54238
ATGCACCTGCTTCCTGAACTCGCCAGCCACCATGCGGTATCAATTCCCGAGCTGCTCGTCAGCCGGGATGAAAGGCAAGC
ACGGCAACACGTCTGGCTCAAGCGCCATCCTGTTCCACTGGTCTCCTTTACCGTGGTTGCGCCTGGGCCGATTAAAGACT
GCGAGGTCACACGCCGAATTTTTAATCATGGCGTGACAGCCTTGCGTGCCTTAGCCGCAAAACAGGGCTGGCAAATTCAG
GAGCAGGCTGCACTGGTTTCCGCCAGCGGGCCGGAGGGCATGTTGAGCATTGCCGCCCCGGCTCGCGACCTCAAGCTCGC
CACCATTGAGCTTGAACATAGTCATCCTCTCGGGCGGTTATGGGATATCGATGTCCTGACGCCCGAAGGCGAAATTCTCT
CCCGCCGCGACTATTCACTGCCGCCTCGCCGCTGCCTGTTGTGCGAGCAAAGCGCAGCCGTCTGCGCGCGTGGAAAAACC
CATCAACTGACCGATTTACTCAACCGCATGGAGGCACTGCTGAACGATGTCGATGCCTGCAACGTCAACTAA
>Cluster_42000
ATGGAGGAAGCCACCTCTTGCAGTCATCTTTTCTTCGCTCTATCCTCTGCCGCTATGAAAACATCCCGTCTCCCTATCGC
CATCCAACAGGCCGTTATGCGTCGCCTGCGGGAAAAACTCGCCCAGGCCAACCTGAAGCTAGGGCGTAACTACCCGGAGC
CAAAACTCTCTTACACCCAGCGCGGAACCTCTGCCGGAACAGCCTGGCTGGAAAGCTATGAAATTCGCCTCAATCCCGTT
TTGCTGTTGGAAAACAGTGAAGCCTTTATTGAAGAAGTGGTGCCGCACGAACTGGCACATTTGCTGGTATGGAAACATTT
CGGGCGCGTAGCGCCACATGGCAAAGAGTGGAAGTGGATGATGGAAAGCGTGCTGGGCGTTCCCGCCCGTCGTACGCATC
AGTTCGAACTGCAATCCGTGCGCCGCAACACCTTCCCCTACCGCTGCAAGTGCCAGGAGCATCAGCTTACCGTACGCCGT
CATAATCGCATAGTTCGTGGCGAAGCCGTCTATCGCTGTGTTCACTGCGGCGAACAGCTGGTTGCGAAATAA
>Cluster_50273
ATGAAGGTTAATAAAAAGAGGCTTGCCGAAATTTTCAACGTGGACCCGCGGACGATTGAACGCTGGCAGTCTCAGGGACT
CCCTTGCGCCTCCAAAGGTAGTAAGGGCATTGAATCTGTATTTGATACTGCCATGGCAATTCAGTGGTATGCGCAGAGGG
AAACTGATATCGAAAACGAAAAGCTCCGCAAAGAACTGGACGATTTGCGTGCGGCAGCGGAGTCAGATTTACAACCCGGC
ACCATTGACTATGAACGCTACCGGCTCACAAAAGCGCAGGCAGATGCGCAGGAACTGAAAAATGCCCGTGAAGACGGAGT
AGTGCTGGAAACTGAACTGTTTACCTTCATTCTGCAACGTGTGGCACAGGAGATTTCGGGGATACTTGTGCGTGTGCCGT
TGACATTACAGCGTAAATATCCGGACATTTCACCATCACACCTTGATGTGGTGAAAACTGAAATCGCGAAAGCCTCCAAT
GTTGCAGCTAAGGCCGGTGAAAACGTGGGCGGGTGGATCGATGATTTCAGACGCGCAGAAGGCAGCTAA
>Cluster_41613
ATGAGGACGATTGAAGCCATGCAGCGACAACTCCTCGGCCTGATTGGGCGGGCAGTGGTGAAAAGCATCAGTGCCGCCAC
GAAATGTCAGACCGTGGATGTGTCCCTGATTGCCGGTGAACCCAAAGCCGGGGTTGAACATCTTGAACCCTACGGTTTTA
CCGCAAGGGCAAACAGCGGTGCGGAAGCGGTGGTGTTGTTTCCGGATGGCGACCGTTCTCATGCGGTGGTTGTTACGGTG
TCGGACCGGCGCTACCGCCTGAAAGGGCTGCAGACGGGTGAGGTGGCTGTCTATGACGATCAGGGGCAGTCCGTGACGCT
GGCCCGGGAGGGGATCGTGGTGGACGGTGCAGGTAAAACGATCACGTTTCGCAATGCGCCTAAGGCACGTTTTGAAATGG
ACCTAGAAGTGACCGGACAGGTGAAAGACCTGTGCGACTCCACCGGCACCACCATGTCAGCGATGCGGCTTGCCTATAAC
GGGCATCGTCACAGAGAGAACGGTCAGGGCAGTAACACCGACAAACCTGATAAAGCGATGGAGGCATGA
>Cluster_11452
ATGACAAATACAGCAAAAATACTCAACTTCGGCAGAGGTAACTTTGCCGAACAGGAGCGTAATGTGGCAGATCTCGATGA
TGGTTACGCCAGACTATCAAATATGCTGATTGAGGCTTATTCAGGCGCAGATCTGACCAAGCGACAGTTTAAAGTGCTGC
TTGCCATTCTGCGTAAAACCTATGGGTGGAATAAACCAATGGACAGAATCACCGATTCTCAACTTAGCGAGATTACAAAG
TTACCTGTCAAACGGTGCAATGAAGCCAAGTTAGAACTCGTCAGAATGAATATTATCAAGCAGCAAGGCGGCATGTTTGG
ACCAAATAAAAACATCTCAGAATGGTGCATCCCTCAAAACGAGGGAGGTTCCCCTAAAATGAGGGACATCCCTCAAAACA
GGGGGACACAAAAGACACTATTACAAAAGAAAAAAGAAAAGATTATTCGTCCGAGAATTCTGGCGAATCCTCTGACCAGC
CAGAAAACGATCTTTCTGTGGTTAAACCGGATGCTGCAATTCAGAGCGGCAGCAAGTGGGGAACAGCAGAAGACCTGA
>Cluster_24047
ATGGACGCAAAGCTGTACATCACTGAGATCATCAATGAATTAACCTTCGTTAAGGACTCTATCCGTGAGGATGACTGCAC
CAAATTGATCGACGCTATTCAGCAAAGCCAGCGCGTTTTCTGCTATGGCCTGGGACGCGCCGGCTTTTCCATGAAAGCCT
TCACCATGCGCCTGATGCATATGGGAAAAGAGGTTTACTTCCTGACGGAAACCATTACGCCGAACTTTGGGCCTGGCGAT
CTGTTTATCGTTTCTTCCGCTTCCGGTGAGACAGCACAACTGGTGGCACTGGCGAAAAAAGCACGTCAGCTTGGCGGCGC
TGTGGCGGTGTTAACCACGAATCGACACGCCACTATCACCGAGTTTGTCGACGTCATCGTGCAAATCAATGCCCCTTCCA
AAAATCAAAAAGATTCTGTTTTCCGCTCGGCACAGCCGATGGCGTCGTTGTATGAGCAAGCTTTGCTGGTGATTGCCGAT
GCATTAGTGATGAAGATGGCTGCTGAAAGTGGTGCGCCGGAAAGTGAACTGTTTAAGCGCCACGCCAATCTGGAATAG
>Cluster_47326
ATGTCTGATGTTTTACGCCCATACCGCGATCTTTTTCCACAAATCGGTCAGCGCGTAATGATCGACGATAGCAGTGTCGT
GATTGGTGACGTTCGTCTGGCTGATGATGTGGGGATCTGGCCGCTCGTTGTGATTCGTGGAGATGTACATTATGTACAGA
TCGGAGCACGCACCAATATCCAGGATGGCAGTATGTTGCATGTCACTCATAAATCCTCGTACAACCCAGATGGCAACCCA
TTAACCATTGGCGAAGATGTCACTGTTGGTCACAAGGTGATGCTCCACGGCTGCACCATTGGCAATCGAGTTTTGGTTGG
GATGGGCTCAATTTTACTTGATGGCGCAATAGTAGAAGATGATGTGATGATTGGTGCGGGTAGTCTGGTCCCACAAAATA
AACGGCTGGAGAGCGGATATCTGTATCTCGGTAGCCCCGTCAAACAGATCCGCCCGTTAAGTGATGAAGAGAAGGCTGGG
TTACGCTATTCCGCGAATAATTACGTGAAATGGAAGGACGAATATCTGGATCAGGGTAACCAGACCCAGCCTTGA
>Cluster_28982
GTGGCGTTAACGCCTGCGCTGCAACCTATTGATGGCGTGGCGGTGAGTTATATTGATGCTGCCGTAGCGTTGGGTAATAC
CATTAACGAAATGGATAAATATTACACCCAGGAAAATTATAAAGACGATGCGTTTGCGAAAGGGAAAACATTCCACTACT
ACTCTCTGGCAGTCATGATTTCAGCGAAACAAATTAATAACCTGATATCGCAAGATAAGTTAGATGCAGAAGCGGCAATG
AAGAAAGTGTCTGAACTGGAAACGCTGGTGGCGCAAGCCAAAGAAGCGGATAAAAGCGGCATGAGTTTCTCGTTTATTAA
TTCGGCAGGCCAGTATCAACTCGAGGCTAAAAAATACGTTCGCCGCATCAGAGATAAAGTCCCGTACTCTGACTGGGATA
AAGAGCAACTTCAGGACGCAAACTCAAGCTGGATGGCCGAAGACTCATTTCCACGAGCGTTATGCGATTACAACGAAATG
GTCGATGAAATATTTCAGTTAATTGTAATCGCGGGGCGGGTTTGTGATGAGCACGGATATGTCACGAAGTCGTAA
>Cluster_48987
ATGAATGACCGTGAAATCCGTTGTTACAGCGGTGAGGTGCGTGCTGAGCGGCATGACGATAACCCGGCGCACATTATCGG
TTATGGATCGGTGTTTGACTGTCGTTCTGAGCTGATATTCGGTTCATTCCGCGAAATCATCCGGCCCGGCGCTTTTGACG
ATGTGCTTGGTGATGATGTACGCGCACTGTTTAACCACGATCCTAATTTTATTCTTGGGCGTAGTGCAGCAGGCACGCTG

AATCTTTCAGTTGATGAGCGCGGATTACGCTATGACATCCAGGCTCCGGAGACACAGACCATTCGTGATCTGGTGCTGGC
CCCGATGCAACGTGGAGATATTAACCAGTCATCTTTCGCTTTCCGTGTCGCCCGTGACGGTGAGGAGTGGTATCAGGATG
AGGACGGGGTTGTTATTCGCGAGATAACCCGCTTTTCCCGTCTGCTGGATGTCAGTCCTGTGACATATCCTGCCTATCAG
GAGGCTGACTCGGCTGTTCGCTCCATGAAAGCATGGCAGGAGGCGCGCAACAGTGGCGCGCTACAGAAAGCCATTAATCA
ACGTATGGCGCGTGAACGCGTCCTGACCCTTCTTAACGCGTAA
>Cluster_24536
ATGAAGAATAAGGCTGATAACAAAAAAAGGAACTTCCTGACCCATAGTGAAATAGAATCACTCCTTAAAGCAGCAAATAC
CGGGCCTCATGCTGCACGTAATTATTGTCTGACATTACTTTGTTTTATTCATGGTTTCCGGGCGAGTGAAATTTGCCGAT
TGAGGATTTCGGATATTGATCTTAAGGCAAAGTGTATATATATCCATCGATTAAAAAAAGGCTTTTCAACAACGCACCCG
CTATTGAACAAAGAAATTCAGGCTTTAAAAAACTGGTTGAGTATCCGTACTTCTTATCCGCATGCTGAGAGCGAGTGGGT
ATTTTTATCACGTAAGGGGAATCCACTTTCTCGGCAACAGTTTTACCATATTATCTCGACTTCCGGTGGTAATGCCGGGT
TGTCACTGGAGATTCATCCGCACATGTTACGCCATTCGTGTGGTTTTGCTTTGGCGAATATGGGAATAGATACGCGACTT
ATCCAGGATTATCTTGGGCATCGCAATATTCGTCATACTGTCTGGTATACCGCCAGCAATGCAGGGCGTTTTTACGGCAT
CTGGGATAGAGCCAGAGGACGACAGCGTCACGCTGTTTTATAG
>Cluster_11133
ATGATGATGCCCGCTCTGCAAAAACTCCCTTTTTCTGGCTTGCCTTTTTCCGGCATTTGCGGATATAGTTTTTCCGCTGC
CGCAAAATCGGCAGCCGGGCGTGGAAACCCGAGCAATTCACAGGCGACATATGACGCGCCATGCGTCTTTTTTTGTGTCG
CAATCAACGCCACAGAGCGCCAGATTATGGTGTGGTGTGTGGTCAGTCGTGCAGATGGCATTCCGTGCATCCTGCCCGCG
TCCGCTCATTATGCCGCAGAGTCAATGGTAGCTCAGGCGGGGCAGCCTCCGGGCTGGCCGGTATCCTGTGAAGCCGGTAT
TTCCACCCCCGTCTGGGCTATCGCCATCGAGCGTGGAAACTCCGGCGATAGCGTTATTTGCTATTCACAGGAGGCTGCCA
TCATGGCTACAACCCTTACCCCCTCACACCCTGAATTTGTCTTTGTGTTTGCGGCTGTCCGTCGCGCAGACCGTCATCCC
CGTATCTGCATGCTTCGCACCGTCGCCGGTGATGAACGCAGCGCCCGCCGTTCCCTTGTCCGTGACTATGTGCTCTCCCT
TGCTGCCCGTCTGCCGGTGGTGGAGGTGTCCCGTGCGTAA
>Cluster_20337
ATGAATAATTTCTTAACCTTCCATGCAGAAGCAACGCCTGACGGCGTAAACATCATGTACCGCAGCAACGATGGCATGAC
AGAACGCGTTGAGGCCGTTTCATATATTGATGCCGTAAATCGTCTGGATGCCGGGGATTATGACGATAAACCAGATGAAG
GTATGTCAATACATCTTGCTATTGCCGATGGCGGCAACCAGGGATATTTCGATTACACATCACAGCATAACGTCATTATG
TGGAGATGGCTGATCGCAACAGTATTCATGCTGGAAATGAGAGAGGAAAACGGCACTGTCAGCATCATTGATGATACTGG
CAATCCTTCCGAAGTGGCTGTTTATTCCAACGGCATCGTCGCCATGCCGCTGTATCCAGTAGCAGAGCGCCTCGCTATGG
CAAACAACATTGAGGGCGCGATGATAGAACGTTTTGGCATCGAGTCTGGCACAGAACGCGCCATCATTTTCTACAGAGCC
ATGATGGATGTGGAACAAGGAGCACTGACTCCATTTGGTCGTGAAACGCTTGCTGAACTTCACAACAGCTTTATAGCTGA
ACTGAATGAAAACGGCATGCCAGCGGAACCAGTGACGCACTGA
>Cluster_34165
ATGAACACAGACACCATCGCCAATTCCAATGCGGATGACCCCACCAAAGCACTGTGTTCGCTGAGCCAAAATTGGTGGCT
TTTTGTGCTGCGCGGTGTCTTGGCATTGATTTTTGCAGCCCTCGCGTTCTGGATGCCACAATCGGCTCTGTTGGCCATGA
CCATCATGTTCGGCGCATTTTCCTTGGTCAATGGCGCGTTCAACTTGTTTGCAGCGGTGCGCCATATCCAGAAAAAAGAG
CGCTGGGGCTGGTTGTTGTTCAGCGGCATTGTCGGCATACTTACGGGCGTCGTCGTCCTGGTTGCTCCTTGGGTCGCCAC
GATAGTCTTGGCTTCTTTTTTGTGGGCTAGCGTGGGCTTCTGGGCGATTTTCACCGGTGTGCTGGAGATATCCGCCGCCG
TTCGGCTGCGTCGGGAAATCAAGGGTGAAATCTGGCTTGCCCTCAGTGGACTGCTCTCGATTATCCTTGGTGCTATCGTC
TTGTGGATATTTTTTACCCGTCCGGTCGAGTCATTCGTGGCCGCAGGCTGGCTGTTGGGTTTCCATGCGGCAGTCTATGG
CGTCACGCTTTTGTTCTTGAGTTGGAGTCTTCGCAAAACGCGCCTGGGGTAA
>Cluster_11346
ATGGCACAGCTATATTTCTACTATTCCGCAATGAATGCGGGTAAGTCTACAGCATTGTTGCAATCTTCATACAATTACCA
GGAACGCGGCATGCGCACTGTCGTATATACGGCAGAAATTGATGATCGCTTTGGTGCCGGGAAAGTCAGTTCGCGTATAG
GTTTGTCATCGCCTGCAAAATTATTTAACCAAAATTCATCATTATTTGATGAGATTCGTGCGGAACATGAACAGCAGGCA
ATTCATTGCGTACTGGTTGATGAATGCCAGTTTTTAACCAGACAACAAGTATATGAATTATCGGAGGTTGTCGATCAACT
CGATATACCCGTACTTTGTTATGGTTTACGTACCGATTTTCGAGGTGAATTATTTATTGGCAGCCAATACTTACTGGCAT
GGTCCGACAAACTGGTTGAATTAAAAACCATCTGTTTTTGTGGCCGTAAAGCAAGCATGGTGCTGCGTCTTGATCAAGCA
GGCAGACCTTATAACGAAGGTGAGCAGGTGGTAATTGGTGGTAATGAACGATACGTTTCTGTATGCCGTAAACACTATAA
AGAGGCGTTACAAGTCGGCTCATTAACGGCTATTCAGGAAAGGCATCGCCACGATTAA
>Cluster_24609
ATGACTATGACAAGACTGAAGATTTCGAAAACTCTGCTGGCTGTAATGTTGACCTCTGCCGTCGCGACCGGCTCTGCCTA
CGCAGAAAACAACGCGCAGACTACCAATGAAAGCGCAGGGCAAAAAGTCGATAGCTCTATGAATAAAGTCGGTAATTTCA
TGGATGACAGCGCCATCACCGCGAAAGTGAAGGCGGCCCTGGTGGATCATGACAACATCAAGAGCACCGATATCTCTGTC
AAAACTGATCAAAAAGTCGTGACCCTGAGCGGTTTCGTTGAAAGCGAGGCTCAGGCCGAAGAGGCGGTGAAAGTGGCGAA
AGGTGTTGAGGGCGTGACCTCTGTCAGCGACAAACTGCACGTTCGCGACGCTAAAGAAGGCTCTGTGAAGGGCTACGCGG
GTGACACCGCCACCACCAGTGAAATCAAAGCCAAACTGCTGGCGGACGATATCGTCCCTTCCCGTCATGTGAAAGTTGAA
ACCACCGATGGCGTGGTCCAGCTCTCCGGTACCGTAGATTCTCAGGCACAGAGCGACCGTGCTGAAAGTATCGCCAAAGC
GGTAGATGGTGTGAAAAGCGTTAAAAATGATCTGAAAACTAAGTAA
>Cluster_54484
ATGAATAGTCTGCTGCCACCGGGTTCAACACCACTGGAGCGCCGACTGGCGCAAACCTGCAGCGGGATTTCTGATCTGCA
GGTGCCGCTTCGTGACTTGTGGAATCCGGCAACCTGTCCGGTCAGCTTCCTGCCTTATCTCGCCTGGGCGTTCTCTGTGG
ATCGCTGGGACGAGGGCTGGACAGAAAGCGTCAAACGCCAGGTAGTGAAGGATGCTTTTTATATTCATCAGCATAAAGGA
ACCACCAGTGCCGTGCGGCGGGTGGTGGAGCCGTTCGGCTTTCTGATCCGCATTATTGAGTGGTGGCAGACCGGAGAGGC
ACCGGGCACGTTTCGCCTGGATATCGGCGTGCAGGACCAGGGCATCACTGAAGATACCTATCTGGAACTTGAGAGGCTGA
TAAGCGATGCCAAACCATGTAGCCGTCACATGATCGGCATGTCCATCAATCTGCAGACCAGCGGCCCGTATTGGTTGGGG
GCAGCCAGTTATCTTGGTGAAGAAATCACGATCTTTCCGTATATCAACGAAACAATTATTTCCGGCGGCACCGCGCATGA
AGGCGGGGCGGTCCATGTTATTGACACAATGAGAGTGAATCCATGA
>Cluster_63637
ATGGCGCATATACAACTGGTCAAACAAACTTCTTCTGGTTTACTTCTCCCGGCGACGCCGGAGAGTTGCGATTTTCTGCA
TCAAATCAAAATAGGTGAGTGGATACACGCAGACTTTAAGCGTGTGCGTAACTACGCATTCCACAAGCGTTTTTTCAAAC
TCCTGCAACTGGGATTCGATTACTGGACTCCGGTCGGTGGGGCGATCACGCCTCGCGAACGAGAACTGCTGTCTGGTTTC
GTTGATTACCTGTGCGAATCAGTTGGTCGGGAACACACGCCAGCCCTGAGTGATGCCGCAGAGCAATACCTTAACACCGT
TGCGACTCGTAGAACCCGGGATACGGCGTTGCTAAAGTCGTTTGAGGCTTTCCGCGAGTGGGTAACCATTCAGGCCGGAT
TTTACACCGAGCATATTTATCCGGACGGTAGCCGTGGGCGTCGGGCAAAATCCATCGCTTTTGCGAATATGGACGAAGTC
GAGTTTCAGCAGGTTTATAAATCTGTACTGAATGTGCTGTGGAACTGGATTCTGTTCCGTAAATTCTCCTCTCAGGAGGA
AGTTGAAAATGTGGCCGCACAACTACTGGAGTTTGCGTAA
>Cluster_35885
GTGAAAGTAAACATCAAGCCAATTACGGGTAACTGGGATTTAGGTTACGCGATGGATAAGCATTTGATTAAGAGTACTTA

TTTAGGTGATAACGAGTATGGCAAGCCTATGTTTCAAAACGATCGCACGGAGGTTGGAGAAGCCGTATACCAGTTAAAAT
ATCAAAATGACATGGACCAAGTTGATGCGCTTGCCCAGTGCCTATTAGTTAATGCTGTGCCTCTTTTTCAAAACATACAA
TTAATCATCCCAATGGCAGCTTCAAATGTTCGAAAAATTCAACCCGTGACAGCAATTACTGACGCTCTGGCATCAAAAAT
GGGAAATAACATGTTTAGCTTCAATGAGTTATTAATCAAAGCACCTGGTGGCGTGTCTTTGAAAAATCTGAACACTAAAG
AAGAGAAAGAAGCTGCTGTTGCTGACGCTTTTTCTTATCAAAACCAAATTGTCGGGGATGGCCCTTTTAATGCCCTCATC
GTTGATGATCTCTTCCATACAGGAGCATCCATGGAAGCTGCAGTTACAGCGTTACGCGGTTATAATAAAATCAATAAAAT
TTATGTAGCAGCACTAACCTGGAGATAA
>Cluster_235
ATGAGTCTCCTTGAACTTTTTAACATGACAACGATGGTTTCCCTTCTTATAAGTGCCTCAGCTTTGATAAGAGAAAAAGA
ATATGGTACCATCGAACAACTTTTGATAACCCCTGTAAAAACCTGGGAGGTTTTTTAGCCAAGATTATTCCTACGGTAG
TCGTAATCGGTGTTTTATCCTTGCTTAGCTTATTTATCATGGTAAAAGGCGTTTTTAAGGTCCCGATAAAGGAAACATC
ATCTTATTTTTTTGGTCACCTGTCTTTATGTGTTTGCCATGTCTTCCCTTGGGATAGCCATCGCAACGATAGTAAACAA
CCTCTCTCAGGCGATGATGGTAATCATAGTGATTCTCGTACCGATGCTTATGATTTCAGGAGCCTGGACTCCCCCAGAAG
CTATGCACCCTGTGATCAGGTCTCTAAGCCTTTTTTCTCCGATGAGGTATTATTTAGAGTTTGGCTACGGGGTTTTACTT
AAAGGGGTTGGGATAGAGTATCTCTGGAAAGACATCTTAGGGATTTCAGCCGTAGGAGGGGTAATCTTTCTTTTATCAGC
CTTAAGATTTAGGAAAAGTTTTGCTAAATAA
>Cluster_44951
ATGCCTGGATTAGTATCATATATATCATCGACTTCATTCGCGAATGAGATGGCGGAAATGCGTCAGCAGGTAATGGAAGG
GCAGATTGGAGGATTTCTCCTGGGAGGGGAGAGAGTTAGAGTTTCTTATATGCCAGATACAGGCCGTTTTTAGCAGAAA
GTGAAGGGCTGGGACTGGTTTATGCAGAATTATTGAATATTGGTTTTAATGATGGAGTTGATGCGCTCAGAAACAGGGTG
TTAAGCGTGCTTCCTGGAATGGTGGCGCAGCGACAAGAGAACTCTTTGCAGGCCAAAATATCGGAATGTACCTTTACTGT
TGATATTGAAAAACTTCACTGTCCTGGTGAGGTGCTTCAATGTCCAATTACACTGGAGCAGCCTGAAAAAGGTATTTTTG
TGAAGAATTCAGATGGTTCAGATGTATGTACTTTATTTGATGCCGCTGCATTTTCTCGTTTGACTGGTGAAGGCTTACCC
CACCCACTGACCCGGGAACCAATAACGGCATCAATAATTGTAAAACATGAAGAATGCATTTATGACGATACCAGAGGAAA
CTTCGTTATAAAGGGTAATTGA
>Cluster_14840
ATGTTCAAATCGATCATGACCGTATCACTGCTGGCCACCGCTATTGCCTCTACCAGCGCAGTGGCCGCAGACAATTCGGC
GGGTGGTATCATTAACTTTACCGGCGCTATTAGCGATACAACCTGTACCATTAACGGCGGTAAAAGCGCAGACTTTACCG
TTGCGCTTTCCCCTATTTCGGTAAAAGATGCGGGCAACACGGTTGGCCTGATCAATAAGAATAAAAAATCTATTGCGCTG
ACTTTCTCAGGTTGTTCACCAGCAGCCGGAACGACCGGTACCCCGCTGAAAGTGTATTTCTCCAGCGCGAATAATATTTC
CACTGACGGTAAATACCTGCTGAATAACAGCGTAAACGAAAGCGATACCAGCGTGGCACGTAATGTCGGTTTTGCGTTAG
TGAAGCCAGGTTCGTCCACGCCAATTACCCTGAACCAGCCTTACACCACGGATATTATGGGTACAGCGACCGCGCCAGAT
TCCGAGACGCTGACGCTGGATGTTTATTATTATAAAACGAACACCGCTGCCGCCACCGTGGGCGAGTTAAGCTCCAACGT
AACGTATACCATTTCCTACCTGTAA
>Cluster_21539
ATGATCATTCGTGATGAAAACTATTTTACTGATAAATATGAATTAACCCGCACACACTCTGAAGTGCTGGAAGCGGTGAA
AGTGGTTAAACCGGGTAAAACGCTGGATCTGGGCTGTGGCAATGGTCGTAACAGTCTTTACCTGGCAGCCAATGGTTATG
ATGTTGACGCATGGGATAAAAATGCCATGAGTATCGCCAACGTCGAGCGCATTAAGTCCATTGAAAATCTGGATAATTTA
CACACCCGGGTCGTTGATCTGAATAACCTCACATTTGATGGACAGTACGATTTTATTCTTTCGACTGTGGTGCTGATGTT
CCTTGAGGCTAAAACCATCCCAGGGCTGATTGCCAATATGCAACGTTGCACTAAACCTGGCGGTTACAACCTGATTGTGG
CGGCGATGGATACCGCTGATTATCCATGTACCGTCGGCTTCCCGTTTGCCTTCAAAGAGGGGGAATTACGTCGATATTAC
GAAGGCTGGGAGATGGTGAAATACAATGAAGACGTCGGCGAGCTGCACCGCACCGACGCCAACGGTAATCGTATTAAACT
GCGTTTCGCCACGATGCTGGCACGTAAAAAATGA
>Cluster_66900
ATGATGAAATCAGACGAACAATATCAGGTTCCCGTGTGGATGCGACCTCTGTTGCCGTTGCTCTGCAATACCGGTGGTAA
TGATCCGGAGGAACTGCTGAATGATACAGAAACCACCGCCAGTGCGAATATTGTCCGTTATGTACTGATAGTTGCAGTGC
GGTCGCAGATTGATCTGCTGCAGCTACTGTACAGGAAAGGACTGTTGCGCACAGAGATACCAGGTGGCTTTTCACCGGAA
GAAGCGCAGGAACTCCTGGATAATCTGGTGCGCAGCCATATCAGCAAGGCGCTGTCGGGTGAGCGAATGGCAGCCCGTGA
CAGAAATGCCGATCTGACCTGGATTCGCCAGCAACTGGTCGATGCCGCCTGGTTTGTCCGTGCCACACTGGAAGCGCATG
GGATGGGCGTCGGAAATGAGAGTCCCTCTGCTCCGCCGGAGACAATGCCGGACATACAGACACGGGAACTGGTTATGTTG
ATCAAGCGACTGGCATCATCGCTGAAAGCGGTGAAACCCGACAGTAGCGTGGTGCGTGAAGCGCAGGACTGGCTGTGCGA
CAGAAAACTTGTGGATATCACAGATATTCTCCGGTGA
>Cluster_6775
ATGTCTCGAATTTTTGCTTACTGTCGGATATCAACGCTGGATCAGACCACCGAAAATCAACGCCGGGAAATCGAAAGTGC
AGGTTTTAAAATCAAACCTCAGCAAATAATCGAAGAACACATTAGCGGCTCAGCAGCAACCAGTGAGCGTCCTGGTTTTA
ACCGGTTGCTTGCTCGCCTGAAATGTGGTGATCAATTGATTGTGACAAAACTGGATCGCCTTGGTTGTAATGCAATGGAT
ATCAGGAAAACAGTGGAACAACTGACCGAAACAGGTATCAGAGTGCATTGCTTAGCATTGGGGGGCATTGACCTGACCAG
TCCAACAGGAAAAATGATGATGCACGTAATTTCAGCAGTCGCTGAATTTGAACGAGACCTTTTACTTGAACGCACTCATT
CCGGGATAGTAAGAGCCCGCGGCGCAGGGAAACGTTTTGGTCGACCACCTGTGTTAAATGAAGAACAGAAACAGGTGGTA
TTCGAACGAATTAAGTCAGGTGTAAGTATAAGTGCCATTGCCCGGGAATTCAAAACCTCGCGGCAAACCATTTTAAGAGC
CAAAGCAAAACTTCAGACACCTGACATATAA
>Cluster_54908
ATGCAGCTTAAACGTGTGGCAGAAGCCAAACTGCCAACCCCATGGGGCGATTTCCTGATGGTGGGATTTGAAGAACTGGC
AACCGGACACGATCATGTCGCGCTAGTCTATGGCGATATTTCCGGGCATACCCCGGTACTTGCGCGCGTCCATTCCGAAT
GTCTGACCGGTGACGCCCTGTTCAGCTTGCGCTGCGATTGTGGCTTCCAGCTCGAAGCGGCATTGACGCAAATTGCCGAG
GAAGGCCGTGGTATTTTGCTGTATCACCGTCAGGAAGGTCGTAACATTGGTCTGCTGAATAAAATCCGCGCTTACGCACT
GCAGGATCAAGGTTACGATACCGTAGAGGCTAACCACCAGTTAGGCTTCGCCGCTGATGAGCGCGACTTCACTCTTTGCG
CTGATATGTTCAAACTCCTTGGCGTCAATGAAGTCCGCTTGTTAACCAATAACCCGAAAAAAGTCGAAATTCTGACCGAA
GCAGGGATTAATATTGTTGAACGCGTACCATTGATTGTAGGTCGTAACCCCAATAACGAACATTATCTCGATACCAAAGC
CGAGAAAATGGCCATTTGCTGAACAAATAA
>Cluster_58421
ATGCCAGCGGAAAACATTGTTGAAGTTCATCATCTTAAGAAGTCCGTCGGTCAGGGGGAGCATGAACTCTCCATCCTCAC
CGGAGTTGAGCTGGTTGTCAAACGTGGCGAGACCATCGCACTGGTGGGCGAGTCGGGATCGGGTAAGTCAACCTTGCTGG
CGATCCTCGCCGGGCTTGATGACGGCAGCAGTGGCGAAGTGAGTCTGGTGGGACAACCGCTACATAATATGGACGAAGAA
GCGCGGGCAAAGTTGCGCGCGAAGCACGTCGGCTTTGTTTTTCAGTCATTTATGTTAATTCCTACCCTTAACGCGCTGGA
AAACGTCGAGCTTCCGGCTCTGCTGCGCGGTGAGAGTAGCGCGGAAAGTCGTAACGGGGCGAAAGCGTTGCTCGAACAGT
TAGGGCTGGGTAAACGTCTGGATCATCTTCCGGCACAGCTTTCCGGCGGTGAACAGCAACGAGTGGCGCTGGCACGAGCC
TTTAATGGTCGACCTGATGTGCTGTTTGCCGACGAACCCACCGGCAACCTTGACCGCCAGACGGGCGATAAAATTGCCGA
CCTGCTGTTTTCCCTCAACCGTGAACATGGCACCACGTTGATTATGGTGACCCACGACCTGCAACTGGCGGCACGCTGCG

ACCGCTGCTTACGGCTGGTGAACGGGCAGTTGCAGGAGGAAGCATGA
>Cluster_44089
ATGAACTCGCATAATATTACTAACGAATCATTGGCACTGGCATTAATGCTGGTGGTGGTGGCAATCTTAATTAGCCATAA
AGAAAAACTGGCGCTGGAGAAAGATATTTTCTGGAGCGTCGGGCGAGCGATAATTCAGCTGATTATTGTCGGCTATGTGC
TGAAGTATATTTTCAGCGTGGATGATGCCAGCCTGACATTATTGATGGTGTTATTTATCTGCTTTAATGCGGCGTGGAAC
GCGCAAAAACGCAGTAAATATATTGCTAAAGCTTTTATCTCATCGTTTATTGCCATTACGGTCGGGGCGGGAATTACCCT
GGCGGTGCTGATTCTCTCTGGGTCGATTGAATTTATCCCGATGCAGGTGATCCCTATCGCCGGGATGATTGCCGGTAACG
CCATGGTAGCGGTGGGGTTGTGTTACAACAATTTAGGGCAACGGGTCATTAGCGAACAGCAACAGATTCAGGAGAAACTG
AGTCTTGGTGCGACGCCGAAGCAGGCTTCAGCGATATTGATTCGCGACAGTATTCGCGCGGCTTTAATTCCGACGGTCGA
TTCAGCAAAAACGGTTGGCTTAGTGAGTTTACCAGGAATGATGTCCGGGCTGATATTTGCCGGGATTGATCCGGTGAAGG
CGATTAAATATCAGATTATGGTGACCTTTATGCTGCTCTCAACCGCCAGCTTGTCGACCATTATTGCCTGCTATTTAACC
TATCGTAAGTTTTATAATTCGCGCCACCAGTTGGTGGTGACGCAATTGAAGAAGAAATGA
>Cluster_55565
ATGAGAAAAGTTGGCAGTACAACTGACACCGCTGATGCTAATGGTGAATACACTAATGGTAATGTCGCTAATGGTATTTC
ACCGACAATAATTAATGCTGAGATGATGAATACCTTTCAGCGTGAGCTGGTAAACGTTGTTGAGGGCGCGGGGCTTGAGC
TTGATCCTGATGATGATTCACAGGTTCTTAAAGCGATTGGTGGCGGTCGCCTGCTTAATATTGTCACCTTTACTAAGTCA
GGGACTTACACTCCAACTAAAGGGACGAAAAGAGTGCGGGTGAAAGTTTGGGGAGCGGGGGGAGGTGGCCAGAATGCTCC
AGTTAGTGTGGGCGCATCTGGAGGTTGTGCTGGTGGATTTTCGGAAGGGTTATTTAATATTTCTGATAACGAAACTATAT
CTGTGACGATAGGTACTGGTGGCAGTGCTGTTGCTGCTGGTGTCTCGTCTAAAGGCGGGAATGGTGGTGATACAAGGTTC
GGGAGTTTAATCTCAGCTACTGGAGGAGTGGTGGCGGGGCTACAGTTCCTGCTGGAGGTATCGGAAGTGGCGGGAACAT
TTTAAATATTACTGGCGGAATATCTCAAGGTGGTCTGTATTATGGTTCTGATGCTTTTATTGGCGGGGCTGGAGGATCTG
CATTTTCATCAACGGGTGGCAATGGCCACTTCGGTAGTTCTGGCGATGATGGCGGTTTCCCCGGCTCAGGTGGTGCCGGG
GGGAATGGTAACTATTCATCAGGAAAAGGAGCGAATGGTTTAGTTTACCTTGAAGAGTTTTCCTGA
>Cluster_42955
ATGTCTGTAGAAGATACTCAACCGCTTATCACGCATCTGATTGAGCTGCGTAAGCGTCTGCTGAACTGCATTATCTCGGT
GATCGTGATATTCCTGTGTCTGGTCTATTTCGCCAATGACATCTATCACCTGGTATCCGCGCCATTGATCAAGCAGTTGC
CGCAAGGTTCAACGATGATCGCCACCGACGTGGCCTCGCCGTTCTTTACGCCGATCAAGCTGACCTTTATGGTGTCGCTG
ATTCTTTCAGCGCCGGTGATTCTCTATCAGGTGTGGGCGTTTATCGCCCCAGCGCTGTATAAGCATGAACGTCGCCTGGT
GGTGCCGCTGCTGGTTTCCAGCTCTCTGTTGTTTTATATCGGCATGGCGTTCGCCTACTTTGTGGTCTTTCCGCTGGCAT
TTGGCTTCCTTGCCAATACCGCGCCGGAAGGGGTACAGGTATCCACCGACATCGCCAGCTATTTAAGCTTCGTTATGGCG
CTGTTTATGGCGTTTGGTGTCTCCTTTGAAGTGCCGGTGGCAATAGTGCTGCTGTGCTGGATGGGAATTACCTCGCCAGA
AGACTTACGCAAAAAACGTCCGTATGTGCTGGTTGGCGCATTTGTTGTCGGGATGCTGCTGACGCCGCCGGATGTCTTCT
CGCAAACGCTGCTGGCGATCCCGATGTATTGCCTGTTTGAAATCGGTGTCTTCTTCTCACGCTTTTACGTTGGTAAAGGG
CGAAATCGGGAAGAGGAAAACGACGCTGAAGCAGAAAGCGAAAAAAACTGAAGAATAA
>Cluster_42208
ATGGAAATCAGTCTGTTGCAGGCATTTGCGTTGGGCATTATCGCCTTTATCGCTGGCCTGGATATGTTTAACGGCTTAAC
CCATATGCACCGCCCGGTGGTCCTCGGCCCGTTGGTCGGACTGGTACTTGGCGATCTGCATACCGGTATTTTAACCGGCG
GTACGCTGGAACTGGTGTGGATGGGGCTGGCCCCGCTGGCAGGCGCACAACCGCCTAACGTGATTATCGGTACTATCGTC
GGCACGGCGTTTGCCATTACTACTGGCGTGAAACCCGATGTCGCGGTCGGTGTCGCCGTACCTTTCGCTGTCGCAGTACA
GATGGGGATTACCTTCCTGTTCTCGGTGATGTCCGGCGTGATGTCCCGATGCGACCGGATGGCGGAGAACGCCGATACTC
GCGGCATTGAACGCGTGAACTATCTGGCACTGCTGGCACTCGGCACTTTCTATTTTCTCTGCGCTTTCCTGCCTATCTAC
TTCGGCGCGGAACATGCAAAAACCATCATTGATGTCCTGCCGCAACGATTAATCGACGGCCTCGGCGTCGCGGGCGGCAT
CATGCCAGCAATCGGTTTTGCCGTGCTGCTGAAAATCATGATGAAAAACGTCTACATCCCCTACTTCATCCTGGGCTTTG
TTGCCGCAGCCTGGCTCAAGTTACCGGTGCTGGCTATTGCTGCCGCTGCGCTGGCGATGGCGCTCATCGACCTGCTGCGT
AAATCTCCTGAACCGACTCAACCTGCGGCACAGAAAGAGGAATTTGAAGATGGCATCTAA
>Cluster_64065
ATGTTCCGTTCTTCTGATTTGTGCTCTGTTTTTAAAATGACAGCATTGCCTGTCATTTTTTTTGTTAATATATCCGCTTG
CTCTGCGACATATCCAGAAAGAACCGCGTCTAACATGGATTCGCAAAATTCAGAGTCTCACGTAATAGAACCAAAGAGTG
GAAACATTTTTAATGCTGATATATGCATTGGAAAAAATAGCGATCTAAAATTATGCAATAGTTATGAAAGGAAGTTTACA
CCGCACCTTCTTTATGATAAACCTGATAATTATGTTCTGAATGGATATTACCCCTCAACCTCATCGGGGAATCTCTATAA
TATCGTTAGAGTTCCTGAATCAGGACCTGAATGGTACTCTATAGCAATTTCAATTATTGCGCTAATTGCATCTGTAGGAG
TTCCTTTGTGGCAGCATTATAAAGGAAGAAATGAAGCTATAAACGAAGGGTATTGGTTACGAGAAGTTATTCTCCCTAAA
ATTAATGGATTAGCTTTTGAAGTTACGGAAGATTTTAAGGATGCGATGGGGCTTCCCCCCTGATGAATTTATATCATTACT
AGATAGCCAACTATTTTCAAAGCTTGGGGAGTTAAGGGATTCATTATATCTATTTAAGGGTTTTTCTAAGTTGGAAAAAG
ATATTGAGTCACTTGATGGTATCTGTGATTTATTTGAGCAAAAGGTTTCTGATAATATTGATTCATCTATTGATACAAGG
ATTTCTGATGTATCAATCTTTCATGCTAGCTTAATAAGGGAGCTCATGAAAATGCATAGAAAAATAGGGTGA
>Cluster_34379
ATGGATCTGTTGTACCGGGTAAAAACGCTTTGGGCCGCGCTGCGCGGTAATCATTACACCTGGCCTGCCATCGATATCAC
CCTCCCCGGCAATCGCCATTTTCATCTGATTGGCAGTATTCATATGGGTAGCCACGATATGGCTCCCCTGCCCACCCGTT
TGCTCAAAAAGCTCAAAAACGCCGATGCGCTGATCGTCGAGGCGGATGTTTCCACCAGCGATACGCCTTTTGCTAATTTG
CCTGCCTGCGAGGCGCTGGAAGAGCGCATTAGCGAGGAGCAACTCCAAAACCTGCAGCACATTAGCCAGGAGATGGGCAT
TTCTCCCTCACTCTTTTCTACCCAACCGCTGTGGCAAATCGCGATGGTTCTTCAGGCGACGCAGGCACAAAAACTGGGGC
TGCGGGCAGAATACGGTATCGATTACCAGCTATTGCAGGCGGCGAAGCAACAACATAAACCCGTGATTGAACTGGAAGGG
GCTGAAAACCAGATTGCCATGTTGCTCCAGCTCCCTGACAAAGGACTGGCGCTGCTGGACGATACGCTGACCCACTGGCA
TACCAACGCACGGTTGCTGCAACAAATGATGAGCTGGTGGCTGAATGCACCGCCGCAAAATAATGATATAACGCTGCCCA
ATACGTTCAGTCAGTCGCTGTACGATGTGCTGATGCATCAGCGAAATCTCGCCTGGCGGGATAAATTACGCGCCATGCCG
CCGGGGCGATATGTGGTCGCGGTCGGTGCACTACACCTGTATGGAGAAGGGAATTTGCCGCAAATGTTGCGCTAA
>Cluster_30745
ATGGTCAGCTATAATGTGACTAATGTGTGGGGGCTAATCGTTTTTTTCCTTTGTAGCTTTGCAGTATTAGCATTTTTTAG
CTTTGGTAAAAGTAACCTTATGAGGCTTATTGCACATTATTTCAATTTTGGATATTCAGACAAAAAATTAAAAAGACTTG
ACCGCGAGTGGCGCGACATTCAACTATTTAAAATAATTAATGGAATCAATGTATCAGGCATTGAAAATGTGAGAATGATA
CAGCAGGGACTGATTGATGGAAAACTAAAAACATCGTATTTTTTCCTTACTCGCATCTGGGGTGACATAACAAAACCACC
ACACATAATTAAAACAATAATTGTAATTCTGGCCAGTATTTTTTACATTCTCCTCGCATGTTACATACACAACGAACAAT
CCGTTATAGTAAGGGATGCCATAGGCATACCATATAAAAACATGATGTACTATGTTTATAGTGACAAAGTTCTTTTATCC
TTCAAAAATAAAGCAGTTGAATTTAATAAAACTTATAGCCTTGCCGATTGCAAGAGACTGCAAAACGTATTTATAAAAGA
CACACTTCCTGAGATCGCCTGCAATAAGCTCTTACAGCTAAACGAGGAGGACTCCGAATGGTTAAGCCAGGAGATTAAAG
ATAATAACAGTCACAAAAAAGCATTATTAATACTATCCCTCGTCTATTTCACTTCAGGTCTGGTTATATTCCTGTCATAT
ACAAAATTCTTTTACGCCAATAAGAAGGTTTTAGAATACAAAGCATCAAATAAAAATCACTCATAA
>Cluster_55641

ATGTTTTCAGCGATCTATCGCTACCGTGGCTTTATTATTGACAGCGTTAAACGGGACTTTCAGTCCCGTTACCAAACCAG
CTTCTTAGGCGCGGCATGGCTGATTTTACAGCCGATCGCCATGATTTCCGTCTATACATTAATTTTTTCTGAATTAATGC
GTGCCCGCCTGGCTGGCATGGATGGTCCTTTTGCATACAGTATCTACTTGTGTTCTGGGGTGTTAACTTGGGGGCTGTTC
ACGGAAATGCTCAACAGTCTGGTCAACGTTTTTCTGACCAACGCCAATATTCTTAAGAAGCTCAGCTTTCCGCGGATCTG
TTTACCGATTATCGTCACTGCTTCGGCATTTATTAACTTCCTGATCATTTTTGGTCTGTTCGTGCTGTTTCTGATCGTCA
CGGGCAATTTCCCTGGCATGATTTTCTTTGAAATCATTCCGGTGCTGATCGTCCAGATGCTGTTCACCCTCGGCCTCGGG
ATCATTCTCGGGGTACTGAACGTTTTTGTCCGCGATGTCGGGCAGTTCGTTAATATCCTGCTTCAGTTTTGGTTCTGGTT
TACGCCAATTGTCTACGTATCCAAAACGCTGCCGGAATGGGTTTCTGGTCTGTTGGCGTATAACCCGATGGCGACCATTA
TCGGTTCCTACCAGAACGTGATGCTCTATCACCAGAGCCCTAACTGGATGGCGCTGCTTCCGGTCACAGTGGTGTCGGTC
ATTCTGTTTTTATTTGCCTGGCGTTTATTTAAAAAACATGCCGCTGATATTGTGGACGAGATTTAA
>Cluster_28064
ATGTCAGTACCTCTCATTCTGACCATACTGGCGGGGGCAGCCACGTTTATTGGCGCGTTTCTCGGCGTTCTCGGGCAAAA
ACCCTCGAACCGCTTACTGGCGTTTTCGCTAGGATTTGCGGCGGGGATCATGTTGCTCATCTCATTAATGGAAATGCTTC
CTGCCGCACTAGCCGCTGAAGGAATGTCGCCTGTGTTGGGTTATGGAATGTTTATCTTCGGTCTGCTTGGCTATTTTGGC
CTGGACCGCATGTTGCCACATGCGCATCCGCAGGATTTAATGCAAAAATCGGTGCAGCCGTTGCCAAAATCGATCAAGCG
CACAGCCATTCTGCTCACTCTCGGCATCAGTCTGCATAACTTCCCGGAAGGGATTGCCACCTTTGTCACGGCGAGCAGCA
ACCTGGAGCTGGGATTTGGTATCGCCCTGGCCGTAGCGTTGCACAATATTCCTGAAGGTCTGGCCGTTGCAGGGCCGGTT
TACGCTGCAACAGGATCGAAACGTACCGCGATTCTGTGGGCGGGGATTTCCGGACTGGCTGAAATTCTCGGTGGTGTGCT
GGCGTGGTTAATTCTTGGCAGCATGATTTCTCCGGTAGTCATGGCGGCAATCATGGCGGCAGTTGCCGGTATTATGGTGG
CGCTCTCCGTTGACGAATTAATGCCGTTAGCCAAAGAGATCGACCCCAATAATAACCCCAGTTATGGCGTGCTATGTGGA
ATGTCAGTGATGGGATTCAGTTTAGTGCTGCTACAAACGGCAGGAATTGGTTAA
>Cluster_59189
ATGCGTTCGATTGCCAGACGTACCGCAGTGGGAGCTGCACTATTGCTTGTCATGCCAGTAGCCGTATGGATTTCTGGCTG
GCGTTGGCAACCTGGAGAACAAAGTTGGCTACTAAAAGCGGCTTTTTGGGTTACTGAAACTGTCACCCAGCCCTGGGGCG
TCATTACACATTTGATTTTATTCGGCTGGTTTCTCTGGTGTCTGCGTTTTCGCATTAAGGCTGCCTTTGTATTATTTGCC
ATTCTGGCGGCCGCAATCCTTGTGGGACAAGGCGTTAAATCCTGGATCAAAGACAAAGTCCAGGAACCACGACCTTTTGT
TATCTGGCTGGAAAAAACACATCATATTCCGGTTGATGAGTTCTACACTTTAAAGCGAGCAGAACGCGGAAATCTAGTGA
AAGAACAGTTGGCTGAAGAGAAAAATATCCCACAATATTTGCGTTCACACTGGCAGAAAGAGACGGGGTTTGCCTTTCCT
TCCGGTCACACGATGTTTGCTGCCAGTTGGGCACTGCTGGCCGTTGGTTTGCTGTGGCCGCGTCGGCGAACGTTAACCAT
TGCTATCTTGCTGGTCTGGGCAACGGGAGTCATGGGAAGCCGCCTGCTGCTCGGGATGCATTGGCCACGCGATCTGGTAG
TAGCTACGTTGATTTCGTGGGCGCTGGTGGCGGTGGCAACGTGGCTTGCGCAACGAATTTGTGGGCCATTAACACCACCT
GCGGAAGAAAATCGCGAAATAGCGCAACGAGAACAAGAAAGTTAA
>Cluster_29461
ATGACGGATTTCAAGTGGCGCCATTTCCAGGGTGATGTGATCCTGTGGGCGGTGCGCTGGTATTGTCGCTATCCGATCAG
CTATCGCGACCTTGAGGAAATGCTGGCGGAACGCGGCATTTCGGTCGACCATACGACGATCTATCGCTGGGTCCAGTGCT
ACGCCCCGGAGATGGAGAAGCGGCTGCGCTGGTTCTGGCGGCGTGGCTTTGATCCGAGCTGGCGCCTGGATGAAACCTAC
GTCAAGGTGCGGGGCAAGTGGACCTACCTGTACCGGGCAGTCGACAAGCGGGGCGACACGATCGATTTCTACCTGTCGCC
GACCCGCAGCGCCAAGGCAGCGAAGCGGTTCCTGGGCAAGGCCCTGCGAGGCCTGAAGCACTGGGAAAAGCCTGCCACGC
TCAATACCGACAAAGCGCCGAGCTATGGTGCAGCGATCACCGAATTGAAGCGCGAAGGAAAGCTGGACCGGGAGACGGCC
CACCGGCAGGTGAAGTATCTCAATAACGTGATCGAGGCCGATCACGGAAAGCTCAAGATACTGATCAAGCCGGTGCGCGG
TTTCAAATCGATCCCCACGGCCTATGCCACGATCAAGGGATTCGAAGTCATGCGAGCCCTGCGCAAAGGACAGGCTCGCC
CCTGGTGCCTGCAGCCCGGCATCAGGGGCGAGGTGCGCCTTGTGGAGAGAGCTTTTGGCATTGGGCCCTCGGCGCTGACG
GAGGCCATGGGCATGCTCAACCACCATTTCGCAGCAGCCGCCTGA
>Cluster_58203
ATGCCGATTATTCAGTCTGTTGAACGTGCGTTGCAGATCCTCGACCTGTTCAACGAGCAGGCCACCGAGCTTAAGATCAC
CGACATCAGCAAACTGATGGGGCTGAGCAAGAGTACCCTCCACTCGCTGCTAAAAACCCTGCAGCTTCACGGCTATATCG
ATCAGAACCCGGAGAACGGCAAGTATCGCCTCGGCATGAAGCTGGTCGAGCGCGGCCATTTTGTCGTGGGCTCCATCGAT
ATTCGGCAGAAGGCAAAAGGCTGGCTGACGGAGCTGTCCCGGCGGACCGGGCAGACCACCCATCTGGGGATCCTGGACGG
GCGTGAAGGGGTCTATATCGAGAAGATTGAAGGCAAGCTGGCCGCCATCGCCTATTCACGCATCGGCCGCCGCCTGCCGG
TGCACGCCACCGCCATCGGCAAGGTGTTGATTGCCTGGCTGGGCGAGGCCGAGCTGAACGCCCTGCTGGAGGGCTATCAG
TACACTACCTTTACGCCCGCCACCCTCGCGTCTCGCGAAGCCTTAATGAGCGCCCTGGCGCAGACCCGCGAGCAAGGCTA
CGCCCTGGACAGCGAAGAGAACGAGCAGGGCGTGCGCTGCGTGGCGGTGCCGGTGTGGAACCACGAGTCCCGCGTCATCG
CCGCCCTGAGCCTGTCGACGCTGACCTCCCGCGTGGACGACGCGGAGCTGGCTAATTTCCGCGAGCAGCTTCAGCAGGCC
GGGCTCGCGCTCTCGCGCGCGCTGGGCTACCCGGCCTGA
>Cluster_5668
ATGCAATTGAGCAGCAGTGAACCTTGCGTGGTGATCCTGACCGAAAAAGAGGTAGAGGTAAGCGTCAATAACCATGCTAC
GTTTACCCTTCCGAAAAACTACCTGGCCGCCTTCGCGTGCAACAATAACGTCATTGAACTCTCAACGTTAAATCACGTAT
TAATCACCCACATCAACCGTAACATCATCAACGATTATCTGTTGTTTTTAAATAAGAACTTAACCTGTGTAAAGCCCTGG
TCGCGGCTGGCAACCCCGGTTATCGCTTGTCATAGCCGTACACCGGAAGTGTTCCGGCTAGCCGCCAACCACAGCAAGCA
GCAACCCAGCAGACCCTGCGAAGCGGAGTTGACGCGCGCATTGCTTTTTACCGTATTGTCTAACTTTCTTGAGCAATCGC
GGTTTATTGCCCTACTGATGTATATCTTACGCAGCAGCGTCCGCGACAGCGTCTGCCGCATTATTCAAAGCGATATTCAG
CATTACTGGAATCTGCGAATTGTCGCCAGTTCGCTATGTTTAAGCCCCAGCCTGCTCAAAAAGAAATTAAAAAACGAAAA
TACCAGCTATAGCCAGATTGTCACAGAGTGTCGTATGCGTTACGCCGTACAGATGTTATTGATGGATAACAAAAATATCA
CTCAGGTGGCGCAATTATGTGGCTATAGCAGCACGTCGTACTTTATCTCTGTTTTTAAGGCGTTTTACGGCCTGACACCG
TTGAATTATCTCGCCAAACAGCGACAAAAAGTGATGTGGTGA
>Cluster_53586
ATGCAAACTGAGCAACAGCGAGCCGTAACACGGCTTTGTATCCAGTGTGGATTATTTCTTTTGCAACATGGTGCGGAAAG
CGCGTTGGTTGATGAGCTTTCCTCACGACTGGGTCGGGCGTTGGGAATGGACAGCGTCGAAAGTTCTATCTCTTCGAACG
CCATCGTGCTGACAACCATTAAAGATGGGCAATGCCTGACATCGACACGTAAAAATCACGATCGCGGCATTAATATGCAT
GTGGTGACTGAAGTCCAGCACATTGTAATTCTGGCGGAGCATCATCTGCTGGATTACAAAGGCGTAGAGAAACGATTTAG
CCAAATTCAGCCATTACGTTACCCAAGATGGCTGGTAGCCTTAATGGTTGGCCTTTCCTGCGCCTGTTTCTGTAAACTCA
ATAACGGTGGCTGGGATGGTGCCGTCATCACCTTCTTTGCCAGTACGGCCGCGATGTATATCCGCCAGCTGCTGGCACAA
CGTCATCTTCATCCACAGATCAACTTTTGCCTCACCGCTTTCGCCGCCACCACCATTTCCGGATTGCTTTTGCAACTTCC
CACTTTCAGCAATACCCCCACCATTGCGATGGCCGCCAGCGTTCTGCTGCTGGTGCCGGGCTTTCCGTTGATAAATGCCG
TCGCCGATATGTTTAAAGGCCACATCAATACCGGACTGGCACGCTGGGCAATCGCCAGTCTGCTGACACTGGCTACCTGT
GTCGGCGTAGTAATGGCACTGACGATTTGGGGGCTACGCGGATGGGTGTGA
>Cluster_550
ATGATAAAACGGACGTTATTAGCGGCGGCCATTTTTAGCGCATTGCCCGCTTATGCCGGGTTAACTTCCATTACCGCGGG
CTACGATTTTACCGATTATTCTGGCGATCATGGCAACCGTAATTTAGCGTATGCTGAACTGGTGGCGAAAGTTGAAAACG

CAACGCTGCTTTTTAATCTTTCCCAGGGGCGTCGTGATTATGAAACTGAACATTTCAATGCCACTCGCGGTCAGGGTGCG
GTCTGGTATAAATGGAATAACTGGCTGACAACCCGAACGGGTATTGCCTTTGCGGATAATACGCCGGTCTTTGCCCGCCA
GGATTTTCGTCAGGATATTAACCTGGCCCTGTTGCCAAAAACGCTTTTCACGACCGGTTATCGCTACACTAAATATTACG
ATGATGTCGAAGTCGATGCCTGGCAAGGCGGCGTATCACTCTATACTGGCCCGGTGATCACCAGCTACCGCTATACCCAT
TATGACTCCAGCGATGCAGGTGGTAGTTATAGCAATATGATTTCCGTGCGTCTGAATGACCCGCGCGGCACTGGTTATAC
GCAACTATGGCTAAGCCGCGGAACAGGCGCTTACACCTATGACTGGACGCCAGAAACACGCTACGGCAGCATGAAGAGCG
TCAGTTTGCAACGTATTCAACCGCTGACTGAGCAACTTAATCTCGGGCTGACGGCAGGTAAAGTGTGGTACGACACCCCA
ACCGATGATTATAACGGTCTGCAACTTGCAGCCCATCTGACCTGGAAATTCTGA
>Cluster_57579
ATGCGCGAAAGGGTATCGCAGCAGTTAAAAGAAATTGAACGGCGGTATGATGTGAAAGTACTGTACGCCTGTGAATCAGG
GAGCCGCGGCTGGGGATTTGCCTCGCCCGACAGCGACTACGATGTACGGTTTTTGTATGTTCACCCGCTGGAATGGTATC
TGCGAGTAGAGTCACCGCGCGATGTTATTGAGCTGCCCATCGACGACGAGTTAGATGTTTCCGGTTGGGAGTGGCGTAAG
GCGCTGGGATTATTGAAAGGCGCGAACCCGACGCTGATAGAATGGCTGGATTCTCCGGTGATTTATCAACAAGATGAAGA
AACGATAACGGCGTTTAAGGCAATGGTCCCAATGTGGTTTTCTCCGCTGCGTGCGCGCTGGCATTACTATTCGATGGCGC
AGAAAAACTTCCCCGGATATTTGCAGGGCGACGAAGTTCGACTGAAAAAATACTTTTACGTGCTGCGTCCGCTGCTGGCC
GTGCGCTGGGTCGAAGCGGGTAAAGGCGTACCGCCAATGCGCTTTGCTGAGTTACTGGCGGGAAGCGAACTTGATGCGCC
GTTACGTGCAGAAATAGACGAATTACTGGAACGTAAACAGCGCGCAGGAGAAGCGGAATATGGTCCGCGCCGTCCATTGT
TACATGCGTTTATTCGTGCGGAACAGGCGCGTGGGAAATTCCACCGCTGTTACCGGATAGCAGAGAAGGGGACGTTAAG
GAACTGGATAGCTTAATGTACCAGACGGTAATGCGCCGGGCATAA
>Cluster_6847
GTGTTTAATTCTGACAACCTGAGACTCGACGGAAAATGCGCCATCATCACAGGTGCGGGTGCAGGTATTGGTAAAGAAAT
CGCCATTACATTCGCGACAGCTGGCGCATCTGTGGTGGTCAGTGATATTAACGCCGACGCAGCTAACCATGTTGTAGACG
AAATTCAACAACTGGGTGGTCAGGCATTTGCCTGCCGTTGTGATATTACTTCCGAACAGGAACTCTCTGCACTGGCAGAC
TTTGCTATCAGTAAGCTGGGTAAAGTTGATATTCTGGTTAACAACGCCGGTGGCGGTGGACCTAAACCGTTTGATATGCC
AATGGCGGATTTTCGCCGTGCTTATGAACTGAATGTGTTTTCTTTTTTCCATCTGTCACAACTTGTTGCGCCAGAAATGG
AAAAAAATGGCGGTGGCGTTATTCTGACCATCACTTCTATGGCGGCAGAAAATAAAAATATAAACATGACTTCCTATGCA
TCATCTAAAGCTGCGGCCAGTCATCTGGTCAGAAATATGGCGTTTGACCTGGGTGAAAAAAATATTCGGGTAAATGGCAT
TGCGCCGGGGGCAATATTAACCGATGCCCTGAAATCCGTTATTACACCAGAAATTGAACAAAAAATGTTACAGCACACGC
CGATCAGACGTCTGGGCCAACCGCAAGATATTGCTAACGCAGCGCTGTTCCTTTGCTCGCCTGCTGCGAGCTGGGTAAGC
GGACAAATTCTCACCGTCTCCGGTGGTGGGGTACAGGAGCTCAATTAA
>Cluster_23879
ATGAAACTGACAACACATCATCTACGGGCGGGGGCCGCATTATTACTGGCAGGAGTTCTGCTGGCAGGTTGCGACCAGAG
CAGCAGCGATGAAAAGCATATTAAAGTTGGCGTTATTAATGGCGCAGAACAAGATGTCGCGGAAGTCGCTAAAAAGGTAG
CAAAAGAGAAATATGGCCTCGATGTTGAACTGGTAGGATTTAGCGGTTCGTTGCTACCGAACGATGCGACAAATCATGGC
GAGCTTGATGCGAACGTCTTCCAGCATCGACCCTTTCTTGAACAAGATAATCAGACGCATGGTTATAAACTGGTAGCGGT
GGGAAATACGTTTGTCTTCCCCATGGCGGGCTATTCCAAAAAAATCAAAACCGTGGCGCAAATAAAAGAAGGTGCGACAG
TGGCGATCCCCAACGACCCGACTAACCTTGGTCGCGCGCTTTTACTGCTGCAAAAAGAGAAGCTAATTACTCTGAAAGAG
GGAAAAGGGTTATTACCTACCGCGTTGGATATAACCGATAATCCACGCCATTTGCAGATAATGGAGCTGGAAGGGGCACA
GTTGCCACGTGTACTGGACGACCCGAAAGTGGATGTAGCGATTATCAGCACCACTTACATCCAGCAAACAGGACTTTCTC
CGGTTAACGACAGCGTATTTATTGAAGATAAAAATTCGCCGTATGTGAATATTCTGGTGGCACGGGAAGATAATAAGAAT
GCAGAAAACGTGAAGGAATTTCTGCAATCTTATCAATCACCCGAAGTCGCTAAAGCGGCAGAAACCATCTTTAACGGTGG
CGCGGTGCCTGGCTGGTAA
>Cluster_18588
ATGGCAGTTGTTAAATGTAAACCGACATCTCCGGGTCGTCGCCACGTAGTTAAAGTGGTTAACCCTGAGCTGCACAAGGG
CAAACCTTTTGCTCCGTTGCTGGAAAAAAACAGCAAATCCGGTGGTCGTAACAACAATGGCCGTATCACCACTCGTCATA
TCGGTGGTGGCCACAAGCAGGCTTACCGTATTGTTGACTTCAAACGCAACAAAGACGGTATCCCGGCAGTTGTTGAACGT
CTTGAGTACGATCCGAACCGTTCCGCGAACATCGCGCTGGTTCTGTACAAAGACGGTGAACGCCGTTACATCCTGGCCCC
TAAAGGCCTGAAAGCTGGCGACCAGATTCAGTCTGGCGTTGATGCTGCAATCAAACCAGGTAACACCCTGCCGATGCGCA
ACATCCCGGTTGGTTCTACTGTTCATAACGTAGAAATGAAACCAGGTAAAGGCGGTCAGCTGGCACGTTCCGCTGGTACT
TACGTTCAGATCGTTGCTCGTGATGGTGCTTATGTCACCCTGCGTCTGCGTTCTGGTGAAATGCGTAAAGTAGAAGCAGA
CTGCCGTGCAACTCTGGGCGAAGTTGGCAATGCTGAGCATATGCTGCGCGTTCTGGGTAAAGCAGGTGCTGCACGCTGGC
GTGGTGTTCGTCCGACCGTTCGCGGTACCGCGATGAACCCGGTAGACCACCCACATGGTGGTGGTGAAGGTCGTAACTTT
GGTAAGCACCCGGTAACTCCGTGGGGCGTTCAGACCAAAGGTAAGAAGACCCGCAGCAACAAGCGTACTGATAAATTCAT
CGTACGTCGCCGTAGCAAATAA
>Cluster_34998
ATGTGTCAGGTATTTGGTGTATCCAGGAGCGGTTATTACAACTGGGTACAGCATGAACCCTCAGACAGAAAACAAAGTGA
TGAGCGGCTAAAACTGGAGATTAAGGTGGCACATATCCGCACTCGCGAAACATATGGAACCCGGCGGCTCCAGACGGAGC
TGGCAGAGAATGGCATCATCGTTGGTCGTGACCGACTGGCACGTCTTCGTAAGGAGCTAAGGCTACGCTGTAAGCAGAAA
CGCAAGTTCAGAGCGACTACGAACTCGAACCACAATCTGCCAGTTGCGCCAAATCTGCTGAACCAGACGTTCGCTCCTAC
AGCACCAAATCAGGTCTGGGTGGCGGACCTGACGTATGTTGCCACACAGGAGGGATGGTTGTACCTCGCTGGCATCAAAG
ATGTTTATACGTGCGAAATTGTCGGCTACGCCATGGGAGAGCGCATGACAAAAGAGCTGACAGGTAAAGCCCTGTTTATG
GCGCTCAGGAGCCAGCGCCCACCTGCCGGGCTAATCCACCACTCCGATCGAGGTTCACAGTACTGCGCATACGATTACCG
GGTCATACAGGAGCAGTTTGGTCTGAAAACATCAATGTCGCGTAAAGGTAACTGTTACGACAACGCTCCGATGGAAAGCT
TCTGGGGAACGCTGAAAAATGAGAGCCTGAGGCACTATCGTTTTAAGAGCCGGAATGAAGCCATCTCAGTAATACGGGAA
TACATTGAGATTTTCTACAATCGTCAGCGTCGTCACTCTCGTCTGGGGAATATCTCCCCGACAGCCTTCAGGGAAAAATA
TCATCAGATGGCTGCTTAA
>Cluster_7102
ATGATTTACATCGGTCTACCGCAATGGTCGCATCCTAAATGGGTGCGGTTGGGGATCACCAGCCTTGAAGAGTATGCCCG
CCACTTTAACTGCGTGGAGGGCAACACCACGCTTTACGCCCTGCCGAAACCCGAGGTTGTCCTGCGCTGGCGTGAGCAGA
CCACAGATGACTTCCGCTTCTGTTTTAAGTTTCCGGCGACCATTTCGCATCAGGCAGCATTACGGCATTGCGATGATTTA
GTGACTGAATTTTTGACCCGCATGTCACCGTTGGCTCCGCGCATTGGGCAATACTGGCTGCAACTGCCTGCCACATTCGG
CCCACGGGAGCTGCCTGCGCTTTGGCATTTTCTCGATTCTCTTCCTGGCGAATTTAATTATGGCGTGGAAGTCCGCCATC
CACAGTTTTTCGCCAAAGGAGAAGAGGAACAAACGCTTAATCGCGGTTTACATCAGCGCGGCGTTAATCGGGTGATTTTA
GACAGCCGCCCGGTTCATGCAGCACGTCCACACAGTGAAGCTATTCGCGACGCTCAACGAAAAAAACCTAAAGTTCCGGT
ACATGCTGTACTGACGGCGACAAATCCGCTGATCCGTTTTATCGGTAGTGATGATATGACGCAAAACCGGGAATTATTTC
AGGTCTGGTTACAAAAATTAGCGCAGTGGCATCAGACCACTACGCCTTATCTTTTTTTACATACGCCAGACATTGCCCAG
GCACCGGAACTGGTACATACCCTGTGGGAAGACTTACGTAAAACGCTTCCAGAGATCGGAGCAGTTCCGGCTATTCCACA
GCAATCTTCTCTTTTCTGA
>Cluster_34937

ATGTTTGTTCCGTTTCTCATTATGTTGCGCGAAGGACTTGAAGCCGCGCTGATTGTCAGTTTGATTGCCAGCTATCTTAA
GCGTACCCAGCGAGGCCGATGGATTGGTGTGATGTGGATTGGCGTGTTGCTTGCCGCTGCGTTGTGCCTGGGCTTGGGTA
TCTTCATTAACGAAACCACCGGCGAATTTCCGCAAAAAGAACAGGAACTGTTGAAGGTATCGTGGCGGTGATCGCCCGTG
GTGATCCTTACCTGGATGGTTTTCTGGATGCGCAAAGTGTCGCGCAACGTCAAAGTGCAACTGGAACAGGCAGTCGATAG
CGCATTGCAGCGTGGAAATCATCATGGCTGGGCGCTGGTGATGATGGTCTTTTTTGCCGTTGCAAGGGAAGGGCTGGAGT
CGGTCTTTTTCCTGCTGGCGGCATTTCAACAAGATGTCGGGATCTGGCCGCCGCTGGGTGCAATGCTCGGTCTTGCTACT
GCCGTGGTGCTAGGCTTCCTGCTCTACTGGGGCGGTATTCGCCTCAATCTTGGTGCATTTTTTAAATGGACCAGCCTGTT
TATTCTCTTCGTCGCCGCAGGGCTGGCAGCTGGTGCCATTCGCGCATTTCATGAAGCCGGATTGTGGAACCACTTTCAGG
AAATCGCCTTCGATATGAGTGCGGTGCTCTCAACTCACTCGCTGTTTGGCACGCTGATGGAAGGGATTTTTGGCTATCAG
GAAGCGCCGAGCGTCAGCGAAGTCGCCGTCTGGTTTATTTATCTCATCCCGGCGCTGGTGGCATTTGCTCTGCCACCACG
CGCAGGGGCGACAGCGTCTCGCTCCGCGTAA
>Cluster_57856
ATGTCTATGCCATTAAGCAATGCCCTTCAGAGTCAAATTATTACAGATAATCACTTCCTTCATCACCCAAAAGTCGACAA
TGAGTTAACACGTAAATATGAACGAGCTCGCCTGGATACGGAAAATATCTATCTTCTGCCTCTTGCTCGTGGCAATAACC
ATAATTACGATGGAAAAAGCGTTGTCGAAATTAGAAAACTTGATATTAGTAAAGAGTCCTGGCCATTTAATTATGTGACA
GAAGCATGCAGAGAATCCGATGGTATTACAACAACAGGACGTATGCTCTATCGAAACTTGAAAATCACCTCAGCTTTAGA
CGAAATATATGGTGGTATATGTAAAAAGGCGCATGCTGCTACAGAATTAGCAGAAGGTTTACGGCTAAATTTATTTATGA
AGAGCCCTTTCGATCCTGTTGAGGACTATACAGTTCATGAAATAACATTAGGCCCTGGTTGCAATGTTCCAGGTTACGCC
GGAACCACGATAGGATATATTTCAACCCTTCCAGTTTCTCAGGCAAAAAGATGGACCAATGAGCAGCCAAGAATTGATAT
CTATATTGATCAGATAATAACTGTCTCAGGGGTAGCAAATTCTTCCGGATTCGCCCTTGCCGCATTACTTAATGCTAATA
TTGGAATGGGAAATGACCCAATAATTGGTATAGAAGCCTATCCCGGTACCGCGGAGATCCATGCAAAAATGGGATATAAC
GTTATCCCCGGTGATGAAGATGCGCCACTCAAACGCATGACTCTGCAACCGTCATCTCTTCCAGAACTGTTTGAATTAAA
AAATGGTGAATGGAATTATATTGGTAAATAG
>Cluster_34156
ATGACCCGTCTGGCTTCGCGCTTTGGCGCAGCAAACCTTATCCGTCGCGACCGCCCGTTAACCCGTGAGGAGCTGTTTCG
CGTGGTGCCCAGTGTGTTCAGCGAGGACAAACATGAATCCCGCAGTGAGCGTTATACCTATATACCTACCATCTCCCTGC
TCGACAGCCTACAGCGAGAAGGCTTCCAGCCATTCTTTGCCTGTCAGACCCGCGTGCGTGACCCCGGTCGTCGTGAACAT
ACAAAGCATATGCTGCGTCTGCGGCGTGAGGGGCAGATTACCGGTAAACAGGTACCAGAAATTATCCTGCTCAACTCCCA
CGATGGCACCAGTTCGTATCAGATGTTGCCGGGACTATTTCGTGCGGTTTGTCAGAACGGGCTCGTCTGCGGTGAGTCGT
TTGGCGAGGTGCGGGTGCCACACAAGGGGGACGTGGTGAGTCAGGTGATTGAAGGCGCGTATGAGGTGCTGGGGATTTTT
GACCGGGTGGAGGAGAAACGGGATGCCATGCAGTCGTTGCTGTTGCCACCCCCGGCACAGCAGGCACTGGCAAAAGCCGC
CCTCACATACCGCTTTGGTGAAGACCACCAGCCGGTGACTGAATCGCAGATCCTCTCCCCTCGCCGCTGGCAGGATGAGA
GCAATGACCTGTGGACCACGTACCAGCGTATTCAGGAGAACCTGATTAAGGGCGGGCTCAGTAGCCGTAATGCCAAAGGA
GGACGGTCACATACCCGTGCCGTTCGCGGTATCGACGGGGACGTGAAACTTAACCGGGCACTGTGGGTGATGGCGGAAGC
CCTGCTCACGCAACTGCAGTGA
>Cluster_42630
ATGATTTTGACTCCCATACGACGATATGGGGCGATGATTCTTATGTTACTCACTCTGGTGTTTTCGAGTGAGGTGTTAGC
GAAGACGCACACAACAACAGCGAGTCAAAAGTCCCACTTAACTAAAGCTAGTAATAAACAGGTAAGCAGTAAACAAGAGT
ATTCTCGCAATAGTGCAAAGAGTAGTTCACTTCCTGATTTGCGAAAATACCCTTCCGGAACACCAAGGAAAAAGGCGTTT
CTCCGGACTGTAATGCCTTACATTACCAGCCAAAATGCGGCCATTACTGCGGAACGTAACTGGCTGATTTCAAAACAGTA
TCAGGGCCAATGGTCACCTGCTGAGCGTGCGCGTCTGAAAGACATCGCCAAACGCTACAAGGTGAAGTGGTCTGGTAATA
CGCGAAAAAATCCCGTGGAATACCTTGCTTGAACGCGTAGACATTATCCCTACCAGTATGGTGGCGACGATGGCTGCAGCA
GAAAGCGGTTGGGGAACGTCGAAGCTGGCGCGCAACAACAACAATCTTTTCGGCATGAAATGCATGAAAGGACGTTGTAC
CAATGCGCCGGGTAAAGTGAAAGGGTACTCACAGTTTAGTTCTGTCAAAGAATCGGTGAGCGCCTATGTCACTAACCTCA
ATACGCACCCGGCTTACTCTTCGTTCCGTAAATCGCGTGCACAACTGCGTAAAGCGGATCAGGAAGTGACTGCCACAGCG
ATGATTCACAAGCTGAAGGGCTACTCGACCAAAGGGAAGAGTTATAACAACTACCTGTTCGCAATGTACCAGGATAACCA
ACGGTTAATCGCGGCGCATATGTAA
>Cluster_25650
ATGAGCATTAAATTAATTGCGGTAGACATGGATGGTACTTTCTTAAGCGATCAAAAAACCTATAACCGTGAGCGGTTTAT
GGCTCAGTATCAGCAAATGAAAGCACAAGGCATTCGCTTTGTGGTCGCCAGCGGGAATCAATATTATCAGTTGATCTCTT
TCTTTCCTGAAATTGCTAATGAAATTGCCTTTGTGGCTGAAAACGGCGGCTGGGTAGTGAGCGAAGGCAAAGATGTTTTT
AATGGCGAGCTGTCGAAGGATGCGTTTACTACTGTCGTGGAACATTTGCTGACGCGCCCGGAAGTGGAAATTATTGCCTG
CGGAAAAAATAGCGCCTATACCCTCAAAAAATATGACGATGCCATGAAAACGGTGGCGGAAATGTATTATCACCGTCTGG
AATACGTCGATAACTTTGACAACTTAGAAGATATCTTCTTTAAATTTGGCCTTAATCTCTCTGACGAACTGATCCCACAA
GTGCAAAAAGCATTACATGAGGCCATCGGCGATATCATGGTACCGGTCCATACCGGCAACGGCAGCATCGATCTGATTAT
CCCCGGTGTACATAAAGCCAATGGCCTTCGCCAACTGCAGAAATTATGGGGAATAGACGACAGCGAAGTGCTGGTCTTTG
GCGATGGTGGTAACGATATTGAGATGCTGCGTCAAGCAGGCTTTAGTTTTGCAATGGAAAATGCCGGCAGCGCGGTCGTC
GCAGCGGCTAAATACCGGGCAGGCTCCAATAACCGTGAAGGCGTACTGGATGTGATCGATAAAGTTCTTAAACACGAAGC
GCCATTTAAGCAATAA
>Cluster_3003
ATGCCCCTGGCGAAAATATTTACAAAGCAAGTTTCTTTATTAACTATGTTTTCAGCGTTAGCTTATGCGATGGCATATTC
TTTTCAGAAAGGTGTTGCATCGTATTATGGCTATCCAGACCTTTTTATTAAAATAGACTTAAATACGCTATTGTATTCGG
CTGTGTGGCTTTTGTTTTTTAGTTTTTTATTTCTCGGCGCCTTATATGCGGCGTTGATGTTTGATTTGAAAGGGAAATAT
TATGTTCCATTTATTGTATTTATAGTTGTTATGCTAATGGCTAATATTTGTATAATAGGATTTCATTGGCCTTTTGGTTA
TTTCTCCGAAACTTCTGTGATGAGTGTTCAGGATATTTTATTATTTGCTATGCTTTTGTTGAATTATCACAATGTGGTGT
CCATGCCAATCCATAGTAAGAGACACGAACATCATATGGAGAGCATTAGAAAATTTGGGAGGCTTGCAGAGTATAAGAAC
GTATTTTATTATATCGATTTAAAAATCCTAAAGTCAGCTTTTCCTTTAGGGGTGATAACGTTGTTGATATTGAGCTATAC
ATTCGGGAAGGTTACTGCATTTAAAAATAACGAATATTATATGGTAGATGGTAGTAATACTAAAGTGTTGCTCAATTCAT
TTGGTAGTGGTTTTGTTGTTGGTGATTGTGAGTCAAATAGCGCAAAATTTGAATTTGTTAATGATTTAAAAGGGATGAGA
TTCACAATCATGTCCTCTAAAAAGGAAATAGCTAAATTAAAAGGGTGTTTCGAAATACGTGTTAAAAATACGGATTAA
>Cluster_9083
ATGTACCAGGTTGTTGCGTCTGATTTAGATGGCACGTTACTTTCTCCCGACCATACGTTATCCCCTTACGCCAAAGAAAC
TCTGAAGCTGCTCACCGCGCGCGGCATCAACTTTGTGTTTGCGACCGGTCGTCACCACGTTGATGTGGGGCAAATTCGCG
ATAATCTGGAGATTAAGTCTTACATGATTACCTCCAATGGTGCGCGCGTTCACGATCTGGATGGTAATCTGATTTTTGCT
CATAACCTGGATCGCGACATTGCCAGCGATCTGTTTGGCGTAGTCAACGACAATCCGGACATCATTACTAACGTTTATCG
CGACGACGAATGGTTTATGAATCGCCATCGCCCGGAAGAGATGCGCTTTTTTAAAGAAGCGGTGTTCCAATATGCGCTGT
ATGAGCCTGGATTACTGGAGCCGGAAGGCGTCAGCAAAGTGTTCTTCACCTGCGATTCCCATGAACAACTGCTGCCGCTG
GAGCAGGCGATTAACGCTCGTTGGGGCGATCGCGTCAACGTCAGTTTCTCTACCTTAACCTGTCTGGAAGTGATGGCGGG
CGGCGTTTCAAAAGGCCATGCGCTGGAAGCGGTGGCGAAGAAACTGGGCTACAGCCTGAAGGATTGTATTGCGTTTGGTG

ACGGGATGAACGACGCCGAAATGCTGTCGATGGCGGGGAAAGGCTGCATTATGGGCAGTGCGCACCAGCGTCTGAAAGAC
CTTCATCCCGAGCTGGAAGTGATTGGTACTAATGCCGACGACGCGGTGCCGCATTATCTGCGTAAACTCTATTTATCGTA
A
>Cluster_40944
ATGATTAAGTGGCCCTGGAAAGTACAAGAATCAGCACATCAAACTGCCCTTCCCTGGCAGGAAGCACTATCGATCCCCCT
TTTAACGTGTCTGACAGAACAGGAACAAAGCAAATTAGTCGCTCTTGCCGAACGTTTTTTACAACAAAAACGGCTTGTTC
CTTTACAGGGCTTTGAGCTGAATTCATTAAGAAGCTGCCGGATAGCACTTCTATTTTGCCTGCCCGTTCTGGAGTTAGGA
CTGGAATGGCTGGATGGTTTTCATGAAGTCTTAATTTATCCTGCGCCATTTGTGGTCGATGATGAATGGGAAGACGATGT
CGGTCTGGTGCATAACCAACGTATTGTTCAGTCAGGTCAGAGCTGGCAGCAAGGGCCTATCGTTTTGAACTGGTTGGATA
TACAAGATTCTTTTGATGCTTCTGGTTTTAACCTGATTATTCATGAAGTCGCTCATAAGCTGGACACCCGTAACGGCGAT
CGCGCCAGCGGAGTTCCCTTTATTCCGTTGCGTGAGGTTGCTGGCTGGGAACACGATCTTCATGCTGCAATGAACAACAT
TCAGGAAGAAATCGAATTGGTTGGCGAGAATGCGGCGAGCATTGATGCTTATGCTGCCAGTGATCCTGCTGAATGTTTTG
CCGTACTTTCTGAATATTTCTTTAGCGCCCCAGAACTTTTTGCTCCTCGTTTCCCTTCATTGTGGCAACGTTTCTGCCAA
TTTTATCAACAAGATCCTTTGCAGAGACTGCATCGCACTAATAATACAGACTCGTTTTCGGCGACGAATGTTCATTAA
>Cluster_41642
ATGAATAATCTGATGGTCATTGATGGTATTGAAGTTCGTCGTGATGCTTATGGGCGTTACAGCCTGAACGATCTGCACAG
GGCTGCCGGTTCTCTGGATAAGCATAAGCCTGCATTCTGGCTCCGCAATGAGCAAACTGAACGTTTAATAAGCGAGTTGC
AGATTTGCAACTCGGTCAATATAGAGCCAGTTAACGTTATTCGTGGTGGAAATAACCAGGGGACGTATGTCTGCAAAGAA
CTGGTGTATGCCTATGCAATGTGGATCAGCCCGTCATTCCATCTGAAGGTGATCCGTACTTTCGACATGGTAACCAGCGC
ACCTGAAAAATTATCCGGACAGGCTGCTGACAAGATGCAGGCTGGCGTGATTCTGCTGGACTTTATGCGCCGGGAGTTAA
ATCTGTCTAACTCTTCAGTGCTTGGAGCCTGTCAGAAACTTCAGGAGGCTGTTGGCTTACCGAATCTGGCACCGCGCTAT
GCCATTGATGCTCCTGCTGATGCACACGATGGCTCAAGTCGCCCTACGCTGTCACTGAGTGCACTGCTGAAACAGTATGG
TATCCGCCTGACGGCTAATCAGGCATATCACCAGATGGTGAAGCTGGGGATCGTCGAGCAGCGCGAACGATACAGCCGTA
CCGCGATTAACAACATCAAAAAATTCTGGTCGCTGACAGCGAAAGGCTGCATGTTCGGCAAGAACATCACCAGTCCCGCA
AATCCGCGCGAGACGCAGCCGCATTTCTTCGAATCCCGATTCTCTGAGCTGTTAAAGCTGCTCGATACCGTTCATTGA
>Cluster_40181
ATGATCGAAACGCTACTTGAAGTGCGCAATTTAAGTAAAACGTTCCGCTACCGGACCGGCTGGTTTCGTCGTCAGACCGT
AGAAGCGGTAAAACCCTTGAGCTTTACGCTACGTGAAGGCCAGACACTGGCGATTATCGGCGAGAATGGTTCGGGTAAAT
CCACGCTGGCAAAAATGCTGGCGGGAATGATAGAACCGACCAGCGGCGAGTTATTGATTGACGATCATCCGCTGCATTTT
GGCGATTATTCCTTCCGCAGTCAGCGTATTCGCATGATTTTTCAGGATCCTTCGACCTCGTTGAATCCCCGTCAACGTAT
CTCGCAAATTCTGGATTTTCCACTGCGCCTGAACACCGACCTGGAACCGGAACAGCGTCGCAAACAGATTATTGAAACGA
TGCGTATGGTTGGGCTGCTACCGGATCACGTCAGTTACTATCCGCATATGTTAGCACCCGGGCAAAAACAGCGTCTGGGT
CTGGCGCGCGCGCTGATATTGCGCCCAAAAGTCATTATTGCCGATGAAGCCCTCGCCTCACTGGATATGTCGATGCGTTC
GCAGTTGATTAATCTGATGCTGGAGTTACAGGAAAAACAGGGCATTTCGTATATTTATGTTACCCAGCATATCGGAATGA
TGAAGCACATTAGCGACCAGGTGCTGGTGATGCATCAGGGCGAGGTTGTCGAGCGAGGCAGCACCGCAGATGTGCTGGCA
TCGCCGCTGCATGAACTCACCAAACGGCTGATCGCCGGTCATTTTGGCGAGGCATTGACGGCGGATGCGTGGCGTAAGGA
CCGTTAG
>Cluster_27363
ATGGAAACGTTTAATAGCCTGTTTATGGTTTCCCCGCTGCTGCTGGGAGTTCTCTTTTTTGTCGCCATGCTGGCGGGATT
TATCGACTCGATTGCCGGTGGCGGAGGGTTACTCACCATTCCGGCATTGATGGCGGCGGGGATGTCTCCCGCTAACGCGC
TGGCAACCAATAAACTGCAAGCCTGTGGTGGCTCTATTTCCGCCACTATCTACTTTATTCGCCGTAAAGTGGTTAGTTTA
AGCGATCAAAAACTCAATATCGCCATGACCTTTGTCGGCTCAATGAGTGGCGCACTGATGGTGCAATACGTTCAGGCTGA
TGTCTTACGGCAGATTTTGCCGATCCTCGTAATTTGTATCGGTCTTTATTTCTTACTGATGCCAAAGCTGGGTGAAGAAG
ATCGCCAGCGCCGGATGTACGGTTTACCCTTCGCGTTAATTGCAGGTGGTTGCGTCGGTTTTTACGATGGATTTTTTGGC
CCGGCAGCCGGATCGTTTTACGCGCTGGCCTTCGTTACGCTGTGCGGATTCAACCTCGCCAAAGCCACTGCTCACGCCAA
ATTACTTAACGCAACGTCAAACATTGGCGGTCTGCTGCTGTTTATTCTCGGCGGCAAAGTGATTTGGGCGACGGGCTTTG
TGATGCTGGTGGGGCAGTTCCTCGGCGCGCGCATGGGTTCAAGACTGGTGTTGAGCAAAGGACAAAAGCTGATCCGCCCG
ATGATCGTTATCGTCTCGGCGGTGATGAGTGCTAAATTACTTTACGATAGCCATGGACAGGAGATCCTCCACTGGTTGGG
GATGAACTAA
>Cluster_55486
GTGACAGAACCGTTAACCGAAACCCCTGAACTATCCGCGAAATATGCCTGGTTTTTTGATCTTGATGGAACGCTGGCGGA
AATCAAACCGCATCCCGATCAGGTCGTCGTGCCTGACAATATTCTGCAAGGACTACAGCTACTGGCAACCGCAAGTGATG
GTGCATTGGCATTGATATCAGGGCGCTCAATGGTGGAGCTTGACGCACTGGCAAAACCTTATCGCTTCCCGTTAGCGGGC
GTGCATGGGGCGGAGCGCCGTGACATCAATGGTAAAACACATATCGTTCATCTGCCGGATGCGATTGCGCGTGATATTAG
CGTGCAACTGCATACAGTCATCGCTCAGTATCCCGGCGCGGAGCTGGAGGCGAAAGGGATGGCTTTTGCGCTGCATTATC
GTCAGGCTCCGCAGCATGAAGACGCATTAATGACATTAGCGCAACGTATTACTCAGATCTGGCCACAAATGGCGTTACAG
CAGGGAAAGTGTGTTGTCGAGATCAAACCGAGAGGTACCAGTAAAGGTGAGGCAATTGCAGCTTTTATGCAGGAAGCTCC
CTTTATCGGGCGAACGCCCGTATTTCTGGGCGATGATTTAACCGATGAATCTGGCTTCGCAGTCGTTAACCGACTGGGCG
GAATGTCAGTAAAAATTGGCACAGGTGCAACTCAGGCATCATGGCGACTGGCGGGTGTGCCGGATGTCTGGAGCTGGCTT
GAAATGATAACCACCGCATTACAACAAAAAAGAGAAAATAACAGGAGTGATGACTATGAGTCGTTTAGTCGTAGTATCTA
A
>Cluster_34480
ATGAAAGCATTCTGGCGTAACGCCGCGTTGCTCGCGGTTTCTCTGCTTCCCTTCTCTTCTGCCAACGCCTTAGCGTTGCA
GGCAAAACAGTATGGCGATTTTGATCGCTATGTCCTGGCCCTCTCCTGGCAAACCGGATTTTGCCAGAGTCAACACGATC
GAAATCGTAACGAACGAGATGAATGTCGCCTGCAAACCGAAACGACCAACAAAGCTGATTTTCTGACCGTACATGGTCTG
TGGCCAGGATTGCCTAAATCGGTTGCTGCCCGTGGTGTTGATGAACGCCGCTGGATGCGCTTCGGTTGCGCTACTCGCCC
AATCCCGAATCTACCAGAAGCGCGCGCCAGCCGAATGTGTTCATCGCCGGAAACCGGATTATCACTGGAAACGGCCGCTA
AACTAAGTGAAGTCATGCCAGGAGCTGGCGGACGTTCCTGCCTGGAACGCTACGAATATGCCAAACACGGTGCCTGCTTT
GGTTTTGATCCGGACGCATACTTCGGTACGATGGTACGCCTGAATCAAGAAATTAAAGAGAGCGAAGCCGGAAAATTCCT
TGCGGATAATTACGGTAAAACAGTGAGCCGCCGTGACTTTGACGCCGCCTTTGCCAAAAGCTGGGGAAAAGAGAACGTGA
AAGCAGTTAAGCTAACGTGCCAGGGTAACCCTGCGTATTTGACTGAAATTCAGATCTCGATCAAAGCTGACGCCATCAAC
GCTCCGCTTTCTGCAAACTCATTCTTGCCACAACCTCACCCAGGTAACTGTGGCAAAACCTTTGTGATTGATAAAGCGGG
TTATTAA
>Cluster_9306
ATGAATGATAATATACCTACAGCGCGAAATCACAAACAATCGACTTGTATTACAGAAAAAAACATGCCTATCTTTTTAAA
CTTCACAGCAGGCAGTATCTTACCTGAGAATGAGCTAGCATCTTTACGTTATATTGTGCAGCAAAATCAAAATGATACTG
TAATCATAAAAGAACGTTATAAAATGGATATCCGTTATATCGAATCAGTCAATGGTTTTACAGTAAATCCTGTATGCAGT
AATCATTTCTCCATATTTATGGCGAGACAAAACACTATTGCTCGCAACCTGGAACAGCAGATCAACAACGGACGAAGTTT
TGCACAAATATCTCAGGATTTTATGCTTCAATTATCTTCAAATATAGGATGGAAAAAAGGGGCCGAAAACGCCCTTAAAA

ATAAAATCCATTCTCATTCATTTGTTGTAAATCCTGATGAATTCTCTTGCGACACACAATTTCTTAAGTGCCCAATAACA
TTATGCGTTCCAGAAAAAGGGGTTTTTGTCAAGAACGCACTGAACTCCAACATATGCACTCTTTATGATAAGTCTGCGTT
CATGAATCTCACAAGAGAACATCTACCCCACCCTCTCAGCAGGGAAAAGATAGTAAAAGAAATGATTATTGAAAGGAATA
TGTGTTATTTTGACACCATAAGTCAGCATTTCATAATTATGGATGCAGACCAACAGAAACAGCATTGTAAATAA
>Cluster_23798
ATGCGTCCAGCAGGCCGTAGCAATAATCAGGTGCGTCCCGTTACCCTGACTCGTAACTATACAAAACATGCAGAAGGCTC
GGTGCTGGTCGAATTTGGCGATACCAAAGTATTGTGTACCGCCTCTATTGAAGAAGGCGTGCCGCGCTTCCTGAAAGGTC
AGGGCCAGGGCTGGATCACCGCAGAGTACGGTATGCTGCCGCGTTCTACCCACACCCGTAACGCGCGTGAAGCGGCGAAA
GGTAAGCAGGGTGGACGCACCATGGAAATCCAGCGTTTGATCGCCCGTGCTCTTCGCGCGGCAGTAGATTTGAAAGCACT
GGGTGAGTTCACCATTACGCTGGACTGCGACGTGCTTCAGGCTGATGGTGGCACGCGTACCGCTTCGATTACGGGTGCCT
GCGTGGCGCTGGCAGATGCGCTACAGAAGCTGGTGGAAAACGGCAAGCTGAAAACTAATCCAATGAAAGGGATGGTAGCC
GCGGTCTCTGTCGGGATTGTGAACGGCGAAGCGGTTTGCGATCTGGAATATGTTGAAGACTCTGCCGCAGAGACCGACAT
GAACGTAGTGATGACCGAAGACGGGCGTATCATTGAAGTGCAGGGGACGGCAGAAGGCGAACCGTTCACCCACGAAGAGT
TACTCACCTTGTTGGCGCTGGCCCGAGGGGGAATCGAATCCATTGTAGCGACGCAGAAGGCGGCGCTGGCAAACTGA
>Cluster_17752
ATGAATTTATTTGATCGTTTGCTGCATCGCCGTCTTTGCAATGAGCAACCTGCTGATGGTGGCGCAGCTCCTGCTGCATC
TGAACCCGCAGCATCAACTAGCGATAATCCGGCACCAGCTGGCGATCCGGCTAAACCAGAAGGCGATAAGTCACAGCCTG
GCACTGAAAGCGACAAGCCTCAGGACGACAAGCCTATCGATGGCGATAAGCCAAAAGAGGAAAAGCCTGGTGAAGACAAG
GATAAGAAGCCGGAAGGCGCTCCGGAGAAATACGAGTTCAAACCAGCTGAAGGTCAGGAACTTGATGCTGCCGCTCTGGA
TCAGTTCGAGCCTATCGCCCGCGAACTGAACCTGACTAATGAGCAGGCGCAGAAGATGGTCGATCTGTACGGCACCAAGA
TTATGCCAATGGTCCAGCAGCAGCAGGCGGAAGCCTGGCAGAAAACCACCGAACAGTGGGCTGCTGACGTTAAGGCGGAC
AAGGAAATCGGCGGAGACAATCTCACCGGTAACCTGAGCGCGGCACAGCGTGCTCTTGCTCAGTTCGGTACACCTGAACT
GAAAGAATATCTGGAAGGCACCGGCCTGGGTAATCACCCTGAGCTGGTTAAAGCCTTCATAAAAGTCGGTAAGGCCATGT
CAGAAGACGGCATGGTAACCGGGAAAGAAAGCGGTCAGCGTAGTGCTGCCGAAGTGCTCTATGGCAAATAA
>Cluster_21990
ATGACAAACGATTTATATATTAACGGTAAAAAGGTTGACAGCTTCGGTGACTGGTCGCCACAGACTGAACATCCAGCTAT
CACTATTCAGCGTAAAGAACACGATGAAAGAATTAAGCTTGAGCAATCGCTAAAGATGTCACCAAAGGAAATCATCTTTG
TGTCTCCTGAGCCTCAAGAAATGCCAGATATCTGCAAGGGTGAAGCACTACTTAATCTTGAAAAGAAATATTACCCACTG
CTGAAAGAACAACGTATTAAACTTGATGACGCTTACAGCAAAGTTACCCTGATGCAATCAGCTATTGAGCCGTCAGAATT
TGAGATTCAGGATGAATTGTCACAGAAGCCGTATGTTTACTTCACATACGAAGACAATGATGGTTTTGGTACTTTTCCAG
AAAACGTCACAGACGTGATTAGTAGTCTTCCTGCTGGACATCGAATTGTGAAGATGGTTAAAGCTTCAAGAGGTGCTGGT
CAGTTTATTTACACGACCAATAAGAGCGATGAAGAACTCCGTGAAATTGCCAGACAGAACATCTTAGCATCACGTAACAA
GCAACTTGAAAATGTTAAGTTGTGTCTCTTAAGAGAGCTTCAGGCTATGAAAGACCTTATTTCTGCATATGAAACGCAGA
AGAAGGTTGCTATGCAGGCTGATATTGAGCAGTTAGTGAAGATTTCTCAGAAATACGCAAAGGCACTTTGA
>Cluster_23549
ATGCAAGAGAACTACAAGATTCTGGTGGTTGATGACGACATGCGCCTGCGCGCGCTGCTGGAACGTTATCTCACCGAACA
AGGCTTCCAGGTTCGAAGCGTCGCTAATGCAGAACAGATGGATCGCCTGCTGACTCGTGAATCTTTCCATCTTATGGTAC
TGGATTTGATGTTACCTGGTGAAGATGGCTTGTCGATTTGCCGACGTCTTCGTAGCCAGAGCAATCCGATGCCGATCATT
ATGGTGACGGCGAAAGGGGAAGAAGTGGACCGTATCGTTGGCCTGGAGATTGGCGCTGACGATTACATTCCAAAACCGTT
TAACCCGCGTGAATTGCTGGCCCGCATTCGTGCGGTGCTGCGTCGTCAGGCCAACGAACTGCCGGGCGCGCCGTCACAGG
AAGAGGCGGTAATTGCTTTCGGTAAGTTCAAACTTAACCTCGGTACGCGTGAAATGTTCCGCGAAGACGAGCCGATGCCG
CTCACCAGTGGTGAGTTTGCGGTACTTAAGGCGCTGGTCAGCCATCCGCGTGAGCCGCTCTCCCGCGATAAGCTGATGAA
CCTTGCCCGTGGTCGTGAATATTCCGCAATGGAACGCTCCATCGACGTGCAGATTTCGCGTCTGCGCCGCATGGTGGAAG
AAGATCCGGCGCATCCGCGTTACATTCAGACCGTCTGGGGTCTGGGCTACGTCTTTGTACCGGACGGTTCTAAAGCATGA
>Cluster_30311
ATGTTGATCGTTTCTGTACTACGCTGCGGCAAGGATTTTACGCCGAAACATGCACAATGGCTTCATAAACAATTTGGTTC
AATGCCCTCAATCTGCTTGACTGATGCTGGAAGTATTGATGGTGTGAATACTGCACCTTTGTTATTTGATTGGCCTGGTT
GGTGGTCAAAAATGGAGTTATTTAACCCAAACCATCCAGTAATCGGGAATCATGACATTTTATATTTGACATAGATGTT
GTCATTACTGGAAAATTGGATATTTTTACAAAAGCTAAAAATTTCACCATGCTCAGGGAGTTTAATCATCATGACCGTGT
TAACTCCTCAATTATGATGATTCCCGTTTCTGCAAAGACAAAAAATATGGGAGGAATTTACTGCCAATCCTGAACAAATAA
TGCAAGAATGCAAAACTGAAGATAAATGGGGTGATCAAGGTTTTATCGGTAGTGTTACTAAGCCAGACTTATGGCAGGAC
ATTATTCCAGATGCAATTAAAAGTTATAAATGCGATATAGCAACAAAATCAATGATTGGATACAATCCAGACCTGGCATC
AAAATCAGCAACTGGGAAAGTACCTGACAGTGTTTCAGTTGTGTGTTTTCACGGTAGTCCACGCCCCTGGCGCACTGGTT
TTAACTGGGTTCCATCTTTTTCTTTAAAAGATACGCTTTATGGCAAAGTAAAAAATCTCAAATTAAAGGTTAAAGGTTAA
>Cluster_40100
ATGCAGTGGCGGAGGTGTAAGGTGATAACGCGTGATTTCTTGATGAATGCCGATTGTAAAACGGCATTTGGTGCCATTGA
AGAATCACTCTTATGGTCAGCAGAACAACGGGCGGCTTCGCTGGCGGCGACGCTGGCTTGTCGACCTGATGAGGGACCGG
TGTGGATCTTCGGTTATGGATCGTTGATGTGGAATCCGGCACTGGAGTTTACCGAGTCGTGCACCGGTACACTGGTTGGA
TGGCATCGCGCGTTTTGCCTGCGTCTGACCGCCGGGCGCGGGACTGCGCACCAGCCGGGACGAATGCTTGCACTGAAGGA
GGGCGGACGCACCACAGGCGTCGCCTATCGACTGCCAGAAGAGACGCTGGAGCAGGAACTAACCCTGCTGTGGAAGCGTG
AGATGATTACCGGCTGTTATCTGCCAACCTGGTGTCAGCTTGATCTTGATGATGGACGCACAGTAAACGCCATTGTGTTT
ATTATGGACCCGCGACATCCAGAATATGAATCTGACACTCGCGCTCAGGTCATAGCGCCGTTGATTGCGGCGGCGAGCGG
TCCGCTGGGAACCAACGCACAATACCTGTTTTCACTGGAACAGGAGCTTATCAAACTGGGAATGCAGGACGATGGGCTGA
ATGATTTGCTGGTATCGGTAAAAAAACTGCTGGCGGAGAATTATCCGGATGGTGTGTTACGGCCGGGATTCGCCTGA
>Cluster_5993
ATGACAACTATTAACAAGCATGGGTTATCTAGAACAATTCCTGAACCTGTAAAACTGGAGGTAAGGCAGCGCTGTGGCTT
TGGTTGCGTTGTTTGTGCTTCTCCCATTGTTGAGTATGAGCATGTAATTCCCACATACGCTGATGCAAAGGAGCACTCTC
CAAATGCAATAGCACTACTTTGTCCTACGTGCCATGCGAAAGTAACGAAACGTCTTTACTCTAAAGAGAAAATAATTAAA
GCAATGGAATCTCCTGCAGCCTTACAAAAAGGGAATGTGGCCGATATATTGGACTTTTCTGATAGGCATCCAACAATAAT
ATTTGGTGGTGCGACGTTTGAATCCTGTACTGTACCAATAATGTTTCAAAATGAGCCATTGTTAACCATTGAAAAAGAAA
ATAATGCTTTTCTTATTTCGGGTAAATTTTACGATAGTCAAGGTATTGTTAATCTTGAATTAATTAAGAATGAGTGGGTT
TGTAGTGCAAGGCATTGGGATGTTCAGGTTGTTGGTCCTAGAATAACTATTATAGAAAAAAGGAAAGGTCCTCGTTTAAT
TATTAAAGTTGATGCGCCTGAAAGATTGATTGTGGAAAGATTAGATATGTTGGTTAAGGGGACTCGGATTGTAGGAGACG
AACACAAGCTAAGAGTCGGTCCACATCAATTTCACAACTGTGGAGTTTCAAATTGCAATATTGGTTTCTGCTTTGGCTAA
>Cluster_57
ATGGATTTTTTGATAGAGGCTTTTAAACAGGCCTTTTTTCTTATCGTTAATTTAGATAGAGAACTTCTTGAGATCATCTT
GCTTTCTTTAAAAGTATCTCTTTCTGCAATTTTATTAGCTTCTTTTTTTGGAGTTCCGTTTGGGGCCTTCTTAGGAATGA
AAGATTTTCCAGGTAAGAGTTTGGTTATAATTTTTTTTAATACCGGGATGGGGCTGCCTCCTGTGGTGGTTGGTCTTTTT
CTTTATATGCTTTTTTCAAGAAGTGGGCCTTTAGGTTTTTTAAACCTCCTTTATACCCCTACCGCTATGGTAATCGCACA

GTTTATCCTGGCTTTTCCTATAGTGGCTTCTATCTCTCAAGCCTCTGTAAGAAAGGTTAGCCCTATAATCAAACTTACGG
CCAAAACCCTTGGAGCTACTCCTTTCCAGATTACCTTGACCTTGATAAAAGAAGCCCGCTATGGGATATTTACAGGGATC
TTGGGTGGATTAGGAAGGGTAATGGCTGAAGTAGGAGCTATTTTGATCGTAGGAGGAAATATCGCCGGTTATACCCGGGT
TATGACTACCACCATAGCTTTGGAAACCGATAAAGGAAACTTCGAGCTTGCCCTTGCTTTAGGTATAATCCTTCTTTCTA
TCTCTTTTTTGATCAATCTTTTGCTATACTATATTCAAAAAAAAGAAATAAGAACCAACCCATGA
>Cluster_14474
ATGAAAAAAATTATCGCATTGATGTTGTTTTTGACATTCTTTGCCCACGCGAACGACTCCGAGCCTGGCAGCCAGTATTT
AAAGGCAGCAGAGGCCGGGGACCGACGCGCACAATATTTTCTTGCCGACAGCTGGTTTAGCTCCGGCGATTTGAGCAAAG
CCGAATATTGGGCACAGAAAGCCGCCGACAGCGGTGATGCTGATGCCTGCGCGCTGCTGGCGCAGATCAAAATCACCAAT
CCGGTCAGTCTGGACTATCCACAAGCAAAAGTTCTTGCAGAGAAAGCGGCGCAAGCGGGCAGTAAAGAAGGTGAAGTAAC
GCTGGCGCATATTCTGGTAAATACTCAAGCGGGTAAACCGGATTATCCAAAGGCGATTTCGCTGTTAGAAAACGCCTCGG
AAGATCTGGAGAATGACTCCGCCGTCGATGCCCAAATGCTGCTTGGTTTGATTTACGCCAACGGCGTGGGCATTAAGGCC
GACGATGACAAGGCAACCTGGTATTTCAAACGCAGCTCTGCAATTTCCCCGAACCGGTTATTCCGAGTACTGGGCGGGAAT
GATGTTCTTAAACGGTGAGGAAGGTTTTATCGAGAAGAACAAACAAAAGGCGCTGCACTGGTTGAACCTGAGCTGTATGG
AAGGGTTTGATACCGGGTGTGAAGAGTTTGAAAAATTAACCAACGGTTAA
>Cluster_67134
ATGCATCTCTTATACGTTGATGAGTCTGGTACACCATCAGACCCTACACTTAATCATTTTGTCCTTTCTGGTATCTCAGT
GTTTGAAAAGCAGACTCATTGGATAGAGCAAGAGATGAATGCTATCGCAGCCAGATTTAATGCTGATGATCCGTATGAAA
GAGAATTGCATGGTTCGCCTATGAGAGGTGGGCGAGATGGCTGGAAAAAATTTCCTTTCGATGACAGAAAACAAGCGATC
AAAGATTGCCTAACACTCCTGACTCAAAAGCATCGGGATATCCGTATATTTGCGTGTGTTGTACCCAAGGGATCCTTAGG
TGGAAAGGACGTAATAACAGAATCTTTTTATCAGATATCGTCTCGTTTCGACATGTATTTGAGCCGCTTACATCACCAAG
GAGCAACATCTCGAGGGGTGGCCATTTTTGATAAATCGTCAACAGAGAAAAGTATTCAGTCTCTTGCTCGTACGTTTAAA
GATACTGGATATGGATATGGAAAACTTCGTAACTTCTCTGAAGTGCCTTTGTTTTTGGATTCCAAAGCGTCAAGGCTGAT
TCAGCTTGCGGATCTTGTATCATACTCAATTTTCAGGAAGTTTGAGCTCAATGATGATGAGTTCTACAAGATTATTGAGA
ATTCCTTTGACTACAATAATGGTAAAGTTCACGGACTTTACGTAGCTCACTAA
>Cluster_37
ATGATAGCAGTTCAAGTTACTACGAATGCAAGTCCAGCAAAAATTAAAAAAACTATCGAATCTTTTGAGAAAATAAATGA
AATAGGAGAGAGTCTGAAAGATAAATACTCTACTTTATATATTTTGGTTTTTGTAAAGCCTCAAGATACCTAACTCCAA
GTTATTGTAAAATAATCGACCCCAGCTATTTCGTGAATGAACTTTGCGATAAAGCAGATGAAGATATGGTCCAGGATATG
ATTGACGCCATTCGACGCCATCATGATTATACATCATTACATCCATGGAGTGATAAAGATTCACTCGAAATTATACTCAA
TATTATTAATCGCAATGCAATAAAACATCGGATGAGTTGCGAAGGCAGTCTGTCTGATATGCTCACTGGTTTAAAAGAAA
TTAATGAAGTAATTACAAAAGGAACAATTCAACGTAAGCAACGTTCAAAATCGATATCTGACTTCAAAGATCAAAGCATG
GTGAAATTTATGAGAGGTGTAATGGATGATTTGTCTGTTATTCAAGCCATAGTGAACAAATCTAAAGTTAACCAAGGTGA
TATGGTTTATATAAGCCATGAAGATATGATTAATATTGATAAATTAAAAGCTAAAATAGCAAGTGATTCATCAGAGATTG
CAAGGCTAAATAATATTGATATAACACTAAATGTTGTTGATTTATAA
>Cluster_19733
ATGCAACGGGAAACGGTCTGGTTAGTCGAAGACGAGCAAGGGATAGCCGATACGCTGGTCTACATGTTGCAACAGGAAGG
TTTTGACGTCGAGGTCTTTGAGCGAGGCTTGCCAGTGCTGGATAAAGCTCGCCAGCAGGTTCCCGATGTCATGATTCTCG
ATGTTGGTTTGCCTGATATCAGCGGCTTTGAATTGTGCCGCCAGTTACTGGCGCTCCATCCGGCGTTACCTGTACTGTTC
CTGACGGCCCGAAGTGAAGAGGTCGATCGCCTGCTTGGGCTGGAAATTGGCGCTGACGACTACGTGGCTAAACCGTTTTC
ACCTCGCGAAGTGTGCGCCAGAGTGCGCACCTTACTGCGTCGGGTGAAGAAGTTCTCGACGCCGTCTCCCGTTATCCGTA
TTGGTCATTTTGAATTGAATGAACCTGCGGCGCAGATCAGCTGGTTTGACACGCCATTAACGCTGACGCGGTATGAGTTT
TTATTGCTGAAGACGTTACTCAAGTCGCCGGGCCGCGTCTGGTCCCGCCAGCAACTAATGGATAGCGTGTGGGAAGATGC
GCAGGACACCTACGATCGCACCGTCGATACCCACATTAAAACGCTGCGTGCCAAGCTGCGCGCCATCAACCCCGATCTTT
CACCGATTAATACTCATCGCGGCATGGGATATAGCCTGAGGGGCCTGTAA
>Cluster_22606
GTGAAAAAACTCATCACAGCGAATGATATTCGTGAAGCACACGCGCGCGGCGAACTGGCAATGTCCGTGGTTCTGCGCGC
CAGCATTATTACCCCGGAAGCTCGTGAAGTGGCTGATCTGTTGGGTTTTACCATCACCGAATGTGATGAATCGATCCCGG
TAACGGCGTCTGTTCCCGCCAGCGCATCAGCCGATAAAACCGAAAGCCAGCGCATCCGTGAAACCATTATCGCCCAACTG
CCGGAAGGTCAGTTTACCGAAAGCCTGGTCGCGCAGCTGATGGAAAAAGTGATGAAGGAAAAACAGTCGCTGGAACAGGG
TGCACTGCAGCCGAGCTTTAAATCGGTGACCGGCAAAGGCGGCATCAAAGTTATTGATGGTAGCAGCGTCAAGTTTGGTC
GTTTCGACGGCGCACAGCCGCACTGCGTAGGCTTGACTGATCTCGTGACCGGAGACGACGGCAGCAGTATGGCCGCTGGT
TTCATGCAGTGGGAGAACGCCTTCTTTCCGTGGACCCTGAACTACGACGAAATCGACATGGTGCTGGAAGGGGAGCTGCA
TGTTCGCCACGAAGGCGAAACTATGATCGCCAAAGCGGGCGATGTGATGTTTATCCCGAAAGGCTCCAGCATCGAATTTG
GTACGACATCCAGCGTGAAATTCCTGTATGTCGCCTGGCCGGCTAACTGGCAATCCTTATGA
>Cluster_62540
ATGTCCCAGACTGAGCGTCGCCATGAGCGGCTGGCGGTTCGGCTGTCCCTCATTATCAGTCGCCTTGTTGCTGGCGAAAC
ACTGGATATGGGCCTTCTTGCAGCAGAGTTTGGTGTATCGATCCGCACCCTCCGCCGGGATTTTCGTGAGCGACTGATGT
ACCTGGATCTGGAATATCGCGACGGCACCTGTCGTCTGCTCTCTGGTAGCCATCAACGCGAACTGGCTGTACTGACTTTT
GCTCGTCAGTCAGGCGTGGAGGCCCTGTTTCCTGACATGGATAACCACATGGTCAGTTCCCTGTTAAGTGGACCCGGTGA
GTCTCCTTGTCTCATATGGGCAGAGTCAGAGTCATTGTCGCCACCCAGGCCAGGCATATTTACACGCCTTGTAAGGGCCA
TATCAGAGCGTCGTCGCGTGACGCTGCTGGCTGAAGGTTGTCGTTGCTCAAGGCTTGCACCATACCGCCTCGTACTCTGC
GGCGGGCACTGGTATCTGACTGGAGAGCACCATCATCGAGTGGCGGTGTTTCCGCTGAATGATATCCATGCGGTTACCTT
GCACCCGGAACTGTTCGTGCCCAACCTTCAGATTCGCAACGTTCTCACCGAACCGGAATTCCTCAGAGCTCTTCCCCATT
TTCACGTTTTTCACTCACTGCTCGCCAGAACCGGCGACGGTCTTTTACCTCATAAGGAATCATGA
>Cluster_6087
ATGAAGGCGTTCGATCTCCACCGTATGGCATTTGATAAAGTGCCTTTTGATTTCCTTGGCGAAGTTGCACTACGTAGTCT
TTATACCTTTGTACTGGTCTTTTTGTTCCTCAAAATGACCGGAAGACGCGGTGTGCGGCAGATGTCGCTGTTTGAAGTTT
TAATCATTCTGACGCTGGGATCAGCGGCGGGAGATGTGGCGTTTTATGATGATGTGCCGATGGTCCCGGTACTTATCGTC
TTTATTACTCTGGCGTTGTTATACCGCCTGGTAATGTGGTTGATGGCGCACAGTGAAAAACTGGAAGATCTTCTGGAAGG
CAAGCCAGTTGTCATTATTGAAGATGGCGAGCTGGCCTGGTCGAAACTCAATAACTCCAACATGACGGAATTTGAGTTCT
TTATGGAGCTACGATTGCGTGGCGTGGAGCAGCTGGGGCAGGTACGTCTGGCGATTCTCGAAACCAACGGGCAAATCAGT
GTCTATTTCTTTGAAGATGACAAGGTGAAACCGGGTTTACTTATTTTACCCAGTGATTGTACGCAGCGTTACAAAGTGGT
GCCGGAGTCGGCGGACTATGCCTGTATTCGTTGTAGTGAAATCATTCATATGAAAGCGGGGGAAAAACAATTATGTCCGC
GCTGTGCAAATCCAGAATGGACGAAGGCAAGTCGGGCAAAACGGGTGACCTGA
>Cluster_39836
ATGATGACAACCTTAACCGCACGACCGGAAGCCATTACCTTCGATCCGCAGCAAACTGCGCTGATCGTGGTGGATATGCA
AAATGCCTATGCCACGCCAGGCGGCTACTTAGATCTCGCCGGGTTTGATGTCTCAACCACTCGCCCGGTCATTGCCAACA
TTCAAACTGCCGTGACCGCAGCGCGAGCGGCAGGGATGCTGATCATCTGGTTTCAAAATGGCTGGGATGAACAGTATGTC

GAGGCTGGCGGCCCCGGCTCACCGAATTTTCATAAATCGAACGCCCTGAAAACCATGCGTAATCAGCCGCAGCTGCAGGG
GAAATTGCTGGCGAAAGGCTCCTGGGATTATCAACTGGTGGATGAACTGGTGCCGCAGCCTGGCGATATTGTGCTGCCGA
AGCCGCGCTACAGCGGTTTCTTCAATACGCCGCTGGACAGCATTTTGCGCAGCCGTGGAATACGCCATCTGGTTTTCACC
GGTATCGCTACCAACGTCTGCGTCGAATCGACGCTACGCGACGGCTTTTTTCTGGAGTATTTCGGCGTGGTGCTGGAAGA
CGCAACACACCAGGCGGGGCCGGAATTTGCACAGAAAGCCGCGTTGTTCAATATCGAAACCTTTTTTGGCTGGGTCAGCG
ACGTCGAAACGTTCTGCGACGCGCTTTCTTCCACGTCCTTTGCTCGTATCGCTTAA
>Cluster_12418
ATGCTGCTCAGTATCATTACCGTCGCCTTTCGCAATCTGGAAGGGATCGTCAAAACTCACGCATCGCTGGCACATCTGGC
GCAAGCGGGTGATATCAGTTTTGAGTGGATTGTTGTTGATGGCGGCTCCTCGGATGGTACGTCGGCGTTTCTTGAAGGGC
TTAGTGGGGAACACCGGTTACGGTTTGTCAGCGAGCCGGATAACGGCATCTATGATGCGATGAACAAAGGCATCGCGATG
GCGCGCGGGCGGTTCGCGCTGTTCCTCAACTCCGGGGATATTTTGCATGCGGACGCGGCACATTTTATCCGCCAGCTAAA
AATGCAGAGCGACCAAACCATGGTCATTGGCGATGCGTTGCTGGATTTCGGCAATGGCAACAAAATTAAGCGCCGGGCAA
AACCGGGTTGGTATATTTATCACAGCCTGCCCGCTAGTCATCAGGCGATATTCATTCCGCTCTCAGGACTGAATGTCTGG
CATTATGATTTGCAATATAAAATATCGTCTGATTACGCGTTGGCAGCCCAATTATACAAGTCCGGTTACGCTTTTAAAAA
GTTGAGCGGGTTGGTATCGGAGTTTTCTATGGGCGGAGTGTCAACGACGAATAATCTGGAATTGTGTCGGGATGCCAAAA
AAGTACAGCGCCAGATATTACACATGCCAAATTTTTGGACCGGTTTGTCAGAATATTTACGTCTGCGCACAACCGCTAAA
ACAAAAACCTTATATGGGAAAACGTAA
>Cluster_11787
TTGTGCAGCGAAATACGCCGGGCGTGGAACCAAAGTCGGGGCTCTGCGGGCGCGCGCACTCTGGCTGAAATGCTGACTCA
AAACGGCGTCCCGATGAGCCGTTACCGTGCCGGACGTCTGATGAAATATCTGAACCTGAGCAGTTGTCAGCCCGGAAAAC
ATCAGTATAAAAATGCCCGTCAGGAGCATACCAGCCTGCCGAATCTGCTTGAGCGTCAGTTCGCAGTACCGGAGCCAGAC
CGGGTATGGTGTGGAGATATTACGTATCTCTGGGCAGGAAATCGCTGGTGCTATCTGGCGGTTGTTATGGATCTTTTTGC
CCGCAGGGTTATCGGCTGGAGTCTGTCAGCGCATGCCGATACCGCACTGATAAGCAGTGCCCTGCGGATGGCCTATGAGA
CGCGTGGCCAACCCCGGGATGTCGTGTTCCATAGCGACCAGGGAAGCCAGTATACAGGCCTTAAATATCAACAACTTCTC
TGGCGTTGCAGAATAAATCAAAGCGTCAGTCGGCGGGGAAACTGCTGGGATAACAGCCCCATGGAACGCTTCTTCCGCAG
TCTGAAAACAGAATGGGTGCCAACGAATGGTTACGCAGGCAAGGACGAGGCCCGGCAGCAAATTAATGATTACATATTGA
ACTACTACAACAGCGTCAGACCTCACCATTATAACGGTGGGCTGACGCCGGAAGAGTCAGAGAACAGATACCATTTTTAC
TGTAAAACCGTGGCCAATATTACTTGA
>Cluster_26311
ATGTCTATCACGGCGCAGTCCGTATACCGTGACACCGGAAATTTCTTCCGTAATCAATTTATGACCATTCTGTTGGTATC
GTTGCTATGTGCGTTTATCACAGTGGTGTTAGGGCATGTTTTCTCACCCAGTGATGCACAGCTTGCGCAGCTCAATGACG
GCGTACCCGTTAGCGGCAGTAGTGGGTTGTTCGACCTGGTTCAGAATATGTCACCGGAACAGCAACAAATTTTGCTGCAG
ACTTCAGCGGCGTCCACTTTTTCAGGATTAATCGGTAACGCCATTCTCGCCGGGGGCGTAATATTAATTATCCAGCTGGT
GTCTGCGGGTCAGAGAGTCAGTGCGCTGCGAGCTATTGGTGCCAGTGCGCCGATATTGCCAAAGTTATTTATTCTGATTT
TTCTGACTACCCTTTTAGTACAGATTGGCATCATGCTGGTGGTCGTTCCGGGGATTATCATGGCCATTTTACTGGCTCTG
GCTCCAGTGATGCTGGTACAGGACAAAATGGGCGTTTTTGCCTCGATGCGTAGCAGTATGCGGCTGACATGGGCGAATAT
GCGTCTGGTGGCACCCGCAGTACTGAGCTGGTTGCTGGCAAAAACACTGTTGCTGCTTTTTGCCTCTTCTTTTGCCGCAT
TAACCCCGGAAATTGGTGCCGTACTGGCGAACACCTTGAGCAACCTGATTTCAGCCGTATTGCTCATCTATCTGTTCCGC
CTGTATATGTTGATTCGCCAATAA
>Cluster_60786
ATGGCTATAGCACTTGTGACTGGTGGCAGTCGCGGCATCGGGCGGGCAACTGCATTACTGTTGGCGCAAGAAGGGTATAC
GGTGGCGGTTAATTATCAGCAAAACCTCCACGCGGCGCAGGAAGTGATGAACTTAATAACGCAAGCCGGTGGCAAAGCAT
TCGTGCTCCAGGCGGATATCAGCGACGAAAACCAGGTCGTTGCGATGTTTACAGCAATCGATCAGCACGATGAACCGCTA
GCAGCGCTGGTCAATAACGCCGGGATCTTGTTTACCCAGTGCACCGTTGAAAACCTTACCGCAGAGCGAATCAACCGAGT
ACTTTCCACCAACGTGACGGGATATTTTCTCTGCTGCCGCGAGGCGGTAAAACGCATGGCGCTTAAAAATGGTGGCAGTG
GCGGCGCTATCGTCAATGTCTCTTCGGTGGCCTCACGGTTGGGTTCGCCAGGGGAATATGTTGATTACGCGGCATCGAAA
GGGGCGATTGATACGTTAACCACCGGACTATCGCTGGAAGTCGCCGCGCAGGGGATCCGCGTTAACTGCGTGCGGCCAGG
GTTTATTTATACCGAAATGCACGCCAGCGGCGGCGAGCCTGGACGCGTCGATCGCGTTAAGTCGAACATCCCCATGCAGC
GTGGTGGACAGGCAGAAGAGGTCGCGCAGGCCATTGTCTGGCTACTAAGTGATAAAGCCTCTTACGTCACGGGAAGTTTT
ATCGATTTGGCGGCGGGAAATAA
>Cluster_51530
ATGAAGCTTCGCCTGTCGGCGCTTGCTCTGGGAACTACGCTTCTGGTGGGGTGTGCGAGTTCCGGTACAGATCAGCAAGG
GCGTTCTGACCCGTTAGAAGGGTTTAACCGCACTATGTACAACTTCAACTTCAATGTATTAGACCCGTATATTGTTCGAC
CGGTCGCTGTCGCCTGGCGTGATTATGTTCCGCAACCTGCGCGTAACGGTTTGAGCAACTTTACCGGCAACCTTGAAGAA
CCTGCGGTGATGGTTAACTACTTCTTGCAGGGCGACCCTTATCAGGGGATGGTCCACTTTACCCGCTTTTTCCTGAATAC
CATTTTGGGAATGGGCGGCTTTATTGATGTTGCAGGGATGGCAAACCCGAAACTGCAACGGACTGAACCTCACCGCTTCG
GTAGTACGCTTGGTCATTATGGTGTGGGTTATGGGCCGTACGTTCAGTTACCGTTCTACGGTAGCTTCACGCTGCGTGAT
GACGGTGGTGATATGGCGGATAGTCTTTACCCGGTTCTTTCCTGGCTGACCTGGCCGATGTCTGTGGGTAAATGGACGCT
TGAAGGGATAGAAACTCGTGCGCAGTTGTTGGATTCCGACGGTCTGCTGCGTCAGTCGTCCGATCCTTATATTATGGTGC
GTGAAGCGTACTTCCAGCGTCATGATTTCATCGCTAATGGCGGTGAACTCAAACCGCAGGAAAATCCGAACGCGCAAGCG
ATTCAGGATGATTTAAAAGATATCGATTCTGAATAA
>Cluster_38894
ATGAATCTTAACAGTATTCCCGCCTTTGATGACAATTACATCTGGGTTTTGAATGATGAAGCAGGTCGCTGTCTGATTGT
CGATCCCGGGGACGCAGAGCCAGTATTAAACGCCATTTCCGCCAATAACTGGCAACCGGAGGCCATATTTCTCACCCACC
ATCACCACGATCACGTTGGCGGCGTAAAAGAACTGGTGGAAAAGTTTCCGCAAATTGTGGTGTATGGACCACAAGAGACA
CAAGATAAGGGAACAACACAGGTAGTCAAAGATGGCGAAACTGCCTTCGTTTTGGGGCATGAATTTAGTGTAATTGCCAC
ACCGGGTCACACTTTAGGACATATCTGTTACTTCAGTAAACCTTATCTATTTTGCGGCGACACGCTGTTTTCTGGTGGGT
GTGGTCGGTTGTTTGAAGGGACAGCATCACAAATGTATCAATCACTTAAAAAGTTAAGTGCGTTACCTGACGACACATTG
GTATGTTGTGCTCATGAATATACCTTATCAAATATGAAGTTTGCTTTGAGTATTCTTCCGCACGATTTGTCCATAAATGA
TTATTATCGTAAAGTTAAGGAGTTACGGGCAAAAAATCAAATAACACTACCCGTAATTCTGAAAAATGAGCGGCAAATTA
ATGTTTTTTTAAGAACGGAAGATATTGATTTAATTAATGTAATTAATGAAGAAACATTATTGCAACAACCTGAAGAGCGT
TTTGCATGGTTAAGGTCAAAGAAAGATAGGTTCTGA
>Cluster_27075
GTGAAAACAATAAGTGTCGTTATTCCTTGCTTTAATTGCATTCAAACTATAGATAAAGCATTGCAATCAGTATACGATCA
AAACTACAAATTTATAGAGATCGTTTGCGTTGATGATTGTTCGACAGATAATACGTATGAGCATATTAAAACAAATCATC
CTTTCGTAAAGCTTGTGAAGAATGACATTAATTTAGGTCCAGCAGCAAGTCGAAATAAAGGTGTTCGCTTATGTTCTGGT
GAGTTTGTGGCATTTTTAGATGCTGACGACATATGGTTTCCTAATAAAACAAATATTCAAATTGAGTTGATGGATAAATA
TGGGTTGGATTTCATCGGTGCATCATTTACCATTAATACTAATGATAATCCAAGCACTGTATCCCAGTTCGAAATTATAA
AGCTTTATAACCTTGCGTTCAAGAATTATTTCCCAACTCCATCAGTAATAATGAAAAAGAATTTGGTTTGCTTTAATGAA

TTACAACGGTTTTCTGAAGACTATGATTTATGGCTAAGGTTAGCTAAAGATAAATGTATAATGGGTTATGTCAAAACACC
ATTAATATCTCTTGGCAAACCAAGTTATGGACACTCTGGGCTCAGTTCAAATTTGATTAAGATGCAACTAGGTGAGATAT
CTTCGATAAATAAAAATCTTAAAGGAAAGTGGATCTGTTTTTTGCCATATCTTGGTCTTTTATTAAATTTATTCGTAGA
TTATGTGTGGTTTTATCCGGAAGTTATAA
>Cluster_22509
ATGTCTTATAAATTATCCAGTCCAATGCTCGTCAGCACAGCGCTGATGGCAATGCTTACCACCGCCAGCCTGACGGCGCA
TGCATCGGTCACCCCGGATCGCACCCGTCTGGTGTTTAACGAAAGCGATAAATCGATCAGCGTGACGTTGCGCAATAACA
ACGAAAAGCTGCCTTACCTGGCGCAAAGCTGGCTGGAAGACGAGAAGGGCAACAAAATCACTTCGCCGCTGGCGGTGCTG
CCGCCGGTGCAGCGTATTGACGCTATGATGAACGGCCAGGTGAAGATTCAGGCGCTGCCGGACATTCACACGCTGCCGTC
CGACCGAGAAAGCCTGTTCTACTACAACGTGCGTGAGATCCCGCCAAAGTCCGGGAAAGCCAACACCCTGCAAATTGCGC
TGCAAACGCGCATCAAGCTGTTCTGGCGACCAAAGGCGCTGGAGAAGATTGACATGAGAAAACCGTGGCAGTTTAAGGTC
ACACTCACGCGCAGTGGCCAGGACTATACCGTCAATAACCCGACGCCGTACCACGTCATCATCAGCGATGCCAGCACACA
GAAGAAAGGGCTGACCGCCGCCGGGTTTAAACCGCTGGTGATGCCACCGAAAACGTCACAACCGTTAAAGGCAAAAATGG
CGAGCGCCCCGGTGCTGACCTATATCAACGACTACGGCGCGCGCATGCCGCTGATCTTCCGTTGTGAAGGGAACACCTGC
AAGGTGGATGAAGATCAAAGCAGCAAAGGTTGA
>Cluster_32461
ATGGGTCTCGCGGTAATGATAGTCTGCGGCATTATTGCTAAACGCCGTGGCTACCCGCTTTCAGAAAGAGCAACCTTCGC
TCAGGCCTGTAAAGCGTTTCTGGATGCCTTACCAAGTTTGCTTCTGGTGTTCATTGTGATGGGCGGCATTCTGGGCGGCA
TTTTTACCGCAACTGAAGCGTCTGCGATTGCCGTTGTCTACACATTTATTCTCTCGGTGCTGATTTACCGGGAAGTAAAA
TGGCGCGATTTACCCAAACTGATTCTGGAATCGGTTGTTACCACTTCGATTGTATTACTGTTGATCGGTTTTTCCGTGGG
CATGTCCTGGGCAATGACCAATGCTGATATTCCTTATATGATCAGCGACGCGTTGATGGGGATTTCTGATAACCCACTGG
TCATATTGCTGCTGATTAATATCGTCTTATTAATTGTCGGCATCTTTATGGACATGACACCGGCTGTACTAATCTTCACA
CCAATCTTCTTGCCGATTGCACAAGAGCTGGGAATGGATCCGGTACATTTCGGTATTATGATGGTGGCTAACCTTTGTAT
TGGGCTGTTAACACCGCCGGTGGGTAGTGCGCTATTTGTTGGTTGTTCTATTTCAGGTGTGAAGATTCAACATTTGATCA
AACCATTGCTGCCATTTTATGCCGCATTGTTAATCGCGTTGATGATGATCACTTACATCCCGCAAATATCCTTGTTCATT
CCACAGCTACTAGGGCTAATGTAA
>Cluster_46848
ATGGCCAATAAAACTGAAAAGCCAACACAAAAGAAACTCCAGGATGCTTCTAAAAAAGGGCAAATTCTAAAAAGTAGAGA
CTTAACCATCTCTGTAATAATGCTTGTGGGTACTTTATATCTCGGATATGTTTTTGATGTGCATCACATTATGTCGATTC
TTGAATACATCCTTGATCATAACGCTAAACCAGATATTTGGGACTATTTTAAAGCTATGGGGGTTTGTTGGTTGAAAACG
AACATTCCTTTTTTGCTGGTTTGCATGTTCACAACAATACTTGTCTCCTGGTTTCAAAGTAAAATGCAATTAGCAACTGA
AGCTGTTAAATTTAAGTTTGATTCATTGAATCCAGTAAATGGCTTAAAGCGTATATTTGGCTTAAAAACCGTAAAAGAAT
TTGTTAAAGCAATTCTTTATATTGTTTTTTTTGCATTGGCGATCAAAGTATTTTGGAGTAATCATAAATCACTGCTTTTT
AAAACTCTTGATGGAGATATCATATCTTTATTATCAGATTGGGGAGAGATGCTATTCCTTCTCATACTGTATTGTCTCGG
CAGTATGATAATTGTCTTAATTTTTGATTTTATCGCTGAATATTTTTTATTTATGAAAGATATGAAAATGGATAAACAAG
AAGTTAAAAGAGAATACAAGGAGCAAGAAGGAAATCCAGAAATTAAGTCTAAACGCAGAGAGCGCATCAGGAAATTCTTT
CTGAGCAATTGA
>Cluster_14548
ATGGCTGAGATGAAAAACCTGAAAATTGAGGTGGTGCGCTATAACCCGGAAGTCGATACCGCACCGCATAGCGCATTCTA
TGAAGTGCCTTATGACGCAACTACCTCATTACTGGATGCGCTGGGCTACATCAAAGACAACCTGGCACCGGACCTGAGTT
ACCGCTGGTCCTGCCGTATGGCGATTTGTGGTTCCTGCGGCATGATGGTTAACAACGTGCCAAAACTGGCATGTAAAACC
TTCCTGCGTGATTACACCGACGGTATGAAGGTTGAAGCGTTAGCTAACTTCCCGATTGAACGCGATCTGGTGGTCGATAT
GACCCACTTCATCGAAAGTCTGGAAGCGATCAAACCGTACATCATCGGCAACTCCCGCACCGCGGATCAGGGTACTAACA
TCCAGACCCCGGCGCAGATGGCAAAGTATCACCAGTTCTCCGGTTGCATCAACTGTGGTCTGTGCTACGCCGCGTGCCCG
CAGTTTGGCCTGAACCCAGAGTTCATCGGTCCGGCTGCCATTACGCTGGCGCATCGTTATAACGAAGATAGCCGCGACCA
CGGTAAGAAGGAGCGTATGGCGCAGTTGAACAGCCAGAACGGCGTATGGAGCTGTACTTTCGTGGGCTATTGCTCCGAAG
TCTGCCCGAAACACGTCGATCCGGCTGCGGCCATTCAGCAGGGCAAAGTAGAAAGTTCGAAAGACTTTCTTATCGCGACC
CTGAAACCACGCTAA
>Cluster_10146
ATGGCTACCAATGCAAAACCCGTCTATAAACGCATTCTGCTTAAGTTGAGTGGCGAAGCTCTGCAGGGCACTGAAGGCTT
CGGTATTGATGCAAGCATACTGGATCGTATGGCTCAGGAAATCAAAGAACTGGTTGAACTGGGTATTCAGGTTGGTGTGG
TGATTGGTGGGGTAACCTGTTCCGTGGCGCTGGTCTGGCGAAAGCGGGTATGAACCGCGTTGTGGGCGACCACATGGGG
ATGCTGGCGACCGTAATGAACGGCCTGGCAATGCGTGATGCACTGCACCGCGCCTATGTGAACGCTCGTCTGATGTCCGC
TATTCCATTGAATGGCGTGTGCGACAGCTACAGCTGGGCAGAAGCTATCAGCCTGTTGCGCAACAACCGTGTGGTGATCC
TCTCCGCCGGTACAGGTAACCCGTTCTTTACCACCGACTCAGCAGCTTGCCTGCGTGGTATCGAAATTGAAGCCGATGTG
GTGCTGAAAGCAACCAAAGTTGACGGCGTGTTTACCGCTGATCCGGCGAAAGATCCAACCGCAACCATGTACGAGCAACT
GACTTACAGCGAAGTGCTGGAAAAAGAGCTGAAAGTCATGGACCTGGCGGCCTTCACGCTGGCTCGTGACCATAAATTAC
CGATTCGTGTTTTCAATATGAACAAACCGGGTGCGCTGCGCCGTGTGGTAATGGGTGAAAAAGAAGGGACTTTAATCACG
GAATAA
>Cluster_62536
ATGAGCTATAACAATCATATCAATTTAACAGATGAATTGTTGGATGAACCTATTTATAGAATTATGCCAATACATCGTTT
TCTTCAATTGCTTGAAGAAAAAAAATTAACTTTAGTAAGACCAATAAAGTGGGATGATCCATTCGAGAATGCCTTATTAA
ATAGTACAATCGAAACTTCTGATGGGGAAACAGGCACATTCTCCGCAAAAGATTCTGTTTATGGTCAATGCTGGACATTT
CATAGTGAAACTGATGCAATGTGGCGGATATATTCCCATGATAAAGATGGTGTAAGAGTTTCAACCACACCAAGGAAATT
ATTAACTGCACTTCAAACAGCAGAACCAACGCACCATAATTTAAAGTGTTTTATAGGCAAGGTTTCTTATCTTCCAGAAA
TCAATCTTCTAGAAAAGCTCATGTCAGTTAATTTATTAGATACTGATGGTTCAGGTATTGCTGAATCACTTTTATATAAA
CGTGTGGAATTTGAACACGAAAACGAAGTTCGTTTAATTTATTCTGGTGATGACAATGCAGTTAATAGTGATATTTTCCA
ATTTACAATTAATCCAGGCGAACTGTTAGATTCGATTCTCTTTGATCCAAGAATGGAGAAAAATTTAAGGCAAGCATACT
TATTGGCAATTAAGGATAAAGGTTTTAAAGCTGAAGTAAAAATATCTACGTTATACGATGCGCCTAAGGGACTGGTATTT
AAATTATAG
>Cluster_41979
GTGAAGTTCAAAGTTATCGCCCTGGCGGCATTAATGGGTATTAGCGGGATGGCAGCGCAGGCTAACGAATTGCCGGATGG
ACCGCATATTGTCACCTCCGGTACGGCAAGCGTGGATGCGGTGCCAGACATTGCCACTCTTGCGATTGAAGTTAACGTGG
CCGCGAAGGATGCCGCTACTGCCAAGAAACAGGCAGATGAGCGCGTCGCACAATACATTTCCTTCCTTGAGCTCAATCAG
ATCGCGAAAAAAGATATCAGCTCAGCGAACTTACGCACTCAGCCAGATTATGATTATCAGGATGGTAAAAGTATCCTGAA
AGGCTACCGCGCAGTGAGAACGGTGGAAGTCACGCTCCGTCAGTTAGACAAACTGAATTCCTTGCTGGACGGCGCGCTAA
AGGCGGGTCTTAACGAAATTCGTTCTGTGTCGCTGGGCGTGGCGCAGCCGGATGCCTATAAAGACAAAGCGCGTAAGGCA
GCGATTGATAACGCGATTCATCAGGCGCAGGAACTGGCGAATGGCTTTAATCGCAAACTGGGGCCGGTATATAGCGTGCG
TTATCATGTTTCCAACTATCAGCCCAGCCCAATGGTGCGGATGATGAAAGCTGATGCTGCACCGGTGTCCGCACAGGAAA

CTTACGAGCAGGCCGCTATTCAGTTTGATGATCAGGTCGATGTAGTCTTCCAGTTAGAACCTGTGGATCAACCACCCGCT
AAAACACCTGCTGCACATTAA
>Cluster_7104
ATGTCTCACCGCGACACGCTATTTTCTGCCCCTATCGCCAGACTGGGCGACTGGACCTTTGATGAACGGGTAGCTGAAGT
CTTCCCGGATATGATCCAGCGTTCCGTACCCGGCTATTCCAATATTATTTCCATGATTGGTATGTTAGCCGAGCGCTTCG
TTCAACCTGGTACGCAGGTTTACGATCTGGGTTGTTCTCTGGGCGCGGCGACGCTCTCGGTGCGTCGCAACATTCATCAT
GATAATTGCAAAATTATTGCCATCGACAACTCCCCGGCGATGATTGAACGCTGCCGTCGTCATATTGACGCCTATAAAGC
CCCTACGCCAGTAGACGTCATTGAAGGTGATATTCGCGATATCGCCATTGAAAACGCATCGATGGTGGTGCTGAATTTTA
CCCTGCAATTCCTGGAACCTTCCGAGCGCCAGGCGTTACTGGATAAAATTTATCAAGGGCTGAACCCCGGCGGCGCGCTG
GTGCTTTCGGAAAAATTCAGTTTCGAAGATGCCAAAGTTGGTGAACTGTTGTTCAACATGCACCACGACTTTAAACGTGC
CAACGGTTACAGCGAACTGGAGATCAGCCAGAAGCGCAGCATGCTGGAAAACGTGATGCTGACCGATTCCGTGGAAACCC
ATAAAGCACGCCTGCATAACGCCGGTTTTGAGCATAGTGAGCTGTGGTTCCAGTGCTTTAACTTTGGTTCACTGGTGGCA
TTAAAAGCAGAGGACGCTGCATGA
>Cluster_67037
ATGTTGTTAAATCGTAAAAATAACTTTATCGCTGCGATTGTTCTGGGGGCGTTTCTCCTGTCTGGCTGTGGTGCGATGAG
CACCGCAATTAAAAAGCGTAATCTGGAAGTCAAAACCCAGATGAGTGAAACCATCTGGCTTGAACCTTCCACTGATCGCA
CTGTTTATCTGCAAATCAAAAACACCTCCGATAAGGATATGAGCGGCCTTCAGGGAAAAATTAACAGCCAGGTGCAGGCG
AAAGGCTATCGTATCGTGAATAATCCTGCCGACGCGCATTACTGGATTCAGGCGAACGTACTGAAAGCCGATAAAATGGA
TTTGCGGGAAGCACAGGGCTTTCTGAGTCGGGGGTACGAGGGGGCTGTTCAGGCTGCGGCGTTAGGGGCAGGCATTACAG
CCTATAACTCCAATTCTGCCGGTGCCACGCTGGGGGTGGGACTGGCTGCCGGCCTTGTGGGAATGGCGGCTGATGCGATG
GTGGAAGATATCAACTACACCATGGTAACTGATGTGCAGATCGCAGAGCGCACTTCCGCGCAGGTGACGACTGACAATGT
GGCTGCGCTGAAGCAGGGAACGTCGGGTTATAAAGTGCAGACCAGCAGTGAAACAGGTAACCAGCATAAATATCAGACGC
GTATTGTGTCCAGTGCCAACAAAGTTAACCTGAAATTTGAAGAAGCCAAACCGGTTCTTGAAGATCAGTTAGCAAAATCT
ATTGCTAATATTCTGTAA
>Cluster_28068
ATGAAATCACCGTTAATCACAACAGGAATGATTTATTTACTGGGGATCTGCAACTTTGCCCAGGCGACTGTCTCCCCCGA
CAGAACACGCATCATTTTTAATGCTTCGAATAAAAGCGCAACGGTTCGCCTGACCAACCAAAGTAAAATCGATCCCTATC
TGGCTCAGTCGTGGATTGAAGATGCCAGCGGTAAAAAAACGCGTGATTACATCTCGACATTGCCGCCGATGGAACGTATT
GAGCCTGATGAACAGGTACAAATTCGCCTGATGGCGCTAGCCTCGTTAAACAACTTACCACAGGACAGAGAAACGCTGTT
CTACTATAACGTACGGGAAATTCCGCCTCGCACTAAAGAACAGAATGTGATGCAAATCGCCATGCAAAGTCGCCTGAAAT
TATTCTGGCGTCCGAAAGCAATCGAATTAAAAGAAGGGCAGATGGTGCCTTTACAAAAAGTCACGATAACACGGACCGCT
GCAGGGCTGACACTCAATAATCCAACGCCTTATCACATCACCGTCGGTTATATCGGTACTAACGGCAAGACACTGATGCC
CGGTGCGGACAGTATTATGGTGGCACCATTTACTTCTGCAACTCAACACCTAAGCAGCCTGCCCTCTACCTTCCAGTTAG
GCTTTGTGGCGGACTACGGCGGGCTGGAGATGTTTAAAGTCGAATGTAATTCCGTTCAATCCCTCTGCCAAAGCTCTCCT
GCTAAGAAGGGGAAAATATGA
>Cluster_37372
ATGAACAAATTTATTAAAGTTGCACTGGTAGGTGCAGTACTGGCTACGTTAACTGCATGTACTGGTCATATTGAAAACCG
TGATAAGAACTGCTCTTACGACTACCTGCTGCACCCGGCAATTTCTATTTCTAAAATCATTGGCGGTTGCGGTCCTACTG
CACAGTAA
>Cluster_40303
ATGGGGGAGGAAAAGAAGAAGAGTGAAATTGCGGAAAGCGTCTCGAAAAACAGGCTGCGACCCGAACATGGCAGTCGCAG
CAAAGACCTCTTAACGACGGAAATCAATCACCATACGGCCACGGATTTTGCCTTCCTCCATCTCGGTAAAGATGGTGTTG
ATGTCCGCTAA
>Cluster_3705
ATGAGTAAAAAAATCGGCCAAAAAGCGCCAGCCAGTGAAGCCCGTGGTGGCGAAAGAACCTGCTCGCACCGCCAAAAATTT
TGGCTATGAAGAGATGTTGAGCGAGCTGGAAGCCATCGTCGCGGATGCTGAAACGCGTTTAGCCGAGGATGAAGCTACCG
CGTAA
>Cluster_14846
ATGACTTTTTCGTGGATTTCCACGGAACGCGAGAAGCAGGTTGTTAAGTTCAATACGTTGCGTGAAGATTTTGCCGGTCA
GGTGTTTCTCCTTCGGCACTTCTCTGGCGTAGTTGCTCCGATCATCGCTGGTCACCATGCCGGTACATTCCGTGGCCTGA
AAGTGTAA
>Cluster_49888
GTGAATATTGAAGAGTTAAAAAAACAAGCCGAAACGGAAATCGCCGACTTTATCGCGCAAAAAATCGCCGAGCTGAACAA
GAATACAGGGAAAGAAGTCTCTGAAATTCGCTTCACCGCACGAGAAAAAATGACCGGGCTTGAAAGTTATGATGTCAAAA
TCAAAATAATGTGA
>Cluster_23990
ATGTCACTGTCAATTGTTCATACCCGCGCAGCCCTGGGAGTAAATGCGCCCCCAATCACTGTTGAGGTACATATCAGTAA
AGGTCTACCAGGCTTAACGATGGTGGGCTTACCAGAAACAACGGTAAAAGAAGCTCGCGATCGCGTGCGCAGCGCCATTA
TCAATAGCGGATTTTTATAA
>Cluster_3612
ATGTTTATCGCCTGGTACTGGATTGTATTGATTGCTCTGGTTGTGGTGGGTTATTTCCTGCATTTGAAACGTTATTGTCG
GGCGTTTCGCCAGGACAGAGACGCACTGCTTGAAGCGCGGAACAAATACTTAAACAGTACGAGAGAAGAGACAGCCGAAA
AGGTAGAATAA
>Cluster_51662
ATGCGAAAACGACACCGATTTAACAGTCGCATGACCCGTATCGTACTGCTCATCAGCTTTATCTTCTTCTTTGGCCGTTT
TATCTACTCGTCCGTCGGTGCCTGGCAGCACCATCAGAGCAAAAAAGAAGCTCAGCAATCCACACTCTCCGTCGAATCAC
CGGTACAACGTTAG
>Cluster_10855
ATGTTAACTGGGGCTTTTCTCTGTCTGCCTTACGGTGGCATGCCCGAGGCAGACAGCCTCAAGCACCCGCAGCAATTCTA
CTTAACTCTCGCTTTACCGCAAACCGTTTTTACCCGATATGGGAATTCCCATATCGTAATGAATTCAGTTCCCTAG
>Cluster_35638
ATGAAAGGTATTGAAATGGAAACTCCCGCGAGCCTTGATTTGACAAGGGTTGCGGCCTTTGCAATTCGCCTTGTGGCGGT
CGCTGTTCTGATTTGGGCTGTGCGTTGGTGGTGA
>Cluster_57707
ATGATGAAACGCCTTATCGTTCTTGTTTTGCTTGCCAGCACGCTGCTCACGGGCTGTAACACCGCTCGCGGTTTCGGCGA
AGACATCAAACATCTCGGCAACTCCATCTCTCGCGCTGCCAGCTAA
>Cluster_32300
ATGTCAAAAACATTCACCAAAATTATATTTATCTTTTCTGTATTCATTTTTAATTATAACGAATCTCAAATTACCGATAC
CGCCGTCAATTTATTTATCCAGCTTTAA

>Cluster_4950
ATGGTTTTTTTTTGTGAGCACATTCCTTCTTATGTAGGAAGAGGCTGTATTATATTCAGTCAACTCTTTTTACTAAAAA
CATTATCTATGCCATCAAAGTTGCCTGCTAG
>Cluster_65227
ATGCTGACAAAATATGCCCTTGTGGCAATCATCGTACTGTGTTGTACAGTACTGGGATTCACGCTGATGGTAGGTGACTC
GTTGTGTGAGTTGAGTATCAGAGAACGTGGTATGGAGTTTAAGGCAGTTCTCGCTTACGAATCGAAGAAGTAG
>Cluster_14833
ATGAACACTAAAATATTCGAAGACAACATTTTATCTCGTAATGATATCGCTGTACGCTACGTCTTTCAGAAAATGTTTTC
CCCACAAGGTACTCTGGTCGCGGTGGAATGCTTAAGCCGCTTTGATAATCTGGTGATGCTGCCAACTTACTGA
>Cluster_9252
ATGACTCGCTACCCGCAGCAGGCGAGTCATTTTTACTCGTTTATCATGCCAGATTACCCGTCATATCAGCGTTTCATCGT
TGGCACGGAAGATGCAATACCCGAAGTAAGACAACCACTGGAGGATTAA
>Cluster_49702
ATGGATCAAACAATTATGGCTATCCAGACTAAATTCACTATCGCCACTTTTATTGGCGATGAAAAGATGTTTCGTGAGGC
CGTCGACGCTTATAAAAAATGGATATTAATACTGAAACTGAGATCAAGCAAAAGCATTCACTAA
>Cluster_3096
ATGAAAAGGGCATTATGTCTGTTAAGTATGGTTTTATCTTTAACTGCCGGGCAAACACATGCAGATATGAACAATTATCT
AGGATATACATCTGGTGAGATTAGCGCATCTATTGGATGCACTATTGAGAGTCTGTACATAGATTTGTGTAATTGCCTGT
TTTTGATATGTTCACTCCAACACCGGAGACAGGCAAATTATGGACGAAAATAA
>Cluster_38971
ATGTTGTTCTTAAATGATAAAGAGCAAATCATCAAGTATAAAGATGAAATGCTGAAAATTAATCCACAAATTACCGAAAT
GGTAGCTAAATACGCTGGGTGTTCTGTAGAGGAAGTAGAAAGATGTGTTGAAAAATATTTTTCTCCTTCTTCTCCGTCTA
CACCTTCCCTTAATGAATTAATCAAATTAAAAGCTAAGGAGATTACTAAATGA
>Cluster_39664
ATGAAGACCTTTGCGCTACAGGGCGACACGCTCGACGCCATTTGTGTCCGGTATTACGGGCGCACTGAGGGCGTGGTTGA
GACCGTGCTCGCCGCAAATCCGGGACTGGCTGAACTGGGTGCGGTGCTGCCACACGGCACCGCCGTCGAACTGCCCGACG
TTCAGACCGCGCCCGTGGCTGAAACTGTCAATCTGTGGGAGTAA
>Cluster_5128
ATGGCGAAAACAAAACAGAAATGGTTATATCAGTTACGGCGGTGTTCATCGGTGAATACACTGGAAAAGATTATCTGTAA
GAACACCTATGATTTACCGGACAGTGAGCGTGAGGCCTTTAATTCAGCAGCAGACCACCGTCTTGCTGAATTAACAATGA
ACAGGTTATATGACAGGGTTCCAAAAGAGGTCTGGGCATATGTCAGGTAA
>Cluster_15805
ATGACGCTTGAGCGAATCAGCGCCTTTATCACGTATTGCATCGCCGTCGTGCTGGCCTGGCTGGGCGATTTGTCCATCAA
GGATGCCTCAACGCTGGGCGGCCTGATGATCGGTGTGCTGATGCTGGCTATCAACTGGTACTACAAACACAAAGCCTACC
AGCTTCTGCGCGACGGGCAGATCTCGCGGGAGGACTATGAATCCATCAATCGTTAA
>Cluster_11505
ATGATGGAAATCAAGGCTCAACAGTTTGTCACATCAACGGGGCGGCAAGTCCTTACTGACAACGGACAACAAGGTATGGG
CGGCGTGGCGGGTATCGGTTCCACGACTGAAAAGCATCAGGGGCGCGTGGCGGAAGCAATTTTTGCGAACTGCGCGGAAC
TGGATAACGACCAGCTTAGCGAGATCATCGAGTGGGTTCGGCTCTATCAGCGCTGA
>Cluster_60521
ATGAACAATCGATCGGCCGTTAGAATACTACGGTTACCAGCGGTTATCCAAAAAACAGGTATGGCACGGGCCACCATCTA
TGACTGGTTGAACCCCAAATCACCACGATACGATGCCACCTTTCCCAAAAAGCGAATGCTCGGCGTGAAATCTGTCGGAT
GGATTGAGGCCGAGATTGATGAGTGGTTATCACAACGCTGTAAACTTATTTGA
>Cluster_64826
ATGATGGTGATAAATATTCACGATAGTCTGGGAATTGGTCGGTTAATTAACCTTGATAATGTCAGTGTAATGGAATCAAC
TGTTAACGGTGATGTTATTTTTGAATTTGTTGATGGTCGAAAACTGGCTGTTCATGTCGGCAACCATCATCACGCAGAAA
ATGTGTTTTGTGAACTTAACGAAATCACAAATGCAACGCCCGTTAATCAATAA
>Cluster_40729
ATGTCAGGTATAACCATCAATATCAATGTGAATGCCCCCTATGTATCCCTGCAGAAATATGCAGAGATAACAGGTATCCC
TCTTAATACATGCAAAAAGATGTTGGCTGACGGTCGAATTATTATCCGCCCCAAACGCGCCAAAATGGAAAAGCCTGAAG
TAAACCTTGTGGCGATGTTAAAAGACGCTTTGGCTAACAGCTAA
>Cluster_54045
ATGCCAACGAAACCGCCTTATCCTCGTGAAGCATATATAGTGACGATTGAAAAAGGAAAGCCAGGACAGACGGTAACCTG
GTACCAACTCAGAGCCGATCATCCTAAACCAGACTCGTTGATCAGTGAACATCCGACGGCTCAGGAAGCGATGGATGCGA
AAAAACGCTATGAGGACCCTGACAAAGAGTGA
>Cluster_6253
ATGAATGAGAACAAAATAAAAAGGCTGGAGCAACTGTTACAGGCACGCCAACGGGAGTTTGCCACTAAATGGGGACGTGC
CGCGCTGCCACATGAACGCCTAACCTATTTTCCACTTGAAGACCTCATTAAAAATAATGGCAAAAAAATCAGGTCTGATA
AGAAACACTTCTTAGAATGCAATGAAGACAAATAA
>Cluster_14259
ATGCAGAGATGGGCTGAAATTTCCACGGCAACTAAAAACCAGTCCCATCAGGCTATTTTATTTGCCATTTTGAACCTGGG
CAGTGCTCGAACTCCTCACGTACCAGTGTACGCTCCGGGTTCTGCGCGCTGTCCGTGTTCAAACTGGCTGCAACAATTAC
GCCAGATGGGCCTCGTTTGA
>Cluster_41361
ATGCAGTTGAAACCTACCAATCAAAAGCTGAACGTAACCCATGTTGATTTTGAAACCTTCGCGGCACTGATTAGATCAGA
GCCACAAAAGGTGAGCACCAGCACCTGGGTTTATGATGTGAAATCCACCAGCGGTGGAAAAGCTATCGGCATTGCCTCTG
GCGATGATTACTTAGTAATAGATCTGATTTAA
>Cluster_18920
ATGGCTAAGCCGCAGTGTACCCAAACTCACCCCAAATATCAGTATCGTTTTATGGCGCTCGATCGCGCTGACCGCCAGGC
AAAACCCTGCAAAATCATTGTTGAGGCCACCAGCGAGCATGAAGCACGTAAGGTGTTGGCACCTCACTACATCCTGTCAC
TGTCCGCCGTTTTGCCAGTTCAGGAGGTGAGCCATGTTTAA
>Cluster_60536
ATGGCTGCAGGATTTATTGCAGATACGGCCAGCCTGCCGCTCATTGTTTCTAACCTGGTGAATATCGTCTCGGCGGACTT
CTTCGGTCTGAGCTTTGCGCAGTACGCCTCCGTTATGATCTCCGTGGATGCGGCAGCGATTGCGGCCACGCTGATTATGC
TGTATCTCTTCTTTCGCAGAGTTATTCCGGCAACGTATTAA
>Cluster_20017
ATGAATACAGAAAATTCACAGGCATTAATTCTTAAATCTGTAAAAGAACTGGCAGCTATTAGCGAGGAATCAGTTATAAA
CACATCGGCGCTTTGCCGCCTTTTAGAAATTGATGCAAATAATGTGCGGCAGCGTGTGTTTCAGACTGGATGCAGCACTT
TTGAAGCAATTACATACTACTGTTCTAAAAAACAATAA

>Cluster_27387
ATGAATTACCAACTGGCAAAACTTTATCGTGGTAAACATTTCGCAGGGTATGGGATTGCAGTTAATGGTGAGTTACTGGA
AGGACAGCTTTCCGCCAGGACAGAGTCACGCGGAGGCGAGCCACCAACAGTCACTGTGACTTTCAGACTGACAGCAGAAC
ATATCGAGAATCAGCCTGTCATTAAACTGAACAGGGTATGA
>Cluster_21826
ATGTCCAACAATGGCTCGTCACCGCTGGTGCTTTGGTATAACCAACTCGGCATGAATGATGTAGACAGGGTTGGGGGCAA
AAATGCCTCCCTGGGTGAAATGATTACTAACCTTTCCGGAATGGGTGTTTCCGTTCCGAATGGTTTCGCCACAACCGCCG
ACGCGTTTAACCAGTTTCTGGACCAAAGCGGCGTAAACCAGCGCATTTATGAACTGCTGGATAAAACGGATATTGACGAT
GTTACCCAGCTTGCGAAAGCGGGCGCGCAAATCCGCCAGTGGATTATCGACACTCCCTTCCAGCCTGAGCTGGAAAACGC
CATCCGCGAAGCCTATGCACAGCTTTCCGCCGATGACGAAAACGCTTCGTTTGCGGTGCGTTCCTCCGCTACTGCAGAAG
ATATGCCGGACGCTTCTTTTGCCGGTCAGCAGGAAACTTTCCTCAACGTTCAGGGTTTTGACGCCGTTCTCGTGGCAGTG
AAGCATGTATTTGCTTCTCTGTTTAACGATCGCGCCATCTCTTATCGTGTGCACCAGGGTTACGACCATCGTGGCGTAGC
GCTCTCCGCCGGTGTTCAGCGGATGGTGCGCTCCGACCTCGCATCTTCTGGCGTGATGTTCTCCATTGATACCGAATCCG
GCTTTGACCAGGTGGTGTTTATCACTTCCGCATGGGGCCTTGGTGAAATGGTCGTGCAGGGTGCGGTTAACCCGGATGAG
TTTTATGTGCATAAACCGACACTTGCGGCGAATCGCCCGGCTATCGTGCGCCGCACCATGGGGTCGAAAAAAATCCGCAT
GGTTTACGCGCCGACCCAGGAGCACGGCAAGCAGGTTAAAATCGAAGACGTACCGCAGGAACAGCGTGACATCTTCTCGC
TGACCAACGAAGAAGTGCAGGAACTGGCAAAACAGGCCGTACAAATTGAGAAACACTACGGTCGCCCGATGGATATTGAG
TGGGCGAAAGATGGCCACACCGGCAAACTGTTCATTGTGCAGGCGCGTCCGGAAACCGTGCGCTCACGCGGTCAGGTCAT
GGAGCGTTATACGCTGCATTCACAGGGTAAGATTATCGCCGAAGGCCGTGCTATCGGTCATCGCATCGGCGCGGGTCCGG
TGAAAGTCATCCATGACATCAGCGAAATGAACCGCATCGAACCTGGCGACGTGCTGGTTACTGACATGACCGACCCGGAC
TGGGAACCGATCATGAAGAAAGCATCTGCCATCGTCACCAATCGTGGCGGTCGTACCTGTCACGCGGCGATCATCGCTCG
TGAACTCGGCATTCCGGCGGTAGTGGGCTGTGGTGATGCAACAGAACGGATGAAAGACGGCGAGAACGTCACTGTTTCTT
GTGCCGAAGGTGATACCGGTTACGTCTATGCGGAGTTGCTGGAATTTAGCGTGAAAAGCTCCAGCGTAGAAACGATGCCG
GACCTGCCGTTGAAGGTGATGATGAACGTCGGTAATCCGGACCGTGCTTTCGACTTCGCCTGCCTGCCGAACGAAGGCGT
GGGCCTTGCGCGTCTGGAATTTATCATCAACCGTATGATTGGCGTCCACCCACGCGCACTGCTTGAGTTTGACGATCAGG
AACCGCAGTTGCAAAACGAAATCCGCGAGATGATGAAAGGTTTTGATTCTCCGCGTGAATTTTACGTTGGGCGTTTGACT
GAAGGGATCGCGACGCTGGGTGCCGCGTTTTATCCGAAGCGCGTCATTGTCCGTCTCTCTGATTTAAATCGAACGAATA
TGCCAATCTGGTCGGTGGTGAGCGTTACGAGCCAGATGAAGAGAACCCGATGCTCGGCTTCCGTGGCGCGGGCCGCTATG
TTTCCGACAGCTTCCGCGACTGCTTCGCGCTGGAGTGTGAAGCAGTGAAACGTGTGCGCAACGACATGGGGCTGACTAAC
GTTGAGATCATGATCCCGTTCGTGCGTACCGTTGATCAGGCGAAAGCGGTGGTTGAGGAACTGGCGCGTCAGGGGCTGAA
ACGTGGCGAGAACGGGCTGAAAATCATCATGATGTGTGAAATTCCGTCCAACGCCTTGCTGGCAGAGCAGTTCCTGGAAT
ATTTCGACGGCTTCTCAATTGGCTCAAACGACATGACGCAGTTGGCGCTCGGTCTGGATCGTGACTCCGGCGTGGTGTCT
GAACTGTTCGATGAGCGCAACGATGCGGTGAAAGCACTGCTGTCGATGGCGATTCGTGCCGCGAAGAAACAGGGCAAATA
TGTCGGGATTTGCGGTCAGGGTCCATCCGACCACGAAGACTTTGCTGCATGGTTGATGGAAGAGGGGATCGATAGCCTGT
CTCTGAACCCGGACACCGTGGTGCAAACCTGGTTAAGCCTGGCTGAACTGAAGAAATAA
>Cluster_21585
ATGAAGCTAACCGATGCGGATAATGCCGCCGATGGCATTTTTTTCCCCGCCCTTGAGCAAAATATGATGGGCGCGGTGTT
AATTAACGAAAATGATGAAGTGATGTTTTTCAACCCCGCCGCAGAGAAGCTCTGGGGGTACAAACGTGAAGAAGTCATTG
GCAATAACATTGATATGCTGATTCCGCGGGATTTGCGTCCTGCGCATCCTCAATACATTCGTCACAACCGTGAAGGTGGT
AAAGCTCGCGTCGAGGGGATGAGTCGGGAGCTGCAGCTGGAGAAAAAAGACGGCAGTAAAGTCTGGACCCGTTTTGCGCT
ATCGAAAGTGAGCGCCGAGGGGAAAGTTTATTACCTGGCGCTGGTACGGGATGCCAGCGGAGAAATGGCGCAAAAAGAAC
AGACCCGACAATTGATTATTGCCGTTGACCATCTCGACCGACCGGTGATTGTCCTCGATCCGGAACGCCATATTGTGCAG
TGCAATCGCGCATTTACCGAAATGTTTGGTTACTGCATTAACGAAGCCAGCGGTATGCAGCCCGATACACTCCTGAACAT
TCCAGAATTCCCTGCTGATAACCGTATTCGTTTACAACAGTTGCTATGGAAAACCGCCCGCGATCAGGACGAATTTCTGC
TGTTGACGCGCACCGGTGAAAAAATCTGGATTAAAGCCTCTATCAGCCCGGTTTATGACGTGCTCGCGCATCTGCAGAAC
CTGGTAATGACATTCTCGGATATCACAGAAGAAAGGCAAATCCGCCAGCTTGAAGGCAATATTCTCGCCGCCATGTGCAG
CAGCCCGCCATTTCATGAAATGGGGGAAATCATTTGTCGTAACATCGAATGTGTTCTCAACGAATCGCATGTTTCGCTGT
TCGCGCTGCGCAACGGGATGCCGATACACTGGGCGTCATCTTCGCACGGTGCAGAAGTTCAAAATGCGCAAAGCTGGTCA
GCGACCATTCGTCAGCGCGATGGCGCGCCTGCGGGGATCCTGCAAATTAAAACCTCGTCAGGAGCAGAAACCAGCGCCTT
TATCGAACGCGTGGCAGATATCAGCCAGCATATGGCCGCGCTGGCGCTGGAACAGGAAAAAAGCCGTCAGCATATTGAAC
AACTCATCCAATTTGATCCGATGACCGGTCTGCCAAATCGCAATAACCTGCACAATTACCTCGATGACCTGGTCGACAAA
GCCGTCTCTCCGGTGGTGTATCTCATCGGTGTTGACCATATTCAGGATGTGATTGATAGCCTTGGCTATGCGTGGGCCGA
TCAGGCATTGCTGGAAGTGGTCAATCGCTTTCGTGAAAAACTCAAACCGGATCAGTATCTCTGTCGTATTGAAGGTGCGT
CTTTGTCCTCGTGAGCCTGGAAAACGATGTCAGTAACATTACCCAGATCGCCGATGAGCTACGGAATGTGGTCAGCAAG
CCGATAATGATTGACGATAAACCATTCCCGCTCACCTTGAGCATTGGCATCAGCTACGACGTGGGTAAAAACCGCGATTA
CTTGCTCTCCACTGCTCACAATGCAATGGATTTTATTCGCAAAAATGGCGGTAACGGCTGGCAGTTCTTCAGCCCGGCGA
TGAACGAAATGGTAAAAGAGCGTTTGGTTTTAGGCGCAGCGCTAAAAGAAGCAATTAGCAATAACCAACTTAAACTGGTT
TATCAGCCGCAAATCTTCGCAGAAACGGGTGAACTGTACGGCATCGAAGCCCTTGCTCGCTGGTACGATCCCCTGCATGG
TCATGTGCCCCCTTCACGGTTTATTCCTCTCGCAGAAGAGATTGGTGAAATCGAAAATATTGGGCGCTGGGTCATCGCGG
AAGCTTGCCGTCAGTTAGCAGAATGGCGTAGCCAGAATATTCATATCCCGGCGTTATCCGTTAACTTGTCTGCGCTGCAC
TTTCGCAGTAATCAGCTGCCTAATCAGGTATCTGATGCAATGCACGCCTGGGGTATTGACGGCCACCAGCTGACGGTGGA
AATCACGGAAAGCATGATGATGGAACACGATACCGAAATCTTTAAGCGCATTCAGATCCTGCGCGATATGGGCATCGGTT
TATCGGTAGATGATTTTGGTACGGGCTTTTCCGGATTATCCCGCTTAGTCAGTCTTCCGGTAACGGAAATCAAAATTGAT
AAAAGTTTTGTCGATCGTTGTCTGACCGAAAAACGCATCCTTGCCTTACTCGAAGCCATTACCAGCATTGGCAAAGCCT
CAATTTAACCGTCGTGGCTGAAGGCATCGAAACCAAAGAGCAATTTGAGATGCTACGCAAGATCCACTGTCGCGTTATTC
AGGGATATTTCTTTTCCCGCCCCCTGCCCGCCGAAGAAATTCCAGGCTGGATGAGCAGCGCGTTACCGCTGAAAATCTGA
>Cluster_20894
ATGAAAATAACGACCGTCGGCGTATGCATAATTTGCGGAATTTTTCCGTTGCTGATTTTGCCCCAATTGCCTGGGACAGT
AACCCTTGCGTTTCTGACTCTCTTCGCCTGTGTACTGGCATTTATCCCTGTTAAAACCGTCCATTATATCGTGCTGACGT
TGCTGTTTTTCGTTGGGGCATATTAGCAGCAAAGCAAATTTTGTGGGCAGGAGAAACCTTAACTGGCGCGACGCAGGAT
GCAATTGTTGAGATCATTGCTACTGACGGCATGACCACTCATTACGGTCAAATTACTCATCTACAAGGTCGACGTATATT
CCCTGCGCCAGGCCTCGTACTGTATGGCGAATATCTTCCGCAAGCGGTTTGTGCCGGACAAGTATGGTCAATGAAACTCA
AAGTTCGTGCAGTTCATGGTCAACTTAATGATGGCGGCTTTGATAGCCAGCGTTATGCCATTGCCCAGCATCAACCGCTC
ACCGGCCGTTTTCTGCAGGCAAGTGTCATTGAACCGAATTGTAGCCTGCGTGCACAGTATCTGGCGTCACTACAAACAAC
GCTGCAACCCTATATGTGGAACGCGGTTATTCTTGGATTAGGTATGGGGGAACGGTTATCCGTCCCCAAAGAAATCAAAA
ATATCATGCGTGATACTGGAACGGCGCATTTAATGGCGATATCAGGATTGCACATCGCTTTTGCGGCGTTGCTGGCTGCA
GGACTCATTCGCGGTGGGCAAGTTTTTCTGCCTGGGTGCTGGATCCACTGGCAAATGCCATTAATTGGTGGAATCTGCTG
TGCTGCTTTTTATGCCTGGCTTACGGGAATGCAACCTCCTGCATTGCGTACCGTGGTGGCGCTTGCCATTTGGGGAATGC
TTAAGTTAAGTGGACGACAATGGAGTGGCTGGGATGTATGGATATGTTGTCTGGCGGCAATTTTGCTGATGGATCCTGTT

GCCATTCTCTCGCAAAGTTTATGGCTCTCTGCCGCTGCGGTCGCGGCACTGATTTTTTGGTATCAGTGGTTTCCCTGTCC
GGAGTGGCAGCTGCCGCCGGTATTGCGCGCGGTTGTTTCCCTCATCCATCTGCAACTGGGAATCACCCTCCTGCTTATGC
CCGTGCAAATTGTCATATTTCATGGCATTAGTCTGACCTCATTTATTGCAAATCTTTTTGCAATTCCCCTGGTGACATTT
ATCACGGTTCCGTTGATCCTCGCCGCGATGGTTGTGCATTTAAGCGGGTCGTTTATTCTGGAACAAGGGTTATGGTTTCT
TGCCGACCGGTCTTTGGCTTTACTTTTCTGGGGGTTAAAGAGTTTGCCGGAAGGGTGGATCAACATTGCTGAACGTTGGC
AATGGCTAACATTTTCCCCATGGTTCTTACTGGTGGTATGGCGATTAAACGCCTGGCGAACGTTGCCAGCAATGTGTGTG
GCTGTAGGCTTGCTGATGTGCTTGCCGCTGTGGCAAAAACCTCGACCTGACGAGTGGCAAGTGTACATGCTTGATGTCGG
GCAAGGGCTGGCAATGGTCATGGCGCGAAACGGCAAAGCGATTCTCTATGACACGGGACTGGCCTGGCCCGAAGGGGATA
GTGGGCAACAACTGATTATCCCCTGGCTCCACTGGCATAATCTTGAACCGGAAGGCGTTATTTTGAGTCATGAACATCTG
GATCACCGGGGAGGGCTGGACTCAATATTGCACACATGGCCGATGTTATGGATCCGAAGTCCGTTAAACTGGGAACATCA
TCAGCCCTGTATGCGTGGCGAAGCGTGGCAATGGCAAGGATTGCGTTTCAGCGCGCACTGGCCTTTACAAGGTAGCAACG
ATAAAGGAAATAACCATTCCTGTGTGGTTAAGATTGATGACGGGACGAATAGCATTCTTCTAACCGGTGATATTGAAGCC
CCCGCTGAACAAAAGATGCTAAGCCGTTACTGGCAGCAAATGCAGGCAACATTACTTCAGGTACCTCACCATGGCAGTAA
TACCTCATCATCGTTGCCATTAATTCAGCGAGTGAATGGAAAAGTGGCACTCGCATCGGCATCGCGCTATAACGCATGGC
GACTGCCCTCAAGCAAAGTTAAACATCGCTATCAACAACAGGGTTATAAGTGGCTTGATACTCCACATCAGGGTCAAATA
ACGGTCAATTTTTCAGCGCAAGGCTGGCGGATTAGCAGCCTCAGAGAGCAAATTTTACCTCGTTGGTATCATCAGTGGTT
TGGCGTGCCAGTGGATAACGGGTAG
>Cluster_42161
ATGAAAATCAAAACTATTTTAACGCCAGTAACCTGCGCTCTGCTGATAAGTTTTTCCGCCCATGCCGCTAACGCCGACAA
TTATAAAAACGTGATTAACCGTACTGGCGCGCCGCAGTACATGAAGGATTACGATTACGACGATCACCAGCGTTTTAATC
CGTTTTTCGATCTCGGAGCCTGGCATGGTCATCTGTTGCCAGACGGCCCGAACACCATGGGCGGCTTTCCGGGCGTTGCG
CTGCTGACGGAAGAGTACATCAACATTATGGCCAGCAATTTCGACCGCCTGACCGTCTGGCAGGACGGCAAGAAAGTCGA
CTTCACGCTGGAGGCATACAGTATTCCCGGCGCGCTGGTGCAAAAACTAACAGCAAAAGATGTGCAGGTTGAAATGACTC
TTCGCTTCGCCACGCCGCGTACGTCACTACTGGAAACCAAAATCACCAGCAATAAACCGCTGGATCTGGTGTGGGACGGC
GAACTACTGGAAAAACTGGAAGCGAAAGAAGGGAAACCGCTTTCCGATAAAACCATTGCTGGCGAATACCCTGACTATCA
GCGCAAAATCAGCGCCACCCGTGATGGCCTGAAAGTCACCTTTGGCAAAGTGCGCGCCACCTGGGATCTGCTGACCTCCG
GTGAATCAGAATATCAGGTGCATAAATCCCTGCCGGTGCAGACTGAAATCAACGGTAATCGCTTTACCAGTATGGCGCAT
ATCAACGGTTCGACGACGCTCTACACCACCTATTCCCATCTGCTGACCGCTCAGGAAGTTAGCAAAGAGCAAATGCAGAT
CCGCGATATTCTGGCGCGTCCGGCGTTTTATCTCACCGCCTCGCAGCAACGCTGGGAAGAGTATCTGAAGAAAGGGTTAA
CCAATCCGGATGCGACGCCGGAACAGACGCGCGTCGCGGTGAAAGCCATCGAAACGCTCAACGGTAACTGGCGATCGCCG
GGCGGTGCGGTGAAATATAACACCGTCACGCCGTCGGTGACCGGGCGCTGGTTCTCCGGCAATCAGACCTGGCCGTGGGA
TACCTGGAAGCAGGCGTTTGCGATGGCGCATTTCAATCCGGATATCGCCAAAGAGAATATCCGCGCGGTCTTCTCCTGGC
AGATCCAGCCAGGCGACAGCGTGCGTCCGCAGGATGTGGGCTTTGTCCCCGACCTGATAGCGTGGAACCTTAGCCCCGAG
CGTGGCGGCGATGGCGGCAACTGGAACGAACGTAACACCAAACCCAGCCTTGCCGCCTGGTCGGTGATGGAAGTGTACAA
CGTCACCCAGGATAAAACCTGGCTGGCAGAGATGTACCCGAAACTGGTGGCCTATCACGACTGGTGGCTACGTAACCGCG
ATCACAACGGCAACGGCGTACCGGAATATGGCGCGACCCGCGATAAAGCCCACAATACTGAGAATGGCGAGATGCTGTTT
ACGGTGAAAAAAGGCGCCAAAGAGGAGACGCTGTCTGGGCTGAACAACTACACCCGCGTGGTGGAGAAAGGCCAGTACGA
CAGTCTGGAAATCCCGGCACAGGTTGCGGCGTCGTGGGAATCGGGTCGTGATGATGCCGCCGTTTTTGGGTTTATCGACA
AGGAACAGTTGGATAAATATGTCGCTAACGGCGGCAAACGTAGCGACTGGACGGTGAAATTCGCGGAAAACCACAGTCAG
GACGGAACGTTGCTGGGCTACTCGCTATTGCAGGAGTCGGTGGATCAGGCCAGCTATATGTACAGCGATAACCATTATCT
GGCGGAGATGGCGACGATTCTCGGTAAGCCGGAAGAGGCCAAACGCTATCGTCAGTTAGCACAACAGCTCGCGGACTACA
TCAACACCTGTATGTTCGACCCGACTACACAGTTCTACTATGACGTGCGTATTGAAGATAAACCGCTGGCGAACGGCTGC
GCGGGCAAACCGATTGTCGAGCGCGGTAAAGGGCCGGAAGGCTGGTCACCGCTGTTTAACGGTGCGGCAACGCAGGCCAA
TGCCGACGCGGTGGTGAAGGTGATGCTGGATCCGAAAGAATTCAACACCTTTGTCCCGCTGGGAACGGCGGCGTTAACCA
ATCCGGCTTTTGGCGCTGATATCTACTGGCGCGGGCGCGTATGGGTGGATCAGTTCTGGTTTGGTCTGAAAGGGATGGAG
CGTTACGGTTATCGTGATGATGCCCTGAAGCTGGCGGATACGTTCTTCCGGCACGCCAAAGGATTAACCGCCGATGGCCC
GATTCAGGAGAATTACAACCCGCTCACTGGCGCGCAGCAAGGCGCACCAAATTTCTCCTGGAGTGCCGCGCATTTGTATA
TGTTGTATAACGATTTTTTTCGTAAGCAGTAA
>Cluster_59312
ATGCAAAGGAAAACTCTATTGTCGGCCTGTATTGCATTAGCTCTGAGTGGTCAGGGTTGGGCGGCAGATATCACAGAGGT
AGAAACCACCACAGGTGAAAAGAAAAATACCAATGTGACTTGTCCGGCAGACCCAGGAAAACTCAGTCCGGAAGAGCTTA
AACGCTTACCCTCTGAATGCTCTCCTTTAGTCGAACAAAACCTGATGCCATGGCTTTCCACAGGCGCTGCTGCGTTAATC
ACGGCCTTAGCCGTAGTGGAACTAAACGACGATGATGATCATCATCATCGCAACAATTCTCCACTCCCACCGACACCCCC
TGATGATGAATCAGACGACACTCCAGTTCCCCCAACTCCTGGCGGAGATGAGATAATACCGGACGATCCGGATGATACGC
CTACACCTCCCAAACCGGTTTCGTTTAATAATGACGTTATTCTCGATAAAACAGAAAAAACGTTAACTATTCGCGATTCA
GTTTTTACTTATACCGAGAATGCTGACGGGACTATATCTCTGCAAGATAGCAATGGTCGTAAGGCAACGATTAATCTTTG
GCAGATTGATGAAGCGAATAACACTGTTGCCCTTGAAGGGGTGAGCGCAGATGGCGCAACGAAGTGGCAATATAATCACA
ACGGTGAGCTTGTTATTACGGGTGATAATGCCACAGTAAACAACAATGGCAAAACCACCGTTGACGGTAAAGATTCCACC
GGTACGGAAATCAACGGTAATAACGGGAAAGTGATTCAGGACGGCGATCTGGATGTCAGCGGCGGCGGTCACGGTATTGA
TATCACCGGTGACAGCGCGACGGTGGATAACAAGGGCACCATGACCGTCACCGATCCGGAGTCCATGGGTATCCAGATCG
ACGGTGACAAGGCCATCGTCAATAACGAAGGCGAGAGCACCATCACCAACGGTGGCACCGGCACCCAGATTAATGGTGAT
GACGCCACGGCAAACAACAACGGCAAAACCACCGTTGACGGCAAGGATTCCACCGGTACGGAAATCAACGGTAATAACGG
AAAAGTTATTCAGGACGGCGATCTGGATGTCAGCGGCGGCGGTCACGGTATTGATATCACCGGCGACAGCGCAACGGTGG
ATAACAAGGGCACCATGACCGTCACCGATCCGGAGTCCATCGGTATCCAGGTTGACGGCGACCAGGCGGTTGTTAACAAC
GAAGGCGAGAGCGCCATCACCAACGGTGGCACCGGCACGCAGATTAACGGTGATGACGCCACGGCAAACAACAACGGCAA
AACCACCGTTGACGGCAAGGATTCCACCGGTACGGAAATTGCTGGCAATAACGGGAAGGTGATTCAGGACGGCGATCTGG
ATGTCAGCGGCGGCGGTCACGGTATTGATATCACCGGCGACAGCGCAACGGTGGATAACAAGGGCACCATGACCGTCACC
GATCCGGAGTCCATCGGTATCCAGATTGACGGCGACCAGGCCATCGTCAATAACGAAGGCGAGAGCACTATCACCAATGG
CGGCACCGGCACTCAGATCAACGGTAACGACGCCACCGCGAATAACAGTGGAAAAACCACTGTTGATGGAAAAGATTCCA
CGGGTACCAAAATCGCGGGCAATATCGGCATTGTAAATCTGGATGGTAGCCTGACTGTTACAGGCGGTGCGCATGGTGTT
GAGAACATTGGTGACAACGGCACGGTTAACAACAAAGGAGATATTGTTGTTTCCGATACTGGATCGATTGGCGTGCTCAT
CAACGGTGAGGGGGCAACAGTATCCAATACGGGTGATGTTAACGTTAGCAATGAAGCGACAGGGTTCAGCATCACAACCA
ACAGTGGGAAGGTTTCGCTGGCAGGCAGTATGCAGGTTGGCGATTTCTCGACCGGGGTAGATCTTAATGGCAACAATAAC
AGCGTGACGCTGGCGGCAAAAGATCTAAAAGTGGTCGGGCAGAAAGCGACGGGCATAAACGTTTCTGGCGATGCGAATAC
AGTGAATATCACTGGTAACGTTCTGGTTGATAAGGATAAAACCGCAGACAATGCGGCGGAATATTTCTTCGATCCATCCG
TGGGTATCAACGTTTACGGCAGTGATAATAACGTGACGCTGGATGGAAAGTTAACTGTTGTATCAGACAGTGAGGTTACT
TCTCGTCAGAGTAATTTATTTGATGGCAGCGCAGAGAAAACGTCAGGTCTGGTTGTGATTGGCGATGGCAATACCGTTAA
TATGAATGGTGGACTTGAACTGATTGGAGAGAAAAACGCGCTTGCAGATGGGTCGCAGGTTACTTCCTTGCGCACAGGAT
ATAGTTATACCAGCGTTATTGTCGTTAGTGGTGAGTCGTCGGTATATCTGAATGGAGATACGACAATCAGCGGAGAATTC

CCTCTGGGGTTTGCCGGGGTTATTCGGGTACAGGATAAAGCTTTGCTGGAAATTGGCAGTGGCGCTACGCTAACAATGCA
GGATATTGACAGTTTTGAACATCATGGGACAAGAACCCTGGATTTGCCCCTATATTTCCAGACATCTGTTATCACTTAA
>Cluster_48628
ATGTGGTACAGGGAAGGTACTATCACATTTACACAGGGTAGTAATACTCTGGTTGGGGTTGGGACAGCCTGGAACGTAAC
AGCTAATGGTGTGTTGCCGGGGATGATCGTCATTGGCCCCGACAATAAGCTGTATGAGATCAAGCGCGTAACAAGCGATA
CGAACATTGTTCTCTCAGAACCTTATACCGGTGAAACTCAGTCTGAAGTTCCGTGCCGAATCATTACTACCTATGAAGGC
GACTTAACACAGTTTAGCGCGCGCTTTACCGCGCTAATGTCGCGTATGTCGGCTGATTCCAAGTCCATGCGTAGTTGGTT
GACTGCTCTGGATGAGGTGACAATCGAGCGTGAAGACGGTACAGAAGTGACCGTTAAGCCGCTGATGCAGATCGTCAACG
AGCACAACGAAAACGTTGAGTGGTATAAAAATAACACTGACGCGATTGATGCCGCTGGTGACAAAGCTCGTGAGGCGGCG
GCCAGTGCTGCCGCAGCAGCAGAAAGCGCCAATACCGCTGGAGAAAAAGCCTCTCAAGCGTCTCAAAGTGCATCCGCTGC
GGCATCCTCACAAAGTGCCGCAAGTGCGAGTGCAACTGCTGCGAAAAAATCTGAAACGAACGCAGAATCAGCATATCTAG
CGGCAGCAGAATCAGCGTCTGTAGCGTCGACCAAAGCATCGGAGGCAGCAACATCTGCGTCCAGTGCCTCTGCATCTAAG
GATGCAGCCAAAGCATCAGAGACTAACGCTTCATCGAGTGCAAACGCGGCTGCATCATTTGCAACAGAAGCTGAAAATTC
GTCTAAGGCAGCAAAAGCGTCAGAGACAAACGCAAAAGCATCTGAAACGGCAGCCGGGCAGAGTGCATCGTCTGCAAACA
GTTCGAAATCTGCGGCAGAAGCGTCAGCAAGCGCAGCCTCAGCAAGTGCGGATAGTGCTTCATCAAGTGCAACTGCGGCA
GGAAAATCTGCGGAAAGCGCGTCGTCGTCAGCGACAACAGCTACCGCAAAAGCGACTGAGGCGACGAATCAGGCTACAGC
CGCTAAAAATTCAGCATCGGCAGCAAAATCCTCTGAAACAAATGCAAAGGCTTCTGAAGATAATGCCGCTTCCTCTAAAG
AGGCAGCTGCTGAATCTGCAAGTTCTGCGTCTGCATCAGCCACCTCGGCATCCGCTTCAAAAGATGAGGCGACCAAGCAG
GCATCAGCTGCGAAGGGGAGTGCTTCAACGGCATCCACGAAAGCAACAGAGGCGGCAGGCAGTGCGACGGCTGCATCTCA
GAGCAAAGCTGCTGCTGAATCCGCTGCGACCCGTGCAGAAGCTGCCGCTGATCGTGCTGAAGAGATTGCCGGTGCCGTTG
CGATGGAAGACGCAAGCCTTACCACCAAAGGTGTTGTGAAACTTAGCAGTGCTGTCGACAGCACCAGTGAATCGCTGGCC
GCAACGCCAAAAGCAGTTAAAGCAGCCAATGACAATGCGAATAGCAGGGTGCCATCTAACCGAAAAGTTAACGGTAAAGC
ACTGACTGCGGATATCACATTAACGCCGAAAGATATTGGTACTTTAAATTCAGTAACGATGTCTTTCTCTGGCGGGGCTG
GGTGGTTCAAACTGGCTACGGTTACCATGCCACAAGCGAGTTCCATCGTTTACATCGCATTGATTGGTGGCGCTGGTTAC
AACGTCGGCTCCCCACATCAGGCAGGCATTTCAGAACTGGTTCTACGAGCAGGCAATGGAAACCCCAAAGGGATTACCGG
TGCTTTGTGGAAGCGTACAGCCGTCGGATTAACGAATTTCGCCTGGATCAACACATCCGGCGATACATATGATATTTACG
TTGAGATTGGCAATTATGCGACTAGTGTAAATATCCATTGGGATTGTACTGCAAATGCGACAGTTTCTATTTATACATCG
CCAACATATTCAGCGAGTAAGCCTTCCAGCGTTACCGATGGTGTTGTTTATACGATGTATAGCACACATCAGAAACCGAC
GCCGTTAGATATTGGAGCACTGCCAACAACCGGAGGAACAGTTTCAGGTCCGTTGTCTGTTACTGGTGGGATCACCGGAA
CATTAAATGGTAATGCAAGTACAGCAACGAAATTGCAGACGGCAAGATCTATCGGTGGAGTTGGTTTCGACGGTTCTGCA
AATATCAACCTTCCAGGTGTAAATACTACGGGTAATCAGAACACCACTGGTAATGCTGCAACTGCTACAAAACTTCAGAC
GGCAAGAACTATCGGCGGCGTGAGCTTTGATGGTACTGCGAATATTAATTTGCCAGGTGTTAATACGACTGGTAATCAGA
ATACAACGGGCAACGCGGCTACTGCAACGAAGTTGCAGACTGCGCGTACTATCAATGGGGTGTCGTTTGACGGCTCGGCA
AATATTTCCTTGTCGCCAGCAAATATAGGTTGCCCGGCATCTCCTACTGGTTGGTTAACTACAGGAAGTAATGGCGGAGC
AATAACAACAGCACAGTTAGTGACGTTATTGCAAAATAATGGAGCATTTAACACAAAGTGCGTGGGCCTATGCCAATAG
>Cluster_1363
ATGTATTCAAGTAGCAGAAAAAGGTGCCCGAAAACCAAATGGGCTTTGAAACTTCTTACTGCCGCATTTTTAGCAGCGAG
TCCCGCGGCGAAGAGTGCTGTTAATAACGCCTATGATGCATTGATTATTGAAGCTCGCAAGGGTAATACTCAGCCAGCTT
TGTCATGGTTTGCACTAAAATCAGCACTCAGCAATAACCAAATTGCTGACTGGTTACAGATTGCCTTATGGGCCGGGCAA
GATAAACAGGTTATTACCGTTTACAACCGCTACCGTCATCAGCAATTACCAGCGCGTGGTTATGCAGCTGTCGCCGTCGC
TTATCGTAACCTGCAACAATGGCAAAACTCGCTTACACTGTGGCAAAAGGCGCTCTCTCTGGAGCCGCAAAATAAGGATT
ATCAACGGGGACAAATTTTAACCCTGGCAGATGCTGGTCACTATGATACTGCGCTGGTTAAACTTAAGCAGCTTAACTCT
GGAGCACCGGACAAAGCCAATTTACTCGCAGAAGCCTATATCTATAAACTGGCGGGGCGTCATCAGGATGAATTACGGGC
GATGACAGAGTCATTACCTGAAAATGCATCTACGCAACAATATCCCACAGAATACGTGCAGGCATTACGTAATAATCAAC
TTGCTGCCGCGATTGACGATGCCAATTTAACGCCAGATATTCGCGCTGATATTCATGCCGAACTGGTCAGACTGTCGTTT
ATGCCTACGCGCAGTGAAAGTGAACGTTATGCCATTGCCGATCGCGCCCTCGCCCAATACGCTGCATTAGAAATTCTGTG
GCACGATAACCCAGACCGTACTGCCCAGTACCAGCGTATTCAGGTTGATCATCTTGGCGCGTTATTAACTCGCGATCGTT
ATAAAGACGTTATTTCTCACTATCAGCGATTAAAAAAGACGGGGCAAATTATTCCGCCCTGGGGGCAATATTGGGTTGCA
TCGGCTTATCTCAAAGATCATCAGCCGAAAAAAGCACAGTCAATAATGACCGAGCTCTTTTATCACAAGGAGACCATTGC
CCCGGATTTATCCGATGAAGAACTTGCGGATCTCTTTTACAGCCACCTGGAGAGTGAAAATTATCCGGGCGCGCTAACTG
TCACCCAACATACCATTAATACTTCGCCGCCTTTCCTTCGGTTAATGGGCACGCCTACGAGCATCCCGAATGATACCTGG
TTACAGGGGCATTCGTTTCTCTCAACCGTAGCAAAATATAGTAATGATCTTCCTCAGGCTGAAATGACAGCCAGAGAGCT
TGCTTATAACGCACCAGGAAATCAGGGACTGCGCATTGATTACGCGAGTGTGTTACAAGCCCGCGGTTGGCCTCGTGCAG
CAGAAAATGAATTAAAAAAAGCAGAAGTGATCGAGCCACGTAATATTAATCTGGAGGTTGAACAAGCCTGGACAGCATTA
ACGTTACAAGAATGGCAGCAGGCAGCTGTCTTAACGCACGATGTTGTCGAACGTGAACCGCAAGATCCCGGCGTTGTACG
ATTAAAACGTGCGGTTGATGTACATAATCTTGCAGAGCTTCGTATCGCTGGCTCAACAGGAATTGATGCCGAAGGCCCGG
ATAGTGGTAAACATGATGTCGACTTAACCACCATCGTTTATTCACCACCGCTGAAGGATAACTGGCGCGGTTTTGCTGGA
TTCGGTTATGCCGATGGACAATTTAGCGAAGGAAAAGGGATTGTTCGCGACTGGCTTGCGGGTGTTGAGTGGCGGTCACG
TAATATCTGGCTCGAGGCAGAGTACGCTGAACGCGTTTTCAATCATGAGCATAAACCCGGCGCGCGCCTGTCTGGCTGGT
ATGATTTTAATGATAACTGGCGTATTGGTTCGCAACTGGAACGCCTCTCTCACCGCGTTCCATTACGGGCAATGAAAAAT
GGTGTTACAGGCAACAGTGCTCAGGCTTATGTTCGCTGGTATCAAAATGAGCGGCGTAAGTACGGTGTCTCCTGGGCTTT
CACTGATTTTTCCGACAGTAACCAGCGTCATGAAGTCTCACTTGAGGGTCAGGAACGCATCTGGTCTTCACCATATTTGA
TTGTCGATTTCCTACCCAGTCTGTATTACGAACAAAATACAGAACACGATACCCCATACTACAACCCTATAAAAACGTTC
GATATTGTTCCGGCATTTGAGGCAAGCCATTTGTTATGGCGAAGCTATGAAAATAGCTGGGAGCAAATATTCAGCGCAGG
TGTTGGTGCCTCCTGGCAAAAACATTATGGCACGGATGTCGTCACCCAACTCGGCTACGGGCAACGCATTAGCTGGAATG
ACGTGATTGATGCTGGCGCAACGCTACGCTGGGAAAAACGACCTTATGACGGTGACAGAGAACACAACTTATACGTTGAA
TTCGATATGACATTCAGATTTTAA
>Cluster_47781
ATGATGACGACCAGAATAGTGGTTGGCCTCACGGCAGGGACGTGTCTGATTTTCTCGCAAAACCTGATGGCCGAGGTCAG
TGTATTCAATCCGGCGCTTCTGGAAATCGACCATCAATCCGGAGTCGATATTCGCCAGTTTAATCGGGCAAACCTGATGC
CCCCAGGTGTTTATAGCGTTGATATTTTTATCAACGGTAAAATGTTTGAACGTCAGGATGTGACATTTGTTCAGGATAAT
CCAGATGCTGATCTGCACGCTTGCTTTATTGCCATTAAAAAAACACTGTCCTCCTTTGGCATAAAAGTTGATGCGCTCAA
ATCGTTCAATGATGTGGATGAGACGGTTTGCCTCGATCCTGCTCCACGTATTGAAGGCTCATCCTGGCAGTTTGACAGTG
ATAAATTGCAGCTGAATATATCCATTCCCCAAATCTACATGGACGCGATGGCTTATGATTACATCAGCCCCACGCGTTGG
GATGAGGGGATTAACGCGCTCACCATCAACTACGATTTTTCTGGTTCACATACACTACGTTCAGATTATGGTTCACAAGA
GACAGATACCAGTTATCTCAATCTGCGCAATGGACTGAATATTGGACCGTGGCGGCTACGTAATTACAGTACTTTAAACA
CCAGCGATGGCCGTGCGGAATACAACTCCATTAGTACCTGGATACAGCGCGATATTGCCGCGTTAAGAAGCCAGATTATG
ATTGGTGATACGTGGACGGCGAGCGATATTTTCGACAGTACGCAAATTCGCGGCGCGCGTTTGTATACTGATAACGATAT
GCTACCCGCCAGCCAGAATGGCTTTGCTCCTGTGGTTCGTGGGATTGCAAAGTCCAACGCCACCGTCATCATTCGGCAGA

ATGGCTACGTGATTTATCAGTCAGCCGTTCCACAAGGTGCTTTTGAGATCACCGATCTCAACACCGCCAGTACAGGTGGC
GATTTGGACGTAACCATCAAAGAAGAAGACGGTAGCGAACAACGATTCACCCAACCTTATGCTTCATTGGCGATTCTTAA
ACGTGAAGGTCAGACAGATGTTGATGTCAGCGTGGGTGAATTGCGCGATGAAGACGGATTTACACCGGACGTCCTTCAGG
CGCAAATACTTCATGGTTTTTCCCACGGGATCACTTTATATGGAGGTATGCAGGCTGCTGAAAATTATGGTTCTGCAGCT
CTGGGTGTCGGTAAAGATCTTGGCGCTTTGGGCGCAATTTCTTTCGATGTGACACATGCTCGTGCGAATTTTAGCCATGA
TGATACAGAAACGGGTCAGTCATATCGCTTTCTCTATTCAAAACGATTTGACGACACAGACACTAGCTTGCGCCTGGTTG
GCTATCGTTACTCCACCGAGGGCTACTATACCCTCAATGAGTGGGCATCGCGGCGCAACAGCCCTGAAGACTTTTGGGAA
ACAGGTAACCGACGTAGTCGCGTGGAGGGAACGCTAACGCAGTCGTTGGGGAGAGATTATGGCAATTTATACCTGACATT
AAGCCGGCAACAATACTGGCATACCGATGATGTCGAACGATTAATGCAATTTGGCTACAGCAGTAGCTGGAAGCGTCTCT
CGTGGAACGTCTCCTGGAGTTATTCCAATACTGCCAGACAGGGGACGGGGAACAACCATGCCAGTGATAACACCAGTGAG
CAGATCTACATGCTCTCTTTATCTGTTCCTTTATCGGGCTGGTGGGGTAATAGTTACGCCACCTATTCTGTTTCGCAAAA
CGATAATTCCGGTAGCTCACATCAACTCGGACTCAGCGGTACGGCGCTGGAAAGAAATAACCTTTCATGGAATTTAATGC
AGTCCTATAACAGTCATGATGATGAGGTTGGCGGTAATATGTCCCTGACCTATGATGGCTCTTATGGCACGGTGAACGGC
AGCTATAACTACAGCCAAAATTCCCAGAGGCTGAATTATGGTATCAGAGGGGGAATTCTGGCACACAGCGAAGGGGTAAC
GTTAAGTCAGGAGTTAGGTGAAACTATTGCTCTTGTTAAAGCACCTGGGGCCGCCGGGTTAGAAATAGATAATATGCGCG
GTGCTGCGACGGACTGGCGCGGCTATACGGTCAAGACACAGCTAAACCCTTATGATGAAAATCGGGTAGCAATCAGCGAT
AACTATTTCTCGAAGTCGAATATAGAACTTGATAATACCGTCGTTACGATGGTTCCCACGCGTGGTGCAGTGGTTAAAGC
GGAGTTTGTGACTCATGTGGGTTATCGCGTTCTCTTCAGAGTGTTAAATGCAAATGGTAAACCGGTACCTTTTGGAGCCA
TTGCTGCGATACAAGATGCAAGTTTGGCAGATTCAGGAATTGTCGGTGACCGTGGCGAACTTTATCTTTCTGGTCTACCA
GAAAAAGGACAGGTTACGTTATCCTGGGGAGAAAACGCCTCAACAAAATGCATCTTCAATTATTCACTTTCGACACCAGA
AAGTGAGAGCGGATTAATTGAACAGGGTGTGACATGTCATTAA
>Cluster_25315
ATGAACAAGAAGATTCATTCCCTGGCCTTGTTGGTCAATCTGGGGATTTATGGGGTAGCACAGGCACAAGAGCCGACCGA
TACTCCTGTTTCACATGACGATACTATTGTTGTTACCGCCGCCGAGCAGAACTTGCAGGCGCCCGGCGTTTCGACCATCA
CCGCAGATGAAATCCGCAAAAACCCGGTTGCCCGCGATGTATCGGAGATTATCCGTACCATGCCTGGCGTTAACCTGACC
GGTAATTCCACCAGTGGTCAGCGTGGTAATAACCGCCAGATTGATATTCGCGGCATGGGTCCGGAAAACACGCTGATTTT
GATTGACGGCAAGCCGGTAAGCAGCCGTAACTCTGTGCGTCAGGGCTGGCGTGGCGAGCGCGATACCCGTGGTGATACCT
CCTGGGTGCCGCCTGAAATGATTGAACGTATTGAAGTTCTACGTGGTCCGGCAGCTGCGCGTTATGGCAACGGCGCGGCG
GGCGGCGTGGTTAACATCATTACCAAAAAAGGCAGCGGTGAGTGGCACGGCTCCTGGGATGCTTATTTCAATGCGCCAGA
ACATAAAGAGGAAGGTGCCACCAAACGCACCAACTTCAGCCTGACCGGTCCGCTGGGCGACGAATTCAGCTTCCGCTTGT
ATGGCAACCTCGACAAAACCCAGGCTGACGCGTGGGATATCAACCAGGGCCATCAGTCCGCGCGTGCCGGAACGTATGCC
ACGACGTTACCAGCCGGGCGCGAAGGGGTGATCAACAAAGATATTAATGGCGTGGTGCGCTGGGACTTCGCGCCTCTGCA
GTCACTCGAACTGGAAGCGGGCTACAGCCGCCAGGGTAACCTGTATGCGGGTGATACGCAGAACACCAACTCTGACGCTT
ACACTCGATCGAAATATGGCGATGAAACCAACCGCCTGTATCGCCAGAACTACTCGCTGACCTGGAACGGTGGCTGGGAT
AACGGCGTGACCACCAGCAACTGGGTGCAGTACGAACACACCCGTAACTCGCGTATTCCGGAAGGTCTGGCGGGCGGTAC
CGAAGGGAAATTTAACGAAAAAGCGGCACAGGATTTTGTAGATATCGATCTTGATGACGTGATGCTGCACAGCGAAGTTA
ACCTGCCGATTGATTTCCTCGTTAACCAAACGCTGACGCTGGGTACAGAGTGGAATCAGCAACGGATGAAGGACTTGAGT
TCCAACACCCAGGCACTGACCGGAACGAATACCGGCGGTGCTATTGATGGCGTGAGTGCCACCGACCGTAGCCCATATTC
AAAAGCAGAAATTTTCTCGCTGTTTGCCGAAAACAATATGGAGCTGACTGACAGCACCATCGTAACGCCGGGGCTGCGTT
TCGATCATCACAGTATTGTCGGCAATAACTGGAGCCCGGCGCTGAACATATCGCAAGGTTTAGGCGATGACTTCACGCTC
AAAATGGGCATCGCCCGCGCCTATAAAGCGCCGAGCCTGTACCAGACTAACCCAAACTACATTCTCTACAGTAAAGGTCA
GGGCTGCTATGCCAGCGCGGGCGGCTGCTATCTGCAAGGTAACGATGACCTGAAAGCAGAAACCAGCATCAACAAGGAGA
TTGGTCTGGAGTTCAAACGCGACGGCTGGCTGGCGGGCGTGACCTGGTTCCGTAACGATTATCGCAATAAGATTGAAGCG
GGCTATGTAGCTGTAGGAAAAAACGCAGTCGGCACCGATCTCTATCAGTGGGATAACGTGCCGAAAGCGGTGGTTGAAGG
TCTGGAAGGATCGTTAAACGTACCGGTTAGCGAAACGGTGATGTGGACCAATAACATCACTTATATGCTGAAGAGTGAAA
ACAAAACCACGGGCGACCGTTTGTCGATCATCCCGGAGTATACGTTGAACTCAACGCTGAGCTGGCAGGCACGGGAAGAT
TTGTCGATGCAAACGACCTTCACCTGGTACGGCAAACAGCAGCCGAAGAAGTACAACTATAAAGGTCAGCCAGCGGTTGG
ACCGGAAACCAAAGAAATCAGTCCGTACAGCATTGTTGGCCTGAGCGCGACCTGGGATGTGACGAAGAATGTCAGTCTGA
CCGGCGGCGTGGACAACCTGTTCGACAAACGTTTGTGGCGTGCGGGTAATGCCCAGACCACGGGCGATCTGGCAGGAGCC
AACTATATCGCAGGTGCCGGGGCGTATACCTATAACGAGCCGGGACGTACGTGGTATATGAGCATTAATACTCACTTCTG
A
>Cluster_42706
TTGTATCTGTTTGAAAGCCTGAATCAGCTGATTCAAAACTACCTGCCGGAAGACCAAATCAAGCGTCTGCGGCAGGCGTA
TCTCGTTGCACGTGATGCTCACGAGGGGCAAACACGTTCAAGCGGTGAACCCTATATCACGCACCCGGTGGCGGTTGCCT
GCATTCTGGCCGAGATGAAACTCGACTATGAAACGCTGATGGCGGCGCTGCTGCATGACGTGATTGAAGATACTCCCGCC
ACCTACCAGGACATGGAACAGCTTTTTGGTAAAAGCGTCGCCGAGCTGGTAGAGGGGGTGTCGAAACTTGATAAACTCAA
GTTCCGCGATAAGAAAGAGGCGCAGGCCGAAAACTTTCGCAAGATGATTATGGCGATGGTGCAGGATATCCGCGTCATTC
TCATCAAACTTGCCGACCGTACCCACAACATGCGCACGCTGGGCTCACTTCGCCCGGACAAACGTCGCCGCATCGCCCGT
GAAACTCTCGAAATATACAGCCCGCTGGCGCACCGTTTAGGTATCCACCACATTAAAACCGAACTCGAGGAGCTGGGTTT
TGAGGCGCTGTATCCCAACCGTTATCGCGTAATCAAAGAAGTGGTGAAAGCCGCGCGCGGCAACCGTAAAGAGATGATCC
AGAAGATTCTTTCTGAAATCGAAGGGCGTTTGCAGGAAGCGGGAATACCGTGCCGCGTCAGTGGTCGCGAAAAGCATCTT
TATTCGATTTACTGCAAAATGGTGCTCAAAGAGCAGCGTTTTCACTCAATCATGGACATCTACGCTTTCCGCGTGATCGT
CAATGATTCTGACACCTGTTATCGCGTGCTGGGCCAGATGCACAGCCTGTACAAGCCGCGTCCGGGCCGCGTGAAAGACT
ATATCGCCATTCCAAAAGCGAACGGCTATCAGTCTTTGCACACCTCGATGATTGGCCCGCACGGCGTGCCGGTTGAGGTC
CAGATCCGTACCGAAGATATGGATCAGATGGCGGAGATGGGTGTTGCCGCGCACTGGGCTTATAAAGAGCACGGCGAAAC
CAGCACTACCGCGCAAATACGCGCCCAGCGCTGGATGCAAAGCCTGCTGGAGCTGCAACAGAGTGCCGGTAGCTCGTTTG
AATTTATCGAGAGCGTTAAATCCGATCTCTTCCCGGATGAGATTTACGTTTTCACACCAGAAGGGCGCATTGTCGAACTG
CCTGCCGGTGCAACGCCCGTCGACTTCGCTTATGCGGTGCATACCGATATCGGCCATGCCTGCGTGGGCGCACGCGTCGA
CCGCCAGCCTTACCCGCTGTCGCAGCCGCTTACTAGCGGTCAAACCGTTGAAATCATTACCGCACCGGGTGCTCGCCCGA
ATGCCGCGTGGCTGAACTTTGTCGTCAGCTCGAAAGCGCGCGCCAAAATTCGTCAGTTGCTGAAAAACCTCAAGCGTGAT
GATTCTGTAAGCCTGGGCCGTCGTCTGCTCAACCATGCGTTGGGTGGTAGCCGTAAGCTCAATGAAATCCCGCAGGAAAA
TATTCAGCGCGAGCTGGACCGCATGAAGCTGGCAACGCTTGACGATCTGCTGGCAGAAATCGGCCTTGGTAACGCAATGA
GCGTGGTGGTCGCGAAAAATCTGCAACATGGGGACGCCTCCATTCCACCGGCAACCCAAAGTCACGGACATCTGCCCATT
AAAGGTGCCGATGGCGTGCTGATCACCTTTGCGAAATGCTGCCGCCCAATTCCTGGCGACCCGATTATCGCCCACGTCAG
CCCCGGTAAAGGTCTGGTGATCCACCATGAATCCTGCCGTAACATCCGTGGCTACCAGAAAGAGCCAGAGAAGTTTATGG
CTGTTGAATGGGATAAAGAGACGGCGCAGGAATTCATCACCGAAATCAAGGTGGAGATGTTCAATCATCAGGGCGCGCTG
GCAAACCTGACGGCGGCAATTAACACCACGACCTCGAATATTCAAAGTTTGAATACGGAAGAGAAAGATGGTCGCGTCTA
TAGCGCCTTTATTCGTCTGACCGCCCGTGACCGTGTGCATCTGGCGAATATCATGCGCAAAATCCGCGTGATGCCAGACG
TGATTAAAGTCACCCGAAACCGAAATTAA

>Cluster_42098
ATGGCTATGTTCACACCTTCATTCTCAGGACTCAAAGGTCGGGCGCTCTTTTCACTGCTTTTTGCGGCACCGATGATTCA
TGCAACAGACTCTGTAACGACCAAAGATGGCGAAACAATCACTGTTACAGCAGATGCAAATACCGCAACTGAGGCAACCG
ATGGTTATCAACCTCTGAGCACCTCTACGGCGACATTAACCGATATGCCGATGCTGGATATCCCGCAGGTGGTCAATACG
GTTAGCGATCAGGTTCTGGAAAACCAGAATGCGACAACGCTGGATGAGGCGCTTTATAACGTCAGTAACGTGGTACAGAC
CAATACATTAGGCGGGACTCAGGATGCTTTTGTACGCCGTGGGTTTGGCGCAAACCGGGATGGCTCCATCATGACCAACG
GCCTGCGAACTGTACTTCCTCGCAGTTTCAACGCCGCCACAGAACGTGTGGAAGTGCTAAAAGGTCCGGCCTCCACGCTG
TATGGCATTCTCGATCCTGGTGGATTGATTAACGTCGTGACCAAGCGCCCGGAAAAAACATTCCATGGTTCTGTCTCAGC
CACGTCCTCCAGTTTTGGCGGAGGCACTGGGCAACTTGATATCACAGGTCCCATTGAAGGCACTCAGCTGGCATACCGCC
TGACGGGGGAAGTGCAGGATGAAGATTACTGGCGAAACTTCGGTAAAGAGCGCAGTACATTTATTGCCCCGTCACTCACC
TGGTTTGGTGATAATGCAACAGTAACTATGCTCTATTCCCATCGGGACTATAAAACTCCGTTCGATCGTGGAACGATTTT
CGACCTTACGACGAAACAGCCCGTAAACGTTGATCGAAAAATACGTTTGACGAACCGTTTAATATTACAGATGGAAAGT
CCGATCTGGCGCAACTCAACGCAGAATATCATCTCAATAGCCAGTGGACAGCGCGCTTTGATTACAGCTACAGCCAGGAT
AAATACAGCGATAATCAGGCGCGTGTTACCGCGTATGATGCAACGACAGGAACACTGACACGGCGTGTTGATGCAACTCA
GGGATCTACCCAGCGTATGCATGCTACTCGTGCGGATCTGCAAGGGAATGTTGATATTGCCGGATTCTATAATGAGATTC
TGGGTGGGGTGTCATATGAATATTATGATCTTCTGCGTACAGATATGATTCGCTGTAAAAAAGCTAAAGATTTCAATATC
TACAACCCTGTTTATGGTAATACCAGCAAATGTACAACGGTTTCGGCGTCGGACAGTGATCAGACGATCAAACAGGAGAG
CTACTCAGCGTATGCACAGGACGCACTCTATCTGACCGATAACTGGATTGCCGTCGCCGGGATCCGCTATCAGTATTACA
CGCAGTATGCGGGTAAAGGCCGTCCTTTTAATGTCAATACTGACAGCCGCGATGAACAATGGACTCCCAAACTGGGGTTA
GTCTACAAACTGACGCCATCGGTATCCTTATTTGCCAATTATTCGCAAACATTTATGCCGCAGTCGTCAATTGCCAGCTA
CATTGGCGATCTTCCACCGGAATCATCTAATGCTTACGAAGTCGGGGCAAAATTCGAGCTATTCGATGGTATCACCGCAG
ATATTGCGCTGTTTGATATCCATAAACGTAACGTGTTGTATACCGAAAGTATTGGTGATGAAACCATTGCCAAAACGGCA
GGCCGTGTTCGTTCAAGAGGGGTAGAAGTCGACCTTGCGGGAGCATTAACTGAAAACATTAATATCATTGCCAGCTACGG
CTATACCGATGCTAAGGTTCTGGAGGATCCTGATTATGCAGGGAAACCATTGCCGAATGTTCCTCGTCATACCGGTTCGC
TATTCCTGACCTATGACATTCATAACATGCCAGGCAATAACACACTGACGTTTGGCGGTGGTGGACATGGCGTAAGCCGT
CGTTCGGCAACCAATGGGGCTGACTATTATCTGCCAGGCTATTTCGTTGCCGATGCCTTCGCCGCATACAAAATGAAATT
GCAGTATCCGGTCACACTGCAATTAAACGTCAAAAACCTGTTTGATAAAACGTATTACACCTCTTCCATCGCCACAAATA
ATCTGGGCAACCAGATTGGCGATCCTCGTGAAGTGCAATTCACGGTGAAAATGGAATTTTGA
>Cluster_29115
ATGAAAAAAAGACATCTGCTTAGCTTACTGGCGCTGGGCATTAGCACAGCTTGCTACGGCGAAACATATCCTGCGCCCAT
TGGTCCGTCGCAATCGGATTTCGGTGGCGTAGGATTATTACAAACGCCCACCGCGCGCATGGCGCGGGAAGGGGAGCTGA
GCCTGAACTATCGCGATAACGATCAGTACCGTTATTACTCAGCTTCAGTGCAACTCTTCCCGTGGCTGGAAACAACGCTG
CGCTACACCGACGTGCGCACCCGGCAGTACAGCAGCGTCGAAGCGTTCTCTGGCGATCAAACGTATAAAGATAAAGCCTT
CGATCTCAAACTGCGTTTGTGGGAAGAGAGTTATTGGCTGCCGCAAGTGGCGGTTGGTGCGCGGGATATCGGCGGTACGG
GGCTGTTTGATGCGGAATATCTTGTTGCCAGCAAAGCCTGGGGGCCGTTCGATTTTACACTCGGCCTGGGCTGGGGATAT
CTGGGCACCAGCGGTAACGTGAAAAATCCGCTCTGTTCAGCCAGTGATAAATATTGCTATCGCGATAACAGCTACAAACA
GGCGGGATCTATCGACGGTAGCCAGATGTTCCACGGCCCGGCCTCACTGTTTGGCGGCGTGGAATACCAGACGCCCCTTGC
AACCGCTGCGCCTGAAACTGGAGTATGAAGGCAATAATTATCAGCAGGATTTTGCCGGGAAGCTGGAGCAAAAAAGTAAG
TTTAACGTCGGGGCGATTTATCGCGTTACCGATTGGGCCGACGTTAACCTTAGCTATGAACGTGGCAACACCTTTATGTT
TGGCGTCACGCTGCGCACCAACTTTAACGATCTACGTCCTTCGTACAACGATAACGCCCGCCCGCAATATCAACCGCAGC
CGCAGGATGCCATTTTGCAGCATTCGGTGGTGGCGAATCAGTTAACGCTGTTGAAATACAATGCCGGACTTGCCGATCCG
CAGATCCAGGCGAAAGGCGATACGCTGTATGTCACCGGCGAGCAGGTGAAATATCGTGATTCGCGCGAAGGGATCATCCG
TGCTAATCGGATCGTGATGAACGATCTGCCGGATGGGATCAGAACGATCCGCATTACGGAAAATCGTCTTAACATGCCGC
AGGTGACGACGGAAACCGATGTCGCCAGCCTGAAAAATCATCTCGCCGGAGAGCCGTTGGGCCACGAAACGACGCTGGCG
CAAAAACGCGTCGAGCCAGTGGTTCCGAAATCCACCGAGCAGGGCTGGTATATCGACAAATCACGCTTTGATTTCCATAT
CGATCCGGTGCTGAACCAGTCGGTCGGTGGCCCGGAAAACTTTTACATGTATCAGCTGGGCGTGATGGGAACGGCAGATT
TGTGGCTGACGGACCATCTACTGACCACCGGCAGCCTGTTTGCAAATCTTGCCAACAACTACGACAAGTTTAACTACACC
AATCCACCGCAGGACTCGCACTTACCGCGCGTTCGTACCCATGTGCGCGAGTATGTGCAGAACGATGTCTATGTGAATAA
CCTGCAAGCCAACTACTTCCAGCATCTGGGCAACGGCTTCTACGGTCAGGTCTATGGAGGTTATCTCGAAACCATGTTTG
GCGGTGCGGGGGCCGAAGTGTTGTATCGTCCGCTGGACAGTAACTGGGCGTTTGGTCTGGATGCCAACTACGTTAAACAG
CGCGACTGGCGTAGTGCAAAAGATATGATGAAATTCACCGACTACAGCGTGAAAACCGGACATCTGACCGCGTACTGGAC
GCCATCTTTCGCCCAGGACGTGTTGGTGAAAGCCAGCGTCGGGCAGTATCTGGCAGGGGATAAAGGCGGCACGCTGGAGA
TCGCTAAACGCTTTGACAGCGGCGTGGTGGTGGGTGGCTATGCCACGATCACCAACGTTTCGAAAGAGGAGTACGGCGAA
GGGGACTTCACCAAAGGCGTGTATGTCTCTGTACCGCTGGATCTCTTCTCGTCTGGTCCGACACGCAGCCGTGCGGCAAT
TGGCTGGACGCCGCTGACGCGTGACGGTGGTCAGCAACTTGGGCGTAAGTTCCAGTTGTATGACATGACCAGCGACCGTA
GCGTCAATTTCCGCTAA
>Cluster_44179
ATGAACAAATCAGGGAAATACCTCGTCTGGACAGTGCTCTCTGTAATGGGAGCATTTGCTCTGGGATACATTGCTTTAAA
TCGTGGGGAACAGATCAACGCGCTGTGGATTGTGGTGGCGTCGGTCTGTATCTATCTGATCGCTTACCGTTTTTATGGGC
TGTATATCGCCAAAAATGTGCTGGCGGTTGACCCGACGCGTATGACGCCAGCGGTGCGCCATAACGACGGGCTGGACTAT
GTGCCGACGGACAAGAAAGTGCTGTTCGGTCACCATTTTGCGGCCATTGCCGGAGCAGGTCCGTTGGTTGGGCCGGTACT
GGCGGCGCAAATGGGTTACCTGCCAGGGATGATCTGGCTGCTCGCCGGGGTGGTTCTCGCCGGTGCGGTGCAGGATTTCA
TGGTGCTGTTTGTTTCTACGCGCCGTGACGGTCGATCGCTGGGTGAGCTGGTCAAAGAAGAGATGGGGCCAACCGCCGGG
GTGATAGCGCTGGTGGCCTGCTTTATGATCATGGTCATTATCCTTGCAGTACTGGCGATGATCGTGGTGAAAGCCCTGAC
TCATAGCCCGTGGGGAACGTATACCGTTGCGTTCACCATTCCGCTGGCGCTGTTTATGGGGATCTACCTGCGCTATCTGC
GTCCGGGGGCGTATTGGTGAAGTGTCGGTCATCGGTCTGGTATTCCTGATTTTCGCCATTATCTCTGGCGGCTGGGTGGCA
GAAAGTCCGACCTGGGCACCGTACTTTGACTTTACCGGCGTGCAGCTGACCTGGATGCTGGTGGGTTACGGTTTTGTGGC
GGCGGTGCTGCCGGTGTGGTTGCTGCTGGCCCCGCGTGACTACCTCTCTACCTTCCTGAAAATCGGGACGATCGTCGGTC
TGGCGGTAGGCATTTTGATTATGCGCCCGACGCTGACCATGCCTGCGCTGACCAAATTTGTCGATGGCACTGGCCCGGTA
TGGACCGGTAACCTGTTCCCGTTCCTGTTTATCACCATCGCCTGTGGCGCGGTGTCTGGCTTCCATGCGCTGATCTCTTC
CGGCACCACGCCGAAGATGCTGGCGAACGAAGGGCAGGCTTGTTTTATCGGCTACGGTGGAATGTTGATGGAATCCTTCG
TGGCGATTATGGCGCTGGTTTCCGCCTGTATCATCGATCCGGGCGTTTACTTTGCCATGAACAGCCCAATGGCGGTGCTG
GCTCCGGCAGGGACGGCGGATGTGGTCGCTTCTGCCGCGCAGGTGGTGAGTAGCTGGGGCTTTAGCATTACACCAGATAC
GCTCAACCAGATTGCCAGCGAAGTGGGTGAACAGTCGATCATTTCCCGTGCGGGCGGTGCGCCGACGTTGGCGGTGGGGA
TGGCTTACATCCTGCACGGTGCGCTGGGTGGTATGATGGATGTGGCGTTCTGGTATCACTTCGCCATTCTGTTTGAAGCT
CTGTTTATTCTGACGGCGGTAGATGCGGGTACGCGTGCTGCGCGCTTTATGTTGCAGGATCTGCTGGGCGTGGTGTCTCC
TGGCCTGAAGCGGACCGATTCACTGCCTGCTAACCTGCTGGCAACGGCGCTGTGCGTACTGGCGTGGGGCTACTTCCTCC
ATCAGGGCGTGGTCGATCCGCTGGGCGGTATTAACACTCTGTGGCCGCTGTTTGGTATTGCCAACCAGATGCTGGCAGGG
ATGGCGCTGATGCTCTGTGCCGTGGTGTTGTTCAAGATGAAACGTCAACGTTACGCCTGGGTGGCGCTGGTACCAACGGC

CTGGCTACTGATTTGTACCCTGACCGCAGGCTGGCAGAAAGCGTTTAGCCCGGATGCGAAAGTCGGCTTCCTGGCCATTG
CTAACAAGTTCCAGGCAATGATCGACAGCGGCAATATTCCGTCGCAGTATACTGAGTCACAGCTGGCGCAACTGGTGTTC
AACAACCGTCTGGATGCCGGGTTAACCATCTTCTTTATGGTGGTCGTGGTGGTTCTGGCACTGTTCTCGATTAAGACGGC
ACTTGCGGCATTGAAAGAGCCGAAGCCAACGGCGAAAGAAACGCCGTATGAGCCAATGCCGGAAAATGTTGAGGAGATCG
TAGCGCAGGCGAAAGGTGCGCACTAA
>Cluster_61864
ATGATTACTCGTATTCCTCGTAGTTCTTTCTCTGCAAATATTAATAATACAGCCCAGACAAATGAACACCAAACCCTGAG
TGAATTGTTTTATAAAGAACTCGAGGATAAATTTTCTGGCAAGGAGCTGGCGACGCCTCTATTAAAAAGCTTCTCAGAGA
ACTGTAGACAAAATGGTCGTCATATCTTTAGCAACAAGGATTTTGTCATTAAATTTTCCACGTCCGTCTTACAAGCTGAT
AAGAAAGAAATTACGATAATTAATAAAAACGAAAACACGACACTTACTCAAACCATTGCCCCAATATTTGAAAAATACCT
AATGGAAATTTTACCTCAACGCTCAGACACTCTTGATAAACAAGAATTAAACCTAAAATCAGATAGAAAAGAAAAAGAAT
TCCCAAGAATTAAACTTAATGGTCAATGTTATTTTCCGGGGCGACCCCAAAACCGTATAGTATGCCGACACATTGCTGCA
CAATATATTAATGATATTTATCAGAATGTTGATTACAAACCCCATCAAGATGATTACTCTTCAGCTGAAAAATTTCTCAC
GCACTTCAACAAAAAATGCAAAAACCAGACTTTGGCGTTGGTTTCCAGCCGTCCTGAGGGGCGTTGCGTTGCTGCCTGCG
GTGATTTCGGGCTAGTTATGAAAGCATATTTTGACAAGATGGAATCAAATGGCATCAGTGTTATGGCAGCCATATTACTG
GTGGATAACCATGCTTTGACGGTCCGGCTAAGAATAAAGAACACAACTGAAGGATGTACCCATTACGTGGTTTCGGTTTA
TGATCCTAATGTAACTAACGATAAAATAAGAATTATGAGCGAAAGCAAAGAGAATATTAAACACTATTCTCTGATGGATT
TTATGAATGTAGATTATAGCCTCCTGAAATGGTCAAATGATCATGTTATTAATCAATCTGTTGCAATAATTCCAGCACTT
CCGAAAGAACAGCTATTGATGTTAAAAGGATCTGTGGATGAAATAACCCCTCCATTATCACCTGCAACGATGAATTTGCT
AATGGCAATTGGTCAGAATCACCAACTTACGCAACTGATGATTCAACTCCAGAAAATGCCAGAACTACATAGAACAGAAA
TGTTGACTGCCTATAATAGTATTAACCTGCCAGGTTTATATTTGGCTATAAATTATGGTAATGCGGATATCGTTGAGACT
ATTTTCAATTCATTGTCAGAAACAGGATATGAAGGATTACTCTCGAAAAAAAATCTCATGCATATTCTGGAGGCAAAAGA
TAAAAATGGTTTTTCTGGATTATTTTTAGCGATATCACGTAAGGATAAAAATGTTGTAACCTCGATTCTGAACGCCTTAC
CTAAACTGGCCGCAACACATCATTTAGATAACGAACAAGTGTATAAATTCCTGAGTGCCAAAAATAGAACGTCCAGCCAT
GTTTTATACCATGTTATGGCGAATGGTGATGCCGACATGCTGAAAATTGTTTTGAACGCGTTACCTTTGTTAATTCGCAC
ATGTCATTTGACTAAAGAACAGGTACTCGATCTCCTGAAGGCAAAGGATTTTTATGGTTGCCCAGGACTATATCTGGCGA
TGCAAAATGGACATAGCGATATCGTGAAAGTTATTCTTGAAGCATTGCCCAGCCTAGCCCAGGAAATTAACATTTCAGCT
TCCGATATTGTAGATCTTCTGACCGCTAAAAGTCTTGCGCGCGACACGGGTTTGTTTATGGCCATGCAGCGCGGACACAT
GAACGTTATTAATACTATTTTTAACGCATTACCCACTCTGTTTAATACGTTTAAATTCGATAAAAAAAAATATGAAGCCCC
TCCTCCTGGCAAATAATTCTAATGAATATCCCGGTTTGTTTTCAGCGATACAGCATAAACAACAAAATGTTGTAGAGACG
GTTTATCTTGCTTTATCTGACCATGCACGCCTGTTTGGATTTACCGCTGAAGATATTATGGATTTTTGGCAACACAAAGC
CCCACAAAAATACTCTGCCTTTGAGTTGGCTTTTGAATTTGGTCACCGGGTTATTGCTGAATTAATCCTTAATACATTAA
ATAAGATGGCTGAAAGCTTTGGCTTTACGGATAACCCTCGATACATTGCGGAGAAAAATTATATGGAAGCTTTACTCAAA
AAAGCATCTCCCCATACCGTACGCTAA
>Cluster_66981
ATGATCGAATATTTCGAATTGGGTGAGCGCCTCGATTCATCGGGGCGCGATAGCCTTTATCCACAAACGATTTCGCTGCT
TAAAGCATGCGAGAATCATCCTTACGTCACAGTCAGAGAATTACGATTTTCTGAAATAAATGATAACCGCTCGGAATATC
TAATCATTGATGCTGCTGATGGGACTGTGGCTTCTGGTAATCAAGCCCGTATTCGTCGTAAAGAACGATTGGCTATTGAG
GTCAATCCCAAATCAAGCATACCTATCTTGGTTCATGCATTGCGGAAAGATTTCCCTGTTCTTTCTCACCAGCATGCTGG
AGAACCTGGCTCACCACGAATTCTTTGCCTATATGAGGCCTCCTGGAGCGCAGTTGAGCGCAGTTGGACCCCCGAGAGAT
TCCTTGAGCGCATTTTTTGGTGGCTTAGAGAATCGGCCGAACTGCATCTACATCGAGAAGATCAGCCACTTGAGCAACTT
TTTTATTTGAGTCCCTATCAGCTCATTTTGCCAGCCAATTACCCCGATTATCATCACGCGACTGACAATAAGCTCAGTCT
CCAGATGGTCAGTGAGGGGCGACCGATTATTTTAAGGGCTGTACCAGAACAAGATACCTCAAGCGTTAAGCCATTTCGCT
TATTAACTATTGCTGTTCCTCCTGTCGATGTTTCTACGGTGGCTACATACCCGGATAATCTGGGAAAATTAGAAGAACAA
CTTAATGAATGGGGAAGTGAGCTCCTAAAACCACTAACTGACGCTGTTTATGAAGCAATACCAAGTGATGGTATCCGCCC
AACGTCTGGTAAAGGAGAAGGCTTACTTATCCTGCTTTGGATCCCCCGTCTAAGAAACGGAGAAACAGAACGTACGGATG
TGATGGGCTATGTTGTGCAAAGTTCACTCGGTGAACTTGCCACTGCACTAGATATGCTGGCGCCAAAAAATGAGCGAGGA
GTACAGCATCGCGTACGGCTTCTTGGCGGATCAATTAGTACCAAGTGGCGACAATTACCGCTTTTACCAGTTGAAATACG
TTCGGCTATGAAGGCCACACATGCGAGAGACATATCTGCTGTTGATTCAGAGAGTGCAGCTTTTCGAGGTATTTTAGCTG
GAGTTGGCGCTCTTGGTAGTACATTGGCTGATATTTGGATTCGAATGGGGTGGGGGACATGGACTTTTATTGATCCAGAT
CGGCTTCTTCCACATAATTTGTCTCGCCATATTGGGTTTGATTGTCATATTGGAGTCTCAAAACCCCATATTATTCAGCA
TATTGCCAAATCTATTTATCCGCATGAGCCCCTCCCTCAGGCGATCCATAAAAGTATTCTTGATGACGATGATGATATTG
CTAAGGCAATGAATGAGGCAGACTTAGTTGTGGATGTCAGTACCACCTTTGAGGTGCCGAGGACTCTAGCGTTAAAAGAT
GATATACCCAGAATAGTTAGCCTGTTTCTGACTCCATCGGGTCAGTCTAGCGTGATGTTAATGGAGGATGTAGACAGACA
ATGCCGAATTGATGCAATTGAGGGGCAATACTATCGGGCAATTTTGAGCAGTGAGTGGGGAAGTACTCACCTACAGCACA
ATTATGGCGATCGTTGGGTCGGTGGTGGATGTCGGGATATTTCTGTACGTATGTCCAGTGAATGTATTCATGTTCATGCC
GGTATCCTCTCACGCCAACTTCGCCAGACTGCACTCAAAGGAAATGCTCGATTGTGTATCTGGGTATCTGATGAAATATC
AGGCGCGATGGATGCACATGAAATTGAACTCTATTCCGTGGTATCTATAATTTCTGGCGAATGGGTTGTTAAATATGACC
AGGGCTTAGCGCAAAAATTGCAGCACACCCGCTTACAGGCACTGCCTAATGAAACAGGAGGTGCAATTGTTGGCATAACA
GATTTTAAGAACAAAACGATTATTCTGGTTGACGTGCTCCCTGAACCTATCGACAGTAAATCCTCTCCAGCCTCCTTTGT
CCGGGGAGAAGCGGGACAACAAGAAGCATTAGAACGGGTTCACCAGTTGACCGCTCGTGTTGTCGATTATGTTGGCGAAT
GGCACTCCCATCCACAAGGCTTTTCCGCAAAAGCCAGTAATGAAGATGATAATTTAATCAAAAAATTACACCAGAAAATG
CGTGTGGAGGGTTTACCTGCCGTGATGCTGATTGTTGCGGAAAACGATATCAATATTGTAGTCAGATAA
>Cluster_440
ATGTCAACCGGATTACGTTTCACGCTGGAAGTGGACGGCCTGCCACCGGATGCTTTTGCGGTGGTCTCCTTTCATCTGAA
CCAGTCACTCTCTTCGCTTTTTTCCCTCGATCTCTCTCTGGTCAGCCAGCAGTTTCTCTCCCTTGAATTCCAGCAGATCC
TCGACAAAATGGCCTACCTGACGATATGGCAGGGCGATGACGTACAGCGCCGGGTGAAAGGTATGGTGACCTGGTTTGAA
CTGGGGGAGAACGACAAAAACCAGATGCTGTACAGCATGAAGGTGTGCCCACCGCTGTGGCGCACAGGGCTGCGCCAGAA
CTTCCGTATCTTCCAGAATGAGGACATCGAAAGCATCCTCGGCACGATATTGCAGGAAAACGGGGTGACCGAGTGGAGCC
CGCTGTTCAGCGAGCCACATCCTTCCCGTGAGTTTTGTGTCCAGTACGGTGAGACTGATTACGATTTCCTGTGCCGGATG
GCGGCGGAGGAAGGCATCTTCTTTTATGAGGAGCACGCGCAAAAAAGTACCGACCAGAGCCTGGTCCTGTGCGATACCGT
GCGTTATCTGCCGGAGTCCTTTGAGATCCCCTGGAATCCGAACACCCGTACCGAGGTGAGCACCCTCTGCATCAGCCAGT
TTCGCTACAGCGCACAAATCCGCCCTTCTTCCGTGGTGACCAAAGACTACACCTTTAAACGACCCGGCTGGGCAGGGCGT
TTTGATCAGGAAGGCCAGTACCAGGATTACCAGCGCACACAGTATGAAGTGTATGACTACCCCGGACGTTTCAAGGGTGC
CCACGGGCAGAACTTTGCCCGCTGGCAGATGGATGGCTGGCGCAACAACGCAGAAGTGGCGCGCGGAACAAGCCGTTCGC
CGGAGATATGGCCGGGACGGCGAATTGTGCTGACGGGGCATCCGCAGGCGAACCTGAACCGGGAATGGCAGGTGGTGGCG
AGCGATCTTCACGGCGAACAGCCGCAGGCGGTACCGGGACGCAGGGGTTCAGGCACCACGCTGGATAACCACTTTGCGGT
AATCCCGGCAGACCGGACATGGCGGCCACAGCCGTTGCTGAAACCGCTGGTGGACGGCCCGCAGAGCGCCGTCGTGACGG
GACCGGCAGGCGAGGAAATCTTCTGTGATGAACATGGCCGCGTGCGGGTGAAATTTAACTGGGACCGCTATAACCCGTCA

AACCAGGAAAGTTCATGCTGGATCCGTGTGGCACAGGCGTGGGCAGGCACCGGCTTTGGTAACCTGGCGATACCGCGTGT
GGGTCAGGAGGTCATTGTGGACTTCCTCAACGGCGATCCGGACCAGCCGATCATTATGGGGCGTACCTACCACCAGGAAA
ACCGCACCCCCGGCAGCCTGCCGGGGACGAAGACGCAGATGACGATCCGCTCGAAAACCTATAAGGGCAGCGGGTTTAAT
GAACTGAAGTTTGACGATGCGACAGGGAAAGAACAGGTCTACATCCACGCGCAGAAGAACATGAACACCGAGGTGCTGAA
TAACCGCACCACTGATGTGATAAACAACCATGCCGAAAAAATAGGTAACAACCAGGCGATCACCGTTACCAATAACCAGA
TCCAGAACATTGGCGTTAATCAGATACAGACGGTTGGTGTCAACCAGGTGGAAACGGTGGGCAGTAACCAGATTATCAAA
GTGGGATCAAACCAGGTTGAAAAGGTGGGGATCATTCGTGCGCTGACGGTGGGTGTGGCTTACCAGACGACGGTAGGCGG
CATTATGAATACCTCGGTGGCGTTGTTGCAGTCCTCACAGGTAGGGCTGCATAAATCACTGATGGTGGGAATGGGCTACA
GCGTCAATGTGGGGAATAACGTCACCTTCTCGGTGGGCAAGACAATGAAGGAAAACACCGGACAAACAGCAGTTTATTCT
GCCGGTGAGCATCTTGAACTCTGCTGTGGTAAGGCAAGGCTGGTGCTGACGAAGGACGGAAGCATATTTCTCAATGGTAC
GCACATTCATCTGGAAGGGGAGTCGGATGTGAACGGTGATGCGCCAGTGATTAACTGGAACTGTGGTGCCACACAACCTG
TACCGGATGCGCCTGTGCCGAAAGATTTACCCCCCGGAATGCCGGATATGCGGCAATTTTGA
>Cluster_27983
ATGAGTCAAACCATAACCCAGGGCCGTTTACGCATTGACGCCAATTTTAAACGTTTTGTGGATGAAGAAGTTTTGCCTGG
CGTGGAACTGGATGCTGCCGCGTTCTGGCACAATGTTGATGAGATCGTTCACGATCTGGCGCCAGAGAATCGTCAGTTGC
TGGCAGAGCGCGATCGCATTCAGGCGGCGCTTGATGAGTGGTATCGCAGCAATCCGGGGCCGGTAAAAGATAAAGCGGCC
TATAAATCTTTCCTGCGTGAACTGGGCTACCTGGTGCCACAGCCTGACCACGTGACGGTGGAAACCACGGGCATCGACAG
CGAAATCACCAGTCAGGCGGGGCCACAGCTGGTGGTTCCGGCGATGAACGCCCGCTACGCGCTGAACGCGGCGAACGCGC
GCTGGGGCTCACTGTACGATGCGTTATACGGCAGCGACATCATCCCGCAGGAAGGGGCGATGGTCAGCGGCTACGATCCG
CAGCGTGGTGAGCAGGTTATCGCCTGGGTTCGGCGTTTCCTCGATGAATCACTACCGCTGGAAAACGGCAGCTATCAGGA
TGTGGTGGCGTTTAAGGTGGTCGATAAACAATTACGCATCCAGTTGAAAAATGGTAAAGAAACCACGTTACGTACTCCAG
CACAGTTTGTCGGTTACCGTGGCGATACTGCTGCGCTGACCTGCATTTTGCTGAAAAATAACGGCCTGCATATTGAGCTA
CAAATCGATGCCAACGGGCGGATTGGCAAAGACGATCCGGCGCACATCAACGATGTTATCGTCGAAGCGGCCATCAGTAC
CATTCTCGACTGCGAAGATTCGGTTGCGGCGGTTGATGCGGAAGATAAAATCCTGCTGTACCGCAACCTGCTGGGCCTGA
TGCAGGGGACTCTGCAAGAGAAAATGGAGAAGAACGGTCGGCAAATCGTGCGTAAACTGAATGACGATCGTCAGTACACT
GCCGCCGATGGATCTGAAATTTCTCTGCACGGACGCTCGTTGCTGTTTATCCGCAACGTGGGTCATTTGATGACCATTCC
TGTGATTTGGGACAGCGAAGGCAATGAAATCCCGGAAGGCATTCTTGATGGCGTCATGACTGGCGCGATTGCCCTCTATG
ATTTAAAAGTGCAGAAAAACTCGCGCACTGGCAGCGTCTATATTGTGAAACCGAAAATGCACGGTCCGCAGGAAGTGGCG
TTCGCCAACAAACTGTTTTCCCGCGTTGAGACAATGCTCGGTATGGCACCGAATACCCTGAAAATGGGCATTATGGATGA
AGAACGCCGGACCTCGCTGAACTTGCGTAGCTGTATCGCTCAGGCGCGCAACCGCGTGGCGTTCATCAATACCGGTTTCC
TCGACCGTACCGGCGATGAAATGCATTCGGTGATGGAAGCGGGCCCGATGCTGCGTAAAAACCAGATGAAATCGACGCCG
TGGATCAAAGCCTACGAACGTAATAACGTGCTTTCCGGTCTGTTCTGTGGGCTACGTGGAAAAGCGCAAATCGGTAAAGG
CATGTGGGCAATGCCGGACCTGATGGCAGACATGTACAGCCAGAAGGGCGACCAATTGCGTGCCGGGGCAAACACGGCCT
GGGTTCCGTCGCCAACTGCCGCTACGCTCCATGCGCTGCACTACCACCAAACCAACGTACAGAGCGTGCAAGCCAACATT
GCCCAGACCGAGTTCAATGCTGAATTTGAACCGCTGCTGGACGATCTGCTGACTATTCCGGTTGCTGAAAACGCTAACTG
GTCGGCGCAAGAGATCCAACAAGAGCTGGATAACAACGTGCAGGGGATTCTGGGGTACGTGGTGCGTTGGGTGGAGCAGG
GGATTGGTTGTTCAAAAGTGCCGGATATTCACAACGTGGCGTTGATGGAAGACCGCGCAACGCTGCGTATTTCCAGCCAG
CATATCGCCAACTGGTTACGTCACGGTATTCTGACCAAAGATCAGGTGCAGGCGTCGCTGGAGAATATGGCGAAAGTGGT
TGATCAGCAAAACGCTGGCGATCCGGCTTATCGTCCGATGGCGGGGAATTTCGCTAACTCGTGTGCTTTTAAAGCTGCCT
GCGATTTGATCTTCCTCGGCGTGAAACAGCCAAACGGCTATACCGAACCGTTATTACACGCATGGCGTTTACGCGAAAAA
GAAAATCATTAA
>Cluster_14802
ATGTCTGCGGTGTTAGAACTATCTGATACAGATGCGATAGAATATGGAATGGTGAAATTTATCGCAGATAGCATTTCTTC
TAAATTATAA
>Cluster_9787
ATGCTCAAGGGCGATGTTGATCTGTTCAATCATGTGATTGGGGAAACGGATATTGCGGGTTGTGGTTCTGCGGGTCCGGT
TTTTCGATGA
>Cluster_27268
GTGCGACTGGAAGCGCCGGGGCGTGATTATCGGCGCTATCAGATGGAAGAGTACGGCGGTGTGGACGTTCGCCTGTATCG
TATTCCTGACCCGATGGCATTTTTGCGCCAGCAGAAAAACCTGCATCGCATTGTGGTGCAACCGCAATATCTGGGCGACG
GGCTGAACAATACGCTGACCTGGCTGTGGGATAACTGGTACGGCAAATCTCGCCGCGTGATGCAGCGTACTTTCTCTTCT
CAGTCACGGCAGAATGTGACTCAGGCGTTACCCGAATTACAGCTCGGCAATGCCATTATTAAACCTTCCTGTTATGTACA
GAACAGCCAGTTTTCCCCGCTGAAAAAATATCCACTGGTGGAACAGTTCCGTTACCCATTATGGCAGGCTACATCGGTCG
AGCCGCAGCAAGGGTTAAAACTGGAAGGCGCATCCAGCAATTTCATTTCGCCGCAGCCGGGCAACATCTATATTCCTCTC
GGCAAACAAGAGCCGGGACTGTACCTCGTCGAGGCGATGGTGGGTGGGTATCGGGCGACGACGGTGGTGTTTGTTTCCGA
TACCGTGGCGCTTAGCAAAGTGTCAGGTAACGAGCTTCTGGTGTGGATCGCGGGGAAAAAACAGGGTGAAGCGAAGCCCG
GCTCAGAGATCTTGTGGACTGACGGTCTTGGCGTGATGACCCGCGGTGTGACCGATGACAGCGGTACCTTGCAGTTACAA
CATATATCGCCAGAACGTTCATATATTCTGGGTAAGGATGCTGAAGGCGGCGTTTTTGTCTCCGAGAACTTTTTCTACGA
AAGCGAAATCTACAACACCCGCTTGTATATCTTTACCGATCGCCCGCTATATCGCGCAGGCGATCGTATCGATGTTAAAG
TGATGGGTCGCGAGTTCCACGATCCGTTGCATTCATCCCCCATCGTCAGCGCACCGGCGAAGCTTTCGGTGCTGGACGCC
AACGGCAGTCTGTTGCAAACCGTCAATGTCACGCTGGATGCGCGTAATGGCGGGCAGGGGAGTTTCCGCCTGCCAGAAAA
TGCTGTCGCCGGAGGTTATGAGTTACGTCTTGCTTACCGCAAACAGGTCTATAGCAGCAGTTTTCGCGTGGCAAACTACA
TCAAACCCCATTTTGAGATTGGTTTAGCTCTCGACAAAAAAGAGTTCAAAACTGGCGAAGCGGTCAGCGGCAAACTGCAA
CTGCTTTACCCGGATGGCGAGCCGGTAAAAGATGCCCGCGTGCAGTTAAGTTTGCGCGCTCAGCAATTATCAATGGTTGG
TAACGATTTGCGTTATGCCGGACGTTTCCCAGTGTCGCTGGAAGGCAGTGAAACGGTTTCCGACGATAATGGTCATGTGG
CGTTGAATCTCCCCGCCGCCGATAAACCGAGCCGCTATTTACTGACCGTCTCTGCCAGTGACGGCGCGGCGTATCGAGTT
ACCACCACCAAAGAGATCCTCATTGAGCGCGGTCTGGCGCATTACTCTTTAAGTACTGCTGCACAATACAGTAATAGCGG
TGAATCGGTTGTGTTCCGTTATGCCGCGCTGGAATCTTCAAAACAGGTTCCTGTTACGTATGAATGGTTGCGTCTCGAAG
ACCGCACGAGCCACAGCGGAGAGCTACAGTCAGGCGGTAAATCCTTTACCGTCAATTTCGCTAAACCTGGCAACTACAAT
CTGACATTACGCGATAAAGACGGCTTAATTCTCGCCGGGTTAAGCCATGCAGTCAGCGGTAAGGGCAGCACGTCGCATAT
TGGTACGGTAGATATCGTGGCAGATAAAACACTGTATCAGCCAGGCGAAACCGCGAAGATGCTGATTACCTTCCCGGAGC
CAATTGACGAAGCATTATTGACGCTGGAACGCGATCGCGTGGAACAGCAGTCGCTGCTATCGCATCCGGCAAACTGGCTA
ACGCTACAACGTTTAAACGATACCCAGTATGAAGCCCGCGTACCGGTGAGCAATTCCTTTGCGCCTAACATCACTTTTTC
GGTGCTGTATACCCGTAACGGTCAGTACAGTTTTCAGAACGCCGGGATTAAAGTTGCCGTTCCCCAGCTGGATATCCGGG
TGAAAACGGACAAAACCCATTACCAGCCTGGTGAACTGGTCAATGTCGAATTAACCTCGTCGCTGAAAGGTAAACCTGTT
TCTGCGCAGTTAACGGTAGGCGTGGTCGATGAAATGATCTACGCGCTGCAACCGGAAATCGCGCCGAATATCGGCAAATT
TTTCTATCCGCTAGGGCGTAACAATGTGCGTACCAGCTCCAGTCTGTCGTTTATCAGCTACGATCAGGCGCTCTCCAGCG
AGCCGGTTGCGCCTGGCGCGACTAACCGCAGCGAGCGGCGAGTAAAAATGCTGGAACGTCCACGGCGTGAAGAGGTGGAT
ACCGCGGCCTGGATGCCGTCACTCACAACCGATAAACAAGGCAAAGCGTATTTCACGTTCCTGATGCCTGATTCGTTAAC

CCGCTGGCGCATCACCGCGCGTGGGATGAACGGCGACGGGCTGGTCGGGCAGGGGCGTGCTTATCTGCGTTCGGAAAAAA
GTCTCTACATGAAATGGAGTATGCTAACGGTGTATCGCATGGGCGACAAACCGGCGGCAGGACTGTTTATCTTCAGTCAG
CAGGATAACGAACCGGTGGCGCTGGTGACTAAATTTGCAGGTGCTGAGATGCGCCAGACGCTGACGCTGCACAAAGGGGC
GAATTATATTTCGCTGACGCAGAATATTCAGCAATCTGGCTTGTTAAGTGCAGAACTGCAACAAAATGGGCAAGTACAGG
ACAGCATTAGCACAAAACTGTCTTTTGTGGATAACAGCTGGCCCGTTGAACAGCAGAAAAATGTCATGCTCGGCGGTGGC
GATAATGCGCTGATGTTGCCCGAGCAGGCGAGCAATATCCGGTTGCAAAGTAGCGAAACGCCGCAGGAGATTTTCCGTAA
CAATCTTGATGCGTTAGTCGATGAACCGTGGGGTGGCGTGATCAACACCGGTAGCCGTCTGATCCCGCTCAGTCTCGCCT
GGCGTTCGCTTGCCGATCATCAAAGTGCCGCCGCTAACGACATTCGTCAGATGATTCAGGATAACCGTTTGCGGCTGATG
CAACTGGCGGGGCCCGGAGCGCGCTTTACCTGGTGGGGTGAAGATGGCAATGGCGACGCATTCCTCACGGCATGGGCATG
GTACGCCGACTGGCAGGCCAGCCAGGCGCTCGGCGTAACGCAGCAACCGGAATACTGGCAACATATGCTCGACAGTTATG
CCGAGCAGGCGGACAACATGCCGTTATTGCATCGGGCGCTGGTGCTGGCGTGGGCACAGGAGATGAATCTGCCGTGCAAA
ACGTTGTTGAAAGGGTTGGATGAAGCTATCGCCCGGCGCGGAACTAAAACTGAAGATTTCTCTGAGGAAGACACCAGCGA
TATCAATGACAGCATGATCCTCGATACACCTGAATCTCCACTGGCAGATGCGGTGGCAAACGTCTTAACCATGACGTTGC
TGAAAAAAGCGCAGTTGAAGTCCACTGTGATGCCACAGGTTCAGCAATATGCGTGGGATAAAGCAGCAAACAGCAATCAG
CCGCTGGCGCACACGGTTGTGCTGCTCAATAGCGGTGGCGACGCTACTCAGGCGGCCGCTATTTTAAGTGGTTTGACCGC
TGAGCAATCCACTATTGAGCGCGCGCTGGCCATGAACTGGCTGGCGAAATATATGGCGACGATGCCTCCGGTTGTGTTAC
CTGCTCCTGCGGGCGCATGGGCCAAACATAAGTTAACCGGAGGAGGCGAATACTGGCGTTGGGTTGGTCAGGGCGTGCCG
GACATTCTCTCTTTTGGTGACGAATTACCGCCGCAAAACGTGCAGGTCCGCTGGCGTGAACCGGCAAAAACGGCTCAACA
AAGTAATATTCCGGTGATCGTTGAACGCCAGTTGTATCGGCTTATCCCCGGTGAAGAAGAGATGAGCTTTACCTTACAGC
CTGTCACCAGCAATGAGATTGACAGCGATGCGCTGTATCTCGATGAAATCACGCTTACCAGCGAGCAGGATGCAGTTCTG
CGCTATGGTCAGGTGGAAGTACCGCTGCCGCCGGGGGCTGACGTTGAGCGGACAACGTGGGGCATTTCGGTCAATAAACC
TAACGCTGGAAAACAGCAGGGGCAATTGCTGGAAAAAGCGCGAAATGAAATGGGCGAACTGGCCTATATGGTGCCGGTGA
AAGAACTGACGGGAACGGTCACTTTCCGCCATTTGCTGCGCTTCTCGCAAAAAGGGCAATTCGTTCTGCCTCCTGCTCGT
TATGTGCGTTCCTATGCACCTGCACAGCAAAGTGTTGCGCCAGGGAGCGAATGGATCGGGATGCAGGTGAAATAA
>Cluster_66740
ATGTCCGTAAAACAGTATGAAACCTATCTGGCAGAAACTTTTATTGAATGGGTCGGTGGCATCATTCAGCCCGGCGAGCG
CTACCAGTTCAAATCTCCCGATCCCGATAATGCCCTAAAACTGTGGGAAGCATTCGTCTCTCTGGCGGACGGCAATCAGT
TGGAAATTGCGCCAGGGCAATATATTGATTGCCTCTCCTGCAATGGTGTTCAGCTTATTCCTGTACTGCACGGAGCCGAG
GCTCCAGCATTCACAGAAAACTACATTTCTCATTTGCGCGATGAAGTTGCAGGGCGCAGCGGCGTGTTTGCCCAAACAGC
GTTGTTAATTATTCACAATAGTATGCTCGATACCCTGATTAATAGCACGAAAGATGTGGCTGCACCGGATGCTATCTGGT
ATCCAGAAACTTTCAGTCATCAACTGGAAAAGCTCATTACCACTGACAGTAACCGTTCGGAATTGTCCCGCTGTTTGCTG
GAGGATCAACTGGCAACGGTACTGGATGAAGGCGCAACCGTATTTGGTTTCTCGTCTTTGTATCGCTTGCTGGATGATGG
AAATCTGGATTTCTCCGAGCTTCATTTGTTTAAAGATGATGAACTACTGAACTATAGCCGGAAGCAACTACGCACCCGAC
TGAATGAAAACCGGAAATTGTATCGGCAGATAGAAGATTGTGTTGAACGCTATAGTGGACAGCTGGAGAATGTGCTGCCT
GAGTTTAGTACAAAATTTATTCAGGAGCATTTTTACGATAAAAATGACTGGCGTGAGCTCGATTTCTCTGACTACAGGAA
AGAGAAAGAAGAGAATAGCAAACAAAAACTGGTACTGGAGGACGTTTCCGTCAAAAACGGCGAGATTTGGCAACGGGCGA
AAAGCTCTAGTAAGGCAGGTAAACGTGACATCAGTCTGCTGGTGCAGGTGCAGCCGGGGAAATCAACTGCTGAGCTGGAG
TTCCGTTTTCAGGGTAGTGATCTTCAGGATCATCAGATAAAGATTGCCCACAATCGCCAATTAAAGAATGTACCTTTCTG
GAGCACCAGTCGGGCGGGCGGCAAGACTTCCCGCATTAAGGCATCGGTGCCTTTTGACGGGCATCCCTGCTTCTTTAGTC
TCGAGCTGATCAACCGCAATCATTCAGCCGAAGAGTATAAATTTCGCCTGTTGTTAGTCGAGCAGGGACAATTCTGGCTA
AACGATATTCAGCACTTCTACCGTGTTGAGCCAAGTAAAGATCAAATAACCCTGCAACTGGAAGATAACACGCTGCGTAT
TGCCGAGTCTGGTAACCTGATCTATACGTTGGATGACGGGAATGAAGACATCGACTGCCAACATTATGCTCAGGTGAATT
TTGAGACGCTGGCAAATCAAAGTGACCTGGTTCAGTTTGTGCTCGTTTCCGGTGATTCCCGTCTGTCATTTAACGTCGAG
GGGCCGGGAGCAGAAGAAGGGTTAACGCTGCCGTTGCTTTTTGACCAAAGTCGTTTCAATAAGTTGTTCAAAGAAGAGGG
CAATGCAACCTGGAACCGATTAAAAAGTCGCATCATACTCGATAATACTGAACACAAGGTCGTCGGAGTACGACAGCAGT
TGTTGATGCAGGAAGCCTTATTAATTGAGCAACGTTTGCTGGGGACGGGAAATGACGATAGCGAGTTTGCGCTGGATGAA
CTCCTTGCCAGCTATCCTGATCTCCATAACGCCTATGAGCAGTTATTTGCCTACTATCATCGTCGTAACACCCTGCCGAG
TCTGGTATCGTGGTCAGCGGAATATCGCACACTGGTCAGGTACATTGTTACTACGTTTGAACAGTCACTCCAGCAAATCG
GCCTGAGTAGAGCGCTAACAGCGCAGGAAAAACGTCTTCTGCATCTTGGGATCTGCCGCAATGATAGCCATGAGCGCATC
TCACCACTGCACCCATTAGTGCTGGCCTACCATCTACAACTGGCAGACGCTATCGTTACTGAGTCGGAACAGAACGATTC
GGCTTCATTTGCCACACTACCGCCGATCACTCTTGATCGCCTGATGGTTTCCGGTTTAATGCCGTTTGTTTACCACAGTG
AACATGAATACGCGCAACTACAGCCGGTGGAAGAAAACCGCTTCTGGATTGATGTGGTTCCTCAGCGGAAAGTTAGCCAT
GACTATGTTAAACGTCTGGTAAAAGACAAACTCAATGAGTTCACGGACGCTTACGCCCGATTGTTCCAGAGCACTGAAAA
CAGTGCATTGATTATTAATGCCATTAATCAAGGGAACGCCAAAGAACTGTTTCTGGGATTAGTAGAATATTTTAAGCAAG
AAAAAGAGCACGCTATTTCGGTTCATGTGAACTGCTACGATGAACGTTTGCTACCCAATATGTTCGATCACTTTGCCGAA
AGTGGTAGTTATGAGCAGCTTAAAAACGATCTCGATCTGAACAGTGGCGTATGGCGAGCTGAAGCCGATATGTTGATCGA
TCTGCTGCGTAGCCGGTTAACGTTCAGCAAGTTTGTCCTGCCGTCAGAAAGCGACAAGCTGGCCTATGCTCATCTGGCAT
TTTTCACTAATACAGCACCTGTGGATTGTCGCCAGATCCGCATTGAAGATGCGTCCAGCGGAGTATTGTGTCATGGGTTG
ATTGCTGGGGAAGGTGCTGAAACACAGGGGGATGCTTACTTTACCGCGTTTGGACTGCGTGATGTGGATACTGAGCCTTA
CTGCGCCCTGCGTCTTGCCCGACTAATCGGTTGCCTGTGGCAACCCGCACGTCAAAGTAACAGCCAATATCATGGACAAG
GGATCGGCCTGGCTGTGAGTGGCAATTTTAAACAGCTACTCAATTACTCCTACAATAGCGCGCTCTGGACCACCATAATT
GATCCAAAAGTCACTCTCGACTTTTTCACCAGTCAGAAAGATGTGGTGCTTATCCACTATTCTGACCAGTACACCAGTTG
CGCAGGCTACGATGCCGTCACCGTCACGAAGCAGGTTGATTTGTTCCTGCGGTTGCTCCAGACCGGAAGTCCGTCAGGGC
AACCTGCTATTGATAGTCAGCATTTGCTGGCTGAATTTAATGCCTTTAACGGCGAATGGCTGCTGAAAATGCTGCGCTCC
AGTGAAAGAGAGCGTAAAGAGAAGCACGGCATTATAGGTGCGTACAAATTTGTGCAATCTATGTTGCAGCAGTCCGATAT
TTGCTGGATTCCGTTATCGGTGGCCGAAATGATCCGCGTGTCCGGGAATGTTGGACTGAAAATGAAAGAGAGCGATTTGT
CGAGAAATTTACAGGGTTATCGTAAAGGTGCGATCTCTGATGATGTGCTCTTTGTTGGTTTCAAAGAAAACCGCCTCTAT
CTGTTGCCGCTGGAAGTTAAAACAGGGGCGCGGCCTGACTACAGCTATGCGGGCCAACAGGCTCGTGAGCTGAAACGTTA
TTTACAGCAGGACATTCTGGAACCACAAACGCTGGCATCACAATTGTACCGGGCGCTCTTTATTCGTCAGGTGCTGATGC
AGGTGGAGAAACTGCGTCTGTATGGTGTGCTTGACAGCAACAAACTGGCTCCCTTGCTCGAGCGACGCGAATGGTGGTTA
ACGGGCGACTATCAGTTAGGCGAGTTGACCGATTATGCGGACGGCTTCGTCGTGGCGCATGTGGACAGCGCAACCTGCTT
CGATCTCTCTTATAAAGAGACAGCGGAGAATATCTTACAGATAGAAATCCCTTATTCACTGCTGCCTTCGCTGATTGCTG
CTCAGGGGGATGAACCACCGCTGGCGGAACGTTATCGGGTGCCGGAAAAATACCGGCTTAAGCCAGAGTGTGATGCCAGT
CCGACACAACCCGAAGCCGTGGTAGAAGTCACCACGCCGACTACTGCACAACTCGTTTCGAAACCAGAGTCGTTACCGCC
TGCTGTACCGGCGGCAACCCCTGATATTAGCAATACTCCATTACAGATCCTGTTTGGTTATGATGCTGCGCGTCAGAGCC
CGTTACATTGGGAGCCAACCAACACCGCGAAGTTCATGAATACCAATACCGGCATTATTGGAACGATGGGGACCGGTAAA
ACCCAGTTTACTAAATCGCTGGTCACACAGTTGATGCGTAATCAGTCCTGTAATGTTGATGGTAAACCTATCGGTTTGCT
GATCTTCGACTACAAATCAGACTATGTGGATGAAGCTTTTCTTGATGCAACGGGTGCTAAAAAGTACAAGTTATTTAAGC
TGCCTTACAACCCCTTATCTCTGTTTGGCGACACGCCAATGCTACCAATCCACACTGCTGGCGGACTATCGGAAACGATG

GCGAAAGCCTATGGGCTGGGCCATAAACAGCAGCTTAAATTAGAAAATCTGATTTAGAATGCTACGAAGCGGCAGGCAT
TTCGCCGGAGGATCCGACAACATGGTCACGGACTGCGCCGACGATTGAAGATGTATGGCAGCGTTTCCTCGATCAGGAGA
AGGTTGAAGAAGATTCGCTTTACGCAGCACTTTCTAAACTGGCGCGTTACAAGATATTCGAGACAATCCCGGAGAAGATG
ACCAGTTTGTACGAATTGATTGACGGTGTCACTGTGATTGAACTGGCTGGTTACCCGCCAGAAGTGCAAAACCTTGTGGT
TGCACTAACGCTGGATCTTTTTTACGCGCAGATGCAAAAGCGCGGTAAACCAGTAGTGCGAGGGGATTATCGTCAGCTCA
CTAAAATGATTCTGGTGGACGAAGCCGATAACTTTATGCGTCAGGATTTTTCCAGTCTGCGTAAGATCCTTAAAGAAGGC
CGTGAGTATGGCGTTGGTGCGATCCTGTCAACGCAGGAGATCACTCACTTTAAGACCGGGGAAAATAATTATGCCTCCTA
TATTTTAACGTGGGTAATCCATCGTGTTTCCGAGATTAGGAATGCGGATATTAAGGCGGTATTTAACGTCGATGATAAAG
GTGAGCAGGAGTCTTTGATGGGACAGATTCGGCAGCTTGAGAAGCATTTTAGCCTGTATGTGGATGGAGATAAGCGGGTT
AAGAAAATGCGGGATCGTGCATTTTGGGAGTTAATTAAATGA
>Cluster_56413
ATGTTTATCACGAATAAAGCTATTTATGTTACCAATATCATTTCGAGTGCGCACACTATCTATCTGTTTTTCGAGATAT
AAAAAACAACGTGTAA
>Cluster_49645
ATGCGTAAAAGTTATGAAGTCGGTATTTCACCTAAGATTAACTTATGTAACAGTGTGGAAGTATTGACCAATTCATTCGG
GACAGTTATTAGTGGTAGACAAGTTTAA
>Cluster_26953
ATGATTGTTGTGCTAAACGGCTGGTGTGATCTCGCGCACATTAATGAAGGCATAAAAAAAGCCGGCATCATGCCGGCCAA
AAAAATATAA
>Cluster_46074
ATGAAAAACAAAATCATCATGGAGCTACAGGCTCCTTTTTTATTGTTCGCATTCACCCTCAAGCGTATTAACCAACAATT
CAGGGATTAA
>Cluster_41249
ATGAATATGCGGATTATCTTTCTACGCAAGGAGTATTTATCTTTACTCCCGTCAATGATTGCATCTCTTTTCTCTGGTAA
CGGTGCCGCAGCGGTCATTGATTCATGTCAGGGATATGATATCAAAGCGAGCTGTCAGGCCAGCAGGCAAAGCCTTTCAG
GCAATATGCAGGACTGGAGCATCGCGGATGGGCAATGGTTGATTTTTTCGGGTATGGCCAATAATGCCAGCGGTGGCGCA
GTATTTTTGCAGCAAGGTGCTGAATTTACGATATCACCACAAAATGAAACAGGGATGACCCTGTTTGCTGATAACTCGGT
TAGTGGCGAATATAATAATGGTGGGGCTATCTTTGCTAAAGAAAACTCAACGATAAATCTTGCGAATGTTATTTTCGACA
GTAATGTTGCAGGAGGCTATGGCGGAGCAATCTATTCTGCCGGTACCAATGATACCGGTGCCGCCGATTTACGTGTCACT
AACGCCGTATTTCACAATAACATCGCTAATGACGGCAAAGGCGGTGCAATTTATACCATCAATAATGACGTCTATTTAAG
TGATGATGCTTTTAATAATAACCAGGCATATACATCAACAAGTTATAGTGATGGGGATGGCGGCGCCATTGATGTTACGG
ATAATAGTACGGATAACACACATCTTTCAGGAAAAACGATAATTAATAATACCTCCTTTACCAATAACTATGCAGAAGGT
TATGGCGGAGCAATTTATACCAGCAGCACCACATCCCCGTATCTTATTGATATTTCTGTTGATGATAATTATGACCAAAA
CAATGGTGTGATGATTGATGAAAATAACAGCGCCTCGGGATATGACCATTCGGCGACGGCAGCGGCGGGGGGCTTTATGT
ATATCGGTCATAGTGTGGCTGAATTTAACATTGCCGCCGATAAAACGCTGGTAATAGGCAATACCAGCAACGATGGCGCG
ATTGATTCTCTTGCCGGAACGGGAGTCATCGTTAAAGAGGGAGCGGGAGAGTTAGTCCTCAATGCCGACAATAATGCGTT
TACCGGCGAGATGAGTATCCAGAATGGCGAGGTGACGTTGGGGCGCAGCGATGAGTTAATGAATGTCGGCGACACGCACT
GTCAGAGCGATCCGCAGGATTGCTTTGGTCTGATGGTCGGCAGCACTGTTCATTCTGAGTATCAGGCAGAACTGAATGTT
GGCAATACACAACAAACGTTTGTGCACTCATTAACCGGTTTTGCTAATGGCATTTTGAATATCGACGCAGGCGGCAATGT
TACAGTAAACCAGGGCGGTTTTTCCGGCTCGATTCAGGGTGAAGGACAGTTGACGGTAGCGCAAGATGGCAGCTATCTCC
TGACAGGGGCGCAGTCGATGGCATTAACCGGCGATATTGTGGTGGAAGATAACGCCGTATTGTCGCTGGCGGGGAATCAG
GCCGATTTACGGGCAATGCAGAGCGATCCGCAATCGATCGTGCTAAATGGCGGAGTATTGGATCTCTCTGATTTCACCAC
CTGGGATGGCGATAGCTCATACAATGACGGTCTGCAAATCAGCGGCAGCGGGGGACAGTGATTGGCAGTAATGATGTCG
TTGATATCAGCAGTGGCGATGATTTACATATTGGCGGTAGTGATGCCAGCCAGAATGGCGTTTATGTCGTCATTAATGCG
GGGGATCAGCGCGTAAAGCTGGCGAATAACAACGGTTATCTCGGTAATACGCAAATCGCCTCCGGTACGCTTGAAGTCAG
CGATAATTCGCAATTGGGGGACACAAGTTACAACCGTTCAGTGATCTTCACTGATCCCCAACAACACAGTGAAATGGATG
TGACGACTGATGTTGACACTCGCTCGGCTACAACAGGTCAGGGCAGGAATATTGAAATGCGCGCTGATGGCGAAATACAC
GTTGAGGATGGCGTAGATACGCAATGGGGCGGGTTGATGGCGGATAGTACCGGGCAACAACTGGATAGCGTAAGCACGTT
GACTAAAAGTGGCGGCGGTACGCTGGAGTTGACCGCCAGCGGCACTGCGACGTCTGCGGTACGTGTGGAGGACGGAACGC
TAAAAGGTGAAGCGGAGAATATTATTCCTTATGTTTCTTCACTGTGGGTGGGGGAAGACGGTGTTTTTGAAACCGGGCAA
AATCAGGATATTCGCTCAATCGATGCCACCTCTGGCGGCGATATCGACATAACCGATGGTACTGTGTTGCGATTGACGCA
GCAGAATACGAACCAGGCGCTGGATGCCTCGCTGTTTAGCGGCGACGGTACGCTGGTGAATGCCACCGATGGCGTGACGC
TGGCAGGTGAACTTAATACCAACCTTGAAACTGACAGCCTGACTTATCTTTCCGACGTGACGGTTAATGGCAATCTGACC
AATACGTCCGGTGCTGTCAGTCTGCAAAATGGCGTCGCTGGCGACACGCTGACGGTAAATGGTGATTATACCGGCGGCGG
TACGCTACTGCTCGACAGCGAATTAAACGGCGATGACTCGGCAAGTGACCAACTGGTATTGAACGGCAATACCTCGGGCA
ACACGGCCGTGGTAATTAACCCCATTACGGGTATTGGTGAGCCGACATCTACAGGTATTAAAGTAGTTGATTTCGCAGCT
GATCCCGCGCAGTTTCAAAACAATGCGCAGTTCAGTCTGGCAGGAAGCGGCTACGTCAATATGGGGGCGTATGACTACAC
GCTGGTGGAAGATAACAACGACTGGTATCTGCGATCACAAGAAGTCAATCCGACGCCGCCGCCTGATCCAGACCCGACTC
CAGATCCTGATCCCACGCCGGATCCCGACCCAACCCCCGACCCGGAACCTACGCCTGCCTACCAGCCGGTGTTGAATGCC
AAAGTTGGTGGATATTTTAATAACCTGCGGGCGGCAAATCAGGCATTTGTGATGGAGCGACGCGATCACGCTGGTGGCGA
TGGTCAGACGCTGAATTTACGCGTTATCGGCGGGCGTTATCATTACACGGCGGTAGGGCAGTTGGCTCAGCATGAAGACA
CTTCTACGGTGCAGCTTAGCGGCAACCTGTTTAGCGGGCACTGGGGTGATGATGGCGAGTGGATGCTTGGGATTGTTGGT
GGCTACAGCGATAACCAGGGCGACAGCCGCTCGAATATGACCGGAACTCGCGCCGATAACCAGAACCACGGTTATGCCGT
TGGGCTGACCTCAAGCTGGTATCAGCACGGTAATCAGAAGCAGGGGGCCTGGTTGGATAGCTGGCTGCAATATGTGTGGT
TTAACAATGATGTTTCCGAACAAGACGATGGCGTGGATCATTACCACTCGTCGGGGATTATCGCCTCGCTGGAAGCCGGT
TATCAGTGGTTACCGGGGCATGGTGTGGTGATTGAACCGCAGACGCAGGTGATTTATCAGGGCGTGCAGCAGGATGATTT
TACCGCCGCTAACCATGCGCGCGTGTCACAATCGCAGGGTGATGATATTCAGACGCGGCTGGGTTTACACAGCGAATGGC
GTACTGCTGTTGGTGTCACGCCAACGTTAGATCTGAATTATTATCACGATCCCCATTCGACGGGAATTGAAGAAGATGGC
AGCACTATCAGTGACGATGCGGCGAAGCAACGGGGTGAAATAAAAGTGGGAATAACGGGCAATATCAGTCAGCGAGTTTC
GCTGCGTGGTAGCGTGGCGTGGCAGAAAGGGAGTGATGATTTTGCCCAGACGGCAGGGTTTTTGTCGATGACAGTGAAAT
GGTAA
>Cluster_42661
GTGGGTGATGCTCTGTCAGGAATCGACAATAATACCCTACAATGGAATAAAACCGCGGGGGCGTTCAGCGCCAATCACGG
TGCAAATGCCACCAACAAAATCACTAATGTTGCTAAAGGTACGGTTTCTGCAACCCGCACCGATGTAGTAAACGGCTCTC
AATTGTACGACCTGCAGCAGGATGCCCTGTTGTGGAACGGCACAGCATTCAGTGCCGCACACGGTAGTGAAGCTACCAGC
AAAATCACCAACGATAACGTGGCGACCAACACCACCAATATCGCCACTAACACCACCAATATCACTAACCTGACTGATAC
GGTGAATAATCTCGGTGAAGACGCCCTGAAATGGGACGACGCCGCAGGCGCATTCACCGCTGCACACGGTACTAACGCCA
CCAACAAAATCAGCAACGTACAAGCTGGCATAGTCTCCTCTGACAGCACTGACGCCATAAATGGCTCACAACTATATGGT
TTGGCTGATTCATTCACGTCCTATCTGGGTGGTGGTGCTGATATTAGCGATACAGGTGTATTAACCGGGCCAACCTATAG

TATTGGCGGCACTGACTACACTAACGTCGGTGATGCTCTGGCCGCAATTAACACTTCATTTAGTGATTCTCTCGGTGATG
CTCTGCTCTGGGATGCGACAGCCAATGACGGTGCTGGTGCATTCAGCGCCGGTCGCGGGGTAGATAACACCGCCAGTAAG
ATTACTAACGTCGCAAATGGTGCAATCTCTGCCACCAGCAGCGACGCGATTAACGGCTCACAACTCTATACCACCAATAA
GTACATCGCTGATGCGCTGGGTGGTGATGCAGAAGTCAACGCTGACGGAACTATCACTGCACCGACTTACACCATTGCAA
ATACCGATTACAACAACGTCGGTGAAGCTCTGGATGCGCTTGATGAGAACGCGCTGCTGTGGGATGCGACCGCCAATAAC
GGCGAAGGGGCTTACAACGCCAGTCATGACGGCAAGGCCAGCATTATCACAAACGTTGCTGATGGTAACATTGGTGAAGG
TAGCACCGATGCTATCAACGGTTCTCAGCTGTTTAACACCAATATGCTGATCCAGCAGAACAGCGAAGTCATCAATCAGC
TTGCCGGTAACACCAGTGAAACCTACATCGAAGAAAATGGTGCAGGTATTAACTATGTGCGTACCAATGACACCGGTTTA
ACCTTCACCGATGCCAGCGCAGCAGGTATTGGCTCTACCGCTGTGGGGTATAACACTGTTGCCAAAGGCGATAACAGCGT
GGCCATGGGTTATAACTCTTTTGCCGAAGGCCATAGTAGCGTGGCCATCGGTCAGGGCAGCTACAGCGGCGTTGAGACGA
GTATTGCGCTGGGTAGCGAATCCGTCTCCAGCCGTGTGATTGTTAAAGGTTCTCGTAACACCAGCGTATCGGAAGAAGGT
GTTGTGATTGGTTATGACACCACGGATGGCAAACTGCTTGGTGCATTGTCGATCGGTGATGATGGTAAATATCGTCAAAT
CATCAACGTCGCGGATGGTTCTGAAGCCCATGACGCTGTTACTGTTCGCCAGTTGCAAAACGCCATTGGTGCAGTAGCAA
CCACACCAACCAAATACTATCACGCCAACTCAACGGCTGAAGACTCACTGGCAGTCGGTGAAGACTCGCTGGCAATGGGT
GCGAAAACCATCGTTAATGGTAATGCGGGTATTGGTATCGGCCTGAACACTTTAGTTCTGGCTGATGCGATCAACGGTAT
TGCTATCGGTTCTAACGCACGCGCAAATCATGCCGACAGCATTGCTATGGGTAATGGTTCTCAGACTACTCGTGGTGCGC
AGACCAACTACACTGCCTACAACATGGATGCACCGCAGAACTCTGTGGGTGAGTTCTCTGTCGGCAGTGAAGACGGTCAA
CGTCAGATCACCAACGTCGCAGCGGGGTCGGCGGATACCGATGCGGTTAACGTGGGTCAGTTGAAAGTAACGGACGCGCA
GGTTTCCCAGAATACCCAGAGCATTACTAACCTGAACACTCAGGTCACTAATCTGGATACTCGCGTGACCAATATAGAAA
ACGGCATTGGCGATATCGTAACCACCGGTAGCACCAAATACTTCAAGACCAACACCGATGGCGTAGATGCCAACGCGCAG
GGTAAAGACAGTGTTGCGATTGGTTCTGGTTCCATTGCTGCCGCTGACAACAGCGTCGCGCTGGGTACAGGCTCCGTGGC
CAACGAAGAAAATACCATCTCTGTGGGTTCTTCTACCAACCAGCGCCGTATCACCAACGTTGCTGCCGGTGTTAATGCCA
CCGATGCGGTTAACGTTTCGCAACTGAAGTCTTCTGAAGCAGGCGGCGTTCGCTACGACACCAAAGCTGATGGCTCTATC
GACTACAGCAACATCACTCTCGGTGGTGGTAACGGCGGTACGACTCGCATCAGCAACGTTTCTGCTGGCGTGAATAACAA
CGACGCGGTGAACTACGCGCAGTTGAAGCAAAGTGTGCAGGAAACGAAGCAATACACCGATCAGCGCATGGTTGAGATGG
ATAACAAACTGTCCAAAACTGAAAGCAAGCTGAGTGGTGGTATCGCTTCTGCAATGGCGATGACCGGTCTGCCGCAGGCT
TACACGCCGGGTGCCAGCATGGCCTCTATTGGTGGCGGTACTTACAACGGTGAATCGGCTGTTGCTTTAGGTGTGTCGAT
GGTGAGCGCCAATGGTCGTTGGGTCTACAAATTACAAGGTAGTACCAATAGCCAGGGTGAATACTCCGCCGCGCTCGGTG
CCGGTATTCAGTGGTAA
>Cluster_29391
ATGTCACGACGCCATATTTTCACCGAACGGCAGCGAGCAGCGCTGTTCGATCTGCCCACGGACGAACTGTCGCTACTGAA
GTTCTACACGCTGGGCGATGATGACCTGGAAAACATTAGGCAGCGCCGCAGACCGGAAAACAGGATTGGCTTTGCCCTGC
AACTTTGTGCCTTACGATATCCGGGCCGTGCACTGGCTCCTGGTGAGATGATCCCGCGTGAAGTCCTTTCCTTCGTCGGT
GCTCAGCTTGGAGTTCCGGCTGATGCGCTTCTCACTTATGCCACACGGCGCCAAACCCGTCAGCAGCACATGGACACGCT
GCGCGAAATTTACGGCTACAAGACCTTCACGGGCCGTGGTGCCCGTGATCTGCGGGAGTGGACTTTCGGCCAGGCCGAAG
ATGCCAGATCAAACGAGGATCTTGCTCATCGTTTTATTGTGCGGTGTCGGGAAACTTCCACCATTCTGCCCGCAGTATCG
ACAATCGAGCGCTTGTGCGCGGATGCTCTGGTCGCCGCTGAGCGGCGGATTGAAACGCGGATTGCGGAAAATTTAACAGC
GGATGTTCGCGATCACCTGGACAAACTTCTGAGTGAAATGCTCGCCGGCAATATCAGTCGTTTCATCTGGCTTCGCAACT
TCGAGGTTGGTAACAACTCGGCTGCTGCTAACCGTTTGCTCGACAGGCTCGAATTTCTGCGTACCCTGAATATCAATCAT
AGTGCTTTGGCCAGCATACCTGCCCATCGCATTGCCCGGCTGCGTCGGCAGGGTGAACGCTACTTCACCGACGGTTTGCG
TGACATCACTTCGGACCGCCGCTGGGCGATCCTTGCCGTCTGTGTTGTGGAGTGGGAAGCGGCGATTGCTGATGCCATAG
TCGAAACCCATGACAGGATCGTAGGAAAAACCTGGCGGGAAGCGAAGCGCCAGCATGACGAAACAATTTCCGGCTCTAAA
GCCACACTCACGGATACGATCCGTACCTTCACCGCGCTGGAGCTTCGTTGCTTGAGGCCCGCAGTGACGGAACCCCGCT
GGAGATGGCTGTCGCCAGTTCGGTTGCATGGGACCGGCTCGCTCAACTGGTAGCGACAGGGACTCAACTCAGCAACACGC
TAGCCGATGAGCCTCTTGCATATGTCGGGCAGGGATACCATCGCTTTCGTCGTTATGCGCCCCGCATGTTGCGCTGTCTG
AAGCTCGAAGCCGCGCCGGTCGCCGGACCATTGGTAGCAGCAGCTTTGTCGATCGGAGAGATGAAAGGTGTTGCATCGCC
AGAAAGGCGTTTCCTGCGGCCCAGCTCCAAATGGAACCGTCATTTACGAGCTCAGGAAAAAGGAGATACCCGTCTTTGGG
AAGTGGCGGTACTCTTTCACCTCCGGGATGCTTTTCGTTCCGGAGATGTCTGGCTCGCTCATTCGCGCCGCTATGGTGAC
CTCAAGCAGGTACTGGTGCCGATGATCGCGGCGCAGGAAAATGCAAAACTGGCCGTGCCTTCCAACCCACAGGATTGGCT
GGCAGACAGAAAGGCGCGACTCACGATCGCTCTTAAGCGGCTGGCCCGGGCTGCCCGTAACGGCACTATTCCGCACGGTA
GCATAGAAGATGGAACGTTGCGGATCGACAGGTTGACAGCAGACGTGCCGGATGGTGCCGAGGCACTCATACTGGATCTG
TATCGCCGAATGCCGTCCGTTCGGATTACCGACATGCTGCTTGAAGTTGATGCAGCCCTTGGTTTCACAGATGCGTTTAC
CCATCTGAGAACCGGGGCTCCATGTCGCGACCGGATCGGTCTGCTCAACGTCCTGCTCGCTGAAGGGCTCAATCTGGGCC
TGCGTAAGATGGCGGAAGCTACAAACACGCATGATTACTGGCAGCTCTCACGCCTTGCCCGCTGGCATGTTGAAAGCGAA
GCCATGAACCAGGCATTGGCAATTGTGGTGGCCGCGCAGGGTAAACTGCCGATGTCACGCGTCTGGGGGATGGGCACGTC
AGCATCGAGCGATGGTCAGTTTTTCCCGACAGCGCGGCATGGCGAAGCCATGAACATGGTCAATGCCAAATATGGTTCTG
TTCCCGGCCTCAAAGCGTATACTCACGTAAGCGACCAGTTCGCGCCATTCGCTTGTCAGTCGATCCCGGCGACCGTGAGC
GAGGCACCGTATATTCTCGATGGACTACTGATGAACGAGGTCGGTCGCCATGTTCGCGAACAGTATGCCGATACAGCAGG
ATTCACCGACCATTTGTTCGGAGCCAGTAGCCTGCTCGGCTACAATCTCGTTCTGCGAATCAGGGATCTGCCATCGAAGC
GGTTGTACGTATTTAATCCCGATACGACCCCCAGGGAGTTACGCAAGTTGGTAGGTGGAAAAGCCCGGGAGGATCTTATC
GTTGCGAACTGGCCTGATATTTTCCGTTGTGCCGCGACGATGACCGCTGGCAAAATCAGGCCCAGCCAACTCCTGCGCAA
GCTCGCTTCTTACCCACGACAAAACAACCTTGCAGTTGCGCTTCGTGAAGTTGGTCGTATTGAACGGACCCTTTTCATTA
TTGAGTGGATCCTGGATACGGACATGCAGCGGCGTGCTCAGATCGGTCTTAACAAGGGAGAGGCCCACCATGCGCTCAAA
AATGCGCTCCGTATCGGGAGGCAGGGGAAATTCGCGATCGCACGACAGAGGGGCAGCACTACCGAATCGCTGGGCTCAA
TTTATTGACTGCGGTGATCATTTACTGGAATACCGTCCATCTTGGTCATGCCGTCACGGAGCGGCGGAACGAAGGGTTGG
ATGTTCCCCCTGAATTTCTTCCCCACATATCCCCATTGGGCTGGGCGCACATTCTACTGACTGGCGAATATCTTTGGCCC
AAGGAACCGAAAGCTTAG
>Cluster_25899
GTGCTAGAAGAATACCGTAAGCACGTAGCTGAGCGTGCCGCTGAGGGGATTGCGCCCAAACCCCTGGATGCAAACCAAAT
GGCCGCACTTGTAGAGCTGCTGAAAAACCCGCCCGCGGGCGAAGAAGAATTCCTGTTAGATCTGTTAACCAACCGTGTTC
CCCCTGGCGTCGATGAAGCCGCCTATGTCAAAGCAGGCTTCCTGGCTGCTGTCGCCAAAGGCGAAGCTAAATCCCCGCTG
CTGACTCCGGAAAAAGCCATTGAACTGCTGGGCACTATGCAGGGTGGTTACAACATTCATCCGCTGATCGACGCGCTGGA
TGATGCCAAACTGGCACCGATCGCTGCCAAAGCACTTTCTCACACGCTGCTGATGTTCGATAACTTCTATGACGTAGAAG
AGAAAGCCAAAGCAGGCAACGAATATGCGAAGCAGGTAATGCAGTCCTGGGCGGATGCCGAATGGTTCCTGAATCGCCCG
GCGCTGGCTGAAAAACTGACCGTTACCGTCTTCAAAGTCACTGGCGAAACCAACACCGATGACCTCTCTCCGGCACCAGA
TGCGTGGTCACGCCCGGATATCCCACTGCACGCGCTGGCGATGCTGAAAAACGCCCGTGAAGGTATTGAGCCAGATCAGC
CTGGTGTTGTTGGCCCGATCAAACAGATTGAAGCTCTGCAACAGAAAGGTTTCCCGCTGGCGTACGTCGGTGACGTTGTG
GGTACGGGTTCATCGCGTAAATCCGCCACGAACTCCGTTCTGTGGTTTATGGGCGATGATATTCCACATGTGCCGAACAA
ACGCGGCGGTGGTTTGTGCCTCGGCGGTAAAATTGCACCCATCTTCTTTAACACGATGGAAGACGCGGGTGCACTGCCAA

TCGAAGTCGACGTCTCTAACCTGAACATGGGCGACGTAATTGACGTTTACCCGTACAAAGGTGAAGTGCGTAACCACGAA
ACCGGCGAACTGCTGGCAACCTTCGAACTGAAAACCGACGTGCTGATTGATGAAGTGCGTGCTGGCGGCCGTATTCCGCT
GATTATCGGGCGTGGCCTGACCACCAAAGCGCGTGAAGCACTTGGCCTGCCGCACAGCGATGTGTTCCGTCAGGCGAAAG
ATGTCGCTGAGAGCGACCGTGGCTTCTCGCTGGCGCAGAAAATGGTGGGCCGTGCCTGTGGCGTGAAAGGCATTCGTCCG
GGCGCGTACTGTGAACCGAAAATGACTTCTGTTGGTTCTCAGGATACCACCGGCCCGATGACCCGTGATGAACTGAAAGA
CCTGGCGTGCCTGGGCTTCTCGGCTGACCTGGTGATGCAGTCTTTCTGCCACACCGCGGCGTATCCGAAGCCAGTTGACG
TGAACACGCACCACACGCTGCCGGACTTCATTATGAACCGTGGCGGTGTGTCGCTGCGTCCGGGTGACGGCGTAATCCAC
TCCTGGCTGAACCGTATGCTGCTGCCGGATACCGTCGGTACCGGTGGTGACTCCCATACCCGTTTCCCGATCGGTATCTC
TTTCCCGGCGGGTTCTGGTCTGGTGGCATTTGCAGCCGCAACAGGCGTAATGCCGCTGGATATGCCAGAATCCGTTCTGG
TGCGCTTCAAAGGCAAAATGCAGCCGGGTATCACCCTGCGCGATCTGGTACACGCGATCCCGCTGTATGCAATCAAACAA
GGTCTGCTGACCGTTGAGAAGAAAGGTAAGAAAAACATCTTCTCTGGCCGCATTCTGGAAATTGAAGGTCTGCCGGATCT
GAAAGTTGAGCAGGCATTTGAGCTGACCGATGCGTCTGCCGAGCGCTCTGCTGCCGGTTGTACCATCAAGCTGAACAAAG
AACCGATCATTGAATACCTGAACTCTAACATCGTCCTGCTGAAGTGGATGATCGCGGAAGGTTACGGCGATCGTCGTACC
CTGGAGCGTCGTATTCAGGGCATGGAAAAATGGCTGGCGAATCCTGAGCTGCTGGAAGCCGATGCAGATGCGGAATACGC
GGCAGTGATCGACATCGATCTGGCGGATATCAAAGAGCCAATCATCTGTGCACCGAATGACCCAGACGACGCACGTCCGC
TGTCTGCGGTACAGGGCGAGAAGATCGACGAAGTATTTATCGGTTCTTGCATGACTAACATCGGTCACTTCCGTGCTGCT
GGTAAACTGCTGGATGCGCACAAAGGTCAGTTGCCGACCCGCCTGTGGGTGGCACCGCCAACGCGTATGGACGCTGCGCA
GCTGACCGAAGAAGGCTACTACAGCGTCTTCGGTAAGAGTGGTGCTCGTATCGAGATCCCTGGCTGTTCCCTGTGTATGG
GTAACCAGGCGCGTGTAGCAGACGGTGCGACGGTGGTTTCCACCTCTACCCGTAACTTCCCGAACCGTTTGGGTACTGGC
GCGAATGTCTTCCTGGCTTCTGCGGAACTGGCTGCTGTTGCGGCGCTGATTGGCAAACTGCCGACGCCGGAAGAGTACCA
GACCTACGTGGCGCAGGTAGATAAAACAGCCGTTGATACTTACCGTTATCTGAACTTCGACCAGCTTTCTCAGTACACCG
AAAAAGCGGACGGGGTGATTTTCCAGACTGCGGTTTAA
>Cluster_63620
ATGAGCACAAAACCACTCTTCCTGTTACGGAAAGCGAAAAAATCATCCGGTGAACCTGACGTCGTCCTGTGGGCAAGCAA
CGATTTTGAATCGACCTGTGCCACTCTGGACTACCTGATCGTTAAGTCAGGTAAAAAACTGAGCAGCTATTTTAAAGCTG
TTGCCACGAATTTTCCTGTCGTTAATGACCTGCCCGCTGAAGGTGAGATCGATTTTACCTGGAGTGAACGCTATCAACTC
AGCAAAGACTCCATGACATGGGAACTAAAACCGGGAGCAGCACCAGACAACGCTCACTATCAAGGCAATACCAACGTCAA
CGGCGAAGACATGACTGAGATTGAGGAGAATATGCTACTCCCAATTTCTGGCCAGGAACTGCCCATTCGTTGGCTTGCTC
AACACGGCAGCGAAAAACCGGTAACGCACGTTTCACGCGACGGACTCCAGGCATTACACATTGCTCGGGCTGAAGAACTA
CCGGCTGTTACTGCCCTGGCTGTTTCCCACAAAACCAGCCTGCTCGACCCGCTGGAAATTCGCGAACTCCACAAACTGGT
TCGTGACACTGACAAAGTTTTCCCTAATCCTGGTAATTCAAACCTGGGACTGATAACTGCTTTTTTCGAAGCATACCTGA
ACGCTGACTACACCGATCGAGGACTGCTGACAAAAGAGTGGATGAAGGGTAATCGTGTTTCACACATCACTCGCACGGCT
TCCGGTGCTAATGCTGGCGGCGGAAACCTCACCGATCGCGGCGAAGGTTTCGTACACGATCTGACGTCACTGGCGCGCGA
CGTAGCCACTGGCGTACTGGCCCGTTCAATGGATCTGGACATCTATAACCTTCATCCGGCACACGCTAAACGCATTGAGG
AAATTATCGCTGAAAATAAACCGCCCTTTTCTGTTTTCCGCGACAAATTCATCACCATGCCTGGCGGGCTGGATTATTCC
CGCGCCATCGTGGTTGCGTCCGTAAAAGAAGCACCAATTGGGATCGAGGTCATCCCCGCGCACGTCACTGAATATCTGAA
CAAAGTACTGACTGAAACCGATCATGCCAACCCTGATCCGGAAATCGTGGATATTGCCTGCGGTCGCTCCTCTGCCCCGA
TGCCGCAGCGAGTAACAGAAGAAGGAAAACAGGATGATGAAGAAAAACCGCAACCATCTGGAACAACGGCAGTTGAACAG
GGAGAGGCTGAAACAATGGAACCGGACGCAACTGAACATCATCAGGACACGCAGCCGCTGGATGCTCAGTCACAGGTAAA
TTCTGTTGATGCGAAATATCAGGAACTGCGGGCAGAACTCCATAAAGCCCGGAAAAACATTCCATCAAAAAATCCTGTCG
ATGCCGATAAATTGCTTGCTGCATCACGTGATGAATTTGTTGACGGAATTAGCGACCCGAACGATCCGAAATGGGTAAAG
GGGATCCAGACTCGCGATTGTGTGTACCAGAACCAGCCAGAAACGGAAAAAACCAGCCCGGATATGAATCAACCTGAGCC
AGTAGTGCAACAGGAACCGGAAATAGCCTGCAATGCCTGCGGCCAGACTGGCGGGGATAACTGCCCTGACTGTGGTGCGG
TGATGGGCGACGCAACATACCAGGAAACATTCGATGAAGAGAGTCAGGTTGAAGCTAAGGAAAATGATCCGGAGGAAATG
GAAGGCGCTGAACATCCGCACAATGAGAATGCTGGCAGCGATCCGCATCGCGATTGCAGTGATGAAACTGGCGAAGTCGC
AGATCCCGTAATCGTAGAAGACATAGAGCCAGGTATTTATTACGGAATTTCGAATGAGAATTACCACGCGGGTCCCGGTA
TCAGTAAGTCTCAGCTCGATGACATTGCTGATACTCCGGCACTATATTTGTGGCGTAAAAATGCCCCCGTGGACACCACA
AAGACAAAAACGCTCGATTTAGGAACTGCTTTCCACTGCCGGGTACTTGAACCGGAAGAATTCAGTAACCGCTTTATCGT
AGCACCTGAATTTAACCGCCGTACAAACGCCGGAAAAGAAGAAGAGAAAGCGTTTCTGATGGAATGCGCAAGCACAGGAA
AAACGGTTATCACTGCGGAAGAAGGCCGGAAAATTGAACTCATGTATCAAAGCGTTATGGCTTTGCCGCTGGGGCAATGG
CTTGTTGAAAGCGCCGGACACGCTGAATCATCAATTTACTGGGAAGATCCTGAAACAGGAATTTTGTGTCGGTGCCGTCC
GGACAAAATTATCCCTGAATTTCACTGGATCATGGACGTGAAAACTACGGCGGATATTCAACGATTCAAAACCGCTTATT
ACGACTACCGCTATCACGTTCAGGATGCATTCTACAGTGACGGTTATGAAGCACAGTTTGGAGTGCAGCCAACTTTCGTT
TTTCTGGTTGCCAGCACAACTATTGAATGCGGACGTTATCCGGTTGAAATTTTCATGATGGGCGAAGAAGCAAAACTGGC
AGGTCAACAGGAATATCACCGCAATCTGCGAACCCTGTCTGACTGCCTGAATACCGATGAATGGCCAGCTATTAAGACAT
TATCACTGCCCCGCTGGGCTAAGGAATATGCAAATGACTAA
>Cluster_41470
ATGGCGAATTTCTTTATTGATCGCCCCATTTTTGCCTGGGTGCTGGCAATCCTGTTGTGTCTGACAGGTACCCTGGCGAT
TTTTTCATTGCCCGTTGAACAATACCCCGATCTCGCGCCACCGAATGTGCGAGTGACCGCTAACTATCCCGGCGCATCGG
CCCAGACGCTGGAAAATACCGTGACCCAGGTTATCGAGCAAAATATGACCGGCCTCAATAATCTGATGTATATGTCATCT
CAGAGCAGCGGCACCGGTCAGGCATCTGTCACGTTAAGTTTTAAAGCAGGCACCGATCCGGACGAAGCCGTGCAGCAAGT
ACAAAACCAGCTACAATCAGCCATGCGAAAGTTACCGCAGGCGGTGCAAAATCAGGGCGTGACTGTGCGTAAAACCGGCG
ATACCAACATTCTGACCATTGCCTTCGTCTCTACCGATGGTTCGATGGATAAACAGGATATTGCCGATTATGTTGCCAGT
AATATTCAGGACCCGTTAAGCCGCGTGAATGGCGTCGGGGATATCGATGCCTATGGTTCGCAATATTCCATGCGTATCTG
GCTGGACCCGGCGAAACTCAACAGTTTCCAGATGACGGCTAAAGATGTCACCGATGCCATTGAATCACAGAACGCGCAGA
TTGCGGTTGGGCAACTTGGTGGTACGCCTTCCGTCGATAAGCAGGCGCTCAACGCCACCATTAACGCCCAGTCACTGCTG
CAAACACCAGAACAGTTCCGCGATATCACCTTGCGGGTGAATCAGGACGGCTCAGAGGTAAGGCTGGGCGATGTCGCCAC
CGTCGAAATGGGGGCGGAGAAATACGATTATCTTAGCCGCTTCAATGGTAAGCCAGCCTCCGGGCTGGGGGTTAAACTGG
CCTCCGGCGCGAACGAAATGGCGACAGCGGAGCTGGTGCTCAATCGTCTCGACGAGCTGGCGCAGTATTTTCCACACGGA
CTGGAATACAAGGTGGCGTATGAAACCACCTCGTTTGTTAAAGCCTCCATTGAAGACGTGGTGAAAACGCTTCTGGAAGC
TATCGCCCTGGTTTTCCTCGTTATGTATCTGTTCCTGCAAAACTTCCGCGCCACGCTGATACCCACTATCGCCGTGCCGG
TGGTGTTGATGGGAACCTTCTCCGTGCTTTACGCCTTCGGTTACAGTGTCAACACCTTAACCATGTTCGCGATGGTGCTG
GCGATCGGTCTGCTGGTGGATGATGCCATCGTGGTGGTGGAAAACGTCGAACGTATTATGAGTGAGGAAGGACTCACTCC
TCGCGAAGCCACACGTAAATCGATGGGGCAGATCCAGGGGGCACTGGTCGGGATTGCGATGGTGCTTTCGGCGGTATTTG
TGCCAATGGCCTTCTTCGGCGGCACCACCGGTGCCATCTATCGCCAGTTCTCTATTACCATTGTTGCGGCGATGGTGCTG
TCAGTACTGGTAGCGATGATCCTCACTCCGGCTCTGTGTGCCACGCTACTTAAGCCACTGAAAAAAGGTGAGCATCACGG
GCAAAAAGGCTTTTTTGCCTGGTTTAACCAGATGTTTAACCGCAACGCCGAACGCTACGAAAAAGGGGTGGCGAAAATTC
TCCACCGTAGCCTGCGCTGGATTGTGATTTATGTCCTGCTGCTTGGCGGCATGGTGTTCCTGTTCCTGCGTTTGCCGACG
TCGTTCTTACCGCTGGAAGACCGTGGCATGTTTACTACCTCGGTACAGTTGCCCAGCGGTTCAACCCAGCAACAGACCCT

GAAAGTCGTTGAGCAAATCGAGAAATATTACTTCACCCATGAAAAAGACAACATCATGTCGGTGTTTGCCACCGTGGGTT
CTGGCCCTGGGGGTAACGGGCAAAACGTGGCGCGAATGTTTATCCGCCTGAAAGACTGGAGCGAACGCGACAGTAAGACC
GGCACCTCGTTTGCCATTATCGAGCGTGCAACGAAGGCGTTTAACAAGATTAAAGAAGCTCGCGTTATCGCCAGCAGCCC
GCCAGCAATTAGCGGTCTTGGTAGTTCTGCGGGTTTTGATATGGAGTTGCAGGACCACGCTGGAGCGGGTCACGATGCGC
TGATGGCAGCACGAAACCAGTTGCTGGCGCTGGCAGCGGAAAACCCGGAGCTAACCCGTGTGCGCCATAACGGCCTCGAC
GACAGTCCGCAGTTGCAGATTGATATCGACCAGCGTAAAGCTCAGGCGCTGGGCGTTGCTATCGACGATATTAACGACAC
ACTGCAAACCGCCTGGGGTTCGAGCTATGTGAATGACTTTATGGATCGCGGTCGCGTGAAGAAAGTCTATGTGCAGGCAG
CTGCGCCGTATCGCATGCTGCCAGATGACATCAATCTCTGGTATGTCCGAAATAAAGATGGCGGCATGGTGCCCTTCTCT
GCTTTCGCGACCTCACGCTGGGAGACAGGCTCACCGCGTCTGGAACGCTATAATGGTTATTCTGCGGTTGAGATTGTTGG
GGAAGCCGCACCGGGGGTCAGTACCGGTACGGCGATGGATATTATGGAATCGTTAGTGAAGCAGCTGCCAAACGGCTTTG
GTCTGGAGTGGACGGCGATGTCGTATCAGGAGCGGCTTTCCGGCGCGCAGGCTCCGGCGCTGTACGCCATTTCCTTGCTG
GTGGTATTCCTGTGTCTGGCGGCATTGTATGAAAGCTGGTCGGTGCCGTTCTCGGTAATGCTGGTCGTGCCGCTGGGGGT
TATCGGCGCGCTGCTGGCAACCTGGATGCGCGGGCTGGAAAACGATGTTTACTTCCAGGTGGGTCTGTTAACGGTCATTG
GTTTATCGGCGAAAAACGCTATCCTGATCGTCGAGTTTGCTAACGAGATGAACCAAAAAGGCCACGACCTGTTTGAAGCG
ACGCTCCACGCCTGCCGTCAGCGTTTACGCCCGATTCTGATGACCTCGCTGGCATTTATCTTCGGCGTATTGCCAATGGC
AACCAGCACGGGTGCCGGTTCCGGTGGTCAGCATGCGGTGGGTACTGGCGTAATGGGCGGGATGATTTCGGCCACTATTC
TGGCTATTTACTTCGTGCCGCTGTTCTTTGTGCTGGTGCGCCGCCGCTTCCCGCTGAAGCCGCGCCCGGAATAA
>Cluster_20082
ATGCCAAAACGTACAGATATAAAAAGTATCCTGATTCTGGGTGCGGGCCCGATTGTTATCGGTCAGGCGTGTGAGTTTGA
CTACTCTGGCGCGCAAGCGTGTAAAGCCCTGCGTGAAGAGGGCTACCGCGTCATTCTGGTGAACTCCAACCCGGCGACTA
TCATGACCGACCCGGAAATGGCTGATGCAACCTACATCGAGCCGATTCACTGGGAAGTGGTACGCAAAATTATTGAAAAA
GAGCGCCCGGACGCGGTGCTGCCAACCATGGGGGGGCAGACGGCGCTGAACTGTGCGCTGGAGCTTGAGCGTCAGGGCGT
GCTGGAAGAGTTCGGCGTCACCATGATTGGTGCCACTGCCGATGCGATTGATAAAGCCGAAGACCGCCGCCGTTTCGACG
TAGCGATGAAGAAAATCGGTCTGGAAACTGCGCGTTCCGGTATCGCTCATTCAATGGAAGAAGCGCTGGCGGTTGCCGCT
GAAGTTGGCTTCCCGTGCATTATTCGCCCATCCTTTACCATGGGTGGTAGCGGCGGCGGTATCGCTTATAACCGCGAAGA
GTTTGAAGAAATTTGCGCCCGCGGTCTGGATCTCTCTCCGACTAAAGAGCTGCTGATTGATGAGTCGCTGATCGGCTGGA
AAGAGTACGAGATGGAAGTGGTGCGTGATAAAAACGACAACTGCATCATCGTCTGCTCTATCGAAAACTTCGATGCGATG
GGCATCCATACCGGTGACTCCATCACTGTCGCGCCAGCCCAAACGCTGACCGACAAAGAATATCAAATCATGCGTAACGC
CTCGATGGCGGTACTGCGTGAAATCGGCGTTGAAACCGGTGGTTCCAACGTCCAGTTTGCGGTGAACCCGAAAAACGGTC
GCCTGATTGTTATCGAAATGAACCCGCGCGTGTCTCGCTCCTCTGCGCTGGCGTCGAAAGCCACTGGTTTCCCGATTGCC
AAAGTGGCGGCGAAACTGGCGGTGGGTTACACCCTCGACGAGCTGATGAACGACATCACCGGTGGGCGTACTCCGGCCTC
CTTCGAGCCGTCCATCGACTATGTGGTTACTAAAATTCCTCGCTTCAACTTCGAGAAATTCGCCGGTGCTAACGACCGTC
TGACCACTCAGATGAAATCGGTTGGCGAAGTGATGGCGATTGGTCGCACGCAGCAGGAATCCCTGCAAAAAGCGCTGCGC
GGCCTGGAAGTGGGCGCAACTGGCTTCGATCCGAAAGTGAGTCTCGACGACCCGGAAGCGCTGACTAAAATCCGCCGCGA
ACTGAAAGATGCTGGCGCAGAGCGTATCTGGTATATCGCTGATGCCTTCCGCGCGGGCCTGTCTGTAGACGGCGTGTTTA
ATTTGACCAACATTGACCGCTGGTTCCTGGTACAGATTGAAGAGCTGGTGCGTCTGGAAGAGAAAGTCGCAGAAGTGGGC
ATCACTGGCCTGAACGCTGAATTCCTGCGCCAGCTGAAACGCAAAGGCTTTGCCGATGCGCGTCTGGCAAAACTCGCGGG
CGTGCGTGAAGCAGAAATCCGCAAGCTGCGCGACCAGTATGACTTGCACCCGGTTTATAAGCGCGTGGATACCTGTGCGG
CAGAGTTCGCCACCGACACCGCTTACATGTACTCCACTTATGAAGAAGAGTGCGAAGCGAATCCGTCTACCGACCGTGAA
AAAATCATGGTGCTCGGCGGCGGCCCGAACCGTATCGGTCAGGGTATCGAATTCGACTACTGCTGTGTACACGCCTCGCT
GGCGCTGCGCGAAGACGGTTATGAAACCATTATGGTTAACTGTAACCCGGAAACCGTCTCCACCGACTACGACACCTCTG
ACCGTCTCTACTTCGAGCCGGTAACTCTGGAAGACGTGCTGGAAATTGTGCGTATTGAGAAGCCGAAAGGCGTTATCGTC
CAGTACGGCGGTCAGACCCCGCTGAAACTGGCGCGCGCGCTGGAAGCTGCTGGCGTACCGGTTATCGGTACCAGCCCGGA
TGCCATCGACCGTGCGGAAGACCGTGAACGCTTCCAGCATGCGGTTGAGCGTCTGAAACTGAAACAACCGGCGAACGCCA
CCGTTACCACCATCGAAATGGCGGTTGAGAAAGCGAAAGAGATTGGCTACCCGCTGGTGGTGCGTCCGTCTTACGTTCTC
GGCGGTCGGGCGATGGAAATCGTCTATGACGAAGCTGACCTGCGTCGCTACTTCCAGACAGCGGTCAGCGTATCTAACGA
TGCGCCAGTGTTGCTGGACCACTTCCTTGATGACGCAGTAGAAGTTGACGTGGATGCCATCTGCGACGGCGAAATGGTGC
TGATTGGCGGCATCATGGAGCATATTGAGCAGGCGGGCGTGCACTCCGGTGACTCCGCATGTTCGCTGCCAGCCTACACC
TTAAGTCAGGAAATTCAGGATGTGATGCGCCAGCAGGTGCAGAAACTGGCCTTCGAATTGCAGGTGCGCGGCCTGATGAA
CGTGCAGTTTGCGGTGAAAAACAACGAAGTCTACCTGATTGAAGTTAACCCGCGTGCGGCGCGTACCGTTCCGTTCGTCT
CCAAAGCCACCGGCGTACCGCTGGCAAAAGTGGCGGCGCGTGTGATGGCTGGCAAATCGCTGGCTGAGCAGGGCGTAACC
AAAGAAGTTATCCCGCCGTACTACTCGGTGAAAGAAGTGGTGCTGCCGTTCAATAAATTCCCGGGCGTTGACCCGCTGTT
AGGGCCAGAAATGCGCTCTACCGGGGAAGTAATGGGCGTGGGTCGCACCTTCGCTGAAGCGTTTGCCAAAGCGCAGCTGG
GCAGCAACTCCACCATGAAGAAACACGGTCGTGCGCTGCTTTCCGTGCGCGAAGGCGATAAAGAACGCGTGGTGGACCTG
GCGGCAAAACTGCTGAAACAGGGCTTCGAGCTGGATGCGACTCACGGCACGGCGATTGTGCTGGGCGAAGCTGGTATCAA
TCCGCGTCTGGTGAACAAGGTGCATGAAGGCCGTCCGCACATTCAGGACCGTATTAAGAATGGCGAATATACCTACATCA
TCAACACCACCTCAGGCCGTCGTGCGATTGAAGACTCCCGCGTGATCCGTCGCAGTGCGCTGCAATATAAAGTGCATTAC
GACACCACCCTGAACGGCGGCTTTGCCACCGCGATGGCGCTGAATGCCGATGCGACCGAAAAAGTAATTTCGGTGCAGGA
AATGCACGCGCAGATCAAATAA
>Cluster_45685
ATGATTCCACAGATTTCCCAGGCACCCGGCGTCGTTCAACTGGTGCTTAATTTTTTGCAAGAGCTGGAGCAACAAGGTTT
TACCGGCGATACGGCGACAAGTTATGCCGATCGTCTGACAATGTCGACCGACAACAGTATTTACCAACTTCTCCCCGATG
CGGTGGTATTTCCGCGTTCAACCGCAGATGTGGCGCTGATCGCCCGTCTTGCCGCGCAGGAACGCTATTCATCGCTGATC
TTTACCCCCCGCGGCGGCGGCACCGGCACTAACGGTCAGGCGCTCAACCAGGGGATTATTGTTGATATGTCCCGCCATAT
GAACCGCATCATCGAAATTAACCCTGAAGAGGGCTGGGTGCGCGTTGAGGCCGGGGTGATAAAAGATCAACTCAATCAGT
ACCTGAAACCGTTCGGCTACTTTTTTGCGCCGGAACTTTCGACCAGCAACCGGGCAACGCTCGGTGGGATGATCAATACC
GATGCTTCTGGTCAGGGATCGCTGGTCTATGGCAAAACGTCGGATCACGTACTTGGCGTACGTGCGGTGTTGTTGGGGGG
CGATATTCTCGATACGCAACCTTTACCCGTCGAACTGGCGGAAACGCTGGGTAAATCCAATACCACAATCGGGCGAATTT
ATAACACGGTTTATCAACGTTGCCGTCAGCAACGCCAGTTAATCATCGACAACTTCCCCAAACTTAACCGCTTTCTTACC
GGTTACGATCTGCGTCATGTCTTTAACGATGAGATGACCGAGTTCGACCTGACGCGCATTCTGACGGGTTCAGAAGGGAC
GCTGGCCTTTATTACCGAAGCGCGGCTGGATATTACGCCCTTGCCTAAAGTGCGCCGTCTGGTGAACGTCAAATATGACT
CTTTTGACTCCGCGCTGCGTAACGCGCCGTTTATGGTTGAGGCGCGGGCGCTTTCGGTAGAGACGGTGGACTCAAAAGTG
CTGAATCTGGCGCGGGAAGATATTGTCTGGCATTCCGTCAGCGAGTTGATTACCGATGTGCCTGACAAAGAGATGCTCGG
GCTGAACATTGTGGAGTTTGCTGGTGATGATGAGGCGCTGATTGACGAGCGAGTTAATGCTCTCTGTGTGCGGCTTGATG
AGCTGATTGCCAGCCAACAGGCAGGTGTGATTGGCTGGCAGGTGTGCCGCGAGCTGGCGGGTGTTGAACGTATCTATGCG
ATGCGTAAAAAAGCCGTTGGTCTGCTTGGCAATGCCAAAGGTGCCGCTAAGCCAATTCCGTTTGCTGAGGATACCTGCGT
ACCGCCGGAACATCTGGCCGATTATATTGCTGAATTTCGCGCGCTGCTCGACAGCCACGGCTTAAGCTACGGTATGTTCG
GTCACGTCGACGCAGGTGTCTTGCACGTCCGTCCAGCGCTGGATATGTGCGATCCTCAACAAGAGATTTTGATGAAGCAA
ATCTCTGATGACGTAGTGGCGCTGACTGCGAAATACGGTGGTTTGTTGTGGGGCGAGCACGGCAAAGGTTTTCGCGCTGA

ATACAGCCCGGCGTTTTTCGGTGAGGAACTTTTTGCAGAACTGCGCAAAGTGAAAGCGGCATTTGACCCGCATAACCGAC
TCAACCCAGGGAAGATTTGCCCGCCAGAAGGTCTCGATGCGCCGATGATGAAAGTGGACGCGGTGAAGCGCGGTACATTC
GATCGGCAGATCCCCATTGCGGTACGCCAGCAGTGGCGCGGTGCGATGGAGTGTAACGGCAACGGTTTATGCTTCAACTT
TGATGCCCGTAGTCCGATGTGTCCGTCGATGAAGATCACCCAGAACCGGATTCATTCACCGAAAGGGCGCGCAACGCTGG
TGCGTGAATGGCTGCGTTTGTTGGCGGATCGCGGCGTTGATCCACTCAAACTGGAACAAGAACTGCCTGAATCCGGCGTC
AGTTTGCGGACGTTAATTGCCCGCACGCGCAATAGCTGGCATGCGAATAAAGGCGAATATGACTTCTCACACGAAGTCAA
AGAGGCGATGTCGGGCTGTCTGGCCTGTAAAGCGTGTTCGACCCAGTGCCCCATCAAAATTGATGTGCCGGAGTTTCGCT
CTCGTTTTCTGCAGCTCTATCACACCCGTTATTTACGCCCGCTGCGCGACCACCTCGTCGCTACGGTCGAGAGCTACGCG
CCGCTGATGGCACGCGCGCCGAAAACCTTTAACTTCTTCATTAACCAGCCGCTGGTGCGCAAACTCTCGGAAAAACATAT
CGGCATGGTTGATTTGCCGCTGCTGTCGGTCCCCTCGCTACAACAACAAATGGTGGGGCATCGCTCGGCAAACATGACGC
TGGAACAGCTTGAATCTCTCAATGCAGAGCAGAAAGCGCGCACAGTGTTGGTGGTGCAGGACCCCTTTACCAGCTATTAC
GATGCGCAAGTGGTGGCGGATTTTGTCCGTCTGGTCGAAAAATTAGGTTTCCAGCCTGTGTTACTGCCATTTTCGCCAAA
TGGCAAAGCCCAGCATATTAAAGGTTTTCTTAATCGTTTTGCGAAGACGGCGAAAAAGACGGCGGATTTCCTCAACCGTA
TGGCGAAGCTGGGTATGCCAATGGTGGGCGTCGATCCGGCGCTGGTACTTTGTTATCGCGATGAATATAAACTGGCGCTG
GGCGAGGAACGTGGCGAGTTTAACGTCTTACTGGCGAATGAATGGCTGGCAAGCGCACTTGAGTCACAGCCAGTGGCTAC
AGTCAGCGGTGAATCATGGTATTTCTTTGGTCACTGTACCGAAGTTACCGCCTTGCCGGGTGCGCCAGCACAATGGGCCG
CGATATTTGCCCGTTTTGGCGCGAAACTGGAAAATGTCAGCGTGGGTTGCTGCGGCATGGCAGGGACTTACGGACATGAA
GCGAAAAACCATAAAAATTCGCTCGGGATCTATGAGTTATCCTGGCATCAGGCTATGCAGCGACTGCCGCGTAACCGCTG
TCTGGCGACCGGATATTCCTGCCGTAGCCAGGTAAAACGGGTTGAAGGCACGGGGGTACGCCATCCTGTGCAGGCTTTAC
TGGAGATTATTAAATGA
>Cluster_22228
GTGAAGTTTTTTGCCCTCTTCATTTACCGCCCGGTGGCGACGATTTTACTGTCGGTCGCCATTACCCTGTGCGGCATACT
GGGTTTCCGTATGCTGCCAGTCGCCCCGCTGCCGCAGGTCGATTTTCCGGTGATCATGGTCAGCGCCTCGCTGCCCGGTG
CGTCACCAGAAACAATGGCGTCTTCCGTTGCCACGCCGCTGGAGCGCTCACTTGGGCGCATTGCCGGAGTCAGCGAAATG
ACCTCCAGCAGTTCGCTCGGCAGCACGCGCATTATTTTGCAGTTTGATTTTGACCGGGATATCAACGGCGCAGCGCGCGA
TGTGCAGGCGGCGATCAACGCCGCGCAAAGTCTGCTACCCAGCGGGATGCCCAGCCGTCCGACCTATCGCAAAGCGAACC
CGTCGGATGCGCCAATTATGATCCTCACGCTAACCTCCGATACTTATTCGCAGGGTGAACTGTACGATTTCGCCTCGACG
CAGCTGGCTCCGACGATTTCGCAAATCGACGGTGTCGGTGATGTCGATGTTGGCGGCAGCTCGCTGCCCGCCGTACGCGT
CGGGCTGAATCCGCAGGCGCTGTTTAATCAGGGCGTCTCGCTGGACGACGTACGCACTGCCATCAGCAATGCCAACGTGC
GTAAACCGCAGGGCGCACTGGAAGATGGCACCCACCGCTGGCAGATCCAGACCAATGACGAACTGAAAACTGCCGCTGAA
TATCAGCCGCTGATTATTCATTACAACAACGGCGGCGCGGTTCGTCTGGGCGATGTGGCGACGGTGACCGACTCTGTACA
AGATGTACGTAACGCCGGGATGACCAACGCCAAACCGGCCATTTTGCTGATGATCCGCAAGCTGCCGGAAGCCAATATTA
TCCAGACGGTAGACAGCATCCGCGCCCGTCTGCCGGAATTACAATCCACCATTCCGGCGGCGATTGATCTGCAAATTGCC
CAGGATCGCTCCCCTACTATCCGCGCGTCGCTGGAAGAGGTTGAGCAGACGCTGGTAATCTCAGTTGCACTGGTGATTCT
GGTGGTGTTTTTGTTCCTGCGTTCAGGTCGCGCCACCATTATTCCCGCCGTTGCGGTGCCGGTTTCGCTGATTGGTACGT
TTGCGGCGATGTACCTGTGTGGTTTCAGTCTCAATAATCTTTCGTTAATGGCGCTCACCATCGCCACTGGCTTTGTGGTG
GATGACGCCATCGTGGTGCTGGAAAACATTGCCCGTCATCTGGAAGCGGGGATGAAACCGCTACAGGCCGCGCTACAAGG
TACTCGCGAAGTCGGCTTTACGGTGCTGTCGATGAGTCTGTCACTGGTGGCGGTGTTCCTGCCGCTGCTGTTGATGGGCG
GATTGCCGGGTCGCCTGTTACGCGAGTTTGCCGTGACGCTTTCTGTCGCCATTGGTATTTCGCTGCTGGTTTCCCTGACG
TTAACGCCGATGATGTGCGGCTGGATGCTAAAAGCCAGCAAGCCGCGCGAGCAAAAGCGACTGCGTGGCTTTGGTCGCAT
GTTGGTTGCTCTGCAACAAGGCTACGGCAAGTCGCTAAAATGGGTGCTCAATCATACCCGTCTGGTGGGCGTGGTGCTGC
TTGGCACCATTGCGCTGAATATCTGGCTGTATATCTCGATCCCGAAAACCTTCTTCCCGGAGCAGGACACCGGCGTACTG
ATGGGCGGGATACAGGCTGACCAGAGTATTTCGTTTCAGGCGATGCGCGGTAAGTTGCAGGACTTCATGAAAATTATCCG
TGACGATCCGGCGGTGGATAATGTCACCGGCTTTACCGGCGGTTCACGGGTGAACAGTGGAATGATGTTTATCACCCTTA
AACCACGCGGCGAGCGGAGCGAAACGGCCCAACAGATTATCGATCGCTTGCGTAAAAAACTGGCGAAAGAACCGGGAGCG
AATCTGTTCCTGATGGCGGTACAGGATATTCGCGTTGGCGGACGTCAGGCTAACGCCAGCTACCAGTACACATTATTGTC
CGACGATCTGGCGGCACTGCGCGAATGGGAGCCGAAAATCCGCAAAAAACTGGCGACATTGCCAGAGCTGGCTGATGTTA
ACTCCGATCAGGAAGATAATGGCGCGGAGATGAACCTTATTTACGATCGCGACACCATGGCGCGATTGGGAATTGACGTG
CAAGCCGCCAACAGCCTGTTAAATAACGCCTTCGGTCAGCGGCAAATCTCGACCATTTACCAACCGATGAACCAGTACAA
AGTGGTGATGGAAGTGGATCCGCGCTATACCCAGGACATCAGTGCACTGGAAAAAATGTTCGTTATCAATAACGAAGGAA
AAGCGATCCCGCTGTCGTATTTCGCTAAATGGCAACCGGCGAACGCTCCACTGTCGGTGAATCATCAGGGATTATCGGCG
GCCTCGACCATTTCGTTCAACCTGCCGACCGGGAAATCGCTCTCGGACGCCAGTGCGGCGATCGATCGCGCAATGACCCA
GCTTGGTGTGCCTTCGACGGTGCGCGGCAGTTTTGCCGGAACAGCGCAAGTGTTCCAGGAGACGATGAACTCACAGGTGA
TCCTGATTATCGCCGCCATCGCCACGGTATATATCGTACTGGGTATCCTTTACGAGAGTTATGTGCATCCACTGACGATT
CTCTCCACCCTACCCTCGGCGGGCGTCGGAGCGCTGTTGGCGCTGGAGCTGTTCAATGCCCCGTTCAGCCTAATTGCGCT
GATAGGGATCATGTTATTAATCGGCATCGTGAAGAAAAACGCCATTATGATGGTCGATTTTGCGCTTGAAGCCCAACGGC
ACGGTAACCTGACGCCGCAGGAAGCTATTTTCCAGGCCTGTCTGCTGCGTTTTCGCCCGATCATGATGACTACCCTGGCG
GCGCTGTTTGGTGCGCTACCGCTGGTATTGTCGGGCGGTGACGGCTCGGAGTTGCGCCAACCCCTGGGGATCACCATTGT
CGGCGGTCTGGTCATGAGCCAGCTTCTGACGCTGTATACCACGCCGGTGGTGTATCTCTTTTTCGACCGTCTGCGACTGC
GTTTTTCGCGTAAACCTAAACAAGCGGTAACCGAGTAA
>Cluster_44943
ATGAAATCCATGGATAAGTTAACAACGGGTGTCGCCTATGGCACCTCAGCAGGTAGTGCCGGTTACTGGTTTTTACAGCT
GCTCGATAAAGTCACGCCCTCACAGTGGGCAGCAATAGGTGTGCTGGGTAGCCTGGTATTTGGCCTGCTGACGTACCTGA
CAAACCTTTATTTCAAGATTAAAGAAGATAAGCGCAAGGCTGCGAGAGGTGAATAA
>Cluster_39457
GTGACTGGAAAAGAAATCATCCTGGAATATCTGAAAACTCATGAACAATTCTCCCCACATGAATTAGCACTGATCACCGG
AATACCAAATAACAGAATCGCTCAAGCAGCAAGGCATATGGTGAAACAAGGACATTTGAGTGTTGTTGAGCGTAAGTGGA
AGACGGTTATTTATGCAAAACGCAAAGTGAAGAAGGAGCCAATTAAAAGAAATCCAGATGGTACGGGGTGGGGATGTGCA
AATCCAATGACGGCGTTTATTAATAGGGCGCTTATGGAGGTAAGGCAATGA
>Cluster_15983
ATGGCTAACCTACGCAAAGAAGCTCGCGGCAGAGAATGCCAGGTACGTATTTACGGCGTATGCAATGGCAACCCTGAAAC
TACAGTTCTGGCACATTACCGGATGGCTGGAATTTGCGGAACGGGAATGAAGCCTGACGACCTGATCGGCGCATGGGCTT
GTAGCGCGTGTCACGATGAAATCGACCGACGCACCCATAACCTCGACAACAAAGACGCCAGACTTTACCACCTCGAAGGC
GTGATCAGGACGCAGGCGATACTGCTGAAGGAGGGGAAGATTAAGCCATGA
>Cluster_66760
ATGCAGGTACGCAAATATTACCTGAAGCTAAAAACCTGCACGTTAGCCCTTTGTAGGCCAGATAAGACGCGTCAGCGTCG
CATCTGGCATAAACAAAGCGCACTTTGCGGGTCTTTTCCCCTCACCCTAACCCTCACCCCGGAGGGGCGAGGGGACTGTC
TGGGCACATTTTTAGACTTTGTCATCAGTCTGAGCCTGCCATTGGCAGCCTCTGGTGTCCTTTTACGCTACCATGCTAAT
AATCAGCACAATAATAAGCCCAACCACGGAGTTGACCAGCTCCAGTAG

>Cluster_14061
ATGCAAAACACAACTCATGACAACGTAATTCTGGAGCTCACCGTTCGCAACCATCCGGGCGTAATGACCCACGTTTGTGG
CCTTTTTGCCCGCCGCGCTTTTAACGTTGAAGGCATTCTTTGTCTGCCGATTCAGGACAGCGACAAAAGCCATATCTGGC
TACTGGTCAATGACGACCAGCGTCTGGAGCAGATGATAAGCCAAATCGATAAGCTGGAAGATGTCGTGAAAGTGCAGCGT
AATCAGTCCGATCCGACGATGTTTAACAAGATCGCGGTGTTTTTTCAGTAA
>Cluster_17763
ATGACAACTAACAACCACCCGGCGAACGGTCCTGTATCACTCGATCGCCTGCACCAGATAAGCGAAATACTCAGCAAAGC
AGCAGCACAAAGTGACGGAGGCAATCTCGGCTACGCAATGGCTGATGCTGTGAAGGTGATTGATGGAGTGATTGCATCGT
TTAGTGCGGAGCCAGTACCTTGTCCTAAATGTAACGACACCGGAATGGCTGATAGTGGCGGCGTTCAGCCATGGGGAGAG
CCGATACTCATCGAATGTGACTGCCGCGCCGCCATGCTTAAAGGAGATGAAAAATGA
>Cluster_47601
ATGATGATGAACGCTTTCTTTCCGGCAATGGCGCTTATGGTGCTAGTGGGTTGTTCTACACCGTCACCCGTGCAGAAAGC
ACAACGGGTAAAGGTTGATCCTCTGCGTTCGTTGAATATGGAAGCGTTATGCAAGGATCAGGCGGCAAAACGTTATAACA
CCGGCGAGCAAAAAATCGACGTCACCGCCTTCGAACAGTTCCAGGGAAGCTATGAAATGCGCGGTTATACCTTCCGTAAA
GAGCAGTTTGTCTGTTCTTTTGACGCAGATGGCCATTTTTTGCATCTTTCCATGCGTTAA
>Cluster_28835
ATGACGAGAATGAGCGAAACCGAACTACTAAAAATAATCCGTCGCGTTACCGGAATCAGCCAGGAAGCAGGCAAACAGGA
GGCCACGCAGCCGGACAGCGTGATAGCCGAAAATTACGCGCGTGTGGTGGCTGAGGTGATGCGCCGTGACGGTATCGAGC
TTAACGGCGTGGATATACGCAATATACGCACCAGAGTTCTGGAGTTGTTGTCATATCGTCGCCGCGTGGAGATGTACCAG
GAGAAAGAAAAAATTACTTACCACTGGAAGAAGCCGGAGCGGTTACGACGTTAA
>Cluster_42765
ATGGAAGCAGCAAATTTAAGTCCTTCTGGTACAGTATTACCACTGGCAGCCACAATCAATGGAAATAATCTAGTGGATGA
GAAAACAGGAGTGATGCAACCAGAAGGTGGAACAAGTCGTAACGTTAGAATCCTAGCTGGAGTTTTGATAAGTGTAGGTA
CTCTAGCCGCTATCGGTACTGGTATCGCATCACTGTGTGTTGAAGACCCTTCACAAAGATTAGGCTTAGGAATCGCAGCC
GGTGTATTAGGTGGCGTAACTACTGTTGCCGGCGGATTAGCGATGAGATATGCCTAA
>Cluster_54558
ATGACCAAGTCAGAATTGATAGAAAGACTTGCCACCCAGCAATCGCACATTCCCGCCAAGACGGTTGAAGATGCAGTAAA
AGAGATGCTGGAGCATATGGCCTCGACTCTTGCGCAGGGCGAGCGTATTGAAATCCGCGGTTTCGGCAGTTTCTCTTTGC
ACTACCGCGCACCACGTACCGGACGTAATCCGAAGACTGGCGATAAAGTAGAACTGGAAGGAAAATACGTTCCTCACTTT
AAACCTGGTAAAGAACTGCGCGATCGCGCCAATATTTACGGTTAA
>Cluster_21898
ATGAATCTTGATGACATTATCAACAGCATGACGCCTGAGGTATACCAGCGTTTGTCGACCGCCGTTGAACTGGGGAAATG
GCCTGATGGCGTTACGTTGACCGAGGAACAAAAAGAGAACTGCCTGCAACTGGTGATGCTGTGGCAAGCCCGCCATAATA
CCGAAGCACAGCATATGACAATTGACACAAATGGTCAGATGGTCATGAAGAGCAAACAGCAGTTAAAAGAAGATTTTGGT
ATCTCAGCAAAGCCTATTGCCATGTTTAAGTAA
>Cluster_3485
ATGAGCAGAACGATTTTTTGTACTTTCCTGCAACGTGAAGCAGAAGGTCAGGATTTTCAGCTGTACCCCGGCGAGCTGGG
AAAACGCATCTATAACGAGATCTCCAAAGAAGCCTGGGCGCAGTGGCAGCACAAGCAAACCATGCTGATTAATGAAAAGA
AACTCAACATGATGAATGCCGAGCACCGCAAGCTGCTTGAGCAGGAGATGGTCAACTTCCTGTTCGAGGGTAAAGAGGTG
CATATCGAGGGCTATACGCCGGAAGATAAAAAATAA
>Cluster_60745
ATGCGAAAAAAAAATAGAGATGAGCTTGATAAAATCTCCTGCAAATGGTGTTGTGATTAAAAGAAAAATTTCTGATGGATT
GAAAGAAATAGTTTCTCTTAAAGAAAAAATCCTTCTTGAAACCACCGCAAAAATTCAGAGTATTGAGGAAAAGAGAGAAG
AAAAATTTATTCAAGGTTATTATGATGGCTATACAAAAGGCATTATTGATGAGATGGATAATTTTATACCCTTGATAAGC
TTGCTATGCTCAGAACTTGAAAAAAAAGAATAA
>Cluster_13621
ATGGGGTCAGGTGGGACCACCGCGCTACTGCCGCCAGACAAATTCTTTTCTAATCTGCCGAACTTTAACCTAAAAAGTGG
TGCTGATACCCAGAGTCGAACTGGGGACCTCACCCTTACCAAGGGTGCGCTCTACCAACTGAGCCATATCAGCACGCTAA
ATTTGATGCCTGGCAGTTCCCTACTCTCGCATGGGGAGACCCCACACTACCATCGGCGCTACGGCGTTTCACTTCTGAGT
TCGGCATGGGGTCAGGTGGGACCACCGCGCTAA
>Cluster_14847
ATGATGGAGATATTCTGGACCATGCTGGCCAGCCAGGACAGGAAGCGCATTCGTGAGTACATTGCGGAGCAGAACCTGAT
GGCCGCCATTGAACTGGACGAACGGATTGGTTATTCAGCCAGCAGCCTGGCCGGGCAGCCGTATAAAGGCCGCAACGGTC
GTGTGGAAGGTACCCGGGAACTGGTTATTCACCCGCATTTTGTTCTGGTTTATGAGGTCGATAGCCAGTGGGGAAAAGTG
TATATCCTGCGCGTCCTGCATACAGCACAGAAGTGGCCATAG
>Cluster_51327
ATGACTGAAGCCGAACTACTGGGATTAATCCGCCGCGTCGTCGGAATCAGCCAGCAGCATGACGAACAGGCCACACAGCC
GGACAGCGTGACCGCTGAAAATTATGCGTGTTGTTGCTGAGGTGATGCGCCGTGATGGTATTGAGCTTAACGGCGTGG
ATATGCGCAACATACGAACAAGAGTCCTTGAGTTGCTGGCATACCGTCGCCGTTCTCAACAACGGAGGGAGAGCGCGAAA
AATACTTACCAGTGGAAGAAGCCGGAACGGTTGCGGCGGTAG
>Cluster_57835
ATGAGCGGTAAGCGTTATCCCGAAGAGTTTAAAACTGAAGCAGTCAAACAGGTTGTTGATCGCGGTTATTCTGTTGCCAG
CGTTGCAACACGTCTCGATATCACCACCCACAGCCTTTATGCCTGGATAAAGAAGTACGGTCCGGATTCTTCCACTAATA
AAGAACAGTCAGATGCTCAGGCCGAGATCCGCCGTCTCCAGAAAGAGCTGAAACGGGTTACCGACGAACGGGACATATTA
AAAAAAGCCGCGGCGTACTTCGCAAAGCTGTCCGACTGA
>Cluster_48655
ATGAGCGAAAAGAAAACAACTTATTGTCAGGTAGCATTGTCTGATAAGGCCAATGACAAACTTGGAAAGTTTCAAGTGAA
ACTAAAAGAAAAAAATATCAAAATGTCTAAGGCTGAAGTCATAAATACCATTCTGGAACAATTGACAATGGCCGACTTTG
ACAAGGTTATATCTTCTGTCGGGGCTTCCGCTAAGACTCGTGAGAAAATCATGCGTATCTATGAGAACTCTAATATGACA
AAGGAAGATCTCGAAACGCTATTAAGCAGATTAAAATAA
>Cluster_12051
ATGGATTTTTTATCTTCGGCGATACTTTCTGGCATAGTTTATGACATGCTTAAACATCATGTTTCGATTACTGCAACTTC
AATAAAAGAAAAGTTAAAAAATTGGGTTATTGATGAGGCTGTAGCCCCTGCTTTAGCTAAAGAGCTTGAAAAACTGTCTT
TGAACGATGAAATGAGTGAAATAGCAATTGAAAGAAAACTATTGGATAGTAGTGAAATTCAAAAAATACTCTCGTCTATA
AAACCTCATGCAGCAACTGTCATTATTCAAAACCATAGTGGCACTGGTGATAATATTGGTGGAAATAAAATTACGAGGTA
G
>Cluster_42911
ATGAACGACAGTGAATTTCATCGCCTGGCTGATCAACTGTGGCTGACCATTGAAGAACATCTGGACGACTGGGATGGCGA
CAGCGATATCGACTGTGAAATCAACGGCGGTGTACTAACCATTACCTTTGAGAACGGCAGCAAAATCATTATCAACCGCC

AGGAACCACTGCACCAGGTGTGGCTGGCAACCAAACAGGGCGGCTACCATTTTGATCTGAAAGGCGATGAGTGGATTTGC
GATCGCAGCGGCGAAACCTTCTGGGATTTATTGGAACAGGCCGCGACGCAGCAGGCGGGAGAAACCGTCAGTTTTCGTTA
A
>Cluster_53929
ATGGGCTTTCCTTCACCCGCAGCGGACTACGTCGAGCAGCGACTGTCCGTTAACTCGATATGCAATGTTGGTCCTAACAC
CCGCGTTTTCGAAAGGGATGGCGGTTATGTTGTGCTGGATATCTCCCTGAAGCCAAAGCAGGGTAGTCAGGTTCTGATCC
AGCACGGCGGCGGGACTGAGCTTGCCACGTTGAGAGGGAGGGCCCTGATTACTGAAGACGGCGAAGCGATTGAAGGCGAG
GCCCTGGATGATATCACTGTCGCCGGCGTGGTGACACACACCATCATCGATGTGAGAAGTGACAGCGTAGCGGTATAA
>Cluster_7882
ATGTTTATAAGCTTTGATAATCAAGGAGGATTTGTTATATCAATATTAAATAATGCTACAAGTGAAACGGCAAATATTAA
TGTGCTCCGAAAAAACATGACTTCTTTAGATAGTAGTGGTTCATTAATATATTTGCCTGGTGGTGATGTTTATCATATTT
CAGATATCTATAAAATGTCCAGAGGTAGAAAATTGTTTAAATTAAATGTAGAAAAGAAACCTGACATTGATGATATCATC
AACGTGGCTATTTTAGAAACTTCTTATCTACAAATAAAAAAACCAATAATGATGATAGTGATTATATTTTATGTTTAG
>Cluster_55973
ATGAGTAAGAAACTGATTGCCTTATGTGCCTGCCCGATGGGCCTGGCTCACACCTTTATGGCCGCTCAGGCGCTGGAAGA
AGCGGCGGTAGAAGCCGGTTATGAAGTGAAAATTGAAACCCAGGGCGCGGACGGTATCCAGAATCGCCTGACGGCGCAGG
ATATCGCCGAAGCGACCATCATCATCCACTCCGTGGCAGTTACCCCGGAAGATAACGAACGTTTCGAATCACGCGACGTT
TATGAAATCACTTTGCAGGACGCAATTAAAAACGCTGCGGGCATCATCAAAGAAATCGAAGAGATGATTGCCTCTGAACA
ACAGTAA
>Cluster_14483
ATGAAACGCTTTCCGCTATTTCTTTTATTCACCCTGCTCACGCTGTCCACCGTTCCGGCCCAGGCTGATATTATCGACGA
TACTATTGGTAATATTCAGCAGGCGATTAATGACGCTTATAACCCTGACCGTGGGCGAGACTATGAAGATTCGCGCGACG
ACGGCTGGCAGCGTGAAGTGAGCGACGATCGGCGCAGACAATATGACGACCGCCGCCGCCAGTTTGAAGATCGCCGCAGG
CAGCTGGACGATCGCCAGCGCCAGCTTGATCAGGAGCGTCGGCAACTGGAGGATGAAGAGCGGAGAATGGAAGATGAGTA
TGGGCGATGA
>Cluster_4220
TTGTTAACTCCCAGTCAATTCACATTCGTCTTCCAGCAGCCACAACGGGCTGGCACGCCGCAAATTACCATTCTCGGCCG
CCTGAATTCGCTGGGGCATCCCCGTATCGGTCTTACAGTCGCCAAGAAAAACGTTCGACGCGCCCATGAACGCAATCGGA
TTAAACGTCTGACGCGTGAAAGCTTCCGTCTGCGCCAACATGAACTCCCGGCTATGGATTTCGTGGTGGTGGCGAAAAAA
GGGGTTGCCGACCTCGATAACCGTGCTCTCTCGGAAGCGTTGGAAAAATTATGGCGCCGCCACTGTCGCCTGGCTCGCGG
GTCCTGA
>Cluster_11281
ATGGAATCGAAATCAGTCGATACCCGCAGTATATATCGTGAACTGGGTGCAACGCTGAGTTACAACATGCGTCTGGGGAA
CGGTATGGAAATTGAACCCTGCCTGAAGGCGGCTGTGCGCAAAGAATTTGTCGATGATAATCGGGTGAAAGTGAATAGTG
ACGGTAATTTTGTCAATGATTTGTCGGGCAGACGTGGAATATACCAGGCAGGTATTAAAGCCTCATTCAGCAGTACGTTA
AGCGGGCATTTCGGGGTGGGGTATAGCCATGGTGCCGGTGTGGAGTCCCCGTGGAACGCGGTGGCTGGTGTGAACTGGTC
GTTCTGA
>Cluster_61173
ATGGCAGCGAAAATCCGTCGTGATGACGAAGTTATCGTGTTAACCGGTAAAGATAAAGGTAAACGCGGTAAAGTTAAGAA
TGTCCTGTCTTCCGGCAAGGTCATTGTTGAAGGTATCAACCTGGTTAAGAAACATCAGAAGCCGGTTCCGGCCCTGAACC
AACCGGGTGGCATCGTTGAAAAAGAAGCCGCTATTCAGGTTTCCAACGTAGCAATCTTCAATGCGGCAACCGGCAAGGCT
GACCGTGTAGGCTTTAGATTCGAAGACGGTAAAAAAGTCCGTTTCTTCAAGTCTAACAGCGAAACTATCAAGTAA
>Cluster_33698
ATGACCGTACGTATTCTGGCTGTGTGTGGTAACGGACAAGGCAGTTCCATGATCATGAAAATGAAAGTGGACCAGTTTTT
AACCCAGTCGAACATCGACCACACGGTAAACAGCTGCGCGGTTGGCGAATACAAAAGCGAGTTGAGCGGTGCGGATATCA
TCATCGCTTCTACCCATATTGCGGGCGAAATCACCGTGACCGGCAACAAATATGTGGTTGGCGTGCGCAACATGCTCTCT
CCTGCTGATTTTGGCCCGAAACTGCTGGAAGTGATCAAAGAGCATTTCCCGCAGGACGTGAAGTAA
>Cluster_22117
ATGGATGATAATATAAAAAGACCAAGAACAGAAAAAACATTAAAGCAAAAGGTTGCTTTTGCTCAGCTTGAACTTAACCG
CCTTAAGTCTCTGGAGAAATCAGAACGAAAGAAAGTTGAAACAAGGCTAAAGATTATACTTGGTGCGGAAGTAGCTAAAG
CTATGCATTGCGATATCGAACAGGTGGATAAAGAACTCGTTATGGGGATTTTGCTTTCAGCATCTGACCTGAATGATATT
GAAAAAATAACATATATAAAAGCAGGCAGGAAGTTTCTCGCGCAAATGGAGGGAAGACAAAAATAA
>Cluster_2244
GTGAACTGTTTTCAGTTTGTGTGCGGATGTGCTTTCGATAACCCGATTCAGCGCCTGATTATGTTGCGTGTTTTGATGTC
GGGTTCTTCAGACGGTGAAGGCGAGAGAGTTATTGATCATCAGGTGCTTGCTGATTTCTGCTGTTGTTCTAAGCAAGCGA
TATTCAGGGAAACCCTGGCACTGGAAAGAGCTGGTTATCTTCATATCCGAAAAATTGCAACGCTTACTATTGATGCAAAA
GCCAGACTACAACCTGCGCGTGGCTACACAATTCTCATGCCGCGGAAGGAGGTTGTATGA
>Cluster_38343
ATGAGCATGGTTAAACATAAACGAGGGACCTCCTCTACACTCGATGTGCAGCATGAGGCCGAACTGAAGGCGCTGGCGAA
TAAATCCGACGAAGATATTGATTACAGTGATATCCCACCTTCCAGCGATGAGCAATGGTCTAACGCCGAACGCGGTAAGT
TTTATCGGCCATTGAAAACTCAGGCTTCGGTGCGTATTGATGCTGATGTGATGGAATGGTTGAAGCGACCAGGTAAAGGC
TATCAAACTCGCCTGAACGCAATTTTACGTGAAGCGATGCTGCGCGATCAGAACAAAAAATAA
>Cluster_66853
ATGTTGACGAATGACAGGGGGCGGCAGGTGCGGAGGGCGCGGTTGCTTCGTCATATGAAGCAAAGTCACCTAGCTGAATT
AATGGGTGTGGATCAGGCAACCGTGTCGCGCTGGGAGCGGGGCACCCCTTGCATTGTCGGATGGGAGGTGGTCAGCGGTTC
TTCAATTGCTTACCGGGCCTTCCGATTCATCGTACGACGCTGCGCTGAAGCGTCTGGTGCAATCCTCCGCCCACAAAGTC
CATCTGGTAGCGACCGGACACATTGTTTGCTCGCGGCATCTCCGGCCAGGCAAAGGGAATTGCGGATTGACCTAG
>Cluster_66955
ATGTACTGGGCAATCATCACGGTGGTGTTCATAGAAAGCAGTGAACAGGCACGCATCCGCCGCCAACAATATTTGCAACA
GTACAAAATGCCAAATGAGATACATGAAAATGTGGTGATAGAAAACAGTATGATTTCGAGAGAAACAACAGATGACGAAT
CTGACATTAACACCCTGTACCTTGATTCATTTAAAAGTAAAAACAACTGCAAATCTTTTGAAACCAATCCAAATTTACTG
GATAAAAATGGTAAGCCTGTGGAAATAATGGTGTGTGATGTTCAGGAACTGCATTTTTTCGTTCCAAAGAATTAA
>Cluster_64821
ATGGCGCGATGCGTCAGGGAATTGCTCGCGCCTGAATCTGAAATGGTGATATTTATGACGAATCAGGAAATGAAAGAATG
CATTGTCTCTTTATGTGCATGGCATAAAGCACGCGTGGATGAATGTCGTTTTTTTATCGATAAAAAAGATGCGGATATTA
CCTTCGACAATGACGGTGAGAAATGTGTTATCCCTGCACATTCAAAGGAAGCAAACCTTCTGAGACTTGGGGCAAGGCTT
GCCCTTTTCATATTCGAAAAATTTCCTGTAACGCTGAATGTAAAGGATGACGAAGATGACGAGGAATAA
>Cluster_63838
ATGTTTGCAGATGAAGAACTTGTTATGGAGTTACTGGTCAACGCGGGGCAGGCACGTTCTGACGCGATGGAAGCCATACG

TTGTGCCGGGCAAAAAGACTGGCAGGGCGCAACTCAGCTAATGGCCAGTTCTGAATCTGCCTGTCTTCAGGCACATAAGA
TTCAGACCGCATTAATTAGTCAGGATGAAGGCTGTGGCAAAATAAAAGTTAATTTAATTCTGATTCATGCACAGGATCAT
TTGATGAATGCAATATTATGCCAGGATTTAGCCAGGGAAATTATTTCATTAAGAAAAGAACTCCATGCCTGA
>Cluster_19999
ATGCCGGGTTACACAGAGTATGTCCTGGCTGAAGGATCTTTCTCATACGGGCAGGCGGTTGCTGTCATAACAGCTTACCG
CAATGTGTTTATCCAGGATGACCTGGGAATGCATTTCCGTCGGATTATCCGCAACACGGAAGGTCAGCGACGCTGGCGCT
GCCGGAATTCTGAACCCGATGCCGGAAAGGTGCTTAATACGCGGCTCGCCAGTGACGGTCTTCTCAGACAATAA
>Cluster_11893
ATGATTGTCTCTCCCGGCAAATGGGTATCCGAAGAGCAGTTAATTGCGCTGAAAGGAATAAAAAAAGGTACGTTAAAAAA
GGCCCGGGAAAAATCGTTTATGGAAGGAAGGGAATATAAGCATGTCGCTCATGACGGTATGCCATGGGATAACAGTCCAT
GCTTTTACAACCTGGAAGAAATTGATCGCTGGATTGAGCGCCAGGCATCAGCGAGACCAAGACGTCATCTTACTTGA
>Cluster_38474
ATGACCGAGATGGAAAAACAGCTTCTGAGCGCCTTAGAGAGCTTACAGACAAGCTACGAGCAACAGCAGCAGGCGTGGCA
GGACAGCTACAGCAGCTTGCAGCACATGTTCGAGGCTACCTCACAGGCGCTGACGCACAGCGACCGGGTGTGTCAGCACT
TGAGCAGTCAGGTAGAGAGCTTGCGCGCGCAGGTCGAGAGCTTGAGCAGGAACGTCAGCCGCTTGATGCGTTGA
>Cluster_17664
ATGAACAAAATGAAGGTTAAGGCGGCTCCCGGGATGAAGTTCCCGATGGAGGATAACGCCCCGGAAATACATCACCACGGA
AGCGGTGATCGTTGAAAACACCGCTTATTACCGGCGCGCTGTTCAGGATGGTGACCTGATTCTGGTGGCTGAAGAGCCTG
AAACGGCAGAACAGGACACCGGACCAGAAAAAGCGAAGGTGAAGAAAGAGAAACAGGTGGGCTCCGATGAGTGA
>Cluster_6730
ATGAAAGTCATTTCTGTTATTAAATCTAAAATTGCTGTGGCTTCAACCGCACTTTTTGTTTCTGCAAGTTGTTTTGCTGC
TGATGGCGCAACGGGTAGCACTGACTATGCAGGTCAGGCAATGGATTCACTGCTGACTCAGGCAAACGATCTTATTGCAA
AAGTATGGCCTGCTGTTGTCGCTGTCGTTGGTGCGGGTCTTGCCATTCGTTTGTTTAAAAAGTTCTCTTCGAAAGCAGTT
TAA
>Cluster_41386
ATGATTTATTTATGGATGTTTCTCGCCCTGTGTATTGTCTGCGTAAGCGGATACATCGGTCAGGTACTGAACATGGTATC
GGCGATCTCTTCTTTCTTCGGCATGGTGATCCTCGCTGCACTCATTTATTACTTCACCATGTGGTTAACTGGCGGTAATG
AACTGGTGACGGGGATATTTATGTTTCTTGCCCCGGCTTGTGGCTTGATGATTCGCTTTATGGTGGGGTATGGCAGGCGA
TAG
>Cluster_44583
ATGTACGAATTAACTCTATCTCCAGCAGAGATTAAAGAGATCACGAAATACGAGCGATACACAAAACAGCAACACCAGTT
AAGACTGCACGGCATCCCATTTGTAATCGGTCCTAAAAACGAACCAATAGTTCTACGCAGGGATATTCCACACGGACTGA
CAACGATGCCAAAAACATCTGAACTGGTTTCTGCTGAACCCGATTTTGAGGCGCTGAATAATGGGAAGACCAAGAAAAAA
TAA
>Cluster_66917
ATGGGAGAAAATATCAATTATCACAGCCAGCGCATTAATTATTTTCAACACAAAAAGGAAAAAGCAATGAATCTGGTAGA
CGCCTTTGTCAAAAAAGTAATATCCGGGCCATATAAAGAATATGGAAAATGGTGGATAGATGTTGAATACATTTCATGGG
GCGTTCCCGGGAAAACAAGACTGATGTTTGAGTCAAAGGAGCAGGCACTGGAAGTTAAAGAAGGGTACAAATTTTTAACG
TGA
>Cluster_6228
ATGGCAAGGTCCGATTATGATATTATCAATCTGTCTCTGGAACATGAACTGAATGAGTGGCTGGCAGAGAGAGGTTATGC
CGGACTGGTGGATAACCGGAATCGCCTGGCAGAAGTTGTTACCCGCAAATTGCAGGACAGCTTTTATATAAACGTTTCCA
GGGATGCGCTGAACACGGCATACAGTGAACACCCGGAGTGGTTTTCTGGGCTTGTCTCCGGGGATGAGAATTAA
>Cluster_26143
ATGACTACATTATTCAGAAAAGAATATCCGCAAAAAAGTAGGGCGACAGAATTTTTGTTTCTCATTCTGTTTATCGTATT
GATGATACCGATATCCCCTCTAATTTTTGTCTGGGCAATCGGGAAAATAATTGAGCCAGTTACTGAATTGTATAACGACG
TGGTATGGGCGTCATTCAACACACTGCACAATAAAATTAATCCGTATAAGGAAAACTGA
>Cluster_29605
GTGAGGAGGCGTAACGCGAGAGGCGGGATAAGCAGAACAGTCTCAGTGTATCTCGATGAAGATACAAATAACCGACTTAT
TCGGGCCAAAGATCGTAGCGGTAGAAGTAAAACTATAGAGGTTCAGATCCGTCTCAGAGATCATCTGAAACGGTTTCCTG
ATTTCTATAATGAAGAAATATTCAGGGAGGTGATCGAAGAGAACGAGTCAACATTTAAAGAACTTTAG
>Cluster_40905
ATGAAAGAAAATAATGAAACCAGCGTAAAACGATTTATCCGCGTTCCTGATGTTTTAAATCGAGTGGGATTTAGTCGCAC
GACCTTATATGAACGTATTAAGGAAGGAAACTTTCCAGACAGAGTAAAAATAGGACCAAGATGCGTCGCCTTTGTTGAAA
GTGAGATTGATGAATGGATTGAAACTACTATTCGTAACTCACGTCAGAACGAAGCATAA
>Cluster_39301
ATGGGCGGCTGGATTGAACATATCACCACCGAGGGCGAGCGCTGGGATAATCTGGCGTTCCTCTATTACGGCGATCCAAT
GGGCTATGAGCGCATCATTGTCGCTAACCCCCATATTGCGATCACGCCGACACTGGCCTCCGGGCTGCGGTTGCGTATTC
CGGTGATCGGCCCGATGCAGGTGCATAACGTTGATGAGGTGCCGCCGTGGCTCAGGTAA
>Cluster_17771
ATGAGTGACGAAAAAAAAATTTGATTTCAAAAAGCACTGGCTGGGGCTGTCTCCTGATGAGCGTGAAGCATTTGCAGTTGA
AGCCGGTACCACCAGTCATTACATCCAGACGCACCTGACCGGTCGCCGTAAGATGCCGGGTAAGCGTCTGATGGAAGGTC
TTTTTAAAGCGTGTCGCTCCCGCGAATGGACAAAGTCTAAACCTGAATTAGTGCTCTTCTTCTACGACCGATAA
>Cluster_41813
ATGGCTCTGAAGGAGGAGGTTTATTCAACCGACGCAATTTTGCGTACTTGCTTCATAAGCATCACGCAAAAGCTGCAAAA
CAGCATCTTTCCCGGAATCACCATCAAGAACTCGCCGTTCGCTTCTTCCCCTGAAATGATTAACTCCGGTATCATGTGCG
CCTTATGTGATTACAACGAAAATAAAAACCATCACACCCCATTTAATATCAGGGAACCGGACATAACCCCATGA
>Cluster_9799
ATGGAAACCACCGTATTTTTCAGCAACCGCAGCCAGGCGGTCAGACTGCCAAAAGCGGTTGCATTGCCGGAAAACGTAAA
ACGCGTTGAAGTGATTGCTGTCGGACGAACCAGAATCATTACGCCAGCCGGAGAGACATGGGACGAATGGTTCGACGGAC
ACAACGTCAGCGCCGATTTTATGGATAACAGGGAACAGCCCGGCATGCAGGAAAGGGAGTCATTCTGA
>Cluster_48566
ATGTCATACCCGACTAATGTCGTTGCGCTCGTAGAGAGCGATTTTCTGGCCCAGGCTCGTGAAATGATGAAAGATCGTGA
GCAGGCTTTCAACTTGTACGAGTGGGCAATTAAGTGCTTGCATCTTGGCGAGCATCGCGAACTTGTTGAACAGCTTTTAG
GTGAGTTGATCAATGAGGTGTTTGCCTTGAATGTTCAACTACATGGTCGAGAAAATAATCAATCACAATGA
>Cluster_46887
ATGGACATTAACAACAAAGCCCGCATTCATTGGGCATGCCGCCGTGGTATGCGCGAACTCGATATTTCAATCATGCCGTT
TTTCGAACATGAGTACGACAGCTTAAGCGATGACGAAAAACGCATCTTTATTCGTCTGCTGGAATGTGACGATCCGGACC
TGTTTAACTGGCTGATGAATCACGGTAAACCGGCCGATGCAGAACTGGAAATGATGGTCCGACTCATCCAGACACGGAAC

CGGGAACGTGGTCCTGTGGCAATCTGA
>Cluster_24598
ATGCAATCACCATTACGGAATGTGCGTAAGGCGCACGGATTTACTTTGCAGCATGTTGCTGCGGGCGTTCAGGTCAATCC
AGCGACGCTGAGTCGTATTGAAAGACTGGAACAAATTCCATCTATCGAGCTTGCAGAACGTCTGGCCAATTTTTTTAAGG
GAGAAATCAGCGAAATGCAGATTCTCTATCCTGCACGCTTTCAATCTAGTCAAAACCGGAATGAGTTAAAACCACAGGAA
CAGGAGGTAAGCCGTGGGTAA
>Cluster_24619
ATGATCGATCTGGAAAACCAGGAACGGGAGATTATCAATTTAATGTTCAGCCAGGGTATATCCTGGCTAACCGCCGTGCG
TATCAGGCATAAACTATCACTTGCTGAAGTAAGTAAAATGTTAGGTATCTCCATTAATTCTCTAAAACAGATCGAAAAGA
CGGAACGGCTTAGTTCAAACATCAAAAGCAAAATGGCAGGAATTTATGGTTGTCCACCAGAGTTGCTGATATGCCCTTCC
TGGATGACGGCTGAACATAAATGA
>Cluster_29371
ATGACTTATAACATCACTATAGGAAACAAAACTATTGAAATAACTGAAAGCGGATACAACATTTTAAAAGCTATTTTGGA
AATAGAGTTTTCACCACCGACAGTGGTTAGCTTTTGTAGTTTAGGCGGCTACAGCGCACAACACGTAAATCATTGGTTAA
ATCATTTTACATCGTTCGGTGTGCTGGATTATGAAGGGATTAACCACACTACATTTCGTCTTTTGAAATTAAACAAAGAT
TTTGAATTGTTTATCACAAACAATCAATAA
>Cluster_24244
ATGGCACTCCCCCGTATTACCCAAAAAGAGATGACTGAACGCGAGCAGCGTGAACTTAAAACACTGCTGGATCGGGCGCG
GATTGCGCATGGTCGCGTACTGACCAACTCCGAAACTAACAGCATTAAGAAAGAGTACATCGATAAATTAATGGTCGAGC
GTGAGGCTGAAGCGAAAAAAGCCCGCCAATTGAAGAAAAAGCAGGCTTATAAACCGGATCCTGAGGCGTCATTTAGCTGG
TCAGCAAATACGTCGACGCGCGGCAGGCGCTGA
>Cluster_18474
ATGATTGAAGATCCCATGGAAAATAATGAAATTCAGAGCGTGTTGATGAACGCTCTCTCCCTCCAGGAAGTCCACGTTTC
CGGCGATGGCAGCCACTTTCAGGTTATTGCCGTGGGTGAGTTGTTTGACGGCATGAGTCGGGTTAAAAAACAGCAGACGG
TCTATGGCCCGCTGATGGAATATATTGCGGATAACCGCATTCATGCTGTGTCGATCAAAGCGTATACCCCTGCGGAGTGG
GCGCGCGATCGCAAACTGAACGGCTTTTGA
>Cluster_50451
ATGCTGCCGCAGCATAGCGATATTGAAATAGCCTGGTATGCTTCGATACAGCAGGAGCCGAATGGCTGGAAGACCGTCAC
CACACAGTTCTACATCCAGGAATTCAGTGAGTATATTGCGCCACTGCAGGATGCTGTAGATCTGGAAATCGCAACGGAGG
AAGAAAGATCGTTGCTGGAGGCATGGAATAAATATCGGGTATTGTTGAATCGTGTTGATACATCAACTGCACCTGATATT
GAGTGGCCTGCAAATCCTGTCAGGGAGTAA
>Cluster_48871
ATGACTGATGATGATCTGGATACAGAGCAGGAGGAATTTTACTCTATGGAACAATGCATCTTTGATTTCGGCATTATAAA
CTATAATCCCGCCATTACATGGACTGTGCCAGCTGTATTTTTCTTTTTAGCGTTGCTGGCAGGTATGGTTTTCAATATTA
GCAGGAAAAATAATCTAATCGTTTTTGTTTGGGCAATGTCGCTTCCTGTAATAACAGTTTTCATTTTTATATTTCTGGGT
GGTAAGATAAATATATGCTGA
>Cluster_66915
ATGTCAGAAAAGTGGAGTAAAGAATTCGACTCTTGCTTTCAGGTGGGGAGCATAGAAGAAATTGCCGATGCTCTCGAGAA
ACGCTGCGGAAGCGAGGAAAATGCGTTAGCCATGCTGGCAATGACAGCCTTCACACTCATGACCCGACGTGGTATCTGCG
AAATGCAGAACGAGGCCCCCGATGGCAAAGGCATTCGGCTGGAGCTAATCGGCTTCAGGGAAAATGAAACAATACCGGAT
ACGCTTCATTGA
>Cluster_49790
ATGAAGAAATTATCCCTTTCACTGATGCTGAACGTGTCGCTGGCGCTGATGCTGGCACTGTCCCTGATTTACCCGCAGAG
CGTGGCCGTCAGTTTTGTCGCCGCCTGGGCGATTCTGGCGACGTTTATCTCGCTGGTCGCTGCCGGGTGGATTTTTACCG
CGCTGATTTATCTCGTGGCATCGGTGTTCTTCCGGCTGATACGTACGGCCTGCTGTCAGCGTTTTGAGGGGCGGGAACCA
TGTCAAAGCTGA
>Cluster_40352
ATGTTTGCAAAAATGACCAATTTTTTTAAGGGTGTTGATAAGAAAGTTGCAGCTTTATCTTTTTCCGCTGCTTCTGCTCT
TGCTGTATCTCCGTCTTTTGCTGGTACAGTCGATTTTTCATCATTGACTAACAGCATTGATATGTCTTCTGTTTCTGCTG
TTATTCTTTCTATCGGTGTTGCAGGAGTTACAGTTTATCTGACTTTTGCTGGCGTTCGTAAAGTATGGGGAGCCATCAGA
AGTATCTGA
>Cluster_40938
ATGAGTAAAAACGTAAAACAGACTTCTGAAAATGTGGCTTCTACTGCTGCAAAAACGCTGGCTAATCCTAATGCTTCTGC
CATCCAGAAAAGCCTCGCTGGTTCTGCGCTTTCGCAACGTGGGACATCAAACCAGACAAGTGGGCAAATGGAGCATAAGG
CATCATCAGCTCTTGATAATCCACGTTCCAGCAAGTTAACGAAGCAACTTGCAGCCTCTGTTTTGGCACAATCAAACAAA
GGGCGTAAGTGA
>Cluster_24681
ATGTCGCAGACAGAAAATGCAGTGACTTCCTCATCTGGCGCAAAACGAGCATACAGAAAGGGGAATCCGCTTTCTGATGC
AGAGAAACAAAGATTATCAGTGGCCCGTAAAAGAGCTTCGTTCAAGGAAGTAAAAGTATTTCTTGAACCAAAGTATAAGG
CCATGCTCATGCAAATGTGTCATGAAGATGGCCTGACTCAGGCTGAAGTTCTGACCGCACTGATAAAAAGTGAAGCGCAA
AAACGATGCATGTGA
>Cluster_53634
GTGCAGTTTACAACTGACCAACGTAAGCCCTGGTATATACAGGCACTGCGTCCCGATGGTTCGCCATTAACCTTTGGCTA
TGACGTACTGGATCTCCAGGAAAATAATATTGGAGTCGTTGGCCAGGGTAGTCGCCTTTTTATTCGCGTAGATGAAATTC
CCACTGGAATAAAAGTTGCTCTCAATGATGAACAGAACTTATTCTGTACTATTACTTTTCAACACGTTATCGATGAAAAC
AAAACATATATATGCCAGTAA
>Cluster_45311
ATGAACAAAGAGACACAACCCATCGACCGCGAAACGCTGTTGAAAGAGGCCAACAAAATCATTCGTGAGCATGAGGATAC
GCTCGCAGGGATTGAAGCCACCGGCGTCACGCAGCGTAATGGCGTGCTGGTATTTACCGGCGACTACTTTTAGATGAAC
AAGGATTGCCAACAGCCAAAAGCACCGCGGTGTTTAATATGTTCAAACACCTGGCCCATGTGCTTTCAGAAAAAATATCAT
CTGGTTGATTAA
>Cluster_66956
ATGCCACAGCAACATCCCGGACGACTACAGGTACTGGTCGTCGACACGCACTGCAAACGCAGGCTGTTTTCAACGAAAAC
ACAGACAGATCCCGATGAGCTTGCCCGACGCTTCTGCACGCCGGATAACTGCCTTGTGGTGGCACTATGCAACAACCGAT
TTCTGTTTCGCCAGGAACGTGCACCCGGTAGCCACTGCCGCTGGCGCAAGGGTAGCCGCTCCCGCCATCAGCACCTGCAG
GACTGGCTGTCGTAA
>Cluster_13755
ATGAAAAAATGTTGGTTTTATCGGCTGGCGCGGTATGGTCGGCTCCGTTCTCATGCAACGCATGGTTGAAGAGCGCGACTT
CGACGCCATTCGCCCTGTCTTCTTTTCTACTTCTCAGCTTGGCCAGGCTGCGCCGTCTTTTGGCGGAACCACTGGCACAC

TTCAGGATGCCTTTGATCTGGAGGCGCTAAAGGCCCTCGATATCATTGTGACCTGTCAGGGCGGCGATTATACCAACGAA
ATCTATCCAAAGCTTCGTGAAAGCGGATGGCAAGGTTACTGGATTGACGCAGCATCGTCTCTGCGCATGAAAGATGACGC
CATCATCATTCTTGACCCCGTCAATCAGGACGTCATTACCGACGGATTAAATAATGGCATCAGGACTTTTGTTGGCGGTA
ACTGTACCGTAAGCCTGATGTTGATGTCGTTGGGTGGTTTATTCGCCAATGATCTTGTTGATTGGGTGTCCGTTGCAACC
TACCAGGCCGCTTCCGGCGGTGGTGCGCGACATATGCGTGAGTTATTAACCCAGATGGGCCATCTGTATGGCCATGTGGC
AGATGAACTCGCGACCCCGTCCTCTGCTATTCTCGATATCGAACGCAAAGTCACAACCTTAACCCGTAGCGGTGAGCTGC
CGGTGGATAACTTTGGCGTGCCGCTGGCGGGTAGCCTGATTCCGTGGATCGACAAACAGCTCGATAACGGTCAGAGCCGC
GAAGAGTGGAAAGGGCAGGCGGAAACCAACAAGATCCTCAACACATCTTCCGTAATTCCGGTAGATGGTTTATGTGTGCG
TGTCGGGGCATTGCGCTGCCACAGCCAGGCATTCACTATTAAATTGAAAAAAGATGTGTCTATTCCGACCGTGGAAGAAC
TGCTGGCTGCGCACAATCCGTGGGCGAAAGTCGTTCCGAACGATCGGGAAATCACTATGCGTGAGCTAACCCCAGCTGCC
GTTACCGGCACGCTGACCACGCCGGTAGGCCGCCTGCGTAAGCTGAATATGGGACCAGAGTTCCTGTCAGCCTTTACAGT
GGGCGACCAGCTGCTGTGGGGGGCCGCGGAGCCGCTGCGTCGGATGCTTCGTCAACTGGCGTAA
>Cluster_42340
ATGACAAAAGTATTGCTGTCTCATCCCCCGCGCCCGGCGAGCCATAACTCAAGCCGCGCGATGGTGTGGGTGCGAAAAAA
TCTGTTCTCCAGCTTGAGCAATAGCCTACTGACTATTGGCTGCATATGGTTGATGTGGGAACTGCTCCCACCGTTGCTGA
ACTGGGCATTTTTGCAGGCTAACTGGGTTGGCTCAACGCGTGCCGACTGCACAAAAGCCGGTGCCTGTTGGGTCTTCATC
CACGAACGATTTGGTCAGTTTATGTATGGGCTTTACCCACACGACCAACGCTGGCGAATTAACCTCGCATTACTGATTGG
GCTTGTGTCGATCGCACCAATGTTCTGGAAAATACTCCCGTATCGCGGTCGCTATATTGCGGTATGGGCGGTGATTTACC
CACTGATTGTCTGGTGGCTGATGTATGGCGGGTTTCTTGGTCTTGAGCGGGTTGAAACCCGGCAATGGGGCGGGCTGACG
CTAACTTTAATTATTGCATCAGTTGGGATTGCGGGGGCGCTGCCGTGGGGATCTTACTGGCGTTAGGTCGCCGCTCCCA
TATGCCGATTGTGCGTATCTTATCGGTCATTTTTATCGAGTTCTGGCGCGGCGTACCGCTGATTACCGTTCTGTTTATGT
CTTCGGTCATGCTGCCGTTGTTTATGGCAGAAGGCACCAGTATCGACAAATTGATCCGCGCGCTGGTTGGCGTGATCCTG
TTTCAGTCAGCATATGTTGCGGAAGTCGTGCGAGGCGGATTACAGGCGCTGCCTAAAGGGCAATATGAAGCGGCAGAGTC
GCTGGCGTTGGGTTACTGGAAAACCCAGGGGCTGGTTATTCTGCCACAGGCGTTGAAGCTGGTGATTCCTGGGCTGGTAA
ATACCATCATCGCACTTTTCAAAGATACCAGCCTGGTGATCATCATCGGGTTGTTCGATCTTTTCAGTAGCGTTCAGCAG
GCAACCGTTGATCCCGCCTGGTTGGGTATGTCGACGGAAGGGTATGTTTTCGCCGCACTGATCTACTGGATCTTCTGTTT
CAGCATGTCACGTTATAGCCAACATCTGGAAAAACGTTTTAACACCGGGCGTACACCGCATTGA
>Cluster_24512
ATGCAACCTTTTGGCGTACTTGACCGCTATATCGGTAAAACTATTTTCACCACCATCATGATGACGCTGTTCATGCTGGT
GTCGCTGTCCGGCATTATCAAGTTTGTCGATCAGCTGAAAAAAGCCGGGCAGGGGAGTTACGACGCGTTAGGCGCAGGAA
TGTATACCTTGCTGAGCGTGCCGAAAGATGTGCAAATCTTCTTCCCGATGGCGGCTCTGCTTGGGGCGTTGCTTGGTCTT
GGGATGCTGGCGCAGCGCAGCGAACTGGTAGTCATGCAGGCTTCTGGTTTTACCCGTATGCAGGTGGCGCTGTCGGTGAT
GAAAACCGCCATTCCGCTGGTCTTGCTGACGATGGCGATTGGTGAATGGGTCGCGCCGCAGGGCGAGCAGATGGCGCGTA
ACTACCGTGCGCAGGCGATGTACGGCGGCTCGTTGCTCTCTACCCAGCAAGGTTTATGGGCGAAAGATGGTAACAACTTC
GTCTACATTGAGCGGGTTAAAGGTGACGAAGAGTTAGGTGGCATCAGTATTTATGCCTTTAACGAGAATCGTCGTCTGCA
ATCCGTACGCTATGCCGCTACCGCGAAGTTTGACCCGGAACATAAAGTCTGGCGTCTGTCGCAGGTTGATGAATCTGATT
TGACCAATCCGAAACAGATCACCGGTTCGCAGACGGTGAGCGGCACCTGGAAAACCAACCTAACGCCAGACAAACTGGGC
GTGGTGGCGCTGGACCCGGATGCACTCTCTATCAGCGGTTTGCACAACTATGTGAAGTATCTGAAGTCGAGCGGTCAGGA
TGCCGGACGTTATCAGCTCAACATGTGGAGCAAAATCTTCCAGCCGCTATCCGTGGCGGTGATGATGCTGATGGCGCTGT
CGTTCATCTTTGGCCCACTGCGTAGCGTACCGATGGGCGTGCGTGTGGTCACCGGTATCAGCTTCGGTTTTGTCTTCTAC
GTACTGGACCAGATCTTCGGCCCGCTGACGTTGGTTTACGGCATCCCGCCGATCATTGGCGCACTGTTGCCAAGCGCCAG
TTTCTTCTTAATCAGCCTGTGGCTGTTAATGAGAAAATCGTAA
>Cluster_20517
GTGGCTCAGGTAAATGCCGTTATCACCCCCGTTTTCACCCTGTTTTACGGGCAGAAAGATATCACTCACGATATTGCTCC
TTTTGTCCTGAGCGTGACGTATACCGACAGTCTCAAGAGCGAATCGGATGAAATCGACGTGCAGGTGGAAGACACCGCCG
GGCGCTGGCGCGAGGCGTGGTATCCGGGCAAAGGCGATATGCTGATGCTGAAGTTGGGCTATCAGGGCGAAGCGTTGCTG
GACTGTGGAGCGTTCTCCATCGATGAGATTGAGCTGGGCGCACCGCCGGATACGGTATCCATTCGCGGCGTGGCGACGTC
GGTCAACAGCGCTCTGCGTACCGCCTCCAGCGAGGGCTACGAAGAGACGACGCTGGATGCGATTGCCAGCCGTATCGCGC
AGAAGCATGGTCTGAAGCTGGTCGGTCAGATAGAGCCGATTACCATTGACCGGGTGACGCAATACGCGGAAACCGACGTC
GGTTTTCTGAAGCGGCTGGCGGGTGAGTATGGTTATGCGCTGAAAGTCACCACTGGCGAGCTGGTGTTTTCCCATCTGGC
TACACTGCGAAGTAACGCGCCGGTGACGACGCTGACGCCGCAGGATGTAGCGCGCTGGTCGCTGCGCGATACCATCAACC
GCATCTATAAGGATGCAAAGGTGGCGCATCAGAAGAGTAAAGAGAAGACGCTGGTGACGTACCACGCCGACGGCCCGCCG
ACCACGGAGACGCGTGGTAAATCGACCAGCGCCGATACGCTCAAGGTCAATGCACGGGCGGCGGATCAGGGGACGGCGCA
GGTGAAAGCCGAGGCGGCGCTGGATGACCATAACGAGTATCAGCAGACCGGTTCGCTCTCGATGATGGGCAACACGCAGC
TTATCGCCGGGAACAAAATCACTCTCGACGGGTTCGGCGTGCTCTCCGGGGAGTGGCTGATCACCAGCGCCCGTCACTCG
TTTGACCGTTCATCGGGCTACAGCACCGAAATTGAGGTCGGACGTGGCCCGAAAACGGCCTCCGGCGGCGGGAAGAAGAA
AAAACCGGATACGCTGACCGTCTATCACCCGGACGGCCCACCCACCCAGGAGAAAAAATGA
>Cluster_41789
ATGAGCAACGGCATTGTGATCATTGGTTCGGGCTTCGCCGCCCGCCAACTGGTGAAAAATATTCGCAAACAGGACGCCAG
TATTCCATTAACCCTGATTGCCGCCGACAGCATGGATGAGTACAACAAACCTGACCTCAGCCATGTTATCAGTCAGGGGC
AACGTGCCGACGACCTTACCCGCCAGACGGCAGGTGAATTTGCCGAGCAGTTTAATCTGCGCCTGTTTCCGCACACCTGG
GTAACGGATATCGATGCCGAAGCCCATGTGGTGAAAAGTCAGAATAATCAGTGGCAATACGACAAGTTAGTGCTGGCAAC
CGGTGCCAGCGCCTTTGTCCCGCCAGTGCCCGGGCGTGAGTTAATGCTGACGTTAAATAGTCAGCAAGAGTATCGCGCCT
GTGAAACGCAACTGCGGGATGCCCGACGCGTGTTGATTATCGGCGGTGGTTTGATTGGTAGCGAACTGGCGATGGATTTT
TGTCGGGCAGGCAAAATGGTCACGCTAATCGACAACGCTGCCAGTATTCTGGCGTCGTTAATGCCACCGGAAGTCAGCAG
CCGCTTGCAGCATCGGTTGACGGAGATGGGCGTTCATCTGCTGTTGAAATCTCAGTTGCAGGGACTGGAAAAAACAGATT
CTGGCATTCTGGCAACGCTGGACCGCCAGCGTTGCATCGAAGTGGATGCGGTAATTGCCGCCACCGGACTGCGCCCGGAA
ACCGCCCTGGCACGACGCGCCGGGCTGACGATTAATCGCGGCGTTTGCGTCGATAGTTATCTGCAAACCAGTAATGCCGA
TATTTATGCGCTGGGCGATTGCGCGGAAATTAACGGTCAGATATTGCCGTTCCTGCAACCGATTCAACTTAGCGCGATGG
TGCTGGCAAAAAATCTTCTCGGCAATAACACGCCGCTGAAACTCCCGGCGATGCTGGTGAAAATCAAAACGCTGGAATTA
CCGCTGCATCTGGCAGGCGAAACCCAGCGTCAGGATTTACGCTGGCAAATTAATACCGAACGCCAAGGAATGGTGGCGCG
CGGCGTTGACGATGCTGACCAGCTTCGCGCCTTTGTGGTCAGTGAGGATCGGATGAAAGAGGCATTTGGATTGTTGAAAA
CGTTGTCGATGTAG
>Cluster_29071
ATGAAAACCTTCAGCGATCGCTGGCGACAACTGGACTGGGATGACATCCACCTGCGTATCAACGGCAAAACGGCTGCTGA
CGTAGAGCGGGCGCTAAATGCCTCGCAACTCACCCGCGACGACATGATGGCATTGCTATCACCCGCCGCCAGTGGCTATC
TGGAACCACTGGCCCAGCGAGCGCAACGTCTGACTCGTCAGCGATTTGGCAACGTCGTCAGCTTCTACGTCCCGCTTTAT
CTTTCCAATCTTTGCGCTAACGACTGTACTTATTGCGGATTTTCTATGAGCAACCGCATCAAGCGCAAAACGCTGGATGA
AGCGGATATTGCCAGGGAAAGCGCCGCTATACGGGAGATGGGCTTTGAACATCTGCTATTAGTCACTGGTGAACATCAGG

CGAAAGTGGGGATGGATTACTTTCGTCGTCATCTCCCTGCCCTGCGTGAACAGTTCTCTTCACTACAGATGGAAGTGCAA
CCGCTGGCGGAGGCGGAATATGCCGAGTTAAAACAGCTTGGCCTCGATGGAGTAATGGTTTATCAGGAGACATATCATGA
GGCGACTTATGCCCATCATCATCTGAAAGGTAAAAAACAGGACTTCTTCTGGCGGCTGGAAACGCCGGATCGGCTGGGCC
GTGCGGGGATTGATAAGATAGGCCTCGGCGCGCTAATTGGCCTTTCCGACAGCTGGCGCGTTGACTGCTATATGGTTGCA
GAACATTTGCTATGGCTGCAACAGCACTACTGGCAAAGCCGCTACTCTGTCTCCTTCCCACGCCTGCGCCCGTGTACTGG
CGGCATTGGGCCTGCGTCGATTATGGATGAACGCCAGTTAGTGCAAACCATCTGCGCCTTCCGGCTGCTTGCACCGGAGA
TTGAACTGTCACTCTCCACGCGGGAATCACCGTGGTTTCGCGATCGCGTTATTCCGCTGGCAATTAATAACGTCAGCGCT
TTTTCGAAAACGCAGCCAGGTGGCTATGCCGACAATCACCCCGAGCTGGAACAGTTCTCACCGCACGACGATCGCAGACC
AGAAGCAGTTGCGGCCGCGTTAACTGCTCAGGGTTTGCAGCCGGTATGGAAAGACTGGGACAGCTATCTGGGACGCGCCT
CGCAAAGGCCATGA
>Cluster_59937
ATGTACGATTATATCATTGTTGGTTCTGGTTTGTTTGGTGCCGTTTGTGCGAATGAGTTAAAAAAGCTAAACAAAAAAGT
TTTAGTGATTGAGAAAAGAAATCATATCGGTGGAAATGCGTACACAGAGGACTGTGAGGGTATCCAGATTCATAAATATG
GTGCACATATTTTTCATACCAATGATAAATATATATGGGATTACGTTAATGATTTAGTAGAATTTAATCGTTTTACTAAT
TCTCCACTGGCGATTTATAAAGACAAATTATTCAACCTTCCTTTTAATATGAATACTTTCCACCAAATGTGGGGAGTTAA
AGATCCTCAAGAAGCTCAAAATATCATTAATGCTCAGAAAAAAAAGTATGGTGACAAGGTACCTGAAAATTTGGAGGAGC
AGGCGATTTCATTAGTTGGGGAGGACTTATACCAAGCATTGATAAAGGGTTATACGGAGAAGCAGTGGGGAAGAAGTGCA
AAAGAATTGCCTGCATTTATTATTAAGCGAATCCCAGTGAGATTTACGTTTGATAACAATTATTTTTCCGATCGCTATCA
AGGTATTCCGGTGGGAGGCTACACTAAGCTTATTGAAAAAATGCTTGAAGGTGTGGACGTAAAATTAGGCATTGATTTTT
TGAAAGACAAAGATTCTCTAGCGAGTAAAGCCCATAGAATCATCTACACTGGACCCATTGATCAGTACTTCGACTATAGG
TTTGGAGCGTTAGAATATCGCTCTTTAAAATTTGAGACGGAACGCCATGAATTTCCAAACTTCCAAGGGAATGCAGTAAT
AAATTTCACTGATGCTAATGTACCATATACCAGAATAATTGAGCATAAACATTTTGACTATGTTGAGACAAAGCATACGG
TTGTTACAAAAGAATATCCATTAGAGTGGAAAGTTGGCGACGAACCCTACTATCCAGTTAATGATAATAAAAACATGGAG
CTTTTTAAGAAATATAGAGAGTTAGCTAGCAGAGAAGACAAGGTTATATTTGGCGGGCGTTTGGCCGAGTATAAATATTA
TGATATGCATCAAGTGATATCTGCCGCTCTTTATCAAGTGAAAAATATAATGAGTACGGATTAA
>Cluster_33276
GTGAAAGTACTGACTGTATTTGGTACGCGCCCGGAAGCCATCAAGATGGCACCGTTGGTGCATGCGTTGGCAAAAGATCC
TTTTTTTGAGGCTAAAGTTTGCGTCACTGCGCAGCATCGGGAGATGCTCGATCAGGTGCTGAAACTCTTTTCCATTGTAC
CTGACTACGATCTCAACATAATGCAGCCAGGACAGGGCCTTACAGAGATAACCTGTCGGATTCTGGAAGGGCTAAAACCT
ATTCTTGCCGAGTTCAAACCAGACGTCGTGCTGGTTCACGGCGATACTACGACGACGCTGGCAACCAGCCTGGCGGCGTT
TTATCAGCGTATTCCTGTTGGTCACGTTGAGGCTGGTCTGCGCACGGGCGATCTCTATTCGCCGTGGCCGGAAGAGGCTA
ACCGTACATTGACCGGGCATCTGGCGATGTATCACTTCTCTCCAACCGAAACTTCCCGGCAAAACTTGCTGCGTGAAAAC
GTTGCTGACAGCCGAATCTTCATTACCGGTAATACAGTTATTGATGCACTGTTATGGGTGCGTGATCAGGTGATGAGCAG
CGATACGCTGCGTTCAGAACTGGCGGCAAATTACCCGTTTATCGACCCCGATAAAAAGATGATTCTGGTGACCGGTCACA
GGCGTGAGAGCTTCGGTCGTGGCTTTGAAGAAATCTGCCATGCGCTGGCTGACATCGCCACCACGCACCAGGACATCCAG
ATTGTCTATCCGGTGCATCTCAACCCGAACGTCAGAGAGCCGGTCAATCGCATTCTGGGGCATGTGAAAAATGTCATTCT
GATCGATCCCCAGGAGTATTTACCGTTTGTCTGGCTGATGAACCACGCCTGGCTGATTTTGACCGACTCAGGCGGCATTC
AGGAAGAAGCGCCTTCGCTGGGGAAACCGGTGCTGGTGATGCGCGATACCACTGAGCGTCCGGAAGCGGTGACAGCGGGG
ACGGTGCGTCTGGTCGGCACGGATAAGCAGCGAATTGTCGAGGAAGTGACGCGTCTTTTAAAAGACGAAAACGAATATCA
AGCTATGAGCCGCGCCCATAACCCGTATGGTGATGGTCAGGCATGCTCTCGCATTCTGGAAGCGTTAAAAAATAATCGGA
TATCACTATGA
>Cluster_45867
ATGATTTTTTTTAACGATATAAAGCCCACTGGTTGGTTAAAGAATAAGATGGAATGGTATATGAACGGTCATATAGGAGA
ACTGGATAAATTAGTTCCCCATCTTATTACGGAGCATAAAATATATGGAAGAGATCGTATAACTCCACGCACTGTATCTG
CTGAAGATGGCGTTATTAAAAAACAGAATAATCCCGATGATAACATGATGCAATATTATTGGTGGGATAGTGAAACTCAA
TCGAATTGGAAGGATGGATATTGCCGCAGTGCCTACTTGTTAGATAATAAAGAATGGCAGGAAAAAGCGAGCGATTTATG
TTTGGAACTTATCGATACGCAAGATGATGATGGCTATATTGGAATTTATGATTCATCATTAAAATTTAACTGTAAAGCAG
AAAATGGAGAGTTCTGGGCACAAGCTACTGCCTTGCGTTTTGTGCTTGGATGTTATGAGTCCACCCTTAATGAGAGTTTA
TTAACTTCTCTTACCCAAGCCGCACAATGCATTATGGCTGGCTACCCTAAAGAAACGTCACATCCTTTCGTTGTGGAACA
GGGTTTTGCTGGACATAGCCACGGATAACAATAACAGATGTTTTCTACCGCCTTTATGAAATAACCAATAACATTGAAT
ATCTCTCATATTGCTTGTGGCTGTATGAAGATTATTCCGCAGGATGCGTGTCGGAGCAAGATCTGCAATATAAAAATGTC
ATAGACCCTGGTTACCTTCTTAAAGGTCATGGCGTACATACGTATGAGCATATTAGGGCATTAGCCATCGCAGCGAGCCA
ACGGCCGCAGTATCAAACTGCATTAGATATTTTTTTATCCCAGTTGAAATATTATCTTACACCATCTGGCGCTCCCATTG
GTGATGAGTGGATTACAGGACGCACAGCTAATGCCACTTATACAGGCTATGAATATTGTTCTGTTCATGAACTATTTAGC
ACTTATGTTATGTTAATAAAACTGACCACTAATATTGGTCTGGCCGATGATGCTGAGTGGCTATTCTATAATTCTTCGCT
TCGCATTTACCCTGACACAGGACAGTCCACGCGTTGA
>Cluster_35618
ATGAATAATACCGTTTTACTTCGGGTTTCCGGTCGCGAATGGGGCGGCTGGACATCCGTCCGTATCAGTGCGGGCATTAA
CCGTATTGCCCGGGATTTTAATGTTGCCATTACCACCCGCTGGCCCGGCAGCCGGGATTATCAGCCCCGGATAAAAAATG
GTGGGCTGGTTGAAGTGCTTATCGGGGATGAGCCCGTGCTCACCGGATATGTGGAGGCACTACCGCTTCGTTATGACGCC
AGCAGCGTCAGTATGGGGATTGTCGGGCGAAGCAAAACAGCCGATCTGGTTGATTGCTCTGCTTTGCCACTCCAGCAGAG
CGGAAAAAAACCTGCTCAGAATAGTCACTGAACTGGCTGCGCCATTTGGCATCACTGTTGTTGATGCTGGCGTGCCTCAGA
CTGCGGTGATTGATGCACAGCCGGAACACGGCGAAACTGTTGCCGATTGTCTTAACCGGTTGCTGGGGCAGGTTCAGACG
CTGGCTTATGACGATGAATGCGGGCGACTGGTTCTTGGGAAACCCGGAACAGGCAAAGCGGCGACGGCACTGGTGCTGGG
AGAGAATATTCTTTCCTGTGACACGGAAAGAAGCATCAGAGAGCGGTTTTCTGAATATCAGGTCAGTGGGCAGCGCCCGG
GCAACGACGATGATTTGGTGAGGCCACCATTGCCGCAATACGTCAGACCATTCAGGACAATGGCGTGACCCGTTATCGC
CCTTTGTTGATTCAGCAGTCAGGCACAGCAACGACAGCAACCTGTAAGGCCCGTTGTGAATTTGAAGCGCGCCAACGGGC
TGCGCTTACCCGTGAGACAACATATACGGTTCAGGGCTGGCGGCAGGGCAGTGGCGCGTTATGGCGTCCGGGGTTATCTG
TCATCGTTTTCGACCCGCTGAATAATTTTGATAATGATGAACTGGTGATCGCAGAAGTTACCTATAACCAGGACGACCGG
GGCACGACGACTGAATTACGGGTTGGCCCGGCAGATGCTTATCTCCCCGAGCCTGTTACCGCCAGGAAGAAAAAAAAATGT
TGAGGAGGATTTCTGA
>Cluster_28449
ATGAATAAGCGCGATGCAATCAATCATATTCGGCAGTTTGGCGGGCGTCTGGATCGCTTCCGTAAATCGGTCGGCGGCTC
TGGCGACGGCGGTCGTAACGCGCCGAAACGCGCGAAATCCTCGCCAAAACCGGTAAATCGCAAAACCAACGTTTTCAACG
AACAGGTACGCTATGCCTTCCACGGCGCATTGCAGGATCTGAAAAGCAAACCGTTCGCCACGTTTTTAACGGTGATGGTT
ATCGCCATCTCCCTGACGCTGCCCAGCGTCTGTTATATGGTGTACAAAAACGTTAACCAGGCGGCGACGCAATATTATCC
GTCACCGCAAATCACTGTTTATCTGCAAAAAACGCTGGATGATGACGCCGCTGCGGGCGTGGTGGCACAGTTGCAGGCCG
AGCAAGGCGTGGAGAAAGTGAATTATCTTTCTCGTGAAGACGCACTGGGCGAGTTCCGTAACTGGTCTGGTTTTGGTGGT
GCGCTGGATATGCTGGAAGAAAACCCGCTTCCGGCAGTAGCGGTGGTGATCCCGAAACTCGATTTTCAGGGGACGGAGTC

ACTGAATACGCTGCGTGATCGTATCACGCAGATTAACGGCATTGACGAAGTGCGGATGGATGACAGCTGGTTTGCCCGCC
TGGCGGCGTTGACCGGGCTGGTCGGGCGCGTTTCGGCAATGATCGGCGTATTGATGGTGGCGGCAGTGTTCCTCGTCATC
GGTAACAGTGTGCGCCTGAGTATCTTTGCTCGCCGTGATTCTATTAACGTGCAGAAACTGATTGGTGCGACGGATGGTTT
CATCCTGCGTCCGTTCCTGTACGGTGGCGCATTGCTGGGATTTTCTGGTGCATTGTTGTCACTAATTTTGTCAGAAATTC
TGGTGCTGCGTTTGTCATCGGCGGTTGCGGAAGTGGCACAGGTTTTCGGAACGAAGTTTGATATCAATGGCTTATCGTTC
GATGAATGCCTGCTATTGCTGCTGGTATGCTCGATGATTGGCTGGGTGGCAGCGTGGCTTGCCACGGTACAACATTTACG
CCACTTTACGCCTGAATAA
>Cluster_20410
ATGAAGATCAAAGCTGTTGGTGCATATTCCGCTAAACAACCACTTGAACCGATGGATATCACCCGGCGTGAACCGGGACC
GCATGATGTCAAAATCGAAATCGCTTACTGTGGCGTCTGCCATTCCGATCTCCACCAGGTCCGTTCAGAGTGGGCGGGGA
CGGTTTACCCCTGCATGCCGGGTCATGAAATTGTGGGGTGTGTGGTAGCCGTTGGTGATCAGGTAGAAAAACATGCGCCG
GGCGATCTGGTCGGTGTCGGCTGCATTGTCGACAGTTGTAAACATTGCGAAGAGTGTGAAGACGGGCTGGAAAACTACTG
TGATCACATGACCGGCACCTATAACTCGCCGACGCCGGACGAACCGGGCCATACTCTGGGCGGCTACTCACAACAGATCG
TCGTTCATGAGCGATATGTTCTGCGTATTCGTCACCCGCAAGAGCAGCTGGCGGCGGTGGCTCCTTTGTTGTGTGCAGGG
ATCACCACGTATTCGCCGCTACGTCACTGGCAGGCCGGGCCGGGTAAAAAAGTGGGCGTGGTCGGCATCGGCGGTCTGGG
ACATATGGGGATTAAGCTGGCCCACGCGATGGGGGCGCATGTGGTGGCATTTACCACTTCTGAGGCAAAACGCGAAGCGG
CAAAAGCCCTGGGGGCCGATGAAGTTGTTAACTCACGCAATGCCGATGAGATGGCGGCTCATCTCAAGAGTTTCGATTTC
ATTTTGAATACAGTAGCTGCGCCACATAATCTCGACGATTTTACCACCTTGCTGAAGCGTGATGGCACCATGACGCTGGT
TGGTGCGCCTGCGACACCGCATAAATCACCGGAAGTTTTCAACCTGATCATGAAACGCCGTGCGATAGCCGGTTCTATGA
TTGGCGGCATTCCAGAAACGCAGGAGATGCTCGATTTTTGCGCCGAACATGGCATCGTGGCTGATATAGAGATGATTCGG
GCCGATCAAATTAATGAAGCCTATGAGCGAATGCTGCGTGGTGATGTGAAATATCGTTTTGTTATCGATAATCGCACACT
AACAGACTGA
>Cluster_12685
ATGAATCTGGTGATGTCAGATATTACCCGCAACAAGCTGGGTTGCTATATGGCGCAGCAGGCGTCGCTTAACAATATCCC
GGTATCTGCACTGGTATCGCGATTTACCGTAGAACCCTCGGTGCAGCAGCGTTTTGAAAACGCAGTAAAGGAGAGCACTG
AATTTACAAAAAAAATTAACGTGTTCGGTGTGACTGACCAGAAAGGCGAAAAAATCCTCCTGGACACCACCGGGCCGATT
GCGCGCACGAATACCAGTTATGACGGCACAAAACGCCGTAACCCGAATAACGTGGTTGATCTGAAAAACCGCAAATACCA
GTGCGAACAGGTGAACTACGACACGTTTATTCGTATCCGCAGCTTGATGCCTGGGCGGCACACCCTGATTTTCAGTCCC
GCGTCAGCACACAGATTGCCCGGCAGGTGGCGCTTGACCGCATCATGATCGGTTTCAACGGCACGTCTCACGCAGATGAG
TCCAACTTCAGCACTAACAAGCTGCTTCAGGACGTTAACGTGGGATGGCTGGAGCACATCAGAACCGACGCCAGCGAGCG
CGTTATGAATGATGTAACGCTGACCTCCCGCAACATGGACAACACCGTGGCGCACGCGGGTAAATATGCGAACGCTGATG
CACTGGTACAGGACGCGCGCTCATCCCTGCTGGATGAATGGCACAAGGAAGCTGACGACCTCGTGGTGATTATGGGGCGC
AACCTGTTTAACTCGCTGCGTCTGCCCGTGCTGAACAGCATCAGCGGCCAGAATCCCAATGCGGAATTACTTGCCGGACA
GCTCATCCTGTCATCGCGCGCCATTGGCGGGCTGGATGTATTCCTTGCGCCGTTCTTCCCGGATGCAACGATGCTGATCA
CCTCGTTCAACAACCTGTCAATTTACTGGCAGAAAGGAACAATGCGTCGCCTGATGAAAGACGAGCCGGAATACAACCGC
ATCGCCACCTACCAGTCCATCAATGACGCTTATGTCGTTGAAGACTATGGCAAGTGCGCGATGGTCACTGGCCTGAAGTT
CGCCGACAGCTAA
>Cluster_20766
ATGAGTAAAAAAAAACGCTTCGTTAAGCGCAACCCGCGCGGCGATAAGTCCAAAAAAATGAGCATCATCACATTCGGCAA
ACCGGAACCCGTCCTGACCACCGGCACGGATTACCGCGACATCTGGTACGACAATGCCGCCGATCACTTCACCCAGCCGA
TTGACCGGCTGGCACTGGCACAGCTTATCAATCTGAACGGTCAGCATGGCGGCATCATCCACGCCCGGAAAAATATGATT
GTGTCTGATTATCTGGGCGGTGGCCTGACTTACGACCAACTGGAAGCCGCAGCGTTTGACTATACAACCTTCGGGGATAT
TGCGATTGGCAAAATCCGCAACGGATGGGGGGATGTGATTGCCCTGGAACCTTTACCCGGCCTGTATATTCGCCGCCGTA
AAGTCAGGGACAACGCCACAGACCAGCCCGGCGATTATGTGGTATTGCAGGACGGGGAACCGCAGGTATGGCCACAGGAA
GATATCATTTTTATCAAGATGTACGACCCGCAACAGCATATTTACGGGCTGCCCGACTATATCGGCGGTGTACATTCTGC
ATTGCTTAACAGCGAAGCCGTGATTTTTCGCCGCCGTTACTACCACAACGGCGCCCATACTGGCGGCATTCTCTACACGC
GCGATCCCAGTATGACGGATGAAATGGAAGAAGAAATTGAACAGCAGCTGCGTGACAGCAAAGGTATCGGCAACTTCTCC
ACTATTCTGGTGAACATTCCAGGCGGGGATGGGGATGCGATCAAGTTTATTGAAATGGGGGATATCTCTGCAAAAGATGA
ATTTGCCAACATCAAAAACATCAGCGCACAGGACATTCTCAACGCACACCGCTTTCCGGCAGGGCTGGCCGGAATTGTTC
CGCAGAACACAGCTGGCCTGGGGGATGTTGAAAAGGCGGAAAGGATTTACAAGAAAAGCGAGATAGCGCCTATTCAGCGT
CGGTTTATGACTGCCGTGAACAACGATCCCGAAATACCGGAACGTCTGCACCTTCACTTTGATTTAAGTTACACAGAATC
AACGGATAAGGATGCGGCATGA
>Cluster_50272
ATGAGTCAACATCAATATTATCCACAGCTGAAATGGAAGCCTGCTGAATATGAATCTCTGATGCTTTTAGATCAAACTAC
GCTCTCTGGTTTTACTCCGATCATTACCATTCCAGACATAGACTGGGATTATGAAAACGAATGCTACAAGAAGAATTTGA
GTTCTTACTTATCTGACTTCGGTATTAACCTTGCGGCATCCTGGAAAGCCAATCGTCCTGTTTTGCTGGATGTTAAATAT
TTAGATAAACATGGTTCGAGCCGCCATCATCCTCTAGATATGTGTATCCAAGATGCTAGAGTAAATGGTAAGGAAATTAT
CCCTGTTGTTTCTCCCGCATATTCAACAAACTATATACATGCTGTTCAACGCAACTTAATCAATGGGCTCGCTATATCTA
TCACCCCCCAGACATGGCACCAATTCACAAGTCTGGTTAACCACTTAAATATTCATCCTAGTTTAATTGATGTAATCATT
GATTTTGGAGATATTCAAAACGCAACTGATAGTTTAAAACAACAAGCATTAAGCATGGTCAACACATTATCAGGCCAAGC
TCCGTGGAGAAACTTGATTTTATCTTCAACCGCATACCCGGCATCACAGGCAGGGATACCGCAACATCAAGTTCATCATA
TTCCGCGCCATGAATACGATCTTTGGATGTACGTAGTACAGAATTTTAGCAATGGAAGAACGCCAAGTTTTAGTGATTAT
CCCACCGCTAGCTCTACCATTACGAGCGTAGACCCACGCTTCATGTCTCAGTATGTCTCAGTGAGATATTCGAACGATAC
CTCATGGATCTTTGTAAAAGGTACCGCAGTTAAAGGAAATGGATGGGGCCAAACTAAAAACTTATGTACTACCCTTGTTA
GTTCGCCAGAGTATCAAGTCTTTGGCTCCAAATTTAGTTGGGGGGATGATTACATTTACCAAAGATCATTAGGCGCTAAC
AAATCTGGCGGCTCTAAAGAATGGCGTAAAGTTGCACATACGCACCATATTACGTTAGTCGTGAGACAGCTTTATTGGTT
GGCGCAGACTCAGCCTGCCAAGCCTTAA
>Cluster_38946
ATGAATCCGGCATTGCTTGCCACGCTCGGAACACTTGCGGAAACGGCGTCGCTGAAAGCGGATATTTTACCGCCGGTGAG
TAGTGAAAACGCGCCCGCGTTTACCCTGCCGAAAATGGCGGTCGCGGCGGTGGCGGAGCGCGTTCATAGCGCAAAAACCA
GTCAGCAACAGGCGACGCGCCCGCAGGAGAACGATCCGGTGGCGATGCAGGCGCTAATGGCGCTGTTACTTCCGCAACCT
GCCGCGCCGCATCAGGACACGCCGCAGCCGCGAAACGTCGCAACATCGCCGGTTATCCAGCAATTGACGAAAGCGGTGGT
GCAAAACGCGGCGCAACGCCCGACGCAGCAGCAGGAACTCACGCCGTTGCCGCCGCAGTTGCAGGAGCTGATCAGTCAGT
TGCCGCAGGCGAAACCCGAACAGCAGGCCAGACTGGCGACTTACGCCAGTGAAGATTTACATGCCATTGCGCCGACGCAG
CCGCGCGTCTCAACACCGCCAGCTCGCCCGAAACCTGAACTAACCCGTGTGACCGCGCGCCCCGCAGGTCGAGCGTAAAAC
GGAAAAAGTGCCGGACAGCGAACCGGTTATTGCGCGTGCGGTGTTGCAGGTTAAGACGCCAGAGTTGGTCAGCGATCATC
AGGAGATTGTCGCCAAACCAGCCACGCTTTCGATGGACGAACTGGGCGAAAAACTGACGACGCTGTTGAAAGATCAGATC
CACTTTCAGCTCAACAAACAACAGCAGATCTCCACCATCCGTCTCGATCCACCGTCGCTTGGCAAGCTCGAGATCGCCGT
ACAACTCGACAACGGCAAACTGATGGTGCACATCGGCGCGAACCAAAGTGAAGTCTGCCGCGCGTTGCAGCAGTTTAGCG

ACGATCTCCGCCAGCATCTGACGGCGCAAAATTTTATGGAGGTGAACGTACAGGTTTCCTCCGAAGGGCAGTCGCAGCAG
CAACAACAGTCGGGCCATCAGCAGGAAGAGGTGAGTGCCGCCTTACAGCTTGATGATGCGCCTCAATTTCAACAGAACGA
ATCCGTTTTGATCAAAGTGTAA
>Cluster_41530
ATGAAAACGATGCTGGCAGCTTATTTACCAGGAAATTCGACCGTCGATCTGCGGGAAGTTGCGGTGCCGACGCCGGGGAT
TAACCAGGTACTGATCAAAATGAAATCCTCCGGGATTTGCGGAAGCGATGTCCACTATATCTACCACCAGCACCGCGCTA
CGGCGGCGGCACCCGATAAACCGTTATACCAGGGATTTATCAACGGTCATGAACCGTGCGGGCAGATTGTGGCGATGGGG
CAAGGCTGCCGCCATTTTAAAGAGGGCGACCGCGTGCTGGTGTATCACATTTCTGGCTGTGGTTTTTGCCCGAACTGCCG
TCGCGGTTTTCCTATTTCTTGTACTGGCGAAGGAAAAGCGGCTTACGGCTGGCAGCGTGACGGCGGCCATGCTGAATACC
TGCTGGCGGAAGAAAAAGACCTGATCCTCCTGCCGGACGCGCTGAGCTATGAAGATGGTGCGTTTATCAGTTGCGGCGTT
GGTACAGCGTATGAAGGGATTTTGCGCGGCGAAGTTTCCGGCAGCGATAACGTGCTGGTGGTCGGTCTGGGGCCGGTCGG
CATGATGGCGATGATGCTGGCGAAAGGTCGCGGTGCAAAAAGGATCATCGGCGTTGATATGTTGCCGGAACGTCTGGCGA
TGGCGAAACAGTTAGGAGTGATGGATCACGGCTATTTAGCGACCACCGAAGGTCTGCCGCAGATAATCGCCGAACTCACT
CACGGCGGCGCGGATGTTGCGCTTGATTGTTCCGGTAATGCCGCAGGTCGCTTACTGGCACTGCAATCCACCGCTGACTG
GGGACGCGTGGTTTACATTGGTGAAACCGGAAAAGTCGAGTTCGAGGTTAGCGCCGACCTGATGCACCATCAACGGCGGA
TTATCGGCTCCTGGGTGACCAGTCTGTTCCATATGGAAAAATGCGCCCACGATTTAACGGACTGGAAACTGTGGCCGCGT
AACGCCATTACCCACCGCTTCTCGCTGGAACAGGCGGGAGATGCTTACGCGCTAATGGCGAGCGGCAAATGCGGGAAAGC
CGTGATCAACTTCCCGGATTAA
>Cluster_57144
ATGTTTACTAAACGTCACCGCATCACATTACTGTTCAATGCCAATAAAGCCTATGACCGGCAGGTAGTAGAAGGCGTAGG
GGAATATTTACAGGCGTCACAATCGGAATGGGATATTTTCATTGAAGAAGATTTCCGCGCCCGCATTGATAAAATCAAGG
ACTGGTTAGGAGATGGCGTCATTGCCGACTTCGACGACAAACAGATCGAGCAAGCGCTGGCTGATGTCGACGTCCCCATT
GTTGGGGTTGGCGGCTCGTATCACCTTGCAGAAAGTTACCCACCCGTTCATTACATTGCCACCGATAACTATGCGCTGGT
TGAAAGCGCATTTTTGCATTTAAAAGAGAAAGGCGTTAACCGCTTTGCTTTTTATGGTCTTCCGGAATCAAGCGGCAAAC
GTTGGGCCACTGAGCGCGAATATGCATTTCGTCAGCTTGTCGCCGAAGAAAAGTATCGCGGAGTGGTTTATCAGGGGTTA
GAAACCGCGCCAGAGAACTGGCAACACGCGCAAAATCGGCTGGCAGACTGGCTACAAACGCTACCACCGCAAACCGGGAT
TATTGCCGTTACTGACGCCCGAGCGCGGCATATTCTGCAAGTATGTGAACATCTACATATTCCCGTACCGGAAAAAATTAT
GCGTGATTGGCATCGATAACGAAGAACTGACCCGCTATCTGTCGCGTGTCGCCCTTTCTTCGGTCGCTCAGGGCGCGCGG
CAAATGGGCTATCAGGCGGCAAAACTGTTGCATCGATTATTAGATAAAGAAGAAATGCCGCTACAGCGAATTTTGGTCCC
ACCAGTTCGCGTCATTGAACGGCGCTCAACAGATTATCGCTCGCTGACCGATCCCGCCGTTATTCAGGCCATGCATTACA
TTCGTAATCACGCCTGTAAAGGGATTAAAGTGGATCAGGTACTGGATGCGGTCGGGATCTCGCGCTCCAATCTTGAGAAG
CGTTTTAAAGAAGAGGTGGGTGAAACCATCCATGCCATGATTCATGCCGAGAAGCTGGAGAAAGCGCGCAGTCTGCTGAT
TTCAACCACCTTGTCGATCAATGAGATATCGCAAATGTGCGGTTATCCATCGCTGCAATATTTCTACTCTGTTTTTAAAA
AAGCATATGACACGACGCCAAAAGAGTATCGCGATGTAAATAGCGAGGTCATGTTGTAG
>Cluster_41172
ATGCATAACTCCCCCGCAGTCACCAGCGCGAAATCGTTTGACCTGACCTCGACGGCATTTTTAATCGTTGCCTTTCTCAC
CGGTATTGCGGGCGCTCTGCAAACCCCGACACTCAGTATTTTTCTTACTGATGAAGTACATGCCCGTCCGGCGATGGTGG
GATTCTTCTTTACCGGCAGCGCAGTCATTGGGATTCTGGTGAGTCAGTTTCTCGCCGGGCGCTCTGATAAGCGCGGCGAT
CGCAAATCGCTGATTGTCTTTTGCTGCCTGTTAGGCGTGCTGGCCTGCACCCTTTTTGCCTGGAATCGCAACTACTTTGT
TTTGCTATTCGTTGGCGTCTTTCTTAGCAGCTTTGGCTCAACCGCTAACCCGCAAATGTTTGCCCTTGCCCGTGAACATG
CTGACAAAACCGGACGTGAGGCGGTGATGTTCAGCTCTTTTTTACGCGCTCAGGTTTCACTGGCATGGGTCATTGGCCCA
CCGCTGGCTTATGCCTTAGCGATGGGTTTCAGCTTTACGGTAATGTATCTGAGCGCAGCGGTAGCGTTTATTGTTTGCGG
CGTGATGGTGTGGCTGTTTTTGCCGTCGATGCAAAAAGAGCTTCCGCTGGCGACCGGCACGGTTGAAGCGCCGCGCCGTA
ACCGTCGCGATACGTTGCTGCTGTTTGTCATTTGTACATTGATGTGGGGCTCGAACAGCCTGTACATCATCAACATGCCG
CTATTTATTATCAACGAACTGCATCTTCCCGAGAAACTGGCCGGTGTGATGATGGGGACCGCCGCCGGGCTGGAAATCCC
GACCATGTTGATCGCCGGATATTTTGCCAAACGTCTGGGTAAGCGCTTCTTAATGCGCGTTGCTGCCGTGGGTGGCGTCT
GTTTTTACGCAGGAATGCTGATGGCGCATTCACCTGCCATTCTGTTGGGCTTGCAGCTGCTAAATGCTATTTTTATTGGC
ATTCTGGGCGGCATTGGGATGCTCTATTTTCAGGATTTGATGCCCGGTCAGGCAGGTTCAGCCACCACGCTCTATACCAA
CACTTCGCGCGTGGGCTGGATCATCGCAGGATCAGTGGCGGGCATCGTCGCCGAGATCTGGAATTATCACGCTGTGTTCT
GGTTTGCGATGGTGATGATTATCGCCACTCTGTTTTGCTTACTGCGGATTAAAGATGTTTAA
>Cluster_50098
ATGCCAGACAGAATGGGCTTTTTTGCTCATGAAAATTCTGTCATTTCCGAATTAGAGGCAGATCACAAAATGGACAAGAA
GCTGAAAATCGCCGATATCGCCGCACGTACCGGGCTATCGCCGAGTACGGTTTCGCGGGTGCTGGCTGGCAAAGCGAATA
CCAGTGCACGCGCTCGCGAACAGGTGCTGGCCTGCGCGCGTGAGTTGGGCGTGATGGATGGCATGGCAGCAGGTCGTATG
TTGCTGAACAACCTGATTATTTTCGCGCCGCAACGCGCTTTTGACCAACGATCTGATATCTTTTATTACCGTGTGATCCA
GAGCATCAGCAAAGCGTTATCGCCGCATGAAGTGCGGCTGCGTTACTGCGCGCTGGAGGAGTTTGACAGCGATCCCGCGC
TATTTCTCTCGCGTATGAATGAAGCAGATACGCAGGCGGCGATCCTGCTGGGGATTGACGATCCGCACATCCATGATCTG
GCCGCCGATCTCGCTAAGCCTTGTGTGCTGATCAACTGCCGTGATGAACGAATGCGCCTCCCGGCGATTGCGCCGGACCA
TCGGTTAATTGGCGCGTTCGCCGCCCGCTATCTGTTTGAAATGGGTCACCGGGAGGTGATGAATGTGATGTGCCTGCGCC
GCTACACTATGGAGCAGCGGTTTGCAGGGATTAAAGAATCATGGCGTCAGCATAATCTGGCCTTTCACCATGACCGTCAT
TTGCTGAATATCAGCAGTTTTAGCGCTAAAGAAGCGGAAGAAAAGGTCGGAGCGTGGCTGGATGCCGCCAATGGCAAGAC
GTTGCCGACGGCGTTTCTGGTCGGCGGGGATTTTATGGCGGCGGGAACGGTAAATGCGCTACATCGTCGTGGTCTGCGCG
TACCACAGGATGTCTCGGTGATGAGTATCGACGGCTTTAACCTTGCGGCAATCGAGGATGTTCCCCTGACGGCTGTGCAT
GTGCCACGCGATGAGCTAGGCAATGAAGCGGTGCAAATGTTGCAACAGCGGCTGATTCGCCCGGATGCTCCGGTGGGCTC
GCTGCTGCTGCACGGACGATTGGCGGTGCGGGAGTCAGTGCGGCGGGTTCGCCCAGGAAAAGGGAACACCGCCGTGGAAC
GGGAAGGGCTGTACGACACTCAGCTGTCGATTCCTAGCGCTCGCTTACCGTGA
>Cluster_25325
ATGGATACGTCACTGGCTGAGGAAGTACAGCAGACCATGGCAACACTTGCGCCCAATCGCTTTTTCTTTATGTCGCCGTA
CCGCAGTTTTACGACGTCAGGATGTTTCGCCCGCTTCGATGAACCGGCTGTGAACGGGGATTCGCCCGACAGTCCCTTCC
AGCAAAAACTCGCCGCGTTGTTTGCCGATGCCAAAGCGCAGGGCATCAAAAATCCGGTGATGGTCGGGGCGATTCCCTTC
GATCCACGTCAGCCTTCGTCGCTGTATATTCCCGAATCCTGGCAGTCGTTCTCCCGTCAGGAAAAACAAGCTTCCGCACG
CCGTTTCACCCGCAGCCAGTCGCTGAACGTGGTGGAACGTCAGGCCATTCCGGAACAAGCCACGTTTGAACAGATGGTTG
CCCGCGCTGCTGCACTTACCGTCACGCCGCAGGTCGACAAAGTGGTGTTGTCACGGTTGATTGATATCACCACTGACGCC
GCCATTGATAGTGGCGTATTGCTGGAACGGTTGATTGCGCAAAACCCGGTTAGTTACAACTTCCATGTTCCGTTGGCTGA
TGGTGGCGTTCTGCTGGGGGCCAGCCCGGAACTGCTGCTACGTAAAGACGGCGAGCGTTTTAGCTCCATTCCGTTAGCCG
GTTCCGCGCGTCGTCAGCCGGATGAAGTGCTCGATCGCGAAGCGGGTAATCGTCTGCTGGCGTCAGAAAAAGATCGCCAT
GAACATGAACTGGTGACTCAGGCGATGAAAGAGGTACTGCGCAAACGCAGTAGTGAGTTACACGTTCCCTCCTCTCCACA
ATTGATTACCACGCCGACGCTGTGGCATCTCGCAACGCCCTTTGAAGGTAAAGCGAATTCGCAAGAAAACGCACTGACTT
TGGCTTGTCTGCTGCATCCGACCCCCGCGCTGAGCGGTTTCCCGCATCAGGCCGCGACTCAGGTTATTGCTGAACTGGAG

CCGTTCGACCGCGAACTGTTTGGCGGCATTGTGGGTTGGTGTGACAGCGAAGGTAACGGTGAATGGGTGGTGACCATCCG
CTGCGCGAAGCTGCGGGAAAATCAGGTGCGTCTGTTTGCCGGAGCGGGGATTGTGCCTGCGTCCTCACCGTTGGGTGAGT
GGCGCGAAACGGGCGTCAAACTTTCTACCATGTTGAACGTTTTTGGATTGCATTAA
>Cluster_24768
ATGATAAAGAATAATCTTGGCGTAGCGGTAATTGGTTCGAAACAATATGCCGTGAATTTATTATGGGGAAGTTCTCAGGA
TACAGAAACAACAAATCAGGCTCTGAATAAGAGCCTGACGTTGATGTCCTCAAAGCTATATTCCGTTATTGGACGATTTC
AGGGTGAACAGTTTGCCGTAGGAGATAAAAATATTGGCCACAAAAGAGGCCAGGTGACCTTACTGTCTGCAATTGATTTC
GATGGCAGTTCTTTTTGTGGGCTGTTTCCTGCTGATAATGAATTATGGCTTGTCATCGGTGTTGATAAAGATGGAATGGT
TCACTTTGACAAGTCCTTTCACAGTAAGGATGATGCAAAAAAATTCTTTTTCGACCATGTGGCATATGGATACCCGTGGG
ACAGAACATACAGTCCTTCAGATGTTGGAGTCGGTGAAAGCAGGAGTATTTCTGAGCTTTCACTAATCAAAGGGAAGAAA
TTAAAGGAAAAAGGTGCCGGTCAACTAATGCCATTGCTGTTATCTGGCGTTGGAATTATAATATTCTTTGTCATCATATA
TAATTTTATGAACTTATGGTTAAGTGGCAGAGATAAGGATATTCTTAACGAAGAACCATCTCCGGCCGTCAGTGAGGAAG
TCAGAAATATCCCGTGGGAAGGGAAAAGCAAACCGGGCTCATTGTTTTCTAAATGTATAAAAGAAATGGATTCATTCCGG
TTCCAGGCAGCATCCGTTCCAGGCTGGATGCCTGAAAATAAAGTAACCTGTAATGACTCTGATATAAACTTTAGTGTTTA
TCGTTCTGGCGGACTTAATATATGGTATGAAAATCCGGTCCTGTTTTTTCACAGTGGAGACATCCCAGACATTACCAGAA
ATGATAATGATAAAATAACATTTACATGGCCTCTTGAAACGGTCAGGTATGACAGTTCGATACTCGACGTGCATACCTTG
AATAAGACTCAAGAAATAATAAGGTATCTTTCACGTCAGTTTGAGAATAGCTGGATAGAGATAAAATTTGAACCGTCCGT
GCCGGACGGCAATACAGAACGAGTGGGTTTTTCTTTTTCATTTAACCATGATCCGAGAATCTTACTCCCAATTCTTTCTG
AAGTAAATGGACTGATTATCACTAATATCGATTATGATTTTTCAGGAAGTAACTGGAACGTGAAAGGTGTTTTTTGGGGG
AGAGCTTAG
>Cluster_20541
GTGAGTATCAGTTTTGAGCAACTGGTGGGATTGTACAGGCAGATTACTTTCGGTAACGATATGGCCGAAGGTACGCTGGT
GCTTACCCCAGAATCCTGCGAGTTGCTCAACGCTTTGCTTGAGGACACCGATACATATGGGATCTCTCTGGCACAAGGTG
AAGTAGTGGCTGGCCAGCAGGTGTCGCTTTTTGTGAACGCCCCAAAGACAAAGCTAGGTCTGCTATGCCGTAATTTGGCG
GCTTTGTTAAAGTCGCCAAAACACCAAAGCGAGGAACCTTCCCGCTACTACCTGATCGACAGCCAATTTTATAGTTCGGA
TGCGCCAACATCTGTAATTGAAAACTACCGCACTATCTTAACATTTCTGCGCCTGCTTAAAGAAAAGTCGGCATACTTTG
ATACCAGTGCATATGAATGCGTTTTTTTCCGCGCCGATGTATTTAAATTACCTATACGTTACAGTGCAGAAACCGTAGAA
AATCTTGATAAAACGACACTCGATGAACTCATTCAGCAGTTTTCTGACGACACACACAAAGAGCAGAAACTCTCGCTTTT
AATTGAAAGCATCCAGTTAATCGGTCAGGAAACGGAAAACAATAAAGTCTTTGAGTATGCACTGAAGAACATTGATAAAT
TAAAAGTTGAGTTCGATAAGGGTTATCGCCTCTTCACTTCTGGGTTCTCATACGAAAAGGTTCTGGATGAACTTAGAACC
GCCAAAGTTGAAGAAATGGGACGTATCCATAAAGTCTTTTCAGATATCCAAAACCAGATACTGGGTATCCCAGTCGCAAC
CATTATCGTCGCGACACAAATCAAGAAAGCAAGTGGTGATGTCTATCAAACCATTATCAATAGCGCAGTTTTTTTAGGGG
CCTTTGTTTTTGCTACGTTGGTTATGTTGACTTTGTTCAACCAGTTACAGACGTTAACTGCTATCAAAGAAGAACTTCGA
CATAAAAAGACTCAGGTTGATAAAAAGTATGCATCAATAAAAAATGACATTGATAGTATCTTTTGCTCTGTACGTAAACG
TCTTTGCCGTCAGGTTATCGCGTTCTGGTTTATTATTATGTTCCTTGTTGTCGCTATTATATCGACAGGATTAATTTACC
TATACTGCACATACCCTGAAACATGGAATATCTTGGTTACAAAAGTGATGTCATGGCGGCATCACTTGAGTAATAAGCCC
ACTCCTTAA
>Cluster_34007
ATGATGTCTGGAACGAATAAGCTCAGCGACAGGAAACTAAAGGCGCTGCAAGGAACGCATAGGGATCGTGTTGAAATGCT
TGCTGACGGAGAGGGGTTAGGGGTCAGGTTATCAACTAAAGGGCATGTGAGCTGGGTTTTTTCATATCGCCTTGGAGGGC
GTGAGAGTTCAGTTATTCGTCTTACTCTAGGGAATTACCCTGATATTAGTCTGAAAGATGCCAGGATGTGGCGTGAGCGT
TGCCGTCAATGGTTGGCTAATGGTCTTGATCCAAAAATTGAACTCCAACTTTTTACTGAAGAATCCCGTGCTCCTGTTAC
TGTTCGAGATGCGTTGGAATATTGGTTGGTCAATTATGCAAGACATAAGCGCAAAGATGAGGAATTGATTAGGGCGCAAT
TCTGCAAACATGTCTACCCCAGAATAGGCCATTATCCGTTATCACGGTGCGATACTCGCCATTGGGTAAAATGTTTTGAT
GAGATTCGTGCTATAAGTTCAAAAACGGCGGGGCGTATGTTTCAACTCTCCAAGCAGGCATTGCGGTTTTGCAATGTTCG
GCGTTATGCCATCAGTGATGCCCTTGTTCATTTAACGTTAACCGATGTAGGGGAATCTGCCGGACAGCGCGACCGTGTTT
TGAGTGATGCCGAACTATCAGATCTTTGGTATTTTGCAAAGGAGAAACATCGGGATATTTATTTTTCTAGATTAATAAAG
TTGCTTATTGTCTTTGGGGCTAGAACTGTAGAGATTCGCCGTTCTCATTGGATTGAATGGGATTTTAATGAATGGGTTTG
GATTGTTCCAAGAAATAACAGTAAAAGTAACAAAAAGATTATTCGGCCAATTCCCGAAGGTATAAGAGACTGGTTAAAAG
AGCTAAAACAGGAAACCGGGAGGACGGGCTTACTTTTGGGTGAGGAGAGAACGCATAGAGCTGTTTCTCTGAAAGGTCGC
AGAATGTATAAAATATTCGGACATAATGAAAAATGGACGCTACACGATTTAAGACGGACATTTTCAACTGGGTTAAACAA
CATGGGGGTAATGCCTCATATTGTCGAGTCATTATTAGGTCACGTATATAATGCTGGCTCCGCAGAATTTAACTATAATC
TTAGCCAGTATATCCCATTCAAATTAAAGGCTCTAAATGACTGGCTTGAATGTTTAGAAAAAATGGTTAAAGATAAATAA
>Cluster_33766
ATGAGCGAGTTAAATTATCTGGAAAAACTGCTAGATGGGATAGAGGTGGAATGGAAAGCCCTTGGAGATGTCACGAGTGT
CTTGCGGGGAAAAAGGCTAACTAAAAATCTGCTTTCAGCAGAAGAAAAATTCCCGGTATTTCATGGCGGCTTAGAACCGT
TAGGATATTACAATAAGTCGAATCGTCCGGCTAATACCGTTATGATTATCAATGTTGGCGCTTCAGCAGGTACTGTTGGC
TATAGCACCGTTGACTTCTGGTCCTCTGATGGATGCTTTTGCTTAGAGCACAATGAGCTATTAAATAATAGGTTTTTATA
TTTCGCACTGATTGGTTATCAAAGTTTGCTGAAATCAAAGGTGAGAGTTGCGGGCATTCCAACACTTGATGCTATCGTAG
TTAATAAACTACTTATCCCCATCCCCTGCCCAGAAAACCCGGAAAAATCCCTTGCCATCCAGTCTGAAATCGTTCGAATT
CTGGATAAATTTACTGCACTTACCGCTGAGCTTACCGCTGAGCTTACCGCTGAGCTTAACATGCGTAAAAAACAGTACAA
CTACTATCGCGACCAATTGCTGACTTTTAAAGAAGGTGAGGTTGAGTGGAAAGCTTTGGGGGAAATTGGTGAAGTTCGTA
TGTGTAAACGAATCCTAAAATCACAAACATCTTCTGAGGGAGAGATTCCATTTTATAAAATTGGGACATTTGGTAAGGAA
CCCGATTCCTATATATCTAGAAAACTATTTAATGAATTTAAAGAAAAATATAGTTATCCTAAAGTTGGTGAAGTATTGAT
TTCTGCCAGTGGAACCATCGGCAGGACTGTTATATTTGATGGTAGGGAATCATATTTCCAAGATAGCAATATTGTCTGGA
TTGAAAATAATGAAAAAATAGTTTTAAATAAATATTTATTCTATTTCTATAAAATAGCAAAATGGGGAATATCTGAAGGT
GGAACTATTAAAAGACTTTATAATGATAACTTAAGAAAACTTATGATACCAGTACCATTTCCTGATTCTCCTGAAAGATC
GCTAGTTGAACAACAAAAAATAGTGAAATTACTGGATAAATTTGATGCTTTGACCAACTCCATCACCGAAGGTCTTCCGC
GTGAAATCGAGTTGCGCCAGAAACAATACGAGTATTATCGTGATTTACTGTTCAGTTTCCCGAAACCTGATACTGTCAGT
AATTAA
>Cluster_28192
ATGAAGTTTGTTGATGAAGCATCGATTCTGGTCGTTGCAGGTGATGGCGGTAATGGTTGCGTGAGCTTCCGCCGCGAAAA
GTATATTCCGAAAGGCGGCCCGGATGGCGGCGACGGCGGTGACGGCGGTGACGTATGGATGGAAGCCGACGAGAACCTGA
ACACGCTTATCGATTATCGTTTTGAAAAATCTTTCCGCGCAGAGCGTGGTCAGAATGGCGCAAGCCGCGACTGTACCGGT
AAGCGCGGTAAAGACGTGACGATTAAAGTGCCTGTAGGTACGCGTGTTATCGACCAGGGTACCGGTGAAACCATGGGCGA
TATGACCAAACACGGTCAGCGTCTGCTGGTTGCTAAGGGCGGCTGGCACGGTCTGGGCAATACCCGTTTCAAATCGTCAG
TTAACCGTACACCACGGCAGAAAACTAACGGTACGCCGGGCGATAAGCGTGAGCTGCTACTGGAGCTGATGCTGCTGGCT
GACGTCGGTATGCTGGGGATGCCAAACGCGGGTAAATCGACATTTATCCGCGCAGTATCGGCGGCTAAACCGAAAGTGGC
GGATTATCCGTTTACCACTCTGGTGCCAAGTCTTGGTGTGGTACGAATGGACAACGAAAAGAGCTTCGTTGTTGCCGATA

TTCCAGGACTGATTGAAGGCGCTGCAGAAGGCGCAGGTCTGGGCATTCGCTTCCTGAAGCACCTGGAGCGTTGCCGCGTC
CTGTTGCACCTCATCGATATCGATCCGATTGATGGCACCGATCCGGTTGAAAACGCGCGTATTATTATCAGCGAGCTGGA
AAAATACAGCCAGGATCTGGCGGCGAAACCGCGTTGGTTAGTCTTCAACAAGATCGACCTGCTGGATAAGGCAGAAGCCG
AAGAGAAAGCGAAAGCGATCGCTGAGGCGCTGGGTTGGGAAGATAAATATTATCTGATCTCTGCGGCGAGCGGACTGGGC
GTGAAAGATCTCTGCTGGGATGTGATGACCTTTATCATTGAAAACCCGGTCGTGCAGGCTGAAGAAGCGAAACAGCCAGA
GAAAGTCGAATTCATGTGGGATGATTATCATCGCCAGCAGCTTGAAGAGATTGCTGAAGAGGATGATGAAGACTGGGATG
ACGACTGGGACGAAGACGACGAAGAAGGCGTTGAGTTCATTTACAAGCGTTAA
>Cluster_12270
ATGTTTCCATTAAATGATCTGTCGTTAAAAACTCAGTCGGTTCAATTAAATAAAATCACATCTAATACTGAATCTACGAT
AAAACAACACGAGTTAGTATCTGATGATGCAATGATAAATGAATTATCAAGTGAGTTATTAAGTTGTCTTGGAAATGGTA
AGTTTACACCAATTAGTGAAGATAGCAAATTACTGAATATGCTGTCTGAATTTAAATTATTGAGAGAGCAATGTTTCAGG
TGGGGTAATTATACTCTATTGTTTGAAAATTATGGGGCTTATGATAAGACGGGGTCTATCACGATAGAAAAAAGTCAGGG
GGAGGGGACTTTACCCATTCGACATAAATTAGAGTTTATATCGACCAATATTGCAGAGTTGCTGGACAAGTTAACCAAAA
TTACAGATACCAGACTTTGCAAAGATTTCAGTGACTGGGCTAGTTCAGTCAAAGAAGGCGCATCCAATGACTTCAAAGAA
AATGTGGATAGGGCATTGCTGAGAATGTTTAAATGTGTTGAGCTCCACAATAATGAACTTGACTTATCATACCTATTTTT
GGGTTCTGTACCGCCTCTTCCCGAGTGGATTGAAATGATTAGCCTTATTCATAATGAACTTGATTCAATACATGTGCCCG
AATCATGCAAAGAATTAGAAGTCGATGTCAATAACCTTACAGAATTTCCACAAGTACCTGATGGAATTACCCTGATCTCC
GTAAATAATAACCTGATATCGCATATTGACTCATTTCCGCCAAAGGCTAAGATAATTTCTATTTGTCACAATAAGCTATC
GAAAATACCAACAATACCAGACACCGCTAAGGTTTTTGATTGTAGTGAGAATAATATAAAGGAAATTAGATGGTTCCCCA
AAAATCTGAAAGAAGTGCATATTGAATATAATAAGATTGAGGTTATTCCTGCGATACCTGGCAATTTAAAATTACTTTTC
ATGGAATGTAATCCTATTAAAGAGGCATTTTTAATGCCATGGACTCTTACAGGGATTTGCTATGAAATATCGCAGCGGAA
ATATATTGTTACGAACCCCGATGATTATGATAAATATTCCGATATGGTTAAAAAGTATGTAATAGATGGTGAGGATCACC
TAATTAAATATTATATGTAA
>Cluster_33172
ATGATAAATGGACTTAATAATGACTCCGCATCTTTAGTTTTAGATGCTGCAATGAAAGTTAATTCTGGGTTTAAAAAAAG
CTGGGATGAGATGTCATGCGCTGAAAAGTTACTTAAAGTACTTAGTTTTGGTTTATGGAATCCAACGTACAGTCGTAGTG
AAAGACAATCATTTCAAGAGTTGTTAACCGTTTTAGAGCCTGTATATCCACTTCCCAATGAATTAGGCAGAGTATCTGCT
CGTTTTTCAGATGGCTCATCCTTAAGAATTTCCGTCACTAACAGCGAAAGTATTGAAGCCGAGATTCGCACACCAGATAA
TGAAAAGATTACTGTGCTCCTGGAGTCAAACGAACAAAATAGGTTATTACAATCTTTACCCATCGATCGCCACATGCCAT
ACATTCAGGTTCATCGTGCCTTATCTGAGATGGACCTGACTGATACTACCTCAATGCGCAATCTACTTGGTTTTACGTCA
AAACTATCAACAACCTTGATTCCTCATAATGCTCAAACAGATCCGCTTTCCGGGCCTACACCATTCAGCTCTATCTTTAT
GGATACATGTCGAGGACTCGGCAATGCAAAGCTTTCACTCAATGGTGTTGATATACCTGCAAATGCACAAATGTTGCTTC
GCGATGCACTAGGACTTAAAGACACACACTCATCACCTTCCCGGAATGTTATAGATCATGGTATTTCTCGCCATGATGCA
GAGCAAATAGCAAGAGAAAGCAGCGGCAGCGATAATCAGAAAGCTGAAGTTGTGGAATTTTTATGCCATCCAGAAGCAGC
AACGGCCATATGCTCGGCTTTCTATCAATCTTTCAATGTGCCAGCCTTAACGTTGACACATGAAAGGATCTCTAAAGCCA
GTGAATACAATGCGGAAAGATCGTTAGATACACCTAACGCTTGCATTAACATCAGTATCTCTCAATCATCAGATGGAAAC
ATTTATGTTACCAGCCATACCGGGGTTCTGATAATGGCGCCAGAAGACCGCCCCAACGAGATGGGCATGTTGACGAACAG
GACTTCTTATGAAGTGCCGCAAGGTGTGAAATGTACAATCGATGAAATGGTAAGAGCGCTACAACCAAGGTATGCCGCAT
CTGAAACGTACTTACAAAACACTTAA
>Cluster_22675
ATGGTTTTGCAATCCACGCGCTGGTTGGCGCTCGGCTATTTCACATACTTTTTAGTTACGGCATTTTTCTACCTTTCTG
GAGCGTCTGGCTTAAAGGGATTGGTTTAACGCCAGAAACCATCGGCCTGTTATTGGGGGCGGGTCTGGTTGCCCGTTTCC
TCGGGAGTTTGCTCATCGCGCCCCGCGTCAGCGATCCTTCCCGCCTGATTTCCGCCTTGCGCGTGCTGGCACTGCTGACA
CTTCTCTTTGCTGTCGCCTTCTGGGCGGGGGCGCACGTAGCGTGGCTGATGCTGGTGATGATTGGCTTTAACCTCTTTTT
CTCACCGCTGGTACCGTTGACCGATGCACTGGCGAATACGTGGCAAAAGCAGTTCCCGCTTGATTACGGCAAAGTGAGAC
TGTGGGGCTCGGTGGCGTTTGTCATTGGCTCGGCGCTGACGGGCAAACTGGTCACTATGTTTGATTATCGGGTGATCCTC
GCGCTGTTGACGTTGGGCGTGGCATCCATGCTGCTCGGCTTTCTCATCCGTCCGACGATTCAGCCACAAGGGGCAAGCCG
CCAGCAGGAGAGCACCGGTTGGTCTGCGTGGTTGGCGTTGGGTCGCCAGAACTGGCGCTTTCTGGCCTGCGTTTGTTTAT
TGCAGGGGGCACATGCGGCCTATTACGGTTTTAGCGCCATTTACTGGCAGGCAGCTGGCTACTCGGCCTCCGCGGTGGGG
TATTTGTGGTCGCTGGGCGTGGTGGCGGAAGTCATTATCTTTGCGCTGAGTAATAAACTTTTCCGCCGTTGTAGTGCACG
CGATATGCTGTTGATCTCGGCGATTTGCGGCGTAGTGCGCTGGGGCATTATGGGAGCAACGACGGCGTTGCCGTGGTTGA
TAGTGGTGCAAATTCTGCATTGCGGTACCTTCACGGTCTGCCACCTGGCTGCCATGCGCTATATTGCTGCTCGCCAGGGT
AGCGAAGTCATCCGTTTACAGGCGGTTTACTCTGCCGTCGCGATGGGCGGCAGTATCGCCATCATGACCGTTTTCGCCGG
TTTCCTGTATCAATATCTCGGCCACGGCGTGTTCTGGGTGATGGCGCTGGTGGCGCTTCCGGCAATGTTTTTGCGCCCGA
AAGTTGTTCCCTCATGCTGA
>Cluster_66977
ATGTTGGGTCATATTAGAACTTTCTTTCATGACTTGCAGGAATGGGGATGGATACCTGTACGATTCAGCCCAGAAAGAGT
TTTCAGGGCACCGCGTAGCTTGACTTCCCTGGTTGGACCAGAGCCTCGTATTGTGGCCGATGATATGTGGTGTAAATTAC
TTCACGCCGGACAGAACCTGCAGGAGTCGGATTTACCAAACTGTGTTGCATATCCTTATTTTTATCCACTGGAGATGGTC
AGAGCACTCAGCGTTCTCTGGTTATTTGGAGGCCTCAGACGTGATGAAATTCTACGTATGCAGTGTGGGTGTATCCGCTG
GCAACAGCCAGAAGAAAATAATGTATCCCGTATCTGTCTGATAGATGTACCCGTTAGCAAGACTTACGCGGCATTCACAA
AGCTAGTTGACCCTATTATCGGGGAATATATCGAACAATGGGAACTTGTTCGTAACCCCCACCCCCTCCAGGAAGATTCT
AAAACAGGTGAAAGCGTCCGTTTTTTGTTCATGCACAGGGGGAAACGGGTACACAGTAGTTATATTAATAATTCGCTAAT
CCCTTTGCTATGCAGAAAGGCTGGTATACCTGAGCAGGATGCCAGAGGTAAAATCACCAGCCATCGGGCAAGAGCCACTA
TAGCCAGTCAGCTTTACAACGCCAGGGAACCACTTGATATTTTTGAGCTACAGAAATGGTTGGGGCATTCTTCACCTGAA
TCTACCCGACATTATGTCGAAATTACACCGACGAGGTTGGCAGGATCACTGGATAAAGCTGGTTACTTTGAATGTAATCG
CCGCATGGTTTCAGTTCTCATTGATCAAGACGCGATAGCTGAAGGGCTGGTAGCATCCGGGAAGCCATGGCGTTATTATG
ATCTTGGACACGGTTTCTGTACTTATGACTTTTTTGAGCAATGCCCCCATCGGATGGCATGTGCAAAATGCTCATTTTAT
ATGCCAAAATCTTCATCTGAAGCACAATACTTGGAAGGTCGCCAGAATCTACTCAAGCTGATGCAGGAAATCCCTTTGAC
TGATGACGAACAAGCCGCTGTAGAAAACGATATACAGGCAGTGGACAAACTTTTGAATAAGCTCACTGGGGGCGCGACCC
CGGGAAATAAGAGAAAGTAA
>Cluster_7258
ATGAAAATCACTATTTCTGGTACCGGATATGTTGGCCTGTCAAATGGTATTCTGATTGCTCAGAGCCACGAAGTGGTGGC
ACTGGATATCATCCAGACGAAAATCGAGATGCTGAATCAGAAACAATCCCCGATTGTGGATAAAGAGATCGAAGAGTATT
TGGCGACTAGGTCGCTAAACTTCCGTGCAACGACTGATAAGTGTGATGCCTACTTAGATGCGGACTACGTTATTATCGCG
ACACCAACTGATTATGATCCAAAGACTAATTACTTTAATACCTCAAGTGTCGAGTCTGTTATTCGCGACGTGGTTGCAAT
TAATCCAGATGCAGTAATGGTAATTAAGTCTACCATCCCAGTAGGTTTTACTAATTCTATCAAAGAAAAACTGGGCACCG
AAAATATTTTCTTTTCCCCAGAATTCCTGCGTGAGGGTAAAGCACTATATGACAACCTTTACCCATCTCGTATTGTCATT
GGTGAGCGTTCAGAGCGCGCCGAGCGTTTTGCAGCTCTGCTACAGGAAGGGGCACTAAAGAAAGATATTCCGACCCTATT

TACCGACTCGACCGAAGCCGAAGCTATTAAGCTGTTCGCTAATACCTATCTGGCGCTGCGTATAGCCTACTTTAATGAGC
TGGACAGCTACGCTGAAACTCTAGGCCTGAATTCACTACAGATAATTGAAGGGGTATGCTTGGATCCGCGAATAGGAAAC
CACTATAACAACCCGTCATTTGGCTACGGCGGTTACTGTCTGCCTAAGGATACCAAGCAGTTGCTGTCCAACTATGAATC
GGTACCGAATAACTTAATCGGTGCGATCGTCGAAGCTAATCGCACTCGTAAAGACTTATTGCCGACTCTATTCTCGCGC
GCAAGCCTAAGGTGGTGGGTGTTTATCGCCTGATTATGAAGTCGGGTTCGGATAACTTCCGCGCGTCCTCCATTCAGGGG
ATCATGAAGCGTATTAAGGCCAAGGGTATCACGGTAATTATTTATGAACCGGTGATGCAGGAAGATGAGTTTTTAACTC
CCGCGTGGTACGTGACTTCGAAGCCTTTAAGCTGGAGGCAGACGTGATTATCTCCAACCGTATGGCAGAAGAGCTGGTGG
ATGTTGGAGATAAAGTCTATACCCGTGATCTGTTTGGTAATGATTAA
>Cluster_17154
ATGATGCAGATAACAATTCCAAAAAGTAATGATTTTAGACGTTTCTTTAATCCCGTTATATTAATTTTAATGTCAATCGT
TTTAAACACTTTTTTTCAATTATATACCAGTGAAGAAGAATGGAATTCATTCTTTATATTTAATCAAAAACCTGATTATA
CGGTGTTTTTTATATCTTTAGTTTTTATTTGTTTTTCGTATTTATTTAATTATCAAAAA
CATAGCAACCATAGAAGCCACTACTTATACAACTTTTCTTTTTATAATGGGTTATTAAAGTTTGTTTTTTATTCAAATGT
AATAAGTTTAATTGTAACCCAGTATTACATGCACGAAGCTAATATATATAATATATATATTAGCGGTGGTGTCTCTGCTC
AAACAATAGAGTCTCAGATTGCTTCATCTCCGCTGGGAATACATGGAATATCTGTAATGTTGGGCTATTTTGGTATTATT
ATGTATGGTATATCTCGTTTGATAAAAGATAATCGAGCTATTGTTTTTATAAGTATATTGGTTATAATAATCAAATTTAT
TAGTTATGCGAAATTGCAATCTTTATTATATGTTTTATTAGCTTTGATGCTATTTTCCAGACGGAGAATATCTATTAATA
AAGGAGTTATAATAGGAGGTGTAGTCATTTTTTTATTTTCATTTACGCGCATAATTCGAAATCCTGAACAAAATTTATCA
TTGAGTTTTGATTTTTTATTTAGATTTGTTGGCGGTTTTTATTTCGGTTCACCTGTTGTTAATTTTAACTATGTTATTCA
AAATAATATACAGGATGTGTTTTATTTCTTCAATTGGTTTTTACCTCAAAAAATCATTCCAGCGTCACCAATTAGCTTGG
AGTTTCCAGATGTTACCTCACCTATAGGTTTTGTAGGAAGTGCTTATGTTTCATTGGGTATATTCAGCGTAATATATGCC
TATTTGGTTGGGTTCATAATTCAATTAATATATTTACAGCGAAATACGTCTCCTTTTGCATTTGTTTTTCAACCATTTGT
CATTATGGCCTGCATATTTGCGATGATGTATAATAATTTTGTAAATATGATATTTTTCATATTGCCGCTTATATTTTCAT
ATTTAGTGGTTATAAAAACTATATGCGCTAGAAAGGAAGATGTTTAA
>Cluster_20143
ATGTTTGAACCAATGGAACTTACCAATGACGCGGTGATTAAAGTCATCGGCGTCGGCGGCGGCGGCGGTAATGCTGTTGA
ACACATGGTGCGTGAGCGCATTGAAGGTGTTGAATTCTTCGCGGTAAATACCGATGCACAAGCGCTGCGTAAAACAGCGG
TTGGACAGACGATTCAAATCGGTAGCGGTATCACCAAAGGACTGGGCGCTGGCGCTAATCCAGAAGTTGGCCGCAATGCG
GCTGATGAGGATCGCGATGCACTGCGTGCGGCGCTGGAAGGTGCAGACATGGTCTTTATTGCTGCGGGTATGGGTGGTGG
TACCGGTACAGGTGCAGCACCAGTCGTCGCTGAAGTGGCAAAAGATTTGGGTATCCTGACCGTTGCTGTCGTCACTAAGC
CTTTCAACTTTGAAGGCAAGAAGCGTATGGCATTCGCGGAGCAGGGGATCACTGAACTGTCCAAGCATGTGGACTCTCTG
ATCACTATCCCGAACGACAAACTGCTGAAAGTTCTGGGCCGTGGTATCTCCCTGCTGGATGCGTTTGGCGCAGCGAACGA
TGTACTGAAAGGCGCTGTGCAAGGTATCGCCGAACTGATTACTCGTCCGGGTTTGATGAACGTGGACTTTGCAGACGTAC
GCACCGTAATGTCTGAAATGGGCTACGCAATGATGGGTTCTGGTGTGGCGAGCGGTGAAGATCGTGCGGAAGAAGCTGCT
GAAATGGCTATCTCTTCTCCGCTGCTGGAAGATATCGACCTGTCTGGCGCACGCGGCGTGCTGGTTAACATCACAGCGGG
CTTCGACCTGCGTCTGGATGAGTTCGAAACGGTAGGTAACACCATCCGTGCATTTGCTTCCGACAACGCGACTGTGGTTA
TCGGTACTTCTCTTGACCCGGATATGAATGACGAGCTGCGCGTAACCGTTGTTGCGACAGGTATCGGCATGGACAAACGT
CCTGAAATCACTCTGGTGACCAATAAGCAGGTTCAGCAGCCAGTGATGGATCGCTACCAGCAGCATGGGATGGCTCCGCT
GACCCAGGAGCAGAAGCCGGTTGCTAAAGTCGTGAATGACAATGCGCCGCAAACTGCGAAAGAGCCGGATTATCTGGATA
TCCCAGCATTCCTGCGTAAGCAAGCTGATTAA
>Cluster_29023
ATGACGCGTAAACAGGCCACCATCGCAGTGCGTAGCGGGTTAAATGACGACGAACAGTATGGTTGCGTTGTCCCTCCCAT
CCATCTTTCCAGTACCTATAACTTTACCGGATTTAATGAACCGCGCGCATGATTACTCGCGGCGCGGCAACCCAACGC
GCGATGTGGTTCAGCGTGCGCTGGCAGAACTGGAAGGCGGTGCCGGTGCCGTACTTACTAATACCGGCATGTCCGCGATT
CACCTGGTCACTACCGTCTTTTTGAAACCGGGCGATCTGCTGGTTGCGCCGCACGACTGCTATGGCGGCAGCTATCGCCT
GTTCGACAGCCTGGCTAAACGTGGTTGCTATCGCGTGTTGTTTGTTGATCAAGGCGATGAACAGGCATTACGGGCAGCGC
TGGCAGAAAACCCCAAACTGGTACTGGTAGAAAGCCCAAGTAATCCATTGTTACGCGTCGTGGATATTGCGAAAATCTGC
CATCTGGCAAGGGAAGTCGGGGCGGTGAGCGTGGTGGATAACACCTTCTTAAGCCCGGCATTACAAAATCCGCTGGCATT
AGGTGCCGATCTGGTGTTGCATTCATGTACGAAATATCTGAACGGTCACTCAGACGTGGTGGCAGGCGTGGTGATTGCTA
AAGATCCAGACGTTGTCACTGAACTTGCCTGGTGGGCAAACAATATTGGCGTGACGGGCGGCGCGTTTGACAGCTATCTG
CTGCTACGCGGGTTGCGAACACTGGTGCCGCGTATGGAGCTGGCGCAGCGCAACGCGCAGGCGATTGTGAAATATCTGCA
AACCCAGCCGTTGGTGAAAAAACTGTATCACCCGTCGCTGCCAGAAAATCAGGGGCATGAAATTGCCGCGCGCCAGCAAA
AAGGCTTTGGCGCAATGTTGAGTTTTGAACTGGATGGCGATGAGCAGACGCTGCGTCGTTTCCTGGGTGGCTGTCGTTG
TTTACGCTGGCGGAATCATTAGGGGGAGTGGAAAGTTTAATCTCTCACGCCGCAACCATGACACATGCAGGCATGGCACC
AGAAGCGCGTGCTGCCGCCGGGATCTCCGAGACGCTGCTGCGTATCTCCACCGGTATTGAAGATGGCGAAGATTTAATTG
CCGACCTGGAAAATGGCTTCCGGGCTGCAAACAAGGGGTAA
>Cluster_37098
ATGGCAACACTAAAAGACATCGCAATCGAAGCTGGCGTATCCCTGGCGACAGTATCCAGGGTCTTAAATGACGATCCGAC
ATTGAATGTGAAAGAAGAGACGAAACATCGCATTCTCGAGATCGCCGAAAAGCTGGAGTACAAGACCAGTAGTGCCCGTA
AACTCCAGACAGGTGCAGTCAACCAACACCATATTCTGGCTATCTACAGCTACCAGCAGGAGCTGGAGATCAACGATCCT
TACTATCTGGCGATCCGCCACGGCATTGAAACCCAGTGCGAAAAGCTGGGCATCGAGCTCACCAACTGTTATGAACACAG
CGGCTTACCAGACATTAAAAACGTCACCGGTATTTTAATTGTCGGCAAACCCACGCCCGCCCTGCGCGCCGCTGCCAGCG
CGTTGACCGACAATATCTGTTTTATCGACTTTCACGAACCCGGCAGCGGTTACGATGCGGTGGATATCGATCTGGCACGC
ATCAGTAAAGAAATCATCGACTTCTATATCAACCAGGGCGTTAATCGTATTGGTTTTATTGGCGGTGAAGATGAGCCTGG
CAAGGCGGATATTCGTGAGGTCGCCTTTGCGGAATATGGCCGACTGAAACAAGTGGTACGCGAAGAGGATATCTGGCGCG
GCGGTTTTTCCAGTTCGTCGGGTTATGAACTGGCAAAACAAATGCTGGCGCGGGAAGACTATCCGAAGGCACTGTTTGTT
GCTTCCGATTCCATTGCTATCGGCGTACTGCGGGCAATTCATGAACGAGGCCTGAACATCCCACAGGATATTTCGCTTAT
CAGCGTTAACGATATCCCCACCGCGCGATTTACCTTTCCGCCGCTCTCCACCGTGCGCATCCATTCCGAGATGATGGGAA
GTCAGGGCGTTAACCTGGTGTATGAAAAAGCCCGCGATGGTCGCGCGCTGCCGCTGTTAGTCTTCGTTCCCAGCAAATTA
AAACTGCGCGGCACGACCCGTTAA
>Cluster_43155
ATGGCAACACGAATTGAATTTCACAAGCACGGTGGCCCGGAAGTACTTCAAGCCGTAGAGTTCACTCCTGCCGATCCGGC
GGAGAATGAAATCCAGGTCGAAAATAAAGCCATCGGCATCAATTTTATCGACACGTATATCCGCAGCGGCCTTTACCCGC
CACCATCGCTACCCAGCGGATTAGGCACCGAGGCGGCAGGCATCGTGAGTAAAGTCGGCAGTGGTGTAAAGCATATTAAG
GCAGGCGATCGCGTAGTCTATGCACAATCGGCATTAGGCGCTTACAGCTCTGTGCATAACATTAATGCGGATAAAGCGGC
GATTCTGCCTGCGGCAATTTCTTTTGAGCAAGCTGCGGCATCCTTCCTGAAAGGCTTAACGGTTTATTATCTGCTGCGCA
AAACCTATGAAATTAAACCCGATGAACAGTTCCTGTTCCACGCGGCTGCTGGCGGCGTTGGCTTGATTGCCTGCCAGTGG
GCAAAAGCCCTTGGCGCGAAACTTATCGGCACCGTAGGAACCGCGCAAAAAGCGCAGAGTGCGCTAAAAGCGGGCGCGTG
GCAGGTTATTAACTATCGTGAAGAGAATCTGGTCGAGCGATTGAAAGAGATCACCGGTGGTAAGAAAGTGCGCGTGGTGT

ACGATTCAGTGGGCAGAGACACCTGGGAACGGTCGCTGGATTGCCTGCAACGCCGCGGCTTAATGGTGAGTTTTGGCAAC
TCATCAGGTGCGGTTACCGGTGTGAACTTAGGCATTCTCAATCAGAAAGGCTCGTTGTATGTGACACGCCCTTCCCTGCA
AGGCTATATCACCACGCGGGAGGAATTAACCGAGGCCAGTAATGAACTGTTCTCTTTGATTGCCAGCGGTGTGATTAAGG
TCGATGTCGCCGAGCAGCAGAAATATCCGCTGAAGGATGCGCGGCGTGCGCATGAGATTCTGGAAAGCCGGGCGACGCAG
GGTTCCAGCCTGTTGATTCCATAA
>Cluster_157
ATGCGTATCTTTGTAGATGACGGTTCTACCAACATCAAACTGGCCTGGGTTGAAGACGGTGCAGTGAAAACGTTAATTAG
TCCGAATAGTTTTAAACCGGAATGGTCTATGACGCTGCAGGGACTGACTGATGCCACTATACCCGCTTCCGCAAACTATG
AGATTGATGGAGAGAAATACAGCTTTGATCAATTGAGTCCGGATGCAGTGCGCACCACAGAAACGCGTTATCAGTACAGC
GACGTTAATGTGGTTGCTATTCATCATGCGTTGATGCAGTCAGGTATTGCTCCGCAACCTGTTGATATTATTGTTACTTT
ACCTTTAGGTGAGTATCTGGATGAGAACGATCAGCCAAATCTGGCTAATATCGAACGTAAGAAAAATAATGTCAGACGAG
CTGTAACAGTTCAGGGGCGTGAAAACTTTACCATTCGTAAAGTCAGTGTCTTGCCTGAGTCCGTGCCTGCCGGTTTTGAC
GTGCTGAAAGATCTGAATGATCTGGAGTCGCTGTTGATCGTTGATATCGGTGGAACGACCCTTGATATTGCACATGTCCG
CAGCAAAATGTCCGGTTTCACAAAAACTTATTGTGACCCCAAGACAGGCGTTTCCATCATTACTGAAGCAGTTAAGCATG
CGATGGATGTGAATGTGAGTACACGAACCAGTTCTTATTTTGCAGATCGTCTAATCCAGAGTCGGGATGACAATGAATTC
CTGACTCGTTATATCCCGAATGCAGAACAGCGTGAGCAGATCCTGACAGTATTACGTGACCGGGAAAAAAAACTGCAGCA
TCGTGTGGCGGATTCAGTAGGACGCTTTGCTGGTTTTACTCATGTTATGGTTGTTGGCGGTGGTGCCAGCCTTGTGGCTG
GGGCAGTAAAAAAAGCGACAGGAGTGGGTGATAACCGTTTCTTTGTCAGTGACAATCCTCAGTTCGATCTGGTGTTGGGA
ATGATGGCAATGAAGGGATGA
>Cluster_26419
ATGCGTAAATATCGTCTAAGTGAAGAACAGCGAGCCTTTAGTTATCAGGATGATGGCACTAAAAAAAGTGTGTTATTACG
GCAGATTATCGCCATGAGCGATTTTAACGATGTGATAGCAGGAACCGCTGGCGGCTGGATCGATTGCGAAACGGTGTTAG
CACAGGAAGGAAATTGCTGGATTTACGACCAGAATGCTATTGCGTTCGGCGGAGCGGTGATTTCCGGTAATACGCGCATC
ACCGGCACCAGCGTACTGTGGGGAGAGGTTTACGCAACGGATAACGTCTGGATCGATAATAGTGAAATCAGTCAGGGTGC
ATATATAAGTGACAGCGTCACCATTAGTGACTCATTAGTTTGCGGTCAATGTAGAATATTTGGTCACGCCTTAATTGATC
AACATTCTATGATCGTCGCCGCACAAGGCTTAACGCCCGACCATCAACTCCTGCTACAAATTTATGATCGCGCGAGGGTT
AGCGCCTCGCGTATTGTCCACCAGGCACAAATTTATGGCGATGCCGTCGTCCGGTATGCTTTTATTGAACATCGCGCCGA
ATTTTTTGATTTTGCCAGTATTGAAGGTAATGAAGAGAACAACGTCTGGCTATGCGATTGCGCTAAAGTTTACGGTCACG
CACAGGTGAAAGCTGGCATAGAAGAAGATGCCATTCCGACGATTCATTACAGTTCACAGGTGGCGGAATATGCCATTGTG
GAAGGTAATTGTGTGTTAAAACATCATGTCTTGATTGGCGGAAATGCCGTGGTACGTGGCGGACCGATTCTGCTCGACGA
GCACGTTGTAATACAAGGTGAAAGCCGTATTACAGGTGCGGTGATAATTGAAAACCATGTGGAGTTAACCGACCACGCCG
TCGTTGAAGCCTTTGATGGCGACACGATTCATGTCCGTGGTCCAAAAGTTATTAACGGCGAGGAGCGCATTACGCGAACG
CTGCTGGCAGGGTTATTGTGA
>Cluster_40833
ATGAGTAAAGAGAAGCAAACAGTTAACGGGGATGTGGGTAACGTTGTCAGCGGTGATGTTACCATTCATAACTATTCTGC
TGACATACTTCCTTCGGCCCAGCAACCAATTTCGTTGCTGCAAAAACGCGATTTACACAGATTGATGGATGAATTGGTTG
ATCTGGGGGAAAGTAAGCGCGAGCTATGGATGACGATCCACACGAAACTTAATACCAAAACAGTTAATGAAATGACTGCG
GCTGATTATCATGGTGCGGTTGAGATCCTTCAGAGATATGCACAACAGATCCAGAATATGAAGGACTGTAATCTTCTGGT
CAGCAAAATAATGGCGCTTACTGACCCTGGGTATCGTCTTGACCGTGACAGATACTGTCTGAAGCATTTCGGTACAACCC
ATCTTAAAGGCCTAGACAAAGAGCAGTTGCAGGCTGTGTTTGGTTATTTTGATGATCTGCTGAATATTCGTGATGAGAGT
AAAACTCTATCACCTCCATCGGAAAGCGGTGCAAAATCAGTCGCAACTGTACCAACCAGAAGCCGGAAGCCTCATGTAGT
AGTGTTGGGGGGAGTGTTAGTTCTTGTAGCAATATTTTTCGTGGGAATGATTGTGTTCGCTGGTAAATGGGGAAATGCCA
AAGCAGTAGCTTCTACTGAAACCAGCGTTTTTAACATTAAAACCAACGATAAAATAATAGAAGATTCCATCCCCGCACTG
CGCAGCATGTTTCCAGGGTTAAATAAGTACTCAGATGATTTCCATTCGGTTTCAAGCTACAAGCAAAAAACGGGCTGGCA
TACCCTTAAATTTACTGTATCAGCAAAGGCATCCGTCCCCGAAAATTATAGTGTTAAGGGCAAGGTTTGTTACATTAACA
TAAGCCCTGATGGTAGTTATGCTCGGGTTTTGACTGCTCCATGTCGTTCACTGCTACTTGATCAGCAAAGCACTCCTGAC
AGTAAATACCGTTTTATTCTTAAATAG
>Cluster_46974
ATGTTATCTTCATTAAATGTCCTTCAATCCAGCTTCAGAGGAAAGACAGCCTTATCAAATAGTACACTTCTCCAGAAAGT
TTCTTTTGCTGGAAAAGAATATCCTCTGGAACCTATTGATGAAAAAACCCCTATTCTTTTTCAGTGGTTTGAAGCAAGGC
CAGAGCGATACGAAAAAGGAGAAGTACCAATATTGAATACCAAAGAACATCCGTATTTGAGCAATATTATAAATGCTGCA
AAAATAGAAAATGAGCGTATAATCGGTGTGCTGGTAGATGGAAATTTTACTTATGAACAAAAAAAGGAATTTCTCAGTCT
TGAAAATGAATATCAAAATATAAAAATAATCTACCGAGCAGATGTGGATTTCAGCATGTATGATAAAAAACTATCTGATA
TTTACCTTGAAAATATCCATAAACAAGAATCATACCCTGCCAGTGAGAGGGATAATTATCTGTTAGGCTTATTAAGAGAA
GAGTTAAAAAATATCCCAGAAGGTAAGGACTCTTTGATTGAGTCATATGCAGAAAAAAGAGAACATACTTGGTTTGATTT
TTTCAGGAATTTGGCCATGTTGAAGGCTGGAACTTTGTTTACAGAGACGGGAAAAACTGGATGCCATAACATATCGCCCT
GTAGCGGATGTATATATCTTGATGCCGACATGATTATTACCGATAAATTAGGAGTCCTGTATGCTCCTGATGGTATCGCT
GTGCATGTAGATTGTAATGATGAGATAAAAAGTCTTGAAAATGGTGCGATAGTTGTCAATCGTAGTAATCATCCAGCATT
ACTTGCAGGCCTCGATATTATGAAGAGTAAAGTTGACGCTCATCCATATTATGATGGTCTAGGAAAGGGTATCAAGCGGC
ATTTTAACTATTCATCGTTACACGATTATAATGCTTTTTGTGATTTTATTGAATTTAAGCATGAAAATATTATACCGAAT
ACCAGTATGTATACCAGCAGTTCATGGTAA
>Cluster_63786
ATGACCCAACCCGTTCTGGACATTCAACAACTGCATTTGAGTTTACCCGGTTTTAACGGTGATGTTCACGCGCTCAACAA
TGTGTCCTTGCAGATTAACCGCAGTGAAATTGTCGGTCTGGTGGGAGAATCCGGCTCAGGTAAATCAGTCACCGCAATGC
TGATTATGCGTCTGCTACCGACGGGCAGTTATTGCGTACATCGGGGGCAGATTTCACTGCTGGGAGAAGATGTTCTTAAC
GCTCGGGAAAAACAGCTTCGTCAGTGGCGCGGCGCACGAGTGGCGATGATCTTTCAGGAACCGATGACCGCCCTCAATCC
GACACGTCGAATAGGTCTTCAGATGATGGACGTAATCCGCCATCATCAACCAATAAGTCGTCAGGAAGCCAGAGCTAAAG
CGATTGCCCTGCTGGAAGAGATGCAAATCCCGGATGCCGTGGAAGTTATGTCGCGCTATCCGTTTGAGCTTTCAGGTGGT
ATGCGCCAGCGGGTAATGATTGCGCTGGCATTCTCCTGCGAGCCGCAATTGATTATTGCCGACGAACCGACTACGGCGCT
GGACGTCACGGTACAGTTGCAGGTACTGCGTTTGCTTAAACATAAAGCCCGCGCCAGTGGAACTGCGGTACTGTTCATCA
GCCATGATATGGCCGTGGTGTCGCAACTGTGCGATAGCGTTTACGTGATGTATGCCGGAAGCGTAATTGAAAGCGGCGTG
ACGGCAGACGTTATCCATCATCCCCGGCATCCGTATACCATTGGTTTGCTACAATGCGCACCAGAACATGGAGTACCACG
CCAGCCATTACCAGCCATTCCAGGGACGGTACCAAACCTCACCCATTTGCCTGACGGCTGCGCTTTTCGCGATCGTTGCT
ATGCGGCAGGTGCACAGTGTGAAAACGTCCCGGCGCTGACAGCGTGTGGTGACAACAACCAGCGCTGCGCCTGTTGGTAT
CCGCAGCAGGAGGTCATAAGTGTCTAA
>Cluster_32623
ATGATACGTTTCGCTGTGATTGGTACGAACTGGATCACTCGCCAGTTCGTCGAGGCCGCCCATGAGAGCGGTAAATACAA
GTTAACCGCCGTATATTCCCGCAGCCTTGAACAGGCCCAGCACTTCGCCAATGATTTTTCTGTCGAGCATCTGTTTACGT
CGCTGGAAGCGATGGCAGAAAGCGATGCCATTGACGCGGTGTATATTGCCAGCCCGAATGCCCTGCACTTTTCCCAGACA

CAACTTTTCCTTAGCCATAAAATTCATGTGATTTGCGAGAAACCGCTGGCGTCAAATCTGGCGGAAGTGGATGCCGCCAT
TGCCTGTGCGCGGGAAAATCAGGTGGTGCTGTTTGAGGCATTTAAAACGGCCTACCTGCCGAACTTTCACCTGTTACGCC
AGGCGCTGCCGAAAGTCGGCAAACTGCGTAAAGTCTTTTTCAACTATTGCCAGTATTCCTCGCGGTATCAGCGTTACCTG
GACGGAGAGAATCCCAACACCTTCAATCCGGCATTCTCCAACGGTTCGATTATGGATATCGGCTTTTACTGCCTGGCGTC
GGCGGTGGCATTATTCGGTGAACCGAAAAGCGTGCAGGCAACCGCCAGTTTGCTGGCAAGTGGTGTGGATGCCCACGGCG
TGGTGGTGATGGATTACGGTGATTTCAGCGTTACGTTGCAGCACTCAAAAGTGAGCGATTCTGTCCTGGCAAGCGAGATT
CAGGGCGAAGCGGGAGCGCTGGTGATTGAAAAACTCTCTGAATGCCAGAAAGTCTGCTTTGTACCGCGTGGCAGCCAGAT
GCAGGATCTCACCCAGCCACAGCATATTAATACCATGCTCTACGAAGCAGAGCTGTTCGCTACCCTGGTGGATGAGCATC
TGGTGAATCATCCAGGGCTGGCGGTCAGTCGTATCACCGCCAAACTGCTGACTGAGATCCGCCGCCAGACTGGGGTGATT
TTTCCGGCAGATAACGTAAAACTATAA
>Cluster_46495
ATGGTTGACATGAAAATTACTATCTGGGAAGGCTGTGACCTCACAGTTGCTGCTGCAATTAAATTATTTGTAGAGCGTAG
TTATCCGTACTCCATATTTTCACGGAGGTATCCAATACTGCGTCTCATACAGGAAGTTATTGATCCAGCAGTAGCTGCTT
ATGTAAATTCCCTGCCACCAAGTCATAAAGACTACATTTCTGGAGTAAATCTAGGATTTAGTGATGTACTTAAAAAAGTG
GGCTTAAACGTCGTAGTACAGGCACAAAGACTTCTTTTGCGTAATTTCGTTCTGACTTTAGATCAACAAAATTCTCTTGA
TAAATGCTTTACCGCTACGATTGAGTCTCTTATTGAATTAGTCAGTGAGTGCGCATCCAAGCGGCCGAAGAAGTCCTCTC
CTGCAAAAGGAGTAACTATAAATTCACAGCGTAAGCTCGGTTATTGCGAGTTCTGCGGTAATCTAACCGAATTTTCCAAG
TTGATCAGTGAGATATCAGAATTTGTTGCTAACGATAATGAGTTCCCCGTTCATGAAAAACAGGTCCTTAGTCATCGATA
TTGTTCTGACCATAGGCCCAAACTCACCAATGGTCTATGGAATCCCAAATACAGACAAGGAAAGCGGTCTCTCGAACAGT
TCAATAAAGAATTAATAAGGCTTAGACGGCAATGCGCTAAACCGCATAAAGCTAACGCAAACACAGGGGATATATTGATT
GATACCTATTTTCATGAGTGGATGCAGGGTCAAACTTTAACCCCAGCAGATCTAGATAAACTTAGGAATATCGCCAGACG
AATGGTTGACTCCAAATTCACTGATACTAAGAAAAAAATAATCATATTAAAACACCAAGGTTTTAACCAGTTTGAAACCA
CACAGGTTTTAGAAAAAAATCACTACACAACTATGACAAAGCAGGCTGTGTCCAAGGCCTTAGCGTCTGTGAGGAAAGAA
TTTTATATTTAA
>Cluster_22162
ATGCACTGCAAGGGGGCGTGTATGAAACCACTGCTGGATGTGTTGATGATCCTCGATGCCTTAGAAAAAGAGGGCAGTTT
TGCGGCGGCGTCGGCCAAACTCTACAAGACGCCGTCTGCTCTGAGTTACACCGTTCACAAGCTGGAAATCGACCTTAATA
TTCAATTGCTTGATCGTAGCGGACACCGCGCTAAATTCACCCGTACCGGAAAAATGTTATTAGAGAAAGGGCGGGAAGTT
CTGCATACCGTGCGAGAACTGGAAAAGCAGGCTATCAAACTGCATGAAGGCTGGGAAAATGAGCTGGTGATTGGCGTGGA
CGACACGTTTCCTTTTTCTCTGCTTGCGCCTCTCATTGAGGTTTTTTATCAACATCATAGCGTGACGCGCCTGAAGTTTA
TCAATGGCGTACTCGGTGGTTCCTGGGATGCACTGACTCAGGGGCGAGCGGATATTATTGTTGGCGCGATGCATGAGCCA
CCATCTTCCAGTGAGTTTGGTTTCTCGCGGTTGGGCGATCTTGAACAAGTCTTTGCGGTCGCGCCTCATCATCCGTTAGC
CCAGGAAGAAGAACCGTTAAACCGTCGTATCATTAAACGATATCGGGCGATTGTGGTGGGGGACACTGCGCAGGCAGGCG
CTTCTACGGCCTCACAACTTCTCGACGAACAAGAAGCCATTACCGTTTTCGATTTTAAAACCAAGCTGGAGCTGCAAATT
AGCGGCCTTGGCTGCGGCTATTTACCCCGTTATCTGGCGCAACGTTTTCTCGATAGTGGCGCGTTAATCGAGAAGAAAGT
GGTCGCCCAAACTCTCTTTGAACCTGTCTGGATTGGCTGGAATGAACAGACCGCAGGGCTTGCCAGCGGCTGGTGGCGAG
ATGAAATTTTAGCAAATAGTGCGATCGCCGGTGTTTATGCAAAATCTGATGACGGAAAATCAGCCATTTAA
>Cluster_13537
ATGAATGGTGAATATTTTGTACGCCTCGCATTACATACTCTGAAATGCACTCAAAAAGATTTAGCAAGCCACTTAGGTGT
TTCCTCAACACAAATTAGCAAGTGGAAAAAAGGGGAACACATGTCCGCTGATATGGAAAAAAAGTTCAGAAATATTACAC
AAATAGGAGATTATTCTCCTCAACTAGTTGAATGGACGGGCTCAGTCGCGAATGCTGAGAAATGGGATCGACTTATCCAT
TTTCTTGCTCGACGAGCTATGGACGATGCTGAAACGGGTTATATAACACAACTTCTTACTGACGAAGATGGTTTTTTGGT
TGAAGAAACAATTGATGTATTAAAGCGAATAGGTTTTCCTACACCTCTTTCCTTTCCTGAAGGACTTAATATTGACGACG
ATAATGCTGATGAAGAAGAGGCTTTTTGGGAGATATTAGAGAGCAATGCTCATTGTTCTGTAATTAATGATATTTATCAT
GCTCTGAATGATGTTTATGGTTTCTACATAGCATATGTTGATGAGTTGATTCAGGACGACGATTTAGATGTCTATTCAAG
CGAAGCCATCAATATTCAGAGTTCATTGATATCACTTGCTGCATGCAAAATTGAAATTGATACTCCTGTTGCGTCAAACT
TTAAAGAATTTCGTTATAGAGTTAAAAAAGACTACGAAAATTGGTTGAATCAACTCAAGATGATGGCATTCAGGGCGGGA
ATTCCGTTAAGAGCCGAACTTCTGGAAATGGTTTATAACACCGCTGACCAACTTAGCGTTGCAGCAGAAGCCGAACGTTT
CGATTTCAATAAATCTCGCATTCATCCAGATATATACATGAACGAGATCCTTACTGGCATGAGAATAATTCACCAAGTGC
TTCCGGTAATAATGCAAAAGTTAGAAATTACAGATTTCAAACTAGATGAAACAGATTTATGCCTGGGTAAATAG
>Cluster_51980
ATGAAAAATGTTCTGGTCACCGTCCCCAGCTTTTCAGCACGCTGTGTTTCCGCCAGCAAGTTGCTAAGAGAGAATAATTT
TAATCTTATTATTAAGAATAATGTGGAGCATCTGCTTAAATCAGAATCCACTGCATTACGAGAATCTATTTGCGCGGTCA
TCGCCGGAAAAGATGGTTATCAGGCGGACACGCTATCATTGCTGCCTGGTGTGAGAATAATATCGAGATTCGGGACGGGG
ATAGACAATATTGATCTTCGTGCGGCACAGCAATCCGGTATTGTTGTCAATAATGCGGTTGGGATTAATTCAAATGCGGT
TGCGGAATTTATCATTGGACTTATCTTCGCGAGCATGAGAAATATCCCTGGCAGCTATCATGCGATGCAAAATGGCTACT
GGGGTGAGTCGCATGGCTGTGAATTACAAGGGAAACGGATTGGCCTGGTTGGCTACGGTAATATAGGTAAAACTCTGGCG
AAAAGGTTATCCGGTTTTGATGTTGAGCTTTTAGCTTTTGACAAACAACCCGACTATCAATTTGCTGACAAAGCTGGAGT
TCAGTTTGTATCAATTGAAGATATCTTTATGCAGTCGCATGTCATCATTGTTCTTTTGCCTTTTTCTTCTGAGCTGGAGA
ACTTCATTAGTCATAAATATTTGTCGATGATGCGCAATGGTGCTCTGATCATCAATGCCGCAAGGGGAAAGTTGCTGGAT
GAGGGCGCTTTACTTCAGGTGATTGAAGAACGAAATGTGTTTGCGGCGCTGGATGTGTTTAGCTCTGAACCGTTGGCGCA
ATTTAGCCCACTGCTACATGCCAAAAATATCATTACAACGCCGCATATCGCGGCCGCCACCGTTGAGTCATACCAGCAAA
CCGGGATACACGTTGCCCAGTCGATGATTGATTACTTCGCAGGAAGGGAGATAAAAAACGTGTTGTGA
>Cluster_20078
ATGCAGATCCTGTTGGCCAACCCGCGTGGTTTTTGTGCCGGGGTAGACCGCGCTATCAGCATTGTTGAAAACGCGCTGGC
CATTTACGGCGCACCGATATATGTCCGTCACGAAGTGGTGCATAACCGCTACGTGGTCGATAGCTTGCGCGAGCGTGGGG
CTATCTTTATTGAGCAGATTAGCGAAGTACCGGACGGCGCGATCCTGATTTTCTCCGCACACGGTGTTTCTCAGGCAGTA
CGTAACGAAGCGAAAAGCCGCGATTTGACGGTGTTTGATGCCACCTGTCCGCTGGTGACCAAAGTGCATATGGAAGTCGC
CCGCGCCAGCCGTCGTGGTGAAGAATCTATTCTCATCGGTCACGCCGGGCACCCGGAAGTGGAAGGGACGATGGGCCAGT
ACAGCAACCCGGAAGGGGGAATGTATCTGGTCGAATCGCCAGACGATGTGTGGAAACTGACGGTCAAAAACGAAGAGAAG
CTCTCCTTTATGACCCAAACCACGCTGTCGGTGGATGACACGTCTGATGTGATCGACGCGCTGCGTAAACGCTTCCCGAA
AATTGTCGGTCCGCGCAAAGATGACATCTGCTACGCCACGACTAACCGTCAGGAGGCGGTACGCGCCCTGGCAGAGCAGG
CGGAAGTAGTACTGGTGGTTGGTTCGAAAAACTCCTCCAACTCCAACCGTCTGGCGGAACTGGCCCAGCGTATGGGCAAA
CGCGCATTTTTGATTGACGATGCGAAAGATATCCAGGAAGAGTGGGTAAAAGGCGTGAACTGCGTTGGCGTGACTGCGGG
CGCATCGGCTCCGGATATTCTGGTGCAGAATGTGGTGGCACGTTTGCAACAGCTGGGCGGTGGTGAAGCCATTCCGCTGG
AAGGTCGTGAAGAAAACATTGTTTTCGAAGTGCCGAAAGAGCTGCGTGTCGATATTCGTGAAGTCGATTAA
>Cluster_58401
ATGCGTCAGACTAAAACCGGTATCCTGCTGGCAAACCTGGGTACGCCCGATGCCCCCACACCTGAAGCGGTAAAACGCTA
TCTGAAACAATTTTTAAGCGACAGACGCGTGGTTGATACCTCACGGTTGTTATGGTGGCCATTGCTGCGCGGCGTGATTT

TGCCGCTGCGCTCGCCGCGTGTGGCGAAGCTGTATGCCTCTGTCTGGATGGAAGGTGGCTCGCCGCTGATGGTTTACAGC
CGCCAGCAACAGCAGGCGCTGGCACAACGTTTACCGGAGATGCCCGTAGCGCTGGGAATGAGCTACGGCTCGCCATCACT
GGAAAGCGCCGTAGATGAACTCCTGGCAGAGCATGTAGATCATATTGTGGTGCTGCCGCTTTATCCGCAATTCTCCTGTT
CTACGGTCGGTGCGGTATGGGATGAACTGGCACGCATTCTGGCGCGCAAACGTAGCATTCCGGGGATATCGTTTATACGT
GATTACGCCGATAACCACGATTACATTAATGCACTGGCGAACAGCGTACGCGCTTCTTTTGCCAAACATGGCGAACCGGA
TCTGCTACTGCTCTCTTATCATGGCATTCCCCAGCGTTATGCAGATGAAGGCGATGATTACCCGCAACGTTGCCGCACAA
CGACTCGTGAACTGGCTTCCGCATTGGGGATGGCACCGGAAAAAGTGATGATGACCTTTCAGTCGCGCTTTGGTCGGGAA
CCCTGGCTGATGCCTTATACCGACGAAACGCTGAAAATGCTCGGAGAAAAAGGCGTAGGTCATATTCAGGTGATGTGCCC
GGGCTTTGCTGCGGATTGTCTGGAGACGCTGGAAGAGATTGCCGAGCAAAACCGTGAGGTCTTCCTCGGTGCCGGCGGGA
AAAAATATGAATATATTCCGGCGCTTAATGCCACGCCGGAACATATCGAAATGATGGCTAATCTTGTTGCCGCGTATCGC
TAA
>Cluster_43522
ATGCCTAACATTACCTGGTGCGACCTGCCTGAAGATGTCTCTTTATGGCCTGGTCTGCCTCTTTCATTAAGTGGTGATGA
AGTGATGCCACTGGATTACCACGCAGGTCGTAGCGGCTGGCTGCTGTATGGTCGTGGGCTGGATAAACAACGTCTGACCC
AATACCAGAGCAAACTGGGCGCGGCTATGGTGATTGTTGCCGCCTGGTGCGTGGAAGATTATCAGGTGATTCGTCTGGCA
GGTTCACTCACTGCGCGGGCTACGCGCCTGGCCCATGAAGCGCAACTGGATGTCGCGCCGCTGGGGAAAATCCCACACCT
GCGCACGCCGGGGTTGCTGGTGATGGACATGGATTCCACCGCCATCCAGATTGAATGTATTGATGAAATTGCCAAACTGG
CCGGAACGGGCGAGAGGGTGGCGGAGGTAACCGAACGGGCGATGCGCGGCGAACTCGATTTTACCGCCAGCCTGCGCAGC
CGCGTGGCGACGCTGAAAGGCGCTGACGCCAATATTCTGCAACAGGTGCGTGAAAATCTGCCGCTGATGCCAGGCTTAAC
GCAGCTGGTACTCAAGCTGGAAACGCTGGGCTGGAAAGTGGCGATTGCCTCCGGCGGCTTTACTTTCTTTGCTGAATACC
TGCGCGACAAGCTACGCCTGACAACCGTGGTAGCCAATGAACTGGAGATCATGGACGGTAAATTTACCGGTAATGTGATC
GGCGACATCGTAGACGCGCAGTACAAAGCGAAAACTCTGACTCGCCTCGCGCAGGAGTATGAAATCCCGCTGGCGCAGAC
CGTGGCGATTGGCGATGGAGCCAATGACCTGCCGATGATCAAAGCGGCAGGGCTGGGGATTGCCTACCATGCCAAGCCAA
AAGTGAATGAAAAAACGGAAGTCACCATCCGTCACGCTGACCTGATGGGGGTATTCTGCATCCTCTCTGGCAGCCTGAAT
CAGAAGTAA
>Cluster_21091
ATGAAAATTGCGCTGGTTATTTTCATCACCCTTGCCCTGGCGGGCTGTGCGCTGTTATCACTCCATATGGGAGTGATCCC
CGTGCCGTGGCGCGCGCTGCTGACCGACTGGCAGGCCGGACGCGAGCATTATTATGTATTGATGGAGTACCGACTGCCGC
GCTTGCTGCTGGCACTGTTTGTCGGTGCAGCCCTCGCCGTGGCGGGCGTGCTGATACAGGGGATTGTGCGCAACCCTCTG
GCATCACCGGATATTCTCGGCGTTAACCATGCCGCCAGCCTGGCCTCTGTGGGGGCTCTACTTCTTATGCCGTCACTGCC
CGTGATGGTGCTGCCGCTGCTGGCCTTTGCGGGCGGCATGGCGGGGTTGATATTACTGAAGATGCTGGCAAAGACCCACC
AGCCGATGAAGCTGGCGCTCACCGGCGTGGCGCTTTCTGCATGCTGGGCCAGCCTGACGGATTATCTGATGCTCTCGCGC
CCGCAGGATGTGAACAACGCCCTGCTGTGGCTGACCGGCAGCTTATGGGGCCGTGACTGGAGCTTTGTGAAGATTGCCAT
CCCGCTGATGATTTTATTTCTGCCGCTGAGCCTGAGTTTTTGCCGCGATCTCGACCTCCTTGCACTCGGCGATGCGCGCG
CCACCACGCTCGGTGTGTCGGTGCCCCATACCCGATTCTGGGCTTTGTTACTAGCTGTCGCCATGACATCTACCGGCGTG
GCCGCCTGCGGCCCGATTAGCTTTATTGGTCTCGTGGTGCCGCATATGATGCGTAGCATCACCGGTGGACGTCACCGCAG
ACTGCTGCCTGTTTCAGCCCTGACAGGTGCGTTGCTGTTGGTGGTTGCCGATCTGCTGGCGAGAATTATTCATCCCCCAC
TGGAGCTCCCGGTTGGCGTGCTGACCGCCATTATCGGTGCGCCGTGGTTTGTCTGGTTGCTTGTGAGAATGCGATAA
>Cluster_55161
ATGATAAAAAAACCTGTGATTGGGATCAGCGGTTGTTTGGGCGGTTCTGCTGTTCGTTTTGATGGTGGTCACAAAAGAGA
TGACTTTTTAATGGACAAATTAGTGGAATGGGTAACATTCAGACCAGTATGTCCGGAAATGGCTATAGGGCTGCCAGTTC
CGCGTCCTGCTCTACGACTTGTGCGCTCGAAGCAAGGAAATATACGGATGTGTTTCAGCCACGACCAGAATGAGGATGTG
ACAGAGAGAATGACAGAGTTTAGTCGTTCTTATATGGACAAATTAAAGGATGTATCGGGGTTTGTGGTTTGTGCTAAATC
TCCCAGCTGTGGCATGGAGCGCGTGCGTGTCTATGATGAAAATGGTAATCGAGGTCGTAAAGATGGAGTGGGACTATTTA
CGAGCACTTTGATGGAAAAGTTTTCCTGGCTACCGGTTGAAGAGGATGGGCGATTACATGATCCAGTGCTTCGTGAGAAT
TTTATTGAAAGAGTTTTTGCTTTGCATGAGCTCAATCATCTTTACAAGGAGAAATTATCAAGAAGAGAGTTATTAGCTTT
TCATAGTCGTTATAAGCTTCAGTTGTTGGCGCATAGCCAGGCAGGCTATAAAGATATGGGACCATTTGTGGCTGCAATAC
ACGAGTGGGCGGACCTTGAATCATACTTTGAGGTGTATCGTGATAATCTGATGGCGATTCTCAGAAAACCAGCATCACGT
AAAAATCACACGAATGTGCTGATGCATATACAGGGGTATTTTAGTAATTACTTAAGTACACGCCAGCGTAAAGAGTTGAG
CGAGGTTATACTTAACTATCGCTTTGGCACATTGCCTCTTCTTGCGCCGTTGACTCTGCTGAAGCATTATCTGGGTGAGT
ATCCTAATGATTACTTGCTTACACAGAATTACTTCGATCCCTATCCGGAAGAACTGGCTCTAAGACTGATGGTGAATTAA
>Cluster_5819
ATGGGATTGTTTACGACCCGCCAGTTACTCGGTTATACCGAACAAAAAGTGAAATTTCGTGCGCTGTTTCTGGAGCTGTT
TTTCCGCCGTACGGTGAATTTCCATACCGAAGAGGTGATGCTGGACAAAATTACCGGAAAAACGCCGGTGGCGGCCTATG
TCTCCCCGGTTGTTGAAGGAAAAGTGCTGCGTCATCGCGGTGGTGAAACCCGCGTGTTACGTCCGGGCTACGTCAAGCCG
AAACACGAATTTAATTACCAGCAGGCGGTTGAGCGTCTTCCCGGTGAAGATCCGGCTCAACTGAACGACCCGGCCTACCG
TCGTCTGCGTATCATCACCGATAACCTCAAACAGGAAGAGCATGCCATTGTCCAGGTGGAAGAAATGCAGGCGGTGAATG
CCGTGCTGTATGGCAAATACACCATGGAAGGGGATCAGTTTGATACTGTCGAGGTGGATTTGGACGCTCTGAAGGAAAT
AACATTGAGCAGGCCGACGGTAAAAAATGGTCTGAGCAGGACCGTGATACGTTTGATCCGACGCATGATATTGACCTCTA
CTGCGATCAGGCCAGCGGTCTTGTGAATATTGCCATTATGGACGGTACTGTCTGGCGTCTGCTGAATGGTTTTAAGCTGT
TCCGCGAAAAACTGGATACCCGTCGCGGCTCAAATTCACAACTCGAAACGGCAGTGAAAGACCTGGGGGCGGTGGTGTCT
TTCAAAGGGTATTACGGCGATCTGGCCATTGTGGTGGCGAAAACGTCTTATGTGGCAGAGGACGGTACCGAAAAACGTTA
TCTGCCGGAGGGCACGCTGGTCCTGGGAAATACGGCTGCAGATGGGATCCGTTGTTACGGTGCCATTCAGGATGCGCAGG
CGTTGTCCGAAGGTGTGGTGGCTTCTTCCCGTTACCCGAAACACTGGCTGACCGTGGGCGATCCGGCCCGTGAATTTACC
ATGACGCAGTCCGCACCGCTGATGGTGCTGCCGGATCCGGATGAGTTTGTGGTGGTGCAGGTGAAATAA
>Cluster_53499
ATGCAAGTGAAAACACAGTCCTGCGTTGTTGCGGGCAAGAAAACTGTTGCCGTTACCGAGCAGACGATAGATTGGAATAA
TAATGGAACATTAGTACAAATAACCCGAGGTGGAATTTGCGGTTCCGATTTACATTATTATCAGGAAGGAAAAGTAGGTA
ATTTCATGATAAAGGCACCGATGGTGTTAGGTCATGAAGTTATCGGTAAAGTTATTCATAGCGACTCATCAGAATTACAT
GAAGGGCAAACGGTAGCCATTAATCCGTCTAAACCGTGCGGTCACTGCAAATACTGCATTGAACATAACGAGAATCAGTG
TACAGATATGCGTTTTTTTGGCAGTGCCATGTATTTCCCTCATGTTGATGGTGGTTTTACCCGTTATAAAATGGTCGAAA
CGTCGCAATGTGTCCCTTATCCGGCCAAAGCTGACGAAAAGGTTATGGCTTTTGCCGAACCTTTAGCCGTCGCGATTCAT
GCCGCACATCAGGCCGGCGAGTTACAGGGCAAGCGAGTATTTGTTTCCGGTGTTGGGCCCATTGGCTGCCTGATTGTCAG
TGCAGTGAAAACACTGGGGGCCGCGGAAATTGTCTGTGCCGATGTGAGTCCCCGTTCCCTTTCGCTGGCCAAAGAGATGG
GTGCGGATGTGCTCGTAAACCCACAAAACGACGACATGGATCACTGGAAAGCGGAAAAAGGCCATTTCGATGTCAGCTTT
GAAGTGTCCGGTCATCCTTCATCAGTGAATACCTGTCTGGAGGTCACTCGTGCACGCGGCGTAATGGTGCAGGTAGGGAT
GGGAGGCGCGATGGCAGAATTCCCAATGATGACGTTGATTGGTAAGGAGATTTCACTCAAAGGCTCTTTCCGTTTTACCA
GCGAATTTAATACCGCAGTGTCATGGCTGGCGAATGGCGTTATCAATCCACTGCCTTTACTGAGTGCTGAATATCTCTTC
ACTGACCTGGAAGAGGCGCTACGTTTCGCCGGTGATAAAACCCAGGCAGCAAAAGTCCAGCTTGTTTTCTAA

>Cluster_9813
GTGAAAAGTGAATCTTTTGTTTCGGTTGTTTTAAGTACGCACGATTTATCGAGTGAATTAACAAATAAATTAAAGCAGAT
TCAGTCTTTACTGGACGATAATTACACTGATTATGAAATTTTAATTGTTTCTAAAGGACCGTATCGTGATGTACCGAGAA
CATCTGCGATCGACAATATATTAAAAACCATTCCGAGCGTGCGCTTTCTGCAACTTTCGGGCTGCGTTCATGATGATGTG
GTGCGGGCTGCTGGTCTTGAGAATGCAATTGGTGATTTTGTGGTTTTATATGATCATCAGACTGATCCTGTTTATGTCAT
TCATCAAGCAGTGGAAGAGTGTAAGTCCGGGTACGATGTTGTTGTGGGGGTCTCAACGCAGTCGCTTCCGTGTGCGTATC
GTATTATGCGGGATACGGCGTCGCATATATTAAAAGCGATCGATTACCATATTCCTAAAAACTCTACAGGTTTGCGCTGT
CTCAGTCGTCGTGCGGTAAATGCAGTAACAGATACTGGGCGTTTCCACCACCAGTTCTATATGCGCATTCAAAAAACGGG
CTATCCCGTCAGCGAACTGACTTATCAGCCCGTTAGCGAAACGCATATTAAACGTTCTATCACATATGGTTTTCGCAATT
TAGTTCGTTTGATGGTCTTCAACTCTTCTCGTCCTTTGCGCTGGATGTCGATTGTTGGCACTGTAGGTAGTTTGAGCGCT
CTGCTTTTCGCGGTATACAGTATTCTCGTGAACCTGATTAATGGCCATGTTGTAGAAGGTTGGACTACTACAGTCTTGTT
TATGTCCACTTTGTTTATGCTGCAATTTATTATGTTGGCCTTCTTTGGGGAATATCTCAGTCGTTTGCTCGATGACCGAA
GCGAACAGGCAGCATATTCCGTTGTGTTTGAGAAAAACAGTGCAGTAATGATTAACAAAGACCGCGTTAATGTGTTGAAT
GATGCGACTGTGGAAGAGGCTAATCTGGTGCAAACCGGACGTGACCGTTAA
>Cluster_15412
ATGAAAACTGTAAACGTAGCTTTACTGGCACTCATAATTTCAGCAACATCCAGCCCTTTTGTTTTAGCTGGTGATACCAT
TGAAGCGGCGGCAACAGAGCTTTCAGCCATTAACTCGGGCATGTCGCAATCGGAGATTGAGCAGAAGATTACCCGCTTTT
TAGAACGCACCGACAACAGCCCCGCTGCATATACCTATTTGACTGAACATCACTACATCCCTTCTGAAACACCTGATACC
ACTCAGACTCCCCCTGTCCAGACAGATCCTGACGCAGGACAAAAAACCGTTGCCGCTACAGGTGATGTACAGACAACCGC
CCGTTATCAGAGCATGATCAACGCCCGACAGTCTACGGTAACTGATGCCCAGCAAACGCAAATTACAGAGCAACAGGCGC
AGATCGTAGCCACACAAAAAACGCTCGCCGCGACTGGAGATACGCAAAATACCGCGCATTATCAGGAGATGATTAATGCC
AGGCTGGCGGCTCAAAATGAGGCTAATCAGCGCACTACCACGGAACAAGGGCAGAAAATGAATGCACTGACAACCGATGT
GGCAGCACAACAGCAAAAAGAAAGGGCTCAATACGATAAACAAATGCAAAGTCTGGCGCAGAAGTCTGTCCAGGCACATG
AGCAAATTGAAAGTCTGAGACAAGATTCCGCACAAACGCAGCAACAGTTAACCAACACCCAAAAACGGGTTGCAGATAAC
AGCCAGCAAATTAACACGCTCAATAACCATTTCAGTTCGCTAAAAAACGAAGTTGAGGACAATCGTAAAGAAGCCAATGC
GGGAACTGCATCTGCCATCGCTATCGCCTCACAACCACAGGTGAAAACCGGTGACTTGATGATGGTCTCAGCGGGAGCGG
GAACCTTTAACGGTGAATCTGCGGTGTCTGTCGGAACATCTTTTAATGCCGGAACGCATACGGTACTTAAAGCAGGTATT
TCTGCGGATACACAATCTGATTTCGGTGCGGGTGTCGGCGTGGGATATTCGTTCTAA
>Cluster_41489
GTGACCGATAAAACCTCTCTTAGCTACAAAGATGCCGGTGTTGATATTGACGCGGGTAATGCTCTGGTTGGAAGAATCAA
AGGCGTAGTGAAGAAAACGCGTCGTCCGGAAGTGATGGGCGGTCTGGGCGGCTTCGGTGCGCTGTGTGCATTGCCGCAAA
AATATCGTGAACCCGTGCTGGTTTCCGGCACTGACGGCGTAGGTACCAAGCTGCGTCTGGCAATGGACTTAAAACGTCAC
GACACCATTGGTATTGATCTGGTCGCCATGTGCGTTAATGACCTGGTGGTGCAAGGTGCGGAACCGCTGTTTTTCCTCGA
CTATTACGCAACCGGAAAACTGGATGTGGATACCGCTTCAGCGGTGATCAGTGGCATTGCGGAAGGTTGTCTGCAATCGG
GCTGTTCACTGGTGGGTGGCGAAACGGCGGAAATGCCGGGGATGTATCACGGTGAAGATTACGATGTCGCTGGTTTCTGC
GTTGGCGTGGTAGAAAAATCAGAAATCATCGACGGCTCTAAAGTCAGCGATGGCGATGTGCTGATTGCACTCGGCTCCAG
CGGTCCGCACTCGAACGGCTATTCGCTGGTGCGCAAAATTCTTGAAGTCAGCGGTTGTGATCCGCAAACCACCGAACTTG
ATGGTAAGCCATTAGCCGATCATCTGCTGGCACCGACCCGCATTTACGTGAAGTCCGTGCTGGAGTTGATTGAAAAGGTC
GATGTGCATGCTATTGCGCACCTGACCGGCGGCGGCTTCTGGGAAAACATTCCACGCGTATTGCCAGATAATACTCAGGC
AGTGATTGATGAATCATCCTGGCAGTGGCCGGAAGTGTTCAACTGGCTGCAAACGGCAGGTAACGTTGAGCGCCATGAAA
TGTATCGCACCTTCAACTGCGGCGTCGGGATGATTATTGCCCTGCCTGCTCCGGAAGTGGACAAAGCCCTCGCCCTGCTC
AATGCCAACGGTGAAAACGCGTGGAAAATCGGTATCATCAAAGCCTCTGATTCCGAACAACGCGTGGTTATCGAATAA
>Cluster_64417
ATGAGTGATTACGTAAATACTGGCGTGGAGGCGTGGAAACCTAATTTTTATATATCTGCTGATAATAAAAACATCACGGA
TAAAATAAGAAAAGGTTTGATCAATATCACGCTGACAGATTACGGCGGTTCCAGTAAACAGACAGACGAACTCCGCGTAG
CAATAGTTTCTGAAACTCTGAAAATTCCGGCCCGTGGAGTGAAAATTAGCATTGGGCTCGGTTTCGGTAATCAGATAATT
GATAAAGGCATATACATCGTTGACGGTGCTAGCAGTGGCGGTGAACCCCGGGTAGTTGATTTCACAGCAAAAGCGGCTCC
GATGAACGCGGCAAAGGGCTCTCCAACAGTTCAGAGCAAAAAGACGCGCTCATGGAGCGACGTCACCGTCAGCGATATCG
TTGCGACAATAGCGAGCGATAACGGGCTAAAACCTCGCGTATCAAAGCGGTTTGCAGACAACATTATCACGCAGCTCGAT
CAAGTCGGTGAGTCAGATATGCACCTGATGTCGAGACTAGCAACACGATTCGATGCGGTGAGCAAACCCGCAGGAGGGTA
CTGGATATTCCTGCCGCGTGGCGCGGGCGAATCTGTTAACGGAGCACCGCTTCTGCACTACACGCTCACGCGTCGTGATA
ACTCCAGCTGGGGTTATTCGAGAAATGGACAAAACGGCGATAGTGGCGGAGGTGGCGAAAATTCGGAGCCGACATATCTG
ATCAAATACCACGATACGGCAACAGGGCAGATCAAGGAGCTGCGCACAGGCAGTGGTGGTGATCCTGTCATTGAATGGCC
GGCAGTTGAGCCGTCGCTCGACGCAGCGAAAGAGGCCGCGCCGGGGCTAAAAGGAGGTGCGGCTAAAAAAGAGTTTTCCA
TGACGTACACAACGCCAGCAACGCTCGATTTAGTATCGCTGACTGCAGAATGCAAAGTCACAACACAAGGGTTCGGGACA
GAGGAGGACCGAGACTGGACAATCAATACGTTGACGCTGACGCTGGCTGAAAACGGATTCTCTGCGCGACTGTCGCTGGA
GTAA
>Cluster_7943
ATGAGCAAAAAACGCAACAAGCGCCAGCAGCCGCCGCGCCCCCCAAACCACACCGCCGCACCGGCGCAGAGCATGGAAGC
ATTCACCTTTGGTGAGCCAACGCCGGTACTCGACCGCCGCGATATTCTCGATTATGTCGAGTGTATCGATAACGGCCAGT
GGTACGAGCCGCCGGTGAGTTTCTCCGGGCTGGCGAAAAGTATGCGCGCCGCCGTGCACCACAGCTCACCGATTTACGTG
AAGCGTAACATTCTGGTGTCGACCTACATCCCGCACCCGCTGTTATCCCGTCAGGACTTCACCCGGTTCGCACTCGACTA
TCTGGTGTTTGGCAATGCGTTTATCGAAGAGCGTCGCGGCCTGACCGGCAAGCCGTTAAAACTGGAAACCTCACCGGCGA
AATACACCCGCCGTGGCATCGAGGATGACGTGTACTGGTATATTCAGTCCTACACGCAGCCGCACCAGTTCGCGCCCGGC
TCCGTCTTCCACCTGCTCGAGCCCGATATTAATCAGGAGCTTTACGGGATGCCGGAATACCTGAGCGCGCTCAATTCAGC
CTGGCTGAATGAATCGGCGACCCTGTTCCGTCGCAAGTATTACCAGAACGGCGCGCATGCGGGTTACATCATGTATGTGA
CCGACGCCGCGCAAAGCAGCACCGACGTCGAGGCACTGCGAAAGGCGATGCGCGACTCAAAAGGACTCGGCAATTTTAAG
AATCTGTTTTTTTACGCGCCGAATGGTAAAGCAGACGGAATTAAAATTGTGCCACTGAGCGAAGTCGCCACGAAGGATGA
CTTTTTTAATATCAAGAAAGTCAGCGCCGCTGACCTGCTTGACGCACACCGCATCCCATTCCAGCTTATGGGCGGTAAGC
CAGAGAACGTCGGCTCAGTGGGTGACGTTGAGAAGGTGGCAAAGGTCTTTGTGCGTAACGAGCTGACCCCGCTACAGGCA
CGGTTCATGGAGTTGAACGAGTGGGCGGGTGAAGAAATTATCCGCTTCGAAAAATATAGCCTCGGCGACGACGAGTAA
>Cluster_28730
ATGAGTGAAAAAACAGAAAAGCCCACACCCAAAAAACTAAGGGATCTAAAAAAGAAGGGCGATGTAACAAAAAGTGAAGA
GGTAATGGCTGCAGTGCAGTCATTAATCTTATTTTCATTTTTTTCTTTGTATGGCATGAGTTTTTTTGTTGATATAGTTG
GGTTAGTTAATACGACAATAGACTCGCTAAATAGACCGTTTTTGTATGCCATTCGAGAAATATTAGGTGCGGTATTAAAT
ATATTTTTATTATATATTTTGCCAATTTCTTTGATTGTCTTTGTTGGAACTGTTACGACTGGTGTATCACAAATAGGATT
CATCTTTGCGGTTGAAAAAATAAAACCATCGGCTCAGAAGATTAGTGTAAAAAATAACCTGAAAAATATTTTTTCTGTAA
AGAGCATTTTTGAGCTACTTAAATCAGTATTTAAGTTAGTGATAATTGTTCTCATTTTTTATTTTATGGGGCATTCATAT
GCAAATGAGTTTGCTAATTTCACAGGACTGAACGCATATCAAGCTCTTGTCGTTGTTGCCTTTTTTGTTTTTCTTTTATG

GAAAGGCGTGCTATTCGGATATCTACTCTTTTCAGTATTTGATTTCTGGTTCCAGAAGCATGAGGGACTGAAGAAAATGA
AAATGAGTAAAGATGAGGTGAAACGAGAAGCCAAGGATACTGATGGTAACCCTGAAATTAAAGGGGAGCGCCGTCGCCTT
CATTCCGAGATACAAAGTGGAAGTTTGGCTAATAACATCAAAAAATCAACCGTTATTGTTAAAAACCCGACTCACATTGC
GATTTGCCTATACTATAAACTTGGGGAGACTCCATTACCTTTAGTTATTGAAACAGGAAAAGATGCCAAAGCTCTACAGA
TCATTAAACTGGCTGAACTCTATGATATTCCTGTGATTGAAGATATTCCTTTAGCAAGAAGTCTCTATAAGAATATACAT
AAAGGACAATATATAACAGAAGACTTTTTTGAACCTGTGGCACAATTGATTCGTATTGCGATAGACCTTGATTATTAA
>Cluster_23526
ATGACTAAGCCCATCGTTTTTAGTGGCGCACAGCCCTCAGGTGAATTGACCATTGGTAACTACATGGGTGCGCTGCGTCA
GTGGGTAAACATGCAGGATGACTACCATTGCATTTACTGTATCGTTGACCAACACGCGATCACCGTGCGCCAGGATGCAC
AGAAGCTGCGTAAAGCGACGCTGGATACGCTGGCGTTGTATCTGGCTTGTGGTATCGATCCTGAGAAAAGCACCATTTTT
GTTCAGTCCCACGTGCCGGAACATGCGCAGTTAGGCTGGGCACTGAACTGCTATACCTACTTCGGCGAACTGAGCCGCAT
GACGCAGTTTAAAGATAAATCTGCGCGTTATGCCGAGAACATCAACGCTGGTCTGTTTGACTATCCGGTGCTGATGGCTG
CGGACATCCTGCTGTATCAAACTAATCTGGTGCCGGTGGGTGAAGACCAGAAACAGCACCTGGAACTGAGCCGCGATATC
GCCCAGCGTTTCAACGCGCTGTACGGCGATATCTTTAAGGTGCCAGAGCCGTTTATTCCGAAATCTGGCGCGCGCGTAAT
GTCGCTGCTGGAGCCGACCAAGAAAATGTCCAAGTCTGACGATAACCGCAATAACGTTATCGGCCTGCTGGAAGATCCGA
AATCGGTCGTGAAGAAAATCAAACGCGCGGTCACTGACTCCGACGAGCCGCCGGTAGTTCGCTACGATGTGCAGAACAAA
GCGGGCGTTTCCAACCTGCTGGATATCCTTTCTGCGGTAACGGGCCAGAGCATCCCGGAACTGGAAAAACAGTTCGAAGG
CAAGATGTATGGTCATCTGAAAGGTGAAGTGGCTGATGCCGTTTCCGGTATGCTGACTGAATTGCAGGAACGTTACCACC
GTTTCCGCAATGATGAAGCCTTCCTGCAACAGGTGATGAAAGACGGCGCAGAAAAAGCCAGCGCACACGCTTCCCGTACG
CTAAAAGCGGTGTACGAAGCGATTGGTTTTGTGGCGAAGCCGTAA
>Cluster_22225
ATGAAAATTCCCTCATTACAGTCCAACTTCAACTTTTCCGCCCCGGCAGGATACTCTGCTCCCATTGCTCCTAATCGTGC
TGAAAATGCCTATGCGGATTACGTTTTGGATATAGGTAAGCGAATACCACTTTCCGCAGCAGATTTAAGCAACGTATACG
AAAGTGTAATACGCGCCGTCCATGACAGCCGTAGCAGGCTTATCGATCAGCATACAGTCGATATGATCGGCAACACTGTA
CTTGATGCTTTGAGCCGATCACAGACATTTCGTGATGCCGTAAGCTATGGCATTCATAATGAGAAGGTACACATTGGTTG
CATTAAATACAGAAACGAATACGAGCTTAACGAAGAATCTTCTGTCAAAATTGATGATATTCAATCACTAACCTGTAACG
AATTATATGAATATGATGTCGGGCAAGAGCCAATTTTCCCCATTTGCGAAGCAGGAGAAAACGATAACGAAGAGCCTTAT
GTCAGTTTTAGTGTTGCGCCAGATACTGACTCTTATGAGATGCCATCGTGGCAGGAAGGACTGATTCACGAGATTATTCA
TCATGTTACTGGATCTAGCGATCCATCTGGAGATAGTAATATAGAGTTAGGACCCACCGAGATTCTCGCACGTCGTGTCG
CTCAAGAACTGGGATGGAGTGTTCCCGACTTCAAAGGATATGCAGAGCCAGAACGTGAAGCTCATCTTAGGTTACGTAAC
CTGAATGCCCTTCGACAGGCTGCCATGAGGCATGAAGAGAATGAGAGGGCTTTCTTCGAAAGGCTGGGTACGATCAGTGA
CCGATATGAGGCGAGTCCTGATTTCACAGAGTATTCCGCTGTGTCTAACATAGGATACGGATTTATCCAGCAACATGATT
TTCCTGGATTGGCTATCAACGATAATTTACAGGATGCAAATCAGATCCAACTGTATCATGGCGCCCCTTATATTTTTACA
TTTGGGGATGTGGACAAACACAATCAGCGATGA
>Cluster_58516
ATGATTTACGTCTCTCGCCGATTACTCATCACCTGTTTGCTGCTGGTTTCCGCCTGTGTGGTTGCAGGTATCTGGGGATT
ACGCAGCGGTGCCGTCACGCTGGAAACCTCGCAGGTATTCGCCGCGCTGATGGGCGATGCGCCGCGCAGTATGACGATGG
TGGTCACCGAATGGCGTTTACCACGCGTGCTGATGGCGCTGTTGATTGGCGCAGCACTGGGCGTCAGTGGCGCGATTTTT
CAGTCGCTGATGCGTAACCCGCTCGGCAGCCCTGACGTAATGGGCTTTAACACCGGGGCGTGGAGCGGCGTGCTGGTGGC
GATGGTGCTGTTTGGTCAGGACCTGACGGCTATCGCGCTGTCAGCAATGGTGGGCGGCATTGTCACTTCGCTGCTGGTCT
GGCTGCTCGCCTGGCGCAACGGCATCGACACCTTTCGGTTGATTATTATCGGTATCGGCGTTCGCGCCATGCTGGTGGCC
TTTAATACCTGGCTGTTGCTGAAAGCGTCTTTAGAAACGGCGCTAACAGCAGGTTTGTGGAATGCCGGATCGCTCAACGG
CCTGACGTGGGCAAAAACCTCGCCTTCCGCACCCATCATTATATTGATGCTCATTGCCGCCGCCTTACTGGTACGCCGGA
TGCGCTTGCTGGAAATGGGCGATGATACCGCGTGTGCGCTGGGCGTCAGCGTCGAACGTTCGCGTCTGTTAATGATGCTG
GTTGCAGTGGTGCTTACCGCTGCGGCAACAGCGCTTGCCGGGCCGATTTCCTTTATTGCTTTAGTCGCACCGCACATTGC
CCGACGCATTAGCGGCACCGCTCGCTGGGGGCTAACCCAGGCGGCGCTATGCGGGGCGCTGTTACTGCTGGCGGCCGATC
TCTGCGCCCAACAACTGTTTATGCCGTATCAACTTCCGGTTGGCGTCGTTACCGTCAGCCTCGGCGGTATTTACCTTATC
GTCTTGTTAATTCAGGAGTCTCGCAAAAAATGA
>Cluster_38992
ATGCAAAAACGTTATCTGGGTAAATCCGGACTCGAAGTCTCCGCCCTTGGGCTCGGTTGCATGGGCTTAAGCCACGGCTA
CGGCCCGGCGACCGATACCCGTCAGGCTATCGAACTTATTCGTGCTGCGGTTGAACGTGGCGTCACCTTCTTCGATACCG
CCGAAGTATATGGTCCGTATCTGAATGAAGAAGTGGTCGGCGAAGCCTTAAAACCGTTTCGTGACCGCGTGGTAATTGCC
ACTAAATTCGGTTTTACCTTCGGCGATGATAATAAGCAGCAGATTTTAAACAGCCGTCCGGAACATATCCGTGAAGCCGT
TGAAGGATCATTACGTCGTCTGAAAACAGATGTTATTGACCTGCTGTATCAACACCGTGTTGATCCGGATGTTCCTATTG
AAGATGTTGCAGGAACGGTGAAAGACCTCATCGCAGAAGGTAAGGTCAAACATTTCGGCCTGTCTGAAGCAGGAGCACAA
ACCATTCGTCGTGCACATGCGGTACAACCTGTCACCGCCCTGCAAAGCGAATATTCCATGTGGTGGCGTGAGCCTGAACA
GGAAATTCTGCCGTTGCTGGAGGAACTGGGAATTGGTTTTGTCCCCTTCAGCCCACTAGGTAAAGGTTTCCTGACAGGGG
CGATTAAGCCGGGAACCACTTTCGGCAAAGATGATTACCGCAGCACCGTACCGCGTTTCGCAACACAAGCCATTGAAGCC
AATGAAAAACTGGTCACCTTGTTAGGTGAACTGGCTGCGGAGAAAGGGGTAACGTCTGCACAAATCGCACTGGCATGGCT
ATTAGCACAAAAGCCGTGGATTGTTCCCATCCCTGGCACCACTAAACTAAACCGGCTGGAAGAAAACCTGGCCGCTGCTG
ACATTGTTCTTTCGCAGAAAGATACGCAGCAGATAAGCGAGGCGCTGGAAACAATTAAAATCGTCGGTGAACGTTACTCG
CCTGAACATCAGGCACGCGTAGGCCGCTAA
>Cluster_191
ATGGAAAAAAATACTTAGCCAAGAAGAGATAGATGCACTACTTTCTGGGCTTTCTGAAGGTAAGATAGATACCACCCCAGA
GGTCAAAGAAGAGGTTAAGGCTAAGCCTTTCGACTTTAGAAACTATACCATATCTACCCGTCTTAAGATCCCAGGGCTTG
AGGTTATCAACGAACAACTTGCTCGAAGTTTAAGGATGCAGCTTTCTACGCTTTTAAGAGAAATGTTGGATATCTCGAAT
TTACCCCTTCAGATGGAAAGGTTCAAAGATTTTTTAAACAAAATTCCTGTACCTACCTCCATCCATATCTTTAAATTAGA
ACCTCTTAAAGGACAATCTCTTCTTATTATAGATGCTACTTTGGCTTTCCTGTTTATTGAAAGGTTTTTAGGGGGGGAAA
GGAAACATATCAAGGTTGAAGGAAGAGAGTTTACACCAATAGAACAAAGACTTCTGAAAAAAGTGGTGGACCTTATTTTT
AACGAGTTAGAAAAGGCTTGGAAAAACATCTATCCTGTAAAACCTAAATATATTAGAGGTGAAGTAAACCCACAGTTTGC
AAGGGTACTTATGCCTGAAGAAACTGTGGTAGTTACTGGTTATACGTTAGAGCTTGAATCTCTCAGCGGTAAAATACTTT
TTTGTTATTCTCTAAGCACCTTACAACCAATAAAAGATAAGCTTTATTCTCCTTATCAGTTAGAAGAATATATAGACTTA
ACCTGGAAAAAGAACTTAGAAAACTACATTCTTAATAGTAGTGTACAACTAAAAGCTATTTTAGGAAAAGGCACCATTAC
CTTAGAAGATTTAGTAAACCTTGAGGTAGGAGATGTGGTAATTCTCGATAAAAAAAATAGAAGAACCTATAGAGGTTTATA
TAGAAGGGGTTCCTAAAATTTTAGGCAAATTAGGGGTCTTTAAAAACCACCTTGCTATTCAGGTAGACAAATTTTTGTCT
TCAGAAAATCCAGAAAATACAGAAAAATAA
>Cluster_65962
ATGAGTAGTTGGCTTGCGATGCTGCAAGATGCCGCAGAGATGTTTGTGTTTCTCGCCGTCGAGCTTTCGTTGCTGTTTAT
AGTGATTAGTGCCGGTGTCAGCCTGATAAGACAAAAGGTGCCAGACCATAAAATCCAGCAGATGATGGGGGCCAGAAAAG

GGAGAGGCTATCTCCTGGCTGCTCTGTTGGGAGCCGTTACCCCGTTCTGTAGTTGCTCGACAATACCCATGTTACGTGGA
TTGTTGTCAGCGAAAGCCGGGTTTGGTCCGACTCTCACTTTTTTATTTGTTTCACCATTACTTAATCCCATTATCGTCGG
ACTAATGTGGATGACCTTTGGTTGGAAAGTCACCTTGTTGTACGCGATTATCGCAGCCGGCGTCTCCGTTCTTGCCAGTA
TTATCCTGGATTCTCTGGGGTTTGAACGTCATATCATTGCCAGTAAAAGCTCATCAGCAAATTGTTGTGCTCCAGCAAAA
ACTTCGCCGGGAACGACATATACGCCCATAAAAGTGAGTTGTTGTAGTCCAGCGGCTAAAGCCATTGAGAACCCCGTAGT
TACCTGTTGCAGTACCAAAGCTGTAGTAAGTATTAATCCCATAAAACTAGCCACCAAAGATGCGTTGCAACAGTTTAAAG
ATGTACTGCCATATCTTTTGTTAGGAGTATTAATAGGATCTTTTATTTATGGCTTTATTCCCTCAGCGTGGATTGCCGCT
CATGCAGGAGCAGATAATCCCTTCGCCATCCCATTAAGCGCCGTTGTCGGTATTCCGCTGTATATCCGGGCTGAGGCAGT
TATCCCTCTGGCATCTGTTCTGATGACAAAAGGAATGGGTCTGGGAGCATTAATGGCATTAATCATCGGCAGTGCCGGCG
CAAGCCTGACGGAAGTGATATTACTTAAATCAATGTTCAGAATGCCGATGATAGCTGCATTCCTGACGGTTATATTAGGT
ATGGCTATCTTGATGGGCTATTTGACTCAATTGCTATTTTAA
>Cluster_39914
ATGAGATGGTATCCATGGTTACGACCTGATTTCGAAAAACTGGTAGCCAGCTATCAGGCCGGAAGAGGTCACCATGCGCT
ACTCATTCAGGCGTTACCGGGCATGGGCGATGATGCTTTAATCTACGCCCTGAGCCGTTATTTACTCTGCCAACAGCCGC
AGGGCCACAAAAGTTGCGGTCACTGTCGTGGATGTCAGTTGATGCAGGCTGGCACACATCCCGATTACTACACCCTGGCT
CCCGAAAAAGGGAAAAATGCGCTGGGCATTGATGCGGTACGTGAGGTCACCGAAAAGCTGAATGAGCACGCACGCTTAGG
TGGTGCGAAAGTTGTCTGGGTAACCGATGCTGCCTTACTAACCGACGCCGCGGCTAACGCATTGCTGAAAACGCTTGAAG
AGCCACCAGCAGAAACGTGGTTTTTCCTGGCTACCCGCGAGCCAGAACGTTTACTGGCAACATTACGTAGTCGTTGTCGG
TTACATTACCTTGCGCCGCCGCCGGAACAGTACGCCGTGACCTGGCTTTCACGCGAAGTGACAATGTCACAGGATGCATT
ACTTGCCGCATTGCGCTTAAGTGCCGGTTCGCCTGGCGCGGCACTGGCGTTGTTTCACGGAGATAACTGGCAGGCTCGTG
AAACATTGTGTCAGGCTTTGGCATATAGCGTGCCATCGGGCGACTGGTATTCGCTGCTAGCGGCCCTTAATCATGAACAA
GCTCCGGCGCGTTTACACTGGCTGGCAACGTTGCTGATGGATGCGCTAAAACGCCATCATGGCGCTGCGCAGGTGACCAA
TCTTGATGTGCCGGGCCTGGTCGTCGAACTGGCAAACCATCTTTCTCCCTCGCGCCTGCAGGCTATACTGGGGGATGTTT
GCCACATTCGTGAACAGTTAATGTCTGTCACAGGCATCAACCGCGAGCTTCTCATCACCGATCTTTTACTGCGTATTGAG
CATTACCTGCAACCGGGCGTTGTGCTACCGGTTCCTCATCTTTAA
>Cluster_41824
ATGAGTCAGAATACGCTGAAAGTTCATGATTTAAATGAAGATGCGGAATTTGATGAGAACGGAGTTGAGGTTTTTGACGA
AAAGGCCTTAGTAGAAGAGGAACCCAGTGATAACGATTTGGCCGAAGAGGAACTGTTATCGCAGGGAGCCACACAGCGTG
TGCTGGACGCGACTCAGCTTTACCTTGGTGAGATTGGTTATTCACCACTGTTAACGGCCGAAGAAGAAGTTTATTTTGCG
CGTCGCGCACTGCGTGGAGATGTCGCCTCTCGCCGCCGGATGATCGAGAGTAACTTGCGTCTGGTGGTAAAAATTGCCCG
CCGTTATGGCAATCGTGGTCTGGCGTTGCTGGATCTTATCGAAGAGGGCAACCTGGGGCTGATCCGCGCGGTAGAGAAGT
TTGACCCGGAACGTGGTTTCCGCTTCTCAACATACGCAACCTGGTGGATTCGCCAGACGATCGAACGGGCGATTATGAAC
CAAACCCGTACTATTCGTTTGCCGATTCACATCGTAAAGGAGCTGAACGTTTACCTGCGAACCGCACGTGAGTTGTCCCA
TAAGCTGGACCACGAACCAAGTGCGGAAGAGATCGCAGAGCAACTGGATAAGCCAGTTGATGACGTCAGCCGTATGCTTC
GTCTTAACGAGCGCATTACCTCGGTAGACACCCCGCTGGGTGGTGATTCCGAAAAAGCGTTGCTGGACATCCTGGCCGAT
GAAAAAGAGAATGGTCCGGAAGATACCACGCAAGATGACGATATGAAGCAGAGCATCGTCAAATGGCTGTTCGAGCTGAA
CGCCAAACAGCGTGAAGTGCTGGCACGTCGATTCGGTTTGCTGGGGTACGAAGCGGCAACACTGGAAGATGTAGGTCGTG
AAATTGGCCTCACCCGTGAACGTGTTCGCCAGATTCAGGTTGAAGGCCTGCGCCGTTTGCGCGAAATCCTGCAAACGCAG
GGGCTGAATATCGAAGCGCTGTTCCGCGAATAA
>Cluster_57004
ATGAATAAATTCACCGGTGTTTTACTATTAGGCACGGCGTTACTGGCGGGATGTGTCGACCGGGAAGGGTACTATAACAG
CGTTAGGGAAGAAGAGAGCCATGGACTGACGTCTCTGCGGGGGCAACCTGCATTACGTTACAGCGATGATTGGTCAAGAT
GGCCGAGAGTGTACGGCGCTACAGCCTTATACCCGCTGTATGCCTCCGCGTATTATAAATTAGTACCCGAGCCAAAAGAT
AAGGATCGAACCTCGCTGGCCTGGCAGGCGTATGGTTTGCAGCAAACCCGAACAGCTGAAGCCTACGATAGTCTGATTAA
AGGTTCCGCGACGGTTATTTTTGTTGCACAACCGTCGGAAGGACAGAAAAAACGTGCAGAAGAAGCGGGTGTTAAACTGA
AATATACCGCTTTCGCCCGCGAAGCCTTTGTCTTTATCGTTGATATTAATAACCCGGTAAATTCTCTCTCTGAGCACCAG
GTTAAAGATATTTTTAGCGGCAAAACTAGCCGCTGGAATAAAGTAGGTGGTAGTGACGAACATATAAAAGTCTGGCAGCG
CCCTGAAGATTCTGGAAGCCAAACGATTATGAAGGGGTTGGTTATGCAAGACACCCCAATGCTGCCAGCTAAAAAATCCA
CTGTGATTGATCTTATGGGCGGTTTAATTACTGAAGTTGCCGACTATCAAAACACGCCATCTTCCATTGGGTACACCTTC
CACTATTACGTCACTCGTATGAATGACAATATGCTCAAAATGCGCAAGCAGATTAAACTTTTGGCTATAAATGGCGTTGC
GCCTACCGAGGAAAATATCCGCAACGGCACTTATCCATACATTGTGGATGCCTATATGGTGACGCTTGAAAATCCCACGC
CGGAAACGCAGAAATTTGTTGACTGGTTTATAAGTCAGCAGGGGCAACAGTTGGTAGAGGATGTGGGGTATGTGCCGCTG
TATGAAGCATCCCCCGAATCATCAGGACAATAA
>Cluster_48627
ATGGAGCATTTAACACAAAGTGCGTGGGCCTATGCCAATAGTGCAACCATACCAAATAGTGAAACTGGTTGTGGCGTTAT
TCCATTGGCAGGAGCTGTTATAGAGGTATTTAATAACGGTAGTAGCTCAAACAATTATACGATCCGTATAACAACGGCCA
CAACGACGAGTGTCTCTGGTGCTCTCACTAATGCGGAGTTTATCTATGTATTTAATGGCACAGATTATTCTCCGGGATGG
CGAAGAGTATATAACACGAAAAACAAACCAACAGCCTCTGATGTCGGTGCATTACCTCTTACCGGTGGTACATTATCTGG
AGGTTTGACATCTTCTGGCGAGATCATTTCAAAATATGCAAATGGTTTCCGCATTGCTTACGGTAGCTTTGGGTTCTTTA
TCCGTAATGATGGATCGAACACATATTTCATGCTAACAGCATCAGGAGACACATTAGGTTCATGGAACGGTTTGCGACCT
ATTACAATTAATAATACCAGCGGTGCGGTATCAATTGGTAATGGACTAAATGTGACTGGTGGCGTAAATGGTAGTTTGAA
CGGTAATGCTTCAACAGCTACGAAGTTGCAAACAGCGAGAAACATCAATGGTGTTAAGTTTGATGGCTCAGGCGATATCA
ACATTAATACACTGGTATCTCGTGGCCGAGTTACGGCATTAAGCGGCTCTACTCAAGGCACTGCTGGCATTCAAATGTAC
GAGGCGTACAACAATAGCTACCCGACCACGTATGGCAACGTATTGCACATGAAAGGTGCGAGTGCTGCTGGTGAGGGCGA
GTTGCTTATTGGCTGGAGTGGTACGAGCGGTGCACATGCGCCAGTTTTCATTCGCTCACGAAGAGATACCACAGATGCGG
CATGGTCAGCGTGGGCGCAGGTATATACTGCTAAGGATTCAATCCCTGGTGTGAATACAACCGGTAATCAGAATACTACT
GGTAATGCCGCAACAGCCACAAAATTGCAGACAGCAAGGAAAATTGCTGGTGTGGCGTTTGATGGCTCTGCCGATATTAC
TTTGACTGCGGCTAACCTTAATGCTTATACGAAAACAGAGGTAACAAACCTTCTAAGTTCCTATGCAAGCAGATCATCAC
TGACAGGCTATAGTGGCAACCTGGATATTATTGCTGAAACACTGGTTGTCAAATCAGGCGGTAGTGGAGGGTTTGCTATA
TGGGATATTGGCACAACTACTAGCGGTGCCAATATGTACATTGATCCAAACCCAGGTATCAATACAGTTTGGCGTTCAAC
ATCTTCAAGGCGCTATAAAAAGGATATTGAAACATTACAAGATCGATATGCTGATGAACTTTTGTCATTAAGACCTGTTT
GGTATCGTTCAATTTGTCGAGGTGACCGAAAGGATTGGGGTATTACGGCCTTATTGCTGAAGAGGTTGGTGAGATTGCC
CCGCAATATGTCCATTGGCGTGAACCAACAAATAATGATTCTCCAGAAGATATTTCCTCAAATGGTATGGTCGCTGAAGG
AGTGATGTATGAGCGTTTGGTTGTACCACTCATTCATCATATTCAGCAATTGACCAAAAGGGTTGAGGAGCTTGAAACGA
AGTTAAATTCACCTAAAGAATAA
>Cluster_27578
ATGAATACTCTCCCTGAACATTCATGTGACGTGTTGATTATCGGTAGCGGCGCAGCCGGACTTTCACTGGCGCTGCGCCT
GGCTGACCAGCATCAGGTCATCGTTCTAAGTAAAGGCCCGGTAACGGAAGGTTCAACATTTTATGCCCAGGGCGGTATTG
CCGCCGTGTTTGATGAAACTGACAGCATTGACTCGCATGTGGAAGACACATTGATTGCCGGTGCTGGTATTTGCGATCGC

CATGCAGTTGAATTTGTCGCCAGTAATGCACGATCCTGTGTGCAATGGCTAATCGACCAGGGGGTGTTGTTTGATACCCA
CATTCAGCCGAATGGCGAAGAAAGCTACCATCTGACTCGTGAAGGTGGACATAGTCACCGTCGTATTCTTCATGCCGCCG
ACGCCACCGGTAGAGAAGTAGAAACCACGCTGGTGGGCAAGGCGCAGAACCATCCGAATATTCGCGTGCTGGAGCGCAGC
AACGCGGTTGATCTGATTGTTTCTGACAAAATTGGCCTGCCGGGCACGCGACGGGTTGTTGGCGCGTGGGTATGGAACCG
AAATAAAGAAACGGTTGAAACCTGCCACGCAAAAGCAGTGGTACTGGCAACCGGCGGTGCTTCAAAAGTTTATCAGTACA
CCACCAATCCGGATATTTCTTCTGGCGATGGCATTGCTATGGCGTGGCGCGCGGGCTGTCGGGTTGCCAATCTCGAATTT
AATCAGTTCCACCCTACCGCACTGTATCACCCACAGGCACGCAATTTCCTGTTAACGGAAGCACTACGCGGCGAAGGCGC
TTATCTCAAGCGCCCGGATGGCACGCGTTTTATGCCCGATTTTGATGAGCGCGGCGAACTGGCCCCGCGCGATATTGTCG
CCCGCGCCATTGACCATGAAATGAAACGCCTCGGCGCGGATTGTATGTTCCTCGACATCAGCCATAAGCCCGCCGATTTT
ATTCGCCAGCATTTCCCGATGATCTATGAAAAACTGCTCGGGCTGGGCATTGATCTCACGAAAGAACCGGTGCCGATTGT
GCCTGCTGCACATTATACCTGCGGTGGTGTAATGGTTGATGATCATGGGCGTACGGACGTCGAGGGTCTGTACGCCATCG
GCGAAGTGAGCTACACGGGATTACACGGCGCGAACCGCATGGCCTCGAATTCATTGCTGGAGTGTCTGGTCTATGGCTGG
TCGGCGGCGGAAGATATTACCAGGCGTATGCCTTATGCCCACGGCGTCAGTACGTTACCGCCGTGGGATGAAAGCCGCGT
TGAGAATCCTGACGAACGGGTAGTAATTCAGCATAACTGGCACGAGCTACGTCTGTTTATGTGGGATTACGTTGGCATTG
TGCGCACAACGAAGCGCCTGGAACGCGCCCTGCGGCGGATAACCATGCTCCAACAAGAAATAGACGAATATTACGCCCAT
TTCCGCGTCTCAAATAATTTGCTGGAGCTGCGTAATCTGGTACAGGTTGCCGAGTTGATTGTTCGCTGTGCGATGATGCG
CAAGGAGAGTCGGGGGTTGCATTTCACGCTGGATTATCCGGAACTGCTCACCCATTCCGGTCCGTCGATCCTTTCTCCCG
GCAATCATTACATAAACAGATAA
>Cluster_43202
ATGATTAAAAAACTATTACCTTTACTGGTTTTAAGCACAATTTCCGCCACAACCGTTGCCGCTACACCGCCTAATACGTT
GGTTGTAGCCCAGGGGCTGGATGATATTGTGAGTCTGGACCCGGCAGAAGCTAATGAACTCTCAAGTATTCAGACTGTAC
CAAGTCTGTATCAACGCGTAGTACAACCGGACCGTAATGATCCGACGAAAATCACTCCGATTCTGGCGGAAAGTTGGCAG
GCTGACCCCGCAGCCAAAACGCTAACCATAAAACTGAAAAGCGATGCGAAATTTGCCTCTGGTAATCCGGTACGCCCGGA
AGATGTGATTTACTCTTATGTCCGGGCTGTCACACTGAATAAATCCGGTGCGTTTATTCTCAATGTGCTGGGATGGCAGC
CGGAAAATATCGCCAGCCAGTTGAAGAAAATCGATGACCATACCGTCCAGGTGCAATGGAGTGCCGACGTCAGCCCGGCG
CTGGCACTGAATATTCTCTCCACACCAATCGCCTCAATTGTTGACGAGAAGCTGGTTGCGCCAAACGCCAAAAATAACGA
CTTTGGTAATGAATGGCTGAAGATGCATTCCGCTGGCAGCGGCGCGTTTAAAATGCGTACTTACCAACCGCATCAGGCCA
TCGTGATGGAAGCCAACGCCAGCTCACCAACCGGCGCCCCGAAACTGAAAAACGTCATCATCAAAAATGTTCCCGATCCG
GCAACGCGTCGCCTGCTGATCCAACAAGGTGATGCTGATATGGCTCGTGATCTGGGAGCCGATCAAATCGATGCATTACA
GGGTAAACCTGGCGTAAAAGTGATGAGCATTGCCTCCGCCGAACAGAACTACCTGGCCTTCAATACCGGTAACAAGGATA
ACCCGCTGATGAGTAATCCTGCGTTCTGGGAGGCCGCTCGCTGGTTGGTCGATTACGACGGCATCACCAAAAATCTCTTA
AAAGGACAATATTTTACCCATCAAAGTTTCCTGCCCGTGGGCTTTCCTGGAGCACTGGAAGAAACACCGTTTACCTTTAA
TCCAGCCAAAGCAAAAGAGATCCTCGCTAAAGCCGGAATCAAAGACCCGCACTTCACGCTTGATGTCGAAAATAAACCGC
CATTTATTACCATCGCGCAGTCAATTCAGGCCAGCTTTGCTCAGGGCGGTGTAAAAGTTGATTTGCTGCCAGCCGCAGGG
AGTCAGGTCTACTCTCGCGTTCGCGCCCACCAGCATCAGGGGGCCATTCGTATGTGGTTGCCTGATTATTTTGACGCACA
TTCCAACGCCAGCTCTTTCGCCTATAACGATGGCAAATCCAGCACCGTTGCCGGGTTAAACGGCTGGAAAATCCCGGAAC
TGAACAAAGAAACGCTGGCGGCCATCGCTGAACCTGATAGCGCTAAACGTCTTGATTTGTATAAGAAAATGCAACAAGAA
CTACAACGTAGTTCCCCGTATGTCTTTATTGATCAAGGCAAAACGCAAATTGTCATGCGTGATAATGTGCAGGGGTATCA
ACAAGGGTTGAATGCCGATATGGTCTGGTTTGATCAGGTAACCAAATAA
>Cluster_38754
ATGAGCCAGCAAGTCATTATTTTCGATACCACATTGCGCGACGGTGAACAGGCGTTACAGGCAAGCTTGAGTGTGAAAGA
AAAACTGCAAATTGCGCTGGCCCTTGAGCGTATGGGTGTTGACGTGATGGAAGTCGGTTTCCCCGTCTCTTCGCCGGGTG
ATTTTGAATCAGTGCAGACCATCGCTCGCCAGGTTAAAAACAGCCGTGTATGCGCGTTAGCTCGCTGCGTGGAAAAAGAT
ATTGACGTGGCAGCCGAATCCCTGAAAGTCGCCGAAGCCTTCCGTATTCATACCTTTATTGCCACTTCGCCAATGCACAT
CGCCACCAAGCTGCGCAGCACGCTGGACGAAGTGATCGAACGCGCTATCTATATGGTGAAACGCGCCCGTAATTACACCG
ATGATGTTGAATTTTCTTGCGAAGATGCCGGACGCACACCCATTGCCGACCTGGCGCGAGTGGTCGAAGCGGCGATTAAT
GCCGGTGCCACCACCATCAACATTCCGGACACCGTGGGCTACACCATGCCATTTGAGTTCGCCGGAATCATCAGCGGGCT
GTATGAACGCGTACCCAACATCGACAAAGCCATTATTTCCGTACATACCCACGACGATCTGGGCCTGGCGGTCGGCAACT
CACTGGCGGCGGTACATGCCGGTGCACGCCAGGTGGAAGGTGCAATGAACGGGATCGGCGAGCGTGCCGGTAACTGTTCC
CTGGAAGAAGTCATCATGGCGATCAAAGTTCGTAAGGATATCCTCAACGTCCACACCGCCATTAATCACCAGGAGATATG
GCGCACTAGCCAGTTAGTTAGCCAGATTTGTAATATGCCGATCCCGGCAAACAAAGCCATTGTTGGCAGCGGCGCATTCG
CACACTCCTCCGGTATCCACCAGGATGGCGTGCTGAAAAACCGCGAAAACTACGAAATCATGACACCAGAATCTATTGGT
CTGAACCAAATCCAGCTGAATCTGACCTCTCGTTCGGGGCGTGCGGCGGTGAAACATCGCATGGATGAGATGGGGTATAA
AGAAAGTGAATATAATTTAGACAATTTGTACGATGCCTTCCTGAAGCTGGCGGACAAAAAAGGTCAGGTGTTTGATTACG
ATCTGGAGGCGCTGGCCTTCATCGGTAAGCAGCAAGAAGAGCCGGAGCATTTCCGTCTGGATTACTTCAGCGTGCAGTCA
GGTTCTAACGATATTGCCACTGCCGCCGTCAAACTGGCCTGTGGCGAAGAAGTCAAAGCAGAAGCCGCCAACGGTAACGG
TCCGGTCGATGCCGTCTACCAGGCGATAAACCGCATCACTGACTATAACGTCGAACTGGTGAAATACAGCCTGACCGCCA
AAGGTCACGGTAAAGATGCGCTGGGTCAGGTGGATATTGTCGCCAACTACAACGGTCGCCGCTTCCACGGCGTCGGCCTG
GCCACCGATATTGTCGAGTCCTCCGCCAAAGCCATGGTGCACGTACTGAACAATATCTGGCGTGCCGCAGAAGTCGAAAA
AGAGTTGCAACGCAAAGCTCAACACAACGAAAACAACAAGGAAACCGTGTGA
>Cluster_64421
ATGGCTATTTCTCAAAACTTTAAAACGCAGGTTGTATTTGGGGGAAGAATAGACCCGTCGTTTCGGCGCGGTACAACAGA
ACTCAACGATGCAATTCGGCAGACGTCGTCTACTGTCGGAAAATTAACGAAAAGCCAGGATAAATTAAAAGACAAAATCG
CAGCAATGAAACTGGCGGGTAAAGACGTTTCTGATTTATCTGCTCAGTATCAAAAGCTGGACCGTCGGATAAAAGCGACA
ACACAGGACCAGGAAGCTCTGAATACTCAGCTCGCCAAAAAACAGCGGCTGGAGAAGTGGACGGGGCGCGCAAAAGGTGC
TGCAAAATGGGGAGGGCGAGCCGCCGCAGGTGCGGTGAGGACCACGGGACGGGGTATCAAATGGGGGACGCTGGGGGCCG
TGGGTCTGCTCGGCGGCGCGGCTGCTGGCGCGTTGGCAATGAATGCAGAGACGTCAGAAAAACTCGGTCTGGCGAAGTCC
TACGGTGTTGGCATCGAAAAATACGCTGCGTGGGAAAATATTGGTAAAGCTGCAGGGCTGAATGGCGAAAACGTAGGAGA
TCTGTCGGAGGAGTTGACTAATAAAATCGGGGAGGTTGGAAACGAAAAAAACCTGAACCCCATGTTGTTTCAAATTGGCC
TGACAAAAAAACGGATGGCTAAATGGGATCGAGAAAAACAGTTCAATGAAGTTATGCGGCGTATCTCTGAAATGAAGGAT
GATCAACAGGCGGCGAGTCTGGCTGATCAGCTCATGGGGGGGGAAGCTAATAAAATAATGACTTATATGCGGGCGACTGG
TAAAAGCTGGGAGCAGACTCTGTCGGACGCTCAAAAATCAAACCTGCTAACGAAAGAAGGGGCTGAGGGTGCAGCGCGCG
CGCACGTATCCGTCACGAATCTGTGGGGGGCAATCACATCGGGGCTGGCAGATACTCTCGGGAAAATCGGAGGGGAACTC
GCGCTGACGTTTGACGCTATGCGAGACGGGCTGGCATCCTGGTTTAAAGAGAATCAAGGGGGGGTTGTTGATAGTATTAA
AGAATGGGTTAAGCCAGAAAATATGAAAAAAATGTGGGAGGGGATAGTCAGTTTTGGCGAGGCGTGCGTTAAATTCGGAA
AAATAATTTGGGCAGTCGTAAAAAAACTGGAGTGGTTAATACCGGACGAGAAAACAGACGAGGAGCAGCGCGTATATAAC
GAAGAATATAATAAGGCGTATCAGGAATTCATGGATGGCGGAGGAAAATATTCACCAAACCCAGGAATGGCGGCGGATAA
TGTTGCAAAAGCAAAAGCCGCCGAAGCCGTGGATAATATGCGACACCCTGAACGTCTTGCTCGTGCAAAATCACAGGCTG
AATCACTGCTCTCGTTTGCTAACCTGTTTACTGGATTAATAAATAAAGGCTCAACGCCTGAATCACAAAACTCAGCAACG

TTGAATATTGACGCGCTGAGACAAGCAGTCGCAACTCCAGCGCCAGAGCAAAACAATAAAATTGAAATCAATATTATCGG
AGCGGCAGATCCACAATCAACACAGCAGGCTGCTGCATCGGGTGTGCTCGACGGGCTGAGACAAGTTGCGAGTTCGTACA
ACCGTGGTGCGATGTTTGATAAACCAGCGGCGGCGGGGTGA
>Cluster_3301
GTGAGGAACACGCTGAAACAGGCCGTCGTGCTGTGGGGAATGGTGTTACTGCTGGTGCTGTGGTCGGTGTTTATCAGTCC
GTCTGGCGTACTGATATGGGCCGGGGCGGCGGCTATCGTTCTGACGGTTGCCGCGTTGCTGATTTACCGGCGCAGGCAGG
CGTGGACGGAGATGACCGGCGATGCCGGGTTGTTATCGCTGCCGCCGGAAACGTACCGACAACCGGTAGTGCTGGTCTGT
GGCGATATGTCGGCGCATCTGTTCACTGACAGCCCGGTCCGGCAGGTTTCAGAAGGGCTGTATCTGCATGTTTCTGATGA
AGAACAGCTTGTGGCGCAGGCGGAGCGATTACTGACCCTTCGCCCGGCGTGGGCATCGCAGCTTGCCGTGGCATATACCG
TCATGCCCGGCATGTACCGGGATGCGGCGGTTCTGACCGGACAACTGCGCCGGTTCGCCCACAGTATGGCGACAGTGCGT
CGCCGGGCGGGCGTAAACGTCCCCTGGCTTCTCTGGAGCGGGCTGTCCGGCTCGCCGTTGCCGGAGAGAGCGAATTCACC
GTGGTTTATCTGTACCGGCGGCGAAATTCATGTAGCGACGTCTGCTGAAACCGCCTCCCCCGCGCAGTGGCTCACGCAGA
CCAGCACACAGGAGCGCAGCCAGCAACTCTGTTACCTGCTGAAAGCCGAAAGCCTGATGCAGTGGCTGAATCTTAATATG
CTGGCGGCACTGAACGGCCCGGAAACGAAATGTCCGCCACTGGCGATGGCCGTGGGGCTGGTCCCCTCGTTGCCTGCGGT
GGATAACAACCTGTGGCAGTTGTGGATCACCGCCAGAACCGGCCTGACGACGGATATCGCGGACACCGGCACAGACGCCA
CGCTGCCGTTTCCGGATGCCCTGTTACGGCGGTTGCCGCGTCAGTCGGGCTTTACCCCGCTGCGACGAGCCTGCGTGACC
ATGCTGGGCATCACCACCGTGGCGGGTATCGCCGCGCTGTGTCTGTCAGCCACGGAGAATCGCCAGTTATTACGGCATAT
CGGTGATGATCTGCACCAGTTTTATGCCGTTCCGGCGGAGGAATTTATCACCAAAGCCCGTCGTCTGTCGGTGCTGAAAG
ACGATGCGATCATGCTCGATGGGTATTACCGGGAAGGAGAGCCACTGCGTCTCGGTCTGGGGTTATACCCCGGTGAACAA
ATCCGCCAGCCGGTATTACGCGCCATTCGTGACTGGCGTCCGCCTGAGCAAAAAATGGAAGTGACAGCCTCGCTTCAGGC
GCAGACCGTGCGTCTTGACAGTATGTCGCTGTTTGACGTCGGACAGGCCCGCCTGAAAGACGGCTCGACAAAAGTGCTGG
TGGACGCACTGGTGAACATCCGGGCAAAACCGGGCTGGCTGATCCTCGTGGCCGGATATACCGACGCCACCGGCGACGAA
AAAAGCAATCAGCAGTTATCGCTGCGACGTGCCGAAGCGGTGCGCAACTGGATGCTGCAGACCAGCGACATCCCGGCCAC
CTGTTTTGCCGTACAGGGACTGGGCGAGAGCCAGCCTGCGGCGACCAACGATACGCCACAGGGCCGGGCAGTCAACCGGC
GCGTCGAAATCAGTCTTGTTCCGCGTTCTGACGCCTGTCAGGACGTGAAATAA
>Cluster_27881
ATGAATATTTTCACAGAGGCTGCAAAACTCGAAGAGCAAAATTGTCCGTTTGCGATGGCGCAAATTGTTGATAGCCGAGG
CTCAACCCCCCGCCATTCTGCACAAATGTTAGTGCGCGCCGATGGTTCTATCGTCGGTACAATTGGTGGCGGAATGGTTG
AGCGGAAGGTGATTGAAGAGTCGCTTCAGGCATTGCAGGAACGTAAGCCGCGATTATTCCATGGACGTATGGCTCGTAAC
GGTGCGGATGCTGTCGGGTCAGATTGTGGAGGTGCAATGTCAGTATTTATCAGCGTCCATGGTATGCGTCCACGTCTGGT
GTTGATTGGTGCGGGGCATGTCAACCGGGCGATAGCCCAGAGTGCGGCGCTATTAGGATTTGATATCGCCGTTGCTGATA
TTTATCGCGAAAGCCTCAATCCTGAACTATTCCCACCATCAACCACGCTTCTCCATGCTGAGTCGTTTGGTGCGGCAGTG
GAAGCACTGGATATTCGCCCTGATAATTTTGTCCTGATTGCCACGAATAATCAGGATCGTGAAGCCCTCGACAAACTCAT
TGAACAACCCATTGCATGGCTGGGGTTGCTGGCAAGTCGCCGCAAGGTTCAGCTTTTCCTGCGCCAATTGCGTGAGAAAG
GCGTGGCTGAAGAACATATTGCCCGTTTACATGCGCCCGTTGGTTACAACATAGGTGCGGAAACGCCGCAGGAGATCGCC
ATTAGCGTGCTGGCAGAAATATTACAGGTGAAAAATAACGCGCCGGGTGGGCTGATGATGAAGCCTTCTCATCCTTCCGG
ACACCAGCTGGTGGTGATTCGCGGTGCGGGGGATATCGCCAGTGGTGTAGCGTTACGTCTGTATCATGCGGGTTTTAAAG
TGATCATGTTGGAAGTGGAAAAACCGACAGTGATTCGTTGTACCGTGGCGTTTGCCCAGGCCGTGTTCGATGGCGAAATG
ACGGTCGAAGGCGTCACCGCCCGCCTGGCAACCAGCTCTGCGGAAGCGATGAAACTTACCGAACGCGGATTTATCCCTGT
GATGGTAGATCCCACCTGTTCATTGCTTGATGAACTGAAACCGCTTTGCGTGGTGGACGCTATTCTGGCGAAACAGAATT
TGGGAACGCGGGCAGATATGGCACCAGTAACAATCGCGCTTGGGCCGGGCTTTACTGCAGGGAAGGATTGTCATGCGGTA
ATTGAAACAAATCGCGGGCACTGGCTCGGTCAGGTGATTTACTCTGGTTGTGCGCAGGAGAATACCGGTGTTCCCGGCAA
TATTATGGGGCATACCACCCGACGGGTGATCCGTGCTCCTGCTGCAGGCATTATGCGATCCAACGTGAAATTAGGCGATC
TGGTGAAAGAGGGCGATGTCATTGCCTGGATTGGTGAGCATGAAATTAAAGCACCGTTGACGGGGATGGTGCGTGGCTTG
TTGAACGACGGACTGGCCGTGGTCGGTGGTTTTAAAATTGGTGATATCGATCCTCGTGGTGAAACGGCTGATTTCACCAG
CGTTTCTGATAAAGCTCGGGCGATTGGCGGCGGCGTACTTGAGGCGTTAATGATGTTGATGCATCAGGGCGTTAAAGCGA
CAAAAGAAGTGCTGGAAGTGGCTTAA
>Cluster_65655
ATGACAGTATTCAACAAATTTGCTAGAAGTTTTAAATCTCATTGGTTGTTGTATCTTTGTGTTATTGTTTTTGGTATTAC
GAACTTAGTCGCCTCTTCCGGCGCGCATATGGTTCAGCGATTGCTGTTCTTCGTCCTGACCATCCTGGTTGTAAAACGTA
TATCATCCCTTCCGCTTCGCCTGCTTGTTGCCGCACCATTTGTGTTACTGACTGCGGCAGACATGAGTATTAGCCTCTAT
TCATGGTGTACCTTTGGTACAACTTTCAATGATGGATTTGCGATTAGTGTGCTCCAGAGTGATCCGGATGAAGTTGTCAA
AATGCTGGGGATGTATATCCCTTATCTATGTGCCTTTGCTTTTTTATCCCTTCTTTTTTTGGCAGTAATAATAAAATATG
ATGTTTCCTTGCCGACAAAAAAAGTGACAGGAATATTATTGCTGATTGTCATTTCGGGCAGTTTATTTTCCGCTTGTCAA
TTTGCTTATAAAGATGCAAAAAATAAAAAAGCGTTCAGTCCATATATACTAGCGTCGCGATTTGCTACCTATACGCCGTT
TTTCAATCTCAACTATTTTGCTTTAGCAGCGAAAGAGCATCAAAGATTACTCTCAATTGCAAACACGGTGCCGTATTTTC
AATTATCAGTCAGGGATACAGGTATTGATACCTACGTGTTGATTGTGGGGGAGTCTGTACGTGTCGACAATATGTCTTTG
TATGGATATACACGCTCTACGACACCGCAAGTTGAAGCACAAAGAAAACAGATCAAACTGTTTAATCAAGCAATAAGCGG
CGCACCTTACACTGCGCTGTCGGTTCCCCTTTCTTTAACTGCTGATTCTGTTTTGAGTCATGACATTCATAATTACCCCG
ACAACATTATTAATATGGCTAATCAAGCAGGATTTCAGACTTTCTGGCTAAGCTCGCAATCCGCTTTTCGGCAGAATGGT
ACAGCAGTTACCAGTATCGCCATGCGCGCCATGGAAACAGTTTATGTCAGAGGATTTGATGAATTGTTGTTGCCGCATTT
ATCGCAAGCATTACAGCAAAATACGCAGCAAAAGAAACTGATTGTTCTTCATTTAAATGGAAGCCATGAACCGGCTTGTA
GCGCCTATCCGCAATCCAGCGCCGTGTTTCAACCGCAGGACGATCAGGATGCCTGCTATGACAACTCCATTCATTACACA
GATAGTTTGCTAGGTCAGGTTTTTGAATTATTAAAAGATCGCCGCGCCTCGGTCATGTATTTTGCCGACCACGGCCTGGA
ACGTGACCCTACGAAGAAGAACGTCTATTTTCATGGAGGCAGGGAGGCTAGCCAGCAGGCATATCATGTCCCGATGTTTA
TCTGGTATAGCCCCGTTCTTGGGGATGGCGTGGATCGCACAACGGAAAACAACATCTTTTCGACAGCTTACAATAATTAC
CTTATTAATGCGTGGATGGGGGTAACAAAGCCGGAACAGCCGCAAACGCTTGAGGAAGTGATTGCACACTATAAAGGAGA
CTCACGGGTTGTGGATGCAAACCATGATGTTTTCGATTATGTGATGCTCAGAAAGGAGTTTACAGAGGATAAGCAAGGTA
ACCCCACCCCTGAAGGGCAGGGTTGA
>Cluster_57879
ATGAAACCAGAAGATTTCCGCGCCAGTACCCAACGTCCGTTCACCGGGGAAGAGTATCTGAAAAGCCTGCAGGATGGTCG
CGAGATCTATATCTATGGCGAGCGAGTGAAAGACGTCACTACTCATCCGGCATTTCGTAATGCGGCAGCGTCTGTTGCCC
AACTGTACGACGCGCTGCACAAACCGGAGATGCAGGACTCTCTGTGCTGGAACACCGACACCGGCAGCGGCGGCTATACC
CATAAATTCTTCCGCGTGGCGAAAAGTGCCGACGACCTGCGCCAGCAACGCGACGCCATCGCTGAGTGGTCACGCCTGAG
CTATGGCTGGATGGGCCGTACCCCAGACTACAAAGCTGCTTTCGGTTGCGCACTGGGCGCGAATCCGGGCTTTTACGGTC
AGTTCGAGCAGAACGCCCGTAACTGGTACACCCGTATTCAGGAAACTGGCCTCTACTTTAACCACGCGATTGTTAACCCA
CCGATCGATCGTCATTTGCCGACCGATAAAGTAAAAGACGTTTACATCAAGCTGGAAAAAGAGACTGACGCCGGGATTAT
CGTCAGCGGTGCGAAAGTGGTTGCCACCAACTCGGCGCTGACTCACTACAACATGATTGGCTTCGGCTCGGCACAAGTGA
TGGGCGAAAACCCGGACTTCGCACTGATGTTCGTTGCGCCAATGGATGCCGATGGCGTGAAATTAATCTCCCGCGCCTCT

TATGAGATGGTCGCGGGTGCTACCGGCTCGCCATACGACTACCCGCTCTCCAGCCGCTTCGATGAGAACGATGCGATTCT
GGTGATGGATAACGTGCTGATCCCATGGGAAAACGTACTGATCTACCGCGATTTCGATCGCTGCCGTCGCTGGACAATGG
AAGGCGGTTTTGCCCGTATGTATCCGCTGCAAGCCTGTGTGCGCCTGGCAGTGAAACTCGACTTCATTACAGCACTGCTG
AAAAAATCACTCGAATGTACCGGCACCCTGGAGTTCCGTGGTGTACAGGCCGATCTCGGTGAAGTGGTGGCGTGGCGCAA
CACCTTCTGGGCATTGAGTGACTCGATGTGTTCTGAAGCGACGCCGTGGGTCAACGGGGCTTATTTACCGGATCATGCCG
CACTGCAAACCTATCGCGTACTGGCACCAATGGCCTACGCGAAGATCAAAAACATTATCGAACGCAACGTTACCAGTGGC
CTGATCTATCTCCCTTCCAGTGCCCGTGACCTGAACAATCCGCAGATCGACCAGTATCTGGCGAAGTATGTGCGCGGTTC
GAATGGTATGGATCACGTCCAGCGCATCAAGATCCTCAAACTGATGTGGGACGCCATTGGCAGCGAGTTTGGTGGTCGCC
ACGAACTGTATGAAATCAACTACTCCGGTAGCCAGGATGAGATTCGCCTACAGTGTCTGCGCCAGGCACAAAGCTCCGGC
AATATGGACAAGATGATGGCGATGGTTGATCGCTGCCTGTCGGAATACGACCAGAACGGCTGGACTGTGCCGCACTTGCA
CAACAACGACGATATCAACATGCTGGATAAGCTGCTGAAATAA
>Cluster_49738
GTGGCGATATTGAGCGCAATTCGACGCTGGCATTTTCGCGATGGTGCGTCGATTCGGGAAATAGCCCGACGAAGCGGCCT
GTCCAGGAACACCGTTCGCAAGTATTTGCAAAGCAAGGTGGTTGAACCGCAGTACCCAGCGCGAGACAGCGTTGGCAAGT
TAAGTCCTTTTGAGCCCAAGTTAAGGCAGTGGCTCTCCACCGAGCACAAAAGACAAAGAAGCTGCGCAGAAACCTGCGC
AGCATGTACCGGGATTTGGTCGCTTTGGGCTTTACCGGGTCTTATGACCGAGTGTGTGCCTTTGCCCGACAGTGGAAAGA
TTCCGAACAGTTCAAGGCGCAAACCTCGGGCAAGGGTTGTTTCATCCCCTTGCGCTTTGCTTGTGGCGAAGCCTTCCAAT
TCGATTGGAGTGAGGACTTTGCCCGCATAGCGGGCAAACAGGTCAAACTTCAGATTGCCCAGTTTAAGTTGGCCCACAGC
CGGGCCTTTGTGCTTCGGGCTTACTACCAGCAAAAACATGAAATGCTGTTTGATGCCCACTGGCATGCCTTTCAAATCTT
CGGTGGCATTCCCAAGCGCGGCATCTACGACAACATGAAGACCGCTGTGGATTCGGTGGGGCGTGGCAAAGAGCGCAGGG
TCAATCAGCGGTTCACTGCCATGGTCAGCCACTACCTGTTTGATGCGCAGTTCTGTAATCCAGCATCGGGTTGGGAGAAA
GGCCAGATTGAGAAGAACGTGCAGGATTCCCGCCAACGCCTGTGGCAAGGGGCACCAGACTTTCAAAGCCTTGCTGATTT
GAATGTGTGGCTTGAGCATCGCTGCAAAGCGCTGTGGTCTGAGCTGCGCCACCCCGAATTGGACCAAACCGTGCAAGAGG
CCTTTGCCGATGAACAAGGCGAGTTGATGGCGCTACCCAATGCCTTTGATGCATTCGTGGAGCAAACCAAGCGAGTCACT
TCAACCTGCCTTGTTCACCACGAGGGCAATCGCTACAGCGTTCCTGCCAGTTACGCCAACAGGGCCATCAGCCTTCGGAT
TTATGCAGACAAGCTGGTGATGGCTGCCGAAGGCCAACACATTGCCGAGCATCCAAGATTGTTTGGCAGTGGCCACGCTC
GGCGTGGCCACACACAATACGACTGGCACCATTACTTGTCTGTGCTTCAGAAGAAACCTGGGGCGTTGCGCAATGGTGCG
CCATTTGCTGAATTGCCACCCGCGTTCAAGAAGCTTCAATCCATCTTGCTGCAACGCCCCGGCGGTGACCGTGACATGGT
GGAAATTCTGGCCCTTGTATTGCACCACGATGAAGGTGCGGTACTCAGTGCTGTGGAATTGGCATTGGAGTGTGGCAAGC
CATCGAAGGAGCATGTGCTTAATCTGTTGGGACGTTTGACCGAAGAACCTCCACCCAAACCGATTCCAATTCCCAAGGGG
TTAAGGCTGACATTGGAACCACAGGCCAACGTGAACCGCTATGACAGTTTAAGGAGAGCCCATGATGCAGCATGA
>Cluster_29233
ATGTCTGAACGAGAAACACGGGGAGCCAATGAGGCTATTGATTTTAACGATGAACTGAGAAATCGCCGCGAAAAACTGGC
GGCACTACGTCAGCAAGGTGTGGCGTTTCCCAATGATTTTCGCCGCGACCATACCTCTGACCAGTTGCACGAAGAGTTTG
ATGCGAAGGATAACCAGGAACTGGAATCCTTAAACATTGAAGTCTCGGTTGCTGGCCGAATGATGACCCGTCGTATTATG
GGGAAAGCCTCCTTTGTAACGTTGCAGGATGTCGGTGGCCGTATTCAACTGTACGTTGCAAGAGATAGCCTGCCAGAAGG
TGTTTATAACGATCAGTTTAAAAAATGGGATCTGGGTGACATTATCGGTGCCCGCGGTACGCTGTTTAAGACGCAAACGG
GTGAGCTTTCCATTCACTGTACTGAGCTGCGCCTGCTGACTAAAGCACTACGTCCTTTACCAGATAAATTCCATGGTCTG
CAGGATCAGGAAGTCCGTTATCGTCAACGTTATCTGGACCTCATCGCTAACGATAAATCCCGTCAAACGTTTGTTGTCCG
TTCAAAAATTCTGGCCGCTATCCGTCAGTTCATGGTCGCGCGCGGCTTTATGGAAGTAGAAACCCCGATGATGCAGGTAA
TTCCAGGTGGGGCATCTGCTCGCCCGTTTATTACCCATCATAACGCTCTGGATTTAGATATGTACCTGCGTATCGCGCCG
GAGCTGTATCTGAAACGTCTGGTTGTAGGCGGTTTTGAACGGGTATTCGAAATCAACCGTAACTTCCGTAATGAAGGTAT
TTCTGTTCGCCATAATCCTGAGTTCACAATGATGGAACTCTACATGGCATATGCGGATTACCACGATTTGATTGAACTGA
CGGAGTCACTGTTCCGCACCCTGGCACAAGAGGTACTGGGTACCACTAAAGTCACTTATGGCGAGCATGTGTTTGATTTC
GGCAAACCGTTTGAAAAACTCACCATGCGCGAAGCAATCAAAAAATATCGTCCAGAAACCGATATGGCGGATCTGGATAA
TTTTGATGCTGCTAAAGCATTAGCTGAATCTATCGGTATTACGGTAGAGAAAAGCTGGGGGCTGGGACGTATTGTTACGG
AGATCTTTGATGAAGTGGCAGAAGCACATCTGATTCAGCCAACCTTTATTACGGAATATCCGGCAGAAGTGTCCCCGCTG
GCGCGCCGTAATGATGTTAACCCGGAAATCACCGACCGTTTTGAATTCTTCATCGGTGGTCGTGAAATTGGTAATGGTTT
TAGCGAATTAAACGACGCAGAAGATCAGGCTGAACGTTTCCAGGAACAGGTTAACGCTAAAGCTGCAGGTGACGACGAAG
CCATGTTCTATGACGAAGATTACGTGACTGCGCTGGAATATGGTCTGCCGCCAACCGCTGGTCTGGGTATTGGTATCGAC
CGAATGATTATGCTGTTTACAAACAGTCATACTATTCGCGACGTTATTCTCTTCCCGGCGATGCGCCCACAGAAATAA
>Cluster_40823
ATGGCGAGGGGTATCTGGGTTTCACCCGATGAATTTGTTGCTTTTTCTGAGCCTCAGAAATCACTGACCGCGCAGATTGC
CTCCCGCAGCCGCGCGATCGACTTTTACGGACTGGGCATGTATCTGCCCAATCCTGATCCCATTCTCAAGGCTCAGGGAC
GGGATATCCGTATCTACCGCGAACTGCGCACCGACCCGCTGGTCGGGGGCTGTATCCGCAGACGTAAAGCAGCGCTCAAA
TCGCTGGAGCGTGGACTGGAGCGTGGTCACGCCTCTGCCCGGGTCTTCCGTTTCATCCGCGACATGCTCGACGATCTGGA
TCTGTCCCGCATCATCGGTGAAATGAGCGATGCCGTGCTCTACGGGTATCAGCCCTGTGAAATCATGTGGGGCCGTTCGG
TCAGGTCGTGGGCGGTGACGGATATTGTCGGCAAACCGCCTGAGTGGTTTCAGTTTGATACGGACAACTGCCTGCGCTTC
CGGGCGCGTGATGCGGGTGTGGAGGGTGAGCTGCTGTCACCGTCAAAATTCGTGGTGCCGGCACAGGATGCCTCGTATGA
CAATCCTTACGGTTTCCCGGACCTGTCCATGTGCTTCTGGCCGGTCGCCTTCAAGAAAGGCGGGATGAAATTCTGGCTCC
GCTTTGCCGAAAAGTTTGGCTCCCCGTGGGTGATCGGTAAGCACCCGAGGGGTGCAAATGATGCAGAGATTGAAAAACTG
CTGGACTCCATGGAGCAGATGGTGGAGGACGCGGTGGCCGCCATCCCCGATGACAGCAGCATCGAACTCAAAGCCGCGGA
TGGTAAGGCGGACAGCAGCGAGGTATTCCGCGAGCTGATCACACTGTCACGCAGTGAGATCTCCATTGCATTACTTGGTC
AGAATCAGACCACGGAAGCGAACAGTAATAAGGCCTCTGCACAGGCCGGGCTGGAGGTAACGGCTGATATCCGCGATGCG
GATGCGGACATCATTCAGGCAGCAGTGAATCAGGTTATCAGAACGGTGGTCACCCTGAACTTCGGCGATGTGCCGTGTCC
GGTCTGGGCCATGTGGGAACAGGAGACCATTGATGACACCCGCGCCACCCGCGACGAAAAACTCACCCGGGCGGGTCTGC
GTCTGACCCCGCAATACTTTAAGCGTGAGTACCAACTGCAGGACGGCGATATTGACGAGACATCACCGTCGGAACGCCAG
AATAACATGCTGCCGCTGTCATTTGCCGAGGCGATTGATGCCGATATTCAGGCTCAGCAGCAGCTTGACGACGCGCTGGA
CATTCTGATGAACGGAGGTGTGTTAAATGGCACGCTGGAACCCGTCCTGGCACCTCTGTTTAAGCGGGTCGAAAACGGGG
TTAACCCGACTGAGCTGCTGGGCGAACTGGCGGAGCTCTACCCTCAGATGAACACGGACGATCTGCAGGAACGGCTGGCC
CGTATTCTCTTTGTGGCAAATATCCGGGGGCGTCTGCATGAGCGTGACAACGGCTGA
>Cluster_4034
ATGCCTGATAACAGCGCAGCTATCGTTATCGATATTGGCACCACCAATTGCAAAGTCACCTGCTTTTCCTGCCTGGACGC
AACGACGTTGGGCGCGCATAAATTCGTGACGGCAAAGCAGATCTCCCCACAGGGCGATGTCGATTTCGATATCGACGCCC
TCTGGCAGGAGGTCCGCCAGGCGATAGCGCAACTGAACGCCGCTTCGCCGCTGCCAGTCAGACGGATCAGCATTGCCAGT
TTTGGCGAATCAGGCGTGTTCCTTGACGAGCATGGCGAGATCCTGACGCCAATGCTGGCATGGTATGACCGTCGCGGTGA
AGAGTATCTGGCAACGCTTAGCGAGGCAGACAGTGCAGCACTTTATGACATCTGCGGTCTACCACTACACAGCAATTACT
CTGCCTTCAAAATGCGCTGGTTGCTGGAACATTACCCGCTGCGTAATCGCCGCGGCCTGCGCTGGCTACATGCGCCGGAA
GTGCTGCTCTGGCGGCTGACTGGCGAACAGCGCACGGATATCACCTTAGCCAGCCGCACGCTGTGTCTGGACGTGCGCAA

AGGCGAATGGTCAGCGAAAGCGGCGGCGTTGTTACACGTTCCCTGTACGGCATTTGCGCCATTGGTGCAGCCAGGCGAGC
ACGCCGGATGGGTCAGCGAGTCACTTTGCAAGACGCTTGGGTTCTCGCAACCGGTCAGCGTGACGCTGGCCGGACATGAC
CATATGGTGGGTGCGCGAGCGTTGCAGATGATGCCAGGCGATATCCTTAACTCGACGGGGACCACGGAAGGCATTCTGCA
ACTGGATACACAACCGACGCTGGATGAACAGGCCAAACGCTGACAAGCTGGCAAACGGCTGTTACTCACTTGCCAACCAGT
TCACCCTGTTTGCGTCGCTGCCCGTGGGCGGTTTCGCGCTGGAGTGGCTGCGCAACACGTTCCGGCTAACCGATGAGGAG
ATCGCCGCATCACTTACTCGCGGACATGCTGATTATCTGGCGGGGAATTGGTTGCTCGATGACATTCCCGTCTTTATTCC
ACATCTTCGCGGTTCGGGTTCGCCCTATAAAAATCGCCATACCCGTGGATTATTTTATGGGCTTGGCGATACGTTAAGTA
TCGACATGTTAATTGCCAGCGTATCACTGGGATTAACCATGGAATTTGCCAACTGCTTCGCCTGTTTTAACGTGCCTGGC
ACCAGCGCGTTAAAAGTGATCGGTCCGGCAACCCATAATCCTCTTTGGCTGCAATTAAAGGCGGATATTTTACAGCGTCC
GGTTGAAGCAATTGCATTTAACGAGGCGGTTTCTGTCGGAGCATTATTAACCGCCGCACCGGATATTCCACCGCCGCCAG
TCGCTATAGCCCAACGTTTGTTACCGAATCGGGCGAGATACCATCAATTACAGCGTTATCAGCACAAATGGAAAAGCTGG
TATCAGTTGAAATTACAACAAGAAGGCGTGATGCCATTACATCATCGGGAGGAACACTATGTTGAATAA
>Cluster_27511
ATGCCAATACACGATAAATCCCCTCGTCCGCAGGAGTTTGCTGCGGTCGATCTTGGTTCAAACAGTTTTCACATGGTCAT
AGCCCGTGTGGTAGATGGTGCCATGCAGATTATTGGCCGCCTGAAACAGCGGGTGCATCTGGCGGACGGGCTGGGGCCAG
ATAATATGTTGAGTGAAGAGGCAATGACGCGCGGTTTGAACTGTCTGTCGCTATTTGCCGAAAGGCTACAGGGGTTTTCT
CCTGCCAGCGTCTGTATAGTTGGTACCCATACGCTGCGTCAGGCGCTGAACGCCACTGACTTTCTGAAACGCGCGGAAAA
AGTCATTCCCTACCCGATCGAAATTATTTCCGGTAATGAAGAAGCCCGTCTGATTTTTATGGGCGTGGAACATACCCAAC
CGGAAAAAGGTCGCAAACTGGTTATTGATATTGGCGGCGGTTCTACAGAACTGGTAATTGGTGAAAATTTCGAACCGATT
CTCGTTGAAAGCCGCCGGATGGGTTGTGTCAGCTTTGCCCAGCTTTATTTTCATGGCGGGGTCATCAATAAAGAGAATTT
TCAGCGCGCTCGCATGGCGGCAGCACAAAAACTGGAAACGTTAACCTGGCAATTTCGAATTCAGGGCTGGAACGTGGCAA
TGGGCGCTTCCGGTACCATAAAAGCCGCCCATGAAGTGTTAATGGAAATGGGCGAGAAAGACGGGATAATTACCCCGGAA
CGTCTGGAAAAACTGGTAAAAGAAGTTTTACGGCACCGTAATTTCGCATCGCTGAGTTTACCAGGTCTTTCCGAAGAGCG
GAAAACAGTCTTCGTTCCTGGACTGGCGATTTTATGCGGTGTGTTTGATACTTTAGCCATCCGTGAACTGCGCCTTTCTG
ACGGGGCGTTACGCGAAGGCGTACTGTATGAAATGGAAGGACGTTTCCGTCATCAGGATGTGCGTAGTCGCACTGCCAGC
AGCCTCGCCAACCAGTATCACATCGACAGCGAACAGGCCCGACGGGTGCTGGATACCACTATGCAAATGTACGAACAGTG
GCGGGAACAGCAACCGAAGCTGGCGCATCCACAACTGGAGGCGCTACTGCGATGGGCCGCCATGCTGCATGAGGTCGGGT
TGAATATCAACCACAGCGGTTTGCATCGCCACTCCGCTTATATTCTGCAAAACAGCGACTTGCCGGGTTTTAATCAGGAA
CAGCAGCTGATGATGGCGACACTGGTGCGCTATCACCGTAAAGCGATTAAACTCGACGATCTGCCGCGCTTTACCTTGTT
TAAGAAGAAACAGTTCCTGCCACTGATTCAGCTATTGCGCCTTGGCGTATTACTCAATAATCAACGTCAGGCAACCACCA
CACCGCCAACATTGACGCTGATTACTGATGACAGTCACTGGACACTGCGTTTCCCGCATGACTGGTTTAGTCAGAATGCG
CTGGTACTGCTTGATCTGGAAAAGGAGCAAGAATACTGGGAAGGCGTGGCTGGCTGGCGGTTGAAAATTGAAGAAGAAAC
TACACCAGAAATCGCCGCTTAA
>Cluster_43465
ATGGGTGCCAATGGTTTACTAGCAGTCTTCGTCATACTTATTGCTTGGTATACTCTCTTAACGGATGAGAGACGTGTTGA
TTTAAGGTTACGCATATCTAAATTTAATTTAGTATTCATTATCTTTTTTATATCAACAATATTAACAGTTATATATTCTA
AGGTTTTGCTAAGTGTTTTCCCGATAAAACCGATCCCTTGGATACTCGGTTTTAACGAAGATACATTGGCTTTTACTTGT
CTTTGTATCATAATTATATTTTTTGGAATTAAAGTACAGGGCAAAATACTACCAAAAGCAAACCTTACCTGTTGGATCAG
TGTTTCAGAAACATATCTGCGCGCAAAAAAAATAGAACAACTAGGATATCTTTTTGATAAATATCACGAGCAGCTATTCA
ATATAATCAGCAATAAAAAATGGTATGTTCGCGTACATAATTACTTAGCCCCTTCATTATCTCCCATTGAAATGGATGAA
GAGAAGGTAAAAAAATTACGATTCAAAAAAGTCAGGAGATTTATGTCAAAGTTTTTCCCTTATGAAGACAAAAGACAAAA
TGATATTCAATTAAATATATCCAATCTTCTGAAATCAAAGGTTTTTTCACATTATCTAATTGATACTTACCCTCACGTAG
CTATGAAGGCCACCTGCTTACAGCTTAGATATAATTGTGAGTATAATACAAATTTCTTTACGTATTTAATATCTAACCCA
AACAGCATTATGTATAGGGAGTTACGAGACAACCAAAATCGTTCATATACAGGGGAGTATGCACTCGACGAAAGTAATGC
CTTGCTTAATTTTTATTTAAATGATATTCGCATGGCGATAGATTTAGAAATATGGAAACCTGTGGGTAATTATGTCATTA
GCTACATAAAAAAACAAAAAGGGAGTTCTTGCTTTTACAATCATCCAGATAATTACTATTCAAGTAGTGATGAGCGTTGG
GAATGCCCAATCTTCGTTGGATTGACGTTTTTCGATGTTATGGTATCTACAGCAATATTCAAACGCTCAAAAAACAATAT
GTGGCTCATGTATTATCGTTGTTTCCTAAAGGAAATACTTGAGAGCTATGAAAAATCTAGCAGCATAGATGTAAATAGAG
AGTTCCCCATGCGATTTGATTATTTAATATATGAGCTAATCAGCCGTTGTAATATATGGGCAGGAGCAACAGAACACCTC
AATTATGATAACTGGACAACAGAAGATAAAGAGCAATCTCCAGAGTTCTTTGCCTCTAAAACCTTGGGGGAAATGATGTA
TCTGATTATTACTTCAGAAAAAATGCATAACAATCAAAAGACATATCTTTTAGAAATCATTATTAAAAGAATGGACTCTC
TTGATAAAAAGAAGAAATCTGCTTACTCCAAGGAAATCTTCAACAATCTTATCCGAGCATTCTCCCCTGCTTCCATTGAT
ATAAATGCAGTAAATAAATTACGGCAACTTTATAAAGGTGTTGTTCATGTTCTAAAAAACAAAAATTCAACTTTTGAAGT
TGAACTATCAAAATATCCTGACCAATAA
>Cluster_27190
ATGGTCAGCTCAACGACTCCGTCTTCCGGGGAATACTTGTTGGAAATGAGCGGTATCAACAAGTCTTTTCCTGGTGTTAA
GGCACTTGATAGCGTTAATTTAAAAGTCCGACCACATTCTATCCATGCATTAATGGGGGAAAACGGTGCAGGAAAATCGA
CATTATTAAAATGCCTGTTTGGTATTTATCAAAAAGACTCCGGCACCATTTTATTCCAGGGTAAAGAGATCGATTTCCAT
TCCGCCAAAGAAGCACTGGAAAATGGTATTTCGATGGTACACCAGGAGTTAAACCTGGTATTACAACGTTCGGTGATGGA
CAACATGTGGCTGGGGCGATATCCCACCAAAGGCATGTTTGTCGATCAGGACAAAATGTACCGCGAAACCAAAGCGATTT
TTGATGAACTGGATATTGATATCGATCCGCGTGCGCGCGTCGGTACATTATCTGTTTCGCAAATGCAGATGATCGAAATT
GCCAAAGCGTTTTCCTATAACGCGAAAATTGTGATTATGGATGAACCGACTTCTTCGTTAACCGAAAAAGAGGTCAATCA
TCTGTTCACTATTATTCGTAAATTAAAAGAGCGCGGCTGCGGTATTGTTTATATCTCGCATAAAATGGAAGAAATCTTCC
AGTTATGTGATGAAGTTACCGTATTGCGCGACGGTCAGTGGATCGCTACCGAACCGCTGGCAGGGCTGACGATGGACAAG
ATCATCGCCATGATGGTTGGGCGTTCTCTTAACCAGCGTTTCCCTGACAAAGAAAACAAGCCGGGCGAAGTGATTCTGGA
AGTGCGTAACCTGACGTCGCTGCGCCAGCCGTCGATTCGCGATGTCTCGTTTGATCTGCATAAAGGGGAGATCCTCGGTA
TTGCCGGTCTGGTGGGAGCGAAACGTACCGATATTGTTGAGACGTTATTTGGTATTCGCGAGAAATCGGCTGGCACCATT
ACGTTGCACGGCAAAAAGATCAATAACCATAATGCCAACGAAGCCATAAACCACGGATTTGCACTGGTAACTGAAGAGCG
CCGCTCAACGGGAATTTATGCCTATCTGGATATTGGCTTTAACTCGTTAATTTCCAATATTCGCAATTACAAAAACAAAG
TTGGCTTGCTGGATAATTCGCGGATGAAAAGCGATACCCAGTGGGTGATTGATTCGATGCGGGTAAAAACACCGGGGCAT
CGGACGCAAATTGGTTCGCTCTCCGGTGGTAATCAGCAAAAAGTGATTATTGGTCGCTGGCTGTTAACGCAACCAGAAAT
ATTAATGCTCGATGAACCGACGCGCGGTATTGATGTTGGGGCTAAGTTTGAGATTTATCAGTTAATTGCCGAACTGGCGA
AGAAAGGCAAGGGGATTATTATTATTTCCTCCGAAATGCCAGAGTTGTTAGGGATAACAGACCGTATTCTGGTCATGAGC
AATGGTCTCGTTTCCGGAATTGTCGATACAAAAACAACAACGCAAAGCGAAATTCTGCGTCTTGCGTCTTTGCACCTTTA
A
>Cluster_53510
ATGTCTGTAGATACCTTACGCGAAACATTTCATATAAACCGCATTAGCACTTCTGATTTGCAGGATTTCTTGACTGCGCA
AACCTACAGACCAGAAATTACGCAAGCAAAAAATCATATTCTTAGCTTGAAAAATTGCTCACTGCAGGCGGTATGCACAA
AACCAATTCAGCAAGGAAAGTCCCCTAAGTATGCGGAGAAGGGCTTAAAGTGCATTAAACCTAAAAATACTAATGACATG

TTAGTTTCGATTGATGACATTGATTGGATTGACTCGTCAAAAAAAGATCAAATTCAAAAACAGAAGCTCGGATATGGTGA
CATTGTCATTACACGTTCAGGTTCAGGAACTATTGGCCGGGCATCGATTTATTGCTATAGCGAAGAAGCGTATACGAATG
ACCATCTTTTTGTTGTTCGCCCTGACAAGGCTGATAGTCATTATATTTGCTCATTTTTAAATAGCTTCCATGGCCAACGC
TTATTGGAAGCAGGGGTATCCGGTTCTACCGGGCAATTAAATCTTTCTAATGAACACATTAAGTCTATTCCGCTGTTTAG
ACCTGATGATAAAGTACAAAAATACATCGGGGATAAAGTTCGCCAAGCCGAACAGTTGCGCGCTTGGGCGAAGCGCCTTC
GCACATCTGTTGACGATCATCTTAACAGTCTAAATCTGCCTATCAACGAGCCCCCCGCACTGTTAAACCGAGTCAGCGCG
CAAACAATGGAGGACCGACTCGATCCACGCCCTTATCGGACTCATTACCTATGTTTAGTGAGTGAAATTGAAAAGCTACC
TCATGACAGTATCGGCACTTTAGTTGCATTGGCATCAGGCTACCCAGTATCCAGTAACGACTTTTTGGAAAACTCAGGCA
TACCTTTAGTTCGCATCCGCAATATCGGTTTTGATGATTTTATTGGTTTAGATACTGGTGTTAGCCAAGACGTATATCAG
GATGCGACTAAGTATCAAGCTAAAGACAAAATGATCGTTGTCGGTATGGACGGTATTTTTCGCTCGCAGTTTTTCATATC
TGACGAGCTACCTATGCTAGTTAATCAACGGGTTGCAATGCTCTCACCTCAAAATATTCGTGGTGAACTTTTAACACATT
GGCTAAATCGCCCCGAAGGACAAATGCACTAAATCAATGGGCAGTTAAAACAACTGTAGAACATACATCTCTATCTGAT
ATTGGCCGTGTATTAATTCCTCGCCTAGATAAAAGCTTAGAAAATAAGCTTGCTGATTATTTGCTCAATGCGAGGCTGGC
TTATCGTTATGCCAAATTTTTAACACAAGTAGCCAAAACCCTGGTCGAAACCCTAATCGAAGGCCAACTGACCGAGCGGC
AATTGATCGAAGCCCAACAAGCCCTGGAAGATGGCGACAACAGCTTAGATCAAGCGATTTTAAGTAAACTAAGTGCTGAG
GGTTATGCCATAGAAGGCGCCACACCGCTATTTAGCGATGTGGATGAGTTATACAGTTTGCTGGAAGAGGCGGCGCAAGC
GGAGGCTGAAGAGTAA
>Cluster_53596
ATGAAACATTTAAATGATTTCCCCAAAAGAGATAGAAATCACAGAATTGAAAGACTTGCAATATCAGCTTTTGAACATTT
CCTTAGTGATTCGAATGATTTCTTTATTCAAAGTAAGGATATAAATGATTACGGCGTAGACTATCAACTCGAGATCACAA
CAAACGGTCAAGCAACTAACATTAGACTGTTTGTTCAACTAAAAGGTACTGAACAAGAGTTAAATAATGATGGTTCGGTT
AGCATATCTGTTAACAGAGCCAATCTCAACTATCTATTAATGCACCCTTATTCGTTCTATGTGTGTTTTCATGCGCCAAG
CGACACTCTAAAAATAACATTCGCGGACAGCGTGATTCGGAGTTATGAACAATCTGAAAAAAAATGGATTGAGCAAGAAA
CTATAACTGTAAGATTCATTGATAATATATCTGAGTCTCGTTTGCACGAAATTGCAGAGGTGGCTAGGCTTAATGCTTTA
GATAATCGTAATGCAAGGATAGAATTTATAACAAAGCCTATTTCAAATGTTAGTGCAGTTATAAATAAAACCATACCGAA
GATACATATACCAGAAGATAAAAAACTAGCCCATGAATTGCTTAATTCGTTATATGAAAATAATCATGATGATGTAATAA
GTGCTAATTTTGATGAATTCTTAGCTATTTTCGGTCCTGAACATCCGGCTATGATATTTCCATACATGTCAGAAATAAAT
AAAGCCATGGACAGAGTTAATGGTAATAAAAAAAGAGTATTGGATGGTATCCAATACCTCAGGAATAGAATTAATGAGGG
TTACATAACTAAGGGAAGTTTATACTATTCAATAGGTAATGGATTTACAGCTCTTGGCAAGGATGAAGAGGCTATTCAAG
AATATAATGCTGCTCTTAATAATATGACTGGGGAAGAAAAATTAGCTGCACAATGTTACAAAAACATGGGTAGTAGCTAC
TCAAATCTCGGAAATGAGAATGAAGCATTTGAATGCTATAAGAAAGCACTTGAATTAGATCCAAACTTAAACGAGGCTAA
TTTAGCTGTTGGAATAATCCATAATCATAGGGGTAATTATGAACTAGCATTAGAATATTTAGACATGGTGATGTTTCCAG
GAATTTATCAGCATAAAATAATACATGTTACAAAGTGGAGAGTGAATACTCTTTTTAATATGGGAGATTATCGCTCTGCA
TATAGAGAAATCAACAACCTATTAACCAGAAATAATAATGAAGAGGACTTATGGAAATGGTGCTTAATGCTAGTTGGAAC
ATTCTGTCGAACTTCGACTATTTCAGCCAAGCTAAGTTTGCCTTTTTTGAATCGTTTTTAGAAATGTACCCGAATCACT
CTGAAGCATCTAGAGAAATTCTATTAGTAAAAAATTACTTAAGAGTAAATGATAATGTAACTAAGTATTCTTATCTTGAT
TTTAAGAATGAATTTGAATTTAGAATAAATAATATCAGCAGTGAAGATAGGGCTTTCTTATGGGATCGTATAGGTCATTG
GGCACAGGATGAAGACGAGTGGGAAGATGCTGAGATGTATTTTCGTAAAGCTTATGAAATGGCAGGGGGTGAATATGGAT
ATTGTCTCGGTGTGGCTCTGAATCATCTTGGTCGCTTTAAAGAAAGCTTGCCCATTTTGATTGAACAAGCGGAAAAAATC
CAACCTGATGATTTTAGCTGGTTTCAAGTCGCTATTGCCTACGAATTTCAAGGGTATATTCAGGAGGCTATTAACTCTTA
CAAGAAATGTTTAGCAATCAATGAGAAAAACGAACTAGCATTGTTCAATCTTGGAGGCATTTACTGGAATTCGAGAAATT
ATGATGAAGCACGACATATCTGGAAACTAGCAGTAGAATATTATCCTGACCATGAGCTTTCGCGTAAGCTTCGAAAGGAT
ATTCCATTTATTTTAGATTGA
>Cluster_29166
ATGAGTCAAATTCACAAACACACCATTCCTGCCAACATCGCAGACCGTTGCCTGATAAACCCTCAGCAGTACGAGGCGAT
GTATCAACAATCTATTAACGCACCAGATACCTTCTGGGGCGAACAGGGAAAAATTCTCGACTGGATCACACCGTACCAGA
AGGTGAAAAACACCTCCTTTGCCCCCGGTAATGTGTCCATTAAATGGTACGAGGACGGCACGCTGAATCTGGCGGCAAAC
TGCCTTGACCGCCATCTGCAAGAAAACGGCGATCGTACCGCCATCATCTGGGAAGGCGACGACGCCAGCCAGAGCAAACA
TATCAGCTATAAAGAGCTGCACCGCGACGTCTGCCGCTTCGCCAATACCCTGCTTGAGCTGGGCATTAAAAAAGGTGATG
TGGTGGCGATTTATATGCCGATGGTGCCGGAAGCCGCGGTCGCGATGCTGGCCTGCGCCCGCATTGGCGCGGTGCATTCG
GTAATTTTCGGCGGCTTCTCGCCGGAAGCCGTTGCCGGACGCATTATCGATTCCAGTTCACGGTTGGTGATCACTTCCGA
CGAAGGTGTGCGTGCCGGGCGCAGCATTCCACTGAAGAAAAACGTCGATGACGCACTAAAAAACCCGAACGTCACCAGCG
TAGAGCATGTGGTGGTACTGAAGCGTACTGGCGGGAAAATTGACTGGCAGGAAGGGCGCGACCTGTGGTGGCACGACCTG
GTTGAGCAAGCGAGCGATCAGCACCAGGCGGAAGAGATGAACGCCGAAGACCCGCTGTTTATTCTCTATACCTCCGGTTC
TACCGGTAAGCCAAAAGGCGTGCTGCACACTACCGGCGGTTATCTGGTGTACGCGGCGCTGACCTTTAAATATGTCTTTG
ATTATCATCCGGGCGACATCTACTGGTGCACCGCCGATGTGGGCTGGGTTACCGGACACAGTTATTTGCTGTATGGCCCG
CTGGCCTGCGGCGCGACCACGCTGATGTTTGAAGGCGTACCGAACTGGCCGACGCCTGCCCGTATGGCGCAAGTGGTGGA
CAAGCATCAGGTCAATATTCTCTATACCGCGCCCACGGCGATTCGCGCGCTGATGGCGGAAGGCGATAAAGCGATCGAAG
GCACCGACCGTTCGTCGCTGCGCATTCTCGGTTCCGTGGGCGAGCCAATTAACCCGGAAGCGTGGGAGTGGTACTGGAAG
AAGATCGGCAACGAGAAATGCCCGGTGGTCGACACCTGGTGGCAGACCGAAACTGGCGGTTTCATGATCACGCCACTGCC
TGGCGCTACCGAGCTGAAAGCCGGTTCGGCAACACGTCCGTTCTTCGGCGTGCAACCAGCGCTGGTCGATAACGAAGGTA
ACCCGCTGGAGGGGGCCACCGAAGGCAGTCTGGTGATCACCGACTCCTGGCCGGGTCAGGCGCGTACGCTGTTTGGCGAT
CACGAACGTTTTGAGCAGACCTACTTCTCCACCTTCAAAAATATGTATTTCAGCGGCGACGGCGCGCGTCGCGATGAAGA
TGGCTATTACTGGATCACCGGGCGCGTGGACGACGTGCTGAACGTCTCCGGTCACCGCCTGGGGACGGCAGAGATTGAGT
CGGCGCTGGTGGCGCATCCGAAAATTGCCGAAGCCGCTGTGGTCGGTATTCCGCACAATATTAAAGGTCAGGCGATCTAC
GCCTACGTCACGCTTAATCACGGGGAGGAACCGTCACCAGAACTGTACGCAGAAGTCCGCAACTGGGTGCGTAAAGAGAT
TGGCCCGCTGGCGACGCCAGACGTGCTGCACTGGACCGACTCCCTGCCTAAAACCCGCTCCGGCAAAATTATGCGCCGTA
TTCTGCGCAAAATTGCGGCGGGCGATACCAGCAACCTGGGCGATACCTCGACGCTTGCCGATCCTGGCGTAGTCGAGAAG
CTGCTTGAAGAGAAGCAGGCCATCGCGATGCCATCGTAA
>Cluster_39390
ATGAACCTGACGACTCTGACCCACCGCGATGCGTTGTGTCTGAATGCGCGCTTTACCAGCCGTGAAGAGGCCATCCACGC
GTTGACTCAACGTCTTGCTGCTCTGGGGAAAATTTCCAGTACTGAGCAATTTCTGAAAGAAGTGTATCGCCGTGAAAGCC
TTGGCCCGACCGCCTTAGGTGAAGGGCTGGCTGTGCCGCATGGCAAAACTGCTGCGGTAAAAGAAGCGGCGTTTGCGGTC
GCCACACTCAGCGAGCCGCTTCAGTGGGAAGGCGTGGATGGCCCGGAAGCGGTTGATTTAGTGGTGTTGCTGGCGATTCC
CCCCAATGAAGCGGGTACTACGCACATGCAACTGCTGACAGCGCTGACCACGCGCCTGGCGGATGATGAGATACGGGCGC
GTATACAGTCGGCGACGACGCCTGATGAATTGCTCTCGGCGCTGGATGACAAGGGAGGCACGCAACCCTCAGCTTCTTTT
TCCAACGCGCCAACTATCGTCTGCGTAACGGCCTGCCCGGCGGGTATTGCTCACACATACATGGCTGCGGAGTATCTGGA
AAAAGCCGGACGCAAACTCGGCGTGAATGTCTACGTTGAAAAGCAAGGTGCTAACGGCATTGAAGGGCGTTTAACGGCAG
AGCAACTCAATAGTGCAACCGCCTGTATTTTTGCGGCTGAAGTCGCCATCAAGGAGAGTGAGCGTTTTAACGGCATTCCC

GCGCTTTCTGTGCCTGTTGCCGAGCCGATTCGCCATGCGGAAGCGTTGATCCAACAAGCACTGACCCTCGAGCGTAGCGG
TGAGACGCGTACCGTACAGCAAGATACGCAACCGGCGAAAAGCGTCAAAACGGAGCTGAAACAGGCGCTGTTAAGCGGGA
TCTCTTTCGCCGTGCCGTTGATTGTCGCGGGGGGTACGGTGCTGGCGGTCGCGGTATTACTGTCGCAAATCTTCGGGCTA
CAAGATTTGTTTAATGAAGAAAACTCCTGGCTGTGGATGTACCGCAAACTGGGCGGAGGGCTGCTCGGGATTTTGATGGT
ACCGGTGCTGGCAGCCTACACCGCCTATTCACTGGCTGATAAACCGGCGTTAGCGCCAGGCTTTGCGGCTGGACTTGCCG
CCAACATGATCGGTTCCGGGTTTCTCGGTGCTGTCGTTGGCGGATTGATAGCCGGTTATCTGATGCGCTGGGTGAAAAAT
CACTTGCGTCTTAGCAGTAAATTCAATGGATTCCTGACTTTTTATCTCTACCCGGTGCTCGGTACGTTGGGAGCGGGCAG
TCTGATGCTGTTTGTGGTGGGGGAACCTGTCGCCTGGATCAATAACTCGCTTACCGCCTGGCTGAACGGTCTGTCAGGAA
GTAACGCGCTGTTGCTGGGTGCCATTCTCGGTTTTATGTGTTCCTTTGACCTTGGAGGGCCAGTGAACAAAGCCGCTTAT
GCATTCTGCCTGGGCGCAATGGCGAACGGCGTTTACGGCCCGTATGCCATTTTCGCCTCCGTCAAAATGGTTTCGGCATT
TACCGTAACCGCTTCCACGATGCTCGCACCGCGCCTGTTTAAAGAGTTTGAAATTGAGACCGGGAAATCCACCTGGCTGT
TAGGGCTGGCTGGTATTACCGAAGGGGCGATCCCGATGGCGATTGAAGATCCGCTGCGGGTTATTGGTTCGTTTGTGCTG
GGCTCTATGGTAACGGGTGCTATCGTCGGCGCGATGAATATCGGCCTTTCGACACCCGGTGCCGGCATTTTCTCGCTCTT
TTTACTTCATGATAATGGCGCGGGCGGTGTTTTGGCGGCAATTGGCTGGTTTGGTGCGGCATTGGTGGGGGCTGCAATCT
CGACTGCAATTCTCCTGATCTGGCGGCGTCACGCGGTTAAGCATGGCAACTATCTGACTGATGGCGTAATGCCATAA
>Cluster_19902
ATGAGTGGCGCTTTTAACAACGATGGTCGGGGCATATCTCCCTTAATTGCAACCTCCTGGGAGAGATGCAATAAGCTGAT
GAAACGGGAGACATGGAACGTACCACACCAGGCTCAGGGCGTGACATTTGCTTCTATTTATCGGCGTAAGAAAGCGATGC
TGACGCTCGGGCAGGCTGCGCTGGAAGATGCCTGGGAATATATGGCACCGCGAGAGTGTGCGTTGCTTATCCTCGATGAA
ACCGCCTGCATTCTCAGCCGTAATGGCGATCCGCAAACCTTGCAGCAGCTAAGTGTACTGGGATTCAATGACGGCACGTA
TTGCGCCGAGGGAATTATTGGTACTTGTGCGCTATCGTTAGCGGCTATCTCTGGTCAGGCCGTGAAAACGATGGCCGATC
AACATTTCAAACAGGCACTCTGGAACTGGGCCTTTTGCGCAACGCCGTTGTTTGACAGCAAGGGCCGATTGACGGGAACA
ATAGCGCTGGCGTGTCCGGTTGAGCAAACTACCGCAGCTGATTTGCCGTTGACGTTGGCAATCGCACGCGAGGTCGGAAA
TTTACTGCTGACGGACAGTTTGCTCGCTGAAACTAACCGTCATTTAAATCAACTTAATGCCCTGTTAGAAAGTATGGATG
ATGGCGTGATTAGCTGGGACGAGCAGGGTAATTTGCAATTTATCAACGCCCAGGCGGCGCGAGTCTTGCGCCTTGACGCG
ACGGCAAGTCAGGGAAGGGCAATCACTGAACTCTTAACGTTACCCGCCGTATTGCAACAAGCAATAAAACAGTCACATCC
GCTCAAACACGTAGAAGCCACCTTTGAAAGTCAGCATCAGTTTATTGATGCGCTGATAACCCTTAAACCGATAATAGAAA
CGCAGGGAACCAGCTTTATTTTATTGCTCCATCCTGTGGAACAGATGCGGCAGTTAATGACCAGTCAATTAGGAAAAGTC
AGCCATACCTTCGCTCATATGCCACAGGACGATCCGCAAACCCGCCGCTTGATTCATTTTGGTCGCCAGGCGGCACGCAG
TAGCTTTCCTGTCCTGCTTTGTGGAGAAGAGGGCGTGGGCAAGGCACTGCTAAGTCAGGCAATTCATAATGAAAGCGAGC
GTGCAGCGGGGCCTTATATCGCCGTCAATTGTGAGTTATATGGTGATGCGGCGCTGGCGGAAGAATTTATTGGTGGCGAT
CGCACGGATAATGAAAATGGCCGTCTGAGTCGGCTGGAACTGGCGCACGGCGGCACGCTGTTTCTTGAAAAGATTGAATA
TCTGGCGGTGGAGTTACAGTCTGCTTTGCTTCAGGTTATCAAGCAGGGGGTTATCACGCGACTGGATGCGCGGCGTTTAA
TACCCATTGATGTCAAAGTGATTGCCACAACGACCGCGGACCTCGCAATGCTGGTGGAACAAAATCGTTTTAGTCGCCAG
CTGTATTACGCGCTGCATGCATTTGAAATTACCATCCCGCCACTGCGTATGCGGCGTGGCAGCATTCCTGCGCTGGTGAA
TAACAAATTACGCAGCCTTGAAAAACGCTTCTCTACGCGGCTGAAAATTGATGACGATGCCCTCGCTCGCCTGGTTTCCT
GTGCATGGCCTGGCAACGATTTTGAACTTTACAGCGTCATCGAGAATCTTGCTCTGAGTAGTGATAATGGACGCATTCGC
GTCAGTGATTTGCCGGAACATCTGTTTACCGAGCAGGCGACAGATGATGTCAGCGCCACCCGCCTTTCCACCAGTCTGTC
ATTTGCGGAAGTTGAAAAAGAGGCAATTATTAACGCAGCCCAGGTCACAGGCGGTCGCATTCAGGAAATGTCGGCTTTAC
TTGGAATCGGCCGCACCACGCTGTGGCGGAAAATGAAGCAACATGGCATTGATGTGAGTCAGTTTAAGCGCCGTGGATAG
>Cluster_21960
ATGCTACCAAAAGCCGCCCGCATTCCCCACGCCATGACGCTTCATGGCGATACGCGCATCGATAATTACTACTGGCTGCG
GGACGATACGCGTTCTCAGCCGGAAGTCCTGGACTACCTGCAGCAAGAAAATAGTTACGGTCATCGGGTGATGGCCTCGC
AACAAGCCTTGCAGGATCGCATATTAAAGGAAATCATCGACCGCATTCCGCAAAGAGAAGTTTCTGCTCCCTACATCAAA
AATGGCTACCGCTATCGGCATATTTATGAACCAGGCTGTGAATATGCTATCTACCAGCGTCAATCGGCATTCAGCGAAGA
GTGGGATGAGTGGGAAACATTGCTCGATGCCAACAAGCGCGCGGCGCACAGTGAGTTTTATTCGATGGGCGGAATGGCGA
TTACGCCCGATAACACCATTATGGCACTGGCAGAAGATTTTCTTTCCCGACGCCAGTACGGCATTCGTTTTCGTAATCTG
GAAACAGGTAACTGGTATCCGGAACTGCTGGATAACGTTGAACCCAGCTTTGTCTGGGCGAATGACTCCTGGACTTTCTA
CTATGTTCGCAAGCATCCGGTGACGCTGCTGCCTTATCAGGTCTGGCGTCACGCCATCGGTACGCCAGCATCGCAGGATA
AACTGATCTACGAAGAAAAAGACGATACCTATTACGTCAGCCTGCATAAAACGACCTCGAAGCACTATGTAGTCATTCAT
CTGGCCAGCGCCACCACCAGTGAAGTTCGCCTGCTGGACGCGGAAATGGCCGATGCCGAGCCGTTTGTTTTTCTGCCGCG
CCGCAAAGATCACGAATACAACCTTGATCACTACCAGCATCGTTTTTATCTGCGTTCCAACCGCCACGGCAAAAACTTTG
GCTTATACCGTACCCGTATGCGTGATGAGCAACAGTGGGAAGAGTTAATTCCGCCACGTGATAACATTATGCTGGAAGGG
TTTACGCTGTTTACCGACTGGCTGGTGGTTGAAGAGCGTCAGCGCGGGTTAACCAGTTTGCGGCAAATTAACCGCAAGAC
CCGGGAAGTCATTGGCATTGCCTTTGATGATCCGGCCTATGTGACCTGGATTGCCTACAATCCAGAACCTGAAACCGCGC
GATTGCGTTATGGTTATTCTTCCATGACCACACCAGATACTTTGTTTGAACTGGATATGGATACCGGTGAGCGTCGTGTA
TTAAAACAAACGAAGGTTCCTGGTTTTGATGCGGCGAATTACCGCAGTGAACACCTGTGGATAGTCGCCCGTGATGGCGT
CGAAGTTCCGGTTTCGCTGGTCTATCATCGCAAACATTTTCGCAAAGGACACAACCCGCTGCTGGTGTATGGCTACGGTT
CTTACGGCGCAAGTATTGATGCCGATTTCAGTTTTAGCCGCTTGAGTTTGTTGGATCGTGGATTTGTCTACGCCATTGTC
CATGTTCGTGGTGGTGGCGAGCTGGGGCAACAATGGTACGAAGACGGTAAGTTTCTGAAGAAGAAAAATACGTTTAATGA
TTATCTTGATGCCTGCGATGCATTGTTAAAACTGGGCTATGGATCACCTTCGCTCTGTTATGCGATGGGCGGGAGTGCGG
GGGGCATGTTGATGGGCGTTGCGATTAATCAACGCCCGGAATTATTCCACGGCGTTATCGCCCAGGTACCGTTTGTTGAT
GTTGTAACAACGATGCTTGATGAATCAATTCCTCTTACCACTGGTGAGTTTGAAGAGTGGGGGAATCCGCAGGATCCGCA
ATATTACGAGTACATGAAAAGCTACAGCCCGTATGACAACGTCACCGCACAGGCTTATCCGCATTTACTGGTAACGACCG
GTTTACACGATTCTCAGGTGCAATATTGGGAACCGGCAAAATGGGTGGCTAAATTGCGCGAGCTGAAAACCGATAACCAT
CTTTTATTGCTCTGTACCGACATGGACTCAGGCCATGGCGGTAAATCTGGCCGCTTTAAATCGTACGAAGGCGTAGCGAT
GGAATATGCTTTTCTGGTCGCGCTGGCGCAGGGAACATTACCCGCTACGCCTGAGGATTAA
>Cluster_24442
ATGAGTCAGCTTGATGAGGCCATCCTTGATGCACTGACACACGTTACTTTCCCAAAGGGTTTTGCACAGGCAGAACCCGC
ATGGGTTGTCACGGTAGACGGTGTTGATTACCCACTCTGGAAAACAGATGCCCTGGTAGTCGGCAGTGGTGCAGCCGGGC
TACGTGCAGCTGTGGAACTTAAACGCCGCCAGCAAAATGTGTTAATCGCCACCGCCGGGTTATATATGGGGACGTCGGCA
TGTTCTGGCTCTGATAAACAAACACTGTTTACCGCCGCTACCGCGGGCAACGGCGACAACTTCGCCAAACTGGCAGAGGC
ACTGGCGAGCGGTGGGGCGATGGATCACGACACCGCTTATGTCGAAGCGGTAGGTTCTCTGCACACTCTTGGCGGGTTGC
AATATCTTGGTCTGGAATTACCGGAAGATCGCTATGGCGCGATTCTTCGTTATCAAACCGACCATGACGAAGCCGGGCGT
GCAACCTCGTGTGGGCCGCGTACCTCAAGGTTAATGGTGAAAGTGCTGCTGGAAGAAGTACAGCGTCTTGCTATCCCAAT
GTTGACCAGTGCAACAGTGATTAAACTGCTGCATCAGCGTGACGAAAACGGTGAAGACCGTGTGGCGGGGCCAATCCTCG
CGACCGGTCATCGCGCCCATAACCCATGGGGGCTGGCAATTGTGACTGCACCCAATGTGGCACTGGCAACAGGAGGGCCT
GGCGAGCTTTATCGCGACAGTGTGTATCCACACAAATGTTTCGGCTCGCTGGGGCTGGCGCTGGAGGAAGGTCTGACGTT
AACGAATCTGACTGAAAGCCAGTTTGGTATAGGCACACCGCGCAGCACATTTCCGTGGAATTTGTCCGGCACCTATGTAC

AGGTGATCCCATATATCTATTCCGTGGATGCTGGGGGCAACGAGTATAACTTCCTCGCAGATTACTATCGCACCACCCAG
GAGTTGGCTTCAAACATCTTCCGTAAAGGCTATCAGTGGCCATTCCACGCCACGCGGGTAATGGATTTTGGCTCCAGCTT
GTTAGATATGGCGGTGGCGCAAGAGCAGCAATCAGGGCGTCAGGTATTTATGGATTTCAATCGCAATCCTGAACCTGTGC
CGGGTGACCTGCCGTTCTCATTAGATCGACTGGACGACGACGTTCGCACGTATCTGGAAAATAACGACGCTCTGGCACCA
TCGCCCATCGAACGCCTGCAACGAATGAATCCTCTGTCTATCTCGCTGTATAAGATGCACGGTTACGATCTCATCACGCA
GCCATTGCAGTTTGCCATGAATAATCAGCACATGAATGGCGGCATTGAAGTCGATATCTGGGGACAAACATCTCTGCCCG
GTTGTTTTGCGGTGGGGGAAGTTGCTGGCACACACGGCGTCACCCGACCTGGCGGCGCGGCATTGAATGCCGGGCAGGTT
TTTGCTGTTCGTCTGGCGCGCTTTATTGGTTGCACGCAAAAGCGGAATATTGATGGAGATATTGCACAGCTGGCAGCTCC
GGCACTGGCTTCTATAAGAGAGATCATTACTCAGGCGCACGATAACGGGACCGGGATGCCGTTGTCGGTTGTGAGAGAAA
AAATTCAGGCACGAATGTCTGACCATGCGGGATTTATTTGCCATGCCGATAAAGTCCGACGCGCCACTCGTGATGCCCTG
CTATTGAGTGAATTTGTGCAACGGCATGGATTGGCAATCAAACATGTGGGTGAAGTTGCCGAGCTGTTTATGTGGCGGCA
TATGGCGCTGACTTCTGCCGCGGTATTAACACAATTAACGCATTATATTGATGCTGGTGGTAGCCGTGGGGCGCGTA
TGGTTATAGATCCACAAGGCAAATGCCTACCACAAACTCGTCGCGGCGCAAAAGAAGAATGGCGTTTTCGCTCTGAACTC
GCTGAAGACAAAAATCACAAATTAACAATTCAATATTCGCAAGGTTCTTTTATTACCGAAGTAAAGTCTTTACGTATGCA
ACCGTGTATTAACGGTATTTACTTTGAAAAAAACTGGCCAGACTTTTTAAAGGGAGACATTTACACACAATAA
>Cluster_27360
GTGAAACACTACGCCATACAACCTGCCAACCTCGAATTTAATGCTGAGGGTACACCTGTTTCCCGAGATTTTGACGATGT
CTATTTTTCCAACGATAACGGACTTGAAGAGACGCGTTATGTTTTTCTCGGGGGTAACCATTTAGAGGCACGTTTTCCTG
AACATCCACATCCTCTGTTTGTGGTAGCAGAGAGTGGCTTCGGCACCGGATTAAACTTCCTGACGCTATGGCAGGCATTT
GATCAGTTTCGCGAAGCGCATCCACAGGCGCAATTACAACGCTTACATTTCATTAGTTTTGAGAAATTTCCCCTCGCCCG
TGCGGATTTAGCGCTGGCGCATCAACACTGGCCGGAGCTGGCCCCGTGGGCGGAACAGCTTCAGGCGCAATGGCCTTTGC
CCCTGCCCGGTTGCCATCGTTTATTGCTCGATGAAGGCCACGTGACGCTGGATTTATGGTTTGGCGATATAAACGAACTG
ACCAGCCAACTGGACGATTCGCTAAATCAAAAGTAGATGCCTGGTTTCTGGACGGCTTTGCGCCAGCGAAAAACCCGGA
TATGTGGACACAAAATCTGTTTAACGCCATGGCGCGACTGGCGCGTCCTGGCGGTACGCTGGCGACCTTTACGTCTGCCG
GTTTTGTCCGCCGCGGTTTGCAGGAAGCCGGATTCACTATGCAAAAACGTAAGGGCTTTGGGCGCAAACGGGAAATGCTT
TGTGGGGTGATGGAACAGACATTACCCCTCCCCTGCTCCACGCCGTGGTTTAACCGTACGGGCAGCAACAAACAGGAAGC
GGCAATTATTGGAGGCGGTATTGCCAGCGCGTTGTTGTCACTGGCGCTATTGCGTCGCGGCTGGCAGGTAACGCTTTATT
GCGCGGATGAGGCTCCCGCACTGGGTGCTTCCGGCAATCGCCAGGGGGCGCTGTATCCGCTTTTAAGTAAACACGATGAA
GCGCTAAACCGTTTTTCTCTAATGCGTTTACTTTTGCTCGTCGGTTTTACGATTTATTGCCCGTTAAATTTGATCATGA
CTGGTGCGGCGTCACGCAGTTAGGCTGGGATGAGAAAAGCCAGCATAAAATCGCACAGATGTTGTCGATGGATTTACCCG
CAGAGTTGGCTGTATCCGTTGAGGCTAATGCGGTTGAACAAATTACGGGCGTTGCGACAAATTGCAGCGGTATTACTTAT
CCGCAAGGTGGCTGGCTGTGTCCGGCGGAACTGACCCGTAATGTGCTGGAACTGGCGGAACAGAAAGGTTTGCAGATTCA
TTATCAATATCAGTTACAGGATCTATCCCGTAAGGATGACTGTTGGTTGTTGAATTTTGCAGGAGATCAGCAAGCAACAC
ACAGTTTAGTGGTACTGGCGAACGGGCATCAAATCAGCCGTTTCAGCCAAACGTCATCTCTGCCAGTATATTCGGTGGCC
GGTCAAGTCAGCCATATTCCGACAACGCCGGAACTGGCGGAGCTGAAGCAGGTGCTGTGCTATGACGGTTATCTCACGCC
ACAAAATCCGGCGAATCAGCACCATTGTATTGGTGCCAGTTATCTCGCGGCAGCGAAGAGACGGCGTACAGTGATGAAG
ATCAGCAGCAGAATCGCCAGCGGTTGATTGATTGTTTCCCGCAGGCACAGTGGGCAAAAGAGGTTGATGTCAGTGATAAA
GAGGCGCGCTGCGGTGTGCGTTGTGCCACTCGCGATCATCTGCCAATGGTAGGTAATGTTCCCGATTATGAGGCAACACT
CGTGGAATATGCGTCGCTGGCGGAGAAGAAAGATGAAGCGGTAAGCGCGCCGGTTTATGACGATCTCTTTATGTTTGCTG
CTTTAGGTTCGCGCGGTTTGTGTTCTGCCCCGCTGTGTGCCGAGATTTTGGCGGCACAGATGAGTGAAGAACCGATTCCG
ATGGATGCCAGCACGCTGGCGGCGTTAAACCCGAATCGGTTATGGGTGCGGAAGTTGTTGAAGGGTAAAGCGGTTAAGGC
GGGATAA
>Cluster_1577
GTGCCGGAACTGAACAACAGCGTCAGCAGGCCACCGACAGCCAGCATGAAGAATAAATCCTGGTTCAGGATGCAGGCGGG
TGGTCAGGGTGAGGCGGATATTTATATTTATGACGAGATTGGTTTCTGGGGAGTTACCGCGAAGCAGTTTGTCAGCGATA
TGAATGCCCTGGGTGATATCACCCACATTAATCTCCACATCAACTCACCGGGTGGCGATGTCTTTGAAGGCATCGCCATT
TTTAATGCCCTGAAAAATCACGGTGCGGCCATTACCGTGTATGTGGATGGCGTTGCCGCCTCGATGGCATCCCTGATTGC
GATGGCCGGTGACACGGTCATTATGCCGGAAAATGCCTTCATGATGATCCATAAACCCTGGGGGATCAGCGGTGGTGATG
CGGAGAAAATGCGCACTTATGCCGAACGTCTGGACAAACTTGAGTCGGTTATGGTGCCGGTATATGCGCAGAAAACCGGA
AAAACTACCGATGAAATTGCCGCCATGCTGGCGGATGAGACCTGGATGTCCGGTGCCGAGTGTCTGGCACACGGATTTGC
AGACCAGGTGACGCCAGCCGTTAAGGCAATGGCATGTATTCAGTCAAAACGTACAGAGGAATTTAAAAAGATGCCGGAAT
CCATCCGAAATATGATCACGCAGCCATACAACAGTGCCCCGCGTGATACCACAGTGACAATCCCTGCACCGGCGGTAACA
GAACCATCACCGGTACCGGCAGTGTCTGATGAGGCGACCATTCGCGCCCGCGTTATGGCAGAACAGAAAGCCCGCATGTC
AGGCATTAACGATCTGTTTGCCATGTTCGGCGGTCGCTATCAGACGCTTCAGGCACAGTGCGTGGCTGATCCTGACTGTT
CGCTGGAAATGGCCCGTGAACGTCTGCTGAATGAAATGGGCAAGGAGTCCTCGCCGACCAACAAAAATACACCGGCTCAT
ATTTATGCCGGAAACGGCAATTTTGTGGGGGACGGGATCCGCCAGGCGATGCTGGCCCGTGCCGGATTTGAAAATGTCGA
GAAGGATAACGCCTATAACGGGATGACCCTGCGTGAATGGGCTCGCATGTCACTGACGGAGCGCGGTATTGGGGTGGCCA
GTTATAACCCCATGCAGATGGTCGGGCTGGCGCTGACGCACAGCACCTCTGATTTTGGCAATATTCTGCTGGATGTGTCG
AACAAGGGGCTGATCCAGGGCTGGGAGGAATCAGAAGAAACCTTCCAGAAGTGGACCCGTAAGGGACGCCTGTCAGACTT
CAAAACAGCGTATCGCGTGGGGATGGGCGGTTTTGGTTCTCTGCGCCAGGTTCGTGAGGGGGCGGAGTATAAATACATCA
CCACCTCAGATCGCAAGGAGACCATTGCACTGGCCACTTACGGGGAGATTTTCTCCATCACCCGCCAGGCCATTATCAAT
GATGATCTGAATATGCTGGTGGACGTGCCGATGAAGATGGGGCGTGCGGCGAAGGCAACGATTGGTGACCTGGTTTACAA
GGTGCTGACGGATAACCCGAAACTGTCAGACGGTAAGGCGCTGTTCCATGCCGATCACAAAAATATTGCCACCGGTGGGA
TTTCCGTTTCCGGACTGGATGCGGCCCGTCAGATGATGCGCCTGCAGAAAGAAGGCGATCGCGCCCTGAATATCCGTCCG
GCCTTTATGCTGGTACCGGTGGCACTGGAGACGGTGGCGAACCAGACCATCAAATCGGCCAGTGTGAAAGGGGCGGATGC
AAACGCCGGTGTCATTAACCCCATCCAGAACTTTGCTGAGGTGATTGCAGAAGCGCGTCTTGATGCGGCAGATCCGAAAA
CCTGGTATCTGGCGGCGGCACAGGGCACTGACACCATTGAAGTGGCCTGGCTGGATGGTGTGGACACGCCATACATTGAT
CAGCAGGAAGGTTTCACCACTGACGGCATTGCCACAAAAATCCGTATTGATGCCGGAGTGGCACCACTTGACTGGCGCGG
GCTGGTGCGTTCGTCGGTGGCCTGA
>Cluster_12318
ATGAAAGACAATAATCCTGCGGATAACCTGGCCTGGCGCGTCATCTGGCGCCAGCTTATCTCCAGCGTTGGCAGTCAGGC
CAGGATGCTGCGACGCAGTATGCTGGCGCTGTTGCTGGCGGCATTCATGCAGGGGATCGCCTTTGCCTGTCTTTATCCGA
TCATTGATGCGCTGTTACGGGGAGACGCGCCGCAACTTCTTAACTGGGCTATGGCCTTCAGCGTCGCCGCAATTGTGACG
CTGGTGCTACGCTGGTATGGCCTGGGCTTTGAATACCGTGGTCATCTGGCGCAGGCTACCCATGAGTTGCGCCTGCGACT
TGGCGAGCAGTTACGCCGCGTGCCGCTGGAGAAGCTCCAGCGCGGCAGGGCGGGTGAAATGAACGCCTTGCTGCTGGGCA
GCGTGGATGAAAACCTCAATTATGTTATTGCGATAGCCAATATTTTGCTGCTCACCATTGTCACGCCGCTGACGGCGTCG
CTGGCGACATTGTGGATAGACTGGCGGCTGGGGCTGGTGATGTTGCTGATCTTCCCTCTGCTGGTGCCGTTTTATTACTG
GCGCCGCCCGGCGATGCGGCGACAAATGCAGACGCTGGGGGAAGCGCACCAGCGTCTGAGCGGCGATATCGTTGAATTTG
CTCAGGGGATGATGGTATTGCGCACCTGCGGCAGCGATGCCGATAAAAGCCGGGCGCTGCTGGCGCATTTCAATGCGCTG

GAAAACTTACAGACCCGCACTCACCGTCAGGGCGCTGGCGCAACGATGCTGATCGCCAGCGTCGTGGAGTTGGGCCTACA
GGTGGTGGTGTTATCCGGGATCGTCTGGGTAGTGACGGGCACTCTGAACCTCGCCTTTTTGATTGCCGCCGTCGCGATGA
TTATGCGCTTCGCAGAACCGATGGCGATGTTTATCAGCTACACCTCGGTTGTGGAACTGATCGCCAGCGCCCTGCAACGG
ATTGAGCAGTTTATGGCGATAGCACCGCTTCCCGTCGCAGAGCAAAGCGAGATGCCGGAACGTTACGATATCCGCTTTGA
CAACGTCAGCTATCGCTACGAAGAAGGCGACGGCTACGCGCTTAATCATGTTTCTTTGACGTTCCCGGCAGCCAGTATGA
GCGCGCTGGTGGGTGCCTCCGGCGCAGGCAAAACTACGGTCACCAAACTGTTAATGCGCTATGCCGATCCGCAGCAAGGG
CAGATTTCTATTGGCGGCGTCGATATTCGCCGCCTGACGCCGGAACAGCTCAATAGCCTGATTTCTGTTGTTTTCCAGGA
TGTCTGGCTGTTTGATGACACGCTGCTGGCGAATATCCGTATCGCGCGCCCACAGGCGACGCGGCAGGAGGTAGAAGAGG
CCGCCCGCGCGGCGCAGTGCCTTGAGTTTATTTCCCGTCTTCCGCAAGGCTGGCTGACGCCAATGGGAGAGATGGGCGGC
CAGCTATCGGGCGGCGAGCGCCAGCGGATTTCCATTGCCAGAGCGTTATTGAAAAACGCGCCGGTCGTCATTCTCGATGA
ACCGACTGCCGCGCTGGATATTGAAAGCGAGCTGGCGGTGCAAAAAGCGATCGATAACCTGGTTCACAACCGGACGGTGA
TTATCATCGCTCACCGTTTATCCACCATCGCCGGGGCCGGAAACATTCTGGTGATGGAAGAGGGACAGGTGGTTGAGCAG
GGTACTCATGCGCAATTGCTCTCACATCATGGACGTTATCAGGCGCTGTGGCAGGCGCAAATGGCCGCGCGCGTGTGGCG
CGACGACGGGGTTTCCGCGTCTGGAGAGTGGGTGCATGAGTGA
>Cluster_25281
ATGATCCACGCCTTTATTAAAAAAGGGTGTTTTCAGGATTCGGTCAGTTTAATGATTATTTCACGAAAACTCAGCGAATC
AGAAAATGTTGATGATGTTTCCGTAATGATGGGTACGCCCGCCAATAAAGCGTTATTAGATACTACAGGTTTCTGGCATG
ACGATTTTAATAACGCCACGCCGAACGATATTTGCGTGGCAATTCGTAGCGAAGCGGCGGATGCGGGGATCGCGCAGGCG
GTTATGCAGCAGCTTGAAGAGGCGCTAAAACAACTGGCGCAGGGTTCAGGCAGCAGCCAGGCGTTGACGCAGGTGCGTCG
CTGGGACAGTGCCAGTCAGAAATTACCCGATGCCAATCTGGCGCTGATTTCAGTGGCTGGTGAGTATGCGGCGGAGCTGG
CGAACCAGGCGCTGGATCGCAACCTCAACGTGATGATGTTCTCCGATAACGTCACGCTGGAAGATGAAATCCAACTTAAA
ACCCGCGCGCGGGAAAAAGGCTTGCTGGTGATGGGGCCAGACTGCGGTACGTCGATGATTGCCGCCACGCCGCTGGCTTT
TGCTAACGTGATGCCGGAAGGCAACATTGGCGTCATAGGCGCTTCCGGTACCGGGATTCAGGAGCTGTGCTCACAGATTG
CGCTGGCTGGGGAGGGAATTACTCACGCGATTGGTCTTGGCGGGCGCGATCTCAGCCGTGAAGTGGGCGGCATCAGTGCG
CTGACGGCGCTGGAAATGCTCAGTGCAGACGAGAAAAGCGAAGTGCTGGCATTTGTTTCAAAACCACCTGCCGAAGCTGT
GCGTCTGAAAATTGTTAATGCCATGAAAGCAACTGGCAAACCGACGGTGGCGCTGTTTTTAGGTTATACCCCGGCGGTGG
CCCGCGACGAGAATGTCTGGTTTGCCTCCTCGCTGGATGAGGCCGCTCGTCTGGCTTGTCTGCTTTCACGCGTCACTGCG
CGACGTAACGCGATAGCGCCTGTCAGCAGCGGATTTATTTGCGGTTTGTATACCGGCGGTACGCTGGCTGCTGAAGCGGC
GGGATTACTTGCCGGACACCTTGGCGTGGAAGCCGACGATACCCATCAACATGGCATGATGCTGGACGCCGACGGACATC
AGATCCTCGACCTGGGCGATGATTTTTACACCGTCGGGCGTCCCCATCCGATGATCGACCCGACCTTACGCAACCTGTTA
ATTGCCGATCTCGGCGCTAAACCGCAAGTGCGCGTGTTGCTGCTTGATGTCGTGATTGGCTTCGGTGCGACCGCCGATCC
TGCCGCCTCGCTGGTGAGCGCCTGGCAAAAAGCCTGTGCCGCGCGTTCAGACAATCAACCACTGTATGCCATTGCCACGG
TGACAGGCACTGAACGTGACCCGCAATGCCGCTCGCAGCAAATCGCCACGCTGGAAGATGCGGGGATCGCGGTAGTGAGT
TCGCTACCGGAAGCCACCTTGCTGGCTGCGGCGTTAATTCATCCGCTCTCGTCAGCCACACAGCAACACACACCGTCATT
ACTGGAAAACGTCGCCGTGATTAACATCGGATTACGCAGCTTTGCGCTGGAGCTACAAAGCGCCAGCAAACCGGTTGTGC
ATTACCAATGGTCGCCAGTCGCCGGCGGCAATAAAAAACTGGCTCGTTTATTAGAACGTTTGCAATAA
>Cluster_1914
ATGAATATAAAGACGCTTAACGTCAGCCTTTTGTCTTTTTCTATTATTACAGCATTGTTTCCATTGAACGCGATGGCAAC
AAAATTAACCATAGAGCAGCGCCTTGAATTGCTTGAAAATGAATTGTCGCAAAATAAACAAGAGCTGAAAGCAACACAGA
ATGAACTGGGAGTATATAAATCCCGACTTTCGACCTTACAAAAAAGCATCACGGAAAACAAATATAAATCAGCCTCGCTT
GCTGAAATATCAGCGACATCTCCCGTCGCTGATAACATCAAAAATGAAAACGGTGAACAGAACTCGTTTACCGCAGCGCA
TACTATAAATGGATCGCAGCAAGTTGCCGTTATTGAAAGTAAAGGCGACAAAACCACTATCGAAAGCGTGACCCTGAAAG
ATATCAGTAAATATATAAAGGATGATATTGGGTTCAGCTATCAGGGGTACTTCCGCTCAGGCTGGGGTACTGGTAATCAC
GGCTCACCACAAACTTATGCAGCGGGTTCTCTGGGACGTTTTGGTAATGAGATGAGCGGTTGGTTTGACCTGACCTTAAA
TCAGCGTGTTTATAATCAGGACGGTAAAACGGCAAATGCGGTCGTTACCTATGATGGCAACGTGGGTGAGCAGTATAACG
ATGCCTGGTTTGGTGACAGTGCCAATGAAAATATCATGCAGTTCAGCGATATTTACCTGACAACGCGAGGTTTTTTACCC
TTCGCACCAGAGGCAGACTTCTGGGTCGGCAAACATAAACTCCCGCAATATGAGATCCAGATGCTGGACTGGAAAACCTT
AACCACGGATGTCGCCGCGGGTGTGGGGATTGAAAACTGGGCACTTGGTGTAGGGCTGTTTGATATGTCCTTAAGCCGTG
ATGATGTCGATGTTTACTCTCGTGATTTTTCGCGTACCAGCCAGATGAATACCAATTCTGTGGATGTTCGTTATCGCAAT
ATCCCGTTATGGGATGATGCGACATTATCATTAATGGCTAAATATTCCGCACCTAATAAAACGGATCAACAACAAGATAA
CGAAAATGACAACAGTTATTTTGAAATGAAAGATAGCTGGATGCTGACTTCTGTTTTACGGCAAAACCTGCAACGCGATA
CGTTTAATGAATTTACGTTACAGGTTGCCAATAATTCCTATGCCAGCAGTTTTGCCAGTTTCTCAGATGCCAGTAATACG
ATGGCGCATGGTCGCTATTACTATGGTGATCATACCAATGGGATCGCCTGGCGTTTAATCTCTCAGGGCGAGATGTATCT
TACTGACAATATTATTATGGCTAACGCGCTTGTCTATTCTCACGGCGAAGATGTTTATAGTTATGAAAGTGGTGCTCATA
GTGATTTTGACAGTATTCGCACTGTAATAAGACCGGCCTGGATCTGGAATACATGGAATCAGACGGGGCTTGAATTAGGC
TGGTTTAAGCAACAGAACAAAACTCAGCAGGGTGTAACACTAAATGAATCGGCTTATAAAACGACACTCTGGCATGCATT
GAAAGTGGGTGAAAGCATTTTAGGTTCACGACCAGAAATTCGCTTCTATGGAACGTATATCAATATTCTGGATAACGAAT
TATCGAATTTTAAGTTTAATGAGAACAGCAAAGACGAGTTTATGGCCGGCATCCAGGCGGAAGTCTGGTGGTAA
>Cluster_28881
ATGGAATTTTCGTTTTCACCCAAACTTCTTGTTGTTGCTGTCGCCGCAGCTCTTCCTCTCATAGCCAGCGCAGCAGATAC
CCCGTCAACCGCCACCGCACGCAAAGGCTTTGCCGGATACGATCACCCAAACCAGTATCTGGTTAAACCGGCGACCACTA
TTGCCGACAACATGATGCCGGTGATGCAGCATCCTGCACAAGACAAAGAAACCCAGCAGAAACTGGCGGAACTTGAGAAA
AAAACCGGTAAGAAACCGAACGTGGTTGTTTTCTTGCTGGATGATGTGGGCTGGATGGACGTTGGCTTTAACGGCGGCGG
CGTGGCGGTGGGTAACCCTACACCAGATATCGACGCCGTTGCCAGCCAGGGGCTGATTTTAACTTCGGCGTATTCTCAAC
CGAGTTCTTCCCCAACCCGCGCCACGATTCTCACCGGACAATACTCCATCCACCACGGCATTCTGATGCCGCCAATGTAC
GGGCAACCGGGCGGTCTGCAAGGGTTAACCACGCTGCCGCAGTTGCTGCACGATCGGGGCTACGTCACCCAGGCTATCGG
GAAATGGCATATGGGGGAAAACAAAGAGTCGCAGCCGCAGAACGTTGGCTTTGATGATTTCCGCGGCTTTAACTCGGTGT
CCGATATGTATACCGAATGGCGTGATGTTCATGTCAACCCGGAAGTCGCCCTGAGCCCGGATCGTTCTGAATACATCCAA
AAGCTGCCCTTCAGCAAAGATGACGTTCATGCCGTGCGCGGCGGTGAACAGGAAGCCATTGCCGACATTACGCCGAAATA
TATGGAAGATCTGGATCAACGCTGGATGGAATATGGCGTTAAGTTCCTCGACAAGATGGCGAAGAGTGATAAGCCTTTCT
TCCTCTATTACGGCACTCGCGGCTGTCACTTCGATAACTACCCGAACGCCAAATATGCGGGTAGCTCTCCGGCGCGCACC
TCTTACGGCGACTGCATGGTGGAGATGAACGATATCTTCGCCAATCTGTATAAAGCACTGGAGAAAAACGGTCAGCTTGA
TAACACGCTGATTGTGTTCACCTCTGATAACGGTCCGGAAGCCGAAGTTCCGCCGCACGGTCGCACGCCGTTCCGCGGTG
CGAAAGGTTCTACATGGGAAGGCGGCGTTCGTGTGCCAACCTTCGTTTACTGGAAAGGCATGATCCAACCGCGTAAATCT
GACGGCATTGTCGATCTGGCGGATCTCTTCCCTACGGCGCTGGATCTGGCGGGGCATCCAGGGGCGAAAGTAGCGAATTT
AGTACCGAAAACCACCTTTATCGATGGTGTGGACCAGACATCTTTCTTCCTGGGAACAAATGGTCAGTCTAACCGTAAGG
CCGAGCATTACTTCCTCAACGGTAAACTTTCTGCTGTGCGTATGGATGAGTTCAAGTATCACGTCCTGATTCAGCAACCT
TACGCTTATACCCAGAGCGGATATCAGGGTGGATTCACCGGCACAGTAATGCAAACGGCGGGATCGTCGGTGTTTAACCT
GTACACCGATCCGCAGGAAAGCGACTCTATCGGCGTGCGCCATATCCCAATGGGTGTACCGCTACAGACCGAAATGCATG

CGTATATGGAGATCCTGAAAAAATATCCACCACGCGCGCAGATTAAATCTGATTAA
>Cluster_62971
ATGTGTTCAATTTTTGGCGTATTCGATATCAAAACAGACGCAGTTGAGCTGCGTAAGAAAGCACTCGAGCTGTCACGCCT
GATGCGTCATCGTGGCCCGGACTGGTCCGGTATTTATGCCAGCGATAACGCCATTCTCGCCCACGAACGTCTGTCAATTG
TTGACGTTAACGCGGGGGCGCAACCTCTCTACAACCAACAAAAAACTCATGTGCTGGCGGTAAACGGTGAAATCTACAAC
CACCAGGCACTGCGCGCCGAATATGGCGATCGTTATCAGTTCCAGACCGGATCTGACTGCGAAGTGATCCTCGCGCTGTA
TCAGGAAAAAGGGCCGGAATTTCTCGACGACTTGCAGGGCATGTTTGCCTTTGCCCTGTACGACAGCGAAAAAGATGCAT
ATCTGATTGGTCGCGACCATCTGGGGATCATCCCACTGTATATGGGCTATGACGAGCACGGTCAGCTGTATGTGGCCTCA
GAAATGAAAGCCCTGGTGCCAGTTTGCCGCACGATTAAAGAGTTCCCGGCGGGGAGCTATTTGTGGAGTCAGGACGGCGA
AATCCGTTCTTATTATCATCGCGACTGGTTCGACTTTGATGCAGTGAAAGATAACGTGACCGACAAAAACGAGCTGCGTC
AGGCACTGGAAGATTCCGTTAAAAGCCATCTGATGTCTGACGTTCCTTACGGTGTGCTGCTCTCGGGTGGTCTGGATTCC
TCAATTATTTCCGCGATCACCAAGAAATACGCCGCCCGTCGCGTGGAAGATCAGGAACGCTCTGAAGCCTGGTGGCCGCA
ATTACACTCCTTTGCTGTAGGTCTGCCGGGTTCACCGGATCTGAAAGCAGCCCAGGAAGTGGCAAACCATCTGGGCACGG
TGCATCACGAAATTCACTTCACTGTACAGGAAGGTCTGGATGCCATCCGCGACGTGATTTACCACATCGAAACCTATGAT
GTGACAACTATTCGCGCTTCAACACCGATGTATTTAATGTCGCGTAAGATCAAGGCAATGGGCATTAAAATGGTGCTGTC
CGGTGAAGGTTCTGATGAAGTGTTTGGCGGTTATCTTTACTTCCACAAAGCACCGAATGCCAAAGAACTGCATGAAGAGA
CGGTGCGTAAACTGCTGGCCCTGCATATGTATGACTGCGCGCGCGCCAACAAAGCGATGTCAGCCTGGGGCGTGGAAGCA
CGCGTTCCGTTCCTCGACAAAAAATTCCTTGATGTGGCGATGCGCATTAACCCGCAGGATAAAATGTGCGGTAACGGCAA
AATGGAAAAACACATCCTGCGTGAATGTTTTGAGTCATACCTGCCCGCAAGCGTGGCCTGGCGGCAGAAAGAGCAGTTCT
CCGATGGCGTCGGTTACAGTTGGATCGACACCCTGAAAGAAGTGGCGGCGCAGCAGGTTTCTGATCAGCAACTGGAAACT
GCCCGCTTCCGCTTCCCGTACAACACGCCAACCTCAAAAGAAGCGTATCTGTACCGGGAGATCTTTGAAGAACTGTTCCC
GCTTCCGAGCGCCGCTGAGTGCGTGCCTGGCGGTCCTTCCGTCGCGTGTTCTTCCGCTAAAGCGATTGAGTGGGATGAGG
CGTTCAAGAAAATGGACGATCCGTCTGGTCGTGCGGTTGGCGTTCACCAGTCGGCGTATAAGTAA
>Cluster_63856
ATGTCCGTTTTAATTTATGGTGCGCTGACTGATGGCGCAGGCATCCCCATGTCCGGATGCCACATTATTCTGAAATCCCG
GGTGAACACATCAGAAGTGGTGATGCGCACTGAAGCTGATGTGGTGACCGGAAATAACGGGGAGTATTCGTTTGAGGCCC
GGACCGGAAAATATCGCGTGTATCTGAAACAGGGCTGGCGCGACGAGTACTGTGTTGGCGACATTGCTGTATACGATGAC
GCAAAGCCCGGCACGCTGAACGACTTTCTGACCGCTCCCGATGAAGGCGACCTCAAACCCGACGTGGTGAAACGCTTTGA
GAGAATGGTGGCACAGGCGCAGCAGAGCGCCGAATCCGCAGCGGAAAGCGAACAACAGGCCGGGCAACATGTCGCTGATG
CGCAGAAGATAAAGGAAGACTGTCAGACGCTGGCGGATAACGTACAGCTGAACGCCACCGCGGTTGCCGAAGATAAACAA
CACGTTGAACATCTGGCTGCAGAAGTTGAGCAGAATGCTGGACAGATGCAACAGGGTGTGCAGAGTGTTACAGATGCAGT
AAAGCAGGCTCAACAGGCAGCAGATGATTCTGCATCCAGCGCAGAGGAATCAAAAAACAATGCGGATAACGCTGCCCGAA
GCGAACAGAGCGCAAAAAGTCACGCTGATAATGCAGCCCGAAGTGCGCAAAACGCGAAAAGTCACGCCGATAATGTTGCA
GGAAACACACTGCAGACCGCACAGGATGTGACGGCAACCGCAGCAGCACGTGATGATGCTGAGCGCTTTGCGGAGAACGC
CAGACAGGATGCAACGGCCACAGCCTGTGACAGAAAGGCCACTGCGGAAGATGTGAAAAGTGCGGGGGAAAGCGCGGCCT
CATCTGAACAGAGCGCACGGGTAGCCGCAGGTTATGCCCGCGCAGCTGAGCAGGCAAAAAACGACATTGATGTCCTGCTG
GCGAACACCCTGAAAACGTCCGGTAATCTGTCAGAGATTGCGGCAGCAGGTGAACAAGCACAACAGGAGTCACGTGATAA
TCTGGGGCTAAAAAGCGCGGCCACGATGGAACCACAGGCAGATATTTACGACTGCACAGACGGTCGTCTTGCTGTCCCCG
GCATGTCCGGATTCGGGAAAATATTTACTGTCAAAGACAGAGTGTATTTTCAGTCAGGGTCAATGTTCCTCTCGTGGGCA
AAAAACGCTCTCCCCGGACGTTATGCCGTGAGTACGAGGCCGGAAAACGGGGTCGTAATAGAGGGGGAACTATTTATTGG
TGTTGTGGACGTCATCTGGACGAGTGCCGATACCAGTGGTGAAGCAGAAAAACAACCTAAAAATATTATTTTCTACGGTG
TTAATGGTGGCGTTCATGTGAATTGGTACTCGCCCACTGGCAAACTGATGGAGTGGGAATCCCTGAAATACACGCCGTCG
TCCGTTGTCGCTACGCTGTCCGGGATTACCCCCGGAACGACATATGACGGATACGGTATTGGCAGTCTTGTACTGGCGGG
GTACAAGGGAACTTACAGCTCAGATTCAAATCGTCAGATAAATCGTGGTGACCTGTATCCCGGCTCACGTCTTATGATTA
TTGAGTTTAAGTGTGTCGGGATAACAGACTACCCCGCGATTCAACAAACCAGCGATATTTCTGTGTATGCAGGCGGTGCG
TTTTCGGGGACATACCGGGCGTTGTCGGGCTCACGGCTGGGGAGTGCGGGTTCTGATGGCATCATGATTGGTCTATTTAT
CCGGGTTTATTAG
>Cluster_55655
ATGAGACGTAGAGATTTGCTTAGAAGCATGGCATTATCACCTTTGGTTATAAGCATTCCTTCAAAAGGTAAAGAAGGTTT
TAGATCTCATCCTGAAAACCTTGGATGCCAAATAAATACAGACTCAGTATCACCTGAAATGTTTGGTGCAAAATCTGATG
GTGTTTTCAATAATAAAGAGGCTATTGAAAAAGCCCTACAATATGCTTATATTAATGGATTAAGTTGTACTTTTTTAAAA
GGCCAATATTACAGCGAAGATATTTCTATTGACTTCCCTATAAGAATTGTTTTACAAAACGGATGTTACTTAAATTTTGA
GCTTTCTGTCATTGGTGGGCTCTTTTATTCTGAAAGCGAATCTATAATAAACCAAGATTGGAAAGCTTTTTCTCGCGGAA
CTCGCTCTTTTTCTAATAAAAATTCTAAAATGAATAAAGGTGATATGGTTTCAGTGTCATTAGATTATCGGCATGGTGGT
AGTGCTCAAATTGGTAATGAAAATGGCATAGATATTCTGAATGTTATTGATATTAATGAAAATGATTTTATAGTTAAAAA
TGGAACTAGATTTCCATATAGTTACCCAATCATAAGTAAACTTAAAAGCGTTATTGCTTTTAAAGGCGAAATACCAAAAG
ATTCATATATAATTAATGGTGACTTCATAAATAAATTTAGCCCCGGAGATATTATTCGTATCGAAAATGTAGACGGTACA
GATAGTGTTGGTGGCGGTAAGTATTATTTCGAATATGTTGAAGTCAAAAGCGTTAATAACTCTCATATCATTTTGAGTGC
CCGAACTGTATATTCACACACTAATCCGTGGTTAATAAAGACTAATTTTATAAACCATGTTAATTTAACAGGTGAAGGGC
GCATAAAAAAACTTGTTTTAAGGAATATTAAAGACGTAAAGCTTTCTTCTTTATTTATTAACCGCCTTATTATATCTAGC
TGCTATGGAGTTAACGTTAGTAATTTAACTTTAACTGGTCTTGGCGAACCGTCTACAGTCAACATAACTTATTGTTTCGG
GAAAAGTATTTTCCAGAACCTACAAATTTCAAATTCTGAAAGTAACAGTGATAATTCAACCTTAAAAATTATGAGTTCAC
CTCAGATTATTTTATCGAATATTACTATAAGTGACTCAAATTCATCCTCAAAAAAACAAAGTAATTATTCACTTTTTGTC
GATTCTTTATACACACCATATTCCTGCTGGAACGATAATATAATTGTTAATAATGTCATTTGTGAGCGGCCAAGGTCACA
TTTCAAACGTGGAGTATGGTTTTATGGTCTACGACGTTCAGTCATTGACTCGATCGTCGGGGCTGATGTTTTTCTTCAGG
GGTCAGTTGACTGTTATTTTAAAAATTTCAATGTATCAAACTATATGTTAGAAATTAAGGATTTAGTTAGATGTGATGTG
ACTGCTAAATGTATGAGCGCAGTTACTAGAGGTGGTATAGACAATGTATTAAGGCTAGAATTTGATGGACGTTTAACCGG
AGATTTTTCGGACAAGAGTCATTACTGTAAATTTACTGCGGGAACATTAGATCCTGAAAAAGGGGTTAATTATTCACAAG
GAGATGGTAATTATCTTTATTTCAACTCTCCAACTTTAAATTCTACTGATAATAAAATAAATTTAAGCGTAGAGTTTCAA
AAAAACTTAATTATTGAACCAACTAGATTATCGTTGCTTAGACAAAGAGAGTCTTTGAAGATGGGTAAAGATGTTTCAAA
TATCTATATTAAGAATAACTTTCAAAAAATTTAA
>Cluster_39367
ATGGCTGCGCAAGGGTTCTTACTGATTGCCACGTTTTTACTGGTATTAATGGTGCTGGCGCGTCCCTTAGGTAGCGGGCT
GGCGCGGCTGATTAATGATATACCTCTTCCTGGTACGACGGGCGTTGAACGCGTACTCTTTAGCGCGCTTGGCGTCTCTG
ACCGCGAGATGAACTGGAAGCAGTATCTCTCAGCCATTCTTGGCCTGAATATACTGGGGCTGGCGGTGCTGTTTTTTATG
TTGCTCGGTCAGCACTATCTGCCGCTTAATCCACAGCAGTTGCCGGGGCTGTCGTGGGATCTGGCGCTCAATACCGCCGT
CAGCTTTGTCACGAATACCAACTGGCAATCTTATAGCGGTGAAACCACGTTGAGCTATTTCAGCCAGATGGCGGGCTTAA
CGGTGCAAAACTTTCTTTCTGCCGCCAGCGGGATTGCGGTGATTTTTGCGCTTATTCGCGCGTTTACCCGCCAGAGCATG
AACACGCTGGGTAATGCGTGGGTAGACCTGTTGCGGATTACGTTATGGGTGTTAACCCCCGTAGCATTGCTGATCGCACT

GTTTTTTATACAACAAGGTGTGCTGCAAAACTTTTTGCCTTATCAGGCTGTGACGACCATTGAAGGAGCGCAACAGCTAT
TACCAATGGGGCCAGTAGCTTCTCAGGAAGCGATCAAAATGCTCGGAACTAACGGCGGTGGCTTTTTAATGCCAACTCG
TCGCATCCGTTTGAAAACCCAACCGCACTGACCAACTTCGTGCAAATGCTGGCGATCTTCTTGATCCCAACAGCGCTGTG
CTTTGCCTTTGGTGAAGTGGCGGGCGATCGTCGCCAGGGGCGCATGTTGCTGTGGGCGATGTCAGTGATTTTTGTCATCT
GCGTAGGCGTAGTGATGTGGGCAGAAGTTCAGGGTAATCCTCATCTGCTGGCGCTCGGCGCGGACAGCAGCATCAATATG
GAAGGTAAAGAGAGCCGTTTCGGCGTGCTGGTCAGTAGCCTGTTTGCGGTCGTGACTACGGCGGCTTCCTGCGGCGCGGT
GATTGCGATGCATGATTCGTTTACCGCTCTTGGTGGCATGGTGCCGATGTGGTTGATGCAAATTGGTGAAGTGGTATTCG
GCGGCGTCGGTTCCGGCCTTTACGGCATGATGCTGTTTGTCCTGCTGGCGGTGTTTATTGCCGGGCTGATGATTGGTCGT
ACACCGGAATATCTGGGTAAAAAAATCGACGTACGCGAGATGAAACTGACTGCACTGGCGATTCTGGTCACCCCGACGCT
GGTGCTGATGGGCGCGGCGCTGGCAATGATGACCGACGCCGGACGTAGCGCCATGCTCAACCCTGGCCCGCATGGTTTTA
GCGAAGTGCTGTACGCCGTGTCATCCGCCGCTAACAACAACGGCAGCGCCTTTGCCGGATTAAGCGCCAACTCTCCATTC
TGGAACTGTTTACTGGCGTTGTGCATGTTTGTCGGGCGCTTCGGGGTGATTATCCCGGTGATGGCAATTGCCGGTTCGCT
GGTGAGTAAAAAGAGCCAGCCCGCCAGCTCCGGCACGCTGCCAACGCACGGCCCGCTGTTTGTTGGCCTGTTAATCGGCA
CCGTGTTGCTGGTTGGCGCACTGACCTTTATCCCTGCCCTGGCGCTTGGTCCGGTGGCGGAATATCTCTCCTGA
>Cluster_39632
ATGAATAAATCCCGTCAAAAAGAATTAACCCGCTGGTTAAAACAGCAAAGCGTCATCTCCCAACGTTGGCTGAATATTTC
TCGTCTGCTGGGCTTTGTGAGCGGCATATTGATCATTGCCCAGGCCTGGTTCATGGCGCGAATTCTGCAACATATGATTA
TGGAGAATATTCCCCGTGAAGCCCTGCTGCTTCCCTTTACGTTACTGTTTCTGACCTTTGTACTGCGCGCATGGGTGGTC
TGGTTACGCGAACGGGTGGGTTATCACGCCGGGCAGCATATCCGCTTTGCCATCCGCCGTCAGGTTCTCGACCGTCTGCA
ACAAGCAGGGCCAGCGTGGATTCAGGGTAAACCTGCGGGGAGCTGGGCGACGCTGGTACTCGAGCAAATTGACGATATGC
ATGATTACTATGCTCGCTACCTGCCGCAAATGGCGCTGGCAGTGTCGGTGCCGCTGCTGATTGTGGTGGCGATCTTCCCC
TCTAACTGGGCTGCGGCGCTCATTCTGCTGGGCACTGCACCGCTAATTCCGTTGTTTATGGCGCTGGTTGGAATGGGGGC
TGCCGATGCTAACCGACGTAACTTTCTCGCTCTTGCTCGCTTAAGTGGGCATTTCCTCGATCGCCTGCGCGGCATGGAAA
CATTGCGTATTTTTGGTCGTGGTGAAGCTGAAATTGAAAGTATTCGTTCTGCTTCGGAAGATTTCCGCCAACGGACAATG
GAAGTGCTACGGCTGGCGTTTTTATCCTCCGGCATTCTCGAATTTTTTACCTCGCTGTCGATTGCTCTGGTGGCGGTCTA
CTTTGGTTTTTCCTACCTCGGCGAGCTGGATTTTGGTCACTACGATACTGGCGTGACGCTGGCTGCGGGTTTTCTGGCCC
TGATCCTTGCGCCAGAGTTTTTCCAGCCATTACGCGATCTCGGTACGTTTTATCATGCTAAAGCCCAGGCTGTTGGTGCA
GCTGACAGTTTGAAAACGTTTATGGAAACCCCGCTCGCCCATCCGCAGCGCGGTGAGGCGGAATTAGCATCGACCGATCC
GGTGACCATTGAAGCCGAGGATCTGTTTATCACGTCGCCGGAAGGTAAAACGCTGGCCGGACCGCTGAATTTTACTTTGC
CAGCAGGCCAACGAGCAGTGTTGGTTGGTCGCAGCGGTTCAGGTAAAAGTTCACTGCTGAACGCGCTTTCTGGTTTTCTC
TCATATCAGGGATCGCTACGAATCAACGGGATAGAATTACGCGATTTATCACCGGAATCATGGCGTAAACATCTCTCCTG
GGTTGGGCAAAACCCACAATTACCGGCAGCAACATTACGGGATAACGTACTACTGGCGCGACCTGATGCCAGCGAACAAG
AGTTACAAACAGCGCTGGATAACGCCTGGGTCAGTGAGTTTCTACCGCTCCTGCCGCAAGGGGTTGATACGCCTGTTGGC
GACCAGGCTGCCCGCCTTTCCGTGGGGCAGGCGCAGCGCGTGGCGGTGGCCCGTGCGTTACTAAATCCCTGTTCGCTATT
ACTGTTGGATGAACCCGCTGCCAGCCTTGATGCTCACAGTGAACAGCGCGTAATGGAGGCGCTGAATGCCGCCTCTCTGC
GCCAGACAACGTTAATGGTCACCCACCAGTTAGAAGATCTTGCTGACTGGGATGTCATTTGGGTAATGCAGAATGGTCAG
ATTATTGAGCAAGGACGTTACGCGGAATTAAGTGTGGCTGGCGGCCCATTCGCCACATTACTGGCCCATCGTCAGGAGGA
GATTTAA
>Cluster_29664
ATGCAACCGTCCAGAAACTTTGATGATCTTAAATTCTCCTCTATTCACCGCCGCATTTTGCTGTGGGGAAGCGGTGGTCC
GTTTCTGGATGGTTATGTACTGGTAATGATTGGCGTGGCGCTGGAGCAACTGACTCCGGCGCTGAAACTGGACGCTGACT
GGATTGGCTTGCTGGGCGCGGGAACGCTCGCCGGGCTGTTCGTTGGCACATCGCTGTTTGGCTATATCTCCGATAAAGTC
GGACGGCGCAAAATGTTCCTCATTGATATCATCGCCATCGGCGTGATATCGGTGGCGACGATGTTTGTCTCATCCCCCGT
CGAACTGTTGGTGATGCGGGTACTTATCGGCATTGTTATCGGTGCGGATTATCCTATCGCCACCTCAATGATCACCGAGT
TCTCCAGTACCCGTCAGCGGGCGTTTTCCATCAGCTTTATCGCCGCGATGTGGTATGTCGGCGCGACCTGCGCCGATCTG
GTCGGTTACTGGCTTTATGATGTGGAAGGCGGCTGGCGCTGGATGCTGGGTAGCGCAGCGATTCCCTGTTTGTTGATTTT
GATTGGTCGATTCGAACTGCCTGAATCTCCCCGCTGGTTGTTACGCAAAGGGCGAGTAAAAGAGTGCGAAGAGATGATGA
TAAAACTGTTTGGCGAACCGGTGGCTTTCGAGGAAGAGCAGCCCGCAGCAAACCCGTTTTCGCGATCTGTTTAATCGCCGC
CATTTTCCTTTTGTTCTGTTTGTTGCCGCCATCTGGACCTGCCAGGTGATCCCCATGTTTGCCATTTACACCTTTGGCCC
GCAAATCGTTGGTTTGTTGGGATTGGGGGTTGGCAAAAACGCGGCGCTGGGGAACGTGGTGATTAGCCTGTTCTTTATGC
TTGGCTGTATTCCGCCGATGCTGTGGTTAAACACCGCCGGACGGCGTCCATTGTTGATTGGCAGTTTTGCCATGATGACG
CTGGCGCTGGCGGTTTTGGGGCTGATCCCGGATATGGGGATCTGGCTGGTAGTGATGGCATTTGCGGTGTATGCCTTTTT
CTCTGGCGGGCCGGGTAATTTGCAGTGGCTCTATCCTAATGAACTCTTCCCGACGGATATCCGCGCCTCTGCCGTGGGCG
TGATTATGTCCTTAAGCCGTATTGGCACCATTGTTTCGACCTGGGCACTGCCAATCTTTATCAATAATTACGGCATCAGT
AGCACCATGCTGATGGGGGCGGGTATCTCGCTATTTGGCTTGTTGATTTCCGTAGCGTTTGCCCCGGAGACTCGAGGGAT
GTCACTGGCGCAGACCAGCAATATGACGATCCGCGGGCAGAGAATGGGGTAA
>Cluster_25079
ATGCGTTACAATCAATTTTCACATTACAAACGCTCCGACACTTTAGCCGGGGAGTTTATGAGATACAAGCCCAATCAATA
CATAATCTGTGATTCTTACGGTAACTACTATTTAAGGATCACGCTGCCTGTGTATATGCAGCCCTTTTTTGAAGGAAAAA
GGACGTTTGTCAGAAGTCTACACACAAGTAATCTTCGTGTTGCACGTAGAAAGCGTGATCAGATTGCAGATGAATACCAT
TGTCTACGGGAGAGTGTCGCCCCTGTAAACAGCACAATAGAGAACACGCTGGAACTGTTACGCAGTAAGGCTAAATACGC
CAAAACAGCTACCAGAATGCAAGATACAGCGTCTTCGTGTCCGTCATTGCTTAAAATTCTTGAAATCTACCTGACAATTA
ACAGCACGAAGAAGAAGCCAGCCACTTTAGCTAAGGCAAGAAAAGCGGTAGAGATGTTTCTCTCCTACCGTAAAAAGCCT
GATATTGCATTGCAAGATGTAAGCCGCACCACTGTTACAGGCTGGATTGAACACATGCAAAAAACCCTTTCACAACAATC
AATTGCAAATTATATCAGCCCAATGGCCCAGCTATGGGAATTAGCTTCATCACGTTACCACGATGCGCCAGAAAGGGCGC
TCTCCCCCTGGCGAGGGCATAGGCTTGATGTGGCACAAAGTAGAGAGAGCTACGAGGCATTTTCTAACAAAGAGCTATTG
CAGGTGTTGCAAGTATTTTCCGGTGATTCAGCAGAAAACAAAGAAATGATGGCTTTGTGTCTTATCGGTTTATATACAGG
TATGCGGATCAATGAGATAGCAAGTCTCACAATAGACGATGTGAAAGAGATCGAAGGTGTGCTGTGTTTTGAAATCACAC
AGGGAAAGACGAAAGCTGCGGCACGTGTTGTGCCTGTACATAGCCTTATCACTCCGTTGGTGTTGTCGCTGCGTGAAAAG
CCTCACAATGGCTTTTTGTTCTATCACGCCAGCATTACAGAGCGTGCTGATGGTAAACGTTCCACGTGGCACACGCAGAG
ATTTACAAGAGCTAAACGAAAGGCTTTAGGGGAAAAGGGAACAGAAAGGAAGGTGTTTCATTCTCTGAGACACGGAGTAG
CACAGCTTCTTGATCGAAATCAAATTCCAGAAGACAGGATCGCCCTTCTCCTGGGCCATACACGCGGCAATACAGAAACA
TTCCGCACATATAGCAAGAATGCAGCTTCTCCGGTAGAGCTTAAAAACTATATTGAGCTTCTACGCTACCCTGAAATAGA
GAAAGGCTTATCAATCAATAAAAAATCAAATTTAAGGCGTAAAACAACACCATAG
>Cluster_45562
ATGGGATTAATGGCCTCATTCGAACGTGGAATGGAACGTTTTCTTGTTCCAGTTGCTATCAAGTTAAACTCACAAAAACA
TGTTGCAGCGGTGAGAGATGGATTCGTTTTTACGTTTCCAATTATCATGGCAAGTTCATTAATTATATTAATTAACTTTG
CCATATTATCGCCAGACGGTTTTATTGCCGGATTACTGCATCTGAATAGCATTTTCCCCAACCTTGAAAAGCACAAGCT
ATTTTTACTCCGGTAATGAATGGTTCTGTAAATATCATGTCAATTATGATTGCTTTCCTGGTCGCCAGGAATATGGCGAT

TAGCTATGAGCAAGATGATCTTTTATGCGGATTAACGGCAATAGGAGCATTTTTTATTGTTTATACGCCATATCAGATGA
TAGATGGGCAAGCATTCCTGACGACCAAATATCTCGGCGCGCAGGGGTTGTTTGTTGCTGTTATCGTTGCATTGATCACC
AGTGAAATATTTTGTCGCTTAGCTCGAAACCCAAAAATCACCATAACGATGCCGGCAGCTGTACCGCCTGCGGTAGCGCG
TTCATTTAAAGTTTTATTGCCAATATTTTTTGTCATGGTGTTCTTTTCCGCACTTAATTATTGCCTGACACTGATATCCC
CGGCAGGACTAAACGATCTTATTTACACATTAATCCAGACGCCGCTCAAACATATGGGAACGAATATCTTTGCGGTAATT
ATCCTGGGGGCTGTGGGTAATTTCCTGTGGGTGCTGGGGATCCACGGACCTAATACCACATCGGCAATTCGAGAAACCGT
TTTTTCTGAGGCTAATCTGGAGAATCTCTCCTGGGCCGCTCAACATGGCACTACCTGGGGCGCGCCATATCCGATTACCT
GGACTTCTATTAATGATGCATTCGCCAACTGCGGCGGTTCAGGTATGACGTTGGGGTTATTGTTGGCTATTTTTATCGCT
TCTAAGCGTGCGGAATACCGTGATCTGGCAAAAATGTCATTTATCCCCGGTATTTTCAATATCAATGAACCGATAATGTT
TGGCCTTCCTATTGTACTTAACCCCATCATGATGGTGCCGTTTATTATGGTTCCCATTGTTAACTGTGCCATTGGTTACT
TCTTTGTTTCGATGGAAATTATTCCACCGGTTGCTTATGCCGTGCCCTGGACTACGCCCGGACCTTTAATTGCTTTCCTC
GGAACCGGGGGGAACTGGCTGGCTTTACTGGTTGGTTTTTTATGTTTAGGTGTGGCGACAATGATCTATTTACCTTTTGT
TATTGCCGCCAACAAAGTCAATAACATGACAACTAACGGATAA
>Cluster_16412
ATGAACATTCAACCGATCGTAACATCCGGAATCACCACACAAAACAATCGACATCATCACGCAGAACAAACGTCCCCTAC
ACAAATACCGCAATCCGAATTACCTAATGGATGCGAAACGGGATTTGTTGTTCATATCCCAGAGGATATGCAGCGACATG
CACCGGAATGCGGTGAAACAACAGCTCTACTGAGCTTGATAAAAGATGAAGGTCTGCTCTCTGGGCTGGATAAATATCTT
GCACCTCATCTTGAAGAAGGCTCTGCAGGAAAAAAAGCATTGGATATGTTTGGTTTATTCAATGTCTCTCAGATGGCATT
AGAAATACCCAGCACCGTTCCGGGTATCTCTGGTAAATATGGTGTCCAGCTAAACATTGTAAAACCAGATATTCATCCTA
CATCAGGTAATTATTTTTTACAGATATTCCCTTTGCATGATGAAATAGGTATTAATTTTAAAGACCTTCCTGGTCCATTA
AAAAATGCATTAAGCAACAGCAATATACCAACCACTGTATCGACTGCTGCATCCACTATTGCATCAGCCACTACTTCGAC
GGTAACCACCGCGTCAAAAGACCCAATACCATGGTTTGGATTAACAGCTCAAGTAGTTCGTAATCATGGTGTGGAACTTC
CTATAGTCAAAACTGAAAATGGATGGAAGCTTGTTGGAGAAACTCCTCTTACTCCTGATGGCCCCAAAGCAAATTATACT
GAAGAGTGGGTGATCAGACCGGGAGAAGCAGATTTTAAATATGGTGCATCTCCACTACAGGCAACTCTAGGGCTGGAGTT
TGGCGCACATTTCAAGTGGGATTTAGATAACCCTAATACTAAATATGCCGTTCTTACCAATGCTGCCGCAAATGCGCTTG
GTGCTGTAGGGGGATTTGCAGTATCCAGATTTACTGGTACAGATCCAATGTTAAGTCCTCATATCGGTGCAATGGTTGGG
CAAGCAGCGGGGCATGCCATACAGTATAATACCCCCGGATTAAAGCCAGACACTATTTTATGGTGGGCAGGTACTACTCT
TGGACTGGCTGATTTAAACAAGGCCGAGTTTGGAGAGGCCAGATTCACTGACTATCCTCGTATATGGTGGCATGCAAGAG
AAGGTGCCATTTTCCCAAATAAAGCAGATATTGAACATGCCACAGGGGCTGATATACGCGCAATGGAAGAAGGTGTATCT
GTTGGACAACGGCATCCAAATCCAGAGGATGTGGTCATCAATATCGAAAGCAATAACTCACCACATCATAACCCATCAAA
TTATGTTGATACCGTTGATATAATCCAAGAAACAAGAGTCTAA
>Cluster_22238
ATGTTTTCAGAAGTCATGCGTTATATTCTCGACCTCGGCCCTACGGTGATGCTGCCGATTGTCATCATTATGTTTTCTAA
AATATTAGGCATGAAGGCAGGCGATTGCTTTAAAGCAGGTCTGCATATCGGGATTGGCTTTGTTGGCATTGGCCTTGTGA
TTGGCTTAATGCTGGATTCCATTGGCCCGGCGGCGAAAGCGATGGCGGAAAATTTCGACCTGAATCTGCATGTGGTCGAT
GTCGGCTGGCCGGGCTCTTCACCCATGACCTGGGCGTCGCAAATTGCGCTGGTGGCGATTCCGATTGCGATTCTGGTTAA
CGTGGCGATGTTACTGACCCGTATGACGCGGGTGGTAAATGTTGATATCTGGAATATCTGGCATATGACCTTCACCGGCG
CGTTGCTGCATCTGGCAACCGGTTCATGGATGATAGGGATGGCGGGTGTGGTAATTCACGCGGCGTTTGTTTATAAGCTC
GGCGACTGGTTTGCCCGCGATACCCGAAATTTCTTTGAGCTGGAAGGCATTGCTATTCCGCACGGTACGTCGGCGTATAT
GGGGCCGATTGCGGTGCTGGTCGATGCTATCATCGAGAAAATCCCAGGCGTTAACCGAATTAAATTTAGCGCCGATGATA
TTCAGCGCAAATTTGGCCCTTTTGGCGAACCGGTCACCGTGGGTTTTGTGATGGGGCTGATTATCGGCATCCTCGCGGGT
TACGATGTCAAAGGTGTATTGCAACTGGCGGTAAAAACGGCGGCAGTCATGCTGCTGATGCCACGGGTGATTAAACCCAT
CATGGATGGTTTAACGCCCATCGCTAAGCAGGCCCGTAGTCGTTTACAGGCGAAGTTCGGCGGTCAGGAGTTCCTGATTG
GCCTTGATCCGGCGTTGCTGCTGGGACATACGGCGGTGGTATCGGCAAGCCTGATTTTTATTCCACTCACCATTTTAATT
GCTGTTTGTGTGCCGGGTAATCAGGTGCTGCCGTTTGGCGACCTTGCCACCATCGGCTTCTTTGTGGCGATGGCGGTCGC
GGTGCATCGTGGAAATCTGTTCCGCACCTTAATCTCGGGTGTCATCATTATGAGCATCACCCTGTGGATCGCGACGCAAA
CTATTGGTTTGCACACCCAACTGGCGGCTAATGCTGGCGCGTTAAAAGCCGGGGGTATAGTGGCTTCAATGGATCAGGGC
GGTTCTCCCATTACCTGGTTACTGATTCAGGTTTTCTCCCCGCAAAATATTCCCGGCTTCATTATTATCGGCACAATTTA
TCTTACCGGTATTTTCATGACCTGGCGTAGAGCTCGAGGCTTTATTAAACAAGAGAAAGTCGTTCTTGCAGAATAA
>Cluster_23389
ATGTTCGGCATAATTATATCTGTCATCGTATTAATTACGATGGGCTATTTGATCCTGAAAAACTACAAACCTCAGGTGGT
GCTGGCTGCCGCAGGTATCTTCCTGATGATGTGCGGTGTCTGGTTAGGGTTCGGTGGTGTGCTCGATCCCGCCAAAAGCA
GCGGCTACTTGATCGTCGATATTTATAATGAAATCCTGCGCATGCTGTCCAACCGCATTGCTGGATTGGGGCTGTCGATT
ATGGCAGTGGGCGGTTATGCCCGCTATATGGAGCGCATAGGGGCAAGTCGCGCGATGGTGAGCTTGTTAAGCCGCCCGTT
AAAACTCATTCGCTCGCCGTATATTATTCTGTCGGCAACTTACGTCATCGGCCAAATCATGGCGCAGTTTATTACCAGCG
CCTCCGGTCTGGGTATGTTGCTGATGGTCACCTTATTTCCGACGCTGGTGAGTCTGGGAGTAAGTCGTCTCTCTGCGGTG
GCGGTTATCGCAACCACCATGTCCATTGAGTGGGGGATTCTGGAAACGAACTCCATTTTTGCTGCCCAGGTCGCAGGAAT
GAAAATTGCCACTTACTTCTTCCACTACCAGCTTCCGGTCGCCTCTTGCGTCATTATCTCGGTGGCGATCTCCCACTTTT
TCGTGCAACGCGCTTTCGACAAAAAAGATAAAAATATCAATCACGAACAGGCAGAGCAAAAAGCTCTCGATAATGTCCCG
CCGCTCTATTACGCCATTTTACCGGTGATGCCGTTAATCCTGATGCTCGGCTCGCTGTTCCTTGCCCACGTCGGGCTGAT
GCAGTCAGAACTGCATCTGGTGGTGGTGATGTTACTGAGTTTGACGGTGACGCTGTTTGTTGAGTTCTTCCGCAAGCATA
ACTTGCGCGAAACAATGGACGATGTGCAGGCGTTTTTTGACGGCATGGGTACGCAGTTTGCCAACGTGGTAACGCTGGTG
GTCGCGGGTGAAATATTTGCGAAAGGCTTAACGACGATTGGCACTGTCGATGCGGTTATCAGGGGGGCGGAGCATTCTGG
TCTGGGCGGTATTGGCGTGATGATTATTATGGCGCTGGTCATTGCCATTTGTGCCATTGTGATGGGCTCAGGCAATGCGC
CGTTTATGTCATTTGCCAGTCTTATTCCGAATATCGCAGCCGGACTACATGTACCAGCGGTTGTAATGATTATGCCGATG
CATTTTGCCACGACGCTAGCGCGCGCTGTTTCGCCGATTACTGCGGTGGTGGTCGTTACGTCAGGAATTGCAGGCGTTTC
GCCTTTTGCGGTAGTGAAGCGGACAGCGATCCCCATGGCAGTCGGTTTCGTGGTGAATATGATTGCCACTATCACGCTAT
TTTATTAA
>Cluster_41868
ATGAGTTCTCAATTTACGACGCCTGTTGTTACTGAAATGCAGGTCATCCCGGTGGCGGGTCATGACAGTATGCTGATGAA
TCTGAGTGGTGCACATGCACCGTTCTTCACGCGTAATATTGTGATTATCAAAGATAATTCCGGTCACACTGGCGTAGGGG
AAATTCCCGGCGGCGAGAAAATCCGTAAAACGCTGGAAGATGCGATTCCGCTGGTGGTGGGTAAAACGCTGGGTGAATAC
AAAAACGTTCTGACGCTGGTGCGTAATACTTTTGCCGATCGTGATGCCGGTGGGCGCGGTTTGCAGACATTTGATTTGCG
TACCACTATTCATGTAGTTACCGGGATAGAAGCGGCAATGCTGGATCTGCTGGGGCAGCATCTGGGGGTAAACGTGGCAT
CGCTGCTGGGCGATGGTCAACAGCGTAGCGAAGTCGAAATGCTCGGTTATCTGTTCTTCGTCGGTAATCGCAAAGCTACA
CCGCTGCCGTATCAAAGCCAGCCGGATGACTCATGCGACTGGTATCGCCTGCGTCATGAAGAAGCGATGACGCCGGATGC
GGTGGTGCGCCTGGCGGAAGCGGCGTATGAAAAATATGGCTTCAACGATTTCAAACTGAAAGGCGGTGTACTGGCCGGGG
AAGAAGAAGCCGAATCTATTGTGGCACTGGCGCAACGTTTCCCGCAGGCGCGTATTACGCTCGATCCTAACGGTGCCTGG
TCGCTGAACGAAGCGATTAAAATCGGTACATACCTGAAAGGTTCGCTGGCTTATGCAGAAGATCCGTGTGGTGCGGAGCA

AGGTTTCTCCGGGCGTGAAGTGATGGCAGAGTTCCGTCGCGCAACTGGCCTGCCGACCGCAACCAATATGATCGCCACCG
ACTGGCGGCAGATGGGCCATACGCTCTCGTTGCAATCCGTTGATATCCCGCTTGCGGATCCGCATTTCTGGACGATGCAA
GGTTCGGTACGTGTGGCGCAAATGTGTCATGAATTTGGCCTGACCTGGGGTTCACACTCCAACAACCACTTCGATATTTC
TCTGGCGATGTTTACCCATGTTGCCGCTGCCGCACCGGGTAAAATCACCGCGATTGATACGCACTGGATCTGGCAGGAAG
GTAATCAGCGCCTGACCAAAGAACCGTTAGAGATCAAAGGCGGTCTGGTTCAGGTGCCAGAAAAACCGGGGCTGGGTGTA
GAAATCGATATGGATCAAGTGATGAAAGCGCACGAGCTGTATCAGAAACACGGGCTTGGCGCGCGTGACGATGCGATGGG
AATGCAGTACCTGATCCCCGGCTGGACGTTCGATAACAAGCGTCCGTGCATGGTGCGTTAA
>Cluster_14703
ATGCATGTGCTTAACATTCTCTGGGTGGTATTCGGCATTGGTCTGATGCTGGTACTGAATTTGAAGTTCAAAATCAATTC
AATGGTGGCCTTGTTGGTGGCGGCGCTGTCCGTCGGGATGCTGGCGGGCATGGATTTGATGTCGCTGCTGCACACCATGA
AAGCGGGCTTCGGCAACACGCTGGGGGAACTGGCTATCATCGTGGTGTTCGGTGCGGTCATCGGTAAATTGATGGTCGAC
TCCGGCGCGGCTCACCAGATAGCGCATACGCTGCTGGCACGTCTCGGTCTGCGTTATGTACAGCTGTCGGTGATTATCAT
CGGCCTGATTTTCGGTCTGGCGATGTTCTATGAAGTGGCCTTTATCATGTTAGCACCGCTGGTTATTGTTATTGCCGCCG
AAGCTAAAATTCCGTTCCTGAAACTGGCGATCCCGGCAGTAGCAGCTGCCACTACCGCACATTCACTGTTCCCACCGCAG
CCGGGTCCGGTGGCGCTGGTGAATGCTTATGGCGCGGATATGGGGATGGTTTATATCTATGGCGTACTGGTGACGATCCC
AAGTGTCATCTGCGCAGGTCTGATCCTGCCGAAGTTCCTCGGCAATCTTGAGCGCCCAACGCCATCATTCCTGAAAGCAG
ATCAACCGGTAGATATGAATAATCTGCCCTCTTTCGGCGTTTCGATTCTGGTGCCGTTGATCCCGGCGATCATTATGATC
TCTACCACCATCGCCAATATCTGGCTGGTAAAAGATACCCCTGCGTGGGAAGTGGTTAACTTTATCGGTTCCTCGCCGAT
TGCAATGTTTATTGCGATGGTGGTTGCATTCGTACTCTTTGGCACCGCGCGCGGGCACGATATGCAGTGGGTAATGAACG
CTTTTGAAAGCGCGGTGAAAAGCATTGCAATGGTGATTCTGATCATCGGTGCAGGTGGCGTGCTGAAGCAGACCATCATC
GACACCGGCATCGGCGACACCATCGGCATGCTGATGTCCCACGGCAATATCTCGCCCTACATCATGGCATGGCTGATCAC
CGTACTGATTCGTCTGGCGACGGGTCAGGGTGTGGTTTCAGCGATGACCGCCGCCGGGATTATCAGTGCAGCAATCCTTG
ATCCAGCAACTGGTCAGCTGGTTGGCGTGAATCCGGCGCTGCTGGTACTGGCGACGGCTGCGGGTTCCAACACCCTCACC
CACATTAACGATGCCTCATTCTGGTTGTTCAAAGGTTACTTTGACCTGTCAGTAAAAGACACGTTGAAAACCTGGGGTCT
GCTGGAGCTGGTCAACTCCGTGGTTGGGCTGATTATTGTGCTGATTATTAGCATGGTAGCGTAA
>Cluster_54093
ATGCAAGTTTCAGTTGAAACCACTCAAGGCCTTGGCCGCCGTGTAACGATTACTATCGCTGCTGACAGCATCGAGACCGC
TGTTAAAAGCGAGCTGGTCAACGTTGCGAAAAAGTACGTATTGACGGCTTCCGCAAAGGCAAAGTGCCAATGAATATCG
TTGCTCAGCGTTATGGCGCGTCTGTACGCCAGGACGTTCTGGGTGACCTGATGAGCCGTAACTTCATTGACGCCATCATT
AAAGAAAAAATCAATCCGGCTGGCGCACCGACTTATGTTCCGGGCGAATACAAGCTGGGTGAAGACTTCACTTACTCTGT
AGAGTTTGAAGTTTATCCGGAAGTTGAACTGCAGGGTCTGGAAGCGATCGAAGTTGAAAAACCGATCGTTGAAGTGACCG
ACGCTGACGTTGACGGCATGCTGGATACTCTGCGTAAACAGCAGGCGACCTGGAAAGAAAAAGACGGCGCTGTTGAAGCA
GAAGACCGCGTAACCATCGACTTCACCGGTTCTGTAGACGGCGAAGAGTTCGAAGGCGGTAAAGCGTCTGATTTCGTACT
GGCGATGGGCCAGGGTCGTATGATCCCGGGCTTTGAAGACGGTATCAAAGGCCACAAAGCTGGCGAAGAGTTCACCATCG
ACGTGACCTTCCCGGAAGAATACCACGCAGAAAACCTGAAAGGTAAAGCAGCGAAATTCGCTATCAACCTGAAGAAAGTT
GAAGAGCGTGAACTGCCGGAACTGACTGCAGAATTCATCAAACGTTTCGGCGTTGAAGATGGTTCCGTAGAAGGTCTGCG
CGCTGAAGTGCGTAAAAACATGGAGCGCGAGCTGAAGAGCGCCATCCGTAACCGCGTTAAGTCTCAGGCGATCGAAGGTC
TGGTAAAAGCTAACGACATCGACGTACCGGCTGCGCTGATCGACAGCGAAATCGACGTTCTGCGTCGCCAGGCTGCACAG
CGTTTCGGTGGCAACGAAAAACAAGCTCTGGAACTGCCGCGCGAACTGTTCGAAGAACAGGCTAAACGCCGCGTAGTTGT
TGGCCTGCTGCTGGGCGAAGTTATCCGCACCAACGAGCTGAAAGCTGACGAAGAGCGCGTGAAAGGCCTGATCGAAGAGA
TGGCTTCTGCGTACGAAGATCCGAAAGAAGTTATCGAGTTCTACAGCAAAAACAAAGAACTGATGGACAACATGCGCAAT
GTTGCTCTGGAAGAACAGGCTGTTGAAGCTGTACTGGCGAAAGCGAAAGTGACTGAAAAAGAAACCACTTTCAACGAGCT
GATGAACCAGCAGGCGTAA
>Cluster_39588
ATGCAAAATAAATTAGCTTCCGGTGCCAGGCTTGGACGTCAGGCGTTACTTTTCCCTCTCTGTCTGGTGCTTTACGAATT
TTCAACCTATATCGGCAACGATATGATTCAACCCGGTATGTTGGCCGTGGTGGAACAATATCAGGCGGGCATTGATTGGG
TTCCTACTTCGATGACCGCGTATCTGGCGGGCGGATGTTTTTACAATGGCTCCTGGGGCCGTTGTCGGATCGTATTGGC
CGCCGTCCGGTGATGTTGGCGGGAGTGGTGTGGTTTATTGTCACCTGTCTGGCAATATTACTGGCGCAAAACATTGAACA
ATTCACCCTGTTGCGCTTCTTGCAGGGCATAAGCCTCTGTTTCATTGGCGCTGTGGGATACGCCGCAATTCAGGAATCCT
TCGAAGAGGCGGTTTGTATCAAGATCACCGCGCTGATGGCGAACGTGGCGCTGATTGCTCCGCTACTTGGTCCGCTGGTG
GGCGCGGCGTGGATCCATGTGCTGCCCTGGGAGGGGATGTTTGTTTGTTTGCCGCATTGGCAGCGATCTCCTTTTTCGG
TCTGCAACGAGCCATGCCTGAAACCGCCACGCGTATAGGCGAGAAACTGTCGCTGAAAGAACTCGGTCGTGACTATAAGC
TGGTGCTGAAGAACGGCCGCTTTGTGGCGGGGGCGCTGGCGCTGGGATTCGTTAGCCTGCCGTTGCTGGCGTGGATCGCC
CAGTCGCCGATTATCATCATTACCGGTGAGCAGTTGAGCAGCTATGAATATGGTTTGCTGCAAGTGCCTATTTTCGGGGC
GTTAATTGCGGGTAACTTGCTGTTAGCGCGTCTGACCTCGCGCCGCACCGTACGTTCGCTGATTATTATGGGCGGCTGGC
CGATTATGATTGGTCTGTTGGTCGCTGCTGCGGCAACGGTTATCTCATCGCACGCGTATTTATGGATGACCGCCGGGTTA
AGTATTTATGCTTTCGGCATTGGTCTGGCGAATGCGGGACTGGTGCGATTAACCCTGTTTGCCAGCGATATGAGTAAAGG
TACGGTTTCTGCGGCGATGGGAATGCTGCAAATGCTGATCTTTACCGTCGGTATTGAAATCAGCAAACATGCCTGGCTGA
ACGGGGGCAACGGACTGTTTAATCTCTTCAACCTTGTCAACGGCATTTTGTGGCTGTCGCTGATGGTTATCTTTTTAAAA
GATAAACAGATGGGAAATTCGCACGAAGGGTAA
>Cluster_25572
ATGAAATGTAGCTGGCGCGAAGGCAATAAGATCCAGTTGCTGGAAAACGGCGAGCAATATTATCCCGCGGTGTTTAAGGC
GATTGGCGAGGCGCAAGAACGCATCATTCTTGAAACGTTTATCTGGTTTGAGGATGACGTCGGCAAACAGCTGCATGCGG
CGCTACTGGCAGCAGCGCAACGCGGGGTGAAAGCGGAAGTCTTGCTGGATGGCTACGGTTCGCCGGATCTCAGCGATGAG
TTTGTCAATGAACTGACGGCAGCTGGCGTGGTATTTCGCTACTACGATCCCCGCCCGCGCCTTTTTGGTATGCGCACCAA
TGTGTTTCGCCGGATGCATCGCAAAATTGTGGTGATCGACGCGCTTATCGCCTTTATTGGCGGGCTGAATTACTCCGCCG
AGCATATGTCCAGCTACGGTCCAGAGGCCAAACAGGATTACGCGGTACGCCTTGAAGGGCCGATTGTTGAAGATATCCTC
CAGTTTGAACAGGAAAATCTGCCAGGACAGAGCGCGGCCCGACGCTGGTGGCGACGTCATCACAAAGCGGAAGAAAATCG
CCAGCCGGGAGAAGCGCAGGTATTGCTGGTCTGGCGCGATAACGAAGAACATCGCGATGATATCGAACGTCACTATCTGA
AAATGCTCACTCAGGCGCGGCGAGAAGTGATTATCGCCAACGCCTACTTCTTCCCCGGCTATCGATTTTTACACGCCTTG
CGTAAAGCGGCACGGCGCGGGGTGCGGATCAAACTGATCATTCAGGGCGAACCGGATATGCCGATTGTCAGAGTCGGTGC
GCGTTTGCTGTATAACTATCTGGTTAAAGGCGGCGTTCAGGTGTTTGAGTACCGCCGCCGTCCGCTACATGGCAAAGTGG
CATTGATGGACGATCACTGGGCGACAGTAGGGTCCAGTAATCTCGATCCGCTCAGTTTGTCACTGAATCTCGAAGCAAAT
GTCATCATCCACGATCGTCATTTTAACCAGACGCTGCGCGATAATCTGAACGGCATTATCGCCGCAGATTGTCAGCAGGT
CGATGAGACCATGCTGCCGAAACGCACCTGGTGGAACCTGACCAAAAGCGTGCTGGCGTTCCACTTTTTACGCCACTTCC
CGGCGCTGGTCGGCTGGCTTCCGGCGCACACGCCACGTCTGGCGCAGGTTGACCCGCCCGCACAACCGACAATGGAAACG
CAGGATCGGGTAGAAACTGAAAACACGGGGGTAAAACCCTGA
>Cluster_3131
ATGGCAAAAATCGCTAAGAAGCTCACTGACACTGAAATCAAAAGCACCAAGCCAGCCGATAAAGAAATCAACTTGTTTGA

CGGTGATGGTCTGATTCTACGAATCGCTCCTTTGGCGAAAGGAGGCAAGAAAAATTGGTATTTCAGGTATGCAGTACCAG
TGAGCAAGAAAAGAACCAAAATGAGCCTTGGGACATATCCTCACCTTACCCTTGCAAGAGCCAGAGCCTTACGTGATGAA
TATCTCTCCTTTCTGGCAAATGGTGTTGATCCCCAAATCCATAACAACGATAAGGCGAAGGCATTAAAGAGTGCTACTGA
GCACACTCTCCAAGCCGTAGCGCGGAAATGGTTAGATGAGAAGGTAAAGACATCAGGTATCTCACAAGACCATGCAGCAG
ACATCTGGCGCAGCTTAGAGAGAAATGTCTTTCCCGGTCTGGGTAATGTCCCTATCAATGAGATCCGACCTAAGCTCTTA
AAACAACACCTTGATCCTATTGAGCAACGAGGCGTATTGGAAACTCTACGCCGTATCATTTCACGTCTGAATGAAATCTT
CCGGTGGGCAGCTACTGAAGAACTTATTGAGTTCAACCCGGCTGACAACCTTGGTCAAAGATTCAGTAAACCAAAAAAGC
AAAATATGCCTGCCCTTCCCCCAAGCGAATTGCCAAGGTTTATGGAATCTTTGACGAATGCGTCAATCCGGTTGGAAACA
CGTATGCTAATTGAATGGCAATTGTTGACATGGGTTCGTCCGGGTGAAGCCGTTCGCGCAAGGTGGTCTGATATTGATAC
AACCAACAGCATTTGGAACATTCCTGCTGATTTCATGAAAATGAAAAAGCTTCACAAAGTTCCTTTGAGTAAAGAAGCTT
TGCGCATCCTTGAATTAATGAAATCAATAAGTGGGCATAGAGAATGGGTTTTCCCCAGCATAAAAGCGCCTCTTAATCAT
ATGCATGAACAAACAGCCAACGCAGCTATCATCCGAATGGGGTTCGGAGGCGAGCTTGTAGCTCACGGTATGCGTTCTAT
TGCACGAACAGCGGCAGAGGAGTCTGGTAAATTCAGAGCTGAAGTTCTTGAGGCAGCGCTTGCCCACTCGAAAAAAGATG
AAATTATCGCAGCATACAATCGTGCAGAATATCTGATAGAGCGACAGAGTTTGATGCAATGGTGGAGTGATTACGTTCAA
GCTCAAAGATCAAATGCTCTGGTAGCCTAA
>Cluster_42810
TTGTATCAGGACCGCGCCATGAAACAGATAACCTTTGCTCCCCGTAATCACCTGCTCACCAATACCAATACCTGGACGCC
CGACAGCCAGTGGCTGGTATTTGACGTGCGTCCTTCTGGGGCGTCGTTTACCGGCGAGACCATTGAGCGTGTGAATATCC
ATACCGGCGAGGTCGAGGTTATCTATCGCGCGTCACAGGGCGCACACGTCGGCGTGGTGACCGTTCATCCGAAGTCAGAG
AAGTATGTCTTCATCCACGGCCCGGAAAATCCTGATGAAACATGGTATTACGACTTTCATCATCGGCGCGGAGTGATTGT
TGAAAGCGGCAAGGTGAGCAATCTCGATGCAATGGATATTACCGCGCCATATACGCCAGGAGCGCTGCGCGGCGGCAGTC
ATGTACATGTCTTTAGCCCAAACGGTGAAAGGGTGAGCTTTACCTACAACGACCATGTAATGCATGAACTCGATCCGGCG
CTGGATTTGCGAAATGTCGGTGTTGCTGCACCGTTTGGCCCGGTCAACGTACAAAAGCAGCATCCGCGTGAATACAGCGG
TAGCCACTGGTGCGTGCTGGTAAGTAAAACCACGCCCACGCCACAGCCTGGCAGTGATGAAATCAATCGTGCTTATGAAG
AAGGATGGGTAGGAAATCACGCACTGGCGTTTATTGGCGATACGCTTTCGCAAAAAGGCGAGAAAGTGCCGGAGCTGTTT
ATTGTCGAGTTGCCGAAAGATGAAGCTGGCTGGAAAGCGGCAGGTGAAGTGCCGTTAAGTGGAACGGAAACAACCCTGCC
CGCGCCACCGCGTGGCGTCGTGCAGCGACGTTTAACCTTTACGCACCATCGTATTTACCCGGGCCTGGTTAACATTCCGC
GCCACTGGGTGCGTTGTAATCCTCAAGGAACACAAATTGCGTTTTTAATGCGTGATGATAACGGCATTGTGCAACTGTGG
CTTATCTCGCCACAGGGCGGTGAACCACGCCAGTTAACCCATAATAAAACGGATATTCAGTCTGCATTTAACTGGCATCC
ATCAGGCGAATGGCTGGGATTTGTGCTGGATAATCGGATTGCTTGTGCCCATGCGCAAAGCGGCGAGGTTGAGTATTTAA
CCGAAAACCACGCCAATCCACCTTCTGCGGACGCCGTGGTTTTCTCGCCGGATGGTCAATGGTTGGCGTGGATGGAGGAC
GGTCAGTTGTGGATCACCGAAACTGATCGCTAA
>Cluster_20092
ATGTCCGAAGATATCTTTGACGCCATCATCGTCGGTGCAGGGCTTGCCGGTTCGGTTGCCGCACTAGTGCTCGCCCCGCGA
AGGTGCGCAAGTGTTAGTTATCGAGCGAGGCAATTCCGCAGGTGCCAAGAACGTCACCGGCGGGCGTCTCTATGCCCACA
GTCTGGAACACATTATTCCTGGTTTCGCTGACTCCGCCCCCGTAGAACGCCTGATCACCCATGAAAAACTTGCGTTTATG
ACGGAAAAGTCAGTGATGACTATGGACTACTGCAATGGCGACGAAACCTCGCCATCCCAGCGTTCTTACTCCGTTTTGCG
CAGTAAATTTGATGCCTGGCTGATGGAGCAGGCCGAAGAAGCGGGCGCGCAGTTAATTACCGGGATCCGCGTCGATAACC
TCGTACAGCGCGATGGCAAAGTTGTCGGCGTAGAAGCTGATGGCGATGTGATTGAAGCGAAAACGGTGATCCTTGCTGAT
GGGGTGAATTCCATCCTTGCCGAAAAGCTGGGGATGGCAAAACGCGTTAAACCGACGGATGTGGCGGTTGGCGTAAAGGA
ACTGATCGAGTTACCGAAGTCGGTTATCGAAGACCGTTTTCAGTTGCAGGGTAATCAGGGCGCGGCTTGCCTGTTTGCGG
GATCACCCACCGATGGCCTGATGGGCGGTGGCTTCCTTTATACCAATGAAAATACCCTGTCGCTGGGGCTGGTTTGTGGT
TTGCATCATCTGCATGACGCAAAAAAATCGGTGCCGCAAATGCTGGAAGATTTCAAACAACATCCGGCCGTTGCACCGCT
GATTGCGGGCGGCAAGCTGGTGGAATATTCCGCTCACGTAGTGCCGGAAGCGGGCATCAACATGCTGCCGGAGCTGGTCG
GTGACGGCGTGCTGATTGCCGGTGATGCCGCCGGAATGTGTATGAACCTCGGTTTTACCATTCGCGGTATGGATCTGGCG
ATTGCCGCCGGGGAAGCAGCAGCAAAAACTGTGCTTTCAGCGATGAAAAGCGACGATTTCAGTAAGCAAAAACTGGCGGA
ATATCGTCAGCATCTTGAGAGTGGCCCGCTGCGCGATATGCGTATGTACCAGAAACTACCGGCGTTCCTTGATAACCCAC
GCATGTTTAGCGGCTACCCGGAACTGGCGGTGGGCGTGGCGCGTGACCTGTTCACCATTGACGGCAGTGCGCCGGAACTG
ATGCGCAAGAAAATCCTCCGCCACGGCAAGAAAGTGGGCTTCATCAATCTGATCAAGGATGGCATGAAAGGAGTGACCGT
TTTATGA
>Cluster_41878
ATGGAAACGACTCAAACCAGCACGATTGCGTCGAAAGACTCTCGTAGTGCCTGGCGCAAGACAGACACCATGTGGATGCT
GGGCCTTTACGGCACGGCAATCGGCGCGGGCGTGCTGTTCCTGCCAATCAACGCTGGTGTTGGCGGTATGATCCCGCTGA
TCATCATGGCTATCCTTGCGTTCCCGATGACGTTTTTTGCTCACCGCGGCCTGACTCGCTTCGTACTGTCTGGTAAAAAC
CCGGGCGAAGACATCACCGAGGTTGTAGAAGAACACTTTGGTATTGGCGCAGGTAAACTGATTACCCTGCTCTACTTCTT
CGCTATCTACCCGATCCTGCTGGTTTATAGCGTGGCAATCACCAATACAGTTGAAAGCTTCATGTCTCACCAGCTGGGTA
TGACGCCACCGCCGCGTGCGATTCTGTCGCTGATCCTGATCGTGGGTATGATGACCATCGTTCGCTTCGGTGAGCAGATG
ATCGTTAAAGCGATGAGCATTCTGGTATTCCCGTTTGTTGGCGTACTGATGCTGCTGGCTCTGTACCTGATCCCGCAGTG
GAACGGCGCAGCACTGGAAACGCTGTCTCTGGACACTGCATCTGCAACCGGAAACGGTCTGTGGATGACCCTGTGGCTGG
CAATTCCGGTAATGGTGTTCTCGTTCAACCACTCTCCGATCATCTCTTCTTTCGCCGTTGCGAAGCGTGAAGAGTACGGC
GATATGGCAGAACAGAAATGCTCGAAGATCCTGGCATTCGCACACATCATGATGGTGCTGACCGTAATGTTCTTCGTCTT
CAGCTGCGTACTGAGCCTGACTCCGGCAGACTTGGCTGCGGCTAAAGAGCAGAACATCTCGATTCTGTCTTACCTGGCTA
ACCACTTTAACGCGCCGATCATCGCGTGGATGGCTCCGATTATCGCGATTATCGCTATCACCAAATCCTTCCTCGGCCAC
TACCTGGGCGCACGTGAAGGCTTCAACGGTATGGTGATTAAATCTCTGCGTGGTAAAGGTAAGTCTATCGAAATCAACAA
GCTGAACCGTATCACTGCGCTGTTCATGCTGGTAACGACCTGGATTGTTGCCACCCTGAACCCGAGCATCCTGGGTATGA
TTGAAACCCTGGGCGGCCCAATCATCGCGATGATCCTGTTCCTGATGCCGATGTACGCAATTCAGAAAGTACCGGCAATG
CGTAAGTACAGCGGTCACATCAGCAACGTATTCGTTGTCGTGATGGGTCTGATTGCAATCTCCGCAATCTTCTACTCTCT
GTTCAGCTAA
>Cluster_63035
GTGTCAAAGGCAACCGAACAAAACGACAAGCTCAAGCGGGCGATAATTATTTCAGCAGTGCTGCATGTCATCTTATTTGC
GGCGCTGATCTGGAGTTCGTTCGATGAGAATATAGAAGCTTCAGCCGGAGGCGGCGGTGGTTCGTCCATCGACGCTGTCA
TGGTTGATTCAGGTGCGGTAGTTGAGCAGTACAAACGCATGCAAAGCCAGGAATCAAGCGCGAAGCGTTCTGATGAACAG
CGCAAGATGAAGGAACAGCAGGCTGCTGAAGAACTGCGTGAGAAACAAGCGGCTGAACAGGAACGCCTGAAGCAACTTGA
GAAAGAGCGGTTAGCGGCTCAGGAGCAGAAAAAGCAGGCTGAAGAAGCCGCAAAACAGGCCGAGTTAAAGCAGAAGCAAG
CTGAAGAGGCGGCAGCGAAAGCGGCGGCAGATGCTAAAGCGAAGGCCGAAGCAGATGCTAAAGCTGCGGAAGAAGCAGCG
AAGAAAGCGGCTGCAGACGCAAAGAAAAAAGCAGAAGCAGAAGCCGCCAAAGCCGCAGCCGAAGCGCAGAAAAAAGCCGA
GGCAGCCGCTGCGGCACTGAAGAAGAAAGCGGAAGCGGCAGAAGCAGCTGCAGCTGAAGCAAGAAAGAAAGCGGCAACTG
AAGCTGCTGAAAAAGCCAAAGCAGAAGCTGAGAAGAAAGCGGCTGCTGAAAAGGCTGCAGCTGATAAGAAAGCGGCAGCA
GAGAAAGCTGCAGCCGACAAAAAAGCAGCAGAAAAAGCGGCTGCTGAAAAGGCAGCAGCTGATAAGAAAGCAGCGGCAGA

AAAAGCCGCCGCAGACAAAAAAGCGGCAGCGGCAAAAGCTGCAGCTGAAAAAGCCGCTGCAGCAAAAGCGGCCGCAGAGG
CAGATGATATTTTCGGTGAGCTAAGCTCTGGTAAGAATGCACCGAAAACGGGGGGAGGGGCGAAAGGGAACAATGCTTCG
CCTGCCGGGAGTGGTAATACTAAAAACAATGGCGCATCAGGGGCCGATATCAATAACTATGCCGGGCAGATTAAATCTGC
TATCGAAAGTAAGTTCTATGACGCATCGTCCTATGCAGGCAAAACCTGTACGCTGCGCATAAAACTGGCACCCGATGGTA
TGTTACTGGATATCAAACCTGAAGGTGGCGATCCCGCACTTTGTCAGGCTGCGTTGGCAGCAGCTAAACTTGCGAAGATC
CCGAAACCACCAAGCCAGGCAGTATATGAAGTGTTCAAAAACGCGCCATTGGACTTCAAACCGTAA
>Cluster_18783
ATGAAAACCTCTCTGTTTAAAAGCCTTTACTTTCAGGTCCTGACAGCGATAGCCATTGGTATTCTCCTTGGCCATTTCTA
TCCTGAAATAGGCGAGCAAATGAAACCGCTTGGCGACGGCTTCGTTAAGCTCATTAAGATGATCATCGCTCCTGTCATCT
TTTGTACCGTCGTAACGGGCATTGCGGGCATGGAAAGCATGAAGGCGGTCGGTCGTACCGGCGCAGTCGCACTGCTTTAC
TTTGAAATTGTCAGTACTATCGCGCTGATTATTGGTCTTATCATCGTTAACGTCGTGCAGCCTGGTGCCGGAATGAACGT
CGATCCGACAACGCTTGATGCGAAAGCGGTAGCGGTTTACGCCGATCAGGCGAAAGACCAGGGCATTGTCGCCTTCATTA
TGGATGTCATCCCGGCGAGCGTCATTGGCGCATTTGCCAGCGGTAACATTCTGCAGGTACTGCTGTTTGCCGTACTGTTT
GGTTTTGCGCTCCACCGTCTGGGCAGCAAAGGCCAACTGATTTTTAACGTGATCGAAAGTTTCTCGCAGGTCATTTTCGG
CATCATCAATATGATCATGCGTCTGGCACCTATTGGTGCATTCGGGGCAATGGCGTTTACCATCGGTAAATACGGCGTCG
GCACACTGGTGCAACTGGGGCAGCTGATTATCTGTTTCTACATCACCTGTATCCTGTTTGTGGTGCTGGTATTGGGTTCA
ATCGCTAAAGCGACTGGCTTCAGTATCTTCAAATTTATCCGTTACATCCGTGAAGAACTGCTGATTGTACTGGGGACTTC
ATCTTCCGAGTCGGCGCTGCCGCGTATGCTCGACAAGATGGAGAAACTCGGCTGCCGCAAATCGGTGGTGGGGCTGGTCA
TCCCGACAGGCTACTCGTTTAACCTTGATGGCACATCGATATACCTGACAATGGCGGCGGTGTTTATCGCCCAGGCCACT
AACAGTCAGATGGATATCGTCCACCAAATCACGCTGTTAATCGTGTTGCTGCTTTCTTCTAAAGGGGCGGCAGGGGTAAC
GGGTAGTGGTTTTATCGTGCTGGCAGCAACGCTCTCTGCGGTGGGCCATTTGCCGGTAGCGGGTCTGGCGCTGATCCTCG
GTATCGACCGCTTTATGTCAGAAGCTCGTGCGCTGACTAACCTGGTCGGTAACGGCGTAGCAACCATTGTCGTTGCTAAG
TGGGTGAAAGAACTGGACCACAAAAAACTGGACGATGTGCTGAATAATCGTGCGCCGGATGGCAAAACGCACGAATTATC
CTCTTAA
>Cluster_39001
ATGTCGATCAAAACCAGTAATACAGATTTTAAGACACGCATCCGTCAGCAAATTGAAGATCCGATCATGCGCAAAGCGGT
GGCAAACGCGCAGCAGCGTATCGGGGCAAATCGGCAAAAAATGGTCGATGAATTGGGGCACTGGGAGGAGTGGCGCGATC
GGGCCGCCCAGATACGTGATCATGTTCTGAGTAATCTCGACGCTTATCTGTACCAGCTCTCAGAAAAAGTGACACAAAAC
GGCGGTCACGTCTATTTTGCAAAAACCAAAGAAGACGCCACCCGCTACATTTTACAGGTTGCCCAACGCAAAAATGCCCG
GAAGGTGGTGAAATCTAAATCAATGGTGACCGAAGAGATTGGTGTCAATCATGTGTTGCAGGATGCTGGGATTCAGGTGA
TTGAAACCGATCTGGGTGAATACATTCTCCAGCTGGATCAAGATCCGCCCTCTCATGTTGTGGTCCCGGCAATTCATAAA
GATCGCCATCAGATCCGTCGGGTGCTACACGAACGTCTGGGCTATGAGGGGTCGGAAACGCCTGAAGCAATGACCTTATT
CATCCGGCAAAAAATCCGCGAAGATTTCCTCAGTGCTGAAATAGGTATTACCGGCTGTAATTTCGCGGTGGCAGAGACCG
GTTCGGTATGCCTGGTGACCAATGAAGGTAATGCGCGAATGTGTACCACGCTGCCTAAAACGCATATTGCAGTGATGGGA
ATGGAGCGTATTGCCCCCACGTTTGCCGAGGTAGATGTATTGATCACCATGCTGGCGCGCAGTGCCGTTGGTGCACGTTT
GACGGGATACAACACCTGGCTGACAGGACCGCGCGAAGCGGGGCACGTTGATGGTCCTGAAGAGTTTCATCTGGTTATTG
TCGATAACGGGCGTTCTGAGGTGCTGGCCTCTGAATTTCGGGATGTGCTGCGCTGTATTCGCTGCGGGGCTTGTATGAAT
ACTTGTCCGGCATATCGCCATATTGGCGGTCATGGATATGGCTCTATTTATCCAGGGCCAATTGGTGCGGTGATTTCTCC
GCTACTTGGCGGCTATAAAGATTTTAAAGATTTACCCTACGCCTGCTCTTTATGCACTGCTTGTGACAACGTGTGTCCGG
TGCGTATTCCGCTGTCAAAACTGATTTTGCGTCATCGTCGGGTGATGGCTGAAAAAGGGATCACCGCAAAAGCAGAGCAA
CGGGCGATAAAAATGTTCGCTTATGCCAATAGTCATCCAGGATTGTGGAAAGTCGGGATGATGGCCGGCGCTCATGCGGC
AAGCTGGTTTATCAATGGCGGCAAAACACCACTCAAATTTGGCGCGATTAGCGACTGGATGGAAGCACGCGATCTTCCTG
AAGCTGACGGAGAGAGTTTCCGTAGTTGGTTTAAGAAACATCAGGCGCAGGAGAAAAAGAATGGATAA
>Cluster_24644
ATGCCAGATTTTTTCTCCTTTATTAACAGCGTCCTTTGGGGATCGGTAATGATTTACCTGCTCTTCGGCGCAGGTTGTTG
GTTCACTTTTCGCACCGGATTTGTGCAGTTTCGCTACATCCGCCAGTTTGGCAAAAGTCTTAAAAATAGCATTCATCCAC
AGCCAGGCGGTTTAACCTCATTTCAGTCATTGTGTACCAGTCTTGCGGCGCGCGTGGGTAGCGGCAACCTGGCTGGCGTT
GCGCTGGCTATTACCGCCGGTGGACCAGGTGCCGTCTTCTGGATGTGGGTTGCCGCGTTTATCGGCATGGCGACCTCGTT
TGCCGAATGTTCCCTTGCACAACTTTATAAAGAACGTGACGTTAATGGGCAGTTTCGTGGCGGACCGGCATGGTATATGG
CGCGCGGACTGGGGATGCGCTGGATGGGCGTTCTGTTCGCCGTCTTTTTGCTCATCGCCTACGGCATAATTTTCTGCGGA
GTTCAGGCGAACGCCGTTGCGCGAGCCCTGAGTTTTTCTTTTGATTTTCCCCCGCTGGTGACAGGTATTATTCTCGCTAT
CTTTGCTCTGCTGGCGATCACTCGCGGTCTTCATGGCGTCGCCCAGCTCATGCAGGGGTTTGTCCCGTTGATGGCGATAA
TCTGGGTACTGACCAGCCTGGTGATTTGCGTAATGAATATCGGGCAACTTCCCCACGTCATTTGGTCTATTTTTGAGAGT
GCTTTTGGCTGGCAGGAAGCTGCAGGCGGCGCGGCGGGATATACCTTAAGCCAGGCGATTACTAACGGTTTTCAGCGCAG
TATGTTTTCCAATGAGGCGGGAATGGGGTCGACGCCAAACGCGGCAGCGGCAGCTGCGTCCTGGCCTCCGCATCCGGCAG
CGCAAGGAATTGTCCAGATGATTGGCATTTTTATCGACACCCTGGTCATCTGCACGGCAAGCGCCATGCTGATATTACTG
GCGGGTAACGGCACAACCTACATGCCGCTGGAAGGTATTCAGCTTATCCAGAAGGCGATGCGGGTGCTAATGGGTTCCTG
GGGTGCGGAGTTTGTTACCCTCGTGGTTATTCTGTTTGCCTTCAGCTCCATCGTTGCCAACTACATTTATGCCGAAAACA
ATCTCTTCTTTTTACGCCTGAACAACCCTAAAGCGATCTGGTGTCTGCGGATCTGCACCTTCGCAACGGTCATCGGCGGC
ACCTTGCTAAGTTTTCCGCTGATGTGGCAACTGGCAGATATCATTATGGCCTGCATGGCTATTACCAATTTGACCGCCAT
ATTATTGCTCTCGCCTGTGGTTCATACCATTGCCAGTGATTATCTACGCCAGCGTAAACTCGGCGTGCGCCCGGTGTTTG
ATCCGTTGCGTTATCCGGAGATCGGTCGCCAGCTTTCTCCAGACGCGTGGGATGATGTTTCGCAGGAGTAA
>Cluster_29504
TTGATTCGTTTTATTATTTCGCTTCTGTTTTTGTTCTTTTCTTCGCTTTCTTTTGCTGAGGAGAAAGAAGATCCGGATAA
GCTTGTTCAGTGTTTTGGCCGTTTCCACTGCAGAACAGCTTATTCAGATGCGGGGGCATCGATTAAATTAGCCAGGGGAG
CTGGTCATATTTATTGTGGTTCCGGTAGTTACGATGCGTGCTCCGGAATAATTCATTATTGCGGTAATAGGCTATCAGAC
TCGTCTCAAGCGCCTTCCACCGTTGGGGTTTTTGTCCATGCCCGGTGGTTCGCATATAAAGTCTGGGATTTAGAGAAGCG
AGAGTGGAGTACCTGCACTCTTCAATATCAGGGTGGTGAGGGGGATTTGACACCTGAATATAATAAGCCGCCAACTGACG
AGATTTGTCTTTCTCGTCCGGTTCTTGATGGTATGACTTTTCCTGGTGTTTATAAAGGTGATGACGGGACCCGTTATGTT
AATGTGCAGGGGTGTATTTATGAGGCAACCGGCAATCCTTTTATTTGTAATCCCGATGATGATACGGTTTGTACTGCCAA
CTGGAAACCAGTTGCTGTAGATCCTGCATTCTCCGATGATAAGGAGGATGGCGGTAGCTCAGGTGGCGGCAGTTCCGGTG
GTGGTAACTCTGGCGGCGGTAGCTCAGGTGGTGGCAGTTCTGGCGGTGGTAGCTCAGGTGGCGGCAGTTCCGGTGGTGGT
AACTCAGGCGGTGGTAGCATAGGTGGTGGCAGTTCTGGCGGTGGTAGCTCAGGTGGCGGCAGTTCCGGTGGTGGTAACTC
AGGCGGTGGTAGCATAGGTGGTGGCAGTTCCGGTGGTGGTAACTCAGGCGGCGGCAGCTCAGGTGGTGGCAGTTCCGGTG
GTGGTAACTCAGGCGGCGGCAGCTCAGGTGGTGGTAGTTCCGGTGGCGGTAGCTCAGGCGGTGGCAGTTCCTCCGGCGAC
GGCTGGCTTGGTGATATTCTTGGCTGGTTGCAGCGTATTTGGGTTTCAGTGAATGATTTAAATAATGCTTTTTCTGTTTC
TCAGGATGATATTACCAGTGCGGTTAGTTCTTATGATTCACAGTTAAATGAATTAATTCATTCTGTAACGCCTGAACAGG
ATGATATGTCTTCATTTGCTGACAGGCTTTTCAGCCTTCTTCCTGATGAGTGGCTTTATATTAATTTTGATAAATTACTT
TTAACTGGTGAACTGCGTCGTTCTGGTGCTCAGCCAGTTCAGATTGAATTTCCGGGAATAAATGGGTTGTATTTTACAAT

TGACACATCTGATTTTGCTTCAGTTTATGATGACATTATCAGGCCTTTATTAGAGTATGCTCTTTATGCATTAACGACTT
TGCGTTGTTATTTTATTGTTCGTCGTGTTCTTTTTGTTGACAGGGGGTAA
>Cluster_509
ATGAGCCAGTCACTGTTTAGCCAACCGTTGAACGTTATTAACGTCGGTATCGCCATGTTTAGCGATGACCTGAAAAAGCA
GCATGTAGAAGTGACTCAACTCGACTGGACGCCGCCGGGGCAGGGCAATATGCAGGTGGTGCAGGCGCTGGATAACATTG
CCGATTCGCCGCTGGCAGACAAAATCGCCGCCGCTAACCAGCAGGCGCTGGAGCGCATTATCCAGTCGCATCCGGTGCTG
ATTGGTTTTGATCAGGCGATTAACGTGGTGCCGGGCATGACGCCGAAAACCATTCTTCACGCCGGGCCGCCGATCACCTG
GGAAAAAATGTGCGGCGCGATGAAAGGCGCGGTCACCGGGGCGTTGGTGTTCGAAGGGCTGGCGAAAGATCTCGATGAGG
CGACTGAACTGGCGGCCTCCGGGGAGATCACCTTCTCGCCGTGCCACGAACACGACTGCGTGGGATCGATGGCGGGCGTT
ACCTCGGCCTCGATGTTTATGCACATCGTGAAAAACAAAACCTACGGCAACATCGCTTATACCAATATGAGCGAGCAGAT
GGCGAAGATTTTGCGTATGGGTGCCAACGATCAGAGCGTGATCGACCGCCTCAACTGGATGCGCGATGTGCAGGGGCCAA
TGCTGCGCGACGCGATGAAAATTATCGGTGAGATCGATCTGCGCTTAATGCTGGCGCAGGCGCTGCATATGGGCGATGAG
TGCCATAACCGCAATAACGCCGGGACGACGCTGCTGATTCAGGCACTGACGCCGGGGATCATTCAGGCAGGCTATTCCGT
CGAGCAACAGCGCGAAGTGTTCGAGTTTGTCGCCAGCAGCGACTACTTCTCCGGCCCGACGTGGATGGCGATGTGTAAAG
CGGCGATGGATGCGGCGCACGGCATCGAATACAGCACCGTGGTCACCACCATGGCGCGTAACGGCGTCGAGTTCGGCCTG
CGGGTCAGCGGCCTGCCGGGGCAGTGGTTTACCGGCCCAGCGCAGCAGGTCATCGGCCCGATGTTTGCTGGTTATAAGCC
GGAAGATTCGGGGCTGGATATCGGCGACAGCGCCATCACAGAAACCTACGGTATTGGCGGGTTTGCCATGGCGACCGCGC
CTGCAATCGTCGCACTGGTGGGCGGCACGGTGGAAGAGGCTATCGATTTCTCCCGTCAGATGCGCGAAATCACCCTTGGC
GAAAACCCCAACGTCACCATTCCGCTGCTCGGTTTTATGGGCGTGCCGTCGGCAATCGACATCACCCGCGTGGGTAGCAG
CGGCATTCTGCCGGTGATCAACACCGCCATCGCCCATAAAGATGCGGGCGTCGGCATGATTGGCGCGGGCATTGTGCATC
CACCATTCGCCTGCTTCGAGAAAGCCATTCTCGGCTGGTGCGAACGTTACGGCGTCTGA
>Cluster_27019
TTGAATATCTCCTCTGCTTTACGCCAGGTTGTTCACGGCACTCGCTGGCACTCTAAACGCAAGAGCTACAAAGTGTTGTT
CTGGCGCGAGATAACCCCGCTTGCTGTTCCTATCTTCATGGAGAACGCCTGTGTCCTGTTGATGGGGGTCCTGAGCACTT
TTCTGGTCAGCTGGCTGGGAAAAGATGCGATGGCCGGCGTGGGGTTGGCGGACAGCTTCAATATGGTCATTATGGCTTTT
TTTGCAGCTATCGATCTTGGTACTACTGTCGTTGTAGCATTTAGTCTCGGTAAGCGAGATCGACGACGAGCGAGGGTGGC
GACGCGGCAGTCATTGGTGATCATGACGTTGTTTGCCGTACTGTTGGCAACGCTAATTCATCATTTTGGCGAACAAATTA
TTGATTTCGTCGCGGGTGATGCCACGACAGAAGTTAAAGCACTGGCGTTGACTTATCTGGAGCTGACCGTACTCAGTTAT
CCAGCAGCTGCCATCACTCTTATTGGTAGCGGGGCACTTCGTGGTGCAGGGAATACGAAAATACCGCTACTGATTAACGG
TAGCCTGAATATTCTTAATATTATTATTAGCGGCATATTGATTTACGGCCTTTTCTCCTGGCCGGGACTGGGATTTGTCG
GGGCTGGGTTGGGTTTAACCATTTCTCGTTATATTGGCGCAGTTGCAATTTTGTGGGTGCTGGCGATTGGTTTTAATCCT
GCGCTAAGGATTTCGTTAAAGAGCTATTTTAAACCGCTGAATTTTAGCATTATCTGGGAAGTCATGGGGATTGGTATTCC
AGCGAGTGTCGAATCAGTGTTATTTACCAGTGGTCGGTTATTAACCCAAATGTTCGTTGCCGGGATGGGGACCAGCGTTA
TTGCCGGAAATTTTATCGCGTTCTCAATTGCGGCTCTTATCAACTTACCCGGAAGTGCGCTCGGTTCTGCTTCTACGATC
ATTACAGGCCGAAGGTTGGGGGTAGGGCAGATAGCGCAAGCAGAGATTCAGTTGCGGCATGTGTTCTGGCTGTCCACTCT
TGGATTAACGGCCATCGCCTGGCTAACGGCTCCCTTTGCCGGGGTTATGGCATCGTTTTACACCCAGGATCCACAGGTTA
AACACGTCGTTGTGATTCTGATTTGGCTAAATGCTTTATTTATGCCTATTTGGTCCGCCTCATGGGTGCTACCCGCTGGA
TTTAAGGGTGCTCGTGATGCCCGTTACGCCATGTGGGTTTCGATGTTGAGCATGTGGGGTTGCCGGGTTGTAGTCGGTTA
TGTGCTGGGAATCATGCTTGGCTGGGGTGTGGTTGGTGTCTGGATGGGAATGTTTGCCGACTGGGCTGTGCGGGCCGTGC
TGTTTTACTGGCGAATGGTTACTGGACGTTGGCTGTGGAAATACCCTCGATCCGAGCCGCAAAAGTGTGAAAAAAAGCCA
GTTGTGTCGGAATAA
>Cluster_22650
ATGGTACCTGTGGTCGCGCTTGTCGGGCGCCCTAACGTAGGAAAATCCACGTTATTTAACCGTCTAACTCGCACCCGAGA
TGCGCTGGTTGCGGATTTCCCGGGTCTGACTCGTGACCGTAAGTACGGTCGTGCGGAAATTGAAGGCCGTGAGTTTATCT
GCATTGATACCGGCGGGATTGATGGCACAGAAGACGGTGTAGAAACCCGCATGGCGGAGCAGTCGCTGCTGGCGATTGAA
GAAGCGGACGTCGTACTGTTTATGGTGGATGCGCGCGCGGGCCTGATGCCGGCGGATGAAGCGATTGCTAAACATCTGCG
CTCCCGTGAAAAACCGACCTTCCTGGTGGCAAACAAAACTGACGGTCTGGATCCCGATCAGGCAGTGGTTGATTTCTACG
CGCTTGGTTTAGGTGAAATCTACCCGATCGCCGCGTCTCACGGTCGTGGCGTATTAAGTCTGCTGGAGCACGTGCTGCTG
CCGTGGATGGAAGATCTCGCACCGCAAGAAGAAGTCGACGAAGACGCCGAATACTGGGCGCAATTTGAAGCGGAAGAGAA
CGGCGAAGAAGAAGAGGAAGACGACTTCGACCCGCAAAGTCTGCCGATCAAACTGGCGATTGTGGGTCGCCCGAACGTAG
GTAAGTCTACACTCACTAACCGTATTCTTGGTGAAGAGCGCGTTGTTGTTTACGACATGCCTGGCACGACGCGTGACAGT
ATCTACATTCCAATGGAACGCGACGGACGTGAGTATGTGCTCATTGACACCGCTGGCGTACGTAAACGCGGCAAAATCAC
CGATGCGGTAGAGAAATTCTCGGTAATCAAAACGTTGCAGGCCATTGAAGATGCCAACGTGGTGATGTTGGTGATTGATG
CGCGTGAAGGTATTTCCGATCAGGATCTCTCCTTGCTGGGCTTTATTCTCAATAGTGGGCGCTCACTTGTGATTGTAGTG
AACAAGTGGGACGGCCTGAGCCAGGAAGTGAAAGAGCAGGTGAAAGAGACGCTGGACTTCCGTCTGGGCTTTATCGATTT
TGCTCGTGTGCACTTTATCTCTGCCTTGCACGGCAGTGGTGTTGGTAACTTGTTTGAATCAGTACGTGAAGCGTATGACA
GCTCCACCCGTCGTGTGGGGACCTCTATGCTGACGCGCATCATGACGATGGCTGTTGAAGATCACCAACCACCGCTGGTA
CGCGGTCGTCGTGTGAAGCTGAAATACGCCCACGCCGGGGGTTATAACCCGCCGATTGTGGTGATTCACGGCAATCAGGT
GAAAGACCTGCCTGATTCCTACAAGCGTTACCTGATGAACTACTTCCGCAAATCGCTGGACGTAATGGGATCGCCGATTC
GTATTCAGTTCAAAGAAGGGGAAAACCCGTATGCGAACAAGCGTAACACCCTGACGCCAACCCAGATGCGTAAACGTAAG
CGTCTGATGAAGCATATCAAGAAAAGTAAGTAG
>Cluster_23371
ATGAAGCAAGGTTTGCAACTCAGGCTTAGCCAACAACTGGCGATGACGCCACAGCTCCAACAGGCAATTCGTCTGTTGCA
GTTGTCGACGCTGGAACTTCAGCAGGAGCTACAGCAGGCGCTGGAGAGCAATCCGCTGCTTGAGCAAATCGACACTCATG
AAGAAATCGACACCCGCGAAACGCAAGACAGTGAAACACTGGACACCGCCGACGCGCTCGAACAAAAAGAGATGCCGGAA
GAGCTGCCGCTCGATGCCAGTTGGGACACCATTTACACCGCTGGTACACCATCCGGCACCAGCGGTGACTACATTGACGA
CGAGCTGCCGGTCTATCAGGGCGAAACGACACAGACCTTACAGGATTACCTGATGTGGCAGGTCGAGTTGACACCGTTTT
CCGACACTGACCGCGCTATTGCTACCTCTATCGTCGATGCCGTTGATGACACCGGTTATCTGACTGTCCCGCTGGAAGAT
ATTCTCGAAAGTATGGGCGATGAAGAGATTGACATCGACGAAGTTGAAGCCGTCCTTAAGCGGATCCAACGGTTTGATCC
GGTCGGTGTGGCGGCAAAAGATCTGCGTGACTGCCTGCTGATCCAACTCTCCCAATTCGATAAGACCACGCCATGGCTGG
AAGAGGCCAGACTGATCATTAGCGATCATCTCGATCTATTAGCCAATCACGACTTCCGCACTTTAATGCGCGTCACGCGT
CTGAAAGAAGATGTGCTGAAAGAAGCCGTCAATCTGATCCAGTCGCTCGATCCGCGCCCCGGGCAGTCGATCCAGACTGG
CGAACCTGAGTATGTCATTCCAGATGTGCTGGTGCGTAAGCATAACGGTCACTGGACGGTAGAACTCAACAGTGACAGCA
TTCCGCGTCTGCAAATCAACCAGCACTACGCCTCGATGTGCAATAACGCGCGCAACGATGGTGATAGCCAGTTTATCCGC
AGCAATCTGCAGGATGCCAAATGGTTGATCAAGAGTCTGGAAAGCCGTAACGATACGCTACTGCGCGTGAGTCGCTGTAT
CGTTGAACAGCAGCAAGCCTTCTTTGAGCAAGGCGAAGAATATATGAAACCGATGGTACTGGCTGATATCGCCCAGGCCG
TCGAGATGCATGAATCGACGATATCTCGCGTGACCACGCAAAAATACCTGCATAGTCCACGAGGCATTTTTGAACTGAAG
TATTTCTTTTCCAGTCACGTCAATACTGAAGGCGGGGGTGAAGCCTCTTCCACAGCGATTCGCGCACTGGTGAAGAAATT
AATCGCGGCGGAAAACCCAGCGAAACCGTTGAGCGACAGCAAGTTAACCTCTTTGCTGTCGGAACAAGGTATCATGGTGG

CACGCCGCACTGTTGCGAAGTACCGAGAGTCTTTATCCATTCCGCCGTCAAACCAGCGTAAACAGCTCGTTTGA
>Cluster_21196
ATGTTTAAGAATGCATTTGCTAACCTGCAAAAGGTCGGTAAATCGCTGATGCTGCCGGTATCCGTACTGCCTATCGCAGG
TATTCTGCTGGGCGTCGGTTCCGCGAATTTCAGCTGGCTGCCCGCCGTTGTATCGCATGTTATGGCAGAAGCAGGCGGTT
CCGTCTTTGCAAACATGCCACTGATTTTTGCGATCGGTGTCGCCCTCGGCTTTACCAATAACGATGGCGTATCCGCGCTG
GCCGCAGTTGTTGCCTATGGCATCATGGTTAAAACCATGGCCGTGGTTGCGCCACTGGTACTGCATTTACCTGCTGAAGA
AATCGCCTCTAAACACCTGGCGGATACTGGCGTACTCGGAGGGATTATCTCCGGTGCGATTGCAGCGTATATGTTTAACC
GTTTCTACCGTATTAAGCTGCCTGAGTATCTTGGCTTCTTTGCCGGTAAACGCTTTGTGCCGATCATTTCTGGCCTGGCT
GCCATCTTTACTGGCGTTGTGCTGTCCTTCATTTGGCCGCCGATTGGTTCTGCAATCCAGACCTTCTCTCAGTGGGCTGC
TTATCAGAATCCGGTAGTTGCGTTTGGCATTTACGGTTTCATCGAACGTTGCCTGGTACCGTTTGGTCTGCACCACATCT
GGAACGTACCTTTCCAAATGCAGATTGGTGAATACACCAACGCAGCAGGTCAGGTTTTCCACGGCGACATTCCGCGTTAT
ATGGCGGGTGACCCGACTGCGGGTAAACTGTCTGGTGGCTTCCTGTTCAAAATGTACGGTCTGCCAGCTGCCGCAATTGC
TATCTGGCACTCTGCTAAACCAGAAAACCGCGCGAAAGTGGGCGGTATTATGATCTCCGCGGCGCTGACCTCGTTCCTGA
CCGGTATCACCGAGCCGATCGAGTTCTCCTTCATGTTCGTTGCGCCGATCCTGTACATCATCCACGCGATTCTGGCAGGC
CTGGCATTCCCAATCTGTATTCTTCTGGGGATGCGTGACGGTACGTCGTTCTCGCACGGTCTGATCGACTTCATCGTTCT
GTCTGGTAACAGCAGCAAACTGTGGCTGTTCCCGATCGTCGGTATCGGTTATGCCATTGTTTACTACACCATCTTCCGCG
TGCTGATTAAAGCACTGGATCTGAAAACGCCGGGTCGTGAAGACGCGACTGAAGATGCAAAAGCGACAGGTACCAGCGAA
ATGGCACCGGCTCTGGTTGCTGCATTTGGTGGTAAAGAAAACATTACTAACCTCGACGCATGTATTACCCGTCTGCGCGT
CAGCGTTGCTGATGTGTCTAAAGTGGATCAGGCTGGCCTGAAGAAACTGGGCGCAGCGGGCGTAGTGGTTGCTGGTTCTG
GTGTTCAGGCGATTTTCGGTACTAAATCCGATAACCTGAAAACCGAGATGGATGAGTACATCCGTAACCACTAA
>Cluster_7305
ATGAAGTTCTGGCGACCCGGTATTACCGGCAAACTGTTTCTGGCGATTTTCGCCACCTGCATTGTCTTGCTGATCAGTAT
GCACTGGGCGGTGCGTATCAGTTTTGAGCGTGGCTTTATTGATTACATCAAGCATGGTAATGAACAGCGATTACAACTGT
TAAGTGATGCGCTTGGCGAGCAGTATGCGCAGCATGGCAACTGGCGCTTCCTGCGCAACAATGATCGCTTTGTCTTTCAG
ATCCTGCGTTCATTTGAACACGATAATTCGGAAGATAAACCCGGCCCGGGTATGCCACCGCACGGCTGGCGTACCCAGTT
CTGGGTGGTTGATCAAAACAACAAAGTGCTGGTTGGTCCGCGAGCGCCGATTCCACCTGACGGTACACGGCGACCCATTC
TGGTCAACGGTGCGGAAGTTGGCGCGGTGATCGCCTCCCCCGTTGAGCGGTTAACGCGCAATACTGATATCAATTTCGAT
AAACAACAGCGGCAAACCAGCTGGTTGATTGTCGCCCTGGCAACGTTACTCGCGGCACTTGCCACTTTTCTGCTGGCGCG
CGGTTTACTGGCACCGGTAAAACGACTTGTCGATGGCACGCACAAACTGGCGGCGGGCGATTTCACTACCCGCGTAACGC
CCACCAGTGAAGATGAACTGGGCAAACTGGCGCAAGACTTCAACCAGCTTGCCAGCACACTGGAGAAAAACCAGCAAATG
CGGCGCGATTTTATGGCCGATATTTCTCACGAACTGCGTACGCCATTAGCGGTGCTGCGCGGTGAACTGGAAGCCATTCA
GGATGGCGTGCGTAAATTCACGCCGGAGACGGTGGCGTCTTTACAGGCGGAGGTCGGTACACTGACCAAACTGGTTGACG
ATCTCCATCAGTTGTCGATGTCTGATGAAGGCGCTCTCGCCTATCAAAAAGCACCGGTAGATTTGATCCCACTGCTGGAA
GTGGCGGGCGGCGCATTTCGCGAACGATTCGCCAGTCGTGGCCTGAAACTGCAATTTTCCCTGCCAGACAGTATTACCGT
ATTTGGCGATCGCGACCGTTTAATGCAGTTATTCAATAACTTACTGGAAAACAGCCTGCGCTACACTGACAGCGGCGGCA
GCCTGCAAATCTCTGCCGGGCAGCGCGACAAAACGGTGCGCCTGACCTTTGCCGACAGTGCGCCAGGTGTCAGTGACGAT
CAGCTACAAAAATTGTTTGAACGTTTTTATCGCACCGAAGGTTCCCGCAACCGTGCCAGCGGCGGTTCCGGGCTGGGGCT
GGCGATTTGCCTGAACATTGTTGAAGCACATAATGGTCGCATTATTGCTGCCCATTCGCCTTTTGGCGGGGTAAGCATTA
CAGTAGAGTTACCGCTGGAACGGGATTTACAGAGAGAAGTATGA
>Cluster_27519
ATGTTACCTTCTCAATCCCCTGCAATTTTTACCGTTAGCCGCCTGAATCAAACGGTTCGTCTGCTGCTTGAGCATGAGAT
GGGGCAGGTGTGGATCAGCGGCGAAATCTCTAATTTTACACAACCAGCCTCCGGTCACTGGTACTTTACGCTTAAAGACG
ATACCGCGCAGGTGCGCTGTGCGATGTTCCGCAACAGCAACCGCCGGGTCACCTTCCGCCCACAGCACGGGCAACAGGTT
TTAGTTCGCGCCAATATTACGCTCTACGAGCCGCGCGGCGATTATCAAATTATCGTCGAAAGTATGCAGCCGGCCGGTGA
AGGATTGCTGCAACAGAAGTACGAACAGCTTAAAGCGAAGTTGCAGGCTGAAGGTTTGTTCGATCTGCAATACAAAAAAT
CACTCCCCTCCCCTGCGCATTGCGTTGGTGTGATCACCTCAAAAACCGGTGCTGCGCTACATGATATTTTGCATGTGTTA
AAACGTCGCGATCCTTCTCTGCCGGTGATCATCTACCCCACCGCCGTACAGGGGGATGATGCGCCGGGGCAAATCGTTCG
TGCCATTGAACTGGCGAATAAGCGCAACGAGTGTGACGTGTTGATCGTTGGCCGTGGCGGTGGTTCGCTGGAAGATTTAT
GGAGTTTTAACGACGAACGCGTAGCGCGGGCGATTTTTGCCAGCCGCATTCCGGTAGTGAGTGCCGTCGGGCATGAAACC
GATGTGACAATTGCTGACTTTGTTGCTGATCTACGTGCGCCTACACCTTCCGCTGCGGCAGAAATGGTCAGTAGGAACCA
GCAGGAGTTATTGCGGCAGATTCAGTCAGTTCAGCAGCGCCTCGGTATGGCGATGGATTATTTCCTCGCTAACCGTACCC
GCCGCCTTACTCTACTTCACCATCGCCTGCAACAACAGCATCCCCAGTTGCGTCTGGCGCGTCAGCAGACGGCGCTGGAG
AGACTTCAGCAACGAATGAATCTGGCGATTGACAGTCAGATTAAGCGGACGAATAAGCGGCAGGTTCGGCTTTTACAACG
CCTGAATCAACAAAATCCCCAACCACGCATTCACCGGGTGCAAAGCCGCATTCAGCACTTGGAACATCGTCTGGCTGAAC
ATGTGCATTCACGCCTGAGCGCCATGCGCGAACGCTTCGGCAATGCAGTAACACATCTTGAAGCCGTCAGCCCTCTGTCC
ACACTGGCGCGTGGTTATAGCGTGACCACGGTCACAGACGGTAAGGTACTGAAAAAAGTGAAACAGGTGAAAACAGGCGA
TGTCATGACCACGCGACTGGAAGACGGTTGGGTGAAAAGTGAAGTAAAAGGGATCACATCTGCAAAGAGAGCGCAAAAGA
AAAAGCCCGACTAA
>Cluster_9132
ATGAGTCACATCACAACGGAAGATCCAGCAACTTTACGCCTGCCCTTTAAAGAGAAACTCTCTTACGGTATTGGCGACCT
GGCCTCTAACATCCTGCTGGATATCGGTACGCTTTATCTTTTGAAGTTTTATACCGACGTTCTGGGGCTGCCAGGCACCT
ATGGCGGCATTATCTTTTTGATTTCAAAATTCTTTACTGCGTTTACCGATATGGGTACCGGCATTATGTTGGATTCCCGA
CGCAAGATCGGTCCAAAAGGTAAGTTCCGTCCTTTTATTCTGTATGCGTCATTCCCGGTCACCTTACTGGCGATCGCCAA
CTTTGTCGGCACACCGTTTGATGTCACCGGTAAAACGGTGATGGCCACTATTCTGTTTATGCTTTACGGACTGTTTTTCA
GCATGATGAACTGCTCCTACGGCGCAATGGTTCCCGCTATCACCAAAAACCCCAACGAACGCGCCTCACTGGCGGCATGG
CGTCAGGGCGGCGCTACGCTGGGCCTGCTGCTGTGCACGGTGGGATTCGTGCCAGTTATGAATCTTATCGAAGGTAATCA
GCAACTTGGCTATATCTTCGCCGCCACGCTGTTTTCACTGTTTGGCCTGCTGTTTATGTGGATCTGCTACTCGGGCGTGA
AAGAGCGTTATGTCGAAACCCAGCCTGCTAATCCGGCGCAAAAGCCGGGCCTGCTGCAATCTTTCCGCGCAATTGCGGGT
AACCGCCCGCTGTTCATTCTGTGCATTGCCAACCTCTGCACTTTAGGGGCGTTTAACGTCAAGCTCGCCATCCAGGTCTA
TTACACCCAGTACGTGCTTAACGATCCCATCCTGTTGTCGTATATGGGATTTTTCAGCATGGGCTGTATTTTCATCGGCG
TATTCCTGATGCCTGCCTCAGTCAGACGTTTTGGCAAGAAGAAAGTTTATATCGGCGGCCTGCTGATTTGGGTGCTGGGC
GATCTGCTCAACTATTTCTTCGGCGGCGGTTCGGTCAGCTTCGTGGCGTTCTCCTGCCTGGCGTTCTTTGGCTCAGCGTT
TGTTAACAGCCTGAACTGGGCGCTGGTTTCCGACACCGTCGAGTACGGCGAGTGGCGCACCGGCGTGCGTTCGGAAGGAA
CGGTCTACACCGGTTTTACCTTCTTTCGCAAAGTGTCTCAGGCGCTGGCTGGTTTCTTCCCCGGCTGGATGCTGACGCAA
ATTGGCTATGTGCCGAACGTCGCACAGGCTGACCACACCATTGAAGGGTTACGCCAGTTGATCTTCATCTACCCAAGCGC
ACTGGCGGTAGTCACCATTGTGGCGATGGGTTGCTTCTACAGCCTGAACGAGAAGATGTATGTCCGCATTGTGGAAGAGA
TAGAAGCCCGTAAACGCACGGCGTAA
>Cluster_12404
ATGAATAAAATAACTTGCTTCAAAGCATATGATATACGTGGGCGTCTTGGTGCTGAATTGAATGATGAAATAGCATATAG

AATTGGTCGCGCTTATGGTGAGTTTTTAAACCTCAAACTGTAGTTGTGGAGGAGATGCTCGCTTAACAAGTGAGAGTT
TAAAGAAATCACTCTCAAATGGGCTATGTGATGCAGGCGTAAATGTCTTAGATCTTGGAATGTGTGGTACTGAAGAGATA
TATTTTTCCACTTGGTATTTAGGAATTGATGGTGGAATCGAGGTAACTGCAAGCCATAATCCAATTGATTATAATGGAAT
GAAATTAGTAACCAAAGGTGCTCGACCAATCAGCAGTGACACAGGTCTCAAAGATATACAACAATTAGTAGAGAGTAATA
ATTTTGAAGAGCTCAACCTAGAAAAAAAAGGGAATATTACCAAATATTCCACCCGAGATGCCTACATAAATCATTTGATG
GGCTATGCTAATCTGCAAAAAATAAAAAAAATCAAAATAGTTGTGAATTCTGGGAATGGTGCAGCTGGTCCTGTTATTGA
TGCTATTGAGGAATGCTTTTTACGGAACAATATTCCGATTCAGTTTGTAAAAATAAATAATACACCCGATGGTAATTTTC
CACATGGTATCCCTAATCCATTACTACCTGAGTGCAGAGAAGATACCAGCAGTGCGGTTATAAGACATAGTGCTGATTTT
GGTATTGCATTTGATGGTGATTTTGATAGGTGTTTTTTCTTTGATGAAAATGGACAATTTATTGAAGGATACTACATTGT
TGGTTTATTAGCGGAAGTTTTTTTAGGGAAATATCCAAACGCAAAAATCATTCATGATCCTCGCCTTATATGGAATACTA
TTGATATCGTAGAAAGTCATGGTGGTATACCTATAATGACTAAAACCGGTCATGCTTACATTAAGCAAAGAATGCGTGAA
GAGGATGCCGTATATGGCGGCGAAATGAGTGCGCATCATTATTTTAAAGATTTTGCATACTGCGATAGTGGAATGATTCC
TTGGATTTTAATTTGTGAACTTTTGAGTCTGACAAATAAAAAAATTAGGTGAACTGGTTTGTGGTTGTATAAACGACTGGC
CGGCAAGTGGAGAAATAAACTGTACACTAGACAATCCGCAAAATGAAATAGATAAATTATTTAATCGTTACAAAGATAGT
GCCTTAGCTGTTGATTACACTGATGGATTAACTATGGAGTTCTCTGATTGGCGTTTTAATGTTAGATGCTCAAATACAGA
ACCTGTAGTACGATTGAATGTAGAATCTAGGAATAATGCTATTCTTATGCAGGAAAAAACAGAAGAAATTCTGAATTTTA
TATCAAAATAA
>Cluster_40035
ATGGAATTATCCTCACTGACCGCCGTTTCCCCTGTCGATGGACGCTACGGCGATAAAGTCAGCGCGCTGCGCGGGATTTT
CAGCGAATACGGTTTGCTGAAATTCCGTGTACAAGTTGAAGTACGTTGGCTGCAAAAACTGGCCGCGCACGCAGCGATCA
AGGAAGTTCCTGCTTTTGCTGCCGACGCAATCGGTTACCTTGATGCAATTGTCGCCAGTTTCAGCGAAGAAGATGCAGCA
CGCATCAAAACCATCGAGCGTACCACTAACCACGACGTTAAAGCGGTTGAGTATTTCCTGAAAGAAAAAGTGGCGAATAT
CCCGGAACTGCACGCGGTTTCTGAATTCATCCACTTTGCCTGTACTTCGGAAGATATCAATAACCTCTCCCACGCATTAA
TGCTGAAAACCGCGCGTGATGAAGTGATCCTGCCGTACTGGCGTCAACTGATTGATGGCATTAAAGATCTCGCCGTTCAG
TATCGCGATATCCCGCTGCTGTCCCGTACCCACGGTCAGCCAGCCACGCCGTCAACCATCGGTAAAGAGATGGCAAACGT
CGCCTACCGTATGGAGCGCCAGTACCGCCAGCTTAATCAGGTGGAGATCCTCGGCAAAATCAATGGTGCGGTCGGTAACT
ACAACGCCCACATCGCCGCTTACCCGGAAGTTGACTGGCATCAGTTCAGCGAAGAGTTCGTCACCTCGCTGGGTATTCAG
TGGAACCCGTACACCACCCAGATCGAACCGCACGACTACATTGCCGAACTGTTTGATTGCGTTGCGCGCTTCAACACCAT
TCTGATCGACTTTGACCGTGACGTCTGGGGTTATATCGCCCTTAACCACTTCAAACAGAAAACCATTGCTGGTGAGATTG
GTTCTTCCACCATGCCGCATAAAGTTAACCCGATCGACTTCGAAAACTCCGAAGGAAACCTGGGGCTTTCCAACGCGGTA
TTGCAGCACCTGGCAAGCAAACTGCCAGTTTCCCGCTGGCAGCGTGACCTGACCGACTCCACCGTGCTGCGTAACCTCGG
CGTGGGTATCGGTTATGCGCTGATTGCGTATCAATCCACCCTGAAAGGCGTGAGTAAACTGGAAGTGAACCGTGACCATC
TACTGGATGAACTGGATCACAACTGGGAAGTGCTGGCTGAACCAATCCAGACAGTTATGCGTCGCTATGGCATCGAAAAA
CCGTACGAGAAGCTGAAAGAGCTGACTCGCGGTAAGCGCGTTGACGCCGAAGGCATGAAGCAGTTTATCGATGGTCTGGC
GTTGCCAGAAGAAGAGAAAGCCCGCCTGAAAGCGATGACGCCGGCTAACTACATTGGTCGCGCCATCACCATGGTTGATG
AGCTGAAATAA
>Cluster_26674
ATGAACTTTCCATTAATTGCGAACATCGTGGTGTTCGTTGTACTGCTGTTTGCGCTGGCGCAAACCCGCCACAAACAGTG
GAGTCTGGCGAAAAAAGTGCTGGTGGGTCTTGTGATGGGTGTGGTTTTTGGCCTTGCCCTGCATACCATTTATGGTTCTG
ACAGCCAGGTACTTAAAGATTCTGTACAGTGGTTTAACATTGTTGGTAACGGCTATGTTCAACTGCTGCAAATGATCGTT
ATGCCGCTAGTCTTCGCCTCTATTCTGAGCGCGGTTGCCCGTCTGCATAACGCATCTCAGTTAGGCAAAATCAGTTTTCT
GACCATCGGTACGCTTTTGTTTACCACGCTGATTGCAGCGCTGGTCGGTGTGCTGGTCACCAACCTGTTTGGTTTGACGG
CTGAAGGTCTGGTTCAGGGTGGTGCAGAAACTGCACGTCTGAACGCCATCGAAAGTAACTATGTTGGTAAAGTCTCTGAC
CTGAGCGTTCCGCAGCTGGTCTTGTCCTTTATCCCGAAAAACCCGTTTGCCGATCTGACCGGAGCCAATCCGACGTCAAT
TATCAGCGTGGTAATTTTTGCCGCATTCCTCGGCGTAGCTGCGCTGAAACTGCTGAAGGATGATGCGCCGAAAGGTGAAC
GCGTCTTAACCGCTATCGATACCCTGCAAAGCTGGGTGATGAAACTGGTTCGCCTGGTCATGCGGTTGACCCCTTACGGT
GTTCTGGCGCTGATGACCAAAGTGGTTGCAGGTTCTAACCTGCAAGACATCATCAAACTAGGAAGTTTCGTTGTTGCGTC
CTACCTCGGTCTGCTGATTATGTTTGCAGTGCATGGCCTTCTGCTGGGCATTAATGGCGTGAGCCCGCTGAAGTACTTCC
GTAAGGTATGGCCAGTACTGACGTTTGCCTTTACCAGCCGTTCCAGTGCTGCGTCTATCCCACTGAATGTGGAAGCACAA
ACGCGTCGTCTGGGTGTTCCTGAATCCATCGCCAGTTTCGCCGCCTCTTTCGGTGCGACCATTGGTCAGAACGGCTGCGC
CGGTTTGTATCCGGCAATGCTGGCGGTGATGGTTGCGCCTACGGTGGGCATTAACCCGCTGGACCCGATGTGGATTGCGA
CGCTGGTCGGTATTGTTACCGTTAGTTCCGCAGGCGTTGCCGGTGTCGGTGGTGGTGCAACTTTCGCCGCACTGATTGTG
CTGCCTGCGATGGGCCTGCCTGTAACGCTGGTGGCATTGTTAATCTCCGTTGAACCGCTTATCGACATGGGGCGTACGGC
GCTAAACGTTAGTGGCTCGATGACCGCTGGCACGCTGACCAGCCAGTGGCTGAAGCAAACCGATAAAGCCATTCTGGATA
GCGAAGACGACGCCGAACTGGCACACCGTTAA
>Cluster_63590
ATGAAGCGACTTAAAAATGAACTTAATGCGCTGGTGAATCGGGGTGTCGACAGACATCTGCGCCTCGCCGTAACCGGACT
AAGCCGCAGCGGCAAAACAGCGTTTATCACTGCGATGGTCAATCAGTTGCTCAATATTCACGCCGGAGCACGCTTGCCGC
TGTTAAGCGCGGTGCGTGAAGAGCGCCTGCTGGGCGTAAAACGCATTCCTCAGCGTGACTTTGGCATTCCGCGCTTCACA
TATGATGAAGGACTGGCGCAGTTATATGGCGATCCTCCCGCCTGGCCGACGCCAACGCGCGGCGTCAGTGAAATCCGCCT
GGCGCTACGTTTTAAATCGAACGATTCGCTGCTACGCCACTTCAAGGACACCTCCACGCTGTATCTGGAGATTGTGGATT
ATCCCGGCGAATGGTTGCTCGACCTGCCGATGCTGGCGCAGGACTATTTAAGCTGGTCACGCCAGATGACGGGCTTACTC
AATGGTCAGCGCGGCGAATGGTCGGTCAAATGGCGAATGATGTGCGAAGGGCTGGACCCGCTAGCGCCTGCCGACGAAAA
CCGGCTGGCAGACATTGCCGCCGCGTGGACCGATTATCTCCACCACTGTAAACAGCAGGGGCTGCACTTTATTCAGCCTG
GGCGCTTTGTCTTGCCGGGAGATATGGCAGGTGCGCCCGCGCTGCAATTCTTCCCGTGGCCGGATGTCGATGCCTGGGGC
GAGTCCAAACTGGCGCAGGCCGATAAGCACACCAATGCCGGAATGCTGCGCGAGCGGTTTAATTATTACTGCGAGAAGGT
GGTGAAGGGGTTCTATAAGAATCATTTTCTGCGCTTTGACCGCCAGATTGTGCTGGTTGATTGCCTGCAACCTCTCAACA
GTGGGCCACAGGCATTTAATGATATGCGTCTGGCACTGACGCAGCTGATGCAAAGTTTCCACTACGGGCAGCGTACCTTG
TTCCGGCGTTTGTTTTCGCCGGTTATCGATAAGCTATTGTTTGCTGCCACTAAAGCGGACCATGTGACCATCGATCAGCA
CGCCAATATGGTTTCATTACTGCAACAACTGATTCAGGATGCCTGGCAAAATGCGGCGTTTGAAGGGATTAGTATGGATT
GCCTGGGGCTGGCCTCGGTGCAGGCGACCACCAGCGGCATTATTGACGTCAACGGCGAGAAAATTCCGGCATTGCGCGGT
AACCGGCTCAGCGATGGTGCGCCACTCACCGTTTATCCCGGTGAAGTTCCTGCGCGTTTGCCCGGTCAGGCGTTCTGGGA
CAAGCAAGGGTTCCAGTTTGAAGCGTTTCGCCCGCAGGTGATGGATGTCGACAAACCGCTGCCGCATATTCGTCTTGATG
CCGCGCTTGAATTTTTAATAGGAGATAAATTGCGATGA
>Cluster_39188
ATGCTAAAAATCTTCAATACTCTGACACGCCAAAAAGAGGAATTTAAGCCTATTCACGCCGGGGAAGTCGGCATGTACGT
GTGTGGAATCACCGTTTACGATCTCTGTCATATCGGTCACGGGCGTACTTTTGTTGCTTTTGACGTGGTTGCGCGCTATC
TGCGTTTCCTCGGCTATAAGCTGAAGTATGTGCGCAACATTACCGATATCGACGACAAAATCATCAAACGCGCCAATGAA
AATGGCGAAAGCTTTGTGGCGCTGGTGGATCGCATGATCGCCGAAATGCACAAAGATTTTGATGCTTTGAACATTTTGCG

CCCGGATATGGAGCCGCGCGCGACGCACCATATCGCCGAAATTATTGAACTCACTGAACAACTGATCGCCAAAGGTCACG
CTTATGTGGCGGACAACGGCGACGTGATGTTCGACGTCCCGACCGATCCGACATATGGCGTGCTGTCGCGTCAGGATCTC
GATCAGCTGCAGGCAGGCGCGCGCGTTGACGTGGTCGATGACAAACGTAACCCGATGGACTTCGTTCTGTGGAAGATGTC
GAAAGAGGGCGAACCGAGCTGGCCGTCTCCGTGGGGCGCGGGCCGTCCTGGCTGGCACATTGAATGTTCGGCAATGAACT
GCAAGCAGCTGGGTAACCACTTTGATATCCACGGCGGCGGTTCTGACCTGATGTTCCCGCACCACGAGAACGAAATTGCC
CAGTCCACCTGTGCACACGATGGCCAGTATGTGAATTACTGGATGCATTCCGGTATGGTGATGGTTGACCGCGAGAAGAT
GTCTAAATCGCTGGGTAACTTCTTCACCGTGCGTGATGTGCTGAAATACTACGATGCGGAAACTGTGCGTTACTTCCTGA
TGTCGGGTCACTATCGCAGTCAGTTGAACTACAGCGAAGAGAACCTGAAACAGGCGCGTGCGGCGCTGGAACGTCTCTAC
ACTGCGCTGCGCGGCACAGACAAAACCGTTGCGCCTGCCGGTGGCGAAGCGTTTGAAGCGCGCTTTATTGAAGCAATGGA
CGACGATTTCAACACCCCGGAAGCCTATTCCGTGCTGTTTGATATGGCGCGTGAAGTGAACCGTCTGAAAGCAGAAGATA
TGGCAGCGGCGAATGCAATGGCGTCTCACCTGCGTAAACTTTCTGCCGTATTGGGCCTGCTGGAGCAAGAACCGGAAGCG
TTCCTGCAAAGCGGTGCGCAGGCAGACGACAGCGAAGTGGCTGAGATTGAAGCGTTAATTCAACAGCGTCTGGATGCCCG
TAAAGCGAAAGACTGGGCGGCGGCAGATGCGGCGCGTGACCGCCTTAACGAGATGGGGATCGTGCTGGAAGATGGCCCGC
AAGGGACCACCTGGCGTCGTAAGTAA
>Cluster_53563
ATGGTGGTCACCAGTTCACGCAAAGCCGCTGTGCGCTACAAGCTGGCGTTTGATAAATACATTAGCGAGAACAATTACCA
GAAAATCAGCGCGATGGTGGCGTTTTCCGGGGAAGTTGAATTCCAGGAAGGCGACCCTAACAGCCCGGCATTGTTAAACC
AGAAATTTACTGAAATAAACATGAACCCCGGCCTGAAAGGGCGGGATATGCGCAAAGCCTTCGACAGCGATGATTATCAG
GTCATGCTGGTTGCCAATAAATTCCAGACCGGTTTTGACCAGCCGAAGCTGTGCGCGATGTACGTGGATAAAGCCCTTGG
CGGCGTCGAGTGCGTGCAGACGCTCTCCCGCCTGAATCGCACCTATCCGGGCAAAGCGGAATCAGGCACTTTTGTCCTCG
ATTTTTATAACGATCCAGACGAGATCCTCGGCGCATTCCAGCCCTATTTCCAGACGGCGGAACTGACCGATGTCAGCGAT
CCGCAGCAGGTATTCGATCTGTTTGAAAAACTGCGCGCCACGCAGATTTTTCAGTGGCATGAAGTCGAGCAGTTCTGCAA
GGCGTACTTCATCAAAAATAAATCGAATGCGGCGATCAGTAATATCTGTAAACCGGCGGTCGAACGCTGGAAAAAGCGTT
ATCTGATGGCAATAGACGCCTATTTGACCGCCAAAGAGATGTTCGAGCGCACTAAAAAAACGGGCGATACGGTGTTAATC
ACCAATGCGGAAAACAGTTTTAAAGCCTGTAAGCAGGAGAAAGATCGTCTGGAGATTTTCAAGAAAGATCTCGGCAGCTT
TGTTCGCTTTTATGAGTTTATGTCGCAAATCGTTGAGTACGATGACAAGGAGCTGGAAAAGCTAAGCCTGTTTGCGCGCC
ATTTACGCCCACTGCTGCATGAGCAACGTCTGGAAGAGGATGAAATTGATCTCAGTAATGTGGAGATGAGTCACTATCGT
TTATCGAAAATCCATGAGCAAGATTTACGCTTACAGGAAGAAACGCCTGACTATACGCTGGATGCAAGTAATAATATTGG
TACGGCAAAAGCGAAAGATAAAAAAGAGGAGTTTTATCTCAGGTTCTTTCTCGGATGAATGAATTATTCATTAGTGACC
ATTTGACCGACAAAGATATGATCAGCAATGCTTTTGCGGTGTATAACAAAATAACTGAAAATGAAAATGTTATGACACAA
ATTAAAAACAATCCGCCAGAACAAGCGATTCTTGGTGATTTCCTCCAGGCAGTTGATAATGCTGTAATGGACAGTAATGA
CGCTCGGCAAGAATTTATGTTGCAATATTTATCCGATCCAGCTCGGGCAAAGAGCTTTGCTTATCTGATGTTTGATATGC
TAACGGGGAAAATTGCTTTACCAACATAA
>Cluster_21974
ATGACAAGTCTGGTTTCCCTGGAAAATGTCTCGGTTTCTTTTGGCCAACGCCGCGTCCTCTCTGATGTGTCGCTGGAACT
TAAACCTGGAAAAATTTTGACTTTACTTGGGCCAAATGGCGCAGGTAAGTCGACACTGGTACGGGTAGTGCTCGGGCTGG
TAACACCCGATGAAGGGGTTATCAAGCGCAACGGAAAACTGCGCATCGGCTATGTACCGCAGAAGCTGTATCTCGACACC
ACGTTGCCACTGACCGTAAACCGTTTTTTACGCTTACGCCCTGGCACACATAAAGAAGATATTTTGCCTGCACTGAAACG
TGTCCAGGCCGGGCATCTGATTAACGCACCGATGCAAAAGCTCTCGGGTGGCGAAACGCAGCGTGTACTGTTAGCGCGAG
CATTGTTAAATCGACCGCAATTATTAGTGCTGGATGAACCCACTCAGGGCGTGGATGTGAATGGTCAGGTGGCGTTATAT
GACCTTATTGACCAACTGCGTCGTGAACTGGATTGTGGCGTTTTAATGGTATCTCACGATCTGCATCTGGTAATGGCAAA
AACCGATGAAGTGCTTTGCCTGAATCACCACATTTGTTGTTCCGGCACACCGGAAGTTGTTTCCCTGCATCCGGAGTTTA
TTTCTATGTTTGGTCCTCGTGGTGCTGAACAACTGGGTATCTATCGCCATCATCATAATCATCGTCACGATTTACAGGGA
CGAATTGTTTTGCGTCGGGGAAATGATCGCTCATGA
>Cluster_25803
ATGTCTGCCAAAAGACGGCTTCTTATTGCGTGTGCCCTGACAACGATTATCTATCATCTTCCTGCATATTCTTCATTAGA
ATATAAAGGGTCCTTTGGCTCAATAAATGCAGGTTATGCTGACTGGAATAGTGGCTTCGTTAACACTCACCGTGGTGAAG
TATGGAAAGTGACTGCGGACTTTGGAGTGAATTTTAAAGAAGCCGAGTTTTACTCATTTTATGAAAGCAATGTGCTTAAC
CATGCCGTAGCGGGGCGTAATCATACCGTTTCTGCTATGACTCATGTCAGACTCTTTGACTCCGATATGACTTTCTTTGG
CAAGATCTATGGTCAATGGGATAACTCATGGGGTGACGATCTTGATATGTTTATGGTTTGGGCTACCTCGGCTGGAATG
GTTCATGGGGGTTTTTTAAACCGTATATAGGATTGCATAATCAATCTGGTGACTACGTATCAGCTAAATATGGTCAAACG
AATGGTTGGAATGGTTATGTTGTTGGCTGGACAGCAGTATTACCATTTACGTTATTTGACGAAAAAATTTGTTTTATCTAA
CTGGAATGAAATAGAACTGGACAGGAACGATGCTTACACGGAGCAGCAATTTGGCCGGAACGGGTTAAATGGCGGTTTAA
CAGTGGCCTGGAAGTTCTATCCTCGCTGGAAAGCCAGTGTGACGTGGCGTTATTTCGATAATAAGTTGGGCTACGATGGT
TTTGGCGATCAAATGATTTATATGCTTGGTTATGATTTCTAA
>Cluster_7264
ATGTCTCATTTGATTGACTTGAAAGATGTGGGTGTTGAATTTCCCATTTACGACGCTAAAAGTAGATCGTTTAAATCGCA
GGTGGCAAATTTAACCCGTGGTGTAATTTCTTCAAAAAAAGAAAGCAGCACAGTTACGGTTAAATCATTGGACGGTTTGA
CATTTAGTCTGAAAGAAGGTGATAAACTCGGACTTATTGGAAATAATGGTGCGGGAAAATCTACGCTACTGAAACTCTTG
GCTGGTATTTATGAACCAACATCCGGCACAATTAAACGAAAGGGTAAGATAGTTCCTCTGTTGGACATCGCTCTAGGGAT
GGATGATGATTCCACAGGATACCAAAATATAAAGCTACGTGGTTTATTACTAGGTATGACGAATGCAGAAATCGAGTCCA
AAATAGAAGAGATCGCTTGTTTTACTGAACTAGGTGATTATCTGAATCTACCAATTCGTGTCTACTCTACCGGCATGCGC
GTGAGATTAGCATTCGCAGTATCAACTTCAGTTGATCCAGATGTACTTCTACTCGACGAAATTATTGGTACTGGCGATGC
TTCCTTCTTAAATAAAGCAAAGAAAAGATTCGAGGAATTGCAGGAGAGGGCAAAGGTAGTTGTGTTGTCTTCGCATGACA
ATAATGTCATTCTGAAAACTTGTAATAAAGTCCTGTGGTTAGAGGGCGGTAAACCAAAGATGATTGGAGATCCGGAAGAA
GTACTAAATAAATATATGGATAGTGTGATTTAA
>Cluster_67042
ATGGAAAACCAGGAATGTTTCTGCAAGGATGATGTACGGAATCCCGGTGCTTTGCCTTTGCTGTCTCTGGCACCATTACA
ACGTGACATTATCAAAATCGTGGCATTTCTGGCGATGACAGGTGACCACATTGCTACAGCATTTCATGTGGGGACTCCCT
GGCTTAACCTGACCGGGCGGTGCGCATTCCCACTTTTCGCACTGATGGCGGGCTGCAACCTGGCGGGGCGGCAGATACGA
CAGGCTGCCGTAAATCGCCTGTGGCTGATGGCAGTGCTGGCGCAACCGGTATTCTGGCTGGCATTTTGTGGTACAGGAAA
TCAGTGGTGGGAACTGAACATTCTGTTCTGCTTTGCCGTGGTGATGCAGGTGGTCTGTTTCTGGCAACGCCCTGATGCCA
TAAAAGGTGCCATAACCGGAACCATGCTGGCACTGTATCTGCCGTTCAGTACTGCCAGTTATGGCTGGCAGGGACTGGTG
ATGCTGCTGGCAGCTGTATCCGTCTGGCAGGTGCCGGAGCAGTACCAGGGGCGGTTATTTTTACTGTGGCTGTGCTCTGT
TGTATGGCTGAACGGTGCAAACGGGGCAGTCCCCGTGCTGGCCGGTATGGGCATGACACTGCTGGTATTGTGTCTGGTGC
ATGAGTATGTACCTTCATCATCAAAGCGCCTGCAGATAAGCCGGTGGTTTGCAGAAGGTTATGTCCTGCATCTGCTGATT
ATTGCCGTCATTGTTTATGGGCTGCAGACTTAA
>Cluster_14382
ATGTTACGTATTGCGGACAAAACGTTTGATTCACATCTGTTTACCGGCACAGGGAAATTCGCTTCTTCACAACTGATGGT

GGAGGCGATCCGCGCTTCCGGCAGCCAGCTGGTGACACTGGCGATGAAACGTGTCGACTTGCGCCAGCACAACGACGCTA
TCCTCGAACCGCTTATCGCGGCGGGTGTGACCCTGCTGCCAAATACATCCGGGGCGAAAACAGCGGAAGAAGCCATTTTC
GCCGCCCATCTGGCTCGTGAAGCGTTAGGCACAAACTGGTTAAAATTAGAGATTCACCCTGACGCCCGCTGGCTGTTGCC
CGATCCCATCGAAACCCTGAAAGCCGCCGAAACGCTGGTACAACAGGGATTTGTCGTGCTGCCTTACTGCGGGGCCGATC
CGGTATTGTGTAAACGTCTGGAAGAAGTCGGCTGTGCAGCGGTGATGCCGCTCGGCGCGCCGATTGGCTCGAATCAGGGA
CTGGAAACCCGCGCCATGCTGGAGATTATTATCCAGCAGGCCACAGTGCCGGTGGTTGTCGATGCTGGCATCGGCGTTCC
CAGCCATGCCGCGCAGGCGCTGGAAATGGGGGCCGACGCGGTGTTAGTGAATACGGCAATTGCCGTCGCGGACGATCCCG
TCAACATGGCGAAGGCATTTCGTCTGGCGGTAGAAGCAGGCCTGCTGGCACGTCAGTCCGGACCGGGCAGCCGCAGTCAT
TTTGCTCATGCCACCAGCCCGCTGACTGGATTTCTGGAGGCATCGGCATGA
>Cluster_29660
ATGAAGATTATTACTTGCTATAAGTGCGTGCCTGATGAACAGGATATTGCGGTCAATAATGCTGATGGTTCGTTAGACTT
CAGTAAAGCCGATGCCAAAATAAGCCAATACGATCTCAACGCTATTGAAGCGGCTTGCCAGCTAAAGCAACAGGCAGCAG
AGGGGCAGGTGACGGCCTTAAGTGTGGGCGGTAAAGCCCTGACCAACGCCAAAGGGCGTAAAGATGTGCTATCGCGCGGT
CCGGATGAACTGATTGTGGTGATTGATGACCAGTTCGAGCAGGCACTGCCGCAACAAACGGCGAGCGCACTGGCTGCCGC
CGCCCAGAAAGCAGGCTTTGATTTGATCCTCTGTGGCGATGGTTCTTCCGACCTTTATGCCCAGCAGGTTGGTTTGCTGG
TAGGCGAAATCCTCAATATTCCGGCAGTTAACGGCGTCAGCAAAATTATCTCCCTGACGGCAGATACCCTCACCGTTGAG
CGCGAACTGGAAGATGAAACAGAAACGTTAAGCATTCCGCTGCCTGCGGTTGTTGCTGTTTCCACTGATATCAACTCCCC
ACAAATTCCTTCGATGAAAGCCATTCTCCGCGCGGCGAAAAAGCCAGTCCAGGTATGGTCGGCGGCGGATATCGGTTTTA
ACGCAGAGGCAGCCTGGTCAGAACAACAGGTTGCCGCGCCGAAACAGCGCGAGCGTCAGCGCATCGTGATTGAAGGCGAC
GGCGAAGAACAGATCGCCGCATTTGCTGAAAATCTTCGCAAAGTCATTTAA
>Cluster_29002
ATGCGACATCCTTTAGTGATGGGTAACTGGAAACTGAACGGCAGCCGCCACATGGTTCACGAGCTGGTATCTAACCTGCG
TAAAGAGCTGGCAGGTGTTGCTGGCTGTGCGGTTGCAATCGCACCACCGGAAATGTACATCGATATGGCGAAGCGTGAAG
CTGAAGGCAGCCACATCATGCTGGGTGCGCAAAACGTGGACCTGAACCTGTCCGGCGCATTCACCGGTGAAACCTCTGCT
GCTATGCTGAAAGACATCGGCGCACAGTACATCATCATCGGCCACTCTGAACGTCGTACTTACCACAAAGAATCTGACGA
ACTGATCGCGAAAAAATTCGCGGTGCTGAAAGAGCAGGGACTGACTCCGGTTCTGTGCATCGGTGAAACCGAAGCGGAAA
ACGAAGCGGGCAAAACTGAAGAAGTTTGTGCACGTCAGATCGACGCGGTACTGAAAACTCAGGGTGCTGCGGCATTCGAA
GGTGCGGTTATCGCTTACGAACCTGTATGGGCAATCGGTACTGGCAAATCTGCAACTCCGGCTCAGGCACAGGCTGTTCA
CAAATTCATCCGTGACCACATTGCTAAAGTTGACGCTAACATCGCTGAACAAGTGATCATTCAGTACGGCGGCTCTGTAA
ACGCGTCTAACGCTGCAGAACTGTTTGCTCAGCCAGACATCGACGGCGCGCTGGTTGGCGGTGCTTCTCTGAAAGCTGAC
GCTTTCGCCGTGATCGTTAAAGCTGCAGAAGCGGCTAAACAGGCTTAA
>Cluster_54581
ATGAATACTGCTCGTCTGAACCAGGGCACGCCATTGTTGCTCAATGCAGTAAGCAAACATTACGCGGAAAATATCGTCCT
GAACCAACTGGATTTACATATTCCGGCAGGTCAGTTTGTGGCGGTGGTGGGCCGCAGCGGTGGTGGCAAAAGTACCCTGC
TGCGCCTGCTGGCAGGTCTGGAAACGCCAACCGCAGGCGATGTGTTAGCGGGCACCACACCGCTGGCTGAAATTCAGGAA
GACACGCGAATGATGTTTCAGGATGCGCGTCTGCTACCGTGGAAATCGGTGATTGATAACGTTGGGTTAGGCCTTAAAGG
CCAGTGGCGCGATGCCGCACGTCGAGCGTTGGCTGCGGTAGGACTGGAGAATCGCGCAGGGGAATGGCCTGCCGCACTTT
CTGGCGGGCAGAAACAGCGAGTGGCGCTGGCAAGAGCGTTGATTCATCGACCGGGATTATTGTTGCTTGATGAACCGCTC
GGGGCGCTGGACGCCTTAACGCGACTCGAGATGCAGGATTTGATTGTGTCACTTTGGCAGGAGCACGGCTTTACCGTACT
GTTGGTGACGCATGATGTGAGCGAAGCGGTAGCGATGGCTGACCGGGTGCTGTTAATTGAAGAGGGAAAAATTGGTCTGG
ATTTGACAGTGGATATCCCCCGTCCGCGTCGATTAGGGTCGGTGCGATTAGCGGAGCTGGAAGCGGAGGTGTTGCAACGG
GTGATGCAACGTGGTGAATCTGAAACTCGCCTGAGAAAACAGGGGTAA
>Cluster_26912
ATGAGTGCCATTGAAGTTAAGAACCTGGTGAAAAAATTCCACGGTCAGACGGTGCTGCACGGTATCGACCTCGAGGTAAA
GCCTGGCGAAGTAGTGGCAATTATCGGTCCGAGTGGTTCCGGTAAAACCACGTTGCTACGCAGCATAAATCTGCTGGAAC
AACCGGAAGCGGGAACCATCACCGTTGGCGATATCACTATTGATACTGCACGTTCATTAAGTCAGCAAAAATCGCTGATT
CGCCAGTTGCGTCAGCACGTCGGGTTTGTCTTCCAGAACTTTAATTTGTTTCCGCATCGTACGGTGCTGGAAAACATTAT
TGAAGGACCGGTGATCGTCAAAGGCGAACCGAAAGAAGAGGCCACGGCGCGCGCTCGCGAGCTGTTAGCGAAAGTTGGGC
TGGCCGGTAAAGAAACCAGCTATCCACGTCGTTTGTCTGGCGGTCAACAGCAGCGAGTTGCGATTGCGCGTGCGCTGGCA
ATGCGTCCTGAGGTGATTTTGTTTGACGAGCCAACGTCGGCGTTGGATCCAGAGCTGGTGGGCGAAGTCCTGAACACCAT
CCGCCAGCTGGCGCAGGAAAAGCGCACAATGGTGATTGTGACGCACGAAATGAGCTTTGCCCGGGATGTTGCGGACCGGG
CGATCTTTATGGACCAGGGGCGGATAGTCGAGCAGGGGGCCGCAAAAGCGTTATTTGCCGATCCTCAGCAGCCTCGCACC
CGCCAGTTCCTGGAGAAGTTTCTGCTGCAATAA
>Cluster_40922
ATGAATACACCAGATGCTGATCTATTACACTTATTTGAATCATTAACTACCGAGCAAAAGATTGATATCGCAAGACGCTT
ACTACAAAACAAAGAAATAAAATCAACGACAGTTATTTCTTCTGAGGAAGATAAAGCTATTTCGTTTTCACAACCTTATG
TATTTGCTTTTACTTCCAACTTTCGTGTGTTCTCTGATCTTGACATATCCAAGAATGAATTCCGTGTATTAACCTATATT
TTGGAGTATATGGAGTTTGGAAATCTGATCAATCTTTCACAATCATCGCTGTTAAAGCACTGAATATCGCCTCCGGTAA
TATGTCTAACATTTTTAAAAAACTCAGACAGAAAGGAATACTTGTAGAGCATCATGGTCATCTCTATATCAACTCTAACA
TTTTCGCAAAAGGGATGAGTCATCAACTGGATCAGAAACGTAAAGAACAGTTGAGCAACGCTCGCAATCTTTGCGCAGAA
ATAGAACAGTATCCAGAAAGTTATTCAGCTATGTCAAAAAGTGAAAGAAGAACCCTATCCTTAGAAACTGAGACCAAAGA
AATCTATACTGACGCATTTGTTTTCAGTAAGAAGAAAAAGAATAACGCTGTTCCTAATCCCAATAACAGCATACATATTG
ATAAACCACGAAAAGTATATAAAAGAACAAAGGAAAACGAGGAAGCAAGGAGGAACTCTGTTGACGCAGACTCTCATATG
CAGAAAGTATCCTGA
>Cluster_38587
ATGAAACAAAACATTAAGTTAATGGCAGTGACCGCCATATTGTCTTCCACTCTGGTCTTGTCTGGTTGTGGAGCGATGAG
TACAGCAATCAAGAAACGTAATCTTGAAGTGAAAACCCAGATGAGTGAAACCATCTGGCTTAAACCGTCCTCGCAGAAAA
CCGTATATCTTCAGATAAAGAATACCTCCGACAAAGACATGTCCGGTCTGCAGGCGAAAGTGACTAAGGCGCTTCAGGAT
AAAGGGTATACTGTGACATCCGCGCCGGACAGCGCGTATTACTGGATACAGGCCAATGTCCTCAAAGCCGATAAAATGGA
TCTGCGCACAGCCCAGGGATTCCTGAGTCAGGGGTATCAGGGGGCAGCAATGGGAGCTGCGTTAGGGGCAGGTATTACGG
CCTATAACTCCAACTCTGCGGGGGCGACATTAGGTGTGGGTCTTGCTGCCGGTCTCGTGGGTATGGCCGCTGATGCGATG
GTTGAAGATATTAACTACACCATGGTCACAGATATTCAGATTTCTGAAAAAACGAATGCCACTGTCCAGACCGATAATGT
AGCTGCGCTGAAACAAGGAACATCCGGATATAAGGTTCAGACCAGTACGCAGACAGGCAATCAGCATAAATACCAGACGC
GTATTGTCTCTTCCGCTAATAAAGTTAATCTCAAATTTGAAGAAGCCAAACCCGTTCTTGAAGATCAACTGGCGAAATCA
GTTGCTAATATCCTGTAA
>Cluster_48965
ATGAGAAAAAATAATACGGCAATAATATTCGGCAGCCTGTTTTTTTCCTGCAGCGTGATGGCCGCAAACGGTACACTGTC
CCCCACCGTGGTGCCAATGGTGAACGGTGGTCAGGCCAGTATTGCCATCAGCAATACCAGCCCGAATCTGTTTACCGTTC
CCGGTGACCGGATTATCGCCGTGAACAGTCTGGATGGTGCCCTGACCAATAATGAGCAGACCGCCTCCGGCGGTGTGGTG

GTGGCCACCGTCAACAAAAAGCCCTTTACGTTCATTCTGGAAACAGAGCGTGGTCTGAACCTTTCCATTCAGGCTGTTCC
CCGTGAAGGCGCGGGGCGTACCATTCAGCTGGTCAGTGACCTGCGCGGAACCGGAGAAGAAGCCGGTGCGTGGGAAACGT
CCACGCCTTACAAATCCCTGCTTGTGACCATCAGCCAGGCCGTCCGTGGCGGAAAATTGCCCGCAGGCTGGTATCAGGTC
CCGGTGACAAAGGAAACCCTGCAGGCCCCGGTGGGGCTGTCTTCAGTGGCAGATGCCGTATGGACGGGGAATCACCTGAA
GATGGTCCGCTTTGCCGTGGAAAATAAAACGCTGTCTGCCCTGAATATCCGGGAAAGTGACTTCTGGCAGCCGGGAACCC
GTGCCGTGATGTTCAGCCAGCCTGCCAGCCAGTTACTGGCAGGTGCGCGCATGGATGTGTATGTCATCCGTGACGGGGAG
GGCAACTGA
>Cluster_58207
ATGGAGGCTCGTGTTACCGTTGCTGGCATGGGGCTGGTGATGGAGGTGCAGGATTACTTCGATGGCGAGGCCGATAGACT
GGCGAAGGCGTGGCTGGCCGAGTACACACCGCAAATCAAGTCGTTGAAAGATGAGCGTAAAGAGGCCTATCGTCAAATCG
TCGAAATGAGTACCGAACCGCAGGATGTGGATCTGGTCAGGCCGGCGAACAAGTTTGAAATGACCAGGGTGCGTGAAGGT
GAAAAGGAAGCTGACCTTCCAGTCTGGAAACACCATTTGTTGTGTGACGAAAGCGGGAACTATCCGGCTCTGTTGAACCA
TTGGGAAACCAAGGTTTTTGAGATCGAAACCAAACGTGAAGGATTTGCTTTCTGGTATCGTAATCCACAGTACACAGGGC
AGTCGTCACTGGGAATCGCTTATGTTGAAGCTGAACAGTACAAGATTGTTCGTCCCGATTTCCTGTTCTTTGCCGAACAG
GATGGCAAAATGGTTGTGGACCTGGTAGATCCACATAGCCTACATCTGGCTGATGCTTTGCCCAAACTGGAAGGACTTGC
GCTATATGCCGAACATCATTCTGATGCTTACAGGCGAATCGAATCTGTCGCCGAAGTAAAGGGTAAATTACGAGTGTTAG
ATTTGAAACGGCAGGATGTGCAGGATGCTGTTGCTACCGCTGAAAATGCAGAAACGTTATTTAGTAGCGGACTTGCTGAT
GACTATCAGTAA
>Cluster_29321
ATGTTAGATCAAGTATGCCAGCTTGCACGGAATGCAGGCGATGCCATTATGCAGGTCTACGACGGGACGAAACCGATGGA
CGTCGTCAGCAAAGCGGACAATTCTCCGGTAACGGCAGCGGATATTGCCGCACACACCGTTATCATGGACGGTTTACGTA
CGCTGACACCGGATATTCCGGTCCTTTCTGAAGAAGATCCTCCCGGTTGGGAAGTCCGTCAGCACTGGCAGCGTTACTGG
CTGGTAGACCCGCTGGATGGTACTAAAGAGTTTATTAAACGTAATGGCGAATTCACCGTTAACATTGCGCTCATCGACCA
CGGCAAACCGATTTTAGGTGTGGTGTATGCGCCGGTAATGAATGTAATGTACAGCGCGGCAGAAGGCAAAGCCTGGAAAG
AAGAGTGCGGTGTGCGCAAGCAGATTCAGGTCCGCGATGCGCGCCCGCCGCTGGTGGTGATCAGCCGTTCCCATGCAGAT
GCGGAGCTGAAAGAGTATCTGCAACAGCTTGGCGAACATCAGACCACGTCCATCGGCTCTTCGCTGAAATTCTGCCTGGT
GGCGGAAGGACAGGCGCAGCTGTACCCGCGTTTCGGGCCAACGAATATTTGGGATACCGCCGCTGGTCATGCTGTGGCTG
CAGCTGCCGGAGCGCACGTTCACGACTGGCAGGGTAAACCGCTGGATTACACTCCGCGCGAGTCGTTCCTGAATCCGGGG
TTCAGAGTGTCTATTTATTAA
>Cluster_18307
ATGGAACAGGTTATTACAAAGCGCCGCTATTACGATATTGGTCTACAGATTGAAGAGCTGCTGTATTCAGGCGTATTTAA
GGCCGGAGAACGCTTACCTTCAGAACGTGAACTGAGCGAGCGGTTTAATACCAGCCGAACTACCATTCGTGAAGCCATCA
TTATGCTGGAACTGAAAGGCGTATTGAATGTGAAGCAAGGGTCAGGGATTTTCTTTGTCGACTCCACCGATAAGCTCAAC
CAGAAATCATTAATGCCCTATTCAGAAATTGGCCCGTTTGAACTGTTACAGGCACGTCAGGTAATTGAGAGCAATATTAC
CGGTTTTGCTGCCAGCCAGATCAGCTTTAACGAGTTACAAGAGTTGAAGAAAATCATTGGATTACAGGAAAAAGCCATCG
CTGCGGAAAGTGATAAATTTGAAGACCTGGATCACCGATTTCATAGCATTATTGCCGAAGCGACGCAAAATCGGGTACTC
ATCAAACAAGCCGCTGAATTATGGCGGGCAGTGCGCACAGAAAACCCCCGCTGGAAGAAACTTAACTACAAGTACCTGCA
TGAAAAGCATTTGCGTCTGCAATGGCTGGAAGATCACCGGGCTATTTTCTTGGCTCTGCAACAGAAAGATTCAGAGCTCG
CCAGGGAAGCGTCCTGGAGACATCTGGAAAACAGCAAAAATGAACTGATCAAAATCTTCAAACAAGACGCTTCCATCAGC
GATTTTGATGACTTTTTCTTCGCTCGTTAA
>Cluster_25010
ATGGACCGCTTGATTTATACCGCCCTGAGCGGGGCTTCTCAGACCCTGTACGAGCAGCAGATCAGCGCCAACAACCTGGC
AAACGTCAACACCAACGGTTTTCGCGCCGATATGGCAATGGCGACCAATGACAAGGTGAAAGGCGGCGGCTTCGACACGC
GCTATATGGCGCAGGAAGGGGTCAGCGGCGTGAACGACAGCACTGGTGTTGCGGAGAAAACCGAACGCCCGCTGGACGTG
GCGATTCAGGGTTCAGGTTACATTGCTGTGCAGGATAAAAACGGCAACGAAGTGTATACCCGCAACGGCAACATTCAGCA
GGATGACCAGGGGCAACTGACTATCGACGGCAATCTGGTGCTGGGAGATAACGGGCCGATCATTCTGCCGCCCAACGCCA
TTGCTTCGTTTGGCAGCGACGGCACGCTCTCCGTGACGCCCGATGACGGTGACGTGACCGCGACGATGGATATCGACCGC
CTGAAGCTGGTGGATATCCCGGTCGCGAACCTGGCGAAAAATCCTGAAGGGATGCTGGTGACTGCCGACGGCGTTCCGGC
CCAGCGCGATGAAAACATCAAAGTCAGCGGCGGTTTTCTCGAGGGCAGCAACGTCTCGGCGGTGAGCGAGATGATGTCGT
CCATCGCCATGAACCGCCAGTTCGAAGCGCAGATCAAGATGATGAAAACCGCCGAAGATATCAGCGACGCGGGCAACCGC
TTATTGCGCGGCTCGTAA
>Cluster_22649
ATGTTGCGTTGGTCTCCAGTGCTTTTTTCTATTATATGGGCAAGTGCTTCATGGGCATCAGAAGGAAAGGTGGAACCAAC
AAATATCAGTTCTACTGATAGCCCTAATACACTTCCTGATTTAGGTAGTAGTGAAAAATCAAACAGCAAAGATGAAAAAG
GTGCTTTAGGCGGTAAAGGTCAGGGTTATTTTATAAGCTCCGCCACGCAAGGTTTTGAAAATCTCACGCCCGAGGCCCTG
AAGGCTCAGGCTGAAAGCTATGCGAAAGGGCAGGTGACGTCCACTGCTCAGTCTTATCTTGAGAACGTCTTGTCGCCTTA
TGGTTCTGTCAGAACCAGCTTGTCTATAGGAGAAGGTGGCGACCTGGATGGCAGCACTTTTGACTACTTTGTCCCCTGGT
ATGATAACCAGGAAACACTCGTTTTCAGCCAGTTCTCTGCACAACGTAAAGACGACCGAACCATCGGTAACATTGGGGTT
GGTGTACGCCAGAACATTGGTAGCTGGTTGCTTGGCGGCAACCTGTTTTATGACTATGACTTTACTCGCGGTCATCGTCG
CTTGGGGCTGGGTAGCGAAGCCTGGACCGACTATCTGAAGTTTTCCGGAAACTATTACCATCCACTTTCAGACTGGAAAG
ATTCAAAAGATTTCGACTTTTACGAAGAACGACCAGCGCGCGGCTGGGATGTCCGGGCTGAAGGGTGGTTGCCAGCATGG
CCACGGTTTGATCTCTAA
>Cluster_32012
ATGGGTAAACTCACGGGCAAGACAGCACTGATTACGGGCGCATTGCAGGGAATTGGCGAAGGAATTGCCAGAACTTTTGC
GCGTCATGGCGCAAACCTAATCTTGCTGGATATCTCCCCTGAGATCGAAAAGCTGGCGGACGAACTGTGTGGTCGTGGTC
ATCGCTGTACGGCGGTTGTCGCCGATGTGCGTGACCCGGCGTCGGTAGCCGCAGCTATTAAGCGCGCTAAGGAAAAAGAA
GGGCGCATTGATATTCTGGTGAATAACGCAGGCGTTTGTCGTCTGGGCAGTTTCCTCGATATGAGCGATGAAGATCGCGA
TTTCCATATTGATATCAATATAAAAGGCGTATGGAACGTCACGAAGGCGGTGCTGCCGGAGATGATTGCCCGCAAAGATG
GCCGCATTGTGATGATGTCTTCAGTCACTGGCGATATGGTGGCAGACCCGGGCGAAACGGCGTACGCCTTAACGAAAGCG
GCGATTGTTGGCCTGACTAAATCGCTGGCGGTGGAGTACGCGCAGTCTGGTATTCGCGTTAACGCCATCTGCCCTGGATA
CGTTCGCACGCCAATGGCGGAAAGCATTGCCCGCCAGTCGAACCCGGAAGATCCAGAATCGGTGCTGACTGAAATGGCGA
AAGCAATCCCGATGCGTCGCCTCGCCGATCCGCTGGAAGTCGGCGAACTGGCGGCCTTCCTCGCATCGGATGAATCCAGC
TATTTAACCGGTACACAGAATGTGATTGATGGCGGCAGCACACTGCCGGAGACGGTTAGCGTCGGCATCTGA
>Cluster_55041
ATGCACTCTGAACGCGCACCGTTTTTCCTCAAACTGGCGGCCTGGGGCGGCGTTGTTTTCCTACATTTTCCCATCCTGAT
AATCGCCGCCTATGCGTTTAACACTGAAGATGCGGCGTTTAGTTTTCCACCGCAGGGCCTGACGCTGCGCTGGTTTAGCG
TGGCAGCACAGCGTAGTGATATTCTTGATGCCGTGACACTGTCACTTAAAGTGGCGGCGCTGGCGACATTAATTGCGCTG
GTGTTAGGGACGCTGGCAGCTGCCGCGCTGTGGCGACGAGACTTTTTCGGCAAAAACGCCATTTCGCTGTTACTGCTGCT
GCCCATTGCGCTGCCGGGCATTGTCACTGGTCTGGCGTTATTAACCGCCTTTAAAACCATCAATCTGGAGCCGGGATTTT

TCACCATCGTGGTCGGTCATGCGACTTTTTGTGTAGTTGTGGTGTTTAACAATGTCATCGCCCGTTTTCGCCGCACCTCC
TGGAGTCTGGTTGAGGCGTCAATGGATCTTGGGGCCAATGGCTGGCAAACCTTCCGCTACGTAGTGTTGCCGAATCTCAG
TTCGGCGTTACTGGCAGGAGGAATGCTGGCGTTTGCCTTGTCGTTCGATGAAATCATCGTTACGACCTTTACGGCAGGTC
ATGAACGAACGTTACCGTTGTGGTTGCTCAATCAGCTTGGGCGACCGCGTGATGTACCGGTAACTAACGTGGTGGCACTG
CTGGTTATGTTGGTAACAACCTTGCCGATCCTGGGGGCCTGGTGGCTAACCCGCGAAGGCGACAATGGTCAATAA
>Cluster_6826
ATGAAGATTGAATATACGCCAGAACGTGGGCGGGGATTCGTTCGCCCTGGTGAAACTGAAAAACAACAAAATTGGGGTTT
TTCAGGTATAAAAAAAGCGGCCCCGAAATGGAGCCGCCTTTCTGAACAGATAACCCGCTGCGCCGTTTGTGTGTGTGATC
CCAAACATAAGCACGGGGATGATAGCCGCTATCAGGCTGGTGGGCAATGCAATCAGTCTGGTTCAGTTCGTTGCCATACC
TGCAATGAGCGCTTTTCCCTGTACTCTTTAAGGAATTGCTCAAGGGCAAAAGCACATGGTGCGAATCTTTCTGATTCATG
CTCTATCTTTCTGCGCCGTCTTTTCCGTGCCGGTGATAATGTTTTGGTCAATTCTTTATCGGTCATTGTGTTGTCCTGCA
TAGCAATGCGCCGTAATACCTCACACCACGGCGCTGATAGTGATTATTCTGATTCTTTGGCCTTGCGGCGCTGGCGGCGT
TTGATCTCGCCTTCAAGTGCCGAAACGATAAATTGCCCTGTACTTTCGCCATCTTCCTTTAAATTTTCTACAGCATCAGC
TATTGCGTGTGGTACTCGAGCCTCAAGCTTTTTGGATTTTGCATTAACTGCTTTTGTTGCCATATCTGATTTCCTCGTTA
TTAGGTGGCTGACAGTATACACACAAGAAAACGGAAAAACACTATTGACGTGGCTGACACCCGTTACTAATATAGTGGCT
GACACCTTTATCAAGGTAATCGATGAAAAGACAAAGCCCCGCAAGTGTCGTTACCACTCGCAGGGCTTCTAA
>Cluster_7928
ATGGAATCACGTAATTCATATGAAAACAAAATAGATGAGATTTCATCTTTGTCAGAGTCAAAGGAACACCCCATAGATAT
TCAAGAAAAAAAAGATGCGTTTGTGAATGAGTTCAAGGGGGTATTATTTGATAAAAATACAAGGTCTTCAGAGCTTCTGT
TTAATTTTTATGAGTGTTGCTATAAGTTTTTACCAAGAGCTCAGCCTCAAGATAAAATCGATAGCTATAATTCAGCACTG
CAAGCTTTTTCCATTTTTTGTTCATCTACGTTGACACATAATAATATAGGCTTTGATTTCAAATTATTTCCTGAAGTCAA
GCTGTCTGGAGAACATCTTGAAACGGTATTCAAATACAAAAATGGCGATGATGTCCGGGAGATAGCCAAAATTAACATTA
CTCTCCAAAAAGAAGAGGGTGGTTTATATAATTTACGTGGATTGGATTTTAAGGGGTGCTTCTTTTCTGGACAGAACTTC
AGTAACTATGATATTCAATATGTGAACTGGGAACGTCATTGTTTGATGTTGATACTCCGTGTATTTTTAATGCGCCTGC
TTACAACAAGAGTAATGAAAAATCATTAAAACCTGTGAGCGAAAACGGTTTAAGTGGAGTATTGACTGATCGTAATAATA
AAATAAAACTCATCACGGGCGTGGCACCATTCGATGATATTTTATTTATGGATGATGACTTTGATGATAGTTCCTCTGAG
GATGATCCCGTTGAGAATAGTCCTGTTGTGACTAGTCCCGTTGTATCAAGTTCTAAAAGCAGTTTTCAATGA
>Cluster_32336
ATGTCTGTGGTAATTAGTGATGCATGGCGTCAGCGTTTTGGTGGCACAGCGCGTCTGTATGGTGAAAAAGCATTGCAACT
GTTTGCTGACGCGCATATTTGTGTGGTTGGTATCGGTGGTGTCGGTTCCTGGGCGGCGGAAGCGCTAGCGCGCACGGGGA
TTGGCGCAATCACGCTTATCGATATGGATGATGTGTGCGTCACCAATACCAATCGGCAAATTCACGCTCTGCGCGATAAC
GTCGGGCTGGCAAAAGCGGAAGTAATGGCGGAGCGTATTCGCCAGATTAACCCGGAGTGCCGTGTAACGGTGGTGGATGA
CTTCGTGACGCCGGATAATGTAGCGCAATATATGAATGCCGGTTATTCGTACGTGATTGATGCTATTGATAGTGTACGGC
CCAAAGCGGCGCTGATTGCTTATTGTCGGCGCAATAAAATCCCGCTGGTCACGACCGGTGGCGCTGGTGGGCAGATTGAC
CCGACGCAGATTCAGGTTACCGATCTGGCGAAAACGATTCAGGACCCGTTGGCGGCGAAGTTGCGCGAGCGCCTGAAAAG
CGATTTTGGCGTAGTGAAAAACAGTAAAGGTAAGCTCGGCGTGGATTGCGTGTTCTCTACCGAAGCGCTGGTGTACCCAC
AGTCAGACGGCACCGTCTGTGCGATGAAAGCCACGGCGGAAGGGCCAAAGCGGATGGATTGTGCCTCAGGATTTGGCGCG
GCGACGATGGTGACCGCCACTTTTGGCTTTGTTGCGGTTTCTCATGCGCTGAAGAAGATGATGGCGAAAGCGGCGCGTCA
GGGTTAA
>Cluster_58255
ATGATATCATTAAAAGCTCCGCACAATAATTTGATGCCATATACGCAACAAAGCATACTTAATACGGTTAAAAACAATCA
GTTACCAGAGGATATCAAAAGCTCCCTGGTTTCCTGTGTCGATATATTCAAGGTTTTGATTAAACAATATTATGATTATC
CTTATGATTGTCGTGATGATTTAGTCGATGACGACAAACTCATTCATCTCATGGCTGCCGTACGAGACTGTGAGTGGTCA
GACGATAACGCACTCACCATAAATGTGCAGTTTAATGATTTCCCTGGATTTTATGACTGGATGGATTACCCTGATCACCC
GGTTAAGTTTGTTTTTCACATACTTGAAAATCAAAAAGGGACAGTTTGGGTATATGACCAGGATGATGCGTTTCTTGATA
TTAAAGCAAACGTGCAGGCTGGACGCTTTACCGGGCTTAAGAAATTAGTGCAGTTTATCGATTCAGTGCGAACAGATTGC
AAATGTATTCTTCTTGAATACCATATGCCTTTGCTGAGAATTTTCCCTAAAGGGAAAGAGTGTATGCATGTGGAAAAATG
GCTTCGCGAAATGTCGTCTATTCCGGAGACAGACGCGCCCATTAAGCAGGCGCTTGCGCATGGATTATTATTACACTTAA
AAAATATCTACCCTGTTTTTCCTGAATCACTGGTGATGTTATTGCTCTCTGTTCTGGACGTAAAAACATACAGAGATGAT
GCGCGATTAAATGAATGGATATCTAATAGAGTGCAGGAATTAGGTGACAGATATTATCCTGTAAATAAGCACGTTAAAAT
CCGCTACACTCTTTAG
>Cluster_5641
ATGCTTCCATCGGAAACGATGATCTGGCAGCCTGAGTTCACAGATAAAACACTCTCCAGGAAACCCGGGGCGGTTCACTA
TACGCCATCAGAGCAGTTTTATCCGCCTGGCGCACCAATCCCGTGGCCATCAGATACCGTTCCGTCTGGCTATGCCCTGA
TGCAGGGGCAGACTTTTGACAAATCTGCATACCCGAAACTTGCAGCCGCTTATCCGTCAGGCGTGATCCCTGATATGCGT
GGCTGGACGATTAAGGGCAAACCCGCCAGTGGTCGTGCCGTATTGTCTCAGGAACAGGACGGCATTAAATCGCACACCCA
CAGCGCCAGCGCATCCAGTACGGATTTGGGGACGAAAAACACATCGTCGTTTGATTACGGAACCAAATCCACGAATAACA
CCGGGGCGCATACGCACAGTCTGAGTGGCTCTACGGGGTCTGCCGGTGATCATACTCATGGTAATGGTATTCGTTGGCCA
GGAGGCGGCGGTTCTGCGTTAGCATTTTATGATGGCGGTGGGTTCACTTATGTCCAGGATTCACAGTATCAAGTAAGCCC
GGGGACTTCTTCCCGTAGATCGTATTATCAACGTATTCAGACACAGTCAGCAGGTGCTCATACCCACTCGCTGTCTGGTA
CTGCAGCAAGTTCTGGCGCACATGCACATACTGTAGGTATTGGTGCGCATACGCACTCCGTTGCGATTGGTTCACATGGA
CACACCATCACCGTTAACGCTGCTGGTAACGCGGAAAACACCGTCAAAAACATCGCATTTAACTATATTGTGAGGCTTGC
ATAA
>Cluster_27198
ATGCTGGCGTTGTCGCAATCAGCAGTGAGCGCAGCCTTGACCGACCTGGAAGGGCAGCTTGGCGTTCAACTGTTTGATCG
CGTGGGGAAAAGACTGGTTGTTAATGAACACGGGCGGCTGCTCTATCCGCGAGCGTTGGCACTGCTTGAACAGGCGGTTG
AAATCGAACAACTGTTTCGCGAAGACAACGGCGCGATTCGTATCTATGCCAGTAGTACCATCGGTAACTACATTCTGCCT
GCAGTTATCGCCCGTTATCGCCATGATTATCCGCAGTTGCCGATTGAACTTAGCGTTGGGAATAGCCAGGACGTGATGCA
AGCGGTGCTGGATTTCCGCGTTGATATTGGCTTTATTGAAGGACCGTGCCACAGCACTGAAATCATTTCTGAACCGTGGC
TGGAAGACGAGCTGGTGGTTTTCGCCGCGCCGACTTCGCCGTTGGCTCGTGGTCCGGTCACCTTAGAACAGCTGGCCGCT
GCGCCGTGGATCCTGCGTGAACGCGGTTCCGGCACGCGGGAGATTGTCGATTATCTGTTGCTGTCACATTTACCGAAGTT
TGAGATGGCGATGGAATTAGGTAACTCCGAGGCAATCAAACATGCGGTGCGTCATGGGTTGGGAATTAGTTGCCTGTCGC
GACGGGTGATTGAAGATCAATTGCAGGCAGGCACATTAAGTGAAGTTGCGGTCCCTCTGCCGCGCCTGATGCGTACGTTG
TGGCGTATACATCATCGGCAAAAACACCTTTCCAACGCGCTACGGCGCTTTCTGGACTATTGCGATCCCGCGAATGTACC
GCGTTAA
>Cluster_4780
ATGCTGATGCTGCACCGGAGCGCTCGCGTCAGTGACGTTGCCAGAACGCTCTGCTGCGCCCGTTCCTCCGTCAGGCGCTG
GATTAACTGGTTCACGCTGTCGAGTGTTGAAGGTCTGAAATCATTACCCGCAGGACGTTCCCGCCGCTGGCCGTTTGAGC
ATATCTGCACATTGTTTCGTGAACTGATAAAACACTCTCCCGGATATTTTGGCTATCAGCGTTCACGCTGGAGTACAGAA

CTGCTGGCAATAAAAATCAACGAGATAACCGAGTGCCAGTTACATGCCGGAACCGTTCGCCGCTGGTTGCCATTAGTCGG
GCTTGTGTGGCGCAGGGCTGCGCCAACCCTGCGTACCCGTAACCCGCATAAAGCAGCAATTCACAAAGCACTGGACGAAT
GTAGTGCGGAGCATCCGGTCTTTTATGAAGATGAAGTGTATATCTATCTTAATCCCAAAACCGGTGCGGACTGGCAACTT
CGCAGACAGCAAAAACATGTGGTAACACCGGGGCAGAATGAAAAATACTATCTGGCTGGTGCTTTACATAGTGGAACGGG
CAAAGTCAGCTATGTGGGTGGCAACAGCAAAAGCTCAGCCTTATTTATCAGTCTGTTGAAACATCTGAAATTGACATACC
GACGGGATAAACCTATCACGCTCATAGTGGACAACTACATCATCCATAAAAGTCGGGAAACACAGCGCTGGCTGAAAGAA
AATCCGAAGTTCAGAGTGATTTATCAGCCAGTTTACTCGCCGTGGGTGAATCATGTTGAGCGGTTATAG
>Cluster_68
ATGAGAGCTGTCGCCGGTGTGCTTGTTCTTCTTGCCACTGCATCACTGCATGCGGCTCCCGACGATGTACTGGTCCCCAA
CTATGCCCGGGAAGAAGGCTGGCAGTGGTACAACCCGAATGCGGAAGAGATTCAACCGGAGCCTGCGCCAGAGCCGCCCC
TTCAGGAACGTCTGGCCAGCATGACGCCATCTGAGCAGAAAAAGGTCCTGCAGCAGGCGACCCGTGAAGCGCTGGATACG
GCCATTCTGTATCCGTCTGCGGAGAACTTCCGCCGTTTTATGATACTCCAGAATTTCTGGACTGACCGGGCGACAGATTT
TACCCAGACATCAAAGCTGGCGCGCCTGAAATATCCGGAACTCGACTACAACGTGAAACGTAGTCACTACAACGGTTCTG
TGGAAGCGCGTCTTACCGAAGAGAGAAAGGTTCAGAATGCGGTCATCCGTCAGGTGGCACAGCGCTATGGTCTGTTCTTC
TTCTACCGGGGGAATAATGCCGTCGACAACCTGATGGCCGGTGTCATCCGCGCGTTCTGCGAAGACCGGGGCATTACACT
TATGGCCGTGAGTGTGGACGGTAAAATCAGCGACCAGCTGCCGCAGAGCCGTCCGGACAGCGGGCAGGCTGAGCAGATGC
GCGCCACACATTTCCCCGCGACCTTCCTGGTCGATCCGAAAACGCACCAGTGGCAGCCTCTTGCATGGGGATTTATGAGC
CACGATGACCTGGACCGCCAGATGGTCAACGTTCTTACCCATTTTAAACCTGACTACTGA
>Cluster_29207
ATGCAAACCATCACCATCGCCCCACCCAAGCGCAGCTGGTTCTCGCTTCTGAGCTGGGCCGTTGTTCTCGCCGTGCTGGT
CGTCTCGTGGCAGGGCGCGGAAATGGCCCCGCTCACGCTGATTAAAGATGGCGGCAACATGGCGACGTTCGCCGCCGACT
TCTTCCCGCCCGATTTCAGCCAGTGGCAGGATTACCTCACCGAAATGGCCGTCACGCTGCAAATCGCCGTCTGGGGCACC
GCGCTGGCGGTGGTTCTCTCCATCCCCTTCGGCCTGATGAGCGCTGAAAACCTGGTACCGTGGTGGGTCTACCAACCTGT
ACGCCGCCTGATGGACGCCTGCCGCGCCATTAACGAAATGGTCTTCGCCATGCTGTTCGTGGTCGCCGTCGGCCTCGGCC
CGTTCGCTGGCGTGCTGGCGCTGTTTATCCACACCACCGGCGTACTTTCCAAACTGCTTTCCGAAGCGGTAGAAGCGATT
GAACCCGGCCCGGTGGAGGGCATTCGCGCCACCGGAGCCAACAAGCTGGAAGAGATCCTCTACGGCGTACTACCGCAGGT
CATGCCGCTGCTGATTTCCTACTCTCTCTATCGCTTCGAATCCAACGTCCGCTCGGCGACCGTCGTCGGTATGGTTGGCG
CGGGCGGAATTGGCGTCACGCTGTGGGAAGCGATTCGCGGTTTCCAGTTCCAGCAAACCTGCGCCCTGATGGTGCTCATC
ATCGTCACGGTCAGCCTGCTGGATTTCCTCTCTCAACGGTTGCGTAAGCACTTTATCTGA
>Cluster_37810
ATGAGTATGGTTGAAACTGCCCCGAGTAAAATTCAGGTTCGTAATTTGAACTTCTACTACGGCAAATTCCATGCCCTGAA
AAACATCAACCTGGATATCGCTAAAAACCAGGTAACGGCGTTTATCGGGCCGTCCGGCTGCGGTAAATCGACGCTGCTGC
GTACCTTCAACAAAATGTTTGAACTGTACCCGGAGCAGCGTGCGGAAGGTGAAATTCTGCTTGATGGCGACAACATCCTG
ACCAACTCTCAGGATATCGCACTGCTGCGTGCGAAAGTGGGCATGGTGTTCCAGAAACCGACGCCGTTTCCGATGTCCAT
CTACGACAACATCGCTTTTGGCGTTCGTCTGTTTGAGAAGCTCTCCCGTGCCGACATGGACGAGCGCGTGCAGTGGGCAT
TGACCAAAGCCGCATTGTGGAACGAAACCAAAGATAAATTGCACCAGAGCGGTTACTCTCTCTGGTGGTCAGCAACAG
CGTCTGTGTATTGCGCGTGGTATCGCCATTCGCCCGGAAGTGCTGCTGCTCGACGAACCGTGTTCGGCGCTCGACCCTAT
CTCTACCGGGCGTATTGAAGAGCTGATCACCGAACTGAAGCAGGATTACACCGTGGTGATCGTCACCCACAACATGCAGC
AGGCTGCGCGTTGTTCCGACCACACGGCGTTTATGTACCTGGGCGAATTGATTGAGTTCAGCAACACGGACGATCTGTTC
ACCAAGCCAGCGAAGAAACAAACAGAAGACTACATCACCGGTCGTTACGGTTGA
>Cluster_29309
ATGAATGACCAACCCGTTCTGTTCAGCAGAGCGGCGGCTTCTTGCCGTCTGACATTGAACCGTGAAGATAAATGTCATGC
TATTAATGAAGAAATGATCGAATCTCTCGACCATTATTTAAATGAAATTGAAAACGACACCACATTACGATTAGTCGAAT
TAACGGCAACCGGCGATAAATTCTTTTGTGCGGGCGGCGATATTAAATCGTGGTCGGCCTATTCGCCATTAGATATGGGC
AGAAAATGGATTAAACGCGGTAATGACGTTTTTAATCGCCTGCGCAACTTACCCCAATTAACCGTTGCGAATCTCAACGG
TCATACCATCGGTGGCGGTATTGAACTTGCCTTGTGCTGCGATATTCGTATTGCCCGCCCCGGCGCGAAATTCTCTAACC
CGGAAGTGATGCTCGGCATGGTGCCTGGCTGGATGGGCATTGAACGTGTGCTTAATCAGGTCGGTCCGGTGGTCGGTCGC
CAGATGCTGATGCTGGGTAAACGCCTGACGGCGCAGGAAGCCCAGGCGGCGAATTTAATTGATGAAGTGGTAGAAAAAGA
ACAGGTAGAAAGCTGGATGGCGAATCAGTTAGCGCAGCTGGAAAAATGTGGTCCGGTGGCACTGGCGCATATTAAGCAGC
TGATTCTGGCGCTGGAAAATAAACACGCCGATTACCCGCACCAGCTACTGGCTGGATTAATGTCCGCCACTCAGGATTGC
CAGCAAGCGACGCGCGCCTTTGCCGAAAAAGCCGGCGTGAGCTTCCATAATCAATAA
>Cluster_20300
ATGAACGCAGCACTATGGATCAGCAAAACCGGCCTCTCGGCGCAGGATGCTGAAATGAGCGCGATTGCCAACAACATCGC
CAACGTTAACACCACCGGCTTTAAGCGCGACCGGGTGATGTTTCAGGATCTGTTTTACCAGACCCAGGAAGCGCCGGGCG
CGATGCTCGATCAGAACAACATTATGCCGACCGGGCTGCAATTCGGTGGCGGCGTACGCATTGTCGGCACTCAGAAAACC
TTCACCGAAGGCAACGTCGAAACCACCGACAACGCGATGAATGTCGCCATTATGGGGCAGGGGTTTTTGCAGGTGCAAAA
GGCCAACGGCGATATCGCTTATACCCGCGACGGCAACTTGCAGGTGAACGCTGACGGCGTACTGACCAACTCGCAGGGCT
TGCCGTTGCAGCCGGAAATCGACGTTCCGGCGGGGGCGACCAACGTCGCGTTTGGTGAGGATGGCACGGTGACGGCGATC
TTACCCGGCGACAGCGACGTGACTGAGTTAGGGCAACTGACGCTGGTGAACTTCGCCAACCCGGCCGGGCTTTCTGCCGA
GGGCGACAACCTCTATCTGGAAACCGCCGCCAGCGGACAGCCGACCGAAGGCGTACCGGGCGAAGATGGGCTTGGCACCT
TGCAGGATAATGCGCTGGAAGGCTCGAACGTGGATATCGTCAACGAAATGGTGGCGATGATTACGGTGCAACGCGCGTAC
GAGATGAACGCCAAAATGGTGTCAGCAGCGGACGATATGTTGCAGTACATCAGCCAGACGCTGTAA
>Cluster_43458
ATGGCCTATCGTTCCGCACCGCTCTATGAAGATGTTATCTGGCGAACGCATCTCCAGCCGCAGGATGCTGGGCTTGCACA
AGCCGTGCGGGCGACGATCGCCGAACATCGTGAACATTTGCTGGAGTTTATCCGCCTGGATGAACCCGCCCCACTTAACG
CCATGACGCTGGCGCAATGGTCATCACCTAATGCGCTAAGTTCTCTACTCGCGGTCTATTCCGATCATATCTACCGCAAC
CAACCTTTGATGATCCGCGAAAACAAACCGCTGATCTCACTGTGGGCGCAATGGTATATCGGCCTGATGGTACCACCATT
AATGCTGGCGCTGCTGACTCAGGAAAAAGCATTAGATGTGTCGCCGGAACATGTCCACGTTGAGTTTCACGAAACCGGAC
GCGCCGCCTGTTTCTGGGTCGATGTGTGTGAAGATAAAAACGCAACACTACATTCGCCGCAGCAGCGAATGGAAACGTTA
ATCAGCCAGGCGCTGGTTCCGGTTGTGCAGGCACTAGAAGCAACGGGAGAGATTAACGGCAAACTTATCTGGAGCAATAC
CGGTTATTTGATCAACTGGTATCTGACTGAGATGAAACAGCTGCTCGGCGAGGCGACGGTTGAATCGCTGCGCTATGCCC
TCTTTTTTGAGAAAACGCTTACTACCGGTGAAGATAATCCACTCTGGCGTACCGTGGTGCTACGCGACGGCCTGCTGGTG
CGCCGCACTTGCTGCCAGCGTTATCGCTTACCGGATGTGCAGCAATGTGGCGATTGTACGCTGAAATGA
>Cluster_12705
ATGATTCTGTATAAATATATAGATAAGACATCATTGGAACGATTTTTTAAAGATGGTTACATCTCTATAAAATTCACTCC
ATATAGTGAGTTTAATGATCCTTTTGAAAGCTATGGCTATGAACTTTCATTAGATGGTGAATCGCTTGAACCATTAGCTA
TGAGGGGGGAGATCAATCAAAATGTTGCCTGCTTATGCCTTTCTAAAATCCTCTCAATGTACTGATGTGGTCACATTAT
GCTGATAAACATCAGGGGTTTGTTGTAGCAATTGACACAGAAAGGGCAGGGTTTGATGATGAAGAAAATGCCTAATTAC
GGCACAGAAAGGTGACATTAATTATTTAGAGAGTCGAATAAAAAGTAAACTAAAAATCAGTCAAGAAAACATTTATAGCG

CTGATGTAATTAGTAAATTATTGCTTACTAAATCATTGCATTGGAAATATGAAGAAGAAGTTAGAATAATAAAGCGAGTG
GGGCTATTACAAAAGCAAGAAAATATTTTAATTGACAAGATAACTGATTTAAGTTCGGTAACAGCTATCTACATTGGGAT
TAACAATAAAGACATTGACAAAATCATTAAGAAAAGCAGTGTGTTAGAAGGGTTGGTTTTAAACAAGACTGTGCGATTAT
ATCAGTGTGAATTCAAGAAAAGTACATGGGATCTCACCATAGAAGATTATGAATATACAGAATTCCCACATGCTATGCAG
AGGTGGGGATTTGAATCTTTAGAGAAGATTGTAAGATATATAGAACGTAATCACATAGGTGATTGA
>Cluster_49700
ATGAGTACAGCACTCGCAACGCTGGCAGGGAAGCTGGCTGAACGTGTCGGCATGGATTCTGTCGACCCACAGGAACTGAT
CACCACTCTTCGCCAGACGGCATTTAAAGGCGATGCCAGCGATGCGCAGTTCATCGCATTGTTGATCGTCGCCAACCAGT
ACGGCCTTAATCCGTGGACGAAAGAAATTTACGCCTTCCCTGATAAGCAGAACGGCATCGTTCCGGTGGTGGGCGTTGAT
GGCTGGTCCCGCATCATCAATGAAAACCAGCAGTTTGATGGCATGGACTTTGAGCAGGACAATGAATCCTGTACATGCCG
GATTTACCGCAAGGACCGTAATCATCCGATCTGCGTTACCGAATGGATGGATGAATGCCGCCGCGAACCATTCAAAACCC
GCGAAGGCAGAGAAATCACGGGGCCGTGGCAGTCGCATCCTAAACGGATGTTACGGCATAAAGCTATGATTCAGTGTGCC
CGTCTGGCCTTCGGATTTGCTGGTATCTATGACAAGGATGAAGCCGAGCGCATTGTCGAAAATACTGCATACACTGCAGA
ACGCCAGCCGGAACGCGACATCACTCCGGTTAACGATGAAACCATGCAGGAGATTAACACTCTGCTAATCGCCCTGGATA
AAACATGGGATGACGACTTATTGCCGCTCTGTTCCCAGATATTTCGCCGCGACATTCGCGCATCGTCAGAACTGACACAG
GCCGAAGCAGTGAAAGCTCTTGGATTCCTGAAACAGAAAGCCACTGAGCAGAAGGTGGCAGCATGA
>Cluster_36019
ATGTCGTTCTGGAAAGTTGCAGCAGCGCAATATGAGCCTCGTAAAACCTCACTCACCGAACAGGTAGCTCATCATCTGGA
GTTTGTGAGGGCCGCCGCCAGGCAACAATGTCAGCTGTTAGTTTTTCCATCGCTTTCTTTACTGGGGTGTGATTATTCGC
GACGTGCCCTTCCTGCCCCACCCGATCTCTCACTGTTAGACCCGCTTTGTTATGCCGCAACGACCTGGCGAATGACCATC
ATTGCCGGCCTTCCCGTTGAATATAACGATCGCTTTATTCGTGGAATTGCGGTGTTCGCACCGTGGCGAAAAACGCCTGG
GATCTACCATCAGAGTCATGGTGCATGTCTGGGCCGTCGTTCCAGAACCATCACGGTGGTCGATGAACAACCGCAAGGTA
TGGATATGGACCCGACCTGTTCACTGTTTACTACAGGGCAATGTCTGGGAGAACCCGACCTGCTGGCGTCGGCCCGTCGC
TTACAATTTTTTCACATCAGTACTCGATTGCCGTGCTGATGGCAAATGCCCGTGGTAACAGTGCACTATGGGATGAATA
TGGTCGTCTCATCGTTCGCGCCGATCGTGGTTCATTATTGTTAGTCGGTCAGCGTTCATCACAGGGTTGGCAAGGCGATA
TCATTCCATTACGCTAG
>Cluster_58
ATGAGTTTAAAACTGGTAATTTCTAACCTTTGGAAGGGATATGGGGACAAAACCGTTTTAAAAGACCTATCCTATGTGTT
TGATGAGAAAAAGGTCTACGTAGTAATGGGGCCTAATGGAAGTGGAAAAACTACCTTTTTAAAAATCTGTGCTTTACTTG
AAACACCTGATAAAGGAGAGGTCCATTACTACAATTCAACCAATAAGATTTACCTAAAGATATTAATCTTCTTAGAAAA
ATTACTTATGTATTTCCTGAGGTAGGTATTTTCAATACTTCAGTTTTTGATAACGTAGCCTATGGATTAAAAATAAGGGG
TTTTAACAAAAAAGAGATAAACTCTAAAGTAGAAGAAGTGCTTGATTTCGTAGGACTGTCGCATAAAAAAAAGCAAAACG
CATTAACCCTTTCAAGTGGAGAGGCAAAGAGGATGGGCCTTGCAAGGGCGATGGTGATAAAACCTGAGTTTCTCTTTTTA
GACGAACCTACAGCTTTTGTAGATGCTGAGAACGTAAAGCTTATCGAAAGACTTTTAATTCAGATCAAAGAACAAAAAAC
CACTACCATAGTTATAGCCACCCACGATAGTCCCTTTGCCTTTACGGTTGGAGATGAGATCTTAAAGTTAGAAAACGGAA
AAGTTTTTGAGGTAAGCTAA
>Cluster_11606
ATGAGAAATATTATGAAAAAAGTACTCATTCTTGGTGCTGGCGGTCAGATAGCCCGCCATGTGATTAATCAACTGGCAGA
TAAACAAACCATTAAACAAACTTTATTTGCTCGTCAGCCAGCAAAAATACACAAACCCTATCCAACTAATAGCCAGATTA
TTATGGGGGATGTACTGAATCATGCAGCACTGAAGCAGGCCATGCAGGGACAGGATATTGTGTATGCCAACCTTACGGGG
GAAGATTTAGATATTCAGGCAAATTCAGTGATCGCTGCGATGAAAGCCTGTGATGTTAAACGTCTGATCTTTGTTCTTTC
CCTCGGCATTTATGATGAAGTTCCAGGAAAATTTGGCGAATGGAATAATGCAGTTATTGGTGAGCCCCTAAAACCATTCC
GCAGGGCTGCGGATGCAATTGAGGCTTCTGGACTGGATTATACAATTCTTCGCCCTGCCTGGCTGACTGATGAAGATATT
ATTGATTATGAGATTACCTCACGCAATGAACTATTCAAAGGTACGATCGTATCGCGTAAGAGTGTCGCCGCATTAATCAC
AGATATCATCGATAAACCTGAGAAACACATTGGTGAGAATATAGGCATCAATCAACCTGGTACTGATGGTGATAAGCCAT
TCTTCATGTAA
>Cluster_5124
ATGATTCAAACACGTAATCAATATCTGCAGTTTATGCTGGTTATGCTGGCTGCATGGGGCATTAGTTGGGGAGCCAGATT
TGTCATGGAGCAGGCCGTTCTGCTTTATGGATCAGGAAAAAACTATTTGTTCTTCAGTCATGGTACTGTTCTGATGTACC
TGCTGTGTGTTTTCCTGGTATACCGCCGTTGGATAGCTCCGCTACCGGTCGTTGGTCAGCTGCGCAACGTTGGCGTACCG
TGGCTGGTCGGTGCGATGGCCGTGGTGTATGTCGGTGTATTTCTGCTCGGTAAGGCGCTGGCTCTGCCTGCTGAGCCATT
TATGACGAAACTTTTTGCCGATAAGTCCATACCTGACGTGATCCTGACGTTGCTGACCATCTTTATCCTTGCCCCGTTGA
ATGAGGAAACGCTGTTCCGGGGGATTATGCTGAACGTCTTCCGTTCACGGTACTGCTGGACGATGTGGCTGGGGGCGCTG
ATAACGTCGTTGTTGTTCGTCGCCGCGCACAGCCAGTATCAGAACCTGCTGACACTGGCAGAACTGTTCCTGGTGGGGTT
GATTACATCAGTGGCCAGGATCAGAAGTGGTGGCCTGCTGCTGCCGGTATTGCTGCATATGGAAGCAACCACGCTGGGTT
TACTGTTTGGTTGA
>Cluster_61334
ATGAACGTTTTCTCGCAAACTCAACGCTATAAGGCGTTGTTCTGGTTATCGTTATTTCATCTGCTGGTGATCACCTCCAG
TAACTATCTGGTTCAGCTTCCCGTCTCCATTTTGGGTTTCCATACCACCTGGGGCGCGTTTAGCTTTCCGTTTATTTTTC
TTGCTACCGACCTGACCGTGCGTATTTTTGGCGCACCGCTGGCCCGACGCATTATCTTCGCGGTAATGATCCCTGCGTTA
TTAATCTCCTACGTCATCTCGTCGCTATTCTATATGGGTTCCTGGCAGGGATTCGGCGCACTCGCCCACTTCAACCTGTT
TGTCGCCCGTATCGCCACCGCCAGTTTCATGGCCTACGCGCTGGGGCAAATCCTCGACGTGCACGTTTTTAACCGCCTGC
GTCAGAGTCGCCGCTGGTGGCTGGCACCGACAGCGTCCACACTGTTCGGTAACGTCAGCGACACGCTGGCCTTTTTCTTC
ATTGCCTTCTGGCGTAGCCCGGATGCCTTTATGGCTGAACACTGGATGGAAATCGCGCTGGTCGATTACTGTTTCAAAGT
GTTAATCAGTATCGTTTTCTTCCTGCCAATGTATGGCGTATTACTCAATATGCTGTTGAAAAGACTGGCAGATAAATCCG
AAATCAACGCTTTGCAGGCGAGTTAA
>Cluster_20982
ATGAATATCTACGAAAGAATTGATGGCAGCAAATACCGAAATATCTGGGTAGCTGGCGACCTGCACGGATGCTACACGAA
CCTGATGAACAAACTGGATACGATTGGATTCGACAACAAAAAAGACCTGCTTATCTCGGTGGGCGATTTGGTTGATCGTG
GTGCAGAGAACGTTGAATGCCTGGAATTAATCACATTCCCCTGGTTCAGAGCTGTACGTGGAAACCATGAGCAAATGATG
ATTGATGGCTTATCAGAGCGTGGAAACGTCAATCACTGGCTGCTTAATGGCGGTGGCTGGTTCTTTAATCTCGATTACGA
CAAAGAAATTCTGGCTAAAGCTCTTGCCCATAAAGCAGAAGAACTTCCGTTAATCATCGAACTGGTGAGCAAAGGTAAAA
AATATGTCATCTGCCACGCCGATTATCCTTGTGACGAATACGAATTTGGAAAGCCAGTTGATCATCAGCAGGTAATCTGG
AACCGCGAACGAATCAGCAACTCACAAGACGGGATCGTGAAAGAAATTAAAGGCGCGGACACGTTTATCTTTGGTCATAC
GCCAGCAGTGAAACCACTCAAATTTGCCAACCAGATGTATATCGATACTGGCGCAGTGTTCTGCGGAAACCTCACATTGA
TTCAGGTACAGGGAGAAGGCGCATGA
>Cluster_29316
ATGGCTTATCGCGACCAACCTTTAGGTGAACTGGCGCTCTCTATTCCTCGCGCTTCGGCTCTGTTTCGTAAATATGATAT
GGATTACTGCTGTGGCGGTAAGCAGACGCTGGCGCGCGCGGCGGCACGTAAAGAACTGGATGTTGACGTCATTGAAGCCG

AACTGGCAAAGCTTGCTGAACAACCGATTGAGAAAGACTGGCGTAGCGCCCCGCTGGCGGAAATCATCGACCACATTATT
GTGCGCTATCACGATCGCCATCGCGAGCAGCTGCCGGAGCTGATCCTGCAAGCGACTAAAGTCGAGCGCGTTCACGCCGA
CAAACCAAGTGTGCCAAAAGGGCTGACAAAATACCTGACTATGCTGCATGAAGAGCTTTCCAGCCATATGATGAAAGAAG
AGCAAATTCTCTTCCCGATGATTAAACAAGGCATGGGCAGCCAGGCAATGGGGCCAATCAGCGTAATGGAAAGCGAGCAC
GATGAAGCGGGCGAACTGCTGGAAGTGATCAAACACACCACCAATAACGTCACACCGCCGCCGGAAGCCTGCACCACCTG
GAAAGCGATGTATAACGGCATTAATGAACTGATTGATGACCTGATGGAGCACATCAGTCTGGAAAACAATATACTATTCC
CGCGCGCGCTGGCGGGTGAGTGA
>Cluster_1676
ATGAGCAACACTACTGATTGGGGAAGCATGATAGCCAACATAGTTATGGCTACTACCGCTGTCTGGGCTGCATTCAAAGC
TAATGACTGGTTTAAAACCAAGCGTCTTGAGAGTGCTGAAAACTTCTATAAAGAAGTACACAATCTCCATAAACGTTACT
ATTCAGCTATCCAATACGTTGAGGATTCAAGAAGAATATTTGATAGTCAGTATGGGTTGCATTCGCCAATATCTGAACCT
AAAGACAAGTCATTGCAATGGGAGGTTAAATCGAACTCTTACAAAATCATCGCTGAACTAGATTATCTGGCATCGTCACT
ACAAATGATGGATGCAATGGGTAACAAACTTAGCCCTAAATCGGCTTCACTGGTTAGTGATATGATGGAAGCACATGGAC
GATTCTGTAATGATGTGATGAACTTCAATGACTCAGTTTGGAAGGTTATAAGGGCAGGTATAACTGAAGAGAACATCCCA
CATACGCGAGGCTATGTGAACGGCGATTACATGGCTATCCTTACCAGTATGGATGAACCTGTAAGAATATCTTTTAAGTT
AAAAGAAATCAGTTTTAAAGATTTTTACCTACATGAAACTACTATGCAGTCAGTAATAACGAGGCTGAAATCGCTTATCG
CCAGAACAGGTAAAAACAGGACATAA
>Cluster_60139
ATGGCAGTTTCAGCAAAGTACGACGAATTTAATCACTGGTGGGCAACAGAGGGCGACTGGGTTGAAGAGCCTAACTATCG
CCGTAATGGTATGAGCGGGGTGCAATGCGTCGAGCGCAACGGCAAAAAGCTGTATGTAAAGCGCATGACGCATCATCTGT
TTCATTCCGTACGTTATCCGTTCGGCCGACCAACGATTGTCCGTGAGGTTGCGGTAATTAAAGAACTGGAACGGGCCGGT
GTCATCGTGCCGAAGATCGTTTTTGGTGAAGCGGTGAAAATTGAGGGTGAATGGCGAGCGTTGCTGGTGACTGAAGATAT
GGCGGGGTTCATCAGCATTGCTGACTGGTATGCCCAGCATGCAGTATCGCCTTATTCTGACGAAGTACGGCAAGCCATGT
TGAAAGCAGTGGCGCTGGCGTTTAAGAAAATGCATAGCATTAATCGTCAGCATGGCTGTTGTTATGTTCGCCATATCTAT
GTGAAAACAGAAGGTAATGCAGAAGCTGGTTTTCTTGACCTGGAGAAAAGTCGTCGCCGCTTGCGTCGCGATAAAGCGAT
TAATCATGACTTCCGCCAGCTGGAAAAATATCTGGAGCCAATCCCCAAAGCGGACTGGGAGCAGGTGAAAGCGTATTACT
ACGCCATGTAA
>Cluster_26023
ATGACGCAAGGCGCAGTGAAAACAACGGGTAAGCGTTCGCGCGCAGTAAGCGCGAAGAAAAAAGCGATTCTTAGCGCAGC
ACTGGACACTTTTTCACAATTCGGTTTTCACGGCACAAGGCTGGAGCAGATCGCGGAGTTGGCGGGTGTTTCAAAAACCA
ATCTGCTGTATTACTTTCCGTCAAAAGAGGCGCTGTATATAGCCGTACTGCGGCAGATTCTCGATATCTGGCTGGCACCG
TTAAAAGCGTTTCGAGAAGATTTCGCCCCGCTGGCGGCGATCAAAGAATACATCCGTCTGAAGCTGGAAGTTTCCCGCGA
TTATCCGCAGGCTTCGCGCCTGTTCTGTATGGAGATGCTGGCAGGTGCACCGCTGTTAATGGATGAACTGACGGGCGATT
TGAAGGCATTAATTGATGAGAAATCGGCGCTCATTGCCGGTTGGGTTAAAAGCGGCAAACTCGCGCCGATTGACCCGCAG
CATTTGATTTTTATGATTTGGGCTTCCACTCAACATTACGCCGATTTTGCCCCTCAGGTGGAGGCGGTGACAGGCGCGAC
GTTGCGCGATGAGGTGTTTTTCAATCAAACGGTTGAAAACGTGCAGCGGATTATTATTGAGGGGATTCGACCACGTTAA
>Cluster_31800
ATGGGTGGCGCGGTAAATCAGACTACGATCAATAATGGAGTGCTACAGGTTTATGGCGCGGCGAATGATCCCACGATTAA
AGGCGGGCGCTTAATTGTTGAAAAAGATGGGATTGCCGTCTTTGCCGTTATAGAAAAGGGAGGACTACTGGAGGTTAAAG
CAACAGGTGATTTCAAAATATTCGTGACGGACACCGGTGCCAGCCCGGCAGCAGGAGATAGCCTTACACTGGTAACAACG
GGCGGCGGTGATGCTGCATTTACGTTGGGCAATGCCGGAAGCGTTGTTGATATCAGTACGTATGAATATACCTTGCTGGA
TAATGGCAACCATAGCTGGAGTCTCGCAGAGAATCGCGCACAAATGACCCCTTCAACCACGGATGTGCTGAATATGGCGG
CAGCACAACCGCTGGTATTTGATGCAGAACTGGACACCGTGCGTGGGCGTCTTGGTAGCGTAAAAGGCGTTAATTACGAT
ACGGCGATGTGGAGTTCGGCAATTAACAGCCGCAACGACGTGACCACTGATGCAGGCGCTGGCTTTGAGCAAACATTGAC
GGGGCTGACGCTCGGTATCGATAGTCGTTTCTCCCGTGAAGAAAGCAGCACAACTCAGGCGGGTGTAGTCTGGACGTTCT
GA
>Cluster_22103
ATGCAAACATTTAAAGATGTATTTATAGAACTTGGTGATAAGTCAATAATAGATTTTATTTCTAAGGTTACTGATAACGT
TAAATCCTATTGGAGTCGATCATATGAAAGTGAGGAAAACTCAAAATATTTAGGCGAAATTGCTTTTAGCTTTAAAAGGT
CAGGTGATAACAGATTACCTGATGCTGGTCTAAGTATTTTCCAGAAAGAAAATAATATTTGGTATATTCCTAATGTTGTA
CCTTTAGAAAAAGGACAGCTAAACTACGATGAATATAATACAATAATCACGGATTTTATAACTCGTGCTTAAAACCAGT
TGCACTTGAGTTAAAAATTGATATTAAAATAACCTCGGATTCCTTAACTGCAGAAGATGTTGTAGGGGTTTACGCAGGAA
AACTATTAAAACAATTTTCATCTTTAGCGAATAAATCAACAGGAAGTTCACATCCAAATGATCAAGAAAGATGGTTTGCA
TTTATTGTAGAAACATGTAAAAAAGATAAATATGTGAATACTTCTGATCTTGTCCGAGTATTATGTGAACAAGGCTGGAG
TGAAGATTCAGCACATAAGCTAGTTATAGAGTATGAGTTTGCAAGGGACTTAATCACGTACATGGAGCAATGA
>Cluster_35974
GTGTTCGCTGAATACGGGGTTCTGAATTACTGGACCTATCTGGTTGGGGCCATTTTTATTGTGTTGGTGCCAGGGCCAAA
TACCCTGTTTGTACTCAAAAATAGCGTCAGTAGCGGTATGAAAGGCGGTTATCTTGCGGCCTGCGGTGTATTTATTGGCG
ATGCGGTATTGATGTTTCTGGCATGGGCTGGAGTGGCGACATTAATTAAGACCACCCCGATATTATTCAACATTGTACGT
TATCTTGGTGCGTTTTATTTGCTCTATCTGGGGAGTAAAATTCTTTACGCGACCCTGAAGGGTAAAAATAGCGAGGCCAA
ATCCGATGAGCCCCAATACGGTGCTATTTTTAAACGCGCGTTAATTTTGAGCCTGACTAATCCGAAAGCCATTTTGTTCT
ATGTGTCGTTTTTCGTACAGTTTATCGATGTTAATGCCCCACATACGGGAATTTCATTCTTTATTCTGGCGGCGACGCTG
GAACTGGTGAGTTTCTGCTATTTGAGCTTCCTGATTATATCTGGTGCTTTTGTCACGCAGTACATACGTACCAAAAAGAA
ACTGGCTAAAGTTGGCAACTCACTGATTGGTTTGATGTTCGTGGGTTTCGCTGCCCGACTGGCGACGCTGCAATCCTGA
>Cluster_33852
ATGTTACAGGTATACCTAGTCCGCCACGGTGAAACGCAGTGGAACGCCGAGCGACGTATTCAGGGCCAGTCTGACAGCCC
GCTGACTGCCAAAGGTGAGCAACAGGCGATGCAGGTGGCGACCCGTGCCAAAGAGCTTGGCATTACGCATATCATCAGTA
GCGATTTAGGACGCACCCGCCGCACGGCGGAAATCATCGCCCAGGCCTGCGGCTGCGATATTCTCCTTGATCCTCGTCTG
CGTGAATTAAATATGGGTGTACTGGAAACAAGAAATATCGATTCACTCACCGAAGAAGAAGAGAACTGGCGTCGGCAGCT
GGTCAATGGCACCGTTGACGGGCGTATTCCTGAAGGCGAGTCAATGCAGGAGCTCAGCGATCGCGTTAATGCGGCACTGG
AATCTTGCCGGGACTTACCGCAGGGAAGCCGACCGCTACTGGTGAGCCATGGTATTGCACTGGGATGCCTGGTGAGTACG
ATTCTCGGATTACCAGCATGGGCAGAGCGCCGCTTACGTCTGCGTAACTGTTCTATTTCGCGCGTGGATTATCAGGAAAG
CTTGTGGCTGGCGTCTGGCTGGGTGGTTGAAACCGCAGGGGACATCTCGCATCTGGACGCCCCTGCATTAGATGAGCTGC
AGCGTTAA
>Cluster_26063
GTGAAGCTATACATTTACGATCACTGCCCTTACTGCCTCAAAGCCCGCATGATTTTCGGCCTGAAAAATATCCCCGTCGA
ATTACATGTTCTGCTCAACGACGACGCAGAAACCCCCACCCGGATGGTCGGTCAAAAACAGGTTCCCATTCTGCAAAAAG
ATGACAGCCGCTATATGCCAGAAAGCATGGACATCGTTCACTATGTCGATAAACTCGACGGCAAACCGTTACTGACCGGC
AAACGTTCCCCTGCCATTGAAGAGTGGCTGCGCAAGGTCAATGGCTACGCCAACAAACTGCTGTTGCCGCGTTTTGCCAA

ATCGGCATTTGATGAGTTTTCTACTCCTGCCGCGCGCAAATATTTCGTCGACAAGAAAGAGGCCAGCGCGGGTAATTTTG
CCGACCTGCTGGCCCACTCTGACGGTCTGATTAAGAATATCAGCGATGATTTACGTGCGCTGGACAAACTGATCGTCAAA
CCGAACGCCGTGAATGGCGAACTTTCGGAAGATGATATTCAGCTATTTCCGCTCCTGCGTAATCTGACGCTAGTAGCCGG
AATCAACTGGCCAAGCCGCGTTGCTGATTACCGCGATAATATGGCGAAACAGACACAAATCAATTTGTTATCATCAATGG
CGATTTAA
>Cluster_21271
GTGGCAAAGAATTACTATGACATCACCCTCGCCCTTGCCGGTATTTGTCAGTCGGCACGCCTGGTGCAACAACTCGCTCA
CCAGGGGCATTGTGATGGCGATGCGCTACACGTCTCACTCAACAGTATTATCGATATGAACCCCAGCTCAACGCTGGCGG
TTTTTGGCGGTAGCGAAGCCAACCTGCGCGTCGGGCTGGAAACCTTGCTCGGCGTACTCAATGCCAGCAGTCGCCAGGGC
TTAAACGCCGAATTAACCCGCTACACACTCAGTCTGATGGTGCTTGAGCGCAAACTCTCCTCAGCGAAAGGTGCGCTCGA
CACTCTGGGCAACCGGATCAACGGCCTGCAACGCCAGCTCGAACACTTCGATTTACAGTCCGAAACGCTGATGAGCGCGA
TGGCTGCCATCTATGTTGATGTTATCAGCCCGCTTGGCCCGCGCATTCAGGTCACTGGTTCCCCTGCTGTACTGCAAAGC
CCACAGGTGCAGGCGAAAGTTCGCGCAACCCTGCTGGCAGGCATTCGCGCCGCCGTGCTCTGGCACCAGGTCGGCGGCGG
TCGTCTGCAACTGATGTTTTCTCGTAATCGCCTGACCACTCAGGCAAAACAAATTCTTGCTCATTTAACCCCGGAGTTGT
GA
>Cluster_64451
ATGAAAACCCTCTATGAGGACTGGCCTGAAACTTTCGTTAGCCGATTAGATATGTTACGAGCTCTGGATGATCGCGGCTC
GACCCGGAGGCTCTATCTTGAGCGGACCGGGGCAATATTTGACGCTCTGGCAGAAGAGATACGTACAGCCGTTACCAGGC
ATCCTGAAATCGACGCGAGTGAACTTGATATCGGGCCGCTGTATCGCTATTACAAACGTGGAGAAAAGGGGAATCCGCTG
GCTGACTTACTTATTGAGTTGGCTCCACCAACTTGTGAACGGGTTCGTATATCTCCTGAAGTGTACACAATCCCGTATCT
GTTTTTTGCACTGTTGATAGCGCAAGGCGCTGACAACGATGCCCGCGATTTTTTCAATATGATGATGCGACCGTTAATCA
TCGCCTACCGTTTCAAACAACTGGCGCGTTACCTTGGCACTAAGGGCGGAGGACGACCACAGCACAGATTAAAAAGCGAA
GCCATTGAACTGGCTGATCGTTTTTTTACTGAAAACCCGACAGCGCCATTATCGCGTGGTGTGCAATACATATCCGGTAT
TTTTGTGGCGAAATACTCTGACCCACCTGCAGCGTCGACGATTAGAAAATGGTTAATTTCAATTTACAGGAGTGATAAAT
AA
>Cluster_20770
ATGGCACTTTCTCCCGCGCAGCGTCACAGCCAGCGTATTGCGATGGAACAAAAGCTGAAACGCAGCCAGGCACTGGAAAC
CACGGAAAGCATGCACTTGCTGATTAAGGCGCTGGAAACGGATGTGGAACATGCCCGTAACCTGCCAACAATCGCGGATC
GCGTTGAGTTTAAACGGGATGTGTTGCTGCCGCGCTGGACACCGACTGTGGAAGCGTATCTGGAAAGCGGGCAGGTGTAC
GCCAACCCGGTATTTGCCTGGTGTGTTATCTGGCTGTTTGACGTGGGCGATCTGGATAAGGCGCTGGACTGGGCTGACAT
TGCAATCAGCCAGCAGCAGGCAACCCCGGATCGGTTGCGCAGTAATTTTCCCACGTTCGTGGCCGATACGATGCTGGCCT
GGGCGCAGGAAACATCCGGGCGCGGGGAAAGTGTTGAGCCGTATTTTTCCCGCACATTTGAGCGTGTCGCCAGTACCTGG
CGGCTGCATGAACAGGTCACGGCCAAGTGGTTCAAGTTTGCCGGGCTGGAGCTGCTGCGCGGCGAAGATGGCCAGACAAC
GGCTGCGGGCGTGGACGACATTGAAACGCTGGAGAAAGCCGATCAACTGCTGGCCACCGCAGAAAAATACTATTTAAAAA
TCGGCGTCAGAACGGCACGGCAGACGATTGCCGCCCGTATCCGAAAACTGACGCAGGGGTAA
>Cluster_42615
ATGGCGACAGGAAAGTCCTGCTCTCGCTGGTTTGCGCCTCTTGCGGCGTTATTAATGGTAGTTAGCCTGAGTGGGTGTTT
TGATAAAGAAGGCGATCAGCGTAAAGCGTTTATCGACTTCCTGCAGAATACAGTGATGCGTAGCGGTGAACGTCTACCAA
CCCTGACTGCCGATCAGAAAAAACAGTTTGGTCCTTTTGTCTCTGATTACGCGATTCTGTATGGTTATTCTCAGCAGGTG
AATCAGGCGATGGATTCCGGTCTGCGCCCGGTTGTAGACAGCGTTAACGCAATTCGCGTACCGCAGGATTACGTTACGCA
AAGCGGCCCGCTGCGTGAAATGAACGGTTCGCTGGGTGTGCTGGCGCAACAGCTGCAAAATGCGAAGTTGCAGGCCGATG
CTGCACACTCTGCGTTAAAACAGAGTGATGACCTGAAACCGGTCTTTGATCAGGCATTCACCAAAGTGGTAACGACGCCA
GCTGATGCATTGCAACCGTTGATTCCGGCTGCACAAACTTTCACGCAACAACTGGTGATGGTTGGAGACTACATTGCTCA
GCAAGGTACTCAGGTGAGCTTTGTCGCAAATGGCATCCAGTTCCCGACTTCACAGCAGGCAAGTGAATATAACAAACTGA
TTGCGCCATTACCGGCACAACATCAGGCGTTTAATCAGGCCTGGACTACAGCAGTTACTGCAACCCAATAA
>Cluster_17736
ATGGTCAGCGCAGAAAGTAATGAAGATGGTGATGATCCAATAGCTGAGTTCACAGGTGGCTGGCATGGGTTTAATGGGAA
CCAGAGTGGCTCTCCTACAGCCAGGCATGTTTCTCTTCAGGTATGGGTAGATGGGAAGATGGTCAATGATACATTTAAGG
GGTATACAAATGATTTAGTGATAGAAACCACGTCTAACATACAGGGCTTTAACACCAAAAAGAAAGATGGTTCTGGTAGA
GAGATTATTCAACAGAAAGTAAAAATGAAATTTAATGAAGGACGTGCAGATGTGCATATCAAAATGGTTCCCCTTGAAAA
GGTGAGGATACACACTTACTACGGACTACAAACTGCACTTTCCCAACTGTATGCTAATAGTTCAATTCGTTATGAATCAG
GTGACTCTCAAGAATGGAAAGACACCTCCAGCGGACCAAATGATAGTGGCCCTAAGGGTAAATATCCAAATGTTTACCGC
ATGACTGCAAAGAACAAACATAATGATGAAGTTGTGGTATGGTTAGACACTAAATACGGATTAGGGAAAAGATTAAATGT
GAGTGATAAGTATCCTTCTGCTTTCTTTGTTGGATATAAAAGTTACTTCAATTTAATAAATGGTATTCCTTTAATTATTT
CTACAGGACAATCGGCGGAAGTTCACGGAGGCTTAGAGTGGATAAGATCAAATTAA
>Cluster_47361
ATGGGTCAGAAAGTACATCCTAATGGTATTCGCCTGGGTATTGTAAAACCATGGAACTCTACCTGGTTTGCGAACACCAA
AGAATTCGCTGACAACCTGGACAGCGATTTAAAGTACGTCAGTACCTGACTAAGGAACTGGCTAAAGCGTCCGTATCTC
GTATCGTTATCGAGCGTCCGGCTAAGAGCATCCGTGTAACCATTCACACTGCTCGCCCGGGTATCGTTATCGGTAAAAAA
GGTGAAGACGTAGAAAAACTGCGTAAGGTCGTAGCGGACATCGCTGGCGTTCCTGCACAGATCAACATCGCCGAAGTTCG
TAAGCCTGAACTGGACGCAAAACTGGTTGCTGACAGCATCACTTCTCAGCTGGAACGTCGCGTTATGTTCCGTCGTGCTA
TGAAGCGTGCTGTACAGAACGCAATGCGTCTGGGCGCTAAAGGTATTAAAGTTGAAGTTAGCGGCCGTCTGGGCGGCGCG
GAAATCGCACGTACCGAATGGTACCGCGAAGGTCGCGTACCGCTGCACACTCTGCGTGCTGACATCGACTACAACACCTC
TGAAGCGCACACCACTTACGGTGTAATCGGCGTTAAAGTGTGGATCTTCAAAGGCGAGATCCTGGGTGGTATGGCTGCTG
TTGAACAACCGGAAAAACCGGCTGCTCAGCCTAAAAAGCAGCAGCGTAAAGGCCGTAAATAA
>Cluster_35345
ATGTCTGCTTTGTATGAACGCTCACAGCTGACGCAGGTGATGATTTCATCTGCCCCGGCGACTGCTGAAACTATGGATAA
GGCGGAATATCTGCGCCTGGATTGCACCATCAAGGAAGTCCAGTTCACCGCCGGTCAGAAACAGGATATTGATGTGACCA
CGCTCTGCTCCACCGAGCAGGAGAACATCAACGGTCTGGGGGCGTCGTCTGAGATTTCCATGTCGGGTAATTTTTATCTG
AATCAGGCCCAGAACGCCCTGCGTGATGCCTATGACAATGACGCGTTGTATGCGTTTAAGGTGCTGTTTCCGTCCGGTAA
GGGCTTTAAGTTCCTGGCGGAAGTGCGTCAGCACACCTGGTCATCCGGTACCAACGGCGTGGTGGCAGCAACGTTTTCAC
TGCGTATGAAAGGCAAACCGGTGTCCTTTGTGGTACCGCTGGCGTTTGTGAAAAAATCTGGATAAAACACTTACCGTGAAT
ACAGGTGCGCTGCTGACAATGTCAGTCAGTGCCAACGGGGGAACGCCGCCGTATAAATACGCCTGGAAGAAGGATGGTCA
GCCGGTTGACGGGCAGACGACAGACACCTTCAGTAAGCCAGGTGCGCAGTCCGCTGATGCGGGGAAATATACCTGCGTGG
TGACCGATTCGGCAGAGAAAGCACAGAGTGTGACGTCTGTTGAATGCACCGTGACAGTGAGCGCAGCCGCCGGATAA
>Cluster_24540
GTGAGTAATAAAAACGTCAATGTTAGGAAATCGCAGGAAATAACATTCTGCTTGCTGGCAGGTATCCTGATGTTCATGGC
AATGGTGGTTGCCGGACGCGCTGAAGCGGGAGTGGCCTTAGGTGCGACTCGCGTAATTTATCCGGCAGGGCAAAAACAAG
TGCAACTTGCCGTGACAAATAATGATGAAAATAGCACCTATTTAATTCAATCATGGGTAGAAAATGCCGATGGTGTAAAG

GATGGCCGTTTTATCGTGACGCCACCTCTGTTTGCGATGAAAGGAAAAAAAGAGAATACCTTGCGTATTCTTGATGCAAC
AAATAACCAATTACCACAGGATCGGGAAAGTTTATTCTGGATGAATGTTAAAGCGATTCCGTCAATGGATAAATCAAAAT
TGACTGAGAATACGCTACAGCTCGCAATTATCAGCCGCATTAAACTGTACTATCGTCCGGCTAAATTAGCGTTGCCACCC
GATCAGGCCGCAGAAAAATTAAGATTTCGTCGTAGCGCGAATTCTCTGACTCTGATTAACCCGACACCCTATTACCTGAC
GGTAACAGAGTTGAATGCCGGAACCCAGGTTCTTGAAAATGCATTGGTGCCTCCAATGGGCGAAAGCACGGTTAAATTGC
CTTCTGATGCAGGAAGCAATATTACTTACCGAACAATAAATGATTATGGCGCACTTACCCCCAAAATGACGGGCGTAATG
GAATAA
>Cluster_10123
ATGGTGAAAAAAGCGATAGTGACAGCGATGGCTGTAATCAGCCTCTTTACTCTGATGGGATGTAATAACCGGGCCGAAGT
CGATACGCTTTCTCCAGCACAGGCTGCCGAACTGAAACCGATGCCGCAAAGTTGGCGCGGCGTGCTGCCGTGTGCTGATT
GCGAAGGAATCGAAACCTCTCTGTTCCTCGAAAAAGACGGAACATGGGTGATGAATGAGCGTTATCTCGGTGCTCGTGAA
GAACCTTCCTCCTTCGCTTCCTACGGTACATGGGCGCGAACCGCTGACAAGCTGGTATTAACCGACAGCAAAGGTGAAAA
GTCATATTATCGGGCGAAAGGCGATGCGCTGGAGATGCTCGATCGTGAAGGCAATCCGATTGAATCGCAGTTCAACTATA
CGCTGGAACCGGCACAATCTAGTTTACCTATGACGCCGATGACCCTGCGAGGCATGTATTTTTATATGGCTGATGCGGCG
ACCTTCACTGATTGCGCGACCGGAAAACGTTTCATGGTAGCGAATAACGCAGAGCTGGAGCGTAGCTACCTGGCTGCGCG
CGGTCACAGTGAAAAACCGGTGTTACTGTCAGTAGAAGGTCACTTTACGCTTGAGGCTAATCCGGATACTGGTGCGCCAA
CCAAAGTATTAGCGCCCGATACGGCGGGTAAATTTTACCCGAATAAGGATTGCAGTAGTTTGGGGCAGTAA
>Cluster_16011
ATGCTTCGCCTGACCGTGGCAGCGGATGACGGCAGTGAGCGGCTGGTCAGCACGGCCCGGACGACGGAAACCACATACCG
CTTCAGGCAGCTGGCGTTGGGGCGTTACACGCTGACAGTCCGGGCGGTAAATGCCCGGGGACAGCAGGGCGATCCGGCGT
CGGTATCGTTCCGGATTGCCGCACCGGCAGCGCCGTCACAGATTGAGCTGACGCCGGGCTATTTTCAGATAACCGCCACG
CCGCATCTTGCGGTTTATGATCCGACGGTACAGTTTGAATTCTGGTTTTCTGAAACGAAGATTTCAGATGTCTCACAGGT
GGACAAATCTGCCCGCTATCTTGGTACGGCACTGTACTGGGTAGCCTCCGGCCAGAATATTAAGCCGGGCCATGATTATT
ACTTTTATGTTCGCAGTGTGAATACCGTTGGCAAATCGGCATTCGTGGAGGCTGTCGGCCAGCCGGGTAATAATCCTGAA
GAGTATCTCAATTTTTTTGAAGGGAAAATAAACAGCACCCTGCTGGGGCAGGAGCTGAATGATCGTATTAATGCCTCTGC
ATTGCGCAGTGAAGTTGAGCAACTGGAGGATGAGGTCAATCAGCGACTGGAGAGTGATATTGCCGGGGTGACCCAAAAAA
TCGGGGAAACTGAAAACAGCCTCACGCAGCTGGTTGCTAAAAAAATGATGAGCAGTCACTGGCCATATCCCAGGTGA
>Cluster_53915
ATGTTATATGCATTTAAGCTGGGCAGAAAACTGCGCGGCGAGGAACCTTATTGCCCTGAAAAAGGCGGGAAAGGTGGCAG
TTCTGATAAAAGCGCAAAGTATGCAGCAGAAGCTCAGAAGTATGCCGCAGACCTGCAAAATCAGCAGTTCAACACCATCA
TGAACAACCTGAAGCCGTTTACTCCTCTGGCTGATAAGTATGTCGGCAGCCTCGAGAACTTATCGTCTCTGGAAGGGCAA
GGTCAGGCACTTAACCAGTATTACAACTCTCAGCAGTATAAAGACCTTGCAGGTCAGGCTCGCTATCAGAGTCTGGCGGC
AGCGGAAGCAACAGGTGGATTGGGTTCCACCGCAACCAGTAATCAGTTAGCAGCAATCGCACCAACGCTTGGTCAGCAAT
GGCTATCTGGACAAATGAACAATTACAACAACCTGGCAAATATCGGTCTTGGCGCTCTTCAGGGGCAGGCAAACGCCGGG
CAAACATATGCCAACAACATGAGTCAGATTTCACAGCAAAGCGCGGCGCTGGCTGCGGCAAACGCCAACCGACCGTCAGC
ATTGCAGCAGGGTGTTAGTGGTGCTGCATCCGGTGCGCTTTTGGGTGGTGGCATAGCCAGTGCTCTCGAGCTATCAACTC
CGTGGGGTGCTGGTATTGGTGCTGGTCTTGGTCTGCTTGGTTCACTGTTTTAA
>Cluster_32576
ATGGGTATTAAGGGAATGTGGAAAGATCTTCGCACATCACCAGTTGACACACTGGTCAGATGGCAGGAGCAACGCTTGTT
GTGGTTGTTAATGGCCGTTGCGATGGGCGCGTTAATTATCCTGGCGCACTCTTTCTTCCAGATCTATCTCTACATGGCAC
CCTGTGAGCAATGTGTATACATTCGCTACGCCATGTTTGTGATGGTTATTGGAGGATTGGTTGCAGCGATCAACCCAAAG
AACATCATCCTAAAGCTAATTGGCTGCGTGATGGCATTTTACGGCAGCATTTTGGGACTGAAGTTTTCGCTCAAACTGAA
CGATATCCACCATGCAGTACATAATCCGGATCCCGATTCACTGTTTGGCGTACAGGGTTGCTCTACCGATCCAACTTTTC
CCTTTAACCTGCCGCTGGCACAGTGGGCACCAAATTGGTTCAAGCCTACCGGGGATTGTGGCTATGACGCACCAATCGTT
CCTGATGGCGTCACACTGAGCAGCACGCAACAGTGGTTTGTGGAAATGTATCAGCAATCTGAAGGCTGGTATCTGCTACC
ACCATGGCATTTTATGAATATGGCTCAGGCTTGTATGCTGGCTTTTGGTATGTGTCTGGTGCTGCTGGTCATTATGAGTG
GCGCATGGGCACTTAAAATCATCAGAGGCTAA
>Cluster_5
ATGAAAAGGTTACTTTTTTACCGGAGAGGAGGATTAGGTGATACCCTTCTTACGTTTCCGGTTCTTGAGGTTTTAAAAAG
TCAAGGTTATAAAATAACTGTCATAGGGGTTAAAGCCTATTATCAGTTAGCTCAAAAAGTAGGATGGGTAGACGAGATCT
ATGAGGACCTTTATCCGCAGGTGTTAAACCAACCCTATGATTTAAAAATTTCTTCTCTAAAACAGAAGGGTTCGACCCT
TTCCCTTCAGAAAGGATATGGCTGGTAGACTATTACTTTAGTTGTTTAAACCTACCTAAAAAGTTTTCTCAAATTTTACC
TGTCGAAGGATCAACTGAAAGCATACTAAAAAACAAGGTAGTTCTTCATCCAGGAAGTGGTTCCTCTAAAAAAATCCCTG
ATTTTTTGCTTTTTTCGATGATAGAAAAATTTTTAAAAGAAAAAAAATTTGACTATATTTACATAGTAGGAGAGGCTGAC
AAGTGGATAAAGGATTTTACCTCAAATTTTTGGGAAGTAGAGGACATTGAGGTTTTAGCTAAGGCTTTAAAAACCGCTCG
AGGGTTTATAGGGCTTGACAGTGGGATATCACACTTTGGCAAGTTATTTAGGAGTAAAAAGTTTTATCTTTTTTGGCCCC
ACAGACCATATTGTATGGCGTCCGATAGGCCCTAA
>Cluster_48651
ATGAGCAAATACATCATCAACGTATCGTTTCAGACACGCGTAAATAAAACCACGCGCACGTTGGAAATCGCTGAGTCGTT
CGGGCTTGGCCTGGACGAAAAAGAGTGGACGCTTTACGACAATCTGGAGCTGGAAGTGAAGCAGGGCGATGTGGTGTACA
TCACCGGCCAATCCGGTTCCGGCAAATCCGTTGTGCTGCGCGAGTTGCAACGACAGATGAAGGATGAAGGGCTGTCTGTA
GCCTCCATCGATGATTTTACCTTCGATAATGAGGTTAACGTCATCGATCAGTTGGGCAAAACGACCAGTGATGCGCTTGG
GTTGTTATCTATGGCTGGTCTGAACGATGCATATCTGTTTGTTCGCAAGCCTTCTGAAATGTCAGACGGCCAGAAATATC
GTCTCAAGATTGCCAAACTGATTGAGTCAGGCGCTAAAGTGTGGGCTGCTGACGAGTTCGGCGCTGTTCTCGACCGTGTA
ACCGCTCAGGTTGTGGCGTCTAACCTCCAGCGTGCCGCTCGAAAGGTTGGTGCGACGGTAATGGTGGCGACGACTCACGA
AGACCTGAAGAACGCGCTGCGCCCGGATATGCAGATCACCAAGCACTACAAAGAACGCGTGAAGGTGGAATATCACAATG
GTAGTCACGATGAGGTCCATTCATGA
>Cluster_20805
ATGTTTGAGTATTTACCCGAACTGATGAAAGGGCTGCACACCAGCCTGACGCTAACCGTTGCCTCGCTGATTGTGGCACT
GATTCTGGCGTTGATTTTTACCATCATCCTGACGCTGAAAACGCCGGTGCTGGTGTGGCTGGTGCGGGGTATATCACAC
TGTTTACCGGTACGCCGCTGCTGGTGCAGATCTTCCTGATTTATTACGGGCCGGGCCAGTTCCCGACGTTGCAGGAGTAT
CCGGCACTGTGGCATTTGTTGTCAGAACCGTGGTTATGTGCGCTGATTGCGTTGTCGCTGAACAGTGCGGCGTATACCAC
GCAGCTGTTTTACGGTGCAATTCGTGCGATCCCGGAAGGTCAGTGGCAGTCCTGTAGCGCCCTGGGAATGAGCAAAAAAG
ATACGCTGGCGATCCTGCTGCCGTATGCCTTTAAACGCTCGCTCTCTTCTTATTCCAACGAAGTGGTGCTGGTGTTCAAA
AGTACCTCTCTGGCTTACACCATTACGCTGATGGAAGTGATGGGATACAGCCAGTTGTTGTACGGACGCACCTACGATGT
CATGGTGTTCGGCGCGGCAGGGATTATTTATCTGGTCGTTAACGGCCTGCTGACGCTGATGATGCGTCTGATCGAGCGCA
AAGCACTGGCGTTTGAGCGAAGGAATTAA
>Cluster_11407
ATGACCCCAATAAAAATATTTATGACTATTGCTGATTTCAAACGGCCTAAATTGGAGCTTCCAAACGGGGCAAACAAACT

ACTACTGCACTCTTGCTGTGCTCCATGTTCCGGTGAAGTGATGGAGGCGCTTCAGGCCTCGGGAATCGACTACACCATCT
TTTTCTACAACCCGAACATTCATCCTCAGAAAGAGTATTTAATTCGTAAGGATGAAAATATTCGCTTTGCTGAACAACAC
GGCGTGCCGTTTATCGATGCTGATTACGACACCGACAACTGGTTTGAACGTGCCAAAGGAATGGAATGGGAGCCTGAGAG
GGGGATCCGTTGTACCATGTGTTTTGACATGCGTTTTGAGCGGACAGCGTTGTACGCTGCTGAAAATGGTTTCAGTGTGA
TCAGTAGTTCACTGGGCATTTCACGCTGGAAAAATATGCAGCAGGTTAACGAGTGTGGGCGGCGAGCTGTTGCGCATTAT
CCGGGTATGGTGTACTGGGATTATAACTGGCGCAAGCAGGGCGGCTCGTCCCGTATGATTGAAATCAGCAAGCGCGAAAA
ATTCTATCAGCAGGAATATTGTGGCTGTGTGTATTCTCTGCGCGATACCAATCTACACCGCAAATCTCAGGGACGCCCTC
TTATCAAAATTGGCCAACTCCACTACGGAAAAGAAGAGAAGGAGTGA
>Cluster_49720
ATGAATACCCAATATTTACAGTATGTTCGTGAGCAACTTATGGCAGCTACTGCTGACTTGAACGGAGCAACGAAAGGCCA
GCTCGAAGCCTGGCAGGAGCATGCACAATTTGATACTGGTACATACAAACGAAAGAAGCCGCGCATTCTGGATGTGGTAA
CTGGCAAGATGATTACGCTGGATAATACGCCGACTTCCGGTAAGCAGTCGTACGCAAAAGGTTCATCCATTGCTTTGGTC
AGCCCGGTTGAATTCTCAACCTCTTCATGGCGCCGCGCGGTTTTGTCTCTCGATGAACATCAGAAAGCATGGTTGCTTTG
GTGTTACAGCGAAAGCGTTCGATGGGGGCATCAGGTCACCATAACGCAATGGGCATGGAGCGAGTTTAAAGATTTGTTAA
GTAACAGAAAAATTGCAGGTAAGACACTGGATCGCCTGAAGACGTTAATCTGGCTGGCTGCACAGGATGTGAAGAGCGAA
CTTGCAGGGCGTGAGGCCTATGAATACCAGACACTGGCATCATTGGTGGGAGTGACAACAAAAAACTGGTCCGAGACATT
TACTGAACGCTGGGTTGCAATGAAGCACATTTTTCTACAGCTTGATAGTGATGCTTTATTGCTTGTGACGAGAACACGTT
CAAAACAAAAGGCAGCATTTTTACAGCAAAATATTGCAAAACTGGATTAA
>Cluster_40172
ATGAAAACCGTTAGGGAGTCCACAACGTTGTACAACTTTCTCGGATCGCACAATCCATACTGGCGGTTGACGGAAAGCAG
CGATGTTTTGCGCTTTTCTACCACCGAAACCACAGAACCTGAACGTACGTTGCAGTTATCTGCCGAACAGGCTGCTCGCA
TCAGGGAAATGACGGTCATCACCTCCAGCCTGATGATGAGTCTGACCGTCGATGAAAGCGATCTTTCTGTGCATCTGGTA
GGACGAAAAATCAATAAACGGGAATGGGCTGGCAACGCGTCTGCATGGCATGACACACCAGCAGTTGCTCGTGATTTATC
ACACGGACTTTCCTTTGCTGAGCAGGTAGTTTCTGAAGCACATTCCGCAATAGTGATTCTCGACAGCCGGGGGAATATCC
AACGCTTCAATCGGTTATGTGAAGATTACACAGGGTTGAAAGAACATGACGTCATTGGGCAAAGCGTGTTTAAACTGTTT
ATGAGCCGTCGTGAAGCTGCGGCATCCAGGCGCAATAACCGTGTATTTTTTCGAAGCGGCAATGCATATGAAGTCGAACT
GTGGATACCAACACGTAAAGGCCAGCGGCTGTTTCTGTTTCGCAATAAATTTGTCCACAGCGGCAGTGGCAAGAACGAAA
TAAACCATTTTCACCGCCCTGAAACAGGTTCTCCAGGAACTTAA
>Cluster_14906
ATGAGTGAAAAAATCGAAAAGAAGATTGAGACAGCTAAATCCTTCGAAAGACAACTCTATGCAGAAAATGAACGGTCTAA
GAAAAATGCATGGCGTGTTGCAGTGGCAGCATCATTACTGTCCTTATGTCTTGGGGCTGCAATTTGTGTTCTTGTGCCAT
TAAAAGAAAAAGAGCTTGCTATTGTGGCTGTTGGCGAAAAAACAGGCCGTACAGAACTGATTACTCAGGTTAAACAGGAA
AAGATCCTGCAATCTGAAGCGCTTGGGCGGTATTTTGTAAACACGTATATTACTTTACGTGAAGGTTATAATTATCCGTC
TCTTCAGTATGATTATGAGACTGTACAGCTATATAGCTCGAATACAGTAAAGGACGATTATCTTCGTCTGTATAACAGCG
ATATGGCTCCTGATAAAATTTATCATAATAACGGTTCTTACGTTGCGGTTGAGGTCATCTCCAATATTATTTCTGACGCA
ACCGCGCCAGATAAACTGGCCTCTGTGCGTTTTAAAAAAACGACGAGAAACTTCACGACCGGACAGGTTTCAGTTTCATA
CTGGACGGCTCGTGTGACATATCGTTTTGAACCAGAAAAGTCGGTTAAAAGTTCCAGTCGTGAGCTTAATCCTCTCGGTT
TTACGGTAACCAGTTACCAGACAGATCGGGAAGTCAGGGGGGAATGA
>Cluster_37325
GTGCCCACACTACGTTTCCCCTTCCATCTGGCGAATCATAATAAAGATGCTGCCATCAATATATTGATTATATTTATAAG
CATAGGGTCGATTATTTTTAATGTGAATTATTTCCATACAACTATAGTTAAAAATGGACAGATCATTAATCAACCAACAA
ACGCATTTCAGTCTGATTTCAGCCTTGCCGCTTTATGGCGTAACGAAAATCATGCTGGGGTAAAAGATGCAAATCCCGTG
GCAGTTAATCAGGAAACACCTAAGCTCTCTATAGCGCTTAATGGCATTGTACTCACATCGAATGATGAGACTTCTTTTGT
CTTAATTAATGAGGGGAGCGAACAAAAGCGATACTCCCTCAACGAAGCCCTTGAGTCAGCGCCGGGAACGTTTATTAGAA
AAATAAATAAAACCAGCGTTGTGTTTGAAACTCACGGGCATTACGAAAAAGTAACTCTCCATCCAGGGCTACCTGACATC
ATCAAGCAGCCTGATTCAGAAAGTCAAAATGTACTCGCTGATTATATTATCGCCACGCCTATACGTGATGGAGAACAGAT
ATACGGGCTTCGATTGAACCCTCGTAAGGGCCTCAATGCATTTACCACCAGCTTGCTACAACCAGGGGATATCGCCCTCA
GGATCAATAATCTTTCGCTGACCCACCCTGATGAGGTTTCACAGGCATTAAGTTTACTGCTAACTCAACAAAGTGCTCAG
TTTACAATTCGTCGCAACGGCGTACCCCGCTTGATAAATGTTTCCGTCGGGGAACTTACAGGAATGAATGGACTGCGTCA
TGAAAGGACTCAATAA
>Cluster_35922
ATGGATAATTTTCTTGCTCTGACCTTAACGGGTAAAAAACCGGTTATCACCGAGCGAGAAATCAACGGCGTTCGCTGGCG
CTGGCTGGGCGATGGTGTGCTGGAACTGACGCCATTAACGCCACCGCAAGGCGCACTGGTGATTTCAGCGGGAATACACG
GTAATGAGACGGCACCTGTGGAGATGCTGGACGCGTTGCTTGGCGCGATATCTCACGGCGAGATCCCGTTACGTTGGCGG
TTGCTGGTGATCCTCGGGAATCCTCCTGCGCTGAAGCAAGGGAAACGTTATTGCCATAGCGATATGAATCGAATGTTTGG
CGGTCGTTGGCAGCTATTTGCTGAAAGCGGAGAAACCTGTCGGGCGCGCGAACTGGAACAGTGCCTGGAAGATTTTTATG
ACCAGGGCAAAGAATCTGTGCGCTGGCACCTTGATCTACATACCGCAATTCGTGGCTCCTTGCATCCGCAGTTCGGTGTA
TTACCGCAACGCGACATTCCCTGGGACGAGAAATTTCTGACGTGGCTGGGTGCGGCGGGGCTGGAGGCGCTGGTGTTCCA
TCAGGAACCTGGTGGTACGTTTACCCATTTCAGCGCCAGACATTTTGGCGCGCTGGCCTGTACGCTGGAACTTGGCAAAG
CGTTGCCCTTTGGGCAAAACGATCTTCGCCAGTTTGCAGTAACTGCCAGCGCAATTGCTGCGCTGCTATCTGGTGAGAGT
GTCGGTATCGTGAGAACACCGCCGCTCCGTTATCGGGTGGTTTCGCAAATTACTCGCCACTCGCCGTCCTTCGAAATGCA
TATGGCAAGTGACACGCTGAATTTTATGCCGTTTGAGAAAGGAACATTGCTGGCGCAGGACGGAGAGGAACGTTTTACCG
TAACCCATGATGTAGAGTATGTGTTATTCCCTAATCCGTTGGTAGCGTTGGGATTACGCGCGGGATTAATGCTCGAAAAA
ATAAGCTAA
>Cluster_5543
ATGCAATACAACCCCTTAGGAAAAACCGACCTTCGCGTTTCCCGACTTTGCCTCGGCTGTATGACCTTTGGCGAGCCAGA
TCGCGGTAATCACGCATGGACACTGCCGGAAGAAAGCAGCCGTCCCATAATTAAACGTGCACTGGAAGGCGGCATAAATT
TCTTTGATACCGCCAACAGTTATTCTGACGGCAGCAGCGAAGAGATCGTCGGTCGCGCACTGCGGGATTTCGCCCGTCGT
GAAGACGTGGTCGTTGCGACCAAAGTGTTCCATCGCGTTGGTGATTTACCGGAAGGATTATCCCGTGCGCAAATTTTGCG
CTCTATCGACGACAGCCTGCGACGTCTCGGCATGGATTATGTCGATATCCTGCAAATTCATCGCTGGGATTACAACACGC
CGATCGAAGAGACGCTGGAAGCCCTCAACGACGTGGTAAAAGCCGGGAAAGCGCGTTATATCGGCGCGTCATCAATGCAC
GCTTCGCAGTTTGCTCAGGCACTGGAACTCCAAAAACAGCACGGCTGGGCGCAGTTTGTCAGTATGCAGGATCACTACAA
TCTGATTTATCGTGAAGAAGAGCGCGAGATGCTACCACTGTGTTATCAGGAGGGCGTGGCGGTAATTCCATGGAGCCCGC
TGGCAAGGGGCCGTCTGACGCGTCCGTGGGAGAAACTACCGCACGACTGGTGTCTGATGAGGTGGGGAAAAATCTCTAT
AAAGAAAGCGATGAAAATGACGCGCAGATCGCAGAGCGGTTAACAGGCGTCAGTGAAGAACTGGGGGCGACACGAGCACA
AGTTGCGCTGGCCTGGTTGTTGAGTAAACCGGGCATTGCCGCACCGATTATCGGAACTTCGCGCGAAGAACAGCTTGATG
AGCTATTGAACGCGGTGGATATCACTTTGAAGCCGGAACAGATTGCCGAACTGGAAACGCCGTATAAACCGCATCCTGTC
GTAGGATTTAAATAA
>Cluster_57285

ATGAAGAAATCCATCACCACGCTTGATCTCAATCTGCTGCTCTGTCTGCAACTGCTGATGCAGGAGCGCAGCGTGACCAA
AGCGGCGAAGCGGATGAACGTGACCCCTTCAGCGGTGAGTAAGTCGCTGGCAAAGTTAAGAGCGTGGTTTGACGACCCGC
TCTTTGTGAACTCACCGCTGGGCCTGTCGCCCACACCGCTAATGGTCAGCATGGAGCAAAATCTGGCGGAATGGATGCAA
ATGAGCAATCTGCTGCTGGATAAACCGCACCACCAGACACCGCGCGGCCTGAAGTTTGAGTTGGCGGCGGAATCGCCGCT
GATGATGATCATGCTTAATGCGCTGTCGAAACGGATCTACCAACGTTACCCGCAGGCGACCATCAAACTACGTAACTGGG
ATTACGATTCCTTAGATGCCATTACTCGTGGTGAAGTGGATATTGGTTTTTCCGGTCGCGAAAGCCATCCTCGCTCGCGG
GAGCTGTTAAGCTCGCTACCGTTAGCCATTGATTATGAAGTGCTGTTTAGTGATGTACCCTGCGTCTGGTTACGCAAAGA
TCATCCGGCACTGCATGAAACGTGGAATCTGGACACCTTTTTACGTTATCCGCATATCAGCATTTGCTGGGAACAGAGCG
ACACCTGGGCGCTGGACAATGTGTTACAGGAGCTGGGGCGCGAACGCACGATTGCTATGAGCCTGCCGGAATTTGAGCAG
TCACTGTTTATGGCAGCGCAACCCGACAATCTGCTACTGGCGACCGCGCCGCGCTACTGTCAGTACTACAATCAACTCCA
TCAACTGCCGTTGGTTGCTCTTCCTCTCCCGTTTGACGAAAGCCAGCAAAAAAAGCTGGAAGTTCCTTTTACCCTGCTGT
GGCATAAACGGAACAGCCATAATCCGAAGATCGTCTGGTTACGGGAAACCATTAAAAACCTTTACGCGTCGATGGCATAA
>Cluster_60594
ATGAGTGAAGCACTACTGAACGCGGGACGTCAGACGTTAATGCTGGAGTTGCAGGAAGCAAGCCGTTTACCGGAACGTCT
GGGCGATGATTTTGTTCGCGCCGCCAATATCATCCTGCACTGTGAAGGCAAAGTGGTGGTTTCGGGAATTGGCAAATCGG
GCCACATTGGTAAGAAAATCGCCGCAACGCTTGCCAGTACCGGCACTCCGGCTTTTTTTGTCCATCCGGCAGAAGCGCTG
CACGGCGATCTGGGGATGATCGAAAGCCGCGATGTGATGCTGTTTATCTCTTACTCCGGTGGCGCGAAGGAACTGGATCT
GATTATTCCGCGTCTGGAAGATAAATCTATCGCGCTGCTGGCGATGACCGGCAAACCGACGTCACCGCTGGGCCTGGCGG
CAAAAGCGGTGCTGGATATCTCCGTAGAACGCGAAGCCTGCCCGATGCACCTTGCGCCGACCTCCAGCACCGTCAATACC
CTGATGATGGGTGACGCGCTGGCGATGGCGGTCATGCAGGCGCGCGGATTTAATGAAGAAGATTTTGCCCGCTCCCACCC
AGCCGGGGCACTGGGCGCTCGCTTGCTGAATAAAGTGCATCATCTGATGCGCCGTGACGATGCCATCCCACAGGTGGCGT
TAACCGCCAGCGTGATGGATGCGATGCTGGAACTCAGCCGCACCGGTCTGGGGCTGGTGGCGGTATGTGACGCTCAACAA
CAGGTACAAGGCGTCTTTACCGACGGCGATTTACGTCGCTGGCTGGTTGGCGGCGGCGCACTCACCACGCCAGTCAATGA
AGCGATGACGGTCGGCGGCACCACGTTGCAATCGCAAAGTCGCGCCATCGACGCCAAAGAGATCCTGATGAAGCGCAAAA
TCACTGCCGCACCGGTGGTGGATGAAAACGGCAAACTCACCGGCGCAATAAACCTGCAGGATTTCTATCAGGCCGGGATT
ATTTAA
>Cluster_29190
ATGGGCTTTACCACAAGAGTAAAAAGCGAAGCGAGCGAGAAGAAACCGTTCAACTTTGCGCTGTTCTGGGATAAATACGG
CACCTTTTTTATCCTGGCGATCATCGTCGCCATTTTTGGTTCGCTGTCGCCAGAATATTTTCTGACCACCAATAATATTA
CCCAGATTTTTGTGCAAAGCTCCGTGACGGTACTGATCGGCATGGGGGAATTCTTCGCTATCCTGGTCGCCGGTATCGAC
CTTTCGGTTGGCGCGATTCTGGCGCTTTCCGGTATGGTAACCGCTAAATTGATGTTGGCAGGTATTGATCCGTTTCTCGC
GGCGCTAATTGGCGGTGTACTGGTTGGCGGCGCACTGGGGGCGATCAACGGCTGCCTGGTCAACTGGACGGGACTACACC
CGTTCATTATCACCCTTGGCACCAACGCTATTTTCCGTGGGATCACGCTGGTGATCTCCGATGCCAACTCGGTATACGGC
TTCTCATTTGACTTCGTGAACTTCTTTGCCGCCAGCGTAATTGGGATACCTGTCCCCGTTATCTTCTCGCTAATTGTCGC
GCTCATCCTTTGGTTTCTGACAACGCGTATGCGGCTCGGGCGCAACATCTACGCACTGGGCGGCAACAAAAACTCGGCGT
TCTATTCCGGGATTGACGTGAAATTCCACATTCTGGTGGTGTTTATCATCTCCGGTGTTTGTGCAGGTCTGGCAGGCGTC
GTCTCAACTGCACGACTCGGTGCCGCAGAACCGCTTGCCGGTATGGGTTTTGAAACCTATGCCATTGCCAGCGCCATCAT
TGGCGGCACCAGTTTCTTCGGCGGCAAGGGGCGCATTTTCTCTGTGGTGATTGGCGGGTTGATCATCGGCACCATCAACA
ACGGTCTGAATATTTTGCAGGTACAAACCTATTACCAACTGGTGGTGATGGGCGGATTAATTATCGCGGCTGTCGCCCTT
GACCGTCTTATCAGTAAGTAA
>Cluster_60861
ATGAGTCATCAACTTACCTTCGCCGACAGTGAATTCAGCAGTAAGCGCCGTCAGACCAGAAAAGAGATTTCTTGTCCCG
CATGGAGCAGATTCTGCCATGGCAAAACATGGTGGAAGTCATCGAGCCGTTTTACCCCAAGGCTGGTAATGGCCGGCGAC
CTTATCCGCTGGAAACCATGCTACGCATTCACTGCATGCAGCATTGGTACAACCTGAGCGATGGCGCGATGGAAGATGCT
CTGTACGAAATCGCCTCCATGCGTCTGTTTGCCCGGTTATCCCTGGATAGCGCCTTGCCGGACCGCACCACCATCATGAA
TTTCCGCCACCTGCTGGAGCAGCATCAACTGGCCCGCCAATTGTTCAAGACCATCAATCGCTGGCTGGCCGAAGCAGGCG
TCATGATGACTCAAGGCACCTTGGTCGATGCCACCATCATTGAGGCACCCAGCTCGACCAAGAACAAAGAGCAGCAACGC
GATCCGGAGATGCATCAGACCAAGAAAGGCAATCAGTGGCACTTTGGCATGAAGGCCCACATTGGTGTCGATGCCAAGAG
TGGCCTGACCCACAGCCTGGTCACCACCGCGGCCAACGAGCATGACCTCAATCAGCTGGGTAATCTGCTGCATGGAGAGG
AGCAATTTGTCTCAGCCGATGCCGGCTACCAAGGGGCGCCACAGCGCGAGGAGCTGGCCGAGGTGGATGTGGACTGGCTG
ATCGCCGAGCGCCCCGGCAAGGTAAGAACCTTGAAACAGCATCCACGCAAGAACAAAACGGCCATCAACATCGAATACAT
GAAAGCCAGCATCCGGGCCAGGGTGGAGCACCCATTTCGCATCATCAAGCGACAGTTCGGCTTCGTGAAAGCCAGATACA
AGGGGTTGCTGAAAAACGATAACCAACTGGCGATGTTATTCACGCTGGCCAACCTGTTTCGGGCGGACCAAATGATACGT
CAGTGGGAGAGATCTCACTAA
>Cluster_35460
GTGGATAAGTCGTCCGGTGAGCTGGTGACACTGACACCAAACAATAACAACACCGTACAACCTGTGGCGCTGATGCGTCT
GGGCGTCTTTGTACCGACCCTTAAATCACTGAAGAACAGTAAAAAAAATACACTGTCACGTACTGATGCCACGGAAGAGC
TGACACGTCTTTCCCTGGCCCGTGCTGAGGGATTCGATAAAGTTGAGATCACCGGCCCCCGCCTGGATATGGATAACGAT
TTCAAGACCTGGGTGGGGATCATTCATTCCTTTGCCCGCCATAACGTGATTGGTGACAAAGTTGAACTGCCTTTTGTTGA
GTTTGCAAAACTGTGTGGTATACCTTCAAGCCAGTCATCCCGCAGGCTGCGTGAGCGCATCAGCCCTTCCCTGAAGCGCA
TTGCCGGTACCGTGATCTCGTTTTCCCGTACCGATGAGAAGCACACCCGGGAATACATCACCCATCTGGTACAGTCAGCC
TACTACGATACTGAGCGGGATATTGTTCAGTTACAGGCCGATCCCCGCCTGTTTGAACTGTACCAGTTTGACAGAAAAGT
CCTTCTCCAGCTTAAGGCGATTAATGCCCTGAAGCGACGGGAGTCCGCCCAGGCACTCTACACCTTTATAGAGAGCCTGC
CCCGGGATCCGGCACCGGTATCGCTGGCGCGGCTGCGTGCACGCCTCAATCTGAAGTCTCCTGTATTTTCCCAGAACCAG
ACGGTCAGACGGGCAATGGAGCAGCTGCGCGAGATTGGATATCTTGATTACACGGAGATCCAGCGGGGGCGGACAAAACT
CTTCTGCATTCACTACCGGCGTCCCCGGTTAAAAGCGCCGAATGATGAGAGTAAGGAAAATCCGTTGCAACCTTCACCTG
CGGAAAAAGTCAGTCCGGAGATGGCGGAGAAGCTTGCCCTGCTTGAGAAACTGGGCATCACGCTGGATGACCTGGAAAAA
CTCTTCAAATCCCGCTGA
>Cluster_38940
ATGCAGGTCGGACTTACATCTTCATCACTCGCCACCGGCGGCGCACATAGCGCTACGGTATCGTCGTCAACCGTTGCGCC
CACTCAGGCGGTGCGGCAAAAACTCCCGGCGACGGCGAGTGAATATCCGGCCTCGCCGCTGATTACCACTCGCCCGCAGC
GTTACAGCGTACAGCTTAACGACCAGCTCACCACGTTGCAGCAGGCGGATCACTATCTCGGGCAACTGGAACAGCAGTTG
CTCGATTATCGTCATAGCCAGCGTAAGGGCGGGCAGGCACAGAGTACGGCGCTGATGCAGATGCTGGACAAACGCGCCGC
GCTTTCCGGCGGCGCGGTGGACCGTCAGTTACAACCAGTGTTGCAGGGCGAAGCGCGGGTGACCTTCCACTCGCCGGATC
TGGCGAATCTGGTACACAATCCCACACCGGGAACGCGCATGTTCAGTGTCTCTGACGGGCGGCAGACCCAGCTTTCGGCG
GTGATGCTGTCTGAGGATGACAGCGCCGGGCAGTATCAGACGCGCCTGACCAACGCCCTGCGCCGCGTGGGCGTGCAGGT
ACATCAGCAAGCGGACGGCATCAGTTTCTCCACCACCGAAAAACAGTGGCCTGACATCGAGCGCACCTTAAGCGTGCGCA
CCGATGGTGATAAGTCGGCGTTTATGCCACTAAAAACCTTTGCCGAACCGTCGCAGGCCGAGCGTTTAGCGCAACGTTTG
CAACAGGGGGGGGCGGGGATTTCCCAGACGTTAGAAGGCATTAATCAGCAGCGCGCGCAGATGGCGGTGCAGCAGGAAAA

AGCCCGCCAGTTGATCGACGGCATGTCACGCTTCCCGCAAACGGAAAACGCGGTGCAGGCATCGGAGAACCTCGGCGGCG
TGCTGGATAGCGCCAATCATAACTATCAGGTTTTATTGCAGGCGGTGAACGGTCAGGCGCGAATTTCCAGCCAGACGGTG
AGGAATTTATTGGGGTAA
>Cluster_15797
GTGCACTCAGAAGAATTAAAAAATACACTTGGATTAGCTATTCAAATCGGGAAGCTAGCATCTGATCAAAGTGTATTAAA
ATTCTTTGAGCTTATCGATTTTGATGTTATTCGTGATATACGCGACTTAACTGAGATGGTTAATAGCCACGGGTTTCTAA
AAGATAAAATATCTGAGACTGATTTTCTAATAGCGAACGCTGAATTGGAGAACCACCATGTTAATGGTGTGGAGCTAATA
CCATATGGGTCTGAATTCTATCCTCTTAGTTTAGCTTTTACTCCTAATCCACCATCAATTCTCTACATAAAAGGGGATAA
AAGTATACTCAAAGAATTGCCGGGTGTCGCAATTGTAGGATCCCGTGATACCTCCCCTGCAGGGGAAGAAATAACGAGGC
GGATCACTAATCAGATAGTGTCTGCTGGGTATATTGTTGTAAGTGGGTTAGCAATAGGCACTGATGCGAATGCCCACAAG
GCAACGTTACAGGCAAAAGGAAAAACCATTGCTGTCTTGGCGCATGGTCTTGAGGAGGCTAAGCCGAAGCAAAACAGTCG
ACTTGCTCAAGAGATTCTGGATAAAGGTGGTGCATGGATTTCTGAGTATCCTATGGGTCGGCCGGCCCAGAAACAATCAT
TTGTACAAAGAAATCGGATACAAGTTGGATTATCTGCAGGTTCAATTTTAATCGAGGCTGCGTTAAATAGCGGAACGATA
ACTCAAGCTGAGTTTGCAAATAAGGCGAAAAGACCAGTGTTTGCGGTTGTACCTCACCTACCTAATAATCCGCTTAATCT
TAATTGTGAGGGAACAGTTGATTTAGTTAAGAGCAACATGGCTAGAGCGCTAAAAACAAAACGGGATTATGATGATGTGA
TTATGATCATAAATGAGTCCCGGGAGTATTTGTTAGAGCTAAAATGGCCAGGGAAACAGAGTACATTAGACTTGATTTAA
>Cluster_23776
ATGATCATCGTTACCGGCGGCGCGGGCTTTATCGGCAGCAACATCGTTAAAGCCCTGAATGATAAAGGCATCACCGATAT
TCTGGTGGTGGACAACCTGAAAGACGGCACCAAGTTTGTGAACCTGGTGGATCTGGATATCGCGGACTATATGGATAAGG
AAGACTTCCTGATCCAGATTATGGCTGGCGAAGAGTTCGGCGATGTCGAAGCGATTTTCCACGAAGGCGCGTGCTCTTCC
ACCACCGAGTGGGACGGCAAGTATATGATGGATAACAACTATCAATACTCCAAAGAGCTGCTGCACTACTGTCTGGAGCG
CGAAATCCCGTTCCTGTATGCCTCTTCCGCAGCCACTTACGGTGGACGCACCTCCGACTTTATTGAATCCCGCGAATATG
AAAAACCGTTGAATGTCTACGGTTACTCAAAATTCCTGTTTGATGAATATGTTCGCCAAATCCTGCCTGAAGCGAACTCA
CAGATTGTTGGCTTCCGTTATTTCAACGTTTATGGACCGCGTGAAGGCCATAAAGGCAGCATGGCGAGCGTCGCTTTCCA
TCTCAACACCCAGCTTAATAACGGTGAATCACCGAAGCTGTTCGAAGGTAGCGAGAACTTCAAACGTGACTTCGTCTATG
TGGGCGATGTGGCAGATGTAAACCTGTGGTTCCTGGAAAATGGCGTTTCCGGCATCTTCAACCTCGGCACCGGTCGTGCG
GAATCCTTCCAGGCAGTAGCAGATGCTACGCTTGCTTATCACAAGAAAGGCCAGATCGAATACATTCCGTTCCCGGATAA
ACTGAAAGGCCGCTACCAGGCGTTCACTCAGGCAGATCTGACGAATCTGCGCGCGGCGGGTTACGATAAACCGTTCAAAA
CCGTTGCCGAAGGCGTAACGGAATACATGGCCTGGCTGAATCGTGACGCATAA
>Cluster_40929
ATGATTATTTCAATTAAAAACAACTGGGAAAAAGCAATAAAATTACATACACATTATTTAAATAATTTGATATGCAACTT
TTTAAATAAATCCATTCCTAGTTCTGAAGGACGAGACGAAGCTAAAAAATTTATTCTAGATGTGCATCACCTATTAATAC
TTGCCGGTAAACAAGATATATTGGAGAGTATTGATATATATAGAGAGTACAAACACAGATTGTCAATAACGCCGGATATA
TCGAAGCAATTCGACAAAGAAATAAGATCTATTTTTAATTACAATAAGTTTGTTAGGAAAACAGGATCATGGAATGCATA
TACTCTATGTAAAAAATCAATAAGCAGAACATGTCCGTATTGCAATCAAGCATATGCTTTTTCGATACAAAAAAAGAATC
GCGGTTTTCGTCCTACATTAGATCATTTTTATAGTAAGGATGACTTTCCTCACCTTGCATTGGTAATTAACAATTTAATC
CCAAGTTGTAGTTCATGTAACTCAAGCCTTAAAGGCACAATTGATTTCTTCGCAAATGAACATTTAAACCCACTGTGGGA
TGATGAAGTTATAAATTTCGTTTTAACTCATGAGGATGGTGTTTTAACATTAATAGACGGAATGATTTCATCACCAGAAA
AAATAAAAATAAACATATCTTACGATACTACAAATATTTCAATTAAAAACACAGTAAATACTTTTTTGATAAACGAAAGA
TATCAAGAAATGACAATAGAAGCAATAGAATTTGCAACTGCCAAATTAAATCTTGAATATGCCGTATCTACTGGAATATC
ATATTTCAAAAATAACTCAGAAAGTAGTATATTAAGATTTGACAAAAGAGAATATAAAAAATATTTGCTCGGTAAATTAT
ATTTAGATATATATAATAAAATGCCAAATGCAAATATAGTTAACACTCGTTAA
>Cluster_66876
ATGAGTGAAAAAGAACATGACATGACAAACGACGGTGGAGAGAGCGTAACGTACACGCTGCGGAACATTCCCGCCGACAT
CGACCGGGTGATCACCGACCTGGCAACTCACGCCCGTAAGCCAAAGGCGACGTTTCTGCGTGAGTTCCTAGAAGACAGCT
TCCGGGACGTCATCGACGGTTTCGCCCTGAAAAACCCGCTCATCGCCTCTCTCGATGACGAGCTGGCCAGTTATCTGGGC
GCCGAGGTCATGGAGAAACGCTACCAGAGCCATTACATCACCCGCTGGAACCAGGAATACCAGAAGCTGCTCGGGATCTC
GACGGAAGAGGAGCTTCGCCGGGTGGTGCTGACCAACACGCCCTTCCTGCACGCACGTGCCGACCAGGTGCTTAAGGGCT
GGAAAAAGATCCCGCGCGGGATCTCCCTGACCTTTTCCCTGTTTGCCGAGATCGCCGGCCGGGACAGGGAAACCATTGAC
AGTGCCTGGAACAGGATTTTCTACTCACAGCTGAGGGAGAAAAAGCACCGCTTTTACCGGGATATTGATGTTATTCGTGC
CCTGAAGAAACAGCATGCCGTCTGCGGCTACAGCTGGACCAGGGATGACATCACCGTGCGTATTTACCGCCCGGATGACT
ACGGCTACGGTGCCTGGCGGGTGCTGCTGGTACTGGATGAAAGTGTCATTACCCAGACGTGGAACATCCCGTTCCCGGAA
CTTGACGGACGTCGCTTTACCACTGACCCGGGCTATGACGCCCTGATCAGCACCGCTCCCGACAGCTGGGATAAGGCCTT
CCGTTTGTGGACGGGATTTGTGAGCTTCATCTTTATAGCAATGGCGTGGAAGAAGATCAGAATCCGACCCCGCTGCCGG
CCGTCGCTGAGGCGCTGATTGAAGCGGTGGTGCACGATCTACTGTAA
>Cluster_22308
ATGATTGAATTTAGCCATGTCAGCAAACTGTTCGGCGCACAAAAAGCCGTTAACGATCTCAATCTCAATTTTCAGGAAGG
GAGTTTTTCGGTGCTGATTGGCACATCTGGCTCCGGCAAATCCACCACCCTGAAAATGATTAATCGCCTGGTGGAGCATG
ACAGCGGCGTGATCCGCTTTGCCGGAGAAGAAATTCGTTCGCTGCCAGTGCTGGAGCTGCGCCGCCGGATGGGCTATGCC
ATTCAATCTATTGGCCTGTTTCCCCACTGGAGCGTGGCGCAAAACATTGCCACCGTGCCGCAATTACAAAAATGGTCGCG
GGCGCGGATCGACGATCGTATCGACGAATTAATGGCGCTACTGGGGCTGGAGCCGGATTTGCGTGAGCGTTATCCGCATC
AGCTTTCCGGCGGTCAGCAGCAACGTGTGGGAGTGGCCCGCGCGCTGGCTGCCGATCCGCAAGTCTTGCTGATGGATGAG
CCATTCGGCGCACTGGACCCGGTAACGCGTGGCGCGTTGCAACAAGAGATGACGCGCATACACCGTTTGCTGGGGCGTAC
CATTGTGCTGGTCACTCATGATATTGATGAGGCGCTACGACTGGCAGAACATCTGGTGTTGATGGATCACGGTGAAGTCG
TGCAGCAGGGCAATCCTCTGGCGATGCTGACTCATCCGGCGAATGATTTTGTCCGCCAGTTTTTTGGTCGTAGTGAGCTG
GGTGTACGCCTGCTTTCGTTACGTAGCGTGGCGGATTACGTGCGTCGCGAAGAACGGGCAGAAGGTGAGGCGCTGGCAGA
AGAGATGACGCTACGCGATGCGCTCTCCCTGTTTGTCGCGCGGGGATGTGAAGTGCTGCCCGGTGGTGAACACGCAGGGCG
AGCCTTGCGGCACGCTGCATTTTCAGGATCTGCTGGAGGAGGCGTAA
>Cluster_39098
ATGAGACTCAGGAAATACAATAAAAGTTTGGGATGGTTGTCATTATTTGCAGGCACTGTATTGCTCAGTGGCTGTAATTC
TGCGCTGTTAGATCCCAAAGGACAGATTGGTCTGGAGCAACGTTCACTGATACTGACGGCATTTGGCCTGATGTTGATTG
TCGTTATTCCCGCAATCTTGATGGCTGTTGGTTTCGCCTGGAAGTACCGTGCGAGCAATAAAGATGCTAAGTACAGCCCG
AACTGGTCACACTCCAATAAAGTGGAAGCTGTGGTCTGGACGGTACCTATCTTAATCATCATCTTCCTTGCGGTACTGAC
CTGGAAAACCACTCACGCTCTTGAGCCTAGCAAGCCGCTGGCACACGACGAGAAGCCCATTACCATCGAAGTGGTTTCCA
TGGACTGGAAATGGTTCTTCATCTACCCGGAACAGGGCATTGCAACCGTGAATGAAATCGCTTTCCCGGCGAACACTCCG
GTGTACTTCAAAGTGACCTCCAACTCCGTGATGAACTCCTTCTTCATTCCGCGTCTGGGTAGCCAGATTTATGCCATGGC
CGGTATGCAGACTCGCCTGCATCTGATCGCCAACGAACCCGGTACTTATGACGGTATCTCCGCCAGCTACAGCGGCCCGG
GCTTCTCAGGCATGAAGTTCAAAGCTATTGCAACACCGGATCGCGCCGCATTCGACCAATGGGTCGCAAAAGCGAAACAG
TCGCCGAACACCATGTCTGACATGGCAGCGTTCGAAAAACTGGCCGCGCCTAGCGAATACAACCAGGTGGAATATTTCTC

CAACGTGAAACCAGACTTGTTTGCCGATGTGATTAACAAGTTTATGGCTCACGGTAAGAGCATGGACATGACCCAGCCAG
AAGGTGAGCACAGCGCACATGAAGGTATGGAAGGCATGGACATGAGCCACGCGGAATCCGCCCATTAA
>Cluster_24054
ATGGTTAAGCCACTGGCTGTCGTCGCGGTTGGCGGCAATGCGCTCATTCAGGACGAGCAACGCAATAGTATTCCCGATCA
ATATGTTGCAGTGATGGAAAGCGTGCAACATATCGTTGATATGGTTGAAGCCGGATGGGACCTGGTACTAACCCACGGTA
ATGGCCCGCAGGTGGGCTTTATTCTGCGCCGCTCTGAACTCGCCAGTAACGAAGTTTCTCCGGTTCCACTTGATTACGCC
GTGGGTGATACACAAGGTGCAATTGGCTACATGTTCCAGAAAGCGCTGCATAACGAATTGGCTCGCCGTGGCATAAACAA
ACCGGTAATTGCCCTGGTGACACAAACGCGAGTCAGCCCACATGACGATGCTTTCGCCAGCCCCAGTAAACCAATTGGCG
CGTTTCTCGATGAAGCAACGGCCCAACAACGCCAACAACAACTCGGCTGGACGCTGATGGAGGACGCCGGGCGTGGTTGG
CGGCGTACAGTTCCCTCTCCTGCACCACTGGAAATTATTGAGCACGACACCATCGCTCACCTGGTGCGCCAGGGATATCT
GGTTATTGCCTGCGGCGGCGGCGGTATTCCGGTGGTGCGAGACGGGCAACAACTGAAAGGTGTAGAAGCCGTGATCGATA
AAGATCTGGCCTCCGCGCTGCTCGCCAGTCAATTAGGCGCAGATCTGCTGGTGATCCCCACCGGTGTAGAAAAAGTGGCG
ATTAACTTTGGTACACCACAACAACAGTGGCTCGACGCTATCAGCGTTGCCGAAGCGCAAACGCTGTTGCGGGAAGGTCA
GTTTGGTGTCGGCAGTATGCAACCCAAAGTGGAAGCCATTGTTGATTTCATCAATGCCAGCCAGCAACAAGGCAAACAGG
CCAGCGGCCTGATTACTTCACCGCAAACCATAAAAGCAGCCCTGGCGCATCAGAGCGGTACATGGATAACCCTTTAA
>Cluster_34188
ATGTCGCAGAATAATCCGTTACGCGCTCTTCTTGATAAACAGGATATCCTGCTGCTGGATGGCGCGATGGCGACGGAGCT
GGAAGCGCGAGGGTGTAACTTAGCCGACAGCCTGTGGTCAGCCAAAGTGCTGGTAGAAAACCCGGAGCTTATCCGCGAAG
TGCATCTTGATTACTACCGGGCGGGGGCGCAATGCGCGATCACTGCCAGCTATCAGGCGACGCCGGCGGGCTTCGCCGCG
CGAGGTCTGGATGAAGCGCAGTCGAAAGCGCTGATTGGCAAAAGCGTGGAGCTGGCGCGTAAAGCCCGTGAAGCGTATCT
GGCAGAGAACCCGCAGGCGGGAACGCTTCTGGTGGCGGGATCCGTCGGGCCTTACGGCGCGTATCTGGCGGATGGCTCTG
AATACCGTGGCGATTATCATTGTAGCGTTGAGGCATTTCAGGCGTTTCATCGCCCGCGCGTGGAAGCCTTGCTGGATGCC
GGGGCCGATCTGCTGGCCTGCGAAACCCTGCCGAATTTTTCCGAGATTGAGGCGTTGGCCGAGCTGTTGACCGCATATCC
GCGTGCGCGGGCGTGGTTCTCATTTACCCTGCGCGACAGCGAACACCTGAGCGACGGTACGCCGCTGCGTGACGTGGTTG
CGTTGCTGGCGGGTTATCCGCAGGTGGTGGCGCTAGGCATTAACTGTATTGCGCTGGAAAACACCACCGCTGCGTTGCAG
CATTTACACGGTTTAACGGTGCTGCCGCTGGTGGTGTATCCGAACTCGGGCGAGCATTACGATGCCGTGAGCAAAACCTG
GCATCATCACGGTGAACATTGCGCGCAGCTGGCGGATTATCTGCCGCAGTGGCAGGCCGCTGGCGCACGGTTGATTGGCG
GGTGCTGTCGCACCACGCCTGCGGATATCGCCGCGTTAAAAGCGCGAAGCTGA
>Cluster_17496
ATGATTACTTTTGATCAGGCAACCGTTGACAGCTCTGGTGCCTTTCTCATCGGGGAGCTGGAGCGACTCGACCAGACGCT
GAACCTGCCACTGGTGGGGTACACCTGGACCCGCGATATTCAGTTGCGTGAAGATGTCTCTATCGCAGATGACATTTCCA
GCTGGACGAATACCAGCTTCGCCGCTGCGGGTACTGGTGCAAATCCGAATGGCAAAAACTGGGTAGGCAAAGACTCAACC
GCTATTGCTGGCGTAAACGTGGATATCGGCAAATCCGGTAACCCGCTGAACCTGTGGGGGATGGAACTTGGCTGGACGGT
CATAGAATTGCAGGCTGCTCAGCAGGTCGGACGCCCCGATCGATACGCAGAAGTATGACGGGATGCAACTGAAATGGCAGA
TGGATAACGATGAACAGGTGTATGTTGGCGATTCCGCATTAAACCTGAAAGGCCTTGTTACCCTGAACGGTGTTCCTGTC
AACAACGCTGCCAAAACGTGGGCAACCTCAACACCGGACGAAATCCGCGCAAGCATTAACCAGGTGCTGTCTGATGCGTG
GGCCGCTTCCGGTTACTCTGTGGTTCCGCGTGATTTGCTGATCCCGCCTGAGCAGTTTGCTCTGTTGTCCAGCATCATCG
TTTCATCTGCGGGTAACCAGTCCCTGTTGACGTACCTTCAGACCAACACCATCAGCTATCACCAGAACGGTGTTCCGCTG
AATATCCGCGCGGTTAAATGGCTGAAAGGCCGTGGTGTGGGGAATAAGGATCGCATGGTTGCGTACACCAACGATAAAAA
ATACGTCCGCTACCCGCTGGTTCCGCTTCAGAGCGTGCCGGTGCAGTATCGCGGTCTGTACCAGATTGCGACCTACTACG
GCAAGCTGGGTGCAGTCGAGCCAGTGTACAAAGAAACCATTTCGTACGTTGATGGCATTTAA
>Cluster_21914
GTGTCAGATCAGATTATCGCCCGCGTCTCGCAATCCCTTGCCAAAGAACAGTCACTGGAAAGCCTGGTCCGACAGCTTCT
GGAGATGCTGGAAATGGTCACTGATATGGAATCAACCTACCTGACCAAAGTGGATGTCGAAGCGCGCCTGCAGCATATAA
TGTTTGCCCGTAACAGCCAGAAAATGCACATCCCGGAGAATTTTACCGTCTCGTGGGATTACTCGTTATGCAAACGCGCC
ATTGATGAAAACTGCTTTTTCAGCGATGAAGTCCCTGACCGTTGGGGCGACTGTATTGCGGCACGCAATCTTGGCATCAC
CACATTTCTGAGCACGCCAATTCACTTACCGGATGGATCATTCTATGGCACGCTTTGCGCCGCCAGTAGTGAGAAGCGCC
AGTGGAGTGAACGCGCGGAACAGGTTTTACAGTTATTCGCCGGACTGATTGCACAATATATTCAAAAAGAGGCACTGGTT
GAACAGCTGCGCGAAGCCAATGCTGCGCTGATTGCGCAATCGTATACCGACTCGTTAACCGGGCTACCGAATCGGCGGGC
GATTTTTGAAAACCTGACGACGCTGTTTTCTCTCGCCCGACATCTTAACCATAAGATAATGATCGCGTTTATCGACCTGG
ATAACTTCAAATTAATCAACGATCGTTTTGGTCATAATAGTGGCGATCTGTTTCTCATTCAGGTTGGCGAGCGCCTTAAT
ACGCTCCAGCAAAATGGCGAAGTTATTGGTCGTCTCGGCGGTGATGAGTTTTTGGTTGTTTCACTGAACAACGAGAATGC
GGATATTTCGTCTCTGCGAGAACGTATTCAACAGCAAATACGTGGAGAATATCACTTAGGTGACGTTGATTTGTATTATC
CCGGTGCCAGTCTTGGCATAGTAGAAGTCGATCCTGAAACGACCGATGCAGACAGTGCCCTGCATGCTGCCGATATCGCG
ATGTATCAGGAGAAAAAACACAAACAGAAAACACCTTTTGTCGCGCATCCAGCGCTACATTCATGA
>Cluster_22237
ATGAAATCAGTGGTGAATGATACTGATGGTATCGTGCGCGTAGCAGAAAGCGTCATTCCTGAAATTAAACATCAGGATGA
GGTGCGGGTAAAAATTGCCAGCTCGGGCTTATGTGGTTCCGATTTACCCAGGATATTTAAAAATGGTGCACATTATTATC
CAATAACGTTAGGCCATGAATTTAGTGGCTATATTGATACGGTGGGATCCGGTGTTGATGATTTACACCCCGGCGATGCG
GTTGCCTGTGTGCCGTTATTACCCTGTTTTACTTGTCCAGAGTGTCTGAAAGGGTTTTATTCCCAGTGCGCAAAATATGA
TTTTATTGGCTCACGGCGTGATGGTGGATTTGCCGAATATATTGTCGTTAAGCGAAACAATGTCTTTGCACTACCTGCGG
ATATACCTATTGAGGATGGCGCTTTTATTGAACCGATTACCGTTGGCCTGCATGCGTTCCATTTAGCGCAAGGATGTGAG
AATAAAAACGTTATTATTATTGGTGCCGGAACCATTGGCCTGCTGGCAATTCAGTGCGCTGTCGCGCTGGGAGCAAAGAG
TGTGACGGCTATCGACATTAGCTCAGAAAAACTGGCACTGGCAAAATCTTTCGGTGCGATGCAAACATTTAACAGCCGTG
AAATGAGCGCGCCGCAAATACAGGGCGTTTTACGCGATCTGCGCTTTAATCAGCTTATCCTCGAGACGGCTGGCGTCCCG
CAAACCGTCGAACTGGCGGTAGAGATTGCCGGTCCTCATGCCCAACTGGCGCTGGTAGGCACGTTGCATCAGGATCTGCA
TTTAACATCTGCAACGTTTGGCAAAATATTGCGTAAAGAGCTGACGGTTATCGGCAGTTGGATGAACTACTCCAGCCCTT
GGCCGGGGCAGGAGTGGGAAACGGCGAGCCGGTTGCTGACAGAACGTAAGTTAAGCCTGGAGCCATTAATCGCTCACCGT
GGAAGCTTTGAAAGCTTCGCCCAGGCGGTGCGTGACATCGCTCGTAATGCCATGCCGGGCAAAGTGTTGCTCATTCCCTG
A
>Cluster_35151
ATGGCTAACGCCTGGCTCAGATTATGGCATGACATGCCAAATGACCCTAAGTGGCGAACAATTGCCAGGGTGTCAGGGCA
GCCAATTGCAACAGTGATGGCAGTGTATATCCACCTCCTGGTGAGCGCGTCACGAAATGTCACGCGAGGTCACATTGATG
TCACGACAGAAGATTTGGCAAGTGCGCTCGACGTGACAGAAGAGGTAATTGATTCAATTTTGCAGACGATGCAGGGGCGG
GTACTTGATGGTGATTTAATCACTGGATGGGAAAAACGCCAGGTGCTTAAAGAGGACAACGGCAATATTTCGCAAACCGC
AAAATCTCCGGCAGAGCGCAAGAGGGCGCAGCGTGAGAGGGAAAGAAAGCGGGAACAAAATGGCGATTGTCACGGCGCGT
CACGAAATGTCACGCACATGTCACGACAAGTCACGACAGATAAAGATACAGATACAGAATTAAACCCCACACATAACGCG
CGCATGCGCGAGAGTGCTCCAACCGGTGAGTCGCATGGTGCGCCGTTGCAGACAGCCGAACCTGAATACCTGGACGGCCT
GAGCGAACCGATCGGGAAATTTTCGATGACTACTGTCTGGCAGCCGTCGTCGGATTTTCGACAACGGGCAGCAGTGTGGG

GTATGGCTCTGCCTGAGCCGGAATTTACACCTGCAGAGCTTGCCGCATTCCGGGATTACTGGATGGCGGAGGGGAAGGTT
TTCACGCAGGTTCAGTGGGAGCAGAAATTTGCCCGCCACGTGCAGCACGTCAGGGCACAGGTAAAACCAGTCAGCAAGGG
GGTAAGCCATGCAGCATCAGGTGGCACGGCATCACGGGCAGTTCAGGAAATCCGGGCAGCACGCGAACAGTGGGAACGTG
ACAACGGATTTATCAGCAACGGAAACGGCCTGGAAGCTGTGGGAGCTTATGGGGGAGGTGTATTCGAACCGCTGGACACA
GAAGAACGGGGCCGCACCTTCGAAGCTCTGGATTGCCCAGATTGGCGCGATGACTGA
>Cluster_61683
ATGTTTCAGCAGCAAAAAGACTGGGAAACAAGAGAAAACGCGTTTGCTGCTTTTACCATGGGACCGCTGACTGATTTCTG
GCGTCAGCGTGATGAAGCAGAGTTTACTGGTGTGGATGACATTCCGGTGCGCTTTGTCCGTTTTCGCGCACAGCACCATG
ACCGGGTGGTAGTCATCTGCCCGGGGCGTATTGAGAGCTACGTAAAATATGCGGAACTGGCCTATGACCTGTTCCATTTG
GGGTTTGATGTCTTAATCATCGACCATCGCGGGCAGGGACGTTCCGGTCGCCTGTTAGCCGATCCGCATCTCGGGCATGT
TAATCGCTTTAATGATTATGTTGATGATCTGGCGGCATTCTGGCAGCAGGAGGTTCAGCCCGGTCCGTGGCGTAAACGCT
ATATACTGGCACATTCGATGGGCGGTGCGATCTCCACATTATTTCTGCAACGCCATCCAGGTGTATGTGACGCCATTGCG
CTAACTGCGCCAATGTTTGGGATCGTGATTCGTATGCCGTCATTTATGGCACGGCAGATCCTCAACTGGGCCGAAGCGCA
TCCACGTTTCCGTGATGGCTATGCAATAGGCACCGGGCGCTGGCGCGCGTTGCCGTTTGCTATCAACGTACTGACCCACA
GCAGACAGCGATATCGACGTAACTTACGCTTCTATGCTGATGACCCAACGATTCGCGTCGGTGGGCCGACCTACCATTGG
GTACGCGAAAGTATTCTGGCTGGCGAACAGGTGTTAGCCGGTGCGGGTGATGACGCCACGCCAACGCTTCTCTTGCAGGC
TGAAGAGGAACGCGTGGTGGATAACCGCATGCATGACCGTTTTTGTGAACTCCGCACCGCCGCGGGCCATCCTGTCGAAG
GAGGACGGCCGTTGGTAATTAAAGGTGCTTACCATGAGATCCTTTTTGAAAAGGACGCAATGCGCTCAGTCGCGCTCCAC
GCCATCGTTGATTTTTTCAACAGGCATAACTCACCCAGCGGAAACCGCTCTACAGAGGTTTAA
>Cluster_54944
ATGAAAAAGTTAGTAGCCACAGCACCGCGTGTTGCTGCGCTGGTTGAGTATGAAGATCGGGCGATTTTAGCTAATGAAGT
GAAGATCCGCGTGCGTTTCGGCGCACCGAAACACGGAACGGAAGTGGTCGACTTCCGCGCCGCCAGCCCGTTTATTGATG
AAGACTTTAACGGCGAATGGCAGATGTTCACTCCGCGTCCGGCAGATGCGCCGCGCGGCATTGAGTTTGGCAAATTCCAG
CTTGGCAACATGGTGGTTGGCGACATTATCGAGTGCGGCAGCGACGTTACCGACTACGCGGTGGGCGACAGCGTATGCGG
CTACGGCCCGCTCTCCGAGACGGTCATCATTAACGCAGTGAATAACTACAAGCTGCGCAAAATGCCGCAAGGCAGCTCCT
GGAAAAACGCCGTCTGCTACGACCCGGCGCAGTTTGCCATGAGCGGCGTGCGCGATGCCAACGTGCGCGTAGGGGATTTT
GTGGTGGTGGTAGGGCTTGGCGCAATCGGTCAAATTGCCATCCAACTGGCTAAACGCGCTGGCGCTTCTGTGGTGATTGG
CGTCGATCCTATCGCCCATCGCTGTGATATTGCCCGTCGCCACGGCGCGGATTTCTGCCTTAACCCCATCGGCACTGATG
TAGGTAAAGAGATCAAAACGCTGACCGGCAAGCAGGGTGCCGATGTGATTATCGAAACCAGCGGCTACGCCGACGCGCTG
CAATCGGCGCTCCGCGGTCTGGCTTATGGCGGCACCATCTCCTATGTCGCGTTTGCCAAGCCGTTTGCCGAAGGTTTTAA
CCTCGGACGCGAAGCGCATTTCAATAACGCCAAAATTGTCTTCTCTCGCGCGTGCAGCGAACCGAACCCGGATTATCCGC
GCTGGAGCCGCAAGCGTATTGAAGAAACCTGTTGGGAACTGCTGATGAACGGTTATCTCAATTGCGAAGATTTAATCGAC
CCGGTGGTGACCTTTGCCAATAGCCCGGAAAGCTATATGCAGTATGTCGACCAGCATCCGGAACAGAGCATCAAAATGGG
CGTTACGTTTTAA
>Cluster_51315
ATGAACATAACAAAAAGCGCCCCGTTACCGGAGCGCCCTTGCGAACAATTAACCTGCTGCGAAAAAAATGGATCAGTGCA
GGGGAATTATATCAACCGTGTGGCGAAGCGCCACAATTGCCGGATAACAGGCAAAACAAAGGCCACCCGCTACGGTGGCC
CCTCGACACAAGCTACACGTTATCCCCAACGCATGAGCATCACCAACAATGCCACATTTGCGGCTGGTGGGCAATGTGAT
CAGTCAGATTTGGTTCGTTCCAAGGTTTGCAACGAGAGCTTTTTCCTGTGCTCTTTAAGGAATTTCTCAAGAGCAAACGA
ACAAGGTGCGAATCTTTCTGATTCATGTTCTATCTTTCTGCGCCGTCTTTTCCGTGCCGGTGATAATGTTTTGGTCAATT
CTTTATCGGTCATTGTGTTGTCCTGCATAGCAATGCGCCGTAGTTACTCACACCACGGCGCTGGTGATGGTTACTCCTGC
TCTTTGGCCTTGCGCCGCTGGCGGCGTTTGATCTCGCCTTGCATTGACGTAATGATGAATTGTGATGTGCTTTCGCCTGA
TTCTTTCACTGATTCCATGGCGTTTACTATTTCGTGTGGTACACGAGCCTTTAGAGCTTGTGATTTTGCGTTTGTTGTAC
CCGTTGCCATTTCTATTCCTCTGGTGGATTGGTGGGGGACAGTATACACAGAAAAAGCACAAATAAAACTCTTGACGTGG
GCCACACCTAAAACATATAGTGGGCCACACCTTAAAATTATCAAGGCGCATAAATGCAAAGCCCCGCAAGTGTCGTTACC
ACTCGCAGGGCTTCTAACCACCAACGATAGCAGAAGTATCGAGGTAGCTATGAGAAATTATACCAGACACCCGCAAGGGC
GGGACTCGCACAACCTGAATAAATACATCTGGCGTTTTATCGCCCTGAGCACGGCACAACCGCGCGTGATTACCATTGAG
GCCACCAGCGAACAGGAAGCACGCCAGCAATCTCCTGATGGCTGCGTGATGGTATTCGCCGCCCGTATTCGTCAGGAGGT
GCGCCATGTGCAATAA
>Cluster_40579
ATGAAAAATTCAAAAGCAATATTGCAGGTGCCGGGCACAATGAAAATTATTTCAGCAGAAATACCTGTGCCTAAAGAAGA
TGAAGTTTTGATTAAAGTGGAATATGTCGGTATTTGTGGTTCAGATGTACATGGTTTTGAATCAGGCCCGTTTATTCCGC
CTAAAGACCCAAATCAAGAAATTGGCCTGGGTCATGAATGCGCCGGGACGGTTGTGGCTGTGGGAAGCCGCGTGCGCAAA
TTTAAACCGGGGGATCGGGTAAATATCGAACCTGGCGTTCCTTGCGGTCACTGTCGTTACTGTCTGGAAGGCAAATATAA
CATTTGCCCGGACGTTGATTTTATGGCGACACAACCCAACTACCGCGGCGCATTAACGCACTATCTGTGTCATCCGGAGA
GCTTTACTTACAAACTGCCAGACAATATGGACACGATGGAAGGGGCGCTGGTGGAGCCTGCCGCAGTCGGGATGCATGCC
GCGATGCTGGCAGATGTTAAACCTGGTAAGAAGATAATTATTCTGGGAGCGGGTTGTATTGGTTTGATGACGTTGCAAGC
GTGCAAATGCCTGGGAGCAACGGAAATTGCCGTCGTTGATGTGCTGGAAAAACGTCTGACAATGGCGGAACAGCTCGGCG
CGACAGTGGTTATTAACGGCGCAAAAGAAGACACTATTGCACGCTGTCAGCAATTTACCGAAGACATGGGTGCAGATATT
GTTTTTGAAACAGCGGGTTCTGCGGTCACCGTTAAACAGGCACCTTATCTGGTCATGCGCGGCGGTAAAATTATGATTGT
TGGTACTGTACCCGGCGATTCGGCAATCAATTTCCTCAAAATCAATCGCGAAGTCACTATCCAGACGGTATTCCGCTATG
CCAATCGTTATCCGGTCACGATTGAAGCTATTTCCTCAGGGCGATTCGATGTGAAATCGATGGTGACGCATATTTACGAT
TATCGGGATGTACAACAGGCATTTGAAGAGTCAGTTAACAACAAACGCGACATTATTAAAGGCGTTATTAAAATTAGCGA
TTAA
>Cluster_23620
ATGGAAACCCCTCAACCCGATAAAACGGGCATGCACATTCTGCTCAAGTTGGCCTCGCTGGTAGTGATCCTCGCGGGCAT
TCACGCAGCGGCAGATATCATTGTGCAGCTATTACTGGCGCTGTTTTTTGCCATCGTTCTCAACCCGCTCGTCACCTGGT
TTATTCGTCGGGGGGTACAACGCCCCGTTGCCATTACGATTGTGGTTGTGGTGATGCTGATCGCACTCACCGCGCTGGTC
GGCGTACTGGCGGCATCGTTTAACGAATTTATCTCTATGCTGCCGAAGTTTAATAAGGAGCTGACGCGCAAACTTTTTAA
ATTGCAGGAGATGTTGCCTTTTCTTAATTTGCATATGTCGCCAGAGCGAATACTACAGCGGATGGACTCGGAAAAAGTGG
TTACCTTCACCACGGCGCTAATGACCGGGCTATCCGGGGCAATGGCGAGCGTGCTTTTGCTGGTGATGACCGTAGTTTTT
ATGCTGTTTGAAGTGCGCCACGTCCCTTACAAAATGCGTTTCGCGTTGAATAATCCACAAATTCACATCGCCGGACTACA
CCGCGCACTTAAAGGCGTTTCGCATTATCTGGCGTTAAAGACGCTACTCAGTTTATGGACAGGCGTAATCGTCTGGCTGG
GGCTGGAGCTGATGGGGGTGCAGTTTGCGCTGATGTGGGCAGTACTGGCGTTTTTGCTCAACTACGTGCCCAACATCGGT
GCGGTAATTTCCGCCGTACCGCCAATGATTCAGGTGCTGCTGTTTAATGGTATTTACGAATGTATTCTGGTCGGCGCATT
GTTTTTAGTGGTCCATATGGTCATCGGCAATATTTTAGAACCACGGATGATGGGCCATCGCCTGGGGATGTCCACCATGG
TGGTATTTCTTTCATTGTTAATTTGGGGATGGCTGCTCGGCCCGGTAGGGATGCTACTTTCGGTGCCATTAACCAGCGTG
TGTAAAATCTGGATGGAAACCACCAAAGGCGGCAGCAAACTGGCGATTCTACTGGGGCCAGGCAGACCGAAAAGTCGGTT
ACCGGGATGA

>Cluster_21357
ATGACGCAGCCATTGGTCGGAAAACAGATTCTCATTGTTGAAGATGAGCAGGTATTTCGCTCGCTTCTGGATTCGTGGTT
TTCCTCATTGGGAGCGACAACGGTACTGGCGGCTGATGGGGTGGATGCCCTTGAGTTGCTGGGAGGTTTCACTCCAGACC
TGATGATATGTGATATCGCGATGCCACGAATGAACGGGCTTAAACTGCTGGAACATATACGCAACAGAGGTGACCAGACA
CCAGTTCTGGTGATATCTGCCACTGAAAATATGGCAGATATTGCCAAAGCGTTACGTCTGGGCGTTGAAGATGTTTTGCT
GAAACCGGTTAAAGATCTGAATCGCTTGCGCGAGATGGTTTTTGCCTGTCTCTATCCCAGCATGTTTAATTCCCGCGTTG
AGGAAGAGGAAAGGCTTTTTCGCGACTGGGATGCAATGGTTGATAACCCTGCTGCAGCGGCGAAATTATTACAGGAACTA
CAACCGCCAGTTCAGCAGGTGATTTCCCAATGTCGGGTTAATTATCGTCAATTAGTTGCCGCGGACAAATCCGGCCTGGT
GCTTGATATTGCCGCACTTTCGGAAAACGATCTGGCATTTTATTGCCTTGATGTCACCCGAGCTGGACATAATGGCGTAC
TTGCTGCCTTGTTATTACGCGCATTGTTTAACGGATTATTACAGGAACAGCTTGCACACCAAAATCAACGGTTGCCAGAG
TTGGGCGCGTTATTGAAGCAGGTAAACCATTTACTCCGTCAGGCCAATCTGCCGGGGCAGTTTCCGCTATTAGTTGGCTA
TTATCATCGCGAACTGAAAAATCTCATTCTGGTTTCTGCGGGTCTGAATGCGACGTTAAATACCGGCGAACATCAGGTGC
AAATCAGTAATGGTGTTCCGTTAGGCACTTTAGGTAACGCTTATTTGAATCAATTGAGCCAGCGATGCGATGCCTGGCAA
TGCCAAATATGGGGAACCGGTGGTCGGCTGCGCTTGATGTTGTCTGCAGAATGA
>Cluster_38914
ATGTCTGAACTTATTGCAACTGCCGCTTCCAGCATTAACGCTTTTACTCTGGCAAAGCGTGTCGCCGCCTTTAACGTACC
GGTGCTCATTCAGGGCGAAACCGGCGCGGGGAAAGAGTGCGTGGCGAAATATATTCACACTGTAGCCTTTGGTGAAAATG
ATAACGCGCCCTATATCGGCGTGAACTGTGCGGCGATCCCGGAAAATATGCTGGAAGCGACCTTATTTGGCTATGACAAA
GGCGCATTTACCGGCGCAATTGCCAGCGTGCCAGGAAAAATGGAGCTGGCGAATAACGGCACCTTATTGCTCGATGAAAT
TGGCGATATGCCGCTGGCATTACAGGCCAAAATATTACGCGTATTGCAGGAACAGCAGGTTGAGCGATTAGGCAGCAACC
GACAAATCAAACTCAATTTTCGCCTGATTGCCTGCACCAATAAAAATCTGGAACAGGATGTGACTGCTGGACGTTTTCGT
GAAGATCTTTATTATCGTCTGGCGGTTATTCCTATAACCATGCCGCCACTGCGTGAACGTCTGAACGATATTATTCCGCT
GGCAGAGTCGTTTATTAAAAAATATGCCACGGTGCTGGTGAAAAATATCACCCTTTCAGAATCTACCCGCCGGGCGCTAC
TTAATTACCGCTGGCCCGGAAATGTTCGCCAACTGGAAAACGCCATTCAGCGGGGAATGATTTTAAACCGCGACGGCGTG
ATTTACCCGGATGCCTTAGGCCTGCCGGAAACTGACATTGCCGACCACAGCGAACTGCAATGGCCTGTTCAGCCCGCCGT
CCACATTGCCGAAACCAGCGATTTAGGCCAGCACGGACGAAGCGCTCAGTATCAATATATCGCCGACCTGATGCGTAAAT
ATCAGGGCAACCGCAGCAAAATCGCCGACCTGTTAGGCATTACCCCGCGCGCACTGCGCTATCGACTGGCCTCCATGCGC
AAGCACGGTATCGAAGTTTTCTCCTGA
>Cluster_48789
ATGTTTGGTGCTATTGCTGGCGGTATTGCTTCTGCTCTTGCTGGTGGCGCCATGTCTAAATTGTTTGGAGGCGGTCAAAA
AGCCGCCTCCGGTGGCATTCAAGGTGATGTGCTTGCTACCGATAACAATACTGTAGGCATGGGTGATGCTGGTATTAAAT
CTGCCATTCAAGGCTCTAATGTTCCTAACCCTGATGAGGCCGTCCCTAGTTTTGTTTCTGGTGCTATGGCTAAAGCTGGT
AAAGGACTTCTTGAAGGTACGTTGCAGGCTGGCACTTCTGCCGTTTCTGATAAGTTGCTTGATTTGGTTGGACTTGGTGG
CAAGTCTGCCGCTGATAAAGGAAAGGATACTCGTGATTATCTTGCTGCTGCATTTCCTGAGCTTAATGCTTGGGAGCGTG
CTGGTGCTGATGCTTCCTCTGCTGGTATGGTTGACGCCGGATTTGAGAATCAAAAAGAGCTTACTAAAATGCAACTGGAC
AATCAGAAAGAGATTGCCGAGATGCAAAATGAGACTCAAAAAGAGATTGCTGGCATTCAGTCGGCGACTTCACGCCAGAA
TACGAAAGACCAGGTATATGCACAAAATGAGATGCTTGCTTATCAACAGAAGGAGTCTACTGCTCGCGTTGCGTCTATTA
TGGAAAACACCAATCTTTCCAAGCAACAGCAGGTTTCCGAGATTATGCGCCAAATGCTTACTCAAGCTCAAACGGCTGGT
CAGTATTTTACCAATGACCAAATCAAAGAAATGACTCGCAAGGTTAGTGCTGAGGTTGACTTAGTTCATCAGCAAACGCA
GAATCAGCGGTATGGCTCTTCTCATATTGGCGCTACTGCAAAGGATATTTCTAATGTCGTCACTGATGCTGCTTCTGGTG
TGGTTGATATTTTTCATGGTATTGATAAAGCTGTTGCCGATACTTGGAACAATTTCTGGAAAGACGGTAAAGCTGATGGT
ATTGGCTCTAATTTGTCTAGGAAATAA
>Cluster_48595
ATGGATAAGGCTAACAAACAAGTCAAAGCAACAAAGTCAGTGCCGACTAAAAAAACAGCCCTCCCCCCAAAAGCTAAGTC
TGTAAAGCCAGCGGCAGAAAAGAATAAAACTGAAGGTATGACATATCCGAAAACCAGTGCGGATGGACACGCTGGAGAGT
ACTTGTTTGCCTATTGGATTTCTCGTTATTTCAGATGGCCTTGTCGATTGCTAGATATTGATATGGGATTGGATGCTCAG
GTTGAAATATATGAAAATAATCTTTCGACAGGTTTTTTTATCGGCGTTCAGATAAAAACCACCTCACGCAAAATGAAGAG
AAGGTTAAGCGTACAAATACCTTATAAAAATATAACCTATTGGGGAAGTTGTGATTTTCCTGTGGTAATTGTTTTGATAT
GCCTACACGATGATAATGAATTAGACGAACCTGCTATTTATTGGAAGCATTTAGATAAAAAAACAATTTCTAAAATAAAG
GATAAAGCATTAAGAAGTGACTCTGGATGTGCTCCTGTAACTTTCAATAAGGATGATTATCTGTCTGAATGGGCAGACAA
ACAAAAGTGGGTCGACATGTGGCTTACAGAAGAAGATAATGAGATAATTGAGCTTGCAAAGCGAGTAAACAAGACGTTGC
TAGAGATAGGTGTTCCTCTTGATAAATTCGGAGACGAGTATGACCGCAGCACCTACCTGTGTAGCCCTGAGCGGGTAGTA
CCAGACTTGAACTCTATGATGAATGAATATGAAAAAATAAATAACCATATACGTGTAAAGCGTCGATTAGTAGAATTCGA
TCCTGTAGTGTCGATTTGTTCACAAAATTATGAAAAGTATCTACCTAAAATCCTTGAATATTTTGAAGATGTGGTCGCAC
GTAGTGTCGGTTCATACTCGATTGCAGAACATTTCGAACCATACAGTGATCGTAATCATATTCTGAATCGAATAATTATA
GAGAAAGCCCCTAATTCAAGATAG
>Cluster_26660
ATGTCACATCTCGCAGAACTGGTTGCCAGTGCGAAGGCGGCCATTAGCCAGGCGTCAGATGTTGCCGCGTTAGATAATGT
GCGCGTCGAATATTTGGGTAAAAAAGGGCACTTAACCCTTCAGATGACGACCCTGCGTGAGCTGCCGCCAGAAGAGCGTC
CGGCAGCTGGTGCGGTTATCAACGAAGCGAAAGAGCAGGTTCAGCAGGCGCTGAATGCGCGTAAAGCGGAACTGGAAAGC
GCTGCACTGAATGCGCGTCTGGCGGCGGAAACGATTGATGTCTCCCTGCCGGGTCGTCGCATTGAAAACGGCGGGCTGCA
TCCGGTGACCCGTACCATCGACCGTATCGAAAGTTTCTTCGGTGAGCTTGGCTTTACCGTGGCAACCGGGCCGGAAATCG
AAGACGATTATCATAACTTCGATGCTCTGAACATTCCTGGTCACCACCCGGCGCGCGCTGACCACGACACTTTTTGGTTT
GACGCTACCCGCCTGCTGCGTACCCAGACCTCTGGCGTACAGATCCGCACCATGAAAGCCCAGCAGCCACCGATTCGTAT
CATCGCGCCTGGCCGCGTTTATCGTAACGACTACGACCAGACTCACACGCCGATGTTCCATCAGATGGAAGGTCTGATTG
TTGATACCAACATCAGCTTTACCAACCTGAAAGGCACGCTGCACGACTTCCTGCGTAACTTCTTTGAGGAAGATTTGCAG
ATCCGCTTCCGTCCTTCCTACTTCCCGTTTACCGAACCTTCTGCAGAAGTGGACGTCATGGGGAAAAACGGTAAATGGCT
GGAAGTACTGGGCTGCGGGATGGTGCATCCGAACGTGCTGCGTAACGTTGGCATCGACCCGGAAGTTTACTCTGGTTTCG
CCTTCGGGATGGGGATGGAGCGTCTGACCATGTTGCGTTACGGCGTCACCGACCTGCGTTCATTCTTCGAAAACGATCTG
CGTTTCCTCAAACAGTTTAAATAA
>Cluster_26462
ATGAAGGCTGCAGTTGTTACGAAGGATCATCATGTTGACGTTACGGATAAAACACTGCGCTCACTGAAACATGGCGAAGC
CCTGCTGAAAATGGAGTGTTGTGGTGTATGTCATACCGATCTTCATGTTAAGAATGGCGATTTTGGTGACAAAACCGGCG
TGATTCTGGGACACGAAGGTATTGGTGTGGTGGCAGAAGTTGGCCCTGGTGTTACTTCATTAAAACCAGGCGATCGTGCC
AGCGTGGCGTGGTTCTACGAAGGATGCGGTCATTGCGAATACTGTAACAGTGGTAACGAAACGCTCTGCCGTTCAGTTAA
AAATGCCGGATACAGCGTTGATGGCGGGATGGCGGAAGAGTGCATCGTGGTCGCCGATTACGCGGTAAAAGTGCCAGATG
GTCTGGACTCGGCGGCGGCCAGCAGCATTACCTGTGCGGGGGTCACTACCTACAAAGCCGTTAAGCTGTCAAAAATTCGT
CCAGGACAGTGGATTGCTATCTACGGTCTTGGCGGTCTGGGTAACCTCGCCCTGCAATACGCGAAGAATGTCTTTAACGC
CAAAGTGATCGCCATTGATGTCAATGATGAACAGTTAAAACTGGCAACCGAAATGGGTGCAGATTTAGCGATTAACTCAC

GCACCGAAGACGCCGCCAGAATTGTGCAGGAGAAAGCCGGTGGCGCTCACGCAGCGGTGGTGACAGCGGTAGCTAAAGCT
GCGTTTAACTCGGCGGTGGATGCTGTCCGTGCAGGTGGTCGTGTCGTGGCTGTCGGTCTGCCGCCGGAGTCTATGAGCCT
GGACATCCCACGGCTGGTGCTGGATGGTATTGAAGTGGTCGGTTCGCTGGTCGGCACGCGTCAGGATCTAACCGAAGCCT
TCCAGTTTGCCGCCGAAGGCAAAGTGGTGCCGAAAGTCGCCCTGCGTCCGTTAGCGGACATCAACACCATCTTTACCGAG
ATGGAGGAAGGCAAAATCCGTGGCCGTATGGTGATTGATTTCCGTCGTTAA
>Cluster_47673
TTGGATTTTAAACATCTTACTCAATTTAAAGATATAATTGAACTGGACAAGCGCCCCGTTAAACTTGATGAACGGGAAAC
GTTTAATGTCTCATGGGGTATTGATGAGAACTACCAGGTTGGGGCTGCGATTTCAATTGCTTCAATTCTTGAAAATAATA
AACAAAACAAATTTACCTTTCACATAATCGCTGATTACTTAGACAAAGAGTATATTGAATTATTATCACAATTAGCAACG
AAGTATCAAACAGTAATTAAATTATATCATATTGATTCTGAGCCATTGAAGGCGCTACCTCAATCAAATATCTGGCCAGT
ATCTATTTATTATCGTTTGCTTTCATTTGATTATTTTTCTGCGCGATTGGATTCATTATTATATCTTGATGCTGATATCG
TCTGTAAGGGTTCATTGAACGAGTTAATAGCATTAGAGTTTAAAGATGAATATGGGGCAGTGGTAATTGATGTAGATGCT
ATGCAAAGTAAAAGCGCTGAGCGTTTGTGTAATGAGGATTTTAACGGTAGCTATTTTAACTCTGGTGTAATGTATATTAA
TTTACGGGAATGGTTAAAACAAAGACTAACGGAAAAATTCTTTGATCTATTATCAGATGAGTCAATTATAAAAAAATTAA
AGTACCCGGATCAAGATATTTTAAACTTAATGTTTCTACATCATGCTAAAATATTACCGAGAAAATATAATTGTATTTAT
ACTATAAAGTCAGAATTTGAAGAAAAAAATAGTGAATATTACACCCGGTTTATTAATGATGACACTGTCTTCATACATTA
TACTGGTATAACTAAGCCATGGCATGATTGGGCGAACTACGCCTCTGCAGATTATTTTCGTAATATTTATAATATATCAC
CATGGAGAAATATACCTTATAAAAAAGCTGTTAAAAAACATGAGTACAAAGAAAAATATAAACACTTGCTTTACCAGAAA
AAATTTCTCGATGGTGTTTTTACAGCAATTAAATATAATGTTATGAAAGGTTAA
>Cluster_38568
ATGTTTGAAGAAACAATCCGCGCACTAAAAAAGCTTGGCGACGAAAAATCTACCGTCGCATTTTCCACAGACGATAGCGA
TTACTTTGATCGCGAGTGTCCCGCACCAGAATGCTTAGCTCAGTTCAAGGTTCTGATGGAAGATTGGAAGTCTAAGGTAA
GGGATGAGGAGGTCTTTTGTCCGTTCTGCGGTCATACCGCTGATGCTCAGAAGTGGTGGACGCAGGAACAGCTGGAACAT
GCGCGGGACGTTGCTCTGGCGAAGGTTAAGGCGACTTTAGGTGGTGCACTTCGCCTTGACGCACAGAGATTCAATCAGAG
GCAGCCCAAGGGGGGGATTCATCTCGATGAGCATGAAGGTGAACAGCATACCTCAGCATGTCCCGATCCCCTATGCTGCGG
CGGCACCCATGCGTCTTAAGATTGCCTGTGGAGAATGCGGATGTCGGTATGCCGTTGTCGGGGCGGCGTATTTCTGTCCG
TCTTGTGGGGCAAACGCTGCAGAGCTTGTCTTCGAACTCACTGCCCAAGGCATCCGGCAGTCGCTTGAAGCGGTTGACGC
CATTAGAGCCGCTATCTCGGACGCAGACACTGCTGAGAACACGTGCCGTCTGATCGTTGAGAGCGCTTTGCAGAACTCAG
TCACGGCATTTCAGAGGGTTGCAGAAGCGCTTCACGCGCGCATAGCCCCGACGGTGAAGACACGACGCAATGCATTTCAG
AATCTTTCCGAGGGCTCAGCTCTTTGGGAGGCCGCGATAGGGGGAGGTTACGATAAGCATCTTACCACCGTCGAGATGAA
ACAACTGACAACGGCCTTCCAACAACGTCATCTGCTTGCTCATACACAGGGTGTTGTCGACGATGACTACATCCGGAAAA
CCGGTGACACACGATACAAATTAGGACAGCGGTTGGTGATCAAGCGTGAGGCTGTGGCTGAAGCGCTTAATCTAATCGAG
CAATTGACGCGTGGCATCCGTCAGGACGCCAAGGATAAAGGGTGA
>Cluster_24994
ATGAGCGAATTGACAATAAATAAGAGCAACAGCTACCTCGAACAGGCGGCGATCCTGCTGCTGTGTCTGGGCGAAGAAGC
GGCGGCAACGGTGATGCAAAAACTCAGCCGTGAAGAAGTGGTGCGCCTGAGCGAAAACATGGCGCGCCTTTCCGGCGTCA
AAACCAGCATGGCGCGCAAGGTGATCAATAACTTCTTTGATGAGTTCCGCGAACAGAGCGGGATCAACGGCGCGTCGCGA
TCGATGTTGCAGGGCATTCTCAACAAAGCGCTAGGCACCGAGATCGCCAGCAGCGTGATCAACGGCATCTACGGCGATGA
GATCCGCTCGCGGATGGCGCGCCTGCAATGGGTAGAACCACGCCAGCTGGCGATGCTGATTTCCGAAGAACATCTGCAAT
TGCAGGCGGTGTTCCTCGCGTTTCTGACGCCGGAAATCTCGGCAGCGGTGCTCTCTTATCTCAACGAATCGGTGCAAAAC
GAAATACTCTATCGGGTGGCGAAACTCAACGACGTCAACCGCGACGTGGTGGATGAGCTGGACCGCCTGATTGAACGTGG
CCTGTCGGTACTTTCGGAACATGGCTCGAAGGTAAAAGGCATCAAACAGGCGGCGGATATCGTCAACCGCTTCCAGGGCA
ATCAGCAGGTGATCCTCGACCAGATGCGCGAACGCGACGAAGAGGTGTTGGGGCAGATCCAGGATGAGATGTACGACTTC
TTTATCCTCAGCCGCCAGAACGAAGAAGTGCGTCGGCGTCTGCTTGACGAAGTGCCGATGGAAGACTGGGCGGTGGCGCT
GAAAGGCACCGAAGCCCTGCTGCGCCGCTCGATTTACGCGGTAATGCCAAAACGTCAGGCGCAGCAACTGGAAGCGATCA
CCGCGCGTCTGGGTCCGGTGCCGGTCAGCCGTATCGAACAGATCCGCCGCGAAATCATGGGCATCGCCCGTGAGCTGGAA
GAAGCGGGCGAAATTCAGCTACAGCTCTTTGCGGAACGGACGGCGGAGTAA
>Cluster_28962
ATGGCAACCATTGCATTTCTCGGGCTGGGGCAGATGGGTAGCCCAATGGCTAACAATCTATTGCAAAAGGGCATTCATT
GCAGGTATTTGATGTCAATGCACAGGCCGTCGATGTGCTAGTTACGCAGGGGGCCACTGCGGCACAAACCCCAGCGGAAG
CAGCAGCAGGCGCTGAATTTATCATCACTATGCTTCCCAATGGTGACATTGTTCGACAGGTTCTGTTGGGGGAAAAAGGC
GTCTGTGAAACTGTTTCATCTGACGCGTTAGTGATTGATATGTCGACTATTCATCCCTTACAGACCGATGCATTAATACG
GGAGTTACAAGAAAAAGGCATCAGTATGATGGACGCTCCCGTGGGCAGAACGTCCGTGAATGCGATTGATGGCACATTGC
TGATTTTAGCGGGTGGCACTGATGAGCAGATTGCACGTGTACGCCCGATTCTCATGTGCATGGGTAATGAGCTGGTCGAG
GCTGGCGGCCCAGGAATGGGGATCCGCGTGAAGCTAATTAACAACTATATGAGCATTGCGCTTAATGCGCTGTCCTCCGA
AGCCGCTGTGTTATGTGAGTCACTGGGATTAAATCTTGATGTCGCAATTAAGGTGATGAGTGGTACGGCAGCAGGGAAAG
GACATTTTACGACCACCTGGCCTGGCAAAGTGCTGAAAGGCGATCTTTCTCCCGCCTTCATGGTGGACCTGGCATTGAAA
GATCTGCGTATTGCAGTTGACGTCGCTCGGGAAACGGGGGCGCCGCTCAATATGGGGATTGCAGCAGAAAGTTATTACGC
TGCAGCAAGCCAAGACGGCAAAGGGTGGCAGGACTGGAGCGCCCTGCTAAATCAGGTTCGCCAGCAGGCTGGTCTTTAA
>Cluster_24287
ATGAAACTGCTCAGTTTTTCTGTAGATAACGAAGAAAAGTATGGTGCCATCGTATCCGATGGAATTATTGACTTGCATAA
ATTATTTCCCGAACTTCCCGACCTGAAATCATTCGTTGCCAGTCAACATTCATTAGACATCGCTTTGTTTGCAGGTAAAA
AAGCCGATTACCATCAGGGCGAAGTGACTTTTCTGCCCGTAATTACTCGACCAAATAAAATCATCTGCGCAGGTATGAAC
TACCGGGAAAAAATGTGCAGAATTTAATGAAAAAGGTGGCGATCCGACGCTATTTATCCGTTTTGCTGATACTCAGACGGG
CCACCGTTGTGGGTTAATAAAACCGACCCTGAGTAATGAATTTGATTATGAAGGCGAACTGGCGGTAATTATAGGTCGCC
AGGGTTCTCACATTCCTGCTGATGAAGCATTTAACTATATTGCCGGGTATAGCTGTTATATGGATGGTTCCGTTCGTGAC
TGGCAGCACAGCTGGTTTACTGCCGGTAAAAATTGGCCTGCAACAGGTGGATTCGGGCCATGGATGGTTACGCCAGATGA
AATTCCTGACCCTCAAAATCTGAATATTATCACCCGTCTTAACGGCAAGGAAGTGCAGAATGATAATACACATAGCATGC
TGCACTCTATTAAGGATTTGATTGCCTATATCAGTACTTTCACCACCTTATCGCCCGGTGACGTGATATTAACAGGCTCT
CCGGGTGGGGTCGGTAAAAAACGCTCTCCACCGCTCTTCTTGTGGCCTGGTGATATTGTTGAAGTTGAGATTTCAAGAAT
TGGTTGCCTGATAAATCACATTATAAGCGACAACGAAGAGTATCCGGTCACTTTACAATCAGAACTCGATAACGCCTGCT
GA
>Cluster_24531
ATGGCTTCCAGTAAATCGCTACAGCAGGCAATTGCCAATATAAAAATCTGGCACAAGGGTGAACAGCGCGCGCCGCATAA
ACCATTGTTATTGCTATACGTATTAGCGGGATACCTGAATGGACATCCGCGCCTTTTCGATTATGGCTCGGAAATCTACG
AACCTCTGCACAGTTTACTGGAACGTTTTGGGCCACAGCGTTCACAGTACCGACCTGATATGCCGTTCTGGCGATTACAA
GGCGACGGATTCTGGCAATTACACAATGCCGGGCTTTGCTCAACAGCAGGGAGCAGCCGACAACCGCCAGTGAAGGAACT
GACCGAACACCGCGTTGCAGGCGGTTTCGATGAACAGCACTACGCCCTGGTAACCGGCAATAAAAAACTCATCAATACCC
TGGCGCAACAGATACTCGAAGCGCATTTTACCGAGAGTATTCAGGAGGAAATTGCTGACGAATTGGGTTTTGACCTCCAG

CAAATCCGTAAACAGCGCGACCCGTTATTTCGCAAAAATGTATTACGCGCTTATAACTACCAGTGCGCGATATGTGGCTT
TAATATGCGCCACGACGACACCACCGTCGCGTTGGAAGCGGCGCATATAAAATGGAAACAGCAAGGAGGTCCTTGTGAAA
TTCCCAATGGCCTGGCGCTTTGCGCAATTCACCATAAAGCCTTTGATAAAGGATCGATTGGCCTGGATGAAAATATGCGG
GTACTGGTCTCGGATGCGGTAAACGGCGGAGGGATCGTTGAGAGATTATTCTGGGATTTTGACGGGAAAACTATTGCCTT
GCCGCAGGTACGTAAAAATTACCCTTTTGAGGGGTTTGTAGAGTGGCATCGGAAAGAGGTTTTTAGGGGGTGA
>Cluster_25848
ATGGCTGAAGCGCAAAATGATCCCCTGCTGCCGGGATACTCGTTTAACGCCCATCTGGTGGCGGGTTTAACGCCGATTGA
GGCCAACGGTTATCTCGATTTTTTTATCGACCGACCGCTGGGAATGAAAGGTTATATTCTCAATCTCACCATTCGCGGTC
AGGGGGTGGTGAAAAATCAGGGACGAGAATTTGTCTGCCGACCGGGTGATATTTTGCTGTTCCCGCCTGGAGAGATTCAT
CACTACGGTCGTCATCCGGAGGCTCACGAATGGTATCACCAGTGGGTTTACTTTCGTCCGCGCGCCTACTGGCATGAATG
GCTTAACTGGCCGTCGATATTTGCCAATACCGGTTTCTTTCGCCCGGATGAAGCGCACCAGCCGCATTTCAGCGACCTGT
TTGGGCAAATCATTAATGCCGGACAAGGGGAAGGGCGCTATTCGGAATTGCTGGCGATAAATCTGCTTGAGCAATTGTTA
CTGCGGCGCATGGAAGCGATTAACGAGTCGCTCCATCCGCCGATGGATAATCGGGTACGCGAGGCTTGTCAGTACATCAG
CGATCACCTGGCAGACAGTAATTTTGATATCGCCAGCGTCGCACAGCATGTCTGCCTGTCGCCGTCGCGTCTGTCGCATT
TGTTCCGCCAGCAGTTAGGGATTAGCGTATTAAGCTGGCGCGAGGACCAGCGCATCAGTCAGGCGAAACTGCTTTTGAGC
ACCACCCGGATGCCTATCGCCACCGTCGGTCGCAATGTCGGTTTTGACGATCAACTCTATTTCTCGCGAGTATTTAAAAA
ATGCACCGGGGCGAGTCCGAGCGAGTTCCGTGCCGGTTGTGAAGAAAAAGTGAATGATGTAGCCGCCAAGTTGTCATAA
>Cluster_65646
ATGAAGCCTTTTTTGCGCTGGTGTTTCGTTGCGACAGCACTTACGCTTGCAGGATGCAGTAATACTTCCTGGCGTAAAAG
TGAAGTCCTCGCGGTACCATTGCAACCGACTTTACAGCAGGAAGTGATTCTGGCACGTATGGAACAAATCCTTGCCAGTC
GGGCTTTAACCGATGACGAACGCGCACAGCTTTTATATGAGCGCGGAGTGTTGTATGATAGTCTCGGTCTGAGGGCATTA
GCGCGTAACGATTTTTCGCAAGCGCTGGCAATCCGACCGGATATGCCTGAAGTATTCAATTACTTAGGCATATATTTAAC
GCAGGCAGGCAATTTTGATGCTGCCTATGAAGCGTTTGATTCTGTACTTGAGCTTGATCCAACTTACAACTACGCGCACT
TGAATCGCGGGATCGCATTATATTACGGCGGTCGTGACAAGTTAGCGCAAGATGATCTGCTGGCGTTTTATCAAGACGAT
CCCAATGATCCTTTCCGTAGTCTGTGGCTTTATCTCGCCGAGCAGAAGCTCGATGAGAAGCAGGCTAAAGAAGTGTTGAA
ACAGCACTTCGAAAAATCGGATAAGGAACAGTGGGGATGGAACATTGTCGAGTTCTACCTGGGCAACATTAGCGAACAAA
CGTTAATGGAAAGGCTCAAGGCGGACGCAACGGATAACACCTCGCTCGCTGAGCATCTCAGTGAAACCAACTTCTATTTA
GGTAAGTACTACCTAAGTCTGGGGGATTTGGACAGCGCCACGGCACTGTTCAAACTGGCGGTTGCCAACAACGTTCATAA
CTTTGTTGAGCACCGATACGCATTGTTGGAATTATCGCTCCTGGGCCAGGACCAAGATGACCTGGCAGAATCGGACCAGC
AATAG
>Cluster_11795
ATGCCACTGCTGGATAAGCTGCGTGAGCAGTACGGGGTCGGACCGCTATGCAGCGAACTGCATATTGCCCCGTCAACGTA
TTACCACTGTCAGCAACAGCGACATCATCCGGATAAACGCAGTGCCCGTGCGCAGCGCGATGACTGGCTGAAGAAAGAGA
TACAGCGCGTATACGATGAAAATCACAAGGTATACGGTGTGCGTAAAGTCTGGCGTCAGTTGTTACGGGAAGGTATCAGA
GTGGCCAGATGCACTGTGGCACGTCTCATGGCGGTTATGGGACTTGCCGGTGTTCTCCGGGGTAAAAAGGTCCGTACGAC
CATCAGCCGGAAAGCCGTTGCCGCAGGCGACCGCGTAAACCGTCAGTTCGTGGCAGAACGACCTGACCAGCTGTGGGTGG
CTGATTTTACTTACGTCAGCACATGGCAGGGCTTCGTCTATGTGGCGTTCATCATTGATGTGTTTGCCGGATACATTGTG
GGGTGGCGGGTCTCACCGTCCATGGAGACGACATTCGTGCTGGATGCACTGGAGCAGGCGTTATGGGCCCGTCGACCGTC
CGGCACGGTCCATCACAGTGATAAAGGTTCTCAGTATGTATCGCTGGCCTACACACAGCGGCTTAAGGAAGCCGGATTAC
TGGCATCAACAGGAAGTACAGGCGACTCGTATGACAACGCGATGGCGGAGAGCATCAATGGTCTTTACAAAGCGGAGGTA
ATACACCGTAAGAGCTGGAAAAACCGTGCAGAAGTGGAACTGGCCACACTCACGTGGGTGGACTGGTATAACAATCGACG
ATTGCTGGAAAGGCTGGGCCATACTCCTCCGGCAGAAGCAGAAAAAGCTTATTATGCTTCCATCGGAAACGATGATCTGG
CAGCCTGA
>Cluster_47941
ATGAAAGGTATTATCCTGGCGGGCGGTTCCGGCACCCGATTGCATCCGATTACGCGCGGCGTATCGAAGCAACTGTTGCC
GATTTACGATAAGCCAATGATTTACTATCCGCTGTCGGTGCTGATGTTGGCCGGTATCCGCGAAATTCTCATCATCACTA
CGCCGGAAGATAAAGGTTATTTCCAGCGCCTGCTGGGCGATGGTAGTGAGTTCGGTATCCAGCTGGAATACGCCGAACAG
CCCAGCCCGGACGGTCTGGCGCAGGCCTTTATCATCGGTGAAACCTTCCTCAACGGTGAACCTTCTTGTCTGGTGCTGGG
CGATAACATCTTCTTCGGTCAGGGCTTCAGTCCGAAGCTGCGTCATGTTGCGGCGCGCACGGAAGGGGCGACGGTATTTG
GCTATCAGGTGATGGACCCGGAACGCTTTGGCGTGGTGGAGTTTGACGACAATTTCCGCGCTATCTCGCTGGAAGAAAAG
CCAAAACAGCCGAAGTCAAACTGGGCGGTGACCGGGCTTTATTTCTACGACAGTAAAGTCGTGGAGTACGCAAAGCAGGT
GAAGCCGTCGGAGCGTGGTGAACTGGAGATTACCTCCATCAACCAGATGTACCTCGAGGCGGGCAACCTGACCGTTGAAC
TGCTCGGGCGCGGATTTGCCTGGCTGGACACTGGCACTCACGACAGCCTGATTGAAGCCAGTACCTTTGTGCAGACAGTA
GAAAAACGCCAGGGCTTTAAGATTGCCTGCCTGGAAGAGATCGCCTGGCGTAACGGCTGGCTCGATGACGAGGGTGTGAA
GCGTGCTGCCAGTTCATTAGCGAAAACTGGCTACGGCCAATATCTGCTGGAGTTACTTCGTGCCCGTCCGCGCCAGTATT
GA
>Cluster_23427
ATGCCTTGGATCCAACTGAAACTGAACACCACCGGCGCAAACGCGGAAGATCTTAGCGATGCGCTGATGGAAGCGGGTGC
CGTTTCTATCACTTTTCAGGATACCCACGATACGCCAGTATTTGAGCCGCTGCCGGGCGAAACGCGCCTGTGGGGCGACA
CCGATGTGATTGGTCTGTTCGACGCTGAAACCGATATGAACGACGTGGTGGCGATTCTGGAAAACCATCCGCTGCTCGGC
GCGGGCTTCGCGCATAAAATCGAACAACTGGAAGATAAAGACTGGGAGCGCGAATGGATGGATAATTTCCACCCAATGCG
CTTTGGCGAACGTCTGTGGATCTGCCCAAGCTGGCGTGATGTGCCGGACGAAAACGCCGTCAACGTGATGTTAGACCCAG
GGCTGGCGTTTGGTACGGGTACCCATCCAACTACCTCTCTGTGCCTGCAATGGCTCGACAGCCTCGATTTAACCGGTAAA
ACAGTCATCGACTTTGGCTGCGGTTCCGGCATTCTGGCTATCGCGGCGCTGAAACTGGGTGCAGCAAAAGCCATTGGTAT
TGATATTGATCCGCAGGCGATTCAGGCCAGCCGCGACAACGCTGAACGTAATGGCGTTTCTGATCGTCTGGAACTCTACT
TACCGAAAGATCAGCCAGAAGAAATGAAAGCCGACGTGGTGGTCGCTAACATCCTTGCAGGTCCATTACGTGAACTGGCA
CCGTTAATCAGCGTCCTGCCAGTTTCAGGCGGTTTGCTGGGCCTTTCCGGTATTCTGGCAAGCCAGGCAGAGAGCGTTTG
CGAAGCTTATGCCGACAGCTTCGCTCTGGACCCGGTCGTGGAAAAAGAAGAGTGGTGCCGCATTACCGGTCGTAAGAATT
AA
>Cluster_9741
ATGAATCAAAATCCATTCTCATTCTATGACTTTCTTGGATATTTAATACCAGGTGGTTTTTTTATCCTATTAATGTATTT
CTGTGGTTTGACATTCGATCTAGATATTGTTATTGACTTAACTGAATTGCTCAGAGGTCAAAGTCAAATCTTTGGCATTT
TGAACTATGGTTCAATAGTAATCATATCTTACATAGCTGGACACTTTATATCTATCACATCCGCATTTTTTATTGAAAAA
TATATGAACGAAGAATTGCAATATCCATCAGAATATCTATTTAATAAGTTATCAGATGACAAAAAAAACGTCTTAACACC
ATCAAGTGACAAACAGAACGAAGACACAAAGAGAAAAATAAAATACTGCATAATCAAAGTAATGTTATTCCCGATAATAC
CATGGGATTACGCCACTCAAAAACTATGTTATTCACAATCATTGCCATTTCAATTAGCTAATACCACATGGCTAATGATC
AAAGAAGGTTATGAAAAAAAATTCAGAATAGACGATAAATTATTAAACATCAGGACAGGTCTTCATGATGATCTTTTCAG
ATTGGCTTATCATTACGTCTATGAATTTTCTAATCAACATCAAACAAAAATTCAAAACTATGTTGCATTATATGGTTTTT
GCAGAAATATTTGTTTAGTATTTGTAATTACATTTGGATATCATTTCTAACATTAATTTATCGTGCTATAGAAGGTGGG

GCGGTGTCTTATATTTCTATTTCGACGCTACTTAGCTTTTTCTTCGTCTATGTTTTTTATGTTGGATTCGTTAAGTTTTA
TAGACGATATACTTTAGAGGTATTAATGGCATTTGCTGTACTTCAGAGCAAAGATGCTATTAATTAA
>Cluster_20431
ATGAGTGTGCTCATTAATGAAAAACTGCATTCGCATCGGCTGAAATGGCGCTGGCCGCTCTCGCGTCAGGTGACCTTAAG
CATTGGCACGTTAGCGGTTTTACTTACTGTATGGTGGGCAGTCGCAGCGCTGCAACTGATTAGCCCGCTGTTTTTGCCGC
CACCGCAACAGGTACTGGCAAAACTGCTCACCATTGCCGGGCCGCAGGGATTTATGGACGCCACGCTGTGGCAGCATCTG
GCCGCCAGCCTGACGCGCATTTTGCTGGCGCTACTCGCGGCGGTGTTGATCGGTATTCCGGTCGGGATCGCCATGGGGCT
AAGCCCGACGGTGCGCGGCATTCTGGATCCGATAATCGAGCTTTATCGTCCGGTGCCGCCGCTGGCTTATTTGCCGCTGA
TGGTGATCTGGTTTGGTATTGGCGAAACCTCGAAGATCTTACTGATCTATCTAGCGATTTTTGCGCCGGTGGCGATGTCG
GCGCTGGCGGGGGTGAAAAGCGCGCAGCAGGTTCGCATTCGTGTCGCCCAGTCGCTGGGTGCCAGCCGTGCGCAGGTGCT
GTGGTTTGTCATTTTGCCCGGCGCGCTGCCGGAAATCCTCACCGGATTACGTATTGGTCTGGGTGTGGGCTGGTCTACGC
TGGTGGCGGCGGAGCTGATTGCCGCGACGCGCGGTTTAGGATTTATGGTTCAGTCAGCGGGTGAATTTCTCGCGACTGAC
GTGGTGCTGGCGGGGATCGCGGTGATTGCGATTATCGCCTTTCTTTTAGAACTGGGTCTGCGCGCGTTACAGCGCCGCCT
GACGCCCTGGCATGGAGAAGTACAATGA
>Cluster_36573
ATGTCTCAGGTTCAATTTTCTCATAACCCGCTGTTTTGCATAGATATTATCAAGACTTATAAACCTGATTTCACGCCACG
AGTGGCCTTTATTTTAGGTTCCGGGCTCGGCGCGCTGGCCGATCAGATTGAGAACGCTGTCGCAATTTCCTACGAAAAGC
TGCCGGGCTTCCCGGTAAGTACGGTACATGGTCATGCGGGGGAGTTAGTGCTGGGCCATCTCCAGGGCGTACCGGTGGTA
TGTATGAAAGGTCGCGGACATTTCTACGAAGGTCGTGGAATGACCATTATGACTGACGCAATTCGTACCTTTAAGCTGCT
GGGCTGCGAGCTACTGTTCTGCACCAATGCGGCAGGCTCGCTGCGTCCGGAAGTGGGGGCAGGCAGCCTGGTCGCATTGA
AAGATCATATCAACACCATGCCCGGTACGCCGATGGTGGGTCTTAACGATGATCGTTTGGAGAGCGCTTCTTCTCACTG
GCAAATGCCTACGATGCGGAATACCGCGCACTGTTACAAAAAGTGGCGAAAGAAGAGGGTTTCCCTCTGACGGAGGGCGT
GTTCGTCTCGTATCCGGGGCCGAATTTCGAGACTGCGGCGGAAATTCGCATGATGCAAATTATTGGTGGGGATGTTGTTG
GTATGTCTGTGGTGCCTGAGGTTATTTCAGCTCGCCATTGCGACCTTAAAGTCGTTGCGGTCTCTGCGATTACCAATATG
GCGGAAGGTTTAAGCGATGTGAAATTGTCACATGCGCAAACGCTGGCGGCTGCGGAGCTCTCTAAGCAGAACTTCATCAA
CCTTATTTGTGGCTTTCTGCGCAAAATTGCCTGA
>Cluster_66741
ATGACGATTATTGAAGCTATCATAAATGTATTAAAGGAACATGGAAAACCTTTGTCACATAAGGATATTTATGATTGCAT
CATAAATAAAGAGTATTATTCATTTGGTGCAAAAGATCCTGTGGCAGTAGTGCGGGGGGAAATTAGAAAACATTGCTATG
GCATTGATTTTCCTAGTGCATCACCACGAAAAATATTTATTGAAGTTAAATCTGTAGGGCAATCAAAGCCATTATACGAC
CTATGGCAGGGACAAGCATCAAATGCTAGCCCTCTTAAAATTGAAAAGGTAACAAAAGATCAACTACCAGAAGAGATAAT
TCATGGGAAGTATCTTGAGCATATTAAAACTATTAAAGAACAATTGCTTGATGCTATTAATTCTTCGGATCCAGCTTTCT
TTGAGAGACTTGTTGTTACACTTTTATTAAAAATGGGTTATGGGTGGCACGAGTCTGAGGCAGGCAAGGTTGTTGGTGGC
GCTGGGGATGAGGGCATAGATGGAATTATTAATGAAGATAAATTAGGTTTGGAAAAAATATATATCCAAGCTAAAAGATA
TAATAGTAAAAAGGTACCACCTAGCGAAATTAGAGAGTTTATTGGTTCGATGAACCAAAAAGGAGCACATAAAGGGGTAT
TTTTTTCATCTTCATGTTTTACCGAACAGGCAATTAATAGTGCAGAAAAAGCACAAGGCATGAACATCACTCTTGTTGAT
GGTGAACAGTTATGTGAATATTTAGTCCAAAATGGCATGGGGGTTGCGAAGGTAAGTACATATGAAATCTATGAAATAGA
TAAAAATTTCTTTGATTTATAA
>Cluster_20532
ATGAGCGTGACGAGCAGTGAGCTGGCGTATTGTCTGAGTCTTCCACCAAAGCGAGCCATCGGCTATCTGGAGTCCAAAGG
CTATACCCTGTCGTGGGACTGGGAAGAAGTTTGGCAGGAGGCCCACGCCCGCGCCTTTACCGTGGCGAAGGTGACAAAGC
TGGATGTACTGGAAGATATCCGCCGTGCACTGGAGCGGTCACTGGCAGAGGGCAAAACCGGCGCGTGGTTCCGTAAGGAG
CTGGAACCAGTGCTGAAAGAGAAAGGCTGGTGGGGTGAGAAAGAAATCACCGACCCGGACACCGGGGAAGTGACCATCAT
CCAGCAAGGCAGTACCTGGCGGCTTGATACCATCTACCGCACCAATATGACGGTGGTTTACAGTGCCGGGCGCTGGGCCG
AACAGATGGAAAACGTCGACGATCGTCCATATGGGATGTACTGTGCCATTTGCGACAACCGCACCCGTCAACCCCACCTC
CTGCTGCATCAGATGGTTATGCGTCTTGATGATCCATTCTGGCAGTCATTCTACCCGCCTAACGGCTGGGGGTGCCGCTG
TACAGTGATAGCAATGACTGAGGCGGATGTCCGCCGCCGCGGGCTGAAAATCACGGCCTCCGGCGATGCGCTGGGGGAAA
CGATGAAACTGATATCGATGCAAACCGGAGAATTGCAACCGGTGGCCACCTTTACTATTGGCAATACGATTATCACCCCG
GATGCGGGCTGGTCATACGCGCCGGGCGCGGCGTACCGCCCGGACTTAGCCAAATACCAGGGCGTGCTCGCGCCGCTGGC
GCAACAGGAGCTGGCACCATGA
>Cluster_6075
ATGGGAAGTGGATGGCATGAATGGCCGCTGATGATCTTCACGGTCTTCGGGCAATGTGTAGCAGGTGGTTTTATCGTTCT
GGCTTTGGCGCTGCTCAAAGGCGACCTGCGAGCAGAAGCCCAGCAGCGTGTTATCGCCTGCATGTTTGGTTTATGGGTGC
TGATGGGCATTGGCTTTATCGCCTCTATGCTCCATCTTGGTTCACCAATGCGCGCTTTTAACTCGCTCAACCGGGTAGGG
GCTTCAGCACTCAGTAACGAAATCGCCAGCGGTTCGATCTTTTTTGCCGTAGGCGGCATCGGCTGGTTGCTGGCAATGCT
GAAAAAGCTGTCACCGGCATTGCGTACGCTGTGGCTGATAGTGACGATGGTTCTTGGCGTCATCTTTGTCTGGATGATGG
TGCGTGTGTATAACAGCATTGATACCGTTCCGACCTGGTACAGCATCTGGACGCCGATGGGCTTCTTCCTGACGATGTTT
ATGGGCGGCCCGTTACTGGGTTACCTGTTGTTGAGTCTGGCTGGCGTCGATGGCTGGGCGATGCGTCTGCTGCCAGCAAT
TTCTGTACTGGCACTGGTAGTGAGTGGCGTGGTGTCAGTGATGCAGGGCGCAGAGCTGGCGACTATTCATAGTTCTGTGC
AGCAGGCCGCAGCGCTGGTGCCGGACTATGGTGCGCTGATGTCCTGGCGGATCGTGCTTTTGGCCGTTGCCCTGTGCTTG
TGGATTGCACCACAGCTAAAAGGTTATCAGCCTGCGGTTCCGCTACTTTCTGTATCATTCATTCTGCTTCTGGCAGGGGA
ATTAATCGGTCGCGGCGTATTCTATGGTTTGCATATGACCGTGGGGATGGCCGTCGCAAGCTAA
>Cluster_4166
ATGAAAAAGCGTCGGAAGGAACCGGAAACGCTACGTGAACACTGTCGCCATATTTTTGGTGATGAGCCGCCAGTGCTCTG
TGTATGGGAAACGGAATTTGATTACGCAGATGCCGAACTCAAGGCGCTGGCGGCTAAAGAGTGGCAGCAGATCAGCGAAT
GGGATTTGAGTGCTTACTATGTACTCAATCTCGTTTATAACGAACCGATGCAAATAGAGTTGTTTCGCTATCTGTTTCCG
CTCTGCCTGGCGCAATGGCATGAAACCGTTTTAGCTGGCGGTTATGGTGATCACTTCGAAGAATCCTTAATGAAGGCATT
GTGTCGGCCCTATCTCTGGCAGGAGATGATGAATGCTTCCCAACGCCAGCAGGTACGGCAATTTTTACTGGACACCGCGT
TGCAGCGTATGGATAACGAGCGTGGTTTCAATAACGTTCTTTGCTGGCTGGCGGTGTTCAATACTCTGGGTGGGGCCGCG
CCGTTAATTCGTTCTCTCTGGTCGCGCTGGTGGGCGCTGGATACGCCAGGAAAAGCGGTGTGTGCTATTCAGTATGCCGC
GCATCTAATTTATCCCATAGAAGCCAATCCGTTATGGTCGCAAGAATGGATAGGCTGGGGGCATCCATTAGGCCATAAAG
ATGGCTGGTCCTCTGATAATCGCGCTTTTCTTCGCCAAATGTTAACACCTGAAATGATTGTGGCTGGCGTACAGGCAGCG
GCTGAAATTCTGCGTGGTGAACCGGAAGGCGCAATGGCGGCGCGAATTGCACAGGATGCGTATGAGGCAATGGATATCCT
CACTATCCAGATTGAAGATTTACTGCGTGACTTGTCTTGTGATGAGTCTGGTCATGCGTTGGAGTAA
>Cluster_67103
ATGAAAAAGACTCTGATTGCACTGGCGCTGGCTGCCTCTGCTGTATCTGGTATGGCGCATGCCTGGACGTCTGGTGACTT
CAATGGCACGGTCGATATCGGTGGTAGCATTGATGCAACTAATTATCGCCAGAAATGGGAATGGGAAGTTGGTACGGGGC
TTAATGGATTTTATAATGCTCTGAGTGAGTTGACAAATAATGATACAAAACTGACTATTACTGTTACTGGTAATAAGCCA
ATTTTGTTAGGCCGAACCAAAGAAGCATTTGTAACGCCGACAGCAGGTGGCTGGGGTGGCATCCCTCAGATTGCATTTAC

CGACTATAAAGGAGCACCGGTAGAGCTCAAAAACCCTGATGGTGAAACTAATAAAGGTTTAGCATATTTTGTTCTGCCGA
TGAAAAATGCAGATGGTACTGAAGCGGGTTCCGTGAGAGTGAATGCATCTTATGCTGGGGCTGTGGTTGAAAAGACTTCT
GATAGTTCGGCTAAATTGTTCTCTGCTTATGCCGGTGAGGTAATTCGCATCTTCAATGGCGGTCTTCCGGTGAATGTTAC
CCAGTCTGAACTGCAGTCAGGTGTTGCTGCTGCTGATAGAACAGCATTGTTTGGTAGTCTGGGGAAAGCTGATTTGGTAG
CGCAGTTTCAAGCTGTTATTCCTGGGGCTACTGTGACTGATGGGACGAGCTCAGAACATTGTACAATGAGACTATTGGCC
TCAGCTGCCTATGCACTGGGTATTGCAGATGGTCAGACTATTGAGGCAACTTTTAATCAGGCTGTAACTACCAGCACTCA
GTGGAGCGCTCCGCTGAACGTAGCAATTACTTATTACTAA
>Cluster_53507
ATGAGTCTTTATTTATCTGCGGCACTGGCCAGTAATCGTAAAGGCCGTTTTTTGCAAACTGTTGCGGGGGCTACTCCCTT
AACGAAGGATTGGATTAGCTCGCCACCAGCCAGCGGTTTGTTAATAATGCAAGCAGAAGAGTTGACTGACGCTAATACTA
TGCAGCACCTGTATCACTGGGCTATGCAGGCAGGTTGTGCCGCTTTGGTAATAAACCTGAAAGCAGAACAATTTACCTTA
TTAGCGCAATTACCCTATCCACTGGATTGGCAGTTAGTACCAGCGAGCTTGAGGAGGCAGGAACCGGGGCTAACCGCTTT
GCTAGCCAGTGAAACAGACCAGGCTATTGCTGGCTTTACTGGCAGCGCAGATCGCTACCAGCACCAGGCCGGTGATGTAG
TGCATACCCGCTACATACGAAAACACAGCAACAGTGGGCTATTAGCCTTTACCACGTTACCTTTGTGGTCGCTGACTTTA
CTCGATCATAGTGAGCTCCTGGTGAGCTGGTTGAATTGGTTTGTGGACCATGCAGGCATTGCTGAACGAATAATCGAACC
TAAAGCGCCATCAACTGACTACACGCCAGATAAGCACGATCTGGTTGTATTGCTATTACTTTACGCTGGCGGTGGCATGA
ACTTACAAGCGTTGTCTGAGCATAATGCGGTTAAGCTGATGTTTGATGTTAATAGCCTTGATATTGTCAAGCGGGGTGAG
ATGTTGCGGCAGCACGATTTTATCGACGACGCGGGCATAACAGCTACCGGTAAAACATGCCTACAAGCCTCGCAATATTG
GGCTTATGCCCCATTACTTGGCGAGCAGCTACACACAGGAACGCTATAA
>Cluster_32007
GTGAGTACATTAGAACAAACAATAGGCAATACGCCTCTGGTGAAGTTGCAGCGAATGGGGCCGGATAACGGCAGTGAAGT
GTGGTTAAAACTGGAAGGCAATAACCCGGCGGGATCGGTAAAAGACCGTGCAGCGCTTTCGATGATTGTCGAGGCGGAAA
AGCGCGGCGAGATTAAACCGGGTGATGTGTTGATCGAAGCCACCAGTGGTAACACCGGCATTGCGCTGGCGATGATTGCC
GCGCTTAAAGGCTATCGCATGAAATTGCTGATGCCCGACAACATGAGCCAGGAACGCCGTGCGGCAATGCGTGCCTACGG
TGCGGAACTGGTTCTGGTCACCAAAGAGCAGGGCATGGAAGGTGCGCGCGATCTGGCGCTGGAGATGGCCAATCGTGGTG
AAGGAAAGCTGCTCGATCAGTTCAATAATCCTGATAATCCCTTCGCCCATTACACCACCACCGGGCCGGAAATCTGGCAG
CAGACTGGCGGGCGCATTACCCATTTTGTCTCCAGCATGGGAACAACCGGCACTATCACCGGCGTTTCACGCTTTATGCG
CGAACAATCCAAACCGGTGACCATTGTTGGCCTGCAGCCGGAAGAAGGCAGCAGTATTCCGGGCATTCGCCGCTGGCCTG
CGGAATATCTGCCGGGGATTTTCAACGCTTCTCTGGTGGATGAGGTGCTGGATATTCATCAGCGCGATGCGGAAAACACT
ATGCGCGAACTGGCGGCGCGGGAAGGGATTTTCTGTGGCGTTAGCTCCGGCGGCGCGGTTGCCGGAGCACTGCGCGTGGC
AAAAGCTAACCCCGGCGCGGTGGTTGTGGCGATCATCTGCGATCGCGGCGATCGCTACCTTTCTACCGGGGTGTTTGGGG
AAGAGCATTTTAGCCAGGGGGCGGGGATTTAA
>Cluster_24967
ATGAAAGCCACGTCGGAAGAACTCGCCATTTTTGTTTCAGTCGTCGAAAGCGGCAGCTTTAGCCGGGCAGCGGAACAATT
AGGGCAAGCAAACTCAGCGGTAAGCCGGGCGGTGAAAAAGCTGGAGATGAAACTTGGCGTTAGCCTGCTTAATCGGACCA
CGCGGCAACTTAGCCTGACGGAAGAAGGCGAGCGTTATTTTCGTCGCGTACAGTCAATTTTGCAGGAGATGGCAGCGGCA
GAATCAGAAATTATGGAGACGCGTAATACACCGCGTGGACTGTTACGGATCGATGCCGCAACTCCAGTGGTGCTGCACTT
TCTGATGCCGTTGATTAAGCCTTTTCGTGAACGCTATCCGGAAGTCACTTTATCGCTAGTCTCCTCCGAAACGATTATTA
ATTTGATCGAAAGAAAAGTGGATGTCGCGATACGCGCCGGTACGTTAACGGATTCCAGCTTACGTGCCAGGCCGTTATTT
AACAGTTATCGAAAAATTATCGCCTCCCCCGATTATATTTCCCGCTACGGAAAGCCAGAAACGATCGACGATTTAAAGCA
ACATATTTGCCTGGGATTCACTGAACCCGCTTCCCTCAATACCTGGCCGATAGCCTGTAGCGATGGACAATTACATGAGG
TGAAGTACGGTTTGTCATCCAATAGTGGGGAAACACTGAAACAGCTTTGCCTGAGTGGCAACGGGATTGCGTGTTTGTCC
GACTACATGATCGACAAAGAAATCGCTCGCGGAGAATTGGTGGAGTTAATGGCAGATAAAGTGTTGCCAGTAGAAATGCC
ATTCAGTGCAGTCTATTACAGCGACCGTGCGGTAAGTACGCGCATCCGGGCTTTTATCGATTTCCTTAGCGAGCATGTAA
AAACAGCTCCCGGAAAAGCTGTCAGAGAGGCTTAA
>Cluster_26120
ATGTATTACGGGTTTGATATTGGTGGAACAAAAATTGCGCTAGGCGTGTTTGATAGCGGTCGGCAGTTGCAGTGGGAAAA
GCGGGTGCCGACACCGCGTGACAGCTATGACGCATTTTTAGATGCAGTGTGCGAGCTGGTAGCCGAAGCTGACCGGCGTT
TTGGCTGTAAAGGTTCTGTCGGCATCGGTATTCCGGGAATGCCGGAAACAGAAGATGGTACGCTGTATGCCGCCAATGTC
CCTGCTGCCAGCGGTAAACCGCTGCGTGCCGACCTGAGCGCACGTCTTGATCGCGATGTACGCCTTGATAACGATGCCAA
CTGCTTTGCCCTTTCTGAAGCATGGGATGATGAATTTACTCAATATCCACTGGTGATGGGGTTGATTCTCGGCACCGGCG
TTGGCGGCGGGCTGATTTTCAACGGTAAACCAATTACCGGTAAAAGCTATATTACCGGCGAGTTTGGCCATATGCGTCTG
CCGGTTGATGCGTTAACCATGATGGGGCTGGATTTCCCGTTACGCCGCTGCGGCTGTGGTCAGCATGGCTGCATTGAAAA
TTATCTGTCTGGTCGCGGTTTTGCGTGGCTGTATCAACACTATTATCATCAACCGTTGCAGGCTCCTGAGATCATTGCGT
TTTATGATCAAGGCGATGAGCAGGCAAGGGCGCACGTTGAGCGTTATCTGGATTTATTAGCGGTTTGTCTGGGAAATATC
CTGACCATTGTTGACCCTGACCTGGTCGTCATTGGTGGTGGCTTATCGAATTTCCCGGCAATCACAACGCAACTGGCGGA
AAGGTTGCCTCGTCATCTCTTACCTGTAGCTCGTGTTCCGCGCATTGAACGCGCGCGCCACGGTGATGCGGGGGGAATGC
GTGGTGCGGCCTTCCTACATCTAACCGATTAA
>Cluster_5965
ATGCGACTATTTAACTGGCGACGTCAGGCGGTGTTAAACGCCATGCCACTGGTCAAACCTGACCAGGTACGTACACCGTG
GCATGAATTCTGGCGACGATTTCGCCGTCAGCATATGGCGATGACCGCCGCATTATTCGTTATTTTATTGATTGTGGTGG
CCATTTTTGCACGCTGGATCGCTCCCTATGACGCCGAAAATTATTTGATTATGACAATCTGAATAACGGACCTTCTTTG
CAGCACTGGTTTGGCGTCGATTCACTGGGGCGTGACATTTTCAGCCGTGTCCTGGTTGGTGCGCAAATCTCGCTGGCGGC
GGGCGTGTTTGCCGTGTTTATCGGTGCGGCGATCGGGACGTTGCTGGGCTTGCTCGCTGGATATTATGAAGGCTGGTGGG
ATCGGCTGATCATGCGCATTTGCGATGTGCTGTTTGCCTTCCCGGGTATTTTACTGGCGATCGCTGTTGTTGCGGTGTTG
GGAAGCGGCATTGCTAACGTGATTATTGCAGTCGCCATTTTTTCCATCCCCGCGTTTGCCCGCCTGGTGCGCGGCAACAC
GCTGGTGTTGAAACAGCAAACCTTTATTGAGTCAGCACGCAGTATTGGTGCCAGCGATATGACCGTTTTGTTGCGTCATA
TCCTGCCTGGGACCGTCTCTTCTATCGTGGTGTTTTTCACCATGCGCATTGGTACCTCGATTATCTCTGCCGCCAGCCTC
TCATTTCTCGGCCTCGGTGCGCAGCCGCCGACACCAGAGTGGGGAGCAATGCTCAATGAGGCTCGAGCGGATATGGTTAT
CGCGCCGCATGTCGCTGTTTTTCCGGCCCTGGCTATTTTTCTGACCGTACTGGCGTTCAATTTGTTGGGCGATGGTTTAC
GCGATGCGCTGGATCCGAAAATTAAAGGATAG
>Cluster_20140
ATGACTGATAAAATCGCGGTCCTGTTGGGCGGGACCTCCGCTGAGCGGGAAGTTTCTCTGAATTCTGGCGCAGCGGTGTT
AGCCGGGCTGCGTGAAGGCGGTATTGACGCGTATCCTGTCGACCCGAAAGAAGTCGACGTGACGCAACTGAAGTCGATGG
GTTTTCAGAAAGTATTTATCGCGCTACACGGTCGCGGCGGTGAAGATGGTACGCTGCAGGGCATGCTCGAGCTGATGGGC
TTGCCTTATACCGGCAGCGGAGTGATGGCATCTGCGCTTTCAATGGATAAACTACGCAGCAAACTTCTGTGGCAGGGGGC
CGGTTTACCGGTCGCGCCGTGGGTAGCGTTAACCCGTGCAGAGTTTGAAAAAGGCCTTAACGATAAGCAGTTAGCAGAAA
TTTCTTCTCTGGGTTTGCCGGTTATCGTTAAGCCGAGCCGCGAAGGTTCCAGTGTGGGAATGTCAAAAGTAGTAGCAGAA
AATGCTCTACAAGATGCATTAAGATTGGCATTTCAGCACGATGAAGAAGTATTGATTGAAAAATGGCTAAGTGGGCCGGA

GTTCACGGTTGCGATACTCGGTGAAGAAATTTTACCGTCAATACGTATTCAATCGGCAGGAACCTTCTATGATTATGAGG
CGAAGTATCTCTCTGATGAGACACAGTATTTCTGCCCCGCAGGTCTGGAAGCGTCACAAGAGGCCAATTTGCAGGCATTA
GTGCTAAAAGCATGGACGACGTTAGGTTGCAAAGGATGGGGACGTATTGACGTTATGCTGGACAGTGATGGACAGTTTTA
TCTGCTGGAAGCCAATACCTCACCGGGTATGACCAGCCACAGCCTGGTGCCGATGGCGGCACGTCAGGCAGGTATGAGCT
TCTCGCAGTTGGTAGTACGAATTCTGGAACTGGCGGACTAA
>Cluster_39882
GTGAAAAAATTACGTATCGGCGTAGTGGGGTTAGGCGGCATCGCGCAAAAAGCGTGGTTACCGGTGCTGGCGGCAGCGTC
TGACTGGACGTTACAAGGTGCGTGGTCGCCTACGCGCGCCAAAGCCTTGCCAATTTGTGACAGCTGGCGCATTCCTTATG
CTGATTCGTTATCCAGCCTTGCCGCCAGTTGCGATGCGGTTTTTGTGCATTCCGGCACCGCCAGCCACTTTGACGTGGTC
AGTACGTTACTCAATGCGGGGGTACATGTCTGTGTCGATAAACCGCTGGCAGAAAATCTGCGCGATGCTGAACGGCTGGT
GGAACTGGCGGTGCGGAAAAAACTGACGTTGATGGTCGGTTTTAACCGTCGTTTCGCACCACTCTACGGTGAGTTAAAAA
CGCAACTCGCCACCGCGTCCTCGCTAAGAATGGATAAACATCGTACCAATAGCGTCGGGCCACACGATCTTTATTTCACA
TTGCTGGATGATTATTTGCATGTGGTTGATACCGCGCTGTGGTTGTCTGGCGGCAATGCAACTCTCGAAAGCGGTACGTT
GCTGACCAATGAGTCCGGCGAAATGCTGTTTGCCGAGCACCATTTTTCGGCCGGTCCTTTGCAGATCACCACCTGTATGC
ATCGCCGTGCCGGAAGCCAGCGTGAAACCGTGCAGGCCGTGACTGACGGTGCGCTCATCGACATTACGGATATGCGCGAA
TGGCGTGAGGAGCGTGGGCAGGGCGTAGTGCATAAACCGATTCCTGGTTGGCAGAGTACGCTTGAGCAACGTGGATTTGT
CGGCTGTGCGCGGCACTTCATTGAATGCGTGCAAAATCAGACAGTTCCGCAAACCGCCGGCGAACAGGCCGTGCTGGCGC
AACGTATCGTTGACAAGATCTGGCGCGATGCGATGAGTGAATAA
>Cluster_38942
ATGTTATCTATTAACACCAATAACGCCTCAATGGCCGCCGTTAATGCAATCAGCAAAAGCAGCTCTTCTCTGTCTACATC
TATGGAACGCCTGGCGACAGGTAACCGGATTAACTCTTCTGCCGATGACGCAGCGGGCAAACAAATTGCTAACCGTCTGA
CGGCACAGTCCAGCGGTATGGGCGTGGCGCTGAGCAACATCAATGATGCCACCGCGATGCTGCAAACCGCCGACAGCATG
TTTGACGAAATGTCTGACGTGCTGGGTCGTATGAAAGACCTGTCAACTCAGGCAGCGAACGGCACCTACAGCGACGATGA
CCTGCAGGCGATGCAGGACGAATACGACGAACTGGGCCAGCAGATGTCTGACATGCTGCAAAACACTACTTACGGCGGCA
CCAACCTGTTCGGTGTTTCCGGCACCAGCAACACCGGTACTGACGGTCTGTTCCAGAGCGCAGTAACTTTCCAGGTGGGC
GCGGAATCTTCCGACACCATGACCGTGAATATTTCAAGCCAACTGAACACGCTGGTGACTGACCTTTCTGCTATCAGCAA
CTCCTTTAGTGCGGATCAGGCAGACACCACTGGCACCGCAGGCGTTTCTGGCGGTACTGAACTGACTGCGTCTGGCTCGG
CTAACCAGATGATCACCAGCATTTCTACCGCGATGGATGACGTTTCGCAGATCCAGTCCAAACTGGGTGCGTCTATCAAC
CGTCTGAACGACACCGCCAACAACCTGACCAGCATGCAGGACAATACCGAAGTGGCTATCGGCAACATCATGGATACCGA
TTACGCGACGGAAGCGTCCAATATGACCAAACAACAGGTGCTGATGCAGACCGGTATCACCATGCTGAAACAGTCCAACA
GCATGTCCAGCATGGTTTCCAGCCTGCTGCAGTAA
>Cluster_27991
ATGAATCAAACTGTCGCGGTGACGGGCGCTACCGGGTTTATCGGTAAATATATTATCGATAACCTGCTCGCCCGCGGCTT
TCACGTTCGCACATTGACGCGTACTGCCCGCGCTCACGTCAATGATAATCTCATCTGGGTGCGCGGTTCGCTGGAAGATA
CACATTCGCTTAGCGAACTGGTTGCCGGAGCCAGCGCGGTGGTCCATTCGCGCCGGGCAAGTGCGCGGGCACAAAGAAGAG
ATTTTTACCCGCTGTAACGTTGACGGCAGCCTGCGACTGATGCAAGCGGCAAAAGAGAGCGGCTTTTGCCAACGTTTTCT
GTTTATCTCTTCGCTGGCGGCGCGCCATCCCGAACTCTCCTGGTACGCAAATTCCAAACACGTCGCCGAACAACGACTGA
TCGCAATGGCTGACGAAATTACGCTGGGCATTTTTCGCCCGACAGCCGTGTATGGTCCCGGCGATAAAGAGTTAAAACCG
CTGTTTGACTGGATGCTGCGCGGCCTGCTGCCACGACTTGGTGCGCCAGATACACAACTCTCTTTCCTGCACGTCACCGA
TTTTGCGCAAGCAGTGGGTCAGTGGTTAAACGCCGAAACTGTACAGACGCAAACCTATGAATTATGCGATGGCGTCGCTA
GCGGCTATGACTGGCAACGCGTACGACAGCTTGCCGCCGAAGCCCGTTGTGGTTCTGTGCGGATGGTTGGCATTCCTCAG
CCGCTACTCACCTGCCTTGCGGATATCAGTACTGCGTTGAGTCGCCTGGCAGGTAAAGAACCGATGCTGACCCGCTCGAA
AATTCGTGAATTAACCCACGCCGACTGGTCGGCAAATAATAACCGTATTTCTGAAGATATTAATTGGTTTCCCGGGATTA
GCCTGGAACACGCATTACGCAACGGGCTATTTTGA
>Cluster_19864
ATGGCTGCCATTAATACGAAAGTCAAAAAAGCCGTTATCCCCGTAGCGGGATTAGGAACCAGGATGTTGCCGGCGACGAA
AGCCATCCCGAAAGAGATGCTGCCACTTGTCGATAAGCCATTAATTCAATACGTCGTGAATGAATGTATTGCGGCTGGCA
TTACTGAAATTGTGCTGGTTACTCACTCATCTAAAAACTCTATTGAAAACCACTTTGATACCAGTTTTGAACTGGAAGCA
ATGCTGGAAAAACGTGTAAAACGTCAACTGCTTGATGAAGTGCAGTCTATTTGTCCGCCACACGTGACTATTATGCAAGT
TCGTCAGGGGCTGGCGAAAGGCCTGGGACACGCGGTATTGTGTGCTCACCCGGTAGTGGGTGATGAACCTGTGGCTGTTA
TTTTGCCTGACGTTATTCTGGATGAATATGAATCCGATTTATCACAGGATAACCTGGCAGAGATGATCCGTCGCTTTGAT
GAAACGGGTCATAGCCAGATCATGGTTGAACCGGTTGCTGATGTGACCGCATATGGCGTTGTGGATTGCAAAGGCGTTGA
ATTAGCGCCGGGTGAAAGCGTACCTATGGTTGGCGTGGTTGAAAAACCAAAAGCGGATGTTGCGCCGTCTAATCTCGCTA
TTGTGGGTCGTTACGTACTTAGCGCGGATATTTGGCCGTTGCTGGCAAAAACACCTCCGGGTGCTGGTGATGAAATTCAG
CTCACCGACGCAATTGATATGCTGATCGAAAAAGAAACGGTGGAAGCCTATCATATGAAAGGGAAGAGCCATGACTGCGG
TAATAAATTAGGTTACATGCAGGCCTTCGTTGAATACGGTATTCGTCATAACACCCTTGGCACGGAATTTAAAGCCTGGC
TTGAAGAAGAGATGGGCATTAAGAAGTAA
>Cluster_54923
ATGCCTTACGATAGCGTATACAGCGAAAAGCGCCCGCCGGGCACGCTGCGTACCGCCTGGCGCAAATTTTATAGTGACGC
CTCTGCGATGGTCGGCCTGTACGGCTGCGCGGGGCTGGCTGTACTGTGTATTTTTGGCGGCTGGTTTGCGCCTTACGGCA
TCGACCAGCAATTTCTCGGTTATCAATTATTGCCGCCGTCATGGTCGCGCTATGGCGAAGTTTCTTTCTTCCTGGGGACT
GACGACCTCGGACGCGATGTTTTAAGCCGTTTACTGAGCGGAGCTGCGCCAACCGTGGGTGGCGCATTTGTGGTGACGCT
TGCCGCGACGATCTGTGGCCTGGTACTGGGCACCTTCGCCGGGGCGACACACGGCCTCCGCTCAGCGGTGCTTAACCATA
TTCTGGATACCTTACTGGCGATCCCTTCGCTGCTGTTGGCAATTATCGTTGTAGCGTTTGCCGGACCGAGTTTGTCCCAC
GCTATGTTTGCTGTCTGGCTGGCATTGCTGCCGCGTATGGTACGTTCGATTTACAGCATGGTGCATGACGAACTGGAAAA
AGAGTACGTTATCGCCGCCCGTCTGGATGGCGCATCAACGCTGAATATTCTCTGGTTTGCTGTGATGCCAAACATCACCG
CCGGGCTGGTCACCGAGATCACCCGCGCACTGTCGATGGCAATTCTCGATATCGCCGCGCTGGGCTTTCTCGATCTCGGC
GCACAACTCCCCTCGCCTGAATGGGGAGCGATGCTCGGTGATGCGCTGGAACTGATTTATGTCGCCCCGTGGACTGTCAT
GCTGCCAGGTGCGGCAATTATGATTAGCGTGTTGTTGGTTAACCTCCTTGGCGATGGCGTTCGTCGTGCGATTATTGCGG
GGGTGGAATAA
>Cluster_23301
ATGACTATGAAAGTTGGTTTTATTGGCCTGGGGATTATGGGTAAACCAATGAGTAAAAACCTTCTGAAAGCAGGTTACTC
GCTGGTGGTTGCTGACCGTAACCCAGAAGCTATCGCTGACGTGATTGCTGCCGGTGCAGAAACAGCCTCTACGGCTAAAG
CGATCGCTGAACAGTGCGACGTCATCATAACCATGCTGCCAAACTCCCCTCATGTGAAAGAGGTGGCGCTGGGTGAGAAT
GGCATTATTGAAGGCGCGAAGCCAGGTACGGTATTGATTGATATGAGTTCTATCGCACCGCTGGCAAGCCGTGAAATCAG
CGAAGCGCTGAAAGCGAAAGGCATTGATATGCTGGACGCCCCGGTGAGCGGCGGTGAACCGAAAGCCATCGACGGTACGC
TGTCAGTGATGGTGGGCGGCGACAAGGCTATTTTCGACAAATACTATGATTTGATGAAAGCGATGGCGGGTTCCGTGGTG
CATACCGGGGAAATCGGTGCAGGTAACGTCACCAAACTGGCAAATCAGGTCATTGTGGCGCTGAATATTGCTGCGATGTC
AGAAGCGTTAACGCTGGCAACCAAAGCGGGCGTTAACCCGGATCTGGTGTATCAGGCAATTCGCGGTGGGCTGGCGGGTA

GTACCGTGCTGGATGCCAAAGCGCCGATGGTGATGGACCGCAACTTCAAACCGGGCTTCCGTATTGATCTGCATATTAAG
GATCTGGCGAATGCGCTGGATACTTCTCACGGCGTCGGCGCGCAACTGCCGCTCACAGCTGCGGTTATGGAGATGATGCA
GGCACTGCGTGCAGATGGTTTAGGAACGGCGGATCATAGCGCCCTGGCGTGCTACTACGAAAAACTGGCGAAAGTCGAAG
TTACTCGCTAA
>Cluster_138
ATGGTCTGCGAATTGTCAAGGATTAGCAATCATTTACTTTGGTTAGCTACTCATGCCCTTGATATAGGTGCGATGACAGT
CTTTCTTTATTGTTTTAGAGAAAGAGAACAGATCCTCTGGTTTTTAGAAGAAATTTGCGGAGCAAGGCTTACCTTTAGTT
ATCCAAGAATAGGAGGAGTAAGGATTGATGTAGAAGAAAAAACCTTAGAAAAAATAGACAGGTTTATCGATGGATTTTTT
CAAAAAATAGATGAATACGAGGGTTTGCTTACAGAAAACAGGATTTGGATAGCCCGTACCAGAGGGGTTGGAGTTATAAA
TCCTGAAGATGCTATAGCTTATGGTCTTACAGGTCCCAGTCTTAGGGGTTCGGGAATAAACTATGATGTAAGAAAACATT
TCCCTTATGACGCATACAGTGAGGTAGATTTTGAGGTCCCGGTAGGAAAACACGGAGATACCTATGATAGATATTTATGT
CGTGTCCAAGAGATGAGGCAATCTGCTAAAATAGTTAAACAATGTATTCAGAAAATGCCAGAAGGAAGTATCCTGGCAGA
AAACGCTATAGAGTTAGACCTTCCCCCTTCTGGTAAAAAAAAGCTTCATCCAGAAGGTTGGTCTACCGGTGCTTTTATTT
CTTTTTTAAAAGAGAAGGAACCTCTTTTACCTGAAGGGGAAATCTATTCAGCAGTAGAAGGGGCAAAAGGAGAACTTGGG
TTTTTTATCATAAGTGATGGGTCAGGGAGACCTTACCGGGTAAGGATTAGGGTGCCCTCTTTTATACATATTTCAGCCAT
TCCTAAGCTTGCAGAGGGTCAGTTTTTGGCAGACGTGGTAGCCATCATCGGAACCCTTGATATCGTTATGGGAGAGGCTG
ATAAATGA
>Cluster_24916
ATGAGCGGCTTACCTCTTATTTCGCGCCGCCGACTGTTAACGGCGATGGCGCTTTCTCCGTTGTTATGGCAGATGAATAC
CGCCCACGCGGCGGTTATTGATCCCAATCGTATTGGCACTGGAGTGGTTGCCGGTGGAGTTACTGCTGGCGCTCGGCA
TCGTGCCTTACGGCGTGGCGGACACCATCAACTATCGCCTGTGGGTCAGCGAACCGCCATTGCCGGACTCAGTGATTGAC
GTTGGTTTGCGTACAGAACCCAACCTTGAACTGCTGACCGAAATGAAACCGTCGTTTATGGTCTGGTCGGCAGGATATGG
CCCTTCACCAGAAATGCTGGCACGTATTGCGCCGGGGCGCGGATTTAACTTCAGCGACGGCAAACAGCCACTGGCGATGG
CGCGTAAATCACTGACGGAAATGGCAGATTTACTTAACCTGCAAAGCGCAGCGGAAACGCATTTAACGCACTATGAAGAC
TTTATCCGCAGCATGAAACCCCGCTTTGTGAAGCGTGGTGCACGCCCGTTATTGCTGACGACGCTTATCGATCCGCGCCA
TATGTTGGTCTTCGGTCCAAACAGCTTGTTCCAGGAAATTCTCGATGAGTACGGCATCCCAAATGCCTGGCAAGGGGAAA
CCAACTTCTGGGGCAGTACCGCCGTCAGTATCGATCGTCTGGCAGCGTATAAAGACGTTGATGTGCTCTGTTTTGATCAC
GACAACAGCAAAGACATGGATGCGCTAATGGCAACCCCGCTGTGGCAGGCCATGCCGTTTGTTCGCGCCGGACGCTTTCA
GCGCGTTCCGGCGGTCTGGTTCTATGGTGCGACGCTTTCTGCAATGCACTTTGTGCGCGTTCTGGATAACGCCATCGGAG
GTAAAGCGTGA
>Cluster_2242
ATGATGACGATTGACCAACGTGAGAAACAAACAAGACTACAGGCGCGAATGGATGAGTTACGGGCAGAAATGGATGAGTT
ACGGGCAGAGATTGCATTTGCTCAGAAGGGCGAAAAGCCATGGCCTTATCGTTCCTGCCTGATGCGTGAAGGTCGCGGAT
ATTGCGAAAAACACGGTAAATATCGTACGCATATACTGGTGTGGATCGATCGTAATGGCGAGGACAGAGAAAAAATTTCA
TGCTGCCCTGACTGCTTGATCGCTGAGGCCAGTGATTTGACCATGGAACTGTCGTCCCTCAAGGCGGAAGAACTGACTGA
TAACGCCGGAATTGCTCTGCGTTTTCGGGACTGCGAGTTTGATAATTATCTGGAGGTTAATCCTGACGCAGCCAGAAATC
TTGCGGCCTGTCGCCGCTATGCGGAGAACTGGCCAGATATGCTGGAGAACGGTACCAGTCTTGTTATGACCGGCAGTTGC
GGTACCGGGAAAAATCATCTGGCGGTATCAATGGCAAAACACATCATCCGTAACTATCTGGCCAGTGTGGAGATCACCGA
CGTGATGCGCCTTACCCGGGCTGTGAAAAACTGCTGGCGGAATGACAGTGAAAAAACAGCGGATGACGTCATTGAGCATT
ATGCGTCACTGGATTTGCTGATTGTCGACGAAGTCGGCGTTCAGTTTGGCAGTGCGGCTGAAATGGCCATTTTGCAGGAA
ATTATCAATGCCCGGTATGAGGGTATTTTGCCAACTATCCTGATCAGCAACCTTTCACCGGAAGAATTGTGGGCGTTCAT
CAGTCCCCGGATTGCCGACAGGATCACCGATGGCGGGCGCAACTGGTTGTCGTTTAACTGGCCCAGCTACCGTTCTCGTA
TCGGAGGTGTTGCCGCATGA
>Cluster_26380
ATGGCTTTTCAGGTAAAAATTCATCAAATTCGGGCTTTTGTTGAAGTGGCTCGTCAGGGCAGCATTCGCGGAGCGAGCCG
AATGTTGAATATGTCGCAACCGGCACTGAGTAAATCTATTCAGGAGCTGGAAGAAGGGTTGGCGGCACAACTCTTTTTTC
GCCGTAGTAAAGGCGTGACGTTAACTGATGCCGGTGAAAGTTTTTATCAGCACGCCAGTCTTATTCTTGAAGAGCTGCGC
GCAGCGCAAGAGGATATTCGCCAACGACAAGGGCAACTGGCAGGGCAAATTAATATCGGCATGGGGGGCCAGTATTTCCCG
CAGTCTGATGCCAGCTGTCATATCTCGTTTTCATCAGCAGCATCCGCAGGTAAAAGTACGCATTATGGAAGGGCAACTGG
TGTCGATGATTAATGAATTGCGCCAGGGAGAACTGGATTTCACCATCAATACCTATTATCAGGGACCCTACGACCACGAA
TTTACTTTTGAGAAATTACTGGAAAAGCAATTCGCGATCTTTTGCCGCCCGGGACATCCCGCCATTGGTGCCCGTTCGAT
CAAACAGTTACTGGACTACAGCTGGACAATGCCGACGCCACACGGCAGCTACTACAAACAGTTGAGTGAATTGCTTGACG
ATCAGGCGCAAACGCCACAGGTCGGCGTAGTCTGCGAGACGTTCTCAGCCTGTATCAGTCTGGTGGCAAAAAGCGATTTT
CTCAGCATACTACCTGAAGAAATGGGCTGCGATCCCTTGCACGGACAGGGGCTGGTGATGTTGCCGGTTAGCGAAATTTT
ACCGAAAGCGACCTATTATTTGATTCAGCGGCGTGATAGTCGCCAGACACCACTGACCGCGTCATTAATCACGCAATTTC
GGCGAGAATGCGGCTATCTGTAA
>Cluster_12409
ATGATTAATTTGAAAGCAGTTATTCCGGTAGCAGGTCTGGGGATGCACATGTTGCCTGCCACGAAGGCAATCCCTAAAGA
GATGCTGCCGATCGTAGACAAGCCGATGATTCAGTACATTGTCGACGAAATTGTAGCTGCCGGTATCAAAGAAATCGTTC
TGGTGACTCATGCGTCTAAGAACGCGGTTGAGAACCACTTCGACACCTCTTACGAACTCGAATCACTCCTTGAGCAGCGT
GTTAAGCGCCAGCTTTTAGCCGAGGTACAGTCTATCTGTCCTCCTGGCGTGACTATTATGAACGTTCGTCAGGCTCAGTT
ACTGGGTCTGGGTCATTCCATTCTTTGTGCTCGCCCGATTGTTGGGGATAACCCGTTTGTCGTCGTATTGCCTGACGTCG
TTCTGGATACAGCGAGTGCGGACCCGTTGCGTTATAACCTGGCTGCCATGGTTGCCCGTTTTAATGAAACCGGGCGCAGT
CAGGTGCTGGCTAAACGCATGGATGATGATCTGTCCGAATACTCTGTTATTCAGACCAAAGAGCCTTTGGACAACGAAGG
CAAAGTCAGCCGTATCGTTGAATTCATTGAAAAACCCGATCAGCCACAAACGCTTGATTCCGATCTGATGGCCGTAGGGC
GCTACGTGCTTTCTGCTGATATCTGGGCTGAGTTGGAAAAAACTGCTCCAGGTGCCTGGGGACGTATTCAACTGACTGAT
GCTATTGCAGAGTTGGCTAAAAAACAGTCTGTTGATGCCATGCTGATGACCGGCGACAGCTACGACTGCGGTAAGAAGAT
GGGCTATATGCAGGCATTCGTTAAGTATGGGCTGCGCAACCTTAAAGAAGGGGCGAAGTTCCGTAAGAGCATCAAGAAGC
TACTGAGTGAGTAG
>Cluster_35718
ATGGTTCAGCGTATTACTATTGCGCCGCAAGGCCCGGAGTTTTCCCGTTTTGTGATGGGCTACTGGCGATTGATGGACTG
GAATATGTCCGCCCGCCAGCTGGTCAGTTTTATTGAAGAGCATCTGGATCTCGGCGTGACCACCGTGGACCATGCTGATA
TTTATGGTGGCTATCAGTGCGAAGCGGCGTTTGGCGAGGCACTGAAACTGGCACCTCACCTGCGTGAACGGATGGAAATC
GTCAGTAAATGCGGTATCGCGACGACCGCGCGTGAAGAAAACGTCATTGGTCATTACATCACTGACCGCGATCACATCAT
TAAGAGCGCCGAACAGTCGCTAATTAATCTCGCGACCGATCATCTGGATTTGCTGTTAATCCACCGACCAGACCCGTTAA
TGGATGCCGATGAAGTGGCGGACGCGTTCAAACATCTGCATCTCAGAGCGGCAAAGTGCGTCATTTTGGCGTATCGAACTTT
ACGCCTGCGCAATTTGCCCTGTTGCAATCACGTCTGCCGTTTACCCTTGCCACTAATCAGGTGGAAATATCCCCGGTGCA
TCAGCCGTTACTGCTGGATGGCACGCTCGACCAACTACAACAACTGCGTGTTCGTCCGATGGCGTGGTCCTGCCTTGGTG
GTGGTCGTCTGTTTAATGATGATTATTTCCAGCCGCTGCGTGATGAACTGGCTGTGGTGGCAGAGGAGTTAAACGCGGGC

```
TCGATTGAACAGGTGGTTTACGCCTGGGTATTACGTTTACCATCGCAGCCGCTGCCAATTATCGGTTCAGGTAAAATTGA
GCGCGTACGGGCAGCTGTCGAAGCAGAAACACTGAAAATGACCCGTCAACAATGGTTTCGTATCCGTAAAGCGGCACTGG
GGTACGACGTACCGTAA
>Cluster_55239
ATGCGTCTTCTTCATACCATGCTGCGCGTTGGCGATTTGCAACGCTCCATCGATTTTTATACCAAAGTGCTGGGCATGAA
ACTGCTGCGTACCAGCGAAAACCCGGAATACAAATACTCACTGGCGTTTGTTGGCTACGGCCCGGAAACCGAAGAAGCGG
TGATTGAACTGACCTACAACTGGGGCGTGGATAAATACGAACTCGGCACTGCTTATGGTCACATCGCGCTTAGCGTAGAT
AACGCCGCTGAAGCGTGCGAAAAAATCCGTCAAAACGGGGGTAACGTGACCCGTGAAGCGGGTCCGGTAAAAGGCGGTAC
TACGGTTATCGCGTTTGTGGAAGATCCGGACGGTTACAAAATTGAGTTAATCGAAGAGAAAGACGCCGGTCGCGGTCTGG
GCAACTAA
>Cluster_40025
GTGGCTCTGGAAAGGCAATTAAATGGAGGTGTCGATTTTTTAAGGAGTGTTAATAACTATTTTCAGAGTGTCATGGCAGA
ACACAGAGAAAATAAAACAAGTAATAAAATATTAATGGAAAAAATAAATTCTTGTGTATTTGGAACGGATTCTAATCACT
TTTCTTGCCCGGAGTCATTTTTGACATACCCGATAACGCTGGACACACCTGCGAATGGAGTGTTCATGAGAAACTCACAA
GGTGCTGAGATATGCTCTCTATATGATAAGGACACGTTAGTGCAACTTGTTGAAACTGGTGGAGCTCATCCTCTGAGTCG
AGAACCTATAACAGAATCAATGATTATGAGAAAAGATGAATGTCACTTTGATTCAAAAAAAGAATCCTTTGTTGCAAGTG
ATGCTTAA
>Cluster_30655
ATGGCACTGCTGAATATTTTTGATATCGCCGGGTCGGCGTTAACTGCCCAGTCCCAGCGCCTGAACGTGGCGGCCAGTAA
TCTGGCGAATGCTGATAGCGTGACCGGTCCCGATGGACAGCCATATCGGGCAAAACAGGTGGTATTCCAGGTTAACGCTG
CACCAGGTGCTGCGACAGGCGGCGTAAAGGTTGCCGATGTTATAGAAAGTCAGGCCCCGGACAAACTGGTTTATGAACCG
GGTAATCCGCTGGCGGATGCAAAGGGCTACGTGAAAATGCCAAACGTTGATGTCGTCGGAGAGATGGTTAACACCATGTC
GGCGTCACGCAGTTATCAGGCCAATGTTGAAGTGCTCAACACGGTGAAAAGCATGATGCTGAAAACCCTTACGCTCGGTC
AATAA
>Cluster_4769
ATGTATACAGAATCAAATGTTAAGCTTTCCTGTAACGCCCTCTGGCAAAAGTGTGTCCTTGCAACTCTGTATCGGGCTGC
AGCTACACATCCTGAAATTAAATTATTACAATTTAAATTAATACCAGGGCAGGGCATTAATAAGGCCATTCCCTCTCTGA
TGGCTGATTTTACTGGTGAACTGCATAGACTCATTTCTGAATATTTGCAGTGCAAGACAGTCCGGGCTGAGACCGCCACA
GGCACTGTGACGACGCCAGCGATTGTAATCACACTCGATATAATTAAACACCGTTTCCCGCATTATTTCCCGGCTGCTAA
AGCGTTCCCCGTGGATACATTCCACCTTCAGCGAATGAAAGAAGGTAAGCGTCAACGGAGCACCGTATTGACGCTTATTT
ATTGGTGA
>Cluster_7961
ATGAATAAATTTTATCAGGGTGTTTTTTATCCGGAAGCACTTAAAGAAGCATATATTTCTGCCGGAGCGTGGCCGGAGAA
TGCCGCCAGTGTGAGCGATGAGGTCATGGCTGTTTATAGCCAGTATCCACCAAAAGAGAAAATGCTCGGGACGGATAAAA
ATGGTTATCCTGTATGGGTTGATATCCCAGCCCTGTCTGCAAAACAACTGGTCGCTCAGGCTGGGCAAATGCAACGCGAA
TTACGCACCGAAGCAGAGTCTGAGATTTGCTGGCGACAGGATGCTGTTGATTCTGGAATTGCCAGCGCAGAAGAAATTAA
CGCCTTAGCTGAGTGGAAAAAATACCGGGTATTACTTATGCGCGTTGACATCTCCAAAGCACCGGATATCGAGTGGCCGG
TTAAGTATGAGTAA
>Cluster_6684
ATGGGGCACTGGCTTGCTGGCAGACTCATGAAAGAGCTGGGGCTGGTCAGTTGCCAGCAGCCGACTCACCGGTATAAGCG
TGGCGGTCATGAGCACGTTGCTATCCCGAATCATCTTGAGCGACAGTTCGCCGTAACGGAACCAAATCAGGTGTGGTGCG
GTGATGTGACCTATATCTGGACGGGTAAGCGCTGGGCGTACCTCGCCGTTGTTCTCGACCTGTTCGCAAGAAAACCAGTG
GGCTGGGCCATGTCGTTCTCGCCGGACAGCAGGCTTACCATGAAAGCACTGGAAATGGCATGGGAAACCCGTGGTAAGCC
CGTCGGGGTGATGTTCCAGGCGATCAAGGCAGTCATTATACGAGCAGGCAGTTCCGGCAGTTACTGTGGCGATACCGGAT
CAGGCAGAGTATGA
>Cluster_7228
ATGAAAACTTTTATCAAAACTTTACTCGTTGCTGTAACTATTCTGTTCTCTGTCTTCGCTACTGCGAAACAAGTAAAACT
GCCAAATAACATCAAATACGTTAATACTACAGAAGCGTTTTCCTGTACCGAAATTGACGGTATGAATTGCCAGACAAAGA
ATCAGTTCAACTATAAAGATAACAGCTATGTTTTCGTGCTTGAACGTGGTGGTGCCTGGTGCTACGACTACACTGTCTCG
GTAGTTAACCTGAAAACCGGGAAAGCACAAATGATTGAATACGGGGACAATCAACTGTGCTCAGGTAGCAACAAACCGTT
CTTCGAAATCAAAAATGGCGTACCGACGGTAGGAGTCATCGACACATCCGGAAAACCTGTCGTTGTAGCCCAGGACAAAC
TTAAAATCTAA
>Cluster_7952
ATGAAAACGATAGTCGTGTTGTTAGTGCTGGCCGTGGCCGGGCTGCTCTGGATGCGGCACGAAAACACCAGTCTGACCCG
TTCCTTTGAGCGGGCGAATAAAGTCGCCGGTGAACAAAAAACGGTGATTACCATGCTGAAAAGCCAGCTTAAAACGGCCT
CCCGTATCCGTGAGGAAAATGAGACCGCTCAGGTCTTACTGCGCGGTGAGCTCATCGATGCCGGAGCGCGGGCGCAACGC
CGGGAACAGACCATTACGAGGTTACTCAATGAAAACGAACAACTTCGCCGCTGGTATAGCGCTGATTTGCCTGATGCTGT
GCGCCGGTTGCACCAGCGCGCCGCCTGTGCCGACGCCGGTGATTGTTTACAACGCATGCCCGAGGGTCAGTCTTTGCCCG
ATGCCGGGAAGTGA
>Cluster_33870
ATGAAATCCGTTATTACGATTTCCGCCAGCCTGGCGATTAGCCTGATGCTGTGCTGCACGGCGCAGGCAAACGACCATAA
AATCCTCGGCGTCATTGCAATGCCGCGAAACGAAACCAACGATCTAGCGCTGAAACTTCCTGTTTGTCGCATTGTTAAAC
GCATACAGCTCTCAACCGACCATGGCGATTTGCAGTTAAGCGGTGCATCGGTTTATTTCAAAGCCACCCGTAGCGCCAGT
CAGACCCTGAATATTCCTTCAGAAATAAAAGAAGGGCAAACCACTGACTGGATCAACATTAACAGCGATAACGACAATAA
ACGCTGCGTCTCAAAAATCACCTTTTCTGGTCATACGGTGAACTCATCGGATATGGCCACGCTGAAAATTATCGGCGACG
ACTAA
>Cluster_41607
ATGATTTATTTCTCAAAATCGACTAATGGTTTTTTCTTTGATGGTATAAACAGCGACATGCCTGCTGACATTGTTGAGAT
AAGTACAGACTTATACAATGAATTAATTGCCGGACAGCAGGAAGGGGGGGAATTAATCACGTCAGATGAAAATGGTTTAC
CGGTACTGAAATCTCCGGCGATTGATTATGTCGCACGTGCTGAAAATCAGCGAATGCAGTTACTTGCTCATGCCGATAAT
GTCACAGCTGACTGGCGGGTGGAATTAATGCTTGGTGATATCAGCAGTACAGATAAAGAAAAACTATCTGCCTGGATGGA
CTACAAAAAAGAAGTAAAAGCCGTCGACACTTCGACGGCTCCTGAGATTAGCTGGCCTGAGTTACCGGAGGTGTAG
>Cluster_17543
ATGACCACTATTACCAAAGAGCGACTGCTGACAATCAAGCAGTGGCGCGAAACATACGGACCGGGTAGCAACGTTGTACT
GCCAGCAGAAGAAGCGGAAGAACTGGCACGAATTGCTCTGGCATCGCTGGAAGTCGAGCCGATAGGTTTCCGTTGCAGGC
GCAATGATAACCTTGGTGATTGGAGTTACGTATATCATCGAGAGCCAGATGATTTTGAGCGCAAACATTTAGTGATAGAG
GGCATTTACGCCGCCCCTCCAGCGCAAGTAGTACCGAAAGAAAAACCAATGCCTAATCCTCTTAGCATGTACGCGGTTGA
TGCTGTTGCCGCTATTGCAGAGGTGAGAGGCTGGAACGCCTGCCGTGCCGCTATGCTTCAGGGGAAAGGAGACTGA
>Cluster_55471
```

ATGGCGGATAAAGAACTTAAATTTTTGGTTGTGGATGACTTTTCCACCATGCGACGCATAGTGCGTAACCTGCTGAAAGA
GCTGGGATTCAATAATGTTGAGGAAGCGGAAGATGGCGTCGACGCTCTCAATAAGTTGCAGGCAGGCGGTTATGGATTTG
TTATCTCCGACTGGAACATGCCCAATATGGATGGCCTGGAATTGCTGAAAACAATTCGTGCGGATGGCGCGATGTCGGCA
TTGCCAGTGTTAATGGTGACTGCAGAAGCGAAGAAAGAGAACATCATTGCTGCGGCGCAAGCGGGGGCCAGTGGCTATGT
GGTGAAGCCATTTACCGCCGCGACGCTGGAGGAAAAACTCAACAAAATCTTTGAGAAACTGGGCATGTGA
>Cluster_62526
ATGTTTTCTTACGTGATGCTGGGTACTAACGATCTACAGCGCGCCATAAGGTTCTACGATCCGCTGATGGAATTGCTTGG
ACATCCAAAGGCAGGCCGTAATGAAGAAGGCGCATCCTGGGGACGTTCAGCAATAACCATACCATCGGATTGTGCGTCG
GCAGGCCCTTTAACCAGCAACCAGCGAGCGTGGGTAATGGCACGATGGTCGCACTGAATACCCACTCGTTTGAACACATC
CAGCAGTTGTACGAACTTGCTATCAGTCTGGGCGGCAGGGATGAAGGTACACCGGGGCACAGGCCACAATACGGACAGGG
ATTCCATAGTGCTTATGTACGCGATCCGGATGGCAATAAACTGGCGTTTGTATTCTACGATTTGGTGGAATAA
>Cluster_16617
ATGTCTGTTATTCGTAGATTAGCTGCGGTTTTACGTCAAAGTGACTCTGGTATCAGCGCCTTTCTTGTGACCGCCCCGAG
AAAGTACAAAGTTTACAAAATCCCTAAGCGTACGACGGGGTTTAGAGTCATTGCCCAGCCTGCCAAAGGGCTAAAAGATA
TCCAACGAGCATTTGTTCAGCTCTATAACTTCCCTGTTCATGATGCTTCAATGGCCTATATGAAAGGGAAGGGAATTCGT
GATAATGCTGCAGCACACGCTGGCAACCAGTATCTCCTGAAGGCGGATCTGGAGGATTTTTTAACTCAATTACACCAGC
AATTTTTTGGCGGTGCATTGAAATGTCATCTGCGCTGACACCTCAATTTGAACCCCAAGATAAGTTTTTAAACCACCGTT
AA
>Cluster_17735
ATGTCAGCCCAGCTAACCAGTGAGTCTTTAAATCAGTGGCTTAGTATGGGTTCTCTGGCTGCAGTGATCGCAGGAGTTCC
ACCAGAGGTGGCGCTTGGTGCTTTATCAGGCGCGGTAATATTTATTACCTCGGCCGTTGAGTATCCAATTCGCCGCCGGG
TTCTCCTGTCGATGCTCAGCTTCCTCTGCGGGCTTCTATTCTACAAACCAACTGCCTCAATTCTTATCGGCGTAGCAAGC
CTGATCCCAACTATCACGCAGGACTCTTTTGAGAAAGGGATCGTCTTCTCTGCTGGCGCGTTCGTGTCGGCAATCGTCGC
AGTACGTATTGGTATCTGGCTCTATCACCGCTCCGACAATCCACGCGATTTAATCCCGGGGAGAAAAGACGATGACAACT
CATGA
>Cluster_26309
ATGTCTACAACACATAACGTCCCTCAGGGCGATCTTGTTTTACGTACTTTAGCCATGCCCGCCGATACCAATGCCAATGG
TGACATCTTTGGTGGTTGGTTAATGTCACAAATGGATATTGGCGGCGCTATTCTGGCGAAAGAAATTGCCCATGGTCGTG
TAGTGACCGTGCGGGTTGAAGGAATGACTTTCTTACGGCCGGTTGCGGTCGGCGATGTGGTGTGCTGCTATGCACGCTGT
GTCCAGAAAGGGACGACATCAGTCAGCATTAATATTGAAGTGTGGGTGAAAAAAGTGGCGTCTGAACCAATCGGGCAACG
CTATAAAGCAACAGAAGCATTATTTAAGTATGTCGCGGTTGATCCCGAAGGAAAACCTCGCGCCTTACCCGTTGAGTAA
>Cluster_60043
ATGAGTAAGACACTGAACATTATCTGGCAATATTTACGCGCTTTCGTCCTGATTTATGCCTGCCTGTATGCAGGCATTTT
CATTGCTTCCCTGCTACCGGTAACCATTCCGGGCAGCATCATCGGGATGCTGATCCTGTTTGTCCTGCTGGCCTTGCAAA
TTCTTCCGGCAAAATGGGTCAATCCGGGGTGCTACGTACTGATTCGCTATATGGCGCTATTGTTTGTGCCGATTGGCGTA
GGCGTCATGCAATATTTTGATTTGCTCCGCGCACAGTTTGGCCCGGTAGTGGTTTCCTGTGCAGTCAGTACGCTGGTGGT
TTTTCTGGTGGTGAGCTGGAGTTCGCAACTGGTACACGGTGAACGTAAAGTCGTAGGTCAGAAAGGATCAGAAGAATGA
>Cluster_1788
ATGAACAGAACGATTCTTGTCCCTATCGATATTTCCGATTCAGAATTAACTCAACGCGTGATTAGCCACGTTGAGGCAGA
GGCAAAGATTGATGATGCAGAGGTTCATTTCCTGACGGTAATACCTTCACTGCCCTACTATGCCTCTCTGGGTTTAGCGT
ATTCCGCAGAATTACCGGCAATGGATGACCTGAAAGCGGAAGCCAAATCGCAACTGGAAGAGATCATTAAAAAATTTAAA
CTGCCAACCGACAGAGTGCATGTCCATGTTGAGGAAGGCTCGCCCAAAGACCGCATTCTGGAAATGGCGAAGAAGATCCC
CGCTCATATGATCATCATTGCTTCCCATCGACCGGATATCACCACTTATCTGCTCGGTTCCAACGCCGCAGCTGTAGTGC
GTCACGCAGAGTGCTCCGTGCTGGTTGTGCGCTGA
>Cluster_28562
ATGGACAACAAAATATCAACCTATTCACCGGCATTTAGTATTGTGTCATGGATAGCTCTCGTTGGTGGTATCGTTACCTA
TCTGTTAGGGCTATGGAATGCAGAGATGCAGTTAAATGAAAAAGGATATTATTTTGCCGTACTGGTATTAGGACTGTTTT
CTGCAGCGTCTTATCAAAAGACCGTTCGGGACAAGTATGAAGGCATACCGACCACCTCCATTTATTATATGACCTGCCTG
ACTGTCTTTATTATCTCTGTTGCGTTACTGATGGTAGGTCTGTGGAATGCGACGTTATTACTCAGCGAGAAAGGTTTTTA
TGGACTGGCTTTCTTCTTAAGCTTGTTTGGTGCAGTAGCGGTGCAGAAGAATATTCGTGATGCCGGAATAAACCCACCAA
AAGAAACACAGGTTACCCAGGAAGAATACAGCGAATAA
>Cluster_39515
ATGTTTCTGAAAACCGAACAATTTGAATATAACGGTGTGTCCGTCACGCTTTCCGAGCTGTCTGCGCTGCAGCGTATCGA
GCATCTTGCCCTGCTGAAACGACGGGCAGAACAGGCTGAAGCCAGCGGTAACCTGCAGGTGAGCGTGGAAGACCTTGTCA
GAACCGGCGCGTTTCTGGTGGCGATGTCCCTGTGGCATAACCATCCACAGAAAACGGAGTCACCATCAATGAATGAGGCT
GTGATGCAGATCGAACAGGAGGTGCTCACCACCTGGCCTGCTGATGCCATTGCCCGGGCGGAAGACGTGGTGTTGCGTCT
GTCCGGGATGAGCGGGGCTGTTCATGTGGATACGGATATCACCGAAGTGGCGAAAAATAACGCGCTGACTGATGATGATT
TTTCTGCGGGAAAGTCTTCGACGGCGAGCTGA
>Cluster_52781
ATGCAAGAAATTATGCAATTTGTTGGCCGTCATCCCATACTGAGTATCGCCTGGATCGCGTTACTGGTGGCGGTTCTTGT
GACTACGTTTAAGAGCCTGACCTCGAAAGTGAAGGTGATTACTCGTGGTGAAGCTACGCGTCTGATCAACAAAGAAGACG
CTGTGGTTGTGGATTTACGTCAGCGTGATGACTTCCGTAAAGGCCATATCGCAGGTTCTATCAACCTGTTGCCGAGCGAA
ATCAAAGCCAACAATGTTGGTGAGCTTGAGAAGCACAAAGACAAACCGGTTATCGTGGTAGATGGTTCTGGCATGCAGTG
CCAGGAGCCTGCAAACGCGCTGACGAAAGCTGGTTTTGCGCAAGTATTCGTACTGAAAGAAGGCGTCGCTGGCTGGGCTG
GCGAAAACTTGCCTTTGGTGCGCGGCAAATAA
>Cluster_9757
ATGAATAAAATGACTGTACTATTACTTAGCGCAACTATCATTTCAGGTTGTACTTCTTCCGTACCATTGATAAAGAAAAC
TCAATCAGGAAAACCTGAGGGGGTTTATCAAAATACGACAAAAGATAAAGTCAAAGATGCCCTTGTGAATTACTGCAATA
GTAGAGGGTTGATAATTTACAACGCGGATAACAGCAGTGTTATATGTGGTAAAGAACTGGAAGGCGGCTCTGCTGTTTTT
GGACAAATGTTAATCGGCAATGCCTATTCAACAACCCCGGTATCAAAAGTCAGATTTACTATCGCTCAAGTTAATAACGA
TACAAAAGTGTGGGCCGATATGTGGATGGAAACTCAAATGGCAATGGGGCAAGTACAACAAATGTCTATAACAGACAACG
CAAGCAAAAACACTATCCAACAACGTCTTGATGAATTAAAACCTTAA
>Cluster_24882
ATGAGAGAATTAGCCACTTTTCTCTGGAAATGCGTCCTCTGGATTCTGTTGACCTATGCAGCGACAAAACTGTTTAAACC
GATGAAGCATGCTGACGTACTCACAACAGCGGGGGTGCTCTCGACTATATCAGGCATCCTGTTTGGTTTTGTTCTGGCTG
CAATATCTATATTTAGTAGTGCAAACAGCGATAAGGAAGGAGCAATTAATGCCCTTAAGCAAAACAACGTGTTACCAACT
CTGGTAAATAGATTACTTTCAACAGGGTTAACTCTCATCGTCGCATGTATATTTCCATTGATAGCGATGTTTCTACCTGA
TGATGTTATTGTTGCGGGCAAACCTATTGATTTCCTGTTCATACTGTTAGGCTTATCCTCCCTTATAATTTCGCTATATA
CCTTCGGAAGGTGTTGGTTAGTGTTACGAAAAATCTTCCCCCACTTGTGA

>Cluster_47591
ATGATTCGGGAAGCGCAACGTTCAGAACTCCCCGCGATCCTCGAACTGTGGCTGGAAAGTACAACCTGGGGGCATCCCTT
TATAAAAGCGAATTACTGGCGTGACTGCATTCCGCTGGTGCGGGATGCCTATCTTGCCAACGCGCAAAACTGGGTCTGGG
AAGAGGACGGTAAGCTTCTCGGTTTTGTCAGCATTATGGAAGGCCGATTTCTGGCAGCAATGTTTGTCGCACCGAAGGCC
GTCAGACGCGGTATTGGTAAGGCGCTGATGCAGTATGTGCAGCAGCGCTATCCCCATCTGATGCTGGAGGTTTATCAAAA
AAATCAACCGGCGATAGATTTTTACCGTGCGCAGGGTTTTCACATTGTCGATTGCGCATGGCAGGATGAAACCCAACTAC
CCACCTGGATTATGAGCTGGCCGGTGGTTCAAACGCTGTAA
>Cluster_3095
ATGGGAGAGAGAATCATCACTTTCTCTGTGAATCAGATGAATAAAATTATTCTGAATACGGTCACCCGACAGAGCACTAA
AGATATTAGTTCATGGAAAAGTGATAAACGAAAAGTTTATCCCTCACGATTAATCAATATGGGAATTGATAAGCATTGCT
CCGAAAATAACACGAATATTACGGGTGTTGTTCGTCAACGCGTTTTTACTCTGATAGCGGAGGACTTTGGAATCAAATTA
GATTCTAATGCAGCACAAAGCTCTATAACTCATATCGTCAATGGAAACGGCTGGTTTAGTAAAAAATTAGCTTCCCTTTG
TGAGGGGATGAGTAGAGATGATAAGAATAAGACCAGAGAGATACTGGAAAATAAGCTGGCAGACATATTTTTTGAGAAAC
ATATTGATAGCAAAATAGACATAAAAAATTACAGGGTAGCGTAG
>Cluster_8956
ATGCGTAACTTCGATTTATCCCCACTGATGCGTCAATGGATCGGTTTTGACAAACTGGCCAACGCACTGCAAAACGCCGG
TGAAAGCCAGAGCTTCCCGCCGTACAACATTGAGAAAAGCGACGATAACCACTACCGCATTACCCTTGCGCTGGCAGGTT
TCCGTCAGGAAGATTTAGAGATTCAACTGGAAGGTACGCGCCTGAGCGTAAAAGGCACGCCGGAGCAGCCAAAAGAAGAG
AAAAAATGGCTGCATCAAGGGCTTATGAATCAGCCATTTAGCCTGAGCTTTACGCTGGCTGAAAATATGGAAGTCTCTGG
CGCAACCTTCGTAAACGGTTTACTGCATATTGATTTAATTCGTAATGAGCCTGAACCCATCGCAGCGCAGCGTATCGCTA
TCAGCGAACGTCCCGCGTTAAATAGCTAA
>Cluster_215
ATGGCAATAGATAGGAAAAGGCTTTTAAGAAGGCTTTGGGCAATAGCAGATAGGCTTTTTGGTAAAGAAAAAGAAGAAAA
AGTATATAGTTATGTTTCTTTTGTTTATGAGAAAGAAAGATTGCGAGAATTAACAGATGAAGAGCTTGAAGAAATGGTTA
AAGCTTTTGTAGAAATGCTTTCAGAAAATGAATGCCCTTCTGCACCTAATGAATGGAGACTTATTAAAGTTTTGCAAAAA
AAACTTGATTGGACAAATGAGCATTTATTGAATTACATTAAAAAATATGCTCGTGTTGATCATCCGAGATTTTTGACTCA
ATATGAAGCAAAGATTGTAATTGCTGCTATGATGACTATATTAAATGGAAGAAAGAAGAGAAGAAAAAATAGAAGTGTGA
GAAATGAAAAAGTTGTATAA
>Cluster_24888
ATGGGACTGATGACACGCATTGCCGATAAAACCGGCGCGCTCGGCAGCGTCGTTTCCGCGATGGGCTGCGCCGCCTGCTT
TCCAGCCCTCGCCAGCTTCGGCGCGGCCATCGGGCTGGGCTTCTTGAGCCAGTACGAGGGACTGTTCATCAGCCGCCTGC
TGCCGCTGTTTGCCGCGCTGGCCTTCCTGGCGAACGCGCTGGGTTGGTTCAGTCATCGGCAATGGCTGCGCAGTCTGCTC
GGCATGATCGGCCCGGCCATCGTGTTTGCGGCCACGGTCTGGCTGCTCGGCAACTGGTGGACGGCGAACCTGATGTACGT
CGGCCTGGCCTTGATGATTGGGGTGTCGATCTGGGACTTCGTGTCGCCGGCGCATCGCCGTTGCGGACCGGACGGCTGCG
AACTCCCCGCCAAGCGCTTGTGA
>Cluster_35627
ATGAAAATCAGGCAAAGTCAGACCAGTGCCACTTACCTTTTACCGGATCCCGGAGAGCTGAATCGCCGGATAAAGATCCG
CCTTCGGGTGGATGAACCCACCGCTGATTTTGGTACGGAGCCAACGTATCCGGAGTCGTTTGATGTCTGGGCAAAGGTGG
CTCAGCCAGGCGCTGCCGCTTATCAGGGCTCAGTGCAGACAGAAAATGTCGTTACGCATTATTTTACAATCCGTTTCCGT
CACGACATCACGGCAGATCATGAGGTGGTTTATTACGGTCAGCAGTACCGGATCCGGCGAATACGCGACCTGAACGGTCA
GCGACGTTTTTTATTACTGGAATGTGAGGAACTGCGTACAGCGCGACGATGGGGTGAATGCCATGAATCAGACAGCATTT
TTACACGTCGACTTTAA
>Cluster_12346
ATGGTACGATTCATTCCATTCTTCGTTCTTTCTGAGCATTGCGTTCAGGATAGTCAGCAGCTTGCGTATACAGGTAACCA
GCGCCACTTTTTTGGGTTTTCCTGCGGCAAGCAGTCGCATGTAAAACACCTTTATTACCGGGTTAAAGCGGGTTGCGACA
AGCGCTGCCATGTACAGGGCTGTTCGCACACCAGCCCTGCCGCCAAAGATGGTTCGTCGCCCTCTCATCGTGCCCGAGTC
CCGGTTTACTGGAACCATACCGACAAGCGCACTGATTTCACGCCTGGAGAGGTTACCCTGTTCGGGTACTTCCGCCAGCA
GCGCAGCAGCCGTCATGGTGCCAACACCTTTAATACTGCTCAGTCGCTTAGCAAGCTCTTTGAAGTGGCTATGGATATGG
CTGTTCATGTCAGCATCTAG
>Cluster_64939
ATGGAGATACGCGTATTTCGCCAGGAAGATTTCGAAGAGGTCATCACCCTTTGGGAGCGTTGCGACTTGCTGCGTCCGTG
GAACGATCCGGAAATGGACATCGAGCGTAAGATGAACCATGACGTCAGTTTGTTTTTGGTCGCTGAGGTAAACGGTGAAG
TGGTCGGAACGGTGATGGGCGGTTATGACGGGCATCGCGGGTCTGCTTATTATCTTGGCGTGCATCCAGAGTTTCGTGGG
CGTGGGATTGCCAATGCGTTGCTTAATCGGCTGGAGAAAAAGCTGATTGCTCGTGGCTGCCCGAAAATTCAGATCAATGT
GCCGGAAGATAACGACATGGTGCTCGGAATGTATGAACGCCTGGGATATGAACACGCCGACGTGCTGAGTCTGGGTAAGC
GTTTGATTGAAGATGAAGAGTACTGA
>Cluster_27698
ATGGGTTCATGGGGAATGGAGGCTCTCGAAAGCGACGAAGGGCTGGATTTGATTAATTGGGTAGAGGAACAACTGCAAGA
TGATTCGACCTTTGATGCCGAGTCAATTGTTCAACGCTTAAGCCAGCATGAAGATTTGTTCGGATTTCAGGGAGATGAAG
AATTTCTCTACGATAATAATGTTATCGGTCTCGTCGAACTTATCATTCAAAAAGCAGCCGGGGAAAAAATCACATCGAGT
AAACAAATTGATCAACTCGACAGTTATCGGCTCACGTCGATTTTCAGCAAAAAACTCCAGGGCCGTTTACAAACAATCGA
CGACACACATGAGTGGATAATGCTTTTTGAGGGGCGAGCACGCGAAAAGGCAAAAGCATATCTGATTGAGATTACTGATA
AATTAAGAGTAGTAAAGACAACAGCCTGA
>Cluster_156
ATGGCTGATAATCGTAAGTGCTCTTTTTATATTTATCCGGAACGAAATGCAGCTGATCGGGTAGCTGACAGGTTTCTTGA
AAAATTGCCTCAGAAAGAACGTGGTCGGGCAATGCGGGCCATGATGTTGTGTGGTGCTGCATTGATGAAACAGGATGAAC
GATTGCCGTTCCTGATAGCTGAATTCCTGACAGACAGCACGTCTATGCAGGATATTCAACGAATTATCAGTAGTACGCTA
CCACAACAGGAAAATGGTGAAGTTGTTCGTTTGCTGGAGGCATTTCTGCAGTCTGCCGGTAATAATGCAAAAACGATCCT
GCCTGCGGTTGATTCTGCTACGCAAGAAATATCTGCTCCAGTCGATCAGAATCTCCTGGAAACAAGAAACAACATTAAGA
ACTTGTTTCCGGATGATGAATAA
>Cluster_2761
ATGCGTAATATTCAACAGGTTTTAGAGCGCTGGGGTGGCTGGGCAGCGAGTGAAGGTGGTAGCGTCTACTTTCCTCCTGT
TGCAGCCGGGTTTAAGAATCTGCTACCCGCGACGCAGTCTGGAAGGCTGAAATGCAGTGACAATGACGGTCTTATCATCA
ACTCCGCTATGAGCTGCCTGAAGAAAAAAGATCCGTATCTGTGCACGCTCCTTGAGTGGCATTACGTCCAGGCCATGCCC
GTGCGGGCGATGGGTGAGAAGCTCGGCGTATCTCACACCCACGTTCTGAAGAGGCTTCAGGCGGCAGAGGGATTTATTGA
CGGTTGCTTAGCCATGCTGGATGTGGTGCTCGAAATGGATCAGTCGGTTCAGTCAAAGCCTCAGGCTATCAGGACTTTGC
GTAGGAGCTGCTCGGCGGCATAA
>Cluster_51758
ATGAAAAAATTTATCGCCCCCTTGTTGGCTTTACTGGTTAGCGGATGTCAGATTGATCCTTATACTCACGCGCCAACCTT

GACCAGCACCGACTGGTATGATGTTGGTATGGAAGATGCGATATCGGGCAGCGCCATTAAAGATGACGATGCATTTAGCG
ATTCACAGGCGGATCGCGGTCTATACCTTAAAGGATATGCCGAAGGGCAAAAGAAAACTTGCCAGACCGATTTTACTTAT
GCCCGGGGACTTTCTGGTAAAAGCTTTCCTGCCAGCTGCAATAATGTTGAAAATGCCAGCCAACTGCATGAAGTATGGCG
AAAAGGGGCTGATGAAAATGCCAGCACAATACGATTAAATTAA
>Cluster_46759
ATGAGTAAGATTTTAGCGAATCATCCACGATTAAGTAAATTTGTATTTGGCTTATTCGGTGCTGCTGGAATGATTTGGCT
TAATAAAGCCTTCCAGGGCGATGAATTATTTCAACTGATAACATCACTGCTTGCTTTAGATACGTTTGTGTACTTTTGCA
TCTACAGTCTGATTAACCCAATACCTGAGGCAAATGATTTTCTTTGGGATTATAAAGTCCATCAACGAAAATATTCATCC
AATATTTCTCATAATGATTTTGTCAGCAATGTAAGGAATAGCAGAAAAATTGTTCGGATACTTATCCTGGTGGGAATAAC
ATTTATTAATGTTATCGCTCTGCTTACTAAACTCAAAATGTGA
>Cluster_46614
ATGTCCTTAAAGTATGCACATGAAAAATTTCATACTGCAGTACTTACTCTTGCGGGCCATGGAAGTATTCAAGAACGCTT
GATTAATTCGTATGTTTTTAGCCTTGGGCATCTTAAAACAGCCGATGATATTCCAAATGCCTTACAAAGCAGGTTCGATG
AATTATGTAAGGAACTGACTAAGTTTGATGCCACTGGCGATGAGGGGCGAGTTCAAGCAACAGTATCAAAACTTAACGAT
TTCGAGATCAATAAACTGATCGAAGATATTGTCAGTCTTAACGATGATATTTGCATGAAATTGGCTCTGACGGATGAGAC
TTACCAAGACATACACCAAAGTTAA
>Cluster_25546
ATGGTGACGAAAACTATCACTGAACAGCGTGCGGAAGTACGTATTTTTGCCGGTAATGATCCGGCTCATACCGCCACAGG
CAGCAGCGGGATTTCCTCGGCAACACCGGCACTGACGCCCCTGATGCTGGATGAGGCCACCGGGAAACTGGTGGTCTGGG
ACGGACAAAAAGCCGGTAGTGCGGTTGGCATACTGGTACTGCCGCTTGAAGGCACAGAGACGGCGCTGACCTATTACAAG
TCGGGGACCTTTGCGACGGAGGCAATCCGCTGGCCTGAAAGTGTGGATGAACACAAAAAGGCCAACGCCTTTGCCGGCAG
TGCCCTGAGTCACGCGGCGCTGCCGTAA
>Cluster_61425
ATGAACATGGTTGAGAATATGCTCGATCAGGCATTCAAAAAGCTTAATCCTCACGAGCATCCTGTTCTGCACTCTGACCA
GGGATGGCAGTATCGTATGAGAAGATATCAAAATATCCTTAAAGAACATGGTATTAAACAAAGCATGTCCAGAAAAGGCA
ATTGTCTGGATAATGCTGTGGTGGAGTGTTTCTTTGGAACCTTAAAGTCGGAGTGTTTTTATCTTGATGAGTTCAGTAAT
ATAAGCGAACTGAAGGATGCTGTTACGGAATATATTGAATACTACAACAGCAGAAGAATTAGCCTGAAATTAAAAGGTCT
GACTCCAATTGAATATCGGAATCAGACCTATATGCCTCGTGTTTAA
>Cluster_58424
ATGAAATATAGTTCAATATTTTCGATGCTTTCATTTTTTATACTATTTGCCTGTAATGAGACAGCTGTTTACGGTTCTGA
TGAAAACATTATTTTTATGAGGTATGTGGAAAAATTACATTTAGATAAATACTCTGTTAAAAATACGGTAAAAACTGAAA
CAATGGCGATACAATTAGCTGAAATATATGTTAGGTATCGCTATGGCGAACGGATTGCAGAAGAAGAAAAACCATATTTA
ATTACGGAACTACCAGATAGTTGGGTTGTTGAGGGAGCAAAGTTACCTTATGAAGTTGCGGGTGGTGTATTTATTATAGA
AATTAATAAGAAAAATGGATGTGTTTTGAATTTCCTACATAGTAAATAA
>Cluster_3374
ATGTTCGGTCTGAGGAAAGTAAATCGAAACACTCATACGTTTGAAACCACTTTTCAAAATTGGAAAGAAAATGGCGAAGA
TGTTGCATTATTAATGCCCGAATTTAGTGCTAAATGGTTACCGATAGCAGAAGAGAGTGGCAGCTGGTCTGGATGGGTAT
TGCTTGGAGAAATATTTCCCTTAATTTCATCTGAGCTCGCTGGGATGGCTTTAATGCCAGAAACGGAAAGGTTAATAGGG
GAATGGCTCAGTTTATCCAGTTCCCCGTTAAATCTAAAGCATCCTGAACTAAAATATAATCGTTTGCACGTAGGTAAAGT
ATTTGATGGAGTATTGAACCCTGCTCAACCACTAATAAGAATATGA
>Cluster_13469
ATGGACTGGTATCTGAAAGTACTAAAAAATTATGTCGGTTTCCGGGGTCGTGCGCGGCGCAAAGAGTACTGGATGTTTAT
TCTGGTCAACATCATCTTTACGTTCGTGCTGGGGCTGCTGGATAAAATGTTAGGCTGGCAACGCGCTGGTGGCGAAGGCA
TCCTGACGACAATCTACGGTATTCTGGTGTTTTTACCGTGGTGGGCAGTTCAGTTCCGCCGCCTGCACGACACCGACCGC
TCGGCGTGGTGGGCACTGTTGTTCTTAATCCCGTTTATCGGCTGGCTTATTATCATCGTCTTTAACTGCCAGGCGGGTAC
GCCAGGCGAAAACCGCTTTGGACCAGATCCGAAACTGGAACCTTAA
>Cluster_48640
ATGAATATTTCATGGCAAACAGAAATTGCGATCTACCGCTTTGGCGCAAAGAACGTTTACGGTGAGGCGCAATTACAATT
CGTCAGGAAGACGAATGTCGGCGTGGTTAAGTTCGAACAGAGTAATGAGAAATCGTCAGTTCGTGCTGATAGCTCTGGTA
GTCGTGGTAAGGCGAGTCTGGAGCTGTTTGATGCTGTGTTAGTTGTCCCTTTGGAAGCGGCTGTACAACTTGATGATGTA
CTGATCCTTGAAGGTCAAAAATTAAAGGTGTCCAGTGTTCATCGCCGCTGGGGGCTACGAGGAAGACCTGGGCATCTTGA
AGTAGGGGCGAATATATGGGTCTGA
>Cluster_18586
ATGGAAACTATCGCTAAACATCGCCATGCTCGTTCTTCTGCTCAGAAGGTTCGCCTTGTTGCTGACCTGATTCGCGGTAA
GAAAGTGTCGCAGGCTCTGGATATTTTGACCTACACCAACAAGAAAGCGGCTGTACTGGTCAAGAAAGTTCTGGAATCTG
CCATTGCTAACGCTGAACACAACGATGGCGCTGACATTGACGATCTGAAAGTTACGAAAATTTTCGTAGACGAAGGCCCG
AGCATGAAGCGCATTATGCCGCGTGCAAAAGGTCGTGCAGATCGCATCCTGAAGCGCACCAGCCACATCACTGTGGTTGT
GTCCGATCGCTGA
>Cluster_47090
ATGTTAAAGCATAAAATTCAAACAATATTTGAAGCTCTGTTATACATAATGCTTACTTATTGGTTAATAGATGCTTTTTT
TGCTTTCAATAAGTATGACTGGATGCTTGAGTCGGGAGACAATATTTGCAGTATACCTTCCGTTTCAGGCGAAGATCGTA
TATTGCAAGCAATGATTGCAGCATTTTTTCTTCTTACGCCACTTATTATTCTTATCTTAAGAAAACTCTTTATGCGGGAA
ATGTTCGAGTTTTGGGTATATGTATTTTCGCTCGGTATTTGTCTGGTTTGTGGTTGGTGGCTTTTTTGGGGACGGTTTAT
ATTTTGCTATTAG
>Cluster_64435
ATGACCACAGAAACGTATAGCCCGGATGATTTTATTCTGGGACCCGATCTTGTTGTGACGACCGTCGGTCATTTTCAGGG
CGGGATCAACGTACCCCGGCTGACACCGATTATGCTCGACGCTACTGCCGGGACGTTTAAAGTCTGGGACGGCTCAATTG
GTAAGGCGGTTGGTCTCACAGCCACAGCAGTTAATACAGGCTCCAGTTCTGCAGACTGCTCGTACTACAAATCAGGATCG
TTTCGTTATACGGCGATTAACTGGGGTACGGTTACTGACGTCGCTAAACGCAAATCAGCGTTTGCTGGTACGCCAGTCAG
CGTTGGCTGA
>Cluster_43572
ATGAAGATCCTACCAGCTGAACCGGGATTATATTTTTTCGCATCTTTTGAGTCCGGTGCTCACGTTTACAAAGTAGTGGC
CTGGTCAGAAAATGAGAATGGTGGTTTAGATCCACAGTGTGTGTTAACGGCCTTATATGACAAGAGCAGTTCCAGCCAGC
AATGGGAACTTCACTCAACTTCAGCATCTGCATATCAGCACAGCAATGACGGCAGACCATTTATGTATTTTATCACCTAC
TTCACCAGCAATGGCGGGATACCTGAACTTTATAACGCAATAAACACACTAGCCGAACGATGGGGATACCCATTAATTGA
TTTACCATAA
>Cluster_38475
GTGAGTCACGCGATTGAGTTTATCGAGACCCCGATGTTTACCCGTCAGATAAAACAGATCGCCACGGATGAAGAACTCAG
GGAGCTGCAAAAGACGCTGATTGAGTCGCCGGACAAAGGCGATCTCATTCAGAAAACCGGCGGGCTGCGCAAAATCAGGA

TGGCGACGGGCAACCAGGGAAAAAGCGGCAGCGTGAGGGTGATTTACTTTCTGGCCACCGCCGAGGTGATCTATCTGGTG
ATGGCCTACCCGAAAAGCACCAAAGACAGCCTGACGGACGCGGAAAAAGCCGCGCTGAAGACGCTGACCCAACAACTGAA
AGACGAGGTGTGA
>Cluster_20777
ATGACCCTGAGTGAAAAGCAGCAGCTGTTTACCGTCATGGTGGCCAACCTGATCCACTGGGCAGAAGAACGCGGCTACCG
GCTGACGTTTGGTGAAGCGTACCGCACCCCGGAACAGGCGGCGCTGAACGCGAAGAAGGGCAGCGGTATTTCGAACAGTC
TGCACACCCAGCGTCTGGCCGTGGATTTTAACCTGTTTGTGAATGGCCAGTACCAGACCCGTACAGAGGACTATCTGCCG
CTGGGTGAATACTGGGAATCACTGGGCGGAAGCTGGGGTGGGCGCTTCAAATCCAGACCGGATGGTAATCATTTCAGCCT
GGAACATAACGGGGTGCGCTGA
>Cluster_44152
ATGAAAAAAGCACTGCAAGTCGCAATGTTCAGTCTGTTTACCGTTATTGGCTTTAATGCCCAGGCTAACGAACATCATCA
TGAAACCATGAGCGAAGCACAACCACAGGTTATTAGTGCCACTGGCGAGGTAAAGGGTATTGATCTGGAAAGCAAAAAAA
TCACCATCCATCACGATCCGATTGCTGCTGTGAACTGGCCGGAGATGACCATGCGCTTTACCATCACCCCGCAGACGAAA
ATGAGTGAAATTAAAACCGGCGACAAAGTGGCGTTTAATTTTGTCCAGCAGGGCAACCTTTCTTTATTACAGGATATTAA
AGTCAGCCAGTAA
>Cluster_53884
ATGGTGAAAAAGACTATTTATGTTAATCCTGACCGCGGACAAAACAGAAAAGTATCTGATAGAGGTCTTACATCTCGAGA
CAGGAGGAGAATAGCGAGATGGGAAAAAAGGATAGCATATGCATTAAAAAACGGTGTAACACCTGGATTTAATGCTATAG
ATGACGGTCCTGAATATAAGATTAATGAAGAACCAATGGACAAAGTTGACAAAGCATTAGCAACACCATTTCCTCGCGAT
GTCGAAAAAATTGAAGATGAAAAATATGAGGATGTCATGCACAGAGTTGTTAATCACGCTCACCAACGAAATCCAAATAA
AAAATGGTCATAG
>Cluster_29089
ATGTCAGTTCTGTATATCCAAATTCGTCGTAATCAAATTACTGTTCGCGATCTTGAAAGCAAACGTGAAGTGTCAGGAGA
TGCTGCCTTCAGTAACCAGCGCCTGTTAATCGCCAATTTCTTTGTTGCAGAAAAAGTTCTGCAAGATCTTGTTCTGCAAC
TCCACCCACGTTCAACCTGGCATTCTTTTTTGCCAGCAAAACGTATGGATATTGTTGTGAGCGCGCTGGAAATGAATGAG
GGCGGTTTGTCACAGGTTGAGGAACGCATTCTTCATGAAGTGGTCGCAGGGGCAACGTTAATGAAATATCGCCAGTTCCA
CATCCATGCGCAATCAGTGGTACTCAGTGATAGTGCGGTCATGGCAATGCTTAAGCAGAAATAA
>Cluster_43194
ATGAATAAAGGGCTTCTCACGCTACTGCTGTTATTTACCTGTTTTGCGCGCGCTCAGGTTGTAGACACCTGGCAATTCGC
CAATCCGCAACAACAGGAACAGGCGTTAAATATTGCCAGCCAGTTACGTTGTCCGCAGTGCCAGAATCAGAACTTACTGG
AATCCAATGCGCCGGTGGCAGTCAGTATGCGCCATCAGGTTTACACCATGGTGGAAGAGGGGAAAAGTGAAGTCGAAATC
ATTGGCTGGATGACCGAACGCTACGGAGATTTTGTTCGCTATAACCCACCGTTAACGGGTCAGACGCTGGTGTTGTGGGC
GCTGCCAGTAGTGTTGTTACTGCTGATGGCACTGATCCTCTGGCGAGTGAGGGCGAAGCGATGA
>Cluster_30107
ATGGATAAGCAATCACTGCACGAAACGGCGAAACGCCTGGCTCTTGAGTTACCCTTTGTCGAGCTTTGCTGGCCTTTTGG
CCCGGAGTTCGATGTTTTTAAAATTGGCGGCAAGATTTTTATGCTGTCGTCGGAGCTACGCGGCGTCCCCTTTATCAATC
TGAAGTCCGATCCACAAAAATCCCTGCTAAATCAGCAAATATACCCAAGCATCAAGCCTGGGTATCACATGAATAAAAAG
CACTGGATTTCGGTGTATCCCGGCGAGGAAATCTCCGAAGCGTTACTTCGCGATCTGATCAACGATTCGTGGAATCTGGT
GGTTGATGGTCTGGCTAAACGCGATCAAAAAAGAGTGCGTCCTGACTCAACCGTAAATCAATAG
>Cluster_48638
GTGTTTATTGAAGCGTTTGCAAGTTTGATGCAGAAGGCAAAGATTGGCACGGTCGGGACTGACATTTTCTGTCACTACTT
GCCTGCCAATGTGAAATCTGGCGTCCTGCTTATTAACCCAAATACCGGTATCAGCATCGATCATGAGCTACAGGGCTTTT
ATCTCGAATCATTCACAATCATTGTGCGTGGTTCGTCAATTACTACGACGGTTGAGAAAGCCAATAAAATCATTGAGATG
TTTCCAGTAGAGGAAACGGAATCTGGTGGTGTTTATTTTCGACTTGTACGACCGATGGCGATGCCAATCATTTATCCCAA
AAATGATGGGGCATTAATAGAAGCAGGCATTCCTGTTGAATTTGCTGGCTATTTATTGAATTAA
>Cluster_49927
ATGTTTCTGAAACAGGGCACGTTTAATTATGAAAAGCAGTCCGTGGTGCTCAGTGAGCTGTCCGGGCTGCAGAGAATTGA
ATATCTGGCGTTTGTTCAGCAGCGAACGGCAAAGTTTGATGCCGAAGAGGGAGAACTGCCGGAGGCTGAACGACAGATTG
CTTTTCTGCGGATGGGGATGGATATCAATGCCTGGCTGGTTTCCCGCTCACTGTGGAATGCGGAACAGTCTCAGGATGTT
GAGACGCTTTGCGCATCCGTTATTACAACATGGTCGTATGATGCCCTGGGAGCGGGGGCGGAGATGGTTCTGTCGCTGAG
CGGTATGGGAGCCATTGAGAATGCCGGGGATTTGGAGCATGAGGTGCTGACGCCGGAAAAGTCCTGA
>Cluster_29870
GTGAAGCAATTTCTTACTGCTATGTTCTTATTCATATCTTTTGGGGCTACAGCAGAGTGCTGGGTCGTTGGAGATATGCG
CGGAATAAGCTATTCAGAACGAAATAATTTCCATCCGGAAGAAGATGGTTTTAGTGGAACATTCATCATTAAGACAAACG
GTGAAGATGCCAGCATCACATATTCTGGGACAGATGCGGGCGGCATGGCTTACAAAGCATTGTCTAAAAACTCCATCATA
GGAATCGGCGCGAATGGCGAAACTCAACGCGTTATCGACTCATGGGTAATACATCCTACTGGAACAGTTTTAATGTCAAA
AACCATTTCCGGTTATGGAAATATGGATTCAACCAAAGCTTTTGTTGGAAAAGTAAAAAGAAAATGTTAG
>Cluster_7598
ATGACACGCAGAACTCAGTTCAAAGGCAATTCACGTTCTCGTCGTCGTGAGCGTTTAAAGGCAAAGGCATTAGCTAACGG
CGTGCTGGCCCGCGAAGAAGCAATAAGTTCAGAAGTATTACACCGCCCTACTCTAAGCAGAGCGCAGATTCAGGCTAAAG
GTACTCACGAAACGCCTGAGCGCATAGAAGACGCTAAGCCAATTAAGTTCATGGCACAGGACGTGATCTGGCAACAGAAA
GAATACAGACGCAATCTGGAGCGAGCGGCCATTGTGTACGCGAATGAGTTTGGACATAAGCAACCAGAAACTGGTGTATG
TCTTCCAAACGTAGCCATTTACGCGGCAGGCTACCGGAAATCCAAACAACTGACGGCGAGGTGA
>Cluster_50984
ATGTATGATTTTTATAAGCATCGTGACCATTTGGTAAGATTTGACGGTAAGGTCTGGAAAGACGTAACACAAACCAATAA
GCCCAAGTTTACCCACCAGCAATTAGCCTCAAGATTTGATATCTGGGCTGAAGCACAAAAAGATAATGGTGATTTCGAGT
TTATCAGAGGTTTTGATTATGCTTACGAAAGCGACCTTAAAGACGCCATTGAATTAATGGAGAACTTCAACTCTATTAAA
AATAGTGACAAGCACAAAGCCAACGGTAACAGATACGATAAAGATTTAATCATTGATGCAATCACCAAAGGACAATCACA
AAGTTATATTTCTGAGAAATACGGCTTATCACGTAGTCAATACTACAAGATTAAAGCGTCTGTGTAA
>Cluster_49728
ATGGCAACAAAAGAAGAAAATCTGAATCGTCTTCGTCAACTGGCTGGCCTGCTGGGGCGCGAGGCGGATATGTCGGGGAG
TGCTGCGGATATTGCGCAACGTGTGTCTGAGTGGGAAGAGGAGCTTGCTGTTTCCCCGGAGGGCATTATGCACTCTGATG
AGAGCGGGGCTGATCAAAATCACACAGACGATGGTGAGCAGTTGAACAACACGGATGCTCCGGATGATGTTAAAGCCGTC
CGGGTACGGAAGTGCCTGCAAGTAATGGGGTATTGCCCGGAGACAGGTCGTCCCGTTGAGCTGGCGTTACGGGGTATGCG
TGTTCTGGTGCCATCATCACTGGCAACGGCCATGATACAGCACGGAACGGCTGAATATGCGTGA
>Cluster_55560
ATGAGCTCACGTTCCAGTTATACAACCCAAACGGAGGGCTATAACGTGGGATTTCCGTCGCCAGCTGCTGACTATGTTGA
ACGGCGTCTGACCGTTGATTCACTCTGCGGTACCGGCCCCAATACTCGGATAGTACAAACAGAAACCGGCTATGCCGTAG
TGGATTGCTTCGTAAAACCAAAGCAAGGAGATACCGTTTTAATTCAATACGGCGGCGGCACTGATTTTGCAAAAATTATG

GGCCGGGCATTTATTACACGAGACGGTGAAGCGTTGGAAGGTGAGGCCCTGGATGATGTTACAGTTGTCGGGGTAGTGAC
ATTCGTTATCAATCGGATAGGGAAGGATGATGATGATTATCCAGTAATATGA
>Cluster_40801
ATGTACCCCAATCTGGCAAAGGCCTACCCCACCAATAAATTACCGGATTTACGCGGTGAATTTATCCGTGGCTGGGATGA
TGGGCGAGGAATTGATGCGGCACGCGCTTTATTGAGCATTCAAAACGGGATGTTGGAAAAACACCGCCATATTGTTGTAG
CTAACGATGGATATGACACAAAAGATGAATGGGAGTTGGCTACGATTTTCAAAAAAACATATACACAAGGCAGGGGGCTT
GATGCCACAAATACAGGAGGGAGTTTGATTCCATCACCAACGCTTCATTCACGAGGAAGTATTGGTAACACAGGTGGTAG
TGAAACCCGCCCCCGCAATATTGCATTTAACTATATCGTGAGGGCGGCTTAG
>Cluster_65583
ATGAAATACCGCATCGCTTTAGCTGTTTCTCTCTTTGCTCTTAGTGCCGGTAGTTATGCCACTACCCTGTGTCAGGAAAA
GGAGCAAAATATCCTTAAGGAGATCAGCTATGCCGAAAAACACCAAAACCAGAATCGTATTGACGGTCTGAATAAAGCCC
TGAGTGAAGTCCGGGCCAACTGTTCAGATAGCCAGCTGCGTGCCGATCATCACAAGAAAATCGCAAAGCAGAAAGATGAG
GTGGCGGAACGCCAGCAAGATTTAGCCGAGGCGAAGCAAAAAGGCGATGCCGATAAGATTGCCAAACGCGAACGGAAACT
GGCAGAAGCGCAGGAAGAGCTGAAAAAGCTGGAAGCGCGCGACTACTAA
>Cluster_61157
ATGTGGTTACTTGACCAGTGGGCAGAGCGCCATATAGCAGAAGCGCAAGCGAAAGGTGAGTTTGATAACCTGGCAGGTAG
CGGCGAACCATTGATACTGGATGATGATTCTCACGTGCCACCGGAATTACGTGCGGGGTATCGCTTGCTGAAGAATGCCG
GTTGCTTACCGCCAGAACTTGAGCAACGGAGAGAAGCAATTCAGCTTCTGGATATTCTCAAAGGTATCCGTCACGATGAT
CCGCAATATCAAGAGGTTAGCCGTCGATTGTCATTACTGGAATTGAAGCTGCGACAAGCTGGATTGAGTACCGATTTTTT
ACGCGGCGATTATGCTGACAAGTTGTTGGACAAAATCAACGATAACTAG
>Cluster_44066
ATGCAACGAATCATTTTAATCATCATTGGCTGGCTGGCGGTAGTGCTGGGTACGCTGGGCGTGGTATTACCGGTATTACC
GACGACGCCGTTTATCCTGCTGGCGGCCTGGTGCTTTGCCCGTTCTTCCCCGCGCTTTCACGCCTGGTTGCTGTACCGCT
CATGGTTTGGCAGCTATCTACGTTTCTGGCAGAAACATCATGCGATGCCGCGCGGCGTCAAACCGCGGGCGATTTTGCTT
ATTTTGCTCACGTTTGCCATTTCTCTGTGGTTCGTCCAGATGCCATGGGTGCGCATCATGTTGCTGGTAATTCTCGCCTG
TTTGCTTTTCTATATGTGGCGAATTCCGGTGATTGATGAAAAGCAAGAAAAGCACTGA
>Cluster_19276
ATGCAACAGCTCGGCCTGAAGTCTCCTGTGCGCCTGAAGAAATACCGGTCTTACCGGGGAAATATGGGACTCGCTGCAGA
AAATATCCTTCAGCGACAGTTCAAAGCGGAAGCTCCCTGTGAGAAATGGGTGACAGATATCACCGAATTCAGGGCTGGCG
GACAAAAACTGTACCTGTCCCCGATACTTGATTTGTTTAACGGGGAAATCGTGGCGTGGGAAACGGCCTGCAGGCCTACA
GAGGAGCTGGTGAAGCGGATGTTAAATAAGGGGCTGGAAAGCCTTGCTGAAGGGGAGAAACCGCTGCTCCACAGTGATAG
TAATAATGCCGGTATCAGTTTTTATCATCACTCTGTTTGCTGTTTAACCAGACTGGTGTGA
>Cluster_18984
ATGAATATGGAAACTAATTCACATACAACAAGTCCATACATTCAGCTTATAGAGCAAATTGCAGTTCTACAGCAGGAAGC
AAAGCGACTGCGAGAGCAGGAAGTTCAAAGTGTAATTGAGTCGATTCAGAAGCAGATTACTTATTACAATATAACCTTAC
AAGAGCTGGGATATACTAATGTGCCTGATGATGGACTCGCTCGCCGGAACTCATCGAAAGGTGTTTACTACCGCAATGAA
GAAGGGCAGACCTGGTCGGGCGTAGGCCGACAGCCACGCTGGCTTAAAGAAGCACTGTTGAATGGAATGAAGAAAGAAGA
TTTTCTTGTGAAGGACACTGAAGAAGAAATAATACCGCTGAAAAATATTTAA
>Cluster_15382
ATGGATGAATACTCACCCAAAAGACATGATATCGCACAGCTTAAGTTTCTCTGTGAAACCCTGTATCATGACTGCCTTGC
AAACCTTGAAGAAAGCAATCATGGCTGGGTTAACGACCCAACCTCGGCGATCAACCTCCAGTTGAATGAACTGATTGAGC
ATATTGCGACCTTCGCACTTAATTACAAAATTAAGTATAATGAAGACAATAAGCTCATTGAGCAGATCGACGAATATCTG
GATGACACCTTTATGTTGTTCAGTAGTTATGGTATTAATATGCAGGATCTTCAGAAATGGCGGAAGTCAGGTAATCGACT
ATTCCGTTGTTTTGTCAATGCGACGAAAGAGAATCCTGCGAGTTTATCTTGTTAG
>Cluster_1657
GTGTCTCAGCTTTGTCCCTGTGGTAGTGCTGTCGAGTATAGCCTATGTTGCCACCCTTATGTGTCTGGTGAAAAGGTTGC
ACCTGATCCAGAACATCTCATGCGTTCGCGTTACTGCGCTTTTGTGATGCAAGACGCAGATTATTTAATAAAGACCTGGC
ATCCCTCTTGTGGAGCAGCGGCGTTACGTGCCGAATTGATGGCCGGATTTGCGCATACTGAATGGTTGGGATTGACCGTA
TTTGAACATTGTTGGCAGGACGCCGACAATATCGGATTTGTCAGTTTTGTTGCTCGATTTACCGAAGGTGGTAAAACAGG
TGCAATAATTGAACGTTCGCGATTTTTGAAAGAAAACGGTCAATGGTACTATATTGACGGTACACGTCCGCAGTTTGGTC
GCAACGATCCCTGCCCTTGTGGTTCAGGTAAAAAATTTAAAAAGTGCTGCGGCCAATAA
>Cluster_2361
ATGTTACCAACAAGTGGCTCTTCAGCAAATCTTTATTCATGGATGTATGTATCAGGAAGAGGTAACCCTTCGACTCCGGA
ATCAGTAAGTGAACTTAATCATAATCATTTTCTTACTCCTGAATTACAAGATAAACTTGATGTTATGATCTCTATATACT
CAAATGCCAGAAATAGTAATGAGCTTGAGGAAATTTATCAAGAGCTAAGTGCTTTTGTAAGTGGGCTGATGGATAAGAGA
AATAGTGTATTTGAGGTGAGAAATGAAAATACTGATGAGGTTGTCGGAGCACTGAGGGAGGGGATGACGATAGATGACAG
GGATGGTTATATCAGGGAGCTTTTTTTTCTGTATTCATTGAAAGTAAAAATTGAGGAAAGTAGACAAGGTAAAGAAGGTT
CTAAATGTAAAGTTATGGTCTGTTATGTCCGCATCACTCTTCAGAGCTATATGGTGATCTACGAGCAATGAAATGTCTT
GTGGAAGGATGCAGTGATGATTTTGATCCTTTTGATATTATTAGGGTGCCGGATCTTACTTACAACAAAGGAGCTTTACA
ATGTGGATGA
>Cluster_9737
ATGTTTTGCGAAGAAAAAGTAGCTCAAATGGCTGCATACCTACTTCTTAAGAGAGGTGGGCGCATGGCATATCTGAAATT
GATGAAGCTGCTCTATCTGTCTAACCGCCAGTCGATTTTGAAGCATGGCAGGATGATCGGCGAAGATAGCCTTTACTCTA
TGAAATTTGGACCAGTCATGTCGAATACGCTGAACTTGATTCGCGGTAAGGCTGAAGGCATTGGTGACTACTGGTACAAC
TTGATAGAGACGAACGGGCATAATGTATCGTTGCGTTCAGATCCGAGGGAAATGGATGCAGACGAGGTCTTTGATGAATT
GAGTCGTGCAGATATCCGGATTTTAGATGAAATCTATTCTCGGTATGGGCATATGAACCGATTTGATCTCGCAAATATGA
CGCATTTAGAAAGCGTTTGTCCAGAGTGGCACGATCCTGGCAATTCTCGTAAGCCTATAGACCTGAAAGAAATGCTGATC
AGTGAGGGTAAAAGCGAGGATGAGGCTAATCGCATAATTGGCAAAATGGAAGAATCTCAGAAACTTAAGGAATTTTCTTT
GCAATTATCATGA
>Cluster_42833
ATGTCTGAAAAAATTGCAGGTGGTTACGTTACTGGGGAGCCTGCGCAAAGGCTCATTTAATGGCATGGTTGCACGTACCCT
GCCGAAAATTGCTCCGGCGAGCATGGAAATCAATGCGTTACCTTCCATTGCCGACATTCCCCTGTATGACGCTGACGTAC
AGCAGGAAGACGGTTTTCCAGCAACAGTTGAAGCTCTGGCGGAACAGATCCGTCAGGCTGACGGTGTGGTGATCGTCACG
CCGGAATATAACTACTCGGTACCGGGTGGGCTGAAAAATGCCATCGACTGGCTTTCCCGCCTGCCGGATCAACCGCTGGC
TGGTAAACCGGTATTGATTCAGACCAGCTCAATGGGCGTGATTGGCGGCGCGCGCTGTCAGTATCACCTGCGCCAGATTC
TGGTTTTCCTCGATGCAATGGTGATGAACAAGCCAGAATTTATGGGCGGCGTGATTCAGAACAAAGTTGATCCGCAAACC
GGGGAAGTGATTGATCAGGGTACGCTGGACCACCTGACCGGGCAATTGACCGCATTTGGTGAGTTTATTCAGCGAGTTAA
GATCTAA
>Cluster_15816

ATGCTTATTGGCTATGTACGCGTGTCAACAAATGACCAGAACACCGATTTGCAACGTAATGCACTGAACTGCGCGGGATG
TGAGCGGATTTTTGAGGATAAAATCAGTGGCACTAAGTCCGACAGACCGGGGCTTAAAAAACTGCTCAGGACACTATCGG
CAGGAGACACTCTGGTTGTCTGGAAGCTGGACAGGTTGGGGCGCAGTATGCGGCATCTTGTTACGCTGATAGAAGAGTTG
CGTCAGCGTGGCGTGAATTTCCGAAGCCTGACTGACAGTATTGATACCAGCACCCCAATGGGCCGTTTCTTTTTTCATGT
CATGGGGGCCCTGGCTGAAATGGAACGCGAACTGATAGTTGAACGTACCATGGCAGGGCTGGCTGCAGCTCGTGCCAAAG
GCAGAGTAGGTGGACGCCGTCCTAAGTTGACCACCGAACAGTGGGCACAGATTGGGCGTTTACTCGAGGCCGGAGAATCA
AGACAGCGTATTGCACTGATTTTTGATGTAGGCGTTTCTACCATTTATAGAAAATTTCCGGCAAATAAGAGCAATGAATC
CCCCTGA
>Cluster_21261
ATGCCCAAAATATCATCAGTTGTATCATCATGTTACCATCTGTTCAGTGAACATCAACAACTTTCAAATGAAACAACAAT
GACGAACTCCGTCTCCAGAAGAATTGTTCATAAAGAATATGGTATATCTTTAAAATCCGTTCCTGTATGGTTGGCTACAG
CTAAAACTCCCCTTGCTCTACTCAATGGCAGACATACAAGAAGTCACTCATTTATTATTGCAGGGACCCCAGGAATGGGA
AGCAGGAGCGGAGCCCAATACTATGCCATAAACAGTGATGATAAACGCTCCCGAATAGACATTGACTCTTTATTTTTAAA
AAAGTTAAATAATGCGCGAAATCAAAATAAATTTCCAATCGATGTAAAAGAAACGCTTATAAAACTACAAGGGCAAAAAT
TCACATGCATTGAAGATTTTCATAAAAAGTATAATGAAACCAGGTTAAAGGCCAATACCAATATCCAACAAGAACAAATT
GCAGATGAAGTAAAATCACTTACATATTTGATTCCTTCAGAAAAAAAGAGATGTGGATATATAAAAACAATGGAAAAGAT
AATGCAAAACCAAACTTAG
>Cluster_44739
TTGACGTTTCGCGGGAACCTGCTGCTGTATTCACTGTGGTATGAACTTCACCCGTTTGAGAACCTTCGCCGCAAGGGGCA
TCACTGCCACGGCCTGACGCACCTGTCCTGGGCAGAACGCAAAATTTTTACCGGGTTTTGGGTAAAAAATTTTGTGGGC
TGTTCATTTACCTTACCCTGGACAGTCAGTGCGGCAGGTTGTGGCTGTATCAGCCCCCGGCGACAGGGACTCATACGGGT
GTGTGGCACCCGGCAGGCAATGAGCAGGGCCCCGCCGCAACGACGGAGAGCGCGTGCGCGAAACGGCGTTGCCCCTTACA
TCCCGGCGGCCTTTTGTCGGGATGCCGGGAGCGCAGGCCACGGCGCGACAGAGGCGGCGCGACCGGAACGGTCGCTGCAA
GCCAGGGTTTCGCGTCAGGGATAGAAGCCCGCCAGGGCGGAGACAGCTCCGCTGGCTCCGTTCACGACAGCCCGCCCCGC
CAGGGGGACGCCCGGAAAAGCCATAAACAGGAGAGGAACGGAAAGAAGAGGAAGAGGAAAAGAAGAAGAACAATAACAAG
CACGGTAACCACAGAAAAATAA
>Cluster_39870
ATGAAAAAAAGCCTGCTTGGTTTAACCTTCGCTTCCTTGATGTTCTCTGCCGGTTCAGCGGTTGCCGCCGATTACAAAAT
TGACAAAGAAGGTCAGCACGCCTTTGTTAACTTCCGCATCCAGCATCTTGGCTATAGCTGGTTATATGGCACCTTCAAAG
ATTTCGACGGTACTTTTACCTTTGATGAAAAAAATCCAGCTGCCGATAAAGTGAATGTGACAATTAACACCACCAGCGTC
GATACTAATCACGCCGAACGCGATAAACATCTTCGCAGTGCGGATTTCCTCAATACCGCAAAATATCCACAGGCAACATT
CACCTCCACCAGCGTGAAGAAAGACGGTGACGAACTGGATATTACCGGCGATCTGACGCTGAATGGTGTAACCAAGCCCG
TCACACTGGAAGCGAAATTAATTGGTCAGGGTGACGACCCGTGGGGCGGTAAACGTGCAGGCTTTGAGGCCGAAGGCAAA
ATCAAGCTCAAAGACTTCAATATCAAGACAGATTTAGGTCCAGCGTCTCAGGAAGTGGATCTGATTATTTCAGTGGAAGG
CGTACAGCAGAAGTAA
>Cluster_60655
ATGCCCCTCTTACACCTGCTCCGCCAGAATCCGGTGATTGCTGCCGTTAAAGATAATGCCAGCCTGCAACTGGCAATCGA
TTCTGAATGCCAATTTATTTCCGTGTTGTACGGCAATATCTGCACCATCAGTAATATCGTTAAAAAGATTAAGAACGCCG
GGAAATATGCTTTTATTCATGTTGATTTGCTGGAAGGCGCGTCAAATAAAGAAGTCGTTATTCAGTTTTTGAAACTGGTG
ACCGAGGCGGACGGCATTATCAGTACCAAAGCCTCAATGTTGAAAGCGGCAAGAGCGGAAGGTTTTTTCTGTATTCATCG
CCTGTTTATTGTTGATTCAATTTCGTTTCACAACATTGATAAGCAAGTTGCGCAATCGAATCCGGATTGTATTGAGATCC
TGCCAGGCTGTATGCCCAAAGTGCTGGGCTGGGTGACAGAGAAAATCCGCCAACCGCTGATTGCCGGTGGGCTGGTGTGC
GATGAAGAAGATGCGCGTAATGCGATTAACGCGGGTGTCGTGGCGCTTTCCACCACGAATACCGGGGTCTGGACGTTAGC
GAAAAAATTACTTTGA
>Cluster_34955
ATGGCTAAACCAGCGCGAAGACGATGTAACCGTAAAAGAGAAGATTTAACTGTTAAAAGGATATTTGAGTTACTAAGTTT
CGATAAATCTACCGGGGTATTTAGATGGAAAGTTCCCACTCAGGGAAGGATAGCATTAAATAGTGTTGCTGGAACTTTTG
ATTCCAACGGTTATTCAATGATCATGATAGATGGGCGTAGATATAAAACTCACGTCTTAGTTTTTTACATAACTCATAAT
CGTTGGCCTGCTGGTCAAATTGACCACGTTAATGGAATTAGGACCGACAATAGGCCAGAAAATTTAAGAGAATGCCTGCC
AATAGAAAATTCAAGAAATATAAGGATCCGAAAGAATAGCAAATCAGGTTGCAGAGGGGTTACTTGGCACAAACGACAGA
AAAAATGGAATGTTAGGCTAGGATTCCATGGCAAGAGTAAACACTTCGGATGCTTTGATGATCTGGAGTTAGCGGTACTA
GTTGCTGAAGAAGCCCGAGATAAGTATTACGGTGATTTTTCCGGCAACGAAAGGAGCACTTATGCGAATCTATCGAAGGA
AATGTAA
>Cluster_36273
ATGCCATTAACCTCAGATATTAATTCATCTTCGTTCCATCTTGGAATGGAGGTTCTTCGTGCTCAAGTTGCAGCCACTGG
GCGTGGAGAATTTACAATGGGTGGTGAAACTGTCAGAATTGAATATAGTCCAACAGATGGGCGCTTTCTGGCCAGCGATG
GCACTGGGGGATTATTTACTGAATTATTGCTTTTAGGGTTCAATAATGGGCCTCAGGCTCTTGGCGAGAGAATGTTAAGT
ATGATTACACAATCGCAAGAGAGTCTTCAGGATAAAATATCTCAATGTAAGTTTTCTGTTAATCCAGATGACCTTCAGTG
CCCGCCAGAGTCTGCTCAATGTCCCATTACATTGGAGATACCAGAGGAAGGGGTGTTTATTAAGAATTCAGGGGATTCAG
TTGTATGTAGTTTATTTGATGTTACTGCATTTTCTCGGTTAGTTAGTGAAAAATCACCTCACCCACTGACACGAGAGAAA
CTAACGGCATCAATGGTTGTAAGTGCTGATAAATGTTTTTATGACCATGGTAAGGGTAGCTTCGTTATAAAAGATAGTTA
A
>Cluster_39819
ATGGGGGCAGGAAGGCTGGTCAAAGAAAGGAAAAAAAGTATCCGGGGGCATTTTTACACCTATAAGAAATCGGTAAAAGG
GCGAAAAAAGAAACGTTCGAAAGGTCAGCGCTGTTTAAACAAAACAGACAAAGAACAGAGCAAATCAGCAAAAGAAGCCT
GGTTGATCTTCAGCAGAACAAATGACTTCAGAGCGCGAGAAATCATCAAGTTATACAGTCGGCGAATGCAGATAGAGCAA
AACTTTCGTGATGAAAAGAACGGACGCTTCGGGTTTGGTCTTCGGGCCAGCAAAAGTCGTTCAACAGGGAGAATTCTGGT
TCTGAGCCTGCTGGCAACGTTGAGCACTATCGTAATGTGGTTACTCGGTTATCACGCTGAAAATAAAGGGTTACACCTGA
AATATCAGGTTAACAGCATTAAATCCAGGCGGGTTATCTCATATCTGACGTTAGCGAAGAATGTTCTTCGACACTCGCCA
TTTATTTTAAGACGAACAGTACTGAGCACAGTTCTAAACCATCTGGCTAGAACCTACCGAAATATAGTGCTGGTTTATTA
G
>Cluster_20754
ATGGATTTCAACAGTGGTGGGCGAAAAGTTATCGAGAGATTGGTTGAAGCATATGGTTTCACCACACGGCAGGCGCTAGC
CGATCACTTGGGTGTATCGAAAAGCACCCTTGCCACTAGATACATGAGAGATATTTTCCCATCAGATTGGGTAATTACAT
GCGCTTTACAAACAGGAGCATCACTGACCTGGCTAGTTAACGGTAATGGTCCTGTTTTTGATATAGAAAGGCAGGACATT
CTAAGATTGCATAAGCAAAAAATAGTTGATGGAAATCTATATGATGCCGGAACTGTTTTTTTTGATAAAACAATACTACC
TAAAAATATTGTCAGCCCCATTGTTATCGAAGAAAATAACAACATATTCATTGCTGATTCATTTTTTGAAGATATTACCG
ATGGCAAGTGGCTTCTGAGTATTGATGAAAAATTTAGTATAAGAAATGTATCGCGTATACCTAATAACTCAGTTAGGGTG
ACTAAGGATGATATTTCCTTTGACTGCCCTATAAAAGATCTGAATTTCAAAGCGAACATAAAAAAAATCATTCATCTAA

>Cluster_24457
ATGACCCTACGCAAGATTCTGGCACTCACCTGCCTGCTGTTGCCGATGATGGCTTCCGCACATCAGTTCGAAACCGGTCA
GCGAGTGCCGCCGATTGGCATTACCGATCGTGGTGAGTTGGTGCTTGATAAAGATCAGTTTAGCTACAAAACCTGGAACA
GCGCGCAGCTAGTGGGAAAAGTGCGAGTACTGCAACATATTGCTGGTCGCACCTCTGCAAAAGAGAAAAACGCGACGCTG
ATTGAAGCGATTAAATCAGCGAAGTTACCGCACGATCGTTACCAGACCACCACCATTGTTAACACCGACGACGCGATTCC
GGGTTCAGGGATGTTTGTGCGCAGCAGTCTGGAGAGTAATAAAAAGCTTTATCCCTGGTCGCAGTTTATTGTTGATAGCA
ATGGCGTCGCACGCGGTGCCTGGCAGCTGGATGAAGAAAGTTCCGCTGTAGTGGTGCTGGATAAAGACGGTCGCGTGCAA
TGGGCCAAAGACGGGGCGCTTACTCAGGAAGAGGTGCAGCAAGTGATGGACCTGCTGCATAAATTAATTAATAAATAG
>Cluster_48864
ATGGAAATAAGCGCTCGTAACTCATCCACAAAAATTATTGCCATTATTATTCTGTCACTCACGGCTCTGGTCTTTATTCT
GGGGTTTGCTGTTACCTGGCTGGCGGTGACTAACAGAACACTCATTCATGAGCAGCGTACGGTCGTCACCCCGATGACCT
ATAACGCGCCTTTCGCGGTATCTGAAACAAAAGCGGATACCGAGTTTTTCCGGATGATGACGCTGTCCTTCCTGGCCCTG
CGTCTTAACGTCTCCCCCGAAACGGTAGATTCAAACCACGCTTTTCTGATGTCTTTTGTCGAACCAGAAGTCAGAGAGGA
ATTTAAAAAAGTTCTGCAGGAAGAGGCTGCACAGATAAAGGCCAGTGATGTGAATTCCACATTCTACACCACGGAAATCA
ATGTCTATCCCGTAGACGGGCGCGTGGATGTCAGGGGGGTGCTGAAAATGTGGATCGGCAACTCAAAACCCACCACGGAA
TTAAAAAACTATCGTCTCCGCCTTAAGTACGCCGGTGGCTTTACCCGCATTGGCCGTTTCTATGAGGTAACCAATGAAAA
ATAA
>Cluster_15962
ATGAAACGAATGACACTAATTGAGATGGATGGATTTCTGAAAGGTAAATGCATCTCATGTGATTTAAAGGTTAACGAAAC
AAATGCTGAATATATGGTGCGTAAATTTGCTGAAGCTGAGGCCAAGTGCGCGGCGCTGGCAGCGGAGAATGCGGGGCTGA
AGTCTGGCGCTATGGACGAAATCAAGGTTATCAACCGTGGAGGGCAGGCATATTGCGTAAAAGATGGAGTGCAAGTTAAT
CCCATGTATGCAAGAGGGTGGAATGACTATCGCGCAAAGTTTCTGCAATCAGACACCCCAGCTACCGATGCTTTTCTGGC
TGAAATTCGTGCAGAAGCACGCAACGAGGGGATTAACTATACCGCCAGCCGTCTTGCTGCTGCTTTCAACCACGGATTTA
TCAATAAGTCTTTGCGTGAAGTTTTCGACGTTACACGCATGATTCTGTCAGCGAAAGAAGAGTTGGCTAATGAACTGCAC
CCGATTGATGGCCTGTCCGGTGAATATGCGGAGAAATCCCTTGAAGAATGGGCGGAACAGATTCGCAAAGGAGGCAACCA
GTGA
>Cluster_36288
ATGGATAAGAAAAAAGGCGGAGCGGATCAGGTGGTTATAGTGATGACCTACAAGCAAGCCCTGCGCGTCGCCGCACGTGA
GTCGAAAGCCGTCCGTCGTTCGCTGGTCGACAAACTTGAATCAATGCAACAGCAGTTGCAACAAAAAACGACCACGAAGA
AATCACCGGATGGCCTTGAAGAATTCCGTAAGGCACGCGCATTGAAAATGACCGTCGACACAATGAAAGACCTCTTCGAC
TTCCTCCCTCACCTCGCACCGGAAGCTAAACAGGTCGTTGCCGCCAGCCTGATTAATCCCGTCGTCGGCTTTAGTGCAAT
CCCTCTTCCGGTCATTGATGAACATCACTACTCTGCGTCGGAGGTTGGCACCATGCTTGGCATTTCAGCCAACAAGGTAG
GGCGCATTGCCAACACCTACATGCTCAAAACAGAAAAGTACGGGAAGTGGTTCATCGACAAATCGGCGCACAGTGACAAG
CAGGTCGAGACATTTCGCTACAACGAGGCGGGAGTGCACAAGATCGAGGAACTCATCGAAGGAGAACGAAAGGCTGCATG
A
>Cluster_822
ATGAACGTGAGTAAATATGTCGCTATCTTTTCCTTTGTTTTTATTCAGTTAATCAGCGTTGGTAAAGTTTTTGCTAACGC
AGATGAGTGGATGACAACGTTTAGAGAAAATATTGCACAAACCTGGCAACAGCCTGAACATTATGATTTATATATTCCTG
CCATCACCTGGCATGCACGTTTCGCTTACGACAAAGAAAAAACCGATCGCTATAACGAGCGACCGTGGGGTGGCGGTTTT
GGCCTGTCGCGTTGGGATGAAAAAGGAAACTGGCATGGCCTGTATGCCATGGCATTTAAGGACTCGTGGAACAAATGGGA
ACCGATTGCCGGATACGGATGGGAAAGTACCTGGCGACCGCTGGCGGATGAAAATTTTCATTTAGGTCTGGGATTCACCG
CTGGCGTAACGGCACGCGATAACTGGAATTACATCCCTCTCCCGGTTCTACTGCCATTGGCCTCCGTGGGTTATGGCCCA
GTGACTTTTCAGATGACCTACATTCCGGGTACCTACAACAATGGCAATGTGTACTTTGCCTGGATGCGCTTTCAGTTTTG
A
>Cluster_53936
ATGATTAAAAATGACAAGGCATGGATAGGAGACTTGCTGGGCGGACCGCTCATGAGCAGGGAAAGCCGCGTCATTGCCGA
ACTGTTGCTAACCGATCCCGATGAACAGACATGGCAAGAGCAAATTGTTGGCCACAACATTTTACAAGCCTCTTCTCCTA
ACACCGCAAAACGTTACGCGGCAACAATCAGGCTTCGCCTGAACACGCTGGATAAAAGCGCGTGGACATTGATTGCCGAA
GGTAGTGAACGGGAACGCCAACAACTTCTGTTTGTGGCTCTGATGCTACATTCGCCGGTAGTTAAGGATTTTCTGGCTGA
AGTGGTGAACGATCTGCGCAGGCAGTTCAAGGAAAAGTTGCCTGGCAATAGCTGGAACGAATTTGTGAATAGCCAGGTTC
GCCTACATCCGGTACTCGCCAGCTACTCAGATTCATCTATTGCAAAAATGGGAAACAATCTGGTGAAGGCGCTTGCTGAA
GCGGGTTATGTGGATACGCCCCGCAGACGTAACCTGCAGGCAGTTTACCTTTTACCGGAAACTCAGGCAGTGTTACAGCG
CCTGGGACAACAGGACTTGATATCTATTCTGGAGGGAAAACGGTGA
>Cluster_62532
ATGAAAGTTACATGGACCGTCACGCCTGTGGGGTATCAGCGCATCGCGAAACGATGCCCCTTCATGCAACATTAAACGGGA
CTTCACGCCGTCAGGTGCATTTCGGGTTAATTCGCAAAAGAAGGTGCTGGACGTGTGGAGTATCTATAAATGCACACACT
GTGATTACACCTGGAATATCTCCCTGTTTTCGCGTCTGCACGTCAGCAAAATTGACCGCCAGCTTTATCATCGGCTAATG
ACAAACGATGCCGCCACGGTTCAACATTTGCCTGTGATATCGCGACGCTGAAGCGGAACAACGCCGAGCTATCGGGTCG
GCCTGATTTTACCGTTCAGGAGCGCTGGCCGTTAAGCATCACCGCCCACCCACGGGTTCGCGTTAGTGTCAGGATCTCCC
GTTCATTTCAGGTAAGTCTGCAGTCGATTTTGAAGCAACAGCTCATGCTGAGCGCTGGTGAAATTCTGAGAGGGTTGGAG
TCAGGGCAGATCAGCGGCGTTACGATGAAGATGCTTAAATCGAGAAAGCTAAAGGCAGCGGAATATGAATTCCAGCTGTC
TGCGGAAACGTTATATGCCAGGCGCAGGATTGTTCTGACGCATCGTTAA
>Cluster_46757
ATGCTCAGGACATTCATCGTAATTATATTTATTTTCAATTTATCAGGCTGTGGATATAACGATATTCAAACCTATGATGA
GCAGGTTAACGCCTCCTGGTCTGAGGTATTAAATCAATATCAACGTCGCGCCGATCTTATTCCTAATCTTGTCGCCAGTA
TTAAAGGTTATTCCAACCATGAAAAAGACGTTTTGGAAGCCGTAACATTGGCTCGTAGCCAGGCTAATCGCGCCAGCAGC
GATCTTCAGCAAACTCCGGGAAATGAACAAAAATTACAAGCCTGGCAACAGGCTCAAGCGCAGGTGACTCGCACTCTCGG
TCAGTTAACGATAATTAGTGAGCGTTACCCTGAGTTAAAGGCTCAAGAGCTCTATCAAAACTTAATGGTACAGCTCGAAG
GAAGTGAAAATCGAATTGCCGTTGCGCGAGGAAGATATATTAAAGCCATTGAACAATATAATGTTACTATCCGCAAATTC
CCCGCTGTATTAACCGCAAAGGTCATGGATTACACACCGAAGAAAAATTATTTGCCGGATGATGTCGCCGCTGTGAGTAA
AGCACCAACAATAGATTTAGCCAGAATGCTAGTGCTCATTAG
>Cluster_60258
GTGCTGCCTTTGGTAATCCCCCGTACCCGTGGTAACCCCCAGACCGGCACGCCTGCCACCGATCTGGATGATGACTACTT
TGACATGTTGCAGGAGGAGCTTTGCAGCGTTGTGGAGGCCTCCGGTGCCAGCCTGGAGAAGGGGCGGCATGACCAGCTGC
TTACCGCGCTTCGTGCGCTGCTGTTAAGCCGCAAGAATCCGTTTGGCGATATCAAATTGGACGGCACGGTGCAAAAGGCT
CTCGAAAACCTTGGTTTGGGAGAAGCGGCTAAAAGAAATGTAGGGACAGGGGCGAATCAGATACCTGATATGGGTAGTTT
TATGCTTTCTGCTTCAGTTCCTGGATATCAAAAATTGCCATCCGGTTTAATTATTCAATGGGGGCCAATTGATGTTCCGC
TGACGTCTCAGGACACAGTAACCTATTTTCCGATTGCATTTCCGAATAGATGTCTGCGGGTATTTGCGACTCAGGATTAC
ACTCCCGGCAGTGCAAATGTTGGCTATATAGCTTGTGCCGGTTATAACCAGGACCCGGTGAAATTTATTTCCAGAGCAGG

CGTACCCGGTATCGGTGCTTCATTTTTCGCATTAGGGTGTTAA
>Cluster_1580
ATGGCAATAAAAGGTCTGGCGCAGGCCATGAAAAATCTGGATGCAATTGACCGCCGTGCCGTTCCCCGGGCTGCCGCCAC
GACACTTAACCGTGTGGCGGAGTCCATCATCGCGAAAACGGCCTCTTCGGTTGCCAGGGAGCTGGCGGTTCCGCGCCGTC
TCATCCGTGAGCGTATCCGCCTGCAACGGGCTAGCGCAGACAGGATTTATGCGAAGGTCATCATCAACACCGGTAATCTG
CCCGCCATAAAACTGGGGACGGCCAGCGTGCGGCTTTCCCGCAGAAAGCGACGAAAGAAAGGCGAGCGTTCGGTCAAGAA
AGGGGGCGGCAGTGTGCTGATTGTGGGGAAAAGACGGATCCCGGACGCCTTTATCACCCGGCTGGCTAACGGACGCTGGC
ATGTGATGCAGCGTATGCCGTGGGCACCATCGTCCACCGGTGCTGACAGCAAAGGGAGGCCGAAACGCTACCGTCTGCCG
ATTGAAGTGGTGAAAATTCCGACTGCCGGACCGCTGGCAGAAACTTTTGAACGTGAACGGGACCGGATGTACCGGGAAAA
ATTACCGGCGCAGATGATGAAAGCCATGACGCATCAGTTACGCCTGGTGCTGAAAAGAAAATGA
>Cluster_25974
ATGGAAACGGTTGGTCAGCGTATAAAAGCTCTGAGAAGAGTTACCGGAACGTCCCAGAAAGAATTGGGTAAATTTTGTGG
AGTAAGCGACGTTGCTGTGGGGTACTGGGAGAAAGACATCAATACCCCTGGTGGGGAGGCACTTTCGAAATTAGCGAAGT
TCTTCAATACGTCAATAGATTACATTCTTTATGGTGCTGAGTTTGAAGGCAAACTCGTCACAAACATGCGCAGAGTTCCT
GTAATATCGTGGGTTCAGGCTGGGCAGTTTACTGAGTGCAGGGCAGCAGAAGTGTTTAGTGAAGTGGACAAGTGGGTAGA
TACATCATTAAAGATTGGTGATAACTCATTTGCATTAGAGGTTAAAGGTGACTCCATGACTAACCCTAATGGCCTCCCAA
CAATACCAGAAGGCGCAACAGTGATTGTAGATCCAGATGCAGAACCTCGTCATGGAAAAATAGTCATCGCTCGACTTGAT
GGAACAAACGAAGCTACAGTAAAAAAATTAGTCATCGATGGCCCTCAAAAGTTTTTAGTGCCATTAAATCCTCGGTATCC
CAACATCCCTATCAATGGTAATTGCCTTATCATTGGTGTAGTCAAAGGAGTTCAATACGAACTCTAA
>Cluster_21772
ATGGCATTCAGAATGAGTGAACAATCACGTACTGTAAAAATTTATAACCTGCTGGCCGGAACTAATGAGTTTATTGGTGA
AGGTGACGCATATATTCCACCTCATACAGGGCTGCCAGCTAATTCTACAGATATCGCCCCACCGGAAATTCCTGCTGGCT
TTGTGGCAGTTTTTAACAGTGAAAATGAATCGTGGAATATTGTTGAAGACCATCGTGGTAAAACGGTCTATGACGTGGCC
TCGGGGGACGCGTTGTTTATTTCTGAACCCGGACCGCTACCAGAGAATGTCACCTGGTTGTCGCCAGCAGGGGAGTATCA
GAAGTGGGACGGCGTATCCTGGGTGAAGGATGAGGAAGCAGAAAAACTGTTTCGGATACGGGAAGCGGAAGAGAAAAAGG
CAAGGTTGATCCAGGAGGCAACAGATAACATCGCAATTCTGCAGGATGCCGTTAATCTTGAAATAGCAACAAACGAGGAA
AATTCACAACTGGATTCCTGGAGAAAATACAGAGTATTAGTGAGTCGAATTGACACCAGTACAGCTCCGGATATCGTATG
GCCAGAGCTGATGAATCAGGGTTATGTGCGGGAGGACGAGCAGATAACTTCAGACTGA
>Cluster_50271
ATGAAAGATCAAGATGTTAGGTTCGCGGTGCATCATAAGCTTTTGAAAGAATCGCATTTAGATCCAGACTGCCTTGTGGT
CGATGAATTTTCCATATCCCTTGGCGCCAGTAGAGCAGACATTGCTGTAATAAATGGTGTTATACACGGGTACGAGCTCA
AAAGTGAATATGACTCTTTGGAGCGTTTGCCTCTTCAAATCAAGCATTATTCTTCTGTAATGGACAAGGTTACTCTTGTC
GTAGCTGAGAAACACCTTGAGGGAGCATTAAAGTTAATCCCAGGTTGGTGGGGCGTTAAAACGGTTTCTGTTGGGCCAAA
AGGCGCCATTCTTATAAAGCACATGCGTGGAGAAAAGCTTAATCGAAACCATGACACATTGATGCTCGCTCAATTGCTTT
GGAAAGATGAATGTATCGACGTACTTGAACGATGGGGCTATTCCAAAGGAATCAAAAGCAAGCCCCGATTTGAGTTATGG
AATATTATTGCGGAAAATATTCCAATAGCGAATCTCAGGCCTGAAGTCAGAACAGCCTTAAAGAAACGCGTAGGCTGGAA
AGTTAAGGCTTGGCAGGCTGAGTCTGCGCCAACCAATAAAGCTGTCTCACGACTAACGTAA
>Cluster_28511
ATGCAAATAGTCATGTTTGACAGGCAGTCAATATTTATTCATGGAATGAAAATCAGTTTACAGCATCGTATTCCAGGAGT
GAGTATTCAGGGGGCCAGTCAGGCAGACGAGTTATGGCAAAAGCTGGAAAGTTACCCTGAAGCCTTAGTGATGCTCGATG
GCGATCAGGATGGTGAGTTTTGCTACAGGTTGCTGCAAAAAACCGTGGTGCAATTTCCTGAGGTTAAGGTGTTAATTACG
GCGACGGATTGCAACAAACGGTGGTTACAGGAAGTTATTCATTTTAATGTGCTGGCCATTGTGCCTCGTGATTCAACCGT
CGAGACATTTGCGTTGGCGGTCAATAGTGCGGCGATGGGGATGATGTTTCTGCCTGGAGACTGGCGAACGACGCCGGAAA
AGGACATTAAAGATCTCAAATCGTTAAGCGCCCGCCAGCGGGAGATTTTAACCATGTTAGCGGCGGGCGAATCAAATAAA
GAGATCGGCAGGGCGCTGAATATCAGTACCGGAACGGTAAAAGCGCATCTGGAATCACTGTATCGCCGTCTGGAAGTCAA
AAATCGCACCCAGGCGGCAATGATGTTAAATATCTCCTCCTGA
>Cluster_35746
TTGAAAAGTAACTTTATAGAAAAGGATAATACTGAAAAGTCAATTTGGTGTTCGCCGCAAGAAATTATGGCTGCTGATGG
TATGCCTGGATCTGTTGCTGGTGTTCACTATCGAGCAAATGTTCAAGGCTGGACGAAGCGAAAAAAGGAAGGTGTCAAGG
GGGGGAAAGCTGTTGAATACGATGTGATGTCGATGCCCACCAAAGAACGAGAGCAAGTTATTGCGCACTTGGGGTTATCC
ACACCGGATACTGGTGCTCAAGCCAATGAGAAACAGGATTCTTCGGAACTGATAAACAAATTAACCACAACCTTAATCAA
TATGATTGAAGAGCTGGAACCAGATGAAGCACGCAAGGCACTGAAACTTTTAAGTAAAGGGGGGTTGTTGGCGTTAATGC
CTCTTGTATTTAATGAACAGAAACTTTACAGCTTTATAGGATTTTCACAGCAAAGTATTCAGACGTTGATGATGCTAGAT
GCATTACCTGAAGAAAAACGCAAAGAGATTTTGTCAAAGTATGGGATTCATGAACAGGAAAGTGTTGTAGTACCTTCACA
GGAACCACAGGAGGTAAAAAAAGCCGTATAA
>Cluster_986
GTGCTGATTGGCTATGTAAGGGTATCAACAAATGACCAGAATACAGACCTGCAACGAAACGCTCTTGTTTGTGCAGGATG
TGAACAAATATTTGAAGATAAATTAAGCGGAACAAGGACAGACAGACCTGGATTAAAACGCGCTTTAAAGCGCCTTCAAA
AAGGTGACACACTTGTTGTCTGGAAACTGGATCGCCTCGGGCGAAGCATGAAACATTTGATTTCTCTCGTAGGGGAATTA
CGAGAGCGAGGGATTAATTTTCGCAGTCTTACTGACAGTATTGATACGTCATCTCCAATGGGGCGTTTTTTCTTCCACGT
TATGGGTGCCCTGGCTGAGATGGAACGAGAACTAATCATCGAGCGAACGATGGCTGGACTTGCTGCCGCCAGAAATAAAG
GCCGTATTGGTGGGCGACCACCTAAACTAACCAAAGCGGAATGGGAGCAGGCCGGGCGTTTATTAGCACAAGGAATCCCC
CGCAAGCAGGTTGCATTGATCTACGATGTGGCCCTGTCAACTCTGTATAAAAAACACCCCGCGAAACGAACGCATATAGA
AAACGACGATCGAATCAATCAAATCGATCGGTAA
>Cluster_30753
ATGAAAACGTCACCGTGGAATAAAGACCGTACCATCGGTCAAAAAAGGCCACTTCAGATATCTCATATCTGGGGGATCCG
GATTAGACTTGAGTTGGAAGGTAAAACACGCGATCTAGCTCTGTTCAACATGGCTTTGGACACTAAGCTACGGGGCTGTG
ATTTGGTCAAACTCAAAGTATCAGATGCTGCATATGGCAGTTCTGTCTCAAGCAGAGCAACGGTGTTGCAACAGAAAACC
GGCAGCCCTGTCCAATTTGAGATAACCAAAGGGACAAGGGAAGCGGTTACTGCATTGATAAAGCTCGGCAACTGGCATAG
TAAAGGCTTCCTGTTCCGCTCTCGGGTCGGAACTAACCACCATATATCAACCCGGCAATACAACCGAATTTTTCATGGGT
GGGTAGAAAAGCTTGGTCTCGAAGATTCGCTGTACAGCACACATTCCATGAGAAGAACAAAACCTTACTTAATCTACAAG
AAAACCAAGAGTCTCCGGGTGATCCAACTTCTGTTGGGCCATAAGAAACTGGAAAGCACAGTCCGTTATTTGGGCATTGA
AGTCGATATAGAAGGTCTCTTTTAA
>Cluster_37473
ATGTATCGGATAGTTCTGGGGAAAGTTTCGACCTTAAGCGCAGCTCCACTGCCACCGGGTTTACGCGAGCAAGCACCGCA
AGGTCCACGACGCGAACGCTGGCTGGCGGGGCGTGCATTGCTTTCGCACACGCTTTCCCCGCTACCGGAGATCATCTATG
GCGAACAAGGCAAACCTGCATTTGCGCCGGAAATGCCGCTATGGTTCAACTTAAGCCATAGCGGTGACGATATCGCCCTG
CTGTTGAGTGATGAAGGCGAAGTCGGCTGCGATATCGAAGTGATTCGCCCGCGCGCCAACTGGCGCTGGCTGGCGAACGC
CGTATTCAGCCTCGGGGAACACGCTGAGATGGACGCCGTGCATCCTGATCAGCAACTGGAAATGTTCTGGCGCATCTGGA

CGCGCAAAGAAGCCATCGTTAAACAGCGTGGCGGCAGCGCCTGGCAAATCGTCAGCGTAGACAGCACCTATCACTCCTCG
CTGTCAGTCAGCCATTGTCAGCTTGAAAATTTAAGCCTTGCGATCTGCACCCCTACTCCCTTTACGCTCACCGCCGACAG
TGTGCAATGGATCGATTCAGTTAACTGA
>Cluster_20184
ATGCGCCCCACTCAACGTAATCTGATGAAAAAATCAATTCTGCTTCTGTCGTTGCTATTCAGCACCACTTTCCCTGTGGT
TGCAGGACAGGATGTTGACATTGTTGCAAATATAAAAAATAACACCTACCAAAGTGGCATCAGCAACAACGGAAATATTG
ATTTAGGTGTCGTTGGCGTGGGCGATTTTACCGGAAACATTTCAGCCGAAAATTATCACCCCGGTGGAAAAGATTTCACT
ATTACAGTACAAGACTGCACACTTCAGGGCACAGGGAATGTGTTAAATCAGTTACATATTAATTTTCGCGTACTTAGTGG
CGTAATGGCGACAGGTTCCAGTCAAATATTCGCTAATGAAGACAACGCCGGGGCTAAAAATGTTGGGGTAGTTATATTTT
CTATTCAGGACCCGGCAAATACCTTCAACGTTCTTAGTGCCACTGGTAGCGCACGTTCCGTTTATCCGGTCATGTCATCA
GCACTAAATAATTCATCCTGGAAATTTAGTACCCGAATGCAAAAAATCGATCCAGCACTGAATGTCACCTCTGGCCCACT
GATAAGCCATGTGTTAGTGGATATCTACTACGAATAA
>Cluster_3663
ATGGCTGTAATTCCAATGTCTTACTCCCCTGCTACTGTAGCTCGTCGTTTTTCGATACTGGATGGAGTAACTATTCAGGG
CGTGCTTTACCAGATCATCTGGGATCCAAAGACTCCGTTTGCTGCGGTAATCGAAGCTGCACCTTCTGTTATTGATGGTG
ATGTTCGCCATAAGGTTGTTGCCACTCTGGAGCTTCAGCGTCGTCCCCGGCTTGAAGGCGTGTTTGTCCAGAAATTCTGG
GAAGAACAGGATGTTGCTCAGATTGAAGGGATTGTTGTCGATGGCGCTGTACGTGATGTTGGTCTTGCAACGTTTGTTTA
TGAAACGATAGTCACAAAAGCGGGCGTTGTACTATTAAGTGATAACGAGCAATATGAAGGAGGTAAAGCTCTCTGGCAGC
ATATTGCGCGTCGTTCAACGAATCTGAAGGTGTTTATTTTAGATACTGATTCCGCACGGTATTACCCGTTTGATGGCGAA
CGGATCAGTTATGACGGGAAAAGCATCCCGGAATCAGAAATCTGGAGTGAACATCCTGAGCGAAACAGACACGCTGTCGT
TCTTGTGGCTGAATCTGTTAATGGTAAAGCCGCATAA
>Cluster_60346
ATGAGCCTGATCCGCACTGAAACCAGAGACACAAAACGCGCAGCTGACCCACTGCACGACCTGCGTAGCAAGCCGTTTTC
TGAGTGGGGCGAAGATGAAATCAGGCGTTTTAATCTGATAGACGCGCTTCTTGAGTTTGTCTACACAGACACATCCAGCC
CGTTCGGTATCGGCATGACCTTCGATTACACGGAGTGCTGGGAAATTGGTGTTCGTGACGACTGCCTGGTAATGACACGC
GTTAAGCCGGTGCATCCAGAATATGCGAAGCACTGGAACATGAAAGGCGTCATGAATGACAAAACCCGCTTCCACGCCGA
CAAGTGGGTAGGGTACAGCAAGGTGCTTGCCTGGGTGAGCTTATCGCACAAAGACACCTTCACGGGTGCGAAACGGTTTC
AATACTTCCAAACGATGTACGACATGGAACGGCAAATCAACGCAAACCTGCCGGTTGGCGGCCTTCCCAACGTTGACACA
GAGCGAACTGGCAAACTGTTCCAACGCGATGACTTTTCTGAGGATAGCCATGCCAACGATCCGAAGTTGGTTGGTGATGA
TTACGTCCCCCAGGCACCGGAGCAAATCAACTGA
>Cluster_60535
TTGCTGCTTGTCGGTTTCTTCTTTCTGGAGCCGCAGGGAATCCTGGTCAGTGCGATAGCGGCCGCTGGCGCAGCAGTGCT
GTTTGTGGTGGCGAAAAGAGGTCATTCGATCAACACCGGAAAAGTCCTGCGCGGTGCGCCATGGCAGATCGTTATTTTCT
CACTGGGCATGTACATCGTGGTCTATGGCCTGCGCAATGCAGGGTTCACGGAGTACCTGTCTGGCGTGCTGAATCTGCTG
GCAGACAAGGGGTTATGGGCAGCGACGTTCGGCACCGGCTTCTTGACGGCATTTCTCTCATCAGTGATGAACAATATGCC
GACGGTGCTGATTGGTGCGCTGTCGATTGACGGGAGTACGGCGACTGGCGTCGTCAAAGAGGCAATGATTTATGCCAATG
TGATTGGCTGTGATTTAGGCCCGAAAATCACCCCGATTGGCAGTCTGGCAACTCTGCTGTGGCTGCATGTGCTTGCCCAG
AAAAATATGACAATCACTTGGGGATATTACTTCCGTACCGGCATTGTCATGACTGTGCCGGTGCTGTTTGTCACTCTGGC
CGCGCTGGCGTGGCGGCTCTCTGTCACTTTGTAA
>Cluster_44470
ATGGAAGACGAAACGTTGGGATTTTTTAAGAAAACATCTTCATCTCATGCTCGCCTGAATGTGCCTGCGCTGGTGCAGGT
GGCGGCGCTCGCCATTATTATGATCCGTGGCCTCGACGTGCTGATGATTTTCAATACGCTGGGCGTGCGCGGTATTGGCG
AGTTCATTCATCGCAGCGTACAAACCTGGAGTTTAACGCTGGTCTTTTTAAGCAGTCTGGTGCTGGTTTTCATTGAGATC
TGGTGTGCGTTTTCACTGGTGAAAGGGCGTCGCTGGGCGCGCTGGCTATATCTGCTGACACAAATCACCGCCGCAAGTTA
CTTGTGGGCGGCTTCGCTGGGGTACGGTTATCCGGAGCTGTTCAGCATTCCCGGTGAATCAAAACGTGAAATCTTCCATA
GCCTGATGCTGCAGAAGCTGCCGGATATGCTCATCCTGATGCTGCTGTTCGTTCCCTCGACCAGTCGGCGGTTCTTCCAG
TTGCAATAA
>Cluster_22387
ATGAAGACCATTCTACCTGCAGTATTGTTTGCCGCTTTCGCCACCACTTCCGCCTGGGCGGCAGAAAGCGTCCAGCCACT
GGAAAAAATCGCGCCTTATCCACAAGCTGAAAAAGGCATGAAGCGTCAGGTGATTCAGTTAACCCCGCAAGAAGATGAAT
CTACCCTGAAAGTAGAACTGTTAATCGGTCAGACGCTGGAAGTCGATTGCAATTTGCATCGTCTCGGCGGGAAGCTGGAA
AGCAAAACGCTGGAAGGCTGGGGCTATGATTATTATGTCTTTGATAAAGTCAGTTCCCCGGTTTCGACGATGATGGCCTG
CCCGGATGGCAAAAAAGAGAAGAAATTTGTCACCGCGTATCTGGGCGATGCCGGAATGCTGCGTTATAACAGCAAGCTGC
CGATCGTGGTGTATACGCCAGACAATGTGGATGTGAAGTACCGCATCTGGAAGGCGGAAGAGAAAATTGACAACGCGGTA
GTCCGCTAA
>Cluster_3228
ATGCGAATTGGACACGGTTTTGACGTACATGCCTTTGGCGGTGAAGGCCCAATTATCATTGGTGGCGTACGCATTCCTTA
CGAAAAAGGATTGCTGGCGCATTCTGATGGCGACGTGGCGCTCCATGCGTTGACCGATGCATTGCTTGGCGCGGCGGCGC
TGGGGGATATCGGCAAGCTGTTCCCGGATACCGATCCGGCATTTAAAGGTGCCGACAGCCGCGAGCTGCTACGCGAAGCC
TGGCGTCGTATTCAGGCGAAGGGTTATACCCTGGGCAACGTCGATGTCACTATCATCGCTCAGGCACCGAAGATGTTGCC
GCACATTCCACAAATGCGCGTGTTTATTGCCGAAGATCTCGGCTGCCATATGGATGATGTTAACGTGAAAGCCACTACTA
CGGAAAAACTTGGATTTACCGGACGTGGGGAAGGGATTGCCTGTGAAGCGGTGGCGCTACTCATTAAGGCAACAAAATGA
>Cluster_23565
ATGGGAATAACGGCTCCTACTCCCCTAACGTCTGAACATAATCTGGCTGACTTTTGCTGTTCCGATCACGGGATGAATGA
ATGGCTAAAAAAGAAAGCGTTAAAAAATCACAGCTCCAGTCTGTCTCGTGTTTACGTTATCTGTATTGCCAATACCCGCC
AGGTAATCGGCTATTACTGCCTTTCTACAGGCAGTATTCAACGTAATTTGGCCCCTGGAGCCATGCGACGTAATGCTCCC
GAATCGTTGCCTGTTGTCGTATTAGGCAGACTGGCAATTGATCAAGCCTGGGCAGGTAAAGGGCTGGGCGTCGCATTGTT
GAAAGATGCAGTCTATCGCACAATGTCCATAGCTCAACAGGTAGGGGTTCGGGCGCTCATTGTTCATGCCTTAGACGATT
CAGTACGCAATTTCTATCTGAAGTATGCCTTTGTACCTTCACCATTCCAGTCACTTACCTTACTGTATCCCATTACTCTG
GAGTAA
>Cluster_127
ATGATAAGACAAGAAAACTTCGTCTACTCTTTTAAAATACAAGAAATGCAGCTTTATAAAATAAAGGAAGATGTTATAAT
TACCACCAAAGACTACTTATCAAGGAGGTTTAACTTGAAGTCCTGGATTATTTATGTATTTGGGAGATGGATATTACTTT
CTGGAATAGCTGGGGCCTTGATGCAGTATCTATTAAGTGATATGTTAAAAATCCATACCATACCCGCTTTTTTACTCAAC
CAATTCATCTTAGCTAATGTATTCTGGTTTATAGATAAAGCCATCTTTAAAGGTTATTTTAGTATTCCACATATTACTCT
CTGGCAGATAGAAAAAAACGTAAAGTGCGCTGATTGCGGGACTGTTGGCGAAGGTTATAGAGTAGTAAAAGCCAAAAACT
ATGATCGTTTGAACGACCCCGAACCCGAATTTAGATGTCCAACCTGCAGAGAACGTAAACTTGAAGAATTAAGAAAAAGA
GGTATAGAGGTGTAA
>Cluster_11024

ATGAACAGGGAAATTCAGCAGCGGCTACAGTGGGTAAAACTCTATGAAACGTCCGGTGATGCGGGATTTGTATGCCGTCG
CTGTGGCATTTCTCGGCCCACATTGCGTAAAATGGTGGCGCAGATATCTGGCTCAGGGCATCGCTGGTCTGGAAAGCCAC
AGCCGTCGCCCCAAGCGTTCACCATCAACGAAAACCGGCACTTGCGAAGTCGCTCTGATTCTGGAGTTACGTTCTCAGCG
AAATCTGGGGGCAAGACGTATTCAAAGCGAGTTAAAACGACTTCATTCAATCTCACTGGCAATAGCAACAATCCACAAAG
TCCTTTTCCAGAATCAGGTTTGAACCGCCCCGGGTTTCCTGGAGAGTGTTTATCTGTGAACTCAGGCTGCCAGATCATC
GTTTCCGATGGAAGCATAATAAGCTTTTTCTGCTTCTGCCGGAGGAGTATGGCCCAGCCTTTCCAGCAATCGTCGATTGT
TATACCAGTCCACCCACGTGA
>Cluster_8462
ATGACAAATAATGATACAACTCTACAGCTTAGCAGTGTTCTTAACAGGGAATGTACGCGAAGCCGCGTCCACTGTCAGAG
CAAAAAACGCGCCCTGGAAATCATCAGCGAGCTGGCGGCGAAACAACTTAGCCTGCCGCCTCAGGTGGTTTTTGAAGCTA
TCCTGACGCGGGAAAAAATGGGCAGTACCGGTATCGGCAATGGTATTGCCATTCCGCATGGCAAACTGGAAGAAGATACT
CTGCGCGCCGTTGGCGTTTTCGTTCAGCTCGAAACGCCTATAGCTTTCGATGCCATCGACAACCAACCGGTGGACTTACT
TTTTGCCCTGCTGGTGCCGGCAGACCAAACTAAAACGCACCTGCATACTCTGTCGCTGGTGGCGAAACGTCTGGCGGACA
AAACCATCTGCCGCCGTTTACGCGCAGCCCAGAGCGATGAAGAGCTGTATCAAATCATTACGGATACCGAAGGTACTCCG
GATGAAGCGTAG
>Cluster_48998
ATGCGTATCTTTGTTTTTTTTATATCTGCACTTTTATCTTTTAACTTGGCTGCGGAAGAGTGTAAGTTCAGCTTTAATGA
GTCAGAATTAATCTCTTCTATAGGTATTGCACCAGTTAAGCAAGAGATAATAAAGGATGAAGGAATAACTAAGCGGCAAT
ATGAATTCAGAAGAGAATTATCTTCTGAAGAAATGCTTAGTGATGACGCTGATGAAAAATATGAGCCGCAGTTTTATATA
TCTGTTTATAATCCATCATGCCCACAAAAGGTTATTGTTTGGTTTTTCAAAGACAATAAAAACACAATGGTTTAAGTAA
TGAGGTCCTTGCTGGTAGAGCATTCAAGTATTTAACTGGTGTTAATGAAAGTATTTTTGAAAATAAAATGAAAAAGTTTT
TAAAGGTACAGTCATTTGAATCTTTTGATGAAAGGACAGATTCTAAATTTATAAAAAGTGGTGATATTTATTCCATTGAT
GTTCAACTCAGATAG
>Cluster_37408
ATGAAAACGCCCCTGGTTACCCGGGAAGGGTATGAAAAACTCAAACAAGAGCTTAATTATCTCTGGCGTGAAGAACGCCC
GGAGGTCACAAAAAAGGTGACCTGGGCCGCAAGTCTGGGCGACCGCAGCGAAAATGCTGACTATCAGTATAATAAAAAGC
GTCTGCGTGAAATCGACCGTCGCGTGCGCTATCTCACTAAATGCCTGGAAAATCTCAAAATCGTCGATTACTCCCCTCAG
CAGGAAGGCAAAGTCTTTTTTGGCGCGTGGGTGGAGATTGAAAACGACGATGGCGTGACTCACCGTTTCCGTATTGTCGG
CTACGATGAAATTTTTGGCCGTAAAGATTACATCTCTATCGATTCCCCGATGGCCCGCGCATTGCTGAAAAAAGAAGTCG
GCGATCTGGCGGTGGTGAATACCCCTGCCGGGGAAGCGAGCTGGTATGTTAATGCTATCGAGTACGTGAAACCGTAA
>Cluster_10315
ATGAACATTATTGAAGCTAACGTTGCTACCCCGGACGCTCGCGTCGCCATCACCATCGCGCGTTTCAACAACTTTATCAA
TGACAGCCTGCTGGAAGGTGCAATTGACGCACTGAAACGTATCGGTCAGGTAAAAGATGAAAACATTACCGTTGTTTGGG
TGCCTGGTGCCTATGAGCTGCCGCTGGCGGCGGGTGCACTGGCTAAAACCGGTAAATACGACGCGGTGATTGCGCTGGGT
ACGGTTATTCGTGGTGGCACTGCCCACTTTGAATATGTCGCTGGTGGTGCAAGCAACGGCCTGGCGCATGTTGCCCAGGA
CAGCGAAATTCCGGTTGCTTTTGGGGTTCTGACCACTGAAAGCATTGAACAAGCGATCGAACGTGCTGGCACCAAAGCTG
GCAACAAAGGTGCAGAAGCTGCACTGACCGCGCTTGAAATGATTAATGTATTGAAAGCCATCAAGGCCTGA
>Cluster_12486
ATGCTAAGTAACGATATTCTGCGCAGCGTGCGCTACATTTTGAAAGCCAATAATAATGACCTGGTGCGTATTCTGGCGCT
GGGTAATGTCGAAGCCACCGCGGAACAGATCGCCGTCTGGCTACGTAAAGAAGACGAAGAGGGTTTTCAGCGTTGTCCGG
ACATTGTTTTGTCGTCATTCCTCAATGGCCTGATTTATGAAAAACGCGGCAAGGATGAGTCTGCTCCGGCACTGGAGCCG
GAACGTCGCATTAATAACAACATCGTGCTGAAAAAATTACGCATCGCGTTTTCGCTGAAAACCGATGACATTCTGGCTAT
CCTCACCGAACAGCAGTTCCGCGTTTCGATGCCGGAAATTACAGCGATGATGCGCGCACCGGATCATAAAAACTTCCGCG
AATGCGGCGATCAATTTTTACGTTATTTTCTGCGTGGACTGGCAGCGCGCCAGCATGTGAAGAAAAGCTAA
>Cluster_24403
ATGATGAATCGAGTAATTCCGCTCCCTGATGAGCAGGCAACATTAGACCTGGGCGAGCGGGTAGCGAAAGCCTGCGATGG
CGCAACCGTAATCTATCTGTATGGCGATTTAGGTGCAGGTAAAACCACCTTTAGCCGGGGCTTTTTACAGGCTCTGGGTC
ATCAGGGTAATGTCAAAAGCCCCACTTATACGCTGGTCGAACCCTATTCGCTCGACAATTTAATGGTTTATCACTTTGAT
TTGTACCGCCTTGCCGATCCCGAGGAGCTGGAGTTTATGGGGATCCGCGATTATTTTGCCAACGATGCCATCTGCCTGGT
GGAGTGGCCACAACAAGGTACAGGTGTTCTTCCTGACCCGGATGTCGAAATACACATTGATTATCAGGCACAAGGCCGTG
AGGCGCGCGTGAGTGCGGTTTCCTCTGCGGGTGAATTGTTGCTGGCGCGTTTAGCCGGTTAA
>Cluster_7953
ATGTACAAGCCGAAGAGTCTGCGAAAAGCCTTAACTGACGCTGTGCCGGTGCTGGCACGAAACCCCGATATGATGCGTGT
CTTTATCGACAACGGGAATCTTGCCTCGACGCTGGCGACGTCGCTGTCGTTTGAGAACCGGTACACGCTTAATGTGGTGG
TGACCGATTTCACGGATGATATCGAGCTGTTACTCGTCCCGATTCAGGCATGGTTGCGCATCCATCAGGCTGACATTATG
ACGACCGATGAAGGGCGTAAAAAGGGATTCACCTATTTTGCCGATATCAACGACAACGACAGCACCGATATCAGTATCAG
CCTGATGCTGACCGAGCGCACTATCGTTAAAGAGCAGGGGGATAAGCTCCACGTCGAACAGGCTGAAGAGCCGCAGCCGC
CGGAGCCCGTTACCCGGCCAGTTGAACTGTATGTTAACGGTGAGCTCGTGAGTCGCTTGCATGAATGA
>Cluster_65662
ATGCAAGCTATTCCGATGACCTTACGCGGCGCTGAAAAATTACGCGAAGAGCTGGATTTTCTGAAATCTGTGCGCCGTCC
TGAAATCATTGCTGCTATCGCGGAAGCGCGTGAGCATGGCGACCTGAAAGAAAACGCCGAATACCACGCAGCTCGTGAAC
AGCAGGGTTTCTGCGAAGGCCGTATTAAAGACATCGAAGCCAAGCTGTCGAACGCGCAGGTGATTGATGTCACCAAAATG
CCCAACAATGGGCGCGTTATTTTTGGTGCTACCGTAACGGTGCTGAATCTGGATTCTGACGAAGAACAGACTTATCGCAT
CGTTGGCGATGACGAAGCTGACTTTAAACAAAACCTGATTTCTGTAAACTCGCCTATTGCTCGTGGCCTGATCGGCAAAG
AAGAAGATGATGTTGTGGTCATCAAAACGCCGGGCGGCGAAGTAGAATTTGAAGTAATTAAGGTGGAATACCTGTAA
>Cluster_4799
TTGCGCTGGTTACCTTTTATTGCCATTTTCCTTTATGTCTATATTGAGATTTCAATCTTTATTCAGGTTGCCCATGTATT
GGGGGTATTGCTGACCCTCGTGCTGGTTATATTCACGTCAGTTATCGGGATGTCACTGGTACGTAACCAGGGCTTTAAGA
ATTTCGTGCTGATGCAGCAAAAAATGGCGGCGGGTGAAAACCCAGCGGCGGAGATGATTAAAAGTGTTTCGCTGATCATT
GCCGGTTTGCTGCTTTTATTACCGGGCTTTTTCACCGACTTCCTCGGTCTTCTGCTTTTATTACCGCCGGTGCAAAAGCA
TCTGACAGTGAAGTTGATGCCGCATTTGCGCTTTTCTCGTATGCCAGGCGGCGGTTTTAGCGCCGGGACCGGTGGCGGTA
ATACTTTTGATGGTGAGTACCAGCGAAAGGATGATGAGCGCGACCGCCTTGATCATAAAGACGATCGCCAGGATTAA
>Cluster_23446
ATGTTCGACGTACTAATGTATTTGTTTGAAACCTATATTCACACAGAAGCTGAGTTGCGTGTGGATCAAGACAAACTTGA
ACAGGATCTTACCGACGCAGGATTTGATCGAGAAGATATCTACAATGCCCTGCTTTGGCTGGAAAAGCTTGCGGATTATC
AGGAAGGACTGGCAGAACCGATGCAACTGGCCTCTGATCCACTCTCCATGCGTATTTATACCCCTGAAGAGTGTGAAAGG
CTGGATGCCAGCTGTCGTGGTTTTCTGCTTTTCCTTGAGCAAATTCAGGTGCTTAACCTTGATACCCGTGAAATGGTGAT
AGAGCGAGTGCTCGCGCTGGATACCGCAGAGTTCGACCTGGAAGACCTGAAATGGGTAATCCTGATGGTGTTGTTCAATA
TTCCGGGCTGCGAAAATGCGTACCAGCAAATGGAAGAATTACTCTTTGAAGTGAATGAAGGTATGCTGCATTAA

>Cluster_23764
ATGCTAAACATCGTACTTTACGAACCAGAAATTCCGCCAAATACTGGCAACATTATCCGTCTTTGCGCTAATACCGGCTT
TCGTCTGCATATCATCGAACCAATGGGATTTGCCTGGGACGATAAGCGCCTGCGCCGCGCGGGGCTGGACTATCACGAGT
TTACCGCCGTTACGCGTCATCATGACTATCGCGCGTTTCTCGAAGCAGAAAATCCCCAGCGCCTGTTTGCCCTCACCACG
AAAGGTACGCCTGCTCACAGCGCCGTAAGCTATCAGGATGGCGATTATCTGATGTTCGGCCCGGAAACACGCGGCCTGCC
AGCGAGCATTCTTGATGCCCTGCCCGCTGAACAAAAAATTCGTATTCCAATGGTGCCGGACAGTCGCAGCATGAACCTGT
CCAATGCGGTGTCGGTCGTGGTGTATGAAGCCTGGCGGCAGTTGGGGTATCCTGGAGCGGTGTTGAGAGATTAG
>Cluster_11068
ATGGCGGTGATTGGTTATTGCAGGGTATCAACACAAGACCAGACTGTAGACAACCAGAAACTACAGATAGAGAAGGTTCA
CAAGGTCGATAAATGGTTTATTGATGAGGCTGTCTCAGGAGCTACAAAGGCCACCAGCAGAGATGGTTTAGAGACATGA
TGAACTATGTAAGGTGTGACGATACGTTAGTTGTGGTAGCTATTGACCGTTTAGGGCGTAATACCGTTGATGTGCTCTCT
AACGTGGAGTCACTACAGACTAGAGGTGTGAGGGTTGTTTCTCTACGTGAAGGTTTTGACCTGTCTACACCAATCGGTAA
AGCAATGCTTACTATGATGGCTGGTTTAGCTGACCTTGAAAAAGACCTTATCAGTGAACGTAGAAAGGCTGGTATTGAAC
GTGCTAAAGCTGAAGGGGTACATATGGGGAGGCCTGCAAAAGCTTCTTCAGAGGCTGTACAGACGCTTATTTCACAAGGT
AAGACACGGTTGCAGATTCAGGAAGAATTAGGAATCTCTAAAGCAACTTATTACAGACTTGCTAAATAA
>Cluster_40889
ATGAAGAAGATTTTACTCGTTGTAGGCGCAACTCTGGCACTGGCGGGATGTGGTGAGAAAGGTGATTTTGAGAAGGCGAT
TAACGCGAAGATATCAAAATCTAAGGTGTGCTACTCACTACAGGACAATGGCCGCGTGTTTAACAAAGGTTTCCCTGTTC
GTATCAATCGTGGATATCGCTCTGACGGATACAATGAAAATGATGAAATTTTAAAAGGTATGGCTAATCAGGGATTGTTT
GATGTTTCGCAGGAATCCAACAGTCTTAGCAACATTGACGTTCTGGAAGTAACAGATAAGGGGCAAGAGGTTGAGTTTTG
GGATCGCAAAAAATGGAGCCTGTGTGGGTCATCGTGCCGTGGCTGAAATTAAAGAATGGACTGAAGCCGATGCAAGTGGCA
TGAAGATTGTTCGTGTGTCGTATACGTGGAAACTTGTTGATATTCCTACATGGGTAGATAAAAGTGCATTCGCTGGCGTC
AGGGGAATGACTGAACCTGCTGAAGGTCTTATTTCTCTTGTAAAGACCAGTAATGGTTGGAAAGCAAACTAA
>Cluster_18
ATGGATAAGGTGTTATTAATAGGTTTTAGGGCGGTAGGCAAAACTACCGTAGGTAGACTCCTTAGTAGAGCTTTAAATTG
GGATTTTATAGACACAGACCAAGAGGTTCAAAACATTTCCGGGAAAACGATAAAAGAGATAGTCGAAACCGAAGGATGGG
AGACTTTTAGGAGGATTGAAAAAGAGGTTTTGGTGAGATTGGTTGATAGAAAAAAAATAGTGATTGCTTTAGGTGGGGGA
GGAATTTTACATCAACAAGAAATCAAAGCTTTAAAATCAAAAGCATTGGTTGTATGGCTTAAAGCCTCACCTGAAACCAT
TATAGAAAGAATGCTTAAAGATGAAAAAACCCTAAAAGAAAGACCTAAGCTTACCTCTGAAGATTTAACTTCAGAGGTTC
TTCAAGTTTTAAAATTGCGAGAGCCTTTATATCGTGAGTTTGCAGACTTAAGTATAGATACCGAGCAAAAGTCTCCAAAA
GAGATACTGGATTTAATCCTTAAACAATTGAAAAACCAAAATATGGTAGGGGAAATTATATGA
>Cluster_19619
ATGAAGACCAAAAGAAAGAGCTTTGATCAATTCTTATGGCATGATATTGATTTTGAAGATGATGCTGCAAAACAGGAAGA
ATACCTTGAATCTGCATTGCCACTTATTGGCCAGATTGTTATGTATTTCAATGCGCTAGAGAAAGAATTAGATAAAGTCA
TTTATGAAGGATTCAGCGATCGGTCAGATCAGTTAGGAATAATAATTGTGCATAACATGAATTATGCAGCAAAAGTCGAG
TTATTTAGACGTCACTCCGAATTATTCCATTTGGGTGTTGGGCAGCATGTTGATATGTTTGAAGGTCTTATAAAGGATCT
CAAGGAATGTGGACGGTTACGAAACATTACTGTACATGCTGACTGGGATAGCACTGATGTCGAAGGATATACATATTTAG
GCATCAAAATAACTAACAATGGAATATCTCAGGAATATATCCAGTTTGACCTGGGGTCGTTGGAAAGTATCCTTCAATTG
ATTTTCAGTACGAGATATAAGTTGAGTGATTATTCAGACTTAAGGGGAGATATACTGGCTTCACATTAG
>Cluster_42023
ATGTTACGCGATAAATTTATTCACTATTTTCAGCAATGGCGTGAACGCCAGTTAAGCCGTGGCGAACACTGGCTGACACA
ACACCTGGCGGGGCGTTCTCCGCGTGAAAAAGGCATGTTACTGGCAGCGGTAGTGTTCCTGTTTAGCGCCGGATATTACG
TTCTCATCTGGCAGCCGTTGAGCGAACGGATTGAGCAACAGGAGACGATGTTGCAGCAGCTCGTGGCGATGAACGCGCGA
CTGAAGAGCGCCGCGCCGGATATTATTGCGGCGCGAAAATCCGGCACGACAACGCCAGCACAGGTATCGCGGGTCATCAG
CGACAGCGCTTCCGCGCATTCTGTGGTCATCAAACGGATTGCCGAGCGTGGGGAGAATATTCAAGTCTGGATAGAACCTG
TGGTGTTTAATGACCTGCTGAACTGGTTAAACGCGCTGGATGAAAAATATGCGCTGCGAGTGACACAAATTGATGTCAGC
GCAGGTGAGAAGCCTGGGATGGTGAATGTGCAGCGGCTGGAGTTTGGGCGGGGTAAAGTATCAGGAGATTAA
>Cluster_1547
ATGATATACCCGGAAATTACAGGCAAAAGCGGTGAGCATTTACGCCTGAAAACGCTGGAAAGTGTCTGGATCCAGGGGAA
ACTGCGTATGTGGGGGCGTTGGTCGTATATTGGCGACGGTAAGACGGGAAATATGTTCAACCAATTACTGACCTCTAAAA
AGCTGACAAAAACGGCAATTAACGAGGCGCTCCGGAGGATGAAAAAAGCGGGTCTGGACAAACCTGAACTTGAGGCTTTT
TTGCGGGATATGATCAACGGCAATCAAAAAAGCTGGCTGGCACATTGTACCGATTCAGAGGCGTTAATAATCGACAGGGT
TATTGGTGAAGTACTGGCAGGTTATCCCGGGCTGCTCAATGTTCTGAGTCAGCGTTATGTGGGGCGGGGGATGACTAAGC
GCAAAATGGCTGAACTGCTGAATGATGCACATCCGGAATGGAGTTTAAGAACCTGTGAAAGACGCATTGAGCATTGGCTA
AAGGTGGCAGAATTTATTTTGTACAAACCAATGGTTATGGCTTTTGGTATAGAGAAAAAAGTTATTGCTTTTTGA
>Cluster_44893
ATGAAAATCACCCCTGAACAGGTTTGTGAGGCTCTGGATGCCTGGGTATGCCGACCAGGAATGACACAGGAGCAGGCGAC
GATATTAATCACGGAAGCATTCTGGGCTCTGAAAGAACGCCCGAACATCGATGTTCAACGCGTCACGTTTAATGATGGCG
AGGTTGATCAACGGGCGCTGTGCGTTAACCGGGTGAAGATATTCGAACGCTGGAAAGCTATCGACACCAGGGATAAGCGG
AAAAAATTCACGGCGCTGATTCCGGCAATTATGGAGGCTATCCGAATTAGTGATTTCAGGCTGTATCGTGAGATCAGTGA
TGGAAAAAGCATTACGTACATGATCGCCGGATTAAACAAAGAATATGGCGATGTGGTGGAGTCCGGGCTGCTTTTTGCGG
ATCCATCTGTTGTGGAACGTGAGACTGACGAGCTTATAGAAAAAGCTATTGCTTTCAAGCATGCGTATCGTCAGCAATAT
CAATATTACTTTGCAGATAAACAAATGTCTGCCAGGGGTTCGTATGAGTATCGATGCACTACGATGGGCTAA
>Cluster_26916
ATGATGGTGCAGCACCTGAAAAGACGGCCATTAAGCCGCTATCTTAAAGACTTTAAACACAGCCAGACCCATTGCGCGCA
TTGCCGTAAATTACTCGACCGCATTACCCTCGTTCGCGACGGCAAAATAGTGAATAAAATCGAGATTTCCCGCCTGGACA
TGCTGCTTGATGAAAATGGCTGGCAAGTGGAACAAAAATCATGGGCGGCATTGTGCCGATTCTGCGGTGATTTACATTGC
AAAACGCAGAGTGATTTTTTCGATATTATCGGCTTTAAGCAATTTCTTTTTGAGCAAACTGAAATGAGCCCAGGTACGGT
GCGTGAATATGTCGTTCGTCTGCGCCGTTTGGGTAATCATCTACACGAGCAAAATATTACCCTCGAACAGCTGCAGGACG
GTTTCCTTGATGAAATCCTCGCCCCGTGGCTGCCCACCACCAGCACCAACAATTACCGCATTGCGTTACGGAAGTATCAA
CACTATCAGCGTCAAACGTGTACCGGACTTGTGCAGAAATCCTCCAGCCTGCCTGCTTCTGATATATATTAA
>Cluster_28844
ATGACAACGCTGACCATTAACCGGAAGCCGAAAGGCATTTACGGCACGCCGCAGAAAACGACGCAGGCGGCACAAGAGCA
GGATAAAACCACATCGGCGCATAAAGTGATACCCGGCAATCAGAACGCACAGCAGAAGCCCACAGGGGCGACACCGTGGC
AGCATATGACCAAAAGGCAGCGCAAAAACCGCCGACGCGTTAACCGCCTCACTGAGATGTGGCCTGACTTATTCAGCCAT
AAAGCACCGAAGCCGCTTAAGGTGGGGATATTCGACGACCTGATGCAGGATATCGCCGTCAGGAGGCTGGCATTCGGGCC
GGGGGTATTACGTGCGACGCTGGCATCCTATGCGCAGTCTCCGCGCTATTACCGCGCCCTGATAGCTGGTGGCGCACGTT
ACGACCTGAAAGGCCAGCCATGTGGGGAAGTGACACCACAGGAACAGCAGGAGGCAGAAACACGGCTGATGGTGCTGAAT
GAGAAGCACAAGCGCCAGCGCCGGGCAGCAAAGGGGGATACATGCCAGTGA

>Cluster_17509
ATGAAAGAAATAAAATTAACGCCAGAAATGGTGCTTTCTGTTGTTGATTACAATCCATCATCAGGCGACTTTCACTGGAG
ATGGAGGCAGGGGAGAGAGAGGACCACTTTGACATGGAACTCTCGTTTTGCTTTCAAGAAATGCTCATCAATAAATTCTG
ATGGGTATTTAATGATTATGATTAATGGTAAAGCATACCCTGCTCACAGACTGGCATGGTTGATTGTTTATGGCACCATG
CCCGATGGTTTTATTGATCACATCAACAGGGTAAGAACAGATAACCGGATATCAAATCTTCGTCTTGTCACTCATTCCGA
AAATATGCAGAACAGGAAAATTCAGAAGAATAATAAATCTGGATACCGTGGCGTGTCTTGGGATGCTAAGTACGGGAAAT
GGAGAGCAAGAATTAATGCGTCTGGAAAGTGTATTAACCTTGGATACCATGACACTGCCGAACTTGCCGCTGCGGCTTTT
GAGGCCGCCAGAATTAAATATCATACCGTTTAA
>Cluster_5159
GTGATCGCTGAATTAACGGCAGCAATGACGGCTATTCGTGAAACAGCCCAGATTGCAAAACTAATGAACGAGGCAAAAAC
TCAAGCTGAAGTAAATGCGGCTATTGGTGAGCTGAACTCAAAGCTTGCGTCTATTCAGCGCGAATGCGTGTCTCTCGTTG
AACTGGTGGGCTCTTATCAAGAAATAAATGCTTCTCTCAAAGCTAAAATTGCAGAATTCGAAAACTTTGAGGCTCAGACG
GAAGGCTATATCCTTAACCAACTTGAGTCGGGTACTTTTGTATACTCGAAGGAGGTAATCGTGAACGGTGGCAGCATAAC
CATGCATCTTTGCCCAAAATGTTTTGGACAAAAGATAGTATCGATACTTCAACCATTCCCGGTTAGCGAAGATGAGCTTT
TTCATAAAAGCAGGTGCCTCCACTGTGAAAATAAGTTTCTGATGAATAAAAATCCGGATTACGTATCGCCTCCATCCATT
GAGGAGTTGTCCAGAAAACTTAACGGCAATCTGTAG
>Cluster_52339
ATGCTAATTCGAGTAGAAATTCCCATTGATGCGCCGGGTATTGATGCCCTGCTGCGTCGTTCATTCGAAAGTGATGCGGA
AGCGAAGCTGGTTCACGATCTGCGTGAAGATGGCTTTCTGACGCTGGGGCTGGTGGCGACAGATGACGAAGGTCAGGTCA
TTGGCTATGTGGCATTTAGTCCGGTTGATGTGCAGGGCGAAGACCTGCAATGGGTCGGCATGGCACCGTTGGCGGTAGAT
GAAAAATACCGTGGACAGGGGCTGGCACGCCAACTGGTCTATGAAGGACTCGATTCGCTTAATGAGTTCGGCTATGCCGC
AGTGGTGACGCTGGGCGATCCGGCGCTTTACAGCCGTTTCGGCTTTGAACTGGCGGCACATCACGATCTGCGTTGCCGCT
GGCCGGGCACTGAAAGTGCCTTCCAGGTACATCGTTTAGCGGATGACGCGCTGAACGGTGTAACTGGTCTGGTTGAGTAT
CACGAGCACTTCAATCGCTTTTAA
>Cluster_3297
ATGGCCGACAGTTTCCAGAATGAAGTTCCCGCCGCTCGTGTAAATATCAAGCTTGACCTGCATACAGGCAATGCTAAAAA
GAAAGTTGAACTCCCCCTCAAGCTTCTTGCTGTTGGCGATTACAGTAACGGAAAAGAGCAACGTCCGCTGTCCGAACGGG
ACAAGATTGATATCAATAAAAACAACTTCAACAGTGTCATGGCTGAGTTTTCGCCTGCGGTTAATTTAACAGTAGAAGAC
ACACTAAGCGGAAGCGGTAATGAGCAAAATATCGCGCTTGAATTTAAAAGCCTGAAAGATTTTGAACCCGAACAGGTAGC
AAAAAATATCCCACAGCTTCGTGTATTACTCGCTATGCGCAATTTATTACGTGACCTGAAATCCAATCTGCTGGATAACG
CAACGTTTCGCCGTGAACTGGAAAATATTCTGAAAGACCCAACGCTCAGTTCAGAACTGCGTGATGAACTGGCGAAATTG
CGCCGCAGGAGAACGTTTAACCCGAAGTGA
>Cluster_20875
ATGCAGCATTTAAAAAATATTACTGCGGGTAATCCCAAAACTGTTGCCCAATATCAACTGACAAAAAATTTTGATGTTAT
CTGGTTATGGTCTGAAGACGGAAAAAAATGGTATGAGGAAGTAAGTAATTTTCAGGAAGACACGATAAAGATTGTTTACG
ACGAGAATAATATAATTGTCGGCATCACCAGAGATGCTTCAACGCTTAACCCTGAAGGTTTTAGCGTTGTTGAGGTTCCT
GATATTACCGCAAACCGACGTGCTGATGACTCAGGTAAATGGATGTTTAAGGATGGTGCCGTGATTAAGCGGATTTATAC
GGCAGACGAACAGCAGCAACTGGCAGAATCACAAAAGGCAACTTTACTTTCCGAAGCTGAATCCGTGATTTTGCCGCTGG
AGCGCGCTGTCAGGCTGAATATGGCAACAGATGAGGAGCGCAGTCGACTGGAGGCATGGGAACGCTACAGTGTTCTGGTC
AGCCGTGTGGATCCTGCAAATCCTGAATGGCCGGAAATGCCGCAGTAA
>Cluster_44255
ATGGCTATCACTGGCATCTTTTTCGGCAGCGACACCGGTAATACCGAAAATATCGCAAAAATGATTCAAAAACAGCTTGG
TAAAGACGTTGCCGATGTCCATGACATTGCAAAAAGCAGCAAAGAAGATCTGGAAGCTTATGACATTCTGCTGCTGGGCA
TCCCAACCTGGTATTACGGCGAAGCGCAGTGTGACTGGGATGACTTCTTCCCGACTCTCGAAGAGATTGATTTCAACGGC
AAACTGGTTGCGCTGTTTGGTTGTGGTGACCAGGAAGATTACGCCGAATATTTCTGCGACGCATTGGGCACCATCCGCGA
CATCATTGAACCGCGCGGTGCAACCATCGTTGGTCACTGGCCAACTGCGGGCTATCATTTCGAAGCATCAAAAGGTCTGG
CAGATGACGACCACTTTGTCGGTCTGGCTATCGACGAAGACCGTCAGCCGGAACTGACCGCTGAACGTGTAGAAAAATGG
GTTAAACAGATTTCTGAAGAGTTGCATCTCGACGAAATTCTCAATGCCTGA
>Cluster_63037
ATGCAACTGAACAAAGTGCTGAAAGGGCTGATGATTGCTCTGCCTGTTATGGCAATTGCGGCATGTTCTTCCAACAAGAA
CGCCAGCAATGACGGCAGCGAAGGCATGCTGGGTGCCGGCACTGGTATGGATGCGAACGGCGGCAACGGCAACATGTCTT
CCGAAGAGCAGGCTCGTCTGCAAATGCAACAGCTGCAGCAGAACAACATCGTTTACTTCGATCTGGACAAGTACGATATC
CGTTCTGACTTCGCTCAAATGCTGGATGCACATGCAAACTTCCTGCGTAGCAACCCGTCTTACAAAGTCACCGTAGAAGG
TCACGCGGACGAACGTGGTACTCCGGAATACAACATCTCCCTGGGTGAACGTCGTGCGAACGCCGTTAAGATGTACCTGC
AGGGTAAAGGCGTTTCTGCAGACCAGATCTCCATCGTTTCTTACGGTAAAGAAAAACCTGCAGTACTGGGTCATGACGAA
GCGGCATACTCCAAAAACCGTCGTGCGGTACTGGTTTACTAA
>Cluster_7587
ATGACTATCAAATCAAATACGCCATCACACGACAAGGACTGCTGGCAAACGCCGCTTTGGCTTTTTGATGCACTGGATAT
TGAGTTTGGATTCTGGCTGGATTCAGCTGCGAGCGACAAAAATGCTCTGTGCGCTCACTGGCTAACTGAGGCCGACGACG
CGCTAAATTCTGAGTGGATAAGCCACGGTGCAATCTGGAATAACCCACCGTACAGCAATATCAGGCCGTGGGTGGAAAAA
GCCGCTGAGCAGTGCATACAACAGCGACAGACGGTAGTGATGCTTGTGCCAGAGGATATGTCAGTCGGATGGTTCAGCAA
GGCTCTGGAGAGTGTCGACGAAGTTCGCATTATCACTGATGGACGGATTAATTTTATCGAACCATCGACAGGGCTGGAGA
AGAAGGGAAACAGCAAAGGCTCCATGCTGCTGATTTGGCGACCGTTCATCAGTCCTCGACGAATGTTTACTACTGTATCC
AAAGCGGCATTGATGGCGATCGGGCAGGGCGTCAGGATGGCGGCATGA
>Cluster_16406
ATGGGCAAAGGTGGCGGCAAGGCGCACACACCGCGTGAGGCGAAAGACAATCTCAAATCCACGCAGATGATGAGCGTGAT
TGATGCGATTGGTGAGGGACCGATAGAAGGCCCGGTGAAAGGCCTGCAGAGTATTCTGGTGAACAAAACCCCGCTGACGG
ACACGGACGGTAATCCCGTGATACACGGTGTGACGGCGGTCTGGCGCGCCGGGGAGCAGGAGCAGACACCACCGGAAGGC
TTTGAGTCCTCCGGAGCTGAAACCGGACTGGGCGTGGAAGTGACGAAGGCAAAACCGGTGACGCGCACCATTACGTCCGC
GAACATTGACCGCCTGCGGGTTACCTTCGGGGTGCTGTCACTGGTGCAGACCACGTCAAAGGGCGACCGTAATCCTTCCT
CTGTCCGGATTCTGATTCAGTTACAGCGTAATGGCCGCTGGGTGACGGAAAAGGACGTCACCATTAACGGCAAGACCACC
TCACAGTTCCTGGCCTCGGTGATTCTGGATAATCTGCCTCCCCGGCCCTTTAACATCCGGATGGTCAGGGAGACGGCGGA
CAGCACCACGGACCAGCTGCAGAATAAGACGCTGTGGTCGTCATACACCGAAATCATCGATGTGAAACAGTGCTACCCGA
ACACGGCCATTGTGGGGCTGCAGGTGGATGCGGAGCAGTTCGGCGGCCAGCAGATGACGGTGAACTACCATATCCGCGGT
CGCATCATCCAGGTGCCGTCAAACTATGACCCGGAAAAACGCACGTACAGTGGTATCTGGGACGGCAGTCTGAAACCAGC
ATACAGCAACAACCCGGCCTGGTGCCTGTGGGACATGCTGACTCACCCGCGCTACGGCATGGGAAAACGTCTGGGGGCGG
CGGATGTGGACAAGTGGGCGCTGTATGCCATCGGGCAGTACTGCGACCAGACGGTCCCGGATGGTTTCGGGGGGACAGAG
CCGCGGATGACCTTTAATGCGTACCTGGCACAACAGCGTAAGGCGTGGGACGTTCTCAGTGATTTCTGCTCTGCGATGCG
CTGTATGCCGGTATGGAACGGTCAGACGCTGACGTTCGTTCAGGACCGCCCGTCGGATGTGGTGTGGCCGTACACCAACA

GCGATGTGGTGGTGGATGATAACGGCGTGGGATTCCGCTACAGCTTCAGTGCCCTGAAGGACCGGCACACGGCGGTGGAG
GTGAATTACACCGACCCGCAGAACGGCTGGCAGACCTCCACGGAACTGGTGGAAGACCCGGAAGCCATACTGCGCTACGG
ACGCAACCTGCTGAAGATGGACGCGTTCGGCTGTACCAGCCGCGGTCAGGCCCACCGTGCCGGACTGTGGGTGATAAAGA
CCGAACTGCTGGAAACGCAGACGGTGGATTTCACGCTCGGGTCTCAGGGGCTGCGGCACACACCCGGTGACATCATTGAA
ATCTGTGATAACGACTATGCCGGGACCCTGACCGGCGGACGTGTCCTGTCCATTGATGCTGCCACCCGCACCCTGACGCT
GGACCGTGAAGTGACACTTCCGGAGACCGGTGCCGCCACGGTGAACCTGATTAACGGCAGCGGTAAGCCGGTGAGTGTGG
ACATCACCGAACACCCCGCGCCGGACCGGATACAGGTCAGTACCCTGCCTGATGGTGTGGAGACATACGGGGTGTGGGGA
CTCTCCCTGCCGTCACTGCGCCGTCGCCTGTTCCGCTGTGTCTCCGTCCGGGAAAACACGGACGGCACCTTTGCCATCAC
GGCGGTGCAGCACGTACCGGAAAAAGAAGCCATCGTGGATAACGGTGCCCGCTTTGAGCCGCAGTCAGGTTCCCTGAACA
GCGTCATCCCACCGGCAGTGCAGCACCTGACGGTGGAGGTGAGCGCAGCTGACGGCCAGTATCTGGCGCAGGCGAAATGG
GACACGCCGCGGGTGGTGAAGGGTGTGCGCTTCAGTCTGCGCCTGACCAGTGGTAAGGGAACGGATGCCAGACTGGTGAC
CACCGCCATCACCGCAGACACGGAGCACCGTTTCAGCGGCCTGCCGCTCGGGGAATACACCCTGACGGTGCGGGCGATAA
ACAGCTATGGCCAGCAGGGTGAACCTGCCACCACCACCTTCCGGATTGCCGCACCGGCAGCACCGTCGCGGATTGAGCTG
ACGCCGGGCTATTTTCAGATAACCGCCACGCCGCATCTTGCCGTTTATGACCCGACGGTACAGTTTGAGTTCTGGTTCTC
GGAAAAGCGGATTGCGGATATCAGGCAGGTTGAAACCGCAGCCCGCTATCTTGGCTCGGCGCTGTACTGGATAGCTGCCA
GTATCAATATCAAACCGGGCCATGATTATTATTTTTATATCCGCAGTGTGAATACTGTTGGCAAATCGGCATTTGTGGAG
GCTGTTGGCCAGCCGAGTGATGATGCATCCGGCTATCTGGATTTTTTCAAAGGAGAGATAGGGAAAACCCATCTGGCTCA
GGAGTTGTGGACGCAGATTGATAACGGTCAGCTTGCGCCTGATCTGGCTGAAATCAGGACGTCCATTACGAATGTCAGCA
ATGAAATCACGCAGACCGTCAATAAAAAACTGGAAAATCAGAGCGCGGCAATCCAGCAGATACAGAAAGTTCAGGTTGAT
ACAAATAATAACCTGAACAGCATGTGGGCCGTGAAACTGCAGCAGATGAAGGACGGACGCCTTTATATTGCGGGTATCGG
AGCCGGTATTGAGAATACGCCAGCAGGTATGCAGAGTCAGGTGCTTCTGGCTGCTGACCGGATTGCGATGATTAATCCTG
CGAATGGCAACACAAAGCCGATGTTTGTTGGTCAGGGCGATCAGATATTCATGAACGAAGTGTTCCTGAAATACCTGACG
GCTCCCACCATTACCAGTGGCGGTAATCCTCCGGCATTTTCCCTGACACCGGACGGGCGGCTGACGGCGAAAAATGCCGA
TATCAGCGGTAACGTGAATGCGAACTCCGGGACGCTCAACAACGTCACGATTAACGAAAACTGTCGGGTTCTGGGAAAAC
TGTCCGCCAACCAGATTGAAGGCGATCTCGTTAAAACAGTGGGCAAAGCTTTCCCCCGGGACTCCCGTGCACCGGAGCGG
TGGCCATCAGGGACCATTACCGTCAGGGTTTATGACGATCAGCCGTTTGACCGGCAGATTGTTATTCCGGCGGTGGCATT
CAGCGGCGCTAAACATGAGAAAGAGCATACTGATATTTACTCCTCATGCCGTCTGATAGTGCGGAAAAACGGTGCTGAAA
TTTATAACCGTACCGCGCTGGATAATACGCTGATTTACAGTGGTGTTATTGATATGCCTGCCGGTCACGGTCACATGACA
CTGGAGTTTTCGGTGTCAGCATGGCTGGTGAATAACTGGTATCCCACAGCAAGTATCAGCGATTTGCTGGTTGTGGTGAT
GAAGAAAGCCACCGCAGGCATCAGTATCAGCTGA
>Cluster_48596
ATGAAGGCTCTGATGGTTCGAACTGACTTCTCTCTTGGGGAGTCGGCTCTAAAAGCAGAAAACGCGGTGAAGATTGCCAG
AGAAGCTGGCTACACCGCTGTAATTTCGGCAGATAGCATGAATATTGCGAGTGTTATTCCACTACAACGTGCCGCTGGTG
ACGACATGGCGGTTATTTGTGGTGTGAAACTAAACATTGTTGATGATCCCACATACGAGCACCGGGCTAAACTTGCTAAA
GAGTCTATGGGATGTATGGAATCATTAGAGCGTGGGCGGAACTACTCCTTTACCGCTCTAATTAAAAATGAGCAGGGATA
TCGAGACATCTGCGAACTAATGACGGCGGCCAACACACGAGAACAGTTCTACTTTGTACCGCGTCTCTCGCTCGAACAGT
TGGTTTCTACATATGCCAAAGGCAACATCATCCTGCTCACTGCCGACATCGGTAGCGTGTTCCAACGCAACGATTTTGCA
AAAATCATAAGCTCACTGATTACAGCGGGTGGAAAAGACAACTTCTATAGTGTGGTTTATCCGCACCCTACCCCATTCTA
CGACCAGATTAACGTCCGGGCGATGAAAGTCGCCAGCGCACTGAAAATAGAGCCAGTAGCGTTCTATCCCGCTTATTACG
AATCGATCGACGATGCAGACATTAAAGACATTGCGCACATGGTTACGAACAACATCAAAATCGACCAGCCGCATCGTCTG
CGTATCCCCCACCAGCGAGATAACGCCGTCAATGGTCGCCGCCATCTCCTTGAGGCGCTGAAAGCCTTCTCCGTTCGCAT
GGATGTGCCGGTAACAGCTGCAATGGCCTCAACAACGCAGGATACCATTATCGATGCCTGCACATGGCGCTGGCATGAAT
TGCCACCAGCACTGCCAAAGATGGCAGACGACGAGCCTGCAACGCTGATGAAACTGGCCGTTGCTGGGCTGCGTAAACGT
CTTACCACAAAAGAGTTTGGCTACACACCACCGGCTTCTGAGAACAGGGTTTATGTTGAGCGACTTAAGTACGAAATGGA
CACGCTTACTCGCCTGGGATTTTGTGGTTACTTCCTGATGGTACGCGATCTGATGAATCACAGCCGTGAAACTGGCATTC
CTGTCGGGCCTGGTCGTGGTTCCTCCGCTGGCTCTTTGGTGGCATGGTGCATAGGCATAACCAACGTCGACCCTATCCGT
CACGGTCTTCTGTTTGAACGTTTCATCAACCCTGAGCGTCTCGACTTGCCAGATGCGGACTTGGACTTCAGCCAGGCACG
TCGCCATGAGGTGATCGAGTATCTGAATGAACGCTACGGCGAAGATTACGTTGCAGGCATTCCGAACTTCACCTACCTGG
GCGCGGCCTCTGCACTACGTGACACCGCTCGTATTTATGGTGTGGAGTCCGCAGATATGGCGGTATCAAAAGAACTGAAG
AACGTCGAGGATGATAGCCTTCCATTGGAAGAGCTGCGCGAACAACTGGCAAGCCTCGACAAATACGCGACAAAATATCC
TGATGCATTCAATGCAGCCTGCAAGTTACAAAGCCTTATGCGTGGCTATGGTAGACATGCGGCAGGGATGATCGTAGCAG
GTGTTCCTCTGACAGAACGAACACCGGTTGAACGACGTGGTGACGCGCGTTGTATCGCATTTGACAAGCGTTACTGCGAG
GCTATGGGCCTAATTAAGCTGGACGTACTTGGCCTGGCGACTCTCGATTTGCTCGATAGTGCAAAACGCTACATTAAAGA
GAACACAGGTGAGGATATCAATCTTGATGCCATTTCTCTTGAAGATCGCAAGGTGCTGGATGGTTTTGCTGCTGGGTACA
CTCAAGGTGTTTTCCAGCTTGAATCAGGCCCAATGCGCAAGCTGCTTAAAGATTTAGGTGGTGGAATTGAGCCAATGAGC
TTTAAAACGGTCGTTGCTACGACTGCGCTCTTCCGGCCGGGGCCAATTCAGTCAGGCATGTTGGATGATTATGTTTCTGT
CGCCAAAGGCTTTATGACGCCGGAATCATTACACCCCGTTCTTGATGAACTTACCGCAGAAACAAATGGCGTGATTCTTT
ATCAGGAACAGACGATGAACGCGACTCGATTGCTTGCGGGCTTCACAATGGCTGAAGCTGACGGCGTTCGTAAAGCGATC
GGCAAAAAAGACATGGAGAAAATGAAGAGCATGGGTGAGAAGTTCATCGTTCAGGCTCAAGCTGGCTGGATAGACGTTGA
GCTGGAAGATGGCACTACACAGCGCATTCACCGTGCGGAACATTTTAAATGCGAAGACGGAACACTGAAAACTGTCGAAG
AGGCGCTTGAACACGGCGCAAAACTACCTATAAACGCAGTACGCGTGACAGCGTCACACCCAGGGCTATCAGAGATGAAA
GCGAAGGAGATCTGGACCGCATTCGAAAAAAATGGTGCCTACCAGTTCAACAAATCACACTCTGTTGCTTACTCCTTGAT
CAGTTATCAATCTATGTGGTTGAAAACTCATTATCCCGCAGAGTTTTTCGCTGCTGCTCTCACTATTCTTGGCGAAGATA
AACACCAGGGGCTGGTTAAAGATGCGCTGACCTATGGTATTCGCGTATTGCCACCAGACGTTAACGTGTCATCCAACCGA
ATCGAGATCCGCACGCTTGAAGATGGCAGCCAGGTGCTGTATGCGCCCTTCTCTGCTGTGAAGGGGTGTTCTGAGAATGG
CTGCCAAGCCATCATGAGAGCGCGAGAAAAAGTTGGCGGCAAATTCGAGTCACTGGCGCAATTCGAAGAAGCTGTCGAGA
AACGTGCCTGTAATAGTCGAGTGCGCGAATCGCTGCAAAAAGTAGGGGCGTTTGCATCCATCGAGCCAGGTAGTCTGCCA
GCAACTGATCCAGAGCGCCTACGCGACCAGGCTGAATTGATGGGAAACCTTGTCATAGACGCAGTTAAAGCCTCACGTCC
GTTTGAGATGAACCCCAAACGTTCGGCTGAGATTAACGTGCTCATGACACGGATGGCGGCTGAAATGGGCTTAGGTGATG
AACTAATCCGCCCCAGCATTGGTATTAAGCCGAAAATCATGATCATTCTGGACAATGCGAACGGCAATGATGCTCGAACC
GGTTACTTTATGGAGAACGGATACGACGATTTTAAGGCCAAGCTATTGACGGCTGGAGATTTACGCATGGGCGATCTCTA
TGTCACAGGCGTTTGCAAGAAGGTTAAAGACAAAGAAAAAGACTACACCAAAGACGAGATCGGCCAGTTCACCGACTTTA
TGCGTGAAGAGATCAATCTGGTGCGTCCAACCTATGTGCTGACGTGTGGCAGCCGGGCGACATCGCTCTTCAACAACAAG
AGCAAACCATCCGACCTGGTTGGACGCAAAGAGTATCTGCCGGAGCTGGATGTGACTGTTTTCTACGGATTTAACCCGAA
TATTTTGTACTTTCGCCTAGAGGAAGGCGAAAAGCTGGAAGCAATTCTTGCAGAGGTAGCGGAGACTATTAGCAAATGA
>Cluster_25144
ATGCACTCCTGGAAAAAGAAACTTGTAGTATCACAATTAGCATTGGCTTGCACTCTGGCTATCACCTCTCAGGCTAATGC
AGCGACCAACGATATTTCTGGTCAAACTTATAATACTTTCCATCACTACAACGACGCCACCTATGCTGATAACGTTTACT
ATGATGGTTATGTAGGCTGGAATAACTATGCCGCTGATAGTTATTACAACGGCGATATCTATCCGGTCATTAATAACGCT

ACCGTTAACGGCGTGATTTCTACCTACTATCTGGACGATGGTATTTCTACTAATACCAACGCCAATAGCCTGACAATCAA
AAACAGCACTATTCACGGTATGATTTATTCCGAGTGCATAGCAACTGATTGTTCTAATCGTGCGGATGACTACTACCATG
ACCGTCTGGCATTGTCTGTTGATAACTCGACTATCGATGACAACTACGAACATTATACTTACAACGGTACTTATAATAAT
GCCGCCGACACGCATGTTGTGGATGTTTACAACATGGGCACGGCTATCACTCTGGATCAAGAAGTTGATCTGTCTATCAC
CAATAACTCTCATGTAGCAGGTATTACGCTGACTCAGAGTTATGAGTGGGAAGATATCGACGACAACACTGTCAGTACTG
GTGTAAACAGCAGCGAAGTGTTTAACAACACCATCAATGTTGCGAACTCTACTGTCACTTCAGGTTCATGGTCTGATGAA
GGAACAGAAGGTTGGTTTGGTAACACTGGCAATGCCAGCGATTACAACGGCATTGACTGGAATGCTGATGATGTAGCATT
AGCGATTATTGCACATCCAAATGCTGACAACGCGATGCAGACGACTGCAACGTTTGATAATTCTACCCTGATGGGCGACG
TTTTCTTCTCCAGCAACTTTGATGAAAACTTCTTCCCGAATGGTGCTGACAGTTACCGTGACGCGGATGGTGATCTCGAC
ACTAATGGTTGGGATGGCACCGATCGTCTGGATCTGACCCTGAACAATGGCAGCAAATGGGTCGGTGCGGCAATGTCCGC
ACACCAGGTTGTCACTGCTTACGGTAGCACTGATTACAACATCGGTACCGAAGAGACTGCAACGCTTATCGATATCACCG
CGAACAGCTTGTGGCCGTCATCGACTGTTGGCGTTGAGAACGATGATTCAGCATACAACGAATATGAGCATATTATTGGT
AACGAAGTTTACCAGAGCGGCCTGTTCAATGTGACCCTGAACACCGGTTCACAGTGGGATACCACCAAAACTTCTCTGAT
TGATACTCTGAGCATCAACAGTGGTTCAACTGTTAATGTTGCTGATTCAACGCTGATTTCTGACTCTATCTCTCTGACTG
GTCTTTCTGCGCTGAACATCAACGAAGATGGTCATGTTGCAACTGATTCACTGACTGTCGACAACAGTACCGTAACCATT
TCTGATGAAGTTTCTGCTGGCTGGGCTGTAGGTGATGCGGCTCTGTATGCCAACAACATCAAAGTGACTAACGACGGTAT
TCTGGATGTAGGTAACACTGCGGCGAATGCTCTGCAGGTTGATACTCTGAACCTGACCAGCACTACTGATACCAGTGGTA
ACATTCACGCTGGTGTGTTCAACATCGAAAGCAACCGCTTCGTGCTGAATGCTGACCTGGTCAACGATCGTACAAACGAT
ACCACTAAAGCTAACTACGGCTACGGCACCATCGCGATGAACTCTGATGGTCACCTGACCATCAATGGTAACGGCGATAA
CGACAACACTGCTTCTATCGAAGCTGGTCAGAACGAAGTTGATAACGCGGGTGACCGTGTAGCTGCGGCAACCGGTAACT
ACAAAGTTCGTATCGACAACGCAACTGGTGCAGGTTCTGTTGCTGACTACAACGGTAACGAACTGATCTACGTTAACGAC
AAAAACAGCAACGCAACCTTCTCTGCTGCTAACAAAGCTGACCTGGGTGCATACACCTATCAGGCTGAGCAGCGTGGTAA
CACCGTTGTTCTGCAACAGATGGAGCTGACCGACTACGCTAACATGGCGCTGAGCATCCCGTCTGCGAACACCAATATCT
GGAACCTGGAACAAGACACCGTTGGTACTCGTCTGACCAACTCTCGTCATGGTCTGGCTGATAACGGCGGCGCATGGGTA
AACTACTTCGGTGGTAACTTCAACGGCGACAACGGCACCATCAACTATGATCAGGATGTTAACGGCATCATGGTCGGTGT
TGATACCAAAATTGACGGTAACAACGCTAAGTGGATCGTCGGTGCGGCTGCAGGCTTCGCTAAAGGTGATATGAATGACC
GTTCTGGTCAGGTGGATCAAGACAGCCAGACTGCCTACATCTACTCTTCTGCTCACTTCGCGAACAACGTCTTTGTTGAC
GGTAGCTTGAGCTACTCTCACTTCAACAACGACCTGTCTGCAACCATGAGCAACGGTACTTACGTTGACGGCAGCACCAA
CTCCGACGCTTGGGGCTTTGGTTTGAAAGCCGGTTACGACTTCAAACTGGGTGATGCAGGTTACGTGACTCCTTACGGCA
GCGTTTCTGGTCTGTTCCAGTCTGGTGATGATTACCAGCTGAGCAACGACATGAAAGTTGATGGTCAGTCTTACGACAGC
ATGCGTTATGAACTGGGTGTAGATGCAGGTTATACCTTCACCTACAGCGAAGACCAGGCTCTGACTCCGTACTTCAAACT
GGCTTACGTCTACGACGACTCTAACAACGATAACGATGTGAACGGTGATTCCATCGATAACGGTACTGAAGGGTCTGCGG
TACGTGTTGGTCTGGGTACTCAGTTCAGCTTCACCAAGAACTTCAGCGCCTATACCGATGCTAACTACCTCGGTGGTGGT
GACGTAGATCAAGACTGGTCCGCGAATGTGGGTGTTAAATATACCTGGTAA
>Cluster_66924
ATGAACATAAACCGTTTAATCTTCACTATCGAAGACTGTCTGAACACGCTATCCCGTTTCTCTGTCGCAAGCAGTTTTGT
TGAGTATTGCGATTTACGTACTGTAATTGGTCTGGACAGACAGGATCGTGAACGTCGCCCATGGCTGAATTCGCCCTATA
TTGCAGCCACAAAGCGTGGAGAATATCTCTCTGTCTTTGAAGTCAGCGGTGCGTTCAGGGAAATGGATGAAGCATCCGAC
CAGACTGGCCCAGGTTCACTGGAATCGCTGATCACCAGCATGAGTGACAGCCTGAATACGGCATATAAAAACAGCGGTCA
TAAAATCAGCTGCGTTTTCGAGCGTGACCCTGAAATGGGGAAAGAGGAAATCGAAGACATGGTCGCCCCACAAAAACGCT
CACTGGCTAATACAGGTATCCAGTTACAGGATGTGGTCGATGAGAAAGTCACAACACTGTCCCCATGGCTGGTCCGGGAG
CGTTGCTGGCTGGCGATCTGGAGTGGACCCGACCTTATCAGCAACAGCGATCGCACTGCCCATGATGAGCTTGTCAGAAG
ACTGGCTGAACGGGTACCGAAAGCACGCTTTGCCCAGTCCCCCTGGCAGTGGACCTTATCTGCACTGAAAATCCCGCCACG
AAGCTTTTCTTGATAATGTGGAACAGGCACTGAGACATTCATCTGATGGCCTGATTCTCCGGCTTCTGGATATACACGAA
GTCGGGCGTGAAATACGTCGTCAGACAGAACGGTACAGCACACCGCGTAACTGGCAACCCCACCTGCCTGAAGATGCCCA
GCCAGCAGGATATCGCTGGACTGACGATGAATCCGTACTACATGCTCCTTCATTGCATTTGCAGTTATTCAACACGCAGG
TGACCACACAGGGCAACCTGGTTCAGGCGGGCGGACTCTGGCACGGAATGGTATCGATTACTTTACCTCCGCAAAATCTG
CAGACGTTTAACGAACTGGTCCGTGCCGTCCCCCGCGCAGTCCCCTGGCGCATCAGAATGGATCTGATGCCCGGCGGGAT
GAAGGCTCTTAATCTGAAGAAAACGCTTCTGACTTACAGCAGCTTCATTTCTGCCGTTCGTCCAATGTATGAATCCGTTA
TGACCCTGGCGGCAACGGATGAAAAAGAACCTGTCTGCATAATGACCATCATGGCCTCCACGTGGGGAAAAACACGTGAA
ATATGCGCACGAAACCAGGCCATTCTGAAATCGGCCATTGAAGGCTGGGGCGTATGTGGCACCACGACAACATTTGGCGA
TCCCCGGCGCGCCTGGGTGAATACCATTCTCGCGGCATCCGGAGGCTCCGGCCCGGTTCCCCTGTATCCCCCGTTGTCAC
ATGCCATCTCGCTTTTCCCGCTTAACAGGGCAGGCTCTGTCTGGCGAGGCAAAGGGAATCTGATGTTACATACAGAAGAC
GGTTCCGCATTTGAGGTTGGTCTGGCGTCATCCCAACAAAATAAACATACAGAACTGGCACCAGGCGATCCGGGACTGGG
TAAATCAGTTCTGATTAATACGTTGTCGGAAATACAGATATCATCTGCTCAGAAAAACCTGCCCTTCATTGCCTATATCG
ATAAGGGATACAGTGCTCAGGGACTGGTTCAGCTTATTCGCGACAGTCTTCCTCCGGAAAGAAAAGACGAAGCTGTCGGT
ATTATTCTGAGCAATGATCCGGAATACACCCGTAACCTTTTTGACGTGATGTACGGCGCTAAAAAGCCTATTACGCCGGA
AAAGAATTTCATGTCATCGGTACTGTGTGCTCTCTGTGTCGATACCGGTACCGGTCAACCCTGTAACCCCGGAGACACCC
GACAAATTATCAACCAGCTTATCGAGCTGGCTTTTAAAGAGTACGGTGAAAATAATCCGCGACTTTATCGCGCCTCAACG
GAAGAACTGGTGGATTCAGCACTGCAGGACTCGGGACTGTATGAAAAACATGATGCAACCTGGTGGGCCAGATCAACCTG
GTTCGAAGTCCGGGATATGCTCCACAATGCCGGGTATATCATGGCGGCTCAGCGGGCACATTATCAGGCAATGCCCCAGC
TTCCCGAAGTGTCCTCGATGCTGGGCCATACCAGTCTCAGGGATGTTTTCGGTACTGTTCAGCGTGATGGTTCAAACGAA
CTTCTGCTGGACTACATCCGCCGCGCTCTCGAGCAGGGACATAACGACTACCCCATGATATCCGGGTATACCCGGTTTAT
GATCAACCCGGAAACCCGGGTCATCGCTGTCGATCTGAATAATGTTGCCGGTGACAAAACGCCTGCAGGCAGGCTTAAAA
CCGGCATCATGTACCTGCTTGCAGGTCAGATCGCCGGTGGTGACTTCACCCTGCCACAATACAGGGATGAAGTGTTAAAG
CAGCTTCCACGGGAATACCATGAAATCGCTCTTAAACGTATTAACCAACTTGATCAGGAGGTTAAGACAAAAGTTTATGA
TGAACTGCATAACGCCCGGGGCATTGACTTCATCTGGGAAAATCTGGATACCCAGGAGCGTGAGCAACGCAAGTTTGCTA
TCCGCACGGTTCTGAGTACGCAGTATCTGAGGGACTACCCGGAAAGCGTTCTTAAGTCAGCAAATACTCTCTGGTTACTG
CGCTACAAACCCGAAGATATCCCCGTACTGAGGGATAACTTCAACGTTCCTGAATTTATGCTGAAACGCTTCCTGAAAAT
GCCGGAAGGCCCGGCACCAGATGGTAGTGGTGTTCCTGTTCTGGGAGTATTCAGGGTAAAAAGTGGCACGCTTGCGAGAA
TACTCAAATTTACCGTCGGTCCACTGGAGCTATGGGCGCTGAACTCATCGCCAAAAGACAGTGCCCTGCGTAAAACACTG
ACAAATAAGTTAGGTTCAGTTCGGGCACGTAAGATTCTGGCCGAAAACTTCCCCAGAGGTTCTGCAACGTCATTAATTGA
GCACCGGGCGGGCCAACATAACAGTGACAATGTTATTGAAGAACTGGCCTCAGAACTCATCCGAAAACAGGGATACAATC
TCTGA
>Cluster_38994
ATGTCACGTTATAAAACAGGTCATAAACAACCACGATTTCGTTATTCAGTTCTGGCCCGCTGCGTGGCGTGGACAAATAT
CTCTGTTCAGGTTCTTTTTCCACTCGCTGTCACCTTTACCCCAGTAATGGCGGCACGTGCGCAGCATGCGGTTCAGCCAC
GGTTGAGCATGGGAAATACTACGGTAACTGCTGATAATAACGTGGAGAAAAATGTCGCGTCGTTTGCCGCAAATGCCGGG
ACATTTTTAAGCAGTCAGCCAGATAGCGATGCGACACGTAATTTTATTACCGGAATGGCCACCGCTAAAGCTAACCAGGA

AATTCAGGAGTGGCTCGGGAAATATGGTACAGCGCGCGTCAAACTGAATGTCGATAAAGATTTCTCGCTGAAGGACTCTT
CGCTGGAAATGCTTTATCCGATTTATGATACGCCAACAAATATGTTGTTCACTCAGGGGGCAATACATCGTACAGACGAT
CGTACTCAGTCAAATATTGGTTTTGGCTGGCGTCATTTTTCAGGAAATGACTGGATGGCGGGGGGGAATACTTTTATCGA
TCATGATTTATCCCGTAGTCATACCCGCATTGGTGTTGGTGCGGAATACTGGCGTGATTATTTGAAACTGAGCGCCAATG
GTTATATCCGGGCTTCTGGCTGGAAAAAATCGCCGGATGTTGAGGATTATCAGGAACGCCCGGCGAATGGCTGGGATATT
CGTGCTGAGGGCTATTTACCTGCCTGGCCGCAGCTTGGCGCAAGCCTGATGTATGAACAGTATTATGGCGATGAAGTCGG
GCTGTTTGGTAAAGATAAACGCCAGAAAGATCCACATGCGATTACCGCTGAAGTGAATTACACGCCAGTGCCTCTTCTGA
CCCTGAGTGCCGGGCATAAGCAGGGCAAGAGTGGTGAGAATGACACTCGCTTTGGCCTGGAAGTTAATTATCGGATTGGC
GAACCTCTGGAAAAACAACTCGATACAGACAGCATTCGCGAGCGTCGAATGCTGGCAGGCAGCCGCTATGACCTGGTTGA
GCGTAATAACAATATCGTTCTTGAGTATCGCAAATCTGAAGTGATCCGTATTGCTCTGCCTGAGCGTATTGAAGGCAAGG
GCGGCCAGACGGTTTCCCTGGGGCTTGTGGTCAGCAAAGCAACTCACGGTCTGAAAAATGTGCAATGGGAAGCGCCGTCT
TTGCTGGCCGCAGGCGGAAAAATTACGGGGCAGGGCAATCAGTGGCAAGTGACGCTCCCGGCTTATCAGGCAGGCAAAGA
CAATTATTATGCGATTTCAGCGATTGCCTACGATAACAAAGGCAATGCCTCGAAACGTGTGCAGACAGAAGTAGTTATTA
GCGGAGCTGGTATGAGCGCCGATCGTACGGCGTTAACGCTTGACGGTCAGAGCCGTATTCAAATGCTTGCTAACGGTAAT
GAGCAAAAGCCGCTGGTGCTGTCTCTGCGCGACGCCGAGGGCCAGCCAGTCACGGGCATGAAAGATCAGATCAAGACTGA
ACTAACCTTCAAACCGGCTGGAAATATTGTGACTCGTACCCTGAAGGCCACTAAATCACAGGCAAAGCCAACACTGGGTG
AGTTCACCGAAACTGAAGCAGGGGTGTATCAGTCTGTCTTTACTACCGGAACGCAGTCAGGTGAGGCAACGATTACTGTT
AGCGTTGATGACATGAGCAAAACTGTCACTGCAGAACTGCGGGCCACGATGATGGATGTGTCAAACTCCACCCTGAGTGC
TAACGAGCCGTCAGGTGATGTGGTTGCTGATGGTCAGCAAGCCTACACGCTGACACTGACAGCGGTGGACTCCGAGGGTA
ATCCGGTGACGGGAGAAGCCAGCCGCCTGCGACTTGTTCCGCAAGACACTAATGGTGTAACCGTTGGTGCCATTTCGGAA
ATAAAACCAGGGGTTTACAGCGCCACGGTTTCTTCGACCCGTGCCGGAAACGTTGTTGTGCGTGCCTTCAGCGAGCAGTA
TCAGCTGGGCACATTACAACAAACGCTGAAGTTTGTTGCCGGGCCGCTTGATGCAGCACATTCGTCCATCACCCTGAATC
CTGATAAACCGGTGGTTGGCGGTACAGTTACGGCAATCTGGACGGCAAAAGATGCTAATGACAACCCTGTAACTGGCCTC
AATCCGGATGCACCGTCATTATCGGGCGCAGCTGCTGTAGGTTCTACGGCATCAGGCTGGACGGATAATGGCGACGGGAC
CTGGACTGCGCAGATTTCTCTCGGCACTACGGCGGGTGAATTAGACGTTATGCCGAAGCTCAATGGGCAGGACGCGGCAG
CAAATGCGGCAAAAGTAACCGTGGTGGCTGATGCATTATCTTCAAACCAGTCGAAAGTCTCTGTCGCAGAAGATCACGTA
AAAGCCGGTGAAAGCACAACCGTAACGCTTATTGCAAAAGATGCACATGGCAACGCTATCAGTGGTCTTTCCCTGTCGGC
AAGCCTGACGGGTGCTGCGTCTGAAGGGGCGACCGTTTCCAGTTGGACCGAAAAAGGTGACGGTTCCTATGTTGCTACGT
TAACTACAGGCGGAAAGACGGGCGAGCTTCGTGTCATGCCGCTCTTCAACGGCCAGCCTGCAGCCACCGAAGCCGCGCAG
CTGACTGTTATTGCCGGAGAGATGTCATCAGCGAACTCTACGCTTGTTGCGGACAATAAAACTCCAACGGTTAAAACGAC
GACGGAACTCACCTTCACCGTGAAGGATGCGTACGGGAACCTTGTCACCGGGCTGAAGCCAGATGCACCGCAGTTTAGTG
GTGCCGCCAGCACGGGGACAGAGCGACCTTCAGCAGGAGACTGGACAGAAACAAGTAATGGGGTCTACGTGGCGACCTTA
ACGCTGGGATCTGCCGCGGGTCAGCTGTCTGTGATGCCGCGAGTGAACGGCCAAAATGCCGTTGCTCAGCCACTGGTGCT
GAACGTTGCTGGTGACGCATCTAAGGCTGAGATTCGTGATATGACGGTGAAGGTTGATAACCAGCTGGCAAATGGACAGT
CGACTAACCAGGTAACCCTGACCGTTGTGGACAGCTATGGTAACCCGTTGCAGGGGCAAGAAGTTACGCTGACTTTACCG
CAGGGTGTGACCAGCAAGACGGGGAATACAGTAACAACCAATGCGGCAGGTAAAGCGGATATTGAGTTGATATCAACCGT
TGCAGGGGAACTTGAAATCGCGGCCGCGGTGAAAAACTCTCAGAAGACGGTCACGGTGAAATTCAACGCGGATGCCAGCA
CCGGTCAGGCAAACCTGCAGGTAGACGCTGCTGCTCAAAAAGTGGCAAACGGCAAAGATGCCTTTACGCTGATGGCGAAC
GTTGAGGATAAAAATGGTAACCCTGTTCCAGGGAGCCTGGTGACCTTTAATCTGCCCCGGGGTGTCAAGCCGCTTACAGG
CGATAATGTCTGGGTGAAAGCCAACGATGAGGGGAAAGCAGAGTTACAGGTGGTTTCAGTGACTGCCGGAACGTATGAGA
TCACGGCGTCAGCAGGAAATGACCAGCCTTCGAATGCGCAGTCTGTAACGTTTGTGGCCGATAAGACTACGGCGACCATC
TCCAGTATTGAGGTGATTGGCAACTATGCACTGGCGGACGGCAAAGCCAAACAAACGTATAAAGTTACGGTGACTGATGC
CAATAACAATTTGTTGAAAGATAGCGAAGTGACGCTGACCGCCAGCCCGGCTTCGTTAAACCTGGAACCGAATGGCACTG
CGACAACGAATGAGCAAGGGCAGGCTATTTTCACCGCCACCACTACTGTTGCGGCGACATATACACTCTCGGCGCAAGTG
AGTCAGAACAACGGTCAGGTATCAACGAAAACCGCCGAAGCTAAATTCGTCGCGGATGATAAAAACGCGCAGCTCACCGC
ATCATCTAATGTGCCTTCGCTGGTGGCGGATGGGAAGTCGACTGCGAAGCTGGAGGTGACCCTGATGTCGGCAACTAACC
CCGTTGGCGGGAATATGTGGGTCGACATTCAGACGCCGGAAGGGGTGACGGAGAAGGATTATCAGTTCCTGCCGTCGAAA
AATGACCATTTCGTGAGCGGAAAAATCACGCGTACATTTAGTACCAACAAGCCAGGTGTCTATACGTTTACATTTAACGC
CCTGACGTATGGCGGGTACGAAATGAAGCCAGTGACGGTGACCATTACCGCGGTGGATGCCGATACGGCAAAGGGCGAGG
AGGCGATGAAATAA
>Cluster_52772
ATGAACAAAATATTTAAAGTTATCTGGAATCCGGCGACAGGCAGTTACAGCGTCGCCAGCGAAACGGCGAAGAGCCGTGG
TAAAAAATCAGGGCGCAGTAAGCTGTTAATTTCTGCATTAATTGTCACCAGTGCAATTTTTACTCCAGGAGCATACGCAG
GACTCTCTCTCGACGGCGGTGACATTTTCGAATCGACTCCATTGACCAATTACTGGCTTGCTATTGGTCAAGGGTCTGTA
GCAACCACCAATAATGGGGGAACTGACGGAGTTGCTATGGCAATAGGATTGAAAGCCAAAGCACTCGGGGCGGGAAGTAC
AGCATTTGGCTATGATGCTGAAGCAAGCGGAGATCGAGCAATAGCGTTCGGACAATTAACAGAAGCATCAGGCAATAGAA
CAATAGCAATGGGTAGTGGAGCAACTGCCACTGGTGACCATTCTCTGGCGCTAGGGGGGGCAACTAAGACTCTAGGTATG
TATTCCGTGGCAATAGGCCGGGATGCTACTACAGATAGCGACTACGCGCTTTCTATGGGGCATATGGCGAAGGCGAATGG
ACTATACAGCCTGGCAATGGGTGCAGGAAGTGCTACTTCGAACGATAACGCCATCGCGATTGGGAAAAAAACGCAAGCCC
AGGGAGTGAATTCTATAGCCTTGGGTAATGCAAGCCAGGCATCGGGCTACTCCAGCCTTGCAATAGGTGAGTTAAGTGAG
ACCGGTGCTGAAAATGCTATTGCACTGGGAAAACTCAGTAACGCAAGTAAAATCAATTCAATCGCCCTGGGTAGTAACAG
CACTGCATCTGGTGAGGGCTCCGTTGCGCTGGGAGAGAATAGTTTTGCTGGAGGCATAAACAGTCTTGCTTTGGGTAGTC
AATCCAATGCAAACGGTGATAACGCAGTTGCCCTCGGTGTAGGATCTGTCGCAGCGCAAGACAATACCGTTTCGGTAGGG
AACAGCACAACACAACGCAAAATTACCAATATGGCTGCCGGTCAAATAAGAAATGGCAGTACTGAGGCCATTAACGGTTC
ACAACTTTATGGACTAAGTGACTCAGTCGCAGCCCGACTCGGGGGTGGTGCTGGCGTAAATGAAGATGGAAGTATAAATG
CGCCAAGCTACAAGCTGAAAAGCAACATCTACAATAATGTGGGTGATGCGCTTTTAGGAATCGACAATGACACTCTGCAC
TGGGACAAAACCAATAAAGCATTTAGTGCCAGCTATCTCGCCAAAAACGCTGATGACTCCCTAAAGGAGCGCTCAGACCA
AAACAAAATCATTAATGTTGCAAAGGGTACTATTTCCGCCACCAGCACCGATGTAGTAAACGGCTCTCAATTGTACGACC
TGCAGCAGGATGCGCTGTTGTGGAACGGCACAGCATTCAGTGCCGCACACGGCACCGAAGCCACCAGCAAAATCACTAAT
GTTGAAGATGGTACTATTTCAGACACCAGTAAAGATGCGGTTAACGGTTCCCAGTTGAAAGAAACCAAAGACGATGTGGC
CACCAACACAGCCAATATCGCCGATAACACTGCTGATATTGCAACCAACACTACCAATATCGCCGATAACACTGCTGATA
TCGCCACTAACACCACGGATATTGCAACCAACACTGCCAATATCGCCGATAACACTGCCAATATCGCCACTAACACTACG
GATATTGCGACTAATACCGCCGATATAGCTACAAACGCTAGCAGTATCACTACTCTGAACGCGGACGTTGATACCCTGGA
AAAAGATGCCCTGCTCTGGAATGGCACAGCATTCAGCGCCAAACACGGCACCGAAGCTACCAGCAAGATCACCAATGTTA
CCGCTGGCGACCTGACTGCTGGCAGCACTGACGCCGTTAATGGCTCCCAGCTCAAAACCACCAACGATAACGTGTCGACC
AACACCACCAACATCACCAACCTGACGGATTCCGTTGGCGACCTTAAGGACGATTCCCTGCTGTGGAACAAAGCGGCTGG
CGCATTCAGCGCCGCGCACGGCACTGATACCACCAGCAAAATCACTAACGTTAGAGCTGGCTCGGTTTCTAAAAACAGCA
CTGACGCCATTAACGGCTCACAACTTTATGGCGTAGCGGATTCATTTACGTCATATCTTGGTGGTGGTGCTGATATCAGC
GATACGGGTGTATTAACCGGGCCAACCTACACTATTGGTGATACTGACTACACTAACGTCGGTGATGCTCTGGCAGCCAT

TAACACATCATTTAGCTCATCACTCGGCGACGCCCTACTCTGGGATGCAACCGCAGGTAAATTCAGCGCCAAACACGGCA
TTAATAATGATCCCAGTGTAATCACTGATGTTGCAAACGGTGCAGTCTCGTCCACCAGTAGCGACGCCATTAACGGTTCG
CAACTTTATGGCGTTAGTGACTACATTGCCGATGCTTTGGGCGGGAACGCTGTGGTGAATGCAGACGGCAGTATCACTAC
ACCGACTTATGCCATAGCGGGCGGCAGTTACGACAATGTCGGTGACGCGCTGGAAGCGATCGATACCACGCTGGATGACG
CCCTGCTGTGGGATGAGACAGCCAATGGCGGTAACGGTGCATTTAGCGCCGCTCACGGGAAAGATAAAACTGCCAGTGTA
ATCACTAACGTCGCTAACGGTGCAGTCTCTGCCACCAGCAGCGATGCCATTAATGGCTCACAGCTCTATAGCACCAATAA
GTACATCGCTGATGCGCTGGGTGGTGATGCAGAAGTCAACGCTGACGGAACTATCACTGCACCGACTTACACCATTGCAA
ATACCGATTACAACAACGTCGGTGAAGCCCTGGATGCGCTTGATAATAACGCTCTGCTGTGGGATGCAACAGCCAATAAC
GGCGCAGGTGCTTACAGCGCCAGTCATGACGGCAAGGCCAGCATTATCACAAACGTCGCTGATGGTAACATTGGTGAAGA
TAGCACCGACGCTATCAACGGTTCTCAGTTGAATGCAACCAACATGATGATCCAGCAGAACAGCGAAATCATCAATCAGC
TTGCCGGTGACACCAGTAAAAACTACATCGGAGATAACGGTGCAGGTATTAACTATGTACGTACCAACGACAGCGGCTTA
GCGTTCAACGATGCCAGCGCAGCAGGTATTGGCTCTACCGCTGTGGGGTATAACACTGTTGCCAAAGGCGATAGCAGCGT
GGCAATCGGTCATGGCAGCAACAGCATCGTTGAGACGAGTATTGCGCTGGGTAGCGAATCCGTCTCCAGCCGTTTGATTG
TGAAAGGCTCCCGTAATACCCGCGTTACGGAAAATGGGGTCGAGATCGGTTATGACACCACGGATGGCGAACTGCTTGGC
GCGTTGTCGATCGGTGATGATGGTAAATATCGTCAAATCATCAACGTCGCGGATGGTTCTGAAGCCCATGACGCTGTTAC
TGTTCGCCAGTTGCAAAACGCCATTGGTGCAGTAGCAACCACACCAACCAAATACTATCACGCCAACTCAACGGCTGAAG
ACTCACTGGCAGTCGGTGAAGACTCGCTGGCAATGGGCGCGAAAACCATCGTTAATGGTAATGCGGGTATTGGTATCGGC
CTGAACACTTTAGTTCTGGCTGATGCGATCAACGGTATTGCTATCGGTTCTAACGCACGCGCAAATCATGCCGACAGCAT
TGCTATGGGTAATGGTTCTCAGACTACCCGTGGTGCGCAGACCAACTACACTGCCTACAACATGGACGCACCGCAGAACT
CTGTGGGTGAGTTCTCTGTCGGCAGCGAAGACGGTCAACGTCAGATCACCAACGTCGCAGCAGGTTCGGCGGATACCGAT
GCGGTTAACGTGGGTCAGTTGAAAGTAACGGACGCGCAGGTTTCCCAGAATACCCAGAGCATTACTAACCTGAACACTCA
GGTCACTAATCTGGATACTCGCGTGACCAATATCGAAAACGGCATTGGCGATATCGTAACCACCGGTAGCACTAAGTACT
TCAAGACCAACACCGATGGCGCAGATGCCAACGCGCAGGGTAAAGACAGTGTTGCGATTGGTTCTGGTTCCATTGCTGCC
GCTGACAACAGCGTCGCACTGGGCACGGGTTCCGTAGCAGACGAAGAAAATACGATCTCTGTGGGTTCTTCTACCAACCA
GCGCCGTATCACCAACGTTGCTGCCGGTGTTAATGCCACCGATGCGGTTAACGTTTCGCAACTGAAGTCTTCTGAAGCAG
GCGGCGTGCGCTACGACACCAAAGCTGATGGCTCTATCGACTACAGCAACATCACTCTCGGTGGTGGTAACGGCGGTACG
ACTCGCATCAGCAACGTTTCCGCTGGCGTGAATAACAACGACGCAGTGAACTATGCGCAGTTGAAGCAAAGTGTACAGGA
AACGAAGCAATACACCGATCAGCGCATGGTTGAGATGGATAACAAACTGTCCAAAACTGAAAGCAAGCTGAGTGGTGGTA
TCGCTTCTGCAATGGCAATGACCGGTCTGCCGCAGGCTTACACGCCGGGTGCCAGCATGGCGTCTATTGGTGGCGGTACT
TACAACGGTGAATCGGCTGTTGCTTTAGGTGTGTCGATGGTGAGCGCCAATGGTCGTTGGGTCTACAAATTACAAGGTAG
TACCAATAGCCAGGGTGAATACTCCGCCGCACTCGGTGCCGGTATTCAGTGGTAA
>Cluster_53938
ATGAATATTGAACAGATTTTTAAAAAACCTCTAAAACGAAATATAAACGGGGTAGTCAAAGCAGAGCAAACCGATGATGC
CAGCGCGTACATCGAGTTAGATGAATATGTCATCACCCGCGAACTGGAAAACCATCTTCGCCATTTCTTCGAATCCTATG
TTCCTGCCACTGGCCCGGAAAGGATCCGTATGGAAAACAAGATCGGCGTATGGGTTTCAGGCTTCTTCGGTTCAGGTAAA
TCGCACTTTATTAAGATTCTTTCTTATCTTTTATCTAACCGCAAAGTTACACATAACGGTACGGAACGTAATGCTTACTC
CTTCTTTGAAGAGAAAATCAAAGATGCATTATTCCTTGCCGATATTAACAAAGCGGTGCATTACCCGACTGAAGTCATTC
TGTTCAATATTGATTCGCGTGCCAACGTAGATGACAAAGAAGATGCCATTCTTAAAGTCTTCCTGAAAGTTTTCAACGAA
CGCATTGGATACTGCGCTGATTTTCCGCATATTGCCCATCTTGAGCGCGAGCTGGATAAACGCGGTCAGTATGAAACCTT
TAAAGCCGCGTTTGCCGATATCAATGGCTCGCGCTGGGAAGACGAGCGCGACGCTTACTACTTCATCAGCGATGACATGG
CACAAGCATTAAGCCAGGCCACGCAGCAGAGTCTTGAAGCCTCCCGCCAATGGGTGGAACAACTCGACAAAAACTTCCCG
CTGGATATCAATAATTTTTGCCAGTGGGTAAAAGAGTGGCTGGATGACAATGGTAAGAACATCCTCTTTATGGTGGATGA
AGTCGGTCAGTTCATTGGCAAAAATACGCAAATGATGCTGAAGCTGCAGACTATTACTGAAAACCTTGGGGTAATTTGCG
GTGGCCGCGCATGGGTTATCGTGACTTCGCAGGCCGATATCAACGCGGCAATCGGTGGTATGAGCAGTCGCGACGGACAG
GACTTCTCCAAGATCCAGGGGCGCTTCTCTACACGCCTGCAACTTTCCAGCTCTAACACATCAGAAGTTATCCAGAAACG
TTTGTTGGTAAAGACTGACGAAGCAAAAGCGGCGCTGGCAAAAGTGTGGCAAGAGAAAGGCGATATCCTGCGTAACCAGC
TGGCTTTTGACACTACAACAACTACTGCACTACGTCCTTTTACCAGCGAAGAAGAGTTCGTTGACAACTACCCGTTTGTC
CCGTGGCACTATCAGATTCTGCAAAAAGTGTTTGAATCTATTCGGACGAAAGGTGCAGCGGGTAAACAATTGGCCATGGG
TGAGCGTTCTCAGCTGGAGGCATTCCAGACGGCGGCGCAGCAAATCTCAGCGCAAGGGCTGGATTCTCTGGTGCCTTTCT
GGCGCTTCTATGCCGCCATTGAGAGCTTCCTGGAACCTGCCGTTAGCCGCACCATCACTCAGGCTTGCCAGAATGGCATT
CTTGATGAGTTCGATGGCAACCTGCTTAAAACGCTGTTCCTGATCCGCTATGTGGAAACGCTGAAAAGCACCCTGGATAA
CCTGGTCACATTGTCTATCGATAGGATCGATGCCGATAAAGTTGAGTTGCGCCGCCGGGTCGAAAAAAGTCTCAACACGC
TTGAACGCCTGATGCTCATTGCGCGCGTTGAAGATAAATATGTGTTCCTGACCAACGAAGAAAAAGAGATCGAAAACGAG
ATCCGTAACGTTGATGTCGATTTCTCTGCGATCAACAAAAAACTGGCATCGATCATCTTTGATGACATTCTGAAAAGCCG
TAAATATCGTTATCCGGCTAACAAGCAAGACTTTGATATCAGCCGCTTCCTGAACGGGCATCCATTAGACGGCGCAGTGC
TTAACGATCTGGTGGTGAAGATCCTGACCCCTAAAGATCCGACTTATTCGTTCTATAACAGCGATGCGACCTGTCGCCCT
TATACGTCAGAAGGCGACGGCTGTATTTTGATTCGTCTGCCCGAAGAGGGCCGTACCTGGAGCGATATTGATTTAGTCGT
CCAGACTGAAAAGTTCCTCAAAGATAACGCCGGGCAACGTCCGGACAGGCTACCCTGCTCTCAGAAAAAGCGCGTGAAA
ACAGCAACCGGGAAAAAATTACTCCGTGTTCAGTTGGAATCACTACTTGCAGAAGCAGATGTCTGGGCGATTGGCGAGCGC
TTACCGAAAAAATCCTCCACGCCATCGAACATTGTCGATGAAGCCTGCCGTTACGTGATTGAAAACACCTTCGGCAAGCT
GAAGATGCTGCGGCCTTTTAACGGCGACATCTCCCGTGAAATTCATGCATTACTGACGGTTGAGAACGACACCGAACTGG
ATCTCGGTAACCTCGAAGAGTCCAACCCCGACGCCATGCGCGAGGTAGAAACCTGGATCAGCATGAATATCGAATACAAT
AAACCGGTGTATTTACGCGATATTCTGAACCATTTCGCGCGTCGCCCTTATGGCTGGCCCGAAGACGAAGTGAAACTGCT
GGTAGCCCGTCTGGCCTGCAAAGGTAAATTCAGCTTCAGCCAGCAAAACAACAACGTCGAACGAAAACAGGCGTGGGAAT
TATTTAATAACAGCCGCCGCCATAGCGAATTGCGTCTGCATAAAGTTCGCCGTCATGATGAAGCGCAGGTGCGTAAAGCC
GCGCAAACCATGGCTGACATCGCTCAGCAGCCGTTTAACGAACGGGAAGAGCCGGCGCTGGTTGAACATATTCGTCAGGT
TTTTGAAGAGTGGAAGCAAGAGCTGAACGTATTCCGCGCCAAGGCAGAGGGCGGAAACAATCCGGGGAAAAACGAGATTG
AATCCGGTCTGCGCCTGCTTAATGCCATTCTTAATGAGAAAGAAGATTTTGCCCTGATCGAAAAAGTCTCATCGCTGAAA
GATGAACTTCTGGATTTCAGCGAAGACCGTGAAGATTTGGTCGACTTCTACCGTAAGCAATTCGCCACCTGGCAAAAACT
GGGTGCTGCGCTGAATGGCAGCTTTAAATCTAACCGCAGCGCGCTGGAAAAAGACGCCGCAGCGGTTAAAGCGCTGGGCG
AGCTGGAAAGCATCTGGCAAATGCCGGAACCTTATAAGCATCTCAATCGCATCACGCTGTTGATTGAACAGGTCCAGAAC
GTCAACCATCAGTTAGTCGAACAGCATCGCCAGCACGCCCTCGAACGCATTGACGCCCGCATCGAGGAAAGCCGTCAACG
CTTGCTCGAAGCGCACGCCACGTCAGAACTGCAAAACAGCGTTCTGCTGCCGATGCAAAAAGCCAGAAAACGCGCTGAGG
TCAGCCAGTCTATTCCGGAAATTTTGGCGGAACAGCAAGAGACAAAAGCGCTGCAAATGGATGCAGATAAAAAGATTAAC
CTGTGGATCGACGAGCTGCGTAAAAAGCAAGAAGCACAACTCCGGGCAGCAAATGAAGCCAAACGCGCTGCCGAGTCAGA
ACAGACTTATGTTGTGGTGGAAAAACCCGTTATCCAACCGGTACCGAAAAAAACGCATCTGGTGAATGTCGCCAGTGAGA
TGCGTAATGCCACCGGTGGTGAAGTTCTAGAAACGACCGAACAGGTGGAAAAGGCGCTCGACACGTTACGCACAACGCTG
CTGGCCGCCATTAAAGCAGGCGATCGCATTCGCCTTCAGTAA
>Cluster_61345

ATGAGCGGAAAACCGGCGGCGCGTCAGGGTGACATGACGCAGTATGGCGGTAGCATTGTTCAGGGTTCAGCCGGGGTACG
TATTGGTGCCCCCACCGGCGTGGCCTGTTCGGTGTGCCCCGGCGGAGTGACGTCCGGCCATCCGGTCAATCCCCTGCTCG
GTGCAAAGGTCCTTCCCGGTGAAACCGACATCGCCCTGCCCGGCCCGCTGCCGTTCATCCTCTCCCGCACCTACAGCAGT
TACCGGACAAAAACGCCCGCGCCGGTGGGGAGCCTCGGCCCCGGCTGGAAAATGCCTGCGGATATCCGCTTACAGCTGCG
CGATAACACACTGATACTCAGTGATAACGGCGGCAGAAGCCTGTATTTTGAGCACCTGTTTCCCGGTGAGGACGGTTACA
GCCGCAGCGAGTCACTGTGGCTGGTGCGCGGCGGCGTGGCGAAACTGGATGAAGGTCACCGGCTGGCCGCACTCTGGCAG
GCGCTGCCGGAAGAACTCCGCTTAAGTCCGCATCGTTATCTGGCGACAAACAGTCCGCAGGGGCCGTGGTGGCTGCTCGG
TTGGTGTGAGCGGGTGCCGGAAGCGGATGAGGTGCTGCCTGCGCCGCTGCCGCCGTACCGGGTACTGACCGGGCTGGTGG
ACCGCTTCGGGCGCACACAGACGTTCCACCGCGAAGCCGCCGGTGAATTCAGCGGCGAAATCACCGGCGTGACGGATGGT
GCCTGGCGTCACTTCCGGCTGGTACTGACCACGCAGGCGCAGCGGGCAGAAGAAGCCCGGCAGCAGGCCATTTCCGGCGG
GACGGAACCGTCCGCTTTTCCTGATACCCTGCCGGGTTACACCGAATATGGCCGGGACAACGGCATCCGTCTGTCTGCCG
TGTGGCTGACGCACGACCCGGAATACCCGGAGAATTTACCTGCCGCGCCGCTGGTGCGCTATGGCTGGACGCCACGCGGC
GAACTGGCGGTGGTGTATGACCGTAGTGGCAAACAGGTGCGCAGCTTTACTTACGATGATAAATACCGGGGCCGGATGGT
GGCGCACCGTCACACGGGCCGGCCGGAAATCCGTTACCGTTACGACAGCGACGGGCGGGTGACAGAACAGCTAAACCCGG
CAGGCTTAAGCTACACGTATCAGTATGAGAAAGACCGCATCACCATCACCGACAGCCTGGACCGCCGTGAAGTGCTGCAC
ACGCAGGGCGAAGCCGGGCTGAAGCGGGTGGTGAAAAAGGAACACGCGGACGGCAGCGTCACGCAGAGTCAGTTTGACGC
CGTGGGCAGGCTCAGGGCACAGACGGATGCCGCAGGCAGGACAACAGAGTACAGCCCGGATGTGGTGACGGGCCTCATCA
CGCGCATAACCACGCCGGATGGCAGGGCATCGGCGTTTTACTATAACCACCACAACCAGTTAACGTCAGCCACCGGGCCT
GACGGGCTGGAATTGCGCCGGGAATATGATGAATTGGGCCGTCTGATTCAGGAAACTGCCCCTGACGGCGATATCACCCG
CTACCGTTATGATAATCCACACAGTGACTTACCCTGCGCAACGGAAGATGCCACCGGCAGCCGGAAAACCATGACGTGGA
GCCGTTACGGTCAGTTGCTGAGCTTCACCGACTGTTCCGGTTATGTAACCCGTTATGACCATGACCGCTTCGGGCAGATG
ACGGCGGTGCACCGCGAGGAAGGGCTGAGTCAGTACCGCGCATACGACAGCCGTGGACAGTTAATTGCCGTGAAAGACAC
GCAGGGCCATGAAACGCGGTATGAATACAACATCGCCGGTGACCTGACCGCCGTCATTGCCCCGGACGGCAGCAGAAACG
GGACACAGTACGATGCGTGGGGAAAGGCCGTCCGTACCACGCAGGGCGGGCTGACGCGCAGTATGGAATACGATGCTGCC
GGACGGGTCATCCGCCTGACCAGTGAAAACGGCAGCCACACCACCTTCCGTTACGATGTACTTGACCGGCTGATACAGGA
AACCGGCTTTGACGGCCGCACACAGCGTTATCACCACGACCTGACCGGCAAACTTATCCGCAGCGAGGATGAGGGTCTGG
TCACCCACTGGCACTATGACGAAGCAGACCGCCTCACGCACCGCACCGTGAAGGGTGAAACCGCAGAGCGGTGGCAGTAT
GACGAACGTGGCTGGCTGACAGACATCAGCCATATCAGCGAAGGGCACCGGGTGGCGGTGCATTACAGGTATGATGAGAA
AGGCCGGCTGACCGGTGAGCGTCAGACGGTGCATCACCCGCAGACGGAAGCACTGCTCTGGCAGCATGAGACCAGACATG
CGTACAACGCGCAGGGGCTGGCGAACCGCTGTATACCGGACAGCCTGCCCGCCGTGGAATGGCTGACCTACGGCAGCGGT
TACCTGGCAGGCATGAAACTCGGCGACACACCGCTGGTGGAGTACACCCGCGACCGCCTGCACCGGGAAACGCTGCGCAG
CTTCGGCCGTTATGAACTCACCACCGCTTATACCCCTGCCGGGCAGTTACAGAGCCAGCACCTGAACAGCCTGCTGTCTG
ACCGCGATTACACCTGGAACGACAACGGCGAACTCATCCGCATCAGCAGCCCGCGCCAGACCCGGAGTTACAGCTACAGC
ACCACCGGCAGGCTGACCGGCGTTCACACCACCGCAGCGAATCTGGATATCCGCATCCCGTATGCCACAGACCCGGCAGG
TAACCGCCTGCCCGACCCGGAGCTGCACCCGGACAGCACCCTCAGCATGTGGCCGGATAACCGTATCGCCCGTGACGCGC
ACTATCTTTACCGGTATGACCGTCACGGCAGGCTGACAGAGAAAACCGACCTCATCCCGGAAGGGGTTATCCGCACGGAT
GATGAGCGGACTCACCGGTACCATTACGACAGTCAGCACCGGCTGGTGCACTACACGCGGACACAATATGAAGAGCCGCT
GGTCGAAAGTCGCTATCTTTACGACCCGCTGGGCCGCAGGGTGGCAAAACGGGTGTGGCGGCGTGAACGGGACCTGACGG
GCTGGATGTCGCTGTCACGGAAACCGCAAGTGACCTGGTACGGCTGGGACGGCGACCGGCTGACCACGATACAGAACGAC
AGGAGCCGCATCCAGACGATTTATCAGCCGGGGAGCTTCACGCCACTCATCAGGGTCGAAACTGCCACCGGTGAGCTGGC
GAAAACGCAGCGCCGCAGCCTGGCGGATGCCCTTCAGCAGTCCGGCGGCGAAGACGGTGGCAGTGTGGTGTTCCCGCCGG
TGCTGGTGCAGATGCTCGACCGGCTGGAAAGTGAAATCCTGGCTGACCGGGTGAGTGAGGAAAGCCGCCGCTGGCTGGCA
TCGTGCGGCCTGACCGTGGAGCAGATGCAAAACCAGATGGACCCGGTGTACACGCCGGCGCGAAAAATCCACCTGTACCA
CTGCGACCATCGCGGCCTGCCGCTGGCGCTCATCAGCACGGAAGGGGCAACAGCGTGGTGCGCAGAATATGATGAATGGG
GCAACCTGCTGAATGAAGAGAACCCGCATCAGCTGCAGCAGCTTATCCGGCTGCCGGGGCAGCAGTATGATGAGGAGTCC
GGCCTGTATTACAACCGCCACCGCTATTATGACCCGCTGCAGGGGCGATATATCACTCAGGATCCGATTGGACTGAAGGG
GGGATGGAACCTGTATGGATATCAATTGAATCCGATATCAGACATCGACCCCCTGGGTTTATCTATGTGGGAGGATGCAA
AATCGGGGGCATGTACTAATGGTCTTTGCGGCACACTATCCGCTATGATAGGTCCAGATAAATTTGATTCTATAGATAGC
ACCGCATATGACGCCTTAAATAAAATAAATAGCCAATCTATTTGCGAAGATAAAGAGTTCGCTGGTTTAATATGTAAGGA
TAATAGTGGCAGATATTTCTCAACAGCACCTAACCGAGGAGAAAGAAAAGGATCATATCCATTCAATAGCCCTTGCCCTA
ATGGTACTGAGAAAGTATCAGCTTATCATACTCATGGTGCAGATAGTCATGGAGAATATTGGGACGAAATATTTTCAGGT
AAAGATGAGAAAATAGTTAAAAGTAAAGATAACAATATCAAGTCATTTTATTTAGGTACGCCCAGTGGTAATTTTAAAGC
AATAGATAACCACGGGAAGGAAATAACAAACAGAAAAGGATTACCTAATGTCTGCAGAGTTCATGGTAATATGTAA
>Cluster_62870
GTGAATGTCACCGGGGCGGAGGGCTATTACAACGCCACGCTGGACTATGGTGACGGCTGCACCACCGTGACGGACGGCAA
GGGCATTCACCGTTATTACTATGATCCTGACGGCAATATTCTGCGGGAAGAAGCGCCGGACGGCAGCACCACCACGTATG
AATGGGATGAATTCCATCACCTGCTGGCCCGTCACTCCCCTGCCGGGCGGGTGGAGAAGTTTGAATACAACGCCGCACAC
GGTCAGTTAAGCCGTTACACGGCGGCAGACGGCGCGGAGTGGCAGTACCGCTATGATGAGCGCGGCCTGCTCAGCAACAT
CACCGACCCAGCCGGGCAGACGTGGACACAGCAGTGTGATGAACGCGGCCTGCCGGTAAGTCTGGTGTCGCCACAGGGCG
AAGAGACCCGGCTGGCGTACACGCCGCAGGGGCTGTTGTCGGGAATATTCCGCCAGGATGAACGGCGTCTGGGCATAGAG
TACGACCACCACAACCGGCCGGAAACACTCACCGACGTGATGGGCCGTGAACACCACACCGAATACAGCGGTCACGACCT
GCCGGTGAAGATGCGCGGCCCCGGCGGTCAGTCAGTGCGGTTACAGTGGCAGCAGCACCATAAACTGAGCGGCATTGAAC
GTGCTGGAACCGGCGCGGAAGGATTTCGCTATGACCGCCACGGCAACCTGCTGGCGTACACGGACGGCAATGGTGTTGTC
TGGACAATGGAGTACGGCCCGTTCGATTTGCCGGTGGCGCGAACGGACGGTGAAGGCCACCGCTGGCAGTACCGCTACGA
TAAAGACACGCTGCAACTGACAGAAGTCATCAATCCGCAGGGCGAGTCATACCGTTATATTCTGGACAACTGTGGACGGG
TGACGGAAGAGCGTGACTGGGGCGGCGTGGTCTGGCGTTACCGCTATGACGCCGATGGCCTCTGTACCGCCAGGGTTAAC
GGCCTGGAGGAAACCATCCTCTACAGCCGGGATGCCGCAGGCCGCCTGGCAGAGGTCATCACGCCGGAAGGCAAAACGCA
GTATGCCTATGACAAATCCGGCAGGCTGACGGGTATCTTCAGCCCGGACGGTACATCACAGCGCACCGGCTATGACGAAC
GCGGGCGGGTGAATGTCACCACTCAGGGCCGACGGGCCATTGAATACCACTACCCCGATGAACACACCGTTATCCGCTGT
ATCCTGCCACCGGAAGATGAACGCGACAGACACCCCGGTGAATCCCTGCTGAAAACCACGTACCGTTATAACGCCGCCGG
AGAACTGACGGAGGTCATTCTGCCGGGGGATGAGACGCTGACGTTCAGCCGTGATGAGGCGGGACGTGAAGTGTTCCGGC
ACAGTAACCGGGGTTTTGCCTGTGAGCAGGGCTGGAATGCAGCCAGCCAGCTTGTCACCCAGCGCGCCGGATTTTTCCCG
GAGGAAACCACATGGGGCGGGCTGCTCCCCTCACTGGTACGGGAGTACCGTTACGACAGCGCGGGCAATGTGTCGGCTGT
CACCAGCCGGGAAGATTACGGACGGGAAACACGGCGGGAGTACCGGCTGGACCGGAACGGTCAGGTCACGGCGGTGACAG
CCTCAGGCACCGGGCTGGGCTATGGCGAAGGCGACGAGTCCTATGGCTATGACAGTTGTGGCTACCTGAAGGCGCAGTCT
GCGGGCAGGCACCGGATAAGTGAAGAGACTGACCAGTATGCCGGAGGCCACCGGCTGAAACAGGCCGGAAACACGCAGTA
TGACTATGACGCCGCAGGCCGGATGGTCAGCCGCACCAGACACCGTGACGGCTACCGCCCGGAAACAGAGCGGTTCCGGT
GGGACAGCCGTGACCAGCTGACCGGGTATTGCAGCGCACAGGGTGAGCAGTGGGAATACCGCCACGACGCCAGCGGCAGA
CGAACAGAGAAACGCTGCGACCGGAAGAAAATCCGCTTCACGTATCTGTGGGACGGCGACAGTATTGCGGAAATCCGGGA

ATACCGCGATGATAAACTGTACAGCGTACGGCACCTGGTGTTTAACGGCTTTGAGCTGATAAGCCAGCAGTTCAGCCGGA
AACGGCAGGCGCATCCGTCGGTGGCCCCGCAGTGGGTGACGCGGACGAATCATGCGGTGAGCGACCTGACGGGCCGTCCG
CTGATGCTCTTTAACAGTGAAGGTAAAACCGTCTGGCGACCGGGACAGACCAGCCTGTGGGGGCTGGCACTCAGCCTGCC
CGCAGACACCGGCTACCCGGACCCGCGCGGGGAGCTGGACCCGGAAGCCAACCCCGGCCTGCTGTATGCGGGACAGTGGC
AGGATGCGGAATCAGGGCTGTGCTATAACCGGTTCCGGTATTACGAGCCGGAAACCGGAATGTACCTGGTGAGTGATCCG
CTGGGGTTGCTGGGCGGGGAGCAGACTTACCGGTATGTGCCGAATCCTTGTGGGTGGGTTGATCCGCTGGGATTGGCTGC
AAGTTCTAAAATCAGCAGTTTGATGGACTATATTGGCGATGGTCGTCGTGTTAGTGGGCATACGGGTTTCCTGGATGGGG
TTCGTTTATCACGTAGTCAAATAAACAATATTGCTAAAGAAATGGAGAAGCTAGGAATTAAAGTAATAAGGAAAGCAGAT
AAATATTTGCCACCAAATGCTAGGGCAGCTTTTGATTATGGCCTTCGCAATATTTATCTTAGGAAAAATGCTACCTTATA
TGAGGTGTATCATGAAGTGATTCATGCCAAGCAATTTGCGAAAATTGGACGAGAAGCATACGAAGCACTAGGACGTTTAT
CTAGGGAGGAACACGTTCTAAATGAAATATTAAAAGTAAAAATTTATTCAATGAAGCGGAAATAGCTCATGCCATAAAA
TATGTTGAGGGATTGAGAGAAAAATTCATGATGGGACTAACAAATTGA
>Cluster_38832
ATGTCGCTACCAATGCATCGCACATTTGTTTTAACTGGAATAATATTTGCGTTATCGGCAGTGTATTCATTGAGCTATGC
ACGAGATGAATTTAACCTTCGTATCCTTGAGTTGGATAGCCCACTTGAAAATACACAAGTACTGGCAGATTTCATTAATA
ATAATAACTTAACTCCAGGTGTATATCTTACCTCTGTCATGTGGGGGCAAGATTCTCTTGATAAGAGAAATATTACATTT
GTTCTTTCTTCGGATAAAAAAAGTCTCATTCCTCGTTTTACAAAAGCGGATCTCCGTGAATTCGGTCTTAAAGTTGATGT
GATTCCTGCGTTAAAAGTAATGAATGATGATACTGAGGTTGGAGATATTGCACAGATTATTGATGGCGCACGTTATGATT
TCCAGTTAGACAGTCAAACGCTGTGGCTGCGGATCCCTCAAATCTATCAGAATGCAATAGCCGCAGGTAGTATTGCACCG
AAATACTGGAATGATGGTGAGAGTGCGGCTTGGTTAAGTTATTATGCCAGTGGTTCCCGGCAAAATAGTGATGGTGATAA
CCTGAGTTCAAATTGGTTGAATTTAAATTCAGGTATTAATTTAGGCGCATGGCGGCTACGAAATAATACAGTTTACAATG
AATCCAATTGGGAATCGATTAGCACCAGTTTGCAACGCGATATCAAAGCATTAAGAAGTCAGATGGAAATTGGGCAGACT
TTCACAAATGGTGATCTTTTTGACAGTGTGCAAATGACAGGTATTAAACTTGAAACTGATACCAGTATGTTACCTGACAG
CGAACAAGGCTTTGCACCAGTAGTTCGTGGTATCGCTAACAGCGATGCGCAAGTTGTGATTAAACAAAATGGCTATGTCA
TTTACCAAACATGGGTATCAGCAGGGCCATTTGAAATTAAAGACTTAAGTCAGGTAACGGCAGGATCTGATCTGGAAGTC
ACCATCAAAGAAACAAATGGGCAAGAACACAGTTTTATTCAGGCCAGTTCAACGGTTCCAATTCTACAGCGAGAAGGCGC
ATTAAAATATAGTCTTGCAGCTGGAAAATATCGTGACAGTGATAATAATGCTGAGACACCTGTTTTTGGTGTTGCAACCG
CGATATATGGCTTGCCCTATGGAATAACGATTTATGGCGGCATACTTGGTGCCTCAATGTATCATTCTGGTGTGACAGGG
ATTGGCGCTGATTTAGGCCGTCTGGGGTCGGTATCTGTTGATATCACCGCAGCAAAAACGAAGTTTGATGATGGGCGTGA
TGATGCAACAGGGCTGTCATGGCGTGCACAGTATGCGAAAGATTTTCCAGACACTGATACGACAGTAACTCTGGCAAGTT
ATCGTTATTCCACATCACAATTTTACACTTTCCAGGAAGCACTTGATCAACGTGATACACCTGATGACAAGGGTATTTAC
AGTTATCGGCAGACAAATAATCGGCGTAATCGTTTACAAATTAATCTTTCACAAAATATTGGTCGTTGGGGCTCGGTCTA
CCTTAATGGTTATCAACAGGATTATTGGGGAATGCATGGGGCCGAACGCAGTATAGGAATGGGGTACAGTACGACCTGGA
ATAATATTAACTGGTCTGTTAATTACACACTAACGAAAACGCCAGGAATGACTGGCGAGCAGCAATTTTCATTAACACTC
AATATTCCTCTCTCGCTGGCTGCCAGATTCATGGGCGATGTATAACGTAAACCGTTCCGATAAAAGTAATACATCACA
TCAATTGGGAATTGGCGGTACTGCTCTACAAGATAACAACCTGAGTTATAATTTACAGCAAAGTTATACTGATAATAATG
TGGGTTATGGTGCCAGCATGAATGGACGTTATCGTAGCTCAGTGGGTGAATTCGGTCTGGGATATAGCTATGATAAAAAT
AGTCGTCAGTGGAATTACAGTGCGCAAGGTGCGGTTGTTGCTCATGCTCATGGAGTAACATTGGGGCAATCAGTGCAGGA
TTCATTCGCGATCGTGCATATTAATGAGGGTGCCAATGTAAAAGTACAAAATGCGCAAGGCGTTTATACTGATTTTTGGG
GAAATGCGATTGTTCCCAATATGACGAATTATCGACATAACGCTATTACTGTGAATACTCAGGGGCACGATAGTCTTGAT
ATTTCTGATGCCACACAAGATGTTATTCCTTCGAAAGGTGCTGTTGTCGGTGTCGATTTTGATGCACGTAGCGGAATGCG
AGCCTTATTGACATTAGTCCATAATAAAGAACGCGTCCCCTTTGGGGCATTGCTGACATTAGGGAATAGCACAGCGATTG
TTGGTGAAGATGGAGAGGTTTATATTACAGGTGTGCAAGAATCAATGACATTTACGGTACAGTGGGGAAAAGAGATTAAC
CAGCAATGTACAGGAGTTATCACTGTACCAGAAAAATACACCACGGGTATCTATAAAGCCACAATGGATTGTCGATAA
>Cluster_3304
ATGGGTATTCAGAATTTCGATCATTCACACCACAAGCTCAAAATTCGTGGCCTGCAAAGTCCGGTGGATGTGCTGACCTT
CGAAGGCCGCGAACAACTGAGTACCCCGTTCCGCTACGACATTCAGTTTACCAGCAGCGATAAAGCCATCACGCCGGAGT
CGGTGCTGATGCAGGACGGCGCATTCAGCCTGACGGCACCGCCGGTACAGGGGATGCCGGTACAGACTGCCCTGCGCACC
CTGCACGGCGTCATCACCGGCTTTAAACACCTGTCATCGTCACAGGATGAAGCCCGTTACGAAGTGCGCCTTGAACCCCG
AATGGCGCTGCTGGCCCGCAGCCGCCAGAACGCCATTTACCAGAACCAGACGGTGCCGCAAATAGTCGAAAAAATCCTGC
GCGAGCGGCACCAGATGCGCGGTCAGGACTTCGTGTTTAACCTCAAAAGCGAATACCCGTCCCGTGAACAGGTGATGCAG
TACGGTGAGGACGACCTGACGTTTATTTCACGCCTGCTGTCGGAAGTGGGTATCTGGTTCCGCTTTGCCACCGACGCCCG
GCTGAAAATTGAGGTTGTCGAGTTTTACGACGACCAGAGCGGCTATGAGCGCGGACTGACGCTGCCGCTTCGCCATCCTT
CAGGGCTGTTTGATGGTGAAACGGAAGCCGTATGGGGGCTGAACACCGCATACAGCGTGGTGGAAAAAAGCGTCACCACG
CGGGACTACAACTACCGCACCGCCACAGCAGAGATGATGACAGAGCAGCATGACGCCACCGGGGGCGATAACACCACCTA
TGGCGAAGCCTACCACTACGCTGACAACTTCCTGCAAAAAGGCGACAAGGAGGCAGCGGAAAGCGGCGCGTTTTATGCCC
GTATCCGTCACGAACGCTACCTGAATGAACAGGCCATTCTGCAGGGCCAGAGCACCAGTTCACTGCTGATGCCGGGGCTG
GAAATCAGGGTTCAGGGCGACGACGCGCCTGCGGTATTCCGCAAGGGCGTGCTGATTACCGGCGTGACGGCCAGCGCGGC
CCGTGACCGCAGCTATGAACTGACGTTCACGGCGATACCCTACTCAGAACGCTACGGCTATCGCCCGGCGCTTATCCCCC
GCCCCGTAATGGCCGGAACACTCCCGGCGCGGGTCACCAGTACGGTGAAGAACGACATCTACGCCCATATCGATAAGGAC
GGGCGCTACCGGGTCAATCTCGACTTTGACCGTGATACGTGGAAGCCGGGCTACGAAAGCCTGTGGGTACGCCAGTCGCG
CCCGTATGCGGGAGACACCTACGGCCTGCACCTGCCGCTGCTGGCGGGAACGGAAGTCAGCATCGCCTTTGAGGAAGGCA
ACCCGGACCGACCGTATATCGCCGGGGTAAAACACGACTCGGCACACACCGACCATGTGACCATTCAGAACTACAAGCGC
AACGTGCTGCGCACCCCGGCGAACAACAAAATCCGCCTCGATGATGAGCGCGGCAAAGAGCATATCAAGGTCAGTACCGA
GTACGGCGGCAAGAGCCAGTTGAATCTGGGTCATCTGGTGGATGCCGGAAAACAACAGCGCGGGGAAGGCTTTGAACTGC
GTACCGATATGTGGGGCGCGGTGCGGGCGAAGAAAGGGATTTTTATCAGCGCCGATGCGCAGGATAAGGCGCAGGGGCAG
GTGCGCGAAATGGCTCCGGCGATGGCAATACTGGATGGCGCACAGTCACAGATGCAGTCGCTGTCCACTGATGCGCAGAC
GACGAATGCCGATCCGGCAGATCTGTCATCGCAGATAGCGCTGTTGCAGCAAAGTGTAAAAGATCTGACGCAGGCCGTGA
TCCTGCTAAGTGCACCCAAGGGCGTGGCGATCGCCAGCGGCGAGCACCTGCAACTGGCGGCGTCGAAGAATGTGATAGCG
AACGCAGGCAACAACGCAGATATCGGCGTAGTAAAAAATATGTTTATCGGTGTGGGTCAGGCGCTGAGCGTGTTTGTGCG
TAAAGCGGGCATCAAGCTGTTTGCCAATAAAGGGGCGGTCTCGGTACAGGCACAGAACGATCTGATGGAACTGTTGGCAC
AGAAATCGATAGAGATCACCAGTACGGAAGATGAGATCAAGATCACCGCGAAGAAGAAAATCACCCTTAACGGCGGTGGC
AGCTACATCCGGCTGGATGCCTGCGGGATAGAAGCGGGAACGCCGGGAGAATATAACGTTAAGGCCGGATATTACGGGCG
TAAACCAAAGGCGAAGCTGACGCCGGAGCTGATGGCATTTCCGGTGATTAAGTCTGAGGATTTAATCAGAGCTTTATTT
TGCTGGATGAAAATACTGGGCAACCACTTATTAATTGGCCCTATGAACTAGAACTGGAAAGTGGGCTAAAAATGAGTGGG
ATTACTGATGAAAATGGAAATACAGAACTAATATCCTCTGATAAGGAGGAGGTCGTTAATATTAGTGTGTTTGAACCCGA
TGAGTTTTTAGACGATGACATTAATTAA
>Cluster_16078
ATGGCTATCAAAAAAACCGAACTCTATTCTTCCCTGTGGGCAAGCTGCGACGAACTGCGTGGCGGGATGGACGCCAGCCA

GTACAAAGACTATGTACTGACCCTGCTGTTTATGAAGTACGTTTCCGATAAGTATAAAGGCGATCCTTACGGGATGATCG
TGATACCCAAAGGAGCCAGCTTTGACGATATGGTTGCGCTGAAAAATGACAAAGAGATCGGCGATAAAATCAACAAAATC
ATCCACAAGCTGGCGGAAGAGAACGGCCTGAAGGGCGTCATCGACGAAGCCGACTTTAACGACGAAGATAAACTCGGCAA
AGGCAAGGAAATGATTGACCGCCTGAGCAAGCTTGTCGGTATCTTTGAAGGGTTAGATCTCTCCAGCAACCGGGTCGAAG
GCGACGATCTGCTGGGCGATGCCTATGAATATCTGATGCGTCACTTCGCAACCGAATCCGGCAAGAGTAAAGGACAGTTT
TACACTCCGGCGGAGGTGTCACGTATTCTGGCGAAGGTGATTGGTATTACTCCCGATACGCCGCGAGATGCAACGGTTTA
TGACCCAACCTGTGGCTCTGGTTCGCTGCTGTTGAAAGTGAATGATGAAGCCCGGCGCGGACTGTCGATTTTCGGCCAAG
AGATGGATAACGCTACCAGTGCGCTGGCGCGGATGAACATGATTCTGCATAACAATGCCACCGCGAAAATCTGGCAGGGC
AATACGCTGAGCGATCCGCAGTGGAAAGAGGCGAACGGCAAACTGAAAGCCTTCGATTTTGCCGTGGCGAACCCGCCTTT
TTCCAATAAAAACTGGACCAACGGGCTGACGCCAAAAAAAGATCCGTTTGAACGCTTCGGCTGGGGTATTCCGCCGGAAA
AAAACGGCGATTATGCTTTTTTGTTGCATATCATTAAAAGCCTGAAAAGTACCGGCAAAGGCGCGGTAATCCTGCCCCAC
GGCGTACTGTTTCGCGGTAACGCCGAAGCGAATATCCGTGAAAACCTAATCAAGCAGGGCTATATCAAAGGCGTTATTGG
TTTGCCAGCCAACCTGTTTTACGGCACCGGCATCCCGGCGTGCATCATTGTGATTGATAAAGAGCACGCCCACAGCCGCA
AAGGCATTTTTATGATCGACGCCAGTCGGGGCTTTATCAAAGATGGCAACAAGAACCGCCTGCGTAGCCAAGATATTCAC
CGTATTGTGGACGTGTTCAACCATCAGCGAACGGTGCCAGGCTACAGCCGGATGGTGCCCTCCAGCGAGATCGCCGGGAA
TGATTACAACCTGAATATTCCTCGTTATATCGAGAGTGGAGAGCCGGAAGATTTGCACGATCTGACCGCGCACTTACAAG
GCGGTATTCCAGTGCGCGATGTAGATGCTTTGAAGGATTATTGGCGGGTATTCCCTGCCCTGCGTAATGTGCTGTTTGCT
GACGACCACCCCGGTTACTGCCGGGCGCAAGTGGACGCTCAGCAGGTGAAACCGACCATTCTGGCTCATCAGGAGTTTAA
GGATTTTGCCACCCGCAGCCTGCTACCGTTTAAGTCGTGGGTAAAAGAGGCAGGTCTGGAAGAGATCCGCAAAGGAGATA
AACCCAAAGCGCTAATCCACGATATCAGCGAAATGCTATTGGCGCAGTACGCCAACAGTGAGTTGCTGAACAAATACAGC
GTGTACCAGATCCTGATGGATTACTGGGCTGATGTCATGCAGGACGATGTGTATGTGATTATGCAGGATGGCTGGCAAGC
CGCCGCGCAAATCCGCGAGCTTCAGCCGGTGAAAGGCAAAGATGGCAAAAACATCTGGAAAGAAACGCACGATTTCGAGT
TTACCAAACGGCGCTACAAGGCCGATGTGCTGCCCCGTTCGCTGGTGGAAATTCGCTGTTTCCCGGAACTGCTGGATGCG
CTGAACCGCGCGCAGGAGCGTAGTGAAGAAGCCAGTCGTCAGCTAGTGGAATTCATCGAGGAGCAGGCAGGGGAAGAGGA
TCTGCTGGCTGAGGCGAAAAACGATAAAGATAAGGTGACGCAAAAGCTGGTGAATGCCCGTCTGGCACAGTTGAAGAAAA
CCCCTGCCGACCCGGATGAGCTGGCAGTGCTGAACCGCTGTCTGGCGCTGATTAAGGCCGAGGCCAGTGCGAAAAAAGCG
CTAAAAGCCGCACAGAAAGCGTTGGATCGAGCGGTATTTAAACATTATCCCACGCTGGATGAAGCAGCGATTAAAACGCT
GGTAGTGCAGGATAAGTGGCTGGCAACATTGCAGGCAGGGATCAAAGCGGAGATAGAGCGTATTACCCAGCAACTGGCGT
CGCGCGTAAAGGAGCTGGAAGAGCGTTATGCTGAACCGTTGCCAGCACTTGCAGCCGCCGTTGAGGCGTTGAGCGAGAAA
GTGGCCGGGCATTTGCGGGCGATGGGGCTGGAGTGGTGA
>Cluster_709
ATGAAAATACCCACTACTACGGATATTCCGCAGAGGTATACCTGGTGTCTGGCCGGAATTTGTTATTCATCTCTTGCCAT
TTTACTCTCCTTTTTAAGCTATGCGGAAAGTTATTTCAACCCGGCATTTTTATTAGAGAATGGCACATCCGTTGCTGATT
TATCGCGCTTTGAGAGAGGCAATCATCAACCTGCGGGAGTGTATCGGGTGGATCTCTGGCGTAATGATGAGTTCATTGGT
TCACAGGATATCGTATTTGAATCGACAACAGAAAATACAGGTGATAAATCAGGTGGGTTAATGCCCTGTTTTAACCAGGT
ACTCCTTGAACGAATTGGCCTTAATAGCAGTGCATTTCCCGAGTTAGCCCAGCAGCAAAACAATAAATGCATCAATTTAC
TGAAAGCTGTACCTGATGCCACAATTAACTTTGATTTTGCAGCGATGCGCCTGAACATCACTATTCCTCAGATAACGTTG
TTGAGTAGCGCTCACGGTTACATTCCGCCTGAAGAGTGGGATGAAGGTATTCCTGCTTTACTCCTGAATTATAATTTCAC
CGGTAACAGAGGTAATGGTAACGATAGCTATTTTTTTAGTGAGCTCAGCGGGATTAATATTGGCCCGTGGCGTTTACGCA
ACAATGGTTCCTGGAACTATTTTCGCGGAAATGGATATCATTCAGAACAGTGGAATAATATTGGCACCTGGGTACAGCGC
GCCATTATTCCGCTGAAAAGTGAACTGGTAATGGGAGACGGCAATACAGGAAGTGATATTTTCGATGGTGTTGGATTTCG
TGGTGTACGGCTTTATTCTTCCGATAATATGTATCCTGATAGCCAGCAAGGGTTTGCCCCAACGGTACGTGGGATTGCCC
GTACGGCGGCCCAGCTAACGATTCGGCAAAATGGTTTTATTATCTATCAAAGCTATGTTTCCCCCGGCGCTTTTGAAATT
ACAGATTTGCACCCGACATCTTCAAATGGCGATCTGGACGTCACCATCGACGAGCGCGATGGTAATCAGCAGAATTACAC
TATCCCGTATTCAACAGTGCCAATTTTACAACGCGAAGGGCGTTTCAAATTTGACCTGACGGCGGGCGATTTTCGTAGCG
GTAATAGTCAGCAATCATCGCCTTTCTTTTTTCAGGGCACGGCACTCGGCGGTTTACCACAGGAATTTACTGCCTACGGC
GGGACGCAATTATCTGCCAATTACACCGCCTTTTTATTAGGACTGGGGCGCAATCTCGGAAACTGGGGGGCAGTGTCGCT
GGATGTGACCCATGCGCGCAGTCAGTTAGCCGACGACAGTCGTCATGAGGGGGATTCCATTCGCTTCCTCTATGCGAAAT
CGATGAACACCTTCGGCACCAATTTTCAGTTAATGGGTTACCGCTATTCGACACAAGGTTTTTATACCCTTGATGATGTT
GCGTATCGTCGAATGGAGGGGTACGAATATGATTACGATTACGACGGTGAGCATCGGGATGAACCGATAATCGTGAATTA
CCACAATTTACGCTTTAGCCGTAAAGACCGTTTGCAGTTAAATATTTCACAATCACTTAATGACTTTGGCTCGCTTTATA
TCTCTGGTACCCATCAAAAATACTGGAATACTTCGGATTCAGATACCTGGTATCAGGTGGGGTATACCAGCAGCTGGGTT
GGCATCAGTTATTCACTCTCATTTTCGTGGAATGAATCTGTAGGGATCCCCGATAACGAACGTATTGTCGGACTTAATGT
TTCAGTGCCTTTCAATGTTCTGACCAAACGTCGCTACACCCGGGAAAATGCGCTCGACCGCGCTTATGCCTCCTTTAACG
CCAACCGTAACAGCAACGGGCAAAATAGCTGGCTGGCAGGTGTAGGTGGGACCTTACTGGAAGGCCACAACCTGAGTTAT
CACGTAAGCCAGGGTGATACCTCGAATAATGGGTATACGGGCAGCGCCACGGCAAACTGGCAGGCCGCTTACGCTACGCT
GGGGGTCGGGTATAACTACGACCGCGATCAACATGACGTTAACTGGCAGCTGTCTGGCGGTGTGGTCGGGCATGAAAATG
GCATAACGCTGAGCCAGCCTTTAGGGGATACCAATGTTTTGATTAAAGCGCCTGGCGCAGGCGGTGTACGCATTGAAAAT
CAAACTGGCATTTTAACCGACTGGCGCGGCTATGCGGTGATGCCGTATGCCACGGTTTATCGGTATAACCGTATCGCGCT
TGATACCAATACGATGGGGAATTCCATCGATGTTGAAAAAAATATTAGCAGCGTTGTGCCGACGCAAGGCGCGTTGGTTC
GTGCCAATTTTGATACCCGCATAGGCGTGCGGGCGCTCATTACCGTTACCCAGGGCGGAAAACCGGTGCCGTTTGGATCA
CTGGTACGGGAAAACAGTATCGGAATAACCAGTATGTGGGTGATGACGGGCAAGTTTATTTAA
>Cluster_17465
ATGCCAGAACATTCTTTTCTCCGTTTACGGGGGCTTTCGTGGTGGATTGCTCTGGCAATTTCTGGAAGTCCATTTAATGC
CCTGGCAGACGATACCATTCAGTTTGATGGCCGATTTCTTGATTTAAAAGGCAATACCAAAATTGATTTGGGCCGTTTTT
CGCAAAAAGGCTATGTAGAACCGGGAAAATATAACTTACGCGTTCATGTAAATAATCAGCCGTTACCTGACGACTACGAT
ATCTACTGGTACGCAACTGAAAACGATCCGAATAAATCATATGCCTGCTTATCGCCAGAGCTGGTTGCCCAGTTTGGTCT
GAAGGAAGATATTGCGAAGAATTTGCAATGGATCCGCGATGGTCAGTGTCTGAATACGGCACTTTTAGCAGGTACAGAAA
TTAGCGGCGATTTAGGCCAGTCTGCACTCCTTGTTTCTGTACCGCAGGCTTATCTGGAATATACCGACAGCGAGTGGGAC
CCGCCTTCGCGCTGGGATGACGGTATCCCTGGGCTTATTGCCGATTACAGTATCAACGCGCAAACCCGACATGAAAATGG
GGGTGATGACACTAACGACATCAGCGGTAATGGTACGGTCGGCGTTAACGTTGGGCCGTGGCGCTTACGAGCTGACTGGC
AGAGCGATTATCAGCATACCCGCAGTAACGATGACGGCGACACTGACGATAGCGGTACGCAAAAAAACTGGGAGTGGAGT
CGTTACTACGCCTGGCGCGCCTTGCCGTCATTAAAAGCCAAACTTTCGCTGGGCGAAGATTATCTCAACTCCGATATTTT
TGATGGCTTCAGTTACATTGGCGGCAGCATCAGTACCGATGATCAAATGTTACCGCCAAACCTGCGCGGCTACGCCCCCG
ATATTTCAGGTGTTGCACACACTACCGCTAAAGTGACCGTAACCCAAATGGGCCGTGTCATTTACGAAACTCAGGTGCCT
GCAGGTCCATTCCGCATTCAGGATATTGGCGATTCCGTCTCTGGCACGCTGCATGTCCGCATTGAAGAACAAAACGGCCA
GGTACAGGAATACGACGTCAGCACGGCGTCCATGCCGTTCCTGACTCGCCCCGGCCAGGTACGTTACAAAGTGACAATGG
GGCGTCCACAAAACTGGGACCATCAGGTTGCAGGTAGCTTCTTCTCGGGCGGCGAGGCATCGTGGGGGATTGCCAATGGC
TGGTCGCTCTACGGCGGTGCGTTAGCCGATGAAAACTACCAGTCGGCGGCGCTGGGCCTCGGTCGTGACCTGGCGCTGCT

AGGCGCGTTAGCGTTTGATGTCACTCACTCTCGTGTTCAACTCGACGATAACAGCGTGTATGGCAACAAAACGCTGGACG
GCAACTCTTATCGCGTCAGCTATGCCAAAGATTTTGATGAACTCAACAGTCGGGTGACGTTTGCTGGATACCGCTTCTCT
GAAAAAAACTACATGACCATGAGCGAGTATCTGGATGCGAACGACGACGACCGGGCGCGCACCGGTAACGACAAAGAGAT
GTATATGGTCACCTATAACCAGAACTTCACGGATGCGCGCGTTTCGGTCTATCTCAATTATTCCCACCATACCTACTGGG
ATCGCCAGGATCAGACCAACTACAACATGATGCTTTCTCATTATTTCAATTTAGGAAGCCTTCGTAACTTGAGCGTTTCT
CTGACTGGTTATCGCTACGAATACGATAAAAGTGCCGACAAGGGGGTTTATCTGTCCTTAAGTTTGCCGTGGGGCGACAA
CAGCACTATCAGTTACAACGGCAACTATGGCAGCGGTGCCGACAGCAATCAGGTGAGTCTGTATCACCGCATTGATGACG
CCAGCCACTACACGTTAAGCGCCGGAACCAGCGAAAACCACAGCAGCGTGGATGGCTACTACAGCCACGATGGCACTCTG
GCAAAAGTTGATCTCAGTGCTAACTACCATGAAGGGCAATACACCTCAGCAGGGATCTCTTTACAGGGCGGGGCAACGCT
GACCGCTCATGGCGGGGCACTGCATCGTACACAAAATATGGGCGGCACACGTCTGTTAATCGACGCTGATGGTGTTGCTA
ATGTGCCGGTTGAAGGTAATGGTTCCGCCGTTTATACCAACATGTTCGGCAAAGCGGTGGTGGCAGATGTGAATGATTAC
TACCGCAACCAGGCTTATATCGATTTGAACAAACTGCCGGAAAACGCCGAAGCCACGAAATCAGTGGTTCAGGCAACGCT
GACGGAAGGCGCTATTGGCTACCGTAAATTTGCGGTAATCAGCGGTGAGAAGGCGATGGCAGTGCTGCGCTTGCAGGATG
GCAGCCATCCGCCATTTGGGGCGGAAGTGAAAAACGATAATCAACAACAAGTAGGGTTGGTTGATGACGAAGGTAATGTT
TATCTGGCAGGCGTTAAACCGGGTGAACATATGACCGTTTTCTGGGAGGGGGAATCTCACTGCGATATCAGCCTGCCAGA
CCCGTTACCGAATGACCTGTTCAATGGCTTGTTGTTACCATGCCAGCAAAAGGGGGGAAGTTCTCCCGTCATACCGCATG
ATATTCAACCCGTCATTCAGGAGCAGACACAACAGGTGACACCAATGGAACCGCCGATGTCTGTTTCATCGAACCAATAA
>Cluster_27319
ATGGCTACAATTCATGTAGACGGCAAAGAATACGAGGTCAACGGAGCGGACAACCTGCTGGAAGCTTGTCTGTCTCTGGG
CCTTGATATTCCTTACTTTTGCTGGCATCCGGCGCTGGGAAGCGTCGGTGCTTGCCGCCAGTGTGCGGTGAAGCAATACC
AAAACGCGGAAGACACGCGTGGTCGCCTGGTGATGTCCTGTATGACACCGGCTTCCGATGGCACCTTTATTTCCATTGAC
GACGAAGAAGCGAAACAGTTCCGTGAAAGCGTGGTCGAGTGGTTAATGACCAACCACCCGCACGACTGTCCGGTATGTGA
AGAGGGCGGTAACTGCCATCTTCAGGATATGACTGTGATGACCGGACACAGCTTCCGTCGCTACCGTTTCACCAAACGTA
CCCACCGTAATCAGGATTTGGGGCCATTCATCTCTCACGAAATGAACCGCTGCATCGCCTGCTACCGCTGCGTGCGTTAC
TACAAAGATTACGCTGACGGCACGGACCTGGGCGTTTACGGTGCGCACGACAACGTCTACTTCGGTCGCCCGGAAGACGG
CACGCTGGAAAGCGAATTTTCCGGTAATCTGGTCGAAATTTGCCCGACCGGCGTATTTACCGACAAAACGCACTCCGAGC
GTTACAACCGTAAATGGGATATGCAGTTTGCGCCGAGCATCTGCCAGCAATGTTCCATCGGCTGTAACATCAGCCCCGGC
GAGCGTTATGGCGAACTGCGTCGTATCGAAAACCGTTACAACGGTACGGTAAACCACTACTTCCTCTGCGACCGTGGTCG
TTTCGGTTACGGTTACGTCAACCTGAAAGATCGTCCGCGTCAGCCAGTACAGCGTCGTGGCGATGATTTCATTACCCTCA
ACGCCGAACAGGCAATGCAGGGCGCAGCAGATATTCTGCGTCAGTCGAAGAAAGTGATCGGTATTGGTTCTCCGCGTGCC
AGCGTGGAAAGCAACTTTGCGCTGCGTGAGCTGGTGGGCGAAGAAAACTTCTATACCGGTATCGCTCACGGTGAGCAGGA
ACGTCTGCAACTGGCGCTGAAAGTACTGCGTGAAGGCGGCATTTATACTCCGGCTCTGCGCGAAATCGAATCTTACGATG
CGGTACTGGTGCTGGGCGAAGACGTTACCCAGACCGGCGCGCGCGTCGCGCTGGCAGTGCGTCAGGCGGTGAAAGGTAAA
GCGCGTGAAATGGCGGCAGCACAGAAAGTGGCTGACTGGCAGATTGCGGCAATCCTCAACATCGGTCAACGTGCGAAGCA
TCCGCTGTTTGTTACCAACGTTGATGACACCCGTCTGGATGATATCGCGGCGTGGACTTACCGCGCACCGGTTGAAGATC
AGGCGCGTTTAGGTTTTGCCATCGCCCATGCGCTGGACAACTCCGCACCGGCAGTTGACGGCATAGAGCCTGAGCTGCAA
AGCAAAATCGACGTCATCGTGCAGGCACTGGCTGGTGCGAAGAAACCGTTGATTATCTCCGGGACGAACGCTGGTAGCGC
TGAAGTGATTCAGGCTGCGGCTAACGTGGCGAAAGCCCTGAAAGGTCGCGGCGCTGACGTCGGTATCACCATGATTGCCC
GTTCCGTCAACAGCATGGGGCTGGGCATTATGGGCGGTGGATCGCTTGAAGAAGCGTTAACCGAACTGGAAACCGGACGC
GCCGATGCGGTGGTGGTGCTGGAAAACGATCTGCATCGTCACGCTTCCGCTACCCGCGTGAATGCTGCGCTGGCTAAAGC
GCCGCTGGTGATGGTGGTTGACCATCAACGCACAGCGATTATGGAAAACGCCCATCTGGTACTTTCCGCCGCCAGCTTTG
CTGAAAGCGACGGTACGGTGATCAACAACGAAGGCCGCGCCCAACGTTTCTTCCAGGTTTACGATCCGGCTTATTACGAC
AGCAAAACTGTCATGCTGGAAAGCTGGCGCTGGCTGCACTCGCTGCATAGCACCCTGCTGAGCCGTGAAGTGGACTGGAC
GCAGCTCGACCACGTGATTGACGCTGTTGTGGCGAAAATCCCGGAACTGGCAGGTATCAAAGATGCTGCGCCGGATGCGA
CATTCCGTATTCGTGGGCAGAAACTGGCCCGTGAACCGCATCGCTATAGCGGTCGTACCGCCATGCGCGCCAATATCAGC
GTTCATGAGCCGCGTCAGCCGCAGGATATTGACACCATGTTTACCTTCTCGATGGAAGGTAACAACCAGCCGACCGCGCA
CCGTTCGCAAGTGCCGTTTGCCTGGGCGCCAGGCTGGAACTCCCCGCAGGCGTGGAACAAATTCCAGGACGAAGTGGGCG
GCAAACTGCGTTTTGGCGATCCGGGCGTGCGTCTGTTTGAAACCAGCGAAAACGGTCTGGATTACTTCACCAGCGTACCG
GCACGCTTCCAGCCGCAGGACGGGAAATGGCGTATCGCGCCGTACTACCACCTGTTTGGCAGCGATGAATTGTCACAGCG
TGCTCCGGTCTTCCAGAGCCGTATGCCGCAGCCGTACATCAAACTCAACCCAGCGGATGCCGCGAAGTTGGGGGTGAACG
CAGGTACACGCGTCTCCTTTAGTTACGATGGCAACATGGTCACGCTGCCGGTTGAAATCTCCGAAGGGCTGACGGCAGGG
CAGGTGGGCTTGCCGATGGGTATGTCCGGCATTGCTCCGGTGCTGGCTGGCGCGCATCTTGAGGATCTCAAGGAGGCACA
ACAATGA
>Cluster_35249
ATGACCCCAGCAGGAAACACGCCTGGCAATATCAACCTGGGTAATGATGTCACCGTCAATGTCAACGACGCCTCTGGATA
TGCAAAAGGAATCATTATTCAGGGCAAAAACAGCTCCCTGACAGCTAACCGACTCACAGTAGATGTTGTTGGTCAAACCT
CTGCCATCGGCATTAACTTAATTGGTGACTATACCCATGCTGACTTAGGCACAGGCAGCACCATTAAGAGTAACGATGAC
GGCATCATTATTGGGCATAGCTCAACACTAACAGCCACTCAATTCACCATTGAAAACTCGAACGGTATAGGCCTAACCAT
CAATGACTATGGCACCAGTGTCGATCTTGGAAGCGGAAGTAAAATCACGACCGATGGAAGTACAGGTGTTTATATCGGTG
GTCTCAACGGCAATAACGCCAATGGTGCTGCGCGTTTTACGGCTACAGACCTGACAATCGATGTTCAGGGCTACAGCGCC
ATGGGGATAAACGTACAGAAAAACTCTGTTGTCGATCTCGGAACAAACAGTACCATTAAAACCAATGGCGATAATGCTCA
CGGCCTCTGGAGCTTTGGCCAGGTTAGCGCGAATGCACTCACTGTTGATGTAACTGGAGCCGCGGCCAATGGCGTCGAAG
TTCGTGGTGGTACAACCACTATCGGTGCAGATAGCCATATTTCTTCCGCGCAGGGCGGTGGCCTCGTCACCAGTGGTTCA
GACGCGATAATCAATTTTACTGGCACGGCAGCGCAACGAAACAGCATCTTTTCCGGCGGTTCTTATGGTGCCTCGGCCCA
GACGGCAACGGCTGTTGTCAACATGCAAAATACCGATATTACAGTTGATCGTAATGGCAGTCTGGCGCTGGGTTTGTGGG
CGCTCAGCGGCGGTAGAATAACCGGAGACAGTTTGGCTATCACCGGCGCGGCAGGAGCCAGGGGAATTTATGCCATGACC
AACAGCCAGATCGACCTCACGAGCGATCTGGTCATTGATATGAGTACACCCGACCAGATGGCCATCGCAACGCAACATGA
CGATGGTTATGCCGCCAGCCGCATCAACGCCTCGGGTCGTATGCTTATCAACGGTAGCGTTCTTTCCAAAGGTGGGCTAA
TCAATCTGGATATGCACCCTGGGTCGGTTTGGACAGGTTCCTCCCTCAGCGATAATGTCAATGGCGGAAAACTGGACGTT
GCAATGAATAACAGCGTCTGGAACGTAACAAGTAATTCTAATCTCGACACGCTGGCGCTGAGCCATTCAACTGTCGATTT
TGCCAGCCACGGGTCAACTGCCGGCACATTTGCCACATTAAACGTAGAGAACCTGAGCGGTAACAGTACCTTTATTATGC
GTGCTGATGTTGTTGGCGAGGGTAATGGCGTTAATAATAAAGGGGATTTATTGAATATCAGCGGGAGTAGTGCTGGTAAT
CACGTATTGGCTATCCGCAACCAGGGCAGCGAGGCCACAACGGGAAATGAAGTTCTGACAGTGGTAAAAACCACTGACGG
CGCGGCCTCGTTCAGCGCGTCTTCTCAGGTTGAGTTGGGGGGATATCTGTACGATGTGCGTAAAAATGGCACTAACTGGG
AGCTTTACGCTTCCGGGACAGTTCCGGAACCGACTCCTAATCCTGAACCCACACCAGCTCCCGCTCAGCCTCCCATAGTC
AACCCCGATCCTACGCCTGAACCCGCTCCCACGCCTAAACCCACCACGACCGCAGATGCTGGCGGCAATTATCTCAATGT
CGGTTACTTATTGAACTATGTTGAAAACCGTACGCTGATGCAACGGATGGGTGACCTGCGAAATCAGAGTAAAGACGGTA
ATATCTGGTTGCGCAGTTATGGGGGAAGCCTGGACTCCTTTGCCAGTGGCAAACTGAGCGGCTTTGACATGGGTTACAGC
GGTATCCAGTTTGGTGGGATAAACGTCTCTCTGATGTAATGCCGTTGTATGTCGGTCTGTATATTGGCTCAACACATGC

ATCGCCGGACTATAGCGGAGGCGACGGTACCGCACGTTCAGACTACATGGGAATGTACGCCAGTTACATGGCACAAAACG
GTTTTTACAGCGATCTCGTTATAAAAGCATCGCGCCAGAAAAATAGTTTCCACGTACTGGACAGTCAGAACAACGGCGTT
AACGCCAACGGCACTGCGAATGGAATGAGCATCTCCCTGGAAGCCGGGCAGAGGTTCAACCTGTCCCCTACTGGTTATGG
GTTCTATATAGAGCCGCAAACCCAGCTTACATACAGCCACCAGAATGAGATGACTATGAAGGCGAGTAATGGCCTCAATA
TACATCTGAATCACTACGAATCGCTGCTGGGGCGTGCCAGCATGATACTGGGGTATGACATCACCGCAGGCAACAGCCAG
CTGAATGTCTATGTGAAGACTGGCGCTATCCGCGAGTTTTCAGGGGATACCGAATATCTGTTGAACAACTCCCGGGAGAA
GTACAGTTTCAAAGGTAATGGCTGGAATAACGGCGTGGGAGTCAGTGCACAGTATAACAAACAGCACACATTCTATCTCG
AAGCGGATTACACGCAGGGTAACCTCTTTGATCAGAAGCAAGTCAACAGAGGATATCGCTTCAGCTTTTAA
>Cluster_25588
ATGAAGGGGAGCAAAGAAAAATCTATGCCGGATGTTCTGGACTCTGCCCAGTTGGTACGTATTGAAGCTGTACACCGAGG
ATTCCTTTATCAGCACTTGTATGCCGTTGGCTGCTTGCTGCTGGCACAGAAAGCCAGCGTGGAGACAGTAACCGTTGAGC
TGGATGAGGATATTGAGCTCAACTCCAGGCAGGAGCGCATATATGTTCAGGTTAAAACCCGCCTGAAACCCATCATTCCC
AGCGATGTGTTTGACGCTCTCGCGCGGTTTGCGGAGCTGCGTAACGAGCACACTGATGGGCGCCGCCAGGAAAGCGCTTC
TTTCGTTATCGTCGCCAACCAGGCGCCAGGTCCACATCTGCAGAAGATGATTGAGGATAAGAGACTTCCTGCGGATGTCC
GTTTTATCTGGCCCCAGTCAACCGCTGAGCGCCATCCTGCACTTCCCCCTGCCTGGGACACCGTGGCTGATGCGGCCGCG
TGGTGCATCGCGCAAGCAGAGCAACTGAATTTTTCGTTATTGTCGCCAGAATCTCTGATCTGGAAGCTGGCCGGTCTGGT
CCAGCTCGCCGCCACCGGAGGTGATGCTGACGGACAGCATGCGTTTTATACCCGGGATCTTCCTGCCCTGTTCGAACAGC
TCATCGTTCAGTTACAGGACTTCCCTGCACCGCCGACACTCTACCGACCACAAAGAGAGGAACCCTCTTTTGTGTCGGAT
GTGCGGATACGCATCATTTGCGGCTTGTCCGGTGCAGGTAAAACAGCTTGGGCAGCACAGACCGCCCAGCACAGTACCCA
GGTGTGTGCGTATTACGATGCTGGCGACCTTCCAGGCCCTGCACTTGCCAGTACGCTGGTTCGTGAGCTGGCAGCCCGGT
TTGCGACTCAGCAGCAAGGTGGCCTGCGAAGAATACTGCTCCCTGGTGCCAGTGGCGTTGAAGCGCTGAGAACGTTCGAC
ACCTTCTTGCAGCAACGGAACGACAACCTGCTGCTGGTTCTGGATAACGCGCATCGTATCCCTGTGGAGAACTTGCGGGA
TGTTCTGCACGCCACAACCCGTATCCATTTTGTGCTGCTGTGCCAGCCCCATGACAATGTCCGTCAACTGGAGGCGATGA
CTGGACTTCAGCGTGAATCGTTACAGGGATGGGATATCGACACAGTGGCTGCCGCAGTGGCCGACCTCGGTGGGCACGCC
AGTGCGCTGGGATATGAGCAGCTACGAAGCTACACCGGAGGGTTGCCGCTGTATGTCGAAAGCGCGGCGAGAGTTGCCGC
AGAGGAATACAATGGAGATATAGACACGCCGTGTGCTGAACTGCGGCAACAGGAAAACAGCACGGAAACCGCGCAGGAAG
TCATTCTCAGCCGTGTCTATCAGGGGTTTGAGCCACTGGTCCAGAACGCTCTGGCGTTGTTCAGTCTGTCCGATGTGGGG
CTAAGCCGTGATGAAGTTTCAGGGTACCTTGCCCGCTCATTGAATATCCCTTCAAACGGGGCAGCCACATTAATTAAAAA
AATGCGGGCCACCGGAACCATTGAAAACTATGGAAACCAGACGTTAAAGGTACACGATGCTGTCAGGGCGCTGGGGCTGC
AACACCTTACCATGATGGACGTGGCTATCGTAAATAACGCGTTACTGGCGTTAAAAGATCTTTTGATTGATAGCCTTCAG
CAAGAACGCGACACCTCGCGATTTTCTCTTCTGACGCAGATTTACATCAAGCTTAACGATGTCATGACGCTCATCGCTCT
ATCTGGTGAAGAGCTATTTTATGAGATGGGCATTACTGTCGACATTATGGAAAGCCTGAAACAGGCGACAGCTTCGGACT
CTCTGGCGCCTTTACAAAGGTTCTGGGCACTCGATGGGCTCGTTTTTTCCGAGCTTAAAGACGGTGCTTCTGGGCAAATT
GCACAATGGCTGGAAGCGATGGGAGCCTTGCTTGCAGAACATGAGTTTGGTTACCGGGAACGCACAGCCTACACCATGAA
ACGGATGCTTTTCTTATCAGAAAAGGGAGACCTTAGTGAAGTGCAAACGCTGGCTGAAGATGCCCGCCCCAGCCTTCCGG
ATGAAGAGCATGCGCGTATTTTTGACTATAACCATGCTATCGCGCTCTGGAGACTGAAAAGATATAAACAAGCAGAAACA
CTGTGTCTGAGCGTAGTGAATCGCTACTACACGTTATTTGGTATTACCCCACAGGATGTCATGGGTAAAAACTCGGATGT
GCTGTGGGCAATCATCAACCAGCCTGAAAATGTTCACGAGCACATCAAACATCTGGCCGATGCCCTAGAGTTGCTCGCCA
GAATTAATGACGCGCAGGGAAAGGTGTCACCGTTTCTGCGCATTCACGCGATGAAATTTTACAACATGACCGCAGCCCCC
GAATCTCTGGTCCGGGTAGGACAGGATCTAGCTGATGAGTTTGTCGCAATAAAGGACTATGTCGGTGCCAGAGAGGTGAT
GGAGCAATACGTCCTGCCAGTCGTCAATGAAGCCGGTCTGGTACAGCGACTGGTTCAGGTCCGAAGCCAGTACGCCGTCA
TACTGGCACTTGCAGGCGAACATGCACAGGCTGAAGCGGAGATGCGTCGGCTAGCCCCTTTTTTGAAGGGTTAA
>Cluster_66982
ATGTTTGCACTGGCTGATATCAACAGTTTCTACGCCTCATGTGAAAAAGTTTTCCGCCCGGACCTTCGCAACGAACAGGT
CATCGTACTCAGCAATAACGATGGCTGTGTGATCGCACGCAGCCCAGATTATGTGGAATTGCAAGTAACGTTGTAG
>Cluster_35023
ATGAACATCATTGATGGAAATGGGGCTTCCGAAGTTCTTAGATTATCAGCGTTACATGCAAACAAAGTAGTGAAAACACA
GGTACAAAAAAAGCCGCCCCTTGAGCGACTCGATTTGCATACGATGTGGTGCGAAGGCCGGACTCAAACATGCGATTTAA
>Cluster_6407
ATGATGAACATCGAAGAACTGCGTAAAATTTTTTGTGAAGATGGCCTCTATGCTGTGTGCGTTGAAAATGGAAATCTTGG
TAAGCGTAAACTGACCGCCGTATGTAGCCATCAGACGAGAATTGGTAACTTAGACGCCCATCTGATATAG
>Cluster_15967
ATGCGCAATGAAATAGCCATCAATCACCAGATGCTTCGTGCTGCACAGAACAAAGCAGTAATAGCCAGATTTATTGGTGA
TTCCAAAATGTGGCTTGAAGCAAATAAAGCGATGAAATCAGCTATCAACCTTCCGTGGTATCGCAGGAAATGA
>Cluster_23791
ATGGCTAAAGGTATTCGTGAGAAAATCAAGCTGGTTTCTTCTGCTGGTACTGGTCACTTCTATACCACTACGAAGAACAA
ACGTACTAAGCCGGAAAAACTGGAACTGAAAAAATTCGATCCAGTTGTTCGCCAGCACGTGATCTACAAAGAAGCGAAAA
TCAAATAA
>Cluster_17640
ATGCGTGTAGCGTTTATCGGTTTATTACCATACCCGACTCGTTTTTGGGCTTCTGCGCTAATTGCAAAGCCACATGCCCT
GATGGCTGACAGCATCATCCCGGCACAAAAGCGCCATACCGGTATTGCTGCTGCACGACGCGAAGCAAAGAAACGCAGGA
GAGCAAAATGA
>Cluster_43570
ATGTCTGGGATGAGAAAAGCATCGGAACATCGTTCTCCGATAAAACCCGCCATGAGTTACGCTCTGTTCCAAGATTTTAT
CAATGCGGGGCTGGCAGCTCAGAAGAGCGATGAGGCTAAGTCTGCTGTTAAAAATCATCGGATTACCAATCAGAACTGA
>Cluster_10669
ATGGCTAAGAATTTTAGCTACGCCAGTCACAGGGATAATTTATGCGCTGCGTCACAAAAACTGCTACACTCCGCTCCCTC
ATGTCATTGTGGTTTTTGTCATTTTCCTTTTCAGTATCTCCCTGAAAACTACACCGGTAACGGTCGGGGCGGTTCGGAGT
AG
>Cluster_15661
ATGCGAAAAACGCAGGAAAATAAACGCGTTAATCACCGAAAAGATGTGCTACGTGACCAGTTTTATCAGGGGGTTAATCC
AGCAATAGCTGTGCCACTGAGAGAAATACTTAACAGGTACAAAACTTCGGAGAAGTCAAAATGA
>Cluster_15090
ATGATTATCAAAACGTTAAAAATGAGCGCACGAAAGCGAAAATTGATGAAACGTTCGCTCACTATTTACCAGGTAAATTT
ATGGGATTGTAGCGTAAAAAAAGACAATTTCGCAGTCTTGCGCCGCATTGATTAG
>Cluster_45924
ATGCGCGCCATGTTAGAGGATGAGCGTTGTTCTTTAGCTGCCATTTCATCAATGACGGCTCGCTCCTCGAGGGACAGCCG
AAGTGCCAGTCTTGGACCTGTGGCGGTGTTACGCGGAATGCGTGATCTGGTATCGTGA
>Cluster_12322

ATGGATAACTTGCGCTTCTCTTCTGCGCCGACAGCAGATTCCATTGATGCATCGATCGCTCAACACTACCCGGACTGCGA
ACCTGTCGCGGTTATCGGCTACGCCTGCCATTTTCCTGAATCGCCGGATGGCGAAACGTTCTGGCAAAATCTGCTGGAAG
GTCGTGAATGCAGCCGACGCTTTACGCGCGAAGAGCTTCTGGCCGTCGGTCTGGATGCCGCCATCATTGACGATCCTCAT
TATGTCAATATCGGTTCGGTGTTAGACAACGCCGACTGCTTCGACGCCACCCTGTTTGGCTATTCGCGACAGGAAGCGGA
GTCGATGGACCCGCAGCAGCGCCTGTTTTTGCAGGCGGTCTGGCATGCGCTGGAACATGCCGGTTATGCCCCCGGCGCCG
TCCCCCATAAGACCGGCGTTTTCGCCTCTTCCCGGATGAGTACCTACCCCGGTCGCGAAGCATTGAACGTGACAGAAGTC
GCGCAGGTAAAAGGTCTGCAATCTCTGATAGGCAATGATAAAGACTATATTGCCACCCGCGCCGCGTACAAACTCAACCT
GCACGGCCCGGCGTTATCGGTACAGACCGCCTGCTCCAGCTCGCTGGTTGCCGTGCATCTGGCCTGTGAAAGCCTGCGCG
CTGGCGAATCCGATATGGCGGTTGCCGGCGGCGTGGCGCTCTCTTTCCCCCAGCAGGCAGGCTACCGCTACCAGCCCGGA
ATGATTTTCTCTCCTGATGGTCACTGTCGTCCCTTTGACGCCTCGGCTGAGGGCACCTGGGCCGGTAACGGTCTCGGCTG
CGTGGTGCTGCGTCGCCTGAGAGACGCGCTGCTGTCAGGCGATCCGATTATCTCGGTGATCCTCTCCAGCGCGGTCAACA
ACGACGGCAACAGAAAGGTCGGCTATACCGCCCCTTCCGTCGCAGGGCAACAGGCAGTCATCGAAGAGGCGTTAATGCTG
GCGGCCATCGACGACAGGCAGGTAGGTTACATTGAAACCCACGGCACCGGCACACCGCTGGGCGACGCGATTGAAATTGA
AGCGTTACGCAACGTCTATGCGCCTCGCCCGCAGGATCAGCGCTGTGCGCTCGGTTCCGTGAAAAGTAACATGGGCCATC
TGGATACCGCGGCGGGCATTGCCGGACTGCTGAAAACCGTTCTGGCAGTCAGTCGCGGGCAAATTCCTCCCTTACTGAAT
TTTCACACCCCCAACCCGGCGCTGAAACTTGAAGAGAGCCCCTTTACCATACCGGTGTCGGCACAGGCATGGCAGGACGA
AATGCGCTATGCGGGCGTCTCCTCCTTTGGTATTGGCGGCACCAACTGCCATATGATCGTCGCCTCGCTGCCCGACGCGC
TCAACGCGCGCCTCCCCAATACGGATAGCGGCAGAAAAAGTACCGCGCTGCTGCTCAGCGCCGCCAGCGACAGCGCGTTG
CGGCGGCTGGCGACGGATTATGCCGGGGCGCTGAGAGAGAATGCGGATGCCAGCTCTCTGGCCTTCACAGCCCTGCACGC
GCGCCGTCTCGATCTCCCCTTCCGCCTGGCGGCGCCATTAAACCGTGAAACCGCCGAGGCGCTCAGCGCCTGGGCTAGTG
AGAAATCGGGGGCGCTGGTTTACAGCGGCCACGGCGCCAGCGGCAAGCAGGTGTGGCTGTTTACCGGCCAGGGCTCGCAC
TGGCGCACTATGGGTCAAACGATGTACCAGCACTCAACGGCGTTTGCCGACACGCTGGATCGCTGTTTTTCCGCCTGTAG
CGAAATGCTCACGCCGTCACTGCGCGAAGCGATGTTTAACCCCGATTCGGCGCAGCTGGACAATATGGCCTGGGCGCAGC
CGGCGATTGTCGCGTTTGAAATCGCGATGGCGGCGCACTGGCGTGCTGAAGGACTGAAGCCAGACTTCGCCATTGGGCAT
TCCGTCGGTGAATTTGCCGCTGCCGTTGTCTGCGGACACTATACGATTGAACAGGTCATGCCACTGGTTTGTCGGCGCGG
CGCGCTAATGCAGCAGTGCGCAAGCGGCGCGATGGTGGCGGTATTTGCAGACGAAGACACGCTGATGCCGCTGGCTCGCC
AGTTTGAGCTGGATCTCGCCGCCAACAACGGTACGCAACATACGGTATTTTCCGGGCCGGAAGCCCGTCTCGCGGTATTT
TGCGCCACGCTCTCGCAGCATGACATTAACTATCGTCGCCTGAGCGTAACCGGTGCGGCGCACTCCGCTTTACTGGAGCC
GATACTCGATCGGTTCCAGGACGCCTGCGCGGGACTGCACGCGGAGCCGGGGCAAATACCGATTATTTCCACGCTCACCG
CCGACGTCATTGATGAGTCAACGCTCAACCAGGCGGATTACTGGCGCCGACACATGCGCCAGCCGGTGCGTTTTATCCAG
AGTATTCAGGTGGCGCATCAGCTCGGCACCCGCGTTTTTCTGGAGATGGGGCCCGATGCCCAGTTGGTTGCTTGCGGGCA
GCGCGAATACCGCGATAACGCATACTGGATAGCCAGCGCCCGGCGTAACAAAGAGGCGAGCGATGTCCTCAATCAGGCCC
TGCTCCAGCTTTACGCTGCCGGCGTCGCCCTACCGTGGGCCGACCTGCTGGCGGGCGATGGACAACGTATCGCTGCGCCA
TGTTATCCGTTTGATACTGAACGTTACTGGAAAGAGCGCGTCTCCCCGGCCTGCGAGCCTGCCGACGCAGCGCTGTCTGC
CGGGCTGGAGGTGGCGAGTCGCGCCGCGACAGCGCTCGATCTCCCTCGCCTGGAAGCGCTTAAACAGTGCGCCACGCGAC
TGCACGCCATCTACGTCGATCAACTGGTACAACGCTGTACCGGCGATGCCATAGAGAACGGCGTGGACGCCATGACCATC
ATGCGCCGTGGACGTCTGCTGCCCCGCTACCAGCAGCTACTCCAGCGCCTGCTGAATAACTGCGTGGTCGACGGCGATTA
CCGCTGCACCGACGGGCGATACGTCCGCGCCCGCCCCATTGAACATCAACAGCGGGAATCACTGCTGACGGAACTTGCCG
GTTATTGTGAAGGTTTTCAGGCTATTCCCGACACCATCGCCCGTGCCGGCGATCGGTTATATGAAATGATGAGCGGCGCG
GAAGAACCGGTGGCGATTATCTTCCCGCAAAGCGCCTCCGACGGCGTGGAAGTGCTGTATCAGGAATTCAGCTTTGGCCG
CTATTTCAACCAAATCGCCGCCGGGGTATTACGCGGCATTGTCCAGACGCGTCAGCCCCGCCAGCCGTTGCGTATTCTTG
AAGTTGGCGGCGGAACCGGCGGCACCACCGCGTGGCTGCTGCCGGAACTCAACGGCGTTCCGGCACTGGAGTACCATTTC
ACCGATATCTCGGCGCTGTTCACCCGTCGCGCCCAGCAGAAATTCGCCGACTATGATTTTGTGAAGTATAGCGAGCTGGA
TCTCGAAAAAGAGGCGCAGTCTCAGGGTTTCCAGGCACAGTCTTACGATCTTATCGTGGCAGCGAACGTGATTCACGCCA
CCCGCCATATTGGCCGCACGCTCGATAATCTGCGCCCCCTGCTCAAGCCGGGCGGGCGCCTGCTGATGCGCGAAATCACC
CAGCCAATGCGTCTGTTTGACTTCGTTTTCGGCCCGCTGGTTCTTCCGCTACAGGATCTCGACGCCCGCGAAGGTGAGTT
ATTCCTCACCACCGCTCAGTGGCAACAACAGTGCCGCCACGCCGGATTCAGCAAAGTGGCGTGGCTACCGCAGGATGGCA
GCCCGACCGCCGGGATGAGCGAACATATCATTCTCGCCACGCTGCCCGGTCAGGCGGTTAGCGCCGTAACATTCACCGCG
CCATCAGAACCCGTGTTGGGGCAGGCGCTGACGGATAACGGTGATTATCTCGCCGACTGGTCTGATTGCGCAGGTCAGCC
CGAACAGTTTAACGCCCGCTGGCAGGAGGCCTGGCGTCTGCTTTCACAGCGTCATGGCGACGCTCTGCCTGTGGAACCGC
CCCCCGTCGCCGCCCCGGAGTGGCTGGGGAAGGTTCGCTTAAGCTGGCAAAACAAAGCCTTTTCCCGCGGTCAGATGCGC
GTTGAAGCCCGTCATCCTGCTGGCGAGTGGCTGCCGCTATCGCCCGCCGCGCCTCTTCCTGCGCCGCAAACGCATTATCA
ATGGCGCTGGACGCCCCTCAACGTCGCCAGCATTGACCATCCGCTTACCTTTAGCTTCAGCGCCGGTACGCTTGCGCGCA
GCGACGAGCTGGCGCAATACGGCATCATTCACGATCCGCACGCCTCTTCACGACTGATGATTGTTGAGGAGAGCGAGGAT
ACGCTGGCCTTAGCGGAGAAAGTGATAGCAGCGCTCACCGCCAGCGCAGCCGGATTGATTGTGGTTACTCGCCGCGCGTG
GCGAGTCGAGGAAAATGAAGCACTCTCTGCATCCCATCACGCGCTATGGGCCTTGCTTCGCGTCGCGGCCAACGAACAGC
CGGAACGGTTGCTTGCCGCCATCGATCTCGCCGAAAACACCCCGTGGGAAACGCTGCATCAAGGGTTGAGCGCAGTCTCA
CTATCACAGCGCTGGCTCGCCGCACGGGGTGACACCCTTTGGCTCCCTTCACTGTCGCCCAATACGGGATGCGCCGCTGA
ATTACCGGCAAACGTGTTTACCGGCGATAGCCGCTGGCATCTGGTGACCGGAGCGTTTGGCGGATTAGGCCGCCTTGCCG
TGAACTGGCTCAGAGAAAAAGGGGCGCGACGCATCGCCCTGCTGGCGCCGCGCGTGGATGAGTCATGGCTACGCGACGTG
GAGGGCGGGCAGACGCGCGTCTGCCGTTGTGATGTGGGCGATGCCGGGCAACTGGCCACGGTTCTTGACGATCTGGCGGC
CAACGGCGGCATTGCCGGAGCGATTCATGCCGCTGGCGTATTGGCTGACGCGCCCTTGCAGGAGCTTGATGACCACCAGC
TGGCTGCCGTTTTCGCGGTAAAAGCGCAGGCGGCAAGCCAGCTGTTGCAAACCCTGCGCAACCACGACGGACGCTATCTT
ATTCTCTACTCTTCCGCTGCCGCCACCCTCGGCGCGCCGGGTCAGAGCGCCCATGCGCTGGCCTGCGGCTGCCTGGACGG
GCTGGCCCAGCAGTTTTCCACCCTTGATGCGCCGAAAACGCTCTCTGTCGCCTGGGGCGCATGGGGAGAAAGCGGTCGGG
CGGCCACGCCGGAAATGCTGGCGACGCTCGCCAGCCGAGGTATGGGCGCGTTAAGCGATGCCGAAGGCTGCTGGCACCTG
GAACAGGCGGTGATGCGCGGCGCCCCGTGGCGACTGGCGATGCGCGTTTATACCGACAAAATGCCCCCGTTACAACAGGC
TCTGTTTAACATCAGCGCCACAGAAAAAGCCGCAACGCCGGTCATTCCTCCTGCTGATGACAACGCCTTTAACGGCAGCC
TGAGCGATGAAACAGCGGTGATGGCATGGCTGAAAAAGCGGATTGCGGTTCAGCTAAGGCTGAGCGATCCGGCGTCACTG
CATCCAAACCAGGATCTGTTGCAACTCGGCATGGACTCGCTGCTCTTCCTTGAACTCAGTAGCGATATTCAGCACTACCT
GGGCGTACGCATCAATGCGGAACGGGCGTGGCAGGATCTGTCTCCTCATGGACTCACGCAGCTTATCTGTTCTAAGCCAG
AGGCGACGCCTGCCGCTTCGCAGCCGGAAGTGTTGCGGCACGACGCCGACGAGCGTTATGCGCCCTTCCCTTTGACGCCC
ATTCAGCACGCCTACTGGCTGGGGCGAACCCACCTCATTGGCTATGGCGGCGTCGCCTGTCACGTCCTGTTTGAGTGGGA
TAAACGCCACGATGAGTTCGATCTCGCCATACTGGAGAAAGCATGGAACCAGCTCATCGCACGCCACGATATGTTGCGTA
TGGTGGTTGATGCCGACGGGCAGCAGCGAATCCTGGCGACAACGCCGGAGTATCACATCCCGCGTGACGATCTGCGCGCG
CTTTCCCCGGAAGAACAGCGCATCGCGCTGGAAAAACGGCGGCATGAACTGAGCTATCGCGTTTTGCCTGCCGACCAGTG
GCCTCTTTTTGAGCTGGTGGTCAGCGAAATCGACGATTGCCATTACCGTCTGCATATGAACCTCGACCTTTTGCAGTTTG
ATGTGCAGAGTTTTAAAGTCATGATGGACGACCTGGCGCAGGTCTGGCGCGGTGAAACGCTGGCGCCGCTCGCTATTACC
TTCCGTGATTATGTGATGGCTGAACAGGCGCGCCGACAGACATCGGCATGGCACGATGCCTGGGATTACTGGCAGGAAAA

ACTGCCGCAACTGCCCTTAGCGCCAGAGCTGCCGGTGGTTGAGACGCCCCCGGAAACGCCACACTTCACCACCTTCAAAT
CGACGATCGGCAAGACAGAATGGCAGGCCGTGAAACAGCGCTGGCAGCAGCAAGGCGTCACACCGTCTGCCGCGCTGCTC
ACGCTGTTTGCCGCTACCCTTGAGCGCTGGAGCCGTACCACAACATTTACGCTGAACCTGACGTTCTTCAATCGCCAGCC
GATCCATCCGCAAATCAACCAGTTGATTGGTGATTTTACCTCCGTCACGCTGGTTGATTTTAACTTCTCAGCGCCGGTGA
CGTTGCAAGAGCAGATGCAACAGACCCAACAGCGCCTCTGGCAAAACATGGCGCACAGTGAAATGAACGGTGTTGAGGTG
ATCCGTGAGCTGGGCCGCCTGCGCGGATCACAACGTCAACCGCTGATGCCGGTAGTGTTTACCAGTATGCTGGGGATGAC
GCTGGAAGGCATGACTATCGATCAGGCGATGAGCCATCTGTTCGGCGAACCCTGCTATGTATTCACGCAAACGCCGCAGG
TCTGGCTGGATCATCAGGTCATGGAGAGCAACGGCGAGTTGATGTTTAGCTGGTACTGCATGGACAACGTGCTGGAACCC
GGCGCTGCCGAGGCGATGTTTAATGACTATTGCGCCATCCTGCAAGCCGTCATCGCCGCCCCTGAAAGCCTGAAGACTCT
CGCCAGCGGCATCGCCAGGCACATTCCCCGCCGACGCTGGCCGCTGAACGCGCAGGCGGACTACGACCTGCGGGATATTG
AGCAGGCGACGCTCGAATACCCCGGCATCCGGCAGGCCAGAGCGGAAATAACCGAACAGGGCGCGTTGACGCTGGATATC
GTGATGGCCGACGATCCTTCGCCATCAGCGGCGATGCCTGATGAGCACGAACTTACCCAACTGGCGCTGCCGTTGCCTGA
GCAGGCGCAGCTTGATGAGCTGGAGGCGACCTGGCGCTGGCTGGAGGCGCGTGCGCTACAGGGGATCGCGGCTACGCTAA
ATCGTCACGGCCTGTTTACCACGCCGGAGATCGCCCATCGCTTTAGCGCAATAGTACAGGCGCTGTCCGCGCAAGCGTCT
CACCAGCGTCTGCTGCGCCAGTGGCTACAGTGTCTGACGGAAAGAGAGTGGTTAATCCGCGAAGGTGAAAGCTGGCGCTG
CCGCATTCCGCTCAGCGAGATTCCTGAGCCTCAGGAAGCGTGCCCGCAAAGCCAATGGAGCCAGGCGCTGGCGCAGTATC
TGGAAACCTGCATCGCCCGGCACGACGCCCTCTTCTCCGGGCAGTGTTCTCCGCTGGAATTGCTGTTCAACGAGCAGCAT
CGCGTTACCGACGCGCTGTATCGCGACAACCCCGCCAGCGCCTGTCTGAATCGCTATACCGCGCAGATTGCCGCCTTGTG
CAGCGCAGAACGGATTCTGGAGGTTGGCGCCGGAACCGCAGCCACTACCGCGCCGGTGCTGAAGGCCACGCGGAACACGC
GACAGTCGTACCACTTCACGGACGTCTCCGCGCAGTTCCTCAATGACGCCAGAGCCCGTTTCCATGATGAATCGCAGGTG
TCTTATGCCTTGTTCGACATCAACCAGCCGCTGGATTTCACCGCCCACCCGGAGGCGGGTTACGACCTGATCGTTGCCGT
CAATGTGCTCCACGACGCCAGCCATGTCGTCCAGACGTTGCGCAGATTAAAACTGTTGCTGAAAGCCGGCGGACGTTTGC
TGATCGTTGAAGCGACGGAGCGAAACAGCGTATTCCAGCTGGCGAGCGTGGGCTTTATTGAGGGATTAAGCGGATACCGC
GATTTCCGCCGCCGGGATGAGAAACCGATGCTCACCCGCTCCGCATGGCAGGAGGTTCTCGTTCAGGCCGGGTTTGCAAA
CGAGCTGGCGTGGCCCGCGCAGGAATCGTCGCCGCTGCGCCAGCATCTGCTGGTAGCGCGTTCGCCTGGCGTAAATCGCC
CGGATAAAAAAGCCGTGAGCCGCTATTTACAGCAGCGCTTTGGCACCGGTCTGCCCATTTTACAGATCCGGCAAAGAGAA
GCGTTATTTACGCCGCTGCATGCCCCGTCTGATGCGCCGACTGAGCCAGCCAAACCCACGCCAGTTGCCGGGGGGAATCC
GGCGCTGGAAAAACAGGTGGCTGAACTCTGGCAATCGCTGCTGTCTCGCCCCGTGGCAAGGCATCACGACTTTTTCGAAC
TGGGCGGCGACAGCCTGATGGCGACAAGGATGGTCGCGCAGCTAAACCGGAGAGGGATTGCCAGGGCTAACCTTCAGGAT
CTGTTCAGCCATTCGACGCTGAGCGACTTCTGCGCCCATCTACAGGCGGCTACGTCAGGAGAGGACAACCCGATACCCCT
TTGCCAGGGCGACAGTGAGGAAACCCTGTTTGTCTTCCACGCTTCAGACGGCGATATCAGCGCCTGGCTGCCGCTCGCTA
GCGCGTTGAACAGGCGCGTTTTCGGCCTGCAAGCAAAATCGCCGCAGCGCTTTGCCACGCTCGACCAGATGATCGATGAG
TATGTCGGGTGCATCCGTCGTCAGCAGCCTCACGGCCCTTATGTGCTGGCGGGTTGGTCGTATGGCGCGTTTCTTGCGGC
GGGCGCCGCACAACGCCTGTACGCCAAAGGCGAGCAGGTTCGGATGGTGTTAATCGATCCCGTGTGCCGACAGGATTTCT
GTTGCGAAAACCGGGCGGCCCTGCTGCGCCTGTTAGCCGAAGGACAAACGCCTCTGGCACTGCCCGAACATTTCGACCAG
CAGACGCCCGACAGCCAGCTTGCCGACTTTATCAGCCTCGCTAAAACGGCCGGTATGGTGTCGCAAAACCTGACGCTGCA
AGCGGCAGAAACGTGGCTCGACAACATCGCGCATCTGCTGCGTTTACTGACTGAGCATACGCCGGGCGAAAGCGTTCCGG
TCCCCTGTCTCATGGTGTATGCCGCCGGGAGCCCCTCGCGCTGGACGCCAGCAGAAACCGAGTGGCAGGGCTGGATAAAC
AACGCCGACGACGCTGTGATTGAAGCCAGCCACTGGCAAATCATGATGGAAGCCCCCCACGTTCAGGCTTGTGCGCAACA
CATTACGCGCTGGCTTTGCGCAACCTCAACGCAACCGGAGAACACGTTATGA
>Cluster_15663
ATGAAATCAAAAATCAAATCGCTACTGGTCGCTGGTTATAACCACGGCTGGTTAAGTATTTCGTTTGTCGATTTCTGGTT
TAAAAATCTCAATCTGAGGGAATCATGA
>Cluster_28780
ATGAAACGCACTTTTCAACCGTCTGTACTGAAGCGCAACCGTTCTCACGGCTTCCGTGCTCGTATGGCTACTAAAAATGG
TCGTCAGGTTCTGGCACGTCGTCGTGCTAAAGGCCGCGCTCGTCTGACCGTTTCTAAGTAA
>Cluster_21435
ATGGAAGGTAAGTTAATTTTTTGTTCCGTTGCAATACTACGGTTTCAGTCTGATTACGATGAAACCGCTGCCGTTCCATT
GTTCTCACTCGCTGAGAGAGAATCCTTTTGTTCCTCTGTTTTAAACATTGATGATAGCTAG
>Cluster_30982
ATGGGTGCGAAAATTTTAATAGCAAAACCGCAATATCGGGATAACGCTACAGGAGAAGGCGATTATTCTATTTTCCGGAA
ACAAGGTGAATATTCAAAAATTCCTGTCAAATTGCCTTTCGCCCTGAAAAATGCATAG
>Cluster_62577
ATGACACAGCAGCGTTGTTACTACCACCCACTGCGGCGCGTACGGACTCGCTACAGTGTAGGGGCCAAAATATTTCTACT
GCTCTGGGCTGCAGGAGTGGGAGGCACGCTGTACAGCCTGTACAGCGTGCTGGCGTACTGA
>Cluster_13929
ATGAACATTTCGGAAGTCGATCTGCGTAAACTGACGGTCAGCGATCCGTTCCTCGGTCAGTACCAACAACTGGTCCGCGA
CGTGGTGATTTCTTATCAGTGGGATGCCTTGAACGATCGTATCCCAGAAGCGGAACCCAGCCATGCGATTGAAAACTTTC
GCATTGCCGCCGGACTTCAGGAGGGTGAATTTTACGGGATGGTGTTTCAGGACAGCGACGTCGCCAAATGGCTGGAAGCG
GTAGCCTGGTCGCTGTGCCAGAAGCCGGACGCCGAACTGGAAAAAACCGCCGACGAGGTAATCGAACTGATCGCCTCCGC
CCAATGCGAAGACGGCTATCTCAATACTTACTTTACGGTAAAAGCACCCGAAGAACGCTGGAGCAATCTTGCGGAGTGTC
ATGAACTTTACTGCGCCGGTCATCTGATTGAAGCCGGAGTCGCCTTCTTCCAGGCCACGGAAAACGACGCTTGCTGGAG
GTGGTTTGCCGTCTGGCCGATCATATCGACCGCGTATTTGGTCCAGATGAAGACAAGCTGCAGGGTTATCCCGGTCATCC
GGAAATTGAACTGGCGCTGATGCGTCTCTACGAAGTCACCGAAGAGCCGCGCTACCTGGCGCTGACGAACTATTTTGTCG
AACAGCGTGGTGTGCAACCGCACTATTACGACCAGGAATATGAAAAGCGCGGGCAGACCTCGCACTGGCACACCTACGGC
CCGGCGTGGATGGTGAAAGACAAAGCCTACAGCCAGGCACATTTGCCCCTTGCGCAACAGCAAACCGCCATCGGTCACGC
GGTACGTTTTGTCTATCTGATGACCGGCGTCGCGCATCTCGCGCGTTTAAGTCACGATGACAGCAAGCGTCAGGACTGCC
TGCGGCTGTGGAACAATATGGCCCAGCGTCAGTTATATATTACCGGCGGCATTGGCTCACAGAGCAGCGGTGAAGCGTTC
AGCAGCGATTACGATCTGCCGAATGACACGGTATACGCTGAAAGTTGCGCCTCCATCGGCCTGATGATGTTTGCCCGACG
AATGCTGGAAATGGAAGGCGACAGTCAATATGCCGATGTGATGGAGCGCGCACTGTACAACACCGTGCTCGGCGGCATGG
CGCTGGACGGCAAACATTTCTTCTATGTGAATCCGCTGGAAGTACATCCAAAATCGCTGAAATTCAACCATATCTACGAT
CACGTTAAACCGATCCGCCAGCGTTGGTTTGGCTGCGCTTGTTGTCCGCCAAATATCGCCCGCGTGCTGACCTCGATTGG
TCATTATCTCTACACGCCGCGTGAAGATGCGTTGTATATCAACATATACGCAGGAAACAGCATGGAAGTGCCGGTAGAAA
ATGGCACACTGCGCCTGCGGGTTAGCGGGAACTATCCGTGGCAGGAGCAGGTGACAATTGCGGTCGAATCGCCCCAGCCG
GTGCGTCATACGCTGGCTTTACGTCTGCCGGACTGGTGCACACAGCCGCAGATCATATTGAATGGGGAAGAGGTCGAGCA
GGATATTCGTAAAGGGTATTTGCACATTACCCGCGAATGGCAGGAGGGCGATACGCTGAATCTGACTTTGCCGATGCCGG
TACGCCGCGTTTACGGTAACCCGCTGGTGCGTCACGTCGCCGGAAAAGTGGCGATTCAGCGCGGCCCGCTGGTGTATTGC
CTGGAACAGGCCGACAACGGCGAGTCACTGCATAATCTGTGGCTGCCCACCGATGCGCCATTTACGACATTTGAAGGCAA
GGGATTGTTTAGCCATAAGATCTTAATCCAGGCACCGGGTTACCGGTATGAACAGAGCAATCCAGAGCAGCAACCGCTGT

GGCATTACGACAGCGCGCCAGCCAAACGCCAGCCGCAAACTCTGACCTTTATCCCGTGGTTTAGCTGGGCTAACCGGGGC
GAAGGCGAAATGCGGATCTGGGTGAATGAGGAAAAGCATCGCCATCCGGAGGTTGGATAA
>Cluster_32975
ATGTCACGTCCGCAATTTACCTCGTTGCGTTTGAGTTTGTTGGCTTTGGCTGTTTCTGCCACCTTGCCAACGTTTGCTTT
TGCTACTGAAACCATGACCGTTACGGCAACGGGGAATGCACGTAGTTCCTTCGAAGCGCCTATGATGGTCAGCGTTATCG
ACACTTCCGCTCCTGAAAATCAAACTGCTACTTCAGCCACTGATTTGCTGCGTCATGTTCCTGGAATTACTCTTGATGGT
ACCGGACGAACCAACGGTCAGGATGTAAATATGCGTGGCTATGATCATCGCGGCGTGCTGGTTCTTGTCGATGGTGTTCG
CCAGGGAACGGATACCGGACACCTGAATGGCACTTTTCTCGATCCGGCGCTGATCAAGCGTGTTGAGATTGTTCGCGGAC
CTTCAGCATTACTGTATGGCAGTGGCGCGCTGGGTGGAGTGATCTCCTACGATACGGTCGATGCAAAAGATTTATTGCAG
GAAGGACAAAGTAGTGGTTTTCGTGTCTTTGGTACTGGCGGCACGGGGGACCATAGCCTGGGATTAGGCGCGAGCGCGTT
TGGGCGAACTGAAAATCTGGATGGTATTGTGGCCTGGTCCAGTCGCGATCGGGGTGATTTACGCCAGAGCAATGGTGAAA
CCGCGCCGAATGACGAGTCCATTAATAACATGCTGGCGAAAGGGACCTGGCAAATTGATTCAGCCCAGTCTCTGAGCGGT
TTAGTGCGTTACTACAACAACGACGCGCGTGAACCAAAAAATCCGCAGACCGTTGAAGCTTCTGATAGCAGCAACCCGAT
GGTCGATCGTTCAACAATTCAACGCGATGCGCAGCTTTCTTATAAACTCGCCCCGCAGGGTAACGACTGGTTAAATGCAG
ATGCAAAAGTTTACTGGTCGGAAGTCCGTATTAATGCGCAAAACACGGGGAGTTCCGGCGAGTATCGTGAACAGATAACA
AAAGGAGCAAGGCTGGAGAACCGTTCCACTCTATTTGCCGACAGTTTCGCTTCTCACTTACTGACATATGGCGGTGAGTA
TTATCGTCAGGAACAACATCCGGGTGGCGCGACGACGGGCTTCCCGCAAGCAAAAATCGATTTTAGCTCTGGCTGGCTAC
AAGATGAGATCACCTTACGCGATCTGCCGATTACCCTGCTTGGCGGAACCCGCTATGACAGTTATCGCGGTAGCAGCGAC
GGCTACAAAGATGTTGATGCCGACAAATGGTCATCTCGTGCGGGGATGACTATCAACCCGACCAACTGGCTGATGTTATT
TGGCTCATATGCTCAGGCATTCCGCGCCCCGACGATGGGCGAAATGTATAACGATTCTAAACACTTCTCGATTGGTCGCT
TCTATACCAACTATTGGGTGCCAAACCCGAACTTACGTCCGGAAACTAACGAAACTCAGGAGTACGGTTTTGGGCTGCGT
TTTGATGACCTGATGTTGTCCAATGATGCTCTGGAATTTAAAGCCAGCTACTTTGATACCAAAGCGAAAGATTATATCTC
CACGACCGTCGATTTCGCGGCGGCGACAACTATGTCGTATAACGTCCCGAACGCCAAAATCTGGGGCTGGGATGTGATGA
CGAAATATACCACTGATCTGTTTAGCCTTGATGTGGCCTATAACCGTACCCGCGGCAAAGACACCGATACCGGGGAATAT
ATCTCCAGCATTAACCCGGATACCGTTACCAGTACCCTGAATATTCCGATCGCTCACAGTGGCTTCTCTGTTGGTTGGGT
CGGTACGTTTGCCGATCGCTCAACACATATCAGCAGCAGCTACAGCAAACAACCTGGCTATGGTGTGAATGATTTCTACG
TCAGTTATCAAGGGCAGCAGGCGCTCAAAGGCATGACCACTACTCTGGTATTGGGCAACGCCTTCGATAAAGAGTACTGG
TCGCCGCAAGGCATCCCACAGGATGGTCGTAACGGAAAAATTTTCGTGAGTTATCAATGGTAA
>Cluster_22017
GTGAGCATGGATATAAGCGATTTTTATCAGACATTTTTTGATGAAGCGGACGAACTGTTGGCTGATATGGAGCAGCATCT
GCTGGTTTTGCAGCCGGAAGCGCCAGATGCCGAACAATTGAATGCCATCTTTCGGGCTGCCCACTCGATCAAAGGAGGGG
CAGGAACCTTTGGCTTCAGCGTTTTGCAGGAAACCACGCATCTGATGGAAAACCTGCTCGATGAAGCCAGACGAGGTGAG
ATGCAACTCAACACCGACATTATCAATCTGTTTTTGGAAACGAAGGACATCATGCAAGAACAGCTCGACGCTTATAAACA
GTCGCAAGAGCCGGATGCTGCCAGCTTCGATTATATCTGCCAGGCCTTGCGTCAACTGGCATTAGAAGCGAAAGGCGAAA
CGCCATCTGCAGTGACCCGATTAAGTGTGGTTGCCAAAAGTGAACCGCAAGATGAGCAGAGTCGTAGTCAGTTACCGCGA
CGAATTATCCTTTCGCGCCTGAAGGCCGGCGAAGTCGACCTGCTGGAAGAAGAGTTGGGGCATCTGACAACGTTAACTGA
CGTGGTGAAAGGGGCGGATTCTCTCTCGGCAATATTACCGGGCGACATCGCCGAAGATGACATCACAGCGGTACTCTGTT
TTGTGATTGAAGCCGATCAGATTACCTTTGAAACAGTAGACGTCTCGCCAAAAATATCCACCCCACCAGTGCTTAAACTG
GCAGCCGAACAAGCGCCAACCGGTCGCGTGGAGCGGGAAAAAACGACGCGCAGCAGTGAATCCACCAGCATCCGTGTAGC
GGTAGAAAAGGTTGATCAATTAATTAACCTCGTCGGCGAGCTGGTCATCACCCAGTCCATGCTTGCTCAGCGCTCCAGTG
AACTGGACCCGGTTAATCATGGTGATTTGATTACCAGCATGGGGCAGTTACAACGTAACGCTCGTGATTTGCAGGAATCA
GTGATGTCGATTCGCATGATGCCGATGGAATATGTCTTTAGTCGCTATCCCCGGCTGGTGCGTGATCTGGCGGGAAAACT
CGGCAAGCAGGTAGAACTGACGCTGGTGGGCAGTTCCACTGAACTCGACAAGAGCCTGATAGAACGCATTATCGACCCGC
TGACCCACCTGGTACGCAATAGCCTCGATCACGGTATTGAACTGCCAGAAAAACGGCTCGCTGCAGGTAAAAACAGCGTC
GGAAATTTAATTCTGTCTGCCGAACATCAGGGCGGCAACATTTGCATTGAAGTGACCGACGATGGGGCGGGGCTAAACCG
TGAACGAATTCTGGCAAAAGCGGCCTCGCAAGGTCTGACCGTCAGCGAAAACATGAGCGACGACGAAGTTGCAATGCTGA
TATTTGCACCTGGCTTCTCCACGGCGGAGCAGGTCACCGACGTCTCCGGGCGTGGCGTCGGCATGGACGTCGTTAAACGT
AATATCCAGGAGATGGGCGGTCATGTCGAGATCCAGTCGAAGCAGGGTACTGGCACTACGATCCGCATTTTACTGCCGCT
GACGCTGGCAATTCTTGACGGCATGTCCGTACGCGTTGCGGATGAAGTTTTCATTCTGCCGCTGAATGCTGTCATGGAAT
CACTGCAACCCCGTGAAGCCGATCTGCATCCACTGGCTGGCGGCGAGCGGGTGCTGGAAGTGCGGGGTGAATATCTGCCC
ATCGTCGAACTGTGGAAAGTGTTCAACGTCGCGGGCGCGAAAACCGAAGCCACCCAGGGAATTGTGGTGATCTTACAAAG
TGGCGGTCGCCGCTACGCCTTGCTGGTGGATCAATTAATTGGTCAACACCAGGTTGTGGTTAAAAACCTTGAAAGTAACT
ATCGCAAAGTCCCCGGCATTTCTGCTGCGACCATTCTTGGCGACGGCAGCGTGGCACTGATTGTTGATGTCTCCGCCTTG
CAGGCGATAAACCGCGAACAACGTATGGCGAACACCGCCGCCTGA
>Cluster_38499
TTGCAAAATGCCGGTCATGCCAAAATAGATGAAATGTTGGTTGCTGCTCATGCCGGCTGTTTTGACGTATTAGAGTCAGT
CATTTTACATCGGAATATTAAAGCGATTTTGGCTAATCTAAGCGCGATTGAGCGTATTGAACCTTGGGATGAGAATAGGA
AGGTTCCAGGAGGCACTGATGGTTTGTTGAATCATCAAACATCCTTGTACGACTATTTGAGTACACAGGTGAAGATGCA
ACTTACAACAACTATGAAAACGTTATTTCTATATTAGAACAACACGGAGTTAAATATGATGAGATTAGACCAAAATGTGG
TCTTCCCTTATTAAGGATAATGGATTTATCCCCAAATGATAGATATATATTAGACATTCTCATTGATTACCCGGGTATAA
GAACGTTAATTCCAGAACCAAAATATTCAGCATTCCCGGTTAGTGTAAGTGATTCTGTTGGCATTGAAACAAATAGCTTT
CCCGTACCATCAGAAGAATTACCCATTGTTGCTGTATTTGACACTGGGGTAAGCCCCATCGCGGCAACAATTACTCCTTG
GGTAGTGAGTAGGGAAACATACGTAATTCCTCCTGATACGAGTTATGAACATGGGACTATGGTGTCTTCATTGATATCAG
GCGCTCATTTTTTAAATGACAATCATCCATGGATTCCTGATACAAAATCTAAAATCCATGATGTTTGTGCTTTAGATGAA
AATGGATCTTATATATCAGATTTAATTCTGAGGCTAGCAGATGCTGTAAATAAAAGACCAGATATAAAAGTCTGGAATTT
ATCTTTGGGAGGCGGACCATGTAATGAGCAGATGTTTAGTGATTTTGCGATGGAGTTAGATCGGCTCAGCGATAAATTTG
GTATTTTGTTTGTAGTTGCTGCAGGTAATTATGTAGATGAACCTATACGTACATGGCCAAATCCTGATCCGCTTGGAGGT
GCTGATTTAATTTCCTCTCCTGGAGAGTCAGTCCGAGCACTAACAGTTGGTTCAGTTTCTCATATGGAAGCTAATGATGC
TTTAAGTGAAATTGGAACACCGACACCATATACTCGTCGTGGCCCTGGGCCTGTATTTACTCCAAAGCCAGATATAATCC
ATGCTGGCGGTGGGGTTCATAGACCTTGGAATGTAGGAGCAAGCAGTTTAAAGGTCGTAGGGCCAGATAATAGGCTTTGC
TCTAATTTTGGTACTAGTTTTGCTGCTCCAATTGTGGCAAGTTTAGCTGCGCATACATGGCAGAGAATAGCCACTAATTC
AGACTTTAATGTTTCACCATCATTGATTAAAGCATTATTAATTCATTCCGCTCAATTATCTTCTCCTGATTACTCGCCAA
GTGAAAGACGCTATTTGGGAGCGGGAATTCCTAATGAGGTTATTGAGACCTTATATGATAGTGATGATAGGTTTACTCTG
ATTTTCCAAACATTCTTGGTTCCTGGGGTGAGGTGGAGAAAGGAAAACTATCCCATACCATCGGCACTTATTCAAAATGG
AAAATTTAAAGGTGAGATTGTAATTACTGCTGCATATGCACCACCACTGAACCCTAATGCCGGCAGTGAATATGTTCGCG
CGAACGTAGAGCTAAGTTTTGGCTTAATTGAGAATAATACTATAAAAGGAAAAGTACCTATGGAAGGAGAAAACGGTCAA
TCTGGATATGAGAGAGCTCAAATTGAGCATGGTGGAAAGTGGTCACCAGTAAAAATTCATCGCAAGGCATTTAATAAAGG
AATTACTTCGGGTAACTGGGCTCTTCAAGCTAAAACAACGTTGAGAGCGAATGAACCGGCCTTAATGGAGCCTTTACCTG
TAACTATTGTAGTAACTTTAAAATCATTAGATGGAAACACACAAGTTTATGCTGATGGCGTAAGAGCTTTAAATGCTAAT

AACTGGGCTCACTATCCATTGCCTGCTCGTGTGCCAGTTTCCGTATAA
>Cluster_28720
ATGAATAAACTCTTAAATATATTTAAAAAAGCAGAGTCATATCACGATCTTATTCTGGCTCTCTTCTTCTTTATGGCTGT
AATGATGATGATTATTCCACTACCAACAGTTGTGGTGGATATCATTATCGCGATTAATATTTCGACAGCTTTACTTTTAT
TAATGCTCTCAATCTATATAAAAAATCCTCTTGAACTAACTTCTTTCCCCACAATCCTGTTGATTACGACGTTGATGCGC
CTGTCGCTCAGTGTTAGTACAACCAGACTTATTCTGCTGCATCACGATGCGGGTGACATTATTTACTCCTTCGGTAACTT
TGTTGTTGGCGGTAATATTGTTGTTGGCCTGGTTATTTTTACTATTATCACCATTGTTCAATTTATGGTTATTACAAAAG
GGGCCGAGCGTGTTGCTGAAGTGAGTGCTCGTTTTTCCCTTGATGGTATGCCAGGAAAGCAAATGAGTATTGATGGTGAT
ATGCGCGCAGGCGTTATTGACCCGCTTGAGGCAAAAGTTCTTCGCTCCCGAGTGCAAAAGGAAAGCCAGTTTTATGGTTC
AATGGACGGGGCGATGAAGTTTGTAAAAGGGGATGCTATTGCTGGTATCATTATTGTTTTAGTTAACCTTTTTGGTGGCG
TGCTCATTGGGATGTGGCAATTTGACATGCCATTTAGTGAGGCACTTAGCCTGTTTTCTGTATTGTCTGTCGGTGATGCT
TTGGTTGCCCAGATCCCTGCGCTTATTATTTCTGTCACCGCGGGCGTGGTTGTTACTCGTGTGCCCGGTGAAAGCGAAAA
AGAAGAAAACCTTGCAGGTGATATTGTTCAACAGGTTTCTGTTAATAGTCGCCCTTTTTTAATCAGTGCCGCACTAATGC
TCGTAATGGCGATTATTCCGGGCTTTCCTACATTGGTCTTCTTATTTCTGGCGGTTTGCCTGTTGGGGATAGCCTGGAAA
TTACAGAAGAAAAGAACATTTGGAACTGGCAATAATAAGGATGCTATGGGAGCTGATTTGTCTAATAGCCAAAACATCTC
ACCGGGCGCTGAGCCATTGATTTTAAACTTAAGTAGTAATATTTATAGTTCAGACATTACACAGCAAATTGAGGTCATGC
GTTGGAATTTCTTTGAGGAAAGTGGAATTCCATTGCCTAAGATTATTGTTAATCCGGTTAAAAATAATGATAGCGCAATA
GAATTTTTGCTCTATCAAGAGTCAATATACAAAGATACTCTTATAGATGATACTGTCTATTTTGAGGCTGGGCATGCAGA
GATATCATTCGAATTTGTCCAGGAAAAGCTTTCGACTAACTCTATCGTATATAAGACGAATAAGACTAATCAACAGCTCG
CTCACCTTACAGGTATGGATGTTTATGCAACAACAAATGATAAGATAACGTTTTTGCTAAAGAAACTTGTATTGTCTAAT
GCCAAAGAGTTCATCGGCGTACAAGAAACGCGTTATTTGATGGACATCATGGAGAGAAAATATAACGAGCTTGTGAAAGA
GCTGCAGCGCCAGCTTGGTTTGAGCAAAATTGTTGACATTCTACAACGTCTCGTAGAGGAAAATGTCTCAATTAGAGATC
TGAGAACTATCTTTGAGACGCTTATTTTTTGGTCAACAAAAGAAAAGGATGTGGTTATCTTGTGCGAATACGTTCGTATC
GCCCTGCGTCGGCATATTTTAGGTCGCTATAGCGTTAGCGGTACACTTCTGAACGTTTGGCTTATTGGCTCTGATATTGA
AAATGAGCTACGAGAGTCGATCAGACAAACGTCATCAGGTTCGTATCTGAATATCTCACCGGAGCGAACTGAGCAGATAA
TTGGCTTCTTAAAAAATATCATGAATCCAACGGGGAACGGCGTCATTCTGACCGCTTTAGATATCAGGCGCTATGTGAAG
AAAATGATTGAAGGTTCGTTCCCGTCAGTCCCCGTGCTCTCTTTTCAGGAGGTTGGGAATAATATCGAACTTAAAGTATT
AGGAACGGTAAATGATTTCAGAGCATGA
>Cluster_27154
ATGACTCAAGTCGCGAAGAAAATTCTGGTGACGTGCGCACTGCCGTACGCTAACGGCTCAATCCACCTCGGCCATATGCT
GGAGCACATCCAGGCTGATGTCTGGGTCCGTTACCAGCGAATGCGCGGCCACGAGGTCAACTTCATCTGCGCCGACGATG
CCCACGGCACGCCTATCATGCTTAAAGCTCAGCAGCTTGGTATCACCCCGGAGCAGATGATTGGCGAAATGAGTCAGGAA
CATCAGACTGATTTCGCAGGCTTTAACATCAGCTATGACAACTATCACTCGACGCACAGCGAAGAGAATCGCCAGTTGTC
AGAACTTATCTACTCTCGTCTGAAAGAAAACGGTTTTATTAAAAACCGCACCATCTCTCAGCTGTACGATCCGGAAAAAG
GCATGTTCCTGCCGGACCGTTTTGTAAAAGGCACCTGCCCGAAATGTAAAGCGCCAGATCAATACGGCGATAACTGTGAA
GTCTGCGGCGCGACCTACAGCCCGACTGAACTGATTGAACCGAAATCGGTGGTTTCTGGTGCTACGCCGGTAATGCGTGA
TTCTGAACACTTCTTCTTTGATCTGCCCTCTTTCAGCGAAATGTTGCAGGCATGGACCCGCAGCGGTGCGTTGCAGGAGC
AGGTGGCAAACAAAATGCAGGAGTGGTTTGAATCTGGTCTGCAACAGTGGGATATCTCCCGCGACGCACCTTACTTCGGT
TTTGAAATTCCGAACGCGCCGGGTAAATATTTCTACGTCTGGCTGGACGCACCGATTGGCTACATGGGTTCCTTCAAGAA
TCTGTGCGACAAGCGCGGCGACAGCGTAAGCTTCGATGAATACTGGAAGAAAGATTCCACTGCCGAGCTGTATCACTTCA
TCGGTAAAGATATCGTTTACTTCCACAGCCTGTTCTGGCCTGCCATGCTGGAAGGCAGCAACTTCCGTAAGCCGACCAAC
CTGTTTGTTCACGGCTATGTGACGGTGAACGGCGCGAAAATGTCCAAGTCTCGCGGCACCTTTATTAAAGCCAGCACCTG
GCTGAATCATTTTGACGCTGACAGCCTGCGTTACTACTACACTGCGAAACTCTCTTCGCGCATTGATGATATCGATCTCA
ACTTGGAGGATTTCGTTCAGCGTGTGAATGCCGATATCGTGAACAAAGTGGTTAACCTGGCGTCCCGTAACGCTGGCTTT
ATCAATAAGCGTTTTGACGGTGTGCTGGCAAGCGAGCTGGCTGACCCGCAGTTGTACAAAACCTTCACTGATGCCGCTGA
AGTGATTGGTGAAGCGTGGGAAAGCCGCGAATTTGGTAAAGCTATCCGTGAAATCATGGCACTGGCTGACCTGGCTAACC
GCTATGTCGATGAACAGGCTCCGTGGGTGGTGGCGAAACAGGAAGGCCGCGATGCCGACCTGCAGGCAATTTGCTCGATG
GGCATCAACTTGTTCCGCGTGCTGATGACTTACCTGAAACCGGTACTACCGAAACTGACCGAGCGCGCAGAAGCATTCCT
CAATACAGAACTGACCTGGGATGGTATCCAGCAACCGCTGTTGGGCCATAAAGTGAATCCATTCAAGGCGCTGTATAACC
GCATCGATATGAAACAGGTTGAAGCCCTGGTGGAAGCATCAAAAGAGGAAGTAAAAGCCGCTGCCGCGCCAGTAACTGGC
CCACTGGCAGATGATCCGATTCAGGAAACCATCACCTTTGACGACTTCGCCAAAGTTGACCTGCGCGTGGCGTTGATTGA
AAACGCAGAGTTTGTAGAAGGTTCTGACAAACTGCTGCGCCTGACGCTGGATCTCGGCGGTGAAAAACGCAATGTCTTCT
CCGGCATTCGTTCTGCTTACCCGGATCCGCAAGCACTAATTGGTCGTCACACCATTATGGTGGCTAACCTGGCACCACGT
AAAATGCGCTTCGGTATCTCCGAAGGCATGGTAATGGCTGCTGGTCCTGGTGGGAAAGATATCTTCCTGCTAAGCCCGGA
TGCCGGTGCTAAACCGGGTCATCAGGTGAAATAA
>Cluster_64084
ATGAGTACCACAACACGAAAATTTAAAACCGTTATCACCGATACAGGTGCCAAAAAATTAGCTCAGGCAGCCGCGCCAGA
TGGTAAGCCTGTCCGCCTGACTCATATGGCCGTGGGCGACGGTGGCGGCACGTTGCCCACACCAGACAGTAAGCAGACCC
GTCTGGTGCATGAGGTGTGGCGACACACTGTTAATCGCGTCATCCTGGACGCAACACATCAGAACCGCATTATTGCGGAG
CTGGTTATTCCTCCTGAAACGGGCGGATTCTGGATCCGGGAAATTGGTGTATTTGATGAGCACGGCGATTTAATCGCGGT
GGGCAATACTGCCGAAAGTTACAAACCAACCGTTGCCGAAGGGTCCGGACGTGCACAAACATTTCGCACCATTCTGACCG
TATCCAGCACTGCCACCGTGGCGCTTACCGTGGATAACACCATGGTGATGGCCACAGTGGATTACGTGGATGACAAACTG
AAAGAGCATGAACAGTCACGACGTCACCCGGACGCCTCGCTGACCGCAAAAGGCTTTGTTCAACTCAGTAGCGCCACTAA
CAGCGTGTCTGAAACGCAGGCTGCAACGCCGAAAGCGGTTAAGGCCGCCTATGACCTTGCTAACGGGAAATATACCGCAC
AGGACGCCACTATAGCGCGAAAAGGCCTTGTTCAGCTCAGTAGCGCCACCAACAGCGATTCTGAAACGCTGGCGGCAACA
CCAAAGGCGGTAAAGGCAGCATATGACCTTGCTAACGGGAAATATACCGCACAGGACGCTACCACAGCGCGAAAAGGTCT
TGTCCAGCTCAGTAGTGCCACCAACAGCACGTCTGAAACATTGGCCGCGACATCGAAAGCGGTTAAGGCGGTAATGGATG
AAACAAATAAGAAAGCCCCATTAAACAGCCCGACGCTGACCGGAACGCCAACAACACCAACTGCGCGACAGGGAACGAAT
AATACCCAAATCGCAAGCACGGCTTTCGTTATGGCTGCGATTGCCGCCCTTGTAGATTCGTCACCTGATGCACTGAACAC
GCTGAACGAGCTGGCTGCGGCGTTGGGCAACGACCCGAATTTTGCGACCACCATGACTAACGCGCTTGCGGGTAAGCAAC
CGAAAGATGCCACCCTGACGGCGCTGGCCGGGCTTGCTACTGCGGCAGACAGGTTTCCGTATTTTACGGGGAATGATGTC
GCCAGCCTGGCAACTCTGACAAAAGTCGGGCGGGATATTCTTGCGAAATCGACCGTTGCCGCCGTTATCGAATACCTCGG
TTTACGAGAACTCGGCACAAGCGGGGAGAAAATACCGTTACTCAGTACAGCGAATACCTGGACCAATCGACAAACATTCA
GCGGTGGCCTTTCTGGGGAACTGTCCGGCAATGCCGCTACTGCAACAAAGCTGAAAACAGCACGAAAAATTGCTGGAGTT
GGTTTTGATGGTTCTAGCGATATTTCAATTAGTGCCAAAAATGTCAATGCATTTGCACTCCGACAAACAGGTAATACTGT
TAATGGTGATACATCCGTTGGATGGAATTGGGATAGTGGTGCATATAACGCCATGATTGGTGGTGCATCTGCATTAATTC
TTCACTTTAATATAAATGCTGGTAGCTGTCCTGCCGTACAATTCCGTGTGAATTATAAAAATGGTGGCATTTCCTACAGG
TCGGCTCGTGATGGTTATGGGTTTGAATTAGGTTGGTCAGATTTCTATACCACGACACGAAAACCTTCAGCGGGAGATGT
TGGTGCATATACGCGGGCAGAATGTAACTCAAGGTTTATTACAGGTATTCGCCTTGGCGGTCTGTCATCTGTTCAGACAT

GGAATGGTCCCGGCTGGTCTGACAGGTCAGGTTATGTCGTTACGGGTTCAGTTAACGGAAACCGTGATGAATTAATTGAT
ACAACACAGGCAAGGCCAATTCAGTATTGCATTAATGGGACGTGGTATAACGCGGGGAGTATTTAA
>Cluster_2740
ATGAACGCTGAAACTATTAAAGATTTCCTCGTCTCGCTTGGCTTCAGTGTGGATGATGCAGGAGCGAAAAAGTTCGGTTC
TGTCCTCGCCGGTACAACTGCAAATGTCATCAAAATGGGGCTGGCTGTTGAAGGAGCTGCGTTGTCCGTGGTGGCCTTCA
CGGCTAAGATCGCCTCCGGCCTGGATAATCTTTACTGGGCGTCACAGCGCACCGGCGCGACAGTCCAGGGAATTCAGTCT
ATTGGCTATGCGGTTTCGCAGGTTGGCGGCAGCGTGGACGCTGCGCGATCTTCTCTGGAAAGCCTATCCCGGTTTATTCG
TAACAATCCCGGTGCAGAAGGCTTTCTGAATCGCCTGGGCGTACAGACCCGTGATGCCAGCGGTAACATGCGTGACATGG
CCGCTATTTTTACGGGCGTTGGACAGAAACTCAGCAGCATGCCGTATTACCGGGCTAACCAGTATGCGCAGATGCTGGGC
ATTGACGAAAATACCCTTATGGCGATGCGCCGGGGTGTGGGTGGCTTCTCCGGGCAGTACAGCGCAATGGCGAAAGCTAT
CGGCTTCAATGCTGACGAGGCGGCCAGAAGCTCCAACAAATTTATGACCTCCCTGCGTGAGTTTGGCGCGATGGCAGGCA
TGGCCCGTGACAAAATCGGCTCTAATCTTGCGGGGGGGCTTGCGGGTTCGCTGGACACCCTGCGCCGCCATATCCTGGAC
AACTTCCCTCGTATCGAGCAGACCCTGACGAAAGCCATAAAAGGCATTCTGGCGCTCGGGGATATTATCGGGCGGCTGTT
CTTCAGACTGATTGAGGGGACATCAGGCCTTATCACCTGGTGGCAATCGCTGGATAAGCAAACGCGGGAGTTGATCTCGC
TGTTTGGCGCACTGACGATTGCGCTGCGCATTCTGAACAGTACGTTCTGGATGTCGCCGATTGGCCTCATTACCGCGCTG
GCGGCGGGTATTGCCCTCCTGTGGGAGGACTATCAGACCTGGAAGGAAGGCGGCGACAGCCTGATTGACTGGGGCAAGTG
GAAACCGGAGGTCGATGCCGCGCTGAAGATGGTTCGTGACCTTAAAACGACCGTTAACGACCTGGTGAAAGCGCTGGCGA
AACTGCTCAATATTGACCCCAAATCATGGTCCCTGAAGTGGGATTTCAGCAACTTCATCGACCAGATGGGCGAATTCAGC
AAAATGCTGAACATGATCGCCGACCTGCTCAACGCTATCAAAGATGGCCGCTGGGCTGATGCCGTCAGCATCGGCAAACA
GATACTTAATCAGGGCAGCGAAAATCCGTCAGCGATGCCGATGGTTACAGACAGCGCTAACAGTACTGCCGACTGGATTA
AAGAGCACTGGGGATTCGATCCCCGCAGTGTGGGCCGGACGGTACGCGGCTGGTTTGGTGATGATGAGCCGGAACAACAT
GCACAGGCTACGAAACGAGGAGAACGGAATAACAATCCGGGAAACCTTAATTTTGCTGGTCAGGCAGGGGCTTCTCTTGA
ACGCCCGGGCGGGCGATTTGCCAGATTTGAAACTGCTTTTGATGGATTACGGGCTCTTGCTCGTCAGTTAATGCTGTACG
CCGGACGGGGAATAAACAGTGTGGAGAAAATTATCTCTACCTGGGCACCTGCGTCTGATAATAACAACACAACTGCGTAT
ATCAGGGCTGTATCGCAACGACTGGGAGTGGATCCCCGGGCTGCCCTGAATATGAGCGATCCGCAAACCATGTCAGCATT
GATGAGCAGCATTATCCAGCATGAGAATGGAAGAAATATCTATTCTCGGGAGCTGATTAATAAGGCTGCCGTGGCGGGAA
TTAGTGGCAAAGTGACAGAGGTTAACCAGCAAAATACCTACCACATTTACGGTGGTGGAGATCCGCACGCTGTCGGTAAT
GAGGTTGCACGTCGGCAACAGTCTGCAAATGCTCAGGTCATGCGAAGTAATCAGGTGAGGGTGGGTTAG
>Cluster_22220
ATGAAACCCACTTTATATACTGCTACTGGTGAGTGCGTTACGCCAGGCCGTGAACTGGGCAAAGGCGGCGAAGGTGCGGT
TTATGATATCGAGGAGTTTGTCGATAGCGTCGCCAAGATTTATCACACGCCGCCACCCGCCTTAAAACAGGACAAACTTG
CCTTTATGGCTGCGACAGCTGACGCGCAGTTGTTGAATTATGTCGCCTGGCCGCAGGCAACGCTTCACGGTGGACGAGGC
GGAAAAGTTATCGGTTTTATGATGCCAAAAGTTTCTGGTAAAGAACCGATTCATATGATCTATAGCCCGGCACATCGTCG
CCAGAGTTACCCTCATTGTGCGTGGGATTTTCTACTCTATGTTGCGCGCAATATTGCTTCATCTTTTGCTACGGTTCACG
CGCATGGGCACGTCGTGGGTGACGTAAACCAGAACAGCTTTATGGTAGGTCGCGACAGCAAAGTGGTGTTGATTGATAGC
GACTCCTTTCAGATTAACGCCAATGGCACGCTGCATTTATGCGAAGTCGGCGTGTCGCATTTTACGCCGCCAGAGCTGCA
AACCATGCCGTCATTTGTCGGCTTTGAACGCACTGCGAATCACGATAATTTTGGCCTTGCGTTGCTGATATTTCACGTCT
TGTTTGGTGGGCGGCATCCTTATTCTGGTGTGCCGCTTATCTCTGATGCGGGGAATGCGCTGGAGACGGATATTGCTCAT
TTCCGTTATGCCTACGCGTCAGATAATCAGCGGCGTGGTTTAAAACCGCCGCCACGATCGATTCCGCTGTCGATGTTACC
GGGCGATGTTGAAGCCATGTTTCAGCAGGCATTCACGGAAAGTGGCGTGGCAACCGGGCGTCCGACGGCAAAAGCGTGGG
TAGCGGCACTTGATTTACTACGCCAACAGTTAAAGAAATGTACCGTTTCGGCAATGCATGTTTACCCCGCTCATTTGACT
GACTGCCCGTGGTGTGCGCTGGATAATCAAGGCGTTATCTATTTTATTGATCTCGGTGAAGAGGTCATTACCACCGGCGG
TGATTTTGTGCTGGCGAGAGTCTGGGCGATGGTGATGGCATCAGTAGCACCGCCAGCATTGCAACTGCCATTACCCGATC
ATTTCCAACCGACTGGCAGGCCGCTTCCTTCAGGCCTGTTACGGCGCGAATACATCATTCTGATTGAGATCGCACTGTCA
GCGTTATCGCTGTTGCTTTGCGGCCTTCAGGCAGAACCGCGTTATATTATTTTGATTCCTGTGCTGGCGGCTATCTGGAT
TATTGGCAGCCTGACAAGCAAAGCGTACAAAGCAGAAATCCAGCAACGCCGTGAGGCATTTAATCGTGCGAAAATGGACT
ATGACCATTTAGTCAGCCAGATCCAGCAGTTGGGCGGGCTGGAAGGTTTTATCGCCAAACGGGCGATGCTCGAAAAAATG
AAGGACGAAATTCTCGGGTTACCGGAAGAAGAGAAACGCGCTCTGGCAGCACTTCACGACACCGCAAGGGAACGGCAGAA
GCAGAAGTTTCTGGAGGGATTTTTTATTGATGTTGCCTCTATTCCCGGCGTTGGCCCTGCGCGTAAAGCGGCGTTACGGT
CCTTTGGTATTGAAACAGCAGCGGATGTTACCCGACGGAGCGTTAAGCAAGTAAAAGGTTTTGGTAATCATCTGACCCAG
GCGGTTATCGACTGGAAAGCGAGCTGTGAACGCCGTTTTGTGTTCAGGCCGAACGAAGCGGTAACGCCTGCAGACAGACA
AGCGGTAATGGCAAAAATAGCCGCCAAACGACATCGGCTGGAATCGGCGCTGACTGTCGGTGCGGCAGAGTTGCAGCGAT
TCCGCCTTCATGCGCCAGCACGGACCATGCCGTTGATGGAACCGCTACGTCAGGCGGCTGAAAAACTGGCTCAGGCGCAG
GCAGATTTAAGCCGTTGCTGA
>Cluster_39657
ATGACCATCACCACAGACACCACTCTTTTACACGACCCGCGTCGTCAGGCGGCGCTGCTGTACTGGCAGGGGTTTTCCGT
GCCGCAGATTGCCGCCATGTTGCAGATGAAACGCCCGACGGTGCAGAGCTGGAAACAGCGCGACGGCTGGGACAGCGTTG
CCCCCATCAGCCGTGTCGAAATGAGTCTGGAAGCGCGGCTGACCCAGCTCATCATCAAACCGCAGAAAACCGGCGGTGAC
TTCAAGGAAATTGACCTGCTCGGACGCCAGATTGAACGACTGGCACGGGTCAACCGCTACAGCCAGACCGGCAACGAAGC
AGACCTTAATCCGAACGTCGCTAACCGCAACAAAGGCGGGCGTCGCAAACCGAAAAAGAATTTTTTCAGCGACGAGGCCA
TCGAAAAGCTGGAGCAGATTTTCTTTGAGCAGTCTTTCGAATATCAGTTGCACTGGTATCGCGCCGGGCTTGAGCACCGC
ATCCGCGATATCCTGAAATCCCGCCAGATTGGCGCGACGTTTTATTTTCCCGCGAGGCGCTGCTGCGCGCCCTGAAAAC
CGGTCATAACCAGATTTTTCTGTCGGCCAGTAAAACGCAGGCGTATGTGTTCCGCGAATACATCATCGCCTTTGCCCGGC
TGGTTGACGTTGACCTGACCGGTGACCCGATTGTCCTGGGCAATAACGGCGCAAAACTGATTTTTCTCGGCACCAACTCC
AACACCGCACAGAGCCATAACGGCGACCTGTACGTCGACGAGATTTTCTGGATCCCGAATTTTCAGGTGCTGCGTAAGGT
GGCATCAGGTATGGCCTCACAGAGTCACCTGCGATCGACCTATTTCTCCACCCCGTCCACGCTGGCGCACGACGCCTACC
CGTTCTGGTCGGGTGAACTGTTCAACCGGGGACGCGCCAGCGCCGCCGAACGCGTGGAAATCGACGTCAGTCATAACGCC
CTTGCCGGTGGGCTTCTCTGTGCGGACGGCCAGTGGCGGCAGATTGTCACCATTGAGGACGCGCTGAAAGGCGGCTGCAC
GCTGTTCGACATTGAGCAGCTCAAACGCGAAAACAGCGCCGACGATTTTAAAAACCTGTTCATGTGTGAATTTGTTGACG
ACAAGGCGTCGGTGTTCCCGTTCGAGGAGCTGCAACGCTGTATGGTCGACACGCTGGAAGAATGGGAAGACTATGCGCCG
TTTGCCGCCAATCCGTTCGGCTCCCGCCCGGTATGGATTGGTTACGACCCGTCACACCGTGGCGACAGCGCCGGATGCGT
GGTGCTGGCACCGCCGGTGGTGGCCGGTGGCAAATTCAGAATACTTGAGCGTCACCAGTGGAAAGGCATGGACTTTGCCA
CTCAGGCGGAATCCATCCGCAAACTCACCGAAAAATACAACGTCGAATACATCGGTATTGATGCCACCGGCCTCGGTGTC
GGCGTGTTCCAGCTCGTGCGCTCGTTCTATCCCGCCGCGCGCGATATCCGCTACACGCCGGAAATGAAAACCGCAATGGT
GCTCAAGGCAAAAGACGTTATTCGCCGTGGCTGTCTGGAATATGACGTCAGCGCCACCGACATCACCAGCTCGTTTATGG
CTATCCGCAAGACCATGACCAGCAGCGGACGCAGCGCCACCTATGAGGCCAGCCGCAGCGAGGAAGCCAGCCACGCCGAC
CTCGCCTGGGCGACCATGCACGCCCTGTTAAATGAGCCACTCACCGCCGGTATCAGCACCCCGCTGACATCCACCATTCT
GGAGTTTTACTGA
>Cluster_20508

ATGCGTAGTTTTAGCCAACTGTGGCCGACTCTCAAGCGCCTGTTAGCGTACGGTTCACCGTGGCGTAAACCGTTGGGGAT
TGCGGTCCTGATGATGTGGGTTGCGGCGGCGGCAGAAGTCAGTGGGCCGCTGCTTATCAGCTATTTTATCGACAATATGG
TAGCGAAAAATAACCTGCCGTTGAAAGTGGTTGCAGGGCTGGCGGCGGCGTATGTTGGGCTGCAACTGTTTGCAGCCGGG
CTACATTACGCGCAGTCGCTGCTGTTTAATCGGGCGGCAGTAGGCGTAGTGCAACAGTTGCGTACCGACGTGATGGATGC
TGCGTTACGCCAGCCATTAAGCGAGTTTGATACCCAACCCGTCGGGCAGGTGATTTCCCGCGTCACTAATGACACTGAAG
TGATCCGCGATCTCTACGTTACCGTAGTGGCAACTGTCCTGCGTAGCGCCGCACTGGTGGGCGCGATGCTGGTGGCGATG
TTCAGCCTCGACTGGCGAATGGCGCTGGTGGCGATAATGATTTTCCCGGTGGTGCTGGTGGTAATGGTGATATATCAGCG
TTACAGCACGCCGATTGTCCGTCGTGTGCGCGCTTATCTGGCGGATATCAACGACGGCTTTAACGAAATCATCAATGGCA
TGAGCGTTATCCAGCAGTTCCGTCAGCAGGCGCGATTTGGCGAACGTATGGGTGAGGCCAGTCGTTCACACTATATGGCG
AGGATGCAAACCCTGCGCCTCGACGGTTTTCTGCTGCGTCCGCTGCTGAGTCTGTTTTCATCGCTCATTCTTTGTGGCTT
GTTGATGCTGTTTGGCTTCTCCGCCAGCGGCACCATTGAAGTGGGCGTGTTGTATGCGTTCATCAGTTATTTGGGGCGAC
TTAACGAACCCTTAATCGAACTGACCACGCAGCAGGCGATGCTGCAACAGGCTGTTGTTGCTGGTGAGCGCGTTTTTGAG
CTGATGGACGGACCGCGCCAGCAATATGGCAATGATGATCGCCCGTTGCAGAGCGGCACCATCGAAGTCGATAACGTGTC
ATTTGCTTATCGCGATGACAATCTGGTGCTAAAGAACATTAATCTCTCTGTGTCTTCGCGCAACTTTGTAGCGCTGGTCG
GGCATACCGGCAGTGGCAAAAGCACCCTCGCCAGTTTATTGATGGGCTATTACCCGCTAACGGAAGGTGAGATTCGCCTT
GATGGTCGTCCATTAAGTACGCTAAGTCACAGCGCGCTGCGCCAGGGCGTGGCAATGGTGCAGCAAGATCCGGTGGTGCT
GGCGGATACCTTCCTCGCCAACGTGACGCTGGGACGTGATATCTCTGAAGAACGCGTCTGGCAGGCGCTGGAAATCGTGC
AGCTGGCGGAGCTGGCGCGTAGCATGAGTGATGGTATTTACACGCCGCTTGGCGAGCAGGGGAATAATCTCTCAGTCGGG
CAAAAGCAACTGCTGGCACTGGCGCGCGTGCTGGTGGAGACGCCGCAAATCCTGATCCTTGATGAGGCAACCGCCAGCAT
TGACTCCGGGACTGAACAGGCGATTCAACATGCTCTGGCGGCGGTGCGTGAACATACTACGCTTGTGGTGATTGCTCACC
GCTTATCGACTATTGTTGATGCCGACACCATTCTGGTGCTTCATCGTGGGCAAGCCGTGGAGCAGGGCACTCACCAGCAA
CTGCTGGCGGCCCAGGGACGATACTGGCAGATGTATCAACTGCAACTTGCGGGCGAAGAGCTGGCAGCCAGCGTGCGTGA
AGAGGAATCATTGAGCGCCTGA
>Cluster_16906
ATGTTAACGGCATGTATATCATTTGGGGTTGCGATGACGACGAACACGCATTTTAGAGGTGAAGAATTGAAGAAGGTTTG
GCTTAACCGTTATCCCGCGGACGTTCCGACGGAGATCAACCCTGACCGTTATCAATCTCTGGTAGATATGTTTGAGCAGT
CGGTCGCGCGCTACGCCGATCAACCCGCGTTTGTGAATATGGGGGAGGTGATGACCTTCCGCAAACTGGAAGAACGCAGT
CGCGCGTTTGCCGCTTATTTGCAACAAGGGTTGGGGCTGAAGAAAGGCGATCGCGTTGCGTTGATGATGCCTAACTTATT
GCAATATCCGGTGGCGCTGTTTGGCATTTTGCGTGCCGGGATGATCGTCGTAAACGTTAACCCGTTGTATACCCCGCGTG
AGCTTGAGCATCAGCTTAACGATAGCGGCGCATCGGCGATTGTTATCGTGTCTAACTTTGCCCACACTCTGGAAAAAGTG
GTTGATAAAACCGCCGTTCAGCACGTTATTCTGACCCGTATGGGCGACCAGCTATCTACGGCAAAAGGCACGGTAGTCAA
TTTCGTTGTTAAATACATCAAGCGTCTGGTGCCGAAATACCATCTGCCAGATGCCATTTCATTTCGTAGCGCCCTGCACA
ACGGCTACCGGATGCAGTACGTCAAACCAGAACTGGTGCCGGAAGATTTAGCTTTCCTGCAATACACCGGCGGCACCACT
GGTGTGGCGAAAGGCGCGATGCTGACTCACCGCAATATGCTGGCGAACCTGGAACAGGTTAACGCGACCTATGGTCCGCT
GTTGCATCCGGGCAAAGAGCTGGTGGTGACGGCGCTGCCGCTGTATCACATTTTTGCACTGACCATTAACTGCCTGCTGT
TTATCGAACTAGGGGGGCAGAACCTGCTTATCACTAACCCGCGCGATATTCCAGGGTTGGTAAAAGAGTTAGCGAAATAT
CCGTTTACCGCTATCACTGGCGTTAACACCTTGTTCAATGCGTTGCTGAACAATAAAGAGTTCCAGCAGCTGGATTTCTC
CAGTCTGCATCTTTCCGCAGGCGGAGGGATGCCTGTTCAGCAAGTGGTGGCAGAACGTTGGGTGAAACTGACTGGACAGT
ATCTGCTGGAAGGTTATGGCCTGACCGAGTGTGCGCCGCTGGTCAGCGTTAACCCGTATGATATTGATTATCATAGTGGT
AGCATCGGTTTACCGGTGCCGTCGACGGAAGCCAAACTGGTGGATGATGATGATAATGAAGTACCACCGGGTCAACCAGG
TGAGCTTTGTGTCAAAGGACCGCAGGTGATGCTGGGTTACTGGCAGCGTCCGGATGCTACCGATGAAATCATCAAAAATG
GCTGGTTACACACCGGCGATATCGCGGTGATGGATGAAGAAGGATTCCTGCGCATTGTCGATCGTAAAAAAGACATGATT
CTGGTTTCCGGTTTTAATGTCTATCCCAACGAGATTGAAGATGTCGTCATGCAGCATCCTGGCGTACAGGAAGTCGCGGC
TGTTGGCGTACCTTCCGGCTCCAGTGGTGAAGCGGTGAAAATCTTCGTTGTGAAAAAAGATCCATCGCTTACCGAAGAGT
CACTGGTGACCTTTTGCCGCCGTCAGCTCACGGGCTACAAAGTACCGAAGCTGGTGGAGTTTCGTGATGAGTTACCGAAA
TCTAACGTCGGAAAAATTTTGCGACGAGAATTACGTGACGAAGCGCGCGGCAAAGTGGACAATAAAGCCTGA
>Cluster_10216
ATGGCTCAACGTGATAATCTGTATCGCCGTAGCAGCGGTATTTATGTTGTCCGTATTGCAGTTCCCGCCCGATACCGTCT
CTACACGGGACAGCGTGAGATTCACGCTTCAACAGAAACAAACGACATCAGAAAAGCGCGAGCGATTGCCGCAGTTCTAT
TGGCTGTCTGGCAGCGATGTCTGGGTGAGTATCAAAAATTGGATAGAGAAAAGTTGGTAAGCAGTGCGCCTGCTTTGGCT
GGAGAAGGAATGATCAGCCTCGCTGAATTTTGCGGTTTAACGAGCGCTTCCCTGCAACAAGTGGCGCAGCGACTACTTGA
TACGAATATCCCACTTTTTTGGCTTGCTGATGGGCATCCTGCCTACTATGTAGAAGATCTTTCACTCGTTGATAAGGATG
ATCCTGAGGCTTCTGGATTTATCATCGATAGTGCCATTAACATCGGGATCAGAGGTTCTCTGAACGGTTATATCCAGCCT
TTGGTTACCTCACGTATATTACGTCAGATGATCGCTGGCGAGCCGCTAGACGCAGAAACCGCATTTAAATCCTCAGAGGC
TTCATCCAAAGCCGCATGGTTCGTTGACTTTCCCGGTGTCGAAATCTCGCCTGGTTCACTAATGATTCATCGTGTGCAAG
CCGAGCGCTTACGATTGTTTTGGCTGTCATATAGCCCCTCACTATCTCCTGCAACTACTGAGAAGCCAAAAGCAATATCC
ACTGCAATATTGTCCCCATCATTGCCGGAGAATGAGTATGTAAATCGCAAATACTACTCGGTGAAAATGAGCGAGTTATG
TGAAAAATATATTCGCTACAAGCGCGGGGGAAATTCACGGAAAAGGCAGAGAATCGAGTCCGGGAGGGTTTTAGCCTAC
TGGTTGAAGTCATGGGTGATCCAAAGCTTGTAAAAGTAGATCGTGACTATCTCCGCGAGTATGAGAGCTTGCTAAGGTCG
ATACCTGCACGGCGTGATTTAGCTAAAATCCGATACAGAATCAATGACATTCATGAGTTGATGGTTAAAGCAAGAGAGAA
TGGCGACCCCTCTAATGAGCGATAACACCGTTCGTAAATATATGCGGGTTATCTTCGAAGCTTTCAGGTGGGCAGATGGAG
AAGGTATTTTTATTAAGTCTCCTGCGAATCAATTCTTCGCACCTGTAGAAAATGAAAAGATGGATCAGGACTTCAAAGCT
GATTTCACTGATGAGGATTTGCACGCTATTTTTAACCGCCCTTGGTTTAAACGTGGAACTGTGGATAGAAATAGCGGGGG
GCGCTTCCATCATTACCGTGCATTTCATTACTGGTTGCCACTCATCGGATTCTTTACCGGAGCAAGGATTAATGAGTTAT
GCCAGCTTTATCTGGATGATATTAAACAGGATGCTGCCGGATTTTATTTCTTTGAAATCAGCAACGAGCGTGTCGATCAG
TCACTGAAAACGATTAATTCACGAAGAAAAATACCGTTGCATCCGACATTGATCAAACTTGGATTAATCCGCTATTGCGA
AGCATTGAAGAAAGCAGGTCATGCGCGTTTATTTCCTGAGCTTCCTTACCACTCTATAAAAGGATACGGAGATAAGGCTT
CGGACTGGTTTAACCGTTCGTTGCTGAAAGTGCAGTTAGGATTTGAAAAGGGCAGTAAGAAATCTTTCCATTCATTCCGT
CACACGTTTTCAACCAGGCTCAAACAAGCAGGAATTGAACCGCCCCGGGAATCCTGGAGACTAAACTTCCTGAGAAAGAG
GTAA
>Cluster_38447
ATGTCAACCGGATTACGTTTCACACTGGAAGTGGACGGCCTGCCGCCGGATGCTTTTGCGGTGGTTTCCTTTCATCTGAA
CCAGTCACTCTCTTCGCTCTTTTCCCTCGATCTCTCCCTGGTCAGCCAGCAGTTTCTCTCCCTTGAATTTGCGCAGGTGC
TGGACAAAATGGCCTACCTGACGATATGGCAGGGCGATGAAGTACAGCGCCGGGTGAAAGGCGTGGTGACCTGGTTTGAA
CTCGGGGAGAACGACAAAAACCAGATGCTGTACAGCATGAAGGTGCACCCGCCGCTGTGGCGTGCCGGTCTGCGCCAGAA
CTTCCGTATCTTCCAGAACGAGGACATCAAAAGCATCCTCGGCACGATGTTGCAGGAAAACGGGGTGACCGAATGGAGTC
CGCTGTTCAGCGAGCCGCATCCTTCCCGTGAGTTTTGTGTCCAGTACGGTGAGACTGATTACGATTTTCTGTGCCGGATG
GCGGCGGAGGAAGGCATCTTCTTTTATGAGGAGCATGCTTACAAAAGTACCGACCAGAGCCTGGTGCTGTGCGACACAGT
CCGCCATCTGCCCGAATCTTTTGAAATCCCATGGAACCCGAACACCCGTACCGAGGTGAGCACCCTCTGCATCAGCCAGT

TCCGCTACAGCGCACAAATCCGCCCTTCTTCCGTGGTGACCAAAGACTACACCTTTAAACGCCCCGGCTGGGCCGGACGT
TTTGAACAGGAAGGCCAGCACCAGGACTACCAGCGCACGCAGTATGAAGTGTATGACTACCCCGGACGTTTCAAGAGCGC
CCACGGGCAGAACTTTGCCCGCTGGCAGATGGACGGCTGGCGAAACAACGCAGAAACCGCGCGGGGAATGAGCCGCTCGC
CGGAGATATGGCCGGGACGACGAATTGTGCTGACGGGGCATCCGCAGGCGAACCTGAACCGGGAATGGCAGGTGGTGGCA
AGTGAACTGCACGGCGAACAGCCACAGGCGGTGCCAGGACGGCAGGGAGCGGGGACGGCGCTGGAGAACCATTTTGCGGT
GATCCCGGCAGACAGAACATGGCGACCACAGCCGTTGCTGAAACCGCTGGTCGACGGCCCGCAGAGCGCTGTCGTGACAG
GACCGGCAGGCGAGGAAATCTTCTGCGACGAACATGGTCGCGTGCGGGTGAAGTTCAACTGGGACCGTTATAACCCGGCA
GACCAAGACAGTTCGTGCTGGATCCGTGTGGCACAGGCGTGGGCAGGCACCGGTTTTGGCCACCTGGCGATACCGCGTGT
GGGTCAGGAGGTGATTGTAGACTTCCTCAACGGCGATCCGGACCAGCCGATCATTATGGGGCGCACCTACCACCAGGAAA
ACCGCACCCCCGGCAGCCTGCCGGGAACAAAGACGCAGATGACCATCCGCTCCAAAACGTACATGGGCAGCGGATTTAAT
GAGCTGAAGTTTGATGATGCGACAGGGAGAGAACAGGTCTACATCCACGCGCAGAAGAACATGGATACCGAAGTGCTCAA
CGACCGTACCACCACCGTAAAACACGATCACCGCGAAACCGTAAAAAATGACCAGACGGTCACGATCCAGGAAGGTAACC
GCCTTCTTACGGTGGAAAAAGGCCACAAGATCACCGGAGTACTGAAAGGGTCTTTATCTGAGGATGTCTTTCAGGACAGA
GGCACGATTGCCGGTTCGGTGCATGTTGACGCTGTAAACAATGGTGGCGAAGGCGACGGTATACAGGCTTATACGGCGAT
TAAGGAAATTTTGCTGGCTGTGGAGGAAAGCAAAATTGCGCTGACGCCGGATGGCATTCAGCTACAGGTCGGGGAATCGA
CGGTAATCAGGCTGTCGAAGGATGGCATCACCATCGTGGGCGGTTCTGTTTTCATCAACTGA
>Cluster_63879
ATGAGTATATCCAGACAACAACTGAATAATATGGCCCGGGCTGTCAGAACTGCGTTGCAGCCGCTGGTAAGGGCATTGCC
AGTAACGGCAGTTGAATGGGCAGATCAAAATTATTACCTGCCTAAAGAATCGTCATATGGTGAGGGGGAATGGAAAACCC
TGCCATTTCAGGTCGCTATCATGAATAGCATGGGTAACGACCAGATCCGTACCATTAATCTGATTAAATCGGCGCGCGTG
GGCTACACCAAAATGTTGCTGGGTGTGGTCGGGTATTTTATAGAGCATAAATCCCGTAACAGCCTGCTTTTCCAGCCTAC
TGATTCTGCCGCTGAAGATTTTATGAAAGCGCATGTGGAAGCAACATTGCGGGATGTCCCCTGCCTTAAAGCGTTGTCTC
CGTGGCTGGGCAGAAAACATCGTGACAACACGCTCACCCTGAAGCGTTTTTCCTCCGGTGTGGGGTTCTGGTGCCTGGGG
GGAGCTGCTGCCAAAAATTACCGTGAAAAATCTGTGGATGTGGTCTGCTATGACGAACTCTCCTCGTTTGAACCGGATGT
GGAAAAAGAAGGTTCGCCGACGCTGCTTGGTGATAAACGTATTGAAGGCTCGGTATGGCCTAAGTCCATACGCGGTTCAA
CGCCAAAAATTAAAGGCTCCTGTCAGATTGAGAAAGCCGCGAATGAATCTGCGCATTTCATGCGGTTTTATGTTCCTTGC
CCTCATTGCGGGGAGATCCAGTATCTGAAGTTTGGTGATGATGCGACGCCGTTTGGCCTGAAATGGGAGAAGGGTAAACC
GGAAACGGTGTATTACCTGTGTGAACATAATGGCTGTGTGATCCGGCAGTCGGAACTTGACCAGATCGACGGACGCTGGA
TTTGTGACAATACCGGGATGTGGACGCGTGACGGTCTGGCATTTTTCAGTGCCAGTGATGAGGAGATGCCGCCACCGCGC
TCCATCACGTACCACATCTGGACGGCCTATAGCCCGTTCACCACCTGGGTGCAGATTGTCTACGACTGGCTGGATGCGCT
GAAAGATCCGAACGGCGTCAAGACATTTATTAACACCACGCTGGGAGAGACCTACGAAGAGGCTGTGGGTGAAAAACTCG
ATTACCAGGCACTGATGGATAAGGTTGTGCGTTACACGGCTGCGGTGCCTTTTCGGGTGGTTTATCTGACGGCGGGCATT
GACTCTCAGCGAAACCGTTTTGAGATGTATGTCTGGGGATGGGCTCCGGGAGAGGAAGCCTTTCTGGTGGATAAAATCAT
CATTATGGGGCGTCCTGATGAGGAAGAGACGCTGTTACGTGTGGATGCGGCGATCAACAAAAAATACTGCCATGCAGATG
GTACCGAAATGTCCATTTCCCGTGTCTGCTGGGACATCGGGGGGATCGATGGTGAAATAGTTTATCGGAGATCAAAAAAA
CACGGTGTTTTTCGGGTGTTGCCGGTAAAAGGCGCTTCTGTCTATGGCAAGCCAGTGATCACCATGCCGAAAACTCGCAA
TCAGCGGGGTGTTTATCTGTGTGAGGTGGGGACGGACACCGCAAAAGAAATTCTCTATGCCCGTATGAAGTCCGCTCCCA
CGCCTGCGGATGAAGCCACGTCGTATGCCATCCGTTTTCCTGATGATCCGGAGATTTTTTCGCAGACAGAGGCGCAGCAA
CTGGTGGCGGAAGAGCTCGTGGAGAAGTGGGAAAAAGGGAAGATGCGTCTGCTGTGGGATAACAAAAAGCGGCGTAACGA
AGCGCTGGACTGCCTGGTGTATGCCTACGCGGCATTACGTGTGTCCGTGCAACGCTGGCAGCTTGATCTGGCTGTACTGG
CAAAATCCCGGGAGAAGGAAACTGTCCGGCCAACCCTTGAAGAACTGGCAGCAAAACTGTCCGGAGGAGTGAATGGTAAC
TATTGA
>Cluster_28804
ATGTGTGGAATTGTTGGCGCGATCGCGCAACGTGATGTAGCAGAAATCCTTCTTGAAGGTTTACGTCGTCTTGAATACCG
CGGATATGACTCTGCCGGTCTGGCCGTTGTTGATGCAGAAGGTCATATGACCCGCCTGCGTCGCCTCGGTAAAGTCCAGA
TGCTGGCTCAGGCAGCGGAAGAACATCCTCTGCATGGCGGCACCGGTATTGCTCATACTCGCTGGGCGACCCACGGTGAA
CCTTCAGAAGCTAACGCGCATCCGCATGTTTCTGAACACATTGTGGTGGTGCATAACGGCATCATCGAAAACCATGAACC
GCTGCGTGAAGAGTTAAAAGCGCGTGGCTATACCTTCGTTTCTGAAACCGACACCGAAGTGATTGCCCATCTGGTGAACT
GGGAGCTGAAACAGGGCGGTACTCTGCGTGAGGCTGTTCTGCGTGCTATCCCGCAACTGCGAGGTGCGTACGGTACAGTG
ATCATGGACTCCCGTCACCCGGATACCCTGCTGGCGGCACGTTCTGGTAGCCCGCTGGTGATTGGCCTGGGTATGGGTGA
AAACTTTATCGCTTCTGACCAGCTGGCGCTGTTGCCAGTGACTCGTCGCTTTATCTTCCTTGAAGAGGGCGATATTGCGG
AAATCACTCGCCGTTCGGTAAACATCTTCGATAAAACTGGCGCGGAAGTAAAACGTCAGGATATCGAATCCAATCTGCAA
TATGACGCGGGCGATAAAGGTATTTACCGCCACTACATGCAGAAAGAGATCTACGAACAGCCGAACGCGATCAAAAACAC
CCTTACCGGGCGCATCAGCCACGGTCAGGTTGATTTAAGCGAGCTGGGACCGAACGCCGATGAACTGCTGTCGAAGGTTG
AGCATATTCAGATCCTCGCCTGTGGTACTTCTTATAACTCCGGTATGGTTTCCCGCTACTGGTTTGAATCGCTGGCAGGT
ATTCCGTGCGACGTCGAAATCGCCTCTGAATTCCGCTATCGCAAATCTGCCGTGCGTCGTAACAGCCTGATGATCACCTT
GTCACAGTCTGGCGAAACCGCGGATACCCTGGCGGGCCTGCGTCTGTCGAAAGAGCTGGGTTACCTTGGTTCGCTGGCAA
TCTGTAACGTTCCGGGTTCTTCTCTGGTGCGCGAATCCGATCTGGCGCTGATGACCAACGCCGGTACGGAAATTGGCGTG
GCCTCCACCAAAGCATTCACCACCCAGTTAACTGTGCTGTTGATGCTGGTGGCGAAACTGTCTCGCCTGAAAGGTCTGGA
TGCCTCCATTGAACATGACATCGTGCATGGTCTGCAGGCGCTGCCGAGCCGTATTGAGCAGATGCTGTCTCAGGACAAAC
GCATTGAAGCGCTGGCAGAAGATTTCTCTGACAAACATCACGCGCTGTTCCTGGGCCGTGGCGATCAGTACCCAATCGCG
CTGGAAGGTGCATTGAAGCTGAAAGAGATCTCTTACATTCACGCTGAAGCCTACGCTGCAGGCGAACTGAAACACGGTCC
GCTGGCGCTGATTGATGCCGATATGCCGGTTATCGTCGTTGCACCGAACAACGAATTGCTGGAAAAACTGAAATCCAACA
TTGAAGAAGTTCGCGCGCGTGGCGGTCAGTTGTATGTCTTCGCCGATCAGGATGCGGGTTTTGTGAGCAGTGACAATATG
CACATTATTGAGATGCCGCATGTGGAAGAGGTGATTGCACCGATCTTCTACACCGTTCCGCTGCAACTACTGGCTTACCA
CGTTGCGCTGATCAAAGGCACCGACGTTGACCAGCCGCGTAACCTGGCAAAATCGGTAACGGTTGAGTAA
>Cluster_40985
ATGCGATATATAGCAGGCATTGATATCGGCAACTCATCAACAGAGGTTGCGCTGGCAACGCTCAGTGCAACTGGAGAGTT
GTCGTTCGTGAGTAGCACCCTGGCGGAAACCACCGGAATTAAAGGTACGTTGCGTAACGTCCATGGTATTCAGGAGGCGC
TCGCACAGGCAACGAAAAAAGTCGGCATCAATGTTAGCGATATTTCGCTTATTCGTATCAACGAAGCAACTCCGGTCATT
GGTGATGTGGCGATGGAAACCATCACTGAAACTATCATCACCGAATCAACCATGATCGGTCACAACCCGAAAACTCCGGG
CGGAGTGGGGCTGGGCGTAGGACTCACCATCACTCCGCAAGAGTTATTAACATGCCCGGCGGACACACCCTACATTCTGG
TGGTGTCATCGGCATTTGATTTTGCCGATATCGCAACGATGATAAACGCCTCCGTACGAGCCGGATATCAGCTGACTGGC
GTTATTCTGCAACGCGACGATGGCGTACTGGTCAATAACCGCCTGGAAATTCCTCTCCCTATTGTTGATGAAGTCCTCTA
CATCGATCGTATTCCGCTCGGCATGCTGGCTGCAATTGAAGTTGCCGTTCCCGGGAAAGTTATCGAAACCCTGTCTAACC
CGTATGGGATTGCCACCGTTTTCGCCCTCAATGCTGAAGAGACTAAAAATATTGTTCCTGTCGCGCGGGCGCTAATCGGC
AACCGTTCAGCAGTGGTAGTGAAAACGCCTTCTGGCGACGTTAAAGCCCGTTCGATACCCGCCGGAAATATCGAACTGCT
TTCTGCCGGGCGCACAACGCGGGTTGATGTAGCTACTGGTGCCGATGCCATTATGAAAGCGGTCGGTGAGTGCCCAAAAC
TGGAAAACGTGACGGGAGAACCTGGCTCCAACATCGGCGGAATGCTGGAACATGTTCGCCAGACCATGGCTGAACTGACC

AACAAACCCAGCAACGAAATTTTTATTCAGGATTTGCTGGCGATAGATACCTCTGTCCCCGTCAACGTAACCGGTGGTCT
GGCAGGTGAATTTTCACTGGAACAGGCTGTGGGTATTGCCTCTATGGTGAAATCCGACCGTCTGCAAATGGCGATGATCG
CCAGTGAAATTAAGCAAAAACTGCATGTTGATGTTCAGGTCGGAGGCGCAGAAGCAGAAGCCGCCATTCAGGGGGCCTTG
ACTACCCCTGGAACGACACGTCCACTGGCAATTCTGGACTTAGGTGCAGGCTCTACCGATGCCTCCATCATCAATCAGAA
AGGGGAAATAGTTGCCACTCACCTTGCGGGAGCGGGCGACATGGTAACGATGATTATCGCTCGTGAGCTGGGCTTAAATG
ATCGTTATCTGGCAGAAGAAATCAAAAAGTACCCGTTGGCAAAAGTAGAAAGCCTGTTCCACTTACGCCATGAAGATGGC
AGCGTTCAGTTCTTCCCTACTCCCCTATCCCCTCATGTATTTGCCCGCGTTTGTGTGGTGAAACCTGACGAACTGGTGCC
TATTCCCGGTGATTTGACGCTGGAAAAAGTACGCGCTGTCCGTCGCAGTGCGAAAGAGCGTGTCTTCGTAACTAATGCCC
TGCGTGCGCTGCGTCAGGTAAGCCCTGCCGGAAATATTCGCGACATTCCGTTCGTCGTTCTGGTAGGTGGCTCTTCGCTG
GATTTCGAAGTACCGCAATTGGTCACCGACGCGCTGGCACATTATCGACTGGTCGCCGGGCGGGGGAATATCCGTGGTAG
CGAAGGTCCGCGCAATGCGGTAGCTACTGGGCTCCTCATCGCCTGGCACAAGGAGTCGATTCATGGAAAGTAA
>Cluster_53907
ATGGCAGAAAAAAAAGATGACTGACTGGCATCGCAAGGTGCTGTGCAACTTTGATAATGCCTGGTCAGCAACGCAGGATAT
GCGTGAGCAGATTATTGAGGCTCAACGTTTCGTCAGGGTGTCCGGCGCACAGTGGGAAGGCAGCACAAACGCTGGTTACT
CATTTGATGAAGGCAGGTTTGAGCATTATCCGCGTTTTGAACTGAATAAGATTGCCCGTGAATGTGATCGCATCATTGGC
GAGTATCGACAGAATCGCATCAGCGTTAAATTCAGGCCGAAGGACGATAAGGCATCGGAAGCGTTAGCCGAAAAGATGAA
CGGCAAATTCCGCGCTGACTATCAGGAAACATCCGGTGGCGAAGCGTGTGATAACGCATTTGATGATGCCGTAACGGGTG
GATTCGGTTGTTTCCGCATGTGTGCCGATTACGAAGATGAAATGGATCCGAGTAACGAGCAGCGACGCATCAGCCTTCTT
CCTGTTTACGACCCAGCGACATGCGTCTTCTTCGATCAGGACAGCAAGCAATATGACCGTTCTGATGCTATGTGGGCTAT
GGAAATGTTCTCCATGACGCCTAAAGCGTTCGAGGCTGAATACCCTGATTCCATCGCGGCAAGCCTTTCTCGTGATGACA
CTGGTACTCAGTATGACTGGTCAACGCCCGATGCCATCTATGTTGGACGCTACTACGAAGTTCGCATAGAGAAGGTGAAG
CTCACGGCGTGGCGCAACCCTGTTAGCGGAGAAACGGCAATTTATGATGAAGAGCAAATCAAAGATATTGTCGACGAGCT
GACCGATGGTGCATTCGAACTGATTGGCGAGCGAACGGTGAAGAAGCGCCGCGTTTATTGCGGTCTTCTGTCTGGCGCTG
AATGGCTGGAAGAACCTAAGCGTATTCCGGGCGAACATATTCCTCTCATCCCGGTATATGGGCGTCGCTCATTTGTTGAT
AATCAGGAGCGAATCGAAGGCCACGCAGCAAAAGCGATGGATGCACAGCGTCTTGAGAACCTGATGGTTTCCATGATTGC
AGATAACGCTACTCAGGCTGGCGGTGATGGCATTCCTGTAGTTGATGTTGACATGATTCCTGGTCCTCTCGCCACTCATT
GGGCGGAGCGCAACAAAAAGCGCCCGGCGTTCCTGCCGATGGTCAGTCTGAAAAACAAAAACGGAGATATTACTGCGCAG
GCTCAGGTCAGCAGTTATACGCCTCCGACACAAATGCCTCCAGCTCTTGCGGGCTATTGCAGTACACCGGAACGGCTAT
TCAGCAAATTACAGGTGCGTCGCAGATTGAGAACATGCCGAGCAACGTCGCCACCGATACCGTTGATAGCATCTTTAACC
GGATGGACACGCAGTCCTATATCTACATGGACAACATGGCTAAATCCATGCGTCGCGCTGGCGTTGTGTGGCTTTCTATG
GCGCGTGAGGTCTATGGCAGTGATACGCCGATGCGTATCGTTAATGAGGACGGCAGCGATGACGTGGCGTTGATGACTGG
TGAAGTGGTTGACCGTCAGACAGGGCAGGTTATCGCGCTTAACGACCTTTCGCAGGGTAACTATGAAGTGACTGTCGATG
TCGGTCAGTCGTTCGCTACTCGCCGTGATGCAACGGTTAAGTCGTTACTTTCCATGCTGGCACTTATCCCACCAGGAACG
CCGAAGCACGATCTTGTATCGTCGATGATTCTCGACAATATGGACGGCGAAGGGATGGACGACCTTAAAGAATACAACCG
CAATCAGTTGCTTCTGTCTGGCGTTATCAAGCCGAGAACGCCTGAAGAACAGCAGATGGTTGAACAGGCGAAACAACAAC
AGGCCAGTCAGCCAGATCCGGCTATGGTTGCTGCGCAAGGTCAGCTTCTTGCTGGTCAGGCTGAATTGCAGAAAGCGCAG
AACGAGCAGGCAGCCATTCAGGTTAAAGCATTCCAGGCACAGACTGATGCTCAGGTTGCAGCGGCAAACGTTGTGAAAAT
CCTCGCATCTGCCGATAGCCAGCAGAAATCTGATATCCGCGAGGCTCTGAAACTGCTCGGACAGTTCCAGCAACAGCAAG
GAGATAATGCCCGTGCTGATGCAGAGCTTGTCCTGAAAAGTCAGGCACAGGGCCATGCGCAGCGCATGGACATCAGCAGC
ATCCTGCAAAAATCAACTCAGCAACAACCACAGCAGTAA
>Cluster_58080
ATGGCGCGTTCCAAAACTGCTCAGCCAAAACACTCACTGCGTAAAATCGCAGTTGTAGTAGCCACAGCGGTTAGCGGCAT
GTCTGTTTATGCACAGGCAGCGGTTGAACCGAAAGAAGACACTATCACCGTTACCGCTGCACCTGCGCCGCAAGAAAGCG
CATGGGGGCCTGCTGCAACTATTGCGGCGCGACAGTCTGCTACCGGCACTAAAACCGATACGCCGATTCAAAAAGTGCCA
CAGTCTATTTCTGTTGTGACCGCCGAAGAGATGGCGCTGCATCAGCCGAAGTCGGTAAAAGAAGCGCTTAGCTACACGCC
GGGTGTCTCTGTTGGTACGCGTGGCGCATCCAACACCTATGACCACCTGATCATTCGCGGCTTTGCGGCAGAAGGCCAAA
GCCAGAATAACTATCTGAATGGCCTGAAGTTGCAGGGCAACTTCTATAACGATGCGGTCATTGACCCGTATATGCTGGAA
CGCGCTGAAATTATGCGTGGCCCGGTTTCCGTGCTTTACGGTAAAAGCAGTCCTGGCGGCCTGTTGAATATGGTCAGCAA
GCGTCCGACCACCGAACCGCTGAAAGAAGTTCAGTTTAAAGCCGGTACTGACAGCCTGTTCCAGACTGGTTTTGACTTTA
GCGATTCGTTGGATGATGACGGTGTTTACTCTTATCGCCTGACCGGTCTTGCGCGTTCTGCCAATGCCCAGCAGAAAGGG
TCAGAAGAGCAGCGTTATGCTATTGCACCGGCGTTCACCTGGCGTCCGGATGATAAAACCAATTTTACCTTCCTTTCTTA
CTTCCAGAACGAGCCGGAAACCGGTTATTACGGCTGGTTGCCGAAAGAGGGAACCGTTGAGCCGCTGCCGAACGGTAAGC
GTCTGCCGACAGACTTTAATGAAGGGGCGAAGAACAACACCTATTCTCGTAATGAGAAGATGGTCGGCTACAGCTTCGAT
CACGAATTTAACGACACCTTTACTGTGCGTCAGAACCTGCGCTTTGCTGAAAACAAAACCTCGCAAAACAGCGTTTATGG
TTACGGCGTCTGCTCCGATCCGGCGAATGCTTACAGCAAACAGTGTGCGGCATTAGCGCCAGCGGATAAAGGCCATTATC
TGGCACGTAAATACGTCGTTGATGATGAGAAGCTGCAAAACTTCTCCGTTGATACCCAGTTGCAGAGCAAGTTTGCCACT
GGCGATATCGACCACACCCTGCTGACCGGTGTCGACTTTATGCGTATGCGTAATGACATCAACGCCTGGTTTGGTTACGA
CGACTCTGTGCCACTGCTCAATCTGTACAATCCGGTGAATACCGATTTCGACTTCAATGCCAAAGATCCGGCAAACTCCG
GCCCTTACCGCATTCTGAATAAACAGAAACAAACGGGCGTTTATGTTCAGGATCAGGCGCAGTGGGATAAAGTGCTGGTC
ACCCTAGGCGGTCGTTATGACTGGGCAGATCAAGAATCTCTTAACCGCGTTGCCGGGACGACCGATAAACGTGATGACAA
ACAGTTTACCTGGCGTGGTGGTGTTAACTACCTGTTTGATAATGGTGTAACACCTTACTTCAGCTATAGCGAATCGTTTG
AACCTTCTTCGCAAGTTGGGAAGGATGGTAATATTTTCGCACCGTCTAAAGGTAAGCAGTATGAAGTCGGCGTGAAATAT
GTACCGGAAGATCGTCCGATTGTAGTTACTGGTGCCGTGTATAATCTCACTAAAACCAACAACCTGATGGCGGACCCTGA
GGGTTCCTTCTTCTCGGTTGAAGGTGGCGAGATCCGCGCACGTGGCGTAGAAATCGAAGCGAAAGCGGCGCTGTCGGCGA
GTGTTAACGTAGTCGGTTCTTATACTTACACCGATGCGGAATACACCACCGATACTACCTATAAAGGCAATACGCCTGCA
CAGGTGCCAAAACACATGGCTTCGTTGTGGGCTGACTACACCTTCTTTGACGGTCCGCTTTCAGGTCTGACGCTGGGCAC
CGGTGGTCGTTATACTGGCTCCAGTTATGGTGATCCGGCTAACTCCTTTAAAGTGGGAAGTTATACGGTCGTGGATGCGT
TAGTACGTTATGATCTGGCGCGAGTCGGCATGGCTGGCTCCAACGTGGCGCTGCATGTTAACAACCTGTTCGATCGTGAA
TACGTCGCCAGCTGCTTTAACACTTATGGCTGCTTCTGGGGCGCAGAACGTCAGGTCGTTGCAACCGCAACCTTCCGTTT
CTAA
>Cluster_58216
ATGAAATTTGATAAACCCGCAGGGGAAAACCCGATCGATCAGCTGAAGGTTGTCGGTCGTCCCCATGACCGCATCGACGG
ACCGCTGAAAACTACCGGCACGGCACGCTACGCCTACGAATGGCATGAAGAAGCCCCCAACGCCGCCTATGGCTATATCG
TCGGTTCCGCCATTGCCAAAGGACGCCTCACCGCCCTTGATACGGACGCCGCGCAAAAAGCGCCGGGCGTACTGGCTGTC
ATTACCGCCAGTAACGCCGGGGCACTCGGCAAAGGCGACAAAAACACCGCCAGGCTGTTAGGCGGCCCCACTATTGAGCA
CTATCATCAGGCCATTGCGCTGGTAGTGGCCGAGACCTTCGAACAGGCGCGAGCGGCGGCCTCGCTGGTGCAGGCACACT
ATCGCCGTAATAAAGGAGCTTACTCCCTGGCGGACGAAAAACAGGCCGTCAATCAGCCGCCGGAAGACACGCCCGACAAA
AACGTCGGTGACTTTGACGGGGCTTTCACCTCCGCTGCGGTGAAGATTGATGCTACCTACACGACCCCGGACCAGAGCCA
TATGGCGATGGAGCCGCATGCCTCGATGGCCGTCTGGGATGGAAATAAGCTTACCCTCTGGACCTCAAATCAGATGATTG

ACTGGTGCCGCACCGATCTGGCAAAAACGCTGAAAGTTCCCGTGGAGAATGTGCGTATTATCTCCCCGTATATCGGCGGA
GGGTTTGGCGGCAAGCTGTTCCTGAGAAGCGATGCGCTGCTGGCGGCCCTCGCCGCCCGAGCGGTGAAACGTCCGGTTAA
AGTGATGCTCCCCCGCCCCTCTATTCCCAATAACACCACGCACCGCCCCGCCACCCTTCAGCACTTGCGTATCGGTGCCG
ACCAGAGCGGGAAAATCACCGCTATCTCACATGAAAGCTGGTCTGGAAACCTGCCCGGCGGCACGCCGGAAACGGCGGTA
CAGCAAAGCGAATTACTCTACGCCGGGGCGAATCGTCATACCGGCCTGCGGCTCGCCACGCTTGATTTGCCGGAAGGGAA
CGCCATGCGTGCGCCCGGCGAAGCCCCCGGTCTGATGGCGCTCGAAATCGCGATCGACGAACTGGCGGAAAAAGCGGGCA
TCGATCCCGTCGAGTTTCGCATCCTGAATGACACTCAGGTTGACCCCGCCGACCCGACGCGCTGCTTCTCTCGCCGTCAG
CTTATCGAGTGCTTGCGCACCGGAGCGGATAAATTTGGCTGGAAGCAGCGCAACGCCACACCCGGACAGGTGCGCGACGG
GGAGTGGCTAGTCGGCCACGGTGTTGCGGCGGGCTTTCGCAATAATCTGCTGGAAAAATCGGGTGCTCGGGTTCACCTCG
AACAAAACGGCACCGTTACCGTAGAAACGGACATGACCGACATTGGCACCGGCAGCTACACCATTCTGGCCCAGACGGCA
GCGGAAATGCTTGGCGTACCGCTGGAGCAGGTTGCGGTTCACCTCGGCGATTCCAGTTTCCCGGTTTCTGCGGGTTCTGG
TGGACAATGGGGCGCGAATACCTCCACCTCCGGCGTTTACGCCGCCTGTATGAAGCTTCGCGAAATGATTGCCTCGGCAG
TCGGGTTTGATCCTGAGCAGTCGCAGTTTGCCGACGGCAAGATTACCAACGGTACCCGAAGCGCCACGCTACATGAAGCC
ACCGCAGGCGGCAGACTGACAGCGGAAGAGAGCATTGAATTCGGAACACTGAGCAAAGAGTACCAGCAGTCGACCTTTGC
CGGGCATTTTGTGGAGGTCGGCGTGCATAGCGCGACGGGAGAAGTTCGGGTCCGGCGTATGCTCGCTGTGTGTGCTGCAG
GACGCATCCTGAATCCGAAAACTGCGCGCAGCCAGGTCATTGGCGCAATGACTATGGGCATGGGCGCGGCACTGATGGAG
GAGCTGGCGGTGGATGACCGTTTGGGCTACTTCGTTAATCACGATATGGCGGGGTATGAGGTGCCGGTTCATGCGGATAT
CCCAAAACAGGAGGTGATTTTCCTGGATGATACCGACCCCATATCCTCCCCGATGAAGGCCAAAGGTGTCGGTGAGCTGG
GCCTGTGCGGCGTGAGCGCGGCTATCGCCAACGCGGTGTATAACGCCACCGGTATTCGGGTACGGGATTATCCCATCACT
CTGGATAAGCTGCTCGATAAGCTGCCGGATGTGGTTTAA
>Cluster_24154
ATGCCCGTTGCCCACGTTGCCTTGCCCGTTCCGCTTCCTCGTACCTTTGACTATCTGCTGCCAGAAGGCATGACGGTTAA
AGCCGGGAGTCGCGTACGCGTGCCGTTTGGCAAACAACAGGAGCGCATCGGGGTTGTGGTGTCAGTGAGCGATGTCAGTG
AGCTGCCGCTTAACGAGCTGAAAGCGGTAGTTGAAGTGCTGGACGTTGAGCCGGTATTTACTCACTCCGTCTGGCGATTA
CTGCTCTGGGCGGCAGATTACTATCATCATCCGATTGGCGATGTGCTGTTTCATGCCTTGCCGATTTTACTGCGCCAGGG
GCGGCCTGCGGCGAACGCGCCGATGTGGTACTGGTTTGCCACTGAACAAGGCCAGGCGGTGGATCTTAACAGCCTGAAAC
GCTCACCCAAGCAACAACAGGCACTGGCAGCGTTACGGCAAGGCAAAATCTGGCGTGATCAGGTTGCCACGCTCGAATTT
AATGATGCCGCGTTGCAGGCGCTGCGCAAAAAAGGTCTGTGTGATTTAGCAAGTGAAACACCAGAGTTTAGCGACTGGCG
AACGAACTATGCCGTTTCTGGTGAGCGGTTGCGGTTGAATACCGAACAGGCCACCGCCGTTGGCGCAATTCATAGCGCGG
CAGATACTTTTTCTGCCTGGCTGCTGGCGGGCGTTACCGGTTCCGGTAAAACGGAGGTTTATCTCAGTGTACTGGAAAAC
GTGCTCGCTCAGGGCAAACAGGCGCTGGTGATGGTGCCGGAAATCGGCCTGACACCGCAAACTATCGCCCGTTTTCGTGA
ACGTTTTAATGCCCCGGTGGAAGTTCTGCATTCCGGCCTGAACGACAGCGAGCGTCTTTCGGCGTGGTTGAAAGCGAAAA
ATGGCGAGGCGGCGATTGTCATCGGCACCCGCTCCGCGCTGTTTACGCCGTTTAAAAATCTCGGCGTGATTGTTATTGAT
GAAGAGCACGACAGTTCCTACAAACAGCAGGAAGGCTGGCGCTATCATGCCCGCGACCTGGCGGTGTATCGCGCGCATAG
CGAGCAAATCCCAATTATTCTTGGCTCCGCAACGCCCGCACTGGAAACGTTATGCAACGTGCAGCAGAAAAAATACCGCC
TGCTGCGCCTGACCCGTCGGGCGGGGAATGCGCGCCCGGCAATTCAACATGTGCTGGATTTAAAAGGTCAGAAGGTGCAG
GCGGGGCTGGCTCCGGCGTTAATCACCCGTATGCGCCAGCATTTACAGGCCAACAATCAGGTTATCCTCTTTCTTAACCG
CCGGGGTTTTGCGCCTGCGCTACTGTGCCACGACTGTGGTTGGATAGCCGAATGCCCACGTTGCGATCACTACTACACGC
TGCATCAGGCGCAACAGCATTTGCGCTGCCACCATTGCGACAGTCAGCGTCCGGTGCCGCGCCAGTGCCCTTCTTGCGGT
TCCACACACCTGGTACCCGTGGGGCTGGGCACCGAACAGCTTGAACAGACGCTCGCGCCGTTGTTCCCTGGTGTGCCCAT
TTCTCGTATCGACCGCGATACCACCAGTCGCAAAGGCGCGCTGGAACAACAACTGGCGGAAGTACATCGCGGTGGCGCAC
GGATATTGATCGGTACACAAATGCTGGCGAAAGGTCACCACTTTCCGGATGTGACGCTGGTGGCGTTACTGGATGTGGAC
GGCGCGCTGTTTTCTGCCGATTTCCGCTCGGCAGAGCGTTTTGCCCAGCTTTATACCCAGGTGGCAGGCCGTGCCGGGCG
TGCGGGTAAACAGGGCGAAGTAGTTCTGCAAACGCACCATCCTGAACATCCGCTGTTGCAAACGTTGCTTTATAAAAGCT
ACGACGCCTTTGCCGAACAGGCACTGGCTGAACGACGCATGATGCAGCTGCCGCCGTGGACCAGCCATGTGATTGTGCGT
GCGGAAGATCATAACAATCAGCACGCGCCATTGTTCCTGCAACAACTGCGTAATCTCATTCTCGCCAGCCCACTGGCAGA
CGAGAAACTGTGGGTTCTCGGTCCGGTTCCGGCGCTGGCCCCTAAACGTGGCGGTCGCTGGCGCTGGCAGATATTGTTGC
AGCACCCTTCCCGCGTGCGCTTACAGCACATCATCAATGGTACGCTGGCGCTTATTAATACAATACCGGATTCCCGCAAG
GTGAAATGGGTGCTGGATGTCGATCCGATTGAGGGTTAA
>Cluster_53566
ATGTCATCAACTAATTTCAGCCAGATTGCAGCCTTCCTGTGGTCTGTCGCCGACCTGTTACGCGGCGATTTTAAACAATC
ACAATATGGGCGAATTATTCTCCCTTTCACTCTTCTTCGTCGTCTGGAATGCGTTCTCGAAACGACGAAAGACGCGGTCA
TTGCCGAGGCTCAAAAAGTAAAAGCAATGAAACTGCCGGAGGAAGCCCAGGAGAAAATGATCCTGCGAGCGACCAATGGC
CTCACGTTCTTCAATGCCTCAGCGATGGATCTCAGCAAAATGGGGCAAAATGGCATACAGGACAACCTGGAAAACTACAT
CCAGTCGTTCTCTTCGGATGCCCGCGAAATTTTCGAACACTTCAAGTTCAGCGAGTTTGTCGGACAGCTGGCGGATGCGA
ACTTACTGTTCAAAGTGGTGCAAATATTCGCTAAAGCAGATTTATACCCGGAGCACGTCACCAACCACGATATGGGGCTG
GTATTTGAGGAGCTGATCCGACGTTTTGCCGAGAGTTCTAACGAAACCGCAGGGGAGCACTTTACCCCGCGTGATATCGT
CAATCTGACCACCTCGCTGGTGTTTTTCGATGATGATGACGCGCTCAATAAAGACGGCATCATCCGCACCATTTACGACC
CAACAGCGGGAACCGGCGGCTTTCTCTCCTCCGGCATGGAGTTCGTCCACCAACAAAACCCCAATGCCGTGATGCGCGCG
TTTGGTCAGGAGTTAAATCCGGAATCCTACGCCATCTGCAAAGCCGACATGCTGATTAAGGGCCAGGATGTCAGCCTTAT
CAAACTCGGCAACACCCTGTCCAACGATCAGCTCCCCGCAGAAAAATTCGATTACATGCTTTCTAATCCACCTTTCGGCG
TGGACTGGAAAAAGATAGAAACCGACATCAACGACGAGCACAAACTCAAAGGTGCTGATGGTCGCTTTGGCCCCGGCCTG
CCCCGAGTCTCCGACGGTTCGTTGCTGTTTCTGCTGCATCTGATCAGCAAAATGCGCGATGCCAAAAGCGGTGGCGGACG
GATCGGCATCATTCTTAATGGTTCGCCGTTATTCACCGGCGGCGCAGGCAGCGGTGAAAGCGAGATCCGCCGCTACATTC
TGGAAGCGGATTTGCTGGAAGGCATTATCGCCCTGCCCACCGATATGTTTTACAACACCGGTATCGCTACCTATGTTTGG
GTGCTGTCTAACAAAAAAGCGCCAGAACGCAAAGGTAAAGTCCAGCTAATCGACGGCAGCAACCTGTGCGGCAAGATGCG
TAAATCGCTGGGATCAAAACGTAATATTCTCGGGGAGGAGGATATTGGTTTAATCACCCGCACGTTTGGCGATTTCGAAC
CCGTTGCCACCACTACGCTTGCGGCGTTAGGCCTGGAGAAAGCACCCGAACAAAAATCCAGCCGGGGACGCCAGCCCGCA
ACAACCAAAACCGAGGCGGCAAAAACCTTTGCCAGCAAAGTCTTTCACAGCACCGAGTTTGGCTATCGTCGCATCACCGT
TGAGCGCCCGTTACGCCTGTCGGCTCAGATAAGTGATGACGCCATTGCCACATTGCGCTTTGCGCCGAAGCCGTTTAACG
CGCCGATGGAGCAACTGTATGACGCCTTTGCCTTCCACTGGCAGGACGGTAATTACGGCGACTTAACAGCGGTAGAAAGT
GAAGCCCGCGCCATTCTTAAAGCCGATTTTAGCGAGCTGAAAGAGAAGCAAATTAAAGATCTGCTCGACAGCAAATTATG
GCTGGCCCAGCGCGGGTTAATGGAAAAAGCGCGGCGTATTCAGGCGGCGATGGGTATGCAGGCGGGCGGCAAAGACACCG
TTAGCAATGACTTTAATCAGTTCCAGTTGACGCTGAAAGAAGCGCTCAGAACCGCAGGCGTGAAGCTGGATGCGAAAGAG
AACAAGCAGTTTATCGATGCTATCACCCGCAAAAATCCGGATGCCGAGCCGGTTATCAGTAAGGTACTGAAAGAAGCGGC
CCAACCGCTGTACGGCGCGTTTGAGTACCACGGAAAGGTTGTGGAGTTTGAGTCGGATGGCGACCTGCGCGATAACGAGA
ATGTGCCGCTGAACCCGGCGGTATCTACCAGCGAGTTGATCGAGGGGTACTTCAAAGCAGAAGCATTGCCGCACGTCGCG
GACGCGTGGATTAACGCCGATAAGCGTGACGCGAAAGATGGAGATATCGGGATTGTGGGGTATGAAATTCCGTTTAATCG
TCATTTTTATATTTATCAGCCGCCAAGGCCGCTGGAGGAGATTGATGCGGATCTGGATGCGATAAGTGCCGAGATTATGA

AACTGTTGCAGGAGGTACATTCCTGA
>Cluster_6219
ATGGTTCATAAATCTGACAGTGATGAATTAGCTGCATTGAGGGCAGAAAATGCCCGTCTGGTCTCATTACTTGAAGCTCA
TGGGATTGAATGGCGACGTAAACCGCCGATCTCTGTGCAGCGCGTTTCTGTATTATCCACCAATGAGAAGGTTGCATTAT
TTCGTCGGTTGTTTCGCGGGCGTGATGATGTATGGGCACTTAGATGGGAAAGTAAAACCAGCGGCAAATCAGGGTACTCT
CCTGCCTGCGCTAACGAATGGCAGGCGAGAATATGCGGTAAGCCCCGGATAAAATGTGGGGACTGCGCTCACCGTCAGTT
GATTCCTGTATCTGATCTCGTCATCTACTACCATCTGGCAGGTACCCATACTGCCGGGATGTATCCGTTGCTGGAAGATG
ATTCCTGTTATTTTCTGGCTGTTGATTTTGATGAAGCTGAATGGCAGAAGGATGCATCCGCATTCATGCGATCCTGTGAT
GAGCTGGGTGTACCCGCTGCGCTGGAAATATCCCGTTCACGTCAGGGAGCGCATGTCTGGATATTTTTTGCTTCACGAGT
TTCGGCCCGCGAAGCTCGCCGTCTGGGGACTGCTATTATTAGTTATACGTGTAATCGAACTCGACAGCTGCGATTGGGTT
CTTATGACCGATTATTTCCTAATCAGGATACGATGCCAAAAGGGGGATTTGGTAATCTCATAGCATTACCTCTGCAAAAA
AGACCGCGTGAATCGGGGGGAAGTGTTTTTGTTGATATGAATTTCCAGCCTTATTCTGATCAATGGGCTTTTCTTGCATC
GGTGATCCCGATGAATGTGCAGAATATTGAACCGACGATATTACGGGCTACAGGAAGTATCCATCCTCTGGATGTGAACT
TTATCAACGAAGAAGACCTGGGTACGCCATGGGAAGGGAAAAAATCATCAGGAAACAGACTGAATATTTCTATTGCAGAA
CCGCTGAAAATCACGCTGGCAAACCAGATCTATTTCGAAAAAGCGCAATTACCTCAGGTGCTGATTAACCGACTTATTCG
GCTGGCAGCATTTCCGAACCCTGAGTTTTATAAGGCTCAGGCAATGCGTATGTCAGTCTGGAATAAGCCCCGTGTTATAG
GTTGTGCGGAGAATTACCCGCAACACATTGCGTTGCCCCAGGGATGTCTGGACAGCGTATTATCTTTCCTTAGGGACAAC
AATATTGCTGCAGAATTAATCGATAAACGATTTGTCGGGACGGAATGTAATGCCGTTTTTATGGGAAACCTCAGAGCGGA
GCAGGAAGAGGCCGTTTCGGCATTACTCCGTTATGACACTGGTGTGCTTTGTGCGCCAACGGCTTTTGGTAAGACAGTTA
CCGCAGCGGCAGTGATTGCCAGACGGAAAGTGAATACACTGATACTGGTACACCGGACTGAATTGCTGAAGCAGTGGCAG
GAGCGTCTCGAGGTGTTTCTGCAGGTTGGTGACAATATTGGTATTATCGGGGGAGGTAAACATAAACCCTGTGGCAATAT
TGATATTGCGGTGGTGCAGTCCATATCCAGACACGGAGAAGTTGAACCTCTGGTCAGGAATTATGGGCAAATCATTGTGG
ATGAGTGCCATCATATTGGCGCGGTTTCATTTTCTGCGATTCTGAAGGAAACGAATGCCAGATATCTGCTTGGCCTGACG
GCAACACCAATCCGACGGGATGGTCTGCATCCCATTATTTTTATGTACTGTGGTGCCATTCGCCATACAGCGGTCCGCCC
GAAGGAAAGCCCACATAATCTGGAGGTACTGACCCGTTCCCGTTTTACATCTGGTCATTTACCATCGGATGCGAGAATCC
AGGATATTTTCAGAGAAATTGCTCTGGATCATGACAGAACGGTGGCGATAGCTGAAGAAGCCATGAAAGCTTTCGGGCAG
GGGCGAAAAGTTCTGGTACTGACTGAACGTACAGATCATCTGGATGAGATAGCATCAGTGATGAATTCACTGAAATTGTC
CCCCTTTATTCTCCATGGACGACTATCGAAAAAAAGCGTGCGATGCTGATATCCGGGCTGAATGCTCTTCCTCCTGATT
CTCCCCGTATTTTGTTGTCAACAGGCAGACTTATTGGTGAGGGATTTGACCACCCTCCGCTGGATACGCTGATTCTTGCC
ATGCCTGTGTCATGGAAAGGGACATTACAGCAGTATGCGGGACGTCTACACAGAGAGCATACCGGTAAAAGCGATGTCAG
GATCATTGATTTTGTGGATACCGCGTATCCAGTGCTGCTCAGAATGTGGGATAAACGCCAGCGGGGTTATAAGGCGATGG
GGTACAGGATTGTGGCTGATGGTGAAGGGCTTTCATTTTAA
>Cluster_17789
ATGGAAGGGAATTTTAGATTAAAAACCAGCGTTAGCTGCATCCTGTTATCAATGTGTGCTGCTTTTCCTGTGCAGGCCGA
TAACTCTGGCATATGGCAAAGCGAGAACCAAACCACAGGTAAGCTTAACGGTACACCGCCGACGGCAGAAAGTGTGTCTG
TTCCAGTATATCAGGGAAGTACGAAACTTGATCCTGCAACTACGACTGCCATTGTTGCAACGGCATTGCCGAAAGCATTC
AGTGCTGACACGACACCGGAAAAACTGTATCTGACGAATCCATCAGATGCCGAAGGGGATCGTTTTGATGATATACCACA
GCTCTCATGGCTAAATGATGTTGCCCCAGATGTCAGTCTGGTCTGGGCTAAAGCGGATAAACCCAATGAGCCCCTCAATC
CACAACCGAATATAGCCCTCTCTTTTTGTGAGCAAAATCTTTCCGGGCAGTTAGTAGTGTGGCCAAAAGTCGATAGCAAT
AGTGCGGCAACGCTGTTACTGCATACCACATCAGGTGTACCGGATAATGGGCCTGTGTCGTTAGCGAATACCAAATTCCC
GTTGAATGTGGCGGCTGCGGTGGCAACGCCGATTGCCGCCAGCATAAGTGCAGCGAAAGCGAAAGTTGGGGAGACCATTA
CGTTGACTCTGACGGTGAAAGATTGTGATGGCAATCTGAAAGCGGGCACGCCTTTTACTGTTAGGCGCGGCGTACCCACT
AACCGTCAGAATGTTGCAAATGATACTGGTGCCCCCGTTTCGCTGGGCGGTACAGTTATGACCGACACCACCACGGAATA
CAAAGGGGTAACGGGGGAAGGTGGCATCGCGACCATCGCGGTAACGCAAAATGAGGGGATAGGGGTAAAAACACCGCTGT
ACATTACTGCTACCGGCATGAGTACTCCCTTGACGCAAGATGTCACTTTTACCGTGATCACCAGCCCGGACAGCACGCTC
GCCAATATGTGGGGCCATATGAGTGAAACGTTTAAGGCCAGTAACGGTATTGTGTTTAAGCGCCCCAGACTTGCTACTGA
GGTACAAAACCCCTTTAAGAGTGTCTTAGAATCAAATGAATCCTGGGTTCTGGTCAGGAATAATCACCTTGAAACAGCGG
TTGAGAAAAGGGGGGGATGTGGCCCTGGAGGATCACTCAATGTTCCAACGCAACAGAACCTGAACTCGTTGATCGATGCT
TATCCTGATGGGGTGAAGGCCAAAACAGGTTTGCCTGTAAGCGCTAACTACCATTCAAAAACGTCAGACTCAGCCGTTGT
TAGTAGTCGTAATTTCCTGATGGTTAATCTGAGTAATAGAAGCACGACTTCTGATCAGTTTGTCCCTGATCCAGTAACAG
TAAACGGCTTTTTGGTAATGTGCCAGCAGACTACGAATCCGAAAATTACGAAATATAAGCTTATAAATCCGTCGGATATG
GTTTCAGTTGTTGGTTTCAACCATGGAAGTAACAGTAGAGGTACTTTTAACGCATTCAAAGGGAAAACAGTTGGTAAACA
GTCAGTGAAGGTCCGTCTTCAGGCTGTGGATGGTAGTGGGAAAGGTGTTCCATATGCACCGGGAACAATGACATATCGTT
ATCCAATAAGCCGTCAGGGGAATAAGAGTGGGGTTGCCGATATGACTATAGTCAATAGTACGCAAAAGACTCATAGTATT
GATGAGATGAGGTATTATTATGCGACAGCGGATAAGTCTGGTTTCTTTGAATTTACATTGGCACAAAATACAAACGGTTT
GGGAAGCCTCCACGATATATATATCCAGAATGACAAGTCGGATCTGAGTGAGCGAACAGCTCAAGGATCAATGCCAGTCA
TTTTTGAGACAATTACCAGCCCGGATACGCCTGACGCTGAGTTTTGGGGGTATATGGAAGATACGTTAACGCTGAATGGT
CGGACATTTAATCGCCCGAAACTTTTTAGTGAATTACCAAATGCGGGGGATTCATATAAGTTTGCAAGCGATCGGTTAGG
TATGCAGGTCGCAGAAAACTGGGCAATGGTAACGTCATCGCAAGCAGCTATAGGTAGTGGTGGTTGTGCCGCAGATAAAT
ACCCGACAGTTGCCGATTTAAGTGCGCTTCGCGCTGAAGTTGATTTTCTGCATGTATATTATCTGAAAGGTGGATGGCCT
GCGGGGAACGGCAATAAAGGGTACTGGACTAATAATCCGACTTCCATTACTCAATGGATGAATATGCTGACGGGAGGATT
AGAGTACGGCGCCCAGTCATCTCTCCAGATTTGCGCCCAGTAA
>Cluster_28660
ATGAAAATTAGCGACGGAAACTGGTTGATTCAACCTGGCCTCAATTTGATTCACCCGCTTCAGGTGTTCGAGGTTGAAGA
GCAGGGTAATGAAATGGTGGTCTATGCTGCCCCCCGTGATGTGCGTGAACGTACCTGGCAGCTTGATACACCTTTATTTA
CGCTGCGCTTTTTCTCCCCACAGGAAGGTATTGTTGGTGTACGGATTGAGCATTTTCAGGGGGCGCTGAATAACGGTCCT
CATTATCCGCTCAATATTTTGCAGGATGTGAAGGTCACAATCGAAAACACAGAACGTTATGCTGAGTTTAAAAGTGGCAA
CTTAAGCGCGCGTGTCAGCAAAGGTGAGTTCTGGTCACTGGATTTTCTGCACAACGGCGAACGTATTACCGGTAGTCAGG
TGAAAAATAATGGCTACGTGCAGGACACGAATAGTCAACGAAATTATATGTTTGAGCGGCTGGATCTTGGCGTTGGCGAA
ACAGTTTACGGTCTGGGAGAGCGCTTTACTGCCCTGGTGCGCAATGGCCAGACGGTAGAGACCTGGAACCGGGACGGCGG
CACAAGTACTGAACAGGCGTATAAAAATATTCCGTTCTATATGACTAACCGTGGTTATGGGGTACTGGTCAATCATCCTC
AATGCGTCTCTTTTGAAGTGGGATCGGAGAAAGTCTCCAAAGTGCAGTTCAGCGTTGAGAGTGAATATCTCGAATACTTT
GTTATCGACGGCCCGACGCCGAAAGCGGTACTTGATCGTTATACCCGTTTTACTGGTCGTCCGGCGCTGCCGCCCGCGTG
GTCCTTCGGCCTGTGGCTAACCACTTCATTTACCACCAACTACGACGAAGCGACGGTAAACAGCTTTATCGATGGTATGG
CGGAACGCAATCTGCCACTGCATGTTTTCCACTTTGACTGTTTCTGGATGAAAGCCTTCCAGTGGTGCGATTTTGAGTGG
GACCCGCTGACTTTCCCGGACCCGGAAGGGATGATCCGCCGCCTGAAAGCGAAAGGGCTAAAAATCTGCGTCTGGATTAA
CCCCTATATCGGGCAAAAATCCCCTGTCTTTAAAGAGTTACAAGAGAAAGGCTATTTACTCAAACGCCCGGACGGTTCGT
TATGGCAGTGGGATAAATGGCAGCCAGGTCTGGCGATTTATGACTTTACCAATCCGGATGCCTGCAAATGGTACGCCGAC
AAACTGAAAGGTCTGGTCGCGATGGGCGTTGATTGCTTTAAGACCGACTTTGGCGAACGTATTCCAACCGATGTTCAATG

GTTTGATGGTTCCGAGCCGCAGAAAATGCATAACCATTATGCGTACATCTACAACGAACTGGTGTGGAACGTGCTCAAGG
ACACCGTTGGTGAGGAAGAAGCCGTCTTGTTTGCCCGCTCGGCCTCCGTCGGTGCGCAGAAATTCCCGGTACACTGGGGT
GGTGACTGTTACGCTAACTACGAATCAATGGCGGAAAGCCTGCGCGGTGGTTTGTCTATTGGCCTTTCAGGTTTTGGTTT
CTGGAGCCACGATATCGGCGGCTTTGAAAATACCGCTCCGGCGCACGTTTACAAACGCTGGTGCGCGTTTGGTTTGCTTT
CCAGCCATAGCCGTTTACATGGCAGCAAATCTTATCGTGTGCCGTGGGCCTACGATGATGAGTCCTGTGATGTGGTGCGC
TTCTTCACGCAACTGAAATGCCGCATGATGCCGTATCTGTATCGTGAAGCTGCACGTGCAAACGCGCGGGGTACGCCGAT
GATGCGGGCCATGATGATGGAGTTCCCGGACGATCCGGCTTGTGATTACCTTGACCGTCAATACATGTTAGGCGACAACG
TGATGGTTGCGCCTGTGTTCACTGAAGCGGGCGATGTGCAGTTCTACCTGCCGGAAGGTCGCTGGACACACCTGTGGCAC
AACGATGAACTCGACGGTAGTCGCTGGCATAAACAGCAGCACAGCTTCCTGAGTCTGCCCGTTTATGTGCGTGATAGCAC
CCTACTGGCGCTGGGCAACAACGATCAACGTCCCGATTACGCGTGGCACGAAGGTACGGCATTCCACCTCTTTAATCTGC
AAGACGGGCATGAAGCCGTCTGTGAAGTGCCCGATGCTGACGGTTCGGTGATCTTTACTTTAAAAGCAGCACGTACTGGC
AACACGATTACTGTGACTGGTACGGGCGAGGCGAAGAACTGGACACTGTGCTTGCGCAATATTGTGAAAGTAAATGGTCT
GCAAGGCGGTTCGCAGGCTGAAAGTGAGCAGGGACTGGTGGTGACTCCTCAAGGGAATGCGCTGACAATTACGTTGTAA
>Cluster_28456
ATGTCGACTCCTGACAATCACGGCAAGAAAGCCCCTCAATTTGCTGCGTTCAAACCGCTAACTACGGTACAGAACACCAA
CGACTGTTGCTGCGACGGCGCATGTTCCAGCACGCCAACTCTCTCTGAAAACGTCTCCGGCACCCGCTATAGCTGGAAAG
TCAGCGGTATGGACTGCGCCGCCTGTGCGCGCAAAGTGGAAAATGCCGTGCGCCAGCTTGCAGGCGTGAATCAGGTACAG
GTGTTGTTCGCCACCGAAAAACTGGTGGTGGATGCCGACAATGACATACGGGCACAAGTTGAATCTGCGGTGCAAAAAGC
GGGCTATTCCCTGCGCGATGAACAGGCCTCCGACGAACCACAAGAATCACGCCTGAAAGAGAATCTGCCGCTGATTACGC
TTATCGTGATGATGGCAATCAGTTGGGGGCTGGAGCAGTTCAATCATCCGTTTGGTCAACTGGCGTTTATCGCGACCACG
CTGGTTGGGCTGTACCCAATTGCTCGTCAGGCATTACGGCTGATCAAATCTGGCAGCTACTTCGCCATTGAAACCTTAAT
GAGCGTAGCCGCTATTGGTGCGCTGTTTATTGGCGCAACGGCTGAAGCTGCGATGGTGTTGCTGCTGTTTTTGATTGGCG
AACGACTGGAAGGCTGGGCCGCCAGCCGCGCGCGTCAAGGGGTCAGCGCGTTAATGGCGCTGAAGCCAGAAACCGCCACG
CGTCTGCGTAACGGTGAGCGTGAAGAGGTGGCAATAAACAGCCTGCGGCCTGGCGATGTGATTGAAGTTGCGGCAGGTGG
GCGTTTGCCTGCCGACGGTAAACTGCTCTCACCGTTTGCCAGTTTTGATGAAAGCGCCCTGACCGGAGAATCCATTCCTG
TGGAGCGCGCGACGGGCGATAAAGTTCCTGCAGGCGCCACCAGCGTAGACCGTCTGGTAACGCTGGAAGTGCTGTCAGAA
CCGGGTGCCAGCGCCATTGACCGGATTCTGAAACTGATTGAAGAAGCCGAAGAGCGCCGCGCACCTATTGAGCGGTTTAT
CGACCGTTTCAGCCGTATTTACACGCCAGCGATTATGGCCGTCGCCCTGCTGGTAACGCTGGTGCCGCCGCTGCTGTTTG
CCGCCAGCTGGCAGGAGTGGATTTATAAAGGGCTGACGCTGCTGCTGATTGGTTGCCCGTGTGCTTTAGTTATCTCCACG
CCCGCGGCGATTACCTCCGGGCTGGCGGCAGCAGCGCGTCGTGGGGCGTTGATTAAAGGCGGCGCGGCGCTGGAGCAGCT
GGGTCGTGTTACCCAGGTGGCGTTTGATAAAACCGGTACGCTGACCGTCGGTAAACCGCGCGTTACCGCGATTCATCCGG
CAACGGGTATTAGTGAATCTGAACTGCTGACACTGGCGGCGGCGGTCGAGCAAGGCGCGACGCATCCACTGGCACAGGCC
ATTGTGCGCGAAGCACAGGTTGCTGAACTCGCCATTCCCACCGCCCAATCACAGCGGGCGCTGGTCGGGTCTGGCATTGA
AGCGCAGGTTAACGGTGAGCGCGTGTTGATATGCGCTGCCGGAAAACATCCTGCTGATGCATTTGCTGGTTTGATTAATG
AACTGGAAAGCGCCGGGCAAACGGTAGTGCTGGTAGTACGTAATGATGACGTGCTGGGTGTCATTGCGTTGCAGGATACC
CTGCGCGCCGATGCTGCAACTGCTATCAGTGAACTGAACGCGCTGGGTGTCAAAGGGGTGATCCTCACCGGCGATAATCC
ACGCGCAGCAGCGGCAATCGCCGGGGAACTGGGGCTGGAATTTAAAGCGGGCCTCTTGCCGGAAGATAAAGTTAAAGCGG
TGACCGAGCTGAATCAACATGCGCCGCTGGCGATGGTCGGCGACGGTATTAACGACGCGCCAGCGATGAAAGCTGCCGCC
ATCGGGATTGCAATGGGCAGTGGCACGGACGTGGCCCTGGAAACCGCAGATGCAGCATTAACCCATAACCACCTGCGCGG
TCTGGTGCAAATGATTGAACTGGCACGCGCCACTCACGCCAATATCCGCCAGAACATCACTATTGCGCTGGGGCTGAAAG
GCGTTTTCCTCGTCACCACGCTGTTAGGGATGACCGGCCTGTGGCTGGCAGTGCTGGCAGATACCGGTGCGACGGTGCTG
GTAACGGCGAATGCGTTACGGTTATTGCGCAGGAAATAA
>Cluster_8246
ATGCGAATAACCACTCTGGCTTCCGTAGTCATTCCCTGTCTCGGATTTTCAGCCAGCAGCATAGCTGCTGCAGAGGATGT
GATGATTGTCTCGGCATCCGGCTATGAGAAAAAGCTGACTAACGCAGCCGCCAGTGTTTCTGTGATTAGCCAGGAGGAAT
TGCAGTCCAGCCAGTACCACGATCTGGCGGAGGCTCTGAGATCAGTAGAGGGTGTGGATGTTGAAAGTGGTACGGGTAAA
ACCGGAGGGCTGGAAATCAGCATCCGAGGAATGCCAGCCAGTTACACGCTGATACTGATTGATGGTGTTCGTCAGGGCGG
AAGCAGTGACGTGACTCCCAACGGTTTTTCTGCCATGAATACCGGGTTCATGCCCCCTCTGGCCGCCATTGAGCGTATTG
AGGTTATCAGAGGGCCGATGTCCACACTGTATGGCTCTGATGCGATGGGCGGTGTGGTGAATATCATTACCAGAAAGAAT
GCAGACAAATGGCTCTCTTCCGTCAATGCAGGGCTGAATCTGCAGGAAAGCAACAAATGGGGTAACAGCAGCCAGTTTAA
TTTCTGGAGCAGTGGTCCCCTTGTGGATGATTCTGTCAGCCTGCAGGTACGCGGTAGCACACAACAGCGTCAGGGTTCAT
CGGTCACATCACTGAGCGATACAGCAGCCACGCGTATTCCTTATCCCACGGAGTCACAGAATTATAATCTTGGTGCACGT
CTTGACTGGAAGGCGTCGGAGCAGGATGTGCTCTGGTTTGATATGGATACCACCCGGCAGCGTTATGATAACCGGGATGG
GCAACTGGGGAGTCTGACGGGGGATATGACCGGACCCTGCGCTATGAGCGAAACAAAATTTCAGCTGGCTATGATCATA
CTTTCACCTTCGGAACATGGAAATCGTATCTGAACTGGAACGAGACAGAAAATAAAGGTCGTGAGCTTGTACGCAGTGTA
CTGAAGCGCGACAAATGGGGGCTTGCCGGTCAGCCGCGGGAGCTTAAGGAATCGAACCTTATCCTGAATTCATTACTGCT
TACCCCTCTGGGAGAATCTCATCTGGTTACGGTGGGGGGCGAGTTTCAGAGCTCGTCCATGAAAGACGGAGTTGTCCTTG
CCAGCACAGGTGAAACTTTCCGGCAGAAAAGCTGGTCGGTATTTGCTGAGGATGAGTGGCATCTCACGGATGCACTTGCG
CTGACTGCGGGCAGCCGCTATGAACATCATGAGCAATTCGGGGGACACTTCAGTCCGCGTGCATATCTGGTCTGGGATGT
GGCAGATGCCTGGACGCTGAAAGGCGGTGTGACCACGGGATATAAGGCACCCAGAATGGGGCAGCTACATAAAGGGATTA
GTGGTGTGTCCGGGCAGGGAAAAACAAATCTACTTGGTAACCCCGACCTGAAGCCGGAAGAGAGCGTCAGTTATGAGGCT
GGGGTGTATTACGATAACCCCGCCGGTCTGAATGCCAATGTCACAGGTTTTATGACTGACTTCTCCAACAAGATTGTCTC
TTATTCCATAAATGATAACACCAATAGCTATGTAAACAGCGGAAAGGCCCGGTTGCACGGTGTGGAATTTGCCGGCACAT
TGCCGCTGTGGTCAGAGGATGTCACGCTGTCACTGAATTACACCTGGACCCGAAGTGAACAACGTGATGGTGATAACAAA
GGTGCGCCGCTGAGTTATACCCCTGAACACATGGTGAATGCGAAACTGAACTGGCAGATCACCGAAGAGGTGGCATCATG
GCTGGGTGCCCGTTATCGCGGGAAAACACCACGTTTCACCCAGAATTATTCGTCACTGAGCGCTGTACAGAAGAAAGTGT
ATGATGAGAAAGGAGAATACCTGAAAGCCTGGACGGTGGTGGATGCAGGTCTGTCGTGGAAGATGACGGATGCCCTGACG
CTGAATGCTGCGGTGAATAACCTGCTCAACAAGGATTACAGTGACGTGAGCCTGTACAGTGCCGGTAAGAGTACGCTGTA
TGCCGGTGATTACTTCCAGACGGGATCATCAACAACAGGATATGTGATACCTGAGCGAAATTACTGGATGTCGCTGAACT
ATCAGTTCTGA
>Cluster_45954
ATGAGTACCACAACACGAAAATTTAAAACCGTTATCACCGATACAGGTGCCAAAAAATTAGCTCAGGCAGCCGCGCCAGA
TGGTAAGCCTGTCCGCCTGACTCATATGGCCGTGGGCGACGGTGGCGGCGCGTTGCCCACACCAGACAGTAAGCAGACCC
GTCTGGTGCATGAGGTGTGGCGACACACTGTTAATCGCGTCATCCTGGACGCAACACATCAGAACCGCATTATTGCGGAG
CTGGTTATTCCTCCAGAAACGGGCGGATTCTGGATCCGGGAAATTGGTGTTTTTGATGAGCACGGCGATTTAATCGCGGT
GGGCAATACTGCCGAAAGTTACAAGCCAGCCGTTGCCGAAGGGTCCGGACGTGCACAAACATTTCGCACCATTCTGACCG
TATCCAGCACTGCCACTGTGGCGCTTACCGTGGATAACACCATGGTGATGGCCACAGTGGATTACGTGGATAACAAACTG
AAAGAGCATGAACAGTCACGATGTCACCCGGATGCCTCGCTGACCGCAAAAGGCTTTGTTCAACTCAGTAGCGCCACTAA
CAGCGATTCTGAAACGCTGGCTGCAACGCCGAAAGCGGTTAAGGTCGCGTATGATCTTGCTAACGGAAAATATACAGCGC

AGGATGCCACAACAGCGCGAAAAGGCCTTGTCCAGCTTAGTAGTGCAACCAACAGTACATCTGAAACGCTGGCGGCAACA
TCAAATGCAGTAAAAGCTGCCTATGACAATGCTGAAAAACGTCTGCAGAAAGCTAAGAATGGTGAGGATATCTCTGATAA
AGACACCTTTACGAAAAATATCGGTGCCTGCCGTGCATATAGTGCAGAGCTGAATATTGGTGGAGATAGTGAAGCATGGA
CAACTGCGCAGTTGATTTTTTGGCTAGAGAGTCAGGGGGCATTTAACCATCCTTACTGGATGTGCAAAGGCTCATGGGCT
TATGCAAATAATAAGGTCATTACAGATACAGGTTGCGGAAGTATTTGTCTTGCAGGTGCTGTTGTGGAAGTTATTGGCAC
CCGCGGCGCAATGACCATACGCATTACCACACCGAGTACATCCAGCGGTGAAGGCATCCCTAATGCTCAATTTACTTATA
TTAATCATGGTGATGCTTATGCTCCTGGCTGGCGAAGGGACTATAACTCCAGGAATAAGCCAACAGCATCAGAGATCGGG
GCGTTACCGTCAGATGGGACAGCAGTATCGTCAGTTAATCTGGCTTCAAAAGGTCGGCTGACCGCCCTGACAGATAATAT
GCAGGGGGCCACAGGTCTGGAGTTATACGAGGCGTATAACAACGGATATCCAACAACGTATGGAAATATCATTCACCTGA
AAGGGATGACAGCCGTTGGCGAAGGCGAATTACTCATCGGCTGGAGTGGTATAAGCGGTGCTCATGCTCCGGCATTTATT
CGTTCACGACGGGATACGCCCGACGCAAACTGGTCGCCGTGGGCGCAGCTTTACACCTCGGCTCATCCTCCTGAAGAGTT
TTATCCAGTCGGTGCACCGATTCCGTGGCCATCAGATACCGTTCCGTCTGGTTATGCCCTGATGCAGGGGCAGACTTTTG
ACAAATCTGCATACCCGAAACTTGCAGTCGCTTATCCGTCAGGCGTTATTCCTGATATGCGTGGCTGGACGATTAAGGGC
AAGCCCGGCAGTGGTCGTGCCGTATTGTCTCAGGAACAGGACGGCATTAAATCGCACACCCACAGCGCCAGCGCATCCAG
TACGGATTTGGGGACGAAAACCACATCGTCGTTTGATTACGGTACTAAAACGACCAGTTCATTTGATTACGGCACAAAAA
CTACGAATAGCGCTGGAAATCATTCACACAATATACCTGTTGGTCACACTGGCGCGGGGAATGGTGTATCAGCCGGTTAT
AACGCTGCGTTAGGTACTGGTACCACGTCGAGCGCAGGCGAGCATGCTCACAATGTATATATCGGTGCCCATAACCACAC
TATCGGCATTGGTGCTCATGCCCATTCTGTCATTATTGGTCCCCACGGACACACCATCACCGTTAACGCTACGGGTAACG
AAGAAAACACCGTAAAAAACATCGCATTTAACTATATTGTGAGGCTTGCATAA
>Cluster_20780
ATGAAACAGTTAGATTTTACATTAAGCCTTATTGATAAATTGTCCCGCCCGTTAAAACAGGCGCAGGGCAACGTAACGGG
CTTTGCTGAAAAATCAAAAGAAGCCTTTATGCAGATTGGTGGCGGCGTTCTGGCGCTGGCGGGTACAGGGATGGCCATTA
AAGGTGCGCTGATGCCCGCCATTGAAATGTATGACGCGCTGAATGATGCGGCTGCAAAAGGGATCGATGATTCTGCGCTT
AAGGCCGTTCAGCGTGATGCGCTGCGCTTCAGTACAACCTACGGTGCCAGTGCGGTGGAGTTCGTCCAGTCCACGGAAAG
TATAAATGCGTCCATTGCCGGGCTGACCGGGAATGAACTGCCGAAAGTGACGAAAGTCGCCAATACCCTGGCGTTTGCGC
TCAAATCCACCGCAGCGGAAACCGCTGAGTTTATGGGGCAGATGTTCGGTAACTTCTCCGCCGATGCCAACCGGCTGGGA
AAGGTTGAGTTTGCTGAACAGCTGGCCGGAAAAATGGTGTACATGCGCAAAACCTTTGGCACGGAAATGGCCGCTATCAA
GGATTTGATGGAAGGGGCGCGCGGTGTGGGTACCAACTACGGCGTGGGGCTGGATGAACAGCTGGCCGTGCTGGGACAGC
TTCAGCGGACACTGGGAACGGAAGCCAGCAGCGCTTATGAAGGCTTTATGACCGGAGCCATTGAGGGCGGTAAAAAGCTG
GGGCTGTCCTTCACGGATTCCACCGGCAAAATGCTGTCCATGCCTGAAATGCTGATCAAGTTACAGGGCAAGTATGGCAA
AAGCCTGGAAGGGAATCTGAAAGCCCAGGCTGAACTGGATGCGGCATTTGGTGACAGTTCGGCGGTGGTGAAACACCTTT
ATGGCAATGTGGCGCTGCTTCAGCGCAATATCACAGAACTGGGCAGTACTGATGGTCTGAAGCGTACCCAGGAAATGGCG
CAGAAAATGGTGAAGCCGTGGGATCGGTTTGTCGCAATCCTGAAGGCTATCCAGACCGTCATTGGACTGACGCTGATCCC
GGTGCTCTATCCGGTTCTGAACCGACTGGCCGATATGGGGCAGACTTTTGCCCGCTGGATGCAGCTATTTCCCAACATTG
CGCGTGTTGTTGGTTATGCGGCAATGGCCTTACTCAGCTTTGCGGCTGTGGGCGCAGTCGCCAACATCGTGATGGGTGTC
TCCAGATTCATCATGATGGGGTTGCGTGGGATCTGGGTGGCGCTGACGGCTGTCACGAAGGTTCACACCGCTGCTGTCTG
GCTGGGGAACATTGCCGTGGTTGCCTGGAACGCCACGCTGAAATTTCTGCGCGGTACGTTGCTGGCGGTTCGTATGGCGG
CAATGATGGCCGGGATAGGTATCAATCTGATGAGCTGGCCGATCCTGCTGGTGATTGGTGCCGTTGCCTTACTTGCTGCC
GGTTGCTGGCTGCTGATTAAACACTGGGACACGGTAAAGAACGCGGTCATGGATACCGCTGCATTTCAGACATGCGCCAG
GGGGGTGGCATGGCTTGCCGGGGTGTTTGCTTCAGCCTGGCAGTTTATCAGCGAAGGCTGGAACAGTTTTATTTCCATGC
TGACCGGGTTTTCTCCTTCCCAGGCATTAAGCGGGCTGGCCACGGGCATTGTGTCATTGTTTGATAATGTCTGGTTGTCC
GTTAAAAACGGCTTTCTGAAATCATGGAACTGGATTGTAAGTAAATTAAATAACATTCCGGGTGTTGATATTGAACTGGC
GGGTGCGGCTTCACCGGATATCGCTAAAAATGAAGCGGCAGTATTTCCAGTGCCGGAATTGAAGCAATCAGCGAAAGCTG
ACGGTGCTCTTGCTGCGGTGACACAAAATAATTACGCCCCAGACCGGCTTTTAACCGGAGGCGAATTAAAAGGCATTGAA
AAGGGCGGGATCACCAAAACGATTAACAGTAATGCCAAATCCGTAACCGACAATAGCCGGAAAATTGACACGGTAAACAT
TTATCCGAAAGAAATGATTACACCCGGTCAGTTAATGGAATTTCAGGAGCTGGGCGCATGA
>Cluster_42708
ATGAAAGGTCGCCTGTTAGATGCTGTCCCGCTCAGTTCCCTAACGGGCGTTGGCGCAGCGCTTAGTAACAAGCTGGCGAA
AATCAACCTGCATACCGTACAGGATTTGCTCTTACACCTTCCTCTGCGCTACGAAGATCGCACACATCTCTACCCCATCG
GTGAACTACTGCCGGGTGTTTATGCCACGGTGGAAGGCGAAGTGCTGAACTGCAATATCTCCTTCGGCAGTCGGCGGATG
ATGACCTGCCAGATCAGCGACGGTTCCGGCATTCTCACTATGCGCTTTTTCAACTTCAATGCGGCAATGAAAAATAGCCT
GGCGACAGGCCGCCGCGTGCTGGCCTATGGCGAAGCAAAGCGCGGCAAATATGGTGCGGAGATGATCCACCCGGAATACC
GCGTGCAGGGCGATCTCAGCACGCCAGAGTTACAGGAAACGCTCACACCGGTTTATCCAACTACGGAAGGCGTAAAGCAG
GCTACGCTGCGTAAATTAACCGACCAGGCGCTGGATCTGCTCGACACATGCGCCATTGAAGAACTCCTGCCGCCGGAACT
GTCACAAGGAATGATGACGCTACCGGAAGCGTTGCGCACTTTGCACCGCCCGCCGCCGACGCTACAGCTTAGCGATCTCG
AAACCGGGCAGCATCCGGCACAACGCCGGCTGATTCTGGAAGAGTTGCTGGCGCACAACCTCAGCATGTTAGCCTTACGT
GCCGGAGCACAGCGTTTTCATGCCCAGCCGCTGAGCACCAATGACGCGCTGAAAAATAAACTCCTCGCCGCCTTACCGTT
CAAGCCAACGGGCGCACAGGTACGCGTAGTGGCGGAGATCGAGCGCGATATGGCGCTGGATGTGCCAATGATGCGCCTGG
TGCAGGGCGATGTAGGTTCCGGTAAAACGCTGGTCGCCGCCCTCGCCGCGTTGCGTGCGGTTGCCCACGGCAAACAGGTG
GCACTGATGGCACCAACCGAATTACTCGCCGAGCAGCACGCCAATAACTTCCGTAACTGGTTTGCACCGCTCGGTATCGA
AGTAGGCTGGCTCGCCGGAAAACAGAAAGGTAAAGCACGACTGGCTCAGCAAGAAGCCATCGCCAGCGGTCAGGTGCAGA
TGATTGTTGGTACGCACGCCATTTTCCAGGAACAGGTGCAGTTTAACGGCCTGGCGCTGGTGATTATCGACGAACAGCAT
CGTTTTGGCGTGCATCAGCGTCTGGCGTTATGGGAGAAAGGTCAGCAGCAGGGCTTCCATCCGCATCAGTTGATCATGAC
CGCCACGCCCATCCCCCGCACGCTGGCGATGACTGCGTATGCCGATCTCGATACCTCGGTGATAGATGAGCTGCCACCAG
GCCGCACGCCAGTGACTACGGTCGCTATTCCTGATACCCGCCGTACCGATATCATTGACCGCGTGCGCCACGCCTGTATG
ACTGAAGGGCGTCAGGCGTACTGGGTTTGTACGTTGATTGAAGAGTCGGAATTACTGGAAGCGCAGGCGGCGGAAGCGAC
CTGGGAAGAGTTGAAACTGGCGCTACCAGAGTTGAATGTTGGCCTGGTACACGGACGGATGAAACCCACAGAGAAACAGG
CGGTGATGACATCGTTTAAACAAGGCGAGCTACACCTGCTGGTTGCCACTACTGTCATTGAAGTCGGTGTTGATGTACCC
AATGCCAGTCTGATGATTATCGAAAACCCGGAGCGTCTGGGTCTGGCGCAGTTACACCAGCTTCGCGGGCGTGTAGGTCG
TGGCGCGGTGGCTTCTCACTGCGTGCTGCTCTACAAAACGCCGCTCTCCAAAACCGCGCAAATCCGCCTGCAGGTACTAC
GCGACAGCAACGATGGTTTTGTGATTGCACAGAAAGATCTGGAAATTCGCGGCCCTGGCGAATTGTTAGGTACACGTCAG
ACGGGTAATGCTGAATTTAAAGTGGCGGATTTACTGCGCGATCAGGCGATGATCCCGGAAGTTCAGCGCCTGGCGCGACA
TATTCACGAACGTTACCCACAACAAGCAAAAGCCCTGATAGAACGCTGGATGCCAGAAACAGAACGCTACTCGAATGCGT
AA
>Cluster_51671
ATGCTGCGATGGAAACGCTGTATTATTCTAACATTTATCTCTGGTGCTGCTTTCGCGGCGCCAGAGATAAATGTTAAGCA
AAACGAATCGTTACCTGATTTAGGTAGCCAGGCAGCACAACAGGATGAACAAACCAACAAGGGTAAATCGCTGAAAGAGC
GCGGAGCCGATTACGTCATCAACTCCGCCACGCAAGGGTTTGAAAACTTGACCCCTGAGGCGCTGGAATCTCAGGCCAGA
AGCTATCTGCAAAGTCAAATCACCTCAACCGCACAATCTTATATTGAAGACACACTCTCTCCCTACGGTAAGGTCCGTTC

GAACCTCTCCATTGGTCAGGGCGGCGATCTGGATGGCAGTTCCATCGATTATTTTGTTCCCTGGTACGATAATCAAACCA
CTGTTTATTTCAGCCAATTTTCTGCGCAACGAAAAGAAGATCGTACGATCGGGAATATTGGCCTTGGGGTAAGGTATAAT
TTTGATAAATATCTATTGGGTGGAAATATATTTTATGATTATGACTTTACCCGTGGACATCGCCGTTTAGGTTTAGGCGC
CGAAGCCTGGACGGATTATTTAAAATTCTCAGGCAACTATTATCACCCACTTTCTGACTGGAAAGACTCTGAAGATTTCG
ACTTTTATGAAGAACGCCCTGCGCGCGGCTGGGATATTCGCGCTGAAGCCTGGCTACCCGCTTACCCGCAGCTTGGCGGA
AAAATTGTCTTCGAACAATATTATGGGAATGAAGTCGCGCTTTTTGGTACCGATAGTCTGGAGAAAGATCCCTTCGCTGT
CACGCTGGGTGTGAAATACCAGCCCGTTCCGCTCATTGTTGTCGGTACAGATTTCAAAGCTGGCACTGGCGATAATACCG
ATCTTTCTGTTAACGCAACGCTGAACTACCAGTTTGGCGTCCCGCTCAAAGACCAGCTTGATCCTGACAAAGTGAGCGCG
GCTCACTCGCTCATGGGTAGCCGTCATGACTTTGTTGAACGTAACAACTTTATTGTTCTGGAATACAAAGAGAAAGATCC
CCTCTATGTCACTTTGTGGCTGAAAGCCGACGTCACGAATGAACATCCCGAGTGCGTGATTAAAGATACGCCCGAAGAAG
CCGTCGGTCTGGAAAAATGTAAGTGGACCATTAACGCACTCATTAATCATCATTACAAAATCGTTGCTGCCTCCTGGCAG
GCGAAAAACAATGCTGCCCGCACGCTGGTGATGCCGGTTATCAAAGAGAATACTCTGACAGAGGGTAACAATAACCACTG
GAACCTGGTGCTGCCTGCCTGGCAGTACAGTTCCGATCAAGCCGAACAAGAAAAACTCAATACCTGGCGAGTACGTCTGG
CGCTGGAAGATGAAAAGGGCAACCGACAGAACTCTGGCGTGGTGGAAATCACCGTTCAGCAGGACCGTAAAATAGAGTTG
ATTGTTAATAACATCGCGAACGTGCCAGAAGAGAACAACCACAGCCACGAAGCCAGCGCTCAGGCAGATGGCGTTGATGG
TGTGGTGATGGATCTCGATGTAACCGACAGCTTTGGCGATAACACCGACCGCAATGGCAGTGCGCTGCCGGAAGATAACC
TCACGCCGCAGCTTTACGACGCGCAGGACAAACGAGTGACGTTAACCAACAAGCCTTGCTCGACCGACAACCCCTGCGTT
TTTATCGCTAAACAAGATAAAGAAAAGGGCACTGTCACCCTCTCCAGTACCTTACCCGGCACCTATCGCTGGAAAGCAAA
AGCCGCGCCCTACGATGACAGTAACTATGTGGATGTCACTTTCCTCGGGGCAGAAATTGGTGGGCTAAATGCTTTTATCT
ATCGAGTGGGGGCGGCTAAACCCAGCAACCTGATAGGTAAAGATAAAGAACCGTTGCCGCTTAACAACATTTATCGATTT
GTTTTATGGCGCGACAACAATAAAGACGGTGTCTTCCAGCAGGTCGAAAAACTGACTGACGAAGAGATGGCGCAGTACGA
CTACAAGTGGGAATTTACCGGCAAGAGCATCAATGGTGAAGTGGGTGCACAGGCGAACACTTCCAATGAGGACATTGTGA
TTCCGGCCACTAACCGTGAAGCGGCGCAAACCTATGGCGCACAAGCGGGAGATGGCTTGCAGGGATACGGTTTACGCGTG
CTGTATACCAAAAAATAA
>Cluster_20204
ATGGCGCGTTCCAAAACTGCTCAGCCAAAACACTCACTGCGTAAAATCGCAGTTGTAGTAGCCACAGCGGTTAGCGGCAT
GTCTGTTTATGCACAGGCAGCGGTTGAACCGAAAGAAGACACTATCACCGTTACCGCTGCACCTGCGCCGCAAGAAAGCG
CATGGGGGCCGGCTGCAACTATTGCGGCGAAGCACTCTGCTACTGCGACTAAAACGGATACACCAATTGAAAAAACGCCG
CAGTCCATTTCGGTTGTGACCCGGGAAGAGATGGATATCAAACAACCCGCAACGGTAAAAGAAGCTCTGGATTATACGCC
AAGTGTCTTTTCAACGCGTGGTAGTTCAACAACGTATGATGTTGTTTCTATACGTGGATTTACTACAGCCAGTACAGTAA
ATACTAACCAATATCTGGATGGCTTAAAACTTCAGGGGGATAACTACTCTGAAGCCTCTATTGATCCTTATTTCCTTGAG
CGTGTGGAATTACTCCGTGGCCCAGTATCCGTACTGTATGGTAAAAGCCATCCGGGCGGAGTAGTGAGCATGGTAAGCAA
ACGGCCTTCAACGGATCCGATTAAAGAGATCCAGTTCAAAATGGGAACGGATAATCTTTGGCAGACCGGATTCGATTTTA
GTGATGCAATAGATGATGATGGCGTTTGGTCATATCGTTTGACGGGATTAGGACGTAGTCAGGATGCGCAACAAAAGTAC
GCAAAAACTACAAGGTATGCAGTAGCTCCCTCATTCAGTTGGCGTCCTGATGATAAAACGGATTTCACTTTCCTGAGTAA
TTTCCAGAGTGATCCCGATGCGGGCTATTATGGTTGGTTACCGCGCCAGGGAACTGTTGTTCCCTATCAGGATGCAAATG
GTAGCTGGCACAAATTGCCAACAGATTTCAATGAAGGCGAGAAAGATAATAAGATGTCTCGTCGTCAACAATTAATTGGC
TACAGTTTTGCTCATGAATTTAATGATACTTTTACTGTACGTCAGAATCTGCGCTATACCCGCATTCATACACTCTACGA
TGCAGTATATGGCAATGGTTACATCGCGCCAGCGCAAATTAGCCGTGCTTATGTACGTTCAGACGAAGATTTAAATTCGT
TTACTGTTGATACGCAACTACAGTCCAACTTTGCTATCGGTGCTGTAGCGCATACTTTGCTTACTGGCGTGGATTACCTG
CGAATGCGTAACGATATTGATGCAAACTATGGTACTGCTAATCCGATTAGCATGACGGATCCACAGTACGGTAACGCCAA
TATCAATATCAATTTCCCATATGCGGTAATTAACCGTCAGGAACAGACAGGCCTTTATATTCAGGATCAGTCTGAGTGGA
ATAAGTGGGTATTGACGCTGGGTGGTCGTTACGATTTTGCGAAAACCTCTACCTATACTCGTTCAACGAGTTCTTTGGAT
GAAATTAACGATCAGCAGTTCACCTGGAGAGGTGGTATTAATTATCTGTTCGATAATGGTATTTCACCTTATTTCAGTTA
TAGCGAATCCTTCGAGCCAGTATCTGGAGCGACTAAAGAAGGTAAACCGTTTGATCCTTCTCGCGGTAAGCAGTACGAAG
CAGGTGTCAAATACGTACCAAAAGATACGCCTGTCACTGTCACTGCCTCGGTATTCCAATTAACGAAAGACAAAAATCTG
ACTGCAGATCCAAGTAATACTTTGTTTAGCGTACAAGGGGGGGAGATTCGTTCTCGTGGCTTTGAGATTGAGGCCAAAGC
AGCTGTCAACGCTAATATCAATGTGACTGCGGCATACAGCTACACCGATGCTGAATACACCAAAGATACACTGTACAAGG
GTAAACGCCCGGCAGAGGTCCCCCGAAATATGGCTTCGCTGTGGGCAGATTACACTTTCCACGAAACAGCTCTTAGTGGT
CTGAGTCTTGGTGCAGGGGCACGTTATTTTGGCGACACCTCAAGTTTCTACACTACGGGTGATAAGACCAATCAGACATT
CAATGTTGGGGGGTATACCTTAGTTGATGCAATGATAAAATATGATCTTGCTCGTTTCGGTATGCCTGGTTCTTCTGTTG
GTGTTAACGTAAATAACCTGTTCGACCGCGAATATGTCTCCAGTTGCTATCGTGATTATGCATGTTACTGGGGCGCAGAA
CGTCAGGTCGTTGCAACCGCAACCTTCCGTTTCTAA
>Cluster_19794
ATGAGTTTTAACGCAAAGGATATGACCCAGGGCGGTCAGATTGCGTCCATGCGTATCCGCATGTTCAGTCAGATCGCCAA
TATCATACTTTACTGCCTGTTTATTTTTTTCTGGATCCTAGTTGGTCTGGTTTTATGGGTAAAAATAAGCTGGCAGACGT
TTGTGAATGGCTGCATTTACTGGTGGTGCACCACGCTGGAAGGCATGCGGGATTTAATCAGGTCCCAGCCGGTCTATGAG
ATCCAGTATTACGGCAAAACCTTCCGGATGAACGCGGCTCAGGTACTGCATGATAAATATATGATCTGGTGCGGTGAGCA
GTTATGGTCGGCATTCGTTCTGGCGTCTGTCGTGGCCCTGGTTATCTGCCTGATCACCTTCTTTGTTGTCTCCTGGATTC
TGGGGCGTCAGGGTAAACAACAGAGCGAAAATGAAATCACAGGTGGTCGTCAGCTGACTGACAATCCGAAAGAGGTGGCT
AGGATGCTGAAAAAAGATGGCAAGGATTCCGATATCCGGATTGGCGATCTGCCGATTATCCGGGATTCTGAAATCCAGAA
CTTCTGTCTGCACGGCACTGTCGGGGCCGGTAAGTCGGAGGTTATCCGTCGTCTGGCCAACTACGCCCGTCAGCGTGGTG
ATATGGTGGTGATTTATGACCGTTCGGGGGAATTTGTTAAAAGTTACTATGATCCCTCCATCGATAAAATCCTGAATCCG
CTGGATGCACGCTGTGCTGCCTGGGATTTGTGGAAGGAGTGCCTGACACAGCCAGATTTTGATAATACCGCAAATACTCT
GATCCCGATGGGGACAAAAGAGGACCCGTTCTGGCAGGGTTCAGGACGTACCATTTTTGCGGAAGCGGCATACCTGATGC
GTAATGACCCCAACCGCAGCTACAGCAAACTGGTGGACACACTGCTTTCCATCAAAATTGAAAAGCTGCGTACCTTCCTG
CGTAATTCACCGGCGGCCAACCTGGTGGAAGAGAAAATCGAGAAAACGGCCATATCCATCCGTGCTGTGCTGACCAACTA
CGTGAAAGCCATCCGTTACCTGCAGGGGATTGAGCATAACGGTGAGCCCTTCACCATCCGTGACTGGATGCGTGGTGTCC
GGGAAGATCAGAAAAACGGCTGGCTGTTTATTTCGTCGAACGCCGACACCCATGCCTCCCTGAAGCCGGTGATCTCCATG
TGGCTGTCCATTGCTATTCGTGGTCTGCTGGCAATGGGAGAAAACCGTAACCGTCGTGTGTGGTTTTTCTGTGACGAGTT
ACCCACGTTACACAAACTGCCGGACCTGGTGGAGATCCTGCCGGAAGCCCGTAAGTTCGGTGGCTGTTATGTGTTTGGTA
TCCAGTCCTATGCCCAGCTGGAAGATATCTACGGTGAGAAAGCGGCTGCCACGCTGTTTGACGTCATGAACACCCGTGCC
TTTTTCCGTTCTCCCAGCCATAAGATTGCAGAGTTCGCTGCCGGTGAAATTGGTGAGAAAGAGCACCTGAAAGCCAGCGA
GCAGTATTCCTACGGTGCTGATCCGGTACGTGACGGGGTATCGACCGGTAAGGATATGGAGCGTCAGACGCTGGTCAGTT
ATTCCGACATTCAGTCTCTGCCGGATCTGACCTGTTATGTCACCCTGCCCGGACCGTATCCGGCAGTAAAACTCTCCCTG
AAATATCAGACACGACCGAAGGTCGCTCCGGAGTTTATTCCGCGTGACATCAACCCGGAAATGGAGAACCGTCTGAGTGC
CGTACTTGCCGCAAGGGAAGCAGAAGGTCGTCAGATGGCCAGCCTCTTCGAACCGGATGTCCCGGAGGTTGTTTCCGGAG
AAGACGTGACTCAGGCTGAACAGCCGCAACAGCCGGTGTCTCCTGCCATCAACGATAAGAAGTCAGATGCAGGTGTGAAT
GTTCCGGCAGGGGGGATCGAGCAGGAGCTGAAAATGAAACCGGAAGAAGAGATGGAACAGCAACTGCCACCCGGGATCAG

TGAATCCGGTGAAGTGGTGGATATGGCCGTTTATGAGGCATGGCAACGGGAGCAGAATCCGGATATCCAGCAGAAGATGC
AGCGTCGTGAAGAGGTGAACATCAATGTGCATCGGGAGCGCGGGGAGGATGTTGAGCCGGGAGATGATTTCTGA
>Cluster_39476
ATGGCCGACGAAAACAGACTCAATTCCATTCTGTGTAAGTTTGACGCAGACTGGATGGCGAGCGATGAAGCCAGAACCGA
GGCGACAAATGACCTGTATTTTAGCCGAGTGTCGCAATGGGATGACTGGCTATCAAACTACACCACCCTGCAATATCGCG
GACAATTCGATGTTGTTCGCCCGGTGGTCAGGAAACTGGTCGCAGAGATGCGCCGGAACCCTATCGACGTTCTCTTCCGA
CCCAAAGACGGCGCTAATCATGATGCAGCCGATGTGTTGATGGGGATGTATCGTACTGATATGCGCCATAACACGGCAAA
GATTGCCGTTAACGTTGGCGTTCGTGAGCAGATAGAGTCCGGCGTTGGTGCATGGCGTCTGGTCACACAGTACGAAGACA
ACGACCCAACAAGCAACAATCAGGTAATCAGACGCCTGCCAATTCATGAAGCCTGCTCACACGTCATATGGGACGCCAAC
AGCAAGCAGATGGATAAGAGCGACGCTAAGCACTGCACGGTGATTAACGCCTTGTCGCGCAATGGCTGGAAAGAGTTCGC
AGAGGATTACGGTATTGATCCGGACACCTTGCCATCTTTCCAGAATCCGAACGATACATGGCTGTTTCCGTGGGTATCGA
ATGATGTCGTCTACGTCGCTGAGTATTACGAGGTCGAAGAGAAGAAAGAGAAGGTCTTCATCTACCGCGACCCGCTGACA
GGTGAGCCGGTCAGCTATTACCAGCAGGATATCAAAGACGTCATCGACGACCTGGCTAATCGTGGATTCATTAAGGTAGC
AGAGCGTAAGGTCAAGCGTCGGCGTGTGTATAAGTCGATCATCACCTGCACGCAGATACTGAAAGACCGCGAGAAGATAG
CCGGAGAGCATATTCCAATCGTTCCAGTGTATGGCGAATGGTCATTCGCTGGTGACAAGGAGTGCTACGAAGGAGTGGTA
AGGCTGACGAAAGACGGTCAGCGCCTTCGTAACATGATCATGTCGTTCAACGCCGATATCGTTGCTCGTTCACCGAAGAA
GAAACCGACCTTCTTCCCTGAGCAAGTCGAAGGCTACGAATACATGTACGGTGGAAATGATGACTATCCGTACTATCTGC
AGAACAGGACCGATGAAAACGGTAACGACCTGCCGATTGGTCCAATCTCCTACATGGAAAACCCTGAAGTGCCGCAAGCC
AACGCTTACATGCTTGAGGCTGCCACCAACGCAGTGAAAGAGGTGGCTAGTCTTGGCGTGGATGCGCAGGCGGCAAATGG
TCAGGTCGCTTTCGATACCGTCAATCAACTGAACATGCGGGCAGACCTTGAGACATACGTGTTTCAGGATAACCTGGCTA
CCGCAATGCGACGTGATGGCGAGATTTATGCCTCAATGGTCAACGATATTTATGACGTTCCTCGTCATGTAACGCTGACA
CTTGAAGATGGAAGCGAGAAAGACGTTCAACTCTATGCGCAAGTTGTCGATTACCAGTCCGGCAATGTGGTCACACTCAA
CGACATTCGTGGTCGATATGAGTGCTATACAGACGTTGGGCCATCCTTCCAGAGTATGAAGGAACAGAACCGCGCAGAGA
TTCAGGAGTTGCTCACCAAGGTTCCGCAAGGTACTCCAGAGTTCCAGATGCTGATGCTGCAATACTTCACGCTGCTTGAC
GGTAAAGGCGTCGAGATGATGCGAGAGTACGCGAACAAGCAACTGGTAATGATGGGGCTGAAGAAACCAGAAACACCTGA
AGAGATGGAGATGGTGCAACAGGCACAACAACAGCCGCAGCAGCCATCAGCAGAGCAAATTCAGGCGCAGGGTATCCTTC
TGCAAGGTCAGGCTGAATTGCTCAAGGCAGAGAACCAACAGGCGCAGATTCAGGTTGAAGCTGCCAAGGTTGAAGCTCAA
AACCAACTCAACGCCGCGAAGATTGCAGAAATCTTCAACAATATGGATCTCGACAAGCAGGCAGAACTGCGTGAGTACCT
CAAGCTCGTAGGTCAATTCCAGCAACAGCGCAGCAAAGATGCTCGTGCTAACGCTGAGCTGCTTCTTAAAGATGCAGACC
AGACTCATTCACAACGCATGGATTTCGCGAATCTTATGCGTCAAGTTCAAATCCCCTCCGGCGGAGTAGCCGAGACACCT
CAATAA
>Cluster_63553
ATGTCTGAATTTGATGCCCAGCGCGTTGCCGAACGTATTGATATTGTGTTAGATATCCTTGTGGCAGGCGATTACCACTC
TGCGATCCATAATCTCGAGATACTTAAAGCAGAACTCTTGCGCCAGGTTGCTGAATCTACGCCAGACATCCCGAAGGCAC
CGTGGGAGATTTAA
>Cluster_15790
ATGGCTGATGCAATGGATCTCGTACAGCAGCGCGTTGAAGAAGAACGCCAGCGTCATATCCGTGCTGCCCGCGCCAAAAC
GCCGGGCGTATCCCGCGTGCTTTGCATTGAGTGTGAAGCACCAATTCCGCCAGCACGACGTCGCGCCATTCCGGGTGTGC
AGCTTTGCATTACCTGTCAGGAAATCGCAGAGCTGAAAGGCAAACATTACAACGGAGGTGCTGTATGA
>Cluster_21993
ATGCTACCTAGGTACAAATTTTATAAACTGGCATCACATCTAATCAGACAAGATACCCGCTCAAAAGAATATGAAGATGT
TACGCTTGACCGTAACAAGAAAAGACTAAAACCACATCAAATTGAGCAGTTACGCCATGAATTAAAGTCACTGGATATCG
AGCTTGATTATAGTGACCCGACTCAATTTAAACGGGCTTATGAACTCATTAAGAATTGGGGGGAAGAATGA
>Cluster_17634
ATGGCTTTTAAACTTCTTAGTGTTACCGAAGCAATATACCAGCCGCCCGGAGAACGCCACGAATACAGGATGAATGACGG
TAGCGCGGTGGTTGAGTTCCCCGGATATCCGGGGGCTTCCCGGTGGCGGTTCTATGACAGTGTGGGACGTCGGATTATTA
AAAGAACCGTGCACAACAATATGAAGGCTGCCGTAGAACGTCATAAGAGAAGGTTTAACTGCAAATGA
>Cluster_31790
ATGCATAAAATCGATTTTGAAGTATTAATCCACTCTGAAAACGCATTAATTAAAGCACGCGAAATGAATGCGTTGTTAAT
TAAATTAATTGAAGTGCCGGAAGGTGGCGAAGATCATTCATCATTAATGTTTGCCGCTGTTCAGACATTATTGACACCTG
TTATTGATGAACTCAATAAGGCTATGGCAATTCACGAGAATAATAAAGCGCCCCACACCGGAGAATAA
>Cluster_19622
ATGACCTACAACCAAATGAGGTGCCAGCACTGGCCGTTACTGGGCTGTGGGAGATGGATGACCTACAGCTTTTGTATTTA
TTTCAGAGTGTTATGTTATGCGAAATCTCGTAATGCTATGATTTATAGTGTATTTTCGTATTACTTGCGGCTCTTGATGC
GAAGAGATGGCGGGGCTTTAGCCGTGCCAGACGATGAGCATCAAGATCAGATTGTGAATGTGATTCATCACTGA
>Cluster_46724
ATGATTTGTCCACGTTGTGCCGATGAACAGATTGAAGTGATGGCGAAATCGCCGGTGAAAGATGTCTGGACGGTATATCA
GTGCCAGCATTGCCTTTATACCTGGCGCGATACCGAACCGCTGCGCCGTACCAGCCGCGAACATTATCCCGAAGCGTTCC
GCATGACGCAGAAAGATATTGATGACGCGCCAATGGTGCCGAGCATCCCGCCGCTGCTGGTGGAAGGTAAGCGTTAA
>Cluster_20760
ATGCACATGACACGTCAGGATTTACCCGCAACGAAATCAGGTACTGACCTTCTGGCCATGCTCACCAAAGCCACACAGGA
AGGTAAAGCCGCTGCTGCTGATCTGTGTTCCACCCGTCTGGATAAGCTGGCCACCCATGCAGCCAATGAAGGTTTAAGCG
CAACTGAAGTCATTGAGTTAATCCGCGAAGAAGCCGCAGCGATTTGCAGTAAGGGCGGTGCAGCATGGCAGTAA
>Cluster_14845
ATGAAATGTAATGATTACCAGTCAATTAAAAAGGAATTGCGAGAATATGTTAATAAAATACGAGTGAATACTAATTATTT
CCGAAGTGATTTTATTGCTGAATGGGCGTTGCTTACTACTATTGCTTGTTGGGGAGCTCCGGAAGGGGGGGGTAAGGTTT
ATATCATTTATGATAACCCTTGCTTTCTTTTCTTCAAAAATATATATACCAAGAAGAAAACGTACAACGCCTGA
>Cluster_11172
ATGCGAGTTGATGAACTTGTCCAGTTTTTTGGTTCTGTTCAGAGGGTTGCCGATTTTTATGGGATAACCCGCGAAGCTAT
TTACATGTGGCGTAAGCGTCCTGGTGAAATAGTTCCGAAGGGGAGGGCTGCGGAAGCTGCTGCATACTCCAAGGGGAAAT
TATCTTTGAACCCAGAACTTTACAAAAAGAAGGATACCACTCAGGGCGAAGGAAAGGGGGATTCATGA
>Cluster_38682
ATGAACTATGCCGGACACGAAAAACTTCGCGCCGAAGTGGCGGAGGTGGCTAATACCATGTGTGACCTTCGGGCCAGGCT
GAACGATATGGAGCATCGCTGTCGCTTTGATTCCGATGTGCTTGTTGAACGCCTGACACGCCAGACGCTGTATCGCGCCA
ATCGCCTGTTCATGGAGGCATATACCGAAATTCTTGAACTGGATGCGTGCTTCAAAGATTAA
>Cluster_5983
ATGACCCCTAACATTTCAATAACTCTGAATACGCCACACGTCACAATCGAGCGTTATAGCGAACTTACTGGTCTTTCAAT
CGACACAATCAACGATATGTTGGCGGATGGTCGCATTCCTCGGCATCGTCTTCGTAAAGACAAGAAAAGAGAAAAGGTAA
TGATCAACCTTGCTGCTCTTACCGTTGATGCACTTACTGATTGCAATGTTGTATTCAACTAG

>Cluster_67090
ATGGTGAGATACTACGGGGGCCTGAGTCCGGTGACGCGGCGGTTACTGAAAGATTTTGTGTACGCCATAACGGAGACAGA
GAGAAAAACGGCGATGCAAATCAGGTGGAGAGGTATGTATCAGAGGTTAGTGAAGGTTGACCCGCTGAAGTGTGTGCTGT
GCAGAAGTCAGAGGCGTTTTACGGGACTGAAGAGGGTTACCGTCTGGCGGAATGCATGA
>Cluster_59265
ATGATTGCACATCACTTCGGAACTGATGAAATACCACGTCAGTGTGTGACTCCTGGCGATTATGTTCTTCATGAAGGCCG
GACATATATTGCCTCGGCAAACAATATTAAAAAGCGAAAACTATATATTCGTAACCTGACCACAAAAACATTCATTACTG
ACCGCATGATTAAAGTCTTCCTCGGTCGTGATGGTTTACCTGTAAAGGCGGAGTCATGGTGA
>Cluster_17627
ATGGACAAGCAGACCATTAACGCCGTCCGTTCCCGTGCCCGTGTGGCCATCCACACCACTGGTGGTCGTGTAACGGGACA
ACATCACCGTTTACCTGTGGTTTATGCCTGCCCGCCCGTGGGTGGTCCTGTATGGCCGGTAACGGAAATCATCCAGACAT
TCAGCGGCCTGCGCCGCGTGGTGAAAACATCCTGTATCGACGGCTGCACCGTCGTATGGCAATGA
>Cluster_64820
ATGACGAGGAATAAGCAACAAGCACCGGAAATAACAGCGGAGGTGTTGATTAAAGACAGCGCCGAATGCCTGGCATCAGT
ACCTGCATTGATTCAACAGGCGAATGCAAAAAATCTCCGTCGTGCAGCTTCGTTTATTCGTGCAGGCATTGCCCGCCAGA
TGATGATTGCGCAGATGTGTATGCGCGAAGCACAGCGTCTGGAACATATGGAGGCAAATAGTGATTAA
>Cluster_48412
ATGAATCACGCCGCAATCTCTTATGACGATATTGTCCGCCTGAAGCATCTTCGTAACGTGGGGGAGTTTGTCACCGGAAT
GGCTGTTCTTCAGGATTGCCACGAAAAACCTGCCGGTGCTCAATGTGAGCAACTGGCTTCCCTGATTTATCTGATGACAG
AGCAGCTTGATGGCGTGGTACAGCGTTGCCAGGATGACCTGATGAACATGGAGGTGGTGCAATGA
>Cluster_67022
ATGGCAGATGAAATCGAACGTTTTCAGTCTCATCAGGCGATGCTGGAAGCCGATCGCATACAACGTATCCGGCAGCAGTT
AACGGGCATCAGTGCCCTGTTCTGCGATGACTGTGGTGCGCCGGTCCCGGCAGAGCGTCGCCGGCTGATACCGGGTGTGC
GGAAATGCGTGGGGTGCCAGGAGGTACAGGAGATTCAGGCCCGCCATTACAGGAGGTGGCAGTGA
>Cluster_42343
GTGAATCAGGCCATCCAGTTTCCGGATAGGGAAGAGTGGGACGAGAATAAAAAATGTGTATGTTTTCCCGCTCTCGTGAA
TGGTATGAAACTGACATGTGCGATCTCTGGCGAGAGTCTGGCGTATCGCTTTACTGGAGATACGCCAGAACAGTGGTTAG
CGAGTTTTCGTCAGCATCGCTGGGACCTGGAAGAAGAAGCGGAAAACTTAATTCAGGAACAAAGTGAAGATGATCAAGGG
TGGGTCTGGTTACCTTGA
>Cluster_49990
ATGATGTACAGCAAATACGATGAGGCGCAGTTCCACTTGAGGCTTACGCACGAGTTGCACGCTAAGATTAAGCAACGTGC
AAAAATGAATAACAGGTCTATCAATTCCGAAATTGTGGCTACGATGGAAGAATCACTCTCCAAACCATCACCTGTAAGCG
GGTATCGTGATGAAGAAGAGAGGCTGGCCTCATTAATCTCGGAACGAGTAAAAGAAGTTGTGGCTGATATCCTTAGAAAA
GAAAAAAACCCGCAATTAA
>Cluster_36238
ATGAAATACCTGGTTACGGGTGCCGCTGGTTTCATCGGCTTCCACGTTAGCAAACGCCTCCTTGAGGCTGGCCATCAAGT
AGTCGGTATTGATAATCTGAACGATTACTACGACGTCAGCCTTAAACAAGCTCGTCTGGAATTACTGGCACAACCTGGTT
TCCAGTTTCATAAAATTGATTTAGCCGACCGCGAAGGCATGACCGATCTCTTCGCATCCGGGCATTTCGAACGTGTGATT
CATCTCGCCGCATAG
>Cluster_67052
ATGACTTCAAAATCCTCATTGATAAAATTCGTTATGACCATCACTACTATCGGTATCGATCTTGCTAAAAATGTGTTCGC
TGTTCATGGGGTTGACCAAAATGGCAAAACCGTTCTGGTGAAATCCAGAGTAACCCGCGCAGCTCTTCCCGGGCTGATTG
CAAGTTTACCGCCCTGTGTTATCGGGATGGAGGCGTGCTCCGGGGCTCATTACTGGGCAGACTGTTTCTGCAATATGGTC
ATGAGCCACGTCTGA
>Cluster_54404
ATGAAAACTATCAATACTGTTGTTGCTGCTATGGCTCTTTCAACTCTGTCATTTGGCGTGTTCGCGGCGGAACCGGTAAC
GGCATCCCAGGCACAGAACATGAATAAAATCGGCGTGGTTTCTGCCGATGGCGCATCCACCCTCGATGCCCTGGAAGCGA
AACTGGCTGAGAAAGCCGCGGCAGCCGGTGCCAGTGGATACAGCATCACTTCCGCCACCAACAACAATAAATTAAGCGGT
ACTGCGGTAATTTATAAGTAA
>Cluster_52904
ATGTCCCATACCGGGGGGAAAGAAGGAACGTATCCTGGCACCCGCGCCAGCGCAACCATGATTGGCGACGATGAAACCAA
CTGGGGCACAAAATCGCTGGCGATACTCGACATGCCATTTACCGCAGTACTGGACACAATATTGCTACCGTGGGATGTGT
TCCGCAAAGACAGCTCAGTGCGCTCGCGCGTTGAAAAAAGCGAAGCCAACGCACAAGCCACCAATGCGGTTATTCCACCG
GCCAGAATGCCTGATAACTGA
>Cluster_40771
ATGTTCCGTTGTCCGCTTTGTGGCGCATCTGCCCGTATCCGCACCAGTCGTCCGGAAAATGATTCAAACACCGTGCGGCA
AAAGTATTACCAGTGTAACAATCTGGAATGCGGCGTATGCTTCTCAACACTGGAAGCTTTCCATAAATTCACATCAAAAC
ACGCCTCCGGCGTTCACTCTTCAGAAGGTATCCCGTGGCATGAGCTGCCAGCTTCACACAGGGGAAACAATCAGATGAGT
TTGCCTTTATCTCAGAATTAA
>Cluster_199
ATGTTTTCACGCCGTTTCCTCATTCAGTTCACTTTGGTGGTTCGGCGGCTTCGACTTGTATCAGTAGGCAGTGCTTCGCC
GCACCCCAAAGGGAACTTACTGACCCGTATTGCCGACATCCTGTCCCGCCACGGTCCCGACGCATGGTTTAGAGTCGCGC
CGTTCGACTTGTGGCTAAAGATACAATTTGAGTTGTATTGTGTAAACCGCAAACGTGGTATTTTGTGGGGTGTGAATACT
ACTTTATTGATATTTAAGTGA
>Cluster_22814
ATGATCCGCATTGAAATTCTTTTCGACCGCCAGAGCACAAAAAACCTCAAATCAGGCACACTTCAGGCGCTACAGAATGA
AATCGAACAACGTCTGAAACCACACTACCCGGAAATCTGGCTGCGTATAGACCAGGGCAGCGCCCCGTCTGTATCTGTCA
CAGGAGCCCGCAACGACAAGGATAAAGAACGTATCCTGTCTCTGCTGGAAGAAATCTGGCAGGACGACAGCTGGCTGCCT
GCAGCATGA
>Cluster_6821
ATGAAAAATAATAAACTGGCGACCAGTGAATTAAGAAGCCTTGCCAACAGCGCAATATCTTTAAACAACGCAGCAAACGA
AGTAATGAATATGTGGCTTCAGTCGCTGGTGAATAATGAACGCGATGAATCAACTGCAAGATTAATATTAGCCGTTTCTG
AATTACTGGATAGTTCAACCAGCAAAATAGAGAGCATATCAGAGAAGATTAACGGCTTATCAGGTCACGCTATCGGGGAA
TAA
>Cluster_50009
ATGGCAGAGATTATTCCCATGACTGAAGAACAGAAATTCCAGTTAGAGATTTACAAACTGGTCATGAACCAGAACGCAGC
CGCAGAAGAAGCATTTCAGTTCATTGGTACTGACGAGTTGAAGCTTGAGCTATTCAAAATTCACTTCCAGTCAGGCGGCG
CTAATTCAGATATCACGATCCGCACATTTGAAGCGGTGCGTAAATCGAAGGAAGTGTTAGACCTGTTCACCACCGGAGCA
TAA

>Cluster_31021
ATGTTGGGTTCTGTCCTTGCGTCCGCATGGCGCGCCGGTTCCTTACGTCTGGTTTCGGTAAATGCGCTCTTTGTCGCACC
AGCGCAAGCATTTACAGTTCCGGAAGTGGGAAAACGTCAATTAAATGAAATCAATCGGATCCGCTACGAACTGGAATTAA
TGGCAGTTGCTCTGGCTGTCGAAAACCTCACTATGCAAAAAAACGTTGCTTTGTTGTGCCAACAATACAGCCGATAA
>Cluster_53928
ATGCACCGTCAGTCAGACATCTATCAGGCTTTCCGCGAGTCGGTATTGCGCAACTCCAAAGGCTACCAGTATCTGCATAC
AAAGGATTTTGTGTCAGCGCTGCTCCGGCGTGGGCTGCACTTTACCGACTCAGAGGCTAATGCGTGGATATCTAGAGAGC
AGACGTACTTTGTCGACAAAACCACCGACCATAGCGAAAACCGTTTGTGGATGATGGCTAACATGGGGAGGGTTTTGTAA
>Cluster_48602
ATGACGATCACTATCTACGGACGAGATAACTGCTCATACTGCAAACGTGCGGTCGAGCTGGCGAAGCAACTAAAGGGACA
TGGCTACGGTGATTATGAGTACATCGACATCACCACTGCCGGTATCGACAAGGAAAAACTTAGTGAAATTGTTGGTAAAC
CGGTAGAGACTATCCCCCAGGTACTGATCGATGGCCAACCGATTGGCGGATACACAGAACTGGCTGCATACGTCAGCACC
CTCTGA
>Cluster_1315
ATGTCGCAGACAGAAAATGCAGTGACTTCCTCATCAGGTACAAAGCGAGCATACAGAAAAGGTAATCCTCTGACGCTTGC
AGAACGTCAGCAGGCATCCCTGGCAAGAAAACGAGCCACACATAAAGAACTCAGAGTGTTTATTCCTGCTGCGTTAAAAG
CGCAATTGCAGGAGATGTGTGAAGCTGAGGGTGTTACTCAGGCTGAGATGATTGCCGAACTGATAAAGCAGAAGAGTGCT
TTTAGCTAA
>Cluster_44018
ATGCCGAAGAAAAATGAGGCTCCCGCCAGCTTTGAAAAGGCGCTGAGCGAGCTGGAACAGATTGTAACCCGTCTGGAAAG
TGGCGACCTGCCGCTGGAAGAGGCGCTGAACGAGTTCGAACGCGGCGTGCAGCTGGCACGTCAGGGGCAGGCCAAATTAC
AACAAGCCGAACAGCGCGTACAAATTCTGCTGTCTGACAATGAAGACGCCTCTCTAACCCCTTTTACACCGGACAATGAG
TAA
>Cluster_24267
ATGCTGGAACTACTTTTTGTAATTGGCTTTTTTGTCATGCTGATGGTCACCGGTGTTTCGTTGCTGGGTATTATCGCCGC
GCTGATTGTGGCGACGGCCTTTATGTTCCTCGGCGGTATGCTGGCATTGATGATTAAGTTGCTGCCGTGGCTGCTGTTGG
CGGTTGCGGTGGTGTGGGTGATCAGGGCGATTAAAGCACCAAAAGTGCCGAAATATCAGCGTTATGACCGCTGGCGTTAC
TAA
>Cluster_17631
ATGAACGCGAAACGCTTTAATCAGCTATATCCGCCAGGGACTCGCTTCATGCACATTGCACATCGGGCAGTGCGTGGTGG
TCGGGTGGTAAAAACTGTTGCACCTGCCAGAGATTTTAAATGTGGTTGTGTGGTTGAAATTAATGTCGAGCCTTATTTCG
TTAAAGTCGAAACACTGAAAGCACCGCATTAA
>Cluster_39337
ATGGCGACGATTAAATGTGTGTACTGCAAAAAAGAAGTCACTGAACTGGATTTTCAACAAGCAAGCTTATTGCAGACTGA
TGAATATAAGGAGTGGTGCGTAAATTTAATTTTGCTCTGTCCGCACTGCGAGCAGGCTTATAACGCCTTTATTCCCACTA
TGGAATTAACCCCGGCAACGGAGGTAGGCGCATGA
>Cluster_5991
ATGCAGGACTATTTTTTGGAGTCTTTGAAGCTCCAGCGCATTGATTTTTTTCTTAAGCTTGTAGCGGCTAGTGAGTGTAG
TGATGAAGAGAAGGGGCTGGCTTTGCAGTGGGTTTCTGAACTAACAGATGAACTCATGGCAAAAATCAGAACCCACGAAT
ACAACCGCTCAATGGATGTCATCAGTTGA
>Cluster_25066
ATGAAATGGTTTACACCGAAACACGTCGTAGAGGCATTTAAAAAAGGTGAGCTTACACGCCATCAAGTAGTAATGAATCG
TAATATGGCAAGGAGTCGAGGTTATCCAGAGCGAGCAGCTTGCTTCAATGAAGCACTCAAGATTATTGATGAACTAAGAA
AAAATGAAAAAGAATCTGAAACAGAGTAA
>Cluster_43633
ATGAGTTATCCCTGGAGCGTGAGCGCCAGCTGGAAATACAGGAACGGACACTGGATGGCCCTTCGCTGGGATGGTAATCA
GAGACAGGCTAAAATGTTGTCAGCGCGTTTCCGGTGCATTTTTTGGGATAATGGTTGGTTCGGGGGCACCGGGAACGCGC
TTCAGTCAGACCTTGCTGAAAACACAGGAACGCGTTAA
>Cluster_50967
ATGACAGAAACAACCTCACAACAAGATGATTATGAAGGCATTTGTCTGGAGCCTGATTCATTTGCGGTAAATGTTTATCA
TTTATTGCATGCAACACAGGTATTACATATTTCCAGTAATCACGAAACGAAAACACTCGGCAGCGAAATTCTGAATTTTG
CATGTGAGTATGCAAAAGCTGCTGCCGAAAATAATTAG
>Cluster_18919
ATGTCAACAGTAATACAGCGTGATAAACAGCCCAAAGGCAGGGGAAAGGCACCAGCTTTCCAAGTGAGGATCGCCCCAGA
ATTGAAAGAGCAATTCGAGGCCGCCGCACGAGATGCAGGTATGAGTTTAGCTAATTGGATGAAAACCTTGGGGCGCAAAG
AACTCCAGCGGCTGGGTATTGAGCCGAAAGCCTGA
>Cluster_61201
ATGTACGTTTGTCTTTGTAATGGTATCAGCGATAAAAAAATTCGTCAGGCTGTGCGCCAGTTTTCCCCTCACTCGTTCCA
GCAATTAAAAAAATTTATTCCGGTCGGCAATCAGTGCGGTAAATGTGTCCGGGCCGCGCGTGAAGTGATGGAGGATGAAT
TAATGCAGCTGCCGGAGTTTAAGGAGTCCGCATAA
>Cluster_41780
ATGCTGATTCTGACTCGTCGAGTTGGTGAGACCCTCATGATTGGGGATGAGGTCACCGTGACAGTTTTAGGGGTAAAGGG
CAACCAGGTACGTATTGGCGTAAATGCCCCGAAGGAAGTTTCTGTTCACCGTGAAGAGATCTACCAGCGTATCCAGGCTG
AAAAATCCCAGCAGTCCAGTTACTAA
>Cluster_59108
ATGTATCTGCATAACAAAAACAATATGCGCCATTTTTGCATTACTAATAAGAAAAAGCAAAAAACATCGATATTCATTTT
TGGTCAATGGGTTTTCTGGCCTATCGTTGTTATTGTGCTACTGGTTATCATTTTCATCTTGCCAGCATATTGGAGCGTGA
TCAATTTTGATCAGCTGTGA
>Cluster_6723
ATGAGTCCACTCTGGTATCAGAAATGGCTACGTCTCAATGAAGTCAGGCGACAGATGCTGAATGAACATTACGATGTCAC
CACAGCAGCCTATGCTGTCGGTTACGAAAGCTATCCCATTTCTGTCGGGAATATTCGCGGATGTTTGGAGAGTCACCCAA
GAGAGATATTACCAGGTTAA
>Cluster_15992
ATGAGGGACAAACCCACGAATCATAAACGCGGTAAAAACCCGGTGTGCATCGTTTTTGATTATTCCCGCACACTCACGCA
GAAGGAATTCCCCGTCGGGCTACGATCATGGTTAATGCGGGAATACGGCGACGATACAGCGCATGATGTGTCAGGCTTGA
ATACCTTTATCCGTTAA
>Cluster_31224
ATGGAAGGTAAAAATGGCCTGAAAATAGTGTGCGATAGCGGTGCGGGGTTTTCGACGGAGCAAGCTGTTTTCCTTGCCAA
CAATCTTGCAAAAAAAATGCGATCGCTAAAACAAAAGTGCAATTTGCGGCAATATAGTTATTCCATGACCGTGCAATCTT

GCATTCAGTGTAAATAA
>Cluster_26947
ATGAGCTTTATGGTTAGTGAGGAAGTTACGGTTAAAGAGGGCGGCCCGCGGATGATTGTCACTGGATACTCCAGCGGTAT
GGTTGAGTGTCGTTGGTATGACGGTTACGGGGTCAAGCGGGAGGCTTTTCATGAAACCGAGCTTGTTCCGGGGGAGGGGA
GTCGTTCTGCGGAAGAAGTTTGA
>Cluster_3651
ATGAAACGTGTTTGTAATACCAGTACGCTGGCAATGTGCGAAATGCCGGCGCTGGTATTGTGGAAATCAGCCCTGTCCCT
GGTGCTGGCGACACTCCCGCTCATCTTTCTGCTGACACCCTCTGGTCAGCCAGCGGACTATCGTCCTTTCCTGACCGATT
CGTCATTCCATTTTTATTGTCTGTAA
>Cluster_17650
ATGACGCGTCAGCACTGGACCCACAGAACGCCACGTAAAGCCGCGCGCTGTGTTCTGGCCCTGTTTCTGGTGATGTTGCT
TCCGGTGGGATGCACAGACATTAACAGAGCGGGCCAGCTGTTTGACGCGGCGGCGCAGGTCTGCCGGATTATCGATGGTA
TCCGGCAGTGTTCACAGAACTGA
>Cluster_25043
ATGTTTAAGCCGACAGGAACACCACAACCTCAAAAACGCTACAAAGATGCCCACGGAGCACTCGTTACTGTCGAAAGCGT
GTCTCACAACCGAGTGACGTTTTATCGCGACGGGTATCAATCGCCATGCGTACAACCGCTGGCGCGCTTCATGAAGGAGT
TCGCGGAGGTTAACAAATGCTAA
>Cluster_44777
ATGCCCCGGAAAGAAGCCAGGCTGTTCTTCCGGTTGCTGAAGCGGCAATATGAAAAAGCGAGAATCGTTCTGACGTCAAA
TAAAGGATTCGCCAACTGGGGGGAAATGCTCGGCGATAATGTACTGGCAACAGTGATCCCTGAGCATCTGCTGCATCACT
CAACCACGCTGAATATTAAGGGGGGAAAGCTACCACCTGAAGGAAAAACGTAA
>Cluster_5505
ATGAATCTGCCTGTAAAAATCCGCCGTGACTGGCACTACTATGCGTTCGCCATTGGCCTTATATTCATTCTTAATGGCGT
GGTGGGGTTACTGGGATTTGAAGCAAAAGGTTGGCAGACCTATGCCGTCGGTCTGGTGACGTGGGTGATTAGTTTCTGGC
TGGCGGGGTTGATTATTCGTCGTCGCGATGAAGAAACTGAAAACGCCCAATAA
>Cluster_25933
ATGACGCGACAGGAAGAACTTGCCGCTGCCCGTGCGGCACTGCATGACCTGATGACAGGAAAACGGGTGGCAACGGTACA
GAAAGACGGACGGAGAGTGGAGTTTACGGCCACTTCCGTGTCTGACCTGAAAAAATACATTGCAGAGCTGGAAGTGCAGA
CCGGCATGACACAGCGACGCAGGGGACCTGCAGGATTTTATGTATGA
>Cluster_38617
ATGGCGAAAACAAAACAGGAATGGCTGTATCAGTTACGGCGATGTTCATCAGTCAATACGCTGGAAAAAATTATTCATAA
AAACAGAGACAGTTTATTAAACAGTGAACGTGAATCATTTAATTCAGCAGCAGACCACCGGTTAGCGGAATTAATCACCG
GAAAGTTATATGACAGGATACCTAAAGAGATATGGAAATATGTAAGGTAA
>Cluster_11888
ATGTCAAATAAAATGACTGGTTTAGTAAAATGGTTTAACGCTGATAAAGGTTTCGGCTTTATTTCTCCTGTTGATGGTAG
TAAAGATGTGTTTGTGCATTTTTCTGCGATTCAGAATGATAATTATCGAACCTTATTTGAAGGTCAAAAGGTTACCTTCT
CTATAGAGAGTGGTGCTAAAGGTCCTGCAGCAGCAAATGTCATCATTACTGATTAA
>Cluster_26132
ATGCAGAAACGAGAACCCGTCATCATCGCGCCAGACTATACCAATGATGAACTTTATGAGTGGATGCACCAGAAAATTAA
TGCAGCGCAGGATCTGAAATGGGCTAATGAAGTCAGGGCTAAGCAGGCTGAAAATCTGTCCTCTCTGGAGCAGGATATCA
CCAATCTGGAAAAAGCAGCGGCATTAACCATTGCCAGAATGATTACATACCCGCGTTAA
>Cluster_1575
GTGAGCACAGAAGAAGCCAGAGAAATGATACAGCGGTACCGTGAAGCGGAAATGGCCGTACTGGAGGGGAAGTCTGTCAC
CTTCAACGGACAGCAACTGACGCTGGAAAGCCTTTCTCAGATCCGCGCCGGACGTCAGGAGTGGGAACGCAGGCTTGCCG
CGATGGTGAGCCGCAGGCGGGGAAAACCAGGATTTAAACTGGCGAGGTTTTAA
>Cluster_14978
ATGAAAAAATATCTGCTTTTTGCTTTGCCGTTCTTCGTTGTTGGTTGTAGTGAAGAAGTAAAAAGTGTGGATTGGTGGGG
GCAGCATCTTACGGAAGCTAAACAAAAGCAGGCAGAGTGTGAGAAGTCTGGCTCTGATTCTCAGAACTGTAAGAATGTTA
AACAAGCATTGTTTATTCAGTCTCAGAAGGATGCTCCAGTCCCTACATTCGATTAA
>Cluster_35783
ATGATGCTGAAAATTAAACCCGCAGCGGGAAAAGCCATCCGTGACCCGCTCACGATGAAATTACTGGCTCCGGAGGGAGA
AGAAAAGCCCCGTAACAGTTTCTGGATACGTCGCCTTGCAGCCGGTGATGTGGTTGAAGTCGGGAACACCGAAAACACGG
CGGATGATACCGACGCTGCGCCGAAAAAACGGAGTAAATCGAAATGA
>Cluster_17523
ATGGAACAACGCATAACCCTGAAAGATTATGCAATGCGCTTTGGGCAAACCAAGACAGCTAAAGATCTCGGCGTATATCA
AAGCGCGATCAACAAGGCCATTCATGCAGGCCGAAAGATTTTTTTAACTATAAACGCTGATGGAAGCGTTTATGCGGAAG
AGGTAAAGCCCTTCCCAAGTAACAAAAAAACAACTGCATAA
>Cluster_19677
ATGCGCCTCCAGCATATCCAGCACCTGATCCCACTGGGTAATTTCCACCGGACGCGTCATCGACGGTACTGGCGGCTGGG
CTTTACGTTGCGCGACAAAATAACCCGAACGCGGCTACGGAGTGATGAGGTTCATCGTCTCCAGCGTCTGATACGCCTGC
TGCACGGTACTGATGCTGACGCCGTGCTCCTGACTTAA
>Cluster_60267
ATGCAACACGAACTACAGCCTGATTCACTGGTTGATTTGAAATTCATCATGGCTGATACTGGCTTTGGTAAAACCTTCAT
CTATGACCGGATTAAGTCAGGCGACCTGCCAAAAGCCAAAGTTATCCACGGGCGAGCAAGATGGTTATATCGTGACCATT
GTGAATTCAAAAATAAGCTCTTAAGCCGCGCCAATGGGTAA
>Cluster_62810
ATGGTTCATCAACATTACGGAACGCAGACCGTTAATCGCGGTGCGGTCATGCCAGGAATGCTGGTCAAACACAAAGATGG
TACCTGGACTGCATCAGCTAATTTACGCGGACGGCTTTATCTGCATCGCGGCATCGAGCGCACTTATACCCGTGATTTGC
TCGTGGAAGTTTTTCTCGACGGACGCGGTAACGGCCTGAATCGCTAA
>Cluster_1559
ATGGTTACAGTCGCTGAACTGCAGGCGCTGCGTCAGGCGCGCCTTGATTTATTAACCGGTAAACGGGTGGTGTCTGTCCA
GAAAGATGGTCGCAGAATTGAATATACGGCGGCTTCTCTGGATGAGCTTAACCGGGCGATCAATGATGCGGAGTCGGTAC
TGGGGACAACCCGGCGTCGCCGTCGTCCGCTGGGAGTGAGGTTATGA
>Cluster_41208
ATGCACCAATCTGGTTCTGTTTCTCTTTGTCGTTCCGCAATATCTGTTCTGGTGGCTACAGCGTTATATTCACCCATAGC
ATTGGCATCAACTGTTGAGTATGGTGAGACAGTTGATGGTGTTGTCCTGGAAAAAGATATCCAGCTGGTTTATGGGGCCG
CCAATAATACGAAAATCAATCCTGGCGGAGAACAGCATATATAA
>Cluster_23824
ATGAAAAAAGAAAGTAGGCTACCACCGACGTTCCGTGGCAGAAACGGCGATGTTCCGTATCAAAACCTTGCTGGGCGGAC

ACTTGAGCCTGCGAGACTACGATGCGCAGGTGGGGACGCGCAGGTGGGCGAAGCGATGGCGATGGTTAAAGCGCTAAACC
GGATGACGTTGTTGGGTATGCCGCATAGTGTCCGGATCGCATAA
>Cluster_13484
ATGTCCCCTGTTTCATCAATTGGTAATGATATTAGTAGTGATTTAGTCCGTCGAAAAATGAATGACCTTCCTGAAAGCCC
CATAGGAAATAACCTCGAAGCACTAGCACCAGGTATAGAAAAACTAAAACAGACCTCTATTCAAATGGTCACTTTACTTA
ACGCGTTACAACCAGGTGGAAAATGCATTATCACTGGTGATTTTCAAAAAGAATTAGCGTACTTACAAAATGTAATTCTT
TATAATGACTCGTCTCTTCGTCTGGATTTTTTAGGTTATAACGCCCAAATTATTCAACGTTCGGACAATACCTGCGAACT
TACCATTAATGAACCGTTAAAAAACCAGGAAATATCTACAGGCAATATCAATGTTAATTGCCCATTAAAAGATATTTACA
ATGAAATCAGGAGGTTGAACGTAATCTTTAGTTGTGGGACTGGAGATATAGTTGATCTATCCTCTCTGGACTTACGTAAT
GTCGATTTAGATTATTATGATTTCACAGATAAACATATGGCTAATACTATTTTAAATCCTTTTAAATTGGATTCAACAAA
TTTTACTAATGCCAACATGTTTCAGGTTAATTTTGTTAGTTCAACACAAAACGTCACAATCTCCTGGGATTATTTACTAA
AAATAACGCCTGTTTTAATAAGCATTAGCGATATGTATTTGTCGAAGAAAAAATCAAGTTTGTCGAAAGTTGTTTAAATGAG
CTTGGAGACATTACCGAAGAACAATTAAAAATTATGAGATTTGCAATTATAAAGTCTATACCAAGGGCAACTCTTACAGA
TAAATTAGAAAATGAATTAACAAAAGAAATATATAAAAGCTCATCGAAAATTAACAATTACTTGAACAGAATTAAATTAC
CAGAGATGAAAGGATTCTCATCAGAAAAAAATAGATTATTACATCGATATAATCATTAAAGATTATGAAAGCGTCAAAGAA
AATGCTTATCTTATCGATCCAAAAATTAATTACAATACAGATTTAAACATAGAGGATTCTAGCTCAGAGGAATTCCTTTC
AGATAATACACTCGAGAAAGACGAAAATTCCCCTGACAATTGCTTTGAGGTTGTAAAATATAACACATATGAAGCATATA
ACTCAGAGAATCTATATTTTACCAGAGAGGAATATACGTATGATTACGACCTTTTAAATGCAATATAG
>Cluster_54891
ATGACAACATTACTTAACCCCTATTTTGGTGAGTTTGGCGGCATGTACGTGCCACAAATCCTGATGCCTGCTCTGCGCCA
GCTGGAAGAAGCTTTTGTCAGTGCGCAAAAAGATCCTGAATTTCAGGCTCAGTTCAACGACCTGCTGAAAAACTATGCCG
GGCGTCCAACCGCGCTGACCAAATGCCAGAACATTACAGCCGGGACGAACACCACGCTGTATCTCAAGCGTGAAGATTTG
CTGCACGGCGGCGCGCATAAAACTAACCAGGTGCTGGGGCAGGCGTTGCTGGCGAAGCGGATGGGTAAAACCGAAATCAT
CGCCGAAACCGGTGCCGGTCAGCATGGCGTGGCGTCGGCCCTTGCCAGCGCCCTGCTCGGCCTGAAATGCCGTATTTATA
TGGGTGCCAAAGACGTTGAACGCCAGTCGCCTAACGTTTTTCGTATGCGCTTAATGGGTGCGGAAGTGATCCCGGTGCAT
AGCGGTTCCGCGACGCTGAAAGATGCCTGTAACGAGGCGCTGCGCGACTGGTCCGGTAGTTACGAAACCGCGCACTATAT
GCTGGGCACCGCAGCTGGCCCGCATCCTTATCCGACCATTGTGCGTGAGTTTCAGCGGATGATTGGCGAAGAAACCAAAG
CGCAGATTCTGGAAAGAGAAGGTCGCCTGCCGGATGCCGTTATCGCCTGTGTTGGCGGCGGTTCGAATGCCATCGGCATG
TTTGCTGATTTCATCAATGAAACCAACGTCGGCCTGATTGGTGTGGAGCCAGGTGGTCACGGTATCGAAACTGGCGAGCA
CGGCGCACCGCTAAAACATGGTCGCGTGGGTATCTATTTCGGTATGAAAGCGCCGATGATGCAAACCGAAGACGGGCAGA
TTGAAGAATCTTACTCCATCTCCGCCGGACTGGATTTCCCGTCTGTCGGCCCACAACACGCGTATCTTAACAGCACTGGA
CGCGCTGATTACGTGTCTATTACCGATGATGAAGCCCTTGAAGCCTTCAAAACGCTGTGCCTGCACGAAGGGATCATCCC
GGCGCTGGAATCCTCCCACGCCCTGGCCCATGCGTTGAAAATGATGCGCGAAAACCCGGATAAAGAGCAGCTACTGGTGG
TTAACCTTTCCGGTCGCGGCGATAAAGACATCTTCACCGTTCACGATATTTTGAAAGCACGAGGGGAAATCTGA
>Cluster_42729
ATGAGGATAGTGTATTTTGTCAATGCAGCGTGGTACTTCGAACTGCATTGGTTGGACCGGTCAGAAGCGGCAATTAGCAA
AGGGTATGAAGTTCATCTTGTAAGTAATTTTGCTGATGACGCCATCAAAAATAACCTGGAAAAAAAAGGTATAAAATGTT
GGGATATTGAGCTTAACAGATTTTCTAAAAATGTGTTTAAAAACATTTCAATATTCACTGCATTTAGAAAGATCTGTAAG
CAAATTAAACCCGATCTTATTCATCTAATTACTATAAAACCAATCCTTTTTGGTGGATTGTATGCCCGGGTTGCAGGCAT
CCCTTTTGTCGTGAGCTTTGTGGGATTAGGGAGATTGTTTGGCAATAAACGAGGTTGGTTGAATAAATTCATTTTCAACT
CAGTACTGTCAATTTATAGTCTTTTGCTATCAGCAAAAAGCCCAGCGCAAGTAATATTTGAGCATAATGCTGACTTTGCT
GAGCTGAGTAAATATATTAAATTTGATGAAAATAACATCCACATCATTGAAGGAGCCGGGGTAGACAGGTTTCGCTTTAG
TTATCAGCCAGAACCAACTCAGAATAATTTCTCGGTATTATTTGCCAGCCGTTTGCTATGGTCAAAGGGGCTCGGTGAAG
TAGTCGAGGCCATCGGTAAGGTTAAACAAAATGAAAATAACACAACCTTATACGTTGCGGGAATTCTCGATGACAAAGAT
CCGGATAGAATTGAATTAGATCAAATTTTGCAATGGGAAAAAGAAGGAAAAATCATTTGGTTAGGTCGAAGAGATGACAT
CCAAAATTTAATCAGAAATAGCCATGTCGTGATTTTGCCAACTAAATATTCAGAAGGCGTGCCAAGAATAATCATTGAAG
CGTGTGCTATGGGCCGAAGTTGCATTGTCGGAAATGTACCCGGCTGTAAAGCCATAATTAAAGATAATTTCAATGGATGT
GTACTAAAAACACATAGCGCTAATGAGATTGCGGAGAAAATTGAATATCTTAGAGATAACATGGAAATAAGAAAAAAATT
CGGTCTACGCTCAGCCGAAATTGTAAAAGAAAGATTCAGCAAAGAAATTGTTATAGATAAAACTTTGCGCCGAATAAATA
CCTGTGACGGAAGATCACTTCGCAGAATAAATAAATCCTGGTGTCCCTGTTGA
>Cluster_3307
ATGCCTGTCCGACTTAATACCATTCCCGACGCAGAATCCTATCCCGCACCGCCAGCCCGCATTAAATGGCTGTCAGCCTT
ATTACTGATGCTGTTTGTTGGTGTGGCAATAACCTCATTATTCGCCAGCGATGAACTGGCAAAAAACGGCCCGCATTTCT
GGGGGCTGGCCTGCGGTGTGCCTGCCTTTATCTGGTCGCTGGTTGCTTCAGTTCGCTGGTTGTTTTTATCACGCAGTAC
ATTCGGGCTGATGCCTGGAATAAGCGCCGGGAAGAGGTGATTTTGCAGGAAACGCGACGGGGACGACGGGCGTTGCAGAT
ACTTAGTTTTTCCGTACAAACCGCGCTGAATGGTGATTCCGTTGCAGAAACTACAACAGCATTTCTGGCGCGTCAGCAGG
TGTTGAATACTTACGCTGATTTGCGCGGAGAAGATACTGTGCGTCGCAGTGTCATTCCCGTATTAACCAATAAACCGGTT
ACCGGGCGTTTATCCGAAATTATTTCCCGCCTTTTTATTGATATCAGACCGCAATTGTTTCTGCTGCCCCCTGATTTTCA
TATCAACGTACTACTGGAAATTGATGCGCCGCTTTCAGGGGCCTCCGTGAGAACTATCTGGCAGGACGAATGGCAAAAAG
CCGGACTCCCGGAAGCCCGTCTGTTTTCTGCCCCGGAGCCGGGGCTGGCCGCCGTGGACGACTGGCTGGACAACTTTATT
CAGGAAAAGGCCGTACTGCTGGTGATTTCCGTGTATCTGGAACCGAAGACTCCGGAACGGACGGCAGAATCCTCCACCGC
GCTGACGCCGCTGGCCCTGCTGCATCGTCCTGAACGTATCACAGATACAGAGATGATGGCGTCAGGCATTGCACAGGCGC
TGGACTGGATGCCGGTGCAGCCAGATGCCATATCCGGCATATGGACTGCCGAACTGGACAGGGAACAGCGTACCGCGTTG
TTATCCCTTAACCAGCCGTTTACTCAGGAAGCCCTGATGTACGAGCTGGATGCCTTTCTGGGGCGCTCCGGACCGGCAGC
GCCGTGGCTGTCCGTCGCTGCGGCAACGCTGGCGGCAATACAATCACAACACCCCCAACTAACGCTCAGCGGCGTTCAGG
GCGGGCATTATTCATGGGCGACCGTGGTGTCGCCATTCGTTTCCCCACAGGAGGCATCATAG
>Cluster_202
GTGAAACATCTGATTAATCTTACCGATTTTGATGATAAGACCATAAAAAGAGCTTTAGAAGAAAAAAAACTCTTACAGGA
GATAACCAGAAAAGTAGAGGAGGAGTTTTATCAAGACGTTGAGTTTTTAAACCTTAGCAAGGCAGACCTTTATCCTAAAG
TAAGCCAACATCTTTCAGAAATGGCTGACCTGGCTTTCAAGGTGGTTGAGAAAGATAAAGCTTACGTAAAATATGGGAGT
GTTTATTTTGATATTTCCAGGCTTGAGGATTACGGGAAGCTTTCTAAGGTTGATCTTTCTCGTTTAAAACCTGGAGCGAC
CATAGACGTTGAAGAATATGATAAAGAAGAACCCTTTGACTTTGCCTTACTTAAAAGAGTGCATATTCTTGAGTTAAAAG
AAGGATATTTTGTAGACACACACATCGGTAAAGTCAGACCTACCTGGCATATCCACTGTGCTGCTTTAATCCTCAAATAT
TTAGGAGAGGAGATAGATTTCTTTACAAGTGGAATAGACCTTCTTTTCCCTCATCATGAAAACACCAGGGCGGTTTTAAA
GGCAATTACAGGAAAGGAAGTTGCTCGTTACTGGCTTCACACCGGGATAGTTTTTTATCAGGGGAAAAAGCTTTCTTCAG
AAAATAGGATTACTGTCGAAGACATTAAGGCAAAGGATTTAACGGAAGAGAACTAAGGTTTTACTTCTTAAGAACCCAT
TACCGAAATCCCTTTAATTTAGTTGGAAAGGACTTGAGGAAAGTGCAAAAATACTGCAAAAGATTGACCAGCATCTTTG
TTTGGTAGAGGCTTCTGCAAGAAAGGACAAAGAAGATCCCTCTACTTGGTCTATTTTAGAAAATTTTGGCCAAAGTTGGG
AAAAGGCCTTAAAAGAGGATTTAAACACCCCTATGGTTATCTCAGAACTTATCTCTTTTTTGAAAAAACTCTATCCCTAT

```
CTAAAGCAAGGTATTAGTGTTGAGTTTAAAGAAAAGGTGTTTGAAAATCTTCAAACTCTTAACAAGGTGTTAAAGGTTTT
AAGGTTTCCTAAAAAAGTGATGGATGAGGAAGTACTTAGGTTGGTGCGGTTAAGAGATGAAGCCAGAAAAGAAGGAAATT
TTGCAGAAGCAGACCAGATAAGAGAAAAATTACTTGCTAAGGGATTTTGGGTATTTGACCTTCCCTGGGGTACGGAGGTG
GTAAGTTTAGAGGTATGA
>Cluster_25147
ATGTTTAAGTCTTTTTTCCCAAAGCCGGGAACGTTTTTTCTCTCGGCCTTTGTTTGGGCATTGATTGCCGTTATCTTCTG
GCAAGCCGGTGGGGGTGATTGGGTGGCGCGTATCACCGGCGCTTCCGGGCAGATCCCGATTAGCGCCGCGCGTTTCTGGT
CGTTGGATTTCCTGATTTTTTACGCTTACTACATTGTTTGCGTAGGACTTTTTGCATTGTTCTGGTTTATCTATAGCCCG
CACCGTTGGCAATACTGGTCAATACTCGGTACTGCGCTGATTATCTTCGTCACCTGGTTTTTGGTGGAAGTCGGGGTTGC
CGTCAACGCCTGGTATGCGCCGTTCTATGATCTGATTCAAACGGCGCTAAGTTCGCCGCATAAAGTCACCATCGAACAAT
TTTACCGCGAAGTGGGCGTCTTTCTGGGGATTGCGCTGATCGCGGTGGTGATCAGTGTGCTGAACAACTTCTTTGTCAGT
CACTACGTGTTCCGCTGGCGTACGGCGATGAACGAATATTACATGGCGAACTGGCAACAACTGCGTCATATCGAAGGGGC
CGCGCAGCGTGTGCAGGAAGACACCATGCGTTTTGCTTCAACGCTGGAGAATATGGGCGTCAGCTTTATCAACGCTATCA
TGACGTTGATCGCCTTCCTGCCGGTGCTGGTAACGCTCTCCGCACATGTGCCGGAGCTGCCGATTGTCGGGCACATTCCG
TATGGTCTGGTGATTGCCGCTATCGTCTGGTCGCTGATGGGGACCGGATTACTGGCAGTGGTAGGGATCAAACTGCCGGG
GCTGGAGTTTAAAAACCAGCGTGTAGAGGCTGCCTACCGTAAAGAGCTGGTTTATGGTGAAGACGATGCCACGCGCGCGA
CGCCGCCTACGGTACGCGAGCTGTTTAGCGCCGTACGGAAAAACTATTTCCGCCTCTATTTTCACTATATGTATTTCAAT
ATCGCCCGCATTCTCTATTTGCAGGTCGATAACGTTTTCGGTTTGTTCTTGCTGTTTCCGTCAATTGTTGCCGGTACGAT
TACACTCGGCCTGATGACGCAGATTACTAACGTTTTTGGTCAGGTTCGCGGTGCTTTCCAGTACCTGATTAACTCATGGA
CCACACTGGTTGAGTTGATGTCTATCTACAAACGTCTGCGCAGCTTTGAACATGAGCTGGATGGTGACAAAATTCAGGAA
GTAACCCATACCTTGAGCTAA
>Cluster_39686
ATGCCCCGCTTCACATATATTTGCATTAATAAAATAATTGTAATTATAAGGTTAAATATCGGTAATTTGTATTTAATAAA
TACGATCGATATTGTTACTTTATTCGCCAGGTGCTCCCTTTTAATTAACTGTTTTAGCGGAGGATGCGGAAAAAATTCAA
CTCATTTGTTAATTTTTAAAATTTATTTTTATTTGGATAATCAAATATTTACTCCGTATTTGCATGAAAACCATGCGAGT
TACGGGCCTATAAGCCAGGCGAGATATGATCTATATCAATTTCTCATCTATAATGCTTTGTTAGTATCTCGTCGCCGACT
TAATAAAGAGAGAGTTAGTGTGAAAGCTGACAACCCTTTTGATCTTTTACTTCCTGCTGCAATGGCCAAAGTGGCCGAAG
AGGCAGGTGTCTATAAAGCAACGAAACATCCGCTTAAGACTTTCTATCTGGCGATTACCGCCGGTGTTTTCATCTCAATC
GCATTCGTCTTCTATATCACAGCAACCACTGGCACAGGTACAATGCCCTTCGGCATGGCAAAACTGGTTGGCGGCATTTG
CTTCTCTCTGGGGTTGATTCTTTGTGTTGTCTGCGGAGCCGATCTCTTTACTTCCACAGTGTTGATTGTTGTTGCCAAGG
CGAGTGGGCGCATCACCTGGGGTCAGTTGGCGAAAAACTGGCTAAATGTTTATTTTGGCAACCTGGTCGGCGCACTGCTG
TTTGTACTTTTAATGTGGCTTTCCGGCGAGTATATGACCGCAAATGGTCAATGGGGACTAAACGTCCTACAAACCGCCGA
CCACAAAGTGCACCATACTTTTATTGAGGCCGTCTGTCTTGGTATCCTGGCAAACCTGATGGTATGTCTGGCAGTATGGA
TGAGTTATTCTGGCCGCAGCCTGATGGACAAAGCGTTCATTATGGTGCTGCCGGTCGCGATGTTTGTTGCCAGCGGTTTT
GAGCACAGTATCGCAAACATGTTTATGATCCCGATGGGTATTGTAATCCGCGACTTCGCATCTCCGGAATTCTGGACCGC
TGTCGGTTCTGCACCGGAAAATTTTTCTCACCTGACCGTAATGAACTTCATCACTGATAACCTGATTCCGGTTACGATCG
GTAATATTATCGGCGGTGGTTTGTTGGTTGGGTTGACATACTGGGTCATTTACCTGCGTGAAAACGATCACCATTAA
>Cluster_51147
ATGATTCAACGGTCTTTAGGCGCTAGAACTTGTTTATATATTTTTCTTTTTACTGCGTTTGTTAGTTACCTAAATGCTTT
GTTAGGTGGTAAGTTTAATGGAGATTTTTCTGGAGTTAATATTAATTTAGATTCCATTCAATTATTGGGAGTCTTTATTC
TTACTTGTGCTCCATTTTTATTTCTATGGTATGTTTATTCTCTTTTTAATCGGATTAAATTTAAAGAACTAGATGCTGGA
CAGTTAGGTTTTAGAGTAAGTGTTTTTTTTAAATTGTTTGTTTTTTGGTCATTTTTTGTTACCATAAATTATGGTGTTGG
TATAATGGCCAGAAGTGAGTATAGCGTGCCAGCAGCTATCTCTTATATTATCTATTTTACGAATCGTATTGATGTTTTTT
ATTTAGGCGTTTTATTCATTCTATTGTATCAGGGGAGATTTCTTTTTTTTTGGATCTTTATTCTCTGTGTTTTGGGAGTG
ATTAGAGGAGGGATTGGGGTTTTTTTATACGTAAGTATAGCATTGATACTTCGTTATAATATTACTTTGGGTAAGTTTTT
TTTCAGAAGACCCTTGCTTTGTTTCTTGGCATTAATCATATCTCCTTTCGTTGTTGATTCATTGTTTTATATTAGAGAGC
TTCTCCGTGGAGATTACATAAATGAGCAATTTACTTTCTATCAGTTAATTATTGGAAAGTTAATAGGACGTTTTTCCTCT
TTTTCTAATTTAGGTATGATATTCCAAAATGAGGGCTATTTTTTGCAAAATATTTTTATTATGGACCAGTTTTATTTTAT
TAAGCACTTTTTTTCTGCTTTTATAGGTCAGTCGATAATTCCAGATGTTATTCCTGAGAGATTATTGATTAATATATTTG
GTGGTGATTTATTTGATAAGTCTTATATGGTGGGATTGTCTGGGAATATGTATATCTCTTCTATGATAAGTCCGACTATA
ATGATGATTAATTTGATTTTAATTTTTATATCTGTTATATGTACATTTATTGTTGCAGGATTAATAGGATTTAAATATAG
TCGAGAGTATGCCTTTGCTTTATTGTTATATCCTTGTATGAGTGGTTCTGCTCAAGAATTTGCAAAGTTGCTATTAAGCA
TGTTATTTATTATGTTAATTCTTGCTTCTTGTAACATCAAATTAAAAATTAAGAGAGGCTTTGCTAGAGGTGAGAGGGTA
TGA
>Cluster_66725
ATGAGCAAAATATCAGGTTGGAATTTTTCTCAAAACATTACATCAGCCGACAATTGTAAACAAAAAAATGAAGACTTAGA
TACCTGGTATGTGGGAATGAATGATTTTGCCCGAATTGCCGGAGGGCAGAATAGCAGAAGCAATATTCTTTCTCCCAGAG
CATTTTTGGAGTTTTTGGCTAAGATATTTACCCTGGGTTATGTGGATTTTAGCAAACGCTCCAACGAAGCGGGTAGAAAT
ATGATGGCTCATATTAAGTCCTCATCTTATATCAAAAATAATGATGGTAGTGAGATAATGAAGTTTGTTATGAATAATCC
TGAAGGGGAGCGAGCGGATTTATCTAAGGTGGAAATAGAAATTACACTTGCCTCAGCTTTTAATAATGGGATTCGTGAAG
GACATACAGTCATTATATTTACACAACCTGATGGTTCGACTAATCGTTATGAAGGGAAGTCCTTTGAAAGAAAAGATGAG
AGTTCATTACACCTGATTACTAACAAGATTCTGGCGTGTTACCAACGCGAAGCTAACAAGGAAATAGCTCGTCTATTAAA
TATTCCGCAGGAGTTAAATAATTCTCAGGACTTGAATAACTCGCAGGTGAGTTGTAAAGATTCAGTTGATTCTACGATTA
CGGATTTATTAGAAAAACCATTGAATAATGCATTATTAGCAATAAGGAAAGAACATCTGCTATTAATGCCTTATGTATGT
AATGAATCGATTTCATACTTGCTTGGTGAAAAAGGGATACTGAAAGAAATAGATGGCCTTAACGCAGTAAATAATTATCT
TCTTAATAATAAGAAAGCTACTGACAATGAAATTAATGACATCAAAGTTAACCTGTCTCATATTCTTATTGATTCCTTAG
ATGATGCAAAAGTTAACCTTACACCGGTCATCGATTCGATTCTGGAGATTTTTTTAAAATTCCCATATATTAATGATGTA
AGAATACTGGATTGGTGTTTTAATAAAAGAATGCAATATTTTGGTGATTCTGAAAAGATAAAGTATGCATGCTCCGTGAT
AAATCATATTGATTTTAGCCGCGATCAATCTAAAGATTTTAGCTGCGATCAATCTAAAATTAAAATAGCTGAGACCTTAT
TTTTCAATCTCGATAAAGAACACTATAAAATAGCCGTAAATTACAGGAATTGA
>Cluster_4618
ATGAACAGTTTAACGGCAAATAACCGTTTGTCGCAACAGCTGGTGGTCAGTGTCGCTGAACACCTGTTGTTACGGCATGA
ATGCAGATTACCAAATCACCTGGCTGTAAGTAACCACAGAGAACTTTACCTGACTGTGGGGGGCGAGTTGTGCAGGAACT
TAACCGCTGGTTTCGTGACGGAAGAGGGCTTTATGTCCATGTTATTCGTTGGGAGCCAGAAACACAGCGCGTTATCTATC
TTCGCAAAGACTACCCGCATGAGTGCTTTAGTCCTTTGTGGAAATTCAGGCGTGATTTTGTTGAGTGTGAAGGACCACCA
GCACATTGATTCTGCCATTCCGGGACGTTACACTGTTCAGGCACCTTATAAAGCGGGTGCCGGGATTGGCGTCCTGGAGA
AGGCTACCGCGTACAACCGCGTAGATGCGGTTTTTTTGTACGCATTATTTTGTCACGCCCAAATTATGGTGGGGCGTACA
GGGGCATCGCAAGATGCGCCGGTAAGGGTAGCCGCCGGTAACGCCAACTCTGTACGTCTCACCACCTCTATGATTGGCGT
CTTATGTGGTGAGTTTTTTAAGCTTGCTACCGAGGCTGCCATTATGGCTACGATCCCAACCTTTGTTCACCCTGAAATCA
```

CGATCATCAATGGTCGTGCTGTCACTACATCTCTTGCAGTTGCTAACTACTTTACTAAACGGCATGAGCGGGTTTTAGAT
AGAATTCGAAACCTTGAATGCTCCGCTGAATTTGCTGAACACAATTTTGTGTTAAGTGGTTATATCGACGCTTCAGGCCG
CAAACTACCTTGCTATCAAATAACCCGCGATGGCTTTGCGTTTCTTGCTATGGGTTTCACGGGTAAACGTGCTGCCCAGT
TCAAAGAGGCATACATCAATGCCTTTAACCAGATGGAGAAACAGCTTTCAAAGCCCTCTGTACCGAGCGACGTTGCACAT
AACGCCAGCGTTCTCTATTCCTACATTTCATCAATTCATCAGGTCTGGTTGCAGCAGCTTTATCCCATGCTGGAAAAAGC
TGAATCACCGCTGGCTGTAAGTCTGTATGACCGAATTAACGATGCGGCATTTCTTGCCCGTCTTATTCATTCGTCGCTGA
ACTCTTCAGAGGTAAGGGGGCGCAAATGA
>Cluster_40807
ATGGCTGAGATAAACAGCACTGCGCAAGTCACATCAGCGTTAACCGGCGTCAGCGATGTGCTGACACCGGTGTTCACTTT
GTGGTATCTGCAGAAAAACATCACCTCTGATATCGCGCCTTATGTCACCCGTGTGACCTGGAGCGATAACATCAAAAATG
AGTCCGATACCATTGAGGTGGAGCTGGACGACACCGATGGCCGCTGGCTGGATAAATGGTATCCGGGCAAGGGTGACACG
CTGACGCTGAAAATGGGCTATCAGGGCGAGAAGCTGCTGTCCTGCGGTACGTTCTCTATAGACGAGATCGAAGTGAGTTC
GCCCGCTTCCGTTGTTTCTATCCGTGGGGTGGCCACCTCGGTTAACAGTGCCCTGCGGACTAAAACCAGTCGTGGTTTTG
AGAACACCACGCTGGCAGCTGTTGCGGGGCGGATTGCCAGAAAGCACCGACTGAAACTGGTGGGCAGCATTGAGTCCATC
AGAATCGACCGGGTGACCCAGTATGCTGAAACCGACGTGGGTTTTCTGCGCCGGCTGGCCAGCGAGTATGGTTATGCAGT
GAAAGTGGTCAGTGACCAGCTGATTTTTTCTCATCTGGCCACACTGCGCAGTCAGGAGCCGGTCAGGCAGTTAAAACCGC
AGGATGTGGCCCGCTTTTCCCTGCGTGACACCATCAACCGGGTCTATAAATCTGCAAAGGTAAAACACCAGAAAAGCAGC
AGTAAAAAACTGATCGTCTACGAAGCTGATGGTGGTACCCGTGAAAGCGACAAAAAGCTCAAAGGTGGTAAGGTTACCAG
CGCTGACTCACTTCAAGTTAACAGCCGCGTCAGCGACCCGGACAGTGCCCGGATTAAAGCGGATTCAGCACTGGCCAGAC
ATAACGAATACCAGCAGAACGGCTCCCTGACGCTGACGGGAACACCTCAACTGACAGCAGGCAACAAAATTGAACTGGTG
GGTTTTGGGCAGTTATCCGGGCCATGGCTCATAACCACTGCCCGCCATGCGTTTGACCGTAACAGCGGCTACACCACAGA
GCTGGAAGTGGCACGGGGGCCAGTCACAAGAGGGAAAAAACAAAAAACTCAGAAACTCACGGTTTATCACCCGGATGGCA
GTACATCGACGGTGATTAAGGAGAAGAAAAAATGA
>Cluster_36201
ATGCATTCGGAAAATATTGCAGCTTACATCGGGCTGGATGTACATAAAGAAACGTTGGCGGTTGCTATTGCTGCACCTGA
ACGGCTTGGCGAAGTTCGTTATTATGGTACGATTAACAACGAAGCCCAGGCTGTACGTCGATTATTTCAGAAATTGCAGG
GACTTTACGGAAACATTCTTTCCTGCTATGAGGCTGGCCCCTGTGGTTTTGGGCTATATCATCAACTCACAGCGATGAAT
ATAAAATGCCAGGTTATTGCCCCTTCCCGGATACCAAAATCACCGACAGACAGAATCAAAAATGATCATCGGGACGCAAT
TTCTCTTGCCAGGTTACTGCGGGCTGGTGAACTTACCCCTGTCTGGATCCCTGACCTGACTCATGAAGCTATGCGGGATC
TAATCAGAGCCCGTGCAGCAGCGAAACGAGACAGCAGAGTTGCCCGACAGCGAATTCTGAGCATGCTATTGCGAACAGAC
AAACGTTATGCAGGGAAGCACTGGACCGGCAAACATCGAACCTGGTTAGCCAATCAATCATTTAGTCAGCCATCTCAACA
GATTGCTTTTCAACACTATTGTCAGAGTCTGGAGCAGATTGAAGATCGCATACTTCAACTCGACCAGGAAATATCCCGCC
TGTTACCTGAGTGGTCTCTGTGCAATCTGGTCTGCCAGTTACAGGCCCTTAAAGGTGTTGGTCAGCTAACCGCAATAACC
CTAGTTGCTGAACTGGGTGATTTTTCACGATTTTCCAGTCCCAAACAACTCATGGCTTTTCTGGGACTGGTTCCAGGGGA
ATATTCCAGTGGAAACAGCATTCGTCCCAGGGGAATAACAAAAGTTGGAAACAGCGAACTGAGACGTCTGCTTTACGAAG
CCGCCTGGTCTTATCGTACACCTGCAAAAGTTGGTGCATGGCTTATATATTACCGACCGGATTCTGTAACACAATATTCC
AAAGATATTGCATGGAAAGCTCAACAACGATTGTGTTCTCGTTACCGAAGTCTGACAGCAAAAGGGAAAAAATCACAAGT
AGCCATTACGGCGGTGGCTCGTGAGTTGACTGGATTTATGTGGGATATTGCACTTGCTGCCCAATCACCATTCAGTCAGC
AGAAGCAAAATTAA
>Cluster_22493
GTGAAAATCCTTGTTGATGAAAATATGCCTTATGCCCGCGACTTATTTAGCCGTTTGGGTGAGGTGACCGCGGTTCCCGG
GCGTCCCATCCCCGTGGCTCAACTGGCAGACGCGGATGCGCTGATGGTGCGTTCGGTCACGAAAGTGAATGAATCTTTGC
TGGCAGGAAAACCCATTAAATTTGTTGGCACTGCCACCGCGGGGACCGACCATGTCGATGAAGCATGGTTAAAGCAGGCG
GGAATTGGTTTTTCCGCTGCACCTGGCTGTAATGCGATTGCGGTAGTGGAATATGTTTTCTCCTCCCTGCTGATGCTTGC
CGAACGCGATGGATTTTCACTGCACGAGCGTACCGTGGGGATCGTGGGCGTTGGTAACGTTGGTCGTCGTTTACAGGCGC
GACTGGAAGCTTTAGGGATCAAAACCTTACTTTGCGATCCGCCCCGCGCCGACCGTGGGGATGAGGGGGATTTCCGCTCG
CTGGATGAATTAGTTCAGCATGCGGATATTCTGACTTTCCATACGCCACTCTTTAAAGATGGTCCGTACAAAACGCTGCA
TCTGGCGGATGAAAAACTGATCCGCAGCCTGAAACCGGGAGCGATCCTGATTAACGCCTGCCGTGGCGCAGTCGTCGATA
ATACCGCCCTGCTGACCTGCCTGAGCGAAGGCCAGAAGTTAAGCGTAGTGCTGGATGTCTGGGAAGGCGAACCGGAACTT
AACGTGGAATTGCTGAAAAAAGTGGATATCGGCACGCCGCATATCGCAGGCTATACCCTGGAAGGTAAAGCACGCGGTAC
TACGCAAGTGTTTGAAGCTTATAGCAAGTTTATTGGGCATGAACAGCACGTTGCGCTGGATACATTACTGCCCGCGCCAG
AGTTTGGTCGCATTACGCTGCATGGCCCGCTCGATCAACCGACGCTGAAAAGGCTGGTGCATTTGGTGTATGATGTGCGC
CGCGATGACGCACCGCTGCGTAAAGTCGCCGGGATACCGGGTGAGTTCGATAAACTGCGCAAAAACTATCTTGAGCGCCG
TGAATGGTCATCTCTGTATGTGATTTGTGATGACGCCAGTGCGGCATCATTGCTGTGTAAACTGGGTTTTAACGCCGTTC
ATCATCCGGCACGTTAA
>Cluster_59936
ATGATCTATCTTGTAATTAGTGTCTTTCTCATTACAGCATTTATCTGTTTATATCTTAAGAAGGATATATTTTATCCAGC
CGTATGCGTTAATATCATCTTCGCACTGGTCTTATTGGGATATGAAATAACGTCAGATATATATGCTTTTCAGTTAAATG
ACGCTACGTTGATTTTTCTACTTTGCAATGTTTTGACATTTACCCTGTCATGTTTATTGACGGAAAGTGTATTAGATCTA
AATATCAGAAAAGTCAATAATGCTATTTATAGCATACCATCGAAGAAAGTGCATAATGTAGGCTTGTTAGTTATTTCTTT
TTCGATGATATATATATATGCATGAGGTTAAGTAACTACCAGTTCGGGACTAGCTTACTTAGCTATATGAATTTGATAAGAG
ATGCTGATGTTGAAGACACATCAAGAAATTTCTCAGCATACATGCAGCCAATCATTCTAACTACTTTTGCTTTATTTATT
TGGTCTAAAAAATTTACTAATACAAAGGTAAGTAAAACATTTACTTTACTTGTTTTTATTGTATTCATCTTTGCAATTAT
ACTGAATACTGGTAAGCAAATTGTCTTTATGGTTATCATCTCTTATGCATTCATCGTAGGTGTTAATAGAGTAAAACATT
ATGTTTATCTTATTACAGCTGTAGGTGTTCTATTCTCCTTGTATATGCTCTTTTTACGTGGACTGCCTGGGGGGATGGCA
TATTATCTATCCATGTATTTGGTCAGCCCTATAATCGCGTTTCAGGAGTTTATTTTCAGCAAGTATCTAACTCTGCCAG
TTCTCATGTCTTTTGGTTTTTTGAAAGGCTGATGGGGCTATTAACAGGTGGAGTCTCTATGTCGTTGCATAAAGAATTTG
TGTGGGTGGGTTTGCCAACAAATGTTTATACTGCTTTTTCGGATTATGTTTATATTTCCGCGGAGCTAAGCTATTTGATG
ATGGTTATTCATGGCTGTATTTCAGGTGTTTTATGGAGATTGTCTCGAAATTACATATCTGTGAAAATATTTTATTCATA
TTTTATTTATACCTTTTCTTTCATTTTTTATCATGAAAGCTTCATGACTAATATTAGCAGTTGGATACAAATAACTCTTT
GTATCATAGTATTCTCTCAATTTCTTAAGGCCCAGAAAATAAAGTGA
>Cluster_48447
GTGAAAAACTATAAAGTTATTACCCCGCTTTTTCCTACCTATGCACAGGTTAAAGCTATGATGAAAGCCGTCTCAGGCTA
TTCCCTGAAAGCGGTACGCAATATGATTACCGCCATTCGCGATCAAACCGGTACGCCGCAAAAACCCGTTGACTGGTCGG
AACCTGACCTGTGGATCAGCGAACGCTTAACGGGCGAAGATGCCGACATCGCCCGTCGTATTTGGGATACAGATAATCAC
ATATTGAACCCGCGCCACAGCTACGGCTGCTACTTATTCCTCAACTATCCCCAGTTTGAGCTGATGGAAAGCACGCCAGA
TGATACCTGGCAGCCCACCTCGCGCGGGCAAAAATTCCTGCAAGATGACGAAAAAACGCTGCGTTCACTGGATGACCAGG
AAGGCATTCTGCAACTGCTTGAGCTGCTGGCTGGCCGAGAAATGTCGCGCCGCGCCGATTTATTGCCGGAATGGCAGGCC
TTTTTGCATCAGCATTCGAAATTCGCGTCTGCAAGTTCAGTGAAAAGCACGCTCTACTCCCGTCTCTATAACCTTATTGA

CCGTGGCATGGTTACTCGCGAAGGTATGAGCTATTGCATCACCGATACCGGACGCGCCTGGCTTGGAAAAGCGTTGCCAG
CACGAAAAAGCGATCCGCGTAAAGAGCTGCTGGAAGCGGTGAAACGCTACAACGAGCAGCAAAAAGAGCTGCTGCGCGAA
CAAATCAGCACAATGAACCCGTACAAATTTGAACAACTGGTCGGTCAATTACTGGAAGCAATGGGCTACGAGCAGGTCGA
AGTGACCAAAGCCTCAGGCGATAAAGGTGTGGACGTGATTGGTCAGGTGCAGGTCGGCATCACCACTATTACCGAAGTGG
TGCAGGTCAAAAGAATGCAGAACACCATCACTCGACCGTATATCGACCAGTTGCGCGGAGCGTTGCCTTACCACAAGGCC
ATTCGCGGTACGCTCATCACCACCGGGAAATTTGCAGCGAAATGTGCAGAAGCGGCGCTCTTTCCGGGGGCCGCGCCCAT
TACGCTTATCGACGGCGATCGCTTACTGGAACTGTTAATCGAAAATAACGTCGGCATTCGCCGCAGCAATGCAGTGGAAT
TGCTGGATGTGGATTTGCAGTTGTTTGATGAGCTGGAGATGGATTAA
>Cluster_17474
ATGCTCACCGTTAAGCAGATTGAAGCAGCAAAGCCGAAAGAAAAACCATACCGCCTACTCGATGGTAATGGCCTGTACCT
TTATGTCCCTGTATCAGGGAAAAAGGTATGGCAGCTTCGCTACAAGATTGACGGTAAGGAGAAAATCCTGACTGTCGGAA
AATATCCGCTTATGACTTTGCAGGAAGCAAGGGATAAGGCATGGACTGCGAGGAAAGACATCTCGGTTGGCATCGATCCG
GTAAAGGCGAAAAAGGCTTCGTCTAACAACAATTCATTTAGTGCGATTTACAAGGAATGGTACGAGCACAAGAAGCAAGT
CTGGTCAGTAGGCTATGCAACTGAACTTGCCAAAATGTTTGATGACGACATTTTACCCATCATCGGCGGTCTTGAGATTC
AGGATATTGAGCCGATGCAACTGCTGGAAGTAATCCGCAGATTTGAAGATCGCGGTGCAATGGAGCGAGCCAACAAAGCC
CGCAGAAGATGCGGCGAGGTTTTTCGTTACGCTATTGTCACCGGAAGGGCTAAATATAACCCGGCACCTGACCTTGCAGA
CGCCATGAAAGGATACCGCAAGAAGAACTTCCCGTTCCTTCCAGCAGACCAGATGCCTGCATTCAACAAAGCACTGGCAA
CATTTTCAGGAAGTATCGTATCGCTCATTGCGACAAAAGTTTTACGTTATACAGCCCTAAGAACGAAAGAGCTTCGTTCC
ATGCTATGGAAGAACGTCGATTTTGAAAACAGGATTATCACCATCGACGCCAGTGTGATGAAGGGACGCAAAATTCATGT
GGTCCCGATGTCAGACCAGGTGGTTGAACTTCTCACTACGCTAAGCTCAATCACCAAACCAGTCTCAGAGTTTGTTTTTG
CCGGGCGCAACGATAAGAAGAAGCCAATCTGCGAGAACGCGGTATTGCTTGTGATCAAACAAATCGGCTATGAAGGTCTG
GAAAGCGGTCACGGATTCAGGCATGAATTCAGCACGATTATGAACGAGCACGAATGGCCTGCTGACGCTATTGAAGTGCA
ACTGGCACATGCCAACGGCGGATCTGTGCGTGGGATTTACAACCATGCTCAGTATCTCGATAAACGCAGAGAAATGATGC
AGTGGTGGGCGGACTGGCTTGATGAAAAGGTAGGGTGA
>Cluster_3438
ATGTCTGTAATTAAGATGACCGATCTGGATCTTGCTGGGAAACGTGTATTTATCCGTGCGGATCTGAACGTACCAGTAAA
AGACGGGAAAGTAACCAGCGACGCGCGTATCCGTGCTTCTCTGCCGACCATCGAACTGGCCCTGAAACAAGGCGCAAAAG
TGATGGTAACTTCCCACCTGGGTCGTCCTACCGAAGGCGAGTACAACGAAGAATTCTCTCTGCTGCCGGTTGTTAACTAC
CTGAAAGACAAACTGTCTAACCCGGTTCGCCTGGTTAAAGATTACCTCGACGGCGTTGACGTTGCTGAAGGTGAACTGGT
TGTTCTGGAAAACGTTCGCTTCAACAAAGGCGAGAAGAAAGACGACGAAACCCTGTCCAAAAAATACGCTGCACTGTGTG
ACGTGTTCGTAATGGACGCATTCGGTACTGCTCACCGCGCGCAGGCTTCTACTCACGGTATCGGTAAATTCGCTGACGTT
GCGTGCGCAGGCCCGCTGCTGGCAGCTGAACTGGACGCGCTGGGTAAAGCACTGAAAGAACCTGCTCGCCCGATGGTGGC
TATCGTTGGTGGTTCTAAAGTATCTACCAAACTGACCGTTCTGGACTCCCTGTCTAAAATCGCTGACCAGCTGATTGTTG
GTGGTGGTATCGCTAACACCTTTATCGCGGCACAAGGCCACGATGTGGGTAAATCCCTGTACGAAGCTGACCTGGTTGAC
GAAGCTAAACGTCTGCTGACCACCTGCAACATCCCGGTTCCGTCTGATGTTCGCGTAGCAACCGAGTTCTCTGAAACTGC
ACCGGCTACCCTGAAATCTGTTAACGATGTGAAAGCTGACGAGCAGATCCTGGATATCGGTGATGCTTCCGCTCAGGAAC
TGGCTGAAATCCTGAAGAATGCGAAAACCATTCTGTGGAACGGTCCGGTTGGCGTGTTCGAATTCCCGAACTTCCGCAAA
GGTACTGAAATCGTGGCTAACGCTATCGCAGACAGCGAAGCGTTCTCCATCGCTGGCGGCGGCGACACTCTGGCAGCAAT
CGACCTGTTCGGCATTGCTGACAAAATCTCCTACATCTCCACTGGCGGCGGCGCATTCCTCGAATTCGTGGAAGGTAAAG
TACTGCCTGCAGTAGCGATGCTCGAAGAGCGCGCTAAGAAGTAA
>Cluster_44617
ATGAGTAGTAATATACATGGAATTAGTTGCACTGCAAATAATTATTTGAAACAAGCCTGGAACAATATAAAAAATGAGCA
CGAAAAAAATCAAAAATATTCAATCACGCTTTTTGAAAACACACTGGTGTGTTTTATGCGGTTGTATAAAGAAATCAGAC
GTCAAAAAGCTGAAGACTACATTCCATGCCTTGGAATGCGATTCACTAGAAAAAGAATTTGAGGAAATGCAGAATGATAAT
GATTTATCATTATTTTTGAGAACATTGCGTACTAATGATACAGAAACTTATTCAGGGGTTTCAGAAGGTATTACATATAC
TATACAATATGTTCGAGATATTGATATTGTTAGAGTGTCCTTGCCGGGCAGAGGTTCAGAGTCTATCACAGATTTTAAAG
GTTATTATTGGTATGGCTTTATGGAATATATTGAAAACATTAATGCGTGTGATGATGTTTTTTCTGAGTATTGTTTAGAT
GATGAAAATATGAGTATCCAGCCAGAATGGATAAATACGCCGGGAATATCTGATTTAGATACTGGTATTGATTTGTCTGG
TATATCTTTTATTCAGAGTGAAATTAATAAAACTTATGGATTAAAATACGCTCCAGTAGATGGCGATGGCTATTGTCTGT
TAAGGGCCATACTGGTTTTAAAAGAACATGAGTATTCATGGGCGCTGGGCAGCCATAAGACGCAAAAGCAAGTATACGAA
GAGTTTATTAAAATTGTTGATAAACAAACGATCGAGGCTCTTGTTGATACGGCATTCAATGATCTCAGGGAAGATGTAAA
GACATTATTCGGCGTTAATCTACAATCTGACAACAAAATTCAGGGGCAGGGTGGTTTTTTATCATGGAGTTTTTTGTCTT
TTAAAAAAGAATTTATTGATAGTTGCTTGAATGACAAAAAAATGCATACTGCATTTACCCGAATTTATATTTAATGATAAC
AAGGTACGCCTTGTTTTAGACACCGACCCAGAGCAGAGGGTTAATGAGGTGAAAAATTTCCTCACTGCTCTTTCAGATAG
CATTTGCTCATTATTTATTGTTAATAGTAATATGGCATCAATCTCCTTGGGGAATGAATCCGTTTCAACAGATGAAGATC
TTGAGTATGGTTATTTAATGAACACTGGCAATCATTATGACGTTTACCTCCCTCCTGAACTTTTTGCTCAGGCTTACAAG
TTAAACAATAAGGAAATGAATGCGCAACTCGACTATTTAAATCGTTATGCAATTTACTGA
>Cluster_20142
ATGATCAAGGCGACGGACAGAAAACTGGTAGTAGGACTGGAGATTGGTACCGCGAAGGTTGCCGCTTTAGTAGGGGAAGT
TCTGCCCGACGGTATGGTCAATATCATTGGCGTGGGTAGTTGCCCGTCGCGTGGTATGGATAAAGGTGGGGTGAACGACC
TCGAATCCGTGGTCAAGTGCGTACAACGCGCCATTGACCAGGCAGAATTGATGGCAGATTGTCAGATCTCTTCGGTATAT
CTGGCGCTTTCTGGTAAGCACATCAGCTGCCAGAATGAAATTGGTATGGTGCCTATTTCTGAAGAAGAAGTGACGCAAGA
AGATGTGGAAAACGTCGTCCATACCGCGAAATCGGTGCGTGTGCGCGATGAGCATCGTGTGCTGCATGTGATCCCGCAAG
AGTATGCGATTGACTATCAGGAAGGGATCAAGAATCCGGTAGGACTTTCGGGCGTGCGGATGCAGGCAAAAGTGCACCTG
ATCACATGTCACAACGACATGGCGAAAAACATCGTCAAAGCGGTTGAACGTTGTGGGCTGAAAGTTGACCAACTGATATT
TGCCGGTCTGGCATCAAGTTATTCGGTATTGACGGAAGATGAACGTGAACTGGGTGTCTGCGTCGTTGATATCGGTGGTG
GTACAATGGATATCGCCGTTTATACCGGTGGGGCATTGCGCCACACGAAGGTAATTCCTTATGCTGGCAATGTCGTGACC
AGTGATATCGCTTATGCCTTTGGCACACCGCCAAGTGACGCCGAAGCGATTAAAGTTCGCCACGGTTGTGCGCTGGGCTC
CATCGTTGGAAAAGATGAGAGCGTGGAAGTGCCGAGCGTAGGCGGTCGTCCGCCGCGGAGTCTGCAACGTCAGACCCTGG
CAGAGGTGATCGAGCCGCGCTATACCGAGCTGCTCAATCTGGTCAACGAAGAGATATTGCAGTTGCAGGAAAAGCTTCGC
CAACAAGGGGTTAAACATCACCTGGCGGCAGGCATTGTATTAACCGGTGGCGCAGCGCAGATCGAAGGTCTTGCAGCCTG
TGCTCAGCGCGTGTTTCATACGCAAGTGCGTATCGGCGCGCCGCTGAACATTACCGGTTTAACGGATTATGCTCAGGAGC
CGTATTATTCGACGGCGGTGGGATTGCTTCACTATGGGAAAGAGTCACATCTTAACGGTGAAGCTGAAGTAGAAAAACGT
GTTACAGCATCAGTTGGCTCGTGGATCAAGCGACTCAATAGTTGGCTGCGAAAAGAGTTTTAA
>Cluster_45290
GTGAGTCACTGGGCTACATTCAAACAAACAGCAACAAATTTATGGGTGACCCTTCGGCACGACATTCTCGCGCTGGCCGT
CTTTTTAAATGGATTGCTTATTTTTAAAACAATCTATGGTATGTCGGTCAATTTGCTTGATATTTTCCATATCAAAGCAT
TTTCAGAGTTGGATCTCTCCTTGCTGGCAAACGCCCCACTATTTATGCTCGGCGTCTTTCTTGTCCTGAACTCCATTGGC
TTACTGTTCCGGGCAAAGCTCGCATGGGCAATCAGTATCATTTTGTTGTTGATAGCGCTAATTTACACCCTGCATTTTTA

TCCCTGGCTGAAATTTAGTATTGGATTTTGCATTTTTACGCTGGTGTTTTTGCTGATACTGCGCAAAGACTTCTCCCACA
GTAGCGCCGCAGCCGGGACAATTTTTGCATTTATTAGTTTCACGACATTACTGTTTTACTCAACCTACGGTGCGCTTTAT
TTAAGCGAAGGTTTTAATCCGCGAATAGAAAGTTTGATGACCGCGTTCTATTTTTCGATAGAAACCATGTCAACCGTCGG
CTATGGCGATATTGTCCCTGTTTCTGAATCAGCACGATTGTTTCACTATTTCGGTCATTATTTCCGGCATTACCGTTTTTG
CCACATCTATGACCTCAATTTTTGGTCCGCTTATCCGCGGGGGATTCAACAAACTTGTAAAAGGAAACAATCATACAATG
CATCGTAAAGATCATTTTATTGTTTGCGGACATTCGATTCTCGCCATCAATACGATCCTGCAACTGAATCAACGCGGACA
AAACGTAACGGTTATCAGCAACTTGCCTGAAGATGATATCAAGCAACTTGAGCAACGCTTAGGCGATAACGCTGATGTTA
TCCCCGGTGACAGTAATGACAGTTCAGTATTAAAGAAAGCGGGAATCGATCGTTGCCGGGCCATTCTGGCGCTGAGTGAT
AACGATGCAGATAACGCGTTTGTTGTACTCTCGGCAAAAGATATGAGCAGTGATGTCAAAACAGTTCTCGCCGTCAGTGA
TAGCAAAAACCTGAATAAGATTAAGATGGTACATCCGGATATCATTCTCTCACCGCAACTGTTTGGCAGCGAAATTCTGG
CGCGAGTTTTAAATGGTGAAGAGATTAATAATGATATGCTCGTTTCAATGTTGTTGAACTCAGGTCATGGTATTTTCAGC
GATAACGATGAACAAGAAACGAAAGCTGACAGTAAAGAATCGGCGCAAAAATAG
>Cluster_62527
ATGAAGCCATTTTCGGGGCGCCTTGGCAGGTATGGCAGCGTGCTATTATTTCCACTGGCGATGATCATCTACGATTTTTC
AGCCTACCTGACAACGGACCTGATTCAGCCGGGCGTCATTAACATTATAAATGAGTTACAGGCGGATGTTACGCTGGCTC
CAGCATCGGTCAGTTTGTATATGGCAGGCGGACTGGCGCTACAGTGGCTCCTGGGGCCACTCTCCGACAGGATTGGCCGC
CGCCCGGTCCTGCTCATGGGTGCAATGATCTTTGCCTTAGCCTGCCTGAGCATGCTGTTTGTCACCTCGATCGAACAATA
CTTCGTCGCCCGCTTTATTCAGGGCACCAGCATCTGTTTTATCTCCACCGTTGGTTATGTATCAATACAGGAGGCCTTTG
ATGAAAAAGATTCCATCAGGATCATGGCTGCGCTGACCTCCATTGTTCTGCTCGCCCCAGTTATCGGGCCGGTGGCCGGC
GCTGCGCTGATGGCGTTCATTCACTGGAAACTGCTCTTTGCCATCATCGGTGTGTTTGGTCTGGTAGCATGGTTCTTACT
ACTGTTCAAAATGCCGGAGACAGTTATATCTCAGGGAAGATCATTCAAACCGGGTGAAGTGCTGTCAGATTTCATCGCGG
CATTTCGCCATCCAGTGGTGCTGACCGGCTCACTGGCAGTCGCTTTTGGTAACCTGCCCATCATCACCTGGGTGGCGCTC
TCCCCCGTGATCCTGATTGAGCACGGGGGGATGTCTCCCGGTACCTATGCCTGGACACAGGTGCCCGTGTTTAGTGGGAT
CATTATCGCCAGTGCGATCGTAGCTAATTTTATCAAAGACCCGACTTCACCGCGTTTTATCTGGGGTACTGTTCCGATAC
AGCTTGCTGGCTTGCTGGCTTTGCTGGTCGGCAACCTGCTATGGCCACACGTCTGGCTATGGTCAGTACTGGGAACGAGC
TTTTATGCCCTGGGTATTGGACTGCTCTATCCGGTGCTGTTTCGCTTTACGCTCTTTTCTCATAACCTGCCAAAAGGCAC
CGTTTCTGCCACGCTGAATATTCTGGCGCTCGGCATAATTGCCCTTAGCATTGAAATAACCCGCTGGGTTTACTTCCATG
CTGGCGGCAGGATTACTTTCCACTGCGTGGCGCTGCTGGCCGGTATAGCCGTCATCATATCAGTTGCCCGCCTGCTTAAA
CTGCGTGGGCAGCATCAGAGCCAGTCAGTATCACCGGTAGGCTGCCAGAAGCAATAA
>Cluster_41746
ATGAGCAGCAATAAAGAGTTAATGCAGCGCCGCAGTCAGGCGATTCCCCGTGGCGTTGGGCAAATTCACCCGATTTTCGC
TGACCGCGCGGAAAACTGCCGGGTGTGGGACGTTGAAGGCCGTGAGTATCTTGATTTCGCGGGCGGGATTGCGGTGCTCA
ATACCGGGCACCTGCATCCGAAAGTGGTTGCGGCGGTGGAAGCGCAGTTGAAAAAACTGTCGCACACCTGCTTCCAGGTG
CTGGCTTACGAGCCGTATCTTGAGCTGTGCGAGATTATGAATCAGAAGGTGCCGGGCGATTTTGCCAAGAAAACTCTGCT
GGTTACGACCGGTTCCGAAGCGGTGGAAAATGCGGTGAAAATCGCCCGCGCCGCCACCAAACGTAGCGGCACCATCGCCT
TTAGCGGTGCGTATCACGGGCGCACGCATTACACGCTGGCACTGACCGGCAAGGTGAATCCGTACTCTGCGGGCATGGGG
CTGATGCCGGGTCATGTTTATCGCGCGCTTTATCCTTGCCCGCTGCACGGCATAAGCGAGGATGACGCTATCGCCAGCAT
CCACCGGATCTTCAAAAATGACGCTGCGCCGGAAGATATCGCCGCCATCGTGATTGAGCCGGTTCAGGGCGAAGGCGGTT
TCTACGCCGCGACGCCAGCCTTTATGCAGCGTTTACGCGCGCTGTGTGACGAGCACGGGATCATGATGATTGCCGATGAA
GTGCAGAGCGGCGCAGGGCGTACCGGCACGCTGTTTGCGATGGAGCAAATGGGCGTGGCACCAGATCTCACCACCTTTGC
GAAATCGATCGCAGGCGGTTTCCCGCTGGCGGGCGTCACCGGGCGCGCGGAAGTGATGGACGCCGTCGCACCGGGCGGAC
TGGGTGGCACCTATGCCGGTAACCCGATTGCCTGCGTAGCGGCGCTGGAAGTGTTGAAGGTGTTCGAGCAGGAAAATCTG
CTGCAAAAAGCCAACGTTCTGGGGCAGAAGCTGAAAGACGGATTGCTGGCGATCGCCGAAAAACACCCGGAGATCGGCGA
CGTACGCGGGCTGGGGGCGATGATCGCCATCGAGCTGTTTGAAGATAGCGATCACAGCAAACCGGACGCAAAACTCACCG
CCGAGATCGTGGCACGCGCCCGCGATAAGGGTCTGATTCTTCTCTCCTGCGGCCCGTATTACAACGTGCTGCGCATCCTT
GTACCGCTCACCATTGAAGACGCTCAGATCCGTCAGGGTCTGGAGATCATCAGCCAGTGTTTTGCTGAGGCGAAGCTGTA
G
>Cluster_25220
ATGAAGATAGCAACGATAAAAACTGGGCTTACTTCACTGGCGATGCTGCCGGGACTGGTAATGGCTGCACCTGCGGTGGC
CGATAAAGCCGACAATGCGTTTATGATGATTTGTACTGCGCTGGTGCTGTTTATGACCGTTCCGGGGATTGCCCTGTTTT
ATGGAGGGCTGATTCGTGGCAAAAACGTGCTGTCGATGCTGACGCAGGTGACCGTGACGTTTGCACTGGTCTGTATTCTC
TGGGTGGTTTACGGCTACTCGCTGGCGTTTGGCGAAGGCAACAACTTCTTCGGCAACATTAACGGGTTGATGCTGAAAAA
CATTGAACTGACGGCGGTGATGGGCAGCATTTATCAGTATATCCACGTGGCGTTTCAGGGATCGTTTGCCTGCATTACCG
TAGGCTTGATAGTTGGGGCGCTGGCGGAACGAATCCGCTTCTCAGCTGTGTTGATTTTTGTGGTGGTATGGCTGACGCTC
TCTTACATTCCGATTGCGCATATGGTCTGGGGCGGCGGTTTGCTGGCTTCTCACGGGGCACTGGATTTCGCGGGTGGCAC
GGTGGTGCATATTAACGCCGCAATCGCCGGTCTGGTGGGCGCGTATCTGATAGGCAAACGCGTGGGCTTCGGTAAAGAGG
CGTTTAAACCGCACAACCTGCCGATGGTCTTCACCGGGACTGCTATTCTCTATATCGGTTGGTTTGGCTTTAACGCCGGG
TCAGCGGGCACGGCGAATGAAATCGCGGCACTGGCATTTGTGAATACTGTGGTCGCAACTGCGGCGGCAATTCTTGGCTG
GATCTTCGGTGAATGGGCGCTGCGTGGTAAGCCTTCACTGCTGGGAGCGTGTTCTGGCGCGATTGCCGGTCTGGTCGGCG
TGACGCCAGCCTGTGGTTACATTGGGGTTGGCGGCGCGTTGATTATCGGCGTGGTAGCTGGTCTGGCGGGCTTGTGGGGC
GTTACCATGCTCAAACGCTTGCTGCGGGTGGATGATCCCTGCGATGTCTTCGGTGTGCACGGCGTTTGTGGTATTGTCGG
CTGTATCATGACCGGGATTTTTGCCGCCAGCTCGCTGGGCGGCGTGGGCTTCGCTGAAGGTGTGACGATGGGCCATCAGT
TGCTGGTACAGCTGGAAAGCATCGCCATTACGATTGTCTGGTCTGGTGTGGTGGCATTTATCGGCTACAAACTGGCGGAT
TTGACGGTTGGTCTGCGTGTACCGGAAGAGCAGGAGCGAGAAGGGCTGGACGTCAACAGCCACGGCGAGAATGCCTATAA
CGCGTAA
>Cluster_59550
ATGGCAAGCAGTAACTTGATTAAACAATTGCAAGAGCGGGGGCTGGTAGCCCAGGTGACGGACGAGGAAGCGTTAGCAGA
GCGACTGGCGCAAGGCCCGATCGCGCTCTATTGCGGCTTCGATCCTACCGCTGACAGCTTGCATTTGGGGCATCTTGTTC
CATTGTTATGCCTGAAACGCTTCCAGCAGGCGGGCCACAAGCCGGTTGCGCTGGTAGGCGGCGCGACGGGTCTGATTGGC
GACCCGAGCTTCAAAGCTGCCGAGCGTAAGCTGAACACCGAAGAAACTGTTCAGGAGTGGGTGGACAAAATCCGTAAGCA
GGTTGCCCCGTTCCTCGATTTCGACTGTGGAGAAAACTCTGCTATCGCGGCGAACAACTATGACTGGTTCGGCAATATGA
ATGTGCTGACCTTCCTGCGCGATATTGGCAAACACTTCTCCGTTAACCAGATGATCAACAAAGAAGCGGTTAAGCAGCGT
CTCAACCGTGAAGATCAGGGGATTTCGTTCACTGAGTTTTCCTACAACCTGTTGCAGGGTTATGACTTCGCCTGTCTGAA
CAAACAGTACGGTGTGGTGCTGCAAATTGGTGGTTCTGACCAGTGGGGTAACATCACTTCTGGTATCGACCTGACCCGTC
GTCTGCATCAGAATCAGGTGTTTGGCCTGACCGTTCCGCTGATCACTAAAGCAGATGGCACCAAATTTGGTAAAACTGAA
GGCGGCGCAGTCTGGTTGGATCCGAAGAAAACCAGCCCGTACAAATTCTACCAGTTCTGGATCAACACTGCGGATGCCGA
CGTTTACCGCTTCCTGAAGTTCTTCACCTTTATGAGCATTGAAGAGATCAACGCCCTGGAAGAAGAAGATAAAAACAGCG
GTAAAGCACCGCGCGCCCAGTATGTACTGGCGGAGCAGGTGACTCGTCTGGTTCACGGTGAAGAAGGTTTACAGGCGGCA
AAACGTATTACCGAATGCCTGTTCAGCGGTTCTTTGAGTGCGCTGAGTGAAGCGGACTTCGAACAGCTGGCGCAGGACGG

CGTACCGATGGTTGAGATGGAAAAGGGCGCAGACCTGATGCAGGCACTGGTCGATTCTGAACTGCAACCTTCCCGTGGTC
AGGCACGTAAAACTATCGCCTCCAATGCCATCACCATTAACGGTGAAAAACAGTCCGATCCTGAATACTTCTTTAAAGAA
GAAGATCGTCTGTTTGGTCGTTTTACCTTACTGCGTCGCGGTAAAAAGAATTACTGTCTGATTTGCTGGAAATAA
>Cluster_24441
ATGAATAAAATAAAGAATTATCGATGGCATATGATTGCCCTCGTATGCTTTATCACTGTAATCAATTATCTGGACAGAAC
GGCGTTAGGTATTGCGGCTCCAACGATTATGGAGACAACCGGAATAACCAAAGAACAATATTCATGGATTGTCAGTGCAT
TCCAGTTGGCCTATACATTGGGGCAACCGATAATGGGGTTCTTTATTGATACCGTGGGTCTGAAGTTAAGTTTTGCGATA
TGTGCCGCAATCTGGGGCCTGGCGACAATGGGGCATGCCTTGACAGGAACATGGTCAGGTCTGGCATTTATGCGCGCCCT
GATGGGTTTCAGCGAAGCATCGGCAATTCCTGCGGGTGTAAAAACCGCATCAACATGGTTCCCGGCAAAAGAGCGTGGCG
TGGCGACAGGTGTTTTCAATATGGGCACCTCACTCGGCGCGATGCTTGCTCCACCGTTGATTGCCTGGTGCATTATGTTT
CATAGCTGGCAATTTGCGTTTATTGTCTCAGGTAGCCTTGCTTTACTCGCGGCTTTATTTTGGTTCTTTTGTTATAAAGA
TCCAAAAGATGCTAAACGCCTTTCTGATGAAGAGCGCCACTATATTGAGTCTGGGCAAGAACAGCATCTTAAAACAGATA
AGAAAGAAAAAACGTCAATCAAGCATATCCTCAGCCAACGTAATTTCTGGGGGATTGGCATCGCGCGTTTTCTCGCTGAC
CCGGCATGGGGAACCATTAACTTCTGGGTACCGATTTTCTTCGTCGAAACGCTGCATTTTAGCCTGAAAGAAATTGCCAT
GTTCGTCTGGCTACCTTTCCTGCTGGGCGATCTCGGCTGTTTAGCCAGTGGTTTTGTCGCGAAGTTCTTCCACGATCGCG
GCGTGAGTTTAATTAACTCACGAAGAATTACCTTCACTATTGCAGCCGTCATTATGATGACGATTGGTCTGGTGAGTATT
GTCGAAAATCCCTACATCGCCGTATTACTGATTAGCATTGGCGCATTCTCGCATCAATGTCTTTCTACTGTTGCAGCAAC
TCTGGGCGGTGACCTATTCAAAAAAGACGAAGTAGCTACCGCTGTGGGTATGGCAGGAGCCTGTGCATGGAGCGGTCAGT
TGATTTTCAACCTGTTTATCGGGGCATTCGTTCACATTATTGGCTTCGCGCCGTTCTTTATTGCCCTGGCTTTCTTTAAC
ATTATTGGCGCCATTGCGCTGTGGATGCTTATCAAAGTTAAAGACGAAGAACCACAAGTACAGTTAGCGACAAGCTAA
>Cluster_22342
ATGGACATAATGAGAAGTGTTGTGGGGATGGTGGTGTTACTGGCAATAGCATTTTTGTTGTCAGTGAATAAAAAGAGCAT
CAGTTTGCGCACGGTTGGAGCCGCGTTGCTGCTACAAATCGCCATTGGCGGAATCATGCTCTATTTTCCACCAGGAAAAT
GGGCGGTAGAACAGGCGGCATTAGGCGTTCATAAAGTGATGTCTTACAGTGATGCCGGTAGCGCCTTCATTTTTGGTTCA
TTGGTCGGGCCGAAAATGGATGTCCTGTTTGATGGCGCGGGCTTTATCTTCGCCTTTCGCGTACTCCCGGCGATTATCTT
CGTCACTGCGCTCATCAGCCTGCTGTACTACATTGGCGTGATGGGGCTGCTAATCCGCATTCTTGGTAGTATTTTTCAGA
AAGCCCTCAACATCAGCAAAATCGAATCTTTTGTTGCGGTTACCACTATTTTCCTCGGGCAAAATGAGATCCCAGCGATC
GTTAAACCGTTTATCGATCGCATGAATCGCAACGAGCTATTTACGGCAATTTGTAGCGGGATGGCGTCCATTGCCGGTTC
GATGATGATTGGTTATGCCGGAATGGGCGTACCAATTGACTACCTGTTAGCGGCATCGCTGATGGCGATCCCTGGTGGTA
TTTTGTTTGCACGTATTCTTAGCCCAGCCACCGAGCCTTCGCAGGTGACATTTGAAAATCTGTCGTTCAGCGAAACACCG
CCAAAAAGCATTATTGAAGCTGCGGCGAACGGTGCAATGACCGGGCTGAAAATTGCAGCCGGTGTGGCTACGGTGGTGAT
GGCGTTTGTCGCAATTATTGCGCTGATCAACGGCATTATCGGCGGAATTGGCGGCTGGTTTGGTTTTGCCAATGCCTCTC
TGGAAAGTATTTTTGGCTATGTGTTGGCACCACTGGCGTGGATCATGGGTGTGGACTGGAGTGATGCCAACCTTGCGGGT
AGCCTGATTGGGCAGAAACTGGCGATTAACGAATTTGTCGCTTACCTGAGTTTTTCGCCATATCTGCAAACGGGCGGCAC
GCTGGAAGTGAAAACCGTGGCGATCATCTCCTTTGCGCTGTGTGGTTTTGCTAACTTTGGTTCTATCGGTGTTGTCGTTG
GGGCGTTTTCGGCTATTTCGCCAAAACGTGCTCCGGAAATCGCCCAGCTTGGTTTACGGGCGCTGGCGGCGGCGACACTT
TCTAACCTGATGAGCGCGACCATTGCCGGGTTCTTTATTGGTTTAGCTTGA
>Cluster_30102
ATGAAAAAAATCGCGCTTATTATCGGCAGCATGATCGCGGGCGGTATTATTTCTGCGGCAGGTTTTACCTGGTTTGCAAA
GGAGGAACCGCCCGCAGAAAAAACGTCGACCGCAGAACGTAAAGTCTTATTCTGGTACGACCCGATGTATCCCAATACGC
GGTTCGATAAACCAGGGAAATCGCCGTTTATGGATATGGATCTGGTGCCGAAATATGCCGATGAAGAGAGTTCTGCGTCT
GGTGTGCGCATTGACCCGACTCAGACGCAGAATCTGGGGGTGAAAACGGCGACCGTCACGCGCGGGCCGCTGACTTTTTC
CCAGAGTTTCCCGGCGAATGTCAGTTACAACGAGTATCAGTATGCCATTGTGCAGGCCCGCGCCGCCGGGTTTATCGACA
AGGTGTATCCGCTTACCGTGGGCGATAAAGTGCAAAAGGGCGCGCCGCTTCTCGACCTGACCATTCCCGACTGGGTGGAA
GCGCAGAGTGAGTATTTACTGCTGCGCGAAACCGGCGGTACGGCGACCCAGACTGAAGGCATTCTTGAGCGACTGCGACT
GGCGGGAATGCCGGAGGCTGATATTCGCCGCCTGATCGCCACGCAAAAAATCCAGACTCGCTTTACGCTCAAAGCGCCCA
TTGATGGCGTGATCACCGCGTTTGATCTGCGCGCGGGAATGAATATCGCCAAAGATAACGTGGTCGCGAAAATTCAGGGT
ATGGACCCGGTGTGGGTCACTGCTGCGATCCCGGAGTCCATCGCCTGGCTGGTGAAAGATGCCTCGCAGTTTACGCTCAC
CGTTCCGGCGCGACCTGATAAAACACTCACCATCCGCAAATGGACGCTGCTACCTGGCGTGGATGCTGCGACCCGCACGC
TGCAGTTGCGTCTGGAAATCGACAACGCCGACGAGGCGCTAAAACCGGGCATGTACGCCTGGTTGCAACTCAATACCGCC
AGCGAACCGATGCTGCTCATTCCGTCACAGGCGCTGATTGATACCGGCAACGAACAGCGGGTGATTACCGTTGATGCTGA
CGGGCGCTTTGTACCGAAACGCGTTGCCGTTTCCAGGCATCGCAAGGCGTCACCGCGTTACGCTCTGGTCTGGCGGAAG
GTGAAAAGGTGGTTTCCAGCGGCCTGTTCCTGATTGATTCTGAAGCCAATATTTCTGGCGCACTGGAGCGGATGCGCTCT
GAAAGTGCTACCCATGCGCATTGA
>Cluster_43123
ATGAAAACAACAGCTCTACGTCTTTATGGTAAACGTGATTTACGCCTGGAAACCTTTGACCTTCCTGAAATGCAGGAGGA
TGAAATCCTCGCAACGGTGGTCACTGACAGCCTGTGCCTCTCTTCCTGGAAAGAGGCCAATCTGGGTGAAAACCATAAAA
AAGTACCCGACGATGTGGCGACCAATCCCATCATCATCGGCCACGAATTTTGCGGCGATATTCTGGCCGTGGGTAAAAAG
TGGCAGCACAAATTCCAGCCGGGTCAGCGTTATGTGATTCAGGCCAACCTGCAACTCCCCGACCGCCCGGATTGCCCCGG
CTACTCCTTCCCGTGGGTAGGCGGCGAAGCGACGCATGTGGTTATTCCCAACGAGGTCATGGAACATGATTGCCTGCTGG
CATACGACGGCGAAACCTATTTTGAAGGCTCGCTGGTTGAACCGCTTTCCTGCGTGATTGGCGCGTTCAACGCCAACTAT
CATCTTCAGGAAGGTAGTTATAACCACACGATGGGGATTCGCCCGCAAGGGCGCACGCTGATACTCGGCGGCACCGGCCC
GATGGGGCTGCTGGCGATTGATTATGCGCTACATGGACCCGTTAACCCGTCGCTGCTCGTCATTACCGATACCGACAACG
ACAAATTGAGTTATGCGCGCAAGCACTATCCATCAGAACCGCAAACACTGATTCATTATCTCAATGCCGCCGATGCAGCA
TTTGATACGTTAATGGCACTGAGTGGCGGTCACGGCTTCGATGATATTTTCGTCTTTGTGCCTAATGAAGGACTGGTGAC
TCTCGCCTCTTCCTTGCTGGCGACAGATGGTTGTCTGAATTTCTTCGCCGGACCGCAGGATAAGCACTTCAGCGCGCCAA
TTAATTTCTACGATGTGCATTATGCATTTACCCACTACGTGGGCACGTCAGGCGGCAATACCGACGACATGCGCGCAGCG
GTCAAATTGATTGAAGAGAAAAAAGTGCAGGCCGCAAAAGTGGTAACACATATTCTTGGGCTGAATGCCGCGGGCGAAAC
TACGCTTGAATTGCCTGCCGTCGGCGGCGGCAAAAAGCTGGTGTATACCGGGAAATTCCTGCCGCTGACGTCACTCACGC
AGATTCAGGATCAAGCACTGGCGGCGATTCTGGCGCGTCATCAGGGGATCTGGTCCGGTGAGGCGGAGCAATACCTGCTC
GCCCATGCAGAGGCAATTTCCCATGATTAA
>Cluster_23134
ATGGCACTGTTTTACTATCAGGCGCTGGAGCGTAATGGTCGCAAAACCAAAGGTATGATTGAGGCGGATTCCGCGCGTCA
TGCCCGCCAGTTGTTGCGCGGTAAAGATCTCATTCCCGTGCACATTGAAGCCCGGCTGAATGCTTCGGCAGGGGGAATGT
TGCAGCGTCGGCGACACGCACATCGTCGCGTGGCGGCGGCAGATCTGGCGCTCTTCACGCGCCAACTGGCAACGCTGGTA
CAGGCAGCAATGCCGCTGGAAACCTGCTTACAGGCGGTCAGTGAGCAAAGTGAAAAACTGCATGTGAAAAGTCTCGGAAT
GGCGCTGCGCAGCCGGATTCAGGAAGGTTACACCCTGTCGGACAGCCTGCGCGAACATCCCCGCGTCTTTGATTCCCTGT
TTTGTTCGATGGTCGCTGCCGGAGAAAAATCCGGACATCTCGACGTGGTGCTCAATCGCCTGGCGGATTACACCGAACAG
CGGCAGCGTCTGAAATCACGCCTGCTGCAGGCCATGCTCTACCCGCTGGTACTGCTGGTGGTGGCAACGGGCGTGGTCAC

TATTTTGCTGACGGCAGTAGTGCCGAAAATTATCGAACAGTTTGATCATCTCGGACACGCGCTGCCTGCCTCCACCCGAA
TGCTCATCGCTATGAGCGACGCGTTACAGGCCAGCGGCGTGTACTGGCTGGCGGGTTTGCTGGGGCTTCTGGTGCTGGGG
CAACGGCTACTCAAAAATCCTGCGATGCGCCTGCGCTGGGATAAAACCTTGCTGCGTTTGCCCGTGACGGGGCGTGTTGC
GCGCGGACTGAATACGGCGCGTTTTTCCCGCACATTAAGCATCCTCACCGCCAGCAGTGTTCCGCTGCTGGAAGGCATTC
AGACCGCCGCCGCCGTGTCGGCAAATCGTTATGTCGAGCAACAACTGCTGCTGGCGGCAGATCGCGTCCGCGAAGGAAGC
AGCCTGCGCGCCGCGCTGGCGGATCTGCGCCTGTTCCCGCCGATGATGCTGTACATGATCGCCTCCGGCGAACAGAGCGG
CGAGCTGGAAACCATGCTTGAACAGGCCGCGGTCAACCAGGAACGGGAATTTGATACCCAGGTGGGGCTGGCGTTAGGGC
TGTTTGAGCCGGCGCTGGTGGTGATGATGGCGGGCGTGGTGCTGTTTATCGTCATCGCCATCCTCGAGCCGATGCTGCAA
CTGAACAATATGGTTGGAATGTAA
>Cluster_13843
ATGTCAATTGACTTTACCCCAGGTATAATAAATACATATCACGGCGATATTTATAACTGCACAACAAATACCGATAATGT
TAAAACGCCAGACACACCGAAGTGGCCTTGTGATAATTGGGAAGAACAACAACCCATTAATTCCACATTCTCTGGAGAAG
GATACAACCCTGAACAATTTGATTTAGCGCAACAACAACTCCAACAAATTAATGCATGCCACACGAACACCACATATACA
AATGCAGACTACTCAAAAGTTGTGGTTCAACTTGTAAGTCTTATTAATAACATTGAAACGATAAGTTCGACACCACTTAC
GCAACAGACACAATCAATACTTAATCAGATTCATAACATTAGATACGAAAAGGATAAGGATTCTGTATGTCAAATCATTG
TTGTCGTTAATCCAGAACCCAACAATCCAATCATAACTAAAATTCTCGTAGAAGAAGGAATACCCGAAATATTCAGTGTC
CAAACCGTTTCTTCCGATAATAAAAATTTTGCTGGGCAACGAGCTGACTTACCAACTGATATAAGAGACATTCAAAGCTT
GTACTTAAAAATGGCGAAGCTGTATATTGAGCACAGTGAAAATGAAAATAGGATGGAAGCCCTTGCTGGATGCGACTTTA
TTGATTTCAACATGACAGGGCAAGATATGTCCAGGCTAGTCCTAACCTTATCAAATTTTTATTTTGAAGATCTACTTAAT
ATAAATTTCACTGACGCAAATTTATTTAACACTATTTTTTCACACAAGGAAAATCCCATCCCCAAACTACATAAATATGA
ACAGCATCTTGACAAACAAATCAATGGCTTGTTTTCTACATTGTTAACTATTAATGACAACTCTTTACGCGCGAAAGCGG
AAATTGCTTCGAGAATAATTGATTTCTTAGAGGCAAAGGTAGTGAATCTTTCATTTGATGACATATTAAAATATAAGCAA
GAGTTCAAAAAAATATGTTATAAACAACTTCAAGAGTTTACAACACCCTCTCTATACAATAAAATTCAAAAATGGGCAAC
AATGTCTAAAAATGAATTCATAGCATTTCATTACGAGACACTTCAACCCGAAAAGATTTCATACCCCTTTTATTTAAAGA
GAGATCTGCCTAATGAAAAAGATATAAATTATGGGATGGAAATAGAAATACCATCTGGCAAAAGAATTCGCTTATCAAAT
CATTATCAGAATATAATACCATAA
>Cluster_41469
GTGAGTAATAATGACACACTGCCAAACATAATAGACGGACAAACACCTCTGTTGTTCTCTACACGCCCTTTTATTCCTAC
CCAAAAAGCGTTGCATGGTATTCGTTTTGATTTTAATGACGGAGCCCGCGTTAAACTTGGCCCTGGTCAGTGGCAGGTAA
ATTTAATTGATGACGACTCCGGCAATATATTACTGACCTGTGAGACTGATCAAGGCTGTGTGATGAGTAATAAAAAGTAC
TTCATCAACTTTCGCATCCAGGTATTTAAAGACGGTGAAACGACACCTGTGCTGGACGTAGTTCAGGATCTGCGTGATAA
AACTGTCATTATCCACTTTCCCATCGGCACTTTGGAGGAGCTCCTTGGTTGGTTTCCCTATGCTGAACGTTTCCGCCAGA
AGCATCAGTGTCAGTTGGAATGTGTGATGACAGAGGAAATCATTGAACTGCTACGCGACCAGTATCCGCAAATCACTTTT
TCCACTCGTGAAAGCATTCAAACCCACAGCCCCTACGCCAGCTACCGCATCGGACTGTTTTTCAAAGGCAATGATACTCA
TCAACCGGTGGATTTCCGCCAGGTCGGCTTTAGTCGTATCGCTGCTTATATCCTGGGAATTTTGCCGCATGAAGAGCCGC
CGTTACTGAAACTTGATGCTGTGCGCAGCATTGACGAACCCTACGTTTGTATCGCCGTTCAGTCTTCCTGCCAAGCGAAA
TACTGGAACAACGGCTATGGCTGGTCGCAGGTAATTTCTGAACTGAAAACGCTGGGCTACCGAGTGCTGTGCATCGATAA
AGAGGCGCACCATGGTTATGGTTTTGTCTGGAATCACATCCCGAACGGCGCGGAGGATTTCACTGGCGCGCTGCCGCTAC
AGCAACGGGTGGATTTGCTTCGCCATGCGGCCTTTTTTGTTGGCCTGAGCAGCGGCTTGAGTTGGTTAGCGTGGAGTTGT
CACATCCCGGTGGTGTTGATTAGTGGGTTTACCAATCCTCTAAGTGAGTTCTATACACCGTGGCGCGTATTCAATTCTTA
TGGCTGCCATGGTTGCTGGGACGATATGCGCATCGATTTTGATCACCAGGATTTTCTGCAATGTCCCAGGCACAAAGGTA
CTGAGCGGCAGTATGAGTGCTCCCGGCTGATCACCGGTCAGCAGGTCATCCGCATTATTCATCAATTGCATCACAGCCTG
AGCGAGGAACACGCGCTCTTAATCTTAAAGGACTAA
>Cluster_61031
ATGGCAACACTAACCACCACCCAAACGTCACCGTCGCTGCTTGGCGGCGTGGTGATTATCGGCGGCACCATTATTGGCGC
AGGGATGTTTTCTCTGCCAGTGGTCATGTCCGGGGCGTGGTTTTTCTGGTCAATGGCGGCGCTGATCTTTACCTGGTTCT
GTATGCTGCATTCCGGCTTGATGATTCTGGAAGCTAACCTGAATTACAGAATCGGTTCGAGTTTTGACACCATCACCAAA
GATTTGCTGGGCAAAGGCTGGAACGTGGTCAACGGCATTTCCATTGCCTTTGTGCTCTATATCCTGACCTATGCCTATAT
TTCTGCCAGTGGTTCGATTCTGCATCACACCTTCGCAGAGATGTCACTAAACGTCCCGGCACGGGCGGCGGGTTTTGGTT
TTGCATTGCTGGTAGCGTTTGTGGTGTGGTTGAGCACTAAAGCCGTCAGTCGCATGACAGCGATTGTGCTGGGGGCGAAA
GTCATTACCTTCTTCCTCACCTTTGGTAGCCTGCTGGGGCATGTGCAGCCTGCGACATTGTTCAACGTCGCCGAAAGCAA
TGCGTCTTATGCACCGTATCTGTTGATGACCCTGCCGTTCTGTCTGGCATCGTTTGGTTATCACGGTAACGTGCCAAGCC
TGATGAAGTATTACGGCAAAGATCCGAAAACCATCGTGAAATGTCTGGTGTACGGTACGCTGATGGCGCTGGCGCTGTAT
ACCATCTGGTTGCTGGCGACGATGGGTAACATCCCGCGTCCGGAGTTTATCGGTATTGCAGAGAAGGGCGGTAATATTGA
TGTGCTGGTACAGGCGTTAAGCGGCGTACTGAACAGCCGTAGTCTGGATCTGCTGCTGGTCGTGTTCTCAAACTTTGCGG
TAGCGAGTTCGTTCCTCGGCGTAACGCTGGGTTTGTTTGACTATCTGGCAGATCTGTTTGGTTTCGACGACTCGGCTGTG
GGCCGCTTGAAAACGGCATTGCTGACCTTTGCCCCGCCAGTTGTGGGGGGGCTGTTGTTCCCGAACGGATTCCTGTACGC
CATTGGTTATGCTGGTTAGCGGCTACCATCTGGGCGGCAATTGTTCCGGCGCTGTTAGCCCGTGCATCGCGTAAACGCT
TTGGCAGCCCGAAATTCCGCGTCTGGGGTGGCAAGCCGATGATTGCGCTGATTCTGGTGTTTGGCGTCGGCAACGCACTG
GTGCATATTTTATCGAGCTTTAATTTACTGCCGGTGTATCAGTAA
>Cluster_11020
ATGAGTGATACGCTTTTAACCGAAAAAATCCTTACCGGAGAAAATGTTTTGCGTGCTGCAATCGCCCGCATTGAGTGGAT
TTTTGAAACATTTCCGTCGGTATGTCTGTCTTTTTCAGGTGGAAAGGATTCCACCGTGCTTTTCCATCTTGTGGCAGAGG
TGGCCCGCAGGAGAAAACGTCATTTCTCTGTTCTGTTCATTGACTGGGAGGCCCAGTATCGGTGCACCATTGAACATATT
CAGAAGATGCGGGAAATGTACCATGATGTGACGGAAACCTTTTACTGGGTGGCACTTCCCCTGACCACGGTAAACGGCGT
CTCGCAGTTTCAGCCGGAGTGGATATGCTGGGAGCCAGGAGTGACCTGGGTTCGCCAGCCACCGGAAGAGGCCATTACGG
ATATGACATATTTTCCCTTTTTCCGGTATGCCATGACGTTTGAAGAATTTGTTCCGGCATTTTCTTCCTGGTTTGCCGGT
AACCGGTGTGGAGTGGCAGTGCTGACCGGTGTTCGTGCGGATGAATCCCTCAATCGCTTTATGGGGCTGGTGTCTCAGCG
CAAACTGAGATATGCCGATGATAAGCCCTGGACCACGGCATCGCCTGAAGGGTTTTATTACACCATGTATCCGTTGTATG
ACTGGAAAGCCCGCGATATCTGGATATATAACGCCAGAGCTTGTGCCATTTACAATCCTCTGTATGACCTGATGTACCGT
GCCGACGTGCCGTTACGCAACATGCGTGTCTGTGAACCTTTTGGTCCTGAACAACGTAAGGGGCTGTGGCTTTACCATGT
TCTGGAGCCGGAAACCTGGGCCAGGATGTGTGAGCGGGTGTCAGGCGCTGCCAGCGGGGCGCTTTATGCCAATGAAAGCG
GTGCCTATTTTGCCCTGCGTAAACGTATATCAAAACCAGCCCATCATACCTGGCGCAGCTATGCGATGTTCCTGCTGGAT
GTGATGCCGGAAAGAACGGCAGAACATTACCGTAATAAAATTGCTGTCTACCTGCGCTGGTATCAGACGCGGGGCTTCCC
GGATGACATCCCGGATGAACAGGAGAATGACCTGGGGAGCCGGGATATTCCGTCCTGGCGGCGTATCTGTAAGACACTTA
TAAAGAATGATTTCTGGTACCGGACCCTCTCCTTCAGTCCGAACAAACCCCGGCACTATGAACGTTATCTGCAGCGTATG
AAAGAAAGGAGGAAGGAATGGGGGATTCTGTGA
>Cluster_36949
ATGGCAAAGGTATCGCTGGAGAAAGACAAGATTAAGTTTCTGCTGGTAGAAGGCGTGCACCAAAAGGCGCTGGAAAGCCT

TCGTGCAGCTGGTTACACCAACATCGAATTTCACAAAGGCGCGCTGGATGATGAACAATTAAAAGAATCCATCCGCGATG
CCCACTTCATCGGCCTGCGATCCCGTACCCATCTGACTGAAGACGTGATCAACGCCGCAGAAAAACTGGTCGCTATTGGC
TGTTTCTGTATCGGAACAAACCAGGTTGATCTGGATGCGGCGGCAAAGCGCGGGATCCCGGTATTTAACGCACCGTTCTC
AAATACGCGCTCTGTTGCGGAGCTGGTGATTGGCGAACTGCTGCTATTGCGCGGCGTGCCGGAAGCCAATGCTAAAG
CGCACCGTGGCGTGTGGAACAAACTGGCGGCGGGTTCTTTTGAAGCGCGCGGCAAAAAGCTGGGTATCATCGGCTACGGT
CATATTGGTACGCAATTGGGCATTCTGGCTGAATCGCTGGGAATGTATGTTTACTTTTATGATATTGAAAATAAACTGCC
GCTGGGCAACGCCACTCAGGTACAGCATCTTTCTGACCTGCTGAATATGAGCGATGTGGTGAGTCTGCATGTACCAGAGA
ATCCGTCCACCAAAAATATGATGGGCGCGAAAGAAATTTCACTAATGAAGCCCGGCTCGCTGCTGATTAATGCTTCGCGC
GGTACTGTGGTGGATATTCCGGCGCTGTGTGATGCGCTGGCGAGCAAACATCTGGCGGGGGCGGCAATCGACGTATTCCC
GACGGAACCGGCGACCAATAGCGATCCATTTACCTCTCCGCTGTGTGAATTCGACAACGTCCTTCTGACGCCACACATTG
GCGGTTCGACTCAGGAAGCGCAGGAGAATATCGGCCTGGAAGTTGCGGGTAAATTGATCAAGTATTCTGACAATGGCTCA
ACGCTCTCTGCGGTGAACTTCCCGGAAGTCTCGCTGCCACTGCACGGTGGGCGTCGTCTGATGCACATCCACGAAAACCG
TCCGGGCGTGCTAACTGCGCTGAACAAAATCTTCGCCGAGCAGGGCGTCAACATCGCCGCGCAATATCTGCAAACTTCCG
CCCAGATGGGTTATGTGGTTATTGATATTGAAGCCGACGAAGACGTTGCCGAAAAAGCGCTGCAGGCAATGAAAGCTATT
CCGGGTACCATTCGCGCCCGTCTGCTGTACTAA
>Cluster_41982
ATGTCTAAGATTTTTGATTTCGTAAAACCTGGCGTAATCACTGGTGATGACGTACAGAAAGTTTTCCAGGTAGCAAAAGA
AAACAACTTCGCACTGCCAGCAGTAAACTGCGTCGGTACTGACTCCATCAACGCCGTGCTGGAAACCGCTGCTAAAGTTA
AAGCGCCGGTTATCGTTCAGTTCTCCAACGGTGGTGCTTCCTTTATCGCTGGTAAAGGCGTGAAATCTGACGTTCCGCAG
GGTGCTGCTATCCTGGGCGCGATCTCTGGTGCGCATCACGTTCACCAGATGGCTGAACATTATGGTGTTCCGGTTATCCT
GCACACCGACCACTGCGCGAAGAAACTGCTGCCGTGGATCGACGGTCTGCTGGACGCGGGTGAAAAACACTTCGCAGCTA
CCGGCAAGCCGCTGTTCTCTTCTCACATGATCGACCTGTCTGAAGAATCTCTGCAAGAGAACATCGAAATCTGCTCTAAA
TACCTGGAGCGCATGTCCAAAATCGGCATGACTCTGGAAATCGAACTGGGTTGCACCGGTGGTGAAGAAGACGGCGTGGA
CAACAGCCACATGGACGCTTCTGCACTGTACACCCAGCCGGAAGACGTTGATTATGCGTATACCGAGCTGAGCAAAATCA
GCCCGCGTTTCACCATCGCTGCCTCCTTCGGTAACGTACACGGTGTTTACAAGCCGGGTAACGTGGTTCTGACTCCGACC
ATCCTGCGCGACTCTCAGGAATATGTTTCTAAGAAACACAACCTGCCGCACAACAGCCTGAACTTCGTATTCCACGGTGG
TTCCGGTTCTACTGCTCAGGAAATCAAAGACTCCGTAAGCTACGGCGTAGTGAAAATGAACATCGATACCGATACCCAAT
GGGCAACCTGGGAAGGTGTTCTGAACTACTACAAAGAAAACGAAGCTTACCTGCAAGGCCAGCTGGGCAACCCGAAAGGC
GAAGATCAGCCGAACAAGAAATACTACGATCCGCGCGTATGGCTGCGTGCCGGTCAGACTTCGATGATCGCTCGTCTGGA
AAAAGCATTCCAGGAACTGAACGCGATCGACGTTCTGTAA
>Cluster_35855
ATGGTAAACGTGAGGGAGGATGAACTTTTCAAACTCATCGAGCAAGGCATCAAAGGCAACGCAAATGCATTTACACTCCT
ATGCAGAAAGATGATTAACAACATCCGTAAAAATGATGAGGCTTTAGCTTCTAAGCTGGCTTCGTTAGTTGCTGAGGGAA
CAGTATTACGTGGGGCATCTAGTAAGGCTCCTATGCCTGTGGATGGAGACTCCAGACGAAACCTTTTGCAGGAAACATCT
GTCAACGCAATTGTTGAAGAACCTGTATGGAATACGGATATCTCAAAGAAGCTGGAGTCAATTGTTAACGAAAGAGAGAA
TGCGGTTTCTTTGTTCAAAGCTGGTCTTGAACCTGTGAAGACGGTTTTACTTTCTGGCCCGCCAGGCGTAGGCAAAACAA
TGTCCGCACATTGGCTTGCTGCAAAGTTAAATTTACCTCTTTTAACATTGGATCTCTCATCTGTAATGAGTTCTCTTTTG
GGAAAAACAGGTAATAATATTAAATCCGTTATGGATTATGCCAAAGAGAAACCATGTGTCCTTTTGCTTGATGAATTTGA
TGCTGTTGCAAAAAGACGTGATGATGACAGGGACGTTGGAGAATTAAAACGTTTAGTTACTGTACTCCTACAGACCATAG
ATGAGTGGCCAGCGACCTCGCTTCTTGTTGCTGCGACTAATCACCCTGATATCTTAGATCCGGCTGTTTGGCGTCGATTT
GAGCACATCCTTAAATTTAATATGCCATCAGTAGAACTGATAGATAGATATTTAGTCAATCATAATATTGAACCTGAACT
ATCTAAAAAACTTGCACCATTATTAGATGGCATGTCCTTTGCCATCATTAACCGTATTTTGAATTTCTCCAAAAAGAATG
AGGTACTTAGAAATATTCCATTTGAAAGCTCATTAATAGAAGCCGTAATTACGGAGAGGGTTTCATTGGATGAGTTTAGC
GATAATGATCTCAATATAATTAAATATCATTTTGACGGTTTTTCAAATAGGAAGATAGCTGAATTAGTTGGTGTTTCACA
TCCAACAATCGCGAATAAATTACAAAAGTGGGGGATTAAATGA
>Cluster_19903
ATGAAAAAATTGATCAATGATGTGCAAGACGTACTGGACGAACAACTGGCAGGACTAGCGAAGGCGCATCCATCGCTGAC
ACTGCATCAGGATCCGGTGTATGTCACCCGAGCTGATGCCCCCGTTGCAGGGAAAGTCGCCCTGCTGTCGGGTGGCGGCA
GCGGACACGAGCCGATGCACTGTGGCTATATCGGTCAGGGGATGCTTTCGGGAGCCTGTCCGGGCGAAATTTTCACCTCA
CCGACGCCCGATAAAATCTTTGAATGCGCCATGCAAATTGATGGCGGCGAAGGTGTACTGTTGATTATCAAAAATTACAC
CGGCGATATTCTTAACTTCGAAACAGCAACCGAGTTACTGCACGATAGCGGCGTAAAAGTGACCACTGTGGTCATTGATG
ACGACGTTGCAGTAAAAGACAGTCTTTATACCGCCGGGCGGCGCGGCGTTGCCAACACCGTATTAATTGAAAAACTCGTA
GGTGCAGCGGCGGAGCGTGGCGACTCACTGGACGCCTGTGCGGAACTGGGGCGTAAGTTGAATAATCAAGGCCATTCAAT
AGGTATCGCTCTCGGTGCCTGTACCGTTCCTGCCGCGGGCAAACCTTCTTTTACCCTGGCGGATAATGAGATGGAGTTTG
GCGTCGGCATTCATGGTGAGCCAGGTATTGACCGCCGCCCCTTCTCTTCCCTTGATCAAACCGTCGATGAAATGTTCGAC
ACCCTGCTGGAAAATGGCTCATACCATCGCACTTTGCGTTTCTGGGATTATCAACAAGGCAGCTGGCAGGAAGAACCACA
AACCAAACAACCGCTCCAGTCTGGCGATCGGGTGATTGCGCTGGTTAACAATCTTGGCGCAACTCCGCTTTCTGAGCTGT
ACGGCGTCTATAACCGCCTGACCACACGTTGCCAGCAAGCGGGATTGACTATCGAACGTAATTTAATTGGCGCGTACTGC
ACCTCACTGGATATGACCGGTTTCTCAATCACCTTACTGAAAGTTGATGACGAAACGCTGGCACTCTGGGACGCCCCTGT
CCACACCCCGGCCCTTAACTGGGGTAAATAA
>Cluster_20859
ATGCGCCAGGAAACCCGCTTTAAATTTAATGCCTACCTGTCCCGTGTTGCCGAACTGAACGGCATCGACGCCGGTGATGT
GTCGAAAAAATTCACCGTTGAACCGTCGGTCACCCAGACCCTGATGAACACCATGCAGGAGTCCTCTGACTTTCTGACCC
GCATCAATATTGTGCCGGTCAGCGAAATGAAAGGGGAAAAAATTGGCATCGGTGTCACCGGCTCCATCGCCAGCACCACC
GACACCGCCGGTGGCACCGAGCGTCAGCCGAAGGACTTCTCGAAGCTGGCGTCTAACAAGTACGAATGCGACCAGATTAA
CTTCGATTTTTATATCCGCTACAAAACGCTGGACCTGTGGGCGCGTTATCAGGATTTCCAGCTCCGTGTCCGTAACGCCA
TTATCAAACGCCAGTCCCTTGATTTCATCATGGCCGGTTTTAACGGCGTGAAGCGTGCCGAAACCTCTGACCGCAGCAGC
AATCCGATGCTGCAGGATGTGGCGGTCGGCTGGCTGCAGAAATACCGCAATGAAGCCCCGGCGCGCGTGATGAGCAAGGT
CACTGACGAGGAAGGCCGCACCACCTCTGAGGTTATCCGCGTGGGCAAGGGCGGTGATTATGCCAGCCTCGATGCACTGG
TGATGGATGCGACCAACAACCTGATTGAACCGTGGTATCAGGAAGACCCTGACCTTGTGGTGATTGTGGGGCGTCAGCTA
CTGGCGGACAAGTATTTCCCCATCGTCAACAAGGAGCAGGACAACAGCGAAATGCTGGCCGCTGACGTCATCATCAGCCA
GAAACGCATCGGTAACCTGCCGGCGGTACGCGTCCCGTACTTCCCGGCGGATGCGATGCTCATCACGAAGCTGAAAAACC
TGTCCATCTACTACATGGATGACAGCCATCGCCGCGTGATTGTGGAAAACCCGAAACTCGACCGCGTGGAGAACTACGAG
TCAATGAACATTGATTACGTGGTGGAAGACTACGCCGCCGGTTGTCTGGTGGAAAAAATCAAGGTCGGTGACTTCTCCAC
ACCGGCTAAAGCGACCGCAGAGCCGGGAGCGTAA
>Cluster_1034
ATGGATAGCGTCCACCATATTTGGTGTCCACTATTCTCTCAGGAATTTCAGGATCTGCCAGACGGTGCTGAGACGACGCT
TACTCTGGCTCAGGGGCTGTGGGAGCTGATAGACAACAGCCAGCTTGCGGATGAGATGGCGGAGATGAAGACCACCATCA
CCGAAACCCGCAATGAAATCACACAGACGGTCAGTAAAACGCTGGAAGACCAGAGCGCCACCATTCAGCAGATACAGCGC

GTGCAGAAGGACACAAATGATGACCTTGCTGCACTTTACATGCTGAAGGTACAGAAAACAAAAAATGGCATACCCTATGT
TGCCGGTATTGGAGCGGGGATTGAGGATACTGATGGCCAGCCCCTGAGCAACATACTGCTGCTGGCTGACCGTATTGCGA
TGATTAACCCGGAGGACGGCAACACCACGCCGTTATTTGTGGCGCAGGGGAATCAGTTGTTCATGAACGATGTGTTCCTG
AAGCGGCTGTTTGCGGTGAGTATCACCTCGTCCGGCAATCCCCCGACGTTTTCCCTGACGCCGGAAGGGAAGCTGACAGC
CAGGAACGCGGATATCAGCGGAGCAATTACCGCGAATACCGGCACGCTCAATAATGTCACCATTAACGAGAACTGTGTCA
TCAGAGGGAAACTGTCTGCAAACCAGATTGAAGGCGACCTGGTGAAGACGGTGGGGAAAGCCTTTCCCCGGAATAACAGT
TATGCCAGCGGGACGGTAACCGTCACAGTTTACGATGACCAGGGCTTCGACCGGCAGATTATCATTCCCCCGGTGCTGTT
TCGCGGGACGAAACACCAGAATTTCAACAGCCCGAATCAGCAGTCGTACTGGTATTCCACCTGTAAGCTGCAGGTGCTGA
AGAACGGGGTTGAGATTTTCCATGAACCGGCAACGGATGTCAGCCGGGTGTTCTCATCGGTGATAGATATGCCGGCAGGG
CGGGGTCATGTCACCCTGACGTTTAATGTGTCGTCGGCCGGTGCGAACAACTGGACGCCGACAACGTACATCAGTGATTT
ACTGGTTGTGGTCATGAAAAAATCCACGGCAGGGATCAGTATCAGCTGA
>Cluster_4936
ATGAATATACTATTTACGGAATCATCACCGAATATAGGTGGTCAGGAATTACAGGCTGTTGCTCAAATGAAGGCCCTGAA
GAAAATGGGGCATTCAGTTCTGCTTGTCTGCAGGGAGAACAGCAAAATTGCTTTTGAAGCCAGTAAGTTTGGAATTGATA
TCACATTCGCGTTATTTCGAAACAGTCTTCACATTCCTACTGTATGGAGATTACTCGGAATAGTTCATAGTTTTCAGCCC
GATGCAATCGTTTGTCACAGTGGACATGATAGTAATATTGTTGGTTTGGTACGGTTTTTTACCTGGAAACATCCATTCAG
AATTATCAGGCAAAAGACATATTTGACACGAAAAACAAAATTTTTTTCAATAAATCATTTTTGCGATGAGGTGATTGTTC
CCGGAACAAATATGAAGACACATCTGGAGCAGGAAGGATGTCGAACTCGGGTTACTGTTGTGCCTCCTGGCTTTGATTTC
CAGGAATTATACGTTGATTCCCGAAACAGTTTGCCTCCGAGTGTTCTTTCCTGGCTGGCGTCCCGAAGAGGATGCCCCGT
AATTGCTCAGGTAGGAATGTTGCGCCCGGAAAAAGGGCACGAATTTATGTTGAATTTACTGTTCCATTTAAAAATGAATG
GACGACAGTTCTGTTGGTTGATTGTGGGGGCTGGTTCACCTGAACTGCGGGAACATTTACAGTATCAGATTGACAGTATG
GGCATGCATGATGATGTTTTTATTGCTGACAATGTTTTTCCTGCCGCCCCCGTATATCGGGTTGCCAGTCTGGTGGTTCT
GCCTTCAGAAAACGAATCTTTTGGTATGGTGCTGGCAGAAGCATCGGCATTTTCTGTGCCTGTAGTGGCCACTCAGATTG
GTGGAATCCCTGAGGTTATTCAGAACAACCAGACCGGGACATTGTTACCAGCAGGTAATAAGCACGCATGGATGTGCGCC
CTGAATGATTTTTTTAATGACCCTGGGCGTTTTTATCAGATGGCTCGCCTGGCAAAACAGGATATAGAAGAGCGGTTTGA
TATTAATAAAACTGCGTTAAAAATACTCACATTAGCGAAGCAAAAGTAA
>Cluster_37378
ATGAAGACGTTTCCTCTGCAAAGCCTGACGATTATTGAGGCGCAGCAAAAGCAGTTTGCGCTGGTGGATAGCATTTGTCG
CCATTTCCCCGGCAGCGAGTTTCTTACTGGCGGTGATTTAGGCTTAACGCCAGGACTGAATCAACCGCGCGTTACCCAGC
GTGTGGAACAGGTGCTGGCTGATGCATTTCACGCACAGGCTGCGGCGCTGGTGCAGGGCGCGGGGACTGGCGCGATTCGC
GCCGGGCTGGCGGCTTTGCTCAAACCGGGGCAGCGTCTTCTGGTGCATGACGCGCCTGTTTACCCGACGACACGGGTTAT
TATTGAGCAGATGGGGCTGACGCTTATTACTGTTGATTTCAATGACCTGTCGGCACTGAAGCAGGTCGTCGACGAGCAAC
AACCGGATGCGGCGCTGGTGCAGCATACGCGCCAGCAGCCGCAGGACAGCTACGTGCTGGCAGATGTGCTGGCAACGTTG
CGCGCGGCAGGTGTTCCAGCGTTAACCGATGACAACTATGCGGTGATGAAGGTGGCTCGAATCGGCTGTGAATGCGGCGC
GAATGTCTCGACATTTTCCTGCTTCAAGCTATTTGGGCCAGAGGGTGTTGGTGCAGTGGTCGGCGATGCTGATGTTATCA
ACCGTATTCGCGCCACGCTTTACTCCGGCGGTAGCCAGATCCAGGGCGCACAGGCGCTGGAAGTATTGCGTGGTCTGGTG
TTTGCGCCAGTGATGCACGCGGTGCAGGCAGGGGTATCTGAACGGTTGCTGGCTTTGCTTAACGGTGGTGCGGTGCCGGA
AGTGAAAAGCGCGGTGATTGCTAATGCGCAGTCGAAGGTGTTGATTGTCGAGTTTCATCAGCCGATTGCCGCCAGAGTGC
TGGAAGAGGCGCAAAAGCGCGGTGCCTTGCCTTACCCGGTGGGTGCAGAGTCGAAATATGAAATCCCGCCGCTCTTTTAT
CGCCTTTCCGGAACGTTTCGCCAGGCGAATCCACAATCAGAACATTGTGCGATTCGCATTAACCCGAATCGCAGCGGTGA
AGAGACGGTGCTGCGGATTTTGCGTGAGAGTATTGCCAGTATTTAA
>Cluster_51157
ATGCTACCACTAGTAAAAGTTTCATTGCCAGAATCCAATATATTAATGCCTTCACTCGAAAAAGTTCTTTATATAGTGGTAT
GATTGCTGAAGGAGATAGCGTATATACTTTCGAAAGGCTATTTTCTGAAAAGTTTGGTTTACAAAATGGAATTGCAATGA
GCAGTGGAACTGCAGCTTTGCATACAGCACTTGTTCTTTCTGAGGTTGGTATCTCTGACGAAGTCATCACCACAAGTATG
ACCGCTGAGCCAACTAATACGACTATTTTGCAAACTGGAGCAAAGCCTATTTTTGCGGATGTCGACGTTGAGAATGGTAA
TATATTAGTTAGTTCTATAGAGGAAAAAATTACAGAAAAAACAAAAGCAATATTAGTAGTACATTATGCAGGTTATCCTG
TTGATATAAAGAGAATCAGAAAACTAGCAGATAAACATAATCTGTTACTGATAGAAGATTGTGCTCATGCTTTAGGAGCT
ACATTTGAAAATAAATATGTAGGGACGTTTGGTGATTTTTCAATTTTTTCGTTTCAAGCTATTAAACATTTAACAACGAT
TGATGGTGGTTTTTATCCATGAAAGATGGAAAAAACTACGAATAGCAAAACAAGTAAGATGGTTTGGTTTAGAAAAAG
GAGTTGATAGAACACAGTCTAATATAACTCGACAGGGTTATAAATATAATATGAATAATGTTGCAGCAGAAATAGGTATA
TCTCAATTAAAAACAATAGATATTAGACTCAATAAATATAAAGAAAATGGAAAATATTACAATGAAAATATAGCTAAAAT
CTCAGGGTTAAAAAGCGCAAAAATATTAACAGGAGCAGAGCCATCTTTTTGGCTGTATACGCTTCTATGTGAAGAGCCTG
ATAATATTATTGAGTGTTTAAAAAAAATTGGGGTTATGGCTTCGAAGTTACATCGACCAAATCATTTGCATCAAATTTTC
GGGAATGGTGTTCAAAAATTACCTTATCTTGATGAGTTTTATAAGCAGATGGTTCATATACCTTGTGGGTGGTGGGTTCG
AGAAGAAGATCGTGAGAATATTATAAATTGCTTGTCTCTAGGTTAG
>Cluster_11037
ATGAGTACAGAAATGAAAACGGGGCTGGTGCTGTCCGGTGGCGGGGCGGTGGGCGCTTATCAGGCGGGAGTGGTTAAGGC
ACTGGCAGAGTGTGGTACACAGATCAGCATGGTTTCAGGGACCAGCATTGGCGCATTCAATGGTGCCATTATCGCGGCCT
CTCCCGATCTGTCAGAAGCTGCCGTACGCCTGGAGGCGCTCTGGGAGCATCTGGGGAATAATCAGGTGCTGTCGGTAAAC
AGATTGGTTTACTTTTCATTGCTGAAAAAATTGTTCCAGGCAATGAACCTCTGCCAGATCCCCGGACGTGCAGGAGCACT
GCTTACGACGCTTCTTCGCCATATATCGACAATCAACGGGTTTGACAATCTGATGGCTCAGCCGTTGTTGTCAGATGAGC
CCCTGACAGCGCTGATGGATCATTATCTTGATACTGATGCTCTGGCAGACGGGCTACCGCTGTATGTGTCGCTGTACCCC
ACAGAAGGGGGCATGCAGGATATTATTGACTGCATTCGTGCTGAACTGGGTGTCGGAACCACGAAAAACGCCGTTTTTCA
GCATATCCAGAGCCTGCCCCGCGGACAGCAGAAAGAGGCTCTGCTTGCGTCAGCCGCGCTGCCCCTGCTGTTCCGTCCCC
GTGAGGTTCAGGGGACAATGTTCGGTGATGGTGGTATGGGAGGATGGCGAAATATGCAGGGAAATACCCCTGTGACGCCT
CTGGTCGATGCCGGATGCAATATGGTGATTGTGACGCATCTGAGTGACGGTTCTTTATGGGATCGCCAGGCTTTTCCGGA
CACCACAATCCTTGAGATCCGTCCCCGGAAAAGGCTGAAATATGCAGGTGATGGTGGCAACAGCGGCGGTCTGCTCAGTT
TTACATCGGCACATACCGACGCCTGGCGTCAGCAGGGCTATGAAGACACGATGCTGGCGATGGAGCATATCCGGAAACCG
CTGGCAGCACGTCAGGCACTGACCCGGTCAGAGGCGGTATTGCAGAAAAGCCTGGATATAACGGAAGAGGCAGATTTGGC
ACTGAGAAACGCGATGGCCCGGATTAAATAA
>Cluster_22329
ATGATCACCATTCGTGATGTAGCGCGTCAGGCTGGCGTCTCTGTGGCAACGGTTTCCCGGGTGCTCAATAACAGCACGCT
GGTCAGTGCCGATACGCGTGAAGCAGTAATGAAAGCCGTGAGTGAGCTGGATTATCGGCCAAACGCCAATGCCCAGGCGC
TGGCAACTCAGGTTAGCGATACCATTGGCGTGGTGGTGATGGACGTTTCTGATGCGTTTTTCGGCGCGCTGGTAAAAGCG
GTGGATCTGGTCGCCCAGCAGCATCAGAAATACGTGCTAATCGGCAATAGCTATCATGAAGCGGAAAAAGAGCGTCACGC
CATTGAGGTGTTAATTCGCCAGCGTTGTAATGCGTTGATTGTTCACTCAAAAGCATTGAGTGACGATGAACTGGCACAAT
TTATGGATAACATTCCCGGTATGGTGTTAATCAACCGCGTTGTGCCGGGGTACGCCCATCGTTGCGTTTGCCTGGATAAT
CTCAGCGGTGCCCGAATGGCGACGCGCATGTTGCTGAATAACGGTCATCAACGTATTGGTTATCTTTCTTCCAGCCACGG

CATTGAAGATGACGCCATGCGTAAAGCAGGCTGGATGAGTGCGTTGAAAGAGCAGGATATTATTCCGCCGGAAAGCTGGA
TTGGCACTGGCACGCCGGACATGCCGGGCGGTGAGGCGGCGATGGTTGAACTGCTGGGGCGCAACCTACAACTAACCGCT
GTATTTGCTTATAACGACAATATGGCCGCTGGCGCACTGACAGCATTAAAAGATAATGGCATTGCGATTCCGTTACATCT
CTCAATCATTGGTTTCGATGATATTCCCATCGCCCGTTACACCGACCCGCAATTAACGACTGTGCGTTATCCCATTGCTT
CAATGGCTAAATTAGCTACCGAACTGGCCTTGCAGGGGGCAGCAGACAATATTGATCCTCGTGCCAGCCACTGTTTTATG
CCGACGTTAGTGCGTCGCCATTCTGTCGCAACACGCCAGAATGCGGCGGCGATCACTAACTCAACAAATCAGGCGATGTA
A
>Cluster_60288
ATGGATTTATCGCTATTAAAAGCGTTGAGCGAGGCAGATGCGATCGCCTCCTCGGAACAGGAAGTGCGGCAGATCCTGCT
GGAAGAAGCGGATCGCCTGCAAAAAGAAGTGCGATTTGATGGTCTGGGATCGGTGCTGATCCGCCTGAATGAATCGACAG
GTCCGAAGGTGATGATCTGTGCGCATATGGACGAAGTGGGATTTATGGTGCGCAGCATCTCCCGCGAAGGGGCGATTGAT
GTGCTGCCGGTTGGCAACGTACGCATGGCTGCCCGCCAGCTGCAGCCGGTGCGCATCACCACCCGTGAAGAGTGCAAAAT
TCCAGGCCTGCTTGACGGCGACCGGCAGGGGAATGACGTCAGCGCCATGCGCGTGGACATTGGTGCGCGCTCCTATGACG
AAGTGATGCAGGCGGGAATTCGTCCCGGCGATCGCGTCACGTTTGATACCACTTTTCAGGTTCTCCCTCACCAGCGAGTG
ATGGGGAAAGCCTTTGATGACCGCCTCGGTTGCTATCTGCTGGTGACGTTACTGCGCGAACTGCACGACGCCGAACTACC
TGCGGAAGTGTGGCTGGTGGCAAGTTCCAGCGAAGAGGTGGGATTACGCGGCGGGCAAACTGCCACCCGCGCGGTGTCGC
CGGACGTCGCCATTGTGCTTGATACCGCCTGCTGGGCGAAAAACTTTGATTATGGCGCGGCTAACCATCGCCAGATTGGT
AACGGGCCGATGCTGGTGTTAAGCGACAAGTCGCTGATTGCGCCGCCAAAACTTACCGCCTGGGTCGAAACCGTGGCGGC
AGAAATTGGCGTGCCGTTGCAGGCAGATATGTTCAGCAACGGCGGCACGGACGGCGGGGCGGTGCACTTAACCGGCACCG
GCGTGCCCACAGTGGTGATGGGGCCAGCAACCCGCCATGGACATTGCGCCGCATCGATTGCCGATTGCCGCGACATTTTG
CAGATGCAGCAACTTTTATCTGCCCTTATTCAACGTCTTACGCGTGAGACGGTTGTTCAACTGACGGATTTCAGATGA
>Cluster_14125
ATGAAAGTTATGCGTACCACCGTCGCAACTGTTGTCGCCGCGACCTTATCGATGAGCGCTTTCTCTGTGTTTGCAGAAGC
AAGCCTGACAGGTGCAGGTGCAACCTTCCCTGCGCCGGTGTATGCCAAATGGGCTGACACTTACCAGAAAGAAACCGGTA
ATAAAGTTAACTACCAGGGTATCGGTTCTTCCGGTGGCGTAAAACAGATTATCGCTAATACCGTTGATTTTGGTGCCTCT
GACGCGCCGCTGTCTGACGAAAAACTGGCTCAGGAAGGTCTGTTCCAGTTCCCGACCGTGATTGGCGGCGTGGTGCTGGC
GGTTAACATTCCAGGGCTGAAGTCTGGCGAACTGGTGCTGGATGGTAAAACCCTCGGCGACATCTACCTGGGCAAAATCA
AGAAGTGGGATGATGAAGCCATCGCCAAACTGAATCCGGGTCTGAAACTGCCTTCACAAAACATTGCTGTAGTACGCCGC
GCAGATGGCTCCGGGACTTCCTTCGTCTTCACCAGCTACCTGGCGAAAGTGAACGAAGAGTGGAAAAACAACGTTGGTAC
TGGCTCTACCGTAAAATGGCCGATCGGTCTGGGCGGTAAAGGTAACGACGGTATCGCCGCGTTCGTTCAGCGTCTGCCGG
GTGCAATTGGTTATGTTGAATATGCTTACGCGAAGCAGAACAACCTGGCGTACACCAAACTGATCTCCGCTGATGGTAAA
CCGGTTAGTCCGACCGAAGAAAACTTCGCTAATGCAGCAAAAGGTGCAGACTGGAGCAAAACCTTCGCTCAGGATCTGAC
CAACCAGAAAGGCGAAGATGCATGGCCTATTACCTCTACCACGTTCATTCTGATCCACAAAGATCAGAAGAAACCAGAAC
AAGGCACAGAAGTGCTGAAATTCTTCGACTGGGCGTACAAAACCGGGGCTAAACAGGCGAACGACCTGGATTACGCCAGC
CTGCCGGATAGTGTAGTTGAACAGGTTCGCGCTGCGTGGAAGACCAATATTAAAGACAGTAGCGGTAAGCCGCTGTACTA
A
>Cluster_36836
ATGACAACATTTATTCAGCTTCATTTGTTAACCGCTTACCCTGCTGCCAACCTTAACCGTGATGATACCGGTGCGCCAAA
AACTGTGGTGCTGGGTGGAGCAACGCGTCTGCGCGTTTCCTCGCAAAGTCTGAAACGTGCGTGGCGCACTTCTGCACTTT
TTGAACAGGCACTGGCGGGCCATATTGGTATTCGCAGTGGGCGTATTGCGCGTGAGGCGGCAACTATCCTGATTGAGAAA
GGAATCGAAGAGAAAAAAGCCATCGAATGGGCGGCAAAAATTGCAGATTATCTTGGGAAAGCTAAAAACGACAAAAAACC
AAAAGATCCGCTCACTAACGCCGAAACTGAACAGTTAGTCCATATCAGCCCGGCAGAATTTGACGCCGTAAAAGTGCTGG
CCCATCAGCTTGCAGAAGAAAAGCGCGCGCCAAAAGAGGAAGATCTCGCGTTGTTACGCAAAGACCGTATGGCGGTAGAT
ATTGCCATGTTTGGTCGTATGCTGGCGAATAAACCCGAGTTTAATGTTGAAGCCGCCTGCCAGGTAGCGCACGCATTTGG
TGTCAGTGAAACGATTGTCGAAGATGATTTTTTCACCGCCGTTGATGATTTGCGCCAGGCTTCTGAAGATGCCGGTGCCG
GGCATCTTGGTGAAACCGGATTCGGTTCTGCACTGTTCTACACCTATATCTGCATTGATAAAGATCTGCTGGTCGAAAAC
CTTGGCGGTGACGAAGCGTTAGCTAATCAGACCATACGTGCCTTTACGGAAGCCGCACTTAAAGTCTCCCCAACCGGCAA
ACAGAACAGCTTTGCCAGCCGTGCCTACGCCTCCTGGGCGCTGGCAGAAAAAGGCACCGATCAACCGCGTTCTCTGGCGG
CAGCTTTCTATGAACCCATTAATGGCACCCGTCAGTTAGAGGTGGCGGTGCAGCGTATTACAACTCTTCGCGAAAATATG
AATACGGTCTACGAACAGAAGACCGATTACGCAAGTTTTGACGTGATGAACAAACAGGGAAGCATGAAGGACGTGCTGGA
CTTTATCTGCGCGTAA
>Cluster_58677
ATGCTGGAACTGAATTTTTCCCAGACGTTGGGCAACCATTGCCTGACTATTAATGAAACGCTGCCCGCCAATGGCATCAC
TGCTATCTTTGGCGTCTCCGGTGCCGGAAAAACTTCGCTGATTAACGCCATCAGTGGACTGACGCGCCCGCAAAAAGGGC
GGATTGTCCTCAATGGGCGGGTACTAAATGATGCCGAAAAAGGTATCTGCCTGACGCCGGAAAAGCGTCGCGTTGGCTAT
GTTTTTCAGGATGCGCGGCTGTTCCCGCATTACAAAGTGCGTGGCAATCTGCGCTACGGCATGTCGAAAAGTATGGTCGA
TCAGTTCGATAAGCTGGTGGCGCTTTTAGGCATTGAACCGTTGCTTGACCGTTTACCAGGCAGCCTGTCCGGAGGCGAAA
AACAGCGCGTGGCGATTGGTCGGGCTTTGCTGACAGCACCGGAATTGCTGTTGCTGGATGAACCGCTGGCGTCACTGGAT
ATTCCGCGTAAACGCGAACTGTTGCCTTATCTGCAACGGCTGACACGGGAAATCAACATTCCGATGTTGTATGTCAGCCA
TTCGCTGGATGAGATCCTCCATCTGGCAGACAGAGTGATGGTACTGGAAAACGGTCAGGTGAAAGCCTTTGGCGCGCTGG
AGGAAGTGTGGGGCAGTAGCGTGATGAATCCGTGGCTGCCGAAAGAGCAACAAAGTAGCATTCTGAAAGTGACGGTGCTT
GAGCATCATCCGCATTACGCGATGACCGCGCTGGCGCTGGGCGATCAGCATTTGTGGGTCAATAAGCTGGACGAACCGCT
GCAAGCTGCGCTACGTATCCGCATTCAGGCTTCCGATGTTTCTCTGGTTTTACAACCGCCGCAGCAAACCAGCATTCGTA
ACGTATTGCGGGCAAAAGTTGTTAATAGTTATGACGACAACGGCCAGGTGGAAGTGGAACTGGAAGTCGGCGGTAAAACG
CTGTGGGCGCGTATCAGCCCGTGGGCCAGGGATGAACTGGCGATCAAACCTGGCCTGTGGCTGTACGCGCAAATTAAAAG
TGTGTCGATAACCGCCTGA
>Cluster_20752
ATGGCAGTACGAAAACTCGACACAGGTAAATGGATATGCGAATGCTACCCTGCTGGGCGCAGCGGGCGGCGTGTGCGTAA
GCAGTTCGCCACCAAAGGCGAAGCACTGGCTTTTGAGCGTCACACAATTGATGAGGCAGAGACTAAGCCTTGGCTTGGCG
AATCGGTAGACCTTCGAACTCTACAGGATATCGTTGAACTCTGGTTCAAACTGCACGGCAAATCCCTGACCGCTGGCGAG
CATGTTTACGACAAGTTGATACTGATGGTCGATGCTCTCGGAAATCCCCTCGCCACCGATCTAACATCCAAAATGTTCGC
GCACTATCGCGATAAGCGCCTGACGGGTGAAATCTACTTCAGCGAAAAATGGAAGAAAGGAGCCAGCCCGGTAACTATCA
ATCTGGAGCAAAGCTATCTGAGTGGGGTCTTTAGCGAGTTAGCCCGACTCGGTGAATGGACAGCACCAAATCCACTGGAG
AACATGCGTAAATTCACCATTGCCGAAAAAGAAATGGCCTGGCTGACACATGAACAAATCACAGAGCTTCTGTTCGACTG
TCAACGCCAAAGCCCCCTGCTCGCTCTTGTCGTCAAAATCTGCTTGAGCACCGGAGCACGTTGGCGCGAAGCTGTGAACC
TGACACGTTCTCAGGTCACGAAATACCGAATCACATTTGTCAGAACTAAGGGCAAAAAAAACCGCAGCATTCCGATCAGC
AAAGAGCTGTATGAGGAAATTGTTGCCCTAGACGGCTTCAAATTATTTACTGACTGCTACTTCCAGTTTTAGCCGTGAT
GGATAGAACCTCGATCGTACTACCACGCGGGCAACTTACCCACGTTCTACGCCATACGTTCGCAGCACATTTTATGATGT
CCGGCGGGAACATTCTTGCTTTGCAAAAAATCCTGGGCCACCACGATATAAAAATGACTATGCGCTATGCACATCTTGCC

CCTGATCACCTTGAAACAGCACTGCGTTTCAATCCATTAGCCACACTTTCCACTAAAGCAACGAGCCTTGAAGCAAACAG
CTAA
>Cluster_26280
GTGCGGGTATTACTTCGACCTGTTCTGGTACCGGAACTCGGGCTGGTGATCGTTAAGCCGGGCCGTGAATCCATGCCGGT
ATTCCACAATACCCGGGTATTGGTGGAGCCGGAACCGAAAAGCATGCGTAATCTGCCGTCCGGGGTCGTTCCTGCCGTTC
GCCAGCCGCTGGTGGAAGACAAAACATTGCTGCCGTTTTTCAGTAACGCACGGGTGATTCGTGCTGCTGGTGGTGCTGGT
GCATTGTCTGACTGGCTGTTGCGCCATATTAAATCCTGCCAGTGGCCACACGGCGATTATCATCACAGCGAAACCGTCAT
TCACCGTTATGGTACCGGCGCAATGGTGTTGTGCTGGCACTGCGACAACCAGCTGCGTGACCAGACATCCGAATCACTCG
AGCAACTTGCTCATCAAAACCTGTCAGCATGGATGATTGACGTCATCGGTCACGCAATAAGCGGTACGCAGGAGCGTGAA
TTATCTCTGGCTGAATTATCCTGGTGGGCGGTCCGCAATCAGGTGGCGGACGCGCTACCGGAAGCGGTATTACGTCGTTC
GCTGGGGTTGCGTGCGGAAAAAATCCGCTCAATGTACCGTGAAAGCGACATCGTACCGGGAGAGCAGACCGCCACCAGCA
TACTGAAGCAGCGCACAAAAAATCTTGCGCCGCTGCCTCACGCCCACCAGCAACAGAACCCACCACAGGAAAAGACGGTG
GTCAGCATTGCCGTTGATCCTGAGCCTCCGGAATCTTTCATGAAACGACCTAAACGTCGCCGCTGGGTTAACGAGAAATA
CACACGCTGGGTGAAGACTCAGCCGTGTGCGTGTTGTGGTAAGCCAGCCGACGATCCCCATCACCTGATTGGTCATGGTC
AGGGCGGAATGGGGACAAAATCTCACGATATTTTCACGCTACCGCTGTGTCGGGAGCATCACAACGAGCTTCATGCGGAT
CCGCTGGCGTTCGAAGAAAAGCATGGCTCTCAGGTTGATTTAATTTTTCGTTTTCTTGATCACGCCTTTGCAACTGGCGT
GCTTGGGTAA
>Cluster_59933
ATGGGAAAAAGCATAGTCGTTGTTTCTGCGGTCAATTTTACCACTGGCGGTCCATTTACCATTTTGAAAAAATTTTTGGC
AGCAACTAATAATAAAGAAAATGTCAGTTTTATCGCATTAGTCCATTCTGCTAAAGAGTTAAAAGAAAGTTATCCATGGG
TTAAATTCATTGAGTTTCCTGAGGTTAAAGGGTCGTGGCTAAAACGTTTGCACTTTGAATATGTAGTTTGTAAAAAACTT
TCAAAAGAGCTGAATGCTACGCATTGGATTTGTCTGCATGATATTACGGCCAATGTCGTCACTAAAAAAAGATATGTGTA
TTGTCATAACCCTGCACCTTTTTATAAAGGAATTTTATTCCGTGAAATTCTTATGGAGCCTAGCTTTTTCTTATTTAAAA
TGCTATACGGGCTGATATATAAAATAAACATTAAAAAAAATACTGCAGTGTTTGTTCAACAATTCTGGATGAAAGAAAAA
TTTATCAAGAAATATTCTATAAATAACATCATTGTCAGTCGGCCAGAAATTAAATTATCTGATAAAAGCCAACTTACTGA
TGATGATTCTCAATTTAAGAATAACCCTTCTGAGTTGACAATATTTTACCCTGCTGTTCCACGAGTATTTAAAAATTACG
AGCTTATTATTAGTGCAGCAAGGAAATTGAAAGAACAATCCAATATTAAATTTCTGCTTACTATCAGTGGTACAGAAAAT
GCGTATGCAAAATATATTATCAGTCTTGCAGAAGGACTGGATAATGTTCATTTCCTCGGGTACTTGGATAAAGAAAAAAT
CGATCATTGTTATAATATTTCAGATATAGTTTGTTTTCCCTCTAGGTTAGAAACATGGGGATTGCCGTTGTCTGAAGCTA
AAGAGCGAGGTAAGTGGGTATTAGCATCAGATTTCCCATTTACTAGAGAAACTCTTGGTAGTTATGAAAAGAAAGCTTTT
TTTGATTCTAATAACGATGACATGTTAGTTAAACTTATTATTGACTTCAAAAAAGGTAACCTCAAAAAAGATATCTCTGA
TGCAAATTTCATTTATCGTAATGAAAATGTATTAGTTGGGTTTGATGAACTAGTTAATTTTATTACTGAAGAACATTGA
>Cluster_26345
ATGAAGTATATAGCAACATCTGTAGTGGCAATGCTGCTCTTATCGGGTTGTGATAATACGCAAAGTAACAATTCATCCCC
GTCAGAAACAGAAGTCGGCGTTGTTACGCTCAAATCTCAACCGGTTTCGATAGTCAGTGAATTAACCGGACGCACCAGTG
CTGCGCTCAGTGCTGAAGTACGTCCGCAGGTTGGGGGAATTATCCAGAAACGCTTATTTAAGGAAGGTGATCTGGTCAAG
GCTGGACAGCCTCTCTACCAGATTGATGCGGCCAGTTATCAGGCTGCATGGAATGAAGCCCGGGCAGCATTACAACAAGC
ACAGGCACTGGTAAAAGCCGATTGCCAGAAAGCGCAGCGTTATGCCCGACTGGTGAAAGAGAATGGTGTTTCACAACAGG
ATGCTGATGATGCTCAGTCTACCTGTGCACAAGATAAAGCCAGTGTAGAGGCGAAAAAAGCCGCACTGGAAACTGCGCGC
ATTAATCTTGACTGGACCACGGTAACCGCACCGATTTCGGGGCGTATTGGTATTTCGTCGGTAACCCCTGGCGCACTGGT
GACCGCGTCGCAGGATACAGCGTTAACGACTATTCGTGGTCTGGATACAATGTATGTCGACCTCACTCGCTCCAGTGTCG
ATTTATTACGTCTGCGTAAACAGTCACTGGCGACCAACAGTGACACCATGAGCGTCTCACTTATTCTGGAAGATGGCACA
ACCTACAGCGAAAAAGGGCGTCTGGAACTCACCGAAGTCGCGGTGGATGAGTCTACCGGTTCGGTGACATTACGGGCAAT
TTTCCCCAATCCACAACAGCAGTTATTACCGGGAATGTTTGTTCGCGCTCGTGTCGATGAAGGCGTGATGGAAAACGCTA
TTCTCGCGCCTCAACAGGGCGTTACGCGCGATGCTAAAGGCAATGCAACTGCGCTGGTGGTGAATAAAGACAATAAAGTA
GAGCAGCGAACGCTCGAAACGGGAGAAACGTATGGTGATAAATGGCTGGTGCTGAACGGCCTGCACAACGGCGACCGACT
GATTGTTGAAGGTTCTGCCAAAGTCACTTCAGGGCAGACCGTCAAGGCTGTTGAAGTTCAGGCTAATGGAGGCAACGCCT
GA
>Cluster_41773
ATGATTTGGCAACTTACCGACGATAAACGCTGGTCGGCGCTACGTCAGCGTTTTAGCTGGGTTGAGGAGATGCATCACAC
GCCACAAGACCCTGAGCATCATGGTGAAGGCGACGTCGGCGTACATACCGAAATGGTGCTTAACGCCCTCATTACTCTGT
CAGAATTCCAGCAACTCCCCGCTCAACAGCAGGAAGTTTTATGGGCGGCGGCGTTATTGCATGATGTTGAAAAGCGCAGC
ACTACCGTGCAGGAAAACGGACGTATTCAGTCTCCTGGTCATGCTCGTCGGGGCGAACTGACGGCCCGACAAATTTTATG
GCGCGATATCCCAACGCCGTTCGTGCTGCGCGAGCAAATTGTTGCACTGGTACGTCTGCATGGGTTACCGCTATGGTTAC
TGGAACGTCCGGAACCGGAGCGTTTGTTGCTCACGGCTGCAATGCGCATCGACACGCGTCTGCTCGCTTTGCTTGCTCGT
GCCGATCTGCTTGGTCGCCAAAGCCCGGATCAGCAATCAATGCTGGAACGCATCGATCTGTTCGAGCTGTTTTGCCATGA
GCAACAATGCTGGGGAAAAATGCGCCCGTTCGTTTCTGATTCCGCACGCTGGCATTACCTGACCCATGAGCAAAGTTCTC
CTGATTTTGTTCCCTGGGAGGCTGAACCTTTTGAGGTCATTTTGCTGTGTGGCTTACCGGGTATGGGCAAAGATCGCTAT
ATCAATGAACAGTGCCAGGGAATGGACGTTATCAGCCTTGATGATATGCGGCGGCGAATCAATGCCAGTCCGGACGACAA
AACCGCTACCGGGCGTATTGTGCAACAGGCAAAAGAAGAGGCGCGCGTTTTTTTACGCCAGAAAAAGCCGTTCATCTGGA
ATGCAACGAACATTACCCGCCAGCTACGCAGCCAGCTCATCAGTCTGTTTACGGCTTACGGCGCACAAGTAAAAATCGTT
TATCTGGAAGTGCCATGGGCGCAATGGAAGCAGCAAAATGCCAGACGCGAGTATGCAGTTCCTGAAGCGGTCATGATGCG
GATGGCCTCAAGGCTGGAAGTTCCTCAACTTGATGAAGCACATAGCGTGGAGTATCGGGTGATTGAAAGGTAA
>Cluster_55636
TTGAGAGTTCTACACGTCTATAAGACCTACTATCCCGATACCTACGGCGGTATTGAGCAGGTCATTTATCAGCTAAGTCA
GGGCTGCGCCCGCCGGGGAATCGCAGCCGATGTTTTTACTTTTAGCCCGGACAAAGAGACAGGTCCTGTCGCCTACGAAG
ACCATCGGGTCATTTATAATAAGCAGCTTTTTGAAATTGCCTCCACGCCGTTTTCGTTGAAAGCGTTAAAGCGTTTTAAG
CAGATTAAAGATGATTACGACATCATCAACTACCATTTTCCGTTTCCTTTCATGGATATGTTGCATCTCTCGGCGCGGCC
TGACGCAAGAACGGTGGTGACCTATCACTCGGATATTGTGAAACAAAAACGGTTAATGAAGTTGTACCAGCCGCTGCAGG
AGCGATTCCTCGCCAGCGTAGACTGCATCGTCGCCTCGTCGCCCAACTACGTGGCCTCCAGCCAGACCCTGAAAAAATAT
CAGGATAAAACCGTGGTGATCCCGTTTGGTCTGGAGCAGCATGACGTGCAGCACGATCCGCAGCGGGTGGCGCACTGGCG
GGAAACCGTCGGCGATAACTTCTTCCTCTTCGTCGGCGCTTTCCGCTACTACAAAGGGCTGCACATTCTGCTGGATGCCG
CCGAGCGTAGCCGGCTGCCGGTGGTGATCGTCGGGGGCGGGCCGCTGGAGGCGGAAGTGCGGCGTGAGGCGCAGCAACGC
GGGCTGAGCAATGTGGTGTTTACCGGCATGCTCAACGACGAAGATAAGTACATTCTCTTCCAGCTCTGCCGGGGCGTGGT
ATTCCCCTCGCATCTGCGCTCTGAGGCGTTTGGCATTACGTTATTGGAAGGCGCACGCTTTGCAAGGCCGCTGATCTCTT
GCGAGATCGGTACAGGTACCTCTTTCATTAACCAGGACAAAGTGAGTGGTTGCGTGATTCCGCCGAATGATAGCCAGGCG
CTGGTGGAGGCGATGAATGAGCTCTGGAATAACGAGGAAACCTCCAACCGCTATGGCGAAAACTCGCGTCGTCGTTTTGA
AGAGATGTTTACTGCCGACCATATGATTGACGCCTATGTCAATCTCTACACTACATTGCTGGAAAGCAAATCCTGA
>Cluster_59047

ATGGCAGCCAGACCGCGTAAGAATAATGTTTCAGTCCCTAACTTATATCCGCTTTATAGTAGGAAGGTAAATAAAGTCTA
TTGGCGGTATAAGCATCCAGTGACAGGAAAATTCCATGCTTTGGGAACAAACGAAGCTGAAGCTATCGCTATTGCCACTG
AGGCCAACACTCGCCTGGCTGAACAAAGAACCCGGCAGATTCTGGCTATCAGTGACAGGATCGCAACCAGCAAAGGAAAG
GCTATCACAACGTCAACCTGGTTAGATCGCTATCAAGCGATCCAGGATGACAGGCTTAAAAGTGGTGATATAAGACTCAA
CACCTATAAACAAAAAGCTAAACCAGTATCCTTGCTCAGAGAACGAGCAGGAATGAAGTTGATCTCAGCCGTTGATGTCA
GAGATATAGCACAATTGCTTGATGAGTATATCGCCGCTGGGCGGCCAAGAATGGCACAAGTAGTTCGCTCCGTCCTGATT
GATGTTTTCAAAGAGGCGCAGCACTACGGAGAAGTACCTCCTGGCTATAACCCAGCATTAGCCACTAAACAACCAAGAAG
AAAAATTACCCGACAACGGCTGAGCCTTGAAGAATGGAAAAAAATCTTCGATATCGCAGACGCTACTCATCGTTACATGG
GAAATGCCATGCTGTTAGCGCTGGTTACTGGTCAGCGGTTAGGTGATATTTCGCGTATGAAATTTAGCGATATTTGGGAT
GATCATCTTCATGTCATTCAGGAAAAGACCGGGAGCAAAATCGCAATTCCTCTTTCTCTTCGCCTCAATGCGATTAACTG
GAGTTTACGCGATGTAGTAGCCCGCTGCCGTGACTATGCAGTCAGTGCATACTTAGTGCATTTTTTTCGTTCAACTTCAC
AAGCAGAGCGCGGAGCGCAGGTTAAAGCCAATACATTGACGATGAATTTTAGTAAAGCGAGAGATTTAGCAAGAATTGAC
TGGGGAGAGGGTTCACCTGCGACATTCCATGAGCAAAGGTCTTTATCTGAACGTCTGTATAAAGAACAGGGGTTAGACAC
ACAAAAACTACTTGGTCATAAAACACAACAACAAACCGATCGTTATCATGATGATCGAGGTAAAGGATGGAGCAAAGTAG
CGTTGTGA
>Cluster_38697
ATGGCTAAGCAAGATTATTACGAGATTTTAGGCGTTTCCAAAACAGCGGAAGAGCGTGAAATCAAAAAGGCCTACAAACG
CCTGGCCATGAAATACCACCCGGACCGTAACCAGGGTGACAAAGAGGCCGAGGCGAAATTTAAAGAGATCAAGGAGGCTT
ATGAAGTTTTGACCGACTCGCAAAAACGTGCGGCATACGATCAGTATGGTCATGCTGCGTTTGAGCAAGGCGGCATGGGC
GGCGGCGGTTTTGGCGGCGGCGCAGACTTCAGCGATATTTTTGGTGACGTTTTCGGCGATATTTTTGGCGGCGGACGTGG
TCGTCAACGTGCGGCGCGCGGTGCTGATTTACGCTATAACATGGAGCTCACCCTCGAAGAAGCTGTACGTGGCGTGACCA
AAGAGATCCGCATTCCGACTCTGGAAGAGTGTGACGTTTGCCACGGTAGCGGTGCAAAACCAGGTACACAGCCGCAGACC
TGTCCGACCTGTCATGGTTCTGGTCAGGTGCAGATGCGCCAGGGTTTCTTTGCCGTGCAGCAGACCTGTCCTCACTGTCA
GGGCCGCGGTACGCTGATCAAAGATCCGTGCAACAAATGTCATGGTCATGGTCGTGTTGAGCGCAGCAAAACGCTGTCCG
TTAAAATCCCGGCAGGGGTGGACACTGGAGACCGCATCCGTCTTGCGGGCGAAGGTGAAGCGGGTGAACACGGCGCACCG
GCAGGCGATCTGTACGTTCAGGTTCAGGTTAAACAGCACCCGATTTTCGAGCGTGAAGGCAACAACCTGTATTGCGAAGT
CCCGATCAACTTCGCTATGGCGGCGCTGGGTGGTGAAATCGAAGTACCGACCCTTGATGGTCGCGTCAAACTGAAAGTGC
CTGGCGAAACCCAGACCGGTAAGCTGTTCCGTATGCGCGGTAAAGGCGTCAAGTCTGTCCGCGGTGGCGCACAGGGTGAT
TTGCTATGCCGCGTTGTTGTCGAAACACCGGTAGGTTTGAACGAGAAGCAGAAACAGCTGCTGCAAGAGCTGCAAGAAAG
CTTTGGTGGCCCAACCGGCGAGCACAACAGCCCGCGTTCAAAGAGCTTCTTTGATGGCGTGAAGAAGTTTTTTGACGACC
TGACTCGCTAA
>Cluster_44922
ATGTCAGGCTCCCTTATTCCGAACGCCCACCGCGTCCGTCCCGCACATTCCAGCAACGCATCTGGAGGTAACGTGCGGGT
ATTACTTCGACCTGTTCCGGTACCGGAACTTGGGCTGGTGGTGCTAAAACCGGGCCGTGAATCCATGCAGGTATTTCATA
ACCCTCGAGTTCTGGTGGAGCCGGAACCGAAAAGCATGCGCGGTCTGCCGTCCGGAGTCGTTCCTGCTGTTCGCCAGCCG
CTGGCGGAGGATAAATCATTACTGCCATTTTTCAGCGATGAGCGGGTGATTCGTGCTGCTGGTGGTGCTGGTGCATTGTC
TGACTGGCTGTTACGCCACATTAAATCCTGCCAGTGGCCTCATGGTGACTACCATCACAGTGAAACCGTCATACATCGTT
ACGGTACCGGCGCGATGGTGTTGTGCTGGCACTGCGACAACCAGCTGCGTAACCAGACTTCCGAATCACTCGGGCAGCTT
GCTCACCAAAACCTGTCAGCATGGATGATTGATGTCATCCGCCACGCAATGAATGGCACGCAGGAGCGGGAATTATCGCT
GGCTGAATTATCCTGGTGGGCAACCATAAATAACGTAGCGGACGCACTACCGGAGACGGTGTTACGTCGTTCACTGGGAT
TACGCGCGGAAAAAAATTCGCTCAGTATACCGCGAGAGCGACATCGTGCCGGGAGAGCAGACCGCCACCAGCATGCTGAAG
CAGCGCACAAAAAAATATTGCGCTACTGCCTCACGCCCACCAGCAAAACCCGCCACAGGAAAAGACGGTGGTAAGCATTGC
CGTTGATCCGGAGTCACCGGCTCAGTATCTCCAGCGCCAGAAACCACGACGGGAAGAGATGCCTGTATACACGCGTTGGG
TAAAAACGCAGAAATGCATGACGTGTGGTAATCAGGCAGATGATCCGCATCACATCATTGGTCATGGACTGGGAGGGATG
GGAACAAAGGCTGATGATTTGTTTGTTATTCCGCTGTGCCGTAAATGCCATAGCGAACTACACGCCGGGGTAAAAGATTT
TGAAGAAAAACACGGCAGCCAGCTGTTGTTGCTGATTCGTTTTTTAATGCACGCGAGAAATTCGGGTGTCCTGAAGTGGA
AAGCATGA
>Cluster_28234
ATGCAAAGCGTTACCCCAACATCGCAATACCTGAAGGCGCTCAATGAAGGCAGCCATCAACCCGACGACGTTCAAAAAGA
GGCCGTCAGCCGCCTGGAAATTATTTATCAGGAACTCATCAATAGCACGCCTCCAGCCCCCAGGACGAGTGGGCTAATGG
CGCGGGTCGGTAAGCTGTGGGGTAAACGCGAAGACACAAAGCATACGCCAGTGCGTGGCTTATATATGTGGGGCGGTGTA
GGACGCGGGAAAACCTGGCTGATGGACCTTTTCTATCAAAGCCTGCCGGGAGAGCGGAAACAGCGTCTGCACTTTCACCG
TTTTATGCTGCGGGTGCACGAAGAGCTGACCGAATTACAAGGCCAAAGCGATCCGCTGGAAATTATTGCCGATCGCTTTA
AAGCAGAAACTGACGTGCTCTGTTTTGACGAATTTTTTGTTTCTGATATTACCGATGCCATGCTACTTGGCGGTCTGATG
AAAGCCCTGTTCGCCCGTGGTATTACCCTGGTAGCGACGTCAAATATTCCTCCGGACGAACTTTATCGAAATGGCCTGCA
ACGTGCGCGTTTTTTGCCTGCAATCGATGCCATTAAACAGCATTGTGATGTAATGAACGTGGACGCTGGTGTTGATTATC
GACTGCGTACACTCACTCAGGCGCATCTGTGGCTTTCGCCACTCAACGATGAAACCCGGACGCAAATGGATAAACTATGG
TTGGCGCTGGCGGGGGTGAAACGAGAAAATTCACCGACGTTAGAAATTAACCACCGGCCGTTGGCGACAATGGGCGTCGA
GAACCAGACGCTGGCAGTTTCTTTTACTACCTTGTGCGTCGATGCCCGCAGTCAGCATGACTATATTGCGCTCTCACGTC
TGTTTCACACAGTGATGTTGTTTGATGTGCCGGTCATGACGCGGTTGATGGAGAGCGAAGCGCGGCGCTTTATTGCGCTG
GTGGATGAGTTTTACGAGCGCCATGTCAAATTAGTGGTGAGTGCAGAAGTGCCGCTGTATGAAATTTATCAGGGCGAGCG
GCTGAAGTTTGAGTTCCAGCGTTGCCTGTCACGTCTGCAAGAGATGCAAAGCGAAGAGTATTTGAAGCGCGAGCATTTGG
CGGGGTAA
>Cluster_14345
ATGGACCGCATTATTCAATCACCGGGTAAATACATCCAGGGCGCTGATGTGATTAATCGTCTGGGCGAATACCTGAAGCC
GCTGGCAGAACGCTGGTTAGTGGTGGGTGACAAATTTGTTTTAGGTTTTGCTCAATCCACTGTCGAGAAAAGCTTTAAAG
ATGCTGGACTGGTAGTAGAAATTGCGCCGTTTGGCGGTGAATGTTCGCAAAATGAGATCGACCGTCTGCGTGGCATCGCG
GAGACTGCGCAGTGTGGCGCAATTCTCGGTATCGGTGGCGGAAAAACCCTCGATACTGCCAAAGCACTGGCACATTTCAT
GGGTGTTCCGGTAGCGATCGCACCGACTATCGCCTCTACCGATGCACCGTGCAGCGCATTGTCTGTTATCTACACCGATG
AGGGTGAGTTTGACCGCTATCTGCTGTTGCCAAATAACCCGAATATGGTCATTGTCGACACCAAAATCGTCGCGGGCGCA
CCTGCACGTCTGTTAGCAGCGGGTATCGGCGATGCGCTGGCAACCTGGTTTGAAGCGCGTGCTTGCTCTCGTAGCGGCGC
GACCACCATGGCGGGCGGCAAGTGCACCCAGGCGGCGCTGGCACTGGCTGAACTGTGCTACAACACCCTGCTGGAAGAAG
GCGAAAAAGCGATGCTTGCCGCCGAACAGCATGTAGTAACCCCGGCGCTGGAGCGCGTGATTGAAGCGAACACCTATTTG
AGCGGCGTTGGTTTTGAAAGCGGCGGGCTGGCGGCGGCTCACGCGGTGCATAACGGCCTGACCGCTATCCCGGACGCGCA
TCACTATTATCACGGTGAAAAAGTGGCATTTGGTACGCTGACGCAGCTGGTTCTGGAAAACGCACCGGTTGAGGAAATCG
AAACCGTAGCTGCACTTAGCCATGCGGTAGGTTTGCCAATAACTCTCGCTCAACTGGATATTAAAGAAGATGTCCCGGCG
AAAATGCGAATTGTGGCAGAAGCGGCATGTGCAGAAGGTGAAACCATTCACAACATGCCTGGCGGCGCGACGCCAGATCA
GGTTTACGCCGCTCTGCTGGTAGCCGACCAGTACGGTCAGCGTTTCCTGCAAGAGTGGGAATAA
>Cluster_22574

ATGAGCATTGAGATTGCCAATATTAAGAAGTCTTTTGGTCGCACCCAGGTGCTGAACGATATCTCACTGGATATTCCTTC
TGGTCAGATGGTCGCATTGCTGGGGCCGTCCGGTTCCGGGAAAACCACGCTGCTGCGGATTATCGCCGGGCTGGAGCATC
AAACCAGCGGGCATATTCGCTTCCACGGCACCGACGTTAGCCGCCTGCATGCGCGCGACCGTAAAGTCGGTTTCGTGTTC
CAGCATTACGCGTTGTTCCGCCATATGACGGTATTCGACAATATCGCTTTTGGCCTGACGGTGCTACCGCGTCGCGAGCG
CCCGAATGCGGCGGCGATCAAAGCGAAAGTGACAAAATTGCTGGAGATGGTGCAGCTTGCCCATCTGGCAGACCGCTATC
CTGCGCAGCTTTCCGGCGGCCAGAAACAGCGTGTGGCGCTGGCGCGCGCGCTGGCTGTAGAACCGCAAATTCTGCTGCTT
GATGAACCGTTTGGCGCGCTGGATGCGCAGGTGCGTAAAGAGTTGCGTCGCTGGCTGCGTCAGTTACATGAAGAGCTGAA
ATTCACCAGCGTGTTTGTGACCCATGACCAGGAAGAAGCGACCGAAGTAGCCGATCGTGTAGTGGTGATGAGCCAGGGCA
ATATCGAGCAGGCTGATGCACCGGACCAGGTATGGCGCGAACCAGCGACCCGTTTTGTACTCGAATTTATGGGCGAAGTT
AACCGCCTGCAGGGAACCATTCGCGGCGGGCAGTTCCATGTTGGCGCACATCGCTGGCCGCTGGGCTATACACCTGCGTA
TCAGGGGCCAGTGGATCTCTTCCTGCGTCCGTGGGAAGTGGATATCAGCCGCCGTACCAGCCTCGATTCGCCGCTGCCGG
TACAGGTACTGGAAGCCAGCCCGAAAGGCCACTACACCCAATTAGTCGTGCAGCCGCTGGGGTGGTACAACGAAGCGCTG
ACGGTCGTGATGCACGGCGACGACGCCCCGCAACGTGGCGAGCGTTTATTCGTTGGTCTGCAACATGCGCGGCTGTATAA
CGGCGACGAGCGTATTGAAACCCGCGATGAGGAACTTGCTCTCGCACAAAGCGCCTGA
>Cluster_57276
ATGAAATTTACCGTAGAACGTGAGCATTTATTAAAACCGCTACAACAGGTGAGCGGTCCGTTAGGTGGTCGTCCTACGCT
ACCGATTCTCGGTAATCTGCTGTTACAGGTTGCTGACGGTACGTTGTCGCTGACCGGTACTGATCTCGAGATGGAAATGG
TGGCACGTGTTGCGCTGGTTCAGCCACACGAGCCAGGAGCGACGACCGTTCCGGCGCGCAAATTCTTTGATATCTGCCGT
GGTCTGCCTGAAGGCGCGGAAATTGCCGTGCAGCTGGAAGGTGAACGGATGCTGGTACGCTCCGGGCGTAGCCGTTTTTC
GCTGTCTACCCTGCCAGCGGCGGATTTCCCGAACCTCGATGACTGGCAGAGTGAAGTCGAATTTACCCTGCCGCAGGCAA
CGATGAAGCGTCTGATTGAAGCGACCCAGTTTTCTATGGCGCATCAGGACGTTCGCTATTACTTAAATGGTATGCTGTTT
GAAACCGAAGGTGAAGAACTGCGCACCGTGGCAACCGACGGCCACCGTCTGGCGGTCTGTTCAATGCCAATTGGTCAATC
TTTGCCAAGCCATTCGGTGATCGTACCGCGTAAAGGCGTGATTGAACTGATGCGTATGCTCGACGGCGGCGACAATCCGC
TGCGCGTACAGATTGGCAGCAACAACATTCGCGCCCACGTTGGCGACTTTATCTTCACCTCCAAACTGGTGGATGGTCGC
TTCCCGGATTATCGCCGCGTTCTGCCGAAGAACCCGGACAAACATCTGGAAGCTGGCTGCGATCTGCTCAAGCAGGCGTT
TGCTCGCGCGGCGATTCTCTCTAACGAGAAATTCCGCGGCGTACGTCTTTATGTCAGCGAAAACCAGCTGAAAATCACCG
CCAACAACCCGGAACAGGAAGAAGCGGAAGAGATCCTCGACGTTACCTATAGCGGTGCGGAGATGGAAATCGGCTTCAAC
GTCAGTTATGTGCTGGATGTTCTGAACGCGCTGAAATGCGAAAACGTCCGCATGATGCTGACCGATTCGGTTTCCAGCGT
GCAGATTGAAGATGCGGCCAGCCAGAGCGCGGCTTATGTTGTCATGCCAATGAGACTGTAA
>Cluster_25151
ATGGAAAAACTGCGGGTAGGAATCGTTTTTGGTGGTAAATCAGCGGAACATGAAGTGTCTCTGCAATCGGCAAAAAACAT
TGTCGATGCCATTGATAAAAGTCGCTTCGATGTTGTGCTGCTGGGCATTGATAAACAAGGGCAATGGCACGTCAGCGATG
CCAGCAATTATCTGCTAAATGCAGACGATCCTGCCCATATTGCGTTGCGCCCTTCGGCGACCAGCCTTGCGCAGGTGCCA
GGTAAACATGAGCATCAGCTTATCGACGCACAAAACGGTCAGCCGTTGCCGACGGTGGATGTCATTTTCCCGATTGTCCA
CGGTACGCTGGGCGAAGATGGTTCCTTGCAGGGAATGCTGCGGGTCGCCAATTTACCGTTTGTAGGTTCTGATGTTCTGG
CTTCAGCGGCCTGTATGGATAAAGATGTCACCAAACGTCTGCTGCGCGATGCCGGGCTGAACATTGCGCCATTTATTACC
CTGACGCGCGCTAATCGTCACAACATCAGTTTTGCCGAAGTGGAGTCTAAACTGGGGTTACCGCTGTTTGTAAAACCGGC
TAATCAGGGCTCTTCTGTTGGTGTCAGCAAAGTAACCAGTGAAGAACAGTACACAATTGCCGTCGATCTGGCGTTCGAGT
TCGATCATAAAGTGATCGTTGAGCAAGGGATCAAAGGTCGTGAAATCGAATGCGCAGTTCTTGGCAACGATAACCCGCAA
GCAAGCACCTGTGGCGAGATCGTACTCACCAGCGATTTCTATGCCTACGACACCAAGTACATTGACGAAGATGGCGCGAA
AGTGGTAGTTCCGGCAGCCATTGCGCCAGAAATCAACGATAAGATCCGGGCGATTGCCGTTCAGGCTTATCAGACGTTGG
GATGCGCAGGCATGGCGCGTGTAGACGTGTTTTTAACCCCAGAGAACGAAGTGGTGATCAACGAGATCAACACCCTGCCT
GGCTTCACCAACATCAGTATGTATCCGAAGCTGTGGCAAGCCAGCGGTCTGGGTTACACCGACCTGATCACGCGTTTGAT
TGAGCTGGCGCTGGAGCGTCACGCTGCGGATAACGCACTGAAAACCACAATGTAA
>Cluster_26079
GTGATTAAATTTCTCTCTGCATTAATTCTTCTACTGGTTATCACGGCGGCTCAGGCTGAGCGTATTCGCGATCTCACCAG
TGTTCAGGGGGTAAGGCAAAACTCACTGATTGGCTATGGCCTGGTGGTGGGGCTGGATGGAACCGGTGACCAGACAACCC
AGACGCCGTTTACCACACAAACGCTTAATAACATGCTCTCACAGCTGGGAATTACCGTTCCGACGGGCACCAATATGCAG
CTAAAAAACGTCGCTGCGGTAATGGTGACGGCGTCACTTCCGCCGTTTGGACGCCAGGGGCAAACCATCGATGTGGTGGT
ATCTTCCATGGGTAATGCCAAAAGCTTGCGTGGCGGTACGTTGTTGATGACACCGCTTAAGGGCGTTGACAGTCAGGTGT
ATGCGCTGGCGCAGGGCAATATTCTGGTTGGTGGCGCTGGAGCCTCCGCTGGCGGTAGCAGTGTTCAGGTGAACCAACTG
AACGGTGGACGGATCACCAATGGTGCGGTTATTGAACGTGAATTGCCCAGCCAGTTTGGCGTCGGGAATACCCTTAATTT
GCAACTTAACGACGAAGATTTCAGCATGGCGCAGCAAATCGCTGACACCATCAACCGCGTGCGTGGATATGGCAGCGCCA
CCGCGTTAGATGCGCGGACTATTCAGGTGCGCGTACCAAGTGGCAACAGTTCCCAGGTCCGTTTCCTTGCCGATATTCAG
AATATGCAGGTTAATGTCACCCCGCAGGACGCTAAAGTAGTGATTAACTCGCGCACTGGCTCGGTGGTGATGAATCGCGA
AGTGACCCTCGACAGCTGCGCAGTAGCGCAGGGGAATCTCTCGGTTACGGTCAATCGTCAGGCCAATGTCAGCCAGCCAG
ATACACCGTTTGGTGGTGGGCAGACTGTGGTTACTCCACAAACGCAGATCGATTTACGCCAGAGCGGCGGTTCGCTGCAA
AGCGTACGTTCCAGCGCCAGCCTCAATAACGTGGTGCGCGCGCTCAATGCGCTGGGCGCTACGCCGATGGATCTGATGTC
TATTTTGCAATCAATGCAAAGTGCGGGATGTCTGCGGGCAAAACTGGAAATCATCTGA
>Cluster_20326
ATGAGTGACAGCCAGACGCTGGTGGTAAAACTCGGCACCAGTGTGCTAACAGGCGGATCGCGCCGCCTGAACCGTGCCCA
TATCGTTGAACTTGTTCGCCAGTGCGCGCAGTTACATGCCGCCGGGCATCGGATTGTTATTGTGACGTCGGGTGCGATTG
CCGCCGGACGTGAGCACCTGGGGTACCCGGAATTGCCAGCGACTATCGCTTCAAAACAGCTGCTGGCGGCGGTAGGGCAG
AGTCGACTGATTCAACTGTGGGAACAGCTGTTTTCAATTTACGGCATTCATGTCGGGCAGATGTTGCTGACCCGTGCTGA
TATGGAAGATCGTGAACGCTTCCTGAACGCCCGCGATACCCTGCGTGCGTTGCTCGATAACAATATCGTTCCGGTAATTA
ATGAGAACGATGCTGTCGCTACTGCAGAGATTAAGGTCGGTGATAACGATAACCTTTCTGCGCTGGCGGCGATTCTGGCG
GGTGCCGATAAACTGTTGCTGCTGACCGATCAAAAAGGTTTGTATACCGCTGACCCGCGCAGCAATCCGCAGGCAGAACT
GATTAAAGATGTGTACGGCATTGATGACGCACTGCGCGCGATTGCCGGTGATAGCGTTTCAGGCCTCGGAACTGGCGGCA
TGAGCACCAAATTGCAGGCCGCTGACGTGGCTTGCCGTGCGGGTATCGACACCATTATTGCCGCGGGCAGCAAGCCGGGC
GTTATTGGTGATGTGATGGAAGGCATTTCCGTCGGTACGCTGTTCCATGCCCAGGCGACTCCGCTTGAAAACCGTAAACG
CTGGATTTTCGGTGCGCCGCCTGCGGGTGAAATCACGGTAGATGAAGGGGCAACTGCCGCCATTCTGGAACGCGGCAGCT
CCCTGTTGCCGAAAGGCATTAAAAGCGTGACTGGCAATTTCTCGCGTGGTGAAGTCATCCGTATTTGCAACCTCGAAGGT
CGCGATATCGCCCACGGCGTCAGTCGTTACAACAGCGATGCATTACGCCGTATTGCCGGACACCACTCGCAAGAAATTGA
TGCAATACTGGGATATGAATACGGCCCGGTTGCCGTTCACCGTGATGACATGATTACCCGTTAA
>Cluster_48574
GTGTCACTCCTTTTGAATCGTGAGCATACGAACGGTCAGGTAACAAACGCATCGTATGCAAAAGTTATTGAGACGGTGCT
TAAAAGCGGCGTGCAGGCTGATGATCGCACAGGCACTGGTACTTTAAGCACCTGCTACGTTCCCTCTTACTACATGCTTA
CTGGTGGGACTGTGCCGCTTATTTCTGGAAAGGCGGTAAATCTTAAGCCACTGCTTGTCGAACTTGAGTGGTATCTGAAA
GGCACGGGCAACATCCAATTTCTCAAGGATAACGGCGTTAAGATTTGGGATGCATGGGCCGATGAGAATGGCGATTTGGG

GCCGGTTTACGGTAAGCAGTGGCGTCGATGGGAAGATACCCGCATCGTGAGCCATAGTGAATATCTGAGCAAGATCGATA
CTTTCCGTGAACGCGGGTACAAAGTCGAGGGATACCTGGGTATCAGTGAAGATCGCGTAGTGCTGTCCCGTGAAATCGAT
CAGCTACAGCGTATTGTCGATACACTGCGCACGAACCCCACCGATCGTCGTATCATGCTTAACGCATGGAACGTAGGCGA
GCTTGAGGATATGAAACTGCCACCTTGCCACTTTGTCTTCTCTTTGTGGAGTCGTGAGCTTGATTTTGAAACCCGTTTAA
CGATGGCAACTGACATTGGTCTTCAACACAGTCGCCTCGGTTATGAGTCTATCTACACCAAGATGCTATACGATCTGGAG
ATGGACGGCAGTGTTACTGAAACTGAACTGGATGAACTTGGAATCCCCAAACGCATCCTCAACTCCTGCCTCGTACAGCG
TAGCGTAGACACTTTTGTTGGTATGCCATTCAATATTGCTGGCTATGGCATTCTCACTCATTTTCTCGCGAAGATTACGG
GTCACATGGCCGGTGCATTTGTACATTTTGGCTTTGACGTGCATTTGTACAACAACCACATGGAAGGTGTGTGTGAGCTA
ATGAAACGACAGGCTCCAGAGCATTCAGATCCGGTCGTTATTTTCCCTCATGAATGGTCTGAGCTGGATGATTTCAAATG
GGACGATATTCTGATTCTTGGCTATGACCCTCTACCGTGGATCAAGGTTCCAGTGGCGGTGTGA
>Cluster_29216
TTGAATCTGATGCGCTTTTTACGCCAACCAATATCACTACGTCAACGGCTGATATTGACCATCGGGGCCATTTTGTTGGT
GTTTGAGCTGATCAGCGTCTTCTGGCTATGGCATGAAAGTACCGAGCAGATTCAGCTGTTTGAGCAGGCGCTACGCGACA
ATCGCAACAACGATCGCCACATCATGCGTGAGATCCGCGAGGCGGTCGCCAGCCTGATTGTCCCCGGCGTCTTTATGGTT
AGCCTGACGCTATTTATCTGCTATCAGGCGGTACGCCGCATCACCCGACCGCTGGCGGAGCTGCAAAAAGAGCTGGAAGC
GCGCACCGCCGACAACTTAACGCCCATTGCCATTCATAGCGCCACCCTCGAAATCGATGCGGTGGTTTCGGCATTAAACG
ATCTGGTCAGTCGCCTGACCAGCACGCTGGATAACGAAAGGTTGTTTACCGCTGACGTCGCGCACGAACTGCGAACGCCA
CTGGCGGGGGTGCGTTTGCATCTGGAACTGCTGGCGAAAACGCATCACATTGATGTAGCACCGTTAGTGGCACGACTTGA
TCAGATGATGGAGAGCGTCTCCCAGCTGCTGCAACTGGCGCGTGCCGGACAGTCATTTTCTTCCGGTAATTATCAACATG
TAAAACTGCTGGAAGATGTCATTCTCCCCTCGTATGACGAACTTAGTACCATGCTCGACCAGCGCCAGCAAACCCTGCTA
TTGCCAGAGAGCGCCGCCGACATCACCGTTCAGGGTGATGCAACCCTGCTGCGGATGTTATTGCGAAACCTGGTAGAAAA
CGCCCATCGTTATAGCCCACAAGGCAGCAACATTATGATTAAGCTGCAAGAAGATGGCGGAGCGGTCATGGCCGTTGAAG
ATGAAGGACCGGGTATTGATGAGAGTAAATGCGGGGAGTTGAGTAAAGCGTTTGTACGAATGGACAGCCGTTATGGCGGG
ATTGGTCTGGGGTTAAGTATTGTCAGCCGCATTACACAGTTGCATCACGGGCAGTTTTTCCTGCAAAACCGGCAAGAGAC
TTCCGGCACGCGGGCCTGGATACGGCTGAAAAAGATCAGTACGTGGCAAACCAGATATAA
>Cluster_28767
ATGGAACATTTCGACGTGGCGATTATTGGCCTCGGCCCGGCAGGGTCGGCGTTGGCACGAAAGTTAGCAGGAAAAATGCA
GGTGATCGCGCTGGATAAAAAGCACCAGTGTGGTACTGAAGGTTTCAGCAAACCTTGTGGCGGTCTGCTGGCACCGGACG
CGCAGCGATCTTTTATTCGCGATGGACTGACGCTTCCTGTCGATGTCATCGCCAACCCGCAGATTTTCAGCGTCAAAACC
GTCGACGTCGCCGCATCGCTCACGCGTAACTACCAGCGAAGCTATATCAATATTACTCGCCATGCTTTCGACTTGTGGAT
GAAATCGCTGATCCCCGCCAGCGTTGAGGTTTACCACGACAGCCTGTGTCGAAAAATCTGGCGTGAGGATGATAAATGGC
ATGTCATTTTTCGCGCAGACGGCTGGGAGCAGCATATTACCGCCCGCTATCTGGTCGGTGCCGATGGTGCCAACTCGATG
GTGCGGCGACATCTCTACCCGGATCATCAGATTCGTAAATATGTCGCCATCCAGCAGTGGTTCGCAGAGAAACATCCGGT
ACCGTTCTACTCCTGCATCTTTGATAATGCGATAACTGACTGTTACTCATGGAGTATCAGCAAAGACGGTTATTTTATCT
TTGGCGGTGCCTATCCGATGAAAGACGGTCAGGCGCGTTTCGCGGCGCTGAAAGAGAAAATGAGTGCCTTTCAGTTCCAG
TTTGGTAAGGCGGTGAAAAGCGAAAAATGCACGGTGCTGTTTCCCTCACGCTGGCAGGATTTTGTCTGCGGCAAGGACAA
CGCCTTTCTGATTGGCGAAGCGGCGGGATTTATCAGCGCCAGCTCGCTGGAGGGGATTAGCTATGCGCTGGATAGCGCAG
AGATTCTGCGTTCGGTATTACTGAAGCAGCCAGAGAAGCTCAATACCGCTTACTGGCGCGCCACTCGCAAACTGCGTTTA
AAACTCTTCGGCAAGATAGTAAAAAGCCGATGCCTGACCGCACCGGCTTTAAGAAAGTGGATTATGCGCAGTGGAGTGGC
GCATATTCCACAGTTGAAAGATTATCCAACGCGCTTCACATCGCCCACCAGCATGATGTAA
>Cluster_40114
ATGATCCCGTTGATGTTGATCTGGCGCTACTTCCGTACCCGCAGGCTGCATTTCACCGATCGCGAGGCACTGGAAAACTG
GCAGGCAAAAAGGCTGCATCAGTTCCGGCAAAATGTCTTATCTCATAGCCCGTGGTTTCAGCGTTATCTGACGTTGCCCT
TTAATCAGTGGCCAATGATGGATAAAGCGTTAATGATGACGCACTTCGATGAGATGAATACCGCAGGTTTAAAGCGCGAC
GAACTTCTCGATTGTGCAATGCGCAGTGAGCAGAGTCGTGATTTCAAACCCTGCGTTGGTAAGTTCAGCGTTGGGCTTTC
ATCAGGTACATCCGGGCGTCGTGGACTTTTTGTTGTTAGCCCGCACGAGCAACAGATGTGGGCCGCCGGTGTGCTGGCAA
AAGTGTTGCCCGATGATTTATTTGCCAAAGAGCGGGTGGCGCTGTTTCTGCGCGCGGACAACAACCTGTATCAAAGCGTG
AATAATCGCTGGTTAAGTCTCGATTTTTACGATCTGCTAGCGCCATTTCAGGCGCAACTCAAACTTCTCCAACAGCGAGC
GCCCACGATTATTGTCGCGCCAGCTCAGGTATTACGCGCACTGGCGCTGGAGGTGATGGCCGGCGAATTGACGCTAAAGG
TCAAAAAAGTGATTTCGGTGGCGGAAGTTCTGGAGCCGCAGGATCGGGAATTACTGCGCAACGTGTTCCACAATGTGGGG
GAAATTTATCAGGCGACAGAAGGCTTTCTCGCTTCCACCTGCCGGTGTGGAACGTTACATCTCAACGAAGAGTTTGTTCA
CATTGAACCGCAATGGCTGGATGAGCACCGTTTCGTGCCGATCGTCACAGACTTTACCCGCACCACACAGCCGATAGTGC
GTTATCGACTGGATGATGTGCTGGTGGCAAGCGAACAGCCATGCCCCTGTGGCAGTGCAACAATGGCGATTGCGCGTATC
GAAGGGCGGCAGGATGACCAACTGCAACTACCAACAAGATCCGGCGATATTCAGACCGTTTTTGCCGATGCCTGTAGTCG
TGTGCTGGCAATGACACTGCCGCTAACAGTCGATTATCGCCTGCTACAAACCGGGTCAGCACAATTGACGTTAATCGCCG
ATTGCGAGCTGTGGATGCTGGAGCATTGCCGGGAGGCGTTGAATGCCTTGTTTATACGCCAGGATATCGCTGTCGGGCAA
TTAATTTGGTCGCAGGAACTGCAAACTGTGCCAGTCTCTTTTACAGCCAAGCGGCGGCGCATTGTGCGTCAGTGGGGGCG
GGAATGA
>Cluster_28002
ATGCTAAATTTATTTGTTGGCCTTGATATATATATACGGGGCTTTTGTTATTACTTGCTCTGGCATTTGTGTTGTTCTATGA
AGCAATCAATGGTTTTCATGACACAGCGAATGCGGTGGCAACCGTTATTTATACCCGTGCCATGCAACCACAGCTTGCTG
TAGTCATGGCGGCATTTTTTAACTTTTTGGCGTATTGCTTGGCGGGCTTAGCGTTGCCTATGCCATCGTCCATATGTTG
CCAACCGATTTGTTGCTGAATATGGGGTCAACTCACGGTCTGGCGATGGTCTTTTCCATGCTGCTGGCGGCGATTATCTG
GAATCTGGGAACGTGGTTCTTTGGTTTACCGGCCTCCAGTTCGCACACTTTGATTGGTGCGATTATCGGCATCGGTTTAA
CCAACGCGCTGTTAAATGGTTCGTCGGTGATGGATGCGTTAAACCTGCGCGAAGTCACCAAGATTTTCTCTTCACTCATT
GTCTCCCCCATCGTGGGGCTGGTGATTGCGGGGGGGCTGATTTTTCTGCTGCGTCGCTACTGGAGCGGGACGAAAAAGCG
TGACCGTATTCACCGCATTCCGGAAGATCGCCAAAAGAAAAAAGGCAAACGTAAGCCGCCATTCTGGACGCGTATTGCCC
TCATTGTTTCCGCTGCGGGCGTGGCGTTTTCGCACGGCGCGAACGACGGGCAGAAAGGAATTGGTCTGGTGATGCTGGTG
CTGGTGGGGATTGCTCCCGCCGGGTTTGTCGTCAATATGAATGCGTCCGGCTATGAAATTACCCGTACCCGCGATGCCGT
CACCAACTTCGAACACTACCTGCAACAGCATCCTGAACTGCCGCAGAAGTTGATTACGATGGAACCTCCATTGCCTGCGA
CATCGACTGATGGCACGCAAGTAACAGAGTTTCACTGTCATCCGGCAAATACCTTTGATGCGATTGCGCGCGTTAAAACG
ATGCTGCCAGGCAATATGGAAAGTTACGAGCCGTTAAGCGTGAGTCAGCGCAGCCAGCTGCGCCGCATTATGCTGTGCAT
CTCTGATACCTCCGCGAAGCTGGCGAAACTGCCAGGCGTCAGTAAAGAAGACCAGAACCTGCTGAAAAAACTGCGCAGCG
ATATGTTAAGCACCATTGAGTACGCTCCGGTGTGGATCATCATGGCAGTAGCTCTGGCGCTCGGCATTGGCACCATGATT
GGCTGGCGTCGTGTAGCGATGACCATCGGTGAGAAGATTGGTAAGCGCGGTATGACGTATGCGCAAGGCATGGCGGCACA
AATGACGGCGGCAGTGTCTATCGGCCTTGCCAGTTATATTGGGATGCCCGTCTCCACAACACACGTACTCTCATCTGCCG
TAGCAGGAACGATGGTGGTGGACGGCGGTGGGTTACAGCGTAAAACGGTAACCAGCATCCTGATGGCGTGGGTATTTACT
TTACCGGCGGCGATTTTTCTTTCTGGAGGGTTGTACTGGATAGCATTGCAGTTGATTTAA
>Cluster_64783

ATGTTTTATATTGATAACGACAGCGGCGTAACCGTCATGCCGCCCGTATCCGCCCAGCGTAGTGCTATCGTTCGCTGGTT
TTCAGAAGGTGACGGGAATAATGTTATCACATGGCCCGGCATGGACTGGTTTAATATTGTGCAGGCGGAGTTATTAAACA
CGCTGGAAGAAGCCGGTATTCAACCGGATAAAACAAAATTAAACCAGCTTGCACTGTCCATTAAATCCATTATGAGCAAT
AACGCGCTGCTGATAAAAAATAACCTCAGCGAAATTAAAATTGCCGGGGCATCAGCACAGCGTACAGCACGTGAAAATCT
GGATATCTATGATGCCAGCCTGAACAAAAAAGGACTCGTTCAGCTAACCAGTGCCACTGACAGCCCCAGTGAAACGCTGG
CAGCCACCGCAAAAGCGGTGAAAATTGCGATGGATAATGCCAATGCCCGTCTGGCAAAAGACCGGAACGGAGCAGATATT
CCCAATAAGCCGCTGTTTATCCAAAACCTCGGTTTACAGGAAACGGTAAATCAGGCTTCTGGTGCGTTGCAGAAAAACCA
GAATGGCGCAGATATTCCTGATAAAAGATTATTCCTGCGCAATATTGGAGCAACAAATTCAACAACCATGTCTTTTAATG
GTGGTGCAGGATGGTTCAAACTGGCAACTGTAACAATGCCACAAGCCAGTTCCGTGGTTTACATAAGCCTGATTGGTGGT
GCAGGGTATAACGTTGGCTCCCCGCAGCAAGCAGGCATCTCTGAGCTGGTTCTGCGTGCCGGAAATGGAAATCCAAAGGG
TATTACTGGTGCATTATGGCGACGGACCTCGGTTGGATTTACTAATTTTGCATGGGTGAATACATCCGGTGATACCTATG
ACATTTATGTAGAAATAGGTAATTACGCCACGGGTGTTAATATCCAGTGGGATTATACAAAGGATGCCACTGTACAAATT
CATACATCACCAACTTATACAGCGAATAAACCGACAGGCCTGACAGATGGGACTGTATATGTAATTTACAGTTCGCACAT
AAAACCGACTGCTGCTGATGTTGGGGCGTTGTCATTATCTGGTGGTCAATTGAATGGTGCACTGGGTATCGGAACATCCA
GTGCTCTTGGCGGTAACTCGATTGTTTTGGGTGATAATGACACGGGCTTTAAACAAAATGGCGACGGTAATCTGGACGTT
TATGCTAATAACGTCCATGTTATGCGCTTTGTCTCCGGCAGCATTCAAAGTAATAAAACCATAAATATTACAGGGCGTGT
TAACCCCTCGGATTACGGTAACTTTGATTCCCGTTATGTGAAAGATGTTCGCCTGGGTACGCGTGTTGTTCAATTGATGG
CGCGTGGTGGTCGTTATGAAAAAGCCGGACACGCAATTACCGGATTAAGAATCATTGGTGAAGTAGATGGCGATGATGAA
GCCATCTTCAGACCAATACAAAAATACATCAATGGCACATGGTATAACGTCGCACAGGTGTAA
>Cluster_28863
ATGGGTTCCACCTCGTCGCTGTATGCAGCCATTGATCTCGGTTCGAATAGTTTTCATATGCTGGTTGTGCGCGAGGTGGC
TGGAAGCATCCAGACGCTGACGCGAATTAAACGCAAAGTGCGTCTGGCTGCTGGCCTGAATAGCGAAAATGCCCTCTCTA
ATGAAGCAATGGAGCGCGGTTGGCAATGTCTGCGCCTGTTTGCTGAACGTCTGCAAGATATCCCTCCCTCGCAAATTCGC
GTTGTCGCTACGGCGACATTACGACTTGCCGTCAATACGGGTGATTTTATTGCCAAAGCGCAGGAAATCCTCGGTTGTCC
GGTACAGGTGATCAGCGGTGAAGAGGAAGCGCGTCTGATTTATCAGGGCGTTGCTCACACCACTGGCGGTGCCGATCAGC
GCCTGGTGGTGGATATCGGCGGAGCCAGTACTGAACTGGTAACCGGCACGGGTGCACAAACCACCTCTTTGTTCAGCCTG
TCGATGGGCTGCGTTACCTGGCTGGAACGCTATTTTGCCGATCGTAATCTGGGGCAGGAAAATTTTGATGCTGCAGAAAA
AGCGGCACGCAAAGTGTTACGTCCGGTTGCCGATGAATTACGGTATCACGGCTGGAAAGTGTGCGTTGGCGCTTCCGGCA
CCGTGCAGGCGTTACAGGAAATCATGATGGCACAGGGGATGGATGAACGCATTACCCTGGAAAAGTTGCAGCAACTGAAA
CAGCGCGCCATTCATTGCGGTCGGCTGGAGGAACTGGAGATTGACGGGCTGACGCTGGAACGTGCGTTAGTGTTCCCGAG
TGGTCTGGCGATCCTGATAGCCATTTTTACCGAACTGAATATTCAGTGTATGACCCTGGCGGGCGGTGCGCTGCGTGAAG
GCCTGGTCTACGGTATGTTGCATCTTACCGTCGAGCAGGATATTCGCAGTCGTACGCTGCGTAATATTCAGCGCCGCTTT
ATGATCGATATTGATCAGGCACAGCGCGTAGCCAAGGTTGCGGCTAACTTCTTCGATCAGGTGGAAAATGAATGGCATCT
TGAAGCAATAAGCCGCGATTTGCTCATCAGCGCCTGTCAGCTTCATGAAATCGGCCTGAGCGTTGACTTCAAACAAGCGC
CGCAACACGCTGCTTATCTGGTGCGTAACCTGGATCTTCCCGGTTTTACCCCCGCACAGAAAAAACTGCTGGCGACGCTA
CTGCTCAACCAGACTAATCCGGTCGATCTCTCATCGCTGCATCAGCAAAATGCCGTACCACCGCGCGTCGCAGAACAACT
CTGCCGTTTACTCCGCCTGGCTATCATTTTTGCCAGCCGTCGCCGTGACGATCTCGTGCCAGAGATGACATTACAGGCTA
ACCATGAACTGTTGACCTTGACGCTTCCGCAAGGTTGGCTAACCCAACATCCGCTGGGTAAAGAGATTATTGATCAGGAA
AGCCAGTGGCAGAGCTATGTCCACTGGCCGCTGGAAGTGCATTAA
>Cluster_64789
ATGTTTGAAGATGCTTTAAATGCCGTTAATTCTGTCCGGGATAAAATCGGCGGTGGTAAGAAAACAACCGGCAAAGGCAC
GTTCCGTAACGTGCCGTTTCTCGTCATTGAGGAGCAAAAACAGGCTGGCGGACGTCGTCTGGTTAAACGCGAGTACCCGT
TACGTGATACCGGCGGTGTCAATGACCTGGGGAAAAAACTTCGCTCCCGCACATTCAGCATCTGCATTCTGAACAGCAAC
GCAGAGACTGCCAGAGATGAAGCGGGTGCGCTGATGGATGCTCTTGATGCACCGGGCAGCGGCGAACTGGTGCATCCAGA
GTTCGGCACTGTGGACGTCATGGTGGATTCATGGGAATGCCGTACTAAAGCGGATGAACTGAATTATTACGCGTTCACCG
TTACCGTTTATCCGTCGTTGCAGGATACAGCCCCGGACGCAGAAACAGACACCAGTACAGCCGTATCTGCGCAGGCCGTT
GCTGTAACCGGTTCGCTGGGGGATACGTTGTCCTCTGTCTGGCAGACCGTAAAAGATGGCACAGCGGCGGCAACAGCCGT
AATGGATGCCGTCACCGGTGTCATCGATGATATCTGTGATGCGGTGGATAATCTGGGGATCACCCAGACTGTCAGCGGCC
TGATGGGGTTACTTTCTGCAATGAAAGGTTCTGTGACCGGCCTGATAAATAAACCAGCCATGCTGACATCTTCACTGATG
GGGGCGTTATCCGGCGTTTCATCGTTATGCGATACCCGAACAGCATTTTCCACCTGGAACCGTCTGGCGCAGCGGTTCGA
AAGCCGACATGCCTCCACCACTGACAGACAAGGTGCCATTACCACTTCATACAGCAGCCCGGTTGCAGAAAAAAATATTG
CCACACTGAACTACGTCATGCTGGCAGCAGCACAGACATACCGGGCGGAGGCAGCAAGCCAGGCACTGACTGCGGCACTG
GATTCCAGCCGTCAGATGGATAATGCCGCCCGCGCACCGGCGCTGGATGTATCAGTCACAACAACCGGCATCGCCAGTGG
TGCCAGCAGCACATCGTCCACGGTCACACAAAGCCAGTTGCAGTTAACCGGGATAACCCCGGACGGCGGCTTTTCACCAG
TATCCTTTTCAGACAGTAGCACAGCCACGCCCCCGGTATTTGAAAGCGTGTCCGATATCGAAAAAACAACTGCCATGCTA
GGGGCGGCGCTGGATAGCGTCATTCTGACGGCATCTGAGCAGGGTTTTTCGACAGACAGTGTTCAGCTTACGCAACTGCG
TCTGCTGGTTGTTGCCGACCTGGAAAAACGCGGGCTGCAACTGGCGGGTAGTGAATCACACCACCTGCCAGAAACGCTCC
CGGCAATGGTTGCACTGTACCGGTTCACCGGAAACAGCCGGAACTGGCAACGGCTGGCCCGCAGGAACGGTATCAGCAAC
CCGTTATTTGTTCCCGGTGGTGTCAGTATTGAGGTGATTAATGAGTAA
>Cluster_43384
ATGGAGTTTAGTGTAAAAAGCGGTAGCCCGGAGAAACAGCGGAGTGCCTGCATCGTCGTGGGCGTCTTCGAACCACGTCG
CCTTTCTCCGATTGCAGAACAGCTCGATAAAATCAGCGATGGGTACATCAGCGCCCTGCTACGTCGGGGCGAACTGGAAG
GAAAACCGGGGCAGACATTGTTGCTGCACCATGTGCCAAACGTGCTTTCTGAACGCATCCTCCTTATTGGTTGCGGCAAA
GAACGTGAGCTGGATGAGCGTCAGTACAAGCAGGTTATTCAGAAAACCATTAATACGCTGAATGATACTGGCTCAATGGA
AGCGGTCTGCTTTCTGACTGAACTACACGTTAAAGGCCGTAACAACTACTGGAAAGTGCGTCAGGCTGTCGAGACGGCAA
AAGAGACGCTCTACAGTTTCGATCAGTTGAAAACGAACAAGAGCGAACCGCGTCGTCCGCTGCGTAAAATGGTGTTCAAC
GTGCCGACCCGCCGTGAGCTGACCAGCGGTGAACGCGCGATCCAGCATGGTCTGGCGATTGCCGCAGGGATTAAAGCCGC
GAAAGATCTCGGCAATATGCCACCGAATATCTGTAACGCTGCTTACCTCGCCTCTCAGGCGCGCCAGTTGGCTGACAGCT
ACAGCAAGAATGTCATCACCCGAGTTATCGGCGAACAGCAGATGAAAGAGCTGGGGATGCATTCCTATCTGGCGGTCGGT
CAGGGTTCGCAAAACGAATCGCTGATGTCAGTGATTGAGTACAAAGGCAACGCGTCGGAAGATGCACGCCCAATCGTGCT
GGTGGGTAAAGGTTTAACCTTCGACTCCGGCGGTATCTCGATCAAGCCTTCAGAAGGCATGGATGAGATGAAGTACGATA
TGTGCGGTGCGGCAGCGGTCTACGGCGTGATGCGTATGGTCGCGGAGCTGCAACTGCCGATTAACGTTATTGGCGTGCTG
GCAGGCTGCGAAAACATGCCTGGCGGGCGTGCCTATCGTCCGGGCGATGTGTTAACCACCATGTCCGGTCAAACCGTAGA
AGTGCTGAATACCGACGCCGAAGGCCGCCTGGTACTGTGCGACGTATTAACTTACGTTGAGCGTTTTGAGCCGGAAGCGG
TGATTGATGTGGCGACGCTGACCGGTGCCTGCGTGATCGCGCTGGGTCATCATATTACTGGTCTGATGGCGAACCACAAT
CCGCTGGCCCATGAACTGATTGCCGCATCTGAACAATCCGGTGACCGCGCATGGCGTTTACCTCTGGGTGACGAGTATCA
GGAACAGCTGGAGTCCAATTTTGCCGATATGGCGAACATTGGCGGTCGTCCTGGTGGGGCGATTACCGCAGGTTGCTTCC
TGTCGCGCTTTACCCGTAAGTACAACTGGGCGCATCTGGATATCGCGGGAACCGCCTGGCGTTCTGGTAAGGCGAAAGGC
GCAACCGGTCGCCCGGTGGCGTTGCTGGCACAGTTCCTGCTAAACCGCGCCGGGTTTAACGGCGAAGAGTAA

>Cluster_53541
ATGCAATATTTAAAAAAACGCTTTTCTTTGATTGAACTGAATGTGTTTATTCCAGCAATATTATTTATTGCGGTAATCAT
TCTGTGCTTGACGATATATCCGCAAGATACCAGCCGGTATATAAATAAAATACATCATTTTTTAACCTGGGAAATGGGCG
GGATTTTTCTGGTCATGACTTTTCTGGTTGTACTTTGTTGTCTGTGGCTGGCCTTCTCACGTTATGGCGATATTTTGTTA
GGTCAGTCGGGAGAAAAACCTGACTTCAGCTTGTTAACCTGGCTTGGACTTATTTTTACTTCTGGAACGGGGGGCAGCTT
GTTATATCTGGCCTCTGTAGAGTGGATTTGGATCATTCAGCAACCGCCTTTTGGCGCAACAGCAGGAAGCGCTCAGGCTG
CTCGTTGGGCCTCTGCTTATGGCATGTTCCATTGGGGGCCGTCCGCATGGGCATGGTATTTGATTTGCGCCGTTCCTATT
GGTTGGTTTATGCATGTTAAGAAAACAAACTCATTAAAGGTCAGTGATTTATGTCGTGGGTGTTTGGGAGCGCGTGCGGA
TGGTTTTTGCGGGCATTGTGTGAATTTTTTCTACATGTTTGGTTTGCTCGGCGGCGCGGTAACGTCTCTGGCGCTGGGAA
CGCCGATGATTTCTGCCGTATTTTGCCATGTGTTCCATCTGGATCCTGCCGGGCAGTTTATCAATGTCATGGTTATTTTT
ATCTGGACGCTAGTACCATTATTCATTCTCTTTTTTGGACTTAAAAAAGGTGTGGCATGGGCCAGTAACTGGAATATTCG
TGCTGACATTTTGATGCTACTGGCAATACTGATTTGTGGACCAACGGCATTTATACTTAATCAATCAATTGATGGTCTCG
GCCTGATGCTGCAAAATTTTGTAGCGATGAGTTTAAGTACCGATGCTATTGGTCGTAGCGGATTTCCACAGATGTGGACC
GTATTTTATTTTTCATGGTGGGTCGTGTATGCCATCCCATTCGGTTTATTTATCGCCCGTATTTCAAAAGGAAGGACGAT
CCGGCAATTGATTGTATGTGGAACTCTGGCAGGTTCATTGGGATGCATGGTTTTTTACATGGTACTGGCTAATTTCGGCT
TATCGTTGCAGACAACTCATGTCATCGATTTTGTTCCCATACTTAACGAACAAGGGCGAGGCGTTGTCGTTTCTCGTTTA
CTGGAGCAGCTACCCGCAAGTCAGGTGTTTTTGGTTGCTTTTGGGGCTATAGCATTAATTTCATATATTACCGGACACTG
TACTGTGGGTTATGCCCTCGGTTTTGCGACGCAAAAACGGGCAGATAGTGAGAGTGAACCGGCATTCTGGAACGTGGCAT
TTTGGTTGATTATGACTGGAATCGTCGCCATCACACTCTATCTTCTTGATGCGCAGAGTTTGCAGCCGCTACAAACGGTC
TCTATCCTGGCCGGACTACCGCTTTGCGGCGTAGTGTTTATTTTATTGAAGAGTTTTTTGACACAGCTTGCGGCTGAAGA
GAAAACCGCGAGAGATGAATAA
>Cluster_11957
GTGTCCACTGCAAACCAAAAACCAACTGAAAGCGTCAGTTTGAACGCTTTCAAACAACCGAAGGCGTTCTATCTCATCTT
CTCGATTGAGTTATGGGAACGTTTTGGTTATTACGGCCTACAAGGAATTATGGCTGTTTACCTGGTTAAACAACTGGGTA
TGTCTGAAGCGGATTCAATCACCCTTTTCTCTTCCTTTAGTGCCCTGGTTTATGGTCTGGTCGCTATCGGCGGCTGGTTA
GGTGACAAGGTACTGGGTACTAAACGCGTAATTATGCTCGGCGCTATTGTGCTGGCGATTGGTTATGCTCTGGTTGCCTG
GTCTGGTCACGACGCCGGTATCGTTTATATGGGTATGGCGGCTATTGCGGTCGGTAACGGCCTGTTTAAAGCTAACCCGT
CTTCTCTGCTTTCTACATGCTATGAGAAAAACGACCCGCGTCTGGACGGTGCATTCACCATGTACTACATGTCCGTCAAC
ATCGGCTCTTTCTTCTCTATGATTGCTACGCCGTGGCTGGCCGCGAAATACGGCTGGAGTGTTGCGTTTGCGTTGAGCGT
TGTAGGCCTGCTGATCACTATCGTTAACTTCGCCTTCTGCCAACGCTGGGTTAAACAGTACGGTTCAAAACCAGACTTCG
AGCCTATCAACTACCGTAACCTGCTGCTGACCATTATTGGTGTTGTGGCACTGATCGCTATCGCCACCTGGCTGCTGCAC
AATCAGGAAGTTGCGCGTATGGCGCTGGGCGTTGTTGCCTTCGGTATCGTGGTTATCTTCGGTAAAGAAGCCTTCGCGAT
GAAAGGTGCTGCGCGTCGTAAAATGATCGTTGCCTTCATCCTGATGCTCGAAGCCATTATCTTCTTCGTGCTGTACAGCC
AGATGCCAACGTCACTGAACTTCTTTGCGATTCGTAACGTTGAGCACTCCATTCTGGGTCTGGCCGTAGAACCTGAGCAG
TATCAGGCACTGAACCCGTTCTGGATCATCATCGGTAGTCCGATTCTGGCCGCTATCTATAACAAGATGGGCGATACCCT
GCCGATGCCAACCAAGTTTGCAATCGGCATGGTGATGTGTTCTGGTGCGTTCCTGATTCTGCCGCTGGGTGCGAAATTCG
CGTCTGACGCTGGTATCGTGTCTGTAAGCTGGCTGGTCGCAAGCTATGGCCTGCAGAGCATCGGGGAACTGATGATCTCT
GGTCTGGGTCTGGCAATGGTTGCACAACTCGTTCCGCAGCGTCTGATGGGCTTCATTATGGGTAGCTGGTTCCTGACCAC
TGCCGGTGCAAACCTGATTGGTGGTTATGTTGCGGGTATGATGGCTGTGCCGGATAACGTTACCGACCCGCTGATGTCAC
TGGAAGTTTATGGTCGCGTATTCTTGCAGATTGGTGTCGCTACTGCCATTATTGCAGTACTGATGCTGCTGACCGCGCCT
AAACTGCACCGTATGACGCAGGATGACGCTGCAGACAAAGCGGCGAAAGCTGCTGTAGCGTAA
>Cluster_26026
ATGGCTATTAGCACACCGATGTTGGTGACATTTTGTGTCTATATCTTTGGCATGATATTGATTGGGTTTATCGCCTGGCG
ATCAACGAAAAACTTCGACGACTATATTCTGGGCGGTCGTAGTCTTGGGCCATTCGTGACGGCATTATCGGCGGGTGCGT
CGGATATGAGCGGCTGGCTGTTAATGGGGTTGCCAGGCGCTGTTTTTCTTTCCGGGATTTCCGAAAGCTGGATCGCCATT
GGTCTGACATTAGGCGCGTGGATCAACTGGAAGCTGGTGGCCGGGCGGTTGCGTGTGCATACCGAATACAACAATAACGC
CTTAACATTGCCGGATTATTTCACCGGGCGCTTTGAAGATAAAAGCCGCATTTTGCGCATTATCTCCGCGCTGGTTATTT
TGCTGTTCTTCACCATTTATTGCGCTTCGGGCATTGTGGCAGGCGCGCGTCTGTTTGAAAGTACCTTTGGCATGAGCTAC
GAAACGGCTCTGTGGGCGGGGGCTGCGGCGACGATCCTTTACACCTTTATTGGCGGTTTCCTCGCGGTGAGCTGGACTGA
CACTGTACAGGCCAGCCTGATGATTTTTGCCCTGATCCTGACGCCGGTTATCGTGATCATTAGCGTCGGTGGGTTTGGCG
ACTCGCTGGAAGTAATCAAACAAAAGAGCATCGAAAACGTGGATATGCTCAAAGGGTTGAATTTTGTCGCCATTATCTCG
CTGATGGGGTGGGGGCTGGGTTACTTCGGGCAGCCGCACATCCTGGCGCGTTTTATGGCGGCAGATTCTCACCACAGCAT
TGTCCATGCGCGTCGTATCAGTATGACCTGGATGATCCTCTGCCTGGCAGGGGCGGTGGCTGTCGGCTTCTTTGGTATCG
CTTACTTTAACGAGCACCCGGCGGTAGCAGGTGCGGTAAACCAGAACGCTGAACGCGTGTTTATTGAACTGGCGCAAATT
CTGTTTAACCCGTGGATTGCCGGGATTTTGCTGTCGGCGATTCTGGCGGCGGTAATGTCAACGTTAAGTTGCCAGCTGCT
GGTATGTTCCAGTGCGATTACCGAAGATTTATACAAAGCGTTTCTGCGTAAACAGGCCAGCCAGAAAGAGCTGGTGTGGG
TAGGGCGAGTGATGGTGCTGGTGGTGGCGCTGGTGGCGATTGCGCTGGCGGCAAACCCGGAAAACCGCGTGCTGGGCTTA
GTGAGCTACGCGTGGGCAGGCTTTGGCGCAGCGTTTGGTCCGGTGGTGCTGTTCTCGGTGATGTGGTCACGCATGACGCG
CAACGGTGCGCTGGCGGGGATGATCATCGGTGCGCTGATGGTTATCGTCTGGAAACAGTTCGGCTGGCTGGGTCTGTACG
AAATTATTCCGGGCTTTATCTTCGGCAGTATCGGGATTGTAGTGTTTAGTTTGCTGGGTAAAGCGCCGTCAGCGGCGATG
CAAAAACGCTTTGCCGAGGCCGATGCGCACTATCATTCGGCTCCGCCGTCACGGTTGCAGGAAGGGTAA
>Cluster_8556
ATGAGAATTCACTCACCGTACCCCGCCAGTTGGGCGCTGGCACAACGAATTGGTTATCTCTATTCAGAGGGCGAGATTAT
TTATCTCGCCGATACGCCATTCGAGCGGTTACTCGATATTCAACGTCAGGTTGGCCAGTGCCAGACCATGACCAGCTTGT
CACAGGCTGATTTTGAAGCTCGGCTGGAAGCGGTATTCCATCAGAATACCGGTGAGTCGCAACAGATTGCGCAGGATATC
GATCAATCCGTCGATCTTCTCTCGCTTTCGGAAGAGATGCCCGCAAATGAAGATCTCCTGAATGAAGATTCAGCGGCACC
GGTTATCCGCTTGATCAATGCGATTTTGAGTGAGGCCATCAAAGAAACCGCCTCTGATATCCACATTGAAACCTATGAAA
AAACAATGTCGATCCGTTTTCGCATCGACGGCGTTTTGCGGACAATTTTACAGCCAAACAAAAAACTGGCGGCACTGCTT
ATCTCCCGAATTAAGGTCATGGCTCGTCTTGATATCGCCGAAAAACGTATTCCACAGGATGGAAGAATTAGTTTGCGTAT
CGGGCGACGTAACATAGATGTCCGCGTATCCACACTGCCGTCCATCTATGGTGAACGCGCCGTACTCCGCCTGCTGGATA
AAAACAGCCTCCAGCTTTCATTGAACAACCTGGGGATGACGGCAGCGGATAAGCAGGATTTAGAAAATCTCATTCAGCTT
CCGCACGGTATTATCCTGGTGACAGGGCCGACAGGCTCCGGTAAAAGCACCACGCTCTACGCCATCCTTTCGGCGCTGAA
TACTCCCGGCCGCAATATTCTGACGGTAGAAGATCCCGTGGAATATGAGCTGGAAGGCATTGGGCAAACGCAGGTGAATA
CCCGTGTGGATATGTCTTTCGCTCGCGGCCTGCGCGCCATACTTCGCCAGGACCCGGATGTCGTCATGGTGGGGGAAATT
CGTGATACAGAAACCGCGCAGATTGCGGTTCAGGCCTCGCTCACCGGCCATCTGGTACTCTCAACACTCCACACTAACAG
TGCATCAGGCGCAGTGACCCGGCTCCGCGACATGGGCGTCGAATCATTCCTGCTTTCGTCTTCCCTGGCAGGGATTATCG
CGCAACGTCTGGTTCGTCGCCTGTGTCCGCAATGCCGACAATTCACGCCCGTATCACCCCAACAAGCGCAGATGTTTAAA
TATCATCAGCTCGCGGTGACAACAATTGGCACTCCCGTAGGCTGCCCTCATTGCCATCAATCCGGCTATCAGGGGCGCAT
GGCGATCCACGAAATGATGGTGGTGACGCCGGAATTACGGGCCGCTATTCATGAAAATGTGGATGAACAAGCACTGGAGC

GACTAGTCCGGCAACAACACAAGGCCTTAATCAAAAATGGCCTGCAAAAAGTGATAAGCGGTGACACCTCCTGGGATGAG
GTTATGCGCGTCGCCAGTGCCACGCTGGAGAGCGAAGCATGA
>Cluster_41603
ATGTCTGAATTTGAAGTGTTGGCACAGCATCTGCTGAAGGAAGCCGAGGCGGAAGAAAAGCTGCGACAGGAAAATGATAA
AAAGCTTATCGAGAAAGTGCTGGAAATCTATGATCAGAAGTATGTGGCTGAATTACTGAGAAAAGTCGGAAAAAATGAGT
GGAGCCGTGAAACCATCAACCGCTGGATTAATGGCAAGTGTTTACCAAAATCGTTGACTTCAGTAGAAGAGTCTCTTCTG
CGTAAGATGCTACCAGAACCACCTGCAAACCATCCGGAATATGCTTTCCGCTTCATTGACTTATTTGCTGGAATTGGAGG
GATACGGAAAGGTTTTGAGGCCATTGGAGGCCAGTGTGTTTTTACCAGTGAATGGAATAAAGATGCTGTGCGTACATACA
AGGCCAACTGGTTTAACGATGAACAGGTGCATAAATTCAATCTCGATATTCGGGAAGTCACGCTGAGTGATAAAACCGAT
GTATTGGAAACGGATGCTTATGCATATATTGATGAGCATGTGCCGGATCATGATGTGCTTCTTGCGGGGTTCCCGTGCCA
GCCATTTAGCCTTGCTGGTGTTAGTAAGAAAAACTCACTTGGTCGTGCGCATGGTTTTGAATGTGAAGCACAAGGAACAC
TTTTTTTTGACGTGGCACGTATTATACGAGCAAAAAAACCGGCAATTTTTGTGCTGGAAAATGTCAAAAATCTGAAGAGC
CATGATAAGGGTAAAACTTTCAAAGTCATTATGGAGACCCTTGACGAGCTGGGCTACGAAGTTGCCGATGCTGCAGAAAT
GGGAAAAAACGATCCAAAGATTATCGATGGGAAGCATTTTTTGCCTCAGCATCGTGAACGCATTGTTCTGGTCGGATTTA
GACGAGATCTGAATATTCACAAGGGGTTTACCTTGCGTGATATCAGTCGTTTTTATCCGGAACACCGCCCATCATTTGGT
GAATTGCTGGAGCCTGTAGTCGACAGTAAATATATACTTACGCCAAAACTATGGGAGTACCTTTATAACTACGCCAAAAA
GCATGCAGCCAAGGGGAATGGTTTTGGCTTTGGGTTGGTCAATCCTGAGAATAAGGAAAGCATTGCACGCACACTTTCTG
CCAGATATCACAAAGACGGATCAGAAATTCTTATTGATCGTGGTTGGGATATGGCAACGGGAGAGGCTGACTTTATGAAT
GAGAGTAACCAGGCTCGTCGACCACGCAGGCTGACTCCCCGAGAGTGCGCACGCCTTATGGGGTTTGAAAAACCTGGAGG
GAAACCATTCCGTATTCCGGTTTCAGATACTCAGTCATATCGGCAGTTCGGTAACTCCGTTGTGGTGCCAGTATTTGAAG
CCGTTGCCAGACTTCTGGAACCCTATATCCTCAAAGCTGTTTCTGCTGATGCTGGTAAGACTGGGCAGCCTTGA
>Cluster_21057
ATGATATCTGATAACAAAGTTACCTATCATGAGGTTGACTTCCTGCTGCCAGCACAGAGGTTTAACATTCAGTTTTCCTA
TGTTTCCCAGAAGGGGCTGCCATTTATCCGGGAGTTTGTGCTGAGACTTGTTCATGTAGCTTCGATGAGCAAAGCACAAA
TAGCAACCTATTTCGGCCTGACCCACCGTGAGACAGAGGAGGCGATTTCCGATCTTGTGCAGCGTGGCGAATTGACGCTC
TCCTCAGACGGCAGACTGGCCTTAACAGACAAATCAAATGGCTATTTTTCAGAGGTTGGCGAGATACCGCATCTTTCAAC
TATTCAGGACAGTGGCTGTACTTTATCCTTTGATTTAGCCACATTTAGTTGCTTCAGCAACCAGGCTTTTCAGGACCACT
GGAGGGGAGGGGTAACATTAAAGGTTGATGACAACAACATTTCTCAAAGTGAGAAACTGGTTGAGAAGCACTTTCAATAC
CAGTTTAATCAAATCCTGGATAAAGGTTACCTGTCTCACCTGCAGGTTCAGGTCGGAAAAGAACAACCTTCTATCTATAC
CGTTAATTCTGTTAATAAGCTGCGGCAAATACCACTCCGTTTAACAACGGAATTTAAAATGGATAGCGATGGTAAGGCTG
TCGAGCGTGAAGACTATGAACAGTTAAATAGTTCGGAATGTGTTCATGAGTTAATGGCTGTTGAGATAGCCAGGTTATCA
CGGCATAACAATACCATGAGTATTTTTAAGGCCATGCAATCCATTAACGATGACCTTACGCTTAAGCTGTTTGACTCTAA
AACGAATCGACTTAATCCGTTGTTTATCAAAGATATGCAAGCACTGGAAGAATACTCGTCCTCAGGCAGAACGACTTTTT
TAGGTCCGGTTTATTCTAAAAACAACTGGGAAAAGCTGCAGAAAGCTCTGGCACCAGCGCTCAATCAGCGTATCAGAGAT
AAAACTGATTATGGAGGTAGCCCGTTCATCTGGGTGGCACCATCAGATCCCTACTGGTCAAAAAGCATTCGATTTATCTC
AAGCCTGAGCAATTTTCTTAGCAAGTCGGCTACGAAGGATAAGCAGCTGTATAAACCGGTGCTGTACGTACCTGTCCGGG
ATGCAGCTGATGCTCGGGTTGCCAGACAGTGGAAGTATGAACTTGGGCAATTCCATGAGTACGCTAAAGGACTAGTTGAG
GGGCTGTTAGATGGTAATGTTGAGATCCTTCATCTTGAGGATGAGTTATCTGCCGTTATCTATCATATTTCTCAACCCGA
AATATTGCCTGTAACGATGCCACTGGGCTTTATCACAACGAACAAAGAAACGGTTAAGATTATTGGTACTCTGGTTACTG
AATATATCAAAGGCACCACTGGTTTTGATAAGTTTCATGACTGTGGCGCAATTTCGGCAATGATTAAGAAAGGTTGA
>Cluster_55661
ATGATAAAAATTGCACGCATTGCCGTGACATTGGGCTTGCTTTCCTCACTGGGAGCCCAGGCTTACGCGGCCGGGTTAGT
GGTAAATGATAACGATCTGCGAAACGACCTTGCCTGGCTTTCCGATCGCGGGGTCATCCATCTGAGCCTGTCGACCTGGC
CGCTGAGCCAGGAAGAGATCGCCCGGGCGCTAAAAAAGGCCAAACCGTCCTATTCTTCTGAGCAAGTGGTGCTGGCCCGT
ATCAATCAGCGACTGTCTGCTTTAAAAGCCGATTTTCGGGTCACCGGTTATACCTCAACCGATCAGCCGGGCACTCCGCA
GGGGTTTGGTCAGACACAGCCGGCAGATAATTCGTTAGGCCTGGCGTTTAACAACAGCGGACAATGGTGGGATGTCCACC
TCCAGGGTAACGTCGAAGGGGGGGAGCGGATCAGCAACGGATCGCGTTTCAACGCCAACGGTGCCTACGGTGCGGTGAAA
TTCTGGAACCAGTGGCTCTCTTTTGGCCAGGTACCACAGTGGTGGGGACCGGGCTATGAAGGAAGCCTGATCCGCGGGGA
TGCGATGCGGCCGATGACCGGCTTCCTGATGCAGCGGGCAGAGCAGGCGGCGCCAGAGACCTGGTGGTTACGCTGGGTGG
GTCCATGGCAGTACCAGATCTCCGCCAGCCAGATGAATCAATATACCGCTGTGCCTCATGCCAAAATTATCGGCGGACGT
TTTACCTTCACGCCGTTCCAGTCATTAGAATTAGGTGCGTCGCGTATTATGCAGTGGGGCGGGGAAGGGCGACCGCAATC
CTTCAGCAGTTTCTGGGATGGATTTACAGGGAAAGACAATACCGGAACGGATAACGAGCCGGGGAACCAACTGGCCGGAT
TTGACTTTAAGTTTAAACTGGAGCCAACCCTCGGCTGGCCAGTGAGTTTCTACGGTCAAATGATTGGTGAGGATGAATCT
GGTTATCTACCATCGGCAAACATGTTCCTGGGAGGTGTCGAAGGCCACCACGGCTGGGGTAAAGATGCGATAAACTGGTA
TCTTGAAGCACATGATACGCGCACTAATATGAGTCGAACCAATTACAGCTACCGTCACCATATTTATAAAGATGGATATT
ATCAGCAAGGCTATCCGCTCGGCGATGCGATGGGCGGGGATGGTCAACTCATCGCCGGTAAGATTGGGCTTATTACAGAA
GATAATCAGCGCTGGAGCACGCGTTTGGTATACGCCAAAGTTAACCCGGAGAATCAGTCGATCAATAAAGCATTCCCTCA
TTCTGACACCTTGAAGGGTGTACAGCTGGGATGGAGCGGAGATGTTTATCAGTCGGTCCGTTTGAATACTTCACTGTGGT
ACACCAACGCTAACAACAGCGACAGCGATGACGTTGGGGCCAGCGCAGGGATAGAAATACCGTTTAGTTTATAA
>Cluster_18685
ATGCAGGTTTTACATGTATGTTCAGAGATGTTCCCGCTGCTTAAAACCGGCGGTCTGGCTGATGTTATTGGGGCATTACC
CGCAGCACAAATCGCAGACGGCGTTGACGCTCGCGTACTGTTGCCTGCATTTCCCGATATTCGCCGTGGCGTGACCGATG
CGCAGGTAGTATCCCGTCGTGATACCTTCGCCGGACATATCACGCTGTTGTTCGGTCATTACAACGGGGTTGGCATATAC
CTGATTGACGCGCCGCATCTCTATGATCGTCCGGGAAGCCCGTATCACGATACCAACTTATTTGCCTATACCGACAACGT
ATTGCGTTTTGCGCTGCTGGGGTGGGTTGGGGCAGAAATGGCCAGCGGGCTTGACCCATTCTGGCGTCCTGATGTGGTGC
ATGCGCACGACTGGCATGCAGGCCTTGCGCCTGCGTATCTGGCGGCGCGCGGGCGGCCGGCGAAGTCGGTGTTTACTGTG
CACAACCTGGCCTATCAAGGCATGTTTTATGCACATCACATGAATGACATTCAATTGCCATGGTCGTTCTTTAATATTCA
TGGGCTGGAATTCAACGGACAAATCTCTTTCCTGAAGGCCGGTCTGTACTATGCCGATCACATTACGGCAGTCAGCCCAA
CCTACGCTCGCGAGATCACCGAACCGCAGTTTGCCTACGGTATGGAAGGTCTGTTGCAACAGCGTCACCGTGAAGGGCGT
CTTTCCGGCGTACTGAACGGCGTGGACGAGAAAATCTGGAGTCCAGAGACGGACTTACTGTTGGCCTCGCGTTACACCCG
CGATACGTTGGAAGATAAAGCGGAAAATAAGCGCCAGTTACAAATCGCAATGGGGCTTAAGGTTGACGATAAAGTGCCGC
TTTTTGCAGTGGTGAGCCGTCTGACCAGCCAGAAAGGTCTCGACCTGGTGCTGGAAGCCTTACCTGGTCTTCTGGAGCAG
GGCGGGCAGCTGGCGCTACTCGGCGCGGGCGATCCGGTGCTGCAGGAAGGTTTCCTTGCGGCGGCAGCGGAATACCCCGG
CCAGGTGGGCGTTCAGATTGGCTATCACGAAGCATTTTCGCATCGCATTATGGGCGGCGCGGACGTCATTCTGGTGCCCA
GCCGTTTTGAACCGTGCGGCTTAACGCAACTTTATGGATTGAAGTACGGTACGCTGCCGTTAGTTCGGCGCACTGGTGGG
CTTGCTGATACGGTTTCTGACTGTTCTCTTGAGAACCTTGCAGATGGCGTCGCCAGTGGGTTTGTCTTTGAAGATAGTAA
TGCCTGGTCGCTGTTACGGGCTATTCGACGTGCTTTTGTACTGTGGTCCCGTCCTTCACTGTGGCGGTTTGTGCAACGTC
AGGCTATGGCAATGGATTTTAGCTGGCAGGTCGCGGCGAAGTCGTACCGTGAGCTTTACTATCGCTTGAAATAG
>Cluster_9282

ATGAATACCCAGTATAATTCCAGTTATATATTTTCGATTACCTTAGTCGCTACATTAGGTGGTTTATTATTTGGCTACGA
CACCGCCGTTATTTCCGGTACTGTTGAGTCACTCAATACCGTCTTTGTTGCTCCACAAAACTTAAGTGAATCCGCTGCCA
ACTCCCTGTTAGGGTTTTGCGTGGCCAGCGCTCTGATTGGTTGCATCATCGGCGGTGCCCTCGGTGGTTATTGCAGTAAC
CGCTTCGGTCGTCGTGATTCACTTAAGATTGCTGCTGTCCTGTTTTTTATTTCTGGTGTAGGTTCTGCCTGGCCAGAACT
TGGTTTTACCTCTATAAACCCGGACAACACTGTGCCTGTTTATCTGGCAGGTTATGTCCCGGAATTTGTTATTTATCGCA
TTATTGGCGGTATTGGCGTTGGTTTAGCCTCAATGCTCTCGCCAATGTATATTGCGGAACTGGCTCCAGCTCATATTCGC
GGGAAACTGGTCTCTTTTAACCAGTTTGCGATTATTTTCGGGCAACTTTTAGTTTACTGCGTAAACTATTTTATTGCCCG
TTCCGGTGATGCCAGCTGGCTGAATACTGACGGCTGGCGTTATATGTTTGCCTCGGAATGTATCCCTGCACTGCTGTTCT
TAATGCTGCTGTATACCGTGCCAGAAAGTCCTCGCTGGCTGATGTCGCGCGGCAAGCAAGAACAGGCGGAAGGTATCCTG
CGCAAAATTATGGGCAACACGCTTGCAACTCAGGCAGTACAGGAAATTAAACACTCCCTGGATCATGGCCGCAAAACCGG
TGGTCGTCTGCTGATGTTTGGCGTGGGCGTGATTGTAATCGGCGTAATGCTCTCCATCTTCCAGCAATTTGTCGGCATCA
ATGTGGTGCTGTACTACGCGCCGGAAGTGTTCAAAACGCTGGGGGCCAGCACGGATATCGCGCTGTTGCAGACCATTATT
GTCGGAGTTATCAACCTCACCTTCACCGTTCTGGCAATTATGACGGTGGATAAATTTGGTCGTAAGCCACTGCAAATTAT
CGGCGCACTCGGAATGGCAATCGGTATGTTTAGCCTCGGTACCGCGTTTTACACTCAGGCACCGGGTATTGTGGCGCTAC
TGTCGATGCTGTTCTATGTTGCCGCCTTTGCCATGTCCTGGGGTCCGGTATGCTGGGTACTGCTGTCGGAAATCTTCCCG
AATGCTATTCGTGGTAAAGCGCTGGCAATCGCGGTGGCGGCCCAGTGGCTGGCGAACTACTTCGTCTCCTGGACCTTCCC
GATGATGGACAAAAACTCCTGGCTGGTGGCCCATTTCCACAACGGTTTCTCCTACTGGATTTACGGTTGTATGGGCGTTC
TGGCAGCACTGTTTATGTGGAAATTTGTCCCGGAAACCAAAGGTAAAACCCTTGAGGAGCTGGAAGCGCTCTGGGAACCG
GAAACGAAGAAAACACAACAAACTGCTACGCTGTAA
>Cluster_13826
ATGCTGAATGACTTTTCGCAAATAGATAACGATTTAGGCCTGAATATCATCTATTTCCTGCAACATGGTCACTGGAAGGT
AAATGATCGTAGTCACCAGATGGAGAAAGTATGGTTTTACAATAGTGAACCTTCTATAGATATTCAAGAATATAATAGAT
TCGCCGACAATACTACTGACACGTTTATCTTTACAATTATACCAGATAATAACCATGTGTTAAAATTATCGTCTCCCATT
ACCGTTACTGTTGAGTGTAAGGGCGGTTATTATTTTATTAATTCCTCTGGAGATAAATCAGATGTAATTTATAAAGTTGA
TGGGCTTTCTATCATAGCGAGAAATTTCTTTACTCTTCTTAGTGGTAATTTCAAACCTGACTGGCGTTGGGATGTCTCTA
AAGAGACGTTTACTAAGGAAAAGTTTGATAGCTATGTTAAATCTGTGTTTTCAAAAATAGACTTTTATAAACAGTGTGGT
GTGATTAACCCACAAAATGCCAACACGGCTTATTTTGGCGATACAGATGGAAGAGTAGGCGCTGTACTTTATGCATTGCT
CGTTTCAGGGCATATAGGCATCAGGGAAAAGGGTTGGAGTTTATTATGTGAATTATTAAAACACGAAGACATGGCTTCAT
CTGCATACAAACATAAAAATAATAAAGTGCTCTACGATTTATTAAATACGAGAGACATGATCTTGAATGAGTTGCATCAA
CATGTTTTTTTAAAGGACGATGCGATAACCCCTTGTATCTTCCTTGGGGATCATACAGGCGATAGATTTAGTACTATTTT
TGGTGATAAATATATCCTTACTTTGCTAAATTCTATGCGAAATATGGAGGGTAATAAAGATAGCCGTATTAATAAAAATG
TCGTCGTTCTGGCTGGAAATCATGAAATCAATTTTAATGGTAACTATACTGCACGACTAGCAAATCATAAACTCTCTGCC
GGTGATACGTATAACTTAATTAAAACGCTCGATGTTTGTCATTATGACTCAGAAAGGCAAGTTTTAACCAGTCATCACGG
AATAATAAGAGATGAAGAAAAAAAATGTTATTGTCTCGGTGCTTTGCAGGTTCCATTTAATCAGATGAAAAATCCTACTG
ATCCTGAGGAATTAGCCAATATTTTTAATAAAAAACATAAAGAACACATGGATGATCCTCTATTCCATCTAATCCGAAGC
AATACTTTGAAACCCACAGCTGTATATGCTGATTATATTAATAACACTACTGATTTCAGACCAGCACGTGAACGTATCTT
CATATGTGGGGAGACACTCAAAGGTGAAGACCCCAGCAAATACATTAGACAAAAATATGGGCATCATGGGTCCGGGGTAG
ATCAGAATCAACAATTTGATAATGGTATTATGGGGCTAAATTCCCTGAAGGAAGCACGTGATAAAAATAATAAAATAATT
TATTTCTCAGGGCTTAGTTGTTTTCAATCTCATTAA
>Cluster_21523
ATGTCAGTACCCGTTCAACATCCTATGTATATCGATGGACAGTTTGTTACCTGGCGTGGAGACGCATGGATTGATGTGGA
AAACCCTGCTACAGAGGCTGTCATTTCCCGCATTCCCGATGGTCAGGCCGAGGATGCCCGTAAGGCAATCGATGCAGCAG
AACGTGCACAACCAGAATGGGAAGCGTTGCCTGCTATTGAACGCGCCAGTTGGTTGCGCAAAATCTCCGCCGGGATCCGG
GAACGTGCCAGTGAAATCAGTGCGCTGATTGTCGAAGAAGGGGGCAAGATCCAGCAGTTGGCTGAAGTCGAAGTGGCTTT
TACTGCGGACTATATCGATTATATGGCGGAGTGGGCACGGCGTTACGAGGGCGAGATTATTCAAAGCGATCGTCCAGGAG
AAAATATTCTTCTGTTTAAACGTGCGCTTGGCGTGACTACCGGCATTCTGCCGTGGAACTTTCCGTTCTTCCTCATTGCC
CGCAAAATGGCTCCCGCTCTTTTGACCGGTAATACCATCGTCATTAAACCCAGTGAATTTACGCCAAACAATGCGATTGC
ATTCGCCAAAATCGTTGATGAAATAGGCCTTCCGCGCGGCGTGTTTAACCTTGTACTCGGGCGTGGTGAAACCGTTGGGC
AAGAACTGGCGGGTAACCCAAAGGTCGCAATGGTCAGTATGACAGGCAGCGTCTCTGCAGGTGAAAAGATTATGGCGACT
GCGGCGAAAAATATCACCAAAGTGTGCCTGGAATTGGGGGGTAAAGCACCGGCTATCGTAATGGACGATGCCGATCTTGA
ACTGGCAGTCAAAGCCATCGTTGATTCACGCGTCATTAATAGTGGGCAAGTGTGTAACTGTGCAGAACGTGTTTATGTAC
AGAAAAGCATTTATGATCAGTTCGTCAATCGGCTGGGTGAAGCGATGCAGACGGTTCAATTTGGTAACCCCGCTGAACGC
AACGACATTGCGATGGGACCGTTGATTAATGCCGCGGCGCTGGAAAGGGTTGAGCAAAAAGTGGCGCGCGCAGTAGAAGA
AGGGGCGAGAGTGGCGTTGGGTGGCAAAGCGGTAGAGGGGAAAGGATATTATTATCCGCCGACATTGCTGCTGGATGTTC
GCCAGGAAATGTCGATTATGCATGAGGAAACCTTTGGCCCGGTTCTGCCGGTGGTCGCATTTGACACGCTGGAAGAGGCT
ATCTCAATGGCTAATGACAGTGATTACGGCCTGACCTCATCAATCTATACCCAAAATCTGAACGTCGCGATGAAAGCCAT
TAAAGGGCTGAAGTTTGGTGAAACTTACATCAACCGTGAAAACTTCGAAGCTATGCAAGGCTTCCACGCTGGATGGCGTA
AATCCGGTATTGGCGGCGCAGATGGTAAACATGGCTTGCATGAATATCTGCAGACCCAGGTGGTTTATTTACAGTCGTAA
>Cluster_20637
ATGTCATCGTTGCAAATTTTGCAAGGCACGTTTCGTCTTGGCGACACAAAAACGCTGCAATTGCCTCGGCTAACGTTAAA
CGCGGGTGATAGTTGGGCGTTTGTCGGTTCGAATGGAAGCGGGAAATCGGCGCTGGCCCGCGCGCTGTCGGGGGAACTTC
CGCTTTTGAAAGGTGAACGGCAAAGCCAGTTTTCCCACATCACTCGTCTCTCCTTCGAGCAATTGCAAAAGCTCGTCAGC
GACGAATGGCAGCGGAATAACAGCGATATGCTCGGCCCTGGCGAAGATGACACCGGGCGTACCACGGCTGAGATCATTCA
GGATGAAGTAAAGGATACTCCGCGTTGCATGCAACTGGCGCAGCAATTCGGTATTACCGCCCTCCTCAACCGACGCTTTA
AATACCTTTCCACTGGCGAGACGCGAAAAACCCTGCTGTGTCAGGCGCTGATGTCGGAGCCTGACTTGTTGATCCTTGAT
GAACCGTTCGATGGCCTGGATGTTGCCTCACGTCAGCAGCTGGCTGAGCTACTCGCCTCGTTACATCAGTCCGGTATTAC
TCTGGTACTGGTGCTCAATCGCTTCGATGAGATCCCGGAATTTGTCCAGTTTGCTGGCGTGCTGGCGGATTGCACCTTAG
CGGAAACTGGCGCTAAAGAAGAACTGCTCCAACAAGCACTCGTTGCGCAACTGGCGCATAGCGAACTGCTTGAAGGTGTG
CAAATGCCGGAGCCGGATGAACCTTCAGTACGTCACGCCTTACCCGCCAACGAACCGCGCATTGTGCTGAACAATGGCGT
GGTTTCTTATAACGATCGCCCCATTCTTAATAACCTTAGCTGGCAGGTGAATCCAGGCGAACACTGGCAAATTGTCGGGC
CAAATGGCGCGGGAAAATCGACATTATTAAGCCTGATTACTGGCGATCATCCGCAAGGTTACAGCAACGATTTGACGCTT
TTCGGACGACGTCGCGGCAGCGGCGAAACCATCTGGGATATCAAAAAGCATATCGGTTACGTCAGCAGTAGTTTGCATCT
GGATTACCGGGTCAGCACTACTGTGCGTAATGTGATTCTTTCTGGCTATTTTGATTCGATTGGCATTTATCAGGCCGTTT
CGGATCGCCAGCAAAAACTGGTGCAGCAGTGGCTGGATATTCTCGGCATTGATAAACGCACGGCTGACGCTCCTTTCCAT
AGTCTTTCCTGGGGACAGCAGCGTCTGGCGCTGATCGTCCGTGCGCTAGTGAAACATCCGACGTTGCTTATTCTCGATGA
ACCACTACAGGGGCTTGATCCGCTCAATCGCCAGCTTATCCGCCGTTTTGTTGATGTGCTGATTAGCGAAGGTGAAACGC
AATTGTTGTTTGTTTCGCACCACGCTGAAGATGCGCCTGCCTGTATTACCCATCGTCTTGAGTTCGTGCCGGACGGTGAC
TTTTATCGCTATGCGCTGACAAAAATAAACTGA
>Cluster_66951

ATGAAAAAATTACACCAGCGTTCAATGAAGCTGGCGGTGCTCCCCTGCATGATCGCCGTCGCTCTTTCCATCTCCGGATG
TACTTTCAGCGAAATCAATAAAATGCAGAAAAAAGCACAGGAAGACTCAGCACATGCACGAGAAAAGGTATCAGCCCTTT
CTGCCCGCAAATCACAGGCTCTTACCTGGCTCGATAATCAATGGATAAACCCTGTTCCGGTCGCTCAGGTATCAAGAGAG
AAAAAACAAACAGCTCCGGCCTGCTACATCACGCAGGCAAGAAAAGGAGAGATCACTCTACAGGAACTGGGGCAACGTAT
TACTGCCGTATGCGGCATCCCCGTAATCATCACCCCTGACGCAGCCAATTCAACTCTTGAAGGAGGCGCTACCCACCAGA
TGACTGGGACACTACCAGCACCTGATGAAAATGGTCGTCTGCCGTTAAGCAGCCTGGGCAGCACAACAATGACTACCTCC
ACTCAGCCATTAACGCTGAATAACCTCATGTGGCAGGGAGATATCAATGGTCTTCTGGATCTGATGGCCAGCCGAAGCGG
TCTGTACTGGCGCATGGATAATGGTCGGATTGTATTCTATCTGACTGAAACCAGAACGTATCCACTTCATATGCTGAACA
CCAAAACCAGCAGCAGTTCCAGCGTCAGCTCTGGCTCAACAAGCACAATGGGGGCAACAGGAGGCCAGGATAACTCAGCA
TCCGGTGATGCTACGTCCTCTCAGAGCACAACCGTCGGTCAGGAATACGACCTGTATGAAGACATCCGGAAAACTATTGA
AGCGATGCTGACACCAGAAAAAGGCCGTTACTGGTTATCTGCATCGAGCTCAACGCTGACAGTCACTGATACTCCTGCTG
TCCAGGAAGCCGTCGCACGATATGTGGACGAACAAAACAGTATTATGAACCGCCAGGTAGCTCTGAACGTACAGGTCCTG
AGTGTCAGCAACACCAGAAACGAGCAGTTCGGTCTGGACTGGAACCTTGTTTACAAATCGCTACATTCCGCCGGAGCAAC
GTTGAACAATGCAAGCGGAGATTTTACAGGCGCTACATCTGCAGGCGTATCAATTCTGGATACGGCAACAGGGAATGCCG
CCAAATTCAGCGGTTCCAGTCTTCTGATTAAAGCACTGAGTGAACAGGGCGATGTCAGTGTTGTGACTTCACAAGAAAGC
ACTGTCACAAACCTGACGCCAGTACCTATCCAGATGGCAGATCAGACGGTTTACGTCGCCCAGTCAGCAACAACAACGAC
TACAGATGTCGGAGCAACAACAACACTAACCCCGGGCATGATCACCACCGGATTCAATATGACCCTGCTGCCTTTAATTC
AGAAAACGGGCAATCTCCAGTTACAGATGAACTTTAATCTGTCAGATCCCCCAACTATCCGTAGCTTTACGTCAAAAGAC
GGAAACAGTTACATAGAAATGCCATATACCAAACTGCGTTCACTGAGCCAGAAGGTCAATCTGAAAGAAGGGCAATCACT
TGTCGTTACTGGTTTCGATCAGAACAATACGACGACAAGTAAAGCCGGTACGTTTACGCCAGCAAATCCATTATTTGGTG
GTTCACAAACCGGGAAAAATGAACGCAGCACGCTTGTGATCATCATTACCCCGACTTTCCCGTCAGGAGGCAATAATGGC
TGA
>Cluster_41119
ATGTACGATTTTAATCTGGTGTTGCTGCTGCTTCAGCAGATGTGCGTTTTTTTAGTCATTGCGTGGTTAATGAGTAAAAC
GCCATTATTCATACCGTTAATGCAGGTCACGGTTCGTCTGCCGCATAAATTTCTCTGCTACATCGTCTTTTCCATCTTCT
GCATCATGGGCACCTGGTTTGGGTTGCACATTGACGATTCTATTGCCAATACCCGTGCGATAGGTGCGGTAATGGGCGGC
TTACTCGGCGGTCCGGTCGTCGGTGGGCTGGTTGGTCTGACCGGCGGGTTACATCGATATTCGATGGGGGGCATGACCGC
GCTAAGTTGCATGATCTCGACCATCGTTGAAGGATTGCTCGGTGGCATGGTACACAGCATCCTGATCCGTCGCGGGCGCA
CTGATAAAGTCTTTAACCCCATTACCGCCGGTGCCGTCACGTTCGTCGCTGAAATGGTGCAAATGTTGATTATCCTGGCG
ATCGCCCGACCTTATGAAGATGCGGTGCGTCTGGTGAGTAATATTGCTGCGCCAATGATGGTCACCAATACCGTCGGCGC
GGCACTGTTTATGCGTATATTGCTCGATAAACGCGCGATGTTTGAAAAATACACTTCGGCTTTTTCTGCCACTGCGCTGA
AAGTGGCGGCCTCGACGGAAGGCATTTTGCGCCAGGGGTTTAACGAAGTGAACAGCATGAAAGTGGCACAGGTGCTGTAT
CAGGAGCTGGATATTGGTGCAGTCGCGATTACCGATCGAGAGAAATTGCTGGCCTTTACCGGAATTGGTGACGACCACCA
TTTACCCGGCAAACCGATTTCTTCGACTTATACCTTAAAAGCGATTGAAACCGGTGAAGTGGTCTACGCTGATGGCAACG
AAGTGCCTTACCGTTGCTCTTTGCATCCGCAATGCAAACTGGGGTCGACGCTGGTCATTCCGTTGCGTGGTGAAAATCAG
CGAGTGATGGGTACCATCAAATTGTATGAGGCCAAAAACCGTTTATTCAGTTCAATCAACCGCACGCTGGGCGAGGGGAT
TGCGCAACTGCTTTCGGCGCAGATCCTCGCCGGGCAATATGAGCGGCAAAAAGCGATGCTCACCCAGTCAGAGATCAAAC
TGCTTCACGCCCAGGTGAATCCCCATTTTTTGTTTAATGCGCTTAACACCATTAAAGCGGTGATCCGCCGCGACAGCGAA
CAGGCCAGCCAGCTGGTGCAGTATCTTTCCACTTTTTTCCGCAAAAACTTAAAGCGGCCTTCGGAGTTTGTTACTCTCGC
CGACGAAATTGAACATGTGAACGCTTATCTGCAAATTGAAAAGGCGCGCTTCCAGTCGCGGTTGCAGGTCAACATTGCTA
TTCCGCAAGAATTATCCCAGCAGCAATTGCCCGCGTTTACCCTGCAACCGATAGTGGAAAACGCCATTAAACATGGGACA
TCACAACTGCTGGATACAGGGCGAGTGGCAATCAGCGCCCGACGTGAGGGGCAACATTTGATGCTGGAGATCGAAGACAA
TGCCGGTTTGTATCAACCGGTAACCAATGCCAGTGGGCTGGGGATGAATCTGGTGGATAAGCGTTTACGTGAACGGTTTG
GCGATGACTATGGGATAAGCGTCGCCTGTGAGCCTGATAGTTACACCCGAATAACGTTACGACTACCATGGAGGGACGAG
GCATGA
>Cluster_25679
ATGTTTTGTGTGCAATGTGAACAAACTATCCGTACTCCGGCAGGAAACGGCTGCTCATACGCGCAGGGGATGTGTGGTAA
AACGGCGGAAACCTCTGACCTTCAGGATTTACTCATCGCGACGCTGCAAGGGCTTTCGGCCTGGGCAGTGAAAGCACGTG
AATACGGCATCATCAACCACGATGTAGACAGCTTTGCGCCACGTGCCTTTTTCTCAACCCTGACCAACGTTAACTTCGAT
TCTCCGCGTATTGTCGGCTACGCTCGTGAAGCGATTGCCCTGCGCGAGGCGCTGAAAGCACAATGCCTGGCTGTAGATGC
CAACGCCCGCGTCGATAACCCGATGGCTGACCTGCAACTGGTGAGCGACGATCTCGGCGAGCTGCAATGTCAGGCAGCAG
AATTTACCCCTAACAAAGATAAAGCGGCGATTGGCGAAAACATTCTCGGCCTGCGTCTACTGTGCCTGTATGGCCTGAAA
GGTGCGGCGGCCTATATGGAACACGCGCATGTTCTCGGTCAATACGACAACGATATTTATGCCCAGTACCATAAAATCAT
GGCGTGGCTGGGGACCTGGCCTGCCGATATGAACGCGCTTCTTGAGTGTTCAATGGAAATCGGCCAGATGAACTTCAAAG
TGATGAGCATTCTGGACGCAGGCGAAACCGGTAAATACGGTCACCCGACGCCAACTCAGGTCAACGTCAAAGCGACGGCG
GGTAAATGCATTCTGATTTCCGGTCACGATCTCAAAGATCTCTACAACCTGCTGGAACAGACCGAAGGCACGGGCGTTAA
TGTCTACACCCACGGCGAAATGCTGCCTGCGCATGGTTACCCGGAGCTGCGTAAATTCAAGCATCTGGTCGGTAACTACG
GCAGCGGCTGGCAGAATCAGCAAGTGGAGTTCGCTCGTTTCCCAGGCCCCATCGTGATGACCTCGAACTGCATCATCGAC
CCAACCGTAGGCGCTTATGACGATCGTATCTGGACCCGCAGCATCGTTGGCTGGCCGGGTGTGCGTCATCTGGATGGCGA
AGATTTCTCTGCAGTCATTGCCCAGGCGCAACAGATGGCGGGCTTCCCGTACAGCGAAATTCCGCACCTGATCACCGTGG
GCTTTGGTCGCCAGACACTGCTTGGCGCAGCCGATACGCTGATTGATCTGGTGAGCCGTGAAAAACTGCGTCATATCTTC
CTGCTTGGCGGCTGTGACGGCGCACGCGGCGAGCGTCACTATTTCACCGATTTCGCCACCAGCGTGCCGGATGACTGCCT
GATCCTGACCCTCGCTTGCGGTAAATATCGCTTTAATAAACTTGAGTTTGGCGATATCGAAGGTCTGCCGCGTCTGGTAG
ATGCTGGTCAATGTAACGATGCTTACTCAGCAATTATTCTGGCGGTAACGCTGGCAGAGAAACTGGGCTGCGGAGTCAAC
GATCTGCCGCTGTCGCTGGTGCTCTCCTGGTTTGAACAGAAAGCGATTGTCATTCTGCTGACGCTGCTTTCTCTGGGTGT
GAAAAATATCGTCACCGGCCCGACTGCACCTGGTTTCCTGACACCAGACCTGCTGGCGGTGCTGAACGAGAAATTCGGCC
TGCGTTCTATCACCACTGTTGAAGAAGACATGAAGCAACTGTTGAGCGCGTAA
>Cluster_5981
ATGAATTCATTTGCCATCGTTATACTCTTATTAGCATTTCTCGCCCCTGTTCTAGCTGTAATATTATTTAAACAAAGTAA
AAAACACAAAGCGGCTATAGATAACCTGACAGCTAACAACATAGCCCTTTCTAACCAACTGATTGAAAATCAAGAAAAAC
TAGCACAGACCGTACGAGAATTATCAGAGCTTGAAGGGCGAGCAGCACCATTGTGGCAATACGAAGAATTGCACAGCGCA
GTAATGGATGCAGAGAATAAGATAAAAAATGCGGACTCAATAGCCAAGCAAAAAATAGATGAAGCCCAAATTAAGGCGGC
TAAAACAGTAAACGAAGCAAATTATCAAGCTCAGATGACAATAAGCAACGCTAATAGCGAAGCTATAGCAATCACCAAAG
ACGCTCGCGATGCACGCCTGAAAGCCAAAGAACGTCTTGATAATGCTAATAATAAAGCAAATGAGCTAATCTCAAATGCT
AATGACAATGCAGTAAAAATTATTTCTGATGCAGAAGAAAGAGCAAAGGAGATTGCTGGTTCAGCATATGAAGCTAAAGA
GTTTGCAGAAAAAATATGAAGCAGTTGCAAAATCAATGAAAAATAAAATTGAAGGTTATGGTGATGAATGGATCATCCCTA
ACCGTAGTGTACTTGATGAGCTGGCAGAAAATTATGAATTTACAGATGCAGGCAAGGAATTACAAAAAGCCAGAGAGTTA
ACCAAATCATTAATAAAAACTAATAAAGCTGCTTCGTGTGATTATGTTGAGCAAAACAGGCGTAATACTGCCATAAACTT
TGTTTTGGATGCCTTCAATGGAAGAGTTGACAGTATTTTATCAAAAGTCAAACACAACAATTTTGGAAAACTTTCCCAAG

AAATAAAAGATGCATTTCAACTAGTAAATTACAATGGCTCTGCCTTTAGGTCCGCAAAAATAAGTGACATCTATCTTCAG
GCAAGACTCAACGAGCTAAAATGGGGTGTAGCAGTTAATGAAATTATGCTCGAAGAAAAAGAAGAGCAAAGAAGGATTAA
AGAACAGCTTCGTGAAGAGGAAAGAGCTCGTAGAGAATATGAAAAAGCGATAAAAGAAGCTGAAAAAGAAGAAAAAGCTA
TTCAGCAAGCTATAAATAAAGCAACGAAAGAACTTATGCTTGCAAATGAAGAACAACGCTTAGCTTTAGAGCAAAAAATA
GCTGAACTACAGTTAAAATATGAAGAAGCTGAAGCTAAAAATCAACGAGCTATTTCTATGGCTCAACAAACCAGATCAGG
CCACGTCTATATCATTAGTAATATTGGTTCATTTGGCGAAGATGTATATAAAATTGGAATGACACGCCGCCTTGAACCAC
TTGATCGTGTTCGTGAACTCGGAGATGCTAGTGTTCCTTTTTCGTTCGATGTTCATGCGATGATTTATAGTGATGATGCA
CCATCATTAGAAAACCATCTGCATAAAGTTTTCAACGAAAAGCAGGTCAATAAAATTAACTCACGAAAAGAGTTTTTTAA
CGTAAATTTAAAAGAAATCAAATCAGTTATTGAAGACATGAACATCAACGCCCACTGGACAATGTTTGCAGAGGCGAAAG
AATATAAAGAGTCTCTAGCTATTGAGCAGGAGCGCAAAGCAGCCACTTCCGCCAACGATGAACTACATGTTGCTTAG
>Cluster_6269
ATGAATCACAAGGACTGGGATTTTGTTAACCGTCGGCTGGTGGCAAAAATGTTGTCTGAGATGGAGTATGAGCAGGTTTT
CCACGCGGAATCTCAGGGCGATGACCACTACTGCATTAACCTGCCGGGAGCACAATGGCGCTTTATCGCTGAACGTGGTA
TCTGGGGCTGGCTGTGGATTGATGCCCAAACGTTGCGCTGCACGGACGAACCAGTACTGGCGCAGACGCTGCTGATGCAG
CTAAAGCCGGTGCTGTCAATGAGTGATGCCACTGTTGCAGAGCATATGCAGGATTTGTATGCCACGCTACTGGGCGACCT
GCAACTGCTGAAGGCCCGTCGCGGACTGAGTGCCAGTGACCTGATTAATCTTGATGCCGATCGTCTGCAATGCCTGCTGA
GCGGTCATCCTAAATTCGTTTTTAATAAAGGTCGCCGTGGCTGGGGCAAAGAGGCGCTGGAACGATATGCGCCAGAGTAT
GCCAACACCTTCAGACTGCACTGGCTGGCGGTAAAACGTGAACATATGATCTGGCGCTGCGACAACGATCTGGATATTCA
GCAGTTGTTGACTGCCGCAATGGATCCGCAGGAGTTTACACGCTTCAGTCAGGTCTGGCAGGAAAACGGGCTGGATCACA
ACTGGCTGCCGCTGCCGGTACATCCGTGGCAGTGGCAGCAAAAAATCGCCACCGACTTCATCGCTGATTTTGCCGAAGGC
AGGATGATGTCTCTCGGCGAGTTTGGCAACCAGTGGCTCGCCCAGCAGTCGCTGCGTACCCTGACCAACGCCAGCCGCCG
GGGAGGGCTGGATATCAAGCTGCCGCTGACCATCTACAACACCTCATGCTACCGGGGGATCCCTGGCAGATATATCGCTG
CCGGACCACTGGCTTCACGCTGGCTGCAACAGGTTTTTGCGACCGACGCCACCCTGGTGCAAAGCGGCGCAGTGATCCTT
GGTGAACCGGCTGCAGGCTATGTGTCCCATGAAGGCTATGCCGCGCTTGCCCGGGCTCCCTATCGCTACCAGGAAATGCT
TGGTGTTATCTGGCGGGAGAATCCGTGCCGCTGGCTGAAACCGGATGAAAGTCCGGTTCTGATGGCAACACTGATGGAGT
GCGACGAAAACAATCAGCCGCTGGCAGGCGCATATATCGACCGCTCCGGACTGGACGCTGAAACCTGGCTTACGCAATTG
TTCCGGGTGGTTGTGGTTCCGCTGTATCACCTGCTTTGCCGCTACGGTGTCGCGCTTATTGCACACGGACAAAATATAAC
GCTCGCCATGAAAGAGGGGGTACCACAGCGTGTTCTGCTGAAAGACTTCCAGGGCGATATGCGGCTGGTGAAAGAAGCGT
TTCCCGAAATGGACTCTTTGCCTCAGGAGGTGCGTGATGTCACATCCCGTCTGAGTGCGGACTACTTAATCCATGATTTG
CAGACGGGTCACTTCGTGACAGTACTGCGTTTTATTTCGCCACTGATGGTTCGTCTTGGCGTACCTGAAAGGCGATTTTA
TCAACTGCTGGCAGCAGTGTTGAGTGATTACATGAACAAACATCCACAAATGTCAGAGCGTTTTGCGCTTTTCTCACTCT
TCAGGCCACAAATCATTCGCGTGGTGCTGAACCCGGTAAAACTGACCTGGCCGGACCAGGATGGCGGCAGTCGCATGCTG
CCGAATTACCTTGAGAATCTGCAAAATCCGCTGTGGCTGGTAACTCAGGAATATGAATCATGA
>Cluster_37962
ATGCTAAACGAACGCCAGTTAAAGATTGTCGATCTGCTGGAGCAACAGCCGCGCACGCCTGGCGAGCTGGCGCAACAGAC
TGGCGTTTCAGGCAGGACCATCCTGCGTGATATTGACTATCTCAACTTCACCCTTAACGGCAAAGCCCGCATTTTCGCCA
GTGGCAGTGCGGGCTATCAGCTGGAAATCTTCGAGCGCCGCAGCTTTTTTCAGTTGCTGCAAAAGCACGATAACGACGAT
CGGCTGCTGGCGCTGTTATTACTGAATACTTTCACTCCCCGTGCGCAACTCGCCTCGGCGCTTAATTTGCCAGAAACGTG
GGTAGCAGAGCGTCTGCCCCGGTTAAAACAGCGTTATGAACGCACTTGTTGCCTGGCCAGCCGCCCTGGTTTGGGCCATT
TCATTGATGAGACAGAAGAGAAACGCGTTATCTTGCTGGCGAACTTGCTGCGCAAAGATCCGTTTTTAATTCCGCTGGCG
GGCATAACACGAGACAACCTTCAGCATTTATCCACGGCCTGCGACAACCAACACCGCTGGCCGCTCATGCAGGGTGATTA
TCTCTCCAGCCTGATTCTGGCGATTTACGCCCTGCGTAATCAACTGACCGATGAGTGGCCGCAATATCCCGGTGACGAGA
TAAAACAAATCGTTGAACATAGCGGTCTGTTTCTTGGTGATAACGCTGTAAGAACCCTGACGGGTTTGATAGAGAAACAG
CATCAGCAAGCGCAGGTAATTTCAGCCGATAATGTGCAGGGGTTGCTGCAAAGGGTGCCGGGCATCGCGTCATTGAATAT
TATTGATGCGCAGCTGGTTGAGAATATTACCGGGCATTTATTACGTTGCCTTGCCGCACCAGTGTGGATTGCTGAGCACC
GCCAGAGCAGCATGAATAACCTGAAAGCCGCCTGGCCTGCGGCCTTTGATATGAGTCTGCACTTTATTACGCTACTGCGT
GAACAGCTCGATATTCCCCTTTTCGACAGCGATCTGATCGGTTTGTATTTTGCCTGTGCGCTGGAGCGGCATCAAAACGA
ACGCCAGCCGATCATTTTGCTCTCGGACCAGAACGCGATTGCCACTATTAATCAGCTCGCCATTGAGCGCGATGTTTTAA
ATTGTCGGGTAATTATTGCCCGTAGCTTAAGCGAACTTGTTGCCATTCGCGAAGAGATTGAGCCGTTATTGATCATTAAC
AACAGCCATTATTTACTGGATGACGCGGTAAATAATTACATCACCGTAAAAAATATCATTACGGCTGCCGGTATCGAACA
AATAAAACATTTTCTGGCGACGGCATTTATTCGCCAACAGCCGGAGCGTTTTTTCTCTGCCCCCGGAAGTTTTCATTATT
CGAATGTACGCGGTGAAAGCTGGCAACATATTACCCGGCAAATTTGTGCGCAATTAGTCGCACAACACCATATTACCGCC
GATGAAGCACAACGCATCATCGCCCGCGAAGGCGAAGGTGAAAACCTGATTGTTAATCGCCTCGCCATCCCACATTGCTG
GAGCGAACAGGAGCGACGTTTTCGTGGATTTTTTATTACCCTCGCCCAACCAGTTGAGGTGAATAACGAAGTCATTAACC
ATGTCTTGATCGCCTGCGCCGCCGCCGATGCGCGTCATGAGCTAAAAATATTTAGCTATCTGGCAAGCATATTGTGTCAG
CATCCGGCGGAGATTATTGCCGGGTTAACAGGATATGAGGCATTTATGGAGTTACTTCACAAGGGGTGA
>Cluster_2904
ATGGATGCCCTGCAATTATTAACCTGGTCGCTGATCCTCTATCTGTTTGCCAGTCTGGCTTCGCTGTTTTTACTCGGTCT
GGACAGACTGGCTATTAAGCTTTCCGGCATCACATCGCTGGTGGGCGGCGTGATTGGCATCATCAGCGGAATTACGCAAT
TACATGCTGGTGTAACTTTAGTCGCCCGTTTTGCCACCCCTTTTGACTTTGCCGATTTTACCCTGCGAATGGATAGCCTC
TCGGCATTTATGGTGCTGGTTATCTCCTTGCTGGTGGTGGTTTGTTCGCTCTATTCATTGACTTATATGCGCGAATACGA
GGGCAAAGGCGCGGCGGCGATGGGCTTCTTTATGAATCTTTTCATCGCATCGATGGTTGCCCTGCTGGTGATGGACAACG
CTTTTTGGTTCATCGTGCTGTTTGAAATGATGTCGCTGTCTTCCTGGTTTCTGGTCATTGCCAGGCAGGATAAAACGTCG
ATCAACGCTGGCATGCTCTACTTTTTTATCGCCCACGCCGGATCGGTGCTGATTATGATCGCCTTCTTGCTGATGGGGCG
CGAAAGCGGCAGCCTCGATTTTGCCAGTTTCCGCACGCTTTCACTTTCTCCGGGGCTGGCGTCGGCGGTGTTCCTGCTGG
CCTTTTTCGGTTTTGGCGCGAAAGCCGGGATGATGCCGTTGCACAGCTGGTTGCCGCGCGCTCACCCTGCCGCACCATCG
CACGCTTCGGCGTTGATGTCTGGCGTAATGGTCAAAATCGGTATTTTCGGCATCCTGAAAGTAGCGATGGACCTGCTGGC
GCAAACGGGTTTGCCGCTGTGGTGGGGCATTCTGGTGATGGCGATCGGCGCAATCTCCGCGCTCCTGGGCGTGCTGTATG
CGCTGGCGGAACAGGATATCAAACGGCTGCTGGCCTGGAGTACCGTCGAAAACGTCGGCATTATTTTGCTGGCAGTCGGT
GTGGCGATGGTCGGTCTGTCACTGCACGACCCGCTGCTCACCGTGGTTGGACTGCTCGGCGCACTGTTTCATCTGCTCAA
CCATGCGCTGTTCAAAGGGCTGCTATTTCTCGGCGCGGGCGCGATTATTTCGCGTTTGCATACCCACGACATGGAAAAAA
TGGGGGCACTGGCGAAACGGATGCCGTGGACAGCCGCAGCATGCCTGATTGGTTGCCTCGCGATATCAGCCATTCCTCCG
CTGAATGGTTTTATCAGCGAATGGTACACCTGGCAGTCGCTGTTCTCACTAAGTCGTGTGGAAGCCGTAGCGCTACAACT
TGCGGGTCCTATTGCTATGGTAATGCTGGCAGTCACTGGTGGGCTGGCAGTAATGTGCTTCGTAAAAATGTACGGTATTA
CTTTCTGTGGTGCGCCGCGCAGTACACACGCTGAAGAGGCACAGGAAGTGCCAAATACGATGATCGTCGCCATGCTACTG
CTCGCGGCACTCTGCGTATTAATTGCGCTTAGTGCCAGTTGGCTGGCACCGAAGATAATGCATATTGCCCATGCGTTTAC
CAATACCCCTCCCGTCACTGTCGCCAGCGGAATAGCACTTGTACCCGGCACGTTTCATACACAGGTCACCCCCTCATTAC
TGTTGCTGTTACTACTGGCGATGCCTTTGCTGCCTGGCCTTTACTGGCTGTGGTGTCGTTCGCGCCGCGCAGCGTTTCGT
CGCACAGGAGATGCCTGGGCATGCGGCTAG

>Cluster_28385
ATGGATAACGTTGAACTTTCACCGGCAACACGCTGGGGGATGATTGCTACCGGATTACTTCAGGGGCTGGTTTGCTACCT
GCTGATCGCCTGGCTTGCCGGAAAAAATCTCAGTTGGATTGTTTATGGCGTGCCTGTGACCGTCGCGTTTTCATCCGTTT
TACTCTTCTCCGTGATCTCCTTTAAACAGAAGCGCCTTTGGGGATGGCTGGCGCTGGTGTTTATTGCCACGCTGGGAATG
AGCGGCTGGCTGAAGTGGCAAACTGATGGCATGACCCCCTGGAGAGCTGAAAAGGCGCTTTGGGATTTTGGCTGCTATCT
GCTGCTGATGGCAATGTTGTTGCTACCGTGGATACAACAAAGCCTTCGCATCCGCAATGACAGTAGCCGCTATCGCTACT
TTTACCAGTCAGTATGGCATAACGTACTTATATTACTGGTGATTTTTCTCGCCAATGGCCTAACGTGGCTGGTACTTTTA
CTGTGGAGTGAGTTGTTTAAACTTGTTGGCATCACGTTTTTCAAAACGCTCTTTTTTGCAACCGACTGGTTTATTTATCT
CACGTTAGGTCTGGTTACCGCGCTGGCGGTGATCTTCGCGCGAACACAGTCACGTTTAATCGACTCTATTCAAAAGTTGT
TCACGTTAATCGCCACGGGGTTGCTGCCGTTAGTATCATTGCTAACCCTGATATTTATCATCACCCTGCCGTTTACGGGC
CTGAGCGCGATTTCTCGCCACATTTCCGCCGCCGGGTTGCTGTTGACGCTGGCCTTTTTGCAATTGATCTTAATGGCTAT
TGTCCGCGATCCGCAAAAATCGTCACTTCCCTGGACGGGGCCGTTGCGTTGCCTGATTAAAACCGCTCTGCTGGCTGCTC
CGCTGTATGTGTTCGTCGCCGCCTGGGCGTTATGGCTGCGGGTCGCTCAGTACGGCTGGACTGTCGACCGCTTGCAGGGC
GCGCTGGCGGTGCTGGTGTTACTGGTATGGTCGCTGGGGCATTTTGTCAGTATCGTCTGGCGTAAAGGGCAAAACCCCCT
TGTTCTTCAGGGCAAAGTGAACCTTGCGGTTTCGTTATTGGTGTTGGTAATACTGGTGCTTCTTAATTCTCCGGTGCTGG
ACAGTATGCGCATTAGCGTGAACAGTCATATGGCACGTTATCAGAGTGGCAAAAACACGCCAGACCAGGTAACTATCTAC
ATGCTCGAACAGAGCGGTCGCTATGGACGTGCTGCGCTTGAGTCGCTGAAAAGCGATGCCGGGTTTATGAAAGACCCGAA
ACGTGCGCGGGATCTGTTGATGGCGTTAGATGGAGAGCAACATCTTCAGCAACAGATATCGGAAAAAGTATTAGCCGAAA
ATGTGTTAATTGCCCCTGGTTCTGGTAAACCGGATGCGACATTCTGGTCGGCCTTAATCCAGGATCGCTATAACGTGATG
ACCTGTATTGAAAAAGACGCCTGCGTACTGGTCGAGCAAGATCTGAATAGTGATGGCAGGGCAGAGCGGATCCTCTTTGC
CTTTGATGATGAAAGATACATTGTCTATGGCTTTGATCCTGACAAGAAAGAATGGCAAGAGCTTACGATGAGTTTACTTC
CGCGTGATATAACGAAAGAAAAATTACTCACAGCTGCTAAGGATGGAAAACTGGGAACGAAGCCTAAAGCGTGGCGCGAT
CTTGTAGTGGATGGTGAAAGGCTGGATGTGAATCTGAATGAGTGA
>Cluster_18233
ATGCCAATAATAAACAAATCGGCATCAAATTATGTTGAATATATTTCAAAAAACAATCCCCCTTATTTATCAAAAAAGAG
AGATGCCAGTATAAATCTAAATGGAAAGGTTAGTGATTGCAATGGGGAAATCATATGGTGTCGACATATAGCATCATATT
GGAGTGAGTTTTTTTGTAGTAATTCAGGAAAGATAGACTACGAAACATTTTCATCCCCTCAATTATTAAGCAAAGCTATT
GTAATTCAGGAAAACAAGGGGACAAATAATATTAAGGGTGATGTGTATTTTGTTGAAAATGAATCTTGGGAAGCGTTAT
ATATAACCTTTTTCTTCAACTTGAGAAAGAGAATAAATCTCACACATCATTAGAAGTTCATTCTCCCGGGCATGCAATGG
CTCTGGGCATTAAAATAAAAAACGACAAGGAAAATAAGTTTGTTATTAATTTCTATGATCCCAATCAGACCGCGACGCAT
AAACGAGTATTTTTTTGCACTAATAACATTTGTGATATAATAAACCTGACAGCATATGATTTTCTTAGTGAGCAATGTCT
GAAATGTTATGGGCTAAAAGAAGACACACTGTCACTTTTTGTGGATAAAACAAAATCGAATGACAACAATAATGTGTTTA
TAAAAAAATTACCTGATAATATTCTCCAAGGTGTTGTTATTAATTTTGCTATGGGAGCAGGACTAAGAGAGATAATAAAA
AAGGTATATAACGATACTCGATTTACAGATCTTACAAAATCTCAGATGAAAATCTTGTGTGAATCAAAAAATGTTAATAA
TGTTCCTGGGTTGCTTTTAGCTCTACAAAATGGACATGATAATGTAATTGATGAATATGGTACTTTAATTAAAAAGTCAA
ATCTTAATAAAGAAGAATTAATACATATATTATCTGCAAGAACTTTGGATGGAACCATACCTGGTTTATATCAGGCCCTT
CAAAATGGACATGCTCAAGCTATTAAGAGTTATGGTAACTTAGTTCTGGATACTATTGATAAAAATATAGATTTAGAGTA
TTTGCTTTCAGCATTTAAATATGAGGCGCATAGCTCTAATAAATATACTCCTGGATTATTTTCTGCATTTAAAAATGGTC
ATGCAGACGCAATAAAAGCATATTGTGACGTACTTGGCAATAGTAATTTGACAAGAGGTGAGATAATCAGGATGCTTGAG
GCGAGAAATTATGATGGTGCACCAGGATTACTTCTTGCGTATCAAAATGGAGATATTAATACGATTCAGTCTTTTTTTGA
CTCACTAATAATGTTAGATATATCAAAGGATTTTATCGAAGAGTTATTGACTGCAAAACATTATGATTTTACGGGGTTAT
CATTAGCGATCAGTCATAGACATGATCATGTTGTCAAATTATACGGAAAGTTATTTAAAAAACTTGATACCAGTCCATAT
AAAATGTCAATTATCCTAGCATTAGCAATTGATTGCGAGAGAAATAATGCAAACATAATTATTGATAGTGAGTACAAGTC
AAATAAGGCAGTAAAGGAATATGTAGAAATTTTAAAAGAATTCAATATATGCCCAGAAAAGGTAGCAGAATACCTAAGCG
AGTTCAGCGGAAAACATTTCCTCGATGTATATAATTATTATTCCAATTGA
>Cluster_43508
ATGACAAGCCTTTTGGCCGCAGAGGTAGCGAAAAGAAGAACCTTTGCAATTATTTCTCACCCGGACGCCGGTAAGACTAC
CATCACCGAGAAGGTGCTGCTGTTCGGACAGGCCATTCAGACCGCCGGTACAGTAAAAGGCCGTGGTTCCAACCAGCACG
CTAAGTCGGACTGGATGGAGATGGAAAAGCAGCGTGGGATCTCCATTACTACGTCTGTGATGCAGTTTCCGTATCACGAT
TGCCTGGTTAACCTGCTCGACACCCCGGGGCACGAAGACTTCTCGGAAGATACCTATCGTACCCTGACGGCGGTGGACTG
CTGTCTGATGGTTATCGACGCCGCAAAAGGTGTAGAAGATCGTACCCGTAAGCTGATGGAAGTTACCCGTCTGCGCGATA
CGCCGATCCTCACCTTTATGAACAAACTTGACCGTGATATCCGCGACCCGATGGAGCTGCTCGATGAAGTTGAGAACGAG
CTGAAAATCGGCTGCGCACCGATCACCTGGCCGATTGGCTGCGGCAAGCTGTTTAAAGGCGTTTACCACCTTTATAAAGA
TGAAACCTATCTCTATCAGAGCGGTAAAGGCCACACGATTCAGGAAGTCCGCATTGTTAAAGGGCTGAATAACCCGGATC
TCGACGCTGCGGTTGGTGAAGATCTGGCACAGCAGCTGCGTGACGAACTGGAGCTGGTGAAAGGCGCGTCTAATGAGTTC
GACAAAGAGCTGTTCCTTGCGGGCGAAATCACCCCGGTATTCTTTGGTACTGCGCTGGGTAACTTCGGCGTCGATCATAT
GCTGGATGGCCTGGTGGAGTGGGCGCCTGCGCCGATGCCGCGTCAGACCGATACCCGTACCGTAGAAGCGAGCGAAGATA
AATTTACCGGCTTCGTATTTAAAATTCAGGCCAACATGGACCCGAAACACCGCGACCGCGTGGCGTTTATGCGCGTGGTA
TCCGGTAAATATGAAAAAGGCATGAAGCTGCGCCAGGTGCGCACCGCGAAAGATGTAGTGATCTCCGACGCGCTGACCTT
TATGGCAGGCGACCGTTCGCACGTTGAAGAAGCGTATCCGGGCGATATCCTCGGCCTGCACAATCACGGCACCATTCAGA
TCGGCGATACCTTTACCCAGGGTGAGATGATGAAGTTCACCGGTATTCCGAACTTCGCGCCAGAACTGTTCCGTCGTATC
CGCCTGAAAGATCCGCTGAAGCAAAAACAGCTGCTCAAAGGGCTGGTACAGCTTTCCGAAGAGGGGGCGGTGCAGGTGTT
CCGTCCGATCTCCAACAACGATCTGATCGTTGGCGCGGTTGGTGTGCTGCAGTTTGATGTGGTGGTAGCGCGCCTGAAGA
GCGAATACAACGTTGAAGCAGTGTATGAGTCAGTCAACGTTGCCACCGCCCGCTGGGTAGAATGTGCGGACGCGAAGAAA
TTCGAAGAGTTCAAGCGTAAGAACGAAAGCCAACTGGCGCTTGATGGCGGCGATAACCTCGCTTACATCGCTACCAGCAT
GGTCAATCTGCGCCTGGCGCAGGAACGTTATCCGGACGTTCAGTTCCACCAGACCCGCGAGCATTAA
>Cluster_34663
ATGAAACGAACGCCTGTCCTGATTGATGTGAACGGTGTTCCGCTTCGGGAGAGCCTCAGCTACAACGGGGGCGGCGCAGG
ATTTGGCGGGCAAATGGCGGAGTGGTTGCCACCGGCGCAGAGTGCCGATGCAGCCCTGTTGCCCGCGTTGCGTCTGGGGA
ATGCCCGGGCAGATGATCTGGTGCGCAATAACGGGATAGCGGCCAATGCGGTGGCCCTGCATAAGGATCATATTGTCGGG
CATATGTTTCTGATCAGCTACCGTCCGAACTGGCGCTGGCTGGGGATGCGGGAGACTGCAGCAAAAAGTTTTGTCGATGA
GGTGGAGGCGGCCTGGTCGGAATACGCCGAAGGGATGTCTGGCGAGATCGACGTGGAAGGGAAACGCACGTTTACGGAAT
TTATCCGTGAAGGTGTGGGCGTTCATGCGTTTAACGGCGAAATCTTTGTGCAGCCGGTCTGGGATACGGAGAGCACGCAA
CTGTTTCGTACGCGTTTTAAAGCCGTGAGTCCGAAACGGGTGGACACGCCAGGACACGGTATGGGGAACCGTTTTCTGCG
GGCCGGTGTGGAGGTCGATCGATATGGCCGTGCCGTTGCGTACCATATCTGTGAGGATGATTTTCCGTTCTCTGGTAGTG
GACGATGGGAACGGATCCCGCGTGAACTTCCCACCGGGCGTCCGGCCATGCTGCATATTTTCGAGCCGGTGGAGGACGGG
CAGACCCGTGGGGCTAATCAGTTTTACAGCGTCATGGAACGGCTGAAGATGCTGGATTCCCTGCAGGCAACACAGCTTCA
GTCGGCCATTGTGAAGGCGATGTATGCAGCGACGATTGAAAGTGAACTTGATACCGAAAAGGCCTTTGAATATATCGCCG
GTGCGCCGCAGGGGCAGAAGGATAATCCGCTTATTAATATTCTGGATAAGTTCTCCACCTGGTATGACACGAATAACGTG

ACGCTGGGCGGTGTCAAAATTCCGCACCTTTTCCCCGGTGATGATCTGAAACTTCAGACTGCGCAGGATTCAGACAATGG
ATTTTCGGCGCTTGAACAGGCGCTGCTGCGGTATATCGCCGCCGGTCTTGGCGTTTCCTACGAACAGTTGTCCCGGGATT
ACTCGAAGGTCAGTTATTCAAGTGCCCGTGCCTCCGCCAATGAGTCGTGGCGCTATTTTATGGGGCGGCGAAAATTTATT
GCGTCCCGGCTGGCCACGCAGATGTTTTCCTGCTGGCTGGAAGAGGCTCTTCTTCGGGGGATTATTCGTCCGCCACGGGC
GCGTTTTGATTTTTATCAGGCGCGATCAGCCTGGTCACGGGCTGAGTGGATTGGTGCCGGAAGAATGGCCATTGACGGGC
TCAAGGAGGTTCAGGAATCAGTGATGCGCATTGAGGCCGGACTGAGCACGTATGAGAAAGAGCTGGCGCTGATGGGCGAG
GATTATCAGGACATTTTCCGCCAGCAGGTCAGGGAATCTGCTGAGCGGCAAAAAGCCGGACTCTCACGTCCGGTGTGGAT
AGCGCAGGCGTATGAGCAGCAGATAGCGGAGAGTCGCAGGCCGGAAGAGGAGACAACACCACGTGAGACGTAA
>Cluster_21932
ATGGAATTCTTAATGGACCCCTCAATTTGGGCGGGGCTACTCACGCTTGTTGTTCTCGAAATTGTGCTGGGTATCGATAA
CCTGGTCTTCATCGCCATTCTTGCTGACAAACTGCCGCCAAAACAACGCGATAAAGCGCGTTTGCTGGGATTATCACTGG
CGCTGATTATGCGTCTGGGGCTGCTGTCGCTGATTTCATGGATGGTCACGCTGACCAAACCGCTATTTACCGTCATGGAT
TTCTCCTTCTCCGGACGCGACCTGATTATGTTGTTCGGGGGGATATTCTTGCTGTTCAAAGCAACAACCGAACTGCATGA
ACGACTGGAAAACCGCGATCATGATTCCGGTCACGGTAAAGGCTACGCCAGTTTCTGGGTGGTCGTCACACAGATTGTCA
TCCTTGACGCCGTCTTCTCGTTGGATGCGGTAATTACGGCAGTAGGGATGGTTAACCATCTGCCAGTGATGATGGCGGCG
GTAGTGATTGCGATGGCGGTTATGCTGCTGGCATCGAAACCGCTGACGCGATTTGTTAACCAGCATCCAACGGTGGTGGT
GCTCTGTCTGAGCTTCCTGTTAATGATAGGTCTGAGTCTGGTGGCAGAAGGTTTCGGTTTCCACATTCCGAAAGGTTACC
TGTATGCCGCGATTGGCTTCTCGATCATCATCGAAGTGTTTAACCAAATTGCGCGTCGCAACTTTATTCGTCACCAGTCG
ACTTTGCCGCTGCGAGCGCGTACTGCCGATGCCATCCTGCGTTTGATGGGCGGGAAACGTCAGGCCAATGTTCAGCACGA
TGCCGATAACCCGATACCGATGCCGATCCCGGAAGGTGCGTTTGCCGAAGAAGAACGTTACATGATTAACGGTGTATTGA
CGCTGGCGTCGCGTTCTCTGCGCGGGATCATGACGCCGCGCGGTGAAATAAGCTGGGTTGACGCTAATCTCGGGGTCGAT
GAAATCCGTGAGCAACTGCTCTCTTCACCGCACAGTCTGTTCCCGGTATGTCGCGGTGAACTGGATGAAATCATCGGTAT
CGTACGTGCTAAAGAACTGCTGGTGGCGCTGGAAGAGGGCGTTGATGTGGCGGCGATTGCTTCGGCGTCTCCGGCGATTA
TCGTCCCGGAAACCCTCGATCCGATCAACCTGCTGGGCGTACTGCGTCGTGCTCGCGGGAGCTTTGTTATCGTGACCAAC
GAGTTTGGTGTGGTACAAGGTCTAGTCACGCCGCTGGATGTGCTGGAAGCCATTGCGGGTGAATTCCCGGACGCTGACGA
AACGCCGGAAATCATTACCGACGGTGACGGCTGGCTGGTAAAAGGCGGTACAGATTTGCATGCCTTGCAGCAGGCGCTTG
ATGTTGAGCACCTTGCCGATGACGATGATATCGCGACGGTCGCGGGCCTCGTGATCTCGGCAAATGGTCACATTCCCCGT
GTGGGCGATGTGATTGATGTAGGGCCACTGCATATCACCATCATTGAAGCCAATGATTATCGTGTTGATCTGGTTCGCAT
TGTTAAAGAGCAACCGGCGCACGATGAAGATGAGTAA
>Cluster_56251
TTGATCCCGGACGTATCACAGGCGCTGGCCTGGCTGGAAAAACATCCTCAGGCGTTAAAGGGGATACAGCGTGGGCTGGA
GCGCGAAACTTTGCGTGTTAATGCTGATGGCACACTGGCAACAACAGGTCATCCTGAAGCATTAGGTTCCGCACTGACGC
ACAAATGGATTACTACCGATTTTGCGGAAGCATTGCTGGAATTCATTACACCAGTGGATGGTGATATTGAACATATGCTG
ACCTTTATGCGCGATCTGCATCGTTATACGGCGCGCAATATGGGCGATGAGCGGATGTGGCCGTTAAGTATGCCATGCTA
CATCGCAGAAGGTCAGGACATCGAACTGGCACAGTACGGCACTTCTAACACCGGACGCTTTAAAACGCTGTATCGTGAAG
GGCTGAAAAATCGCTACGGCGCGCTGATGCAAACCATTTCCGGCGTGCACTACAATTTCTCTTTGCCAATGGCATTCTGG
CAAGCGAAGTGCGGTGATATCTCGGGCGCTGATGCCAAAGAGAAAATTTCTGCGGGCTATTTCCGCGTTATCCGCAATTA
CTATCGTTTCGGTTGGGTCATTCCTTATCTGTTTGGTGCATCTCCGGCGATTTGTTCTTCTTTCCTGCAAGGAAAACCAA
CGTCGCTGCCGTTTGAGAAAACCGAGTGCGGTATGTATTACCTGCCGTATGCGACCTCTCTTCGTTTGAGCGATCTCGGC
TATACCAATAAATCGCAAAGCAATCTTGGTATTACCTTCAACGATCTTTACGAGTACGTAGCGGGCCTTAAACAGGCAAT
CAAAACGCCATCGGAAGAGTACGCGAAGATTGGTATTGAGAAAGACGGTAAGAGGCTGCAAATCAACAGCAACGTGTTGC
AGATTGAAAACGAACTGTACGCGCCGATTCGTCCAAAACGCGTTACCCGCAGCGGCGAGTCGCCTTCTGATGCGCTGTTA
CGTGGCGGCATTGAATATATTGAAGTGCGTTCGCTGGACATCAACCCGTTCTCGCCGATTGGTGTAGATGAACAGCAGGT
GCGATTCCTCGACCTGTTTATGGTCTGGTGTGCGCTGGCTGATGCACCGGAAATGAGCAGTAGCGAACTTGCCTGTACAC
GCGTTAACTGGAACCGGGTGATCCTCGAAGGTCGCAAACCGGGTCTGACGCTGGGTATCGGCTGCGAAACCGCACAGTTC
CCGTTACCGCAGGTGGGTAAAGATCTGTTCCGCGATCTGAAACGCGTCGCGCAAACGCTGGATAGTATTAACGGCGGCGA
AGCGTATCAGAAAGTGTGTGATGAACTGGTTGCCTGCTTCGATAATCCCGATCTGACTTTCTCTGCCCGTATCTTAAGGT
CTATGATTGATACTGGTATTGGCGGAACAGGCAAAGCATTTGCAGAAGCCTACCGTAATCTGCTGCGTGAAGAGCCGCTG
GAAATTCTGCGCGAAGAGGATTTTGTAGCCGAGCGCGAGGCGTCTGAACGCCGTCAGCAGGAAATGGAAGCCGCTGATAC
CGAACCGTTTGCGGTGTGGCTGGAAAAACACGCCTGA
>Cluster_48212
ATGAAAAGAGTTCTGACGGCGCTTGCCGCCACACTCCCTTTCGCAGCTAACGCCGCGGATGCTATTAGCGGGGCCGTAGA
GCGCCAGCCAACGAACTGGCAGGCGATTATTATGTTCCTGATTTTCGTCGTGTTTACGCTCGGCATTACCTACTGGGCAT
CAAAACGCGTACGTTCTCGTAGCGACTACTACACCGCAGGCGGCAATATCACTGGCTTCCAGAACGGGCTGGCGATTGCC
GGGGACTATATGTCCGCCGCCTCATTCTTGGGGATCTCCGCGCTGGTGTTTACCTCCGGCTATGACGGCTTAATTTACTC
GCTGGGCTTCCTGGTGGGCTGGCCGATCATTTTGTTCCTGATTGCCGAACGTCTGCGTAACCTGGGGCGCTACACCTTTG
CCGATGTGGCCTCTTACCGTCTGAAACAAGGGCCGATTCGTATTCTTTCGGCCTGTGGTTCTCTGGTGGTGGTGGCGCTT
TACCTTATCGCCCAGATGGTGGGCGCAGGTAAACTGATCGAGCTGCTGTTTGGCCTTAACTATCACATTGCGGTGGTGCT
GGTCGGCGTGCTGATGATGATGTACGTCCTGTTCGGCGGCATGCTGGCGACCACTTGGGTACAGATTATCAAAGCCGTGC
TGTTGCTGTTCGGTGCCAGCTTTATGGCCTTTATGGTGATGAAACACGTCGGCTTTAGCTTCAACAATCTGTTCAGTGAA
GCGATGGCGGTACACCCGAAAGGTGTCGACATCATGAAGCCGGGCGGGCTGGTGAAAGATCCGATCTCCGCGCTCTCTCT
GGGTCTGGGACTGATGTTTGGTACGGCGGGCTTGCCGCACATTCTGATGCGCTTCTTTACAGTCAGCGATGCCCGCGAAG
CACGTAAGAGCGTGTTCTACGCCACCGGGTTTATGGGCTACTTCTATATTCTGACCTTTATTATCGGCTTCGGCGCGATC
ATGCTGGTTGGTGCGAATCCGGAATATAAAGACGCGGCGGGCCATCTGATTGGTGGTAACAACATGGCGGCCGTTCACCT
GGCGAATGCAGTGGGCGGCAACCTGTTCCTCGGTTTTATTTCAGCGGTTGCTTTTGCCACCATTCTTGCGGTGGTTGCTG
GTCTGACGCTGGCGGGCGCATCAGCGGTTTCGCATGACCTGTACGCTAACGTCTTCAAAAAAGGCGCGACCGAACGTGAA
GAACTGCGGGTATCGAAAATCACCGTGCTGATTTTAGGTGTGATTGCCATTATCCTCGGCGTGCTGTTTGAGAATCAGAA
CATCGCCTTCATGGTAGGCCTGGCGTTTGCCATCGCAGCGAGCTGTAACTTCCCGATCATTCTGCTTTCTATGTACTGGT
CGAAACTGACCACTCGTGGCGCGATGATGGGTGGCTGGCTGGGGCTGATTACCGCAGTGGTACTGATGATCCTCGGCCCG
ACGATTTGGGTACAGATCCTTGGTCACGAAAAAGCCATCTTCCCGTATGAATACCCGGCGCTGTTCTCTATCAGCGTGGC
ATTCCTCGGCATCTGGTTCTTCTCGGCAACCGATAACTCAGCGGAAGGTGCGCGCGAGCGTGAACTGTTCCGTGCACAGT
TTATCCGCTCCCAGACCGGCTTTGGCGTTGAGCAAGGCCGCGCACATTAA
>Cluster_28713
ATGCCTATTGGTAACCTTGGTAATAATGTAAATAGCAATCATTTAATTCCCCCTGCGCCGCCACTACCTTCACAAACAGA
CGGCGCGGCACGGGGAGGAACTGGTCATCTAATTAGCTCTACAGGAGCATTAGGATCTCGTTCATTGTTTTCTCCCCTGA
GAAATTCTGTGGCTGATTCTGTCGATTCCAGAGATATTCCAGGACTTCCTGCAAACCCATCGAGGCTTGCTGCAGCTACA
TCTGAGACATGCTTGCTTGGAGGATTTGAAGTTCTCCATGATAAGGGGCCACTTGATACTCTTAATCAGCAAATTGGACC
CTCTGCATTTCGTGTTGAAGCGCAGGCAGATGGTACTCATGCCGCTATTGGAGAAAAAAATGGTTTGGAGGTTAGCGTTG
CATTAAGTCCTCAAGAATTGCAAAGCTTGCAATCTATTGATATTGAGGGGAAAAACAGATTTGTTTTTACCGGGGGACGT

GGCGGTAGTGGGCATCCGATGGTCACTGTCGCATCAGATATCGCGCAAGCTCGTACGAAAATACTGGCCAAATTAGACCC
AAACAATCATGGAGGACGTCAACCCAAGGACGTTGATACGCGTTCTGTTGGTGTTGGAAGCGCTTCGGGAATAGATGATG
GCGTTGTTAGCGAAACCCATACTTCAACAACAACTTCCAGAGTTCGTTCAGATCCTAAATTCTGGGTTTCTGTCGGCGCA
ATTGCTGCTGGCTTAGCGGGGCTGGCGGCAACTGGTATTGCACAGGCGTTGGCTTTGACACCGGAACCGGATGATCCTAC
AACCACCGATCCTGATCAGGCCGCAAATGCTGCAGAAAGTGCGACAAAAGATCAGTTAACGAAAGAAGCATTCCAAAACC
CTGATAACCAGAAAGTTAACATCGATAAGAACGGAAACGCTATTCCGTCTGGGGAATTAAGAGATGATGTTGTTGCACAA
ATAGCAGAACAAGCTAAAGAGGCTGGTGAGGCGGCCAGACAGCAGGCTCTTGAAAGCAATGCACAGGCGCAGCAGCGATA
TGATGATCAGCATGCCAGACGTCAGGAGGAATTACAGCTTTCATCGGGTATTGGTTACGGCCTCAGCAGTGCATTGATTG
TTGGTGGGGGAATTGGTGTTGGTGTAACGACTGCGCTCAATAGACGAAATCAGCCGGTAGAACAGACAACTACAACAACA
CATACGGTAGTGCAGCAGCAGACCGGAGGGAATACCCCAGCACAAGGTGGCACTGATGCCACAAGAGCAGAAGACGCTTC
TCTGAATAGACGTGATTCGCAGGGGAGTGTTGCATCGACACACTGGTCAGATTCCTCTAGCGAAGTGGTTAATCCATATG
CTGAAGTTGGGGAGCCTCGGAATAGTCTATCGACTCGTCAGCAAGAAGAGCATATTTACGATGAGGTCGCTGCAGATCCT
GGTTATAGCGTCATTCAGAATTTTTCACGGAATGCTCCAGTTACCGGAAGGTTAATGGGAAGCCCAGGGCAAGGTATCCA
AAGTACTTATGCGCTTCTGGCAAGCAGCGGCGGATTGCGTTTAGGTATGGGAGGATTAACGAGTGGTGGCGAGAGCGCAG
TAAATACTGCAAATGCCGCACCAACGCCGGGACCAGTACGTTTCGTTTAA
>Cluster_65120
ATGACAGTGGCAAATTATAATTCTCTTGTCCAGAAAACTTTCTGCGAAAATGCAATTCGTTCCGTTGTCATGATTGATGA
CGATTTTCTGACGTATTCTGAATCAATCAGGGCGTTGAATAACGAAGTTGATTTAGACTACAACAAAATTGACTCATCTA
AACGAGCCGCTACTCTTGAGAGCTTTTTTCAATCTAAAAATATGATTTGTGATGTTGACAATGGTTCTGTTAATTTCGAT
GTGGATCGGATTAGAAAATCAGATCTTATTATTGTAGATTATCATCTTGATAATAATGCACCTGATAAAACACTTAAACT
ATTACAAGATTTGAAAGACTCCGATCATTTAAAAATGATTGTAATATATACTAGAGAGAATTTAGAAACGGTTTGGATGC
AGATATCATCGACTCTCAAAGGTGCTCTGGATATCAACAGCTTGATCATTGACTACGATAATGAAGATGTCCAAAGTTAT
TGGGAAGACGTTGTATTACCGAACTTAAATGATAATGGTAATAAAGCTCTCACAAGAGATGAAACAATAGCCTATATTAA
AGACAGTAAGCCTTGTAGAAGAATTAAAAGATTAATACATGATGATGCTGTGTTGGAGGATCAAAAGGATAAAAACTTCA
TTGCAAAAATGATTGCAGAATATGCTGTGTCTAGAAATGCAATTATTTCTAGCAACACATCTGGCAATGTCATTCGGGGT
GATGAAAGCGGAGTAAAATGGATTCAATGTGGTAATATCTTTGTCTCCCTATTTCATAAGGTTCAAGATGATCATGAAAA
CGATGGAGATAGGATTTGGCAAACTCTCAATGATTCTCTCATTGAATGGAAACCATCTTATTATCAGTTAATAAAATCTG
AAATTCAGAATGCAATCGAAGCTGAGGCTTTATCTTTTGTAAATCATTTGGCTAACGATCATTACGGTCAAGCTGCGTGG
TTAAATGAGATATTAAAATCAGACTCGCCTGATATTAGATGTAGAAATATTGACTTTGTATTTGGTAATTTATCAGAAGA
GCTTTATCAAAGACTTAAAAATAATAATACGCTGGATGAATTTATCAAAAGTGTTTTTGATAGCTATTCAAATGAATACG
CTAACAGCGGAGTTGCTGCATTGCTCCAATATTGCTCTTCAAAAATGGATCTGCCATCAAATAATGATACTTATCACGAA
ATGTATCATGCTTTAAATATGAATTTGTCTTCAAAGAATTTTGAAGATGGTCATATTTCTACTGGCACTATTTTCTTTGA
TACAGAGTCGAACAAATGGTATTTATGTGTATCTGCGGCATGTGATTTGGTTCCTACTCAGTGTAACGACCCTCACCATG
TAAGATTAAGTCCGCACAGGCTCATTAAAGTTCTGGAGCTTTTTAACGCCAGTCAGAGTAAAGCATTGCCATTTGCTGAA
CATTCGAAATATATATATGTAATGCATAAAAATCAAAGAAAATATCTCTCTATTTTCGAAGGGGATAAAACGCTTCCTGT
TGTTGATTATATGGTGGTGTTGAATCATGGAACAACAGTTGATGGCGAAGAAAAAATATTATTTCTGCCGTGTTTTTAA
>Cluster_427
ATGATCCAGATTGATCTTCCCACGCTGGTAAAACGGCTGAACCTGTTCTCCCGCCAGGCGCTGGAGATGGCCGCCTCTGA
ATGTATGAGTCAGCAGGCAGCGGAAATTACCGTCAGCCATGTGCTCATTCAGATGCTCACCATGCCACGCAGTGACCTGC
GGGTTATTACCCGCCAGGGCGATATTGGCATGGAAGAGTTGCGCCAGGCGCTGACGGTAGAGAACTACACAACCGCCCGT
TCTGCGGACAGCTACCCGGCGTTTTCCCCGATGCTGGTTGAGTGGCTGAAAGAGGGCTGGCTGCTGGCGTCGGCTGAGAT
GCAGCACAGCGAACTGCGCGGCGGCGTGTTGCTGCTGGCCCTGCTGCATTCGCCGCTGCGTTATATACCGCCTGCTGCCG
CCCGGCTGTTGACCGGCATTAACCGTGACCGTCTGCAACAGGACTTTGTGCAGTGGACACAGGAGTCGGCGGAATCAGTC
GTGCCGGATGCAGACGGTAAAGGCGCAGGCACAATGACGGACGCCTCTGACACCCTGCTTGCCCGCTATGCCAAAAACAT
GACCGCAGACGCCCGTAACGGCAGGCTTGACCCGGTACTGTGCCGCGACCACGAAATCGACCTGATGATCGACATTCTCT
GCCGCCGCCGTAAAAACAACCCGGTGGTGGTGGGCGAAGCGGGCGTGGGCAAAAGCGCACTGATTGAAGGGCTGGCGCTG
CGCATCGTGGCAGGCCAGGTGCCGGACAAGCTGAAAAACACCGATATCATGACCCTTGACCTGGGCGCATTGCAGGCCGG
GGCGTCGGTGAAGGGTGAATTCGAAAAACGTTTCAAAGGGCTGATGGCGGAGGTCATTTCCTCCCCGGTGCCGGTCATTC
TGTTTATCGACGAAGCACATACCCTGATTGGCGCGGGCAACCAGCAGGGCGGGCTGGATATCTCCAACCTGCTCAAACCG
GCGCTGGCGCGCGGCGAGCTGAAAACCATCGCCGCCACCACCTGGAGCGAGTACAAAAAATACTTCGAAAAAGATGCCGC
CCTGTCGCGCCGCTTCCAGTTGGTGAAGGTCAGCGAACCCAACGCTGCCGAAGCCACCATTATTCTGCGCGGTCTGTCGG
CGGTCTATGAACAGTCTCACGGCGTGCTGATTGATGATGACGCCTTGCAGGCCGCTGCGACATTAAGCGAGCGTTATCTC
TCCGGGCGTCAGTTACCGGACAAAGCGATTGATGTGCTGGATACCGCCTGCGCCCGTGTGGCCATCAACCTGTCGTCGCC
GCCGAAGCAAATCTCGGCGCTGACCACTCTGAGCCACCAGCAGGAGGCGGAAATTCGCCAGCTTGAGCGCGAGCTTCGCA
TCGGACTGCGTACCGACACATCACGGATGACCGAGGTGCTGGTGCAGTATGATGAAACGCTGACGGCGCTGGATGAACTG
GAAGCGGCCTGGCACCAGCAGCAGACGCTGGTCCGGGAGATTATTGCGCTGCGCCAGCAGTTACTGGGCGTGGCAGAGGA
CGATGCGGCGCCGTTGCCGGACGCAGATACCGTGGAGGATACGCAGCCAGAGTCAGAGTCAGAACAGGATAAATACCGGT
GCCGTACCGGCTGA
>Cluster_26016
ATGGCAATGTTCGGTTTTCCTCACTGGCAGTTAAAATCGACCTCTACAGAGAGCGGCGTGGTCGCGCCGGATGAACGATT
GCCGTTTGCTCAAACGGCAGTAATGGGCGTTCAACACGCGGTGGCGATGTTTGGCGCGACGGTGTTAATGCCAATTCTGA
TGGGGCTGGATCCCAATCTTTCCATTTTAATGTCGGGGATCGGCACGTTACTGTTTTTCTTTATTACCGGCGGGCGCGTG
CCCAGTTATCTCGGTTCCAGCGCGGCTTTTGTCGGCGTGGTGATTGCCGCGACGGGCTTTAATGGTCAGGGAATTAATCC
GAATATCAGCATCGCGCTGGGCGGATTATCGCCTGTGGGCTGGTTTATACCGTCATTGGCCTGGTGGTGATGAAAATCG
GTACGCGCTGGATTGAACGACTAATGCCGCCGGTTGTGACGGGTGCGGTAGTGATGGCGATTGGCCTGAACTTAGCGCCT
ATCGCAGTGAAAAATGTATCGGCTTCGGCCTTTGATAGCTGGATGGCGGTAATGACGGTGCTGTGTATTGGGCTGGTGGC
CGTATTTACCCGTGGCATGATCCAGCGATTATTGATTCTCGTAGGATTAATTGTTGCCTGTCTGCTGTATGGCGTGATGA
CCAACGTTTTAGGGCTGGGAAAAGCCGTCGATTTTACTCTCGTCAGCCATGCTGCCTGGTTCGGTCTGCCACATTTTTCC
ACTCCTGCTTTTAATGGTCAGGCAATGATGCTGATTGCGCCCGTGGCGGTGATTCTGGTAGCGGAAAACTTAGGTCATCT
CAAGGCGGTCGCCGGGATGACCGGACGCAATATGGACCCGTATATGGGGCGGGCATTCGTAGGCGATGGGCTGGCGACGA
TGCTTTCTGGCTCTGTCGGCGGTAGCGGCGTAACCACCTACGCCGAGAATATTGGTGTGATGGCCGTGACAAAAGTCTAC
TCGACGCTGGTGTTTGTTGCCGCTGCCGTCATCGCCATGCTGTTGGGCTTTTCGCCGAAATTTGGCGCGCTGATCCATAC
TATTCCGGCCGCGGTGATTGGTGGTGCATCAATTGTTGTGTTCGGGCTGATTGCCGTCGCAGGCGCACGGATCTGGGTAC
AAAACAGTGTGGATTTAAGCCAGAACGGTAATTTGATTATGGTCGCAGTAACCTTAGTGCTGGGTGCGGGTGATTTTGCT
CTCACATTGGGCGGTTTTACGTTGGGAGGGATCGGTACAGCCACCTTTGGCGCGATTTTACTCAATGCGTTGTTAAGCAG
AAGGTTGGTTGACGTTCCACCGCCAGAAGTTGTTAATCAGGAGCCATAA
>Cluster_63674
ATGATTATCAACCAGGTACCTATAAAAATAAAAATCTTCATCTTTTTATTTTCATGCATCTCTATTATATTTTTGTTACT
GCATGCAAATAATGGAATATACATAACACAAACAACACAAATAAGTTATAGTGTTTTCATTATTGGGCTTTTTTTCATAA

ACCTGATGATTTTTATTTTTCTATTGCTTTACTATGTTTCTAATCAGAGACAAAGTTATCTCTTAATTCTTTCATTCGCG
TTTTTGAGCAACACGTATTATTTATTAGAAGTGGCTATTATTTCTTTATCTCCGTTAGGTAACGATTTATCTACAATCTA
TCAGAAATCAAATGATATCGCAATATATTATCTATTCCGTCAGTTCAGCTTTATATCTATAATCTTTCTGGCTGTTTATT
CCACCAATGTTAAAAATAAAAGTGTTTTAGAAGATAAAAGAAACATAATAATTGTTGTTTTGTCAATATTAATTCTTTTT
ATTACTCCGTTTGTAGCAAAAAAATCTAAGCAGTGACAATATAAAATATAGTCTTAATATTATACAATACTCGCTGAATCG
TCATTTGCCGACGTGGAATATCGTGTACACCAAAATAATATCAGTATTTTGGCTTGTATTACTTATCAGCTCATGCATCA
GCATACGTAATTACTCAAAAATATGGTTGTGTATAATACTTATTAGTATAGTGTCAGTATGCAATAATCTAATTTTATTG
TATTTTATTGATAAATCCCATCCTGCATGGTACATGACAAAATTTCTTGAATTGATATCAATGATTTATATCATTTCAAC
ACTCATGTATTATATTTTCAGGAAATTAAATCATGCTAATCATATGGCAATTCATGATCCACTAACGAATACATACAATA
GAAGATACTTTATTGACTCATTGAAGAATATATCAAAACACCATGATTTCTCAGTAATAATGTTAGATATTGACAGTTTC
AAAAGCATCAATGACAAATGGGGGCATCATATGGGTGATCAAGTCATAGTAATGGTTACCAGAATAATAAAAAAATCCAT
CAGGAAAGAGGATATATTAGGGCGCTTAGGCGGTGAGGAGTTCGGTATTATCATTAAGGTAATACTCAAAAGCTCTTGC
TATCAATTGCAGAGCGAATCAGAAAAAACATTGAAGAGCAATGCTCGGAAAAATTATTATCGCATGGACCTGAGAAAATA
ACTGTCAGTATTGGTTGCTTTACTTCAAAAGAGAATAATCTCAGCCCATCTGAAATGTTAGTCAATGCCGATAAAGCGTT
ATATCAAGCCAAAAGAACCGGAAAAAACAAGGTGATAATTCACTCAAAATAA
>Cluster_23073
ATGAGTGATATATCCCGGCAAGAATTTCAGCGTCGCCGTCAGGCCCTGGTAGAGCAAATGCAACCCGGCAGCGCCGCGCT
GATTTTTGCTGCACCAGAAGTAACACGTAGCGCCGACAGCGAATACCCCTATCGTCAGAACAGTGACTTCTGGTACTTCA
CCGGCTTTAACGAACCGGAAGCGGTGCTGGTGCTGATTAAAAGCGATGACACTCATAACCACAGCGTTTTGTTTAACCGC
GTTCGCGACCTGACGGCAGAGATCTGGTTTGGTCGTCGCTTAGGCCAGGATGCCGCGCCAGAAAAACTGGGCGTTGACCG
CGCACTGGCATTCAGCGAAATCAATCAGCAACTTTATCAACTAATTAATGGTCTGGATGTGGTTTACCACGCCCAGGGCG
AACATGCATATGCTGATGAAATCGTGAACAACGCGCTTGAGAAACTGCGTAAAGGTTCGCGGCAAAATCTCACCGCACCG
GCAACGATGATCGACTGGCGTCCTGTTGTTCATGAAATGCGCCTGTTCAAATCACCAGAAGAGATTGCCGTACTTCGCCG
CGCGGGTGAAATCACCGCACTGGCGCATACACGGGCGATGGAAAAATGCCGTCCGGGGATGTTCGAGTACCATCTGGAAG
GCGAAATTCACCACGAATTTAACCGCCACGGTGCGCGTTACCCTTCCTATAACACCATTGTTGGCAGCGGTGAAAACGGC
TGCATTTTGCACTACACCGAAAACGAGTGTGAACTGCGTGACGGTGACCTGGTGTTGATTGACGCGGGTTGTGAATACAA
AGGTTACGCTGGCGACATTACCCGCACCTTCCCGGTCAACGGCAAATTCACTCAGGCTCAGCGTGAAATCTATGACATTG
TGCTGGAGTCTCTTGAAACCAGCCTGCGCCTGTATCGTCCGGGAACTTCCATTCAGGAAGTCACTGGTGAAGTGGTGCGC
ATCATGGTTAGCGGTCTGGTAAAACTCGGCATCCTGAAAGGTGAAGTTGATGAACTGATCGCTCAGAACGCCCATCGTCC
TTTCTTTATGCATGGCCTTAGCCACTGGTTAGGGCTGGATGTTCATGACGTTGGTGTTTATGGTCAGGATCGCTCGCGTA
TTCTGGAACCGGGTATGGTACTGACCGTAGAACCAGGGCTGTATATTGCGCCGGATGCAGAAGTGCCAGAACAGTATCGC
GGTATCGGCATTCGTATTGAAGACGATATTGTGATTACCGAAACCGGTAATGAAAACCTCACCGCCAGCGTGGTGAAAAA
GCCGGAAGAAATCGAAGCGTTGATGGCAGCTACGAGAAAGCAATGA
>Cluster_25640
ATGTCCAGAATCAATAAATTCGTACTTACAGTCAGTCTGCTGATTTTTATCATGATTTCAGCAGTTGCCTGCGGGATCTA
CACTCAAATGGTAAAGGAACGGGTGTATGGCCTGAAACAGTCCGTTATTGATACTGCTTTTGCGGTGGCAAATATTGCTG
AATATCGGCGTAGTGTGGCAATTGATCTGATCAACACGCTAAATCCTACGGAGGAACAGCTGTTGGTTGGCTTACGCACA
GCTTACGCCGACTCGGTTTCACCCTCTTATTTGTACGATGTCGGTCCTTATCTGATTTCCAGTGACGAATGTATTCAGGT
AAAGGAGTTCGAGAAAAATTATTGTGCAGATATTATGCAGGTTGTTAAGTATCGACATGTCAAAAATACAGGGTTTATCT
CTTTTGATGGTAAAACCTTCGTCTATTACCTCTATCCGGTAACTCACAATCGTAGTCTGATATTTTTGCTTGGTCTGGAA
CGTTTTTCTTTACTGTCAAAATCGCTGGCGATGGATAGCGAGAACCTGATGTTCTCCCTGTTCAAGAACGGTAAATCGGT
GACCGGTGATGAATATAACGCTAAAAACGCCATCTTCACCGTTTCGGAAGCAATGGAGCACTTCTCCTATTTGCCGACCG
GATTGTACGTATTTGCTTATAAAAAAGACGTTTATTTTCAGGTATGTACATTGATTATTTTCTTTGCCGCATTGGTGGCA
GTGATATCGGGTGCCAGTTGCCTCTATCTGGTACGCAGAGTGATTAATCGTGGTATTGTGGAGAAAGAAGCCATTATTAA
TAACCATTTTGAACGCGTACTGGATGGCGGGCTTTTCTTTTCGGCTGCCGATGTCAAAAAACTCTACAGTATGTATAACT
CGGCATTCCTGGACGATCTGACCAAAGCGATGGGCAGAAAATCCTTTGACGAAGATTTAAAAGCGCTGCCGGAAAAAGGC
GGTTATTTATGCCTGTTTGACGTCGATAAATTCAAAAACATTAACGACACCTTCGGTCATTTGCTGGGCGATGAAGTGTT
GATGAAAGTGGTGAAAATCCTTAAATCACAGATCCCGGTAGATAAAGGTAAAGTTTACCGCTTCGGAGGTGACGAATTTG
CGGTGATTTATACTGGCGGCACGCTGGAAGAGTTGCTATCGATTCTAAAAGAAATCGTTCATTTCCAGGTGGGAAGCATT
AATTTAAGCACCAGTATCGGTGTAGCGCATTCAAATGAATGTACTACCGTTGAACGTTTGAAAATGCTGGCGGATGAGCG
GCTGTATAAGAGTAAGAAAAACGGCAGGGCGCAGATTAGCTGGCAGTAA
>Cluster_26415
ATGGCGAACAGCATCACGGCGGATGAGATTCGGGAACAGTTTTCGCAGGCAATGTCAGCCATGTACCAGCAAGAAGTTCC
GCAGTACGGCACGCTGCTGGAACTGGTAGCTGATGTAAATCTGGCGGTGCTGGAAAACAATCCTCAACTGCACGAAAAAA
TGGTAAATGCAGACGAGCTGGCGCGACTGAATGTTGAACGTCATGGGGCGATTCGCGTTGGGACTGCACAAGAGCTTGCT
ACTCTTCGGCGGATGTTTGCCATTATGGGGATGTACCCGGTGAGCTATTACGATCTCTCGCAGGCAGGGGTGCCGGTACA
TTCGACAGCATTTCGGCCCATTGATGATGCTTCTCTGGCGCGTAATCCCTTCCGCGTTTTTACCTCGTTACTCCGTCTTG
AGCTTATCGAGAACGAATTTTGCGCCAGAAAGCGGCGGAGATTCTGCGTCAGCGCGATATCTTCACCCCACGTTGTCGA
CAACTGTTAGAGGAATATGAGCAGCAGGGCGGTTTTAACGAAACACAGGCACAGGAGTTTGTGCAGGAAGCCCTGGAAAC
GTTTCGCTGGCACCAGTCAGCAACGGTAGATGAAGAAACCTATCGCGCCTTGCACAACGAACATCGGTTGATTGCTGATG
TGGTCTGTTTTCCTGGATGCCATATCAACCATCTGACGCCACGTACGCTGGATATTGACCGGGTGCAGTCGATGATGCCT
GAATGCGGAATTGAACCCAAAGTTCTGATCGAAGGGCCGCCGCGCCGCGAGGTGCCGATTTTACTACGCCAGACCAGCTT
TAAAGCACTGGAAGAGACGGTGTTGTTTGCGGGGCAGAAACAGGGCACGCATACCGCGCGCTTTGGTGAAATTGAGCAGC
GTGGTGTGGCATTAACGCCGAAAGGGCGACAACTGTATGATGATCTTCTGCGGAACGCTGGAACCGGGCAGGATAATCTC
ACTCACCAAATGCATTTACAGGAAACCTTCCGCACTTTTCCTGACAGTGAGTTTTTAATGCGTCAGCAAGGGCTGGGATG
GTTCCGGTACCGTCTGACGCCTTCGGGTGAGGCGCATCGTCAGGCGATTCATCCCGGAGACGATCCACAGCCCTTAATAG
AACGTGGTTGGGTAGTGGCGCAACCCATTACCTATGAAGATTTCTTGCCCGTTAGCGCGGCGGGGATCTTCCAGTCAAAT
CTGGGTAATGAAACGCGGGCATGCAATCACGGTGATGCCAGTCGCGAAGCATTTGAGCAGGCGTTGGGGTGTCCGGTTTT
GGATGAGTTCCAGCTTTATCAGGAAGCGGAGGAACGCAGTAAACGTCGCTGTGGTTTGCTTTAA
>Cluster_1913
ATGTCAGGATTTAAAAAAGGTTTTTTATGGGGCGGCGCGGTAGCCCGCGCATCAGTTGGAAGGTGGCTGGAATGAAGGAGG
AAAAGGCATCAGCATCGCTGATGTGATGACTGCTGGCGCTCACGGGGTGCCGCGTGAAGTGACAGAAGGCGTTATCGACG
GGCTTAATTATCCCAATCATGAAGCAATTGATTTTTATCATCGCTATAAAACAGATATTCAGTTATTTGCCGAGATGGGA
TTCAAATGCTTTCGAACTTCCATTGCCTGGACACGAATCTTTCCGCAAGGTGACGAACAGGAGCCGAATGAAGAGGGTTT
ACAATTTTATGATGATCTGTTCGATGAATGCCTTAAGCAGGGAATGGAACCTGTGGTGACGCTTTCACATTTTGAGATGC
CTTATCATCTGGTGACAAAATATGGTGGCTGGCGAAACCGTAAACTGATCGACTTTTTTATCCGCTTCGCATCAACGGTC
TTCACGCGCTATAAAGAAAAAGTAAAGTACTGGATGACGTTTAACGAAATCAATAATCAGGTGAATTTCAGCGAAAGCCT
GTGTCCATTTACTAATTCCGGTATCTTGTATTCGCCAGAGGAAGATATCAATGAGCGCGAACAAATAATGTACCAGGCGG
TACATTACGAGTTAGTTGCCAGTGCCCTGGCGGTACAGACTGGAAAATCGATCAATCCTGAATTTAACATTGGCTGTATG

ATCGCCATGTGTCCCATCTATCCTCTGACGTGTGCACCCAACGATATGATGATGGCCACGAAAGCAATGCATCGTCGTTA
CTGGTTTACTGATGTTCATACTCGTGGATATTATCCGCAACATATGCTGAATTACTTTGCCAGGAAAGGATTCAACCTCG
ATATCACACCAGAAGATAACGCGATTCTTGCCAGTGGTTGTGTCGACTTTATCGGCTTTAGCTACTACATGTCTTTTACG
ACACAATTTTCGCCCGATAACCCGCAACTGGATTATGTTGAACCACGAGATTTGGTCAGCAACCCTTATATCGATACATC
CGAATGGGAATGGCAAATTGATCCGGCAGGGCTACGTTATTCACTCAACTGGTTCTGGGATCATTTCCAGTTGCCGCTGT
TTATTGTCGAAAATGGATTTGGTGCGGTTGACCAGAGACAAGCTGACGGCACGGTGAACGATCACTATCGCATTGATTAC
TTTGCTTCCCATATTCGGGAAATGAAAAAGCGCTACGGAATGATTTATGTCGATAAAGACAACGAAGGGAAGGGAACGCT
GGAACGGATACGTAAAGCGTCGTTTTACTGGTATCGGGATCTCATCGCCAACAATGGCGAAAATATTTGA
>Cluster_18411
ATGAGTACTTCAGATAGCATTGTATCCAGCCAGACAAAACAATCGTCCTGGCGTAAATCAGATACCACATGGACGTTAGG
CTTGTTTGGTACGGCAATCGGCGCCGGGGTGCTGTTCTTCCCTATCCGCGCAGGTTTTGGCGGACTGATCCCGATTCTTC
TGATGTTGGTATTAGCATACCCCATCGCGTTTTATTGCCACCGGGCACTGGCGCGTCTGTGTCTTTCTGGCTCTAACCCT
TCCGGCAACATTACGGAAACGGTGGAAGAGCATTTTGGTAAAACTGGCGGCGTGGTTATCACGTTCCTGTACTTCTTCGC
GATTTGCCCACTGTTGTGGATTTATGGCGTTACCATTACCAACACCTTTATGACGTTCTGGGAAAACCAGCTCGGCTTTG
CACCGCTGAATCGCGGCTTTGTGGCGCTGTTCCTGTTGCTGCTGATGGCTTTCGTCATCTGGTTTGGTAAGGATCTGATG
GTTAAAGTGATGAGCTATCTGGTATGGCCGTTTATCGCCAGCCTGGTGCTGATTTCTCTTTCGCTGATCCCTTACTGGAA
CTCTGCAGTTATCGACCAGGTTGACCTCGGTTCGCTGTCGTTAACTGGTCATGACGGTATCCTGATCACCGTCTGGCTGG
GGATTTCCATCATGGTTTTCTCCTTTAACTTCTCGCCAATCGTCTCTTCCTTCGTGGTTTCTAAACGTGAAGAGTACGAG
AAAGATTTTGGTCGCGACTTCACCGAGCGTAAATGTTCACAAATCATCTCTCGCGCCAGTATGCTGATGGTTGCAGTGGT
GATGTTCTTTGCCTTTAGCTGCCTGTTTACGCTGTCTCCGGCGAATATGGCTGAAGCGAAAGCGCAGAATATTCCAGTGC
TTTCTTATCTGGCTAACCACTTTGCGTCCATGACCGGTACCAAAACAACGTTTGCGATTACACTGGAATATGCCGCTTCC
ATCATCGCACTCGTGGCTATTTTCAAATCTTTCTTCGGTCACTATCTGGGGACGCTGGAAGGGCTGAATGGTTTGATTCT
GAAGTTCGGTTATAAAGGTGACAAAACCAAAGTGTCGCTGGGTAAACTGAATACTATCAGCATGATTTTCATTATGGGCT
CCACCTGGGTTGTTGCCTACGCCAACCCGAACATCCTCGACCTGATTGAAGCCATGGGCGCACCGATTATCGCATCACTG
CTGTGCCTGTTGCCGATGTATGCCATCCGTAAAGCGCCGTCTCTGGCGAAATACCGTGGTCGTCTGGATAACGTGTTTGT
TACCGTGATTGGTCTGCTGACCATCCTGAACATCGTATACAAACTGTTTTAA
>Cluster_41539
ATGAGCCATAAACCTGCGCATTTATTATTGGTCGATGACGATCCGGGATTGCTGAAACTGCTTGGCCTGCGCCTGACCAG
CGAAGGCTACAGCGTGGTCACGGCGGAAAGTGGCGTTGAAGGATTACGGGTACTGAATCGCGAAAAAGTAGATTTAGTCA
TCAGCGACCTGCGGATGGATGAAATGGATGGTATGCAGCTATTTGCTGAAATTCAGAAAGTGCAGCCGGGAATGCCAGTA
ATTATTCTTACCGCGCATGGTTCTATTCCCGATGCCGTTGCAGCAACACAGCAGGGCGTTTTTAGTTTCCTCACCAAGCC
TGTCGATAAAGACGCGCTATATCAGGCAATTGACGATGCGCTGGAACAATCCGCGCCAGCCACCGACGAACGCTGGCGCG
AGGCGATTGTCACCCGCAGCCCGCTGATGCTGCGTTTGCTGGAACAGGCGCGGCTGGTGGCGCAATCAGACGTTAGCGTT
TTGATTAACGGCCAGAGCGGCACCGGGAAAGAGATTTTCGCCCAGGCTATCCACAACGCCAGCCCGCGCAACAGCAAACC
ATTTATTGCTATTAACTGTGGCGCATTGCCCGAGCAATTGCTGGAGTCGGAGCTGTTTGGTCATGCGCGTGGTGCGTTTA
CTGGCGCTGTCAGCAATCGCGAAGGTTTATTCCAGGCGGCGGAAGGCGGTACGCTATTTCTCGATGAGATTGGCGATATG
CCCGCACCGTTGCAGGTCAAACTGCTGCGCGTGTTGCAGGAGCGTAAAGTGCGCCCGCTGGGCAGTAACCGCGATATTGA
TATCGACGTGCGGATTATTTCTGCCACTCACCGCGACCTGCCAAAAGCGATGGCGCGCGAGGAATTCCGCGAAGATCTCT
ATTACCGCCTCAACGTTGTCAGCCTGAAAATTCCGGCACTGGCGGAGCGCACAGAAGACATTCCGCTACTGGCAAATCAC
CTGTTGCGCCAGGCGGCAGAGCGACATAAACCGTTTGTCCGCGCGTTCTCCACCGATGCGATGAAACGCCTGATGACCGC
GAGCTGGCCGGGTAATGTGCGCCAGTTGGTCAACGTGATTGAACAGTGCGTGGCGCTGACCTCATCTCCGGTGATTAGCG
AGGCATTAGTAGAGCAGGCGCTGGAGGGTGAAAATACGGCGCTGCCAACCTTTGTTGAGGCACGTAATCAGTTTGAACTC
AACTATTTGCGTAAGCTGCTGCAAATTACCAAAGGCAACGTCACCCACGCGGCGAGAATGGCGGGGCGCAACCGGACAGA
ATTTTATAAACTGCTATCCCGACACGAGCTGGATGCAAACGATTTCAAGGAATGA
>Cluster_66739
ATGTACCCTATCGCAACTGAGTTAAAGGTGGGTAACAATCAACTGGATAGCTACCTGCCAGTACGCAATAAAAATAATGA
TGTTAACTGGCAATTTGTTACTGGCCTGGTCCTTAGCTATGCCCTGAAATGCAAAATTGACGCCTACGATCCTGACCAAT
TCCGTGAAGATTGTAAAACACATATGCAGGAGCTGCTTGATGAGCCTGCATTCTGGTCCGTGCTGGAACGGATGTATTTT
TCCAGCCAGGACATTTTCCGTGTCTCCCCACTTTTTTTACTTTTTCACGCCCAGTTTAATGGTGAAAAAATAAGTGCGGG
TTCAATGGCAGATAAACGACTTGGCACGTTGTTTGCCAATTTAATGGGCGATTTCAGCCTTCGTTACCCCATACAGGACA
GACTGAACTTCATTGAGCAGCAAATGCTGAATAAGCTCAATGAAAAAATAAAGCAGTTGGAGAAAGGGCCGTTTGCCGAA
GAGCAACCATATCTTCCGTATATGGTAAAGTCTTTTCAGTCTGATCTGGCATTTCTGGCAGAACATCCACAGTACCTTTT
ACAGGAATTGACCAACACCCTACGTTTGTACGCCTTTAGCTGGTGCGCTCAACTGGCGCTCAATATTGACAATTGGCAGG
ACGGCGAACCACAAAGCAAATCACTGTTTTTTATTCTTGATAGCGAAAAAGCGAGCTCCGAACGGGAAAAGGTGAAACGT
TATGGCTATAAGCTGTTCGCCAGCCAGAGCGAAAAACTGTTCCCGGTGCTCTCTGCTCTAGAGGTTTTGCAGTGGGGAAA
AGGACAGAAAAAGCGTCCGTTATGGCAGATTTATCAAGACACTCTGAATGATTCAGACTCATCTGCCCGAGTACTAAATG
ACCTTAATGTTTATTTGCAGGATTTTATTGTGGATCGAGGCCTCCCGCTTCGTGACCGCGCAACAAACCTAGAAAGTGCT
TTCAAACAGCTATTATCTGTTGCCGTAGAGCAATTCCAAGATAAAAAAACTAACCGTGCGGATATAAATAAGAACTACGT
TAAGGAGCTGGAAAGCCAGATTTGCACTGACTTCATTCAGGTCCGTGGTCGGTCAGGGAAAGTGTTGGTGCTGAATCAGG
ATCGTCTTCTGTTGCTGACCAATCTCACTGTGGGCAAAAATGACAAGCTCCGCCTACACGAACTGCTGCGTGGTTTTGAA
CAACGCGGTTTTTATCTGGATAACCAGTCGTCGCAAACGCTGGTGGCGTTTTATGAACGCATGGGAAATGTAGAGCGAAT
GAGTGATAGTGGAGACGCCGTGTATGTCCGTAAAACAGTATGA
>Cluster_24376
ATGCTAGTTGTAGAACTCATCATAGTTTTGCTGGCGATCTTCTTGGGCGCCAGATTGGGGGGAATAGGTATTGGTTTTGC
AGGCGGATTGGGGGTGCTGGTTCTTGCCGCTATTGGCGTTAAACCCGGTAACATCCCGTTCGATGTCATTTCCATTATCA
TGGCGGTTATCGCCGCTATTTCTGCCATGCAGGTTGCTGGCGGTCTGGACTATCTGGTTCATCAGACAGAAAAGCTGCTG
CGCCGTAACCCGAAATACATCACGATCCTCGCACCGATCGTGACCTATTTCCTGACTATCTTTGCTGGTACTGGCAACAT
CTCTCTGGCGACACTGCCAGTTATCGCTGAAGTTGCGAAGGAACAAGGCGTTAAACCTTGCCGTCCGCTGTCTACTGCAG
TGGTATCCGCGCAGATTGCGATCACCGCATCGCCAATCTCAGCGGCAGTGGTTTACATGTCTTCCGTGATGGAAGGTCAT
GGCATCAGCTACCTCCATCTGCTCTCCGTGGTCATCCCGTCCACCCTGCTGGCGGTTCTGGTGATGTCCTTCCTGGTCAC
TATGCTGTTCAACTCCAAACTCTCTGACGATCCGATTTATCGCAAGCGTCTGGAAGAGGGCCTGGTTGAACTGCGCGGTG
AAAAGCAGATTGAAATCAAATCCGGTGCAAAAACGTCCGTCTGGCTGTTCCTGCTGGGCGTAGTTGGCGTGGTTATCTAT
GCAATCATCAACAGCCCAAGCATGGGTCTGGTTGAAAAACCACTGATGAACACCACCAACGCAATCCTGATCATCATGCT
CAGCGTTGCAACTCTGACCACCGTTATCTGTAAAGTCGATACCGACAACATTCTCAACTCCAGCACCTTCAAAGCAGGTA
TGAGCGCCTGTATTTGTATCCTGGGTGTTGCGTGGCTGGGCGATACTTTCGTTTCCAACAACATCGACTGGATCAAAGAT
ACCGCTGGTGAAGTGATTCAGGGTCATCCGTGGCTGCTGGCCGTCATCTTCTTCTTTGCTTCTGCTCTGCTGTACTCTCA
GGCTGCAACCGCAAAAGCACTGATGCCGATGGCTCTGGCACTGAACGTTTCTCCGCTGACCGCTGTTGCTTCTTTTGCTG
CGGTGTCTGGTCTGTTCATTCTGCCGACCTACCCGACGCTGGTTGCTGCGGTACAGATGGATGACACGGGTACTACCCGT
ATCGGTAAATTCGTCTTCAACCATCCGTTCTTCATCCCGGGTACTCTGGGTGTTGCCCTGGCCGTTTGCTTCGGCTTCGT

GCTGGGTAGCTTCATGCTGTAA
>Cluster_4564
ATGAAAACCCGTACACAACAAATTGAAGAATTACAGAAAGAGTGGACTCAACCGCGTTGGGAAGGCATTACTCGCCCATA
CAGCGCGGAAGATGTGGTGAAATTACGCGGTTCAGTCAATCCTGAATGCACCCTGGCGCAACTGGGCGCGGCGAAAATGT
GGCGTCTGCTGCATGGCGAGTCGAAAAAAGGCTACATCAACAGCCTCGGTGCACTGACTGGCGGTCAGGCGCTGCAACAG
GCGAAAGCGGGTATTGAAGCAGTCTATCTGTCGGGATGGCAGGTAGCGGCGGACGCTAACCTGGCGGCCAGCATGTATCC
GGATCAGTCGCTCTATCCGGCAAACTCGGTGCCAGCTGTGGTGGAGCGGATCAACAACACCTTCCGTCGTGCCGATCAGA
TCCAATGGTCCGCAGGCATTGAGCCGGGCGATCCGCGCTATGTCGATTACTTCCTGCCGATCGTTGCCGATGCGGAAGCC
GGTTTTGGCGGTGTCCTGAATGCCTTTGAACTGATGAAAGCGATGATTGAAGCCGGTGCAGCGGCAGTTCACTTCGAAGA
TCAGCTGGCGTCAGTGAAGAAATGCGGTCACATGGGCGGCAAAGTTTTGGTGCCAACTCAGGAAGCTATTCAGAAACTGG
TCGCGGCGCGTCTTGCAGCTGACGTGACGGGCGTTCCAACCCTGCTGGTTGCTCGTACCGATGCCGATGCGGCGGATCTG
ATTACCTCCGATTGCGACCCGTATGACAGTGAATTTATTACTGGCGAGCGTACCAGTGAAGGCTTCTTCCGTACTCATGC
GGGCATTGAGCAAGCGATCAGCCGTGGCCTGGCGTATGCGCCATATGCTGACCTGGTCTGGTGTGAAACCTCCACGCCGG
ATCTGGAACTGGCGCGTCGCTTTGCACAAGCTATCCACGCGAAATATCCGGGCAAACTGCTGGCTTATAACTGCTCGCCG
TCGTTCAACTGGCAGAAAAACCTCGACGACAAAACTATTGCCAGCTTCCAGCAGCAGCTGTCGGATATGGGCTACAAGTT
CCAGTTCATCACCCTGGCAGGTATCCACAGCATGTGGTTCAACATGTTTGACCTGGCAAACGCCTATGCCCAGGGCGAGG
GTATGAAGCACTACGTTGAGAAAGTGCAGCAGCCGGAATTTGCCGCCGCGAAAGATGGCTATACCTTCGTATCTCACCAG
CAGGAAGTGGGTACAGGTTACTTCGATAAAGTGACGACTATTATTCAGGGCGGCACGTCTTCAGTCACCGCGCTGACCGG
CTCCACTGAAGAATCGCAGTTCTAA
>Cluster_28371
ATGGATGAATTCAAACCAGAAGACGAGCTGAAACCCGATCCCAGCGATCGTCGTACTGGTCGTTCTCGTCAATCTTCTGA
ACGTTCTGAGCGTACTGAACGTGGCGAACCGCAGATCAATTTTGATGATATTGAACTTGATGACACTGACGATCGCCGTC
CGACTCGTGCGCAAAAAGAGCGCAATGAGGAACCGGAAATCGAAGAAGAAATTGACGAATCCGAAGATGAAACCGTGGAT
GAAGAGCGCGTAGAGCGTCGTCCGCGTAAGCGCAAAAAAGCAGCCAGTAAACCCGCTTCTCGTCAGTATATGATGATGGG
CGTCGGCATTCTGGTTCTACTGCTGTTGATCATCGGTATCGGTTCTGCGCTAAAAGCCCCCCTCGACCTCTTCCAGCGCTC
AAACCGCGTCTGGCGAGAAGAGTATTGATCTTGCAGGCAATGCGACCGATCAGGCGAATGGCGTGCAGCCTGCGCCGGGA
ACCACGTCTGCGGAAAATACTCAGCAGGATGTTTCTCTGCCGCCGATCTCTTCTACGCCGACTCAAGGGCAAACCCCGGC
GGCAACGGATGGTCAACAACGTGTTGAAGTGCAGGGTGACCTGAACAATGCGCTGACCCAGCCACAAAATCAGCAACAGT
TGAACAATGTGGCGGTCAATTCTACATTGCCGACTGAACCCGCGACTGTCGCGCCTGTTCGCAATGGCAATGCATCGCGT
GACACGGCGAAAACGCAAACCGCTGAACGTCCGGCCACTACGCGCCCAGCTCGTCAGCAGGCGGTGATTGAACCGAAAAA
ACCGCAAGCAACCGTGAAAACGGAACCGAAGCCGGTAGCACAGACGCCGAAGCGTACTGAACCAGCTGCTCCTGTGGCGA
GCACGAAGGCACCGGCTGCGACTTCTACGCCAGCAACAAAAGAGACGGCGACTACGGCTCCAGTACAGACGGCATCCCCG
GCGCAAACCACGGCAACACCAGCCGCTGGAGGGAAGACCGCAGGTAATGTGGGTTCGTTGAAATCGGCACCGTCCAGCCA
TTACACTCTGCAGCTGAGCAGTTCCTCTAACTACGACAACCTGAACGGTTGGGCGAAGAAAGAGAATCTGAAAAACTACG
TTGTCTATGAAACGACGCGTAATGGTCAGCCGTGGTATGTCCTGGTTTCTGGCGTGTACGCTTCGAAAGAAGAGGCGAAA
AAAGCGGTATCTACATTGCCAGCCGATGTCCAGGCCAAAAACCCGTGGGCGAAACCGCTGCGTCAGGTACAGGCCGATCT
GAAGTAA
>Cluster_62665
GTGCTGGAACGGCTGTCGTGGAAAAGGCTGGTGCTGGAGCTGCTACTTTGCTGCTTCCCGGCTTTCATCCTGGGTGCATT
TTTTGGTTACCTGCCCTGGTTTTTGCTGGCATCGGTAACGGGACTGCTTATCTGGCATTTCTGGAATTTATTGCGCCTTT
CATGGTGGCTGTGGGTGGATCGTAGTATGACCCCGCCACCGGGGCGTGGTAGCTGGGAACCGCTACTATACGGCTTACAC
CAGATGCAGCTGCGAAATAAAAAACGCCGCCGTGAACTGGGCAATCTGATTAAACGCTTTCGTAGCGGCGCGGAGTCGCT
GCCCGACGCAGTGGTGCTGACCACTGAAGAGGGCGGTATTTTCTGGTGTAACGGTCTGGCGCAACAAATTCTTGGTTTGC
GCTGGCCGGAAGATAACGGGCAGAACATCCTTAACCTGCTGCGTTACCCGGAGTTTACGCAATATCTGAAAACGCGTGAT
TTTTCTCGCCCGCTCAATCTGGTGCTCAACACCGGGCGGCATCTGGAAATTCGCGTCATGCCTTATACCCACAAACAGTT
GCTGATGGTGGCGCGTGATGTCACGCAAATGCATCAACTGGAAGGGGCGCGGCGTAACTTTTTTGCCAACGTGAGCCATG
AGTTACGTACGCCATTGACCGTGTTACAGGGTTACCTGGAGATGATGGATGAGCAGCCGCTGGAAGGCGCGGTACGCGAA
AAAGCGTTGCACACCATGCGCGAGCAGACCCAGCGGATGGAAGGGCTGGTGAAGCAGTTGCTGACGCTGTCGAAAATAGA
AGCCGCACCGACGCATTTGCTCAATGAAAAGGTTGATGTGCCGATGATGCTGCGCGTTGTTGAGCGTGAGGCTCAGACTC
TGAGTCAGAAAAAACAGACATTTACCTTTGAGATAGATAACGGCCTCAAAGTGTCTGGCAACGAAGATCAGCTACGCAGT
GCGATTTCGAACCTGGTCTATAACGCCGTGAATCATACTCCAGAAGGCACGCATATCACCGTACGCTGGCAGCGAGTGCC
GCACGGTGCCGAATTTAGCGTTGAAGATAACGGACCGGGCATTGCACCGGAGCATATTCCGCGCCTGACCGAGCGTTTTT
ATCGCGTTGATAAAGCGCGTTCCCGGCAAACCGGCGGTAGCGGATTAGGGTTAGCGATCGTGAAACATGCGGTGAATCAT
CACGAAAGTCGCCTGAATATTGAGAGTACAGTAGGAAAAGGAACACGTTTCAGTTTTGTTATCCCGGAACGTTTAATTGC
CAAAAACAGCGATTAA
>Cluster_32894
ATGCCATTAGTCATTGTTGCTATCGGTGTAATCTTGTTATTACTCCTGATGATCCGCTTCAAAATGAACGGTTTCATCGC
TCTCGTCCTCGTGGCGCTTGCTGTGGATTAATGCAAGGAATGCCGCTGGATAAAGTTATTGGCTCCATCAAAGCCGGTG
TCGGCGGAACGCTCGGTAGCCTTGCCCTGATCATGGGTTTTGGCGCAATGCTGGGCAAAATGCTGGCAGACTGCGGTGGC
GCACAACGTATCGCCACCACGCTGATTGCTAAATTTGGTAAAAAACACATCCAGTGGGCGGTGGTACTGACCGGTTTTAC
CGTTGGTTTTGCCCTGTTCTATGAAGTGGGTTTCGTGCTGATGCTGCCGCTGGTGTTTACCATCGCGGCTTCCGCGAATA
TTCCACTGCTGTATGTTGGTGTACCAATGGCGGCTGCACTGTCTGTGACCCACGGCTTCCTGCCACCGCATCCGGGTCCG
ACTGCGATTGCCACCATTTTCAATGCCGATATGGGTAAAACCCTGCTGTACGGTACTATTCTGGCAATCCCGACCGTTAT
TCTCGCCGGTCCGGTTTACGCTCGCGTGCTGAAAGGTATCGATAAGCCAATCCCGGAAGGTCTGTACAGCGCGAAAACCT
TCAGCGAAGAAGAGATGCCAAGCTTTGGCGTCAGCGTCTGGACTTCTCTGGTGCCGGTTGTGCTGATGGCGATGCGTGCA
ATTGCCGAAATGATCCTGCCGAAAGGTCACGCTTTCCTGCCGGTGGCGGAGTTCCTCGGTGACCCGGTAATGGCAACGCT
GATTGCCGTGCTGATTGCTATGTTCACCTTTGGTCTGAACCGTGGTCGTTCAATGGATCAGATTAACGACACGCTGGTTT
CTTCCATCAAAATCATCGCGATGATGCTGTTGATCATCGGTGGTGGCGGTGCGTTCAAGCAGGTGCTGGTAGACAGCGGC
GTGGACAAATACATTGCTTCCATGATGCACGAAACCAACATTTCTCCGCTGCTGATGGCCTGGTCGATTGCCGCCGTACT
GCGTATCGCGCTGGGTTCCGCAACCGTTGCGGCAATCACTGCGGGGGGTATCGCGGCACCACTAATTGCAACGACGGGTG
TTAGCCCGGAACTGATGGTTATTGCGGTTGGTTCCGGTAGTGTGATTTTCTCTCACGTGAACGATCCGGGCTTCTGGCTG
TTCAAAGAGTACTTTAACCTGACTATCGGCGAGACCATCAAGTCCTGGTCGATGCTGGAAACCATTATCTCGGTGTGCGG
TCTGGTAGGCTGTCTGCTGCTGAATATGGTGATTTGA
>Cluster_53700
ATGGCTGATTATCAGGGTAAAAATGTCGTCATTATCGGCCTGGGCCTCACCGGGCTTTCCTGCGTGGACTTTTTCCTCGC
TCGCGGTGTGACGCCGCGCGTTATGGATACGCGTATGACACCGCCTGGCCTGGATAAATTACCCGAAGCCGTAGAACGCC
ACACGGGCAGTCTGAATGATGAATGGCTGATGGCGGCAGATCTGATTGTCGCCAGTCCCGGTATTGCACTGGCGCATCCA
TCCTTAAGCGCTGCCGCTGATGCCGGAATCGAAATCGTTGGCGATATCGAGCTGTTCTGTCGCGAAGCACAAGCACCGAT
TGTGGCGATTACCGGTTCTAACGGCAAAAGCACGGTCACCACGCTAGTGGGTGAAATGGCGAAAGCGGCGGGGGTTAACG

TTGGTGTGGGTGGCAATATTGGCCTGCCTGCGTTGATGCTACTGGATGATGAGTGTGAACTGTACGTGCTGGAACTGTCG
AGCTTCCAGCTGGAAACCACCTCCAGCTTACAGGCGGTAGCAGCGACCATTCTGAACGTGACTGAAGATCATATGGATCG
CTATCCGTTTGGTTTACAACAGTATCGTGCAGCAAAACTGCGCATTTACGAAAACGCGAAAGTTTGCGTGGTTAATGCTG
ATGATGCCTTAACAATGCCGATTCGCGGTGCGGATGAACGCTGCGTCAGCTTTGGCGTCAACATGGGTGACTATCACCTG
AATCATCAGCAGGGCGAAACCTGGCTGCGGGTTAAAGGCGAGAAAGTGCTGAATGTGAAAGAGATGAAACTTTCCGGGCA
GCATAACTACACCAATGCGCTGGCGGCGCTGGCGCTGGCAGATGCTGCAGGGTTACCGCGTGCCAGCAGCCTGAAAGCGT
TAACCACATTCACTGGTCTGCCGCATCGCTTTGAAGTTGTGCTGGAGCATAACGGCGTACGTTGGATTAACGATTCGAAA
GCGACCAACGTCGGCAGTACGGAAGCGGCGCTGAATGGCCTGCACGTAGACGGCACACTGCATTTGTTGCTGGGTGGCGA
TGGTAAATCGGCGGACTTTAGCCCACTGGCGCGTTACCTGAATGGCGATAACGTACGTCTGTATTGTTTCGGTCGTGACG
GCGCGCAGCTGGCGGCGCTACGCCCGGAAGTGGCAGAACAAACCGAAACTATGGAACAGGCGATGCGCTTGCTGGCTCCG
CGTGTTCAGCCGGGCGATATGGTTCTGCTCTCCCCAGCCTGTGCCAGCCTTGATCAGTTCAAGAACTTTGAACAACGAGG
CAATGAGTTTGCCCGTCTGGCGAAGGAGTTAGGTTGA
>Cluster_64436
ATGCCGTGGAACAATTTTCCGCACCTCGCCGCTAGGGCGTTCAATCAACCGCTTTTGCTGGAGCCCGCCTACGCGCGGGT
ATTTTTTTCTGCGCTGAGCGACCGGTTCGGTACCGGGCGACTGATTGATACAGCGTCAGGAGAGGTAATGAACAGCGACG
AAATGAACGCGCTCGCGATGGGGTGGGACAGCAGCGAGCGAACACGCCAGAAATCGTATCGCGTGGAGCGTGGTATAGCC
GTTCTGCCGGTTACCGGGACGCTGGTTCATAAATTGGGTTATATCAATCCGGTCAGCGGGATGAGTGGTTACGACGGAAT
CGCAAAACGCCTGCAGCAGGCGATTTCTGATCCCGATGTTAAGGGGATCCTGCTGGATATTGATTCCCCTGGCGGTGAGG
TCGCCGGCGCGTTTGATACCGCTGATTTAATCGCCCGGGCGCGAGAGCAAAAACCGGTGTGGGCGCTGGCCAGCGATACG
GCCTGCAGCGCCGCATATTTGCTGGCGTCAGCGTGTTCGCGCCGGCTGATAACGCAGACCGGCACGGTTGGTTCAATCGG
TGTCCTGATGGCTCACCGCTGCGTCGAAAAGGCGCTGGAGATTGCCGGCGTTGACGTGACGCTGATTTACGCCGGCGCGC
ACAAAGTCGACGGGAACCCGTATTCCCAGCTGCCCGACGACGTTCGCGACGAATTCCAGCTGAGTATTAACAGCACACGC
GAGCAGTTCGCGCAAAAAGTCTCGGATTATACCGGGCTGAAAAAATCCAGGGTGCTGGCCACAGAGGCCGCAGTATTTAT
CGGCGCGGACGCGATTAAATCTGGTCTCGCTGATCAACTCGTTAATTACGCGGACGCTATCGCAGTGATGGCCGACGCAC
TGAAACCAAAAACGGAGCGATTTATGCCAGGTACAACAGAAACCACGGCGGAGACCACGACCACAGAACAAACCGCGGCT
ACGACTACGGTCGCGCCGGTTGAGTCCAACGCGGAGCAGATTCGCGCGGACGCCGCATCGAGCGAACTGGCACGCGTGAT
GGCCATCATCAACTGTCCCGAAGCTGTTGGGCGCGAGGCGCAGGCAAAAGCGCTCGCTGCCGTCCCCGGGATGACGGTCG
GGCAGGCGCAGGCTGTCCTCGCGGCAGCACCGCAAACAGCGCAGGCGCGGACAGAAACGGCGCTCGATACACTCATGAGC
ACTGAATCACCGGAAACTATTCAGGATGCCGGCAGCACCACGGCAACAGGAACAACCGCAAACGTCTCGATGCTGGTCGC
GGCAGGGCGTTCAATTTTAGGGGATGAATAA
>Cluster_26995
ATGTTCAACGAGCTGTCAGGGTGTTTTCACGCCGTCGGAAATGGCACTTTTTTCACTTCAAAAATTCGGAGAAAAGATGG
CAATGAATTCAACTGGGGTTATGACTGTGGTTCTACCAGCGAAAAAGCCATAAATAACATAATTGACTCATCTCATATGG
AGTTTAACAAAAATGATAGTATAGACATGATGGTCATTTCTCATTTTGATGATGACCATGTTAATGGGTTGATTACTCTA
TTAAGGAAGCACACAATAAAAAGATTGGTGCTTCCTTATAGTGATTGGACTCAGGCTATTAGAGAAATATCTATAGGTGG
ATCCCATGGGATATCACCTTCTGTAGCACTTTTTCAGTTAAATCCAGCACTATGGTTATATAATAACGATCTATCAAATC
GGGTGGAGAAAATAATATTAGTACAAGGCAATGGCGGCCCAAAAAATCCTGATAAGTTGATAACTCCCGAGATTTTCCCA
AAAAATTCTGATGAGCCTTTAATACTAAGAGATAACTTAGATGAGTTAAAAGATATAGAACAAGAAAGACATATAAATGA
TGCTAAGGATAATGTTTTTTTTGATTTAGGTATGCCAATCAATAGTAGTGGAATAAAATTGTCAACAATTAACCATATGC
AGCCTGTTAGTGTGCTCGATGGTATATTTGAGTTTGTATTTTATAATGCCGAAAAGTCATTTAAAAAACTCAATCTTGTA
TATGAGAAAGATGAGGAATTTTATGCCATCAAGTCTCATTTTAAACTTGCTGATGCTAAGAAAGATATCCAGCAAACAAT
TGAGAGTATAAACCTACACTGTGGTATTAACTCCAGTATTGATGTAAAGTGGCGGGAGAAGTTAAAGGCTTGCTACCAGA
AACATTTTGGTAGCACCAATAAAGCTCAAAACAATATTTCTTTGTGTATGTATGCTTCACCTAAAATTACCCCACATGGC
TTCCATTTCATCTTGGGTAAATGTAAGAAAAGCTACAATATTCATTCTTCTGGTTTGATATTTACAGGGGATATTAATCT
GACAACTCCTGTTCTAGATGATTTTGAGTCCCATCTGGGAACTGCTCGATGGATGAATTGTGGATTGTTTCAGATTCCTC
ACCATGGTTCTGCCAGTTGCTGGGAAAAAGGACATACTAAAAGGATCAAACCTGCTTATTTTGTTCAATGTGCTACTCCT
ACAGTAAAACATCCCAGTCAGTTTGTTTTAGATGATTTGAATAGTGAAAAGAGCATTGTTTATAATGCATCTCGACAAAA
CCCAGTACATTTTTCATATGTTTTTTTACAATGA
>Cluster_23382
ATGAGTCAGAATGTTTATCAATTTATCGACCTGCAGCGCGTTGATCCGCCAAAGAAACCGCTGAAGATCCGCAAAATTGA
GTTTGTTGAAATTTACGAGCCGTTTTCCGAAGGCCAGGCCAAAGCGCAGGCTGACCGCTGCCTGTCGTGCGGCAACCCAT
ACTGCGAGTGGAAATGCCCGGTACACAACTACATCCCAAACTGGCTGAAGCTCGCCAATGAGGGGCGTATTTTTGAAGCG
GCGGAACTGTCGCACCAGACAAACACCCTGCCGGAAGTTTGCGGACGTGTCTGCCCGCAAGACCGTCTGTGCGAAGGTTC
CTGCACCCTGAACGATGAGTTCGGTGCGGTGACCATCGGCAACATTGAGCGTTATATCAATGATAAAGCGTTCGAGATGG
GCTGGCGTCCGGATATGTCCGGTGTGAAACAGACCGGTAAAAAAGTGGCGATTATCGGCGCAGGTCCGGCGGGTCTGGCG
TGCGCGGATGTCCTTACGCGCAACGGCGTAAAAGCGATCGTCTTCGACCGTCACCCGGAAATTGGCGGCTTGCTGACCTT
CGGTATTCCGGCTTTCAAGCTGGAAAAAGAGGTAATGACGCGCCGCCGTGAAATCTTCACCGGCATGGGTATTGAATTCA
AACTCAATACCGAAGTGGGCCGCGACGTGCAGCTGGACGATCTGCTGAGTGATTACGATGCCGTGTTCCTTGGCGTCGGG
ACTTATCAGTCAATGCGCGGCGGGCTGGAAAACGAAGACGCCGATGGCGTGTACGCAGCGCTGCCGTTCCTTATCGCCAA
CACCAAACAGTTAATGGGTTTTGGTGAAACCCGCGACGAACCGTTCGTCAGCATGGAAGGCAAACGCGTGGTGGTCCTTG
GCGGTGGCGACACTGCGATGGACTGCGTGCGCACGTCTGTACGCCAGGGGGCAAAGCACGTTACCTGTGCCTATCGTCGT
GATGAAGAGAACATGCCGGGTTCCCGCCGCGAAGTGAAAAACGCGCGGGAAGAAGGCGTAGAGTTCAAATTCAACGTCCA
GCCGCTGGGTATTGAAGTGAATGGCAACGGCAAAGTCAGCGGCGTAAAAATGGTGCGCACAGAAATGGGCGAACCGGATG
CCAAAGGCCGTCGCCGCGCGGAGATTGTGGCAGGTTCCGAACATATCGTTCCGGCAGATGCGGTGATCATGGCGTTTGGT
TTCCGTCCACACAACATGGAATGGCTGGCAAAACACAGCGTCGAGCTGGATTCGCAGGGGCGCATTATCGCCCCGGAAGG
CAACGACAACGCTTTCCAGACCAGCAACCCGAAAATCTTTGCTGGCGGCGATATCGTCCGTGGTTCCGATCTGGTCGTTA
CCGCCATTGCCGAAGGTCGCAAAGCGGCAGACGGCATTATGAACTGGCTGGAAGTTTAA
>Cluster_21614
ATGCCGCCTGATTACAACCTGCTGGAATATCATCGGGGGACGATCACAGCCCCGGCCGGGTGCGGTAAAACACAAATCAT
TGCGGATACCCTAGCTCTGTATACGGGAGCCAGGCCGGTACTTATCCTCACGCACACCAACGCGGGTGTGGCAACACTGC
GTCTGCGTATGCAACGTGCCGGTGTTTCTACAGCGGCCTACCGTATCGCCACTATTGATGGATTTGCCATGCGTCTAGTG
GCGATGTTTCCAGCGCGTAGTGGGCTCAACGCTGAGGTCCTTCAACTGCGCAATCGCGGAACTGACTATCCCGCTATACG
ACTGGCTGCCTGCCAGTTGCTGCAGGCAGGACATCTCAATGATGTTCTGCCGGCCAGTTACGTTCACCTCATCGTGGACG
AATACCAGGACTGCAATCTGGTGCAACATGCAATGGTAACCGCGTTATCAACAGTGCTGCCTGCCTGTGTACTCGGTGAC
CCTATGCAGGCTATTTTTGGCTTTCGGGGTAATCAGCTCGTGAACTGGGACGCACATGTACTCGCTCACTTTCCACTGAT
TGGTGAGCTCAATCATCCATGGCGCTGGGCTAACTCAGGGGCGCCGGAACTTGGTCGCTGGCTTCTGGACTGCCGGAGGA
TGTTGCTGAATGGCAATGCCATTGACCTTCGCCAGGTGCCTGCTGAGGTAGCCTGGGTACCGTTACAGGCAGAAACTGCT
GTTCAGCAACGTCTTACTGCCGCCCAGACCCGAGCGGTTACCGCTACCGGGCAGGTACTGGTGATTGGAAACAGCTACGA
CACTCGCGGGCGCCAGCAGATGGCCAGCCAGACCCCGGGTGCGACGACCGTCGAAGCTGTCGATTTGGTTGACCTTACCG

ACTTTGGTGCAGGGTTTAACCTGGAGGCAGCGGATGCGTTGACGCGCTTGGTAAAGTTTGCCGCAGGCGTCATGACCCAG
GTTGGCCCCGCAGCATTGCTGCCCCGGCTGGAAACGCTACGAAGAGGAAGAGCCCGCAATCCGCCGACGGTCGCAGAAGC
AGCGTTAGTGGCATTTGCAGAGAGACCAGATTTTACGCTGGCCGCAGAGGCGCTGCGCCTTCTGACTCGTCAGGACAATG
CGCGAGTCTATCGGCCAGAGGTTTTGCGCTGCTGTTTGCGCGCCCTGCAGTCGGCTGCTGCCGGAGGCTGCACTTTTGCA
GACGCCACGATAAACGAGCGCGAACGCAATCGCCACGAGAGTCGACCGTTGCCCCGGCGTGTAATCGGCAGCACATTGTT
GCTAAAGGGACTGGAAGGGGAGGTGGCAGTCATCACATCCCCGGAAGAGATGGATGCACAACACCTCTATGTCGCTATGA
CGCGGGGAGCTTCGCGGCTGGTTGTGTGCTCGCTAACTCCCATATTGGCGGCCCGCTAA
>Cluster_66780
ATGACGCGTTATCAACATCTGGCGACTCTACTTGCCGAGCGGATTGAGCAAGGGCTGTATCGTCACGGGGAGAAATTGCC
GTCGGTGCGCAGCTTAAGTCAGGAGCACGGCGTCAGCATCAGCACCGTGCAACAGGCGTATCAGACGCTGGAAACGATGA
GGCTCATCACTCCGCAGCCGCGTTCGGGTTATTTTGTCGCACAACGTAAAGCCCAGCCGCCTGTACCGCCGATGACGCGT
CCGGTGCAGCGCCCGGTGGAAATTACCCAGTGGGATCAGGTGCTGGATATGCTGGAGGCGCATAGCGACAATTCCATTGT
TCCGTTAAGCAAAAGCACGCCGGATGTCGAAACGCCCAGCCTGAAACCGCTGTGGCGTGAGCTAAGCCGGGTGGTGCAGC
ATAATCTGCAAACCGTTCTCGGTTATGACTTGTTAGCCGGTCAGCGAGTGTTGCGGGAGCAGATTGCCCGCCTGATGCTC
GACAGCGGCTCGGTGGTCACCGCCGATGACATCATCATCACCAGCGGCTGCCATAACTCGATGTCGCTGGCGTTAATGGC
GGTGTGTAAACCGGGCGATATTGTCGCGGTCGAATCTCCCTGTTATTACGGTTCGATGCAGATGCTGCGAGGCATGGGCG
TGAAAGTGATTGAAATCCCAACCGATCCAGAAACTGGCATCAGCGTTGAAGCACTGGAACTGGCGCTGGAACAGTGGCCG
ATTAAAGGCATCATTCTGGTGCCAAACTGTAATAATCCGCTGGGATTTATTATGCCGGACGCACGCAAACGGGCCGTTCT
CTCTCTCGCTCAGCGTCATGATATTGTGATTTTTGAAGATGATGTCTACGGCGAACTGGCAACGGAGTATCCGCGCCCGC
GGACCATCCATTCATGGGATATCGACGGGCGAGTGTTGTTGTGCAGCTCGTTCAGTAAAAGTATTGCTCCAGGCCTGCGC
GTGGGTTGGGTCGCACCGGGGCGTTATCACGATAAACTGATGCATATGAAATACGCCATCAGCAGCTTTAATGTGCCGTC
CACGCAAATGGCGGCGGCAACCTTTGTGCTGGAAGGTCACTATCATCGCCATATCCGGCGGATGCGGCAGAGCTATCAGC
GCAATTTGGCGCTTTATACCTGCTGGATACGGGAATATTTTCCCTGCGAAATCTGTATTACGCGCCCGAAAGGCGGATTT
TTACTGTGGATAGAATTACCTGAACAGGTCGATATGGTCTGCGTTGCGCGGCAGCTGTGCCGCATGAATATCCAGGTGGC
AGCAGGCTCGATTTTCTCGGCTTCCGGCAAATACCGTAATTGTCTACGCATCAACTGCGCTTTGCCGCTCAGCGAAACCT
ATCGCGAAGCACTAAAGCAAATTGGCGAGGCCGTGTATCGGGCAATGGAATAA
>Cluster_16092
ATGCAGAACGGAATTGCACACAACGGACTGATACTTCTCTGGCAGATGAGAGAGGGGAAGCATTTATGTCCGGGAAAAAC
CACTTTATCTGACCATGCTGTTTTGTACCTGCCGGTATCCACATTTGTGGGTACCGTCATTAATGAGAAGGGCTGGCAGG
CCGTCAAATCCGGCGCAATGGCAACCGACACGGTTGTGAATACCGGCGCGGAAGGGGGACCAGATGCAGAAAATGGTGAT
ACCGGGCAGTTTGTTCGCGGAAATGCCGTACGTACCACCATCAATAAAAATGGTCGTCAGATTGTGGCTGTTGAAGGAAC
AGCAAATACCACTGTGGTTTATGCCGGCGGCGACCAGACGGTACACGGGCATGCGCTGGATACCACACTGAATGGCGGTT
ACCAGTATGTGCACAACGGAGGCACAGCCTCTGACACGGTTGTAAACAGTGACGGCTGGCAGATTGTCAAGGAAGGTGGT
CTGGCGGATTTCACCACCGTTAACCAGAAAGGCAAACTGCAGGTGAACGCCGGTGGTACAGCCACGAATGTCACCCTGAA
GCAGGGAGGCGCACTGGTCACCAGTACGGCGGCAACCGTCACCGGCAGCAACCGTCTGGGCAATTTCACTGTGGAAAACG
GTAATGCTGACGGTGTTGTTCTGGAGTCCGGTGGTCGCCTGGATGTACTGGAGGGCCATTCAGCCTGGAAAACACTGGTG
GATGACGGCGGAACCCTGGCAGTGTCTGCCGGTGGTAAGGCAACAGATGTCACCATGACATCCGGTAGTGCCCTGATTGC
AGACAGTGGTGCCACTGTTGAGGGGACCAATGCCAGCGGTAAGTTCAGTATTGATGGCACATCCGGTCAGGCCAGCGGAC
TGCTGCTGGAAAATGGCGGCAGCTTTACGGTTAATGCCGGAGGACTGGCCAGCAACACCACTGTCGGACATCGTGGAACA
CTGACGCTGGCTGCCGGGGGAAGTCTGAGTGGCAGAACACAACTCAGTAAAGGCGCCAGTATGGTACTGAATGGTGATGT
GGTCAGTACCGGCGATATTGTTAACGCAGGGGAGATTCGCTTTGATAATCAGACGACACAGGATGCCGTGCTGAGCCGTG
CTGTTGCAAAAGGCGACGCCCCGGTAACGTTCCATAAACTGACCACCAATAACCTCACCGGCCAGGGCGGCACCATCAAT
ATGCGTGTTCGCCTTGATGGCAGCAATGCCTCTGACCAGCTGGTGATTAATGGTGGTCAGGCAACCGGCAAAACCTGGCT
TGCGTTCACAAATGTCGGAAACAGTAACCTCGGGGTGGCAACCACCGGACAGGGTATCCCGGAAATACGATTTCAGGAAA
ATTCATGCAGTAATGGGAAAGGACTCTCATTGCTGTTCACCGCAGTTCTGAATTATTGA
>Cluster_58190
ATGTCCATACGTTTAGAAGGTTATATGCAAACAACACAACAAAATGCGCCACTGAAGCGCACAATGAAAACGCGTCACCT
GATTATGCTTTCCTTGGGCGGCGTGATTGGCACAGGATTATTCTTCAATACCGGGTACATCATTTCCACCACTGGAGCGG
CGGGAACGCTGCTGGCCTATCTGATTGGTGCGCTGGTGGTCTGGCTGGTTATGCAGTGTCTGGGCGAGCTGTCGGTCGCG
ATGCCGGAGACCGGAGCGTTTCACGTTTATGCCGCGCGCTATCTTGGTCCGGCTACCGGGTATACCGTGGCCTGGCTTTA
CTGGCTGACCTGGACCGTGGCGCTGGGTTCGAGCTTTACCGCCGCTGGATTCTGTATGCAGTACTGGTTTCCACAGGTGC
CGGTATGGGTCTGGTGCGTGGTGTTCTGCGCGATTATTTTTGGTCTGAATGTTATCTCCACGCGCTTTTTTGCCGAAGGG
GAGTTCTGGTTCTCGCTGGTCAAAGTGGTCACTATCATCGCCTTTATCATCCTCGGTGGGGCGGCGATTTTCGGCTTTAT
TCCGATGCAGGATGGCTCGCCCGCGCCGGGGCTGAGTAATATCACGGCAGAAGGCTGGTTCCCGCACGGTGGCTTACCGA
TTTTGATGACTATGGTGGCAGTGAACTTTGCTTTTTCGGGTACCGAGCTTATCGGCATTGCCGCCGGTGAAACGGAAAAC
CCGCGCAAAGTTATCCCGGTAGCGATTCGTACTACCATCGCGCGACTGATTATTTTCTTTATCGGCACCGTGTTTGTGCT
GGCAGCGCTGATCCCGATGCAGCAGGTGGGCGTGGAGAAAAGCCCGTTTGTGCTGGTATTTGAGAAAGTAGGGATCCCGT
ACGCCGCTGATATTTTTAACTTCGTGATCCTGACGGCTATTCTTTCTGCAGCGAACTCCGGGTTATATGCCTCCGGGCGC
ATGCTGTGGTCGTTGTCGAATGAACGTACGCTACCGGCCTGTTTTGCGCGAGTAACGAAAAACGGCGTGCCACTGACGGC
GCTGTCGGTCAGTATGCTCGGTGGTGTGCTGGCGCTGTTTTCCAGCGTGGTGGCCCCGGACACGGTATTTGTTGCGCTGT
CGGCAATCTCCGGGTTTGCGGTGGTAGCGGTGTGGCTGAGTATCTGCGCCTCGCATTTTGTTTTTCGTCGCCGTCATCTG
CAACAAGGTAAGGCATTGAGTGAATTACATTATCGCGCGCCGTGGTATCCGCTGGTGCCAGTATTAGGTTTTGTGCTGTG
CCTGGTGGCCTGTGTTGGGCTGGCATTCGATCCAGCGCAGAGAATTGCGTTGTGGTGCGGGTTACCGTTTGTTGCGTTGT
GCTATGGTGCTTATTTCCTTACTCAACCCCGAAACGCAAAACAGGAGCCAGAACATGTCGCAGAATAA
>Cluster_42159
ATGTCTGATACCAAACGTAATACAATCGGCAAATTCGGCTTGCTCTCGCTGACTTTTGCCGCCGTTTACAGCTTTAACAA
CGTTATCAATAATAATATTGAGCTTGGACTGGCCTCGGCACCGATGTTTTTCCTCGCGACGATTTTTTATTTTATTCCCT
TCTGTCTGATCATCGCAGAATTTGTTTCGTTAAATAAAAACTCAGAAGCCGGTGTCTACGCGTGGGTAAAAAGTTCGCTG
GGCGGGCGTTGGGCATTTATTACTGCCTATACCTACTGGTTCGTAAACCTGTTCTTTTTCACCTCACTGTTGCCGCGCGT
TATTGCTTATGCTTCGTATGCCTTCCTCGGCTACGAATATATTATGACGCCGGTTGCCACCACCATTATCAGTATGGTGC
TGTTCGCCTTCTCCACCTGGGTTTCCACCAACGGGGCGAAAATGCTGGGGCCAATTACCTCCGTCACTTCAACGCTGATG
CTGCTGTTAACGCTCTCCTACATTTTACTGGCAGGTACGGCACTGGTTGGCGGCGTACAGCCTGCCGATCCGATCACCGT
TGACGCGATGATCCCGAACTTCAACTGGGCGTTCCTCGGCGTTACCACCTGGATCTTTATGGCCGCAGGTGGCGCGGAGT
CCGTCGCGGTGTACGTTAACGACGTCAAAGGCGGTTCGAAATCGTTCGTTAAAGTGATCATCCTCGCCGGGATTTTTATC
GGCGTACTGTATTCCGTTTCCTCGGTGCTGATTAACGTCTTCGTCAGCAGCAAAGAGTTGAAATTTACTGGCGGATCGGT
GCAGGTATTCCACGGCATGGCGGCGTATTTTGGTCTGCCGGAAGCGTTGATGAATCGCTTTGTCGGTCTGGTGTCCTTTA
CCGCGATGTTCGGTTCCCTGCTGATGTGGACCGCAACGCCGGTGAAAATTTTCTTCTCCGAAATCCCGGAAGGCATCTTT
GGTAAGAAAACCGTCGAACTTAACGAAAACGGCGTTCCGGCGCGCGCAGCGTGGATCCAGTTCCTGATCGTCATCCCGCT
GATGATTATCCCGATGCTCGGTTCCAATACCGTGCAGGATCTGATGAATACTATTATTAATATGACTGCCGCAGCGTCCA

TGCTTCCGCCGTTATTCATCATGCTGGCTTACCTGAATTTACGCGCCAAATTAGATCACCTGCCACGCGATTTCCGCATG
GGCTCCCGCCGCACCGGTATTATCGTTGTTTCAATGCTGATTGCGATATTTGCCGTAGGGTTTGTCGCTTCGACATTCCC
GACTGGCGCGAATATTCTGACCATCATTTTTTATAACGTCGGCGGTATTGTTATCTTCCTCGGCTTTGCGTGGTGGAAAT
ACAGTAAATATATAAAGGGATTAACGGCTGAAGAGCGCCATATTGAAGCGACGCCAGCCAGCAATGTTGATTAA
>Cluster_17665
ATGAGTGAAATTCAGTTTGACACCATTTCGGGCAGTATCCGTAAGCCCGGTGTGCATTTTGAGTTTAATACCCGGCTGGC
CGTTAACACGTTGCCGGGTAACGAACAGCGTGTTCTGGTGATTGGCCCGATGCTGTCAGGCGGCACCGCCACGCCCCTGA
ATGCGGTTTCCGTGTATTCCGAAGACGAAGCGGATTTGTATTTCGGGGCCGGTTCACTGGCCGCAGCAATGGCGCGCGCG
GCCATTAACGCCAACAGCTATCTGCAACTGGATGTTATCGGTATTGCAGACGGTGGTGCAGGAAAAGCGGCAACAGGCGC
AGTTAAAGTCACCGGCACTGCAACCAGCAGCGGTACGCTGTCGGTATGGATTGCTGGCGAGCAGATCGCCATTAATGTGG
ACGTCGGGGATGAACCGTCGAAAATCATTCCGGCACTGGTGACGGCAATGACGCAGACGCCATCGCTTCTGGTCACGGGG
GAATACAAATCTGACACCTCTCAACTGACAGTGACCGCCCGGACCAAAGGTACCTGGGGGAATGATATCACCCTGTCGGT
ATCCACCACTGCAACCGGCCTTGTAGTGGCTACCACGCCGATGGCGAGCGGCGAAATGGACCCGGATATTCAGCCCGCAC
TGGATGCGGTCTTTGCTGCCGGTCACAACATTCTGATTTGTCCGTTCAGTACCACACCAGCCCTTGCCGCCCTGAAGCAG
CACCTGGAGAAAACCGGGAACGCGATGGAACAGCGTGGCGCGATTGGTTGTGCGGGCTGGACAGGTAGTCTGGGAAACGG
GATCACCCTTGCAGCCGGTGTGAACAGCGGGCGCGTGTCTGTCCCCTGGTATCGTGGTTCTGTGATGCTTCCTGCCGTGC
TGGCCGCCATCTACGGCGCAGTAATGGCGAGCGAAGAAGACCCGGCACGCCCGCTGAACTCGCTGGCGCTGTCCGGGCTG
GATGTGGTCGCCATGTCACAGCGCGAAAGCCGTAACGAGCAGGAAAGCGCCCTGCATAACGGCCTGACACCGGTTGAGGT
TGGACCGGGTAATACGGTGCAGATTGTGCGTGCGGTCAGTACCTACACAGTGAACGCTCAGGGTGTGGCTGATGTCTCGT
TACTGGATATCACGTCCATCAGAACGCTGGACTACACCCGCAAGGCGTGCCGCGAACGTATCAGTCTTCGCTTCCCGCGT
GAAAAACTCAGCACCCGAACCATCGCAAAAGTGGAAAGCGAACTGTATGACGTGCTGCTCAAGCTGGAGGAGTCTGAAAT
TCTGGAGAATGTGGAAGCGAATAAGGCAAAACTGCGCGTTCAGCGAAACGGGAAAGATGCAAATCGCCTGGACTGCGCGA
TCCCTGCCGACGTGGTTAACGGCCTGCATGTGTTTGCCGGTCGCATCGACATGATTTTGTAA
>Cluster_56275
ATGTCAGTATTTCCAGAAAGTTTTTTATGGGGCGGCGCGCTTGCCGCCAACCAGTCTGAAGGTGCGTTCCGTGAAGGTGA
CAAAGGTCTGACCACTGTCGATATGATCCCACACGGCGAGCATCGAATGGCGGTGAAACTGGGGCTGGAAAAACGTTTTC
AGTTGCGAGATGACGAGTTTTATCCCAGCCATGAGGCGACGGATTTTTATCATCGTTATAAAGAAGATATCGCCCTGATG
GCAGAGATGGGATTCAAGGTTTTCCGTACCTCAATTGCCTGGAGCCGTCTCTTTCCGCAGGGCGATGAAATCACGCCCAA
TCAGCAGGGCATTGCTTTTTATCGTTCTGTCTTTGAAGAGTGTAAAAAGTACGGTATCGAACCGCTGGTCACGTTGTGCC
ACTTCGATGTGCCGATGCATCTGGTCACCGAATATGGCTCCTGGCGTAACCGCAAGCTGGTGGAGTTTTTCAGCCGCTAC
GCCAGAACCTGCTTTGAAGCATTTGATGGTCTGGTGAAATACTGGCTAACCTTCAATGAAATCAACATTATGTTGCATAG
CCCGTTCTCCGGCGCGGGTCTGGTGTTTGAAGAAGGTGAAAATCAGGATCAGGTGAAATATCAGGCCGCGCATCACCAGC
TGGTTGCCAGTGCGCTAGCCACCAAAATCGCCCATGAGGTTAACCCGCAAAATCAGGTGGGCTGTATGCTGGCGGGCGGT
AACTTCTACCCTTACAGTTGCAAGCCGGAAGATGTCTGGGCGGCGCTGGAGAAAGACCGGGAAAACCTGTTTTTTATCGA
TGTGCAGGCGCGGGGCACGTATCCGGCTTACTCTGCCCGCGTATTCCGCGAAAAAGGGGTAACCATCAACAAAGCACCGG
GCGATGATGAAATCCTGAAAAACACCGTCGATTTTGTCTCTTTCAGCTATTACGCCTCGCGCTGCGCCTCGGCGGAGATG
AACGCCAACAACAGCAGTGCGGCGAACGTGGTGAAATCGCTGCGTAATCCGTATCTACAGGTGAGCGACTGGGGCTGGGG
AATTGATCCACTCGGTCTGCGTATCACCATGAATATGATGTACGATCGTTATCAGAAGCCGCTGTTTCTGGTGGAAAACG
GCCTGGGCGCAAAAGATGAATTTGCTGCCAATGGCGAGATTAACGACGACTATCGCATCAGCTACTTACGCGAACATATC
CGCGCAATGGGCGAAGCGATTGCAGACGGCATTCCGCTGATGGGCTACACCACATGGGGCTGTATTGATTTAGTTTCCGC
CTCTACGGGTGAAATGAGCAAACGCTACGGCTTTGTCTTTGTTGACCGTGACGACGCAGGCAACGGTACGCTGACGCGCA
CGCGTAAGAAATCATTCTGGTGGTATAAAAAAGTGATTGCCAGTAATGGGGAAGATTTAGAGTAG
>Cluster_28958
ATGTCCGCTGAACACGTACTGACGATGCTGAACGAGCACGAAGTGAAGTTTGTTGATTTGCGCTTCACCGATACCAAAGG
TAAAGAACAGCACGTCACTATCCCTGCTCATCAGGTGAATGCCGAATTCTTCGAAGAAGGCAAAATGTTTGACGGCTCCT
CGATTGGCGGCTGGAAAGGCATTAATGAATCCGATATGGTGCTGATGCCAGACGCATCCACCGCAGTGATTGACCCGTTC
TTCGCCGATTCTACCCTGATTATCCGTTGCGACATCCTTGAACCTGGCACCCTGCAAGGCTATGACCGTGACCCGCGCTC
TATTGCGAAGCGCGCCGAAGATTACCTGCGTTCTACTGGCATTGCCGACACCGTACTGTTCGGGCCAGAACCTGAATTCT
TCCTGTTCGATGACATCCGTTTCGGATCGTCTATCTCCGGTTCCCACGTTGCTATCGACGATATCGAAGGTGCATGGAAC
TCCTCCACCCAATATGAAGGTGGTAACAAAGGTCACCGTCCGGCAGTGAAAGGCGGTTACTTCCCGGTTCCGCCGGTAGA
CTCTGCTCAGGATATTCGTTCTGAAATGTGTCTGGTGATGGAACAGATGGGTCTGGTGGTTGAAGCCCATCACCACGAAG
TAGCGACTGCTGGTCAGAACGAAGTGGCTACCCGCTTCAATACCATGACCAAAAAAGCTGACGAAATTCAGATCTACAAA
TATGTTGTGCACAACGTAGCGCACCGCTTCGGTAAAACTGCGACCTTTATGCCAAAACCGATGTTCGGTGATAACGGTTC
CGGTATGCACTGCCACATGTCGCTGTCTAAAAACGGCGTCAACCTGTTCGCAGGCGACAAATACGCAGGTCTGTCTGAGC
AGGCGCTGTACTATATTGGCGGCGTAATCAAACACGCGAAAGCGATTAATGCCCTGGCAAACCCGACCACCAACTCTTAC
AAGCGTCTGGTCCCGGGCTACGAAGCACCGGTAATGCTGGCTTACTCTGCGCGTAACCGTTCTGCGTCTATCCGTATTCC
GGTGGTTTCTTCTCCGAAAGCACGTCGTATCGAAGTACGTTTCCCGGACCCGGCGGCTAACCCGTACCTGTGTTTTGCTG
CCCTGCTGATGGCCGGTCTTGACGGTATCAAGAACAAGATCCATCCGGGCGAAGCCATGGACAAAAACCTGTATGACCTG
CCGCCGGAAGAAGCGAAAGAGATCCCACAGGTTGCTGGTTCTCTGGAAGAAGCACTGAACGAACTGGATCTGGACCGCGA
GTTCCTGAAAGCCGGTGGCGTGTTCACTGATGAAGCGATCGATGCGTACATCGCTCTGCGTCGCGAAGAAGATGACCGTG
TGCGTATGACTCCGCATCCGGTAGAGTTTGAGCTGTACTACAGCGTCTAA
>Cluster_44897
ATGAAAAGACTATATTATACCCATCATGATAGTCGAACCTTAAAACTTGCTATAGAAGGTGATGTTTCTGATAATGCGAA
TATTTTTACAGTAATTGTAGGTAAAAATGGCGTTGGAAAAAGCAGATTGTTATCTCAGATTGCTAAAGACTGTATTCAAG
ATATTCATTTCTTGAAAACTTTTAATCCATTATTTGACAGAGAAATAACACCAAAGTTAATTGCCGTATCTACCAGCCCA
TTCGATAAATTCCCTCCAGGGCGTAGGTCATTAAATCACCAAAGGAAAGAAAATAATTATCGCTATGTGGGTATGCGTGG
AGAAGGCCCCTTCGGCGTATCAAATGCCGTATCCCTTTTATCTTCAGCAGCGAGAGGTTGGCTAGAGAAACTTGCTTATT
CAGAAAATCCTGCAAATTTACTCGATGTTTTTCATTCTATTGATTTTGAGCCCCATATAGAGTTTGTATTTAAACCAACT
TACAACGGACCGGACACTCCTGACTATAACTTCATTGGTGTATATTCAGATGAGATATTAACGGAAGTTAAGGAGTTATA
TTTAAAATACAATATAAAAATTGACAGGAAGAATATTGAGAGTCTCTTAAAGTTAAACTCCGAATCACGTCATGATATTT
ACGAAGCACTGGTTGAGATTAATCGATTTGAACCTGAACGTAGGGCTGTAACCTTGTCAGTCGATTTTACCAATGGTGAA
ACATTGGTTGGCTCGACTTATGCGAATAATTATTTTTCTAGATCAATTCTTAAACTTATGAACGCTGGCTTCATGAGATT
GATGGATATGCGACTCATAAAAAAAAGCTATGGTCCCATGTCACTTAAACGTGCGAGCTCGGGAGAACAATGTTTATTAG
TGATGTTGCTTGGAATTGCAGGGCATATAACTGATGGTTCTATAATACTAATTGATGAACCGGAAATAAGCTTGCATCCT
CGATGGCAGGAAGAGTTTATCATTATGTTAACAAAAGCATTTTTAACTTACTCAGGTTGTCAATTCATAATTGCAACTCA
TTCACCACAAATAATATCCAATTTGCCGAATAAAGGTTGTTATATAACATCTTTGTCGAAAAGTTATCTTTATAAAGCAA
AATACTTTAGCAACCGGTCTGCAGACTATCAACTTGCGGAACTATTTGATGCCCCTGGCATTATGAATGAATACATTGCC
AGATTAGCTTTCAATTTACTTTCAAAAGTTAAGACTGCTAAGTTAGTAACAGATGATAATTGGCGTGAATTAAAACAACT
CCAGCTTCTCCTTGAAAACGTTAATAGTAATGACCCTATTAATGAACTCGTAAATTCCGTATCTCAGGTGTGTGAAATTT

ATGCCAAAAATTAA
>Cluster_37541
ATGGAGATGTATTTTAAAAGAATGAAAGATGAGTGGACCGGACTTGTCGAACAGGCAGATCCGCCCATTCGTGCTAAAGC
CGCGGAAATTGCCGTTGCGCATGCTCATTATCTGAGTATCGAGTTTTATCGAATTGTCCGCATCGACCCGCATGCCGAAG
AATTCTTGAGTAATGAACAAGTTGAGCGGCAGTTGAAGAGTGCGATGGAACGCTGGATTATTAACGTGCTTTCTGCCCAG
GTTGACGATGTCGAAAGGCTAATACAAATCCAGCATACCGTCGCGGAAGTGCATGCCCGCATAGGAATTCCGGTAGAAAT
TGTCGAGATGGGGTTTCGGGTGCTGAAAAAAATCCTCTATCCGGTGATCTTCTCTTCGGATTATTCCGCCGCAGAAAAAC
TTCAGGTCTACCATTTCTCGATTAACAGTATTGATATCGCGATGGAAGTGATGACTCGCGCGTTTACCTTTAGTGACAGT
AGTGCCTCAAAGGAAGATGAAAACTATCGTATCTTCTCGTTACTGGAAAACGCCGAAGAAGAAAAAGAACGGCAAATAGC
CTCAATACTTTCATGGGAAATAGATATTATCTATAAAATCCTGCTGGATTCTGATTTAGGCAGTAGTTTGCCTTTAAGCC
AGGCTGATTTTGGCCTGTGGTTTAACCATAAAGGTCGACATTATTTTAGTGGCATTGCCGAAGTAGGCCATATCTCCCGT
CTGATTCAGGATTTCGACGGTATTTTCAATCAAACCATGCGTAACACCAGGAATTTGAATAACAGAAGTCTGCGGGTGAA
ATTTTTATTACAGATAAGAAATACCGTATCGCAAATTATTACCTTGCTGCGTGAATTGTTTGAAGAAGTATCGCGCCACG
AAGTCGGTATGGATGTACTGACGAAATTACTTAACCGCCGTTTCCTGCCGACTATCTTCAAACGCGAAATTGCCCATGCC
AACCGGACCGGTACACCGCTGTCAGTGCTGATTATTGACGTTGATAAATTCAAAGAGATCAACGATACGTGGGGCCATAA
CACTGGCGATGAAATTCTGCGTAAAGTCTCTCAGGCCTTTTATGACAACGTCCGCAGTAGTGATTATGTTTTCCGCTACG
GGGGCGATGAATTTATCATTGTTTTGACTGAAGCTTCTGAAAACGAAACGTTACGTACCGCAGAACGTATTCGCAGTCGG
GTGGAGAAAACCAAACTGAAAGCCGCAAACGGCGAGGATATTGCCCTCTCACTTTCCATCGGTGCCGCCATGTTTAATGG
TCATCCTGACTATGAGCGCCTCATTCAAATAGCCGATGAAGCTCTGTATATCGCCAAAAGACGAGGTAGAAACCGTGTTG
AACTCTGGAAAGCCAGTCTTTAG
>Cluster_20229
ATGCTGAGTTTTCTCTGGGATTTGGCTTCGTTCATCGTTGCACTGGGTGTACTTATCACCGTGCATGAATTTGGTCATTT
CTGGGTTGCCCGGCGTTGTGGTGTTCGCGTTGAGCGTTTCTCAATAGGGTTTGGTAAGGCGCTCTGGCGGCGAACTGATA
AGCTCGGCACCGAATATGTTATCGCCCTGATCCCGTTGGGCGGTTATGTCAAAATGCTGGATGAGCGCGCAGAACCGGTC
GTTCCGGAACTCCGCCACCATGCCTTCAATAATAAATCTGTCGGCCAACGAGCGGCGATTATTGCCGCAGGTCCGGTTGC
AAACTTCATTTTTGCTATCTTTGCCTACTGGCTGGTTTTTATTATTGGTGTGCCTGGCGTACGTCCGGTGGTTGGTGAAA
TAGCAGCCAATTCGATAGCTGCGGAAGCACAAATTGCACCAGGTACGGAACTAAAAGCCGTAGATGGTATCGAAACGCCT
GATTGGGATGCCGTGCGTTTGCAGTTGGTCGATAAAATTGGCGATGAAAGCACTACCATTACGGTAGCGCCATTTGGCAG
CGACCAACGGCGGGATGTAAAGCTCGATTTACGTCACTGGGCGTTTGAGCCTGATAAAGAAGATCCGGTATCTTCGTTGG
GGATTCGTCCTCGTGGGCCGCAAATTGAACCTGTACTGGAAAATGTGCAGCCAAACTCGGCGGCAAGCAAGGCAGGTTTG
CAAGCAGGCGACAGGATCGTTAAAGTCGATGGTCAACCCTTAACGCAGTGGGTGACCTTTGTGATGCTTGTCCGGGATAA
CCCGGGTAAATCCTTAGCGTTAGAAATCGAAAGACAGGGGAGTCCCTTGTCTTTGACATTAATCCCGGAGAGTAAACCGG
GTAATGGTAAAGCGATTGGTTTTGTCGGTATTGAGCCGAAAGTCATTCCTTTGCCTGATGAGTATAAAGTTGTACGCCAG
TATGGGCCGTTCAACGCTATCGTCGAAGCCACGGACAAAACGTGGCAGTTGATGAAGCTAACGGTCAGTATGCTGGGAAA
ATTGATCACCGGTGATGTGAAACTGAACAACCTCAGTGGGCCGATCTCTATCGCCAAGGGGGCTGGGATGACAGCGGAAC
TCGGGGTAGTTTATTACCTGCCGTTTCTTGCGCTTATTAGCGTGAACTTAGGGATAATTAACCTGTTTCCGTTGCCCGTA
CTTGACGGGGGGCATCTGCTGTTCCTTGTGATCGAAAAGATCAAGGGCGGACCGGTATCCGAGCGGGTTCAAGACTTTTG
TTATCGCATTGGCTCGATTCTGCTGGTGCTGTTAATGGGGCTTGCACTTTTCAATGATTTCTCTCGGTTATGA
>Cluster_53539
ATGGTGGACATGATTAATGAAAGTGCACGGCAAACGCCAGTCATTGCACAAACGGACGTTCTGGTTATCGGTGGCGGTCC
GGCAGGATTAACGGCGGCGATAGCGGCGGGGCGGCTAGGTGCCAGAACCATGATTGTTGAGCGTTACGGCTCACTAGGCG
GCGTATTGACGCAGGTTGGTGTGGAAAGTTTTGCCTGGTATCGCCATCCGGGGACGGAAGATTGTGAAGGGATTTGTCGT
GAATATGAAGGCCGCGCGCGAGCATTGGGCTTCACGCGACCAGAACCTCAGTCAATTAGCGAAGTTATAGATACTGAAGG
ATTTAAAGTTGTTGCCGATCAGATGATAACGGAAGCTGGCGTTGAGCCGTTATATCACTCCTGGGTTGTGGATGTGATCA
AGGAAGGGGATACGTTATGCGGTGTTATTGTCGAGAACAAATCAGGACGAGGGGCAATTCTGGCGAAAAGAATCGTCGAT
TGCACGGGGGATGCTGATATTGTCGCTCGTGCAGGCGCGCCCTGGACGAAACGGGGAAAGGACCAACTGATGGGCGTCAC
CGTGATGTTCAGTTGCGCAGGTGTTGATGTGGCACGCTTTAACCGTTTTGTTGCGGAAGAACTTAAGCCGACCTATGCGG
ATTGGGGCAAAAACTGGACGATTCAAACCACGGGTAAAGAAGACCCGATGTTTAGCCCGTATATGGAGGATATTTTTACC
CGCGCGCAACAGGATGGTGTGATTCCAGGTGACGCCCAGGCGATTGCCGGAACCTGGTCGACCTTTTCTGAAAGCGGTGA
GGCTTTCCAGATGAATATGGTGTACGCCTTTGGTTTTGACTGTACCGATGTCTTCGATTTAACCAAAGCTGAGATTGCCG
GAAGGCAGCAAGCATTATGGGCAATTGACGCACTACGCCACTATGTTCCGGGCTTTGAAAATGTACGGTTACGCAATTTT
GGTGCCACGCTGGGGACGCGTGAATCACGGCTTATTGAGGGGGAAATACGTATTGCTGATGATTACGTCCTTAATCAGGG
GCGTTGTTCGGACAGTGTAGGGATTTTCCCGGAATTTATTGATGGTTCCGGTTATCTCATTTTGCCAACGACCGGGCGTT
TCTTTCAGATCCCTTATGGTTGTCTGGTGCCGCAAAAAGTGGAGAACCTTTTGGTCGCCGGTCGCTGTATTTCCGCAGGC
GTAGTTGCACATACTTCTATGCGTAACATGATGTGTTGTGCCGTTACCGGTGAGGCCGCAGGTACTGCCGCCGTGGTTTC
GCTACAGCAAAATTGCACCGTGCGTCAGGTTGCTATCCCTGATTTGCAAAACACGCTGCAACAGCAGGGCGTACGTCTGG
CATAA
>Cluster_63652
ATGGCTGATATATCAACACCCAACCTCGACTATAACGATATGCTGGAGGCGTGGGATATTAACGACGCACTGATGGGCGG
TACGCTTGAAATGCGCAGGCAGGGGGAAAATTATCTCCCAAAATGGCCTAACGAAGATGAAGACGCTTATAAAAAACGCC
TGTCTGTGGCTACGCTACTTCCTGTGTATGAAGAAAGCATCAAGCAAAATATTGGGCGCATATTTGCAGAGCCGACAGTA
TTGAGTGAGGAAACGCCGGCAAAAATCAGGGAATATGCAGAAAATATCGACATGGAGGGGAGCCGACTGGATGTGTGGGC
GCAGCAATTTTTCAGCCTCGCATTTCAGTATGGTGTGGCACATGCGCTGGTGGACTATCCACGAACGGATATGAAAGAGA
TTCGTACAAAAGCCGATGAAAACGCGACCGGTGGTCGTCCGTACGTTACGATGCTGAATCCACGCCAGGTTATTGGATGG
AAATCGAAAGTTGAAAAAGGGAAAGTTGTTCTCACTGATTTGCGTATAAAAGAGGTCATCATTATTGATGGTGATGATTT
CGGGCAGAAGAAGGTGGAGCAGATCCGCCATATTATGCCCCGTCGAGTTGAAATTTATCGACGTAGCGAAGGCACTAATG
GCGAATCTGTCTGGACGCTTCATGAGTCATGGAATACCAGCCGTGATGATATTCCTCTGGTAACACTGTACACGAAGAAA
ACAGGATTTATGCGTGGTACACCGCCATTGCTTAATCTTGGCTTGCTGAATATCAAGCACTGGCAAAGCCAGAGCGAGCA
GGACAATATTCTTCATGTTACCCGCGTTCCATTGCTGGTGGCCTACGGGCTGGACAGGAATGAAGAACTGACGGTTGGTG
CATCCACCGCTACGATTTTTGAGGACAGAACAAAAAATGGCCTCGAATATGTTGAACATAGTGGCGCAGCGATAGAATCT
GGCGAAACATCACTTGAGAAGCTGGAAAATCAGATGCGTCATGCCGGCGCTAAACTTCTGCGTGCTGAAAATACATCCAC
CAAATCTGTTGATCAGACTAATGAAGAGCGAATGCAGGAAAACTCGCCGCTTTATACAATGGCGAACTCCCTCGAAGATG
CCCTCGATAATATTCTCCAGATAATGGCGGAATGGATCGGAGAGAGTTGCGGTGGCAATGTGGATGTGCGCACTGAACTG
GATGTATCTGCCCAGGTGTTTGACTCATCCTCCGCGCTGGCTGTTCAGTCATTGCGTCAGGGCGGTGATATACGTCAGAT
TGATGCGGTTCGGGTGTTGCAGGCGTTGAAATTTATCGATCAGGATTCCCGTCCAGAGGAAGTGATCGATGAATTAAAAA
ATCAGAGTGTAATGCTGATGGAAATCAATGATGCAAACCGTGAATGA
>Cluster_29873
ATGCCTGATATAACTCCAAATGTTGTAATTGGGATGCCTTCTCAACTCTTCACTATGGCTCGTTCTTTTAAAGCCGTAGC
TAATGGCAAAATTTATATCGGTAAAATTGACACTGACCCGGTAAATCCTGAAAACCAGATTCAGGTTTATGTGGAGAACG

AAGACGGCTCTCACGTTCCTGTATCGCAACCAATCATCATTAACGCTGCTGGTTACCCGGTATATAACGGACAGATTGCC
AAGTTCGTAACTGTGCAAGGCCATTCTATGGCTGTATATGATGCGTATGGTGCGCAGCAGTTCTATTTTCCTAATGTGCT
GAAGTATGACCCAGAACAATTTAAAGATACACTTCATTCTGCGAGCGGAGCGGATAATGTTGGATATAGGGGTTATACTG
TTAGTGAGTATCTTTCACACACCGCATTGAGTTTTGGTAACTTAAACGATTGTCTGTCTCATGACAAATACAAGATAGGA
GATAAAGTATTTGTACATGGATATAATTATCCATCAGATGGAGGAATGGCTTCATTTGAGGTTGTGTCATCAGATGAACC
AGTTGGAATAAAAACTTACGATGGTAAGTTTAAGCTAAAGCGAACATCTGGGCATAATTATCTTGAGTTAATAATAAAAA
ATGGTGCGACGCCAAAGATAATTGCCCATAGAGGATGGGCAGGAATAACGCCAAATGAATATCAGGGAGCATTGACTGCG
CCTGTTTATTTCGTTCCAGAAAATACAAGGGCATCAATTAGATTTTGTGCTGAAAGAGGAGCTTTTGGTGTTGAAGGGGA
TACCAAAATAACATTAGACGGAGTTCCTGTCATATTCCATGATGAGACAGTTGATAGAACTACAAATGGTACTGGTGCCG
TATCAACATTTACTTTTTTAGAGCTAAAGGCCCTCGATGCTGGATCGTATGTTTCTGGTATTTATGCAGATGAGAAAATA
CTCAATTATGATGAATGGATTAGAGAATGCAAGCGTCGCGGAGTAATGCCATTTGTTGAGTGGTCTGCGCCAATGAGTGT
CACTCAAGCTGATAATTTTCTTGAAACTATAAATAAATATTATGGCACAAAGCCGACGAATGTCTTTGTTTATTCAACAT
ATCCTGCCGTTCTTGAACTGTTGCGTTCAAAAAATGCTTATATTGGACTTGGTGTTATACCATCTTACGGTGTCGCACCA
ACTACAGAACAATTGGAGCTGGCGTATAAGCTTGGCAACTGTGGTATATCTCTATCAGGTGGAATAATTACGAATGCCGC
TGTTGTAAATTCAGTTAAGAAATATGGTTTGTTGTTGATTTATGCAATTGCTAACACCCCGGCAAGAATTGATAATTGCA
TATCTGCAGGCGTTGATATAATGATTACTGATTGCTATAGAGGGTAA
>Cluster_40993
ATGAATAACTCTGAATTAGAAACACTGATCCGCAATATTCTGCGTGAACAACTTGTCCCCGCAACACCACGAATGCAAAG
AAACGCTATTTTCCAGACTGTTGATGAAGCGGTCTGCGCAGCACACCAGGCCTTTTTACGCTTTCAACAGTGCCCGCTTA
AAACCCGTAGCGCCATTATCAGCGCCATTCGCGAAGAATTAATGCCCCGCCTTACCGAACTGGCAGAAGAAAGCGCCAAA
GAAACGGGGATGGGAAATAAAGAAGATAAGTACCTAAAGAACAAAGCAGCACTCAATAATACGCCAGGAATTGAAGATTT
AACCACCACTGCACTAACCGGTGATGGTGGCATGGTTTTGTTTGAATACTCCCCGTTTGGGGTTATTGGCTCGGTCACGC
CAAGCACCAACCCCACGGAAACCATCATTAACAACAGCATCAGCATGCTGGCAGCAGGTAATAGCGTCTACTTCAGTCCA
CACCCGGGAGCCAAATACGTTTCGTTAAAACTGATTACCACAATCGAAGACATCGCGTTTCGCTGTAGTGGCATTCATAA
CCTGATCGTTACCGTATCTGAACCGACCTTTGCTGCCACCGAACAGATGATGGCGCACCCTAAAGTCAGCGTTCTGGCGA
TTACCGGCGGCCCGGGGATCGTCGCCATGGGAATGAAAAGTGGCAAGAAAGTGATTGGTGCCGGAGCGGGTAACCCTCCG
TGTATCGTCGATGAAACAGCGGATATTGTGAAAGCCGCCGAGGACATCATCAACGGCGCTTCATTTGATTACAACCTGCC
GTGTATCGCTGAAAAAAGCCTGATTGTCGTGGCGTCAGTGGCAGATTATTTAATTCAACAGATGCAAAGTTTCGGCGCAC
TTCTTCTTAACCATGAACAAACGGAGAAATTGCGTGCAATTTGCCTGCCAGACGGTAGCGCCAACAAAAAACTGGTTGGG
AAAAGCCCATCAGCCTTGCTGGAAGCAGCGGGCCTCCCTTTGCCCGCAAAAGCGCCGCGCCTACTGATTGCTGTTGTTGA
CGCCGATGATAGTTGGGTTACGTGTGAACAACTGATGCCAATGCTGCCCATTGTGAAGGTCAACGATTTTGACAGCGCTC
TCGCCCTGGCGTTGAAAGTGGAAGACGGCTTGCACCACACGGCAATTTTGCACTCGCAGAACGTCTCACGGCTCAACCTT
GCCGCGCGCGTTATGCAAACCTCAATCTTTGTCAAAAACGGCCCCTCCTGGGCGGGTATTGGCGTCGGCGGCGAAGGCTT
TACGACCTTTACCATTGCAACACCGACCGGAGAGGGTACAACGTCAGCACGGACTTTTGCCCGCTCACGACGCTGCGTGT
TAACCAGCGGCTTTTCCATTCGCTAG
>Cluster_60553
ATGGGGCAATCATCGCAACCACATGAGTTAGGCGGCGGGCTGAAGTCACGCCACGTCACCATGTTGTCTATTGCCGGTGT
TATCGGCGCAAGTCTGTTTGTCGGTTCCAGCGTCGCCATCGCCGAAGCGGGCCCGGCGGTATTACTGGCCTATCTGTTCG
CCGGGCTACTGGTGGTTATGATTATGCGGATGTTGGCGGAAATGGCGGTTGCCACGCCCGATACCGGTTCGTTTTCCACC
TATGCCGATAAAGCCATTGGTCGCTGGGCGGGCTATACCATCGGCTGGTTGTACTGGTGGTTTTGGGTACTGGTTATCCC
GCTGGAAGCCAACATCGCCGCCATGATCCTGCACTCATGGGTTCCAGGCATTCCCATCTGGTTATTTTCCCTCGTCATTA
CCCTCGCCTTAACTGGCAGTAACTTATTAAGCGTTAAAAACTACGGCGAATTTGAGTTCTGGCTGGCGCTGTGCAAAGTC
ATCGCTATCCTGGCCTTTATTTTCCTTGGTGCAGTCGCAATTAGCGGTTTTTACCCGTATGCCGAAGTGAGCGGGATCTC
AAGATTGTGGGATAGCGGCGGCTTTATGCCCAACGGTTTCGGTGCGGTATTAAGCGCGATGTTGATCACCATGTTCTCGT
TTATGGGCGCAGAAATTGTCACCATTGCCGCCGCGGAATCCGACACGCCGGAAAAACATATTGTCCGCGCCACCAACTCG
GTTATCTGGCGTATTTCTATCTTCTATTTATGTTCTATTTTTGTCGTCGTGGCATTAATTCCGTGGAATATGCCCGGACT
AAAAGCCGTCGGTTCTTATCGCTCGGTACTGGAATTGCTCAATATTCCCCATGCGAAATTAATCATGGACTGCGTGATAT
TACTTTCCGTAACCAGTTGCCTGAACTCGGCGCTGTATACCGCGTCAAGGATGCTCTACTCCTTAAGTCGTCGCGGTGAT
GCTCCCGCGGTAATGGGTAAAATCAACCGCAGTAAAACCCCGTACGTGGCGGTGTTACTCTCCACCGGCGCGGCATTCTT
AACGGTGGTGGTGAACTATTACGCGCCTGCGAAGGTATTTAAATTTCTGATCGACAGCTCCGGCGCTATCGCCCTGCTGG
TTTATTTAGTCATCGCCGTTTCACAGTTGCGGATGCGCAAAATTCTGCGAGCAGAAGGAAGCGAAATTCGCTTGCGAATG
TGGCTTTATCCGTGGCTCACCTGGCTAGTCATCGGCTTTATTACCTTTGTGTTGGTAGTGATGCTATTCCGTCCGGCGCA
ACAGTTAGAAGTGATCTCCACCGGCTTATTAGCGATAGGGATTATCTGTACCGTGCCAATTATGGCTCGCTGGAAAAAGC
TGGTATTGTGGCAAAAAACACCCGTTCATAATACGCGCTGA
>Cluster_23541
ATGCATATCAACATTGCCTGGCAGGACGTAGATACCGTTCTGCTGGATATGGACGGCACGTTGCTCGACCTCGCCTTCGA
TAACTATTTCTGGCAAAAGCTGGTGCCTGAAACATGGGGCGCGAAAAACGGAGTTACGCCACAGGAAGCGATGGAATATA
TGCGCCAGCAATATCACGACGTACAGCATACGCTAAACTGGTACTGTCTTGATTACTGGAGTGAGCAACTGGGTCTGGAT
ATCTGTGCGATGACCACCGAGATGGGACCGCGTGCCGTACTGCGTGAAGATACCATTCCGTTTCTTGAGGCACTGAAAGC
CAGCGGTAAGCAGCGAATTTTGCTCACCAATGCGCATCCGCACAACCTGGCGGTAAAACTTGAGCATACCGGTCTGGACG
CACACCTTGATTTATTACTTTCCACCCACACATTTGGTTATCCGAAAGAGGATCAGCGGTTATGGCATGCGGTGGCCGAA
GCTACGGGTCTTAAAGCTGAAAGAACGCTGTTTATTGATGACAGCGAAGCGATTCTCGATGCTGCCCGCGCAATTTGGTAT
TCGTTACTGCCTCGGTGTGACTAATCCTGACTCCGGGATTGCCGAGAAACAGTATCAACGCCATCCGTCACTGAATGACT
ACCGCCGCCTGATCCCCTCGCTAATGTGA
>Cluster_37725
ATGAATACCAGAGCTCTTTTTCCCCTGCTGTTCACTGTGGCATCATTCTCCGCCTCCGCCGGCAACTGGGCTGTCAAAAA
CGGCTGGTGTCAGACCATGACGGAAGATGGTCAGGCGCTGGTAATGCTGAAAAATGGCACGATTGGTATTACCGGCCTGA
TGCAGGGATGCCCGAATGGTGTACAGACGCTCCTGGGCAGCCGTATCAGTATTAACGGTAACCTGATCCCCACATCACAA
ATGTGTAATCAGCAGACGGGATTCAGGGCTGTTGAGGTGGAAATCGGACAGGCGCCGGAAATGGTCAAAAAAGCCGTTCA
CTCCATAGCAGAGCGTGATGTGTCCGTTTTACAGGCATTTGGTGTACGAATGGAATTCACCCGCGGTGATATGCTGAAGG
TCTGTCCGAAATTTGTCACATCACTTGCCGGTTTTTCCCCGAAACAGACGACCACTATTAATAAAGATTCCGTCCTGCAG
GCTGCTCGGCAGGCATACGCTCGGGAATATGACGAGGAAACAACAGAAACCGCTGATTTTGGCTCTTACGAAGTAAAAGG
CAATAAGGTTGAGTTTGAAGTATTCAATCCTGAAGACCGTGCGTACGACAAAGTGACCGTCACGGTTGGTGCTGACGGTA
ATGCCACCGGCGCCAGCGTTGAATTTATCGGAAAATAG
>Cluster_66854
ATGAACAAGCTCCAACGCGAGGCCGTGATCCGAACCGCGCTCGAACTGCTTAACGACGTGGGCATGGAAGGTCTAACGAC
GCGCCGACTGGCTGAGCGCCTCGGGGTGCAACAGCCAGCGCTCTACTGGCATTTCAAGAACAAGCGTGCGTTGCTCGACG
CACTTGCCGAAGCCATGCTGACGATAAATCACACGCATTCGACGCCAAGGGATGACGACGACTGGCGTTCGTTCCTGAAG
GGCAATGCATGCAGTTTTCGACGGGCGTTGCTCGCTTATCGCGATGGCGCGCGTATTCATGCCGGGACGCGGCCAGCCGC

GCCGCAGATGGAAAAAGCCGACGCGCAGCTTCGCTTCCTTTGCGATGCTGGCTTTTCGGCAGGTGACGCGACCTATGCGT
TGATGGCAATCAGCTACTTCACCGTCGGCGCTGTTCTTGAGCAGCAAGCTAGCGAGGCAGACGCCGAGGAGCGGGGCGAA
GATCAGTTGACCACCTCAGCGTCTACGATGCCGGCGCGCCTACAGAGCGCGATGAAAATCGTCTACGAAGGCGGTCCGGA
CGCGGCATTCGAGCGAGGCCTGGCTCTCATCATCGGCGGTCTTGAAAAAATGAGGCTCACTACGAACGACATTGAGGTGC
TGAAGAATGTTGACGAATGA
>Cluster_3300
ATGAAAAAAGAGACGGATATCAATATCGACGCGCTGCTGCGCGACACGTTTCTGACGGTGGTGGAACTGCGTCAGGGAAC
CACCGTGCGTCACGGTATGGAGCTGTATCGCCACTGCCAGCGGCAGGTGGAGGGGGTCCGCGAACGCCTGAAAGATGCCG
GGTTCAGCCGGGAGAACGTGGAGCACATTACTTACGCACAGTGCGCCCTGCTGGACGAAACAGTCCTGAGTCGCAGCGGC
ATGGACGACGGTCAGGCCATCTGGATGAAGGATCCACTCCAGGCGCATTTCTTCAACACCCTGCAGGCAGGCGAGCTGCT
GTACGAGCGGATGAAGCAGGTGTTGCAGGAGCCCGCGCCTGCACAGGCAGTGCTGACCTGCTTTCACCGTGTCCTGCTGC
TGGGATTTCGCGGGCGTTATCAGGATCCGGTGGCCCCGGAGCGTGACCAACTTATTTCGACGCTCAACGGGCTGGTCGCG
CCATTTGGCGTGCTGCCGGAAACGGCGGTACTGAACGTGCCGTTATCCCCCCGTCAGCACCCTTTGCTGCATTCGCCGTT
TTTCTGGCTGGTAACGCTGACGCTGCTACTGGCCGGTGTCTGGTGGGGACTGCACCACTGGCTGAACGTGCTGGTGGATG
AATTGCTGCCGCAAAGCCTGCGGTAA
>Cluster_44903
ATGAATAACCAGTATTTACAGTTTGTGCGTGAGCAGCTCATTATCGCCACCGCTGATTTGAGTGGGGCAACAAAAGGTCA
GCTTGAAGCCTGGCAGGAGAATGCCATGTTCGATACAGGGCGTTACAGGCGTAAAAAAATCCGGTACCGCGATGAAGTGA
CTGGAAAAATGATAACGCGGGATAATCCACCAATCCCGGGAAAGCAATCGCTGGCGAAGGGGACGTCAATTCCTCTGGTC
AGTCCGATTGAGTTTTCGACATCATCGTGGCGGCGGGCTGTTCTGTCTCTTGAAGAACATCATAAAGCCTGGTTGTTGTG
GTGTTACAGCGGGAGTATTTGTTGGGAATATCAGATCGCGATAACACAGTGGGCGTGGAATGAATTTAATACTCAATCCG
GTACCAGAAAAATTGCAGGGAAAACGCAGGAACGCCTGAAAAAATTAATCTGGCTGGCGGCGCAGGCAGTAAAAGCAGAA
CTTTTTGGTGGGGAAGGTTATGAATACAAGGAGCTGGCATTACTGGTGGGAGTGACAACTAAAAACTGGTCCAAAACATT
TACTCGTCACTGGGTTGCAATGAAACACATTTTTCACCGACTGGATAGTGAGGCTTTATTGTTTGTAATGAGAACGCGTT
CAAAACAAAAGGCGGCATTTTCAAAGCAAAGTGTTGCAAAAGTAGATTGA
>Cluster_20819
ATGTTCAGAAAATTAGCAGCTGAATGTTTTGGTACTTTCTGGCTTGTTTTTGGCGGCTGTGGTAGTGCCGTACTGGCCGC
AGGCTTCCCCGGAATTAGGCATTGGTTTTGCCGGCGTGGCGTTGGCGTTCGGTCTGACCGTTCTGACAATGGCCTTTGCTG
TTGGTCATATTTCTGGTGGTCATTTTAACCCGGCGGTCACTATTGGTTTATGGGCTGGCGGACGTTTCCCGGCAAAAGAA
GTCGTTGGCTACGTAATTGCCCAGGTTGTCGGCGGTATTGTTGCGGCGGCGCTGCTGTATTTAATTGCCAGTGGTAAAAC
GGGTTTTGACGCGGCAGCCAGCGGTTTTGCTTCTAACGGTTATGGCGAGCATTCACCAGGCGGTTATTCCATGCTTTCCG
CGCTGGTAGTTGAACTGGTATTGAGTGCAGGTTTCCTGCTGGTGATCCACGGCGCAACTGACAAATTCGCTCCTGCTGGT
TTTGCGCCAATTGCCATTGGTCTGGCCTTAACCCTGATTCACTTAATCAGTATTCCGGTGACTAACACTTCCGTTAACCC
GGCGCGCAGTACTGCGGTTGCTATCTTCCAGGGCGGATGGGCATTAGAACAACTGTGGTTCTTCTGGGTGGTGCCAATTG
TCGGCGGTATTATCGGTGGTCTGATTTACCGGACTCTACTGGAAAAACGCGATTAA
>Cluster_40846
ATGACAGACCAGAATAAACACATTGAGAAACTGAAAAAGTTGCTGGCGCTGGCCGCATCCGGCAACCCGCACGAGGCCGC
TCTGGCACTGCGCCGAGCCCGTAAACTGATGGATGTTCACGGCATCACACATTCCGACATTGCTATGAGTGATATTGATG
AAACCATCAGTCACTACTGGCCGACAGGCAGTCTCCGTCCACCGCGCTACATGCTGGGCCTGATGAACATCATCCGCGAG
GTATTTGGTGTTAACTCCATCATTCACCCCGGCACGCATCCGTCTGTGGGGTTCTACGGCAACCGGGAACGTGCGGCACT
GGCTGCATACACCTGGGAAGTGCTGGCCCGTCAGCTGAAAAAGGCGCGTCAGCAGTATATCAGTGCACAGAACAAAAGAA
TAAAAACCGCCACCCGCACCAGCCGTGGAGACCAGTTTGCCGAAGGCTGGGTGCTGGCCGTTATCAGTGAAATACAGTCC
TTTGCCCTGACCGATGATGAGCGTGAACTGATGCAGCAGTGGCTGGAACATAAATACCCGCAGACGCAAACCACCAGGGC
GCGTAAACCGGGAAGAAGCCGCAATGGCGACGCCTCGCGCTATGCGGGGTTTCGTGAAGGGCAGAACGTCAGACTGCACC
GCCCGGTCAGTGGGCAGGAACAACAGAAACTGGAGGCCAGATGA
>Cluster_44747
ATGTGTGCGCTGGATCGTAGAGAAAGGCCACTTAACAGTCAATCTGTAAATAAATACATCCTTAACGTTCAGAATATCTA
CAGAAATTCTCCCGTTCCGGTTTGTGTCCGTAACAAAAACCGGAAAATCCTTTATGCCAATGGGGCTTTTATTGAACTCT
TTTCCAGAGAAGATAAACCCTTATCCGGAGAGAGTTATATACGTCTGCAGGTTGAAATTTTTCTTTCATCACTTGAACTG
GAATGCCAGGCTCTTGGACATGGCTCTGCATTTGTCGTCGTTTTAATTTCATGGCGAAATCTATCAGATAAGGATGGA
GAATGTTTCTTTTTATAATGACGAATCTGTTGTTTTATGGCAAATTAATCCGTTTCCTGATTATCCATTTTTTGCGTTAA
ATCAGAGTGGAAGTAATACAAATACTTCTGATAAATTAACGATATGGAATGATCTTTCTCCAGGGACATTGGTTGTTTTC
TCTTTTTATATGCTGGGTGTTGGTCACGCAACAATTGCCAGAGAGTTGGGTATTACAGACAGAGCATCTGAGGATCGAAT
TAAACCAGTTAAACGGAAAATAAAAGAATTTTTTGAACACTTTGATTTATTCAGAGTGTCATGTATCTATAAAGGAGAAA
TAGATTCGCTATTAAGTATAATTCGTGAATTTTATGGTGTTAAGTAA
>Cluster_54948
ATGAAACTGCAAGGGGTAATTTTCGATCTGGATGGTGTAATCACCGATACCGCGCATCTGCATTTCCAGGCGTGGCAGCA
GATTGCCGCTGAAATTGGCATCAGCATTGATGCGCAGTTTAACGAATCCCTAAAAGGGATCAGCCGCGATGAGTCTCTGC
GGCGCATTCTGCAACACGGGGGCAAAGAGGGCGACTTTAACTCGCAGGAGAGGGCGCAACTGGCGTATCGCAAAAATCTG
CTCTATGTCCACTCACTACGCGAGTTGACGGTCAACGCTGTTCTACCCGGCATTCGCTCTTTGCTGGCAGATCTCCGTGC
ACAGCAGATCTCGGTTGGGCTGGCTTCTGTCTCCCTGAATGCGCCGACGATTTTAGCGGCGCTGGAGCTGCGCGAGTTTT
TCACCTTCTGCGCGGATGCTTCCCAACTTAAAAACTCGAAACCGGACCCGGAAATCTTTCTCGCCGCCTGTGCAGGGCTG
GGCGTGCCGCCGCAGGCATGTATCGGCATTGAAGATGCGCAGGCGGGCATTGACGCCATTAACGCCAGCGGTATGCGCTC
GGTGGGGATCGGCGCGGGCTTAACCGGGGCGCAATTACTGTTGCCTTCAACGGAATCACTCACCTGGCCGCGGTTATCGG
CCTTCTGGCAAAACGTATAG
>Cluster_56090
ATGAAATCAGTTATGGCTGAGTTTATAACCACTGAAACGGTGACCGAGGCGCACTATATATCGAATAATTTTACTCGCGG
AATCCTGCATCAATACTCTAAGGAAACACAACATGTACACCTAAATACACATCGACGTGCACAAATATACTACGGCAACT
GGAGGGCAGGACATGTAAAGTGGGCCCACCGCTACGGTGTTCCAACTTTACCACTCCCTATTCGCCGCATCGCGGCTCAA
GGGCGTCCTGCTCTGCGAGGCTGCCAGGCTACCGCCTGTATGGAGAGCTATATGAAAGTTACTCAGACTAGAAAAAATAG
TACGCCAATAAAAGTTTATTGTTTACCCGAAGAAAAAATTCTCATCCAAGAAAATGCAGAAGCATCTGGATTAAGTGTTG
CCTGTTTTGTTCGTAGAGTTGCACTGGGTTATCAGGTTGATTCTATCGTAGATATAAAACAGGTTAATGAACTTAGCCGA
GTAAACGCTGATCTTGGAAGACTTGGCGGATTACTTAAATTATGGCTCGCAAATGATCCTCGAACAGTTGATTTTTCACC
CACACTGATAAGAACCTTGCTTGCGAAAATAGAATCAACTCGGCAAGAACTAAGGAACATTATGGATAAAATCTTGGATA
AATAA
>Cluster_27824
ATGGAACGAAATAAACTTGCTCGTCAGATTATTGACACTTGCCTGGAAATGACTCGCCTGGGGCTGAACCAGGGGACAGC
GGGTAACGTCAGTGTACGTTATCAGGATGGGATGCTGATTACGCCTACAGGTATTCCATATGAAAAACTGACGGAGTCGC
ATATTGTCTTTATTGATGGCAACGGCAAACATGAGGAAGGAAAACTCCCCTCAAGCGAGTGGCGTTTCCATATGGCGGCC

TATCAAAGCAGACCGGATGCCAACGCGGTTGTTCACAATCATGCCGTTCATTGTACGGCAGTTTCCATTCTTAACCGACC
GATCCCCGCTATTCACTACATGATTGCGGCGGCTGGCGGTAATTCTATTCCTTGCGCGCCTTATGCGACCTTTGGAACAC
GCGAACTTTCTGAACATGTTGCGCTGGCTCTCAAAAATCGTAAGGCAACTTTGTTACAACATCATGGGCTTATCGCTTGT
GAGGCGAATCTGGAGAAAGCGTTATGGCTGGCGCATGAAGTTGAAGTGCTGGCACAACTTTACCTGACGACCCTGGCGAT
TACGGACCCGGTGCCAGTGCTGAGCGATGAAGAGATTGCCGTAGTGCTGGAGAAATTCAAAACCTACGGGTTACGGATTG
AAGAGTAA
>Cluster_52771
ATGAACCTAAAGAAAACACTGTTAAGCGTGTTAATGATATTGCAACTTTGCTTATTGGTAGGGTGTGACTATATTGAAAA
AGCGAGTAAGGTCGACGATCTCGTTACACAGCAAGAGTTACAAAAAAGCAAAATTGAGGCGCTTGAAAAACAACAAACTA
CCATCATAAACAGTACCAAAACGCTCGCTGGCGTGGTGAAGGCAGTTAAAGACAAACAGGACGAATTTGTCTTTACAGAA
TTTAACCCGGCACAAACCCAATACTTCATTTTAAATAACGGTTCTGTTGGTTTGGCAGGGAAAATACTGTCTATTGACGC
AGTAGAAAACGGCAGCGTTATTCGTATTTCACTGGTTAACTTATTAAGTGTTCCCGTATCAAATATGGGTTTCTACGCGA
CATGGGGGGGAGAAAAACCCACCGACATCAACGCATTAGCAAAATGGCAGCAATTGCTATTTAGTACCTCAATGAACTCC
TCCCTGAAATTATTACCAGGTCAATGGCAAGACATTAATTTGACGCTAAAAGGTGTCTCGCCCAACAACCTCAAATATCT
GAAATTAGCCATCAACATGGCAAATATTCAGTTCGACCGTCTTCAACCTGCTGAATCTCCACAGCGGAAAAACAAAAAAT
AA
>Cluster_34507
ATGAAATCTTTACAGGCTCTGTTTGGCGGCACCTTTGATCCGGTGCACTATGGTCATCTAAAACCCGTGGAAACGCTGGC
GAATTTGATTGGTCTGACGCGGGTCACAATCATCCCTAATAATGTTCCTCCGCATCGTCCCCAGCCGGAAGCGAACAGCG
TGCAGCGTAAACACATGCTTGAACTGGCGATTGCCGACAAGCCATTATTTACTCTTGATGAACGCGAGCTAAAGCGCAAT
GCCCCCTCTTACACTGCGCAAACACTGAAAGAGTGGCGGCAGGAACAAGGACCGGACGTGCCGCTGGCGTTTATTATTGG
TCAGGATTCACTGCTGACCTTTCCGACCTGGTACGAATACGAAACGATACTCGACAATGCACATTTGATCGTCTGTCGGC
GTCCAGGTTACCCACTTGAAATGGCGCAACCGCAATACCAGCAATGGCTGGAAGATCATTTGACACATAACCCGGAAGAT
CTTCACCTTCAGCCTGCCGGTAAAATTTATCTGGCTGAAACGCCGTGGTTTAACATCTCGGCGACCATCATCCGCGAACG
TTTGCAAAACGGTGAATCATGTGAGGATTTATTGCCGGAACCGGTACTGACTTACATTAACCAACAAGGCTTGTATCGCT
GA
>Cluster_18979
ATGTCTCAATTAATGACCATTGGCTCACAGCCAATATTTTTAATTATTGTATTTTTTCTGTTATCATTACTGCCAATATT
TGTTGTTATTGGTACTTCATTCCTGAAAATTTCTATTGTACTGGGGATATTGAAGAACGCATTAGGCATTCAACAGGTAC
CACCAAACATGGCGCTAACATCAGTGTCTTTGATACTGACAATGTTTATTATGTCTCCGATAATATTACAGATAAATGAT
AATATTTCTCAGGAACCAATCAATTATACCGACTCTGATTTTTTTCAAAAAGTTGATGAGAAAATATTATCACCATATCG
CGGTTTTTTAGAAAAAAATACAGAGAAAGACAATGTAGAGTTTTTTGAACGTGCAGCTCAAAAAAAATTGGGTAATGAAA
CTATCTTAAAAAAAGACTCTCTATTTATACTGTTACCGGCTTTCACGATGGGGCAGCTTGAAGCTGCATTCAAGATAGGT
TTTTTGCTCTATTTACCCTTTATTGCGATAGATTTGATCATTTCCAATATCTTATTGGCCTTGGGTATGATGATGGTATC
GCCAGTAACAATTTCGATTCCTTTTAAGATCTTGTTATTCATTCTTGTTGGAGGCTGGCAAAAATTATTCGAATTTCTGT
TGGTGGTGAATTGA
>Cluster_22035
TTGATCAACGTTCTACTTGTTGATGACCACGAACTGGTGCGCGCAGGGATACGACGCATTCTGGAAGATATAAAGGGTAT
AAAAGTCGTCGGTGAGGCATCGTGCGGTGAAGACGCCGTTAAATGGTGCCGGGCAAATGCCGTTGACGTGGTGCTAATGG
ACATGAGTATGCCGGGCATTGGCGGTCTTGAGGCGACGCGTAAAATCGCGCGTTCCACAGCTGATGTCAAAATCATCATG
CTTACCGTCCATACAGAAAACCCTTTACCAGCGAAAGTCATGCAGGCCGGAGCTGCGGGCTACCTCAGCAAAGGCGCGGC
TCCGCAGGAAGTCGTGAGTGCGATTCGTTCTGTCTATTCAGGGCAGCGTTACATTGCTTCTGATATCGCTCAACAAATGG
CGTTAAGCCAGATCGAACCAGAAAAAACAGAAAGCCCATTTGCCAGTTTGTCTGAACGTGAATTGCAGATTATGCTGATG
ATCACCAAGGGCCAGAAGGTCAATGAGATCTCAGAACAGCTCAATCTCAGTCCGAAAACGGTGAACAGCTACCGCTATCG
TATGTTCAGTAAACTAAACATTCATGGCGATGTTGAGCTGACTCACCTGGCAATTCGCCATGGTCTGTGTAATGCGGAGA
CATTATCAAGTCAGTGA
>Cluster_24447
ATGTATCTTGATACCAGCAACGTACAGAGCCTGAAAGAAAAGCTGCTGTTATGCGCGGTTAATGAATTTGCCGAATATGG
TTACGAAGGGGCCAGAGTCGATAATATCGTCAAGGCCGCGGGGTGCAGTAAACAGACGGTTTATCACCACTTTGGTAATA
AAGAAAATTTATTTATCGAAGTACTGGAATATACCTGGAACGATATTCGACAAAAAGAAAAGGCCCTTGATTTCTCAGAT
CTTCCGCCACAGAAAGCCATTGAGAAAATAATCGATTTTACATGGGATTATTATATTTCTAATCCCTGGTTTTTAAAAAT
TGTGCATAGTGAAAATCAAAGTAAGGGTGTGCATTATGCAAAATCACAGCGTCTTTTAGAAATTAACCATGCGCACTTGC
AGTTAATGGAATCCTTATTGGATGAAGGGAAGAAAAACAATATTTTTAAGCCAGATATCGACCCATTGCAGGTGAATATT
AATATTGCTGCGCTTGGGGGATATTATTTGATCAACCAGCATACGCTTGGTCTGGTTTATCACATCAGTATGGTTTCACC
CCAGGCACTGGAAGCCAGACGTAAGGTCATCAAAGAGACAATCCTTAGCTGGCTTTTGGTTGACCCTTCATCTACCGCGC
GCGAATAA
>Cluster_54870
GTGATTCAGACCTTTTTTGATTTTCCCGTTTACTTCAAATTTTTCATCGGGTTATTTGCGCTGGTCAACCCGGTAGGGAT
TATTCCCGTCTTTATCAGCATGACCAGTTATCAGACAGCGGCAGCGCGAAACAAAACTAACCTTACAGCCAACCTGTCTG
TGGCCATTATCTTGTGGATCTCGCTTTTTCTCGGCGACACGATTCTGCAACTTTTTGGTATATCAATTGATTCGTTCCGT
ATCGCCGGGGGCATCCTGGTGGTGACGATAGCGATGTCGATGATCAGCGGCAAGCTTGGCGAGGATAAACAGAACAAGCA
AGAAAAATCAGAAACCGCGGTACGTGAAAGCATTGGTGTGGTGCCATTGGCGTTGCCGTTGATGGCGGGGCCAGGGGCGA
TCAGTTCTACCATCGTCTGGGGTACGCGTTATCACAGCATTAGCTATCTGTTTGGTTTCTTTGTGGCTATTGCATTGTTC
GCTTTATGTTGCTGGGGATTGTTCCGCATGGCACCGTGGCTGGTACGGGTTTTACGCCAGACCGGCATCAACGTGATTAC
GCGTATTATGGGGCTATTGCTGATGGCATTGGGGATTGAATTTATCGTTACTGGTATTAAGGGGATTTTCCCCGGCCTGC
TTAATTAA
>Cluster_11458
ATGAATGAGCTGATAAATAGCAATGCCATCAAAATGACAAGCATTGAAATCGCTGAGTTGGTGGGAAGTCGTCATGACAA
GGTGAAACAATCCATTGAACGACTGGCGGTTCGAGGTGTGATCCGAAATCCCCCAATGGTGGTTTTCGAAAAAATCAATA
ACTTAGGATTACTTCGTGGCGTAGAGGCTTACGTTTTTGAGGGCGAACAAGGTAAGCGCGACAGCATTATTGTCGTTGCC
CAGTTGTCGCCGGAATTCACCGCTCGCCTTGTTGACCGCTGGCGAGAACTCGAAGGGGCAACCGCGAAAATACCACAAAC
CTTTTCTGAGGCATTGCGCCTTGCGGCCGACCTTGAAGACCAGAAGGCTGAACTGGAGAAACAGCTTGCTCTCGCAGCAC
CTAAAGTTGAGTTTGCCGATCGCGTTGGCGAGTCCAGCGGAATTTTGATTGGAAACTTTGCAAAGGTTGTTGGTATTGGT
CCAAACAAACTGTTTGCGTGGATGCGCGATCACAAAATCCTTATTGCTTCAGGCTCCCGGCGCAATGTGCCAATGCAGGA
ATATATGGATCGTGGCTATTTCACAGTGAAAGAAACAGCGGTCAACACAAATCACGGAATACAGATATCGTTCACCACAA
AAATCACCGGGCGTGGTCAACAGTGGCTGACAAGAAAGCTGCTAGATAACGGAATGCTTAAAGTAACAGGGGAGGCTGCT
TAA
>Cluster_66971
ATGGGCGAGTTAAAAACAACCCATACGATAATGACAATGACAATAAGTACCGCGAAAAAATCAGTCAACATTAGTCAATC

CAATGAACTGACAGAAGCAGCTTACTACTTGCCGTTGCAAGCCAAGAGGGTGCTATGGTTATGCCTTATTCAGTGCTATC
CACTGAAAGATGATCCTGATTCGGTATCGCCAGTATTCACAGTTCTTGTTGCTGATTATCAAAAGTATTTCAAAGTGTCT
GTTGATACAGCGTCATCTGATGTTAAAAAGGGCGTCACTGCACTGGCTGAAAGTAGTGTTGTGTTTTATCCCAAAGACGG
AGAGTTTGACGAGATCAAACGGCCCTGGCTGGCTGAGGCTGGGCTAAAAAAAGGGCGCGGAAAATGGCAAATTGAATTTA
ATTACAAAGTCATGCCCTATCTGATGGGACTGACCAGCCAGTTCACCACGTATTCACTCTATGACTGCGGAAAAAATAAAC
AGTGTTAGGGTCATTCGACTGTATGAAAGCCTGTGCCAGTACCGTTCCAGCGGAGTATGGGTTACTACTCAGGAATGGCT
GTCAGAGCGATTCATGCTTCCTGAATCGCAAAGATCAAACTTCGCTGAAATGAAGAGAACGTTTATCAATCCGGCACTGA
AAAAGATAAACGCAAATACACCACTTAAAGCCGCAATGACACAGAATGAAGATGGCAGGTTAGTCTTCACCATTGTTGAT
TCGAAAAACTAA
>Cluster_20681
GTGAAAGGTTTAATTGGTGTACTCGGTGGAATGGGGCCGGCAGCTACCGTCGACCTGTTTAATAAATTTGTAACTTTTAC
CGCTGCACAATGTGATCAGGAACATATTCCTTTAATTATTTCTTCGATCCCGGATATTCCTGACCGTACCGATGCATTAA
TGCATCATGGGCGTTCACCATTACCCGTTATGCGTGATTATATGCATAAGCTGGAAGATGCCGGGGCAGAATGTATTGTT
ATTCCCTGTAATACCGCGCATTACTGGTTCAAAGAATTAAAAGATGCTTGTCATATTGATATATTAAGTATCGTAGAAAC
TACGATCAATGAAGTTCGCGCCTGTGGTAAAACCCGAATTGGCTTGTTAGCCACCAATGCCACGCTATATATGGGTTTAT
ACCAAAAAGGAATTGAGTCGCTGGGATTTACCTGTGTCAGCCCCGATGCCGACGGTCAGAAAAAAGTGATGGAAAGTATT
TATTCCTTGAAAGCTGGCAATATAGCGCACGCACAAAAGTTGATGAATGAGCAGGCTGAAATATTATTTTCTCGCGGTGC
AGAAATCCTTGTTCTTGGTTGTACAGAAATTCCGGTTATTCTGGCGCAAGCCGTTAAAGAGCAGACTTCCCGCTATATTG
ACTCAACGGCGTCACTCGTTCGTGCCGGAATAAAATGGTACGAAAATCGTGTCGGTAAACATTATCTTTTAACCCAATAA
>Cluster_25616
GTGATTGAATTTAAAAACGTCTCCAAGCATTTTGGTCCAACCCAGGTGCTGCACAATATCGATTTGAACATTGCTCAGGG
CGAAGTCGTGGTGATTATCGGGCCGTCCGGTTCCGGTAAATCGACCCTGCTGCGCTGCATCAACAAACTGGAAGAAATCA
CCTCCGGCGATCTGATTGTCGATGGCCTGAAGGTTAACGATCCGAAAGTTGATGAGCGCCTGATTCGCCAGGAAGCGGGT
ATGGTGTTCCAGCAGTTTTACCTCTTCCCGCATCTGACGGCGCTGGAAAACGTCATGTTTGGCCCGCTACGCGTGCGTGG
CGCGAATAAAGAAGAGGCGGAAAAACTGGCGCGTGAGCTGCTGGCGAAAGTCGGTCTGGCAGAGCGTGCACATCACTACC
CTTCCGAACTTTCTGGTGGTCAACAGCAGCGTGTGGCGATTGCCCGCGCGCTGGCGGTGAAGCCGAAAATGATGCTGTTT
GATGAACCGACTTCCGCTCTTGACCCGGAACTGCGCCATGAAGTGCTGAAAGTTATGCAGGATCTTGCTGAAGAAGGGAT
GACGATGGTAATCGTGACCCACGAAATCGGTTTTGCCGAGAAAGTAGCTTCGCGCCTGATCTTTATCGACAAAGGCCGGA
TTGCAGAAGATGGCGATCCGCAGGTGTTGATCAAGAACCCGCCGAGCCAGCGCTTGCAGGAATTTTTGCAGCACGTCTCT
TAA
>Cluster_17153
ATGATTTCTGTAATTATGTGTATAAATAAATATGATTCATATGTAATGCCTGCGATAAATAGCATATTGAATCAAACTTA
TAAAAAATATGAGTTAATAATTGTTGCTAATGGCAATGAAGCCAGTGACATTGCCCGTGTCTTAAAAAATAACATAAAAC
AAACAGAGAATGTTAATATTATTGAAACCAATATAGGGCAATTGGCTTTTTCTTTAAATTTGGCAATATCCAAAGCTAAA
TATGATATCATCGCGAGGATGGATTCTGATGACATAGCCTTACCTAACCGACTTCAAAAGCAAATTAAATATCTTATAGA
TAATGAACTTGATTTAATTGGAACCTCAATAAATCTAATTGATGAAAATGATGGTTTTTAGGTGTAAGGCTTTACCCCA
GACAAAAAATTAATAAATAGATATATAATGTTTCGAAACTGTTTCGCACATCCTTCAATAATGTTCAGAAAAGATTTGTTT
CTTAAAACAAGAGGGTATAATGGTGGTTTTAACTCTGAAGATTATGATTTGTGGTTAAGAATGCGAAGCTTAAAACCACG
TTGGGATAATATGGAAGAGCCTTTGTTAAATTACCGTGTTCACTCAAATTCAACACAGAAGTCTAAACTGGCATATTATG
AATGTGCTGGGTATTCTTTGCGAGAATTTCTTAAAAAGAAAAGTATAATGAGTTTTCTTTCTTGTACATATCATTTTATT
AAAGCATTAGTTAAATAA
>Cluster_5621
ATGGCTGATATAGCAACAATGAGAATGAAGGCTCTGCACTTCTGGGATAAACACGGTATTTCTGCGGCTTCTGAAGCCTT
TGGCGTGTCCTGCCGCACGCTTTACTGGTGGCGTCAGTTACTGAACAAGGGAGGACCTGAGGGGCTAATCCCGCACAGTA
AGGCACCTCTGGTGCGACGAAAAAAGCACTGGCATCCCGATGTGCTGAAAGAGATTCGACGCCTCAGGACAGAGCTGCCA
AACCTCGGTAAAGAGCAGATTTTTGTTCGCCTGAAGCCCTGGTGCGCACAGCGCCATCTGACCTGCCCGAGTGTTTCCAC
CATCGGCAGAATGATCGCCACCGCACACGATAAAATGCGGATGGTACCAGTACGTCTGAGCTCGCGGGGTAAGGCTCTGC
TTGTCAAAAAGCGAACCGCCAAACCCCACCGGCCAAAGCACTACCGTCTGGTAAAGACCGGTGAGCTCATTGGGATGGAC
GCCATTGAGCTCAGAATGGGTGAAATGCGTCGCTATATCATTACCATGATCGACGAACGTGAATTTGGCAAAAAAATTACA
GGCAGGCAGAAAAAGTGTGTCAGGAGGGCATAAATACAGATATGAGTATAACGGTCAAACAATGTACTGGAGTGGACAAG
GTCGTCGGCCTAATGTTATTGCAGAAGCAATAAAAGCCGGAGGTTCTCTTGATGATTTTCTTATATCTCAGGATACATCA
TCTTGTTCTGATTATGAGTGA
>Cluster_5842
GTGGCGCTGCCCTTTGCTGCTGACGCCTGTCTGGTCGCCTCATCTTTTGAAGCAGACGCAGATGATGCCGATGACGCCGC
CGAACTGGCGGACGATGCGGCAGCCGTTTTTGAGGATTCTGCGCTGGTTTCCGACGCTTTCGCGTTCGTCTCGGATGTCT
TCGCTGCGGAAGCAGACCTCGCTGCTGCGCTGGCCTGTACAGCGGCTTCGCCAGCCTTCGTTGTGGCTGTTGAAGCAGAC
GATGCAGCACTTTCTGCCGATTTTCCGGCGGCGGTGGCACTGGCTGAGGCCTGCCCGGCACTTGTTGACGCGGCACTGGC
AGATAATGCAGCCGCTGTTTTTGAGTCTGCCGCAGCTGAGGCGCTCTGTCCCGCTGCCGTTTCAGAAGACCTGGCGTTCG
TCTCGGACGTTTTTGCCGCCTTCGCAGAATTTGCTGCCGCCGTTGCCGAGGAAGCTGCGCTGCTGGCGCTCGAGGCTGCG
TTCGTTTCTGATGATTTTGCCGCCTCTTTTGAAGCCGACGCATCCCGTGCTGAAGTGGCTGCTTCTGACGCTTTCGTGGT
CGCGGTGGATGCAGAAGTGGCTGCTGATTGTTGTGACGCTGCGGCATTCGTTGCTGACGTTTTCGCGGCACTGGCGCTGG
TAACTGCCGCGCTTTTTGATGACTCTGCAGCAGCAGCACTTTTTGCTGCTTCACGGGCCTTTGTCGATGCCGTTCCTGCG
CTGGAAGACGCTGACTGA
>Cluster_1370
ATGTATCCCGTCGACCTTCATATGCATACCGTTGCCAGCACACATGCATATAGCACATTGAGTGATTACATTGCCCAGGC
CAAACAAAAGGGCATTAAACTTTTTGCGATCACCGATCATGGCCCGGATATGGAAGATGCTCCGCATCACTGGCACTTCA
TTAACATGCGTATCTGGCCGCGAGTGGTTGATGGGGTAGGGATCCTGCGCGGCATCGAAGCTAACATTAAAAATGTTGAT
GGTGAAATTGATTGCAGCGGTAAAATGTTTGACTCGCTGGATCTGATTATTGCCGGTTTTCATGAGCCGGTTTTTGCACC
ACATGACAAAGCGACCAATACACAAGCGATGATCGCCACTATCGCCAGCGGCAATGTGCATATAATAAGCCATCCCGGAA
ATCCCAAATATGAAATAGATGTGAAAGCCGTTGCTGAAGCAGCCGCGAAACATCAGGTGGCGCTGGAAATCAATAATTCT
TCATTTTTACACTCACGTAAGGGCAGTGAAGACAACTGTCGTGCGGTAGCCGCAGCGGTACGTGATGCTGGAGGTTGGGT
GGCATTAGGCTCGGATTCTCACACCGCGTTTACCATGGGGGAATTTGAAGAGTGTCTTAAAATCCTCGACGCGGTAGATT
TTCCGCCAGAGCGCATTTTGAATGTTTCTCCGCGCCGCTTACTGAACTTCCTTGAATCTCGCGGTATGGCACCGATTGCG
GAATTTGCAGACCTTTAA
>Cluster_53921
ATGTTGTCATCCAATAAAAATAGTGAAGCCCGGCAGTGCGCGAACACAAACCGGGCCTCTATGTCAGTAACCGTATGCAA
GGAAACTAACATGAATATTGTAGCAAAATCAGATTACAACTTCCACGGAGTTGAGTTGGTGCCCACCCGTGATATGCATG
GTGTTTGGTTTACATCATCTAATATTGCATCTGCACTTAAATATGCAAATAGCCGTGCAGTAACAATGATTTATAACAAG
TATAGCGATGAGTTTAGCGCCGGAATGACTCAGGTACTCGAAGTGAGTACCTCAGGAAATTATCGCAAAAAAGTGCGAGT

TTTCTCACTACGCGGTGCCCACCTAATCGCGATGTTTGCTCGCACTCCGGTAGCCAAAGAGTTCCGCCGCTGGGTGCTGG
ATATTTTGGATCGGCAGGCAGAATGCTCACCGATTGCAAAACAGTTTACTGACGAAGAACTGGTTAATCTCTGCTACTTA
CAATTGTGGATGGAGAAGAGTCAACAAATGTGCAAACACATCTACCCAGGAATGAAGCAAATTGGTTCTGAGCTTTCAGG
AAGAATTTACGATATTGCATATGAGACTCGCTATATGTCAGAAGAAACCAAGAAATCACTTCTTCGTGAAATGAAGAATC
TTGATACCAACAATTTTGTCGTAAAGAACGCTCAGCCAATGCTGACAAAACTGCGCGGCGAGGAATGGATTCATTGA
>Cluster_1606
ATGCAGGATATTCACGAAGAAAGTCTGAACGAGTCGGTTAAATCAGAGCAGTCACCGCGGGTGGTGCTCTGGGAAATTGA
CCTGACGGTGCAGGGCGGTGAGCGGTATTTTTTCTGTAATGAGCTGAATGAAAAAGGGGAGGCGGTCACCTGGCAGGGGC
GGCAATATCAGGCATACCCGATTGACGGCAGTGGCTTTGAGATGAACGGGAAGGGCAGCAGTGCCCGCCCGTCGCTGACG
GTGTCGAATCTGTTCGGTCTGGTCACCGGAATGGCGGAGGACCTGCAGAGCCTGGTGGGGGCCACGGTGGTCCGCCGCCG
GGTGTATGCCCGTTTTCTGGATGCGGTGAATTTTGTGGCGGGCAATCCGGAAGCGGACCCGGAGCAGGAGCTGAGCGACC
GCTGGGTGGTGGAGCAGATGTCAGAGCTGACGGCCATGACAGCCTCGTTTGTGCTGGCAACACCGACGGAGACGGACGGA
GCGCTGTTTCCCGGTCGCATCATGCTGGCGAACACCTGTATGTGGGATTACCGGGGAGATGAATGCGGGTATAACGGTCC
TGCGGTGGCGGATGAGTTCGATAAACCCACCACCGATATCCGTAAGGACAGATGCAGCAAGTGCATGCGCGGGTGTGAGA
TGCGCGGCATGGTGGCTAATTTTGGCGGTTTCCTTTCCATTAACAAACTTTCGCAGTAA
>Cluster_20116
ATGTTAAAACTGACTGATATCACCTGGCTTTACCACCATTTGCCGATGCGTTTTAGCTTAACGGTGGAACGCGGCGAGCA
GGTGGCTATCCTCGGGCCAAGCGGCGCGGGTAAAAGTACCCTGCTTAATTTGATCGCCGGTTTTCTGACGCCAGCCAGCG
GTTCGCTGACTATCGACGGCGTAGATCACACCACGACGCCGCCGTCACGCCGTCCGGTGTCGATGCTGTTTCAGGAGAAC
AACCTGTTCAGCCACCTGACGGTCGCGCAGAACATCGGGCTGGGGCTAAATCCGGGGTTGAAACTAAATGCGGCGCAGCA
GAAGAAAATGCACGCGATTGCCCACCAGATGGGGATTGATAATTTAATGGCGCGGTTACCTGGCGAGCTTTCCGGCGGTC
AGCGACAGCGCGTGGCGTTAGCGCGTTGTCTGGTACGCGAACAGCCCATTTTATTGCTCGATGAACCGTTTTCCGCACTC
GATCCGGCGCTACGTCAGGAGATGCTGACGCTGGTGAGTTCGAGCTGCCAACAGCAAAAAATGACGCTCTTGATGGTGTC
GCACAGTGTGGAAGATGCGGCGCGGATCGCCACGCGCTCGGTGGTGGTCGCCGACGGGCGTATCGCCTGGCAGGGTAAGA
CCGATGAGTTGTTGAGCGGTAAGGCGAGTGCTTCGGCGCTGTTGGGAATTAAAGGTTAA
>Cluster_23209
ATGTTATCCTGCCTGTCACCTACACTTGCCATTGCAGAGCAGCCGCATGGATTTATTGATTATCGTCATGAATATCTAGA
TGATACTCGCACCCACGCGGATCGCGTCGAATTTGGTACATTTTTTAGTAATGGTATCGGTTTGATGGGGGAATTACGTT
ATAACACTGACGAAGGAGACAAAGATAAATGGGATCCTTCACAATTTGGTAATAATGGAACGGGTTTATCCATTGTATAC
CGATTTAAGCCACTGGATGACAAAAAAATTCTGGTTAGAGCCTATGTTCTGGCTTGATACCACGCAATATTGGTCAACTTA
CGAATATGGTCTTTCTGCGGGTTATGATTTCAGCAAAGAGTGGAAAGTTTCGGGTCGTTTTCGTTATGACATGGATAAAG
CCACCGATAAATCCAAAGGTTATGGCAACGATGATCGTAATAACCGCCGTTACGATGTGTGGATCGACTATCGACCACAA
AAAACCAACTTCCAATATCAGTTAAACCTGGTCTATTACAATAATGGTTATTTAACCTGGAATGATGGCCATAAAAACTA
CACCGCATCATTTAAAGTAGGGTACAAAATGGGATCGTGGATACCCTATATGAGTATTGCAGATTATAAAGGTGTGGATA
AAACATCAAGTAATCGCCAAATCCGCTGGCGCTGGGGTCTGACTTATACTTTCTAA
>Cluster_44868
GTGAACGGAGGTGATTATTTTTATAATAACCTTTCATTCACAGTCACCAGGTACAACGGCATCATGGCAACTGACTCAAC
ACAATGTGTAAAAAAAAGCCGTGGCCGCCCAAAAGTGTTCGACAGGGATGCCGCGCTTGATAAGGCCATGAAATTGTTCT
GGCAACACGGTTATGAAGCGACTTCTCTTGCGGACCTCGTCGAAGCGACCGGAGCCAAAGCGCCCACGCTATACGCGGAA
TTTACCAACAAAGAGGGGTTATTTCGTGCCGTTCTCGACCGCTATATCGATCGTTTTGCCGCTAAGCATGAAGCACAGCT
GTTTTGTGAAGAGAAAAGCGTGGAGTCTGCGCTGGCTGACTATTTTGCTGCCATCGCCAACTGCTTTACCAGCAAAGACA
CCCCGGCAGGCTGCTTCATGATCAACAACTGCACCACCCTCTCCCCAGATTCAGGAGATATCGCCAATACGTTGAAATCA
CGCCATGCGATGCAAGAGCGCACTTTGCAGCAGTTTTTATGTCAACGACAAGCGCGCGGGGAAATCCCGACCCACTGTGA
CGTGACACATCTGGCAGAATTCCTTAATTGTATTATTCAGGGGATGTCGATCAGCGCACGCGAAGGTGCATCGCTGGAAA
AACTAATGCAGATTGCCGGAACGACTTTGCGTTTATGGCCCGAACTGGTGAAATAA
>Cluster_40140
ATGACCCTTGATTTACCTCGCCGCTTCCCCTGGCCGACGTTACTTTCGGTCTGCATTCATGGTGCTGTTGTGGCGGGTTT
GCTCTATACCTCGGTACATCAGGTTATTGAACTACCTGCGCCCGCGCAGCCGATTTCTGTCACGATGGTTGCGCCTGCTG
ATCTTGAACCGCCACAAGCCGTTCAGCCACCACCGGAGCCGGTGGTAGAGCCAGAACCGGAACCTGAGCCGATCCCTGAA
CCGCCAAAAGAAGCTCCGGTGGTCATTGAAAAGCCGAAGCCGAAACCTAAGCCAAAACCGAAGCCGGTGAAAAAGGTGGA
GCAGCCAAAACGCGATGTCAAACCCGTAGAGTCGCGTCCGGCATCACCGTTTGAAAATACGGCACCCGCGCGCCCGACAT
CAAGTACGGCAACGGCTGCAACCAGCAAGCCGGTTACCAGTGTGGCTTCAGGACCACGCGCATTAAGCCGTAATCAGCCG
CAGTATCCGGCACGAGCACAGGCATTGCGCATTGAAGGGCAGGTTAAAGTTAAATTTGATGTCACGCCAGATGGTCGCGT
GGATAACGTACAAATCCTCTCAGCCAAGCCTGCAAACATGTTTGAGCGTGAGGTGAAAAATGCGATGCGCAGATGGCGTT
ATGAGCCGGGTAAGCCAGGCAGTGGGATTGTGGTGAATATCCTGTTTAAAATTAACGGCACTACCGAAATTCAGTAA
>Cluster_38948
ATGCTACAGGATGTATTCGAGTCAGAAGAATTTGCTGCCACGCTGGTATTAACTCCGCAGCAGGAGAGCCACTACTTGCA
GGCCTATTTACCGTTGGTGCGGTAAAGTGGTGCGGCAACTTGCCCCGCAGTGTAATTGCATTATTGACCGTCAGGATATGG
AACAAACGGCGTTAATGGGGCTGTTGAACGCCATTCGTCGCTATGGGCTGCCGGACGAAGGTTTTGCGGGCTATGCCGTG
CACCGTATTCGCGGTGCGATCCTGGATGAGCTGCGCGCTCTCGACTGGCGACCGCGCCAGCTGCGACAGAAATATCATCA
GATGAAAGACCTGATTCGCGAAACGCGTAAAAAACTGGGGCACGAACCGGAGTGGCAGGAGCTGGCTGAGGAAGGGATCT
CGCATGAAGATTATCTCGAATATCAGCAACTGGAAGGGGCGGAAACGCTCGCCAGCCTGGACGAACTGCTGAACGGCGAA
GGGCCTGGTATTCCGCTTGCTGGCCGGGAACTGGAAGATCAGTTTGTCACCCAGCAAATGTTGCAACACGCGCTTTCACA
ATTAAGCGAAAAAGAGCAACTGATCCTCTCCATGTATTACCAGCATGAGATGAATCTCAAAGAAATTGCGCTGGTATTAG
GGCTAACCGAGGCCCGAATTTGTCAACTGAATAAACAGATCGCGCAGAAAATTCGTGATTTTGTCTATCCGAATTAA
>Cluster_1685
ATGGCTATCCGTGATGAAAATGAAATGATTATTGAAAGCCGACCAAGTGCACTTGATATTCACGAATTAACTTTGCGTGA
ATCTTTTGATAGAGCTTGGCGGGAATCACACGAAGTAAATAAAGGTCCATATATTCCACCAGAACCATTTGAGATTCCAA
GAGTTGAAATTGATTTTTCGATGAATGAGGAATGTGAGCTTGATTTGAAACTAAAGCTGCAACGCAAATATTTCAAAAAC
CTTCGAGACTCTCAAAAAGCTATGAATCGTGTTTACGAAAAACTTCAGTCCAAACTTGAAAATGAAATTGACGAAATGGC
TATTTTGATGGAGCTTCAGACAAATACCTACGATTATTATACAAGACTAGACCGTGACGGTTGGGGATATGACCATTCAG
AAATTGGTAAAGTGATTGCAGATTTACCAGAAGGCCAAAGACTTATTCGCACAGAAAGAGCTGTCAGAGCGCCACATAAA
CTGACTTTAATTATTTCTGATAAATCACAAGCTGAGCTTGAAGAAATGGCTAGAAAGCGACTGATGAAGGTAAGGGAAAC
CGAGATTAATTTGGTGAAATCAACTCTGGCTAATTTGATTCGTGACCATCAAAAAATCGCTAAGGAATACAAACAAGAGC
TTGCACAGATTAGTAGCTTCAATGACTTTTTGAGTGTTCCACGAGAATTTAGTAAGGTTATTTAA
>Cluster_2126
ATGATGAAGATACAATCGCGTAAAATCTGGTACTACCGTATAACCCTCATCATCCTGTTGTTCGCTATGCTGCTCGCATG
GGCGCTGCTTCCCGGCGTCCATGAGTTTATCAATCGCAGCGTTGCGGCGTTTGCCGCCGTGGACCAACAGGGTATAGAAC
GCTTTATTCAGTCTTACGGCGCACTGGCGGCAGTTGTCTCGTTCTTGTTGATGATTTTACAGGCCATTGCTGCACCGCTA

CCTGCGTTTTTGATCACCTTTGCCAATGCGTCGCTGTTTGGCGCGTTCTGGGGCGGCTTGCTGTCGTGGACCAGTTCGAT
GGCCGGCGCGGCGCTGTGCTTTTTTATCGCCAGAGTGATGGGCCGCGAAGTGGTGGAAAAATTAACCGGCAAAACCGTGC
TTGACAGTATGGACGGCTTTTTCACTCGCTACGGCAAACACACCATCCTGGTCTGTCGGTTATTGCCTTTTGTCCCTTTC
GATCCAATCAGCTATGCTGCCGGTCTGACTTCAATACGTTTTCGCTCGTTTTTTATCGCCACCGGGCTTGGTCAATTACC
GGCGACTATTGTTTATTCCTGGGCGGGCAGCATGTTAACAGGCGGTACTTTCTGGTTTGTCACCGGACTGTTTATTCTGT
TTGCCCTGACCGTGGTGATTTTTATGGCGAAGAAAATATGGCTTGAGCGCCAGAAGAGGAATGACTGA
>Cluster_51729
ATGAGCGAGCAGTTAACGGACCAGGTCCTGGTTGAACGGGTCCAGAAGGGAGATCAGAAAGCCTTTAACTTACTGGTAGT
GCGCTATCAGCATAAAGTGGCGAGTCTGGTTTCCCGCTATGTGCCGTCGGGTGATGTTCCCGATGTGGTACAAGAAGCTT
TTATTAAAGCCTATCGTGCGCTGGATTCGTTCCGGGGAGATAGCGCTTTTTATACATGGCTGTATCGGATTGCTGTAAAT
ACAGCGAAAAATTACCTGGTTGCTCAGGGGCGTCGTCCACCTTCCAGTGATGTGGATGCCATTGAAGCTGAAAACTTCGA
AAGTGGCGGCGCGTTGAAAGAAATTTCGAACCCTGAGAACTTAATGTTGTCAGAAGAACTGAGACAGATAGTTTTCCGAA
CTATTGAGTCCCTCCCGGAAGATTTACGCATGGCAATAACCTTGCGGGAGCTGGATGGCCTGAGCTATGAAGAGATAGCC
GCTATCATGGATTGTCCGGTAGGTACGGTGCGTTCACGTATCTTCCGAGCGAGGGAAGCTATTGATAACAAAGTTCAACC
GCTTATCAGGCGTTGA
>Cluster_41432
ATGAAATCGCTGCGTTTAATGTTATGCGCTATGCCGTTGATGCTGACCGGCTGTTCCACGATGTCGTCAGTTAACTGGTC
GGCCGCTAACCCGTGGAACTGGTTTGGATCATCCACCAAAGTGAGCGAGCAGGGCGTGGGTGAATTAACGGCGTCCACAC
CACTGCAAGAACAAGCCATTGCTGATGCGCTTGATGGCGATTATCGCCTGCGCAGCGGAATGAAAACCGCGAACGGCAAC
GTGGTGCGCTTTTTTGAAGTGATGAAAGGCGACAACGTGGCGATGGTTATTAACGGTGATCAAGGCACGATCAGCCGCAT
TGATGTGCTGGATAGCGATATTCCTGCTGACACCGGTGTTAAAATCGGTACACCGTTTAGTGACCTTTACAGCAAAGCAT
TTGGCAATTGCCAAAAGGCCGACAGTGATGATAATCGTGCCGTCGAATGTAAAGCCGAAGGCAGTCAACATATTAGCTAC
CAGTTCAGCGGGGAATGGAGTGGCCCTGAAGGGTTAATGCCTTCGGACGATACCCTGAAAAACTGGAAAGTCAGTAAAAT
TATCTGGCGGCGTTAA
>Cluster_13223
ATGAACAATAAACATAAACATGCAGGTCATATTATCACTATTGAAGGCCACAGCTTTAAGTCTAATGAGTCTGGCATGTG
GAATCTTACAGAAATATGGAAAATCCTCGGATTACCTAACAATAAGCTACCCTCTCAGTGGCGCGGCAAGGTAACAAAGA
GGCTCACCGACATGCAAAAAATGCACGTCGAGAAGATTGGTATTGAAAGCATAACCTACGCAGACAAACAGGCTACCCTC
AAGTATGCAGGATGGGTCTCTGAAGATTTCGAGGACATGGTCTATGCAGCCTTTGAGGCTATCCTTGAGATGCCTGAAGT
TGCCACGGTCGTTGCTAATAAGATGGTCGAACTTGGCTATCACGCTGAAGCTGAGCTATTGGAACGTCATAAGGACGACT
ATAGAGAAGCTATGCAGACCCTTAAAAAGATTGGTAAACGTGTTGACGGCAGGAAGCCTGAGCGCATCTACAAGGCCGTA
CTTAACGGTAATCTGACGAAATTTCAAGGGTTATCCATGATTTCTCGCAGCAGTGTCTGGTACGCCCGAACAGTGAATCT
ATGA
>Cluster_34173
ATGGACATCGATTTCAAGAAGTTGGCTCCCTGGAACTGGTTCAAGAACGAGCAGCAAGAGCAGCAGACCGCCTCTTCCCT
GCCGGTGCAGCGCAATGACCTGCCAGCGGCGAGCGGGCCAGTCAGCCCGCTCCTGCAACTGCATCGGGAAATCGACCGGC
TGTTCGATGACGCATTCCGGGGCTTCGGTTTTCCGGCGTTGAACATGCCGCAGTGGCCATCCGATTGGTCGGGCATGCTG
AAGCCGGCCCTGGACATCCAGGAAACCGACAAGCAGTACAAGATTGCCCTGGAAGTGCCCGGTGTCGAGGAGAAGGACAT
CCAGATCACCCTCGACAACGACGTGCTGATGGTGCGTGGCGAGAAGCGCCAGGAACAGGAGAAGAAGGAAGGTGGCTTCC
ACCGTGTGGAGCGCTCCTACGGCAGCTTTCAGCGTACCTTGAACCTGCCTGACGACGCCAACCAGGATTCGATCAAGGCA
TCGTTCAAGAACGGGGTGCTCACGGTCACGATCGACAAGCGCGAGGTCAGCGCGCCGAAGCAGGGACGCTCGATCCCGAT
CAACGGCTGA
>Cluster_19847
ATGGGGGTGGGCATTGCGCCTACAAAAACGCTGGCTAAAAGTGCACAGTGGGCAACAAAGCAATGGCCACAGTTTTCCGG
TGTGGTCGCGCTGACGGCAGAAAACCGTAATTGGATCTTGAAGCTACTGGGGCTGCAGCCAGTTGGTGAGGTCTGGGGAG
TAGGACGCAGACTGACGGAAAAGCTGAATGCGCTGGGTATTAACACAGCACTGCAACTGGCGCAGGCTAACACGGCATTC
ATCCGGAAAAACTTCAGCGTCATTCTTGAGCGTACGGTACGCGAACTCAACGGCGAGTCCAGCATATCCCTGGAAGAAGC
ACCACCGGCAAAACAGCAGATTGTCTGTAGTCGCAGTTTTGGTGAACGAATCACAGACAAAGATGCCATGCACCAGGCTG
TTGTTCAGTATGCAGAGCGGGCCGCAGAGAAACTACGTGGGGAGCGTCAGTATTGTCGGCAGGTGACGACATTTGTACGG
ACATCCCCCTTTGCAGTAAAAGAACCCTGTTACAGCAATGCCGCTGTGGAAAAGCTTTCATTGCCACACAGGACAGCCGG
GGCATTATTGCCGCCGCATGCAGAGCCTTAA
>Cluster_21263
ATGCCGATGAATACTACAAGTACGTCTTTCAGCTCTTTTGGTATAAGTTGCCATAGAGAAAACTCCTTCAGGAACTCTTT
CCTAGGGAAGAATGATGAGGTCATAAAATGTTCAATGGGAGAACGGACGATTCACTTTTCTGTTCGCAAATTTAGCGGCA
ATATACTGGATACAGTGAACAGGCAGAATACTAAAGATATTAATGGGTGGATAAAAGATGAACGGATAGTATATCCCTCA
AGGGTGATCAACCAGGAGATTGATAATTACTGTTTTCAAAAAAATGCAAAAATTTCCACTGAAGAGAGGCAAAGGGTCTT
TTCTCTTGTGAGCCAGAGATATCAACTAACTCTTGATGTTAAGGCAGCACAAAGTTCTATTAATCATGTGATAATGGGGA
ATGCTTCTTTTGGCAAGAAAATAGATACTCTTTGTGATAGTATGAGCCGGGATGTAAAAAATCGTACGGCAGATTGCATA
GCAAACTTGCTTGCAGACAAGTTTTACCAGAAACATATCGAGCCGGATATTGATATTGTAAAACTACGAAATGAGATCCC
AGATTATTTGAGGTGTGCTATACAGGCATAA
>Cluster_4642
ATGAAGGGGCTTGAGAATGCCATCCGCAATCTGAACAGCCTTGATACCCGTATGGTGCCACAGGCCAGCGCATGGGCGAT
AAACCGTGTGGCACAGAAAGCGGTCTCGGTTGCCACCCGGCAGGTTGCCGGGAATACCGTTGCGGGAGATAACCAGGTGA
AAGGGATCCCCCTGAAACTGGTACGTCAGCGTGTCCGGGTGTTTAAAGCCAGTCCGTCAGGAAAAATGACGGCCAGGATC
CGCGTTAACCGGGGCAATCTGCCCGCCATTAAGCTGGGGACAGCCCGGGTCAGACTGGCCCGGCGTGGTGGAAAACTGCA
GTACCGTGGCAGTGTGCTGAAGGTGGGTAAATATCTTTTCCGGGATGCGTTTATTCAGCAACTGGCGAATGGTCGCTGGC
ATGTGATGCGGCGTATTGATGGCAAAAATCGTTACCCCATTGATGTGGTGAAAATCCCGCTTTCCGGACCGCTGACACAG
GCATTTGAAGATGCCCGCGACCGCATCATTGCTGCGGAAATGCCGAAACAGCTGGGGTATGCACTGAAACAACAACTGAG
GTTATGGCTGACCCGATGA
>Cluster_66316
GTGAATCTGATGACGACGATAACAGGCGTTGTGCTGGCAGGCGGTAAAGCCAGACGAATGGGCGGCGTAGATAAAGGATT
GCTTGAATTAAACGGCAAACCATTATGGCAACATGTCGCTGACGCGCTTATGACGCAGCTCTCTCACGTCGTGGTTAATG
CCAATCGCCATCAGGAAATCTATCAGGCAAGCGGTCTGAAAGTGATTGAAGATTCACTGGCGGATTACCCAGGCCCTCTG
GCAGGAATGCTTTCAGTAATGCAGCAGGAAGCAGGCGAGTGGTTTTTGTTTTGTCCGTGCGATACTCCTTATATTCCCGA
GGATTTAGCAGCCCGGCTTAATCATCAGCGCAAAGATGCGCCTGTCGTGTGGGTCCACGACGGTGAACGCGATCACCCGA
CTATTGCTCTGGTAAACCGCGCAATTGAGCCTTTATTACTGGAATATCTGCAAGCAGGAGAACGCCGGGTAATGGCATTT
ATGCGTCTGGCTGGCGGTCATGCGGTTGATTTCAGCGATAATAAAGATGCATTTATTAACGTGAATACGCCAGAGGAGCT
TGCCCGATGGCAGGAAAAACGATGA
>Cluster_5710

ATGTCCTTGATTAACACCAAAATTAAACCTTTTAAAAACCAGGCATTCAAAAACGGCGAATTCATCGAAATCACCGAAAA
AGATACCGAAGGCCGCTGGAGCGTCTTCTTCTTCTACCCGGCTGACTTTACTTTCGTATGCCCGACCGAACTGGGTGACG
TTGCTGACCACTACGAAGAACTGCAGAAACTGGGCGTAGACGTATACGCAGTATCTACCGATACTCACTTCACCCACAAA
GCATGGCACAGCAGCTCTGAAACCATCGCTAAAATCAAATATGCGATGATCGGCGACCCGACTGGCGCCCTGACCCGTAA
CTTCGACAACATGCGTGAAGATGAAGGTCTGGCTGACCGTGCGACCTTCGTTGTTGACCCGCAGGGTATCATCCAGGCAA
TCGAAGTTACCGCTGAAGGCATTGGCCGTGACGCGTCTGACCTGCTGCGTAAAATCAAAGCAGCACAGTACGTAGCTTCT
CACCCAGGTGAAGTTTGCCCGGCTAAATGGAAAGAAGGTGAAGCAACTCTGGCTCCGTCTCTGGACCTGGTTGGTAAAAT
CTAA
>Cluster_17657
ATGAGCAGCGGTGAACTGGATATTAAGATTGATTTAAGCGCGTATAACACCACGCTGGGTAAACTGATTCGTTCCGTGAA
GGACCGGCGCGACCTGATGACGGCGCTTGCGGGTTCCATGCTGGATGCGGTTGAGACTAACCTGGAACAACAGGGTCGCC
CGAAATGGATGGGCTGGAGTCCGGCTTATGCGAAGCGGCGCGGCCCCGGGCAAATCCTTCAGAAGTCCGGGCGACTGGCC
GCCAGTATCCGCTCTGCGGTTAACAATAACGAGGCCACGGTCGGGACAAACGTCCGTTATGCCCGTATCCACAACGAAGG
CGGCGAAATCCGCCATCAGGCACGGACGCAGAACCTGTATTTTAAACAGTACAAAAACGGCAGCGTCAGCACCCGCTTTG
TGAAAAAGCGCAACAGTAATTTTGTGCAGAGCGCAACGGTCGGAGCGTATACGGTCAACATGCCCGCGCGCCCCTTCCTT
CAGCTTGTGCAGGACGACATCGACGAGCTGGAGAACACCGCAAACCGCTATTTTGCGCGTGTGATTGACTGA
>Cluster_29513
ATGAAAGATATGAAAGCTGTTGTGGTTTTTACCGGGAAGGATTTGAACATAATGAGAACCGAGGGGGGGTCAGGCTACTG
GCATGCCAGAACAGACAGGCTCAACGATGCCGATTACCTCATAGCAGTAAGAAACAGAAGGGAAACGTGGGCAGTAAAAG
ACCTGGAACACGGCACAGCATTCCTGATCGCCAAAATTACCGGGTGTTTTAAAAGCCCTGATTACGACGACAGGAACGTC
ATAACCTTTGATGAATACGCAGAAATTCACACTCCAAAAGCATGGAAAATGCTAACCGATGGTCAGCGCTATCCGGTTGC
TTACCTGAGCGCACAGGAAGCTTTTTTACGTATTGGGGTAACACCGGAACAACTGGAATGGAAGAAATTCCATCCTTCAT
CCCCATCGGTTCCTAATACAGTGATTCCTGGTCTGGCTGAAGAGAAAACAGAAAAACTCTCCCTTAACGAGGCAATTGAA
AGAGCCAAAAAGGATATTAGCAACGCAACAGGGATTGATAGCTCAGCCATAACAATAAGCATCAAGATCTGA
>Cluster_67064
ATGAAAAAAGCATTCTTATTAGCATGTGTTTTTTTTCTCACTGGGGGCGGGGTTTCTCACGCTGCGGTTCAGAAAACCAT
ATTCAGTGCTGACGTGGTGGCCTCGGTCTGTCATGTGGTGGTGGATGCGGACAGCACCGGCAACAGTGGCCGGCTGACGT
TCGGGACTTACCGTAAGTCCACGGGGGCGTCTGTACCGCCGCGTGACTTCACGGTGCGTCTGTATGAGTCCGGCGCCACG
GTTCAGGGTTGCTCTGCGTTTCTTGCCGGGCAGGTCGCCACCCTGGATTTTGGTAACCCGGGACAACTGGACGCTGCCGG
CGTGGTCACCCGCGGTGCCGGTGATGGTATTCGCGTGGATGTACGGGCTGTTGATGCACAGGCTGATTATCGCGGACGTC
TGACGCAGGATAACCATTCCGTGAAATATCCGGTGGATTTTGCCGCTAAGGGCCAGTTTCGTTTTCGTGCGCAGCCGGTG
TTTCCGGCTGATGTGAAGGCGGGAGAGTATTCCGGGGCGCTGACTTTTGTTGTCACTTATCAGTAG
>Cluster_22033
ATGCAATTTAATATCCCTACGTTGCTTACACTGTTCCGTGTCATCCTTATCCCATTCTTTGTATTGGTCTTTTATTTGCC
TGTCACCTGGTCGCCATTTGCCGCCGCGCTCATTTTCTGCGTCGCGGCGGTGACTGACTGGTTCGATGGTTTTCTGGCAC
GCCGCTGGAACCAGAGTACCCGGTTTGGTGCTTTCCTTGACCCTGTCGCAGATAAAGTTCTCGTGGCTATCGCCATGGTG
CTGGTAACCGAGCATTACCACAGCTGGTGGGTGACCTTACCGGCAGCAACGATGATCGCCCGTGAAATTATTATTTCTGC
GCTACGCGAATGGATGGCGGAGTTGGGTAAACGCAGTAGCGTGGCTGTCTCCTGGATTGGGAAAGTGAAAACCACTGCCC
AGATGGTGGCGTTAGCCTGGCTGCTGTGGCGTCCGAATATTTGGGTTGAGTACGCCGGTATTGCACTTTTCTTTGTGGCT
GCGGTACTGACTCTGTGGTCAATGTTGCAATATTTGAGCGCTGCGCGTGCAGATTTGCTTGATCAGTGA
>Cluster_66
ATGCCAAGATATGCGATGGTTATAGATGTAGAGAAATGTGTAGGGTGTATGGCTTGTGTTATAGCGTGTAAAAAGGAGAA
TAAGGTAGCAGATGGTTATTTTAGGACAAGGGTGGTAGAAGAGGTTAGAGGAGAATTTCCCAACCTCAGGATGGAACTCA
GGTCAGAACTTTGTAATCATTGTGAAATTGCTCCTTGTATAGATGCTTGTCCCACCAGGGCTTCTCACAGGGCAAAAGAT
GGAACCGTTCAAATAGACCATAAAAAGTGTATTGGATGTAAGGCCTGTATTTTGGCCTGTCCTTATGATGCAAGATACAT
TCGTCCTGATGGGACAGCTGATAAGTGTACCTTTTGTCAGCACAGAATAAAAGAAGGAAAATTACCTGCTTGTGTAGAAA
CTTGTTTGGGTAAATCGAGGATCTTTGGAGATTTAGACGACCCTGAAAGTGAAGTGGCTAAGATTTTAAGGACTCACGAT
GCTGCTGTAAGGCTTGAATGGGCAGGTACTAAACCTCGTGTTTTTTATATTAACAAGAGATTCCCAGGGGGGTTTTAA
>Cluster_3906
ATGATCCTGCTTATAGATAACTACGATTCTTTTACCTGGAACCTCTACCAGTACTTTTGTGAACTGGGGGCGGATGTGCT
GGTTAAGCGCAACGATGCGTTGACGCTGGCGGATATCGACGCCCTTAAACCACAAAAAATTGTCATCTCACCTGGCCCCT
GTACGCCAGATGAAGCCGGGATCTCCCTTGACGTTATTCGCCACTATGCCGGGCGCTTGCCGATTCTTGGCGTCTGCCTC
GGTCATCAGGCAATGGCGCAGGCATTTGGCGGTAAAGTTGTGCGCGCCGCAAAGGTCATGCACGGCAAAACCTCGCCGAT
TACACATAACGGTGAGGGCGTATTTCGGGGGCTGGCAAATCCACTTACCGTGACACGCTACCATTCGCTGGTGGTGGAAC
CTGACTCATTACCAGCGTGCTTTGACGTGACGGCCTGGAGCGAAACCCGCGAGATTATGGGGATTCGCCATCGCCAGTGG
GATCTGGAAGGTGTGCAGTTCCATCCAGAAAGTATTCTTAGCGAACAAGGACATCAACTGCTGGCTAATTTCCTGCATCG
CTGA
>Cluster_41605
ATGCTTATTGGCTATGTACGTGTGTCAACAAATGACCAGAACACAGATCTACAACGTAATGCGCTGAACTGTGCAGGATG
CGAGCTGATTTTTGAAGACAAGATAAGCGGTACAAAGTCCGAAAGGCCGGGATTGAAAAAACTGCTCAGGACATTATCGG
CAGGTGACACTCTGGTTGTCTGGAAGCTGGATCGGCTAGGGCGTAGTATGCGGCATCTGGTCATTCTGGTTGAGGAGTTG
CGCGAACGTGGCGTTAATTTTCGCAGCCTGACGGATGCTATTGATACCAGCACACCGATGGGGCGTTTTTTCTTTCATGT
GATGGGTGCCCTGGCTGAAATGGAACGAGAACTGATTGTTGAACGAACAAAAGCTGGACTGGAAGCTGCTCGCGCACAGG
GACGAATTGGTGGACGTCGTCCCAAACTTACACCAGAACAATGGGCGCAGGCCGGGCGATTAATTGCATCAGGCGTTCCT
CGCCAGAAGGTGGCGATCATCTATGATGTTGGTATATCGACACTGTATAAGAAGTTTCCGGTCGGAGATAAATGA
>Cluster_50453
ATGAATACAGCTATAACTTTTTCTAACGAGTCACCAAAAGTCTTTTTCAAAGATGGTCGCCTGGTTACCACCTCTCAAGC
TGTTGCCGACTACTTCGATAAACAACATAAACACGTTTTAGCTAAGATTGACACACTCGACTGCTCTCCCGAATTTACAT
CAGCCAACTTTTCGGCCGATGTTCAGACTGTCGAAATTGGCAACGGCGCAGAACGTGAATCACGATGCTACCTGATCACA
AAAGACGGCTTCATGTTCCTCGTAATGGGATTTACTGGCAAAAAAGCAGCCAGACTAAAAGAAGCTTACATTGAAAAATT
TAACGCAATGGAAGAAGAACTCCACAAACGTCCTCCTGCAGCACAAAACTCCCCCGCCCCAAATAATGGATGCGCATTAC
TGATCCACTTCGATAAACACGGTCAGGTCGAGTTCACAGAAAAAGTCCCCGCCGATGCGATGGTATGCACGCTGGAACGG
TTTAAATTTTATCTGGAGCAACATGGGTGGATCGTTGCCCGTAAAGAACAATTGGTGGAAAGGTTGATGCGGTTTTAA
>Cluster_9571
ATGATTAAGGTGTATGGCGTACCCGGATGGGGCTCTACAATCAGTGAGCTGATGCTAACACTGGCTGATATTCCTTATCA
GTTCTTTGATGTCAGTGGTTTTGACCACGAGGGAGCTTCACGCGAGTTATTGAAAACCCTGAACCCGTTGTGTCAGATTC
CGACACTGGCGCTGGAAAATGACGAGATTATGACGGAGACAGCGGCGATTGCATTGATGGTTCTCGATCGTCGCCCGGAC
CTTGCGCCACCGGTTGGGCGCGCCGAACGTCAGCTGTTTCAACGGCTATTGGTCTGGCTGGTTGCTAATGTCTATCCAAC
ATTCACTTTCGCCGATTACCCTGAGCGTTGGGCTCCTGACGCACCTGAACAGTTAAAGAAGAATGTTATTGAATATCGAA

AATCGCTTTATATCTGGTTGAATTCGCAGCTCACTGCTGAACCTTATGCGTTTGGTGAGCAACTAACGCTGGTGGACTGT
TATCTTTGCACTATGCGCACATGGGGGCCTGGGCATGAATGGTTTCAGGATAATGCCACGAATATCAGTGCGATTGCTGA
TGCTGTATGTCAGTTACCGAAATTACAAGAAGTATTAAAAAGGAATGAAATTATTTGA
>Cluster_29033
ATGAAAGCTTCACTGGCGTTGTTCAGTCTTCTGACGGTATTTACCAGTTACTCCTTAAAATCTCCCGCCATTCAACCAAC
CGTGGTACAAATCCAGGCCAACACCAACCTTGCCATCGCGGACGGTGCCAGACAGCAAATCGGCAGCACGCTATTTTACG
ATCCGGCGTATGTGCGGCTTACCTATCCTGGCGGTGATGTCCCGCAAGAACGCGGTGTTTGTTCTGATGTGGTGATCCGA
GCATTGCGCAGCCAGAAGGTCGATTTGCAGAAACTGGTTCACGAAGATATGGCGAAGAATTTTGCTGAGTACCCGCAAAA
ATGGCAGTTAAAGCGCCCGGACAGCAACATCGATCACCGTCGGGTGCCTAATCTGGAAACCTGGTTTACCCGCCACGATA
AAACACGCCCCACCAGCAAGAACCCCGGTGACTATCAAGCGGGCGATATTGTCTCCTGGCGATTAGATAACGGGCTGGCA
CATATTGGGGTGGTGTCAGATGGCTTCGCCCGCGACGGTACGCCGCTGGTGATACATAACATTGGCGCAGGCGCGCAGGA
AGAAGATGTACTGTTTAGCTGGCGGATGGTGGGGCACTATCGGTATTTTGCTAAATAA
>Cluster_35136
ATGGAATTTAAAGATTTACCAAAAGAAATCCAGATAATTGCTGCAACGACACTCGGTGATAGTCTGGTGAAAATTGACCC
GGCATACACCAAAAAAGAAACCATCGATAATATGGTTCGTAATGTGCGCAATGCTTTTTCAGGGCTATATGGTTCTGATA
ATCAAAAACAGGAAAACGATATTGATGAACGGGTAATTTCTGTTTGCCTGAATGGTCATGTTATTTCAGCAATCAGAACA
GAAACGGCAACTGTTTTTGATTATCTCTGCATGATTCAGAGTCTTGCTGATGTTCTGTTGAAATCAAAAGATTTAGAAAA
CGATGCAAATTTACAGGGGCGCACAATAGCACATCCATATGCACATATTTTAAGCTCTGTGGATGTTAAAAATAATACCC
TGCAAGTTGCTTTCGAACATTCACCGCAAGAGTTAAAAACGAAAAAAGACGATGCGGATAAAAATAATAACGATGCACCT
GATTTGTTTATTATCAGCTCAAAGCTGCTGGAAATCATGAAGAGCGAGCTTGAAAAACACAACATCAAGCCCACTTCTGT
AAATTTACGATGGGTAATGAGGATTATTGACGCTTCTCTGCCATAA
>Cluster_58806
ATGACAACCCAGTATGGATTTTTTATTGATTCCAGCCGTTGCACCGGTTGCAAAACCTGCGAGCTGGCCTGTAAAGACTA
CAAAGATTTGACGCCAGAAGTCAGCTTCCGCCGCATTTATGAATATGCTGGCGGCGACTGGCAGGAAGATAACGGTGTCT
GGCACCAGAACGTGTTTGCCTACTATCTGTCGATTTCATGTAACCACTGCGAAGATCCGGCTTGTACTAAAGTCTGCCCG
AGCGGTGCGATGCATAAACGTGAAGATGGTTTTGTTGTGGTCGATGAAGATGTGTGCATTGGCTGCCGCTACTGCCATAT
GGCTTGCCCGTATGGCGCACCGCAATATAACGAAACGAAAGGCCATATGACCAAATGCGATGGTTGTTATGACCGTGTTG
CGGAGGGTAAAAAGCCGATCTGTGTTGAATCTTGTCCGCTGCGGGCGCTGGATTTCGGCCCTATCGACGAGCTGCGTAAA
AAACATGGCGATCTGGCGGCCGTTGCGCCGTTGCCGCGAGCTCACTTTACCAAACCGAATATTGTGATCAAACCCAATGC
CAATAGCCGCCCGACCGGGGATACCACTGGCTATCTGGCAAACCCGAAGGAGGTGTGA
>Cluster_9149
ATGAAACGACGTGACACCATCGTGCGCTACACCGCGCCGGAACGTATCAACCACTGGATCACCGCCTTCTGCTTCATCCT
GGCGGCGGTGAGCGGGCTGGGCTTTTTGTTCCCGTCCTTCAACTGGTTGATGCAAATCATGGGCACACCGCAGCTGGCGC
GAATTCTGCACCCGTTTGTCGGCGTGGTTATGTTTGCCTCGTTCATCATCATGTTTTTCCGTTACTGGCATCACAACCTA
ATCAATCGGGATGATATCTTTTGGGCGAAGAATATTCGTAAGATCGTCGTCAACGAGGAAGTAGGTGACACCGGGCGTTA
TAACTTCGGTCAGAAATGCGTTTTCTGGGCGGCGATTATTTTCCTGGTTCTGCTGCTGGTGAGCGGCGTGATTATCTGGC
GTCCTTATTTTGCGCCTGCTTTCTCAATCCCGGTGATCCGATTCGCGTTAATGCTGCATTCATTTGCCGCAGTGGCGTTA
ATTGTGGTTATCATGGTGCATATCTACGCCGCCCTTTGGGTGAAAGGCACGATTACCGCCATGGTGGAAGGATGGGTTAC
CAGCGCCTGGGCGAAGAAACATCACCCGCGCTGGTACCGTGAGGTCCGCAAGACAACGGAAAAGAAAGCTGAATGA
>Cluster_6900
ATGAACCATCAGGATGAATTGCCGTTAGCGAAAGTCAGCGAAGTTGACGAGGCGAAGCGGCAATGGTTGCAGGGGATGCG
TCACCCCGTTGATACGGTAACCGAACCAGAACCTGCTGAAATCCTGGCTGAGTTCATTCGCCAGCATTCTGCGGCTGGGC
AGCTGGTGGCGCGCGCCGTGTTTCTCTCGCCACCCTATTTGGTTGCAGAAGAGGAACTGTCAGTTTTGCTGGAAAGCATT
AAGCAGAACGGAGACTACGCGGATATCGCTTGTTTGACGGGAAGCAAGGATGATTACTATTACTCAACGCAGGCCATGAG
TGAAAACTACGCGGCGATGTCTTTGCAAGTTGTGGAGCAGGACATTTGTCGCGCCATTGCACACGCCGTTCGTTTTGAGT
GCCAGACGTATCCTCGTCCTTACAAAGTCGCCATGCTGATGCAAGCGCCATATTACTTCCAGGAAGCGCAGATTGAAGCC
GCGATCGCCGCGATGGATGTTGCACCTGAATATGCCGATATACGGCAGGTGGAGTCATCGACGGCGGTGTTGTACTTGTT
TAGCGAGCGATTTATGACCTATGGCAAGGCTTATGGCTTATGCGAATGGTTCGAGGTAGAGCAGTTTCAGAACCCCTGA
>Cluster_10973
ATGAACATAATCAAGCAACCGCTGCGTGTCGGCGTCGGCGGTCCGGTCGGCTCAGGTAAAACCGCGCTTCTGGAAGCCCT
CTGCAAATCCATGCGTGACACCTGGCAATTAGCCGTAGTCACCAACGACATCTACACCAGAGAAGATCAACGCATTCTCA
CTGAGGCCGGAGCACTGGAAGCGGAACGCATTGTCGGTGTCGAAACGGGGGGCTGCCCTCATACAGCCATTCGTGAAGAC
GCATCAATGAATCTGGCAGCCGTGGAAGCACTGAGTGAGAAATTTGGTAATCTCGATCTGATCTTTGTGGAAAGCGGCGG
CGACAACCTGAGTGCGACCTTCAGCCCCGAACTGGCGGACCTTACCATATATGTCATAGATGTGGCAGAAGGAGAAAAGA
TCCCGCGTAAAGGCGGTCCGGGGATCACCAAATCCGACTTCCTCGTGATCAATAAAACTGACCTTGCACCATACGTGGGC
GCCTCCCTCGAGGTAATGGAGCGTGATACTTTACGTATGCGCGGAGAACGCCCATGGGGGTTCACTAACCTGAAAAGTGG
TGAAGGTCTGCAAAACATCATCGCCTTTATCGAAGAGCAGGGCATGCTGGGTAAGTAA
>Cluster_48648
ATGACGGTCGAAAAAGACGAGGTAAAAACTCGCCTGACACCAGCGGAGTGGGCCGAAGCTGAAGCCAAATGGACGTCAGG
CGAATATACACTCTCAAAGCTGGAGGAAGAGTACGGCATTCGTCGTGAAACACTCTCCAGACATTTCAAAAAGCGAGGAT
TAGAGAAAGGCGCGGACTCTGTTGGGAAGATGGTTCGTGAGTCTCTTAAATCTGACGCAGAGCTTCGCGCTAAAGCCCGT
GCGGAAAAGATAGAAGAACGTCGTACACGTTATGACGGCTGGGCGTATGCGTTGGGGCAGATGGTGATGGTCGAAGTCAC
TACGGCCAAACGTGAGGGTAAGCCTTTAGGGGCGATTGAGGATTCTCTCAAGAGCTTACAGAGAGCCAGTAATACCCTTG
CAAAATGCTTTGAAGTTTCGTCCAAAGCATTGGGCATGGATCATGCGGAAAATGACGAGGAAGAAATTCCGAACCTGGTA
TTTGGTGAGCTTACGCCTTCCCAGGTGGCGAAATTACGTCAGGAAGACGACGAGCCTGAAATCATCGATGACGAATTGCT
TGAGACGTTGGAAGAAGAAGCTCTAAGCGAATTTGATGCGACAGATGATGGAAGTGAAGGGGAGGACGAATAA
>Cluster_35706
ATGAAATTGTTCTACAAACCGGGTGCCTGCTCTCTCGCTTCCCATATCACCCTGCGTGAGAGCGGAAAGGATTTTACCCT
CGTCAGTGTGGATTTAATGAAAAAACGTCTCGAAAACGGTGACGATTACTTTGCCGTTAACCCTAAGGGGCAGGTGCCTG
CATTGCTGCTGGATGACGGTACTTTGCTGACGGAAGGCGTAGCGATTATGCAGTATCTTGCCGACAGCGTCCCCGACCGC
CAGTTGCTGGCACCGGTAAACAGTATTTCCCGCTATAAAACCATCGAATGGCTGAATTACATCGCCACCGAGCTGCATAA
AGGTTTCACACCTCTGTTTCGCCCTGATACACCGGAAGAGTACAAACCGACAGTTCGCGCGCAGCTGGAGAAGAAGCTGC
AATATGTGAACGAGGCACTGAAGGATGAGCACTGGATCTGCGGGCAAAGATTTACAATTGCTGATGCCTATCTGTTTACG
GTTCTGCGCTGGGCATACGCGGTGAAACTGAATCTGGAAGGGTTAGAGCACATTGCAGCATTTATGCAACGTATGGCTGA
ACGTCCGGAAGTACAAGACGCGCTGTCAGCGGAAGGCTTAAAGTAA
>Cluster_17782
ATGAACGGTAAATACACTCTGATTTATGCTGATCCTCCTTGGTCATACCGCGACAAAGCAGCCGACGGCGAGCGCGGCGC
CGGATTTAAATATCCGGTTATGAACGTGCTGGATATCTGTCGCCTGCCAGTGTGGGATCTTGCCGCCGACGATTGCCTAC
TGGCAATGTGGTGGGTTCCTACTCAGCCGGTTGAGGCGTTGAAAGTGGTCGAATCGTGGGGCTTCAAGCTGATGACCATG

AAGGGATTCACCTGGCACAAGACGAATAAGCACAAAGGTAACAGCGCGATCGGCATGGGCCACATGACAAGGGCGAACAG
CGAAGACTGCCTGTTTGCGGTGCGTGGGAAACTACCGGCCCGCATGGATGCGTCAATCTGCCAGCACGTCACGGCGCCGC
GCATGGAAAACTCGCGCAAGCCTGACATCATCCGCGAAAAACTGGTGCAGTTGCTGGGCGATGTTCCGCGCATTGAGCTA
TTCGCCCGCCAGTCGTCACATGGTTTCGACGTATGGGGCAATCAGTGCGAAGGCCCGTCGGTGCAGTTGCTACCAGGCTG
CTCTGTTCCGGTTGTAAGGACGGAGGCAGCATGA
>Cluster_34391
TTGCCCTTCCTGTTCCTGGTCCTGTTAACCTTCCGTGCCGCCGCAGCGGACACGTTATTGATTCTGGGTGATAGCCTGAG
CGCCGGGTATCGAATGTCTGCCAGCGCGGCCTGGCCTGCCTTGTTGAATGATAAGTGGCAGAGTAAAACGTCGGTAGTTA
ATGCCAGCATCAGCGGCGACACCTCGCAACAAGGACTGGCGCGCCTTCCGGCTCTGCTGAAACAGCATCAGCCGCGTTGG
GTGCTGGTTGAACTGGGCGGCAATGACGGTTTGCGTGGTTTTCAGCCACAGCAAACCGAGCAAACGCTGCGCCAGATTTT
GCAGGATGTCAAAGCCGCCAACGCTGAACCATTGTTAATGCAAATACGTCTGCCTGCAAACTATGGTCGCCGTTATAATG
AAGCCTTTAGCGCCATTTACCCCAAACTCGCCAAAGAGTTTGATGTTCCGCTGCTGCCCTTTTTTATGGAAGAGGTCTAC
CTCAAGCCACAATGGATGCAGGATGACGGTATTCATCCCAACCGCGACGCCCAGCCGTTTATTGCCGACTGGATGGCGAA
GCAGTTGCAGCCTTTAGTAAATCATGACTCATAA
>Cluster_26322
ATGAGTGATGAACGCTACCAACAGCGTCAGCAGCGAGTGAAAGAAAAAGTAGATGCTCGTGTGGCCCAGGCCCAGGATGA
ACGCGGTATTATCATCGTCTTTACCGGCAATGGAAAAGGCAAAACCACCGCGGCATTTGGTACGGCAACACGCGCAGTTG
GTCACGGAAAAAAAGTGGGCGTCGTGCAGTTTATTAAAGGCACCTGGCCTAATGGTGAACGAAATCTGTTGGAGCCACAC
GGCGTTGAGTTTCAGGTAATGGCAACGGGCTTTACCTGGGATACACAAAACCGAGAGTCTGATACCGCCGCCTGCCGCGA
AGTCTGGCAACATGCAAAGCGGATGCTGGCTGATTCCTCACTGGATATGGTTTTGCTCGATGAACTGACATATATGGTAG
CGTATGACTATTTGCCACTGGAAGACGTGATTCAGGCGTTAAATGAACGTCCACATCAACAGACGGTGATTATCACAGGT
CGTGGTTGTCATCGGGATATTCTTGAACTGGCGGATACGGTAAGTGAATTACGCCCCGTCAAACATGCCTTTGATGCCGG
TGTAAAAGCGCAGATAGGGATTGATTACTAA
>Cluster_47469
ATGAATGAAATCATTTCTGCAGCAGTTTTATTGATCCTGATTATGGATCCGCTCGGAAACCTACCTATTTTCATGTCCGT
ACTGAAACATACTGAACCGAAAAGACGGCGGGCAATCATGGTGCGAGAGTTGCTTATTGCTCTCCTGGTGATGCTGGTGT
TCCTGTTTGCGGGTGAGAAAATTCTGGCATTTCTTAGCCTACGAGCAGAAACCGTCTCCATTTCTGGCGGCATCATTCTG
TTTCTGATCGCCATTAAAATGATTTTCCCCAGCGCTTCAGGAAATAGCAGCGGGCTTCCGGCAGGTGAAGAGCCATTTAT
CGTGCCGTTGGCAATTCCGTTAGTCGCCGGGCCGACTATTCTCGCCACGCTGATGTTGTTGTCTCATCAGTACCCGAATC
AGATGGGGCATCTGGTGATTGCTCTGCTGCTGGCCTGGGGCGGCACCTTTGTCATCCTGCTACAGTCTTCGCTATTTTTA
CGTCTGCTGGGCGAGAAAGGGGTGAACGCACTTGAACGCCTGATGGGATTGATTCTGGTGATGATGGCAACCCAGATGTT
CCTCGACGGCATTCGAATGTGGATGAAGGGGTAA
>Cluster_53944
ATGCAAACTTATAACACCAATCCCGCTTACGAAATGGCTCCCCAGCTCCTGGAGCACTTTAACCAGCATCTGGATTCATT
ATTCGGGGTCTATTCGAAACTGTTACCGTTCAGAATGGATTTTGCTTACCGAAAAAACACCCTCAGCTACAGATGTGCCT
GCAGGTACGCCATGTGCGCGGAGATGCTGCAACTGATTAACGAGGTGGGAGAGAAACTGGTCGGGTATGCCTGGGTGATG
GAATATACAGAACGTAAAGGGCTTCACATTCATTTTGTGGGCTATCTGAACGGCCAGAGCCACAGGAGTTCGTATCTGGT
ATCCAGATTGATGGGAGATATCTGGAGGCGGGTTACCGAGGGTAATGGTTACTACCACTGGTGCCGGTTTAATAAAAATT
ATCCGGTAAACATTAACCACGTCATTCACTATTCAGATCACAAAGCGGTGAATGATTTACGCTATGCCATCAGCTACCTG
GCCAAACGGGAACAGAAAGAATGTGGCATCATCCTGAAATGCAGCGGGCTTCCTGAAAAAAGTAACCGGGGTCGACCACG
ACTGGACAGCCCCCTGCCGGGGATATGTGCTCTGGTGTAA
>Cluster_67044
ATGGTGACCGGAAAATCAGAGAAACAACAGGTGATTGCGTTTATTGATTACGAGAACTGCAGTAATCTGACAGAAATTGC
CTTAAATATATATACAGAGTTAATTATATTTGCCGGCGCGAAACAGAATAATGTGGCACTGCCAGTAAACGCTTTCCCTG
AAGCAGTAAAAGTCACACTCCGGCATGTCAGTGAAGTCTCCCGGAACAATGTTGATTTTCATCTGGTACTGGAGCTGGGG
CAGTGTGTATGCCACTACGGGCCGGAGAAGGAATACCATATTGTTTCCGCTGACAAGGGATATGACGGTATTGTCCGGAC
ACTGCAGGCGCGCGGGATTCGTTGCCGTCGCGTCTCCCCGGATAAGGCGTCATCAGTAAAGATGGCTGCACCCGACATGG
ATATCGTTATCCGGTGGGCAAACAAAATTCAACAGGCAGCGCGGGAGGTTCGTAATATGCCGGTAACAGAAGAGACATTT
AATAATTATTTAAAATCACAGATGCGCGGTGAATGGCGTGATGTCCTGACCAGAGGTATCCGGGATGAACTGGTCCGTCG
CGGGGTAATGAAAATTCAGGGGAATAAAATCATCTGGTAA
>Cluster_5974
ATGCTGGAAAATTTGAATCTCTCTCTATTCTCTCTTATTAACGCGACGCCAGACTCGGCTCCGTGGATGATCTCGTTGGC
GATTTTTATTGCTAAAGATTTGATTACCGTGGTGCCGTTGCTGGCCGTGGTACTTTGGTTGTGGGGGCTTACAGCACAAC
GGCAACTGGTGATAAAAATCGCTATCGCGCTGGCGGTCAGCCTGTTTGTGTCCTGGACGATGGGACATCTTTTTCCGCAC
GACCGACCCTTTGTCGAAAATATCGGCTATAACTTCCTGCATCATGCGGCGGATGACTCATTCCCAAGCGATCACGGTAC
GGTGATTTTCACCTTTGCACTGGCATTTTTATGCTGGCATCGCCTGTGGTCCGGCTCACTTTTAATGGTGCTGGCCGTCG
TCATTGCCTGGTCGCGCGTTTATCTTGGCGTCCACTGGCCGCTGGATATGCTCGGTGGATTGCTGGCAGGTATGATTGGC
TGCCTTAGTGCCCAGATTATCTGGCAAGCGATGGGGCATAAACTCTATCAACGTCTGCAATCGTGGTATCGCGTCTGTTT
TGCATTACCGATCCGCAAAGGCTGGGTGCGTGACTGA
>Cluster_6878
ATGAACAAACACACCGAACATGATACTCGCGAACATCTCCTGGCGACGGGCGAGCAACTTTGCCTGCAACGTGGATTCAC
CGGGATGGGGCTAAGCGAATTACTAAAAACCGCTGAAGTGCCGAAAGGGTCCTTCTATCACTACTTTCGCTCTAAAGAAG
CGTTTGGCGTTGCCATGCTTGAGCGTCATTACGCCGCATATCACCAGCGACTGACTGAGTTGCTGCAATCCGGCGAAGGT
AACTACCGCGACCGCATACTGGCTTATTACCAGCAAACACTGAACCAGTTTTGCCAACATGGAACCATCAGTGGTTGCCT
GACAGTAAAACTCTCTGCCGAAGTGTGCGATCTGTCAGAAGATATGCGCAGCGCGATGGATAAAGGTGCTCGCGGCGTGA
TCGCCCTGCTCTCTCAGGCGCTGGAAAATGGCCGTGAGAACCATTGTTTAACCTTTTGTGGCGAACCGCTGCAACAGGCA
CAAGTGCTTTACGCACTGTGGCTTGGCGCGAATCTGCAGGCCAAAATTTCGCGCAGTTTCGAGCCACTGGAAAACGCGCT
GGCCCATGTAAAAAACATTATTGCGACGCCTGCCGTTTAG
>Cluster_31637
ATGATAAAAGTGTTTATCGTATCCTTAAATGATTCACCGAGAAGAACATCAATCTCACAATTGTGTGAAAAATACGGTGT
AGAATTTGAGTTTGTTGATGCAGTTAATGGCAGAAAATTAGATGATAAATATATCAATAAAATTAATTCTTATGAGTGGA
CAAAGTTAAAATATAAAAGAAAGTTAGGTCCAGCAGAGATTGGTTGTACATTAAGTCATCTCAGTATATACAAAAAAAAT
ATTAATAGTTGGTTCATCGTTTTGGAGGATGATGTCGATTTTGATGAACGATTTGTAAAATTAATCAATACTGATATAAC
GGGTTTAAGGACTGATGCTCTCTATCTTCTTGGTGGACAAGAGGGACTTGAGTCATTTAAAAATGTTATTTTTTCAAGAT
TTGGTGGTATTAAGGTGAATACAGATATAGTCTTTCTTAAGACTATCTGTAGCGAACGTTTTTTATTTCGAACATGTTGC
TATATGATTCATCATTCGTTAGCTAAGAGAATAGTAGACTTCAACCAAAATAAACTTACTTTAGCTGATGATTGGTTTAA
TTTATCAAAGTTTAATATCATAAACAATATATATTATATTGATCTTGTTGCACATCCTAGTGATCTGAGTACTTCTCTAA
TTCATTCAGATAGGCTAAAATTAATTAAAACAAGGCAAAAAATATATAGAACATTCATGAAAAGAATAGTATTAAAATTC
AGGTCTATATTGAGGTTTTTCTGA

>Cluster_53543
ATGAATATTGTATTTATCGGCACCGGCATAATGGGTAAGCCGATGGCTTATAATTTACAACAAGCCGGACATACTCTTTA
TTTTTCAGCGCATTTTGAACCTGCACCGCAAGAGCTTATTGGCGAACGTGGCATTGTTTGCTCTACACCGCGTGAAGTCG
CGCAGGAGTGTGAGGTCATCATCACCATGCTCCCTGATACCCCACATGTTGAGGATGTTCTTTTTCATCCCAACTTTGGC
GTAATACACGGTTTAAGCCACGGGAAAATTGTGATTGATATGAGTTCAATTTCCCCGGTTGCCACCAAAGCATTTGCACA
ACGTATTATCGCGGCAGGCGCGGAATATATCGATGCACCCGTCTCTGGCGGTGAAGTTGGCGCGAAAGCCGGGACGCTTT
CCATTATGGTTGGCGGATGTGAAGAGGTTTATTTGCAGATCAAACCGATTCTGGAATTGATGGGTAAAAATATCACTCTG
GTCGGTAACGTCGGTGATGGTCAAACGTGCAAAGTCGCTAACCAAATTATTGTTGCACTCAATATTGACGCGGTGGCTGA
AGCCCTGCTCTTTGCCAGTAAATCAGGTGCCGATCCCGCCCGCGTACGTGAAGCGCTGATGGGAGGGTTTGCCTCCTCTC
GCGTGCTGGAAGTTCATGGCGAACGGATGATTAAAGGAACATTCGAACCCGGTTTTCGTATTAGTCTGCATCAAAAAGAT
CTGAATCTGGCGCTGGAGAATGCCCGCCTGTTGAATACCCCGCTTCCTAACACGGCGACGACACAGCAGTTATTTAGCGC
CTGCGCCGCGCTCGGTGGTAAAGAGTGGGATCATTCTGCGTTAATTCGGGCGCTTGAAACACAAGCCAATTTCACTATCC
GTAAATAA
>Cluster_43052
ATGAGCTTTTTTCACGCCAGCCAGCGGGATGCCCTGAATCAGAGTCTGGCAGAAGTCCAGGGGCAGATTAACGTTTCGTT
CGAATTTTTCCCGCCGCGTACCAGTGAAATGGAGCAGACCCTGTGGAACTCCATCGATCGCCTTAGCAGCCTGAAACCGA
AGTTTGTATCGGTGACCTATGGCGCAAACTCCGGCGAGCGCGACCGTACGCACAGCATTATTAAAGGCATTAAAGATCGC
ACCGGCCTGGAAGCGGCACCGCATCTTACCTGCATTGATGCGACGCCTGCCGAGCTGCGGATCATTGCACGCGACTACTG
GAATAACGGTATTCGCCATATCGTGGCGCTGCGTGGCGACCTGCCGCCGGGAAGTGGTAAGCCAGAAATGTATGCTTCTG
ACCTGGTGACGCTGTTAAAAGAAGTGGCAGATTTCGATATCTCCGTGGCGGCGTATCCGGAAGTACATCCAGAAGCGAAA
AGTGCCCAGGCCGATTTACTCAACCTGAGACGAAAAGTGGATGCCGGAGCCAACCGCGCCATTACTCAGTTCTTCTTCGA
TGTCGAAAGCTACCTGCGTTTTCGCGATCGCTGCGTCTCGGCAGGCATTGATGTGGAAATCATTCCGGGAATTTTGCCGG
TTTCGAACTTTAAACAGGCGAAGAAATTTGCCGACATGACCAACGTGCGTATTCCGGCGTGGATGGCACAAATGTTCGAC
GGCCTGGATGACGATGCTGAAACCCGCAAGCTGGTGGGCGCGAATATTGCCATGGATATGGTGAAGATTTTAAGCCGTGA
AGGAGTGAAAGATTTCCACTTCTATACGCTTAACCGCGCTGAAATGAGTTACGCGATTTGCCATACACTGGGTGTTCGAC
CTGGTTTATAA
>Cluster_40809
ATGTTTGCCGTACTGGGTGATATTGAGTTTGAGCTGATTACCTACTGGGACGGCTTCGAGGCCACGTTCGGCGTCGATTA
TGCGGAGCATGCCCGCATCGGGGGTAAGCCTGGCCTGCAGTTCGTCGGCGACAGGCTGGACGAAATCCAGATAACTCTGG
TTTTCCATCAGCATTATTGTGTACCCGATGTGGAGCTGGCGAGACTGCGAACAGCCATGAAAGCCCATCAGGCACTGGCG
CTGGTCTTCGGCAACGGTGACTATCGCGGCTGGTTCGTGATTACCGATGTGACTGCAACCAGCGAGCAGACTGACAGTAC
CGGCAACGTGCTGGCTGTCAGTGCCACCGTGTCTCTCCGGGAATACACCGGTGACCCGAAAAATCCTCTGCAACCACCGG
CAATACGCACGAAGCTCCCGGGTGTCGGGGCGGTCTCCGGTGCCATACCTTCACCTTCAGGGGTGGCGCAGTTCATCCGC
AACGGCGTCAACTATGCGAAACAGGCGCAGTCTGTACTCCAGACCACTATCAGCGCCGTTCGGGTGACACAGAAAATGAA
GGATAACCCCGTTGTCGCACTGACCCGTGTGCCGGGGCTGATGAGCGGACTGGGTAATATCTCCGGGGCTCTGGGGAAAA
GTGTTCCGGCGTTTAACGCACTCTCTGAATCCATGCCCGATGCCATCAGTCTGGCCAGAACAGCCAGCGAAGCAGTCACG
TATGTACAGCAGGCACAGTCTGCGCTGAGTGGTGTGGACAAAAGAAATATTGCAGGTGCTCTGGATACCGTTTCCGGGCA
GCTTAACGCCGCCGGCACAGCATTCAACCGCATGTCTCCGGGATTAAGTGCAATGGCCGCCAGAATACTGACGAGGAGTG
TGTGA
>Cluster_26894
ATGCCTGCTGTTGACCTTTCCCAGTTACCGGACCCCGCCATTATCGCGGAGCCTGATTTTGAGGCAATTCTGGCTGACAC
AAAGGCCATGATGATTGCGGCTTATCCCGCCGAACAGCGTGAAGCCGTCTCCGCCGCGCTGGAGCTGGAATCGGAACCCC
TGAACGTTATCGCCCAGATAACAGCGTTTCGTGAAATGCTGTTACGCCAGCGGGTCAATGAGGGGGCACGGGCCTGCATG
TTAAGCCACAGCACCGGGACAGACCTGGACAACCTCGCGGGCAATATGAACACAAAGCGCCTGACCATCACTCCGGCAAC
GGATACCACCGACGCGGTGATGGAGAGTGACACCTCACTGAGACTGCGGGCGCAGCGGGCGTACGACGGCCTGAGTGTTG
CTGGCCCGTCAGGTGCATACGAGTATTTTGCCCGCAGCGCCAGCGGTCTGGTGCGTGATGCGCGGGCCATCAGCCCGTCT
CCGGCAAATGTGACGGTTTCCATCCTGTCCACTGAAGGCGACGGCACAGCAACGGAGGCGTTGCTTAATACCGTTCGCGC
CGTTCTGAATGCAGAGGATACCCGTCCGGTGGCCGACCGCCTGACCGTACAGAGTGCCAGAATCGTGACATGGCGGCTGA
ATGCAAAACTGTACTTTTACCCCGGCCCGGAATCCGAACCTATTCTGGCTGCGGCGGAATCGTCGTTCAGGAAGTGGCTG
GCTGAGCAGGGGCTTATCGGTCAGGACGTGGCGTTGTCCGCCATTGCTGCCGCACTGCATGTGCACGGTGTGCAACGCGT
GGAGATAATCGAACCCACACAGAATATGGCCATCAGCGACATACAGGCGGCGCGCTGTGAGTCGTTCACCATCAGCGAAG
GTGGGCGCAATGAGTAA
>Cluster_59240
ATGAAGCGTGAAGAAATCGCTGATCTGATGGCGTTTGTCGTCGTTGCAGAGGAGCGTAGCTTCACTCGTGCAGCAGCCCG
CCTGAGCATGGCGCAGTCAGCTTTAAGCCAGATAGTGCGTCGTATAGAAGAACGATTGGGATTGCGGCTTCTGACGCGAA
CCACGCGCAGCGTTGTTCCAACTGAAGCGGGCGAGCATCTTTTGTCTGTTCTTGGCCCTATGTTGCATGACATAGATTCA
GCCATGGCATCCCTGAGCGATCTGCAGAACCGCCCATCCGGGACAATACGTATTACTACTGTAGAACATGCAGCAAAAAC
GATATTGTTACCAGCAATGCGCACATTCCTGAAATCGCATCCTGAAATTGATATTCAGCTCACCATTGATTATGGTTTGA
CCGATGTCGTTTCTGAACGTTTTGATGCAGGCGTCCGTCTGGGTGGGGAGATGGATAAAGATATGATCGCCATTCGAATC
GGGCCAGATATACCAATGGCTATTGTTGGCTCACCGGATTATTTTTCTCGCCGAAGTGTTCCAACGTCAGTGTCACAATT
AATAGATCATCAGGCAATTAATTTGTATCTTCCCACATCGGGTACAGCAAATCGCTGGAGATTAATACGCGGTGGACGTG
AAGTTCGTGTTCGCATGGAAGGTCAGCTTTTACTGAATACGATAGACCTGATCATTGATGCTGCAATTGATGGGCATGGA
TTGGCGTATCTACCTTATGATCAGGTTGAGCGGGCTATTAAAGAAAAAAAACTGATACGTGTTTTGGATAAATTCACACC
AGATTTACCCGGTTATCACCTGTACTATCCACACCGTCGACATGCTGGCTCGGCATTCTCATTATTTATAGATAGGCTGA
AGTATAAAGGTGCTGTTTAG
>Cluster_31636
ATGTTTTCAATTATAATACCTAGCTTTAATCGAGAGAAACAACTTCGTAATGCATTGGCCGAACTAAATAAGCAACTCTA
CAAAGAGTTTGAGGTTATTGTTGTTGATGATGCTTCGGACAGTGAATATGATTTGAGCTTGGATCTTTATCATTTTGAAC
TGAAATATTTCAGAAGCAATAAAAATTTAGGACCATCAGGTGCTAGAAATATTGGAGTTAGTTTAGCTCGTTATGATTGG
ATCATTTTTTTGGACGATGATGATTATTTCATGCCGGAAAAACTGATGATCTTGAGGGAGACTATATTAAATAATGATAT
TGACTTTATCTATCATAGAGCTCGTATATTTATGATCAATGAAAAAATTAGTTATATTTCTAGTCAAAAGGATATAGATA
AAATTCCAGGTGAGGTATATCAACATATGCTTTCTGGTAATTTTATTGGGGGCCCTCCAAATTTTGCAATAAAAAAAAGC
CTGTTTAACAAACTTCATGGCTTTTCTGATAATGTACGAGCAATTGAAGATTACGAGTTTCTACTAAGGTTAGTCCGTGA
CGTTAGTAAAAAAAGAATTATTTTTGTAGATAATGTGCTTACTGGGTGTAATTATATAACAAAATCAACCAGCGTCTCTA
AAAATATAAACAATTTAAAACAAGCATGTACATATATATCAGAGGAATATATTCGAGACAACCGCGAATTTAATCTTTTT
AAAATAAATACCTCGTTGATGACTGCACATGCTTTGTTAATGAGTTTAAATCGTAAATCTGCTTTCTTTTATTTTAAAGC
AGGATTGTTATGTTTAATATAAAATATATTGTATCTGGGATTATTTCTTTTATATCTCCAGTTTTGATGATTAAGCTTA
GGGGCAAGGTTTAA
>Cluster_317

ATGCCGCCTCGCCGCTATAACCCTGACACCCGACGTGACGAGCTGCTGGAACGCATTAATCTCGATATCCCCGGCGCGGT
GGCCCAGGCGCTGCGGGAAGATTTAGGCGGAACAGTCGATGCCAACAATGATATTACGGCAAAACTTTTACCGGAAAATT
CTCGCTCTCATGCCACGGTGATCACCCGCGAGAATGGCGTCTTTTGCGGCAAACGCTGGGTTGAAGAGGTGTTTATTCAA
CTGGCAGGCGACGATGTCACCATAATCTGGCATGTGGATGACGGCGATGTCATCAATGCCAATCAATCCTTGTTCGAACT
TGAAGGCCCATCCCGCGTGCTGTTAACGGGCGAACGCACTGCGCTTAATTTTGTGCAAACCCTTTCAGGAGTTGCCAGTA
AGGTACGCCACTATGTCGAATTGCTGGAAGGCACCAACACGCAGTTGTTGGATACGCGCAAAACCTTACCCGGCCTGCGT
TCAGCTCTGAAATACGCGGTACTTTGCGGCGGCGGAGCGAATCACCGTCTGGGGCTTTCTGATGCCTTCCTGATCAAAGA
AAACCATATTATTGCCTCCGGCTCAGTGCGCCAGGCGGTCGAAAAAGCGTCCTGGCTGCACCCGGATGCGCCAGTAGAAG
TCGAAGTAGAGAATCTGGAAGAACTTGATGAAGCCCTGAAAGCAGGAGCCGATATCATCATGCTGGATAACTTCGAAACA
GAACAGATGCGCGAAGCCGTCAAACGCACCAACGGCAAGGCGCTACTGGAAGTGTCTGGCAACGTCACTGACAAAACACT
GCGTGAATTTGCCGAAACGGGCGTGGACTTTATCTCCGTCGGTGCGCTAACTAAACACGTACAAGCACTCGACCTTTCAA
TGCGTTTTCGCTAA
>Cluster_61029
ATGAAATATTCCTTAGGGCCAGTGCTGTGGTACTGGCCAAAAGAGACGCTGGAAGAATTTTATCAGCAGGCCGCCACCAG
CAGCGCCGACGTGATTTATCTTGGTGAAGCGGTATGCAGCAAGCGTCGGGCAACCAAAGTTGGCGACTGGCTGGAGATGG
CAAAATCGCTCGCCGGGAGTGGTAAGCAGATTGTGCTCTCCACGCTGGCGCTGGTGCAGGCATCATCTGAACTGGGCGAA
CTGAAACGCTATGTTGAGAACGGTGAGTTTCTGATTGAAGCCAGCGATCTCGGCGTGGTGAATATGTGCGCTGAACGCAA
ACTGCCGTTCGTCGCCGGGCACGCGCTGAACTGCTACAACGCGGTGACACTGAAAATATTGCTCAAACAGGGCATGATGC
GCTGGTGTATGCCAGTGGAGCTTTCCCGCGACTGGCTGGTGAATCTGCTTAATCAGTGCGATGAGCTGGGCATTCGTAAC
CAGTTTGAAGTGGAAGTCCTGAGCTACGGTCATCTGCCGCTGGCCTACTCCGCCCGCTGCTTTACCGCGCGTTCGGAAGA
CCGCCCGAAAGATGAGTGTGAAACCTGCTGCATTAAGTATCCGAACGGGCGCAACGTGCTGTCGCAGGAAAACCAACAAG
TGTTTGTACTCAATGGCATTCAGACCATGAGCGGCTACGTTTACAACCTCGGTAACGAGCTGGCATCCATGCAGGGCCTG
GTTGATGTGGTCCGCCTGTCACCGCAGGGTACTGACACTTTCGCGATGCTCGACGCCTTCCGCGCTAATGAAAATGGCGC
TGCACCACTGCCGTTGACGGCGAACAGCGACTGTAACGGCTACTGGCGGCGGCTGGCAGGACTGGAGCTGCAAGCCTAA
>Cluster_26591
ATGTCAATTAAAACAATTAAGTATTTCTCAACAATCATTGTAGCGGTAGTTGCGGTTCTTGCCGGATGGTGGCTGTGGAA
TTATTACATGCAATCTCCGTGGACACGCGACGGGAAAATACGCGCAGAACAGGTTTCTATCACCCCACAGGTGTCGGGAC
GTATTGTTGAGCTGAATATAAAAGACAACCAGCTGGTTAACGCGGGGGATCTTCTGCTCACGATCGATAAAACGCCCTTT
CAGATCGCCGAGCTGAACGCACAGGCTCAGCTGGCGAAAGCGCAATCTGACCTCGCAAAAGCCAATAACGAAGCGAATCG
TCGTCGTCATCTCTCACAAAATTTTATTTCTGCCGAAGAACTCGATACCGCTAACCTCAATGTTAAAGCGATGCAGGCCA
GCGTTGATGCCGCACAGGCGACGCTGAAACAGGCACAATGGCAACTGGCGCAAACGGAAATTCGCGCCCCGGTGAGTGGA
TGGGTGACTAATCTCACTACCCGCATCGGCGACTACGCTGACACCGGGAAACCGCTGTTTGCCCTTGTTGACAGCCACTC
GTTTTACGTCATTGGTTATTTTGAAGAAACCAAATTGCGCCATATCCGCGAAGGTGCACCAGCACAAATTACCCTCTATA
GTGATAACAAAACGTTACAGGGTCACGCGTCCAGTATCGGTCGGGCGATTTATGATCAGAGCGTTGAAAGTGACTCAAGC
CTTATTCCGGATGTGAAACCTAACGTCCCCTGGGTTCGCCTCGCCCAACGCGTTCCCGTCCGCTTTACGTTGGATAAAGT
CCCAGGCGATGTCACGCTGGTGTCGGGCACCACCTGTAGCATTGCCGTAGGTCAATAA
>Cluster_24820
ATGAGTATTCAACATTTTCGTGTCGCCCTTATTCCCTTTTTTGCGGCATTTTGCCTTCCTGTTTTTGCTCACCCAGAAAC
GCTGGTGAAAGTAAAAGATGCTGAAGATCAGTTGGGTGCACGAGTGGGTTACATCGAACTGGATCTCAACAGCGGTAAGA
TCCTTGAGAGTTTTCGCCCCGAAGAACGTTTTCCAATGATGAGCACTTTTAAAGTTCTGCTATGTGGTGCGGTATTATCC
CGTGTTGACGCCGGGCAAGAGCAACTCGGTCGCCGCATACACTATTCTCAGAATGACTTGGTTGAGTACTCACCAGTCAC
AGAAAAGCATCTTACGGATGGCATGACAGTAAGAGAATTATGCAGTGCTGCCATAACCATGAGTGATAACACTGCTGCCA
ACTTACTTCTGACAACGATCGGAGGACCGAAGGAGCTAACCGCTTTTTTGCACAACATGGGGGATCATGTAACTCGCCTT
GATCGTTGGGAACCGGAGCTGAATGAAGCCATACCAAACGACGAGCGTGACACCACGATGCCTGCAGCAATGGCAACAAC
GTTGCGCAAACTATTAACTGGCGAACTACTTACTCTAGCTTCCCGGCAACAATTAATAGACTGGATGGAGGCGGATAAAG
TTGCAGGACCACTTCTGCGCTCGGCCCTTCCGGCTGGCTGGTTTATTGCTGATAAATCTGGAGCCGGTGAGCGTGGGTCT
CGCGGTATCATTGCAGCACTGGGGCCAGATGGTAAGCCCTCCCGTATCGTAGTTATCTACACGACGGGGAGTCAGGCAAC
TATGGATGAACGAAATAGACAGATCGCTGAGATAGGTGCCTCACTGATTAAGCATTGGTAA
>Cluster_7661
ATGAGTAGTTTGTTGTTGTTTAACGATAAGAGTAGGGCACTGCAGGCGGATATCGTCGCCGTGCAGTCGCAGGTGGTTTA
CGGCAGCGTGGGCAACAGCATTGCCGTGCCTGCTATCAAACAGAACGGCCTGAATGTCTTTGCCGTGCCGACGGTATTGC
TGAGCAATACGCCGCATTATGACACTTTCTACGGTGGTGCGATTCCGGACGAATGGTTTAGCGGCTATTTGCGTGCGCTT
CAGGAGCGTGATGCGCTGCGCCAACTTCGTGCTGTAACCACGGGCTATATGGGAACGGCATCGCAAATCAAAATCCTTGC
CGAGTGGCTGACTGCGCTACGCAAAGACCATCCTGACCTATTGATCATGGTCGATCCGGTGATTGGCGATATTGATAGCG
GAATTTATGTCAAACCTGACCTTCCCGAAGCGTATCGACAATATTTACTGCCGCTGGCGCAGGGAATTACCCCCAATATC
TTTGAGTTGGAAATCCTGACCGGTAAAAATTGCCGCGATCTCGACAGTGCCATTGCTGCCGCAAAAAGTCTGCTTTCAGA
CACATTAAAATGGGTGGTGGTTACCAGCGCCTCCGGTAATGAAGAAAATCAGGAGATGCAGGTTGTGGTGGTCACTGCCG
ACAGCGTGAATGTCATTTCCCATTCACGGGTAAAAACCGACCTGAAAGGGACTGGCGACCTGTTTTGTGCTCAGCTCATC
AGTGGCTTGCTGAAAGGGAAGGCGTTAACCGATGCAGTGCACCGAGCGGGGTTGCGCGTACTGGAAGTGATGCGCTACAC
CCAGCAGCATGAGAGCGATGAATTGATTTTGCCGCCGCTGGCGGAAGCATAA
>Cluster_33993
ATGACGCTATTTACAACCTTACTGGTGTTAATTTTCGAGCGCCTGTTTAAGTTGGGCGAGCACTGGCAGCTTGATCATCG
TCTTGAAGCGTTCTTTCGGCGGGTGAAACATTTTTCTCTCGGGCGCACGTTAGGCATGACCATTATTGCGATGGGCGTGA
CTTTTTTACTGTTACGCGCATTGCAGGGAGTATTGTTCAACGTTCCCACGCTACTGGTGTGGCTGCTGATTGGTTTGCTG
TGTATTGGCGCAGGTAAAGTTCGTCTTCATTATCATGCTTATCTGACAGCTGCTTCACGTAATGATAGCCATGCCCGTGC
CACGATGGCTGGCGAACTCACCATGATTCACGGCGTCCCGGCAGGCTGCGACGAACGTGAGTATTTGCGTGAGCTGCAAA
ATGCATTGCTGTGGATTAACTTTCGTTTTTATCTTGCACCGCTGTTCTGGCTGATTGTGGGGGGAACCTGGGGACCCGTT
ACGCTGATGGGGTATGCGTTTTTGCGTGCATGGCAATACTGGCTGGCGCGATATCAGACGCCGCATCATCGTTTACAGTC
CGGCATTGATGCCGTGCTTCATGTACTGGATTGGGTGCCGGTTCGTCTTGCGGGTGTGGTATATGCCTTGATCGGTCATG
GTGAGAAAGCGTTACCGGCCTGGTTTGCTTCGCTGGGTGATTTCCATACTTCGCAGTATCAGGTGTTAACGCGTCTGGCG
CAGTTCTCTCTGGCGCGTGAACCGCATGTCGATAAGGTGGAGACGCCGAAGGCAGCGGTTTCAATGGCGAAGAAAACCTC
GTTCGTGGTCGTGGTGGTGATTGCACTACTGACGATTTACGGGGCGTTGGTGTAA
>Cluster_962
ATGAAGAAGATTGATTACAAGTCCATACCAAAACCAATAGACTCAGCATCAGAGCGAAAAAAACACAAAAAAGAGGCTGA
AAAATTAGCAAATTATATCAGTTTTATTAGAAACAATGCTCACGGCGATGGCGACAAGAAGTTGCTTTCAGATGCTCGGA
CCCAAGCGTTCGGTATACTTCGTAAGCAGATGCAATATCGTCTTCATCCTGGCTACATAATTGAAATTCGCCCTACTGAA
AGACAATTGTTCTTATTAAACTCTGTCTTTGACTTTGTAAACGTAGTTGGAGATCTTATCGACAGGTCTGTAGATAAGGA
TCCTGATACAAATAGTTTTCTTCTAACAAATAAAGAATACTTATATGGTAAATTCGGTATAAACGGATGGCAAAAATATG
TACGCTTCTTACGTGCATTCGTTGATGCGTATAAAAATTCCGACATGATTTATACATATTTGCCTGGTGGTGATAATTGT

ACGCTTTCGGCAGACAATAGAATATTCATACCTGTACTTGGTCTTGGGCTGATTAATGCAGTAAATGAAAAAGATGTTAT
AATTGTTAAGCAGTGGCGAAAATATGAGGGATATAGATATCTGCCATGCTTTGATATATTAAAAATACAGAATAAATTCT
ATGTAAAAATTAAATATAAAGAAGATATTTTCTTCCTCAGAAAAAACATAGATCTTTTACAAGAACTTTCTGGTACAATA
GATGTCATCTTAGGTTCTAGGTTTATAAAAGAACTGAAAGAAAGCAGGTCTTTCTCTCGTGTAGAGATAAGCGAGTCAGA
GTTATGGGGTATATCAAATAATCCTGTAAACCACGCTCACCAACGAAACCCAAACAAAAAGTGGTCATAA
>Cluster_41031
ATGAGTATAGTGGTTGCACGCTTGGCTGGAGGTTTAGGAAATCAGATGTTTCAGTATGCGAAAGGTTATGCTGAGTCAGT
TGAGCGAAATAGCTATCTTAAATTAGATTTGAGGGGGTATAAAAACTATACACTACATGGTGGTTTTCGTTTAGATAAGT
TGAATATAGATAATACATTTGTCATGTCAAAGAAAGAAATGTGTGTCTTCCCTAATTTCATAGTGCGTGCCATTAATAAA
TTTCCAAAATTATCTTTATGTAGCAAAAGGTTTGAGTCAGAGCAATACTCAAAGAAGATAAATGGCAGCATGAAAGGAAG
TGTGGAGTTTATAGGTTTTTGGCAAAATGAGAGGTATTTTTTAGAGCATAAAGAAAAATTAAGAGAGATATTTACACCTG
TCAATATAAATTTGGATGCTAAAGAATTAAGTGATGTAATTCGATGCACCAATTCAGTCTCCGTGCATATACGCCGCGGA
GATTATGTATCGAACGTCGATGCGTTAAAAGTACATGGGTTGTGTACTGAAAGATATTATATTGACAGCATTCGTTATTT
AAAGGAAAGGTTTAATGACTTGGTTTTTTTTGTATTTAGTGATGATATCGAATGGTGTAAGAAACATAAAAATGAAATAT
TTTCAAGGAGTGATGATGTGAAGTTTATTGAAGGGAATACTCAAGAGGTAGATATGTGGTTAATGTCAAATGCTAAATAT
CATATAATAGCTAATAGTTCATTTAGTTGGTGGGGGGCGTGGCTAAAAACTATGATTTAGGTATTACAATTGCGCCAAC
GCCTTGGTTTGAGAGAGAAGAACTTAATAGTTTTGATCCATGCCCAGAGAAATGGGTTCGAATTGAAAAATAA
>Cluster_4
ATGAGTTTAACCATAGCTTTAGCCATAGACTTTAAAAAGTTAACCCCAAATGTGATAACCTCTGGCCTTTTCTTGGCTGA
CCATTTGCCAGAGGTTTCTAACATCATACTTTTTCATGCCATCGAATACTTTCTAACTCCTCCTGCCTACCTTCAACCTT
ATTTAGAAAAGGAAAAACAAAAGTTAGACCAAGATTTAAAGGAACTTGTTAGTTCCTATTTAGACGAAAAATTCAAAAAA
CGATTTACTTTTCAGACTAAAGTCATTTTAGGTAACTTTTGGTATTCCCTTACCAATTTTATCGAGCAGGTCAAACCCTT
TGTAGTAGTTTTAGGGTATGTCCCCCATTTTCTAAAAGTCCCTACCGCAGAGAAAATGATCGAAAGGCTTAACTGTAATT
TCTTAGTGGTTAAAGAAACCTCTCTTACCAAGATCCAAAGATTAGGTTGTCTTATAGATTTTTCTGAAACAAGCGAATCC
TGTTTAAAAATGGCTTCTCTTTTAAGCAAAAAAAACGTCTTAGAAATATCTTCCCTAAACGTAATACAACCGCCTGATTT
TCTAAAAAAAACTGTGTTAAACAATGACCCTATCAAAGAAGAACTTGAGCAAAGATATACTTTATGGAACAAACTTATAG
CCAAATTTAGTGATGAACAGGAGTTTGTCCAAAAAATAAAGTTTGAAGTAACAGTAGGTAACAGGCTTGAAAAAATAGAA
GAATTTGTTAAAAACCAGGCAATCGACCTGTTAATCATGGGAAAAAAAGGTAAAATGGTTCAAAAAGGGTTAGGTTCTAT
TGCCAGAGAAGTCGTTAGAAGGATTGAAATACCTATGTTGATAGTAGATCAAGTTTTTTTATAG
>Cluster_13121
ATGCGTAAATATCCTTTAAGTTTACTGAAGGATAAAAATATTGTGACTTTTTTTGATTTCTGGGGAAAAACCCGACGCGG
CGAGAAAGACGGGGGCGACGACTATCACCTTCTTTGCTGGCATTCGCTGGATGTAGCCGCGATGGGCTATTTAATGGTTA
AAAGAAATTGCTTCGGGCTGGCTGATTATTTTCGTCAATTAGGGATTTCTGACAAGGAACAGGCGGCTCAATTTTTCGCC
TGGTTGCTGTGCTGGCACGATATTGGAAAATTTGCCCGCTCTTTTCAACAACTTTACCTGCCCCCTGAACTCAAGATTCA
GGAAGGTGCCAGAAAAAATTACGAAAAAATCTCTCATTCAACGCTGGGCTACTGGTTGTGGAATCATTATTTAAGTGAAT
GTCAGGAGTTACTTCCTTCATCTTCGCTCTCTCCGCGCAAACTTAGGCGAGTGATAGAAATGTGGATGCCCGTGACGACA
GGGCATCATGGTCGGCCCCCTGATCGTATGGATGAATTGGATAATTTTCTGCCTGAAGACAAAGCTGCAGCACGAGATTT
TCTCCTTGAAATCAAACCACTGTTTCCGCTCATAGAGATCCCCGCATTCTGGGATGATGATGAGGGCATTGAACTTATAA
AACACCTTTCCTGGTATATCTCTGCAACCGTCGTGCTCGCAGACTGGACGGGTTCGTCAACGCGATTTTTTCCACGCGTC
GCACACCCAATGGATATTAAAGGTTACTGGCAGAAAACTTTAATTCAGGCTCAAAACGCCTTAACCGTCTTTCCTCTAAA
AGCAAAAGTCGCGCCTTTCAACGGAATTAATACGCTGTTTCCTTTTATTGAGAATCCGACACCGTGA
>Cluster_33601
ATGCAAAAACAGCATAACGTCGTAGCGGGCGTGGATATGGGGGCAACGCATATCCGCTTTTGTCTGCGGACAGCAGAAGG
TGAAAAGCTACACTGCGAAAAAAAGCGGACCGCAGAAGTTATTGCTCCCGACCTGGTGTCGGGTATCGGCGAAATGATTG
ACGAGCAACTCAGGCGCTTTAACGCTCGCTGTCATGGTCTGGTGATGGGATTTCCGGCGCTGGTCAGTAAAGATAAACGC
ACCATTATTTCTACGCCTAACCTGCCGTTAACAGCGGCAGATTTATATGATCTCGCCGATAAGCTCGAAAATACGCTGAA
TTGTCCGGTTGAGTTTTCCCGCGACGTTAACCTGCAACTTTCCTGGGACGTAGTAGAAAACCGCCTTACGCAACAACTGG
TTCTGGCAGCCTATCTCGGTACGGGGATGGGGTTCGCAGTGTGGATGAACGGTGCGCCGTGGACGGGTGCACACGGTGTG
GCAGGCGAACTGGGCCATATCCCCCTGGGAGATATGACCCAACACTGCGCGTGTGGCAATTCTGGGTGCCTGGAAACCAA
TTGCTCTGGAATGGCGCTAAGACGCTGGTACGAACAACAGCCTCGAAATTACCCATTGAGCGATCTTTTCGTCCATGCGG
AAGACGCCCCTTTCGTCCAGAGCTTGCTTGAAAACGCGGCACGAGCCATTGCCACCAGCATTAATCTGTTCGATCCCGAT
GCGGTGATTCTGGGCGGTGGCGTGATGGATATGCCCGCCTTCCCACGCGAGACTCTCATTGCCATGACCCAAAAGTACCT
GCGCCGTCCACTGCCGTATCAGGTCGTGCGCTTTATTGCCGCCTCATCTTCTGACTTTAATGGCGCTCAGGGTGCAGCAA
TATTGGCGCATCAACGTTTTTTGCCACAGTCCTGTGCTAAAGTCCCATGA
>Cluster_33565
ATGATCAATATAGATCTCAGAGGTTTGACCCCGGCACCGGTTACCCCTTTCAATCGTGATGGTAGTATTGATTTTGATGC
TATCCAGCGTCTGGGAAGCTGGCTGGGTAGTATTGACGGCGTTAAAGGGTTAGTTGTACTTGGTCATGCTGGTGAAGGTA
CTTTCCTGACACAAGATGAACAGGCCGCTGTGATCCGTGCTTTCGTAAAATCCGTAGATGGCAAGCTGCCTATCATTGCC
GGTATCACCGGTGAAGGGACTGACGTCGCTGCTAAAGAAGCCAAACGTGCCGTGGAAGCTGGTGCTTCAGCAGGCCTGTT
ATATCCTTCTCATGGATGGTTGCGTTTCGGCTACCAGGACGGCGCACCACAAGATCGCTACCGCGTTGTCTATGAAGAAA
GCGGCCTGCCCTTGATCCTGTTTCAGTATCCTGATGTGACTAAAGCAACCTATAATCTGCAAACACTGCTGGATATTTCC
GCACAACCCGGTGTTTTTGCTATGAAAAATGGTGTACGCAATATGCGTCGTTGGGATACAGAGATCCCGGTTATCCGCCG
CGAGCGTCCGGAATTACAGATCCTCAGTTGCCATGATGAATATCTGGTACACACTGCTTTTGACGTAGATGGTTTCCTTG
TCGGCTATGGCAATATTGCGCCAGAACCGCTCATCGAGATGATCAAAGCAGGTAAAGCTGGCGATTATAAAAAAGCACGC
GCGATACATGACCGTCTGCTGCCAGTAACGAAGTGCGTATATCATCGCGGTTCCCATATGGAAGGAACCGTCGCCCTGAA
ACACGCGCTGGTTGCTCGCGGTATTCTCGAACATGCTACTGTTCGTTCACCGCTATTACCGCTGGAGCATGGTGCCGAAG
AAGAAATTCATGCTGCTATGCGTGCAGCTGACCTGCCGCGCGTTAAATAA
>Cluster_9134
ATGTCGTTAATTAAGGTGCCTTGTATGCAAATCACCAATATGCATTGCAGTGGACAGACCGTTTCACTTGCCGCGGGGGA
TTATCACGCGACCATTGTGACCGTCGGTGCTGGTCTGGCTGAATTAACCTTTCAGGGTTGTCATTTAGTCATCCCGCACA
AACCCGAAGAGATGCCGCTGGCGCATCTGGGTAAAGTGCTTATTCCGTGGCCGAACCGGATCGCCAACGGCTGTTACCGC
TATCAGGGGCAGGAATATCAGTTACCCATTAATGAACATAGTTCAAAAGCCGCTATTCACGGGCTGCTTGCCTGGCGAGA
CTGGCAAATTAGTGAATTAACCGCCACCAGCGTTACCCTGACAGCTTTTCTGCCGCCCAGTTACGGCTATCCGTTTATGC
TGGCTTCACAAGTTGTTTATTCGCTCAACGCACACACGGGTCTTTCCGTTGAGATCGCAAGCCAGAATATCGGCACTGTC
GCAGCGCCTTATGGCGTGGGTATTCATCCTTATTTAACCTGCAACTTAACATCTGTTGATGAATATCTGTTCCAGCTTCC
GGCAAACCAGGTTTATGCCGTCGATGAGCACGCGAATCCGACAACGCTTCACCACGTTGATGAGCTGGATTTAAATTTCA
CGCAGGCGAAAAAAATAGCCGCGACAAAAATCGACCACACCTTTAAAACCGCTAATGATTTGTGGGAAATGACCATTACC
CATCCACAGCAGGCGCTTTCGGTAAGCCTTTGTTCCGATCAACTCTGGGTGCAGGTTTATAGCGGTGAAAAATTACAGCG
TCAGGGACTGGCAGTTGAACCTATGAGTTGCCCGCCTAACGCCTTTAATTCAGGTATCGACTTATTATTACTGGAATCAG

GAAAACCGCACCGCCTGTTTTTAATATTTACGGACAGAGAAAATAA
>Cluster_22280
ATGAGTAGCAAGATCCTCGGTAATGTCTGGGATGCATGCGCAGCATATGGCGTCAAAGGTGCAAAACTGATGATTATGGC
GCGCCTGGCTGATTATTCGAATGATGACGGGGTGTGCTACCCGGGTGTTGAAACCATATGTCGACAGCTTGGATTGGGAG
AAAGTACAGTCAGAACGGCAATCTCCGAACTGGAGGCTGATGGCTGGCTGACGCGTCAGTCACGCCGCAAAGGTAACCGT
AATACGTCCAATCTTTATCATCTGAATGCTGATCGGCTTGAGCAGCTTGCCAGAACTGAGCGGGATAAGGTTGCAGAACT
GAAACAGCAGCGCAGACTTTCAGTATTACGTGACCCTTCAGATTCTGAACCTTCAAAATCTGAACCGTCAGAATCTGTAT
GTTCAGGCGTGTTTGACCCTTCAGATTCTGGCAAAAATACGCGTTTGACCCTTCAGAATCTGACTCCAGATCCACAAGGT
TTAAAACATGAACCACCAGTAAATTCAAAACATGAACCGCAAGATATTGGCGCATCCGCTGACGCGTCTGCACCAGCGCG
TTCTGCCAGACAGGAATATTCACCGGAATTTGAACAGGCCTGGCAGGAATATCCCAAACGTGCTGGTGGCAATTCCAAGT
CAGCAGCCTTCAAAGCCTGGAAAGCCCGTATCAGGGAGGGAATAAAACCGGAGACCATGCTTGATGGCGTGAAGCGGTAT
GCCGCCTGGGTACGTGCTACAGGAAATACCGGCACACAGTTCGTGAAGCAGGCTGCGACGTTCTTTGGACCCGATCGTCA
CTTCGAAGATTACTGGCAACAGCCAGCCGCTCACGGAGGTGGGCGACAGCGACAGGTCGATGTCCTGGCTGGCCTGGGAG
CCATGTCTGACAAATTCGGTAAATCCAGTAACAAATTGACATTCTGA
>Cluster_27206
ATGTCTGAATTAAAAATTCCCCCTGAATTATTACAAATTTCCCCGGAAGTGCAGGACGCTTTAAAAAACAAAAAACCGGT
TGTGGCGCTGGAATCGACCATTATTTCTCACGGGATGCCGTTCCCACAAAATGCCCAGACCGCAATTGAAGTTGAAGAAA
CTATTCGTAAACAGGGCGCAGTACCTGCCACTATCGCCATTATCGGTGGCGTGATGAAAGTGGGTTTAAGCAAAGAAGAA
ATTGAATTACTGGGTCGTGAAGGGCATAACGTGACCAAAGTTAGTCGTCGCGATTTACCTTTTGTTGTTGCCGCCGGAAA
AAATGGCGCAACCACTGTGGCTTCAACGATGATTATTGCGGCGCTTGCCGGAATTAAAGTATTTGCCACCGGGGGAATTG
GTGGTGTTCATCGCGGGGCGGAACATACCTTCGATATTTCTGCCGATTTGCAAGAACTGGCAAATACTAATGTCACCGTT
GTTTGTGCCGGGGCGAAATCTATTCTCGATTTAGGCTTAACTACCGAATATTTAGAAACTTTCGGTGTACCGCTAATTGG
CTATCAGACCAAAGCTCTGCCTGCGTTTTTCTGCCGCACCAGCCCGTTTGACGTGAGCATTCGTCTCGACAGCGCCAGCG
AAATTGCCCGTGCAATGGCGGTGAAATGGCAAAGCGGGCTGAACGGTGGCCTCGTGGTAGCGAACCCGATCCCGGAACAG
TTTGCTATGCCGGAACACACTATCAATGCGGCGATCGATCAGGCGGTAGCTGAAGCTGAAGCGCAGGGTGTTATTGGTAA
AGAAAGTACGCCATTCCTGCTGGCGCGCGTTGCTGAGTTGACCGGCGGTGACAGCCTGAAATCCAACATCCAGCTGGTGT
TCAACAACGCCATCCTGGCGAGTGAAATAGCCAAAGAATACCAGCGTCTCGCGGGTTAA
>Cluster_25826
ATGAACACGTTTTCTCAAGTCTGGGTATTCAGCGATACCCCTTCTCGTCTGCCGGAACTGATGAACGGTGCGCAGGCTTT
AGCTAATCAAATCAACACGTTCGTCCTCAATGATGCCGACGGCGCACAGGCAATCCAGCTCGGCGCTAATCATGTCTGGA
AATTAAGCGGCAAACCGGACGAGCGGATGATCGAAGATTACGCCGGTGTCATGGCTGACACTATTCGCCAGCACGGCGCA
GACGGTCTGGTACTGTTGCCAAACACCCGTCGCGGCAAATTACTGGCGGCGAAACTGGGTTACCGCCTTAACGCGGCGGT
GTCTAACGATGCCAGCGCTGTTAGCGTGCAGGACGGTAAAGCGACAGTGAAACACATGGTTTACGGCGGTCTGGCGATTG
GCGAAGAACGCATTGCCACGCCGTATGCGGTACTGACCATCAGCAGCGGCACGTTCGATGCGGCTCAGCCAGACGCGTCA
CGCACTGGCGAAACGCACACCGTGGAGTGGCAGGCTCCGGCTGTGGCGATTACCCGCACGGCAACCCAGGCGCGTCAGAG
CAACAGCGTCGATCTCGACAAAGCCCGCCTGGTGGTCAGCGTTGGTCGCGGTATTGGCAGCAAAGAGAACATTGCGCTGG
CAGAACAGCTTTGCAAGGCGATAGGTGCGGAGTTGGCCTGTTCTCGTCCGGTGGCGGAAAACGAAAAATGGATGGAGCAC
GAACGCTATGTCGGTATCTCCAACCTGATGCTGAAACCTGAACTGTACCTGGCGGTGGGGATCTCCGGGCAGATCCAGCA
CATGGTTGGCGCTAATGCGTCGCAAACCATTTTCGCCATCAACAAAGATAAAAATGCGCCGATCTTCCAGTTCGCGGATT
ACGGCATTGTTGGCGACGCCGTGAAGATCCTTCCGGCACTGACCGCAGCTTTAGCGCGTTGA
>Cluster_53483
ATGGAAAACAAACCAACACTGGTTATCGCTCTCGGCGGTAATGCCCTGCTTAAACGAGGCGAACCACTCGAAGCAGAAAT
CCAACGTAAAAATATCGATCTTGCGGCTAAAACTATCGCCCAACTGACTCAACACTGGCGGGTTGTACTGGTTCACGGGA
ATGGCCCACAAGTTGGACTTCTGGCACTTCAAAATAGCGCCTATGCCCATGTCGCTCCCTATCCACTCGACATTCTGGGG
GCCGAAAGCCAGGGGATGATCGGCTACATGCTACAACAGGCGCTGAAAAATCAACTACTACAACGTGAAATCAGCGTGCT
GCTGACGCAGGTTGAAGTGGATGCCAACGATCCCGCCTTCAGTAACCCAACTAAGTACATCGGCCCAATTTATGACCACG
CTCAGACTCAGGTACTACAGGCAGAAAAAGGCTGGGTTTTCAAAGCAGATGGTCACTCTTTCCGCCGGGTCGTACCTTCT
CCACAACCTAAACGCATCGTCGAGCGGGATGCCATCCAGACACTGATCGCCCACGATCATCTGGTGATCTGTAATGGTGG
CGGTGGTGTTCCGGTGGTTGAAAAAGCCGATGGATACCATGGGATTGAAGCGGTTATCGACAAAGATCTTTCAGCCGCGC
TACTGGCAAGTCAGATCCATGCTGACGCCTTGTTGATCCTCACTGATGCGGACGCAGTATATCTCGACTGGGGCAAGCCA
ACCCAACGCCCGCTGGCCCAGGTCACGCCGGAATTACTCAACGAAATGCAGTTTGATGCCGGGTCTATGGGGCCGAAAGT
CACCGCCTGCGCAAAGTTTGTTAGTCAGTGCCGGGGCATTGCAGGGATCGGCTCTCTTGCAGATGGTCCGGAAATTCTCG
CCGGAGACAAAGGCACTCTTATTCGCCTTGATACCCCCCATAACCACGCTTGA
>Cluster_23022
ATGGCCGCCGTTAACTTACGTCATATTGAAATTTTTCATGCGGTAATGACCGCCGGAAGCCTGACTGAGGCGGCACACCT
GCTACACACCTCACAGCCAACCGTCAGCCGCGAACTGGCGCGCTTTGAGAAGGTGATCGGGCTGAAATTGTTTGAGCGCA
TACGTGGACGATTACATCCTACCGTGCAAGGACTGCGTCTGTTTGAAGAAGTGCAACGATCCTGGTACGGACTGGATCGC
ATTGTCAGCGCCGCAGAAAGTCTGCGCGAGTTTCGCCAGGGAGAACTGTCTATTGCCTGCCTGCCGGTCTTTTCGCAATC
TTTTTTACCGCAGCTCCTGCAACCCTTTCTGGCACGTTATCCCGATGTCAGCTTAAACATCGTGCCCCAGGAATCACCGC
TACTTGAAGAGTGGCTCTCGGCCCAGCGTCATGATTTAGGACTCACTGAAACGCTCCATACGCCTGCGGGAACAGAACGT
ACCGAATTACTCTCTTTAGATGAAGTGTGTGTGTTACCTCCGGGCCATCCGCTGGCGGTAAAAAAGGTATTAACGCCGGA
TGATTTTCACGGTGAGAACTACATCAGCCTTTCCCGTACTGACAGCTATCGCCAGTTGCTGGATCAATTGTTTACTGAGA
ATCAGGTTAAACGACGCATGATCGTAGAAACCCACAGCGCCGCGTCAGTCTGCGCAATGGTACGGGCGGGGGTAGGCGTT
TCGGTGGTTAACCCGCTCACCGCACTGGATTATGCGGCAAGCGGTTTAGTGGTGCGGCGGTTCAGCATTGCGGTTCCATT
CACCGTCAGCCTGATCCGCCCCCTGCACCGCCCGTCATCAGCGCTGGTGCAGGCGTTTAGTGAGCATTTACAAGCGGGAT
TACCGAAACTGGTCACTTCTCTTGACGCTATTTTGTCGTCAGCTACGACAGCATAA
>Cluster_15050
ATGAAAACAATTGAAGGGATTATTCCGGTAATGCTAACGCCTTTTACGGCGGATAATAAAATTGATTACCTGGGTCTTAA
ACGGCTAATTGACTGGTATATCGAAAACGGTGCCGACGCTTTATTTGCCGTGTGTCAGTCCAGTGAAATGCAGTATTTAA
CTTTAGAAGAGCGCGTGGAGCTGGCGAGCTTTGTGGTTGAATATAGCCAACAACGAGTACCGGTTATTGCGTCCGGGCAT
ATTTCTGATGATCTGGAACAGCAAATTCAGGAGCTCAATGCTATGGCGGCAACGGGCATTGATGCGCTGGTGTTAGTGAC
GAATCATCTTGATCCACATAACGAAGGCACCACGGTATTCCTCCGTACGCTTGATGCGCTATTGATGTCATTACCCGAAA
GTATGGCGCTGGGTCTGTATGAATGCCCGGCCCCGTATCGCCGCTTATTAACCGATGAGGAACTGCTTTACTGCGCACAA
AGCGGGCGTTTTCAAATCTTAAAAGACGTCAGTTGTGATCTACCTACAGTGACGCGACGGGTGCAATTAACCCAGCACAC
GCCAATGACGATTGTTAATGCTAATGCTGCCATTGCCTGGGAGGCGATGCTTGCAGGTTCGAAAGGTTTTTGTGGCGTCT
TTACCAACTTTCATCCTGACTTGTATCACTGGCTTTGGCTTGAAGGAAAACAACACCCTGAAGAGGCAAAACAACTCGCC
TGGTTTTTATCGCTGAGTGCCGTCAGTGAAAATATTGGCTATCCGAAAAATGCCAAAATATTTCATCAGCAGCGAGGAAC
ATTTGACTGTGTTCATTGCCGGGTAACGGCTGACGATGTGCTGAAAAAATATTGGGGGCTGGAGGTGGTACTAAAACAAA
TTGCTGATGCCGCAGATTTCCAGCGTCAACAACTCAAATATTAA

>Cluster_14742
ATGAGTCGTTCACAAAATTTACGCCACAATGTGATTAACCAGGTCATTGATGATATGGCGCGGGGTCATATCCCTTCCCC
ACTTCCATCGCAAAGTGCGCTGGCGGAAATGTACAACATCAGCCGCACCACAGTGCGTCATATTCTCAGCCACTTACGCG
AATGCGGCGTCTTGACGCAGGTTGGAAACGACTATGTTATTGCGCGTAAACCTGACCATGATGACGGTTTCGCCTGTACT
ACCGCTTCAATGAGTGAACAAAACAAAGTGTTTGAGCAGGCTTTTTTCACGATGATAAACCAGCGCCAGTTACGCCCAGG
GGAAACGTTTTCTGAACTGCAACTGGCTCGGGCCGCGGGTGTCAGCCCGGTTGTAGTAAGAGAATATCTTTTAAAATTCG
GCCGTTATAATCTTATTCACAGCGAAAAGCGCGGCCAGTGGAGCATGAAGCAATTCGATCAATCCTACGCCGAGCAGCTG
TTTGAGCTACGCGAAATGCTCGAAACACATTCACTTCAGCACTTTCTCAACCTGCCTGATCATGATCCCCGCTGGCTGCA
GGCGAAAACCATGCTCGAACGTCATCGGCTACTGCGTGACAACATCGGCAATAGCTTTCGCATGTTCTCGCAACTTGACA
GAGATTTTCACTCCTTACTGCTTTCAGCTGCCGACAATATCTTTTTTGATCAATCGCTTGAGATCATCTCAGTCATCTTT
CATTTTCACTATCAATGGGACGAAAGCGATCTTAAACAGCGCAATATCATTGCCGTCGATGAGCATATGACCATTCTCAG
CGCTCTGATTTGCCGCAGTGACCTTGATGCCACCCTTGCGCTCCGTAACCATTTGAATTCAGCTAAACAATCAATGATTC
GATCCATTAATGAGAACACGCGTTATGCTCATTAA
>Cluster_35561
ATGGGTAAAACAATCAGTATAAAGGTCCTGTTCGGTATTTATCTTCTATTAATGGCAGGCAAAGTTTTTGCCTTCAGTTG
TAATGTCGATGGCGGGTCAAGTATTGGAGCAGGTACAACCTCTGTTTATGTTAATCTCGACCCTGTAATACAGCCGGGCC
AGAATCTGGTTGTAGACTTGTCTCAGCATATAAGTTGCTGGAATGATTACGGCGGCTGGTACGACACTGATCATATAAAC
CTGGTACAAGGCTCAGCTTTCGCAGGCTCCCTACAGTCATATAAAGGATCGCTGTACTGGAATAATGTGACCTACCCCTT
CCCGTTGACGACAAATACCAACGTATTGGATATTGGTGATAAGACACCGATGCCATTACCGTTAAAACTCTATATTACCC
CAGTTGGGGCGGCCGGTGGAGTTGTCATAAAAGCGGGGGAAGTCATTGCCCGTATTCATATGTATAAAATTGCAACATTA
GGAAGCGGAAATCCGCGTAATTTTACGTGGAATATTATTTCAAATAACAATGTTGTTATGCCAACAGGCGGCTGCACCGT
TGATAGTCGTAATGTCACCGTGGATCTCCCGGACTTTCCTGGAAGCGCAGAAATACCCCTTGGCGTATATTGCTCAAGCG
AACAAAAGCTCTCATTTTATCTTTCGGGTGCCACCACTGACAGTAGCCGACAAGTGTTTGCTAACACAGCACCTGATGCA
ACAAAAGCATCGGGAGTGGGTGTCACTCTAATGCGTAATGGAAAAATACTGGCTACGGGAGAAAATGTCTCCCTGGGGAC
AGTAAATAAAAGCAAAGTACCTCTGGGTTTAAGCGCAACCTATGGACAAACAGGAAACAAGGTTTCAGCCGGAACCGTCC
AGTCAGTCATTGGTGTCACTTTTATCTATGAATAA
>Cluster_19637
ATGGCTAACTCACCAGTTCTTGAACTTCAAGCACTAGCGTGCGATCCCGACTCCGACATTTTATCTGTACTCATGAAGGC
CAAAATGATTGCAGTGAAGCTCGGCCTCAGCGATTTGACCGAGTGGATACAGCTCGAATTAAATGGCTATCCCAGCTTTG
ATACGGTTCCTGAATACAGGAAAGGTCAATCGCAGTTAAAAGCATATAGTCCCCAACATCAGAGCTGGGTTCCGCTTGTG
GTCAACTTCAACGACGCGGAATTAAATGCAATGGTATCAACGTTTCACATTACAGAATCAATATCATCTATAAAAAAGAT
GAGCAATAAAGGAGAAAACCCTTTTTTACCTCTCCCGGATTGGATGGCTGATACATTAGCTCAAAGTATCGGACAAAAGA
CTGAGATGGTATGGGTTATAAGCGCTGGTGTACTAGAACATGTAATTACTACCGTTAGGAATAAGATCCTCGACTGGTCT
TTAGACTTAGAGTCAAAGGGTATTTTAGGCGAAGGGTTACAGTTTTCATTAAGAGAAAAAGAGGTAGCGCCAATGACTGT
CCATAACGTGACCAACATTCATGGCAACGTAAACAATGCTGGCGTTATTGGCTCTGGTAACGGGGACATTTCACAAAATA
ATACTGTCAACGCTGGAGACTTCAACTCGCTTGAAAAGCAGCTCAAGGATTGGGGCGCTAATGATGAAGATATTAAGAGC
CTTCATCAAGCTATACAAGAATCACCCGCCCCAACTACCCCGGACAATTTCAGTGATAGCCTTGGGAAGTGGGTTGTAAA
TATGATTGGTAAAGCATATGCGGGGACGCTAAATATTGCAGCGTCTGCTGCACCAGCTCTACTAACTAATGCAATTTGTC
ATTACTACAACATTCCTGTTTGA
>Cluster_66976
ATGATGCAATCTGAACATACAGCGCCTTGTCCGACAACATCACTCAGTCTTCCTGCCCTACTTTGGGATACCCGATCGGA
GATCTCTGAAAGCGAGCTTGCGGCACTGGATACATTGGTCGACCACTTCCAGCAAGGAGGTAAAAACTGGTCACCTGATA
TACAAAAACGGCTTTCCCGCCTTCTTTTACCACTACGCGACACGCTAACAAAAATGCATGCGGCCAAGGCCCCCTACAAT
TCATCTATTCACGATATAGTCCTAGAGATGCAGCGCATCCGGAAAACATACTGGGCCTGGACACAAGAAGAGTGGTTAGA
AGTTATTTGCAACAGCGAAGGGGAATTTCGTCGACGGTTTGGAGCCAGCGGCAACTGTCGGCAATATGTTATAGCGTTAG
CATGGTTGCTATGTGGGTTTGAGCGGCTGGAACACTGTGGCATATTCTATCAATATCGGCTCTGTCTTAAGGTTTTTGGC
CGACAGAGCACCGATTTTGCCGTCAGCCAGCTTGACAATATGATGCAGGTTCTGGGATATGTGCCTCGTGATAGTCGGAA
CAACGGCATCAGAAATGCCATGTGTATGGCAATGCTTCTGCAAAGGGATGCTCAACTAGACCATATCACCGTGACCACGT
TGCAACAGATTGCTGCAACGTGCCCGGATTCCCTCCGGGAAGCCTCAGCTACTCTATCGCGTATACTGGCTGCATCAGGG
ACTATTGAGGAAGGGGTTGATTACCGAATAACACAACGTCGCCGTCCTCCCCGCGAATATAATGCAACAGCTGATGTACC
AACTAAATGGCTGGTATGGTGCAAACGGTGGCGTGCTACCTCAGTACTTCGCCCTTCATCTATTTTGTCCGGTTGGTACG
TTTTGCTAAAGTGTGGTCAGCTGGTTAGCTGA
>Cluster_31957
ATGTCAGCCAAAGTGTGGGTTTTAGGGGATGCGGTCGTAGATCTCTTGCCAGAATCAGACGGGCGGCTACTGCCTTGTCC
TGGCGGTGCGCCAGCTAACGTTGCGGTGGGAATCGCCAGATTAGGCGGAACAAGTGGGTTTATAGGTCGGGTCGGTGATG
ATCCTTTTGGTGCGTTAATGCAAAGAACGCTGCTAACTGAGGGTGTCGATATCACGTATCTGAAGCAAGATGAATGGCAC
CGGACATCCACGGTGCTTGTCGATCTGAACGATCAAGGAGAACGTTCATTTACGTTTATGGTCCGCCCCAGTGCCGATCT
TTTTTAGAGACGACAGACTTGCCCTGCTGGCGACATGGCGAATGGTTACATCTCTGTTCAATTGCGTTGTCTGCCGAGC
CTTCGCGTACCAGCGCATTTACTGCGATGACGGCGATCCGGCATGCCGGAGGTTTTGTCAGCTTCGATCCCAATATTCGT
GAAGATCTATGGCAAGACGAGCATTTGCTCCGCTTGTGTTTGCGGCAGGCGCTACAACTGGCGGATGTCGTCAAGCTCTC
GGAAGAAGAATGGCGACTTATCAGTGGAAAAACACAGAACGATCGGGATATATGCGCCCTGGCAAAAGAGTATGAGATCG
CCATGCTGTTGGTGACTAAAGGTGCAGAAGGGGTGGTGGTCTGTTATCGAGGACAAGTTCACCATTTTGCTGGAATGTCT
GTGAATTGTGTCGATAGCACGGGGGCGGGAGATGCGTTCGTTGCCGGGTTACTCACAGGTCTGTCCTCTACGGGGTTATC
TACAGATGAGAGAGAAATGCGACGAATTATCGATCTCGCTCAACGTTGCGGAGCGCTTGCAGTAACAGCGAAAGGGGCAA
TGACAGCGCTGCCATGTCGACAAGAACTGGAAAGTGAGAAGTAA
>Cluster_37736
ATGGGTTCCACCAGAAAGGGGATGCTGAACGTTCTGATTGCCGCCGTGTTGTGGGGAAGTTCAGGGGTCTGCGCGCAATA
CATCATGGAGCAAAGCCAGATGTCGTCGCAGTTTTTGACTATGACGCGTTTGATATTCGCCGGTTTGATTCTACTGACGC
TGTCATTTGTTCATGGCGATAAAATCTTTTCTATTATTAACAATCATAAAGATGCCATTAGCCTGCTGATTTTTTCCGTG
GTTGGCGCGCTAACTGTACAGCTCACTTTTTTGCTAACCATCGAAAAATCGAACGCAGCCACGGCAACGGTGCTGCAATT
CCTCTCACCGACGATTATCGTCGCCTGGTTCTCACTGGTGCGTAAATCGCGCCCGGGCATTCTGGTTTTCTGCGCTATTT
TGACATCGCTGGTCGGGACTTTTTTATTGGTGACACACGGTAATCCGACGTCATTATCGATCTCTCCTGCCGCGTTGTTC
TGGGGCATTGCCTCGGCATTTGCTGCTGCATTCTATACCACCTATCCCTCAACGCTAATTGCCCGCTATGGCACGTTACC
AGTCGTCGGCTGGAGTATGCTGATTGGCGGTCTGATTCTGTTGCCTTTTTATGCCAGACAAGGAACAAACTTTGTCGTTA
ACGGCAGTTTGATTCTGGCGTTTTTTTATTTGGTGGTCATTGGTACGTCCCTGACATTTAGTCTGTACCTGAAAGGAGCA
CAATTAATTGGCGGTCCAAAAGCCAGCATTTTGAGCTGTGCAGAACCATTAAGTAGCGCGCTACTCTCTTTGCTGTTGCT
GGGGATCACGTTCACATTACCGGACTGGCTGGGAACGCTGCTGATTCTGTCATCGGTGATTTTGATTTCAATGGATTCCC
GTCGCCGCGCCAGAAAAATAAATCGTCCGGCGCGGCATGAGTGA
>Cluster_19916

ATGTCTCTGTTTCACTTAATTGCCCCATCGGGTTACTGCATTAAACAGCACGCCGCGCTGCGTGGTATCCAGCGCCTTAC
CGACGCGGGGCATCAGGTCAATAACGTCGAGGTTATTGCCCGTCGTTGTGAGCGTTTTGCGGGTACGGAAACGGAGCGTC
TGGAAGATCTCAATTCTCTTGCCAGACTGACTACCCCCAACACCATCGTGCTGTCTGTACGCGGCGGTTACGGTGCCAGT
CGTTTACTGGCAGATATTGACTGGCAGGCACTGGTGGCTCGCCAACAACATGACCCGTTACTCATTTGCGGACATAGCGA
TTTTACCGCCATTCAGTGCGGTCTTCTGGCGCAAGGCAATGTCATCACCTTTAGCGGCCCCATGCTGGTAGCAAACTTTG
GCGCGGATGAACTAAATGCCTTTACCGAGCACCACTTCTGGCTGGCGTTACGCAATAAAACGTTCACCATTGAATGGCAA
GGGGAAGGCCCGACATGTCAGACCGAAGGCACGCTGTGGGGAGGCAATCTTGCGATGCTGATTTCACTGATTGGTACACC
GTGGATGCCAAAAATTGAGAACGGTATTCTGGTGCTGGAAGATATTAACGAGCACCCTTTCCGCGTCGAACGTATGCTGT
TGCAGCTCTATCATGCTGGAATTTTGCCACGTCAGAAGGCGATTATTCTCGGTAGCTTTAGCGGCAGCACGCCTAATGAT
TATGACGCAGGTTACAACCTGGAGTCAGTGTACGCGTTTTTGCGTTCCCGCCTGTCGATTCCACTCATTACCGGCCTCGA
TTTTGGTCATGAACAACGCACGGTTACGCTGCCGTTGGGCGCACATGCCATTCTGAATAACACCCAGGAAGGCACTCAGT
TGACGATTAGCGGTCATCCTGTTCTTAAAATGTAA
>Cluster_29122
ATGAAAATGAATAAAAGTCTCATCGCTCTCTGTTTATCAGCAGGGTTGCTGGCAAGCGCACCTGGAATCAGTCTTGCCGA
TGTTAACTACGTACCGCAAAACACCAGCGACGCGCCAGCCATTCCATCTGCCGCGCTGCAACAACTCACCTGGACACCGG
TCGATCAATCTAAAACCCAGACTACTCAACTGTCGACCGGCGGCCAACAACTGAATGTCCCTGGCATCAGTGGTCCGGTT
GCTGCGTACAGCGTCCCGGCAAACATTGGTGAACTGACACTGACGCTGACCAGCGAAGTGAACAAACAAACCAGCGTATT
TGCGCCGAATGTGCTGATTCTTGATCAGAACATGACCCCATCAGCCTTCTTCCCCAGCAGTTATTTCACCTACCAGGAAC
CCGGCGTGATGAGTGCAGATCGGCTGGAAGGCGTTATGCGCCTGACACCGGCGTTGGGGCAGCAAAAACTTTATGTTCTG
GTCTTTACCACGGAAAAAGATCTCCAGCAGACCACTCAACTGCTCGACCCGGCTAAAGCCTATGCCAAAGGCGTCGGTAA
CTCGATCCCGGATATTCCCGATCCGGTTGCTCGTCATACCACCGATGGCTTACTGAAACTGAAAGTGAAAACGAACTCCA
GCTCCAGCGTGTTGGTAGGACCACTATTTGGTTCTTCCGCTCCAGCTCCGGTTACGGTAGGCAACACGGCGGCATCAGCT
GTGGCTGCACCCGCTCCGGCACCGGTGAAGAAAAGCGAGCCGATGCTCAACGACACGGAAAGTTATTTTAATACCGCGAT
CAAAAACGCTGTCGCGAAAGGTGATGTTGATAAGGCGTTAAAACTGCTTGATGAAGCTGAACGCCTGGGGTCGACATCTG
CCCGTTCCACCTTTATCAGCAGTGTAAAAGGCAAGGGGTAA
>Cluster_42738
ATGGTGACGGTGTTCGGCATTCTGAATCTCACCGAGGACTCCTTCTTCGATGAGAGCCGGCGGCTAGACCCCGCCGGCGC
TGTCACCGCGGCGATCGAAATGCTGCGAGTCGGATCAGACGTCGTGGATGTCGGACCGGCCGCCAGCCATCCGGACGCGA
GGCCTGTATCGCCGGCCGATGAGATCAGACGTATTGCGCCGCTCTTAGACGCCCTGTCCGATCAGATGCACCGTGTTTCA
ATCGACAGCTTCCAACCGGAAACCCAGCGCTATGCGCTCAAGCGCGGCGTGGGCTACCTGAACGATATCCAAGGATTTCC
TGACCCTGCGCTCTATCCCGATATTGCTGAGGCGGACTGCAGGCTGGTGGTTATGCACTCAGCGCAGCGGGATGGCATCG
CCACCCGCACCGGTCACCTTCGACCCGAAGACGCGCTCGACGAGATTGTGCGGTTCTTCGAGGCGCGGGTTTCCGCCTTG
CGACGGAGCGGGGTCGCTGCCGACCGGCTCATCCTCGATCCGGGGATGGGATTTTTCTTGAGCCCCGCACCGGAAACATC
GCTGCACGTGCTGTCGAACCTTCAAAAGCTGAAGTCGGCGTTGGGGCTTCCGCTATTGGTCTCGGTGTCGCGGAAATCCT
TCTTGGGCGCCACCGTTGGCCTTCCTGTAAAGGATCTGGGTCCAGCGAGCCTTGCGGCGGAACTTCACGCGATCGGCAAT
GGCGCTGACTACGTCCGCACCCACGCGCCTGGAGATCTGCGGGCACTGTTGCAAAGTTAG
>Cluster_27642
TTGATTACAATTACATTCGACAAACTAATCACCTACATATCTTCAGTTGGTACTTTCATTTCAGCAATTGCTGCATTATA
TGCAATATGGTTAACGATATTTCAAAGAAGGCTCTCTTATAAACCCAATTTAGTGATAGACACTTTAAATGTCAAGATGA
ATGTTAACGATTTCAACGGTTTTCATGTTGATATCTTACAAGCCCCTACGCTTCCACAAACAAAATTTTCAAACATAGGA
TTGGGGGCGGCAATTTCACTTAGATATCATTGGGATTTTAATTATAAAAAAAACATAAATCTATACTTAGAGTTGTTTTC
GAAAAAATTCAAAAAAGAAGATAAATCTTTTACAACCGACTCCCAGTATGGAATGTTCAAAATAATAAAAGAAAGAAAAG
TATATATGTACAATACTTTATCGACACCTTACGATATTGATTTTTCGTTACCATATAGTATAGATAAGAAGTCAAAAAAC
ATTTTTGTACCAACCGCGGCAATTGATATTCTATTGAATATAGCATATCTTTCACATAGACTTAAATTAGTCGGTTCTTC
ATCATTCACTGGTCCAAAATTAATTATTGAATATCAAGATATAGAGGGTAAAACTAAAAAAGTCACTTGGCAAACAAAAT
TGGAACGTGGAGTGGCACGTTTCAGTGGTGATAATATGGAGGCAGATTTTGCACTACGGTTTACACCTGTACCAGAGAAA
TGGACTACAAAAGGTCTAGAGAAGATACGCAAAAGCGCCGCAAACGCCATGTTCAGATAA
>Cluster_27532
ATGTCCGAAACAAAAAACGAACTTGAAGACCTGCTGGAAAAAGCAGCAACCGAACCGGCGCACCGCCCGGCCTTTTTCCG
TACTCTACTGGAATCCACCGTCTGGGTGCCTGGTACGGCGGCGCAGGGCGAGGCTGTGGTTGAAGATAGCGCGCTTGATT
TACAGCACTGGGAAAAAGAAGACGGCACCAGCGTCATTCCTTTTTTACCTCGTTAGAAGCACTTCAACAGGCGGTTGAA
GGCGAACAGGCATTTGTCGTTATGCCCGTTCGCACATTGTTTGAGATGACCCTTGGCGAAACGCTCTTCCTTAATGCCAA
ACTGCCAACCGGTAAAGAATTTATGCCGCGTGAAATCAGTTTGCTGATTGGTGAAGAAGGGAATCCGCTGAGCAGTCAGG
AAGTTCTGGAAGGCGGTGAATCGCTGATATTATCGGAAGTCGCAGAGCCGCCAGCGCAAATGATTGATTCACTTACCACC
TTATTTAAAACCATTAAGCCGGTGAAGCGTGCTTTTATTTGTTCAATTAAAGAGAACGAAGAGGCACAGCCTAATTTACT
TATTGGCATTGAAGCCGATGGTGATATCGAAGAAATTATTCAGGCGGCGGGAAGTGTAGCGACCGATACATTACCTGGCG
ATGAACCCATCGATATTTGTCAGGTGAAAAAAGGGGAAAAAGGAATTAGCCACTTTATTACCGAACATATTGCGCCATTC
TATGAACGTCGCTGGGGTGGTTTTTTGCGTGACTTTAAACAGAACCGGATAATCTAA
>Cluster_61814
ATGATGAATCCATTAATTATCAAACTGGGCGGCGTACTGCTGGATAGTGAAGAGGCGCTGGAACGTCTGTTTAGCGCACT
GGTGAATTATCGTGAGTCACATCAGCGTCCGCTGGTGATTGTGCACGGCGGCGGTTGCGTGGTGGATGAGCTGATGAAAG
GGCTGAATCTGCCGGTGAAAAAGAAAAACGGCCTGCGGGTGACGCCTGCTGATCAGATAGACATTATCACCGGAGCACTG
GCGGGAACGGCAAATAAAACCCTGTTGGCATGGGCGAAGAAACATCAGATTGCGGCCGTAGGTTTGTTTCTCGGTGACGG
CGACAGCGTCAAAGTGACCCAGCTTGATGAAGAGTTAGGTCATGTTGGACTGGCGCAGCCAGGTTCGCCTAAGCTTATCA
ACTCCTTGCTGGAGAACGGTTATCTGCCGGTGGTCAGCTCCATTGGCGTAACAGACGAAGGGCAACTGATGAACGTCAAT
GCCGACCAGGCGGCAACGGCGCTGGCGGCAACGCTGGGCGCGGATCTGATTTTGCTCTCCGACGTCAGCGGCATTCTCGA
CGGCAAAGGGCAACGCATTGCCGAAATGACCGCCGCGAAAGCAGAACAACTGATTGAGCAGGGCATTATTACTGACGGCA
TGATAGTGAAAGTGAACGCGGCGCTGGATGCGGCCCGCACGCTGGGCCGTCCGGTAGATATCGCCTCCTGGCGTCATGCG
GAGCAGCTTCCGGCACTGTTTAACGGTATGCCGATGGGTACGCGGATTTTAGCTTAA
>Cluster_9139
ATGAGCCTTACCGAACTAACCGGTAACCCGCGGCACGACCAACTCCTCATGCTGATCGCCGAGCGTGGGTATATGAATAT
TGATGAGCTGGCAAATCTGCTGGATGTCTCCACGCAGACGGTCCGCCGGGATATTCGTAAATTAAGCGAGCAAGGCCTGA
TTACGCGCCATCACGGTGGCGCGGGTCGGGCTTCCAGCGTCGTTAATACGGCGTTCGAGCAGCGTGAGGTTTCGCAAACC
GAGGAAAAAAAAAGCGATTGCCGAAGCGGTGGCAGACTATATTCCTGATGGTTCAACAATATTTATCACCATTGGTACGAC
TGTTGAGCATGTTGCCCGGGCGTTACTTAACCATAATCATTTGCGGATAATCACCAACAGCCTGCGTGTGGCGCATATTC
TTTACCACAACCCGCGCTTTGAAGTGATGGTGCCCGGCGGTACGTTGCGCTCTCATAATAGCGGGATCATTGGCCCTTCA
GCGGCGTCCTTTGTGGCTGATTTTCGTGCTGATTATCTGGTAACAAGCGTTGGGGCGATTGAGAGCGATGGCGCGTTGAT
GGAGTTTGATGTAAACGAAGCTAACGTGGTGAAAACGATGATGGCGCACGCGAGAAATATTCTGCTGGTCGCCGATCACA
CTAAGTATCATGCTTCGGCGGCGGTTGAAATTGGTAACGTGGCACAGGTCACTGCGCTCTTTACCGACGAGCTGCCGCCC

GCTGCGCTAAAATCACGCTTACAAGACAGCCAAATTGAAATCATTCTTCCCCAGGAAGACGCGTAG
>Cluster_39255
ATGCTGGTTGCCGCAGGACAGTTTGCTGTTACATCTGTGTGGGAAAAGAACGCTGAGATTTGTGCCTCGTTGATGGCGCA
GGCGGCGGAAAACGAGGTTTCGCTGTTTGTCCTGCCGGAAGCATTACTGGCGCGTGATGATCATGATGCGGATCTTTCGG
TTAAATCAGCACAGCTGCTGGAAGGCGAATTCCTCGACCGTTTACGGCGAGAAAGTAAACGTAACATGATGACGACAATT
CTGACGATTCATGTTCCTTCAACACCGGGGCGCGCGTGGAATATGCTGGTGGCGCTACAGGCAGGAAACATCGTCGCCCG
TTATGCCAAACTGCATCTCTATGATGCATTTGCCATTCAGGAATCACGCCATGTTGATGCTGGTAATGAAATAGCTCCGT
TACTGGAGGTGGAAGGGATGAAGGTCGGTCTGATGACCTGTTATGACTTACGCTTTCCAGAGCTGGCGCTGGCACAGGCA
TTACAGGGAGCTGAAATCCTGGTACTTCCTGCCGCCTGGGTTCGTGGGCCGCTCAAAGAGCATCACTGGTCAACGTTGCT
TGCCGCTCGTGCGCTGGATACCACCTGTTATATGGTGGCGGCGGGGGAGTGCGGGAATAAAAATATCGGGCAAAGCCGGA
TTATAGATCCCTTTGGCGTCACCATTGCGGCAGCGTCAGAAATGCCTGCACTCATTATGGCGGAAGTGACGCCCGAACGT
GTGCGTCAGGTACGCGCGCAACTGCCCGTCTTAAACAACCGTCGCTTTGCGCCGCCGCAATTATTATGA
>Cluster_36440
ATGTTGGAATCAAGCAAAGTTCCCGCCCTGACACGGGCTATTGATATTCTCAATTTGATTGCCCGCATTGGCCCCTGTAG
TGCTGCGACGATCATTGACACATTGGGAATCCCTAAAAGCACGGCCTATTTGCTGCTTAATGAACTCAGGCGTCAGCGTT
TTCTTAGCCTTGATCACCAGGAAAATTTTTGTTTGTGGACCAGGCTGGTGGAGCTTTCCGGTCATGCGTTAAGCAAGATG
GATCTCCGGGAACTGGCTCGACCGCGTTTGACGCAACTGATGGATACCACCGGGCTGTTATGTCACCTGGGGATTATTGA
TAACGGCAGCGCTTATTACATTTTGAAAGTGGAGTCATCAGCCACTATCAGCGTGCGTTCCCATGAAGGAAAAAGCCTTT
CGCTTTATCGCTCCGGAATAGGTAAATGCCTGCTTGCCTGGCAACCTGCAGCGGTACAGCAAAGTATTATCGAAGGGCTG
GTCTGGGAGCAGGCGACACCCACAACGATTACTCATCCACAGCAACTTCACGAAGAACTGGCGCGTATTCGCCGTCAGGG
CTGGAGCTATGACAACGGCGAAGATTACGCCGATGTCCGTTGTGTCGCTGCGCCGGTGTTTAACGCCAATAACGAGCTTA
CCGCTGCGATCTCGGTGGTCGGTACCCGCTTACAAATCAATGAAGAATACCGTGATTATCTGGCGGGCAAGGCCATTGCC
TGCGCCAGGGATATTTCACGTCTGTTGGGATGGAAAAGTCCCTTTGACTTACAAGCCTCATAA
>Cluster_59772
ATGATTGAATTATTATTTCCCGGTTGGTTAGCCGGGATCATGCTCGCCTGTGCCGCGGGTCCGCTGGGTTCGTTTGTAGT
CTGGCGTCGTATGTCTTATTTCGGTGATACGCTGGCTCATGCCTCATTACTTGGCGTCGCGTTTGGTTTGTTGCTGGACG
TGAATCCATTCTATGCGGTGATTGCCGTTACGCTGCTGCTGGCGGGCGGTCTGGTATGGCTGGAGAAGCGTCCACAGCTG
GCGATCGACACGTTATTAGGGATTATGGCGCACAGTGCCCTGTCGCTGGGCCTGGTGGTCGTGAGTCTGATGTCTAATAT
TCGTGTTGATTTGATGGCTTACCTGTTCGGTGATTTGCTGGCAGTGACGCCAGAAGATCTCATCTCTATTGCGATTGGCG
TGGTCATCGTGGTGGCTATTTTGTTCTGGCAATGGCGCAATTTGCTGTCAATGACGATAAGCCCGGATCTGGCGTTTGTT
GATGGTGTGAAATTACAGCGAGTGAAATTGTTGTTGATGCTGGTGACGGCATTGACGATTGGTGTAGCGATGAAATTTGT
CGGTGCGTTGATTATTACTTCGTTGCTGATTATTCCTGCTGCTACTGCGCGTCGCTTTGCCCGCACGCCGGAACAGATGG
CTGGTGTCGCTGTTTTGGTGGGGATGGTGGCAGTGACTGGCGGTTTAACCTTTTCCGCGGTTTACGATACGCCGGCGGGT
CCGTCGGTGGTGCTATGTGCGGCACTGTTATTTATTCTCAGTATGATGAAAAAGCAGGCCAGCTAA
>Cluster_25909
ATGATGCATCTTTACTGGGTGGCGCTAAAAAGCATCTGGGCGAAAGAGATCCATCGCTTTATGCGTATCTGGGTGCAGAC
GCTGGTGCCGCCAGTCATCACCATGACCCTTTACTTTATTATCTTCGGTAACCTGATTGGTTCGCGTATTGGCGATATGC
ATGGCTTCAGCTACATGCAGTTCATCGTACCGGGGCTGATCATGATGTCGGTGATCACCAACGCCTACGCTAACGTCGCG
TCATCATTTTTTGGTGCCAAATTCCAGCGTAATATTGAAGAGCTGCTGGTAGCGCCGGTTCCGACTCATGTCATTATTGC
CGGATATGTCGGTGGTGGCGTGGCGCGTGGTCTGTTTGTTGGTATTTTGGTGACGGCAATTTCACTGTTTTTTGTGCCGT
TTCAGGTGCATTCGTGGGTATTCGTTGCCTTAACCCTGGTGCTCACTGCGGTGCTATTCTCCCTTGCGGGTTTGCTGAAC
GGCGTGTTTGCCAAAACGTTCGATGACATCAGCCTGGTGCCAACCTTTGTGTTAACGCCACTCACGTATTTGGGCGGGGT
GTTTTACTCCCTGACTTTGTTGCCGCCGTTCTGGCAAGGGCTGTCGCACCTGAACCCAATCGTTTATATGATCAGTGGTT
TCCGCTACGGCTTCCTCGGTATCAATGATGTTCCGCTGGTCACTACCTTTGGCGTACTGGTGGTCTTTATTGTGGCGTTT
TATTTGATCTGTTGGTCGCTGATCCAACGTGGACGTGGTTTGCGTAGCTAA
>Cluster_39414
ATGGCTGTAACTAAGCTGGTTCTGGTTCGTCATGGCGAAAGTCAGTGGAACAAAGAAAACCGTTTCACCGGTTGGTACGA
CGTGGATCTGTCTGAGAAAGGCGTAAGCGAAGCAAAAGCAGCAGGTAAGCTGCTGAAAGAGGAAGGTTACAGCTTTGACT
TTGCTTACACCTCTGTGCTGAAACGCGCTATTCATACCCTGTGGAATGTGCTGGACGAACTGGATCAGGCATGGCTGCCC
GTTGAGAAATCCTGGAAACTGAACGAACGTCACTACGGTGCGTTGCAGGGTCTGAACAAAGCGGAAACCGCTGAAAAGTA
TGGCGACGAGCAGGTGAAACAGTGGCGTCGTGGTTTTGCGGTGACTCCGCCGGAACTGACTAAAGATGATGAGCGTTATC
CGGGTCACGATCCGCGTTACGCGAAACTGAGCGAGAAAGAACTGCCGCTGACGGAAAGCCTGGCGCTGACCATTGACCGC
GTGATCCCTTACTGGAATGAAACCATTCTGCCGCGTATGAAGAGCGGTGAGCGCGTCATCATCGCCGCACACGGTAACTC
GTTACGTGCGCTGGTGAAATATCTCGATAACATGAGCGAAGAAGAGATTCTTGAGCTGAATATCCCGACCGGCGTGCCGC
TGGTTTATGAGTTTGACGAGAACTTCAAACCGCTGAAACGCTATTATCTGGGTAATGCTGACGAGATCGCGGCGAAAGCA
GCGGCGGTTGCTAACCAGGGTAAAGCGAAGTAA
>Cluster_24426
ATGACTGAAGCACAAAGACATCAAATCCTCCTGGAAATGCTCGCACAATTGGGCTTTGTGACCGTTGAGAAAGTCGTTGA
GCGTCTGGGAATTTCGCCCGCCACTGCACGACGCGATATCAATAAACTTGACGAAAGCGGCAAACTGAAAAAAGTGCGCA
ATGGCGCAGAAGCTATTACCCAACAGCGCCCGCGCTGGACGCCGATGAATCTGCATCAAGCGCAGAATCACGATGAAAAA
GTACGTATTGCTAAAGCGGCCTCGCAGCTGGTTAATCCGGGCGAAAGCGTAGTCATCAACTGCGGCTCCACCGCGTTTCT
GCTTGGGCGGGAAATGTGTGGCAAGCCAGTGCAAATCATCACTAATTATCTACCACTGGCAAACTACCTGATCGATCAGG
AACATGACAGCGTGATCATTATGGGCGGACAGTACAACAAAAGCCAGTCCATCACCTTAAGCCCGCAGGGCAGCGAAAAC
AGTCTCTATGCCGGGCACTGGATGTTTACCAGCGGCAAAGGACTGACAGCAGAAGGGTTGTATAAAACCGATATGCTGAC
GGCAATGGCAGAGCAGAAGATGCTGAGCGTGGTAGGGAAACTGGTGGTACTGGTTGATAGCAGTAAGATTGGCGAACGCG
CGGGAATGCTTTTTAGCCGTGCCGATCAAATCGATATGCTGATCACCGGCAAAAATGCTAACCCGGAAATCCTGCAACAG
CTGGAAGCGCAAGGGGTCAGCATTATGCGTGTTTAA
>Cluster_55367
GTGACCAAACTCAAACTTCTGGCACTTGGAGTGCTTATCGCAACGTCTGCAGGCGTAGCGCACGCTGAAGGTAAATTTTC
CCTGGGCGCAGGCGTAGGTGTCGTTGAACACCCATATAAAGATTACGATACCGATGTTTACCCAGTACCGGTAATCAACT
ATGAAGGCGATAACTTCTGGTTCCGTGGCTTAGGTGGTGGTTACTACCTGTGGAATGACGCAACGGATAAACTTTCAATT
ACCGCTTACTGGTCGCCGCTTTACTTCAAAGCGAAAGACAGTGGCGATCACCAAATGCGTCACCTGGATGACCGTAAGAG
CACCATGATGGCTGGTCTGTCTTATGCTCACTTTACCCAGTACGGTTACCTGCGTACCACCCTGGCTGGCGATACCCTGG
ATAACAGCAACGGCATCGTCTGGGATATGGCCTGGTTGTATCGTTACACCAACGGTGGCCTGACCGTGACTCCGGGTATT
GGTGTGCAGTGGAACAGCGAAAACCAGAACGAATACTATTATGGCGTATCGCGCAAAGAGTCCGCTCGCAGCGGTCTGCG
TGGCTATAACCCGAACGACAGCTGGAGCCCTTACCTGGAGCTGAGCGCCAGCTACAACTTCCTCGGCGACTGGAGTGTTT
ACGGTACCGCACGCTACACCCGTCTGTCTGATGAAGTTACTGACAGCCCGATGGTGGATAAATCCTGGACTGGCCTGATT
TCTACCGGGATCACCTACAAATTCTGA
>Cluster_62517

ATGGATATTCCACGTATTTTTACCATCAGTGAAAGCGAACACCGCATCCACAACCCGTTCACCGAAGAGAAGTACTCCAC
GCTGGGTCGCGTGCTGCGCATGAAACCGGAGACCCGAATTCTGGACCTCGGCAGCGGCTCGGGCGAGATGCTCTGCACCT
GGGCCCGGGATCATGGCATTACGGGGACTGGCATCGACATGAGTTCGCTCTTCACCGCGCAGGCCAGACGCCGCGCGGAA
GAACTCGGCGTCAGCGAACGCGTACATTTTATTCATAATGATGCGGCGGGTTACGTCGCCAATGAAACATGTGACGTAGC
AGCCTGCGTCGGCGCGACCTGGATTGCCGGAGGCTTTGCCGGGACAGTCGAACTGCTGGCGCAGAGCCTTAAACCGGGCG
GGATCATGCTCATCGGTGAACCTTACTGGCGTCAGCTTCCGGCAACGGACGAGATAGCCCAGGCCTGCGGCGTCAGCTCG
ACAAGCGATTTCCTGACGCTGCCAGGACTTGTGAGTGCATTCGATGAACTCGGCTACGACGTAGTGGAAATGGTGCTGGC
AGACCAGGAGGGCTGGGACCGATATGAAGCTGCGAAATGGCTGACCATGCGCCGCTGGCTGGACGCCAATCCTGACGACG
ACTTCGCCGCCGAAGTCAGGGCCGAGCTAAACATCTCGCCGAAACGTCACGTAACGTACGCGCGGGAATACTTTGGCTGG
GGCGTGTTCGCGCTCATCGCCAGGTAA
>Cluster_321
ATGGCCTACAGCAAAATCCGCCAACCAAAACTCTCCGATGTGATTGAGCAGCAACTGGAGTTTTTGATCCTCGAAGGCAC
TCTCCGCCCGGGCGAAAAACTCCCACCGGAACGCGAACTGGCAAAACAGTTTGACGTCTCCCGTCCCTCCTTGCGTGAGG
CGATTCAACGTCTCGAAGCGAAGGGCTTGTTGCTTCGTCGCCAGGGTGGCGGCACTTTTGTCCAGAGCAGCCTATGGCAA
AGCTTCAGCGATCCGCTGGTGGAGCTGCTCTCCGACCATCCTGAGTCACAGTATGACTTGCTCGAAACACGACACGCCCT
GGAAGGTATCGCCGCTTATTACGCCGCGCTGCGTAGTACCGATGAAGACAAGGAACGCATCCGTGAACTCCACCACGCCA
TAGAGCTGGCGCAGCAGTCTGGCGATCTGGACGCGGAATCAAACGCCGTACTCCAGTATCAGATTGCCGTCACCGAAGCG
GCCCACAATGTGGTTCTGCTTCATCTGCTAAGGTGTATGGAGCCGATGTTGGCCCAGAATGTCCGCCAGAACTTCGAATT
GCTCTATTCGCGTCGCGAGATGCTGCCGCTGGTGAGTAGTCACCGCACCCGCATATTTGAAGCGATTATGGCCGGTAAGC
CGGAAGAAGCGCGCGAAGCATCGCATCGCCATCTGGCCTTTATCGAAGAAATTTTGCTCGACAGAAGTCGTGAAGAGAGC
CGCCGTGAGCGTTCTCTGCGTCGTCTGGAGCAACGAAAGAATTAG
>Cluster_41798
ATGAGCAATTTATTAGGCCCCCGTGACGCCAACGGCATTCCGGTCCCGATGACGGTGGATGAATCCATCGCCAGCATGAA
GGCGTCGTTACTGAAAAAAATCAAACGTTCTGCCTACGTTTATCGCGTGGACTGCGGCGGCTGTAACGGTTGCGAAATCG
AAATTTTCGCCACGCTTTCGCCGCTGTTCGATGCAGAACGCTTCGGCATTAAAGTCGTCCCGTCACCGCGTCATGCGGAT
ATTTTACTGTTTACCGGCGCGGTCACCCGTGCAATGCGATCCCCTGCGCTGCGTGCGTGGCAGTCCGCGCCGGACCCGAA
AATCTGTATCTCTTACGGTGCCTGCGGTAACAGCGGCGGGATCTTCCACGATCTCTACTGCGTGTGGGGCGGTACGGATA
AAATCGTTCCAGTGGATGTTTATATCCCTGGCTGCCCGCCAACGCCTGCCGCCACGCTGTACGGCTTTGCGATGGCGCTC
GGCCTGCTGGAGCAGAAAATCCACGCCCGTGGGCCGGGTGAGCAGGATGAACAACCGGCGGAGATTCTGCATGGCGATAT
GGTGCAGCCGCTGCGCGTGAAAGTGGATCGCGAAGCACGCCGCCTGGCGGGTTATCGTTACGGTCGCCAGATTGCCGATG
ATTTCCTGACGCAATTAGGGCAGGGCGAAGAACAGGTTGCACGCTGGCTGGAAGCGGAAAACGATCCGCGTCTGAACGAG
ATTGTCAGCCATCTGAATCATGTTGTTGAAGAGGCGCGTATCCGATGA
>Cluster_35163
ATGGAGACTGTTTTTGACGCACTGAAAGCAATGGGAAAAGCCACATCCATAGAACTTGCTGCGCGACTTGATATCAGTCG
TGAAGAAGTGCTGAACGAACTATGGGAACTGAAAAAGGCTGGTTTTGTTGATAAAAGCGCGTACACCTGGCGTGTGGCTG
ATAACAACGTTCAGCAGGAACAGCCAGCGCAGGCAGAACTGCCGGAAGAAATCACCACAGCAACAGTAGCGAAAATCTCA
GAGTGCGATTTAACCGCGACGATTGAACAACGAGGACCACAAATGGCTGATGAGCTGGCTACATTGTTTGGTACCACATC
ACGCAAAGTGGCTTCAACGCTGGCAATGGCAATCAGCAAAGGTCGTCTGATTCGCGTAAATCAGGGCGGTAAATTTCGTT
ACTGCATACCGGGCGATAATTTACCAGCAGAGCCGAAAGCAGCATCGGTAGCGGAAACTGATGGTAAGGCCTTTCCTCAT
ACCGCAGGTGTTGCGTTACCAGTACAGGAGGCTGCAACACAGGAAGATATTAAAACAGAAACTGTGGCGGACATTGTGCA
GTCGCTGCCATCGTTTACTGAAACGCGAGCGGATGACCTGGTTTTACCATCACTGCATATGGCAAACCGCGAACTGCGTC
GGGCGAAAAATCATGTCCAGAAGTGGGAGCGAGTCTGCGCCGCGCTGCGGGAGCTGAACAAGCACCGGGATATTGTTCGA
CAGATTACTGATTCTTCCCGCCGTGTTGTATCGGAAAAGTGA
>Cluster_11575
ATGGAAAATATAATGAACAATCCGGTTATCGGTGTCGTAATGTGCAGGAACAGGCTTAAGGGTCATGCGACCCAGACTCT
GCAAGAAAAGTACCTGAATGCCATCATCCACGCAGGCGGCTTGCCAATTGCGCTACCACATGCGCTGGCGGAACCGTCAT
TACTTGAACAACTTTTGCCGAAACTCGATGGCATTTATCTTCCTGGTAGTCCCAGCAATGTGCAGCCGCACCTATATGGT
GAAAACGGCGATGAGCCTGACGCCGATCCCGGGCGTGATCTTCTGAGCATGGCGTTAATTAATGCCGCACTCGAAAGGCG
CATCCCCATTTTCGCCATCTGCCGGGGTTTACAGGAACTGGTGGTGGCAACCGGTGGTTCGTTGCATCGCAAGCTGTGCG
AACAGCCTGAATTGCTGGAACATCGGGAAGATCCCGAACTGCCGGTGGAACAGCAATATGCACCGTCGCATGAAGTTCAG
GTTGAAGAGGGGGGATTACTGTCTGCGTTGTTACCTGAATGTAGCAACTTTTGGGTAAACTCTCTGCATGGGCAAGGGGC
GAAGGTCGTTAGCCCACGGTTGCGTGTTGAAGCTCGCTCGCCGGATGGCTTAGTTGAGGCGGTTAGCGTCATCAATCATC
CTTTTGCGCTGGGCGTACAGTGGCACCCGGAATGGAACAGTAGCGAGTACGCGCTTTCGCGTATATTGTTCGAGGGCTTT
ATCACCGCTTGTCAGCACCATATCGCTGAAAAACAGCGACTCTGA
>Cluster_17776
ATGAATACCGGTATCTATTTCGACATCAGCAATGAGGACTACCACGACGGTGACGGAGTGAGTAAGTCACAACTGGATAT
GGTAGCCAAGAACCCTGCCCTTCTGAAATGGGTCAAGGCAGCGCCGGAAGATGAAGAGAAAAAATCGGCACTGGACATGG
GTACCGCCCTGCACTGCCTGCTACTGGAGCCGAAGGAATTCGATAAGCGTTTCATCGTGGCGCCACAGTTCAACCGCCGC
ACAAATCAGGGAAAAGCAGACGAAGAAGCATTCCTGAAAGACGTTGCCGACATGGGTATGACGGTGATGGATGCCGAGCA
GGGCCGGAAGCTGAAACTGATGCGCGACAGCGCAATGGCTCACCCGGCGGCGCGCTGGATGCTGGAAGCGCCAGGACACT
GTGAAGCATCGATGTACTGGAATGACGAAGATACTGGCGAACTGTGCCGAATCCGTCCGGATAAATGGCTGAACGAGCAC
AACGTGATCGTCGATGTGAAAAAGGTTGCAGACATGGATCGCTTTGCCCGTCACATCGAGGAATTCCGCTACCACGTCCA
GGACGCCATGTACCGCGAAGGCGCACTGAAAGTAACCGGGCAGCCACACGGATTCTTCTTTCTGGCTGTGAGCGAAACCA
TCGACTGCGGTCGATACCCTGTCCGCGTGTTCGAGCTGGATGCGCCAGATGTTGACGCCGGGCATCAGTTATTCCGCCGG
GATCTGAACACCTATCACGAATGCCGCATCAGCGATGAATGGGGCGGCGTGGAAATTATTAAACGCCCTGAATGGGCACG
AAAACAGGATATGTACGTATGA
>Cluster_66878
ATGGGTTGCATGAAGGAATATATGCAGGATCTGGAAGCTGAACGGTTTGATGAATGGCTTGAGGAGAATTACCCGGATGT
TAATCCCAACTCTGAAGAATGGGAGCAGGCCGCGAACCTGTACTGCTGGGAGCAGGAAGCGCTGGCCGATCAGGCTCAGT
GGGAACATGAACACGGGCTCTTTGTGGCTTCCCTGAACAATGTTCATCTGCGTTACATACACGCAAAGGAGGAGCTGAAG
AAGCTGTATACCCTACTCGATAAGGAGCAGCCTGAACTGGTGTACAGGATGTCTTTCGTCCATGCCGTGACTGTAATGGA
GGCGTACCTGATGTACTGCGCCAGGGCTCTGCTTGAGCATGACTGGCCCCTTAAACGTTTCCTTAATGAGTACTATCTGA
AATCCGCACCTAAGGTCACTAATAAAGATAAAACCGCCGCCCGCACAATGGATGTTGAACTGTTCAGGCCTGCAGCCCGA
AATTATGTTTCCCGAATGACATTTCATAACGTTAAAACCATTGAGCGCTATTTCGGGGCCGTGCTGCACATCCCTCCCGT
CTGGCCAACTGAGCCACTGGGCATCATCTCAGACTGGCGAAATGATCTTGTACACCGCAACGGCGTGGATGAACATGATG
TGCCACGGGGGATATCAGCACAGCAGCTGCAGAATACCCTGCAGAAAGTATCGGACCTGATAGAGGCTGCTGACATATCC
CTGCGTCAGGAAGTTGACTATTTCGGTAACTGGCGTAATGAGGAAAACCGGGCAATCATCGCATCAGCACTGAATATATC
GCCAGTCGGAGAGTCACACTGA
>Cluster_4620

ATGAGTATGGAGCTGATGGTTAAAGCGATGAAAATTCGAGTGGGAAATCCATTGCGAAAACTGGTTCTGATCAAGCTGGC
TGATAATGCCAGCGATCAGGGGGAGTGCTGGCCCAGCTACCAGCATATTGCTGATCAGTGCGAGATTAGCAAACGTTCTG
TGATGAATCATATTGCGGCCCTTTGTGAGTCCGGGCTGGTAAAAAAAGTCACCCGGAAAGGTGAAAAAGGTAACTCAAGT
AATATCTATCTCCTTCATCTGGATGGTGCAGGAGATTCACTAGGGGGTAGTGCAAATAATTCACTATCTGGTGCAGCAAA
TTCACCAGGTAGTGCAGGAGTTGCACCAGGGGGTAGTGCAGGAGATTCACCCAGAACCAGTCACTCTTTTGAACCAGTCA
AAGAACCAGTCAATGAACCAATAGCTGTTGGTGCATCTGCTGATGAGTCTGTGCGAGTTCGTTCAAACCGACCGGAATAC
TCTCCGGAGTTTGAGCAGGCATGGCTGGTATATCCCAAACGTGCTGGTGGCAATTCAAAATCTGCAGCCTTCAAAGCCTG
GAAAGCCCGTTTGAATGAGGGGGTAAACCCCGAAACCATGCTGGAAGGTGTGAAACGCTACGCGGGCTGGGTATCTGCGA
TGGGTAACAGCGGCACACAATTTGTGAAACAGGCTGTCACGTTCTTTGGTCCGGATCGTCATTTCGAAGAATCCTGGGAA
GTTCCTGCGGTATCTGCAGCCAGACGCGAGGACCCGTACTTCAAAGCCAGTTACGACAATGTGGACTACAGCCAGATCCC
GGTAGGATTCAGGGGGTGA
>Cluster_66938
ATGAAAGCACTCACCTCTCTCATTTTGCCAGTAATACTGCTTACTGGTTGTTCTTCTTCCCCTACCATCTCGCCAGGTGG
GGTCAATACGCCACCACCTGACATTAATGCATGGCTTAAACCACGAAGTGAAAAGCCGGATAGTCTGTCAGAAACACGCT
GGAAAATGCTGACCGACGCAGGTAAAACGCTTGGATTTCGTGGTGGCAAGGCTCAGCGTTCATGGGAGCTGATTCAGGCA
CTCAACGCCCGGGAAAGCACACTTAATGCCTTATACGATTTCCGGCCACTGATTAGCCCGGAAGGCTGGCTGCCACCGGT
TATTGATGAAGCACAGGACGTAGCTCATATCACGCCAGATCAAATCCGGACCTCTTCCCGGGTCTGGACCATCATTCGCC
CTGAACGCTTTGTCAGCAATCCACCGGGCTGGCGTGACTGGCTTCTCCGGGGGCTTTCAACAACAGCCACTCCGGGTACA
GAAGGCAGCGTCGTTCCAGAAGACAGCGTGCAACGAAAAGTGTGGGAAACCGCATTACGGCAGGGATGGCAGGAAGGAAG
ACAAAACGCCGACCTGACGCTTGAAGCCAACCAGAAAACCCTCACCCGTGATTACCGGGGCATGATGCTGTACTCACTTC
TCTGGCGTCAGGGCATGATCACCCGTCCGGATGTTTCTGATCAAATGCAAACCGTTACAGGTGACGGGAAAAAACTGGTC
ACTGGAGATCGTGTTCGTCGCCTTAAAAATCATGCGGAATTCAATCTTCAGAAATCTCACTGGCGTCCGCTTATAGGCAC
TGAAGGAGGCTCCCGATGA
>Cluster_19428
ATGCCGATGTTTCTCTTTTTACGCCGTTTACGCCATTCACCCGCCGCGTTTTGCGGGTTGATAATTATCCTGTTATTGAT
GCTAATTGCGCTCTTCGCGCCGTGGCTGGCACCTCACGATCCTAACTGGCAGGATGCGGCGGCGCGGTTACAAGCACCGA
GTCGTCAGCACTGGCTCGGCACTGACAGTTACGGGCGAGACCTACTTTCACGTTTAATCTACGGCAGCCGCCCGGCACTG
GGGCTGGTGGCATTGGTGACAATCATTACACTCCCGGTCGGTCTGCTGATTGGCATTTTAGCCGGGTACTACGGCGGCTG
GCTGGAACGCGTGCTGATGCGTTTTACTGACGTAGTAATGTCAATGCCGCGTTTAATTCTGGCTTTTGCTTTTGTTGCCA
TGCTGGGGCCGGGGCTGGTTAACGGCGCACTGGCGCTGGCATTAACCACCTGGCCTGCTTATGCGCGTCAGGCACGGAGT
GAGATCCAACGTCTGCGACACAGTGATTATCTTGCCGCCGCAGAAATGATGGGCATCCGCGGCAGGCGTCTGCTGGCTGG
TCATATTTTGCCATTGTGCCTGCCATCTGCCATTGTCCGTCTGGCGCTGGATTTAGCGGGTATCATTCTGGCGGCTGCTG
GCCTGGGTTTTCTTGGGCTTGGTGCTCGCCCGCCAATGGCGGAATGGGGAGCCATGATCGCCGACGGTATGCAGGTCATT
TTTGATCAATGGTGGATTGCCGCCGCTCCCGGCGCAATTATCTTAATTGCCAGTCTGGCCTTTAATCTGCTGGGTGATGG
CTTGCGCGATGTTCTGGAGCCTCAACATGACTGA
>Cluster_23814
ATGTTTGCCGATATGAAAAGTATGGTGATGAAAGCCTGGCATGAACATTACGCCTTGCTGGCGATCAACTGTATGAATCT
GGAAAGTGCTCACGCAGCTATCCGCGTGGCGGAGAAGAATCGCGCTCCGATCATTCTCAATCTCTATCAGGGGCATCTGG
CGCATTTTCCTGCACCCGTCGCGGCGGCAGTGGTTAAAACGCTCGCGGAATCTGCCTCAGTACCGGTCGCTCTGGCGCTT
GATTATGGCAAAAATCCGGACTGTATTCGTCAGGCGTTTCGTGCTGGTTTCAGCGGATTAATGATTGATGCTTCTGCTTT
TCCCCTGGAGGAAAACGTGCGCCAGACCAGGGCGGTGGTGGAACTCGCCGCCAGCGCGCAGCTCTGCGTGGAAGGTGAAC
TGGGCCATCTGGCAGATGCGCCGCGTTATGACCAGGCCGCCAATGCAGACATGATGACGCAACCCACTGATGTGGAACCG
TTTATCGTGCAGACAGGCATTGATCTACTGGCCGTTTCGGTAGGCACGGCGCACGGCATGTACGCGCCTGGTGTTGTACC
TGCTATTGATTTTCAGCGTCTGGCGGAGATTTTCCATTGCTCGAGCGTGCCTCTGGCATTACATGGCGGTAGCGGTACGC
CGTTCGATCAGCTCCAGCTCTGTACGACTCTGGGGGTGGCAAAAATCAACGTGGGGGCGGCCATTTTTGAACGGGGGAAA
TCCGCGCTCCTGCATACCCTCTGTCACGATATATCCATTGAACTGGTGGACGCGCTGAAAGCTATGGAACTGGCCTTCGA
AGAAGCGATTACACCGTATTTACAGGCGAGCGGCGCTATCGGCAAAGCCTGA
>Cluster_48588
ATGAAGAAAGGTTTACTTGCAGTTGCTCTGGCGGCTATTTGCACAATGGGTCTTACTGGCTGTGATCGCGTGGAGCCTGG
ATACGTTGGCATCAAAGTAAACAAATTAGGTGAAGACAAAGGGATCGGTGAAGTGGTTGGCGTTGGTCGCCAATGGACAG
GTCTTAACACCGAACTTTACGTATTCCCGACCTTCAAACAAATGAAGACCTACGACGAGCCGTTCACATTCCAGATGAGT
GACGGTACTGCTATTGGTCACAAAATTGGCGTTGCGTATCTGGTTAATCGTGACAAGGTAACGACGGTGTTCCAGACCTA
TCGCAAAGGCGTAGACGATATCACCGAATCAGATCTGCGTCAGAAAATTGCCGACTCTCTAAACCGTTTGGCCAGCCGTA
TGACCACTGACTCATTTATCGACGGTGGTAAGGCGCAATTGCTGGACAACGCACTGAAAGATATTCAGAAAGAGATGTCT
CCGGTTGGTATTGAGGTACTGAGCCTGTCATGGGTTGGAAAGCCTGATTACCCAAAAACCGTCATTGAATCTATCAACGC
CAAAGTAACGGCTAACCAGCGTACTCTGCAACGTCAGCAGGAAGTTGAACAGCGTAAAGCTGAGGCGAATATGCTACGTG
AACAGGCTAATGGTGAAGCTGATGCTATCCGTGCTCGTGCGCAAGCAGAAGCCGACGCCATTCGTCTGCGCGGTGAAGCT
CTGCGTCAAAACCCGAACGTTATGGAGCTGGAAGCCATCAATAAATGGAATGGCCAGTTACCGCAGTACATGACTCAAGG
GGCTAACACTCCTTTCATTACAGTGAAATAA
>Cluster_27447
ATGTTTGCAGTTTCATCAAGACGCGTGCTGCCGGGCTTTACCTTAAGCCTCGGCACCAGTCTGCTGTTTGTGTGCCTGAT
TTTGCTGCTGCCGCTCTCCGCGCTGGTGATGCAGCTGGCTCAGATGAGCTGGGCGCAGTACTGGGAGGTGATCACCAACC
CGCAGGTGGTCGCGGCCTACAAAGTGACGCTGCTGTCAGCGTTTGTCGCGTCGATTTTTAACGGCGTTTTCGGTTTGCTG
ATGGCGTGGATCTTAACCCGCTATCGCTTTCCGGGCCGCACGCTGCTTGATGCGCTGATGGATTTACCCTTTGCGCTGCC
AACGGCAGTCGCCGGTTTGACGCTGGCCTCGCTCTTTTCCGTGAACGGTTTTTACGGTGAATGGCTGGCGAAGTTTGATA
TCAAAGTTACCTATACCTGGCTGGGGATTGCGGTGGCGATGGCTTTTACCAGTATTCCGTTTGTGGTGCGTACCGTGCAG
CCGGTGCTGGAAGAGTTAGGCCCGGAATATGAAGAAGCGGCGGAAACGCTTGGTGCAACGCGCTGGCAGAGTTTCTGCAA
AGTGGTGCTGCCGGAGCTTTCTCCGGCGCTGGTGGCGGGCGTGGCGCTGTCGTTTACCCGTAGTCTTGGTGAGTTTGGCG
CGGTGATTTTCATCGCCGGGAACATCGCCTGGAAGACGGAAGTGACGTCGCTGATGATTTTCGTTCGCTTACAGGAGTTT
GATTACCCGGCGGCGAGCGCGATTGCTTCGGTGATCCTCGCGGCATCCCTGCTGCTGCTGTTCTCAATTAACACTCTGCA
AAGTCGCTTTGGTCGGCGTGTGGTAGGTCATTAA
>Cluster_58128
ATGGCGTTCAAATTCAAAACCTTTGCGGCAGTGGGAGCCCTGATCGGATCACTGGCACTGGTAGGCTGCGGTCAGGATGA
AAAAGATCCAAACCACATTAAAGTCGGCGTGATTGTTGGTGCCGAACAGCAGGTTGCAGAAGTCGCGCAGAAAGTTGCGA
AAGACAAATATGGCCTGGACGTTGAGCTGGTAACCTTCAACGACTATGTTCTGCCAAACGAAGCATTGAGCAAAGGCGAT
ATCGACGCCAACGCCTTCCAGCATAAACCGTACCTTGATCAGCAACTGAAAGATCGTGGCTACAAACTGGTCGCAGTAGG
CAACACTTTTGTTTATCCGATTGCTGGTTACTCCAAGAAAATCAAATCACTGGATGAACTGCAGGATGGTTCGCAGGTTG
CCGTGCCAAACGACCCAACTAACCTTGGTCGTTCACTGCTGCTGCTGCAAAAAGTGGGCTTGATCAAACTGAAAGATGGC
GTTGGCCTGCTGCCGACCGTTCTTGATGTTGTTGAGAACCCCAAAAATCTGAAAATTGTTGAACTGGAAGCACCGCAACT

GCCGCGTTCTCTGGACGACGCGCAAATCGCTCTGGCAGTTATCAATACCACCTATGCCAGCCAGATTGGCCTGACTCCGG
CGAAAGACGGTATCTTTGTTGAAGATAAAGAGTCCCCGTACGTAAACCTGATCGTGACGCGTGAAGATAACAAAGACGCC
GAGAACGTGAAGAAATTCGTCCAGGCTTATCAGTCTGACGAAGTTTACGAAGCAGCAAACAAAGTGTTTAACGGCGGAGC
TGTTAAAGGCTGGTAA
>Cluster_39915
ATGTTTTTAGTCGACTCACACTGCCATCTCGATGGTCTGGATTATGAATCTTTGCATAAGGACGTGGATGACGTTCTGGC
GAAAGCCGCCGCACGCGATGTGAAATTTTGTCTGGCAGTCGCCACAACATTACCGGGTTATTTACATATGCGGGATCTGG
TAGGCGAACGTGACAACGTCGTATTTTCTTGTGGCGTGCATCCACTCAACCAGAATGATCCCTACGATGTAGAAGATTTA
CGCCGTCTGGCGGCAGAAGAGGGTGTTGTAGCGCTGGGTGAAACCGGGCTGGATTATTATTACACACCGGAAACTAAAGT
ACGTCAGCAAGAGTCCTTCATTCATCATATCCAGATTGGTCGTGAACTGAACAAGCCGGTTATCGTCCATACCCGTGACG
CCCGCGCCGATACGCTGGCAATTCTGCGCGAAGAAAAAGTGACGGATTGCGGTGGCGTACTACACTGTTTTACAGAGGAC
AGAGAAACGGCGGGTAAATTACTGGATCTCGGATTTTACATCTCCTTTTCCGGCATTGTGACCTTCCGTAATGCGGAGCA
ACTGCGCGATGCTGCGCGATATGTCCCCCTGGATCGGTTACTGGTGGAAACTGACTCACCTTACCTTGCGCCGGTACCGC
ATCGAGGAAAAGAGAATCAACCTGCGATGGTTCGTGACGTTGCAGAATACATGGCTGTGTTGAAAGGTGTTGCCGTTGAA
GAACTGGCGCAGGTAACCACCGATAACTTCGCCCGTCTGTTTCACATCGACGCTTCCCGCCTTCAATCCATCCGTTGA
>Cluster_59730
ATGGAGATTACCACTCTTCAAATTGTGCTGGTATTTATCGTAGCCTGTATCGCAGGTATGGGATCAATCCTCGATGAATT
TCAGTTTCACCGTCCGCTAATCGCGTGTACCCTGGTGGGTATCGTTCTTGGGGATATGAAAACCGGTATTATTATCGGTG
GTACGCTGGAAATGATCGCGCTGGGCTGGATGAACATCGGTGCTGCAGTTGCGCCTGACGCCGCTCTGGCTTCTATCATT
TCTACCATTCTGGTTATCGCAGGTCATCAGAGCATTGGTGCAGGTATCGCACTGGCAATCCCTCTGGCCGCTGCGGGCCA
GGTACTGACCATCATCGTTCGTACTATTACCGTTGCTTTCCAGCACGCTGCGGATAAGGCTGCTGATAACGGCAACCTGA
CAGCGATTTCCTGGATCCACGTTTCTTCTCTGTTCCTGCAAGCAATGCGTGTGGCTATTCCGGCCGTCATCGTTGCGCTG
TCTGTTGGTACCAGCGAAGTACAGAACATGCTGAATGCGATTCCGGAAGTGGTGACCAATGGTCTGAATATCGCCGGTGG
CATGATCGTGGTGGTTGGTTATGCGATGGTTATCAACATGATGCGTGCTGGCTACCTGATGCCGTTCTTCTACCTCGGCT
TCGTAACCGCAGCATTCACCAACTTTAACCTGGTTGCTCTGGGTGTGATTGGTACTGTTATGGCAGTGCTCTACATCCAA
CTTAGCCCGAAATACAACCGCGTAGCCGGTGCGCCTGCTCAGGCAGCTGGTAACAACGATCTCGATAACGAACTGGACTA
A
>Cluster_9479
ATGACATTACCAACAACTATTTATTCATTCCCCGCATATTTACCTCGTTTTTCGTCCACCGACAAACCAGCAAAACTAAA
ATTTCATCAATACGCACGCGCTACATTACTGTCAAATCGTGGAAGAGATCATAATTGCGATGGCAGACGTACTGTTGAGA
TCCACAAACTAGATCTCTCAGACTGGCAAGCATTCAATAAACTAGCGATGGCGTATAATGCTTACGATGGAATAACAATG
AAGGGTGATAATTCTTTTGGATGGAACCATGAAGCTACATTAGATAATATTCACGCCCAGAAATACAATAAAGCTTACGC
GGGTGCGCGGTTAACCGCAGAACTGAAATATTTATTGCAAGATGTCGAATCCTTCGAACCCAACAGTAAATACACAATAC
ACGAGGTTGTTTTGGGACCTGGTTATGGTACTCCAGACTATACCGGCCAAACGATAGGATACGTTGTAACCCTTCCTGCC
CAAATGCCTAATTGCTGGTCATCTGAACTACCAACAATCGATCTTTATATCGACCAACTTCGCACTGTTACAGGCGTTTC
TAATGCCCTCGGATTCATCATTGCTGCATTGCTAAACGCGTATTCTGATTTACCCCATGATTTGAAAATTGGTCTGCGTT
CCTTATCAAGTTCAGCCGCAATCTACTCGGGTTTGGGTTTCGAGCGAGTGCCGCAGGAGCGAGACATCAGTTGCGCTCGT
ATGTATCTAACGCCAGCAAACCATCCTGACTTATGGACACAAGAGAATGGTGAATGGATCTACTTGCGAAACTAA
>Cluster_5368
GTGACGCAACCCAGTGCTCCGGCTCCCCAAATCGTCATCGACAGTCACGACGACAAAGCCTGGCGCGACACGCTGTTAAA
AGTGGCGGCCATTTTATGTGAACGCCAGCCGGATTCACCGCAGGGCTACCGTCTGCGCCGCCATGCGCTTTGGCAGAACA
TCACCAGTACGCCCCAGGCGGAAAGCGATGGCCGTACTCCACTGGCTGCGGTCTCCGCCGATATGGTGGCAGATTACCAT
GCTCAGCTCGGTAGTGCCGATATGGCGCTGTGGCAGCAGGTGGAGAAAAGCGTACTGCTGGCTCCTTACTGGCTGGATGG
TCACTGCCTTTCTGCACAGACGGCGCTGCGTCTGGGTTACAAACAGGTGGCAGACGCCATCCGCGATGAGGTCATCCGCT
TCCTTGAGCGTCTGCCCCAGCTTACCGGGCTGCTGTTTAATGACCACACACCGTTTATCAGTGAGCAGACGAAACAATGG
CTGGCAGCTTCGCCCGACGCCAAAGTTGCACCGGTTGCGCAAATCGGTGAGGAATCGAAGGCAGCCAGAGCCTGTTTTGC
TGAGCAGGGTCTGGAGGCGGCGCTGCGATATCTGGACATGCTACCCGAAGGCGATCCCCGCGATCAGTTTCACCGCCAGT
ACCTTGCCGCACAGTTGACGGAAGAGGCGGGGCTGGTACAGCTTGCGCAGCAGCAGTACCGGATGTTGTTCAGGATGGGA
TTGCAGATGATGGTGGCAGACTGGGAGCCGTCCCTGCTTGAGCAGCTAGAACAAAAATTCACGGCAGAACAATAA
>Cluster_55243
GTGGCGCGGATAAACCGTATTTCGATCACGCTCTGTGCTTTACTTTTTACCACCCTGCCTTTAACGCCTATGGCCCATGC
TTCAAAGCAAGCCAGGGAGAGTTCTGCTACCACTCATATCACCAAAAAAGCAGATAAAAAGAAAAGCACGGCAACCACCA
AAAAGACCCAGAAAACAGCGAAAAAAGCCGCCAGTAAAAGTACGACCAAAAGCAAAACCGCTTCTTCCGTTAAAAAATCT
TCCATTACCGCTTCTAAAAACGCCAAAACTCGCAGCAAACACGCCGTCAATAAAACGGCCTCAGCCAGCTTTACCGAAAA
GTGTACCAAACGTAAGGGCTATAAATCGCATTGTGTGAAAGTCAAAAATGCCGCGTCAGGAACTCTTGCCGACGCGCACA
AAGCGAAGGTGCAAAAAGCTACGAAAGTGGCAATGAATAAACTGATGCAGCAAATTGGTAAGCCATATCGTTGGGGTGGC
AGCTCACCGCGTACCGGTTTTGATTGCAGCGGCCTGGTTTATTACGCTTATAAAGATTTGGTGAAAATTCGTATTCCGCG
TACGGCGAATGAAATGTATCACCTGCGTGATGCAGCGCCAATCGAACGTAGTGAACTGAAAAACGGCGACCTGGTCTTTT
TCCGTACTCAGGGACGCGGCACAGCCGATCATGTCGGCGTGTATGTCGGCAACGGCAAATTTATTCAGTCACCGCGCACA
GGTCAGGAAATTCAAATCACTTCTCTCAGTGAAGACTACTGGCAGCGCCACTATGTTGGCGCTCGTCGGGTAATGACCCC
AAAAACACTTCGCTAA
>Cluster_23423
GTGCCGAGAAAATCGTCTAACCTACTGAAACGCAGATTAAAAATTGCAGAAATTGTTGCCGCCCAGGGTGAAATCAAAGT
TGATGACTTATCTGCGCAACTTGGTGTCTCTGGCGTGACGATCCGCGGCGATCTCAGTTATCTGGAGCAACAGGGATATT
TAAAGCGATCATTTGGCGGTGCTATTGCGACACCACTCTCCTCTGAACCAGAAGCGATACAACCGATCGCGCCATTGTCT
CTGGCACAACAAATGGAAATAGCACGACATTGCGCACGATTAATTCGCGATCGTGACACGCTATTTCTTGGACATGGGAC
AATCTGCCGTAAGATCATTCCATTGCTCAGCGAAGTGAAAAAACTGCGTCTGATCACCAACGATCCCGAACATGCGCTGC
TGGCAAATCAGTTTATTGATGGCGAAATAATACTGGCTGGCAGTGAAATGTTGCGCCCCGATACCGCACTGGCCGGAAAA
CAGCTTGAACAGGCACTTCAGCATTTCACCATTACACATAGCATTCTGGAAATCAGCCATCTGGATGCTGATGGTACTTT
AAATATCAACGTTCCCAGACTCGCCGCTGCCTGGCAGCTCTGTTTCGATCATGCCCAGAATAAGACGGTTATCGTTGCCG
CCGATCCTGCGCCATCAACGGCTGCTATCGGGCATTTGCATCAGGCAGAAAATGTGATCGTCAGCCGCAGCATTAACGAA
CAATATCAGCAACAATTGCAGTCGGCTGATTTCGTTATCCTTTATACCAGTAATGAGTGCTTTACCTGGTCCAATCGCGA
AAGGCTTCAGGAATGA
>Cluster_12324
GTGACGCAATCTGCAATGTGCATCCCGCTGTGGCCCGCCCGGAACGGCAATACTGCGCATCTGGTCATGTGCCCTTTCGC
TGGCGGCAGCAGTAGCGCGTTTCGCCACTGGCAAGCTGAGCAACTGGCTGATTGCGCGCTTTCTCTGGTGACCTGGCCGG
GGCGCGATCGCCTTCGCCATCTGGAACCGCTCAGAAGCATTACACAACTGGCGGCACTGCTGGCGAACGAGCTGGAAGCA
TCCGTATCGCCTGACACGCCGCTTTTACTCGCCGGGCACAGCATGGGGGCGCAGGTGGCGTTTGAAACCTGCCGACTTCT
GGAGCAACGGGGGCTTGCGCCACAAGGACTGATTATTTCCGGGTGCCATGCCCCGCATCTGCATTCTGAACGCCAGCTCA

GCCATCGCGATGATGCCGACTTTATCGCTGAGCTGATAGACATTGGCGGATGTTCTCCTGAACTGCGGGAAAACCAGGAA
TTAATGTCGCTGTTTCTTCCTCTTCTGCGCGCTGATTTTTACGCCACCGAGAGCTATCACTACGACTCGCCCGACGTCTG
TCCGCCGCTGCGCACGCCTGCGCTGTTATTGTGCGGCAGCCACGATCGCGAAGCCTCCTGGCAGCAGGTCGATGCCTGGC
GTCAGTGGCTGAGCCACGTTACAGGCCCGGTGGTGATTGACGGCGATCATTTCTATCCCATTCAACAAGCCCGGTCCTTT
TTTACGCAGATTGTCCGCCATTTTCCCCACGCATTTTCTGCAATGACCGCGTTGCAAAAACAGCCCAGTACTTCAGAAAG
GTGA
>Cluster_48568
ATGAGTTCGGTTGAAAATGTAATGACAAATGATGATCTGGATGAGCTGACAGCCATGTTGCAATCACTTGATGAACCAGT
AAAAAAAGCTGCACAGGTTGAAAATACTGATGATATTGACGATCTGCTTCTCGGCCTCGATGCTGGCGTAGCCATGAGTT
CTGATGATGTTGCCGAAGAACTGTTCAATGAAGAAAAAGCAGGTGATTTCAGCTCCGCTTTAAATGAGTTGGAGTTAGCT
CATGAGCCTATAAACGTAATTAAGGCTGAAAGTGTTGAATCTGCCGAAAACGAGCCAGAACAATTGGGGTTTATTGAGGT
TGAAGAGTGTGTTGAGGTTAATGATGAATTAAAAGTTCAACAGTCAAATGATAGCAATACAAATAAAAAAGCTCGTACTG
CAAGAGGTCCTCGTTTTACTTTAAGTGATAAAGATGATTTGTTTTTCAATAAAGCAGGCTTAGAAAAAGATATTTTTCTTA
GACGCTTACGAAAACGCGCCTGTCAAAGCAAAGGATAAGATATTAAACCTTCTTAATTGGTTTAGCGGAGGTCCAGATAT
TAGTGTTTACACGGTAATTTCCATGAGACACCTTCTCACAGAAAAGAAGGCTACAAGTAATAGTATTAAGATTGCTTTAA
TGAGCAATCCAGAAAAACCGTATCCGCTTAACACTGCGTCAACTCAGGCTGGGCAAATGATGGCTGTATTTCCAGCTACA
GGAATTGCTGTTAGAGACGGTGGAAATCTAACATTGAACGAAGAATCACCGATCGTTAAGAAGTTTGTCGCGGAGTACAC
TATTGGATGA
>Cluster_64792
ATGGCTGGAAATCAGCGTCAGGGGGTGGCGTTCATCCGTGTCAATGGCATGGAGCTTGAAACGATGGAAGGTGCCTCCTT
CACACCTTCCGGCATCACCCGTGAAGAAGTGACCGGCTCGCGGGTTTATGGCTGGAAAGGTAAACCCCGCGCCGCAAAGC
TGGAATGCAAAATTCCGGGCGGTGGTGATATCGGTCTGGATGAAATCATCGGCTGGGAAAATATCACGGCAGAGTTTCAG
GCAGATACCGGGGAAACCTGGATGATGGCGAATGCCTGGCAGGCAGATGAACCGAAAAACGACGGCGGCGAAATTTCGCT
GGTACTGATGGCAAAACAAAGCAAACGCATTGCATAA
>Cluster_39818
ATGGTGGAGAAGAATGGCACGCTGTCGAAAAAATCCAGACTGGGCGAAGCGTTCAGCTATGTACTGAATCAGTGGGATGC
CCTCTGTTATTACAGTGATGACGGTCTGGCGGAGGCGGATAATAATGCTGCGGAAAGAGCGCTTCGTGCAGTCTGTCTCG
GAAAGAAAAACTTTATGTTCTTTGGCAGCGATCACGGCGGCGAGCGTGGAGCACTGTTGTACGGGCTGATCGGCACCTGC
CGTCTGAACGGTATCGATCCGGAAGCGTATCTGCGCCATATCCTGAGCGTACTGCCGGAATGGCCTTCCAACCGAGTTGA
TGAACTCCTGCCATGGAACGTAGTACTCACCAATAAATAA
>Cluster_30588
GTGACCTTTGAGCAGAAAAAAGCCCGGGCCATTGCCCTGATGGACAGCAAAAAGATGTGGCGGAGCAATTATGCCCCTCC
TCTTCTGCGTATTCTCTGGCGGCTGGGTATCCGGCTTCCGCCATTACCCTTTATGCCGTTCTGGCAGGTCACGGTGCTGA
CGGGGGGCTTATGGGGCACTTCCTGGGGATGTGCAATGTGGTTTATCTACTGGGGGCCGTCAGGAATGGTAGCAGGTGAG
GCCATTATTATAAGTATCACGAGTGGTTTTTGTCCGGACTGTGCATGGCCTCTTTCCACTGGTGGCGCAGGAAAGTTAA
CCGGCTGCCACCATGGGATGATGTGTAA
>Cluster_37497
ATGTCATTTCTGTTACCCATCCAATTGTTCAAAATTCTTGCTGATGAAACCCGTCTGGGCATCGTTTTACTGCTCAGCGA
ACTGGGAGAGTTATGCGTCTGCGATCTCTGCACTGCTCTCGACCAGTCGCAGCCCAAGATCTCCCGCCACCTGGCATTGC
TGCGTGAAAGCGGGCTATTGCTGGACCGCAAGCAAGGTAAGTGGGTTCATTACCGCTTATCACCGCATATTCCAGCATGG
GCGGCGAAAATTATTGATGAGGCCTGGCGATGTGAACAGGAAAAGGTTCAGGCGATTGTCCGCAACCTGGCTCGACAAAA
CTGTTCCGGGGACAGTAAGAACATTTGCAGTTAA
>Cluster_17475
ATGATAACAATGATGAAGTATTACAAAGATGAAAATAACGTCGTATATGCCTACGATGCGTATGGCACACAGGATGCTTT
TATCAAAGAAGGGCTTGTGCCAATCACCAGAAGTGAGGCTATGGAAATTATTAATCCCCCACCGACTCATGAACAACTCA
TCCAGGCTGCTGAAAATGAGCGACAGCGATTACTGTCCGCCGCTGATGCAATTATGCTCGACTGGCGCACAGAGCTGATG
CTGGGTGAAATCAGCGATGCCAACAGAGCTAAACTTTCGGCGTGGCTTTTATATAAGAATCAGGTTAAGGCTGTTGATGT
AACAACCGACCCTGAGCATGTTAACTGGCCTGTCATCCCGGAACTTTAG
>Cluster_27646
GTGGCCAAAAAATTGTGCTGATATTCGCTTTTACGATAACAAAAAGCCAGAACTGGAAAATGGGGTGCGTTTCTATTTCAA
AACCTTTGGTTTTCCTGTTGAAGCTCAGGTTGTGGAGTTTGTCCCACCGATGGAAGGCAAACCTGCTCGTCTGGCCTGGC
ACGGATGGTCCGGTGAAGAAAATACTACTCAACGTCTCGATGTACATCATGCATGGTTACTGGAGGATCTTTCCAGTAAC
CGGGTACGTATCCTGACGCAGGAAACACAGAACGGAGTTCCGGCAAAAGAACTGGCAAATACTCGCCCGAACCCAATGCT
GAATGGTCACCAGGCATGGCTTGATGGCCTAGTTGAAGCAGCAACGAAATAA
>Cluster_44350
ATGGATTATTCACAGTTAAGTGATTTTGAAATTAACGTGGCGGTATTCGAAGCCATTCATAACGGATCGCCGGATTACAA
AGAAGGTGAGAATGGCGATATGGTGTTTGTCTCATTTGAGGGAGACATTGTAAACGGAGACGCAGTTGAAGTAGAAGTTG
AGCGCGGATCCTTTAACCCATGCGCAAACCCAGCAGACTCATGGCCGATTATTGAAAAATACAGGATTAGCATTATCAAT
CTCGATGAAGACGAGTGGGGTGCACGCGGTGTGGCCTACTGTAAATCTAAGCGAGCTATACATGAAAATCCCCTTCGCGC
CGCCATGATTGTCTTTCTCATGATGCAGAGAATCCAATAA
>Cluster_20789
ATGGAAACTTCTATTTTCGAATCAAAAAGAGGTGTTGAGTATCAACTAGTTAATCACTCAGGCCTTGAAATTGAAGTTCG
TCTTGATGGCAAGGCCGTTGGTAGCATTGTGTTTAGAGAGGTTGAGCTTCCACATCCACCTTATGTACTGTATCACATTA
CGAATTTAGGCCTTGATAAATGCAAAAAGCAAGGTGTTGGGAGAGCTTGCCTGTTGTTCCATAAGGAAATTTATAGCGCT
CCACTCAGTGCCGGCAGAGATGACGGTATCCTGGAAGATGATGGAAGTCACCTTACAGGGGATGGGGTTGGTTTTATAGC
CAAAATGAGACAAGAAGGAATAGTCTGCCCTAAAACAGAGTAA
>Cluster_3509
ATGAAATCATTAACCACGGAAACAGTGCTGGATATTCTGATTACATGGCTGCAGGACAATATCGACTGCGAATCAGGAAT
TATCTTCGACAACGATGAGGATAAAACAGATTCGGCAGCACTGTTGCCCTGTGTCGGCGGCAGTTCACTGACTTTCAGAT
TCTCACAATTAATAACGGACAGGCTTTTTATATTGACCGGTAGCCCTGTAAGACCGGTCAGCTTATTACAGTTCTCTATT
GTTATCGCTGTGATTTCTTCCGGTAATGAGGCAAGAACTCCATGTCACAATTTTTTATATGCAGACAACCACTTTCCCGT
GCATCTTCCAGTTGCCGGGACGTAA
>Cluster_15979
GTGGCTGACTGGCAAATACCAATCATCATTCTTGCCGGAGCTTCGCTGGTTGCTGGCTTTATCCTGCTGAAGAAGCATAA
AGACCGTGATCAAAAAGTCGAAGTTCTCTATGGGTATCCGGCGAACAGCACAACATGGCTGACTATTTACCACTACCGAA
AATCAGGCCGTTGGGTATTCGAATGGGATGATCTGTTTGCTGAAAAGCGACCAAAGTCATGGGGAGACATCAGCGAATGC
ATGATGTTTGAAGAAAGAAAATCCGGCGCAACCCGAGAAGAGTTTAACGAAGCGTGGAGGCGATTAAGTGAGAGGGGGTA
TCAATGA
>Cluster_7968

ATGTTTTCTATAAAACCAGGACCCAGAAATTTACCTATCGACAACCCCACATTGTTATCATGGAACATTACTGACGGGGA
TCTAAATTCCAAATTAAATACATTAGAATATCTAAACTGTATAACAAATATTATTAATTCTTGTGGAGTTTACCCTCAAG
GATTAAAAGACAGAGAAATTATATCAACTTTTCACGCAGAAAAAGTTATTAATGATCTGTTAAAAAACGATTATAAAATT
TCCCTTTCTCCAGATACAACTTACCGAGAGTTGAATAAAGCAGCACAGCGTAGCATTACAGCGCCAGACAGGATAGGAGA
AAGAAAAACATAG
>Cluster_48989
ATGCTGTTAAAAATGGAAGAGATTAAGCTTCAGCTCCGTCTGGATGATGATTTCTCTGATGAAGATGAGTTGCTTGAACT
GCTTGGTAAGGCCGCTCAGAGTCGTACGGAAAACTTCCTTAACCGTAAGTTGTATGCAACCGCAGATGACAGGCCTGCGG
ATGATCCTGATGGGCTTGTGATATCTGATGATGTGAAGCTGGCGCTTCTGCTACTTGTCAGCCATTTCTACGAAAACCGC
TCAACGGTTACAGACGTTGAGAAAATGGAGTTGCCAATGAGTTTTAACTGGTTGGTTGTTCCTTATCGCCTTATACCACT
ATGA
>Cluster_42473
ATGGATCAGTTCGAATGTATTAACGTTACCGACGCGCACCAGAAGTTGCAGGAAAAAGAGGCGGTGCTGGTCGATATTCG
CGATCCACAGAGTTTCGCAATGGGGCATGCGACCCAGGCTTTCCATTTAACTAACGACACACTGGGCGCGTTTATGCGTG
ATAACGACTTTGACACACCGGTGATGGTGATGTGTTATCACGGCAATAGCAGCAAAGGCGCGGCGCAGTATCTGCTGCAA
CAGGGCTACGATGTGGTCTACAGCATTGATGGCGGCTTTGAAGCCTGGCAGCGCCAGTTTCCCGCAGAGGTGGCGTACGG
CGCGTAA
>Cluster_34021
ATGTTTGTACCTAAGCGTGGTGATATTTGCTGGCTGGATTTTGAGCCGCAGAAAGGAAAAGAGATCGGAAAGTATCGCCC
GGCACTCATACTGTCCCATGAGCAATATAACCGACTCACCGGGCTTGTCATATGCTGCCCCATAAGTACGAGCATACGAA
ATGCTCCCACAGAAGTTCCGGTAACAAATCTGGACGCCCCCAGCGTTGTAGCAACCACACTCATTCAAACCCTTGACTGG
CGTCAGCGGAAAGCGCGCTTAATCACAGAGGCAGAAGATGGGGTATTTAATGATGTGCTTGAAAGAGTCATTACCCTGAT
TGGCGGAGAGCATCTGCTTAAGTAA
>Cluster_24674
ATGCCTTACTCGGCGAAACGATATCAGACAGAAAACGGTGAGGTTCCATACACCGACTGGATGAAAAAATTAAGACGTAA
AGACCAGACCGCAGCGTTAAAGGTGGATTCCAGAATTGCCCGCGCTATGAGCGGAAATTTTGGAGATCATAAATTCGAAA
GGGACGGTGTCTGGGAATTACGAGTTGATTACGGTCCTGGTTACAGGGTTTATTATTCAATCGAAGATGGCGAAATAATT
CTTCTGCTCATTGGAGGGAATAAAAAAACTCAGACAGTCGATTTAGATAAAGCCGTTAGTTATCTGCAAGATTTTAAAAA
GAGGTCAAAAAATGACAGCCACTAA
>Cluster_15788
ATGGCTATTGAAGGCGCTGCGGCGACTGTTCCATTAAGCCCCGGTGAACGCCTGAATGGACTTAATCATATTGCGGAATT
AAGAGCGAAAGTTTTTGGCCTGAATATTGAGTCAGAGCTTGAGCGGTTTATTAAAGATATGCGTGATCCACGGGATATCA
ATAATGAACAAAATAAACGGGCACTGGCTGCCATATTCTTTATGGCAAAAATTCCAGCTGAACGTCATAGCATCAGCATT
AATGAGCTGACCACTGACGAAAAGCGGGAGCTGATTAAAGCAATGAATCATTTTCGTGCAGTGGTGAGCTTATTTCCCAG
ACGGCTAACCATGCCGAATTAA
>Cluster_66874
ATGAAAAATTTGTTCGACACCGAGGGAACGGTTCTATTCCCATTCCAGACTGAAATCCATTTTATCTGGACGATTTACAC
CACAGCTAAAACCCTTTACGTCAGTGCCTGTGGGCACATCGGACAGAAGCAATACTGGAGCGCCTGTCTCTGTATTTTGC
TTCTTATGGCCTACGTGGGTCTCTGTGCTGCGGTGGTCTGGGCTGTAGTGCCCTGGCTTAAGGCCTTTATTGTGTCGAAA
TTTGCGGTTTCAGCACTCTGGGTCACGCCAGTAAAGAGTTTGTTGCTCAGGGGTATCGGTAAGATGGTGGGGGCCATCTG
CCGAAAGTTTACTCTATTAAAGTAA
>Cluster_64797
ATGTCAGGCACTTCGCTTAATTCACAACGGCTGGACACTTCTCGCATCACTTGCACGGCGATTATTAAATGTCTTCGCCC
GGTCTACCGCCGCGCCGGTATCGCCTTTACGCGTGGCGAAAACACAGTGGAAGTCACCGAAGAACAACTGGCGATCATCC
GTGCCGACAGCGTGTTGTCTGTTGTATCGACAACGGCAGCAGAAACGCTCGCTGAGGCTGGGGGGCTGGACGTTCTGGGT
GTGGGCGATCTGAACGCCCGTATCCGTGCGACCGTTGCCGGTCTGGATCAGGCAAACCCGGAACACTTTACCGCAGGGGG
GGAACCCAAAGTGAAAGCGGTCAGTGCGGTGCTGGGGGAAAACGTCACTGCGGCACAAATCAAAGCAGCACTGGCTGAGG
CGCAAGCATGA
>Cluster_14899
ATGACAGCACAGATAGCGGCTTACGGTCGGCTGGTGGACGACCCGCAGGTAAAACAGACCAGCAAGGGCACACCGATGAC
GCTGGCGCGTATGGCGGTATCACTGCCATGCAGTCAGGCACAGGACGGACAGGCGACGTTATGGCTATCGGTCATCGCAT
TTGGTAAGCAGGCCGACTTCCTGGCTAAACATCAAAAAGGCGACGTTGCCAGCGTATCCGGCACGATGCAGGTCAGCCAG
TGGACCGGACAGAACGGGGAGACGCGTCAGGGTTATCAGGTTATTGCAGACAGCGTAATCAGTGCCCGTGCGGCACGTCC
TGGCGGGAACAGACGCAAAACCACAGGCGCACAGGGTAATCAGCCACCAGCGGGAGGCGATGACCCTTACGGTGATGATA
TTCCGTTCTGA
>Cluster_24998
ATGAAAACGGTCATCACCTTCGGCACCTTCGATGTGTTTCATGTCGGTCATCTGCGCCTTTTACAACGTGCCCGCACGCT
GGGCGAACGGCTGCTGGTCGGCGTATCGTCCGACGCCTTAAATATCGCCAAAAAGGGCAGAGCACCGGTTTACCCTCAGG
ATGACCGCATGGCGATCATCGCCGGGCTGGCGTGCGTGGATGGCGTATTTCTGGAAGAGTCGCTGGAGCAGAAAGCGGAG
TATCTGCGGGGGTACCGTGCGGATATTTTAGTGATGGGCGATGACTGGGCAGGAAAGTTTGACAGCTTCGCGTATATTTG
TGAGGTGGTCTATTTCCCCCGCACGCCGTCGGTGTCGACGACGGGGATAATTGAGGTGATTAGGGGGAATTATATAAAGT
ACTAA
>Cluster_50258
ATGAAAAACGAAACCTTCGGTGCTCGCCTCTTACATAGGCGTAAAAAATTAAAACTGTCTCAGGCCGCATTAGGTAAGCT
GGTCAAAGTGGCTCACGTAACAATTTCTCAATGGGAAAGAGATGAAACACAGCCGGCGGGGAAAAGATTATTCGCACTGA
GCCAAGCGCTTCAGTGCTCACCGACTTGGCTTCTTTTTGGGGATGAAGATAAACAACCAGGCGAACCGATCCCGAATAAT
CAGCCAGCCATTCTGACAGAAGATCAAAAAGAGTTACTTCAACTGTTCGACGCACTGCCTGACTCAGAGCAAAAGGCCCT
GTTGTCAGAGATGCGTGCTCGAGTTGAAAATTTCAACAAACTTTTTGAAGAACTACTTAAAGCTCGCAAAAGAAGCGCAA
ACAAATAA
>Cluster_54486
ATGAATGCGTCATATGCAGTTATTGAAAATGGGATGGTTGTGAATGTCATTGTCTGGGATGGCGAGGCTGAATTCACAGT
GCCGGATAATCAGCAGCTCATTAATATTTCTGATATCAGTGAGCAGCCCGGAATCGGCTGGGTTTATTCAGACGGGGGAT
TTACTGCACCACCCACCCAGGAACGTTCTCATGATGAACTGGTAGCTGACGCTGAACAGAAAAAACAGTCGCTGTTAGAC
GCAGCAATGGCCAATATCAGCGTGATTCAGTTAAAGCTGCAGGCCGGGCGCAAACTGACGCAAGAGGAAACTACCCGACT
TAACGTTGTGCTGGATTATATCGAGGCTGTGACGGCAATAGATACCAGCACCGCACCTGATATCATTTGGCCTGTTTTCC
CTGCAAGCAGATAA
>Cluster_58981
ATGCTCGATAAGCTCGACGCCGCCTTACGTTTTCAACAAGAGGCGCTCAATCTGCGCGCCCAGCGTCAGGAAGTGCTGGC
AGCAAACATCGCCAATGCCGATACCCCTGGTTATCAGGCGCGCGATATCGATTTTGCCAGTGAACTTAAAAAAGTCATGC

AACGTGGACGGGATGCAACCAGTGTGGTTGCACTGACGATGACCTCAACGCAACACATTCCGGCGCAGGCGCTGACGCCT
CCTACCGCAGAACTGCAATACCGTATTCCGGACCAGCCTTCGCTTGACGGTAATACCGTCGATATGGATCGCGAACGCAC
CCAGTTTGCCGATAACAGCCTGCAATACCAGATGAGCCTTAGCGCGTTGAGCGGGCAAATCAAAGGCATGATGAACGTTT
TACAGAGCGGAAATTAA
>Cluster_31213
ATGATATGGAAACGAAAAATCACCCTGGAAGCACTGAATGCTATGGGGGAAGGAAACATGGTGGGATTGCTGGATATTCG
CTTTGAACATATTGGTGATGACACCCTTGAAGCGACAATGCCAGTAGACTCACGGACAAAGCAGCCTTTCGGGTTGCTGC
ATGGAGGTGCATCCGTGGTACTGGCCGAAAGTATCGGTTCCGTTGCCGGTTATTTATGTACCGAAGGTGAGCAAAAAGTG
GTTGGTCTGGAAATCAATGCTAACCACGTCCGCTCGGCACGAGAAGGGCGGGTGCGCGGCGTATGCAAACCGTTGCATCT
CGGTTCGCGTCACCAGGTCTGGCAGATTGAAATCTTCGATGAGAAAGGGCGTTTGTGCTGTTCGTCACGATTGACGACCG
CTATTTTGTGA
>Cluster_57904
ATGACATCCCGACGAGACTGGCAGTTACAGCAACTGGGCATTACCCAGTGGTCGCTGCGTCGCCCTGGCGCGTTGCAGGG
GGAGATTGCCATTGCGATCCCGGCACACGTCCGTCTGGTGATGGTGGCAAACGATCTTCCCGCCCTGACTGATCCTTTAG
TGAGCGATGTTCTGCGCGCATTAACCGTCAGCCCCGACCAGGTGCTGCAACTGACGCCAGAAAAAATCGCGATGCTGCCG
CAAGGCAGTCGCTGCAACAGCTGGCGGTTGGGTACTGACGAACCGCTATCACTGGAAGGCGCTCAGGTGGCATCACCGGC
GCTCACCGATTTACGGGCAAACCCAACGGCACGCGCCGCGTTATGGCAACAAATTTGCACATATGAACACGATTTCTTCC
CTCGAAACGACTGA
>Cluster_9807
ATGGAAAAGTTTTCATTGGAGTTCAAAAAGGTAGTATCAGAAAAGTACCTGGAGGGAGAGTTGAGTCTGAAATCTGTAGC
AAGGATGTACGGTATCAGTCCCTGCACAGTAAGAAAATGGGCTTATGCCTATCGGGAACATGGGATAAGCATACTTACGG
GTAAAAAAGGACGCTATTCTGCTGAGTTAAAACTCACAGTAGTAAAAGAAGTCGTGGATGACGGTTTTTCTGTTCGTGAA
GTAACGGTAAAGCACGGAATACCAGCCTTTGGTACTGTATGCAACTGGCTTGAAAAATATAGAAAATATATAGCGAAGAT
GCTTTCATTCGAAAAAATAAAAATAGTATTCCTGTGCCGGATAAAAGCGCCATTTCAGCACCACCACTACCAACATTAA
>Cluster_28439
ATGACCATTCAGTTTATCTCTGCGGAGGAGATAATTTATTTCCACGATAAGTTGCTTAGCGTTACACCAGGTGTAACAGG
AATGTCTGATCCAGGGCGAGCAGAAGCGCTTTTGTACCGGGTACTGAATAAGTACGAGTATGAAGGCGTTTCCGATCTTT
GGCTGCTGGCGGCGATGCATCTATTGGCAATCTCGCGCGGTCATATTTTTAATGATGGTAATAAGCGCACCGCACTGTTT
ATCACGCTATTATTCCTTAAACGTAACGGCCTCATACTTGCTGCAAACCCAGAGTTTGTGGAATTGACGGTTGATGTGGC
GGCGGGAAAAGTATCGTTAGAAGATATCGTTACCCGCTTGCGTCAACACGGAACCTGA
>Cluster_28698
ATGAATACATTACCCGACACTCACGTACGGGAGGCATCGGGCTGCCCGTCTCCCGTCACCATCTGGCAGACACTGCTCAC
CCGACTGCTGGACCAGCACTATGGCCTCACGCTGAATGACACACCGTTCGCTGATGAACGTGTGATTGAGCAGCATATTG
AGGCAGGGATTTCACTGTGTGACGCGGTGAACTTTCTCGTTGAAAAATACGCGCTGGTACGTACCGACCAGCCGGGATTC
AGCGCAGGAGCCCCATCGCAGTTAATCAACAGCATTGATATTCTCCGGGCTCGCAGGGCGACCGGCCTGATGACCCGGAA
CAATTACAGAATGGTAAATAACATTACCCAGGGCAAGCATCCGGAGGCAAAACGATGA
>Cluster_19972
ATGAAAGACATCGATACTGAAACCCGGAATAACACCGCGGCGGATGATGTGACGGCAGGTGAGGATATGGCTGTCGAACG
TGGCGTAAAACTGACCCGACCAATTGAGCGTGGTGGCGAAAAAATCACGTATGTGGAGATCACCGGGGCTATTGAACAGG
CTGGATCCCTGCGCGGTCTGTCGCTGTCTGATGTGCTGAATCTGAAAGCGGATACCATGTTCACGCTGTTGCCTCGCGTG
ACCTCGCCACGACTGGATGAAGTGATGATTAAAAAAATGTCTTCACGCGATTTTATTCAGTTGTGCGCTGTGGCTGTAAA
TTTTATGAGCGAGCCAGACTCTGGCGCGAAGAGCGTGCAGGAGACGGCAGCGTAA
>Cluster_63338
ATGACCATGTACGCAACGCTTGAAGAAGCCATTGACGCTGCACGCGAAGAATTTCTTGCAGACAACCCCGGCATCGACGC
CGAAGATGCGAATGTGCAACAGTTCAATGCCCAAAAATACGTTTTGCAGGACGGCGACATCATGTGGCAAGTTGAGTTTT
TTGCCGACGAAGGGGAAGAAGGTGAATGTTTACCTATGCTTAGCGGTGAAGCCGCGCAAAGTGTTTTTGATGGCGACTAT
GATGAGATAGAGATACGCCAGGAGTGGCAGGAAGAGAATACATTACATGAATGGGACGAGGGGGAATTTCAGCTTGAGCC
ACCGCTGGATACCGAGGAAGGACGCGCAGCAGCTGATGAGTGGGATGAACGTTAA
>Cluster_22108
ATGCGAAAAATAAAAACAAAAATCTACTTGCCTACAGCTTTGACAGGAAACATGGATAAACACTATATCCCTGTTTTACA
GGATTTAAGAAAAACGATATACAGCGACAGAATGTTATCAAGGCTTGCAGATAGCGGCAATATTGTTATCCATAGTTCAG
TTGGGTATCCGGTTGCTAAATACAAAAACACGGGGATTAGCATAGGTATAGAGCCATTGAATCCAATGATAAGACAAGAT
CTGACTCTTGGATATATAGTGGTCGTCAGAAACGGGAAAACATCACAGGAGATTAATGGTTTACTTAACAGGTCATTGCT
AAAAGCAATAAGCACTTTTAAGGATCACATCAACGAATATGAATCAGTAAAAATCAAAATGCGATAA
>Cluster_24053
ATGACTACAACGCTTTTAGCCGTTAACGGAACGCTGATGCGCGGCCTGGAACTCAACCCAAATATGCAAAAAGCGGGCGG
TATTTTTGTCCGTGAAGACCGAACTGACGCCCATTACCGGTTGTGGAGTATTAATGATCGCCATCCGGGAATGATTCGCG
TGAATGAAGGAGGAACACACGTCGACGTAGAAATTTGGCAATTACCGCTGGCCAGTTTTGCCGCTCTGTTAATGAGCGAA
CCCGCTGGGCTGGCAATTGGCAAAATCAAACTTGCCGATGGTAGCGAAGTGCTGGGCGTACTGGCAGAAAACTGGCTGAC
CGAAGGCCAACGGGAAATTACAGAACTGGGTAGCTGGCGCAAATACACCGGGCATTTTCATACTGTGTAA
>Cluster_39340
ATGAAAGAACCGCAACAGCCAGAAATCTTTGCTGACCTTGCGCACCACGTCGCAAACACATTGAAAGAAATGGTTGGAAT
GGAAGATGATATCGCTGAACAGATTGCCGTCGCTGTGGCTAATCACATGATGCAGGCATGGGGCGGACAAACCATTTACC
TGCCCAAAGGCATGGTCTTCATGACATCGGCGCGGGACTATAAAATCTGGCAGGAGTGTGATGGACGCAACTACCGCGAA
CTGGCGAAGAAGTACAACCTGACTTTGCAGTGGATCTACAAAGTGGTGAAAAAGATCCAGCGTTCTGAAAGCGCCCGCCG
ACAGCTCCCATTATTCTCTATCATTGATGAACCTGAAGCAGTAAAATCACCCCGGAAGTGA
>Cluster_43395
ATGGGAAGAAAGAAAAAGAACAACTTAAAACGTATTGAAAAAAACAGTAACTTCAATTCAACAAGACAACGTCCACCGAC
ACACACCAATAACGAAATTCCAGGGGAAGAGATAATATTTATTCTTGAAACAGAATCCCCTGAATTAAGCGATGAAGAAT
TAGAAATAATCCAGTCAATGCCAGGCATTTACTTTCGCCCAAGAACAAGAAAACAAACTATCATTTCTGATGGCTCGCTG
AGGCGGTGGCAGCTTTTAGGGTTTAGCTCCTATCATCATTTTTTGGGATTTAACGCTTTCAATAATTGTGTAGGTGGTGC
ATCGGAAGTCGATCTGATGGCTCATTTAGAAGATACATTAGCTTCTAACAAACGTTCTAACTAA
>Cluster_46632
ATGACTTATACGGTAAAATTCAGGGATGATGCGCTCAAGGAATGGCTGAAACTGGATAAGACCATTCAACAACAGTTTGT
GAAAAAACTAAAAAAATGCAGCGAAAATCCTCATATACCGTCGGCAAAACTAAGAGGGCTAAAGGACTGCTACAAAATTA
AACTACGTGCATCGGGATTTCGTCTGGTTTATCAGGTGATTGACGATATGTTAATTATTGCGGTTGTTGCGGTAGGAAAA
CGAGAACGTAGTAACGTTTATAATCTCGCCAGTGAGAGAATGAGATAG
>Cluster_39472
ATGACTGAACAAGAAATGCCGAGATACCAGTGCCACAAAAAAGTTCGCGCCCTGAAGATTGGCTCTATAGAACATAAGCC

AAACCCAGATCAGTCTGGTAAGACTGGCTCTTCTAGTTATGGGGCAATTATTCATCCGGATGATAAGAAATACGCAGCAT
TTGATGTTAGCGCGGAATATATCTGTAAGCACCGACCAATGTCTGGAGGCTATTACGTTGTCTATGAGGATGGATATGAA
TCATATTCTCCTGCTGAGGTATTTGAGTCTGGATATTCAAAATTATAG
>Cluster_17112
ATGCAACAAGAAGCGTTAGGAATGGTAGAAACCAAAGGCTTAACTGCCGCCATAGAGGCCGCAGACGCCATGGTGAAGTC
GGCAAATGTTTTGCTGGTCGGCTACGAAAAAATAGGTTCCGGGCTGGTGACCGTCATCGTTCGTGGTGATGTCGGGGCGG
TAAAAGCGGCAACAGACGCGGGTGCCGCAGCTGCGCGTAACGTCGGCGAAGTAAAAGCCGTACACGTTATTCCCCGCCCA
CATACCGATGTCGAAAAAATCTTACCAAAGGGAATTAACCCATGA
>Cluster_47067
ATGAATAACCATTTTGGTAAAGGCTTAATGGCGGGATTAAAAGCAACGCATGCCGACAGTGCGGTTAATGTGACAAAATT
CTGTGCCGATTATAAACGCGGTTTTGTATTAGGCTACTCACACCGGATGTACGAAAAGACCGGAGATCGCCAGCTTAGCG
CCTGGGAAGCGGGTATTCTGACGCGCCGCTATGGACTGGATAAAGAGATGGTAATGGATTTCTTTCGTGAGAATAATTCC
TGTTCTACGTTGCGCTTTTTTATGGCCGGTTATCGCCTCGAAAATTGA
>Cluster_42997
ATGGATAAGGCACTTTTTGAGCGGTTGACTCACAGCATGGCCCAGATGAATGAAATTATTGAAGGAACGCGACAGCCTTC
GCGGACTTTTGAAGTTGATGCTATGAAAATCAAAGAAATACGCCGCGCTTCAGGGTTGTCACAATCTAAATTTGCCGATC
TTATTTCTGTAAGCGTAGACACGTTGCGTAACTGGGAGCAGGGGCGACGTTCACCGACCGGGCCTGCAAAGGCTTTGCTG
CGAGCGATTGCCAATGATCCGCAACATGTGCTGCAAGCACTTAACCGTTAG
>Cluster_22098
ATGAAGATAGAACTTATTACGACAAAACAGTTTATTGAACAAGCCGAATATTATTTCAGGAGCTATATGGACGGATTGCG
AAGGAATGCACCAGATGATTTTTATTATTTTATCAACAATAAATACAATATGAATGACATCATGGAAAGTATCATTAAAA
AAACGAGATATCATTTTTACGATGACTCAGAAGAAGGTAAAAGGAATCGGATTTATGGAGAAGTAAGCCACAGTAAGGTA
AAGCAACACCTTCGTCAGCTTTGGATTATCTATAAGTGTGTATACCGCTAA
>Cluster_45241
ATGGATGAAGAATATCGTAAAGACCTGCAATTGTGGTTTGGCCTGACGCATGCGTCGTTCTGCGTGATGCCGAGAGTATT
CATGGAGGCTATGCCGCAAGAATGGCAAGAGAAGATGGCGCAGTTGCTTTTTGAGTATGGCGACACGATCAAAACGGATG
TCTGCGGAGTTCACAGCTGTTTCGTTACTGCCAAAGACGGCAACAACCGCTTTATGAGGATGCCAGAAGATATTCTGAAC
TATCGTCATCCCCGGCGTGAGTTCATCGAATCATTTCTGAAGAAGTAG
>Cluster_49774
ATGAAAAATATATTGCTTAATAACTGGCTGAAGATTTCAGTCATGAAAAATGGTGATTTGTCGCTAGCTGATATTAAACG
CGATAAAAACACTGGGGATATGGTGGAATCAACTATAGCCATTTATGCGGATAAATTAAATCTCCTGTCTGATGTGGTCA
ATTTACTTGTTAAACGCGCTGTATTTCACAAGCAAATCTCCTCCGTGGATGAACTGACGAAATTAACGACAGAAATTGCC
AGCTATTGCGCTGATGAATTTAAAAAACTGAACGACAAAAGGAGCTGGTAA
>Cluster_55785
ATGTTTACTATCAACGCAGAAGTACGTAAAGAGCAGGGTAAGGGTGCGAGCCGCCGCCTGCGTGCCGCTAACAAGTTCCC
GGCAATCATCTACGGTGGCAAAGAAGCGCCGCTGGCTATCGAGCTGGATCACGACAAAGTCATGAACATGCAAGCTAAAG
CTGAATTCTACAGCGAAGTTCTGACCATCGTTGTTGACGGTAAAGAAATCAAAGTTAAAGCTCAGGACGTACAGCGTCAC
CCGTACAAACCGAAGCTGCAGCACATCGACTTCGTTCGCGCTTAA
>Cluster_28667
ATGGACAAAGGCTATGAAGCCGAATGGCTGGAGGCCTGTCTGAAAAACCGCCGTTACGAACCTCATATCCAGTCAAGGAA
AGAGGAGTCGGAAGCCATCAAAAGCACGGATTTTAAAGCTCACCGCTGGGTCGTGGAGAGAACGCACAGTTGGATGAATC
GCTTCCGTCGGGTGTTGACTCTCTGGGAGAAGAAAGTCGAAAATTATGTGGCAATGCTGTATCTTACCTGCGGCGTCATT
GTCTGGAATAAAATCCTATTGGGATAG
>Cluster_11446
ATGGTAACCATTGTCTGGAAAGAATCCAAAGGTACGGCAAAAAGCCGCTACAAAGCTCGCAGAGCAGAACTTATTGCCGA
GCGACGCAGTAATGAAGCACTGGCGCGAAAAATTGCGCTAAAGCTCTCTGGTTGCGTCAGAGCAGACAAAGCAGCATCAC
TCGGAAGCCTTCGCTGCAAGAAGGCAGATGAATGCAGTGGAAGTATTTGCCTGCCAAACGTAGCCATTTACGCGGCAGGC
TACCGGAAATCAAAACAACTGACGGCGAGATGA
>Cluster_10546
ATGTCAGATAACACTATTAAAATCATACCTCAGCACATGACCGCCACATCAGTTCTTATTACGCCTGATCGTGCTGAAAC
AATCATTACTTTTTACCGCCATGAATTTGAGCATCACATGCAGTCTGATGAGCAAGGAAAGAATAGCTTCCAAGTAAAAG
TTGAGTTGATCCCTAACATGTCAGTCTCAATGAGCCCGGATCAAGCTGTTGCATTAGTAAAATCATTACAGGTAGCTCTC
AGGGATAATGGGCTATGGAAAGACTGA
>Cluster_11724
ATGGGGGAATTTGGAGGTATGTCCCATGGTGAGGATTACTCATCAGCACCAAATAGCATTAAGAAATGTGCAAAAAAATA
TGGTTTCGAGCCAGAACCAAGTAAAGATCTGTCATGCAAAACCTCCTGGCTATCAACTACTGATGCGGCTCTTGCAACTG
GTGCTACAATTGGCGCAGGGTATGTTATATATAGAGTGATTCGATTTATACCTTCACTTGCACCACCTCTTTGGTGGTCA
ATACCTGCAAACGTTGTTACTCCATGA
>Cluster_48086
ATGGTCACAGTTAACCTTCATTGCCCCCGTTGCCAGTCCGTTCAGGTTTACCGTCACGGGCAGAACCCAAAAAGACATGA
TCGATTCCGCGGCCGTGACTGTCACCGGGTATTTCAGCTCAGTTACTGTTACGAGCCCCGTAAACCGGGAGTTAAAGACC
AAATCGCTGAAATGACTTTCAACGGTGCCGGGGTTCGCGATACTGCCCGTACGCTGAAAATCGGCATTAATACAGTCATC
CGAACTTTAAAAAACTCTCGCCACGAAGAATAA
>Cluster_48664
ATGACTGATTTCACCATTACCACCAAAGCACAAAATGTATTCCTCGAATCATGGCTGGACTTGCCGGAAACAGAACAGCA
AGAAATGGATCATGTGGATTATGATGAGCAGGTAAGCACGCGATTCTTCCACTTTGAGGGATGCGTTTACGACATTGCCG
ACTTCATGCGAGATGACCGCTTCCCTGAATGGCACGCAAGCTATCCACTAAACGCCTTTGCCATGCTGATGATCCGTGTG
GATGATTCAGGGGATACCATCGACATCGGTCTGCTCCACTGA
>Cluster_66859
ATGGGAAAAAAATCAGGAAGAAAGGAAAACTCCCGTCATTGTGGTGAAAAAACGTCGTACTTTTTCACCACCGTCCTTATC
CGAAAAAACAGATATTATCACACCTGTATTTACTGAACAGACGGCAGAATCAGCCCCCGCCGGAATAAATTCATCTGCCG
TCGAAACACATATCCCCGAAGCGCCAGCCAGGAAGAAGAAAAAGAAAAGACACCGTTTTCCACGTCCCTCCCACTGGACC
CGTGAGGGCACTGTTGCAAATAGTCGGTGGTGA
>Cluster_1274
ATGGACAATAACACCATTTCTCTACAGGAGTTGCTCGACAGCATTTCCAGGCTTCGGGAAGACGTGAATACCCTTACCGT
CGCCTTCTCATATCTGGCATTCTCAATTCCAAGGGAACAGATGCAATCAACGCTGGCATCAATCCAGTTTGAATCATGCA
ATCCCAAATGGTCTCAGGAACAACAAGACTCTTTCAGGCGGCTTGCTGTATTACTGGATGAAAAATATGCTGGTAAAATT
ACCATTTCGGCGGACTCTTCAGAGAACCCGTAA
>Cluster_58953

ATGTTCAGACCTTTTTTAAACTCTCTTATGCTCGGCAGTTTGTTTTTTCCTTTTATTGCCATTGCTGGAAGCACCGTGCA
AGGGGGCGTGATCCATTTTTATGGCCAAATTGTGGAACCGGCATGTGACGTCAGCACCCAGTCATCACCCGTAGAAATGA
ACTGCCCACAGAATGGTTCTATTCCGGGCAAAACCTACTCCAGCAAAGCGTTAATGAGCGGCAATGTCAAAAACGCGCAA
ATAGCCTCAGTAAAAGTGCAGTATCTTGATAAACAGAAAAAGCTGGCTGTAATGAACATCGAATATAACTAA
>Cluster_36075
ATGACGATCACAAAACAACGAGTAGAAAAAATCATATATCGCCATGAAATGGGACTGAACAGCGATATCACTGCCGAAGA
GGTTTATGACCTGGCTGTACTGGCGCTGAATTTATCAAATATCGCAAACCTGAAGCGATACGAGCTTGATATGGATGGTT
GCGACTCGTGCGGTCAGGATTGTGGCGCAGATATGACTGAAGATCCTGATGGTGATTATGTCCTGTTTGATGACGTGGTT
AAGTTGTTTGAATTTGATACAACCACTCAAAAGTTAGAAATCCCGGCAAAGGAGGCTGCCAGTGAGCAAGATTGA
>Cluster_20536
ATGATGGATAAAGTACGCCGTGAGTCGATGCGCTGGAATCTGATGCTGGCGCTGAACAAGGCGCGGCCCTATACCAGCAA
TGAACGGTTTTTACTGGCGGTGATGCAGGCGATTTACCCGGATGCCACCGCGCTGGAGCTGCGGCGCGAGCTGGACTATC
TGGCCGACCGCCGGATGCTGGAACTGGTGAAGGAGCCGTCCGGCACCTGGTTCGGTGATTTAACCCGTCTCGGTGTTGAT
GTGGTTGAGTACACTGTGGACTGCGGCCCCGGCATCGCCCGCCCGCAAAAGTACTGGAGCGAATGA
>Cluster_35759
ATGAATAATGGTGTATTTTCAAAATCAGAAGAGCTTGCTGCCCTGCTGATTGCCGGTGGTTTGCCGAAAAAAGAAGTGCA
TGACTTTGTGAATTTTGTATATGAGACGTCTCATGAAAAAGCTATTGATGAGGTCATGGCGTACCGGTTTGGCATTATTG
CTGAGTCACTGAGTCCTCACTGGATTATTGAAAATCTCCGCGCTATTGCTGATGGCAACGATAAATCAGCAGCCGTTGCT
GCACTGGATGTGTTACGGCAAATGAGTCTGAAATTTTATCCTGAATTTATTCTGAGGGCCGCAAAATGA
>Cluster_35594
ATGTATGAGCTACTATTTCACCCCGAAGCGGAAAGCGAAATTTATGACCTCGAACCTATTATGCAAGCAAAGGTACTTAA
GGGACTAGAAAAGCTGGAAGCGAAAGGGCCGGAACTCCGCTATCCAGATACAGGCATTATCCAGGATGGTTTGTTTGAGT
TGCGCGTAGGAAGAAAAGACATTTCAAGAACATTTTTTGCTTACGCTAAAGGTCGAAAGATCTATATTTTAAGAACGTTT
ATTAAGAAGACCCCTAAGACTCCGAAGGCCGAAATTGCTCTGGCGACGTCAAGATGGGAGGAACTGAAAGATGGCTGTTA
A
>Cluster_66404
ATGAAGGCAATACAATGTTGTTATTTCCTTGCTTTAGTCTCCAGTCATTTAATGGCTGGAGTTATGGGTGATGATAATAT
ATTTTTAAGTAATTCGGATTCAGCTGCTTTATATTGTGTGGCTAAAATATTTCCAGATGGAGAAAATATTTTAATATATA
AAGTAAATATAATTAATGCTAGTTCAAAAAAAGATGTTTTTTTTTATGAAAGCCCAACATCAACCCACAATGACATAATG
GGGGGCGGGGGAATGTCATATGCCTATGAATATGATGTAAAATATAATATATGTCATTTGAGAAAAAAGAGCAGAATGAG
GTAA
>Cluster_66537
ATGAATGGCACTATTTATCAGCGGATAGAAGACAATGCGCATTTCAGGGAGTTAGTCGAAAAACGGCAACGGTTTGCCAC
CATCCTGTCGATTATTATGCTGGCAGTTTATATCGGCTTTATTTTACTGATCGCCTTCGCGCCCGGCTGGCTGGGCACGC
CGCTGAATCCGAACACCAGCGTCACACGCGGTATTCCAATTGGTGTTGGAGTGATTGTGATCTCCTTTGTTCTCACCGGT
ATCTACATCTGGCGGGCGAACGGCGAATTCGACCGTCTTAATAATGAAGTCCTGCATGAGGTACAAGCATCATGA
>Cluster_48099
ATGACGAAAATTATTGCAGTAACCGCATGTCCTTCAGGTGTTGCCCATACCTATATGGCAGCAGAGGCGCTGGAAAGTGC
CGCGAAAGCGAAAGGCTGGGAAGTGAAAGTAGAAACCCAGGGGTCAATTGGTCTGGAAAATGAACTGACTGCGGAAGATG
TGGCGAGCGCTGATATGGTGATTCTGACCAAAGATATCGGCATCAAGTTTGAAGAGCGTTTTGCTGGCAAGACCATCGTG
CGCGTCAACATCAGCGATGCAGTAAAACGTGCTGACGCCATTATGAGCAAAATTGAAGCTCACCTGGCGCAAACCGCTTA
A
>Cluster_23976
ATGAGTGAAGCCAGAATTACACCTGATGAAGTCAGGGCACATCTTCGACTTGATGATGATTTATCCGGTGAAGGCGAACT
TCTGAAAATGTATACCGATGCGGCGCTGGAAGCCTGCCAGAAGCATATCGGGAAACGTTTTGAAGACGGGCTGGAATTTA
CCCCGGCAATGCGTGTTGGTTGCCTGATGTACATCGCTTTCCTGTACGAGAACAGGGAAGCAGTTTCACCAGTGGAGCAG
TCTGAACTGCCTATGGCTATTTCTGCGCTCTGGTCGGTTTATCGTGATGTGGGGGTGTACTGA
>Cluster_34630
ATGACACAAACAAGTTACAGCAAACTATCACAGCGCGACGTTGATCGCGCAGAAACAGATTTACTCATCAACCTGTCAAC
GCTTACCCAGCGCGGTCTGGCAAAGATGATTGGCTGTCATGAATCGAAGATAAGCAGAACAGACTGGAGGTTTATTGCTT
CGGTCTTGTGTGCTTTCGGAATGGCATCAGACATCAGTCCGATTAGTAGGGCTTTTAAGTATGCGCTTGATGAAATCACA
AAGAAAAAACGCCCGGCGGCAACCGAGCGTTCTGAACAAATCCAGATGGAATTCTGA
>Cluster_49701
ATGAACGCTTATTACATTCAGGATCGTCTTGAGGCTCAGAGCTGGGCGCGTCACTACCAGCAGATAGCCCGTGAAGAGAA
AGAGGCAGAACTGGCAGACGACATGGAAAAAGGCCTGCCCCAGCACCTGTTTGAATCGCTATGCATCGGTCATTTGCAAC
GCCACGGGGCCAGCAAAAAAGCCATTACCCGTGCGTTTGATGACGATGTTGAGTTTCAGGAGCGCATGGCAGAACACATC
CGGTACATGGTTGAAACCATTGCTCACCATCAGGTTGATATTGATTCAGAGGTATAA
>Cluster_6334
ATGAGTATTGATCGCACTTCGCCTCTGAAGCCTGTAAGCACCGTTCAACCGCGCGAAACCACTGACGCGCCGGTAACGAA
CAGCCGGGCGGCAAAAACAACCGCCTCCACCAGCACCAGTGTGACGTTAAGCGACGCGCAAGCAAAACTGATGCAACCCG
GCAGCAGTGATATCAATCTTGAACGTGTCGAAGCGTTAAAACTGGCGATTCGTAACGGTGAACTAAAAATGGACACCGGC
AAAATTGCCGATGCGCTGATCAACGAAGCGCAGCAAGACTTGCAGAGTAACTGA
>Cluster_67007
ATGAGCGCATTTTCTTTTAACACCACTTATCAGCCAATCGGTGATCAACAGAAAGACGCAATCGCACAAATCGACATCAT
GCAGAGTCGTGCTGTTCAGGCAAATCTTGCATATCAGAGCAACCAGGATGCGGAAACGCTGATGAAACAGTTAGGGCAAA
TCAATGATGAACTGGGGGCGCTGTTGGATAGCCTGGAAAATCACACGCCAGTTATCAGAAAAAAAGCCAGTGATTTGAGT
GCACTGATGATGCTTTTCAGCCAACAGACCGGTCAGCCCATGACCAGTCCGGTGTGA
>Cluster_14953
ATGAAATGCTCATCTGTTTTTACTTCAACCACTAATCATGTTTTCACATTCGAACGAGTAACGCTTTGCACTATTATCCT
TATGCATAAGGATACGGGACAACAGTATGTTGTCCTATTTACTGATAACAATAAAATTCGTGACTATAAAACCGGAATTG
TTCCTCAGTTTGGCGAACTTAAGCAGAGTGATGTTGATTTAGTCCTTTTCTACAGGGACGAATATGAAAAATATTTTGAT
TCGTTAAAAGATGGCGATGAATGCTTGAGTTTTAAAGATTTTATTGAGTGCCTTCGTTAA
>Cluster_39330
ATGAAACCCACCTCTACTTCCGGCGCACGTATCCTGCGCGTACTCAAAGCCCTGAAAGGCTACTCGCTCACTGGGTTGTC
CAATGGCGAATTGGCGGCGGCGTTGCATGAATCCCCGGCCAATATCACCCGCGCCCGCTGACACTCTGATCGAAGAGGGAC
TGGTACAACGGCTGGAAAATGGGCGCTTCGCCATGAGTATGGCGATGCTGCAAATCGCCCACGCCCACGCGACGGAAGTC
ACACGCGCTCAGGACAGGCTGAATGAAATGAATCAGCGGTTGCTGGCTGGCAGTCGCTAA
>Cluster_37500
ATGCAGTTGGAAGAAGTCGCTAAAGCGCTGAAAGAGCTAGGTCACCCGACCCGTTTGTTCATATTTAAACATCTTGTCAA

AGCCGGGGAACAAGGGTTGCCGGTCGGTGAGTTGCAAAAGCAGCTTGGGATACCTGGCTCCACACTCAGCCACCATATAT
CAGCACTGGTATCGGTAGGACTGGTCAAACAAAACCGTGAGAGTCGGACGCTGATGTGTGTTTCGCAATACGCAATGCTG
GAAGCGATAATTGAATTCTTACGCGAGGAGTGCTGCGTAAATAGCAAAATTGCCTGA
>Cluster_3136
ATGACAAAAACAGTATCAACCAGTAAAAAACCCCGTAAACAGCATTCGCCTGAATTTCGCAGTGAAGCCCTGAAGCTTGC
TGAACGCATCGGTGTTACTGCCGCAGCCCGTGAACTCAGCCTGTATGAATCACAGCTCTACAACTGGCGCAGTAAACAGC
AAAATCAGCAGACGTCTTCTGAACGTGAACTGGAGATGTCTACCGAGATTGCACGTCTCAAACGCCAGCTGGCAGAACGG
GATGAAGAGCTGGCTATCCTCCAAAAGGCCGCGACATACTTCGCGAAGCGCCTGAAATGA
>Cluster_28165
ATGGAAACACTCACCGCCATCAGCCGTTGGCTGGCAAAACAACATGTTGTCACCTGGTGTGTGCAGCAGGAAGGGGAACT
GTGGTGCGCCAATGCTTTTTATCTTTTTGATGCGCAGAAAGTCGCCTTCTACATTTTGACGGAAGAAAAAACGCGTCATG
CGCAGATGAGCGGGCCGCAGGCGGCAATTGCCGGAACGGTAAACGGTCAGCCGAAAACGGTAGCGTTAATTCGCGGCGTG
CAGTTTAAAGGTGAGATCCGCAGACTGGAAGGCGAGGAAAGCGACCTCGCACGCAAGGCGTACAATCGTCGCTTTCCGGT
TGCCAGAATGCTGTCGGCCCCGGTATGGGAAATCCGGCTCGATGAAATCAAATTCACCGACAACACGCTGGGCTTTGGTA
AAAAACTGGTCTGGCAACGGTAA
>Cluster_12687
ATGAGTATGGTTGCCAGAACTGAACCCAGACCCGCAGAGGACGACATCACCGATACCGATGATGGCGATACCCGCATTTC
AGCGGGTGCATTCTGGCCGGATATTGTGCTGCGCGAGCTGCGTCTGGCGGTACGACTGCCGGGCCGCGTGACCACCTCCC
GCCTGCTGCATACTGCCACCGGGGCTGTGGCACACGTTACCCGCGAGCTGGAAGCATGGCAGCAGGAACAGCAGGCAGCT
GGCCATCAGACGCTGGCCGATGTTCCGGCACCCGTAATTAACGGAGAAAGCGTCAATCTCTGGCACTGGCGCAATGCTGT
TTATACCGCCACGCGCGCCCTGATTCTGGAGCGTTACCGCGATGCGGACACAACGGACAAGGGCGACCGCCGGGCGGACG
CACTGGATATACAGACATCGGATTTGTGGCGCGATGTGAGCTGGGCCATCTCTGACATTCTGTGCCGCCCGCGAATCTTT
GCGGAATTGTGCTGA
>Cluster_14966
ATGAGACATTTTCTTAAGGCTCTTTCCTTTGTTGTTTCCCTGTTACCAGCTACATTGTGTGCGGCTGAAATCCAGGGGAA
AGTTATTCGCGTTCTCGATGGCGACACCATAGAAGTTAAAACACTACCCGCGAAAATTGTTGTGTATGAAGTTCCGATCA
GGGTTCGATTGATAAATATCGATGCTCCTGAAAAGAAACAGGCTTTCGGGCACTGGTCAGCAAATCAGCTTAAAGCTCTG
CTCGCAGGACAATCAGTTACCGTCTCTTATACGCAGACAGACCGCTACGGACGCATTATCGGTCGTGTGTTCACGACGAA
CGGCACGGATGCCAGCCGCTTTATGGTTCAGTCTGGCGCTGCATGGGTATATGAACGTTACAACACAGATAAATCATTAC
CGGCTTTGCAACGGGAAGCGCAGGAACAAAAGCGTGGTTTATGGGCTGACAGTAATCCGGTTCCACCGTGGGAATGGCGA
CATAAACAGAACTGA
>Cluster_59656
ATGCGTAAATTAACTGCACTGTTTGTTGCCTCTACCCTGGCTCTTGGCGCGGCTAACCTGGCCCATGCCGCAGACACCAC
TACCGCAGCACCGGCTGACGCGAAGCCGATGATGCACCACAAAGGCAAGTTCGGTCCGCATCAGGACATGATGTTCAAAG
ACCTGAACCTGACCGACGCGCAGAAACAGCAGATCCGCGAAATCATGAAAGGCCAGCGTGACCAGATGAAACGTCCGCCG
CTGGAAGAACGCCGCGCAATGCATGACATCATTGCCAGCGATACCTTCGATAAAGTAAAAGCTGAAGCGCAGATCGCAAA
AATGGAAGAACAGCGCAAAGCTAACATGCTGGCGCACATGGAAACCCAGAACAAAATTTACAACATCCTGACGCCGGAAC
AGAAAAAGCAATTTAATGCTAATTTTGAGAAGCGTCTGACAGAACGTCCAGCGGCAAAAGGTAAAATGCCTGCAACTGCT
GAATAA
>Cluster_7572
ATGAGAAAGACTTTCTTAACCCTGCTTTGTGTCAGTAGCGCCATAGCTCATGCTGCTGATGAAGACATCACCTTTCACGG
GACATTATTGTCACCGCCAACCTGTAGCATCAGCGGTGGTAAAACCATTGAAGTCGAATTTCGCGATCTCATTATCGACG
ACATCAACGGCAACTATGGTCGTAAAGAGGTGCCATATGAACTCACTTGTGACTCGACGACCCGTCATCCAGATTGGGAG
ATGACCCTCACCTGGACTGGTACACAAACCAGTTTTAATGATGCAGCAATTGAAACTGATGTACCCGGTTTTGGTATCGA
GCTTCAGCACGATGGTCAGCGTTTTAAACTCAATACTCCGCTTGCTATCAACGCCACTGATTTTACGCAAAAGCCGAAGC
TGGAGGCCGTACCGGTAAAAGCGAGTGATGCTGTGTTAAGCGATACGAATTTTTCGGCTTACGCAACATTACGGGTGGAT
TATCAATGA
>Cluster_60461
TTGTCTGAAGTCGAATTTAGCCACGAATACTGGATGCGTCACGCGCTGACGCTGGCGAAACGTGCCTGGGATGAGCGGGA
AGTGCCGGTCGGCGCGGTATTAGTGCATAACAATCGGGTAATCGGCGAAGGCTGGAACCGCCCGATTGGTCGCCATGATC
CCACCGCACATGCAGAAATCATGGCCCTGCGGCAGGGTGGTCTGGTGATGCAAAATTATCGTCTGATCGACGCCACGTTG
TATGTCACGCTTGAACCATGTGTAATGTGTGCCGGAGCGATGATCCACAGTCGCATTGGTCGCGTGGTCTTTGGTGCGCG
TGACGCGAAAACTGGCGCTGCGGGATCTTTAATGGATGTGCTGCATCATCCGGGTATGAATCACCGAGTGGAAATTACGG
AAGGAATACTGGCGGATGAGTGCGCGGCGTTGCTCAGTGACTTCTTTCGCATGCGCCGCCAGGAAATTAAAGCGCAGAAA
AAAGCGCAATCCTCGACGGATTAA
>Cluster_25594
ATGGCTAAGACCCCGCGCAGTCGCAATGCAGACGATAAAGTCATTCAGGGTAAGGAACTTGATTCTTACATCAAAGCCGA
GCTACAAGCCATGGTGCAAGAGGGACTGGAAAAATCCCCAATTCAGCCATCCACGTTACATGCCCGTCTTAAAGCCAAAG
GAATAGTTAAAGGTGGCATTAGTACCCTCACCTCTCGAAAAATTATCATCAATAATTACAGGCTTCGCCAGCATAACAAC
TATGAAGATGGTAAACGAGAGTTGACAACAGATGAAAAGGCTAAATTGGCTATGGGGCGAACCGGCGCTGCTTATATAAG
TAAGGCAAATCGTCTTGATGCTGAAATTATAGCATTGAAAGCTAAATATGAACGAAATGTACTGGCGCTCGCCGACATCA
TTGAGTATATCGACAACGCAACTCCTATCAAAGTTGAGGACATGCTTGCAGACGATCTCATTCGAGAACTTGCTGAGCGC
AAAAGAAATTCAAGAATGAGCTAA
>Cluster_24239
ATGAACATTACGCTCGCCCGCATTGATGACCGCCTGATCCACGGCCAGGTCACTACCGTCTGGTCTAAAGTGGCTAACGC
CCAACGCATTATTATCTGCAATGACGAAGTTTATAACGATGAAGTTCGCCGGACATTATTGCGTCAGGCTGCGCCACCGG
GAATGAAGGTTAACGTGGTCAATATTGAAAAAGCCGTCGCGGTTATCACAACCCGCAATATCAGGACGAAACCGTTTTT
TATCTTTTCACTCGCCCACAAGATGCCTTAGCAATGGTTCGCCAGGGGGTAAAAATAGGTACCCTGAATATTGGCGGTAT
GGCCTGGCGTCCTGGTAAGAAACAGTTAACCAAAGCAGTTTCATTAGATGACGATGATATTAACGCATTTCATGAGTTAA
ATAATCTTGGCGTCATACTGGATTTACGCGTCGTAGCGTCAGATCCCTCAATCAATATTATCGACAAAATAAACGAACAG
TTAATCGCAAATTAA
>Cluster_60584
ATGACAGAATCAACATCCCGTCGCCCGGCATATGCTCGCCTGTTGGATCGTGCGGTACGCATTCTGGCGGTGCGCGATCA
CAGTGAGCAAGAACTGCGACGTAAACTCGCGGCACCGATTATGGGCAAAAATGGCCCAGAAGAGATTGATGCTACGGCAG
AAGATTACGAGCGCGTTATTGCCTGGTGCCATGAACATGGCTATCTCGATGACAGCCGATTTGTTGCGCGCTTTATCGCC
AGCCGTAGCCGCAAAGGTTATGGACCTGCGCGTATTCGCCAGGAACTGAATCAGAAAGGTATTTCCCGCGAAGCGACAGA
AAAAGCGATGCGTGAATGTGACATCGACTGGTGCGCACTGGCGCGCGATCAGGCGACGCGAAAATATGGCGAACCTTTGC
CAACTGTCTTTTCAGAAAAAGTTAAGATCCAGCGTTTTCTGCTCTATCGTGGCTATCTGATGGAAGATATCCAGGAGATT
TGGCGAAATTTTGCCGACTGA

>Cluster_20751
ATGTCCGGCATTACGGATTTTTTTTCAGTTGTGGCTGCAGTTGGGGCAGTTGTAATCATCCCTGCTGCATATCAACCTAT
TTATCAATCATCTAAAACAAAAAACTATGAGTCTTTGGTAAAAGTTGATTGGGGCAATGAAGGTGACATCTCTACTTCTT
ATCCACATTTCATACATATACAGTTAGAGAAAGCAGAAGGTAATTGGACTTTTTCAGGAGAACTCACCTGCACAATTAAT
CCCGAAGAAAAGTTCGTCATTCAGGGTAAACTGAGATGTCTTAGGACTATAGATGCTGTAATACATCGTACTATTGGCTG
GCGGGCTATTGAGATTGGAAAGGCTAAAATCAAGTATGACCGCCGCACAAATCAGTGTCGATTTATCCCACAAAAGGTTG
CTCAGAAGAAACTTGAGACTGATTGCGAACGTGCAATCTTCAGCACCAGCCTCCCTTTATGGGGAAATGACAAATCTCCG
GTATAA
>Cluster_48572
ATGAAGATGATAGCTGCTGTTGGCCGTAACTATGAGATCGGCATAGCGAACGAACTGCCCTGGCGTTGTTCTACCGATCT
GAAGCTATTTAAGAGACTCACCAAAAAAGCCACTGTCGTTATGGGGCGTAAAACGATGGAAAGTCTCAAACGCCCTCTTC
CAGAGCGTCATAACCTCGTTTTGACGCGCTCTCATGGCTTTGTACCAAATGGATTCTACCCTGCTGGTGTGGATGATGTG
TTGCGATTACCAGAGCCTGTGTGGGTGATTGGCGGGGAACAAATTTACTCGCTATTCATGCCGCATGTTGAAGAGATTTG
GCTCTCCCACATCGGCGTTGATGTGCCAAACGCCGATGCATTCTTCCCGGCAAGCATGATGCGTAATTTAGGCTTTGTGC
CTGTTGAAACAGTTTTTACCCAACGAGCCAGCGAGGAAGAGCCTGGCTTTTCGCAGATCGTATACAGAAGGTCGTAA
>Cluster_19996
ATGCATAAATCAGAAAATGAAAAAATAGATTTTATTCTGGATGAGCATGAGCGGATATCCAGAAAACAGGGTTCTTTGTG
TATGGCACTGGGTAAGGGCTTTTTAGTTATAGCTATTCTTTGTGGTGTTGCCACATTCTGTGTTTCTGGCCTGTATCTGA
AATCCCTGTGCCTGTCCGTTGCCTGCTGTTGTGGGGTATTCAGTCGTATTTTTAATTATTACAGCGTACCGGAGGAATCA
CGCATGGTACTCAGTCGTCAGGAATTACTCCGGCTTATGCGTCTGACTGAAGACGTACCAGATGTGAGACAGGAACTGCT
TAACCGTTTGTTGTCCGGTAAAAAACTGACTGGTCGGGATGAACAGGATATCCGGCGTCTCTGGCAGGAGAAAGTGGATG
CAATGCAGGAGTCTGCCACCCGTCAGCGTGAACAGGATACTATTCGGAAGTTTACTGAAGGGAATAAATCCGAATGA
>Cluster_18422
ATGCCAAATATTGTTTTAAGTCGGATTGATGAACGCTTGATTCACGGTCAGGTCGGCGTTCAGTGGGTCGGATTTGCGGG
GGCAAATCTGGTGCTGGTAGCCAACGATGAGGTTGCCGAAGATCCGGTACAACAAAACCTGATGGAAATGGTACTGGCAG
AAGGGATCGCCGTACGTTTCTGGACGCTGCAAAAAGTCATCGACAACATTCATCGCGCCGCCGATCGACAGAAAATCCTG
CTGGTTTGTAAAACACCGGCCGATTTCCTGACGCTGGTGAAAGGTGGCGTTCCGGTGAATCGCATTAACGTTGGCAATAT
GCACTACGCCAATGGCAAGCAACAAATCGCCAAAACGGTTTCTGTGGATGCGGGTGATATCGCAGCATTTAACGACCTGA
AAGCCGCTGGGGTGGAATGCTTCGTTCAGGGCGTCCCGACAGAGCCTGCTGTGGACCTCTTTAAATTACTTTGA
>Cluster_64468
ATGGAAAACCCAGAGGAATTACAAAAAGAGATTTTGACCTGGGCGGCAAGGGCTGGGCAGGAACTCGTCACGATTGAAAT
CTGCCGGGCCTGGTTCAGTCAGGGACGCAATGATGAGTTGAGACTACATGAATTTGAGGACGCGGACGGCAATGTGGACT
GGAGAGCCATCAACAACAATCGGCAGAAAATCTTTCGCTGGTTACGTGGCGAGACAACGGCGGCGCGCCGAAAAACTCAG
GTGCTGGCCAGTGTGATGAAAGCCGTGCTACCCGCAGAACGGCGGGCGCGTCTGGAGTCGCCGGGCGATCCCGTTTTGCT
GGCAACGCTGGCGGCAAAAGAAGGGGTGGAAGCGATTAACGCTGTACATCTCCACTTCGCGCCAGAGTTAACTATCCAGG
AAATTGACGAAGCAATAGCGGCGCTGGTAGCGACGCGAGGAGCAGTGATACGCACTGCGCAAGACCACCATGCATGA
>Cluster_54155
ATGAAACTTCAGGTATTACCGTTAAGTCAGGAAGCCTTTAGTGCTTATGGCGACGTAATCGAAACGCAGCAACGGGATTT
TTTCCATATTAACAATGGCCTGGTGGAGCGTTACCACGATTTGGCGCTGGTTGAGATTCTTGAGCAAGACCGTACGCTTA
TCAGCATTAACCGCGCGCAACCGGCGAATCTGCCGCTGACCATTCACGAACTCGAACGTCATCCGCTGGGTACTCAGGCC
TTTATCCCGATGAAAGGTGAGGTGTTTGTGGTGGTCGTGGCGTTAGGTGACGACAAACCAGACCTGTCAACGCTGCGGGC
GTTTATCACCAACGGCGAACAGGGAGTGAATTACCATCGTAACGTCTGGCATCACCCACTTTTCGCCTGGCAGCGCGTCA
CCGATTTTCTGACCATCGATCGCGGCGGCAGTGACAACTGTGATGTTGAAAGTATTCCTGAACAGGAACTCTGTTTTGCG
TGA
>Cluster_41351
ATGCAAGTTTTTATCATGCGTCACGGCGACGCAGCCCTCGATGCCGCCAGTGATTCGGTTCGTCCTCTGACCACTAACGG
TTGTGACGAATCTCGCCTGATGGCGAACTGGCTGAAAGGTCAAAAAGTGGAAATCGAACGCGTTCTGGTGAGCCCGTTCC
TGCGAGCCGAGCAAACACTGGAAGAAGTAGGGGATTGTCTGAACCTGCCTTCCAGTGCAGAAGTTCTGCCGGAGTTAACG
CCCTGCGGCGATGTTGGTCTGGTCGGCGCCTATTTGCAGGCGTTGACCAATGAAGGTGTTGCTTCGGTACTGGTGATCTC
CCACTTACCGTTAGTTGGGTATCTGGTTGCCGAGTTATGTCCGGGCGAAACGCCGCCTATGTTTACCACTTCAGCGATTG
CCAGCGTAACGCTTGATGAAAGCGGAAAAGGCCAATTCAACTGGCAAATGAGCCCTTGTAATTTGAAGATGGCAAAAGCA
ATTTAA
>Cluster_48662
ATGAATTTTATCGCTACTGTAAACGCACCCGCACATGGCAATATCGCTGTAACGTTCTCTGACATTGAAAAACGAGTACT
TGGTGCATGGCGCGACAATGAGACGGTAGAACTGTCAGCACAAGAAAAATGCATTATTGCACGCGACATCATTGGCAATC
GTCGTTACTCGCGGGTATTTGAGAAAGCGTATGTGGTAAATTCTGGATTCGGAACGTTCGTCTTTCCGGTGCGCTCCGGG
CGATTCTGCCAGTCCAAGCTGATTGAGTTCGCTACGCAGATTTCTTTCTGGATTAAAACTCAATCGTCGTTCAAATTTTC
CGACGATGAAGCAGTATCGCAGGGGATGCGGATCGCCAACAATGCGATTAAATGCAAAAACATTACGTATACCGCTGGCG
TTGACACATGGAAACTGTTTTGCGCTAACTTTATGCTGAATGTATACGCAAGCAACCGCATCCACATCCTTGATGGCGTG
TAA
>Cluster_17732
ATGATCTGGGCATTCATCAAAGCGTACTGGAAACAGTTGCTTATAGTGTCGATGCTTGCTGCTCTTGTCATCAGCGGCGT
GGCTGCCTGGAATGTCCACGGTGACCGCCAGTACTATGCCGGGTATGCGCAGGCGCAGGAAGACCAGAAACAGGCTGATG
ATAAAGCCCGTAATCACGATGAACAGGAGAAAGCTACCAATGAACGTGAAGCGCAGCAGAGGATCGACAAGGCGCGCAAT
GATGCTCTTGATGCTGCCGCTCGCGCTGGCAGGTTGCAGCAACAGCTCGTTGCCATCCGTGAGCAGCTCAGGCAGTATAA
CTCCACTGTCGGCACTGGGGCGTCAGCCGCAGATACCGGAGTTTTGCTTGCCGACGTGCTCAGCAAATCTCTCGAGCGAA
ACCGACAGCTGGCAGAGTACGCTGACCGGGCCGCAGAAGCCGGACGAGTCTGTGAAAAGCAGTACGATTCGCTGACCCGG
TGA
>Cluster_163
ATGGCATTGATGAGAAGTTTTCTGCAGCGGGGCCAGGCATTACTGCAGGACAGTCTGATCCCCCTTCTGGCTCGTACAGG
CCTGGCCGCCACATTCTGGCTGTCAGGGCAAAGTAAGGTAGAGGGGTTGGTTCTGGATATACTGGGAGGGATGCCACCGG
AACTGGATGTTCCGCATATTACCCGTTCGGCGGTTGCGCTGTTTGCCACAGAGTACCGACTGCCGTTTCTGGCACCGGAG
ACTGCTGCACTGCTTGCAGCAGGAGCCGAACATATTTTTCCGGTTCTGCTTGTCCTGGGACTGGCAACGCGTTTTTCCGC
ACTAGTGCTGGCAGGAATGACGATAGTGATCCAGGTGTTTGTCTATCCCGATGCCTGGCCGGTTCATGCCACCTGGCTTA
CCGCCCAGATGTATTTACTGGCGTATGGCGGCGGAAAAATATTCCCTGGATAACCTGCTGAAACGTACGTACCGGCGTAGG
CACCAGAATGTGATGCAGAAAAGGAATAACCCTATGATGACGCTATAA
>Cluster_67131
ATGAAAAAGACAATTACTGCAGCTTTCGTGTCTTCTGTTCTGGCTGTATCAGCAATGACCGTTGCTCATGCAGCAACCAG
CACAACTGTAAATGTTATAGGAACTGTCAACACCGCAACCTGTGATGTGGTTGCAAATACCCAGTCCGTAAATCTGGGTA

ATGCTAAGCCAGCAGAATTTACCACAATAAATGCAGCAGTTGCCTCAACGAAACAGCAGTTTGCTATTTCGTTACAAAAC
TGCTCCGGCGCCGGCACTGGACAAGCCCAACTGAAAGTGACCGGGCCTGTTTCAAGTGCAGGGAGTACTTATTTCTCTAA
AGACCCTTCCTCGGTAGTAGCTATTAGTTTGACTGACAAAACTAGCTCAAGCGAAGTATCTAATGGTGCGCTAATTAATC
TGGGGAGCGCAAATGCTCCTGACGGCACTCTGAATAGCATCCAGATGCCGTTTGAGGTCGCTCTTAAATCTTCGACAACT
TCCCCAAAAGGTGGTGTAGTTGTTGAGGCACCATTAACTTTCTCATTTGTATATAACTAA
>Cluster_67079
ATGCACCGCCTTGATGAGGTGGTTGATACGCTGCTTGTCCTGCAGAAGAAGCACCGGATACGGTTTGATGTCTGGCAGGT
CGTTAAACGGGATCACGCCATTATTTCGTTCTTCGATCAGGGGATGAATCCTGCAGTGCCCAGGGTTGCCTACTGGACCC
CCTTTCGTTACCCGCTGCTACTGAATCTGGCAAGCCTGTTCGATAATGAACTGGCTGAAAAAGCGTGGCGTGCCCGCCTC
GAAGCCCATGATGGAAGGTCCTCATCTCTGTTCAGTGAGGTCTGCAGTGAGCTGCTTGCGCGCGTTCATACCCTGGGGGA
CCGCCGTTACATTGAATTGATTACCGATGCGTTGTCCTGGGCGATGACCCACTTTGACGAACTGGGATATAACTGCAAAA
CCGGTAAACAAAAGCTGCAGATCATGCCCAATATGGTTGGATTTCAGTTCGTATTGCGCGGTATCTGTTCCCGCCTGGGA
GCACCAAATCGCAAAGCCGACATCGTTGGTAACCGGCTCATTTAA
>Cluster_55162
ATGAAGTTATGGCCTGTGTTGACTGGCATTGCACTCTCTTTCACTCTTATAGCATGTAAGGCCCCGACACCACCTAAAGG
TGTGCAGCCGATTACAAATTTTGACGCCAACCGCTACCTCGGAAAATGGTATGAAATAGCTCGCCTCGAGAACTGGTTCG
AACGTGGTCTGGAACAAGTCAGCGCTACTTATGAAAAACGGAACGACGGAGGGATTCGCGTACTTAACCGTGGATACGAT
CCAACGAAAAACAAATGGAGCGAGAGCGAAGGTAAAGCATACTTTACTGGAGATACTAAAACTGCAGCGTTGAAAGTTTC
GTTTTTTGGCCCTTTCTATGGTGGCTATAATGTAATCAAGCTGGATGATGAGTATAAGTATGCTCTTGTCAGTGGTCCGA
ACAGAGAATACCTATGGATTCTGGCAAGGACCCAAACTATTCCAGATAATGTAAAAGCAGACTATGTGCGTACCGCTCAA
AAGTTGGGATTCAATGTCAATGAACTATTATGGGTTAAACAATAA
>Cluster_48679
ATGAAGGTCGAAAAAATAGATGTTCTTTCCTTTGTGCTGACGGATCTGGAACGCCTCGATCCGGTTCGAGTAATGATTGA
AAACTACGAACCTGGTAAGGGAAGAATCACCATCATCTGCTACGGAAAAGCGTGGACTGCGGCTTGGTTTGCTATGGGCG
GTGATGATGTGCAGACGTTCATTAAGCGGGTCAGCAACGAGTATCTAATCGGCTATTTCGACTCTCAACTGCGAAGCACG
GTCGACGATGACAACGATGCAAATCTGCTTTTCGTGAAGTCAGAAATCATAAAGTTACGAAGAGAGAGAGAAATCGACGC
CGTACTGGCTCGTGAAATGTGGGACGAGGCGGAAAACGCCGATGACGTAAAAGAAAGCTGCTGTTGTTTCGGCGTCGGTA
ACAAACTGCTGAATCTCTTTGGTGATGATCCGTGGTATGCCGACTGGCCAACGGTACCAAACCCGAAATACCAGTATCTG
GAACGCATTGTGAACGCGGTACGTGAAGGGCTGAAAGAGTTAGATAAGTTAGAGGCATCGTAA
>Cluster_25461
ATGAAAACGATTGAAGTTGATGATGAACTCTACAGCTATATTGCCAGCCACACTAAGCATATCGGCGAGAGCGCATCCGA
CATTTTACGGCGTATGTTGAAATTTTCCGCCGCATCACAGCCTGCTGCTCCGGTGACGAAAGAGGTTCGCGTTGCGTCAC
CTGCTATCGTCGAAGCGAAGCCGGTCAAAACGATTAAAGACAAGGTTCGCGCAATGCGTGAACTTCTGCTTTCGGATGAA
TACGCAGAGCAAAAGCGAGCGGTCAATCGCTTTATGCTGCTGTTGTCTACACTATATTCTCTTGACGCCCAGGCGTTTGC
CGAGGCCACGGAATCGTTGCACGGTCGTACACGCGTGTACTTTGCGGCAGATGAACAAACGCTGCTGAAAAATGGTAATC
AGACCAAGCCGAAACATGTACCAGGCACGCCGTATTGGGTGATCACCAACACCAACACCGGCCGTAAATGCAGCATGATC
GAACACATCATGCAGTCGATGCAATTCCCGGCGGAATTGATTGAGAAGGTTTGCGGAACTATCTAA
>Cluster_62815
ATGCAACCACTTACATACCAACAGACTAGCGGATTTAGCCCGACTGCGGTGATAAATCGTTCTCAAACAAAACAGGCGCC
AGGCCACGAAAAAATCCGTGATGCCGTTCGCGCCTGGTCGGCTGCAGATAATCAGGATGTTGTTGCCGCACTCATTGTGA
ATGAGTATCGGGAGCAGGGCGGCGGCACCATCGATTTCCCTGATGATGTCAGCCGTGCACGCCAGAAGCTGTTCCGCTTC
CTCGATAACAAATTCGATTCTGAAAAATACCGAAATAACGTGCGTGAACTGACCCCGGCAATTCTGGCGGTACTACCGCT
GGAATATCGCGGCCACCTGGTTGAGCAGGATAGCTTCATGGCTCGGCTGGCTGAAATGGAAAAGGAACTCAGTGAGGCAA
AACAGGCTGTCATTCTCAACGCACCACGCCACCAGAAACTGAAGGAAATTAGTGAAGGTATTGTGTCGATGTTTCGTGTG
GACCCAGATCTGGCTGGTCCATTGATGGCGATGGTTACTACCATGCTGGGGGCGATATGA
>Cluster_25086
ATGGAATGTCAAAACCGTTCTGATAAATACATCTGGTCTCCCCATGACGCCTACTTCTATAAAGGACTATCTGAACTGAT
TGTGGATATCGACAGATTAATTTATCTATCGCTGGAGAAAATCAGAAAAGATTTCGTGTTTATCAATCTCAATACGGATT
CTTTAACTGAGTTTATAAACCGTGATAATGAGTGGTTATCCGCGGTAAAGGGGAAACAGGTCGTATTGATTGCGGCCAGA
AAGTCAGAAGCCTTAGCAAATTATTGGTATTACAACAGCAATATTAGGGGCGTGGTATACGCTGGACTGAGTCGTGATAT
TAGAAAAGAACTGGCCTATGTGATTAATGGCAGGTTCCTGAGAAAAGATATTAAGAAAGATAAAATCACTGACCGGGAAA
TGGAAATTATCCGCATGACGGCTCAGGGAATGCTGCCTAAATCGATTGCCAGAATTGAAAATTGTAGTGTGAAGACAGTG
TATACCCATCGGCGGAATGCAGAGGCCAAGCTGTACTCAAAATTATATAAGTTGGTTCAGTAA
>Cluster_47587
ATGAAAAAGGTTCTGTTTGCCTTATCTGCGCTTGCGCTGACCTCTACTTCCGTATTCGCAGCTGATGCGGGCGACGGTAC
TGTTAAATTCACCGGTGAGATTGTTGACGCCGCTTGTGTTGTGTCTCCAGACACTCAAAAACAAGAAGTTGTTCTGGGTC
AGGTGAACAAGTCTGTATTTACTGCTATGGGTGATAAATCCACAGCAACGCCGTTCAAAATCAAACTGGAAAACTGCGAT
ATCTCCACTTTCAAAAATGTGGAAATCAGCTTTAACGGCGTTGGCGATGCAGACAACAGCAAACTGGTTTCTGTAAGCAC
TGAACCAGGTGCCGCAACTGGCGTAGGTATCGGTATTTATGATAACACCAATACCCTGGTTGACCTGAATACTGGTAAAT
CTGCTACTGTCCTGAAAGAAGGTCAGACCGTTCTGTATTTCACCGCTAACTATGTCTCTACCAAAGATGCAGTAACCACG
GGTTACGGTAACGCTGAAGTCGACTTCAACCTGACTTACAACTAA
>Cluster_15666
ATGAGAAATATAAGCAACATAGACGAATTAAGAAATAAATTATCATATGATGATGTTTTAGGTGTACTTAGATGGAAAGT
GTCTCCTTCTAATAATGTTAAGGTTGGCAGTGTTGCTGGTTACATTAGGAGTGATGGTTATAGGATGTTAACTATCGATG
GTGTAATTACTTATGCTCACCACGTTATATGGATGATAAATAATGGTGAGATTCCATTAGGGTATAAAATTGATCATATA
AATGGAGTAAGGTCAGATAATAGGCTATCTAATCTTCGATTGGTTACACACCAACAGAATGCACAGAACCAAAAGAGAAA
AATAACCAATTCGTCAGGAGTGACTGGAGTGTATTTCAATAAAGAGAGATGCAAGTGGATTGCGAATATATGCGTAGATG
GGCGCACCAAATATCTTGGAATATTTGCTAGCATCATTGACGCTATTGCAGAAAGGAAGAGGGCAGAAAAAGAACTGGGT
TTTCATGAAAACCATGGCAGACCATAA
>Cluster_55572
ATGGATCAATTTGGTCAGAATGCCTTTGCACCTGGCATGAAGAGTTCAGTGTTTGTGCCGGATCAGTTAATTGCCGGTAC
GCTGCAACTGGTCACTGACACCGGCACGATCACCGGCGGTGTGTATAAGCGTGGCACTGTGCTTGGCATGATCACCGCCA
GTGGCAAATATACAGCCAGTGTGAAAACCGCAACGGATGGTAGCGAGACGCCAGCGGCCATTCTGGTTGATGATGTTGAC
GCTTCCACCCATGGCGATCAGTCCGGCGGCCTGTACCTGATGGGCGAGTTCAACCAGAATCATATTATTTTTGACGATTC
CTGGACTGCGCCGGAACTGAAAACAGCACTACGTCCGCTGGCCATCTTCCTGAAAGACAGCGCCCAGGCACCTTTACCAG
CGTTCCGAAAGCAGCCAGCAACTGGCTGGCACCATGAACACGCTGATCGTAGTGCTATGTACCGGAGCCATGACGGATGC
TGCTGCAGATTACAACCCAACCAGTCGTAA
>Cluster_35361
ATGAATCGTATTGAACATTATCATGACTGGTTACGTGACGCCCACGCAATGGAAAAGCAAGCCGAATCTATGCTTGAATC

CATGGCCAGCCGTATAGATAATTATCCTGAACTACGCGCTCGTATTGAACAACATCTTAGTGAAACCAAAAACCAGATTG
TTCAACTGGAAACTATTCTTGATCGTAATGACATTTCACGTTCAGTCATTAAAGATTCCATGAGTAAAATGGCTGCGCTT
GGGCAGTCAATCGGTGGTATATTCCCTTCTGATGAAATAGTCAAAGGCTCTATTAGCGGATATGTCTTCGAGCAATTTGA
AATCGCCTGTTACACCTCACTATTAGCAGCAGCAAAAAATGCCGGTGATACAGCTTCAATTCCAACCATCGAAGCGATTT
TAAATGAGGAAAAGCAAATGGCCGACTGGCTGATTCAGAATATTCCGCAAACAACTGAGAAATTTTTAATTCGCTCTGAA
ACTGATGGCGTAGAAGCGAAGAAATAA
>Cluster_2750
ATGAATAATGTATTTCTGTACCGGATGCCTGTTGGTATTGCCGGGGCTGTCTCTCGCCCGCAGGACTTAACCGTCGAACC
GGTGGTCCTTAAATCCGATAACGCCTTCGCTGCCTATGGGCTGGCTGGTAAATACGATGATGACGGTTTTTCGTGCCGC
TGGCAGATGGTGATACCGCAGACAAGGTGAAGGGGATCTACGTGCGCCCTTATCCGACCACGTCGCAGCCGGACATGGTT
CGCCAGGTGGGAACAGGCAAGAACTTCCCGGGCGACGCCATGAAGCGTGGCTACGTGACCGTTAATCTCGGTTCTGATTT
TGATGCCAGCACTATCAAAAAAGGCGACCCGGTATACGTTGTCGTCTCCACTGATGAATCCATCAAAGTGCCGCTGGGTG
GATTCATGTCCACGTCAGTCAGTGGCAAAAATGTGGTGCTGACCAACGCTGAATTCACAGGTGCCGGTGATGCTAACGGC
AATGCAGAAATTTCCTGGAAGATTTAA
>Cluster_6675
ATGTCTGACCGCGCCACTACCACAGCTTCCTTAACGTTCGAGTCGCTTTATGGCACACATCACGGCTGGTTGAAAAGCTG
GCTGACGCGCAAACTCCAGTCTGCTTTTGATGCAGATGACATTGCCCAGGACACTTTTTTGCGGGTAATGGTCAGCGAAA
CGCTCTCGACGATCCGCGATCCTCGCTCCTTCCTCTGCACTATCGCCAAACGCGTGATGGTGGACCTGTTTCGCCGAAAC
GCGCTGGAAAAAGCGTATCTGGAGATGCTGGCGCTTATGCCGGAGGGGGGAGCGCCTTCACCTGAGGAACGCGAAAGCCA
ACTCGAGACCCTACAACTCCTCGACAGCATGCTGGACGGGCTAAACGGCAAAACACGTGAAGCGTTTCTGCTTTCGCAAC
TGGATGGTCTGACATACAGCGAGATTGCGCACAAACTCGGTGTTTCCATCAGCTCCGTGAAAAAATACGTGGCGAAAGCC
GTCGAGCACTGCCTGCTGTTCCGTCTGGAGTATGGGTTATGA
>Cluster_206
GTGCAAAAGAATGGCGATACCTTGTCCGGTGGGCTTACTTTTGAAAACGACTCAATCCTTGCCTGGATTCGAAATACTGA
CTGGGCGAAGATTGGATTTAAAAATGATGCCGACAGCGATACTGATTCATACATGTGGTTTGAAACAGGCGACAACGGCA
ATGAATATTTCAAATGGAGAAGCAAACAAAGCACCACAACAAAAGACCTGATGACGCTGAAATGGGATGCACTAAATATT
CTTGTTAATGCCGTCATTAATGGCAGCCTTGGAGTTGGTACGACGAATGCGTTAGGTGGTAGCTCTATTGTTCTTGGTGA
TAATGATACCGGATTTAAACAGAATGGAGACGGTATTCTTGATGTTTACGCTAATAGTCAGCGAGTATTCCGCTTTCAGA
ATGGAGTTGCTATTGCTTTTAAAAACATTCAGGCAGGTGATAGTAAAAAAATCTCGCTATCCAGCTCCAACACCTCCACA
AAGAATGTAACGTTTAATTTATGGGGTGCTTCAACCCGTCGGTAA
>Cluster_57878
ATGCAATTAGATGAACAGCGCCTGCGCTTTCGTGACGCGATGGCCAGCCTGTCGGCAGCGGTAAATATTATCACCACCGA
GGGCGACGCCGGACAATGCGGGATTACGGCAACGGCCGTCTGCTCGGTCACGGATACACCACCATCGCTGATGGTGTGCA
TTAACGCCAACAGTGCGATGAACCCGGTTTTTCAGGGCAACGGTAAGTTGTGCGTCAACGTCCTCAACCATGAGCAGGAA
CTGATGGCACGCCACTTCGCGGGCATGACAGGCATGGCGATGGAAGAGCGTTTTAGCCTCTCATGCTGGCAAAAAGGTCC
GCTGGCGCAGCCGGTGCTGAAAGGTTCGCTGGCCAGTCTTGAAGGTGAGATCCGCGATGTGCAGGCAATTGGCACACATC
TGGTGTATCTGGTGGAGATTAAAAACATCATCCTCAGTGCAGAAGGTCACGGACTTATCTACTTTAAACGCCGTTTCCAT
CCGGTGATGCTGGAAATGGAAGCTGCGATTTAA
>Cluster_20878
ATGGCAATGCCGCGCAAACTCAAGTTAATGAACGTCTTTCTGAACGGCTACAGCTATCAGGGCGTTGCAAAGTCCGTCAC
GCTGCCAAAACTGACCCGTAAGCTCGAAAACTATCGCGGTGCGGGAATGAACGGCAGCGCACCGGTAGACCTCGGCCTTG
ATGACGATGCGCTGTCAATGGAGTGGTCGCTCGGGGGCTTCCCGGATTCGGTTATCTGGGAGCTTTACGCCGCAACCGGT
GTGGATGCTGTGCCGATTCGTTTTGCAGGCTCTTACCAGCGTGACGATACCGGCGAAACGGTGGCCGTCGAGGTGGTCAT
GCGTGGACGTCAGAAAGAAATCGACACCGGCGAGGGTAAACAGGGAGAAGACACCGAGTCGAAAATCTCCGTGGTCTGCA
CCTATTTCCGGCTGACGATGGACGGTAAGGAGCTGGTCGAAATCGACACCATCAACATGATTGAGAAGGTGAACGGCGTC
GACCGGCTGGAGCAACACCGCCGCAATATCGGCCTGTGA
>Cluster_46583
ATGAACTCAGTATTCTTTTCACCCGCAAGTAAAAGTTTTTATCTGCAAGAATTGTTTCCAGAATATGAGGATGCGGGAAC
GCTTCCTGATGATGTTATTGAAATTACCAGAGAAACATATGAGCAATTTCTTGGGCTGCATCCAGAAGGGAAAGAAATTG
GCGCTGACAGTTCAGGACGGCCAATATGGATTAATTCCCCGCCGCCTTCAAAAGAGGATGAGATGCTGGCGGCTGAAATG
AAAAAGATATCTTTGGTTTCAGAAGTCAATACCTACATCAATACCCATCAGTGGCCTGGCAAAGCTGCTATTGGTCGTCT
GAAAGGTTACGAGCTGGAACAATATAATTTATGGCTGGATTATCTGGACGCACTGGAACTGGTTGATACCTCCAGTGCTC
CAGATATTGAATGGCCTACGCCTCCGGTAGTTCAGGCCAGATGA
>Cluster_2665
ATGAGTATGTCAACATCCACTGAAGTCATCGCTCATCACTGGGCATTCGCTATCTTTCTTATCGTTGCCATTGGCCTGTG
TTGCCTGATGCTGGTAGGCGGTTGGTTTTTAGGCGGTCGCGCACGCGCGAGGTCGAAAAACGTGCCGTTTGAATCCGGTA
TCGACTCGGTCGGCTCCGCCCGCTTACGCCTGTCCGCCAAGTTTTATCTGGTGGCCATGTTCTTCGTTATCTTCGACGTT
GAAGCGCTGTATCTGTTCGCATGGTCAACCTCTATCCGCGAAAGCGGCTGGGTAGGCTTTGTGGAAGCTGCAATTTTTAT
TTTTGTGTTACTGGCAGGTCTGGTTTATCTGGTGCGTATTGGCGCGCTGGACTGGACGCCCGCGCGTTCACGCCGCGAGC
GTATGAACCCGGAAACGAACAGTATCGCTAATCGTCAACGCTAA
>Cluster_20176
ATGTTAGGTTGGGTAATTACCTGTCACGATGACCGGGCGCAAGAGATACTGGATGCGCTGGAGAAAAAACATGGGGCACT
TCTTCAGTGCCGGGCCGTGAATTTCTGGCGCGGATTAAGCTCTAATATGCTCAGCCGCATGATGTGCGATGCCTTGCATG
AAACGGACTCTGGTGAGGGCGCTATCTTTTTAACCGATATAGCCGGAGCGCCACCGTACCGCGTGGCTTCATTATTAAGC
CACAAACACTCTCGTTGCGAAGTGATTTCTGGTGTCACGTTACCGTTAATTGAACAGATGATGGCTTGCCGTGAAACCAT
GACCAGTTCAGCGTTTCGCGAGCATATTGTCGAACTGGGTGCGCCGGAGGTGAGTAGTCTTTGGCACCAACAACAAAAAA
ATCCGCCTTTCGTCCTCAAACATAATTTGTATGAGTATTAA
>Cluster_48247
ATGCCGTTAAGTCCCTACCTCTCTTTTGCCGGTAACTGTGCCGACGCAATTGCTTATTATCAACGTACGTTGGGCGCTGA
ACTGCTCTATAAAATCAGCTTCGGCGAAATGCCAAAATCAGCGCAGGACAGCGCCGAGAACTGCCCTTCCGGAATGCAAT
TTCCCGATACCGCCATCGCTCATGCCAACGTGCGCATTGCCGGAAGCGACATCATGATGAGCGATGCCATGCCGTCAGGA
AAAGCCAGCTACTCCGGCTTTACGCTGGTGCTCGATTCGCAACAGGTCGAAGAAGGAAAACGCTGGTTTGACAATCTTGC
CGCTAACGGAAAAAATCGAAATGGCCTGGCAGGAAACTTTCTGGGCGCATGGCTTTGGCAAAGTCACCGATAAATTTGGCG
TACCGTGGATGATTAATGTCGTCAAACAACAACCAACGCAATAA
>Cluster_24774
ATGTTTAGTTTATCATTAATTACAGGTGTTGCAGCATGTGTTCTCAATGCCAGTTCTGTGAATAATATTGAGCCTGCACT
ATTACTGTCAGTTATGACTGTCGAAGGTGGTAAACCTGGTAGTGTATCCATTAATAAGAATGGAAGCCATGATTTAGGTA
TTATGCAGATAAATACCCATGCATGGTTAAAACTTATCAGTAAGAGTTTTTTTAACGGAGATGAGAGGTCTGCTTATACC
AAACTTAAGGATGATGCATGTTTTAACATATCAGTAGGGGCCTGGATCCTCCGCAGATCTATTATATTCGAGGGTGGTAA

TTTATGGGAAGGTGTGGGCCGATATCATTCGGCCACACCAGAGTATAAATATAGATATATAGATAAAGTGAAGAATGTCT
ATGGTCAAATAACATACAGCCAATCAATACATCAAAGGAATATGTAA
>Cluster_60737
ATGAAGCCACGAAATATTAATAATAGCCTACCACTGCAACCATTAGTTCCTGATCAGGAGAACAAAAATAAGAAAAATGA
AGAGAAATCCGTTAATCCAGTTAAAATCACAATGGGGTCTGGTTTAAATTATATTGAACAAGAATCTCTTGGAGGAAAAT
ATCTAACACATGATTTGTCAATAAAGATAGCGGATATTTCTGAAGAGATAATTCAGCAAGCAATATTATCTGCTATGAGC
ATATATAAATTTTCGATAACAGATGATTTAATGAGTATGGCTGTAAATGAACTCATAAAACTGACCAAAATAGAGAATAA
TGTAGACCTGAATAAATTCACTACTATATGCACAGACGTTCTATCCCCCCGCGTCACCAGACATAATAAAGAAAAAAACA
AACGACATTCTACCCTTCTCAAAAATCCCCTTTTTAATTTTCATTGA
>Cluster_22155
ATGCAACGTATGATGCTTATCGGCCCCAGCCAGTGCGGAAAAACGTCACTCACGCAATGTCTGCGTGGCGAAAATATCCA
TTATCAGAAAACGCAGGCAATCGTCTGGTCCCCGACCACCATTGATACACCAGGCGAATACCTCGAAAACCGCAGCCTCT
ACAGTGCATTGTTGGTCAGTGCCTGCGAAGCAGACATTATCGCGCTGGTACTTAATGCGGACGCACCGTGGTCACCTTTT
TCGCCAGGCTTTACCTCGTCAATGAATCGCCCGGTCATTGGAGTAGTAACCAAAGCAGATTTAGCCAGTATGGAACAAAT
ATCTTTGGTGAAATGTTGGTTACGGGAAGCTGGAGCGCACAACGTGTTAATAACCAGTGCGGTTAATAACAATGGAGTTA
CAGAACTTTTTGCCTTGCTGCATACAGAAGAAGGCTGTCGCTAA
>Cluster_17677
TTGTTGTTTTATCGCGTTGGGGTATTAAGAATGAATAAATTTTATAAAGGCTCTTTCTATCCGGAAGCACTAAAAGATGT
ATATATCAGCGCCGGTTCATGGCCTGAAAATGGCGCTGATGTTGATGATGAAACAATGGCAATTTACACAGGCGTAGCGC
CAGAAGGCAAAACGCTGGGGGCTGATAAAAATGGTAATCCTGCGTGGATTGATATCCCGCCACTCTCCGCTGAACAACAG
ATTATTCAGGCTGAACAGAAAAGAACGGTATTGCGTTCTATGGCTGATAAGGAAATAGTCTGGAGACAGGATGCTTTTGA
TGCGGAAATCGCGACGGCAGAAGAAACCGCCGCGTTATCTGAATGGAAAAAATACCGGGTCTTGCTGATGCGCGTTGATA
CATCAAATCCCGTCTGGCCTACGCCTCCGGGGGAGCAGGCCAATTGA
>Cluster_12034
ATGGCAGAGAATGTTGCACATATTTCGCGGTTCAGAAAAAAGAAAGAGAATGCCTATAATGTATCCAACAACCAAGTAAC
AGGAAGTGGTAATGATGGAGGTGATGGCATGAATGACGACCTTGAACGGCGGGTCAGTTGCCTGGAAAGTGATGTGACTG
AAATCAAAAACAACCTGATTACTCTCACCACAAGAAGCGAATCCTTTGCTACCAAATCTGATGTACTGGAGATTCGCGAA
GGGTTGAGGCTTGAGATGGCAGAATCGCGCCAATCCCTGAAGTCTGAGATGGCTGATTTACGCCAGTCTCTGAAAGTTGA
GATGGCTGAGCATCGTACTGAACTTCAGAAATCATTTGCAAATCAGACTTGGTTACTCACAGGTATTGTCCTGTCTGCGA
TGGCCGTGCTTGTGGCTGTGGTTACTGTTATTAAGTAA
>Cluster_49469
ATGATGAATAGCTCCATTAAATCCTTTTCCCTGCTGGCGGTTATATTACTGGCTGGCTGTAGCTCACCCGCTTCCCGCAT
CGCAGATTGTCAGGCGCAGGGCGTCAGCCATGACACCTGTTATCTCGCAGAACAGCAGCGCCAGACGGCTATTTTAAGTG
CTTCTGAAGCGCAGGCATTTAAAAATGCAGAAGCCGCACAACATGCCCAGGCGGCAAAAAAAGCCATTTATAAAGGATTT
GGCATGACCTTTAGAATGAGTAGTAAGAACTTTGCTTATCTCAATGATTCATTATGTGCCATAGATGAAGATAATGCCGA
TGCCACGGTTTATCAGTCGGGTCTGTATAATGTCATTGTTTATCATCACACAGGAAAAGTCGCCTTAATGAAAGAAGGCC
AGTTTGTGGGTTATTTAAAATGA
>Cluster_39396
ATGATCCCATCTCAAATCTCTTTCAACACCCTTCCTGTCAATGCAACATACGCTTCAGAGACAACAGTTGACACACAAAA
ATTCTCTGACATCCTCTACTCAGCGGGGTGTTCCCTAAAGGAACTGGCTTATCTGAGCAGATGTCTGGCATCAATTCATC
CGACTATCGCCAAAAACATTTATGAAACAGAAAATCTCAGCGATCAAAAATTACTACATATTGATTGCCGTAGTACAAAT
GAAATCAAGATAAATATCTTTTTTGGTCAACAACGAGAAGGATTAATTGAAATTAATTCTGACACTGTCATTTTTTCGAT
ATTGTCTTCATTAATTGACTCCGCCATTTCTAAATTTCAACACCAAATCCCCGTTAACGGTTCAGTTAATCGTGAACTAT
TGTATGAGGATTATACTGATTAA
>Cluster_35174
ATGAAAATGACTGAATTAACAAAAGAACAATTAATCGAAGAAGCCAAATTAAAAATAGCGATTACGAAATGCCACCCCAA
TTCAGGGATGGCGCGAGTAGAGGGCGAGTTATTCAAAATTGCACTGGCATCGCTGGAAGCAGAGCCGATAGCGTGGCGAT
ATCGCTACGTGAAAAAAGGTGTTATGGACTCTCAGGGGGAGTTGTGGGTTGGTGACTGGAAATATGTACCGAAAAAAGAG
GATTGTAACGACAGGCCGAACTATGAAATTCAGGCCTTATTCACTGCCCCACCAGTCCCTGTTACATCAGAAGAACTGGT
TAAAGCTGTGCACTTTTATGAACAACTAAAACGCGAAAATCCACCAGCATCCGGCAACCAGATTAATGGGTTAACTATGC
CGGTTAAACGACCAGCCGACTGA
>Cluster_740
ATGTTCCTGAAAACCGAATCATTTGAACATAACGGTGTGACCGTCACGCTTTCTGAACTGTCAGCCCTGCAGCGCATTGA
GCATCTCGCCCTGATGAAACGGCAGGCAGAACAGGCGGAGTCAGACAGCAACCGGAAGTTTACTGTGGAAGACGCCATCA
GAACCGGCGCGTTTCTGGTGGCGATGTCCCTGTGGCATAACCATCCGCAGAAGACGCAGATGCCGTCCATGAATGAAGCC
GTTAAACAGATTGAGCAGGAAGTGCTTACCACCTGGCCCACGGAGGCAATTTCTCATGCTGAAAACGTGGTGTACCGGCT
GTCTGGTATGTATGAGTTTGTGGTGAATAATGCCCCTGAACAGACAGAGGACGCCGGGCCCGCAGAGCCTGTTTCTGCGG
GAAAGTGTTCGACGGTGAGCTGA
>Cluster_45790
ATGATACTTTGGTCTGTGAAAAAAGAAACCGATATCCGGCGTGGAAGGCATTGTGTTTTCCTGATGCATGTTCACCTGGT
CTTTGTCACCAGATACCGACTCCAGATTTTTGACCATGACGCGACAGAAAAACTACGCACTTACTTTTCAAATGTATGTG
CTGATTTTGAAGCTGAACTTGTTGAAATGGATGGCGAACCAGATCACGTCCATTTGTTAATTAACTATCCTCCCAAACTG
GCGATATCCAGTCTGGTAAACAGCCTCAAAGGCGTATCGGGTAGGTTACTGCGACGAGATCGACCAGATATTGCAGTCAG
GTATTACTACAAAGGCGTTTTGTGGAGTCCTGGCTATTTTGCCAGTAGCTGCGGAGGTGCGCCAATATCCGTCATCCGCC
AATACATTGAACAACAGCAAACACCTGGTTAG
>Cluster_62530
ATGGATAACTATACGCTGCATCGTGGCAGACTTATCGACCATATCCAGCTCGTGGTAAACAACTTTGCCGCAAGCAAAGC
GTTTTACACTGCTGTTCTTGCGGAACTTAAGATCCCCATGGTTATTACCGATGACGATTATATCTGGGCTGACGAACTGG
TCATTTCGTCGGTAAAAAGTTCGGAGTCTGGCAGAGTGCCTACAGGACGGCACCATCTGGCCTTTCAGGCCGCAGACCGT
GAATCGGTGAACGCGTTTTACCACGCAGCACTAAATCATGGCGGACGTGATAACGGCGCACCTGCCGAACGAACCTATCA
TCCCGGATACTATGCCGCTTTTGTTCTGGATCCGGACGGTAACAACATCGAAGCTGTTTACCATGGTGAGGGCGATCGCA
GTGCCCGCTCTGTAGAAATCAGCTTCACTGTCTGA
>Cluster_29501
ATGAATCAAATTTATGACAGTTTTCCGTTTCCTTCCCCTGAGGGTTTTACTGCAGGTATGACAATGGCGGGATTTTTTAT
TCACGCCACTGTCATAAATGATGAGGAGTTAAAACCCACGGGTTCTCATTATAACCCTGAGGCTAAACCCAAATATGCCA
TTGTTATTGGCTATCCGTATGAGGACCGAAAATTAAAAGTCCGCCGTGAGGAGCATGAGCGATTTTCCTGTACTGAGGAG
GATTTTAAATTTTTTAATTCATGTCCTGATTTGCAGGGCAAACCTGTTTATCTCACGGTGGATGTTAACTCATGGTCGAC
AGGCTCTGAACGTCATGGTGTCTGGTATCGTTTTATTTCCGGCTCCATGAAACGTTTTGACGGTCAGCCGCTGGGTGCAC
TGCCCGCAGGTAAAGAAAAAGGCTGA

>Cluster_63641
ATGGGTTTTCGTAGCGCATCAATTCTTACGTTGATTATTAGTGGGATTATTATCGGGTGCACTGATGCTGTATCGACAAA
TTATCATGACCGTACATCATATTACTCCGATAAAGCAATAGAGACACAGTATGTGAGTTCATCTGAACGTACTTCTGATG
TTAGTGAGGATATCCGTCTGTATGCCCATCAAATCAAGAGCGCCATCGAAAAACAGTTCGGGGATGCGAGTAAGTATTCA
GGAAAAGAGTGTACACTGAGAATGCATATGGCCCCGAATGGCCTTCTACTGGAGGTTAAAAGAGAAAGTGGAGACCTCGA
TTTATGTCGTGAAGCGATGAATGCGATAAAGAATGCTGATATACCTGCCCCCCCTTCGCCGGAAGTATATAAAGTATTTC
AAAATGGGGTGCTGGATTTTAAACCCTGA
>Cluster_6145
ATGGAACTGACGACTCGCACTTTACCTGCGCGGAAACATATTGCGCTGGTGGCACACGATCACTGCAAACAAATGCTGAT
GAGCTGGGTGGAACGGCATCAACCGTTACTGGAACAACACGTACTGTATGCAACAGGCACTACCGGTAACTTAATTTCCC
GCGCGACCGGCATGAACGTCAACGCGATGTTGAGTGGCCCAATGGGGGGTGACCAGCAGGTTGGCGCATTGATCTCAGAA
GGGAAAATTGATGTATTGATTTTCTTCTGGGATCCACTAAATGCCGTGCCGCACGATCCTGACGTGAAAGCCTTGCTGCG
TCTGGCGACGGTATGGAACATTCCGGTCGCCACCAACGTGGCAACGGCAGACTTCATAATCCAGTCGCCGCATTTCAACG
ACGCGGTCGATATTCTGATCCCCGATTATCAGCGTTATCTCGCGGACCGTCTGAAGTAA
>Cluster_62873
ATGCAGTTCACTTTTAATGAAGGCCACATTCAACTGCCGTCTCAATGGCAGGATCAGTCAATGCAGGTCCTGGTGTCCAC
AGATAACAGTGGCATCAACCTGGTCATTACCCGCGAAGCCGTTCCACAGGGAACCCTGACACCAGAGCTGTACCAGGAAA
CGCTGGCGCTGTACCAGGGCAAACTTGATGGGTACACCGAACATGCCTGCCGGGAAATCACTCTGGCAGAGGCCCCGGCC
TGGCTGCTGGATTATTCCTGGAACGGTCCGGAAGATGAGGGGAATCAGGGGCGGATTAGCCAGATAGCCGTATTCCAGCG
GCGTGGAGATACGCTGCTGACCTTCACTTTCAGCACCTCCTTATCCCTGAAGAACAGTCAAAAGACGATGCTGCTGGAGG
TGATAAAGAGCTTCACCCCGTTGCCACCGGAGAATGACATCCAAAAGGACCAGCCGCGCTGA
>Cluster_10136
TTGACTACTAACACTCATACTCTGCAGATTGAAGAGATTTTAGAACTTCTGCCGCACCGTTTCCCGTTCTTACTGGTGGA
TCGCGTGCTGGATTTTGAAGAAGGTCGTTTTCTGCGCGCAGTAAAAAATGTCTCTGTCAATGAGCCATTCTTCCAGGGCC
ATTTCCCTGGAAAACCGATTTTCCCGGGTGTGCTGATTCTGGAAGCAATGGCACAGGCAACAGGTATTCTGGCGTTTAAA
AGCGTAGGAAAACTGGAACCGGGTGAGCTGTACTACTTCGCTGGTATTGACGAAGCGCGCTTCAAGCGCCCGGTCGTGCC
TGGCGATCAAATGATCATGGAAGTCACTTTCGAAAAAACGCGCCGCGGCCTGACCCGTTTTAAAGGGGTTGCTCTGGTCG
ATGGTAAAGTAGTTTGCGAAGCAACGATGATGTGTGCTCGTAGCCGGGAGGCCTGA
>Cluster_44684
ATGCTGGAGGCGACCCAGGTCAGAGGAGATTTCGTTAAAGCTGTATCAAAAGCCTTCCCGAAAAAAGTCGGTACGTGGGG
TAACACGGAAACACCAAACGGTACGGTTACAGTCACCATCAGCGATGATCATAACTTTGACCGCCAGATTATTATTCCGC
CCATTATTTTTAACGGTATAGCGTATGACGATCCGGGGAGCGGAAATAACCCAGGAGGCACGCGATACACGGGTTATGGT
TTTGAAGTTCGCAAAAACGGCGTATTAATCGCATCCAGAGAAACTAAAGGGGCCATTCCCGGTAGTTACAGTGCAGTTAT
TGATATGCCGAGTGGCAGGGGAAGCGTCACTCTGGAGTTTAAGATTTTCCAGAAAGGCAATCAGGGGGCAGGCAATATCA
CCGACTGTACGGTGATTGTGACCAAAAAAGCCGCTTCCGGCATCAGTATTCGTTGA
>Cluster_10986
ATGCAATTAAATACCAGACAGGCCCGGATTTTTAAGCTTGCCAACTTACTGGGCACCGGCAAGCCCGTTTCCGCTGCCGA
CATTATAACCAGTCTGGAATGCTCTGAGCCGACATTAACCCGGCCCTCAAAGAGCTACGCGAGTCATATTCTGCGGAGA
TCAAATACAGCAAGGCAGGTCATTCCTATCATCTCGTTAATCCGGGCCAGTTGGATAAAAAGACCCTCCGCCGGATGAAT
GAAGCGCTCGCACAGAATGCTGAACTGAAAACCGGTGAGTCCACAGGGAAGGTCGTACTCGATAAGGATAAAAAAACAGC
CGTGTCATTATCGCTACGCATGCGGATTCTAAGAAAAATTGACCGTCTGGCTGCGCTGAGTGACTCGACCCGAAGTGAAG
CGGTAGAGAAGCTGGCTCTGCACTCCGTTGATGAACTGATAAAAGAATACAGCGCTAAAAAGTCCTGA
>Cluster_32824
ATGCCACGTCGTCGCGTCATTGGTCAGCGTAAAATTCTGCCGGATCCGAAGTTCGGATCAGAACTGCTGGCTAAATTTGT
AAATATCCTGATGGTAGATGGTAAAAAATCTACTGCTGAATCTATCGTATACAGCGCGCTGGAGACCCTGGCTCAGCGCT
CTGGTAAATCTGAACTGGAAGCATTCGAAGTAGCTCTCGAAAACGTGCGCCCGACTGTAGAAGTTAAGTCTCGCCGCGTT
GGTGGTTCTACTTATCAGGTACCAGTTGAAGTCCGTCCGGTTCGTCGTAATGCTCTGGCAATGCGTTGGATCGTTGAAGC
TGCTCGTAAACGCGGTGATAAATCCATGGCTCTGCGCCTGGCGAACGAACTTTCTGATGCTGCAGAAAACAAAGGTACTG
CAGTTAAGAAACGTGAAGACGTTCACCGTATGGCCGAAGCCAACAAGGCGTTCGCACACTACCGTTGGTAA
>Cluster_34617
ATGGAACTGGCTGTTGTTACCGGGCAACGAGTTGGTGATTTATGCGAAATGAAGTGGTCTGATATCGTAGATGGATATCT
TTATGTCGAGCAAAGCAAAACAGGCGTAAAAATTGCCATCCCAACAACATTGCATGTTGATGCTCTCGGGATATCAATGA
AGGAAACACTTGATAAATGCAAAGAGATTCTTGGCGGAGAAACCATAATTGCATCTACTCGTCGTGAACCGCTTTCATCC
GGCACAGTATCAAGGTATTTTATGCGCGCACGAAAAGCATCAGGTCTTTCCTTCGAAGGGGATCCGCCTACCTTTCACGA
GTTGCGCAGTTTGTCTGCAAGACTCTATGAGAAGCAGATAAGCGATAAGTTTGCTCAACATCTTCTCGGGCATAAGTCGG
ACACCATGGCATCACAGTATCGTGATGACAGAGGCAGGGAGTGGGACAAAATTGAAATCAAATAA
>Cluster_18874
ATGTCAGAACAAAACAACACCGAAATGACTTTCCAGATCCAACGTATTTATACCAAGGATATTTCTTTCGAAGCGCCGAA
CGCGCCGCACGTTTTCCAGAAAGATTGGCAACCAGAAGTTAAACTTGATCTGGATACGGCATCTACTCAACTGGCAGATG
ACGTTTACGAAGTGGTACTGCGTGTTACCGTAACGGCCTCCCTGGGCGAAGAAACCGCGTTCCTGTGTGAAGTTCAGCAG
GGCGGTATTTTCTCCATCGCGGGTATCGAAGGCACCCAGATGGCACATTGCCTGGGCGCATACTGCCCGAACATTCTGTT
CCCGTATGCTCGTGAGTGCATCACCAGCATGGTATCCCGCGGTACATTCCCGCAACTGAACCTTGCGCCGGTTAACTTCG
ATGCGCTGTTCATGAACTATTTGCAGCAGCAGGCTGGCGAAGGTACTGAAGAACATCAGGATGCCTGA
>Cluster_26041
ATGAAAAACAAATTGTTATTTATGATGTTAACAATACTGGGTGCGCCTGGGATTGCAGCCGCAGCAGGTTATGATTTAGC
TAATTCAGAATATAACTTCGCGGTAAATGAATTGAGTAAGTCTTCATTTAATCAGGCAGCCATAATTGGTCAAGCTGGGA
CTAATAATAGTGCTCAGTTACGGCAGGGAGGCTCAAAACTTTTGGCGGTTGTTGCGCAAGAAGGTAGTAGCAACCGGGCA
AAGATTGACCAGACAGGAGATTATAACCTTGCATATATTGATCAGGCGGGCAGTGCCAACGATGCCAGTATTTCGCAAGG
TGCTTATGGTAATACTGCGATGATTATCCAGAAAGGTTCTGGTAATAAAGCAAATATTACACAGTATGGTACTCAAAAAA
CGGCAATTGTAGTGCAGAGACAGTCGCAAATGGCTATTCGCGTGACACAACGTTAA
>Cluster_12406
ATGTTTATTGCATCAGATAAATTTAGAGAAATTATCAAGTTAGTTCCATTAGTATCAATTGATCTGCTAATTGAAAACGA
GAATGGTGAATATTTATTTGGTCTTAGGAATAATCGACCGGCCAAAAATTATTTTTTTGTTCCAGGTGGTAGGATTCGCA
AAAATGAATCTATTAAAAATGCTTTTAAAAGAATATCATCTATGGAATTAGGTAAAGAGTATGGTATTTCAGGAAGTGTT
TTTAATGGTGTATGGGAACATTTCTATGATGATGGTTTTTTTTCTGAAGGCGAGGCAACACATTATATAGTGCTTTGTTA
CACACTGAAAGTTCTTAAAAGTGAATTGAATCTCCCAGATGATCAACATCGTGAATACCTTTGGCTAACTAAACACCAAA
TAAATGCTAAACAAGATGTTCATAACTATTCAAAAAATTATTTTTTGTAA
>Cluster_43670
ATGTCTGAATCTGTACAGAGCAATAGCGCCGTCCTGGTGCACTTCACGCTAAAACTCGACGATGGCACCACCGCCGAGTC

TACCCGCAACAACGGTAAACCGGCGCTGTTCCGCCTGGGTGATGCTTCTCTTTCTGAAGGGCTGGAGCAACACCTGTTGG
GGCTGAAAGTGGGCGATAAAACCACCTTCTCGTTGGAGCCAGATGCGGCGTTTGGCGTGCCGTCACCGGACCTGATTCAG
TACTTCTCCCGCCGTGAATTTATGGATGCAGGCGAGCCAGAAATTGGCGCAATCATGCTTTTTACCGCAATGGATGGCAG
TGAGATGCCTGGCGTGATCCGCGAAATTAACGGCGACTCCATTACCGTTGATTTCAACCATCCGCTGGCCGGGCAGACCG
TTCATTTTGATATTGAAGTGCTGGAAATCGATCCGGCACTGGAGGCGTAA
>Cluster_7588
ATGAAGCAAACCTTTCTGCTTCGCAACGAAGCAATCAGAAATAACGCCATAGACGCCATTCTCTCACTACCCATCGACGA
CAAGTCACCCCACGAAGTCCACGTTAAAGAACCCAAGCGCAGCAAAGCGCAGAATGACCGTATGTGGCCGATGCTGAACG
ATGTTTCGCGTCAGGTGCTATGGCATGGTCAACGGCTGGCGCCGGAAGACTGGAAAGACCTGTTCACTGCCCTGTGGCTT
AAGACCAAAAAACTGGAGCAACGAAGTGTGCCTGGTATCGACGGTGGCGTTGTCATGCTTGGCGTGCGTACCAGCAAAAT
GCGGAAGGCCAGCATGACTGAGCTTATCGAAATCATGTTCTGGTTCGGCTCAGAGCGCAACGTGCGGTGGAGTGATGACT
CCTGGCGAGAGTATGAATGGTCACAACGAAAAGGGAGAGCTGCATGA
>Cluster_21295
ATGAGCGATGTACCTTTCTGGCAAAGTAAAACCCTGGACGAAATGAGCGATGCGGAATGGGAGTCGTTGTGTGATGGTTG
CGGTCAGTGTTGCCTGCATAAACTGATGGATGAAGACACCGACGAAATCTACTTCACTAACGTCGCCTGTCGCCAGCTCA
ATATTAAAACCTGTCAATGTCGGAACTACGAACGTCGTTTCGAGTTTGAACCCGACTGCATTAAATTAACCCGTGAAAAT
CTGCCAACATTCGAATGGCTGCCAATGACCTGCGCTTATCGTTTGCTGGCGGAAGGTAAAGGTTTACCTGCGTGGCATCC
GCTACTTACTGGTTCGAAAGCGGCAATGCATGGTGAACGTATCTCTGTGCGTCATATCGCAGTGAAAGAATCAGAAGTGA
TTGACTGGCAGGATCATATCTTAAATAAACCTGACTGGGCGCAGTGA
>Cluster_66949
ATGCGTCCAGGTAAATTACTCATTATTCCATCCATATTTTTCTTCTCCGGTTTTTCATTTGCAACAACACAACCTCTTGT
CACAATAGGAGAACTTGAAGCACAACAAAACCGGAATATTCTTTTACAGGCAAAAGTTCAGGGCGCACAGCTACAGAAAC
AACTCGAAGAATCAGATATTACTTCATCATCTGAAACTGTTTCAGGTTTCTCTGGTGTGACTGCCTCTTTGCCATCAGTT
TCAGAGCAGCCGACATCTAAAAACAGAAAGGAATTGCCCATCATTATGGAAATAAACGGCAAAGATAAACGTCTTAATGC
CGTTCTTCGCATGGCTGATGGACGTCAGACAAGCGTCACCACTGGCAGCCAGCTTCCAGGCACATCCGTCACAGTGAAGT
CGATCTCATTGTCTGGCGTCACGTTAAGTGATGGCACCACCCTTACTTTCTGA
>Cluster_43374
ATGATCATCGGAAATATTCATAATCTTCAGCCGTGGCTACCCCAGGAGTTACGCCAGGCGATTGAGCATATCAAAGCACA
CGTTACGGTAGAAACGCCAAAGGGCAAGCACGATATCGAAGGCAATCGTCTGTTTTATCTTATCTCGGAAGATATGACCG
AGCCGTACGAAGCTCGCCGTGCGGAGTACCATGCCCGCTATCTCGACATTCAGATTGTGTTAAAAGGTCAGGAAGGCATG
ACCTTCAGCACACAACCTGCAGGTACGCCGGATACCGACTGGTTAGCTGATAAAGACATCGCATTTTTGCCGGAAGGCGT
TGATGAGAAAACAGTTATCCTTAATGAAGGTGATTTTGTTGTGTTTTATCCGGGGGAAGTGCATAAACCGCTGTGCGCAG
TGGGCGCGCCAGCCCGGGTTCGCAAAGCAGTAGTGAAGATGCTGATGGCGTGA
>Cluster_24438
ATGCAAGTTATTCTGCTTGATAAAGTAGCAAACCTGGGTAGCCTGGGTGATCAGGTAAACGTTAAAGCGGGCTATGCTCG
TAACTTCCTGGTACCGCAGGGTAAAGCTGTTCCAGCTACCAAGAAAAACATTGAATTCTTCGAAGCACGTCGCGCTGAAC
TGGAAGCTAAACTGGCTGAAGTTCTGGCAGCAGCTAATGCTCGCGCTGAGAAAATCAATGCACTGGAAACTGTTACCATC
GCGTCTAAAGCTGGCGACGAAGGTAAACTGTTCGGTTCCATCGGTACTCGCGACATCGCTGACGCTGTAACTGCAGCTGG
CGTTGAAGTGGCTAAGAGCGAAGTTCGTCTGCCGAACGGCGTTCTGCGTACCACTGGCGAACACGAAGTGAGCTTCCAGG
TTCACAGCGAAGTATTCGCGAAAGTGATCGTAAACGTAGTAGCTGAATAA
>Cluster_29098
ATGCAACGTCAATATAAAAAATACATTCTTCTTTTTTGGGGTGTCATGGATGTTATCGCGATAGCCACATATCTGTTCTA
CTCGCTTGGTAATGGTCGTATCCCTTTTATTCCGATATCGTTCATTCACTTTCTTTACTTAAGGATATCGGTGTTGAAG
GCGGTTTTTACGCTTATGCAGTCGCCACTGTTACGCTACAAATAGTATTATTGCTCTCTTTATTCTTTTCTGCGCAATGT
TTTTTAAGACAAAAATTATTTAGTTTCTCTTTTTTGGCTATTCAGGAGTTTATGCGTTTTGCTACCGTAAGCTGCTCAAT
CTCCGTAATACCGTTGTTACTTAATTATTTTTATTCACAGGATCTGGTCTTAAATATTTCGTTGTTTATCTTCTCAGAGG
TCACAAAAGTGACAACGATTATCTGGTGTAAACGACAGGGGAAAGTGTGA
>Cluster_35633
ATGGCAAAAGTGGCTGACGGGATCCGCTACGCCGAACGTGTTGTTGCAGGAGAAATTGTCGCTGGTGAATTTGTCCGTCT
GGCCTGCCAGCGTTTTCTTGATGATCTGAAGTACGGCGAAGAGCGGGGGATTTATTTCAGTGAACCCCGTGCGCAGCACA
TCCTGAATTTCTACAAATTTGTGCCCCATGTAAAAGGGGCGCTGGCAGGTCAGCCCATTGAACTGATGGACTGGCATGTT
TTTATCCTCATTAATATTTTTGGTTTTGTCATTCCGCTGGTGAATGAAGAGACCGGGGAAGTTGTCATGCGCAGCGATGG
CAGCGGACGCCCGGTGATGGTGCGCCGTTTCCGGACAGCGTACAACGAAGTCGCCCGTAAAAACGCAAAATCAACCCTGT
CATCAGGTATCGGTCTGTATATGACGGGGGCAGATGGTGAAGGCGGGGCTGAGGTGTATTCAGCCGCAACCACGCGTGAC
CAGGCCAGAATCGTGTTTGAAGACGCCAAAAAATATGGTCAGAAAAGCCCGATCGACACTCGGGCGGTTGTTTGATTTCAA
CAAGCTGGCGATTTACCAGGAGCAGAGCGCATCAAAATTTGAACCGCTTTCCTCGGATGCAAACAACCTGGATGGTCTGA
ACATCCACTGCGCCATTATTGATGAGCTGCATGCACATAAAACCCGCGACGTGTGGGACGTTCTGGAAACGGCAACCGGT
GCCCGTCTGCAGTCCCTGTTATTTGGTATCACCACGGCTGGCTTTAACAAGGAAGGGATTTGTTACGAGCAACGCGATTA
CGCCATCAAGGTATTGCGAGGCTATAACAGCGACGTGGAGGGCGCGGTAAAAGACGACTCCTACTTTGCGATTATTTACA
CCCTCGATGAGGGAGATGATCCGTTTGATGAAACGGTCTGGCAGAAAGCGAATCCCGGCCTGGGCATCTGTAAACGCTGG
GATGATCTGCGTCGCCTGGCGAAAAAAGCGAAAGAACAGGTCTCTGCGCGGGTGAATTTTTTTACCAAACACATGAATGT
GTGGGTAACAGCAGAGTCTGCCTGGATGGACATGATTAAGTGGGAGAAGTGCGAATACATTGCTCCACAGCATGAGCTGA
AAACATATCCCATGTGGGTCGGCGTCGACCTTGCTCATAAGATTGATATCTGTGCGGCGGCAAAACTCTGGCGAACGGAT
AACGGGCATGTTCATGCCGATTTTAAATTCTGGCTTCCGGAAGGACGGCTGGAACGATGCTCACGGCAGCAGGCAGAACT
TTACCGGAAGTGGGCGGAGATGGGTAAGCTCATCCTGACGGATGGTGATGTTATCGATCATGCTCAGATCAAAAGTGACC
TACTGGAATGGATTGGCGGTGAAAACCTGAGGGAACTGGGATTTGACCCGTGGAGCGCAATGCAGTTCAGTCTGGCGCTG
ACTGAAGAAGGTATACCGCTGGTGGAAGTTCCGCAGACGGTCCGCAATCTGTCAGAGGCCATGAAGGAAACGGAATCACT
GGTCTATGCCGGGCGTTTCCACCACAGCAATCATCCGGTCATGAACTGGATGATGTCTAACGTTACGGTAAAACCGGACA
AAAACGACAATATCTTCCCGAATAAATCCACGCCGGAAGCCAAAATCGACGGCCCTGTTGCGATGTTTACAGCAATGAGC
CGGATGCTGGTCAATGGCGGTGAACCGGAGCCGGATCTGTCTGAACATCTGGTCAGCGTTGGTATTCGCTCGCTTTAA
>Cluster_50603
ATGAATCAGTCACTTACCCTTGCCTTTTTGATTGCAGCCGGGATTGGTCTGGTAGTCCAGAACACGCTGATGGTGCGCAT
CACCCAGACCTCCTCTACCATTCTCATCGCCATGTTGCTGAACTCACTGGTGGGGATTGTGCTCTTTGTTTCTATTTTAT
GGTTTAAACAAGGCATGGCGGGGTTTGGCGAACTGGTGTCCAGCGTGCGCTGGTGGACACTTATACCTGGCTTGCTGGGA
TCGTTTTTTGTCTTCGCCAGTATCAGCGGATATCAGAATGTGGGAGCGGCGACAACTATTGCAGTACTGGTCGCCAGCCA
GCTGATTGGCGGTCTGGTGCTGGATATCTTCCGCAGCCATGGCGTACCACTTCGGGCGTTGATCGGCCCAATTTGTGGCG
CAATTTTACTTGTGGTTGGCGCCTGGCTGGTGGCCAGACGCTCATTTTAA
>Cluster_39293
ATGAGGCCGGTGTATCTGGCGTTCTATAAAGGGCGAGCCGATCATCATGGGTTCGCACGTCTTTCCGACTGGCTGACGCG

GCTGGTGACGCGGGGTGAGTATTCTCACTGCGAGCTGGCTGTTGACCAGGGTAACGGCGAGTTTCTTTGTTATTCGTCGT
CGGTGCGTGACAAGGGGGTGCGCTGTAAGCAAATGCCGCTGCCACTCGATAAATGGGATCTTATCGCGCTAAATGCGCTA
CCAACCAGTGTGGAGCAGTTCTTCCGTCGAAACGACGCCATGAAATATGACTGGCTGGGGGCTGTGGGATTCGTTGTTTT
TAATCGTGGACGGGAGAACAAATATTTCTGTAGCGAGTTTTGCGCAGATTTTCTTGGACTGGTCGATAGCTGGCGTTATT
CACCCAATATGCTCCACGCGCTGGCCAGCAGTATTCAGACTATGAAGTTATAA
>Cluster_7954
ATGAATGACTTTAAACCCTTTGACGATAAGCTCGCGGGGCTGATAGCGGCGCTGTCACCTGCTGCCCGTCGCAGAATGGC
CGCAGATATCGCGAAGACCCTGCGAGCCCGTCAACAGCGCCGGATTAAAACGCAGAAAGCCCCGGACGGGACACCTTACG
CCGCCCGAAAACGCCAGCCGGTAAAAGCCAAAAAAGGCCGGGTTAAGCGGGAAATGTTCGCCAAACTCCGCACCAGTCGC
TTTATGAAAGCTACGGCGGGGAATGACGCGGCGGTCGTGGAATTTACCGGTAAAGTACAACGGATGGCGAATGTGCATCA
GTACGGCCTCAAGGATAAGCCCGGGCGAAACAGTACGCCGGTACAGTATGATGCCCGACCATTGTTAGGTTTCCAGAGTG
ACGACTTGGCTATTATTGAAGTTATAATAGCCAAGGGCTTCGATATCTAA
>Cluster_19444
ATGAAAAAGATTGCATTTGGCTGTGATCATGTCGGTTTCATTTTAAAACATGAAATAGTGGCACATTTAGTTGAGCGTGG
CGTTGAAGTGATTGATAAAGGAACCTGGTCGTCAGAGCGTACTGATTATCCGCATTACGCCAGTCAAGTCGCACTGGCCG
TTGCTGGCGGAGAGGCTGATGGCGGGATTTTGATTTGTGGCACTGGCGTCGGTATTTCGATAGCGGCGAACAAGTTTGCC
GGAATTCGCGCGGTCGTTTGTAGCGAACCTTATTCCGCGCAACTTTCGCGGCAGCATAACGACACCAACGTGCTGGCTTT
TGGTTCAAGAGTGGTTGGCCTCGAACTGGCAAAAATGATTGTCGATGCGTGGCTGGGCGCACAGTACGAAGGCGGTCGTC
ATCAACAACGCGTGGAGGCGATTACGGCAATAGAGCAGCGGAGAAATTGA
>Cluster_18794
ATGGGGAACGAAAAGAGCTTAGCGCACACCCGATGGAACTGTAAATATCACATAGTATTTGCGCCAAAATACCGAAGACA
GGTGTTCTACAGAGAGAAGCGTAGAGCAATAGGCTGTATTTTGAGAAAGCTGTGTGAGTGGAAAAGTGTACGGATTCTGG
AAGCTGAATGCTGTGCAGATCATATCCATATGCTTGTGGAGATCCCGCCCAAAATGAGCGTATCCGGCTTTATGGGATAT
CTGAAAGGGAAAAGCAGTCTGATGCTTTACGAGCAGTTTGGTGATTTGAAATTCAAGTACAGGAACAGGGAGTTCTGGTG
CAGAGGGTATTACGTCGATACGGTGGGTAAGAACACGGCGAAGATACAGGATTACATAAAGCACCAGCTTGAAGAGGATA
AAATGGGAGAGCAGTTATCGATTCCCTATCCGGGCAGCCCGTTTACGGGCCGTAAGTAA
>Cluster_48925
ATGAGGAAATATATTCCACTGGTATTATTTATCTTTTCATGGCCGGTATTGTGTGCAGATATTCATGGACGGGTTGTTCG
TGTACTGGACGGTGATACCATCGAGGTCATGGACTCACGGAAGGCAGTGCGGATCAGGCTGGTAAATATTGATGCGCCGG
AAAAGAAACAGGATTATGGCCGGTGGTCAACGGACATGATGAAATCACTGGTTGCAGGAAAAACGGTTACCGTCACATAT
TTTCAGCGAGATCGTTATGGTCGTATTCTCGGGCAGGTTTATGCACCAGACGGAATGAATGTTAATCAGTTTATGGTCCG
GGCCGGGGCTGCCTGGGTATATGAGCAGTACAACACAGATCCCGTGCTGCCTGTCCTTCAGAACGAAGCCAGACAACAAA
AACGTGGGCTCTGGTCAGATGCTGATCCGGTTCCCCCCTGGATATGGAGACACCGTAAATAA
>Cluster_34168
ATGTCTGCATTGACTCCGTGGGACCCCTTCCGGGAACTGGATGAATTGCAAAACCGCCTGGCGACGATGTTCGGACGAAT
ACCCCAGCGACAGGGCGCCCGTACCGGCAACGAAGCCATGACCACGGCGGACTGGGCTCCAATGGCGGACATCAGCGAGG
ATGAGAACGCATTCCTCCTCAAGCTGGATCTGCCGGAGGTCCCCAGGGATGCCGTGCGCGTCAGCGCGGAAAACGGTGTG
CTCACCATCAGCGGCGAGCGCAAACTGGAAAAAGAGGAGCAGGGCAAGAAGTTCCACCGCATCGAACGTGCCTATGGCCG
CTTTGTGCGCAGCTTTGTCTTGCCTGACAACGTTGATCCGACCAAGGTGACGGCTTCCATGAAAGACGGCGTGCTGGAAG
TGCGGCTTGTCAAGGCCGAGCAAGCCAAACCGAAACAGATTGAAATCTCAGTCAACTAA
>Cluster_52862
ATGAAAAAAATAATACTGAGTATCATTCTCATTTCAAATTGCTGTAATGCTTCCGATTGTTTTGAAATTACAGGAAAAGC
ATATAACATAGATCCATTAATATTAAAAGCAATTGCATGGAATGAATCAAAATGCAAAAGCGGAATAAAAAGCAAAACAA
ACAAAAACGGAACGTATGATATAGGTATAATGCAAATTAATTCATCTCATTTAGATTTATTAAGCAAATTTAATATATCT
GAAGATGATTTATTGAATAATGCCTGTATAAACATATCTGTTGCTGGTTATATCTTAGCCTCTAACATAAAATCCAGAGG
GAATACATGGGATGCAGTTGGTGCGTATAATGCAGGTTACTTTAATACACCAAATGCAGTAGAACTCAGAAGGCAATACG
CAATGAAAATATATAAAACATATAATAAATTAAAGAATAATGAGCAAATTATCGATTAA
>Cluster_23535
ATGAACATGCTCTTTGACTGGTGGTTCGCCACATCACCCCGCCTCCGCCAGTTTTGCTGGGCAGTCTGGTTGCTGGTGTT
AGTTACGCTCATTTTCTGTCATTGACACACCATGAAGAGAGCGACGCATTAATTCGACTACGGGCAAATCATCACCAGC
AGTGGGCTGCACTGTATCGCCTGGTAGACACCACTTCCTTCAGCGAAGAAAAAACGCTGCCCTTTTCGCCACTGGATTTT
CAGTTATCCGGCGCGCAACTGGTTTACTGGCATCCATCCGCGCAGGGAGGTGAGTTGGCGTTGAAAACGCTGTGGGAAGC
AGTGCCGTCGGCATTTACACGGCTGGCAGAGCGCAACGTCAGCGTGAGCCGTTTTTTGTTAAGCGTGGAAGGTGATGACC
TTTTGTTCACGCTACAACTGGAGACGCCGCATGAGGGTTAA
>Cluster_50039
ATGAAACAAATGACACTAATTGAGATGGATGGATTTCTGAAAGGTAAATGCATCCCATGTGATTTAAAGGTTAACGAAAC
AAACGCTGAATATCTTGTGCGTAAGTTCGGTGAACTTGAATCAAAACTAGAAACGGCGTTGCGGGAGTGTAGTTCTGCTG
GAATCACGATTGATAACCTTGAGGCCAAGTGCGCGAAGATGGCTGCTGAAAATACCTCGCTTAAGCAATCTGAGAAGGAA
TTTAATGACTTTTGTCGTGAGGAGTTTAGCGAATGGGAAGATGATGTTACTGAAACCCCAGCCACCGATGCTTTCCTGGC
TGAAATTCGTGCGCAGGGCGTGGAGATGTTTGCGGAGTGTGCATACACACTTGAACATCATGATCACGCAGTAGCCTTCG
CCGCTGAGCTTCGCAAAGGAGGCAACCAGTGA
>Cluster_26665
ATGAGTAAAAACGATATCATTATCAGAACTCATTATGAGTCTCCTCTTAGAATGCACATCGATAGCGACATACTAACGCC
TTCATCAGAGCCTATTAATCAATTTGCGCGCCAGCTCATCACCCTACTTGATACCTCTGATTTAAGTTCGATGCTGTCAT
ACTGTGTTACTCAGGAATTTACCGCAAGCTGTCGAAAAATATCACACAATTGTTATTCCACTGCCCTTTTTATCATTAGC
TTTACCACTTCACCCATCCATGCAGAAAATACACTCATTACATTACACTATAAAAAAGAAATCATTTCCTTATTTCTGGA
AACCACGCCTATTAAAGCTAACCATTTGCGAAGCATACTGGATTATATTGAACAGGAACAGTTAACTGCCGAAAAACGTA
ACCATTGCATGAAACTGTCCAAAAAAAAATTCATAG
>Cluster_46839
ATGATAAACGATCTGAAGAGTATATTGTTAAAATCCTCAGAGGAAGTTGATGTTTTTATCAAAATTTTTGAAAGTTGGAG
AAAAAAATTACCGGCAATATCAGGTCCTGTAAATCTCTATATACCTACCAGATTCAAAGATAAGTATCTTGAAGTAGAGT
CATATTTTGTCGACAAATCAATATGGAATGTCCATATTACATTTCATGATGATAAGCGTTTTGTGTTTTTTACCGATCAA
TTTATTGCTGAATTTAGTCCGCGCAGGAATTTGTGGATAATTGTGAGCAATATTTAATTAATAATCATTGTTTCTCCCCTGA
CAAGGTTAACGAAATCTGCGAACAAGCCAGACATTATTTAGTTGAAAAAATGTTTGAAACACATTCTTTGGATATGAATA
ATTCTGTTTTGGCAAGCCCTGAGGACTTATAG
>Cluster_43573
ATGAACATCCCTACTCATCTGAAACACAAACCGATTATCGCTGTAGAAAACTATGATCGAATTGATGGACCTTATGCTGA
TGACACTGATGCTATGGGGCTTTCCGTTGGAATTGCTCAATGGAACACCCCCGGGTATACCGAGCTATCAGCAAAAGTGT
GGCGCAACACCGGAGAAAAGTGGTCACGGCAATCAGAAGAATTACCTCTTCATCGTGTTATCGACTTGGCAACGCTTATC

TGCATAGCAATGGATTACAGTAAACATGGCCGCTTATCTTCCAGCGACGACTTTCCGGTCAGTCGTTCAGGAGGCAATCC
TGAGTTATCTCGTCACATTGATTTAATGAAAAACGAGCTAAAAGAGAATCACGAGCATTTAGATGAGAGTCTTAAGCGTT
TAGCAGCAGAACTAAAACGTTTAGGTTATTAA
>Cluster_58212
ATGGCAACCCCAGCAACTGTATCCATAGAACCCACTCTGGCAGCTATCAGAGCTCGCTGGTGTATTAATTCAAGTAAAAC
AACTCAATCCTTTAACGATCCTGCGTCCATGGAAGAGGTTGTCGAGTATCTCAAAGGAACATACTCAGCTCTTCGCAAGT
CTGTCGCATGCGCCAAACTGAAAATTTTACATCTTAAACAAAGAATGCAAAATGCTACTAACTTTCTCGCGCGTCTGATG
TCATGTAAAAATCAGGCATCCAGATCGCATCACAGTACGGCTAAATCAGCTAAAAGTGCCTTATCATCAGATTCAGGTGA
TGGTAGTGACCCCGACCCCGAGCCCGAAACGTTTCCTTCTGCCTTCATTACTACCCCTACTAATTCAATAATGCTTAAAG
CTTTCTTTGCCAATATCTCAATCACTGAGGTGGCAAAATGA
>Cluster_43027
ATGACCATACAGCAATGGTTATTCTCATTTAAAGGGCGTATTGGACGCCGTGATTTCTGGATTTGGATAGGCCTGTGGTT
CGCAGGCATGCTGGTGCTTTTCTCACTGGCGGGTAAAAATTTACTCGATATTCAGACCGCGGCGTTTTGCCTTGTGTGCT
TGCTTTGGCCAACAGCGGCAGTAACCGTTAAGCGCTTGCATGATCGCGGGCGTTCCGGCGCATGGGCATTTCTGATGATT
GTGGCGTGGATGCTGCTGGCGGGTAACTGGGCAATTTTACCGGGTGTCTGGCAATGGGCGGTGGGGCGTTTTATCCCGAC
ATTGATTCTGGTGATGATGCTTATCGATCTTGGTGCGTTTGTTGGCACACAAGGCGAAAATAAATATGGTAAAGATACCC
AGGATGTGAAGTATAAAGCCGACAATAAATCAAGTAATTAA
>Cluster_26168
ATGTTTTATAGTCCATCTTTAAACATTTTTGTGAATCCTGCACTTAAGGATGATTACATTAATGCAAATTCATGGCCAGA
TGATGCTCTGGCTGTCAGTGATGATGTTTATAATGAATTTGCAATCAATACGCCTCCAGATGACAAAATTCGTGTTGCAG
GAAAAAATGGATTACCCACATGGGCACTAATACCTCCACCATCACATGAAGAACTTATTCAACAGGCAGAATCAGAAAGG
CAATTATTGCTTAATCAGGCCAACGAATACATGAACAGTAAGCAATGGCCCGGTAAAGCCGCTATTGGTCGTCTGAAAGG
TGATGAACTGGCGCAATATAATTTGTGGCTGGATTATCTGGACGCACTGGAGCTGATCGATACTTCCGGTGCGCCAGATA
TTGAATGGCCTACGCCTCCGGCAGTTCAGGCCAGATGA
>Cluster_17525
ATGATTAAAGAACGTATTTCTTATATCATTCCGATTGCGATGGATGAAGGCAACCCAGTAACTCCAGTTCTTATCTATGA
AATGGATAAAGACTCCCATGAAGTGGATCTATCATTTGGCGCTTTTTTTATCGGCCTTAAGGCTACAAAAAAATATTCCA
TAGGCATTGAGGTTTTCAATGCTCAAGAAATACCGATACCAATTGACACAAAACTGTACTCCAACCATAAGTTTTTTACG
GTAGCAGAAGCCAACGATGGAGAAACCATCGTATCAACTTCTATGAGAATTAACTTCCCAAGGGTGAAAATTATCAAGCC
TGGGATATTCGAAGTTAGAGCATCACTGGTTAATCCTGATAAAGGCGAAGTAATTGATGTAAAAAGTTCGTTTTTTGACG
TGAAGATAACAGGATCAGTTCGCGATGAGTTTAGATAA
>Cluster_13762
ATGAGTGAGATAGTAATACGCCACGCAGAAACACGGGATTACGAGGCCATCAGGCAGATTCACGCCCAGCCGGAGGTGTA
TTGCAACACACTACAGGTGCCTCATCCTTCCGATCATATGTGGCAGGAGCGACTCGCCGATCGTCCCGGCATCAAGCAAC
TCGTCGCCTGTATTGATGGAGACGTCGTGGGCCATCTCACCATTGACGTGCAACAGCGCCCACGCCGCAGTCATGTTGCC
GATTTTGGTATCTGTGTCGACTCTCGTTGGAAGAACCGCGGCGTCGCCAGCGCCCTGATGCGAGAGATGATTGAAATGTG
CGACAACTGGTTGCGGGTAGATCGCATTGAACTAACCGTGTTTGTCGATAACGCCCCGGCAATTAAGGTCTATAAAAAAT
ACGGCTTTGAAATTGAAGGGACTGGTAAGAAGTACGCATTGCGTAATGGTGAATATGTCGATGCATATTATATGGCGCGG
GTGAAGTAA
>Cluster_17463
ATGAAACGACTACTTTTGGCCGGGCTGGGAGGAGTGCTGCTTACGGGCAGTGCTCTTGCCGGTGAAACGAAAAGTGTGCC
ATTGACATTGCGCGTACTGGTAGACCCACCACCTCCTTGTACCGTAAAAGGCAGTAACGTCGATTTTGGTGATGTGATCA
TCAAAAAAAATCAATGGTACTGAATATCGCAAAGATGCAGGTTATACCCTGAACTGTGCCAATAGCCTGAGCGATGACCTT
CAGATGCAGTTTAAGGGCACAACCACCACGATCAATGGCGAAACGGTGTTAAGCACGGGAATTACCGGGTTTGGCATTCG
TATTGAAAATGCAGCAGATAACTCTCTGTTTGCGATTGGCGACAATACCTGGACTCCATTTAGCGTCAGTAGCCAGCCAC
AACTAAAAGCAGTGCCAGTCAAACAGAATGGCGTGACGCTGACAGGCGCACAGTTTAACGCCAGCATGACAATGGTGGTG
GATTACCAATGA
>Cluster_33396
ATGAGCTATGCAGCTAAAAAACTGCTTGACCGCTATTGGGATCGCCGACTACCTGTCGATCCGTTTAAGCTTGCTAAAGC
GTGGGGTGCTCGTGTTGAAGCACTTGAGGAATCGGCTTATAACAACGATGGGTTAAGCGGTCTGGCTGTCATCAAAAAGG
GTGTCCATAGGATTTATTTTGATTCTAGTGAACATTCTAATCGTCAACGTTTTACTGTTGCACACGAGTTAGGTCATCAT
GTGCTGGGGCATACCCAGGACGGAGAGTACCATCGGGATAATGTCGGGAATTATTCAACAGGTGCTCGTGATTACCGTGA
GGTTGAAGCAAACAAGTTTGCTGCTGAGTTGTTAATGCCTGAGTCTGCTATACGTCAATTGGTATCCAGAGAAGGTATTG
ATAGCACTTTGAGATTAGCAAGCATCTTTAATGTATCAGAAGCCGCTATGCATTGGCGCTTAAAGAGCTTAGGAATGGCT
TATTAA
>Cluster_1611
ATGAAAAATAAATCAGGTAATTCTTATTATAGAGAAGCGGTCGATATTCATAGCTCACTTGAGCTTTCATTTATATTGCA
TAAATATCATTTGCGTGTAGATGAGAAGAAATCTCGAAATGTATTCAGCGCCATACGTGATGAGCTCATCCTTGATGGGG
ATTCACGACAAAACTTAGCTACTTTCTGTCAAACATGGATTGATGATGAAATCCGTGAGTTGCTTAATTTATCTATTGAT
AGAAACATGATTTATAAAGATGAATATCCACAAATGGCGGAACCTGAAGGTCGCTGTGTGCGTATGCTTTCCGATTCGTG
GAGTTTCCCCGATTCGCGACACACCTTAGGTTGCTCAACCATTGGTTCATCTGAAGCTGCTATGCTCGGCCGCTTGGCGT
TAAAATGGCAGTGGTGTAAAAAACGCGAAGTACAAGGTAAATCAACCGAACCTGATTTGCGGTCCTGTGCAAATATGCTG
GCATAA
>Cluster_66448
ATGGCTTTAAATCTTCAAGACAAACAAGCGATTGTTGCTGAAGTCAGCGAAGTAGCCAAAGGCGCGCTGTCTGCAGTAGT
TGCGGATTCCCGTGGCGTAACTGTAGATAAAATGACTGAACTGCGTAAAGCAGGTCGCGAAGCTGGCGTATACATGCGTG
TTGTTCGTAACACCCTGCTGCGCCGTGCTGTTGAAGGTACTCCGTTCGAGTGCCTGAAAGACGCGTTTGTTGGTCCGACC
CTGATTGCATACTCTATGGAACACCCGGGCGCTGCTGCTCGTCTGTTCAAAGAGTTCGCGAAAGCGAATGCAAAATTTGA
GGTCAAAGCCGCTGCCTTTGAAGGTGAGCTGATCCCGGCGTCTCAGATCGACCGCCTGGCAACTCTGCCGACCTACGAAG
AAGCAATTGCACGCCTGATGGCAACCATGAAAGAAGCTTCGGCTGGCAAACTGGTTCGTACTCTGGCTGCTGTACGCGAT
GCGAAAGAAGCTGCTTAA
>Cluster_34161
ATGAAATCAACACGCCTTAACCCATTTCAGGTCTTACGTGACCAATTGGCCATCATGAGTTTTTACGAGCGCTTCGAGCA
GGTCGTCGCGCTCGTGCTGTCGGCGGTGATCGCCGTCATCATCGTGGTGTCGCTGTTCCAGCTCATCTCCATCGTCTTCA
CGCTGCTGGTTCTAGATGCCTTCAATCCCCTGGACCACGAGGTATTCCAGAGCGTGTTCGGCATGATCATGACGCTCTTG
ATCGCGATGGAGTTCAAGCATTCCATCGTGCGCGTGGCGCTGCGTCGGGACAGCATCATCCAGGTCAAGACGGTGGTCCT
GATCGCGCTGATCGCACTCTCCCGCAAGTTCGTGATCCTCGACTCCAACGCGAGCCCCGCAAAGATAGCCGCGCTGGCTA
GCGCCACGCTGGCTCTGGGTGCGACCTACTGGCTGCTGCGCAAGCGCGGCGACCGCGCCGCCGAGACCTCTGAGCATGAC
CCGTCATCATCCCAGTGA

>Cluster_21035
ATGAACATTGTCGATCAACAAACCTTTCGCGATGCGATGTCCTGTATGGGCGCGGCGGTCAATATCATCACTACGGACGG
TCCAGCCGGGCGCGCCGGGTTCACCGCCAGCGCCGTCTGCAGCGTGACCGACACGCCGCCCACATTACTGGTGTGCCTGA
ATCGTGGGGCGTCCGTCTGGCCGGTATTCAATGAAAATCGAACGCTGTGTGTAAATACACTTAGCGCCGGGCAGGAGTCG
CTTTCAAACCTTTTTGGCGGCAAAACGCCCATGGAACACCGCTTTGCCGCCGCCCGCTGGCAGACTGGTGTGACCGGATG
CCCGCAACTGGAAGAGGCACTGGTTTCGTTTGACTGCCGTATCAGCCAGGTGGTGAGCGTCGGCACCCACGACATTTTGT
TTTGCGCCATCGAAGCGATTCATCGTCACGCCACACCCTACGGGCTGGTGTGGTTCGATCGCAGTTATCACGCGCTGATG
CGCCCTGCTTGTTAA
>Cluster_58449
ATGGTTACTTTCCACACCAATCACGGCGATATTGTCATCAAAACTTTTGACGATAAAGCACCTGAAACAGTTAAAAACTT
CCTGGACTACTGCCGCGAAGGTTTTTACAACAACACCATTTTCCACCGTGTTATCAACGGCTTTATGATTCAGGGCGGCG
GTTTTGAACCGGGCATGAAACAAAAAGCCACCAAAGAACCGATCAAAAACGAAGCCAACAACGGCCTGAAAAATACCCGT
GGTACGCTGGCAATGGCACGTACTCAGGCTCCGCACTCTGCAACTGCACAGTTCTTCATCAACGTGGTTGATAACGACTT
CCTGAACTTCTCTGGCGAAAGCCTGCAAGGTTGGGGCTACTGCGTGTTTGCTGAAGTGGTTGACGGCATGGACGTGGTAG
ACAAAATCAAAGGTGTTGCAACCGGTCGTAGCGGTATGCACCAGGACGTGCCAAAAGAAGACGTTATCATTGAAAGCGTG
ACCGTTAGCGAGTAA
>Cluster_14324
ATGAAATACGATACTTCCGAGCTTTGTGACATCTATCAAGAAGATGTTAACGTCGTGGAACCGCTGTTCTCCAACTTTGG
CGGACGGGCGTCGTTTGGCGGACAAATAATCACGGTAAAATGTTTCGAGGACAACGGGTTGCTGTACGATCTGCTCGAAC
AGAATGGCCGTGGTCGTGTTCTTGTCGTCGATGGCGGTGGTTCTGTTCGTCGCGCACTGGTCGATGCTGAACTGGCGCGT
CTGGCAGTACAAAATGAATGGGAAGGTCTGGTCATTTACGGCGCGGTGCGTCAGGTAGATGACCTGGAAGAGTTGGATAT
CGGCATCCAGGCGATGGCGGCAATTCCGGTTGGTGCCGCTGGCGAAGGCATTGGCGAAAGCGATGTCCGCGTCAATTTTG
GCGGTGTCACCTTCTTCTCCGGCGACCATCTTTATGCCGACAATACCGGGATTATTCTTTCTGAAGATCCGCTGGATATT
GAATGA
>Cluster_25445
ATGATTATCGGTAATCTCAACCATTTGTCGCTGGCGGGGCTTCCTGCCTGGGTGAGAAATATTCTGCTGCGCCCGGAGTG
TTCGTTATCGGCGTTATCCACCAGAGAGGACGGTCGCTGGCAACCTGAAGGATGTCGGTGGTTCTGCACGCTGGGAACTT
CAGACACGCAACCCGCCGAACTCCGCCATACGGAGTATCACCATCTATGGGCCGATATACAGGTGGTGATTACTGGTTGT
GAGGGCATTAATGCGGGAACGCGGCCCATCGCTCGCGAAAACGATGAAGAACGTAAGCCCGATCTGTTTATCGCCCCATC
CCCTGAAAACAGTGTCGCCATCACCCTACACGCCGGTGATTTCGCCGTCTTTATGCCTGAGGAACCTCACCAGGCGCTGT
GCGCAATTGGCACGGCTACTTCAATACGCAAAGCGGTCTTTAAAGTACCGCGCGACATGCTGGAAGCCTAA
>Cluster_47272
ATGGAATCGCTCTCAGAGAGAACCTCAACAGGCTACCAGCAAATCCATGACGGTATTATTCACCTGGTCGATAGCGCCCG
GACGGAAACGGTACGTAGCGTTAACGCGCTGATGACCGCGACATACTGGGAAATTGGCCGACGAATTGTCGAATTTGAAC
AAGGTGGCGAGGCCAGGGCTGCGTATGGTGCGCAGCTAATCAAGCGACTATCAAAGGATTTAAGTCTAAGGTATAAGCGT
GGTTTCTCTGCAAAAAACTTACGCCAAATGAGGCTTTTTTACCTCTTTTTTCAACATGTTGAAATTCGCCAGACAGTGTC
TGGCGAATTAACACCATTGGGAATTCCGCAGACACCATCTGCAGAATTCCCATCGGCAAAGATTTGGCAGACGCTGTCTG
CCAAATTGAGAGGCTGTTTGATATTTCCACAGCTTTTGTATTGCATCTCCTATTTTCGGTTTGTATATTCATGCAAATAA
>Cluster_39670
ATGCTCAAACCCGACAGCCTGCGCAGGGCGCTGACTGATGCCGTCACGGTGCTGAAAACCAGTCCAGAGATGCTTCGGAT
ATTCGTGGATAACGGGAGTATTGCCTCCACGCTGGCGACGTCGCTGTCGTTCGAAAAGCGTTACACGCTCAATGTGATTG
TGACCGACTTTACCGGTGATTTTGACCTGCTCATCGTGCCGGTGCTGGCGTGGCTGCGGGAAAATCAGCCCGACATCATG
ACCACTGACGAAGGTCAGAAAAAGGGCTTCACGTTTTATGCAGACATCAACAATGACAGCAGCTTTGATATCAGCATCAG
TCTGATGCTGACCGAGCGCACGCTGGTCAGCGAGGTTGACGGTGCGCTGCATGTGAAGAATATCCCGGAACCCCCGCCGC
CGGAGCCGGTCACCCGCCCGGTGGAGCTTTATATCAATGGCGAACTGGTGAGCGAGTGGGATGAATGA
>Cluster_60533
ATGAAAATACGTTCACTAAGCCGGTTTGTACTGGCAAGTACAATGTTCGCCAGCTTTACAGCCTCTGCGGTCCCCGGACT
CTGGCAACAGGGGTACGGTCAGGGCAATGCGGAATACAGTGTGACGGATGCCAGCGGAAAGATGTTTACCATCAACTGCA
CGGGAAATCCGGACCAGAACGGTATTTACCAGCACTCGGTTTTTCTGATCCTCGCCGGGGATAAAACGGTCAGCTCGCAT
GATGACAGTACCGGCATCACAGTGGTGATGGACCATAAGCAATATGCCATTCCCTCAACCCTTGGCTGGCGTAACGGAGA
TAATGCCTGGTTCAGCTTCATCATGGATATCCGTAAGGCCCGGCAGTTCGACGTCTACGTCAATGACCAGAAAGTTGGAT
CGTTTAACCCTGATGTTCGGAATGCACAAAAGGTCCTGCCAACATTAGCGGACTGCACTAACGACTGA
>Cluster_61085
ATGAAACGGATAATAACAGGATGCCTGCTGCTGAACTTCGCCATGGCCGCTCAGGCAGAGTGTAATATCTCCAGTAGCAT
ACAAAATATCGACTATGGCAAACGAAGTGCTGCCATGCGCCAAGTTGACCGGGGTAAAACAACACAATTAGCAGACAGAA
CAATCACGCTGGTAATGCAATGCGATCAGGACGCTCATATTCGTGTTCAGTTGAATACCGCTAATATTTCCAACAACGGA
TTTGGATTTGGCCCGAATGGTTCATTAAATCTGATCGCCAGCGATGCATTTTCAGGTAGTAACAATCTCGACCTGGCGTT
AGCCAGTGGCAAAAATGACAATCCGGGCAGCACAGGAACAGCATCAATTAGGGAAGGTGCGAATAAGCGGGGAAATTCTT
CTCGGCTGACTCAGTCATTTCATTTCTTCATGTTTGAGCCGATTTTTTCTCCCGTAAATGCCTTGAATCAGCCTATTTAG
>Cluster_56609
ATGACAAACCTGACACTGGATGTAAACATTATCGATTTCCCATCAATACCTGTGGCGATGTTGCCGCACCGCTGTAGCCC
TGAATTGCTCAACTACAGCGTGGCGAAATTTATCATGTGGCGTAAAGAGACGGGGCTTTCTCCTGTTAACCAAAGCCAGA
CTTTTGGCGTCGCCTGGGACGACCCTGCCACCACCGCACCGGAAGCGTTTCGCTTTGATATCTGCGGCAGCGTTAGCGAA
CCGATTCCCGATAATCGTTATGGTGTGAGCAATGGTGAACTTACCGGTGGACGTTATGCCGTGGCCCGCCACGTTGGCGA
GCTGGACGATATTTCACACACGGTATGGGGCATCATTCGCCACTGGCTGCCTGCAAGCGGCGAGAAAATGCGTAAAGCAC
CGATTCTGTTTCACTACACCAATCTTGCCGAAGGGGTGACAGAGCAGCGACTGGAAACGGATGTTTATGTGCCGTTGGCG
TGA
>Cluster_22053
ATGTTCACGATTAAAACAGATGATCTCACCCATCCAGCAGTGCAAGCATTAGTGGCTTACCATATTTCCGGCATGCTGCA
GCAGCCTCCCCCCGAAAGCAGTCATGCTTTAGATGTGCAAAAATTACGTAACCCGACAGTGACATTCTGGTCAGTATGGG
AAGGCGAACAACTCGCAGGAATTGGTGCGCTGAAGTTGCTGGATGATAAACATGGCGAGCTGAAATCAATGCGTACCGCG
CCTAATTATTTACGTCGTGGTGTCGCCAGTCTGATTTTACGCCACATTTTGCAGGTCGCCCATGACAGATGCCTTCATCG
CCTGAGCTTAGAAACGGGTACACAGGCTGGATTTACGGCCTGCCATCAACTTTATTTGAAGCATGGTTTCGTTGATTGCG
AACCGTTTGCTGATTATCAACTTGATCCACACAGTCGATTTTTGTCATTGACGCTATGCGAAAATAATGAATTGCCATGA
>Cluster_53822
ATGCGTTTACATGTTAAATTAAAAGAGTTTCTTTCGATGTTTTTTATGGCCATATTATTTTTCCCTGCATTCAATGCTTC
TTTGTTTTTTACCGGAGTTAAACCTCTATATTCAATAATAAAATGCTCCACGGAAATATTTTATGATTGGAGAATGTTGA
TTCTGTGCTTTGGTTTCATGTCGTTTTCCTTTTTAAATGTTCATGTAATTTTACTTACGATAATAAAATCATTCTTAATC
AAAAAAACGAAGGTAGTCAATTTCGCCACAGACATAACAATACAGCTAACTCTTATATTTTTACTAATAGCTATCGTAAT

AGCACCTTTAATAGCTCCTTTTGTCACAGGATATGTCAATGCTAATTATCACCCCTGCGGTAATAATACGGGAATTTTCC
CAGGAGCAATCTATATTAAAAATGGCATGAAATGTAATAATGAATATATTTCTCGCAAGGAAGATTCAGCAGTTAAATAA
>Cluster_54439
ATGGGTGTGTATAAGGCAAGGCGATTCAGCCAAAGCACGAAAAAACTTGGTATTCACGATAAAGTTCTCATGGCTGCCGC
TGAAGAAGTGATGCAAGGCATATGGGAGGCTGATTTAGGTAGCGGTGTCATCAAAAAACGGTTACCCCTCCAACAAGGAA
AGAGTGGTGGAGCCAGAACAATTATCTTTTTTAAATCAGCAAATCATGTGTTTTTTTATGATGGTTGGTCCAAGAGTGGT
CTAAGCAGTAAAGGAACCAAAGAAATCGAAGATGATGAACTGGCAGCCTACAAAAAAATGGCAAATGCATTTCTGGCCTT
TAGCAATAAGCAAATAGAGGATTTGATCGAAACTGGCTTCTTGATTGAGGTGAAAAATGAGCGATAA
>Cluster_26020
ATGCCAAAATCCGTAATTATTCCTGCTGGCAGCAGCGCACCGCTGGCCCCCTTCGTTCCCGGCACGCTGGCTGATGGCGT
GGTGTATGTCTCCGGTACGCTGGCTTTTGATCAACATAACAACGTGCTGTTTGCCGATGACCCAAAGGCGCAAACCCGCC
ACGTTCTGGAAACCATCCGCAAGGTCATCGAGACGGCGGGTGGCACGATGGCGGATGTGACCTTCAACAGCATCTTTATT
ACCGACTGGAAAAATTACGCCGCGATTAACGAAATCTACGCCGAGTTTTCCCGGGTGACAAACCGGCGCGATTCTGCAT
TCAGTGCGGACTGGTAAAACCTGACGCGCTGGTGGAAATCGCCACAATTGCGCATATCGCCAAGTGA
>Cluster_50037
ATGGCACATTCGATTACTGTAAGACTAAACAAGCCCGCAAGAGAGTTTCAGGCCGGGGAAAATATCGGATTCAACATCCG
TGCTGGCGTTCAGTATTACGATCGCCAGACAAAAAAGAAAGAATGGACAAACTACAGCGCCGTTGTATTTGCCAAGCCGG
GAGCGCAAGCGGATTACTACCGTAGTGTTCTTGTTGAAGGTGGCATTGCTGAAATTACCGGAGAAAACATCAGGGTTGAT
GTTTATCAGGGGCAAAATGGTCAATCAATCACTCTTGAATTACTGAATGCAAAGATTGGATTTGCAACTTCAGGAAACAG
CCAACAGCAACAAAGTAGCAATCATCAAAATCATCCTGAACACGACGATTCAATTCCCTTCTAA
>Cluster_22304
TTGCTGAAACCGCAGCGGACAGACGGCGGTCATCGGCTGTTCAACGATGCCGATATTGACCGGATCCGCGAGATCAAACG
CTGGATCGACAACGGTGTGCAGGTCAGCAAAGTAAAAATGCTGCTCAGTAATGAAAATGTTGATGTGCAGAACGGCTGGC
GCGATCAGCAAGAAACATTACTGACTTACCTGCAAAGCGGCAATCTGCATAGCCTGCGAACGTGGATCAAAGAGCGCGGT
CAGGATTACCCCGCCCAGACACTCACCACACATCTGTTTATTCCTCTGCGCCGACGGCTTCAGTGCCAACAACCGACTCT
CCAGACGCTGCTGGCGATCCTCGACGGCGTACTGATCAACTACATCTCTATTTGCCTGGCTTAG
>Cluster_17647
ATGTCTGAACCCTTATCAGGCGGCGGAGCTGTGGCAGTCACCATCGGTGGTGCCAGTGTGTTTGGCCTTCTCACCAATAC
CGATTTCGGGGTCGTGGTGGGCGCGTTTGCCGGGGCGCTTTTTGTTGTCACTCAGCAGAAAGAAATCCCGGTATGGCGAA
TGGCTATTCACCTGCTCGTCGCGTTTGTGGTTGGCGTTCTGGGGGCTGGCGTGGCTGCGTCACTGATGCAGTGGCTGACC
CACTACAACGACAAACCGCTTGATGCATTGTGTGCGGTCGGTGTCTCGGCGTTATCCATCAAGATACTGACCTTTCTTTA
TCAACAGGAAATTTCATCGCTGTTCGGCCTGTTTTCCAGACTGCGCGGCGGAGGGGGTGGAAATGGAAAGTAG
>Cluster_26519
ATGAAATTTCAGGCCATTGTATTAGCAAGTTTTCTTATCATGCCTTATGCGTTGGCAGATGACCAGGGGGGATTAAAACA
GGATGCAGCGCCACCGCCGCCTCATGCAATAGAAGATGGCTATCGCGGAACCGATGATGCAAAAAAAATGACCGTTGATT
TCGCAAAAACCATGCACGATGGCGCTTCGGTTTCACTACGCGGTAATTTGATTTCTCACAAAGGAGAGGACCGTTACGTT
TTTCGCGATAAGAGCGGTGAAATTAATGTTGTTATTCCTGCGGCCGTCTTTGATGGACGAGAAGTTCAGCCGGACCAGAT
GAGCAACATTAGCGGCAGTCTGGATAAGAAAAGCGCGCCGACCGTCGTTCGGGTCACTCATTTGCAGAAATAA
>Cluster_44724
GTGGACGATGAACGGAAAAGGAAAAAATACACACTTTATCTGCATCCGGAAAAAGCTGCAGACTTTCAAACTTTGGAAGC
GATAGAGTCCGTTCCGCGTTCTGAGCGTGGTGAGCTGTTCCGGAATGCATTTATTTCTGGAATGGCGTTACACCAACTTG
ATCCTCGCCTGCCTGTATTGCTGACAGCCATTTTGAGCGAGGAGTTTTCAGCAGATCAGGTTGTAACCTTACTCAGTCAG
ACGACTGGATGGAAGCCTTCTCAGGCCGATATCAGAGCTGTGTTGACTGAACTCGGTGCTTCGCAATCTGTTGAAAAAAT
GCCTCCTTCTGCTACTGATTCGGTGCAGGAGGCAATGAATGATGTGCGTCTTAAAATGAAAAAATTATTCTGA
>Cluster_20310
ATGTACCAAGTACAGGACGGCTATTCGCAATATAAAGAGATCGATCTCGCGGCGCGTACTGCGGCGGCATCGCCTCTTGA
ACTGGTGCTGGTGCTGTTTTCTGGCCTGATGGACGAGCTGGAACGCGCCAAAAGCCATATTGAAGGGCGGCGTTTCGAGA
AGAAAGCCCAGAGCATCAATAAATGCATCGACATTCTCAATGCACTCACCAGTTCGCTGGAATTTGAAACCGGCGGCGAG
CTGGTGGTGAATCTTTCGCGCCTCTATGACCACTGCGTTTATCGCTTGTATGAGGCCAGCGGCGAACTGTCGGCAGAGAA
AATCGATGAAGTGATGCTGATCTTAAGCAACCTGCGCGAAGGCTGGGAAGGGCTTTCGGGCAAGCTGGGTTAA
>Cluster_4785
ATGAAAGGTGATTGGGCGGACAGTCATCGCGATGAAGTTGACGATTTTTACAGACATCAGGCTGATATTAAATATTATTC
ATTTCTCGCAGGCCCAGCTTATCGTCTGAATGACTATATCAGTTTTTATGGTTTGGTGGGGATATCTCATACTAAAGCGA
AGGGAGATTATGAGTGGAGAAACAGTGTGGGTGCTGATGAGTCTGATGGATATTTAAGTGAGTCTGTATCAAAAAAATCC
ACTGATTTTGCTTATGCAGCTGGTGTTATTATTAATCCGTGGGGCAATATGTCTGTAAATGTTGGGTATGAGGGAACTAA
AGCAGATATATATGGAAAACATTCGGTCAATGGATTTACTGTTGGAGTTGGTTATCGTTTTTAA
>Cluster_2748
ATGGCGAAAGAAAAGCTGGTTACCATCCATGTTCACACCCCGTTTACGCTGACGCTCGGCGATCAGTCAAAACAGGAGTT
TGGCCGGGGACGGCATAACGTACCGGAAGAGGTCGCGTCGCACTGGTTCACCCAGGCGCACTCTGAGCTTTCCGAAAGCG
TGATTAGCGACACCGATGATCTGCAACCCATTATCGACGGCCTGCAAGCGCAGATTGCCGACAAAGATAAGCTGATTGCC
GATCTTAAAGATGCATTGCTCAAACTGCAGGAGCAGAACGACAGCCTGCAGGCGCAGATTACTGCCGCCCCGGACTGGCGG
TAATGGGGCTAAAGATGTCAAAGAATCAAAGTCTGCCAGCGGTAAGTGA
>Cluster_33541
ATGGCCAATAATACCACTGGATTCATCCGAATTATCAAAGCTGCTGGCTATTCCTGGAAAGGTTTACGCGCTGCATGGAT
CAACGAAGCGGCATTCCGTCAGGAAGGCGTAGCGGTATTGTTAGCGGTGGTTATCGCCTGCTGGCTGGATGTGGACGCGA
TTACCCGCGTGCTGCTTATCAGCTCCGTGATGCTGGTGATGATTGTGGAAATTCTCAATAGCGCCATCGAAGCTGTGGTT
GACCGAATTGGCTCTGAATACCATGAGCTTTCCGGACGCGCTAAAGATATGGGATCCGCCGCAGTGCTGATTGCCATTAT
CGTCGCCGTGATTACCTGGTGCATCCTGTTATGGTCGCATTTTGGATAA
>Cluster_7540
ATGGCACAACCTGCCGCTATTATTCGTATAAAGAACCTTCGTTTGCGTACGTTTATCGGAATTAAGGAAGAAGAAATTAA
CAACCGTCAGGATATTGTTATCAATGTGACGATCCACTACCCCGCCGATAAAGCGCGCACTAGCGAAGATATCAACGATG
CGCTGAATTATCGCACCGTAACGAAAAACATTATTCAGCATGTAGAGAATAACCGTTTCTCTTTGCTGGAAAAATTAACT
CAGGATGTGCTCGATATCGCACGTGAACATCACTGGGTGACGTATGCTGAAGTGGAGATCGATAAACTGCACGCGCTGCG
CTACGCCGATTCGGTATCCATGACCTTAAGCTGGCAGCGTTAA
>Cluster_30721
ATGGAATTTAAAGATTTACCGTCTGATGTACAGAAAACAGCAGCTCATACATTGCATTCTGTGCTGCGAGAAATCGGGAA
AGATATTGCAAGCGAGCCAGCAAAAGATCTGGCCCGGAAAATCAAGACCGCTTTCGTTGAGCTTTATAATGTTGGCACTG
ACTCTGAAACTGTCGAGACCAAGACCGTAAGTTCACCAATATTCTCACTTGAGCCAGGCGTATTATCAGGTGAAATATGC
ACCGAGATTTCCAGCGAACTGCTTCCGGTAATACGAGAAGCAATTTGCCGTCGCGGTTTGGATGGAAGTTACGATCATGA

CGTCCTGCAAGTTCTCAGGACAATGGTGACTTCACTGGGGATTTGA
>Cluster_17749
ATGTCAGGTGAAAAAGCAAGATACCGCGTCCTGCGTTTATCTCACATTCATAACAACCTCTGGCCAGAAGGCTCTGAAGT
TGAGTATGACGGCGAACCAGGTACGGCGCTGGAGCCACTGAACGATGCGGCGAAATTGGCAAAAGAGATCGCCGAAAAGC
GTAAAGGAAGATCGGCTTCTGTAGTTATCAATGTTCCATCTCCAGCTGCGGTTCATCAAGTAGTGGAGGAAGTGAAATCT
GATGGCACTGGCGCGGTAAGTGAAGACCTTACTCTGCTTCGACAGCAGTATGAGGATTTACTAGGCAAGAAGCCGGGCAA
TAAAACGGCTGAAACTCTGGCAAAAGAGATCGCCGAAAAGCGTGCCGAACTGGGCGTCTAA
>Cluster_66792
ATGCCGCACTTTATCGTTGAATGCAGTGACAACATCCGCGAAGAAGCCGACCTGCCGGGGTTGTTCGCCAAAGTGAATCC
GACGCTGGCAGCCATGGGTATTTTTCCGCTGGCGGGTATTCGCAGCCGCGTGCATTGGGTCGATACCTGGCAGATGGCCG
ACGGGCAGCGTGATTATGCCTTCGTGCATATGACGTTGAAAATCGGCGCAGGTCGCAGCCTGGAAAGCCGTCAGCAGGCG
GGTGAAATGCTGTTTGAACTGATTAAAACGCACTTCGCCGCCCTGATGGAGAGCCGCCTGCTGGCGTTGTCGTTTGAGAT
TGAAGAGCTGCATCCGACGCTGAATTTTAAACAAAACAACGTGCACGCATTGTTTAAGTAG
>Cluster_679
ATGCTTCATACTGCCAATCCCGTCATCAAACACAAAGCCGGTTTGCTCAATCTGGCTGAAGAACTCAGCAACGTATCAAA
AGCCTGTAAAATCATGGGCGTTTCGCGTGATACGTTTTATCGTTACCGCGAACTGGTCGCTGAAGGCGGTGTGGATGCGC
AGATTAATCGTAGTCGCCGCGCTCCTAACCTTAAGAACCGTACCGATGAGGCAACTGAACAGGCTGTTGTTGATTACGCC
GTCGCTTTCCCGACACATGGTCAGCACCGGGCCAGCAACGAGCTGCGTAAACAGGGGGTTTTTATCTCCGACAGTGGTGT
CCGTTCTGTCTGGTTACTCCATAACCTTGAGAATCTCAAAAGACGATACTGA
>Cluster_41067
ATGGGCGCTGCCATTACCAAAGCCCTGGATATGGTTGAGGAGCGGAAACGTGAATATCGTGCCAATGGTATTTCCTATTA
TCGTCCGTGGATTTTCCTGATCACCGATGGCGCACCAACCGATGAGTGGCAGGCTGCCGCCAACAAAGTGTTTCAGGGGG
AAGAAGATAAGAAGTTTGCCTTCTTTACCATCGGTGTTCAGGGGGCGGATATGAAGACGCTGGCCCAAATCAGCGTCCGT
CAGCCTTTGTCTCTGCAGGGATTACAATTCCGTGAACTGTTTAGCTGGTTATCCAGTTCGCTGCGTTCGGTTTCCCGCTC
CACGCCGGGAACGGAAGTTGTGCTGGAAGCGCCAAAAGGCTGGACGTCAGTGTGA
>Cluster_67139
ATGTCAGATCAAAAAAATAATTTTGACCTTCAAAAACAGACACTTGAATCAAAATGGGGAAAGGAAGCATTAAGCATGGG
CTGGTCAGCTATTCCTACATCCCTTTTCTTCTTACAGGGTGCACTCAATATGACACCCACAACATTCAATGTTTTACTCC
ATCTAATTATCCACTGGTGGAAACCCTACGATTGGCCACACCCCTCTCAGGAGAGCATTGCAGTTAGAATGGGTGTTTCA
TCAAGAACAGTACAACGCTCACTCCAAGAGCTTGAAAACTACGGATTGCTTACAAAAATTCGTACAAGTAAGGACAACCC
TAAGTACAAAGGAAGAAACATTTACGATATGTCTCTTTTGGTTAAGTCACTAAATATTCTTACTCCTGACCTTAACGCCA
AACTGAACGAAAAATAA
>Cluster_64796
ATGAGCTATGCCTCTCTTGACGACATGGTAAAGCGTTACCAGCGCAGCAATCTGGACAAACTGACCGCTGATAAATCGGA
CAACTTTGAACCAGATGATGCACTGATTACGCAGGCGTTACAGGATGCCAGCGCGCTTATCGACGGTTATATCAGTCCCC
GTTACCAGCTGCCATTGTCAGTGGTTCCTGTAACGCTGGTTCGTGTTTGTTGCGATATTGCTTTTTATTACCTGTGTGAT
GAACAGGCAACAGAGCAATGCACACAACGTTACAAAGACGCCATCAGGTGGCTGGGGGTGGTTCTCGACGGAAAATTACC
GCTTGGCGCAGATGTGCATGGTGTTGAACCGGTCAGCGAAAATCTGGTTGAGGTGCAGTCTGACGCAGCCATTTTCTCCC
GCAACCAGAAGGGGTTTATCTGA
>Cluster_1009
ATGGGGAGGCCTGACTGGCGCGCCATGCTTGCCGGGATGACATCCACCGAATATGCCGACTGGCGACATTTTTACCGCAC
GCATTATTTTCACGATACCCAGCTGGATATGCATTTTTCCGGGCTGACGTACGCTGTACTCAGCCTGTTTTTTTGCGATC
CGGATATGCATCCCTCTGATTTCAGTCTGCTTGTCCCCCGGCATGAGGAAGAGCAGGTGGAGAGGCCGGATGAGGACAAA
ATGCTGATGCAGAAAGCGGCAGGACTTGCCGGAGGCGTCCGGTTCGGTGGGGACGGAGGGCGCGATATTTTATCGTCTGC
GGATGTGGCGGATGTCATGGTGGATGATGCCGCATTAATGATGGCTTCAGCGGGGATTTCCGGAGGTGTGAGATATGTCC
CAGCCGGTTGGTGA
>Cluster_54668
ATGAACAATTTAATTATTACGACCCGACAATCACCAGTACGTTTACTGGTTGATTATGTTGCCACAACCATCTTGTGGAC
ATTATTTGCGTTGTTCATATTCTTATTCGCCATGGATCTGCTGACGGGTTATTACTGGCAAAGCGAGGCCAGAAGCCGAC
TTCAGTTCTATTTTTTGCTGGCAGTGGCGAATGCCGTCGTGTTAATTGTCTGGGCGCTGTACAATAAGCTGCGTTTTCAA
AAACAGCAGCATCATGCAGCCTACCAATATACGCCGCAAGAATATGCAGAGAGCTTAGCAATACCTGATGAGCTCTATCA
GCAACTACAAAAAAGCCACAGGATGAGCGTACACTTCACCAGCCAGGGGCAAATAAAAATGGTTGTTTCAGAAAAAGCGC
TAGTCCGGGCATAA
>Cluster_12890
ATGGCTATTGAACGTACTTTTTCCATCATCAAACCGAACGCGGTAGCAAAAAACGTCATTGGTAATATCTTTGCGCGCTT
TGAAGCTGCAGGGTTCAAAATTGTCGGCACCAAAATGCTGCACCTGACCGTTGAACAGGCACGTGGCTTTTATGCTGAAC
ACGATGGAAAACCGTTCTTTGATGGTCTGGTTGAATTCATGACCTCTGGCCCGATCGTGGTTTCCGTGCTGGAAGGTGAA
AACGCCGTTCAGCGTCACCGCGATCTGCTGGGCGCGACCAATCCGGCAAACGCACTGGCTGGTACTCTGCGCGCTGATTA
CGCTGACAGCCTGACCGAAAACGGTACCCACGGTTCTGATTCCGTCGAATCTGCCGCTCGCGAAATCGCTTATTTCTTTG
GCGAAGGCGAAGTGTGCCCGCGCACCCGTTAA
>Cluster_25007
ATGTCATTTACTGATATCTATCAGATTTCCGGCTCGGCGATGACGGCGCAAACGCTACGTCTGAATACCGTTGCCAGTAA
CCTGGCGAATGCCAATGCGCCCGCCAGCAGCGAGGCGCAGGCATATAAAGCGCGCAGCCCGGTGTTTGCGGCGGTGTATC
ACCACAGTTTGCTGGCGGGTACACATCGTCATGCCATTGACGGTGCCAGTGTGCAGGTGCAGGACGTGCTGCAAACCGGC
GGGGCTGTGAAACGCTATGAACCACATTCGCCGCTCGCCGATGCTAACGGGGATGTCTGGTATCCCGACGTCAACGTGGT
GGAACAGATGGCGGACATGATGTCGGCTTCGCGCGATTTCGAAACCAATGTCGATGTGCTCAACAACGTGAAAAGTATGC
AGCAAAGTTTGCTGAAACTGGGAGAAGCCTGA
>Cluster_19636
ATGGTTACACGGACAGACCAGCTACTAAGAAAAGCTATGAGGGGCTATCTGGAACACCTTGGCAACATGCCGCTGGCGGA
ACGGATTACAAATGCTGCTGTATTCGAAGATGTGTATCGCTTTTTGTTGAATGGTTCCGGGCGGCTGGAGCTGCGCCGCG
CTGACGTGGCTGCCGTCCGTGTCTTCCTTTGGAACTATCAATACCGCCGCTGTGCTGTGACAGGTAAGCCGCTGCGCCTT
GCGTCTGCCGTTCTGGATCACTGCCACCGAACCGGGCGCGTGCGTGCTGTGGTGCATCGTTCTGCTAACGCTGCTGAGGG
AGGCCGCTACGTTGGGCGGACGTGTGGTGTTAGCGTTACGAATCTAAGGGCTATGATTGGCAGCTATCGCAAGCAATACA
CTGGTATTGGCATGTTATATCCATAA
>Cluster_63905
ATGAAAATCAAACATGAACATATCCGCATGGCGATGAATGCCTGGGCGCATCCGGACGGCGAAAAAGTACCGGCTGCGAA
AATTACCAAAGCGTATTTCGAGCTGGGAATGACGTTCCCGGAACTGTATGACGACAGCCATCCGGAAGCCCTGGCTCGCA
ATACTCAGAAAATTTTCCGCTGGGTGGAGAAAGACACCCCTGATGCAGTTGAAAAAATTCAGGCGTTGTTACCAGCGATC
GAAAAAGCAATGCCTCCTCTGCTGGTGGCCCGAATGCGCAGCCACAGTTCAGCCTGGTTTCGGGAGCTGGTGGAGACGCG

GGAACGACTGGTGAGAGACGCTGATGATTTTGTCGCAGTGGCGATCGCTGGTTTCAATCAGATGAATCGTGGTGGCCCGG
CAGGAAACACTCTGGCTGTGCATTAA
>Cluster_43888
ATGCCTGCCCCCGTCGTCCTGATCCTTGCGGCCGGGCGTGGAGAGCGCTTTCTCGCCTCCGGGGGAAATACCCATAAGTG
TATCGGCTGGCGTCAGTCCCCGGAGGTTGCGCCTTATCGCTGGCCATTTGAAGAAAACGGGAGAACTTTCGACCTTGCGA
TTGAACCGCAGATTACGACTAATGATCTGCGTCTGATGTTGAGGCTGGCTCTTGCCGGCGGAGGAATAACAATTGCCACT
CAGGAAACTTTCAGGCCATATATTGAAAGCGGTAAGCTTGTATCGCTGCTTGATGACTTTCTTCCACAATTTCCGGGCTT
CTATCTGTATTTCCCACAGCGTCGCAATATTGCACCAAAGCTCCGCGCCCTGATTGACTACGTCAAAGAATGGCGGCAGC
AATTGGTTTAA
>Cluster_63642
GTGGGATTAAAGAAAATCGTTATGTTGACTTTTTGGGTCGGTTTTGTTGCGGGATGCACACCTTTACACCCTTCAGATTG
CCACAAAACTACTGCTACAGGTAGTTGCAGTTCAGGACGCTGGGATGATCAGGATGAATGGGGGGCGCAAGCGCGGGGAA
TCAGAGCTGCAATTAATGCCAAACTTGATGAGCCGCATAACTGGAAAGGGAAAAAATGCAGGTTGCATATGGAATTCTCT
CAGGATGGCACGGCGTTAAAAATATCTACCAGTAACGGTGATAAAGCCTATTGCGAAGCGATAAAGTCCGCAGCTCATAA
AGCCAAATTTCCGGCCTTCAACAATCCGGAAGTCTACAGAGATTTTCAGAAATCTGGCTTTGACATGCGAGGTTAG
>Cluster_61102
ATGACCTGGGAATATGCGCTAATTGGGTTAGTCGTCGGCATCATTATTGGTGCTGTGGCCATGCGTTTTGGTAATCGTAA
ACTACGCCAGCAACAGGCGTTGCAGTACGAACTGGAAAAGAATAAAGCTGAACTGGACGAGTATCGCGAAGAGCTGGTTA
GCCACTTTGCCCGCAGCGCGGAATTACTGGATACCATGGCGCACGACTATCGCCAGCTGTATCAGCACATGGCAAAAAGC
TCCAGCAGCCTGCTGCCGGAACTGTCTGCTGAAGCAAACCCGTTCCGTAATCGTCTGGCAGAGTCTGAAGCCAGCAACGA
TCAGGCACCGGTGCAGATGCCTCGCGACTATTCTGAAGGCGCATCCGGCCTGCTGCGTACTGGCGCGAAGCGCGACTAA
>Cluster_9976
ATGATTCAGTATGTTCTTGCATCGCTATTCACAGGGAAGCAACAGTTAAAAACCATGAAACAGGCAACAAGAAAACCGAC
GACACCCGGAGATATTCTTCTCTATGAATATCTGGAACCGCTCGATTTGAAAATCAATGAGTTAGCAGAGTTGCTGCATG
TTCATCGTAATAGCGTCAGTGCACTGATCAATAACAATCGTAAACTCACCACTGAGATGGCATTCCGTCTGGCGAAAGTT
TTTGATACCACAGTCGATTTTAGGCTAAACCTCCAGGCGGCAGTTGATCTTTGGGAAGTTGAAAACAACATGCGCACCCA
GGAAGAATTGGGACGGATTGAAACAGTGGCTGAATATTTGGCACGCCGTGAAGAGCGTGCAAAAAAGGTCGCGTAA
>Cluster_35841
GTGGAAAAAACAGTTAATAGCGATTTGCATTTAGGTCAGTACTGGAGAATAGACATTGTATGCGATCCAGAACTTCGAGA
TGAGGTTGAGCAATATTTTTCTCTACACGATGTTGGTTTAGCAAAATTGAAGTTTTTTCCGTCGAAAATCCATATAAAC
TTGCTCTGTTCTTTGATTTCGCGAAGAAGGGTGTGGAGGTTGCAAAAGCCATTATGGGACTCTTAGACCGAAATGATATT
GAAATAACGATGTATCGTGCTACGGACAGTAGCCCGCAGTCTGTAAAAAGCATCAAATTGCGTAAGTCTGAAGACGTAGA
GAAATGTAAAGATTTGCTTAGTACGTGTGTCGCAATTGGCGTTCAACGAAATAAGGAAAAAGAGGGGAGCGAATGA
>Cluster_13711
ATGAGGGTTAAACGCTGGTTGTTGGCAGGTATTGCATTGTGCCTTTTAACCGGTATGCGTGACCCTTTTAAACCGCCGGA
AGATCTATGCCGGATTAGCGAACTTAGCCAGTGGCGCTATCAGGGGATGGTAGGGCGAGGCGAGCGCATCATCGGTGTAA
TAAAAGACGGGCAAAAGAAATGGCGACGGGTGCAGCAAAACGATGTGCTGGAAAACGGCTGGACAATTTTACAGCTGACG
CCAGACGTACTAACGCTGGGTACCGGGACAAACTGCGAACCGCCACAATGGTTGTGGCAACGGCAAGGAGATACAAATGA
AGCAATGGATAGCCGCACTACTGTTGATGCTGATACCCGGCGTACAGGCGGCAAAGCCGCAAAAAGTGACGCTGATGGTG
GATGA
>Cluster_65205
ATGGCGTTACGAAATAAAGCGTTCCATCAGTTACGACAGCTTTTTCAGCAGCACACGGCTCGCTGGCAGCACGAGTTACC
TGACCTGACCAAACCACAGTACGCGGTGATGCGCGCCATTGCTGATAAACCTGGTATCGAACAGGTGGCACTGATAGAAG
CAGCTGTCAGCACCAAAGCAACGTTAGCAGAAATGTTGGCAAGAATGGAAAATCGTGGCTTAGTCAGACGTGAACATGAC
CCCGCTGATAAGCGGCGACGCTTTGTCTGGTTGACTGCCGAAGGCGAAAAGATACTTGCTGCGGCGATACCCATTGGTGA
CAGCGTGGATGAGGAATTTTTGGGGCGTTTGAGTGGAGAAGAGCAGGAGCTGTTTATGCAGCTGGTGCGCAAGATGATGA
ACGCATAG
>Cluster_28383
ATGAAAGAAAAACCTGCTGTTGAGGTTCGACTGGATAAATGGCTATGGGCTGCCCGTTTTTATAAAACCCGCGCGCTGGC
CCGTGAAATGATTGAAGGCGGTAAGGTGCATTACAACGGGCAGCGCAGCAAGCCGAGCAAAATCGTCGAGCTGAATGCCA
CGCTCACTCTGCGCCAGGGAAATGACGAACGCACGGTGATTGTAAAGGCGATTACTGAACAGCGTCGCCCCGCCAGCGAG
GCAGCCTTGCTGTATGAAGAGACTGCGGAAAGTGTAGAGAAACGCGAAAAAATGGCGCTGGCACGTAAACTTAATGCCTT
AACCATGCCGCACCCGGACCGACGCCCGGACAAAAAAGAGCGCCGCGACCTGTTACGATTTAAACACGGCGACAGTGAAT
AA
>Cluster_30544
ATGAACCGACATACACAAATACGCCAGGTCGTACTGGCACGCCTTCAGGAACAGTGTGGAGACAGCGCCACGTTTTTTGA
CGGACTTCCGGCATTTGTTGATGCGCAGGAACTGCCTGCCGTGGCGGTGTGGCTGAGTGATGCTCAGTACACCGGAAAAA
TGACGGATGAAGATGACTGGCAGGCTGTTCTGCATATTGCTGTCTTCATCCGGGCACAGGCACCGGATTCAGAGCTGGAT
ATGTGGATGGAGAGCACCATTTTCCCGGCCCTGAATGATGTACCGGCACTTTCCGGACTCATCGACACCCTGATCCCACT
CGGTTTTAACTATCAACGTGATAATGAGATGGCCACCTGGGCGATGGCGGAAATCACGTACCAGATCACGTACACGAATT
AA
>Cluster_9723
ATGCAAGTCTTTTCCAGTGATGTCTACTTCACTGTAGTCACTAATGCATTATTAGCATCCCAAAAAGAATATTACAGCGA
TTTAGTTGCGTTGGTTGATCTTGGGCATTCATTCGTTGTTATTGATGAGCATCAACATCGAAATCTCAAACCAAATACAG
AACCTGTAAACATCCTTTTAAGCAACAATTTCATCAGGATCAATAATAATATTAGATTATCTGATTTAACGCATTTCCTG
ATTTCGAATCTTCATACACAAAATGTGTATTCCACACAAGAAGCACTTACCCATGATGATATAGATATCCTGCGGTTATG
TGTTTCTTACTCGTTAAAACAAGTAGCCATTATTAAAGGAATTGATTATAAAACAGTATCATATCACAAAATAAGAGCCC
TAAATAAACTTAACATAAAAGGTACTGTTGAGTTGTTTATAGCACTCTGTGAATGGGATAAACATTATTTAAACTCCAA
TCTTGCGTAAGAGAAAGTTGA
>Cluster_15796
ATGCTTTTACCCGTAAGTATTACATCGTGTGGCTTGTATCACCCCTACTGGCATTTGGGAGAGCGCAACCCTCATAGCGA
TGAACTATCAAGGCAGATGATGGATTTTAAGGATGAAAATAATCGTAATCATCAAAGGGCCATTAACCGTTTTTCTCGAT
TAGCTATAGATAAGGTTGGGCGGTTAGTAATCAGAGGTGGCGGTGGGCGTTTTGTTGAGTCTCCTTTTTCGATAGCAGTT
GTCCCGTCTCACACTGAAGGCAGAATTTCACCGGCTTTGATTGAGGTCGCCGAGCGAATCGTAAATGTTCACCGTCGGGG
CATTGTTGATTGCTGTTTGGAAAGAACACGCACTGTTCCTAGCGCACATCGTGAAGGAGGAGATCGCTCAATTACTGGTC
ACATGTCTAGTATCAGAGTTAGGGGTGGTAACCTCATGGGGCGAAATGTGTTACTACTAGATGATGTTAAAACAACAGGC
GGTAGTTTGTCTGCGTGTTTTTATTTACTGGAGTCTGCAGGAGCTGGAGTTATAATGCCATTAGCTCTGCTAGAAACTGC
AAACTATGAGGAGTAG
>Cluster_42268

ATGAGTAAAGCCAGACGTTGGGTTATCATTGTGCTATCACTGGCGGTTCTGGTGATGATCGGCATTAATATGGCCGAAAA
AGACGATACCGCCCAGGTGGTCGTCAACAACAATGATCCCACCTATAAAAGCGAGCATACGGACACGCTCGTCTATAACC
CAGAAGGGGCACTAAGCTATCGATTGATTGCTCAACACGTTGAATATTATTCCGATCAGGCCGTTTCGTGGTTTACGCAG
CCGGTACTTACCACGTTTGATAAGGATAAAATCCCGACATGGTCCGTAAAAGCAGATAAAGCCAAGCTGACCAATGACCG
GATGCTCTATTTATATGGACACGTTGAAGTCAACGCACTCGTGCCGGACTCTCAACTTCGCAGAATCACGACGGATAACG
CGCAGATCAATCTGGTGACGCAGGATGTTACCTCTGAAGACCTCGTCACGTTATACGGAACAACATTTAACTCCAGCGGT
CTGAAAATGCGCGGCAACTTACGCAGCAAGAACGCCGAGCTGATTGAAAAGGTTAGAACATCCTATGAAATTCAAAACAA
ACAAACTCAGCCTTAA
>Cluster_47783
ATGAAGCGTAATATTATAGGCGGTGCATTCACTCTGGCATCTCTAATGCTGGCCGGGCATGCACTGGCAGAAGATGGTGT
TGTTCACTTCGTCGGTGAAATTGTCGACACTACTTGTGAAGTTACCTCCGATACAGCCGATCAAATTGTCCCACTGGGTA
AAGTCAGTAAAAATGCATTTTCAGGTGTAGGTAGTCTGGCGTCGCCACAGAAGTTCAGTATTAAACTTGAAAATTGCCCG
GCAACGTACACTCAAGCAGCCGTTCGTTTTGATGGTACAGAAGCGCCTGGCGGCGACGGCGACCTGAAAGTGGGTACGCC
GCTTACAGCAGGCAACCCTGGTGATTTTACCGGTACAGGACAAGCGATTGCGGCAACCGGCGTTGGTATTCGTATTTTTA
ACCAGTCCGATAATTCGCAGGTTAAACTTTATAACGACTCTGCTTATACCGCTATCGATGCTGAAGGCAAGGCTGAAATG
AAGTTTATTGCACGCTATGTGGCAACCAATGCGACCGTAACGGCTGGTACGGCGAACGCGGATTCACAATTTACTGTCGA
ATATAAGAAATAA
>Cluster_20918
ATGCGTGTCATCACCCTGGCGGGTAGTCCTCGCTTTCCTTCTCGCTCCAGCTCCTTGCTGGAATATGCGCGGGAAAAACT
AAATGGCCTGGATGTAGAGGTTTATCACTGGAATTTGCAAAACTTCGTCCCGGAAGATCTGCTTTATGCTCGTTTCGATA
GTCCGGCACTCAAGACCTTCACCGAACAGCTGCAACAGGCCGATGGGTTGATTGTCGCCACGCCTGTGTATAAAGCCGCC
TATTCCGGTGCACTGAAAACCCTGCTCGACCTGCTGCCAGAACGTGCGTTGCAAGGCAAAGTGGTGCTGCCGCTGGCGAC
AGGCGGCACCGTGGCCCATCTGCTGGCGGTCGATTATGCCCTTAAGCCAGTTTAAGTACGCTGAAAGCCCAGGAGATCC
TGCACGGCGTGTTTGCCGATGACTCACAAGTAATTGATTACCAACACAAACCCCAGTTCACGCCAAATCTGCAAACTCGT
CTTGATACCGCGCTAGAAACTTTCTGGCAGGCGTTGCACCGCCGCGATGTTCAGGTTCCTGACCTACTGTCTCTGCGAGG
TAATGCCCATGCGTAA
>Cluster_45624
ATGACTGACTACCTGTTACTGTTTGTCGGAACTGTACTGGTCAATAACTTTGTACTGGTCAAGTTTCTCGGTCTCTGTCC
GTTTATGGGGGTTTCCAAAAAACTGGAAACCGCGATGGGCATGGGGCTGGCAACAACGTTTGTGATGACGCTGGCGTCTA
TTTGCGCCTGGCTTATCGATACGTGGATTTTGATCCCACTTAATCTGATTTACCTGCGCACCCTGGCATTTATTCTGGTG
ATTGCTGTGGTCGTGCAGTTCACCGAGATGGTGGTGCGCAAAACCAGCCCGGTGCTTTACCGTCTGCTGGGGATTTTTTT
GCCGCTTATCACCACCAACTGTGCAGTGCTCGGCGTGGCGTTGCTGAATATCAATCTCGGGCACAATTTCTTGCAGTCGG
CGCTGTACGGTTTTTCCGCCGCTGTCGGTTTCTCGCTGGTGATGGTGCTCTTCGCCGCCATCCGCGAACGCCTTGCTGTG
GCTGATGTCCCGGCACCTTTTCGCGGTAATGCCATTGCGTTAATTACCGCAGGTCTTATGTCTCTGGCCTTTATGGGCTT
TAGTGGTTTGGTGAAGTTGTAA
>Cluster_1866
ATGTCGGATACCACCGAACTGGTTGATTTAGCCGTAATCTTCCCTGATCTGGAGATCGAATTGAAATACGCCTGCGCTGA
TAACATCACAGGTAAAGCTATTTATCAGCAAGCGCGTTGTCTGCTACACAAGGATGCGATTACCGCGCTGGCGAAAAGTA
TCAGTATCGCCCAGCTGTCAGGCTTACAACTGGTGATTTACGATGCGTATCGCCCCACAACAAGCACAGGCGATGTTGTGG
CAAGCCTGCCCAGACCCGCAATATGTTGTTGATGTGACGGTCGGTTCTAATCACAGCCGTGGCACGGCGATCGACCTGAC
GCTTCGTGATGAGCACGGGAACATCCTCGATATGGGCGCAGGGTTCGATGAAATGCACGAGCGCTCCCATGCCTATCACC
CTTCCGTCCCGCCCGCCGCTCAGCGCAATCGGCTGTTGCTGAATGCGATAATGACTGGTGGCGGTTTTGTCGGTATCTCC
AGCGAATGGTGGCACTTCGAATTACCCCAGGCAGCGAGTTACCCTCTGCTCGCTGATCAATTCACCTGTTTTATATCCCC
TGGCACACAACACGTCAGTTAA
>Cluster_62803
ATGGTAAATCAGCAACAGCAACAGATTAACGAAAATCTAAAAATTGAGGCAATCCGTTGGTATCAGAAGCTGCAGGAGAT
TACTTATCTTGAAGCAGGACAACACATGCGGGCTTTAAATCAGCTCATGTGGCAAATCCCTAGTTTCGTAATTGCTGTCA
ATGGTGGGTTGTGGTATGCAACAACACTGGCAAATGAAAGTTCCCTATGGATTATCTTTGCAGTGTTAGCATTGTTTGAT
TTCACAACAATGATTACACTTTATCGCTTAAGGTCTTTAATTGGTTCGAAAATAGCACTACAAAAAAACATTGAAGATCC
TTCAAAAAACTATATTTTAAATGAAATTACTAGAGACGCTGGATTTAAATTGCCCTCTAGCTATGATAATAAGGAAACTG
GTTACATAGTAGTGTCGTGCTGGGCAGTAATGCTTATTGTTTGCTTCTTTGTTAATATTGCTGGAGTATATCATCCAAAC
TTGTTTTCAAAAGACATTTCACATAATACATACAAAGCAACAATTAAAAATATTGAGTCAGGATTATTTATCGAAGCAAA
ATCAGGATTATCAAAGTGA
>Cluster_67016
ATGGACCTGAGCGCCCATCGCTCAGCCCTGAAGTTCACATCCCTGACATTTGTCATTCTGCTGTTTTTCCTCGGACTAAG
CCTGTCCGCCAACGTTCTTGCCTGGTTACGTATTGAAGACCTGGAGCGGGGACAAAAAGAAACCTATATCCCGATGTTTT
TTAATCAGCCGTTTTCGCTTTCACGCAATGTGGCAGATGCCAGCTATCTGCAGCAGGTGGCTGAATCTCTGATGTTTCTG
CGGCTTAACGTCACGCCGGAGTCGATTAAAGCACAGCATCAGGCATTACTGCGTTACTTCGACACGGCTGCACGTCCGCA
GATGAAAGCGATCCTGGACGCAGAAGCCAGTCAGATCATCACCAACAACGTCTCTTCGGCGTTTTACATGACGGGTATTG
ATGTGTACCCGCAAGCCGGTGTGGTGGATTTTCGCGGGACACTGAAAACGTGGATAGGTAAGCGGGAAGCTCTGCCGGAA
GAGAAAGCGTACCGGCTGGAGCTGGGTTACCGGGATGGCATGACGACAATCAGGTCATTTAAGGAACTGGTGAATGAAAA
AGAGAAAACCGCACAGTAA
>Cluster_25222
ATGAAACTCGTGCACATGGCCAGTGGTTTAGCGGTTGCGATTGCGTTGGCGGCTTGCGCAGATAAAAGCGCGGATATTCA
GACGCCAGCACCGGCTGCAAATACGTCTATTTCAGCAACACAACAACCTGCTATCCAGCAACCGAATGTCTCCGGTACCG
TTTGGATCCGTCAGAAAGTCGCACTGCCGCCTGATGCCGTGCTGACCGTGACACTTTCTGACGCGTCGTTAGCCGATGCG
CCGTCGAAAGTGCTGGCGCAGAAAGCGGTGCGTACCGAAGGTAAACAGTCACCATTCAGCTTTGTTCTGCCATTTAACCC
GGCAGATGTTCAGCCGAACGCGCGTATTTTGTTGAGTGCGGCGATTACCGTGAATGACAAACTGGTATTTATTACCGATA
CCGTTCAGCCGGTGATCAACCAGGGCGGAACTAAAGCCGACCTGACATTGGTGCCGGTGCAGCAAACCGCCGTGCCGGTT
CAGGCCAGCGGTGGCGCAACGACTACCGTACCATCAACTTCACCAACCCAGGTGAATCCGTCTTCGGCAGTTCCAGCTCC
AACGCAATATTAA
>Cluster_6814
ATGATGAGTATGGAAATCCGGTCATCGGAAATCACCACCAGCGGCAGCAACACGCTTACAGGCTACGTTGTTCGCTGGGA
TAACCTTTCAGAGCTGCTATGGGGGGAGTTTTACGAAAAATTCCAGCGGGGGGCGTTTACTGAATGGCTTGCGGTGGGTA
ATGACGTTCGCGGCCTGTATGAGCATGACCACAGCATGTTACTGGGGCGCACCCGTTCCGGTACGCTGAGACTGGAAGAG
GATGACACAGGGTTACGTTTTGAACTGATCCCACCGGATACCAGCACAGGGCGGGACGTTATCGAACTGGTTAAACGTGG
TGATATATCCGGCATGAGCTTTGGCTTTCGCTCCCGTAAGGATGTATGGGATACCACAACAGATCCATGCGTGCGCACCG
TGCTGGTGGCGGAACTGTACGAAATTACCGTTACATCCGTACCGGCTTACCCTGATTCCGGCGTGGAGCTGGCCCGCCGC
TCCCTGTACGAGCAGCACCCCGAAAAAATGCCGCGTGCGGATAATCGCCGCTGGTGGGCGGATTTAGCGGGGGTGTGA

>Cluster_42440
ATGAGCAAATCATTACAAAAACCCACCATTCTGAATGTTGAAACTGTAGCCCGTTCCCGACTGTTTACCGTCGAAAGCGT
GGATCTGGAGTTCAGCAATGGCGTGCGGCGTGTTTATGAACGAATGCGTCCAACCAACCGGGAAGCAGTGATGATTGTGC
CGATTGTGGACGATCACCTGATCCTGATCCGCGAATACGCAGTGGGAACTGAATCCTACGAATTAGGTTTTTCGAAAGGA
TTAATTGATCCGGGTGAAAGCGTCTTTGAAGCCGCTAACCGCGAGCTAAAAGAAGAGGTTGGATTTGGTGCGAACGATCT
GACTTTTTTGAAGAAGCTCAGCATGGCACCGTCTTACTTTTCCAGCAAAATGAATATCGTGGTAGCGCAAGATCTCTACC
CGGAATCACTGGAAGGCGATGAGCCAGAGCCGTTACCACAGGTGCGCTGGCCGCTGGCGCATATGATGGATTTGCTGGAA
GCCCCTGACTTCAACGAAGCGCGTAATGTCAGCGCGCTGTTCCTCGTGCGCGAATGGTTGAAAGGGCAGGGGCGAGTGTA
A
>Cluster_59939
ATGAATGTGATTAGAACTGAAATTGAAGATGTGCTAATTCTGGAGCCAAGAGTATTTGGTGATGATAGAGGTTTCTTTTA
TGAGAGCTTTAATCAATCAGCATTTGAACATATTCTAGGCTATCCGGTCAGCTTTGTTCAAGACAATCACTCACGTTCAT
CAAAAAATGTACTCAGAGGCCTTCACTTTCAACGCGGCGAGTACGCACAAGATAAACTTGTACGCTGCACTCATGGAGCA
GTTTTTGATGTTGCTGTTGATATTCGACCCAATTCGGTATCCTTTGGTAAATGGGTTGGTGTTCTGCTTTCAGCTGATAA
TAAGCAGCAGTTGTGGATACCAAAAGGGTTTGCTCATGGCTTTTTGGTTCTGTCTGATATCGCTGAATTTCAATATAAAA
CTACAAACTATTATCATCCTGAAAGCGATTGTGGAATATGTTGGAATGATGAACGCATTGCAATTGATTGGCCCCAAACA
TCAGGGTTAATCCTTTCGCCAAAAGATGAAAGGCTCTTTACGTTAGATGAGCTTATCAGATTAAAATTAATTGCATGA
>Cluster_48801
ATGTCCACCCATGACAGTCTGATTGAACTGACTGACACACTTATCCAGCAGAACGGTTACCAGGGGTTCAGCTATGCTGA
CCTGGCGAAGACGCTGGGTATCCGTAAAGCCAGCATTCACTACCATTTCCAGACAAAAACAGACCTGGGAATAGCCTACT
GTGAATATAAAAAAGCCGGTTTTCTGGCGCTGGAAGCCACCCTGCAGCAGTTACCTGCCGGGAAAGCCCGCCTGAAGGGC
TATATGGAGGCCTTTCTGGCCTGCGCGGACACCGGCCAGATGTGCGGGGTGTACGCAATGATGTCGGACTGTAACCTGTT
TGAGCCTCCCCTGCAGGAGGCCGTTAACCGGTTGGCACAGACGGACCTGCGCATTCTGACGGCGGTCCTGCTGTCCGGAA
AGGAAAGCGGAGAACTGTTTTTTTCTGCCCCGGCAGAAGATGTTGCTATGATTGTGGCCAGTTCGATAAAAGGCGCCCTG
ATGCTGAACCGCATACCGCCTCACGACGCCTGCATCCGCACGATGAAAGCGCTGGAGCAGTTGCTCTGCCGGGTCTGA
>Cluster_31941
ATGAGTAAGTTTGCCAAAACAGCCATTGCTGCGGCGATGGTCATGGGCGCAGTTGTTTCCAGTTCTGCTTTTGCAGCAGG
CAACAACGGTACCGCGCGCTTCTACGGCACCATTGAAGACTCCCCGTGCTCTATCGTTCCAGACGATCACAAACTGGAAG
TCGATATGGGTGACATCGGTGCGGCTAAACTGACCGGCGGTGGCACTACCACACCGAAAGATTTCCAAATCCGTTTACAG
GATTGTGTGTTTACCACTGAAACAAACATGGAGACCACGTTCACCGGCACTAACTACGCGGGGCCAGCCAACGCAGATAA
CTATGCGTTATTCAATGTTGACAGCGGTGTTGCCATGAACCACGTGAGCCTGGTAATCGGCGATACCCATGGCAAAGGTT
ATAAAGTGGGCGAAAAGATTGTTCAGCCAATCGTGATGGATACTTCAACCGGCAAGGGTAAAGACAAGCAAACGCTGAAC
TTTAAAGCCTGGCTGGTGGGTGAAACCGATGCGCCGGATCTCGGCCAGTTCGAAACCCTGACCACATTCCAGATTACCTA
TCTCTGA
>Cluster_19621
ATGAGGACTTTTATCTACTGCCGCGTTTCAACACGCGAGCAGGATACGCAGAATCAGGTGCTGGCTATTGAGCGTGCCGG
GTATAAGGTGCCGGAGAGCAGGGTAATCAGCGAGGACATTTCCGGCAGCGTTCAGGCTATGCAGCGCCCTAAGTTCCGGG
CGATGGTTGAGCACAAGCTGGAAGCGGGAGATCAGCTTGTCGTGCTGAAACTGGACAGGCTGGGGCGTGACAACATCGAT
GTGCAGCAGACCATCGACATGCTGTTAGAACGCGGCGTGAAGGTGGTGTCTCTTGACCTGCCAGCGCGTGATCTGACTTC
ATCTGAGGGCAAGCTAATCCGCCAGATGTTTGGAGCCTTTGCTGAGTTCGAGCGAAACCGTATCCGTGAGAGGACGCTGG
ACGGGCTGGAGAAGGCGAAAGCCAATGGCGTTAAGCTGGGACGCAAGGAAGCCACCGACACCACTCAAAAGGTGCAGGAG
CTGCGGAGTGAGGGGCTGTCTCAATCGAAGGTAGCGAAGCATCTCGGCGTTAGCGTGAGCACCGTTAAACGTCACTGGAA
CAAGGGTTAG
>Cluster_7109
ATGGCGAACTGGCAATCTATCGACGAGCTGCAGGATATTGCATCGGATTTACCGCGATTTATCCACGCATTAGATGAACT
TTCCCGCCGTCTGGGGTTGAATATCACTCCGCTGACTGCCGATCACATTTCTTTGCGTTGCCATCAAAACGCCACTGCTG
AACGCTGGCGTCGCGGGTTTGAACAGTGTGGCGAGCTTTTGTCAGAAAATATGATCAATGGCAGACCGATTTGTCTGTTT
AAATTGCATGAACCGGTACAGGTTGCGCACTGGCAGTTTTCTATTGTGGAGCTACCGTGGCCCGGGGAAAAACGTTACCC
ACACGAAGGTTGGGAACATATTGAAATCGTTCTGCCGGGTGATCCAGAAACGCTGAATGCCCGTGCGCTGGCATTGCTTT
CTGATGAAGGACTCAGCCTACCGGGGATCTCAGTGAAAACCAGTTCGCCAAAAGGTGAGCATGAGCGATTACCGAATCCG
ACGCTGGCGGTCACCGATGGCAAAACAACTATTAAGTTTCATCCGTGGTCCATTGAAGAGATCGTTGCCAGCGAGCAGTC
GGCGTAA
>Cluster_42008
ATGAATACGTTGACTTTCCTGCTTTCAACGGTCATTGAGCTGTATACCATGGTGCTGTTATTACGCATCTGGATGCAGTG
GGCTCATTGTGATTTTTACAACCCCTTCTCACAGTTTGTAGTGAAGGTAACGCAGCCGATTATCGGGCCACTGCGCCGCG
TTATTCCGGCAATGGGACCCATTGATAGCGCCTCGCTGCTGGTTGCCTATATTCTCAGTTTCATTAAAGCCATCGTGCTG
TTTAAAGTGGTGACCTTCCTGCCAATCATTTGGATTGCCGGTTTATTAATTCTGCTGAAAACCATCGGCCTGCTGATTTT
CTGGGTCCTGCTGGTGATGGCGATTATGAGCTGGGTAAGCCAGGGTCGTAGCCCGATTGAATACGTGCTGATTCAGCTGG
CCGATCCGCTGCTGCGCCCGATTCGCCGCTTGCTACCGGCAATGGGTGGGATTGATTTCTCGCCGATGATCCTCGTTCTG
CTGCTGTATGTCATCAATATGGGTGTCGCAGAAGTCTTGCAGGCGACCGGAAATATGCTGCTGCCGGGGCTGTGGATGGC
GTTATGA
>Cluster_48625
ATGTACGGAAGCAAGATATATCGATCTGACGGGAAGGTCTGGATGTCTCCTTCTTTGTCTCCTATCGTTTTTCAGAGAAA
GCAGGTTGTTTCTTTGTCTGGCGGGACAGAGTTTAACACCCAAATATCTCCAGATCGTTCACCGATGATTTTTGTTGCCT
ATTCGAAAGCTGTTTCATTAATAGCCAACAGAATAGTGCGTAACAACCAGGTGATTTACAGTTTCGGAGGTCAGGGTAGC
GACTCTAGTGCCACGATATACGTATTTTCGAAAGGTATAGCCAAAAAAGAAACTTGGGGCATGAGTTTTTTCAATGCGCG
AGGAGAGGAGATCTATAATACGGCAAATATCCCACTTTCATTTACCTTTCTTAATAATACGGAGTGGAACAGTTCTGGAG
GGCATGTTTTTGATTACCCGCCAGCGATAATCCCAACATATGCAAATGTGTTCGCGGTTCCAGTACCGGGCGGGGCCATG
ACAATGGTTTATGGATATGCGGCATATGGAAATACTGTTAGTTCCATATTCGTAAACCAACTTAATGGCGGCCATAGTTT
TAGCGTAAATGGCAGAGTACCGGTAATAAATAGAAATCTGTATAACTGA
>Cluster_983
GTGCTGGCGAGCCTCGGTGGGCTGGTTTCCTATGCGGCAAAGGTTCATTTCAAAGAAGCAGGCAACGCCATGAATGAATT
AATTGCGAATCATGACTTCGACTTTCGCCAGTTAGTTACCGCAGCAGAAGGTCAACCGGTAACTGACACCTTCCAGATTG
CCAGGGCATTTGGTAAACGCCATCAGCATGTGATTAGGGCTATTAAATGTTTGAGATGTTCTGAGGAATTCTCGACAACC
CATTTTTGGGCCGTCGAGAAAATCAATGACTTAGGTATTTTTGACAAGAAACAGATTTACTACCGCATGGACTTTAGTGG
CTTCGTTATGCTGGTTATGGGATTTAACGGGGCAAAAGCCGATGCTGTTAAAGAAGCCTATATCAATGCGTTTAACTGGA
TGTCAGCAGAACTCCGTAAGTACAGCGAAAGTTATGAAGCAGAACGTAACGCCGTAATGCTGGAGTACATGAAAGAGAAG
GATGTCGCCAGCATGTCAGGCCGTCTGCTCAATCGCTGGGGGAGAACGAAAAAACCTCAATTGCTTGCAAAGCTGGAACG
TCTGGAGAGACAGGGACAGTTTTTATTACCGGGATTCGATAAAGGTATTCAAGCCTGA

>Cluster_43149
ATGAAAGCGTTAACGGCCAGGCAACAAGAGGTGTTTGATCTCATCCGTGATCACATCAGCCAGACAGGTATGCCGCCGAC
GCGTGCGGAAATCGCGCAGCGTTTGGGGTTCCGTTCCCCAAACGCGGCTGAAGAACATCTGAAGGCGCTGGCACGCAAAG
GCGTTATTGAAATTGTTTCCGGCGCATCACGCGGGATTCGTTTATTGCAGGAAGAGGAAGAAGGGTTGCCGCTGGTAGGT
CGTGTGGCTGCCGGTGAACCGCTTCTGGCGCAACAGCATATTGAAGGTCATTATCAGGTCGATCCTTCCTTGTTCAAGCC
GAATGCTGATTTCCTGCTGCGTGTCAGCGGGATGTCGATGAAAGATATCGGCATTATGGATGGCGATTTGCTGGCAGTGC
ATAAAACTCAGGATGTACGTAACGGTCAGGTCGTTGTCGCACGTATTGATGACGAAGTTACCGTTAAGCGCCTGAAAAAA
CAGGGAAATAAAGTCGAACTGTTGCCAGAAAACAGCGAGTTTAAACCAATTGTCGTTGACCTTCGTCAGCAGAGCTTCAC
CATTGAAGGGCTGGCGGTTGGGGTTATTCGCAACGGCGACTGGCTGTAA
>Cluster_21779
ATGAGTAATTCGTTGTTACCACCATCAGCCAGCAGTTTCATGCGTTGTGCCGAAGCCGTCGGAACACGCATTACAGACAT
TCCGGTAGACCTCAATACGCTGTGGTCGCCGGATACCTGCCCGGTGCATCTGCTGCCTTATCTCGCCTGGGCGTTTTCCG
TTGACCGCTGGGATCGCAACTGGCCGGAAGAGACAAAGCGACAGGTAATTCGTGATGCATGGCTGATACACCGACACAAA
GGGACCATCAGCGCACTGCGAAGAGCCGTGGAGCCTCTCGGCTACCTGATTGAAGTAAAGGAGTGGTGGCAACTCAACGA
GGAGCCAGGAACATTTCGCATTGTTGTCGGAGTACTTGATCAGGGCATCACCGATGAAATGTATCAGGAACTTGAGCGCC
TTATTGCGGATGCAAAACCAGTAAGTCGCCATCTGACGGGGCTGGCGATCAGCCTGAGTGTGAACGGAAAGATTTTCGTT
GGTACGGGATGCTATCACGGCGATGCCCTGACGGTTTATCCCTACACCCCGGAGTCCATTATTGTCGAAGGGGATTATTT
CCCTGCCCCGGCCATTCATTTAATTGATAATCTGAGAGTAAACGCATGA
>Cluster_6227
ATGCGTACACGAATTGACTATCTGGCGGATAAATACTGTTTTACAGAACGGAACGAATCCCCCCGTCTTCGCCGGCAGTG
GCAGGATGTTCTGGAGGAGTGCCGGCAGACAGAGGCCGGGCCAGAAGAACGGTTGCGTATTGCACTGCTGAATGTGGATT
ACGTCACCAGTTTTGAACTGCCTTTTCGCTTGCTGCTTACCCGTACACCGCAATTGATTGCCGCGCTGCGTGAAGAATGG
GGCATCAGCCAGAAAAATGTGGTGTTTAACGATAAACGGTTTGGCTGCGTGTACAGCCTGAAGGCCAGCCTTTCTGGTGT
GCCGGATACATACCGGTATCATCTGTCTCACCGTATTCGTCGCGTGGTTGGAAATGAAAACACATCATTGCCATATCAGC
AGGTTGCCCGGGAAGTGAAAGCGCCCCGTGAACGGCTGAAGTATGCACTGGAGGCGGGTTTACTGGTGACTGCACTGGAC
GGCCTGTTCTGGTTTGGCAGTCAGCGTATTGCGGCTGATGTTCTGAGGCTGAGAAAGGCCGGAATGCCGGTGGTGACCAC
GACCGTGGAGGTGCACGATAACCTGACGGGAACAACCCGCAAAGTACCGGCATACCATCTCTGA
>Cluster_17064
ATGCGTCTGACAGCAAAACAGGTGACATGGCTGAAAGTTTGCCTACATCTTGCCGGATTGTTGCCGTTTCTCTGGCTGGT
CTGGGCGATCAATCACGGTGGACTAGGTGCCGATCCGGTGAAAGATATTCAGCATTTTACCGGTCTCACTGCACTGAAAT
TTTTGCTTGCGGCCTTGTTAATCACACCTCTGGCACGCTACGCAAAACAGCCGTTATTGATACGCACTCGCCGCCTGTTA
GGCTTATGGTGCTTTGCCTGGGCGACACTGCATTTAACCAGTTACGCATTGCTGGAGTTGGGCGTGAATAATCTGGCGTT
ATTAGGTAAGGAGTTAATTACCCGGCCTTATTTAACGTTAGGAATTATCAGCTGGGTTATTTTGCTTGCTTTAGCGTTCA
CTTCAACCCAGTCGATGCAGCGAAAACTCGGCAAACACTGGCAACAGTTGCATAACTTCGTCTATCTTGTCGCGATCCTG
GCCCCAATACATTATCTGTGGTCAGTGAAGATTATTTCACCGCAGCCACTCATCTACGCTGGGCTGGCTGTACTGCTTTT
AGCCTTACGGTATAAGAAGTTGCTTTCCCTTTTCAACCGGTTACGCAAGCAGGCCCACAACAAATTATCTCTATAA
>Cluster_10530
ATGACCACTCAACTCATCCCTGTGTTTGAAGGTACAATCTCAAATGAACCTACGCTTCTGGTGAACGCGCGTGATTTGCA
TGGATTTTTAGAGGTAGGTAAAGATTTTTCTAACTGGATACGGGCCAGGCTAAACGAGTATGGATTCGTAGAAAATTTAG
ACTACATTCTATTTTCGCCAAATTTGGCGAAAACTCCAGGCCGCCGCCGCAAGGATTACCACCTCACCCTCGACACAGCC
AAAGAGCTTGCGATGGTTGAACGTAACGAAAAAGGCCGCCAGATACGCCGATACTTCATCGCGTGCGAAAAGAAACTTCG
CAACATGCAGCCGCAGATCCAGCAGCAATTCACAGACGAGGAAATCATCCTCCTTTGCTACATGCAGGTACAGATGGAGA
ATGCACAGGACATCTGCAAACGCCTGTACCCGATAATGAAGGAACTTAACTCATCATACGCGAGTAAACTGTATGACATT
GCGTTTGAGACCATCTACATGGTGACGAAGAACAGAGACGCGCTACTGAGGGAGGTAACACGTCTCGACATGTCAAGTTC
CGTTATCCAGCGGGCCATGCCAATGCTGAAAAGCCTGCGGGCAAGACAATTCGAGTTTTAA
>Cluster_20211
ATGCTCGTCTATTGGCTGGATATAGTCGGCACAGCGGTATTTGCTATCTCTGGCGTTTTGTTAGCCGGAAAATTGCGTAT
GGACCCTTTTGGTGTTCTGGTATTGGGCGTGGTTACCGCAGTAGGCGGCGGAACAATTCGCGACATGGCGCTGGATCACG
GCCCGGTATTTTGGGTGAAAGATCCCACCGATCTGGTCGTTGCAATGGTCACCAGCATGCTGACCATCGTGCTGGTGCGC
CAGCCAAGACGCTTACCAAAATGGATGTTGCCGGTGCTGGACGCCGTTGGTCTGGCGGTGTTTGTCGGCATTGGCGTGAA
TAAAGCCTTTAATGCGGAAGCCGGTCCGTTAATCGCGGTTTGTATGGGCGTCATTACTGGCGTTGGCGGCGGGATCATGC
GTGATGTACTGGCCCGCGAAATCCCCATGATTTTACGTACAGAAATCTACGCAACTGCCTGTATTATCGGCGGTATTGTC
CACGCTACGGCTTATTACACATTTTCCGTACCACTGGAAACAGCCAGTATGATGGGCATGGTCGTGACGCTATTGATTCG
GCTGGCGGCTATTCGTTGGCATCTTAAGCTACCGACGTTTGCGCTGGATGAGAATGGTCGTTGA
>Cluster_17747
ATGGCTTCTGTTATCGAGATCTGCAACCGCGCGCTGAGCAATATCGGCAACAGTCGTAGCATTAACAGCCTGAATGAAGC
CAGCAAAGAGGCCGGGCAGTGCTCCCTGCATTTTGATGCATGTCGTGATGCTGCTCTGGCTGACTTCGACTGGAACTTTG
CTACCAAACGCGTGGCGCTGGCTGATACCAATAACCCGCCTCCTGACTGGCAGTACGCTTACCAGTACCCATCTGATTGC
GTCCGTATAACTGAGATCATGCCGACCGGCATACGTAATCCTACCGCTGCGCAGCGCATTGAATATGTTGTCGGTTCCAA
TGAGGAACTGACAGGTAAGCTCATTTACACCGATCAGCCGAAAGCGTGGTTGAAGTACGTGGCGCGGGTTACTGACGTCA
ATATGTATGATGCCATTTTTATGGAGGCGCTTTCCTGGCGTCTGGCTGCTGCAATTAATATGGCGCTGACCGGTAGCGCA
GATCTCGGTAACAATGCACTGACGATGTACAACCGTGTGATCCTGAGCGCTGGCTCACATAGCCAGAACGAATCACAGGA
GCCACAACCGCCGGTAGATGAGTTCACAGCAGCGAGGTTGTCATAA
>Cluster_20191
ATGTCTAAAAAATTAGGTTTTGCGCTGTCCGGTATTATGTTGGCAATGGCTGCTGGTACTACATTTGCTGCTAACGATAT
GGACGGCGGTCAATTAAATATTAGCGGTCTGGTCGTTGATAACACTTGTGAAGCTCGTGTTGATGGCGGTAACAAAGACG
GTCTGATCCTGCTGCAAACCGCTACTGTTGCTGAAATTACTGATGGTGTACTGGATACCACTGTAGGCGCTAAAGCTAAA
CCATTCAGCATTACTATTGACTGCAGCAAAGCTAACCCGGCTCCGGGTACTACTGCAAAAATGACCTTCGGTTCTGTTTT
TTTTGGTAACAGCAAAGGAACACTGAACAACGACATGTCTATTAACACCCCGTCTGACGGCGTAAATATCGCTCTGCACA
ATATTGAAGGTTCTACTATTAAACAGGTTCAGGTCAATAATCCGGGTGATGTTTACAGCAAAACTCTGGATTCAACGTCT
AAATCTGCAACCTACGATTTTAAAGCATCCTATGTTCGTGCGGATGCAAGCAAAGCAGCTACTGCCGGTTATGTAAAAAC
CAACTCAGCATATACCATTACTTACCAGTAA
>Cluster_53922
ATGACTTTGAAGAATGTAGCAAACATCGGATCCGTTGTCACGGATAAAACCATTGACAGCCAAAGTCTTCTGATGATGGT
TAATGAAGCTCGCAAGTTATGTGGAGAGCCATCAGTACGTAACAACAAATTCATCGAGAAAATTGAGGATGAATTGGAGG
GCGAGACCTACACAAAAAGTGTAGGTCGGAAAAACGGGGCTGACATTGATGTTATCTCCATGACTATCAAGCAGGCGCTT
CGTGTTGCTGCTCGCGAATCTAAAGCAGTTCGCCGAACACTTGTAGACAAACTTGAAAGTATGCAAGAAGCGCACATTAA
AAGCGGTAAATCGTCGAGTGGACTTGTTGAGTATCGCCAGGCTCGAACATTGAAAATGACGGTTGAAGCTGTTACCAATC
TGTTAGATTTGATGCCAAATCTTGCGCCGGAAGCAAAGCAGACTGCGGCAGCAAGCATAATCAACCCGATAGTTGGTTTT

AATGCAATACCTCTTCCAGCAATAGAAGAGCATTACTACTCAGCAGGGGAGGTTGCAGAGCAGCTTGGAGTAACGGCAAA
CAAGATTGGTCGCATTGCTAACGCAAACAACTGA
>Cluster_40896
ATGATAGAAAATAAGATACAAACGGAAGTAAAGAAATCAAGAAGAGGTCTACCTCGCCATGTAAAAAACCCATTCCTTAA
TGATACAAATATACATACAAAAACAGGTATCAGACGTATCACAACAGGAAAAGACCGCTTAGCCGTAGTAAATGAAAATA
CAGGAGAACAGGTTGGGCATGGTGGTTTCTTTCAGTCAATGGAAGTAGACAAGACACAGTTCGTGAAGCTTTATGTGGAT
GGCGTATCAGCTATTGAGGGTTTATCCTCATCAGGTAAGAAAGTATTTAAGATACTGTATCTTGCTATTAGAGACAATAA
AGATACAGATACGATATTAATGTCATTCGATATTGTTGATCAAGAGATTGTAAAAATATCAAGAACGACATATTTCAAAG
GGATGAAAGAACTTGCTGACAAGAAATTTATTGCTGAGACCATGATACAGAACTATTATTTTATTAATCCAGACTATATG
TTTAATGGTGATCGCTTAACCTTCATGAAAACTTATTACTTGAAGGACAAAAAAGCTAAAACTAAAGAGAACAAAATTAA
TTGCCAAGGCAAAACGCAGTAG
>Cluster_24732
ATGGCAAATAATATGTCACTCCGTCAGATATGCCATGCCACCCGGTATGTTGTTGCCCAGGGCCTGTGTTGGTTGTTCTG
GTGTCTGAAATACGCCGTCATTCTGCCGCTGGCCACAATGGCTCTGCTGGCGTTACTGGTTTTATGGAAGGACAACACCA
CACCGGGGAAATTGCTGGTTCAGGAGATCGAGTTTGTAAGGGGCAGAACGCCAGCCGGGCTGTATCCGGTCCGATACTGC
TCGAACAGTTTTGGTTCCGATTTAGCGATGCCTCCGTCTGTAACGTGCGGTTTTCGCCGGGGGGATGTTGCAGAATATGC
CAGGGAGACTGACCAGTCTCTGATGCAACTGTTCACCGCATTATGGGCAACGCTGGCACTGATGTATGTCTCGCTGGCGG
TGATCTCCGGAAAATATCCGATGCGACCCGGAAAAATGAAATGTGTCCGGGTCGCAACTGCAGATGAACATCTGAAGGAA
GTTCTTACGGAAGATGCTTCCCTGCCGGAAAAAATCAGGAAATGCCATGTTTATTTCCCTGATGACCGAACAAACAGAAG
CAACGGAGATAAAAATGAACATGCGTAA
>Cluster_42410
ATGAGCGATAAATTCGGCGAAGGGCGCGATCCGTATCTTTATCCAGGCCTTGATATCATGCGTAACCGGTTGAACATCCG
CCAGCAGCAGCGGCTGGAACAGGTCGCTTACGAAATGACGGCGCTGCGTGCTGCGACCATTGAGCTTGGTCCGCTGGTGC
GCGGTTTACCGCATTTGCGCACTATCCATCGCCAGCTGTATCAGGATATTTTCGACTGGGCGGGGCAACTGCGTGAAGTT
GATATTTATCAGGGCGATACGCCGTTCTGCCACTTTGCTTATATCGAAAAAGAGGGCAATGCCCTGATGCAGGATCTGGA
GGAAGAAGGTTATCTGGTTGGCCTGGAGAAAGCGAAGTTCGTCGAGCGGCTGGCGCATTACTATTGTGAAATCAACGTGC
TGCATCCCTTCCGGGTGGGAAGTGGTTTGGCACAGCGGATCTTCTTCGAGCAACTGGCGATTCATGCCGGATATCAACTG
AGCTGGCAGGGTATCGAAAAAGAGGCCTGGAATCAGGCAAATCAGAATGGGGCAATGGGGGATCTCACCGCGCTGCAGAT
GATATTTAGCAAAGTGGTAAGCGAAGCCGGGGAATCTGAGTAA
>Cluster_57003
ATGATGAATATAATCAGACTTTATATTTATCCGTTATTTCTCGGCTTTGTTATTGTGCCGTTGTTGGTGTGGCCGATGGT
GGTTGCGCTGGCTGCTTGTATTATCACGCTGAGTTTTCTGGGGGAAATGCTGTTCTCAATCCCGCTCATGATCAGGCGTA
TTTCACTTCTGCAATTACAACTTTGGTTATCGGCTGAATATGCGCTTTTCTTCTGTGTAATGTGTTGCGTTGGTTGGCAA
TTTACTCGTAGAACTCAATCTGAATTGAAAAGCAGGCTCCATTGTTGGTTGGTATTCGCACCGGTCTATTTATGGTTGAT
CCTCTGGAATATCATTTTTTACATCGCGCCAGCGAAAATTGCGTTGCTGGAAAGTATCCGCAATTTCTTTCTGCTTGTTG
TCTGGCTACCCCTGAATTTTTCGCCATTTTGGCCTCAGCCGTGGACGGATTTTATCGGTCCAATAAGCGCACAGCTTGGT
TTTGCGTTGGGATATTATTGCCAGTGGTGCAGCAAAAATAGATGCCAGATACGGAAGTTGAGTGAGTGGGGAACGTGCTT
AAGTTTTGTTACGTTGGGTATGATGGCGTGGGCTTTGCGGTAA
>Cluster_29351
ATGGTCACTAAAAAACAATCTCGCGTTCCAGGTCGTCCCAGACGTTTCGCTCCTGAGCAGGCTGTCTCTGCGGCAAAAGT
GCTTTTTCACCAAAAAGGTTTCGATGCTGTCAGTGTTGCTGAAGTTACTGATTATCTTGGTATTAACCCCCCGAGCCTCT
ACGCGGCTTTTGGCAGTAAGGCTGGGTTATTTAGCCGTGTACTCAATGAATATGTCGGTACGGAAGCTATTCCGCTTGCC
GATATTCTTCGTGATGATCGTCCGGTAGGCGAGTGCCTGGTTGAGGTATTAAAAGAAGCGGCGCGCAGATATAGCCAAAA
CGGCGGCTGCGCTGGCTGTATGGTTCTTGAAGGTATTCATAGTCATGATCCACAAGCGCGTGATATTGCCATTCAATATT
ATCACACCGCAGAAACGACCATTTATGACTATATCGCCAGGCGGCATCCACAAAGCGCACAATGTGTGACTGATTTTATG
AGTACCGTGATGTCAGGGCTTTCTGCGAAGGCACGAGAGGGGCACTCTATCGAACAACTCTGTGCAACAGCAGCCCTGGC
GGGGGAAGCGATAAAAACTATTCTCAAGGAGTGA
>Cluster_3305
ATGGCAGATAAATTCGAAAAGAAAAAAGAAACCACAAATAAATCAGCAAAAAAAACACTAACGAAGAAGAGCGAAAAATT
AACTCCAGCAAAAGACACCGCACCAAAACAAGTGAATGTAGATCCAGCTATAAGATTGAAACAAATTCGTCAACTTGTCC
GTAATAATAATAAATCCATCATGGGAGAGGATTATATTATTTGTCAAATCTATAAAGAATCTAGATTTAAGCAATTTGCA
GGTAAGAATAAACATAATGCGAAAGGCTTAATGCAAATGCAACGAAATGCAGTGAGGCAGGTTTTTAAATATCGACAACA
AAAAATAAAAGGAAGAATGACGACTGATAAAGAGACAAATGAAGCTTTCGCTAATGCTGATACATTTTATAAATCTGATA
AAATATTTGATGAAAAAGAAAATATTAAGATTGGCACAGAGTATTTGCAATACTGGATAGATAAAGAAGCAACAATTGAG
GAGGCATATCGTGGAACAGATGAAGCATACTATTCTGTTATAAAACCTTGCGCTGAAAAACTGGCTAAAGATCCTGATAA
CATACAAATACTAATGGAGGGAATAGGAAGATAG
>Cluster_25235
GTGAAAACCACCCTGCTACTGGAAAAACTGGAAGGACAGCTCGCCACGCTGCGTCAGCGTTGTGCCCCGGTAGCGCAATT
TGCCACGCTAAGTGCCCGTTTTGACAGGCACCTTTTTCAGACCCGTGCGACAACACTACAGGCTTGTCTCGATGAGGCGG
GCGATAACCTGGCTGCGCTTCGTCATGCGGTTGAGCAGCAACAGCTGCCGCAAGTCGCCTGGCTGGCAGAACATCTGGCG
GCGCAACTGGAGGCTATAGCGCGTGAAGCCACAGCCTGGTCGCTACGCGAGTGGGATAGCGCACCACCGAAAATTGCCCG
CTGGCAGCGTAAACGTATTCAGCATCAGGATTTTGAGCGGCGGCTACGTGAGATGGTTGCCGAACGTAGAGCCCGTCTGG
CACGAGCGACCGATCTCGTGGAACAGCAGACGCTGCATCGTGAAGTGGAAGCCTATGAAGCGCGCCTGGCACGCTGCCGC
CATGCGCTGGAAAAAATCGAAAACAGGTTAGCGCGTTTAACCCGCTAG
>Cluster_18765
ATGATTTTTCTCATGACGAAAGATTCTTTTCTTTTACAGGGCTTTTGGCAGTTGAAAGATAATCACGAAATGATAAAAAT
CAATTCCCTGTCAGAGATCAAAAAAGTAGGCAATAAACCCTTCAAGGTGATCATTGACACCTATCACAACCATATCCTTG
ATGAAGAAGCGATTAAATTCCTGGAGAAATTAGATGCCGAGAGAATTATTGTTTTGGCACCTTATCACATCAGTAAACTA
AAAGCTAAAGCGCCTATTTATTTTGTCAGCCGTAAAGAAAGTATCAAAAATCTTCTTGAGATTACTTATGGTAAACACTT
GCCCCATAAGAATTCACAACTATGTTTTTCACATAATCAGTTCAAAATTATGCAACTGATTCTGAAAAACAAAAATGAAA
GCAATATCACGTCAACGCTCAACATTTCGCAACAAACATTAAAGATTCAGAAATTCAACATTATGTACAAGCTGAAATTA
AGACGTATGAGCGACATCGTCACCCTGGGTATCACATCTTATTTTTAG
>Cluster_36739
GTGACGTCACCTTCCGGACAGCAAGAAACATTTAAAATATTAATCAATCCAGAACATCTACGGCAAACACTTGAGCGTAA
ATCTCTTACTGCTGTTCAGAAATCACAATGTGAAATCATCACCCCCAAAAAACCTGGCGAAGCGATTATTCATGCTTTTA
ATGCCACCTACCAGCAGATCAGAGAAAATATGTCTGAATTTGCACGTTGCCATTATGGGTATATACAAATCCCTCCAGTG
ACAACTTTCCGCGCCGACGGACCAGAAACTCCCGAAGAAGAAAAGGGTTACTGGTTTCATGCTTATCAACCCGAAGATCT
TTGTACCATCCATAATCCAATGGGAGATTTGCAGGATTTTATTGCATTGGTTAAAGATGCTAAAAAATTTGGTATCGATA
TCATTCCTGATTATACCTTTAACTTTATGGGAATTGGGGGTAGTGGTAAAAATGACCTGGATTATCCCTCTGCTGATATA

CGAGCGAAGATCAGTAAAGATATAGAAGGTGGTATCCCTGGCTATTAG
>Cluster_4635
ATGTCAAATGTGAGTGGGATCGGTGATGCTTATTACTGGAGTGTTTTAAAATCGCCGAGGCCTTTGGGCTTCACCGGGA
CACAGTAAAAAAACGGCTCCTCGCGGCCAACACTCCTGTGGCTGCGACTGTCAGGGGGAACCCCGTTTACGCCCTGCAGC
ATGTCGGGCCAGCCCTGTTTAGTGTGAAGCATGAGGCAGCAGACTCTGTTCATGATCCATCCCGTATGGAGCCGAAAGAG
AGAAAGGACTGGTACCAGTCTGAAAATGAAAGGATCAAGCTGGAAAAGGAGCAGCGAAAACTCATCCCAGTTGATGAAGT
AGTCATCGTCTATTCGTCCATGAGAAAGGCTGTCGTCCAGGTTCTGGAGACAATTCCGGATGTTCTTGAACGCGATTGCG
CCCTGACTCCTCAGGCCGTCGGCGTTGTACAGCAGGCCATTGATGACCTGCGATACACTCTTCAGGAAAAATCCTACGAG
GCTTGTGCTGCTGAATTAATTCCTGATGAGGAAGGAGAGAGTCTCTAG
>Cluster_35757
ATGTTTTTTAAAACGTCAAATCCCACAGCACTGCTGGCATGGGACCAGTTTATGGCGGACTGCCTGAAACTACGCGAAGA
AGCCCGCCATCTTGATAAGGTTCTGGACTGTGGTTGCCGGTCAGTATTCAGTACAGGTATTGGTGGGCGTCATTTTCATG
GTGTGAATTTTCCGGGGAATGAGCGCCCCTTCTCGCGGGAGCTGTGGACCGTACAACGGCCCGCATCAGGTAACAGTTGC
AGGCCCAGAACTTCGCGGATACCTGCTCATCTCAGGGGGCAGGCAAGAGAGCTGGCGAAAATATGGCAGGAGAATATTCC
GGTCACTTACGCCCGCACAGACGCTCTTTTACCCGCACTGGGACTGGATTTCAGCGCAACAATATTCGGGCCGCTCCAGT
GGTTCCGTGTTGGTGATGTGATTTACGTCATGACGGGGATGACACCCGCGCAAGGCCGCATGACTGAAATCCTTTCTGAC
GAATTTATCCGGGCGCAAAAACAGGCGGAGGTAACTAATGGAAAACAGTGA
>Cluster_14136
ATGTCTGAATTTATTACGGTAGCTCGCCCCTACGCCAAAGCAGCTTTTGACTTTGCCGTCGAACACCAAAGTGTAGAACG
CTGGCAGGACATGCTGGCGTTTGCCGCCGAGGTAACCAAAAACGAACAAATGGCAGAGCTTCTCTCTGGCGCGCTTGCGC
CAGAAACGCTCGCCGAGTCGTTTATCGCAGTTTGTGGTGAGCAACTGGACGAAAACGGTCAGAACCTGATTCGGGTAATG
GCTGAAAATGGTCGTCTTAACGCGCTCCCGGATGTTCTGGAGCAGTTTATTCACCTTCGTGCCGTGAGTGAGGCTACCGC
TGAGGTAGACGTCATTTCCGCTGCCGCACTGAGTGAACAACAGCTCGCGAAAATTTCTGCTGCGATGGAAAAACGTCTGT
CACGCAAAGTTAAGCTGAATTGCAAAATCGATAAGTCTGTAATGGCAGGCGTTATCATCCGAGCGGGTGATATGGTCATT
GATGGCAGCGTACGCGGTCGTCTTGAGCGCCTTGCAGACGTCTTGCAGTCTTAA
>Cluster_18808
ATGTGGAGAAATAGTATACGTTGCCTGTTGCCCTTTTTAGCTCTGGCATATTTTCCCGGCGCTGAAGCCAAAATAAGCCT
CGGTGAAGCAGGAAAACTGACGTTTTCTCTTTCAGTCGTGGCCAAAGGGTGTGAGTTTGACGCCACTGATTTAGAAGTTG
TCATGGGGAAAATGATGTTAATGAAGCCAGTCACGGTCGGAAGAGTCATAAACGAAAAAAATTTCGTAATCGAATTAAAA
GATTGTGACGGAATATCAAAAGCTAAAGTAACAATGGACGGCCTTCCTGACACCAATGATAACTCTCTTTTTGCTCTGGA
TGCCGGAGGCGCAACGGGAATAGCCCTGCAAATTGTTGACGGCAAGGGCACAAAACAGATTCCGAAAGTCGCTGGTGGAA
CGGCGATAGAGTGGCCAGTAAATGGAACAACAACCCAACTGAACTACAAAGCGAGTTATGTCGTTGTAAACGCCAATGCA
ACGTCTGGACATGCAAATGCCATGGTCAATTTCTCCGTGGAGTATGAGTAA
>Cluster_41938
ATGGGGCAATGGAAAATGATGTTACCCTGGCAACAGGGAACAATTCTTGATACAAGTATCAAAAATGCGTTGCAGTTTTG
TAGCCGAATTATTTAGTCACCGGCTATCGTGGTAATGAACTGCACGAACGTTATGCGAACCAGAGCAATATCACTATTA
TCCACAACCCAGATTATGCGCAGGGTTTACTGACATCAGTAAAGGCCGCAGTACCCGCGGTGCAAACAGAACATTGTTTT
CTCACCCACGGAGATATGCCAACCCTCACCATCGATATTTTTAGAAAAATCTGGTCGTTACGAAATGATGGCGCAATACT
GCCACTCCATAATGGCATCCCCGGCCATCCGATTTTAGTGTCAAAACCATGCCTGATGCAGGCAATACAGCGACCCAATG
TCACCAATATGCGTCAGGCACTCCTTATGGGAGAACATTATTCCGTCGAAATAGAAAATGCAGAAATAATTTTAGATATT
GATACTCCGGACGACTTTATTACAGCGAAGAAAAGGTATACTGAAATTTAG
>Cluster_233
ATGGGTAATAAAGGTGCCATGACCATCCGTGTAACCACCCCGAGCATGTCCAGCGGTGGCGGAATTACTAACGCTCAATT
CACTTATATTAATCATGGTGATGCTTATGCTCCTGGCTGGCGAAGAGACTACAACACGAAAAACCAGCAGCCTGCATTTG
CTTTAGGGCAGACAGGAAGCACTGTTGGAAATGACAAAGCTGTTGGATGGAACTCAAATAGCGGCGTTTATAATGCGACT
ATTAGTGGAGCGTCAACATTGATTCTTCACTTTAATATGAATGCGGGGAGTTGCCCGGCGGTTCAGTTCCGTGTGAATTA
TAAGAACGGCGGTATTTATTATCGTTCAGCGCGAGATGGTTATGGCTTTGAAGCTGACTGGTCAGAGTTTTACACCACAA
CCCGCAAACCCTCTGCGGGAGATGTTGGAGCATATACGCAGGCAGAATGTAACTCAAGGTTTATTACAGGTATTCGCCTT
GGCGGTCTGTCATCTGTTCAGACATGGAATGGTCCCGGCTGGTACTGA
>Cluster_42150
ATGGTTGAGGATATTTTGGCTCCAGGGTTACGGGTCGTGTTTTGCGGTATCAACCCTGGTCTTTCATCCGCCGGAACGGG
TTTTCCCTTTGCTCATCCGGCAAACCGCTTCTGGAAGGTGATATATCAGGCCGGGTTTACTGACCGTCAGTTGAAGCCGC
AGGAGGCACAGCATCTGCTGGATTATCGTTGTGGCGTCACCAAACTGGTAGACCGTCCAACGGTGCAAGCCAATGAAGTG
TCAAAGCAGGAGCTGCACGCAGGCGGGCGTAAGCTAATTGAAAAAATAGAAGATTATCAGCCGCAGGCGTTGGCGATTCT
GGGCAAACAAGCATATGAACAGGGATTCAGCCAGCGCGGTGCACAGTGGGGAAACAAACGCTCACCATTGGTTCGACGC
AGATTTGGGTGCTGCCAAATCCCAGCGGTTTAAGTCGCGTTTCACTGGAGAAACTGGTTGAAGCGTATCGCGAGTTGGAC
CAGGCGCTGGTGGTGCGTGGCTTGTGA
>Cluster_38667
ATGAAAAAGTGGATGTTAGCAATCTGCCTGATGTTTATAAATGGGAGCTGCGAAGCCGCCGATTGCTTTGATCTTGCAGG
TCGGGATTACAAAATAGACCCGGATTTACTGAGAGCGATATCATGGAAAGAATCCCTTTACCGGGTTAATGCCATCGGTA
TTAATCCGGTAACGGGATATGGCAGCAGACTGATGCAGGTAGATTCCCAGCATTTTAACGAACTGGCCCGCTATGGAATT
AAGCCGGAACATCTGACAACAGATCCCTGCATGAACATTTATACCGGTGCTTATTATCTGGCAATAGCCTTTAAAAAATG
GGGCGTCTCCTGGGAGGCCGTTGGTGCATACAATGCCGGATTCAGGAAGACCGAACGCCAGAACCAGAGACGTCTTGCCT
ACGCATCAGAGGTTTACCGGATTTATACCGGGATAAAAAGCAGTAAAGGTATCCGGATTCCGGCCACGAAGAAATCACTT
CCGGAAATTAACAGTGTGCAGAAAAATTAA
>Cluster_47350
ATGGCTCACATCGAAAAACAAGCTGGCGAACTGCAGGAAAAGCTGATCGCGGTAAACCGCGTATCTAAAACCGTTAAAGG
TGGTCGTATTTTCTCCTTCACAGCTCTGACTGTAGTTGGCGATGGTAACGGTCGCGTTGGTTTTGGTTACGGTAAAGCGC
GTGAAGTTCCAGCAGCGATCCAGAAAGCGATGGAAAAAGCCCGTCGCAATATGATTAACGTCGCGCTGAATAACGGCACT
CTGCAACACCCTGTTAAAGGTGTTCACACGGGTTCTCGCGTATTCATGCAGCCGGCTTCCGAAGGTACCGGTATCATCGC
CGGTGGTGCAATGCGCGCCGTTCTGGAAGTCGCTGGGGTTCATAACGTTCTGGCTAAAGCATATGGTTCCACCAACCCGA
TCAACGTGGTTCGTGCAACTATTGATGGCCTGGAAAATATGAATTCTCCAGAAATGGTCGCTGCCAAGCGTGGTAAATCC
GTTGAAGAAATTCTGGGGAAATAA
>Cluster_20771
GTGTGTCACTGTGCCGTGGAAACCGGTCAGTCCGCCTTTTTCGGGGGAATTATGTTTAGCGGAAAACCGCTGGATTATCA
GGATGAGCCGCTGACGAATAACGGGTTCTGGCCGGATCTGAATCTGAAGGACTTTCAGGCGCAGCGGTCACTGCCAGCTG
ATATCGACGCGGACACCATCAGTCAGGCGCTGCTGGCCGCAGTCGCGGAGGTGAATGCCGAGCTGGAAAACGTGGAGGCC
AGCTGGAAAGCAAAAGGCCACACGCTGGCAGCAGATGTTCCGGGCGTAAAAATGGGCGGGCTTAACAGTCTGTGTGCCCA
GTACATGAAAGCGGTATTTGCCAGGGCAAAAGCGGATCTGCTGGGGGAGTTCGCCACTGTAGGGCGACGTGAAACCCATC

CGGGACAGGAAAGTATGGAAACCCGCGCCGGGTTACTGGCTGAGGCTTCAGTGGTTATCCGCCGCATGAAGGGGCTTAAA
CGTGCAACGGTGAAAAAAGTATGA
>Cluster_20809
ATGCGCTATAGCAAATTGACCATGCTTATCCCTTGCGCACTGCTACTCAGTGCCTGTACCACCGTCACGCCAGCTTATAA
AGATAACGGCCCCCGCACCGGTTCCTGTGTCCAGGGGGGGCCTGATAGCGTGGCTCAACAATTCTATGACTACCGCATTC
AACATCGCAGCAATGACATTACCGCCCTGCGCCCCTATTTGAGTGACAAACTGGCAACCCTACTTAGCGATGCCAGCCGC
GATAATAGTCATCGTGAATTGTTAAGCAGCGATCCGTTCTCCAGTCGAACCACCTTGCCAGACAGCGCCCACGTAGCCAG
TGCATCGACTATCCCTAATCGTGATGCCCGTAACATTCCATTGCGCGTGGATCTCAAGCAGGGTGACCAGGGCTGGCAGG
ATGAAGTGTTAATGATTCAGGAAGGCCAGTGTTGGGTCATCGACGATGTGCGCTACTTAGGCGGTAGCGTTCATGCCACA
GCGGGTACGCTCCGTCAGTCCATCGAGAATCGCTAA
>Cluster_47987
GTGTTTGATATCGGTTTAGCGAACTGCTATTGGTGTTCATCATCGGCCTCGTCGTTCTGGGGCCGCAACGACTGCCTGT
GGCGGTAAAAACGGTAGCGGGCTGGATTCGCGCGTTGCGTTCACTGGCGACAACGGTGCAGAACGAACTGACCCAGGAGT
TAAAACTCCAGGAGTTTCAGGACAGTCTGAAAAAGGTTGAAAAGGCGAGCCTCACTAACCTGACGCCCGAACTGAAAGCG
TCGATGGATGAACTACGCCAGGCCGCGGAGTCGATGAAGCGTTCCTACGTTGCAAACGATCCTGAAAAGGCGAGCGATGA
AGCGCACACCATCCATAACCCGGTGGTGAAAGATAATGAAGCTGCGCATGAGGGCGTAACGCCTGCCGCTGCACAAACGC
AGGCCAGTTCGCCGGAACAGAAGCCAGAAACCACGCCAGAGCCGGTGGTAAAACCTGCTGCGGACGCTGAACCGAAAACC
GCTGCACCTTCCCCTTCGTCGAGTGATAAACCGTAA
>Cluster_49500
ATGTCTTCCCGCAATAATCCGGCGCGTGTCGCCATCGTGATGGGGTCCAAAAGCGACTGGGCTACCATGCAGTTCGCCGC
CGAAATCTTCGAAATCCTGAATGTCCCGCACCACGTTGAAGTGGTTTCTGCTCACCGCACCCCCGATAAACTGTTCAGCT
TCGCCGAAAGCGCCGAAGAGAACGGTTATCAGGTGATTATTGCGGGCGCAGGCGGCGCAGCGCACCTGCCTGGCATGATT
GCCGCCAAAACGCTGGTGCCGGTGCTGGGCGTGCCAGTACAGAGCGCCGCACTGAGCGGTGTCGATAGCCTCTACTCCAT
CGTACAAATGCCGCGCGGCATTCCGGTGGGTACGCTGGCGATTGGTAAAGCTGGCGCGGCAAACGCGGCGTTACTGGCAG
CACAAATTCTTGCGACTCATGATAAAGAACTGCACCAGCGTCTGAATGAATGGCGCAAAGCACAAACTGATGAAGTGCTG
GAAAACCCGGACCCGCGAGGTGCGGCATGA
>Cluster_60240
ATGACGGGGGGAGTTATGAGTCAAAAGTTTGTAGTGGGCGCAGGGCTGCTGGTATGCAGTGTCTGTAGTTTATCTGCTAT
GGCTGGTAGTAAACCCGTGGATTTAATTCTGCGCGTGTTGGTCGATGCTCCGCCACCCTGTTCAATCAAGGGCAGCCAGG
TAGAGTTCGGCAATATGATTGCTGATAACGTTGATGGCACCAATTATCGGCAAGATGCGAAATACACGTTGAACTGCACA
AACAGTCTTGCTAACGACTTGCGGATGCAACTTAAGGGCAATACCTCAACGATTAACGGCGAAACGGTACTCAGTACCAA
TATCACCGGGCTGGGCATTCGGATAGAAAATAGCGCCGATAACTCATTGTTTGCCGTCGGTGAAAATAGCTGGACGCCAT
TTAATATCAATAACCAGCCACAGTTGAAGGCTGTACCTGTGAAGGCAAGCGGGGCGCAACTTGCTGCGGGCGAGTTTAAC
GCCAGTTTGACCATGGTGGTGGACTATCAATGA
>Cluster_39441
ATGAATCACTTAATGGTTGACCTTGAAACAATGGGCAACGGGCCATACGCGCCAGTTATTTCTATTGGGGCAGTATTCTT
TGACCCGAATACCGGAGAAACAGGAGAAGAGTTCTCGGTAAATATCTCGCTTGAGTCATCAATGCGATATCGGGCGCGTC
CTGACGCTTCAACGATTTTATGGTGGATGGAACAGAGTGAAGAAGCCAGAAAATCGCTAACCAGCAACACTCAAGAGCTT
TCAACGGCTCTTTCATGGTTATCTGAATTCATCATAAAGAACGCTAACCACAAATTCGTTCAGGTTTTGGGGAATGGAGC
ATCATTTGACTGCGTTATTCTCCGCAACAGTTATTCGCTGACAGGGCAGCCAGTTCCGTGGCAGTGGTGGAATGACCGCG
ACGTAAGAACAATCGTCGAGCTTGGAAAGGTAATAGGATTCGACCCTAAGCGAGATATGCCATTCAAAGGAACTCGCCAC
AACGCGCTTGATGATGCCATCCACCAAGCCAAATACGTTTCAGCGATCTGGAAAAAGTTAGCTAAATAA
>Cluster_10641
ATGATACCTGTATGGAGCACGGCATGTCCGGACTGGGCAGAGCGCCTGAAAAAGGGGCTGTCGATTATTCCGGCTCCGAT
TTATCCGGACCAGGCTGCACATGCACTGGCGATTTTTAAACAACTGCGAATTGTGGATGCACCGGGTAGCCCGACATTCG
GGGAGTCCTGTGCACCGTGGGTGTTTGACCTGGTGGCGGCCCTGTTTGGCTCCTACGATGCGCAGACCGGTGTTCGCCAT
ATCAAGGAAGTGTTTATCCTTATCCCCAAGAAAAACAGTAAGTCCACGCTGGCCGCGGGGATCATGATGACGGCGCTGTT
ACTGAACTGGCGGCAGGCGGCGGGCTACACCATTCTGGCCCCGACCGTGGAGGTGGCGGCTAACGCCTTCAACCCTGCCA
GGGATATGGTACGACGGGACGATGATCTGGATGACCTCTGTCAGGTGCAGACACATATCCGGACCATCACCCATCGGGTG
ACGGACACCACCCTGAAGTGGTGGCAGCCGATCCGAATACGGTGTCCGGTATCAAGTCCGTGGGGACACTGA
>Cluster_46774
GTGGACGATTTGACCGCACAAGCCCTGAAAGATTTACTGCACGCTACTGCGATGCGTGGCATGAAGAGCATAAAAGCTG
GCCGTTAAGTGAGGAACTGTACGGTGTTCCTTCACCGTGCATTATTTCAACTACCGAAGATGCCGTATACTGGCAACCCC
AGCCGTTTACCGGGGAACAAAATGTAAACGCGGTTGAACGCGCTTTTGATATTGTGATACAACCCACAATTCATACCTTT
TATACGACTCAGTTTGCCGGGGATATGCACGCGCAGTTTGGCGATATCAAACTGACATTGCTGCAAACCTGGAGTGAAGA
CGACTTCCGTCGGGTGCAGGAAAATCTGATTGGTCATCTGGTGACTCAGAAAAGGCTAAAACTGCCGCCCACGCTATTTA
TCGCGACGCTGGAAGAAGAGTTAGAGGTCATCTCGGTGTGTAATCTCTCCGGTGAAGTGTGCAAAGAGACGCTGGGAACA
CGTAAACGGACGCATTTAGCCTCAAATCTTGCGGAATTCCTTAATCAACTTAAGCCTCTTCTGTAA
>Cluster_38802
ATGGTTATAGATATGAAACATACTGTAGAAGTAATGATCCCCGAAGCGGAGATTAAAGCGCGTATCGCCGAACTGGGTCG
TCAGATTACTGAGCGTTACAAAGACAGCGGCAGCGATATGGTGCTGGTGGGTCTGCTGCGAGGCTCATTTATGTTTATGG
CGGACCTGTGCCGTGAAGTTCAGGTATCTCATGAAGTCGACTTTATGACCGCCTCCAGCTACGGTAGCGGCATGTCCACC
ACCCGTGATGTGAAAATCCTCAAAGATCTGGATGAAGATATCCGTGGCAAGGACGTGCTGATTGTTGAAGATATCATCGA
CTCGGGGAATACACTGTCGAAAGTGCGTGAGATCTTAAGCCTGCGCGAACCGAAGTCGCTGGCGATTTGTACGCTGCTGG
ATAAACCGTCCCGTCGTGAAGTGAACGTCCCGGTAGAATTTATCGGTTTCTCGATCCCGGATGAGTTTGTGGTGGGTTAC
GGCATTGATTACGCACAGCGTTACCGTCATCTGCCGTATATCGGTAAAGTGATTCTGCTGGACGAGTAA
>Cluster_4314
ATGGGGCTATTTACCCAACTGGAAAAATTTGACCAAAAGCTTACGCGCGGTTATGCCCGGTGGGGGCGCTGGGTATGGCG
GACGTTAATTGTCGTTCCGGTACTGGTCGTGTTGTGGAATATAGGACAGGCAGTATGGGGAGGACCAAGAGGTGGAGTGA
TTCTGGAAATCCACAGTGAAATTGACCGCCCGATACTGGGATTCAGTGTTAACGGCGTGGCGGGCGCGAATGCTTTTGCA
AATGGTGGCGGTTCAACAACCTGTTGTGGTGATGTTAGTGGGGATACGGCAGAGGTTATCTGGACATTAAGTACTACTCG
TACACAGTATAATGCTGGAATGCGTCTGGAGAAACGGAATATGACGTTACCTCTCCCCAAAAGAGAATGGGGAGAAGATT
TTCTGCATGTGCATTTTATGCCTGGCGATAAAGTGCTGCTGGGGTGGAGTAAAGATTCTTTTTCTCCGTATGAAGATCTT
CACAACGGGGGGTACAAGACCCGCGTTCGGCAGGATGTGAAAGATAAACTTTATGGTACAGGGAAAATGAATTGA
>Cluster_2734
ATGAGCAAGCGTTCGTCAATAGATACAACAGAAGAACAAAAGGCAATTGAGACTATAGGCGCTGATTTTATTTCGTCATA
TTATAAAGATAGATATGAGGAAGCAAAATCAACATATCGTCGTCTTGAGGACAAAGTTAACTACCTTCTTGCTATTTTAG
CTGTAGAAACCTCTGCTCTACTGGCTGTATTTGGGACTGTTGATTTCAAAAGTGGGCTAAAGAGTAATTGGTTTTTAACA
TTATCTATACTTTGTGCATATCTGTGCTTTGTTTGTATTGTTATGTGCTTTTTTTTCCTATGGCGTACTTGGGGACTGAA

AGATATACCAAAAATGCCTATTCATAAGCGAAAAGAAGAGCATGATTTTTTATTTTACGGTGATAAAATTAAAACGCTTA
GATACCATATGTTTCAGTATGGTAAAGCTGTTGAATTGATCGAAAGTCTTCATAAAGAAAAGGCAGTTTACGCTCGTTAT
CTTTTTGAAGGAATTGCGTTATCTTTCGGTTTTTTCATATTATCGGTTACTTTTCTTTTGATAAATAAGGCATAG
>Cluster_7864
ATGATAAGAAAAGAAAGCGGCCTTTCATTAATTGAAGTAATGATTGGCGTCCTTTTTTTTTCGATTGTTTCTCTTTCTGT
ATATAACTATTACAACAGGAGCGCAGAACAACATTATATGAATAATGTCTCTGTCGTTATGTGCAGATATGCTGATGCTC
TTAGCTACTATTTACAGGATAGCACAGCAACCAATGGTAACGGTAGTGTATCATTATCTACTCTGGTTGCTTCTGGATAT
TTGCCACCAATGCAAGAAACGTCTGGTGTTGGAGAATATGAAATAAAAATGTATGTCGATAAGAATAAGAATGGTCTGAT
AGCATTATATGGTGGAGAGTATTATTCACTTACCTCTGAGAACATAAAAAAATTTCTGGGGATTCGTGGTGCATCTGCTA
AAGATAATAAAATCATATCTGCAGGTGGGTATTATTCATTGCCTGTATCTGACTTTACAGGTTTTCGCACTGTTAATTTA
AGAGGATTAATGCTCATTCCTTCCATTCAGACATCAGGGCAAAATTGTGACAGAGGTGGATATGATTTTTGA
>Cluster_9552
ATGGCAAAGTACAACGAAAAAGAGCTGGCGGATACGAGCAAGTTTTTAAGTTTTGTCCTCCGACACAAACCGGAGGCGAT
TGGTATCGTACTGGACCGTGAAGGATGGGCGGATATCGATAAGCTCATACTTTGTGCCCAAAAAGCGGGCAAGCGGTTAA
CCCGTGCATTGCTGGATACTGTGGTTGCCACCAGCGATAAAAAGCGTTTTAGTTATTCCAGTGATGGTAGGTGCATCCGC
GCGGTTCAGGGGCATTCGACTTCGCAGGTCGCTATTTCCTTTGCCGAAAAAACACCGCCACAGTTTCTCTACCACGGTAC
GGCAAGCCGTTTCCTTGACGAGATAAAAAAACAGGGGCTGATTGCAGGTGAGCGTCACTACGTACATCTCTCTGCTGATG
AAGCAACGGCACGCAAAGTGGGAGCAAGACACGGATCGCCGGTTATTTTAACCGTCAAAGCACAGGAAATGGCGAAACGA
GGCCTCCCTTTCTGGCAAGCGGAAAACGGGGTCTGGCTGACATCAACGGTTGCGGTTGAGTTTCTTGAGTGGTAA
>Cluster_17461
ATGAACAAACTGAAGAAATATCAACGTATCAGGGTGCAAACCCTTCTGTTGGGCGGTGTGCTGGTGCTGGTGAGTCATTC
AGCACTGGCAGCTAATAATTTGCATTTTTACGGCAACCTGATGAGCAAAACCTGTACTCCGGTGGAACAGGCTGGAACGT
TGGCAGAGGTACAGTTTGCTGATATCAGCTACAAAGATTTGATGGTGCGCGGTCAGTCCGATCGTGTACCGGTGGTTTTT
CGCCTGAAAGATTGCCATGGGCCAGCGCAGTACAGCGTAAAAGTTACTCTGACCGGAACAGAAGACAGTGGGCAACCGGG
ATTCCTCGCTCTGGATGGTACCTCCGGCGCACAGGGCGTAGGCATTGGTATGGAAACTACTGATGGTACTGCTGTTGCTA
TTAATAACACGACGGGAGTGACCTTTGTGCTGAATGATGGCAATAACAGTCTTAATTTCAGAGCATGGTTACAGGCAAAA
GAGGGGCGTGATGTAACACCTGGTGATTTTACCGCCCAGTTAACCGCAACGTTTGAGTATATTTAA
>Cluster_3116
ATGCCGGACCAGCGTCACCACATGTTGTTCGCCACCATGTGGAATACCGGGATCCGTATCGGTGAAGCCCGGACACTCAC
CCCGGAATCCTTCGACCTGGATGGTCTGCGCCCTTTTGTGCGGGTGCTGTCGGAGAAAGTGCGCCTGGTGCCGCTGACGG
ATGTCAGTTTTGTGCGTCAGATGGAGAGCTGGATGGTGACCACCCGCCCCCGCCGGCGGGAGCCGTTATGGCCGGTCACG
GATGAGACAATGCGCAACTGGCTGAAACAAGCGGTGAAGCGGGCTGAAGCTGATGGCGTGCATTTTTCGATTCCGGTCAC
ACCACATACCTTCCGGCACAGCTATATCATGCACATGCTCTATCACCGCCAGCCCCGGAAGGTTATTCAGGCGCTGGCCG
GTCACAAGGATCCGCGCTCGATGGAGGTGTATACGCGGGTGTTCGCGCTGGACATGGCCGCCACACTGGCTGTGCCGTTC
ACCGGTGACGGGCATGATGCCGCGCAGATCCTGCGGACACTCCCCCCTCTGACGTGA
>Cluster_40142
ATGAAGCAGTTTCTTGATTTTTTACCGCTGGTTGTCTTTTTCGCGTTTTACAAGATTTATGACATCTATGCGGCTACTGC
GGCGCTCATCGTCGCCACGGCGATTGTGCTTATATATAGCTGGGTTCGCTTTCGTAAGGTTGAGAAGATGGCCCTGATCA
CTTTGTTCTGGTGGTCGTCTTCGGTGGCTTAACGCTGTTCTTCCACAATGATGAGTTTATTAAATGGAAGGTTACTGTC
ATTTATGCCCTGTTTGCGGGTGCCCTGTTAGTCAGCCAATGGGTGATGAAAAAGCCGCTAATTCAGCGGATGCTGGGTAA
AGAACTCACCCTGCCGCAATCGGTATGGTCGAAGCTGAATCTGGCCTGGGCTGTTTTCTTTATCCTTTGCGGTCTGGCAA
ACATCTACATCGCATTCTGGCTGCCGCAAAATATTTGGGTCAACTTTAAAGTCTTTGGCCTGACCGCCCTTACCTTAATC
TTCACATTGTTAAGCGGTATCTATATCTACCGCCACATGCCGCAGGAAGATAAATCCTAA
>Cluster_59063
ATGCCATTTGCTTGTTATTTCTTCATTTTCATAAACGTTGGTTTGGGAGAAGGCTCTGCATTACCTGTTGGTGTCCCGGT
TCCGTGGCCTTCAGCCACTCCGCCAACAGGCTGGCTGAAATGCAATGGTGCCGCTTTTTCTGCTGAAGAATACCCGGAAC
TGGCAAAGGCTTATCCGACAAATAAATTGCCTGATTTACGTGGTGAGTTTATTCGTGGCTGGGATGACGGGCGGGGGATT
GATACAGGTCGCTCTATTTTAAGTATTCAGGGGTATGCAACGGAGGATCATGCTCATGGATTACCGTCAAGATCCACGAT
TGTGACTGATGCAACGATTAATTTCTATTTTGATGAAATATGGGTAAATAGTGGCACTGACATTATCAAAAGAGGAAACA
CAAACGATGCCGGATTACCAGCCCCGGATTATGGAACCTTTAAAACATATAAACAATCAGTGGATGGTTTAGGTGCCGCA
GCCTCAGAAACGCGTCCGCGTAATATTGCATTTAACTATATCGTGAGGGCAGCCTGA
>Cluster_21849
ATGACGTATCAACAAGCTGGACGCATTGCTGTTTTGAAAAGGATTTTGGGTTGGGTGATTTTTATACCTGCATTGATCTC
TACATTGATTTCTTTGCTGAAATTTATGAATACCCGGCAGGAAAACCAGGAAGGCATTAATGCGGTCATGCTCGACTTTA
CTCATGTCATGATCGATATGATGCAGGCGAATACGCCTTTTTTGAATGTGTTCTGGTACAACTCTCCGACGCCCAATTTT
AATGGTGGCGTGAACGTTATGTTCTGGGTGATTTTCATCCTGATTTTTGTCGGACTGGCGTTGCAGGATTCCGGAGCCCG
GATGAGTCGCCAGGCGCGCTTCTTGCGGGAAGGTGTTGAAGATCAACTCATTCTGGAAAAAGCCAAAGGGGAAGAGGGGC
TAACGCGTGAACAAATTGAGTCACGTATTGTTGTTCCGCACCATACTATTTTCCTGCAGTTTTTCTCGCTGTATATCCTG
CCGGTTATTTGTATTGCTGCAGGTTATGTGTTCTTTTCTCTGCTTGGGTTTATTTAA
>Cluster_51154
ATGAAGATCATAAATACAAAAATTGAAGATGTAAAAATCATTAAACCAATTATTCATGAAGATGAAAGAGGATTTTTTAT
GGAGAGTTGGAATCAGAAAGTTTTTAATGAAGTAATTGGGAAAACAATCTTTGTGCAGGACAATCACTCGAAATCATATA
AAGGTACTCTTAGGGGGTTACACTTTCAAACCAGTAATCCGCAAGGAAAATTAGTAAGAGTAATAAATGGAGAAGTGTTT
GATGTTGTTGTTGATTTGAGACATCAGTCAAAGACTTTTGGAAAATGGGTAGGAGTTTATTTATCGTCTGTGAATAATTA
TCAACTTTGGATTCCTGAAGGGTTTGCACATGGTTTCTATGTAGTGAGTGACCATGCGGAGTTTGTTTATAAATGTACAG
ACTATTATAATCCTTACTCGCAACAGACACTGTCATGGGATGATCCTGAGTTGGGTATTCTATGGCCATTAGACACTGCT
CCAATTTTATCGGAGAAGGATAAGTCAGGTAGTAGCTTTAAAAATTTGAAAGGTCTATTTTAA
>Cluster_9432
ATGAGTGCCAATGAAAACAACCTGATTTGGATCGATCTTGAGATGACCGGTCTGGATCCCGAGCGCGATCGCATTATTGA
GATTGCCACGCTGGTGACCGATGCCAACCTGAATATTCTGGCAGAAGGGCCGACCATTGCAGTACACCAGTCTGATGAAC
AGCTGGCGCTGATGGATGACTGGAACGTGCGCACCCATACCGCCAGCGGGCTGGTAGAGCGCGTGAAAGCGAGCACGATG
GGCGATCGGGAAGCTGAACTGGCAACGCTCGAATTTTTAAAACAGTGGGTGCCTGCGGGAAAATCGCCGATTTGCGGTAA
CAGCATCGGTCAGGACCGTCGTTTCCTGTTTAAATACATGCCGGAGCTGGAAGCCTACTTCCACTACCGTTATCTCGATG
TCAGCACCCTGAAAGAGCTGGCGCGCCGCTGGAAGCCGGAAATTCTGGATGGTTTTACCAAGCAGGGGACGCATCAGGCG
ATGGATGATATCCGTGAATCGGTGGCGGAGCTGGCTTACTACCGCGAGCATTTTATCAAGCTGTAA
>Cluster_20471
ATGAATACTAACGTTTTTCGACTGCTCCTGCTGGGAAGCCTGTTCAGCCTTAGCGCCTGTGTGCAGCAAAGCGAAGTGCG
ACAGATGAAACACAGCGTCAGCACGCTGAACCAGGAGATGACGCAACTCAATAAAGAAACGGTCAAAATCACTCAGCAAA
ACAGGCTGAATGCAAAATCCAGCAGTGGGGTTTACCTTCTGCCTGGAGCGAAAACACCAGCAAGACTGGAAAGCCAGATC

GGTACTTTACGTATGTCGCTGGTGAATATTACGCCTGATACAGATGGCACCACCCTGACGCTACGTATTCAGGGCGAGTC
GAATGATCCCTTGCCCGCATTCAGCGGAACTGTTGAATATGGGCAAATTCAGGGAACAATAGACAACTTTCAGGAAATCA
ATGTGCAGAATCAATTGATTAACGCCCCTGCCAGCGTCCTCGCCCCCAGCGATGTTGATATTCCGTTACAGTTAAAGGGT
ATGTCTGTGGATCAGTTAGGCTTTGTACGTATCCACGACATTCAACCTGTTATGCAGTAA
>Cluster_62547
ATGAATACCGCATTATCCCCGCTGTCATCTCAGCCTGAACTTTTTCCACTGGCGGTTATGGCTCAACATGATTACCTGCT
TACCGCGACATCCGGATTAACACCCTATGCTCAACGGACCATCCGACGCGCTACCAATCTGCTGGATAAATATCTGCGCC
AGCCCGGTATATCCTTTACCTCAAGTACTGCTGCCCGTGACTGGCTTCGGTTACAGCTGGCAGGACAAGAACGGGAAGTG
TTTATGGTGCTCTATCTCGATAACCAGCATCGTTTACTGGAGAGTGAAACGCTATTTGCCGGTTCGATTAACCATGTACA
GGTCCATCCCCGTGAGGTAGTGAAATCAGCGCTTCGTTTCAATGCTGCGGCCGTTGTGTTGGCGCATAACCATCCATCCG
GTGATCCGGAGCCCAGCCAATGTGACCGCAACATTACGGGCAGGCTTAAAGAAGCGCTGGCACTGGTTGATGTGAACACT
CTCGATCATCTGGTTATCGGTTCAGATGGAATTGTGTCGTTTGCAGAGCGAGGCTGGATATGA
>Cluster_15804
ATGAAAGTGCGTGCGCATCAGTATGACACGGTGGACGCACTTTGCTGGCGTCATTACGGGCGCACGCAGGGTGTCACGGA
GCAGGTACTGAAGGCAAATCCGGGGCTTGCCGAATACGGCCCCTTTTTACCTCACGGGCTGCAGGTGGAGCTGCCGGACA
TTCCGACAACCACCACCGTGCAGACCGTCCAGCTATGGGACTGA
>Cluster_49572
ATGGGTGAGATTAGTATTACCAAACTGCTGGTAGTTGCGGCGCTGGTCGTTCTGCTGTTTGGGACTAAGAAGTTACGTAC
GCTGGGCGGAGACCTTGGAGCGGCCATTAAAGGGTTCAAGAAGGCGATGAATGATGACGATGCTGCGGCGAAAAAGGCG
CAGACGTTGATCTTCAGGCTGAAAAGCTCTCTCATAAAGAGTGA
>Cluster_37352
ATGGCAATCCGAAAACGTTTATTGCGGGCGCAAAATGCCCGGCCTGTCAGGCGCAGGATTCAATGGCGATGTGGCGCGA
AAATAATATTGATATTGTTGAATGTGTTAAGTGCGGACATCAGATGCGAGAAGCAGACAAAGAAGCCCGCGATCACGTTC
GCAAAGATGAGCAAGTGATCGGGATTTTTCATCCGGACTAG
>Cluster_26266
ATGAGCAGCTTATTTTTAACCGAAGATGAATTGCTAATATTAACGGGCTGCAAATATGCAAGCCACCAGCGAAAATGGTT
AATGGAAAACGGGCTTCCGTTCTATACCAATCGTAGTGGCAAACCGATTGTCAGCCGGGATCTATTTACCTGCAATAAAA
CTTTACCACCACGCGAGGTAGAGCCGAATTTTGGTGCGATCTGA
>Cluster_61905
ATGAATATATCATATGTGAATAGCAACAAAACGACATCTTTACCTGTTGAGCTAGATGCACTAAATAATAAAGATATCTC
TTATGCCAAAGATTTCTTTTTATACATAGAAACACAGTTAAAAATTGCAAAAGATTTTTTAGACCTGGAGAAGAAGTATC
AAGTTCTATTGCAAGTAAAGTTTTTCACGCATTTATTGATTTAG
>Cluster_727
ATGAATAAAGAACAATCTGCTGATGATCCCTCCGTGGATCTGATTCGTGTAAAAAATATGCTTAATAGCACCATTTCTAT
GAGTTACCCTGATGTTGTAATTGCATGTATAGAACATAAGGTGTCTCTGGAAGCATTCAGGGCAATTGAGGCAGCGTTGG
TGAAGCACGATAATAATATGAAGGATTATTCCCTGGTGGTTGACTGA
>Cluster_21004
ATGCTAAAACAGCAAGATATGACAGAAACCGCCGCCGCAGTCCTTCATTTCTTACCTGCTGACAAGTGGGTAACGCCACG
CATGATGACGAGAACTACCGGAGTAAGCGAAGCCCGGTGCCAGTTAATACTGACTCAGTTAGTTCTGGCGGGTCTGGCGA
AGGATAACGGCGGGTACGGGAATAAATTCAGACGCTGCCAGTAA
>Cluster_29431
ATGACAGATCCACTCAAAGCCCTTTTCGGCAAGCCTGATTACTCCCATATCGTAAGGGACACCACGGCCACTATCAGCAT
CACAGCCGCTGAAATGGCCGCTGTTTTGGAGGCTTACGACAGGGGTATAGACACGCTGGACGGCACCACCAGAACGGCGC
TGGATTCCGTTATATCGAAACTCAAGGACGAGGTATGGCCATGA
>Cluster_35704
ATGCAGGTTAATTGTTTTTTTAATAGCGAAATTTTCTATTCATTCGTGATCAAGATCACGATGCTGTCGCAATGTAAAAA
CATTGCAAGAATCAGGTTAAAATCATTTAAATTTACACACGCAACAAATATTGACCTACAAAACATTACACTGGCTATTT
TTCAGAAACTGGACTATCTCACTAGTAAAAACGCTAAATAG
>Cluster_18912
ATGTCTATCTATGACCCGAAATTAGCCGCAGGCAACCCACAGCGCGCCCACCGCCCAATGCTGACCCGTCAGGATCTTGA
AACGGCAAAGGCACGCAATAACTTCGATCGCCAGAGTCGCAACCTGCAGCAACTCAACGAATCAAATGCACGGTTCTGGA
GTGAACAGAATGAGAAAGCCAAACGTTAA
>Cluster_11621
ATGTACAAAAAGCCAGTAAAACGACTGAAAATCAACTTTTTAGGGTGTCAGTCAGATGTTAATCAAGTTTGTTCCAGATT
GTTCGCCGTTGTTCGTCGTGGTGTCTCGTTGTTTCACGTTGTGTCAGTTCTAAAAAATCGCAAAAAAAATTATATTTCTC
TGGCTATTGGCAGTGTGGTTACGTTTTTGTGA
>Cluster_53910
ATGGCACGAACAATGCTCTATAAGCCTGGCAACATGATCACCTGTGGTCAGTTTGCTGTCGATTACATCATTGTTGATGA
CGAAGAAGTTAAATCTCACCTGAAAAAAGGTTGGGTAAAAACTCCTGAAGAAACCGCAACGAAGCAAAAAGTGGCTAAGG
CGGAAGAAGATGGCGAAAACGAAGGGTGA
>Cluster_29499
ATGAGCAGGAAAAGTATTGGTATAAGCAACGAGAGATATCTGAGAATAGAAAGAGCAGCCGTAGATATCACAGCCAAAAC
AGGAAAAGTCACAAAATGGTCGGAGATCGTGAATTATCTGATAGATGAATATTTAAACGATGCAAAACAAGATATGATTT
CGAAAAATGAAATTACACAAAAAAAATAG
>Cluster_7936
ATGTCACGCGATGAATTAAGGATTGTTTTAGGTGCAATGATTCCAAATATGGATGAAGGTTTTGAAATTAAAACCCGTGA
CGGCGCGATACTTCGCGTTGACCCTGAGTGGGATTGCTGCAAAGAATTTAAAGATGGATTAAAGGCCGAAATCATCAAAC
AGTTAAAAAGCAAACCTGCTGTTGTATTTGGATATAGTTAA
>Cluster_10561
ATGACGTTCACAGTAAAAACCATTCCTGACATGCTCGTTGAGGCATATGAAAATCAGACCGAGGTAGCCAGAATACTGAA
CTGTAGTCGCAACACGGTCAGAAAATATATTGGCGATAAAGAAGGCAAACGGCACGCCGTCGTCAACGGTGTCCTTATGG
TCCATCGTGGATGGGATAAGGGTAAAGACTCTGATGCGTGA
>Cluster_9793
GTGAACAAACAGCAACAAACTGCGCTGAATATGGCGCGATTTATCAAAAGCCAGAGCCTGACACTGCTTGAAAAACTGGA
TGCTCTGGATGCCGACGAACAGGCGGCCATGTGCGAACGACTGCACGAACTCGCGGAAGAACTCCAGAACAGCATCCAGG
TTCGCTTTGAGACCGAAAGTGAAACAGGAACATAA
>Cluster_24524
ATGAAACAAGAAATGATTATCAGCAAAGACGGGGAAACGATGATTGTTAGTGATGAAAACCTCATTAAGGTTGCCGCACT
GTTGGGAGTTACTCCCGAAAGTCTTCGTTCTACCACTGCTCAATTATTTAAATCGGCTGATGCGGTTAGTCTTGCTGATG

CCCTTAAATCTAAAATTAAACAACTGAAAGGAAAATAA
>Cluster_10637
ATGAAGAAATTACAGGTAACGGTAAAACCTTTTCAGGGAACAATTCCGTTCCGTGTTTTGCAACATGGCCGTGTTCTGCT
TGAAGAGGTGTTCAGAGGTAAATGCACTGAATGTTATTCACGAACATATGAAGTGAATGCCACGCATGAAGAATTCACCG
TTGAGTGTGTGATGAATACTGATAAATGCCGAATGGTATCCGCTGAATTACAGCCAGTGTGTTGA
>Cluster_995
ATGATGGCCCGAAATATAAAAATGGCAACGGATGCGCAGAACTGGTTACAGGCGCGCGGGAGTCATGTAAATGAATCATA
TCTCGGCGTGGCGCGTCCGATTCTTGAAATCACTTACCCACCGGTGGAACTGGTAAAAAACGCGGTCAGAATTATGGAGC
ATAAGTCCGGGGTGGCCCGTTCTGTATGGACGGCCCGTCTTAATGGTTGCCAGATTATCTGGAGATAA
>Cluster_48419
ATGACAGGCGAAAATCGTGGCCGGGGAAGGCCGCGAAAGACAGAAAGTACCTATGCAGATACTCGTAATGATCTGATCCG
CAGTGGCCTTGAACTGCTGACGCAGAATGGTTTTCTTGCGACGGGAGTCGATGCCATTGTTAAAAATGCCAATGTCCCTA
AAGGCTCATTTTATTACTACCTTAAGAGTAAAGAGGATTATGCTGTCAACGACGGATGA
>Cluster_17632
ATGATTGATGATGAAATTGCGGCAGCAATTACTGCTAACAAAGCTGCGTGGAGCTTTCTGGTTAAACAGCTTTCAGAAAA
TGGCTGTCTGAACCTCAGCGCCCTGGATTCTGAATTACGCAACTTCCAGCACGGCCTTCAGGAACGTGGGGAGTTCGTTC
TGGCTGCGGCGCTGGATGAACATCTGCTGTCGATTGAGAACTGGTCGCAAGACGATTAA
>Cluster_30767
ATGGTAAAACCACCTCCCGTGGAGGTGGTTGCTTGCAGTCAGGTGTTTCGGGCCTGCGGCTTTTTGTGTTCCTTGGTAAG
TGGTTACGATTCTTTTGTGTTATTCCTCTCTGCGTCTATTGTCTTGGATAGCCATAAGAGTTCAGATACTGAGAATTTTA
ATTGTGGTGTTCTGTCTCTCGCGCCCGGGTGGGATCATGTCAAAAGCGCAGGATTTTCTGCTGAATACTGTGTAG
>Cluster_9579
ATGTCGCATCTGGATGAAGTCATCGCGCGCGTGGATGCCGCGATTGAAGAGAGCGTCATTGCCCATATGAACGAATTATT
AATCGCCCTGAGCGATGACGCGGAGTTAAGTCGGGAAGATCGCTACACCCAGCAGCAGCGTTTGCGCACCGCTATTGCTC
ATCACGGTCGCAAGCATAAAGAAGATATGGAAGCGCGCCACGAACAGTTAACCAAAGGCGGCACCATCCTCTGA
>Cluster_3661
ATGTGGAAGCCGTTAAGAGATGAGAACCGTTTATCAATTTTGAAGATCTATGGATACACCTGTGACAGACTGGTACGAGA
AGAGGAACGTCAGAACATTACGTCTATTTCTCGTTCACAAGCCTGGAAACTGGAACGTGAAGGAAAATTCCCGCCACGTA
AAACTATTGGAAAGAAATCCTGTGGATGGTTATTAAGTGATCTATTGTGGTGGATTAGTCAGTATTAA
>Cluster_25410
ATGAAATTTATTTACCGCGGCCCGGCCAGCGGCGTGACGTTAGCCGATGGCGCAGAAGTGCTGCTCTGGCCAGAGCATGA
GGTGGAACTGCCGGAAGATCTGGACTATGTGCAGACGCTGCTGGCGCTGAAGCATCTCACACCAGCGCCAGTTGAACCCG
AAGTGGTGCCTGAACCTGAACCACAACCCCTGAAGGGTAAAAAAGAGAAGGAGACAACCAGTGGCAGCTAA
>Cluster_37292
ATGAGTCGATATCAGCATACTAAAGGGCAGATAAAGGATAATGCGATAGAAGCATTACTACATGATCCCTTATTCCGACA
GCGCGTAGAGAAAAATAAGAAGGGGAAAGGCAGTTACATGCGAAAAGGAAAACATGGCAATCGGGGGAACTGGGAGGCCA
GTGGCAAGAAAGTAAATCACTTTTTTACCACTGGTCTTCTGCTTTCAGGCGCTTGTTAA
>Cluster_67071
ATGAATAAGGATTTGATGGTAGCATTGTTAACTTTATGAAAGACCAAGAAGAATCTTTCCGGAAAACATTCTTCAGTAC
CCGTGAAATTGCCGATGCAGTCGGGTTGACAATATATCAGGTTGGGGAGCATCTGGACGCATTGCAGTCATTCAGTGTGG
TGGAAAAAGTAAATTCCGGTCGAGAAAACCCCGGGCAGTGGAGGCTACTCTGA
>Cluster_50405
ATGTCAAATACACAGAAAATTATTAACACTGAAAAATATAACGAGTGGGTGAAAAAATTCTCTGAGCAGATTTTTAAAAT
TACTGGCGACGAGAATGTGGCAAAAAATGAATTGGAACCGTGGACACCTGAAGGAAACGCACCAAATTATTGCTGGTGGG
AGGTTGATCCGGTTGATGCTGCAAATGAAGCCATGAGTTACCACAACGATTAA
>Cluster_50022
ATGGACACCGTTAACGGAATGTGTTCAGACGCACCGCGTGCCAAAAAATGTAAATGCGGAAAATCACCGACAATATTCGA
CATGGAGAACGGGTGCCAAATCTACTGCGCTAACCACGCCGCTGTGGCGGCCGCGAATTATAGCAGTGCGGTAACGGAGT
GGAATAACCTGAAATCTGTTAGAGAGGGAAGTCATGAAAAAACTAACCTTTGA
>Cluster_55055
ATGTTATGTTTTTTGATTTATATCACTCTCCCCTTCATTCAGCTTGTCTATTTCATTTCCTCTGAAAAGAAACTAACTAT
TCACATTGTGCAGATGTTTCACTTGTTATCACAGGTATTCTATAATCTCAAAAAGTTTTTAATGATGGATATGCTCGGAG
TTGGAGATGCAATTAATATTAATACAAATAAAAATATCCGGCAGGTATGCTAA
>Cluster_5177
ATGAAGCAGCGTATTACAGTGACAGTTGACAGCGACAGCTATCAATTGCTCAAGGCATATGATGTCAATATCTCCGGTCT
GGTAAGCACAACCATGCAGAATGAAGCCCGTCGTCTGCGTGCCGAACGCTGGAAAGTGGAAAATCAGGAAGGGATGGTTG
AGGTCGCCCGGTTTATTGAAATGAACGGCTCTTTTGCCGATGAGAACAGGGACTGGTGA
>Cluster_51672
ATGGCACTCAACTGTCCAACCTGCAAAACCCCTCTCGAACGCAATGGCGAGACTGCTCACTGTGCAACTTGCGACAAAGA
CTTTACCGTCCAGGCACTTTGCCCTGAGTGTCATCAGGCGTTACAAGTGTTAAAAGCTTGTGGTGCTGTGGATTATTTCT
GCCAACATGGGCACGGTTTAATATCTAAAAAACGCGTTGAATTTATTATTGCTTAA
>Cluster_60718
ATGAAAAAAATGGGCAGTAATAATTTCTGCAGTCGGACTGGCGTTTGCTGTTTCCGGGTGTTCCAGTGATTACGTCATGGC
GACCAAAGATGGCCGTATGATTTTGACCGATGGAAAACCTGAAATTGATGATGATACCGGGCTGGTGAGTTATCACGATC
AGCAAGGTAACGCGATGCAAATTAACCGTGATGATGTTTCGCAAATTATTGAACGTTAA
>Cluster_20007
ATGTTATTTACCATAACATCCGTTGCCCGCACCGCCCCGGACAGCTTCGCCGCGAACGGGGCGGCAGTCAGCCAAAACCA
ACAACAAATCACCCGAAAAGCTACCGCGATCGCGCCTGATAAATTTTAA
>Cluster_9883
ATGCGTCAGTTTTATCAGCACTATTTTACCGCGACAGCGAAGTTGTGCTGGTTGCGTTGGTTAAGCGTCCCACAACGATT
AACCATGCTTGAAGGACTGATGCAGTGGGATGACCGCAATTCTGAAAGTTGA
>Cluster_35766
ATGTACAGAAAATTCAGTGATGAATGTTTCGGGCCGTCCACGCTGATGAGTGCGATAAAAGTGATTGCCCTTGTGGTTCT
GATAACCATCAGTGCCGTGGTGTATCTTTCTGTCTGTTAA
>Cluster_17761
ATGTCCAAGTGCGACGCTCTGCTTTACTCCATGATAATCGGGTTTGGCGTGGCTGCAGGAATCAGGGTTTACATCGCCTG
GGAATCATTAATTAATCTGGCATGGAGCGCTATCCGTGGCTAA
>Cluster_17740
ATGATTGTTATAATTCTTGTTGTGTTGGCACTTGTCGCGCTTGTCGTAATGAACCGTAAATGTATCATTGAAGATGGGGA

ATTTGCCGTCGCAGTTGTTTTAATATTATCTGGTATAGCAGGGTACATAGGTTTGTCATAG
>Cluster_15238
ATGAAAGTCCTTAACTCTCTGCGTACCGCAAAAGAACGCCATCCAGACTGTCAGATTGTGAAGCGAAAAGGACGGCTGTA
TGTGATTTGTAAATCTAATCCACGTTTTAAGGCCGTTCAGGGTCGTAAGAAAAAACGTTGA
>Cluster_8147
ATGTTGCAACGTAAAACTTTACGTAAATTAACATGGTTAACATTTATGCCACTATTGTTTGTAAATTCATATTTCGTAAT
GCTTCTGAATTTTTCGTGTGATGGTTTTAAATACTATGGTGTTTACTCTTGA
>Cluster_24667
ATGTCAACACGACAGATCGTACAGCTTGCTGATTCTCTTGTCAAAGGCTGGGAAACCAGAATCCCGGCAATGACGTTTAG
TGAGTGGGATCAATTCCGGTGGTGGTTAAACTACCTGCAAGGGTATGAAATGTTTTAA
>Cluster_31469
ATGAGTATATTTATCTCATGGCTTGTTCTGATTATTTCGGTGGTCTGCGCCATTGGGATTATGCGAATTATTAATTCAGT
AAAAAAGATTGAACGCTTTTTCACTGAAGAATAA
>Cluster_14162
ATGAACTACGAGGAAGGGAACAACATTCATACTGAAATTGAATTTTTTTCACTCACTATTTTATTTTTAAAAAACAACAA
TTTATATTGA
>Cluster_61835
ATGGTTTACTCCTATACCGAGAAAAAACGTATTCGTAAGGATTTTGGTAAACGTCCACAAGTTCTGGATGTACCTTATCT
CCTTTCTATCCAGCTTGACTCGTTTCAGAAATTTATCGAGCAAGATCCTGAAGGGCAGTATGGTCTGGAAGCTGCTTTCC
GTTCCGTATTCCCGATTCAGAGCTACAGCGGTAATTCCGAGCTGCAATACGTCAGCTACCGCCTTGGCGAACCGGTGTTT
GACGTCCAGGAATGTCAAATCCGTGGCGTGACCTATTCCGCACCGCTGCGCGTTAAACTGCGTCTGGTGATCTATGAGCG
CGAAGCGCCGGAAGGCACCGTAAAAGACATTAAAGAACAAGAAGTCTACATGGGCGAAATTCCGCTCATGACAGACAACG
GTACCTTTGTTATCAACGGTACTGAGCGTGTTATCGTTTCCCAGCTGCACCGTAGTCCGGGCGTCTTCTTTGACTCCGAC
AAAGGTAAAACCCACTCTTCGGGTAAAGTGCTGTATAACGCGCGTATCATCCCTTACCGTGGTTCCTGGCTGGACTTCGA
ATTCGATCCGAAGGACAACCTGTTCGTACGTATCGACCGTCGCCGTAAACTGCCTGCGACCATCATTCTGCGCGCCCTGA
ACTACACCACAGAGCAGATCCTCGACCTGTTCTTTGAAAAAGTTATCTTTGAAATCCGTGATAACAAGCTGCAGATGGAA
CTGGTGCCGGAACGCCTGCGTGGTGAAACCGCATCTTTTGACATCGAAGCTAACGGTAAAGTGTACGTAGAAAAAGGCCG
CCGTATCACTGCGCGCCACATTCGCCAGCTGGAAAAAGACGACGTCAAACTGATCGAAGTCCCGGTTGAGTACATCGCAG
GTAAAGTGGTTGCTAAAGACTATATTGATGAGTCTACCGGCGAGCTGATCTGCGCAGCGAACATGGAGCTGAGCCTGGAT
CTGCTGGCTAAGCTGAGCCAGTCTGGTCACAAGCGTATCGAAACGCTGTTCACCAACGATCTGGATCACGGCCCATATAT
CTCTGAAACCTTACGTGTCGACCCAACTAACGACCGTCTGAGCGCACTGGTAGAAATCTACCGCATGATGCGCCCTGGCG
AGCCGCCGACTCGTGAAGCAGCTGAAAGCCTGTTCGAGAACCTGTTCTTCTCCGAAGACCGTTATGACTTGTCTGCGGTT
GGTCGTATGAAGTTCAACCGTTCTCTGCTGCGCGAAGAAATCGAAGGTTCCGGTATCCTGAGCAAAGACGACATCATTGA
TGTTATGAAAAAGCTCATCGATATCCGTAACGGTAAAGGCGAAGTCGATGATATCGACCACCTCGGCAACCGTCGTATCC
GTTCCGTTGGCGAAATGGCGGAAAACCAGTTCCGCGTTGGCCTGGTACGTGTAGAGCGTGCGGTGAAAGAGCGTCTGTCT
CTGGGCGATCTGGATACCCTGATGCCACAGGATATGATCAACGCCAAGCCGATTTCCGCAGCAGTGAAAGAGTTCTTCGG
TTCCAGCCAGCTGTCTCAGTTTATGGACCAGAACAACCCGCTGTCTGAGATTACGCACAAACGTCGTATCTCCGCACTCG
GCCCAGGCGGTCTGACCCGTGAACGTGCAGGCTTCGAAGTTCGAGACGTACACCCGACTCACTACGGTCGCGTATGTCCA
ATCGAAACCCCTGAAGGTCCGAACATCGGTCTGATCAACTCTCTGTCCGTGTACGCACAGACTAACGAATACGGCTTCCT
TGAGACTCCGTATCGTAAAGTGACCGACGGTGTTGTAACTGACGAAATTCACTACCTGTCTGCTATCGAAGAAGGCAACT
ACGTTATCGCCCAGGCGAACTCCAACTTGGATGAAGAAGGCCACTTCGTAGAAGACCTGGTAACTTGCCGTAGCAAAGGC
GAATCCAGCTTGTTCAGCCGCGACCAGGTTGACTACATGGACGTATCCACCCAGCAGGTGGTATCCGTCGGTGCGTCCCT
GATCCCGTTCCTGGAACACGATGACGCCAACCGTGCATTGATGGGTGCGAACATGCAACGTCAGGCCGTTCCGACTCTGC
GCGCTGATAAGCCGCTGGTTGGTACTGGTATGGAACGTGCTGTTGCCGTTGACTCCGGTGTAACTGCGGTAGCTAAACGT
GGTGGTGTCGTTCAGTACGTGGATGCTTCCCGTATCGTTATCAAAGTTAACGAAGACGAGATGTATCCGGGTGAAGCAGG
TATCGACATCTACAACCTGACCAAATACACCCGTTCTAACCAGAACACCTGTATCAACCAGATGCCGTGTGTGTCTCTGG
GTGAACCGGTTGAACGTGGCGACGTGCTGGCAGACGGTCCGTCCACCGACCTCGGTGAACTGGCGCTTGGTCAGAACATG
CGCGTAGCGTTCATGCCGTGGAATGGTTACAACTTCGAAGACTCCATCCTCGTATCCGAGCGTGTTGTTCAGGAAGACCG
TTTCACCACCATCCACATTCAGGAACTGGCGTGTGTGTCCCGTGACACCAAGCTGGGTCCGGAAGAGATCACCGCTGACA
TCCCGAACGTGGGTGAAGCTGCGCTCTCCAAACTGGATGAATCCGGTATCGTTTACATTGGTGCGGAAGTGACCGGTGGC
GACATTCTGGTTGGTAAGGTAACGCCGAAAGGTGAAACTCAGCTGACCCCAGAAGAAAAACTGCTGCGTGCGATCTTCGG
TGAGAAAGCCTCTGACGTTAAAGACTCTTCTCTGCGCGTACCAAACGGTGTATCCGGTACGGTTATCGACGTTCAGGTCT
TTACTCGCGATGGCGTAGAAAAAGACAAACGTGCGCTGGAAATCGAAGAAATGCAGCTCAAACAGGCGAAGAAAGACCTG
TCTGAAGAACTGCAGATCCTCGAAGCGGGTCTGTTCAGCCGTATCCGTGCTGTGCTGGTAGCCGGTGGCGTTGAAGCTGA
GAAGCTCGACAAACTGCCGCGCGATCGCTGGCTGGAGCTGGGCCTGACAGACGAAGAGAAACAAAATCAGCTGGAACAGC
TGGCTGAGCAGTATGACGAACTGAAACACGAGTTCGAGAAGAAACTCGAAGCGAAACGCCGCAAAATCACCCAGGGCGAC
GATCTGGCACCGGGCGTGCTGAAGATTGTTAAGGTATATCTGGCGGTTAAACGCCGTATCCAGCCTGGTGACAAGATGGC
AGGTCGTCACGGTAACAAGGGTGTAATTTCTAAGATCAACCCGATCGAAGATATGCCTTACGATGAAAACGGTACGCCGG
TAGACATCGTACTGAACCCGCTGGGCGTACCGTCTCGTATGAACATCGGTCAGATCCTCGAAACCCACCTGGGTATGGCT
GCGAAAGGTATCGGCGACAAGATCAACGCCATGCTGAAACAGCAGCAAGAAGTCGCGAAACTGCGCGAATTCATCCAGCG
TGCGTACGATCTGGGCGCTGACGTTCGTCAGAAAGTTGACCTGAGTACCTTCAGCGATGAAGAAGTTATGCGTCTGGCTG
AAAACCTGCGCAAAGGTATGCCAATCGCAACGCCGGTGTTCGACGGTGCGAAAGAAGCAGAAATTAAAGAGCTGCTGAAA
CTTGGCGACCTGCCGACTTCCGGTCAGATCCGCCTGTACGATGGTCGCACTGGTGAACAGTTCGAGCGTCCGGTAACCGT
TGGTTACATGTACATGCTGAAACTGAACCACCTGGTCGACGACAAGATGCACGCGCGTTCCACCGGTTCTTACAGCCTGG
TTACTCAGCAGCCGCTGGGTGGTAAGGCACAGTTCGGTGGTCAGCGTTTCGGGGAGATGGAAGTGTGGGCGCTGGAAGCA
TACGGCGCAGCATACACCCTGCAGGAAATGCTCACCGTTAAGTCTGATGACGTGAACGGTCGTACCAAGATGTATAAAAA
CATCGTGGACGGCAACCATCAGATGGAGCCGGGCATGCCAGAATCCTTCAACGTATTGTTGAAAGAGATTCGTTCGCTGG
GTATCAACATCGAACTGGAAGACGAGTAA
>Cluster_14715
ATGTCTTCAACAAATGCGCATCAAATAAAAAATAATGATCAAAACTCTTTATGTGGTCTGGGTGACAAAATACGCCGCCT
GACCGCCGGAGTTTGTTTATTTACACAAATTTTTTTCCCCGTCATGGCGACGGCACAAAATGTGGTACATGCTAAACCTC
AGACGACAGTTTCATCTGCCCCCCCTCTAATAGAAAATAATACTGTGCCTTACACGCTTGGTGCGCTGGAATCAGCGCAA
AGCGTTGCTGATCGATTCGGTATTTCACTGGAGGAGCTTCGTCGTCTTAATCAGTTCCGTACTTTTGCTCGCGGCTTTGA
TAACGTGCGCCAGGGTGAAGAGCTGGATGTTCCGGCAACAACCTTGCAGAAAAGTCATGAGCAACAAAATGCCGTACCGC
CTGCGAATGGCGAAAACACGCTGGAGAATCAAATAGCCAGCACTTCGCAGCGAGTTGGCACCCTGCTTTCACAAGATATG
AATAGTGAGCAGGCCAGCGGCATGGCGCGTGGTTGGGCGTCTTCAGAAGCCTCAGGCGCGATGACTGATTGGTTAAATAA
CTTTGGCACTGCGAAAATCTCTCTGGGTGTGGATGAAGATTTCAGCCTGAAAAATTCGCAATTCGACTTCCTGCATCCGT
GGTATGACACACCTGATTATCTGCTCTTCAGCCAGCATACCCTTCACCGAACAGACGATCGTACCCAGATCAACACCGGT
TTGGGCTGGCGTCATTTCACCCCCAGCTGGATGTCAGGCATCAACCTGTTTTTTGACCACGACCTGAGCCGCTATCACTC

CCGCGCGGGTCTTGGCGCAGAATACTGGCGTGATTATCTGAAGTTGAGCAGCAACGCTTATATCGGCCTGACTGGCTGGC
GTAGCGCACCGGAATTGGATAATGACTACGAAGCCCGCCCGGCCAACGGCTGGGATTTACGCGCGGAAGGCTGGTTACCA
GCCTGGCCACAACTGGGTGGAAAACTGGTCTATGAACAATACTATGGTGATGAAGTGGCGCTGTTTGACAAGAATGATCG
CCAAAGTAATCCCCATGCTATTACCGCGGGCCTCAACTATACCCCCTTCCCGCTTCTGACTCTCAGTGCGGAACAGCGTC
AGGGAAAACAAGGTGAAAATGACACACGTTTTGCCGTTGATCTAACCTGGCAACCCAGCAGTTCAATGCAGAAACAGCTT
AACCCGGACGAAGTGGCCGGACGGCGCAGTCTGGCCGGTAGTCGTTATGACCTGATTGATCGCAACAACAACATCGTTCT
GGAATATCGCAAGAAAGAGCTGATTCGCCTGAGTCTGCTGGATCCGGTGAAAGGAAAGTCTGGAGAAATAAAACCGCTGG
TTTCCTCGCTACAGACCAAATATGCCCTGAAAGGCTATAACATCGAAGCCGCTGCGCTGGAAGCTGCCGGAGGTAAAGTC
AGCACGTCTGGAAAAGATATCACGGTTACGCTGCCAGGTTATCGCTTCACTAACACTCCAGAAACCGATAATACATGGTC
GATAGACGTTACCGCCGAGGATGTAAAAGGGAACCTGTCACGGCATGAACAAAGCATGGTCGTCATTCAGGCTCCGACAT
TAAGCCAGAAAGATTCTCTGTTATCCGTTAATCCGCTAACCGTAGCTGCAGATAAAAAATCGACGACCACATTGACCGTT
ACTGCGCACGATTCCGACGGAACTCCGGTGCCGGGGCTGGCGCTGCAAACCCGCAGTGAAGGCGTTCAGGATATCACCCT
GTCTGACTGGACAGATAACGGTGATGGTAGTTACACACAGATGCTGACCGCCGGAACGACATCAGGTTCAGTAACACTGA
CGCCGCAAATTAACGGTGAGAGTGCGGTAAAAGAATCCATTGTCGTTAATATCGTCCCTGTTGTCTCATCCCGCGACCAT
TCATCAATAACAATAGATAACGTATCGTATTATGCCGGAGACGACATCAAGGTTAGGGTGGAACTGAAAGACGACAGCAA
TCAACCGGTTGCATATCAAAAAGAGGAATTGGTAAAAGCCGTTACTGTCGAAAACAGCAAACCTGGCGCCACGATTGTCT
GGCACGAAGAGCAGCCTGGCGTTTATACCGCGAATTATCCGGCCCATAAGCAAGGGACAGCACTAAGGGCACAACTTAGC
CTTCACAACTGGAATGCTCCACTGCAATCGCATATTTATAACATTGAGGCAAACCAGAATAAGGCTCGCGTCGCCACATT
ATCAGCGACGAATAATGACGTTTACGCCGATAAAAAGACATTTAATACCCTCACGATCAACATCACTGATGAGAGTGATA
ATCCCCTGACAAATCATCAGGTCACCTTTAAGAATGAAAAAGGAAGCGCGGAGTTTGTTGAACCGCCGCAGCAAAATACG
GATGCATATGGTGTTGCCACAATCAACATGGTAAGTCAGGTTGCGGAAGAAAATACGATTAGCGCCACGCTGCCAAATGG
TTTCTCACAACGGATAATTGCGAAATTCGTTAGCGATTCGAGTACGCCAAAATTCAAACAACTGGTTGCCGATCCAGATA
CCATTATTGCTGGCAACAGCCAGGGCAGTACTCTGACCGCCACCGTCACAGACTTTCATAACAACCCATTAAAAGATATG
AAAGTGAATTTTGTGGCACCTGGTGGTTCGCAACTGGACAACACGACCGCCACAACAGACCAGTCCGGTATTGTGCGAGT
GCACCTGACCAGTTCAAAAGCTGGTAGCTATTCCGTCGATGCCTCGCTTGAGGCGGATAAAAATATTCACCAGTCGGTCA
CGATCACCGTGGTCCCAAACAGGGAACAATCGGTAATGACCTTAAATGCCGGGTCAGGCAGTGCGATCGCTAACAATACA
AATACCGTTATCCTGACAGCCAGTGTGAAAGATGTTTATGGACACCCGTTGCCGGATGAGGATGTGAAATTTACCTTGCC
AGCCTCTATGACCGGGAACTTCACGCTAAGTAGTGAAAGCGCCCGCACCGATGCAAACGGCGATGCCGTGGTCACATTGC
GTGGCACAAAAGCGGGTGAGTTTACAGTTACGGCGACGCTGACCAGAAATAATACCGTTGCTCATCAGCAAGTCACTTTT
ATTGGGGATACAAACAGCGCGCAGCTCCAGCCGCTGACTGCCTCATTAAATACCATTGTTGCGGGTAACAGTACGGGGAG
TACCCTGACTGCAACGATCCTGGACGCTTACCAAAATCCGCTTAAAGACCAGCTGGTCACTTTCCAGAGTAACGATGTCA
CTCTAAGTGGAACAGAAGTCACCACCAATACACTGGGTCAGGCGACGGTAACAATGACCAGCAATATTGCCGGGCAACAT
AACGTCGTGGTGAACCGGAAAGCGCAAGCCTCCGATAATAAAACGTTTAGTTTATCAGTGCTGCCGGATGAAAGTTCGGC
GAAGGTAATAAGTATAACCGGAGCCGAAAAAACGATAACGGTTGGCGAAAACATCACGCTACGGATACTCGTCCAGGACG
CGTTTAACAATGTAATAGCGGGTCAACGCGTCAGATTAAGTGCGCAGCCAACAGCTAACATTACGATAGGCGATACGGCT
TACACTGATAATAACGGTTACGCGTACGTTAACCTTCTCAGCACCCAGCCTGGGGTTTATCAGGTGACGGCAACGCTGGA
[truncated: 616,057 more chars]
